# Comparative transcriptome profiling uncovers a *Lilium regale* NAC transcription factor, *LrNAC35*, contributing to defence response against cucumber mosaic virus and tobacco mosaic virus

**DOI:** 10.1111/mpp.12868

**Published:** 2019-09-27

**Authors:** Daoyang Sun, Xinguo Zhang, Qingyu Zhang, Xiaotong Ji, Yong Jia, Hong Wang, Lixin Niu, Yanlong Zhang

**Affiliations:** ^1^ College of Landscape Architecture and Arts Northwest A&F University Yangling 712100 China; ^2^ State Agricultural Biotechnology Centre, School of Veterinary and Life Sciences Murdoch University Perth 6150 Australia; ^3^ Institute of Pomology/Jiangsu Key Laboratory for Horticultural Crop Genetic Improvement Jiangsu Academy of Agricultural Sciences Nanjing 210014 China

**Keywords:** *Cucumber mosaic virus*, lignin, *Lilium regale*, NAC transcription factor, petunia, *Tobacco mosaic virus*, transcriptome

## Abstract

Cucumber mosaic virus (CMV) is a highly prevalent viral pathogen causing substantial damage to the bulb and cut‐flower production of *Lilium* spp. Here, we performed an Illumina RNA sequencing (RNA‐Seq) study on the leaf tissues of a virus‐resistant species *Lilium regale* inoculated with mock control and CMV. A total of 1346 differentially expressed genes (DEGs) were identified in the leaves of *L. regale* upon CMV inoculation, which contained 34 up‐regulated and 40 down‐regulated DEGs that encode putative transcription factors (TFs). One up‐regulated TF, *LrNAC35,* belonging to the NAM/ATAF/CUC (NAC) superfamily, was selected for further functional characterization. Aside from CMV, lily mottle virus and lily symptomless virus infections provoked a striking increase in *LrNAC35* transcripts in both resistant and susceptible *Lilium* species. The treatments with low temperature and several stress‐related hormones activated *LrNAC35* expression, contrary to its reduced expression under salt stress. Ectopic overexpression of *LrNAC35* in petunia (*Petunia hybrida*) resulted in reduced susceptibility to CMV and *Tobacco mosaic virus* infections, and enhanced accumulation of lignin in the cell walls. Four lignin biosynthetic genes, including *PhC4H*, *Ph4CL*, *PhHCT* and *PhCCR*, were found to be up‐regulated in CMV‐infected petunia lines overexpressing *LrNAC35*. *In vivo* promoter‐binding tests showed that LrNAC35 specifically regulated the expression of *Ph4CL*. Taken together, our results suggest a positive role of transcriptome‐derived *LrNAC35* in transcriptional modulation of host defence against viral attack.

## Introduction

Viral pathogens are the major constraining factors for crop growth and production worldwide. They occur ubiquitously in various environmental conditions. Disease symptoms resulting from virus invasion often include chlorotic or necrotic leaves, shortened internodes, stunted upright growth and weakened vitality (Galvez *et al.*, 2014; Rojas *et al.*, 2007). Plants have evolved multiple defensive mechanisms against the threats posed by massive virus proliferation. The recognition of foreign virions by plants firstly stimulates the establishment of a basal defence system, which involves the participation of reactive oxygen species, cell wall structural components, and antiviral proteins and compounds (He *et al.*, 2007; van Loon *et al.*, 2006). If viruses break through the basal guard, a second‐line host defence called the innate immune response is activated (Liu *et al.*, 2004).

Numerous virus‐responsive genes have essential roles in the innate immunity system, such as the resistance (*R*) gene (Kang *et al.*, 2005), tobacco *N* gene (Marathe *et al.*, 2002; Whitham *et al.*, 1996), and the genes associated with RNA silencing (Agius *et al*., 2012; Sun *et al.*, 2016a) and translation suppression (Zorzatto *et al.*, 2015). The transcription of these genes is potentially controlled by specific transcription factors (TFs), of which many members of various families, including MYB (Yang and Klessig, 1996), zinc finger (Guo *et al.*, 2004), WRKY (Park *et al.*, 2006), AP2/ERF (Huang *et al.*, 2016), bZIP (Gaguancela *et al.*, 2016), bHLH (Aparicio and Pallás, 2017) and NAC (Huang *et al.*, 2017), have been revealed to be implicated in the response to viral stimuli. In particular, NAC domain‐containing proteins comprise a large TF family in plants and play essential roles in plant growth, development and stress responses (Puranik *et al.*, 2012). Approximately 105 and 75 NAC proteins are present in *Arabidopsis* and *Oryza sativa* genomes, respectively. Based on sequence similarity, NAC proteins from both species are classified into two large groups and 18 subgroups (Ooka *et al.*, 2003). These proteins share five highly conserved subdomains (A to E) within the N‐terminal region or a divergent C‐terminal domain (Hegedus *et al.*, 2003; Kikuchi *et al.*, 2000).

Multiple studies have demonstrated the critical functions of NAC TFs in abiotic stress resistance, such as drought (Tran *et al.*, 2004), salt (Balazadeh *et al.*, 2010) and low temperature (Hao *et al.*, 2011), as well as their disease resistance to fungal and bacterial pathogens, including *Botrytis cinerea*, *Pseudomonas syringae* and *Alternaria brassicicola* (Wang *et al.*, 2009). In addition, a number of studies have also reported the involvement of NAC TFs in plant response to virus infection. For instance, GRAB1 and GRAB2 interact with wheat dwarf geminivirus (WDV) RepA protein, and their expression in wheat cells restrains DNA replication of WDV (Xie *et al.*, 1999). *SlNAC1* overexpression causes an enhanced replication of tomato leaf curl virus by specifically binding to geminivirus replication enhancer (REn) protein (Selth *et al.*, 2005). The mutant of *rim1‐1*, a novel NAC gene, shows reduced susceptibility to rice dwarf virus, whereas its overexpression enhances virus multiplication (Yoshii *et al.*, 2009). Transgenic *Arabidopsis* plants overexpressing NAC TF gene *ATAF2* exhibit decreased proliferation of tobacco mosaic virus (TMV) and up‐regulation of some defence‐associated marker genes (Wang *et al.*, 2009). The interaction between NAC protein TIP and turnip crinkle virus coat protein interferes with the basal defence against virus invasion in *Arabidopsis* (Donze *et al.*, 2014). Another NAC protein, NAC083, interacts with mungbean yellow mosaic India virus replication initiator protein in *Arabidopsis*, but its biological role in virus resistance has not yet been examined (Suyal *et al.*, 2014). A recent study shows that six NAC genes differentially respond to tomato yellow leaf curl virus (TYLCV) infection in tomato plants, and among them virus‐induced gene silencing (VIGS) of *SlNAC61* leads to increased TYLCV accumulation (Huang *et al.*, 2017). These findings indicate that NAC TFs have a conserved biological role in virus resistance mostly through the interaction with viral proteins. However, little is known about the transcriptional regulation of virus‐responsive genes by NAC TFs in plants.

Lily belongs to the genus *Lilium* of the family Liliaceae. It is a summer‐blooming perennial bulbous plant with various food, aesthetic, medicinal and economic values (Wang *et al.*, 2016). The fleshy bulb scales are rich in nutrients and antioxidants (Jin *et al.*, 2012). Viral infection is a frequently occurring disease in lily plants, which affects bulb and cut‐flower production (Ram *et al*., 2000). More than 20 virus species have been reported to be capable of infecting *Lilium* and of these, *Cucumber mosaic virus* (CMV, genus *Cucumovirus*), *Lily mottle virus* (LMoV; genus *Potyvirus*) and *Lily symptomless virus* (LSV, genus *Carlavirus*) are most prevalent (Chinestra *et al.*, 2010). To cultivate new lily varieties with desirable antiviral characteristics, conventional crossbreeding or molecular genetic manipulation cannot be inseparable from the excellent wild germplasm resources. A representative lily species native to China, *Lilium regale*, enjoys a high reputation because of its broad‐spectrum resistance to abiotic stresses, fungi and viruses (Li *et al.*, 2014; Rao *et al.*, 2013). A couple of genes have been identified from *L. regale*, and one example is that *LrP5CS* overexpression confers increased tolerance of transgenic *Arabidopsis* plants to osmotic, drought and high salinity stresses (Wei *et al.*, 2016). With respect to virus resistance, our recent research shows the lowest viral disease incidence of *L. regale* among ten species tested under natural infection in the field (Sun *et al.*, 2016b). *L. regale*'s outstanding antiviral performance is also supported by our mechanical inoculation experiments in which CMV, LMoV and LSV could not elicit some visible symptoms, such as vein clearing, leaf mosaic, leaf curling, necrotic spots or hypersensitive response (HR), in *L. regale* initially. Only mild leaf curling symptom appeared in CMV‐inoculated *L. regale* leaves at the late stage of infection.

Considering the recalcitrance of lily plants to stable transformation, we employed petunia as a heterologous expression model system for studies of antiviral machinery in lilies. In previous work, we constructed a CMV‐induced *L. regale* cDNA library based on suppression subtractive hybridization (SSH), from which a gene termed *LrABCF1* was functionally determined to modulate the resistance to CMV and tobacco rattle virus (TRV) in transgenic petunia (Sun *et al.*, 2016b). However, SSH analysis can only generate a relatively small amount of differentially expressed transcripts. In this study, we used a high‐throughput Illumina RNA sequencing (RNA‐Seq) approach to further investigate the molecular basis of CMV resistance in *L. regale*. A NAC TF gene, annotated as *LrNAC35*, was selected from up‐regulated TFs based on transcriptome data and validated for its crucial role in virus (CMV and TMV) resistance by ectopic overexpression in petunia.

## Results

### Sampling, RNA sequencing and *de novo* assembly

**Figure 1:**
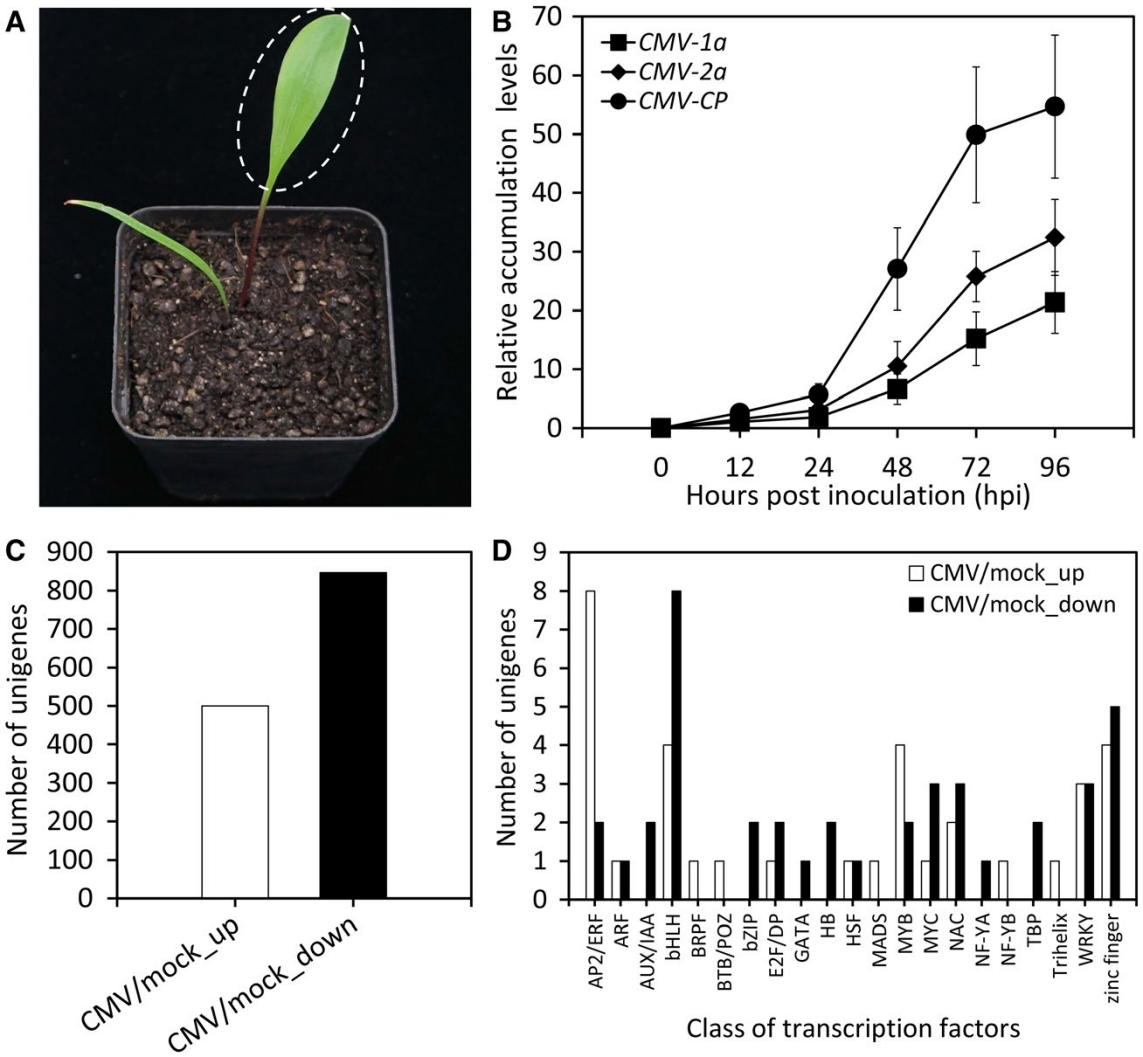
Differentially expressed transcripts in CMV‐infected *Lilium regale* leaves. (A) Representative growth phenotypes of two‐leaf‐stage *L. regale* seedlings propagated from seeds at 4 weeks post‐germination. The second newly sprouted leaves used for CMV inoculation and further RNA‐Seq are marked in a dashed circle. (B) qRT‐PCR analysis of CMV accumulation levels (*CMV‐1a*, *‐2a* and *‐CP*) in the inoculated leaves of *L. regale* seedlings at various hours post‐inoculation (hpi). Expression levels were standardized to *LrGAPDH*. Error bars represent standard error (SE) of the mean from three biological replicates. Number of differentially expressed genes (C) and transcription factors (D) in CMV‐infected *L. regale* leaves by comparison with mock. Up, up‐regulated; down, down‐regulated.

To investigate the host transcriptome response of *L. regale* to CMV infection, the second broad true leaves of seed‐grown plantlets were used for virus inoculation (Fig. 1A). Viral gene expression tests showed a moderate increase in transcripts of *CMV‐1a*, *‐2a* and *‐coat protein* (*CP*) during an initial 24 h of infection, followed by a rather sharp rise at 48 and 72 h post‐inoculation (hpi) (Fig. 1B). The sampling time of *L. regale* leaves for RNA extraction and sequencing, representing a surge of virus replication, was thus determined to be 48 hpi.

Illumina paired‐end sequencing in total generated 154 921 638 and 154 400 476 raw reads for the mock control and CMV‐infected *L. regale* libraries. Correspondingly, 151 810 642 and 150 595 506 clean data were obtained after trimming off the adaptor sequences, and ambiguous and low‐quality reads. *De novo* assembly resulted in a total of 115 826 unigenes. The maximum, minimum and mean lengths of the assembled unigenes are 11 418, 224 and 615.8 bp, respectively (Table 1). The sequences and functional annotation of all assembled unigenes are shown in Fig. S1 and Table S1 (see Supporting information). Their size distribution revealed that 75 601 (65.3%) unigenes ranged from 200 to 499 bp, 22 496 (19.4%) from 500 to 999 bp and 17 729 (15.3%) were more than 1000 bp (Fig. S2, see Supporting information). To corroborate the accuracy of RNA‐Seq data, nine unigenes, comprising one unknown and eight annotated transcripts, were randomly selected and analysed via quantitative real‐time PCR (qRT‐PCR). The qRT‐PCR results for the unigenes tested were consistent with the transcriptome data (Fig. S3, see Supporting information). The reliability of differential gene expression identified by RNA‐Seq was accordingly validated. Principal component analysis (PCA) of transcriptome data showed that principal components 1 and 2 explained 60.8% and 21% of the variance, respectively, and CMV‐infected samples were significantly different from the mock control (Fig. S4, see Supporting information).

**Table 1:** Summary of CMV‐infected *Lilium regale* transcriptome sequencing dataset

Item	Number
Raw reads from mock‐inoculated samples	154 921 638
Raw reads from CMV‐inoculated samples	154 400 476
Clean reads from mock‐inoculated samples	151 810 642
Clean reads from CMV‐inoculated samples	150 595 506
Total assembled unigenes	115 826
Total assembled bases	71 330 030
Maximum length (bp)	11 418
Minimum length (bp)	224
Average length (bp)	615.8
Unigenes against NR database	43 064
Unigenes against Swiss‐Prot database	36 468
Unigenes against COG database	32 211
Unigenes against KEGG database	14 641
Annotated unigenes	47 307
Unannotated unigenes	68 519

### Detection of differentially expressed genes during CMV infection

Comparative transcriptome analysis of mock control and CMV‐infected *L. regale* leaves generated 1346 differentially expressed genes (DEGs). Of all DEGs, 500 unigenes were significantly up‐regulated in CMV‐infected leaves, while 846 unigenes were down‐regulated (Fig. 1C and Table S2, see Supporting information). Gene Ontology (GO) enrichment analysis showed that 663, 418 and 158 DEGs were assigned to three functional categories: biological process, cellular component and molecular function, respectively. The largest number of enriched genes fell into the metabolic process, response to stimulus and single‐organism process (Fig. S5, see Supporting information). We further implemented a pathway‐based categorization of DEGs by searching against the Kyoto Encyclopedia of Genes and Genomes (KEGG) database and found that 219 DEGs were mapped to 58 biochemical pathways. Among these mapped pathways, the metabolic pathway contained the largest number of DEGs (36), followed by secondary metabolites biosynthesis (28) and plant–pathogen interaction (22) pathways (Table S3, see Supporting information).

### Candidate transcriptional regulators involved in response to CMV

**Figure 2:**
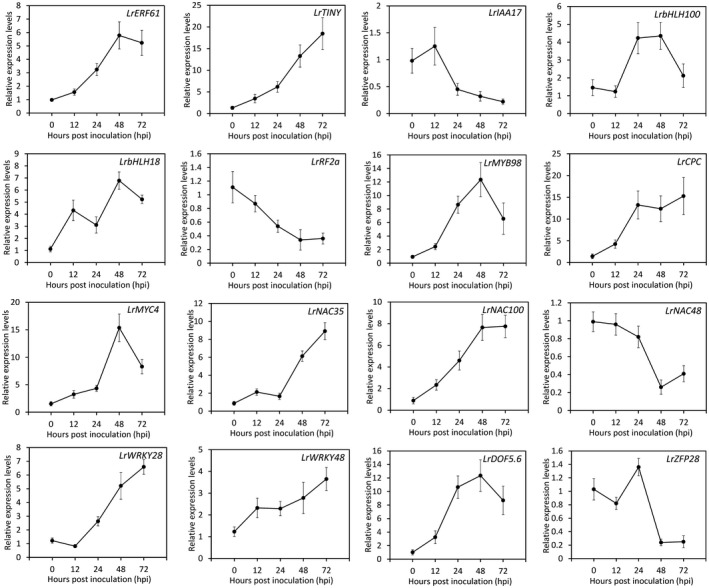
Expression of candidate transcription factors (TFs) associated with *Lilium regale* defence against CMV infection. Sixteen unigenes, classified into nine families, were chosen from up‐ or down‐regulated TFs and evaluated by qRT‐PCR at given time points. Transcript abundances were normalized to *LrActin*. SE of the mean from three biological replicates is shown as error bars.

To understand the transcriptional modulation mechanism of CMV defence in *L. regale*, 74 DEGs encoding putative TFs, of which 34 were up‐regulated and 40 were down‐regulated, were obtained based on gene annotation. These TFs could be classified into 21 TF families, with a considerable number of genes grouped into the bHLH, AP2/ERF, zinc finger, MYB, WRKY and NAC families (Fig. 1D and Table S4, see Supporting information). Fifty‐six DEGs covering the nine TF families that are most probably correlated with the virus response of *L. regale* plants were identified (Table 2). Next, a time‐course expression profile analysis of selected candidate TF genes was conducted. The results show that CMV inoculation leads to a continuous increase in transcript abundances of *LrERF61*, *LrTINY*, *LrCPC*, *LrNAC35*, *LrNAC100*, *LrWRKY28*, *LrWRKY48* and *LrDOF5.6*, and reduction in *LrIAA17*, *LrRF2a* and *LrNAC48* expression levels throughout the infection process up to 72 hpi. An increase followed by a drop occurred when examining the expression of *LrbHLH100*, *LrbHLH18*, *LrMYB98*, *LrMYC4* and *LrZFP28* (Fig. 2).

**Table 2:** Putative transcription factors associated with the defence response of *Lilium regale* to CMV infection

Gene ID	Annotation	RPKM		Log_2_ (CMV/mock)
		Mock	CMV	
*AP2/ERF family*				
Unigene0000389	Ethylene‐responsive transcription factor ERF061‐like	0.362	2.026	2.486
Unigene0000390	Ethylene‐responsive transcription factor ERF061‐like	0.371	2.501	2.752
Unigene0014641	Ethylene‐responsive transcription factor ERF061‐like	0.169	0.957	2.503
Unigene0052014	Ethylene‐responsive transcription factor ERF061‐like	0.140	0.580	2.047
Unigene0052016	Ethylene‐responsive transcription factor ERF061‐like	0.401	1.947	2.281
Unigene0072729	Ethylene‐responsive transcription factor ERF061‐like	0.001	0.8321	9.701
Unigene0073936	Ethylene‐responsive transcription factor TINY‐like	0.001	0.7314	9.515
Unigene0089226	Ethylene‐responsive transcription factor ERF061‐like	0.038	1.575	5.377
Unigene0020351	Ethylene‐responsive transcription factor PLT2‐like	6.224	0.983	−2.662
Unigene0037049	Ethylene‐responsive transcription factor ERF012‐like	0.644	0.001	−9.331
*AUX/IAA family*				
Unigene0090221	AUX/IAA transcriptional regulator family protein	0.979	0.203	−2.272
Unigene0108320	Auxin‐responsive protein IAA17‐like	129.788	48.931	−1.407
*bHLH family*				
Unigene0015592	Transcription factor ABA‐inducible bHLH‐type‐like	0.001	0.495	8.951
Unigene0038912	Transcription factor bHLH100‐like	1.341	6.279	2.227
Unigene0038913	Transcription factor bHLH100‐like	0.727	4.409	2.600
Unigene0111737	Transcription factor bHLH18‐like	4.817	25.620	2.411
Unigene0009308	Transcription factor bHLH75‐like	1.880	0.045	−5.395
Unigene0029409	Transcription factor PAR1‐like	0.414	0.050	−3.064
Unigene0031740	Transcription factor bHLH137‐like	1.508	0.020	−6.255
Unigene0038757	Transcription factor bHLH35‐like	0.832	0.094	−3.141
Unigene0042758	BHLH family transcriptional factor	6.980	1.631	−2.098
Unigene0051700	Transcription factor bHLH35‐like	0.223	0.001	−7.802
Unigene0059321	Transcription factor bHLH30‐like	1.232	0.431	−1.514
Unigene0011609	BHLH family protein	1.397	0.134	−3.379
*bZIP family*				
Unigene0010955	Transcription factor RF2a‐like	5.961	0.724	−3.041
Unigene0027495	Bzip‐like transcription factor‐like protein	1.748	0.459	−1.928
*MYB family*				
Unigene0003524	Transcription factor MYB98‐like	0.139	2.429	4.123
Unigene0006408	Transcription factor RL9	0.122	0.408	1.736
Unigene0007577	MYB‐related protein Zm1‐like	0.226	1.360	2.592
Unigene0054070	Transcription factor CPC‐like	0.604	6.966	3.527
Unigene0003522	Transcription factor MYB98‐like	0.335	0.001	−8.386
Unigene0097039	Transcription factor R2R3‐MYB	1.876	0.135	−3.800
*MYC family*				
Unigene0053370	Transcription factor MYC4‐like	0.001	3.317	11.696
Unigene0076682	Transcription factor ICE1‐like	0.956	0.051	−4.237
Unigene0105852	Transcription factor MYC2‐like	5.815	0.096	−5.917
Unigene0111554	Transcription factor MYC4‐like	5.449	0.796	−2.776
*NAC family*				
Unigene0012070	NAC domain‐containing protein 35‐like	5.611	38.013	2.760
Unigene0025824	NAC domain‐containing protein 100‐like	0.088	0.558	2.656
Unigene0050073	NAC domain‐containing protein 90‐like	1.611	0.112	−3.718
Unigene0093619	NAC domain‐containing protein 21/22‐like	3.284	0.446	−2.881
Unigene0112474	NAC domain‐containing protein 48‐like	8.865	2.983	−1.571
*WRKY family*				
Unigene0001022	WRKY transcription factor 28	0.385	1.892	2.296
Unigene0039394	WRKY transcription factor 28	1.759	12.578	2.838
Unigene0104357	WRKY transcription factor 48	14.561	34.974	1.264
Unigene0042962	WRKY family protein	5.930	2.080	−1.512
Unigene0051891	WRKY transcription factor 53	4.355	0.411	−3.404
Unigene0078584	WRKY transcription factor 51	0.665	0.001	−9.377
*Zinc finger family*				
Unigene0071735	Zinc finger protein WIP3‐like	0.429	1.637	1.932
Unigene0076256	Zinc finger protein RICESLEEPER 2‐like	0.723	2.298	1.668
Unigene0109816	Zinc finger protein ZAT11	0.001	1.402	10.453
Unigene0111263	Dof zinc finger protein DOF5.6‐like	0.240	2.770	3.530
Unigene0018640	Zinc finger protein 7	1.513	0.121	−3.645
Unigene0092120	Zinc finger CCCH domain‐containing protein 28‐like	1.094	0.158	−2.792
Unigene0093967	Zinc finger protein ZNFphex133	0.713	0.151	−2.239
Unigene0027578	RING‐H2 zinc finger protein ATL79‐like	32.849	12.804	−1.359
Unigene0060238	RING‐H2 zinc finger protein ATL3‐like	1.615	0.010	−7.357

### Identification of *LrNAC35*

**Figure 3:**
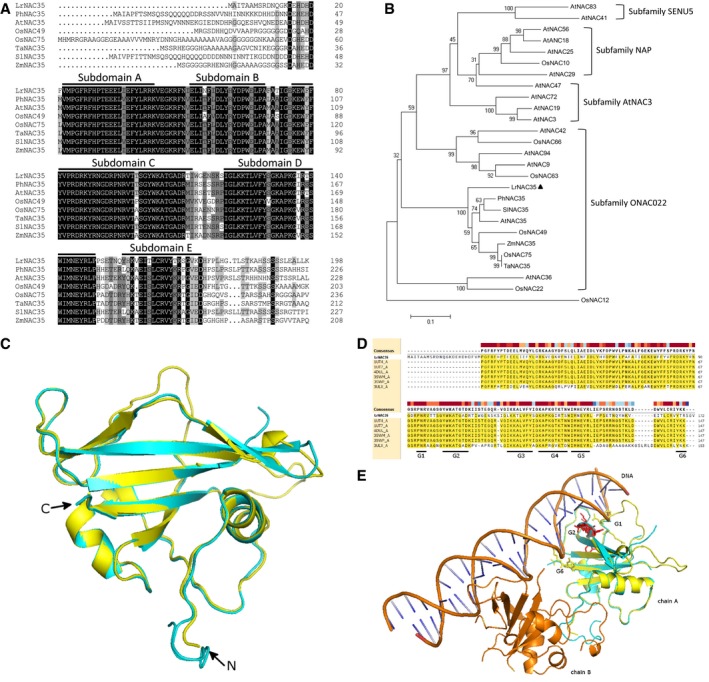
Sequence analysis of LrNAC35 from *Lilium regale*. (A) Alignment of conserved regions of deduced LrNAC35 amino acid sequence with similar proteins, including *Petunia hybrida* PhNAC35 (GBRU01060495), *Arabidopsis thaliana* AtNAC35 (AtLOV1, AT2G02450), *Oryza sativa* OsNAC49 (AJO53625) and OsNAC75 (XP_015631974), *Triticum aestivum* TaNAC35 (CDM85391), *Solanum lycopersicum* SlNAC35 (XP_004230395) and *Zea mays* ZmNAC35 (PWZ31007). Solid lines indicate the conserved subdomains A to E. (B) Phylogenetic analysis of LrNAC35 with the aligned proteins above and other similar proteins including AtNAC3 (At1g02220), AtNAC9 (At1g26870), AtNAC18 (At1g52880), AtNAC19 (At1g52890), AtNAC25 (At1g61110), AtNAC29 (At1g69490), AtNAC36 (At2g17040), AtNAC41 (At2g33480), AtNAC42 (At2g43000), AtNAC47 (At3g04070), AtNAC56 (At3g15510), AtNAC72 (At4g27410), AtNAC83 (At5g13180), AtNAC94 (At5g39820), OsNAC10 (XP_015645677), OsNAC22 (XP_015630174), OsNAC63 (XP_015649454) and OsNAC66 (XP_015628846). LrNAC35 is highlighted by a black triangle. OsNAC12 (EEC79300) belonging to subfamily TERN of NAC proteins served as the out‐group. Boot‐strap values are expressed as a percentage of 1000 replicates and shown at branch nodes. (C) Superimposition of LrNAC35 (cyan) with AtNAC19 (3SWM chain A, yellow). (D) Amino acid sequence alignment of LrNAC35 with the identified homologous protein templates. G1–G6 represents the identified DNA‐interacting residue groups in AtNAC19. (E) The potential DNA‐binding interaction of LrNAC35 (cyan) in superimposition with chain A (yellow) of AtNAC19 (3SWM). The spatial position of monomer chain B (orange) is also displayed. The side chains of the corresponding G1, G2 (red) and G6 residues in LrNAC35 are shown as sticks.

We selected *LrNAC35* (GenBank accession no. MK805884) from the expression‐quantified TF genes above for further functional characterization. The full length of *LrNAC35* cDNA includes a complete coding region encoding 358 amino acids (Fig. S6, see Supporting information), possessing five subdomains A to E. Alignment and the phylogenetic tree showed that LrNAC35 has high homology to NAC49 and NAC75 from *O. sativa*, and NAC35 proteins from *Petunia hybrida*, *Arabidopsis thaliana*, *Triticum aestivum*, *Solanum lycopersicum* and *Zea mays* (Fig. 3A,B).

To investigate the protein structural features of LrNAC35, particularly its DNA‐binding potential, homology‐based protein structural modelling was performed for the NAC DNA‐binding domain. A combination of multiple homologous protein structures (chain A of Protein Data Banks (PDBs) 1UT4, 1UT7, 4DUL, 3SWM, SWP and 3ULX) was identified and used as a template for protein modelling. As shown in Fig. 3C, LrNAC35 harbours a conserved NAC domain (N‐terminal) and a divergent transcription regulatory domain (C‐terminal). The NAC domain of LrNAC35 adopts the typical NAC protein fold, which mainly consists of beta‐sheets. Based on the report on the structure of *Arabidopsis* AtNAC19 bound with DNA (PDB 3SWM) (Welner *et al.*, 2012), six residue groups (G1–G6) have been suggested to have DNA‐binding potential (Fig. 3D). The spatial positions of the corresponding G1, G2 and G6 in LrNAC35 were displayed in superimposition with the chain A of AtNAC19 (PDB 3SWM). The protein model of LrNAC35 resembles the spatial coordination of AtNAC19. The side chains of the residues in G1, G2 and G6 are in close proximity to the bound DNA molecule. Each of these three residue groups inserts into the adjacent grooves of the DNA, similar as that observed for AtNAC19 (Fig. 3E).

### Biotic, abiotic stresses and hormone treatments alter *LrNAC35* expression

**Figure 4:**
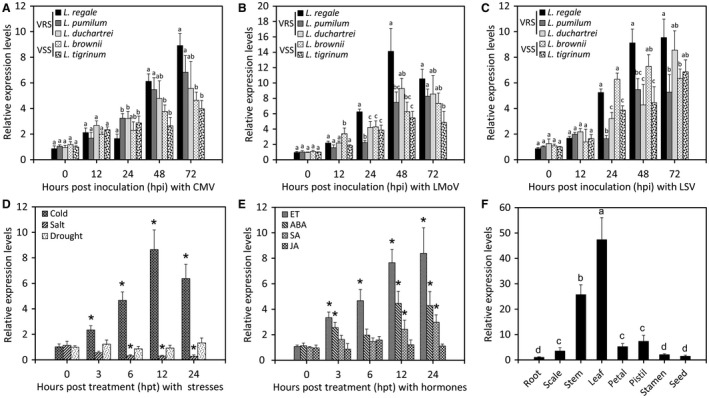
Expression of *LrNAC35* in lily leaves under virus infection, abiotic stress and hormone treatments or in different tissues. qRT‐PCR analysis of *LrNAC35* transcript levels in the leaves of five wild *Lilium* species at different hours post‐inoculation (hpi) with CMV (A), LMoV (B) and LSV (C). VRS, virus‐resistant species; VSS, virus‐susceptible species. qRT‐PCR analysis of *LrNAC35* transcript levels in *L. regale* leaves treated with abiotic stressors (4 °C, 150 mM NaCl and dehydration) (D) and stress‐related hormones (15 μL/L ethylene (ET), 50 μM abscisic acid (ABA), 100 μM salicylic acid (SA) and 100 μM jasmonic acid (JA)) (E) at indicated time points. (F) Tissue‐specific expression of *LrNAC35* in various tissues of *L. regale* plants by qRT‐PCR. Six‐week‐old plantlets of bulb‐propagated wild *Lilium* species were used for virus inoculation, abiotic stress and hormone treatments. Different tissues of *L. regale* propagated from bulbs were collected at 12 or 16 weeks post‐germination. *LrActin* was used as an internal control. Error bars represent SE of the mean from three biological replicates. Asterisks or different letters at the top of columns indicate statistical significance as determined by Student's *t*‐test or one‐way ANOVA test at *P* < 0.05, respectively.

To assess the expression patterns of *LrNAC35* during multiple plant–virus interactions, additional virus infection tests using CMV, LMoV and LSV were carried out in five wild *Lilium* species, including virus‐resistant species *L. regale*, *L. pumilum* and *L. duchartrei*, and susceptible species *L. brownii* and *L. tigrinum* (Sun *et al.*, 2016b). The overall expression levels of *LrNAC35* increased dramatically in all species tested when challenged with three viruses, and in particular *L. regale* displayed the maximum abundances at 48 or 72 hpi (Fig. 4A–C). CMV infection resulted in significantly higher transcript levels of *LrNAC35* in *L. regale*, *L. pumilum* not *L. duchartrei* than in two susceptible species at 48 or 72 hpi (Fig. 4A), while only *L. regale* exhibited higher *LrNAC35* expression than the susceptible ones at 24 or 48 hpi with LMoV (Fig. 4B). A different expression profile was observed after inoculation with LSV, showing that *LrNAC35* transcripts in the susceptible species *L. brownii* surpassed those in the resistant species *L. pumilum* or *L. duchartrei* at 24 or 48 hpi (Fig. 4C).

For treatment with abiotic stresses and plant growth regulators, *LrNAC35* transcripts increased markedly at low temperature but reduced under high salinity (Fig. 4D). A pronounced elevation in transcript levels of *LrNAC35* occurred following treatments with ethylene (ET) and abscisic acid (ABA), while a moderate and delayed up‐regulation was observed upon exposure to salicylic acid (SA) (Fig. 4E). No significant change in the expression of *LrNAC35* was found under dehydration and jasmonic acid (JA) treatments (Fig. 4D,E). Furthermore, *LrNAC35* was constitutively expressed in stems and leaves with higher abundances than in roots, scales, seeds and floral tissues (Fig. 4F).

### Ectopic overexpression of *LrNAC35* alters flowering time in petunia

**Figure 5:**
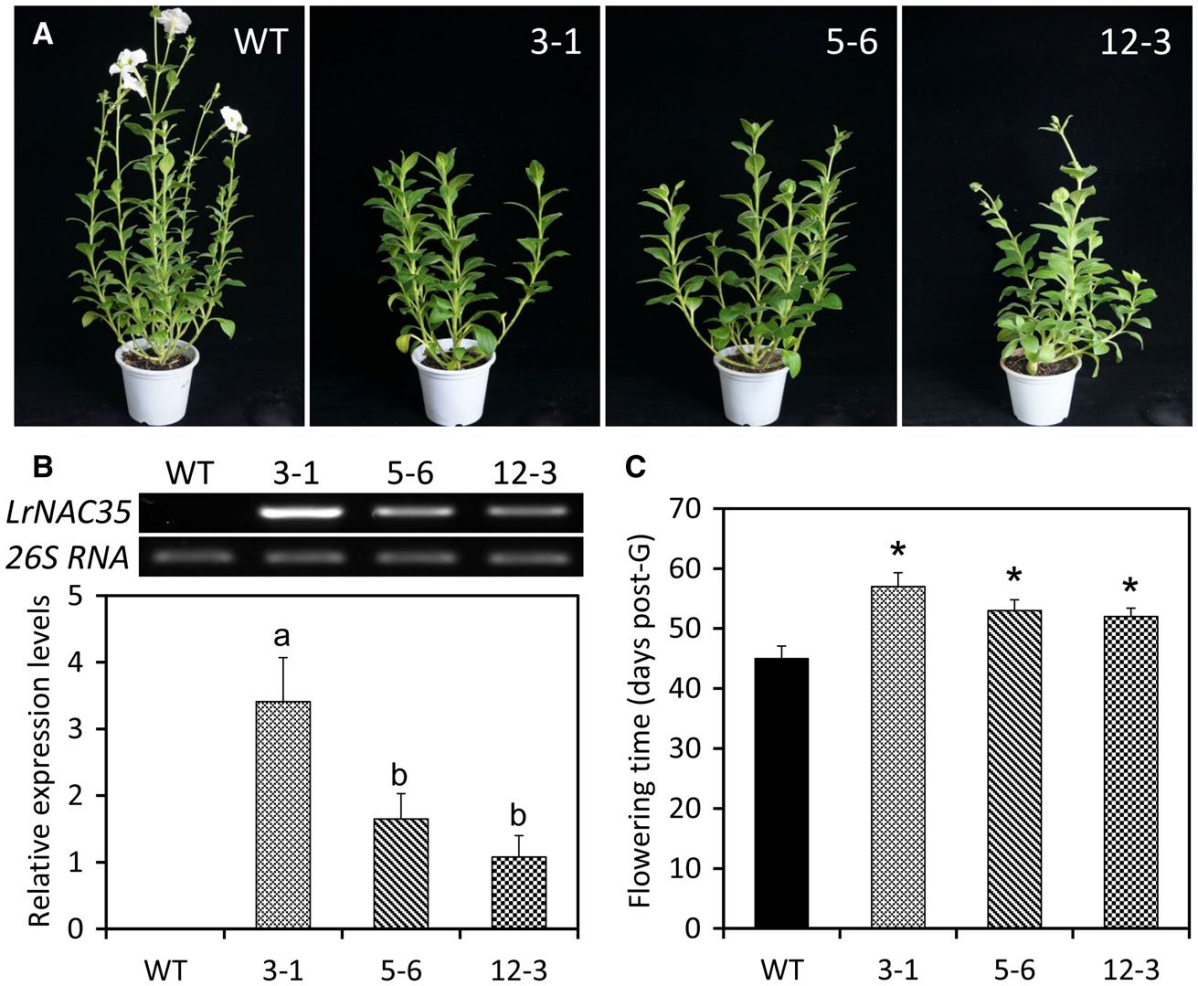
Impact of *LrNAC35* overexpression on flowering time of transgenic petunia plants. (A) Representative growth phenotypes of wild‐type (WT) and *LrNAC35*‐overexpressing lines (3‐1, 5‐6 and 12‐3) at 8 weeks post‐germination. (B) Semiquantitative RT‐PCR and qRT‐PCR analyses of *LrNAC35* transcript abundances in the leaves of WT and transgenic petunia lines overexpressing *LrNAC35*. *26S rRNA* was used as a normalization control. Error bars represent SE of the mean from three biological replicates. Significance of difference was evaluated using one‐way ANOVA test (*P* < 0.05) and shown as various letters. (C) Flowering time of transgenic petunia plants overexpressing *LrNAC35* post‐germination (post‐G) compared to WT plants. Five independent plants for each line were used for the analysis of flowering time. Asterisks denote significant difference based on calculation by Student's *t*‐test at *P* < 0.05.

To elucidate the possible function of *LrNAC35*, we performed a genetic transformation assay in petunia, which is a common model platform for molecular research of floral crops. *LrNAC35* was ectopically overexpressed under the control of the CaMV 35S promoter in petunia. Three transgenic lines (3‐1, 5‐6 and 12‐3) with *LrNAC35* overexpression showed delayed flowering phenotypes compared with wild‐type (WT) plants (Fig. 5A,C). A dual expression analysis using semiquantitative and quantitative methods confirmed the substantial transcription of *LrNAC35* in transgenic lines (Fig. 5B).

### *LrNAC35* affects susceptibility to CMV

**Figure 6:**
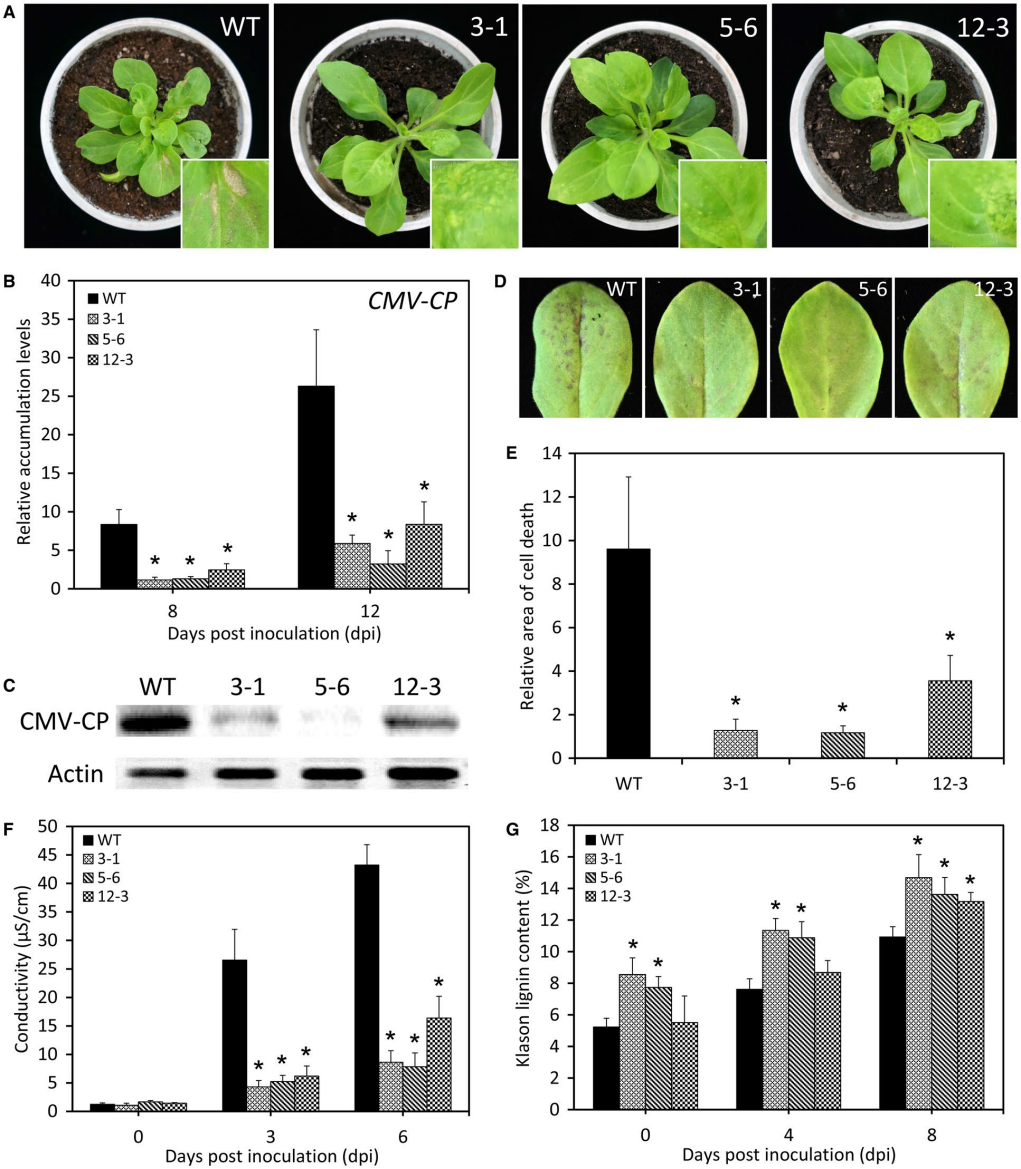
Increased resistance to CMV infection in transgenic petunia plants overexpressing *LrNAC35*. (A) Disease symptoms of wild‐type (WT) and *LrNAC35*‐overexpressing lines (3‐1, 5‐6 and 12‐3) at 18 days post‐inoculation (dpi) with CMV. The magnified views of symptoms in systemically infected leaves with CMV are indicated as the insets. Four‐leaf‐stage seedlings were used for inoculation. qRT‐PCR (B) and western blot (C) analyses of *CMV coat protein* (*CMV‐CP*) transcript and its protein levels in the uppermost leaves of WT and overexpression lines at 8 or 12 dpi with CMV, and the leaf samples at 12 dpi were used for western blots. *26S rRNA* and actin were used as a reference gene and protein, respectively. Disease symptoms (D) and relative cell death area (E) of inoculated leaves of WT and *LrNAC35*‐overexpressing lines (3‐1, 5‐6 and 12‐3) at 6 dpi with CMV. (F) Electrolyte leakage (conductivity) in the inoculated leaves of WT and transgenic lines challenged with CMV at intervals. (G) Klason lignin content in CMV‐inoculated leaves of WT and transgenic lines at different time points. Error bars represent SE of the mean from three biological replicates. Asterisks indicate significant difference as evaluated by Student's *t*‐test at *P* < 0.05.

In view of *LrNAC35*'s prominent induction when viruses invade, the function of *LrNAC35* in the defence response against CMV was investigated. Both WT and transgenic lines were inoculated with CMV. At 18 days post‐inoculation (dpi), *LrNAC35*‐overexpressing transgenic petunia lines showed milder leaf distortion and necrosis than WT plants (Fig. 6A). Accumulation of CMV‐CP at both transcript and protein levels was reduced in systemically infected leaves of overexpression lines compared with the control at 8 or 12 dpi (Fig. 6B,C). In the inoculated leaves, CMV infection elicited a higher level of HR‐like cell death in WT lines than that in transgenic lines overexpressing *LrNAC35*, where relatively smaller lesion areas were observed (Fig. 6D,E). Transgenic lines generated significantly lower levels of electrolyte leakage than the WT control after CMV inoculation (Fig. 6F). There was a notable increase in Klason lignin content in the CMV‐inoculated leaves of three overexpression lines compared to WT lines (Fig. 6G).

For functional validation of *LrNAC35*, we selected a petunia orthologue of *LrNAC35*, designated *PhNAC35*, to perform TRV‐based virus‐induced gene silencing (VIGS) assay. Petunia leaves infiltrated with TRV empty vector or TRV‐*PhNAC35* were subsequently inoculated with CMV, and the mock control was used to differentiate the symptoms triggered by TRV vector and CMV. *PhNAC35* transcripts were markedly down‐regulated in TRV‐*PhNAC35*‐infected petunia leaves. We observed more severe symptoms in CMV‐infected petunia plants with *PhNAC35* silencing. *CMV‐CP* transcript and protein levels were significantly higher in *PhNAC35*‐silenced petunia leaves than those in non‐silenced ones (Fig. S7A–D, see Supporting information), suggesting that *PhNAC35* silencing caused a compromised resistance to CMV.

### *LrNAC35* modulates expression of lignin biosynthesis‐related genes

**Figure 7:**
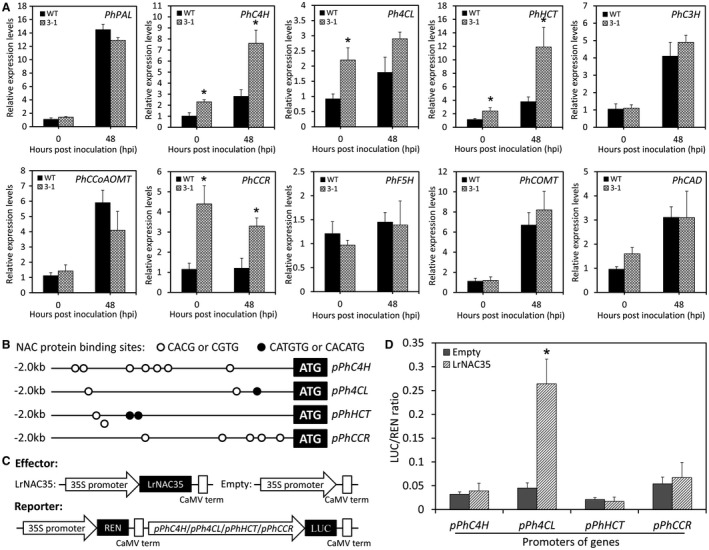
Involvement of LrNAC35 in the lignin biosynthesis pathway. (A) qRT‐PCR analysis of lignin biosynthesis‐associated genes, including *PhPAL*, *PhC4H*, *Ph4CL*, *PhHCT*, *PhC3H*, *PhCCoAOMT*, *PhCCR*, *PhF5H*, *PhCOMT* and *PhCAD*, in the leaves of wild‐type (WT) and *LrNAC35*‐overexpressing transgenic petunia line (3‐1) at 0 or 48 h post‐inoculation (hpi) with CMV. Expression levels were standardized to *26S rRNA.* (B) Graphic representation of petunia *PhC4H*, *Ph4CL*, *PhHCT* and *PhCCR* promoters (*pPhC4H*, *pPh4CL*, *pPhHCT* and *pPhCCR*) with 2.0 kb region upstream of their coding sequences. Hollow and black circles denote two different NAC protein binding sites. (C) Schematic diagrams of the effector and reporter constructs for dual luciferase assay. REN, *Renilla* luciferase; LUC, firefly luciferase. (D) Dual luciferase assay of the *pPhC4H*, *pPh4CL*, *pPhHCT* and *pPhCCR*. The activation was expressed as a LUC/REN ratio. Error bars indicate SE of the mean from three biological replicates. Statistical significance was determined using Student's *t*‐test (*P* < 0.05) and shown as asterisks.

Lignification creates a non‐degradable mechanical barrier to hinder pathogen spread within the host tissues (Moura *et al.*, 2010). To verify if *LrNAC35* can regulate lignin biosynthesis, the expression of the core genes in the lignin biosynthetic pathway were examined. Transgenic petunia lines with *LrNAC35* overexpression showed higher transcript abundances of *cinnamate 4‐hydroxylase* (*PhC4H*), *4‐coumarate: CoA ligase* (*Ph4CL*), *hydroxycinnamoyl CoA: quinate*/*shikimate hydroxycinnamoyl transferase* (*PhHCT*) and *cinnamoyl*‐*CoA reductase* (*PhCCR*) than WT plants at 0 and 48 hpi with CMV. The expression levels of the other structural genes, such as *phenylalanine ammonia*‐*lyase* (*PhPAL*), *coumaroyl*‐*quinate*/*shikimate 3‐hydroxylase* (*PhC3H*), *caffeoyl CoA O‐methyltransferase* (*PhCCoAOMT*), *ferulate 5*‐*hydroxylase* (*PhF5H*), *caffeic acid O‐methyltransferase* (*PhCOMT*) and *cinnamyl alcohol dehydrogenase* (*PhCAD*), remained unchanged between WT and overexpression lines during CMV infection (Fig. 7A).

To investigate the possible transactivation of *PhC4H*, *Ph4CL*, *PhHCT* and *PhCCR* promoters by LrNAC35, the promoter binding sites of these genes were predicted. Two types of NAC protein binding sites with the core CACG and CATGTG (or CGTG and CACATG in the opposite strand, respectively) elements were found in the 2.0 kb promoter regions upstream of their coding sequences (Fig. 7B). Based on the effector and reporter constructs (Fig. 7C), a dual luciferase assay was performed to examine the potential interaction of LrNAC35 with *PhC4H*, *Ph4CL*, *PhHCT* and *PhCCR* promoters in petunia leaves. The co‐expression of *35S*::*LrNAC35* with *pPh4CL*::*LUC* led to a 5.9‐fold rise in firefly luciferase (LUC) activity, whereas no significant change in LUC activity was detected for the *pPhC4H*/*pPhHCT*/*pPhCCR*::*LUC* constructs (Fig. 7D).

### *LrNAC35* affects susceptibility to TMV

**Figure 8:**
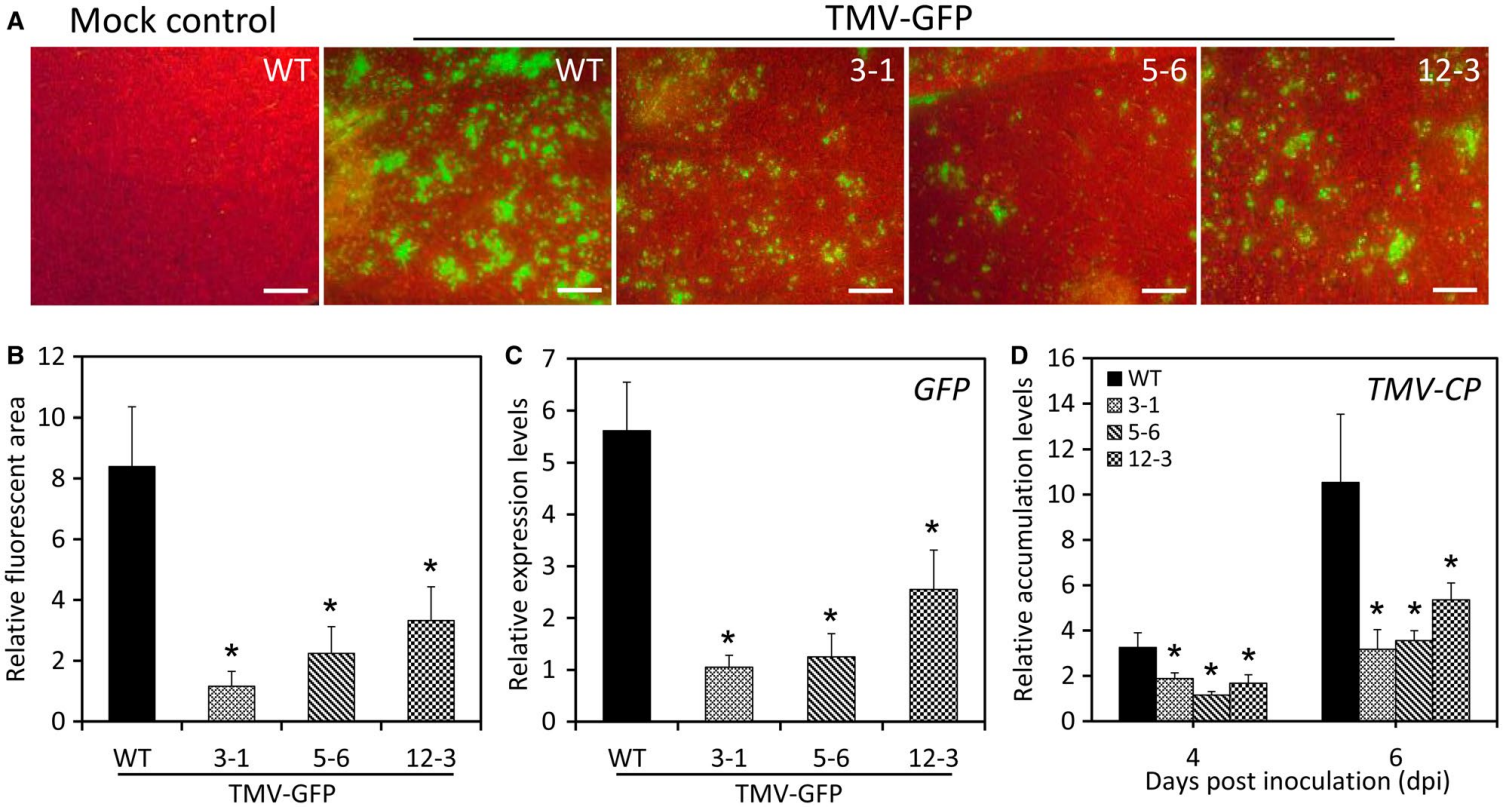
Enhanced resistance to TMV infection in *LrNAC35*‐overexpressing transgenic petunia plants. GFP fluorescent foci (A) and relative fluorescent area (B) in the inoculated leaves of wild‐type (WT) and transgenic petunia lines (3‐1, 5‐6 and 12‐3) at 6 days post‐inoculation (dpi) with *Agrobacterium* bearing no TMV vector (mock control) or TMV‐GFP. Photographs were taken under UV light. Scale bars = 2.0 mm. qRT‐PCR analysis of transcripts of *GFP* (C) and *TMV‐CP* (D) encoding TMV coat protein in the leaves of WT and transgenic plants at 4 or 6 dpi with TMV‐GFP, and the samples at 6 dpi were used for assessment of GFP expression. Error bars represent SE of the mean from three biological replicates. Asterisks denote significant difference as calculated by Student's *t*‐test at *P* < 0.05.

To determine whether *LrNAC35* participates in the response to other viruses, a TMV vector expressing green fluorescent protein (TMV‐GFP) was used to inoculate the transgenic and WT petunia lines. Under UV irradiation, the *LrNAC35‐*overexpressing lines had visibly smaller sizes of fluorescent foci than the WT lines at 6 dpi (Fig. 8A,B). This fluorescence variation was consistent with the measured transcript levels of GFP, which were decreased significantly in transgenic petunia lines compared to WT lines (Fig. 8C). At 4 and 6 dpi, the petunia plants overexpressing *LrNAC35* accumulated lower abundances of *TMV‐CP* transcripts than the WT plants (Fig. 8D).

To further determine the impact of *PhNAC35* silencing on the defence response to TMV, TRV empty vector‐ and TRV‐*PhNAC35*‐infected petunia leaves were challenged with TMV‐GFP. A clearly enlarged fluorescent area and increased accumulation of *TMV‐CP* were found in *PhNAC35*‐silenced petunia leaves compared to empty vector control (Fig. S7E,F, see Supporting information).

## Discussion

The wild lily species *L. regale* displays exceptional resistance to various biotic stress factors and has drawn extensive attention from lily breeders and plant biologists. Interspecific hybridization between *L. regale* and *L. rubellum* (Niimi *et al.*, 1996) or *L. nobilissimum* (Obata *et al.*, 2000) has been performed with attempts to introduce resistance traits of *L. regale* to other species sensitive to fungi or viruses. The *L. regale* × *L. nobilissimum* hybrids show some resistance to fungal disease, including *Fusarium* and *Botrytis*. Analogously, Lim *et al. *(2003) found that *L. regale* is highly resistant to *Fusarium oxysporum*. In recent reports, differentially expressed transcripts in *L. regale* response to *F. oxysporum* have been identified using the SSH method (Rao *et al.*, 2013). Transcriptome‐wide identification has also been performed to characterize the microRNAs (Gao *et al.*, 2017) and genes (Cui *et al.*, 2018a) with significantly changed expression in *Botrytis elliptica*‐infected *L. regale*, and two TFs, *LrWRKY4* and *LrWRKY12*, have been characterized as important regulators of resistance to *B. cinerea* (Cui *et al.*, 2018b). In this study, we employed a comparative transcriptome approach to dissect the antiviral molecular mechanism in *L. regale*.

We screened the DEGs encoding putative TFs in CMV‐infected *L. regale* leaves. One NAC TF gene, *LrNAC35,* was identified to be consistently and significantly up‐regulated upon virus infection. Protein modelling analysis revealed that three residue groups (G1, G2 and G6) of LrNAC35 within the NAC domain seem to be associated with potential DNA binding (Fig. 3E). As reported by Welner *et al. *(2012), G2 has been suggested to be related to DNA binding specificity recognition, whilst G1 and G6 may affect the binding affinity. The results also showed a great resemblance with AtNAC19 in DNA binding, suggesting that the NAC domain is highly conserved in LrNAC35 in comparison with other NAC proteins. LrNAC35 may adopt a similar DNA‐binding mechanism with AtNAC19.

LrNAC35 belongs to the ONAC022 subgroup of the NAC family in plants (Fig. 3B). Several members of the ONAC022 subgroup have been linked with responses to fungal or viral stimuli. For example, *AtNAC94* transcripts accumulate more at fungus‐infected sites with hypersensitive cell death in *Arabidopsis* (Michel *et al.*, 2006). *Arabidopsis* rosette leaves infected with cabbage leaf curl virus display elevated abundances of *AtNAC36* and *AtNAC42* (Ascencio‐Ibánez *et al.*, 2008). Our RNA‐Seq and qRT‐PCR analyses based on CMV infection tests showed that the up‐regulation of *LrNAC35* is probably associated with CMV resistance in *L. regale* (Table 2 and Fig. 4A).

*LrNAC35* transcripts also increased significantly in all resistant or susceptible lily species infected with two other common lily viruses, LMoV and LSV. It is particularly interesting that the expression of *LrNAC35* appears not to be in complete accord with virus‐resistant levels of five species when challenged with LSV (Fig. 4C). This may be explained by the potential genetic variations in *LrNAC35* across different lily species. This hypothesis is supported by several previous studies in which plant sensitivity to viral diseases has been associated with a single‐nucleotide or amino acid polymorphism in the defensive gene or protein (Lee *et al.*, 2010; Ling *et al.*, 2009; Wang *et al.*, 2011). Thus, we performed sequence analysis of *LrNAC35* by amplifying its complete coding sequences and translating the nucleotides to amino acids. Sequence alignment verified that *L. brownii* and *L. tigrinum* had four and one amino acid variations in the amino acid sequences of LrNAC35, respectively, compared to three resistant species (Fig. S8A, see Supporting information). *L. brownii* also exhibited the most polymorphic nucleotide sites (Fig. S8B, see Supporting information). This could perhaps explain the discrepancy between higher *LrNAC35* transcript levels and lower resistance levels in *L. brownii* during LSV infection.

Apart from virus‐caused induction, increased expression of *LrNAC35* under low temperature treatment may suggest the involvement of LrNAC35 in *L. regale* cold tolerance (Fig. 4D). This notion is supported by the report that a mutant of *AtLOV1*, an *Arabidopsis* homologue of *LrNAC35*, exhibits enhanced hypersensitivity to cold, while the *AtLOV1* gain‐of‐function allele displays reduced hypersensitivity (Yoo *et al.*, 2007). An opposite expression of *LrNAC35* was observed in *L. regale* leaves during salt stress (Fig. 4D). The biological role of LrNAC35 in response to high salinity awaits further investigation.

Moreover, we also measured the effects of plant hormone treatments, including ET, ABA, SA and JA, on *LrNAC35* transcripts. These plant hormones have all been shown to affect virus resistance in different plants (Alazem and Lin, 2015). In the present study, expression analysis demonstrated that *LrNAC35* was significantly up‐regulated in ET‐, ABA‐ and SA‐treated *L. regale* leaves (Fig. 4E), providing further support that these hormones may be involved in virus resistance in plants. The crucial involvement of LrNAC35 in *L. regale* response to viruses may be attributed to the interplay between LrNAC35 and these hormones. Future studies will examine the hormone variation in WT and transgenic plants overexpressing *LrNAC35*.

We verified the enhanced resistance to CMV conferred by *LrNAC35* overexpression in transgenic petunia plants (Fig. 6A–F). We also detected reduced fluorescence signals and *TMV*‐*CP* expression in transgenic lines infiltrated with an artificially modified vector, TMV‐GFP (Fig. 8), but no significant difference in disease symptoms was observed between TMV‐GFP‐infected transgenic and WT lines (data not shown). It was speculated that the GFP tag may have repressed the full manifestation of visual symptoms caused by TMV infection. In addition, *LrNAC35*'s function in antiviral defence was validated through the TRV‐VIGS assay of a petunia orthologous gene *PhNAC35* (Fig. S7, see Supporting information). We observed lessened symptoms of HR‐like necrotic lesions and reduced electrolyte leakage in systemically infected or inoculated leaves of overexpression plants with CMV compared to WT lines. Earlier studies have indicated that virus‐induced HR is correlated with increased lignin, which is a major constituent of the plant secondary cell wall (Kimmins and Wuddah, 1977). Lignin deposition perhaps acts as a physical barrier in preventing the spread of viruses (Candela *et al.*, 1994; Nicholson and Hammerschmidt, 1992).

There have been a large number of publications on the role of lignification or lignin biosynthetic genes in plant responses to fungal penetration (Moura *et al.*, 2010; Wang *et al.*, 2018; Zeyen *et al.*, 1995). However, the reports on lignin's function in response to viruses are fewer and sometimes controversial. Tobacco necrosis virus and tomato spotted wilt virus infections increase lignin content in dwarf bean (Kimmins and Wuddah, 1977) and petunia leaves (Quecini *et al.*, 2007), respectively, but Kofalvi and Nassuth (1995) found no significant change in wheat streak mosaic virus‐infected wheat leaves. Application of aminooxyacetate, a competitive inhibitor of phenylalanine ammonia‐lyase upstream of the lignin biosynthesis pathway, to tobacco leaves enlarges the size of TMV‐induced necrotic lesions (Massala *et al.*, 1980). A significant discovery is that synthetic or natural lignin could inhibit the replication and cytopathogenicity of human immunodeficiency virus infection (Nagashima *et al.*, 1992).

Indeed, we detected higher lignin content in *LrNAC35*‐overexpressing plants than WT plants inoculated with CMV (Fig. 6G). It appears likely that lignin is an essential intermediate in transcriptional modulation of antiviral defence by LrNAC35. This hypothesis is further confirmed by the altered abundances of several lignin biosynthesis‐related genes in *LrNAC35*‐overexpressing lines, and a particularly direct activation of *Ph4CL* promoter by LrNAC35 (Fig. 7A,D). However, the specific DNA binding sites recognized by LrNAC35 remain enigmatic. To date, multiple *cis*‐acting elements have been clearly identified in the target gene promoters of NAC members. For example, a complete NAC recognition sequence containing the core elements CATGT or CACG was determined in *Arabidopsis* (Tran *et al.*, 2004). AtNAC19, AtNAC55 and AtNAC72 physically bind to the CATGTG motif of the *ERD1* promoter (He *et al.*, 2005). Two NAC proteins, SND1 and VND7, specifically bind to a secondary wall NAC binding element (SNBE) (Zhong *et al.*, 2010). The binding sequence of ATAF2, an NAC TF, harbours an imperfect palindrome CAAATNNNATTTG (Huh *et al.*, 2012). In a recent study, ThNAC13 was reported to bind to NACRS and CBNAC elements with the core sequence CGT(A/G) and GCTT, respectively (Wang *et al.*, 2017). In the present study, we only identified the CACG and CATGTG motifs in the promoter regions of *Ph4CL* (Fig. 7B). The unanswered question is whether LrNAC35 could bind to these two motifs or to other unrevealed ones. Thus, a deletion analysis of the *Ph4CL* promoter is required to identify the exact DNA binding sites of LrNAC35 in the subsequent studies.

Given the impact of *LrNAC35* overexpression on several lignin biosynthesis‐related genes, we searched the corresponding orthologous unigenes against our RNA‐Seq data in CMV‐infected *L. regale*, and two *4CL* and three *CCR* homologues were identified. Although all these homologues displayed a false discovery rate (FDR) > 0.05, their transcript abundances were increased by 2.0‐ to 4.8‐fold upon CMV infection (Table S5, see Supporting information). We found no orthologous genes of *PhC4H* and *PhHCT* in our transcriptome data, probably due to a very large genome size in *Lilium* (Siljak‐Yakovlev *et al.*, 2003) and an incomplete coverage through the Illumina RNA‐Seq approach.

In summary, our results suggest that *LrNAC35* plays an important role in antiviral defence by mediating the biosynthesis of lignin. The identification of *LrNAC35*, arising from a comprehensive transcriptome sequencing effort, provides a valuable genetic solution to reduce the susceptibility of lilies to viral disease. To find more promising candidate genes, functional screening of massive transcriptome data requires a rapid and efficient approach. For instance, high‐throughput VIGS is an attractive tool for permitting the quick knockdown of gene expression in plants, and a CMV‐based VIGS system has been established in *L. leichtlinii* (Tasaki *et al.*, 2016). Using the VIGS method to unravel the functions of candidate genes in response to CMV or other viruses in *Lilium*, not in heterologous model plants, should be pursued in future work.

## Experimental Procedures

### Plant materials and growth conditions

Wild lily species *L. regale* seeds were planted in small pots filled with sterile soil mix (peat:perlite:vermiculite = 3:1:1 v/v/v) and germinated in a growth chamber at 22/20 °C day/night temperature with a 16/8 h light/dark photoperiod. The second newly sprouted leaves with relatively large sizes were inoculated with CMV for subsequent viral detection, transcriptome assay and expression analysis of candidate genes. Uppermost young leaves of 6‐week‐old plantlets of bulb‐propagated *L. regale*, *L. pumilum*, *L. duchartrei*, *L. brownii* and *L. tigrinum* were used for assessment of *LrNAC35* expression upon CMV, LMoV and LSV inoculation, abiotic stress and hormone treatments. These lily plants were cultivated in the germplasm resource nursery under 25/22 °C day/night temperature and natural photoperiods. Tissue‐specific expression analysis of *LrNAC35* was carried out using rootlets, outer bulb scales, top stems, young leaves, floral tissues at anthesis (petals, pistils and stamens) and mature seeds collected from *L. regale* plants at 12 or 16 weeks after bulb germination. To study the *LrNAC35*'s function in resistance response to CMV, we selected petunia (*P. hybrida* ‘Mitchell Diploid’) as material for genetic transformation experiments. Petunia seeds were sown in a 36‐well tray, and four‐leaf‐stage seedlings were then transferred to pots under identical chamber conditions to those described above. The leaves three to eight from terminal were collected as explant source for genetic transformation.

### Inoculation assay

Viral inoculum of CMV strain LS (CMV‐LS, subgroup II) was obtained from the infected leaves of *Lilium* Oriental hybrids cultivar Sorbonne, while both strains of LMoV and LSV were isolated from the wild species *L. davidii*. Leaf tissues infected with three types of viruses were separately homogenized with kieselguhr in 100 mM phosphate buffer adjusted at pH 7.0 (1:6 w/v) to prepare infectious sap. The sap from leaves of virus‐free plants was used as mock control. The virus preparations were rub‐inoculated onto healthy young leaves of wild lily species, WT and transgenic petunia plants through the mechanical method (Hull, 2009). An agroinjection method with TMV‐GFP plasmids was used to inoculate petunia (Dorokhov *et al.*, 2006). For functional analysis of *LrNAC35* in petunia, virus symptom development was evaluated during the post‐inoculation growth periods. Accumulation levels of CMV coat protein (CMV‐CP), GFP and TMV‐CP in the infected leaves were tested via qRT‐PCR (Sun *et al.*, 2017) or western blot (Jeon *et al.*, 2017). GFP fluorescence was monitored using a Blak‐Ray long‐wave ultraviolet lamp (UV products, Upland, CA, USA; Model B 100AP) and photographed using a Canon EOS 40D digital camera. The cell death and GFP fluorescence at the infection sites were quantified by measuring pixel sizes of the necrotic and fluorescent spots using Photoshop CS6. The calculation of relative cell death and fluorescence area was based on the quantitative data, and the lowest relative data was set to 1.0.

### RNA extraction, library construction and sequencing

*Lilium regale* leaves from 20 seedlings at 48 hpi with CMV or mock were randomly divided into two groups serving as two independent biological replicates. Ten seedlings for each replicate were pooled to prepare RNA samples. Total RNA was isolated using the modified cetyltrimethylammonium bromide (CTAB) method (Li *et al.*, 2011) and purified with RNase‐free DNase I (Promega, Madison, WI, USA) to avoid genomic DNA contamination. The quality and quantity of RNA was assessed using a NanoDrop ND‐2000c spectrophotometer (NanoDrop Technologies, Wilmington, DE, USA). Generation of cDNA libraries and sequencing projects was carried out using Illumina protocols at Gene Denovo Bio‐Technology Co., Ltd (Guangzhou, China). Briefly, mRNA harbouring poly (A) was enriched through magnetic beads with oligo(dT) and randomly chopped into small fragments. These pieces were used as templates to synthesize cDNAs. The resulting cDNAs were subjected to purification and the 3’‐end repair process and then ligated with sequencing adaptors. The ligation products were size‐selected, PCR‐amplified and sequenced on an Illumina HiSeq2000 platform. The raw transcriptome reads were submitted to the NCBI Sequence Read Archive (SRA) database under accession no. SRP193127.

### RNA‐Seq data processing

Raw data/reads from the sequencing machine were yielded through base calling. Before assembly, raw reads were handled by removing reads harbouring adaptor sequences, more than 10% of unknown nucleotides (N) and more than 40% of low‐quality (*Q* value ≤ 10) bases using a Perl script tool. The generated high‐quality clean reads were assembled *de novo* using Trinity software (v. 2.1.1) with k‐mer size parameter set to 25 by default (Grabherr *et al.*, 2011). Clean data were mapped back onto the assembled transcriptome sequences, and the reads for each unigene were counted based on the mapping results. Trinity combined reads with certain length of overlap to create longer fragments not containing N or contigs. Next, these contigs were processed using sequence clustering software TGICL (v. 2.1) (Pertea *et al.*, 2003) to form sequences longer than 200 bp, which were defined as unigenes.

A homology search of all unigenes against the public Non‐Redundant (NR) (released on July 24, 2015, http://blast.ncbi.nlm.nih.gov/), Swiss‐Prot (released on July 24, 2015, http://www.expasy.ch/sprot), Clusters of Orthologous Groups (COG) (released on July 24, 2015, http://www.ncbi.nlm.nih.gov/COG) and KEGG (released on July 27, 2015, https://www.genome.jp/kegg) databases with a cut‐off *e* value of 1 × 10^−5^ was performed for functional annotation, sequence orientation and coding region prediction, using the standalone BLAST (v. 2.2.29). Transcript levels of unigenes were calculated and normalized to reads per kilobase exon model per million mapped reads (RPKM). PCA was conducted using the gmodels package of statistical program R (http://www.r-project.org/). Significant DEGs between mock‐ and CMV‐inoculated *L. regale* samples were identified with a threshold for FDR ≤ 0.05 and fold change ≥ 2.0 in multiple comparisons using edgeR package (v. 3.12) (Chen *et al.*, 2014) with an R environment wrapper (v. 3.2.1). Based on NR annotation results, the GO annotation of unigenes was carried out using Blast2GO (v. 2.3.5) (Conesa *et al.*, 2005) and then the WEGO tool (http://wego.genomics.org.cn/cgi-bin/wego/index.pl) (Ye *et al.*, 2006) was used to perform GO functional categorization of unigenes. To identify the active metabolic pathways in CMV‐infected *L. regale* leaves, a BLAST search against the KEGG database was employed to perform the mapping of DEGs to reference canonical pathways (Wixon and Kell, 2000). A formula was used for calculation of *P* values (Lu *et al.*, 2012), which was gone through FDR correction. KEGG pathways with FDR ≤ 0.05 were defined as significantly enriched.

### Semiquantitative RT‐PCR and qRT‐PCR

Total RNA from lily and petunia tissues was extracted using the modified CTAB method and TRIzol reagent (Invitrogen, Carlsbad, CA, USA), respectively. After genomic DNA elimination, 2–5 μg of RNA samples was reversely transcribed to first‐strand cDNA using a PrimeScript RT reagent Kit with gDNA Eraser (TaKaRa, Kyoto, Japan). PCR amplification was performed using Premix Taq DNA polymerase (TaKaRa, Kyoto, Japan), and the abundances of products were analysed by electrophoresis on 1% agarose gel stained with GelRed (Biotium, Hayward, CA, USA). qRT‐PCR was conducted using the SYBR Green PCR Master Mix (2×) (Applied Biosystems, Foster City, CA, USA) in a LightCycler480 Real‐Time PCR System (Roche Diagnostic, Basel, Switzerland). *Glyceraldehyde‐3‐phosphate dehydrogenase* (*LrGAPDH*) and *LrActin* served as internal controls in *Lilium* species, and the reference gene in petunia was *26S ribosomal RNA*. Data were calculated using the 2^–△△CT^ method (Livak and Schmittgen, 2001). A set of oligonucleotide primers used for analyses of virus accumulation, RNA‐Seq validation and gene expression are listed in Table S6 (see Supporting information). The unigene sequences used for expression or functional analysis in this study are provided in Fig. S9 (see Supporting information).

### Isolation and sequence analysis of *LrNAC35*

A 1366‐bp cDNA sequence of *LrNAC35* containing a complete coding region was selected from up‐regulated TFs in CMV‐infected *L. regale* leaves. To amplify the coding sequences of *LrNAC35* from various *Lilium* species, the forward primer 5′‐ATGGCAATTACCGCAGCCATGAG‐3′ and reverse primer 5′‐TCACTCCCATAGCTTGTCTGGAT‐3′ were used. Its deduced amino acids were obtained by the ExPASy translated tool (http://web.expasy.org/translate/). Proteins homologous to LrNAC35 were found by a BLAST search. The conserved motifs were identified based on comparative NAC domains analysis between *O. sativa* and *Arabidopsis* (Ooka *et al.*, 2003). Multiple protein alignments were performed using DNAMAN (v. 5.2.2) (Wang, 2015). A phylogenetic tree was constructed using MEGA (v. 4.0.2) (Tamura *et al.*, 2007). Homologous templates for protein modelling were identified from the RCSB Protein Data Bank (https://www.rcsb.org/). The modelling process was carried out using the Modeller server (v. 9.20) (Webb and Sali, 2014) based on sequence alignment performed in Chimera (v. 1.12) (Pettersen *et al.*, 2004). A combination of multiple protein structures (chain A of PDBs: 1UT4, 1UT7, 4DUL, 3SWM and 3SWP) was used for the homologous modelling of LrNAC35. Only the NAC domains were used and modelled. The best model was chosen based on the lowest Discrete Optimized Protein Energy (DOPE) values and GA 341 score of 1, which suggest that these models are reliable. The final model was validated by Ramachandran plot analysis using PROCHECK (http://www.ebi.ac.uk/thornton-srv/software/PROCHECK). Molecular visualizations were performed using PyMOL (v. 1.3r1. Schrodinger, LLC, Cambridge, MA, USA).

### Abiotic stress and hormone treatments

To determine the impacts of abiotic stresses and stress‐related hormones on transcript levels of *LrNAC35*, *c*. 20‐cm long stems from the top were cut from 6‐week‐old *L. regale* plantlets and used for the follow‐up experiments. For the cold treatment, the plants were placed into a large glass container with fresh deionized water at 4 °C in cold storage. For the salinity and dehydration treatments, the seedlings were exposed to a NaCl solution with a concentration of 150 mM and no water. For treatments with hormones, the stems were immersed in water without addition and treated with continuous 15 μL/L ET in a sealed chamber, or in water supplemented with 50 μM ABA, 100 μM SA and 100 μM JA. Terminal upper leaves were excised from three individual plants at five time points (0, 3, 6, 12 and 24 h) after the treatments.

### Plasmid construct and petunia plant transformation

The full‐length of the *LrNAC35* open reading frame (ORF) sequence was PCR‐amplified using the forward primer 5′‐ATGGTACCATGGCAATTACCGCAGCCAT‐3′ bearing a *Kpn*I restriction site, and reverse primer 5′‐ATGTCGACTCACTCCCATAGCTTGTCTG‐3′ bearing a *Sal*I restriction site. To generate the overexpression construct, the amplified DNA fragment was inserted into the corresponding position of a modified pCAMBIA1300 vector downstream of the CaMV 35S promoter in the sense orientation. *Agrobacterium tumefaciens* LBA4404 was transformed with the recombinant plasmid by electroporation. *Agrobacterium*‐mediated genetic transformation and regeneration of ‘Mitchell Diploid’ leaf discs were carried out according to a previously described protocol (Sun *et al.*, 2016a). After a continuous hygromycin selection on MS plates and cultivation process in soil mixtures, the *T*_2_ lines 3‐1, 5‐6 and 12‐3, verified as homozygotes, were finally chosen for phenotype and further antiviral analyses of *LrNAC35*.

### Western blot assay

The CMV‐infected leaves were homogenized in liquid nitrogen, and proteins were prepared using a Plant Total Protein Extraction Kit (Sigma‐Aldrich, St Louis, MO, USA). Equal amounts of samples were resolved by 10% sodium dodecyl sulphate‐polyacrylamide gel electrophoresis. The fractionated proteins were transferred to a polyvinylidene fluoride membrane (0.45 μm) on a Mini Trans‐Blot Cell (Bio‐Rad, Richmond, CA, USA). The primary antibody anti‐CMV‐CP was applied to probe the blots, which were then incubated with goat anti‐rabbit IgG secondary antibody conjugated to horseradish peroxidase. The antigen–antibody complexes were visualized using an ECL western blot detection kit (Pierce, Waltham, MA, USA). The actin antibody was used for examination of reference protein.

### VIGS assay

A 310‐bp fragment of *PhNAC35*, the petunia homologue of *LrNAC35*, was PCR‐amplified and cloned into TRV plasmid. The generated TRV‐*PhNAC35* was electrotransformed into *A. tumefaciens* GV3101. The transformed bacteria were cultured, centrifuged and resuspended in the infiltration buffer as previously described (Sun *et al.*, 2017). The bacterial mixture containing TRV1 and TRV2 plasmids in a 1:1 ratio was used to infiltrate four‐leaf‐stage petunia seedlings. For virus inoculation, mock control and CMV or TMV‐GFP were inoculated onto the leaves of petunia seedlings at 5 dpi with TRV empty vector and TRV‐*PhNAC35*. PCR primers to sequence beyond the inserted fragment for silencing were used to examine expression levels of *PhNAC35* in VIGS assay (Table S6, see Supporting information).

### Electrolyte leakage assay

Leaf discs, 8 mm in diameter, were prepared from the leaves of WT and *LrNAC35*‐overexpressing transgenic petunia lines at intervals after CMV inoculation using a hole punch, and thoroughly immersed in 20 mL of deionized water with gentle shaking at room temperature. Conductivity was measured using an Orion conductivity meter (Thermo Scientific, Waltham, MA, USA).

### Measurement of Klason lignin content

The CMV‐inoculated leaves of WT and *LrNAC35*‐overexpressing transgenic petunia plants were harvested, ground into a fine powder in liquid nitrogen and sequentially extracted using a method previously described (Wang *et al.*, 2012). Sulphuric acid (72%) was added to the methanol extract upon air‐dry treatment for hydrolysis reaction. The hydrolysate was diluted with distilled water to adjust the acid concentration to 4% and boiled under reflux for 1 h. Lignin obtained as an insoluble solid residue was filtered, washed with hot water and freeze dried. Determination of Klason lignin content, expressed as a weight percentage of dried cell wall residues, was conducted using the Klason technique (Dence, 1992).

### Dual luciferase assay

The dual luciferase assay was performed using a previously reported method (Chen *et al.*, 2017). The full‐length ORF of *LrNAC35* was PCR‐amplified and introduced into pGreenII62‐SK vector, with the recombinant construct being used as effector. The complete promoter regions of *PhC4H*, *Ph4CL*, *PhHCT* and *PhCCR* were amplified using the primers listed in Table S6 (see Supporting information). Next, these promoter sequences were ligated into pGreenII0800‐LUC vector to generate reporter plasmids, where the promoters drive a *firefly luciferase* (*LUC*) gene and the CaMV 35 promoter drives a *Renilla luciferase* (*REN*) gene. The *A. tumefaciens* GV3101 cells transformed with the effector and reporter plasmids were co‐infiltrated into the petunia plants at the four‐leaf stage. The LUC and REN enzyme activities were tested by a Tecan Infinite M200 luminometer (Männedorf, Switzerland) and represented as the LUC/REN ratio.

## Supporting information


**Fig. S1** Sequences of all assembled unigenes in *Lilium regale*.

**Fig. S2** Length distribution of unigenes in *Lilium regale*.

**Fig. S3** Validation of RNA‐Seq data by qRT‐PCR. Nine unigenes were randomly selected for expression analysis in mock‐ and CMV‐inoculated *Lilium regale* leaves at 48 h post‐inoculation (hpi). *LrActin* was used as a reference gene. Error bars indicate standard error (SE) of the mean from three biological replicates. Significance of difference was calculated using Student's *t* test (*P* < 0.05) and is shown as asterisks. Unigene0000074, *cytochrome P450 86B1*; Unigene0004763, *lecithine‐cholesterol acyltransferase 4*; Unigene0007097, *endonuclease*/*exonuclease*/*phosphatase* *family* *protein*; Unigene0011570, *auxin*‐*induced* *15A*; Unigene0013897, *unknown* *protein*; Unigene0017759, *mannose*‐*specific* *lectin* *3*; Unigene0063829, *argonaute* *1*; Unigene0073681, *ABC transporter G family member 11*; Unigene0079669, *geranylgeranyl* *diphosphate* *synthetase*.

**Fig. S4** Principal component analysis of *Lilium regale* transcriptome data.

**Fig. S5** GO functional classification of differentially expressed genes in *Lilium regale*.

**Fig. S6** Full‐length cDNA sequence of *LrNAC35* and its deduced amino acids. *LrNAC35* cDNA sequence harbours a 1077‐bp open reading frame region encoding a polypeptide of 358 amino acids. The italic bold type and bold type in squares denote the start and stop codons, respectively. The type shaded in grey indicate the conserved region of LrNAC35 protein containing subdomains A to E.

**Fig. S7** Reduced resistance to CMV and TMV infections in petunia plants with *PhNAC35*‐VIGS silencing. (A) Disease symptoms of TRV empty vector‐ and TRV‐*PhNAC35*‐infected petunia plants at 14 days post‐inoculation (dpi) with mock control and CMV. (B) qRT‐PCR analysis of *PhNAC35* expression levels in uppermost leaves of TRV empty vector‐ and TRV‐*PhNAC35*‐infected petunia plants at 14 dpi with mock control and CMV. qRT‐PCR (C) and western blot (D) analyses of *CMV coat protein* (*CMV‐CP*) transcript and its protein levels in the uppermost leaves of TRV constructs‐infected petunia plants at 14 dpi with CMV. *26S rRNA* and actin were used as a reference gene and protein, respectively. GFP fluorescent foci (E) and qRT‐PCR analysis of transcripts of *TMV‐CP* (F) encoding TMV coat protein in the leaves of TRV constructs‐inoculated petunia plants at 6 dpi with TMV‐GFP. Four‐leaf‐stage petunia seedlings were used for VIGS assay, and the seedlings at 5 dpi with TRV constructs were thereafter inoculated with mock control, CMV and TMV‐GFP. Error bars represent SE of the mean from three biological replicates. Asterisks indicate significant difference as determined by Student's *t*‐test at *P* < 0.05.

**Fig. S8** Sequence analysis of *LrNAC35* from five *Lilium* species. Alignment of LrNAC35 amino acid sequences (A) and nucleotide sequences (B) from *L. regale*, *L. pumilum*, *L. duchartrei*, *L. brownii* and *L. tigrinum*. Identical amino acids and nucleotides are shaded in black, while similar ones are shaded in grey.

**Fig. S9** Sequences of unigenes used for qRT‐PCR or functional analysis.

**Table S1** Functional annotation of all assembled unigenes in *Lilium regale*.

**Table S2** Differentially expressed genes in *Lilium regale*.

**Table S3** KEGG pathway annotation of differentially expressed genes in *Lilium regale*.

**Table S4** Differentially expressed transcription factors in *Lilium regale*.

**Table S5** Unigenes associated with lignin synthesis exhibiting increased expression in *Lilium regale*.

**Table S6** Primers used for qRT‐PCR, gene promoter and fragment amplification.
