# Supplementary figures and images for: Comparative transcriptome profiling uncovers a Lilium regale NAC transcription factor, LrNAC35, contributing to defence response against cucumber mosaic virus and tobacco mosaic virus
Source: Mol Plant Pathol. 2019 Sep 27;20(12):1662–81. doi: 10.1111/mpp.12868 (PMC6859495; doi:10.1111/mpp.12868)

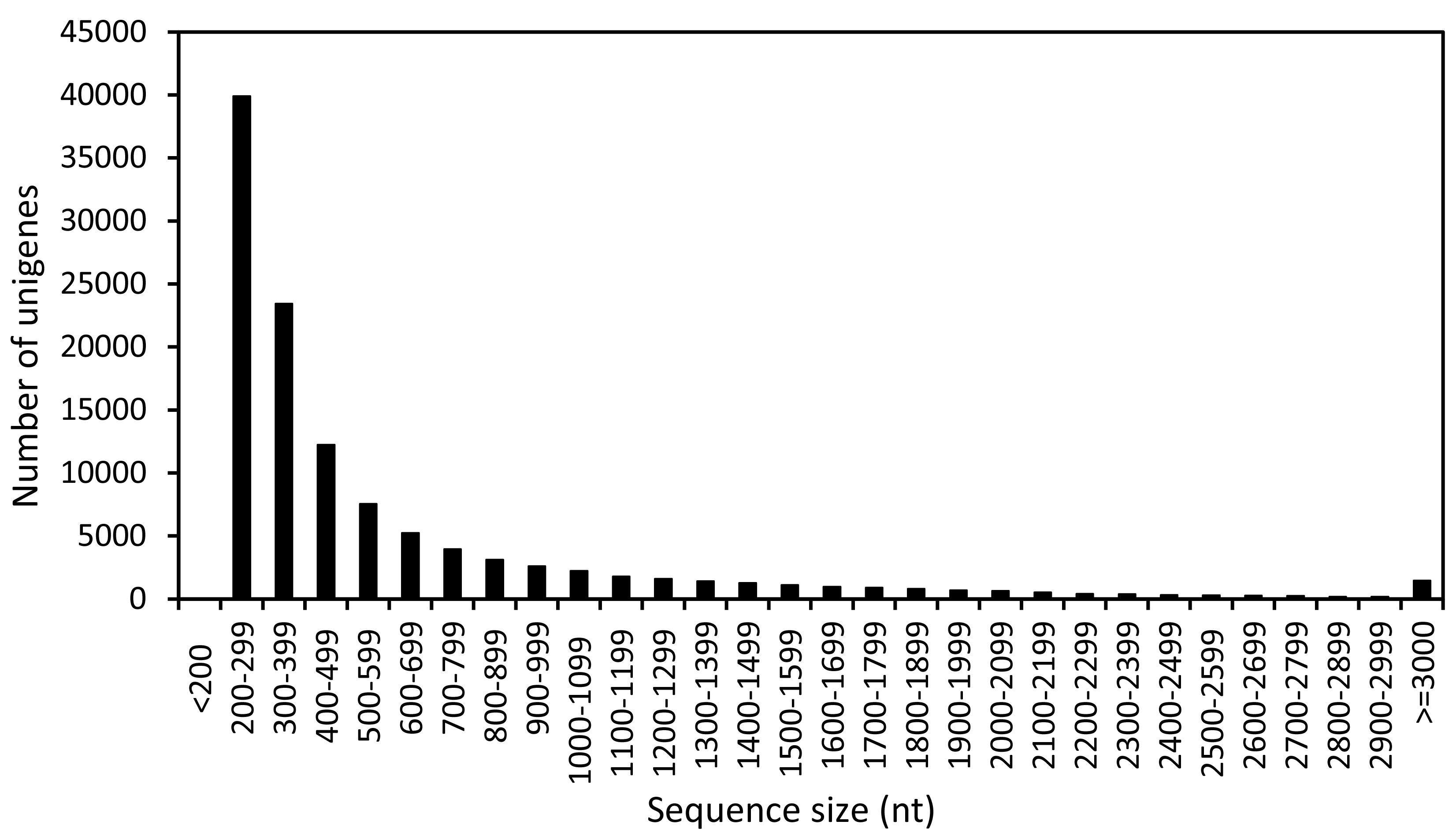

Supplement: Supplementary file 2 — Fig. S2 Length distribution of unigenes in Lilium regale. [file MPP-20-1662-s002.tif]

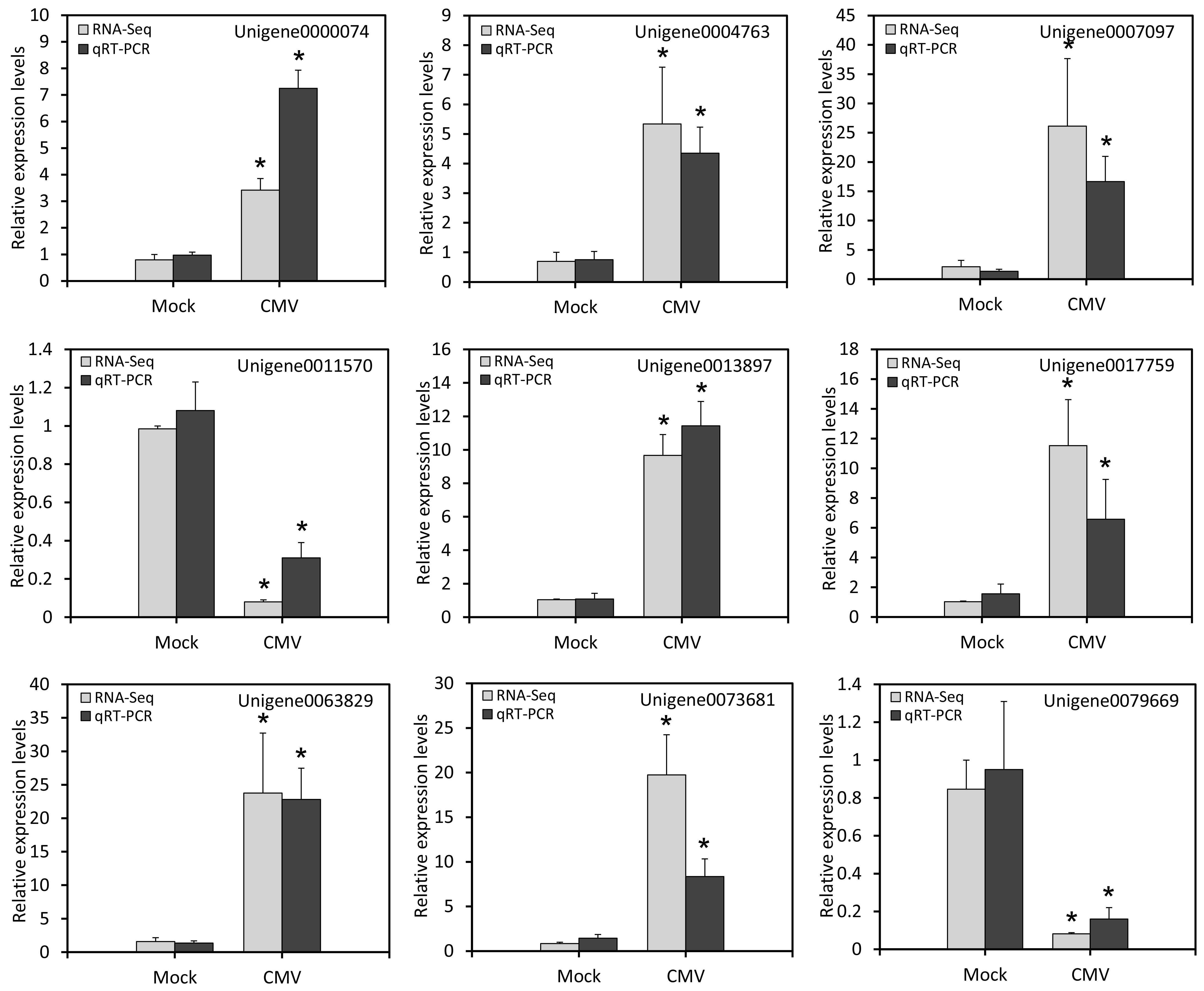

Supplement: Supplementary file 3 — Fig. S3 Validation of RNA‐Seq data by qRT‐PCR. Nine unigenes were randomly selected for expression analysis in mock‐ and CMV‐inoculated Lilium regale leaves at 48 h post‐inoculation (hpi). LrActin was used as a reference gene. Error bars indicate standard error (SE) of the mean from three biological replicates. Significance of difference was calculated using Student's t test (P < 0.05) and is shown as asterisks. Unigene0000074, cytochrome P450 86B1; Unigene0004763, lecithine‐cholesterol acyltransferase 4; Unigene0007097, endonuclease/exonuclease/phosphatase family protein; Unigene0011570, auxin‐induced 15A; Unigene0013897, unknown protein; Unigene0017759, mannose‐specific lectin 3; Unigene0063829, argonaute 1; Unigene0073681, ABC transporter G family member 11; Unigene0079669, geranylgeranyl diphosphate synthetase. [file MPP-20-1662-s003.tif]

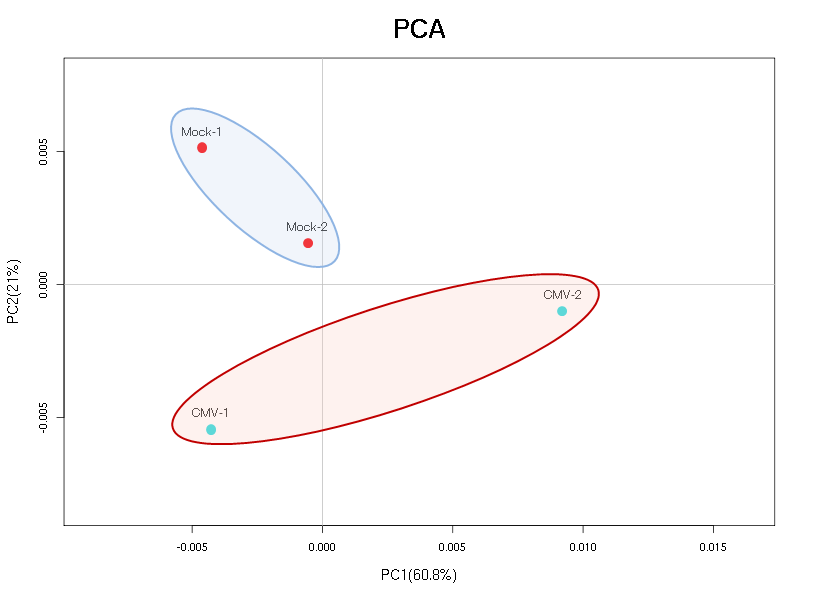

Supplement: Supplementary file 4 — Fig. S4 Principal component analysis of Lilium regale transcriptome data. [file MPP-20-1662-s004.tif]

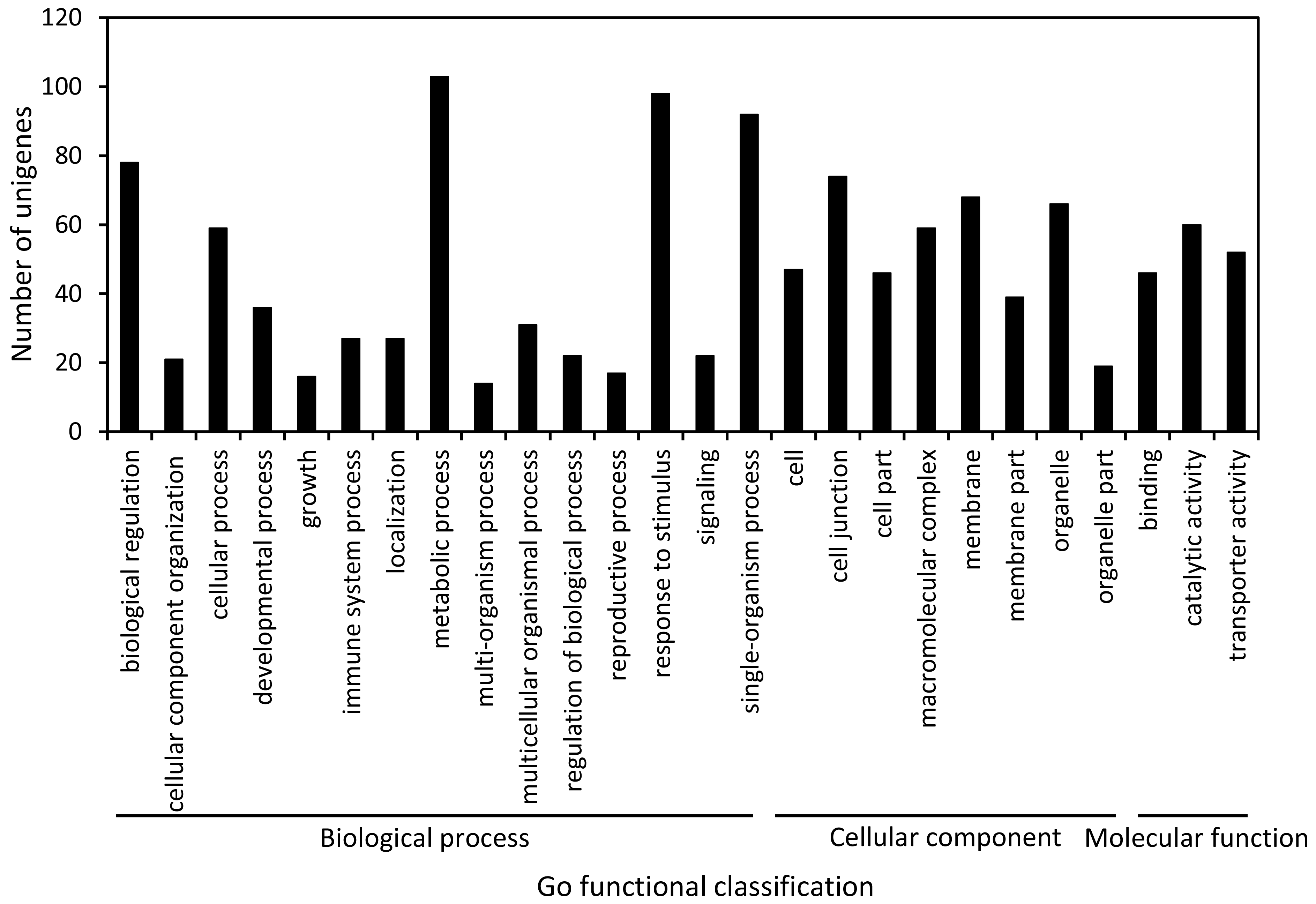

Supplement: Supplementary file 5 — Fig. S5 GO functional classification of differentially expressed genes in Lilium regale. [file MPP-20-1662-s005.tif]

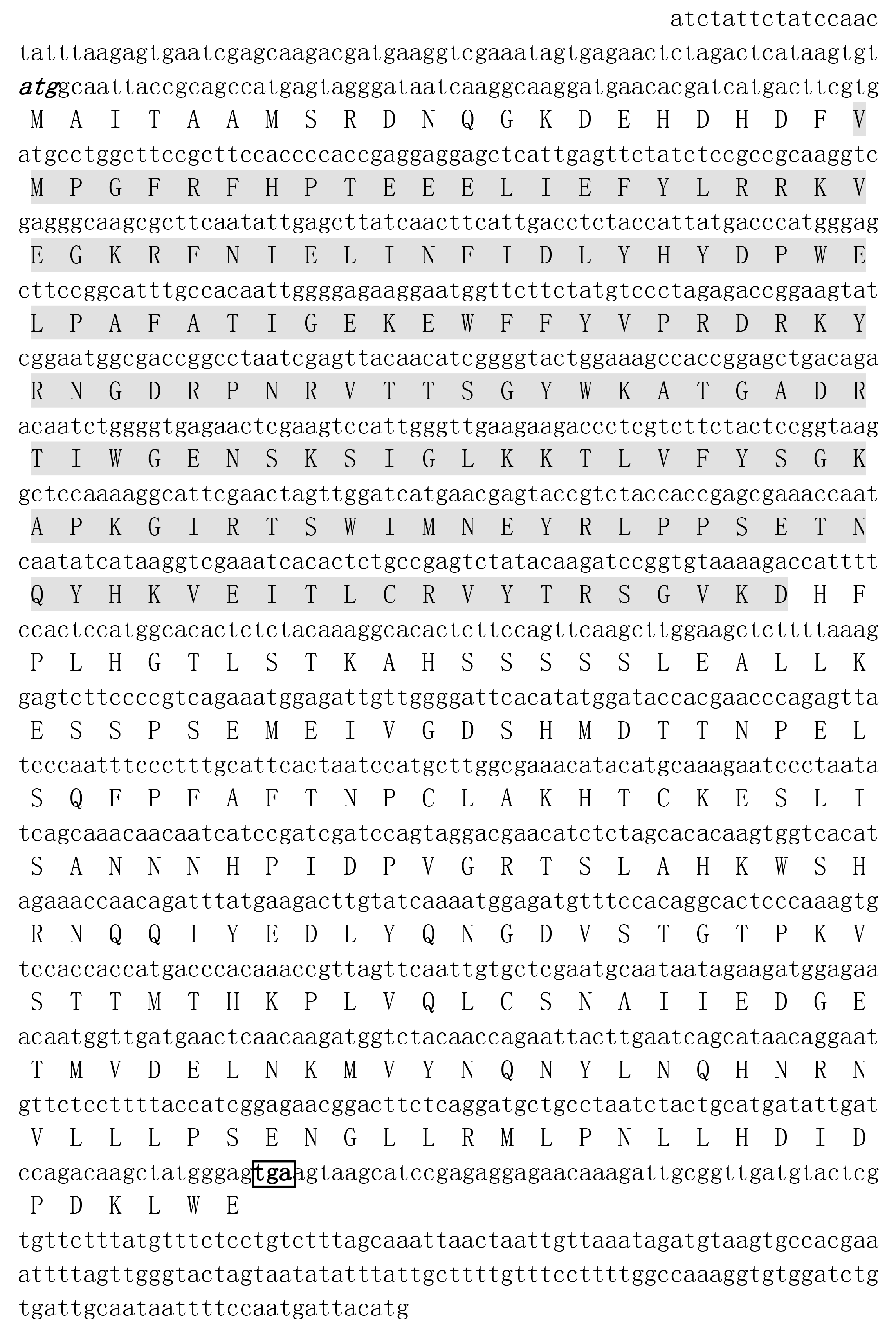

Supplement: Supplementary file 6 — Fig. S6 Full‐length cDNA sequence of LrNAC35 and its deduced amino acids. LrNAC35 cDNA sequence harbours a 1077‐bp open reading frame region encoding a polypeptide of 358 amino acids. The italic bold type and bold type in squares denote the start and stop codons, respectively. The type shaded in grey indicate the conserved region of LrNAC35 protein containing subdomains A to E. [file MPP-20-1662-s006.tif]

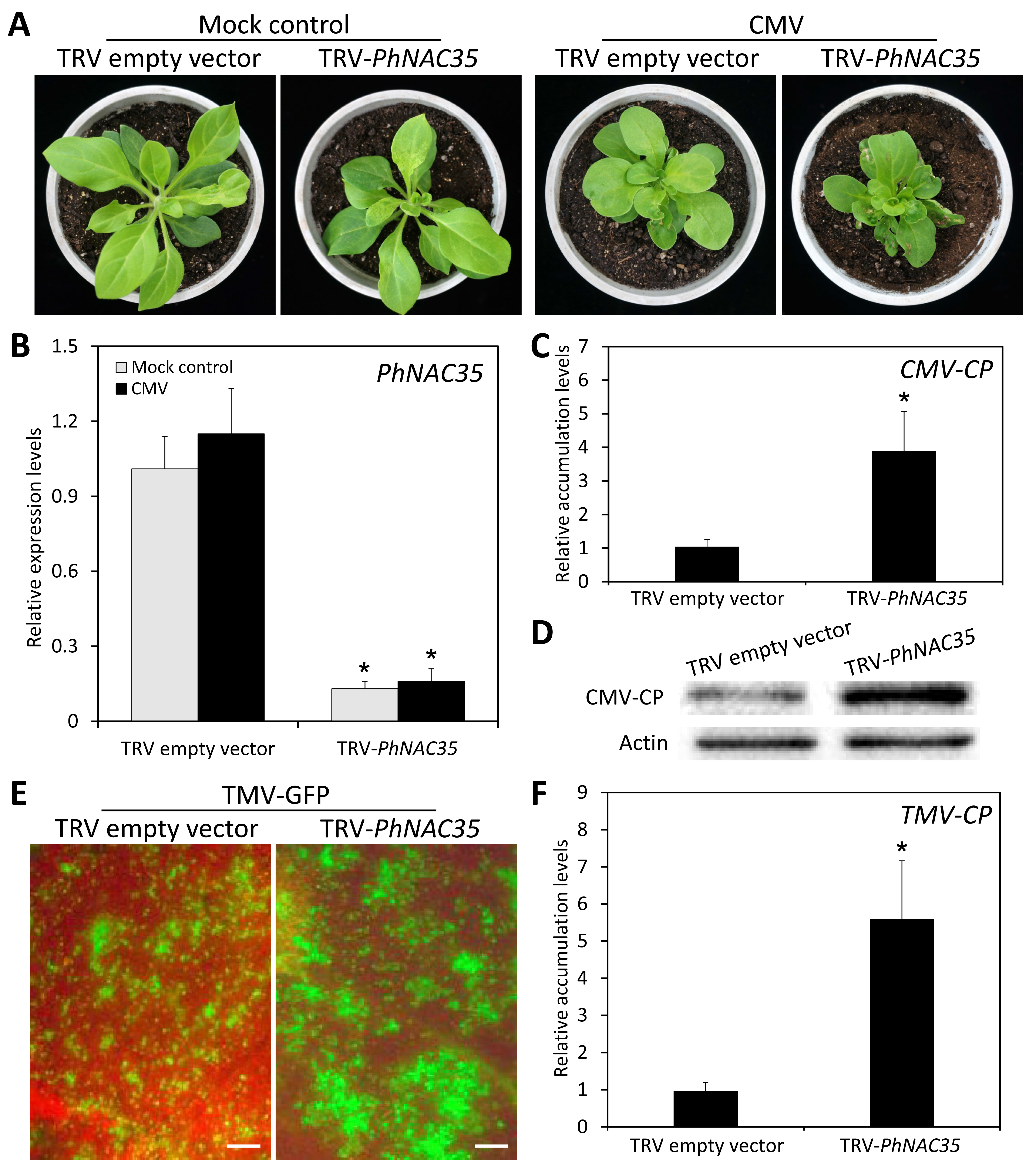

Supplement: Supplementary file 7 — Fig. S7 Reduced resistance to CMV and TMV infections in petunia plants with PhNAC35‐VIGS silencing. (A) Disease symptoms of TRV empty vector‐ and TRV‐PhNAC35‐infected petunia plants at 14 days post‐inoculation (dpi) with mock control and CMV. (B) qRT‐PCR analysis of PhNAC35 expression levels in uppermost leaves of TRV empty vector‐ and TRV‐PhNAC35‐infected petunia plants at 14 dpi with mock control and CMV. qRT‐PCR (C) and western blot (D) analyses of CMV coat protein (CMV‐CP) transcript and its protein levels in the uppermost leaves of TRV constructs‐infected petunia plants at 14 dpi with CMV. 26S rRNA and actin were used as a reference gene and protein, respectively. GFP fluorescent foci (E) and qRT‐PCR analysis of transcripts of TMV‐CP (F) encoding TMV coat protein in the leaves of TRV constructs‐inoculated petunia plants at 6 dpi with TMV‐GFP. Four‐leaf‐stage petunia seedlings were used for VIGS assay, and the seedlings at 5 dpi with TRV constructs were thereafter inoculated with mock control, CMV and TMV‐GFP. Error bars represent SE of the mean from three biological replicates. Asterisks indicate significant difference as determined by Student's t‐test at P < 0.05. [file MPP-20-1662-s007.tif]
