# Supplementary material for: Comparative transcriptome profiling uncovers a Lilium regale NAC transcription factor, LrNAC35, contributing to defence response against cucumber mosaic virus and tobacco mosaic virus
Source: Mol Plant Pathol. 2019 Sep 27;20(12):1662–81. doi: 10.1111/mpp.12868 (PMC6859495; doi:10.1111/mpp.12868)
Supplement: Supplementary file 9 — Fig. S9 Sequences of unigenes used for qRT‐PCR or functional analysis. [file MPP-20-1662-s009.docx]

>*L. regale* NAC domain-containing protein 35_GenBank accession no. MK805884

ATCTATTCTATCCAACTATTTAAGAGTGAATCGAGCAAGACGATGAAGGTCGAAATAGTGAGAACTCTAGACTCATAAGTGTATGGCAATTACCGCAGCCATGAGTAGGGATAATCAAGGCAAGGATGAACACGATCATGACTTCGTGATGCCTGGCTTCCGCTTCCACCCCACCGAGGAGGAGCTCATTGAGTTCTATCTCCGCCGCAAGGTCGAGGGCAAGCGCTTCAATATTGAGCTTATCAACTTCATTGACCTCTACCATTATGACCCATGGGAGCTTCCGGCATTTGCCACAATTGGGGAGAAGGAATGGTTCTTCTATGTCCCTAGAGACCGGAAGTATCGGAATGGCGACCGGCCTAATCGAGTTACAACATCGGGGTACTGGAAAGCCACCGGAGCTGACAGAACAATCTGGGGTGAGAACTCGAAGTCCATTGGGTTGAAGAAGACCCTCGTCTTCTACTCCGGTAAGGCTCCAAAAGGCATTCGAACTAGTTGGATCATGAACGAGTACCGTCTACCACCGAGCGAAACCAATCAATATCATAAGGTCGAAATCACACTCTGCCGAGTCTATACAAGATCCGGTGTAAAAGACCATTTTCCACTCCATGGCACACTCTCTACAAAGGCACACTCTTCCAGTTCAAGCTTGGAAGCTCTTTTAAAGGAGTCTTCCCCGTCAGAAATGGAGATTGTTGGGGATTCACATATGGATACCACGAACCCAGAGTTATCCCAATTTCCCTTTGCATTCACTAATCCATGCTTGGCGAAACATACATGCAAAGAATCCCTAATATCAGCAAACAACAATCATCCGATCGATCCAGTAGGACGAACATCTCTAGCACACAAGTGGTCACATAGAAACCAACAGATTTATGAAGACTTGTATCAAAATGGAGATGTTTCCACAGGCACTCCCAAAGTGTCCACCACCATGACCCACAAACCGTTAGTTCAATTGTGCTCGAATGCAATAATAGAAGATGGAGAAACAATGGTTGATGAACTCAACAAGATGGTCTACAACCAGAATTACTTGAATCAGCATAACAGGAATGTTCTCCTTTTACCATCGGAGAACGGACTTCTCAGGATGCTGCCTAATCTACTGCATGATATTGATCCAGACAAGCTATGGGAGTGAAGTAAGCATCCGAGAGGAGAACAAAGATTGCGGTTGATGTACTCGTGTTCTTTATGTTTCTCCTGTCTTTAGCAAATTAACTAATTGTTAAATAGATGTAAGTGCCACGAAATTTTAGTTGGGTACTAGTAATATATTTATTGCTTTTGTTTCCTTTTGGCCAAAGGTGTGGATCTGTGATTGCAATAATTTTCCAATGATTACATG

>*L. regale* ethylene-responsive transcription factor 61_GenBank accession no. MK805885

CTCAGAACCGAATGCGGGTGTGGCTGGGGACATATGACTCACCTGAGTCGGCAGCCTATGCGTACGATCGGGCTTCCTACAAACTCCGCGGCGTGTACGCTCGCCTCAATTTCCCTGCTCTCCGTGATACCGCCGACGAAGACTGCCCCGAAAAATTGCGTGCGCTGCGAGCTGCCGTCGATGCTAAGATCCAAGCCATCAGCCATCGACTCAGCCAGAAGCGGCGTGCTAACAGGCTCAAAAGAAAAGAGATGCAACAATCCAAGCCTCAGAGCAGCGAGGTACTGAGTGTCTGCCCCTCAAATCACTCAGCCTCATCGTCGGCAACATCAGGGGAGGAGTTGGACTGTGAATGGTCGCTGGCGAGGATGCCATCGTTCGATCCTGAGCTCATCTGGGAAGTACTGGCTAATTAGAGAGCT

>*L. regale* ethylene-responsive transcription factor TINY_GenBank accession no. MK805886 GAAAGGAGCATCAACCCATTATTCTCTCCGTCTTCATCGGCCATAAACCCCTCAAGCAGTAATGACTCTGCAGAGGAGGCCAAGAAGAGCACCAAGACACTGCGCAATGGCAGCAAGCATCCCACTTACCGGGGAGTACGCAAGAGGAAGTGGGGTAAGTGGGTGTCCGAGATCCGAGAGCCGCGCAAGAAATCTCGCATATGGCTTGGCACATTTCCCACACCAGAGATGGCCGCCCGAGCCCATGACGTCGCTGCCCTCAGCATCAAGGGCGCCTCGGCCATCCTCAACTTCCCGGACATTGCTGCCTCTCTTCCCCGCCCAGCCACGCTTTCCCCACGGGATATCCAAGCCGCTGCCTCCAAGGCCGCCACAATGGACCACACCACCGCTGCTCCATTGTCTCCAGATGAGCTTGGGGAGATCGTGGAGTTACCCCAGATGGACGAATGCTTCTTCG

>*L. regale* auxin-responsive protein IAA17_GenBank accession no. MK805887

GGAACCTTCTCTCTCATCAAAAACCCACCCCCACCTTTCTGCTCTCCCACACAAACGACGCCCGCCTCCGTTCCTTCCTTCATGCTCCTCTCTCCAACCCCTCTCACATCCTCCACCTAACCATGTCGCCACCGCTCGAGCACGACTACATCGGCCTCTCTGCCGCCGACGGCGGCACCGCTGGGCGGCCGTCACTCAACCTCAAGGCCACCGAGCTCCGCCTCGGCCCGCCAGGCTCCGAGTCCCCCGACCGTGGCGACACCAAGAAGCCGCTGGGACTGACCCTGGATTTACTCCCTGCTAAGCCCGGCTTCGTCTCCGGCGCGAAGCGTGGTTTCTCGGATGCCAGCGGCGCCTGGGCCTTCGCTGCCGGGGTTGGATCTGATGCTGAGGCGGCTAAGGGCGCCGCCGGTGGCGCCCCCGTGAAGGAGGGGGTGGCAGCGGTTGCTGCAGAGCTGGAGAAGAAGGCTCAGGCTGCAGCTGATCGCGCTCCTGCTCCTAAGGCGCAGGTTGTTGGTTGGCCACCAATCCGGAGCTACCGTAAGAACACGATGGCGACGTCTAACCCCGTAAAGAACAAAGAAGATACCGAAGCAAAGCAAGGAATGGGGTGCTTATATGTCAAGGTTAGCATGGATGGAGCTCCATACCTCAGGAAAGTGGATCTCAATATGTACTGCAATTACAAGGAGCTCTCTTATGCTCTGGAGAAGATGTTCAGCGGCTTTACTGGCCAATGTGGTGGCCAAGGGATTCCAGGCAGGGATGGCCCGAGTGACAGCTGGCTGAAAGATATTCTTAATGGATCTGAATATGTGCTTACTTATGAAGACAAGGATGGCGATTGGATGCTTGTTGGTGATGTTCCATGGAAAATGTTCATCGACTCATGCAGGAGGATGAGAATCATGAAGGGATCAGATGCTATTGGACTTGCTCCAAGGGCTATGGAGAAGTGCAAGAACCGGAACTAGCATCACCCTCGACAGTCATTGAAACCGCGAGCGCCTGTACTCTGGCATTGTCGAGGGAAGGTCTACTATTTTAATTTGGAAATTGCCCAGTATCTATATATGGTTTGTTGTTCTGCATTACTAAAACCTCTATCTAGCTTCCCAGGATTAAGTTTCGTCAGACATTGTTGTCTCTGCTATTTGTATGTTTGCATGGTCGTTACCTATTTGTGTAAATTGTTGGTTGTTTTTTACATTATATTGCGAGTTATGGAAGTAAATTCTCATCATCAATGGTGAGATACTTCCATGCAGGCTTTTTCTTCTAGCTAGGCATGACTGTCAACTGCTCTGAAAATTGGGTTTAGTTGGGCTATTCAAAAAGAGTCATGATATTGTCTATATAATGGATGAGATCTTAGCTATCATGATAATATTGATTCAGTTGAATAG

>*L. regale* transcription factor bHLH100_GenBank accession no. MK805888

TTCCATATAGTGATCAAAATAAGAAGTTGAGCATTCCAACCATCATCTCCCATGTTGTAAAGTACATTCCAGAGCTACAGAAGAGCGTGGAGAAGCTGAGGAGAAGGAAGCAAGAGATAATTTCGAGAGCTTCACGAAATGGAGACACAACCAATCTTCTCGGTGATAGCTTGGTTTACCCAGCTATTTCGGTAACCGGCTTGGATAAAAATGAGGTGATGGTCCAGATAAGCATTATTAGCAAAGCCACTATGGCTCCTTTATCTGAAATTCTGAAGGTTTTGGAGAAGGAGGGACTTCAACTGATAAACGCATCAACATTCACCACTCAAGATGATAAAACATTCTACACTCTCCATCTCCAGGCAAAGGATTATGTAACCATTATGGGCGAATGCTTATGCGAGTTGTTAACAAAAACTATGAAGGAACAAAGGAAATTAAGA

>*L. regale* transcription factor bHLH18_GenBank accession no. MK805889

CGATATTCTCTTAAAGATTCAGAATGGCACAATGGTTCTCGGATTCGGGAATGGACCTGCCTAGTCTAGTCCATCCATGGGAGCCAAGCTCGCTGGAGCTCACTGACCAACAGCATATTTGGGATAATATTCATCCATCCCTGTCTCCAGAGAGCTATAGTCCACTGCCGGCCACCGCCTTCAGCACCTCATCGAACACTAGCAGCACCGAAAGGCCGAAGAAAATCTTGAGGACTGACAGTTGGAGCTCATTCCCCGCGGAGCGAGATTCGTTTCCTAGCATACTTTCATTCGGTAATCCGAGCTCTTCAAAGGGTCACTCCGACTTATATGCCAACCTAATTAGAGGAGCTGTGAAGCCCAAGGAGGAGGGGAGCTACGAAGCAGTGGCAGCTGGACAAGTTCTGAAGAAGGTGAAGCCAGTGACTAGGCCACCCTCCCACAATCAAGATCATGTAGTGGCCGAGAGGAAGCGGCGAGAGAAGCTTACCCAGCGATTTATCGCCCTCTCAGCCGTCGTTCCCGGACTAAAGAAGATGGACAAGGCTTCGGTTCTCGGAGATGCGATCAAGTATCTGAAACAACTCCAAGAGAAGGTGAAAACTCTCGAAGACCAGACTGCAAAGAGGACGGTGGAGTCGGCAGTACTCGTAAAGAAGTCCCAGCTCTCTACAGATGATGATACTTCATCTTGTGATGATAGTTCTGACGGGGTTTTATCGGGCGAGTCTACCCTTCCTGAGATTGAAGTCAAGCTATCAGATAGGACTCTGCTCATAAGGATTCATTGCGAGAGACACAAAGGTGTGCTCACTAAGGCACTAAGCGAGATCGAGAATCTCCCACTCACCATTATTAGCACCAATGCCATACCCTTCACGAGTTCAACGCTTGATGTGACCATTACTGCTCAGATGGATGAGGAGCTCTGCATGACAGTGAAGGAAATCATGAAAAAGCTAAGCATAGCATTCAAGCATTTCATGTGAGGAAGCTGGTCGAGTTGTTGAACAACAGGGGTCAAGGGTGGGTAGATGTTTGACGAAGAAGTCCCTGAAAAGGCTGCAATCCTCAGTTCAGAGAGCACCATCTACAGTGGAGTTTGCAGCAAAGATTTCCCTTCTGTTTTTGGCTTTCCTCTCAAGCCATTTTTGCATGGTTTAATCTTTGTGATCATGTTTATATGGGAGGAAAGGTTTGTGCATTTTGCTTCTTTTGGGGTCTTCATTTCCTATGGGAAATGGACATTTTTTTTTTCTTTTTTAAGAATTTTTTTTTTTCAAGTCTTGTGGATGAGAAATAGTTATGCTGTATTATTGCAATAATGTAAAAA

>*L. regale* transcription factor RF2a_GenBank accession no. MK805890 TGGCTAGCTGCCTCCCTCTCATGGTCATGTGATCCTATCCTCTCCTTCACCACCTCCTCCTCCATTACCTTATAGAGAGAGGAGGAGAGGGGGAGAGAGAACTCCAACTCAAATCTTGCATTTTGCTAGAATGCAAGCCCTAGTAGAAGCAGAATACAACATGACCCAACTCCCTCCCAAATCCCCTACCGCCATCACCATGCCAGTCAACCACAGCCACCTCCTACCGCCACCCACGGCCGATCCCTCTTGGACCGACTTCCTCGACTTCTCCTCCATGCGCCGCGGTTCCCACCGCCGCTCAGTCAGCGACTCAGTCGCCTTCCTCGAATGTGTCGAGATTGACGACGATTGTGACAATGACCAAGAATTCGAATTCAATATACTCGACGAAGAGAAGATCACCTCCATGTTCAAAAGCGAAGTGGAGCCACCATCATCCTCTGCCACTATACCGGCATCCACCGCCCAACAAAATTACTCGAAAGAGGAGAAGCAAATGGGCGGGCATACAAACGATAGGCAAAGCTCATGTAAGACGGAGACACAAGAGCTGGCCCAACCATCGGCCGAGACTGATTCGATGGTTGTTGATCCCAAGAGGGTCAAGAGGATCATAGCAAACAGGCAATCAGCACAGAGATCGCGGGTGCGGAAGCTGCAATACATATCAGAGCTCGAACGCAGTGTGACGACATTGCAGACCGAAGTGTCGGCATTGTCTCCTCAGGTGGCCTTCCTCGATCACCAGCGCTCGATTCTAACCGCTGGGAATGGCCACCTGAAGCAGCGAATTGCCGCTCTTGCACAGGACAAAATCTTCAAAGATGCTCATCAGGAAGCACTGAAGGAAGAGATTGAGAGACTGAGGCATGTATACCACCAACAGAACATGAAAAATATGGCTGCTCCGGAGCCTGCCATTTGCGAAGCACTGCTCAGTTGATGTTGCAGTTCACTCAGTCAAGGAAGAAGATGATTCAGTTTGGGTGTGGGATGGTATATGTATTCAATATGTATCTTATCTTCTTTATTCTCCTCTTCTCCTCCATTTTGATTCATTTTCCATCTCAATTTGATCATGGAAATGGTAGTTGTGGGAAGCATTCGGTTGTGTTGGAATATATTGGAAGTTTGTCATTGTGATATGATA

>*L. regale* transcription factor MYB98_GenBank accession no. MK805891

CCTTTAAAGTAACCGACCAACCCCTCTTATCCTCTCCCATCATCAATGGATCTGAACAACTTCGCCGTCCCAATTCCGCCCTTCTATGATCTCCATCATCAAGACCTTCCCTTGGATGCCCTAGCAGAAAGGTTCCAGAACCTCCCCTTTTTAGGAAACGAGAAGAATGCAAAGATTGAAAGTAATAGTGATTGGGCTCCTGGAAAGAAGGAAGACCTGAACTCCTCTCCAAAACACCATTATGTTAAGCGGCCATGGAGTCTCGCCGAAGACAGGTTGTTAGTTAGATTGGTGAGGCAACATGGAGCCGGTAATTGGTCTCATATTGCTCAGATGATGCATGGGAGAATAGGGAAGCA

>*L. regale* transcription factor CPC_GenBank accession no. MK805892

CCCACCAACTGTTCTGCCGGCACCTAAATACCTTATTTAATGGCCTACCACCTCACCCGCCGCAACCCCCCTCCCCTCTGACCCTCATTTCTCTCTCAAGGACTTTGAACCCGTTCCTCTTCTTTTGTATGACATGGCTGGCTTAGATCAGTCTCCTGATGATGAGTTGGTAAATTCCTCCAATGGTCCTGGTGAGGAGAACCCTCAAGAACCGAAGCTCGGTTTCTCAATAGATGAGGAGGACCTCATCGCGAGGATGTATGGTCTTCTTGGACAGAGATGGATTCTGATTGCGGGAAGGATCCCCGGAAGAACAGCTGAAGAGATTGAGAAGTATTGTCTCTCCAAGTATTCTTCTTCTTCCCCGACTGAATGATTGATGAATGAGATATTTTCCTCTTTGCTTTCTATTCCGTGTCCTAGGCGCTGTTGGAAGTCCTCGGCTTCTAGTTATATATGGGATAAATTTGCAATTTCGCTTTGGATATATATATAT

>*L. regale* transcription factor MYC4_GenBank accession no. MK805893

GAGATCAGTAATGACCGATGGCAATAATAGCATCTGATGGTTGGGAAGACAACGATGAGATAAGATTACTTCATTGACAGTAGTGAAAGGATTCATCGACGATAGGGTGCATCATGATATACTTTGATGGAGTTGCTTCGAGTGTGGAATAAGAGATGTGAGTTCTCTCTGGGTTCTTCTTCTTAATTGGTTCGTCCCAATCTGAATCGAGCCAGTTCATAATGGTCTTATTCAGACAGTCAGATCCTAATACAGATCAGGAGGTGGCAGATCATATCAGCTATGACTAATACCGCCAATGATCGAAGTACTCATGATAGCAGTGGCAGGGGTCGGCATCCTCATCAGAGTCATTATTGGAGGGGAGACAATAATACAACACTGAGATCACTACATCGGTGCTCACTTATAGGCATAGCCACCGATGTGAGTGGTGTTTGATGTTAGTCTCTCTTCTGGTAATGGGATTATCAGTATCGCCAGAGGCGACAATATCGACTCGGAGCATTTGGATCTCGATACATCGATATGGAAAATAGAGAGCAATAATGTGGTGAAGCTAGAGAAGTCACGCCCGAGGAAGTACAGGTAAAAGTCGGCCAGTGATTGGAAGAAGCCGCTCAACCATGTTGAGGTGGAGCGACAACAATGGTAGAAGCTGAATCAATAGTTTTACATCCTCCGAGCAGTAGTCAAAGACTGTACCCTAGCTACAAAGGAAAAATCTGGTCTTTGTATCAGGCATAGAGGCAGTAAGATATACTAGATATATAATTACACCAAGTGCACAAAAATTTACTCTGGCCTCTCTATCTCCCTTGGAGGTGGCAGGTACCGCCAATTTTCTGTATGTTGAAAAGGCACACAGGGGAGCGCAT

>*L. regale* NAC domain-containing protein 100_GenBank accession no. MK805894

TGAATGGGCTGTTTGTAAGGTTTTCCACAAGAGCTTGGGTGCAGTCAAGAAGAGCCCAATTGCGAGGTTGAATTCTTCCGATGACGAGCAGCCTCTTATAGATCCAGCAATTTTCGATTCCTTCAGGCCTGAATTGTGCTATGCCAACAATGGCGGTAGCGTAAACTATGGCCTCTCAAGCATCAACGATCAAGTGGGCATTTTCGACTCAACACAAAGATCAGGTTTCTACCCACAAATCCAACCCCCAAATCCTTATTTTCAGCTTCCCCTGTCGGGAGAGAGTTACTTGCAACAGAAACTCTCGATGATGAGACCTCTAGTTTCAACTAATCATACACCATCGGTAACCGGGGTGAGAGGGCATTGTAAGGCAGAACAGTTCTCGAGCCAGTCGATGGTGTCCCAGGACAACGGGCTGAGCACCGATAGGAACAGCGAGATCACGTCGGTAGCATCCAAGCGAGACGTTGGCAGCAGCCGTACCTATGGTGATCTTGACAATCCAGACGAACCGCTTATAAACATGGACAATATGTGGAAGTACTGAACCTTGCAT

>*L. regale* NAC domain-containing protein 48_GenBank accession no. MK805895

GGCGGCTACTGATCTACTGCTCCCCCCAGGATTCAGATTCCACCCCACAGACGAGGAGCTCGTCAGGCACTACCTCTGCCGGAAATGCACCGGCATGCCGATACCTATTCCGATCATTGCTGAGATCGATCTCTATAAGTACAACCCATGGCAACTCCCCGGAATGGCGTTGTATGGAGAGAAGGAATGGTACTTCTTCTCGCCGAGGGATCGGAAGTACCCGAATGGGTCGAGGCCGAACCGTGCAGCGGGGGCAGGGTACTGGAAGGCCACCGGGGCGGATAAGGCGGTCGGGGCGCCGAAGCCGATGGCTATAAAGAAAGCGTTGGTGTTTTATGCCGGGAAGGCTCCGAAGGGGGAGAAAACTAATTGGATTATGCATGAGTATAGACTCGCCGGCGTTGATCGATCTGCTCGGAAGAAACAGAGTCTACGGCTGGACGAGTGGGTACTCTGTCGAATCTACAACAAAAAAGGCGCCACAAATACTCCGGCCAGCCCGGACCGGAAACCGGCCGCTGGTTCACGACCCTTCCGGTCACCGCAGCCTCCGATCACATATTCGCCGAAGCTGGAGATGTGCACGTCGGACGTGCCAACGCCGCCGAGCCTGGACGAGTTCTTCTCCTTCGACACAGCAGTACCGTTGCCTCATCTGCTAACTGACTCGAGCTGCTCGGAGCACATGACGTCGGCGGAGTTCACGTGCGACCGGGAGGTTCAGAGTCAGCTCAACTGGGCGGAGCTGGAGCAGGCCCTCGATATCCCCGGCGGCGATGTGGACCCCACCGCCGGTTTTATGGAAACCGGCGACGCATGGGATCCGCTGCAAGATATCTTCATGTACCTTAGGAAGCCATTCTAAGCTTAAGCAGGGCATCAGGGAGAGACGGCAAAGAGAGTCAAATGCCTTTGAGCCGTTGGATTGTACAAAGACTTTGTACGATGTGGATATGATACTCGGAGTGGGCAACAAATTTGTGATCACGGATTTTTGGGATTTTTAACATTGGCGTGGGAAGGCAAACCCTTCAATATAATTTTGGTATTCTTGTGGGTTGACATAAAACAGAAATCGTAGATGGGTTTGCTATGGGTTAGTTGCGTGCCACCGATTTATATGAATA

>*L. regale* WRKY transcription factor 28_GenBank accession no. MK805896

GCAATCTGAGGAAGAAAAGGGAGAAGAGGCCACATGAACCACGTTTTGCTTTCATGACGGTAAGTGAGGTCTATCATCTCGAAGAGGGATGTGGATGAAGTATGGACAGAAGACCGTCAAGGACAGCCCTTATCCGAGGAGCTACTACCGCTGCACCACACAAAAATGTTTAGTGAAGAAGAGGGTGGAAAGATCATTCCAAGATCCAAAGACTGTGATCACAACATATGAAGGGCAACACACACATCACAGCCCGGAGACTATTCGAGGGAGATCGCATATGTTAGCTTCTCTGCCAATGGTGCCGTCAAGCTTTCTGCAGTATCAATTGATGCAGCTGCACCCAATAGCTCGAACAGGCAGATGAATCCGAGCATCTAAGCTCAAAGTCAGTATGATTTGTGAGGGAGAAGAAGCGCTTCGTCTTAACGAAGAAAGGAAGAGATGGGAGAGAAGAAGCAAACACGAGCAT

>*L. regale* WRKY transcription factor 48_GenBank accession no. MK805897

CACTCCACTTTCCCCATGAAACCCTAGCCCGCCGATTCACTCGAATTGAGCTGATCGGAGCGATGATGGCGGTGAAGCGAGAGGAGGAGATCGAGCTCGCATCGGCGATCTCGCCGTTCACCGAGCAGATTGCGAGCTCATCGTTCGAGTTCTCCGGGATCTTCGACTTCGACGGCGGCGCCGCAGGCGGTGCCGGCAGGAGCTTCATGGAGCTGCTCGGCATCGGCGACTTCCCACAGTCGATGCATGATTTCCCACCGCTAGCTGAGGAGTCGATGACCGCGGGCGCAACTCCAGCGCTGGAGTCGTTTGACACAGTAAATTTTCCCGCCACACCGAATTGCTCATCTATTTCATCTTCCTCGGCTGAAGAGATCAATGACACGAAGTGCGCTGTCAACGGGAATGATGAAGAACGCAATAAAATCAAATCTAAGGTGACGACGAGCAAGAAGGGGCAAAAGCGCACTAGAGAGCCGAGGTTCGCATTCGTGACGAAGAGTGAGGTGGATCATCTCGAGGACGGCTACAGATGGAGGAAGTACGGCCAGAAGGCCGTCAAGAACAGCCCCTTTCCAAGGAGCTACTACCGCTGCACCAGTGCAGCCTGTGGGGTGAAGAAGCGGGTGGAACGGTCGTCGGCTGACCGGACTGTGGTGGTCACAACATACGAAGGGCAACACACGCACCCGAGTCCAGTGGGACCACGCGGAGGCGGTCACCCACCGCCGCCATTTGCGGTCTCTTCGTCAGCATTCGCAGCTCAACCAGCGATGAATCAACCCTCACCATATGTGAGCTCGCTCTTCCCGCAGCAGATGAACTTCTATCCGTCTAATGCTTGTTTTGTTGCCACTGTTGATCAAAGATTTTGCAAGGAGGGGGCGAGTATGTCGAGGGCAGGTCAAAATCCGATGATCGATAATGGTCTGTTGCAGGATGTAATTTGGCCCATCCGAAGCAAGGATGAAGCAGGAGAGTTTGTGTAGATGAGGAAGGTTCGGTATGTTGGTTCTTTCTTTATATTCGTCTAGCTTCGTTTTAGGGTTTATTACTGACTAAATGGAGTCTTGGCGTCCTCCATGTATACACTGATTAGAAGTTGTATTACTCTTACTATTTACTAGCATGTAAGATTATATTCTAGATGTTCAT

>*L. regale* dof zinc finger protein DOF5.6_GenBank accession no. MK805898

CCACTTCACACTGTTGATTCCCCTCTATAAATTCTCTTCTCTTCTCCCCCAATAGACCCCTCTTCATCCTTCCATCATCACAGTCCATTAATCGCTCTATTCAGAATTCTTCTCTCTCCAATGGATATCTGCATGGACTCGTCTGACTGGCTCAAGAGCATTACCCAAGACGAAAGCCCCATTGACTCCTCCTCCCCCTCGATGGATCTAATCCCATGCTCGAGACCCCCAGTCCTAGAACGACGTCTCCGTCCACCGCACGACCACGCCCTCAAGTGCCCTCGCTGTGACTCCATGCACACCAAGTTCTGCTACTACAACAACTACAGCCTCTCCCAGCCCCGGTACTTCTGCAAGACCTGCAGACGCTACTGGACCAAAGGCGGCTCGCTCCGCAACGTCCCCGTCGGTGGCGGATGCCGTAAAAACAAGCGCTCCACCAACAAAAACAAGCAGATCCTCAACCACCCTCAGCCCATCTCTTGCTTCCAAGACAGCCATGGCACTGATCTCCATCTCTCCTTCTCAGCTAACCAGGATACACAGTATCGCAGCCTCGTAAATCTGATGGAATACAAGTATGGCACTCCTATGTTTTTGGGTTCCAACAGTCTGGATTTCGGATTCGGAGGCTCGACGAGCCATTTTATGGGCGGGAGCGGGTTTGATAACATGGGACTAGAGATCACTGATCACTTTAATCCACTCGGTTTCTCGCTCCCGTTTGAAGGGCAGGAGGGGCAGAGTGGCGGAGATGTGAAGCCGGTGGAGAGGGGCTTGTCGCTCGAGTGGCAAGAGCCTGGGTGCGGGGAAATTGGGCGGGAGTCGTTCGGGTATTCAAATGGACTCGGGTTATGGGGTGGGATGGTTAACGGGCACGGATCCTCAGCAGTAATTTGAAGACTAATATGGGACTACTTGTCGACACACCTGATGTGACAGATGATGTGGAAAACTTTGTATTGTCTTATTTTTTTATTAGTGATATATTTTTCCTCGGGTAGTCCATATCTCCCTCCCCTGTGAGGGAAGAGTGGAGTATGATGGCCGTAGAGGAAGTTGGCTTTTAGTCCTTTTATATTCTCCCTTCCTTCTTGCTTTATTTTTTCCCTTGTCATGGAGGTAAGATGTTTGAAATGGAGTTAATTGTAAGACTTTTTTTTGTTGAAGGTTTTGTGTGTTCCCTTTACAATAAATTTA

>*L. regale* zinc finger CCCH domain-containing protein 28_GenBank accession no. MK805899

GCAGAAACATTTGTATATTGTACAATCTTTTGTTAAACTTTGATTTCTAAGACAAAAAATGGCTGTTGTTAACATGAATAGCTTAATGAATGGGAAAGACTCACGGTGGCTGCAACTAGAAGTATGCCGTGAATTTCAACGTAATAAATGTTCACGTAGTGATACAGAATGTAAATTTGCTCATCCAGCTGCTAACGTAGATGTCCAGAATGGCAAAGTAACTGCTTGCTATGATAGCATTAAAGGACGGTGTAATAGAACAAAACCTCCTTGCAAATATTTTCACCCTCCACAACATTTGAAAGATCAACTTTTAATTAATGGAAGAAATCATTTAGCTCTCAAAAATGCCTTAGTGCAGCAAATGGGCTTAGTGGCTAATCAACCAATGGTTCCTGGACAAGTTTCTGCTCCCATGCCAAATCCAGCTTACTTAGCTCAGCTACCTAATACTCAGATTGGAAATGCATTTAATCCTTACTTTAGTCATACCAATCCACTCATGAATCCTTTGTCACAATTTTTTGCTCCCAATCCTAACAATTTGACAATGGCAATGCAACAAACAGTTGTGCAACAAAATCTACAACCACCAGAACGTTTAGATATGGACTTGAGTAAAATTTCCCCTTTCTATTATGAGAATTTGTCATTGCCTGGCTTAATGCCTTTTAAAAGACCTTCGGGTGATAAAGCAGGGTTACCAATGTTTCAACCAAGTGCAGCAGCAGCAGCTTACCAACAATTATTTCAGTTTGTTCCACAACAAGCTGACTATCCCCCTACATCATCTGCCGCAGCTTTATCTGTTATGCCAAAACCACAATCTCAGGCAGTAGCACAACAACAACAACAACAGCAGCAGCAAACTCAACAGTCAATAGCC

>*L. regale* cytochrome P450 86B1 (Unigene0000074)_GenBank accession no. MK805900

GATGGAGAAGAAGTTGGCTAGAGCTCGGAAAGTGATCGATGGCTTTGTAGCTGAGACCATATCAAACCGACGGTCATTGAACACGAGCAATCCAGATTTATTATCATCGTACATCGTTGATAATGATAATAATGTGGACAATGACAAGTTCCTTCGGGACACGGTCGTGAATCTCATGCTAGCAGGGAGAGACACCACCGGCGCAACGCTGGGATGGTTCTTCTGGA

>*L. regale* lecithine-cholesterol acyltransferase 4 (Unigene0004763)_GenBank accession no. MK805901

ACTGGATGCCACTCACACCCCCTACTCTTTAATATTGATGCACCCGTCACTGATTAGCCAGTCCGTGATGGTCGCATCCTGAGTCAGCGAAGATACAAGCATGTCACTATTTTGCCTTGTAAACATGGGCCAAGCAGATCCTTGTGACTCTTAGAACGGTGCTAGTAAACCAAAAAAATACAAATATGTGTCACAGATGTGTGGATTCACATTAAAGAGGAGCGCAAAACACAAAATGTACTTGTTCTCATCTCTCAAGATTCACCAATCTCTACTTTCATCTTACAATGCATATAAATGATGATTGGAGTTCAATACAGTCTGATTTTTCTTCTTGTTAAATATTAAGAACCTATACATGTTACTGACTAAATATAAGTGTCTTCATGTTACTGACTATTGCCTCTTCACACTCTCTCGAACCCTTTCTCCAGGTTGGTTACAATGGTCTAGATATTCACCTACCATTCAATTTAGAGATATTAAAATGGGCCAATGAAACTCGTGAAATTTTGTCACGTGCTAAA

>*L. regale* endonuclease/exonuclease/phosphatase family protein (Unigene0007097)_GenBank accession no. MK805902

ACTCCACCCTTTCACCGGATGAGCAGGAGGGGGGCTTCGGAGGTTCCTGTGACTTTGGGTCTTTTGGGGATTTTGTGTCTGATTGTTCTCTGTTTGACATTCCTCTGTCGGGTCGACGGTTCACCTGGACTAATGGCCAGCACTTTGCTCGGTTGGACCGCTTTCTGACTTCTGATAAGTGGCTCCTTTCCCATCCGATCTCCTATTTTTAGGTTGCTGCAGTAATAGTATCTGATCACTGCCCCATCATACTTTCAGGTTGGGCCCTCCCTCGTCCGACCTTGTTGATCTGATTTGAGG

>*L. regale* auxin-induced 15A (Unigene0011570)_GenBank accession no. MK805903

ATGAGCAGAACGGGTTACCCCTGGATGTCCCCAAAGGCCATTTTGCAGTCCACGTCGGCAAGAAGCGGAGTCGTTTCATCGTCCCCATCTCTCTGCTGCACCAACCGGAGTTCCAGTCCCTCCTCCATGATGCTGAGGAGGAATTCGGATTCGACCACGACGTGGGCCTCACCATCCCATGCGATGATGCTTTCTTCCAATCTCTCACTTCCATCCTCTGTAGAAGCTGAAGAAGACCAGCCATGGATCCCTTCAAGGTCTTTATTTTCTTTTCTTTTTCTAATCGGTTCTGACCCATTTAGCCCCGTGTTGGTTTTTGTGGGATGAACTTGTACATTTGGAGCAGCGTTGCAGTTGAGCGGCCCTTTGAGAGCTAGTTAGATGATACTGGAGGCGAT

>*L. regale* unknown protein (Unigene0013897)_GenBank accession no. MK805904

TTTTTAATGAGAGTAACACCTTTCCACCTACTACTCATAACTTGAGTTTCTTGCTTAAGTCCCCCCGCTCCCACGGTCGCTCTTCCCAAGGATCTTCCGTATCTTCAACCTCCATAGAGTCTGTTGTCATTCCCCTTGACTCATCCTCCTCTCCTCCAGAGCTATCCCCTGTCTTGTTAGTTCTTCCATTGACCCCTTACAAATCAGTCATTGCTTGAGTCCGTTCTTTGTATCGTGACTCTACCTACCGCTCCTACCACTATATTTTCATGCTTTGATCACATCTATACATGCAAGCATTGTCATCTAGCCACTTAGTCATCCTTGTCTATTCCATCGTCAACTCCTACTTCAGAGAATCCTCCACGACGCTGCTACCCATGCGCACACGTCGCCAATGTTTTGGCTTTACTGCAATTACTACTTATG

>*L. regale* mannose-specific lectin 3 (Unigene0017759)_GenBank accession no. MK805905

TGTGTCTTGCGTCCTGTCAACTGTCCTGATTGCCTTCTTTGCATCTCCATCTACTGCGGATGAAAACAACATCCTGCTCACCGGGGAAGTCCTGGGTACTGACAACCAGCTCTCGAACACAGACGGCACCTTCGTCATGCAAGGCGACTGCAACCTAGTTCTTTACAACAAGGCCAATGGCTTTCAATCCAACACTCACGGAAAAGGAGTCAACTGCACCCTCACTCTCAGCGACTACGGCCAGCTAATCATCCGCAGCACCAATGGGACTGCCGTTTGGACCTCCCCCAACACTCACAACTCTAAAAGTGGCAAGTATGCCGCCATCCTCCGTCCCGACGGACAAGTTGCCATATATGGGACAAGCGTGTGGTCGACTCCTGAGTGGTCGTCTAGCGCCAACGATGAGAA

>*L. regale* argonaute 1 (Unigene0063829)_GenBank accession no. MK805906 TATAAAAGTTGAAGTCTCACAACATGGGAATATGCATAGGAAGTATCGTATATCTGGTTTAACAGCCCAACCAACAAGAGAGTTGCAGTAAATGTTCTTTTATATTTATCTTATTTTCAATCATTTTGACTATTCTTGATTCTCTACTCTTATTGTTGTATGTACTTTGTTATGTAATTCTTTTATAATTTTATGGTTGGATAATAGATTTCCTGCTAGTGATCTAGGAACAATGAAGTCTATTGTTTAATACTTTCAAGAAACCTATGGTTTCAAAATTCAAATGACTGTGTGGCCTTGCCTGCAAGTTGGTAATGCACAGAGACCAAATTATCCTCCAATGGAGGTCTGTAAAATTGTTCCAGGTCAAAGGTACTCAAAGAGGTTAAATAAAGATCAAATCAAGGTTAAATAAAGATCACATCAAAGAACTTCTTAAAGTCTCCTGTCAACCGCCAAATCTTCGTGAGAAAAACATTGTGAAGACTGTTGGTCTGAATGCGTACCATAGAGACTGTTACGCACAAGAATTTGGCATAAACATGAGTAATAGTCTTACTTCGGTTGATGCACGAATCCTGCTTCATGGGTTGATTTTGTAGCTAAAATATCGCGGTAGCAGTCAGGAGAGAGACTTTCGACCTGAATTTGGGCAGTGGAATATGGTGAATAAGAATGGGACGAGAATGTGAGACCTCAAACGCGCTCACCGGCTGGGACATTGACCTTCAATTACGAGAGCATGTGTGGCTTTAATGTTTATGTTTTTAAGACTTCTAAAGGAGTTTGGGATGTGCCTTGGATCACCTATTATATTGGACAAGTAATAATAAACAACAAAAGGCGTAGCTACAAGACTAGTAGCATGTAGCTTGATTTGTTGGTATATATTGGCTCATTACAATTATTTGTATATGATGTGATATTTG

>*L. regale* ABC transporter G family member 11 (Unigene0073681)_GenBank accession no. MK805907

ATTCCATTCTTTTCTCGTGCATAATTTAAAAATGCATTATAAACCTGGTCAATTAACTTTGCTTGCAAATCCAGTTTTGCAACTGCATCATGTTGTTTGATTCTGTCAGTTTGTTTTCTCATTTTTTCTCCATTGATCAGGGAATTTTCATGTTGGTTTCCGGGTATTTTAGGCTCCCGAAAGACATACCAAAACCCTTCTGGCGATACCCTATGTCATTCATCAGCTTCCACTTCTGGGCACTGCAGGGTCAATATCAAAATGACCTAAAAGGTCTGGTGTTCGAAAGCCAGTTCCCGAATCAGCCAAGCATATCAGGAGAATATGTGCTTGAGCAAGTGTTTCAGATCGATGTGAACCGATCGAAATGGTTGGACTTGAGTGTTCTTTTCTGTATGATCATCGCATATCGGATCATATTCTTCATCATGATCAAGATAAGTGAGGATGTGACCCCATGGATCCGAGGGTACATTGCCAGAAGGCGGCTTCAACATAAAAGAAGCAACAATGAGAAAATGAATGGATCTGATGATCTTGTCTCCGGAACTCCTTCCCTCAGGGAATATGTAGCTGAACCAGCATCAGCTTCTACTGATATATAGATGAAAAATCAACCTTTACATGACATGATATATAGAATGTTGAATTACTGTTTAATTCCCATTTATAGATTTGATGCCCGGCTCAAAATGAAATTGATCATTTGACTAGAGGTCGTGGTATCAAAACCTTGATCATCAGTAAATGATGCTATTATTGTGTCTGGAGATGTAGAAAACTAAATTATCATAGCTTTTGGTGTTCGTTTTAAATTTAACTTGTTGTATGATGTGGTTTCTTCAGCTCACTAAAGC

>*L. regale* geranylgeranyl diphosphate synthetase (Unigene0079669)_GenBank accession no. MK805908

CAGATTCGAGATGATTATTGCAACTTGTGTTTGCAAGAATATTCTGAAAACAAAAGCTATTGTGAAGATTTAACAGAAGGAAAGTTTAGTTTTCCTATTATACATGCAATCCATACTCACCCTGAAGACAAACAAGTGATTCATATTTTGCGACAGCGAACAAGAAATGTGGACGTGAAAAAGTATTGTGTAAAGTTGTTGGATAATTATGGTTCATTTGAACATACAAGGAAGGTGTTGACACAGTTGGATAGTGACGCTCGTAAGGAAGTTGAAAATCTTGGTGGTAATCCATTGATGATTAAAATATTGGATGGACTTAGAAACTGGTCATATAATTTTGATGAAAAGACAAATAATTAAATGATTTCATTTTAATATTTTTATTTTAATTTCCACCATGACCAACTGTTATGAAGTAGTTATATTTATGTGAATTGTCACATTTTTAGTTGTGTATTAAATATTTGTTGTACCAAAATGTAATGGGGACCCTTTTATTCGTATGTTTATTAATCATCAACTTTAATTAAAGCATTGCTTATACTCTGTTAACCGAATACTATAAGAATTAAAATTC
