# Supplementary material for: Comparative transcriptome profiling uncovers a Lilium regale NAC transcription factor, LrNAC35, contributing to defence response against cucumber mosaic virus and tobacco mosaic virus
Source: Mol Plant Pathol. 2019 Sep 27;20(12):1662–81. doi: 10.1111/mpp.12868 (PMC6859495; doi:10.1111/mpp.12868)
Supplement: Supplementary file 14 — Table S5 Unigenes associated with lignin synthesis exhibiting increased expression in Lilium regale. [file MPP-20-1662-s014.docx]

**Table S5** Unigenes associated with lignin synthesis exhibiting increased expression in *L. regale*.

| Gene ID | Annotation | RPKM | | Log2  (CMV/mock) | FDR |
| --- | --- | --- | --- | --- | --- |
|  |  | **Mock** | **CMV** |  |  |
| Unigene0042594 | 4-coumarate: CoA ligase-like 4 (4CL4) | 1.222(±0.249) | 2.479*(±0.415) | 1.021 | 1.177 |
| Unigene0103052 | 4-coumarate: CoA ligase-like 2 (4CL2) | 0.239(±0.166) | 1.139* (±0.0001) | 2.253 | 0.936 |
| Unigene0064568 | Cinnamoyl-CoA reductase 1 (CCR1) | 57.267(±24.352) | 123.467* (±4.065) | 1.108 | 0.827 |
| Unigene0105931 | Cinnamoyl-CoA reductase 1 (CCR1) | 15.599(±3.599) | 37.934* (±1.638) | 1.282 | 0.703 |
| Unigene0105935 | Cinnamoyl-CoA reductase 1 (CCR1) | 35.993(±11.771) | 73.208* (±4.946) | 1.024 | 0.925 |

*indicates significant difference as determined by Student’s *t* test at *P* < 0.05.
