# Supplementary material for: Comparative transcriptome profiling uncovers a Lilium regale NAC transcription factor, LrNAC35, contributing to defence response against cucumber mosaic virus and tobacco mosaic virus
Source: Mol Plant Pathol. 2019 Sep 27;20(12):1662–81. doi: 10.1111/mpp.12868 (PMC6859495; doi:10.1111/mpp.12868)
Supplement: Supplementary file 15 — Table S6 Primers used for qRT‐PCR, gene promoter and fragment amplification. [file MPP-20-1662-s015.docx]

**Table S6** Primers used for qRT-PCR, gene promoter and fragment amplification.

| **Gene ID** | **Accession no.** | **Forward primer (5’-3’)** | **Reverse primer (5’-3’)** | **Product size** |
| --- | --- | --- | --- | --- |
| For assessment of viral accumulation levels | | | | |
| CMV-1a | LC390165 | TTATAACGATAAGAAGGCGTGGA | ACTCGAAGCATTCCACATATCAT | 217bp |
| CMV-2a | LC390166 | CATCGATCAGCTTTCACTCTTCT | TCTTTCGCTGCTTAATGAAACTC | 212bp |
| CMV-CP | LC390167 | ACAAATCTGAATCAACCAGTGCT | ACGAAGGTTGGGTGGTTAATAGT | 172bp |
| TMV-CP | KJ438787 | TCAGTTCGTGTTCTTGTCATCAG | TCTAGTGTCGAATGCACCTAACA | 247bp |
| GFP | FJ172221 | ATGGCCAACACTTGTCACTACTT | ATTCCAATTTGTGTCCAAGAATG | 260bp |
| LrGAPDH | JZ391059 | TACATCTGGTGTGGTTTGTTGAG | AAAGCCAATTCATTGATAAAGCA | 178bp |
| LrActin | JX826390 | ACAGTGCCAATCTATGAGGGTTA | CTCTAGCTCCTGCTCATAATCCA | 201bp |
| 26S rRNA | AF479174 | AGCTCGTTTGATTCTGATTTCCA | GATAGGAAGAGCCGACATCGAAG | 185bp |
| For validation of RNA-Seq data | | | | |
| Unigene0000074 | MK805900 | ATGGCTTTGTAGCTGAGACCATA | AAGGAACTTGTCATTGTCCACAT | 116bp |
| Unigene0004763 | MK805901 | TCTCATCTCTCAAGATTCACCAA | CATTGTAACCAACCTGGAGAAAG | 202bp |
| Unigene0007097 | MK805902 | CTTCTGATAAGTGGCTCCTTTCC | CCTCAAATCAGATCAACAAGGTC | 132bp |
| Unigene0011570 | MK805903 | TCCATCCTCTGTAGAAGCTGAAG | GCTCCAAATGTACAAGTTCATCC | 135bp |
| Unigene0013897 | MK805904 | GTCCGTTCTTTGTATCGTGACTC | TCGTGGAGGATTCTCTGAAGTAG | 151bp |
| Unigene0017759 | MK805905 | GTCCTGTCAACTGTCCTGATTGC | CATTGGCCTTGTTGTAAAGAACT | 169bp |
| Unigene0063829 | MK805906 | TCCAGGTCAAAGGTACTCAAAGA | CATTCAGACCAACAGTCTTCACA | 141bp |
| Unigene0073681 | MK805907 | ATGTCATTCATCAGCTTCCACTT | GAACACTCAAGTCCAACCATTTC | 175bp |
| Unigene0079669 | MK805908 | TGATTATTGCAACTTGTGTTTGC | AACTGTGTCAACACCTTCCTTGT | 237bp |
| For assessment of gene expression levels | | | | |
| LrNAC35 | MK805884 | TCTCTACAAAGGCACACTCTTCC | CACTTGTGTGCTAGAGATGTTCG | 241bp |
| LrERF61 | MK805885 | AAAGAGATGCAACAATCCAAGCC | TTAGCCAGTACTTCCCAGATGAG | 167bp |
| LrTINY | MK805886 | CATTATTCTCTCCGTCTTCATCG | ATCTCTGGTGTGGGAAATGTGCC | 214bp |
| LrIAA17 | MK805887 | TACCTCAGGAAAGTGGATCTCAA | AATATCTTTCAGCCAGCTGTCAC | 150bp |
| LrbHLH100 | MK805888 | CTTCTCGGTGATAGCTTGGTTTA | GATGCGTTTATCAGTTGAAGTCC | 161bp |
| LrbHLH18 | MK805889 | GACCAACAGCATATTTGGGATAA | TATAAGTCGGAGTGACCCTTTGA | 227bp |
| LrRF2a | MK805890 | AGAAGATCACCTCCATGTTCAAA | ATCAACAACCATCGAATCAGTCT | 200bp |
| LrMYB98 | MK805891 | TAGGAAACGAGAAGAATGCAAAG | GAGCAATATGAGACCAATTACCG | 180bp |
| LrCPC | MK805892 | AGTTGGTAAATTCCTCCAATGGT | TCAATCTCTTCAGCTGTTCTTCC | 166bp |
| LrMYC4 | MK805893 | AGAGAGCAATAATGTGGTGAAGC | TGCCTGATACAAAGACCAGATTT | 207bp |
| LrNAC100 | MK805894 | GGGAGAGAGTTACTTGCAACAGA | TTGTCCATGTTTATAAGCGGTTC | 258bp |
| LrNAC48 | MK805895 | GATACCTATTCCGATCATTGCTG | CTTTCTTTATAGCCATCGGCTTC | 224bp |
| LrWRKY28 | MK805896 | TTTCATGACGGTAAGTGAGGTCT | TCACAGTCTTTGGATCTTGGAAT | 169bp |
| LrWRKY48 | MK805897 | CCGAATTGCTCATCTATTTCATC | CGTACTTCCTCCATCTGTAGCCG | 211bp |
| LrDOF5.6 | MK805898 | TGGAATACAAGTATGGCACTCCT | CATAACCCGAGTCCATTTGAATA | 289bp |
| LrZFP28 | MK805899 | CCACCAGAACGTTTAGATATGGA | GGATAGTCAGCTTGTTGTGGAAC | 191bp |
| PhPAL | SGN_Peaxi162Scf00123g00096 | GACCATTTGACACACAAGTTGAA | GTTCACCGAGTTAATCTCCCTCT | 243bp |
| PhC4H | SGN_Peaxi162Scf00390g00225 | GGAGTATGAGGTGGAAAGTGTTG | GCTATCCATGCTCTTGGTATTTG | 367bp |
| Ph4CL | SGN_Peaxi162Scf00195g01223 | TTAACAAGCTAGGGATCCAACAA | AATCTGTTCGTCCACCTGAATTA | 311bp |
| PhHCT | SGN_Peaxi162Scf00835g00312 | TAAGGAAGATGGCAATACGGTAA | ACCATACCAAATAGGCTTGGATT | 241bp |
| PhC3H | SGN_Peaxi162Scf00220g00211 | ATGGGCCTCATTATGTTAAGGTT | CATCCATCACTCCTTCAAAGTTC | 255bp |
| PhCCoAOMT | SGN_Peaxi162Scf00016g02023 | TTGCCTGTTCTTGATCTAATGGT | AAATTTCAATTCTTGGATCAGCA | 274bp |
| PhCCR | SGN_Peaxi162Scf00332g00433 | TTGCTTCTTGGCTTGTTAAACTC | CACATCCATAGATTGCTTCCTTC | 183bp |
| PhF5H | SGN_Peaxi162Scf00083g00156 | GTTTGGAGGTACAGAGACAGTGG | AACATTAGCCAGTTCCTGTTGAA | 106bp |
| PhCOMT | SGN_Peaxi162Scf00912g00111 | CCATTCAACAAGGCATATGGTAT | GTTCATGGTAATGGTGGAGTGAT | 111bp |
| PhCAD | SGN_Peaxi162Scf00016g02329 | ACAAAGTGGATTAAGAGGTGCAA | TCTTTCTCCCAAGCATAACCATA | 347bp |
| For amplification of gene promoters | | | | |
| pPhC4H | SGN_Peaxi162Scf00390 | CTGTAACACCAGCCTTTCCCCTC | TTTTGTTTGGACAAGTTCTTTTT | 2,000bp |
| pPh4CL | SGN_Peaxi162Scf00195 | GCAGGTCATCACAAAGACGGTAT | CTTGGGTACTTATTGTTCAGTTT | 2,000bp |
| pPhHCT | SGN_Peaxi162Scf00835 | ATATGATTCAAGTCGCTGCGCTC | TGCTCCTGATGAGTCTTGTTTTC | 2,000bp |
| pPhCCR | SGN_Peaxi162Scf00332 | TATGGTCACTCAACTTTCAGAAA | GTTTACTTTCTTAGTGAGAAGAA | 2,000bp |
| For amplification of inserted fragment used for TRV-VIGS | | | | |
| PhNAC35 | GBRU01060495 | TCGGCCTTAGTAATTCTCATCAA | ACTGAGTTCCAATCCCATAATCC | 310bp |
| For assessment of *PhNAC35* expression levels in VIGS assay | | | | |
| PhNAC35 | GBRU01060495 | ACTTCAGGCTACTGGAAGGCTAC | GGGAGAGATGGATGGTCTTCTAC | 242bp |
