# Supplementary material for: Comparative transcriptome profiling uncovers a Lilium regale NAC transcription factor, LrNAC35, contributing to defence response against cucumber mosaic virus and tobacco mosaic virus
Source: Mol Plant Pathol. 2019 Sep 27;20(12):1662–81. doi: 10.1111/mpp.12868 (PMC6859495; doi:10.1111/mpp.12868)
Supplement: Supplementary file 1 — Fig. S1 Sequences of all assembled unigenes in Lilium regale. [file MPP-20-1662-s001.docx]

>Unigene0000001

CAAGCCATGGCCGACCCCGCCGCCTCCCCTGAGCCCCCGATCATCGGCGGCTGGGGCGTC

CGTCGCAAGGTCCAGTACACCGGCCGGAAGCCGCAGCGCGACTCTCAGTCCTCGACCGAC

ACGGCCTCCTCCGAGCCGCCGCCGCCGGTTCCCGCCACCGTCGCCCCAAAGCGGAAGAAG

ACCGCCTCCGCCACCGCCGTCCGCTGGTCCAAGGAGCGGTACGCCATGGCGGAGATGAAG

CTCGTCGAGATCATGCATCAGATGGGGGCGCGCGTGGGGAACCCGATCGGCCGGCATACG

GTGAGGGAGGAGGCGCGGAAGCACATTGGGGACACCGGCCTGCTGGATCACCTCCTCAAG

CACATGGTGGGGGAGCTCGTGGGGGACTGCTCCGTGCGGCTGCGCCGCCGGCACAACGCG

GAGGGGGCGATGGAGTACTGGCTCGAGCCGGGGGAGTGGGCGGAGATGCGGCGTGACGCT

GGGGTGAAGGACCCCTTCTGGGTGCCCCCTCCAGGGTGGAAGCGGGGGGATGGGACCGCC

CCGTGCCCGTGCGGGGAGGAGTGCGATCGGGAGGCGAGTAAGCTCGGCGAGGAGGTCGCC

GCAGTGAAGAGGGATTATGAGAGCTTGATGGAATGGAAGAACAGGGCAGAGGAAGAGATG

GTCAGTATGTCGAGTAGCTTGCAGGTGCTGAAGCAAGAGATGATGTTGATGAGGGAGGAG

AAGCGCCGGAAAGCCAGCTTTGCGGGAACTTGCCATTCCAGTGTCTTGAACAGAACTTAG

ATTCCTTGGTGAGAGCAACTGACGTAACCATCTTCGCAACTGTACATTGATGACTGTCAT

TGGAGAGTCTGATGATTTTCCATATGAAACTAGGTGAAATACATGTATCTGAGTACATAA

GACTGTTACAAACATTACTAAGTAATCCGAGCTTGATGAGTAGGAGTAACTTTTATCTGT

AAGGTGGGGTGATTGGATAGGTGCTGTACTGGAGTGTGGTCTTTCCAAATGCTGCTGTCA

GAATGTCTAGTCGTGTTTACCTGGATCTTTCATGTGTTTGTGTTGGAGTATGCCTTTTGT

TTTTATGTTTCCTCTTTTTTGGTTGTTATTTGACATTTTCCAAACCGATTTTTTCCCTTA

TAATG

>Unigene0000002

AGGATGTTCTGTCCCCGCCTTGCTTGGGACACAAGATGAACAAGGGTTTCGACAGTTAAG

CGAGTCGGCCACTCTTCATTCCACCCTCACTCCGCCATTCCTCTCGCCGGCGGCGCTGCT

CGCCGCCGCCGTCCGTTGCCGCCCTCTCATGTCCTCAAACCCACCGGGACAAATTCTCCG

GCGAGTCACTCCCCCGTCCCACCTCAGCGACGAGTCGAAGCTCCCTCGCAGCTTCTGGCT

GCTCGTCCCCAAGCCATGGCCGACCCCGCCGC

>Unigene0000003

GCAAATAAAGATGGTGATGGTTCTACAGTTGTGGTGGAGCTTTACAAAAAAAAAATGCTA

AAATATGTAGAAGCTCGAACGATGCCCGCCAAATGCGTGGTTATGGTCATTAATTGGAAA

TTGCTCCAGCCGAGAGGATATAACTCTTCTATTTCAGATATTGCAAAAGCTACGAGTATT

TTGATTGTCAAACCTCTCATAATGGATAATTTCAATGATCATCTCTGTATGAAAGTTTCG

GAGGCTTGTGCCCGTGCCAATGGCATCGATTATGGCATGAAGGCATTATGGAAGCATAAC

ATTTACGAGTTAACTCCAAGCATCGCTTCTGCTCATTATTTACTGTTGCATGCTAAAAAG

AACAATGACATTAAGCTCATGGAAAGGATTATGCAAGTTCTGCGAAGAAACTCTTTGCCA

TTGCAACCTGGTACAGCGAATATAGTCTTCAGTACTTGTTTCAACACAAACAACTGGGAT

TTGATATCGAAGTTCTCAGAACTTTTTCTGAAAGCTGGAGTGAAACTACGCTTATCCACG

TTTGACGTCTGGATGGAGTTTGCTGCAAAAATTGGAGATGCTTAGTCTATATGGAAAATC

CAGAAGCTAAGGTCAAAATCAACACAAAAGCTCATGCTCGCAAGTGCCTTTTCATGTGCA

AAGGGATATCTAGTTGAACACAACCTCAAGAGTGCTAGTGCCATCATTCATCTTCTCCAC

CAAGAACTTTATAACAAGAAAAAGCCAGGCATCAAAGATGAACTTCATAAGCTGATCAGT

CAATGGCCAGATGAATTGATGAAGACGAAGGAGGAAGACGATGCTGACGCGGAAGCCACA

TCAGCCTCACGCCACCTCACCCTCCTCCGTCTGCGTCGTCAGTCACCCGTCGTCACGTTC

CGTGACGGCACCGTCAAGTCCATATCAGCAATGTCCGTCACCCTTCCGTGATGGCTCCGT

CACCTTCCATCCTTCCTCCGTTACCGTCCATCAAGTGATCGAGTTTTCTGCCCCGACTGA

CCACGCAGTGAAGACATCTTTCTTCTCCCCCGACTGACCGAGCCTTACAGACCAAGTCCT

CCGTCGAACCCCTTTCTCCAAAACAACAATTGGAGACCAACCCGCACGGGGCAGAACATA

CATTGGGTGAATGCTATAAAGCCAGGGAGGATTGCAGTGAAGCGGCATGGTGGGAGTGGG

AGAGCTTCACATGTGGGAGTGGGAGAGCTATTGTGAAGGGATATTGCGAAGTAGTCATTG

AGAGAGTTTTGATATTTAGAGAGAGATAGTGAGCTTAGGTTGTATTAGTGAGGAGCAGAG

GCATTAGAAGAATCTGTGTGACCGACATATTATAGGCTTCTGTTCGAAGAGATTCTTTGA

TTATAAGGGAATTGTAATTGTCTGTGATTGTGGATTGTTGGCAATTGTCTCCTCTGTAAG

GAAGTTCGAACCCTTTGTAAGCTCTGTTTTTCATTAATGAATGAACTATT

>Unigene0000004

GCGCAGAGCAAACCGATGCAGTTGTCGTCGAATCTCCGGCGCCTCCCCGCCCTCATCTCT

CGTCCCCTCGTCCTCCGCCCACAGCTACACCTCGCCGCCAACATCGTCGCCCCTCTCCCC

GCCGCCGGGCAATGGTCGCCGCCGAGGAGTTTCTCTGTCCAACCCCGTCGTCCTCTCGGA

AATAATCATGCCAGTATGGTCTTTCAGACTGCTCTTCAAAAATGGGATTATGGCCCATGG

TCCACTCCAATTCTGTTGTCTTTTTATTCTTCAGAAGCAAATAAAGATGGTGATGGTTCT

GCAGAAGCAAATAAAGATGGTGATGGTTCTGCAGAAGCA

>Unigene0000005

CCAACGGTATTTGCTGTCCCATCCCTTATCACGTGTTTTCTACCTTTGTTAAATGCCAGA

TGAGAGAGATAGGGCTGAATGTGTTTGCTGGTAGCTGTCTTAATGGATTCCATTCTTCCC

GTTTTTCTGGTGCTTACTAGGCCGAGAGAAGAGGGCACAGGTGAGGATTATTTCAAACCT

CACATGCTAAGCTATGTTGGCTGCTGTGTTAACCCTAATTTCAAACCAAACGTATCAGGT

TTAGGTGAGTTGGGTCTGATCCTGTATATTTAAGCAAAACATATTGGGGCTGTTTATTTG

GGCCTAGATTGCCATTAATACCGAGTTTGATTCGGGTCCAATAATAAGCTGGAACCCAAC

TAAACTGAGATTACCTTTATCCTGAATTAGTGCTTAGGCTGAACCTGATTTGAATTAATC

CAATCCTGACTTGCTTATACTGGGCTGAACCCAATACAGACCCATTAGGCCGGTGCACTC

ATGCTTAACCCGAGTAATAGAGCGGGATGTGCCAGGCTGATTTCTCCGAACCATGTTGGT

GCCAAATCTCAGGTTAGCCTGTGCTGGCCAGGCTAACCCACGCGGGGTAAGTGTTGTATG

TTTGCGGACTCTCGTTCTCCGCGGTCGGAAGCATTAAATTTGAACTGTCATTCCAGACGC

GCATGTCAAGCAAGTATGACGCGGCCTGTCAAGTAAGCGGCCGTTGCGCTTGTTGTCGTG

TTCTGGTGTCCCGGAGCGGGTTGGGCTAGTCAGGCGACCACAGATGGTCTTGAGTTGACC

GAGGCTAGAAGAGCGGGTTGTGTGCTCGTGGTCTGGCCGTGTCGACTTGGGAGATGGGCT

GGACCCATCTAGTCAGGTCGGGACCCGAGTAATTGGTTTCCTGGTCCCCGATAATTGCGG

CATCTTCACTAGTGACCTTAGGAGAGAGCATGGTTGCCCATAACCAATTGCAGTTACTCA

CAAACATATTAGAGGATAATTTGGAGAGAATTGATGATAATCCCTTAAGATATATGTGAT

GTTGCCTTTAGCCTTATACTTTTTATCCACAAATACATTGGAGGTTAAGTTGAAGAGAAA

GTTTTGATGAAAACATTGAAGGAATTGTTGTGGTTGCCTTTATTAAAATCTTATAAGTAC

GCCACATACACCAAATTTCCCCTTTAGCTCATGTGTCAATCCACATAACCAAGTCACCAC

ATCTCTTAGTCCCTTGTGGCAAGCCACATGGCTAGCCACCTAACCAACTTTAGCCATCCC

ACCACACCCATCAATCTGGAGAGTTACATCCGAGACAATCTCCTCCTCTCATCCATTCAA

TCCTATGGCTAGGATCAAACCACTGTAAACCCTAATCCACACCAACCACTCCCATGCCAC

CTCACTGATCCGGCCCATCACTCCCCCTTCAATCTCCACCCTCTATCAAGATTTAACCCA

CGGCCAAGATCCTGCCACGTAGAGGCACGAATCTTCATCCGTTTTCCAAAAGACCATCTT

GCCCCTGCAATCCAATGGTACCTTATGCATTTGCCACTGAAATTACCAACCTGTCCCTCC

AATATAAAAGACCAACTCCCGACTACCGACCCCCATTTCCCTTCTCTCTGATCCGGAGAG

CTCGAAGCTCTCCAGCAATGGCGGACTCCGGCCACCGACGATGCTCCGACCATCTCCGAT

CCTCTTGCATGT

>Unigene0000006

GCCTTCTCAAATTCTCTACAACCTAATGAGGAAGCTATGTCTCAGGTTTGAGCTCTCTGG

GAAGAAGCGACTCCTGCCGGCGGCAGCGCAGAGCAAACCGATGCAGTTGTCGTCGAATAT

CAAGCGCCTCCCCGCCATCCTCTCTCGTGCTACAGTCCGCCGCCCACAGCTACACCTCGC

CGCCGACATCGTCTCCCCTCTCCCCGTCGCCGGGCAATGGCTGCCGTCGAGGAGCTTCTC

CGTCCAATCCCGTCATCCTCTCGGAAATAATCATGCCAAAGCAAATAAATATGGTGATGG

TTCTGCAGAAGCA

>Unigene0000007

GCCTGCCTATAGAGGAGGTTCATTGAAGTAGAACCTCCTCGCTGATGCCGAAGCCTGAAG

CTCTGATAAACTGGAGTTCGGAACAGTGTTTATGTATGTATGGAAACGGTAGCTTAGAGT

AGCTAGGTATGCGTGGAATCATTAATTAAATTTGGAGGAAAGAAGATCTATGAAAGCGTT

ACTAGAACCATTAGTGCGAACTGATCTACTTAATAATGATGTATGCTTAATAATGGATGT

ATTGACTCAATATTAGCCCTCACCAGGGCTGTTAATAAAATCAGTATAAGTGCTCAGAGT

AAGGTCAGTTAGCTAGTACATTAAAACAATGGAAACTGGTAATAAAAGAAAATCTCTTAC

TGATACGGTTTTGGGGCCTGTAGGGATGAATGTATTTACATTGCGATGTGCTCTTCAACC

CGTCAAGGAAAGCATGTAAACTGTCGGAGTTGATAGCAGGGTGGTGATGCCATACAGTCG

GGGACCGGCCGGATTGCCGAGGGCCTGCAGATGGCTTGCTGAGTATCCACACTAAGAGAA

CTGCGAGGTCAGAGTTCTTCCTCTCGCCCTCTAGAACCTCTTTCTCTCTCCTCGTACTCC

TGCCCGCACCTGTTCCGCGTCCTTCTCC

>Unigene0000008

AGGGCTCTTCTACAACTTCAAGGCAGCACAGGGTGAGCAATCCAGCTATGATGTAAGTCT

TTCTCCATCATTTACTTCAGATATACTTTTCAGGATACTACTTTAGTTGGAGAGTTGGGT

GTGGATGGGTCTCTTTTATGTGCCAGATGTGCAGCTCTTAAATACTTTGTGAACAGAAGT

TGTTTAGTTTGACATGGGCATTAAATTTTGGTTAAATTGCGTGCTAAGACAGTCTGGAAC

ACCTTGGTCAAGCTGCACCACAAATTGGTTTGGCTCTACATCATGAGGGGCTCACCTTAA

TATGACACAAATAGGAACTCGTTGGGTTCCACCATCACACGCATTAAACAACGATGCGTC

ATGCAACCGTTTGGTCCACCTGTCTTACTGGTGTGGGCTAGCTAAGAAGACTTGTTGTTA

AGTTGTACATTGGGCCAGCAACTTTCATTAGTGCAGGAAACTATGCCAATTTCTCATTAA

AACCTTCTAACAGCACATATTATACAAATTTCCAAGCAATCAAACTCTCAAAAAGTTAAA

TTCCGTTTATATGAAAAACATATCATCAGTCATTTAATCTTTCAAAGAAGTCTATTTATG

GGCAGGAATTTATGGCTGAAGTAGAGACGAGAGCTGCACTAAACGTAATTGAGGCG

>Unigene0000009

CTTGCTTTAGACACCCCCTACCCTCGAAGTCCATGGTATATAGATTCAGTGATGACACGC

ATTCACCCGTCCCGATGCTTGCTGGTGGCTAAGTGATCAAGGAAAACACATGTCACCCGA

CTGTACTTGGGTTAGGCATTGGCACTGAGATACGAGCTTAGTTGGCAAAAACTTAAGATA

CCCGATAAGATACACATTCTCCATACCATATCAGCATCGACATTTTTTCCTAAAGAAAAC

TGTGTTTCTTATGATACATGATAAAGTAAATATAGTACCTTCTACACGTGTATTGGTTTG

GGGGTGGGAGGTAGTAGCAACTCTTTTCCTCGATTCCTTGCTTTGACTCTCTCACACACA

CAATGTAAGCTCCCAAGTCCCTCATTATTCTTCGCACCATTCTTTTGTCACTCTCTTGTT

CATTAACAATTCCTTGAAAATCTTCTAGCTTTTGTCATGATGTTCTCTACTCCCACTAGT

TTATGTGTTACACATATAAATCATGTTTAATGGGACATTTTGA

>Unigene0000010

AGCGTGGAGGATATTTTGAGATGACTAACCTTCCTCCTCAGGTATCTTCTCCTTTTCATA

TGGTGGACAAATCTGTTCAACCAGGGATAATGTCTTCATAAATATACTCACTCCATGTTT

CTCTATGTTTTATAGGTTTGTTTCACAGTTGAGTAATATTTGAGGTTCAAGTCTTAGTAT

GATAACATTTCAATTAATTTACTTTCTACAGCTAATGAAATTTAAGAAAATATCTAAATA

AGATTGCTTAACTAGTAACTTCATTATCTCTTGTATATTCGTCTGATCTGTATTATACTG

GAAGGTTAATGCAAAATTCTGAAAAAAAACATTTATCCAAGGTCAGGATTGCGTTTCAAC

CGAACCAATGGATCATAAAGAGTCAGTGGCAAAACTGTGTATACAATAGTTTTTTGTACA

CAACATTCATTTACCCTTTTGGAGCCCCCCAGCTGTCTGGTACCAGATAAATGACCGGGT

ACAAAAATCTCAAATTGTCTACTATGGGTCATTATGAGGTGAACTAAACTTAATTAATTA

GACAACGAAACCTAATAAATTAATCAGTAGACCTACTTTCACTGGTTAAACCCAAACTAA

ACCCACTCGAACGGGACCAAACAAAATCAGGTCGATTCGAACTGTACGCCCTGAACCAGA

CCAACCCGACTGACCTGGACTTGCTTTAAACACCCCCTACCCCCGAAGTCCATGGTATAG

ATTCAGTGATGACACGCATTCACCCGTCCCGATGCTTGCTGGTGGCTAAGTGATCAAGGA

AAACAC

>Unigene0000011

TTGTAGTTTCTTTTCGTTGTAATTTTTTTCGGAGCAATTAGTATAATGTTGTTTTTCGTA

CTCCCCGCTTTCGTCTGAATCCCAGAATTAGATAGAATTAGTCATTTTTTTCTTTCAACA

TTTCTCAGACTTTTTTTTTACTTTCTGTCGCTTCACCAATGTTTTAATATATAAAATTAA

TATCGTCTTTTTATGAAAAGACAGTGTTTAGTTCCAACTTTAGGGACTCGACACTCGACT

TGGAATAAAAAAGAAATATTAACCAAAAATACGTCTTTCCATTCCTAAAACCAAAAACGA

AAAAAAAAACATATTTTACTTCAGTCTTTCAATTTTCAGTAAAACCCAAAATACCAAAAA

GAAGAAAACCACTCTTTCCCTCTCTGTATGAATTCCAGAGTAGGGTAGTCGACTTTTCTC

TCTCCTGAGAGAAGGAGATTCAGACCCTACCACCTGAGTGGGTTCGACCTCTCGAGTCAA

CTTCTGAACACGGGGGTATTCATCTACTACAATGATAACTAAAAGCTCCGATCGTCAAAC

ACATCGACGTCACAGGGGAGTTCCGGTTCCCTCTTTATTTAGAAACGCATTATTATCATT

TCTTCTTCTTCCCTATCAAAGTTATTATTTCTATCGTTTACAGGGAAGCAGAGAACAATC

TAGAAGTAACAAAAACTTCTGGAATCTAATTAATTTATAGCTTTTTACGCCTTATTGCGT

GAAACTCAACAGGTACGCAAAAAGACGTTTATTACTAATTGCCTCTTATGCTTAACCCTT

AATGGGTTTTCTCCACTCTATTTTTCTTTTATTTGTGAGAATTCACTAACCAAGTTTGCT

GAGGGGGTTATGGCAAGTCCAACACCCCTTCCACCCCACCAAAAATAAAAGTT

>Unigene0000012

GAAAACAGAAAAAAATTATTTTAATCGGACTTAAGATGAAGAAGTTATAAATAAAAGATT

TTTTAAATAAAAAAATAAAATAATCCCTCGGTGGGCACGGTCTCGGCCCTGACGGCCAAG

CATGGCCGGGTGTTGCACAAGCAGCCACATGTCCGGGGACACTTGGCACACATGCACAGC

CTCGGACCTGTCGGTCCATCTGGGGCCGGATGCCTGTCCTCGGGCCCTGCCAGCCCCATT

GCGGCCTGCACACGTGTGTGGACCTCGCCGGCTGCATTTATTGTGACCAGGATTTTTTTC

TTTCCTCCCTTTGTTTTATTTTGTATTTTAAAAAAAACCCCTCCAATGTTCTTTTATTTT

GCAATCAACCCCCTTTAAAACCCTATAAATAGAGAGTTGAGGCTTAAAATAAGGTTTTTG

GTTAGGATTTCTGTTGGATTTTTGGGTTGGAGTTTTAGTTAGGTTTTTATTCCGGAAAAT

TCGAAAAAAAAAGGCAAAGGAGAGGAGAAAAAAGGAAAGAAAAGTGGAGGAGAGGAATGT

CTAGAGGCAACACTCGAGAGGAGTCGGAGAAGAAGCGGAAGACTCT

>Unigene0000013

GGATGCCTGTCCTCGGGCCCTGCCAGCCCCATTGCGGCCTGCACACGTGTGTGGACCTCG

CCGGCTGCATTTATTGTGACCAGGATTTTTTTCTTTCCTCCCTTTGTTTTATTTGGTATT

TTTTTAAAAACCCCTCCAATGTTCTTTTATTTTGCAATCAACCCCCCCTTTGAAACCCTA

TAAATAGAGAGTTGAGGCTTAAAATAAGGTTTTTGGTTAGGATTTCTGTTGGATTTTTTG

GGTTGGAGTTTTAGTTAGGTTTTTATTCTGAAAAATTCGAAAAAAATGGCAAAGGAGAGG

AGAAAAGAAAAGGAAAGGAATGTCTAGAGGCGACACTCGAGAGGAGTCGGAGAAG

>Unigene0000014

TTCATGTTTAATGGGACATTTTGACTTATAATCCCCGAAACAAACTCAATATTAGGGTTG

GGTAAGAGATCCTAGGTTCCCTCTCACTATTATTCGCTCCGTTCTTGCATCACTCTTGTT

CATCAATAATTTCTTTCAGATCATCTAGCTTTTATTGTGATGTTTTCTCTACTCCCTTTA

GTTTTCTCTACTCCCTTTAGTTTGTGTTATACATATAATTCGTGTTTAATGGGTCATTTT

GACTTAGGATCCCGAAGCAAATCAATATCATGGTTGGGTAAGTGGTTGTAGGTTCCCTCT

CACTAATTATGTGCTCCTTTCTTGCGTCATTCTCTCGTTCACCAACAAGTTCTAGCAATT

CTTCTAGCTTTTGTCATGAATCGCGATGTTTTCTCTACTCCCATCATTTTATGTGTTATA

AATATAATTCATGTTTAATGGGGCATTTGAAGTTAGGGTCCCTGAAACAAACTCAGTATT

AGGGCTGCGTAAGAGGTCATAGATTCCCTCTCACTCTTATTCGTTCCGTTCTTGCGTTAC

TCTTGTTCACTAACAGTTTCTTGCAAATCTTCCAGCTTTTGTTGTGATGTTCTCTACTCC

CATCAGTTTGCGTTATACATATAATTCATGTTTAATGGGACATTTTGGCTTAGGGTCCCT

GAAACAAACTCGATATCAGGGTTGCGTAAGAGGTCTTAATGTTAGGGTCCCAATATTTCT

TCTTGGTGAATTTTTAGCGGAATTTACCT

>Unigene0000015

GAGTGTGTCTTGACTCGCACCTTACCTGTGAGAGTGGGATGGACCAAATGGCTCGTTAGG

TTCAAGTCATCTGTCTCCTGCCGATCGTCGTCGCCATCATCATACTCTACTTATTTTGTT

CAGCACCCCCTCCACCCCACCAAAAATAAAAGTTCCGGTGATATAAGCACAGGCCCCTGC

ACATCAGTGCTTTTTGCTTGGACCGGGAGTGTGTCTTGACTCGCACCTTGCCTGTGAGAG

TATGATGGACTAAATGGCTCATTAGGTTCAAGTCATCTGTCTCCTACCGATCTTTGTCGC

CATCATCATACTCTACTTTTTTTGTTCAGCATATTATAGCTTTCTGTAGTCCAGATCCTT

TTCCATATCAAACTACTTTTTTCTTTGTGCATTCATAAAAGCTGGGCTGCTGCAAATATC

CCCGAGTCTCATATTTGGGATCAAAGGTAGGATAATTTATTTTTTTGTTGAATTTTTAGC

AGAACTTACCCGGGACAGCTCGCGTGCCCTGCAGCAAATTTCCGCCTGGGGCATTGAGGC

GTAAATGCTATCCCCTACCAACCTCCGCCTGGGGCATTGAGGTGTAAATTCTATCCCCTA

CCAACCTGCTCTTTCTGGGATTGGGACCTCTGTCAAGGGTGCAATCCTGGGGCCAGTCCG

CCTACCGAGTCAAGCTCGGTTGGTTCTCCAGCATAATTTTTCAGTGGTGGAGGTGCATAC

ACCTCCTCACCAGGAAAACAAAAACCTGACAGGTTTTTAAAAATCACCCCAATTTATTTT

AGAAGGCATATAAAGTCACTCCATTGTGTGTTATATGCTGAGGTAACCCAAGTATAAATA

ACCTTTAGATCCTATATTTGTTTGGAACTTTGCAGGGCCGGGGTATTCCAAGGAACTTCA

TATCAGATGCTGCTTCAAAGATGCCGTTA

>Unigene0000016

TAGTGTCTGTCAAGTGGGCCTTGATACACGGAGACCATTAAGTTTTTTATTTATTTTTAG

CAGTCACAAATACAAGCCATATTTAGGTTAAGTTACATAATTGATGCACCCTATTATTTC

TCTGATACACAATCTGAAAATCACCACGAGAGAGGCCCTGGAGCGATATGCTCCTCCCTA

TTTTTTCTGATACCGGTAATTTTACCTCCCTTCTTGAGTGTGGTGTCCGAATGCGCTCCT

TGTCGGACTATGTAACTCTCTCTTCTCACTTTTGAACTAGATTTGTCCCCTCTTCTCCTC

CCTCTCTGTCTATTCTTAGTGTGGTATTGAGTCTTGTGTCATTTTAGGTACTTTATTATG

TTAATTTTGTTGGATAACATCAATTTATTTTTTTCTAAAACTATTATTATTTTTCCTTAA

AAATAAAGTGTTTTATGCCTTGTGCAAAAAGGGCGATTCTTGTTGGTGTGCT

>Unigene0000017

TAGTGTCTGTCAAGTGGGCCTTGATACACGGAGACCATTAAGTTTTTTATTTATTTTTAG

CAGTCACAAATACAAGCCATATTTAGGTTAAGTTACATAATTGATGCACCCTATTATTTC

TCTGATACACAATCTGAAAATCACCACGAGAGAGGCCCTGGAGCGATATGCTCCTCCCTA

TTTTTTCTGATACCGGTAATTTTACCTCCCTTCTTGAGTGTGGTGTCCGAATGCGCTCCT

TGTCGGACTATGTAACTCTCTCTTCTCACTTTTGAACTAGATTTGTCCCCTCTTCTCCTC

CCTCTCTGTCTATTCTTAGTGTGGTATTGAGTCTTGTGTCATTTTAGGTACTTTATTATG

TTAATTTTGTTGGATAACATCAATTTATTTTTTTCTAAAACTATTATTATTTTTCCTTAA

AAATAAAGTGTTTTATGCCTTGTGCAAGAAGGGCGATTCTTGTTGGTGTGCCCGGGGCGA

GAATT

>Unigene0000018

CGGGGCGAGAATTTATTTATTCTTAAACTATTATTTTTTCTTAAAAATAAAGTCTTTTAC

ACCTTGTGCAAGAAGTGCGTTTCTTGTTTGTGTGCCTTGAGCCTCGGGCGCTTGGAAGCC

TTTGTACCTTAAGTGTGGCTCGTCATCTTGACTACATTGTTCTCCCCATTTTCTCTGTCA

AGGACATGCCAGTAAATAGTTCGCAGTGTAGCAAACGGATCATGACACAAGTAGGCCAAT

CATCCCTCGGGCACCGGTTGCTATCTCCGGTTCAACTCCCTCTCTTCCAGGATAGAACTA

GGGAAAGTTTCTTTTAAGAAGGATAAAAGAAAAACAATTAATGGGCTACAATAGAAGATC

AATGAGAGCAGCATGAGGAGTGTACAATGGAGTGACCAATGAGCGGTTTTCGCATGAGGA

GCATGAATTGACGAGAATGACAGAGAGAATAAGGTTGAGATGCTAAACGGCTATTTGTTC

TCTAAGTTGTCACTTCACTCGTGTTTCGAAATGCTTACGAGCGCAAAAATGAAAGCCCTT

TAAGGTTTAGACAATGGCACATTTGAAATTTAGTGACCTAATGTTTTCTAAAGGAATATT

GGTTTTGTAGTGTGGGGTTCTAGTGTGTACTCTTCTCTTCTACCTTCAGTCCGGAGCTTT

AACCAATCCATTTTTTTGTCAAAGACCTTAAAAAGGTAAGTGTTTATTGCTTTCTCATAG

TTAATTTATATCCATTCATTTTTTGCTAGGTCAATTATATAATTGATACCAGTTGTATAT

TAGGATTTTTGGAAATCATGTTTTATTACATGTTCCTTCAATATCATTAAGTTGCTTTCT

TGATTCGCTTGTTGTGAGAATGATAAAACAAAATTCCTTATTTATTTTCTCTTTCTTCTC

TAAATTTAGATTTTTTTATGTTGATATGAAATTAATTTAC

>Unigene0000019

TGCAATCCACAACTTCCATCACTCTCTTGCTACAATTCTCCACCATTGTTACCGGTAAGC

CCTTCATCTCTCCCTCAATTTTAATTCACAGCCGTTCATAGATTGTGAGATAGTTTATTA

CAACTTTTTCCATTAATGCGAGCTCTAGATCTTGCCTTCAATTCTAGAGGCCGCCATGTT

TTACCTCAGATCTACGGCACCAGGATGGGGATGGGAGTAGTATATTACATGTGACTACCA

AACTTCTTTCCAAATTGTGCTAAACACACCAATCGGTGGCATGAGAGGAGCCCCTGTAAA

ACCCGTGAGTTACCGCGGAGCGTGGAGACACTTCGAGATCGATGAGGCGGCGGCGCAAGC

TGTGGCTCAGCTTTGACAGGAGATGCATGCTACTAGTACTTTGACTGCCACTGTCGCACT

CGCTACTGCCCCTGCCTTTACCGCGACGGCAGTCCAGGAGGGGGAGTTGATGGAGGATAT

GTCATCGGCGGTGGCGCTGCCATTTAGGATAAGCAATTCTCTGTGTGAGACAGCCCCGAT

GGAGATCACTCGCATCAGGCTGGTTCAGGATACTGCGGCCATGATGTCTGATCCCATGGT

CGCTGATCCGCCCCCGGACGTGGCGGAGGTGGCGGAAATTCCTATGGGAAGGGCGGTGAG

CGACGAAGAGGGCGATGATGATGCGATGTCGGTGGGGGCGGAGCCGTCGGTGGGAAGCCC

TTGCTCGGTTGTTAGTGATACAAGTAGTTTAGCTAGCATGGCTGATGATTTCGCGGCTTT

GGAGGGCTCTTATGAAATTGGTAATGGAAGGAATATTGGTTCTGGTGTGGAGATTATACC

GGGGCCTGTTTTTGAAGCTTTTGATGCTAATTTGTTGAATGGCGGCGCATTTGTGTCTGA

TACGGAGGTGCCGTTTGTTACTGAGACTGCGATTCAGAGTCGGAGCGGTGGTCGGAGTGT

GTTTCTGGTGGATTTTGTACCTTTCTGGGCTTGTGTGTCTTTGTGCGGGAGGAGACCAGA

GATGGAGGATGCAGTGGCCACAATGCCCTGGTTATGTGAGATTCCGCTCCGGATGCTGAC

TTCTGAGCAGATGATTGATGGCTTGGATCCTAGTGTGATTCGGGTACCTGCTCATTTCTT

TGGAGTCTACGATGGTCATGGTGGATCGCAGGTTGCAGACTATTGCCGTGATTATCTCCA

TCTCGCATTGATGGAAGAGCTGAGAAATCGGCAGCAAGACATGAATGGAAGCGGGGTTGA

TTGGCAGAAACAGTGGGAGAAAGCATTCGTCAATTGTTTTCAGAGAGTTGATGATGTGGT

TGGGGGGAGGGTAAGCGAAGGCAGTGCGGGAAGCACCGCGGATTCAGTTGGAGAGAGCAG

CAGTGATCCATGTAGTACACCATCGGAGCCGGTTGCCCCGGAGACTGTTGGGACGACAGC

TGTAGTAGCTGTTATCAGTTCCTCGCACATCATTGTTGCTAATTGTGGGGATTCGAGAGC

GGTCCTATGTCGTGGAAAACAAGCAGTGCCTTTATCGGTGGATCATAAACCAAATAGAGA

GGATGAATATGCACGGATTGAAGCAGAGGGGGGCAGAGTCATCCAGTGGAATGGATACCG

TGTTTTCGGTGTTCTGGCAATGTCAAGGTCGATTGGGGATAGATATCTGAAACCATGGAT

CATCCCAGTACCAGAAGTCAAAATCGTTCCCCGAATGAAAGAAGATGAATGCCTCATCCT

TGCCAGCGACGGCCTCTGGGACGTTATGACCAACGAAGAGGCATGCGACGCAGCCCGCAA

GCGGATACTTCTCTGGCACAAGAAGAACGGTGGGACACCTGTCCCCCCACTTAAGGGCGA

GCAACCAGACCCCGCAGCTCAAGCTGCTGCAGAGTACCTCTCGAAGCTTGCCCTTCAAAA

GGGAAGCAAGGACAACATCACCGTTGTCGTGGTCGACCTCAAAGCCCAGAGAAAGTTCAA

GAGCAAAACCTGACTGGTAAGTCGGCTACAAGTTTGGTTTACCATAATGACTGAACTTAA

AAACTCCCATCTGTGTATTTTATTTTTTTACTTGAGTAATATTTAGGTAGCCCATCTTTC

TCTTTTCTCATTTGGGTTTAAGGGCTGGACTGTGTAAGTAGTACAAAAGTGTCTCTGATA

CCGGTCGCCGTGATCATGTAATTTCTATCTTGAGCTGGCACTGTAAATTTAAGTAGGATC

AAACTGGTGGGAAGGATTTTATACTAACCCCTCCTGGGCAAGTGCTTATTTCCTTTATAT

TTAGTTTCTTAGGGTGTTAGTGCAAATGTTGTGTTTATTTGTTATGTGGCATAATGTACT

TGTGTATAGTTTGTGTACACATATTTTGATTTACTTTGATATGTGAGATTAACTCAAGAG

GT

>Unigene0000020

ATCGACGACCAACTTCCGTACACTGTGTAGCAGAGGCTGAGTCCAAGCATCTGCTGAAGG

AGAAGGAGGGGAGGGGACGAAATCCACTCACGATGTCTGAGTGCATCATTTTTTTCTGGG

ATGTTTCGTTTCATGCTGGGCGAGCTGATGATGTTATGATATTTTTCGAGAAAGAGATAG

AAAAGGCGATGAAGAGGCAGAGGATCCTGTTACAGCACCTCTGGTCGTCGCCGGCGACCG

GTGAGTCTCCTGCAATCTCAGGTACGGTGTGTGCTGCTGGGATACGCGGCATACCAGAGC

AATCCTTGCTACGATATGATGTGGTTATTATCGCCGTGTATCGAACTGCGATTCAAAGTC

GGAGCGGTGGTCGGAGTGTGTTTCTGGTGGATTATGTGACTCTGTGGCCTTGTGTGTCGT

TGTATAGGAGAAG

>Unigene0000021

TGTCAGGCACTCGGGAAAAGTCACATCACTGTCCAATGCAGTTGTCGCAGTATTGGTTGC

GGCGGTATCAGTTGTACTATTTTAAGATTTTTACATAAATATTGAAAAAATATGGCAAAC

TAAAAACAAAATACCAGAAATGCATAAACAGTAAGGACTGATACACACCAATATTGATAT

GAGGCTGATACAGGGCAGTTACAGGCACAATAGGAAACCGATACGTATGATACAATTCTG

AAACTGCTCGATACTGACCGGTACAGGCTTGTATGGCCGATACGGACTGTATCGGACCTG

TTGCACATCGGACTTCCCATGTACAATTATTCAGCCACCTCTTAAATCCGTTTCTGATAA

ATCATAGTAAGTTGTTGCAAGGTGC

>Unigene0000022

CCCCCAAAGATCAAGAGAGCCACCTTGTCCACACAGCAGAGGGAGAGAGAATGGAGTGCT

GCACACCACTGCAGCCACGCCTCCTGCCACTCCTCTCGCCATCGTCCCATCCAGTGTTCA

GCTCTCCCCTCCTCTTCGGCCGGCTGAGGCCGAGCCTGAGGCAGCATCTCGGCCTCTGCT

CTCCCCGTTGCCACAAGATGTTCGTCCCCGGGTTCGGCGAGGGATCGCCGGAATCTCAGG

CTGCGAGGAGTTTGCAGAACTTCTTCACCTTCGTTGCAGTCAAGATTGTGCTTGCCCAAC

TTGAGAGTTATAATCCGGAGGCATACGGTGAGTTGAATGAGTTTCTTAGCAAGAATTCTT

TGAATGATGGGGAGAAGTTCTGCAGTGGATTGATGCGGGAGTCGCCGAGGCACAAGGCTT

TAGCTTTACGCATTTTGGAGGTTCGCTCTGCATATGCTAAAAAAGATTTTGAGTGGGAGA

ACTTGGAGAAATTGGCTTTCAAGATGGTCGATGAATCTAACACGAGGCTTATGAGGGAAT

ATGTCTTGGAGACCAGCTTCATAGAAGATGATGAGCGATGAAGTACCATCAAATGGAACC

CTTTCTGGTCCCTAATAGTGTACCTCTCGTATATATTGACCACATATTTCTATACTTGTT

ATGCATACAAGCTCAACTATGCACTTCATTGTAAAATTACTGCATGTACCTCTCGTATAT

ATTAACCACATATTTCTACACTTGTTATGCATACAAGCCCAACTATGGGCTTCATTGTAA

AAATACTGTCTGTATTTATCTATTATATATACCAAAATAAATTCTGTAAGTATTTCAATC

TGGAAATCGTATTTATACATGTTAATGTCAG

>Unigene0000023

TCCGCCAAAACAAAGAAGTGAAAAAACGAACCAGCAGAAAGATGAGAGCTATTGGTGATA

ATGGAAGGGTGCAGCGGCTGAATGCGAAGGATCCATATCCTATACCAGAAAAGGGATTCT

GGTTGAACCGGCTTCTACTCACTTATCAAAATGGATTCTACTCACTTCTCAAAAGAGCAA

TTTCTCATTGGTGATAGTTTTATTGACAGTTGAGATGGTGACCCTGGCTCAATGGCTCAG

GTTCGAATCATGAAATAGCCTCTCTGCACCTTGCATGTAGGGTAAGGCTGTGTACATCCA

AACCTTCCCAGTACCACGCAGTAGTGGAACCTTGTGCACTGGTTGCCCTTTTTTAGTTTT

ATTGACAATTAGGTTGCATTTTATTGACAATTGGGTGCAACGGAATGCAAAACATTCCAA

TATTCAAGAGGCGTCCATTGTGGTACTTCCACAAAGTCTGCAGTGTCAGATACCAACTGC

ATACATGGTACCTCCAATGCAGTCCATGTTTTCAGGTAAAGTCTCATATAAATTTATTTT

TCTATGCTATAACTTGAATATGGTCTGTTTACATTGGTATTGTTAATTTTGATTTTCTAT

ATAATTTTGATTTTCTCTTTGTGTGCATGAAAATTGGGTGCAACGGAATGCAAA

>Unigene0000024

AAAACATTCCAATATTCAAGAGGCGTCCATTGTGGTACTTCCACAGAGTCTGCAGTGTCA

GATGCCAACTGCATACATGGTACCTCCAATGCAGTTCATGTTTTCAGGTAAAGTCCCATA

TAAATTTATTTTTCTATGCTATAAATTGAATATGGTCTGTTTACATTGGTATTGTTAATT

TTGATTTTATTTGTGTGTGCATGAAAATACTGGCTGGTGGATTCTGCAAACATATGTT

>Unigene0000025

AATTGGGTGCAACGGAATGCAAAACATTCCAATATTCAAGAGGCGTCCATTGTGGTACTT

CCACAGAGTCTGCAGTGTCAGATGCCAACTGCATACATGGTACCTCCAATGCAGTTCGTG

TTTTCAGGTAAAGTCCCATATAAATTTATTTTTCTATGCTATAAATTGAATATGGTCTGT

TTACATTGGTATTGTTAATTTTGATTTTATTTGTGTGTGCATGAAAATACTGGCTGGTGG

ATTCTGCAAACATATGTT

>Unigene0000026

AAAACATTCCAATATTCAAGAGGCGTCCATTGTGGTACTTCCACAGAGTCTGCAGTGTCA

GATGCCAACTGCATACATGGTACCTCCAATGCAGTTCATGTTTTCAGAAAAAAGAAGAAA

GTTCAAGCAAAGGGAAGAGAGGAATACAATCGATAGTGATAGGAAGCATCATACCCTTTT

CGAAGCTTTGTTTCCAAGCAAGATTTTTGGTCATGTGGAATTCTAGTTATCTATTTTTAG

TTGAGCTTATCTATGTGTAGATTTGGAAGGCTTATGTCTTGGTATTGATTATGACTCTAC

>Unigene0000027

GCCGCCATCGGCCCACTCATGTCCCTTCAATGAAGGAGGCTGATCTGCTCCCCTCGTCGG

CGGGGGCCTCCGCCAAGACCCCCACCGCCGACGCCGCCCTCTTCGGCAAAGGCCGTTACA

AGTTCTGGGCCCTCGCCGCCATCATCCTCCTGGCCTTCTGGTCCATGCTCACCGGCTCCG

TCACCCTCAAGTGGTCCGCCGGTAACCTCAACAGCCTCTCCGACGACCTCGACGCCCCTA

TCCACGATGATCTCGACGTCCTCGAGATGGAGGAGAGGGAGAAGGTTGTGCGGCACATGT

GGGATGTGTACACCAATGGACGCCGGATCCGTCTGCCACGGTTCTGGCAGGAGGCGTTTG

AGGCGGCGTATGAGGAGCTTGCTGATGATGACCCGGAGGTGAGAGATGCTGCGGTGGCTG

AGGTTGCGAGGATGTCGACGCGGATGCTAGATCTCGATCCGCTTCCTCACCAGCACCGAA

AGGCTACCGAGGGAGAGCTGAAACTAAGGAACAGAGACAGCCGGGTCGAGGGGAAATCAG

CAACTATGTCATCTGCAGAATCTCACTGATGCGGAGCTGTATTTCTTCTTTACTGTGTCC

TATGTTAATTGATGCTGCGCCTACTCATTTACTCGTGTTTGCCAATGCTGTTTCCTTATT

CAGCAGTAGCATGCAGAAATTGATTTCTTGCTTTGTACATTCGTATTTTGGTTGCATGCA

TATGGTTCCCTTTCTTTGATGCTAAAACTCAATATTTAAAGCTAGTCAGTTTGTAAGATT

TTCACCTCATCTGCTAATATGTTCAAGAATACCATAGGGATTTACAGACAGCTGCCACTA

TT

>Unigene0000028

CCCGCGTCTTCATAGTGTGATGGTGAACATAATGTGACAAGACTTGCAACTCTTAATTAT

AGTCAGGCAGCACTTGTTAAGGAATGAATGATATCCCCATACATATGACGCTGAATAGCC

TGCATAGTTGGGTACCTTGGAAGAGCCAGTCTCTCTCAATCATTAACAGGTCATCAGTAA

AACCTAGTTCACAATGCTGGTTCCTTAAGTAGGGAAAGCGAGATGGTGAACGTAGTGTGA

CAAGGCTTGCGACTCTTAATTATAGCCAGGCGGCACTTGTTAAGGAATCAGTGATATCCC

ATGCAGCACTTGGTAAGGCATCAATGATAGCCCCATAAATCAGACGTTAATGGCCTGTAT

AGTTGAGTACCTTGGAAGAGTCAGGCTCTCAATCATAAAAAGGTCATC

>Unigene0000029

ATATATATATATATATATATATAACCTGAACCCATCAGACTCACTGAAGTATACCCATCT

TGGTTATTGGGTGCTTTCCCTGCAACAACACTACTCCCATAGCCTCCAGTTCAGGTTTTA

TATGGTCAAAGAAAAGATTAGACAGCTCATATCCTTCTTTAAGATTGCTCTGTGCTAAAA

GCTTGTTCTTATGAGTGGTCTTCCCTCTTAACCCATCTATGTGAGTGCCTCTTAACTCAT

CTACTTGTGTCACTTGGCTTTTTCTTAG

>Unigene0000030

AATTTCTTTCAGATTCTAGATTTTCTTATCTTGCCCTATTGGCATCGGTATCGGTCGTGG

TACCGGCTGACCCAAAAGACAATTCAATACGGGTATGGGCTTTATGCAAAGTGAAGTTAG

GGTGTGTTTGGGGGACCAAATTGTGAGTGCGTTGTCAGTTTAGAGTGATTGGAGTTATAA

TTGCTTTGGCTTCCAAATGAGTAGATTTTTGATTTTCTGGATAGACTACTGCAATATAGT

GTCTAGAAGGTATTCTGTATGAGCGGCGTAGAATACATCGCATCAAATGGGAGTCCTAAG

AACTTTTAGATGAAAGAATTCAATGTGCTGCAAAACTATTGGTTATGAGAGGTCTTACTT

GAAATTTGGGATTAATACTATATACATAGAATAGCAGACCTGCCTGAATGAAGTTGGAAA

AAAACACATGCTGAGGCACCAAGACTAGTATAATTATTGGATTTATACCAATAAACAAAA

AAAGTCTTAAGAAATTATTTGATAAGTTGAATAAAAGTTCCAGAAAAAGAATAGGGTTCT

CTTCTTCTAGACATGCTTGGAAAAAGGGCAGACTAAACAAATGAGGTAAAGGAATGCTTG

TCTAAAAATCTCCTGTAAATGCTTGAAAAAAGTGTCATTCTATACAAATGATGAGAACGA

AAAAGAATGCTTGTCCAAAACATGTGATCCTTTTTTGAATACTACATGTGATGCAAGCGC

AGAACATGGTGCTCAGCGAATTCAGCGCCTTGAACAATCCCAACAGTAATTAGATCATAA

CTGAGACCAAAATGATATAATTTTAAAAAATGAAATCACGGTACACACCCTTGCTATCAT

GTGAGCTAAGATGGCTGGTAATTGTGCAATACCACATCAACGT

>Unigene0000031

GGGGGTAAAGGACTCTCAATCTCCCTTTGTTTGTCTCAACCTTTGGGTCCAATATACCGA

TCTCTTCTCTTCTTTGTTGTTTCCTGATTTTAATCACAAACATCTTGGCATGAGGTTGGC

TGGAAACTTGTTTGGCTACTTGCTCATTTTGAAGCATGCAATGTGGAGAGAGCTATGTAA

ATAGGGTGCAAAATGTTTAGTTTTCAGCTAGCCTGGGCTCGTGCTACCTGCGTACTGTTA

ACATGTTATGGGTCACTAGAGGGTATTTCACTAATGGACTTTGATTGAATTCTATTATGG

GTTATTTGAGATATCTGATCAACCGGCAACTCTACTGGCAACTGGGTGTTTGTTGGAGAC

CCCATTAGTAATTGTTGTAAGGTGGCGTTAAGGTTTTGGGTTGTGTTATGGGATACATGT

GAATACCCGCTCGGACACGTGGTGTCACGGTGAATTATCTTTCCTTTTCCCCAAATTGCC

CCTCCTCCTCGAAGGACTGTTGTCTTTCGACATCTCCCTTGTTGGCTGGAAACTTGTTTG

GCTACTTGCTCATTTTGAAGCATGCAATGTGGAGAGAGCTATGTAAATAGGGTGCAAAAT

GTTTAGTTTTCAGCTAGCCTGGGCTCGTGCTACCTGCGTACTGTTAACATGTTATGGGTC

ACTAGAGGGTATTTCACTAATGGACTTTGATTGAATTCTATTATGGGTTATTTGAGATAT

CTGATCAACCGGCAACTCTACTGGCAACTGGGTGTTTGTTGGAGACCCCATTAGTAATTG

TTGTAAG

>Unigene0000032

AAGTAGCCACGCTCCCTTGCGCGCGTGTGGCGAAAGCATTCCCTTTTCTAGGGTTTCGTC

CGCCTCGCCGCTGCAGATCGGATCAGAACTAGGGTTTCCGGGAGCTCAATTGATCCATTA

GCGCGATGTCGGATCGGCTCACTAGAATCGCCATCGTCAGCTCCGATCGCTGCAAGCCGA

AGAAGTGCCGGCAGGAGTGCAAAAAGAGCTGTCCAGTAGTTAAAACAGGAAAGCTTTGTA

TTGAGGTGGCCCCTGTGTCTAAGATCGCGTTTATATCAGAAGAACTGTGCATTGGGTGCG

GAATCTGCGTGAAGAAATGCCCATTTGAAGCCATTCAGATTATCAATTTACCCAAGGATT

TGGATAAAGATACAACTCATCGTTACGGACCAAACACTTTCAAACTTCACAGGTTGCCAG

TTCCTAGGCCTGGACAAGTTCTAGGCTTGGTTGGAACCAATGGTATTGGAAAATCGACTG

CCCTTAAAGTGTTGGCTGGTAAACTGAAACCCAATTTGGGTCGATTTAAGAATCCACCAG

ACTGGCAGGAAATCTTGACATATTTCCGGGGGTCTGAGCTTCAGAACTATTTTACTCGAA

TCCTAGAGGACAATCTTAAGGCCATTATCAAACCACAATATGTTGATCACATTCCAAAAG

CTGTCCAAGGGAATGTCGGTGAGGTGCTTGACCAGAAAGATGAGAGAGATATGAAGGCTG

AGCTTTGTGTCGATCTTGAACTAAATCAGGTCATTGAGCGGAATGTAGGTGATTTATCTG

GTGGAGAGTTGCAGAGGTTTGCAATTGCAGTGGTTGCTGTGCAGAATGCGGAAATATATA

TGTTTGATGAACCATCAAGTTACCTTGATGTCAGACAAAGGCTTAAAGCTGCACAAGTCA

TTCGATCTTTGCTGAGGCCTAACAGTTATGTTATCGTTGTGGAGCATGACCTTAGCGTGT

TAGACTATTTATCAGATTTCATCTGTTGTTTATATGGGAAACCTGGAGCTTATGGAGTTG

TAACTCTACCCTTTTCCGTAAGAGAAGGGATCAACATATTCTTGGCAGGTTTTGTTCCAA

CAGAGAACCTTAGGTTTCGAGATGAATCACTCACATTCAAGGTTGCTGAGACCCCTCAGG

AAAGTGCTGAGGAGATTCAGACATATCAGCGGTACAAGTACCCTACCATGAGTAAAACAC

AGGGAAATTTCAAGCTAGCTGTCATGGAGGGTGAATTTACTGATTCTCAAATCATCGTGA

TGCTGGGGGAGAATGGAACAGGGAAAACTACATTTATTCGGATGCTGGCTGGATTGTTGA

AGCCTGATGATGTGGGAGATTCAGATATCGAGATTCCAGAATTTAATGTTTCCTATAAGC

CACAGAAAATCAGTCCCAAATTCCAACATAATGTGAGGCATTTGTTGCATTCAAAAATTC

GGGATTCATATATGCATCCTCAATTCACCTCTGATGTTATGAAACCCTTGCAAATCGAAC

AATTGATGGACCAAGAGGTTGTAAATCTTTCTGGTGGAGAGTTGCAGAGGGTAGCACTCT

GTTTGTGCCTTGGAAAGCCGGCAGATATTTATCTGATTGATGAACCAAGTGCGTATCTCG

ACTCAGAGCAGCGTATTGTTGCTGCGAAGGTGATAAAGAGATTCATCCTTCATGCAAAGA

AAACTGCTTTTGTTGTTGAGCATGATTTCATTATGGCAACATACCTTGCCGATAAAGTCA

TTGTCTACGAGGGGCAGCCATCAATTGATTGTGTGGCAAATGCACCTCAATCTTTGGTAA

CGGGAATGAACCTCTTCTTATCTCATCTTGATATTACTTTCAGACGGGACCCTACTAATT

ACAGGCCGCGTATCAACAAGCTGGACTCAACTAAAGACAGGGAGCAGAAAAATGCTGGAT

CCTATTATTACCTTGATGACTGATGATCATATGCTGATATTTTTCAAGTCAAGAGGGCGG

CATCCATTATATATTTCATTACGATAGAGAGCTACTGAGTTTTGCCACTCCTGGAGGGGT

TTATTGCAGAGCCTATCAGAATACCCATCTCTTACACTAAGATATGGACATGGATATGAG

ATTACTTAGTGCTGCAGTTTGAGAAATACTTTTTCATATGTTGAGGCTTCCTGCATTTTA

TGACTGTTGTGTAGATGATGTTCGTGTCTTTTCTATAGAGTTTACTTTTTGAATTTTTGA

GAAGCTATGTTTATGTTCAACTATATGTATCTTGTTTTTGGGATTTATGTCTACTCTCCT

AGATGGGTGGAATGACTGGATGAGTATGTATGCTTTGTGATTCCTACCAATTACTGTTGA

CCTACGGACATCAAAACATAATGTTAAAATAGCT

>Unigene0000033

ACATCAACGTTATCTTCACTGCTTTGATGTAAACAATTTTATTTATTCATCTGTCTTGCC

TGTTATGGCTTTGTTTACAAGTAATTGGGCCTGTGATTGTTGCTAACAAGTATTTTGAGC

TTTAGTTTTATGTTGGAATGTATGAATCAAATTGAATGTGAATTTGACAGTCATCATCAT

GACGCACCTTTTTGAAGTGTTCTTGCCTAATCTAATGCTATCTCTACATCATAATAATTC

TTGCAGCCGGCAGATATTTATCTGATTGATGAACCAAGTGCGTATCTCGACTCAGAGCAG

CGTATTGTTGCTGCGAAGGTGATAAAGAGATTCATCCT

>Unigene0000034

TTGTAGCAACTGTTAGTAGTATGGTACTTTTATCATTTTATTGCATAATATTCTGTTGAA

CCTCTTAATTGGAATGGACCATTTCACTTCACGTACCCTATTCTATACTTCTTAATTGGA

ATAGGACACTGCTAGCTGAGATAGTTTCAGGCCCAGTTTGGCCCCCCCTTCTGTTTTTCG

ACTGGGCTTTTAAAAAACTGCACAGTATTTGAATGGATCTCCTGCTCCATTCAGCTGGTT

TCTACCCAACTGCATGCGGACGAAAGCAGAAGCCTGATTTCCTGGCTTTTTGCTT

>Unigene0000035

GCCGAAATCGTCCGCTATAACGAAAACCGCGCCCTTGGGGAGTAACAGAGAGTGAGAGAG

CTTACTGAAATCCCACCATGGCAGACGAGAGTGACAGGAGGAAGTCGAAGAAGAAGCGGA

GGCACAACAAAGACGCAGAGAAGGAGCACGAAACCCCCACCGTTAGCATCGCCGTCTCCG

GCTCCATCATCGACAACGCCCAGTCGCTCGAGCTTGCTACCCTCCTAGCTGGCCAGATAG

CCCGCGCCGCCACGATTTTCCGAGTTGACGAGGTCGTCGTCTTCGACAGCGTACCCGACG

GCTCCGATACCGGTACAGCCGGCGGGGAGGATGAGAGCGGCGCGCGGTTTCTTGCTCGGA

TCTTGGAGTATCTGGAGACGCCGCAGTATCTGCGTCGGCGGCTCTTTCCCATGCATAATA

ATCTGAAATATGTGGGTTTGCTTCCGCCGCTGGATGCTCCTCACCATGTGCGGAAGCATG

AGTGGTCGCATTTCCGGGAAGGTGTAACATTAGAGGGGAACTCTTCACCAAATGGAACAC

TTGTTGACGTTGGATTGAGTAAGAGTGTTGTCGTTGAACAAGTCCTCGAACCTGGACTAA

GGGTGACCGTGGCCATGGGGAGTGATCGTAACCTAGATACAGATTGTTTAAGAAAGGTTG

TTATGCGGTGTAGTCCTAGAGAAGAAAAGGGATTATACTGGGGATATACAGTCCGATATG

CATCAAATTTAAGTCATGCTCTTAAGAGTTGCCCATACAAGAGTGGATATGATCATGTCA

TTGGAACTTCAGAGCATGGTCTTGTGATTAATTCATCAGATCTTGTTTTACCTACATTTC

GGCACCTTTTAATTACCTTTGGTGGCCTCGCGGGGTTGGAAGAGAGCATTGAAGAAGATA

CTAGCCTAAAGGGAAAACAAGTAAGTGATGTATTCAGTGCCTACTTGAATACATGCCCTT

CTCAGGGAAGCAGAACCATTAGGACTGAGGAGGCTATTTTCATCTCTCTCCAATACTTTC

AAGAGCCTGTCGAGCGGGCTGTGAAGCGGTTTGAAGGAAAATAGGTGCCTCACAACGATG

AGATTTTTGTTTAATCTCATGGTATCCAAAGGAAGCTGCTGATGAAATCAAGGTGATTAG

TTAATGGTGGTTTTAGGGCATGTGTGCAACGTCGTGAAAGACAAGTAAGAGACTGGTGAA

ACGTGTCAGAGAAAGCTGGAGAATTTTGAAAGGTACAAGTCCTTGGAAACAAATACGTTG

ATGACAATGTGTTTTTCAGTTGGGTCTATTTTAATATGAGATAGTTCAGTTATGTGTCGG

GAATGTGCAAAAAGAGTTCCTTGCGAATTATAGATGTTCCATGTACTAGTTCGTACATGA

AATACATAGTTTGTCAGAACTGAACCCCATTGTAGACACTGCACGAGGCGGTGGCATTCG

ACGTTATCATCCTGACAAAGCTTTGATTTGCTGATGGGTTATTTGGATTTCTGTTGATCA

TTGCTTCAAGAGGCAAGCCTTTTTCTTCCTCACATGTATGAATCCTACATTACGATTGTT

GTTTGCTGAAGCCATACCCCCGATTGAATTATTATTTTGTTATTTCGATGATCGAAACAA

GTATTTGGTGTATGCTACTGGTTAGCAATTTGAATGATGTGCCTCAGCAGCTGAAGAATT

GAGATATACCAACAATTGATGAATGAGTCTCCATGTGCGACATATTGGTTGGAAATAT

>Unigene0000036

GGCATCGTCACCAACTGGGACGACATGGAGAAGATCTGGCACCACACCTTCTACAACGAG

CTGCGCGTGGCGCCCGAGGAGCACCCGGTGCTGCTCACGGAGGCGCCGCTCAACCCCAAG

GCCAACCGCGAGAAGATGACGCAGATCATGTTTGAGACCTTCAACGTTCCGGCCATGTAC

GTGGCCATCCAGGCAGTGCTGTCCCTTTACGCCTCCGGCCGTACCACCGGTATCGTGCTC

GACTCCGGCGACGGCGTCACCCACAATGTGCCCATCTATGAGGGCTACGCGCTGCCGCAC

GCCATCATGCGTCTGGACCTGGCTGGCCGCGATCTCACCGACTACCTTATGAAGATCCTC

ACCGAGCGTGGCTACTCCTTCGTGACCACAGCTGAGCGCGAGATCGTGCGCGACATCAAG

GAGAAGCTGTGCTAC

>Unigene0000037

GCCAGAGCAGACTGCAGAGCAGTGTTTTAGATTTCTTTTATCGTAGATCAATTCAAAAAC

CCAAATCAACAAAATGTGTGACGATGATGTTGCGCCTTTGGTTGTCGACAACGGCTCCGG

TATGTGCAAGGCTGGTTTCGCTGGTGATGACGCACCACGTGCCGTCTTTCCATCTATTGT

CGGCAGACCACGTCATCAAGGTGTCATGGTCGGTATGGGACAAAAG

>Unigene0000038

CCTCATGAAGATCCTGACAGAGAGGGGCTACAGCTTCACCACCACGGCTGAGAGGGAAAT

CGTGCGTGACATCAAAGAGAAGCTGTGCTACGTCGCCCTGGACTTCGAGCAGGAGATGGC

CACTGCTGCCTCTTCCTCCTCCCTGGAGAAGAGCTACGAGCTGCCTGACGGGCAGGTCAT

CACCATCGGCAACGAGCGGTTCCGGTGTCCGGAGGCTCTGTTCCAACCCTCTTTCCTGGG

CATGGAGTCGTGCGGCATCCACGAGACCACTTTCAACTCCATCATGAAGTGTGAT

>Unigene0000039

TGGGTATGGAGTCCTGTGGTATCCATGAAACTACCTTCAACTCCATCATGAAGTGTGATG

TGGATATCCGTAAGGATCTGTATGCCAACACAGTGCTGTCTGGTGGTACCACAATGTACC

CTGGCATTGCTGACAGGATGCAGAAGGAGATCACAGCCCTGGCACCCAGCACGATGAAGA

TCAAGATCATCGCCCCTCCGGAGCGTAAGTACTCGGTGTGGATCGGCGGCTCCATCCTGG

CCTCCCTATCCACCTTCCAGCAGATGTGGATCAGCAAGCAGGAGTATGACGAGTCCGGCC

CATCCATCGTGCACCGTAAATGCTTCTAAACGGACTGCTGGCAGGTGCACAGCATCTGCT

GCATGAGTCGTCTCACTAGTATACGTTTGCCCTGGCAAATCTATACACCTCATGCTAGCC

TCATAAAACTGGAATAAGCCTTCTTTCAAAAGAAACTTTGCCCTTGGAAAGTCTGTACCT

GATGTTAGATGTGTCAGCACTGGATTGTCGAGCTGGCCCTTGATTTGACCTTGTATTCGA

GTCCACCGTTCCTTCGGTGCACACCTTTTAGAGAAATACCCTGCACAGATCTGCGCGGGG

GGGCTGTGCTGCGTGCCCAGATGTGTACAGGGTATTGAGAGTCGGTCAGGCTTCCATCCA

GTGCGGAGTATTCCAGATTTGGTGTAGAGGCTGGTAGGAGTCCATCCACACCCACTTCTG

TCGCCGCCGTTCTAAGCAAGGTTGGGAAACATATCGCATCCGAGCCTTACCAACCAGCCC

TCCTCCTCCCCTCTGCAACCACCACGGGGCTGAGGTTCCTCAACGCGGGTTGCTGAAACT

TGGCATTGACACCTGTAAATTTATGCATCGTTTTAATTTATGTAAGACTTTTTTTTTTCT

TTTTTTTT

>Unigene0000040

CCCGTGCCCCGCTCCGCCGCCGCCTCGCGCCGCCCGCCCCGGCTCTGACTGACCGCGTTA

CTCCCACAGCCAGCCATGGATGATGATATTGCTGCGCTCGTTGTTGACAATGGCTCCGGT

ATGTGCAAGGCCGGTTTCGCCGGGGACGATGCCCCCCGTGCTGTGTTCCCATCTATCGTG

GGTCGCCCCAGACATCAGGGTGTGATGGTTGGTATGGGCCAGAAAGACAGCTACGTTGGT

GATGAAGCCCAGAGCAAAAGAGGTATCCTGACCCTGAAGTACCCCATTGAACACGGTATT

GTCACCAACTGGGATGATATGGAGAAGATCTGGCACCACACTTTCTACAATGAGCTGAGA

GTAGCCCCTGAGGAGCACCCTGTGCTGCTCACAGAGGCTCCCCTGAACCCCAAAGCCAAC

AGAGAGAAGATGACACAGATCATGTTTGAGACCTTCAACACCCCAGCCATGTATGTAGCC

ATCCAGGCTGTGCTGTCCCTGTATGCCTCTGGTCGTACCACTGGTATTGTGATGGACTCT

GGTGATGGTGTTACCCACACTGTGCCCATCTATGAAGGCTACGCCCTCCCCCACGCCATC

CTCCGTCTGGATCTGGCTGGCCGTGACCTGACGGACTACCTCATGAAGATCCTGACAGAG

AGAGGCTACAGCTTCACCACCACAGCCGAGAGAGAAATTGTGCGTGACATCAAGGAGAAG

CTGTGCTACGTCGCACTGGATTTCGAGCAGGAGATGGCCACAGCTGCCTCTAGCTCTTCC

CTGGAGAAGAGCTATGAACTCCCTGATGGTCAGGTCATCACCATTGGCAATGAGAGGTTC

AGGTGCCCCGAGGCCCTCTTCCAGCCATCTTTCTTGGGTATGGAGTCCTGTGGTATCCAT

GAAACTACCTTCAACTCCATCATGAAGTGTGATGTGGATATCCGTAAGGATCTGTATGCC

AACACAGTGCTGTCTGGTGGTACCACAATGTACCCTGGCATTGCTGACAGGATGCAGAAG

GAGATCACAGCCCTGGCACCTAGCACAATGAAAATCAAGATCATTGCCCCACCTGAGCGC

AAGTACTCTGTCTGGATTGGAGGCTCTATCCTGGCCTCCCTGTCCACCTTCCAGCAGATG

TGGATCAGCAAGCAGGAGTACGATGAATCCGGACCCTCCATTGTCCACCGCAAATGCTTC

TAAACCGGACTGTTACCAACACCCACACCCCTGTGATGAAACAAAACCCATAAATGCGCA

TAAAACAAGACGAGATTGGCATGGCTTTATTTGTTTTTTCTTTTGGCGCTTGACTCAGGA

TTAAAAAACTGGAATGGTGAAGGTGTCAGCAGCAGTCTTAAAATGAAACATGTTGGAGCG

AACGCCCCCAAAGTTCTACAATGCATCTGAGGACTTTGATTGTACATTTGTTTCTTTTTT

AATAGTCATTCCAAATATTGTTATAATGCATTGTTACAGGAAGTTACTCGCCTCTGTGAA

GGCAACAGCCCAGCTGGGAGGAGCCGGTACCAATTACTGGTGTTAGATGATAATTGCTTG

TCTGTAAATTATGTAACCCAACAAGTGTCTTTTTGTATCTTCCGCCTTAAAAACAAAACA

CACTTGATCCTTTTTGGTTTGTCAAGCAAGCGGGCTGTGTTCCCCAGTGATAGATGTGAA

TGAAGGCTTTACAGTCCCCCACAGTCTAGGAGTAAAGTGCCAGTATGTGGGGGAGGGAGG

GGCTACCTGTACACTGACTTAAGACCAGTTCAAATAAAAGT

>Unigene0000041

CGCTTCGCTGTCATTGAGCCATCACCGTATCCACTTTCGGCACATGACAGCTCACCGGAT

CTGCTGTTCACAGCCGAGAACCCGTTTTAAGCCCCCTCCTTCGCTCCCCACCCCATTTCT

ATCGGGGTCTCGGCATCCTCATCGGAGGAAGCAGCCGCTGCTCTCTTCCTCTCCTCCCGC

TGTCCTCGTCAAGCTAAGCTCTTTATTATATAGATCTATAAGAAATGGCTGATGCTGAGG

ATATCCAGCCTTTGGTGTGTGACAATGGTACCGGAATGGTTAAGGCCGGTTTTGCTGGCG

ATGATGCACCAAGGGCTGTTTTCCCAAGTATAGTCGGTCGACCTCGCCACACTGGTGTCA

TGGT

>Unigene0000042

CGCTTCGCTGTCATTGAGCCATCACCGTATCCACTTTCGGCACATGACAGCTCACCGGAT

CTGCTGTTCACAGCCGAGAACCCGTTTTAAGCCCCCTCCTTCGCTCCCCACCCCATTTCT

ATCGGGGTCTCGGCATCCTCATCGGAGGAAGCAGCCGCTGCTCTCTTCCTATCCTCCCGC

TGTCCTCGTCAAGGTTCGGATTTCATTTTCTCGATCTAATTCTCTCTA

>Unigene0000043

CGCTTCGCTGTCATTGAGCCATCACCGTATCCACTTTCGGCACATGACAGCTCACCGGAT

CTGCTGTTCACAGCCGAGAACCCGTTTTAAGCCCCCTCCTTCGCTCCCCACCCCATTTCT

ATCGGGGTCTCGGCATCCTCATCGGAGGAAGCAGCCGCTGCTCTCTTCCTCTCCTCCCGC

TGTCCTCGTCAAGATCTATAAGAAATGGCTGATGCTGAGGATATCCAGCCTTTGGTGTGT

GACAATGGTACCGGAATGGTTAAGGCCGGTTTTGCTGGCGATGATGCACCAAGGGCTGTT

TTCCCAAGTATAGTCGGTCGACCTCGCCACACTGGTGTCATGGT

>Unigene0000044

TCCTCATCGGAGGAAGCAGCCACTGCTCTCTCCATCTCCTCCCGCTGTCCTCGTCAAGCT

AAGCTCTTTATTATATAGATCTATAAGAAATGGCTGATGCTGAGGATATCCAGCCTTTGG

TGTGTGACAATGGTACCGGAATGGTTAAGGCCGGTTTTGCTGGCGATGATGCACCAAGGG

CTGTTTTCCCAAGTATAGTCGGTCGACCTCGCCACACTGGTGTCATGGT

>Unigene0000045

CACCAACTGGGATGATATGGAGAAAATCTGGCATCACACTTTCTACAACGAATTGCGTGT

TGCCCCAGAAGAGCACCCAATCTTGTTGACCGAAGCCCCATTGAACCCAAAGGCTAACCG

TGAAAAGATGACCCAAATCATGTTTGAAACCTTCAACACACCCGCCATGTATGTTGCCAT

CCAAGCCGTACTCTCCTTGTACGCTTCCGGTCGTACCACTGGTATCGTTTTGGACTCTGG

TGACGGTGTCTCCCACACAGTACCAATCTACGAAGGTTATGCTTTACCCCATGCCATCCT

CCGTTTGGACTTGGCTGGTCGTGACTTGACCGACTACTTGATGAAGATCTTAACCGAGAG

AGGTTACAGCTTCACCACCACCGCTGAAAGAGAAATCGTCCGTGATATCAAGGAGAAATT

GTGCTATGTCGCTTTGGACTTCGAACAGGAAATGGCCACCGCCGCCGCTTCCACCTCATT

GGAGAAATCCTACGAATTACCTGACGGACAGGTCATCACCATCGGAAACGAACGTTTCCG

TTGCCCAGAATCTTTGTTCCAACCTTCATTTTTGGGAATGGAATCTTGCGGTATTCATGA

AACTGTGTATAACTCCATCATGAAATGCGACGTTGACATCAGAAAAGACTTATATGCTAA

CACTGTGCTCTCCGGAGGTACTACTATGTATCCAGGTATTGCCGACAGAATGCAAAAGGA

AATTACCGCATTGGCTCCAAGTACAATCAAAATTAAGATCATTGCCCCACCAGAACGTAA

ATACTCCGTATGGATTGGTGGTTCCATCTTAGCTTCTCTGTCCACCTTCCAACAAATGTG

GATCTCTAAACAAGAATACGACGAATCCGGCCCAGGCATTGTCCACCGTAAATGCTTCTA

AGTCATCACCTCCCACAAACATACAAAATCAAAATTACAATCACGCTTTATGTTCATTTA

TACTGAAGAAAACCTATGTGTTTTTT

>Unigene0000046

CACCAACTGGGATGATATGGAGAAAATCTGGCATCACACTTTCTACAACGAATTGCGTGT

TGCCCCAGAAGAGCACCCAATCTTGTTGACCGAAGCCCCATTGAACCCAAAGGCTAACCG

TGAAAAGATGACCCAAATCATGTTTGAAACCTTCAACACACCCGCCATGTATGTTGCCAT

CCAAGCCGTACTCTCCTTGTACGCTTCCGGTCGTACCACTGGTATCGTTTTGGACTCTGG

TGACGGTGTCTCCCACACAGTACCAATCTACGAAGGTTATGCTTTACCCCATGCCATCCT

CCGTTTGGACTTGGCTGGTCGTGACTTGACCGACTACTTGATGAAGATCTTAACCGAGAG

AGGTTACAGCTTCACCACCACCGCTGAAAGAGAAATCGTCCGTGATATCAAGGAGAAATT

GTGCTATGTCGCTTTGGACTTCGAACAGGAAATGGCCACCGCCGCCGCTTCCACCTCATT

GGAGAAATCCTACGAATTACCTGACGGACAGGTCATCACCATCGGAAACGAACGTTTCCG

TTGCCCAGAAGCCTTGTTCCAACCTTCATTCTTGGGAATGGAATCTTGCGGTATCCACGA

AACCGTATACAACTCCATCATGAAGTGCGATGTTGACATCAGGAAGGACTTGTACGCCAA

CACAGTACTTTCCGGAGGTACCACCATGTACCCAGGTATCGCCGACAGAATGCAAAAGGA

AATCACCGCTTTGGCCCCAAGCACAATCAAAATCAAGATCATTGCCCCACCAGAACGTAA

ATACTCCGTATGGATCGGTGGTTCCATCTTGGCTTCTCTGTCCACCTTCCAACAGATGTG

GATCTCCAAACAAGAATACGACGAATCCGGCCCAGGCATTGTCCACCGTAAATGCTTCTA

AGTCATCACCTCCCACAAACATACAAAATCAAAATTACAATCACGCTTTATGTTCATTTA

TACTGAAGAAAACCTATGTGTTTTTTCTTTTTGTACATATCGAGACGATTTTTGCGTCGT

TTTACACGGGGATAAAATAACAGTTCGATTTTTGAAATAAAAAAAATGTATTTATTAGCG

CGTTAATCGTTTCGAAATAAA

>Unigene0000047

CGCTTCGCTGTCATTGAGCCATCACCGTATCCACTTTCGGCACATGACAGCTCACCGGAT

CTGCTGTTCACAGCCGAGAACCCGTTTTAAGCCCCCTCCTTCGCTCCCCACCCCATTTCT

ATCGGGGTCTCGGCATCCTCATCGGAGGAAGCAGCCGCTGCTCTCTTCCTCTCCTCCCGC

TGTCCTCGTCAAGATCTGTAAGAAATGGCTGATGCTGAGGATATCCAGCCTTTGGTGTGT

GACAATGGTACCGGAATGGTTAAGGCCGGTTTTGCTGGCGATGATGCACCAAGGGCTGTT

TTCCCAAGTATAGTCGGTCGACCTCGCCACACTGGTGTCATGGT

>Unigene0000048

CAGCCACACACTAGCCGCAGACCCGCCGCAGTCGTTCAGATTTTCTTCCTCGTAAACCGG

TTCAAAAACCCAAACCAGCAAAATGTGTGACGATGATGTAGCAGCTTTGGTAGTCGACAA

TGGCTCCGGTATGTGCAAAGCCGGTTTCGCCGGAGATGACGCACCCCGTGCCGTCTTCCC

TTCCATCGTCGGCAGACCCCGTCATCAAGGAGTCATGGTCGGTATGGGACAAAAGGACAG

CTACGTAGGCGATGAAGCCCAAAGCAAACGTGGTATCCTCACTTTGAAATACCCCATTGA

ACACGGAATCATCACCAACTGGGATGATATGGAGA

>Unigene0000049

CGCTTCGCTGTCATTGAGCCATCACCGTATCCACTTTCGGCACATGACAGCTCACCGGAT

CTGCTGTTCACAGCCGAGAACCCGTTTTAAGCCCCCTCCTTCGCTCCCCACCCCATTTCT

ATCGGGGTCTCGGCATCCTCATCGGAGGAAGCAGCCGCTGCTCTCTCCATCTCCTCCCGC

TGTCCTCGTCAAGCTAAGCTCTTTATTATACGGATCTGTAAGAAATGGCTGATGC

>Unigene0000050

CACCCTACCAGGAACTGATCACAACCATAGTATTTTAGAACTTATACCGAATGTGCATTT

TTCGTTAAAAAAACAAGGATTGTTTGTAGTTCTGTGAAAAACAAGGATTGTTTGCTTCTG

CACTAAATTATAATTCATTATTGACAGTTGATTTCTGGATCAGCTGCGTGCTGAAAGAAA

TTTAGAAATTTGGATGACCATTTAGTGTGATTGCTTGTTTGATTTCCGACCTCCTCAGTT

TTTAGTTTATGTTGGATGTTTGTTTCAAAATTTAGTTTGAAATGGCAATGAGAATCTACT

ATGTAGCACTACACTTTACTTAATTTCCATACTAAAAAATAATGAGGGAAAAGTTGCTTT

CAGTTCTTTCTCTCACTTTCTCACAGAGATAACTTTTTTCAAAGATGTACACACGTTAGA

GTATGAATTAAATTTAATTCTTTCCATATTAAAATATAAATTTAATATTGTTACTTGACG

GACTTGGACTAAATTTAATATTCCTTCCACATTGTCTATATCTACATCAGTTATAGAGGT

AGATGATGATTGTGGACATTCTCGTTCCTTTGTTCTAGGCATTTTTAATGTTTAACGCTT

GTAACTTTCCTTTCTATAATTGATCTAAATGCCAACTATCCCGTAAATTTTCTGAAGGGG

TACTTCTCACATCTTCTTTGTTCTTGATACACTTCAGACGTAATTAACTTTCATGGAAAA

GTAGTATGTTTACTTGGCCTGCCAGTTGAAACACTTGCTTCCTTTGGTTGGAGCAAAAAT

GGCGTGAATAGTGGTTGCAGAACTAGAAACATGAACACTTCTATGGAGGTGCTGTAACCG

TGTTGGACTCCCTGACACTCCTTGGACCTCTTTTTACACTCCCTGACACTCCTCGGACCT

CTTTTTACGTGGCTATAATTTAACAATAAAAAGCATAAGGCATTGTGGATGTGTCCAACA

ATTTCTTTTCCTAATAAATATTGTTGAACACTGATGGATATTCTATGTTCAATAGTCTAT

GTTTAATCTATCTTGGTTGGTGTTGCATTTTCTATTTTTAGGTTGAGTGGGATACGGATC

ACTAATGAAATATGTATTTTGTTTTGGTGATGCATTTAATCATTGAAGATTGAGTAGGCT

TGTTGTTGGTACTTGAAATTTGCTCTAATATCTTTAGAGTGTTGTGTTCATTATTTAGTT

AATATTTGTTAAAGTTGTTTAAGAATTCTTTTTATTAAGATTGTGGTTAGAATAATTTCA

TGTTTATGTGTATATTTTATACATAATTCTTCGGCTTGAATGACGTTCAAAACTTCAACA

GCTAAGCTCTTTATTATATAGATCTATAAGAAATGGCTGATGCTGAGGATATCCAGCCTT

TGGTGTGTGACAATGGTACCGGAATGGTTAAGGCCGGTTTTGCTGGCGATGATGCACCAA

GGGCTGTTTTCCCAAGTATAGTCGGTCGACCTCGCCACACTGGTGTCATGGT

>Unigene0000051

ATATCCAAAAGATATTTTTCAAAAGATTTACGAACACCTTCAAATCAAAATTTTTTTTGG

GGCAGGGGGGTGGGGTGTTGGTTACCGACAAATAATCTCCCCACGATCAGACAGTACTTA

ACTTTTGTCAACAATACATCTGCCAAGTTAATGATAATTTACAAGAGAGCCTATCAAATA

TAGATAAAATTTAGAGGCAATGGCACAGTTTTGTGGGGCTTGGCAGTGAAGATGACCTGG

CATTTTAGTAGTTGGTCAAAGAAGAAACTCTTTCAAGATATTTGCTACATTTTCCCATCC

ACCATGACCATACTTCATCCAATTAGAGGGAACTGGTTTTTCAAGTTAATAGTAGTTTGC

ATGTAAGAGACCTTCAAATATAGAGTGGATTTAGAAGCAGATCTTCAGAGGCTTGGGTTT

CAAGATGCTTTTCCT

>Unigene0000052

CGCTTCGCTGTCATTGAGCCATCACCGTATCCACTTTCGGCACATGACAGCTCACCGGAT

CTGCTGTTCACAGCCGAGAACCCGTTTTAAGCCCCCTCCTTCGCTCCCCACCCCATTTCT

ATCGGGGTCTCGGCATCCTCATCGGAGGAAGCAGCCGCTGCTCTCTCCATCTCCTCCCGC

TGTCCTCGTCAAGATCTGTAAGAAATGGCTGATGCTGAGGATATCCAGCCTTTGGTGTGT

GACAATGGTACCGGAATGGTTAAGGCCGGTTTTGCTGGCGATGATGCACCAAGGGCTGTT

TTCCCAAGTATAGTCGGTCGACCTCGCCACACTGGTGTCATGGT

>Unigene0000053

CGCTTCGCTGTCATTGAGCCATCACCGTATCCACTTTCGGCACATGACAGCTCACCGGAT

CTGCTGTTCACAGCCGAGAACCCGTTTTAAGCCCCCTCCTTCGCTCCCCACCCCATTTCT

ATCGGGGTCTCGGCATCCTCATCGGAGGAAGCAGCCGCTGCTCTCTTCCTATCCTCCCGC

TGTCCTCGTCAAGATCTGTAAGAAATGGCTGATGCTGAGGATATCCAGCCTTTGGTGTGT

GACAATGGTACCGGAATGGTTAAGGCCGGTTTTGCTGGCGATGATGCACCAAGGGCTGTT

TTCCCAAGTATAGTCGGTCGACCTCGCCACACTGGTGTCATGGT

>Unigene0000054

TCTTCCCGTCGACAGATTGAGGGAGCAAATTCTCTCGATTCTCGAAAGCAACACGAAGAA

TCGGGATCTATCCCGATCCCTAATCGCCTGCATCAACAATTTCTTCTGTAGATTTGTAGA

AGATGGCCGACGGTGAGGATATCCAGCCTCTTGTCTGTGACAACGGAACTGGAATGGTCA

AGGCTGGTTTTGCTGGTGATGATGCACCCAGGGCAGTATTCCCAAGCATTGTCGGCCGTC

CTCGCCACACAGGTGTGATGGTAGGGATGGGCCAGAAAGATGCTTATGTGGGTGATGAAG

CACAGTCCAAAAGAGGTATCCTCACTTTGAAATACCCCATTGAGCATGGAATTGTTAGTA

ACTGGGATGACATGGAGAAGATTTGGCATCACACCTTCTACAATGAGCTTCGTGTAGC

>Unigene0000055

AGCCCAAAGCAAACGTGGTATCCTTACTTTGAAATATCCCATTGAACATGGAATCATCAC

TAACTGGGATGATATGGAGAAAATATGGCATCATACTTTCTACAACGAACTTCGTGTTGC

CCCAGAAGAGCATCCAACTCTGTTGACCGAAGCCCCGTTGAACCCAAAGGCTAACAGAGA

AAAGATGACCCAAATTATGTTTGAAACCTTCAACACACCTGCCATGTATGTTGCCATCCA

AGCTGTATTATCTTTGTACGCTTCCGGCCGTACCACTGGTATCGTTTTGGAC

>Unigene0000056

CAATTCCAGATCTCAGAATCCTCCCCAATCGGCCATCAATCCATGGAGCCCCGATCTCTA

TTCCTCGCCGCCATTCTCGCCGCCGTCTCCCTCACCGCCACCGCCGCTCTCTTCCCCTCC

GATGCCCTCAATTCCGCCGCTCCCAAGGCCATCGCAGATTTGAGGGAGGCGATTGTGAAG

GGGTTGGGGACGCAGGCGGAGGGGGTAAGTTTGACTGGCTTCGACGTGCAGGACGCGCTG

GTGGGTCAGGCGTTGTCGTATGAGTTTTCTATAGAGGTCAATAACGAGGTGATTCCGGTG

AGGTTGCTGGAGGATGTGAGTCGGTGGGATTTTGTGGATCTACCGATTTTTAGATCTGAG

GTGGAGGGAGAGGGGAAGGTGTTGGCGAAGCCGGGGATGAGGACGGATGGGGTTGTGCCG

GTACTTGCGCCGTTTCAACTTGCTGGGCCGATGGAGTTGTGGATTCAGGATGGGGATGAT

ATGCGGCTAGCTCTTCCGCATGATGTGGACGCTGGTAAGCTGAAGAAGGTTATCTTATCA

GATGGTGCAGTGGTGACAGTTAAAGGTGCTAGGTCTGTCAGTCTCCGCCATCCACTTGAA

TTGCCCCTTCCCCTCAACAGAACCAATCATCAAAGCAACCCCTTTGCCTCTGGCCTCCTG

ACCATCGCAGATGCCCTCCGCCAGTCATCCCGCAATGATGAGATACCCCTCCTCTCCCTC

CGCATTGTCGGCCCCACTTCCATCACCTCAACTCCTTCAGAGTCTGTCAGTGATAAGCTT

AAGCTCAAACTCCTCGCTCCGGGTCTCGTTCAGCTGTCCTCCCGCTCAATCCCGTCTGTT

ACTGAAGACATGGACGAATCTGTGAGCACCTTGTGGCCTTTGACTTCACTCAATGGGTCG

GATGCAACATTGATGGGAATTGAGGAACTCCTGGCTTCCGTGCTAAGAAAGAAGGGAAGT

AGTGAAGGGTCGTTCAGATTGGTTAAGGCAGATGTTTCAGCACGAACTTATGTGAAGATG

GGATTTGTTGTGGAGAAGAAGCTCTTGGGCGAAGAGGTTGGCTGGTTTGATTTCCCTGAG

TGGAAGACTAAGCCAGAGAAGGTAAGGGCCCACTATGAAGTATTGGCACGGGTTGAGGAG

AATGGGAAGGTGATCCCTGAGAGAATAGCAGAGGTTCATCCCTTCCAGGTTCAGGAAAGC

TTCGAGGAGAGCCTTCTAACGGGTAATGTGTCAATGTCCCATGTGCCCATAGTCCACCCG

CCGCCAGCTTACTTTACTCTGTGATATTACTTGTTTTGGGAAAATAGTGTTGCGATCGAC

AGCCAAATGTTTTTGAGGTATTTCGTTTTTCTTAATCTACTGTAAATAGAATTGAAGTGC

ATGTTACTTAATAATAAAATTTAATATATATACAACTTTGTTTTTTTGACTGTGGCATAT

TCGTTTTCTAAGTGTGGTTTCTTTAAGTTCGTGGCAGTATATACTTGTGCCTAATATTAT

TTCTTGTATCATGTAATTCCAGATATTTATATTAGTGTATTATACTTTGGTGAGAAAAAA

CTTTTAGCAATGGT

>Unigene0000057

TTTTTTTTCTTAATCTACTGTACATGGAATTGGAGTGCATGTTACTTAATAATAAAATTT

AATATATATACAACTTTGTTTTTTTGACTGTGGCATATTCGTTTTCTAAGTGTGGTTTCT

TTAAGTTCGTGGCAGTATATACTTGTGCCTAATATTATTTCTTGTATCATGTAATTCCAG

ATATTTATATTAGTGTATTATACTTTGGTGAGAAAAAACTTTTAGCAATGGT

>Unigene0000058

TAAAGTCCACCTACGAGTCAATGCCCACCTGGTTTTGGGTTGGATGCTCCTATTCTCATT

AGCGGTGCATATTGTTTTTCAGTCGCATCCACAATCTGGAATTGCGGCCTTAATTCCTAC

ATATTGTGATTTGGAGTCTTTGGCTTGATATTTTGACAGGAGATGAATTTGCACACCAAG

ACTCCCCAAAAGCTAACAGTGCAATTTAGCTACCACTGACAATGGTGATTTTTTTGCTTG

AAAATGAGAGATCTAAAAAATCCCTTTCACAGTACTGTTGTGGATGCTCTTTAACCTTCC

AAATTCTGATTCTTAGGTGAACTTGGAGTTGAATTTGGAATTCTGCCATGATAATATTTG

AATGGTCGACAATTGTATTCAGTGTTTGCAGTCCTAATTTTTGTTATAGATGTCATTTTT

TTTGTCACAAAATATGATGAGTATGATGAGTTGTGAAGCTTTTGTTGTCACGGAAGATCA

AAAGTGTTTTTAGTTATTAGGCTATATAGGGACGGATAATGTGATTTGCATTTA

>Unigene0000059

TAAAGTCCACCTACGAGTCAATGCCCACCTGGTTTTGGGTTGGATGCTCCTATTCTCATT

AGCGGTGCATATTGTTTTTCAGTCGCATCCACAATCTGGAATTGCGGCCTTAATTCCTAC

ATATTGTGATTTGGAGTCTTTGGCTTGATATTTTGACAGGAGATGAATTTGCACACCAAG

ACTCCCCAAAAGCTAACAGTGCAATTTAGCTACCACTGACAATGGTGATTTTTTTGCTTG

AAAATGAGAGATCTAAAAAATCCCTTTCACAGTACTGTTGTGGATGCTCTTTAACCTTCC

AAATTCTGATTCTTAGGTGAACTTGGAGTTGAATTTGGAATTCTGCCATGATAATATTTG

AATGGTCGACAATTGTATTCAGTGTTTGCAGTCCTAATTTTTGTTATAGATGTCATTTTT

TTGGTCACAAAATATGATGAGTTGTGAAGCTTT

>Unigene0000060

GCTGCTTCTCGGCCATGACCACTGTACCAAATGGATGTGAAAAATGCCTTCATGCATGTT

GTTCGTGGAAAGGTACTTATGTTACCCTATGTTGATGATATGATCATTGCAGGGGATGAC

ACCAATGAGATTTAATTTGTAAAGCACCATCTTCCGAAATAATTTCAGATGAAGGAATTG

GGTATCTTCGCTACTTTCTTGGTTTAGAGGCCGACTATTATGCTCATTTAC

>Unigene0000061

ACCAGACCCGACATCTCTCATGCAATCGGGATTGTGAGTCAGTTTGTTGCTGCGCCGCAT

ACTAATCACTATGCAGCTCTTCTTCGGATCCTGAGATATCTACGAGGTACCATGACTCGC

TCTCTGTTGTTCCCTGCTACCTCACGTCTTGAGCTTAGAGCTTACACCGACGCTGATTGG

GCTGGCGATCCCACTACAAGAAAATCCACCACTGGTTTTTGTATCTTCCTAGGAGACTCA

CTGATT

>Unigene0000062

TCACTTTATTGCCGCTTTGCACACAGAGTACTATGCCACTCTTCTCCGTATTCTAAGGTA

CATATGAGGTACCATCACTCACTCTCTATTGTTTCTTGCTACCTCACAACTTGAGCTTTA

AGCTTATATTGGTGCCGATTGGGCCGACGATCATACTACGAGGAAGTCTACCACTGGTTA

GCTGACTTGCTGATTTCTTGGAAGAGTAAGAAGCATGACACAATCTCTCCTCAACATTGA

AGCAGAGTACCGCGCCATGTTGAGTGCTACCAAAGAGATTGTACATTTCAGACAACTCCT

TGCTGATATGGGTGTT

>Unigene0000063

GCTCTGCCATCTCCGGTTCATGAACCTCAATCCCCCCTCCAACAACCCCTTTTTCATCTC

CTACACAATACATGGTCTACAAGTGTTTAAACCGCAAGTAGCCTACTTATATGAGCCACC

TCAACCAATCTCCACCATGCCAATACCTGTTTTGCTAGATCCTGCAGCTCCACACCGATC

CCTTCGATTTCGTCATCAAGCAAAGCGAATGAGATTTACGACAATTGCTACCTACACTCC

TGCCTTTTGAGGTTTTCTCTCTGCAGTCCACAATGTTTAAGAACCACAATCATATCAGGA

GGCTATTCAAAATGTGGAGCGG

>Unigene0000064

CTCCTTTACTTCGATTAGTTCCTCCTTGGGAACATCAACTTTTGTGTGTCCAAACTCCTC

CGGCCGCTCTGCCATCTCGTGTCCCTGAACCTCAATCCACCCCCTCCTCCAAGAGCCCCT

ACTCCTATTCCACTATTACACCATACAGGGTCTACAAATGTTTAAAGCGCAAGTATCCTT

TTGATATAGAGCTACCTCAACCAATCTCCACCATGCGAATGCCTACTCTGCCAGATCCTA

CGACTTCATGTCGATCCCTCCGATTTCATCATCAAACGAATCGACTGGGATTCATGACAG

TTACTATCTACACACCTATCTTTTGAGTTTTTCTCTCTGCAGTCCACAATGTCTAAGAGC

CACAATCATATCAGGAGGCTGATCAGAATGCTGAGTGGCGACAGACGATAACAGAGGAGC

TACGGGCCTTTTCACAGACGCACACCGGGACATCATATCCTGTCCTCCAGGTGTGATGCC

TATTGGAAGCAAATGAGTCTACAAAGTCAAATACTGACAAAAC

>Unigene0000065

TTTAATGCTCGCCCATCTATACTTGGATCTCCTCCCTACTCTACTTCTTCCTCCTCGTCT

CCTTTACTTCGATCGGTTCCTCCTTGGGAACATCAACTTTTGTGTGTCCAAACTCCTCCG

GCCGCTCTGCCATCTCGTGTCCCTGAACCTCAATCCACCCCCTCCTCCAAGAGCCCCTAC

TCCTATTCCACTATTACACCATACAGGGTCTACAAATGTTTAAAGCGCAAGTATCCTTTT

GATATAGAGCTACCTCAACCAATCTCCACCATGCGAATGCCTACTCTGCCAGATCCTACG

ACTTCATGTCGATCCCTCCGATTTCATCATCAAACGAATCGACTGGGATTCATGACAGTT

ACTATCTACACACCTATCTTTTGAGTTTTTCTCTCTGCAGTCCACAATGTCTAAGAGCCA

CAATCATATCAGGAGGCTGATCAGAATGCTGAGTGGCGACAGACGATAACAGAGGAGCTA

CGGGCCTTTTCACAGACGCACACCGGGACATCATATCCTGTCCTCCAGGTGTGATGCCTA

TTGGAAGCAAATGAGTCTACAAAGTCAAATACTGACAAAAC

>Unigene0000066

CTCTGCATCACCAGACCATCTCTCGTGCTATCGGGAATGTGAGTTAGTTTGTTGCTGGTG

CCGCTCTTCTTCGGATCGTGAGATATTTACGAGGCACCATGAATCGTTCTTTGTGCCCTG

CTACCTCATGACTTGAGCTTCGAGCTTACACCGACTCCAACTCGGCTGACGATCCGCCTA

CAAGGAAATCAACCACTGGTTTTTACATCTTCCAAGGAGACTCATTGATCTCCTAGAAGA

TCATGGTCACTCTCTGAAGCAGAAAATCGTGCCATGTCAAGTGCTACCAAAGAAATT

>Unigene0000067

AAGTCTACATCTGAAGCACCAGCACGAACTGTTCTCTACTTCTCTCTTCGAAAACCTCTT

CCTCCTCCTCCTCCGGGGTTAGAAAATCATGGCGGCCGAGGAAGCGAAGCTCCAACTCTT

CCTACAATGGCTGCAGGCCAACGGAGTGGACCTCCGCGGCTGCGCCATCCGCTACTGCGG

CCCTGACCGAGGTTTCGGTGTCTTCTCCACCGCCACCGACACCGACAACAACGGTGTTGT

GATGGTGGCGCCGCTCGATCTCGCAATCACTCCCATGCGGGTGCTCCAGGACCCCGTTGT

GGGACCCAGATGCCGGGCGATGTTCGAGGAGGGGGATGTAGATGATCGGTTCCTTGTTAT

GCTGTATCTCACCGTTGAAAGACTTCGGCCTCACTCGCCTTGGAAACCGTACCTTGACAT

GCTCCCAAGCACATTTGGTAACCCACTCTGGTTCACCAAGGATGAACTTGAGGAGCTGAA

GGGGACTACTTTGTATCGTGCCACCTTGATGCAGAGAAAAAAATTGCAAGCTTTATATGA

TGATAAAGTGAAGGGCCTAGTTGAGGAGCTCTTGTGTCATGAGGGGGAATCCAAAAGTGG

GAGGAAGGTTCAATTTGAGGATTTCCTTTGGGCAAATTCCATATTCTGGACTAGAGCTTT

AAATATACCCTTTCCTCGGTCTTATGTATTTCCCGAGTTACCATCAGGGCAAGACGAAAG

TTCTGTGGGCCTTGATAGGGATTCTGGTGTTTCGGAAACTAATTTTCCTGGTGAAACTGG

ACTGGATGAAAAGCCTGCTGACATTCTTCTAAAAGATAGTGTTAATGGAAACTCCAATTC

AGTTGATGGAGTGACTGTCTGGGTTGAGGGACTTGTGCCTGGTATTGATTTCTGCAACCA

TGGTTTGAAAGCCACAGCAACTTGGGAAGTTGATCAGAGAGGATCTGTAACGGGGGTTCC

TACGTCAATGTACCTTATTCTTGCTGATCAGAGGATTACCGAAGCTGACACAGAGATATA

TATCAGCTATGGTAACAAAGGAAATGAGGAATTGTTGTACCTCTATGGATTTGTCATTGA

TAGTAATCCGGATGATTATCTTATGGTCCATTATCCCATGGAAGCTTTTCAGAATGTTTC

ACTATCTGATAGCAAAGCGAAGCTTCTTGAAATACAGAAGGCTGAGTTGAGATGTCTCTT

ACCTAGAAGTCTGCTTGATACCGGATTTGTATCCGGTGACACTTATCAAGATGAAAATCA

TGGCAAGAAATTGGTCAGTCAGCAGTATAGCTATAGCTGGTGTGGCCAGCGCAAGGCTCC

TTCATATCTAAATCGTCTTGTTTTCCCACAAGATTTCTTGGCAGCATTGCGGACAATTGC

TATGCAAGAACATGAGCTTATTCAGGTCATTTCACTGCTTGAAGAGCTCGGTGGATCCGG

TGAGGAAAGACAACCATCTGATAAGGAGGTGATAGCTGCTGTCTGGGAAACCTGTGGAGA

TTATGGCGCGCTAGAATTGCTTGTTGATCTTCTTCGTGTGAAGCTGACGGAACTTGAAGA

TGGTTCCGGAACAGAACCCTGTGACGCCAAATTTCTCGAGAAATTCAGAGTCGTGGAGCT

GCAAGACGAGACTAGCAGACATAAGACTAGTGAAGATAGTTCGATGAGTCGAAACATATT

GTCGTGTGTTGTTTATAGACGAGGGCAGAAACAGCTCACGAGTTTATTCTTGAAGGAAGC

AGAGCATGTGCTAGAGCTATGTGCAAGTGAAGAACAATGATATATCTCCAACTTATATGG

TATGGAAATGATCATTGTTCCATCTATATGTATTCACAACTTGTTGGCAAATTTACCGGC

TATTCGAATTAACTTCTACACCTTCAAATTTCTTTATTATTATTATTTTTAAGTTGTCTA

GATGTGTGCTTGATAATTGTTTTTGTTGATAGTTTGATTAAACTGCTGACACTCACTTGA

AATTTCTCTTTTATGTATTGTGAAGTCCGAGTATAGTCTATAAAAAT

>Unigene0000068

TCCTCCGAAGCCGGCGACGATGACGCCCCCTTCGATGGCATCGACGACACCACCCCCGTC

TACGTCTGGGGCACCAACATCAGCGTCCAGGACGTCAAAGCCGCCGTCCTCCGCTTCATC

CGCCACTTCCGCGACACTCAATCAGACGGACAAGCCCACATAGACGGCAAGTACATGGCC

GCTGTCCACCGGATCATGGAGCTGGAGGGCGGGGAGTCGCTTGATATTGACGCACATGAC

GTGTTTGATTACGATCCTGACCTATACGGCAAGATGGTTCGGTATCCTCTGGAGGTGTTG

GCCATATTTGATATTGTGCTAATGGATATGGTGGCTCGGATAAACCCCGACTTTGTGCAG

CATATACAAACGAGGATATATAATCTGAAGTCTTCTGTGTGCATGAGGAATCTCAATCCC

TCTGATATTGAGAAGTTGGTGTCGGTCAAGGGGATGATCATTCGGTGCAGCTCGATTATA

CCTGAGGTCAAGGAGGCACTTTTTCGGTGTCTGATTTGCGGGCACTACTCTGAACCTATA

ATGGTTGACCGAGGTCGGATCAATGAACCACTCAGGTGTGGAAAGCAGGAATGTCTTGCT

AAGAACTCCATGACACTGGTTCACAACCGGTGCAGATTTGCGGATAAACAGATAGTAAAG

TTGCAGGAGACGCCAGATGAGATACCTGATGGAGGGACCCCACATACTGTCAGTATTCTG

ATGCATGACAAACTAGTGGATTCTGGAAAACCTGGAGACAGAGTTGAGATCACTGGGGTA

TACAGAGCCATGAGTGTCAGGATAGGGCCGACTCAGAGAACTGTCAAATCTGTATTCAAG

ACATACATAGATTGTCTTCACATAAAGAAAACGGACAAGTCACGGTTGCATGTAGAAGAT

CCCATGCAATGTGATGACTCTTATGCCAGTAAAAGTGTGGGTGAAACTGAAATTCCAGAG

TATCAAACTAAGATCGCCCAGTTGAAAGAGCTTGCAAAGCTTCCAGATATTTATGACAGG

TTGACCAAGTCCCTAGCTCCAAACATATGGGAGTTGGATGATGTGAAAAGGGGTCTTCTT

TGCCAGCTTTTTGGTGGAAGTGCTCTGAAGCTTAAT

>Unigene0000069

GGTCTTCTTTGCCAGCTTTTTGGGGGAAGTGCTCTGAAGCTTAATACCGGGGCTACCTTC

CGTGGAGATATCAATATTCTGCTTGTTGGAGATCCTGGAACTAGCAAGTCACAGCTACTC

CAGTACATGCACAAACTGTCTCCACGTGGAATATACACCAGTGGTAGGGGGAGCTCTGCA

GTGGGCCTTACTGCGTATGTTGCAAAAGACCCAGAAACTGGTGAAACTGTTCTGGAGAGT

GGGGCATTGGTTCTGAGTGATAGAGGTGTCTGCTGCATAGATGAGTTTGATAAGATGTCC

GAAAATGCACGCAGCATGTTGCATGAGGTTATGGAGCAACAAACAGTTTCAATTGCCAAG

GCAGGAATTATTGCTTCCTTGAATGCCAGGACTTCTGTTCTAGCTTGTGCAAACCCAAGT

GGATCTCGTTACAACCCTCGACTCTCTGTGATTGATAATATCCACCTTCCTCCAACATTA

CTTTCTAGGTTTGATCTGATTTACCTTATTCTGGACAAGGCTGATGAGCAATCAGACAGG

CGCCTGGCTAAACATATTGTTGCATTGCATTTTGAAAATCCAGAGAGGTCAGTGCATGAT

GTCATTGACCTTCCTACACTAACTGCCTACATAAGCTACGCAAGAAAGCACATCCATCCC

CGACTCTCCGATGAAGCTGCAGAAGAATTAACACGCGGCTATGTTGAAATGAGGAGACGC

GGTAACTTCCCAGGTAGCAGCAAGAAGGTAATCACAGCGACTGCTAGGCAAATTGAGAGC

TTGATTCGTCTCAGTGAAGCGTTAGCTCGCATGCGCTTTTCTGAATCAGTGGAGATAAGT

GATGTTACTGAGGCGTTTAGGTTGCTTGAAGTTGCAATGCAGCAGTCGGCAACTGATCAT

AGCACAGGGACTATTGACATGGATCTCATTATGACTGGTATATCTGCGAGTGAGAGGATA

AGGCGAGAGAGTCTGGTTGTGACCACCCGTAATTTGGTTATGGAGAAAATGAAAATTGGT

GGACCTTCGACACGTGTGGGGGAGTTGCTAGAGGAGCTAAAGAAGCAAAGCCCGGTGGAT

ATTCATATGAGTGATCTGCGGAATGCCCTCTTGACTCTCATGAGTGAAGGGCTTGTCTCT

TTTCATGGCGATGTTGTTAAGCGGATTTGAAAGTGTAAGCCATCTTATGCTGTGATCCTT

CGGTTAAGTTAAATATGATCCAGAGGTTGGAGTGTCAAGTGTAATTGCTGCATGTGGGTG

AGAGAATTGAACTATGTGGTGTTTTTCTCAGGTTTTATGTATTCTGTTGTGTTTGAGGAT

AGTAATTTTTGAGATATGGATGTATTTAGGATTAATTTCTGCATGCCTATGATGGTGCTA

GAATGCCCAATCTCTGATAATGCTTCTTACCTGGTGTAGTTCCTATTCCAGAAACATAAA

>Unigene0000070

CTTGAAGTAGCAATGCAGCAGTCGGCAACCGATCATAGCACATGTTACTTTAATTTCTCT

TGACTGGTGTTTTAAGTTGAATATATGTAATTTTAATCCAACATTTATGAGCAGGGACTA

TTGACATGTATCTCATTATGACTCGTATATCTATGAGTGAGAGGATTAGGCGAGAGTCTG

GTTGTGACCACCCGTAATTTGGTTATGGAGAAAATGAATATTGGTGGACCGTTGAAACAT

GTGGGAGAGTTGCTAGAGGAGCTAAAGAAGCAAAGCCCGGTGGATATTCATATGAGTGAT

CTGCGGAATGCCCTCTTGACTCTCATGAGTGAAGGGCTTGTCTCTTTTCATGG

>Unigene0000071

TCCTCCGAAGCCGGCGACGATGACGCCCCCTTCGATGGCATCGACGACACCACCCCCGTC

TACGTCTGGGGCACCAACATCAGCGTCCAGGACGTCAAAGCCGCCGTCCTCCGCTTCATC

CGCCACTTCCGCGACACTCAATCAGACGGACAAGCCCACATAGACGGCAAGTACATGGCC

GCTGTCCACCGGATCATGGAGCTGGAGGGCGGGGAGTCGCTTGATATTGACGCACATGAC

GTGTTTGATTACGATCCTGACCTATACGGCAAGATGGTTCGGTATCCTCTGGAGGTGTTG

GCCATATTTGATATTGTGCTAATGGATATGGTGGCTCGGATAAACCCCGACTTTGTGCAG

CATATACAAACGAGGATATATAATCTGAAGTCTTCTGTGTGCATGAGGAATCTCAATCCC

TCTGATATTGAGAAGTTGGTGTCGGTCAAGGGGATGATCATTCGGTGCAGCTCGATTATA

CCTGAGGTCAAGGAGGCACTTTTTCGGTGTCTGATTTGCGGGCACTACTCTGAACCTATA

ATGGTTGACCGAGGTCGGATCAATGAACCACTCAGGTGTGGAAAGCAGGAATGTCTTGCT

AAGAACTCCATGACACTGGTTCACAACCGGTGCAGATTTGCGGATAAACAGATAGTAAAG

TTGCAGGAGACGCCAGATGAGATACCTGATGGAGGGACCCCACATACTGTCAGTATTCTG

ATGCATGACAAACTAGTGGATTCTGGAAAACCTGGAGACAGAGTTGAGATCACTGGGGTA

TACAGAGCCATGAGTGTCAGGATAGGGCCGACTCAGAGAACTGTCAAATCTGTATTCAAG

ACATACATAGATTGTCTTCACATAAAGAAAACGGACAAGTCACGGTTGCATGTAGAAGAT

CCCATGCAATGTGATGACTCTTATGCCAGTAAAAGTGTGGGTGAAACTGAAATTCCAGAG

TATCAAACTAAGATCGCCCAGTTGAAAGAGCTTGCAAAGCTTCCAGATATTTATGACAGG

TTGACCAAGTCCCTAGCTCCAAACATATGGGAGTTGGATGATGTGAAAAGGGGTCTTCTT

TGCCAGCTTTTTGGGGGAAGTGCTCTGAAGCTTAATACCGGGGCTACCTTCCGTGGAGAT

ATCAATATTCTGCTTGTTGGAGATCCTGGAACTAGCAAGTCACA

>Unigene0000072

CAAAACCTCCCAACTAAACCAAATCGTAGTCAAACTCAATCACCATCCGACCCTCACCCG

AACAAATCAGCGGCTCCGATCACACCACCCCCTCCCCCCAACGCCGCTCCTCACCCCCAA

TCTCCCAGTGTTTCTCCTCAATTCAAGCTCAAAGATCCAATCTTTAGGGCAATGGAGATC

CAGACCTCCGGGAAGGCGATCGACACCCTGCTCGAGAAGGTCCTGTGCATGAACATCCTC

TCCTCTGATTATTTCAAAGAGCTTTACCGGTTAAAGACGTATCATGAGGTTATAGATGAG

ATCTATAACCAAGTCGATCATGTCGAGCCATGGATGACTGGTAATTGTAGGGGGCCGTCG

ACGGCTTTCTGTTTACTGTACAAGTTTTTCACCATGAAGTTGACTGTGAAGCAGATGCAC

GGGTTGCTGAAGCATCAGGATTCGCCGTATATCCGGGCGGTTGGCTTTCTCTACTTGCGC

TATTATGCGGAACCAAAATCATTGTGGGGCTGGTTTGAGCCTTACATCAAAGATGATGAG

GAGTTTTCTCCTGGCTCTGATGGTCGAATGACAACCATGGGGGTATATGTGCGCGATCTT

CTTCTTGGGCAGGTCAGTAATTTTCCTTGCTTGTTTTTATATTGTCAAGAACCTTGCTTT

CTGCTCCCTTATTGTAGACTATCCACGTCATTTTCTTTAGCTGATCAATATTTGTCTAAG

GGTGCTGTCATCTACTGTATGCCATGTATTTGTCATTCAGAGTTAACTGTTTGTCATGGT

ACTTGGAAGCTTATTCATTCCAGTACATGACCCATTTGTTGATGTGAGATTCTGAGCTTC

GGTCCCCATATGGAGGTTGCTTTAAGAGGACAGCACATCGCTGTATGTAAGTACTACTTT

GACACCCTTTTCCCGCGCATCCCTGTCCCTGTCCTGCGAACTGTCACTGGTCACCTTGAG

AGAATGAAGCTTCCTACTAAGCATGCTGGAGTAACTGGAGACACCAACCGATGTTCTGAG

GACACTGCTCGTCGCCCCCCTTCTGTGAAAGCTGCCCTATCTGTCTCCTTCGGTCAACGC

GCTCCACACCGAGCCTCAACCAGGGATTCCTCCCCGGTTCGCAGGACCTTACCCACCTCT

CATGGAAAGAAGAACACTGATGAGGTGAGAAAGGCATCTCCCAGCGACAATCGCCATGAG

GGATCTGATCGAGACCGTTCAGACCGGGGCCGGGAGCGCGACCGGGATCGTTATCGGGAA

AGGGAGCGAGAACGGGACAGGGACAGGAAGCATGATCACGACCGGCGTGGCCGTGACAAT

GAGAGATACAGAGACTCCCATCGCAGTGGTAGGGACAGCGGAAGGCGTGACTATGAAAGC

AGCAGCCGGGATGTTGATCGGCGCCGCAGTCGAAGCCGGAGCCGGAGCAGAAGCGTGCAG

GTCCGTGGAGCTAGCGCTGACCGTCCTGCAAGCCCGTTCAGAGATGGGATCAAAGAGAGG

AAGCCGGCTGTTTCTAGCAATCTGGCGAAGCTGATGGATATCTATGGGGACGGAGGCAGC

AAAAGTAGCGATGGAGGCGCAGATAGGCTGCATCCAAGAGATACCAGTACCGAGGAGGTG

ATCAGATTGGGAGGCTCTACTTGGAGGTAAAATGCAATTATCGTCCAGAGAACACCATTT

ACAATATGTAATAGTAGTCTTGGGAGTAAAATACAACTGAAAATTCTTGTACTTGTTAAA

ATTATTGTCAGGCTTAGTAATCTTTAAGCTTAAAGATCGTGAAGGGTGATCACATATTGA

TTCTGGATATTGTGATGAATGTTGAGCTTAAAGATCTTGAAAGGGTGATCACATATTGAT

TCTGGATATTGTGATGAATGTT

>Unigene0000073

TCCTTATCCTTCCTCCAAGCATACCCTGAACTCCTTTTATCTGCGACATTTTTTATCGTT

TTCTTTGCCTACCGTCTAACTCGTTGTAGCTCGGTGATCCCAGTGAATTGGCCGGTTGTT

GGTATGCTTCCCTCGATCCTCGCCAATCTCTATCGTTTTCATGATTGGGTCACGGAGGTT

ACTCGCAAGTCTGGCTGCACCTTCCATTTCCTCGGCCCATGGTTCTCCGCCATGGAGTAT

CTCATCACGTGTGATCCTGCCAACGCCAACCACATCTTCAACATCAACTTCTCCAACTAC

CAGAAAGGTGACGATTTCTCTGACATTTTCGAAATCCTCGGTGATGGTATATTCACTGTC

GACGACGGGCTGTGGAAGAGCCAACGTCAAAAGATGCAACTCCTCTTGTCAGACAAGCGG

TTCCGATTGTTTGTGTTCGATGCCAGCCGAGACAAGGTGGAGAAGGCTCTCCTACCTCTA

CTAACTCTAATTCTTGAGAAAGAAGAGGTAGTTGACTTGCAAGATGTGATGTTGAGGCTG

ACCTTCGATATGACGTGTTACTTGGTATTGGGTGTCGACCCTGGATGCCTTGCGGTCGGT

TTCCCGACGGTCGGATTTGCCAAAGCGATGGATGATGCTATGGATGCCGTGTTTCTCCGC

CATATATTACCCACTAGCTGGTGGAAGCTGATGAGGTTTCTTGGTGTGGGGATGGAGAAG

AAGTTGTCTAGAGCTCGGGAAGTGATCGATGGCTTTGTAGCTGAGACCATATCAAACCGG

CGATCATTGAACACGAGCAATCCAGATTTATTATCATCGTACATCGTCGATAATGATAAT

AATGTCGACAATGACAAGTTCCTTCGGGACACGGTCGTGAATCTCATGCTAGCAGGGAGA

GACACCACCGGCGCAACGCTGGGATGGTTCTTCTGGATGCTGGCAGAGAACCC

>Unigene0000074

GATGGAGAAGAAGTTGGCTAGAGCTCGGAAAGTGATCGATGGCTTTGTAGCTGAGACCAT

ATCAAACCGACGGTCATTGAACACGAGCAATCCAGATTTATTATCATCGTACATCGTTGA

TAATGATAATAATGTGGACAATGACAAGTTCCTTCGGGACACGGTCGTGAATCTCATGCT

AGCAGGGAGAGACACCACCGGCGCAACGCTGGGATGGTTCTTCTGGA

>Unigene0000075

CAATGACAAGTTCCTTCGGGACACGGTCGTGAATCTCATGCTAGCAGGGAGAGACACCAC

CGGCGCAACGCTGGGATGGTTCTTCTGGATGCTGGCAGAGAACCCATGCGTTGAAGAGAG

AGTGTTCGAGGAGTTGAAGGAATGTGTTTCCACGTCTGGTTCTTCAATGGAGCTTTGGAA

GTTGCTTTACTTGCATGCAGCGCTATGTGAAACACTGCGACTGTTTCCACCGGTACCGGT

GGAGAAGAAATGTGTGGTTCGGTCGGAGGTGCTCCCAAGTGGTGCGGTTGTCCGACCTGG

CCTGGAGATATTGTTGTCGGTGTATGCGATGGGGCGGATGGAGGGGGTGTGGGGGAAGGA

TTGCATGGAGTTTAAGCCGGAGAGGTGGATTTCGGGGCAAGGGAAGATGAGGCACGAGCC

AAGCTACAAGTTTTTGTCGTTCAATTCGGGCCCGAGGACGTGCTTGGGGAGGGACGTAGC

TTTTACTCAGATGAAAGCGGTCGCGGCAGCTGTGGTGTACAACTTTCATGTTGAAGTGGT

GAGAGGGCATGTTG

>Unigene0000076

CTCCTCAAATGAGGGAATCCTCGGCTGAGCCAGTAAGATAGTGGCCCTCTGCTTTGGCCT

CGGTGCGCTCTCACTCGCAACCATACTGAATGCTCGTATCAACATTTGGAGCTCTGAAGC

GCTCAACTTCGCCATGACCTCCTCCATGGGATTCTTACCCTTGCCAGAGTTCTCTCCTGC

CATCGATCCAACTCAGATCTACTCTCTACACACGAATCTGAGTTGAATCCTCTTTCTCCT

TCCTCAATATCTGATCTTGCACTCTCAAGATT

>Unigene0000077

GAGTACCCTCATCTGGCTCCTTGTCCCCAGTCAAGTACCCCTCTAGATCCTTTCCGTCTA

GAGTGTACCTTACCCGATGCGACCAGCTCAGATACGAAGCTGGCCCATCGAGTTTCATAT

CGATCGGTGGGAGCTCCACCCTCTGCAGTGACCTTGGTGCGCCCTCACTCGCAGCCATAC

TGAATGTTCGCATCAGCATCTGGAGCTCTACGGCGCTCAGCTTCGCCATGACCTCCTCCA

TGGGATTCTTACCCTTGCCAGAGT

>Unigene0000078

ACAGTCCTCTCCCCCTGAACTGCAACAACGATCTCCTCTTTGATCTGATACACCCTCAGG

TTGTTCCCCACTCCATCATAAGTCCTCTTTAGCTTCTCCCAGACATTCTTGACCCGATCT

ATGCCATCCACTGTGGATGCAATAGACGGAGCCATCGAACTCAGAAGCCAAATGTACACT

GTCATGTGAGTGGATTTCCACTCAGCGCTCCTCGGAGTACTCTCATCTGACTCCT

>Unigene0000079

GTGATTGGTAGATAATAAGGTTTCTAAATTGTGATTGGTAGATAATAAGGTTTCTAAATT

GTGATTGGTAGATAATAAGGTTTTTGAGGATTGGTATATGGATCAGTCACTCTGGAAACT

GATCTGGAACATGTGTTAGGGCCATCTTATTGGCCAGTCTGGTGGTCATATTAAAATAGT

AAACTAAAAATAAGTAGTAAAAATCAGAATCTGACTAAAGAAATTAGTATGGTTTTCACA

GGATTTAGTTTCTGCTTGCAAATCAGCCAAGATGGTGAGGGTCAGTGTCTTGAATGATGC

CCTAAAGAGCATGTATAATGCCGAGAAGCGTGGGAAACGACAAGTTATGATCAGGCCCTC

TTCCAAGGTGATCATCAAGTTTCTTATTGTCATGCAGAAGCATGGTAAGATAATCGGGCA

GTAGTCTTCCTGGCAAACTGTTCTTTTGCTTATTTAATTATTTTACCTGACTTATGTTTT

TTATGCATGATTTGTAGGATACATTGGTGAGT

>Unigene0000080

TTCTGCTCCTTCACCTATACAGTGATAAGTGATGATAAATGAGTGTAAGTACTCGACATT

TGATCCTTCAGAATCAGTTGAAACATGTTAGCTATTTGATGTGTGCAAAGCATTGTTCTT

ACTGATATCAGAGTTGGATTAATTATTACTTAGTGCTTGTCGATTTTTTTTATAATTTGT

ACCCTTCTGTTAATACATTACAATAGTGCATTCTAGATTGGTGGTGTTGTTTGACAGTTG

GGTAATTTCACCAATCCCTGAGTTTGTGTTTACACCTTTTA

>Unigene0000081

AGTGTTCTGAATGATGCTCTGAAGAGCATGTACAATGCTGAGAAACGAGGGAAGAGACAA

GTCATGATCAGGCCATCCTCAAAAGTCATCATAAAGTTTCTAATTGTCATGCAGAAGCAT

GGTTACATTGGTGAGTTTGAATATGTTGACGACCATAGGTCTGGTAAAATAGTCGTTGAA

TTGAATGGGAGGCTGAACAAGTGTGGGGTCATCAGTCCTCGCTTTGATGTTGGTGTCAAA

GAGATTGAACCCTGGACTGCCAGGTTGCTCCCATCGCGACAGTTTGGTTATATTGTGCTG

ACAACATCCGCGGGCATCATGGATCATGAGGAAGCTAGGAGGAAGAATGTTGGCGGTAAA

GTACTCGGTTTTTTCTACTAGTTGATTTTTGTTCTTCGTTCTGGTCAGAACGTTAGAGGT

TAAATACCTGATGTTTTCTTAGAAGAGTGTTTTGAAGATGTGTTTTGTTATGTTATTCCT

TTGAGAAACTTCTGAAGAATTTTGTGATTTAATTCAAATGGTTGATTGTTGTTTTATCTT

GGTTTATTATGATGCATAACTGTTGGTTGTATTGAAAGGGATTATTAATATATGTTCTGC

TCCTTCACCTATACAGCGATAAGTGATGATAAATGAGTGTAAGTACTCGACATTTGATCC

TTCAGAATCAGTTGAAACATGTTAGCTATTTGATGTGTGCAAAGCATTGTTCTTACTGAT

ATCAGAGTTGGATTAATTATTACTTAGTGCTTGTCGATTTTTTTTATAATTTGTACCCTT

CTGTTAATACATTACAATAGTGCATTCTAGATTGGTGGTGTTGTTTGACAGTTGGGTAAT

TTCACCAATCCCTGAGTTTGTGTTTACACCTTTTA

>Unigene0000082

TTGCTTGGGCTGGCGGGCTGCCCAACGGGCTTCGAGCCCTCTGGCGACCTGCATAAAGAC

GCCCCATACATCCGATCTTGCTTCTAGGGTTTCACCGCCGCGCCTCCGATCTCTCGCTCC

CATCAGAGTTTTCATAGGCCTGTGGTGCTACTTTTCTTTGCGAGGATGGTGAGAGTTAGT

GTTCTGAATGATGCTCTGAAGAGCATGTACAATGCTGAGAAACGAGGGAAGAGACAAGTC

ATGATCAGGCCATCCTCAAAAGTCATCATAAAGTTTCTAATTGTCATGCAGAAGCATGGT

TACATTGGTGAGTTTGAATATGTTGATGATCATAGATCAGGTAAAATTGTGGTCGAGCTG

AATGGACGTTTGAACAAATGTGGTGTCATCAGTCCTCGCTTTGACGTAGGTGTCAAAGAA

ATCGAGCCTTGGACTGCGAGGCTGCTCCCATCGCGTCAGTTTGGTTACATTGTGCTGACA

ACTTCTGCGGGCATCATGGACCATGAGGAAGCTAGGAGGAAGAATGTTGGTGGCAAGGTA

CTTGGATTCTTTTACTAGAGGTTTTCGGGAGGTGTTAGTCGAGTTGGTAGTCTCTTTTCC

GATAGTTATGTTGTGGATGTGATTTTGTTTCCATTTCTGAGGAATTCCCAAAGCTTGAAG

GCTGTTTCATTGTTATACCTCTCGTCTTGGTATTGAGTACAATATGGATTTGATTATAAC

TATTCAGAGGATGTTGGTTTTTCTATTCGAAGTATTTGT

>Unigene0000083

AAACGGTATTCGATTACGGAGTGTTCGATTACGGAGAAGCTGATTGAAGGGTTCTTGTGC

GAAAGGGGGTAAACGGTATTTAATCCAATATCTTTTGGATGCGGGAACTCAACATATATT

ATACTTATTTTGATTTCAGAGTGAACTTTATTTCTGTCTTGAGTTACCGCATCATCGGAA

ATGATTTCTGTCTTGCTGCACCATCGGAAATGATTTTTTTGAAATTCTTTTAGCACTTCA

TTTTCTATTTGGGATGGGGTGGGTTCTGACTAAATGACCCTAGTAATCACTAATTTA

>Unigene0000084

AAACGGTATTCGATTACGGAGTGTTCGATTACGGAGAAGCTGATTGAAGGGTTCTTGTGC

GAAAGGGGGTAAACGGTATTTAATCCAATATTTTTTGGATGCGGGAACTCAAGACATATT

ATACTTTGATTTCAGAGTAAACTTTATTTCTGTCATGAGTTGCCGCATCATCGGAAACGA

TTCAAAATATTTCCGTCTTGAGCTGCCGCATGATGTGGCATCATTGGAAATGATTTTTTT

GAATTTCTTTTAGCACTTCATTTTCTATTTGGGGTGGGGTGGGTTCTGACTGACCCCAGT

AATTACTGATTTACGTGTGATAGGCATGATGTCACAATTATTATACCTCTTT

>Unigene0000085

CTTGAGGGGGTGTGGAGTACATCGTAGGGTGGCGGAGGGAGAGAAGTGGTACGAGGTGAC

CAAGAAAGAATCATAGTAACATTTTTTTTCCTTCTAGATTTGGGAGTGGGGAGGATAAGC

TGACTGAAGGAGTTGGAGTGCCCGTTTACGGAGAGGCTGGTTGTAGAGGTTCTTGTGCTA

ACGAAATCAGACCACAGGTGGTCGAAGATGAAAAACCCACACGAGGTCGGGTGCAAGAGG

GGGTAAA

>Unigene0000086

GGTCGAATGTGAAAATTACCCACATGAGGTCGAGTGCAAAAGAGGGTATACGGTATTTAA

TCCAATATCTTTTGGATGCGGGAACTCAAGATATATTATATCTATTTTGATTTCAGAGTG

AACTTTATTTCTGTCTTGAGTTGCCGCATCATCGGAAATGATTTCTGTCTTGCTGCACCA

TCGGAAATGATTTTTTTGAAATTCTGTTAGCACTTCATTTTCTATTTGGGGTGGGGTGGG

TTCTGACT

>Unigene0000087

CTTGAGGGGGTGTGGAGTACATCGTAGGGTGGCGGAGGGAGAGAAGTGGTACGAGGTGAC

CAAGAAAGAATCATAGTAACATTTTTTTTCCTTCTAGATTTGGGGGTGGGAAGGATAAGC

TGACTGAAGGAGTTGGAGTGCTCGTTTACGGAGAAGCTGGTTGAAGAGGTTCCTGTGCTA

ACGAAATCAGACCGCAGGTGGTCGAAGATGAAAAACCCACACGAGGTCGGGTGCAAGAGG

GGGTAAATGGTATTTAATCCAATATTTTTTG

>Unigene0000088

ATATTTTTTGGATGCGGGAACTCAAGACATATTATACTTATTTTGATTTCAGAGTGAACT

TTATTTCTGTCTTGAGTTGCCGCATCATCGGAAATGATCTGTCTTTGAGTTGCGGCATCA

TCAGAAATGATTTTTTTTTGAAATTTCTTTTAGCACTTCATTTTCTATTTGGGGTGGGGT

GGGTTCTGACTAAATGAACCTAGTAATCACTAATTTATATGTGATAGGCATGTTGTCACA

ATTATTATACTTCTTTTATTGGTCGTGCTGAAGTAATCACAATGAATTTATCCGTGTCAA

CATAATTGATACATGTGTTTTTATTATCGTTTCGTGAAATTATAGATAGCTGATCCAAC

>Unigene0000089

ATTATTAGTGTTTTTGCCCTCCATTTCTTTCAACCGATATTTGGATGACCCCCCATTCCC

ATCCATTGTTTTTTCTAAAAACACTTTGAAAAATATTTTTTGGATGCGGGAACTCGAGAT

TGAACTTATTGGCCCTTATTTTTTTCTCACTAATTGGCCCTTATTTATTTCACTATTAGC

GCATAGTCATTTTAGTTCAGCTTCTCTACAAACTGTAAATGTCTAAATGAGTTCGTAGGG

CTGTTGGAAAGCTAAATCTAGATATG

>Unigene0000090

CTCATTCACTTATGGTATGTTTTACTAATCGTTGTTGTCATCTCCATTTCATCTTTCAAG

CCCTTAGCAAATGAGTGCAGAGAGGAAGAAGATGAGTAATGGGGAGAAGATGATAGAACG

TCACCAATCAAACCTTCACATGCACATTCCACTCTTTCACTTATGTGCGGACCCACCATA

TTTAAGTCATTTACGAGAGTAAAATGCTGGGTCTTAATAGGATTTACCCTTAGACTTAGA

CTTTTCCTTTGAGGTGTAACTGACCCTTATTTAGACCCTTAAATTATTTTTAAAATAGAA

TCCGAAGAAATATTTAGGACAAACCAACCCCTGCCGAGCATAAGGGTAGTTGTTTTAAAT

TCAGGTAAATCTAGCACAATTTCCAGGTTGGGTTCTTACAACAATTCCCCCATTTCTGTC

CAATAAGACTTGTTTGATGTAACCCTTCCTTCCCCAAGCATCTTCTATTGAGAGGCCCAT

TTAAATGGTGTGTTGCTTTCTTCCTTTTTAACAAAAATGAGTTTACGAGGGTTTGAACCC

GTGTCTACAACATGGCTTCATATCCAACCATCACACCATTTCACATATTTTCTTCAAACA

ACAAAATAAGTGGTTTGTGCGGTAGGGGGCTGGATGGTGGGTTATAAAAAGTTGATATCA

AATCTTCCCCTCCCATGTGGTGTCTGCATGGGTATTCACTAGCCGTATTTGGTGTACTCT

GAAATTTGATTGTGTTAATTTTCCAATGGTAGCTGCTGTTGTATCTTATGTGCTCTGAGT

AAGAATCTCTAATTAGGAATTGATCTTGGTATTTTTCATTCCGGAGGTTTGGTCCCCACG

TTCACTTGAGTTTGTAGACTTGTACCCCTAGTAACAGTTTCTGTCAATCTGGGATCCAAA

CATGCATCATGAAAAAAAATCTGGTACCCAAAAAGTACATTATGAAAGAACTGGGAACAG

ATTCCTAATTGAGAATTGGTAAAATTTTACTGCACAGATGAGATAGTCTCTGCCACATCT

CAGCTCATGAACTGGTCTTGATTTCACTGCTTATTGTTTCTGCCCCATCTATCCAGGAAG

CACCTTGTGGCGATTTAACCACCCATTTGTTCTGCCATCTATGTGCCCTTTGTCAAGAGT

ACAGGGAGATTCGCGAAAGATCTGATGGCTCGGCTCCATCCACCGTTATCCCTACCGTGA

CAGCTCCTCAGGTGCAGACAATGGAATTAGCTCCGCAGAAGTGATGAATGGTCGATCATG

CTTTATCACTAGTTAACTTTACATCTTGTGATATGCTGTTTTTATAGTGTCCTGCCATAT

GCCTTGTAGCATACGGAGGAAAATGTTTTCTTTCTTTCCAAAATTCCAGTGTTCAGGGAA

GTCCGCAGAATTATTCACATCATTTTAGCTGTGCGCTGGTGTGGGGTACGTTGCTTTGTA

TGTAACGTAATGTATATGTATGCAGAAGTTTGAACATTTTCTTGAATCTCTCCGGCCCTG

TATGAGTATGTCTGTTTTACGGTGCGGCAGTATTTGTGATTCTTGTAACAACAAATGGTT

AATTTTTTTCTCGT

>Unigene0000091

CTGACGCCGTCCCCGGTAGCGTCACCACCCTAATCCGAGCTTCACTTCTCTCCACGCCGT

CCAATCCCACGGTTTTCTCTCCTCCGTGTCGGTTCCGGCGATCCATGCGCGCCACACCTC

CATTTCACGTCCTTTGAGCTGATCTCCATCGAGGGTTTACTTGGAGAGATGGCTGCCTCG

TTGGTTGTCCCTCCGTTGCCGAGGGATGCGGTGAGGCTGAAGAGGTCGCTGCTTGCCGCG

GTCATGACGTGCCCCCTTTGCAACAAGCTACTAGACGAGCCCACCACCATCTCTGAGTGC

CTCCATACGTTTTGCAGGAAGTGCATATTCGAGAAACTCAACGACGAGGAGGAGGATTGC

TGTCCAACATGCAATATTGTTCTTGGAAGTATCCCAGTGGAGAAGCTCAGGCCTGATCGC

ATTTTACAAGATGTAAGATATAAAATATTTCCTTTTAAAAGGAGAAAAGTTAATGCTCCT

GAGGTGCGTATGATATCATTACCTGTCCGAAGAAAGGAAAGATCACTCTCTTCACTGGTG

GTCACCCATCCAGTAGGGACACAGACCGGTTTGACTGGAAGAACTAAAGCTGCTGCTAAT

AGGGCTGCTATCTTGGGTGGATTATCCCCTGCTACTAACGGTTCCAATAAAAGGGAAGAT

GTGAATGCCCAATATTGTTCAAAAAACTCAAAGTCGCCTGAGATTCTGGAAAAGATAACT

AAAAAAAGAAGGCAGAACTCTTCGAATGCGGAAACATCTTATCAAACATCTATTAAAGAT

ATGGTGAAGGGAGATCCCTACATGGATAATGCGAACCTTTGGAAATCTTTAAATTGTCTT

GTTGAGGCTGCAACGGTTAACTCGACCTCGCAAAGTCCTACCAAGGCAGAACCAATCAGT

GTTGGTGATACCAATGTGCATACTCCTAAAACCAAAGACACTGGGCATACAAATAAATCC

AGAATTCAACATAATAAGAATGGTGATATTCCCACATCTTCAGAATCCGTGAAAGTGAAG

AGATCGCAGGGAATCAGCCGGAAAAGAAAGAAACTTCAAACCTCAGCTCAAGCACTTGTT

GATACTGCAGCAGGAAAACACGATAGGAGGATGGGTCCTATTTGGTTTTCACTAGTTGCT

GCTGCTGATCAGGATCGAGGTGCACCAGTGCCTCAGTTGTCTGCAAATTTCTTGAGAATC

AAAGATGGTAGCCTTCCAGCCTCATTCATCCAGAAATACCTTGCAAAGAAACTTAGTCTT

GCAACTGAAACCGAGGTGGATATTTACTGTCAAGGGCAACCAGTGGATCCAATGATGCCT

CTGCATACCCTATTGGATTTGTGGCGGCAAACGGGATCAACCCCTGAATATCATGCAGTC

GGCTCCTCAGCCAAGGATTGCGTTATGGTGCTTGTGTATCGCTGCAAATCTTCTATCTCC

ACGCTAAGCAGCCTCCAGTAATCTTGAGGTCCTGAGCAACCATTTT

>Unigene0000092

TATCATGCAGTCGGCTCCTCAGCCAAGGATTGCGTTATGGTGCTTGTGTATCGCTGCAAA

TCTTCTATCTCCACGCTAAGCAGCCTCCAGTAATCTTGAGGTCCTGAGCAACCATTTTGA

TTATCATCTTGTTATGGTGCTAACGTATAGCCGTAAAGCCCCTTATCCAGATGAACACCA

AAGACAAACTTCTAGAGCTACACACTGATGCCCTCATCCACTACTGAGTCCATTACTGTA

CAGAAGAAGGGTGCATGATGCAATTCTCTTCCGGTTTTCCACATGTT

>Unigene0000093

TGAAGGCCTCCCTGAAACTCCGAGAAGAAGAACTCAAACCCCCACTCATCCGCGCCAAGC

TCCCCATCTCCGTCCTCGGCTTCCCCCTCATCTCCGGCGCCGTCTCCGGTAGCGGCGCAG

CCGACCTCGCCCTTCACCTCCGCACCGCTCTCCCCTCCGGCCCCTCCCTCAAACTCGCCT

ACCGCCCCTCCGCCTCCTCCTTCGCCCTCTCCCTCAAATCCGGCGTCGGCCCCTGGGGCT

CCCCGGATCGCTCGCCCCTCCTCATCTCTGCCCACCTCTCCCTCCCTTGCGCCCGCTCCC

CCGCCCCCTCCTTCACCATCCAATTAAAGCCCCAGATTGGCGACTTCATTCTCCGCAAAT

CCGCCGCCTCCGCCCCAGTCCGGCCGAAAGAGAACGGCGGCGGAGTCTACCACCCCCTCC

CGGTCTCGAATCCCGCCAATCCATCCATTGACAGCCTGCTATCGTCTGGTGCAGTGCTGT

CGGCGATGACGGTGTTGCCGGTGGCGAGTCGGGCGGTGGTGAAGCTGCGGTGGGGGGTGA

ATTTCTCGGAGGCGGCGGTGGCCAGGTGGCGGGGGCTGCCGTATCTGACGGTGGAGAAGA

TTGGGATTGAGATGGTGGAGAGGAAGGTGGAGACGGAGGCGGAGGATGTGTCGGGGGTGG

GGTTGTGGATGAGGAAGGAGGTGGAGGACCTGCGTCGGGAGAGTAAGTTGATAAATGAGA

GTATTGAGGAGATGAGGAGGATGATTGCAGATGGGAAGAGCGGGGATGCTGGTGGCGGGA

GCAGGGTTTCGGCTTTCGCGAGGAGCGACAGCAGCCCGACATTGGCTGCTGTGAGGAGGT

CATAATCGGGGTTTGATTTCTATGTCTTCCATTAATTTTTTCGTTATGTTTAGCAGGTAG

AGCTTATGTAAATAGGGATAAAGTAGGATTTATCTGTAGAATTGGCTCATTGGTGTTCTT

GTGTTCCATACTTTTCGAATAAACGATAGAGTTAATGGCACTACTAGATTTGGAAAAGAT

T

>Unigene0000094

CAGTCTGGTCTTATATGCAGTTTTGCAGTCTGGTCTTATATGCAGTTTTGCAGTCTGGTC

TTATATGCAGTTTATCCCCGTGCAAGGAATTGTGATGATAGGCGGTGGGATAGGTGATGA

GAAGGGAGTATAGCCCTTGTATTACTTAGATTATTATTATTTTTCTTATCAAGCTTAATT

ATTTATGGATTGACTAATTCATGTTTGCAGTTTTAGACTCATACAAATGTGTCGAGTTAA

GTCGTCATATGTATGTTGACTGATTATTGTCGAGTTGAAGGATCCATCAATTCGTAGTAC

TGATACTTGTTTACATCTCTCTCAATTATGGGAGGCATAAGTCACTCCCCTT

>Unigene0000095

ACAGATATATTGTTCTAAAGGATCATTGGAGGAATCAGCCGAATATTTTGAGCGTGCAGT

CTCCAAGATTCAAGAGGAGATTCAAGAGGAACAGCTTGGCCTTTTGGTTCTTGCTCTTTA

TGGAGCTGGTTTTTCACGTATAAATCAGGGTAGAAAACCCGAGGGAATAGAGCACTTGAA

AAGAATTGGAGAGTTGAAGGAGCCAGAGAGTCAGGTGAACAAAGGCTGTTATTACAGAGG

ATTGGTGTTGTTGGCAAGCCAATTGTTCAATGACGGTGATAAATACGAAGCTGCAAAGTA

CCTCCGTAGAGCTGCAGCCTATGATCCTGCTATTAATGCAATGGTGAAAGAATGCGAGGG

ATAAAGCATTTTTACACATTTGGTGTAGTGTTTATCTTATTCTGTGTAAGAGAAACATGG

TGGCTTTGTTTCAGGTTTCTTCCGTACAAATGTCTGAAGTACGGGGCTGAGCCAAGATTT

AGATATTGGTGGTCCGACTGGCTTATGTACAGTACGCAAGGGATTAATGATAATTTATTG

GGGGAATATGAGTTTTTGAGTTATTTGGCCTCAAGAACAGGTGCAGTATATTGACCAACT

GGATACGTTTCATCGTCGGTCAATACAATCAAAAAGGGAAATTTTATCTTAGTTAACAAA

GTTTGGTGGTATGATTGC

>Unigene0000096

CTCCTCGCCACACCCTCCCTCTCCCTTTCCCGCCTCATCCTCGCTGGAGATCCCCCCGCC

CCCTCTCCCGCCCCCTTCTCCACGCCATCCTCCCCCGCATCCGATCAATTCCACGAGGCC

CTCCTCCCCCTCCTCCTCGCCCTCGCCCCCAGAGCCGCCTTCACGGACGGCGTCATCCCG

CAGATCCCCTTCATCGCGTTCGACGACGACGTCATCTGCAGCAACCCGGTCGAGATCGTC

ACGGGCCCGATCGCCGGTGAGATACTAGTGGAGGATGTCGGTATCGGTGGATCGGAGCAG

TCGGACCCGCTGGAGCACCGGAGGAGGCTGCGGTTCAAGCGGATGCCGAATCTCGTGCAG

ACACAGGTGCGCCTCCTCCCCGATCCCGTAGATCCAAAGCTTTTGCGGCCGGAGGTGGGG

TCGCTGGTGCAGCCCTACCTCGCGCCGATGGCGGCCAGCCTCTCACTGATTGCCCCACAC

ATCGGTCGGGGGCGGCCGCGGGCGCTCTGCATCGGTGTCGGTGGCGGTGCCCTCCCCATG

TTTCTAGAATTGAAGTTAGGGTTTGATGTGGTCGGTGTCGAGGCTGATGATGTCGTTCTC

AGCCTTGCTCGGCGGCATTTTGGATTGGTGGAAGGGGAATTTATAAATGTCTTTGTTGGT

GATGGGATTGAACTGGTGAAGCGATTTGCTGAAACTGATGCTGGGGGTTTTGATGTTGTG

ATGGTGGATTTGGACGAGGCTGATGCGGCGAGCGGCATGAGTGCACCTCCGCTGGAGTTT

GTTGAGAAGAGTGTTTTGTTGGCTGCGAGAACGCTGCTCCGCAAGCATGGATTGCTGGTG

GTCAATGTGATCCCTCAAGGGGAGGGCTTCTATAAGGGGTTGGTAGATTCTTTTCGTGAG

ATTTTTGGTGAATTGCATGAACTGGAGGTTGGGAATGAAGAGAACTATGTTCTGATTGCG

AGTAATTATGTCTATGGATCGGATGTGAGGGCAGGAGGTGGAGATTTTTGTTCGAAGTTG

AAGCGAGTCATTGGTGAAGGATACATTGACAAGATCAGGAGGCTTTGAAGGGAAATGCAA

TCATTATGAGTACATAGATTGAGAGGCATGAGTAGAAAATACATAGATTGATCAAAGGTA

AACTGCAGAATACATAGATTGGTCAAAGGTAAATTGCAGAATTTTGGTTTATGATAGAGA

AATTTTGATTTAACTGACTTGTTTGTGATTAGAGTATGGTTTAC

>Unigene0000097

GCCCCACCCATTCCGACTGTGTGTGGAAGGGGCTCTGATCCTAAAGAAGAGAAGCTCTTT

CCTCCTTTCGCTCCCATTTTTCTTCCTTGTCGCCATCCTTTTGGTCGTCAACTACAAGCA

CCCTTCTAACCCTCTCGCTCTTCGCACCCATCATGACGACAACCAAAGCCTCTCCACCCC

TGCTTGCGATCTGTTCACCGGCAAATGGGTAAGAGACTTCCGCCCTCACCGATACACCAA

CTTCACATGCCCCACAATCCCTGGGTCGAAGAATTGCATCAAGTTCGGCAAGGATCAAGA

CTACATTAACTGGAGGTGGCAACCCGAGGCCTGCGACCTCCCACGGCTTGATGCCACTGA

ATTCCTCACATTCATGAGAGGCACTACGCTCGCTTTCGTGGGTGACTCCGTCGCCCGGAA

CCAGATGGAATCCCTCCTCTGCCTCCTCTCTCAGGGCTCGACTCCGATACTAGTCGAGAA

CAACATAGTCAACGGCGAGAAGGATAGATATCCAACCTGGCACTTTCCTTCCCACAACTT

CACCCTCAAGGCCATATGGACGAGGTACTTAGTACAAGAAACTGAGAGAATAGTGAATGG

CTCGGGTTCGGATGTGTTTGACTTGGACTTGGACAAACTCAACCCGATATGGGTAAACAG

GATTCACGAAGCCAACTACATTGTCATATCGGCAGGACACTGGTTTTTCCGGCAGCTCTA

CTTGTACCAGGGCGGAAAAAAGATCGGCTGTGTCTACTGTAACGAGGAAAATGACCTCGG

GACGAAGTTTGCTTTAACGGCGGCTTTGAAACTCGCGTTAGGGTTTCTTAGTGAGCGGGA

GGGGAC

>Unigene0000098

CTCTGCCCGGGCCCACCCTATCCCTTATCTTCACCGGCAATCATTGCCTCCCCCCCTCCC

ATGCATCCCTGGACTGCAGGGGGTCCGAGGGGCATATCGGGATCCCTCGTCCCTTCGGCT

TTGCTCTCCCCACCCCCTCGGGGGACCTCACGGCTCCTCCCTTGGGGGCGGATGGGGCAC

TGGCACATCGGGTTTATCCTATTCGGGGGGCTACTCAGAGTCCCATTTCGGCGGCCCCTT

ATCCGGCCCATCCTGTATCGGGGCCCGAGGGGGCACTCTTGGACGGGGGACCCTCTCTAT

CGGGGCTGAGTCTTCTGCCTTTGTGGTTCAGGAGCATCTATTCGGTTTTCTTCCGACCCT

CAGGGAGGGGGTGGCTTCTCCTTCTGATTCACCGGGGTTCTCTCACCTCTGGGGTGCCTG

GGGGGCGGCATCTATGGGGGGCATCGGTCGGGCACGGGTTTTCTTGATCGACTTTCCCAT

GTCTTTCATCTCTTGGAATGTTTGGGGTTTGGGTTCTCTGGGGCATCGGGGGGCAGTTCG

GCAACTTATCATCGATCAACACCCATACCTTATTTGTCTATAAGAGATGAAGTCCGCGGT

CCCCTCGGATTGGGTCCTGGCCCACTTGGGGGCGGGGCGGATTCGTGTGTGGGAGGGTCT

CGATTCG

>Unigene0000099

CAAAAAGGGATCATTACCGCCCAATCGGAATCGGAAGAAATCATCACCGCCCAATCGGAA

TCGGAAGAAATCATCACCGAGGATGGAATTCTTTGTATCATATTATCCTCACTCACGGTG

AGAGAAGCAGTTAGAACCAGTGTTCTCTCCTCTCGATGGAGACCAATCTGGACCTATCTC

ACCAAGTTCTACTTCAACGCTAAAACCGTGCTTAATGTTCCCACTCCTCCAGGCTGCCAT

ATCTATGGGGATGAAGTGTATTACCTAACAAGGGGAAAGACAAACAATTTCATCCAAGCA

GTAAATTTAGTCCTACAGCAGGTTCAGGGCGGCAGAGTAGACCGCCTTTCTGTCCAGTAT

TTTCTCGATAAAAATCACTCTCACGATCTTGATGCATGGGTCAGATTTGCAGTTGCATCA

AAAGTTAAATTTCTGCGCCTCCAACTGTTTAGGTGCTACTCAAATCCATTGACGAACCGG

TACGATTTTCCATGTCACCTGCTCGGATCATCGCAATCTATAGAGACGTTAAGCCTAATG

GCTTGTTCTTTCAAACTACCATTTACATTTGATGGTCTTTGCCATTTGAGTACATTAATT

CTTGATGCCGTAGATTTGAGTGAAGAGGATTTTGACTGCATATTGTCTAATTGTTCTGGC

ACTCTTGTAAAACTGGCTGTGAGGAATGACTGGCAGCGGGATTCAATAAGCTTTCCTCAT

CACCTAAGTCAGTTGCAATCTTTATTAGTTATAGACTGCCGGAAATTGAGCAGAATAGAG

ATCAATGCCGAAAATCTCCGTGTCCTGAAGTATGAGGGCTTTCCAATTATATTTAGCTTC

CAACAATCTTCGAAGCTCAATAAGGCGGATATTTGTTTTAGAACTGGAGGAACCATTGAC

AACATGTATTTTTATCTCATCAAGTTATCACGCAGTTTGCCTCATCTCCACTTTCTAAAC

TTAAAAATTAGCGGAAATTCTAGGGTGGCTGATTTACCAACAGGCTCAAGTTGTTGCTTC

GGGTATCTAAAGCATTTGGATTTGGAATGGAATGCCAGGGTGAAGTTGAGCCTTGTCCAT

TTAGCTACCCTCCTGGATGCTGCTCCCTACATGGAAGAACTGAAATTGCACTTCTCGTCA

CTCATACAACAACCGCCACCTTATGCATCAAGAAATTTATGGGAGATGCTCCCTGAACAT

CATCATACCCATATTAAGAGAGTTGAAGTTAGAGGGTTTTATTTTGAAACATGGCAAATT

GAGATTGCAGTATATCTTCTT

>Unigene0000100

GCAGCACTTCTGGATGCTGCTCCCTTCCTTGAAAAACTGAAATTATGTCTCTCACAACGT

GTATTAGAACCACCACCGCAAACAATAGGCAAGTTTGGGAAGATGCTACCTAAACATCCT

CATACACATCTCAAGAAGATTCAAGTTAGTGGATTTTATTTCAACACATGGCAAATTGAG

ATTGCATTATATCTTCTTAGAAGTGCTGTTGAACTGGAATCAATGACAATAAAGAAAGGC

AGTACACCTTCTGAGGAAATTCATGAGAGGGGCACCACGATTCTTAAGCAAGAAGTCCCA

GAAAAAGTCAAATTAGTAATTTTATAATGACACATGGTCGAAATCAGAGGTTTGGAGAAG

TTCAGAAGCCCTGGTACCAGCAGTCCTGTATATCTTATGCTTTTTTTTTCTTTTGTTTAT

TTATTGGTGGTTGAAGTGGGAACAGATGGTCAGGGGAAGCACTGCTATTGTTTCTTTCAG

CTGTTATCTCTCTCTTTCTTCATTCAACATGTTGGCTTTTTAAAGTAAGTATATTGTTTG

TATTAATATATGGCATCATTAATCATACATATAGGATATGTTGATGTTTCTGGCCATATT

TGTGATAGGATAATCAACTTACACATACTTGACGGAGAATAATAAACTTCCTTTTAATAA

ATGATTCTAAAATTGATTCTTTATGTTTTTTTTTCTTTCGTGCTGTTCTTGATTTAAGAA

CTTCTCACCATGTTCTTTCGTATCAAAATACTTCATATGAATTTTCTTTTCTTGCAGTTC

TGGCTTTCAGAACTTCTCCTCCGTGTTCTTTCCTATCAAAATACTTTGCTACTGTTGTTG

AATCATTGAATTTTCAATCAAATTCGACAATCGAGAGATACAGATCGGGAGCATATATGA

AGATTAGATTGAGGGGCCTCGGGAGGTTGAGTTTTATATGGCTAAAGACTGTATTGATCT

CAAGAGAGATATTAAGAGTTGCATGATCATGGCAAAGTATTTTGATATGACCGAAGAAGA

TTGTAATTGATCTCAAGAGAGATATTAAGAGTTGCATGATCATGTTGATGCTCCTCAGAT

TGAGGGGTCGGTTGGTAGGTGATGGTGCTCTATCGAGTTGTCGCGCTTCAAGAGGGGATA

TTCACTCTATGGCTTTGTTTTACCCATCCGAACCCCAAATTCTTGTCGGATGGATTTGGT

TCCTTCAGGTTGGGTGGACCCCCTGTTTTATACACTACTTCTCGGGAGGTTGAGTTTTAT

ATGGCTGAGGACCGTAATTGATCTCAAGAGAGCTATTAAGAGTTGCATGATCATGTTGAT

GCTCCCCAGATTGAGGGGTTGGTTGGTAGGTGATGGTGCTCTAACGTGCTGTCGCGCCTC

AAGAGGGGATATTCAATCTATGGCTTTGTTTTACCCATCCAAACCCCGAACTCTTGTAGG

ATGGATTTGGTTCCTTCAGGTTGAGTTGACCCCCGTTTTATAACACTACTTCTTGGATAT

TCGTTTATGAGTAATGTTATGATTACAGAGTTTTATTCATAGAGTTGGGCTACAGAGTAG

GCTCAATGCCATTCAGCATTGATTGCTGTGCTATGCCAGTTCAGTCACATGCCGTATGAC

TTCGGGCCTACTCTGTAGCCCAGCTTTGTGAATGAAACCTGTAGCCGTAGCAATACTCTA

TTTCTGTAGCCCAACTTTGTGAAGAAAGTCTGTAGCCGTAGCATTACTCTATGTTACTAG

TGAGTACCTTTGCATCGGAACCAACGTGCTTAATACAGAGATAGAGCCAAACGGCACAGA

ACTGCACTTCACAGCCAATTGCGGTGTGGGATGAGCTGGTGGGTGGGGGGCAATTCAGTC

CCTTTATTTGCTTCTCAATGCCAACTTTTAGCCTCCTAAATTTATAGCTCAGTCTGGAAT

ATCTGAGTTATCCCTGATTTGCCGAAAGAGAGGATTGCTTCTACTTATTCAGTGGATAAA

ACACTTGGAGAACACATTATGAGAGTTGAATTTACCGAAAATGACGGAAAAAATGGGTAT

ACTTACTACGCCACTTTCTAGTCTCTGGTATTGGTGAACACATTGGGAGAGTTGAATTTA

CTGAAAATGACGGAAAAAATGGGTATACTTAATACTATTTAAGAGACTTTATGTTTCTCT

CATTGTGTGTTCTGGAGAAAAAATTGTGGGGGTCTTCTAGTGAACGCCATCCAAATTCTT

ACATAGTAAGAATGCACAAAAGTTGTATACTATTGTCACAAATTTTTTCACTGGGAGGGT

TTAGTGAACACTTACAACAAATTTTTCACAGTGCCAAGCGGCTTAACGAATGCCCACCAT

AATGTTTTCACATTTTTTTCCTAGTGCTAAGCAGTTTAGAGAACACCTACCATTTGATAA

TACTTGTTTAGAATCCTCGAGTTTGTCTCATGTCGTGTGGTCTGACAAGATTGGGTTCTT

TTTCTGAGTCATAGGATTTAATTCTGAAAACATTGTGTTGAACATTGGATGTTGCTCCAT

GAATGTCGTCATCGGTTTCAGATGTTGCTCTGAAGACAAGATTTTTCAATTTCGTATGCT

GTTCCAAAGAGGTTTCGATGTTGCTCCGAAAATACCTCGGATATAGTAGCTCTGGAGACA

TGTTTACAAGGTACGGATGTTGCTTCGAAGGCCTTGTTGATATAACTTTAACATGTGTAT

TCATCTTATGTATTACTTACTTCAGATGATAGATCATTCTTGGTTCTGCCATGTTCTTCG

ACCATATGACATTCAGTTTATCTTTATTTTATATATGATATCTTCGGCAGTTAGTGGCAG

TTTGATTGTGCACGATCTGAAGGTATTTTAACCGTTCGCCCGAAATCCGAATCCTCGTGA

TAGTTACCCAGTTTCCCATAAAAGAGGTGGTATGGGGACCCGATCATCCACACCATTGTT

TGGCTTCTTCCTCTCTCAAACTCTCATATGTGCCAATGACCTCCTAAGCAATGTTGAAGA

GAATGTCGGAAAGGCATCGATGATGGCGGAGGATAAGACCATTGAGGTCATACTACCATA

GTGAGTTTCTTCTCTGACTTTTCGCTATACTCGTGCACGAATTTGCAGTATTTGTTGTGT

TCCGGAACTAGTTCATTGCGCTCTTCAAGAACTACCAGATTTGATTTAAATTTTGTTAAG

TTTCATTGCCGTAGGTGTTTCATGCGTGTATTTCTGGTTAAAACTCATTTTGGACTTTGG

TTGTGAATTATGTTTCAGTAGTCGTATAGTAGCTTCGATTGCCTGAAGTTCTTGCTCTGG

GTTGTGGGGTCGGTTATTCCATACATTGTGAGTTGTAAGTGTGTGTTAATTTCTTTTTAA

TTTTAAAGATGG

>Unigene0000101

CGCCGTTCATCGACACTCCCGATTGAATTGAAAGCCCTAACCGAGGGCCAAGCAAAGCAT

GGACCCGACCGAAGCCGTTCTCGAAGCCCTAATTTCCAGAGGATGGCAGTTCAGGGATGC

CGACGCGATCCGCGCTCTCATCCGCGCCGCCTCCACCTCCGCCGCCTCATCCTTCGCCCC

CATCTTGGACACCGTCGAGTCGGAACTCCTCAACCTTGATTTGCGATCCTTCGGCGCCAA

GTCGCTCCCCGATGCCGCAACGATCAAGAAATTATCCCACCTCCAGGGTCCCAAAATCCT

GCAGGTGGTTTCTGTGCGTGATATATACCGAAGCAACATTGATGCCGCTTTTAGAGACTC

ACCGCATCACCGGCTTCTTCGATTTGTTCTCACCGATGGGCATAAAGAAGTAACTGCCAT

AGAGTTTTCGCCGATTCCATCTGTTACTGAAGAGATAGTGGTTCCTGGTACCAAGATTCG

TTTGGAAAACAAAATTACCATACATAGTGGAATATTATGCTTGAATCCCAAAGTTGTGAC

GATAATGGGAGGTATTGTCCAATCTCTTTACGAGGAGTGGCAGATGAGTCAGAAATATTC

AGGTTTCTCTCGTTCTTCCTTGAGGCTTTCACAGAATGATGATGATGTTGGCCCTCCTCC

ATTTGAGAAGCTACAAATTCAAGCATATCGTCCTATGTCATCCTCTAAATTTTCTTCTCA

TATGAGTGGAAATATGATCCATAAACAAGTGGGGCGGAATGAGGGATATCAAGAGAGCAC

TGGAGGAACAGAAAAGGTGATGGACGAACCAAAGCCGATCTTGTCAAGAAGTAAGGGAGA

AGACAAACCTGGTACTTCAGAGGCCAGACCAAAGGAAGTGAGTGATGCAGTTCCTATTCA

AAACCAAGCTGCTGCTCAGAAACTTCTGCAGAAAATGAGCCAGCCAGCATATCAGGACAG

GCATTCCAGGGGTCAAAGACAGAGATCAAGGGGAAAGGAAGAAGAGACACCGGTGTTTAC

TCTAGAGGAATGGGAAAGAAGAAAAGGCAACCTGAAACCTGCAGGCATTGGCCGAACCCA

AGATTTTAGCCGAGATGAAGAGCTTGCTCGGCAGTTGCAGAACCAATTGGATTTGGAAGA

CTTTCAACAACTTCAGGGGAATACAGAGCATACAGAAGCAGAGCGTATAAGGATGAGCAT

GTTCAGCTATGGCGGGGCTGAAGAGAGGATGAATGATGGCGGGCGAGAATTTCGGGGAAG

AGGGAGAGGAAGGGGAAGAGGTAGAGGAAGAGGAAGGAATAGGTTTGGTTGACTTTCCGT

TCGAAAAAATACTGGCGGTTATCTTCTTCTGCTGTCAGATAATTGGCCGTTTCCAGGGTG

CTTTTATTGCCGGAGTGACGAAAGCTGAATTGTGTTTGGATAAAGTTTATTTGTCATGCC

AGGTTGCCGTTTCACTGGTTCCAGAGGCGATGATACACTTAAGGTAATGCAACCCTAAGT

AGAATCTGTACATTTGTACCGTGTGGAATCGTCGGTAAAAGGAATTGGTTTTGGTGCTGT

AATTATGTTTGGATAAAGTTTATTTCATGTCTGAACACTGTTTACACCAATTAACGTAGC

CCTGAGAAAATTAATTTGATGTCTGAACTCTCGTTACACAAACTGAAGGAGACCCAAGGC

AACGGAAGCTCTTGTGCGTTTTAGTATCAACTGTTAAGAATGCATGGAAATAATACATCT

GCTTCCA

>Unigene0000102

AATCTCTGGTACCATGTTACTATCCACTACATGCCAAAAGCTAGCTGTTGGGATGAGGGA

TACCATTAACATTAACATCAATATATGAAATGGTTTGTTTCAGTTTAGTCTTTTTTTATA

GATAATATATAGATCTAAAAGAATATGTTTAAATAAGCAGGATCCAGAAAGTGTTATCTA

GTGTTGTATTTCAGTTATACCCATTAAGTTATTACTGAGGTATTGGACACACTTGTACCA

GATCACATAAATAAAG

>Unigene0000103

ATAAAGAATTAACACATAAACATTCAAGTCTACAGCTCGTACAACTCTCAAGATCACCTA

AAATAAAAGAAACGAAAAACACATCAACTAGGCTTCTTTAGTTCTGCCTTATTTCAATTG

CTAATACATGATCTATCTTACTCAAACCGGGGTTGTACAATTCAATCTTGTTGCACCTGT

TCAAACACTTTATTCATCTGGCATAAGTATTTTATTTATGTTTCAGATTAGTTAGTCATA

TCTGCTCATGTAACTTTACAGTTACTACTCAGGACCATAGGAATA

>Unigene0000104

CTGTGTCGGTACTCCGGCAATGGCGACAGGTGGCGGCGGCGTAGACGACGGCGGCGGCAG

CGGCGGCGGCAGCGGCGTAGATGGCGGTAGTGGGGGCGGCGCGATGTCGACCGTTCTCAG

TGACAGGAATCTACCCAGGGCGAATACTATGCCTGGAAGGGCAGAAGTTGCTGAAGCATC

TGAGACGTCAACTAGTCAGTCACATATAGAACGTTCAAGAACTGAGAGAAGGCGACAAAG

TAACCCTAATGCTGATCCAACAACACAATTATTTCTTGATAAGATTTCTGTTAAGAAAAC

GCTTAAGATGTTAAATCGGATAGCAACTGTGAAAGATGATGGAACTGTAGTAGTTGAAAT

TCCAAGCAACATTGAAGCATCAGCACTTGATATTCGGCCTGAGGATGTTTATTGTGAAGT

AACTGATGAAGAATTGTTAGATTCAACTGACCCTCAATATATACCACCACTGCAAATTGC

TATTCTTATTGTGGGTACACGTGGAGATGTGCAGCCATTTGTAGCTATCGGCAAAAGGTT

GCAGGACTACGGCCATCGTGTTCGACTAGCAACTCATGCAAACTTCAAGGAGTTCGTATT

GACTGCTGGTTTGGAGTTTTACCCTCTAGGAGGAGACCCGAAAATTCTCGCCGAATATAT

GGTCAAGAATAAAGGGTTCTTACCTTCTTCACCTTCAGAGATAACTATTCAGAGAAAACA

ACTGAAGGAAATTATATTTTCTTTGCTCCCAGCCTGCAAGGATCCAGATGTTGATTCCGG

TATTCCTTTCAGAGCAGATGCCATAATTGCTAACCCTCCAGCATATGGACATGCTCATGT

TGCGGAGGCACTGAAAGTACCACTCCATATATTTTTCACAATGCCATGGACGCCGACTAG

TGAGTTTCCACACCCTCTCTCTCGTGTAAAGCAACCTGCAGGATACAGACTTTCTTATCA

AATTGTAGACTCCATGATTTGGCTTGGAATACGGGACATGATAAATGATTTCAGGAAAAA

AAAGCTCAAGCTGCGGCCTGTCACATATTTAAGTGGTGCTCAGGGGTCTGGTTCTGATGT

GCCACATGGATATATATGGAGCCCTCATTTAGTCCCCAAACCAAAAGACTGGGGACCCAA

AATAGATGTCGTTGGATTTTGCTTCCTTGACCTCGCATCAAACTATGAACCTCCAGAATC

ACTTGTGAAATGGCTTGAAGCTGGTGACAAGCCTATATATGTTGGGTTTGGTAGCCTTCC

TGTCCAAGAACCAGAAAAAATGACTCAAATTATTGTGGAAGCACTGGAGATCACTGGACA

AAGAGGCATCATTAACAAGGGCTGGGGTGGCCTTGGGAATTTGGCAGAACAGAAAGAATT

TGTGTATCTACTGGATAATGTTCCCCATGACTGGCTATTCTTGCAGTGCAAAGCAGTGGT

GCACCATGGTGGTGCTGGAACTACAGCTGCTGGTCTTAAAGCTGCGTGTCCAACCACTGT

TGTACCTTTCTTTGGTGATCAACCATTTTGGGGAGACCGGGTGCATGCTAGAGGATTAGG

TCCGCCTTATATTCCTATTGACCAGTTTAGCCTACCAAAGCTTGTTGATGCAATAAAGTT

CATGATGAATCCTGAGGTGAAGGAACGAGCGATCGAACTGGCGAAGGCAATGGAAACAGA

AGATGGGGTGAACGGGGCCGTGAAAGCATTTTTCAAGCACCTTCCTAGAAATTGTCAAAC

GCAGTCTCCGCCACCAGAGCCATCAAAATTCCTCCATCCGATCCTCACCCCTATACGGAA

ATGTTTGGGATTTTCATGAGTCTTGTATGTATGTGTACATTTTATTTTATTTATTTGTTT

TTCACTTCACTCCCTCGTTTCTTTTTCCGATATGTAAGGCTCTGGAGTAGCTTACCAACT

TTTTTTTTTTTGTGTGGTTATTTCTCCTCTTAATTTTGTATTTAATTTTGATTCCTTATC

CTCCAATAAGAGGGAGCAAAATAGTTTCAAGATTGACATGAGATTCTATATTTGGGTTTG

TCGTGAGGTAAAGTT

>Unigene0000105

AAGAACTGGACAGGGCACAATTTGGGAGCGAGGGAGAGGGGAAATCACCTTCACTCCCAG

CTCTGCGTCGCCTCTACTTCTTCAATCCCCCGTGAATTCCCTACAATCATCCCTTCACGA

GGGATGTTGGGGAAATCATTGGCATTACCTCTACTGACTTTTGAGTCTGCTGCATGCTGC

CCCTTTTCACCGCCAGTTTTTTCGACTTCAACATCAAGTTTGGCACAGTGGAATCCATCA

CATAGAGTCAAATCTTTTAACAACTCGAAAAGATGGAAGATATTGTTAGCTGATCAAACA

CAGCCAGCAGTTATCCTAGAAGAAGAAAAGGAAAAATGGGACGAATGCAAACAAGCACTA

TCAGCATTCGATTTCACCCCTGCAGAAGCAAGTAACATGTTAGCGAAGGCATTTGGTTGG

AAACACTCACCATATTGGGGCGAAGAGAGGAAGAAAGAGGTTCCAAATAACGAATTGGTA

GTCGAAATCTTGAATTTTCTCAGAAGCTTGGGACTCTCTGACGACGACCTCCACAAATTA

CTCAAGAAATTCCCTGAGGTTCTCGGATGTGCCTTTGATGAGGAAGTGAAAGCGAATGTT

GCGACACTAGAGAAGCAATGGGGGATAAAAGGGAAATCACTTAGGAGTCTTCTTCTACGG

AATCCGAAGGTTTTAGGCTATAATGTCGATTGCAGGGGAGACTGCATGGCTCAATGCACC

CGATGCTGGGTTCGATTTTAGAAGCTTAATGTGCAACTGTACAAATTTGCATAATTTCTG

AAGTCGAATATTCTTTGAGCAAAAATGCATTGTAGATATGTATTTTTCTTTTTCCATAGG

GGACATAAAAATAGCAAACTTGAAAGACACTGGTGGGTCGATGGCTTGGATTGAAACTGA

GTTACTGTATTAACTTAACCCTGTAAAATGTGTAGTCAGGGAAAACATGTTATACATGGA

G

>Unigene0000106

AACCCAAGTCCCTCACTCCTAGGAGTCCAGAACTCTCGCCTTCACCCTCTCTATCTAGGG

TTTAGGACGACGTCGGCGAGAGCAGCAACGAACTCGAGAACCCAACAACGATCATCATCC

TCTTTCTCCAAATAAGGTTCTTCAGTGATCATCCATCCGGCCAAAACTCTCTCCAATGTC

CACAGTATGAATCTGACCCGACTGCAGCGGCCCTGCCAGCAACCAGAGTTTCTCCTTGAC

TAGAGTTCTCCTTCCTTCTACAGCGGTTCCCCCTTGAATCCCATCACTGTTTTGTGCATC

ACTGCTCGTCGGATATCCTGCCCCAAATTCCCCATCTCCACCTTCCATCAGGACCCCTGC

GATATGAGAGCCCCAACAAGTAAGCAATGTGTAACCTGTTGAGAGATAGAGATACACCGA

TGGGTTTTCATGTTGTGCTGTCAATGCATGAGGTCTGTTTCTATTTCTCTTTTTGTTGAG

CCTTGATGGAGGACGATG

>Unigene0000107

CTGGCATCTTGCGACTACTCAAGGTATTCAATCTGTGTTTATATACATTTATGCTTTTCG

GCTTTTAGCTCCCCCACCACTTACTTTCAAGTATGCTTCTGACCCCTAATGAAAATCCAA

GAGAATTACTTGCTCTATGGTTTTGTTTGGTTCGTTGGTTTTGATAAACCACAATGATAA

CGCAACTTTTTGAGGTTTCCAAAACGCAGATTTTGCGTTGCGGAAACCCCGTTGTGGGGA

GTGAACCCCA

>Unigene0000108

CATCAGCAAGATGTTTCTTCGTGGAGACTCGGTTATCATAGTTCTTAGAAATCCAAAGTG

AGGCTCCGCTAGTCACCGGTAACATGTTTCTTATGTAGACTCCATAATCGCAATTAGATA

CCTTGAGTTGTTTCTCCGAATATGGTCGAACATTTAAACACATTATTAAATCTGAGTTTG

TTGAAGTCATACTATTTTTTTCTTTCCAATTAAGATCATCTGTGGGTTTATTTAAATCTA

TAATTCAATATGCAGA

>Unigene0000109

TTTATTGGTTCATAGCTTTTAAATCATGGTGGGACTGCACACACCTTTTATGCTCTACCG

ACACCACAATGAGCAGTACCAGTATTATCTTGCATGTTATATATATTCAGGCTTGTCGGC

TTTTATGCTCCACCTACACTTAGTATTCCACAGTTCTCCAAGGCTTTGATTCAATGGAGT

GGGGCTGCATTGGCTTTCTGGCATCTTGCGACTACTCAAGGTATTCAATCTGTGTTTATA

TACATTTATGCTTTTCGACTTTTAGCTCCCGCAACTCATACTTTCAAGTATGCTTCTGAC

CCCTAATGAAAATCCAAGAGAATTACTTGCTCTATGGTTTTGTTTGGTTCGTTGGTTTTG

ATAAACCACAATGATAACGCAACTTTTTGAGGTTTCCAAAACGCAGATTTTGCGTTGCGG

AAACCCCGTTGTGGGGAGTGAACCCCA

>Unigene0000110

GCTTGTCGGCTTTTATGCTCCACCTACACTTAGTATTCCACAGTTCTCCAAGGCTTTGAT

TCAATGGAGTGGGGCTGCATTGGCTTTCTGGCATCTTGCGACTACTCAAGTAAGGCCCGT

GCCTCACACCTTCGGTGCCAGCATGGTCTCGTGCTTCAAGTGTACCTCTGGAAATGCGCA

TCAATTATCAGCTGGCTCTGTAAAATTTTATTCTACACAGGTAGAATGGTGGTTAGCGTT

AAATTTGCCAAAGTTCAGTTCATTAGTTAACCTTGGAGCTGCTAGATGTGTTTTGAAGTT

TCTGTTCTACCCCTCTTCACGACAACTGTATCTGATCGATCCCATTCATTTGAATTGGAT

CTGACAGTTGTACTGGTTCAGAATTTGAAGTGAGCAATAGCCAGAAAAATGACTATTTGT

ATTACCATTTTCTGGCTTGGCTATAAAAAATTGTTATGATCTTAGTTT

>Unigene0000111

AGTAGTCGGCGCCAGAGAGCAATTCCAGGACGAATCCTGACTTGAAGGGCGGTCGGATCG

AATTGGGATCGTGCACAGGATCATAGATCATTTGGTTTCTTTGGTGCACGGATCTCTTTC

TGTGGTACAGCAATGGAAGATGACACCAATGCTGGAAAGAAGGAAGAGGAGGAGTTCGCC

AAGGGGCCCCTCTCCGTTCTGATGCTCAGTGTGAAAAATAATACCCAGGTTCTCATTAAC

TGCAGGAACAATAAGAAGCTACTTGGTCGTGTCAGGGCATTCGACCGCCACTGCAACATG

GTCCTTGAGAATGTCAGGGAAATGTGGACCGAGGTTCCGAAGACGGGTAAGGGGAAAAAG

AAAGCTCTCCCAGTCAATAAGGATCGGTTCATCAGCAAGATGTTTCTTCGTGGGGACTCT

GTTATCATCGTCCTTCGAAACCCCAAGTGAAGAGAGAATCGTCGACGCTGGATGCTTGGC

CATCCTTATATGTCGTCTAGGACTAAATCTAATTTTGTTGAGCAACATGGACTGTTCTGT

ATCTTGCTGTGATGATTGAAGTGGTTTATTTTCGAGCTCTCTAGATATTTGATGATTTAG

TCCAGACAATTAGTGATTATGCAGAGAACTGAAAATGGGTACTGATGCTTGAGATATGTA

ACATCAGAGTATGGTTTTATGTTTGGGAAGTTTTAGTCCGTTGTGTTTGTTAATTAATAT

ACTTTTACAGTGTGACGTGAATCATAGGATGTGTAGCTACATTATTAAGGATATCTCTTT

AGTAATTACATGCCCGTGTATAATGGCAGAACTGGAGTGTTCGTAGGTAGATGAACTCTA

GGAATATTGAATTATAGTTGACCACACTAGTA

>Unigene0000112

TCTCTCTTTCTCTCTCCTCCCATGGAGCTGGAATCGATGGCGTCGGCGATCGGGGTCTCG

ATCCCCGTGCTCCGCTTCCTCCTCTGCTTCGTCGGCACGATCCCTGTCAGCTTCGTGTGG

CGCCTCATCCCCGGCGCGACGGCGAAGCACCTCTACGCCGCCCTCTCCGGCGCGGTCCTC

TCCTACCTCTCCTTTGGATTCTCCTCAAATCTGCTGTTTCTCGTCCCCATGGCGCTCGGC

TACGCCTCCATGCTGCTGTTCCGGCAGTACAGCGGCGCCTTCACTTTCTTCGCCGGATTC

GGCTTCCTCATCGCCTGCCATATGTATTACATGAGTGGAGATGCTTGGAAGGAAGGCGGA

ATAGATGCGACTGGGGCTTTAATGGTGCTTACACTGAAAGTCATTTCATGCGCAATGAAT

TACAACGACGGCCTATTGAAAGAAGAAGGCTTGCGTGAGGCACAAAAGAAATATCGATTG

GTTCATCGGCCCTCAATAATTGAGTATATTGGTTACTGCCTCTGTTGTGGTAGCCACTTC

GCTGGACCAGTATATGAAATGAAGGATTATCTTGAATGGACAGAAAATAAAGGGATATGG

ACCCGTTCCAAGGATAAGTCGCCGCCATCACCTTATGGTGCCACCCTGCGTGCTGTACTT

CAGGCGGCCATATGTATGGGCATATACTTGTCGTTGTCCCCAAAATATCCACTTACGCGG

TTTAGCGAGCCCATATACCAGGAGTGGAGTTTCTGGCAGCGGTTGTATTACCAGTACATG

TCTGGTTTTTCTGCCCGTTGGAAGTACTATTTTATATGGTCAATATCAGAAGCTGCAATC

ATTATATCTGGCCTGGGTTTCAGTGGATGGTCAGATTCTTCCCCACCAAAGCCAAAATGG

GACCGTGCTAAGAATGTAGACATTCTTGGCGTTGAGCTTGCAACAAGTGCAGTGCAATTG

CCACTTGTGTGGAACATACAAGTCAGCACTTGGCTTCGTTACTATGTCTACGAAAGGCTT

GTTCAGAAAGGCCAAAAACCAGGGTTCCTCCAGTTACTCGGCACACAGACTGTCAGTGCT

GTTTGGCATGGACTATATCCTGGATACATCATATTCTTTGTTCAATCAGCATTAATGATT

GCAGGTTCACGAGTAATTTACAGGTTGCAGCAATCCGTTTCCCCGAAGTATGGTTTACTG

AAAAAGATAATGACTTTTACAAATTTCGCATACACAGTGCTTGTTCTCAACTATTCGTGC

ATTGGTTTCATGGTTCTGAGTTTGCACGAAACGCTAGCGGCCTACAAAAGTGTATATTAC

GTTGGCACCATTGTTCCCATTATAGTTTTCGTGATTGGTAAAGTTGTCAAACCCGCGAGG

CATGCCAAGCCTAAAGCACAGAAGACTCAATAAGAAAGTTTACATACTCTTGACATTGCC

TGGATTAAAATTCATGGACTGCCATATCCCTTTGAGATTTTCGAGTTTGCCAAACATAGA

TAGTTTTCTGCTGTTGTAATGTAGTTTGTTTATTTTATCTCGTACAACTTTATTTTTCTC

ATGAGGAAAAAGTTTCGTAAGTGTATGGTCTGCAATGCGTGTGGAATGTCTGCAATTTTA

ATTTGAGAAATATTGATTTCAACATCGAGAACCTCCACGTAATTGTATATGACACTTGGG

CTCCACTTGAATTTAATCCTC

>Unigene0000113

TAGAAGAAAAAGAAGAAGGCCTGCTCGCTTTCAGCTGGGATTTTTTTAGATAAGAATAGA

AAGAAGTCGGGGAAGTGCTATACGCATATTTACCAATGATTTCTACATTTTTTTCTAAAA

AATCCTCCAATTGATCGTCCTCCACGCTCCTCATCAACGATCGAAACATTGCTTCAGAAC

GCCCGATCAAGACCTGGTTTCTTTATTTATTTATGTATTTTTGTTCTTTTCAGTTCAGTT

GTAAA

>Unigene0000114

GAATTTCTCTCTCCAACGATCGTCCTTCAGCCTTTTTCGTCAGAAGATCGTAACTTTGCT

TCAGAACGCCCGATCAAGATCTGGTTTCTTTCTTTATTTATGTATTTTCGTTCTTTTCAG

TTCAGTTGTAAAAACTTATGTTGTAATAAATTAGGTTATTTTAGTTTTTACTTTCAGTTG

TAATAACTCTTTTCAGTCATTTTTGTTGTAAATCTCGACCCTTTTTTATAAATAGAAAGA

AGTTGTTTCTTATCATCT

>Unigene0000115

AGTCTCTATCAGTCCGCCAGCTCCCGATCTTATCGGGGACGATGACGCGAATATAGGTCA

GACCCCACCTGACCCCATCATCCCTTCCCTTCTACTGCTACCTCCTCGAATCGAAGAAGC

GATCGCGAGGTCAATGGCAGCAGTGGCGATGCCCTCCTCTTTCTTTCGATCCGAGCAGCC

CGGCGCCCTCGACGTCTCCGCCGACCGCGCCGGCAGCAGCGCCGGCTTGCCCTCTCGCCG

CCGACGAGACTTCGTCTTCGTCGTCAATCCTCAAGGCGCCAATGGCCAGACTGGGAGGGC

GTGGAAGAAGCTGCTGCCGTATCTCCGGATGCGCCTCGGGGATCATTGCAATATACGCGA

GTCTGTTACTTCAGGTCCTTCTCATGCTGTGGACATAACCAGGGAGGCAATAAGGGAAGG

GGCAGATGCTGTGATAGCTGTTGGCGGTGATGGCACACTTCATGAGGTTGTTAATGGCTT

CTTTTGGGCTGGAAAGCCTGTCCTTGCTCTTGACAAGGGGCCCGAACATTCAACTGCACT

TGGCCTCATTCCATTGGGGACCGGCTCAGATTTTGCTAGAACATTTGGCTGGAAAAACGA

TCCACATGAGGCAGTAGAACGGATTGCTAAAGGATTGAGGCTGAAAATAGATGTTGGTGT

CATTAATGGAACAAATGGAGAACACCATTTCTTTATTAATGAAGCCGATATTCATTTGAG

TGCTAAAGCAGGTTATTATGCCTCCAAGTATAAAAAATTTGGAAGGTTATGTTATGTTCT

CGGGGCTCTGAGAGCCTTCATTGGTCACAGTAACCGAGACATAAAAATCAAGATCGATGG

AGGAGAGTGGGAAGTATTTCATAAGGCGACTGCCCTTTGCATAGGAAACGGCAAATTCTT

TGGTGGTGGCATGAAGATAACCCCAACAGCTGATCCTTCCAGTGGATACTTTGAGGCAAT

TATTCTTCAGGATTTCAAATGGTACGACTTTGTTCTTAAACTACATAAGCTGTATAACGG

AATGCATCTATCAGAGAGAAATGTATGCTCAAGGAGTGGACGATCCATAGAAGTTGCGGA

GGTAACGAGTAGCGGTGACGTCTACGTACAGTCTGACGGGGAGCATTTGGGATTCCTTCC

AACAAAATGCTCCATTTTACCGGCTGCAATTGAATTTTTCGGCTAATTTGCTCCTCGTGT

GTACGTTCCATTTGCCTGGTTTTAAATGGAGTGTGATGTTGGCCTCACATAATTGATATT

AAAAACCGTGTCTACTGAAAAGATGTTCTTGTTGTTGCCTTCCTATTTAGCTAGCCTCTA

GGTCTCTGGGCATAATAATACTTCATTTTGTTCTTGCTAGATTGAGTTTTGCATATAACT

TCATTTTGTTCTTGATTGAT

>Unigene0000116

GAGGCAATAAGGAAAGGGGCAGATTCTGGCACCCTCACGTGTAAGTGTGAGGACTTGGAT

AGCAACACAGGCGTGTTCTGCAGAGCCGTCATCGCACTCGAGCACGCGTACAAGGTGGTT

AAAGAGCGGGTTGTCCTCCGCTGCCAGTATATGGCGGCAGCTTTGGACGGCGGCAATCCC

GGCGACCGCTGTCGCGGCTTTCCCACATAGGGGGGGTTGAGGAGGCGGACCAGCGACGGA

AGCACCCCCTGAGCGGCGACACCCCTGAGCAGTGGCGATCGTGGCGTTCTTGTCGTCGTT

CCGGATTAGCGTCGCCATCACGTCGAGAGTAGCAGTGCAGGAATCTAGGGTTTCGATCTA

GAGACGTGGACAGAGGAGGTGCATATCGGCGGGTCGAGGAGTGCACCGGAGCGAAGGAGG

AGCTCAGCGTTGGTAGTGAGTTGGTGGAGGGAGGAGGCGACGAAGGTGAGGTCGGACTGG

T

>Unigene0000117

GTTAGGTTTAAGTAACACTCCTCAAATAGAAAAGTGATCGCGCGGGTCAATAGCGGCAGT

GACTATGCCCTTCTTCTTCCGAGCCGAGTTTTTTGGTGCCCACGACGTCCCCGCCAACCA

TGCCGGCAGCAGCGCTGGCTTATCTTTTCGCTGCCGCCGACTTCGTCAATCTGCAAGACA

CCAATGGCCAGACTTGAAGGGCGTGGAAGAAGCTACTGCTGTATCTCCAGGTAAGCCTCA

TAGATCATTATAATATAAACGAACCTGATCCTTCAGGTCCTCCTCTTGTGGTGGACATAA

CAGTGGAGGCAATAAGGAAAGGGGCAGA

>Unigene0000118

CGATCTCGTCTGATCTACCCTCCAAAGGGTGTTATTGGACTGAGACGTGTGAAAACAAAT

GGTTTGGAGGATGTGGTGAAGGTCATCTCATCACCAAACAGTCGGGTGACTGTAATGGCC

TTTGTGGCGAGTCAAGTTATCCACCATGCCTACCTTTCCATACTCATTATCACTGCTGCA

AACCAGAAATGCCTAAGGTAACAAATAGGTGCACAATATGCAAGAACAAGTTGGACTTCG

GCGATGAGTTTGTGTGTTGCAGTGATTGTTCAGAGCCCTACCTCATAGACAAAAATAGCA

AGCTGGGTTATTGTAATACTGGTGCAGAGTTAGCTGTGCAATTGAAACCACAAGAGATAT

TTAAGTGGGTTGCTGGACCATGGATGGAGTGTTCTTCTCCTTGTGATGGCGGAATTCGTT

ACCGAGATGTGGGGTGTTTTGGAAGTACCGAAGATTTGACGGTCAAACACTACCCAGCGG

ATAGCCATAAATGTTCAAGCGAAGAAATGCCTGTCAGACAAGAGCATTGTAACATGAAAA

GCTGTGTGGACTTCAGCAACAGTTATGTAAAAGATAACAGTCAAAGCAGGATGTCTGGAT

GGTTTGTTACATTCCTTGTTCTTCTTGGGCTTGTGGCTGTTGGGGGACTTGGATTTTCAG

GTTACACTTATTACAAGAGGAGAACGTCAGCCCCGAGTGGCTTCGTATATATCATGCTCC

AAGGCTATTCGTAAGAGGATGATTTTCCCTCGACTCAGGTTTGAGATCGGTATATGGCGC

CCGTTTGTGGGCATTGTTTCCTGTACATTGTTTGGTGATTCATTCATACAATTTACTGGC

AGTGTTTGTTGTATCATATCTTAAATCTTAGAAGGTAATGAACTGAGTGAAACTTTGAAC

TCCTGTTAGCAGTTGAGCGTTTGTGCTTCTCATTGCTCGTGTCCACATGTTGCGGATGTC

ATCTTCCTTATTGTTTGTTCACTAAGGTACTCTCGTCAGTTATTTGATGTTGGGGTTCGT

AACATTCTATTTTTCAGCTTACCTCCCTATG

>Unigene0000119

GAGGGTTTCCCTTGCAAAGGAAATTTTAGATGGGCCGGTGGACTGGGCGACACCATTAAG

ATAATTGCTCAGTCCGAGACTCCAAGGCGAATGGAGATGGCTCGAGCCCGGCTTCTGCTC

CCCTCTCTCCTCCTCCTCCTCATTCCGTCGATCTCGTCTGATCTACCCTCCAAAGGGTGT

TATTGGACTGAGACGTGTGAAAACAAATGGTTTGGAGGATGTGGTGAAGGTCATCTCATC

AC

>Unigene0000120

TTTCTTACTCCTCCGATGTCATCCCCTGTTTGCTCTCATAAAAGAACTCCCCTGACAGTG

ATGTTCTAGTGATGTCCTCCTACTTCCCCCATTGACCATATTGCTCTCAATATATAAATC

TGCCACTCCCCTCCCCATCCATCTCCACATCTCCCCCATTACTCAATCCCACCAAAGGGT

CACCATATCTCTCCTTGGATTCCCATTTGCTTGAAGAGCTAGCCCCTCTCCATTGTCACC

AGGTTTGAGGAGCTAGCCCCTCTCCACTCCTGCATGCCAATTTCTTGACCAAGAAAAGCA

TGTAACCGGATCCAGTTACCATCTTTCAGTTTCGACAACTATTTCCATCATTGCCCAAAT

CGGGCATGGAGGAGACATTCGTGCCGCTCCGCGGCATCAAGAACGACCTCAATGGAAGAC

TGAAGTGCTACAAGCAGGATTGGACCGGTGGGTTCAGCGCCGGCATCAGAATTTTGGCTC

CGACCACTTACATATTTTTTGCTTCGGCAATCCCGGTCATCTCATTCGGAGAACAGTTGG

AGAGGAACACAGATGGGCTTATAACGGCTGTTCAGACACTAGTGTCAACCTCGCTTTGCG

GGTTCATTCATTCAATCATCGGTGGGCAGCCTTTGCTGATCCTCGGAGTAGCCGAGCCGA

CTGTGCTTATGTACACCTTCATGTTCAACTTTGCTAAAGATAAGACAGATTTGGGGCCGA

AGCTATTTCTAGCGTGGGCTGGATGGGTATGTGTGTGGACGTCGATTTTGCTCGTCTTAC

TGGCTGTTCTAGGTGCATGTTCGATAATTAATAGGTTTACTCGTATAGCCGGTGAGCTCT

TTGGTCTGCTGATTGCGATGCTGTTTATGCAGCAGGCTATTAAAGGCCTCGTGGATGAGT

TTCGCATACCCAAAAGAGAAAACCCAAAGGCACTAGAGTTCATTCCATCTTGGAGGTTCG

GCAATGGGATGTTTGCTCTAGTTTTGTCGTTTGGTCTTCTATTGACTGCACTTAAAAGCA

GGAAGGCAAGGTCGTGGAGATATGGAACTGGATGGCTGAGAGGCTTGATCGCTGATTATG

GTGTGCCTTTCATGATCCTTGTGTGGACTGCGGTTTCTTATATACCTTCTGGCAGTGTCC

CAAAAGGAATACCTAGGCGCCTTTTCAGTCCAAACCCATGGTCCCCTGGTGCATATGACA

ACTGGACTGTTATGAAGGAAATGCTGAATGTACCTGTTCTCTACATTGTCGGAGCTTTTA

TTCCAGCTACAATGATAGCAGTCCTTTATTATTTCGACCACAGTGTAGCATCTCAACTTG

CACAGCAGAAGGAATTCAATTTGAGAAAACCACCATCGTTTCATTATGACCTACTTCTTC

TGGGCTTTTTGACCTTATTGTGCGGACTTATCGGCATTCCCCCATCAAACGGTGTCATCC

CGCAGTCTCCTATGCATACGAAGAGTCTTGCAACTCTCAAGCATCAGCTGCTTCGTAATC

GACTAGTATCTACAGCGAGGCGAAGTATGCATCAGAATTCAAGCTTGGGGGAACTGTATG

GAAACATGCAACAGGCTTATCAGCAAATGCAAACGCCATTGATTTACCAGGAGCCATCTG

CCCGAAGATTGAATGAACTGAAAGACTCGACCATTCAGTTGGCTTCAAGCTTGGGTAATA

TTGATGCACCGGTAGACGAGTCGGTATTCGATATTGAAAAAGAAATCGATGATCTTTTAC

CAGTGGAGGTTAAAGAACAGCGTTTAAGCAACTTGCTCCAGGCTATAATGGTAGGAGGAT

GTGTTGCAGCGATGCCATTGCTTAAAAAGATCCCAACATCGGTACTTTGGGGATATTTTG

CATTCATGGCCATCGAGAGCTTACCTGGTAATCAGTTTTGGGAAAGGATTCTCCTCATGT

TCACTGCCCCAAGCAGAAGATACAAAGTGCTTGAAGAGTATCACGCCACATTCGTCGAGA

CAGTCCCTTTCAAGACAATCATCATGTTCACACTTTTCCAGACATCATATTTGCTCGCAT

GCTTCGGAATGACATGGATCCCCATAGCCGGAGTCCTTTTCCCTCTTATGATCATGCTTC

TAGTTCCCGTGAGGCAATATTTTCTCCCGAAGCTTTTCAAAGGCGCGCACCTCACAGATA

TAGATGCAGCAGAGTACGAGGAGATGCCTGCACTATCATTCAACCTCGCAACAGAGTTCG

AGGGAGGTATCAGGCGTTCCGACAGTAGGGAAATTCTAGATGAGATGGTCACCAGAAGCC

GAGGAGAGGTCAAGCATATGAGTAGCCCCAAAGTGACTAGCTCAACTGCAACACCAACTA

CCGAACTAACGAGTGTCCGAAGCCCTCGACTATCGGAGAGAGTGTACAGTCCTCGAGTGA

GCGAATTGAGGCAGGAGAGGAGTCCTCTTGGTGGAAGAGGGCCTGTTAGCCCGACTGGAC

CATCAAAGCTGGGGGAGAGCTCTCGAGGTTCGACTCCGGAGTAGAAAGAAAATGAAGTTA

TGGAGTTGTTCCGATGGCAATTATGAGAGGTTTATCCATTGCTAACACATTGATACAAAC

TATATTGTCTTGGTTCGATGTAATATTTGTTTCCGATTAGCGTCGTTCTTTGTGCAGCAT

TCTGCAGGCTGCCACTCTTGTGTAAAATCTGTACCTTCCAATTTTCCGACCTCTCATTGT

CGAGGATATTGTAATCTTGAAATC

>Unigene0000121

GCCGCGGTTCTTCTCCCTTACTGCTCATCTCCAGAATGCAGAGTTCCTGTGCTGGTCCTA

GGGTTAGGGTTCGGATCCCAACCTTCGCCCCCATCAAATACCGGACGATTCCGGCCACCA

GCTTCTCGATTCGAGGGAACCGCCGGGCATTCGGTCCGCTCAGCTGCACAGTCGCCGATG

AGGGGTCTATGGGTTCAGGTGTTACAGGAACAGATAATAGTGATTATTTGGCAAACTCAA

TTGAAGTGAGACAGTGGGAAAGTGGAAAGTCACTAGATGACATTGCAGCAGAACAGGGTA

TTAAAATTAGACGACATGCTTCGTCTAGACCTCCTATGCATGCTGTTGGTCCCTCTGACT

TATGTTTGGAGAACGAGGGAAGCACTCCCCTCAATATTCTAGAGAAAATAATATGGGACA

AGGATGTAGAAGTTAGACAGTTGAAAGAAAGAAATCCTTTGATCATGCTGAAGAAAGCTC

TTGAAGATGCTCCTCCTGTTAGAGATTTTGTTGGGGCATTGAAGGCATCCTATAAAAGGA

CAGATATGCCTGCTTTGATAGCTGAGGTGAAAAAGGCTTCTCCAAGTCGAGGGGTATTGC

GCGCGGATTTTGATCCCGTTGAAATTGCTAAGGCGTATGAAGCTAATGGGGCAGCTTGCT

TAAGTGTTTTGACAGATACAAAATATTTCCAGGGAAGTTTTGAAAATCTAGAGGCTATAC

ACAGAGCTGGAGTTAAGTGTCCTCTTCTTTGCAAAGAGTTCATCATTGATGTTTGGCAAA

TTTATTACGCACGGACAAAAGGAGCAGATGCAATACTCTTAATTGCTGCTGTGTTGCCTG

ATATTGATATCAAATATCTGTCTCAAATTTGCAGAAAGCTTGGTCTTTCCGCACTGGTCG

AGGTTCACAATGAAAGGGAAATGGACCGTGTTCTGAACATAGATGGAATCCGTCTGATTG

GTATTAATAACCGTGACCTGGAAACTTTTGAAGTTGATATCGCTAACACAAGGAAACTTC

TGGAGGGAAAGCGTGGTGAGACCATCCGTCAGAAGGACATAATTGTGGTGGGTGAATCTG

GATTATTTACTCCCGATGACATTTCGTATGTCCAAGATGCTGGAGTTAGAGCAGTTTTGG

TGGGTGAGTCTCTTATCAAGCAACCGGAACCTGGCAAAGGAATAGCAGGACTTTTCGGCA

AAGATATTTCGATATTGATATGACTGTGTTATCTCGATCGGAGTTGTGGTAGTATCTGGT

GTATGTAACAATGTCACCTGCGAAGAGCATAAGGGCGTAGCTTGGTAATTTTGTGGAAGT

ACACGAGGGTGTAGCTTGGTAGTTTTGTGGAAGAGACATTGACTATGGTTTACTGTAAGA

CATGAGAACCTTGGTACAGTTGTTGGTCATAAGTGTTAAAAACTTGGTTGGAAAACTTTG

AAGTTCGGTATGCTGTATGTCTTTATTACCATTATGTTATGACAGTGTTACCTATATCAT

GTATAGGTAGACATGAGCATAGTCTGCAGTGATGAGGCATATTTAGTA

>Unigene0000122

TCAAATCCCAATCTCTCACCCTCCCCTTCGAGTCGTCCTCCAACATCTCCCGCATCGCCG

TTTCGCCCGATGGCGTCTTTCTCCTCGCCGTTGAAAACAACTCCCGCGCCGCGGCGCTAT

CCTCGACCGCATCACCTTCAAGTGTCCCGTCACCGCCCTCCGCTTCAGCTCGGACGGATC

CCTCATCGCCGGCGCCCTCGGCAAGCTCCTGCAGATATGGCGCTCCACCGGATTCCACAA

GGAGTTCTTCCCCTTTCAGCTGGATCGCACCATCCCCGACTGCTCCGGTGCGATACCGCC

CTCGATTGGAGCCCTGATTTCGCTTATTTCCACGTCGGATCAAAAGATCTCACCGTCCGT

TTGATCTCTTTATTCAAAGGTTGCGACAAGCCGTTCATGTTTCTCCACCGGACGATCATC

GGTGCATTCTTCTGCACCGAGAAGAAAACGGCCCAGGTGTACCGGGTGTACACACTGTTG

AAAAATAGAGCCATCTTCACATGGAATTTAGAACGGAGTAACAAGTTTGATAATCCACCG

AGTCCGTAGGGGCGGGAGGGGTTTGATAA

>Unigene0000123

CTCGCCGGCGAGGCTGACGGTATGTGATTGCCATAGAGGGATTGATATGGTGGTGGTGGG

GTTCTTGAATGGGATGTTTGGGCTGTACCAGATACCTGATTTTGTGTGTATTGACTTGCT

TTCGTTATTGAGGGAGAAGATTACCGATGACCATTTTCAATAAGAGGGGGAACTGGTTGT

CGTTCGGGTGTGCGAAGCTCGAACAGCAGCTCGAGTGGGAGTGTCGGTGGGAGAGCTATA

TTTTGAAGCAACAGGGGCATTATTTTGATGTCAATTGTGTGGTTTATTCACCAGATTTGT

AGATGTTGGCAACAGGAGAGATGAGAACAAAATGAAGGCAGTGGAAGATGCTCTCAGCAA

TGACCAGCAACAGAGGGCATTGTTACTTAGCCTTTGTTTGAATGAAGACTCGAAATGAAA

ACAATGTATATTTGCTATTAAACCATTAGATATACAGGCTGTCACTTCATCTATACCTGT

CAAATATTTACAGGGTTACTCCATGCATTTGCTGACCTCTTAGAAAGCTGTCCACATTTG

GAATTTATTCTTCATTGGGGTCAGGAACTTCGCTAGTCTCATGGTCATTTCATTCAGCAG

AATTCAATAAGTCTGCTTCAGGCTTTAAGAACCTTGCAGAAAGCACTACAAAGCTGCATT

GGGATTTGACTGATACATGTTCGTCCAATGAATATTTACTCCAGTATCTTTTCTCTACAA

GGACCAAGATTTGATAGACACTCTTCCAGTTCACAACTTAATCAGACAGTGGAAGATTCT

CTCAG

>Unigene0000124

TTTACAGACGGCCACTTCGACCGCTAGGGTTTTAGCTCCGCCGCCGAGCAACCAAACCCT

AGTGCCGCTCGCCGCCGCAGCCCACCTTCGCCTCTGCCCACCGTCGCCGCCGCTATGAAC

TTCCGCTTCCAAAACCTCCTCGGCGCCCCGTACCGCGGCGGTAACATCGTCATCTCCGAC

GACACCCTCCTCTCCCCCGTCGGCAACCGCGTCGCCGCCACCAATCTCGTCAAATCCCAA

TCTCTCACCCTCCCCTTCGAGTCGTCCTCCAACATCTCCCGCATCGCCGTCTCCCCCGAC

GGCGTCTTCCTCCTCGCCGTTGACAATAACTCCCGCGCCCTCTTCGTCAACCTCCGCCGC

CGCGCCGTTCTCCATCGCATCACCTTCAAGCGCCCCGTCACCGCCCTCCGCTTCAGCCCC

GACGGATCCCTCATCGCCGTCGGTCTGGGCAAGCTCCTCCAGATCTGGCGCTCCCCCGGA

TTCCGCAAGGAGTTCTTCCCCTTCCAGCTGGAGCGCACCATTCCAGACTGCACCGGTGCC

ATCACCGCCCTCGATTGGAGCCCTGACTCCGCTTACCTCCTTGCCGGATCAAAAGATCTC

ACCGTCCGGTTGTTTTCTTTATCCAAAGGTTCAGGTAAGCCGTTCATGTTTCTCGGCCAC

CGGGAGGCCATCGTTGGTGCATTCTTCTGCACTGAGAAGAAAACTGCCCGTGTTTGCCGA

GTGTACACACTGTCGAAAGATGGAGCCATCTTCACATGGAGTTTGGATCGGAGTAGTGAG

TTTGATAATCCACCGTCGCCCGGGACACCTGGGGGTGATAAGGATGTTGATAAGAGAGAG

GCCAACTCTGAGACTCCGTTGAACAAAGCGAAGTGGGAGCTGATGAAGAAGGATTTCTTC

ATGCAGGCTCCGGCGAGGCTGACGGCGTGTGATTACCATGGGGGGGTTGATATGGTGGTG

GTGGGGTTCTCCAATGGGGTGTTTGGACTCTACCAGATGCCGGATTTTGTGTGTATTCAC

TTGCTTTCGATATCCAGGGAGAAGATTACGACGGCGGTTTTTAATAAGAGGGGGAACTGG

TTGTCGTTCGGGTGTGCGAAGCTCGGGCAGTTACTTGTGTGGGAGTGGCGATCGGAGAGC

TATATTTTGAAACAACAGGGGCATTATTTTGATGTGAATTGTGTAGCTTATTCGCCAGAT

TCGCAGATGTTGGCGACCGGAGCGGATGATAACAAAGTCAAGGTGTGGACTGTTTCTTCA

GGTTTCTGTTTCATAACATTCTCTGAACACACTAATGCTGTCACAGCACTCCATTTTATG

GCTAGCAATCATTGTCTATTGAGTGCTTCTCTAGATGGGACGGTCCGTGCTTGGGATTTG

TTTCGATACCGAAATTTTAGGACGTTTACCACCCCTTCTCCAAGGCAGTTTGTTTCTTTG

GCATCAGACGAGAGTGGTGAAGTGATTTGTGCTGGGACTCTTGATTCGTTTGAGATTTTT

GTTTGGTCAATGAAGACAGGGCGTTTGTTGGATATACTTAGTGGTCATGAGGGACCCGTT

CATGGTTTAACATTTGCACCTACAAATGCTGTTTTAGCTTCATCCTCATGGGATAGAACT

GTTCGCTTGTGGGATGTTTTTGATGGCAAAGGTGCAGTGGAAACCTTTCCACATACGCAT

GATGTTCTTACTGTTGTTTATCGTCCGGATGGAAAGCAGCTAGCTTGCAGCACATTAGAT

GGTCAAATTCATTTCTGGGATCCCTCTGATGGACTTTTGATGTACACTATCGAGGGTCGA

CGGGATATAGCAGGTGGGCGGCTAATGACAGACCGAAGATCAGCAGCCAATTCGAGTTCT

GGAAAGTATTTTACTACTTTATGCTATTCTGCTGACGGAAACTATATTTTAGCTGGAGGA

AGCAGCAAATACATATGCATGTATGATGTTGCTGATCAGGTTTTGCTGCGTAAATTTCAA

ATTACCCACAATCTCTCATTGGACGGTGTACTCGATTTCTTGAACTCTAAGAAGATGACA

GATGCTGGCCCGCTTGATTTAATTGATGATGAGAACAGTGATATAGAAGAAGGTATAGAT

CAACAAACACGAGAAAACTTGGGACATGATCTACCTGGATCAATGCCAAATCGTGGGCGG

CCTGTTGCCCGTACAAAATGCTTGAAAATAGCACCAACGGGAAGATCTTGGGCAGCGGCA

ACTACTGAAGGTGTTCTGATGTACTCTATGGATGAATCTTTTATTTTTGATCCCACAGAT

CTTGATATTGATGTGACACCTGAGGCAGTGGAAGAAGCTCTCAACAAGGACGAGCAAAAG

ATGGCATTGTTACTTAGTCTCCGTTTGAATGAAGACTCGATTATCAAAAAATGCATTTTT

GCTGTAAAACCATTGGATATACAGGCTGTTGCTTCATCCATACCTGTCAAATATTTACAG

AGGTTACTTGATGCATTTGCTGACCTCTTAGAGAGCTGTCCACATTTGGAATTTATTCTT

CAATGGTGTCAGGAAATTTGCAAGTCTCATGGTCGTTTTATTCAGCAGAATTCAAGAAGT

CTGCTTCCTGCTTTAAGAACCTTGCAGAAAGCAATTACAAAGCTGCATCAGGATTTGACT

GATACATGTTCGT

>Unigene0000125

ATCTCTTGATGCTCGGAAGCCACAGTCTTTACCAGAGTGCCGCTTTTTTGGACCTGATTC

GGCTGTAAATCCGATGTTAAAGAAATGGAAGAAAAATGGAAGTAAATGGACGAGTGAAAA

AGCATTCCACGAGAATCTTGCTGTTATGCTTGAAACCACGCTGCCGAGACCTCCGAGTGC

CAGCTGCAAGGATGACGAGCCAGTCGATTGTGGGATATGTTATGCTCAGTATTTACCTGT

TGATGATGAACTTGGAGCTAACAGTGGAAGTACGCCGAATTACACCTGCGACAATTATAG

TTGCAGTAGAGCTTTTCACACAGTTTGCTTAAGAGATTGGTTGCGATCGATCACGACGAC

AAGACAGTCGTTCGATGTTTTGTTTGGGAATTGCCCGTATTGCTCCGAACCGGTTGCAGT

GAGAGTAGATGGATACAAATGATCAGACTTAGACTCTGAGTTCTTTTGTTCCTTGCGAGA

TCTATTCAGTTTTGTATGAATTATATACCAAGATGACATCTAATTGTTGAATTTACACAA

TTAGTAGTAACTTCTAATTTCTGTTGAGAACCA

>Unigene0000126

AAGGAAAACCACATGTTTTGGAGTTATCATTGTCTCACAACTATCCAGAATCCGCTCCTT

TAATTGCTGCGGTAGAAGAGATTGGATGGGAACAACATTTGTTGAAATTGGAGGAAGTCC

ATTCCTTTCTTAGCTTCCATGTCCTAGATAAGAAAGGAAAACCACATGTTTTGGAGTTAT

CATTGTCTCACAACTATCCAGAATCCGCTCCTTTAATTGCTGCGGTAGAAGAGATTGGAT

GGGAACAACATTTGTTTAAGTTGGAGGAAGTCCATTCCTTTCTTAGCTTCCATGTCCTAG

ATAAGAAAGGAAAACCACATGTTTTGGAGATATCATTGCCTCACAGCTATTCAGAATCTG

CTCCTTCAATTGCTGTGGTATTGCTGTGGATGTGCCTAACATGTGTGAGTTAGTGTAGTC

AAAGAACTCTAGATTGAAAGATGTCATGCAACAGTTCACCGAGCATCTGCAGACGTTGCA

GGAATTTTGGTCTACCTTAGATGACATCGAAAAGGCTTTCTGGGTTGTGGATACAAAGCA

GCCTTTTAGTGCTATTATCGTATAAGGCAGATTGGCGTTGGAAACGATTGCTATCTTTTG

TTATCTCTTGATGCTCAGAAGCCACGGTCTTTACCAGAGTGCTGCTTTTTTTGGACCTGA

TTCAGCTGTAAATCCGATATTAAAGAAATGGAAGAAAAATGGAAGTAAATTGTACACATT

TTCACTAGTTCACGAACCTTTGCAATGAATGCCCAAATAGATGCTTAGTTTACCATTGGT

TTAAATTGTTCTTTATTGTTGGAACCTAAAAACTTATTTTTTCT

>Unigene0000127

CCTCTTGGTGTCTTTGTGTTTGATTTCTCATTGTGGGTGTGCTGGAGAATCAACTAAATT

TGGCAACTGGTATAAAGAATGTGGGAAACCATTTGCAAGAAATCAGAGACAGCTAAAGAA

ACCAAGAGGAGAGGCGGGTGTTGGGGAAAGCTTGGGAGAGGGAGGATGGGATCAATGAAG

TCCTTCAAGGGTAGGCCGGAGCCGAGGAGGTTAGGGACTCTCGCAATCATCGCAACCATG

CTCGTATCCCTGTTCGTTCTCTCTATGATCAAGGCTCGCTGCAGCACTACTTCTCATTCT

GAGAATTTAGAAGCACCATTGCAGCTTAAAGCAACTACAACTGGTGATGTGAAGATGGTC

ACTGATGAAATCATAGAAGACGAAGATGAAGAGGTGGCTACAACCCATCCGAGCAAACCG

ATATGCTTCGAAAGCAGTCTTAGATCCAATACCTGTGAAGCTGAAGGCGATGTAAGAGTC

GAGGGAAGCACCACCACAATCTACATTCCCACTACAAATAACCAAGAATGGAAGATCAAA

CCTTACCCTCGCAAGGGAGATGCTATAGCCCTCTACCATGTGAAGGAATGGTCTTTTAAA

CCTCTCAGCGGGAATGGACCTCCTCCTGCTTGTACTCAGAACTACACGGTCCCGGCCATA

CTCTTCTCGCTCGGTGGCTTCACCGGCAACATGTTCCATGACTTCACTGATGTTATGGTG

CCCGTCTTTGCCACCGCTTACCAGTTTCGTGGCGAGGTCCGACTACTTATTAGTGATCTG

AAGTCGTGGTGGGTCAGTAAGTTTGTGATGATACTGAGGCAGCTCTCGAATTATGAGGTA

ATTGATGTGGATGCCACTGGGAACGGTGGTGAAGTTCGCTGCTTTCCCAAGGTGATCGTA

GGGCCGATGTTCCATAAGGAGCTAGGAGTGGATGCTGCCAAGACGCCAGATAGGTCCTCG

ATAGTGGATTTCAAGGCTCTTCTCAGGAAGGCACTAGGGCTACAGAGGGTCTCATCTATG

ACAGAAGGCATCCAAGTGGAAGGCATGAGGACGAGGCCACGACTGCTGATCATATCACGG

AAAAACTCGAGGGCTTTCATGAATGTTCGAGGAATGGTGTCAATGGCCACAAGCTTGGGG

TTTGAGGTGCGGGTGGGCGAACCTGAAGTAGGCACGGATGTCTCCAAGTTTGCGAGGCTC

GTAAACTCAGCAGACGTGATGATCGGTGTCCATGGGGCAGGGCTGACCAACATGTTGTAC

CTTCCGGAAGGGGCAGTGGTGATCCAAGTGATCCCTTTCGGAGGGCTCGAGTGGCTGGCA

AGGAGGAGCTTCGAAGAACCCTCTGCAGACATGAAGTTGCATTACTTCGAGTATGTGATT

CAAGCAGAGGAGAGCTCACTCATAGAACAATATCCGAGGGATCATCCTGTGTTTACGGAT

CCCTCCTCAGTGCATAAACAAGGATGGAATGCTCTTAAGGAAGTGTATCTTGTTAAGCAA

AATGTGAGGCCTCATTTAGGGAGGCTTAGGGACACTTTGATGGAGGCACTCAAGGTTTTG

CCTCACACTAGTTCTCACTCAACTTGAGTTGTTGGTCTATCTCTTTGGTGATTATTGTTG

TTATAATGAGGTTATTATTTTTTAAATATTTTTATAATAATTTTTTCCCCTTCTTGTAAA

TGTGGCAA

>Unigene0000128

GCGTCCATACCATTCATCAAAAGATCGTCCCTCGCTATGAGAACTGACAAGAACTAGGCA

ACGAATTCTCGTAGTTAATTGAGGGACGTGGGGGTCGTGCAAGGTTCGATTCCCCTTAGA

CGCGATGTGGCGAAAAAGACTAATTCAACAAGTCGGACCCACACCATGCCCGCATTAAGA

TTTAAAACGTGTCGTATACTTCCAGGAAATGTTTGGAAGAGAGAACTTTCTCTAATACAA

CGCCGCATTCTCCGAAGATTGAGGAACAAGAGGAGATCCTTTAAAAGAAATCTTTCTATG

AGAGAAAATCTAAACAGTAACATCAAATTACAAACTACACGAAAGTTGCCCCTTTATTAT

GGGGATTTACCCATAAGGGAGATGCACAGAGGAATAGAACGAACTTCATATATCCCTTTT

CTACTCAATCAAGAAACAAGATCGGACGTTCTTCCGGTTCGTCTCCATTTTAGTGACACT

CTTCCTCAAGCAAGGCAGCCGATAAGTCATCGAAGGGTTTTTG

>Unigene0000129

TTATGGACCTAACATAGGTCACATCCCTCACGACATAAGATTGAAAGATCCAAACCTTCC

TCTTCTCCGCGGAAACGGACGTGGCCAAAACATTTAAAGAAAAAAAGAAAGAGGCTCGCT

CATAGGGACATCTCTATCCGGAAAGAGGGTAGTCTAGATCTCTTTATAGAGAGAGATCTC

TCTATTATAGATAGATAGGTATCCGCGTGCATAGAGATAAGGATAGGGATCCTCAGATAG

AAATTTATATATAGGAAAAAGTGCACTTTTTGACGACTACTACTATTTATTATACTTAAA

GCAAGGGAATGTAATTGAACCATTTGTACTAAATAATTGGTCGGAGCGTAGACGGGCGAG

CAGAGCCCAGGAAAGAGGTCAGACCGTCATAGAGTAGTCGACTAGAAGTAGACGACTGCT

GGCCTGAGAGAAGGCAGTCTCTCTCGGGAAATATGGCCCAATTTATATTTTTTCTTTTAA

CACGGGCAACATAGGGGAATACTAGAATACGTGGAGATACCTAACTACCAAAACCGGGAA

GAAGTGCTTGAACGAAACCCGAAAGGTGAAGTCGAGCCAAGTCTTCCATAACGATACTAA

AGAAGAAGCCAAGCCGACGAACATAAAAAGACCCGCAGTGTCGACGGGCTGCTCTGCAAT

TCCCTTCCTGATCGGAACTTGTCAACAGAAACTACCTAAAAAGAATAAGAGTAGCTCGGA

TAAAGTCTGCCGATTACCCTTTATTCGTATTTTGTTCACATTGGCCAAGGCCGAAAGGAG

AGAGAACAGTCACCTACCCCTGCCGCGCTGGTCTTGCCTTTGTTCTCATACTCTGTTTCA

TCCCTAACAAAAGATAGGCCCCACCACTTCCTTTACTCAAGTTATAGCCCACCTTTCCAA

AGACTCGAACAAAATAAAGATCGGAATTAGAT

>Unigene0000130

GATAAGTCATCGAAGGGTTTTTGTGAATAATGGAATGGTAAGCATTACTCATTTAAAAGT

GTCCCACGGTGATCTAATATCTTTTCAAGAAAATGACGCGATAACCCGCGGTGAAGAAAT

CCGGAGATCTTTCTATATGGACATCTCAGTTGATCAAATCATAGGCAAATTCCTGCCGGT

CAGAATGTGGAGAAGAACCAAAACAGAATGGTTCCGCCTACTCAAAACTCAGAGGGGGTG

CCGCCTACTACTCAAATCCCGGTTTTTGCAAGAGTTGCGTTCTTCTATGCAAGAAGAAGA

CTTAGAAAGAACAAAGAAGTTTGGATCAGCAAAAGTATGCTTAGGCAGTGCCTTCGCTGA

GCACAACAGAATGAAGAGGAATTTGTTTCATTTCAAATACTTCTTCTTATTGAAGAGAAG

GGAGGATAAAAACCGAAATCTTCCCACTCGAAGAATCAGTCCTTTTGTTTACAAGTCTTC

TTTATATAGTAATTTGACCTCTTGTTCCGGATCCCCATTTACTAGAAAGAGAAGAATCCA

AAGGATCGAACTACCTACTCATTATTTGGAGGTGAATTATAGAACACTAAAAGCTGTGGT

ATTTTATGGACCTAACATAGGTCACATCCCTCACGACATAAGATTGAAAGATCTAAACCT

TCTTCTTTTCCGCGGAAACGGACGTGGCCAAAACATTTAAAGAAAAAAAGAAAGAGGCTC

GCTCATAGGGACATCTCTATCCGGAAAGAGGGTAGTCTAGATCTCTTTATAGAGAGAGAT

CTCTCTATTATAGATAGATAGGTATCCGCGTGCATAGAGATAAGGATAGGGATCCTCAGA

TAGAAATTTATATATAGGAAAAAGTGCACTTTTTGACGACTACTACTATTTATTATACTT

AAAGCAAGGGAATGTAATTGAACCATTTGTACTAAATAATTGGTCGGAGCGTAGACGGGC

GAGCAGAGCCCAGGAAAGAGGTCAGACCGTCATAGAGTAGTCGACTAGAAGTAGACGACT

GCTGGCCTGAGAGAAGGCAGTCTCTCTCGGGAAATATGGCCCAATTTATATTTTTTCTTT

TAACACGGGCAACATAGGGGAATACTAGAATACGTGGAGATACCTAACTACCAAAACCGG

GAAGAAGTGCTTGAACGAAACCCGAAAGGTGAAGTCGAGCCAAGTCTTCCATAACGATAC

TAAAGAAGAAGCCAAGCCGACGAACATAAAAAGACCCGCAGTGTCGACGGGCTGCTCTGC

AATTCCCTTCCTGATCGGAACTTGTCAACAGAAACTACCTAAAAAGAATAAGAGTAGCTC

GGATAAAGTCTGCCGATTACCCTTTATTCGTATTTTGTTCACATTGGCCAAGGCCGAAAG

GAGAGAGAACAGTCACCTACCCCTGCCGCGCTGGTCTTGCCTTTGTTCTCATACTCTGTT

TCATCCCTAACAAAAGATAGGCCCCACCACTTCCTTTACTCAAGTTATAGCCCACCTTTC

CAAAGACTCGAACAAAATAAAGATCGGAATTAGAT

>Unigene0000131

CCCCTCCGCCCTCTCCCTCGACGACCCCTCCTTCCTCTGCCCCCCTGTCGCTGTCGGCTC

CAGCAACACATCTATCTCAACCTGTTGCGTCCCCAACCCCAAGTTCCGCGCCAACACCAC

CGACGACCCGCTCGCCGGCGCCAACATCACCGTTTCCGCCCGCCGCCCGGGCGACTTCGT

CATCTCCTACGACGTCATCCAGGCCTTCCCGTCCAGCTACCTCGCCCTAGTCACGCTCGC

CAACCACCACCCCCTCGGCCGTCTCGACAATTGGCACCTCTCATTCGACTGGACCCGCGG

CGAGTTCATCTACGAGATCAAGGGCGCCTACACCTCCGTCAAGGGCCCCTCCGGCTGCAT

CACCGGCCCACAGGGTCAGTACTACCAGCAGCTAGACTTCACTAATGTCGCCAATTGTCA

ACGCCGCCCGACAATCCTCGACCTCCCAGCCACCATGGCCAACGACTCCGCCACCGGCCG

CATCCCCAACTGCTGCCGCAACGGCACCATCCTCCCTGCCGAAATGGATCCAACCGAAAC

CGAATCCGCATTCCAGATCCAGGTCTTTAAGATGCCACCGGATCTCAACCGCACCACCCT

CTTCCCCCCGAACAACTGGCGGATCACCGGCGGCGAACTCAACTCCGACTTCCGCTGCGG

CCCTCCCGCCCGCGTCTCGCCCACCCAATTCCCCGACCCCAGCGGGCTGCAATCCCACTC

GCTCGCCATCGCCAGCTGGGAGATCGTCTGCAACATAAACAAGCGGAAGGGGTCGACCCC

GAAATGCTGCGTCTCCTTTTCCGCCTACTACAACGACACCGTCGTTCCCTGCAAGACCTG

CGCCTGCGGCTGCCCATCATCGCCATCCCGCACCTGCAGCACCACCGCACCAGCCCTCCT

CCTCCCCCCGCAGGCGCTCCTCGTCCCGTTCGAGAATCGCACGTCCCTCTCGCTCGCGTG

GGCGAAGCAGAAACACTACAATGTGCCCAACCCAATGCCATGCGGGGACTCGTGCGGCGT

CAGCATCAATTGGCATATAAACTCGGATTTTCGACAGGGATGGACGTCGCGGGTGACGCT

GTTCAATTGGGAGGATGTGAGCTTTGCTGACTGGTTTGTGGCGGCGGAGATGGATAGTGC

GGCGTATGCTGGGTTTGAGGCGATGTATTCGTTCAATGGGTCCGCGATGGGGAACAACAC

TCTCTTCATGCAGGGGCTGCCGGGGCTGAATTATCTGGTGGGGGAGGTCGACGGGGAGAA

ATCAGGTGATCCGAGAGTGCCTGGGAAGCAGCAGTCGGTCGTGTCGTTTACGAAGAAGAA

TAAGCCGACGATCAATGTGGTTGAAGGGGACGGGTTCCCGACGAAGGTGTTCTTCAATGG

GGAGGAGTGCTCGTTGCCGGATATAATTCCGGTTAGCGGGGTTTTCAGAGTTGGTAGCAG

TGGCCCCTTGACACTGATCTTGCTTCTGATCTCAATTCTATTGCAATAACATTATTTGTC

AATCGTTTTGTGCTATTTATACTTAGAAATTATGTGAGATTTTATATCATTTACTTAGTT

TTATTATAAAACATTGTATAAGAATTTTGTAACTTCACATTGATATATTTGAGGAGTTGT

TGTTCATCATTTTTAAGATTGATATCTCACAAGTAAATTATGACTGATTGCTTCTTTGTT

AGCTTGTTGAGCATTCTTACCAGA

>Unigene0000132

ACCCACCAGCTCGTTTCCTCTGTTCTCCAGCGCCTGGGTATGATGGCTGCAGGGATCGAC

GGCGGCGATTTCTGGCTCCCTGCTGACTTTCCCGTCGCCGACGATCTCACAGCTGACCGT

CGCCTCAAGGAGTCCGACGACGAGGACTACCTCGCCGATCTTACCCGCCGGATGGCCTTA

GCCTTTCTCCACGACGACGAGGTCCCCCACCCCAAGATGCAATCCATGGCGAGGTCTCCT

CAGTCGACGCTCTGCGACTACCGGATCTGGAACAGAGGAAGTAACAACCTGACGTCACCG

CCGTCGCAGCTCTCGTCGCCACCGGCGGCTTCCTTCGAGCAGAGCCATGACGATGCTTGG

GATCTTCTGACGGCGGCTGCCGGACAGGTTGTACGCCTGCGGCTCGCTGAGGAAGCACGA

CGTCAGAAGTTTAACGAGCGCGGCCTGCTCACCCCGGCGGGAAAGATCTCTGCTCCGATT

GCAGCACCTCCGAAGAGATCAGCCGGCACCGGCGTCTACTACCCCCCGCTTCTCGCGCAG

CAGCAGCAGCTGCAGGTTGAGAGGTTCTACCAGCTGAAGCAGCAGCAAGTCCTGAAGCAT

CAGATCTCCCTGGCAGCTAGCCGGCAGCAGATCAGAAGCGGCGGTCAGCCGGGTGTAGTG

CCGACCTCAACCCCTCACCGGCAGCCGGCGCCGGTGCCGCTCATGCAACATCCCCGTCCA

GGTGTCGGTACTAGGCCTGTCTTCCTCACCGGGCACGGCACACGAAGGGAATCGACTGGA

ACCGGGGTTTTCTTGCCTCGCCGACCGGAGAATCCGTCGGATCTCAGGAAAAAAACAGCT

TGCCCTCCGGTTAGATCAATCCAGTCTCCGAAGCTAAATGGGGACGACATGAGATCCCAG

CCACGAGTTCTCGGAGACTTTCTTGATCCCGAATCTCTGGTGGCAAGAGGCAATCGGTTT

CTCCTCCAGCAACATCAGCATCAGAACAGGTACCAGCATCATGCTCCGGCGCCGGCGATG

GCCGATCATGAGAAGCTCCGTTTACCGGCGGAGTGGACTTACTGAACACTGGACTAACGT

TAGGAAATAATAGTACGAATGTGATGAAAGATGCAGTAGTAGGTTTTTGGTTTTTGCTAT

AGCAATATTGTGATTGTATTTTTGGGGCGGTGAAGGAACCGATGGTTGGTGGAACTAAAT

CAATAAGGTGGAGTAGATAGAGGAATTGGTGGGTTGGGATATAGTTTGCAATAAATAATT

GCTCGCCCTTGTGTTGTATCCCGTTTTGATGTAAACCCGTCCTTTTCCACTGTTTGCTGA

AGTTGGGTTGAAATAACAATGTCTACTCACTTTATGTTATC

>Unigene0000133

AAAAAACAAAAGCTCACGCCTAATCCAAATTTTATTGAGTCCGACCCGTGGGCTGGACTG

GTTGGACCCAAATCTCAAAGATGTCCCAATCAGATGCCTTGATGTCCTCTGAAAGCGTGC

TTGATGTTTTCATCACTTACCCATAAAACTATTAAAAAATAATTTATCTAAAAGGATTTG

TGATGATAAAAGTTCTATTAACATACTAAATAATTATCATTAATCCTCACATCTCTCCGT

GAATCTAGTTGCAGGGGCATTGCACAATAATTTCGGACCCTCTGGCTTAGAGTCCATGTT

CACAACAAGCAGCGCTGCTCGGCGCAGCACCGTTCCAGAGCAGTGTGAAGTGTGAATCA

>Unigene0000134

AATGCACGCAGACGAGTGAGCTGTCCCAAAACATCCCACGTCGGCATATCTTTCGTGGAA

CCACAAAAAAAAGAACATGATGTCCAACGTTGGCATGTAGGCTTTGTGGGACGTAAAAAC

AAAAACTCACATAATCCTAATTTTATTGAGTTCGATCCGTGGGTTGGACGGGTTGGACCC

AAATCTCAAAGATGTCCTAATCAGATGCTTTGATGTCTTCTGAAAGCATGTTTGATGTCC

TCATCAATTAACCTCCTTCTAGCCGATTTACCGCAAGTTCTTGCTTCTACTATCACGAG

>Unigene0000135

AATGCACGCAGACGAGTGAGCTGTCCCAAAACATCCCACGTCGGCATATCTTTCGTGGAA

CCACAAAAAAAAGAACATGATGTCCAACGTTGGCATGTAGGCTTTGTGGGACGTAAAAAC

AAAAACTCACATAATCCTAATTTTATTGAGTTCGATCCGTGGGTTGGACGGGTTGGACCC

AAATCTCAAAGATGTTCCAATCTAATGCCTTGATGTCCTCTGAAAGC

>Unigene0000136

AATGCACGCAGACGAGTGAGCTGTCCCAAAACATCCCACGTCGGCATATCTTTCGTGGAA

CCACAAAAAAAAGAACATGATGTCCAACGTTGGCATGTAGGCTTTGTGGGACGTAAAAAC

AAAAACTCACATAATCCTAATTTTATTGAGTTCGATCCGTGGGTTGGACGGGTTGGACCC

AAATCTCAAAGATGTCCCAATCAGATGCCTTGATGTCCTCTGAAAGC

>Unigene0000137

GGAAAATCATGGCGGATGAAATGAATCGAGCCCTTGCCTATAAAATCCCCCTCAATTTCA

AACCCCCTTTCCATTCTTGTTCGTGGCGCAATTCGAGATCACAAACCCATCCCGCACTGC

TCCCCTCTGCCCTCTTCCCCCAAACCCTAGATTTTGGGGGTGTAGAAAATGGGCAACAGA

AAGGAGGGGGAGAGCAACGAGAAGATCATCAGAGGCCTCCTGAAGCTCCCTGCCAATAGG

AATTGCATCAATTGCAACAGCCCGGGCCCGCAGTATGTTTGTACGAATTTCTGGACCTTC

GTGTGTACAACTTGCAGCGGAATACATCGTGAGTTTACACACCGCATAAAGTCAATATCT

ATGTCTAACTTCACCAAACAAGAAGTTGAGGCTCTTCAAAGGGGTGGCAATCAGTGTGCC

AAAGAAGCTTTTCTGAAGGACTGGGACATGAAGCAGATGAAACTGCCTAATATAAGCAAC

CCTGACAAAATTAGGGCATTTATAAGAGATGTATATCTGAAGAAGAAATATGCTGGGAGA

AGGGTCTCTGATATGCCACCTAGAGACATGCAGAACCTTAAGAAACATGATGAAGAACGA

AGGAGGGCAAGCTCTTATTATTCTTCCCTCCAAAGCCCACCTTATGAAAATCAATATGAT

GGTAGGTTTTATGACAAGCAAACGTGTAGCAGACTAGGTTCAGATCTTAAAGGCAAGATA

TCTAGCTTTGTGCACAGTTCAGGTCATCTACGAGAGCAATTATATGAAGATAGGTCTGCT

AATGGGAGTTTGGGTTCAAGAACTCCGGATTACTCAATATCTAGTTCAGAGAGTTCATTT

AGATATGATGGTCAGTCAACGAATTCTACCATTCCTCATTTGAACCAAGAAACTGCATCT

TCAAGTAGTTTTGGCTCTCCTGATTGTAGCTTGGTTGATGTTGCCCTAGAGCCTGAGCAT

TCCTCTGGTAACAAGCATGTAATCAAATGTATAAATCCTCCGTTATCGCTATCACCTGCT

TCTGTGCAAGATAGGAACCGTGACCTTTTTCATTTACCATTTGTGTATCCACCAATGAAT

TCTCCGGCTCTGCCTCTAGATCTGCTTGCTTCATGGGAAATATCTTCCAAGAGAAAACCT

TCTTTGGTACCGATTTCTGAGAATGATGGATGGATTACCTTTGACGTTACTTGCCCTTTG

AGGTCTGTTTCTGCAAAAAGTAAGAGAGCCTCTGCTGTGGCTCGCCCTGTTGATGAGGCA

CCTAAGAGGGCTGAAGATGCAATACCATCTGTGGATAACAGTTCAAAGGGTTTTTCTGTT

CAATGTTCTATTCCCGAAGTGTCCCGTTTAACTTTGACTGGTAAGGGGTATGCAGGCTTA

CAAGTAGAAAGATCACCTGCAGTTCTGGAAAGCTCTCAGTCATGGACTACCTTTGACGAT

ATTGATGCAAAGACTCCTCATACTTCATTTGGTGATCTATCACATGATATTGAGCCATCT

ATTTTGATAAAAGACTCCCAGATGGCTGTTGATCCATATGCAGAGTTGAAGGTTCCAGAG

TCATCCTACATAGAAGGGCATCAAATGGTACCCGTGGGTGGTATAGATTTGATCACAGGA

TCACTGTTCTCTCCATCAGTGCCATCTTTACTGGGAAGAGCTACTCAGGTGCGAAAGTCG

ACAAACCCATTTGATCTCCCATATGACCCAGATTCTGAAGTCAAGTTTGTGGATGGAGCT

GCCCAGTTGAGGAAATCAAGAAATCCATTTGATCTCCCATATGATTCAAATTCTGAAGTC

GACAATGTGTTTTGCCCGTCGGTGGACATGAGCTCCTTACAAGCAGCAATACCAACTGCA

ATGTGCACTGCCCAAGTCGAAGGCGCAAACCCACCATGGACTCTTTAGCATACACCGGTG

AAAGCACCCATTTTCTCTAAGTTGCCTAGGTGCATGTACATTCCTACCTTCGTGTTGTTG

ATTTACCTATTTAGAAAAATTGACGTGTCTTGGCAGGAATGTACAGCCATAGGGCCTTGT

GCCTCGCTTGACGGGAATCCTTCTGCTTAAGGAAATTCTTAGTGGATTACACTAGACACA

TGCATTGGTTAATTTTCATTCATGTACATAACATAGCTGTGAGAAGTGGATATGGGAATA

TTGAATATATTGTTCATGACATAGTTGAATAGATAAATATGGTTT

>Unigene0000138

TTTAAATAGACCATAAGATTCTCTTCCAAGATAGAGAACAAGATAACTATTTAGTAACAA

GCCACGCCTCATACTGCCATGGCAGTAGCTAAAGCAACAAGAGAGAAAGCTGCTGTCAGC

ATATAATGGCATGATATCTATTCTAGTGAAATCCATGTTTTAAGGATGTGTATCATCCTA

GTCGAGCTCAATTAGCGCAGTGAAGCACCGGGCAGCTGGTATGGCTAACAATCATGGAGA

CGAAGATAGCTTCGTTGCCATCGATATGGAAAGCTTGCTGCTACTTGCTTCTCCGACTGA

CTCGCAGTCTCCTAGTCCAAATCCGAGAGTACAAACATACAAAAGCTTCTCTCGAAAAGG

GTCCAACCAGGAAAGAGAGAAGAAGGCGCTTGAGGTTGGATCCGCCGTCTCAACCTCTGG

TGTGCAAGAAATATGCATAGATTCCAAATTAGCTATTGGTGTGACCGAGGATCAGGGGAC

TACCGGCGTCGTGACACCGACAATCGGGAGGTGTAGGAGGATCGGCAAGCGCTCATCCGC

CCGGATAGATCCGAGGCGGGTTGTCATCATGTTTGCTACACTATCCAGCATGGGAACACT

CATACTACTGTACTTAACTCTCTCCGTTGGTGGTGAAGCCAACCCCCAGTGATTCAAATT

TTCCTTGATCGGTGGTTAATTCACATTCAGCTAGCGATTATCGGTGTGTATGTTGTGTTG

AATCGTTTTACATGGAAGAACTCAGGCTAAAATTGCAGAACAGTGTACATGGCTCAGTAA

TTGCTCTCCTCTTTTTACTTTATTTTTAGCGAACGAAGTTCCTACGGACTGGGGCATCCC

TGTATACTCCAAATTTTTTTAAATTGCTGCGAAAAGGATTTAAGGTGTATTTGTATAGCT

CTTTGATCCAATTCTTTACTTAATAACCCTCACA

>Unigene0000139

AGTTCTTCTGGTCTTGTTAGTTCTTTGCTTCAGGATACCTTGCGGCGTGCGAAGATGGCC

GAGGTTGAGATTCTGAGGGGAAAGCTGGCTGAGGCTAATGTGTTGAGAGAGCAGGCTGCG

CAGAGTGCGGCCGATACTGAGAACCTGAGGTGGAAGATTCGGTCTATGGAGGAAGGCGTG

GCTGAGGCACGCTGAGTTGAAGACCACTCAGGGGGCCCTTCGGTAGGCTGAGGCTGAGGT

GGAGGCGCTGAGGAAGCGGACCGAGGAGGCGGTAGCTGAGGCGGGTGTG

>Unigene0000140

TGGGTGCTAGTTCTTCTGGTCTTGTTAGTTCTACTTTGCTTCAGGAGGCTCTGCGGCACA

CGGAGATGGCCGAGGCTGAGGCTGAGATGTTGAGGGGGAAGCTGGCCGAGGTTGACGTGT

TGAGAGAGCAGGTTGCGCAGTGTGCGGTAGGTACTGAGAACCTGTGGTGGAGGATTCGGT

CTTTGGAGGGTGTGGCTGGGGCATGTCACGAGTAGATGATTGCTCAGGGGGCCCTATACA

GGGCAGAGCAGGTTGCGGCTAAGGTGGAGCTGATGAGGAAGCGGGCTGAGCAGGCTGTAG

CCGCGTTTGAGGTACTGAGGGCTCGGGCTGTGTAGTTTTAGGTGGAGTGTGGGGGCGTTG

AGGCTACGGGCTGAGCGGGCTGAGGCTGACCTT

>Unigene0000141

CAGCTGAGTTTGAGATATTGAGGGCTTGGGCTGCGGAATTTGAGGCAGAGTGCGAGGTGT

TGAGGCTGCGGGCCGAGCGGGCTGAGGCTGATCTTGTGTTGCTGAGGGGACATCTTTCGC

AGGATACAGAGCTTTCCACCCACGTGCGGGAGATGATCACTACTTTGGAGAGCCCTGGTG

GGCCGAGGTTTTAGAGGATGCTGCAGGAGTATCGTTCGATATTGACTGACTTGGAGGCGG

CATAGGGGGTCCCGG

>Unigene0000142

GAGGTGGAGTGCGAGGCGTTGAGTCTGCGGGCCGAGCGGGCTGAGGCTGACCTTGTGTCG

TTGAGAGGATATCTTTCGCAGGATATCAAACTTTATACCCATGTGCAGGAGATGATCGTT

ACTCTAGAGAGTCTTGGTGAGCCGAGGTTTCAGAAAATGTTGCAGGAGTACCGCTCGATA

CTAGCTGACTTGGAGGTGGCACAGGGGGTCCCGGGAGACGGTCGTGATGAGGCTGGCCAG

GAGGGTGAGCAGGT

>Unigene0000143

TTCCTCGCACGTACGGGAGATGATCATACTCTAGAGAGTCCTGGTGGGCCGAGGTTTCAG

AAGATGCTGCAGGAGTACCGCTTGATACTGGCTGACTTTAAGGCGGCACAGGGGTCCCAG

GAGACGGTTATGGTGAGGCTGGTCAGGAGGTTGAGCAGGGGGTGAGCAGGTAGCTGAGCA

GGGGTTGAGGCTGAGCCGGTGTTGCCCCCGAAGCAGGGCTCTGA

>Unigene0000144

GCTGAGGGGACATCTTTCGCAGGATGCGGAGCTTTCCACCCATGTGCGGGAGATGATCAC

TACTCTAGAGAGTCCTGGTGGGCCGAGGTTTCAGAAGATGCTGCAGGAGTACCGCTCGAT

ACTGGCTGACTTGGAGGCGGTACAGGGGTCTCGGGAGACGGTCATGATGAGGCTGGTCAG

GAGGCTGAGCAAGGGGGTGAGCAGGTAGCCGAGCAGGGGGTTGAGGCCGAGCCG

>Unigene0000145

TCTACCTATCTTTAACTTATTTTGATGTTGACGAATGAATGCTATGATATCGGGGAGCAG

GCTCATTTTGTTGCTTACTGCAGTTAGAAAGTTAGACATTATATGGCGTCTTGTGTTACC

CCAACAGTTAGAAAGTTAGACATTATATGGCGTCTTGTGTTACCATGTTGGGGTATGGTG

TCTTGTGTTATGACGTTGGGTAGCAGGTTCATTTTGTTTCTTCTGCAGCTA

>Unigene0000146

TCTACCTATCTTTAACTTATTTTGATGTTGACGAATGAATGCTATGATATCGGGGAGCAG

GCTCATTTTGTTGCTTACTGCAGTTAGAAAGTTAGACATTATATGGCGTCTTGTGTTACC

ATGTTGGGGTATGGTGTCTTGTGTTACCATGTTGGGGTATGGTGTTTTGTGTTATGACGT

TGGGTAGCAGGTTCATTTTGTTTCTTCTGCAGCTAAAAAGTTTAGGCGTTATATGGTGTT

ATGTTACAATGAATTGTGTTGTGATGTCTGATTGT

>Unigene0000147

CCTCCCTCCTCTTCAAAATTCCAAACCCTAAACGAAATCTAGGGTTTCGCCCTCTCCTCC

CCGCTACCTCAAATCAATCTCTCTCCAATGGTGAGCACCCGCAAGAGACCTCACGCCGCC

GACGTCCCGGCTAGCCCCAAGCGGCGGATCGTCAAGGAGGAGCAACCTACGCAGCCCATC

TCTCCTCCCACCGCCTTCGCCGCCGATGAATACCAGAAGCTCCGGGAGCAGCGGATCCGT

GAGAACCAGGAGAGGATGCAGAAGCTGGGGATCGTAGACCTCTCCCGCCAGCTCACCAAA

TCGCACCAACAGCTCGCCTCCGCCCGCCGCGCTAGCACCCCCCGGTCCCCGACCATTCCG

GCCGATACCCCCACACGGCGCCCCTCCCGGCGCTCCTCTCGTTTGCAGAGTGTGACTCCG

GTTACCTACTCGGAGGTTGATGATGTGGGGGAGAGGAAGAAGAGGGGATCGTTAGTTGTG

GAAGGGGAAAGGGAAGAGGTTTACACTGTTGAGCATGAGAAGCTGCTGGGGTCATGTGAG

AATAGCTGGGAGCTGTTTGTTGATGGGTGCGGCCCCGATGGTAAGCGCGTGTACGACTCT

GTGTCCGGGAAGACTTGTCATCAGTGTAGGCAGAAAACTCTCGGTCATCGTACCCAATGC

AGCAATTGCAAGATTGTCCAAGGACAGTTTTGTGGTGATTGTTTGTACATGAGGTATGGC

GAGAATGTCTTGGAAGTTGAGAATAATCCCAATTGGATCTGTCCCGTTTGTCGGGGCATT

TGCAACTGCAGCATCTGTCGAGTGAAGAAGGGATGGGCACCCACCGGCGTTCTTTACAAA

AAGATCACAAGTCTGGGGTTCAAGTCAGTAGCCCATTATCTTATTCAGAGTCGACGTGGC

CAGACGACTGAATCAGGAGAGCAGAGTGATGTCAGAATCTCTTCCAAGAAGTCGCTACCC

TTCGTGGATTCAGAAGGGCCACCAAAGCTGGAGAATTCTTCCCTCCCCGAGGAGAACAGC

TCAAGTCTGGTGGGACCTGGCGACCAGAACACTGCTGAAAAAGACCTGCCAAAGCCTGAG

AATCCCCTCCCAGAGGCTGGCTCAAATCCGGTGGTGCCTGGTGATGAAAAGAGTTCTGAA

TGCAATATGTCACAGATTTCAGATGCAGAATTTGTCCAAACTGTGATTGCTGCTCTGAAG

CTGAAAGATGATCCAACCCCGGACTCTATTGGTGCTAGACTGAGGAATCGGCGTAACAAG

GCATGAGGCCTCTCTACCTATCTTTAACTTAT

>Unigene0000148

GCCGGCGGAGGACGGCGCGGTGGCGACGACGGCGGCGCATGGAAGGTGGCGCGGCGGTGC

TCCGGCGGCTCATCGGCGAGATCCAGGGGATTTGGGAGCTCTTTGGCCCTGACACTGTCG

AGCAAAGATGGGTTTTTCTTGACCACACTGACAGCTTGGCGAAGAATGGCTTTTGGGGTC

TTTTCGTGGACGGTAAATCTGAGTTTACAATGATGGAAGAGAACAATTCCCCTCCAAGCA

AGAAATCCAGACGAGAAAAGAACCGTGATAAAGCAATTATTCCTTCGAGTTTGACTTCGT

TGATGGACCAACACATCTGGAAAGAGTTTCCTGAAGATCTTTTCGAAGCTGTTGCTGCAA

GACTCCCTGTATCGACACTCTTCCGATCTCGATTAGTATGCAAGAAGTGGAACTCTTTCC

CGACTTCCAACAGTTTCTCCCAACACTGCGCGAAAATTGTGCATGCCCATCCGTGGTTTT

ACACCATAACGCATGAAGACGTCAACAATGGAGCGATGTACGACCCGTCCCTGAAGAAAT

GGCACCACCCTTCCATCCCCTTCCTGCCGGAGAAGATGATCATTCTGCCTGTTGCTTCGA

CTGGAGGTCTCATTTGTTTCCTGGACATTGGCCATACGAACTTCTATGTCTGCAATCCGC

TCACCAGCTCGTTCAGAGAGCTCCCACCGAGAACAGTCGGGGTCTGGTCGCGCGTAGCAG

TCGGGATGGTTCTGAACGGGGTGAATGCCAGTTATGGTTACAAAATAATCTGGCTTGGAT

CTGATGGGCATTACGAGATATACGACTCCCTAATGAACATCTGGTTGCGTCCTGGTATCG

TACCTCAGACCATCATGCTCCCTCTGTCGCTGAATTTCAGGTCTCAGGCCCTATCTATAG

GGAGCACGCTCTACTTCATGCGTACCGATCCAGACGGTATCGTGTCCTACGATGCGGCCA

GCGGTGCTTGGAAGCAATACTTCATCCCATCACCGCCGCGCCTGACGGACCACTGTCTGG

CGGAATGTGGGGGGCAGGTTATACTCGTGGGGCTGATATCGAAGAATGCTGCCACGTGTG

TAGGTATCTGGGAGTTGCAGAAAATGACTCTCTTGTGGAAGGAGGTTGACAGAATGCCAA

ACATCTGGTGCTTAGATTTTTACGGTAAGCACATACGGATGACCTGCTTGGGCAACCGGG

GGTTGCTCATGCTGTCGTTGAGGTCGATGCGGATGAACCGCCTGGTGACCTATGATGCGG

TGAAGAGGGAGTGGCGGAAGGTGCCTGAATGTGTGCTTCCCCATGGCAGGAAGAGCCGGT

GGATTGCGTGTGGCACAGCGTTCTACCCCTGCCCTACTGCTACAGCATAACCAGTTCCGT

CACTCTATGATTCGGTTTTTGTTGTTATATGGCATGTATGATTATGTGTGTATGGACCTA

GATATGGTGTGATAAACCCCTGAGTATTGTGGACCTGTGGTAATGGTTTTTTACGGAAAT

AATGGTTTTTATGATGTTATATATTGTGGATCTGTGATAATGGTTTT

>Unigene0000149

AATTAAGTCGACAAATTTTCTCGCGCTGTCGATGCTCCTATGCCTTACATGGACCACGAC

TCCACCTTAAAGCCCTAAAATCCTCAAAATTGCCGTAGCGAATCTCGTCGGAGTCCTCCA

AAATCGAGGATTCAATCACTCCTCTCCACGCCGCCGCCATGGATTTCCTCCACGGGGACG

TGAGGCTGAAGGAGGTGCAAAAGCTCGAAGGCCACACCGATCGCGTCTGGAGCCTCGCGT

GGAATCCGACGACCGGAGCCACCGGCGTCCCGCCGATGCTCGCCTCCTGCAGCGGCGACA

AGACCGTCAGGATCTGGCAGCAGGGCCCCGATGGGTCTTGGATCTGCACGTCTGTCTTGG

AAGATACTCATACCCGCACTGTTAGGTCTTGTGCTTGGTCTCCTAGTGGTAAGCTGCTAG

CAACAGCAAGTTTTGATGCCACAACTGCTATATGGGAGTATGTTGGTGGTGATTTTGAAT

GTGTTGCTACCTTGGAGGGCCATGAAAACGAAGTAAAGAGTGTTTCTTGGAGTGCTTCTG

GGTCCTTGCTTGCCACATGTAGTCGTGACAAGTCTGTTTGGATTTGGGAAATGCAACCTG

GTAACGAGTTTGAGTGTGTTTCGGTGTTACAAGGTCACTCACAAGATGTCAAGATGGTGC

AATGGCATCCATTCGAGGATATTCTAGTTTCTGTCAGCTATGACAACTCTATAAAGGTAT

GGGTGGAGGATGGTGATAGTGATGATTGGCATTGTGTACAAACATTAGACGTAACTAGCA

GTGGCCATTCATCAACAGTTTGGGCAATATCCTTCAACTCCAGTGGAGACAGAATGGTTA

CATGCAGTGACGACGTTACTCTGAAAATATGGGACACGAGCAAGAACCCAACTCAAACAA

GTGGTGATGGCAATGGTTGTTGGAGACATCTCTCCACTTTATCTGGATATCACGAGCGAA

CCATATTTTCAGTTAGTTGGTCAAGCGAGGGTGTAATTGCCAGTGGAGCAGCTGATGATG

CTATATGTCTTTTTGTCGAGAATGAAGAAGACAAAATGCTTGATGGCCCATCCTACAAAT

TGCTCTTGAAGGAAGCAAAGGCACACAACATGGACGTCAACACCGTGCGATGGAACCCAA

AGGACCCTAGTCTTCTAGCCTCAGCGAGTGATGATGGGACTATTAAGATCTGGCAACTCT

CCAAAGTGTCACCTTAACTTATGGTTATTATGCAACCAATATTAAAGATGGCGATCCTCA

CTGCAACTTTTCGATACTGTGAAACCAATGTTATAGATGGAGTTCATACGAGTTCATGCA

GAACCAAGCGTAACTGTTGAAATTCTCCAAGTTTCTGTTGTTTTTATTGGATTTTAGAGA

GAACAACATTTGATTTTTATGTTTTCTGGTAGATGAAGTTGACTGATTCTAGTCTATATG

GGACTGCATTATTATGCGTGGTATTAATAGGTATTAATATACAAGACGGACAATTAT

>Unigene0000150

TGGATGCTTCAACGCCCGTCTCCATCGATCCCTCTTTTTCAAAATCTATTCAACAATTAC

ATATGGGCTTCATCATCGTCGTCATCAGCAACCATGGCGACGGCGGCGGGTGGCTCCGGT

GTCGCTGAGAATCCACCCGAGGACACCGTCAGCGGCGGCGGAGGGGGAAGGGGGACGTGG

AAGCACGCCGCCTTCCATGTCGCCACCACTATCGCCACACCGGCGGCCTATGCTCCGCTC

CCCTTTGCGCTGGCCTCTCTCGGCTGGCCCCTAGGTGTTTTCAGTTTGGTTGGTGGGACA

TTTGCCACATGGTATTCTAGTTTATTGATTGCGTCATTGTGGGAATGGAATGGGGAGAAG

CACATCACCTACCGCTTACTTGCAAAGAGCATATTTGGGTCTTTGGGTTATTGGTCTGTG

ACTCTCTTCCAGCAGGTTGCTTCTCTAGGTAATAATATTGCTATTCACATTGCTGCCGGA

AGCAGCCTTAAGGCCGTCTACAAATACTATCATAGCAATGGTACTCTTACGTTGCAAGAT

TTCATTATTTTCTTTGGGATCTTTGAGCTTCTTCTGTCACAGCTCCCCGATATCCATTCC

CTACGATGGGTAAATGCTCTATGCACAGCCAGCACAATTGGGTTTGCTGGCACAACTATT

GGTGCAACCATTTACGATGGGGAAAAGATTGAACGTAAGTCAATTAACTACGGCATGCAT

GGAAGTTCTGCCTCCAAGATATTCAAAGCTTTTAATGCACTAGGGACGATTGCTTTCTCA

TTTGGCGATGCAATGCTTCCAGAAATACAGAACACCGTAGAAAAACCTGCAAAGAAGAAC

ATGTACAAAGGTGTATCAGCCGCATACGGGATCATTACTGTGACCTACTGGCAACTCGCT

TTCAGTGGCTATTGGGCGTTTGGATCCCATGTTCAACCCTACATTCTATCATCCCTCACA

TCCCCTCAATGGACCATAGTCATGGCAAACATATTTGCGGTGATTCAGATATCAGGATGC

TTTCAGATATATTGCAGGCCAACATATGCATGCTTTGAAGAAAAGGCACAATCTGAAAGT

ACAGGCCATAAGCAGCGGATCAACATGCGGTTGCGCAGACTGGCTTTTACCTCAGTCTAC

ATGGCTATGATTACTCTGATTGCTGCAGCCATGCCGTTCTTCGGGGACTTCGTTTCAATA

TGTGGAGCTGTAGGGTTTACCCCTCTGGATTTCGTGTTCCCGGCTCTGGCGTTTCTGAAA

TCGGGGCGAATGCCTAAGAGGCATGGCCGTCGCTTTGCAGTGCAAATTTTGCACCTGACG

ATTGTGAGTGTGTATTCTATAGTAGCAGTGGTGGGTTGCATTGGTGCTGTCAGGTTCATT

GTAATCGATCTCAAGACCTACAAATTCTTCCATGATATGTGAACTAAAGGAAGTCTGTAC

TCGAACCCCATTTGATTCTCAGTAAATCAGTTTAATTCGAAAATTATGTATATTCACATG

AAACCCTGTTGGACTGATGTTTCTGTTCCGTAAACTATCATCTTTGTATATTCACATAAA

AACCCTGGTTGGATTGATGTT

>Unigene0000151

GAAAAGGAGGCATATGCACAACAACCAGGTGGAATCGATGGCTATGGCCAAATCTTCGTT

GTGTCAGATGACCAAAAACTTGACTGGGGAGATATGTTTTTCCTACGTACCCAGCCTCTA

ACAAGTCGGAAAATGCAGTTCTGGCCTACTCACCCTCCCGCATTCAGGGATACACTGGAA

ACGTACACTTTGGAGTTGAAAGAAGTTGCTTACAAGCTCTTCGAGTTAATTGGCAAGAAC

TTAGGAGTCGAATCCAAGAAGTTTGCTGACCGGTTTGAGACGCAATCAGTGAGGATAAAC

TACTACCCACCGTGCCCCCAAGCTGACAAGGTTTTGGGACTCTCGCCGCATTCTGATGCG

GTTGGCATAACTTTACTCCTTCAAATAAATGAAGTGCACGGATTGCAGATCAAGAAGAAT

GGTGCTTGGCTCCCCATCAAACCACTTCCTGGCGCCCTTGTTGTTAACGTTGGCGATGTC

GTTGAGGTATTTAGCAATGGAAAATATAAGAGTATTGAACATAGAGTTGTTATCGACCCG

AAGAAGGAACGGTTATCGGTGGCCACATTCCACAATGTGAGGAATTCGATGGTTGGGCCT

CTGCCAGAGATTGTGAATGGAGGAGAGGATAATTATATGAGCATGAGCCATTCGGAGTTC

ATGAAGATCTACTTCTCTAGTAAACTCGATGGGAAGAACCTTATTGATTATGTGAAGTTG

AAGAAGGGATAAGTGTTGTGAAAATTTTCAGTGATCCAGATTCCTATATGTGTGATAAGG

GATTAATAAGTTTCCATATCTTGGCTGTTCCCAGTTTGTACTGCAATAAATTACTTCAAA

TTAATTCATCAGGGATGTTAAATCATATGTTATGAACTATATGCATGATATATATAGCTG

AATTGAAAATTACTCAC

>Unigene0000152

TTGAGAAGCTGTTGCAGGAGGAGCACATGGAGGCATAGCAGTTCCAGTTGATGTCTTTAA

CTAATGATGATTTCAACCTCAGGAAGGAAGAGGTGGGAAGGTTCATAGTGAGCCAGGCGA

GAGAGGTTGATGAGTTTGAGGGCGAGAGGGAGAAACTGATTATTGCACATGAAGAGAATA

AGGTTCAGCTTTTTCGTAAGTTTCTGGCAGATGAAGTTGAACTTTAGAAGAAGTTTGCTG

>Unigene0000153

CGGCTTAGAGACATTTTAAAGCATTTGAGCCGAGTAGGGCCTTCCACAGTTATTGGAGAG

CAAGAAGCATCAGAGATGATATCCCCAAAGAAAGATGCCAATTGAAGCACAAGATCCTTC

AGCTCAATGCAATATGCAATCAGATCGTTCATACTTCTCACTGCTTCTATGGAATCTCCG

TGGAGTAGCGCCTCGAGGAAGGGAACAAGCTTTTCATGTGGAACCAGACCTACCTATCAA

TGTTGATTTTCCAAGTTTTACAAAAGAGTAAATTCATAGGATCAAAGTGGTTGAAGTGCA

TGGCTAATAC

>Unigene0000154

GTTTTACAAAAGAGTAAATTCATATGACCAAAGTGATTAAAGTGTGCAGCTAATACTCAC

GAGTTGTTCAACCATGGATACAGTGATCGTCTGTCCCAGAAGCATCCGGTCCACATGGCG

GCTGCATCGTTGAGCTTCGTGAAGCTGAACTTCAAGCACCGAGAGGAAACAGCTCTGGGA

ACGTCGCTCACGTCCTGGACTACCACAATGTAAACCAAGCAACCTCTTTTGTCTTGCTCT

>Unigene0000155

TCCCGCCATGTCCGTGGAAAAGCAAGGAGTATAAATAGAAAATTAATTTGGATAATGTTA

TTCAGATTGGAAATTCGACTTCTTAGAGCTAAAAATGGTTTGGTAATAGAGATCTAATTT

AAATATAAGCCTGTGGTCGTAATGGATTTTTGCTCTTTAGATGGACTACCTAGAGAAGTT

TTACAAGGAACAAACTGCAAATATTCACAAGAGTGTAGAAGAGAAAGAAAATGTTTTTG

>Unigene0000156

AGCAGCTAATTTGGCTGAGGAATAAAGTTACTAAACAAGAGTAACGCTCAAAGACACTCG

AAGAATCCTGTGGGGCCGTTGCCCAGAAATTGCGTGAGACCATGGAAGAAAACATGATTG

TGAGTCTTAGAACCAAGACGAATCACGAAGAGAACCAAGAACAGATGCACTATCTAAAGA

AGTTTTACAAGGAACAAACTGCAAATATTCACAAGAGTGTAGAAGAGAAAGAAAATGTTT

TTGAGAAGCTGTTGCAGGA

>Unigene0000157

CTTAGAGACATTTTAAAGCATCTTGAAGTATAAATAGAAACTTAATTTGGATAATGTTAT

TCAGATTGGAAATTCACCTTCTTAGAGCTAAAAATGATTTGGTAATAGTGATCTGATTTA

AATGTAAGTCTGTGGTCGTAATGGATTTTTACTTTTTAGATGCACTATCCAGAGAAGTTT

TACAAGGAACAAACTGCAAATATTCACAAGAGTGTAGAAGAGAAAGAAAATGTTTTTG

>Unigene0000158

GCATATGAGAGAAGACAATTAGCAGCTAATTTGGCTGAGGAATAAAGTTACTAAACAAGA

TCAACACTCAAAGATGCTCAAAAAGTCCTTTGGGGCCATTGCCCAAAAATTGTGTGAGAC

CATGAAAGAAAATATGATTGTGAGGCTTAGAACCAAGACGCAGTAAGAAGAGAACAACGA

ATAGGTAAATACTGTCAATGGAAAACTATTTATCGGCTTAGAGACATTTTAAAGCATCTG

AACCGAGTAGGGCCTTCCATTATTATTGAAGAGCAATAAGCATCAGAGATGATATCCATG

AGGAAAGATGCCAATTGAAGCACATGATCCTTCGACTTAATGCAATATGCAATCAGATCA

TTCGTGCTTCTCACTGCTTCTACGGAATCTCTGTGGAGTAGCTCCTCGAGGAAGGGAAGA

AGGTTTTCATGTGGAACCAGAACTACCTGTCAATATTGATTTTCAAAGCTGTACAAAAGA

GTAAATTCATATGATCAAGGTGGTTGAAGTGCATGGCTAATACTCATGAGTTGTTCAACT

ATGGATACAGTGATCATCTGTCCCAGAAGCGCCATCCGGTCTAGTAAGTTCTCTGCTTCC

CTGAGATTCCCCTCCCCTGTTTTGGCTATGAGTTTGACAGCATCATTTGTGATATGAATT

CCCTCTCCTTCCACAATCTTCGCCAATTTTAGTGTGACTTCTGCATCCTTGAGCTTTATG

AAGCTGAACTTCAGGCACCGCGAGGAAACTGCTCTGGGGATGTGGCTTACATCCCTGACT

ACCACAATGTAAATTAAGCAACCTCTTTTGTCTTGCTCTATTATATCCAGTAGCTGACTC

CATGAATCATCTAGCAGAGACTGACACTCATCTATAATTAAAGCCATCTGATTAAATACA

ATGCTTTGGATGATGATTTTTACGTTATCGAAGCTCGAAGTTCTGTTTCTAGTGTGTAGT

CCTATGTTGTGCTCACTCTGTGAGCAGCCTCTGTAGGTCAAGCACGGTTTAGAAGGTGAT

GTAGAATTACAGTTCAGAGCTCTGGCGAACACCTTGGCAGCTGATGTCTTGCCACTCCCG

CCTTGTCCGTGGAAAAGCAAGAAGTATAAATAGAAACTTAATTTCAATAATGTTATTCAG

ATTGGAAATTCGACTTCTTAGATCTAAAAATAGTTTGGTAATAGAGATCCGATTTAAATA

TAAGCCTGTGGTCGTAATGAATTTTTGCTTTTTAGATACACTATCTAGAGAAGTTTTACA

GGGAACAAACTACAAATGTTCACAAGAGTGTAAAAGAGAGAAGGTTTTTGAGAAGCTGTT

GCAGGAGCGCAACAGTTCCAGTTGAAGTCTTTAACTAATAATGATTTCAACCTCAG

>Unigene0000159

GTCCGTGGAAAAGATACAATGGCGAGATCCTCCTTGTCTGAATGGCATTGTGCAGGGCTT

TGTTGATCATCTTGTGGCCGACCATGTCTTCAAAAACTTTTGGCTTGTATTTTTGGCTCA

GGCCTTTGCGCGACCCATAATGGAGCTGCACGGTTGAACGGCCTGAGCGAGTTGATTCCA

TATCTTTGAGGACTTGTTTTGGCCGATTGATGGTCTGTTGGCTCACAGGATTAATGGGAA

GTCTGTCCTTCCTACAGATCCCTGCTCGCAGTCTTGATAGGTGGCAGGGGACAGTGACAG

TGGCGCTCATCGTGGTAAGATGCCGGTGCAGCAGCTTGCTAGTTGTAGTTGAGACCGGAG

GAGAAGTACATTTTAGCAGCAACTCAGCATCTTCTCATTTTATATTATTGTTACGGAAGA

GAGATTACTTCTTGGTTTTATGACTTACCTCTCATTATAATTCGCCTAATTAGGGATGCA

CCACATAGAAGACAAATACCATATTTAAACTCTATATTAATATATGTTTTCATTGTAGGG

AAGTTGGGAATCACTTTCCTATAGTTTTTCCCACTCAACAAATTACTGAAAACCAATGAA

GCTAAAAGGTGGTGAGTCAACAAAATTCAATTGTTATTAGCATGTATTGTCCAATTGCCA

CTTGACGA

>Unigene0000160

GTAGATTCACAGTTCAGAGCTCTGGCGAACACCTTGGCAGCTGATGTGTTGCCACTCCCG

CCAGGTCCGTGGAAAAGATACAATGGCGAGATCCTCCTTGTCTGAATGGCATTGTGCAGG

GCTTTGTTGATCATCTTGTGGCCGACCATGTCTTCAAAAACTTTCGGCTGGTATTTTTGG

CTCAGGCGCGACCCGTAATGGGGCTGCACGGTTGAACGGCCTGAGAGTTGATTCCATATC

TCTGAGGACTTGTTTTGGCCGATTGGTGGTCTGTCGGTCTGTCGGCTCCCAAGATTAATG

GGAAGTCTGTCCTTCCTACAGATCCCTGCTTGCAGTCTTGATCGGCGGCGGGGGACAGTG

ACAGCGACAGCGACAGCGACGCTCATCGTGCTAAGATGCCGGTGCAGCATCTTGCTAGTT

GTAGTTGAGACCGGAGGAGAAGTACATTTTAGC

>Unigene0000161

CGCAGCAAGAAGAGAATAAAGAAGTGGTAAATACTGCCAAGGGAAAAATATTTATCTGAC

TTAGAGACATTTTAAAGCATCTTGAAGTATTAATAGAAACTTAATTTGGATAATGTTATT

CGGATTGGAAATTCGACTTCTTAGAGCTAAAAATGGTTTGGTAATAGAGATCTGATTTAA

ATATAAGTCTGTGGTCGTAAAGGATTTTTGCTTTTTAGATGCACTATTAGAGAAGTTTTA

CAAGGAACAAACTGCAAATATTCACAAGAGTGTAGAAGAGAAAGAAAATGTTTTTG

>Unigene0000162

AATGTAAATTAAGCAACCTCTTTTGTCTTGCTCTATGATATCCAGTAGCTGATTCCACGA

ATCATCTGGCAACGACTGACACTCTTCTATAATCAAAGCCGTCTGATTGGATGCCGTGTT

ATGGATGAAGGGTTTTATGTTATTGAAGTTTGAGGTTCGGTTTCTTGTCCGCAGTTCTTG

TGCTCACTCTGCGAACAACCTCTGCAGGTCAAGCATGGCTTAGGAGGTGACGTAGATTCA

CAGTTCAGAGCTCT

>Unigene0000163

CTGCCAAGGGAAAAATATTTATCAGCTTAGAGACATTTTAAAGCATCTTGAAGTATAAAT

AAAAAATTAATTTGGATAATATTATTCAGATTGGAAATTCAACTTCTTAGAGCTAAAAAT

GGTTTAGTAATAGATATCTGATTTAAATGTAAGTCTGTGGTCGTAATGGATTTTTGCTTT

TTATATGCACTATCTAGAGAAGTTTTATAAGGAACAAACTGCAAATCTTCACAAGAGTGT

AGAAAAGAAAGAAAAGGTTTTAGAGAAGTTTTATAAGGAACAAACTGCAAATCTTCACAA

GAGTGTAGAAAAGAAAGAAAA

>Unigene0000164

AAGAAAATATGATTGTGAGGCTTAGAACCAAGACACAGCAAGAAGAGAACAAAGAACAGG

TAAATACCGCCAAGGGAAAACTATTTATCGGTTTAGAGACATTTTAAAGCATCTGAGCCA

AGTAGGGCCTTTCACTGTTATTGGAGAGCAAGAAGAATCAGAGATGATATCCCTGAGGAA

AGATGCCAATTGAAGCACATGATCCTTCGACTTAATGCAATATGCAATCAGATCATTCGT

>Unigene0000165

TGGGGCCGTTGCCCAGAAATTGCGTGAGACCATGGAAGAAAACAAGATTGTGAGGCTTAG

AACCAAGACGCAGCAAGAAGAGAATAAAGAATAGGTAAATACTGCCAAATGAAAAATATT

TATCGGCTTAGAGATTAAAGCATCTTGAAGTATAAATAGAAACTTAATTTAGATAATATT

ATTCAAATTGGAAAATTGACTTCTTAGACCTAAAAATGGTTTGGTAATAGAG

>Unigene0000166

AGCCCCTCCCCGGAGCTCGAATTAGGGATTCCATGGACGACCGCGCCATCGCCGACGACG

AGGACAGCGACCTCGTCACCAAAATGACCACCACCACCCTCTCCACCACCCACCAGCGCA

AGGTCCACTCCTACAGCCAGCCTCTCGGCCGCGACGCCTCCGCCCGCCGCTCCGCCCTCC

GCAAACACAGCCTCGATGAGGACCGCTCAGATCCCCCCGCCACCCCCTCCCCCTTCCACG

ACTCTGACGGTCGCTCCTACTCCTACCCCCACTACCACTCCCCCCCTGCCTCTTACCACC

AGTACTCCTCCTCCGTCGAGGACCTCCGTCTCCCCCGCCCTGTCATCCCCCCACACCTCC

GCAAACCCCCCTCCTCCCTTTCTGATAGCGACGGCGGCCTCACGCTCGAGCGCGCCATGT

CCGAGTACGGCGGCGCCCCCGGCACCCTCCCGGAGTTCACCGGCGCCGGAGGCGGCTCCG

GCATCTTCCGCGTTCCCACCCGTGCCGCCATGCACCCTTCCCGTCCCCCAGCGCTCGAGC

TCCGGCCTCACCCGCTCCGCGAAACGCAGATCGGCGCATTCCTCCGCACCATCGCCTCCA

CACGCACCCACCTCTGGGCCGGGCAGGAGTCGGGGCTCCGGTACTGGAATCTCAACGATG

TGTTCGAAGGCTGGTCGCCGCCATCCACGCCCCCTCCTAAGCGGGGCGATGAGGAGAGCG

CACCCTTCTGGGAGTCCGCACGCACACCACCGACCCTCTGTCTTGCTGTTGATGCGGCTA

ATGGGCTTATATGGAGCGGGCACAAGGACGGGAAGATTCGGTCGTGGAAGATGGACCAGG

GGGTGACTCTGCCGCCACTCGCCAACCAGTTCTCGGTCGACTGGAATGGGCATGGTGCTA

ACGAGGCTTTCAAGGAAGGGCTATCGTGGCAGGCACACCGAGCGCCAATTCTCTCCCTCA

TCATCACCTCTTATGGTGATTTATGGTCTGGTTCCGAAGGTGGATCTATTAAGGCTTGGC

CTTGGGAATCCATTGAAAAATCCCTCTCTCTAACTATGGAAGAAAGACATATGGCTGCAT

TGTTAGTTGAGAGGTCATATGTTGACCTTCGGACGCAGGTCACTGTCGGTGGTGTCTGTT

CCTTACCTGCCGTGGATGTGAAGTATTTGTTATCAGATAATTCTAGATCTAAAGTTTGGA

GTGGTGGTAGCCTATCCTTTGCATTATGGGATGCCCGTTCAAAGGAACTTCTGAAGGTAT

TTGGAATAGATGGCCAAGTTGAGACAAAATTAATAGAACCGTCATCGGCACAAGATCCAT

TTGTGGAGGATGAGATGAAAGTCAAGTTTGTTTCCTCATCGAAGAAGGAAAAATCACAAT

TCAACTTCTTTCAACGCTCACGTAATGCTCTGATGGGAGCCGCTGATGCTGTTAGGAGAG

TAGCAGTCAAGGGGACATTTGGAGAAGATAACCGGAGAACAGAGGCTCTGGTAATAGCAA

TGGATGGAATGATTTGGACTGGATGTACAAATGGCTCACTCGTTCAGTGGGATGGAAGTG

GGAATCGACTACAAGAAGTTCAGCATCACCCACATGCCGTTCAATCCATCTGTGCCTTCG

GCACGCGGCTGTGGGTGGGTTATTTAAGTGGTACTGTGCAGGTAATGGATCTTGAGGGAA

ATTTGCTTGGTGGATGGGTTGCACATAGCAGTCCAGTTATAAAAATGGCTGTTGGAGGTT

CCTATATCTTTACTCTGGCTAATCATGGTGGAATCCGCGGATGGAGCCTAACATCACCTG

GACCTATTGACAATATCTTGCGAGCAGAGTTGGCCAGTAAAGAGCTATCATATACCAAGA

TGGAAAATCTCAAAATCATAGTAGGCACTTGGAATGTTGGACAAGAAAGGGCATCTCATG

ATTCACTAATTTCATGGCTGGGTTCCGTAGCATCAGAAGTTGGATTGGTAGTTGTTGGGT

TGCAGGAGGTGGAAATGGGTGCTGGTTTTCTTGCAATGTCTGCGGCAAAAGAAACTGTAG

GGCTCGAAGGTAGCGCTAATGGACAGTGGTGGTTGGATACTATTGGGAAGACCTTAGACG

AAGGATCATCTTTTGAGCGTGTTGGTTCTAGGCAGCTGGCTGGATTGTTTATTGCTGCAT

GGGCTAGGAAGAATCTGAGGCCACATATTGGCGATGTTGATGCAGCTGCAGTAGCATGTG

GATTTGGACGGGCAATTGGCAACAAGGGTGCTGTTGGCTTGAGGATGAGAGTCCATGATC

GGGTACTGTGTTTTGTTAATTGTCATCTGGCTGCACATTTGGAAGCAGTTAGTCGTCGGA

ATGCCGACTTTGATCATATTTACCGTACTATGGCTTTTAGTCGGCCATCCAATGTACAAC

ATTCTGGATCAGGTGGTGGTGCTTCATCTGTTGTCCAGTCACATCGTGGAGCAAATGCGA

CAAGTTCTAAATCCGAAGATGAAAAACCTGAGTTGTCAGAAGCAGACATGGTGGTTTTCC

TGGGTGATTTCAATTACCGACTTTTTGGCATTTCTTACGACGAGGCAAGGGATATGGTTT

CACAAAGATGTTTCGACTGGCTTAGAGAAAAGGACCAGCTCCGAGCAGAAATGAAAGCCG

GAAAGGTATTTCAAGGAATGCGGGAAGGGCAATTCAAATTCCCTCCTACATATAAGTTTG

AAAGACACACCGTAGGTCTAGCAGGATATGATTCCAGTGAGAAGAAGCGGATCCCTGCAT

GGTGTGACCGAATACTATATCGTGACAGCCGTTCTATTTCAGTGGCTGAATGCTCTCTAG

AGTGCCCTGTAGTTTCTTCAATCTTGCAGTACGAAGCTTGCATGGATGTGAATGATAGTG

ACCACAAACCTGTCAGGTGTATGTTCTCTCTTGATATTGCTCATGTAGATGAGTTGATAC

GGAGGCAAGAATTTGGAGAAATTATTGAAACAGATGAGAAAATTCGGTCTTTACTGGACG

AATTTTCCAATGTACCAGAAACAGTTGTTAGCACGAACAATATCATCCTGCAAAACCAGG

ACACTTCGATCCTCCGAATCACTAACAAAAGTGAGAAGTACAAGGCTACTTTTGAAATTA

TTTGTGAAGGCCAATCCACCATCAAGGACGATGGAAGTGCATCTGACTTCTGTCCAAGAG

GTGCCTTTGGGTTTCCTCATTGGCTACAGGTCCAGCCAGCAGCTGGCATCATCAAGCCTG

GCCAAATTATAGAAGTTACTGTGCACCATGAGGATTTTTATACCCAAGAAGAATTTATTG

ACGGAATCCCACAAAATTGGTGGTGTGAAGATACGAGGGACAAGGAAGTAGTGTTATTAG

TAATTGTGATGGGCAGTTACCAAACAGAGAGTAGAAGTCACCGTATCCACGTGCGCCACT

GCTTCTCAGCAAAGGCCTCATGCAGCGAGACGAAATCAACTTCGAAAAAAAAAACCGCCG

GCAAACCATCTACAAAGGTCTGACTTCGAACCATTTAGCAGCTCCTCAGATGTGATGCGT

GACTTGTGTAGAATGCGGCAGATGTGAATGCAAGAATGCAATAGAGATGGAAAAATGTTT

TCTCTATCTGAGGGGATGGAATATAATGTCAGGGAATCTATTGGTATACATTTCCCTTGA

AAACAAGGATCAAAGACTGCCGCTAACATTTTTTCTGTACATACACCCGTCCCCTGCCTG

TCCTGAATCTTACTTTACCCAGATGAAAAATCGAAGTATCCAGTATCGTGTTGTGGAAAT

ATTCTTTAGACATTTGATGGTTAGAATTTTTTTAGTCGGTGAGTACGCTTGTAGTATTAT

CATCAGAAAGAGAGTGCCAATTCAGATGTATTAAGAGTTGAAGGTTTTTGTTCTTTTTGT

GTATTTGCCTTTTTGTAATGTTGAAGGTGTTCTCCTAGTGTACAACATTGTATGTTAAGT

TTTTGTAATACCATCTCTTGTGCAGAATCTGGATAATATTGTTACAGGAAAATATTGCAC

TTAGTTCTTACCTTTAAAGAGTGTTGTACAGTTCGTATTGCTACATTTGACTTTAGTCCT

GTTAGTCACCTCCTTGTTT

>Unigene0000167

GATCTCTAGAGGGAGGAGGAGGAGGAGGAGGAGGAGGCAGTAATGGCGTCCGGACTTCAT

TTAGACCCGGAAACCGCCCTGGAGCTGGTGAAGAAGGGCTCCACACTGCTCCTCCTCGAT

GTTCCCCAGTTTACGCTCTTCGGCATCGACACTCAGGTGTTCTCTGTTGGGCCAAATTTT

AAGGGCATAAAAATGATTCCTCCTGGTCCACATTTTATATATTATAGATCCTCGAACAAG

GAAAGAAAGGAGTTTTCTCCAACAACTGGCTTCTTCATTACTACTCATGTCTCCGAGATT

CTGGTTCGGAAGTGGGACTTAAAAGAGGAGAGATTGGTCGATGTTCCTGAAGATGAGGAA

TATAGGTACAGTGAGGCTGTGAAACATTTTGAGTTTGATCACCAACTCGGACCCTATGCT

CTAGACCACTACAGAGAGTGGAGAAATCTTTCTGATTTCATCACAAAGAGTACCGTTGAA

CGAATCGAACCCATTGGAAGAGAAATCTCAGTAGTCTATGAATCTGGATTATTTGATGAA

GCTCCTAGTACATCCATGGAGAAATCCTTGGTCAGTCAGTTAAACGACAGCAAGTTTTCA

ACGTATGCTGCCGAAAAGCCTCAAAAGCATGGATGCTTTTACACAACTATTCCTCGTGTT

GTCAAGAATAAAGATATGTCCGCAGTAGAGCTTACTGCACTGAATCTTGACAAAACAAAG

TTACTGGAGAACATCCTAATAAAAGTTTATGGAGGTGCAGAAGATCTACTTTTGGGAGAA

CTGCAATTTGCTTTCATTGCTTTCATGATGGGGCAGTCACTTGAAGCCTTCTTACAATGG

AAATCACTTGTTAGCCTTCTTCTTAGCTGTACCGAGGCTCCCCTTCATACAAGAACTCAG

CTCTTCTCAAAGTTTCTCAGAGTCATATATTTCCAGCTGAAACAAGGTTTTCGTAACACC

AGCGGAGAGGGAAACTCTCTTTTCTTAGATGATGCAATGTTCTCTAAGGACGCATTTTTA

TACCAACTTTGCAAGGATTTTTTCTCATTGGTTCTAGAATCCCGAGTTGTTGATGGAGAT

CTGATATCATGGACAAGGAAACTGAAGAGACTCTTTGAAAAATCACTTGGATTGGATTTT

GACGCACCTAAGGATCTTATCTTTGAAGAAGATGACGAGTTCTCTCCTGTCATTGTACCT

TCAAATGAAGCAAGTCTTGACGACATGTGATGATAATCTTAACATTGACGACTGAATTTC

AATGTTAGTAGAAACGTACGTAAAGAAGACAAAAGTGGTGTCTATTTGTATTAGCTAGGA

ACATATTCTTTCATTTGTATGGTAGATGTGAGGCTAAAAACTCTAAAGATCTCATAGTCC

CCATAAACCATTTTGAAAGTTATCTTTTGGGGAACAAACGAAAAACGAACCCCTCTCTTT

TTTTACAAGATTTATCTAGACCATAGCTATTTTATGCCTTGTCTTATAGCATCTTTATGG

CTTGGCATAAACAACTATTTTATGC

>Unigene0000168

ACTCGCCCAGTATGCTCTTGTGTTCCAAGAGCCTTTACGAGGATATCATCGTGGGGCTTT

TGCTGCAAGGAAACACCCTGACTCTGTAAATTTTCCTGCATTATACCATAAAAAAATATT

AGCTAGATAATATCTTTTTTTTTCCAAAATTATGAATCTCTACTTGGAAAAAGTTGCTTA

CAATTATTTTGATCACTTTTTCAGTTTCCGGATGAACAACTTTTCTAACA

>Unigene0000169

GGACTCCTCCCATCCAGACTCCTCTTACCAAGACCCGTCCCACATTTGTGGGAAAACTTA

GAGAAAAATTAACTATGGCGGCATCAAGTTCCGATCCTCCTCCACAAGGTTCTCCTCAAC

CCGATCCTGTGCGGCTATCAAGTAAATCATGCCACAATATTGTGGCCAAGGCTTTGTTAT

ATATGTCTCTCTCAGAGTATGCATACAGTTGCCATGCCACATGGTACATCTCACGTCTCT

GTGAAAGAAGTCATCAAGGATTATCTTCTACCAGTCCCCGTAGAGGATGAAACACATCTT

AGCAAAGTCATCGGAACTTTTATCAGTTGGCCAGATGAATGGATAC

>Unigene0000170

GAAAAAGTGATCAAAATAATTGTAAGCAACTTTTTCCAAGTAGAGATTCATAATTTTGGA

AAAAAAAGATATTATCTAGCTAATATTTTTTTATGGTATAATGCAGGAAAATTTACAGAG

TCAGGGTGTTTCCTTGCAGCAAAAGCCCCACGATGATATCCTCGTAAAGGCTCTTGGGAC

ACAAGAGCATACTGGGCGAGTCAGGGGAATAGGGGGTCATGTTACTCCCACTCAGTACTG

GGGTGAGTCGCGCAAAAGAGTTCGACGATCAAGAGTCGAAGATTTGGCTTCCCAAATTGA

AGAGTTGAGGAATAAATTGGAAAGTCAAACTACTCAGTCCCAGACTCTTCCCACCCAGAC

TCCTCCCATCCAGACTCCTCCTACCAAGACCCGTCCCACGTTTGTGGGAAGACTTAAAGA

AAAATTAACTATGGCGGGATCCAGTTCCGATCCTCCTCCACAAGGTTCTCCTCAACCCG

>Unigene0000171

AATATTTATATATGCAGCAAAAAGGAAGGACGCTGAGGAAAAATATGTCCCTGAACGACT

CCCTCGGCATGTACTATGGGCCGAAGGTAGAAAAAAGGCAGTTATGGAGATTGAGAAGAT

TATTAGAAAAGTTGTTCATCTGGAAACTAATAAAGTGATCAAAATAATTATAAGTAACTT

TTTCCAAGTAGAGATTCATAATTTTGGAAAAAAAGAAAATATCTAGCTAATATTTTTTAT

TGTATAATGCAGGAAAATTTACAGAATCAGGGTGTTTTTCGGCCGCAAAAGCCCCACAAT

A

>Unigene0000172

CTCAAATGCTCCATTCTCCATACAATGGGAATAAAACATAGAGGACACAAGTATGACATT

TATCGTCAGTACTTGAAGAAGCTTCCCGACACCGAGGAGGCATGGTTGGGTGCATGGTCC

AAAATTTCTGATGTATATCCGAAGTTACAAAAGGATGACTTCGTGAAATACATTAAACAA

AGACGCGACAAAGACTTCAAAGCTTATAGTGAGAAGCAGAAAGAAAGAAGAGCTTTGAAC

AAATACCCTCACCACTTGGGGAGGAAAGGATATGCTCGCTTTGAGCAAGAGTTGTTGCAA

AAAAGGAAGGATGCTGGGGAAAAAAATGTCCCTGAACGACTACCTCG

>Unigene0000173

CCGGGCCTTGCATCGTCCATAAACGGGGGACCGACATCCTCCACGATCCCTGGTTCAACA

AGGATACTGCTTTCCCCTTGACAGAAAGAGATCGGCTTGGACTTCGCGGCCTCCTTCCAC

CTCGTGTTATATCATTTGAGCAACAATATGAACGTTTTATGAATTCATATCGGTCGCTGG

AGCACAATACTCGAGGTGAACCTGATAGCTTTGTATCTTTAGCAAAATGGAGGATTCTTA

ATAGGTTGCATGACAGAAATGAGACTTTGTATTACAGAGCCCTCATTGATAATATAAAAA

ATTTCGCACCAATTATATATACTCCTACTGTTGGTTTGGTTTGCCAAAATTATGGAGGCT

TGTACAGACGGCCACGTGGAATGTACTTCAGTGCAAAGGATAAAGGGGAGATGATGTCTA

TGATATACAATTGGCCATCCCATGAGGTAGACATGATAGTGGTGACAGATGGGAGCCGCA

TTCTAGGCCTTGGAGATCTTGGGGTTCAGGGTATTGGAATACCCATTGGGAAACTTGACA

TGTATGTTGCTGCTGCTGGAATCAACCCACGAAGAATACTCCCAGTTATGCTCGATGTTG

GAACCAACAACCAAAAGCTTCTTGAAGATAAACTCTATTTAGGATTAAGACAACCTAGGC

TGGAAGGTGATGATTATTTATCCATTGTTGATGAATTCGTGGAGGCTGCATTTACACGGT

GGCCAAAGGCAATTATTCAGTTTGAAGACTTCCAAATGAAGTGGGCATTTGAAACTCTTC

AGCGGTATCGAAGAAGGTTTTGCATGTTTAATGATGATATACAGGGCACTGCTGGTGTTG

CATTAGCTGGGCTACTTGGAGCTGTACGAGCACAGGGTCGTCCACTGGAAGACTTTGTGA

AACAGAAAATTGTTGTAGTAGGTGCTGGCAGTGCAGGGATAGGGGTGCTTAATATGGCTA

AACAAGCGATCTCAAGAATGCTGAGTTGCAATGAAGGCATGGATACATCAACAAATAACC

AGTTTTGGCTACTAGATAAATATGGTCTTATCACAAAAGAAAGAAAGGATATCGATCCGG

CAGCTGCTCCTTTCGCCAGAGATCCAGAAGATGGACTTAGGGAGGGAGCTGGTCTTGTAG

AAGTGATAAAGAAAGTGAAACCCCATGTGCTTTTAGGGTTATCTGGAGTTGGAGGTATCT

TCACCAAGGAGGTGCTCAAAGCCATGCAAGAATCGGATTCCTCTCGCCCTGCAATATTTG

CAATGTCAAATCCTACGACAAATGCTGAATGCACTTCAGTTGATGCATTTAAATATGCAG

GAGAGAACATAATTTTTGCTAGTGGGAGCCCTTTTGAAAATGTTCCATTAGAAAATGGAA

AAATCGGATATGTCAACCAAGCAAATAATATGTATCTTTTTCCTGGGATCGGTCTAGGTG

CTCTGCTCTCAGGTGCTCGAATTATTTCAGATGGGATGCTTCAGGCAGCATCGGAATGCC

TTGCTTCGTACATCACAGATGAAGAAATCAACAAAGGGATCATTTTCCCTTCTATTTCTA

GTATTAGACATATTACTACCCAAGTTGGAGCGGCTGTTGTCCGTGAAGCTGTCTCTGAAG

AACTTGCCGAAGGACATGGCGATGTTGGCCTTAAAGATCTAATGAACATGTCGAAGGAGG

AAACTGTGGATTATGTTGCTCGGAACATGTGGTACCCTATTTACAGTCCTCTTGTTCATG

AAAAATGAAAATTTCAACGTCCACCTTTTAGTTGTAAGATTAGAAGAGACCAATTATCTA

TTGAGTTTGGGAATCGATCTGCAAGATCTCCCAAGAAATATTTTTAATTAAATTTATTTT

GTATGATGAGGCGAAACATTTTATATTTGTACTAATTGAACTTGAGGCAACATTTATACT

AATCAATCTGCAAGGTCGCCTAATGTAACTTGTGCGCTTACTGAAATACCTGACCATTAT

ATATAATACAATATTATGTTTGAA

>Unigene0000174

GGGTTTTATTATTTATTTTAATATCCCAAACAGGCGCTGTCCCATCCGTTACTTGTTAAT

TCAGTTGAGTTGTCTGGTTCAGTCATGGCACTTAGTCCATGTGACTTTACAACCAAGTCC

TTTCAAGCAGAGCTACATAAATCTGATTTATTGAAAACATCACAATTTAATTGCGACGTA

GAGGTCCACCGATGTAATACACTGAAAATTCTGCTTGCGCCATTAAATCACGATGCAATT

ATGATTTAAGCTGACTTGTGTGTTAACTTTGGCCCATATTCCCCTTTCTATTGATTCTTC

CTTTGGCAAACAAAAAAGAGACTTTTTTTGTGCTCACGTCCATCCTGCTTAATTTTCCAT

CATTTGTATACAATTTTCTCGATTTTTAGGCAATTTTTACGA

>Unigene0000175

TCCCACTCAGTACTGGGGTGAGTCACATAAAAGAGTTCGACGATCAAGAGTTAAAGATTT

GGCTTCCCAAATTAAAGAGTTGAGGAATAAGTTGGAAAGTCAAGCTACTTAGTCCCAGAC

TCTTCCCATCTAGACTCCTCCCACCCAGACTCCTCCCACCAAGACTCATCCCACATTTAT

GGGAAAACTTAGAGAAAAAATGACTATGGTGGGATCTAGTTCTGATCCTCCTCCACAAGG

TTCTCCCAAACCCGTTCCT

>Unigene0000176

AGAGCAAAAGCAAGCAAGCAAGCAAACACCACCAACAATATTCCTCCTCCTCTCCTCGTG

TTGATGCCTACCCGGCTCGCCGGAACTCTCGTCCGGCTCGACGCAAAGTGGAGCCGCCTC

CGCCGCCTCAGCTCCCGCTTCTTCCGCCACTTCTGGACCCGCCTGCTATCCTGCCCTTCA

ATTTATTCTCCCGCCCGTTACCGTCGTCTCAGGTCCTGTTTCGCAGACCCTCAGTTCTCC

GTACTGGACGAGACAGCGACTGCCAAAGTCTCCACAGTTTCAGGTGATGATTCAGACCTG

GTGGCGCTCAAGATCAGCCTCCTCGGCGACCGTCATATCGGCAAGACAAGCTTCATGGTT

AAATATGTAGGAGATTTGGATCAACACGAGGGCCTACACATGACGGGGTTGAATTTGATG

GATAAGATTTTGTTAGTAAAAGGAGCACGGATTGCGATTAGTATTTGGGATGTTGGAGGT

GATGTTCAATATGTCGATCATGTCCCGATCGCCTGCAAAGATGCTTCGGCGATTCTCATC

ATGTTCGATCTCACAGTTCGCTGTACTCTAAATAAGTATGGCTGGATTCCAATTTTGTTC

TCGGTAGAAATTTAAGTTTATTAGCTGCATTTAACTTGTGGATTAAACATTTCAGCGCGA

TAGACTGGTTTCGACGTGCGAGAAAATGGAATAAGACTGCAATTCCGGTTTTGATTGGGA

CGAAATTTGATGATTTTGTTCAGCTACCAATTGACATGCAATTGACAATTGTATATGAGG

CGAGAGCATGCGCGAGGGCAATGCGAGCGACCCTCTTCTTCTCCAGTGCCACCCACAATA

TAAATGTGAACAAGATTTTCAAGTTCATCACTGCCCAGCTATTTAATTTGCCATGGACTG

TAGAGAGAAATTTGACTATTGGAGAGCCTATCATAGATTTTCTCTAGGATTTCTTTTATT

TTCTTTTCTTCTCTGACCTGTCTTGCCAGTGCCAGTACATACGAAGAGCTGTCTTGCATT

TTTCTTTATTTTAGTGGTGCCATAATCACACAGATTATATAGAAGAGAAACTAATCATGT

GAATCTCTTTTTAATTATTT

>Unigene0000177

CCCCTTCCCGACCACAAAGCTTCCCTTATCCATCTCTTTCCCTCTCTCGTTCAACCTCGC

TGCTGCGTTCTCGGCCTCACTGATCTCCCAGCTTCATCTGTATGCAGAGGCTTGTCTGCC

ATTTCTCATTCTAAAGGGAAGGGTATACTCATACAAACAACGGAAATAAAAGGAAAGCAA

AAAAGGAGAGCTTTCTTCAATCTATTGCTTCTCATGCCTCATGCTTTGGCTATGGATTGT

CATACCATCTATGTAACATGTATCGACACTTCCAGGGTGCTTAGTTTGAGAACCCTTCCA

TCAATGTTCCGAGGAAGACTGGAAATGAGCAACACTAGTAGAAAGCTAAATAGTTTTGTG

GTATGCGGTGCCAGAGGCCCAAGACCCAGACAACCTCGTGTTTGGAAAACTCGAAACAAA

ATTGGTACCATTTCCAAGTCACAAAAGCTTGTTCAATGTATAAAGGAACTATCAAATGTC

AAGGAAGAAGTTTATGGAGCTCTCGATTCATTTATTGCCTGGGAGTTAGAGTTTCCTCTC

ATCGTAGTGAAGAAGGCACTGAAAACTCTTGAGAGTGAAAAAGAATGGAAGAGAATAATC

CAGGTTACAAAGTGGATGTTAAGTAAAGGCCAAGGAAAGACCATGGGAAGCTATTACACA

TTATTAAATGCTTTTGCTGAGGATGGAAGACTTGAAGAAGCAGAGGAACTTTGGACGAAG

ATTTTCTCGGCTAATTTGGAAAGTCTACCCCGTACTTTCTTTATAAAGATGATCTCGATA

TACTACAACAACGGCATGCACGAAAAGATGTTTGAGATTTTCGCTGACATGGAAGAACTC

AGCATCCGACCTGACGGCTCTATCGTAAAGAAGGTCGGTGACGTTTTCCAGAAATTGGGC

ATGCTGGACCACTACAAAAGGTTAATGAACAAGTATCCACCTCTGTCCTGGGAATACCGT

TACTACAAGGGAAAGCGTGTCAGGGTTCGCTCGAAGCAGCCAAACAAACAACTGAACGAG

CACATTAAGGAGATAAATGAACCAGAGGAGGAAGCTTGCGTTACACAGCCAAACGAATCA

GAAGACAAAGCTTGCGTTACGCAGCCAAACGAACAACTGAATGATCACATTAAGCAGACA

AATGAACCAGAGGAGGAAGCATGCGTTAGGCAGCAAAACAAATCAGAAGACAAAGCTAGC

GTTACGCAGCCAAACGAACCAGAGGACCTCGAGTTGTCTCGATAAATCTGCCTGGTTGAG

TCCAAACGATTTCTTATGATACAAATAAACAAACGAAATGGCACAGGAGTATCAATGTCC

AGGGAATCGAGAGAAGGACATGGAAATTGTCGAGGGGGCTTCTTGGTCGAGAGGAATGTG

AAAGTATTTGTGCTGTTTATTACTGAGTTATATGTATGCACATAAAATTTCTGAGGCCTC

CATATATCGTTGCTGGATTGTTGGTTTTTCATAAAATCGCCACTAGATATTAGAAGTTTT

ATTTCGGAGAATTATTCGTCG

>Unigene0000178

ACCAACATCCCTTACATGTTCAAGGTCGGCTCAGGTATTTTCAAACTATACTTTTTTTTT

TTTAATGTCGTATGTATTTATTGATTTGTTTGTATTCTAATTTTTTTGATCATAATATTT

GGAATTTGTTTCTTTATTCCATTGTTTGGGCCTGTTGATATTGAGTTGGCAAGGAGATGT

TCCGATTTGGTGGCAGTTCTATAAAATAGAAATGGGAAAAGCTTTCTTTTGCTTGTAATC

CCAAAATTATTAGAAACCGTAATTCGGACATGAGTCTCGGAACACGGCCACACCTTTATT

TAATGCCAGATAACTTAAATCAGAGTGCATGCGTCAATCATGTTAACATTTTACGGGGTG

GTGTGGCCATCTAATTGTCAATGGTGATTATAATGGTACTTCTTAGCTGGAGGCAAACAA

GTTTGTCAGAAAAGTAATATTTATACGCATATCCGATACACAATTTTCAAATCTGATACT

TACGAGATGTTGAATTACTTATACATAAATGATTCTCAAGGTGTTCAATTCTAAATCTTG

ATATTGTTTCAAATTAGAATTGGGCCTACATTTTGTATTCCAACATTTCTATAGGCTAGG

GAGCTTGCATATGTGCACAACTTATATAATCTGTAAACTGATGATGCTACAATCCCCGTA

ATATTGTGCAATCTTAAAAAAGGAGCAGAAGTTTTGCAAAGTTCGAAAGTCTAACAATAT

GCTACCGTTAAGCAAGTTTTGCAGGCAGAAGTAGACAGCCTTTTGTCCAGGCATATCTGT

GTAGAATGTAGTACTCGTGCCGACAAAATATTTTTTATAAGGAGAAGACGTAGTGATTTA

AGAGGGGAATACCAATATTATTGTTTCAAATA

>Unigene0000179

ACCAACATCCCTTACATGTTCAAGGTCGGCTCAGGTATTTTCAAACTATACTTTTTTTTT

TTTAATGTCGTATGTATTTATTGATTTGTTTGTATTCGAATTTATTTATGATAATATTCA

GAATTTGTTCTCTATTCCATTGTTTGGGCTTGGCTTGGAGCTGTTCGGATTTGGTGGCAG

TTCTATGAAAAAGAAATAGGGAAAATTTTCTTTTGCGTGTGATCCCAAAATTATTAGAAA

GCCTAATTCGTACTTGACTCTTGGAACACGGCCCCGCCTTTATTTAATCCCAGATTAGTT

AAATCAGAGTGCCAGCTAATTGTCAGAGGTAGTTATAATGGTACTCTTCAGTTGGAGGCA

AACAAGTTTGTCAGAAAAGTAATATTTATAGGCATATCTGATACGTGTAAGGAGAGTATT

CG

>Unigene0000180

AGACATGCGCCCTTAGCATCTCAATAAATTCTGGTCACTCCGCGGTCTCTCCAAACCGTT

AGCTGCTCCCATTTCAAGGGTTAGGGTTTGGATTCCATCCCCTCCTCCCCCATCCTCACC

ACCGCATCAATGGCGACTGCCGCCATCACCGCCGCCGTCAACTCCATCCCCTCCAAGAAA

GCGGACCTCGAGAAGGCCTTCGACGCCCTCCAATCCCACTCCTCCGCCCTCGCCTCCTTC

ACCCTTAAATGGAAGGACCTCGAGGACCACATCTCCTCTATCGAGCGCTCCATCCAGACA

CGCTTCACCCAGCTCGACTCCAAATCCTCCTCCTCCGCGGTGCAGCCGTCCGATGAGCCG

GAGTCAGCCGCCCGCCCCGAACTCTCCTCTCTCTGCGCCAACATGGACGCCAAGGGCCTC

CGCTCCTTCATCATGGGCAGCTGCGCCACCGAACTCCCCGACATCCGGGCCGAGCTCGCC

GCTGCCCTCAGATCAGCCCCCGACCCCGCGACACTCGTCCTTGATGCCCTTGATGGGGTT

TACCCCCCTAAAGGGGTTGTACGGGAGAGTGATACTAATGAGCTAAGGCTTCGTAGGTTG

TGCATGAATCTATTGGAGAAGCTCCATGCGGTGGCGCCTGAGATCAAGGTTTCGGTTCGG

GAACGGGCGAAGAAGATTGGGAGGGAGTGGAAGGGAAAGATCCCTGTCGGGGAGAGGATT

GGCAGGTCTGACAGGGGGATTGAGGTGCTGTCTTTTTTGCAGTTTGTGGTGACCTATGGG

CTGTCTGCGGAGTTTAAGGCTGATGAGCTGTTGGAGATACTTGTTTCGGTTTCACGGAGA

AAGCAGACTGTTGATACGATTAAGAGTTTGGGGCTTGAGGATAGCGTGCCAGATTTTATT

CAGAAATTGAGCAACAAGGGAAAGCAAATTGATGCTGTCAAATTTGTGTATGCTTTTAAT

CTTTTGGATAAATACCCTCCAGTGGCCCTTTTGAAAGATTATGTCAAAGAGTCAAAAAAG

GCCGCTGAAGAGGTGCGGAAGAGAAAATATAGCGGTAACCAGTCTGAGCATGATGCAGCT

ATGAAAGAGATTTCTGCTCTGAAAGCCGTAGCCAAAGCTGTTGAGGACTACAAGCTAGAG

GCTGAATATCCGTGTGAGAATCTTCGAAAGCGCATTGCTCAGTTAGAGAAGCAGAAGGCA

GATAAAAAGCGGCCTGCTGTCTCTGCTCCTACCTCAGATGTCAAAACTAAGCAGCAACAG

CAGCAGGGGAAACGACTCCGATCATCTTTAACTACCCCAACAGCTGAAGTATCCCATCCC

CTTCAGCCTCGAGATCAATCTACATTCCTGGATTTGAGCAGGTCTTATGGGCTGCATACG

GGCGGTTCTCTCTATGATCGCCCAGTTCCTGCTATATCTGGGATCTCCGATCCACTTGGT

GGAAACCTGGCTCGCCACTATTCATTGGAGCCCCTTGTGAGCTCTGGCTTGTATGATAGG

CTGCCTGGCAGGTACTCCATGTCAGGTCTGCCATCTTCATCTTATTTTGCGGGTTTGCAA

GAAAGGCCAGATGCTGGTTACTCTCTTCCAGCTCACTTGTCTTATGGCTCTGGATTACAT

GAGAAACCATCTGGCGGTTATTCTCTCCAGTATCCATAGTGTGTTCTGCGTGAAATTTTT

CTGTGCCTTGAGGTTCTCTCTTGAAAGTTCAGGAACTGGAAGTGTTTGGTTTTCAGAGTT

AAACTTGGGTAACTTGTCTTATGCCCATATAAAAGGGCTTGTTTTTCTTTTATTTGTAGT

ATTTATTGGGAATAGTTGAATTTACATTTGGCATTTTGTGAGTTTCGGGGATTGTTTCAT

TGGAGTAGAAATGATATTTTTACTCATGTCAAGTTTGCAGTTATGTTCTTTACTTTTGAT

AGATGGTAAATTAT

>Unigene0000181

AAAAAAATATATTGTGTATTAGGTGCAAAATAATTTTTTTATTAATGTACGTATTTTAAG

TGAATGGTAGAGAGAGATATAAAATATATATAATATATAAAGCGAATATAATATTATTTC

TAATATTATCAGTGAGGAATTATTAATCAAAACGAAATAAAAATATGTTTATATTATCGA

GTGTTTGATGTCGCGTGTGTCCTCCAGACAATGATAAAACTAAAGTAGTTTAGAATTTTA

ACCAATATTAACTTATTTAGGCTATAAATAGATCGCACCAAAAAAATAAATATATATATA

TAATATATTAACTAAAGCAAAAAAAAATCCCATCTCATCAGGCGTTTTTTTTATTTGTTT

TGCCAGATGTCTTTCATGTTCAAATATCTATATTGAAAAAAAGATATGTTAATCTTAGTG

TAAGTGTTCCTAACTGACATCACACATACAATTAATACAATATGCTATGCATTTATATAT

ACTAATATATATATATATATATTTATGTATAGTTATTAGCGATTTTAAATTATTTAAAAA

AAAAAATCCCTAAGAGCTTTTATTATTTTGTGTGATTATCTCACAAATGTGATATAATTT

AGTATATCTATATGTGTATATGTTTCCATGATATATACATGTATCGACGAACGGAATGAG

ATTATTATTAATGTATTTCAAGAATAAATGTAGTAGAGGTCTTTATAAAATTTTTCTTAT

GCAAATAAGATAAAAAAAAAATATTTTATTTTAAAATCCAGTCAAAAAAAATATATAAGA

ATTGGCATTAAAACTAATCATTTGTTTTTATAATAATTTTTTAATTTAAATAGGCTAGTG

AAAAATACCTATGTATTGTGATAACGGATTTTGACTTTTTTCCTTAAACGTAAATTTTCA

AATTTCGTAGGCTTTAAGCTCGAGTGCGATGTGAATTTTTTATATAACAGCCTGTTTTTT

CCTCCAAAAATTAATAATTTATAATTCTATAATAATAACTAAACCATTGTAATAAGATAT

ATTTTTATTTTTTTTATTTTTATTTTTTTTTGGTTATTGTAATGTATTTCTGATCTCAGT

GGCACTGCATGAAGTGTAATCTTAACAGTATTTTTTATTTTTTTTTTTTTTAAAGTTAAG

TTTACCTTTGAATTGTGTTAAAAGCAAAATAATAAAATGCTGCGCTGATTTCGAGTAGTA

GTATTGTACTAGTACTACTCAACAAAGCGTCTAAAAGATTATATATTTTTGTTTGTATTT

TACATATATGTATATATATATATATATGTGTGTGTATATATATATTATATTATTATACTT

TTTTTTTTTGTTTCGTTTATACGAAGAATCCCCTTTTTATTGTGGCACAACCTCACCACC

CCAGGCCAGTCTCATATCACTTCATAGCAATTGTCATTTTTTTTACTTTTGTTATGTAGT

AGATAATGAACAAAAAAATTAATGCAAAATATAAAATTTTAAAATGAAAAATAAAAAA

>Unigene0000182

GTAACGAATGAAACCGAACACATTGTTCTTGACAGAGCTGAATGGGGAAATATGATTCAT

GTAACCGACCCCAAATAATTGGGATTGAGGCTTGGTTGGTTTGGTTTGAAATTAAAATAT

ATATTTTCTACAGAGTCCCCTCAGCCCCCTGACTCTCCACCAATTTATTCATTTGAGGAT

ATTTTGGTCAAATTTTGGCGATTGTTCATATAGTCTTTCACGACGTTGAAGAAAGTCACG

TCAATATCTCTATAGTCTGTGTGTGCGTGCGGTAGTGTGTTTTTTTTATTTTTAAGGACG

GGCATTCCTTTCAGTCAAGTTGGACGGGATTTTCCCTTGTTTATACAAATGCATATATAT

ATATATATTTTGGTCATTAATTTTTACAGAAAAATTCG

>Unigene0000183

CGGTGACGGATGCCAGGTTGATTTCGCTGACCTCTCAAACGTTCCTCGTATTTTATTGTA

TGGTGAAACTGAAATTTTAACAGGAAAAATATCAGTTTTATTTTGGAGAATTTTGATTTT

TACTTGTTCTATTTGTGTTAATCCCTTTCTTGATTTTGAAGTTGCTTGGAATATTTTGGA

AAACAGTTTTAGGTTGAAATAGCAAAAACATAATATTACATGTGAAATCAATGTATTCCT

TATTTTTAAACAGACTTTAAAATTATAGCCAGGGATGACACAAAATGACCGAGATCTATG

GGGATTAATGCATAACGAGGACATTTTATACTGGGGCAAAATTAAATTTCTGCATTGGTT

TCGTACAGGGAACTTTTTATCTAGGGCTTATGACATGAAATCAAGATATTTTTTTTAACT

TTAGTAATTAGTGTGGAGTTTCGAATTATGAAATTTTTTATTCTGGAAATTTATGAGCAT

AGTTGAAATGGATTTGAGCTGTTTGTATTTCCAAGAAATTGGTGGTTCTGATTAATAAGA

ATTCCAAATAAATCGGTTCAGGGGATAATCATATAGGGGAATTTTTGCTGGGAATCATTA

AGTAGA

>Unigene0000184

TACACAAAATTTGTCAACAGCTCACAACCCTTCCATAGTGGTTCTCCCAAAAAACAGCCC

CTCTATCACCTCCCTCAATTCAGCTCCGGCCACCGCCATGGGCATCGCCGCCGCTCTGAC

CCCCGATCAACTCGACTCATACAACAAAGACGGGTTTCTGGTCATCGAGTCCTTCTCGAG

TCCCGAGGAGGTCCGGGAGATGCGGGATCGGATGGCCGAGCTGCTGCGGGACTTCGACGG

CAACTCCTCCTCCATCTTCTCCACCAAGAACCAGAAGCAGCATACGGATAACTACTTCTA

TGATAGCGTGGAGAACATCTCATTCTTCTTTGAGGAGAAGGCGTTTGGGGATGATGGGTG

CTTGAAACAGTCGAAGGAACTCTCTATTAACAAAGTTGGCCATGCACTACATGAACTAGA

TCCTGTCTTCAAGAAAGTCTCATATTCAGAGAAAATCTCGAGCATATTTTCTTCGTTGGG

TTACAAGAGGCCTGCAGTAATGCAGTCTATGTATATATTTAAGCAACCAGGAATTGGTGG

AGAAGTCGTACCACACCAAGATAATTCATTTCTCTATACAGAACCTCCGTCATGCACAGG

GTTGTGGCTAGCGCTAGAAGATGCAACCATAACTAATGGTTGCCTTTGGGCCATCCCCGG

TTCCCACAAGAATGGTCTTGTCAGGAGGTTTATTAGAGACGAAGATGGTGTCCACTTTGA

TCGCCCATCACCATCTTATGATCAAAATGAGTTTGTTCCGCTGGAAATGAAAGCAGGCTC

TTTGGTGGTTATACATGGCGACCTTATACATCAAAGCCTTGAAAACCAGTCTCCCAATTC

AAGACATGCTTTGAGCTTGCATGTGGTGGATACCAATGGATATATTTGGTCGAAAGAGAA

CTGGATTCGGAGAAAAACAGATCCAGCACCCCTGTATGTCTCTTGATTCCAGTAACCACT

TCAGCAACCTTTTCCGACGAGGATTCAAGGTTTGCTCAATGGACAAACTATCTTTCCATT

AAGGAGAGTTCAATCACTTTTCTTGGAACTACATGAGCGATCATATATTAAGGAGAGTTC

AATCACTTTTCTTGGAACTACATGAGCGATCATATACTTGTATAAGGGAGCATAAATATT

GAAATGATATCTAACTTTGTATTACTCATCTAACTTTGTATTACTTATGGAAGTGAGCAT

AAGTATTGAAATGATATCTAACTTTGTATTACTTATAGAAGTGAGCATAAATATTACTTA

TTAATGTGAGCATAAATATTGAAATGACATCTAACTTTGTATTACTTATGGAAGTGAGCA

TAA

>Unigene0000185

TTTGTGTTAATCCCTGTCTTGATTTTGACTGGTTCGGGTCGGCCCAACCCGCCTCACCAT

CTCAGGTTGGGGATTTTCAAAAAAAAAAAATCCCTGTCTTGATTTTGAAGTTGTTTGAAA

TATTTTGGAAAACTGTTTTTAAGGTGAAAATAACAACAACAAAAAATTACATGTGAAATC

AATATATTCCTTTTTTTTTTAAACAAACTTCAAAATTATACCCGGGGTTGACACAAAATG

ACGATATCTATGGGAATTAATGCATAACAAGGAATAGTGGGGCTAAATCAAATTTCTGGG

TAACTTTTTATCTAGGATTTATGATATGAAATCCAGAATTTTATTTTTATCTTTAGTAAT

CAGTGTGGAGTTTCGAATTATGAAAATTTTGGTTCCGGAAATTTATGAGAAAAGTTGTAA

TGAATTTGAGATATTTGTATTTCTAAAAATTTGGTTGTTCTCATTAATATGATCTCCAAA

GAAAATCGTTCATGGGATAATCATGTAGGTGAATTTTTACAGGGAG

>Unigene0000186

CGGTGACGGATGCCAGGTTGATTTCGCTGACCTCTCAAACTTTCCTCGTATTTTATTTTA

TGGTGAAACTGAAATTTTAACAGGAATTTTATCAGTTTTATTTGGAGAATTTTGATTTTT

ACTTGTTTTATTTATGTTAATCTCTATCTTGATTTTAAAGTTGCTTGGAATATTTTGGAA

ATCTGTTTTTAGGTGGAAATGACAAAAAAATTTATATTACATATAAAATCAATGTATTCC

TTTAAAAAAAAAAATAAACTTTAAAATCATACCCGAGGTTGACACAAAATGATTAGATCT

GTGGGGATTAATGC

>Unigene0000187

ATTTATTTAATATACTGGTTCGGGTCGGCCCAACCCGCCTCACCATCTCAGGTTGGGGAT

TTTCAAAAAAAACTTTACGGGTGAATTGCCAATTTAGAGCCTAGTTGGTTCCAAACGCAA

AAGGACCAATAAAATTGGGACACCAATACAAAATCATCTTTTTGTTTAATCACTAAAGCT

TTTTTCCAAAACAAATATCTCAATATAACTACTACTATTTTAGTGATCCGCAAATA

>Unigene0000188

AAAAAAATTTATATTACATATAAAATCAATGTATTCCTTTAAAAAAAAAAATAAACTTTA

AAATCATACCCGAGGTTGACACAAAATGATTAGATCTGTGGGGATTAATGCATAAGGAGG

ACATTTTATAGTGGGTCTCAATCAAATTTCTGCATTGGTTTCTTACAAGCAACTTTTTAT

CTAGGGCTTATGATATAAAATCCAGGATTTTTTTTACACTTTAGTAATCAGTGTGGAGTA

CCTAATTATGAAATTTTTGATTCCGGAAATTGATGAGAAAAGTTGGAATGGATTTGGGCT

ATTTGTATTTCGAAAAAATTGATTGTTCTGATTATAAGAATTCCAAAGAAACCAGTTCAT

GGAATAATCATATAGGTGAATTTTTGCTGGGAGTCATTAAGTAGAGTAAGAAATGAGAGG

AAATACATATATATATATGTATATATATATATATATAAATTAATTAAAGATAATTTTATT

TATGCCCCCTAATTGAGCCCCTATTAACCTCGTTTACATAAGACTAAGAAATATCGCTTG

TACCCCAACTTCATGAAATTTCTCCGTCTCTTACAAAGCATCATTTACTATCAAAATATT

CTTCAACTATAAAAATATGTTGTCCACGCTCAAAATGATTTGTGCTGAAGTATCTAATAT

GTTCTTGAGATTATATCATTTAGTGAGCCCTTATTACCAATATAAATTCCGAATATCATA

ATATGAACTC

>Unigene0000189

ACTGTGGCGCCCTTAAGAGAAGACCCCAAAATAGAATCTGCGCCGGAGAGCCGCCCCTTC

TACGACGCCGCCGGCGAAAGCGGCGGCAGCCAACGAGCAAACGCCCGCACTTCTCTCCGC

CATTCCCCATTAACCAGAAATTCTCCCTCCCTCAGATTCCTCGTACGATTCGATGACCCA

TCCTCCGTCGCGAAGACTCGGCAGCCGTCATGCAAGTAGCAGCACGGAACATCGTGCCGG

AGCTGAGAGGACCACTGTCTCTCTCCAGAGATCTTGCGACGTTTCATTCCACATCCGTTT

CTCTCGCAAAATGGAGGAGCAAATGGAACTACACGGATGAAGGGAGCTTTCAGAAACCAT

CTAAGACTTACATAAGGTACACTGTTCGTCAGAAGCGTGCAGATGCGAAGAAAGCTCTTA

AAAATTTTATTTTCTATGGTAAACCTTCAAAGCTGCACTGTCAGGATGAAGATATTGGCT

GTCACACTGATAGAACCAGTTCCAGTAATTCTGGTAAATCGAAAACTCATAGTTCACCTT

CCTCTGGAAAATATGGTGGAACCAAACGTTCGAAGAATAAATATACTTTCTGTTATGATG

ATGATTATGCGTCTCATGAGACAAATTTTCAAGCTGCTTTTGGTGGCCACAGAGGTTTTA

CATGGTGCTCTTCAGGAGGATTTAATTGGAAATATAAAAGTTGGGAGAGAACACAGAGAA

CGGAATGGAATGAAAGTGATGCTGAGGACAATCCCACTGATACAGTCTTAGATACCCATA

GACGTACGCTGGGTTTATCCTCCACGGGCCCTCTAACTCTAGATGTTGTTAAATGCGCAT

TTCGTGCATCTGCTTTGAAGTGGCACCCTGACAAGCATCAAGGTGATTCACAGGCTGTTG

CAGGGGAGAAATTCAAGCTTTGTGTCGATGCGTACAACGCCCTTCGTGACACCCTCAAAT

CTGCATGAAATATGGGTGGTGATTATAATTTGTCACATTTTTTTATATTATTATTAACAA

TTTTGTCAACACAAACTTTTGTCGAGACCTTATAAGTTATTCTATTTCCACTTTCCTCTT

TGTACTTAGTTTCTGTTCAATTCCAGTTTTGTTTTTGTGGAAAAATTTATTTTGTTTGTG

TTTGAGACAAATCGTGAAT

>Unigene0000190

CGAGTCCTCATTTCAAACAGTTGCTATTTCCCCTGCTCCAAATCCCCAATTCCAGTTCCA

AATTCGATGACAATCGGAACCCTAGCTCCGGCGAACAGCCGCTCCCCCCTACTACCGAAG

CATCACTCCGGCGATGACGCCAAGGAATTCAACGGCGCCTCCTTCTCCGGGGCCGTTTTC

AACCTCTCAACGACGATCGTCGGGGCGGGCATCATGTCGCTGCCGGCGACGATGAAGGTG

CTGGGGCTGGTGCCGGGGATGTTGATGATTGTGTTTATTGCATTACTTGCGGAGAGATCG

ATCGAGATGCTGGTTAGGTTCAGTCGAGCGGCGGAGGCGGTGAGCTACGGCGGGCTGATG

GGGGATGCGTTCGGTAGGTGTGGGAGGATGATGATACAAATCTGTGTGGTGGTAAATAAT

GTTGGAATCATGATCGTCTACATGATTATCATCGGTGATGTGCTTTCGGGGACTTCTTCC

AGTGTTACACATCATTCTGGTATTTTAGAAGGATGGTTTGGAGAGCACTGGTGGAACGGA

CGCTTTTTTGTTCTTCTCGTAACAACCC

>Unigene0000191

AGTCGCCTCGTCAACTGCCCGCCGCGTCCTCATTTCCACTAAGTGCTACAGTCTCGGCTA

CCGTCCGAACGTGTCCGCGTACATCTCCGGTCTCTGCCTTTCCCCACAGCTCCAAATTCG

ATGACAATCGAAACCCCAGCTCCGGCGGACAACAACCGCTCCCCCCTACTACCGAAGCAT

CACTCCGGCGATGACTCCAAGGAATTCACCGGCGCCTCCTTCTCCGGCGCGGTGTTCAAC

CTCTCGACGACGATCGTCGGAGCGGGCATCATGTCGCTGCCGGCTACGATGAAGGTGTTG

GG

>Unigene0000192

TCTTCCTTCCTCTGTTCTTTCCCCAACTTGTAAAACTTCTCCTACCTATAAAATGTCCCC

CATCTCCACCGCCCCATCCCTTCCCCATCTGTCTCTTCCTCTCCGATCCCCACCCCATAT

GGCCCGAATCCTCTCCCAAACCCTAACCCTTCCCCCTCCGCCCTCCCTCCTCGCCTCCCG

CCTCCTCACCCTCCGCCGTCGCTCCCACCGCTCTGACGATCCCTCGCCGCAGTTCATCGA

GATCGATCTCGACGCGGAGCAGGACGTCCCGGGGATCCGTCGCCTCGAGGACGCGATCCA

CGGCATCATCGTACGGAGATCTCAGCCTGATTGGCTGCCCTTCTTGCCTGGAACTTCGTA

CTGGGTGCCGCCCCGGCGGAGGAGAGCGCTGGGGCTTATTGAGCTTGTTGGGAAGCTTGC

ACAGCCGATGGGTGAGGAGGAGGCGATGTCGCTCACTACGCCGCGTGGGTGGCCGTCACT

CGATTACTTCGTTGAAGGAACATTTCCTAATCCTCTGAAAGACAGTAGTAGCACACAAAC

TGATGATGAAGAATGAGATGGAAAAAGAACAGGCGATGAGTCTTTCTGTAGATACTAGAA

GTGGTGCGCTTGACATATGGGAGAGCTGCCCACTTCCCTTTGTATTGTATGTTAACAATG

TTCTTAATGTAGTGCATAATAAGATAGCTGGTCGCTAACTTTCTGTCACTTGTAAATTGA

AAATAAATTCGTGGAATTTATCTGTCTGGGGGATGTTTGCTTGGTTTAAATGTGCTCCTT

ATGAAGGAAACATTCTGGGGCTGTCGAGAAAGGAAGTTATGTTCTTAGTATCGATCTTGA

TGTTACGGTGTATCAGAATGTCATAATGCAGCAGAAAGATGAGTTTTCCACGAGAGGATT

ACTGTGTAGAAGATTAAAATCTGCTTCTACGTGAGCAGAGCCTCGTTATTGAGCTTATTA

ATCGAGAATTGACGGCCTATGATTCATCTTCGATAGGTGCTGGAATTGTTACTGGAAATA

AATATCTCGTCAACTTACACAATGTGGATTCTACTACTCAGCAACAATGTTAAATTTGTC

TCATCATGTATGAATTGTATGAATTGCAATCCTATGTTTGAAGTATCTGTTTGCTGTGCT

AGA

>Unigene0000193

CGGGCACACCACAAATGTGTTTGCGCCCCCAAATGGGTGGCTGCAAATGCGCGGCGGCAC

ATAGAGGTGCCGCCACGGCGGCACCCACACTGGTGGTCTAGGTTTGGATGAAGTTCTGCC

TGTATGGGTCGACAGAGTAGACCTCAAGACGAACATAGCGGAGGCCTTCGCTCGACAAAG

CAAATTTACGTATGGACTCGTGATGGAAGAAATTACCACTCGTGATGAAACTACTACTGT

ATGTACTCATGGTTCTCATCCGTTGCCTAGTGGGGGCCTAAGCATGGATGGATCTCCAA

>Unigene0000194

CGGGCACACCACAAATGTGTTTGCGCCCCCAAATGGGTGGCTGCAAATGCGCGGCGGCAC

ATAGAGGTGCCGCCACGGCGGCACCCACACTGGTGGTCTAGGTTTGGATGAAGTTCTGCC

TGTATGGGTCGACAGAGTAGACCTCAAGACGAACATAGCGGAGGCCTTCGCTCGACAAAG

CAAATTTACGTATGGATTCGTGATGGAAGAAATTACCACCCGTGATGAAACTACTATTGT

ATGTACTCATGGTTCTCGTC

>Unigene0000195

CTCCACTCCCCCCAACAATGTCTCCACCACCCTTCCTCCTCCTACTCCTCCTCCCCGCCC

TCATCTCCACTGCCACCGCCGCCGTCTGCGCCCCCTCCGACCACTCCGCCCTCCTCGCCT

TCCGCGAGGCTCTCTCAGGCCCCAACACCACAGTCTTCTCCACCTGGACTGGCTCCGACT

GTTGCCACCTCTGGCGCGGCGTCACCTGCGACCCCACCAACGGCCGCGTCGGTGACCTCA

CCCTCCGCGGCGAATCCACTGACCCCCTCAGCACCGACCAGTCCAGCATCATGGCCGGCA

AGCTTTCACCGTCCATCTGCCTCCTCGACCGTCTCTCCTCCCTCATTATCTCCGACTGGA

AGCAGATCACCGGGCCCATTCCCGCTTGCATCACCTCGCTCCCCTACCTTCGCATCCTCG

ACCTCGTCGGCAACCACCTCACCGGCCCGCTGCCGGCCGACATCGGTCGACTTTCCCACC

TCACAGTCCTTAACATCGCCGACAATGCAATCTCCGGAAGTATTCCTGCAACTCTTCCAA

ACTTGACCTCCCTCATGCACCTCGACCTCAGCGGCAACCAGATCACCGGCCGGTTCCCGG

CCGGGTTCGGCCTGCTCAGCAAGCTGAGCCGGGTGCTGCTCGCCCGGAATCGGATCACCG

GCGGGATCCCGCTGTCCATCGGGAGGATGGATCGTCTTGCGGATCTGGATCTGTCGGATA

ACCGGATCTACGGGCGGATCCCGTCCGTGCTGGGGCGGATGCGGGTGCTGTCATCCTTAT

ACCTCGACGGGAACCGGATCTCTGGCGGTATTCCTCGGACGCTGCTGACGAATAAGGGGT

TGGGGATTTTGAATGTGAGCAGAAACGCCTTAAATGGCCCGATACCGGATGTGTTCGGGC

CGGATTCGTATTTTATGGTGTTGGACTTGTCGTATAATCGGCTGACGGGTCGGGTGCCGG

CGTCGTTGGGGAAGGCCATGTATGTCGGGCATTTGGATCTGAGCCATAACCATTTGTGTG

GCCCGCTGCCCGGAGGGGAGCCGTTCGACCACCTTGAGGCGCCCTCGTTCGTGGGAAATG

ATTGTCTGTGCGGCGCGCCGCTGCCAGCTTGCAAGTGAGCCGGGTTGCTATAGCTAATAG

CTCTATGGTGGGGTCCATGCAAGCCCGTTACCTGAACTGTATTGTGTAACTGTGTGTGCT

GCTATTTCTTGATTATACGGTAATTATACGAACAAAAGCTATGTACGTGAATTCAGTTTA

GTGTTTACTTCCCAGCAATGTCAGAGTGATTAATCTTCATCAT

>Unigene0000196

ACTCCCTTCCATCCCAAACCCTCTCAGTACTGAGAATCGGAGATGCAGAGATGCTGCAGC

AAGCTCGCCCGCACCCTCTTTTCCTCCTCCTCCAGCCGCCACCTCCCTCCCCTCCCGGCA

AAAATCCACCTCCATGTCTCCCCCACCACTGCCCCATGCATCTCCCGTCTGGGGTTTCGC

CCTCCCCCTTCTCGTCCCCCGGCTGTGTCTCCGGCGATGCAAATGCGACATTTCGCGGCG

AAAGCGAAGGACCGGTCGAGGGCTCCGAGAACGCCAATCGTCTCCAAAGTGAAGAAATAC

AAGATGAAGTGTCCCTC

>Unigene0000197

ACTCCCTTCCATCCCAAACCCTCTCAGTACTGAGAATCGGAGATGCAGAGATGCTGCAGC

AAGCTCGCCCGCACCCTCTTCTCCTCCTCCTCCAGCCGCCACCTCCCTCCCCTCCCGGCA

AAAATCCACCTCCATGTCTCCCCCACCACTGCCCCATGCATCTCCCGTCTGGGGTTTCGC

CCTCTCCCTTCTCGTCCCCCGACTGTGTTTCCGGCGATGCAAATGCGACATTTCGCGGCG

AAAGCGAAGGACCGGTCGAGGGCTCCGAGAACGCCAATCGTCTCCAAAGTGAAGAAATAC

AAGATGAAGTGTCCCTC

>Unigene0000198

TCCGGCGATGCAAATGCGACATTTCGCGGCGAAAGCGAAGGACCGGTCGAGGGCTCCGAG

AACGCCAATCGTCTCCAAAGTGAAGAAATACAAGATGAAGTGTCCCTCGTCAATGAAGTT

TCGATTTAAGACACTGAAGGACGGGACTATCCGGCGGTGGCGTTCGGGGAAGCGGCATAA

TGCACATTTGAAGTCCAAGATAGCAAAACGAAGACTCAGACTACCTGCACTTGTACATCC

AGCTTATGCCAAGGTGATTAAGAAGCTCAACTTCTGCGGATAGAAATATGAAGATCCTCG

TCACTAGCTCCGCGACTGTGTTGAAGCTCATCGGTTGTGTACCATTTGGAATTTCTGGAT

GTATCATTAGAAGAATATTTTGGTTGTATTAATTGCTGATCTGAAAGAGATTTTTTTTTA

ACCTTTTAAATTTATTTATATTTTCTGCTGCTAGAAGAGAATAAGGAACTGAACAGATTG

ACCAGAAATTTTTTATAGTTCTGGCCAAAAGGGTACTTGAATGGGATATCTTACTTACTT

TTGCTGGGACTTCTATTGCTTGTTTGATAGAATATGGGTATAAAGTTAAATCGAT

>Unigene0000199

CACTGAGAATCAGAGATGCAGAGATGCTGCAGCAAGGTCGCCCGCACCCTCTTCTCCTCC

TCCAGCCGCCACCTCCTTCCCCTCCCGGCAAAAATCCACCTCCATGTCTCCCCCACCACT

GCCCCATGCATCTCCCGTCTGGGGTTTCGCCCTCTCCCTTCTCGTCCCCCGACCGTGTTT

CCGGCGATGCAAATGCGACATTTCGCGGCGAAAGCGAAGGACCGGTCGAGGGCTCCGAGA

ACGCCAATCGTCTCCAAAGTGAAGAAATACAAGATGAAGTGTCCCTC

>Unigene0000200

CCTTGTAACACTTACACTCCCACCACACTGGACACTTCAAGAAAATAAACGTTTTATATC

AAATGCCAAACATTACATCTGGGATGACCCTTATCTATTTAAATACTGCCCAGACCATAT

CTTAAGACGTTGTATTCCTGAATGGGAAGTTGACTCGGTCCTATCATTTTGCCATGAGCA

AGCATGCGGTGGACATTATGGTGGCCAAAAAACAGGCCACAAGATACAACAATCAGGCCT

ATATTGGCCAACTTTATTTCATGATGCCCAATTACATAGTTTAAAGTGTGATAAATGCCA

ACGTCTTGGTAAAAATTACAAGAAGGAATGAGATGCCACTCCAACCCATCTTAGCTGTTG

AAATTTTTGACATTTGGGGCATCGA

>Unigene0000201

TGATGCCCATATTTTCAGCCAGACATGTGATAAGTGCCAACACTTAGGAAAGATAACAAA

ACAGAATGAGATGCCCTTTCATCCTATCCTTGCTGTTGAGATTTTCGACACATGGGGCAT

CGATTTTATGGGTCCCTTTCCAAAGTCCAAATTCAATGAGTACATTCTTGTGGCTGTAGA

CTATGTTTCAAAGTGGGTAGAAGTCCAACCATGTCGCACAAACGACCACAAAGTGGTGAT

CAAATTCCTCAAGAATCACATTTTTGCACGCTAT

>Unigene0000202

ATATTGCCCCGATCAGATCCTTCGGCGTTGTATTCCCAATTGGGAGTATGACTCCGTCCT

AGCGTATTGCCATGCCCATACTTGTGGAGGACATTTTAGCGGACCGAAAAATGCTCATAA

GGTCCTTCAGTCAGGTTTATACTGGCCAACCCTATTTCATGATGCCCATATTTTCAGCCA

GACATGTGATAAGTGCCAACGCTTAGGAAAGATCACAAAGCGGAATGAGATGCCCCTT

>Unigene0000203

TTGCCATGCCCATACTTATGGAGGACATTTTAGCGGACCGAAAACTGCTCATAAAGTCCT

CCAGTCTGGTTTATACTGGCCGACCCTGTTTCATGATGCCCATATTTTCAGCCAGACATG

TGATAAGTGCCAACGCTTAGGAAAGATCACAAAGCGGAATGAGATGCCCCTTCGACCCAT

CCTTGCTGTCGAGATTTTTGACACATGGGGCATCGATTTTATGGGTCCCTTTCCAAAGTC

CAAATTCAATGAGTACATTCTTGTGGCAGTTGACTATGTTTCAAAGTGGGTTGAAGCCCA

ACCATGTAGCACGAACGATCACAAGGTCGTTGTCAAGTTCCTCCAGAATCACATTTTTGC

ACGTTATGGAATACCCCGTACCAT

>Unigene0000204

ACCATCATCAGTGATGGTGGAAAACACTTTTGTAACAAAATATTTGAGGCCCTTCTCAAG

AAAAATGGTATCAGACATAACATAGCAACGCCCTACCACCCACAAACCAGTGGACAAGTA

GAAATCTCCAATAGGGCAATCAAGACCATACTTGAGAAAACTGTAAACCGCTCTCGATCA

GACTGGTCAGATAGACTTTCAGAAGCCTTATGGGCTTATCGCACCGCTTTCAAGACCCTA

CTGGTATGACACCGTACAAGCTTGTTTATGGTAAGGCATGTCATTTACCAGTAGAAATTG

AACATAAGGCATACTGGGCTATTAAAACTCTAAACTTTGACACAGACCAAGCAAAGAAAC

TAAGATTATTACAACTAGACGAGTTAGAGGAAATAAGAGACAATGCTTATGATACTTCTA

GAAAATATAAGGCCAAAATGAAAGCTATACATGATCGTTTCATAAAACCAAAGGAATTTC

AAGTAGATGATTATGTTCTTTTATACGACTCCCGACTTCACATATTCCCAGGAAAATTAA

AATCCAGGTGGACTGGACCTTTCCAAATTACAGGAGTCACAGGACACGGAGCAATCGAAA

TCCAAAATCCATATTCAGGAGAATCATCACTTGTTAATGGCCAAAGACTTAAACCATTTC

ATGGCCCCATTCCGCAACCTGGTCCTCCAAACATTGGGGGCAATGTTTGATTTAAGTATA

GGGGGGTGCTGTCACATCTAGCACATCCCTATATTTAGGTCCTACATATATATATATATA

TATGTATATAAATCAAAAAATCAAAAATAAAAAATATGTCATTTACTTTATCATATTAAA

AAAAAATTCAAAAAAAAGTCTTTCATTTTATAAATCATCAAAAAAAAACCAAAAATATGT

CTTTAATTTTATAAATAGAAAAAAATACCAAAAAGATTTTATTATTTTCACCCATTCCGT

TTTACCAAGTCATCTACTGGACTTGGTATGACTTCAACTTGGTTCACTCCATTAATTTAC

ACACAACCAAGTTCCACATCTCTTATTTCTAGATACTGATATAAGTTTAGAAA

>Unigene0000205

AAAGAGTTCCAAGTTGGCGACCACGTACTTTTATATGACTCACGTTTACACATCTTCCCC

GGGAAACTGAAATCCCGGTGGACCGGACCATTTCATGTCACACAAGTCACTGGACACGGA

GCCATCGAAATAGAGGACCCACAAACTGGTCACAACTCCCTTGTAAATGGACAACGCCTG

AAACACTATCATACAAATCAACCTGACGCCATTGCTCCGAACACTGAGGACAGTGTTCCA

TCTTAAGTGTAGAGGGGTGTCGTTACACTCACACCACTTTGTTTATAACCACCTTTTTGA

AAAAAAAAATATAA

>Unigene0000206

CCCATATTTTCGCCCGTTTCGGTATGCCTCGGACCATCATCAGCGATGGTGGCAAACATT

TCTGTAACCGAACATTTGAGGCACTTATGAAGAAGAATGGTATACGCCATAAAGTCGCCA

CGCCCTACCATCCGCAGACTAGCGGACAAGTAGAAATTTCCAATAGGGCGATCAAGACCA

TTTTAGAGAAGACCGTGAACCGCTCCCGATCGGATTGGTCGGAACGACTCCCTGAAGCCT

TATGGGCTTACCGAACTGCATTTAGGACCCCGATCGGAATGACACCTTATCGACTAGTCT

ATGGCAAAGCTTGCCATTTACCCGTCGAAATTGAGCACAAGGCCTACTGGGCTATCAAAG

CCTTGAATTATGATGCCACCCAAGCCCGGGAGTTACGTTTACTACAGCTAAGTGAGCTGG

AAGAGTTGCGAGATGACGCCTACGACTGCTCGAGGCGTTAC

>Unigene0000207

ATATATATATATATATATATGTATATTTTATGCATAAATTTAGAAGTAAATGAGTAAGTC

GCACACCTATGCCATATACGATGTAATATACATGTTATATCCGATTTCGATCCAATAATG

ATATACCTATATAAAATATGTGAAACTTGATAATAATATTGACGAGATTTGTATACAATC

TTTGTCTAGTAACAGTGGCAATTCAATACCATATCAATGCTAAACATCTGAAACTAATGT

TTAGCAATTTATTATGGCAATAATTAGCGTATAGTAGTATACACGATTTTTTTTTTTTAT

AATATATAAATATATATATATATATGTATAATTATACATACATTTTATATATACATAATA

TTATAAACGTATGGTACCAATAAAAATAAATAAATAAA

>Unigene0000208

AGCACACACGAATAGCACCCGCTCGCATACTCCTTCACCGAAAACCCTCTACCATCTCAG

CCGTCCGTCAGAAGTCTCCCTCGTGATCCACCGTACCCGGCCTCCATCTTTCCACAAAAT

TCCCCAATCTCACCCGAGATCTAATCCACCGCCGAGAGAGAACCCCATAGCCTACGCTTT

TCACACCCCCTCGCCGCCGCCAATGTCCGACCGAGGCGGCGGTCCCGCCCACCTCGACGG

TGCCTTCCTCCTCCAGCTCCTCCGACGCCCCCAGCCTGCCACCCCCTCTCCGCCGCCGCA

GCCCCACCCCGACCCCGCCGTCACCGCCGTTGGCAACAGCTACGCCTTCCCTCCCCACAG

CTTCCGCCCTCCCCCTCCGCCGTCGGAGCCCCACCACTTCCAACCCCCTCACTCCATCGT

CCGACCCGCTTTCGCGCCCTTCGTTCCCGACTTCAGCGTCGCCCCCCCAGACTCAGCGCT

CGGCGTTAGGGCTGAGAACCCACCTCATAACTTCCCCGGCTCCCGCCCCTCGTCCAGTGA

TTTGAGGTTTTCACAGAGAGATAGTTTTAGGGGTCCTGGGGCTTATGAAGTCCGCCGAGA

GAGTGCTAGAGTCGAGAATTTACCGAAGAGAGATGGTTTAAGGGATCCCAGACTTGATTA

TGGTGTCCGGCGAGAGAATGCTAGAGTCGAGAATTTACCGCAGAGAGATGGTTTTAGGGG

TCCCAGAACTGATTACGATGTCCGACGAGAGAATGCTAGAGTCGAAAATATACCACAGAG

AGACTGTTTCAGGGGTTTCGGAGCCGATCACGATGTCCGACGTGAGAATGCTAGAGTTGA

TAATTTTCCCCAGAACTTTCAGGGTGATCTCAGGGGTTTAGGTTTGGGAGAGAGGATTCC

ACATAATGGAGCCGGGCGGTGGAAGGCAATGGCGGGGAACGGCGTTGGAGTGGATAGTCC

TGGGCAATGGGGGAAGGGGAGGAGCCCACATCCTGGACCCATGAGGCAGGAGCCGCAACC

GAGGTTTAGGGCTGCTGAGCAGGGGAGGTCACTGGATTCTTTCCACCGACAGAACGGGCC

GCCTGAGTGGGTTACTTTCAAGCGGCCGGATGAGATGCATAATGGGGAGCAAGTTGAGTA

CTACAGGAGGAAAGAGAATGGGTGGTCTGCACCTTTTGGCTCGGAGCGACATGGTGGTCA

AGGTAGCAGGAGCGAATATCGTCCAACTTTCCAGGCTCATCAGCCAGGAGATGCTCAGAG

CAAGTCAGTGGGATCTGTTGATGACGACAGCCTTAAAGTGACAACAAATGGTTCGGTAGA

AGATTCTATCTCTGGAGGGGAGGTGTCAGAGGAACAGGGTGTGTTGGCTGAGCAGCTAAA

GGATGCTCTTATGCTGAAGGATGGTTCCGGTGAGAAGAAATCAAGGAGGTATAAGGACCT

TAGATCAGATTCTTTCAGAGGAAATCATGTATCAAGTCAGCGAACGAGGATCCATAGGAG

GGAGACAGATTGTCGTTTTGACATCAAAATCTTTGCTTCTAACTTCCTTTCAATTTATAA

ATCTCTGATACCAGCAAAAGAGGAGAAAGAAAAGCAAAAACAACTGTTGCTCACACTGGA

GAAATTAGTTGCAAAAGAATGGCCCAGTGGGCAGCTGCACATTTATGGATCATGTGCTAA

TTCATTTGGGTTTTCGAACAGTGACATTGATATTTGCCTTGCAATTGATGGTATCAGTAT

GAGCAAGTCTGAGATTTTGTTGAAGTTGGCAGATATTTTACAAGCCGATAATCTCGAAAA

TGTACAGGCGCTAATTCATGCAAGGGTCCCTATTGTCAAGATGATGGACCCAGTCACTGG

AATTTCGTGTGACATCTGCATTAATAATCTCTTAGCTGTTGTTAATACCAAACTCCTAAG

GGATTATGCACAGATAGATGAAAGATTACCACAGTTGGCGTTTATTGTGAAGCATTGGGC

CAAGTCACGTGGAGTCAATGAAACATATCGAGGAACCCTTTCTAGTTATGCGTATGTGCT

AATGTGCATTCACTTCTTACAGCTACGCATGCCTGCCATACTTCCTTGTTTACAGGAAAT

GGAGGTGACTTATGATGCGACTGTAGATGATACTAGGTGTGCTTACTTTGACCGAGTTGA

AGAACTCAAGGGTTTCGGGGCAAAAAATAAAGAAAATATTGCATACTTGCTTTGGGCATT

CTTTCATTATTGGGCGTATTATTATGACTACACAGATGATGTTATATCTGTTCGCACTGG

GTGCATTATCAGCAAGCGATCGAAGGGCTGGACGAAGCGGATTCTAAATGACCGTCATCT

CATATGCATAGAGGATCCTTTTGAAACCTCCCATGATCTGGGCCGCGTAGTTGACAAGTT

CACCATCAGAATTCTGAGGGAGGAATTCGAGCGGGCTGCGAATGTGCTACAATACGATCC

AAATCCCACCGTGACTCTCTTTCAACCTTATGTCCCACCTACCCATAACTAGCACCAAAA

TTATATTGTTTATAGGCTAGATTTTATGTTTGCCCGGAGCATGTAAATCGCTCAGTTATG

CCCCTCTATTCAGGTAGGGATTTTATATATAGTTTTGTGGTCAGTATCAGGTTTCTGTTA

GGCAATGTGAAGAAGGCTCTTTGGTAGTGTGATTTGTGACTTGTTTTTATGGACGAAGAT

GATGTTTGTTTATAGCCTGCTTGATCCTGGCAAATACATTTGGGTTGGAAGTTGAAATTG

GAATTTGTAGTAGTTTGTG

>Unigene0000209

CCACAGTAGTGGAGGTCACTGCAGCTCAGCAACTGCTTGTCGCCATGGATCCCCGTCTCG

TTTCCCACCATCAACACCTCCTATCAGCCGTCCGCTCCGGCGATCTCGACCACATCCGCC

TCCTCATCGAGGCCCTTGACGGTGGCGCGGCAGCCGCCGCCGCCGCCGCTCAGACGGAGG

CCGGAGAGACGGTACTCTACATCGCCGCCGAGAACAACCTCGTCAGCGTATTCCGCTACC

TCGCCCCACTCTACGACTTAGACACCGCCTCCATCAGATCCCGCCTCGGTCTCGATGCGT

TCCATGTCGCCGCCAAGCTCGGCCACTCCGAAATCGTGAAGGAATTCCTGATCCTTTGGC

CGGGAATCTGCAAGCTATGTGATTCATCAAACACCAGCCCACTTTACTCAGCTGCCATCA

AGGATCACTTGGATGTGGTGAATTCCATATTGGATGTTGATCCAAGTACGATTAGGATTG

TGAGGAAGAATGGGAAAACTTCATTGCATACAGCTGCTCGGATAGGCTACTATAGGATAG

TCAAAGCCCTTTTGGAGAGGGATCCAGGGATTGTTTCGATTGTAGATAAGAAGGGTCAGA

CTGCGCTCCATATGGCTGTCAAAGGAAAGAATACTATCGTCGTGGAGGAGTTGTTGCTTT

CGGATATTTCTGTACTGAATAACCGTGACAAGAAGGGGAACACTGCATTGCATATAGCTA

CACGAAAGTGGCGACCACAGATGGTGGGCCTTCTACTCAGCTATAAATCGATCGAAGTCA

ATGCGGTGAATAATCAGAAAGAGACTGCGATGGACTTGGCTGAGAAGAACCCATACGGTG

AGGATGCGATGGACATCATGGAATGCCTGACTGATGCCGGTGCAAAGAACGCTAGGAACC

TCGGCAAGGTCGATGAAGCTTCAGAGCTGAGGAGAACTGTGAGTGATATAAAGCATGATG

TCCAATCTCAGCTCATACAGAACGACCAAACCAACAAACGGGTAACCGGTATCGCCAAAG

AGCTAAGAAAACTGCACAGAGAAGCTGTCCAGAACACCATCAACTCAGTGACGCTTGTTG

CAGTTCTGATTGCCTCCATTGCATTTATGGCCATATTCAATTTGCCAGGCCAGTATGTTC

AACAAGGTCCCGGTACTGGTGAGGCTAACATCGCTTCCGATAAAAGGTTTCATGTGTTCT

GCCTCTTGAACGCCACTGCTCTGTTCATATCCCTGGCAGTTGTCGTAGTTCAGATCACTC

TGGTTGCTTGGGAAACCGGTGCTCAGAAACAGGTTGTGTCTGTGGTGAACAAGCTCATGT

GGGTCGCCTGTCTTAGCACTTGTGGGGCTTTTCTGTCGTTAGGGTTTGTGGTTGTCGAGA

AGAATTCGTCCTGGATGGCGACCACAATAACTGTTGTTGGAGTACCGATTATGGTAGGCA

CGCTGCTCACAATGAGCTACTATGTTCTTCGACAACATTTCAAATGTGGCAATGATTCAC

AAAGGCGCATAAGGAGGGCGAACAAGAGCAAGTCGCTCTCATGGTCTCATTACTCGGCTT

ATTCAGACCCTAATGATTTCTCTGAGAGTGAGAAGAAGATATATGGTCTGTGATAACCTT

ATGGAAGTCATGTGTACATAGTATGTAGAGTGCTGCATCTTGCTATGTTGTAAATGTCTG

CTTGCGACTTGTGAATGATGAGGTGGTTGTGGGACCCGGGAGGAACTAGCTTCTAGTTTG

GCTAGAATTAAAGGTATATTTGCTATGCCTCAAAGTGATGATATTTGCTTATTGGCGGAG

AATTTATGTAACTGTGGTAAATTTGTGTAACCTAAGGGCTTCTGAATTTTATGAATTGGA

GTTGCGGATAATAATGTGGCTACTTTGTAATTATTTTTAAAATAGCTTGACTTCGTACAT

TACATGCCCTTTGCCTTGACCTCCCCATATCTGTCAACAT

>Unigene0000210

ACTATTGGGTTTAGTTAGAATCCCTATGATGAGAATATCATGTTTGCACTTCACACAACC

CGACTAAGCAAACAAAGCGTCGGATACAACTACATGAGGAAATGCTAGGGTCCTATGGAT

TTCTACTTCTAATACATTATACTATATTATCACGAAATCATTGATCTAAGGTGGTGCTTG

GCATGTATGTATGTCTCAGTTTCTCCTCTTTTGCGGGTAAACACCAAGGGGACAAACTCT

TTAGTTTGCTGATCAGCCTCTAGAAATAATGATATTGTGGTGCCTCCATTTCTAAACTTG

ACTCTTTGATTTATGGGTAAACACCTCGGATTGCCAACCCAATGTTTTAGTTAGCCCGAA

GCTCAAACAAAAGAGAGCGGGTAAGTCTCCCTTGAAGATTGGGGCACTGCTACAAACTGA

CTAACTATGTCAACTGCGTGGGAAATGTTCGGCTTGTTAATGCACAAAATCGACCTAACT

TGCCAATGAAGTGCTTGTAGCGAATGAGATGGGATTAGGGAGGAGCTCTTGTTCAGTAGG

GCGCAACATGACATTCTGTCGAAGAGTATATGCATCGGAGAGATCAACTCGGTCAAGAAT

GCCACAAATGTAATTTTCTCGTGAGAGAATGTCCTAAGGACCTGAATAGATCTCCAGCCC

AAGAAAGTAGCGCTACCAACAACATTATTGCCTGTGATGATCATGTCATCAACATATAGA

A

>Unigene0000211

GTGCTAGTGCATTTTGTTTACTTCATGATGCAATTATATTCTTTTGTTGGTCCGAACAGA

TATGCAGCCACCACTATGATGGTTGCTCAACATCCATGGTTCAAATTACCACGGTTAAAA

TTGCCACAGTTCTGATGCTAAGGATGAGTATATACTTTCCGCAGACCTTTAGTAAGTTAC

ACTACTGTAGAGATTTGCACCTCAAGAATAATGGCATGTTTCAAAATTTCAGTGTAAACC

TCCCTCTATATCCTTAACCAAGTTTGATTATTAATCGAAGAATCACAGTTCCAATATATT

GTCAAGTTATGAAGTCAACTATCTCTGATATTTAAAAGGAGCTTAGAACTTTAAAGGAGA

TTAAATCCACTATTGGGTTTAGTTAGAATCCCTATGATGAGAATATCATGTTTGCACTTC

ACACAACCCGACTAAGCAAACAAAGCGTCGGTTACAACTGCATGAGGAAATGCTAGG

>Unigene0000212

GATCCGTTCTCACTGCCCCTCGCCCCAAGCAAGGTTGATTCTTTGGCTCCGCAATGCCGA

CAGTGAGTGTCAAGTGGCAGAAGGAGCTATTTCCAGCTGTAGAGATTGACACTAGTCAAC

CTCCTCTCGTATTCAAGTGCCAGTTGTTTGATCTGACTGGTGTGCCTCCTGAAAGGCAAA

AAAATTATGGTTAAAGGTGGTCTGCTGAAGGATGATGCAGATTGGTCAGCCTTGGGTGTT

AAAAATGGTCAAAAGTTAATGATGATGGGCACTGCTGATGAAGCCGTGAAAGCTCCAGAG

AAAGGCCCTGTATTTATGGAAGACCTTCCTGAAGAAGAACAAGTGGTCGCTGTGGGTCAC

AGTGCTGGTCTTTTTAACCTGGGAAACACTTGCTACATGAACTCCACATTACAATGTCTT

CATTCAGTCCCAGAGTTGAAAACAGCCTTACTAAGTTATACAAACTCCAGCAGAAGTAAT

GAAGTAGATCAGAACTCCCATCTTTTGACTGCTGCAACTCGTGATCTTTTTAGCGAGCTT

GATAGAAATGTTAAGCCTGTTGCACCGTTGCAGTTTTTATCGGTTTTGAGGAAAAAATAT

CCTCAGTTTGCACAGCAGCAAAATGGCGTCTATATGCAACAGGATGCCGAAGAATGTTGG

ACCCAACTTATGTATACACTTACTCAATCTCTACAGTCATCAGGATCAAGTGAAGTATCT

AGCTCAGTGAAGACACTATTTGGGATTGACCTTGTCAGCAGGGTCCATTGTGTCGAAAGC

GGAGAAGAGAGTTCTGATACCGAATCTGTATATGCATTAAAATGCCACATCTCACAGGAT

GTGAATCACTTGCATGAGGGGATCAAACATGGTCTGAAATCAGAACTGGAGAAAGTTTCT

CCTGCATTGGGACGCAGTGCAATATATTTAAAAGAGTCACGAATCAGTGACTTACCTAGG

TATTTGACTGTTCAGTTTGTCCGCTTTTTCTGGAAAAGAGAATCAAAACAGAAGGCAAAG

ATCTTGAGGAAAGTAGGGTACCCACTGGAGCTGGATGTTTACGATTTTTGTTCAGATGAA

CTTCGCCAAAAACTTCAAGCTCCTCGCCAGGTTCTAAGAGAACTAGAAAATGCTAAGCTT

GGTTTAAAGCCTCAGGACAAGAATGGCAGTTCTAAGGAAACTGACGTCGTCATGGTTGAA

GCAACGGGATCATCTGCTTCTGGGCAGTCATCTAGTACTACATCTGAAGCTGGTTCATCT

GCGAAAAAAACACAGCTAACTGGAATTTATGACCTGGTTGCCGTTTTGACTCACAAGGGA

AGGAGTGCTGACTCTGGCCATTATGTCGCATGGGTAAAACAAGAAAGTGGGAAATGGGTT

GAGTATGATGACGACAATCCTATTCCACAAAGAGAGGAGGATATCACCAAACTTTCTGGT

GGAGGTGACTGGCACATGGCTTACATCTGTATGTATAAAGCTCGGGTTGTTGATGTTTAA

TGCCATTATTAAGTCAGTTTATACACCATTTATACTTTCGAAGCTTAAAACATAGAGTAT

GATTGATGTTGCTTTTCGTTAGTCCCTCTTCCTATATGTTGGACAAAATGGTGAAGACTT

GGAATACTATTTGATGACTCTGTTTCTAAATTCATAGTTGAAAGAATAGTAGTACTTGAA

CTAGAAGTGATCATGTTGCTTTATTGTGTTTTTCTGCTTCCTTTTAGATTTGGTGAATTG

GACATTCAGTCTGTGCC

>Unigene0000213

GATCCGTTCTCACTGCCCCTCGCCCCAAGCAAGGTTGATTCTTTGGCTCCGCAATGCCGA

CAGTGAGTGTCAAGTGGCAGAAGGAGCTATTTCCAGCTGTAGAGATTGACACTAGTCAAC

CTCCTCTCGTATTCAAGTGCCAGTTGTTTGATCTGACTGGTGTGCCTCCTGAAAGGCAAA

AAAATTATGGTTAAAGGTGGTCTGCTGAAGGATGATGCAGATTGGTCGGCCTTGGGTGTT

AAAAATGGTCAAA

>Unigene0000214

ACGTGTGAACGATAACCGGTCCGGTCCAGAACATTCCGGTCTCACGGACAGTCTCCCTTC

TCCGACCGTCATTATTTCCTCCCCCAAGCCCCCCTGCTCGGTCAAACCACAAACCCTAGG

AATCCTCCTCCTCCTCCCCTCACCGGTTACGATGCCGGAGCTACCGGAAGTCGAAGCCGC

CCGCCGCGCTCTAGAGGACCACTGCGTCGGCAAGCGCATCCTCAAATGCACGGCGGCTGA

CGACACCAAGGTCATCGATGGCGTCTCCCGCGCCGACTTCGAGTCCTCCCTCCCCGGCAA

GTCCATCGTCTCCGTCCACCGCAAGGGAAAGAACCTCTGGATCCGCCTCGACTCCCCTCC

CTTCCCCTCCTTCCAGTTCGGGATGACTGGCGCGTTTTACATCAAGGGCGTGGCGGTAAC

TAAGTACAAGAGGTCGGCGGTGAGCGAATCGGAGGAATGGCCGTCGAAGTTCTCTAAAGT

TTTCTTAGTACTGGATGATGGTCTGGAAGTGTCTTTCACCGATAAAAGACGTTTTGCAAA

AGTTCGCCTGCTTGATGATCCAGAGTCTGCACCCCCGATTTCTGCTCTAGGTCCAGATGC

ATTATATGAACCCATGCAGGTTGATGAACTTGCGGGCTTACTGAGTAAAAAGAAAATACC

AATCAAGGCTCTCTTACTTGACCAGGGTTGTATTTCGGGTATTGGAAATTGGGTTGCTGA

TGAAGTTCTTTACCAGGCAAGAATTCATCCTGAACAAACTGCTGCGACCTTGTCAAAAGA

GAGTTGTGAATCATTGCACAAAGCCATCAAAGAGGTTATTCAACATGCGGTCTCTGTTGA

TGCTGATTCTAGACGTTTTCCAATTGAATGGTTGTTTCACTTTCGGTGGGGAAAAAAGTC

TGGGAAGGTGAATGGGAAAAAGATTGAATTCATTGTTGCAGGAGGCAGAACTACAGCCTA

TGTGCCGGAGTTACAGAAGCTGAGTGGCAACGCAACTGTGAAGGCTGCAACTGCTCGGTC

AAAAAAGCCCAAGAAAAATAATGGTGGTAAAGATGGCTCTGACAGTGAGAAAGAGAAAGA

TGAAGCTCAGACTGCGAAGAAAGGTTCTAAGAAGCCACGAAAGAAGGGTGGGGACAAGCC

AAAAAAACCAGAGAAGGGCACGACAAAAAAATTTGGCAAAAGCAGAGGAAGGACCATCAA

AGAAGAGAGCAAAACCTAACAAGGCAAGCCATGATGATGCTGATGAGGATGAGCGACAAA

TACCAGAAAAAGGCACGGGAAAGAAACAGACGTCTGAGGAAGGACCATCAAAGAAGAGAG

CGAAACCTAGCAAGGCAATTATTGATAATGTTGACAAGGATGAGCCACAAAACCCAGAAG

AGGGCACTGAAAAGAAACATGCAAAGGCAGAGAAAGAACCATCAAAGAAGATAACAAAAC

CAAGCAAGGCCAGTCGTGATGATGTTGATGAGGATAAGCCACAAAACCCAGAGAAGGGCA

CTGGTAAGAAACAGGCGAAGGCAGAAGGGCCATCAAAGAAGATAGCGAAACCTAGCAAGG

CAAGTCATGATGATGTTGATAAGAATGAGCCACAAAAATCAGAAAAAGACACTGGAAAGA

AGCGAGCAAAGGCAGAGGAAGAACCATCAAAGAAGAGTGCAAAATCTAGCAAGGCAAGTC

GGGACGATGGTGTTGAGGATGAGCCACAAAAACCAGGAAAGGGCACAACAAAGAAACATG

AGAAGGCAGAGGAAGAACCTTCAAAGAAGAGGGGAAAGCCTAGCAAGGCAGAGGTAGAAC

CATCAAAGAAGAGTGGAAAATCTAGTAAGGCAGGTGTTCAACAAAAGAAATAGGTTTGGG

CAGGTTATCCTTTTGATGCTTACCCTCCTTTATGTTGGATTCGAGTGAAGGCAGGTTGTA

TTTTGTTTACCAGCCATTTGGTTGTGGGGGAATGTGTGCAGTTTATGTTTACTTTCGAAA

GTGGCCCGGGGGGATCTGAACTTTGTAGTTGGTCCAAACAAAGTTATTTTGATTTCGCTA

TATGCAAAA

>Unigene0000215

GTTTTGGGATTCCACCTCTTCAGTTCCACATTCCTTCGCTCGCGCTGGTAAATTGGAGCA

TTAATTATCCCGGAGATTCGAAAGAGATCCCTAGCTGTTCGTGATATGTTAGACAACCCT

ATCCGTTGGCGCATCCTTTTCTTGTTTGACGTGGTTTTATGTGTCGCAATTATATTTAGC

AAGCATAATGTGAAAAACTTGGGATAGGCCATAATATCTTTATACAAATGAACAATGATT

TGTAGATTTTGACAATTTTTAATGCAGGCAAGAATTCATCCTGAACAAACTGCTGCGACC

TTGTCAAAAGAGAGTTGTGAATCATTGCACAAAGCCATCAAAGAGGTCCTTGAAACAGCA

CTGGAA

>Unigene0000216

TATGATATGTTTTGATTCTTGATACATGATACAATATTTGACCAACAATATGGGATCTGA

TACACGTTTTGACAACTTTGGTGGTAGCCAGCCCGTGCATTATAATTGTGTAAATGGTAC

ATATTTCATACTTACTGATGCGTGCTTCTCATTTTTTGACAGGGAAAAAGATTGAATTCA

TTGTTGCAGGAGGCAGAACTACAGCCTATGTGCCGGAGTTACAGAAGCTGAG

>Unigene0000217

AGTTTGACGGCAATAACTTAAGTTATTGAAAGACTCAGATGGAGGACTACTTTTGTAAAA

AAAAAATTTGTACGTGTCACTACTGGACGTGGATGTTGGCAAGGGGAAATTCGATGAACA

AGAATGGAAGGTATTAGATCGCAAGACGTTGGGTGTCATCCAAGGAGACCTATCTGTGGT

CTTCTTCAACATCGCTAAAGAGACCATGATATTAGGTCATATATAGGCATTGGAGAAGAT

GTACGAGAATCCCTCTGTCGCAAACAAAATTTTTCTGATGAAGAAGTTTTTCATTCT

>Unigene0000218

GCACTGCTGAGTATATTAACCAGTTCATCTCTATACTGGCTCAATTATTATCGGTGAAAA

TTACATTCGACGACGAAGTACAGGCGTTGCTATTTTTGTCAGGTATTTCTGATAGCCGGA

ACAACTTGGTGGTGACTTTGGGAAATTCACATGATAATGAGAAGTTGAGTTTCACAAATA

TGTAGGGTTTTATATTTAGTAAGAAGAGTCAACGGAAGATTGCCGATCAGGATGATAATT

CCGATATTGCACTGCGCGTGGAGGATGATAGAAAAGACAATGAAAGAGGAAGGAGCAAGC

GATGTTCGAAGTCTCACAATCATCGTAGCAATCAAGTGGGCCCTTCAAGAGTGAGTGCTA

TAACTGTAATAAGGTTGGTCACATTGCATGTGAATGTATCGTGCCAAGGTGACCCAGGCA

GGGTGACAACAAAGACAAGAGCTAGAAACAAGAAAATTCCTCAGTTAATGTCACGAAGGC

TGAGAAAGCATATGACACACTCATCCTTTCCTTTCTTTGAGGAGTGACTGTGAGTCATGG

ATTGTTGACTTAGACGCGTCATTCCATGCCATGGGCAATCTGAGCAAGCTGCAAAACTAC

GTTAACGGGAGCTAAGGTAAGGTTTATCTCGGAGATGATAAACAATGCGATATTATCGGC

ATAGGCAATGTACAGCTGCATCTATCCAATGGGACAATTTTGAATATGAAGGATGTGAGG

TATGTGTCAAGCCTAAAACATAACCTCATATCAGTTGGCCAACTTGCGGAGAACGGAATG

CGCACCACTTTCATGAATAATGCGTGGAAGGTCACCAACGACTTGCTGGTCATGGCACAT

AGAAAGAAGGAAAACACTTTGTACGTTACAGCTGTAAACAATGGTACTATGCCATTGGTG

GCTAACAATGTGGGATGCGACAAATGACATTGTCGTCTCGGTTACATGAGTGAGAAGGAG

ATGCATGTGATGTTTGTAGATGATAAACTTCCGGGTCTGAAAAATGTAGACTTAAAGTTT

TGCGATCATTGTATCTATGGCAAGCAACGGAGGGTGAACTTTAGGCGTAGTGACAGATAG

TCGAAGTCGGAGAAGTTAGAGCTTGTGCACACAGATGTTTGGAGTCCCGCCCAGGTACCA

TCCTTAGGCAATGCTCTCTACTACGTTACCTTCATTGATGATTGTACTCGCATGCGGTTC

TCCACTTTTTAAATACAACAAACACCTGATTTGTGTTT

>Unigene0000219

ACCTCTATCAGAAGGACTTGTGCGCCACTACTGGGCGTGGATGTTGGCAAGGGAAAGCTT

GATGAACAAGACTGGAAGATTCTCGATCGCAAGACGTTGGATGCCATCCGAGGATCCATG

TCCGAGAAGGTATTCTTCAACATTGCCAAAGAGACCACGAAGTCAGGTCTTATGGAGGCA

TTGAAGAAAATGTATGAGAAACCCTTAGCTGCAAATAAGGTCTTCTTGATGAAAAAACTT

TTCAGCCTCAAGGAGAGAGGTATGAGCGTTGCCGAGTATATCAACCAGTTTAACTTCGTA

CTGGCTCAATTGTTATCGGTAAAGATTACGTTTGACGATGAAGTACAGACATTGCTGATT

TTGTCGGGTCTTTCTGATAGCTGGAACAACCTGGTGGTGACTTTGGGAAACTCACATAAT

AATGAGAAGTTGAGTTTCACAAATATGTTGGGTTTTATACTCGGCGAGGACAGTCGGTGG

AAGATTGCCGGTGAAGATGATAACTTCGGTGCTGTACTACACGTGGATGATGATAGAAGA

GGAA

>Unigene0000220

ATGAACACGACTGGAAGATTCTCGATTGCAAGGCGTTGGATGCCATCCGAGGATCCCTGT

TCGAGAAGGTATTCTTCAACATTGCCAAAGAGACCACGAAGTCAGGTCTTATGGAGGCAT

TGGAGAAAATGTATGAGAAATCCTTAGCTGCAAATAAGGTCTTCTTGATGAAAAAACTTT

TCAGCCTCAAGAAGGAGAGGTATGAGCGTTGTCGAGTATATCAACCAGTTTAACTTCGTA

CTGGCTCAATTGTTATCGGTAAAGATTACGTTTGACGATGAAGTATAGGCACTGCTGATT

TTGTCGGGTCTTTCTGATAGCTGGAACAGCCTGGTGGTGACTTTGAGAAACTCACATGAT

AATGAGAAGTTGAGTTTCACAAATATGTTGGGTTTTATACTCGGCGAGGAGAGTCGATGG

ATGATTGCCGATGAAGATGATAACTTTGGTGCTGTACTACACGTGGAGGATGATAGAAGA

GGAAGAAACAAACATCGTTCAAAATCGTTCAAAGTCTCGCCATCATC

>Unigene0000221

TTCGATGTTTTTTCGACCGGTTGTTCAACGTCGACCGAGGTTACTGCCCTAGTAGTTCGA

GGGTATGTACTCGCCGGCGACGGCCGCCAAGGTCGACGCCGGCTATAGTCGATCTTCCTC

ATCCGCCCGATGCCTCCGCCGCCGTCGCCTCACCCGTCATCGTCGCCTCCGCCGTCGTCT

CCCCTGTCATCGTCGCCTCCGCCGTAGCCGTTGCAATCGCCATCGCCCTCGCCTCGGCCT

CGGCCTCGGCCTCCTCCCGCCCTTCCAAGCCCGCTCCTTTCACCAACCGCATCCCGAGCT

TTGTCAAGAAAATACTCAAAGCCTGTGGGTTTTCAAGAGGTTCTCGGGAAGGATCGGCTG

GGGTGGAATTGGTGACTGAGAGCAGAGAAGGCCAAACTCAAGGGCCACAAATGCATCTGC

AATCGTCAGAGTCCTCAATTGAGGAGAGCAGGAAGGGCGTGGAGGAAGCAGAGATTCTTC

ACGGGTCTGAGATTGAAAGGCAATTTACAAAATCGTTGGTCGCGGTGGAGGAAATTGAGA

CTGCTCACTGGCCAGAGATTGTTCAGTCCCCAGATTTGGTGATCACGACAGAAGATTCCT

CTGCTGAGGAGAGCAGGGAAGGATTGGCTGGGGGCGAATTGGTGAGGGAGGGCATAGAAG

GCCAAGGGCAAGAAATTCACGCCCAATCGTCAGAGTCAGCTGAGGAGAGCAGGGAGGGTG

TGGACGAAGGGGAGACTGTTCACAGGTCAGAATTTGAATCTCAGTTTTCGGAATTGTCAG

TGGAGACTGCTCACTGGCCAGAGAT

>Unigene0000222

GAGAGAATCCGTGGAGAGGGTGTGTGGGAGCATCCAGCAGCTGGGGATGACAGATGTATG

TTTTACTTTTAACACTGCAGGCACAAGTGAAATCCTTTAAATATCTCTTAATTTCTGGTA

ATACATAAAGCCTGCTCCAGCTGATTCGCCAGATTGAAATGAGAAAAGTGAAAATAACAT

AAAGGGTGAATTATTATTTTTACTATACTTAGGCTGCGTTTGTCCTACTTCTCCTATATT

GGGGCTTTTGGTGGTGTCAACTTAAATAACGCTACAGCTGATCACTTCCCACACATCCGG

CTTTTCTCTGCCCCCTAACATAGATATTTTAGATGGAAAGGGGAAGCCACAAGTTCAGTT

TCTCAGCTAAAACATGACGGAGGGCAGAGAGAAGCTGAGCTTTTGGAGAATATCATGTTT

ATTTGATAACTAGTTTAACGAATTCAAAACCCGGTTGAAGTATTGGGGAAGAGATTTTAC

CTACGTAAATCTAGAAAACATTTCCAAGTTTGTATATATGTCTCTGTGCAGTTCCTTGAC

GTGGCCACGATGGTCTCTCTCACCGTGTACGCTAGAAAAGGCCTGTGCTACCTAATGTGT

GACCAACCCGTGCCATCCTACGAGATGCAATGCAAGCACAATGTGTTTATCCTGAGTGGC

CGACAGTCGACAGTCTTCCACATGCAAGCAGTCGTCCTTACCAAGCTCAATATAAATAAG

GATGCCGCTGATATGTTGAATGAGGTAGTTCTCAATATGCAAAATGATGTCGCTGATATG

TTGACTCAGACAGCAAAGCTAGGAGTTTGAGGCTGATATGTTGAATGAGATACTTCTCAA

TATGAGAAGTGATGCCGCTGATATGCTGACTCAGGCAGCAAAGCTAGGAGTTTGGAGGCA

GAAAGAGGGAAAGAAGTGTTGCTGTATCCATTCTGAAGAGTTCTCAGATAGTGATTAATA

GGCCTTTGTGCATATGTAAAATAGAGAAACCACTCTGGTTATGTTATACACTTTGTTTGC

GCTTTATTGGTCTATTCTTTTTATTTGGTGTGCATGAGAAACAATTGCAACTCACAGTGG

GTGGTGGGGACCTTTTTT

>Unigene0000223

CCAGGAGTGGACACAGCAAATGAGAGATATGCTAGAAGCAAGGAAGCGTGGAGACTTTGC

TTTTCGTGAAAAAGACTTCAAGGCTTCAATTGATTGTTATTCTCAGTTCCTTGACGTGGG

CACGATGGTCTCTCCTACCGTGTACGCGAGAAGAAGTCTGTGCCACCTAATGTGTGACCA

ACCCGATGCCGCCCTACGAGATGCAATGCAAGCACAATGTGTTTATCCTGAGTGGCCGAC

AGCCTTCTACATGCAAGCAGTTGCCCTTGCCAAGCTCAATATGCATAGTGATGCCGCTGA

TATGTTGAATGAGGCAGCAGGGCTAGAAGAGAGGAGGCAGAAAGGGGGAAAGAGTTCTTA

GAGCATTGTGAAGTGTTGCTGTATCTATTTTCATGTGTTCGTCAAAGGTTGAGGGAGTGT

AATATTTGTGATTAATAGGCCTTTGTGCATATGTAAAATAGAGAAATCACCTTGGTTATA

TTATACACTTTGTTTGCGCTTTATTGGTCTATTCTTTTTATTTGGTGTGCATGAGAAACA

ATTGCAACTCACAGTGGGTGGTGGGGACCTTTTTT

>Unigene0000224

GTGAAAGTCCCGGCAGTCGATTAGATCCCCCTTTTTCAGCACACGGGCGGCACCTCCACC

AGCTCATCAATCGTCGCCGGAGATCCACCGATGGGGAGCTGCTGCTCCACCTCCCGGCCG

GAGACGCCGCCGGAGAAGCAGAAGCTTCGCAACTCCGCCGGTCATCACCAGCAGCAGAGC

CAGCGCCCCTCATTCTCGATGCGCAACCAGCCGAGCCCCGCCGCCGTCAGCGGTGAGGTC

CCGGCGTTCTCCGAGTTCTCGCTCGCTGAGCTGCGTGCGGCGACGGAGGGGTTCAGCTCC

GAGAACATCGTGTCGGAGAGCGGGGAGAAGGCGCCGAATCTCGTCTACAAGGGGCGGCTT

CAGAATCGGCGGTGGATCGCCGTCAAGAAGTTCACTAAGCTGGCCTGGCCTGATACGAAG

CAATTCGCTGATGAGGCGATCGGGGTTGGGAAGTTGCGGCACCGGCGGCTGGCGAATTTG

ATCGGGTACTGCTGTGACGGGGATGAGCGGCTACTTGTGGCTGAGTATATGCCGAATGAC

ACGCTTGCCAAGCATCTCTTCCACTGGGAAAATCAAACTATCGAGTGGGCTATGAGGTTA

AGGGTTGCATTCTACATTGCTGAAGCCTTGGAATACTGTAGCACTGAGGGGAGGCCTTTG

TACCATGACCTAAATGCATATAGGGTTCTTTTTGATGAGGATGGTGATCCACGGCTTTCT

TGTTTTGGATTTATGAAAAATAGCAGGGATGGAAAAAGTTACAGCACAAACTTAGCTTAT

ACGCCCCCAGAGTATTTAAGAAATGGAAGAGTTACCCCAGAAAGTGTCATATTCAGCTTT

GGTACTGTCCTTTTGGATTTGCTTAGTGGAAAGCATATTCCTCCAAGTCATGCTCTTGAT

ATGATAAGAGGTAAAAACATTCCGCTCCTGATGGATTCACATTTGGAGGGGAAATTCTCA

ACGGAAGAGGCAACCGCACTAGTGGACCTTGCTTCTCGCTGTTTGCAATATGAACCTAGG

GAACGACCAAACACAAAGAACCTTGTTGCTACGCTCGCGCCATTGCAAGCTAAATCAGAG

GTGCCATCCTATGTGATGTTGGGCATTCCGAAGCACGAAGAAGCGCCTCCTACTCCACAA

CACCCACTCTCCCAACTGGGCGAAGCATGTTCCAGGATGGACCTCACCGCCATTCATCAA

ATTTTAGTAACGACTCACTACCGAGACGATGAAGGAACCAACGAGCTGTCATTCCAGGAG

TGGACACAGCAAATG

>Unigene0000225

AAAAGATTCGAACTTTCCCCCTTCTCTCCCAAACCCCAACTCATCAATAATGGCAGCATA

AACATCTCACACAAGACAGCATCCAGAGAGAGAAAGCGTGGCCAGGTGGTAGTGGGGATG

GGCGGCTGCTGCTCCTCCAACCCATCCAAGCAACCCCACCACCAAAACCACCACCAGCCA

ACGCACCGCCTCTCTTTCTCCCTCCACCAGCCGGCAACCCCCGCCGGCGGCGGCGACGTT

CCGGCCTTCTGCGAGTTCCCGCTGTCGGAGCTGCGGGCAGCCACAAAGGGGTTCAGCTCC

GAGAGCATCGTGTCGGAGAGCGGGGAGCGGGCGCCCAATCTCGTCTACAAGGGTCAGTTG

CAGAATCGGTGGATTGCTGTCAAGAAGTTCACAAAGTTGGCTTGGCCCGATGCGAAGCAG

TTCACGGAGGAGGCGCGGGGGCTCGGGAAGTTGAGGCATAGGAGGTTGGCGAATTTGATT

GGTTATTGTTGTGATGGGGATGAGCGGCTTCTTGTTGCAGAGTATATGCCTAATGATACA

CTTGCCAAGCATTTGTTCCATTGGGAAAAGCAAACTATTGAGTGGGTTATGAGGTTAAGG

GTTGCTTTCTACATTGCTGAAGCTTTGGAATATTGCAGCAATGAGGGATGGCCTCTGTAT

CACGATCTAAATGCATACAGGGTTCTCTTTGACGAGAATGGTGATCCACGCCTTTCTTGT

TTCGGTCTCATGAAGAACAGCAGGGACGGAAAAAGTTATAGCACAAATCTTGCATATACA

CCTCCAGAGTATTTAAGAAATGGGAGGGTTACACCTGAAAGTGTGATATTTAGCTTTGGT

ACCGTCCTCTTGGATCTTCTCAGTGGAAAGCATATTCCTCCTAGCCATGCTCTTGATATG

ATAAGGGGTAAAAACATTCTACTCTTGATGGATTCACATTTAGAGGGAAACTTCTCTACA

GAAGAGGCAACTGCACTTGTGGAACTTGCTTCTCGATGTTTACAGTACGAACCAAGGGAC

CGGCCCGATATGAAGAACCTGGTTGCAACAGTAGAACCATTGCAAACTAAAGCAGAGGTG

CCGTCTCATGTAATGCTGGGCATTCCGAAGCATGATGAAGCCCCTCCAACTCCACAGCGC

CCTCTCTCCCCTATGGCTGAAGCCTGTTCCCGAATGGACCTCACTGCCATTCATCAAATT

TTGCTAGCAGCACACTACAGAGACGATGAAGGAACTAATGAGTTGTCGTTCCAGGAGTGG

ACACAGCAAATGAGGGATATGCTGGAAGCAAGGAAGCGCGGGGACGCTGCTTTTCGTGAA

AAAGATTTTAAGATGGCTATTGATAATTTTTCACAGTTCCTAGATGTGGGTACAATGGTC

TCTCCTACTGTATATGCCAGAAGAAGCCTATGCCACTTAATGTGCGATCAACCGGATGCT

GCTCTGCGCGATGCAATGCAAGCGCAATGCGTCTATCCAGAGTGGCCAACTGCGTTCTAT

ATGCAAGCCGCTGCGCTCGCCAAGCTCAGCATGCATAGTGATGCCGCAGATATGCTGAAA

GAAGCTGCGGAACTGGAAGAGAAGAACCAAAAAGGTGGAAAGGGTTCCTAGCATTCCATT

ATCCTCCTGATTTTTTTCTTGCTTTAATGAATTGTAATATTTTTAGGGCTATTGCAAAAT

GTGAAATATGATATTTTGGTTATATTAATGTATTCAGTTTGGCAATTGTTGATTCATATT

TTCCTTGCAGTTTATGAAACAAAAATTACAAAAACATGGGTTGTAACAGAAAAAATATGT

ACCTTTTTTTATTCTATTCTGGGTTGAACTAGAATCCTAATATCTTCTCCTGTAGGCACA

ATGTTACTTGAGGAGTTTAATAATATTCAAGAGTA

>Unigene0000226

GCAGCATACATCCGCAGCATTCTAATGGGAGTCGGTGTAGGGCTCTGGATCCGTCCCTCG

TCGTCGCCCACCGGAACCGTCCGACCGGATCCCTCGCCCTCTAGAGGAGAGGGATCGCGC

GCCGATTTTCGCCGTATAGACCTTCGATTGTCAGGGATTATTGAGCCGATTCCATCACCG

TGACTCCGATTTGAGATCTGTTAATTTCAATTCCGATCGGAGCTTATCCCACACGGGATC

GGATTCGATAGTCATGGATTTGGTGGCTAGTTGCAAGAGCAAGTTGGCCTACTTTAGAAT

AAAGGAGCTTAAAGATGTCCTCTCCCAACTTGGTCTAGCAAAGCAAGGGAAGAAGCAGGA

CCTTGTGGACAGGATTCTAGCTTTGGTTTCTGATGAACAAGCTTCCAAATCCCATGCTTG

GGGAAAGAAAAATTGTATAGGTAAGGAAGGAGTTGCCAAGATTATTGATGACACCTATAG

AAAAATGCAAGCTCCTGGAGCTACAGACTTAGCATCAAAAAGCCACAGCGGCTCCGATTT

AAATCTCGTGAAGCTTAAAGACGAAGTGGCTGATTCCTATCAGATGGATACGAAGGTTCG

GTGCCCCTGTGGTAGCTCATTGATTACAGAATCAATGATTAAGTGTGAAGACCCTTTATG

TCAAGTGTGGCAGCATATTGGCTGTGTTATTATCCCGGAGAATCCTGTCGAGGGTGTTCC

GCCTGAAATTCCTTCTCATTTTTATTGTGAAATTTGTCGAATAAAAAGGGCAGATCCATT

TTGGGTGACATATGGACATCCACTACTACCTCTTAAGATCACATCGCCTGGTCCTGCAGC

AGATGGAACCACTACTCTACAGAATGTGGATCGAACATTTCCACTGTCAAGAGCCGACCG

AGAAATGTTACAAAGAGCTGAATATGATCTACAGGTTTGGTGTATCCTTTTAAATGATAA

AGTGCCATTTAGGATGCAGTGGCCACAACATGCAGATCTGCAAGTCAATGGTGTCCCAAT

TCGAATTACTAATAGGCCAGGTTCTCAGTTGTTAGGATTCCATGGTCGGGACGATGGCCC

AGTAATCACGACATGCAGCCGGGAAGGAATTAACAAGATTTTATTATCAAGGATTGATGC

TCGAGTATTTTGTTTTGGGATCAGAATTGCAAAGAGGCGGACACTCCAACAGGTTCTCGG

CTTAATTCCGAAGGAGGTGGATGGTGAGCGCCTTGAGGATGCTCTAGCACGCATTCATCG

TTGCATTGGTGGTGGAGCTACGACAGAGAATGCAGACAGTGATAGTGACTTAGAAGTTGT

AGCTGATTCCGTCACCGTCAATCTCCGCTGCCCTATGAGTGGATCTAGAATGAGGATTGC

GGGCAGGTTCAAGCCTTGCCCCCACATGGGTTGTTTTGATTTAGAAGCTTTTGTCGAACT

TAACCAGAGGTCTCGGAAGTGGCAATGCCCAATTTGCCTGATGAACTACTCTGTGGAGAA

CGTTATCATTGATCCATATTTCAATCGCATAACTTCCATGATGCTAAAATGTAATGAAGA

TGTAACCGAAGTCGATATCAAACCCGATGGTTGTTGGCGCGCAAAGAATGAAGCCGAATT

CAGGGAGCTCGCTCAGTGGCATTTACCTGATGGTTCCCTCTGTTCGTCATCCGGTCCAAG

TGTGAAACCCGAGTTGGAGCCGTTGAGACTGGTTAAACAAGAAGCTACCTCTGAAGGCCA

CACGAGTTTGAAGATCGGAATCAAGAGAAACCGAAATGGAACATGGGTGGTTAGTAAACC

TGACGACCGGAGGCCTCGGTCTTCTGGAAATCCTGGGCTTGAAAGATTTGGAAGTCAGTG

TCCTGACATCATTGTTCCCATGAGCAGCAGCGCCACTGGGAGTTACCGAGATGGGGAAGA

CCCGAGTGTTAACCAAGACGGAGGGCTTATTGATCTCTCTGTACACAATGGCCATGAGCT

TGATTCTCTCCCTTTGAACTTTGAACCAATTTACGGTGTTGCGAATAGAGCTCTGGCACC

GTCAAAAGATGCAGATGTTATTGTTCTCAGCGACACTGAAGATGACGATGACCTTGTACC

GACGACATCCTCTAAAACCCTCCGTGACACTGTTCCAACTGCTGCTGGTTCTGCAATTCC

TTTTCCTACTGTTCATGGAGTTTCGGAAAGATTCTCTGAAGAACCTGCTACTGGGACAAG

TCTATTCAATAATAATGGTGATGATTTTCCCATCTGGTCGATGCCTTCTGCCCCCCCTGG

TTTTCAATTATTCACAACAAATGTTGATATCCCGGATCCTTTAGTTTCTTATGATTTCGC

TTCTGCTGGGGATTTGGAGGATGATTCGCAAGTCCAGAATATATGTGTATCTCATTCCAA

TACTGAGATTCACGAAGTTGATACTCTAGCGTTTGTCAATGATGATCCCTCGTTGCAAAT

ATTTCTCCCGTCTCGGTCTCCTAGTGTCCATCAGCCTGCCGAGTTAGGTGATCATACCGA

GATGCCTGGTGGTGACCATTCTGATGATTGGACGTCTCTTCGCCTTTCTGCTGGTGGAAA

TCACAATGATTCCACCCATAAGAATGGACCAAATTCGAGCCAGCAAGGAGTGAAGGATAA

ACGGATGGAGTCGTTAGCTGATACTGCTTCTATGTTACTTCTGAGCATGAATGGTGACAA

GTCGAAGAAAGCGACACCAAATGGTGAAAGTTCGAATAGTCCATTCTCGCCCCCTCGACA

GCCGCGGTCTGTGAGGCCCCGATTGTATCTGTCGATTGCGACAGATTCAGATTAATTAAA

CTGACTTTTTGGGTAGGGAGTCTTTTTTTGCAATCTTAAAATACTGGTTGTAGTTCCAAT

GATTTTGGTGATCGATCGTATTTTCCTTTTTATACCAAACTATTAGAAGACAGTAAAGTG

CAGAAGCTTGGCAGTGGCCGATCACTGGAGGAGGGGAATAAATTTATGCGTCTTGAACAG

GTAAAATTTAGTGCCTGATGAGTTTGAACTGTTCGTGTAGGTTCGACTTGTGTAGTAGAA

AATCTCTATGAGCCTCCATTTTGCCCAGGTCAGTACAGTTTTGACCTGATTTGCAGGTGG

TCTGCTGCTAGATGTACAAAGATAATTTATAGAGAATGGGAAGTGGTGCCTTTCTCTGGG

TATACATCAATTTTTGAAGGAAGGGATCATGTACAATATTTGGGTCTGGTCAGTGTAAAG

TCTAGTGTTGAAGTTGTATTTTTTGTTTAACTGAATTATATTGCTTATTTTTGTTTAATT

CTGACTACGTTTGGCTAGTTTTTCTCGTAGAAATCTCTGAATGTGGGAATTCATTGTGTT

TTGTAGTATGTTATTATT

>Unigene0000227

ATCAATGGTTAGAAATTTGATGACTGCAACATGGACACCTCAAACCCAATCTGTAGTGAC

ACAACCAATGCAAGAAGACTGAAATTAGTTTAGTATGTTGTAACGGCTTACTCAAAAAAG

TAGATCTCTCACCTATTTTAGAGGTTTGAAGAGGAAGTATCAGGGGATGCTGAGGACTAA

GGTCCTTCAGTTTTTGTTCACTTGCAGTTCAGTGGAAATGACTAAATTTAGATCAGCAGT

CTGTGGGGAAGAAAAAGATTGAGATGAGGAGAAAATATTACACACTGCTAACCGTGAGTG

TAGAGCTAATTCTGTTGTGATTTCTAGGGGTTATTTTAGGAATTTTATGTATCTAAAATT

TAATGCTATCTACGTTACGGGCAGAGATAATGGTGGTGATGTGAGGGTTAAGTTGGGTAT

GCGTGTCAATCTCTGAAAGTGGGGTTTGGGGGATCGTGGTACACAAGATTTGAACTCGGT

TCACACA

>Unigene0000228

CTTTTGATGGAGGCCACATATTCACCTTGCACCTCTTATCTTTTCAGACAATACTCTTAA

TTGCTGTAAAGCATCTGAGGGACATTTTGTTATAGGGTATGAAAGATAACATATTACCTA

ACAGGCTTACTTGTGTTTGAGAGCCATCAACTTTTAAAACCTCCCAACTAAGATGGACGA

TGGGCTCGTAGATAACCAATCCTTTTTAGATCATGCAGGGACAGGATAGTCTGAGGGGTA

GATAAACTGTTGAGTTGATAGATTGCTTTTGCTAAGGTGTTTGTCTTTCTTTCGAGCTAG

CCAATCCTTCTTATATGCAGTAGGGAGGGAATAGTCTGGCCAACAACTAGTCTT

>Unigene0000229

CTTCTTATATGCAGTAGGGAGGGGATAGTCTGGCCGACAACTAGTCTTATACACCATCCT

TGACTCTCTGCCTGGTCTGTTATCGTGGGGAACTTCAACTCTTGAAAGGTTTCTGGATGT

TTGACACATGGTTTTCGTGCGATCCCACCCATCTACAAGGTATGATGATGAGTTGAGGTC

ACAGTGTTAAGTATGTATGCCAATTGTTTGCATGGACCACTCAGTTTTTCGCCATACGAG

ACCTGAACGTGTTCAATGGGTCTTGAATCTAGATTTATTCACCCCTTCTCATGGGTTGCT

CGCCCTGAATTTGTTTCCTTTAGAAAGGAGACTTGACACCTAGCTACTCGATTAAATCTA

ATTGTCATTGTTTTGTGTTCGACAGTGCTTTTACTTTTTCAACAAAGTTTTTCATATCAG

AGTCAGATTGGTTGGTGG

>Unigene0000230

CCATTTAGTAATTATTTGTTGAGTATAACATAAGTAATGATGAATTAATGACATCATTTG

ATCAAGAGGTTAATGATAATTAGGAAAATAGAGAGAGACAATACCTTGTCCTCGTGATTT

TCTGCAGGTCCGAAGCTCCTTACCAAATTAATCTGTATCAATGGTTAGAAATTTGATGAC

TGCAACATGGACACCTCAAACCCAATCTGTAGTGACACAACCAATGCAAGAAGACTGAAA

TTAGTTTAGTATGTTGTAACGGCTTACTCAAAAAAGTGGTTGATCTCTCACCTAGAGGAA

GTATCAGGGGATGCTG

>Unigene0000231

TATCAAGTTTGGTGGATTGGATAGGTTTTAAGATGAGCCTACCCCCCTCCCCCATCATTC

CCGCTGTCCATGGTTATCAACATGATATAATTTATAACCTGCATCTGATAGTCTCTGTTT

TTTGTTCTGGCTCCTCTCACCCCGAGCTTCTATCGCTTGGAATGATGTTGGCACTGCAAA

TGAGCGCCGTTTGTTGATGGAGTATACAATTACAATGACTTGATCACAACCCTAACAATG

ACGAGGCCAGTATTCTGAATTCAATCTCCCTCGGTTTTGACCTTTTATGTCAATCATCCT

TCCTAACTGACAAGGGTCCTGGGTTGGCTGATGCAGACCAAATGATATAGTCGTCCTTAT

TTTTTGAACCATTTTGTTTTTGTAATGATTTATGTGATGTGTCATAGATCATGCTGCTAG

AACAGTCCTTGAATGGAACATACACTACACTAAAAAATGAAATATAGAGAATGTCATAGA

TCTGGAACGAACTGGTCCAACCCCTTGAACAGTGTTCCGGCCCCCAAACTGACCCAGAAC

AGCACATGGTTCTGTCCCAGCCTTCCTGGCAAAACTTGCCAAATATTTGTTAGTGCTAGT

TATATACGGGAAACTTTTTAC

>Unigene0000232

GCAAAATCAACTCAGGGCCAAGTTCTAGAGCAAGTTGCAGTCCATCTAGCTATAATTCCC

CAGAAGTAGTAATTCTTGTAGTGTATATTTTATTTTCTGTTCTCTTCATTTCACTAAAGA

AAACCTCTTGCAGCTTAGAGTTTCTTCGGAATTCAAGATTCCTTTCAAGTTGAAACCAAT

ATAAGCATTTGGTACACATTTGTTGTTTATGGACTTGTATGAAATGGATGCATGATGGAT

ATACAGGTAAATTCCAACTTACACCGTAGCTCATAGGTGTTTTAAATAGAACATATTTGG

CGTAGCTTACATTTATCAGACATCCATCATAGATGATGTGGAACTTTAACGGGTAGCGGG

TTTATGTGAAAGCTGATAGAAATCTACATTTCTTCCCAAACTTAGTTTTTTTTGATTCTT

GAGGCATGGTGATGCATTTGGGGGCACTTGCTTGGTAGTAGCTACAAGTGGCCTTTAATG

ATGTTTTCTCAATCATGTTTCGAGTTTTTAAAGTGAATATCTCGACCACATTATGTCTTA

TCCTATCTGCCAGTAGGCTATTTGCTTGGTACTGGCAGCCCACGGTGGCTTACTGCAGGG

TTTCTGACATGTTTCAAGTTTTTAATGATGCTTTCTCAATCATGTTTCAAGTTTTTAAAG

TGAATATTTCGACCACATTATGACTTATCCCATCTGTCAGTAGGCTATTTGCCTGGTACT

GGCAGCCCTCGGTGGCTTAATACCGGTTTTCTGACATGTTTTTATTTATTATCTTAGAAT

GCAATATAATTTCTTCTGTACATCTTTATTATTTTCTTGCCTGATATGTTGAGTTTTTTT

AATATTTGAAGGACACTGATTCTGAGGAG

>Unigene0000233

AGAGAATGTCATAGATCTGGAACAAACCGGTCCAACTCCTTGAACAGTGTTCCGGCCCCC

AAACTGACCCAGAACAGCACATGGTTCTGTCCCAACCTTTCTCGCAAAACTTGCCAAATA

TTTGGTAGTGCTAGTTATATACAGGAAACTTTTTACCCAGTAATATATTTTAGTAGTATT

CAAACTACGTGGTAAAGTTGTCAGTTATGTAAAATAGGATTGTTGATCACAAGTTACAGC

ATGTTTTGTTTTGTTCATGTGTAGACTAAGTGGGGATAATGTTCCTCTGAGGGATGCATG

TGTGGATTGGCGGATAGATGGATTTGTCTAATCTGAAAGGAAAATGGGTTCTGGCCATCC

TTGGTGAATTCATCTTTATTGAGAAACTAGAACTAAAATACCAGACACCAAGACAAATTC

TGTGTTTTTTGGTTTGATTTTACAACTTTATTATGTATTTTGTTAGTTGCTGTAAACTTC

AGGTACTGGAACTGGATTAATTTTACAATTGGACTAAGTATTTTACCTGGAACTGGATTA

ATTTTACAATTGGACTAAGTATTTTGTTGATTGTGCGTGGAATTGAGAGGGGTTCAATTT

AATTTAAATAGTAAGATAACTAAAATCAGTCAATGTTTACCTTCCACATTTTCGATATTA

GAAGAATATGTTTCCCATTATGTTTCCCATAATTTGCTCTTGACTGGCACCATCTCGGTA

ACTACTGAGATACTTAAGACACTTGGATGCAAATTCAATGTGTTTGACATGGCCTCATTT

TTAGATTGTTGTATGTCTTCCGCTTGAAAGGGTTGGAGAACAACATGGGTGCACAAGAAA

TGTATTTCATGGGCCCCAGATAGATCATAATGTGCTTTTGTAGAGCAGTTCGAAAAAATA

GATAATGCTAGATAGA

>Unigene0000234

TGCTTGAAAAGAGCATAGATCTCGGTTGCTTGAAAAGAGCATCAAGAAGAGATTCATGAA

CTTGGTCCTTTGAGTCGCAAGGCTTAGCATTTAATAGAGGATGCAATGTTTCACAGCACA

CGATTCATGTTTGCATGGCAACCCCCTTTCAGAGCCATTGCTTACGAATATTATTACTTT

TCTTTATCTTTTTGTATTCAAAGCCACTCCACATATTCACTGTTTATGAATAAAATGTGA

ATTGACACTAATATTCCTAATAGGTGTTTTGCTTCTAGTTATTTATGTTTCCCTTATAAC

TATGTTTTCCTAATGAAGGTGGAAGATGGGGAGAAACAAGAATATGTCTATCGAGTTCAG

TATTTAAGCCATCTCTCTTGGCAGTGGAATTGTGACTTCGGGATTTGTGTTATGGGATAT

GTTTTAAAAAAATATGTATGTAGAGTGTGTGATTCATTCAAAACTTGGAGCTGA

>Unigene0000235

AAAGCAACCGCGACCTCCGGCGATCACCGGCCGGAGACGAAGCGCCCGAATACTCTGAGA

CGAAGGGGGCGAATACTCTCCGCCGCCGATCCCATCGTCTTCTCGAAATGGCGGAACAAC

TCACTGAAGACCAGATCGCGGAGTTCAAGGAGGCGTTCAGCCTCTTCGACAAGGATGGCG

ACGGTTGCATCACTACTAAGGAGCTGGGCACTGTGATGAGATCCCTCGGCCAGAATCCGA

CCGAAGCTGAACTGCAGGACATGATCAATGAGGTTGATGCTGATCAGAATGGGACGATCG

ATTTCCCGGAGTTTCTGAATCTGATGGCACGTAAGATGAAGGACACTGATTCTGAGGAGG

AACTCAAGGAAGCTTTCAAGGTTTTTGACAAGGACCAGAACGGCTTCATTTCTGCTGCTG

AGCTTCGT

>Unigene0000236

GTGGCCCCCAAACATAGTCAGACCAGTATATCACCGAACTGCCGGTCATCTAGGGCTCGC

TCGCTGACTCTTTGGAACCTCCGCTCTTCCTTCACTGAGGATTCCCAGATCTCTTTCCAG

ATCTCACAATGTCGAGCCTTACAATCCCAACTGCAACCGCCTGTATGATCCGAAACAACA

TTCAAAGTCGCCCCTCCCCTAAGCTGTTCAAGAGTCCTTGTTTACCAGGTTCCTCGAAAA

GCATCTCAAAGTCTTTTGGTCTGAAGTCAAGAAACTGCTTCCGAGCATCTGCAATGACTG

TCTACAAGGTGAAGCTCATTGGGCCGGATGGTGAGGAGCATGTCTTCGATGCCCCAGACG

ATTCCTACATCCTTGACTCTGCTGAGAATGCTGGACTGGAGCTTCCTTACTCGTGCCGTG

CTGGGGCATGCTCGTCCTGTGCTGGTCAGTTAACGTCCGGCACGGTTGATCAGTCCGATG

GGTCTTTTCTCGATGAGCCTCAGATGGAGAAGGGGTATGTCCTGACCTGTGTCTCCTACC

CGAAGTCTGACTGTGTCATCCACACACACAAGGAAGGTGAGCTCTATTGATTCCTGGAGT

AACCAAAGTTTCGTTTGTGATATTGCAAAGGGTATTGATAATTTAGATGGGTTGTATCTG

GTTATTTTGTTTCTTTGACAGTTCCCTGCAGCTATGTGTAGTAATTATCACGGGAGTTTG

AACTCGTGTTTAAGTTCACTTATTTGAATGCAATGAACTGGAATATCGGTATCTTGAACA

GTTATCGTTCATGACATCTTATAAGGTGAATTTATGAGACACCAAATA

>Unigene0000237

ACAATTTTGTCCTATCCAAACTCTCTATAACCATTTCGCCGGCTCATCACGCAAGCAGAC

AATCCAAACGCTAAAAAGTTGGGATCTCTGAGGTGAGGAGGTAATCAACGATGGCGATCC

TTCCGCTTCTCTCTCTCATCTCCCTCCTCGTTGCCGCTGTCGCCGCCGTTGTTCCGGACT

CCGTCCTCCGCCTCCCCAGCGAGGCGGGGCTGCTGGCGGAGCAGCGGGAGTTCGATTACT

TCGCCCTCTCCCTCCAATGGCCCGGTACCTTCTGCGCCCGCACTCACTACTGCTGTCCCC

AAAACGGATGCTGCCGCTCGGCTCTTCTGACCGAATTTACCATTCATGGGCTCTGGCCAG

ATTACAATGACGGAAGCTGGCCATCATGCTGCGAAGGTCCAGATTTTGATGTGAAGAAGG

TGCATTCTTTACTCCCAATTCTTGAAAAGTACTGGCCCTCCTTAAGTTGCAGCTCTGCGT

CTCTTTGCCATGGTGGAAAAGGACTATTTTGGGCACATGAGTGGGAAAAGCATGGGACAT

GTTCTTATCCAGTAGTAAAAGATGAGTATAGTTACTTCTCGATGGTCCTCGATCTATACT

TCAAATATAACATTACGGCAATTTTAAGCGATGCCGGTTTCCTAGCCTCCAATGCTGTGA

AATATCCGTTGGGGGACATTGTTTTCACCATTGAGAGAGCTGTAGGGGCATCACCACAAT

TGGTTTGCAGGCATGGTTCTATCCAGGAACTTCGATTGTGCTTTAACAAAGATTTGACGC

CTCGTGATTGCTTAATCGGGGCCAGCGCAAACAGCACATCAAACTCGAGGAGTTCCTGCC

CCAAATATATCAGCTTACCATCATACACACCTAGAGTGCCTGAAGATGATGTCAGTGCAG

CAGCGGGAGCTCAGATAGCCGAAGGATCCGTTAGTCAGATTATGAATTACTTATGGAACT

GGAATTAAGTAATTGGGCAACTATTATGAAATCGAAATCGTTTGCTGCATTATATATATA

TATATATATATATATATATATATATAATACTTGGTTGAACAAATTACGGTTATGGAAGTT

GATATGCATCTGGTTTTTTCCTTATTCTTTTTGGATGTATTCCTTTTATTTTATCAACAT

GTTATTTCGTGATTTATCGTCATTTTCATCTAAATATCAGTGCGATCAACAATATTTGTA

ATATGTACAAATCACAGGTTTACAATCCCCCGAGCACTATTCAGA

>Unigene0000238

TTTCAGTAGAAGTTAGAGAACAGAGATTTCCCCGACAAGGCGAGAAATCAAAACCATTAT

CCTCGAGAAAGAAGAAACGGAAATCAATTCCGCCCTAGCCCCTCCAAACCCCCAGCGCGG

CAGCGATGGAGGCCGCCGGAGGAGCCGCGGTGCCGGCGCCGTTAGAGCAACAGCCGCCTC

CGCCGAAGGCGGACCGTCTCAACTCGGCGCTGCAGCAGCAGCTTAATCTGGACTCGGTGA

AGGCCCGCGCTGATGGCCTCTACAAGGCTGTCTCGCGCATCCTCCACGATTTCGACCTCT

TCGCCCGCAACAACGCTACCCCGAAATGGCAAGATGTTTTGGGACAGTATTCCATGGTAA

ACATGGAACTCTATAGCATCACGGAGGAGATTAAGAAGGTGTCAAAGGCATTCGTGGTGC

ATCCGAAGACTGTCAATGCTGAGAATGCTACTATATTACCTGTGATGTTGTCATCAAAGC

TGTTACCGGAGATGGAAGCAGAGGATAATGCAAAGAGGGAGCAGTTATTATTTGGGATAA

CAAATCTTCCTATAACCACTCAAATTGAAAAGTTAAAGGCTAGAATAGATATGATTGGGA

CGGCCTGTGAGACAGCTGAAAAAATCATAGCTGATGCTCGTAAAAACTATGGACTGGGTT

CACGTCAAGGACCAAACCTCCTCCCATCTTTGGACAAGGTTCAAGCCGCAAAAATTCTAG

AGCAAGAGAATTTACTTAGAGCTGCTGTCAATTCGGGCGAAGGATTGCGAATACCTGCTG

ATCAGAGACACCTCCCATCTACCCTTCCTAGCCATTTAGTTGACCTACTCGCTTTTAGTG

ATGGTGCACAGACTTTCAGCGATGCATCTGGTGCATTCCCCAAGAATGCTCCCCCTTTCC

CTTCCAACAATGTCAACACTCAAGTCCCTGGAGTGCAGGCCCCTGGAGGACAATTAATGG

GAAGACCAGTTCCATCACCGTCGGCTGTCTCAGGGGTTACTTCATTTGAAAACGCGGCTA

CACCTCCTTTGCCCTATGCTAATTCACCTAGATCTAGCACAAATATAATGAATACACCTT

CACCTCAGCAACAGACACAACAGCAACAGCAGAGACAGAAGATTATGCAAATACCTCAAC

ATCAGCAGCAACTCATTGGTCAACAACAATTGAGACAAACATCAACAGCAGGAGTAATGG

GACAGAATGCATTATCCCAACTGCATGATTTCCAGGGGCAAGCACAACAGAAGTTTCAAC

CGATGCCTGGCGCGCATCAGATGCAATTTTCTCAGCCTCTAGCTCCACAGCAGTACCAGA

ATAGACAATTGCAATCCGCACATATACAGCATAATATTGCACAAAGTCAGATGAACCAAG

GAAATCAACTAAGGAACCATCATTTAGGACAGTTCTCTGGTTCTGCTAATAGTGCCTTGT

TCAATGCTGCCCAGACATCACCAAATCCTCAGTTGATAAACAACATGTCAGCTACAATTC

AGTCGCAATCCGTATCACGACAGTATATGTCTGGGGCACATACTCAAAGGACTCATCCAT

CACAAATTCTTAATGACCAAATTTATGGAATGGGTTCCTCGAATCCTGCTAGTATGGCAG

CAATGCAGCAGCAGCAACAGCAACATGGTGGATATGGAAACATGCAAATGAATCCCCAGA

ACCTACAGCAGGGTATGGGCTCACTTCCCAACACAACTACAAATCCCAACTTTCAGCAGC

AGAGGCAACAGAACCAGCAGTGATCGCCAAATATGAAGCAAAAGTCAAGTTGCCCTTGAT

GTTGCTTGCTGCTCCTTAGGAGTATTTTGTTTACAAATTTCTCTCTCTCTCTCTCTCTCT

CTATAAATATTTGGTTATCAGAAATTTAATTTTGGAATTTTATGTCACCTATTGTAATTT

CAAAAGTTAATTATTCTTGTTGCTTGTAATCGTTTGTTGTTCGTAATCGTTTTAAGTAAC

ATAATTTTGA

>Unigene0000239

TGGTATTTTTCGGTGGGATTTTGGTTAGGGCTGAGGGTTTGACTAATTTTCCCCCCTTTC

GATTTGGGGGCAAAATTGGAGCTGGTGGAGGGGGAAGTGAGTCAAATTCGATCTTCTGGC

TGAGTTTTCTCCAAAAGGGTGAGATTCTAGGAGGTGAGAGCAAAGTTGGTTTAGACAATG

GAATGCAACAGAGATGAGGCCCTCAGGGCGAAGGAAGTCGCTGAGCAGAAGTTCTCCGCG

AAGGACATCGTGGGTGCAAAGAAATTCGCGTTGAAGGCCCAGAACCTCTTTCCAGAACTA

GAGGGTATTGGCCAGATGATAGCAACTATGGATGTGTATCTCGCAGCTGAAGCAAAGATC

AATGGTGAGAGTGATTGGTATTCGGTTCTTGGTGTGAGTGCTTCGGCGGATGAAGAAACT

GTGAAGAAACAGTACAGGAAGTTGGCCCTCCAACATCACCCAGACAAGAACAAGTCAACT

GGATCCGAGGGTGCTTTCAAGCTCATCTCGCAGGCCTGGAGCGTGTTATCCGATAAGGAG

CGGAAAATGTTGTATGATCAAAAGAGGTGCACAAAGCCAGCCCAGAAGAGAGCTTCTCAG

CCAAAAAGAGATAATTCTGGTGCAGTCAATGGGTTTTACAATTTCGCCAATAATACATCA

ACTGTAAAAACTCGAAAGAGAAAGGGTGGTGCCGCTAAATCTGCTACCGCGGCTTCGTCT

TGTCCATCAAATCCGACGACATTCTGGACTTCATGCAACAAGTGCAGGATGCAATATGAG

TACCTTAGAGTGTACCTAAATCATAACCTCCTTTGTCCGAACTGCCATGAAGCATTCTTG

GCAATTGAGACAGGGTTTCCATCAAACCGGGCAAATTCTTCTTCGGCGGTACCTCATCAG

GGTCGGCAGAAAGCGAACCATAACTCCACAACGAACAATGCTTATGGTCATGGAAAGGAT

CAAGGCATGGCAAATATGAGCTTCCAACATGGAGCGAATGTTGATCCATATGACAACCCA

AGTTTCCAAGAGGGTGTGTACTCGAGAACCGCAGGTGCTGCTAGCGCATCTGCTTCGACA

TCTGCTGCTGCTCAGGCCGCTAATTTGGTTCATCAGACATATGAAAAAGTGAGAAGGGAG

CGTGAGGAGGCACAGGCAAGGGCTAGGTGGGAGGAGGTCCTACTTCAGAAGAATCACGTC

TCAAAGACGAATGCAAGTGGTTCTAGCGCTAACAGGCCTTCAAAGCGAAGTAAAGGCGCG

AGTGTCGACATCGCTTCTTATTTTAGAGGAAAAGAAACTGAGGCATCAGGTTTCAATGCT

ACTGCCGAGCAGTATGTCAACTACAATATGGGTTATGGAATGCCAGGTGTGAACTATGAT

TTCACTGACTCTGGAAATGCAAGAATGAAAATTAAGCAGTACATATTAAGCAGAGACTTT

GCCCAAATTGATCTCCGAAACATATTAACAGGGAAATCTAAGACGACAATCAATAAGAAA

ATAGAAGAATGGGACTCCACTGCCACTGCAGCAGCAAAAACAGTAGAAGAAGAGAAGGCG

AAACAGAAACCAGAAAAACAGGAAAAGAAAGATGGTTTAATCGAAAAAGAATCTGTTAAG

GTTGATGGTGGTGCTAGTGATCAGAACAAGCTGAATGATTCCCGAAAGAACAATAAGCAG

CATGTGCATGTCAAGAAACTCTCTAACAACTCAGCTGATCCAGTTAAGCAAGTTGTTCAC

CATGTAACTATCGATGTTCCTGATCCAGACTTCATGGAGTTCGACAAAGACCGCTCGGAA

GGCTGTTTCGAAAACGACCAGGTCTGGGCTACATACGATAATGAAGATGGCATGCCTCGT

TTTTACGCTTTTGTTCAAAAGGTTATCTCCTTGAAGCCGTTTAAAATCCGCATGTGTTTT

CTCAACTCGAAGTCCAACAGTGAATTGGGGCCACTGAACTGGGTTGCCTCTGGTTATGCC

AAGACATGTGGGGATTTCAGAATAAGCCGATACGAAATCAGTGATACAGTCAATATGTTC

TCCCACAGAGTGAAGTGTGAGAGAGTCTCGCGTGGGGTTATTCGAATTGTACCCAGAAAA

GGTGATACTTGGGCACTTTACCGAAATTGGTCTCCCGAATGGAACGAGCATACACCGGAT

GATCTGGTATACAAGTACGACATGGTCGAGGTGCTCGATGATTACAACGATGACCAAGGG

GTGGCGGTAACACCTTTAGTGAAGGTTACCGGTTTCAAAACAGTGTTCCATAGGCACATG

GATCCAAAGGAAGTGAAAAGGATCCCGAAAGAGGAGATGTTTCGGTTTTCGCATCAGGTT

CCGTCGTATTTACTTACCGGTGAAGAAGCTCATAATTCCCCAAAGGGTTGTCGTGAGCTC

GACCCCGCAGCTACTCCTTTGGAGCTCCTTCAAGTCATTACCGAGGTGAAAGAAGATGTG

ATGGAGGCTGATCAGCAGCAGAATATTAGTTGATCTTGGATTTTATGTGCAATTGTTGTT

GTCGACTCATATTGAAACTTCAGCTTTTTCTTCTGATTACTCAGTTAGTCGTCATCGGGT

ATCCTAACCTTAGTTTTGTCTGAATATGAGGAAAGTGTGGAAATCGAGACTCGGCGAAGT

TGGTTTTTGGTGGTCAGTTATTTGATGCTAAAGCTCGCAATGATGTTTCCCTGATGTTTC

GTCCTGGTATGTTCTCTAATATTAGTAGTTTTATCAGTTACGGGTTCTAGCAACTGCTCG

ATTAATTGGCTGTATTGACTCGAAAGCAGCTAACATTCATCGTGTATCCTATTCAGTGTG

GTCAGTTTATTGCTTTGTGGATTATTTCTTTGTTTAGTTGCGTCTTTTATCCGATATCTG

TCAAATGTTTGGTTTTGATATATGATTAAGATGGTATCATTTCTTCATTGATGTT

>Unigene0000240

CCCCCTCTCTCACCTCAATCCTCTGAGATCTAGAGAGAGAAAATGGGATCAGAAGCTCCA

CCCCTCACCCTCGACCTCAAGCTCTGCGCCGCCAAGGCCGCCGCCTCCCTCCTCAGGCCG

GCGATCGCCGGCCAGACCCTCTCCTCTCAGCTCGAGGAACTCCTTCGCGACCTCGCCGTG

GAGCGGGCCAAGATCGATGCCTTCAAGCGCGAGCTCCCCAAATCCTCCCTCATCGTCGAC

GACGGTACGTTTCTTCACCTCCAAGTCTTCCTCCCAGCGAATTCTCTAACCTAAAGATGC

GATGTCACTGCATGCAGTGATGAAGAATCTACAGGACCAGCTGGATCAATGCCGGAGTGA

GAGACATCATCTCACCGTGCAATCGGAAAAATTGGGGGTTGAACAAGAGTTGAAGTCTGA

CAAAGATAAGATGAATTGGATGAGCTCGGCACAACTTTGGAGCGATAATTATGATGAATC

CGAACATACCCCCACTATTACTAATATTAACAAATCAGCGAAAAAGAAGGTGGATGAGGA

AATGTGTCTAGGCGGGGGTGCATTTGCGCCGTTTAAGCCACCAGTGAGGAAGGAGGAGAA

GGTTCAGGTGGAGTTGCCTAATTTAGGCTTGTTGACTAGTGGGATTAACTCATCAGCGTG

CTCGCCAACTGTTGTGGAGGCGGAGATCCACTATGGTGGGTTGGAGTGTAAAATGAGGAC

TCCGGTGGCAGCGGCGGCGGCGGCATCTCCCATGCCCAGCCATTTGAATCTGCACTCCCA

GCAGCAGCAACAACTGTCGAGAAAAGCAAGACGGTGTTGGTCGCCGGAGCTCCATCGGCG

ATTCGTTCTCTCCCTGCAGCAGCTTGGTGGTCCTCAAGTCGCTACTCCGAAGCAGATCAG

AGAGTTGATGAAGGTAGATGGTCTCACAAACGATGAGGTGAAGAGTCATTTGCAGAAATA

TAGATTGCACACCCGAAGGGCGCCACCGAATTCTGCTCAAGTGAATCGGCCAATGGGGGG

AGGCTTGTGGGTTCACCACTATTCGGCCTCCTCGCAGCAGAGTGGAAACCAATCTGGGTC

TCCCCAAGGCCCTCTTCAACTTGCTGGTTCGGGTGTAGCTGTCTCCGTCACTGCTGGAGA

TAGCTGCGATGAAGACGGTAGATCGGAGAGCTATAGCTGGAAATGATAGAAATGTGGTGG

AAGGCAATTAGTGAGGTAGCTTCGAGAGCTTACACCTGAACAATCTCCTAGAAGAGAGTG

CTTGATGGATATAGGTGTATAAAATGGTTTGCGGTTTTGCTGATACAATACTGACACCGG

GGTAAATCATACAGGAGATAAAAAGGAGTAATGGAGCTCTTCTCATTAGATTTGGGCAGA

GTTTCAAATTTTCCCTCCTTGTTCTTCGTTTTTCTCCTTTGAATTATATTGTGAGGTTAA

TGTATTATATATTTGAACTTAGTTTCATTTCTCTTTGGACATTACTCTTCAGTTCTCTCT

AGCCAGTGCAGATGGAGATCCTAGCATTTGCATTTTGGGCAATTGTCAGCATTTTGGATT

TTGCCAACTATTTTATTTACTAATTGGC

>Unigene0000241

GCCAATAGAACATGTGATAAGCATCTTCCCACACAGGAGACCGAGCGAGCAGCAAGTTCC

AGCCTGAGCCATGGCGACCCTGTGCCCTAACTTCATCTTCCAGTGTGGTTCTCCATCTCC

TCCCTTCCCCATGATGTGCTCCCCCCACCTCTCCATTACCCGTGCTTTCACCAACATCCA

CGGCTCTAAACCTCGCCACCGCAAAGCCCCTTTCCTCTTATCTACGCCGCCCTCACTTCT

TGCCACCGCCGCCGCTACAGCGTCCTCATTCAACCTATCACCACCTCCGATCGACCACGA

CGATGAAAGCATAATGGCTGCAGGAGCAAAAGCTTTAGAAGGTGGTGGTCTTGAAGTATC

TCGAAATGATGAACAGGCAATAGAAGCTGCACTTAATGGGGTTGCGGTCATGGACCTCTC

ACATTTCGGGCGCATAAGAGTTACTGGAGACGATCGAATTCAGTTTCTTCATAACCAAAC

CACAGCAAAGTTTGATTCTCTCTCTGATGGAGAGGGATGTGATGCAACTTTCGTCACCCC

AACTGCTCGAACAATTGATCTTGCTCATGCTTGGGTTATGAAACAAGCTATAGTGTTACT

TGTCTCACCTTGTACCTGCCAAAGCATAACTGAAATGCTTAACAAGTACATATTTTATGA

TGATAAGGTAGAAGTTCACGACATCACTGAGAAGACATCCTTTTTTGTCCTTGTAGGGCC

TCAGAGTACCCAGGTAATGGAGACTCTGAAACTTGACCATCTTATTGGACAACCATACGG

TACACATCGACATTATAATGTAAACGGGAAACCAGTAACTGTTGGTGTTGGAAGCATTAT

CTCAAAAGATGGTTTTTCATTTTTGTTGTCACCATCCTCCGCTGAATCAGTATGGAAAAC

TCTTTCAAGTCTAGGTGCTACACCCATGGGACGTACAGCATGGGAGAGACTTAGAGTTAT

ACAAGGAAGGCCAGCTCCCGGCAAGGAGTTAACGAAGGACTTCAACGTTTTGGAGGCCGG

TCTCCGGATGGCGGTTTCTCTTGACAAAGGGTGTTACAAAGGCCAAGAGACCATATCTAG

ATTGATCACCTACAACGGTGTCAAGCAGAGGTTGTGGGGCTTCAATCTATCTGGCCCAGC

AGAGCCAGGATCCTCGATTATGGTAGATGGTAAAAAGGTTGGGGTACTGACAAGTTATTC

ACTTGGCAAAAAAGATGGCGATCACATTGGTCTTGGCTATGTGAAGAGGCAAGTTAGTCC

AGGTGAAGAAGTATCCATCGGTGACGTCATAGGAACTGTTATACATGTTCCCTTCTTGTG

CCATTCACTTCCCTAGTTTACGCAAATATGCATGCTATTGATTTTGTCAACGTGTTCGCA

GGAAGTCTTTTAGTCATGTATGATGCAGCGTTTGTTCTTATTTCAGATTTTATACTGACT

ATTAAGTAGCTAAACTTTTGTAGGTCATTTGTGAAGTGCCAATACTTCCGTATCTTATTT

CTCTTTCATGTTTTGTGCTCGATCATTATTTAATCACATCCTTAAACACAATAGTCTTTG

AAGTTGTTATTGCATCTTCTTCGTACTGTCTTCCAAAGTTGTTACCGGAACACTTTATCT

CTGGCCTCTCCGATTTATCTTCATTAGCATCATCCTATTT

>Unigene0000242

GCTGCGGTTCCAAGTCCCCACCCCGTAATTGTCCGAGCCAGAAGCATCCTCAGCCGCGAC

GGCCAGGCCTCACCCGAGTCTTTGAAGGAGATCATCACACTTCTTGATGAGGCGCCGGAG

GAGGATGACACTGCTCTGCTGAAGTATCTTCGGTTGCAGCTTCATAGATCTTATCAGGAG

AAAAGCGCTGAAAAACATGACCAATTTTTGGGCTCTATAGTAATGGCGATTCGGGGTTTG

CCGAAATTCATCGGGAAGTTTGTTTCAGATTTTCCTTTCCATTCACATTTGTCAGTTCCA

TGCGTGGTATGTGAACTGAAAGCTCTGACTGAAGGACAGGAAACACGGACAGACACCCTA

AGAAATGCACTATGCAGTTCTTATCCCTCGCACACTTTCTTTGAACCAGGGAGAACAAAT

GATGTTTCAAAGGTGTTGAAAGCTCTGTTCTTTTCCATTCATCTCTCCGCTGATTGCAGC

CAGAGTTTCTGTGGCAGGAAGTGTACAGCGCATGAACTTTTTGGTAGTCGTTTTGTCGAA

ATAATTACGACTGATTTCTGTCTTGAAACAAAAGATTGTGAATATACCCGGTTTTTTTCA

CAGAATTAGTGCCAACGAACTACTAGAAACGAAGGTTTCTCTTCATGAAATGTTGAAGAA

AGATCTGTATGCCCTGCGAGATC

>Unigene0000243

GCTTCAGCGGCGGCTCCAGGCGTCCGCTCCACTGAAGGATCTTCGGTTTCAGCTTCAGAT

TACGTCTTCGCTCCTCTCTGGTTTTCTGGTGGATTTCTTGATTGACGGGTTGGTTACTTG

TTAGGGGGGAAATAAATGATGTCTCAAAGGTGCTGAAAGCCTTGTTCTTATCCATTCATC

TTTTCCCTGACTGCAGCCAGAGTTTCTGTGGCAGGAAGTATACAGCTCATGAACTTTTTG

GCAGTCATTTCTTCGAAATAATTATGACTAATTTCTGTCTTGAAACAAAAGATTGTGAAT

ATACACGTTTTTTTCTCAGAATTAATGCCAACGAACTACTAGAATTGAAGATTCCTCTTC

ATGAAATGTTGAAGAGGGATCTGTATGCCCTGCAAGATCCCACGGATGGTAGTTCACTCC

GATTTTTGCCTGATCTATCCTGCAGAGCAGACTACCTGATCATAATTTCAGAATGGCCAA

AGCCATCTACAGAGGCTGACTTATCTCACATCCTTGAGGCGATTCCGCTCGAGATGAGGT

CGGAACCCAATTTCCATAATATCAAGCGGTATTACCCGCTTATTTCGATGATAGCGCATA

ATACTTGGGAGAGCATTTCCTTCAGTTACAGCGATAGCGTGCCTGGTTGGCTCAGAAATG

GACCGCCTGCAGTCAATATGGGTTGTTGGTATGATGTCTATGGCCTTCTCAAGCAGTTGA

AGTGTGAGCCTCATGTACTATTTTTCGAAGCAATAGCTTGAAATCCATGCGGAGACTATT

TAAGGGTTTGAGATTGAAGTTGATTGACATAGGAAGACCTGTGAATTCGTGGAAAGCTCA

AAGGAGGAAGCTTTTTTAGCGGAAGATGTCATTACTCAGATAAGCTTGTGCAACGGCTAC

TCAGATAAGCTTGTGCAACGGCAGGCTCGAGTGTGATTGGAAAAGCTACCGTGAGAATTA

CTGACGGCACGTCTCAAATGACAAACATAGGCAATAATGCTATGTTTTGTTTTTCTTTTC

TTTTTTGAATGTCCTCATCCCGAGGAGGTGAGGCGGGTT

>Unigene0000244

TGTGGGGGATGGTGGTGAGGGTGGGGATGAGGAGATGGTTGATGCTGGGACGCCGGTGTC

GAGGGTGTTTGGAAGGGGGTGGGGGACTGGGAATACGCGGCCGATCAGGCAGAGGTTGCT

TGTGGTGGCTAATCGGCTACCGGTTTCGGCAGTGCGGAAGGGCGAGGATTCATGGCAGCT

GGAGATCAGCGCTGGTGGGCTGGTTAGCGCACTTCTTGGTGTAAAGGATGTTGATGCTAA

GTGGATTGGATGGGCGGGCGTTAATGTCCCGGATGAAGTCGGGCAGATAGCACTTACAAA

GGCTCTGGCGGAGAAGAGATGTATACCGGTTTTTCTTGATGAGGAAATAGTTCATCAGTA

TTACAATGGCTACTGTAATAACATCTTGTGGCCACTTTTTCATTACCTTGGGCTCCCACA

AGAGGACCGGCTCGCTACTACTCGGAGCTTCCAGTCTCAGTTCGATGCGTACAAACGTGC

AAACCAGATGTTTGCAGATGTTGTGAATAAGCATTATGAAGAGGGGGATGTTGTTTGGTG

CCATGATTACCACCTAATGTTTCTTCCGAAATGCTTGAAGGACTTCAACAGTAAGATGAA

AGTCGGGTGGTTTTTGCATACTCCGTTCCCGTCTTCGGAAATACACCGAACCCTGCCATC

TCGATCCGATTTGCTGAGATCAGTTCTTGCTGCTGATATTGTTGGTTTCCATACATATGA

TTATGCAAGACATTTTGTGAGTGCTTGTACCAGAATTCTTGGACTTGAGGGAACTCCAGA

AGGGGTGGAAGATCAAGGAAAACTAACTCGAGTAGCTGCGTTTCCCATTGGAATAGACTC

AGATCGGTTCAAGCGAGCACTCGAGCTTCCAGGAGTCAAAGAGCACATCAATGAATTGAA

GCATAGATTTGCTGGTCGAAAGGTAATGTTGGGGGTTGATCGGCTGGATATGATTAAAGG

GATTCCTCAAAAGATTTTGGCATTTGAAAAGTTCCTCGAGGAAAATGCAGGGTGGCGTGA

TAAAGTAGTATTGCTTCAAATTGCTGTGCCAACAAGAACTGATGTACCTGAGTATCAAAA

GCTCACTAGCCAGGTTCATGAAATTGTGGGGCGCATAAATGGGCGATTTGGGACACTCAC

TACGGTTCCTATACATCATCTGGACCGTTCTCTTGACTTTCACGCGCTTTGTGCTCTATA

TGCTGTTACAGACGTTGCTCTTGTAACCTCATTGAGAGACGGGATGAATCTTGTGAGCTA

CGAGTTTGTAGCTTGTCAAGGCTCAAAGAAAGGCGTTCTCATACTAAGTGAGTTTGCGGG

AGCAGCACAATCCCTCGGAGCTGGTGCCCTTTTAGTAAATCCATGGAATATTACAGAAGT

TGCTGCATCTATTGGATATGCATTAAACATGTCGGCTGAGGAGAGAGAAAAGCGGCATAG

ACACAACTATGCTCATGTGACCACGCACACTGCCCAAGATTGGGCTGGAACGTTTGTCAG

TGAGCTCAATGATACGATTGTGGAAGCTCAACTACGGACCCGACAAGTTCCACCTGCACT

GCCTACTAAAGTTGCAGTTGATCGCTACTTGCAGTCCAAGAACCGACTACTCATATTGGG

ATTCAATGCAACATTAACGGAATCAGTTGAATCATCTGGAAGAAGAGGTGGTGATCAAAT

TAAAGAGATGGAGCTTAAAGTGCATCCTGATTTGAAAATACCCTTAACAACACTATGCAA

TGATCCAAAGACAACTGTTGTCGTCCTCAGTGGAAGCGATAGAAGTGTCCTTGACGATAA

CTTTGGAGAATACAACATGTGGTTGGCAGCTGAGAATGGAATGTTCTTACGCCTCACTGA

TGGAGAATGGATGACTACCATGCCGGAGCATCTAAACATGGATTGGGTTGACAGTGTAAA

GCATGTTTTTGAGTATTTCACTGAGAGAACACCTAGGTCGCATTTTGAACACCGGGAGAC

TTCACTTGTGTGGAACTACAAATATGCAGATGTTGAATTTGGAAGACTTCAAGCCAGAGA

TATGTTGCAGCATTTGTGGACTGGACCAATTTCTAATGCAGCTGTTGATGTTGTTCAAGG

TAGCCGATCAGTTGAGGTCCGATCTGTGGGAGTCACAAAGGGTGCTGCAATTGACCGTAT

ATTGGGGGAAATAGTTCATAGCAAAAGCATGGTTACACCAATTGATTATGTTTTATGCAT

TGGCCACTTTCTTGGAAAGGATGAGGACGTCTACACGTTCTTCGAGCCAGAACTTCCATC

TGAGCCAGTAGGATCCACTGTCCGAACGAAGGCCGCTGATGTCATGAAAGCATCTGCTGA

CAGGAGGTTCACGGGAAAGCTATCGAACGGCAAGAACAGCTCGAGGTCATCAAATGGCAG

GATGAACAAAGCCCAATCGGGATCTGAGAAGAAAGTGACAACTAATCATAACACTGTCGG

GGGCTGGCGCCTCCCCCAAGAGATAATGTCGTGGCATGAAGGGTCATCTGTGCTCGACCT

GAAGGGCGATAATTACTTCTCTTGTGCGGTTGGGCGGAAGCGCTCTAATGCAAGGTACCT

TCTTGGGTCTTGTGATGAAGTGGTATCCTTTCTCAAGGAATTGTCGGCAGCTGCAGGGTC

GACACAGAGTGTCAACACATCCTCTGGAGGTTATCTTTTCGCCTGAGGGCGTATACTTTA

TGAGTAACGAAAATTCAGTGGGCGTGTGTACATGAAATCTTCGTGGGATAAGTCATATAT

ATGTGATATATAAAGGTAAACTTGTTAAGGAGACTTCTCGGACATTCTCATGTACTCGTT

AAAGAAGCTTCTAGAATATGATCCTTGTATAACGGTATGTGTGATTGTCGGAGAGTCATC

TTAAAAGGTTATGTAAATGGCATATTATGTACTCTTTAAAGAAACTTCTAGAATATAATC

CATGTATTATGGTATGTGTGATTGTTCAAGTTTCAATGGCTGCGATGAGCAACTTCAGT

>Unigene0000245

CTCATCCGCTCCCGTCGTTCGCCGCTCCTCCTCTCTTGGCGATCAACTATGGCGGAACAG

GTGCTAATCCTCGATCCACACTCAACTGCAATTGCTCTGAATTGTCCAGTCGATTGACTC

TCACTCGATCTGAATCGTGTAGTTGATCTACATGTTCATTCTTATCGACCCACACTCGAT

GGAATGGAAGATCGAAAGTATTGCGAAATTAGTTTCATCAGATCCGTATATCTTGATTAT

GATTGGATTGTTTTT

>Unigene0000246

CTCATCCGCTCCCGTCGTTCGCCGCTCCTCCTCTCTTGGCGATCAACTATGGCGGAACAG

ACGGAGAAGGCGTTTTTGAAGCAGCCGAAGGTGTTCCTCAGCTCTAAAAAGTCTGGCAAG

GGCAAGAGGCCTGGGAAGGGCGGGAACCGCTTTTGGAAGAGCGTTGGGCTGGGATTCAAG

ACTCCCAGAGATGCCATTGAAGGGACATACATTGATAAGAAATGTCCATTCACCGGCAGT

GTCTCAATCCGAGGCCGAATCCTTGCTGGCACCTGCCACAGTGCTAAGATGAACCGAACC

ATCATTGTTCGGCGGAACTATCTTCACTATGTTAAGAAGTATCAAAGGTATGAGAAGCGA

CATTCCAATATTCCTGCCCATTGTTCCCCCTGTTTTCGTGTGAAGGAAGGAGATCATGTC

ATCATAGGCCAATGCAGGCCTTTGGCAAAGACTGTTCGTTTTAATGTGTTGAAGGTTATT

CCTGCTGGATCAACCGGTGGAGGCAAGAAGGCTTTTACTGCAGTTTAAGTTGCTCTAAGT

GGTTGTGTTTCAAAGGTTATTCGAACATTATAGTTGCTGATTTTGATTTGGTGGTTAGTA

TTATGTAATCATTTTGTTTTGGTACTGGTTTAGCACAACTAGTGTTTTGGTTCCGAGTTC

TGATTTTATCGAAGATATCTATTTTGGTCGGATTTCAGTTTATCATTGCTGAGTATGCTA

CTAGGTTTTTGAGTTTTTAAGCTTCAAATTGTCTTTTTCTGATCTCTGAAAAATAGTAAT

GGTATTATGATTACTGGGTTA

>Unigene0000247

TGTCCCCCTCCGATCCTCCCTCTCCGATCCTCTCTCCCTTCAGTCAATCATGGCGGAACA

GACGGAGAAGGCGTTTTTGAAGCAGCCTAAAGTGTTTCTCAGCTCCAAGAAATCTGGGAA

GGGAAAGAGGCCCGGCAAGGGTGGGAATCGGTTCTGGAAGAGTGTTGGTCTTGGTTTTAA

GACTCCCCGCGAAGCAATCGATGGGACCTACATTGACAAGAAATGCCCGTTCACTGGCAA

TGTATCTATCAGGGGGCGTATCCTAGCAGGCACTTGCCACAGTGCCAAAATGAACCGTAC

CATCATTGTGCGCCGGAACTACCTTCACTATGTGAAGAAGTATCAGAGGTATGAGAAGAG

GCACTCCAATATCCCTGCTCATGTATCACCCTGCTTTCGTGTGAAAGAAGGAGACCATGT

TATCATTGGCCAGTGCAGGCCATTGGCGAAGACAGTGAGGTTCAATGTGTTGAAAGTCAT

TCCAGCGGGATCGACTGGCGGGGCTGGGAAGAAAGCTTTTGCTGCAGTCTGAAGCAGTTC

AGGGAATTTAAGGTCACATGACTGATAATTTGAGGTTTTGCAGTTAATTTAAGAGAGAAC

AATGTTGTTTAATTGTTGTTTCTACCGTGTTACTTCTCATCTGGTTTGAGTACTAGTTGT

CTAATGAACATTTTTTCCTAGTCTTTCTATATTTTGAAGGTCTTATTTTTAAATTGTATT

CTAGCTATGATCTAATACTGATCTCATCTATAATGAGATCTGAAAGAAACCAATGGTTCT

TTGCGACTCTGAAATTATACAGGCTTTCTACTG

>Unigene0000248

TGTCCACGTACATGACAACGACAGTTCGCCCCGCACGCAGAGTTGAGCGGTTGGCGAGAC

TTCAATGAAATCCTCTCGTCGAGCCTGAGATGGAGAACCGGCGCTTAGCCGTGATTGCCT

CCCATCTCTCCCAGCAGCGTCCAGGACCGGCGGAGCCGCCGCCGGTCTCCGCCTTCAGCT

GTCACTCCGGCGAGCGGCCGTCGGTGGAGGCCGGTGGAGACTGTGTCTTCTGCAAGATCG

CCAGCGGCGAGTCCCAGGCTTTCATGCTCTATGAAGATGACATATGCTTTTGCATTTTGG

ATTCAAATCCACTGAGCGATGGGTAAGGCACTCCCTTGTCATTCCGAAGTTGCATTTTCC

TTCATTGGAAGTAACTCCGCCTCTTGTAGTAGCTGCTATGTGTTCGAAGGTGCCTTTTCT

AAGCAACGCAATCATGAAAGCTACTCTATGTGATTCATTCAACCTGGTGGTGAACAATGG

ATCTGCTGCAGGTCAAGTAATATTTCATACACATTTCCATATAATTCCTCGTAAGGCGGG

>Unigene0000249

TAAAATCTTCTAAAGCCATATGAGAACATTACTTTGTTAGTTTCATAATTTCTGGAGAGC

TATCTTCTTTTTTTGCCATCAAGAAAATATTTTCCATATTACTTTACAAAATTATTTTCT

AACATGCTTTAGAAGAATGTTTATTAAAATTTATGTATTTTTCTTCTAAAAATAGTTACA

ATAACTTAAATGCCGCGCTATTAAGTTTTGTCCAAAAGTAAAACTTAAATTTGTCCTAGA

TTTTTTCTACTCAGTTTTAATGTGATGATATGAAGTGTATGCATCATGCTATTGTTTGAC

TTGAGTTTATCACAACGTTTGCTATTTATTGTGAGACCTTTTTATTTCTATGACTTATTG

GAAGCTAACAAGTGCAGCTTCAGTTTGTTGGGCTAATCGTGGTAGTTACAGAGCATAACT

GAAGGAATAACTCTGGTTTTCAAGTGAATTGTATTACTTTTTTTACTTGGTTCTTCCATT

CTTGTAATGCCTTCTAACAACTGAGTATAGCATACAGTTAGCTATTTGATGGTCTCTTTG

ACACTAGTATATCTTGTTTCTTTTTGTAGGCACTCCCTTGTCATTCCGAAGTTGCATTTT

CCTTCATTGGAAGTAACTCCGCCTCTTGTAGTAGCTGCTATGTGTTCGAAGGTGCCTTTT

CTAAGCAACGCAATCATGAAAGCTACTCTATGTGATTCATTCAACCTGGTGGTGAACAAT

GGATCTGCTGCAGGTCAAGTAATATTTCATACACATTTCCATATAATTCCTCGTAAGGCG

GG

>Unigene0000250

GGCACTCCCTTGTCATTCCGAAGTTGCATTTTCCTTCATTGGAAGTAACTCCGCCTCTTG

TAGTAGCTGCTATGTGTTCGAAGGTGCCTTTTCTAAGCAACGCAATCATGAAAGCTACTC

TATGTGATTCATTCAACCTGGTGGTGAACAATGGATCTGCTGCAGGTCAAGTAATATTTC

ATACACATTTCCATATAATTCCTCGTAAGGCGGGCGACCAATTATGGCCTTCCGAGAGCT

TTCGGAGGAGGCCGATAAAACGGAACCAGGAGACCCTGGGTCTTGTGGATTGCATCCGTG

GGAAACTTTCTCTTTCTGGGGATAATGGCAAAGATCATGAGTTCATTCTCGGAAGATTAT

AGAGTAAGTTTCTACATGTAATTTATGTAACATCATGTATACAATTTATTTTCGGAGGAC

TAGAATGGAAGAGTGAGATATAAAACATGAGTTGTTTTATCAG

>Unigene0000251

GGCACTCCCTTGTCATTCCGAAGTTGCATTTTCCTTCATTGGAAGTAACTCCGCCTCTTG

TAGTAGCTGCTATGTGTTCGAAGGTGCCTTTTCTAAGCAACGCAATCATGAAAGCTACTC

TATGTGATTCATTCAACCTGGTGGTGAACAATGGATCTGCTGCAGGTCAAGTAATATTTC

ATACACATTTCCATATAATTCCTCGTAAGGCGGGCGACCAATTATGGCCTTCCGAGGAAA

ATTGTAGAGTTTTCGGAGGAGGCCGATAAAACGGAACCAGGAGACCCTGGGTCTTGTGGA

TTGCATCCGTGGGAAACTTTCTTCTCTTCCTGGGGATAATGGCAAAGATCATGAGTTCAT

TCTCGGAAGATTATAGAGTAAGTTTCTACA

>Unigene0000252

ATTATAGAGTAAGTTTCTACATGAAATTTATGTAACATCATGTATACAATTTATTTTCGG

AGGACTAGAATGGAAGAGTGAGATATAAAACATGAGTTGTTTTATCAGCGAAAACTCAAC

ATTGGATTTCTTATTCCTCCATTGTTGATATAAATGAGAGATGTCACACATAAACTCTTA

CATACTTTTCTTTTGTTTATTGTTTCCAACTTATATCACCCATAGTTACATAACCAACTT

AAACTCTATATCGATG

>Unigene0000253

ATGATTCACATTCTTTATGATTCATAGTGAATGATGGTACTTGACGATGATCATAATGAT

GTTCAATCCACTGATCGATCGAGGTCCGTGTTTCATCAGAATTAGTCGTGTATGAGGGTG

TGATAACAATTATTCATCATCCTGAAATAACTCCCACAAAACTCATCAAAAGCATGAGAA

AACGAAGACATTTTGCACCAAATTTCTTTAAAATATTGGATGAATTGATTAAGTATACAA

ACATCATCGATTGATAAAAATGAGTGCAATTCGTTGGAATATATGAATGTTCGATCATAA

ATACTTGATGAAATTGTGGAAATAAAGACAAACATACATATTCTTCATGTATCATAGTTC

CTGTGAGGCACTGATTATGACATATTAGTGGAAGACAATGACTATAACATATTTTATTTG

TCCTTTTGCTTAAATAATTGTTTAGAAATTACAGAAAAATTGATGTATTTATAATATTGT

GTGAATATGCCTGAATTTGTGTGCTATAATAGCTGCTATCATCTGTTGCTATTTTTGTAA

ATGCTTGC

>Unigene0000254

AATGATGTCCAATCCACTTATCGAGGTTCGTCTTTTATCGGAATTTGTCATGAATGATTG

TGATACCAATGATTCATTGACCTGAAATAACTCCATAAAACTCATCAAAAGTATGGGAAA

ATGAAAACACATGTTTTGCACCAAATATCTTCGAAATATTGGATTAATTGATTAAGTATA

CAAACATCATCGATTGATAAATATGAGTGCAATTCATTGGAATATATGTATGTTCGATCG

TAAATACTTGGTGAAAGTGTGGAAATAAAGACAAACATATACACTCTTCAGTCTCCCATG

TCCATTATCAGATCTACTACAAAGGAAGTGTTGGCTGCTTGGAAAGGACTTTATAGTTTT

GGTCTCCAATGTCAATTCTTAGATCCAACCACTGTGTATGCTTTGGTTGCTTTCTACAAG

TTTTGTTCCCCTTGTGAAAGGAACAACTTGATGAGTCTGTCCTTATTTCCATATTTGAAT

CAACACAGAAAAATTGAACAGCTCTTGCTTACCTTGATGGGTTCAGGATCAGAATTAATC

CATCATTGTGGATTCTTGTCTATGAAAAACAAATCAAAATTGCTTGGATCTCAAGGCATG

TCAATGCTATTTAAGCAGCTTCTCTACTTGCAAAGTGTGGATTCTGTTTGAGAGAATTCC

ATCTTGGTTTGTTTGTTTGATCCACCAAGGTTTATATGCATCAATGTTTGTTTATTCCAC

CACCACAAATCTGCATCAACAAGAGGAGAGACTTACGAGTTTTTACTGATGTTTCTTTGC

CTTGCGAGGTAAATGAGGGATTTGAGGAGCTCTTGGAAGAAGCAACAATTGGCAACGATT

GCTGATTCTTGTGTACGATCACGTGACTTGATCGGTTGACAGGAGAGGGTACAAGATTAT

AATGACCACTAGCCTCTCGTCAGATGTGCCAGTTGGTTATTTCTCATGGGCTGAGTATGA

TATTATGGCGCCTTTGCATCCAAAGACTGAAGAAGCCCTTGCTGCTGCTTTTATATCAAA

TTGTGGGGCACGGAATTTTCGCTTGCAGGTCCTTGAGATGCTAGAGAAGTTGGACATTAA

AATAGATTCATATGGTAGTTGTCATCGAAACCGTGATGGCAAAGTGGACAAGGTAGAAAC

TTTAAAGCGCTACAAATTCAGCTTAGCTTTTGAGAATTCCAACGAGGAGGATTATGTTAC

TGAAAAATTCTTTCAGTCCCTTGTTGCAGGAACTGTTCCAATTGTTGTGGGTGCACCGAA

TATCCAAGATTTTGCTCCTTCTCCTGGTTCAATATTGCATATTAAAGAGCTAAAGGATGT

TGATTCAGTTGCGAAGACCATGAGACACCTTGCAGAAAACCCTGATGCCTTCAATCAGTC

AGTAAGTTGGAAATTTGAGGGCCCATCTGATTCTTTTAAGGCACTTGTCGACATGGCGGC

AGTGCACTCATCGTGTCGGCTTTGCATTCATATCGCAACAAAGATCCAAGAAAAAGAGGA

AAAGTCTCCCAAGTTTCAGAATCGCCCTTGTAAGTGCACAGATGGTTCAGAAACCGTATA

CCATTTGTACGTAAGAGAAAGGGGACGCTTCAAGATGGAGAACATCTATTTGAGATCAAG

TAAGCTAACTATGGAAGACCTGAAATCTGCTGTACTTAGCAAGTTTAAATCGCTGAATCA

TGAGCCTGTATGGAAGAAAGAAAGGCCAGAAAGCATCAGGGGTGGTAATGACCTCAAAAT

TCATAGAATATATCCAGTCGGCTTGACCCAAAGACAAGCCTTGTACAGTTTTATGTTTAG

TGACGATGTTGAACTTAAGAAATTTATAGAGAGCAACCCCTGCGCGAAGCTTGAAGTCAT

TTTTGTATAGCTTTGGGCCTGCCTAGCTGAACGACTCTTTTCGAGTTTCTGGTTTGCCAT

TTTTGTAGGATTTTCTAGGCCATTTTAGGATTAGTTTTTGGGTGTGTAATTTAATTATTT

TGCGAAATGTTCTCTTATATTTAGGATTGTTCCACAGTTCAGAGAACGCACGCTCACTCA

TGTAAGTTAAGAGAATGCACACTCATTTATATAATCTAGTTAAGAGTTTATGCCATAATA

TT

>Unigene0000255

AGGATGAGAAGCAACACCTGAACACAATTCCTGTCTTGATTTCATCCCCAAATAAACGCT

GTCTTGATTTCAGCTCCACAAGGCGGTTTGGATAACCATGAAAGCCATGCACATCATTTC

CTCCCTCTCTGTCTCACCGTTTACTCCAACAAGATGGGTGCCCCTCAAGATAAGCTGCAA

GTCTCATAAAAGTTTCTTGGGTGCTGAAGAAGAGGATGCATGTGGAGTGAAGAGCAGCCG

CAGGGTCATCCTCATGCTCCCATGGGTTGGTTTGGGATCGTTGCCGGCTTTGGCGGCCTC

CGGGAAGGGGAAGATCACGAGCCCGTATGACGAGAGGCGACTACTCGAGCAGAACAAGAA

GATTCAGGAGGCTAACAAGGCACCAAAAGACTTTCCGAATTTTGTTAGAGAAGGTTTTGT

GGTCAAAGTAGTGACATCCGAAAATTATGTAAAGTGTGACTCTGGACTTATATATCTGGA

TATAGAAGTTGGTAAAGGTGATTGCCCGAAGGATGGTCAGCAGGTGATATTTCAGTATGT

TGGGTTCAATGAATCAGGCCGTCGTATTGATAGCAGTTATCAGCAGGGTGCGCCTGCGAA

AATCCGACTAGGCAATAAAGCACTAGTTCCAGGGTTTGAAGAAGGAATAAAGGACATGAG

ACCCGGAGGGAAGAGAAGAATCATCATTCCTCCTGATCTTGGCCCTCCAGTTGGCCCATC

TACATTTTTCAGCGCAAAACAGTATGAGGTTTTCGATGTTGAACTATTAGACGTGCAAGA

TTGTCAGCGCAGGACGATAGCATTTTACTCTGATGTTGTATGTAGTTAGACAAACAACAT

TGTTCATACAATTTCTGCAGTTTTCTTTGAACTCGGATCACTAGTTCGTGATACTAATTA

TTTTTACCAACTACTGAATAGTACAGTTATTTGTTCTCAACCCCCCCACCCCACACACAA

CCTAACCAAAGAAAAACACTCAAATAGGAACAGTGAAACACCTGCATAAT

>Unigene0000256

GGAACTAAAATATGGTTTAATAACAATATTCCTTGTGGTTTAAATGTTTTGCACGTATCC

CGCTCTGGTATCGTTTTGCTCACTTACCTATTTTGTCCGTTTGCACATAATCCCTTCTCT

AAGCCTATACTGGTAAATATCTTGTAAGCAACAATTTTACCCTTGTTTTTTACTTCTAGC

TCTTCCGGAGTCCACCGAGCCACCATGGTTTCTATGTATATGCACTGCCGTTGCCTGGTG

GGTGTCAGAGGAGCAAAGGAAGAAATAGGGACACAAATGTAATTTAACAGGGGATATGAC

GACTAAAGCCGACAGAAGGGCTTACTCTGGTAAGATAGAAAACCACCGGGAAGTAAAGTG

AGGAAAAAAAAAAGAACTACATGGGCCTAAGTTCAACCGTGTAAATCACCGTGGTAAATT

GCAATCAAACCTTTCTATAAAATATAAGTCAGACCAGATCTTATTATTAAAGTA

>Unigene0000257

CCCTCAGACCCCAAACCTTAACCAAGCTCCGCCGCATCCGCCCCCTCTCCTCCTCTCCTC

CCCCTGATTCCTCCGCTGATACCTCCTCCTCCGCCGTCCCCGATCCCTCCTCTGCCGTCG

CTGCTGCCGCATCGCCACCTCCACCGTCCTGCGCCTCCCCTTCCAGAGACATCAAGGACC

GCCTCTTCAAGAAAAACCACCTCGACAAGCCGTCCTTCGCCGCCTCCGA

>Unigene0000258

CCCTCAGACCCCAAACCTTAACCAAGCTCCGCCGCATCCGCCCCCTCTCCTCCTCTCCTC

CCCCTGATTCCTCCGCTGATACCTCCTCCTCCGCCGTCCCCGATCCCTCCTCTGCCGTCG

CTGCTGCCGCATCGCCACCTCCACCTCCACCTCCACCGTCCTGCGCCTCCCCTTCCAGAG

ACATCAAGGACCGCCTCTTCAAGAAAAACCACCTCGACAAGCCGTCCTTCGCCGCCTCCG

A

>Unigene0000259

GGATTCGAGGATGGGCGGGGAGTCGTTGGTTGGACCTCCGGGAAATCCTCTCGAGGTTCA

GGGCGGCTGATCTTCATAAGAAGGCTGTCAAAAATTAGTGCAGCTTCTACCATGGCTGTT

AATTTGTTTTTTATTTTGAATAAGTCTAGGAGTATGTTTGGGAGTTTTAGTGGGTGTTTT

CATTTTTAGTTTGTTGAGAATTTTTATGAGAAATCTTAGTATAAATAGAGTCTAAAATTG

AGATTTAATAGAACTTATTCTTTCAGTTTTAAATTTTAGGTTTTGTTTTTCCTCTGTTCA

TCATCGAGA

>Unigene0000260

CCCTCAGACCCCAAACCTTAACCAAGCTCCGCCGCATCCGCCCCCTCTCCTCCTCTCCTC

CCCCTGATTCCTCCGCTGATACCTCCTCCTCCGCCGTCCCCGATCCCTCCTCTGCCGTCG

CTGCTGCCGCATCGCCACCTCCACTTCCACCGTCCTGCGCCTCCCCTTCCAGAGACATCA

AGGACCGCCTCTTCAAGAAAAACCACCTCAACAAGCCGTCCTTCGCCGCCTCC

>Unigene0000261

CCTCCGCCTCTTACCTCGCCGACATCCGTAAACACCTCGCCGAGTTCCGCAACTCCTCCC

GCTCTCCCTCCTCCTCTCCTCCCACCCTCTCCTTCCAAGACCTCTTCAAGAAAAACCACC

TCGACAAGACCTCCGCCGCCGCCGCCGACGACCCCAAGCGCATCGCTCATTCCTTTGACT

CCATCCGAGAGAGCCTACGTAAGTTACCCGGCGTCCAGACCCGGAAACCCAGCTTCCGCG

GCCGCGATAACTTAGCCTCCGGCTTCACGCCCCCCATCAAGCCAGATGAGCTCCCCAGCT

CCATCTTTGGCAAGGAAAAATCCACCGACGATGCCGAGCCGAAGGCTCCAAAGACAGAGT

TCTGGCGGACGTACAGCTACAAGGAGCTCGGGGAGAAGCTCCGCCGGTTACGCCCCGGGG

TCGGAGATAACCGGTTCTCGATCGCCGAGCTGAATGACCGGCTGGTGAAGCTCCACGAGC

TGGAGGAGAAGGAGACGGATTCGAGGATGGGGATGGGCAGTACGACTTTCCGCGACCTCC

GGCAAAGCCTCTACAGGATCAAGGAGGCTGATCTTAATAAGAAGGCGGCTAACCGGGACA

GGCTGTCTATTCTAACTAATATCACTGGGCTGCCGACGCCGGCGTTCATGCTCAAGCCCC

CGCAGGAGGAGCTGTTGGAGAAGTTTAACCATCCGGATCACATGTCGGCGGCGGAGAAGA

TGAAGCTGGAGCTTCAGAAGGTCAGAGATGAGTTTAAGATGTCGGAAAGTGATTGCGGCT

CGACAAGAGTTCAAGTTGCGCTACTTACAACCAAAATCAAGCATTTGTCTTCTGTTTTAC

ATAAAAAGGATAAGCATTCTCGAAAGGGACTCAATGAGATGGTTCAGAAGAGGAAGAAAC

TTCTCAAGTACCTTCGGAGGACGGATTGGGACTCTTACTGCTTTACACTGTCAAAACTCG

GACTTCGGGATTCACCCCAGTACAAGGCGTCTAATTATAAAATCAAAGCCTAAATATTTC

CTACTTTGGTGTCGGGTCCGATTCTGAATGGAATTACGGAGAACTTGATGACTCGCATCA

TAACTTGATGACTCGCATCACATTGTAGACCTACTGGAGTATGTGCTTAGGTCCCCATTT

TTGGGAATAAAATTGATATTTGATCAGTAATTTATTGAAAAGCTTGTCTTGACATCCATG

TAGTGAACTGGATAGTAGGCAGTTCGGTTGCAGTGATGAGTTCCATGTATTTTGTCGGTT

TAGACATTTTGGGTTATTAAGCTTTGTGATGTTATAAATTTGAATGTATCCCAGCTTACA

TTTTAGGTTATTAAGCA

>Unigene0000262

CCCTCAGACCCCAAACCTTAACCAAGCTCCGCCGCATCCGCCCCCTCTCCTCCTCTCCTC

CCCCTGATTCCTCCGCTGATACCTCCTCCTCCGCCGTCCCCGATCCCTCCTCTGCCGTCG

CTGCTGCCGCATCGCCACCTCCACCTCCACCGTCCTGCGCCTCCCCTTCCAGAGACATCA

AGGACTGCCTCTTCAAGAAAAACCACCTCGACAAGCCGTCCTTCGCCGCCTCCGACGACC

CCAAGCGCACCGATCACTCTTTCGACTTAATCCGAGAGAGGCTACGCAAGATCGCCGGCG

TCCAGCCCCCGAAACTCAGCTTCCGCGGCCTCGATGACTTAACCTCCCGCTTCACGCCCC

CCATCAAGACAGATCAGCTCCCCAGCTCCATCTCTGGCAAGGAAAAACCCACCGACGATG

CCGAGCTGAAGGATCGAAAGATGGTGATGGAGCTCAGGGAGAAGTTCGGCCGGTTGTCAC

TCCCCGAGAAGCTTCGCGAGCTGGAGGAGAAGGTGGCGGATTCGATCGCCGAGCTGAATG

ATCGGCTGGTGAAGC

>Unigene0000263

ACATTACCCATCCATCCTCTCCTCAGCTGGCTCCATTCCTCAGCTCCTCCACTGCCACCA

CCATGCCAGAAGCTCCGAAAGCGACCAAGCTTCCAGAAGGTCATCGTCGCGCCCTCGACT

TCATCGAGGATGTGACGACCAACGCCGACAAGGTTCAACGCCGCGTTCTCGCCGAGATCC

TCTCGCAGAACGCCTGCGTTGAATACCTAACCCGCTTCGGCCTATACTCCGTCGACCCAG

AAGCCTTCCGTCGAACTCTGCCGACTGTGACATACGAGGATCTCCAACCGGACATCCTCC

GTATCGCCCACGGGGACAAGTCCCCCATTTTCTCTCGCCGCCCGGTCACCGAGTTCCTCA

CCAGCTCAGGGACATCAGGCGGCGAGAGGAAGTTAATGCCGACGATCGAGGAAGAGCTCG

ATCGTCGACAGAAGCTCTACAGCCTTCTCATGCCAGTGATGAATCAGTTCATCCCAAATC

TTGACAAGGGCAAGGCAATGTACCTCCTCTTCGTGAAATCCGAGGCTAAGACCCCTGGCG

GCCTCCCCGCCCGCCCCGTCCTCACCAGCTACTACAAGAGCCGCCACTTCCTCGAAAGTC

CCCATGATCCCTTCAACGTCTACACCTCCCCTAAAGAAGCTGTCCTCTGCATTGATTCCT

ATCAGAGCATGTACGCTCAGCTCCTCTGCGGCCTCCTCCAGCGCTCCGAGGTCCTCCGCG

TCGGAGCCGTCTTCGCCTCCGGCTTCATCCGGGCCATCCGCTTCCTCGAGAAGCACTGGC

CCGCAATGTGCCGAGACATTCGCACCGGTGATCTCAACGCGGAGATCACCGATCGGGCGG

TGCGTGAGGCGCTCGGCCGCGTGCTCCGTCCGGACCCGGAACTCGCGTCCATCATAGAGG

CTGAGTGCCGGGTGGGGTCATGGCGGGGGATCATCACCCGCCTATGGCCCAACACAAAGT

ACGTCGACGTCATAGTCACGGGCGCCATGGCCATGTACATCCCCACCCTTGATTACTACT

CCGGTGGCCTGCCTCTTGTCTGCACCATGTACGCTTCCTCGGAGTGCTACTTTGGTCTCA

ATCTGAACCCAGTGTGTAAGCCGAGCGAGGTCGCATACACTCTCATTCCCTCCATGGCCT

ACTTCGAATTCCTCCCAGTGGAAGCCTACAATGGCGGCACCGAGCCTCACCATCGCGAAT

TGGTCGGCCTGGTCGACGTCAAGCTCGGACAAGAGTATGAGCTCGTTGTCACTACCTATG

CTGGACTGTATAGGTACCGAGTTGGCGATATACTAAGAGTCACTGGATTCAAGAACAAGG

CGCCACAGTTCAGCTTTGTAAGACGGAAGAATGTGGCACTGAGCATCGACTCCGACAAGA

CTGATGAGGTGGAGCTGCACGCAGCAGTGGGGAATGCGATCACCAACCTCACGCCGTTTG

GGGCGTCGCTGGTGGAGTACACCAGCCAGGCGGACCTGTCGACTATCCCTGGCCACTATG

TGCTGTACTGGGAGCTGCGCTCGGGGGCGATTGCTGTGCCTGCATCAGTTTTCGAAGATT

GCTGCCTGGCGGTGGAGGAGTCTTTGAACAGTGTTTATCGGCAGGGACGGGCGGCAGATG

GATCGATTGGACCGCTAGAGATTAGGGTGGTGGAGGAGGGAACGTTCGATAAGTTGATGG

ATTACGCACTGAGCCAGGGGGCGTCGATTAATCAGTACAAGGCGCCGAGGTGTGTTAAGG

CAGCGGCGGTGGTGGAGCTGTTGGATGGGAGGGTGAAGGAGAAGTACTTCAGCCCCAAAT

GCCCTAAGTGGTCTCCGGTGAACAAGCAATGGATAAAGGAGCAGGGAGAGAGCAAGGTTG

AGGGTTTGGAGAGGTCTTAGCGAGAAGACGAATGAGAAGAGGAGTAAGAAAGAAGCATGA

CTTATTTCTTTTTGTTGTAGTTTTCTTTTAACGGTCATCTTTTCTGTTAGGGTTTTAGTC

AGCTGATCAGTTCATCTCAACTATGTACAAACAAATCCTATTTCAAGGGATGTTTTTCTT

GTTCGCCTCCGCTTTCTCTCTCTCTCAACGTGTTGTTCC

>Unigene0000264

CTCCGATCTCGATTCCATCGAAGCTCTCACCATGCTCAGATCGGCCGGATCCGCCCTCCG

CCGCGCCGTCGAGCGCCCCTCTGCCCACCTCGCCCGTCGATCCTTCCACTCCGATCCACC

GTCCCGCAAGGTCGCTGTGCTCGGCGCCGCCGGCGGCATTGGCCAGCCGCTCTCCCTCCT

CATGAAGCTCAATCCCCTCGTCTCCCACCTCTCCCTCTACGATATCGCCGGAACCCCCGG

CGTCGCCGCCGATGTCAGCCACATCAACACCCGCGCCGAGGTGAAAGGCTACATGACCGA

GGAGCAGCTCGGGGAGGCTTTGGATGGCTCTGACATTGTCATCATCCCGGCCGGGGTGCC

TCGCAAGCCCGGGATGACCCGGGACGACCTGTTTAACATAAATGCCGGGATTGTGAAGGG

ACTGTGTACTGCCATCGCCAAGTACTGCCCCAATGCTCTGGTTAACATGATAAGCAACCC

TGTGAACTCAACTGTGCCAATCGCGGCAGAGGTGTTCAAGAAGGCGGGGACTTATGATGA

GAAGAAGCTGTTTGGTGTGACCATGCTTGATGTTGTTCGGGCAAAGACTTTCTATGCTGG

GAAAGCGAACGTCCCAGTTACTGACGTAAATGTCCCTGTTGTTGGTGGGCATGCTGGTGT

AACCATTTTGCCACTCTTTTCTCAGGCAACTCCAGCAAGCAATTTGTCCGATGAGGTGAT

AAAAGCTCTTACAAAGAGAACACAAGACGGTGGGACTGAAGTGGTTGAAGCAAAGGCTGG

GAAAGGATCTGCCACCTTGTCTATGGCCTATGCTGGTGCTATCTTTGCAGATGCATGTTT

GAAGGGCCTTAATGGGGCTCCTGATATCGTGGAATGTGCTTTTGTGCAATCAAGCATTAC

TGAGCTACCTTTCTTTGCCTCCAAGGTGAGGCTGGGGAAGAATGGTGTAGAGGAGGTTCT

TGGTTTGGGTACCCTCTCCGAGTTTGAGAAGCAGGGCTTAGAAGCTCTCAAGCCCGAGCT

CAAGGCTTCAATTCAGAAGGGGATCGACTTTGCCAATGCGAGTTAAAGAGTCTTTGATTT

TAAGGTATTGTACCAGCTCAGTTTTGCATGCATGTTTTGCTAGAGGGTTTAATCTCTAGA

TGTCATGTTTTATGGATTTTTGCAAACTATTTGCATCGGCTACCAGCCTCGAATAAGGTG

TTCAGGAGAATTAGAGAGACATGTATCATGAATTATGTTAATTTGACCGAGTGATAATTC

CTGATTTAGATTTGGTCCTCATGATTAATTGATGGGATGTATTTATTATTTTTTCCTCTC

TAGTCTGAAAATTGTGGAGCTTATCAATTGGGATTTCGGACTCCATACCTACAGCCTGGG

TATACCCATTATAGACTAGGTTAACTGGTTAACCTATGGA

>Unigene0000265

TGTTATTGTTGGAACCGTCAACGGAGAGTCTCCAAGTAGCGGTTGTGAGTTTTGTCTGGC

ACAAAAGTTCGTGCTTGGCCTGGATGACTGTATCCTCAGATAAGTCTGCGATGAAGTCGG

CCAGTGCTTGAGATTTGATCGCTGTGCGTGGCTGATATGTGACATCGAATTCACTAAGTT

TGATCATCCATTTGGCAAGGCGGTCGGATAGCTCGTGACTGTGTAAGATGGTGCGCAATG

GGTACTTACTGATGACGACGATGGAGTGGCATTAGAAGTAGGGCCGGAGTCTGCATCCTA

TGGCGACTAGTGCAAGAACTAACTTCACCAGTGCACTGTACAGGGTTTCTGGATCCAGGA

GGGATTTGGAGATGTATTATACTGGATGCCGCTTTCCTTCTTCTTCTCGGACGAGCTCGG

CACTGACAGTTGTTTCGAAAACTGCTAAGTATGCATAGAGAACTTCGTCGTCTTGCGGTT

TGGCAAGGAGCAGG

>Unigene0000266

CCTTCAGTTGTTGTAGGGCTTCTTCATATTTTGCTATCCATTCAAACCTGGCTGACTTGC

GAAGGGTGGAAAAGAAAGGCCGACACCGCTCGAAGGACCTTGAAATGAAGCGGCTAAGTG

CTGCCACTCTTCCGTTTAGCCGCTGAACTTCCTTGACGCTTCGTGGTAAGCAGATCTTTT

GGATAGATCTGATCTGATTCGGATTAGCCTCGATTCCTCGCTTCGTGACAAGGAATCCAA

TGAACTTTCCGGATGAAACCCCGAAAGAGCACTTGGCAGGGTTTAGCTTCATATTGTACT

GTTTGAGCACTCGGAATGCTTGGCGAAGGTGGTCGATGTGGTCACCTGATTCTAATGATT

TGACAAGCATATCATCGATGTAGACCTCCATAGTTGTCCCTAGTTGATCTGCGAACATGC

GGTTTACCAACCGCTGATAAGTTGTTCCG

>Unigene0000267

CCTCCTTCTCCTCTGAGCACCGCCTCTCCCCGGCAAGCCTCCCCCTTCGAACAGATCAAA

TCAACCCCAAAATTGAGCTAGAGATTTGCCTCTTCTTCCTGAGTCTTTTCCCCAAATCTA

TTTTGGATTTTATCGAGTATTTTTTGAGATCTCATCGATGGCTACTTCGCCGGAGATCGA

AATCCAATCATTACTAGACCTAAGCATATCCTATCTTCGATCAATCTCATCTCAAGTCTC

CTTTCTTCATCTTCTTTTAGATACTATGTGAGTTGTTCTAATTCGATTTTATTTATGTTT

CAGGTACTGATGGCCGGAACGATGAAGATAATGGTCAAGACATATGGAGCTCAATGATGA

AGCATTAGTAATCAAGCAATATAACCGAAGCTTTATAAAGATGACTTCCTTGGCGGCCAA

GTTTATGCACATGGTGGAGCGGGATAATTCCGTAACGCTCAAGATACTTGGTCATAAAGC

TATATTGGCATGAAATTTGAGGTATGTAGTCTATAAATGGAGGAAGATGAAGGCCATTGG

AAAAAAGTTCCGAACTTGATCCTCTTGGAGGATAAGTCTGTGAGTCGGGTGGTGAAGATG

TGGGATTGCTGAAGCTGTCGATGATGAAGACCATGAAAACTTGATGAAGCTGAAGAACAC

AATTATCCGAATTGATTCTGAGAAGTCATCTATGTTTTATTGCTGGATGTCTCAACTGCA

ATTGTGAGTTCTTATTTATGCTTTAAAGTTTCTAGTGATATTGTAGAACAATGTGATGTT

GAGTTTAGTTCATAT

>Unigene0000268

TTCCTCTTCTCCGCCTGCGAGCACCTCCCTCTGTCGGAGATAGCCCCTCCCAATAGATCA

AATTACCACCAAATGATAGCCATAGAATCACAAGGTATTTGATGGTTGGCTGGAGTGAGG

ATGGGTTGATCTCTCTGGAACATGAAGAAGGAGATGATCTCTCTTGGAGCAGTCGAGATG

TTGCATGTCTATATAGAGGTAAAGTCCATGGCTGCCAAGGACCTTAGAGAGTAAGAAAAG

ATGAAGCAGGGTATGCCGTAAAGATCAAGATATGTGACTATCAAGTAGAGATGGCATAGA

GATGAAGCTGAGTAGTTCTATAAAATGAAGAAAATGAAGACCAGTTGAAAGAAGTTTCGG

ACTTGATCCTCTTGGAGGACAAGTCTGTGAGTCAGGTGGTGAAGCTGTGGGATTGCTGAA

GCTGTCGATGATGAAGACCATGAAGACTTGATGAAGCTGAAGACCCCAATTGTCTGAATT

GATTCTGCGAAGTCATCTATGTTCTATTACTGGATATCTCAACTATAATTATGAGTTCTT

GTTTATGCTTTAAAGTTTATAGTGATGTTGTAGAACAATGTGATGTTGAGTTTAGTTCAT

ATGTGATATTGAGATTGTTGATCTTGTTATGATATCAAGTCTTCTAATCTGTATTGGTTC

TTTAGTAACTGTATAACTAAATTGAGAATTTGTGGAGATGTCTTAGTTTGGAGATCAATT

GTATGTTGTGATCATTGTTAAGAACTTAGAAGTAAGGTACATGACTAAGAGAAGTTGTAA

TAGATAGTCTCAGTAGTTGCGATTGATGATCTT

>Unigene0000269

TCTTCTCCGCCTGTGAGCACCTCCCTCCGTCGGAGATAGCCCCTCCCAATAGATCAAATT

ACCACCAAATGATAGCCATAGAATCACAAGGTATTTGATGGTTGGCTGGAGTGAGGATGG

GTTGATCTCTCTGGAACATGAAGAAGGAGATGATCTCTCTTGGAGTAATCGAGATGTTGC

ATGTCTATATAGAGGTGAAGTCCATGGCTACCAAGGACCCTAGAGAGTAAGAAAAGATGA

AGCGGGATATGTCGTAAAGATCAAGATACGTGACTATCAAGTAGAGATGGCGTAGAGATG

AAGCTGAGTAGTTCTATAAAATGAAGAAGATGAAGACCAGTTGAAAGAAGTTTCGGACTT

GATCCTCTTGGAGGACAAGTCTGTGAGTCGGGTGGTGAAGCTGTGGGATTGCTGAAGATG

TCGATGATGAAGACCATGAAGACTTGATGAAGCCGAAGACCTCAATTATCTAAATTGATT

CTGCGAAGTCATCTATGTTCTATTACTGGATATCTCAACTGTAATTATGAGTTCTTGTTT

ATGCTTTGAAGTTTCTAGTGATGTTGTAGAACAATGTG

>Unigene0000270

TCTTCTCCGCCTGTGAGCACCTCCCTCCGTCGGAGATAGCCCCTCCCAATAGATCAAATT

GCCACCAAATGATAGCCAGAGTATCACAAGGTATTTGATGGTTGGCTGGAGTGAGGATGG

GTTGATCTCTCTGGAACATGAAGAAGGAGATGATCTCTCTTGGAGCAGTCGAGATGTTGC

ATGTCTATATAGAGGTGAAGTCCATGGCTGCCAAGAACCTTAGAGAGTAAGAAAAGATAA

AGCGGGGTATATCATAAAGATCAAGATATATGACTATCAAGTAGAGATGGCGTAGAGATG

AAGCTGAGTAGTTCTATAAAATGAAAAAGATGAAGACCAGTTGAAAGAAGTTTCGGACTT

GATCCTCTTGGAGGACAAGTCTGTGAGTCGGGTGGTGAAGCTGTGGGATTGCTGAAGCTG

TCGATGATGAAGACCATGAA

>Unigene0000271

TTGTAGAACAATGTGATGTTGAGTTTAGTTCATATATGTGATTGAGATTGTTGATCTTGT

TATGATATCAAGTCTTCTAATCTGTATTGGTTCTTTAGTAACTGTATAACTAAATTGAGA

ATTTGTGGAGATGTCTTAGTTTGGAGATCAATTGTATGTTGTGATCATTGTTAAGAACTT

AGAAGTAAGGTACATGACTAAGAGAAGTTGTAATAGATAGTCTCAGTAGTTGCGATTGAT

GATCTT

>Unigene0000272

TGTGATGTTGATTTTAGTTCATATATGTGATTGAGATTGTTGATCTTGTTATGATATCAA

GTCTTCTAATCTGTATTGGTTCTTTAGTAACTGTATAACTAAATTGAGAATTTGTGGAGA

TGTCTTAGTTTGGAGATCAATTGTATGTTGTGATCATTGTTAAGAACTTAGAAGTAAGGT

ACATGACTAAGAGAAGTTGTAATAGATAGTCTCAGTAGTTGCGATTGATGATCTT

>Unigene0000273

CGCTTCCCTTTCTACCATCGCCGCCGATGAAGCTCGCCGGCCTCAAATCTATCGAGAACG

CCCACGACGACTCCATCTGGGCCGCCGCCTGGGTCCCTGCCACCGAATCCCGCCCGGCAC

TCCTCCTCACCGGCTCGCTCGACGAGACGGTGCGGCTATGGCGCCCCGACGAGCTCACCC

TAGCCGGCGCCCCCATCGGGGGGCACTGTCTCGGAGTTGTCTCGGTTGCCGCGCACCCGT

CGGGGGCGGTTGCGGCGTCGGCGTCGCTGGATAGCTTTGTGAGGGTTTTTGATGTGGATA

CGAATGCGTCGGTGGCGACTCTGGAGGCGCCGCCGTCGGAGGTTTGGAACATGCAGTTCG

ATCCCAATGGTACCATTCTGGCGGTAGCCGGGGGTGGAAGCGGTTCCATCAAAATTTGGG

ACACCGCTTCATGGCAGCTGATTGATACGCTATCAGTTCCACGCCCTGAAGGTTCCAAAC

CGGCGGATAAAAGTGGTGGAGGCAAATTTGTGCTCTCGGTTGCATGGAGTCCTGACGGCC

AGCGGTTAGCTTGTGGATCCATGGATGGTTTGATTTCTGTATTCGATGTCTCACGTGCCA

AGTTCCTCCACCACCTTGAGGGGCACTATATGCCTGTCAGGTCCCTCGCGTACTCGCCTG

TAGACCCCCGTGTGCTTTTCAGTGCATCTGATGACACCCATGTGCACATGTACGATGCTG

AGGGAAAGAGCATGATCGGTGCCATGTCAGGTCACACTAGCTGGGTTCTCTCCATAGATG

CCAGCCCTGATGGTGCAGCTATCGCAAGCGGCTCAAGCGACAGGACAGTTCGCCTTTGGG

ATATTGGCATGAGGACTGCGGTGCAGACGATGACGAACCATACGGAACAGGTGTGGGGTG

TAGCATTCCGGCCACCTGGGGGTACAGGTGTACGTGCAGGGCGTCTTGCGAGCGTATCGG

AAGATAAGAGCATCTCATTGTATGATTACTCATGATTAGACGCTCAGTTGTTGGATAAGC

CTTTGTTTCATCAAGAGGCGTAGGATTAACTGGTTTCCTGTCTGTTGTATGATATGTTAG

TTCGAATGACAGACGATATGTTAGTTCGAATGGTGCATTGCTTTAAAGCTTTTGCTCCTG

ATTTCTCAATAGTGGTAAGCTAGGAATCTTTCATTTTGCTGTTTAATCTCGGTTTAACAG

AGGCCATTGTCTGGATATAGAGATGTATGCGTGTCAGCTTGGTTACTGGATACATGAACT

TGTTTACTCTTCATTCATGACTATGAT

>Unigene0000274

CAGCGATCATCAGTGGGAAAAATATATTTCCTCCTCAAAAGCTAACGTAAAGTCACCGTG

GGCTGTCGCAATTTCTGCTAATGCGCGTACAAACGGAAACATAAAGCATCAGGCTCGGCG

GTGAGTTAGATGAAGGGCGTCAAAATGGGAATTTCCAACTCAGGAACAGGGAACGGCATC

CATAAAGATGATAAAAATGAGGTTCCAAAAGAGAAAAAGGCACTTGTTGTTTGTTTCGGG

GAGATGTTGTTAGACTTTGTTCCAACTCAGGAACAGGGAACGGCATCCATAAAGATGATA

AAAATGAGGTTCCAAAAGAGAAAAAGGCACTTGTTGTTTGTTTCGGGGAGATGTTGTTAG

ACTTTGTTCCAACTGTCGCTGGAGTTTCACTTGCTGAAGCACCTGCATTTCAGAAAGCTC

CGGGTGGCGCCCCTGCCAATGTTGCTGTTGGAATATCACGTCTCGGTGGATCGTCGGCTT

TCATCGGCAAGGTAGGAAAGGATGAATTTGGGTATATGTTGGAGGGTATACTGAAGGAAA

ACAAGGTCAACAGTTCTGGTGTTCGATTTGATGTGGGCGCAAGAACTGCACTGGCATTTG

TAACACTCAGAGCTGACGGGGAACGAGAGTTTATGTTTTACCGCAATCCCAGTGCTGACA

TGCTCCTTCAAGAATCTGAACTCGATGTTGATCTTATAAAGCAGGCGTGCATCTTTCACT

ATGGCTCGATCAGTCTAATTGCGGAGCCATGCAGATCCACTCATATGAAAGCAATGGACA

TAGCTAAGAAATCAGGTTGTCTCCTGTCATATGATCCTAATTTGAGGTTGCCACTGTGGC

CATCTACTGATGCTGCTCGTGAGGCCATCATGAGTATATGGGACAAATCAGACATTATCA

AGGTAAGTGAGGACGAAATTTCATTTTTGACTGGAGGAACCCATCCTCATGATGATAAGG

TTGTCTTAGAGAAGCTCTTCCATCCCAACCTTAAACTTCTCCTTGTGTCGGAAGGAAAAG

ATGGCTGCAGATATTACACTAAGAATTTCCGTGGTAAAGTAAATGGTTTGAAAGTTAAAC

CTATTGACACAACTGGCGCTGGTGATGCTTTTGTGAGCGGGTTACTTAGCATTTTTTCTT

CTGATACAAATCTCTATCAGGATGAGAAGAAGCTGAGAGATGCTCTAATGTTTGCAAACG

CATGCGGCGCTATCACTGTCACCGGGAGAGGTGCCATCCCTGCACTGCCTACAAAAGCAG

CAGTCCTGGAATTCCTATCAAAAGCTGCTGTGTAGCTTGCCCCACCTCTCTGTTTGCCAT

GTTGGTTCCTATAGTTCCAGATCAGTGGCATTTATTATACTTCGGATGTAAACGTATATA

TTTGAATCTCCTAATTTGGAAATGTTTTGTAGCCAGAATTTATTTTCTTGATTATCGTGA

TAGAGTAGAGGTATTCCAGAGGTGATGCAGAGTCAGTTGGGTCCTGGTTGGATTGGCTTA

TGATTCTGAACTCTGCTGTCGAAAGAGTAGCTGAAAGCCAAACATGTTCTTTTTGCTCTC

CCCGACAACTTGAGTTTTGAGTTATGTGAGTTCTGTTGATTTTCAGTATGTTATCTACTT

GAACTCTTTATATTAAGCCATAAACGATGAATCATGACTGGGGTCAATCTTATATCTCCT

TAATTACCGAGCTCTATAAGTATCGCAGTTAAGCATAGAAAAAAAAAAGTTTCCTAGCTC

GCCTATATTTCGCGGACCGACAAGCTGCACCGAACATGACAATGGAGAACTTCACATTTA

TGAGGAGCAGTTAGACCTCTTTTTTATGTATTCCAATTCCTTCGTTTACTTTGTATCAAT

TATGAATGTTATTCATTATTATTGATTGTTCTTCTCGTTGTAAACTCACGCACAACATTG

T

>Unigene0000275

GGGTCAATCTTATATCTCCTTAATTACCGAGCTCTATAAGTATCGCAGTTAAGCACAGAA

AAAAAAAAAGTTTCCTACCTCGCCTATATTTCGGGGACCGACAAGCTGCACCGAACATGA

CAATGGAGAACTTCACATTTATGAGGAGCAGTTAGACCTCTTTTTTATGTATTCCAATTC

CTTCGTTTACTTTGTATCAATTATGAATGTTATTCATTATTATTGATTGTTCTTCTCGTT

GTAAACTCACGCACAACATTGT

>Unigene0000276

CCTGCACCAAATGCTATCTACACTCAGCCTTCGTCCTCACGGAACCCCCCTCATTCGAAA

CCCTAACCCTAATCACAATGCCGGGAAACGTCTCCTCACTCGGATCAACTGTTCCTCCAC

AAGGTACGCAGTCCTCGGAGCTGGATTCGCCGGCTTGTCCGTGGCCTGGCACCTCCTCAA

GCATTGTGCAAAGGAGTCTCGTGTGTGCATAGATATCTATGATGAAGCAGGAATTGGTGG

TGGTGCATCTGGTGCCTCCGGAGGACTCCTCCATCCTTATTCCCCTAAAGGTAAGGTATT

ATGGAGAGGTGCCGAGTGCTGGAGCGAATGCTTACAGTTACTAGCAACTGCAGAAAGGGC

AATGGAAGCTAGTCTTTCAAAGAAAATAAATCTGGTTTCACCATGCAGTTTTGATGGACC

AATAGTTTTGAGAAGATGTCCTTCAGGGGGATCTTAAGGCCGGGTACAACCGAGAAAAAT

GTGGAAGTTTTGAAGGAGTGATTAGAATGCTCAGAGTTGCCTCAGTGATTGCTCTCTAGA

AGTCTTAGATGGTGATGCAGCTCAAGTTCTTGTACCCAATCTCTGCCTTCCATATAATTC

CGCGGTTTATATGCCCCGAGCAATGAATATCCATCCAAAGCGTTATCTCCAGGCACTCTT

TTTGGCCTGTCAACATACAACAGATGTGCTATCTACTTTGAATCATGAGGAAAGAGTAAT

AAATTTTCTCAAGAAATCTGTTCATAGTCTTCATGAACTAGAAGGAGAGTACGATGCGGT

GATTGTATGTTTAGGTGCTAAAGTGAACATTGTCCCAGAGCTCTTGGGAAAACTCCCTTT

AAGGACATGCAGAGGTGTTATTACGGAGCTCCAATTGCCACTAGATATGAGCGGAGAATA

CGGTGATGACAACCCATCAATCTTATCTGATGCATGGCTCGCATTCCAAGGGCCACGAGA

TATGCTTTTGGGATCAACGTGGGAGTGGGGATCTGGGAATTATTCTTCTAGTGTGTCAAC

TGAAGAGGCATCCAAGGCTTTCAAAGAGCTTCTACCAAAAGCGTCTGCTGTCTATCCGGC

TGCTCGAAATTGGGCATTTGTTGGAGCAAGGGCCGGCTTAAGGGCCATGCCTCCGGTGAC

TCCCCTTGGGTCCCTTCCACTTATGGGTTGTGTGGACGATGTAATTCGCGACAGAACGAG

GTGCAGGTATTGGTTTATTGGAGGGCTTGGTTCCAGGGGGCTTCTTTACCATGGTTTGCT

AGGAAGACTGATGGCACAAGCTGTGTTATCTTCTGATGAAAATCTTCTCCCCCCGGAACT

GATTTCATGGAAAAAATTGAAACCGTGGAAAGGAATTGCATAGA

>Unigene0000277

AAGTCCAAGGGTTCTGGGTGTTGCTCTATTGATTTTACTATGTGTAGAGGTCATTGCTGC

TGGTAGAGCTGCCATCCATGCCACAAGTGGTGGCGGTGGCGGTGGCGGAGGCGAAGGTGG

TGGGGGTGGCGGAGGAGTAGGTTCTGGTTCAGGGTCAGGATATGGGTCTGGATACGGCTC

GGGTTACGGTAGTGGTGGTGGTAGAGGTTCCGGTGGAGGAGGAGGAGGAGGAGGAGGCG

>Unigene0000278

ACTCACCAACCCACCTTGCCTCGTTTTGAGAGAGAGAGGAGGAGGAGGAGGAGGAGGTGG

GAGATATGGCGGTGACGGCGAAGATTGCGCCGTCGATGCTGTCGTCGGACTTCGCGAATC

TGGCGTCGGAGGCCGAAAGGATGCTCCGATGCGGCGCAGATTGGCTTCACATGGACATCA

TGGATGGGCACTTCGTTCCAAATCTAACTATTGGAGCTCCAGTCATTCAGAGTTTGCGGA

AGCACACCAACGCTTATTTGGACTGCCATCTTATGGTTACAAATCCTCTTGATTATGTGG

AGCCTTTTGCTAAAGCTGGTGCTTCAGGCTTTACATTCCATGCTGAAGTGTCCAAAGAAA

ATTGGCAAGAACTCATCCAAAAGATCAAGACAAGCGGCATGCGACCCGGTGTAGCATTGA

AGCCGGCAACTCCCATAGAAGTGGTGTATCCGTTGGTTGAAAATGAAAATCCTGTAGAAA

TGGTGCTTGTGATGACAGTGGAACCTGGTTTTGGTGGCCAAAAATTCATGCCGGAGACGA

TGGATAAGGTACGAGCACTGAGAAAGAAGTACCCTTCCCTTAATATAGAGGTTGATGGTG

GTTTGGGCCCGTCGACCATCGATATTGCAGCTGCAGCAGGAGCAAACTGTATCGTAGCAG

GAAGCTCAGTTTTTGGCGCTGCAGAACCAGCTCAAGTTATATCCGGTTTAAGGAAGAGTG

TGGAGGCTGCTCAAATTAAGAGCTAATTTTAGCTCAAGAATATTAAGAATACACTTAAGA

CTTTTGACTGCATATTTTTTATTTGTTGGGAATATTTTTTTTTTGTATTGTGTGTGCTTT

AATACATGATTTACCCAAAGCTTATGAAGGGAAATAAATGGTATAGTTATGTTATCTTGT

TAGTACCTGATTTGTTTATTCAATTATGTTGCATG

>Unigene0000279

CTCATTTTCGTGGCCGGAAAGATGGCTAGCGAGAGGGAACCGCTTCTCTCCACCAAACCC

TCCGGGGCTGACCAACTCGTATCTCTCCCATCATCGGCATCCTTCAAAGCCGAAGCCGGC

GACATCGACCAAATACGAGGTGTGAGAGACTTCTGGAGACAGTTCCTGGTCGAGTCGAAA

AAGCTCTGGTATCTCGCCGGCCCGGCCATCTTCACCTCCATATGTCAGTACTCTCTCGGC

GCCATCACTCAGACCTTCGCCGGTCAACTCGGCACCATCGAGCTCGCTGCCGTCTCAGTC

GAGAACTCTGTCATCGCCGGTTTTACTTTCGGCGTCTTGTTAGGGATGGGGAGTGCGCTG

GAAACGCTATGTGGGCAAGCCTACGGCGCCGGCCAACTCGACATGCTAGGCGTCTACATG

CAACGTTCGTGGGTGATACTTTTTGTGGCTGCGCTGGTACTGTCGCCACTCTACATATTT

GCGGGACCATGGTTGAGGCTGATCGGGCAGACAGAAGCCATTGCCGCTGCTGCCGGAACG

TTTTCGCTGTGGATGATTCCCCAACTGTTCGCTTATGCAGTGAACTTTCCCATCGCCAAA

TTCCTGCAGGCTCAGAGCCGGATGATGGCGATGGCGGTGGTGTCTTTTGTGGCGTTGTTG

CTGCACACCTTCTTCAGTTGGCTGCTAATGCTAAGGCTGGGGTGGGGGTTGGTGGGGGCG

GCCGTGGTGCTTAATGCCTCGTGGTGGTTCATAGTCGTGGTACAGCTGGTGTATATTTTT

GGAGGGGCGTGTGGGCGGGCGTGGTCGGGGTTTTCGTGGAAGGCGTTTCAGAGCTTGTGG

AGCTTCGTCCGCCTGTCGCTAGCGTCTGCCGTCATGCTCTGCTTGGAGATCTGGTATTTT

ATGGCATTGATTCT

>Unigene0000280

TGGTATTTTATGGCATTGATTCTTTTTGCTGGGTATCTCAAAAATGCTGAGGTTTCAGTG

GATGCTTTGTCAATTTGTATGAATATCTTAGGCTGGGCAGTTATGGTTGCCATGGGAGCC

AATGCAGCAACGAGTGTGAGGATATCTAATGAACTTGGAGCAGGTCATCCAAGGACAGCA

AAGTTCTCTATTCTGGTCGTTGTTATAACTTCATGTCTGATAGGCCTTGGGCTTTCTCTG

ATTCTGATCATTCTTCGAAAGCAATTTCCGTCTGCATTTACAAGCAGTGAAGATGTCAAA

AAGCTTGTATACGATCTCACCCCATTACTTGCTACTTCTATCATTGTGAATAATGTGCAG

CCTGTTCTTTCAGGAGTGGCAATTGGAGCAGGATGGCAGGCACTGGTTGCATATGTGAAC

ATCGCATGTTATTACATATTCGGAATTCCTCTAGGCCTTATCTTGGGTTATATGCTTAAC

ATGGGTGTGAGGGGCATTTGGTATGGAATGCTCATGGGCACTGTGGTACAAACAATTATC

CTGGTTTGGTTGACATCCCGAACAAACTGGAATAAAGAGGCATCAATTGCTGGGGAAAGG

ATTAGAAAATGGGGAGGAGAAAATGCTAAGCCTGATGGAGTAGGGGAATAAGTTGATGGT

GGAAAGACCCTAAGAGGTAAACTAGAATATATTGTTAGAGACTAATCTAGTCTCGGTAGA

AAATCATGGATTTTATTTCATTTAGATTATGTTTTGTAAACTGCAATATGTTTGCATTCT

ATGAGGTGAGATTGCTTTATTTTCCTAATGCAATACTGCAATCTCAAATGTAAGGATTAT

GATATGTTCTCTCTTGAATAAAAGTAAATGTAATGCATTGAATTCCACGGATAATTTCCT

TGC

>Unigene0000281

GCGGCAAAGTCATACCGCAGTATATGGTTCAGATGTTCTGGGCTTTTGGATTAGTCAACA

TGCTCAAGGACAATCCTGGGCTGGATCCTTGTGCCAAGCAGAACAATGAGTTTGGTGAGC

TGTGCGGTCGTTTTTTCATCAACAAAGTTGACCAGGTGTATAGTTATTGTTGGCTTCACT

CTTATAAGAAGATATTAAAAAAAGATGAAACTGGAGGAGCCAATAATGTCAGAACTGACA

GCTCACCGATTAGAAGGTCCTGATCAATTCGGGCGGGATTCAATCCGAGGTGCCTCTTCT

ATTGAAGTCAGCTGGAAGATTTTCCAAGAAAAGTTTGAAGCCAACTTTAGTTCCCATCCT

GTTTGCGCTAAGATGGTCTGGGAATTTGAGAACCTGAGACAAATAGGAGACATGACAATG

AGACAAATAGGAGACATGACAATCCGATAATGTGAGGCCAAGTTTTTGGAACTAACGCTT

TACCTGACCTATTTGATTAAAACTCCTCTTAAGAAAAATGAAAGCTTCCTCATTGAAATT

GATGACCACGTTAGATATT

>Unigene0000282

TAGCCACTACAACTCCAGTTGGGCAATCCGAAGAAAAGACAATCTTTTGTCGTATCCGAA

TAAAATACCTGGGCATCTATGATAGAATTGTTCAGAGGCTGGTAAGTGTTGTAAGTGTGG

CTAGATGAGGCATCCGAAGAAGAATCCTCCCTAAGGAGGAAAGCATGATAGGATGTGGGT

CTAGCTTATGCACTTCTTCATGGCATAGAGAATGTGTAAGATACGTTATGGTTTATGCTT

TATGGCATGCAAGTTAAAGTAATTTTTTTTTTATACTGGAGCATCACATTCCATTTCTTT

ATATTTGAGA

>Unigene0000283

AAACTCCTCTTAAGAAAAATGAAAGCTTCCTCATTGAAATTGATGACCACGTTAGATATT

AGATATTTGTTGCTGGGTACTGAAGGAGTTTTGAAGACCTTGTGCATCGTGTCGATAAAT

ATGGGGCAATATGAGGAGCATCTAGGGAAAAACTCCAGAAAATAGAAAGTGAATTGTCAC

AAAGTGGGCCCAAGTCCAAGAGCCCCTTCTTTTATAAGGGGCGCCGCGAGATAAGAACAG

ACCTTACATAAAGCCTTCTGAACGGAAGCACAACTGAGGGTTGCAATAACATATGGCGAG

AGCTTAGCCACTACAACTCCAGTTGGGTAATCCGAAGTTGGGCAATCCGAAGAAAAGACA

ATCTTTTGTCGTATGCTCCCTGAATGAAGTACCTGGGCATCTACGATAGAACTGTTCAGA

GGCTGTTAAGTGTTATAAGTGTGGCTAGA

>Unigene0000284

TATCAAAAAAGATGAAGCTTGAGGAGCCAATAATGTCAGAACTGACAGCTCACCGATTAG

AAGGTCCCGATCAATTCGGACAGGATTCGAGCTGCCTCTTCTATTGAAATCAGCTGGAAG

ATTTTCGAAGAAAAGTTTGAAGCCAACTTTAGTCCCCATCCTGCTTGTGCTAAGATGGTC

TGGGAATTTGAGAACCTGAGACAAATAGGAGACATGACAATCCGATAATGTGAGGCCAAG

TTTTTGGACCTATCGCGTTACCTGACCTATTTGATTGAAACTCCTCTTAAGAAAAATGAA

AGCTTCCTCATTGAAATTGATGACCACGTTAGATATTAGATATTTGTTGCTGGGTACTGA

AGGACTAAGTTTTGAAGACCTTGTGCATCG

>Unigene0000285

AAACTCCTCTTAAGAAAAATGAAAGCTTCCTCATTGAAATTGATGACCACGTTAGATATT

TGTTGCTGGGTACTGAAGGACTAAGTTTTGAAGACCTTGTGCATCGTGTCGATAAATATG

GGGCAATATGGGGCAAAATGAGAAGCACTTAGGGAAAAACTCCCGAAAATAGAAAGTGAA

TTGTCACAAAGTGGGCCGAAGTCCAAGAGTCCCTTCTTTTATAAGGGGCGCCG

>Unigene0000286

TGATAGTCTTGGAGTTTGGCATGGCAGACTTGGATGTGTAGGAGAGGCGCAATCTAAGTG

ACATTATTGATTGGAGTATCGTAGGCTTGGAATATAAGAATGATGTCAACACAACATCAA

ATTAGAAACTACATGGAAGCAGATTGAAATGCTTATGGTTAGAATGTGTGTGCATATTGG

GATGTGTGTATTATCCATGGGCATGTGTATTATCCATAGGGTGTGTATATGTGATTTCTC

AAGAGTATGGGTTGTCTTGTGTTGAGTAGGGCATGTACTCATATTGCCTG

>Unigene0000287

TCACACTTATTTAGCAATCTTAATAATTTTAGTTATAGATGATATATTGTGCTTGTTATT

GTTTATACATGTTTAGTGACCTTAATAGTTGTGACTATAAACAGTTTGTTGATGCTCATA

CATATTTTGCTTCCTTGGTAGTTGTAAATATAAATGATAGCTTATGTTTGGTGGTTTTCA

TATATTCTTGTGACCTCAATAGTTGCTAATTGTTGAGGATAGTATATGCTTAGTGTTTGT

GATGTGTATGCTTAACTATAAGATAATAGTGTTTGTAAATGTTGGGTTATGGAGCCAATC

TAATCTAATAACTCTCTTATACTGAATTACGATGGTTTGATCCTAATGAGGTTAAGTATA

TTGCTTTTTGGAGAGTTGAGAAGTTCATTCAGGTTCATTGTTGCATGATTTATGTAGGTC

ACTCTAATATGTGGGAGCATAGACCAAGTGATAGTCTTGGAGTTTGGCATGCGGCAAACT

TGGATGTGTAGGAGAGGCGCAATCTAAGTGACATTATTGAATGGAGTATTGTAGGCTTGG

AATATAAGAATGATGTCAACACAACATCAAATTAGAAACTACATGGAAGCAGATTGAAAT

GCTTATGCTTATGGTTAGAATGTGTGTGCATATTGGGATGTGTGTATTATCCATGTGGGT

GTGTGTATTATCCATGGGTGTGTATTATCCATAGGGTGTGTATATGTGATTTCTCAAGAG

TATGGGTTGTCTTGTGTTGAGTAGGGTATGTACTCATTGCCTGCATGCCTACTCATTTGG

TGTAATGCACATGCGTAAGTGTTAGGTGTACATATTTGAGTGCTGTTATTTCTCTGGTAA

GTAAAAAAATACTTTGAAAATGCCAAACTATAACTCGTTGGTATACCTTTAGCTACAATA

TTTTATGGGTATATATACTCATGTGTGGTAGTT

>Unigene0000288

ACTCACACGCAACGTCCCCCCTTCTTGTAAATCTAGGGTTATACTGAATCATCTCCGACC

ATCCAACAAATGGACGAAACGAAATCAGATAAAAGTGTGAAGGCGCCCAATTTGTTTGAG

AGAGCTAAGGAGGAGGTCGAAGCAATCATGCACAAGGACAAGAAGAAGACCCATGATAAG

GAAACCCACGGCACGAGCGATGACATCGACGAGAGCACGCCGATTGACAAGGTCAAGGGC

CCGAGCGTGTTCGAGCGGGCTAAGGAGGAGATCGAGGCCCTTGTCGAAGCCATCCACCCT

AAGAAGGAAGAGAAAAAAGATGAAGGGGGATTCTTGGAGTCTCTCGGGAGAAGGTTTGAG

AAATTCTGCTCCCCTTTTACTCGAAAGAAAGATTAATGCATATTGGATTGTGTGATATGT

GCATATTTATGGATACTTTCATGTTGGTGATATATTATCTCTTGTTGTGCAATATGACGT

GATTTGTGGGGTCGGCATGGTTTATTTTTAATTATCGGGTTTATTTTTTTGGCAGAGAGT

AGTAGTTGGGCTATTTGGAGAGGAGTGTCTTGATTGTTGAAAGAGGTTTGGTATAGAAAC

ACCCCTCGGATATCCTATTTGGTGATGTATCTTTTAAGAGAGTCATGAAACAATCTTTGT

TTGTATGCAATACATCTTGTGGTGGATTATGCAGTATTTGACCCTGTAATTTGATTTTTA

AGTGTTTCGTTACCCAATTATGTGTTATTTCATGACTTTAT

>Unigene0000289

TATGTGAATCTCATACTCAATAAGAGATCGGGTAAACTTGATTTCTTTCTACGACACCGA

AGGTGTCCCTCCCACCAAGCTTGAATCCTTCGACTTTACTTTAACAGGTGCAGGGTTTAT

GTCTCGAGATACGTACTACTATGGATTCTTCAGTGCTGCCATCAAGCTTCCATCAGGTTA

CACTGCAGGAGTTGTCATCGCATTTTATGTAAGATCGCCCTGCCCTTATTCGTGTTCTTG

AATCATATCAGTGGTCATAACAGAAATTTATTATGCTGAAACGTATGAAGAAACATGTAT

CCTTTCTAGATAAAGCATATAAGTCGTGATATGTGCAGTTGTGCTAATTAATAGTAAGAC

ATGTCCGATACGAATAAGAGTTATGATCTTTGTAACTTGCACTAAGTAATTCATTAAAAG

TATTGTTGTGGGTATCTTCTCACCAAACCATTATATAATTTATGAACTGCATAACCATAT

ATATTAATTGCAGACTAGTGGAAGTATCAATACCTTGTCAGCTGGGGCATGTTCTTGCAG

TTCAGGGTTGGTATTATTATAGCCCAAGATTAGTCTGCCCTTTGATTCCATCCTGCACAA

AGTAACAAGTTTAATCCTCCAGCTTTCACAAACAAAAAGTATCTATGATGAAAGTATAAA

AATCAATTCGAGGCAACCGCCAACTGAGCTGAATAGTTTGCGTTCTCAAATATATCAATC

ATAGCTGAGTAATATGCCAAGCATATTTTATGCCAATCAAATATATCAATCATAGAGTGC

TCATGAAATTTTGATATTCAAATCCTTCGATTAGATAATTATTTCTATCAGTCTTATTTT

GTACTCTGACTACCAAGTATAAAATACTGAGAAAAAGACAAATAAATTTAGAATACTGCC

AGAAATAATCTGACTTGTTAGAGTTTGAGCTTATCTTGTCTGTGGTTGGTGCCGTCCACT

TGGCATAGATGATGATTCCAAAGACTTTATGAATAAGCAGCTCTGAATTCTGCTTCTTAA

ATTAGCGACCAAACACCTGAGAAATCCTTGTGCGAAGGCACATGGTTGGCTCCCACCTAT

TAATATGATAACGCTGAAGATGCCTTGTAATGCTTCTTATTCATTCTTTGTTAAGCTAGC

CACAATACAGACAAAAATTAAGATGTCACATACCTTATGGAATTATGAATTATCTGTCCT

AGATGACTTGTTTGCCTGCCGTACCAGTTCCTAGACTTAGGTCAAAAGCTTGTGTTATGT

GCATACTGATTAAGCAATAATCAAACCTAATCAAATTGATGCTTGACAGATGTCCAATGC

GAATGTGTTCCCGAATAACCACGATGAGATTGACTTTGAGCTATTGGGGCACGAGAAGAG

TAAGGAGTGGGCACTGCAGACTAATGTCTATGGCAACGGCAGTGTCAGTACAGGACGGGA

GGAGAAGTTCAACCTGTGGTTCGATCCCACGGAGGAGTTCCATGAGTACAGTATCATCTG

GAACGATCACCACATCGTGTTCCTTGTGGATAACATCCCTGTCAGAGAGGTTTTCCACAG

TGAGGCGATGGCCGGAGCATACCCTTCAAAGCCCATGTCCTTGTACTCCACCATCTGGGA

CGGGTCACAGTGGGCGACGCACGGAGGAAAGAAGCCGGCCAACTATAAGTTTGCGCCGTT

TGTTGCGACCTTCAAAGATATGGAAATGGCTGGATGTGTGTCGAACCCGAAGGGAAAGTC

TGCTCCCTGCTCTAATGCTGGAGGACAAAATGGCCACCTGAGCCTTGATCCAGTTGAAGG

GGGAGAGTTCAGCAAGCTATCTCAGCAGCAACAGGCAGGGATGGATTGGGTCAGAGGTAG

ATTCATGTTCTACTCTTATTGCAAGGACCCTAATAGATTTCGGGTCATGCCACCAGAATG

CAAGGCTAGCAAATAAGTTCTTCCGAAAAAATAGCTAGAACAAAAATTTCAATATTTTCA

TGACGGGTTATCTGGCTTTAAAGAGTAATTGAGAAGGTCTAGAGAATACACACAAACTTC

AAAGATTGATTGTGATTTATTATGAGAATTGAGCAACTACCTAATCTGATATACTATTCT

TCTACTTGAGTAAAACAGTGTTCTCCCATGTGTTATATTGATAAATCTAGTGAAGATAAT

GTGTTTGTCAAGTTCAAGATTTGTGTTATGGATCAGTAGTTATTCTTCTTTGAGAGCATC

ATTTAGTTTAGA

>Unigene0000290

TTTTAGAAATTCTGTGGCATTTACCTCTTACCAATCCCGCGTTGCCTTGATCCAAAGACA

ATTTTCTCGCAAACTCAAAATTGCAGGGCAAACCCCTTCCCCTACCAGCCCTTCCCTCGT

CTGTTGTCCTGAGTTCCGATTGGCTGTTCCCTTCAACTCTTGCCTCCATCCACATCAGCC

TCCGGAGTTTAGCCGCAGTAATGATTACTGTAGGAGGGGTGAATCACAAGACAGACCGGA

GTTACACACATTACTCCATGATCCAAGTGGTGAAGTTCCATGTATTCAAGGTCTGATACT

TCATCAGGCAGCCGCGGCATACCGGCTTCCGTAACTGCCAAACGTCATCACGACTAAGCA

CTCAAAGCTCTTATACTGAGACTCTTGCACTGGACTCAGTGCATCTCTGTCGAGGCACTG

CATTATCAACTTAATCTCCATGTCGTAACT

>Unigene0000291

TTTTTGTTGTGTTTTGTTTTGAAGAAAATAGAGCATTTTTATTCTTTCTCTTTTGTGATG

TATTACTATATTAATGATCATAACAAGATTCTAGATTACAGCACGCAGAAGTGGAAGTAA

TAGGACGACCTAATATGAAGAACTGTAGGAGGTGACCCCAAAAAGTTACCATACCAAATT

CAAAGGAGGGTTGATTTGACGGATGAAATGATGATGTTATTGAAATACTTGATGTTCTTA

ACAATATCTGAGTGAGAATCAGTCAGCAGATAACTAATGTGTCTTGCCAGAGATACTAGA

AATGTGTAATCCTTCATGAAATTTGATCATGGAGATGGGGACATGGTATCTTCAGTAGTA

ATGTGATTTTCATCCTCTTCTAATTAAGATGATTATAAAATTCAATGATGAAATATGTAT

ATATACAGTCTATGCATATTGTAAAAATGGTTCTAGGTAGCTCAGATGGCTTTGCTCTTT

TGCATGATGCCATTGCAGCCATTTTTTGTTGGGTGTGTGATGTTTCATTTTCTATATAAT

GTATAAATAAAAGGTGAATCTGAGCATTTCTTGCATGGCTTCTCATCTGATTAATGATGT

TGAGTCAAATGTAGGCTGAGGCAATTTTAGAAGTAAAGGCAGCTACCCTTTTTATTACAA

TTTACCTCTTGTTCAGAGCTACATAAATCTCCGATTTTATTTTTGAAAACATTGCGTTCA

GCAGATCAAAGCCCACTGATAATACCACATACATGATGAAATTGTGATTTAATCATTAAA

CCACGACTTGATGATTTGAAGCCACCTGTTGAATGATGTTGAATTATGTGTAGGCGAGGC

AGTTTGATTTCTGATTTAATACCCAATTTTGTGTCAACCTGATTTCATCATCCGC

>Unigene0000292

CACTGATAATACCACATACATGATGAAATTGTGATTTAATCATTAAACCACGACTTGATG

ATTTGAAGCCACCTTTTGAATGATGTTGAATCATGTGTAGGCGAGGCAGTTTGATTTCTG

ATTTAATACCCAATTTTGTGTCAACCTGATTTCATCATCCGCATCAAACCAACCAGAGCT

TTGTTATAACCCATGCGATATATATATATATTATATATATATTGAAAAGTGATGCATGTT

AGAAGCATCTGTTTTACAT

>Unigene0000293

TTTTTGTTGTGTTTTGTTTTGAAGAAAATAGAGCATTTTTATTCTCTCTTCTGTGATGTA

TTATTATATTAATGATCATAACAAGATTCTAGATTACAGCACGAAGAAGTGGAAGTAATA

GAGCGACCTAATATGAAGAACTGTAGGAGGTGACCCCAAAAAATTACCATACCAAATTGG

AAGGAGGGTTGATTAGACGGATGAAATGACGATGATATTGAAATACTTAATGTTCTTAAC

AATTTCTGAGTGAGAATCAGTCATCAGACAACTAATGTGTCTTGCCAGAGAT

>Unigene0000294

GAGGCCTACTGATCCAATGTTAGAGGAGGGAATGGTTCTTCGTCAATTTGTTGAGGCAGC

ATTTCCTGAAAGGGTAATGGAGATAATGGATCAAACATTGCTTCCATCGGAGAATGGAGG

AGACATTGATTCTATTCTTGATGGAAGCTCTGAAAGAGTGAGAGTAGAGCAATGCTTGAT

CTCCATACTTAGGATTGGCCTCTTGTGCTCCAAAGAAGCAGCCAATGAAAGGATGGAGAT

GCGAGATGTCGTCGCTGAAATGCAAGCAATTAGGGCAACATACCTGAAAGGGCTTTGAGA

AACTTGGGGATGAAATGAAACCATAGGTATAAAGCTATTTGTTTTGTCTGATAACGGCTC

GAAGAAAAGATTAGAGGAGATTTCAGTATCCTCTCAGAATTAATTGTTAATAAGACCCTT

TTATAGAAAAACAAAGTTTTATGTATACAGATTTTGGCAACTCAGAGCTGATATATGTTT

AATGCCCTTTATGGAAAGCTGGGAGAGGGCTTTAACTAATATATGTTTATTGCCTTTATG

ATAAGTTGGGAGAGAGCTTTAACTTCCAACTCAGTGCTGATATATATTTAATGCCCTTTA

TGGAACCTGGGAGAAAGCTTTTTAACTCCGATGTGTAAAAAATTCAAGCTCTAAAATAAG

TTTTCTTGCTTTCTAC

>Unigene0000295

CAGCTTCAGCCGTCCAAAACCCGTTGGGGGTCGTTTTCTCCCCTCTCAGACAGCTGACCT

CCTCTCTCCCTGATCCATGGCGCTAAAACCCTAGATCGCATCCCTCCCCTAATTCCGCCC

TCGATCCCCAAAATCTTGCGCTCGGCGATCGCAATCGGAATCAATGACCTCACTCCCTAG

CTCACCCCTTCTCCTCTCCCTCGTCGCCCTGCTCTTCCTTTCCCCCACCGCCCTCTCGGC

CCCTCAGCCTTTCCGTCGGGACCCTGGCCACCCGCAGTGGCACCACGGCGCGTTCCACGA

TGTGCAGGACACGATCCGGGCCGATGTCCGCCATATGCTCCACTCCCGCGCCGAGGTTCC

ATTTCAGGTTCCTCTGGAAGTTAACATTGTTCTAGTTGGTTTCAATGGTGATGGTGGCCA

CAGATACACACTGGATCCGCATAAGCTTGAGGAATTTCTGAGAAGCAGTTTCGCAACCCA

TAGGCCCTCATGTCTTGAAACTGGCGAACCAATTGATATCGAGCATCATATTATATATAA

CACCATACCTGCTGGCCAGCCAGAGTTAATAGCTCTTGAGAAGTCGTTGAAGGATGCAAT

GATTCCCGCTGTGACTGGCAGAGAGAGTGAATATGGAAGGTTGCTTCCTCTTTTTGAAGT

TGATGCAACAGTGGTGGAGCCAATATTTCAAAGATTATACTCTTTTATATTTGATATGGA

AACTGGTGGATACTCCGCAACTGAAATGGACAGACCTGTGCCTGCTGCAATATTTATTGT

AAATTTTGATAAGGTTAGGATGGACCCCAGGAACAACGAAACTGATCTTGAAAGTCTGAT

GTATAGCAAAATTGGCGGCCTTACAGATGAACAGCTAAAAAAGCAAGAGGGAGATTACAT

TTATCATTACCGTTACAATGGAGGAGGAGCAACTCAAGTTTGGCTGAGTTCTGGGAGATT

TGTTGTGATCGATCTATCAGCAGGTCCTTGTACATATGGTAAAATAGAAACCGAAGAAGG

AAGTGTTAGTTATAGAACATTGCCCCGGTTATCAAACTTAATCTTTCCAAGAGGCTCAAA

TGCTGCAAATGTCGCGGCAACACATGATCTTTTAATGGGGCAGCTTGGAGCTCTTATTTC

CACCACCATTGAACATGTTATTGCTCCAGATGTTAGGTTTGAAGCTGTGGACTTGACGAC

GAGGTTGCTTATACCAATAATAATCTTGAGAAATCATAATAGATATGACATCTTTCAAGC

TGGCCATAACTATAGCATAGATATTCAAGCAATTGAGAAAGAGGTTAAAAAGATGGTTCA

TACTGGCCAGGAGGTAATAGTTATTGGTGGTTCATATGCGTTGCATCAGCATGAAAAATT

ATCAATTGCTGTTTCTAAAGCTATGCGAGGTCACTCTCTTCAAGAAACAAAAAAGGATGG

GAGATTTCATGTCCGTACCAGAACATATCTAGATGGAGCCATTCTCAAAGAGGAAATGGA

ACGTTCTGGTGATGTACTTGCCGCTGGTTTACTTGAGGTCTCAGATCCCTCGCTCTCAAG

TAAATTCTTCCTCCGGCAGCATTGGCCAGATGATTCTGATAGTACTCAGGATTCCTATGT

CAAGCACAAGCCACTTTGGGCTTCTTACAGTTCCAAGCGAGGCAAAGAGAAGAAATCGAT

TTTGAGAAAGAAAGAGGGAAATCTGTATCGTACGTATGGGACCAGAGTGATTCCTGTCTT

TGTTTTGTCATTGGCTGATGTTGATGCTGACCTTTTAATGGAAGATGAAAGTCTTGTTTG

GATGAGCAAGGATGTAATCATTGTCCTGCAACATGATAATGAGAAGATACCTCTAAGCTA

TGTCTCAGAAACAAGAAGGCAATACGCATTTCCTGCTCTAGCACAACGCCATATCTTGGC

AGGATTAGCTTCTGCAGTCGGAGGATTGAGTGCGCCTTATGAGAAAGCATCACATATACA

TGAAAGGCCTATTCTCAATTGGCTATGGTCTGCAGGTTGTCATCCATTTGGGCCATTCTC

CAATTCATCACAGATCAGCCAAATGCTTCAAGATGTTGCTTTGAGAAGCACAATATATTC

ACGAGTAGATTTGGCCCTCCGCAAGATTCGGGAGTCATCAGAGGTTGTACAGTCTTTTGC

CTCGGAACATCTTAAGACTCCACTTGGCGAGGCTGTTAAGGGCAAGAAACAGAAATCTAG

CACAGAGCTGTGGGTGGAGAAATTTTATAAAAAACTTACTAATCTACCCGAACCTTTCCC

CCATGAACTAGTTGAACGCTTAGAACAATACCTTGATAAACTTGAAGAACAGCTTGTTGA

CCTGTCTTCTTTGTTGTATGATCACCGGTTAGAGGATGCACATCAAAACAGCACAGACAT

TCTCCAAAGTGCTGTATTTACTCAAAGGTATGTTGAGCGTGTTTTGGACAATGAAAAGGA

GAAAATGAAGTGCTGCAGCATCGAATATAGCTATCCGACTCAGTCCTCCCAGGCTTTCAT

TTATGGGGGAATCCTTCTTGCTGGATTCCTCGTATACTTTCTTGTTATCTTCTTTTCTTC

ACCTGCCCGTTGATACTGTGATGGTGCCGCCCTCGAGAAACACTTACAGCTTCCCTAAGA

TGCAGACTCATGACTGTTCTTGTACCTTGGCCTAGCTAAGATGTTAAAGATGGAAGTTGG

TGAGAGGAAGTAACCAAGTAGTCTGAGGATATGCCTGTTTCTGGGTATAGTCATTTTTTT

CTCTGGATTTCATTTTGTGAGCACAACATTACATCAGTAGTTTCAGACTTGATTATTGGC

AGTTATAAGCTGTAATGTGGTCTCATTCATTGACTGCAGTTGAACTGTGCATAGCTACAC

TTGATTTACAAGTGAACTTGAACAAATCTAGTGTAATGATCTTTTATTTCTCCCTGGCGA

CTTCTTTAACAACCTGAGTCCAGAGAAACACAATGTTTGTAGCAAGTCATTTTTGGTGTA

C

>Unigene0000296

GAAGGCAAAGCAGAAGAAACTAAAGCTTTTTGCTGAAGTCAAGTTTTTGAGAAGAAAATT

GAAGTCCTTCTTGGGAGATAATTCATTAGAAATACCACTAAAATTGAAGAAAGAATCATA

CAGAGTGGCACCTCCATCCCCGGAATATGAGCAGATGGATAATTCTGGTCTTCCTAGCGA

AGCCCATCTGGATGATAGGAACTACAAGATAGCAGAAACTGCAACTCGAGGGACTAGTGC

ATTGTCAGATTTAAATCAGGTATCCAACAGATCAACATCTCCATCTATGAGATATGCACA

GATGTTCAATTCTGGCCTTCCAAGCAACAACTTTTCGGTAGATAGGAACCACAAAGAAAT

ACGAGCTGCAGCTCGAGGGACCACTGCATTGTTAGATTTGAATCGGGCATCAAACAGATC

GACACCCCCGTCCATGAGCAATTCACAGATGCATAATTTGGGTCTTCCTCCGAAAGCACC

TACGGTTGATAGAAACCGCAAAGTAACAGAAGGTGCAATCCGAGGGACCAGTGCATTGTC

AGATTTGAATCAGGTTTCATACAGACCAGCACCGCCGTCTTCCAGATATTCACGTATGAA

TAATTCAGGTCTTCCGCCGAAAGCCCCTCAGGCCAATAGGAATCACAAAGAGACCGCTGC

ATTGTCTGATTTGAATCGAGTATCATACAGAGTGGCGCCGCCATCCATCAGATATGCACA

TAATTCTGGTCTTCCAAGCAAAGCCCATTCAGTAGTTGGGAACCACAAACTAACACAAGC

TGCAGTCCCAGGGAACACAATACTCTTAGATTTGAATCTGGATGCTGAAGAATTGGAGGA

GCAGGAGTCCCTAAGAATAGATAATCTGAAGAGGTTTTCTGTAGGTGGAAGTACTACAAT

GAATGATCATAAACGGTTGCTGTGCAGAGTTGGCGGTAATGAACGTAACCAGTTACCTTT

GAGAGTGTAGAATTAAAGAGTACAGTCATTCTGCCCTTTTCATCCCAGGCATATACCCTG

AAGCTTACTACTTGTTATTGTTAATCTTTAGTTCGGGATAGTTGCATCCCAAATTTACAT

GCAATCTTTTTTGCAAGTGATATTTTGGTTATGGGGTATAATAACTCTTTTACAGGAAAA

AAAAGAGAGGTTGTTTTGAT

>Unigene0000297

AATTCCCTTGTGTTATGTATCCTGAGCTGTAGTGGGATCACAATCACCCCTAGCGGCACA

TTCCGACACGTGCCACCCGCTCGCCCCCAGATTCGAGGGCTCGATGATTCGCCCTCGACA

GTGATAGCTCCGCCTCAATCCATCTCCTCTCCAGCTCCATCGAAATCGCCGCCGACCTCC

AATCCATTCCACCCTCTCCCTTCGATCCTCCAATTTCTCATGGGCTAGCCGCTGTTGTTT

GCTGTTCCTTCTGTCGTCGCCGGAGGGGAGAAGCTATCGGAGATTGCACGGGATAGAGGT

TCCGGATCGAACTGATACCGAATTCGAGATTTGAAGCCCTTCTTGGAAGATAGTAAAGCT

TTGCAACCATGCAAAAGGATAGCGGTGCTAGTGACCCCCCAACTCCCTCTGGTCGCCTCC

GACATCGTAGAAGGAATAGTGAGGTTGATCCAAACAAAGCGAATGGTTCTCATTTACTTG

TTGATGATCGTAACAAATACAAGTCAATGCTGGTTCGCACACATTCATCTGTGTGGATGA

TAGCACTTTTCGTGTTTGTCATCTACATGGGCCATCTCTATATCTGGGCCATGATTGTTG

TGATTCAAATCTTTATGGCAAGGGAACTGTTCAATTTGCTAAGAAGAGCCAATGAAGAGA

GACAACTTCCGGGCTTCAGGCTTTTGAATTGGCATTTTTTCTTCACTGCTATGTTGTTTA

CCTACGGACGCTTTCTCAGCCGGCAGCTTGTTAACACTGTAAATTCAGACCGATTGCTAT

ATCAACTTGTCAGCGGGCTTATCAAATATCAGATGCTTATTTGTTATTTCCTATACATTG

CAGGCTTTGTCTGGTTTATTCTAACGCTAAAAAAGAAGCTGTACAAGTATCAGTTCAGCC

AATATGCATGGACGCATATGATTCTTCTTCTGGTGTTTGCACAATCCTCTTTCACTGTGG

CAAATATATTTGAAGGAATACTCTGGTTTCTGTTGCCAGCTTCGCTTATTGTTATCAATG

ATATTGCTGCTTATTTCTTTGGATTTTTTTTCGGAAAAACACCACTAATCAAACTGTCAC

CTAAGAAAACCTGGGAAGGGTTCATTGGTGCATCTGTCACAACTATAATATCGTCATTTG

TGCTAGCTAACATTTTGGGGCGTTTCCAGTGGCTAACATGTCCAAGAAAGGATTTGTCCA

CTGGGTGGCTTTACTGTGATCCTGGCCCTTTCTTCAAGCCTGAAAATTATGTTTTACCGG

GATGGGTAACTCTGTTGCTTCCTTGGAAGGAAATTCCAATTATGCCTGTGCAGTGGCATG

CTTTAGCTTTTGGTTTATTTGCATCAATCATAGCTCCATTTGGAGGATTTTTTGCCAGTG

GTTTTAAAAGGGCTTTCAAATTTAAGGATTTTGGAGATAGCATCCCTGGACATGGTGGAA

TTACTGACCGAATGGATTGCCAAATGGTCATGGCAGTTTTTGCATACATCTACCATCAAT

CATTCATCGATTATCAGAGCTTCTCAGTTGAGACAATTCTTGATCAGATTCTGAGGAACC

TTACTTTTGAGGAGCAACAAATTCTGTACCTAAAGCTTGGGAAGATGATCCAGGAGAGGC

TGTTGAGGCAATCATGAAGCCCCATTGTGGACCAGATTCCTTGCGTGGCATGTATATAGT

CTGGTGCTTTTAAGAGCTCATTTCAGATAACCCCGTTTTAGCTTCATGTTAAGCATGTCC

ATATGGTGCTGCTGGTAGCTTCCTGCTCACATGCACTGTGATTCAGATGTGTGGTGCACG

CAGTATTGTTTTGCTCCTAGGAAATGGATGTAACATCAAATGCTTGTTCAGCTTGAGTTT

TTGGTAACTTGAATGGTTATGGCATAGATAGGGCTAACAGATTGGACAATTCCCGCGCAT

AATAGACTGTAGGTTGGGTCGGGTGCTGCCACTGGATGGGCAACATATGAGCCTATCTGA

CATTTGAGTATATTTGATACACTTGCAAGCTAGTCGATCTGTATTGTAATGATTTGTGAT

ATTTCTTTGGCTGCTGTCTGAGTGTCTTGCATTTGGAGATTTAATAGTTTGGTACCTTCA

CCATACGGTGGTTATGCAGATGCCAAGATTGATCCCACAAT

>Unigene0000298

CATAAACCCTTGTTCATTCATCGCCACCGCCTGCTAGGGTTTTTGGCGGCTTCGGTGATA

CCACACCGCCGGAACCCTATCCCTCGTCATCGCCTCGTCCAGCATCCGGCGGCGACTTTT

AGTTTAGGGGTTTGAGTTGGATTTGAGATGGGCAGCGGTCCGGCTCCGTTCTCTGAGATA

GGGAAGAAGACAAGAGATCTTCTGACAAGAGACTACAACTTTGATCACAAGTTTACCTTG

ACAACGTTGAGTGATGCTGGACTAGGGCTTACCACTACTGCTGTGAAGGTGGATCAATTG

TTTATTGGCGATATATGTACCAAGTACAAAAGTGGTAGAACAACAGTTGATGTCAAAGTT

GATTCCAGTTCGAATGTCTCAACTGCTGTTACTGTTGATGAAATAATGCCTGGTGCTAAG

GCTTCCTTGAGCTTCAAAATTCCCGATCAAATGTCTGGCAAGGTTGATGTGCAATACCTC

CATGATCATGCAGCCATTAACTCTAGCATTGGTTTGACACCTACACCTTTGCTAGAGTTT

GCTGCTACGATTGGTAGCAAAGAATTTAGTCTTGGTGGTGAGGTTGGATTCAATAGTGCT

TCTGCAAGTTTCACCAAATACAATGCTGGGATTGGATTTAACCAGCAAGACTTGTCGGCT

GCTTTAATTTTGGCTGACAAGGGAGAAACCTTGCGAGCTTCTTACCACCATGTGTTGAAC

CCGATCAGTGGAGCTGCGGTGGCTGCTGAGATGGTACACAGGTTCAACACGTACGAGAAC

AGCTTCAGCATTGGGAGTTCTCACTCCGTCGATCCACTGACGACTGTAAAAACACGGTTC

AGCAACAGTGGTAAGGCTGGCGTTCTGTGTCAGCACATGTGGAGGCCCAAATCTCTTGTG

ACCCTCTCAGCTGAGTATGATCCCAAGGCAGTAACTGCACCGACTAAGGTGGGACTTGCA

GTAGCTCTCAAACCCTGATCTGTTGGTGAGCTGGTCTATACTGTGGCGAGCTTAAATATG

GTGACATAAGAGTCGGCTGGTACATTTCCCTTTCATATTTCGTTTGTGTTATTTATGTAG

TTTTGTAGCTCAAACATTATCTGGTATTTGTAGGGAAAGTTTGGTTGGGGGCTATAATTT

GGTTTCATCATATCATGATTGAGCAGAATGACTCAAACTTTCTTAGGTTTCGCTCTAAAA

ATGGAATGGAATATACCTGGAATGACAAATAATTTGTTAGAATCAATTGTTTATCGGTTT

AACAGTTTTTTCACTGAATTAATTGAA

>Unigene0000299

GATGTGACTACTGTGGTGGATCAATTGGGAGGTTTTTTTTTATGGGGTTTGACTTGTCAA

AGTATTGATGCGATGTTTTCGTGGATGTGACTACTGTGGTGGATCAATTGGGAGGTTTTT

TTAATGGGGTTTGGCTTGTCAAAGTATTGATGTGATGTTTTCGTGGATTACATTGGGAGG

TTTTTTTATGGGGTTTTGCTTGTCAGTTTTCGTGGATGGAATGGAGATAACTATAAAGAA

GACTTTTACATGTCTAAAATCATTTATTATCATAAGTTTCGAGGATTCTTTTTTGCTACA

GTAAAATGTTAATTTCTTTTAACCACTTTTTATTAATTATTTACACCTCAGTTGTTTGAT

TTCCTGAGAAGAAAAGATCCTGTTTAACCGAACAAGTTGATCTTTTTCTGGGTATCTATT

TATTATCAATATTTTTACTAGAGAAAACTTGTTCAAAATATCCGAAGGACAGAGTGTCCG

GCTCCTGCACTTGTTAGAATCATGTTGTGATCAC

>Unigene0000300

CTGCACATGATACAATTTCAGGATTCACTCAAAATAAGCAAGTTCAAATTTGTTAGTATT

TTACATAATAAGAGTTCAATTTTATCCTTAATTTGAATTCTTTACCCCACTAGAGTTCAA

TTTATCAGATTATAGGAACAAATATAAGTGTTTTTGTTGCAATTGAAATAACATTCTGTG

CAAACCAACATCCTTTCCATTTACTATCTTGACGACACTCCTAAATGTTGGTTATAAATT

CTTGTTATTTTCATCGTGGACAATTTTTAGTGAATTTAATTCTCATTAATTGTGTAATAA

ATGGTATTCGTCTTATTTACTCACATGCTTGATCCTTTTTTCCCAAATAAAGTTTTTCAC

TAAACTTTTCTTTTGTGAATTAATTACTAATTTTAATTTTAGATTATTTGGCTCTACATA

TTGTTGAACACATAGTGCTCTTATCATTTTTAACTATGGTATGAATTCTTCACCATGTGG

CTCCAATTATGTTTAGCTCTTTTTATTTTATTATTATTATGAGCATATATAATCATCCTA

ACGAATCTGATCTTATTTATTGGATAGAGAATTGCGAGTTTTATGATTAATTTAAAATAT

GTCAAAATAAAGTTTTTCAACAGCAAAATATCACTTGATGCTTGGTTAATATGGACGTTT

CTTGGTATTGTGTTAACTAGTTTCGACTCTAGAAAGGATTTATTCACCCATGTGACTCAG

ATTATGGTTTAGTTATCTTGTT

>Unigene0000301

CAGAGTGTCCGGCTCCTGCACTTCTTAGAATCATGTTGTGATCACGGTATCTGTATATAT

CGGAAAAGAAGAGGAACTTAATTTCAAAAGCCCTGTGACTCGTTCCTTGATCACATCCCA

TATAGCATGATTCACACCATGATCACATCCCCATATAGTATGATTCACTCCTTGATCAAA

TCTTATTTTGGACGATTCTCAAAAGTTACAATAGGGGAGAGTGTTTTTATTTTTAATTAA

AATCAGCTGATATTTTAATTTTTATCCACAACGTCCAGTTTACATATTCTTGTTCTTGCA

AACAA

>Unigene0000302

CATATTCTTGTTCTTGCAAACAATTAATATTACACATAATACAGTTTCAGGACCCACTCA

AAATAACCATGATTCGGTCGATTATTATTAACTTTTTCATTCACAACTATTAGAGTACAT

ATTATTGTTCTTGAAAACAGTTAGGCACAATATACAGTTTCATGATCCACTCCAAACAAC

ATTATTGTTTTTGAAACCAATACTGCACATGATACAATTTCAGGA

>Unigene0000303

TACTTAGCTTCATATGGAGTTTGTGTCCAAATCATATCATCTCGAGAGAGTACTGAGATG

TATCCCCTACACGTTTCTCATAGCGTATTGTTATCATATATTATATTGATTATCTTTTAA

TAAAGTATTGTATATTGCGTTGTGAATTTAATTTAAAATGACTGGATTAATATCTATTTC

TTTGTTTCACGATTTCCGTGATTATCATCCAATATGTAGTGGCTCGTGGTTGTCACAACG

TACGACATTCAACACTCCACACTAGCGAGACAAAGTCTCTCTCCTCTTTAAAAGTTTCAT

AAGTATCGTACAAAAGAAATTGGAGAGGAATCCCTAATTTCAG

>Unigene0000304

TACTTAGCTTCATATGGAGTTTGTGTCCAAATCATATCATCTCGAGAGAGTACTGAGATG

TATCCCCTACACGTTTCTCATAGCGTATCGTTTATCATATATTATATTGATTATCTTTTA

ATAAAGTATTGTATATTGCGTTGTGAATTTAATTTAAAATGACTGGATTAATATCTATTT

CTTTGTTTCACGATTTCCGTGATTATCATCCAATATGTAGTGGCTCGTGGTTGTCACAAC

GTACGACATTCAACACTCCACACTAGCGAGACAAAGTCTCTCTCCTCTTTAAAAGTTTCA

TAAGTATCGTACAAAAGAAATTGGAGAGGAATCCCTAATTTCAG

>Unigene0000305

AATCCCTAATTTCAGCCAAAATGTAGGGTGTCCCTGAACTAGGACATTATTTGGTGTGTG

TTGGTTGAATCCTTCATTTCTATGGCTGCGTAACAACCCAAACGCATCCAACAATAACAT

GCTCAAGCTTAGTTGGCTAAGGATCGAGTCGAGCTCAAGTAAGAGCCTAGTTCAGACATT

ACATGGAAATGCTTGTTTGAGCCAATGTTTGTTGTATCAGTATCGGCCAGTTACATATCG

CTGTATCAGTATCGGCCAGTGCCGATACATAACGGATCCAGTGTGGCCCGTATCAGCTGG

TAGCAGTTGTACATTTATAAT

>Unigene0000306

GTATCTTAAGCTTCTTTAAGCCCCTGCTCATGGCTTGCATGGCCAGTCTGGCCATCCTAC

CACGATGGAAGACAACCCCTGTCAGCATGTTCGATTGGGTCAAGGGTAGGCGAGCTCAGG

ACGAGAAGCCAACACAAAAATACCATGAAATCGATCTCCCGTTTCCAACATCTCTGCTTG

CCCGAACACATCTGCGAGGCAGGGAGCTAAAATGTTGCTACAAAGCCTCCACCGACGGCT

TCAGTGCGACCAAATTCCACGAATGCTGTGACTTCAAAGGCCCTTGTGTAGTCATAGGCT

ACACCGAGTCCTCCTTCAAGTTCGGGGCCTTCAACCCTGAAGGATATAGAAGCACAGATG

ATTATTATGACTCATTCGACCCCTTCTTGTTTTACTGGAACATGGATAGTGACAGTGATG

TCTTTCCCTTTGTGCTGCCGAAAATCGGTGGGAGCGGCGCAGCACTTTTCGATTATGCGA

GGGGCGGTCCACAGTTCGGAGCAGACGGCCTGCTCATCGGTCCGCCGCTTGCCCCGGTGA

TGGGCGGGTTTGCAGGGCCCGATACGAACTCTGGGGTAGGGGACCTAAGGCAGGCAAAGT

CTAGGTTGGGGTTGTCATATGCTAAGAGGGAGGATGGGAAGGAGTCTTTGTTTGGAGATG

AGGGGAAGGTGAATTTAGTGGAGGTTGAGGTGTTTTGTAGTCCTCAAATAGCAAGTTTGT

ATTAGTCATCAAGTACTATCATTGTAGGCTGTAAATTGGCTTATAAGATGAAATAATTAA

CTATATTTTACCTCTGTGTGGTTTGGCTATTAATTCATCAATTTG

>Unigene0000307

TTTAATAAATTAATCGATATAGACGTGACATTTTACTGACTGCTCATCTATTATATCGTA

AACAAGCTCTCATAATTCACAAACAAATCTAGCCGAATAGATAAGTGGTCACAAATTCAC

CAACAACATCAAACAAATACAATTGTTCAAGCCAAACTTGAATAATGTCACAAATGAACA

TGATCAAACTAATTACCGAACTAAGTAGCATTGAATACTAATTTCTTGTGTGATTGTAAC

ATGCAGGCAGGGAGCTAAAATGTTGCT

>Unigene0000308

GTTTATAGGAGCACATGTTAAATGAGCGGTTATTTTCTCTATCATTCTCATCTTTATAAA

TACCAAGCTATCTAAAAGAAAAATCTTATCTTTATAAATATCAAGCTAGCTAAAAGAAAA

AGGAGAGAGGGAACATTGGTGGAGAGCTCTACTATATTGTAATTGTCCTTCTATTTCAAT

TTTGGTGAATAGTCCTTAAATGAATAGTGTTAGATTTTTTTTTCTTTTGGTGGTTAAATT

CCTAACACTACATCCTTGACATGTGGGATATATTTTTTGTTCAGAACAATTTTTACCCGA

CAAATATAGATAGGGTCTAGTTCTTGTCGGTACTATTCTTTAGAAATAATTGATTTCCTA

TTAACTCATAAATTGA

>Unigene0000309

TCCAGCGCCGATGCTCCGGCGATCCCCTCGAACAGGACGATCTACATTAACAACCTCAAC

GAGAAGACCAAGCTCGACGAACTCAAAAAGTCCCTGCATGCTGTTTTCTCTCAATTCGGA

AAGATATTGGAAGTTCTTGCCTTCAAAACTTTGAAACATAAAGGCCAAGCATGGGTCATA

TTTGAGGATGTTACATCAGCATCTGAAGCACTTAAGAGGATGCAAGGATTTCCTTTCTAT

GACAAGCCAATGAGGATTCAGTATGCAAAGACCAAATCGGATATTATTGCAAAGGCTGAT

GGTACTTTTGTTCCTAGAGAAAGGCGGAAGAGGCATGATGATAAAGATAAAAAGCGCAGA

GAACAACACCATGATGCTAATCATTCTGGTTCGGCTTATCCAGGTGCCTATGGTGCTGCA

CCGCAGCTATCTCAAATGCCTTTTTCTGGAGGGGCCAAGTCTATGCTTCCCGAGGCACCA

GCGCTGCCTAACAACATACTGTTTGTTCAAAATCTACCCCATGAGACTACATCGATGATG

CTACAAATGCTTTTCGGCCAGTACAACGGCTTCAAAGAGGTTCGGATGGTGGAAGCGAAG

CCTGGGATTGCTTTTGTGGAGTACAAGGATGAGATTCAGGCTTCGATTGCTATGCAAGGA

CTACAGAGTTTCAGGATATCGCCTCAGAATCCGATGCTCGTGACATATGCTAAAAAATAG

GTTTTCAAGGCAATCAGGATTGTAGACAGTTTAGCTTCATTGTGTCCAGCATCTCAGTAG

ATATTTTATTGCTTTGCGTTTCTGTCTCTGTATTGGTTTAGGGACATGCTGGATTCCTCG

CTGGATTGTTTAAGGAATTTTGTTGACAAAAGTTTGAAACCGCTACTTACCTTTCTTTGT

TCATTATATAAACATGTCTTGAGAAGCGCTGAATGCTGAGAGTA

>Unigene0000310

GGGGGTTATGGCGGCATCAGTGGGTGCTTCTGATTCTTGGGTTGCGCAGAATTTGTCGAG

GTATCTTGTGAAGGGCGGCGTGGATATCAGGTACGTCGCAGTCGGCAATGAGCCCTTCCT

CTCGAGTTACCAAGGGCAATATCAGTCTCTAGTTCTGCCTGCAATGGTGAACCTTCAGCA

GTCATTAGCGAAAGTGAATCTAGCCGGTTACGTAAAGCTGGTAGTACCTTGCAACGCTGA

TGCCTACGAATCCTCACTTCCTTCTCAAGGATCGTTTCGGCCAGAACTGACTCAGATAAT

TACCGAAATAGTCTCATTTCTCAACTCAAATGGAGCCCCTTTCATAGTCAACATTTACCC

CTTCCTGAGCCTTTATGAGAGTTCAGACTTCCCACAAGATTATGCCTTCTTCGGTGGGTC

CTCTCACCCGGTTGTAGATGGACCAAATACTTACTACAACGCGTTCGATGGAAACTTTGA

CACTTTGGTGGCTGCACTTGGAAAGATCGGCTATGGTCAGATGCCAATCGTGATAGGTGA

GGTGGGGTGGCCCACAGACGGAGCTCCGGGTGCTAATCTAACAGCTGCATT

>Unigene0000311

CATTGGCCTTCAATCAAGGTCTTGTTAGCCATATCCTGAGCAACAAAGGAACTCCTCTAC

GACCTGGTATCCCCCCAATTGATATTTATCTATTCAGCCTCCTCGATGAAGAGCAGAAAA

GCATACTTCCCGGGAATTTCGAGCGACACTGGGGGATTTTCTCCTTCGACGGCCAGGCTA

AGTACCCTTTGAATCTCGGCTTGGGGAATGGAGCGCTGAAGAATGCAAGGGATGTCAACT

ATCTTCCTTCGAGGTGGTGTGTCGCTGACCCGACTAGCGATCTTGACAGTGTCACAAATC

ACATGAGGGTTGCTTGCGGTGCAGCGGACTGCACAACTCTCTCGTATGGAGGGTCGTGTA

ATGCGATAGGCGAGAAAGGGAACATATCGTACGCCTTCAATAGTTATTACCAGTCGCAGA

AGCAGAAGTCGCAGAGCTGCGATTTTGATGGGCTTGGGATGGTGACGTTCCTCGATCCTT

CGATCGGTGAGTGTCGATTTCTGGTCGGGGTGACCGACAGCAACTGCTCTTGCGTTGGTT

GTGGATTCTTCTGTGGAGTATGGGTTCTGACCTTGTGGGCGATGATATATCTGAGGATGT

TGGAGGTCTTTTAGTGTCAGAAATAGTTTTGCAGTAGGAATCGGTGGCGTATCGCTCGAC

TACTTGTGATACAGTGGTAATATCGGTTTTGAACTGTTAGTCATTTTCCTTCTCTTGAGA

AGGAAAACCAAGAAAATTATCAGGAATCCAGAGACAGACTGCATTCTGCACAGAAGAGTC

TGTAGTGTTGTCGAAAAGCGTTATGGTTGTAAGCTGTAAGTTACTTCTACACTGTAGTTG

AAATC

>Unigene0000312

TTGTGCACCAAACCATGGATACAGTTAATATGGAAGAAAACATGACACGACTCACTAACC

AACGACAATATATGAGACCTTGATGTATGAAGAAAAATTATGGAGTCACATGCTAATTGA

TGAGGATCACAAAAAAAACATGTCGATTGTGACATGTTGTAGTTGATGTCTTGCATCTAT

GGCACCTACAGGGTATACATTTGTTGGGCCTATGGAAGTCGATGACTTGCAACTATGACA

TTAACGAGGGAAGACCGTACCAAGTGGTTGTGGATGAGGTAAGTGTTGCTTCATCCATGT

GAGCAAGTGTAAGGAGTGTGCTTCGAGGTGGAGATATGTAGCATGCACCCATATCAGAGT

TGAAACAACTTGTACCGTGTGTGAGTTGTGATGATTTGAAGCTGTCCACATTAATGAATG

TTATAAATAAAGGTAAATATTTAAGTGAAAGGATATTCTCATTTTCTCTATTGTTCTTGA

CTTATGTTGATTTGGTTAGGTTTCTAGATCGACCTTAACAGA

>Unigene0000313

TTTTTATTAGTTTGTCCAAGATTTTTTTTGTTTGGCGAAACTATTAGGAGAGCAAAGTGT

GTATTTGTGCCATATGATGTCGTGTAATAGTCCATCATCGATATCGATGGATGATGGAGA

TGCTTGTTCTTGAGGTACAACCATCACATGGGCGGCGAATAATATTGTAGTGGTATTGTC

AATATGATGGAAATCTAATGTTGAAGCATGATCGAGATGTGAAGAGCCAATGGTGCAATG

TACTCATGTGTCGGACATTGTAGAAAGATCCAACATACAGATAATGATCATAATGGAAGG

TTGACGAAGATCAACAATGATGGAGAGGTTGTGCACCAAACCATGGATACA

>Unigene0000314

GGGGATGACGGCCATACTCTCAACTTGCTTGCCGTGAGAAACCGATTGGACCGACGGGAT

TAGGGATTCGGTTCGGTACGCTGCCTCGCGTGGAAATTAAACTGCACTGGCTAACTCAAG

ATAATCACACTACACTCTCGTGCAAGATTGACTAGTCATGTGTAGCACACATTGAGCCCT

AAATAACAGAGGGCTACAGTTTCTTCTACTTTTGCAAGCTTGATTAGTCATATTTAGGAC

ACATTGAGACTCTAAATAACAGAGGTACACATTAAGTCCTAAATAATAGAGGACTACAGT

TTCATCTACTTATGTAAGCTTGATTAGTCATATTTAGGACACATTGAGACTCTAAATAAC

AGAG

>Unigene0000315

ATCCCGTCGGTCCAATCGGTTTCTCACGGCAAGCAAGTTGAGAGTATGGCCGTCATCCCC

TTCCGTCCCCTTCCCCCGCCGCCGTCCGCCACCTCTAAATCTGCGGTCCCCGCCCACCGC

ATCATCTCAGCTCAGCTCCCACGGTGGCGCCCAACTCCGCTCCGCCGCGCAACCATCCGC

CTCCAACCGCACTCCTCCATCCAAAACAACAACGGCGCTTCCAGCGAGGAAGAGGTGGGC

AACGAGGCGGCCGACGGAAAGGAGGGAAACCGGGAGGAGGAGGAGGAGAGAGCCGCTACA

ACGGCCGCCACTGTGGCGGAGATCAAGGAGCTGATGAAGAAGAGGGAGGAGGCGTCGGCG

GACGACCTGTGGGGAGGGGTGGCGGCGGAGGTGAGGGAGATCGAGTGGCCGGCGTTCGGA

AAAGTTCTCGGCACAACCGGCATCGTCCTCGCTGTAATCGCTGGATCGAGCGTGGCGCTT

CTCACCGTCAACGCCGTGCTCGCCGAGATCTCCGACCGCGTCTTCGTCGGCAGGGGACTC

CAGGACTTTTTTGGATAAGCATCCTTGTCCACCCAATTGTAATGAAGATTTTAGGGTACC

TTTTCCTAAAATTAAAAAGAAATTATCTACTATTTTTCTATCATAAGCTGGTTTTTTTTT

GGAAGTAGCTTACGAAGATATCTTATTTTTCGTCGGACTGATATGTAAATTTGGCATCCA

TAAGCC

>Unigene0000316

GCTGCCACCTCCATTTCTGTGGGCGTGTCTCGTTATCGTAGACCCACTCGCCGGCCGACA

AGTCGCAGCTTTCCGGCACGCTGAAGACAAAGTCGTCAGGTGGTACTTGTTGGCTCTGCA

TCGATGGTGGCGCTACTTGCTCTGCCTGTTCTTCTTTGTGAGTAGTCTGGTTGTGGTTGC

TGCTGTTTAGATGGGTTTCTAGTTGTTGAGATTGTGGAATGTCGCTCCCGGCAAAGTCTT

GGATGCAGATGAACATGGCAAGTGTGAAGATGGAGGCCACCAAGAGAAAGATGGGGACAG

TTGAGGTCTTCAGACATTCTGTCAACATTTGCTGAGATTTGGAGCTGGTAGGGCGGAGGA

TTCCGGCGAGATACTGGGAGAGGGAGAGAGAGAGTTGGTAGGGTTAGACTGATCTGAAGA

GTTGGTAAGGTTAGGCTGATCTGAAGTCCTTTGTTTGTTTGAAGAGGTTTTCTTTCACTT

GCACCCAATCACTTGCACCCAACTGATGTGCTTAACTAGGATTATTCTGCAACTCAAAAT

AAGTTGGAAATAACTGCTCAGTTTGTTTTTGAGCATAAAGTTTGTAATTTCAATAGGAGC

CACAGAGATAATAAGTTATCCATAAATGGAATTTCATTTATAAAATCATTCTGACTACCA

TATATATATATAAAATATTAATTTACTAACTGATTTTGACTCGGTTGGCAGGTGGACTGA

TCTGAGGTTCCATTCTAACAAAGGTTAAAAGTATGAATTCCGAAAGAAAAACACAGTTGT

AAGGAATTATTTTTCTTTGTTAAAAGTTTGAACTCCGAAAGAAAAACACAGTTGT

>Unigene0000317

CTGGAGAACGTCATCCGTGATGCTGTGACCTATACTGAGCACGCCCGCCGCAAGACGGTT

ACTGCGATGGATGTGGTGTATGCCCTCAAGAGGCAGGGGCGTACTCTGTACGGCTTCGGC

GGCTGAAACGATTGATCTTCTGCAAGCTTTAGTGGTACTGGTGTCTGGTGGCTATTTCCT

TGTGTTGTTGAATGTTGATGCTGGTAGGACTTCTGTTGTGTCTCCCGTAAAATTATCAGA

TTTCTCATCTGATATTGAA

>Unigene0000318

GGAGACCCGCGGGGTGCTCAAGATCTTTCTGGAGAACGTCATCCGTGATGCCGTGACCTA

CACTGAGCACGCCCGCCGCAAGACGGTGACTGCGATGGATGTGGTGTATGCGCTCAAGAG

GCAGGGGCGAACTCTGTACGGCTTCGGCGGCTGATGGGTTTGGTTTTCTGCAGGCTTTTG

TGGTGCTGGTGTCTATTTCCTTTTGTTGTTGATTTGGAATTATGGTATGTCTACTGTAAA

ATTATCAGATTTCTCACTGATATTGAAATGGTCAAAGTTTCTTTTGAGTTTGAA

>Unigene0000319

GGAGAACGTCATTCGCGATGCCGTGACTTACACTGAGCACGCCCGGCGGAAGACGGTGAC

AGCGATGGATGTGGTGTATGCGCTGAAGAGGCAGGGGAGGACGCTGTACGGCTTCGGCGG

CTGATGGGTTTGAGTTTCTGCAGGCTTTTGTGGTGCTGGTGTCTGGCGTCTATTTCCTTG

TGTTGAATGTTAATGCTAGTAGGATTTCTGTTATGTCACCTGTAAAATTATCAGATTTCT

CATCTGATATTGAACGGGAGTTGAAAAAAGTTTATTTGAGTTATCTTCTTGTGTGATCCG

TTCTGTTGTGAGATGGTTCTTTTTTCTTTGGTATGGGTCAAAGTCTTGAGGAGGT

>Unigene0000320

CCTTACCAAAGAAAAAAAACTCTCCGCCGGCGGCGGCTTCTGGCCGCGGCTCCGGGTTAG

GGTTACGCTCCAGATCGTGGCCCTCTCCTAAACCATGGCATCCCCCGCCGAGTTCGAGAT

CTCCGTCCTCGGCTTCTGGCGCGTCATGGCCGCCGGCTCCGTTGCCGGCATGGTCGAGCA

CATGGCCATGTTCCCCGTCGATACCCTCAAAACCCGTATGCAGATGCTCGCTGCCTCCTC

CCCCCACCACGACGTCCTCGGCCGCTTCGCCGCCTCCATCCTCCGCTCCGAGGGCGCCTC

CGGCCTCTACCGCGGCATTGCCGCCATGGGCCTCGGTGCAGGCCCTGCCCACGCCGTCCA

TTTCGCCGTCTACGAGCTTGCCAAACACCGCCTCGGCGCCAACACCGCCGACGGCCGCCA

CCACCCCCTCATCCACGCCGCCTCCGGAATCCTCGCCACTACTGCCAGCGATGCGGTTTT

CACTCCAATGGACGTCGTCAAGCAGCGGCTGCAGCTGAGGCGGAGTCCGTACCGAGGAGT

GCGGGACTGTGTGGCGAGGATTCTCACGGAGGAGGGGATCGGGGCCTTTTATGCGTCTTA

TAGAACTACCATCCTCATGAATGCCCCATTCACAGCGGTGCATTTCTCTACTTACGAGGC

GGTTAAGAAAATTCTCAGCGAGGTCAAGCCGGAGAATGCTAGTGAGGAGAGGCTCATGGT

GCATATTACCGCCGGTGGGGCGGCTGGCGCTTTGGCTAGTGCTCTGACGACTCCATTAGA

TGTGGTTAAGACGAGACTGCAATGCCAGGGTGTGTGTGGTGCTGATAAATTCGGTAGCAG

CTCGATTCATATGGCTATCAAGAAGATAGTTGCAAAAGAAGGACCTCAAGCTTTAATGCA

GGGGTTGAAGCCCAGAGTATTGTTCCACACTCCCGCGGCTGCAATATGTTGGTCCACATA

TGAAGCAGTAAAAGGTTTCCTTAAGCGCAGATCTGATGAAGCATGACTACCCTGATGTGA

GATGGTTGTTTTCGTTCTTTCCCATTCGTACGAGTTGCTGGAATGGGTTTTTCATTAGAC

CAGCATGATGATTTGTTGGTTGATTGTTGTTATCGACCATTCTGTGAGAGTAAACATTTG

TTCGGGATTTGGGTTATGTTATACCATAGAGAGCCTAAATTTCTCAGCTTTTTGGAGGAG

ACGGTTGTGTAACTGGCTATTCAGTTTTGTTAGAATTTGTTTTATTTAATCAGAGATGTT

CTAACAAACATTTGTTACGATACTTAAGGCTTCTCTCTTGCTTAGATTAGCTTGTAATTA

ATTTGGTTCAATATGTCTTTAAAATGAAGGGTTT

>Unigene0000321

TCATGCCAATAGCAGTGTAATCAATGACTTATTGGTACATGGAATCAAGGGTGTATGTTG

TACACATGTGGTCTGACTGTTTTCTCGATTGTAAAAATACTTGGGATGATGAATAATGAC

TACGTATGGATAGCAACGAACTGGTTGGCTTATGTTTTGGATTCATCTAAGCTTTTTAAT

CCCCGCATAATGAACTGTATATAAGGGGTCATTGTTTTTAGCCAATATACGCCAGATTCT

AATATCAAACAGGTATTTATATCTAGGTGGAGACATTTACTCAGAGAAGAGAATG

>Unigene0000322

TCATGCCAATAGCAGTGTAATCAATGACTTATTGGTACATGGAATCAAGGGTGTATGTTG

TACACATGTGGTCTGACTGTTTTCTCGATTGCGAAAATGCTTGGGATGATGAATAATGGC

TACGTATGGATATCAACGGACTGGTTGGCTTATGTTTTGGATTCATCTAAGCTTTCTGAT

CCCCGCATAATGAGCCTTATATAAGGGGTCATTGTTTTTAGCCAATATACGCCAGATTCT

AATATCAAACAGGTATTTATATCTAGGTGGAGACATTTACTCAGAGAAGAGAATGCCAGT

TCAAGTTTGAATACT

>Unigene0000323

TCATGCCAATAGCAGTGTAATCAATGACTTATTGGTACATGGAATCAAGGGTGTATGTTG

TACACATGTGGTCTGATTGTTTTCTCGATTGTGAAAATGCTTGGGATGATGAATAATGGC

TACATATGGATAGCAACGAACTGGTTGGCTTATGTTTTGGATTCATCTATGCTTTCTGAT

CCCTGCACAATGAGCCTTATACAAGGGGTCATTATTTTTAACCAATATACGCTAGTTTCT

GATATCAAACAGGTATTTATATCTAGGTGGAGACATTTACTCAGAGAAGAGAATGATAGT

TCAAGTTTGAATACTTACG

>Unigene0000324

AATCCCTCCGCTCCCTCCGCTCCCTCGCCGCCTCCCCCATCTTCTCCCCTAATCCCCACC

GCCCCCATCTCTTCCTCCGCCGCTTTTTCGCCGCCGAGCCCCACCCCGACGATCCCTGCC

CTTCGCCGGACTTCGACAGCGCCGATTTCACCCTCCCCGCCACCTCCTCCTCCACCTCCC

CACAGCCGTCACAGCCCACATGGGACAAGACCTACCGGGAGAAAGCTGACCGTGCTTTGT

TTGGGAACAAAACGGTCCGATCCGTGGAGGAGGATGAGGAGAGGGGGCGGCGGCTCGCCA

GGGCGCTGCTCGACGTAGCGTTGGAGCCGCCGGATGAGGATGAGGAGGAGTCACCGGTGA

AGCTGGAGGACCAGCGGTCGCTCACCGTCGGGATTATTGGCGCGCCGAATGCCGGCAAGT

CTTCGCTGACGAATTTGATGGTTGGAACAAAGGTTGCTGCTGTCTCACGAAAGACCAATA

CAACAACTCATGAAGTCTTGGGAGTGATGACCAAAGGGAATACTCAAATATGTTTCTTTG

ATACTCCAGGCCTGATGTTAGGTCACCATGGATATCCATATAGGTCAGATGTTAAAGTTC

GTGTTGAAAGTGCTTGGAGCTCTCTTGATCTTTATGATCTGATAATAGTTATTTTTGATG

TCCATCGGCAACTAAGCATTCCTGACACGAGAGTGATGAAGTTAATTAAACATCTTGGAG

AACAAGTTCGGCCTGAACAAAAGCGGGTTTTATGCATGAACAAGATTGATTTAGTAGAAG

ATAAGAAAGACTTGCTGACTATTGCTAAAGAATTTGAAGATCTCCCAGGATATGAGAGGT

ATTTCATGATATCTGGACTAAAAGGTTCAGGAGTGAAAGACCTTGTTCAGTATTTGGTGG

ACCATGCCATTAAAAGACCTTGGGATGAAGAACCCACAGTCATGAGTGAAGAAATAATGA

AAAACATATCATTGGAGGTTGTTCGAGAAAAGATGTTGAACTACATTCATCAGGAAATTC

CCTATGCTATTGAGCATCGGTTGATGGATTGGAAGGAACTACGAAATGGTTCTCTGAGAA

TAGAGCAACACTTTATTACCTCAAAACCAAGCCAGCGCCAAATTCTTGTTGGGAAGAATG

GCTCTAAGATAGGGAGGATAGGCATGGAAGCAAATGAAGAGCTGCGGTCTATATTCAAGA

GGGAGGTGCATCTCATACTACAAGTTAGACTTTCTAAAAGGAAGGGTGCATGAATTTTTT

ATTGTCTCCTGTTTACACAATACACATCTATCTGACATCGCATTAAAGGTGGGACACATT

TTTTAGACCAGCTGAGTCTTGGAACTTTGTATGAAGGGAGCAATGTAGCCGAGACTTCAA

TTCGGCCAATCAGGTGCTCGTCTTTGTTGGATTGGAGGCAATACTTTGGTGAGACCAGCC

TAGATTGATTAAGCTTTTGGAAGATGGTGAATCTGTATGTTATTTGTATTGCTATTTATC

AAATAGTTGATTGTCTCGTGTCATCAATATTCAATAGAAGTTACTTTGCTTCTACTGAGT

TGTGTATGAATCAAACTCTCTTGTTCCTGGTTCTAAATTTTATCTAAACGGACGAGAAGG

AACTAATTTGTATGACCCAATTTTCATTCATAATGTCAATGTAAAGATAAGTGTGCTGTA

GCTACAGCTGCAACAAAGCTACAATTAGGTTTTACATAAAATGTCTATACATACCTTGGG

ATGTAA

>Unigene0000325

TATCGATCAAAACCCCTCCCCTCCCATCAAACACCTCACGCGCAGAACCCAAACCCCCAC

TCGATCCCCTCCTCCGTGCTCAAGATGCCGCACCGGTCACGCCCCATGGCCGCCGTCCTC

CTCTTCGTCGGACTGAACGCCGTGCTGATGTCGACCATCTCCCCTGTCTACGACTTCGTC

TGCTTCCTCCCCTACTGGGAGCGCAGGAGGGAGCAGCGCCGAAGGGAGCGTGGAGCTGTT

CTTGAAAATGTGCCTGGATCGACCTGAAGTCTGACTCACCATAGGTTAACTCCCGTGTTC

CAGTTATCTATGTAATTCCGAATCTGTTCATGTTTACCATGGCATAAATAAAAACCTTTT

GCAATTGCAAACTGTCTTGTTGCTGTTTTCTTGTTCACATATTCTCTATTAAGTTGGTGA

TTTTGTTTTTCAAAGTCATTCTGATCCATGTAAAGGATTTGCCACTGTTTGGGCTATAAG

AGGACACTGACAGTCAATTATGGATGATTCCCTTTCTGATAGAGCTGGAATGAACTTGTG

ACGTGCTTTCAGTTCCCAATGGTACAAAGTGTTTCATTCATTGTTTGCTGAACAAGATTA

AACTTTTTTGGTGTGTTTCTTATATGCCTGTCTGGTCACTGCACAACTCATATTTGTCTA

AGTTTGCCCTTTCCCTATTATCCATGTTGTGTCTCCTGTAACGATAGAATAGCA

>Unigene0000326

CAAGTCGATACTTACTCTCACGCTGGAGTGGTTTCGCAGGGCCACGTCCCTGCACTCCGC

TAAGGTGTTCTTTGTTGACAGGTATTCGATCCCTCACTCAAGCCAGGAATTCGTAGACTC

TTCCTGCCGGCCCACTGAAGATCTTGCATCATAACTGACCAACACGGTTACCGAAAACCC

TGGTGTGCTTGATCCCATTGATTATGAAGCTTGTGCCGATGAACCCACCGGTTGTCCATG

ATGAGGCCGAGGACGTTTTCGGAGATCTTCATCCGCCATTTTCTCTCCTTATTTGGCGCC

GATATCTCATGCGAACGAGAGACAGTGGGCGGATTCTCCGCTGCAAAGGAGAGAATTTGG

GTTTTGGGCGTCGGCATTGAAGTCCTAAGGACCATCTACAAATGCTGTGTTGCAGAGCGC

CTGGATTTCAAGAGTTCTGTTGAATCAATTCTTATTGGATCCATTCTCGTCAAACATCAA

ATCCAGGAATAAGGTATTTTTAACCAAATGAGAAACAACTAAAGTCTGGGTAGTCGAGCA

GATGTCACTAATCCTCTGCCAGCTAGGCAGGATAGCGATGAATTGAACAGCAAGAAGTTA

AAAGTAAACATCCAGTTAATGCTTTTCGTTGGTAGTGCCACTAGGGATTTTTGTTGTATT

CATTCAGCTCGTTGGTTAGGGATTGTCGTTAT

>Unigene0000327

GCGCCTATAAAAAGCCCATTTCTTACCATTCTCAAGGTACATGCGCTCTTACACACTCTC

TCAACCTGCTCTCTCCTTCTTTCCTACTTGCATACAAACGAGCCAAAACGCTGGTACTTC

GCTCTCTCACGAGTGATCAGCTATACATCTCCTTCTCAAGTCGATACTTACTCTCACGCC

AGAGTGGTTTCGCAGGGCGACGCCCCTGCATCCCGCTAAGGTGTTCTTTGTTG

>Unigene0000328

GCGCCTATAAAAAGCCCATTTCTTACCATTCTCAAGGTACATGCGCTCTTACACACTCTC

TCAACCTGCTCTCTCCTTCTTTCCTACTTGCATACAAACGAGCCAAAACGCTGGTACTTC

GCTCTCTCACGAGTGATCAGCTATACATCTCCTTCTCAAGTCGATACTTACTCTCACGCT

GGAGTGGTTTCGCAGGGCCACGTCCCTGCACTCCGCTAAGGTGTTCTTTGTTGACAGGGA

TTCGATCCCTCACTCAAG

>Unigene0000329

CCCCCTGTGCAGCGCAGCGAATACCTGCCGGTCCGGAAACAATATCAGGCCGGTCTCTCA

GCTGGGAAGGAAACAGTTTTCGTCCGGCCGGCGCTGTGGATATCGTACCCCTTTAGATCG

ACCAACCCGGCCGAGATCAACTTTGATGTCATAATGTTTGGAGAGAAGCTGGTCAACGTG

CAACTCAGACGTCCTCAGTGAACAAAAGGCTACCAAATGGTGTTTCGCGCTCGATTTTAC

CATTCCTTGTCCTAGACGGTGGCGGCGGTGAGGTGGGGTGAAAGACGTTGAAGGCTTATT

GCTATTGTTGTTTTACTTGGTATCATGGACCGGCTTGACAATGATCAAGAAGGAACAAGC

GTAGCAGGATTCGAAGTTCCAAGATCGCCCGATTCAAGCTACAGCAACCCTATCCCAGGG

AATGAAGACGAAGCTCGCGACCCACCTTTAGTCCCATCTCACTTGCACCATTCCTTGTTG

AGCTACCCTGCTAGCGAAGATGGTTCAACCAGTCTCCCTGCACCGCAAAGTGTTATTCTC

AACCACCTCTACATTGAAAACAGAGAAAGCCCAAGATCAGTGCTAGCTCTGGGGATCACG

CACCGATACCGCTCGAAATACGTTACGGTTGTCCTTTATAAACCGATCCAGAGAAGGTGA

TCTTTGTGTCGGAACATGACGTATTAAGGTGTGCTTTTGGTCCGATTTGGAACCAATATT

ATTCAGATCTTTCATTTTCGGTTGCAGTGTTTCTGAGATTTGTGTACATGTATAGATTAT

TGGTGTTGATCTTTCCGTGATTTATTTACTTAACTATGGGTTACTGAACTAAGGAATTGT

TGTTTGTAGGCTTAGAATGAGTTTAACTATTTGGAATTGTTGAAGACACCTTGGGTTCCG

TTTATTGAGATTTTTT

>Unigene0000330

AACTTGAAATCTTCTTTGTCATTCATGGATGTCTTCATCATGAACTTGGCATCATCCTCC

AACGGCTCCACAATGATCTTGTCGTCATTATTCGATGACTCCACCTTGAGCTTTATCACC

AACCTCCAACGACTGCACATTCATCTTGAGTGTCTCCTCCATTATCGCTCTGTGGCGCCC

CTCTGCACACCACAGGGAGGCCCAAGACACATGAAAGTTTGTTCCTTCCCTAAAAGAGTC

TTTAACTATCAACCAAGGAACTCTTCCTTGCAAATGTATCTTTCTTTCAATCTTGATATT

CAATAAAGACTCTATAACCTCTCTCTAATTAATATTTAAGGAATATTTACCCCTACTCCT

CAACAAACAAAACACTTTCTAGGTTGACTTCGAGGCATT

>Unigene0000331

GGAACACAATTTCAATCAATTTCTTCTTTTATTTATTTTGAGTTTCATATTATATAAATT

AAGTTTTCACTTATTTAATATCTTTTGTGCAAACTCTGATTAGGATGAATTTTTGAGCCT

CCACCTCGAAGATGATTTCTTACGTACCTATCAAATTCTTACAGGAATCAAGCCACAACT

ACATCTTATGTGTATCTGTCTTTCCCGATCAACGTCTAGATATGGAAATCAACTATGGGC

TCCATGTCAACCATTTTGGTGTTCGGATCCTACCTCTCATGATGAGTTGGGATCTTCACG

ATAAGTTGGTGCGTCAATGAGTCATCCCACACTTCCACCCACATCAAGCTCATATATGGA

TATGTCGACGGCTTCTTCTTGTCAATCTTAGTACTTTTCATGATGGACTTCTCATGATGT

GATCGGATCCTCCTTGCTCAACACTCGACACACTAGTACGGTTTGGGATCCTCCATATGG

TGCATTTCTCCACCACTGGGCTTCTCCATATGATGTCTTCATCATGAACTTGGCATCATC

CTCCAACGGCTCCACAATGATCTTGTCGTCATTATTCGATGACTCCACCTTGAGCTTTAT

CACCAACCTCCAACGACTGCACATTCATCTTGAGTGTCTCCTCCATTATCGCTCTGTGGC

GCCCCTCTGCACACCACAGGGAGGCCCAAGACACATGAAAGTTTGTTCCTTCCCTAAAAG

AGTCTTTAACTATCAACCAAGGAACTCTTCCTTGCAAATGTATCTTTCTTTCAATCTTGA

TATTCAATAAAGACTCTATAACCTCTCTCTAATTAATATTTAAGGAATATTTACCCCTAC

TCCTCAACAAACAAAACACTTTCTAGGTTGACTTCGAGGCATT

>Unigene0000332

GTGCTCAACACTCAACACAAGAGTATGGTCCGACACAAGAACATTCCCCACTAACTTGAA

ATCTTCTTTGTCATTCACAGATGTCTTCATCATGAACTTGGCATCATCCTCCAACGGCTC

CACAATGATCTTGTCGTCATTATTCGATGACTCCACCTTGAGCTTTATCACCAACCTCCA

ACGACTGCACATTCATCTTGAGTGTCTCCTCCATTATCGCTCTGTGGCGCCCCTCTGCAC

ACCACAGGGAGGCCCAAGACACATGAAAGTTTGTTCCTTCCCTAAAAGAGTCTTTAACTA

TCAACCAAGGAACTCTTCCTTGCAAATGTATCTTTCTTTCAATCTTGATATTCAATAAAG

ACTCTATAACCTCTCTCTAATTAATATTTAAGGAATATTTACCCCTACTCCTCAACAAAC

AAAACACTTTCTAGGTTGACTTCGAGGCATT

>Unigene0000333

ATGGCAGCTCTGGGAGCCCCAGCTGCACCTCCAAGGAAAACCAGCCCACCACGGCAACAA

TCTTTGTCGTGTTCTCCTAAACATCGTTATTTCGAAAGGGAATAATCCCCCACCCCCTCT

CCGTCCCCAACTCACCGGCTGAATAAACTCCACCACCACCATCAATGGCGATGGCCGATC

CTTCCTACCCTCCCATCTACCGCCAACCCAACACTGATCCACCCGTCACCGACCTGCAGC

AGGGCCAGGACATCCCTCTCATCGACCTGCAGCACGGGCTCGACCCCCGCCGGCTCGGCG

ATGCATGCAGAGAGTGGGGGGTGTTTAGGTTGGCCAATCACGGGATACCAAAGGAGCTTT

CGGCGCTGCTCATGGAGGAGGCGAAGAGTGTTCTGTGTATGCCTTTCGAGACGAAGCAGG

CGATGGCTGAGAAATCAGTTGTTTACTTCTGGGGCACGCCGGTTGTTACGCAGTCGGTGA

AGAGCCTGCACTGGTTTGATGCGCTGCAGGTTTTGCTCGGCCAGCAGGAGGCCGAGGGGG

AGGTGGGTTCTGCGACGTGCTCGTCGGGTTTCAGGTCTTTGGTGGATGAATATGGTTCCC

ATATGGCACGAATAGCAAGGACGGTATTTGATGCTTTGGCAGTTGATCTCAAGTTGGAGA

GCGGGTTATTGACTTCTTACCTTTGTGAATCTGATGGCATATTTCGAGCCTACCGTTACT

TATGTTGCCCCAATACCTCCAACCATATTGCATTCCATGCTCACACAGATAGCTCCACTG

TATCCATTCTGAACCAAGATGAGGTAGGAGGTTTGCAAGTCCTTCATGACGACGTTTGGC

TAGATGTAGCACCAGTGCCTGACACACTTATTGTAAACTTGGGCGATATGATGCAGGCTA

TTAGCGGTGACCAGTATAAGAGCGTGGAACATAGGGTTCTGATGAGATCGACCAAAGAAA

GGATCTCCTTATGCTACTTCAGTTTTCCATCTGATGAGGGTGTTATTGTCAATGCGAAAT

ACAAGGCATTCACCTACAAGGACTTCAGAGACAAGGTGAAGGAAGACATTGAGGGTTCTG

GTTACAAAGTCGGATTAGAGCGCTTTAGGATCAATGATCTGTTATGCTAATCTTATGATC

AACTTCCCTTGTCTTCCTTTGAAATCTTAGTTTCATCTGCAAATCCAAATAATTTTATGT

AAATGTTGTAGTACTTTACCTGATGAATAAGAGTGCTTTATTTAGATAACCCACCTTGTT

TGTTTTAGATTTTGAAAGCATATCTTTCATCC

>Unigene0000334

CCTACAAACTGTGCGGCAAAAAGCAAGCCGCGTGTCGAATGTGGTGCCTCTAAACAACCT

CTCCGCCGGCGAGCTCGCCGCCGTTGAAAACCTTATGGGCGAGCTCGCTGCCGCAGTCCC

CCCATGGATCACTATCAAACCCTAGGTCTCACCAGCGCCGCCACAAAAGACGAGATCAAA

GAGGCCTTCCGCCGCTCCGCCCTGAAATTCCACCCCGATCGCCACTCCAATTCCTCCCCG

GAGATCCAGAAGAACGCTTCCCTCCGTTTCCGCCTCGCCTCAGAGGCCTACGAGATCCTC

GCCGACGACCGCCGCCGCACGGAATACGACCTCCGCCGTCGGGCTGGATGGGGTCCTTCG

GCGAACTACGGGGCGCCGCGCTACGGTTACGGCGGTGGGGGCGGTTACAGAAGGCAGACA

GCGGGGGCGGGTTTCGATGGAGGCTTCATGTTTCAGTACCTCAGTGGACGGCGGTTCCTC

GCCAATCTTGCCTTTGCGAGTGCATTGTTTGGTGCATCTGTTGTTATTCAGAAAAGTGGT

GAAGCAATCTGGAAGATGAACAATTCTGGGATTTTTTTGCAGGAGGGGATTGATGCCTGA

CTCTCTCTTTCTAGTTTTATGCATTGTGGATGTTTTCCTAGGACTGCCGCCATCAAGCGA

CACGATATACCCTCTGATCACGCAGTTTTGTGATATTTTAGAGTAACTTCTGGGACGCCT

ATTTAGGTGTGATTGCTTTTTATTTAAATACCTCTATGTTGAGACAGTTCGTGCTTACCC

GAGTATAGTTCGATATTAGATTTGTCACTATTTTGTGTACGAAGTGCTTAGAAGAATATG

AAGACAAAAGACAGAGAGAGTTTGCGCTAACCCAAGTATTGTACGAAATTAGATTTGTCA

CTATTTTGTGTACGGAGTGCTTAGAAGAATATGAAGACAAAAGACAGGATTCACGATTCC

CGGACCAGAAATGAGTCCTATTACGGAAGTCGTAATAGTGGGAAAAAAAAAAGAAATCAT

ACGACGAAGCTATCGAGTCTATTGAAAAGATGAAAGTACAGAAATAGTGGATGGGATTTT

CCGGATCTCTTTACAGGCTTAAATCGGTTTCCATTACGAGAGGTTCTCTCAGTGTACATA

AACTCATATCGTTCCCCTTGTTTTTATGTGAATATGTAACTATATAAACGACCAAATAAC

AAGCTCCTAATAATTGGGTTGCTGCGTGCAGCCTGTAAGAGGCCTCAAT

>Unigene0000335

AAATCATACGACGAAGCTATCGAGTCTATTGAAAAGATGAAAGTACAGAAATAGTGGATG

GGATTTTCCGGATCTCTTTACAGGCTTAAATCGGTTTCCACTACGAGAGGTTCTCTCAGT

GTACATAAACTCATATCGTTCCCCTTGTTTTTATGTGAATATGTAACTATATAAACGACC

AAATAACAAGCTCCTAATAATTGGGTTGCTGCGTGCAGCCTGTAAGAGGCCTCAAT

>Unigene0000336

TGTGGGAAGTCAATGGCGGGGAGGAGGGATGGGGGCGGGGAGGTGAGGAGGGCGCATATT

GTGGATACATTTGGGTCGTCGGTGGCTGAGCCGTGGTTGGCGGCACATGCAGATCCTTCC

AGGTGTGTTCTACTTGGTGTGAGCTGTGGAGCAAACATAGCAGATTTCGTTGCACGGAAG

GCAGTTGAAGCCGGGAAGCTTTTAGACCCCGTCAAGGTTGTCGCTCAGGTTCTAATGTAT

CCCTTCTTCTTGGGTGGCACCCCCACCCCCTCCGAACTCAAGCTAGCAAACTCCTATTTC

TATGATAAGGCTACATGCTTGCTTGCCTGGAAGCTATTCTTGCCCGAGGAGGAATTCAGC

CTCGATCATCCAGTCGCAAACCCTCTTGTACCAAACAGAAGCCCACCACTCAAATGCATG

CCCCCGACCTTGTCGGTGGTCGCAGAGCACGACTGGATGAAGGATCGTGCGATTGCCTAC

TCACAGGAGCTCCGAAAGGTAAATGTTGACGCCCCAGTCCTAGAGTACAAGGATGCAGTG

CATGAGTTTGCCACCCTCGATATCCTTTTCAAGTCACCACAGGCGCAGGCGTGCGCTGAG

GACATTGCCATCTGGGTCAAGAAGTACATATCGATCCGCGGCCATGAGTTCTCATATTAG

ATGTTTGAAGAAAGGTGGGGGTCCGTTTTCTGTATGTTTTGTAGATTTGAGTCAGGAGCA

TTGGTATATTAGTCGGGATGTATTCTAGGTTTTTTTTTTTTTTGGTTTACCAATGTGGTT

GTGTAGAGATGACTGTTGTTTCAGTTCTTGTGCCGAGTTTGTTAATTTGTGTAATGTAAT

TTGCAATAGTAGAAAATAGTGCCCATTTCCCTTA

>Unigene0000337

CAGTGGAGTGAGAAAACTCTTCTCTCTCCTCGGTATGCCAGAATTACAGGCGACGACGGG

CTCCCTCCCCTCCAGCAGCGAGCGGTCATCCAGCTCAGCTCCTCCGATGGAGATGAAGGA

AGGGATGGAGAGCGATGAGGAGATACGGAGGGTGCCGGATTTCGGCAGCGGCGGTGCGTC

GACGGCGGGGAGGGAGGCGGCGTTGGCGAGGGCGCAGGCGGGGCAGAAGAAGCGGGGGAG

GAGTCCTGCCGACAAGGAGCACAAGCGGCTGAAGAGGTTGCTCAGGAACCGGGTGTCGGC

ACAGCAGGCTAGGGAGAGGAAGAAGGCCTACATGACAGATCTCGAGTCGAAGGTGAAGGA

CCTCGAGGGGAAGAACTCTGAGTTGGAGGAGCGGCTATCCACACTGCAAAATGAGAACCA

GATGCTCAGACAAATATTAAAGAACACAACTGTGAGCAGGAGGGCTTCTAGCAGCAGTGC

CAATGGGGATGGAGCGCTGTAGGATTAATTTTACCACTATGGGGTTTGTTGAGTGTAGAT

TATTAGGTATAGGTTCCATTGATGTACTAAACAATGTAAGATTTATAAATATAAAATTTA

TTTCTGTAATTTTCAGTGTCTTTTTCAGACTAAAATAGAACAAGAAATTGTTAGAAAATA

TTGTCATTCACATCAGAACCACTATGTAAATTGTATAGAAAGTGATGGAGATTTCGACGA

GAGGAGGTTTTATCTGCCGAAAGAACTGGTCATGGTAAATCCCGCCAATGTACCTATAGG

AATTTCTTGTCTATACGACAATCTAGTTTTCCAGTG

>Unigene0000338

AGTGTAGATTATTAGGTACAGGTTCCATTGATGTACTAAACAATGTAAGATTTATAAATA

TAAAATTTATTTCTGTAATTTTCAGTGTCTTTTTCAGACTAAAATAGAACAAGAAATTGT

TAGAAAATATTGTCATTCACATCAGAACCACTATGTAAATTGTATAGAAAGTGATGGAGA

TTTCGACGAGAGGAGGTTTTATCTGCCGAAAGAACTGGTCATGGTAAATCCCGCCAATGT

ACCTATAGGAATTTCTTGTCTATACGACAATCTAGTTTTCCAGTG

>Unigene0000339

AATCTATGGGCCTCCTCGTCTTCCTCACCTGCTTCCGGCGAGCCTATGAGCCGGACGAAT

CGCCGCCGGCGACGCCGGATCCTCTCTCGCCTCTCTGCTCCTCCGCCTCACTGGAGAGGC

CCCGCCGTAAAAGTACCCCCCAGTCATGGCGCCCGTCGCTCAGCGCAATCTCCGAGGATC

GCGTCGGCGTTGCCACCAAGACCACCGAGCCGACGGCTCTTCCTCGTAGTAAGAGTGCCC

GACCTGCAGGGGCATTTTATGGCAAGGATTTTGATCGGTCCCGGAGCGCGAGGCCGGCGG

CGGCCTACACCGTCAGCCTGTGGCGGAGCGTGAGTGGCGGATCCGGAAGGAGAGCTTTGC

CACCTGGCAACTCGGAGAGGCGAGGAATGGGGCAACCTGTGATTGTGCCGGCATTTATTC

CAACAGGCTTTATTTTCTGAATCTAACATCAACTGTAATGCCAATGTACAAAAATGGTAA

TCCATGTATTGCTTGTATTTGTTTTGTAAATATAATACATGGGTTGCTACTAGTTTTTAG

GCTTTGTATGGATTGTGAAGCTACATAATTGTATTTATTTTGTACATATAATAAATGGGT

TGCTATTTGATTCG

>Unigene0000340

GCGGCGTAACCGATCGCGTTACAGGTGACTGATCTGAAGAAGCAGATTTCAGTTGAGATC

TACTTCTCAATCTCTATATATTTCAGTTGTTCCCCATCCTTCTGCTTGCTTCATAGTGCT

GCAATAATCACCCTTTTTTTACTCAATTTTGCTCTTTCCTCAAACAAATCTAATGGGAGA

GGTAGATACGATTATTACATCTGCATCTACTCCCCAAGTAGAACTAGAAGGAGTTATTGC

TGATGCATTTACATCTGCATCCA

>Unigene0000341

GTTCCCATTCACCGAGATTCTCCGCCGTCGCTGCCGGGATTCCCCGCCGTCGCCGCCGGG

ATTCTCCACCGCCATGGCACTTTTCTCCTGCTCGACCTTGTCCTCCCCCTCCGATTCTTC

CCACCTCCAAAACCTCGCCGCCGCCACCCCCTTCCCCGCCCGCCACTCCACCGCCGACTA

CCCCCGAACGCTCATTTTCCCCAAACAACCCCCCTCAATCTCTCTCCGATGCAGCGCTGA

GAAGGACAGCCGGATCCCCCGAAGCTTCGATCTCGGCCTCAATCGGGACATCAACACCGC

CGATCCTTCCAGCGGCGGCGGCAACGGTAAATTCCCCGGCACGGGAGATGGATCCGGCGG

CGGCGGCGGTGATGGAGGCGACGGTAGCGGGGAGGAAGAGGAGTTCGGCCCGGTGCTGGG

GTTCGCAGAGGTGATGCGGGCGGCGGAGGAGCGGGGGGTGAGCCTACCGGATGACATGAT

GGAGGCCGCGAAGACAATTGGGATCCGGAGCTTGATTCTTTTCAGATACTTGGACCTGCA

GGGTTCGCCCTGGCCATTGGCGTTTTTGATTAGGTCTTGCTCGATGCTCCGTGATCGGAT

GCTGGCCGACCCGGCTTTCCTCTTCAAAGTCGCCACTGAGGTTGTTATTGATTCATGTTG

TGCGACTTTTGCGGAGGTCCAAAAGAGGGGAGACTCTTTTTGGGCGGAGTTTGAATTGTA

CGCAGCTGATTTGTTGGTAGGGGTGGTTGTGGATATTGCATTGGTGGGAATGTTGGCACC

ATATGTGCGAATGGGGAAGCCGTCAGTGTCAGCAGGCTTGTTTGGGCCCGTCTCGCGGGT

CTGTGCAGCTTTGCCTAGCAGTGTGTTTGAAGCTGAGAGACCGGGATGCAAATTTTCACT

TCAACAACGAATTGGGACATACTTTTACAAGGGAGTATTGTATGGGTCAGTTGGATTCGT

ATGTGGTATAATAGGCCAAGGCATTGCTAATGCTATTATGACCGCCAAACGGTCTGGAAA

AAAATCAGAGGAAGACGTACCTGTTCCACCTCTTGTGAAAAGCGCTGCTCTTTGGGTGTT

TCATTGCAGGGGTATTTCTAGCTGTTTCTTCAAACACTCGTTATCAGATCGTCAATGGTT

TGGAAAGCCTGGTTGAGAAGTCACCCGTAGCTAAACGTCTTCCTCCTGTCGCGATGGCTT

TCACTGTTGGTGTGCGATTTGCGAACAACATATATGGAGGCATGCAGTTCGTGGATTGGG

CTCGGTGGAGTGGTGTTCAGTGATGATATGCAATTTTGCCTACTCAATGTTCTTTTCAGG

TACTTTAGGTTCCAGTTTGAGGATAATGGTACAGAACTAGTTTCATTGCAATTAGTAAAT

CATAACTATTTATGACATTTTAATGCATTTCTGAGTCAAGCTTATAGGAGTGAGGAATGT

ATCTCCTGTTTATTAATGTTATTGCAAATATAGCATGTGCTTTTAGGAATTGTTCTATCT

AATAAAGC

>Unigene0000342

GAGGTTGTTATTGATTCATGTTGAGCGATTTTTGCGAAAGTCCAAAAGAAGGGAGACTCT

TTTTGGGCGGAGTTTGAATTGTACGCAGCTGATATGTTGGTAGGGGTGGTTGTGGACATT

GTATTGGTTGGAATGTTGGCCACTATATGTGAGAATGGAGAAGCCGTCAGTATCAACAGG

CTTGTTTGGATCCGTCTCCCCGGTTTGTGCAGCTTTGCCTAGTAGTGTGTTTCAAGCTGA

GAGACCAGGATACAAATTTTCACTTCAATAACGAATTAAACGAATTAGGGCATACTTCTA

TAAGGGATTATTGTATGGGTCAGTTGGATTCATATGTGGTATTATAGGCC

>Unigene0000343

ATAAATAAGGCCGAGAAGGTGTCTAAACACAAGAGCAGCAAACCAACCATCTTGGATGAG

GTGGAGGCCCAAAGGGAGACGAAGGGTTGGAGCACTGATTATACTATCTTTGGCGATGAC

GACTATCGACATCATGCTTAAAATCCAGGACAACGATCGTCTGTCAGATGGTAATGGAGA

GGCTCGATACTGATGCGGAGGATGTGGTTCTGTAGTTGTTTAGATTTACTGCTTTAGTAT

GGGTCTTGCTTTCTTGATTGCTCAGTATTGTGAATAGGGAGTTCTCTTTCATGTGTTGCT

GTATGACAGCTCAAACAAGTGGTCCCATATGTAGTCCTTTTAACTATCTAACTCTGATGT

ATGATATTTGGCTGTGAGTACGGTTGTGTTAATTAATGTA

>Unigene0000344

CCTTTTCTCACCCACGGTACTGTCTCTCAAGATTTCTCTAGATCAGGAGAGAGAGAGAGA

TGGGCACCGGATCTTTTCTCAAAGTTGTGGCGAAAAACTTCGATGTTCTTGCCGGGCCTT

TTGTGACACTGGTCTATCCATTGTATGCTTCGGTCAAGGCAATAGAAACCAAATCTCCTG

TTGATGATCAGCAATGGCTCACTTATTGGGTGCTGTACTCTATGCTTACACTCTTCGAGC

TTACTTTTGCCAAAGTTATTGAATGGCTACCATTCTGGACTCATGCAAAACTGATCCTGA

ACTGCTGGTTGGTCCTACCCTATTTCAGCGGCGCTGCCTATGTTTACGAACATTTCGTCA

GGCCATTATTTGTGAAGCAACAAACGGTCAACATTTGGTATGTTCCAGGAAAGAAGGATG

TCTTCAGTAGACCCGATGATGTCTTGACAGCCGCAGAGCTCTATATTCAAGAGAATGGAC

CAGATGCATTTCAGAAACTTATAAATAAGGCCGAGAAGGTGTCTAAACACAGGAGCAACA

AACCGACCATCTTGGATGAGGTGGAGGCCCAAAGGGAGACGAAGGGTTGGAGCACTGATT

ATACTATCTTTGGCGATGACGACTATCGACATCATGCTTAAAATCCAGGACAATGATCGT

CTGTCAGATGGTAATGGTGAGGCTCGATACTGATGCGGAGGATGTGGTTCTGTAGTTGTT

TAGAGTTACTGCTTCTCTTCTTTAGTATGGGTCTTCCTTTCCTAATTGCTCAGTATTGTG

AATAGGGAGTTCTCTTTCCTGTGTTACTGTATGACAGCTCAAACAAGGGATCCTGTATGT

AGTCTTTTTAACTATCTCTGATGTTTTTGATATTTGGCTGTGAGTACGGTTGTGTTAATT

AATGTACGTACTGATATTTGAGATGACAATCTTTTCTGACCACCCACCTTCGAGTAACAT

GGACATTTGGTCAACAGATTGTATGTAGCCAACCTGATTAGGCCTTGTACCAATTGGAAG

AAAGAAAATTGGTGTTGTGAATCCAACTAGCATAATTATCCATCAAGT

>Unigene0000345

TAGCTCGCCGGCCGGTCCAGGCGATCTTCGTCGAAAATTCTCCCACCCGATTCCCGATCC

CCCATCCAAACTCATCCGATCCACAACCATGGCCGCCCTCCTCCCCTTCTTCCTCCTCCT

CCTCGCCGCCTCCCCCTCCCTCGCCCGCGACCCCCACAGCATCACCATCTCCACCCCCGA

TCTCCACCCCACCTCGCTCGCCTGGGACCCCACCGCCCAGCACTTCCTCATCGCCTCCCT

CCACCATCCCACCATCTCCTCCGTCTCCGACGCCGGCGTCGTCTCCCCCCTCCTCTCCTC

CCCCTCCCACACCGCCGCCATCGCCATCGATGACGCCCGCCGCCGCCTCGTCGTCGCCCT

CGCCCACCCCGCCGCCCTTGCAGCCTACGACCTCCGCTCCCCTCTCCCACACACCCAGCT

CTTCCTCTCCTCCCTCCCGGACTCTGCCTCCGTCCCCGGCGGCGTCACCATCGACCCCGC

CACCGGTGACACCTTCGTCACCGCCGGTACCACCATCTACAAGATAGATGTTAATGGTGT

CGCCGTCGTCCTGTCACAGTCGAAGATATTTGCCCAAGTGGTTGATGGAGTGGCCTATGT

TAGAAATGGTTATCTGCTGGTGGTTCAGGCTGGTAGCAGGAGGCTGTTCAAGGTGGATGC

GGAGGACGGAGTGGCAAGAGAGGTAATTGGGGCGGCGACGGAGGCGGAGACGATAGCGGT

GGCGAGCGATGGGTCGGTGGTGGTGGCTGGGGGGACGGTGGTGCGGA

>Unigene0000346

GTTTCTACGTCTACAATTAGTATGCAGACTATTTGATTTGAGTGTGAACGTCGTAGTAAC

ATATATATCTGTGCGCCGGAACACTAGTTTGTGGTGGGGATACTTTCATTAGAAAATGTT

TATGCGTAATCTTTCTAAAGATAATCTAACTATATATTAGTAGGTTGTGTTGTTTTACAG

GATCAAAGTATCGATAGTTACACAATGTATTCGCAATCAATGACAATCAAGCCAACCATT

AGGAAACTAGAAACATTTTGACAGTAACATACGTAATACATTTGAATTGTTTTATATCAT

TCCTATTGAGATCGGTACAAAACCGGTACAGGATTGGTAATCACGTAAACTGGAAGGCCA

ATTGTTGAGATTTAATCACGGTATATCAAACAAAATAAAAATTTGGCTCCCA

>Unigene0000347

ACAAAATAAAAATTTGGCTCCCAAGATTTCTCTCAATGTGGGAACATTTGTGTACCATAT

CATTTTGATACTGGTTCTAGTTTTTATCCTTTGTGAGGGCAAATCATTGTGAAATAACTT

CTATGGGTAGGAAACTTTGTACCCGCAAACAACTTGGAAGATGTACTCTCCCTTGTAAGA

AAATTAGATCCATAAGCCCATAAATGAAGAAAAATTCCTAGCTATTGTCTAAACTACTTG

GGTGCTAGCTCAATGTGAGTCGTATCACATATGAAGAGTTCACCATAATGTCTCTT

>Unigene0000348

CCACATACTCCAAAATGTCCGCCCCAATCTCAAATCAACTCTCCCTCTTCAGCCCGGAGC

TCCAAAATCGAAGATTCAATGCCGGAACGCTCCAGATCCTCGAATCACTCCTCGTCGTGA

GGGACGTCCGATCCCTCACCGAGATCAGAACCGCCCTCCGTGACCTCCTCAGATCCACCG

CCATGTCCTCGATGAGCGAGATCGCGGACAGGTCAGTGGATCAGAAGCTCTGGACCGTCG

AGTTCTTTGTCCGTGCCCTCGCTCTAGTAGGAGACGTCGAAAGTTGTTTGGCTCTGAAAT

ACGAGGCATTGGTTTTACGGGAGACTAAATGTGCTAATGATGAGGGACTCCGGGTTTCGT

ACAAGGAGTGGGTGACTTTTGCCGAGGACTCACTTGATTATGGATTTTACACAATTGCAT

CCAAGGGATTCGAAAGTGCACTTTTATGCATCCAGGCTAACAAAGATGCAGATCCAGATC

CAGAGCTCGATGATATCGTTTTCAAGGAGAATACTATGTACAATATAAAACAACTCAGAG

ACAAGGCCTCGGCATTAGTTGCGTCACATTCTGTTCAAACTCAGTCCGCTGAATATTCGA

AAAGAAAGGCTACTCGAGAGGTCCATAAGAACAGTTCATGCCCAAGCAGCGAGGACCATT

TTGCGAGTTTAACATTCCGAAGTGGGATTAGAAGAAGGAATATGCAGAAGCTGATTCAGA

GTCAAGGCCTCTGTCGAGATTTTCCTGATCCATGAAGAATATGCATCTTCAGTTTTGACA

CCAGAAACTAAGTGCAAACTATTTTCAAGATGTCTGTAAATTCTCGATTACAGAGGTATG

TTACATTTTCTGTTATACAGATGATGTTTAGCTTTAACAATCGATTGCCTGTTGTCGAGA

AAGATAGCAACTGTTTAGTTGGTTACTTAAATTACCATACTAGTAGCTTAACCCCAGATA

GGTTTACGGATATTATGTGATTTTTGGTCCACTTGATACTGGAATATG

>Unigene0000349

CCGCCGGAAACCCTAGCCCCTTACGATCCGCCCACATATCAGCAAACCCTAGCCCCTGGC

TACCAGGACACCGCCGTCAATCTATCCCCCTATCAGCAAACCCTAATTTCCGGCTATCAG

GATACCGCCGCACCTCTCTCCGGCTATCAACTACCACCACCGCCCCCCGAGTCTCAGTGG

CCCTCGGAGCAAAATCAAGCTTACGCCTACCCTTCTTATTACGCCTACGATTATCAGCAA

CAAGCCCCCATCGCTGTCGTAGGCGAAGTTTTCTCCGCCCCGGCTGAAGCCCCGCCGCCC

GAGCAGCCTTCGAATCCACAGCCCGAAAGCCACGTGCCACCGCCGCCCGAGCAGCCATTG

AATCCACAGCCCGAAAGCCACGTGCCACCGCCGCCCGAGCAGCCTTCAAATTCACAGCCC

GAAAGCCACCCGCCACCGCCACCGCCACCCGAGCAGCCTTCGAATCCACAGCCCGAAAGC

CACCCGCCCCCGCCGCCGCCTGAGAAACTGGAACATCCACAGCTGGAAAGCCACACATTG

CCCCCACCGTCTGATCAGCAGTCAATGCCACAGCCGGAAAGCCATGCGCCTCCCCAGCCA

TCCGAGCAACCAGTTAATCCACTATCTGAAGGCCATGTACCACCTGTACAAGCCCGCACT

TTGTTATCTGAGAACGGCCGCAGTGGTGCAGACAAAGATGCATCAGGCGGGGAAGAGGAA

ACTACAAGTCGGCGTAGGAGGCGCAGCCGCTGGGAAACCCCATCGGAGTCTGAGCAGAAT

GCAGATGCCTCCGGTACCACTGTGAAGAAGCGAAAGTCCCGGTGGGCTGAAGAGGAGCCC

CGCCCCCTCATCCAGTTGCCCGACTTCATGAGGGGCTTCACTGCTGACCTTGATCCCGAG

GTTCAAGTGCTTAATGCACGGCTTTTGGAGATTAGTCGTCTCCTCCAGTCAGGCATGCTA

TTGGATGACCGTCCGGAGGGGATGCGCTCGCCGTCACCGGAACCCGTGTACGACAATATG

GGTATTAGGATCAACACACGGGAGTACCGGGCGAGGGAGAGGCTGAATAAGGAGCGGCAC

GAAATCATCTCACAGCTCATCCGGCGCAACCCGTCCTTCCGCCCCCCAGCAGACTACCGG

CCGCCGAAGCTCCATAAGAAGTTATACATCCCTATGAAGGAGTACCCGGGTTACAACTTC

ATCGGCCTCATCATCGGTCCAAGGGGGAACACTCAAAAGCGGATGGAGAAGGAGACCGGC

GCGAAGATCGTGATTCGGGGCAAAGGGTCGGTGAAGGAAGGGAGGCTGGGACAGAAGAGA

GACTTGAAACCAGACCCTGCGGAGAATGAGGACTTGCATGTATTGGTGGAAGCTGATACT

CAGGAGGCTCTAGATGCTGCAGCGGGGATGGTACAGAAGCTGTTGCAGCCTGTGGACGAG

GTTCTGAACGAGCACAAGAGGCAGCAACTGAGGGAGCTCGCTGCACTGAATGGTACCATT

AGGGAAGATGAGTTTTGCAGGTTGTGCGGTGAAGCTGGCCACAGACAGTTTGCATGTCCT

GCTCGGACCTCCACTTTTAAGAGCGACGTTTTATGTAAGATTTGTGGTGATGGAGGTCAT

CCTACTATCGACTGTCCCGTGAAGGGAACAACAGGCAAGAAGATGGATGACGAGTACCTA

AACTTCTTAGCCGAATTGGGTGGTAGTGCACCAGAATCTCTGACTAAACCGGGCTCTGCA

TTATCTATTCTTGGCTCAGATTCTGCTGGAAGTAATCCTCCTTGGGCTAGTGGCAGTACA

GTGCCAACAGGATCAGCTAATGGTCTCAAGAAGGACTACGATGAAACGAACTTGTATATC

GGGTACCTTCCACCTACAATGGATGATGATACCTTGATCCGGCTGTTCACACCGTTTGGA

GTCATTGTGATGGCCAAGGTTATCAAGGATCGTCTCACTGGAACAAGCAAGGGTTACGG

>Unigene0000350

ATTAATCATAGGAGGTATTGATCTTGGTTGAGAAATATAAATATATCTCAAATTATATAC

TACAGATATAAGCACAAGGGGTATTACATAAAGGATTATCCTAATAGGAAGGAAAGTCAC

ACCAACAAAGATAAGGAGAATATGAAAGTTATACATCTTGAATAAATTACACATCAATAG

TCTAATTCTTTTATGTAAAATCATCACCTTAGTTGAGATTGGTAGGATGCCAAAGATCTG

ATGAAGAACTGATGTTGTATGTGTCTATAAAAGAACTGATGGTTATCAGAGAACTGGGAA

TGGAGCATATGATTAAGACTATTAAATATGAGATTCTAGAACACTAATTTTTTGGGCATA

ATCCTATATTATTTATAGGGTATAGATGGGGAGGCCAGTTGCTGGTGAAAAATA

>Unigene0000351

TGATAAATGACATATAAAGTATCTTTATCTGTCTAGATCAAATTTATTCTCTAAATGTTG

ACTGATTATAAATTGATAAGGGGAAGAAGCCACTCTCTACATATGTACTATGAGATCCAC

CCATGGATATGTGGAGCTCAGTAAAGGTACTTACACCAGAGGAGATTATGAAGAGGAATA

AGTTAGATCAGAAGGCTTATATCACAATCTGCTTATGATTAACTCATAATATGGTATATA

AGCTTATGGATTGGATGACAATAAGTGGTATATATGACAAGCTTTAGGTGATCTATCATT

GTAGTGATCAAATAAGTGTGTTATACTTTCAAAAGCTCTTTACCTTCAGATTATAACCAT

ACAAGCTTATGTCAAATCATATCAATAAATTTGATATTATGTACTCAGATTGTGTTAGCC

TTAGAGTTAAGTTTGAAGATTTCTAGAAAGCTAT

>Unigene0000352

TATGGTGACATGGATATATATATGATAATAGTGAGCATGCACAAGTACTGTGAGCATGCA

CCCTATACTATAAGCATGTGCATGTACCTGTGAGTGTGTAAATCTTACTGTAAGCATACA

TCTAATATATGATTTTGAAAATGTTAAAGAGTTGGTTCTGATCTCCAATGTAAATGCCCT

CAGTAATGTTCTACGAGCACCTGTATGGTTCCTTGCTGCTATATAAGGTTATTTAGGGGT

TCAAGAACATATATTTAAAAGCTTTAATTCTTCTATCTCTTTCAACAATAAAGTAAGAGA

GAGATTGGTGATCAAGAGGGTTTAATTATTATGATCAAGATTCATCGGTGAAGTAGTGTA

AGGTTTTCGATTACGAGAGAACTTAGTAGATCGAGAATTTTGATTATGGAGGTAGATCAT

ACCTCATCGATTGGGGGTAATCCTTTGTTTATTATTATTTGGATTTTAATTATACTCTTA

TATTATTCAATTATTAATAGAATACATATGAGGTTACCATCCATGGAAGTAGGTATTATA

TACACCGGATTACATAAATTCTTATGTGTTGTTTACTTTCTATACTTGGTGTGTTATTGC

CTGTTGCTTGTTGTTATTACTTGTTGGGTATTCTTATATGCTTTTCTAGATTGGTAGGAA

GTGGTTATTCTCATCAAGTTGAGATCGGTGTTATTGGATTTGGTTATATTGGTTTTTGGT

TCTTAATTAGGATTATACCAATAAATAGTATTAGATCTTGTAGGTAAAGTTTTTGTGAAA

ATATCTATAAGAGATTTTGTATCTTGGGGAGACTATATTAACGTTCACAAATTCAACAGT

AAGTCATTTAATGGTAAGAATAACTTTAAGGTATGATAAATGACATATAAGGTATCTTTA

TCTGTCTAGATCAAATTTATTCTCTAAATGTTGACTGATTATAAATTGATAAGGGGAAGA

AGCCACTCTCTACATATGTACTATGAGATCCACCCATGGATATGTGGAGCTCAGTAAAGG

TACTTACACCAGAGGAGATTATGAAGAGGAATAAGTTAGATCAGAAGGCTTATATCACAA

TCTGCTTATGATTAACTCATAATATGGTATATAAGCTTATGGATTGGATGACAATAAGTG

GTATATATGACAAGCTTTAGGTGATCTATCATTGTAGTGATCAAATAAGTGTGTTATACT

TTCAAAAGCTCTTTACCTTCAGATTATAACCATACAAGCTTATGTCAAATCATATCAATA

AATTTGATATTATGTACTCAGATTGTGTTAGCCTTAGAGTTAAGTTTGAAGATTTCTAGA

AAGCTAT

>Unigene0000353

GATCATGAACAGCTTCATCAATGATCTCTTTGAGAATATGGCTCAGAAGTCTAGTTTTTA

TAGTTTTATTTTTTTAGATATATATACTTTAGCCGACAAAATGTCTGTGGAGTCGTCGGC

TAAAGTCCAAACTTTAAGCCGACAATATATTTTGTCATCGACTAAAGTCCAAACTTTAGC

CGATGACTATCACTATTTTGTCAGCTAAAGTATGACTGACAATCATGGATTCATCAGCTA

AAGTGCTAACTTTAAGCCGATGATGTATTTTGTCGTCGACTAAAGTCTAAACTTTAGTTG

ACTACTATCACTATTTTTCTCAGCCAAAGTATGGCCGATGACCTTTAGCCGATGAAGTTT

CGTCGACTATTACCTTAGCCAACAAAATTTTCTAATAAGCTGACAAATGAAATTCGTCAG

CTAAAGTGCTTTTTCTGGTAGTGAATACAACGATACATATAAGTCATATTTCATTTA

>Unigene0000354

TTCTAGAAAGCTATATTCTTGTTGGTTTATTTGTTATCAGAATGAGAACCTTTTGTGGAT

TAGATGACCGTACTTTAGAAGGAAAAGTTTATGTGTAAGGATATTTTTCAGACCATACCG

GCCAAGGCTTCTAATAAGAATATGTCATATGTGGATATCCATATATAAGTTCTTGTGGTT

ATAAGAAGTAGAATCATGATTAGCTAGAGTGGTAGTATTAATCATAGGAGGTATTGA

>Unigene0000355

CAAAAATAAAACAGTTGAATGCCCATCAGCATATTGTGTATGTATTTAATTTATTTAAGT

TTCACTAAATGTCTTCTACATATTGTGTGCGCAATTCATTTATTGGAGTTTCATGTGCGT

TTTATTTTTCCTGTGGGTATAAATAAGATACCTTGTTAATCTCCTAATTCAAGTGACTAG

ACCTATGAACCAAAATTTTCTTTTCCTGGTACTCCTTCGTCCAACGCTTTCGGACAGTAA

CCCTTACTCAACTCCAACCTGTTTATGTGAATACAATGCCAGCGCTTTCAGACGGTAATC

CTTACTCAGCTCCCACTTGTTAATGTGAATACAATGCCAGCGCTTTCAGACGGTAATCCT

TACTCAGCTCCCACTTGTTTAAGTTAGTGTTGATCATAATATATGATTTCTTATTATTGG

CTCCCTTAAACATAAATTTGTTCTTCAATTAATGTGCTTTTCTTTCATTACATGATATGA

GATTGTTTTAGTATTTCACATCATATTTAAATACTTACTAAACTCTGTGACTATAGGTTC

CTATTATTGTAGGATCAATATTTAGATAGTACCTACTCCATAAGATTGAGGGTCGCAGAC

AACCAAAGTAACAGTGAAATT

>Unigene0000356

ATTAGAGAAGGGGAGGCTTGTCTCTGTAACATGTTCTAAAATTACCCAATAATGAGAGAA

TTACGGAGTTCCAATTTAGAGTTGGTGGGTCAACGAGGAGTATTTTATATTAATCAACTG

TAAAGAAATACGAACACCGATTGGAGCACTAAGCTCCAAAGAAAATATTTGATGCTCACA

CAATTGGGGCAGATATAGTTCCTTATTGGGAGATCTAATATTTCTCAGCTAGGTTGAAAT

CTAAGTTTATCTATATTTCGGCTGGGCTCAAACTAACAAAACCTGAATATTTTTTGTTTA

TGAGTTTGTCTTGGGTGAGATTATCCAATTTCATATAGAATTTTCGTCATACAGAATTTT

ATCATCACCCAAGAAAACTAATATGCTGGCTAGTAC

>Unigene0000357

ATATTATGTTATTTTATGAAGTCAAAATGGTATTGCTCATTGAGCTCTAATGTTAAACAA

ATATGGTCATACTAGTTAAGCCCTTAATTATAATCGGTACAAGCATTCGAACTAACCTTA

TCTATACCTATAGTGGAGTGCATAACTAAAACTCAAGATACACGAACAATCAAACTAAAA

AAGTACAAAGATTATCTCAGTGCCCAAATAACTATTGATAGAGTTTGTGGAACACTCCTT

GGACTGAGAAAAATTGTGATGTAGATGAGCTCAGAGTCCAATTAGATCAGAGAAAGGGAG

GCTTGTCTCTGTATCATGTTCTAAAATTACCCAATAA

>Unigene0000358

GCCCAGCTAGGGTTTTTCATCCCCTTCTCTCCCACCAGGCGATCTAAGAGATAATCGGGA

CCTAATCTGGTGTTGAGAGCACCGGATCCGTCATGGAAAACGGTGTGAAAACCGTTGATG

AGATGCATGGCAACGGTGATGTGCCGCATATTTCTTTAGATGATAAAGAGGAGAATGAAA

TTTTTGAGGAAGCCATGGAGACGCCTAGAGAAGCTTTTCTTACACCGCATCATCGTGATC

CGCGAGATTCTGTGTTTAGGAGTCTGGGTGGCTTTGGATATGAGAACGAGGAGCCTGGAC

TGGAGGTTGCCGAGTTAGATGGGTTTGGTGATGAGAAAGAGAAGCCTAGGTCGGACGTTG

GCAAGTTTGTTGAGAGGGTAGATGAGTTCGGAGATGAGAATGAAGAGCCTAGCTCGGAGT

TTGGCATAGTCGAAGCTCCTGTTGACAGCGAGGAGACTGCGGTTGCTCCGGTTAGCAACC

TGAGTACTGATGATGAACCACACATGGCTTTGTCGGAGAAGTTTGATGAGTTACCTGATG

TGCAAGCTAGTGGTCAGATTATTGGGAGTCATGAAGCAGTTGATACAGAGCTGGTTGATA

CCCTTGGTTCTGAGAAAACAGGAGAACCTGACAGCTACCCTGAGGCAGCAGCAGAAGTGA

TTGGACAAGCACCAGGAGATGTTGAGGGAGATCATTCTGTGGCTGCGGTTGCAGATGATG

GTGGAAGTGTAGTCGATTATAGCGGTAAGTTTGAAAATGGGGATGTAAACTCTCAGGGGT

CACACTTTTCTGTCCCTGAAAATGGAAAATCTTATGAGGGTGCTTCCGTGAAACCAACCT

CTGTTGTAGATGATGTTTCTGTCAACTTGAATGGTCCTTCTGAAGAACAAACACGAGGGC

TACACGCTGAAGACATTCATTTTTTAGAAGATTGCAAGGACACAAATAATGCAGATGAAG

CTGAAACAAGTGCATCAGTAGCAAAAGAAGATAAAAGAGAAATGATGCAACAAGTTAGAG

AGTCACCTAGCTCCCACAATGATTCTATTATGAGGAAAGAGCTTGAAAAGAAACCTGACT

TGGTGGACGATTTGTCTGAATCAATCTCTTCAAATGATTACAATGACAGCACTCCCAGCC

CGGCAGTCCCACCTGCTAATTCACAGCCCGAACCGAGTTCACCAAATGGTCACAACAGCC

GCAGTCCCAACCAGGCAGTCCCACCTCCTAATTCACACTCTTTGAGTTCACACCCTGAGT

CAAGTTCTTCAAATGGTTACAATGGCAATGTTCCCAGCCCGGCAGTCCTTCCTGCTAATT

CACACCCTGAGTCGAATTCTTCAAATGAGCACAATGATAGCGGTCCCAGCCCGACAGTTC

TACCTGCTAATTCATACCCTGAGTTGAGTTCTTCAGATGATCGCAATGGGAGCACTCCTA

GCTTGGCAGTTCCACCTGTTAATTCACACCTTGGGAATTTGGGAGCTGCCACTCTGCCTT

CTCGTCCGGATGGACTAGGTTCTTCAGTTTCTGCAGGAGCCCCCTCTTTGCTATCTCAAC

CTGCTGGTCGGACTGGCCTGGGGTTTTCGGCTTCTGCTGGTGCAACCAGTTTGCCATCTC

GACCGGCTGGCCTAGGGTCTTCGGCTCCCAAAGGTCCACCTGGTTTGCCGTCTCGTTCGG

CTGGCGTGGGGTCCTTGGCTCCCACTGGTGCACCCAGTTTGCCATCTCGTCCGGCTGGCC

AGGGATCCTCGGCTCCTACAGGTGTACCCTCTTTGCCATCTCATCCTGCCGGTCTTGGGT

CTTCAGCTCCACTATTAGAACCCGCGGCCCGTGCTCTTCAACAACCCAGGGCGACTGGTT

CCCACCTTCAGCGCCCAACTCAGCCTTCTGAAGAAATCGTTAATGATGAAGCAGGGGATA

CCGATGAGACACGTGAGAAACTCCAGATGATCCGGGTGAAGTTCCTACGTCTAGCCCTTC

GGCTCGGCCAAACACCCCACAATGTTGTCGTCGCACAGGTTCTTTACAGACTTGGTCTTG

CTGAACAACTCAGTAGGAACACCAATCGCCCCGGCGTCTTCAGTTTCGACCAAGCTAGCA

TCATGGCGGAGCATCTTGAGACAGGTGGACATGAGCCTCTTGACTTCTCTTGCACGATAA

TGGTCATTGGGAAGACTGGAGTCGGGAAGAGTTCGACCATCAATTCAATTTTTGATGAGA

TTAAGTTTCGCACCGACGCGTTCCAACCAACTACAAAGAAGGTTCAGGAAATAGTGGGTA

CAGTCCAGGGCATCAAGGTTAGGGTTATCGATACACCTGGGCTTTTCACTTCCTCTTCTG

ATCAGCGATCCAATGAGAGGGTCCTCCGTTCAGTGAAGAAATTCATCAGTAAGACCTCAC

CGGACATTGTTTTATACATTGATCGATTGGATATGCAGAGCAGGGATTTTGGAGATGTAC

CCTTGTTACGCACTGTCACTGATGTGTTTGGAGCGTCGATTTGGTTTAATGCCATTGTTG

TTCTGACCCATGCCGCGTCAGCTCCGCCTGATGGACCGAATGGTACTCCATTGAGTTACG

AGATGTTTGTGACTCAGAGATCTCATGTGGTTCAGCAAGCGATTCGCCAAGCTGCCGGGG

ATGTCCGGCTCATGAACCCGGTTTCTTTAGTGGAAAACCATTCAGCATGCAGAATGAACA

GGGCTGGTCAGAGAGTCTTACCCAATGGTCAGGTTTGGAAACCACAGTTACTGTTACTCT

CTTTTGCATCCAAGATATTAGCAGAAGCGAATATGCTTCTTCAGTTGCAGGATAGTCCAC

CTGGAAATACTTTCGGGTCTCGTACAAGAGTTCCTCCGCTACCATTTCTCTTGAATTCTC

TTCTCCAGACAAGGACTCTATTGAAATTACCGGATGAACAGTTTGGTGACGAGGATGGCC

TGGATGAAGATTTGGCTGATGCATCTGATTCAGATGCAGAGTCAGATTACGATGATTTAC

CACCCTTCAAGTGTTTGACCAGGGCGCAGTTAGAGAAGCTGAATAAAGCACAGAAGAAGG

CGTACTTCGAAGAGCTCGACTATAGAGAGAAGCTGTTCTTCAGGAAGCAAATGAAGGAGG

AAAAGAAGCGTCGGAAACTGATGAAGAAAATGGCTGAAGCAGCTGCAAATCTGCCGAGTG

ATTACAGTACTGAGAACTTAGAAGATGAAAGCAGCGGCCCAGCTTCTGTGCCTGTTCCGA

TGCCAGATTATGTGCTACCGGCTTCTTTTGACTCTGACAATCCTAGCCATCGCTACCGTT

TTCTTGATTCCTCGAGCTCGTGGCTTGTTCGACCCGTGCTGGATTCCCAAGGATGGGATC

ATGATGTTGGTTACGAGAGCCTTAATGTTGAAAGGTTGTTCGTTGTTAAAGATAAGTTCC

CGCTGTCTGTTTCTGGTCAGCTTACGAAGGACAAGAAGGAATTCAATCTTCAGATGGAGG

CAGCCAGTTCGATTAAGCACAGTGAAGGGAAAGCCACTTCACTGGTTCTGGATATGCAAA

CTGTAGGGAAAGATGTAGCCTACACATTACGGGGTGAAACAATGTTTAAGAATTTCAGGC

GAAATGACACAACAGCCGGTCTTTCGGTAACCATGCTAGGAGATTCGGTGTCAGCTGGCA

TGAAAATAGAAGACAAACTGATAGTTAGCAAAGCGCTCCGGTTACTCATGAGTGGTGGTG

CAATGCATGGTCGGGGCGAGGTAGCCTATGGAGGACGTTTGGAGGCAACTTTACGAGATA

AAGATTATCCTATTGGGAGGATGTTGTCCACTCTTGCCCTCTCTATTGTGGATTGGCGCG

GGGACATGGCAATTGGCTGCAATGTTCAGTCTCATATTCCGTTGGGTAGAGGAACTAATT

TGATTGGTCATGCAAACTTGAGCAATAGGGGAAGCGGACAAGTTGGTATTCGCTTGAATA

GTTCAGAGCACCTTCAGATCGCTCTGCTTGCACTTGTCCCGGTACTCAGAAATGTCAAGA

GAGTGCTATCAGGTTCTTCTCAGTCAGCGTAGGTGGGATAAGGTGGACAACATAAGCAGC

CATTCAGCAATTACGTGGGACAATCGCTCTAGCCGTTTGCTTTGGTATCTATGTGAGTAA

GACTAAGTTGCCGAGAGTAAATTTTCACTTCTGTAGGTCTTTTAAAGGATCATTTCCCAG

ACAAGTTTGTTTAGTTTGTTTGAAGATAAGTGATTTTCCGTTATTGTAGAACCCTCTTTT

AGGAGAGGAATTTCTTCATGTTCATTCAATGTCGCCTTTGACTAAAATTTCTGTCTTTGA

TTAGTCATGAATTAATGCTTGACTG

>Unigene0000359

CTACAATATTATACTATTAGAGTTCACAAAATACAATATTTATATTAACACTTGTGAGTA

ATAAAGGCTCCGCCTTTCAATTTTTAATCTATTGTTATTTACCGGTTTGTACACGACGTG

TATAGTCTATACATGGCGTTGCTGATAAAATCCGCCATTAATTGCTGTCGCCTGACCGCT

CGACCTAAACATTTGATCGTTTCACAGACGTTAAAGTTCAGTCCTCGTTTGTCGTACTCA

ACTAAATCCGCCGCCATGCTCGAACACGAAGTGGTTCCCGACGTGATCGCCGTTGCGCCC

AGCGACAAAATTGAAGTATCATACCCAAGTGGAGTGGTTGTTAATATGGGTAATGAACTC

ACACCCACTCAAGTAAAAGATGAACCATCTGTAAGCTGGCCTGCTGATCCAAATGCTTTG

TATACTCTTTGTATGACAGATCCTGATGCTCCGAGTCGTAAAGAACACACATATAGAGAA

TGGCATCATTGGTTAGTTGGAAATATTCCTGGTACTGATATCACTAAGGGTGAAACACTT

TCTGAATATGTAGGCTCTGGACCACCACCTGAAACTGGACTTCATCGTTATGTATTCTTA

GCTTATAAACAACCATCAAAGTTGAATTTTGATGAACCCCGTTTAACTAACAGATCTGCA

GAAAAGCGTGAAAAGTTTTCTATTGCTAAATTTGCACTTAAATACAATTTGGGAAATCCA

GTTGCCGGAAATTTCTATCAAGCTCAATATGATGATTATGTGCCAATATTATACAAGCAG

CTTGGTGCTTAAGTTATTACATAAGTATATATTTTCAGTACTCGGCTCACATGTGTAATT

AAATATTTCGAAATATTAAATTTTAAAACAATAGCAATATTATTTATATTTTTATTACTC

AAACAAAATTTGTCTTTATAATAATTATAAGTAATAACATGTATTTTTTTTTTATTAGAA

TCAATTATGGATGTGGTTAAAAATG

>Unigene0000360

AAAATTGGAAAGCTCTCGCGTTCGCGACTTCGCTGTACGTGACTCTTCCGGTTTCCGTGG

TGATTCCTGTCGCGTTTCCGAATTTTCGACCACTCTTGGCAATTTTGTCCATTCGTGTAA

AGTCATGCCGCCAAAGAAATCGAAGAAAGCCAACAAGCACAATCAAGATTGGGCCGACGA

CGACGACGATTCAGCTGTGGATATATTAGCTGCTGCCAAAGTAAATGATGATGTAGCAGT

TGAAGTGCCGGTGCAACCTAATCGTGGAAATAAAACTTCTAAAAAAAACAAGAAAAAGAA

TAAAAATCAAGATTGGGATGATGAATCTGAAGATTTGGTAGATATTTTAGCTGCCTCAGA

AGTAAATGATGATGCAACTACCAATGAACTGCCACAACCTAACCGTGGAAATAAAATTTC

TAAAAAAAACAAAAAGAAAAATAAGAATCAAGATTGGGATGATGAATCAGAAGATTTAGT

TGATCTATTGGCGGACACAAAATTAAATGATGATGAAGTGGTTGTTGAAGAAATCCCCCA

GCCAAATCGCGGAAATAAGATCTCTAAAAAGAATAAGAAAAAGAACAAGAATCAACAAGA

TTTGGACGAAAACTCAGCAGTTGATGATGATGATAACGATGACATTAACATTAAGGTTTC

AACATCGCCGACCGAAGAAGTTGCAGACAATACATCGGCAAAAATTTCGACGGGAAAAAT

TTCGAAAAAAAACAAAAGGAAAAAAAAAGATTTTGATTTAGAAGAAGACGACGATGACAC

ATTGCTTCAAGACAATGTTGTCAGAATTGATGATAATAAATCATTGGACATCGAAGATAA

ATTTATTGATGCTGGTGACGATCTCTCAGCAGTAGAAAAAACTGCCGAAAAAATCAATAA

GGTTTCCGGCGATGTTGAAAATGACAGTACTTCAACAGCTATGCCAGTTGTAGAACAAGA

TGTAGTTGCTGAAGATACTGGTGATGTAGACGAAGAACTGGAAGCAGCTAAGCTTGCAGG

AAAAAAAAAGAAGTCGAAGAAAAATAAAAAGAAGATAAATTATGATGAGGAAATCCTAAT

GGAGGCGCCGAAAGCAGATGTAATAGTGGAAGGCGATAAAAGCGAAAAATCTGCTATAAA

TGTTGAAGAAAAATGCGATATTGACGAGAAAATGCCCGTTGAACAGCCCGTTGATATAGC

TAATGCCAAGAACGCAATGGCGACATTAATTAGCGCTGATGGCGAAAATGACGATAACAC

AGACCAACTCGGGGATGAAGCTGTACCAGAGGTAGTTAAAGTGCCCAAACAGAAATCAAA

AACTAAGGACGTAAGCAAATCCGATAAGCTATCCCATAAAGAGAAGAAGAAAATGAAAAA

AGAGTTGGAGTATCAGAAACAAATGGATTTGTTGACCAAGAAAGGTGGTCAGGGTCACAG

TGAGTTGGGAGCTAACTTTTCGGTGTCACAGATTCAAAAGACAGCGGGTCAATTAGCAGC

CATGGAACACGCAGTTGACATTAAAATTGACAGCTTTAGCATTGCAGCCAAAGGTCAGGA

CTTATTCGTCAACGCTAGCCTGCTAATTGCCCAGGGCCGACGATACGGTTTGGTTGGACC

AAACGGTCACGGAAAAACGACACTACTCAGACACATTGCGGAGCGGCTGTTTGATGTACC

ACCGAGTATTGACATCCTATACTGTGAACAGGAGGTAGTAGCTGATGAAACAACGGCGGT

AAAGGCCGTACTTAGGGCGGACACACGGTGTACAGAGCTGCTCGCTGAGTGCAAAAGACT

CGAGGAAGCTCAAGAACAAGGCACAGGTGAAGACGTAACGGATAGATTAAACGAAGTTTA

TGACGAGCTGAAGGTACTCGGGGCGGACTCAGCTGAACCCCGAGCTCGGCGTATACTTGC

CGGTCTCGGTTTCTCTGCAGCTATGCAGGATCGGGCTACCAAAGATTTCTCCGGAGGCTG

GCGGATGCGTGTGTCCCTTGCTCGAGCCCTATTTCTGGAACCAACTTTGTTGTTACTTGA

CGAACCAACTAATCATTTGGATCTGAATGCCGTTATCTGGCTGGACAATTATTTGCAGGG

GTGGAAAAAAACTCTGTTAGTTGTATCTCACGACCAGAGCTTTTTGGATAACGTATGTAA

TGAAATTATACATTTGGACCAGAAACGATTGTTCTACTACAAAGGTAATTACAGTATGTT

TAAAAAAATGTACTCTCAGAAAAAAAAGGAGACTATCAAGGAGTATGAGAAACAGGAAAA

GCGGCTAAAGGAAATGAAACAACAAGGTCAATCCAAGAAGCAAGCTGAGAAAAAACAAAA

AGAAGTATTGACTCGGAAACAGGAAAAGAACAAGGGCAAACCAGGTAAAAATGCCGGCAT

GGATGATGATGATAACGCTGAACCTACACAGCTATTGCAAAAACCTCGAGATTATAACGT

TAAATTTTCATTCCCCGATCCCAGTCCGTTACAGCCGCCTATACTGGGTCTGCATAGTGT

GAGTTTTGCGTATCCTTCTCAAAAGCCATTGTTTAAGAACGTTGATTTTGGCGTTGATCT

CAACTCCCGGGTAGCTATTGTGGGGCCCAACGGTGTGGGTAAGTCGACGTTCCTTAAGCT

ATTAACCGGTGATTTGCAGCCTACCGTCGGTGAAATGCGCATGAACCATCGAATGAAACT

AGGAAAGTTCGACCAGCATTCTGGTGAACATTTGACAGCCGAAGAGACGCCCGCTGAATA

TTTGATGCGATTGTTCGACTTGCCGTATGAGAAAGCCCGCAAGCAACTAGGTACTTTTGG

GCTGGCTGGCCATGCGCATACCATCCGCATGAAAGATCTGTCTGGTGGACAAAAGGCTCG

TGTTGCCCTGGCTGAACTCTGTCTGAATGCTCCTGATGTCATTATATTGGATGAACCTAC

AAACAACTTGGACATAGAATCTATTGATGCATTAGCAGAAGCAATAAATGATTACAAAGG

AGGTGTCATAATTGTATCTCACGACGAAAGACTGATCCGTGACACTGATTGTACGCTGTG

GGTTATCGAGGACCAAACCATCAATGAAGTGGACGGAGACTTTGATGACTACCGTAAGGA

GCTGTTGGAATGTCTTGGCGAGGTGATCAACAGTCCGAGTATTGCAGCCAATGCAGCTGT

CGAACAATAATGACAATCTGGTGTTGTCAATTATTAATTATGTTTAACAATTTTGGTTGT

GTGCATGAACAGACTCATCAAAATATACTTATAATTATATTGTTGTCTTTGGCGTATTTG

TGATAATGTTATCGTCATTGACGTACTGTAATTCCTATTTACGTGCCTTGTTTACTCACA

ATATATTTTATGAATAAATTGAATAAAAACT

>Unigene0000361

ATTCGGTCATATCCGACTTCTTCTGCTGAAGCATTTTGGCACATTTCCGGCTTGTGATAG

CAACGATTGTCGGCTGGGGATGCTGCTTTACCTTTCTATGGGAACTCATCCCTAACTCGC

TTGAGGACTTAGGGTTTCTTCCCCTTTTCTCGCTTAACAAGTTTTGGTTTTGGGTCGACT

TCAACTTTGACCCGAGTTTAAAACTTGGGTACATATTTGACCCTATAGTGCCCGGTCAGT

CTAACAAAACCTAATCCCGTTTGAGGTGTTTTTGGGCAAACCGAGCCTCTTGTTCTC

>Unigene0000362

CGGATTTGGTAAAGAATTTTACTGGCGCCAAATTCTCATGATTTTTAGCATATGTGGGGA

GAAAACTTTTCAGAGCAGGTTCAAGGACTGATTGAAATTGTTGAAGGACAGGATGATCTT

AATGCACAATTCAGTGTACGTGATCGTTCAGGATTACCAAGATTGCTTTTATTTTCTTTT

GCAAAAACAATATATTAAAAACATAATTACTTCTATTTCTATGAACTTCTGTGTTACTGA

AATATAAAGAAAACTCTTTCTGACTTTGCTTTTATCTGAGACTCTTGCTAAAGCAGAATG

GCTCTGTTATTATGTGTTTCTCTTTATTT

>Unigene0000363

TCAAAGAGAAGCTGAGCTACTAAAGCCTCAAGTGGAAATGGAGTGACATGCATAGGTTTA

TGTTCTTATTCAGAGCTAGTCAGAGCACATAAAATTGGTGGACTCTTAACTTCTCTGTAA

AGACAGATCAGATTTTGTGTCTAAATTGTTGGTGCTCTTGTTCGACTCCCAATATGTGGA

AAATTTGGAGCGAGATGCAGTGCTTAAGGCTCTCATATCCCTGTTGAGGCAAGATGTGAA

AGAGCAGGTTCAAGGACCGATTGAAATTGTTGAAGGGCAGGGTGATCTTAATGCACAATT

CAGTG

>Unigene0000364

AAAGCTGAGCTACTTAAGCCTCATGTGGAACTGGAGCGGCATGCAACAAATGTGATAATA

GAAGGTTTATGTTCCTATTCAGAGAATCTGCAAGATGTTACCAACTCTGAACTCAAATTG

TTCATTGACTTGTAGCATATTTGGGGAAAAAGATCCCCCAGAGCAGCCTTGTTTAAGAAA

CTTGAAAACAACGACGAGAGTATTCATGACAAGGTTATTGTTTT

>Unigene0000365

CAGCGGAGCTGATTGTAGGCCTTTGATTTATGAAGGTTGTGGGTGACTTTGAGGGGATTA

TTAGGGCGGTGAAGGGGCAGAGTACTAAGGCGAAGCAGCTGGCGGCGGAGTTATACCTAG

GATCATGAAGTTTTTCCCGACGCTCGGGAATGATGCAATGGATTGAGTGTTTGGTTTGCT

CGAGGAAGAAGACTTCGCGGGGAGTTTGTTCTTTCTGGTTTTCTTAATGCTTGGAGCTTT

TAGGTAGGTGTGCCTAGGTGTGGCAGTCTATGTGTGACTCCGAGATTTAGATAATTTTGT

TGTTTGCAAAACAAGATATGCGCCTTCTATTTTCTGTCTACAAAAATATGTCTGGAAGGA

AGACAGATGAGGCAAATCTTGATTATGTTGAATGTCTTGTGTTCACATTTCACCACATCT

CATAAGACTCGAAACTCAAGGAACAGTCTCTGTGATTATAAGATTGACCATCTGACAGAC

TTGGGGAGGATTTTTCCAGAAACAATACAGAATTTACTGAGCAGGCTGAGTGCTATTGAG

AAAATTGTAAGATATGCTATGAAGAAACTAGCCTAGGGAATGGCAGAACACAAGTCACCG

ACTAAAGCTAAGACAGAAGATGAAAAGGCTGAAAAGTGGAACAGTAGAAATCAACAAGTT

CTCGGCAGATATTTTATAACATACTGGCTATGTCCCAGAAGGACCAGTAATCATGTCGAG

ATTATGCATTTCAACCTATCATGATTATCTGATTTAGGTCAGAGCACATAAAATTGGTGG

ACTCTTCAACTTCTCTGTAAAGACAGATCAGATTTTGTGTCTAAATTGTTGGTGCTCTTG

TTCGACTCCCAATATGTGGTAAATCTGGAGCGAGATGCAGTGCTTAAGACTCTCATGTCC

CTGTTGAGGCAGGATGTGAAAG

>Unigene0000366

TATGTCATGGGAGAGAATATTATAAGAGAGAATAGGAAGATAAAAAAATTAGGAAAGAGA

ACATTATAGAGAAAATGTGAGGAGATATAAAAATATTGGGAGAAAGAAATTACAAGGAGA

TAGGAAATATAAGGTGAGAGAAAATGGGGAAAAAGAAAATGAAGAGAGAATACAAGGAAA

GAGAAAATGAAATGAGAGAAAAATGAGGAGAGAATGTGAGAGAAGAAAAAAAAAGAA

>Unigene0000367

TATGTCATGGGAGAGAATATTATAAGAGAGAATAGGAAGATAAAAAAAATTAGGAAAGAG

AACATTATAGAGAAAATGTGAGGAGATATAAAAATATTGGGAGAAAGAAATTACAAGGAG

ATAGGAAATATAAGGTGAGAGAAAATGGGGAAAAAGAAAATGAAGAGAGAATACAAGGAA

AGAGAAAATGAAATGAGAGAAAAATGAGGAGAGAATGTGAGAGAAGAAAAAAAAAGAA

>Unigene0000368

ATGGGTGGGAGAGGAAATAAAATGAGAATGAATATGGGTGGGGAGAGAAAGTATGAGATG

AGGTAAAAGCTGGGGAGAGAAAATATGAGGCTAGGGAAAATGTGGAGATAAAAAAAGAGG

AGAGAATATGATAAGAGAAAAAAAAGGAGAGGATATAAGAATGTATGGAGATAAAATGAG

AAGAGAGAAAAATGTGTGGAGAGAGAAATACATGATGAGAGCAGATGAGTGGAGTGAGAA

AATTAGAGGAGAGAGAACAATGAGGAGAGAAATAACGAGGAGAGGGAAAATGAGAGGAGA

AATAAAATAAGGGGAGAAAAAATAGGGAGATAAAATTGGAGGAGAGAGAAAATGAGAAGA

GAGAAAATTTGAGGAGAGCGAAAATATGAGGAGAGAAAAATTATAGAAGAGAATACGAGG

AGAGGGGGATGAGAGGAGAGAATACGAGAAGAGAAAAATTGTGAGGAGAGAGAAAATGAG

AAGGGAGAATATAAGGAGAGAAAGTTAAAAGGAGATACAATAT

>Unigene0000369

TTATGTGCCACCGGCGGCAGCACCGTGCCCATCGCCGGAACATGTAAGTCTCCCCCCTGG

TCAACTCTTGCATGATATCAATACGTGATTATCAACTAGTCTTAGTCTTAGAGGACAATT

CTAACTTGCCCACCTGGATAACATGTCAATTTTGTGTCCACTCTTGTGATCAGTGATAAG

CACTAATTTTGCTGGAAATTCATTTTTCAAACTGAAAAATCGCAAATATTGTTTTCTTTT

TATGTCTTTGTTAGTCATTTTTTTCTTCTTATTACTGCCTGTATTAAATTTTTTTTAATA

ATAATAATAATTGAAATTATGGACACTACATATTGTATTTCGAAGAAAATTTACAGGGTC

TTTCAATAATGGGATAATATCATTTAGACCCCTAATTGGACCCTTGTTGCCATTATTACC

CACAAATTCAAACAATCTCAGTTACACCCTTGACGAATTCAAACAATCTCAAATACACCC

CCCCTAATACTCAAGGGCATAACCATTGGTAATAATAAATGCA

>Unigene0000370

TTATGTGCCACCGGCGGCAGCACCGTGCCCATCGCCGGAACATGTAAGTCTCCCCCCTGG

TCAACTCTTGCATGATATCAATACGTGATTATCAACTAGTCTTAGTCTTAGAGGACAATT

CTAACTTGCCCACCTGGATAACATGTCAATTTTGTGTCCACTCTTGTGATCAGTGATAAG

CACTAATTTTGCTGGAAATTCATTTTTCAAACTGAAAAATCGCAAATATTGTTTTCTTTT

TATGTCTTTGTTAGTCATTTTTTTCTTCTTATTACTGCCTGTATTAAATTTTTTTTAATA

ATAATAATAATTGAAATTATGGACACTACATATTGTATTTCGAAGAAAATTTACAGGGTC

TTTCAATAATGGGATAATATCATTTAGACCCCTAATTGGACCCTTGTTGCCATTATTACC

CACAAATTCAAACAATCTCAGTTACACCCTTGACGAATTCAAACAATCTCAATTACACCC

CCCTAATACTCAAGGGCATAACCATTGGTAATAATAAATGCACCAACTCAACAATTTCCT

CCATAATTGTCCTCCGTTCCTTTATAGTCATTAGAAAGGATTTACGCCTCTATATAACTC

CCTACTTTTTATCTCCTCTCATTTTATTAAGTTTGGTAAGTATGGACAAGGAGAGAATAA

AAGGAGAACGAATATGAATGGACATAGAAAATATGAGGCAAGATAAGTGACGGGGAGAGA

AAAATGTGGTGAGACAGAAAAGTATTAAGATAAAAAATGAGACGAGGGAGAAAATATGAG

GAGTGAGAAAAATTGAGGAAAAAAAAATGAAAGGAGAGAAATTAGGAAGAGAAAAAAAAT

TAGAGGAGAGAAAAGGGGAAGAGAGAAAACTCATTGGGAGAGAAAAATATGAAAAGAGAA

AATATTAGGAGATAAAATGGGAGGAGAGAAGATGAGAAGAGGAAAAATTGTGAGGAGAGA

GAAAA

>Unigene0000371

GAGAAAAAAAATATAAGGAGACAAAATATAAGTAACACAAAATATTATTAGAGAAATATG

AGGTAAGAAAAAAATGAAAATAGGAAATAATAAATAGGAAAATAGGAGGAGAGAACACAT

GAGAGAGAATATATCAGTATAGAGAATAATATAAGATATAATAAAAAAAGTGAAATTTTA

AAGAGAGAACACAATAGAAAAAATATGAGGAGATAAGAGAAAAAAGATATATGGATGAGG

AGATAAGAGACAAAAATGGAAACAGAATATTTGAGAAGACGAAAAAAAATCTTAGGAGAG

TAAAAAGAGAAAATATGAGGAGAGAATGAGAGGAGAGAATATGAGAAGAGGGAAAATGCA

AGGAGAGAAAATGTGAAGAGAGAAAA

>Unigene0000372

AGAAAATAGAAGGAGAGAAAATATTAGGAGAGCTCAAATGAGATAAGAGAGAATGAGATG

AGAGATGAGATTAGAAGAGAATAAATATAAATAGAGAAAAAAAAATTAGGAGTGAAAATA

TGAAAGAGAAAAAAAATGGAAAAAAAGAGGAGAGATTATATGTCAAGGGAGAGAATATAT

AAGTATTAGGAAAGATAAAATTATAGATAAAATGTGAGGAGAGAGAAAATCAGTGGAGAG

AAATATTGGGATAAAGAAATTACAAGGAAATAAAAAATATAAGGAGAGGCAAAATGTGAG

AGAGAAATGAAGAGAGAATACATGGAAAGAGAATATGAGAGAATATGAGGAAAGAAAATG

AGATGAGAGAAAAAAATGAAGAGAGAAAATGAGAGGAGAAAAAAAATATAAGGAGACA

>Unigene0000373

GAATGAGAGAAAAAATGAAGAGATAATATATGAGAGAAAAATGAGGAGAGAATATTTGAG

GAGAGAAAAAATTAAGAGAAAAAAATACGAGGAGAGAATAAGATGTGAGAGAATGCGAGG

AAAATTTTTAGGAGAGAAAACATGAGAATAGAAAATATGTGTAGAGAGAAAATTAAGAGG

AGATACAACATAATGAGGGAAAATATAAGGAGATTGAAAAGAGATTTGAGTACAAGTGTT

ATCCACACACATGATTACTATGTACAATAACAAAAAAT

>Unigene0000374

TAGAGAAAATATGAGGGGAGAGAATGAGAGAAAAGATAAAATGAGAGAAAATAGGAGAAA

ATGAGAAGAGATAATATATGAAGAGATAACCTATATGAGGATAGAGAAAATTTGAGGAGA

GTAAAGAATAAAAAATATGAGAGAATGGGGTGAGAGATAAAATGAGAAGAGGGAAAATGC

AAGGAGAGAAAATGTGAGGAGAAAAATTAAGAGAAGTAAAACATAAGAGTTCCCACATCT

GCACTATAAATAGCATCGGTCCAAAGCTTATGGCTGAGTATTCCTCTCCATTCAGTGCTA

GAAGGCAATGGTGCCCACGTCAAAGTGTGAGTTCCATACTGTCAAAATAAAGCATGGAGC

GAAGCGCTTCGAGGTGTGCCCCTGGTTTTTTAAATTCAAATTCACGTTGGCAGTCCCGAC

TTTTAAATTGTTTTGTATTTTAAAATATTAAAATAATATAAAAGTACCAATGGCCCTAAT

TTTAAACTAATAAAGAATAATGAATGTAGGTAAATCCCTAGCTCCCGTTGGAGCGAGCCA

CTTTTAGATACTATAAATTGAAAGCTTTTTTGTCACATAGGTCCACTAAAAACACTATTC

ACTCTACCATTCCACAGCTCCGGCTGTGGAGGCTCTAAGGACGCAGACAGATAATATAGG

GAAAGAGAAAACAA

>Unigene0000375

GAATGAGAGAAAAAATGAAGAGATAATATATGAGAGAAAAATGAGGAGAGAATATTTGAG

GAGAGAAAAAATTAAGAGAAAAAAATACGAGGAGAGAATAAGATGTGAGAGAATGCGAGG

AAAATTTTTAGGAGAGAAAACATGAGAATAGAAAATATGTGTAGAGAGAAAATTAAGAGG

AGATACAATATAAGGATGAAACTATAAGGTGAGAATGACAGAGGATTTTCGGAGAGAAAA

TGAGGTGAGAGAAATTAGAAGAGAGAATATAAGTAAGATAAAATATTAGGAGAGAAA

>Unigene0000376

AGAAAATGAGAGGAGATGAAAATGTTTGGAGTGAGAAAATGAGAGAGAACTAAGTAAATA

TGAGAGAAATTATAGGAAAGAAATATTCGGAGAGAGAATAGGAGAGAATATTAGAAGAGG

GAAATTGTGAGGAGAGAAAATACAAGAGAGAATTAAGAGGAGATGCAATATGAGGAAAAT

ATGAGAAGTTTTTTGGAGAGATGAGAGAGACTTAGCTGAGAAAAAAAAGTAAGACAAAAT

GTTATGAGAGAAAATACAAGGTAAGAGAATATGAAAAGAGTAAA

>Unigene0000377

GAGGAGAAAATAGGAAGAGAGAAGAACAGAGGAGATAAAATATGAGGAGAGAAAAAGGGA

AGAAAGAAAATATGAGAGAAATTATTATGAGAGAATTTGATGAGAGAAAAATACGAGAAA

ACAAATTATAGAAGAGAATATGAGAGAAGAGGTGGAGAGAATATGAGAAGAGGGAAATTG

TGAGGAGAGAGAAAACAAGAAGAGAAAATTAAGAGGAGATACAATATAAGGAGAGAAATT

ATAGGGAGTGACAAGAAAAACTATTAAAAGAGAATTTAAGGAAGAGAAAATGTTTGGAGT

GACAAAATATGAAGTAAGAGAATAAAAAAGAAAATGAGAGAAAATAGGAGGAGAGAATAT

ATCGGGGGAGAGAATATTAGAAGAGAGAAAATGAGGAGAATGAGAAACTCTAAGGAGAAC

AC

>Unigene0000378

AGAAGAGAAAATATGAGTGAGAAAATGAAAGGATAAACAAATTAGAGGAGAGAATAGGAA

GAATAAACTAGAGAGAACGAATATAAGAGATTATAGAAGAGAAATTACGAGAGTTAATAG

GATGAGAGAATATGAGAAGAGGGAAATTGTGAGGAGAGAATACGAGGAGAGACAAGAGAG

ACTATTGGAAGAGAGAAAATATGAGGAGACAATATTATTAGAGAGAAAATATAAGTAAGG

CAAAATGTTAGGAGAGAAAATACGCGGTAAGAGAAAATATGAAAAGAGAATTAAAAAAAG

AATATGAG

>Unigene0000379

TATAAGTAAGACAAAATGTTAGGAAAAAAAATATGAGATAAGAGAATCTGAAAAAAGAGT

GAAAATGAGAGGAGAGAATATATTGAGGGAGAGAATATTAGAAACGATGAATTGTAAGGA

GAGAGAACACGATAAGGAAAATGTGAGGATAGTTGAAAGAAGATAAAATATGAGAGAATG

TCATGAGAGAATTTGAGAAGAGAAATTGTGAGGAGAGAGAATAAGAAGAGAAATTGAGGA

GAGAAAATGTGAGGAGATAAAAT

>Unigene0000380

GTCAACATCGGAGAGCGGGCTTTTGCGGTATTTCTTGATCTGCTGACAGGGATGATGTGG

GGCGGAAGGTTAGAGTGGGAGGGCGAGGGGGCGGAGTTTCGGCGGACGGTGATAGAGATA

GTGGAGTTGTTGGGTAGGCCCAATGTATCGGATTATTTCCCGTGTCTTGCTTGGTTTGAT

ATGCAAGGAATTGAAAGGGAGACAAGGAAGAGGGTGGCTTGGCTGGATGGGGTCTATGAA

AGGATTATTGATGAGAAGGTGCTGTTGGCAGTGGGAAATGGCGAGGGAGCAAAAGATTTG

TTATCGATTCTTTTGAAGATTAGGGATGATAAGAATCCTGATACGCCGTTGACCACCAAC

CATGTCAAGGGCTTGATCATGGACATCATGGTAGGCGGGACGAGTGTGACCTCGACGACA

GTTGAGTGGGTGATGGTGGAACTGATGCAGAGGCCTGAGGTACTGAAGAAAACCCAGGAG

GAACTGGAAGCAACAGTTGGACCGGGGTTTGCTGTCAATGAATCCCACGTCCCAAAACTG

CATTATCTGCGCGCGGTCATCAAAGAGACCCTGAGGCTCCATCCCGTGGTACCCTTGCTG

GTCCCCCGTGTACCAAGCAGCACATGCATCGTCGGAGGGTACCTCATTCCCGCTGGCACC

CGTGTTGTGACAAATGTGTGGGCAATCCACACAGACCCTGCTGTCTGGGATGACCCATTG

GAGTTCCTCCCCGAGAGGTTCTTGGCAACTGGCATTAAGGAGAGAGAGAATCACAGCTTA

GCCAGCTTTCGCTTCTTCCCATTCGGGGCAGGGCAGAGAAGATGTGCAGGATTGCCATTG

GTGGAGCGGATGTTGCCTGTGGTGGTGGCTACACTTTTGCACTTGTTCGAATGGCGGGTG

CCAGAGGGGATGGAACTCGATCTTTGTGAAAGATCCGGGGTCACTCTCACGAAAGCAAGA

CCGTTCCTTGCTGTCCCGACGCCAAGGTTTTCAAATAGTGTGCTATATGGTTGAGTTGCT

CCCAATTCCTTTGTCATTTTCCTTCAATAGGTTAGGTGTCTGGGTGGTCGTTCAAAGTGT

TGCATTAGTCTAAATTTGTTTCCAACTGTAAAATAATCATGTTCTCCTATCTTAAAATAC

ATTAGGTCTTTCTAGTACAAATCATCCAAGTTTGTAT

>Unigene0000381

ACTCTCTCCCATAGAAACCTTCCCCTATGATTCATATAACACTCTTCCATGGTATCCAAT

TACAACCATGGTATTGTAGTGTTGTCATGCTCTCCATCACTCTGATCACATCCCTATACA

TTTTTCCCCGAAAAGCCAGAAAACCTGTGCTGCCACCTGGCCCCATTGGCCTCCCATTGC

TCGGCAACCTCCACCTCCTCGGCCCGAATCTCCACCACCGCCTCACCCAATACTCCAACT

CCTACGGACCCATCATGAAACTCCACCTCGGCACCAAGCTTTGCATCATCATCACCTCTC

CGGCTATCGCCAAAGAAGTATACAAAGACCACGACCCAATATTCGCCAACCATGATCAAC

CCATTGCAGCACGAGCCATCTCATATGATGGCTCGAACCTCGTTCGGAGCCCCTATGGCC

CCCAATGGCGCATGTTGCATCGTGTCTTCCTTCATGAGCTCCTAAATAGCCGAAGCCTTG

ATAACAGTTGCCACTTTCGGCAACAAGAAGTTGGCAGAATGGTGCAGCAGCTGCAACTGA

AAAACGGCAGCCTCGTCAACATCGGGGAGCGGGCTTTTGCGGTATTTCTTGATCTGCTGA

CAGGGATGATGTGGGGCGGAAGGTTAGA

>Unigene0000382

AAAGCATTCTGCAGCAATGAGTGCAGACAGAAAGAGACGCTGTTTGATGAAGGGTTTAAG

AATGCATCATCAGGTTGATCTGCAGTACTCTGGCGGGATCCTCTAACAGTATCACTAAAC

TATGTAATGGCTGTGGTTTGGATGGTTTGGGTTTGGAGTTGGAGTTTGATTTATTGATGT

TTTCTGCCGTTATATTTAAATTTGGCTTTTCCTATTGTCGTGTAATTTAGGGATTCTCCC

TTCGTCATTTAATTTTGTTTCCACTAATGGTTGGTTTGCTGAGCTATTTGAAGCAGGACA

GTTCTCCGGCGAATTACATGAGGATGGAATATGTTGTTACGATGATCGGATAGGAGTGAT

CGAGAATCTTCTCGTACATGTTGAATGATGTCTGTTGGCTTGATGTTGTGACGTTGAAAA

GAAATCATAGTCAATCTCTAGGGTCTTTTTATAGATCCAACCACAAAGAGAAGTAACAAG

ATAATTTTTTCAATTGATCTAATGGTTGGTTTTTAATCACTTTTAACATGGAGTTTCCTT

CTGAA

>Unigene0000383

GTGAAGGACATTTTCATGAACAGGTTAGAACTATTTTGTATCGCTTAATTGATTATTGTA

CTTCTGTTTATGTTGACATTGCGTTATTTTGCTAAATTGCTATGCACTTGTTTATTGATT

AAATAATTAATATTACCCATTGTTGTTTAACTATTATGCCCAAATTTTAGTAGGATTCAT

CCTTGTCCCTTTTTTTCTAATAGATTCTTTGATCCAAACTTTAGCATTGAAAACAGTGGT

GTCTCCTTGATGAACCAAGGGCTATATTCAAATGTCCCCATTGATGATGAGCTCATAGTT

GAAATAAAGACAATCGTTAATAATTTTGCAAGTATCCATCTTTACTTAACTCCAAACATA

TACCGAAAAAAGTAACTCCATTATTGAAGGTAAAAAAAAGAAAAAGAAAAGTAAACTTTT

AGTTAGTTTACCTTATTTTTCTAAAGACACTGAGTTTTTTTTGGTCAGGTAAGAAAAATA

TGTATACTCCTAATAAGTGTATGAACATAATGTATCATGGGGTTCTAAACGGTGGTAGCA

TCTCCATGCAGTTTTATGAATATTAACAGACAAGAGAGGGCTATGTTTATTGGTTTAGAT

TTCAGACAAATATACTAGAGTTGCATTCTGATTATAATCATTTTGAACAAAAAAAATTGA

CAAATTGTAGCCCTGGCGCATCAGGAATATTAATTTGATTTATAAAGTGCATTAAACTGT

TTAGGTTTGTTAGATAGCATTTGTGCCTCCATGACTTGGCGAACTATATTAGAGACATGT

CGCTTTTATCTGGGTCCCCAATCCTCGTAAGTGGGACACCTCTATTGAAAGTGTTATAAG

CATGATTGATTAATCACGAGGCATTTATTTAGTTTAGTTCTGATTTCTTAAGCCAATTTG

TGTGCTAAAATGGTCAGCGTTATTGGTTAAATGTGAAGGGCTTGGATTTACTGGTTCTCT

ATATTTCAGAGGCGAGAAAGCATTCTGCAG

>Unigene0000384

TCACAGGAAATCCGGCATTCCAGGTTGTCCAATCGTTCTGTTTGGATCAAAACTCAAAAT

TCAGATTCCTACTATGTACCCTACTTCAGTCTCTGCGACCGCCTGGGCAGAGTCCCCTGG

ATCGCCTATAGAGTTTGGGGTCAAGAACAAGAGCTCGCAGCTGGCTCTGATCTCTCCTTC

TCGGAGGTCTCCCATGGCATGTTCGGGCGATACGCGGTCACCCCCAACCCATATGGAGCT

ATCCGAGGATTATACATGCGTGATTGTCCACGGGCCCATTCCAAAGGCAACTCACATCTT

TGATAATTGCATCGTGGAGAGCTGCTGCACTGACCACGGTTACGTTCCTTGGCAGAGAGA

GAATGGGTCCCTAGCTGACCATTCAGTTGATTTCTTGGCCTGTTGTCATGCCTGCAATAA

TAGTATTGGGCCTGGGAAGGATATTTTTATGTACAGTGAAAAAGCATTCTGCAGCAACGA

GTGTCGACACAATGAGATGATG

>Unigene0000385

ATTTCAAAGAATGCGTTGGATATCATGCTGATGAGAAAAAGGTCGTCAGTTAGAGTTAGC

AGCAGAATGAACAGCAATCTCCCTTCCTCTTACTCTGCAACCTCAAGACTGTTGGATTTT

TATGCAAAGCCCTTATCAGACACTGACCTAACAGCAAGCCCAACTTCCATAATCGAAACC

AAGATCTTCTCTCCTTCTTTCAACCCCTTCTTCACTCAAATGAAGCCAAGG

>Unigene0000386

GTTTTTGTATTTTTTTTTTTCCGCTTGTAATAGCAATCTGCCTTTTCCGTTGGGGAATAA

ATTACCAATAAAGAGTGAGAGTCATGCCCCTCCCTTTTTTTTAGCACTCTTTGTGTTTCT

CTACAGCGGAATTATATGCTGAGCCTTGTTTATATGTTACAAATTTCAAAGAATGCGTTG

GATATCATGCTGATGAGAAAAAGGTCGTCAGTCAGAGTTAGCAGC

>Unigene0000387

CAATCATATACTGATGGTCTCTTTTATTTGTAGCGCACTTCAAAGATTCCGAGAGAGATA

CATACCATGCAGATGAGAAAGAGATCATCAATGGCACTCAGCAGCAGCAACAACACCAAT

CCCACCTCCCACTTTGCAACTTCAAGGCTCTTGTTGGGTTTCTCAGCCCGGCCTTCATCA

GATACTGACCCATCAGCAAGCCCAACTTCCATACTTGAGACCAAGCTCTTCTCTCCTACT

GTCAACCCCTTCTTCACCGACACGAAGCCAAGATCCACATGGCGCAGCGCCAAGTCGAGG

GCCGTCGGGCTCGGTATCGTTGACGCCCTGCACAACGAGAAGCCAGAGATTCAAAAGAAC

TGCAGCAATCCAGATAGTCGAGTGGTTTTGGTTGGATCACAGCTTAAGGTTCAGATACCG

TCCATGCATCCCACTTCATTTTCTACGGCAGGGAGGGCTGAGTCCCCTGGATTGCCTGTA

GAATTTGGGGTCAAGAACAAGAACTCGCAACTAGCTCTGCTCTCCCCTGCTCCGAGGTCT

CCCATGTCATGTTCGGTTGATATGTGGCCGCCATCAACCGATATGGAACTATCTGAGGAC

TATACCTGTGTGATTGCCCACGGGCCGAATCCGAGAACTACCCACATATTTGATAACTGC

ATAGTGGAGAGCTGCAGCACTAGCCACAGTTCTGTTTCTTCACCGAGGCAGAAGAGGTCC

TTGGCTGGCGATTCCGTCAGTTTCCTGGCCTTTTGCCATGTCTGCAAGAAGAGTCTTGGG

CAGGGGAAGGATATTTTCATGTACAGAGGTGAGAAAGCATTCTGCAGCAATGAGTGCCGA

CACAATCATCGTGAAGGGATGGAGGAGGCATCATGAGGCTCATCTATGATACTTTATCGA

ATTTCTTGTACCGTTCACATCTTTGTAATGTATGTAAAAAGTGTAATCAGCTTGGAGCAA

GGATTTTGAGTGTTTTTTTTTTCTTTTGAATAGGTTTACCTTAATATGAGAACTTCTATG

GTTGAATCGATTCAAGATATCATCAATATATTTATGATGATGCTCTTATGTTTTTATGTA

CTCTTTCAA

>Unigene0000388

AATATGTGTATAGAAAGAACAACCAATAGGCCTAAACACAGCTGCTGCCGATTTTATCAA

ATATTGCACCGTTTGATAAATTTGTAATTCTATGTAAATCTCTATAATTTTCCAAAAAAC

GTTATTCTATCTAAATCTCTATGGTTTTAGATAGAGAGGGTGTGGCGTTGTGACATTACT

TGGTATTTTGTCATGATGTTTGTGATGACTTTATCAACCACCAAAAGAAAAGAGGGGAGA

GTCATTCCCCTATCTTATCTTGTTGCACTCTTTTTGTCTCTATAGCTCAATTATATGCGG

AGGGTGGCTCTTTATTTCATTCAAGTTTCAAAGATTCTGAGAGAGATAAATATGATGCTG

ATGAGAAAGAGATCATCAATGGCAGTCAGCAGCAGCAACAACAACACCAAACCCTCCTCC

CACTTTGCAACCTCAAGGCTCTTGTTGGGTTTCTCTGCCAATCCCTCAACAGATGCTGGC

CCAGCAGCAAGCCCAACTTCCATACTCGAGACCAAGATCTTCTCTCCAACAGTGAACGCC

TCCTTCAATGATAGGATGTCAAGACCGACTTGGCACAACACGGAGATGAGGGCCGTCGGG

CTTGGTATCATAGACGCCATTAGCAATGAGGAGCCAGAAGTTCACAGGAAGTCCAACAAT

CCAGATAGTCGAATGGTTCTGTTTGGATCACATCTTAAGATTCAGATACCCTCTATGCAC

CCTTCGTCAGATTATGCCGTCGGGTGGGCAGAGCCAGCTGGATCGCCTATAGAGTTTGGG

GTCAAGAACAAGAGCTCAAAGTTGGCTCTGGTCTCCCCTGATCTGAGGTCTCCCATGGCA

TGTTCCGCTAATATGCTGCCACTATCAACCGATATTGAGCTATCGGAGGATTATACCTGT

GTGATTGCCCATGGGCCGAATCCAAGGACCACCCACATCTTTGATAATTGTATCGTCAAG

AGCTGTGGCAATCGCCACAGTTATGTTCCTTCAACGAAGGAGAAGTCCTTGGCCTGCAAT

TCGGTTGATTTCTTGGCCTTCTGCCATTCTTGCAAGAAGAGTCTTGGTCCGGTGAAGGAC

ATTTTCATGAACAGAGGCGAGAAAGCATTCTGCAGCAATGAGTGCCGACACAATCATCGT

GAAGGGA

>Unigene0000389

CTCAGAACCGAATGCGGGTGTGGCTGGGGACATATGACTCACCTGAGTCGGCAGCCTATG

CGTACGATCGGGCTTCCTACAAACTCCGCGGCGTGTACGCTCGCCTCAATTTCCCTGCTC

TCCGTGATACCGCCGACGAAGACTGCCCCGAAAAATTGCGTGCGCTGCGAGCTGCCGTCG

ATGCTAAGATCCAAGCCATCAGCCATCGACTCAGCCAGAAGCGGCGTGCTAACAGGCTCA

AAAGAAAAGAGATGCAACAATCCAAGCCTCAGAGCAGCGAGGTACTGAGTGTCTGCCCCT

CAAATCACTCAGCCTCATCGTCGGCAACATCAGGGGAGGAGTTGGACTGTGAATGGTCGC

TGGCGAGGATGCCATCGTTCGATCCTGAGCTCATCTGGGAAGTACTGGCTAATTAGAGAG

CT

>Unigene0000390

TTCCAGAAAAGTCCCTCTCTCATTCTCAGAATACCCCTCATTCTCTGCTTCAGCCATAAG

TGGAGCAGGATGAACCAATTCGCTTCTCCCAGCGAGATCAGCTCGGCCCTGTCCACCATC

CTTCTCTCCGGCACCAATGTCTTAGACTCCATCTTCGCCCACCTCCCTCCCCCAATCCCT

TTTCCTACTACAGAGCCGTGTCTTGGCTCCGCTGTTTACCTCCGCCAGACAGAGATGCTC

CGACAATTCAGCAGCAGCCAATCCCTGCCCCCCTGGTCTCTACCAAACTACGCCGGATGG

CCCCGCAAGAAGATGTACCG

>Unigene0000391

CGCCGGTAACTCGCGGACCTAATTCGCTGAGCTCGACGACTCCAATGGATCTCTCGCCCC

ACACCTCCTCCGGCAACGGCGGCTTCGCCGCCGGCGCCTCTCTCACCCCCGCTCAAACTC

CGATCTCCAACCCCGCCACCGCCGATCCAGCGGACGATCAGAAGCAGAACCTCAACCACG

TCATCAATTCCATCCAGAAAACCCTAGGCCTCCTCCACCAGCTGTACCTCACCGTCTCCT

CCTTCAACGTCGCCGCGCAGATGCCGCTCCTCCAGCGCCTGAACACGCTTGTCGGGGAAT

TGGATACGATGCAGAAATTGGCGGATAATTGTAATATTCAGGTCCCGATGGAGGTTCTTA

ATCTGATTGATGATGGGAAGAACCCGGATGAGTTCACGAAGGATGTGCTCAATAGCTGCA

TTGCGAAGAATCAGATTACGAAGGGGAAGACTGATGCTTTCAAGAGCATGAGAAAGTATC

TTTTGGAGGATCTGGAACAAGCATTTCCAAATGAAGTTGAAGCTTATAGGGAAATACGTG

CGACTTCTGCTGCTCAATCTAAGTATCAAGAGAGAAGGAGAGAAAGAGAGGAGGAGAGGA

GTGGAGGTGAAGGAAGAGAAGAAGAGAGGGAGGAGAGGAAGAGAAGAAGAGTGGAAAGAG

ATGAAAGAGAAGAAAAGTTCAAGAATCTGCTCAATTTCTCATCAACCCCAGAGGTCAAAT

TGCTGAAAGTTGATTTCGGTTTAAATGAGGAAGCAAGGCGACTCGCCCAATCACAGAATG

CTTTACCCAACGGCGACGCCAAAGTGAAGTCGGAGCATTGACATGTCTTATTTATAGACA

TGGATTTCCGACAATCTGTAATGTATCAAGGACTTTAGCATTTATGCAAGGTTACTTCTA

TATGAACTGTGGCTGATGCTACAGTCTAGAAATGAAAATATCAAGGCAAGTAAAATAGCA

TAAAATTTTCC

>Unigene0000392

TATTTTTATTCCTCTTCTTCTCGGAACTCATTTCTTTAATGGAGCTTGAGGGGAGCTCAA

TAATGGCCGCCGCCTGCTAGGGCTTGAGCAAGACACAAAGGTAAGTTAGATGTTCACTGA

TTTAAGCTTCACTGATTTAAGTTTATAATCGTTCGATAATTTACCGATCCACAGTTAGAT

AAAGTATTCTTCTCCAATTTTCCATTTTCATCGTTTTAGTGCTGATTTGATCTTCTTTGG

TGCGAGATGAGCTAGCGGAGGAGATCAACCTGAAGAATACGTCGTCAGGGATGAAGACAT

CGAGGGATTATGATACATCAGCAACTAGCTGAACTCGGAGGAGATTAT

>Unigene0000393

TATTTTTATTCCTCTTCTTCTCGGAACTCATTTCTTTAATGGAGCTTGAGGGGAGCTCAA

TAATGGCCGCCGCCTGCTAGGGCTTGAGCAAGACACAAAGCTAGCGGAGGAGATCAACCT

GAAGAATACGTCGTCAGGGATGAAGACATCGAGGGATTATGATACATCAGCAACTAGCTG

AACTTGGAGGAGATTATTGCTTGTGCATGACTTGTAGAGGGAGACCAAACCCTCGGCTAC

TTCTTCATGGAGACAAACATTGAATGAGCTTGGTGCTGTTGCTGGTGAAGCTGAGCTGAA

TTTTGTTGATGAAGAACACGAGGTTGAACAGAACCTGATGCAGTTGAGTTTGAAGCTTGA

AGTCGAGCTTGCGAGTTCTTGTTAGCGATGATGCTTGTTGATGATGTTGGGGTGCTGGAC

AACACTTTGATGATGGTGGAGAGCTCGGATGTGATGCTCAGTGATTCGATGGCGAGGACG

GCAGTCGGCGGTGGGATGAATCTGTTGAGGAAGGGGCTAGCGTTCTGGCAGTAATATGAT

GTCGGGGTATAAGCTTGAACCTAAACTCCTGATGCAGATTTTGTAGCCAATGTGACACAA

ATTGTGTAGCTAATATATATATATATAATGAGCCAATGTGTTTATGTGTATAGTTTATGC

ATGTAATGGTTGGATTGTATTACAATCTTGGTGTAAACGTAAATATACGTGAAATGCAAG

TA

>Unigene0000394

TATTTTTATTCCTCTTCTTCTCGGAACTCATTTCTTTAATGGAGCTTGAGGGGAGCTCAA

TAATGGCCGCCGCCTGCTAGGGCTTGAGCAAGACACAAAGCGGAGGAGATCAACCCGAAG

AATACGTCGTCAGGGATGAAGACATCGAGGGATTATGATACATCAGCAACTAGCTGAACT

CGGAGGAGATTATCCGATGAGACTGATAATGATGACAGTGCTTGTGCATGACTTGTAGAG

G

>Unigene0000395

GATTCCACCCTCTCCAAATCCCCCAGATTTGATTCCCCAATGCTCAATTTCGCTCTCCAA

TTCAATCACCGACACTGATCGCACCATCACCGACTCATTCCTCTGCTCAATTGGAGCGAT

AATGGTGGGAATGCCGGAGCGGGGGGCGACGACGATGATGAATGGCGGCTATGCTCAGAT

TCAGAACTTCTGCGGAGGGGAGAGCAGCGAGGAGGAGCTCACCGTACTTCCGAGGCACAC

CAAAGTTGTCGTCACCGGCAATAATCGCACCAAGTCGGTACTCGTCGGGTTGCAGGGCGT

CGTCAAGAAGGCCGTCGGCCTCGGCGGCTGGCATTGGCTGGTTCTGACAAATGGCATAGA

GGTGAAACTGCAAAGGAATGCTCTTAGTGTAATTGAAGCCCCAACTGGTCATGAGGATGA

TGATGAGCTTGAATATGAAACTATGCAATGGAATGGTTCTGACAAGGGTTTTGCAGTTTC

TGATGACGGGCTGTCACATCTACAATGGTCCAGAGCTAGGCCACACAAAGCATCGGCTCA

CAAATCCTTGATTCGGTTCCAGTCTTTTGATTCAGAGTCTAAAGGATCCAACTCGTCCTC

CCGGGGAACCATGATGTCGAAAGTTGATCTGGGCAAGCTAGAAACAACAGCACTTTGGAG

ATATTGGCATCACTTCAACCTAATGGATGCGGCCCCTAACCCTTCCAAGGAGCAATTGAT

TGAGGTGGTTCAGAGGCATTTCGTATCGCAGCAATTGGACGAGTTGCAGGTAATTGCGGG

GTTTGTGAGAGCAGCGAAGAGGCTCAAGACCGTCTGCAATTGACAAAGTGAGACTCTGCT

CTCTTAGTACTAACATATATGTGGTTGGCAGTATATGTAAGATATCTATCCTTGTGTTGG

TGATAGAGACACTAGATGGTCGTGTAACATAGATATGTCCTACTAAAGACCTTTTAATTC

AGATACTGTGCTTCTCATAGCTTAGGTGCACCTGGGTCTTTGAGTTCTTTGGTTTCCGTG

AAACCGTTAGTGACTCATTTCCTAGTACTCGAATCTCTCAGAGCAATTTCACTGACTTAT

GTTTCCAGTTTTCTTTCGAACATAAAACCTCAATAGTAATGGGTATAGAAT

>Unigene0000396

AGCACGACGTAACACCCTCCACCCCCACCGGCACCGCCATGGAGCTCCCCCTCCTCATCG

TCCTCACCGCCGCCGCAATCATCCTCTCTCTCGTCATCTCCGCTATCTACCTCTCCCTCC

GCTGCCGCCGCCGGACTCCAGTCGACCTCGAACAACCCGTTACACACAAACCCGACCCCA

CCGCCCCCCACCGCTTCACCTGGTCAGAAGTCGAAACCCTAACCACCAACTTCTCTCAAT

CCAACATAATCGGCTCCGGCGGCTTCAGCACCGTCTACCTCTCCTCCACCCTCGCCGCCT

TCAAAGTCCACCGCTGCAGCGAGCGCCTCCACCGCGCCTTCCGCAACGAGCTCACCGCCC

TCCTCCGTCTCCGTCACCCCAACATCGTCACCCTCCTCGGCTACTCCGACGACACCCCAG

AAGACGGCGCACTCGTCCTCGAATACGTCCCAAACGGCACCCTCTACGACCACCTCCACG

GGAGCCGCGCACCGCCGCTCCCATGGCCCCGCCGGACGTCCATCGCCGTCCAGCTGGCAA

CAGCGGTACAATACCTACACGACGAGTGCAACCCCCAGCTCGTTCACGGCGACCTGAAGT

CGTCCAACGTGTTGCTAGACAACCATCTAGCTCCAAAGCTTTGTGACTTCGGGTCGGCGC

AAATTGGGTTCTCGGCGGTGGTGAGTGCCGCTGCTGCCAGCGATAGGAAAACAATGATGG

GCTCGCCGGGGTATGTGGATCCGCACTATCTTAGGACGGGGATGGTGTCGAAGAAGAGCG

ATATTTATAGCTTCGGGGTGTTGTTGCTTGAGCTTGTGACGGGAGCTGAGGCGTTTGGGG

AC

>Unigene0000397

AGCAAAATGGTGTGGATTGAAGATACTTTTCAGGAGAGTCAATTGTCTTTGGCCTCATAT

TTTTTAAGGTGGGCCTCAACACAGTTGACTGGTTGCAGCTACAGCTACTGCAATGCCGTC

GTATGCTCACGTTCTTAAGCACCTGGTGCTCTGGTTTCTTCAGCCAAGAAGATGAAAGGG

TTGAGTTATTTTCCCCTTATGTTGAAGAAGACTAACATACTACTAGGAACACAATTGGTC

ATGTAACAACATATTTGATCCAAATTCTTGCACTGGAGTCTTGGCACTGTAATTGGGATA

AAACAATTGCCTCCCCTCTAATAAGGCTCTCTTGGGATTCTGATATCTGCTAAGAACAAG

AGCCTCTGGAAATGATGGCTGTGCGCTCAGGAGCAAGCTCGGAGCATTCGGAATAAAGGG

AATGTCAATTTATGCACTACTTAAAAGGCTCGGATTTGAATCAGTCATATTTCTGCAAAC

TACTCTGGTGAGCATGTATTCAGAGATGAGTTAGCTTTTGATATATTCATTGTACTCGAC

GGAGGAGTGTCATCTGCTGGACCGTTATGATCTCTGTCTACATCAATGATGGGCCAGGCA

AAACTTTAGAGCTCTTTAGATGGATGCAGATGGAAAGCATGGAACGAAATGCGCTTACTC

CGACAGTCTCTCTCTCTGCTACATCTATTGTCACTACTGCCAGGAAAAAGGATCAGGAGA

GGATCTAGTCCTTGACAATGCTTTGGTGATCATGTACGTGAAATGCTGCGACATCAGAGC

TGCTTGTCATAGTACCATCTCTTTAATCAGAAAAGCAGAGGAGGCTCTCAGGCTATTCAT

GAGTTGAAGACAAACCATTATTTTTGCTCATAAACATCGCCTAGCCTCACCTTCATCAGG

GTCCTGAAGGGCCGCAGCATTGGGAGAGCATGGAAGGAATTAAGGCCCGAGGCTGAAGAT

TGGTAGATGATTTATTAGCGTACGACTAATCTGCTTTGCCAAGCAGGATTTCATCAAGAG

AACGCTGGTTCAGGTATTATTTCAGTTCCTTTTGCAATTACGAAAATTGAAAGTCTTTTA

AATTATTAGACCTGTGTCATCCTTTCACTAGTTTT

>Unigene0000398

CTTTCAATTCCTTCTGGGCATTATTCTCACACAAATCAGCCATGGAAGAGACTCGAAACT

TCCTCGTCTTCCCTTTCCTCTTGCTGCTCATCTCCCGCACCAACCTTGCTGATGTCGGCA

CGGCCGCCTCATTCGGGCCACCATATCTGCCGACAGTGTGCTACGGAAGCGACCCTGGAC

AGTTTCCGGCGGACAACCTGTTCGCCGCAGCGGGACAGGGAATATGGGACAATGGTGCTG

CGTGTGGACGGTTGTACCTGGTGAGGTGCCTTAGCTCGGCGACACCAGGAGCTTGCACGG

ATGACGGCAGTGGTGATGGTGGGCCGGCCATTCAGGCGGTGGTGGTCGATCTTGCCGACG

ATCTTAGTTCGGACCCTTCTATGAATGGGACTACCTTGGTGCTCTCTGAAGCTGCGTTCC

AGATGATTGCAAGTTTATCTGCTTCAGAGGTTAACATAGAATTTACACAGATTGATTGAT

GAGTGGAGAACGGTGCAAACCCAGTTGTGAGTTCTTTGAAGATTGTTTTGACAGTTCCGC

AGGGTCTTAAATTCGAAGTTCCAATGAACCATCCAATTATTTTTGTAATCTAAGTTTAAT

TTGTAAATCAAATGGTAATTGAGAATGAGAGTTTTTCTCTGAGAGTTGTGTATTTTTTTT

ATTTTTTTTGTAATGTGAATTATTGGAATCAGGTGGTCTG

>Unigene0000399

AATAGAGCCACCCAATTCCTTCTGGGCATTGTTCTCACACCAAACAGTTATGGATGAGCT

TCGAAACTACCTCCTCCTCTTGCTGCTCGCATCCCGTACCACCCTGGCCAATGTCGGCAC

GGCCGCCTCATTCGGGCCACCATATCTGCCGACAGTGTGCTACGGGAGCGACACTGGGCA

GTTTCCCCCGGACAACCTTTTCGCGGCAGCGGGACAGGGGATATGGGACAATGGTGCTGC

GTGCGGGCGGTTGTACTTGGTTAGGTGTCTTAGCTCGGTGACACCAGGTGCTTGCACGAA

TGACGGTGGTGGTGATGGTGGGGCGGCGATTCAGGTGGTGGTGGTCGACCTCGCCGACGA

TCTTAGTTCGAATCCTTCGATAAATGGGACTACATTGGTGCTCTCTGACTCGGCGTTCCA

GTTGATTGCTAGCTCATCAGCTTTAGAGGTTAACATAGAATTTACACAGATTGATTGATG

AGTCGAGAACGATATAAACCCAGTTGTGAGATCTTTGAAGTTATTTTTTTCCTTTTCTTA

TATTTGGAGGATTGATTGATGAGTGGAGAACGGTATAAACCCAGTTGTGAGATCTTTGAA

GATTGTTTTGACAGTTGAACCGCAGGGTATTTAATTCAAAATTCCAGGGAACCAATCAAT

TCTTTTTGTAATCTGAGTTTAATTTGTAAATAAAATGCTTAATTGTGAATCAAAGTTTTT

CTCGAGGAGTTACTTGTATTTCACTAGTCAATTTAGCCGGATGCTCAGTAATCCCCTGTT

TGGACAAG

>Unigene0000400

TGCATCCCACAGAAGGCGCCATTTTTGTGGCTACAAGGTATGATGGCGTTTTTGAGCACT

CTCCTTTGTTCTCTCTCACTTGAAGCTGCCAATCCTGCAAGCTTTTTCTGAAGGTGCTAT

GCTATTTTTGTCACCGTAGATCTCCTCCGCCAAAGAATGGCGACGGAGTCCACTGTTGAG

CATTCTCCTATGTTTTGTCTCACTTGAGGCTGCCAACTCTGCAAGCTTTTTCTGAATTGA

ATATCCACAAATGCTTAAATCTCCATGCTGATAGCAGTTGCATCCCAGTGAGGCTCCTTG

GTGGAATATAATTGTCATATTGGTTCTATCAAAAGGGGAAAACATTCATGAGCTCCAGAA

AAATATGCATTGAGGATAAGCAATAATTGTTTTCAGGAAAATGCATGACTCAGAAAAGAT

TGCTGTGGTTAAACCTGTTGCTACAAGACCTTATTCCAGTTTCAGGTCCTTCTCAGATTT

CCTGGCAGGTGCCATTGATGCTTCTCCTACAGATTCTCTTGCCGAGACGACAGTCGCCAT

CAGGCCAAAGACTGTCAGGTTTATGCCCTCACAAAACAATCCCCCAGCTGAGCCAGCTTC

ATCCATGGATAATGTATCTGAATCGCCTATTTTTCGTTCATCTGATAATCTATCTATCCC

TACTTCAAGGGAAGACTCTGGATCAAATGTGATATACAAACCTATGGCAAAAGTTGTTTC

GAGGACAACTGCCTCTCTCTTGGCCAAGATGGGCAATGATATATGTCATCAACAACCATT

AGAGGAGCAGACTCAGTTTGAGGTTCCTGACCAAGTAAACCAAAGCCAGTCTACTTTTGA

TATTTATCAGAATATGCCATCAGACATGTTGATAAACCAAGTATATGAGCCTAATATAAG

TGAGTCTTCAAATTTGAGGGCTCCAAACACAGAACTAGACAATATAAGAGATCGGCCTTC

GTACGATGGATATAATTGGAGAAAGTACGGGCAAAAGCAAGTAAAAGGAAGCGAATATCC

ACGAAGTTACTATAAATGCACGCATCCAATTTGTCCTGTGAAGAGGATGGTTGAGAGGTC

AATTGATGGAAAAATAGCTGAAATTGTCTACAAGGGTGAACACAACCACCCTAAGCCTCA

ACCTCCCAAGCGTTTATCGTCAGCTTCACAAGGACAAACTTCTATTGCTAGTGAGCAAGC

GAGAGAGATTGAGGAACCATTGTGGAGTGATCTTCTCATTGAGAAGAACTTGTCTGATAG

TAGAATGGATAGCCACATTGATGTAAATTTTTATGGCGCTCTAGGTTCACCAAAGGTGCA

ATGCTCTCATGACTCACTAATAGGTACAACATATTCGAGCGGGATCATGGCTCCTGATCC

TTCTCAAAGACTTGGCGTGGACTTAGAAGCACGAAGCAAAGGAACTGTTGTCGATGATGG

CTTGCATAGGAGTAAGCGAAGGAAAAACCTTAATCTGGATAGTGAAGCAGGTGCATCAAG

GAACATTACTGCAGAGCTACATTCTGTATTTCAAGCTCCGACAGAATCTGATGTCTCAGC

AGATGGTTTCCGTTGGCGAAAATATGGACAGAAGGTTGTGAAGGGAAATTCATATCCAAG

GTGTTACTACAGATGCACCAGTCCTAAATGTAGCGTGCGGAAATATGTCGAAAGGGCATC

AGGCGATACAAGATCTTTCGTTACAACTTACGAAGGAAAGCACAACCATGATAAGCCAGA

AAAGAAAGTCGATTCGACACACAAGAACGAGAGAAAGCTCGGGTAATGAACTAGGGTGAT

AGGATTTTCGTTTCACAGCTAAACACTTCTAACATGAAAGGGCTTCAGTAATGGAAATGG

TTAACCATGTATTATAAGGAAGAACTCCACTTGAGATGGAGAGTTGGTTATGATTTATCT

TTGTTGACATTTCGGTATTTCTGAGTTGTTACATTTGTTTTCAGAGATGAAGTTGATC

>Unigene0000401

CTACTAAAAATGTGTTTCTTCCTCCTCCTTCACTGCCGGTAGAGATGAGCGGTGAAAGCG

ATGTGATCAGTCGGGCGGCGGAGGCGGCCGATGAGCTTTACGCGGTCCGCGATACATTCT

TCCCCAAGGATCCGTTGGAGAGGGCCGCGAAGATCCAAACCCTAGCCGACGCTGCCCTCT

CACTCCTCGATACCCTCCCCCCAGAGCAGCGGAAATCACCGTCACAACGTGCAGTTTATG

AATTTCTCAGAGGAAAAGTACTGGATGCATTTCCTGATTATCGTAAAGAAGCAGAAGATC

ATTTGTCAAAAGCTGTTAAGTTGAACCCATCTCTTGCAGATGCATGGTTATGCTTAGGTA

ATTGCATTTGGAAGAAGGGAGATCTACCTTCAGCAAAGAATTGCTTTTCTTTAGCGCTTA

GCAAGGGACCAAACAAGAAAATACTTTGTCAGCTGTCAATGCTTGAAAGAAGAATGGCTC

AAGGCACAGATAATGAAGCACAAATTATTGAAGAGAGCATCCTTCATGCAAAAGAAGCAG

TTATGTTGGATATCAAAGATGGAAACTCGTGGTATAATCTTGGAAATGCATGTCTCACAA

GTTTTTTTCATGACTGGGGCATGGGAGCACAGTAAACTTCAGCAATCACTGAAAGCTTAT

CAGAATGCTGAGAAGGACGAAACAATGAAGGCAAATCCTGACCTTTTCTTCAATTGTGCT

ACAGCTAATAAGTATCTTGAAAACTATGAAAGGTCCCTTCGTGGATTTGAAGCTGCTGCT

TTGAAGGATCCTGGCCTTAATGCCGACAGGGAGGTTCAAAAGATAATCACTCTTCTTGAC

AAGCTTGAGAACTCTGTGAAGGGGCAGGCTAGAGCTAAACGCATGGGATCCCTGATGTCA

TCCTTGGCTGGAGCTAATTTGAAATCCTCGCATAGAAAGGTCATGGTAAATCTTCTAGTA

GAGGGGCTGAACAAAACTGTAGCAATTGTTGGGAAGGTTCTCCTCTTCATAAAGCATGAC

AATGTTGCTCCACTGTATTATTTAGCATGCGACCTGGATCGAACTTTATTCATTCTCTCT

GTATATGGATTACACAGCGACGCGATAAAAGAAGGAGATCGAGTGGCCGTACTGGAACCC

TATCACCGGATGATTGATTTCAGATGGAAAGGCAAGCATTACGAATTCAAGTCAATTCGG

GTGGACTTCGTGGAGCAAATTCTGGTGAACGAGAAAGTTCCAGCTCCTCATCACGCCATC

CGGGCTTCCATTCGTGCACAACATAAGCCGTGAAGCAGTTTTACAGTTTACAGTGTGATT

TGATTTGTTATGTATTTTGGATCGCATAGAGATTATGTAAACCAAACTATTTGGTTGACA

AACTAGGGTGGTTAAGATTTGTGCTTGTTGTATCTCTTGGTTACTGGGGCTTGTTGTGTA

TGGAGTTCTTCAGGTCATTTTAAAGATGTGAATAACATGGGGATAAATTGACCTTATAAC

AGTTCAATTATAACAGTTAG

>Unigene0000402

AATACAATCAATGTGAGTTCTGCACCATTTACTGCTACAAAAAAGTTGCAGACCCTCCTC

AGCATATGCTGTGATCAACAGTGGATGGACGAAAGCTTGCCAAGACTAACAAGTCTGCGA

AGGTTGGGTATTCAAACGTTTGGAACTGGAAAGATGTTGTTTCATTCACTTAGAGAACTC

ACGCGTCTTGTTGAGCTGATAATTACAAGTGATAATATTCTTGTAGAGACCATCATTACA

ATGGTATCAAACAATGATCACCTTCACGAACTGTTTCTTCAGGGTAAATTGGAGACACTG

CTACCAGAGATTAACAGATTCCCTCAGAACCTCACGAATCTGAAATTTTGGAACTCTGAA

ATGGAATATGATCCAATGCCAATACTGGAGAAGCTTCCAAACCTAGTTGTTCTAGAACTG

TCTGAT

>Unigene0000403

ATACCTGCCAACTATGATCGGTGGGATTTGTCCACCATTTACTGCTACAAAAAAGTTGCA

GACCCTTCTCAGCATAACGTGTGGACGACAATGGATGGAGGAAAGCTTGCTAAGACTGAC

AAGTCTGCGAAGGTTGGACATTCAGACGCTAAGGTTGGATATTCGAACGCATGGAACTGG

AAAGATGTTTCATTCACTTGGAGAACTCACTCGTCTTGTTGAGCTGTCAATTACAAGTGA

TAATTTTCCAGTAGAGACTATCATTACAATGTTATCAAACAATAATCACCTTCACGTACT

GAATCTTCTGGGTGAATTTGGGACACTGCTACCAGAGATTAACAAATTCCCTCAGAGCCT

CACGAAGCTGACATTTTGGGACAAATTCCCTCAGAGCCTCACGAAGCTGACATTTTGGGA

CTCTGGAATGGAGTATGATCCAATGCCAATACTGGAGAAGCTTCCAAACCTAGTTGTTCT

AGAACTGTCTGAT

>Unigene0000404

ATACCTGCCAACTATGATCGGTGGGATTTGTCCACCATTTACTGCTACAAAAAAGTTGCA

GACCCTCCTTAGCATAACGTGTGAACAACAGTGGATGGAGGAAAGCTTGCCAAGACTAAC

AAATCTGCGAAGGTTGGATATTCAAACGCTAAGATTGGATATTCGAACGCGCGGAACTGG

ATTACAAGTGATAAAGTTCATTACAATGGATGGAGGAAAGCTTGC

>Unigene0000405

TATTACTGCCAGCTCCAATCGATGTGAGTTGTCCACCATTTACTGCTACAAAAAAGTTGC

AGACCCTCCTCAGCGTAACCTGTGGACAACAGTGGATGGACGAAAGTTTGCCAAGACTAA

CAAGTCTGCGAAGGTTGGGTATTCGAACGCTTGGAACCGGAAAGATGTTGTTTCATTCAC

TTAGAGAACTCACTCGTCTTGTTGAGCTGTCAATTACAAGTGATAATGTTCCAATAGAGA

CCATCGTTACAATGGTATCTAACAATGATCACCTTCACGAACTGAATCTTGATGGTAAAT

TGGAGACGCTGCTACCAGAGATTAACAGATTCCCTCAGAACCTCACGAATCTGTCATTCG

ATTGCTCTGAAATGGAATATGATCCAATGCCAATACTGGAGAAGCTTCCAAACCTAGTTG

TTCTAGAACTGTCTGAT

>Unigene0000406

AACCACCCCCCACCTCCACCTCCACCTCCACCACCGGGGCGGAGGCGGAAGAGACCAAGG

CCCCAATTGACCGATCTAGGGTTAGGGTTTTGGAGCCCCTCCCCCTCTTCCAATTCCCAA

AATGACCTCGAAGCCGGACCCTGCGAGTTCCTTCCCTCTGATCCCCCCCACCCTCGTCCC

GGAGCCCGGCGAGATTGACCTCGAGGCCGGCCCCGGCGAGCAGTTCCAGTGCCGGATCTG

CCTCGAGACCGACGGGAGGGATTTCATTGCTCCGTGTAAGTGCAAGGGGACCTCCAAGTA

TGTTCACCGTGAGTGTCTCGATCGCTGGCGGGCTGCAAGGGAAGGTTTTGCATTTGCTCA

TTGTACAACATGCAAGGCTCCTTACTATTTGAGGGTTCATGTCCAAGCCGACAGGAAGTG

GAGAACCTTAAAGTTCCGGTTCTTTGTAACTAGAGATATATTGTTCATCTTTGTGGCCGT

TCAACTTGTAATTTGTGCACTGGCATATCTTGTGTATTTTATTGATAGTTATCAGCAGTA

CTGGCTGCGGTTGGCATGGGGTTTTGAAGCTGGACACAGTTTCTACTATATCTGTGGGGC

GCTAGTGTTTTTTGTTTTGCTTGGGTTGTCTGGATGTTTCATAACTTGTTATGATCGGCG

GATACGGAATGATCTGGCTCAGCCTTGTCGAGAATTATGTCTTTGCTGCTGTCAACCAGG

AATGTGTGCAGACTGCCATATCCCTGGAACACTCTGTATGTGGACTGATTGTACCACTTG

CTTTGAGGGCTGTGCGGGCGCAGCTGGCGAATGTGGATGCGTAGGAGGTGCTGGTGAAGC

TGGCCTACCGCTACTGTTTATAATGGGATTGGTCGTGCTCGGTCTATTCACCATTATTGG

CATATTCTACAGTGTTCTCGTGGCCACAATGGTTGGCCAGAGGATCTGGCAGCGCCACTA

CCATATACTCGCGAAGCGAATGCTGACAAAAGAGTACGTTGTGGAGGACGTGGATGGTGA

GATGACAGATCCAAATTGGAGTCCGCCGTCACTTCCACCTGAGCATGTCCAGCAGCTCAA

GTCTCTTGGACTTCTATAGGTAGAGTAATGGTACTCTCCATGTCATAACATTGAGCTTTT

AATGTAAATTATCTTGTATAAATGGTGAAGAGAAGAACCCTAGCAGCCATACCAAGAAAC

TGATACTGCCCTTTATCCCATTTTTTATGGGTCAGAAGTAATTTACTTTTATATACACTA

ACTGTTTTATTGAGTTCATGGGAATTTCGGAATAACTCTATGGTATTTGAGATTTCTTTT

ACCCTAGTCGAAACAATATGGTTTTCTTTGTAGACATGG

>Unigene0000407

ACACTCTTATAGGCTCGAACAACCTCTCACGCAATACACACAGCCGCCATGACCTACGGC

AGGCTGGAACGGCCTGATTTCTGCCTTCCTCCTTCCTCGAGTGTATTGAATTCACCTAAC

CATGGCCGCCGCCGAGCTATCTTTCTCGTTCTCGGCATTCTCTTCCTTCTCATAGCTGCC

ATTTCCGTAGTCTTCGTAATCCGAACTCGAACCCGGCCCCAGCATTCCCTCCTCCAGCCA

ACTCTAGCCATGTCCCGTAC

>Unigene0000408

ACACTCTTATAGGCTCGAACAACCTCTCACGCAATACACACAGCCGCCATGACCTACGGC

AGGCTGGAACGGCCTGATTTCTGCCTTCCTCCTTCCTCGAGTGTATTGAATTCACCTAAC

CATGGCCGCCGCCGAGCTATCTTTCTCGTTCTCGGCATTCTCTTCCTTCTCATATCTGCC

ATTTCCGCAGTCTTCGTAATCCGAACTCGAACCCGGCCCCAGCATTCCCTCCTCCAGCCA

ACTCTAGCCATGTCCCGTACATGTGCCCTCACCCGCTACCCCTCCCTCTGCATCACCTCC

CTCCTCAACTTCCCCGGCGCGCTTGCCGCTGGCGAGCATGACCTCATCCACATCTCCCTC

AACATGACCCTCAACCACGTGAGCCACGCCCTCTACTCTGCCTCCTCGATCACCGCCGTC

AACATGGACACTCTCACTCGCTCCGCCTATGAGGACTGCATGGAGCTCCTCGACGATTCC

ATCGACCAGCTCACCAACTCCCTCCTCTCCCTCACTGACGACAGCGACAGCAACACCGAC

AACGACGATGTAGTCACCTGGCTCAGCGCCGCCGTCACCAATCAAGACACGTGTAACGAC

GGACTCGACACTATCCCAGAAGGCTATATAAAAAGCCAGATGGTTAACTACCTTCAAGAC

CTTAGTGAGTTGGTCAGCAATTGTCTCGCCATCTTCGCTGCTGCCGGGAAGAACCCAGAA

TTCTCCGGCGTACCCATAGAGAACAGGCGGCGGCGGCGGCTGATGGCGGAGTTTCCGGAC

TGGGTGAGATTGGGCGACAGGAGGTTGCTGCAGGCTCCGGCGATGGGGGTGCAGGCAGAC

TTTGTTGTATCAGCTGATGGTAGTGGTGATTATAAAACGATAACTGAGGCGGTGAAGGCG

GCTCCGGAGAACAGCAGGAGGCGGATTGTTATATATGTGAAGGCTGGGAGGTACATGGAA

AATGTGAAGGTGGGGAGGAAGAAGAAGAATTTGATGTTCATCGGCGATGGGGAGGGGAAT

ACAGTGATTACTGCTTCGCGCAGTGTGGCTGACCACTACACTACCTTCCGCTCCGCCACA

TTCGCGGCTACGGGCGCGGGATTCATAATGCGAGACATGACAATCGAGAACACAGCCGGC

CCCTACAAGCACCAGGCAGTGGCACTCCGCGTGGATGCTGACCACTCCGTAGTCTACCAC

TGCAGCATATACGGTTATCAGGACACCCTCTACGTCCACTCCAAGCGCCAATTCTTCCGT

GAATGCGACGTCTACGGCACCGTCGACTTCATCTTCGGCAACGCCGCCGTCGTCCTCCAA

AACTGCACCATCTCAGTGCGCAAGCCGCTCCCCCAACAAAAGAACACAGTCACCGCACAG

AGCAGAAAAGATCCAAATCAGAACACCGGCATCTCGATCCACGACTGTCGCATTGTGGCG

ACACCAGAGCTGGAGGCGGTCAAATTGAGTTATCCAACATTTCTGGGACGACCATGGAAG

ATGTACTCGAGGGTGGTGGTTATGTTGAGCTACATTGGAGACAATGTTGATCCTGCAGGG

TGGCTGGCTTGGAATGCAACGTTTGCGCTGGATACTCTTTATTATGGGGAGTATATGAAC

TACGGGCCGGGCGCCGCTTTGGGGAAGAGGGTGACGTGGTCGGGGTATCGGGTGATCACA

CTGCCGGAAGAGGCGAAGAAGTTCACTGTCGGGGAGTTCATTTCTGGGACTTCTTGGTTG

CCCTCTACTGGGGTTGCATTCTTGGCTGGTTTGGTAGTTTGAGGGCTATATAGACTATTT

AAATTTTTTTATTTTGTTTATTAATTTTTATGTAAGTCTACATACATATTGTAGAGTTTT

TCTTTTGTTTGTTCATGCAAAAGAGAAATAATAAAAATAAAAAAGGTAAACT

>Unigene0000409

CCTCCGCCAGTTCTACCGCGACAGCACCATCTCCGGCACCGTCGACTTCATCTTCGGCAA

CGCCGCCGTCGTCTTCCAGAACTGCAACCTCGTCGCTCGTCTCCCGATGAGCAACCAACA

GAATCTCGTCACAGCCCAAGGCCGCACCGACCCGAACCAGAACACTGGCACCTCTATCCA

GAACTGTCAGGTAGTGCCGGCTCCTGATCTTGTTCCGGTGAAAGCGACGCT

>Unigene0000410

ACGGACGATATTTCGGAGGCGTCGAACTTGGCCTCGGCCTCGCCTCCTCGTCTCAATCAA

CGAAGCGGTTCCTCTCGGCCTTGGCTCGGGGCCGACGGGGACGAACGACAGGGCGAAGAC

GTCGAACTCGGCCTCGGTCTCTCCTCCTCCTCTCCCTGAAATGGAGAACGGCGGAATCCC

TCAGCTTCTCGAGAAGGTCATCATCGACACGGATCCTGGAATCGATGATACCATGGCTAT

CTTGATGGCCTTCCAAACACCCGAGATTGAAGTCTTAGGTTTGACAACAATATTTGGTAA

TGTTCAGACAGAAGATGCTACTCGCAATGCTTTGCTTCTGGATAGTGTGAGATCGCAGGA

TGTTCTGATGTTCCGGTTGCAGAGGGCTCCCCAGAGCCTCTAAAGGGAGGAAAGCCACGC

GTTGCGGACTTTGTTCATGGATCTGATGGAATGGGTAATTTATTTTTGCCTCCACCACTG

GGAAAGAAAATCGAGAAAAGTGCAGCTGAGTTTTTGGTTGACAAGATTTCTCAGTACCCT

GGTGAAGTATCTATACTTGCATTGGGACCTCTGACAAATTTAGCCTTGGCCATCAAAAGG

GACTCATCTTTTGCAAGCAAGGTGAAAAGGGTGGTGGTACTTGGTGGGGCATTTTTTGCA

GCTGGGAATGTCAATCCGGCTGCTGAAGCAAATATCTATGGAGATCCTGAAGCCGCTGAT

ATTGTGTTCACCTGTGGAGCAGAAATTTCTGTTGTTGGCATAAACATCACTACTCAAGTA

AAATTTACAGACGATGACCTTTCCGAACTGAGAAATTCGAAGGGGAGATTCTCGCAATTC

ATATGTGATACATGCAAGTTCTACAGAGACTGGCATGTCAAATCAGATGGCGTATATGGG

ATTTTCCTCCACGACCCTGTCAGTTTTGCTGCACTAGTTCGCCCAGATCTTTTTACATAC

AAGAAAGGGGTTGTGAGGGTTGAGACACAAGGCATCTGTGCCGGTCACACATTGATGGAC

CAAGGATTAAAAAGGTGGAATTCGGATAACCCTTGGTCAGGTTATGAACCGGTTTCAGTT

GCATGGACAGTCGATGTGCCCGGTGTTCTTGCTTACATTAAAGAACTACTTATGAAATCA

TGAAGACCATATTCTGTGTTGCCTGCCGCGACAGCTGAGTAAGCTGGAATTGTCGTTCAA

TTGTTCGGATCTTGTTTGTCCCCTTTAATGTTTAAGTTGCAAACACTTATGCAAAAACAT

CACAATTTGGTTCAGATTCGTTAAATTTTGGGTGAAATAGTTTGTTGCAAAATCCTTCCC

GTCAGTTTTTATACTCAATTTATTCTCGAAGGTATGAACACCATTTCGAGAATTGGGAAG

GTCAAATATTGATATTCATATAGTTGTTTTGCAATACAGCTGACCTTTATGCTTTTTTGT

GAATTGTTTGAATTTTGAATAATA

>Unigene0000411

GAAGTTAAGACTAGTTTCATGGATATTTATGCGTGGAAAATGAAATGATGTGCTGCTTTT

GGTGAAACTTGTGCGGTGAAGCTAGAGATGTGTTGTGTTTGTGTGTGCAAGTTGCATGTA

CAACTGAGACAACACTAGTTTGTAAGTGAACTGTAGTTAATAGAAGGTTAGTTTATCCAT

TTTGTGTTGGAAAATTTTTGGATGCCACTGATGCACATATTCCGTA

>Unigene0000412

CCCCGCCGAGTACCTCGAATCCATACCACAGAACTAATCGTCCTTCCCCTTTCCCATTTC

AAATACCAACCCCCTCTTCTCTCTCTCCGGCGATGGCTACCGCCCTGCAAGCGGCGGCGG

CGTTCCTCTCCGCCGCTCCGGCTGCCAATTCCCAGAGGTTCCGGTTCGGATCGTTGTCCA

AGCATCCCGCAGGTTGTACTGGGAGTGTTTTACTCGCCAGATCGGGTGGAAGGTCCTCTT

TTAGGCAGCTCAGCGGAAGCAATTTGATCACTTGTGCACTTGGGACTAAACCGGAAGCTT

CTGCGAGTTCGACAACGTCAAAACCCGGTCATGAGGTCCTGTTATTTGAGGCTCTAAAAG

AGGGCCTAGAAGAAGAGATGGACCGAGATCCTCGAGTGTGTATCATGGGTGAAGATGTTG

GACACTATGGTGGCTCATACAAGGTGACCAAAGGTCTAGCCCCGAAGTTCGGAGACCTGA

GGGTTCTCGACACGCCCATTGCCGAAAATTCATTCGTGGGCATGGGCATTGGGGCTGCGA

TGACAGGCTTAAGACCGGTTGTTGAAGGCATGAACATGGGCTTCCTCCTTCTCGCCTTCA

ACCAGATATCCAACAACTGTGGTATGCTCCATTACACATCAGGAGGTCAATTCACCATCC

CAATCGTAATCCGAGGGCCCGGAGGTGTCGGCCGCCAGCTCGGTGCCGAGCATTCACAAC

GCTTGGAGTCATACTTCCAGTCGATTCCCGGGCTCCAGTTGGTCGCTTGCTCGACCCCGT

ACAATGCGAAGGGTCTCATAAAAGCTGCGATTCGCAGTGAGAACCCTGTTGTGTTGTTCG

AGCACGTCTTACTGTATAACCTGAAGGAGAGGATCCCAGACGAGGAATACGTGTTGTCTC

TGGAAGAAGCGGAGATGGTGCGACCGGGCGAGCATGTCACGATTCTCACCTACTCCCGGA

TGAGATATCACGTAATGCAGGCTGCAAAGACGCTGGTGAACAAAGGGTACGACCCTGAGG

TCATTGATATCCGCTCGTTGAAGCCGTTTGATCTTTATATGATTGGCAATTCAGTGAAGA

AGACGCATCGAGTACTGATCGTCGAGGAGTGCATGCGTACAGGTGGGATCGGTTCTAGTC

TGAGGGCGGCTATTATTGATAACTTTTGGGATTACTTGGATGCTCCGATCATGTGCTTGT

CATCGCAGGATGTGCCGACGCCCTATGCTGGGACTCTGGAAGACTGGACCGTGGTGCAGC

CGGCGCAGATTGTGGCTGCAGTGGAGCAGATCTGTATGTAAGCTGTTTTTTATTGATTGC

ATTGAAGTTTGAATTAGTAGCATACTTAAACTTTCAAGACAGATTTGTTTGTATTTTTTT

CTGATTTTTATGTTATCCGTTGGTTATTTTTTGTCTGAATAGTAACTGAGGCTCGTATAA

TTTGGGGCTTCAATGGAAATTGAGTTGGTTAGATTAAAAGTATTGGCTTGCCTGTATGCA

TTTGGATGCAT

>Unigene0000413

GAAGTTAAGACTAGTTTCATGGATATTTATGCGTGGAAAATGAAATGATGTGCTGCTTTT

GGTGAAACTTGTGCGGTGAAGCTAGAGATGTGTTGTGTTTGTGTGTGCAAGTTGCATGTA

CAACTGAGACAACACTAGTTTGTAAGTGAACTGTAGTTAATAGAAGTTAAGACTAGTTTC

ATGGATATTTATGCGTGGAAAATGAAATGATGTGCTGCTTTTGGTGAAACTTGTGCGGTG

AAGCTAGAGATGTGTTGTGTTTGTGTGTGCAAGTTGCATGTACAACTGAGACAACACTAG

TTTGTAAGTGAACTGTAGTTAATAGAAG

>Unigene0000414

ATGAACCTGAGAGTTCATCTCCATGGGCTTCTTGTCCTAGTTTTCATTCTCTCTATCTAC

ACTATCAAGGGCTGCTCACGTCCCCTTCCTGCATTAGAAAAAGGAGGAGCTGAGGCCATA

GTTGATGTACTCAAGCAAATGGGTCACTCGGAAGAGATGGAAACACTGATGGGTTTGGAG

GGGTGTACTGATGATAAAGATGAGGAATGCCTGAAGAGGAGAATGATATCTGAGGCTCAT

CTGGACTACATCTACACACAACATCACAAACCGTAGATCTCAATAGGTGAAGATGAGAAG

CAGTTTATGATGTGTCATGTAACGACATTAAATAGTTATTGTGACTGATTGACCTTAATA

GCAGATCAGATAACTATGAAAGTTTTATTTGGTTTCCCATTTTGACGTTTGCTTATTCAC

AACATGATGTAATGCAAGGAAATGAGGTTTTGAATTGGTGACATAATGTAAGGAAAATGG

TTAGCTGAACTTCTCAATTGATAATGAGATCAATCAGAGGGTAGAATGGCATCTGATAAA

GTCTGAC

>Unigene0000415

TCAATCCCAAACCGAGCGAAAACTCTCGTCTCTCCCCCATCATCGTCTCCCCCTCCTCGC

GAGGCCCGCATGGCCGCCGGGCTCGCTAGCTTCCCCTTCAAATCCGCCGCCGCTCCTGCC

CTATCCCCCGCCTCGAATTCAGGAAAGCCCCTCTTCTATTCATCCTTCTCGCCTCTTCCC

CTCAAGAAACGCGGGCTCTGGTCATGCCAGTTAGTCGGGATAACTGGGACGGTGATGGCG

CGCTACGGCGGCGGCGGTTACCGCCCGCGGCCGTCGGGCGGCAATCGGAACAGGAGGGGG

CAGAGAGATAACCGGGTTGCGGAGTCTGATTATGATGCTGCCCTCGATCTCGACCGGCTC

AAGTCAAATTCTGCAAGACTATTAGATACACAACAGAATATGATTGGTGTAGTATCTATA

TCTGAAGCCATTCGAATGGCAGAAGATGATGAATTGATACTGGCCATAATGTCTGTAGAT

GCTGATCCTCCAGTCCTCAGACTATTTGAAATTGAAGAATACAAAAAGCACAAGTATGAG

CAACTCAAAAAGAAAAGGGATCAACAAAAGAGATCTTCTGCAAACCGTCAGGATATGAAG

GAGCTCAAAATGGGGTACAACATTGACTCGCATGATTATTCTGTGAGGCTGAGGGCTGCC

CAAAAGTTTCTTAAAGATGGAGATAAGGTTAAGGTCATGGTTAATCTCAAAGGTCGGGAA

AACGAGTTCAGAAATATTGCTATTGAACTTCTCAAGCGTTTCCAGAATGATATTGGGGAG

CTGGGAGCCGAGGAGAGCAAGAACTTCAATGACCGAAATGTATATATCATTTTGATGCCG

AATAAAGCCGTTATTCAGAAATCTCAGGAACAAACGAAGAAAAAGGCAGCACCAGGAGTA

GAAATTTCTATATAAATTGAGTTATAGTTCTGGCGGCAGGGTGAAGTCGATACATGAGGA

GATTTTAGATTTGTAACTTTTTGCTAGAATTTATAGTGAAGTTGTGTATTCTGTTGTTTC

AAGCGTTTTTCCTCGTGTTCTTTGTTATTATTGGTATTAGAGCGAGTTCTTACAATAATC

AAGATGTGTGTTAGGTTAATTGGTATTGTGATTAGGATATATGCATTGCTCCATCTTTTA

G

>Unigene0000416

CTATTTGAAATTGAAGAATACAAAAAGCACAAGTATGAGCAACTCAAAAAGAAAAGGGAT

CAACAAAAGAGATCTTCTGGTGAGTGTATCATTCTGTAAAAACTTGTCAGCATGATTCTT

AATGTTGCTTATTGTCTCTGGTGTCATAAATCGACATGCTCATACTGCTATCATTCACAG

CAAACCGTCAGGATATGAAGGAGCTCAAAATGGGGTAAGAGTGTATATCATCCAAGTTTT

ACTCTCATTTGCACGATGATCCTACCATTTCTGTTGTTATTATTGTAAAATTTCCAACTG

TTCTTGCCTCGTTATGTGGCCGGATCTAACAGTCGTATTGTTCATTTACTTCCTC

>Unigene0000417

AACTGCTTAGCCAAAACATTGGTAACTTAGAAAAGCCATTTTCAAATAATTTATATTTTG

TAGAAATCTTGTCTTTTCATGTTCAAGTCTTAGGGGCAACTGCGAAGAGAAAACATTGAT

AGCTGAGGAGAAAAAACATTTTCAAATAATTTACATTCTGTAGATAGCTTGTCTTTACGT

ATAAGTCTTAGAACTGAGAAGACAAAAAAATAGAACCTGTCTAGTAAGCATATGAGCAAG

GTGATTCCATTCCTAAGAGTGTATAAGTTGC

>Unigene0000418

ATATTTTGTAGAAATCTTGTCTTCACGTTCAAGTCTTAGGGGGCAACTGCTTAGCCAAAA

CATTGGAAGAGCCATTTTCAAATAATTTATATTTTGTAGAAATCTTGTCTTCACGTTCAA

GTCTTAGGGGCAACTGCGAGGATAAAAAATTGGTAGCTGAGGAGAAAAAACATTTTCAAA

TAATTTACATTCTGTAGATAGCTTGTCTTTACGTTTAAGTCTTATAACTGAGAAGACAAA

AAAATAGAACCTGTCTAGTAAGCATATGAGCAAGGTGATTCCATTCCTAAGAGTGTATAA

GTTGCATGTGACCATTGTTCATGAGTTGGTTGAACATCGATACAATATCAACAATCCAAG

AAATATTAATAGAATGGAGAGCATGGAGGACAATACTCTGAATTCTCTCTCTCT

>Unigene0000419

AGATGTATGCATAGAGTACTAAAAATTTTCATTATTCATCACAGAGAACACTTTCCAACA

GAGGACCCCAAGTTTCATCGGTTGAGTCACATGTACTAGCTTAGATCGGTGGAGTACATA

CAAGTTTAGACTCAGGTTCCGTGAATGACATAACATCATATCTTCTTTAAGGTTTTGAGC

ATTTTGGGAATCAATTGAAGCTTTCAGGCGAGAAAGTTGATAAAATATAGAAGAGGGATG

AATAGCTGGAAGTCATCTTACATTTCGAGGTGGAAGTGTGTTCTGAATTCTATTGCCAAC

CAAGTTTCACAGGTTGGTATATAATAATTCCCTGTCTAAGCTGTGACCTTAATTTGTACA

>Unigene0000420

GTAGAACCCTTATAGTAATCCATGATCCCCCCTCTCTCAAATCAAACAATTTTGGAAATT

TAGGGTTATTGATTCCACTCAATTGCTGCCGAGCTCGTGGTGATAGACGGCGATGGCTAA

CCCTAATTCGGGGACGCCGGCGAAGCCCTCCGACGTCTCTTTCAAGCGCAAGCGCGGGAT

GTTCCAGAAAGACCTTCAGCACATGATGTATGGATTTGGTGATGATCAGAATCCACTTCC

AGAAACTGTTGCACTTTTGGAGGACATTGTTGTTGAATATGTCACTGATTTAACATCTTT

ATTAAAGCTCTACGAAAAAGGATCTCTTGAGAAATTACAATATGGGATGTGCATGAGGTG

CATAAAGCCCAAGATATCTCATCCAAGAGGGGAAAGATCTTGACTGAAGATTTCTTGTAC

CTAATGCGTAAGGATCCTCCAAAACTGCGGCGATCCACAGAGCTGCTATCCATGCACGAG

GAGCTTAAACAAGCAAGGAAAGCTTTTGAAGTAGATGAAGAGAAGCTGGTTAACACAGAA

TGAAATGGGTGGCAAGTCTGTAGACTCGAGTCTGTTCATCTGTTGGCTTAGATCTTTGTA

AAACTGCCACCCTAGCTTTGCTCACTTGTCTTGTTCATGTCTAATGTGTCTGTTTAAACA

AGATAGATTTTCTCTTTAATGTAGGATACTTGCATACTGGCGCCTTTGAATACATAGAGA

AATATCCATGACATAAACTCGAGGTCAAGGAATCTGATGGTGATTTTTCCATTTCCTCAA

TAAAAATTAAAAGCATTTATCTGAGTGTCACGCTGGTAACATTATGACTGACCGGTTGTT

CGATCCCTTAATATCTACGTGTTATTTGACTGTACCTA

>Unigene0000421

CGCCTGTCCCTTCTCTCTCTTCTGATTTCCCTTTCCTGGTCCAATTAAAAAACCAGTGAA

AGTGATCAGACTTTTGATAGTTTCATTTCTTGAGGTTGCAGAAGTCTTCAATGGAGCTTC

AGTTGTTGCCGCTGTGGGTCTCGGTTTGGTCGCTGATGATTTCTGTGGTGGCGGGGCTGG

GGGTGAACTGGGGCACGATGGCGACCCACCAGCTGCCGCCAAAGACGGTGGTGCGGATGC

TGCAAGATAATGGGATAAAAAAGGTGAAACTATTTGATTCAGACTCGTCTACCATGAACG

CCCTCGCCGGCACCGGCATCGAGGTCATGGTTGCGATTCCGAATGATCAGCTGGATACCA

TGACTGATTATGGATCTGCGAAGGAGTGGGTTGCAGTGAATGTCACCAAGTACAACTTCA

AGGGCGGAGTGAATATCAAATATGTAGCGGTAGGGAATGAGCCCTTCCTAACTACCTACA

ATGGCTCATTTCTCAACACCACATTTCCCGCTCTACAAAACATTCAAAATGCCCTCAACG

ATGCAGGGCTTGGCAATATCATCAAAGCCACCGTCCCCCTCAACGCGGATGTCTACAACT

CCCCTGTCACTGCAGCTTTCCCATCAGCTGGCCGCTTCCGTACCGACATTGCCGACCTCA

TGACCGAAATCGTCCAGTTCCTCCATGCCAACAATGCTCCCTTCACTGTCAACATCTATC

CCTTCCTCAGTCTTTACGGAAACGACAACTTCCCCTTCAACTACGCCTTCTTTGATGGTG

GCGCCGCTCCTGTTGTTGACATCACTACCGGCATCCAGTACACCAACGTCTTCGATGCCA

ATTTCGACACCCTTGTCTCCGCCCTTAAAGAAGTTAATGCTGGCAACCTCCCCATCATTG

TCGGAGAGGTCGGATGGCCGACTGATGGCGACAAGAATGCAAAATCGTCCTATGCTCAAA

AATTCTACAACGGCCTTATAAAGCGTCTCGCCGCCAATCAAGGTACTCCTCTGCGGCCTA

ACCAGTACATCGAAGTGTATCTCTTCGGGCTCATCGACGAGGACGCAAAGAGCATTGCTC

CTGGAAACTTCGAGCGCCACTGGGGCATCTTTCGCTACGATGGAAGGCCCAAATTTGCTA

TGGATTTCTCGGGTCAGGGCTCGAACAGTATGCCCGTGGGAGCTAAAAATGTCGAATACT

TGCCTTCGAAATGGTGTGCTTTTAACCCGAATGCGAAGGATTTGAGTAAACTCGGGGAGA

ACATAAACTACGCCTGCACATTCGCTGATTGCACTTCGCTCGGATATGGGTCGACTTGCA

ATGGATTGGATGCGAATGGGAATGCATCATATGCGTTCAATATGTACTATCAGGTGCAAG

ATCAGAATTACCTGAGCTGTAACTTTGAAGGGCTGGCAATGGAGACAGACCATAATTACT

CGCAGGGGGATTGTGAGTTCATCATTCAGATTGCTGGGTCGATTGAGTATCGTGCACCGT

CTTGCATAGTAGTGGTTGTACTTCTATTAGTTCTGATGATGTGGGTATAAAGATTTTTCA

GGGTGAAAATGCAAGCCTGTGGAGTCTCTCCTCGGGGCTCGTATTCTGGTAGACTGTTAC

TGTTATTCCATTGGATTCATTGGATAAAGTTATTAGGGAGGCTTGTGTTCTGTAGACTGT

GTTACTTGGATAAAGTTATTAGACAGGCTTGTGTTCTGTAGACTGTG

>Unigene0000422

CCATCACGCCACTCCCATTCGCCACACGATCACTGCCGCCATGAACATCGCCGGCGATCT

CCGGACTACCGCTCACGTCGCCGTCCTCGCCTTCCCATTTGGCACCCACGCTGCGCCACT

CTTCGCCCTCACCAACGCCCTCGCCGCCGCCGCCCCATCCGCCACCTTTTCCTTCCTCAG

CTCGGCCCGCTCGCTTGCCGCTCTCCCGCAAGCTAGATCCGTCAATATACATATGTTCCC

CGTCTCCGACGGGTGTCCAAACCCTACGTCAGCGAGCGTGGAGGAGCAGATCGGGATGTT

CCTCGTCGCGACGCCGGGGAACTTCAGGGAGGGGATGGAGGTGGCTGTGGCGGCGGCTGG

CGGTGCGGCGGTGAATTGCGTGGTGAGCGACGCTTTTATATGGATGGCGGGAGAGATGGG

GGTGCCGTGGGTGGCGGTGTGGACCGGGGGGCCTGCGGGACTGGTGGCGCATCTCTACAC

TGACGATCTACGACGCAAAATTGGGGATGGCGATCAAGTTGTTCCTGAGCACGCAGACGA

CCTCCTGGACTTCATCCCCAGTTTCTCCAACATCCGAGTACGCGACCTACCTGAAGGTAT

CGTGCATGGCAACATTGTTTCCGGCCTCTTCCTCTTGCTTCACCGAATGGCCAAAGCACT

ACCCCGTGCCGCTGCTGTCGCCCTCAACACCTTTGAAGGCCTGGACCCCACTGTCGACTC

TGACCTCATGCTCAAATTCAACAACTACCTTCCAGTTGGCCCCTTCAATCTCCTTAGCCT

GCCAAGGTCCGAGCCCAATGACCCCCATGACTGCTTAGCTTTTCTCGATCGACAAGAGCA

AGCTACGGTGGCATACATTAGCTTCGGCACGGTAATGTCACTATCATCGTCAGAGTTGTC

TGAGCTAGCGGAGGGGTTGGAAGTGAGTTGTGTACCTTTCTTGTGGTCGCTTAAGGAGAC

AGCGGTGCAGAATTTGCCCCCAGGGTTTTTGGAGAGGACCAAGGGGAAGGGGCTCGTGGT

GGCGTGGGCCCCACAGGTGGCGGTGCTCCGACACACGGCTGTGGGGGTGTTTGTAACACA

TTGTGGGTGGAACTCAGTGTTGGAGAGTATTACCTGTGGGGTGCCTATGGTGTGTAGGCC

ATTTTTCGGAGACCAAAAGATAAATGCTAGGTTTGTGTCCCATGTGTGGGGGATTGGTGT

CACATTTGAGGGTGGGGACGTGACGAGGAAGGAGACGGTTAGAGTTATGGAGGCGGTGCT

AAACAGAGAGGAAGGGAAGAAGATGCGGGAGAGAGCCAAAGAGCTGATGTTGATGGCGAC

ACGAGCAGTGGGCACTGGTGGAGGGTCGGAGAAAAACTTTCGTACTCTACTGAAAATGGT

TTGTAGGGGTTGAGAAATGAGAACTTCAAATTGTAAGGGTATTGTTGATGTAGCAAATAA

CGTTTATAATCTATAAAAATAGATAATGTTATAGTTACTCTTGATGGGCTGACTAATTCT

TGGAATTGATGTTCCTTCTCATCAACTAACGATAAATGTACAGAGAAACTAATAATGCAG

GCATAAACTTCAAGTGCTATTTAT

>Unigene0000423

CAATAGACCCCTAAGTGGAGAAAGCCCTCACCTCTGCTCGCCGCCGCCGCCGCCGCCCCA

ATCACTCCGGCGATCTCTGATCCTCGTCGGACATATAACTTGAAGAGACATGGCATCTAA

AGGTACCTGTGAGACCAGGGAGCCGATCAATGAGCAAGCAATTGCAAACAAGTACGGTAT

AATGCGCTCCGACCTCAACCAAATCTACTCGAAGATCACAGAGCTCGAGATGGAAGTCAG

TGAGCATTCTCTTGTGATTGGTGCAATCCAGCCACTTGATCAGTCCAGGCGCTGCTACCG

CATGATCGGTGGGGTGTTAGTCGAGAGGACGATCAGGGAGGTCCTCCCTGCAGTCCAGCG

CAACAAAGACGGTCTCGAAGAGATAATTGCTCGTCTCCATGAAGCTTTGGAGAAGAAGAA

AAAGGAGATTTCTGAGTTTGAGTTGAAGCACAAGATCAAAATTAGGAAGTCAGATGGCGA

GGCAAAAGATGATGGAGGCAGGAAGGAAGGTTCTGCTCAAGGAGTTCTTGTGGGTCCCGC

GGGCGAGCAAGGAATGTAGTCGGGGTTGAAGATAAGTTGTTTCTCGTCGGTTTGGTGTCG

GTTGTATTACCGCCGTAGGTGCTTCATTCTCTAATTTGGATTGTTTTACACCAGGATATG

TTGTGTTGTACAATTTGGTGGTTTTATCGATCAGAGTTTGAAAATTCACCTATGAATTGC

AGAAGTCTGGAAGTTATTTTTAGTGTTCAAATTTACGCAGCAGCAACGATAATCAACGTT

TTAGAACTTCCGAGAAGATGATCGAGACGATGATGTATCAGTCAGCGTTTAATGCCACGG

TATTTTAAAACTTTTTTTTTCTTTGGTAAAATACCGAGTATTAATTTGTGAGATTTTTT

>Unigene0000424

TACGTTTCATCTCCAACCGAGAGCCCCGACGAGAGAATCGGCGCTGATGGCGACGGCCCC

CTCTACCAATTCCGCCGCCAAATCGGACGGCGAAGAGCACAACGACGGACAAAACCCTAA

AAACTCTATGGAGGTGGCGAACCCTAACCGGCTTCCGGTGGCGCCTCTCGTCTGCTTATT

GAGGTTCGCCGGCGACTCGGCTGCCGGCGCTTTCATGGGATCCATCTTCGGTTATGGTAC

AGGTTTGGTGAAGAAGAAAGGATTCAAGGGCTCCTTTGCAGATGCAGGATCTTCTGCTAA

GACATTTGCAATCTTGTCTGGAGTTCAGAGCCTTGTTGTTTGCTTTATGAAGAGACTGCG

AGGAGGAAAGGATGATGTCATCAATGCAGGTGTAGCAGGATGTTGCACTGGTCTTGCCTT

GAGCTTTCCAGCTGATCCACTAGCTCTAGTCCAGAGTTGTGTCACATTTGGAGCATTTTC

GTGTATTATGGAAAGGTTCAACAAGCAAGAACAGCCTGCACTAGCATACCCTTCTGTCGG

TAGCAGCATGTCAAGAGCCGACAGTGTTCTTCCACCCTTCGTTCTTCCTCTTCCAAATAA

CATGGTAGAGGGGTTTTCAGCTTTCTGTGATTCCTTAACTAAGTCTAAGAGGGCAACTCG

CCTGTGACTTCAAGCTATCGAGGATGCGGCCTAATATTTATCCGTTAACTCTCCTTTTCA

GTGGTAGCAAATTTTGTATAAATTTGAAGCATGTGGTGACCATAACTTGCTGTTACATCC

CATACGTCAATTTATAATTTTGTTTGAGATCAAAAAAAAAAA

>Unigene0000425

GCTGATGAGGCTTCTGTTGGTTGGACAGGCCGAGGTAGTTACTGGAGGTCAGTTGACGAC

ATAAAGATAAAGATGAGGAAGGACATGGCAAAGGGCAAGGATAAAGTGGGAAAAACATAA

AATAAAGAGGAGGAGCTGTAGGACCTTGATGTTTTTGACCGTGGCATCTCTACTACTTCC

GATTGATATTAGGAATTCAGTCTTAAGTGAAATAAGATTTGCTTACTACGAGACAATGAA

TTGTATGGCTGTGATAATGCTCGTATCTTTTACTGATAGCTCGAATCCAGTGTAAGGCTG

TGATAATGCTCTAA

>Unigene0000426

AAGTTGAAGACTTGAGTGTTGGGTGGAGCCATTAAGGCTTGAGGTGGCCAATGCCATCAA

ACAAGTGGAATTGTCTACTTTGACCCTCCAGCGGTTCTTGGCTGAGTTTGATGGGTTAGA

ACTGCTGCCAGAAGGGACTGAGGCCGAACGACTTTTCTATTGCAGAAGGATGGAGTGGCA

TGCTAGAAAAGCAGATTTAGTGAAGCAAGTTATTGACATGCAACAGTTGGAAGATGGACG

TGCTCAAGAGAGGCTTGCAAAGGACTTGTATACTATCATGGTTAACTCTGAGCTCCAAGC

CGGCTTCTCAGCATGCTCTACGAAGAGGGATACGCCTGGGGCTCGGTTGCGAACATTGTG

CATACACCAAGGTCTGAAAACATTGATGACCTGGAGACAGTTGAGAAGTGTTGTTGAGCT

TCCCATGGGGGGCTCTGAGGGGTTCAACCATGGAGGCCGCCGTCATCATCCTGGAGAAGG

CTTGAGAAGCTGAGGAAGAACTGGCCGCCTAGGAACTGGACTATGTATTTTTTTTTATGT

GGCCCTCGAGAGTAGGACCACCTTTTTGTAACGGTACTTTAGTGAATGGAATATTGTGTT

CACTGTGTTGATTGAATTTGTGCATGATTTATGTATTTTCG

>Unigene0000427

AGTGCGTGGAATCCCTCTCCAAACATGCCAACATCAAGCCTGTCATCACTTCCACTGGTT

TGTATCTCTCTCTACATCCATGGATTAGTCGGAGTACTATGATAGCGTTAGAGCTCAGAA

ATCTGTACATTTTTGTTAGTTACTCGTGTTAGGGATAAATTTATTTTAGTGATATAACCA

CGCTATGACTTACAGTGTGGAAGGAGCTCGAGAAGGAGAACAGGGACTTCTTCGACACAT

ACAACCGAG

>Unigene0000428

CCTCCCCTCCCTCCCCATCTCCCACAGCTCCTTCTTCTCCCAGGCCCACCCATTCCCTTC

TCCAGCCACTATAGCTGCCATCTCCCACGATGCCGAACTCCCGCCCAACCTCATCTCTGC

CATAGACATTCACCACAGAGGCCACCCTATCTTCTCCCGCGTCATTGACACTGATTCCTC

TACTCACTGGTTCCATGGCTCGCCTTTCCGCATCGACGCCCTCGACCGCAATGAACCGGC

GGCACCGCCGCCAGCAGTGATCTCGCCAGAGGAGCTTACACTAAATTGGGTCATCATCAA

TCCTACAACTCAGAGTGTGATAAACGTGTCGAGCCGGC

>Unigene0000429

AGGCACTGGTACACCGGGGAGACGCTGCTGCGATACGCAACAGTGGTGGGCGGTGGCACA

GACAAGGAGTGTACAGTGGTGGCAGTAGTGGCATGCGGGGATGCGGGGCAAGTGAAGGAG

GTGAGCCTGACAGTGGAGGATATGGATGGCATTTATCTCAGCGGGAGGGAGAGCTTGATG

GTGATTAAAGGACTCATGGATGGGGGCAGGACGGTGAAGGAGGGAGAGGCAGAGGCTGCA

AGGCGGTATGCCGATCACATCAGCAGAAGGAGATGGAGGAAGGAACAGAAGCTGTGGCGA

GAAGGAATGCTTGACCTCTGCTGCTCCGGTGCTGGTGTCGCCCTCTTCTTGGTCTTCTTG

TTCTTGGTGGCGTTCCGGTGAATTAAGCGCAAAAAAATATCAGCAGCTGCATTAACTAAA

TAGAGAGTTGTTGGCATAATCAAGCTTCAGAATTTCCACCAATCTTTTTTAACTGGTTCT

AGTATATGTTGCATATGGACCAATAAAACTTATTAGTTTTTTCACAAATATATTTGGATA

ATT

>Unigene0000430

CAAATATCATGTCCAAAGCTGCAGAAAACCAGTGTTCTGCCGCCACCATTGAAGACCTCC

ACCCTGACCTCGTCACGCATGTCCTCCGCCGGCTTGACGGTGGCGCTCTCGCCTCTGCCA

GCTGCGCCAGCTCCCTCCTCCGGTCACTCTCCACCGACTCCGAACTCTGGCGCCAACTCT

GCCTCACATCCTTTCCCTCCCTCCGCCTCCTCCCCTCCCTCCCCATCTCCCATAGTTGCT

ACTTCTCCCAGGCTCACCCATTCCCCTCCCCAGCAGCTATAACCGCTGTTTCCGATGACA

CCCAACTCCCGGCCAACATCATCTCTGCCGTCGACATCCACCACAGAGGCCACCCTACCT

TCTCCCGCATCGTTAACACTGACTCCTCTACACCCTGGTTCCGTAGCTCGCCTTTCCGCG

TCGACGCCCTTGACCGCAAGGATCTGGTGGCACCGCTACCACCAACCCTTTCGCCGGAGG

AGCTTACACTAAACTGGGTGATCATCGATACTACGATTCAGCGTGTGATAAACGTGTCGA

GCCGGCGGATGGTGGCTGTGTACAGGCACTGGTACACCGGGGAGACGTTACTGCGATATG

CGACAGTGGTCAGCGACGGGACGGATAAGGAGTGTGCAGTGGTGGCGGTGGTGGCCTGCG

GGGATGCGGGGCAAGTGAGGGAGGTGAGCCTGACGGTGGAGGATATGGACGGGATATGTC

TCAGCGGAAGGGAGAGTCTGGTGGTGATTAAAGGGCTCCTGGAGGGCGGCAGGACGGTGA

AGGAGGGAGAAGTAGAGGCTGCGAGGCGGTATGCGGATCACATCTGCAGAAGGAGATGGA

GGAAGGAGCGGAAGTTGTGGCGGGAGGGAATACTTGACCTCTGCTGCTCCGGCATCGCGG

TCGCTCTCTTCTTGGGATTTTTGTTCTTGGTGGTGTTCCGGTGAATTATGCGCAAAGGAA

CTACCATTTTTTTTTTTTTAAGACGTCCTAATACTTTGTATTGGATATCAACCAATAAAG

CCTCAATACTCTCTCTGAAATACAT

>Unigene0000431

ATTTATCATGTCCAAAACTGAAGACCACTATTCTTCTGCCACCACCATTGAAGACCTCCA

CCCTGACCTCCTCACGCACGCCCTCCGCAGACTCGACGGTCCCACCCTTGCCACCGCCAG

CTGCGCCACCTCCCACCTCCGCTCCCTTTCCACGCACTCCGATCTCTGGCGCCAACTATG

CCTCACTACTTTTCCCTCCGTCAGCCTCCTCCCCTCCCTCCCCATCTCCCATAGTTGCTA

CTTCTCCCAGGCTCACCCATTCCCCTCCCCAGCAGCTATAACC

>Unigene0000432

TGAAGATGAAGATGATGAAGAGGTAGGACTTCACTTTTGTTAGAGTAGCACTAACAATCT

GACACCGAGAAGCTTGCAAGATTCAAATATAATCTTCTACTTGATAGTGGTTAATATGAA

TTGTCTTTTTTGTTTTACTAAATTTCTAGTATGCATTGGATTGTATTGATTGGATGCCTG

CATTGTTTGGTTGCATAATATATCTGCTGTGTTTTAAATGATGGGTACGATGAGGCTTTA

TTTTTGGCAGGAGAAGGGTGTAGATGATGGT

>Unigene0000433

CACAATTGCAGTCTCAAATCTCTCTTTCAGACACGAGAGAACCCCGATTTAAGGGTGCGG

TCGAACTCTGTGTGTAATTGACTTTTGATGACATGACGTACTTCTGATGGTATGATATCT

GCTTTTGTATTGCTTCTGTAACACAAGGTTGAACTTGGTTTTCCTCGGTGCGGTTGAACT

TTCCAGAGTGCGGTGAACTATGAGCACTCCTTCATGCATTGTATGTTATGGTTCTAATGA

GGTGTTAATGTCCTTCGGTGAACTCCATTTTCTAGGAGGTGTTTCCTTCGGATGAAGTCT

TTGGCCCATGATAATTTTTGGGAGTCGTCATGTGGTTGATGTAGTGGAGTATAGACAGTG

TAATCTAAGGTTCTTAACCCTTGAAATGCCATCACTTCTACTCTTTCTCCGGAATGTCTA

TAGATGGTTTTGGAGGTTGGCTATAATCAATTGTTGAGTTTCTCTTGTATCTCCTTTCCA

CCTCTTCTAAAGATCTTGTGTGTTAATGCTCCAAATGTGGTGCAAACTCCACGTTTTATG

TCATTTTCGAGTTAAATAAACTTTTGAACATTCAGGTGCATAATAGGTTTTGATAAATAC

ACATTAAAGACTATACTTGCTACCTTTAACCGGTCCTTCCATGACTGATCAATATCTGTT

TTTGATACATGTCTTTTGTTTAGTATAGTCCAAGAAATTAGGGTTGCTTGTGAATTAACT

TTAGTAGTTTGCTCGGCCCATCAAGATATAAGTGTTGTTTATTATTGTATGTCATTTTGT

GGTGGGGGTACCACTATTCTGTGTGTTTCCAGTCTGATGTATTAGTTGCATGTCATTATT

GATCAATTGCATCTTAAATTTAAGAATCCTTTTTTCCCTAATGCAGTTTCTTTACCTGGT

TTAGCGAAAATCAACAGAAAAGTCTTGCCGAGGGATTCACTGATGAGGTGGCTGAGATAA

TAAAGGAAGATCTTTGGCCCAACCCTTTGAAGTATTTTAACAATGAGGCTGATGAAGAGG

ATTTTGACGACGATGAAGATGAAGATGATGAAGAGGAGAAGGGTGTAGATGATGGTGAAG

ATGAGGATGATGAGGAGGAAGATGATGATGACGAGGACGAGTAAAATGATAATTGAATAT

GTTTTGAGTATGACCTTCTCAGACTGCATTCATGGTTTGTTGGATCCTTTTCAATTTTAA

GCAGCAGCCTGGGTTATAAGTCATAATAGAGTTGGAGGAAAAAGGGTGACACTATTATAG

CCATTCTTCCTTCCTTAAAAAGACTGTTGTCTCTGATGGGACTTGTGACTACAGTAGGAT

GGATGTTTGAAAGTTCGTTAGATTTTTTTTTCTAATTTCTTATTTAACTTTTGCTGTTTT

AAATTACTCCTGTGGTCTTACTGGAGTTGAGAAGCTTCTTTTTTTTTATCTATGTTGTTC

ATGG

>Unigene0000434

TGAATCTCGTAGGTGTCTCGCGTGATGATCCTATTATCCATGTAGAGTAGTAGAAGCACC

ATGTTGTGTGTTGTGGAGCGAACAAATAAGAAGTCATGAGAACTCTTCACAAACCCTGCC

TACAACACAGCCGCGCGGAAGCAGTCAAACCAATCCCGCGGTGCCTGCTTTAGTCTATAG

ATAGCACGATGCAAGCGACTAACATGGCCAGGAGGAGTAGAGAC

>Unigene0000435

AAACCCTCGTCTCCACCGACCAGGTTTTCCCTCCTCGTCCCCCGTCGCCGATCGCCATGG

ATTCCCAGCCTAACCTCGACGCCGCCCACTCCATGGGCACCACCATCATCGGCGTCACCT

ACGACGGCGGCGTCGTCCTCGGCGCCGATTCCCGCACCAGCACAGGGATGTATGTTGCGA

ATCGGGCATCGGACAAGATCACACAACTCACGGATAATGTTTATGTTTGCCGATCTGGAT

CGGCTGCTGATTCCCAACTCGTCTCTGATTATGTCCGATACTTCCTTCATCAACACACAA

TTCAACTTGCTCAGCCTGCAACTGTCAAAGTCGCGGCTAATTTAGTTAGATTGCTGGCAT

ACCAGAACAAGAGCATGCTGCAGACTGGCCTAATTGTTGGTGGATGGGACAAGTACGAAG

GAGGTCAAATTTACTCTGTGCCCCTTGGTGGGACAATTTTGAGTCAGCCTTTTACAATTG

GAGGTTCCGGTTCTAGTTACTTGTATGGCTTCTTTGATCAAGCGTGGAAGGAAGGGATGA

GCAAAGATGAGGCTGAGAAATTAGTGGTTAAGGCGGTCTCTCTTGCCATTGCACGTGATG

GTGCCAGTGGTGGTGTTGTCCGAACTGTTATTATAAATGCTGATGGCGTTACTAGGAACT

TCTACTCTGGCGATTCTCTTCCCCTGTGGCATGAGGAGCTGGAGCCGGTGAATTCGTTAC

TTGACATACTATCATCGAGCAGCCCTGAGCCAATGAGCACATGAAATGTTTATTCTCTTC

ACTCAATGAGTGCACTCAGAATTACAATTTGCTTGTATCATACACTCTTCGGTCTGAAAT

ATATGCATGACCTGAGACTGACCCTGTAACCCTATTTCTTTGAAACCGGAAGGGTGAATA

TGATTACAGTTTCGATCTTCATGATTTTTTTTCTTGGTTTGCTTGATAGGCAATAATCCA

TTTCTTCTTGGACTACAGACGCCTATCTTCGTGATTTTTCATGTGTATCCATTTCTTCGG

AAGTTT

>Unigene0000436

ACTCAATGAGTGCACTCAGAATTACAATTTGCTTGTATCATACACTCTTCGGTCTGAAAT

ATATGCATGACCTGAGGCCGACCCTGTAACCCTATTTCTTTGAAACCGGAAGGGTGAATA

TGATTACAGTTTCGATCTTCATGATTTTTTTTCTTGGTTTGCTTGATAGGCAATAATCCA

TTTCTTCTTGGACTACAGACGCCTATCTTCGTGATTTTTCATGTGTATCCATTTCTTCGG

AAGTTT

>Unigene0000437

GCGTGGGAGCTCATGGCCGGGCTGGACGACACCAGCCCGCTCAGCCGGTCGGATCCAAAT

CGCTACTTCCCTGCCAGCGGTTGCACATCCTTAGGAAACTCTGACCTCAAGACAGAGCTG

ATAATGACATCGCATATTGTTAGCTCCATGTCTGATTCCGGGACGATATTTACGGAGGCT

AATCAAAATTCCGAAGATGTGATGGTAATTGATTCAATTGTTTCTGATTTCGATCCGGAG

GTGATATCCAGCTTCCAGAAGGCACTCCAGCAGCTGTCTCCGCAAAGCTCATTTAAGTTA

AGCACGTTTGATGATTCGGATTCTACTACACAGACCCCCACACAGACCCCTGAATTCACC

CGCAA

>Unigene0000438

GGCATTGTGAAGGCCAGGGTGACAGCATTCCAGGAAAAGATCAATTCGCGACGGAATAGC

AGTATCTGTGAGGGGGTGAACATCGGGAGTTGTCCACCGGACGGGGAGAGGAAGGTGGTG

TTCTACTTTACGAGTCTGAGGGGGATAAGGAAGACGTACGAGGATTGTTGGACGGTGGGG

GTGATACTTAAGGGATATGGTGTAAAGGTGGATGAGAGGGATGTGTCAATGCACGCAGGG

TTTAAAGAGGAGCTCAAGGAGGCATTGGGCAGATATACGCTTCCAGTG

>Unigene0000439

GGCATTGTGAAGGCCAGGGTGACAGCATTCCAGGAAAAGATCAATTCGCGACGGAATAGC

AGTATCTGTGAGGGGGTGAACATCGGAAGTTGTCCAGCGGACGGGGAGAGGAAGGTGGTG

TTCTACTTTACTAGTCTAAGGGGGATAAGGAAGACGTACGAGGATTGTTGGACGGTGGGG

GTAATACTTAAGGGATATGGTGTGAAGGTGGATGAGAGGGATGTGTCAATGCACGCAGGG

TTTAAAGAGGAGCTCAAGGAGGCATTGGGCAGATATACGCTTCCAGTG

>Unigene0000440

GCACTCACCCAAAACCGCTATAACACCTCTCCTCGGCTCCATCTTACCGTGAAATTACCT

CCACCCCTCCATCTATAACCCTAGTTTCACCCCAGCTCTCTCGATCGCCGGCGATCTCAT

GGCGCCGCCGGATCGCCACCGCCGCCTGCCACTGAAAGACTCCGACGAAACCGAGGCGAA

ACCTTCGAACACCCTCTGGGTCGGCAGTATCTCGCCCCATACCGTCGATTCCGATCTCAT

GGCCATCTTCGCCAACTACGGTGCCCTCGACTGCACCGTCATGCTCGGCATCAGGACGTA

CGCGTTCGTCAATTTCCGTACCATCGATGAGGCTTGCGCTGCCAGGGAGGCCCTCGATGG

ATCCCCGTTCCAAGGCAGCAACATTCGGCTCAAGTTCGCCCTCCCGGCAAGACCGTTTAG

GCACCTGTGGATTGGTGGGTTTGGTCTATCCACTTCAAAGGAACAACTTGAGGCCGCATT

TCTGAAGTTTGGGAAGATTGAAGAATGCAGATTCTTCAGGGACCGGAATTCTGCAATTAT

TGAGTACTACAAGACTGAAGATGCTATTGCTGCCAAGAAAGACATGAATGGAAAGCGTTT

AGCTGGTGAAGAGATACGTGTAGATTTTCTAAGACCCCCACCTTCAAGAAGGGATTTGAC

TGATCATCATGAACCAAGGGATGGGCGCCTCAGGAGCATGGGTCCACCAGAACAATGGCC

AAATAGGCACCAGCAATATGGAGGGCGGAAAGACAGTCCACCTAGTAAAGTTCTCTGGAT

AGGATATCCAGCTACTGTTCGGGTCGATGAACAGATGCTTCATAATGCTATGATTTTGTT

TGGTGAAATTGAAAGTATCAGGTGTTTTCCTGCAAGAAATTATTCTTTTGTACAATTCAG

AAGCATTGATGAAGCTCGACGTGCGAAGGAAGGACTGCAAGGTCGCCTTTTCAATGATCC

GAGGATAACAATTATGTTCTCAAACAGCACCGCGCCAAAGGACGTCTCACAATCTTTTCC

AGGACTTAGAGGGCCAAGACCGGATATGTTCTTTCCAGATGCTCCCTTTGGATCCTTTGA

ATTATTTGGCAATAGTCATCCTGTGGCCCCAAATAAAGCATTGGGAGTCCCTCCACTTCC

TGGACCAAATATATCAACGAGGCAATATCCATCCCAAGGATTTGATCCCCATCACGGAGG

ATCAGAATTCCATGAGTTTGGAAGTGATATGCATAGTTTTCCTGATGGAGATTTCAATGC

TCCTATGCCTCAACCATGGGGTCGGCGATCTCCTCCTGCACCAGGTATACTCTCTTCTGG

TTTGAGTCGGCCCCCTTTTGCGCCCATGCCAGGCGGATGGGATGGATTTGATATGAGAGA

AGCTAAACGGTCAAGGATTGATGGTTCTCTTGCCAATGATACTCTTTTTCACACAAGAAG

GGGGAATGATGAGACTTACGGAGATTTTGGATTTCCTCGCCCTGATAGAGCGTCCTTGGG

TCAAAGCGGACACAATCATACTTCCCGAGGTCATAGTCAGATGCATGCTTCTTCTGACAA

TGACCACTACTGGCGTGGTATTCTTGCTAAAGGTGGAACCCATGTTTGTCGTGCTCGATG

TGTACCCCTAGGAGAGGGCATAGATGCTCCCTTGCCGGAGATCATCAATTGCTCAGCAAG

AACAGGATTGGATATGCTTACAAAACACTATGCTGAAGCCAGTGGTTTTGACGTTGTCTT

CTTTCTGCCAGACAGTGAAGATGACTTTGCTGCTTACACAGAATTTCTTCGGTACCTGGG

TCAGAAAAATCGTGCTGGAGTTGCCAAGCTTGATGATGGAACTACATTGTTTTTGGTACC

CCCATCAGATTTCCTTTCGAAAGTACTGAGCGTTTCTGGTCCTGAACGTCTTTATGGTGT

TGTTCTTAAGTTGCCTCAACAATCATCTGAAATTCAGCCACCAGAACAGGAGATTCCTCT

TCCACCGTCCCATTACGCTGGCAGGCAGCAGGTATATGATTCACATAGAGGTTCTAATTT

TGTTCCTCTGTACGTGGACCAAACAATGAAAGGGGATAATAGTAGGCCTTTGCCTGAGGA

CTCGAGGTTGCAGACAGGTGTTGAAAATCCACAACCAGCACATGGTGTGGTGTCTCATAC

AACCCAGTTATCTTCACAGAATTATGTTAGCGAACCTGCAACTACTTCCCCGGTTGGAAT

ATCTTTGACACCTGAACTACTAGCTACTTTGGCTTCTCTTATACCCAGCACTCTCCAGAC

AGGTTCTACTGGGACCAATCAGATGCCTCTATCTTCTGTGGGGACAACTTCATCTTCTGC

TTATGTTCATGATGGAACAGTTCCACTGCAAGGATGGAGGCAGGACCATCCAGTTACTCC

TGCTGTTTCTTTAGAGCAACAGGGTCATTCACTTCAACAGTTAGGGTATCAATATCCTAA

CTCGGCACCTCTTGGCTCTCAGTTTCCAATGTATACAACCACCCCAAATGGGCCAGATTA

CTCTGTACAGTCTGCTATGGGCTGCACGCAAATTCAAGACCCTTCGCTGAACTTGCCACA

AGCCACTGCTGTTCATACAATGCCAACAAATAACTATGTAATCCCATCTCAAGGTGAACA

GTTCTTTTTGCCCCAGGGCAACCAACAGTACCAGCTTGAACCTTCTTTTGCTTCGCAAAA

CAATTATGGGTTGCTTCAAACAGCGGAACCTGGCGTGCATGCGCCTTTTATTCAGCAATT

AAATTCTAATACATCAACGGCTCAGATTGGACAACCTCAAATAGTTATGCCCTTACCAAC

TGATCAAGCGAATATGGGGTATCCTTCTCAAGGACAACAGTCGCAGACGGTTCTATCAAG

TTTCAGTCAGGGAACATCGGAGGGAGATGCTGATAAGAACCAGAGATACCAGTCAACTTT

GCAGTTTGCTGCCAACTTACTCCAACAGCTTCAACAACAACAAGCTAGCGGTCAAGCTGC

ACAGGGTGGAAATTAACATTGAAAAGTGCTCTCCCTCAGCAGGTCCCTAAAGAGAATCAG

GAAGGATATGCAATGGTGGTTTTATTCTTGTTTCCTTTTCCAGCTGGATCGGTCTTTACG

ATTAGCAAAATGTTGATCGAGATTGCATTACAATAGGTCTAAATTAAATATCCTCCTCTT

ATGTTAATTTCCTTTTAGGTCGTGTATTGTCTCAGAACAAGTGTATCAGCTAGAGCATTG

ACTATGCGGTACTCTGTGAATACTCTTTACTCATGGTTGAATTGGAAGCCACTTATCTCT

ATGGTTTGGATCTTCGATTTTTCTCGAAGATGTTGAACAATCACT

>Unigene0000441

TCTCGTCTCACTCTCGAAGCTCTCCCCATCATCTCCTCCAGCTCAAAACCCTAAATTCCC

CAAACTCTAACCCTAACCCTAAAGATCACATCTTGCATACTCCCCTCCCCATGGCCGCCG

CCCAGGCGAGGGAATCCACTCCGCCGCCGATAATTGGCCGCGCCGGACGCTACACCGTCT

TCATCACCCCGCCGGCGACGCCGCGTCCTTCCGAGCCGCCCCGCCCTCTCGCTTCCACCC

CCATCTCCACCCCCCGGCCGATCCCGAGGAGCCCGGTGCCTCTCCCGCCTAAGACGCCAT

CGCCGCCGCCGCCAGCGGCGGCGCCGCCCGTGGCTGCGCCCCCCGTCCAGGTACCGCCGC

AGCAGTTTAACAAGCCGGCGTCTAGGTCTGGATCGGGATCTCTGGTCGGATTCTTCCGTG

ATGCCGTTGCCAAAGTCCAAGATGTGCATTCGAGCTTGGATGAGTATTTAGCGGACTGGT

TAGGGCTGAATCAGTCGAAGTATCAATGGGCGTTGGATGATTACTATGAGAGCAATGGAG

TGGAAAAGGAGGGTGGTAAACCAAAACAACTTTCTAGTAAAGAGCAAGCTGTATAATAGG

CAGGTCGTCAGTCTAGCGATCAATTGGGGGAGTGCTGGGAATTCAAGTGTATGTACATGG

ATGCCGAGATGGTTCTTCTAGAGCTGGGCCATTGTTGTTGCTATATTTATCTCGACTTGG

ACTTTAACCATCAAGTTGTGGTGGCAGTTAATAGTAATGTTGGGGCTGAATTTTTATTTT

CAAAATTTAATCTGTGGTTAACTTTAGTTTTGTTTACCCCTTCTGCAGGGTAATGTTATA

CAGCATTATTCTGGACCTTCTGTAGAGTGATTCAGTGTAAGTTTATGACATTGGGCTCTC

ATATTGCATGCATCTAATGTCTTGGAGCCTGTGCTCTGGAATCTAATCGAAACTGTGTTC

T

>Unigene0000442

TCTGCGGCGTAACCGATCGCGTTACAGATCTTATGGAAGAGAAAAAGAGATCTGATTCAA

TCCCTCTACTAGATATCATTATCACAAAGGCATTACTCCCCAAGTAGGAGGAGGAGTTAT

TGCTGAGGTATCTCCAACTACTCCCCAAGTAAAACTAAGAGATGTTGCTGAGACATCTAC

ATCTACTCCCCAAGTAGAAGGAGGAGTTATTGCTGAGGTATCTCCAACTACTCCCAAGTA

AAACTAAGAGTTGTT

>Unigene0000443

CGCGTTACAGATCTAATGGAAGAGAAAAAGAGATCTGATTCAATCCCTCTACTAGATATC

ATTATCACAAAGGCATCTACTCCCCAAGTAAAACTAAGAGTTGTTGCTGAGACATCTACA

TCTACTCCCCAAGTAGGAGTTATTGCTGAGGTATATCCAACTACTCCACAAGTAAAACTA

AGAGTTGTTGCCGAGGCATCTCCATCTACTCCCGAAGTAGAAGGAGAAGGAGTTATTGCT

GAGGTATCTCCATCTACTCCCCAAGTAGAAGGA

>Unigene0000444

GGAAAGAACAGTACGAAGAAGATGAAATGTTGATGTTATGAATTGCTCAAGCCATGTGGA

ATATGCCTGGTGGTGGAGGAAATGCACTGTCCAAGGCTCCAGATTCTTTGTTGTCCCATC

ACAGTAAGGCAATTTCTCTACCCAAGCTGCTCGGTGTGTTTTGGAATTAATCCTGAGCAC

TTGTTGAGGAGTCTCAGAACGTGGATGAGGGGTTTCCGAGCTTTTGTTGAGGCCGATACT

TGGGATGTGGTGTCCTTGCTCACTAGTGCTGAGCTTTTGTTGCTGCAGTCTGATATTGAC

>Unigene0000445

TCCTGCCACATGGATGTTGGAAACCACAACCATGGCCCAGGAAGGAATTCTGGGGATTAA

TTTTGCTGAAATATACAAGAATTCAGAATTGTACCAGAAAAACAAAGCTTTGATTAGTGA

ACTGAGCATACCTCCTCCTGGTGTCAAAGATCTTTACTTCCCTACACGATATTCTCAGTC

TTTCCTCACCCAGTGCAAGGCTTGCTTCTGGAAACAGTACAAATCATACTGGCGCAATCC

ATCATACAGTGCCATTAGAATGTTCTTCACAACAGTCATAGCCTTGATGTTCGGGACAAT

CTTCTGGAGGGTTGGATCCAAAACGAGTTCCCAACAAGATCTTTTCAATGCGATGGGTGC

CTTGTATTCTGCAGTTATGTTCATTGGTGTTCAGAATTCTCAAGCAGTGCAGCCCATCGT

AGATGTCGAAAGGACTGTTTTCTATAGGGAAAAGGCTGCGGGAATGTACTCTGCTTTACC

ATATGCATATGCACAGGTCGCAATCGAGATCCCATACACCTTCGTTCAGACCATTTTATA

TGGTATCCTGGTTTACAGCATGGTTGCTTTTGATTGGACTGCAGCCAAGTTCTTATGGCA

CATATTCTTCATGTTCTTCACATTTCTATACTTCACATATTACGGAATGATGGCAGTGTC

GCTTACTCCTAATTCAGACATAGCAGCAGTCGTCTCTGCAGCATTTTATGGTATTTGGAA

TCTTTTCGGAGGATTTGCGATTCCCCGTACTAGGTTGCCTGCATGGTGGAGTTGGTACTA

TTGGATATGCCCAATTTCTTGGAGCGTATATGGATTAATTGGGTCGCAGTTTGGTGACAT

GGACAATGTGATGGAGAATGGTGAAACAGTGAAGGACTTCACGAGGAGATTCTTCGGATA

CAAGTATGAATTCTTGGGAGTCGTTGCTGTTGCGGTGATTGCATTCCCTCTACTATTTGC

GATTGTCTTTGCCTTTGGGATAAAGGTATTGAATTTCCAAAAGAGGTGAGATTTTGTGAA

CCACCAAAGTATTAGAAGATCTGTCTCGCCTAGATGTAAATCCTTAGGGCTTTTTGTCAG

TTAAGACCGCGTAGAAATGTAAATATTTACATTTTTTTTTGGGTTGATGATTAAGGTTGC

TTAGAAATGTAAATTTTTCCAGAGGACAATCCGGTTTGCCACATGTTAATTTTGCACTAA

ACATTTATTTCATTCCTCA

>Unigene0000446

GCAAGTGCCTCGTCGAGCGGCTGATCAGAGTCGCCGAAGAAGACAATGAGAAGTTCTTGA

CGAAGCTCAAGAATCGGCTCGATCGGGTTGGGATCGAGAACCCGACGATCGAGGTGAGGT

TCGAACAGCTGGATATCAGCGCGGAGGCTTATGTGGGGAATCGGGGAGTTCCGACGGTGC

TTAACTTTTTTGTTAACAAAATTGAGGGGATGCTTAGCGCGCTTCGTGTGGTTCGGAGTA

GGAAGAGGACTGTGACGATTCTTAATGAAATTAGCGGAATAATTAAGCCGAACAGGATGA

CTTTGCTGTTAGGGCCGCCGGGGTCGGGGAAGACGACATTGCTGCTGGCTTTAGCAGGGA

AGCTTGATAAAGATTTGAAGGTTTCTGGTGGAGTGACTTACAATGGCCATGGGATGCACG

AGTTTGTTCCGCAGAGGACCTCTGCTTATATCAGTCAGCATGATCTCCACATAGGAGAAA

TGACAGTGCGAGAAACATTGGCTTTCTCTGCTAGGTGTCAGGGTGTTGGCACCCGATATG

AGATGCTGGCGGAACTATCACGAAGAGAGAAGGAGGCGAACATTAAACCAGATCCTGATA

TTGATGTTTACATGAAGGCCACTTCACTAGAAGGGCAGGAAAGTGTGGTAACAGACTATA

TATTGAAGATACTAGGATTGGAGATATGTGCGGATACCATGGTTGGCGATGCAATGATAA

GGGGAATCTCGGGAGGGCAAAAAAAGCGAGTTACAACAGGTGAAATGCTTGTTGGGCCAG

CAAGGGCTTTGTTCATGGATGAAATATCTACTGGTCTCGACAGCTCTACGACCTTCCAGA

TTGTAAATTCCCTTAGGCAGTCAATACACATCCTTGGTGGCACAGCACTGATAGCGTTGC

TTCAACCAGATCCAGAGACTTACAACCTCTTTGATGATATAATTCTCCTATCAGAAGGGC

AAATAGTGTATCAGGGTCCTCGCGAGCATGTACTTGAGTTCTTTGAATCTTTGGGATTTA

AATGCCCAGATAGGAAAGGCGTCGCGGACTTCTTGCAGGAAGTAACATCAAGAAAAGATC

AGCAGCAGTACTGGGCACGTAGCAAGGAACCTTACCATTTCATTCCTGTTAAAGAGTTCT

CAGATGCATTTCTGTCATTTCATGTTGGGCAGAGGCTGGAAAATGAACTCGCCATCCCTT

TTGACAGGAACAAGAACCATCCTGCTGCCTTGACAACTTCCAGATATGGTATTAGCAAAA

AGGAACTGTTGAAAGCTTGCATCTGGAGGGAGCTTCTGCTGATAAAGAGAAACTCTTTTG

TTTATATCTTCAAACTGTGCCAGCTTATATTGATATCAACTATTGCAATGACGGTCTTCT

TCCGTACCAACATGCACCATCGATCATCTGAGGATGGAATAATTTATATGGGCGCCCTTT

TTATTGGGCTTATTACTCATCTGTTCAATGGTTTTGCTGAGCTTGCAATGAGCATTGCTA

AGCTTCCTGTTTTCTACAAGCAAAGAGACCTCTTATTCTATCCAGCCTGGTCATATGCAT

TACCTACATGGATTTTGAAGATTCCAATATCTTTCTTGGAATGTGGTGTCTATGTCGGCA

TGACTTATTATGTTATCGGTTTTGATCCAAATGTCCAAAGACTGTTTAGACAGTTCCTGG

TATATTCTTTGGTTAGCCAAATGGCCAATGGACTTTTCCGGCTCCTTGCTGCATGCGGTA

GAGAAATGGTTATTGCAAACACGTTTGGTGCTTTCGCACAGCTGGTGTTACTGGTTTTGG

GTGGCTTTGTCATATCACGAAAGGATATTAAGAAATGGTGGATCTGGGGTTACTGGTCTT

CGCCTTTGATGTATGCACAGAATGCAGTTGCCGTGAATGAATTTCTTGGGGCAAGCTGGA

GACATGTTTTTAATGCCACTTACAGCACGAAAACATTGGGAGTCCAAATCTTGGAGTCTC

GTGGTATGTTTCCGGAAGCAAAATGGTTTTGGATCGGTGTTGGTGCATTGCTGGCATATG

TTCTCACTTTTAATCTTCTCTTTACTGCTGCACTTGCTTACCTCAACCCCATTGGAAAAG

GTCGAACAGTTATATCAGAAGATGTTTTGAAGGAGAAGCATGCAAACCAAACAGGAGAAA

TCATTGAGCTCTCAGCTGTGGAAGCAAATTCCGGCCATCCATCAAGAGATAGAAGCACCA

GAAGTAGGACTACTATGAGCGAAATCCATACTGAAGATCACCACAACAAGAAGGGTATGA

TACTTCCCTTTGTTCCCTTGTCAATCACCTTCAACGATATCATGTACTCCGTGGACATGC

CACAGGAAATGAAAGCTCAAGGTGTGAGAGAAGATCGTTTAGTACTTCTGAAATCTGTTA

GCGGAGCTTTCAGGCCAGGAATTCTCACTGCTTTGATGGGCGTTAGTGGAGCTGGTAAGA

CAACCCTAATGGACGTATTAGCTGGGAGGAAAACCGGCGGATACATAGAAGGAAACATCT

GTGTATCTGGCTTTCCAAAGAGACAGGAAACATTTGCACGAATTTCGGGATACTGTGAGC

AGAATGACATCCACTCACCTCATGTGACAGTCTATGAATCTCTAACATACTCTGCTTGGC

TTCGGTTACCCGCCGGAGTGTCCTTGGCTACAAGAAAGATGTTCATTGAGGAGATTATGG

AGCTTGTGGAACTGTCCCCACTTAGAGAAGCATTAGTTGGGTTGCCTGATGTCAGCGGAC

TTTCAACTGAACAGCGCAAGAGATTAACAATTGCCGTTGAGCTTGTTGCTAATCCGTCCA

TAATCTTCATGGATGAGCCAACCTCTGGATTAGATGCAAGGGCGGCAGCAATTGTGATGA

GAACCGTCAGGAACACAGTTGATACAGGAAGAACTGTTGTGTGCACAATTCACCAACCTA

GCATAGACATTTTTGAAGCATTTGATGAACTTTTCCTTCTGAAAAGAGGGGGAGAAGAGA

TATATGTCGGCCCACTGGGGCGCAACTCACATGACCTGATTATGTACTATGAGGGAATAA

AAGGAGTCGGTAAAATAAAAGACGGTTACAATCCTGCCACATGGAT

>Unigene0000447

ACCATCAGCTGCTTATTTATTTATTTATTTTTTTAAAAACCAATCTCTTGCCCTGGCCGT

CTCGATCGAAACCCAAACCTCGACAGCACCACCTCCTCACCGTCTCCAGCAACAGCTCCG

GCAAGCTACGGCAATCGCTCCGGCCCCCTTTCCATCACTCCAGATAATTATATATGGATC

CTAGTATGGAGCAGATTCTAATTGAGGGTGAGGTCGCGGCAGAGCATCTCTTTCTGGCAC

GACATCAGCTGATTGAAAATGATAAACTGCGAAATGGCAACAGGGAAGCCCTTACAGCTC

TCCGGAAGAAAGCCCGAACGACAAGAAGTAGTGTGCCATCTCCTTTTGAGGCCATAATGA

AAGAAATTGAGGGGGCTGAGTCTAGACCATTAGTGAGGGAGATTTGTCCCACTTGTGGTA

ATTATGACTCCAAGGAGCCTACATGGATGATGCTACCGGGATCTGATATTTTTGCTCGCA

TGCCATTTCATGCAGCCCACACCATCTTAGAGAAAGATCAGGAACATCTGGATTATGAGA

CTAAGAAGTTGCGGAGTTTAGAGAAGGAGAAAGCCCTTGAGATATCAGAAAGAGGAGGTC

TTGCCGACCGGATCAGTCCTGGTGTCCTCAAGTCTATGGTCACCCTGAAGGACAGTCCTA

TAAAGTTGTAACGCTCCCTCTTTTTTTTTTTGAACTGCTATTGTCTTGGATATGAATGTT

CATTTGCCAGTTTTCATGTTGAAATTATAGTTAATATTGACAAATAATCTTCTATTATCC

TATATTCAA

>Unigene0000448

AATTACTGCTGAATGCATTAGTTGACTGCGATTGCTACTCCAGAGAAGAAGAGAATCAAA

TTGTCGTTTTTGGTGATCCATGGGATTCAGCGGAAGAAGACGAGAAAGAGAGATGTCTGG

GAATACGATGGAGGGTGATGGCCGCAGTGAGAATTTTAAAGGGTGAAGTAGGGATTTTAC

CAATAAAAAGGATCAATCACAGTGCACCAACACCCTGGAGATGTTGCCCCAGGCTAATGT

TATACTAATGATGGGAATGAATTGGCTGTA

>Unigene0000449

AATTACTGCTGAATGCATTAGTTGACTGCGATTGCTACTCCAGAGAAGAAGAGAATCAAA

TTGTCGTTTTTGGTGATCCATGGGATTCAGCGGAAGAAGACGAGAAAGAGAGAATGTCTG

GGAATACGATGGAGGGTGATGGCCGCAGTGAGAATTTTAAAGGGTGAAGTAGGGATTTTA

CCAATAAAAAGGATCAATCACAGTGCACCAACACCCTGGAGATGTTGCCCCAGGCTAATG

TTATACTAATGATGGGAATGAATTGGCTGTA

>Unigene0000450

ATTTCTTTCCTCTGTCTTCATGACCGCTTTCCTCGCAGCTCCTCGAATCCATCCTGGCTG

GCTGACCTCTTGCTGGCTCCACCATCTTGAGCAGATTGTCGTCCTCTCGATGGCCTCGCA

AAAGATCTCCATCATCATAGCTGACTGACCTCTGCATACCACCGAAAGTCACCGAGGCAC

ACCGAATCATCCTCTCTTCTCCCTCGATATAGATCTTTGTCATTATGAGATTT

>Unigene0000451

CCTTCCCCACCTCAAAACCGACCCGGACAAACATCGACAACTGCAACCCCTGTCACCATG

CCGGATGAGCAGCCTCAGTCGTCACCGGCGCCGGTGACGGCGGCGGTCAAGACGGTGGTC

GTTGGAGTCCGCCCCGACCAACAAAGCCGGGACCTCCTCACCTGGTCCCTCCTCAACCTC

GCCCTCCCCGGCGACACCGTCATCGCCCTCCATGCCGCCTCCCCTCCCCCCTCTCACCGC

TCCAAAAACTCCTGCATCTTCGACCCTGCCGCCCTCCTCCCCACCATCCAAATCCTCAAC

TCGATGATCGGCACCTACGAGGGCTTCTGCAACCTCAAACAGATAGACCTCAAGCTCAAG

TTCGTCCGCGGCCCCTCCGCCCGTAACCTGCTCACCCGCGAGGCCAAGTACCTCTACGCT

GCCGCTCTCGTCGTCGGCGTCGCCGACAATGGTTCCGCCTCCGTCGCCAAGCACTGCGCT

AAGAAGCTCCCCGATAGCTGTTCCGTCCTCGCCGTCAACGTCCACGGCAAGGTCATCTAC

CAGAGAGACTCAGCTCTAGAACTTAACCAAAGTAATCAGTTGATGGTGGACAGTCTGACC

GAACAAAGCTGTCCGGTGTGTGCTCGGAAACCGGAAGAGCCAGCAAAGTCACCTCCGGTG

CTGTCCAGCTGTAGAACGTCCCCGGCACCGGAGCGGTCGAGGATCTCGGTGGTTCGATGG

GCGCTGAGCCTCCCCAGCCGGTACTCGACTTCCATAAAACCGCCTTCAATACCTGACGAA

CTGGAGGCGTTTCGAGAGAAGTATGCGCCAATTTGTAGGGTGTTCAGCTACCAGGAGCTG

GCGATGGCAACAACGAATTTCTCTGATGAAAATGTGGCGGGCAGGGGTGGCAGCAGCAAC

GTGTATAAGGGCTACCTCTCCGACGGTAAGCAAGTGGCGGTGAAGATGATGAAGCACACC

GAGGAATCGGTGCAGGAGTTTGTGTCTGAAGTCGAGATTATCACTGCATTGGATCACAAG

AACATTGTGTCTTTGATTGGGTTCTGCATTGACGGAGGCAATGAGAATTTGATGTTGGTC

TATGATTTCTTGTCTCGGGGCACGTTAGAGGAAGTTCTTCATGGTGAAGGAGTGGATGTG

GATGTTCTTGGTTGGAATGAGAGATATAAGATAGCTGTGGGTGTTGCCGAGGCGCTTGAT

TATCTACACAGTGGTTGCAGTTCGGACCTGGTTATTCATAGGGATGTGAAATCGTCAAAT

ATACTTCTCCCGGATGATCTAGAGCCGAAGTTGTGTGATTTCGGGATAGCTAAATGGGCA

TCTTCGTCATCAACACATATATCCCGCCGTGATGTTGCAGGGACATTCGGGTACATGGCT

CCTGAGTATTTCTTGTATGGGAAGGTTGACGAAAAGGTGGATGTTTATGCTTTTGGCGTT

GTGCTTCTTGAGCTCATTTCAGGGAAGGAACCTGTCAGCTTACAATCTCCAAAGAGCCGC

GAGAGCTTGGTTATGTGGGCAAAGCCAATCCTGCAAGATGGAAAATTCAATCAATTGGTG

GATCCAAAATTAGGGGACAAATATGATTATTACCAAATGGAGAGGATGGCTCTAGCTGCT

TCCCTTTGCATCAGACGATTTGCTCAGTCTCGGCCCTGCGTAGCTCTTATCTTGAAGCTA

CTCCAAGGAGGCGATGATTCTCTCAGGTGGGCAAGGTTGGAGATTGGTAGTGGTAGTTCA

GAGGATTTTTCTGGGTGGGACGATGAAACATCGTGTGCAGATTCCGAGATACAGTCCCAT

ATCAACCTTGCATTGCTTGATATAGACGATGAAACAACTTCTGTGTGCAGCTTAGAGCGC

TCTGTCGATTACCTGCAAGGAAGGTGATGATACTCATTGTTAGTAATCGACTTGCCGGTA

GGGTGAATTTGGGTTCTCGATTTTGCTAGGTTTGTATAGTTCTTCGACCTATTGTTTTAT

TTCTCCTTCTAGCCAATGAATTCATATTTTAGAAGGAACTAGGATGTGGTGGTTTGGTGT

GTTCCTATTTTTTTTAAGATTGTAATTATTTCCTTTGGTCATGAAGAACCCTCACCACCT

CAAATCAGTTATGAATGTCCCTGAGTAGTTGTGAATTTCCACCAACGATTTTCCTTCAAT

TTCAAGAC

>Unigene0000452

TGTACTGGGATGAGACTTTGGAGAAAGAGTGTCTTACATTACTAAGAAATTTAGATGTGC

TTATTAACTCAAGAAAACAAACTTAACCCTTTGCAGTAACCAATAATGAGGAAGAACTAC

AGATTAATAGGTATGAAATCTTCAGTAAAGTTTCAACAAAAATAGTGCTGGGAAATGCTT

ACTGTCGAGCAAATTTTGTTATTTTGGAAATGACACAACCGTCCGTCTTAATGTACTCTA

CGTTGCAGATCTTGAAGCTACTCCAAGGAGGCGATGATTCTCTCAGGTGGGCAAGGTTGG

AGATTGGTAG

>Unigene0000453

CCTTCCCCACCTCAAAACCGACCCGGACAAACATCGACAACTGCAACCCCTGTCACCATG

CCGGATGAGCAGCCTCAGTCGTCACCGGCGCCGGTGACGGCGGCGGTCAAGACGGTGGTC

GTCGGAGTCCGTCCCGACCAACAAAGCCGGGACCTCCTCACCTGGTCCCTCCTCAACCTC

GCCCTCCCCGGCGACACCGTCATCGCCCTCCATGCCGCCTCCCCTCCCCCC

>Unigene0000454

GAATTTTTCTCTTTAAAATAAAAGAAGAAAAAATCCAAAGTCTTTCCGGTTTTTATCCTG

GTTGGACTGGAATCTTAACAATTTAATTGCAGAGACCACTAATGTTTTGATGTTCGCTCA

CTTTTCTGGGGTCTTCTATACTTTGCCTAAGAGGAGCACTCTGTCTATTATAACGTGATC

ACGGAGACCATGCTAATAGTTATAGAGTCCGAATCCTTGATGTAAATATTAAGGATTGCC

ATGGAAATGACATATCTTTATCTTTGTTTATGATCTGCTAGTTATGGCTAATTACTGCTG

AATG

>Unigene0000455

GTTGACGGAGGCAGGATGGGGAGGGTTCGGATCGACTGCTTCGCCTTCCTCCTCTTCCTC

CTCATGTCTCATGATGGAATCCTCGCCCTTCAAGATCCAGAGATCGGATCAGCGCGTGTC

GTCTTCCAGACAAACTATGGCGACATTGAATTCGGCTTTTTCCCTCGTGTTGCTCCCAAA

ACTGTTGACCATATCTTCAAACTCGTTCGCATGGGATGCTATAATACTAACCACTTCTTC

CGGGTTGACAAGGGTTTTGTCGCACAAGTTGCTGATGTTGTAGGAGGCAGAGTGGCTCCT

ATGAATGAAGAGCAAAGGCTGGAAGCTGAGAAGACTGTTGTTGGTGAGTTTAGTGAAGTT

AAACACAGAAGAGGAATTCTCTCCATGGGAAGGTATTCTGATCCGAATAGTGCCTCATCT

TCTTTCTCAATTCTTCTCGGAGATGCGCCTCACCTTGATAGGGAGTATGCCATTTTTGGA

AGAGTCACAAAGGGCGATGATACCTTAAGAAAGCTAGAGGAGCTTCCTACTCGTCGTGAT

GGGATCTTTGTGATGCCGACTGAGCGCATCACCATTCTGTCAACGTACTATTACGGTAAG

TCGAGCAATTTGTTTGATGCTTCACAGGTTAAAAACCATATGTTGTTTAGTTCTTTTACT

AAACAAATGCATAATTGAATCTTCTCCGGAGTAATGTATGCCTCTTATGATAGCCCAGTT

CTGTATCTAGGGCTGCATAACGAGCCAAACAAGCTGATCACTAAGGTTGTTCAAGCTTTG

TTCACGGAAGATTGAGACGAGATTGAGCCTAGTTTCAGTTAAGCTTTGTTTGCTGAATCT

TGAGTCGAGCTCGAACATTTATCGGAGTTAAAAACATTATGTTGTTTCCAGACAATTGGC

ACCCATTTATATAGTAAGGGTGGCTCTTGCAAACATTGTGCTATACATACGCTAACTTAC

TTCTTGTAGGGATTAGTTGCCATCTTACCACCTAAACCACCTCCAAAATCATCACTGTCC

TAAATGCAAATATTTAAAATTCAGCGTAAATTATCCGAACTGTTGCATCCTAAACAGGCT

TTTCAAGAGTGATCGTACTTGGTTCACTAACAATAGCGAAAAAATATTTTTTTTTAGGCT

AGCGATGTTTGGTGCCGTGAATGACACAAGCGAGCTAAATACGAAGGAGTATTGAGTGAG

TATCGGATTGCTTAGTTCGCTATGCAGCCCTATCTGTGTCTGAGGAATCCCTATGGAAGT

TTCCTCTCAGATATTTTTCGGTTAAAATTACATTGTGCCATTATGAAAAAAAAGCTTTTA

TTAAAGAGAATTTTTTGGTTAAAAGTTACCGCTTTGGGCAATCTCCAGCCAAGTGTAAAT

CTGGGGATAATTAATTATATATAATGTCAAATTAAGGGTTATTCACTTGGTTATGGTTGA

GGGATTGAATCAAAGTTAAAGATTTTTGGGATGTCGGCATGGGTTATTCACTTGGTTATA

TATAATGTCAAGTCAAGGGTAAATCCTGGGATAATGAATTATATGGTTACTATCCTCAAA

ACAATATTACATCTGACATTGGTATTTGGGACAGCAGTACCTCTGCCTGGAGGAGTTATT

TCATGGGACGGAAATTGTCGGAAACAACCCCCTAATTTAGGATAACTAGCAGGTAATTTT

ATTACAACCTCTCGGATCTGGTATTCCGTTGAGTTCTGTCTTGTGCCATTTCAGCCATAT

TTAGACATTGCCCTTTAGGCATCTTGATTATGTGCTGTTCTGGACCCTGACCCACATGCA

GCACGTTACCCAGATTCAGCAACATTCCCGTTTCGTTCACTATCCATTTTGTATGCTCTC

AACGACAATTAATTCATATTGTACAGAGGTGGTTGGGGTGGTGTTATATGATCGAAAATG

CGAAAATAAAACAGTCAGCAACCAAAATTCAAAGGAAAAAGCAACAAGAAGGAACACAAC

TTTTCACTCTACTATTTTCTCATTCACATATAATCAAACACATCGTTTACATGCCTCACT

CATGAAGATTAAACAAGAATTGAATGAGACCAATCTTGATGTCTGCTGCTGGAAGATATG

TGCAGCTGCTCCACCTCAATGGAAAGCTCCTCTCTGATATCCTGAAAGCGACTGCCTGGA

GCTCTTCTCATTGAGCTCCACCTCCTGGACTATCTTCTCAATACCAAACAATAAATCAAT

CACTTTGAAATCTGTGGCAACCTAATTGCGGACAAAAAGCCATGACGTTGACACTTCATT

TTGACAGCATTTTATGTCTCATGCATCAGTCTTTTGTTCACTTACGTAGATACAGAGTTA

GAGGGATGTGAACGACAAAAATCCATTCTGAAGAGAAGGCTTTCCGAGTCCTTTTTTGAA

ATTGAAAGACAGAGAATCAAATGTCTTCCGTGATTCCTGACAGAAGAGATGTACGTGGAT

GTTTCCTAGAAAACTTGTACATTGTTATATATACTGGCAGCAACAGAGTTGAAATGTAAT

AATGTAATAAGAGCTTTTATGTAATCACAACTTTTTAAACCGCGTTGAATTCAGAAGAAG

CTAAACTTGCAGCGTGACAAGGAACATGTATCATATGATGAAAAAGCTAAACAAAATCAA

CTGGAGAGGATGTTAGCCTCAAGTCAATCCACAAATATATTTTTTAT

>Unigene0000456

ATATATGCTGATACCTGTCGTCACCACCGCGGCAGTGAGCGGCGGCGCATCCGGCCGCGG

TGACCCATTGACTTCTTCGCGGACGACGGTTCCCAGCATATCTGGAAGAAAAATCGCCAA

ACCCGCCTTCCCGATCGGGGCTCTCATAAAAAATCAGCGCGTAACTGGAGGCATCACATC

GGAATTTCTTCCACCGCCGCTCACAAACGTGGCGAGAGTTTCAGTTGAACCGCCTGATGA

AATCAGCTCGTAACTGGAGGCATCATGCCGGAATTTCTTTCACCGCCACTCACAAACATG

GCGAGAGTTTCGGTTGAACCGCCAGATGAATGTCTTCCAATATGGCCCTGCCACTAGCTC

CGACCACACTCGAAACTTCATCGGCATCTTCAGCCCGCGCTTCCAACACCCATCATGCTC

CCTCGATGACTTCTTATCAAGGGAAGACAAGAAAATTGATCCAAAAAAGACAAGATTTTC

TTGATAGATGTCATTCACGCTCGAAATTTAGAAGCAGAGCCAAAAGGAAGAAATGCTACA

AATACGATGAGGTTAACAATTTCAAGATCAACGGCCTCAACTCAAGTCAAAATAAGATGG

TTCTTCCAATATGGCTCAGAAGGCTGGTTAGAAAGATGACACGAAGGGAAAGAATGTGAA

GTAAGAGAGTGACAAAGCTGGTAAGAAAGACGACACAAAGGGGAAGAATGTGAAGTAAGA

GAGT

>Unigene0000457

AAAAGACAAGATTCTTCTACCCGCGCTTCCACCACCCATCATGCTCCCTCGATGACATCT

CATCAAGGGAAGACAAGAAAATTGATCCAAAAAAGACAAGATTTTCTTGATAGATGTCAT

TCACGCTCGAAATTTAGAAGCAGAGCCAAAAGGAAGAAATGCTACAAATACGATGAGGTT

AACCATTTCAAGATCAATGGCCTCAACTCAAGTCAAAATAACTTGGTTCTTCCAATATGG

CTCAGAAGGTTGGCTAGAAAGATGACACGAAGGGAAAGAATGTGAAGTAAGGGAGTGACA

AAGCTGGTAAGAAAGATGACACAAAGGGGAAGAATGTGAAGTAAGAGAGTGACCGGTCGT

ACTTTATTGTTCCATCAACGTGATACACATATGATTCGGAA

>Unigene0000458

CATTGACTTCTTCGCAGACGGTGGTTCCCAGCATACCTGGGAGAAAAATCGCCAAACCTG

CCTTCCCGGTCGGGGCTCTCGTCAAAAATCAGCGCGTAACTGGAGGCATCATACCGGAAT

TTCTTCCACCGCCACTCACAAACGTGGCGAGAGTTCCAGTTGGACCGCCTGATGAAATCA

GCGCGTAACTGGAGGCATCATACCGGAATTTCTTCCACCGCCACTCACAAACGTGGCAAG

AGTTTCGGTTGAACCGCCGGATGAATGTCTTCCAATATGGCCCTGCCACTAGCTCCAACC

ACACCCGAAACTTCACCGGCGTCTTCTGCCCACGCTTCCACCACCCATCATGCTCCCTCA

ATGACATCTCATCAAGGGAAGACAAGAAAATTG

>Unigene0000459

GTTGATCGTCTCTTTACTGTGGGTTACCACTTGCGGTCCCCTCATCTTTAGCATTGGAGT

AGAAGCCCGAGTTTTGAAGGCAAAGGAGTGTTGCATCTAATGATGAAGATGCTAGAGTTT

TGCTGGTGGTTGATCTATTTGGTGAATTGAGTCTTGTGTGACCCATCGTGAGTGTGTAGT

TGGGTCTCTAGTGTGCTTTCTCTCTGTATGGTTGTTGCTTGCGCGAGTGTGTTCCCGATT

GTACCTAGCTAGAGTTATGTGTTAGGGTCTGGGCTAACATGGGTATAGTTGATCGTGCAC

AGAACTGGTGCATGATCCATGATAGGCTCACGGGAATCGCCTGGGCTTCCGACTCATTTC

CAAAGCATGTATGTTCAATATTTCCAAAGCATGTGTGTGCTAGTCATGAGTAGACTAAAA

AA

>Unigene0000460

GTGATTCATCATACCCCCAGCCCCTCTAATCATCAGATTCATTAAGTGATTTAGCTATAA

CTTTCAATCTGCTGGTTGAATCTTTTTGAGATTTGGATAGGGTGTTTGTGGGTTTGTCAC

CATCTTGGGTTTTGGCATAGCCAAAAGCACAATTTTCAGGACGATCCAATTGTTGAATTT

TCCTGAAACAATGATTATGTGATGACTTGTCATTTGTGGGCCACCCGCGGCCACTTTGCC

TCTCGCCGGTTGACCACCGACGGCCTCCTGCTTCCTGTGTGTTTCACCCATAGCCTCCAC

TACTCTCATCACCACTGCCCTTTGTACCTTGCCACCGGTGGTCGCCATTCGTGACTCTCG

CGTTGATCGTCTCTTTACTGTGGG

>Unigene0000461

TCCTCCGCTGGAGAGCCATTCTCTTCCCTGACGCCATCCTCTGCCACCCAACAATGCCGC

AATGGTCATCTGCTCTTCATAGAGGACCACTAAAACTACCCCTCGCCTAGCTCGCTATGT

GTCCCCTCCTCAAGATCTAGGAGAGCCTCATCTGCACCGTCGCCGACCATCACCAATGGC

CTGCCGGCCAACCGTAGGTATGTCTCCGACCTTTATCTCCCTCCTTTTTTACTAATTAAT

CTCTTATATGTGCAGGAGGAAGGATAATCGAAGGGTTGGGGGCTAATCGAAGGGATTTGG

GCACGAAGGTCCTGGATTCACTAATCAAATTGGAGAGGAAGCTCGTGCGGATTAGCTAAA

TTAATGAAGGGAGGATGCGATTGGCTGAAGGAGGACATCAATTGATGAAAGGATGCCTCG

GATTACTGAATTGGAAAATCAGAGAGTTGGACCTGGAGGGAAGGCACATCAATGGTGATC

CAAGCAGGAGTAGGGCTATAAAAGGGGCTATCCTGATGTTGTGTTGGGCGGCTCACAATC

CCTACAGTGAGACCCTTGAGTCCTTTACTACCTATCCACCACCTCTTCTCACCTACATAC

AAGCACAATAAGAGGGACCAAGATGGCAAGAGGAGGAGAAGCAAGTGGGAGAAGCATTGA

AGATGAGGTGCCTCTAAAAAGTTGAAGCTTGCAGTATCAATTTCGAGGTGAGTTCTAGAA

ATGTAGTAGTTATGGTAAATTGTAATGGGATGACTAGAACCCTGTATAGAGAATGTTCAA

GTTAGGCAGGAATAGCTACTCAGTCATAAATGCTCATGTATGGATATCTTTGTTATCAAT

CTAATTCT

>Unigene0000462

TCCTCCGCTGGAGAGCCATTCTCTTCCCTGACGCCATCCTCTGCCACCCAACAATGCCGC

AATGGTCATCTGCTCTTCATAGAGGACCACTAAACCTACCCCCTCGCCTAGCTCGCTATG

TGTCCCCTCCTCAAGATCTAGGAGAGCCTCATCTGCACCGTCACCGACCATCACCAACGG

CCTGCCGACCAACCGTAGGTATGTCTCTGACCATTATCTCCCTCCGTTTTTACTAATTAA

TCGCTTATATGTGTAGGAGGAAGGATAATCGAAGGGTTGGGGGCTAATCGAAGGGATTTG

GACACAAATGTCCTGGATTCGCTAATCAAATTGGATAGGAAGCTCGTGCCGATTAGCTAA

ATTAATGAAGGGAGGATGCGATTGGCTGAAGGAGGACATCAA

>Unigene0000463

CCGCTCTAGGGCAAAATCAAGCCTTCAAGATCCGCCGAATTGCTCTTCAATCCTCTCCAT

ATCAATGCTTCCCCTCGCGATATGCAGGACATCATATTTTGTGTAAAATAATGTCAAGAA

TTTTCATCTCTGGTGATGTCTGCATATCATTGGATATTTTTCGCCATGCATTTGGTATCT

AACGATAGTCAATCAGGGGATATAGCAGAAACTGAGCCCATTCTGTCTCATTCAGAGGAT

ATATTGGAAAATGAGCCCATTCTATCACAGTCAACAACTGCTGGTAGTTCCAAGGACACT

CAATCCTCTTGTGAAATCAGATTGGTGTGTTCAGATGTTGATTGTGAAAACTCAGAAGCT

GAGGAACACTGCAATTTGGTGACTACAGAACAACCACAATGTCGAATTTGCCTTGATAAC

GGAGGCGAGGATTTAATCGCACCTTGCTACTGTAGGGGCACCCAGAAATACGTACATCGA

TCCTGCCTAGATAATTGGAGATCAACCAAGGTAGGTTTTGCGTTTTCACACTGTACAGAG

TGCAAGGCTGTCTTCATACTGCGTGCAAATGTGCCACCGGACCGATGGTGGCTGAGGCTC

AAGTTCCAGCTTCTTGTTGTTAGGGACCACTCACTCATATTCATTATCGTGCAGCTGATA

GTGGGGTTCTTGGGTTTGTTGGTGTACAAGTTTTATGGCGAGGAGCTGCGGGAGATGTTT

GGGTATGAGGAACATCCATATGGATTTTACTCAATGGCTATTTTAGCTATTGTAATGGTC

GGCCTGCTGTATGGATTCTTCATAGCAATAATTTGTGGACGGAGAATCAATGAACGCCAT

TTCCATGTTCTTGCAAAGCAAGAACTTACAAAGGAATATGTAGTGGAAAGTCGCGATGAA

AATAAGAACATGCCTGACCTTGATTCGAGTCAAGTTAGAGAGTTACGAATGTTGGGGCTT

TATTAAACCAAATGTTATATTTTTATGACAATCTTTGATTAGGCCTTTCTCGAATTCCCC

TCTACTACTGTTAGAATGCTTGCCAATGCCGCCCGTACAGATGCCAAATGAGTTGTAATT

ATACATTATCTTTTATTGGACAACATAAATACTGTAGTTTCAAAGAATTACAGGATATAC

TTCGGAACTGGATGTATCTGCTTTTGCCGAGACTGGAGTTGGATCATCTTTTGTTGTTAG

CCGAAACTTTTTATTGTGTTATATGTTATATGGCCGAGAGACTAAATTTATTTATTTGTC

TCCCAACTTCATGTCTGATATGTCGGAGGGCTGTTTTAATTTTGGCATTGAACACCAATA

TGTCTCTTATAAGTGGTCAAGTTGAAGTATACAAAGAAATTCCACCGTATTGTCTC

>Unigene0000464

AAAATTCGTTCTCCCCAAATTTACTCCCGAGAAACCCTAATTCCCCTAAATCCGGTAGCA

ACGGAAAGCGGACGAGCACCGTCGTCCCCCTCGGCGGCTGCATTCACCCGAAAAGGTTTC

TCTACAGATAATTTGTTGGCAATCAAACCACAAACAATGGATGGGACGTTTAATGGTCAG

ATATTGACGGAAAAGCTCACCAAGCTGAACAGTACGCAGCAGAGCATTGAGACTCTATCA

CATTGGTGTATATTTCATAGGAAGAAAGCAAGACAAGTTGTGGAAATATGGGACAGGCAA

TTTCACTGCTCTCCTCGTGGGCAACGTGTCCCTTTTCTGTTTCTTGCAAATGATATATTG

CAGAACAGTAGGCGGAAAGGCTTTGAATTTGCCGGTGAATTTTGGAATGTTCTTCCTGAT

GCACTTAATGATGTTTATAAGAATGGGGACGAAGCTGGAAGAAAAGCTGTGTTACGACTG

GTTGGTATATGGGAAGAGCGCAAAGTTTTTGGTTCTCGTGGGCAAATTCTCAGGGAAGAG

ATTTTGGGAAGAAATGCAGACAATTTAAGCAAAACTGGAAAAACTTCATATAAGTTGAAA

AAACCTGGTGGAGAGATTGTGCAAAAGCTCATTTCAAGCTACGAGTGTGTACATGATAAT

CTTGTGAATGAAGATAATTTGTTTGGAAGATGTCACAATGCCATTAGCTATGTTGAGAAA

ATGGAAAAGGAGATAGCAAATGAGAACTTGGGGAGCATCGGTGGACCTGGAGCTGTGGAA

GAGTTGCAAGAGCAACATGGGGCACTAAGAGAATGCATAGAGCAACTGATGATTGCTGAA

TCATCTAGAGTCAATCTGGTGACCCTTTTAAATGAGGCAACTCGTGAACAGGAGACCATC

ATCGAGCGAATCCGTAACCAACTTCAGGTTACCCAGTTACGATACGAACAAGCAGGCAAC

ATCTACCAGCAGTTACAGACCTTCACCAACGGTCAGCTGCCACTCGAACAGAGGCCAAAC

GAACCTCCGCCGCCCACTGACATCCAACCCACAAGCAGCGACGTAGGAAAGTCGACTCCT

GTCAGATATGTTCAGCAGTCGTCCTTCACCGACAACTCCCCCCAGTCCCAAGACGACCAC

CGCAAATCAGCAGCTGCAGCGATGGCAGAACGCCTTACATCATCAACCTCCTCCGCACAG

ATGCTCAGCTACGTCCTCTCTTCTCTGGCATCGGAAGGTGTCCTCCAGTCAGCCAAAGAA

GATTATCCGTCCGACCATAAACGGTCTAAACTGGAGAACGGCACAACCTCGTACATCCCC

CCACCTCCTCCATCATACCCCCACCCCGATTCTCTGCTCCTACCACCGCCACCGCCGCCA

CCACCCCAGTCATCTCCGATGCAAGATCCAAGCCCCCAACTTCAGGCCCCGCCACCTCCT

CCATACATGCAGACTGCAGGGGCGATGATGAACGTGCCATACACCTACGGCTCCGCCTTG

CCACCGATGATGCCGCCACCGCCACCACCACCGCCATTTGGTGGATACCCGATGGTTGGC

ATGTCTTCGTATTATGGTCCTCCGAATCCTTACCATAACTTTCAGGCATCAGAGGGCGGG

CTATTCAACCATGCGCCACTGCCGCCTACTCCACCGTCGGTGACCCGGCAATGAAGTTTT

ATCTTGAGGTTAGAATCTTGAGTCGGACGGCTTTGTAGGCTCCAATGTGGTCTTGTTGAT

GTATATTTAGTTCCTGAGTTTATACAACTTTTATGTATTACTGTAACCATAGACATATTG

TACATTAACAACAATGGACTTCAGAATTTGCGTCGTTAACTTATAATTTAGAATTT

>Unigene0000465

ATGCTGGTGCACGATTACTTCGGGAATCCGTGGTCGGTTGTTTCTCTGATAGCTGCGATC

GTGTTACTGGTTCTCACTGCGGTCCAGACCATCTTTACTGTATATTCTCCGAGCAAGTGA

AAACTCTTTGTGTGTGATTCAAAAGATGCAGCTACCTTTTCTAAGGTTTCCTTGTTAGTT

GGGGGAGACATAATTTGTAGTTTGGGATTTAAGATTGGTACGAGTTGTGTAGAATTTGTG

GATTGATTGTGGATATCTTCCT

>Unigene0000466

ATGCTGGTGCACGATTACTTCGGGAATCCGTGGTCGGTTGTTTCTCTGATAGCTGCCATC

GTGTTACTGGTTCTCACTGCGGTCCAGACCATCTTTACCGTACTTTGATTCACCACCTGA

ATATGCCCCTTCTTCCCTCCGAGCAAGTGAAAATTCTTTGTGTGCGATTCAGAAGATGCA

GCTACCTATTTCTAAGGTTTCCTTATTAGTTGGGGAAGATATAATTTGTAGTTTGTGTTT

TAAGGTAAGCTAGTAGGAGCTGTGTAGAATTTGTGGATTGATTGTGGATATCTTCCTAAG

CATGTATAAATGATGCGTGTGACCATCTATTTGAAAGAAGGCGGTGTGTGGACAAGGTTT

GCTACCGGATTTCTGTTGCTTTGGCTTATTATAAAACATTAATGGGAAGTTGGATTTGGT

TTGCATCTAAATTTTAATCACAAAATA

>Unigene0000467

TCGGGAATCCGTGGTCGGTTATTTCTCTGATAGCTGCCATCGTGTTACTGGTTCTCACTG

CGGTCCAGACCATCTTCACCGTACTTTGATTCACCTCCTGAATATGCCCCTTCTTCCCTC

CGAGCAAGTGAAAATTCTTTGTGTGCGATTCAGAAGATGGAGCTACCTATTTCTAAGGTT

TCCTTATTAGTTGGGGGAGATATAATTTGTAGTTTGGGTTTTACGGTAAGCTAGTAGGAG

CTGTGTAGAATTTGTGGATTGATTGTGGATATCTTCCTTAACCATGTATAAATGATGCGT

GTGACAAACTATTTGAAAGAAGGCGGGGTGTGGACAAGGTTTGCTGCCGGATTTCTGTTG

CTTTGGCTTATTATAAAACATTAATGGGAAGTTGGATTTGGTTTGCATCTAAATTTTAAT

CACAAAATA

>Unigene0000468

CTTTGGGAATCCATGGTCGGTTGTTTCTCTGATAGCTGCCATCGTGTTACTGGTTCTCAC

AGCGGTCCAGACCTTCTTTACCGTACTTTGATTCACCACGTGAAAATGCCCCTTCTCCCC

TCCGAGCAAGTGAAAACTTTTTGTGTGCGATTTAGAAGATGGAGATATAATTTGTAGTTT

GGGATTTAAGGTAAGCTGGTAGGAATTGTTTAGAAGTTGTGGATTGATTGTGATGTCTTT

CTAAGCATGTATAAATAATGCGTGTGGGAATCTATCTTATGACCAAAGAAGGCGTAAATA

AAGGTGTGGAAGGTTTGCTACTGGATTTGTGTTCTTTT

>Unigene0000469

GAATCTATTACGTGGCGATCACACTTCTCCCCACTCACTCCCTCCCATGGCCAACCGCGG

CCGCCACGCAGCACCGCCGCCAGCGGCGACGACGTCGGCGGCGCGTGTCCCGCTTCGCCT

CCTCCTCCGCGCCGCTTCCGTCGCCTGCGGCGTCCAGTTCGGGTGGGCTCTACAGCTCTC

CCTCCTCACCCCGTACGTCCAGGAGCTCGGCATCCCCCACAAGTGGTCTAGCCTCGTCTG

GCTCTGCGGACCGCTCTCGGGTCTCGTCGTCCAGCCACTCGTCGGACACCTCAGCGACCG

CTGCGCCAGCCCCGCCGGACGCCGCCGCCCCTTCATCGCCGCCGGCGCCGCGATGATCGC

TGTCGCCGTGCTCCTGATCGGCCATGCCGCCGATATCGGGAGGTTGATCGGCGATCCGTT

ACCCGGCGTCGGGGTCCGGCGGCCCGGTGCGATCACGGTGTTTGTGTTGGGGTTTTGGCT

GCTGGATGTCGGGAATAATGCGATTCAGGGACCGTGCCGGGCGCTGCTTGCCGATCTCAC

AGGGAAGGATCACAGACGGACACGTGTTGCAAATGCTTACTTTTCACTCTTCATGGCCTT

GGGTAATGTGCTCGGATATGCTACTGGAGCATATAGTGGCTGGTTCTCCATCCTACCCTT

CACTGTCACCAGAGCTTGCAGTGTAAATTGTGCCAATCTCAAGTCTGCTTTTCTCCTTGA

CGTTGTTCTCCTTGCCATTACTACGTACATTTGTATATCATCAGTGCAAGAGATCCCTCT

AAGTACCGAATATACCGAAGGAACAGAACATCTGCCGAATCATGGCCAAGAGGCTTTTTT

CTGGGAACTGTTTGGCGCATTGAGGTACTTGTCACTACCTGTTTGGATAATCCTGATTGT

TACTGCACTCACATGGACTGGGTGGTTTCCATTTATTCTGTACGACACTGATTGGATGGG

GCGAGAGATCTATAGAGGGGAACCAAATCAAGGGCAAGCTTATCACTCGGGTGTGAGTAT

GGGAGCATTGGGCTTGATGCTGAATTCTGTTATCCTTGGGTTGACATCAATTACGTTGGA

AAGACTTTGCAGGAAGTTGGGAGCGGGACTTGTCTGGGGAATCTCAAACATAGTTATGTC

TCTCTGCTTTGTAGCAATGCTTGTTATCTCTTTTGTGGCTAAAAGTGTAGAGTTTACTGA

CACCGGACATCCTCCTAATGGCATTATTGTCGCCGCAATCGTCGTTTTTGCATTTCTGGG

AGTGCCGTTATCGATTACTTATACAATTCCTTATGCAATGATTTCTACTCGCACAGAGGT

TTTAGGTCTTGGCCAAGGACTGTCAATGGGCATTCTGAATTTAGCAATTGTTATACCTCA

GGTGATTGTAGCTCTTGGAAGTGGACCATGGGATCAGTTATTTGGAGGAGGCAATTCACC

AGCTTTTGCTGTAGCAGCAATGGCTGCTTTTTTCAGTGGACTCATGGCAATCATTGCAAT

TCCCCGATCTTCTGTTTCAAGGACAAGGACTCGAAAGTAAGGTATATTGTGTGTTCCCTG

GTTCTTGTTACTTTTGTAAAAACTGATGAGGAACAGTTGTCACACGAGGAAAATGTAAAT

ATTATCAGCTTTATGGGGTCATAAGGTGCCACTGATATTACGTTATTGTTTCTTTGAGTT

GCCTAACTTTTTGGTTTTCACTTTGTGGTTCCATGATTATCCTTGCTTAAATT

>Unigene0000470

GCTCCCATCAATCCATCCGTCGCCGGTGAAACCATGTCGTCCTCCGCCGCCGTCGCAGCC

CCCACCGCGCCCCGGCGCTACTTCTGCCACCAGTGCGACCGCACCGTCACCCTCACACCG

TCTTCCGCCTCTGACATCGCCTGCCCCGTCTGCCAGGGCGGTTTCCTCGAGGAATTCGAC

CTCCCACCACCTAACCCTAACCCTAACTATAGCCCCGCCGCCTTCCATTCCTTCCACTCC

TCCCCCGTCGATTCCCTCTTCTCCTCCGGCCATGGCCCCAGCATCTTCTTCTCCCCGGCC

TCCGGCCGATTTGACATTTCGGCCTTCCTGGGCCCCGACCCCATCCGCTCTGCCTCCCAA

GCCCCCACCGCTGACGGTGAGCCCTTCAACCCCCTCCTCTTCTTCCAGACCTACATGCAG

AACCTCGTCGCCGGCGGAGCCAACGTCCAGGTCGTCTTCGAGGGTCATGGCGGTGGGCCG

CTCTCCGGAGCCAGCCTCGGCGACTACTTCGTCGGCCCAGGGCTCGAGCAGCTCATCCAG

CAACTCGCCGAGAACGATCCCAACCGCTACGGCACGCCTCCGGCCTCGAAATCTGTAGTC

GACTCCCTCCCCGACATCAAGATCTCCTCCTCCCTCCTCGAGTCTGATGAGGCCCAGTGC

GCTGTCTGCAAGGACACGTTCGAGATCGGGGAGACGGCGAAGCAGATCCCGTGCAAACAC

ATCTACCACAAGGATTGCATCGTGCCGTGGCTGGAGCTGCATAATTCGTGCCCGGTGTGC

AGGTATGAGCTGCCAACTGATGATCCTGAGTATGAGCAGAGGAGGGGGGGTCAAGCCCCG

GCTGGAAGCCAAAGGGTTGCGCCAGATCCTGCAGCTGCAGGTGCGTCGGCCACTACAGCG

TCGCCGGATGCGAACGCTAGGGCGGTGGAGAGGAGGTTTAGGATCTCGCTACCTTGGCCG

TTCAGTGGCTTTGGGACACAAGCCGAGGGGAGTGGTTCTGGCGCTGGAGAGGGATCGGAC

AATCCCAACAACCAAGGCGGCAGAGATGGTCCTGGGGGGAACAGTTCAGCACGTCGGCAA

GAGGATTTGGATTGAGGGAGGTTGGAGGATCTGCCCCTTCTGAGGTACCTGCTTTCCAGG

TTACAGTTTCTGAGGTACCGCTTTCCAGGTTACAGTTTCACCGAGATTACCGAGGGTCAT

AGCTTATACATGCTCTGCTGTCAAGTACTGTTGAAAATAATAATCTAGTTGAATATTTTT

TGGTTTTATTTAGCGAGAGGTTTTGTCCTTTGTAGAACTTGTTGGCGTATCGAGGTTGAG

ATTTGAAGTTGAGTCAACTGGAATTTACTTGAAAAAGCTATGCATCTGGTTATGATATGC

CTCCTATATTTTGTTACTTGAAAAAGCTATGTGACTGGTTATGATATGCTTCTGGTCATG

AGATGCCTCCTATATTTTGTTACTTATAAGAGCTATACATC

>Unigene0000471

TTTGTGTAAAGGTCTTATCGAACCCACAGATATGGAATTCTTTGACATGCACCGACTATA

TATGGATGATGTTAGTCATCTCCAAATTTTGTTGGGACCCCCTTGTCATTGCTGCCCTTC

ATAGACATTGCCATTGAATAGGCCATTCCCTCATGATGATGTGATCTTAGAAGCCCTACA

AACTTCTGCGTAACACTTAGCCGAATGATTGCAGCTGGGGATGCATCAGCATTACCACAT

TTGTGGCCACATCAGAATCCCAACGGCCCTTACAACTTTGATATGCTCTATGCTCACTAT

GTACACCCAATATATAATCAAAAGCCTCATAGATAAAATGTGAAGCATATATTGAACTTT

TGAGTCAAAAACATTGATTGGAGGTGCTCTATAACAAGGTTCCAATGTGCGAGGAAAAGT

GAAAAATAACTAATAATTTTCATGATAGCACCGACATCATGTCTATAAGCTACTATAATC

TTTTTCATTGAAACTCTTAGTTGTCCTCATAAATACACTTTTGTTATGTGTCGTTAATTA

TGTGTGTACTTATCTGTGTTTCTTAGTAATGGGCTTTTATATTTTTATTCTTGGAAGGGA

GGGCTCTTTGAAGTTTATTTTGATCTATACTTGTGTTCAGTGTGAAGAAAAGGTATTATT

TCCATCTTCTGATGGCTGAAAATTTGATTAAATGTACATAAACTTGTTTCTGTTCATATT

GTGTAGGATCTGCCCCTTCTGAGGTAC

>Unigene0000472

ATCCCGACCCTCTCTCATTCTCCCCCTTTCCTCCTCACCGCCGAGAGGGTCGAGTGGAAA

TCACAGCAATAAAAAACAACAATGCTCGATCCTAGAGCCGATCAGCTGCTGTCTCATCCT

CTCCCCCAAAGATTCAGCTATTAGTTACTTCAGACAGCCGCAATTAGTAGCAGGGATAAG

AGATACTAGACGATGGCATCATATGGAGAGGAGGAAGAGAGTCGGGGCTATCTTTTTAAA

ATTGTTTTAATTGGTGACTCGGGGGTCGGGAAATCAAACTTGCTTGCAAGATTTGCTCGG

AACGAATTCTTTTCAAATTCCAAGTCCACTATTGGAGTAGAGTTCCAAACACAAAAGATG

GAGATAGATGGGAAGGAAATCAAAGCTCAGATATGGGATACTGCCGGTCAGGAGCGTTTC

AGGGCTGTCACATCTGCATATTATCGAGGAGCTGTCGGAGCACTGGTGGTTTATGACATA

AGCAGGCGCCAGAGTTTTGATAGTATCGGGCGCTGGCTCAATGAACTGCACACTCATTCC

GACATGAACGTCGTCACAATTTTGGTTGGCAACAAGACCGATCTCATGGATGCAAGGGAG

GTAAGCACAGACGAAGGTCAAGTCCTGGCTGAGGCGCAAGGACTCTTCTTCATGGAGACA

TCAGCCTTAGACTCTTCCAATGTTAAGGAGGCTTTTGAGACGGTGGTGAAAGAGATCTAT

GACATACTGCGCAGGAAGGTTTTCTCCTCTCAAGAGCACAAGAAGACTGAGACGTCGTTA

GGGAACGGGAAGACGGTGATCTTACAAGGGGACACGGAGGCCGATGGCGGGGGTAAAGGA

TTTTTGTGTTGCTCGTCGTAGAGCCGACTTTGTTACCTTTTATGTCTACATTTTTTGGTT

CTTCCTCGGCTTACTAGGTTTGCCTTCTCTCTCAAATGTATAGAATGGAATTGGGGCTCC

TTCAACATTTATGAATGCATAATATCTTTTTCTGCCCCCTTTAACATTGGTCTTATGATC

TCAATTGGTTGGAATTTGTCATTTGTTGTGGAAATGAATCAGCTGTGGATTGGTTTCATG

ATGCTGATTAGATGCTGCAATTTTATCTGTCACAGCCAGATTGTTACATGTTTGAATAAA

AGGCTACATGCAGCATTAGTAGTTTACTAAGA

>Unigene0000473

GACAAAGTTTAATATCATGAGGATATGAGGCTTGGTGAGGAGCAGAGGGTGAGAAAGGTG

GTACTCATGGCAAGTAATTTGATTAAGATAATGAGGATTTTTGGATGAAAAGATTGTTGC

TTTTGAGTGTTTTCCTCTCCTAGTTTCTTTTTATTCTGTTTTGTAACCTTTTTTTTGTTT

GTATTCTTATGTTGGGATGCGAGAATTAACATCCTTTTTTTTATGCATCCTTCTTTTCCT

TTTATGTTGTTATTGCCGATTTGTTTGTTTGATTGATGGTGATTACCTTTGCATTGAAGG

TCGCGACAATGTACAGGTGCAGTTCTTCATGCCTCTCAAAATCCCCTATTTTGAAGAGGC

CTCGAACAAACTGCTACATTTTGTATTTCACTCTACTTTGCTAACTGTCAGCACAAAACA

TATTAGATCCATAAAGGAGGCAATGGCCAGGGTTTGAGAACATCCTTAAGATTTTATAAC

AAGTTGATAAACATGTTAAAGAATAGGAGCATCCAACTTAAAACCACCTGCAAGGCTAGC

CTAATGGATGTGGGACTAAACAAAACAAAATATTGATAAGAAAATTTATCCATAATCTAA

TACACATTCATCATATTTCATTTCGCCATCTTGCTAGAATCACATTTAATCTTATTCCAT

TCAACTTGTTGAACTTTTGCTTCCAACTCCAAATGAAAGTGAAAACTTCTGTCGACTTTT

GAAAATCTTTACTCTCCCTATGTAGGCTTCCAATCTATGACCAGTCATTTAACAGCCGAT

TGCTTTACGTAAGAACCTATGACCAGTCAGTTAGCAGCCAACCTC

>Unigene0000474

TCTCTCCACCGTCTTACTATAAACGCCGACGGCCTCCAAATAGAGAACCACCCCTGAAAA

CCAAGTCCCTTTCCTCCATCCTGGAACAACTGGAAGGAATTTGCTAGGGTTTCTCATAAA

AAGAGAGGAGAGAAACTCAGAGAGACTGATAATGGATTCTCAGAGAAAGGGAGACGAAGG

GGAGCCGATACTCACGGCGACACCTGACAGATTCTGCATGTTCCCCATCAAGTACCCGCA

GCTCTGGGAGATGTACAAGAAGGCGGAGGCGGGTTTCTGGACCGCCGAGGAAGTCGACCT

ATCTTACGATATTCAACATTGGGAAAGATTGACTGATTCTGAGAAGCACTTCATAAGCCA

TGTGCTTGCCTTTTTTGCAACATCCGATGGAATTGTTTTGGAAAACCTAGCTGCAAGGTT

CCTGAAAGATGTCCAGATTCCCGAGGCTCGTGCTTTTTATGGCTTTCAAGTTGCTATGGA

GAATATTCATTCTGAGATGTATAGCTTGCTTCTTGAAACATATATTAGGGATGCCAAGGT

GAAACACAGGTTATTCCATGCTATAGAAAGCATTCCTTCGGTTGCTAGGAAGGCAAAATG

GGCGTTGAATTGGATAGGAAGTTCTAGTTCATTCGCTGAGAGACTTGTTGCTTTCTCTTG

CGTTGAAGGAATATTTTTTTTCAGGAAGTTTCTGTGCCATTTTTTGGCTGAAAAAGAGAG

GAATCATGCCAGGATTAACCTTCTCAAATGAACTTATTTCAAGAGATGAGGGTCTTCATT

GCGACTTTGCATGTCTTTTATACAGTTTGTTGCACAAACCTCTTAATTGGCAAAAGGTGT

ACCAAATTGTCCATGATGCGGTCGAGATTGAAACCGAATTTGTTTGTGATGCCCTTCCTT

GTGCATTGATTGGAATGAATTCTATCCTCATGAGTCAATACATCAAATTTGTTGCGGACC

GCTTATTGGTTTCCTTGGGTTGCCCGAAGAAATATAATGTGGAGAATCCCTTTGAATGGA

TGGAGTTTATATCCTTGCAGGGGAAGGCTAACTTCTTTGAGAAAAGAGTTGGAGATTATC

AGAAGGCTTCAGTGATGTCAGGTCTTCAAGATGGAGCTGAAAATTTCACATTCAAGATGG

ATGAAGACTTCTAGTTCTCTCTCTCTACGTGATGCCGAATGCTTTCGAGAACCTTGGCAT

CAATTTTTTGTCACCATATGTAGTAATATTGGCGAATATGTCTCCCTAGAACTTGGGAAT

TTTAGAAGCTTAGAAATCTATTTTCTTTCAACTTTTGTAATTATATTATTTATCAATGGA

AGTTT

>Unigene0000475

TTTTTTTATTATTATTATTATTATTACTATTATTATCTTATTATTGTTATTGAATTTTGT

TGTTAAGTAAAATAATTATATAACCAAATATTATAAATGGACGATAAAAATTGTCCAATC

AAAAACTATTTAGTAATGTACGAGTATTTATATTTTATATAATATTTTGTTAGTGTATTT

CATAATATGAAAAGTATTATACGATTATAGATGATAATCTCAAAATTATATCAACCTCTA

ATGTAACTAATTAGGTATAACTGATATTACAACCCAGTCCATAATTTTGGCTTTTTAATG

CTTAGAGGTACGTATTACTAGTACCTATTATCAATACCCAATTTAATATGTGTTAA

>Unigene0000476

TCAGAACCCCCGAGGAAAATGCAGCTCCCGCCCCGCGCGCGACTCGGCCCACCCCCCGAT

CTTCAAGCTCGAGTCGCCACCCTCGCCATCAGCTCCTTTCAGGTTCGAGGCTTCCCCTCT

GCGTTCCCATAACCCCATCCCTCGCTTGAGTCTTCCCAGACCTCGATCTCCCTCCCCTGC

AGAGAAGCTCCATTGCAGAAGAACCCCTCAAAGAGAGAGGAGCAGTACACGATGTCGTGG

CAGACTTACGTCGATGATCACCTCATGTGCGACGTTGATGGCCAGCATCTCACGGCCTCG

GCTATTGTAGGGCATGATGGGAGTATCTGGGCCCAGAGCGCCGGTTTCCCCCAGTTCAAG

CCCGAGGAGATCACTGGGATCATGAATGATTTTGCTGAACCCGGGTCTCTGGCACCAACC

GGCTTATATCTCGCGGGAATGAAATATATGGTAATTCAAGGAGAGCCTGGAGCTGTTATC

CGTGGAAAGAAGGGATCTGGTGGTGTGACTATTAAGAAGACAGGCCAAGCCCTTATTTTT

GGTATCTATGAGGAACCGATGACTCCAGGGCAGTGCAACATGGTCGTGGAGAGGATGGGC

GACTACCTTGTTGATCAGGGTCTGTAGATTGGAGACGAATTGATGCTTCCTCCTATCATT

TATATGTGATCATATTTATAAATGATCATATTTTTATTCTTTTTTCTCAATTTTGAGCCA

TAAATATTTCTTGACCATGAACTGGTTCTTGCCAGAGTTCATTTTTCATTTTGGGTAATA

ATCTTGTGTGTAAACAAGAATGTTGATGTATTTTGCTTTGATTGTTTCTATCACATCCTG

TGT

>Unigene0000477

GTCGGCTGTTAGGCAGGGGAAGGAAGTACATTGCCGGTTCATGAGAATGGGTTGTTCAAA

AAATGTAGTAGTTGGATCTGCGCTCGTTGATCTCTATGCTAAGTGCGGTCTTATAGATTA

CGCGTTCAGAGTCTTTGATTGGATCTCAGTGAGAAATCTGATAACATGGAACACGATGAT

CAGTGGATTTGCGCAAAATGGTAATGCGACTCAAGCAATAGAAATGTTTGAACGACTGGT

GGGCGAAAAGATGATTCCCGATTACATTAGTTTTGTGGGGGTTCTTTTTGCTTGTAGTCA

TGCTGGTTTGGTTGAGGAGGGTAGGAGGTACTTCAAATCGATGAGTGAGGATTATGGAAT

CACGCCTGGAATCGAGCATTATAATTGCATGGTTGATTTGCTTAGCAGGTTTGGTCTGCT

AGAAGAAGCTGAGGAGTTGATACGGAAGTCGGTGTGTAGGGATGATTCTTCACTTTGGGC

GGCACTTTTGGGTGCCTGCAGCACGTATTCAGACCCAGCGCTAGCAGAGCGGGTGGCCAA

GAAGATGATGGAATTGGAGCCTAGCTATCATCTGAGCTATGTTCTTCTTGCGAACGTGTA

TAAGACGGTTGGGCGGTGGAATGATGCGATGAAGATTATAAGGACAATGAAGGCGCGCAG

AGTTAGGAAGACACCTGGGAAGAGCTGGATTGATAGTAGGGATGATTTGGAAGGATTGGA

ATAAGTGGAAGAAATTATAAGTTTCGCATGAGGTTTTTGTTATCGAACTGATGTTGGTAA

TTACCTCATTGCAACAAAGACCGATGATCATCAAAGATGAAAAACTTCTTGGATGAATCT

CAAGGGGATCTTGAAGTCAACTTGGTGTGATGAAAGAGAAGGATTCATAAGAAAGTTGAA

GGGCATGATGCATTTGAAAGCCACTCGATGTGTGATGTGAAGTTTCAGTTGACATTGGTT

GTATTTCTGACTGAAATTTTTATGGAGACGGGATGAAGTTGGTTT

>Unigene0000478

ATGCAGAGAATGCAATGCTTGACATGTACATGAAATGTGGGTGTCCGGCATATGCACAAT

GTCTCTTTAAACGGATGTGTATCGTCAATATTGTGTCATGGAACACGATGATCAGTGGAT

TTGGATCTGACGGGCATTGCATGGAAGCAATTGGGTTGTTTGAAAAGATGCAGAGGCTAG

AGGTGTTACCGGATGAAACCACTTTCCTCTCATTGATCTCATCTTGCAGCCATTCGGGTT

TAGTAGAAGAAGGTGTGAAGCTCTTCCAGATGATGACTAGAGACTACGGTATCATGCCTC

GAATGGAACATTATGCAAACATGGTAGACCTATTGGGTCGATCTGGTCGCTTGAATGAAG

CCTACTGTTTCATCAAAGATATGCTGACCGCGCCTGATGAGAGTGTTTGGTTGTGCTTGT

TGAGCTCTTGTAGAGCTCAGCGAGATACTGATATTGGAGAAATGGTAGCTGATCACTTGT

TCAAGTTGAAGCCTGAAGGAAGTGGAAGTTATACTCAACTGCTGAATTTCTACGGGGAAC

TGGGCTTGGTACACAAGGCAGCAAGCGTGAAACAACAGATGAGAGAAGC

>Unigene0000479

CGAGAAGACACACGGTGAAAACCTAATCCCCATCATCCGTTCATCTCCGCGAGACGACCA

GATCCTCCACACAAGAACTAGACCGAACCCTGCGGAGCTCGGAGGAGTGCTGAAGCGTGC

GCGAACCCACAGATCTCAGAAAGTCATGGAGAAATCTCGGAGGAAATCGATTGACCCACA

GAAAGACACTGTTGATGCATATGCTATACAGTGTGAGAAGTGTTTCAAATGGAGACTGAT

TCCAACAAAGGAGGAGTACGAGACTATCCGTCACAGCATCTTGGAGGAACCCTGGATCTG

TGACAGGAAGCCTGATGTATCTTGCGATGATGCTGGTGATATAGAGTACGACACCAGTCG

GCTCTGGGTCATTGACAAGCCTAACATTCCAAAGCCTCCCCCTTCGACCCAAAGAATGGT

GATAATGAGGAAGGATCTCTCGAAAATGGATGCCCTCTATGTGTTGCCCAATGGGAAAAA

AGTTAGGAGTGGGGGTGAGGTGGAGAAGTTTCTGCAAGCATATCCCGAGTACAGAGACCA

ATTCAGTGTGTCGGACTTTGTTTTCACTGTTCCCAAGTTGACGGATGAAATGGTTCCACA

GAAGCGAGAGGTCAGCGCCAAGAAAGCTTCAGCCAGGGCTAATAAGAAAATGAAGATGAC

TGTGTTTGAAGGCTGAGTTTGCTATTCCGGTCGATACCAGTTGTGGCACAAAGTGTGCTG

GTTATTTATGACAGTACTGTTGTATTCGGGCGAAACAAGTTTTGTGTAAACATATGGCAT

TGGAGTATGTATATGGGTCTTAGGATATAACAGTCTGCTGAGGAGCTGTGTGACCCGGAG

GAGAAACAGTTGCTGAGAATTCAGTTTTTCTGTTTGTAATCATGTACCATTAGTAATTTT

GATGTGGATGATTGGATGTATGAGGTTAATGATTACA

>Unigene0000480

ACAGCGCTGGTGGAAACGTTTGTGTCGTGGAAATATTATTACCCCTTTCTGAAGATGAGC

TACCACTCCGTGATGTCCCTCGCCGGCGGCCGCGCAGCCGCGCTCTCCGCCGCCCGCTTC

CCCCGGCTACCGCCCCGCCGATTCTCTTCAACACCGCCTGTACGATGCTCCCCCCTCGAG

CCCCCCGACGTCTCCCGCCTCGCCGAGTCCGCCCGCATCTCCCTCACCCCAAATGAGGTT

GAGGAATTCGCCCCAAAGATTAGACAAGTTGTGGACTGGTTTGGACAACTTCAGGCTGTT

GATCTTGGAAGCATTGAACCTTCGGTTCGAGCCGACACTGAACTTAGCACGAACCCGAGG

GAAGATCTGCCAGAAATATTCGAGAACAGGGATGCTATTATAGCTGCTATCCCAAGCTTC

GATGATCCATATATTAAAGTTCCCAAGGTCATAAACAAGGAATGAGGTTTTCTATTGTAC

TCCAAATAGTTTCCTTTTTTAATTTGAAATTGTGATGGATGACATCACAATTCTTTGTAT

GGTTTTCTCTTTGCTGTTCTACATCTAGCATGAAAAATATACCAGCTCGAAATCCTCGAT

TCCCTCAGGAAATAGTTACAATTTCTTTCTTTTAACTTGGTCATTGTTATGCAACAATAA

CAGTTTATTGATTGGGTGGAACAACTTGTGCAGTCTAACTTAGACTAATCACTAGTTAAA

TCTATCAAGAACTGGCTGTACCATTTTTGTATTTGTTGAATAA

>Unigene0000481

ATGACGCCGATATCATGGATAGGTTTGGTGGTCTAGTAGCGAATTATTTCTTGGATGGAT

TTTTAATTGGGGCCTTCCATGTAGTGAGGAAGGGGATCCCGGTGGTCCTTGCTAGTGGCA

ATGATGGACCAGGTTTTGGCAGCATCTCAAATGTTGCACCTTGGATGATAACTGTGGCGG

CCAGCACCATATTATTTGAATTGGAAGTAACTACTCGCGTTAAAGAAAATACCACGTAAG

AGTCAGAGGCTGAGTCACCACTACCACATCACCTTTGTTGGTCACCTAACATC

>Unigene0000482

ACTAGATGTTAACTTAAAACTACCGAAATTTCAGGCATTGGCTGCTGCAGTTTGCGCGAC

CTTTGGGTGATGGACTATATCGTGTGATTGACATATTTTTTAAGGCTCCCCATGACCCGT

GTTGCTGCCTACAAGGTTTGCTGGGGTAAATGCTATGACGTTGATATCATGGATGGGTTT

AGTGGTCCAGTAGCAAATTATTTCTTGGATGGAGTTTTAATTGGGGCCTTCCATGCATTA

AGGAAGGGGATCCCGGTGGTCCTTGTTAGTGGCAATAATGGACCAGGTTTTGGCAGCATC

TCAAATGTTGCACCTTGGATGATAACTGTGGCAACCAACACCATATTATTGAAATTGAAA

GCAACTACTCGCGTTAAAGCAAATACCATGTAAGCATCAGAGGCTGAGTCACCACTATCA

CATTACCTCCATTGGTTACCTGACACTGTCCTACATTCTGTCAGTCCACATTACTGCCGC

CGTCAGGTTTCTATCTCTAATTTGCCTGACGACTGTCAATCCTCCATATCTATGCATGTC

GTCCGCAATATTGGGAGAGTTGGTGAAGTCCAAACGGCCTGAGATGCCCATGTCTTCCTC

CTCAATTCCACCACCGAAGCAACAATGTCGTTCCTAAAGATAAGGATGGGAGGAATTTGA

AGGGTCAAATAGTTGGGAGCCAGCTATATATATAGTGGAGGCCGAGGCAAAGCATCTCAT

AGTGGGCTAGAAGCGATCCAGCAAGGAGGCGGGGGCATGGTGGTTCTGTGGTCTAGTGGA

GTATAGAGGTTTGAGGAGTGGTCTTTAGTGGTTGTATGTGATTTATGGTCTGTAGTTTGT

GGTTTTTGATTTATGAGCAGTTGTGTGGAGTGATTTATGTGTAGTAAGATTGTGAGGCCA

TTTGTGAAGAGGGTGAGGACAACTTGTGTGACAGGCAAGTTATATTCAACTTATTTGTAA

CTGGAAAGTACAACTCGGTATTCATGATTGCTCTGTAAGACTTTACTTCATATTCTTGCT

AGTTAATGTATATGTGGTG

>Unigene0000483

ACGTTGATATCATGGATGGGTTTGGTGGTCTAGTAGCGAATTATTTCTTGGATGGAGTTT

CAATTGGGGCCTTCCATGTAGTGAGGAAGGGGATCCCGGTGGTCCTTGTTAGTGGCAATG

ATGGACCGGGTTTTGGCAGCATCTCAAATGTTGCACCTTGGATGATAATTGTGGCGGCCA

GCACCATATTATTTGAATTGGAAGTAACTACTCGCGTTAAAGAAAATACCACGTAAGAGT

CAGAGGCTGAGTCACCACTACCACATCACCTTTGTTGGTCACCTAACATC

>Unigene0000484

GTCACCCTTCCGTCATCCTTGCCGTCAGATTCCGCCATCCTCGCTTCGACTCTCTGACTT

TCCCGAGGCCGTTTCCCTCGACATCATCCGAACAGCTATGAAGATAGAGAGGAAGCCGAG

AAGATCACGCCAGGGCTGTGGCTGAGGATAGAATATGTTGAGAAGATTACGTCCAAGGCC

GAAGAGATGATGCAGTATGGATAGAGCTGGAGCCGTTGGGAGCAGATGAGGAGAGCGGTT

TTGGGAGATAAGCTCAAAGGTAAGAGATCGTTGAAGAATGCCCCTATATATATGAGGCCA

GGGGCCAGGGTACTCTACACGGTGGGCTTGGAGCTGATCCAGCAAGGAGGCGGGGGCACG

GTGGCCGGTGGTCCAGTGGACTAGTGAGGAGTTGTCTTGTGGAATTATCTTCAAGTGGTT

TTAGTCTTTTTGGTGA

>Unigene0000485

CGAAAAATCTCGTAGTATCGGCGAACACGTGCGCCGCACATCCGAAACCACGGTGACCGG

AAATCCGGCGATTTCACGTGACAAACACCGCCGACAGATCTCCACCGGCTACGACATGAA

CACTTCAACTCTCGTCTGGTTCGCCCTGTGCTGTTGCTCGTCGTTGGCCGTTCCGATGCC

CGACCGACGGCTGCGGCAGACCGACCGGGTCGTTGTCACCGAGGCTGCTGCGGACGTCGA

CCAAC

>Unigene0000486

CGAAAAATCTCGTAGTATCGGCGAACACGTGCGCCGCACATCCGAAACCACGGTGACCGG

AAATCCGGCGATTTCACGTGACAAACACCGCCGACAGATCTCCACCGGCTACGACATGAA

CACTTCAACTCTCGTCTGGTTCGCCCTGTGCTGTTGCTCGTCGTTGGCCGTTCCGATGCC

CGACCGACGGCTGCGGCAGACCGACCGGGTCGTTGTCACCGAGGCTGCTGAGGACGTCGA

CCAACCGTTCGACCCATCGCTGGACGCCTTGGCGGACGCCAAGTACGACGAAGGCGACGC

CACCACCGACGACAGGGAAAAGCGGCAGTTGTCCTTCGTCCAGGGCAAGCTGTTGAATTT

TTTGAAACCTTTACCGGTGGTCGATGTGACTAGAGAAGAAGATAAATACGGCAACGATGG

TGACAAATTTAACAGGATTGGACGAGGTATAATCGGCGGCGTGGAAACTGTTTCCAATAT

GCTGACCTCGGCGCTTTCGTTTCCAGTGGATACA

>Unigene0000487

ACGAAGGGAGGAGGAGGAGGAGGAAGATGGGGAGGAGCAGTACCTGTTCAAGGTGGTGAT

CATCGGAGACTCGGCGGTCGGGAAATCGAACCTGCTGTCCCGGTACGCGATGAACGAGTT

CAATCTCCACTCAAAGGCGACCATCGGGGTGGAGTTTCAGACTCAGAGCATGGAGATTGA

TGGGAAGGAGGTGAAGGCTCAGATCTGGGATACTGCTGGGCAGGAGAGGTTTAGGGCTGT

TACTTCGGCTTACTACCGCGGCGCGGTGGGGGCGCTGGTTGTGTATGATATCTCACGGCG

GACTACGTTCGAGTCGGTTTCCCGGTGGCTGCAGGAGCTTAATACACATTCTGATACAAC

AATTGCAAAGATGCTGGTCGGCAACAAATCTGATCTGGAGAACATAAGGGATATCTCGGT

GGAGGAAGGTAAGAGTCTCGCTGAAGCAGAAGGGTTGTTTTTCATGGAAACTTCAGCCCT

AGATTCTACAAACGTGAAGACAGCATTTGAGATCGTCATCAAGGAGATATACGGTAATGT

CAGCAGGAAAGCGATAAACTCTGATTCATACAAGAAGGAATTATCACTCAACAGGGTAAG

CATTACAAGCAGTGGAGCTGAGGAATCAAAACAAACTTCAACCAGATTCTCCTGCTGCTA

GGATTAATTGTAAGTACTGGGTGGAAAGGGCTTTATTTCGTGTATCAGTTATTGTTGCAT

AGAAAAAGGGAATTTGTTTGCGATATTGATGAAATTGCATTGAATTTTGGTAAGAAACAG

AATAATATGGTATGTCTCTCTCAAAAGAATAATATTTTATGCCTTCATTAGTTTTTGAAT

TTTTTGTGTGCGTTATAAATGCATTATGTTTTAAGAATAATATGGTATGTCTTTCTCAAA

AGAATAATATTTTATG

>Unigene0000488

GAATATCAAAACAAGCGAGCCCCACCCACTCAGATCCAACCCTAGCTTTCCAAGAACTGC

GATCACGACGCCTGACAATCTTGCCGCAACTGCGCCGGCGAGCAGCCGTCTCCTCCTTCA

ACGATTTCAGGTCCTCTTTCCTTGTCCCCGCAAATCACGTCTCCTCCAACGAGTTCAGGC

TTGACATGACAGAAACATCTGCTGAGAGCCTCCAAGTACTAACAGAATCAGTGGAGCATT

TACATGTGGAAAACCCTCGGTCGATATCCATTGACCATGATCATCATGATGCAGACAATG

TAAGTGACGATGACATCTGGGGTGAAGATGATGACATAAACGACACATCTTTGTTGAATA

GGGAGTGGAAGTTGAGGCATGACCAACATCACAAAATGGGCTATGTCGAGGGCATCACAG

CAGGCAAGCAAGCTTCTGCTCAGGAGGGTTTCAATACTGGCTTTAAGCAATCTGTACATG

CTGGCTACAAATTGGGACTTGCGAGAGGGATTTCAAGCACGCTGGCTAGTCTTTCGGATC

AACTAAAAGAGAAGTTGGCTGGGAACCCGGAGAATAAAGATAGGGTTAGAAGCTTACATG

AATCTTTACAAGCAATATCCACGAAGGATGCAGAGATAATGTTCTATGATAGTATCCGAC

AGAATGCAAGTAGTCAATCTTGTGATCTTTCAGATTTGAGTTCCAATAAGTCGGAGATTC

TTTTCAAGGAGCTCAAACTCTTTGTAGCCGATGCCCCGAAACTTGAAGTGAATTTTGAGT

TTTGTCAAGAAAGGGCTGGATAAGCTTCCTCTTAGACAAAGAAGTATATGTTCAAAGTAG

TATAATTTCATAATAAATTCTGTGTTGTATTATATTTCTTTGTAGCCGAAGCCCCGAAAC

TTGAAGATATTATATTATTAACTCATTTAA

>Unigene0000489

GTTAAAAAAAAATCCATTGCTCGTTAACCCGGTCCGAGACTGGCTCCATCAAGCCACTCT

CACAACTCCTCCCTCGCCGCAGATCGAGATCCAGCTTCTTCTTCATCTCCGGCACCGGCA

CAATGGAATCTTGTCCTTCTATAAAAAACATTCTTCTCCTTGATTCTGAAGGGAAGAGAG

TAGCTGTCAAGTACTATACAGATGATTGGCCAACACTAGCAGCTAAGTTAGCATTTGAAA

AATCTGTATTTACCAAAACTCAGAAGACAAATGCTCGTTCTGAAGCGGAGATAACAATGT

TTGATGGCTACACTGTAGTGTACAAGTTCATCCAGGACCTGCATTTTTTTGTTACTGGAG

GGGATGACGAGAACGAGCTCATTCTAGCAACAGTTCTTCAGGGATTTTTTGATGCAGTTG

GCCTTCTTCTGAGGAGCAATGTCGACAAGAGGACTGCACTTGAGAACCTGGATTTGATCC

TTTTATGCCTCGACGAAATTGTTGATGGAGGTATTGTCTTGGAAACCGAATCGAGCGTCA

TTGCTGGTAAAGTAGCGACACATGGTTTGGAAGGTGCAACATCCTTCTCTGAGCAGACTA

TTTCTCAAGCCCTAGCCACTGCCCGTGAACATTTGGCTAGATCTCTTCTCAAGTGATGCG

GGCCAAATGATTTATTAATGTTCTGTCTGTTACAATGAGTTAGATATGATTGGAACTGTG

GTTGTGCTTTCTTTTTTGCCCGGATGAATTTTTGCATGAACAAATTATTTCTTTTTGTTT

AAATTTATAAGTAGTTAAAAGCTCCGATGGAGCATTTAAGCACTTTTTTGGCTTACATGG

ATGGTTATGGTGAAATGTATTTAGACCTGATGGATTGGTTCATTTCTTC

>Unigene0000490

AAAAGTCGGAGAGAGATCGGGACTCGGATGTCACCGTCCCTATCAGACTTCCAAGGGATT

GCCTGCGGAGGGTATTGGGTATGATTATTGAAGATGGCAGAGAGGATGAGGTGGAGGTGT

ACAAGCTCGGACTAGCGGTGGAGGCAAGGATGGTGAAGTATGAGGAGGAGAGGAGGGAGA

TGGAGAATAACATTGTGAGTTTGATGGAGGAGAATAGGGATATGGTGAGTTTGTTGAGGG

TGGCGGTCGCAGAGAAGGAGGTGGCG

>Unigene0000491

TACTGATGGCTGAGCAACTCCTCTCCACCGGACCTCACCTCAAAGCCCTGCCGGAGAGCT

ACATCCGATCCGAGTCGGAACGACCAAAGCTCTCTGAAGTTCTCGCTCTCGATGACATTC

CGGTGATCGACCTAAGTCATCCTTGTAAGGGACAAATTGTCGCCGCAATCGCTGCTGCCT

GCAAAAGCCATGGCTTTTTCCAAGTGGTGAACCATGGGATCTCCGATGAGCTGATCAACA

CAATGATGACAGTGGCTCGCGAGTTCTTCCACCTCCCTGCTGAGGAGAAAGCGAGACTTT

ACTCCGACGATCCAACACGGACGGTGAGGTTGTCGACGAGCTTCAATATTCGGAAGGAGA

AGGTTCGCAACTGGAGGGACTATTTGCGGCTCCACTGCTATCCTCTGGATAAGTACTTGG

TCGACTGGCCCTCCAAGCCCGCTACGTTCAAGGACGTGACGGGCACCTACTGCAGAGAAG

TTCGCTATTTGGGTTTCCGGCTAATCGGACTGATATCACTGAGTTTAGGCCTCGAAGAGG

ACTATATGGAGAGAGTACTGGGAGAACAGGAGCAACACATGGCTATCAACTACTACCCGC

AATGCCCAGAGCCGGAGCTCACCTACGGTCTCCCAGCACACACTGACCCCAACGGCCTCA

CCATCCTCCTCCAGGACCCAAAGGTATCTGGTCTCCAGGTGCTCAGAAACGATATATGGC

ACAAGGTGGATCCAATACCCAATGCTTTGGTCATCAACATTGGAGATCAACTACAGGCAC

TAAGCAATGGAAAGTATAGAAGCGTGTGGCATCGGGCGGTTGTGAACAGTAATATCGAAA

GGCTGTCGGTAGCATCGTTCCTCGCCCCTTGTAACACTGCAATCATCAGCCCCCCAGAGA

AGCTCATCGGGGAGGGATCGCCAGCTGTCTACAGGGCCTTCACATATGATGAATACTACA

AGAAGTTCTGGAGCAGGAATCTTGATCAGGAGCACTGCTTGGAGCTTTTCAGGAGTTAGT

TGCAACTATCCAAACTGAGGTGCACAAGTTCCAAGTTATTATGGATACATAATTTGAAGG

TTTCTCTGAGTGGAGTGGGACCCTCTAGTTTTGATATATATATATATATA

>Unigene0000492

ACAGATTAACAACCGAAGTATATAACTATGTATTACACGCAAATATAAAAATCTATTGAA

ATTACTTAAACACTCTTAAAAAAGTTGAAATTACTTAAACACTCTTAAAAAAGTCGAGTT

AAAATTATATATCGATCCATGATGATTGTTGATGCAGCGGGTATTATATGTGCTAAATAG

AGTATGTGTCAGAGACTTTGTTCTAATTGATCATCAATCTGTAATTTTTATGTATTTTCG

AGTTAAACATCAGACTTCAGCTATTTT

>Unigene0000493

TGTGTTCAATTTTTTATAAGCTGAATATTTCAGCTTCCGACTGCTCCCGTACGGCCTCGC

CTCGCGTCGTTTTTCCCACTCCCGTTTGAAATTGAACTCTCGAGTCTCCACTCACCGGCG

GTGCTCACCTAGCCTATAAATAGCCTTCCCATCCGGCTAGCCTCCTCAGTTTCAAAGACC

TTCAGATCTCATCTCCTCCACTGAGCTCCATCTCTCTGATCACTCGGCCAAGATGGTCGT

AGCTCAGAAATTGAAGGAGGCTGAGATCACCGAGCAGGATTCTCTGCTACTGACGAGGAA

TCTGCTGCGGATCGCGATCTTCAACATCAGTTACATCCGAGGTCTGTTTCCCGAGAAGTA

CTTCAACGACAAGTCTGTTCCCGCTCTAGAGATGAAGATCAAGAAGCTGATGCCTATGGA

CGCCGAGTCCCGGAGGCTGATCGATTGGATGGAGAAAGGTGTTTACGATGCCTTGCAGAA

GAAGTACCTGAAGACGTTGCTGTTCTGCATATGCGAGAATTTGGAGGGCCCCATGATCGA

GGAGTACGCCTGTGAGTATTTCTCTCTTGTTTTCCTGCCGTACGATCCGAACAGATCCAA

CAGCGATTTCATCATTAGCAGCCGATCCAGTTTAGATCGGACGGTAGTGAGGCTTGGGAG

TCCGTACCGCATGTTCGTCAAACATAGTCACTTAATTTTAATTTAGTTGGTATTTTGCAG

CTGTTGTTTATGTATGATCTGATTAATTAAAACCTATGTTGCTCTTGATGATTTATACAT

TATCCATTGAAACTCGATAATAACGGAGGAATCCTGCCTCCTTGTTGTGCATAGTTCCAA

ATATCTGATTGATACGATTGTGTGTGGACATTCCCCATCTCTTTTATTAGTTATATACTG

CCAGTTATATACTGCCCTCGCCCACCTCTCTCTCTCTGATCTGATCCTGAATCAAATGTT

CGGTGAAATGCCCCGTAGTTTCCTTCAACTACGGGGATGCCAAGGGTGATGAGGTCTCGA

TGAATGTGAGCCGTTCCGGGAACAAGAAGAATGGAGCCACCTTCAAATCTAATGCGACAG

ACATCACCCCCGACCAGATGAGGAGTTCTGCTTGCAAGATGGTCCGGACACTGGTTCAGC

TGATGCGAACTCTGGACCAAATGCCAGATGAGCGCACTATTTTGATGAAGCTTCTCTATT

ATGACGAGACAACACCAGCGGATTATGAGCCCCCATTTTTCAGGTCCTGTTCAGGAAACG

AAGCTAACAATACCTGGACCAAGAATCCGTTGAAGATGGAAGTGGGGAATGTCAACAGCA

AACACTTTATGTTAGCTCTTAAGGTTAAGAGTATTCTTGATCCCTGCGAAGATGAGAATG

ACGATCTTCATGAGGATGAAGAGGTGAGCTTGGGAGCTGATTCTATACAAGAGGACGATT

CTTCTTCTGACAGTGAGATCCACCCGCCTCTTGCCGACCAATTTGTGATAGCTCTGGGTG

GTGGTGAAATAGATGCACAAGAAGATAATGGCATGGTTTCTGAAGCTGAAACTGAAGATC

CGGTTCATGAAGAAGAGCAGACAGCTCGTGTGAAGGAGTGGATCAAGACTAGGCACATGA

GCACAGTTGATTTAACAGACATTCTTTCCAGTTTCCCAGACATATCAATGGCATTGACTG

AAGATATTGTTGAGAGGCTCTTGAAAGAAGGCTTACTATCAAAAATTGGGAAAGATAGTT

ACACCATAGTTAAAGAAAAGGAGCAGCTCAGTCCTAAGATCTCTGAAGTGAAGGAAGAGG

TGGAGATGCAAGAGACCCCTCGCGCAGAGAAAATCTGCAGAACTGCTGATGAGGATTTCA

TATACATGAAGGCTCTGTTTCATGCTCTGCCCATGGACTATGTGACAGTGGCAAAGCTTC

AAAGCAAACTGGGCGGGGAAGCTAATCAGACTACTGTCCGTAAGTTGCTTGACAAGATGA

CAAGGGATGGTTTCCTCACAAACTCTGGCAACAGAAGGCTTGGTAAGCGTGTCATTCACT

CTGAATCGAGCAATAAAAAGCTCCTTGAAGTCAAGAAAGTACTGGAGAACAAGCTAGTGG

ATATGGAAATAGACACCAACGTAAAACCTTTTGAATTTAATGGCGCAGACATTCATTCCA

ACGGACACATGAGAGACGGCTCTACTTTTGGCGGCCTTCACTCGATTGGATCTGATCTTA

CTCGCACACGGCTGCAGCTTGAGTCACATCAGAATGGTTCTGTTTGGAGTGGTCAGGTTG

CTCAAACAAAGGTAGACCAAGGCAATACTACACCAACAAGCAGGGCTGCACCAATTTCCT

CTCTGGAGAGCGGAGTGGCTGGAAAAGACGGTGAGAGGATGAAGAGTAAGGCGTCCGGTG

GAGGGGATGACAGCCATTCCACTCAAGACAAGCGTCCCTGGAAGGCGAGCATGGTGAAGG

AGCCTATTCAGCAATACATGAAGCGCCAGAAGTCCCAGTCTAATCTGTGAGATGAGATCA

AGGAACCTGATATACTCTTATCTAAAGTGCCAGAAGTAACAAGCTCAGTGATTGCTGGAT

GAACTCAGTGCAGGCATACTTAATTTTGTCTGGATATGGTTTGAAACAGTTTAAGAGATC

TATTTCTAGGAGATTTTGTTTTTCCCCTCTAAAGATATGGTAACAGAGATGTATGCAAAA

CATGGTGGTATTTGTTTTAATCCTGATGTGTTTTCGTGGAACACTTGTCTATATAGCATT

AATCAGTTCTGTTGCAAAAGAT

>Unigene0000494

ATCTCGCGTGGGTCAAGAAGCCCGTCTTCGTCTTCATCTTCCCTCCACCGCCATCAAACC

CAAACCCCACCTCCAGCTCTTCCAAAATGGGCGCATCCTCCTCCACTGCTCAACCCCCCG

CACTCCACGAGCAAGAAACCCTCGCTGCATCCACCGTTCCCCTCCCCACCCTCCGGGCCG

CCTTCTCCAATCTCTCCCCCCCAAACCCTAACCCTAACCCCCTCCACGCCGTCTTCTCTC

TCGAATCCGTAACTATCAAATCTGAATCAGCCCCGCCCCACCTAGCCCTCATCCTCCCCC

ACATCGCCCCCTCCATCGTCTCCTACTTCTTCCCCAACCCCGCCGCTCTCACCTGGCTCG

ACTTCCTGCAGGGGTACAACAACTGCTGTTCCCGCACGCCGGCGTCGGCCTCCCTCAACT

CCCTCCTCACCATCTACCACACAGCCTGCGAACGCGCCGGCATCCCATCCAAATTGGAAG

TTCACGATGTTGATAAGATCGGCGGGTCGCTGTCGGCGGCTGATCTGGAGGTAATTTTGT

TGGTGTGCTGGATCATGGCGAGAGGGGAGGGCGGTGGGGTGGTGATCCCGGAAGTGGGGC

ATTTGGTGAGGTCGGCCTTTGTGGAGTGTGGGGCCGTGGCGGATGGT

>Unigene0000495

TGCAAAAAAAGCTTTCTTTGAATTAAAAAACTTTTTATATCATTACCCTGAAAGCATTTA

TATTATTAATGTAAAAAAAGATTTATTTGCTTTAAAACAACGCTTATTAAATTATGATTT

TGAAATATTAAAATACTATTTTAATCGTAAAGAATATATAGCAGTTATTAACCGAGGCGA

GGAAATACTTAAAAAATATCCAGAAACTTTTTCTGCAAGAGAAGTTTTAAATTATATTAG

ACAATCTTTCATTGAATTAAAAATGTTTA

>Unigene0000496

ACATGCACTTAGGTACTCACTAGCATCACCTAATTTATTAACTCTTTCTTTTACACTAGG

GAAGAGAGGAAAACACAAAGAGGTGCAAAACCGACTTTTACACTCCCATTTCCATTTCCT

GATCCATTCCTATACTTCCTATTTGCCGTCCAAAATCTGTGAACCAAACAAGAGCATCCA

TTTCTATTTCAATGGCATTGGAATTGGAAGAAGAGGGGAATTAACAAATCCGTGAGCCAA

ACTATGCCTAGTTTTCACTGTCAGAAAAACTATGACTAGTTTCCTTTT

>Unigene0000497

TTGTTTAGAGTGTGAAGAGGAAGATTTGAAAGAAATTAAAAAAAATTCCAAATATTGTAT

GTGGAAATTGATAAAATTTAACTCGCCGGAATGGGCATTTTTGTTGTTCGGTTGTATCGG

TTGTATCGTCAACGGCGGTTTAGTTCCTATTTATGCATATTTTTATGGACAAGTGTTTGA

GTCTTTAACACTAAAAGGGGAAGAGTTGAAAAGAGAAGCACGGTTTTGGTCTTTTATGTT

TGTCCTACTTGGAATAACGGCTGGGTTGACCATAGTATGTCAAACGTGGTTACTAACATT

CGCTTCGGAGAAGCTAATGATGCGTCTTCGAGCCATGGCATTTAC

>Unigene0000498

ATCCCAAATCCAATTACCATTATCATCAAGATCATGGACAGCAAAAGGATATGCATGGAA

CCTCCATGAATCTCCATCCTCTCTCCCGCCCTCTCCTCTTCTATCCTCGTCCCCCTCACC

TCCTCCCCAAATTCTTAATCCCCACCAGCGTCCACCACCATGGCTTCTCCCCTCTCCTCC

TCCTCCGCCGCTCACAGCGGAGCAGCAAGCGCTGCAGCACCATCGTTGTGAGAGCGACCC

GAAGGGAGTCCCCTTTCGAGGTCCTCGGCATCCCCCCCTCTGCGACCTCTCAAGAGATAA

AGCGGGCTTACCGGAAGCTCGCCCTAAAGTACCACCCCGATGTCAACAAAGAGGCAAATG

CTCAGGAGAGATTCATGCGGATCAAGCATGCATACAATACCTTAATAAATTCAGAAAAGT

ATGACTCCAACAATCGGGGTTCAGATTATTCAAGAACATCTCAAAGGAGCAGAAATCAGA

CTCAACAAGAAGAAGAATTCTATGGGCTTGGTAATTTTTTCAAGGATGTACAGATAACAA

TAGCCGATCTTTTCCGAGACTTACAAGCTGAATATCAAAACTGGGATGCAAGCATTAGCT

CTCAAGAGAAACCTAAAAGCTTGTGGGAGGAGTTGGCAGATATCGGTGAGGAATTTGTCG

AGTTCTTGGAGAAGGAGCTCAATATCAATGATACGAAATCCGAAGCTGAGGATGATACTA

ATGGGCCCTTTAAAGGGTACCCAAATACCAAGTCTAGAGTTCAAGATGAAGGCAATATTA

AAAGTAACATTGAAGAGAATATCGATGATATTGAAGCTGCTCTGGCTCAACTGAAAAAAG

ATTTGGGACTGTAGAAGAACTGCTATTGAAAGAATGTTTCCCAAATTTTTGTCAGGTCAG

TATTCTTTATGTGTAAATCCCTTCGAAGATATCCTTAGAGTGTGCTTGTGTTACAGCTGG

ATTTGTATAAAATTCATCTTTAATGAAATCGGCATTGTAATTACTAATTCTCGACCCTCT

ATGTAGATTAAATTTGTAGGGATTCTTATCACATTTGATCTTGTCGCTGTTCGGGGCGTT

TCAACAATCTTGTCTTAATAGATATTTTTAAGACGACAAAAGTGTAAATCCTAACTGAAT

TGCGC

>Unigene0000499

TGACTTTGACGACATCTTTCATTCGGACCCTTCGATGAATTGTACTACCATGGTGCTCTA

TGAAGCTGCATTCTAGATTATTGCAAGTTCATCAGCTTCAGAGGTCAACATAGAACTCAC

TAAGTAAGTTCTCATCTTACTTCATTCTGTTAGTCATTCTTCTGTTAGTCATGGTTTGTT

AAGCAAAAATTGTTGATTGTCATGGTTGTTAACTCACTAAGTAAGTTCTCATC

>Unigene0000500

GATCTAATGCCAGATTTGCTCAGCGTTCTGGAAGAATCGTCTGCGAAGCTCAGGAAACTG

CTGCCATTGTACCTGCTGCTTCAGAAGCTACCTGGGATTCACTGGTTCTTAAATCCGAAT

TACCCGTCCTAGTCGAATTCTGGGCTCCATGGTGCGGTCCTTGCCGCATGATTCATCCGA

TCGTTGACGAACTATCTAAACAGTATGCTGGTAAGTTCAAATTTTACAAGGTGGATACTG

ATAGCAACTCTGGTGTTGCATCCCGATATGGTATCAGAAGCATACCAACTGTCATTATCT

TCAAAGATGGTGAGAAAAAGGAGGCAATAATAGGTGCAGTTCCCAAAGCAACTTTGACTG

CCAGCATTGAAAAATTCTTGTAGAGATGGCTGATGATGAGGTTTAAGCTGCTTTCCCACA

ATTTCCTTATTTTAGCCTCTGATATCTGAAGATGAATTCTCTACTCTGG

>Unigene0000501

GCAAATATCTAAGTAGGTTTATATTATATTAAAATATGTAAACATTTACATAACTTGTAT

GAGTTATTAAAAAAAATGGTACCTACTATTTAAAAACGACAAATATATAACTTTGTAGAT

ATTTTATTTTATCGATACGGTTTTTATTTATACTACCGTAAGTTATAGTTAATTACTCTC

TTTTATTTTGTATTAATTATGATTTATTATATATTTGCACCTTATTTAAAAATTGCACTA

ACCTTGTCTACATTTTAAAGCCCAATAGCCACACTA

>Unigene0000502

TGGCCGATTTTTCGCGACTCTTTTACTGCAATTTTTGCCATCCCTGAGCCAAATTCTTTT

CAGGAGGCTGTCCGGAGCACAAAATAGCATCATGCCATGATTGATGAGTTTCTGGGCTTT

GCTTGGACTCAAATGTGATATGTTGTGCCTAGTCTTGCTAGACCTGGGGTGTTGTACCTT

ATCCTGCTGTTGACCTTATAGGTTGTAGGTTGGTTTACAAAGGTCAAGGATCGGCATGAT

GACACTACCGAGCTCCAGAAGGCCGGTTGATGGCTCATGGTTCACTCAAGAGCATGGTGT

TGACTATGAGAGAACATTTCCCCTGTTGCTCAACTGACTACTGTGTGCACACATCTTGAA

GTTACTGGAGTCAAACATTGTCCTCTCTGCAAGATGGATTTGAAGAA

>Unigene0000503

AGACATATTTGCCTTGTCCTATAGCATCGACATCCAAAGATATCCATTATATTATCATGG

CTTGCATCATGGATACTCCGCCATAATCTAAAATTTACTGGCGACGCTTGGGTACGTGTG

TCGCATACATACGTGGCGAGTAAACAAAAATCATAGTATTGAATAAGTACTGGATTACTG

GTTGGTACCCCCTAAATGAAGTGTCCCTGCTTCATAGAAAGCTTCATCT

>Unigene0000504

TACTAGAGAACCACCATTTTACATAATAACAGAATTTATGAGTAAAGGAAATCTGTTGGA

TTATTTACGGCAAGGCAATAGGGAAAATATTAATGCGGTTGTGCTTATGTATATGGCTAC

TCAAATAGCTAGTGCAATGAGTTATTTAGAAAGTCGCATGTTTATTCATAGGGATTTGGC

TGCACGTAATTGTCTTGTTGGGGAAAATCACCTTGTCAAAGTTGCTGATTTTGGTCTGGC

CCGATTAATGCGTGATGATACATACACAGCACATGCAGGTGCAAAATTTCCTATAAAGTG

GACTGCTCCTGAAGGCTTAGCTTATAACAAATTTTCAACCAAATCTGATGTGTGGGCGTT

TGGTGTTTTACTATGGGAAATTGCAACATATGGTATGTCACCATATCCTGGAGTTGATCT

AACCGATGTTTACCATATG

>Unigene0000505

AATTATTGGCCTCATGAGTCCTAAAGCTACACATCGTCTTACCCTTGTTCATCTCTTATT

CCATTGATTCTGAAGATATTTGTAGTTTGCTTGATTGCAATACACTGATTTGGCTTGTTT

CATTCATTAATAACTTCTGTCCAGGTGATGAGGCAAACTCATACTTTGTTCCATGGATCA

TATGTGCCACAGGTTTGACAAATTGGAATATTGAATTTCAATTCACTGATCATCGCCCTA

AAACTTGGTAATATACCGGGATTATGAAGGTGCAACACACCAGTAATCTGCTTTTGATGG

CAATTGACGTGCAC

>Unigene0000506

GATGATGTAGATTTATTTGGGTCCGATGATGAAGAGGAGGATGCTGAAGCAGCCAGAGTT

AGAGAAGAAAGACTTAAAGCCTATGCCGCTAAAAAATCAAACAAACCAGCTTTAATCGCT

AAATCTAGTATTGTTCTTGATGTGAAACCCTGGGATGATGAAACGGACATGAAAGAAATG

GAAGCTAAAGTTAGGTCTATCACCAAAGACGGTCTTGTATGGGGAGCTGCAAAATTAGTT

CCTGTAGGGTATGGAATCAACAAACTTCAAATAATGTGTGTTGTGGAAGACGACAAGATC

TCAATTGAAGAACTTAGTGAAGAAATCCAAGAATTTGAGGACTTTGTGCAGTCTGTTGAT

ATCGCTGCCTTCAACAAAATCTAGTGTTATTTTTTATCCAATGTACATTTTAT

>Unigene0000507

GTTCGTTTCAATTTCAGTAATTTGAAGAACTTATAAAATGGATTTGAAGTGTGTATCGAA

TATAAAATGTGACCAAGGAGCAATACGTTCTGTGAGGTTTAATGTGGATGGTGAATATTG

CCTTACGTGTGGCAGTGATAAGAAAATTAAACTCTGGAATCCATTTAAGGAGTTATGTTT

GAAAACGTACGCTGGCCACGGAGACTGTGTAATGGATGCATGTGGTTCATGCGATAGCAG

TGAATTAGCATCATGCGGGGCTGATAAA

>Unigene0000508

TAGTGTTCAATGAAGATGTGCTCTCTTATGATTCGAGCAAGGAGGTTGTGAAGAAGAAGC

AACTACATGTTCTTGTGATGGATGATGAGTTGCAGCTAATGCATGTGTATGAATGTCTTG

ATCTCAAGCAGCCAGAGGCAGAGCAACCAAAATTTTCATTACTGCCGCATACACCAATAT

TAGGACGTCTAGAGTCTCCAAGCCTACCCAACCTTATTCGCTGTCCCTACATTATCTTAG

AGGCATGTTGAGGCATGCTATATGGAATGGAGGTGTGGACATTCAGCTGTAGG

>Unigene0000509

TGAATGATCAGCTTCAACATTAGGTTTGATCCAAAGTCGGTTCGCTGTTTAGCTCCGACC

GAAATTTGGTGGTAAGCTTTCTTGATCTTTTCTTTTACCCAGTGATAGTGAATGACAATC

ACCCTCTCTCCACATCCAAGTTCTCCTTCTGAATTTATTAAGAAGGAATATTAACAAACT

TGAACTTGTTTCCTCAAAACTTATACAATTGATGATTCCGTTTGATTAAAAAAAAACTCG

TTGCGTCCAAACAGAAGAGACTTCCACCGATTTGGTATCAGACACACCTTGTTTGTTGCC

GGATGAATACACATTTTAGAAAAGATGCGATAAATGCGCACGCACTAGGTCATTATGCGC

ACGCAATATTATCCGCGATTGCGCACGCATTCTAGAAGATTGCGTGCGCCCTTCATTCCT

GAGAGCACCTTTCTGAGAGCCTTCCATAATGTGTACGCTGGAACTCCAACTGCGAACACA

TTATGATAATACGCGCGCATATCAAAATAAATACGGGCACATTCTTGCCGCAAGCCTTCT

ATTATTGACTGTACCAAAATACGGGCACATTCTTGCTGCAAGCCTTCTATTATTGATTGT

ACCAACCTGGTTGCCTATGTATCCGCACCTCAGAATCAAGTCTCGCATAGTTCTTGGTGA

TGGTGTGATAGACTCCACACCACTATAGCCCAACAAAAGGATATAGCCAATATTTTCCAA

CAACCGTGCTCCCTCAGAATTTTGAATTCTTAACTGTTAAAAGTGCGAATCTGAAACCAG

TCCACTTATCAACTAAGCCCAGAATTTTGAATTCTTAACTGTCAAAAGTGCGAATCTGAA

ACCAGTCCACTTATCAACTAAGCCATATCTGACTGCTTCTAAAATGTCATTTTAATGACA

ATCTGAAAAGCTTTTATTTAAATAATTAATTTTATTTTCCTCTTTTCTGCCGTCCACCGA

TAAGGCAATAATTCCCCTTAAAAGAACTGCCTCTTTAGAAGCAGAACCACCACACAGCTC

AAAGCATCCTTATAATAAGGGGTGTTCCCACTTGTTATATCTTCTCCCTCTCATTGCCCT

CCGTTTACACCAACACAATCTTAGATGGCGGCAGACAGTGCTGTTTCGTTCGTTGTAAAC

AAGCTCAGCAACCTCCTAATCGAAGAGGCATCTCTACTGGTTGGTGTTAAGGGCGAAGTC

GAGTGGCTGCGCGACCAGCTGGGATGGATGAAGTCCTTCCTCAAAGATGCCTACACCAAT

CGCCGCAGAGACGAGCGAGCAAAGCACTGGGAGAGGGCTGTCAGAGACACCGCTTACCGA

GTCGAGGACATCATCGATTCCTACATGATCGAAATAATTAGCCGCCGTCAGCAACCACTC

AGGTGTTGTGGGATTTTGAAGCCAGGAGAAAGGTACGCTCGCGAACTAAAACACCGCCAC

AAACTGGGGGCTGATATCAAACAACTCAAGGTGAAGATCCAACAGATATCTGAAAACCGA

ACTGCATATGGCCTTGTTGACCTTGGCCAGAGCGCATCAGACTCAACCATGGATGCTGAT

ATACAGGAACGAAGGAGAATATCTCCTAGCTATGGCGACAGTGTCGTCGTCGGCTTTCAG

GAACACAAGCGTAAGATATTGGAAGAAATACTCGATACCAACCGTACTCGACGATGTGTT

AACTCTATCGTTGGTATGGGTGGTCTTGGCAAGACAACCCTTGCCAAGATGGTTTTCAAT

ACTGTTCAAATTACGGAATCTTTCAGTACTCGAGCATGGGTGTCTGTATCACAAGATTAT

ACACTTATCCAACTCTTAAAGAACATAACAAAACAGATCACAGGCAGTGAATCCGAGGGA

TTGGATGAGTCGGCTCTTCGGGAAAGGATCTACCAACTCTTAAAAGGGGAAAGGTACAAA

TCGTTTCCAATGTTTCTTCTTGTTCATTAATTTCTATTGAGTCTTTGATCCAGGCAAGTA

GTCAGAGGTAGGCCATAAATCAGGTCAGGACAGGTTGTATCCCGGTGCTC

>Unigene0000510

GCTCACTACCCGTTCTGGGGAAGTCGCAAGATTTGCAAATCCTAGTATTGAACCCTACCA

ACTCCCCTATCTAAATGTAAAGGAGAGTTGGGAACTATTTCTTAAGAAAGCATTTCCCAT

GACTCATGAAGCAGTACCGGAATGCCCCCGAGGTTTGGAGGATATTGCTGGAAGACTGGT

TAATAAATGTGGAGGTCTGCCTCTTGCGATACTAGTGTTAGGTGGACTCATATCAAGTAA

AAAAATGACTCCTAAGGCGTGGATGAAAGTGGACGAGAGGATGGATTGGGCATCCCCACA

AGATGGACAACAATGTCTCAAGATATTGGCTTTGAGTTACGACGACATTCCAAACCCCAA

CATGAGATCCTGCTTTTTGTACATGGGTGTCTTCCCAGAAGATTCACTGATCCAAGCATC

ACAACTAATCACATTATGGATCGCCCAAGGTTTCATACCACAGTTGCCAAACCATACGAT

GGAGGATACGGCTGTGGATTACTTGGATCAACTGGTACAGAGATCCATGATTGAAGTAGT

CGAAAGAGATTATGATGGAAGCATCATGTTATGCGGGGTTCATGATCTGCTGCGAGACTT

GTGCTTAAGAGAAGCAAGAGAAGATGGATTTCTTCATGTCTGCCGGGGTAGCACTGACCT

TGGTACTAAGGGAATTGAGCACCATCCAGTTCGCCTAGCA

>Unigene0000511

TGCACTATAGGCTTTTTACTGAATAACCTGAACAACAATCCTGGCAAAAAAACAATCTTC

ATGTTATGAGATTCAAGTCTTGGCTGATTAAAGTGGCGGAGAACAATAATGAGGATTCAA

ATATTTTAAGTTTGGGTGTGCGAGCTGGTGTGCGTGAGCTTGTTATGTTATTTATTTTCT

GTCTAGGAACGTCCCTCCCTGTCAGGGGAATAGTTGGAGGTAGGATTCCCTTGACTTGTC

GTGGATCTCCAGCTCCTGTCAGGGCCACTCCTGATTCGTGCAAGACTGTTCCCCCTCCCA

GCGCTCGCATCGCTCTAGAAGGGATAGGATCCAGTGACATAGACATCGTCCCCATAATCC

TCTGCTTAGTGTGCACGGCGGCCAGGGGGCCATGATGATAGCACATGGTACTCTTCTCGC

CCTCAAACCAATGTCGGCTGTGGGTCCCTTGTCCTCGCCTCCGAACAAACTCCACCAGTT

AGGAATTTGTAGCTTTGTGCCACACTAGACCCCTTTGAAGAGGATGTTGCCACATCGGAC

AAATTTGATGTCATCAGATTAGGTTCCATTGTCGGGGTTTCTCCCACATTAATGGCGAGG

ACAGAAAAAAGGCGGGCTACTGGCATGGTGGTTGGCAGATCTGGCCATCAGGCCGCCCAA

TCAGTCCTGGCTACGGTGTTACTCGCCGGGGCCTATATCTGGGGACTCCCGGAGGTGCAC

ATCTCAA

>Unigene0000512

GGACAGGTTGTATCCCGGTGCTCTAGACCCGATTCTAAAAAAATGGGTCTTCACGAGAAA

TCGGGTCGGCCAGATCCAGATCTGGACTTGTGTTAAAATTAAAAAATTATCTTTTAAAAC

AAGAGAAAAATAAAAGAATATGATACAAACTTTTACTTTATTCCATATAGTTAACCAATA

CCATACATAAAAAAAATATTGTTCCTCCGAAATATATAATATACATTTTATATGAACTTT

TTATATGGACATAAATATAGCTATTTAATCGGATCAGATTTTTATCAAACTAGAACAGCC

TAGATTCGGACCAGACCAATTTTTTAATACAATAATCGGGTTGAAATACAGCACTGACCT

GATTAAACGGATCATACTGACCTGATTAAACGGATCATGATGGAGCCTGACCTGGGTCTT

AGAATAGATTGGGATGGAGTCCGAGTCAGGTCTCAGAATCGATCTAATTCATTGCCAGGC

CTATTAGGAGGTACATTAGATTAATGCCTCCAACCCATCTGGAACGGTCCTGAGGGTAAT

TTGGTAATCTTAGAT

>Unigene0000513

ACATAAATATAGCTATTTAATCGGATCAGATTTTTATCAAACTAGAACAGCCTAGATTCG

GACCAGACCAATTTTTTAATACAATAATCGGGTTGAAATACAGCACTGACCTGATTAAAC

GGATCATACTGACCTGATTAAACGGATCATGATGGAGTCTGATCTGGGTCTTAGAATAGA

TTGGAATGGAGTCCGAGTCAGGTCTCAGAATAGATCTAAATTCATTGACAGGCCTATTAG

GAGGTACGTTAGATTAATGCCCCCAACCCACGTGGAACGGCCCTAAGGGTAATTTGGTAA

TCTTAGATGTATTTACTTGGGCCACCACAAGCTAATTTTCATTATGAGATATTTTTCTAT

AAAAGTCAATGATTAGTCATCCTATAAAAGTCAATTTTCGTCCTATAAATGTCAACAATT

AATCATGTGAAAAAAGTCAATGATTAGGTACTATATAGTTTTTCGTCTGGTTTAATTTGA

AATAAAGGTAACCAATGACAGATTTCTCTCATATATTCTTCTATTGATTACGGATTATAT

TCTTCTATTATTGTTTTTTCAAAGTAAGAACTTTTGACCACTTTGAGTTATATATGTAAA

GGTACTTCATTGTATTGGATGATATTTGGACGACCGGAGTATGGCATCTAATAGAAGCAA

T

>Unigene0000514

TTTTTACTGAATAACCTGAACGACAATCCTGGCAAAAAACAATCTTCATGTTATGAGATT

CAAGTCTTGGCTGATTAAAGTGGTGGAGAACAATGAGGATTCAAATATTTTAAGTTTGGG

TGTGCGAGCTGGTGTGTGTGAGCTTGTTATGTTATTGGGGTTGGATATAAAATACTGGTT

TGTGAGAAATTGTTTCAAAGGGAATTTCCCATGACCTACTATAAAATCGTCCTTGCTATT

T

>Unigene0000515

CCTGCATCTGGAACACAAGTCATGTATCAGGAACAAGCTAGTTGGGCAAGCATAGCGTAC

TATGAATTAAATAGTAGAGTTGGAGAACTTTTTCATTGCCAATCTCCTGTTGTGGTTGTG

GATGGTTTCACTAACCCAAGTAATAACTTAAACCGATTTTGTCTTGGACAACTTTCTAAT

GTTAATCGAAATCAAACAATTGAAAACACAAGACGACATATTGGAAGAGGCATTCAATTA

CATTATATTGGCGGTGAAGTATATGCAGAATGTTTATCAGATTCTGCCGTGTTTGTACAG

TCACGTAACTGTAATCACCATCATAATTTTCATCCATTAACTGTGTGTAAGATACCAGCT

GGATGTTCTTTACGCATTTTCAATAATCAACAATTTGCAACTTTACTCACAGAATCTGTA

AATTCTGGTTATGAAGCGGTATTTGAGCTCACTAAAATGTGCACAATTAGGATGTCATTT

GTGAAGGGCTGGGGTGCAGAGTATCATCGACAAGACGTTACCAGCACACCTTGTTGGATT

GAAATACATTTAAATGGACCATTACAATGGTTAGACAAAGTTTTGTGTGCC

>Unigene0000516

AGACCTGATACTTGGACTTCCAGACTCAATGGCCCATGACAGAGGTGCTAGTGTTTTTAT

CTGTTTGAAATATGGGGACTGGATACCTCTAAAAACAACAATGGGACTGGACAAAAGAAA

ATGAAATTTTTTAACATTCATTTCTGAAGCTTGATCCGTGTATAATTAATCCTTGTCATG

TAGGATAGCAGTGTCTATTCAAATTACACCGCTGTAGATGCTCTAAGTTCGTGGTTCAAG

ACTCCAAATGACCAAAATCATCCTATAACCGCCACCTGGTGATCTGGCAATGATAGCCCT

CCAAATTAGATCTAAAGACTACGATGCCCCATAATGTAAATATTAAAGGTGGTCAAAACC

ATATATGTAATTACGTAATGCCATTGTCTAAAACATGTCTTATTAGGAGCATAAACGAAG

TATGCTGGATTTGTATGCAATTTTATTCACAATTATTCTTCATATATATTATTTATGACA

TGCTTATGCTATTCAGAGTTGACTAGTGGTCTTCGTATATACTGTGATGTAGAAAATCTC

ACTCTAGACTTAAATGTTATCATGTGAACAATCTATGGATCATTAGAATCTGTTTAAATT

TCCAAAATTAATATAATTATTTAGAAACTTCTAATAAAAAT

>Unigene0000517

CCAACTATAGTTCTCCCGCCCGCTCCTCCGTCGCCAGCCGCCACTTTCTCCACCATCCAA

TGCCGCCATCACTTGACACCGGCCCCGTCCATCCCGGCCGAGTCCGCACCCTCAAACTCG

GCATCCCCCTGCCGACGCCGTCGAAACCCGGCCCAGTAATCTACTGGATGTTCCGCGACC

AGCGATCCCGCGACAACTGGGCCCTCCTCCACGCTGCCCACCTCGCCTCCCTCTCCGCCT

CTCCCCTCGCCGTCGCCTTCACCCTCTTCCCCCGCTTCCTCGGCGCCCACGCCCGCCAGC

TCGCTTTCATGCTCCGTTCCCTCCGCCTCCTCATCCCCCACCTCACCTCCCTCAACATCC

CCTTCTTTCTCCTCCGCGGCGAAGCCGCCGTCACCCTCCCCCGCCTCATCAACTCCCTCT

CCGCCTCCGCTCTTGTCACGGATTTCTCCCCTTTGCGGCCCGTCAGGGCCGCCAAGGACC

AGATCTGCGAAAGCATCTCGAATCTCGTCACCGTCCACGAGGTCGACGCTCACAATGTGG

TCCCCGTCTGGGCGGCGTCCGGCAAGCTGGAGTACAGCGCTCGGACACTCCGGTCGAAGA

TCCACCGGCTGCTGCCGGAGTATCTAGTTGATTTCCCGGATGTCTCGCCGGTTGCGCCAT

GGAGTGACGCACCCCCGCCGGTGATTGACTGGGATGGGATTATCGATGAGGTTGTAAGGG

AGGCGGGGGATGTGCCGGAGATCGGGTGGTGTGAGCCGGGAGAGGCGGCGGCGATGGAGG

TGCTGAAGGGGAGTAAGGAGGGGTTCTTGACGAGCAGGTTGAAGAGCTATGAGAGTGACA

GGAATGATCCGGTGAAGCCGAAGGGAGTGTCGGGGCTGTCGCCGTACCTGCATTTCGGGC

AGATTTCGGCGCAACGGTGTGCGTTGGAGGCTCGGGCACTGAGGAAGATTGATCCCAAGT

CAGTAGATGCATTCCTAGAGGAATTGATAGTCCGGAGAGAACTGGCAGACAACTTCTGCT

ATTACCAGCCCTATTATGATTCGATGCAAGGTGCATGGGAATGGGCTCGCAAGACATTGT

TAGACCATGCTGCTGATAAAAGAGAGCACATTTACACGTGAGTTCGTTCATTGCTTCTTG

ACTTCATTTGGTTGGATATATTTTTCACTCAATTTCATTTCCTAGGTTGTAGGAAGGAGC

AGCTGGAGAAAGCCACAACACACGATCCTCTTTGGAATGCATCGCAGCTGGAGATGGTCC

ATCATGGAAAAATGCATGGATTCATGCGGATGTATTGGGCCAAAAAGATTCTTGAATGGA

CCAGCGGACCTGAAGAAGCACTTTCAATAGCAATATATTTGAATGATAAGTACGAGATAG

ATGGTAGAGATCCAAATGGCTATGTGGGCTGCATGTGGTCTATATGCGGCGTCCATGACC

AGGGCTGGAAAGAGAGACCCGTGTTTGGCAAAATACGTTATATGAACTACGCTGGCTGCA

AAAGGAAGTTTAATGTAGATACGTATATATCATATGTGAAGAGGCTAGTTAGTGAGACCA

AGAAGAGGAAATTAGACAACACGGTGGGTCCAGTTGATAAGCACATAAAAGTCGCTAAGA

CAAACCCAATCTTAAAGATTATTAGTCGAAATGATGGCAATTAAAGATCGCCTTAATGGC

TAGTTGAGGTGAAGATTCATATTTTTATCATATACAAGGTATGTCAGGTGAAAAATATCA

TACTGGATGTAACTTTGGATTGAAACTGTACTCTTTTGTTTAACTGTATATTTAGTAATA

CTAGGATGTTAAAGGCAATGGACTAATATTTCTTTTTTCTTAATTCATGTCCTTGTATGT

ATGTAAACCAATTGTTGAACAT

>Unigene0000518

GCGTTTTGGGATCTATATTAATTTGATTTATTTAGAATTTTTTTCTCATTCCAGTTTGGT

ATCAGATCATAGGTTGTGGGAAGTTCAAATTATCCGATGGTGTGAGTAGTAGATATTTTG

GGGTCTAAATAAACCGATTGCTAAGTTGGAGTTAGGTTGAGAGTCTATGTCTAGTTGTCA

TGAATACCTCCTTATTTAGAGGAGATTGTTACTTATCCTCTAATGTTGGGAACCTAATTT

TTTTTTTAGGATGGAGG

>Unigene0000519

TTGCTGCTCAGGTGAGGATGATACGTATGCAAGAATAAATCTAAAGACATATCTATGCCT

TGATTTTTGTACAGCTTAACTCAACATGTAATACCTTGTAGATGTTTGGTTGAACCTGAT

ACGACGCTAGGTGAAACTTGTTCAGATACTGCCTAACTAAGGGGTGGGTAGGTAGAGTGT

ATCCCGACTTAAAATACACACTATATATTAGTAGATCCTCGGGATCCTTGGTGCATCCCT

CTATCACCTCCCTTGTTGTCGGAATAGACAAGCTGAATTCATCTGGTATCTCATGCACAT

AAATCATAATTTCCTTCAT

>Unigene0000520

ATCTGAAATTGTACTGGAACGAAGATTTGGGTGACGTTGAATATCAGTTCAAAATTAGAG

ACAGAACGGCTCCCGGATTTTGGACTTCGTCTGGATTTCAAAACGGCACCTTAGTGCAAG

TGACCAGCTACGACCAGTTCGCGGGTATGGTGTACGTCCGGAAGGCGATCAGCAATCACA

TGGTGTTGACGTTCTGCTCGCCGAACACGCAGTTGTACAGCGTGGTGCTGGCCAGGGACA

AGACCCTGGACCCCAGAGACCTGAAGAGTATCGTGAACCACATGCACTTGCAAAAG

>Unigene0000521

CTTATCGTCATTATGAGATTTTCACTGGCCATCTTCTTCGTGGTGTGCTTGGCTCTGATC

AGTCTGCCCACGGCATTGGGCGACAGGAAGGGCTGCCGCATCCGTGACTCCAAAACAAGC

TGCTACTCTGACCAAGAATGCTGTAATGAGAGCCACAAATGCCAAAAACAAGGTTCATCT

TACTCGCAGTGTATATCGATGATGGGATAAAAATCCTCTCCGTTTTGCGTTGCTTAGTTT

CCCAATAA

>Unigene0000522

GGGCAGTTCCTGGTAGTTTTGGCTTTCCATGCTTTCAATGAATCTTTTAAGCCTGAAGAA

TTGTAATTGACACTCCTATGCGTAACGCCCATTGTTGACCTGTTGCAGTACTGTACTGTA

AGTGCACCTACTGCTGGCCGTGTTTTAACTGCTTGCTTTCTGGTTTGAGAGATGCAGTGG

TTCCTTTCACTCCTGGATTAATATCTAAGAAGATAACTCATTAACTGATG

>Unigene0000523

ATGATTTTCCCTAGCACCGGATTACTGATCACATTGATCTTGCAAATTGGTTTATATCGA

TGTGGTCTGCCACCACCATCGAACGAAATCACTCAAAACTTTCTGTGGAATACTGATGCG

GTAGAGTGGAGAATCGGTGAATGGGAACCGTGCACTAGATCTGAAGACACCGGGCTAAAT

GACAACCAGGGAATACGCCGCAGGAAGATCACATGTGTCATGGTTATACATGACGGAATA

AAACTTGAGGAACCAGTCAGAATTGAAGATAGTAGTTGCC

>Unigene0000524

AGAACAACATTATTGTTTTTGTAATTCTCTGATTCGCCCCTGATTCACTCCCAGATCACT

ACAATGTGAGCCAAATTCCATAACATGTTCTTACTTATTCCGCTTTCAAGCCTTCTCTTG

TGTGTAGCACGCCTAATACCGACTTGGGGTACAAGTGAATTATTCATGTGTCATTGCATG

TCTTAAGATTATGATTCATTCTTGAGCTTTAGGTTGTGTCGTGTATTATGT

>Unigene0000525

GTAAATTGGCTAAAAATTTGCGACATTTTTCTTTAGTATTATCTTGTTGAATTTTTTGAT

GCGCCTGTTGAGGTTGAGCTATCGTTTGAGTTGGTGTTGTAGTTGGTGTACCAGCAATGG

AAACATTGTTAGTTGCAACTGAAGGAACAGGTTGAACTCTAAAATTTGCATTATTTTGAG

TAATAGCATTTCCTTGACTAGGTGCTGGGCCAACTCGTAATAAC

>Unigene0000526

TAAAACCCTGTTTCGAAGCATTTCCCACATTATTGAAACTGACATCATCGTAGGCAGCTA

GAAAATGCCCTTCAGTGATGACATCATGAGGCCTCATCGCCTTGCTTGCCACACTCCGGT

ACCATAAAGAGCACATGTGTCGTGCCGCAGCTGATGAAGCAAGATGCATTGACTCGCAGT

GTTGTCTTTGGCATTGACACGGGTGACATCCCGAGACCTGCAAAGCTCTCCAATGTTGCC

GAGAAATGCATCCAAGAAAAGCATATGTGTCGTGTCGCAGCTGATGAAGCGAGATGCATT

GACTCACAGTGTGCATTGACATGGGTGAC

>Unigene0000527

AAATTCAGAGTGTGTCACAGCTCTTATTATGCTGGCAGAAGAGGAAGCTCCAACTATGCT

TAAAGTTGAAAAAATTTTACGTAAAGCATTAAAACTAGCTGAAGCAAACTACAAAAAAAC

ACAAAAATCTCATCATCTGGACTCCAAAATTGAAATGCAACACAGACGAGACCTAAATGT

TTTGATATATATCAAAAGGCGTATAGCTATGTGTTGTAGAAAACTAAGAAAGTTAAAAGA

AGCTGTGAAAATGTTTAGAGACTTAAGC

>Unigene0000528

GTCAGCAGATCTTCTTTAGATAGTCATCCAGACTATTCTGCTTATCAAAAAACTGTAGTT

GTGTTTCCACTAGTGGATACCTCTTGCATGATCGGGATAAATTGGAAACCATGTACATCT

TGGTAATTGAGTCTCTATCTCTAACGGGTGCATAGAGATGATTATTAAGAATATGATTTA

TAAAGGACGAAAAATTTGATCAATATATAATGTTATTAAATAGGTAACTTGTTTGGTTCT

TGTGTCGCAATGTCAGGTTCTTCTACTTACATATTTTTAAACGATCAATAATTTGGTGAA

GCCTTACAAATTTCATGCTTTGAATTTAAGCATATCTCTTGTAATAAGTTTCAAAATAAT

TGAGGGTTATTTTTAATTTTCATGGTTAAATTGGGAGCTTGTATTAAAGTGTCCCTTGTC

TAGGGTGTAGTTGACAGAAGAGTACTAAATAAGCATGATTTAACGCACTGAGCAGATGGA

TTTGTCAAAACATGTCGATTGATACCTTTGTCTACTGCTGTGGTCAACAAAACAGTGAAA

CGCCACTTAGTTTTTTTCAGTGTGTAACTGTTCGACCAATGATTAACGGGTGATGATTTG

AAGGTCAAAAAGTCTAAAGCCTGCAGTCGACGAATGATGTTTTAGTGAGCGAAGTAGCAT

TGACTTCAACTTGCAGCAATATGTTCGATAACTAACAGCTGAAGTTCCTTCCATTTATAA

TACCAGTTCTCTAGATATAGCCCTAGATATTGGTGCATTAACATAAACACCTTTCAGAAC

CTGGAATTTGATAGACTAACAATTTGCTTACTTGCACCCAAAATAATCTAATAAATTACA

TGGTAAACACAACTTTTTTCATTGATAAGATGAAAACATAAAAGCTTGAATGTGAGTGTA

TTGACAGAGTAAACATTAGCATTAAAAATTCAATCAAATGCCCCAATGTCAACCTCCAGT

TCCACACTGTACATATTTTTTTACACATCTACCAACTTGTGGTTGTAATGCACGGTAGAG

CATAAGTGGAGTGGTAAGAGAATCAAGGAGAACTAACATTGTGCCCTATATGTGTTTGTA

CATAAACCAACCAAAAGCAAGACTCTACTTCACCCTTTCAAACCAAACTCATTAAAGTTA

AGATGCTCTATCCACAATCTATACTGTTTCTGCCCAGCTCAGGACTTTATCCCTCCTAGG

AATGACCTACTATTCTTATCCACCTGTTTTGCTTTTCAATTCAACAAATAAGAACGACAA

ATAAGCCACACGAGAGATGTAATATTGCGGATTTCTCATCTGATAGTAGCCAACGACCTA

CGATTATTCTGCTGTGCTATTCTCTTTGTTTTCTCCTTGTTTCTCGCTCTCTCAGGTGCT

AGGGCAACGAGAACCCTTTATTGAAGGAGTCGTTGATGTTATTGGAGGCAGATTAGGGTT

TTGGAGGGCTTTACAGACTGGATGAAGATTTTGAGGAGCTGCATCTGATAGGCTTCACTC

CTACCACCAATTGGCTATGTTACTTTCCCTTTATAAAGTTATCTCCTTATTTCCTCGACT

TTTGACATATAACCTTCATAGCATCCGTGGCTTTATGGCGTTCACGAACCTAGTTCTCTA

ACATCGCTTCCTTCGTCACCTATGATTATTCTGCTGCGCTATTCTAATTAGAAACATACC

TGGCCTGCGAGGAGAAGATTGGAACACCTTTGTGATCCCCCTTGTTTTCTCCTTGTTTTT

CGCTCTCTTGGGTGCTAGGGCGACGAGAACCTTTTCCTGGAGGTATTGTTGATGTTATTG

GAGTCAGATTAGGGTTTTGGAGGGTTTAAAGAATGAATGAAGATTTCGAGGAGTTGCATC

TGATAGGCTTCACTCCTCCTACCACTGATTGGCTATGTTACTTTCCCTTAGTAAAGTTAC

CTCCCTATTTTGCCGACTTTTGGCATGTAACCTTCACAACATCCGCGGCTTTAAGGCATT

CATGAACCTAGTTCACTAACATTGCTTCCTTTGTCACCAAATGAAGTTCTTGTGGATCCG

CTACCTGGCTGACCTAGGTGGCCCAAATTAAAGAGAGAAATGGTAAATAAGAAAGTTGTA

CAACTATCAACTAATGAAGGATCTTTGGGGGTCAAAGATCTTGCTGACACGATGGTGGAA

CTTCAACACAAACTGGCACAGAAATTCTGTGAAGGGAACTCCCTCTGGGCCAAGGTTCTG

CGCAATAAACATCATGACCACCTATCTGTCTATTTTCCACCCCACTGAAATCCTCCGGCC

ACATTTGGAACACCCAAAATTGAAGTTGATATGAAACAGTCCTCCTGGAGATTAGACCGG

ATGGGGAGAGGAGGCTATTCTGGATGCCATTTGCGCCGAGATACTTTTTTCTCGGAAGCA

TTAGGAAATTACTCAGAGATTGTAACACCAAACAATAGTGGATGTCCATTGTGTGGAACT

AAGACATCCTCACGAAGCACTCCATACCCACCTGGAAAGTCATGAATGCCTACAGAAGAT

GGTGTGAAAGATTCATGTTTGTATGTCAAAATATGCATGTTTTAATAATCAACTAGAAAC

GGTGGACCACCTCTTTGTGAGTAGTGAATTAGCTATATTTGTGTGGAATGAACTTGCACC

AATCATGGATCTGAGAGGGATTGTGTTCAAAACTGGTGTAATGGGTGCAACATGGACTAA

TTGGAAACATGAAGAATTGAGAAAAAGACATCCAAATGTTTGTGGCTGTGGTGTTAGGTG

TTTTCGCGGGGAGTAGCGTATGTCATTGCCGAAGGGGTTGGATGTTAGGGTCGAGGAGGT

TGGATGTTAGGGTCGAGGAGGTTGGATGTTAGGGTCGAGGAG

>Unigene0000529

CCGGAGGAAGGCGGCCGGCGGAGGGAGTGCGGCGGAGCAGCTGCCAGTATGACGTTCAAG

CGAAGCCGCGACCGTTTTTACAGTACTCGCTGTTGTGGTTGCTGCCATGTGCGGACCGGC

ACCATAATTTTAGGGACCTGGTACATGGTGGTGAACTTGCTGATGGGCATCTTGTTGACT

GTAGAAGTGACTCATCCAAACGTAGTGCCAACTGTTAATATCCAGTATGAAGTCATTGGA

AATTACTATGCTTCTGAGAGAATGGCTGAAAATGCCTGTGTTCT

>Unigene0000530

AAGTATTCATCATCGTATCATTTTGCGTGGTAAGCTCGCTGCAGTTTACGCCTTTCACTA

AATGTAAAGATGGAAATTCACCCTCAATGTTGCGTATTGACGGATGTACATCATCACCCT

GCAAATTCAAAAAGGGCACCGATGTATACGGAGAATTTGATTTTGATGTTGTGACCGACA

CTGACAATCTTCGAGTCGATGTGGTCGCCAAGGCCCTGGGTGTCACCACCAATTATCCAA

TGCCCGAGCCAAACGCCTGTAAATCCTTAGTCAGCGGAGAGTGCCCAGCTACCCAGGGTG

ATTCTCTTACCTATGCTCTCACGATGCCAGTCCTCAAGAAGTACCCAAAGGTTAAACTTC

ACTTAAAGTTCCAGCTCCTTGACAACGATGATAACATACATGCTTGTTTCGAGTTGGATG

CAAAGGTTGTCTCCTAACAAAAAAAAAAAAAATCCCACAAGTCAATAAAACCACATAAAT

ATCAATTATTAATAATAATAATTATTATGGAA

>Unigene0000531

GTAGGAGCGAGGGGCTCTGTTCGGAAGTTGTGATTGTTCTAGAATAAGGTCTGACGTGAC

ACTGCCACTTCAGCTGACAACATTGGCGGCACTGAGGTAAGTGGGCATAAATAGAACAGC

TGGCATATTGCAGTTGGTTGGATGAGTCGACATGATCAGAATTGGGAAGCGAGTTGATGG

GATCGGCTTATTAGCTCTTAATATGTCAGGTATAATATAAATATGGCATGGTGAAAGCAT

TTGTAGC

>Unigene0000532

TAGCTCATGTCAATGGAAATTTTGATTGTTGCTTATACCGCTTTAGAAGCCATTTATGCC

ACTTTAGAAGCCATTTATGCATTATTCTCATGACTCTGACACGTTATATAGGATTGTGGT

TATCATTTTTGACATTTTATTTACGCATAAATTTTATGTACCTTGAGTATGATTTTGTTT

TCTTGATTGTCGCTTATTTGCATTTTCTGAGGCTGGTCTTATACCAATTGGAAGCCACTT

ATGCATAATGCTCCTAGTTTTTTGTTATATAAGAATCTTTATGGGTATCCTTTT

>Unigene0000533

TGATATTGTATGCACTTTGACGGACATGCAGTTTGTTGTCTTCCACGCCTCTATTTCGTC

TGATGGCCTCATTGCATTGCAGGAGTTGTACATACGCCACAAGGATGGTTGCACTCTTGA

TGCAAAACCGCAGAAGGAGATGGTGGTCAAGTGCCTTGAAGCAGCAATTTGTAGAAGAGT

ATCTGAGGGTTTTAGCTTTGAAATCTGTGGCAGAGACAGAGTAGGTTTGCTATCCGAAGT

GACGAAGGTTCTCAGAGAGCATGGCTTGTCTGTGACAAGAGCCGACGTGACAACGGTCGG

GGAGCAAGCGTTGAATGTGTTCTATGTGAGGGATGGATCCGGGAACTCGGTCGACATGGA

GACCATTGAGGCACTCCGTCGAGAAATCGGACACACCGTGATGCTCAACGTGAAGAGGGC

GCCTTCTTCACGTACCAAGGCTCCTGTGCCCAGTGTTTGGGCCAAAA

>Unigene0000534

ACCGACTCTCAGGACTCACTGTTTCTCAAAATGACCTATCCGAGGACTTGATACCGGACT

TTCTGATTACCTTAAAATAAGTGCACACAACAGACAAATAAGGCTCCCAAGCACCATGTA

ATTAACACAGCAAAAGTCTCTCTCAAGTTGTTTATGAGACTGGACTCTCTCTTTCTCTCA

CACACACAAAAGTAATATCCATTGTTTATGGAAGGTAATTCTGATATGAGGTGAGGAAAA

GTAACTACTGTAAGTTAACGTGTCTT

>Unigene0000535

ATAAATAAAATACCTAATAATACAATAATAGAAGAGGTCAATAAATCTGATTATATTAAT

GATTCAGAACAAATGGAAGATGTTGTACAATTAACTGTTGAATCACCAGATTTAGTTGAC

GATGAAGATCCTAAACCAATTGATGACGAACCTAAACCAGTCTATGAAGAAGAATTGAAT

TCAGTTGATGTAAGTAAAACTGAATCAGTCGATGAAATAAAACCT

>Unigene0000536

AGTCACGACTGCTAGTACTGCTGAAAAATCTGCAACAGATGCTCCACAGGAAGTAGATGA

ATATGAACTTGTTGATCCTATTGATATCCTGACTCCCTTGAATAAGTCCGGATTTTGGGA

CGGAGTGAAAGCTACTAAGTGGTCTGAAAGGAGGGATGCCGCTGCGGAGCTTTCAAAGCT

TGCTTCTGTAAAACGAATCGCTCCTGGTGATTTTACTGAAATCTGTCGGATTTTGAAGAA

GCTAATTACAGATGTCAATCTT

>Unigene0000537

ATGCATATTACAAGTTGAGATACCTACGGTCTGAGTTTTGTGGTGGCCAATATAGTACTG

AAGCACTTCTGTGTAAAAGAAGGGATTTGATGGCTAGAGACATGTCCTAACACAACTTAG

CATAACGACATTACAGGACAGATGATTCGAACCATCGTGGAGTGAGTCAAAAGTATGTAG

TTGCATTCTGGGCTACTAAAAAAGTTTTGGGTAGAGGCAGATGAACCGATGAGAGTATTT

GATCTTGGTATGCCAGAGGAAGCTTCGATAGGGAAATATGTCCCCT

>Unigene0000538

TAGTTCTTCCCTCGGTCGAGGGAAACTCCTGGGCTAGCTGAGCCATCACCTGTGTAATAG

GATCAACTGAATACACAGAAAAATAGGAAAGTAGTATCAAAATCAGATTCCAGCTAAGAG

ATAAGTCAAAATCAGTTAATCAGAAGGCAACATTCGAACCTGGCTCGACCGGCTTGTCCA

GGCCCTCTTCACCCGAAATTCTCATGAGACTGTCTACATGATGGTCGAGGGCACG

>Unigene0000539

CTCTCTCTCTCTCTCTCTCTCTCATCCACATATGGGCAGGTCTCCTTGCTGCGAGAAGGC

TCACACCAACAAGGGAGCATGGACTAAAGAAGAAGATCAACGTCTCATCTCCTACATCAA

AACCAATGGAGAAGGCTGCTGGAGATCTCTCCCTAAAGCCGCCGGTCTCCTCCGCTGCGG

CAAGAGCTGCCGCCTCCGCTGGATCAACTATCTCCGCCCCGATCTCAAGCGCGGGAACTT

CACGGAAGAGGAGGACGAGATAATCATCAAGCTCCATAGCCTTATTGGAAATAAATGGTC

GCTTATCGCCGGCCGCCTACCGGGGAGGACGGACAATGAGATCAAGAACTACTGGAACAC

CCACATCAAGCGGAAACTCCTCAGTCGCGGTCTCGACCCCCAGACCCACCGGCCAATCAC

CGGCTCTCACTACGCCATCGCTGCTCGCCAGCTAGAGGTCACACACGATGCAGCAGCCAT

GACTGCTGCCGCAGATTCGGCGGATGAGAGGCACAGTAGCAGCAGCGATAGCGGCAGTGG

CGGCGTAGAAGAAGACCGCTGCATTGACCTCAATCTTGATCTATCCATAAGCTTGCCTTG

TCCCTCTCCGAAGCAAGAGCTAGCAGTAGCGACCCCGATCATTACGGGGACATACACCCA

GGCTGTGTGCTTGTGCTGCCATTTGGGGTTTCAGAGCAATGGAGCATGTAGCTGCCAAGC

CATGCCAAATTCTAATGTCCTCAGATACTGTAGACCGTTGGAGGATGGGCAGAGTATGTA

TCAGTAGTAGTGTAGGTTGTACTGTGAACTGAGCAGATTATGCTCACAGGAAATTAAGTA

TATTTTCAATTTTCATTCTCTAAAATGTTTTTATTTTATTTTTTTAGGATCATAATGAGG

GGATTGCTAGTTCGGAATGTCGGGCACATATTCAACCACAATAAAAGAT

>Unigene0000540

CCCCCTGCTGCGAGAAAGCTCACACCAACAAGGGCGCCTGGACCAAAGAGGAGGACCAGC

GCCTCATCTCCTACATCAAATCCCACGGCGAGGGCTGCTGGCGCTCCCTCCCCAAAGCCG

CAGGCTTGCTCCGTTGCGGCAAGAGCTGCCGCCTTCGCTGGATCAACTACCTCCGCCCCG

ACCTGAAGCGCGGAAATTTCACTGAAGCCGAAGATGAGCTGATTATCAAGCTACATAGCC

TGCTCGGTAACAAATGGTCTCTCATCGCCGGCCGCTTACCAGGCAGAACAGACAACGAGA

TCAAGAACTACTGGAACACCCACATCAAGCGGAAGCTGCTCAGCCGCGGGGTCGACCCGC

AGACACACCGTCCGGTCGGCGGCGTTGAGATTCCGTCGGCCATACATGAGTCCAACATGA

TCAAACCAACAACTCATTCTTGCGACGAGAGCGGCGGCACCACCAGCTCCGGATTTGACG

ACGAGTACCGGTGCATTGACTTGAATTTGGATCTCTCAATCAGCCTGCCGGAACCGACTC

CGGAGCCCGGGGTGTGCTTGTGCTACCATCTTGGTTTACAGAGCAACGAGGCTTGCAGGT

GCCGAGGGGTTGAAAACCCTAGGGTTATGAGATTCTATAGACCTTGGGAGGAAGGGCAGA

GGATGGTTTGAGTTTTAGTTGAACATGTAATGTAAAGTTGAAAGAA

>Unigene0000541

CATGTAATGTAAAGTTGAAAGAAATTGAAATCAAAGCTGTTATCTTTTTGCCTTTTTGGT

TTTGGGTCTCTTCTTTTGGGAGAAGATGCGAGTTAGAGGATGGAGGTAGGGGGAGTGAAT

GGCTGAGGTGGCAGTTTTTTTGAGTTTTGGAGGAGTGGCATGTGAGGTGTTTTGGGAGTC

GGTAGCCGGACTGGGATTAAGAAAGGGATAGGGTTGTTAGGGCCGGTCGGAGCTCTGCCA

TAGGCTGC

>Unigene0000542

AAAGGGATAGGGTTGTTAGGGCCCGTCGGAGCTCTGCCATAGGCTGCAGTGGAGTGTGGG

GGTGCTCATAGAATATTTGTTTGTGTCATTGATGATAGGGGTGGGGTTTGGACTGGAGTT

CAACTCCCTACTGGCTTATCATTTACTTTTCAGTCGAAACACTATATTTTTCAACATTCT

GGGGATGGGCCTATCTCCAAACTAATCTCCATAATGGAACTTGTATTATTTGAGCAGAAA

TGTAGGAATGAGATGTTATTCGGAGAGTTTGGGACCCATCTCACCTCTTCATTTGGGGAA

ATTTTTTCAACATTTCACATTTTGGGTAGTTGAGGGTTGGAAACTTCTAAGGGCATCTTC

CTTTGTAATTTGAGTTTTGAAATTAGTATAGACA

>Unigene0000543

ATTAGAACACACCGATACTACATTTTGCTAAAAAGTGTGACACTTACATTGAGTTACATA

TAATCAAGGTTTCGAAAAGCTAATGGTGGTCTTTAGACATTTTGTCCTCCCTGAGGCAAG

GCGTAAACCTCGAGGCATACACCTCAAAGTGAACTAAGGCATTTTCATTAATAATTGCAA

AATGTTAAAATGCAAAAAGGTAATAATAAATAAAAACTACTAGTAATTCATAAAATGTAG

T

>Unigene0000544

TTGCAGCACCATTGATGAAGCTATTAACCAAGGAAGGTTTTGCTTGGAACGAGAATGCAG

AAGCCGCCTTCACTTAGCTCAAAAAGGCACTAACCACCCCTCCCATCCTATGTCTTCCGA

AATTCACCGACGGTTTTGTGATCGAGAGCAACGTTAGTGGAATCGGAATTGGGGCGATTC

TCTCATAGAATAAACCGACCCATAGCTTATTTCAGTGAGGCGCGCAAAGAATCGGCTTTA

GCCTTCTCGACTTATGAGAAAGAGATGTTAGCAATCGTCAAGGTCATTCGAAAGTGGCGA

CCATATTTGTTGGGGAGAAGATTCACAGTGAGGACTGACCAAAGAAGCCTCAAATATCTC

CTCGAACAACGAATCACTACGCCGACTCAGA

>Unigene0000545

CATGGTGTTTATAGGTTCTGAATGTTAGATTCCAAGAGCATGGCCTGTGGGATTCGATTC

AACGTACTGGATGTGTTTGTGTCCAAGGTTGAGGATGGTGTGCTGGAATAGTTACATGGG

AGTAAAGAGGGGCACCTGTTCTGTGTGATTCAAAACTTGGCAATAAAGTCTGTTAATAAA

GAGGATTGTTGAGGCACCTTAAGTTACTTGTTACTGTAGTTTTGTGATGGGGCTGGGAGT

TTTGATCAATGCGGGATTTGAAAGGCACAAATAAGCACATTCTGAGTCCGATCAAGGAAT

GTGGAGGAATCCTATCTAGGTTGATCTGATATGACAAAAATCCGATCTGACTAAATGATA

TTAAACACTATTGTATTTTATTGATTTACTGTATAGTATATAATGCACTTTCTAAACAGA

TGAACTTTTGATTGCACACAATTTTTTATGACTTATGATATGAAATGGCTTATTATTTGA

TGAAACAATGGTAGGAGAGAACTTGGTCTTCTGTATTTTTTAAGTTCAAATTATGTTGTT

ATGTCTGGCAATACCCTATTATTCAAATTTCTAGTGTATAAACTAGCACATCTATTACAT

TTGTTGATCAGACCCAGATTT

>Unigene0000546

TCAATTAACCGCAATTTTCTGTTTTCATGGTCGATCATAACATTGTGAGGCTTTACATCA

CGATGCATGATGCCCATACTATGGCAATAGTCCAATGCCTTGAGCAACTCATAAAGATAA

AATCTAATATCATAGTCATTCAGCGTCTGGTAGAGTTGTTTAAAGTCCATATTGTTAACA

TGCTCAAATATTAGAGCAGGTGTACGGGATACAGGGTCTTTAACCACTGCTTGCAGCGAT

ATAAT

>Unigene0000547

GGCACGCATCAACACATGAATGCTGCGGCTGCGGTAGCGTTCAACCCGGCAGCGGCGGTC

GTGAGCCTTTCGGCCGGCTCGTTCCAGTCCGCATCCGGTGGCCAACACGGAGCTCACGGC

CAATACAGCGGCCACGATCACCACTCGACAGCCGCCGCGTCAAATTTCAATTCTCCTCAA

GGGACTTCCCACGGCACGTGGCCCACCACCGGCGCTGCCACTGGATACTATCCTTCGACG

>Unigene0000548

GCTGCGGAGGTTGTGAGGGACTCTGTGACCCCTCTGGTGGCTCGTCTTATGTCGGCGTCG

GTGGCACCGCCGTGCGGCGGCGGTGATACGACGGGATCTTCCAATTCGCTCCAAGGATCT

GGCGATATGATGCGCTGCAAATGCTGGGTAGAGCAGTGTCGCCGGATTGAATATGTTCTT

CTTCTCCGATCAGGAGGGGAGGCGAAGAAGGGGATGCGGAGGCTGTTCCATCCGCCGCTA

TTGATTGGGTTTGTGAGAAGGAGTTTGGGTGT

>Unigene0000549

GTACGCAAAACTGCTGCAGTTTGTGTTGCTAAACTTTATGATATAAATGCTCAACTAGTT

GACGATCAAGGATTTTTAGAACAACTAAAAGAACTTTTATCTGACTCTAATCCTATGGTG

GTTGCTAATGCTGTTGCAGCTTTATCTGAAATGAATGAAGCTAGTATAACTGGTTCTCCA

TTAATTGAAATGAATTCACAAACTATTAATAAACTTTTAACTGCGTTAAATGAATGTACA

GAATGGGGTCAAGTGTTTATTCTTGATTCATTAGCTAATTACAGCCCTAAAGATGATCGT

GAGGCTCAAAGTATATGTGAAAGAATAACACCCCGATTAGCTCACGCTAATGCAGCAGTT

GTTTTATCTGCTATTAAAGTGTTAATGAAACTAATGGAAATGTTACCAACTGATTCCGAC

TTTGTTACTACATTGACTAAAAAACTTGC

>Unigene0000550

TCTCCTCCAGTTCCTGAACCTCATACACTCACTACTGTGGCTCAAGCAGATCCTCTTGTT

AGAAGACAGAGAGTACAGGACCTTATGGCCCAGATGCAGGGTCCATATAACTTCATGCAG

GACTCTATGCTGGAGTTTGAGAACCAGACACTTGATCCTGCCATTGTATCTGCACAGCCC

ATGAATCCAGCACAGAATTTGGACATGCCGCAAATGGTCTGCCCTCCAGTTCATACTGAG

TCAAGACTTGCCCAGCCTAATCAAGTTCCTGTGC

>Unigene0000551

AGACGAAGGACTTTACGTTTGTAATGCTGAAAATGACGTGGGTTCCGAGGAGGCTAAGGC

TTCATTAACCGTACACTCCCCGCCGACATTTGTGACCAGACCCAAAGACCAAAAAGTGGG

CCTCAACGGAATTGCAAATTTTGACTGCGTAGCCCAGGGCAACCCACCACCGTCTGTGTT

TTGGTCGCGTGAAGGGTCACAGGTGCTGATGTTCCCTGGCAACGCATACGGGCATTTTCA

CGTCACTCAAGACGGCACGCTACGCATACAGGGGGCTCAAAAAGAAGACGCCGGTTTCCT

CGTGTGTTCCGCA

>Unigene0000552

GTATACTGTTTTGGAGGCTTGCAAGTATCTTATTTAATTTGGGGTGTAATTCAAGAAAAA

ATGATGTCTGAGCGTTATGGTACAAATGAATCTTCAAAATTTCATGATTCCCAAATGTTG

GTGTTTTTAAATAGAGGTTTATCTACAGTTTTGTCTGGTATATTTTTATTTATGAATGAG

GGAATAAGATCTAAGAAATCACCTCCATTATATAAATACAGCTATTGCACTGTGTCCAAC

ATTATAAGTTCTTGGTGTCAATATGAGTCACTCAA

>Unigene0000553

TCCACTACCCGCCGCCGGCAACACCAACTCTTCCCCTCCGACCTCCTCAAAACCACACCA

AATTGCACCCAAATAGTCACCCGAACCTCCTCTCTTTAATTCTCCTAATGATTTTGCATT

CCATCAAGAGATCTATGAGATCTGATCAATTTATTCTCTGACCAATCTACGAATACCTCC

GATCAACTTCACCTCCATCTAACTTGAGTATCCATGAGCTTTTAGAGCATTCTTAATCTC

TCCCTTCTCCAATATTTTGATCAGTTGGTAATATGTTTCTTTGATTTCCTTTCAGGTGAT

CAGCTGGAGCACCGTAGCAATTTGGAGATCAGTGGAGAGCAGCAAAGGATATTTGTAGAG

GCTTATGTAAGAATGAATAATGGTGGCAGTGTACATGAATCTGATACAGAAGCAAGAAGG

GAGAAGTCAAATCAAGTTAAGCGAGGGCAAGTAAGTACCAATATAGCCTAGAGAGGTAAT

AGGGTCAATTATGGATTGTGGTAGTTAGAAGTTGAGTTGGCTGATACAAACATCGGTGGT

TAGGCAGGCCCAAGGCTACAAGCTTGAATCTACAGTAGATCAGTCTAGAGGTATAAGGTA

GTATTTACTGGTATAGAGAGAGGCAAGAACAAATGGTGATTACTGGCATAGAGCTCCATT

CAGGTAGATATTATGTAAGGAAGGACAGTAATATGGTCGAAGCAGTGGATATATTGATGG

CATTCTTGGCAATCAACGAAGAGATTCAGCAATGATTTCATGGCGATAAAGACACATTCA

GTTCCTTGACACCGAAGGCATTATTGTTTGGTAGTAAAGGCAAGATTCCTTAATAATCAA

GAAAAGTAAGAGGAGTGGCTTATAAGGAATAAATAACCTACAAAGAAGGAAGCATTTATT

AGGAAAGGAGTTTAGCGAGTCATTGGTGGAGAAAGTGTGGTTGAGACTGGTCTCTTCTGG

AAATAGTCAGGAGTTCGTAGTGATCCGTGATAAATCCATGATATTGTGAACATGATCCAT

GAAGCTATTGAAGATGATGCTAAGGCTTGAAAGTGATCATTCCTCAAATTTATGTAAGTT

CTTAGGTTCCAATGTCTGAATATGATTGAGTATGATCTTTAGTGAATAGAGTCAATGTTG

TATACAGATTGATCAACTATTAGTCATAATAAGGAATTGTTAGTTATGATCTCTTATACT

ACCTGTGATCATCTTAAGAATGAATAGAATGTTATGTACTTTTACATATTGCTTTAATTG

AATATATCTACTTTTGGCTATTACATT

>Unigene0000554

TCCACTACCCGCCGCCGGCAACACCAACTCTTCCCCTCCGACCTCCTCAAAACCACACCA

AATTGCACCCAAATAGTCACCCGAACCTCCTCTCTTTAATTCTCCTAATGATTTTGCATT

CCATCAAGAGATCTATGAGATCTGATCAATTTATTCCCTGACCAATCTTTGAATACCTCC

GGTGATCAGCTGGAGCACCGTAGCAATTTGGAGATCAGTGGAGAGCAGCAAAGGATATTT

GTAGAGGCTT

>Unigene0000555

TTAAACTTCTAAGGCTATGATAATGATTTTAAAAATTTCGTTGCTAATAGAGAACATATA

ATACCAAATTTTATGAAACTAGAAAGTATCGTGCTAAAAATATTTTGAAAAAAGCGTAGC

GCAACTCAAAAGTTCTTCCCAAAAAGAAGAAAAGAACTTCCGAAAAAGAAATAGTTCCAC

TACATATTCGTAGATCCTTAACAACATTTTTAGAAGAAAAATAAGCCATAGAAACGAAAA

GGAA

>Unigene0000556

CACGGTGAAGGTTTTAATCCGGCTGCTGAAGGACCTGAGGATTCGGTTCCCCGGCTTCGA

GCCCCTCACCCCCTGGATTCTGGATTTGCTGGGGCACTACGCTGTGATGAACAACCCCAC

CAGGCAGCCCCTGGCTCTCAACATCGCCTACAGGCGCTGCCTTCAGATCCTGGCTGCGGG

GCTCTTCCTGCCCGGATCCGTTGGTATCACCGATCCCTGCGAGAGTGGGAACTTCAGGGT

CCACACGGTCATGACTTTGGAGCAGCAGGACATGGTGTGCTACACAGCGCAGACGCTCGT

CCGGATCCTCTCTCACGGAGGCTACAGGAAGATCCTGGGCCAGGAGGGCGATGCCAGCTA

TCTGGCTTCTGAGATGTCCACGTGGGATGGTGTGATAGTGACGCCTTCAGAGAAAGCTTA

CGAGAAACCTCC

>Unigene0000557

CAATTAGAAAGTGAAATGATGAACTTGGAAAGAATCAATAAGCAATTAAAATCTGAACTG

AACGATACACTAGAAAAATTGCAGAAACAGAGTAAACCTGAATCATCATCAGATACAAAA

TTTAGATTATTGCAACAAGAGTTGGCTGATAAAAATATTGAGTTAGTCAATTTAAAACAC

ATTTTATCCAAACAAAAGAAAATGTATGTTGACCAAAGTGCTGAGTCAGCGCATTTAGTA

AGGCGGGGTGAGCAATATGAGAGTGAAGTGAAACGTTTAAGAAGCAGAGTAGAAGAATTA

AAAAGAGAATTAGCTACTACAGAAGA

>Unigene0000558

TTTCTTTGGTGGCAGAGGACCATTGGATGGAGATCTGATGGATGAGAATGGGACTAGGTA

TTTTTATTATAAGATAAAATATTGGTAATTAGATAGAGAGTGGAATGGATGGGTATGGTG

AAGAAATGGTGGATTGCTAATGTGGTTGGATGATTGGGTGTTTTTTTAATCAAGTTTTGA

TCAGATGGTATGGAAATAAGGAGGTAAAGACTGTGGATTATTGACTTGGTATGTGTTTTC

TCCGATAGTAGAAGGACATTGGATGAAGACCTGATGGATGGGAGTGGGATTGAGTGT

>Unigene0000559

TTTCTGTTAAGGATGCAGACCTATTCCTTGTCATTGTATACAACTGGCATGTACTGCTGT

TCTTCAAATGCTTTCAACTTTTGTCCTGGTTTGTTTTTATCTAAAGCAGATCTATCACCC

TTAGAGTGATATCACCCTTTTACTACTGTGCTGAAAAGATACTGTTCAGATTACACGTTT

TTTTCCTTTATATTTAACAGTCTTCTGATGATTTGTTTGCTCTATACAAAACTGTTCACC

TGTTCTTTTAAAAGAAA

>Unigene0000560

GTATACCTCCTCATATGCCCACCAACATATTCAGGAACATCACCACCCTCAATCTCATAC

CACCAAAACCCAGCACTCCAAGTCAGCATGACTGTACTAACCTCGATGTCACCTCCATCC

TCGACTGTGTTGCCATCTCACCGTGGCTTTGTCAGCCTGCCAACTTGGATACTACACCGT

ACTTCCCCTCCCCCTCCTTAAAGAAACCGAGTCGAAGCTCTATAAAGATCAAAGGAGGAG

CACATTCAAGAGAAACCCGCGAACCTATCCAAGAAGGAATGAAGGGAAGGATATCGTG

>Unigene0000561

GCAGGTCAAGAAAATTGAAATAACCCAACACTCCAAATACACATGTTCTTTCTGTGGAAA

GCAAAGTATGCGAAGATCGTGTGTAGGAATCTGGTCATGTGACAGGTGTCGAAGAACGGT

TGCCGGTGGAGCTTATGTTTATGCCACAACTGCCGCTACGTCTGTGAGATCAGCCATCAG

ACGTCTCAGAGAAATCCATAATGTATAAGTTAACCAATTGACCTGTTTTC

>Unigene0000562

TTATGTTGTTGGGTTAATCGGTTAATGGACCGATTAATTAGTCATTGGCAAATTAAAATC

GATGACCCAAAGCCATGTCATATTGGTCTCCCCTGATACATGATTAAACAAGGATCAATC

ACAATTTTAATTACATTGGCACCGCCAACTTCACCTCTATCTATGTTAGCTTCACTCCTC

ACCGCCTTAGCACCTCTAGGGCATCGTTGATAGTCACCGTCGTGCCCCATCGGTGCTGTC

GTATATGTGTTGTGCTCCCGTCTCTCTATTTTGCTGACATTATT

>Unigene0000563

AGCATCACCGTCATGGGCAAAACCTGCCAACTCACCCCTGAATCTCTTGTTGTGAGCGCA

TGCACCCGCACTGAGATCGCTCCCGGTCGTCAGCTCCACTTTGTTGCAGCAATCGATGTG

AACCTGGAAGTAATTAAAGGATGAGATATGGAACAGTCTGGTTGTTGGGATACAGAGAAA

ATCAGGTTATTGAGAGAGACGAGACTTGTTTTCTAAAGGCTTTGACGGTGTATTCACATT

CAAGTTTTGGGGAGTTTGCCCTTCAACTTACTGTCTAGATGATTCATCTACCTTAGATCA

CCAAACCTAGCAGTTTAGAAGGACTTATAAGAAATCTCACCCTCCACTCAGCTTCGA

>Unigene0000564

AAAAAAATAATGATCTCAAGATTGCTTTTAGATCACAAGAGGAGAGTATGGCAAAAGAAA

AAGAGCTTTATGATTGCGACAAAAAAGAACTGGCTAGCCATTTGGCGTATAAAATGCAGA

TGATTGCAGATTTGAAAACCACACTGGAGCGCACCCAAGATGATTTGCACGTTTCCAAAC

GGAAGTTCAAAACTACTGTGTCCGAGCTGACTAAAGAGTTACAGAAAAAAAACATTGTTG

ATACCTCTGTTGAAATGAAAATGGTCTTAGATAAAATGGTCAAACTGCAGCAAGAAAATG

CAAGGATATGCGAAAAACTAGATTTTGTTGAAGACCACAACAAGCATTTGCTGTGTGAAC

TTAAGAAAAAAAGTAGTATTATGCAAAAACATGGTATACCTAGTAGCAAGAATGGCCTAT

TGTCCGGTCTGAGTGAACAGAAACCAGAACGTTACTGACAATATGTATACTATCAACAGT

CAACTATTTAAAACATTATCACTGTATATCTGCTCTAATTTATAGATATTCATTATTATT

TTTTAACTTTAATACAAACCAAGTATTTAATCTAGTCCTATTATATTATTATCG

>Unigene0000565

ACGGAGATGCGGCAGCACCACAATAAAGCGCTCCATGACCGGGCCGTGCAAAGATTGCAG

GAGAAACAAATTGAGATGGACATGGCTTCGAAGCGGGCTACGGAGCTTGAGAACGGGCTC

GCACGACTTCGTGACGAGGTCATGGCCAGGCGGGCCAAGGCTATAGAAGAGCAGGCCACG

GTGGCCGCCCTACACGCACAGCTTCAGCAGGCAGTTGAGGACCCTGTGCAAGTGGAAGAA

TGCGGGGAGTCGCCCGATTCCGAGTCATTTGGTGTTGACAAGAACTTGGCCGAGCGGCGG

GGTGTATGTAGGGGTTGCCGAGTGCGGCCAGCGGCGCTGGTGATGATCCCGTGCCGCCAT

CTCAGCCTCTGCTCTGAGTGCGAGTCCACCTCATCTGTTAGAATGGCAGTGACCTGTCCT

GTGTGTCACTGCCAGCGGACTGGAAGCGTCCAAGTGTTTTTCTCGTGAAACTTGTCAATG

AGGTGAATGGTGTCAACCAAAAAAAATAATAATAAATTGATAATGTTTAATAAATGATTT

TCCTCTTTCATTTCTGCACTCTTCAGTAAACAAAGGGACAAAAATAAGCAAACTATTACA

CCTCACCTCCCTGTTTCATTGCTGTACTCTTCATGAAATCGGATTTTATATATAGAT

>Unigene0000566

CACCGTCTTCTTCTCCATTGGAGAAAATGAGAATACAAGAAAGCGGCAGAGGCCGGCGGA

GGAAATCCGATTGCACAGCCGGCCACTTCCATTTGTTTCCCTACAGAGCAACAATGAGTC

ACTTTATCTCTCCTCGTCTGGCTTCCCCGGCGAGCTAAATGTCAAGTTCAGTCAAACATA

CGAAGAGATTCGTCAGCTTCTAGCTGATCAGGGTGAGCAGTTACGTCGGGCACTAACGGA

GATGCGGCAGCACCACAATAAAGCGCTCGATGACCGGGCCGTGCAAAGATTGCAGGAGAA

ACAAATTGAGATGGACATGGCTTCGAAGCGGGCTACGGAACTTGAGAACAGGCTCGCACG

GCTTCGTGACGAGGTCATGGCCAG

>Unigene0000567

TGTTTCCCTACAGAGCAACAATGTGTCACTTTATCTCTCCTCGCCTGTCTTCCCCGGCGA

GCTCAAAGTCAAGTTCAGTCAAACCTACGAAGAGATTCGTCAGCTTCGAGCTGATCAGGG

TGAGATGCTGCGACGGGCACTAACGGAGATACGGCAGCACCACAATAAAGCGCTCCATGA

CCGGGCTGTGCCAAGATTGCAGGAGAAGCAATTTGAGATGGACATGGCTTCGAAGCGGGC

TACGGAGCTTGAGAACGGGCTCGCACGGCTTCGGGACGAAGTCATGGCCTGGCGGGCCAA

GGTTATAGAAGACCAGGCCACAGTCGCCGCC

>Unigene0000568

GATTTAGGCTTCTGGGTTTTGTTGAGAGGCGACCAGGAGCGGAAGGGTATGGACATAGGG

CCGCCGCGGACGTTCCTTGATCACTCAGGCGACACCGTCTTCTTCTCCATTGGAGGTAAA

AGGGCTGAGTTGTATTTTCTTGGGTTCGTCAATTTTGATTCCTGAGACAAATTTTGTTTC

TAATAGAAAATGAGAATACAAGAAAGCGGCAGAGGCCGGCGGAGGAAATCCGATTGCACA

GCCGGCCACTTCCATTTGTTTCCCTACAGAGCAACAATG

>Unigene0000569

ATGACCGGGCCGTGCAAAGATTGCAGGAGAAACAAATTGAGATGGACATGGCTTCGAAGC

GGGCTACGGAACTTGAGAACAGGCTCGCACGGCTTCGTGACGAGGTCATGGCCAGGCGGG

CCAAGGCTATAGAAGATCAGGCCACGGTGGCCGCCCTACACGCACAGCTTCAGCAGGCCG

TTGAGGACCCCGTGCAAGTGGAGGAATGCGGGGAGTCGCCCGATTCCGAGTCATTTGGTG

TTGACAAGAACTTGGCCGAGCGGCGGGGTGTATGTAGGGGTTGCCGAGTGCGGCCAGCGG

CGCTGGTGATGATCCCGTGCCGCCATCTCTGCCTCTGCTCTGAGTGCGAGTCCGCCTCAT

CTGTCAGAACGGCAGTGACATGTCCTGTGTGTCACTGCCAGCCGACTGGAAGCGTCCAAG

TGTTTTTCTCGTGAAACTTGTCAATGAAGTGTCCCCTGTTTTTCTCGTGAACGGTGTCAA

CCAACAAAAATAATAATAAACTGATAATGTTTAATAATTGACTTTTATGTTTCATTTCTG

CACTCTTCAGTAAACAAAGGGACAAAAATAAGCAAACTATTACACCTCACCTCCCTGTTT

CATTGCTGTACTCTTCATGAAATCGGATTTTATATATAGAT

>Unigene0000570

GATAGGGTTATTGTTAGTTGTATTTTCAAGGTTATTTTACGTTTAATTGAGTCTGTTGAT

TGTGTATTTGTAGACCATGTCAACTAACTAAAGTGATGTATTTTTGTTTAGTTTAGAGTA

TATTGGTCTAGGAATGCCACTGATATTGGCCTATTGGACTGAATTTCAGAAACTCTGACT

GTGAAAACAAACCAAACAAAACCTGTTTTGATGGGTAGACGCTAATGGTTCGGTTCTGGT

CTCACCCTGGAACAGATCTGAGTTGAACAGTGTGAAAAGCCCCTAGTATTTTGCCTCGGA

TCGTCAAAAATATGTAAGCTGCAACCGTGAGGAGAGCTTCACGGGTAAAGAACAAGGGTG

CTAAGTAAACCA

>Unigene0000571

TATATTGGTTGGTCTAATACCTTGTGGAAAGGAGACAGAAGATAGGAAATGTGGGGTTGA

TTTTGTTAGTATATAAGATATGCATTGCCAAGCAAGGTATATGATACTACTCTTACTATC

ACACCTATATACATACACCTCTACTCATATACCTCATGTACATGTACAATTATGTTTCTT

TAGATGGTATAGAGGTGTGACTAAAGGCCAATTTACTGCTTATGGTTCTGTAGATG

>Unigene0000572

GGCTGAAGATTGGACTGGTTTAGACGAAGATGAAGATGCACATGTCTGGGAAGACAATTG

GGATGATGACAACGTAGAAGATGATTTCTCCAATCAGTTAAGAGCTGAATTAGAAAAACA

CGGATACAAGATGGAAACTTCATAGCTTTGGCTGATACGGAGTGGTGTAACATTGAACTT

TGGATAGACTATATTGAGAAAAGGTGGAAAATGAGAAGACGCTTAGTTCATTATCTGCTT

GGATT

>Unigene0000573

TTATTTACTTCTGAAATCTTTTACTAGCCTACAAAACCTGTATACATGAGAGTTCTTGGT

TACGTGCGACGATTAACATGACATATTAGATTGAAGTCCTTTAAATGTCTATGTATGCTA

TGACATAAGACTACTTTAGATTTCTAATTTATGTTGCAAACTTATATTTATAATAAAATT

AAGTTATCTTAACCAGCTCTGGTCTGACATCTCACGGTTCACATTCTAAATACTTTGTGC

AGATTTAGATATCAAAATATAGTTTAGTTTGAGCCATATATTTGAAGTTCTTTCATATAG

TATAGTATGTTGCATTGACATGCTAGCGAGGACTGTACATGCAAAGTAATGCATCCAAAC

T

>Unigene0000574

CAACTGAAAAATTTTATCAAAAAAAAGCTGATGAGAAATTATGTAATATCAGTGAAGAAT

ACATTAAAGGCGGAATAGAGTATTATGAAATATTAATTCAAAGAAATCTAGCTTTAAGAA

ACATTTTACCTAATGGTGAAAACATGTATTCAAAAGAAGGAGATCATATTGAATTTATTT

CAGAATTATCTGAATTACCCTTAACATACAGAAAAAGGTATCTTGAAAATAAATTAAAAA

ATTACATAAATGAAAATAAAGTAACATCAACATAATGTGATTAAATATGCCAATACTTTT

TTTTTTGATCTTGTTAGTTA

>Unigene0000575

CAGAAGCCAAATCTTGGAAAATATGATGATCCACCTGACTGGCAAGAAATTTTGACTTAT

TTCCGAGGTTCTGAATTACAAAACTATTTCACCAAGATCCTGGAAGATGACCTAAAGGCT

ATCATTAAGCCTCAGTATGTGGACCAGATCCCTAAAGCTGCAAAGGGAACAGTGGGATCA

ATTCTGGATAGAAAGGATGAAACTAAGACACAAACTGTTGTTTGTCAGCAGCTTGACTTA

ACCCATCTGAAAGAAAGAAATGTTGAGGACCTTTCAGGAGGAGAGCTTCAGAGATTTGCT

TGTGCAGTTGTTTGCATTCAGAAGGCT

>Unigene0000576

TCAATCACCGGTACTTTACCACCCTATAAACCCCATCCCCTTTTTAGTATTTAATTTAGT

TGAAAAAAAGAAAGAAAAGAACAATAAACAAAAAAAACAGAAGAAAATAACAAAAAAATA

CAAGTACACTTAAGAGGCCAAACATGCCCTCGTCTTATCCGCAGTCTGTACAAATTGCAG

GCCCGACTAAACTCTAACGCTCGCAACCGCCATTAGGGTTTAGCCATCCCGATTCATTTC

TAAGAACTCATCCCTAGCGGTCGCCCTTGTACACTCACACTGTCCGCCTCTACGACGGCC

CTCACGTCTCCTTGCGAAGGTCCCCGCCCCGACCGTGAACTCCCCCAACATCCATCCTGT

CACTCCCCATGCGAGCTCCATTA

>Unigene0000577

TTTATATAAATAATATAACATGTATGGTGTCACAATTATTTGTACTTTGTGACTTGATTG

ATACTTTTTGTTGTTAAATAATTGTACTTAAAAAAAAACAAATTATATTCACATAAATAC

ACACTTGTGTGAACCAAATTTATTTTAAATAGTACCAAAATTACGGAAATTGCACTGTCC

TGATAGTTTTAAATATATACTTAAATTCTATTTGCCTTACGGATAATATTAAAATGTTAT

TTATAAAGTGACAGAAATT

>Unigene0000578

CTGTGCCCAAGGCAGTCACCCTGCTCACCTGGCCTTGGGGCCGATACTGATTGTCGATGA

CTCATTACAACACCTCATCAGATTTATTTATTTGGTACTTGTTTCTTACTGTGCCAACAT

GTTTATTCTGTTGATGCATCTAATTGCATACTTGATTATCATAAACATTTTTTGCTGAAT

GGCAACAAAAAGAAATAAAAACAGAACAAAATAAAATAGTTACATTTTCTTTTGAGTATT

ACGGTATCATGTAACCTAATCAGATGACTCTTTCCACTGTACATACTGATATTACTGTAG

TTGTGTTTTGCATAGACATTGTCTGATGCATTTGGTCTATTTCATATAATACTATAGTAA

GATTTATGTGT

>Unigene0000579

TGCTTATTTAGACACTTTAAATAGTGCAGATGTTCCTGATAACAGTATATTTGAGTCTCT

ACCTAGATTTTGGGAATCAAATTTCCATTCAAATATGGCTTCTTTAAATATTTTACCACC

AGATAAACTTTCTAGGGTGAGCGAGTATGTCCCTGAAATAATAGCTTACATAGAAACAAT

AATAAAAAATGGTTATGCTTATGAATCTAATGGTTCAGTGTATTTTGATGTTGCTGCATT

TGATTCTCAACCTATTCACCATTATGCTAAGTTGGTTCCAGAAGCATATGGAGATTCAAA

ATCCCTTCAAGATGGAGAAGGTGA

>Unigene0000580

TTCCCGCTGCCGCTCGCTCCGCCCTCCGCCACCTCCTCCGCCGTCTCCACCTCCGCCTAC

GCCTCCTCCTTCTCCTCTCCTTCCCCTCCCTCTATCTCCTCCTCTCCCGCTCCTCCTCCC

CCCTCCTCGACTCCCTCGCCCTCCTCTCCTCCGCCCTCCTCCTCCTCCTCCTCGCCCTCT

CCCTCCACGCCCTCCCGCCGCTCCCCTCCCTCCGCCTCTTCCTCTCCCGCTCCTCCGCTT

TCCTCCTCCCTCGCCACCTCCCCCGCCCGCCTCCAGTCCTCTGGTCCATCG

>Unigene0000581

CAGGCGCCCCCTCCTCTTCCCCGCTGCCGCCCGCTCCGCCCTCCGCCACCTCCTCCGCCG

TCTCCACCTCCGCCTACGCCTCCTCCTTCTCCTCTCCTTCCCCTCCCTCTATCTCCTCCT

CTCCCGCTCCTCCTCCCCCCTCCTCGACTCCCTCGCCCTCCTCTCCTCCGCCCTCCTCCT

CCTCCTCCTCGCCCTCTCCCTCCACGCCCTCCCGCCGCTCCCCTCCCTCCGCCTCTTCCT

CTCCCGCTCCTCCGCTTTCCTCCTCCCTCGCCACCTCCCCCGCCCGCCTCCAGTCCTCTG

GTCCATCG

>Unigene0000582

CCACCTCCTCGCCGCCGTCGCCGCCACCTCCGCCGTTATCCTCCTCATCGCCGTCCTCCT

CCTCGCCCTCTACCTCTACCTCACCCTCCTACGCCGCTCCCAAACCCTCCCCTTCCCTAC

CTCCCCCACTCCCCCTCTCCGCCGCTTCTCCCACCGTCAGCTCCGCGCCGCCACCCGCTG

CTTCTCCCCCTCCCTCGCCCTCGGCCGCGGCGCCTCCGCCGCCGTCTTCCGCGCCGTCCT

ACCCGACGGCAAGTCCGTCGCCGTCAAGCGCCTCGCCCCCTCCTCCCTCGACCGCGAGTT

CCAGAACGAGCTCACCGTCCTCGCCGACCTCCCGCCGTCCCCCTCCGTCGTCGCCCTCCT

TGGCTACTGCCTCGAGGGCCGCCACCGCCTCCTCGTCTACGAGTACATGTGCAATGGCAG

CCTCCAGGAGGCCCTCTTCGGTCCCCGCTCCTCCGATCTCACCTGGGAGCGGCGGTTCCA

GATCGTGCTCGACATCGCCGATGCTCTCGCCTTCCTCCACCTTGAGTGCGAGCCTCCGAT

CATCCACGGCGACATCAAGCCGAGCAATGTACTCCTCGGACCTGATTTCCGTGCCAAGAT

ATGTGACTTTGGACTTTCTCGCTTCAATTCCGTAGTCATTGATCCACCTCTGGCTGATCT

CTTCAGCCAGGAGCTCGGCCCCAGCCAGGAGCTCGGAATCTCCGCACCCAATTTCGGTTA

CTCAACTACTAGTCCCAAAGGGAAGGAGGCGGCAGTGGAGGAGATGAGCGGGAACTTATC

GCCGTTGATCGGAGAATCTAAAACCGGATCAAAGAGGCAATGGGGGAAGGATTGGTGGTG

GAAGCAGGAGGATAGCGGCGAGTTGTGCAGCAAGGACTATGTGACGGAGTGGATAGGGAG

CCAGATTTGTCCGACGAGCAATCCTGATTGGGACGGTGAGACGAAGATCCCTGCTGAGAG

CGGTGCTGGGGCTCAGTTGTCGAGCTCGAGCTCAAACCGAGTGGATAACAGTAACGGGAA

GAGGAAAATGGCGGCGGAGGGCAGCGATGGTTGCGATACGAAGGGCAGGGAGATGAGGGA

ATGGTGGAAGGAGGAGTACTTTGCAGAGATCAGCAACAAGGGAAGCAGCAAGCGCAACAA

GGGCCTGAAATGGTTTCGGGGGCAGAGTGGCCGTGGGGATGAGAGCAGTACTAGTAGTCG

CAATGCAACTCAACCTTTCGATATAAGCTTCCGAAAGGGTTGGAAGAAGAAGAGAGTATC

GGCGGGGAGTATCGACTGGAGTGGTGACCTTAGCACGACGACGAGCATGAGAGGGACTGT

TTGTTACGTTGCCCCTGAGTTCGGAGGGTGTGGGAATTTGATGGAGAAGTCGGATATATA

CAGCTTTGGGGTTCTGATACTTGTGATTGTCTCTGGTCGGAGGCCTTTGCATGTGTTGAC

ATCACCGATGAAGCTCGAGAAGGCTAACCTTATAAGTTGGTGCAGACAGTTGGTGCAGAC

GGGCAACATTTTGGACCTAGTGGATGAGAGGTTGAAGGATTTGTATAACAAGGAGCAGGC

GAGCCTTTGCATCAATTTGGCTCTACGGTGTTTACAGAGGTCGCCGGAGTTGAGACCAGA

CAGTGGGGAAATTGTGAAGATTTTGAAAGGGGAGACTGAGCTCCCGGTTGTACCGTTCGA

GTTCTCACCTTCCCCTCCGATTAGAGCGTCCAGTAAGTCCCGTAGAAAGACTAGTTTGGA

TCCTGAGTAAGCCTTCCATCTCCCACTTTTCTAGGTTAATTTGAGTAAGAGATGAATTCA

TGAGTTTATTTGAATGCTTTGGTTGAATGGACATGGCAATTATGGTAACCTTGTGGAGTG

GAGTATCTGAATTGTAAACTCAAACATCTATGCTTTGATTAACATGTGTGAATATAGTTT

TCAACTGATTGAGGTT

>Unigene0000583

ATTTTCGTCCCTTTTCTCCGGTCTAAACCTGGGTCTGATGTCGTTGAATCGAATGGACTT

GAAGATCATTTGTAACACTGGTACAGACGCAGAGCGAAAGTACGCCAAAGCTATCTTGCC

TGTACGTATTCACGGTAACTACTTGTTATGTAGTATTCTGCTGGGTAACGTCATGGTCAA

CTCAATATTTACCATCCTGTTGGATGACTTGACGTCTGGCTTGGTGGCCGTCATT

>Unigene0000584

ACGTTTTGTTACTCAAAACAACGTGTAAAAGTCTACATACGATCGGCAGTGTCTGTCCGA

GGTCACTGTGAAGGGTACATTATTGCTTTTGACAAACACTGGAATTTAGTAATGGATGAT

GTAGATGAAGTATGGACAAGAAAAAACAAATACAAATCACTAGCATTTGGTGATGCTAAA

AGTCTTGTCGATCCCGTAGAGAAACCGTACACAGTCGTCAGGAGGATTCGAGGACACCAG

GTGTGCCGGAGGCATGTACCCCGATTTTTAGTCCGTGGAGAGCAGATTGTTCTAGTGGCT

AAA

>Unigene0000585

CCACACTGTGAAGGAGGTATTTGTGCAGAAAAATGTTTAAATGGTGGAAAATGTATACAA

AAAGACACATGTCAATGTCCCAAGGGGTTCTATGGCTTACGATGTGAATATTCAAAGTGT

ATTATACCATGTTTAAACGGTGGCAAATGTAAAGGGGTGAATAAATGTAGATGTATATTT

GGTTTTCAAGGAGACCATTGTGAAATTGGTCAACCAATAAAACAATATTATAATTGTAAA

CGACCTTGTCGTCATGGATACTGTACAGCAAATAGAACCTG

>Unigene0000586

CCAATGCCCATTTACAGAAGGGGTCGCAATAATTCAGGCTCAGCTCGGGTCGGCCAACCC

TCAGGATATCCAGATCCGAATATCTGATAAGAATCTATCCGGATCAGTTTAAATTCGGAT

TATCTGTATCCGATCTGGTTTGTCCAAATTTTATCAGCTTATATCCGAATACAAAGAATT

TGAGGAAAGGACTCATTATGCTGACAGCAGATCAAAGATATCATAATTAAGATTACATAC

GTAG

>Unigene0000587

CTTGTCCTTCCTTTCGTTTTCGAAGAAGCTAAGGCCATATCGGCAACTTGCACATTAATT

TACATCCATTGATCTTTTTAAGACAAATTAATCAACAAAAATGACTACCGCTGCTGAACT

CGCTTGCGTTTACTCCGCACTCATCCTCACCGACGATGATGTTGCAGTCACTGGTGAAAA

GATCCAAACAATCTTGAAAGCCGCCAACGTCGATGTCGAGCCATACTGGCCCGGTCTCTT

CGCCAAGGCTCTTGAGGGAATCAACGTTAAGGACCTCATCACCAACATTGGATCCGGTGT

AGGAGCCGCCCCAGCCGGTGGTGCTGCAGTTGCCGCTGCTGCTCCAGCTGGTGGTGCTGC

TGCCCCAGCCAAGGAAGCCGAGAAGAAGAAGGAAGAACCTGAGGAAGAATCTGATGACGA

CATGGGATTCGGTCTTTTTGATTAATTTCCTAAAAAAACAATGGTCCATTTTTTACATCA

ATGTA

>Unigene0000588

ACCGTGGAGAGTTGGGACGGACGGCGGCATGGCAATAACAAGACTCGTGTGCAACACATT

GAAGCTGAGTGCCAAGACGATTATATGAAAATTCGCATCGGTTTCAACGGCTCCTTCGGC

GGTCTGCTGTACTCGTCCGGATATTCATACGACCCGGATTGCGTTTACATCAACGGCACA

GGTCGGGATTACTACGAATTTTACATACAGCTGAACCGTTGCGGTACACTGGGAAAGAAC

TCCAGACAAGAGGACGCCAGGAAAAATCCAGCGAAAAATCTCATGTGGAACACCGTAACC

GTCCAATACAACCC

>Unigene0000589

TTCACTTTTATACTTAAAATATTTAACATTTATACTTGTTTTTACTATCAACAAGTAATT

TCCAAGTATTAAGTATGAATACTGATGTTAATTGTAAATTTTGTACTGGGAATCAAAGCA

GATATGTTTGTCCAAAGTGTGGAGCACCTTATTGTTCCTTAATATGTTATAAATCGTCGA

TTCATTTAGAGTGTTCAGAACAATTTTACAAAGAATGCATTCAAGAAGAATTAGATGTTC

AAGATTCAGACGATGTAGGCAAAAAAGAAATGATGGATATTCTTAAAAGAGTATATGGAG

AAAATGAAGATTCTGAAAGTGATTCGGATGATGAAGAATTCACAGATATTAATGAACGAT

TAAATGGAATTGATCTAAATGATGCTGAGCAAGTTTGGAGTTCTTTAACG

>Unigene0000590

TAGGGAAGATCTCACTACTACAAAAAACAGCTTTAGTAGCGCACATTTAACAATGCTTTT

TTTTAAAAGCGCTGTCTAAAATAAGCTGGGAGCCCTGCTGAACAGATACAGATACAGAAT

ACACAATGACATGGAGCTTGTGAGTTATGTCGTGGTGAGACTGATGGAGTTGGAGCTCGA

TAATATGGGAGGCTATTTAGTACTTTTATCCATTGCACTATAAATAACGCGAACACGTTA

TAGAATAATGTCGACTGTAAGATGTGAAAACTAGAATATGCTTGTGTATATGCATGGAAC

TTATTGTGGGCTGAGTAAGCTATAGTCAGAAAA

>Unigene0000591

CCAGATCTAGAGACCGAGAGCCGCCCACCGGAGCCGGAGAAGAAGATGAAGGTGATCGAC

AAGATTGCCGCCGCTAACGATGACCGCGTCGTCTACTCCTTCGAGTTCTTCACCCCCAAA

CCCCGTGGCAATGTCACCTCCAAGGCCGCCCTCACCGACACCTTCATCTCCACCGTCACT

CAAATGGCCTCCCACGGCCCTTCCTTCTGCGACATCACCTGGCGCCCCACCGACGCCTAC

CCTCCGATCACCCTCGACCTCGCCGCCAGCATGCAGAACTCCCTCAACATCGACACCATG

CTCCACCTCACCTGCGTCAACATGCCCATCGCCCAGCTAGACGACGCCCTCGACACTGCC

CGCTCCCTCGGCGTCCACAACATCGTCGCCCTCCGCGGCGACATCCCCCCCAACCACACC

CCCCACCCCGACGAGCTCACCTGCGCCCTCGATCTCGTCCGCCACATCCGTGCCCGCCAC

GGCGACTACTTCGGCATCGCCGTTGCGGGCTATCCCGAAGCCCATCCCTCCAAGATCCCT

CCCGGCGCACTCACCGCCACCTCCGAAGGCTACGAAAGTGACCTGCATTATCTAAAAGCT

AAAGTTGATGCTGGTGCGGAACTGATTGTGACACAGTTATTTTTTGATGTTGAGTTGTTC

CTGAAATTTGTGAAAGATTGCCGGCGGGTGGGGATTAAGTGTCCGGTTGTGCCGGGGATT

CTGCCGATCACGACGTATCGGAATTTTAAGTTTATGACTGAGACGTGCCGGACGTTCGTG

CCGGCGGAGGTGAAGGAGGCGGTGGAGGCGAATAAGGGGGATGATAAGGCGTTGGCGGCG

TATGGTGTGGTGCAGGCGACAGCAATGTGTCGGCGGATATTGGATAGCGGAGTGCGGGAG

CTGCATTTTTATACCATGAATAGGGAGGGGCCGGCGGTGGCGGTGCTGAGGAATTTGGGG

TTGTTGCGGGGTGGGGTTAAGATTCCGAAGGCGATTGAAGTGGCGAAGGAGAGGGAGGTG

AAGAAGGAGATTGAAGCGGTGAAGGAGAGGGAGGTGAAGAAGGAGATTGAAGCGGTGAAG

GAGAGGGAGGTGAAGAAGG

>Unigene0000592

GCCGCCGTTGCCGTTTCCTCCAGAGCCCTGCCCCTGCTCGCGCTGTCGCATGCGCGCGAT

CCACTCCTCCGTGAGGAGCAGACGACCTCCTGGGGTAGGTGCGTCGCTGGTGCCGTACCG

CTCCTCGACAGAGCGTAGCCGCCCGGTGAGTTCCTCGATCGACATCGTGCGCAGATCGAG

GAGGGTCTCGATGGTGATGGCCACCTGTGTGTACCTCGGCGGCACTACACGGAGGAACTT

CTTGACCACCCTCTCATCCTCCAGTGTGTCCCCGAGCGTGC

>Unigene0000593

CCCTAATTCTGGCCCCGCAAAACCAGGACTCCCACCATTCAGAACCCCCCTCTCCGCCGG

CCGTCGGCGGCGATGCCGCCCCCGGACCCCGGAGAGTTCCGGGTTTCTAAGGTACTATTC

AACCGCTACGAAGTCGGCCGTCTTCTTGGTTGCGGCGCCTTCGCCAAAGTATACCACGCC

CGCAACCTCCGCTCCGGTCAGTCCGTCGCAATCAAGATCATCTCCAAGTCCAAAGCCCTC

CGAAGCAATCTGGCCCCTAACATCCGCCGCGAGATCAGCATCATGCGTCGTCTTCACCAT

CCTCACGTCATCCGCCTCCTAGAGGTACTCGCTTCTCGTTCAAAGATCTACTTCGTCCTC

GAGTTCGCTAAGGGTGGCGAGCTCTTCGCCCGCATCGCTTCCCGTGGCCGTCTCCCAGAG

GATGCATCCCGCCGCCTCTTTCAGCAGCTCATCTCCGCCATCGGATTCTGTCACGCTCGT

GGTGTTTTCCACCGTGACCTAAAACCGGAAAATCTATTGCTCGATGATGCTGGCGATCTC

AAAGTCTCGGATTTCGGTCTCTCAGCAGTCGCAACGGACCAAATCCGCCCCGACGGGATG

CTACATACCCTCTGCGGCACTCCGGCGTATGTAGCACCGGAGATTCTTGCTAAGAAGGGC

TACTCTGGAGCAGCCATTGATATATGGTCGTGTGGGGTGATTCTGTTTGTGCTGAATACT

GGCTATCTTCCGTTCAACGACCCTAATTTGATGGAGATGTATCGGAAGATCTATCGTGGG

GAGTACCGGTGCCCACGGTGGATGTCGCCAGAGCTCAGGGGGCTTATTGCAAGGTTGCTT

GATACCAATCCGGAGACGAGGATTACCGTCGATGGCATTATTGGGGATCCTTGGTTTCGG

AAAGGATTGAAGGAGGAGAGGATGATGGCTCTTATGAGGTTTCATGAAGAGGTTGAGACG

AAGCTGGCTGGCGGTGGGGAAGAGGATGATCGAGATCTGAATGCGTTTGATATCATTTCG

TTTTCGTCTGGGTTTGATCTATCGGGGTTGTTCGGGACGAGTCGGGCTGGGAGGAGCCGG

TTTGTTTCGGATTGGGAGGCGGAGTGTGTGCTTGC

>Unigene0000594

ACGGCTAGAAATAGAAAGAAACCAAACCCCCCTCAACCCCTCGACCTCACCGACAGCAAC

CCCGGCGAGGATGGCTCTCTTCTCCTCCGCCGCCCCCATGATGCTGCTGAACCCCTCTCA

TCCTCCACATCAGCGGAAGAGCTCCACCGCTCTCCGTCTCTCCCCCTCTTTCTCCCCCAA

CAAACTGGCCGCCCGCCGTCTCACCGTCCATGCTTTCTTCAACCCCTTTGAAGACCAACC

CATCATCAGAGACGCCCTCAAGGAGCCTGTGGCTTTCCTGGGTGGGGTGTTTGCTGGCCT

TCTGAGGCTTGATTTGAAGGAGGACCCGCTCAAAGAGTGGATTGCGAGGACGGTTGAGGC

GGCCGGGGTGACGGAGATTGGTTCCGGGGAGGAGGCGGTCGGGGGAGATGATGATGAGCC

CCAGGAGATTCAGATTGAATGAGAGTGATTTCACTTATATGTGTAAGATTTCTTAAATTG

TTGGTTATGTTAGTTGTTTAGTGTTGTTACAAGTAAATTATGGAGTTGGATTTTCGGTCT

GATATGGAAGGTTAATATTTGATTTGTCTTGCTTTTATGGATGTCCATATTAATAGTTGA

TTGTGTCTAACCTTTTATGGATGTCTATAATCGGTGTAAGTTATCGAAGAAGTTGATATT

TCGCCTATCATGCTTTTACTAACATTGATAGTAATTTGTTTAAGTGAGCGATT

>Unigene0000595

GGTGGGGTGTTTGCTGGCCTTCTGAGGCTTGATTTGAATGAGGATCCGCTCAAAGAGTGG

ATTGCGAGGACGGTTGAGGCGGCCGGGGTGACGGAGATTGGTTCCGGGGAGGAGGCGGTC

GGGGGAGATGATGATGAGCCCCAGGAGATTCAGATTGAATGAGAGTGATTTCACTTATAT

GTAGGATGAAGAAGAGAAGGGACTCATTTGCCGATTTTAGTTTTGAGGTTTCCTAAAATG

TAATCTGGCTTGCTGTACAAAACCTATTTCTCGCCAGTAGCAAGTTGGCTAACTAGTTCT

CGTGGTTAGCCGAGTTCCTTTGAGCTTAACCCTCTCAGGCCTCAATATCCGGTTTTCTTC

ACGGACTCTGCACTGGTTCCCTACATTGACGGGGGGCATTATTGATGATTTATGTTCAAT

GATCAGTAGTTATATTTCTGCAGTGATGTAATCACCTCTTTCCTTTGTATGGAACTTATA

TTACCTGTTTACCTGGTTTTCTGCATCAACTTAATCTCCTTTTCTTTGTATGGAAATATG

GATATGTCTAATTGTATCGTATTTTGGTCGATCTTTCTTTTAATAGAAAATAAATTT

>Unigene0000596

TAAATAGAGAGAAGATAAAACTGACTGCTGTGTCGTGTTGCGCTGAACCCGAACCGTCTC

GCACATTTCATGTGATTATAATCCTCCCAGATTTTTTGATCGCTATCGCCTCATCATCGT

CTTCGTGGCCCTAATCCAGGGCTTTGGGGTTTTAAGCCATGGCGGTGAAGGACGAGGTCA

AGGACGAGGAGTTCTTCATGGAGGACGGCATCGATGTTGAAGATGACAGGACCCGGAGCG

GCGGCAGCAGTAGCGAGGGGGAGGAGGCGGACGGCCATGAGTCCGCCTCGGCTGCGTTCC

ATTCGCGGCAGTGGCCTCAGAGTTTCAGGGAAGCAACTGATTCTTACACTATTGCTGCAT

CTCCCTCCTTTGGAACCTTTGCTCGCACACCAAGTCTTAGATACTCGAGTTTTAACCACT

CTCAAAATGAACAAGATTCAGACACGAGTTTGCCCCTTTTGTCGGATAGCGTACCAGAGA

AACAAGAGTCCAGTAAGAACTTGAGAAAATCTTTTAATTTGCCGGCAGCTGAGACATTAT

CTTTCCATCCTCAAGATACTGGAGAAGGAAATCTTGGTCATGGTTGTAGCTTCACTCAGA

CTGTATTTAATGGTGTCAATGTGTTAGCAGGCATTGGTGTTCTTTCTACACCATATGCAG

TGAAAGAGGCTGGCTGGGCGAGTTTGATGTTACTGGTTACTTTTGCAGTTGTTTGCTGTT

ATACCGGCGTTCTTTTGAAATATTGTTTTGAGCACAAAGATGGTATTACAAATTACCCAG

ATATTGGAGAGGCAGCTTTCGGCAAATGTGGGCGGATTTTTATATCTATCATTTTGTACA

CGGAGTTATATTCATATTGCATTGAGTTCATTATATCAGAAGGTGATAATCTTACGAGGC

TTTTTCCTGGGGTTGGTTTAAATTGGGGTGGAATTCATCTGGATTCTTTGCACTTCTTTG

GGATCTTGACTGCTCTCATTGTGTTGCCGACTGTTTGGCTGCGAGATCTTAGAGTCATTT

CTTACCTTTCAGCTGGTGGAGTGGTTGCCACTATTTTGATTTCTCTCTCTGTAGTTGCCG

TTGGTGCCACTGATGGGATGGGATTCCATCAGACTGGAAAAATAGTGAACTGGAGTGGTG

TCCCATTTGCTATTGGTATTTATGGGTTCTGCTACTCTGGTCATGCCGTGTTTCCGAACA

TATATTATTCAATGGCTGATCGAACAAAATTCAACAAGGCTTTGATAGTATGCTTTGCTT

TGTGCACGGTGATATACGGTACTGTGGCTATTATCGGGTATCTCATGTTTGGCGAAACTA

CTTTGTCACAAATAACATTGAACTTACCAAAGAACGCCTTTTTCTCTAAAGTTGCTTTAT

GGACCACGGTTATCAATCCTTTTACCAAATATGCATTATTGCTAAATCCATTAGCCCGGA

GTTTAGAGGAGCTTCTTCCTTCGAGAGTCTCAAATAAAACATGGTGCTCCATTGTTTTGC

GAACTGCTCTAGTTATGTCAACCGTATGCGTTGCTTTTCTTCTGCCCTTTTTTGGTCTTA

TGATGGCTCTGATTGGATCTCTCTTCAGCATAGTTATGGCGGTGATAATGCCAGCGCTCT

GCTTCTTATGGATTGTCGGGAAAAAAGCTACGTCTATTCAGGTGTGCTGCAGCATTGCCG

TTGTCGCATTGGGTACTATTAGTGGAATCCTTGGAACTTACTCTTCAATAGTTAAAATCT

CGAAGAACTACTAAGGGGCATATTTCGAGTGACATACCTTTTGTTAGTGCTCGTATGCAC

AGATAAGATCGTCCAAGTCTAGCAAACCTGTACTAGCAGGATATGTTAGCTTGTTAAATA

ACCTTATTGTTTATCAGGGGCTTCGATAATCTATTGCATCCAGAATTATTCTGACTCTCA

TCCTCCTTCACACTCACATTTCGTCCACCTACTGTCGTGTGCTACAAAATAGTGTGAAGT

TAGCATTATACTTGATTTATGACTTCCCAACAAATTTTAATAAGTTTGATAGTGGAAAT

>Unigene0000597

GGATGGATTGCATGACTACTGTACGACTTCAGTGTGAATGCCAATAAAGTCAGAAAGTCT

CCACTGTTTGTGCTGATTCCTTTTCCTATCGCTGAGAATATATATATTTCTTTTTCCAGC

TAATCTGTATTATTTACTTTGAATTGTCATCTTGTCAATCTTTGTGTGAGCATACATACT

GTATTTTTTTTTGTGGTTGACTTAATGTTTTTTGTCCTTTGGGCCATATTGCAACATGCT

GTCGTTAAGAAATGCCCATTTTAGGCCAGTCCTTTAAGATCTAGGGTCATATATTATATT

ACACATGTCTGAATAATCGGAACTATGTGTGCAATTGTTTTTATTTTACCTGTAATGCAT

TTTGAACGAGGTCAGTTTTTTTACTTCAAAAGAAGTGCTAGCTTATAATCGACTCGTGAC

CATGTCAAGTGCTTTATACCTTGCATGCAGTTAGTGCTAGTTTCATATCTGCTCTCTGTG

GTCAGGACATGCTTCTACATAACCACAACCTCCTTAGACCTTGAATGACCCTAGATTGTC

CCATGAACCAGGTATAAGGAACTTGATACTTCGAAGTACCCAATCTGCTGTCTCTCAGGT

CTCTTAGTGTCATTTCTTACTCTCCCTCTATCATCAGTTTCGTCTTCTTGACTGCAATTT

CTGCCTTGCACATAAGCATATGAGATTGTGCTGTTATTTGTTTCCTGTATTGCCCCATCT

CTC

>Unigene0000598

CTGCACTCTCCATGGCTTCCAAACTCTCCTTCATGCTGTTGGTTTCATGGTTTGCATTTG

CCTTTGCACTTCAGCCAATAAGCACCAACATTAGCCTCGACTCCACACTCCACCCTACAG

TCACCCCTACATCATGGCTCTCCCCCTCTGGCCATTTCGCCTTCGGATTCTATCCCCAAG

GCAGCGGCTACTCCGTGGGCGCATGGCTCGTCACTCCCGGAAACACCACTGTCATATGGA

CAGCTTTCCGAGACAATCCTCCATTTCCTAAATCCGTCTCGTTGAAGCTGACCAATCAAG

GCCTCAAGCTCCTAAACACAATCTCCGGAGGCCGGCTCATCTCTGA

>Unigene0000599

CTGCACTCTCCATGGCTTCCAAACTCTCCTTCATGCTGTTGGTTTCATGGTTTGCATTTG

CCTTTGCACTTCAGCCAATAAGCACCAACATTAGCCTCGACTCCACACTCCACCCCACAG

TCACCCCTACATCATGGCTCTCCCCCTCCGGCCATTTCGCCTTCGGATTCTATCCCCAAG

GCAGCGGCTACTCCGTGGGCGCATGGCTCGTCACCCCCGGAAACACCACTGTCATATGGA

CAGCTTTCCGAGACATCCCTCCATTTCCTAAATCCGTCTCGTTGAAGCTGACCAATCAAG

G

>Unigene0000600

GGATTCTATTCTGAAGGCAGCGGCTATTCCGTCGGCGCATGGCTCGTCTCCGCTCCCGGA

AATACCACCGTCGTGTGGACAGCCTTTCGCGACAACCCAATATTCCCTGAATCCGTCTCC

TTGAAGCTGACCAATCAAGGCCTCAAACTCCTAAACACAATCTCTGGAGACCGGCTTATC

TCTAAAGACATATCCACCACTGCCTATGCTTCCATGCTCGACTCGGGCAACTTTGTCTTC

CACGGTCCCAACTACGAAACACTGTGGCAAACCTTCGATCATCCCACTGATA

>Unigene0000601

ATCGAGCAGATCTTGAGATCTAGCTCCTTTTTCTTCATTGGCCAATTCAATATATTGATT

GATCCGGACGGACGTTCTCTGAAGCATGGAGAGGATAATAGAGCGCAAGGATCATGAGCA

GTGAGGATATACAAGCTTTATAATTTGAGATTCCTTGAGGGCCAAGCTGCAACATGTGAT

GGTGTGGGTAGATTACTTGTTGATGAAGGTCCTTGGATATGAAGGATGCTCAGGTGAAAT

TAATGTTATGTACCTCTATATATTGAAGAAGCCGAAGTCCATTGTCAACTAGTCTGACTT

GCCTCTTGTTAGAGAGAAAGTGAAGGAGGAGTCCGGGAGTTGGTTGTAATACACGAGAGG

AGTTATGGGAGTCGGGATTCCTTGATGCTGAAGAAGTCTACAAAGCATTTGATGAAGTTG

GATACTCTGTGAAGCTGGAATTCCTTTCTTCTCTCTCGGATGATACACATTGACATTGTG

AGTTTATGATGCCTTAGTTTATGATGCTTTAGTCTATGATGTCTTGATATATGTGATTGA

GATGTGTATTGTATGAGTTTGATGTCTAGATTGCTGTTTAGTTATGTTATATGTGAACTT

ATTGATAAGTTGAGAATCAGGCTGTGACACTTAAAAGCCTTGTAATGGATAAGCTTTGTG

GATGCTTTGATTAGATATGTTGGGATAGTTGAGTCTATGCTATGATCTTAGATGAGTTCA

ATTATAATGAATGA

>Unigene0000602

ATCGAGCAGATCTTGAGATCTAGCTCCTTTTTCTTCATTGGCCAATTCAATGTATATTGA

TCTACTTCTTCTCGACCAATACTCTCGCTGGATCTTCTATGCTTAATATTTCCTTAAGGT

AACACTCTGATCTTGAGTTCTAATGAATTTATTTCTTTTCTTATTTCAGATATTGATTGA

TCCGGACGGACGTTCTCTGAAGCATGGAGAGGATAATAGAGCGCAAGGATCATGAGCAGT

GAGGA

>Unigene0000603

GCCGCCCTCCACCGGCGAGCTTATCCCCTCCTATGAAATCAGACCTCGCCCAAATTATAG

CCACAGAGTCGCCAAGCCACCCTGATCATTTACCTCCAATCAATTCCAAAATCTATCGAG

CAGATCTTGAGATCTAGCTCCTTTCTCTTTGTCGTCCAGTTTAATGGAGATTAATCTACT

TCTTAATATTTCCTCTCTCTAGATCTTCTATGCTTAATATTTCCTTAAGGTAACACTCTG

ATATTGAGTTCTAATGAGTTTATTTTTTTCTTCTTATTTCAGATATTGATTGATCCGGAC

GGACGTTCTCTGAAGCATAGAGAGGATAATAGAGCGCAAGGATCATGAGAAATAAGGAGA

TGCGGGTCTTATAACAGTAGATTCCTTTAATGCCAAGCTTTGAGCATGTGATGGCACGGG

AAGAGACTTGAAGGTGAAGGTACTTTGTATTGAAGCAAGGTCACATGGAGATTAAGCAAT

GTGTTCCTATATAAAGATGAAGTCGGAGTTAATTGGAGCTAGTCTGGAGAGGGCTTACAC

TGTGAGCCAGGGAGTGACATTCATCTCTGGAGTTGGTCGGGTGGAGTTATGGGAGTTGGG

CTAGTTCTTTGATGAGGTAGATGCTGATGAAGTGATGCTAAAGATCTCTATGAAGCCATT

CTTTGAAGTTGGATTCTCATCTTTCTCTCTCTCGGATGCTACACACCTCGACTGTGAGTT

CTTCGTCCTTACCTTTGTGAGATGATGTTATGTTAGAGTTACTTGGTTGATTGTATGAGT

TAGTGTTGATGAGTTAGTTGTGATGAACTTGAGTAGTCTGTGATGAATGTTTAGGATGAT

GTTTAAGCTTTATAATGGCTGAACTCTGTAGATGTTTTGATGCTTTAGCTATGTGATAA

>Unigene0000604

GCTTATCCACTCCTATAAAATCAGACCTCGCCCAAATTATAGCCACAGAGTCGCCAAGCC

ACCCTGATCATTTACCTCCAATCAATTCCAAAATCTATCGAGCAGATCTTGAGATCTAGA

TCCCTTCTCTTTGTCGGCTAGTTTAATGGAGATTAATCAACTTCTTCCCGACCAATCCTC

TCTCTAGATCTTCTATGCTTAATATTTCCTTAAGCTTAGAGAGGATAATAGAGCGCAAGG

ATCATGAGCAGTGAGGAGATGCAAGTCTTATAGCAATAGATTCTTTAAAGGCTAAGCTTT

GAGCATGTGATGGCACGGGAAGAGACTTGAAGGTGAAGGTACTTTGTATTGAAGCAAGGT

CACATGGAGATTAAGCAATGTGTTCATATATAAAGATGAAGCCGAAGTTAATTGGAGCTA

GTCTGGAG

>Unigene0000605

CACTTAATATAATTTTGAGTGGCAACAAACTCTCGGGAATGATTCCCAAAGAGTTCGGAA

AGTTGACCGACCTTGAAGTTCTTGATCTTTCTGAGAACAATTTAAGCGGGCCGATACCAC

AACAGTTGGGAGATTGTTCTAAGCTGCTAATGTTGAGTTTGAGAAGTAATAATCTGAATG

GTAGTATTCCTGCACAGATTGGTAAGCTAAAAAGCTTAAGCGAGCTCCTTGATCTGAGTC

ATAATTCTATCACAGGAGAGATACCATCAGAAATTGGAACCTTGGTTGTGCTGACAAATC

TGAATCTCTCACACAATAATCTTACAGGTGATGTACCATCATCTTTTACAAATACACTAA

GTTTGTTATCGATTGATCTGTCTTACAACAATCTAGAGGGACGACTTCCTAAAAGTAAAT

TATTCGAAAGTGCCCCATTGCAATGGTTTGTCCCAAACAAGGATTTGTGTGGTGAAGTTC

CAGGTCTTCCTCAGTGTGTTTTGACTTCTACAAACAGCCAATACTCAAGGAAAGGCCATA

ATGTAATTGTCATTGTCCTTCCTGTCTTAGGAAGCTCGGTTATATTA

>Unigene0000606

CCCATTTATTAAACCCTGGACCCAGAAGGGAGAGCTTGGTGGTCGGCAACTCCTTCGTCT

CCGGCAGCGGCGACTGCGCTCCTCTAACGGATGACTGAAGAAGGTTCTCTGGAAGATACT

CCATGATGAAGGACTGATTTCCCCTCTGCTGGTCAGGATCCAATTTCTACTTTCTCATTG

AAGCCACTGTTTTTGTGGGACTGCTGTGCTGGTCATTTTGAAAACAAAATTTGAGGTTGG

CGATAACGAAAGGTCTTGCGTGGGAGCTAACTCGTAAAGATGCAAATGCCACCATTGCAA

GGACTACCCATTTTTCTCTATCAAGTGTGGAAACTGGAACTCTTGCTGTGTATGCGAGCT

CTGCAAAAAGGAAAAAAGGGAAAGAATGGCTTTAATTTTTCCAAGTTGCAGAAGAAATTA

TTAAACATGATTGCTGCTAAAAATATTTGAAACTCAATATGAAAAACTTGTGGGTGGGAT

TTGGTATACTTTCTTTGCAAAAAGAGAACAAAAAAAGAAAAAAGAACTCGTGATACAATT

TCGGCTCTTCAATTCAAACCCGTAGTATCCCAAAGGATCCTTAAATAAATTGTTGACGTC

GTAGTCTGATCCTGTCACTTAATAGAACCAGTCAGCATACGGTATGAGGTTGACCATTTT

TGTTATGCCATCTGTTGTACTCTTGAGTTATATATTACATTTCAAACAGTGAAAGCAACA

TTTATATACCCTTAAACTTGCTAATTATCCAATGTTATTTTCGTGCTCAATTTTTAAAGA

GATTAGTGAATACCTGTGCACTCACCTTTACAGGGAGACCAGACAGCCGTTTCTGCATTG

TCA

>Unigene0000607

AAAAATATTTGAAACTCAATATGAAAAACTTATGGGTGGGACTTGGTATACTTTCTTTGC

AAAAAAAAGAAAAAAGAACTCGTGATACAATTTCGGCTCATCAATTCAAATCCCTAGTAT

CCCAAAAGGATGCTTAGATAAATTGTTGACGTCGTAGTCTGATCTTCTGTCACTTGATAG

AACCAGTCAGCATACGGTATGAGGTTGACCATTTTTGTGTTATGCCATCTGTTGTACTCT

TGAGTTATATATTACATTTCAAACAGTGAAAGCAACATTTATATACCCTTAAACTTGCTA

ATTATCCAATGTTATTTTCGTGCTCAATTTTTAAAGAGATTAGTGAATACCTGTGCACTC

ACCTTTACAGGGAGACCAGACAGCCGTTTCTGCATTGTCA

>Unigene0000608

TCTAGGGTTTCTTCGTCGTCCCTTGTCGCCGCTGCTGAATTGGTGGCGATCTCCTCCTTC

CCGATTTTAGGGTTTCGTCGTCGTCCGTCGCCGTCCTTTACCTTTCTAACCGAGATCTAC

AACAATGGCTACCCCAGATTGGTCAAATCTCCATACTGATATCCTGTTATTAATTCTCCG

CCGATTGCCGGTAATCGACTATGTCCGATTTGGCGCCGTAAGCGTCTCCTGGAACCGCCT

GGCCGCCTCCAACCTCGTCCCTCCTGCCCCGCAGGTCCCTTGGCTGATGCTTTCATCCCG

CGATTATTACCACCTAGACACTGCCATGTACGATCCCTTCACAAAGAAGCAGCAGTGCAG

GATTCGGCTCAAAATTCACCAATTCTACCGATTCTTCGGCTCTACCTCCGACGGATATAT

GATCTTTCATTTCTACTCGGACATTCTCTTGCTGAATCCTCTCACCGGATCCCGCATCAC

CCTCCCGTCACTGGACACCCTCCCCGGCCGACACGATTGTCTCTCAGAGATAAGATCAGT

TGTTGCTCGCATAAAACCGAATACAGTCATGGCCATTTGCTACACGAGCTTTCATACAGA

AATTGTGGCCTTTTATAAGGCTGGAGACAAAGCGTGGACTTTTCTGTATCAAACTCAGCA

GCTTAATCACATCATGCTTTACAAAGATAGGCTCTATGGGGTCACTGATGATAAGGAATC

TCTATTAAGCTTTGATCTCAGGGAAGATTCGACATGCTCGTTGATAACGGAAGAAGATGT

TACAGATCCGAGTTACAAGTATCTGGTGGAGTCGACCGGAGGTGATCTGTTGAAGGTCCA

CCGTAGCAAAGGTGACATGGTGAATGCAGTCTTTAAGCTAGAATTGAGCAGCAATGAGTG

GCGGAGAGTGAGGAGTATTGGAGACGATGCGATCATGTTATCTTTTCAGTGGCAAGAGTC

GACTCGATGTGTGTGTATCTCCACACAGAACTTACCTGAGCTGCGACCGAACTGCGTATA

TTCGGTTTCAGGTTATCATCTACCTGTGAGAGTGTACAATATTGGAGATGGGAGCACGGA

AGAGATGGATCTTCCCTTAGGTAAGGAGAACTTATATTACGTCCATATAAGGAATTGGTT

CACTCCGAGTCTTTTCTAATAGGGTTGGTGTGAGCACACTCCATTTTGTTTCGGTAATGG

CGACTTATAATTTTGAAAATGACGTAGTTTTTATGCTTTGTTTGTATGAAATGCATTGCT

ATTTCATTAATATTGGTCATATGAAGCACAATATCTT

>Unigene0000609

TCTGCACAGAAGAAAACAGCCAATGGGAGATGGTCGGGGATTCAAAGAAGACCCACCTAT

CGTCCAAGCTTGCCGACGGCACTGCCATTTGCTTGGACGTCGAACAAGACGGAACAATCG

TCTCTAACCCTTGTAAAAGGCAGGCTAGTGGTGAAGAACGTGATAACCAGTGGTTCGATA

TGTTAACAGGTCCTTGGAGTGCAGATGATGCCGAAAATGGGGTTTCTTGTGCTGCCAGTG

AATCAAACTAAGTGACCAATGGAATGGTCGACATCAAATACACCAACATCCAAGTCAGGG

ATTGTAAAACTAAATAATGATGGGGGGATCTCAACGATGAACTAGATTACATATGGATCA

ACGGTGAAATATTGTGTGTGTGAATTTAAACT

>Unigene0000610

AACGAGCTCCGGTGATTCAAATCCGATGGGGATGGAGACAAAGAAGGGAGCGGTGGACGA

GCTCATCGAGCGGGCCGGCGGCTGCGCCATCATCGACGGCGGCCTCGCCACCCAGCTCGA

GGCCCTCGGGGTCGACATCGGCGATCCCACGCTCTGGAGCGCCAGCTGCCTCATCTCGAA

TCCGGATGTTATCCAAAAGGTACATTTGCAGTATCTGGAGGCGGGGGCGGATGTATTGGT

TACTTCTTCTTATCAGGCAACTATCCCTGGTTTTTTGTCCAAAGGGCTGTCTATTGAAGA

AGGAGAACACTTACTAAAAATAAGTGTCAAATTAGCACTTGAAGCCCGGGATAAGTTTTG

GAAAATTGTTCAATGCGGTCCTAAGCATAACTACACTCGTGCTTGGGTGGCAGCTTCTAT

CGGAAGCTACGGAGCTTACCTTGCAGATGGTTCAGAATACGGTGGATATTATGGGGAGGA

TGTGAATGTGGAGAAACTAAAAGATTTTCATAGGCATCGTTTACAAGTCCTAGTCGACGC

TGGTCCAGATTTGCTAGCTTTTGAGACTATTCCCAACAAACTTGAAGCTCAGGCGATTGC

TGAGCTGCTTGATGAAGAGGACGTCCAAATTCCTTCTTGGGTCTGCTTCAGTTCTGTAGA

TGGCGAGCTTGCCTCTTCAGGAGAGGGCTTCCAAGAATGCCTTGAAGTATTAAATAAGAG

CGATAAAGTTAATATCGTTGGAATAAATTGTTCATCACCTCAGTTCATTGAGAACCTCAT

AATCAAATTTAAGAAGTTAACTAAGAAAGCCATAGCTGTTTACCCGAATAGTGGTGAAGT

ATGGGATGGCAAAGCAAAGAGATGGTTGCCATCTGAGTGCTTTGGAGATGGGAGTTTTGC

ATTACTTGCAAAGAGATGGCATGAATCAGGTGCTAGTTTTATAGGAGGTTGTTGTCGGAC

GACGCCATCTACCATAAAAGCAATTTCGAAGGCTCTGAAGAACAGCCAGTAGCAAGAAAC

TATCGGCTACGCTCCTTGGTTGACCGACTGCCAGACTGTCAGATACTTTTAGGCTAGGGA

ATTGATGTTGTGACAGATACACTCATGTTGTCAACGAGAGGATAAATTGTAGCTAACTGA

AGAAAAATTAGTATGAAAAACTATTTTATACGCTCTGTCGATCAAATAGGTTACACATGT

TAGGATAGGGTTGACCTATTTAGAATGAGTCTGACTAACC

>Unigene0000611

GAGTCGATCCTGCTTCAGTTGAGCAGGAATTAGGGTTCTCGAGCAAATATCGAAATTTCA

ATCCTAGGAAACACTTGATTTCTCAATTGCGAACAATGGAGGCAACCCTAGAGCATACCC

TCCATGAAGGCAATCTTCTCCGCCAAGTCAATGCCCTAATCGTCGCCCACCTCCGCGACA

ACAATCTCAGCCAGGCCGCCACCGCCGTCGCCTCCGCCACCATGACGCCGCTCGGCGCGG

AAACTCCTCCAAACAAGCTTATCGAGCTCGTTGCGAAGGGTTTGGCGGTGGAGAGGGACG

AGGCGGCGAGGGGAGTTTTTGCGGCTGCGCAGGTAGAGGGCGGGATACTCACTGGCTATG

GCTCCGTGCCGGTTCGTACCAATGCTGTAGATTTCAGCATCACTGAGGAAACAAAAGGTT

CGTCCAAGAGCTTTCCTAAACATGAGACTCGACATGTTTCAGAACATAAGAATGTTGCAA

GATGCGCAAGATTTAGTCCTGATGGGCGGTTTGTTGCAACTGGGAGTGCGGATACATCAA

TAAAGCTATTTGAGATATCTAAGATCAAACAAATGTTACTTGCCGATAACAGAGATGGGC

CTGTCAGGCCTGTTATTCGGACATTCTATGATCATTTACAGCCTGTCAACGATTTGGATT

TTCATCCACAAAGTACTTTACTAATATCTGGGGCGAAGGACCACACTATCAAGTTTTTTG

ATTTTTCGAAAACAGTTGCAAGGAGAGCTTTTAGAGTTATCCAGGACACCCATAATGTTA

GGTCGGTATCTTTCCATCCTTCTGGGGACTTTCTATTGGCAGGGACAGATCATTCTATAC

CTCATTTGTACGATGTTAATACTTTCCAGTGTTACTTATCAGCAAATGTACAGGAGCTGA

ATGCTAATGCTGCCATCAACCAGGTGCGGTATTCATCTACCGGCAGTATGTATGTCACAG

CTTCGAAAGATGGGTCCATACGAGTGTGGGATGGGGTGACTGCAGAGTGTGTGCGGACAA

TTATTGGCTCCCATGGGTCGACAGAGGCCACTTGTACAAGTTTTACCAAGGACCAAAAGT

TCATTCTTTCTTGTGGGAAGGACTCAACTGTCAAGTTGTGGGAGGTTGGCACAGGAAGAC

TTGTGAAGCAATACTTAGGAGCAGTTCACACGCAGATGCGCTGCCAGGCTGTTTTCAATG

AGACAGAAGAATTTATCCTGTCAATCGATGAGCTGAACAATGAGCTTGTTATTTGGGATG

CATTAACTGCTGAAAAGGTTGCTAGATGGCCATCGAATCATATAGGCCCTCCACGGTGGC

TGGAGCATTCCCCGGCTGAAGCCGCCTTTGTTTCGTGCGGGACTGATAGGTCTGTTAGAT

TTTGGAAGGAGATCCTTTAGTTCTGTAGTTCCACCAGCTGGTGGTGATATGCAATGGTTA

GTACAGTTGCCTCAAGATCTTCTAGTCCCACCAATGGACTTCACACATCTTTGGTATTGG

CCCCTGGGGTAGACAGATGTTTCTTTCTTGCACTCCTTCAGAGATGGCCTTCAAAGTAAA

TGCAACACCAAAAAGAGGAAGTACTCCCCTTTCTCATCATGACTTGATATAGTATCATGG

TTGAGCTTCGCAAAGGGGCTGGTGAATACTTTTGATGAGGAGTGAATCTTCTTGATTTCG

TTTTAGTGGATTCTTTTTTTTGCTGCTGTACTATGAAAACGAATTTTGATGGAATGGGCT

GATGCAATCTCACTTCATGTATCAAGCCTGGATTCTCTTGACCCAAATTTACGGATAATA

TAGGTGGCATCAGTGGATGAGGGCCAAGCTTGTGCGAAGGATCAAAGTCAAAAGCCTTTC

TTTGCTTGCTGTGAATAGAGCTGGAATTTGTGTGTTCATATTGTTAGGAGATGGTAGCAT

CCTAATTTAGGAACACGTAAGCTTTTTTTGGGTAAACATCG

>Unigene0000612

GACCATTTCCTATCCTCCATATCAGTCCTTGGTTTAACCAGTCCTTGGTTTAACAACTTT

GCACCGTATAAGATACCTCTCCAACTTGTGGACATATCAACATTATCTGAAAGGTTAGGC

TGCAGTAGACGTTTGTCTTTAAGATATTTCTCTGGAACAGATCCGCCCATAAGCCATCAT

TATTATATGATAATCTCCATGCAGTTTTAGCCAGGAGAGCCTTATTCATGTATGTTGCTT

TCTTCAATCCCAATACACCCATTGATTTAGGTCTACATACCAAGTTCCATCGATATAGAT

GAATTTTATGCTTTGCTTCAGTATCACCCCATACGAAGTTACGATTAATTTTGTCTAAGC

TATCACATATACTCATAGGAAGTTTGGCAGTTTGTATTGCATAAATAGAGACAACTGTAA

CAGATTGAATTAGGGTCATTCGTTCAACCATGGAAAGAGTATTACTTTTCCAAGTGGCTA

ATCGT

>Unigene0000613

TCGAATCCCGATATCAATCCTTCCGCATCACTCCTCTGCATCATCGTCGCCGTCAATTGC

TTGTTCAGGTACGCCCATCGCCGGCTGCCGTCGCTGGCATTCGAATACCGATTTCGATCC

TTCTGCCTTGTTCCGCTGCATCGTCCTCGCCGTCAATTCCTTGTTCAGGTTAATCTCTTT

TAGGGTCAATGGAAGCCAGGTTCTTTCGGTTTCTGAAGATCGTGGGAGTCGGGTTCAAAG

CCAGATCTGAAGCCGAAGGCCGTAAACTGCTTCTGAAATTGGGTTACAGTCATGAGGTGC

TATTCGACGTCCCTCCTGCTGTCCGAGTGTTCTGTTTCAAACCAAACATAGTCTGCTGCA

CGGGAATAGACAAGCAGAGAGTGCATCAATTTGCGGCTGCTGTTCGGAGCTCAAAGCCTC

CTGAAGTTTACAAAGGGAAGGGTATTATGTACATAGATGAAGTTGTGAAGAAGAAGCAAG

GCAAGAAATCTAAGTGATTAAGTAATTGAGCATGTGGTTAATGGTAATTCGGAAGATAAA

CCGGTAGCTGGTGTATGGTTTCTAAAACATGAAATTGTCAGAGTCCTAAGTTATTTGTTT

TTAGCTTCGCAATTGTTGAAATGTGGAGTCTTGAAGAAAACAGTTTTTCAGTGAATCTGT

TGCTTGTTAGTAAGTATGTTATATCGTCCACTTTCTTTTCAAGATTTACAGTGACACTAT

TGCATCAGGAAACCAGTTTTGTAGTGACGCTGTTGCTATAGGAAACCAGTTTTGCAGTGA

CACTTTGTTAGTAGG

>Unigene0000614

TTTCCTCCTGACATGTTTTGGGTGGGCATCCGGCCTCGTGCTCAACTGTGCAAAGACGCT

GCTCTTCTATCTGGGGCATAATGTAACAAGGGCCGTGTGATTAGCATCAACCTTCGGGTG

CTCCCTGGGGCCACTCCCTATGACCTATCTGAGAATCCCTATCTCTGATCGGCCGCTCCC

CAAGAGCTGCTCGACCGGTTTGATTTGTCAGGTTAGGGATCATTTGGGCCGCTGGCAGAA

CCAGTCCCTGTCCTGGGGGCTGCCTTACTCTGATCAACTCGATGTTGTCCCAGATCCCCA

CCTTCCTCTTATCTTTTCATTGGT

>Unigene0000615

CCTATCTAGGAATCCCTATCTCCGATCGACCGCTCCCCAAAAGCTGCTCGGCCGGTCTGA

TTCGTCAGGTTAGGGGTCGTTTGTGCCGCTGGTAGAATCGGTCCCTGTCTTGGGGGGCTG

CCTTACTCTGATCAACTCGGTGTTATCCCAGATCCCCACCTTCCTCTTATCTTTTCATTG

GTACCCCGAATAGGTGCTTCCGCATCTCGATGGGCTGCGGAGGAGTTTTTTTGCGGGGAT

GGGAGGGTCCCTCAGGTCTAGTTCTGGCTAGCTAG

>Unigene0000616

CCCCACCCTCATCTCCGCCGCCCGCCGCCTCCTCGCCGCCGCCCTCCTCGACGACCCCGC

CAACGCCTTCTTCGCCCTAATCTCGCAGACCGACATCCCCCTCCACTCCTTCGGCTTCCT

CTACCACTCCCTCCTCGCTGACCCCGGCTCTCCATCCGCCAATCCCACTTCCCTCCGCCA

CCGCCATCGCTCGTTCATTGAGATCCTCTCTGGCGACTCCAACCTCTACGACCGGTACAT

CGCCCGTGGAGACACTGTCATGCTCCCTGAAATTCCCTTCGATCGCTTCCGAGTCGGCTC

ACAATTCTTCGTCCTTGCCCGCCGGCATGCTCTTCTCGTCGTCCGTGACCGGAAGCTCTG

GAAGAAGTTCAGATTGCCGTGTCTCAAGTCGAAGGAGGACTCATGCTACCCGGAGGAGCA

CTATTTTCCGACGCTGCTATCAATGCAGGACCTGGATGGGTGTACAGGATATACATTGAC

TAGGGTGAATTGGACGGAGAGCGTTGGGGGTCACCCGCATATGTATCACCCACCAGAGGT

GAGTGGAGAGCTGATAAGGGAGCTCAGGAGGTCGAATTCGACTTATAGCTATATGTTTGC

CCGGAAGTTTTCGCCTGAATGTCTCGATCCTTTGATGGAAATTGCGAGCACAGTTATCTT

CAAGGATTCGTAGCGGTGCGGTGGATGTTGTGGATATGAATTGGGTGAGCTTGGTGGGTT

CATCAATGTGAGGGTTAGAAGAGAGCAGAGGAGGCCAAAGCAGATTCGATCTCTTCCCTC

GTCTGGAAGCAGCATCCATTTGCTTCGTTAGTCACAGCGTCACGTATTCTTTTTTCAAGT

GTGATTAAGATGTCGTAAGATGATATCAGTGAGAAAAGAGAAGACGATGAGTACGATAAG

CGAGGACGACGACAATCGAGTAAGCAAGTGATGGCGGCAATAAGGTGTTTGTACATTGTT

CTGAGTTATATGATACTGCAATATGTGATGATGTTGGTGATATGATATCTATAAATCTTG

CAGTGAAGTGTTGGGCTAGTTTGTACTTTGCTTGTGAGATGGTAAATGGAAAAGTGATTT

CCTCCCCCACTAAGGTGGTGTTGTGTTGCCCTTTGCTTGTGTGATAAA

>Unigene0000617

CTCCGCTAAAGATCACGGGCTTTCTTCTCATCCAAGATCATGGTGACAAGGACAGGATTC

GGCTCACCTTCATCTCAGATTCTCTATTTCACTCATTCGTGTTGAAGTATCGGAAGGATT

TAGGCATCTTCTCTCCGGCAGCAGTGGCTACTGCGTCGTTCTTGCTCCACCGACATAGCT

CATCGCCACATATACTCGTTGGGTTGTTGCTGTCGTCGTCACCCACTTCATGGGTGCCAC

TGCCACCGTTCTCCTGCAACTCCAGCCAGGGAGGTTGAATTTTGGCTAGGAGTTCCAGGG

TTCTGATGAGCTCGTTCCACCTTTCGACGGTGACGAGGAGATTAATGACCAGATCGGCCC

GAAGGGCGACAACCTGGCAGCTGAGTACACCTACTATGCAGCAAACTGATAGCACCGTCG

GAGCTGCTCCATTGCTGATGTGGCAAAGGCTGGCGACTGGCAGCCATGTCTCTAATTTGC

GAGGGGATTCTACAACAACAGCGGTAACTGCCGGTTTGTGCATGTTCCGACGAAGGATGG

CACCGCCAAGCTGCAGTGCCAGAGCTCCTGCTACAGAGGTTCCAGAGCCTGAGTTTCACC

ACCGCCGCCGCCTTCCCCCACTCGCCGACTGGCCCGCTAACGTCGACGACTCCATCTTCG

CCGTCAGCCAATTATATCTGCTTCCTGCTACAGCCGGACTCCAAGAGGGCGGCTGCAGCT

GCAGCCGCTGTATTGATGCTCAACGGTGATGATGCTCGGAGCTTCGTGGGTCGGCCGACG

ATGGGGTAGAGTGAGTTTACAGGGATGCTGAATCCGGGGTCGAGCCAGATCTATTTAACT

TTTCCAGTAGGCTCTACCTTCAGAGAGGGATGTGTCTAGCTGTTTGTTGAAATGCCGTAA

TGGGAAACACCACTATAAATTGTGTTGTTCAGCTAAATGGTTTTATTTATTTTTGTTGTT

TGTAGTATCTACGTTCCGGTTCAGGATGTGAGAATCTCCTACCAGCAGAAAAGGATGTTC

GGCTTCATCAACTTTATCTACCCGGAGACTGTGAAGCTGATACATGTGAAGGGCAACCCA

AACTTTGTCTGTGATGCTCGTGTTCTTGTCAAACCGTATAAAGAGAAGGGCAAGATTCCT

GGAAGCCTAAACATTTCAACGTGGGAGATCTTGCATCACCCAATGGGTTGGATACCGCTG

ATCCATATGTGTATGATCTGCAGATCTGGGTGTCTGGCTTCTGTACAACAGGAGGAAGTT

GGAGCTACAATAGACAATTGAGCTGCAGGGTTGGCGGTTTGCCGACCTGGATCTTGATCT

TGAGTGACATGGCATCATCTCTCTAATAACCACTCTTAATGGTGGTTTCATCTCCACTTC

AGAGCAATCTCCATCACCAGAAGACATGAGCCCTAGTCCTACAGCAGCTACTGCCCTTTC

GATTTCGATCTCGCAGACAGCCAACCGGGTTCTCAATGAAGGCTGTGCTTATGGGTTGCT

CTGCTGTATGTCAGTCAACGACAAATCCAATGAGGCCGACAACGAATTGAAACAACAAAG

GGGAGGCCGACTTTAGTAGAACTTATGCAGATATGCTCACCTCAGTCAGAGCAGGCTCTA

TCTTCAGAGAGGAGGATGTCTAACTACTTAAGGATGTGAGAATCCCCTACCAGCAGAAAA

GGATGTTCGGCTTCATCATCATTATCTACCCAGAGAATGTGAAGCTGATACTTGTGAAGG

GAAACCAACACTTTGTCTGTGAGGCTCGTGTTCTCGTCAAGTAGGAGATCTTGCATCACC

CACTAGGTTCCCGACTTCTGTACAACAGGAGGAAGCTGGAGCTACAACATGCAATTGAGC

TACAGGGTCTACAGTTTGCCGACTTGCATCTTAATCTTGAGGGCCCAGTTCAGGATGTGA

GAATCCCCTACCAGCAGAAAAGGATATTCAACTTCATCACCATTGTCTACCCGGAAGCTG

TGAAGCTGATACTTGTGAAGGGAAATCCACACTTTGTCTGTGAGGCTCGTGTTCTCATCA

AGTGGGAGATCTTGCATCACCCACTGGGTTGGATGTCATTGTTCCATATGCGTATGATCT

GCAGAACCTGGGTTCCCGGCTTCTGTACAACAGGAGGAAGCTGGAGATGCAGCAAGAAAT

TGAGCTATAGGGTCGGCCGTTTTTCGACCTGCATCTTAATCTTTAGGGACATGGCATCAT

CTCTCTAACAACTGTTCTTAATGACAGTTTCATCTCCACTTCAGAGCAATCTCCATAAAT

TGAAGGTATGACTTGAGATTTTGAGTACGCTAATGGTTTTGATTATTATGTTAAGCGAGA

GTTGATGAAATATGTTATTTGTAGACATGAGCCCTAGCCCTACAACAACTACCACCCTTT

CGATCTCGCAGGCGGCCAACCGGGTTCTCAATGGTGGCAGTGCTTATGGGTTGCTCTGCC

GTGTGTCAATCAACGGCGAATCCAATGAGGCTGACAATGAATTAAAACAACAAAGATAGT

GCATTCTAATTTGATTTAATTTTCATTTTACAGTACTTTCCTAAATATAAATGCATCACA

TACACTGATGATCAACATACTCAACTTAAGGATGAGTGGTGTCGTTATGTTATGAATTTT

ATCAACAATTAAAGGGTACGACATACTTTTGGTTGATTGGGGGAGCTTATGTTGATTGGG

GGAGGCCGACTTTAGTG

>Unigene0000618

TCCGACTTATCTGAATTGATTTTCTCCGCAATCCTTGTCACTCCACTCACCGAACCTCGC

CGGCGCACTAATCATGGCCGCCGTCGACGCCGACATCGCCGGAGATCAGTCACCCTCCGC

CGCCCGACTCGCTATGATGGAGCTCGCAAACATGATCTCCGTCCCCATGTCGCTCAACGC

CATCGTCCGCCTCGGCGTCCCCGACGCCATCTGGCAGTCCGGCGCCAACACCCCCCTCAC

CGCCGCCGAGATCCTCCCCCTCCTCCGCCCCGCGCCTCCCCCCTCCGCCGCCACCAACCT

CCAACGCCTCCTCCGCCTCCTCTCTAGCCACGGCGTCTTCACCGAGCACAGCTCGATCAA

TCATCCGAGTTACAGTCTCACCAACATCGGTCAGACTCTCATATCCGGTGAGGGCTCCTA

CGCCCCCTACGTCCTCCAGCACCACCAGGACGCCTTAGTCAAATCCTGGCCGCTGCTGCA

TGAGGCGGTGGTGGACCCGGAGGGGTCGGTGCCATTCCGGAAGGCCAATGGTAAGGCGGC

TTACTTGTACTACGGTGATGATCCAAATGCCAATGAGTTGATGTTGAAGGCTATGTGGGG

AATCTCGGTGCCCTTCATCAAGGCGATGCTTGATGGATATGAAGGAGGTTTCGAAGGTGT

GCGGACGTTGGTGGATGTCGGCGGGAGCTCGGGGGCTTGTTTGGATATGATCATGAAGAG

AGTACCGACGGTTGAGGCTGGGGTTAATTTTGATCTACCGGAGGTTGTCGCCGGCGCGCC

GGAGTTTCCAGGTGTGACCCATGTTGGCGGTGACATGTTCAAATCCGTTCCACGGGGTGA

TGCTATTTTCATGAAGTGGGTGCTGATGACATGGACCGATGATGAGTGCAGGAAGATACT

GAAGAACTGCTACGACGCCCTGCCAGAAGGCGGTGGCAAGGTTATCGCTTGTGAGCCGGT

TGAGCCGGATGTGGCAGATGGGAGCTGCAGAACTCGAGCCCTACTGGCCAGCGATATTTT

TGTCATGGCCATATATAATGCTCAAGGTAGGGGGCGGACCGAGGAGGACTTCAGGCAGCT

CGGGTTGTCTGCTGGATTCCCGGGCTTCAGAGCTATCTATCTTGATCCCTACTATACTGT

CCTGGAGTTCAGTAAGTGATGTCTCACCATTTTTGGTGAACAAGAAGAAACCGATATACT

ACAGATTACATTCATTTGAGGATGTGAACTTTGTTGGTATTCCCCTCTCCTACTTCGTTG

CAGATAGATGTTTTCAGAGGCTCGTTCCCCGTTTGTAAGGGCTTATTTTGTGGCATCTAC

TCCCATGTAGTGCTTATTTGACAGGCGATTACAAGCTGTTGGAAATACTCGGATAATATC

CTAGAATAATCTGAGACATAGGCTGAAGTGTGATGTTGCTCTGATATGTTTGGTGGCACC

GATGATTGTTGATTCA

>Unigene0000619

GGATCTAGCACAAGAGTTGGGGGGACGCAAATTTACATGGCAAAAAAGCAATACCAGAAA

GGAGGGAAATGGTGTATAATAATACTAGAAAGGTGGTAGTGGAGGAAAAGATGACCGATG

TGCTCAATAGGTCCTCGCAAAAAACACACAATGGAAGAGATGTCGTAGTAGAAGTGTAGA

CAAGCAGAGCTGGTAGAAGTGCAGACAAGCAGAGCTGGAAGGGAGGGCGTTTCTAAGGAT

TTTCCAAGCAAAGAGAGCAAGACAAGCAAAGAGAGCAAGACGCTCGTGGTTTTTATTTTC

CAGATTTTTTTCCACATTTGAATTCGCATGGGAAGAATCCATCCTAGGGTGGAAATTAGT

CTGTTAAGTCCAGCTACAGTGAGAGTATCGGAAGGACAAAGAAGCAAAATTAGTAGGTCT

TGGTCCGCCAGGTCAAGGATAAGTAGATCAAATTTAGTTCCGCTGTTCATCACACACCCA

CAATATATTAGATTACGTCAGCACGAGTCTTTTGGGATCTCTCAAAGGTTATTTCCAGAG

GGGAATCATGATTCTTAGTGTTTTTTATGGATGACTTCTCCCATAAAATTTGGGTCTACG

TGAGAAAGACCAACAACCAAGCCTTTGAGATGTTCAAAAAGCGAAAGGTTTTGACGAAGA

AGCGGACTAAGAAGCAGCCTAAGCGCATCTCTGTAACAATAATGGGGAAAGGCTTATATC

AAATAAGTTCTAGAAGTTATGTGAAGGATAATGGTATCGCTCAACGCCTTTTTTGTGAAA

CACACATTGCAACAGAATCACATTATTGAAATGATGAATGACGCCTTGCTCGAGTGAGCA

TGTTGTGTCCTCTTAGATTTTGGACTCAGTTCCAGGCAGAGGTTGTTAACACGACGTACT

ATTTTTTTATATCTTGGACTCAGTAAGAAATTCCAGGCAGAGGATGTTAACACGACATAC

TATTTTGTGGAAAGTTTCATTTGTGACTGTGATTGAAGTCCCCTGCTGCGCTTGTACCAT

TCCAAAGTCACTCGATATGTGAGTTGCATATAAATCTATTTTTATTGTTCTGAAATTGTA

TGATCTTGTTATTTTTTAGGTGTGACCCATGTTGGCGGTGACATGTTCAAATCCGTTCCA

CGGGGTGATGCTATTTTCATGAAGTGGGTGCTGATGACATGGACCGATGATGAGTGCAGG

AAGATACTGAAGAACTGCTACGACGCCCTGCCAGAAGGCGGTGGCAAGGTTATCGCTTGT

GAGCC

>Unigene0000620

ATCCATCACAAAGAAAGGGGGGAGAAAAGAAACAAAGAAAGATGGGAACCAAAATGTTTG

TTGTCAAATCCTCTTCCAAAAAGAAGAGAAATCGTCCTTATAAACAGAGAGAAGAGGGGT

GTCTTGGAAAAGGAAAAGTTAGAAATTACCAAGAATGAAGAGGGATGCTTTGGAACGTGA

GAAGTCACAAGTTAATGTTTGATGCTACTGTTCGCCGTGAATTGTACCTCGTGAATAATA

ACCTGGCTTTAAAATTGACAGTTGGTTTTGACTAGTCGCAACTTACTACTTGGCTTGAGT

TCTCATTTATGTGAACATAAATTAATTGGGACACCCCCCACTATAGGCCAATATCAACAA

GTCCTTGTTGTTCAAAGCAAAAGTAAAATAAATACATTAAAGTATGATGCATAATGCATC

AATTACTCACTTAACCAAATTCGATCACAGATAGACGAGGGACGACGTTCCAGGTATTTA

TCTAAAAATCGTATTTATTGCTGAATAAATAAAACAATTGAATTCTTTGTATTATTTTTT

GAATTTGATAAATTAAAAATTAAAAACAACTCAAAGCACATGGCTTTGCCGTCGGTCTCC

TAGGGCCGATGTTAGCACGACCTGACATCGATTGACGTCAACGGATGGCTGAGTGGCCGA

CGGATTGCTGATGGTGGGGGACAACCATCAATGGCGGCGGGGCTGCTATCTCCCAGTTGT

GCTGCATCAACTACTTGTTCAGTCCATTGTTGTGGAGTTGGCTGTACTCTCGGGCCGAGA

TGCTCTTTACTGGGCAACCTGGCGAAGACAACTGATGAGGTTGAATGGGCTGTTGCGCGG

TACTTGTGCTCAGTCGAGGAGCAGCCCAATTGTGTTTTATAACAGAGTGGCTCTCAGTCA

AGTAAAGA

>Unigene0000621

TTTGAACATATATTAAGTGGGACAAACCCCCCACCATAGGCCAATATCAACAAGTCCTTA

TTGTTCAAAGCAAAAATAAAATAAATATATTAAAGTATGATGCAAAATTAGTGCCGAGAG

TGTTCCCATTAAGGATAAACAACATGAAGTGTCGAGGCCCATTGAGCGGTAGACACGGCC

ACCAATGATGTCGAAGCCGTTGAAGTTGTCGAGGGGAGGGGACACCGGTACTTTC

>Unigene0000622

AGTGGCTCTCAGTCAAGTAAAGACAATGAAGGGGTGAGTTTGATATTTGTGAAATTAAAT

CCTAGAAATTAGCATTTTGGTTTCTATATGATTAGTTCTTGGCGAAGACAAAGAAGGGGG

TGATAATGAAGAGTTGAGATGTTGGAGCTGATGGATAGTTGCGATGACGAGGCTGGGCAG

GATGTCGATGGTGTGGGGTTCCTTGACTTCGACTTTTGGCGGATGTCGTGGAGAGGAGAT

GAAGCCAGTGTTGGGAACGACAACGATGGTGACTAGGGCTTGGTGATGGTAACATGCTCG

TTACGACGGCTTTTGGCGTTTGTGAAGGAGAG

>Unigene0000623

CTTTTGGCGTTTGTGAAGGAGAGGGAGATGGATGCTTATCGAAGATGGCATTGATGTTGA

GGGAGCTCCGAATCTTGATGGTGATTCATGGAGGGGATGATGATGGATCCCTTCTCCTTT

GGATTCCCCGGTTTTTATAGCCTTTTTGGGTTGAATTTAGGAAGTTTCACGGGATGAGAA

AGGAGAGATTTTCTTATTTTCGGGGTAAAATGTGTTCTTCGTCCTTATTCATTCTTAGAC

GTCCCACCTTTTTATACAAATGGTTGTCTACATAAGAAAAATCAAAGTACCGGTCTTTGA

AACCTTCAAATGTGTGTATCTTCAGCTCGGGTGTCAAATTTAGTTATTGTTTCTTCTGTT

AGACGTGTTTTTTTTCGATATTCAGTTTGAGCCTAATTTTGGCTATATCTGGAGGTACGC

AATGTTACACTGTCCATCTAGTAGAAAGGTCATCGACGGATCACTGTAACTTCTAGAAGT

AGGCATGATATAGACATCTGAGCA

>Unigene0000624

CTTGACTCCTTTCTCCTCCTCAGAGGAGAGAGAAAGCGATTCAAACCCTAACCCTAACCC

TAACCCTAGCTCGCCAGATCCGGCCTGGTGAGAATGGGGATGGCTTCCCAGGATGCGATC

AAGCTACTCAGCTCGCTCATCGATGAAGTCGACGACGAGGCGATGAAGCGAACATTCCAG

AACATCCATCAAGGATATCCGACTGAAACATTGGTGCGCTTTCTTAAGGCGAGAGATTGG

AATGTCTCCAGAGCACACAAAATGTTAATCGATTGTTTAAACTGGAGGATACAAAATGAG

ATCGATAACATATTAGCCAAACCTATAATTCCATCTGGTGCATATAGATCGCTACGGGAC

TCACAGCTTATAGGATTGTCAGGATACTCAAAGGAGGGCATTCCTGTCTTTGCGATTGGC

GCTGGCCTTAGCACGTTTGACAAAGCATCAGTCCACTATTACGTGCAATCACACATTCAG

ATTAATGAATACCGAGATCGTGTTGTTCTGCCTGCTGCAACAAAGAAATATGGACGGCAT

ATCGGTACCTGTGTAAAAGTTTTGGACATGACTGGTCTAAAGCTTGCAGCACTGAGCCAA

ATAAAGTTGTTAACCCTTATATCTACTGTTGATGACTTGAATTATCCGGAGAAGACAGAC

ACTTATTATATTGTAAATGCTCCTTATATATTTTCAGCATGTTGGAAGGTTGTGAAGCCT

CTCTTACAAGAGAGGACAAGAAGAAAAGTGCAAGTATTGCAAGGTTGTGGCCGAGATGAG

CTGTTGAAGATAATGGATTATGCGTCCCTCCCCCACTTCTGCAAGCGAGAAGGATCTGGA

TCATCTCGTTACTCAACTCCTGGATGTGACGACTGCTTCTCCTTGGATCATCCCTTTCAC

CAACAGTTGTATACCTACGTCCAGCAGCAAGCATTAGAGCAGCAGGCTGTCATCCCCATT

AAGCAAGGGTCTTTCCATGTAGATATTCCAGATCCAGATCTCCAAGATGAAATGATTGTT

CAGACCATAGAGTGCGAGCTTAACAAGATAGGGAACGCCAACGGCTTAACCCGTTCAATC

AGTAATCTCAAAGTTGACAGTGCATGAGGCTTTGACGCTTTGACGAGGGAATTTCTCACA

TTATAAGAAGTCAAATATGGTTGGATGTCTGAATGTCGGTTTAGTTGGTTGAGAGGGACA

TGGGCGTGTAATGTCAGAAGTAGGATCAAGTTATTAATTGATAGAATTGGGAAGGGGAAG

ATGCCCAGAGCAAAAGGTAAATACTGTATTATTGTACTCCATACCTGCTTTACCTTTATA

TAAATGGTGAAATAATGTATTTCTTAGTGTTGTGATTCAGCTGGCCTTTTGTT

>Unigene0000625

TTTCCACAAGTTTATCTCGCCCTCGGGGCCGCCATACATTTGTGAAAAAAAGCAGGATGG

ATCAATGACGGGGAAAGTGATTCTCGACGACATGGCTTCATTAAGAGAACGATGATCGGT

TTATCGGCTTTTCTGTTCTTCCCAGTCGTTGGCTACCAAGTTTATTATCTTCCATATGTC

ATATGGATACAGGAGGACGCTTTTGTATTGTATTTCTATTGCTAATTTATTTGAAAGAAA

AGATTGATTTTCTATGTATGAAATATATTGGTTTCGGAAAAAAA

>Unigene0000626

TTTCCACAAGTTTATCTCGCCCTCGGGGCCGCCATACATTTGTGGAAAAAAGCAGGATGG

ATCAATGACGGGGAAAGTGATTCTCGACGACATGGCTTCATTAAGAGAACGATGATCGGT

TTATCGGCTTTTCTGTTCTTCCCAGTCGTTGGCTACCAAGTTTATTATCTTCCATATGTC

ATATGGATACAGGAGGACGCTTTTGTATTGTATTTCTATTGCTAATTTATTTGAAAGAAA

AGATTGATTTTCTATGTATGAAATATATTGGTTTCGGAAAAAAA

>Unigene0000627

ATACAAGGTCCCGCGAGGTAACAGGTAATTCCCCAGGTACTAGGGTTTCTCTGGCCATAA

AAAGATCCCTCGTATTGGTGATGGTCGTTGTGTTTCCATTTGGGTGTCACGTCTTCCTAA

CATATTTTCCTCTAGTTTCCAGAGAATCTGATTTGATGTATTATATTGTTTCACTATCGG

TTTGGATCCTGGAATTTCCACAAGTTTATCTCGCCCTCAGGGCAGCCATACATTTGTGGC

AAAAAGCAGGATGGATCAAGGATGGAGAAAGTGACTCTCGACGACACGGCCTCATAAAAA

GTACGATGATTGGTTTATCGGCTTTTCCGTACTTCCCCGTCATTTGCTGCCAAATTTATC

TACTTGGAGGTTTTATTTATGCTTCATGAAGACCAGAGGACGCTTTTTTATTGTATTTCT

ATTGCTAATTTAGTTGAAAAAAAGTGTGACTTTCGATGTATGAAATATATTTCTTTTTGA

AAAAAAAAA

>Unigene0000628

GGATGGATCAATGACGGAGAAAGAGACTCTCCACGACACGGCCTCATAAAAAGTACGATG

ATTGGTTTATCGGCTTTTCCGTACTTCCCCGTCATTTGCTGCCAAATTTATCTACTTGGA

GGTTTTATTTATGCTTCATGAAGACCAGAGGACGCTTTTTTATTGTATTTCTATTGCTAA

TTTAGTTGAAAAAAAGTGTGACTTTCGATGTATGAAATATATTTCTTTTTGAAAAAAAAA

A

>Unigene0000629

ATACAAGGTCCCGCGAGGTAACAGGTAATTCCCCAGGTACTAGGGTTTCTCTGGCCATAA

AAAGTTTCCTTGTATTGGTGATGGTCGTTGTGTTTCCATTTGTGGGTAGCGCCATCCAAA

AATATTGTCCTGGAGAATCTGATTTGTTTCAGTATATTGGTCCACTATTGGTTTGGATCC

TGGAATTTCCACAAGTTTATCTCGCCCTCAGGGCAGTCATAAATTTGTGGCAAAAAGCAG

GATGGATCAATGATGGAGAAAGTGACTCTCGACAACTTGGCCCTATTAAGAGTACGATGA

TTGGTTTATCGGGTTTTCTGCCCCTCCCATACGTTGTCTGCCAAGTTTCACAACTTGTAC

ATGCTTCCGAAGTTTTTGTACGTATATATGCTATCTGAAGACCGGAGGATGCTTTTTTTA

TTGCATTTCTATTGCTAATTTATTTTCCATGAAAAGAGTGATTTTCTATGTATGAAATAT

ACTTGTTTTGAAAAAAAAAAAAAATT

>Unigene0000630

ACCACCACCGCGGTGTACACCGGACGGTATCTGACTATTACCTACCCACAAATCATACCA

ACTTTTTATACCGCAACCCCGGTAAGGCTTATTCCGTACACCTAGAAACGCGGAGCTTTT

GACTGTCTAATATAATAGTAAAAAAAAACCGCATCGCGATTCGACATCATTATCTGAGTA

ACAAGTCGAAGTCATACGCCGTCGCGTCGAAACTCAAAATTCGCTGGAGGCGTACGAACG

CTTCATTATAACAATATAACATAATTAATTTAGCAGTATATAATATCGAAAACTTCACTC

AGCCATGTATAGATTCCAAGACGTGGCTCGTCCAGCGGCGACGGCGGCCAAACATTTTGT

CGGCACACCTGCAATGAGAATGGCATCTTCATTGTGTCCTGGAGAATATTCTGCACCGTC

GGTTGAAACATCGATTCCTGGACCGAAATCAAAAAAACTTCTCGCTGAGCTTTCACAAAT

TCAACTATCCGGTTCGGTTCAACTTTTCGCCGACTACAACAAATCTTTGGGTAACTACTT

GGTTGACGTCGACGGTAATGTTATGTTAGACGTATACACGCAAATATCATCAATGCCATT

GGGTTACAACCATCCGGACATGTTGAAAGCGTTGGAATGCGACGAGAACAAAAAAGCAGT

TGTCAATCGTCCGGCGTTAGGTGTGTTCCCAGGTGCTGATTGGCCAGAGAAATTACGAAA

CACGCTGATGAGTGTTGCACCTAAAGGCATGGACTCTCTGACGACCATGATGTGTGGGTC

TTGTTCCAACGAAAACGCATACAAAAATATGTTCATGAAGTACCAGCGGGACAAGAGAGG

AGGCACTTCAGACTTTACAGACTTGGAAAAAGAGTCATGCATGATTAACATGCCACCAGG

TGCTCCAAAGTTGTCCATACTGTCTTTCAAGGGCGCATTCCACGGCCGCACACTGGGTGT

CTTATCCACCACGCATTCCAAGTACGTACACAAGTTAGATGTCCCAGCGTTCGACTGGCC

AATAGCCAGATTCCCTGTGTACAAGTATCCGTTGCATGAAAATCTCAGAGAAAATGAACA

AGAAGACGAACGTTGCTTGGCAGAAGTAGAGGAACTGATAGAGAAGTGGGAAAAAAAGGG

CAATCCAGTGGCCGGAATCGTGGTGGAACCTATCCAGTCGGAAGGTGGAGACAACCATGC

GTCGCCGTGTTTTTTCCAGCATCTTCAGAAGATTGCTAAAAAGTATAATGCGGGTTTTCT

AATCGACGAGGTGCAGACGGGCGGTGGCCCCACCGGAAAGATGTGGTGTCACGAACATTT

CGACCTGGAAACTTCTCCAGACATTGTTACTTTTAGCAAGAAAATGCAAACCGGTGGATA

CTACTTGAAAAGCGAATTCTTACCCGACATGCCGTACCGTGTGTTCAACACTTGGATGGG

AGATCCGACCAAAATCGTATTACTCGATGCTATATTGAAGGTGATTAAGAGGGAAAATTT

ATTATCAGTGGTCGAAAAGTCCGGCGATATACTTATGTCTGGACTCAGAGAGTTGGAAGC

CGAGTTTCCAGCTCTGATAAACTCTACCAGAGGTCGTGGTACATTCATAACGTTCGACGC

TGCTGATACCAAACTCCGAGAAGATATTGTCAATAGACTTTTAACTAAAGGAGTCCAATC

CGGAGGATGTGGAGTTGTGGGTATCAGAATGCGACCGGCGTTGATCTTTCAACCACATCA

TGCTCACATGTTCCTCGACAGACTTCGCCAGGTGTTGGCCGAAACTAAATAAGTTCCACG

TTGTATTATGTAACTATTATACGTCTGTAGAAAGAGTGAGCATTTTTTTATAATTTTGTG

ATATTATTGAAAAATATGTCTTCCAATGTTAACATAAAGCTTTTTGATTGAAAACGAGTC

TTCCAAAATATTTTACACAATTATTACAAATATTGAATCGTATGTATAA

>Unigene0000631

CCTAATTGCCTGCGAGGGACTGATGTAGATTTTTCAAAACCGGATCAAGTTTCAGAGTCT

CATATGGCCGCTTTCCGTTGATGTATGCGTGGAATTCATCATTGTCGAATTCATAGCCTA

ATGCCTGCAATGTAAGAATTAAACTCCGAGTTGTATATTTAGAAATCAAGAAAAAAATAT

ATTTACATTTGGTTTTCAAGATGTACCTTTAGCCCAGCCATCGTGGTTCCATATTCCTTG

TAAAGATCCAAACACATCTCTGGGACTATGCTTTCATCGATTTGGAGATGGTGCATCATG

TAGTCTGCAATTTTTAACTTAAAATGATAAACTTTACGAATAAGAGATGTCATTATT

>Unigene0000632

TTCAAAGCAATCTTCTACATCCAGCCTACTCAGGACTTTTGTTGCATGTGCTCTGTCAGT

ATTGGTGAAGATCTGAAAGAATTAACCACATTAGGGACACCGATGGTGTGTTATTTTTCC

CAGGGATTGAAGTAGATTTTTCAATACTGGATCAAGTTTCAGAGTCTCATATGGTAGCTT

TCCGTGGATGTATGCTTGGAATTAATCATTGTTGAATTCATAGCCTAATTGCCTGCGAG

>Unigene0000633

ATGATTCTTTCAAAGCAATCTTCTACATCCAGCCTACTCAGGACTTTTGTTGCATGTGCT

CTGTTAGTATTGGTGAAGATCTGAAAGAATTAACCACATTAGGGACACCGATGGTGTGTT

ATTTTTCCCAGGTAGTGTTTATTATGCTTATCATTTTCTACTATCAGTAACGTATTATCG

TATTGATGCAGTCGTTGAATTTTTTTATCTTAAAATAAAATTTTGATATGACATGCTAAT

TTTTGTTGTGGCAGGGATTGAAGTAGATTTTTCAAT

>Unigene0000634

TGATTTATTTTTAGCGGGGTAGTTGATGGACTCATTTTCTGAGTTACTCTCGCTTCAAAT

AGCATGACTAAGATCAATTGATTTGTTGGTGGTTATATAGTCATCACCATTTTGATCTAT

TGATGAAACTGGAGGATTCAGGGTTTCGAAACATATGATTCTTTCAAAGCAATCTTCCAC

ATCCAGCCTACTCAGGACTTTTGTTGCATGTGCTCTGTCGGCATTGGTGAGGGTCTGAAA

GAATTAACCACATTAGGGACACCGATGGTGTGTTATTTTTCCCAGG

>Unigene0000635

AAGCGCCGAGGTCCCAACCTGCACTTCCACTCATTATACAAAGTTGTTCCTACACGACTT

ATTACTATCATATGTAATGGATTTGATGCATATACAATACTTGCAAAGTAAACTATGAAG

GAAGCACTGGCTTACAATGACAGTATGAAGTCCTGCGAACTTTGCTGCAGCTATATTTCG

AACACTGTCATCGAAAAATATCTGCAAGTCCAGAAGTTTTAGTCCAACAATTCTTTTTAA

ACGAGGGAAGTCGAACAGAATAATTTCTTCATCTTACCGTCCTCTCTGGATCAATATTTG

CAATGCGAACAGCGGCTTCCATGGCTTCTAAAGAGGGTTTGCATAGGATCCTCATGCTGT

TTGCCAAGTTATTTTCAGTGGGGTAGTCGATGGAATCATTTTCTGAATCACTCTCGCTAT

AAATAGCATGACCAAGTTCACTTGATTTGTTAGTGGTTACATAGTCATCACCATTTCGAT

CTATTGATGAAACTGGAGGATTCAGGGTTTCGAAACATATTATTCCTTCAAAGCAATCTT

CTACATCCAGCCTACTCAGTACTTTCGTTGCATGTGCTCTGTCAGCATTTGTGAAGATCT

GAAATAATTAACCACATTAGGGACATCGATGGTGCGTTATTTTTCCCAGGGACTGAAGTA

GATTTTTCAAAACTGGATCAGGTTTCAAAGTCTCATACGGCAGCTTTCCGTGGATGTATG

CGTGGAATTCATCAT

>Unigene0000636

GGAGTGAACGCCGTTAACTCGTCGAGGAACTCGAGACCTCGGATACTCTACTCCCGAGCA

CACGATAAACCCCTCAGCACCTCGATAAATCAGTGGTTATTATCAAACCGAATCCCTTCT

ACCCCTCTTCGACGTCTCCTTCCCCCTTCCCTCCCATGGCGATCGCCGCCTTTTCCCCAT

CTGCTCTACAGGGCATCCTTGTTCGCAATCTCTCCTCTCCGCGCAGGAGCTCACGGGTAG

TCCCTCAGGCTGGAGGTCGAATGCTCTCCCTTTCCATGGTTACTCTCCACCCGTCTTGCT

CGCTCGCACAGCAGCGCCTGGACACCCGTCGGATGATCTGCCGTGCTGCTGCTATCTCTG

CGGAGAAAGAAGGTCTCAGTCTGCAAGCAGAAGTAACTACAAAGTGCTTCTTTGATGTTG

AAATTGGGGGAGAGCCTGTTGGTAGGATTGTATTAGGCCTATTTGGGAAGGTCGTCCCAA

AAACTGTTGAGAACTTTCGTGCTTTATGCACTGGGGAAAATGGATTTGGCTACAAGGGTT

GTTCCTTCCACCGCATTATAAAGGATTTTATGATCCAGGGTGGAGATTTCGAACGCGGGA

ATGGAACTGGAGGAAGAAGCATTTATGGGTCTAAGTTTGAGGATGAAGGCTTTACTTTAA

ACCATGTTGGCCCCGGAGTCTTGAGCATGGCAAATGCAGGACCAAATACTAATGGTAGCC

AGTTCTTCATATGCACATTAAAGACCCCGTGGTTGGATAAACATCATGTCGTGTTTGGGC

ATGTTGTGGAGGGGATGGATGTTATACGGAAGCTTGAATCTCAAGAAACTAGCAGGTCTG

ATGTTCCACGACTGCCCTGCAGGATTGTTAACTGTGGCGAGCTGACCATGGATAACTGAC

CTGGCTCAAGTACCTCTTGATTCAGCATAAGATTTTTTATTTCTTTAGTGCATATGAACT

TTACGTTTGCCATTTGTAATTCCTTTTGCTCACTATCTTTCCATATTTCTGTACAAGAAT

GAAGAATTCAGATCTAGTCCATATGATATTAGATATAAAGATCTGTTATCGAATGATCGA

GTCAAGGTAGAGGGTTGTTTATTAGGGAAGTTAGCATATTTAATCATGTTAAAAATGATT

TTGGTTGCTTGCTAATGA

>Unigene0000637

TAACTCCACTCCTTTCCCCTTTCTCTTAGTGCTTGAGATAAAAGCAACAAGCGTCCGAGC

CTCCCCCTCTCTTTCGTTCTCAATCTCGATGATGCTGTCGGCCCGCTCCGACTCCCCGCC

GCTGCTCCCCTGATCCTGACTCCCCGATTCCCACCGCTCTATTTGGTCCAAAACTCCGTA

CATTAGGGCCTTTTTCTCTGCAAGGCTCTTATGCTATGGCTGACCCAGAATCTGTTGCTA

CACTTATTGATTCCACAACAACTAAGATTCAACAGCTTCAACAAGCAATTTCTGAACTTG

AAAGTCGCAGTGCTATATCTTGGAATCTGAAATGGATACAGCTTGAAGAACATTTTCATG

GGCTCGAGAAGTCACTTAAGAAAAGATTTAATGAGTTGGAAGGTCAGGAGAATGAATATG

TAGCCAAGGTCTCCGAGACGCAGGTTATGTTGGAGAAGGAAGAAGCTGTTGTTGTGGCGA

AGGAGCAAGCTTCATTGGAGAGGCTACAAAAGAAAAGAGATGCTGCTTTGTTTGCCATGT

TTGAGAAATGCAATAACCACCCTTCACAACCCATTGATGATGGGGCATGTGATAGCATGC

TGCATTCTGTAGTGGAAGAAAGGTCAGATGTTATGACTGCCAATACTGAACCAGATAATG

TGATGCCTGCTGAAAATCACAATGTGGATGCCATTCCTTGCTCACCACTAAGCAAACTAT

GTCGGGAAATGGATGCCGACGGACTGCATAAGTTCATCTCAGACAATCGCAAGAATCTGG

CCTCGATACGTGAAGAAATTCCAGATGCATTAAGAAGTGCATGTAACCCTTTTAGTCTGG

TTCTGGATTCCTTAAAAGACTTTTACCCTGGAGATATTCTTGGGGTAGATGGCAAGAAGG

ATGCAAATCTTTTGGGTGTGCGCAGAACCTGTCTCATGTTAATGGAGTGTCTTAGCCAAG

TATTTTCAGATGGTGTATCAGGGTTTTCTTCTGATAACCAAATGCTTACCTCAGAGATTA

AAAAGCAAGCGAGAGCAATAGCTAATGAGTGGAAGCCAAAGCTAGGTGATCTTGGCGTTG

ATGCCAGTAGTGGTAACTCATTGGAAGCGCATGCATTTCTGCAACTTCTTGCCACTTTTG

CTGTTGCTTCCGAGTTCGATCAAGATGAGATATGCAAATTGGTACCCGCTGTTTCCAGGA

GGCGTCAAACGGCTGATCTATGCTGCTCACTTGGGTTGTCACAGAAAATGCCAGGTATAA

TCGGTGCACTGGTGAGTAGTGGAAGGCAAATTGAGGCTGTTAATCTGGCATATTCCTTTG

AATTGACAGAGCAATTCGCCCCCGTACCATTACTGAAGGCATTTTTGAAAGAGGCAAGGA

AAGTGTCACAGGTCAAAGCTGGGAATACATCTCCTGGTGCTCAGAATGAGATGAATGAGC

GCGAAATGTCCGCTCTCAAAGCTGTCATCAAATGCATTGAAGATCACAAGCTCGAGGAAG

AATATCCCACCGATCCACTTCAGAAACGGATCCTCCAGCTTGAGAAGGCCAAAGCCGACA

AGAGGAGGGCTGCAGAAACTGCAAAGCCGCAATCCAAGAGGCCCCGTGCTAATGGCAGCA

CATATGCCCCTCGTGTGACTGCATTCCCCGAGAAGAGCTTCTACCGAACACCAGAGAGGC

ACCCATATTCATACGACAGGCAGTATGTGTACCCAGTCGAAGGCCATCCTGCTCCCCCTA

TTCTGGGTTCCACACCGTACGCCATCTCGCCGACGCATACAGCATATTATGGTAATGGCT

ACCAAGTTCCGTATCCAGTCCCTTATTTACACTAAGTGAGGAGGCAGAGGAGATTATGAT

CTTTCCTAATTTCCTAACTAGTAGCTTTAACCCGCCTGAACTCCTTTCAGTGTTGTCATC

TAATGTCCGCCATGTATGGCGAGGCTAAAATTGCTTCGATGGTAAGTAGGTTGTTTGTAT

TAAGAATATTGTGAGTGGCCTAACAGACAAACTTGTAATTTGCTTCTGATTGCTACCATG

ATTTCTTTCCCTCGTTTACTGTCATGTTTTTCTACTGGAAACTGCTAATGAAGTTCTGCA

GGTGACAGATTGCGGCAGTTGCGGAAAGCTCAATTTGACAAGTTTGGCCTCATCACCTTG

ATGTTTTTATTTAAAATTTAATCTAAATATTCCTATTTGCAATAAGAAAATGTGA

>Unigene0000638

GAGGGTCTCGGTCGTCACCTGGAGACTCCGGATCACGCGCACCAGGGACAATACCGAGGA

CAATTCCAGTCTCCACTCGTAGTCACCCTCATAGCAGTGCTGCGGGCTTATGTGGTTCTA

TATCTCGGTTTTATTTGGTTGTCATCGCACTTGAGTTTGTAGTTGTTTGTAGTAGGCTCT

GAAAACATCATAGTATGAGTTTGTAGTTGTTTGTAAGAGGCTCTGAAAATATTGTAGTAT

TTT

>Unigene0000639

AACATCATCTACATCATGTTTCGGCCATGACTAAGCGCCGGAGGGCGGTCACAAGGGGAC

CTACCCCGACACCTTACCTATAAAGACTAAAATGTCTTAGTTTAGATACTGATAACCATG

CTAATAATACTGTAAACCATAGTTACCAAAGTTTCTGTTATATTCAGTAAATATTAAAAA

AACTCCAAGATATTTGTCTTCTGATTTAATTTAATCCCTGAAAAATAATCCAAAGCCATT

AAATCGCTTTAGTCCCAATTTAATTGC

>Unigene0000640

CTTAATGTTTTGATTTTCAAATTGGATGTTGGATTATGTAGTATGGATTATTATTCTTTT

GGTAAAGTCCATGTCTGGTATGCTTTTATTGTCTTTTAGGTCATTTTGTATTAGATAATA

GATATTTAAGCATTTCTCTTATATGATAATCTAAAGTTTTTGTTGTTTTTGATTAATTAA

AATTCTCCTTTTTCCCTTTCCTGAATTCTGATTTTTTAACTTCACCGATTTAATCTTCTG

CCACAAACCGGTAACTATGCTTTGAACTAAGG

>Unigene0000641

TAACTGTGCTCCCCTGACATCAAACTCGTGCTCCCCCTCGCACCACGTCTCCACCATTCC

AGGTTTCGGCTCCGCCACATCTCGCAACCCCGCCGGCTCCGTCTCCGTCTCCGGCGGCGA

TTGCCGTCCGCCATCCCGTTTCTTCCCGAAAGAGAATCCGAGATGAGATGGCTTCCCATC

CTCGAAAATTGTCTGGCATGCAAAAGCAGATCTTAGGCTTATACAGAGGATTTCTTCGGG

CAGCCCGTAGAAAAGCCTCAGAAGAGCGTCACAGAATTGAGTCCCTAGTCACTGCTGAGT

TCCGCCATAATGCAAAGAATGTCGATCGAAAAAACACCCTCTACATTGAGTATCTGATGC

GTCGAGGAAAGAAGCAGCTTGATCAACTCAATGGTCCTGATATTGTTGGATTATCAACTT

TCAAGCTCGCTTCTTCTACAACAACAAAGTTGTCTGCTAAGTCACATAGTGACAAGTCAA

CTTTGAATCATGATGATGTTGCACAAGCATTCTCTTCATAGCCATTTCCTTCTAATGTAA

TCCAGTAATATATGTACCTAAACGGTAGTTGATTTCCGGAACTGTAAATGAAAATTGTTT

TTGTATTATATATGCCAAACTGGCGAAAATGTACTAGGTAGGGAAACCATGTGGTTGGGA

ATTTACTTATATAAATGGAAATAAGTTGGGTCCTATCTGGGGACGTAAATATTTTTGTAA

GTTTGATAGTTTTTTTTACAGT

>Unigene0000642

CGGGGCGTTGTTGTGTGGTTGGGTAAGGTCGGTGGCGCGCTTAACAACACCCCGAACAAA

CAAAAACCAGCGAGCACCGGCGATAGCATACTCCAATGGCTCTCAGTCGTCTTCAACTAC

TCCGGCTCCCGCTCCCCGCCGTCGCCCCCTTTTTTCTCTCCAAAACCCCAATCGTGCTCT

CCTCGATCCGCTCCCTGCGCTCCTCCCGCCGCCTCCTCTGCTCCATTTCGCCCCCTCCAC

CACCGCCACCGCCGCAGGATTCCGTCGCCGGGGAGAAACCGACTTCGATCGTCGGAGATC

TGCTTGATTACCTCAACGAATCGTGGACGCAGTTCCACGCCACCGCCGAGGCGAAGAGGC

AGCTGATTGCTGCGGGATTCCAGCTGCTCAACGAGAACGATGAGTGGAACCTGCAGCCAG

GTGGCCGTTACCTCTTCACCCGCAACATGTCCTGTTTGATTGCATTTGCCGTGGGTGAAA

AGTATACTGTTGCCAGTGGTTTTCATATTATTGCTGCACACACAGATAGCCCATGTTTGA

AACTAAAACCCAGGTCTGCATCTTTTAAATCTGGATTTCTGATGGCAAACGTACAGACAT

ATGGAGCTGGATTGTGGCACACATGGTTTGATAGAGATCTCAGTGTAGCTGGGAGGGTCA

TCATTAGAGCTAGAGATGGTTCTTTCATGCATAAGCTAGTTAAAGTTTGGAGGCCTCTGC

TACGTGTACCGACATTGGCTATACATCTTGACCGCTCAGTGAACACTGATGGGTTTAAAC

CAAATCTTGAGAGTCACCTTGTTCCACTCCTTGCAATGAAAGCTGAGGAGGGACTGACGG

AGTCTGGGGAGAAAATTACCACATGCTTGTCAAATAACGCTCATCATTCGTTGCTTTTGC

AGATTCTCGCCGAGGAATTGGGGTGCCATACTGATGAAATAGTGGGCGTAGAGTTGAACG

TTTGTGACATCCAGCCTAGTTGCCTTGGAGGTGGAAATAGTGAATTTATATTCTCTGGCA

GACTTGACAATCTAGCTTCAAGTTATTGTGCAGTCAGATCTCTTATGGATTCATGCAAGT

CACCAGGAGATTTAACAAATGAGCAGGCTATTAGGATGGTTGCCTTATTTGACAATGAAG

AGGTTGGATCAAACTCATTACAAGGAGCAGGTGCACCTACCATGTTCCAGTCCATGAGAC

GCATTGTTGATTGTTTAGCACCTAAATACGTGGGTGAAAGTTCTACTGAGCGGGCAATAC

GTTCATCTTTCCTTGTCTCTGCAGATATGGCTCATGGTGTGCATCCAAACTTTCCAGATA

AACATGAAGAGCACCACAGACCTGCACTACAGAAAGGGCTTGTTATAAAACATAATGCCA

ATCAGCGGTATGCTACTAGTGCTGTAACAGCGTTTCTTTTCAAAGAGGTGGCAGATGCCC

ATAATATTCCAGTTCAGGAATTTGTTGTAAGAAATGATATGGGCTGTGGATCAACTATCG

GCCCTATACTTGCTTCTGGGGTTGGTATCCGAACTGTAGATTGTGGTATTCCTCAGCTCT

CAATGCACAGTGTGCGGGAGATATGTGCAAAGGAAGATGTTGATATTGCCTACAAACATT

TTGTGGCTTTCTTCAGGACCTTTTCAAGCATTGACCGGAAGTTGTCTGTCGATTTCTAGG

TTATCTTATGGATCATTTTATTGCTATTTGTTTCAGTATCATCCAAGGATTATCATTTAA

TGGTTAAACTTCGAGCTATTTTTGTTTAATTAGCCTTTAGTTTTCCATCCTTGATAAAAC

CACTTGATGTAGTAGTACTAGCAAGCAAGTTTGAGAAGAACTGAACGTTTTCTAACTGCA

CTTT

>Unigene0000643

CCAGTTGTAAATTCCGACCCTTTGCTGCAGCCCACCTTCGTCCCAAACGCCCGCCGCTTT

CCCTCCTTGGCTCGCCGGAGACGATGAGATCGGCGGCGAGGGAGATGCTGAGAAGAAATC

TGGCATCACTCTCAGACCTCACTTCCTCCAGGCCTCTGCGGCGCCCACATCTCGCAAGCT

ACTCATCCGCTTCAGAGGCCCCGGGTGGGAACGGTACCAGAACTGGAAGCGGCAAGAAGC

CCTGGTTTGGCCGGAGGGCGGTCCCGATCGTGCTGCTCAGCCTCACTGGAGGCGTCGCGC

TCAGTGCGATCAATGATCTCGCCATCTTCCACGGCTGCAGCATTAAGGCAATAGAAAAGG

CTAGTCATAACCAACAGATTGTTGAAGCTCTTGGCGAGCCAATTGTTAGAGGCCCGTGGT

ATGATGCATCTCTTGCAGTCGGTCACAGAAGGCACTCAGTATCTTGCACCTTTCCTGTGT

CCGGGCCACAAGGATCTGGTGTTTTTCGATTGAAGGCTATTCGCCCTGGAGAGGATGGTT

GGTTTTCGTTCTTACGGCACCATGACTGGGATATACTAACCATGGACGCTCTTCTACATG

TTCCCACAAAGGACGAGGAAAGCCGTACTGTCCGCATAAATATCTTGGACGGAGTCCTCC

ATCACACTACCGGTGTTGAGTGCATGGATTGCAAGGCTGTGGAAACTGAGACTCCCAAGG

AGTAAGTTGTTTTCCTTGGCCAACCTTCTATATCAGATGTCGCATCTCGCATCTCGCATC

ATCCTTAATAAGGGCACTATAAAGACAAACTTTGCAAAAGATGAAGTTGATAGCGCTCAG

TTGCCATATATAAGCTGAACAAGTTGAGGTAGCATTTTTGTTAGTGTTCAGTGTAGAATT

ACTTTAATATAGGATCCATGTTTCATTCAGTGGTTAGTATTTGCTTTCTGGGAAGTTACA

CCGTTTATTTATGTTCTGTTACTTCATTGGATTTAGACATAACATAGCCACATTTGGCTT

GCTTAATGTGAAAAATGCTCTGTTGCAACGTTG

>Unigene0000644

AAATCAAGAGGAGGTTGAAGCAATTCGCTCCTGCAATTTTTGGCGTGCGAGGAGTGAGAG

CGATGATATCATCGGCGGCGACGGTTTCCCCTTTCCGCTCGCCGGCGACCGCGTTTCAGA

GCGGCCGAGCGGCGGATCGATGCATCTTCTCCGTCCCCAGCAGCAGGACGCCCAGCAGGA

GGAATTTCCAAGTGAGAGCATCATCACGGGTGGACAAGTACTCGAAGAGCGACATAATTG

TGTCTCCGTCGATTCTCTCGGCGAATTTCGCCAAGCTGGGCGAGCAGGTAAAAGCCGTGG

AACTGGCAGGATGTGACTGGATTCATGTTGATGTAATGGATGGGCGTTTTGTGCCAAATA

TTACGATCGGACCCCTTGTGGTTGATGCATTGCGTCCAGTAACTGATCTACCGTTGGATG

TACATTTGATGATTGTGGAACCTGAACAACGAGTTCCTGATTTCATCAAGGCAGGTGCTG

ATATTGTGAGCGTTCATTGTGAACAACAAGCCACTATCCATCTGCATCGGACAATTAATC

AGATTAAGAGTTTAGGCGCAAAAGCTGGAGTTGTTTTGAATCCTGGAACCCCATTAACCG

CTATAGAATATGTGCTTGATGTGGTAGATCTGATCTTGATTATGTCTGTTAATCCTGGTT

TTGGCGGCCAGAGCTTTATTGAGAGCCAAGTGAAGAAAATATCTGATTTGAGAAGAATAT

GTGCAGAGAAGGGCGTCAACCCATGGATTGAAGTGGACGGTGGAGTTACCCCATCGAATG

CTTATAAGGTTATTGAGGCTGGGGCAAATGCTCTAGTTGCGGGTTCTGCAGTCTTTGGAG

CTAAAGATTACGCAGAAGCTATAAAGGGTATAAAGGCCAGCAAAAGGCCTGTAGCAGTTC

CAGCCTGAAGATTGGCAATTTGTCAATGGAAGCTTTATTTATTCCTTCAGTTTGTTCCAA

TGTAAGTTTGATTTAGGTAATAACTGCATGTTCCTTATATATGCATATGCACGAAAAACA

AAAATATGGCTCTGTGATACAAATGCATGGATATGAATTACAAATACAGATTTTTGGAGG

AAAGGTAGCATTTTGCTGTCAAAGATGGCTGTCTGCAACTCGGCATGATTATGTTAGAAA

TCTTTAATGCTGGACTTGGTGCATGTTGTTGACATTTAGCGTTCTAAAATTATAGTACCC

CTGCAAAATATAGGAGTAATGCTATATGTCAAGCTAAAAATCTCCAGAACTATTTGGTCA

AGTGACTGCTGACCTGAAACAAGTCAGGTCACCTAGTCTTAATTTCCAGTTTATTGCAGG

TTCCAGTTT

>Unigene0000645

GGCTTTTACAGATTCATTATCTCGAATATTTTATTGCATAATAGATCATGAAATGAAATA

TAATTATCTTGAAAGGACACATTTGACATGATAATAGCTTAAAGCAAAGCAAAATATTAC

TGGTAAGGTGAGAATGAATATCAATTAATTAAATCCAAACAAAGTTTGAACTCAGAACAG

AAATTAAAAACAATGTTGGAGCATGCAAAATATAGTCAAGTTGAATCAAGTGCCTCCCTG

AGATGGTCTCTCTTGACTCCTCTTGTAATTAGTCCCTAGTTTCTCTGAATTAGGTACTTC

CTGAGATATTCTAGGGTGAAACTGTTTCAAATTATGTTTGTTTGGGCAAGAGTTG

>Unigene0000646

AGAGATGATATAAAAATGAGTGTCTAGTGCTCGGATGTGGATGCTATGTATTGAACATTG

ATTCTGATGCTTAAATTTGTGCAAATTGTTTGTTTGAGTATATTCATTATGACTATTTAC

GGTTCAATTAACTTTGTGTGTCATATGACTTACTACTTACTAGTTGAAAACTAGCGAGGG

CTTGACTGAAGAATCTGAGAAATATGCTGGGGAGACTCTGGGATGCATCAGGCTTTTACA

GATTCAT

>Unigene0000647

ATTATTCGTGATTGCTCATAATATTTCAGAACTTAGAGATGCTCAAGAAGAAACAACCAA

TCAAAATGGTGAGTTAGTCGGTCGCTACTGGGAACTCCTTGGAAGGAAAGCAAATTCTCG

GCTGCAAGCAACTATCGCAGTGCTATCGTATATAGTAATGGGTCTTCTTCCCCCTATCAT

TTACGGATTCTCCTTCCGGAAAAGCGACGACAAGGAGTACAAGCTTACAGCGGTTGCTGC

ATCGTCTCTTGTATGCATTTCACTGCTGGCAATTGGAAAAGCGCATGTTAGTGCACAGAG

AGCTTATATCAAAAGCCTGGCATACTACCTTTCGCTCAGCTATGCAGCCTCTGGCCTTTC

TTATGTAGCTGGCGTGATGATCCACCGGTTTCTTGATCAGCTCGGTTTATTTGATAATAG

TGCAGCAGCACCTGTACCTCCTGCTAATATCTATCCTTATATAGGCTCTACTGTGGAGGC

ATGGGCATCCTCATCAGCATAGGTTGTTTCTTTGATTGTGAGCAGCTTATCTGAAACAAG

ATTCCTTTTATTATTATTATTGTCGCCTGTATTGAAGTACTTGTTTTATTTATGATTTTC

AATAATTTATTATCTCTTGGATTAAGGTGATGTTATTAGTAATATTAGTATAAATCCTGC

ACATTATGTTAGTATGTATTAATAATAGTTCGAATGTACTTCAGGTTCTCCGAACTCATC

ATCTTTGGAAGTA

>Unigene0000648

GCCCACTCAGGCAGCATAGAAGATAAACAGAAAGAAGGGGAGAGATAGGAATCCAAATCT

ATCCATGGAATCAACAATGGGCATCTACTGCAACAGCAGCACCAGACCATTCATCCCATC

TCTCACCTCCAAATCCATGGCTGCCAAGAAGCCTGCAACCATAGTCGGAGCTGCAAGAAA

GGTGTTCCCTGTGGGCAAACCAGGCCCAAGTGAATCAAACGGCACCCCTCCCATAAAGCT

CTTGACGAATGTCGAGAAGCTCAGGCTCCTGACCAAGGCAGAGAAAGCAGGTCTGCTCTC

AGCAGCAGAGAATTTCGGACTCTCGCTGTCAACAGTCGAGGAGCTGGGACTCCTCTCAAA

GGCGGAGGAGCTAGGCGTCCTGTCGGCTGCCACTGATCCTGGCACGCCCAGTGCTCTACT

AAGCCTAAGCCTGGCACTACTAGTCCTTGGTCCTGCATGTGTCTACTTCGTGCCCGAGGA

CCACGCATGGGAGGTTGCAGTGCAGGCTGCAGTGGCTCTGGTCAGTGTTCTCGGAGGGTC

AGCAGCATTCGCTGCCTCCAATTTTGTAGCTAATTTGCAAAGGTCTAGCTGAGCAACTAA

GCTCAGCTCAAGCTACTATCAGCTGTAATTTAGTATAAACAACACTATTTCATCATCTTT

TATCTTAATGCTTGATAATGACTCCATCATAATCAATTAGACGAAATACCAAACACACTG

AACTAAGCCACCAACCAACAAAATTTAGTCCTGCATGATCTCACCCAACCATATAAATTA

AAATCACTGAAGAGTCTACTAACCTTTCCCTGTACCATCATCATAAACCACAAAATGGTA

TTCCTGTCTGATCCGAAGAAAGAAGCCCACCAATATTTGTAAATATAGGACAGTAATCCG

ATCACAAATCCTTTTCCAAAATTGAGAACAGCGTTACCTCAGTTTTTACGGATGAAAGGC

AGAATTCACTGCTTGGAACGATTGCCTTGATTCACCCTGCCAGATCGGGCCATCCTACCA

TCGCCACTCTGGACAGGACGTTGCTGTCCCGCCCGACCATCACCATTGTTCTGGAAGGAG

CGCTGCTGTCCCGCCAGACCATCGCCACTGTTCTGGAAGGGGCGCTGCTGTCCCACC

>Unigene0000649

GAGTGAGAGAAGCAGAGAATCTGGCTCGTACTGTTGCAGCCTCAGGATTGAATTGTCGTG

TCGGTCAAAGTCGTTTCCGAGGTCTCCCCTCTTTCTCTCTTTCCTCGGACGGTCAAATAA

GGACGGAGAGTAGTACTGCGCTCTGTTTCAGCTTTCCAGCGCCCGTGCTAGGGTTTGTGG

AGACTATTATTGATCCAACTATAATGGACGAGGAACCGAGTCATTCCTGGGACAGCGATG

ATGAATATGAGAAGTTTATTCAGAGGATGAACCCACCTAGGGTTGTGATTGATAACGAAT

CCTGCACGAATGCATCGGTTATTCGGGTGGATAGCGCAAATAAGTATGGAATCCTCTTAC

AAGTAGTTCAAGTTCTTACGGATCTGAATCTTATCATCACCAAAGCTTATATAACATCCG

ATGGTGGATGGTTCATGGATGTTTTCAATGTGACTGATCGGGACGGAAAGAAATTGCACG

ATGCGGATGCAGCAAATGGAATAGTGGATTATATTCGGAAGTCGCTTGGAGCAGACTCTT

ATTTCGTACCTGCACGGAGAAGATCGGTGGATCTTGCACCATCGGCCAACCACACATCGA

TCGAGCTAACCGGTACCGACCGTCCCGGTTTGCTGTCCGAAGTGAGCGCGGTGCTCACAG

GTCTTCGATGCAATGTGGTGAATGCGGAGGTTTGGACACACAACACCCGAGCAGCTGCAG

TGATGCAGGTGACGGACGAAACCACCGGTAAAGCCATCTCGGACCCTGAGAGGCTGTCTC

GGATCAAAGAGCTTCTGTGCAATGTGCTCAAAGGAAACAACAAGACTCGAGGAGCGAAGA

CCGTCGTATCGAAGGGCGTTACCCACACCGAGCGAAGGCTCCACCAGATGATGTTCGCTG

ATAGGGATTACGAGAGGTGTGATCAGGAGCCAACGGATGAGAACCGGAAACCAAATGTGA

CAGTCGTCAATTGCTATGACAAGGATTATTCGGTGGTCACGGTTAGATGCAAGGATAGGC

CGAAGCTTCTTTTCGATACGGTTTGCACTTTAACGGATATGCAGTATGTGGTTTTCCACG

GAAATGTTGATGCGGAGGGGCCTCAAGCTTACCAGGAGTACTATATCAGGCACGTAGATG

GGTCTCCCGTCAACTCAGAAGCTGAGAGACAGCGAGTGATTCAGTGCCTCGAAGCAGCAA

TTGAGAGGAGAGTATCTGAGGGTCTGAAGCTAGAGTTGTGCACGAGTGACAGAATGGGGT

TATTATCAGAAGTCACCCGCATTTTCCGCGAGAACAGCCTGACCGTCACAAGAGCAGAAG

TATCGACCAGGGGAGGAAAAGCAATCAACACATTTTACGTGCGAGATGCTGCGGGAAATC

CAGTCGATTCCAGGACCTTAGATACCATACGTAAGGAGATCGGCCAGGCCGTGCTACAAG

TGAAAGGCTACGACGAGCGTCCGAAAACTCCTTCTGGCGATTCTCCTACACGATTCCTCT

TTGGTGGTCTGTTCAAGTCGAGGTCCTTGGTCAATCTCAGGCTGTCGAGGCAGTAATCCT

AGATTCTGCCAGTCCTAATTCCCCTGGCCAGTTGTATACATAACGATTAGACAAGAAACA

TGTATATGAATCCTCTCTGGACGATTGAAAACAATTGGCTATTGGCTTGTGTGAATGAAT

GTAAATATTGTTCCTGAGTTATAAATGAAAATT

>Unigene0000650

TAAAAAATGTATTGGAAAAATTAGTATATAGAAAATTACGCCACATCAGCATATACATTG

TTCATCCCTACCACCTTCCTCCTCTAACCAGAGAATTAATGGCGCCTCCACTCTTTTCAA

ATGTTTACACTGCCACCATTGAAATTATTGAGGGCTAGGGTTAGGTTTAGGGACGGTGCC

AACCCTATAGAAACTAGTGGTGGAGAAGCTTTGATGAAAATGGTTTGCCCCCCCTCTCCT

CCTATAAAAAGGTATCAAGAGCAGCAAGAGAGAATCATCAAGCTCCATTTAAGAGCAGCA

AGCTCCTAGGCATCCCCTACAAAGCTCTAACGAAGCTCCGGTGAAGTTCCGGCGAAGCTC

CGGCGAGGCTCCGGCGAAGCTTGTCAAGCTCCCAAAAGCCCTTAAATACTCTCTAAAGTG

ATCAT

>Unigene0000651

CCCTCTACTACGCCGCGGCAGCGGCGCTCCTCTACTACGCCGTCCTCATCTATCGCTGGC

GCTCTGCCGCCAGCTCCTCCGCTCGCCCCCTACCGGGCCCCACCGGTCTCCCCCTCCTCG

GCAGCCTCCCCTTCCTCGACCCCGAACTCCACACCCACTTCGCCGCTCTTGCCGCCACCC

ACGGCCCCATCTTCCGCCTCCGCCTCGGTGCCAAGGTCGGCGTTATCATCTCCTCCCCCT

CCCTCGCCCGCGCCGTCCTCCGCGACCACGACCCTGTCTTCGCCAACCGCGACGTCCCCG

CCGCCGCCCGCATAATCGCCTATGGCGGCAGCGACATCGTCTGGACCCCCAACAACCCCA

CCTGGCGCATGCTTCGCCGCGTCTGCGTGCGGGAGATCCTCGCCCCCGCGTCGCTCGATG

CTGTGTATCATCTCCGACGACGACAGATGAGAGCCACCGTGGCGAGACTGGCTTCGGGTG

GTGGGAAACCTGTTGATGTGGGGGAGCAGATGTTTTTGACTGTGCTGAATGTGATCACCG

GAGT

>Unigene0000652

GCGGTGAAGGATCTTATTTCGGCAGCCGAGGATGAGGAGGGGTTGAGGGAGTTGCTATCG

AGGCAGAACCAGGCGGGGGAGACTGCGCTGTTTGTTGCGGCGGAGTATGGATATGTTGAT

GTGGTGAATGAGATGATTAAGCACTATGATCCTTCGACTGCGGGGATCAAAGCGAAGAAT

GGGTATGATGCATTGCATATAGCAGCCAAGCAAGGGGACGTAGATATTGTGAAGGAGCTT

CTATTTGCCCTTCCAGAACTCTCCATGACCGTCGACTTATCGAACACTACCGCTTTACAT

ACTGCTGCAACACAAGGCCACACTGAGGTGGTGAATCTCCTATTAGAAGCTGATGGAAGT

CTGGCGTTGATTGCAAAGAGCAATGGTAAAACAGCCCTGCATTCTGCTGCTAGGAATGGG

CATTTGGAAGTTGTCAAAGCTCTGCTAAACAAGGAACCTGCAATTTCTATAAGAACCGAT

AAGAAAGGACAGACTGGACTTCATATGGCAGTAAAGGGAACAAGTCTCGAGTTAGTCGAG

GAGCTTCTTAGATGTGAACCTAGTCTGGTGAATTTGGTTGACAACAAGGGCAACTCTGCA

CTGCATATAGCAGCTCGGAAGGGTCGTATTCCGATTGTAAAAAGACTACTTGAGATTAGT

GAGACGAACACCAAAGCCATCAACAAATCCGGAGAGACTGCACTTGATACTGCGGAGAAG

ATGGGGAATTCAGAGGTTGTTTTAACATTGATTGAGTATGGTGTCCAGAGTGCAAGGACT

ATACAACCCTCGAACCCCGCACGAGAACTAAAACAGACGGTAAGCGACATAAAACACGAG

GTTCACTCCCAGCTTGAGCACACCCGCCAGACACGTAAGCGGGTACAAGGGATAGCTAAA

AAGCTCAGCAAGCTTCACGCTGAGGGCCTCAATAACGCAATCAACTCCACCACTGTAGTC

GCAGTTCTCATTGCAACCGTTGCATTTGCCGCCATCTTCACTGTTCCCGGCCAATACGTC

AATTCTGACAATCTCGCACCAGGGCTCTCCTTGGGAGAAGCCAATATCGCACAGCATACA

TCCTTCATGATCTTCTTCCTATTCGACTCCGTGGCACTATTCATATCCTTGGCTGTTGTG

GTGGTTCAGACTTCTCTTGTGGTTATCGAGAGCAAGGCAAAGAAGCAGATGATGGCAATT

ATTAACAAGTTGATGTGGTTCGCCTGTGTCCTTATCAGTGTCGCATTTCTTGCATTGTCT

TTCATTGTTGTTGGAAGTCAAGAGAGGTGGTTGGCCGTCGGGGTGACGATTATAGGGACA

ACCATACTGGTGACTACATTGGGGATTATGTTGTATTGGGTGATTAAATACCGCATTGAA

GCTAGAAAATTAAGGAATATCAGAAGATCTTCGCTTAGCAGGTCGAGGTCATGGTCTGTC

TCTAATGTATCAGATGTGGATATGTTGAACAATGAGTATAAGCGAATGTATGCCATTTGA

GATGAACAGGTTGTATATTCCACAAGCCATGTAATTACTGTTCTCCGATAGTTATAGTCC

TTGCTCAGATTGAATGGGCAAGTGTTTTGACCAGAGCTCGACAGAATGCTTATTACTTCT

AATATGAGGGAAGCTAACTCCCAGCAGTCAATTTAATTGCGAAAATGATGAGAATCGAGT

AAGGTCTGTATTCCTGTTGTATATTTTTACTGACATTTCTTTATTATCCGATTTGTAGGA

TCCCTCTGCCGGGATTTGACCATATCCTGCAAATACATTCAGAAATATAACATTT

>Unigene0000653

ATAGTTGGTTGGTTATAACAATCCAAATGGTTGCGGTGAAACCCATTTTTAGTTCTGGTT

GCAGGAATTTGTCTTGCGGAGATTGCAAAGGAAGCAATTCTGGGTGAGCTGGTAACTGAG

CTACCATGGCGGGCTCGCCGTCGAGGCAGCAGAGCTTCCGGCGGAAGAAAATGACGAAGC

AGCTGACGGGGAAGCGGGACGATACGCCGCTGCATTCTGCGGCGAGGGCGGGGAATCTCG

CGGCGGTGAAGG

>Unigene0000654

GCCAGAAGAAAACTGAGGCACTACTAAGCAAATCATGGGTGCATCTTAGTAATCTATTTC

CGCTAAACCCTGCCTACTGATCTCCTCCTCTCTCCCCCATCCCTACAATGGCCGCCACCT

GCATCCGATCCATCAGCCGCTGCTCCCTCTCTCCTCTCAGGTCAAACATCGCCAAATCCA

AAGTCGCCGCCCCGTCTCCGTCTTTCATCCGACCCGCCGCCAGAACCACCACTTCTCCCA

CATCTCGTCGATTCTCCACAAGGATTCCGGTGGAACTGGGTTGCTCGCTGTTCCCGCTGC

ACAGCGCGGTTGCGGCGGCGCGGATGACATCGCGGCTGAGCATGGCGAGCTGCCGGGCGC

TCACACAGGGCACATAACACTATCAGTGCTTGAAAGGACTAAAGTTGGTTGTGGACCTGC

TGATTGTGCTTCCAGGAACAGTCAAATGGAATTGATGGCACGTGAAACATTTTACTTCGT

CTATTCGCTGTGTCTGAAGGTGGATCATGGCATTGGTCTAGTACATGTTAACAGTATTAG

TGGAACAAATGCAATATTTTACTGTTTTAAATTTGTGAACACCTTGTAACACATTTTCAT

TAATTTTAGGAAAACATTTAATAGACCACAGCTATCCTGTTTAAATTGATATGCTTGATT

TGTTTCATCATTACATTGTGATATAGCTTTCTAATATATGTATTATTTGTTCAATCCAGA

GATGGGACTCTCAGTTC

>Unigene0000655

TGTTCTTGTATTTGACTGCTCCAGATGGGACTCTCAGTTCCAAGGTGAAATTCCCTATTC

AACAAAACAACTTTGGTACCTTCGTTTAATGTATTGGGTTTTGAAGAATTTGTGATATTT

CACATACATTTCTAATTATTGTAGCAAGAGTAATTTTGTTTTACGTCTGGTGTTGTGCTA

TTTTTATGAACGCTTGGGAGTCCTAGTATATTATCTAATATAAA

>Unigene0000656

TTGTGCTTGTTCTGTAGTTTGAGGAAAAAAGGGTGTGAGCTAAGTGTTTATACATACACT

TCGTTGGTTCTTGGATTCTGTAAGGATGGCTTGTGGGATGAAGCGTATCGGGTGTTGGAG

CAGATGGATGAAGACGGGTGTGTGCCAACAGTGGTTACGTACACCGTGTTGATTAATTTT

CTTTGTAAGGATGATAAGATTAGCGAGGCATTGTGGGTTTATGAGATGATGGGGCGAAAG

GACTGCCAGCCGGATTTGGTTGTATGCAATGTGTTGCTCTATTCGTTGTGTCGGCATAAT

AGGATTTCTGAAGCTGGTGAGCTGATGCAGGCCATGGATGACAAGGGCCTCTTGCTGGAT

CGGTATACCCATTGCTCGCTTGGTGCTGGTCTCTTGAAAGCTGGACATGCCAAGTATTCC

CGAGAGCTTCTGCTTAATGTAATCCCTATGAGCGAGATGGTGGATGCTGTGACATGCAAT

GTCTACATCCATAATTTGTGTCTTGGCAACAGATTGAGAGAGGCGTTATCAATGTTGAGT

CTTATGATGAAGAGGGGCTTTGTTCCGACTAATGCAACACGTAACACTATTCTGAAAGGG

TTTTGTATGGAGAAGAACATTGAAGAAGCAGTGAATTATCTGGATCACGTTGAGTGGGGA

AGCAACAGTTTTGACTTGATTGCTTTCAATACAATTCTGTCTGCAGCATGTAAGCAGACA

AACTCTGCATTGGTCGAGAGGATTCTGCACAGAATGGTGTCCG

>Unigene0000657

ATGTGGATTGCCCACCTTATGGCTCAAACCTTCAGTTTGTGTAAGAGTTTGATGTTAGAA

GATCCCTCCAAAGTTGTGGACCATATTTAATCATCAAGGATTTTGAGGGTTTATTCTATG

GTTACTTCACTGTCACTTTGAACGAGGAAAAATAAGACAAGAGAACTTAACCTCCCAGCG

TGTAGCTTTAACACTGGGAAGTTGGTCAAATGGATCTTTTTTTGAGAATTCCCAAGCCTG

AGCCCGAAAAGGGAGAATTCCCGAGCTCAATGTAGATACAATGGATCTTGATAATATCTC

ATTTGCAGAGGGTTGAAGTTTAGTTTTATTGGTGGCATGGAACGATCATTGCGAAGGCCT

TGTTAAACCTGATACATGTCTTAAAGTCTCGATTCTATAATGAGCTATGGAATGCGCTTC

ACCTGTTCAGATTTGTGGACGCTATCAGTGTATAGATGCTCCTGCACATGTAGAGATCTC

CTGGAGTTGTCATACTTAGCTCAAGGGCGGCAACGGCGCCAATTGATAGGTACAAATGCA

ATGTGATGAGCAAGTACCATCTAGTTTTGTCCATGCCTGTCAATTGTCCGAATCAGGACA

TTCTGAGTTTGGACACTTTCTGAGTTTGGGTAAGAAACTGATAAATCCCTCTTTCCAGTC

GGAGCGGTATATAACCCCCCCCAAACCCAAACTGTTAGGTACCAGGTCAGAGCAAGGGTG

CTAGAGTTTGACCAAAAAAGCAGTGAGTAGATTTCGAACATGACGGGCTACTACCAAATA

AGCATCAAGGACACAGCAAGTCAGTGATTTCACACTCATAAGAGGCAATATTGGTCCTGG

GGGAAAGCTCGTTCCACTGGGGGAAAGCTCGTTCCATGATGTCCATAGCATGGGTCTAAA

CATAATGGGTCTAACAGGAAACCTCTACACCAAGAAGTGAAGAAGTTGTACATAAGCTGC

TCGTTTTCAGCAGATTCTTGGAGTTACGGTTGTCTAACGCTTGAAAACTCAAGTGAAAAA

CATCTGAAGAGAGATCTCCAGTTTTCTGTAAATGTTCTGATCAAGTCTCTTCAAATCAGA

GAAAAATCTTCGACATGGTGTCGTTCCTTTAACATGACGGAATGAAAGGAGAAAATATTT

GTGCCAAGTCTTAGTCGAGGCAGTTGTTAATATCGTCGGTCACAGTAAGTCGTACCATAC

ATTACATTGCAAAGCCTATTTATTGCATATAAAATATTGTTTGTGTAATACTGAATTACT

GGACAAAGGATACTCAGCCGAAGGAAATTTTATATCTTAGGTATGCTATATCTTTGAAAA

ACCTTTCTACTCCACAAAAATTTATACAAAT

>Unigene0000658

AAAACCGCTTATTTAACGTTCAATCATCTGAAGGATACAACGAACTTTCTGGATACAATT

TCTTATAATATAATTATACGCGCCTCTGCAAGGAAAAATGACTTGTCCAAAATGAACAAC

TTGGTGACGGATATGTGTTGCCGTGGTTTGGTGCCTGATGCCATAACATATGGATCCATA

GCTTACGGCCTATGCAAGGCAGGGGATATCAAGGCTGCCCTGCGTTTGGAGGAATCAATG

GCGGACAATGGAGTAGCACCAACTATATCATATTACAACACGATTTTGATTGCTATGTTT

AGGTGGAGAAGATTATGGGATATCTCTCAACTACTGCTTAAGATGGAAATCGTAGGAATT

GAACCAGATGCAGTGTCTCTTGAAGCGTTCAACCGAGCAATGGCAAAAGGTTGCAGGAAG

AGATTTCCAAAAGCCATGAAGGTCCTTGAGACCCTGATCACCAAAGGCCAGAACCGAAGA

GGGTTGGAGGTTGGCTGAAAGACCATGATTGTATCTTTCAACATTTGATTATTGCAATGA

TTTGCCATCGAAGAATCAAAATCATGTGGATTGCCCACCTT

>Unigene0000659

CAGGAGCTGTTTCTGGTGATGATGAAGCCAAGGCCAAAGAATCGATGGTGAAACTCTTCA

AGGCTGCAGAATCTTACCTCATCGCCAATCCTGATGCATATGTTTAGAACCAATTACTGC

ATGTAAGATGCTGGATACCCTTATCCTCGATAAATATATATGGGAGAGATCTATGTATGC

GTTTATGTTCATGATTAAATGGGAGAGATCTATGTTCTAGATAAATAAACTTGTGTGTAT

CTTCGTATACCAACTCAAGTAATTGGGAGAGTTAAATATATGGGAAT

>Unigene0000660

TCAAGGCTGCAGAAGCTTTCCTCATCGCCAATCCCGGTGCATATGTTTAGAACCACTTAC

TGCATGTAAGAGGCTGGCTACCCCTATTCTCGATAAATAGACTTGTGCCTATCTTCGTAT

AGACTTGTGTGTATCTTCATGTGAGCCAGCACAAGTAAATGAGAGAGATCTATGTTCTAG

ATAAATAAACTTGTGTGTATCTTCGTATACCAGCTCAAGTAATTGGGAGAGCTAAAATAT

ATGGGAATTCTGGTCTATCT

>Unigene0000661

CAGGAGCTGTTTCTGGTGATGATGAAGCCAAGGCCAAAGAATCGATGGTGAAACTCTTCA

AGGCTGCAGAAGCTTACCTCATCGCCAATCCTGATGCATATGTTTAGAACCAATTACTGC

ATGTAAGATGCTGGATACCCTTATTCTCGATAAATATATTTGTGTGTCTTCGTATGCGTG

TACGTTCATGTGAGCCAGCTCAATTAAATGGGAGAGATCTATGTTCTAGATAAATAAACT

TGTGTGTATCTTCGT

>Unigene0000662

TGAAACTCTTCAAGGCTGCAGAAGCTTACCTCATCGCCAATCCCGATGCATATGTTTAGA

ACCACTTACTGCATGTAAGAGGCTGGCTATCCCTATTCTCGATAAATAGACTTGTGTCTA

TGTTCGTATAGACTGGTGTGTATCTTCATGTGAGCCAGCTCAAGTAAATGGGAGAGATCT

ATGTTCTAGATAAATAAATTTGTGTGTATCTTCATGTGAGCCAGC

>Unigene0000663

CAGGAGCTGTTTCTGGTGATGATGAAGCCAAGGCCAAAGAATCGATGGTGAAACTCTTCA

AGGCTGCAGAAGCTTACCTCATCGCCAATCCTGATGCATATGTTTAAAACCAATTATTGA

GTGTAAGAAGCTGGCTACCCTTATTCTTGATACATTGACTTGTGTGTATCTTCATGTGAG

CCAGCTCAAGTAATTGGGAGAGTTCTATGTTCTATATAAATAAACTTGTGTGTATACCAG

CTCAGGTAATTGGGAGAGTTAAATATAT

>Unigene0000664

CAGGAGCTGTTTCTGGTGATGATGAAGCCAAGGCCAAAGAATCGATGGTGAAACTCTTCA

AGGCTGCAGAAGCTTACCTCATCGCCAATCCTGATGCATATGTTTAGAACCAATTATTGA

ATGTAAGAAGCTGGCTACCCTTATTCTCGATAAATTGACTTGTGTGTATCTTCATGTGAG

CCAGCTCAAGTAATTGGGAGAGTTCTATGTTCTATATAAATAATCTTGTGTGTATACCAG

CTCAAGTAATTGGG

>Unigene0000665

CAGGAGCTGTTTCTGGTGATGATGAAGCCAAGGCCAAAGAATCGATGGTGAAACTCTTCA

AGGCTGCAGAAGCTTACCTCATCGCCAATCCTGATGCATATGTTTAGCACCAATTACTGA

ATGTAAGAAGCTGGCTACCCTTATTCTCGATAAATAGACTTGTGTGTAACTTCGTATGTG

TGTATCTTCATGTGAGCCAGCTCAAGTAATTGGGAGAGTTCTATGTTCTATATAAATAAA

CTTGTGTGTATACCAGCTCA

>Unigene0000666

TGAAACTCTTCAAGGCTGCAGAAGCTTACCTCATCGCCAATCCCGATGCATATGTTTAGA

ACCACTTACTGCATGTAAGAGGCTGGCTACCCCTATTCTCCATTTATAGACTTGTGTCTA

TGTTCGTATAGACTTGTGTGTATCTTCATGTGAGCCAGCTCAAGTAAATGAGAGAGATCT

ATATTCTAGATAAATAAACTTGTGTGTATCTTCGTATACCAGCTCAAGTAATTCGGAGAG

CTAAAATA

>Unigene0000667

ATTCATCAAAGGTCTTGTGCTTTCGTATCCCACTTAATAGACTCTGATACCACTTGTTGG

AGATATTGGTCCCTATTAGGTAGGTGCATATACTTTTTGTTGTGCTGAATCCAGGGTATA

CTGTTGCAGATCTCTGATCCTGTACCGTTGGAAGCATTTGAAGTCATGATACAAAATCTG

GTTTCACTGTTGTGGTAGTTGTCCCCAACAGATTCATCCACAAATAAAGATGAATCACGA

GCAAGCATCAAATACAATCATGAAGAGTATAATACATGTCATATTTTACCACGGGAATAA

AGATGAAGAAGATTTATTGATAGTATCTGAGAAGTATTGCAATGATAGAACGAATCTACC

CATATAGGAGAAACTTGAAGCTCTCCTTTGCCTTCCTCATTCTAGGGGTATTTTGATGAT

GGAAGATCAAGCTCCTGCTGTGCATTTGGAACCCAGAGCCAAGATCACTCTACATCTCGG

CTCTGAAGTATATTCAGTTAGTTCGGGGCCTGGGAGTCTCTCTCAACAGCTGGCTGCTCT

GAAGGAGGAGAGCATGATCATACTGAAGGACTTCATTACTACGCACAATGAGCCCACCGA

AGTTCCCGATGAGCCAATCGAGGGCTCTTCAGATGAAGCTGAGAACATCGTCAACCCTCC

GAAGAAATCCAAGAAACAGAAATGAAAGCTCAACTCCTGGATCAGAAGGAGAGTCTGACT

TGGTTTTGAATTTATGTTGTAGTTTCATGAAGCTCTCTCTCACAACTAGCCTTGATGTGC

TTACTTAACTATACACGCATCGATATCTTCATGTAAGGGGGTGTATGCACTTATCTGACA

ACTTTTTGTTATTATTTCCAAGAGAGAAATATTTGCATCTCTCTAAAATTCGTCGTTTAT

TGTATTGACTTAATTTACAAGATACCAATATTGCACCTCTCTAAGTTTCTCCTTTATGGG

GATCTAAAGATAACTGTAGTCTTCGTATCT

>Unigene0000668

CTTTCTAGATTGCCTAGAGGCACCAAAGCTGGATAAACTGTGGTTTATACCGTGTCAGTT

TCACCAAGATTTCCAGGGGGTAGTGAATCCATTTCTTAATATTGGATTGTATCTGGGTAA

ATTCTGGACTAGGCATGTTTGTTGTCTTAACAGCAACCACTAGTGTAATAATTTGTCCAA

ACTAGCCAATCATGTCAAAGATAAAATGCGTCAATTGAGACAATAACTGTTTGTTTTTTA

GGGTTCTGCCATGTCAAGTCAAGATGCGCCAATCGAGGCAGTCAGGAGCCATTCCATTGT

CCTGATGTTCTGTCATGTCAAGTTAGGGAACAACAGTCGTGGCAGTGAGCTGTCGATTAG

GGTCCTAGTGTTCTGCCGTGTCATCCATTTTCCCATTATAATTTGGAGCATCTTTCTCAA

TTTGGGTCATGTTTGATGTGACTCCAAATTTTTCCCAATGATCGGTAGGCCAGAATCCAA

ACTTGTGGCCAGAATCCAAAAGCTGTGTGACCGGTAAGCTAATGGAGAACCGAATAATGT

TTGTGATCTGCATCATTCATCAAAGGTCTTG

>Unigene0000669

TTTACTTCATCCACCCAATCAACCCGCCGCCTCTCTCCTCCCCTCTTGCCGCCGCGGTTG

CAGCAACCCTCCGGTGCATGCGGGTCAATCGCCGATCAGCGCTTTGAAAGCTCCGTTCAG

TCCGCGAATCCTATCTCTGATCCTGTACCGTTGGAAGCATTTGAAGTCATGATACAAAAT

CTGGTTTCACTGTTGTGGTAGTTGTCCCCAACAGATTCATCCACAA

>Unigene0000670

ATCCGAACAGCCACCTCCCCGGAAGAAGTGTTCAGGCAGAGAGGAGGGTGGGAGCGCCAA

ATGCGCGTCTGGGGGAAGATGCCACTGCTCGAAGAGGAAGTTGAGGGTGAAGAGATCAAT

TAAGGTGGCTGCAATCAGTAACAAGCTTGCTGATATCCCCTCTGACGATTATTCTTGGAG

GAAATATGGGCAGAAGCCAATCAAGGGCTCTCCACATCCCAGGGGATACTACAAATGCAG

CAGCATGAGAGGTTGTCCTGCAAGGAAGCATGTGGAGAGATGCCTCGAAGAGCCCTCGAT

GCTAGTTGTCACTTACGAAGGCGACCACAACCACCCCAAGCTACTAACACAATCTGCCCA

TGCATAAGAGGCTTTGTAACATCTGATCATTTTATTTGTTAACCATGTTTGCGAGGGGTT

AGTCTTCTCTGTACATTTACTTGTGTGTTGAGAAAAACATGTGGGTGAGTGAGAAATTCA

AAACATTTTTTTGTCACCTAAATTGAGCCAGATCTAAATGTTCGCAGGGACTCATTGTAA

TTTGAAGAATGCACTGATATTTTTTGGGCCCT

>Unigene0000671

GGGAGTTCTGCTGAGCTGCTGAAATCCAAGATGGAAGAGGTTGACTCAGCCAGCAGAGCT

GCTGTGCAGAGCTGCCACAGAGTCCTCACTCTCCTCTCTCAGCCTCATCACCATGATGAG

CTGCAGAGCAGAACCCTCCATGCTCAAACATGGGAGGCTGTTTCACAGTTTAAGAGAGTG

GTATCCATGCTGGGTGACGGTGTGGGTCATGCTAGGGTTAGAGCACTCAACAGATCTCCT

CCTCTCTTCAATCTCAAGTTATTCTCCGACAATCCATTTACTTCTTCTGATTCCAACCCC

AAAATCCTTCATCAAATTTTACATGAAACACCAAAGTTCAGCCCATTATCTCAATTATTT

CAGCAGCAGCAGCAAATGAGTTTTCAGGCTGAGATTTGTAAGAAGAGTAACAGTTGCATC

AACTTCAATTTTGACTGCACGCCGACTGTATCGTCAAGCAG

>Unigene0000672

CGAAGATGATGAGTTCGTCGTCTACGAGATTCTCCCCATACCCTGTCTCATCGATCCAGG

GCGGAGACCGCCTGCAGCAATGATTGCCGTCACCCTCTTCTCGCCGCCGCCGTGCTGGAG

CTGTCTCCGCCAAGGCTTCGATGCCCGCACACCGGCCCTTGTTCGGCCCCGTTTGAATCG

CCGCCTCTTCTCTAGGACAAGGGACTCGTGCCATCAGAAATCTTTGTATCGTAACAGGAA

CATTTCTGGCAAAGTATTGTGCAACCAACTAAAATTAGAGGATGTTGTTGATGATGAAGC

TTGCGAACTGGTTAATGGGGTTGATCTTATAATAGGCGAAAATCAAGATAGCATTCGTGC

TTATTTATTGAAGCCAGTCAAGAACAACAATGAGACTGGGGTGCTGATTCTGTCTGATGT

TTTTGGTTTTGAAGATTCATCTACAAGAGATTTTGCCTATCGTGTCGCCTGCACAGGTTA

CAACGTTCTTGTCCCTGACTTGTTCCATGGGAATCCATGGAAGAAAGGTCGGCCTTCTTC

CGAGTTTGAGCAATGGTTGTCAGGACAATCATTGGAGAAGGTATCGAGAGACATTGACGC

ATCATCAAAGTGGTTCATCGATGAATTTGAAGCAGCCGGACTATCGAAGAAGCTAGGCAT

TGTTGGATTCTCTTATGGCGCTGAACGATTAATCGAGACATTGGCTAGAGATGAAGCGGA

AAATTTTGTCACCGGGGTCTCGTTTTATGGGGCAAACTTGAATGCATCCTTGGCTAAAGG

TATCAAGGTTCCAATCTTGTTCATTTGCGGAGATAATGATCCCCTTTGCCGTGTTAGTGA

ATTAAGGGATATAGAGAAGAGCATTGGAGGCTCAAGATTGGTGGTTTATTCAGGGCGAGG

TCACGGGTTTGCTCACCGGCCAGAATCTCTGGAAGAAGATGAAGATGCAGAGAAAGCATT

TGCTGTAATGAGAAATTGGTTGCAAGATATATTGCTTGCAAGCATGGATGGTGCAGAATA

AGGGTTGAGTGTAAAGAATGGTATCAGGTGCTCAGGGAGATGACTGGATGAATCGAAAAG

GATATGGATGTTATTGGAAGGAACATGAAGACTCTGGAGCTATGAAAGATCATCATGAAG

TCACTGGAAGGGATGGCTAGCAAGTAATGAAGCTCTGGATTCGAAGCTTCTGGAGGTGAT

AATGACATTGAGGTAGAGGCCATATAAGGGAGTACTTGCAGCTGATCATGGGATCTCAGT

CTTCTCTCCCTGGGAGGGAATTATGTGAGAGTAGAATTGTGTGCTAGGTGAGCTGAGAGT

TATGTGATTAATATCTTATGGGAGTGAAAGTGATAGTCTGTATGAGATGAATGTGAGTCT

GTTTGAGATGACGATGATGTATGTGAGC

>Unigene0000673

ATCATTAAGCAACCATTTACTTGATCCTATATGTCGGGAAATTTAGTTTCAATGGTTGAA

GGTTTCTTAAAATAAATGTAACTAATATTTATTTGCTTGTTATATATTTGTTCTTTGTAG

CTTTTTTATTCTTAGACTTGTCAGGTAGCTTTTTTCTTTATGCTAAACACTACATATATA

CCCTTAACTATAATTGATTACTTGACATCTCCATCATCTGCTTTATATTTTTTAAATGAT

CTGCGATGAACAGTCTCTACATGAAAAATTGTGAAATTACCTATAATAAATGATTCATTG

ATTTGAAGCTACACAGATTAAATGGATTGATGGGCTTTGTAATAAATGAAGAATGACTTT

ACTCATGGTCCCTGAAAATTTTGCAGATGCT

>Unigene0000674

CAAGTTTTATGGGAGACAATGAGATTGTGGAGCTCTTTTTTTTTTGCGGTTTTGGGGGTT

GGGGGGTTATCCCCCCAAAAGCAATATTTTGGATTCTTTTGTGGCTGATCACAAGCAGTT

TTGGATTAATGTTGACCAACTGATTCTGTTGGAATTAGATCAGGTTTCTGCATCTGTTCT

TGAAATATCCAACAAGTTCACATTTTCTGTTCTCCAAACTTAACATTAAAGAGTTGTAAC

TAAGCTTGGAAGTATCATATCTTTCAACCCCAAAAGCAATATGTTGGGGCCTGTATTAGT

TTCTGTCTCAGCCATCTGGGGTGATACATAGTGGTCGGTTCGATGTGGTTGCTGTCGGTT

CGATGTGGTTGCTGGTGAAATGACAAACCTCAGTTAAACTACCCACCCATGTCCTTTGTG

ATAAAATTATACTTTTGAGAAGGGTTTTCTGTTTCCTTAGAAACAAGATATTTTGTTTTT

GGGAAAGAAGATCTCAATTGAGGAGAGTGAGTTAATTATACTCTCCATTGTATCTCTTCC

TTGTGCCACCAGAACACAAACCCTAGCTGACTGCCATCTCTCTTTGTTTCCCTCTCATCA

TTAAGCAATCATTTACTTGATCCTTTATGTGGGGAAATTTAGTTTTAATGGTTGAAGATT

TCCTTCTAATTTTCAGATCCCTCAGTAGAATTTATGGGATTTTTGCTTCCTTGTAATG

>Unigene0000675

ATTGCAGAATTTATCTGACCAAGTACTGTGGAGGTCTCCTCTCCTACCATTCTGGAACCC

GCACATGGTGGGAGGGAGGAATTATGTTGTCTGTTGTGGCTGATTACAAGCAGGGGGGAG

GGGGAGATGTTGACCAACTGATTCTGTTAGAGTTAGATCAGGTTTCTGCATCTGTTCTTG

AAATATCCAACAAGTTCACATTTTCTGTTCTCCAAACTTAACATTAAAG

>Unigene0000676

TGACCTTCTCAAAATGACAGACAGCAACAATAACCCTAAAGCGAAGTTCTTAAATCTTAG

TCCTGTACTACGTAATGCATTGCATATGAGACCTGGTGTTAATATAATTGGTGGATCAGA

ATCACAAGACTTTCAGCTAGAAAAACGCATGGACAATATTCTTATAACTGCTGCACAACC

AGTAATTGATGGTTTGCAAAAAAGTGTAAATATTGAGCTTACCATTAATAATGAATGCCG

TGCTTTTGTTTCAACTTTAAGCTACCATATTTCAATGAAATATGGAGATGCTGGTTTACC

AGATAACAGTATAAATATAAAATTAAAAGGCTCTGCTGGGCAGAGTTTCTGTGCATTTAT

GACTAAAGGAGTACATGTTACTTTAGAAGGAGATGCTAATGATTATGTTGGAAAAGGTCT

TTCTGGAGGTGAAATAGTTATTTTCCCACCAAAATCGTCAACATTCCAATCTGAACTCAA

TGTAATAGCTGGCAATGTTTGCTTGTATGGAGCTACTAGTGGAAAAGCATTTTTCAGAGG

TATCGCAGCCGAACGTTTTTCAGTTCGAAATTCTGGTGCTGTAACAGTTGTTGAAGGAGT

TGGTGATCATGGTTGTGAGTACATGACTGGTGGTTGTGCAGTTATTTTGGGTCTAACAGG

ACGTAATTTTGCTGCTGGTATGTCTGGTGGTATTGCTTATATTCTTGATGTTGATGGATC

TTTTAAAAGCAAATGCAATACAGAAATGGTAGAACTGCTTCCTCTTGACCGTAAAGAAGA

TCTAGACTATGTTCAGTCTCTTTTAGAAGAATTTGTTCTTAAAACTGAATCAGTTATTGC

ATCTAAGCTGTTAGATTCTTGGCCCCATTCTGCATCTAAGTTTATTAAAGTTTTCCCTTA

TGAATATCAAAGAGCTTTAAAACAATTGGCTGAAGAAACAAAAGTAACTCAGAGCCACAC

AAATACAACAGAAGCTGTTACTGAACCTAGTGTCCGTGAGCCAAGTGTTCGTGACATTGA

AGAAACAATTAATGATCAAACATTTGAAAAAAAAAGACTTGAAAAGATATTAGATAAAAC

TCGAGGTTTTGTAAAGTATTCGCGAGAAACAATGATGTACAGACCTGCTGAAAAACGACT

CAATGATTGGGACGAAATTTATAATTTCCAACATGTCAGGAAAGGACTTAGGGTTCAAGC

TGCAAGATGTATGGAATGTGGTGTACCATTCTGTCAATCTTCACATGGATGTCCTTTGGG

AAATATCATACCAAAATGGAATGATTTAGTTTTCAATAATAATTGGCAAGAGGCATTAAA

TCAACTTTTACAAACTAATAATTTCCCTGAATTTACTGGACGTGTTTGTCCCGCTCCTTG

TGAAGGCGCATGCGTTTTGGGTATCAATGAACCTGCTGTAACTATTAAGAATATAGAATG

TGCCATTATTGACCATGCTTTTGAACAAGGTTGGATTAAACCTGAACCACCAAAGTTCCG

ATCTGGTAAAACTGTAGCTATTGTTGGATCTGGTCCATCAGGGCTAGCAACTGCTCATCA

ATTGAATAAGGCTGGACATTTGGTAAAAGTTTTTGAGAGGAATGATCGTATTGGTGGATT

ATTGCAATATGGAATTCCAACCATGAAATTATCTAAAGAAGTTGTGCAAAGGAGAATTAA

ACTTTTAGAAGATGAAGGCGTGGTTTTCCATACAAATGTCAGCGTTGGCAAAGATTACTT

AGCTAAAGAGCTCTTGGATCAGTTTGATGCTGTTGTATTATGTACTGGTGCTACATGGCC

ACGAGATCTACCTATACCTGGTCGTCATCTAAACGGTATTTACTTTGCTATGAACTTCTT

AGAATCTTCACAAAAAAAACAGCTCGGTGGGCCAACAGATCTACCCCTATTAGCTAGGGA

CAAAGACATAATAGTGATTGGTGGTGGTGATACTGGTTGCGATTGTATTGCTACTTCATT

AAGACAGAGGGCAAGATCTATTACATCATTTGAAATCCTTCCTGAACCACCAGCAACTAG

AAGTCGAGATAACCCTTGGCCACAATATCCTAGAGTATTTAAAGTGGATTATGGTCACGA

AGAAGTTAAAATTAAGTATGGCAGAGACCCAAGGCAATTTTGCATCATGAGCAAGGAGTT

TGTTGACGATGGTAATGGTAACGTTAGTGGTATTAAGACAGTTAAAGTGAATTGGACTAA

AGATGAAGCTGGACGATGGAAAATGGATGAAGTACCAGATTCTGAACAGATTTTCAAATG

TGATATGGTTCTTTTGGCAATGGGATTCCTTGGTCCAGAACGTACAATTGTCAATGAATT

ATCATTAGCCCTCGATCCGAGATCTAATTACCAGACACCTACTGGTCGTTATGCTACATC

CGTTCCTAAAGTATTTGCTGCTGGAGATTGCCGCCGTGGCCAGTCATTGGTAGTTTGGGC

AATATCTGAAGGACGCCAAGCAGCTAGAGAAGTAGACGCATTCCTCATGGAGCAATCTGT

ATTGCCTGGACCTGGTGGTGTGATATCTATTCAAACTGTTAGTGCTTAAATTAGTACGTT

ATATGTAACTACATATGGACAATATTGAGGGAATCACGTTGGTTTTTTAAAATAATATTT

TAACATTACAGTGTGTTAGTTCTTTTTTCAACTGATTAGGTATCAAAACCATATTATTGT

TATAAATATATATATTTTTTTTTTTACTAATAATTTAAGTATGGTTACGTTATTTTTTTA

AGCTCCTATGGAAACAGTAAATTCCTAAGATCGATTTTCGATTTACTCATAACAATAAAA

AAAACAACACACAAACATATGTGTTTTCCTTATTCATACAATATAGATACATATTATATA

ATTATATAAGTGGAGTGAGTGTGTGTTATAAATACCCATACATTTAGTATTATCTTAAAT

TCCATTTTTCCCCAGCTATTTAAATTTATATCAAAATACGTGAATATTGACAAAAGTGTA

AGAGACTGACTTTCTATTAAGAATTTTATTTTTAAATAATATGTTTGTATGAATATTAAC

ATTTTTAACTTGTGTGTGTATTTTCTATACATTATAAAGCATAGAATGGATAAAAAATGT

ATTCTATTTAGACAAATTGACTCTGGATTAATATTTTTTTACAAACAAAAACATTAAGTA

GATAAAGATTTTTGAGCTATCCACACAGAGCAAAATATACAATTAGGTATATATCAATTT

TCATTGAAAGCTGTCTAAAATATGACATATCACATTCAAATTTAATTTTTGATTAAACAA

AATTTGCTATCACTGATTAATAATTATTAATTATGAATGTGTATACTATAATATAGTAAG

AAGACAATAGAGAATCAGTCTTTGACACTTATAACAAATTTCACACTTGTTAGATTTCAT

ATTCATAAATATTGTTTACAGTATTATCGTCCAATTTGTTGAATGTATTTATTTTTATTA

TTATTTAAATATATTCAATAGCTTACAAACGTAAGATTTTAATATTATTATAATATTATT

GCATATCCTAAACTATTACTACCTACCATACTAAATATATAATTATAAATTATCACTATA

GAATTAATTACTTATGCTTACATAATATGTTATTATTGTTAAAATTTAATTACATAAATA

ATGTCAATTTAGTATGTAGAAATATTGAAAAATTAATTGGTGCGCAGTTAAAATATACTG

TATTATAGCTGAACTATTATGAAATTTATGCTATAAACTTTACTTTTGTGTAAAAGCTAT

TTGCCATTTGAAACATTGTGCCCTTAAAGACAATAATAAATTATAAATGATACTTAGATA

TTTGTTTAATGTGTATTTTTAATATTATTCGATCATTATGTCGATGTTTTGTGCTAAGTA

TAATTATTATTATATATATATAATATTATTATGTAGTCGTTGAATTAT

>Unigene0000677

GAAGTTGGTGTAGGAGTAGTTGCTTCAGGTGTAGCTAAAGGAAAAGCTGAACACATTGTA

ATTTCTGGTCATGATGGTGGTACTGGAGCTAGTTCATGGACTGGTATCAAAAATGCAGGT

CTTCCATGGGAACTTGGTGTAGCAGAAACTCATCAAGTCCTTGTATTGAACAACTTAAGA

TCCAGAGTAGTCGTCCAAGCTGATGGTCAAATACGGACCGGTTTTGATGTCATTGTAGCT

GCTTTGTTGGGTGCGGATGAAATAGGCTTGAGTACTGCACCATTAATTGTACTAGGATGC

ACCATGATGAGAAAATGCCATTTAAATACTTGTCCTGTTGGAATTGCAACTCAAGATCCT

GTTCTGAGGAAAAAATTTGCTGGCAAACCAGAACATGTTATTAACTTCTTATTTATGTTA

GCCGAAGATGTACGAAAGCATATGGCAAACCTTGGTGTTGCCAAGTATCAAGATCTTATT

GGACGCACTGACCTT

>Unigene0000678

TGGCACCTAACAGTGAAGTCATTACTAAGTCGATGGCTAGCAATGCAAAGGCAGCGAAGC

CTCGCAGGCTGTCGATGGGGGGTCTCCAGAGGGCTATGTCCAACCTCTCCTTTGAGCTGA

GCAAAGAGTCCTCCCTCGACTGCAAGCTCCCTCCCATCTCGGAAGTCTCTGAGGCCAAGT

GCGAGTGCTGTGGTATGTCTGAGGACTGCACACCCGACTATATCCGTGGTGTCCGCGACA

AGTTCTGCGGCACCTGGATCTGCGGGCTCTGCTCGGAGGCGGTGAAGGAAGAGGTGGCGA

AGAACGGGGGCACTGATAAAGCTGTAGCCCTAGAAGCACACATGAGTGCATGCAAGAAGT

TTAATAGGATTGGGAGGACGCACCCCTTGCTCTACCAAGCGGAGGCGATGACGGAGATTC

TAAGAAAATGCTCGAAGTCGAGGGCGATGTCGATTAGCCCGAAAGAGAGGGGCGGAGGTA

AGGGAAAGGGAGGGATTGCACGGAGCTCAAGCTGCATCCCGGCTATAATGAAGGATACTA

AGGAGAGGATGACTGCAAATTGAGGTTGGAGCATGGAACTAGACACGTTTATCGATCAAG

AATAAAGAGTAGTATATGATGATCAAACAACCCTAATTAGAGCCATGGATGATATATTGT

GGAGTGATCTTGGACTAGGTCCGACCCTCATTTTTTTCCTGGCTCACCTTTTAATTTATT

TTCACTTGGTCTTTGTAAAGTGTTCTACGTGTAATATTGTCTACTAGGGCGTCTAATCTC

TCTAACGCATGGGTACTGTGTTGTAAGTATTATCCCTGACAGTTGTTTAGTAGTGTGTTT

ACCTTTCTCGATCTCATCTTTACTTTCTCGATCTCATCCATACTGTCGTATAAG

>Unigene0000679

CCCAAAGAGATAACCCAACCTCATTCCTTTCTCCCCCGTCTCTCTTCTCCACCTCACCAT

GCTCCTCACCTCCCCCTCTCTCCTCCGCCTCCCCTCCTCCCTCCCCCACTCTTCCCCTTT

CCTCAATGGGGTGTCTTCCCACCCTCTCCGCATCTCCCTCCCCATCACTCCATCTCCTCT

CTCCCCCCCTCGCTCCGCGTCCCTCACCATCCACGCCATGAAGAAGATGCAGGGCCGAGT

CGTCTGCTCGGCCAACGACAAGACTGTCGCCGTTGAGGTTGTCCGCCTCGCCCAGCACCC

CAAGTACAAGCGCCGCATCCGGATAAAGAAGAAGTACCAGGCGCATGACCCGGATAACCA

GTTCAAAGTCGGCGACTATGTACAGCTCGAGAAGAGTCGACCCATAAGCAAGAACAAGAC

CTTCGTCGCGATCCCGGTTGTGGCAAGGAATGCACCGAAGCCGGTGGAGGCTGTTCCGGC

TTCTCTACTGGGGCTTCCACTTCAGTCGGAAGAGCCGAAGATTGAGTAATTTCACCAACC

TTCTCCTTTTATTTGTTCACTTGTTGTTATCGGTTGTCTAGATTTGGAATTCGGGAATTT

ATGTTTCTGATTTGTATGGTTATCTTGTTGCTTGATTGAATTTGCTGTCTTCAAAATAGG

GAAGTTCAGCTTTAGTAATTGCTTAAATTGGGTATACTTGGTTTGGGGT

>Unigene0000680

TTATTCCATACTTTCCGGCTCCTTTCTCTCTCATCTCAGAGCTCTACCATGACGGACTGG

GCGCCGGTGGTGGTGGGGGTGGTGCTGTTCGTGTTTCTGTCGCCGGGGCTGCTCTTTGAG

CTGCCGGGGAGCCACAACCACGTCGAGTTTGCGAAGATGCGGACCAACGGGAAAGCAGTC

TTCGTCCACACCCTCATCTTCTTTGCCCTTTTCTCCGTCATCATCATCGTCCTCCACGTC

CACATCAACTCCACATGATCCTGCTGCTGCTGTTTCTCGTCATCATGCTTTGCTAGCTAG

TTTTCTGAGTGAGTCCTCCTATGCTTTCTTGAAAGAATAATTGGGAAAAGTAACTTGTTG

AAATATGAATGGGACCTGTTTTGCTTTATGCTTTATTTTATTGTCATTTTTTTTCCTCTA

CTTGCTATTCTTTA

>Unigene0000681

TTATTCCATACTTTCCGGCTCCTTTCTCTCTCATCTCAGAGCTCTACCATGACGGACTGG

GCGCCGGTGGTGGTGGGGGTGGTGCTGTTCGTGTTTCTGTCGCCGGGGCTGCTCTTCGAG

CTGCCAGGGAGCCACAACCACGTCGAGTTCGCGAAGATGCGAACCAACGGAAAAGCAGTC

TTCGTCCACACCCTCATCTTCTTTGCCCTCTTCTCCGTCATCATCATCGTCCTCCACGTC

CACATCAACTCTACATGATGCTGCTGCTGCTGTTTCTCGTCAT

>Unigene0000682

TTATTCCATACTTTCCGGCTCCTTTCTCTCTCATCTCAGAGCTCTACCATGACGGACTGG

GCGCCGGTGGTGGTGGGGGTGGTGCTGTTCGTGTTTCTGTCGCCGGGGCTGCTCTTCGAG

CTGCCGGGGAGCCACAACCACGTTGAGTTCGCGAAGATGCGGACGAACGGCAAAGCAGTC

TTCGTCCACACCCTCATCTTCTTTGCCCTCTTCTCCGTCATCATCATCGTCCTCCACGTC

CACATCAACTCCACATGATCCTGCTGCTGCTGTTTCTC

>Unigene0000683

GGTGGTTTTCAAGTTGTGTTCGAGTCTTTGAATGCAGAAATAGCTGGTTCCGGGTGGTTT

TCAAGTTGTGTTCGAGTCTTTGAATGCAGAAATAGCTGGTTCCGGGTGGTTTTCAAGTTG

TGTTCGACTCTTTGAATGCAGAAGACAATCAAAAGAGGAACAAGGTCTTTGCATCCTTAT

TTTGTAAGTCTGTACAAAGTTTTCTTTAGTATAGTTTCATACAGTATAAAGCCTGATCAG

TTTGATCATAATATGTAACAATTTCTTCCTTAGTTTTGGTACTCTGAGTTTTGAGATTCA

CCCCGTCGAGGAAGGTCAAGGGATCAATCCATGTAAACTTTGTGCCATGTAATCTGTTTG

ATGTGATGATCTCTCTACTGTTGATGAATGTTCTGATTTGATTCTTTAAA

>Unigene0000684

GGAGAGGGAGTGGGAGAGGAGAAAGCCATGGCTGATTTTCGAAAAAGAGCCGTTGCTCTG

TCAACTACGTAATTTTTGTTTCTGATTCTGAGTGAAAGTGTAATAATTGTTCTTACATGA

AACTTTTACTTGATAAATAGCTACAAAATATAATGTAACTACTTAGTATTTAGTCCCAAT

ATGTCACTAAGGAAGATGGGGATGGAGTTCCTTTTGGACACCTATTACAGTAGATTTGAA

CATGACTTGTAGGCAATCGTCTCCAATCCTTGTCTGGTATCACATTGGCTCGTTACATTG

ACCCAGCTTCAAGGTATAATGCTGAACATCAATATGCAAAGCCCTCAACACTTGCTCTGT

GCACCGAGCCACGGATCCCTGTATGTCCAATATACATATGAGTTGTCCGTTTTGTAGGTG

GGCCGACATACATATGAGTTGTCCGTTTTGTAGGTGGGCCGAGCCTTCAGCTAGAGTCCC

CTAATGCTCCGCACACCTCACCATCATGAAGCACTAAACACCTAGGGTAGTCCAAGTCAT

CCATCCAACCTCAGGCATGCTCCCATCCTATGTTGAAGAATCCGTCCATTCAACCTTCGC

ACGATAACCCGGACATGGCAATATGCACCCTATCACGTGCAAATACCATGGGCCTGTTGC

ATCCCCTACGCACAGGTGATAGAGTCGGGATTTATGAGTCAGTGTTTGACGACCCTGCTC

CTACTGTATCTGCTAAACATTCCCACATTTTCACCGTAACTATCCTGTCTAATGGAGTAC

CTAACCTATTTTGGGAGAACTACATTCGAACCTCTATGAATTTTTCTGACCATGTGTCAG

ACATTCTGTGTGTGCAGGCCAGGTCCTGCATCAGAAAATATCTTTGACAGTATGCATGCC

TTGAATGTATGTGCGCATTGTATAAATTCTGGATTGGCGGGAACAGTTTGTGTGCACATC

TACTGAAGGTGTGTGTACGTTGAATAATTTGCACTCGCACATTTATTAAATGTGCGTATA

TCACCCGTGATTGTTGAACTAATATAACCCTTCGATTGAACGGAGGCGACTTCAAGGCTA

TT

>Unigene0000685

GTGGGAGACGGCACGGAGGCCTTAAACCCTAGCCGCCCTAAAATCCCCTTTCTCTCTCCT

ATTCTCTCTCCAGCTGCGCCAGATATTCGAAAGAGATGGCTTTCCGTAGCAAGCTCGGGA

GCTTGATCCCCTCCAGCACCTTGAAAAACTGCCTATCAAATGGGCCAGCTCCCATGATGC

ATCTGCAAAATGCTGTTCGCCACATGTCATCAAAGCTTTTTGTGGGAGGTCTATCTTATA

GCACTGATGACCAGGGACTGAAAGCTGCATTTGACAGCTTTGGAAATATTGTTGAAGCCA

AGGTTATCCTTGACCGAGAATCTGGACGATCTAGGGGGTTTGGGTTTGTAAGCTACGAAG

AAGAGGGGCATGCCAGTGCCGCTCTGTCTAACATGGATGGCCAGACATTGGACGGCCGTA

ACATTCGTGTGAGTCTTGCCAACGAGAGACCTGCAGGTGGTGGCTTTCGCGGTGGCTACG

GTGGCGGTGGCAGCTACAGTGGTGGTGGTGGTGGCAGCTATGGTGGGAGATCTGGTGGTG

GTGGTGGTGATTATGGTGGTGATTTCTGAGAGACTCGACGGTGGCAGTTGGACTGAGGGT

TCTATATTTAAGACTAGATAATGAAATTTTCAGGTACAAGTAGTATCTGGTACTTGGTAT

TCCTTAATATTGTTTCTTGAACAATTCTAGTTGTATTGAGCTTTGTGTGTTTGCATTTTG

CAACGGTGAATGGCAGAGCTATTTGAGCTTGATAGACTCGTAGAGTTTTGGGTGCTACAT

TAGATGAAGATACTATTTTTTTCCGGTTTTCCTGTTAACTTCGGTGAGTACAATTATGAA

CAAAATTTGTCAATAAATTAGTTGGGATGAGGGTTAACATCCCAATTTGATCTATCCCAG

AGTCCCATGTTTTATTGCTCTTTGTAATGAAATCTGAATGGATATTGAGATTTGTTAAAC

ATCAAAAGGATTAATATTGGTCTATGTATGTTTTATTGGAGATTAATTGA

>Unigene0000686

TGGTTAGAAGACAGTACAAGGACTAGTTTGGGTTATCTCAACACAAAGCCAGTGCATGAA

GGGATAATGAGCAATGATTGTCATGATAGTAGCATCTAGACAGTAGCTGAAGGTAAAAGT

CCAGTGAGGGTTGGTGGTCTCTCATCCATTTGTCATAGCAGACCTAGCAGACAGTGAGCA

AATATGAGCATCTACAATGGCTAAGGTAGTGAGGCTAGGACATAGGCAGACATGGTCTAT

CTCAGTAGATAGTCAAGGCTTGTAGTACCTAGAAGCTATAAGGAACATTGGCAAGGTGGA

TGCAATTGTAGTAGTCCAAGACAGACAATCTCTCAAGACAACATAGGCAGATGGAGATAG

TTTAGTGAACAAACAGGCAAGACAATGACACTCCAAGAGTTACATGATGTAGTCTCTCTC

AACCTCTCTTTCTTAATGTGTTCCAAGATGAGACATCTAGGGTGGCTACAAGGTACAGTA

AAGGGGACAAGATAATCTGGATAGGGTGGCTAATATCTCCAGAGGCTATGAGAAGAAGAT

ACTATAAGGATCTAGTGCAAGCAGTCAACTCTGTATAAAGACCCCTCCTCACTCAGTGTA

TCTCCAAGTCTTCCCTTTGTTGATTTCTGAGTTCTTAGGAACAGTGATCAAGAGATACAG

>Unigene0000687

ATGGAGATAGTTCAGTGAACAAATAGGTAAGACAATGACACCCCAAGAGTTACGGGATGT

AGTCTCTCTCAACCTCTCTCTCTTAATGTGTTCCAAGATGAGACATCTAGGGTGGCTACA

AGGGACAATAGAGGGGACAAGAGAATCTGCATAGGTTGGCTAATGTCTTTGGAGGCTGTG

AGAAGAAGATACAATAAGGAGCTAGTGCAAGCAGTCAACTCTATATAAAGACACCTCCTC

ACTCAGTGTATCTCCAAGTCTTCCCTTTGCTGATTTCTGAGTTCCTGGGAACAGTGATCA

AGAGATACAGATAGAGATAGAGTTCTTCTGCATTACCATAGAATAGGAAGTCAGGAGATG

TTCTAGCATCCAGTATAGTTTTAGCCTACAATTGAAGTCAAGGTTTGAAGTTCCTAGCAA

GTAGTTATAGTAAT

>Unigene0000688

AAGAGATACAGATAGAGATAGAGCTCTTCTTCATTACCATAGAATAGGAAGTCAAAAGAA

GTTCTAGCATCCAGTATAGTTCTAGCCTGCAATTGAAGTCAAGGTTTGAAGTTGCTAGCA

AGTAGCTATAGTAATCAAAGTGCTTAGTTTCAGCTTATTGTTAATTGAATGAATCTATTA

GTATAGTGTTTTCAGTTTATGTCAAAGAATCTTAGGTTGTTTAATAGTTTAAACTTCAAT

TTGTGAGTGAAACAAGTATTGTTCAATCTCTTATATTGAGTAGAATGATTAAGCATAGCT

AGTTAAGGTTAATCTAATTATCTCTGATTAGAACTTGTAATCTATCTACCAAATTAAAAT

GTTTGTTT

>Unigene0000689

TGGGTAAGGTGGATGTAATTGTACTAGTCCAAGACAGGCAATCTCTCAAGACAGCATAGG

CAGATGGAGATAGTTCAGTGAACAAACAGGCAAGACAATGACACTCCAAGAGTTACAGGA

TGTAGTCTCTCTCAACCTCTCTCTCTTAATGTGTTCCAAAATGAGACATCTAGGGTGGCT

ACAAGGGACAATAGAGGGGACAAGAGAATCTGCATAGGTTGGCTAATGTCTTTGGAGGCT

GTGAGAAGAAGATACAATAAGGAGATAGTGCAAGCAATCAACTCTATATAAAGACACCTC

CTCACTCAGTGTATCTCCA

>Unigene0000690

AATAACAAATATATGGGCAGAAGGTGATCTCTAGTATAGGTTATAATGAGCGTGATGGGA

ATGAGCATGAAGGGTTAGCAGTATGAAGCTCGAAGTAAGGCAGACATCTTCAATGGCTCT

AGGGAGGCAAGCAATATCAGCAGGCAATGAAGCAACATGGGCATTGTTGGCATGGGTTTG

TGGTAGCAGTCAATGTGCAAGGCATGAGCTGAGGATTAGTCTGGGCTATGGGTGAAGGCA

TTGAAGCATGGCCAATACAAAGAAGGCACAGTCAGGTTAAGCATCTCCAAGAGAAGCTGA

CTATGATGGTGTAGTACCTAGTGATTATGGGTCAGACAATGGTGATACATGGATCAAAGG

CATGGCGGAGCATGAACTAGTCTCTTTGTGCCTAGAAGGTGAAAACAGACATTGGTAATT

CAAAGTAGGGA

>Unigene0000691

AGTAGTAGGCAGTAGTGAGGCATGACTTGACATAGGCAGTGGGCAGTATTGGCATAGCAT

CTAGGCAAGACATCTCTAATACAGATAGTAAGCCCTCTCTCTCTCTCTTAATATATCCAA

TGGGCAGTATATAAGTATAGAGTAGGATGTAGTCTCTCTCAACCTCTCTCTCTTAATATT

TTCTAAGCTGAGACATCTAGGGTGGCTATAGGGGATAATAGAAGGGGAAAGAAGATC

>Unigene0000692

GACCTCCATCTCTGACATGATTCCAGTGACCTCGACCCCCATAAGCACCTCCAAAACCAT

CCCTTCACCTCTCTCTTGCTTTTGATACTAATTCTCTGATTTATTCTAATGATTTTTTTC

TCTTCCTCTTACAGGGATTCAATGGATGAGGAAGGCTCATGTGGCTCAAGCAGTGAAGCA

TAGAGGCTGGACAGTAAAGGCAACATTGGCATGACAACTCATGACAAGCAATCAAAATGT

ACATAGGTCTCTTTAGTGCCAATCCAAATAATAGTGGTTGGGCAGTAACACAAGGGAAAG

GCATGGGCATCAAGGTGAAGCAG

>Unigene0000693

AAGCAATAACAAATATATGGGCAAAAGTTAATCTCCAGTACATGTTATAAAGAGTGTGAT

GGGAATGAGCATGAAGGGCTAGCAGTATGAAGTTCGAAGTAAGTCAGATATCTTCAATAG

CTCTAGGGAGGCAAGCAATATCAGCAGGCAATGAAGCAACATGGGCATTGTTGGTATGGG

TTTGGGGTAGTAGCAGTCAGCGTGCAAGGCATGAGCTGAATATTATTCCGGGCTATGGGA

GAAGGTATTGAAGCATGGCCAATACAAAGAAGGCACAGTCAGGTCAAGCATCTCTAAGAG

AAGCTGACTGTGATGGTGTAGTACCTAGTGATTATGGGTCAGACAATGGTGATACATGGA

TCAAAGGCATGGCAGAGCATAGGCTAGTCTCTCTGTGCATAGAAGGTGAAAACAAACATT

GGTAATTCAAAGTAGGGACATCTACAAATAGTGATGGATGCTAGTAGTAGGCAGTAGTGA

GGCATGACTTGACATAGGCAGTGGGCAGTATTGGCATAGCATCTAGGCAAGACATCTCTA

ATACAGATAGTAAGCCCTCTCTCTCAATATCTTCAATGGGAAATATGCAAGTACAGAGTA

GGATGCAATCTCTCTCAACCTCTCTCTCTTAATGTGTTCCAA

>Unigene0000694

AATAACAAATATATGGGCAGAAGTTGATCTCCAGTAAAGGTTATAAAGAACTTGATGGGC

ATGAGCATGAAGGGCTAGCAGTATGAAGCTTGAAGTAAGGCAGACATCTTCAATGGCTCT

AGGGAGGCAAGCAATATTAGCAGGCAATGAAGCAACATGGGCATTGTTGGCATGGGTTTG

GGGTAGTAGTAGTTAGCGTGCAAGGCATGAGCTGAGGATTAGTCTGGGCTATGGGTGAAG

GCATTGAAGCATGGCCAA

>Unigene0000695

CTCCCCCCTAGTTAGGAAACACCTCTCTCTCTCTCTCTCTCTCTCTCTCTCTCTGCTCAG

TGTGTTTGTGACTTGACCTGCATGCCATAGCAATTATTACTCCCCCTCGTCGTCCTCCTC

CTCCTCCTGTGTATCTCCCGACCAAAGCTCGCCGGCGCCGCCGTTGAGCACGGCGATGGA

CGCCCTCTCCTGTGCAGTACCCCGCATCTCCGTCGCTTTCGCCCTCTTTTTCCTCGCCAC

AACTCTAGGAACCAGTATAATGATTGAAGTCGACGATGGAAGGGAGTTGAGGCTACAATA

TATTGAGTACCCGGAGCAGTCCGGCAGCCGGCGGTTGTTGGTCGGACCGGGATCGTCTCC

GCCGACATGCCGGGCTCGCTGCGGCCGGTGCTTCCCCTGCCGGCCAGTCCACGTCGCTAT

CCAGCCCGGCCGGAGCGTCCCGATGGAGTACTACCCCGAGGCCTGGCGATGCAAGTGTGG

CAACAAGCTCTTCATGCCTTAAAACACAGAGTTTTATATTTTAGCTTTAGCTAGTAGAGA

AAATTGAATGTAATATTAGTTTTGATGAAACAAAAGTACAGAGAGCAAGAAAACCCTAAC

CCTTGATTTTGATGTACATATACTGTGTTTCTTTTGATGAAGGTATGTAGAGAGTGTCCT

CCCAGCTTTGTTGATCATGGAATGGTGTAATAGGAGG

>Unigene0000696

CTTTATCAACTTTTTCTTCATTACCCCTCCCACCCTACCTCTCGAGCTCCACCTCCATCA

ATCTCCGCCCACCCATCTCCAATGGCGAGGAAGAAGATCCGAGAGTACGACTCGAAGCGT

CTCCTCAAAGAGCACCTCAAACGTCTCGCCGGCATCGACCTCCAGATCCTCTCCGCCCAG

GTCACTGAATCAACCGATTTCACCGATCTAGTCAACAACGAGCCATGGCTGTCGTCGATG

AAGCTCGTCGTCAAGCCCGACATGCTGTTCGGCAAGCGAGGGAAGAGCGGGCTGGTGGCG

CTCAATCTGGATCTGGCGCAGGTGGCTCAGTTCGTCAAGGAACGCCTCGGCGTCGAGGTT

GAGATGGGCGGGTGCAAGGCGCCGATCACGACGTTCATTGTTGAGCCGTTTGTGCCGCAC

GAGCAGGAGTATTATTTGTCGATTGTGTCGGATAGGCTGGGGAGTACTATCAGCTTTTCC

GAGTGTGGGGGGATTGAGATTGAGGAGAATTGGGACAAGGTTAAGACGGTGTTTCTTCCT

ACAGAGAAGCCGATGACGTTGCAGGTGTGTGCCCCGTTGATTGCGACACTTCCACTGGAG

GTTAGAGGTAAAATTGGGGACTTCATAAAAGGAGTCTATAATGTGTTCGAAGACTTAGAT

TTTACGTTCCTCGAGATGAACCCATTTACGCTGGTGGATGGGGAGCCCTATCCATTGGAT

ATGCGAGGAGAATTAGATGACACAGCTGCATTCAAGAACTTTAAGAAGTGGGGAAACATA

GAATTTCCGCTCCCTTTTGGCAGAGTCCTTAGCGCCACAGAAAGCTTCATTCATGGCCTG

GATGAGAAGACAAGCGCCTCTCTGAAGTTCACTGTTTTAAATCCCAAAGGACGAATTTGG

ACGATGGTGGCTGGTGGTGGCGCCAGTGTTATATATGCTGACACAGTTGGAGATCTGGGT

TATGCATCAGAACTTGGGAACTATGCTGAGTACAGTGGTGCTCCGAAGGAGGATGAGGTC

TTGCAGTATGCGAGAGTTGTTCTTGATTGTGCTACTGCAGACCCCGATGGTCGGAAGAGG

GCTCTTCTCATTGGAGGAGGAATAGCCAACTTCACGGACGTTGCTACTACTTTCAATGGC

ATTATACGGGCCCTGCGGGAGAAGGAATCTAAGTTGAAGGCCTCGAGAATGCACATATAT

GTTCGGAGAGGTGGTCCCAACTACCAGACCGGTCTTGCGAAAATGCGGGCTTTGGGCGAT

GAGCTAGGTGTTCCACTTGAGGTTTATGGGCCAGAAGCGACAATGACTGGAATCTGTAAG

CAAGCGATAGACTGTGTCATGTCTGCGGCATAACTTCTCGACTTGGGGAAAAGTCGCTTG

TATTGTCGATTGAAACCAAGATTGAGGATTGATATTGTAAGTGTAAAAACATAAATAAGA

AAGGTTTTGCTGGTTTCTTCGAGGTATTGCTTCAATAAGCAAATGTGGGGCTCCTCCTTG

GTTGTGAGTTTTGATATGACAACTTAACTATGATGGTTATGATATTGCGATGCTTGGGTA

GCTCGAATTTGTGCACCAGCTTGATTATCATGCCAAATAAATCTTGTTAGTAGATAAATT

TCAAATTCA

>Unigene0000697

ACAGAAAGATGGGGTCCGAGGAGTACCGCCCGATCTTCGAGACCGAGAGCTCCGGCGATG

GTACCGGCGCCGACGCCGGCGATACCCTCTACGGCGTCCTCAGTCGGCTCGTCGCCGACA

TCATCTCTCCGGAGGCATCGCCGCCGCCACCGGTGATCCAGCGGGTGAGGGCCTCGTTCT

GTAAAACCGCACCCCGCATCCGAGATGCCTCGAAGAATTCCGCTCGAGATCTCCTCGTCT

GGACTCGAAGCGGCAGCCACCTCCGCGCCCTCCTCGTCATCTCGGTCGGGTCAATCACAC

TGATGGCTTTGACAGGGTTGCTTATTTTTATGCTTTTCTTCTTGGTAGCAACGGTCAACG

CTATTATCGTCGCCTTCTTAATATCTTTGGCAGCTGCTGGAGGGTTTTTAGCTCTTTTCT

TTGCATGCTTAACTGCGGTTTACATTGGAGCTTTGTCAGCTGCAATCTTTGTGATCTCCG

CAACAACCATCTCAACAATTATTGGTGTTACTATTGCTACAGGGTGGCTTGGATTCTTCT

GTGTCGTCTGGTTGGCCGCTAAGAAAAGCATCAACCTCACGAAGAAATCGCTGACCATGA

CAAGTTCAGCAATCTCAGCCTATTCCACCGCTCGGCAAGCTAGATCAGAGGCATCGGTGA

AGTCTGCTGATTGAAGGCAATGGGGGTGTCTTTCACATATTTGCAGTTTACTTCTTTGCC

TATAAATAATCTCTGCCTGCTGTTTTCTGTGAATTTGGGCTCACAGCAGACTGTAGTCGT

TCAGTGGACCTTGCATTACTTATGTATCATCCGATATGTATGTTATATGATCTGTCTTGC

AGGTTTAGGTTTGCCATATTATAAACCCTGGACAATCCATGATATATTAATAAAAGTGTG

ACGATATATATCAAAATGTTTGAAATTCGTTTTGCAAATATTGGTCATACGCTATTCGTC

TCCTATAAAGGAGATATCTGTTCCAGACTTTTGACATCCTTAAACACAAGTTTTGTGTTG

ATACTAATGTCATTGGTTGTGTTTAGTGTTGATACTAATGTCATTGGTTATATTGGGCTT

ATGTTATTCGTCTCCAATAAAAGACATATTTGTGCATATGTTTG

>Unigene0000698

AGGACATGGTCTTCACTGTCTACGGCGAGCACTGGCGCAAGATGCGGCGGATCATGACCG

TCCCTTTCTTTACTAATAAAGTTGTTCAACAGTACCGGGCCGGGTGGGAGGAGGAAGCGA

GATTAGTTGTTGAAGATGTTAAATCTAATGCTGAAGCTGCCACTAAGGGATTGGTGCTGA

GAAAGAGGTTGCAGCTTATGATGTATAATAATATGTACCGGATTATGTTTGATAGGAGGT

TTGAGAGCGAGGAGGATCCGCTGTTCTTGAAGCTGAAGGAGTTGAATGGAGAGCGGAGTA

GGTTGGCGCAGAGCTTTGATTATAATTATGGTGATTTTATTCCGATTTTGCGGCCGTTCT

TGAGGGGATACTTGAAGATCTGCAAGGAGGTGAAGGACAGGAGGCTGAAGTTGTTCAAGG

ACTATTTCCTTGATGAGAGGAAGAAACTTGCAAGTACGACACCAGCAGACAACACCCAGC

TGAAATGTGCCATCGACCACATCCTCGACGCTGAGAAGAAGGGCGAAATCAACGAAGACA

ACGTCCTCTACATCATCGAAAACATCAACGTCGCTGCCATTGAAACAACCCTCTGGTCCA

TCGAATGGGGCATCGCTGAGCTCGTCAACCACCCCACCATCCAAAACAAGCTCCGCCATG

AGATCGACACCGTCCTCGGCCCAGGAGTCCAAATCACTGAGCCTGACACCCACAAGCTCC

CCTATCTCCAAGCCGTCATCAAAGAGACCCTCCGCCTCCGCATGGCGATCCCTCTCCTAG

TCCCCCACATGAATCTCCATGATGCGAAGCTAGGTGGTTTCGATATCCCAGCAGAGAGCA

AGATCTTGGTGAACGCATGGTGGCTCGCAAATAATCCCGACCAGTGGGAGAGGCCCGAGG

AGTTTAGGCCGGAGAGGTTCTTGGAGGAGGAGGCAAAGGTGGAGGCGAATGGGAATGATT

TTCGATACCTCCCTTTTGGTGTTGGGCGAAGGAGCTGCCCGGGGATTATATTGGCGCTGC

CAATTCTCGGGATTACGATCGGGCGGTTGGTGCAGAACTTCGAGCTGCTGCCGCCACCGG

GGATGAAGAAACTGGATACGACTGAGAAGGGTGGGCAGTTCAGTCTTCACATTTTGAAGC

ATTCCACTATAGTTGCAAAGCCGCGGGAGTTTTGAATTGTTTTCGGGGTTGTAGGAGTGG

GCTGTTTGGGAATGGGTGGTTTGTGTATTTATGTTGAATTATTAGACCTTTATGAGAAAT

GGAGGTCAATTTGTTGATGAGAATTGGTATGAATGAATAAAAGATTCATAATTTACTTAA

TTATTGCTGAAGGCTACCGGCAGGCAATTTTGTGGGGTGGTTAAGTAGGTGAGCTACCTT

GTATGATTTTGATTTATAAAGATTGTC

>Unigene0000699

TACGCGCTGTGCTTGGGGCCTATCCGACCCATTCGTTCGATTTACCCTCGCGATTCCGCC

GCTACAGGTCTTGGCACTCCTTTTGTCTTCTCCATGGCTTAATCTGACATCTTCGTTCCT

AAGTTATCGGAGGGTTACCAACTTAGATCCCCTACTGTGCAAGAAGCGGCTATCGAGGTC

GCAGAGGGGGCCAACTTTGGGTATATTTTTAGTTATTATTTGATGGATTTTGCTCATACC

GACTGCTATCCCTGAGTACCTTTGCATGGCGAATCTGGCACCGTCTCAGATTTTGTTGAA

CGCCTACAATGCCTTGTTTGCTATGTTGAAGATGTCTGAATCGCGAGGTAGCGCCCTCGT

TTAAGGGCTGCCTAGATCTGAAAGGGCTGGCCAGTTAGTCTGTGGTTCTATCAGGTAGGA

TTTGGACCTATCCGAAGGTGCGGCTCCTCTCCTTTGGCTGTAAGTACTGACTACAATTTT

TCTGAAGTCCTTGATCCTGATAATATATCTTTGAAGTCGTGTCCGACTTTCAAGGATATT

ATATTTTTTGAGACCGGTTGAGCATTCGTGGTACAAGTGGGAAGACACTCTTCCGGCTGA

AGAAGACCTCGGGACCCGTATGCTTTTGATGCTCTTCTGTCTTGGCCTCCGGTTTGAGTA

TTATGACCTGAAAATTCTGGGGGTCATTATCCTGTTGCCCCTGACCGTGGCTCATTCAGG

CCATTGGAGTACTGGGGATCATACTTTGCTGCTACCTCCCAGACTCGGGATGATAGATTG

AGCACTCCGACAACTAGGCTCAATCCGACAACTAGGTAGAGTCCCGCTTGACTTTTGCTG

ATTCTGGCCCTATTGCTGTAACTTTGAAGACAGGGGCTATTCGTGAAAAGCGAAAAGCAG

AAAAATCATCTTCTTCAAGAAGAACTAAGAGGTAGTCTCTTGTAATCCTGCGAGTTCCCT

CCTGATGCTGACAGTGACAGAAAAAGAAGTGCTGCAAGAGGCTGATCATAATGAACTTGT

CTCCAATATTGGCTTGGTTGTGGAATGTATACTGAAGAGTAGCACTGATACTGTCTATGA

CTCTGATACGATAGCTGAAATATTGTGGACGACCAATGCTGATAACACCCTGAATATCAT

TCCGATTGTCAAAATGATGCAAACGCACTGATACTGTCCATGACTCTGATCCGATAGCTG

AAATGTTGTGGACAACCAATGCTGATAACACCCCAAATATCATTCCGGTTGTCGAAATGA

TGCAAACAAGTAACACTGGTGCTATTTCTGATCCTAACATTTAGTTCCACATCGGTTAGG

ATCAATGAGTTTAGTCCCACATTAGTTAGGCCAGCCTTGCAAGCTAACATAAATATGGCA

ACGGGCTACTCCACCTACAACCAATTGGAAATGCCGCGCAACGACATACGGCTTGCTCCC

AGAAGAATAACTGGGAAGCTGTTCAGATTGCCTCCCCACACCCCGAAACTGATCAACCCG

AGTGGGGAGGAAATGCCGCGCAACGACATACTTATAACCGTCGTGAAGGAAAAACACCTG

TCTTTGAGTTTCGTAAAACATCTCATTGATGTTGGCACACTCAATGCCGATCAATCTGAA

GACTTTCGATTAACTCTGAATGAACTAGACCGATGCAAAGAGTACATTTTGTGCTTAGAG

AGTTAACTCTGCAGATCTGAAGAAATAGTTGTGACATCTGAAAAAGGCCGTCATAGATCG

TGTCTTGATAGAGGGACTTCATGACAAAAGGTCACGGTCTCATTCTCGCATGACTGATTT

GGAGAAAGAACTGGGATACTATGAAGAAGATCTTAATCTCATGAAGTACAAAGCATATAG

AGAAGCTGAAGCAATGGGGCATGTAGTACGCGCCCGTGATGCAGAACTTGAAAAACAACT

ATCGTCCATTGATGTACTGAAATCGAAAATTGAGCATATTCGTTCGAAGAAGAATGCTGC

TAATGGGAAGATTTAGGTCTTATTCAGCTTTCTGATCTATCTGCGAAAAGACAATGAAGA

GATAATAATTACTATTTCTGATCAAAGGGACACTACTGACATGATCGGAGACGGTTGAGA

CTAAGGAAGCAAAGTTCAAGGCTTTGCAGATAGAGGTTGATGCGTTGATGGAGCAAATTC

TGAACTATTCTACTACCTTAAACAAGATAGTGGGGGAATCGGACAGGTGTGACCCGATTT

GGCCAGAATCTGACCTGGTCGACCCGTACAACTTTCGCTTGGAATTGTGGCATGCTGAAG

AGGCAAGGCTTCGTGAAAAGGTCACCCCGTTGTGGTCTCAAGAAGTTGGAATGTGCTGAC

ACTGTCAAACCCGAAATGGAAGCCACTTTATACTCTCACTTGGAACACTCCAAGAAAGAA

CAATACCACCATGGGTATCATGCCAGTTTTGCTGAAGGTCGATTAAGCCGAATTGACGGG

ATCTTAGTGCCGGGGAATTCACAATATTTGTCTACGTTTGGATGATTACCTGAGCGATAT

TATTGCAGACATGGAGGGTCTAGGAGACGAGGTTTGGGAAAGACTACTAAGGGAGTTCAA

AGTTGCCTTATGGGATCGTGAAAGCGAAGGAATGTCACCCCTTCGTCTGACTCAGCAGTA

GATTAGTTCTCTTGGTGATGTAACACTTATTTTATTAAACAGTTATTCTGAAT

>Unigene0000700

AAATATAGAAGCTGGAAGGGCCATCTAGGGATGTCCCCTTCACCCTCTCCGACGCCGATG

ATCACCATTTTCATCGTCCCCATTCAACCCCCATCTCTATTGATGCGATGATGACGAAAT

TAACTGCTCAAATCGGGTCAATTTCACCCCCAACAGTCGTGAGGCAGCTTGCATGGCCTG

ATCGGGCAACCAATGCAGCCATTTCTCGTTGGGGTTTGATGAATCGAAGGCGAATCTGAG

CATCTCATCTTCAG

>Unigene0000701

TTTGTGATCGCAATTTCGCTTCTTTGATCTTTTATGCTGCAGGGATTTGTAAAAATCGGA

TCCTTCGAGTTCCAGACTCCTGGATGCTATTAGATCGCTGTTGATTTGGTTTGTTCAATA

TCTGCCCTTTTTTCTGGATTTTATTTTCTCAAAATAAATCGGATATTGATTGGCTGGCTA

CTCCAGGGGTACAATCGCACAATAGGCTCCGGGATGCCTTCCGCCATCTTTTTTTTTGGG

TTTCATAGGCTTGTAAAGTGATTATTTACAAGATACTTTGGTGTGTTGTAAGAATTGATT

GCTTTCTCATATAATAATAAACTATAAAATATGTTGGAGCGAGAAAAATGCCATGAAACC

AAATTTTTTTCACTTTTTTTCCTAACTCAGAATCAAGTTATTGTTACTGATTCCTTTGGA

TGACTCCCAAATTGATAAGGATCCAGTGAAAACCTTGTTGTATTGACATAATATAATACA

ACATAATAATGTGTATCTGGGAACCAAATATCTTGTGCGGAATAGATAAAATAACAACTC

ATTTAATACATACAAATAGGGCTGTCATTTGGAA

>Unigene0000702

TGGTTTGTGGGTTCTGCAAACATGATTTCCTAAAACATGGAGAGCCTTAACACATGTCCC

CAAAAGCATGGGGAACCTACACAACTTGTCCGTTGTCTTCTTTGTTTTCTAGTGTTGTTT

TCCAGATTTTACTATTTGGTTGGTTTGGGTGTAAGACTTACTGTTTTACCCCACTTTTGT

GACCTCGGTGGCTCCCAATTTTAGGCCCCATTTAGTAGGGGTGAATTTTAGTTTCCTCAT

AAGATATGTTGGTTTTGAGTCACCCCTTCATATTGTTGCTAATTAGACTCTCCTTCATTT

TTTATTTGGGAAGTCCTCGAAAATTTTGAAAAATATTAGTTTTTTTAC

>Unigene0000703

AGAAAGGAACTTTTCATAGAACGAAATGCTCAATAGGATCGTTGAATGTTCCATTCAAAC

TCCTCATTGTTGTTGACTTGTTTCTGATAGTAAATTATGAAGATATTGGTCGTATATATA

AATGTGACAAAGAATTACCAAGTTATCTATAGCATATTACGTCCGTGAGACATGTTGATC

AGCTGATTAAATGGATTCGTGCCTTGCTGTCCAGGATCTGCTTATCACATTTATTACCTA

TTTGGTTTGTGGGTTCTGCA

>Unigene0000704

TTGGACCTGGTAGCGTTGATCAGAGATTTATGCGGATCCCTCTCATTCATCTTATGATGC

ACTAAGTTTTGTTTATGGAGTTTCGACAACATTTACTACATTAGCTGAGATGAAGATTAT

ACAATCATATCTTGAAGGATATAGACAGGACTGGGAACTTCCCTTCAAAAAGGACACAAT

GAGCAAAGGTGGTTGGTAAGCAGACAAGTTACGAATTTATTTCTCATTACCTATGTATTT

CTATTACTATATTTTATTGTAGCACAAGTCTGCTTACATCTGACCCTCCCCAGTATCTCA

CATTGGTGGGAGCCTCGTGAACTGGGTTGCCCAATTTTACTATTTACGATACTTAAAAGC

AATGAATATAATTGTTTTGTGAGAACTGGAAGTGGTTTTACATGAAATATATTTCA

>Unigene0000705

GGGATGGGAGCAGGAGAAGCCACGTAAACTGCAGCTCTGCTCCAATGGCGCTGCCTCTCC

GTGCAACTCCGTCCATCACCGCCGCCGCTTGGCAAGACAAGAGCCGCAACCGCCAGGCAT

CAGCTTTTAACGTAATTGCTACCAGCGCCGCCCAGTCTATCTGCTCATCGGCGCAGGCCA

GCGGCCGCTCCCGGTCCATCCGTCCACTCTCTGCAACGCCGCCAGCGTCTGCAGGGAGCG

CATCTGAAGTTCTCACAAGTCCTTTAGAAAAGGTTGAAGTTTTTGATTTAACAGGAAAAG

CAGTACCTATCATTGATCTCTGGAGGGAGAGGAAGGCTGTCGTCGCTTTTGCTCGACATT

TTGGATGTGTTTTGTGTCGTAAACGAGCTGATCTTCTTGCCTCTAAGAAGGAGCTGATGG

ATGCAGCTGGGGTCACACTTGTTTTGATCGGGCCTGGTAGCGTTGATCAGGCAAAGGCTT

TTTCTGAACAAACAAAATTCCAAGGAGAGATTTATGCAGATCCCTCTCATTCATCATATG

ATGCACTAAGTTTTGTTTACGGAGTTTCGACAACATTTACTCCATCAGCTGGTATGAAGA

TTATAAAATCATATCTTGAAGGATATAGACAGGACTGGGAACTTCCCTTCAAAAAGGACA

CAATGAGCAAAGGTGGTTGGTAAGCAGACAAGTTACGAATTTATTTCTCATTACCTATGT

ATTTCTATTACTATATTTTATTGTAGCACAAGTCTGCTTACATCTGACCCTCCCCAGTAT

CTCACATTGGTGGGAGCCTCGTGAACTGGGTTGCCCAATTTTACTATTTACGATACTTAA

AAGCAATGAATATAATTGTTTTGTGAGAACTGGAAGTGGTTTTACATGAAATATATTTCA

>Unigene0000706

TCCATCCGTCCACTCTCTGCAACGCCGCCAGTGTCTGCAGGAGCTGATGGATGCAGCTGG

GGTCACACTTGTTTTGATTGGACCTGGTAGCGTTGATCAGGCTGAGATGAAGATTATACA

ATCATATCTTGAAGGATATAGACAGGACTGGGAACTTCCCTTCAAAAAGGACACAATGAG

CAAAGGTGGTTGGTAAGCAGACAAGTTACGAATTTATTTCTCATTACCTATGTATTTCTA

TTACTATATTTTATTGTAGCACAAGTCTGCTTACATCTGACCCTCCCCAGTATCTCACAT

TGGTGGGAGCCTCGTGAACTGGGTTGCCCAATTTTACTATTTACGATACTTAAAAGCAAT

GAATATAATTGTTTTGTGAGAACTGGAAGTGGTTTTACATGAAATATATTTCA

>Unigene0000707

CCTATCTTCAATACAAGTCATGCAGACGGATGAGAAGAACATGAAGGTTAAAAAGGGCTC

ACTCGCGGTGACTGTGGGGCTCGGAGGAGAGCAGCGGAAGTTCGTCATCCCCATCTCCTA

CCTCTACCACCCACGCTTCAACAACCTTCTCGATGCTGCCGAGGAGACCTATGGCTTCCG

ATGTTCTGGCCCCCTCAAGCTGCCTTGCTCCGTCGATGACTTCCTCCACCTCCTATGGCT

CATCGAGCGTGAAACACTACAGCAAAGCACTTTCAACCGCTGCCGCTACCATCACCACGC

CACTTTCACCTTACACTCTTGCTGAGTTCAAGAAAATATGGTTTCATATGATGTTGGTGA

CCGCCTGAATGACTGTGTCGTACTCCCTTTTACTTGTATGTCATGAAAGTTCAGAATTCA

TATGTTACCCTCTCAGTTTCCGTTTGTGACCGTCTG

>Unigene0000708

CTCCTCCCTCGCTAGCCAGCCATGCAGAGGGACGAGAAGAGCATGAAGGTGAAGAAGGGC

TCACTCGCGGTGACAGTGGGGCTTGCTAGCGAGGAACGGAGGTTCGTCATCCCCATCTCC

TACCTCTACCACCCACGCTTCAAGACCCTTCTCGACTCGGCCAAGGAGACCTACGGCTTC

CGTTGCTCCGGTCCCCTGAAGCTGCCTTGCTCCGTCGATGACTTCCTCCACCTCCTCTGG

CTCATCGAGCGTGAGACACCGCAGCAAAGCTCTTTCAACCGCCGCCACTACCACCACCAC

CCATCTTTCTCCTTGCACACTTGCTGAGAGCAGGATTAGGGATTCTCATGTTGGTGTTTT

TCTTTCAGGCTCTGTGCCATACTCCTTTTTACATGTATGTCATGGAAGTTTAGATTTCAC

TCTCCAAGTCGTAACTCATTAGCCATGTCCCTTTGAAAGGACAAAGGTGCTGCTAAGTGC

ACTTCAATCATTTTGTCACTTCAATCATTTTGTCAAACCGGCTTTTGCTTGTTTATTGTT

TGTCGACAGAAAATGGGGAGTGTATAATTATTTTAATTCTGGAGTTTCAGGTAGGATATT

TATTGAGTGGTGACGTTAATATCAATACCTTATATATTTATTGAG

>Unigene0000709

CGGTAGGTCGGCAATCAATCAATCAATCCCTGCCTTGTGCGCTTCCCCGAAGACTTCATT

CGTCTGACAAGAAATCGCTGATTCTCCCCCCAATTTCTCATGGCGATGGCGATCCCTTCA

GAGCTAACAACCACCCCCTGCACGCACTGTGAGAGGGACATTCCAACATCAAATATCGAA

TTGCATCAAGTACACTGCGCCAGAAATCTTCAGAAATGTACCATATGTGGAGACATGATT

CCCCGGAAGCATGCTGATGAACATTACAGTGATGCTCATGCCCCTGTAGATTGTTCTTTA

TGCAGTGAAAGAGTACCACGTGAAGACTGGTCTGCTCATAAAGGAGAAAAATGCCCCCAG

AGAATGGTTACCTGTGAATATTGTGAGTTTCCACTGCCTGCAGTTGATCTCTTTAAGCAT

CAGGAAACTTGTGGAAATCGGACAGATTATTGTCATATATGTAGCAAGTACATTAGACTG

CGTGAAAGAATCAACCATGAGATCCAGTTTCATAGCAATGCAAATGGCAGTGCAGAATCT

TCCAGGAACAGGTGGCCGGAAAGAGAAGGTCCCCATAGAAGGCCCGCAAGGGACGCGTCG

CGTGGTAAGAAGATATTGTTCACAATTGCAATAACGGGGATGGCCGTTTTACTGGGATCC

ATTTTCCTGCAAAGAAGAGTGGATACACTGCAGCAACAGTGACATCTGCAATCTCCTTGC

ATGTTGGATGCCTGGATTTATTACATGAGAAGAGGTCTCATTTTCACTTGTATGCAATGG

TTTATCTATGTGATACTCAGAAAACAAGGTACCATTTGTTGTGGTGACTGAAAACTTTGA

TTTATGTATCTATTTCTGCTTTGGGAGCCGTCAACTCTGTCTTGCAATATTTA

>Unigene0000710

GGCATCAGTTTTAATAAAACAGGGGGTTTGTCTAAATGAGACCCTAATTCAGGGTTGTAT

AAATAAATTCTTGATAAGATGTGCGAGGGTGTAATTATATACTTAACCATTATATGAGGG

TGTAAGCTGAATACAATCACACTCGCTTAAAAACCTTACAATTCGTTGGGATCAACTCAT

AAATTCCATTGAGGACTGCAAGGAGTAACTCATTTCTTGCTGGTGATTGTAACGAGAAGA

AAATCTCACACA

>Unigene0000711

TCGTGTCAAGACTAAAAAATACAAATTGTGGATATATTGTTATATTGAGCGACATTTTGT

ACCAGACCATACACCAGGCCCTACAGTTTTGGATATGCAGTGATGTCCGCACTCTCTCCA

TTGCTTATATCAAGGTTGACTGCCTTAATATTGCTATTGTCAGGGTGCCGGATTCCGGAG

ATTGGTGGGAGCCTGGGAAGTGGGAGCTCACAAATATTCTTAATGAGGTCTGGTGCCCTT

TCCGCTATGTCGTCCGAAATTATGATAAATGGTTCTTCCTTCTAGTTCACAAGGACATAA

TGAAAATTGCTTGTTCGTCTCATTACCGATATTCTTGATTAAGACTTTTATGAGTTTTCA

TCGTTATGCTAGCGTCTCCTAGCTCTACTCGTTGTAGAAACAGGCCTGCTAGTTGCAAAA

TAAAAAGATTTATATTAGCATCCAGCAATTTCCCAGAAATGCCATTTTTCTCTTGTGCTG

GAAAAATGGCAATTTTGCTCCTTTTTTATAGCATTCCACAATATGTTCTGATGTAAGATC

ATCGATTTTATGAAACTACTGAACTTCCGCATGTTCAGTAGGCACAAACCTGTAGTGATG

TAGATGATCTTATAGTTTTACTACAATCTGCTTATGGAATCCTATTCGAACTAGTCACAT

GGTTAGGCACATATAATCATACTAGGCACTAGCTGCTTGCCTACTTCATCTATCTAGGAC

TAAGTAATGAATAAAAGTCCGTTTGCTTGCTTATGGATGCTCCTAGACAGTTATTAAAAT

CTCATGATACTCGGCACGATACACGATGCGGTAGGGACCGATATGTTTTTAGCGATTCAC

TATAAGAGCGGTGCATCTTACAATACAAGATATGTTACTTACGATATGCCTGTATTCATG

TGGGGAATATGCTCATTTATTTAGTTTTTAAAGGTTATTTTATTCCTTCGCGATTTAATA

GATTTTTATATATTTAGTATATTTTTATTCATTTATTTATGTAGGTTAATATTCTTCGTC

CAAACAGCTAGCTTTTGGTGTAGTGGTTAGTAACGATGCACCCATCTACCATGTTTCGTG

TAGCCATGTATCTTATGATACACGATATGGGGGTGAACAATCCACACGAAATGATACACA

ACTGTTTTCCTTAGATACTATTGGTTAGCTAATAGGTGTTCCAACAAATATTTGTAATTT

TTCCAGGAGCTGTTATTTGTTGTATTTCCGTCTCTTTTTTTTGGCTTATCAGGGGACTGT

TTATCTTTAATCATTGCTATTTCGCTAATTTTTGTCTTATTGTTACTACCAAATCTACTG

CCATTTAGTCATATCATGAGATTACTTTAACTTACGAATAGAAAACCTCCTTACTTGTCA

TAGTTCTGCAAATATTGCATAAATAAAAATTGAATATAGCATTAAGATACTGCTATTTAC

CTTTCGTGGTCACTTCATAGCAAATATCCAACTTATTATGTACTGCAGACTAATTTTTCG

GCCATCAATATGCCTATTTACATTCGACTGACCCGAACTCCTGAACAGAAATTATCCTAT

CTCTCCTGTTTTGCAGGAGTCTTCGGGTAGTGCAATTAAAGTTTTTCACATCCTGTATCT

TCTCCAAGAGGAGACAATTTTTTTTTTTTGTG

>Unigene0000712

TAATCTCACCGTACGGTGACGAGATCCCCGTTCTCCAAATATCGACAACGGCCACCGCCG

CCGCCCGATCTGAAATCACAGTCGTCGGAGCGGAGCAGCAGCAGTAGCGATGGGGTTCGA

GAAGCAGATCTTGAAGGCCGGGGATGGCCCCAGACCCTCCCGCGGCCAAACCGTCACCGT

CCACTGCACCGGATTCGGTAAGAATGGTGATCTTTCTCAGAAATTCTGGAGCACGAAAGA

TCCGGGTCAGACGCCATTCACCTTTAAGATTGGGATGGGTTCGGTTATAAAAGGATGGGA

TGAGGGTGTCATGGGCATGCAACTGGGGGAGGCTGCTCGCCTCCAGTGCTCTCCTGATTA

CGCATATGGACCCAATGGGTTTCCAGCATGGGGCATACAACCGAATTCGGTTTTGATATT

CGAGATCGAAGTCCTCAAAATACAGTAAGTACTTGATCTCCTTCAGTCTCCTTGGCTTCC

AAATACTTATGCTATTGGAAGTGATGGCATCACGATGGTTGGTTGATCCTTGTCTTGCAA

GAATGAACTTCGTAACGACAAAACTTAAATTCTGTTTCAAGTCTTTTCCTTTGAACTCAT

ACTTGTTCTGTAATGCATTCAACATGTGTTTTTAATAACAAAAGTGAGACTTC

>Unigene0000713

TAGGATCACGACTGCAAGCATCACTGAAGTTTTGGGAGATGCAACCTGTACATTTCCATC

TGAAAAAGATTCAAATTAGTAGTTTTTATTTTGGGATGTGGGATATCGAGATTGCAGTAT

ATCTTCTTAGGAAAGCTACCGAACTCGAGATCACTGAAGTTTTGGGAGATGCAACCTGTA

CATTTCCATCTGAGAAAGATTCAAATTAGTAGTTTTCATTTTGGGATGTGGGATATCGAG

ATTGCAGTATATCTTCTTAGGAAAGCTACCGAACTCGAGTCAATGATGATAAAGAAAGTC

AGTACATCCAATGAGGAAAATATTCAGTGGGGGACTAAGGTTCTTACTCGAGAAACTCCA

CTTAAAGTCAAATTAGTGATTTTATAGTGCCACCTGATTGTCGGAGAAGACAAGTTCAGA

AGCCCTGATACTAGCAATTCTGAATTTCTTATGTCATTTTCCCTTTATTTATTTATTTAA

GGTGGTCGGATGGTTCTTTGCATGTGGTTCTAGAAACTCTCCGATATCAGCAATTCTGTA

TTTCTTATGTCATTTTC

>Unigene0000714

GGAGACGGAGGTTCAGGGTCAATGGCGAACGCCGACCTTACCATGAAGCTTCTTATCCAT

GGACCAAGAAAGAAGGTCCTCTTCGCCGAAGGTGGTAAGGAGGTGGTTGACTTCCTCTTT

AGCCTACTCGCTTTGCCTTTGGCCACCATTGTAAATCTTCTAAAGGAGGACAACATGGTG

GGATGCATCGGAAAACTCTACAAGAGCCTACAGAGTCTGGAAGAGCCCTACCTCAAATCC

AAACCTTGCCAGTCAGCTCTACTAAATCCTACTGTATCCTCCACCTCTCTTTTAAATTCT

CTGCTCCTTCCTGCAGCTGCCCCAGCTGCAGCTCCAGGTGCTGCATCAACCCGGTATTAC

AGATGCGGGAACAGCTCTGTCTATGACTCGAGCTACCATAATTACATTACAGAGGTGAGC

GGAACTCTGTGCCCTAACTGTTCTGGGTCTATGAGAGTAGAAGTAACATGCGTTGGTACA

AAACCTGTGAATGAGGCGGCGTCTACAACGGTTAATGATGGTGGGTGCGTTAAGGGAGTT

GTCACCTATACGATAATGGATGATCTCACCGTAAGCCCCATGTCGTCCATATCAAGCATA

ACTCTCCTCAACAAGTTTAAAGTGAATGATTTGAGCTCTCTTGAGGAGAAAACAGTCGCG

CTCAGTCTAGAAAAGGGTTTGGAGCTGCTGAAAGCTTCACTGCAGTCGAAAACAGTTCTA

ACAGATACCTTTCTGAAACAAAGTTGAAGTCGATGCATAGTCTTTAGGATCTTTGGTACA

TCTGCTGCTGTTCGTACAAAAACATCGCCCTGGCTGCACTACCAATAACTTTTTGGTTAT

CTATATGTATTTTGAGTTTGTTCTTACCAGTTAAATATTGGTAACTATATGTATTTTTTG

TTACCATGTAAATATTGTATTGAATCCTTGAACTCGGTTAAGTAATATAAACCTC

>Unigene0000715

TGTGGATGAGATCCTGGTGATTAGCGGTATATTCTGATTGATGCTCATTTGCTGTTTATA

AGTAGATGTTCACATTTGAAGGAAATTTGAATTAATTATTATTATCATAAACGTGATCAT

AAAGAATTTCAGGAATGTCATAATGCATGTTGATGGTTTGATTTGTAGTGGATCCTATTT

TTTGTTAGGAGTAGCTGACAATTAAGTACAAGTTTTCCAAATAAAGGTGTCATTGATATC

CAAAACCTCAGGTTATTCAGGACTTGATCTTGACACTCCTGTTCCGAGGAGCAATTCAGT

GAAACAACCTTTTATGGTTCTCTATCTTCAGTCCTGAGAGCCTTGAGCAATTCACTCTCA

GATGCGATAGATATATAGTGTACATATCTGGAGGCATTATATTTTATTAGCAGTGAATGG

TGGGTATTTTTATTTTATTAGCAGTGAATTGGTGGGGAAGTGTGGACTCATGTTGATCTA

TGAAGCACCAGCACTTCAGTGTCATGGTGTTGTCCGAGAGTGTGTTGTCCGACACTCTCG

GATGAGCCTAGGACAAATATTGGGATGTATTAAATTTTATG

>Unigene0000716

ATGCCCTCCAGCGAGGAAGCAGTGGCGATGGCGGCGGCAGCGGAGGCCGAGGACTACCGC

GTCTGGAAGAAGAACACACCCTTCCTTTACGACCTCGTCATCTCCCACGCCCTCGAATGG

CCCTCCCTTACTGTCCAGTGGCTCCCCTCCCACCGCCTCATCCTCGGCACTCACACCTCC

GACGACTCCCCCAACGCCCTCATGATCGCCGACGTCCTATTCCCCCCCAAAACCGCCGCT

GCCGCTGCCGCTGTCCCTATCCCGGCCGTCGAGATCGTGCAGACGATCCCGCACGACGGA

GAAGTCAACCGCGCCCGCTTCATGCCTCAGAAACCCTCCGTAATCGCCACCAAGACCTGC

GGATCGGATGTTCACGTCTTCGATGCCGGCAGCGGTCCCCTCGTGCTGAGGGGGCACTCG

GCGGAGGGGTACGGGCTGGCGTGGAGCCTGCACAGGGAGGGGTTTTTGCT

>Unigene0000717

ATGCCCTCCAGCGAGGAAGCAGTGGCGATGGCGGCGGCAGCGGAGGCCGAGGACTACCGC

GTCTGGAAGAAGAACACACCCTTCCTTTACGACCTCGTCATCTCCCACGCCCTCGAATGG

CCCTCCCTTACTGTCCAGTGGCTCCCCTCCCACCGCCTCATCCTCGGCACTCACACCTCC

GACGACTCCCCCAACGCCCTCATGATCGCCGACGTCCTATTCCCCCCCAAAACCGCCGCT

GCCGCTGCCGCTGTCCCTATCCCGGCCGTCGAGATCGTGCAGACGATCCCGCACGACGGA

GAAGTCAACCGCGCCCGCTTCATGCCTCAGAAACCCTCCGTAATCGCCACCAAGACCTGC

GGATCGGATGTTCACGTCTTCGATGCCGGCAGCGGTCCCCTCGTGCTGAGGGGGCACTCG

GCGGAGGGGTACGGGCTGGCGTGGAGCCCGCACAGGGAGGGGTTTTTGCTCAGCGGTGGT

TATGATTCTAAGATATGCTTGTGGGATCTAGGTGTGTTGCCGGAGGGGAAGGTGATGGAT

GCCCGGCAGGTGTTTGAGGCTCACACGGAAGCTGTTGAAGATGTTGCCTGGCATGTCAAG

GATGGGAACCTGTTTGGGTCTGTGGGCGATGATCATCGTTTGATGATTTGGGACTTGAGG

GATTCTTCCTTGAAGAAGGCACAGCATTCGATTGTAGCTCATGATGATGAGATCAACTCC

TTAACCTTCAGTCCATTCAATGAGTGGATTCTGGCAACAGCATCTGCAGACACTACTATC

AAGCTGTTTGACTTGCGGAAGTTCACAACGAGCTTGCATATATTTTCCAGTCATACGGGA

CCAGCACTTCAAGTGGAATGGAGCCCGAAGCATCAGACTATACTTGCATCCTCTTCTGAA

GACAAAAGAGTAATGGTTTGGGACATAAACAGGATTGGTGATGAGCAGAACGAAGAAGAC

GCTGAGGATGGACCACCGGAGCTGTTCTTTGTTCACGGTGGTCACACTGCCAAGATCTCT

GAATTCTCATGGAGCCCCGATGAAGCGTGGGTCATTGCTAGCGTGGCGGAGGATAACATT

CTCCAAGTGTGGCAGGTGGCGGAGAGCATATACCATGACGACTACAATTCGCAGGGGGGT

GATGATCCGAATGCTCATATCTAGATTTGCCTTTATTGCTCAGGTATGTGTAATAGCTCT

GTTTTCTCAATTTCTGGCAAATTTTGCATCGTTCGGTTAGCCTTGGTGTTTGGCTTTGCT

AGATGAAAGTGGTTACTGATTGATCATCTTGGAGTTTGGTATTTGTATTTGTATCTTGAC

AAAAAGACTTTTGTAGGATTG

>Unigene0000718

GCAGAAACCGACGAGACGAGCAAGAGATCCAACGGCGATCTCCCCCGCCTGATCGAGATC

TCAGCCGCCGGAGATGGCGGCGGAGAGACGCTGTACGGCGTCCTCCGCGACCTAATCTCC

GCGATCGTCTCGCCGGAGGCGTCACCGCCGCCGCCCGTGATCAAGAGGGCGAGGGCTTCG

ATCTGCAAGAACGCGCCGCGGCTCCAAGAGGCCTTCAGGAATGCTTCTCGTGATCTCATG

GTTTGGACCCGTGGCGGCAGCCCCCTCCGCGCCCTCCTTGTCATCTCGGTTGGGACAGTC

ACTCTTTTGGCATTGACAGGGTTTCTAGTTTTTATGCTTTTTTTCTTGGCAGCAACCTTA

AATGCTATCGTCATCGCCTCCTTAATGTCTTTGGCAGCTGCTGGAGGATTTTTAGCTCTT

TTCTTTGCCAGTGTGACTGCAATTTATATTGGAGCATTGTCTGTTGCGATCTTTGTTCTC

TCCATCACAACTATCTCAACTATATCTGCTGTTGTTATTGCAACAGGGTGGGTGGGATTC

TTTTGGATCATCTGGTTGACTGCCAAGAAAAGCATGGACCTCACGGTGCACTCGTTCACC

ATGACAAGTTCAGCCATTTCATCATATTCTACTGGTCGCCGAGTTATTCGTGATGAATCT

TCAAAGTCTTCTGGTTGAGGAATTGTTCCCATTTGCATCCGAGGACTTTTCCTGTAATTA

TCATGTCTTATCAAAGTTTGCTTTGTATCTGGAGTGGAGTATATGGATGATTGTTTGGTG

AAAGGTTATTTGTTACGTTCTGGCTATCTGACTGTCACAGTAAGTTGTTGTTACTAGTGA

GTAATAGATATGTGTATATCAAGAAAATTGGCATTTGGGAAAACAAAGACTTATGTAACA

TTGTGGTTCTGCTAAATCCATCTTTCATGGATGTTGGGAATTAAGTTTTGCCATTTGAAA

TT

>Unigene0000719

TGCAAATGGCGAGCACCGGCGGCGCAGCGGCGGATTTGGCCGACGTCCCGTCGGAGCATC

TGATGGCGGAGCTCCTCCGCCGCATGAAGTGCGCCTCCAAGCCGGACAAGCGCCTCATCC

TCATAGGTCCACCAGGGTCAGGGAAGGGTACACAGTCGCCCATTATTAAGGATGAATTTT

GCTTGTGTCATTTGGCCACGGGTGATATGTTGAGAGCTGCAGTTGCTTCTAAGACCCCTC

TTGGGATTAAAGCCAAAGAAGCTATGGACAAGGGTGGACTTGTTTCTGATGACTTGGTAG

TTGGAATAATTGATGAGGCTATAAAAAAACCATCATGTCAAAAGGGTTTCATTCTCGATG

GGTTCCCGAGGACAGTTGAGCAAGCCCAAAAGCTTGACGAGATGCTGGAAAAGCGGGGAA

CTAAGTTAGACAAAGTTCTCAATTTTGCAATTGATGATGCCATTTTAGAAGAGAGGATTA

CTGGTCGTTGGATTCATCCATCTAGTGGGCGGTCCTACCACTCAAAGTATGCACCTCCGA

AGGTTCCTGGTGTTGACGATGTCACGGGAGAGCCTTTGATTCAAAGGAAGGATGATACTG

CTGAAGTCCTGAAGTCAAGGCTTGAAGCTTTTCACAGACAAACTGAACCTGTCATTGAAT

ACTATGCCAAGAAGGGAATTGCGACAGAGGTTCATGCTAATAAACCCCCAAAAGAAGTGG

CCGCCGAGGTACTGAAGGCTCTTTCTTAAATGCCAATACCTAGGTCCTATTTATGGTGTG

CAATCTGTAACTTATCACACAATGGTTCCCTTTTGCACTATCAAGGTTGAGGAGTCCTCG

CCAATTTTGTTACTTGTGTTTTTCATCTGAAACAAAGTTTCTTTAATTGTAGCAAATGCG

GTATTTTTGTTTTAAAAATATAAATCTTATATGTTCAACTTGACAGAACGAATAA

>Unigene0000720

GTGGACTACACCATGTTCAGATGGAGGATGATCCTTCGGAGATGGTGCTTAATCGACGAG

CTCTTAAGGTTGTAGGACTCTTCGTCACTGATCCTTTGAAGCTTTCGATGTCAATCACCA

CTCGCAGCTGCTGGAGCCACAATGATTGATTTTTGGTTTTGATTCGTGTGTTCTGAGTTA

TAAACAACTCTAATTGATAACCTGTAAGTCCTATTTTTTAATTCTTGCCCTTTTGCTTTT

TCAAGATGTTAATTGAAATCTGGTCATCTGCTTGTTGTGCGCAGCGGAA

>Unigene0000721

GTTAATCCCAAAGAGTCCCAGATCTCACAACCCTGGCGGCCAAACCCCACCAATACCTAG

CACCGCCGGCGGCGGCGGCATCGGCGTCGTCGTCGTCAGCGACTCCATGTTGACGTCCTC

TGCCGTGCCCGATCAGTGGACTACACCATGTTCAGATGAAGGATGATCCTTCGGAGATGG

TGCTTAATCGACGAGCTCTTAAGGTTGTAGGACTCTTCGTCACTGATCCTTTGAAGCTTT

CGATGTCAATCACCACTCGCAGCTGCTGGAGCCACAATGATTGATTTTTGGTTTTGATTC

GTGTGTTCTGAGTTATAAACAACTCTAATTGATAACCTGTAAGTCCTATTTTTTAATTCT

TGCCCTTTTGCTTTTTCAAGATGTTAATTGAAATCTGGTCATCTGCTTGTTGTGCGCAGC

GGAA

>Unigene0000722

TAGCTACCATCTTCCTCATTTACCCCCTCTCTCTCTCTTGTCATTGAAGCAACACAATCT

CAACAGATGTATCAGCTTGATCTTAATGCCCCTCTCCTCTCAACCAGGCGGCGCTCCGAT

GCTAAGAAATCGAATCACCATTCCCACTCCTCCATGCGCGACACAAGCCGAATTAGGGTT

CCATTCTCATGGGAGAGAGCCCCCGGAGTACCGAAAGATGCAGCAATATCAGCATTTCAC

TCACATTCTCGCCCTGAGCTTAGGCCGCCAAAGCCACCACCCGGCCGCCAATGCTGCAAG

ATGTACACTCCTAATTGCAATGATCTATATAGTGATGATGAAGATGGGGATCATGCATTC

GAGGATAACCCATCATTTTCAGAGTCGTT

>Unigene0000723

TAGCTACCATCTTCCTCATTTACCCCCTCTCTCTCTCTTGTCATTGAAGCAACACAATCT

CAACAGATGTATCAGCTTGATCTTAATGCCCCTCTCCTCTCAACCAGGCGGCGCTCCGAT

GCTAAGAAATCGAATCACCATTCCCACTCCTCCATGCGCGACACAAGCCGAATTAGGGTT

CCATTCTCATGGGAGAGAGCCCCCGGAGTACCGAAAGATGCAGCAATATCAGCATTTCAC

TCACATTCTCGCCCTGAGCTTAGGCCGCCAAAGCCACCACCCGGCCGCCAATGCTGCAAG

ATGTACACTCCTAATTACAATGATGTATATAGTGATGATGAAGATGGGGATCCTGCATTC

GAGGACAACCCATCATTTTCAGAGTCGTTTGGGGTTGCATATAGGCTGAGCGATGCCAGT

GTTCATGATCAGTACACCAGAGTCCCAAGTTTCATCATGAATCGGTTCCTTCCCGCCGCT

GATGCTGTTGCTGGTAATTCCTCCAACCCCAATGTACCAAAGTATTCTAGACCATGCCGT

CGTCTAGAGAGGGAACTGAGCAAGCCCCTATCGAAGCATCAAATCACTATCCATACTACT

AATGCAAAGAATGAATATCATCGATCTCAATCACCACCGCCACCGACACCCTCAACTGAT

GTTCCTTGGAGTTTGAAGCATGTAGTTTGTGGGTACAAAAGTCCAGTTCGAAGCCAGACC

CCTAGAGCCAATGTTGGTGGACTTACTGTGAGGACCAACAGCATCAAGACTGGTGGATTG

AGGATGCCTTTTCTGGATACTAGTAGGTTGCGACTGAGAGGCCGGAGGTCGGATATGGAG

AAGCAGGAGTTTAAGGTTGCAGCGGAAGCGCGAGTTGAGAGTGGGAGAAGCTTGGATAGA

AGAGGAGTTGCTAGGGTTTTTTCAGAGAAGAAGAGACTACCATCTTGGAGTCTGCCGCAT

ATGAGGTCCCCTTCAGAGCCATGGCTTTCACATGCTCTCAACTCTTACAATACATAGTGT

GCATATATATGTTTAGGTTGCCAAGAGGGCCTTGTTTGTTGTATGTAGGCAAAGGTACAT

AGTAAACTTTGCATATAGGTGATGTAAGATGATTATAAGCCCTCTTATTTGTTTAAGTTC

TTTTAG

>Unigene0000724

TCAACTCAGTATCCGGCTGCACAGTCGCCGCCCACGCACTACTCGCAACCGGCACCGCCA

TCTCCGAATTACTATCAACACTCGGGAAGCACCGACAGCGGTTACACCGAAGAGTCCAGC

GACCTGCGATACCGCGAACCGCCAGGCAAACTGTACACGCAATTTGCGGCGCCATCGTTC

CAAAACTCCAAAGAATCGCCACTGCTTCACCAACTCAGAGAGGAGTCGGAAAAGAATCGC

AACAAGTACGCTCCGCCTTCTTATTCTAATCAGTACCAAACGGTGGACGCCGCGTAGATG

AGTAATTACCGCACTATACTTGGCCAATGGATGTCGGTGCTGTATGCTTTCCGCGAGACA

GCTGATGTGCTAATTTTTTATTATTATTAATAATTAACGAAAACTATTTTTCTATCCATA

TATTTGTATAGGTTACACTTTTATAACGAAAGACTATTATTCTTACAATATTATTTATAT

TTTTTATTATTTATAAAATATTTAAATTATATAACCATACCTATATATTATATATATATA

TTATTTAACTAGACTTATATGTCTAGAAAAAAAGTACACTTTGCAGTAGTACCGAGAGAA

GAATAAGAAACAATAGTTTTGAATTAATTGTTCACTTTATCAGGTACATATTATAATATT

AATTATTTAGCTTTTTTAGTTTCTACTAAAAAAAGAAAAAAAGCAATGACACTTTTGAAG

TTCGTTAGGCGTTCAATAAATATATTATATTCAATGTGTACACCAGTCCTTTTGTAATAC

ATTTTATATTATTTTTGCAATAATTATGTACTTATCGTAAAATAATTATATAAAATCTAA

ATTAAATATGTTTATGATTAAGGAATTTAAGTTGGATAATATTATTGAAACTC

>Unigene0000725

AGACCCGTAAATGTGGCTAACTTTGTACTAATTCCGGTGGTTCAAGTCCCATCCTCAGAT

TTAAGGATGGTTTATCGGCCACAGATTGATAGTGTTTCAAAAACCGAACCAATTGTAATG

TGCAAATCGTTAACAAAATCATTTGAAGCTACTCCCGCGGTTCAAGTGCAGTTACAGCAA

CCGTTCTACCTGTACGATGAACGGACGCTTATACCGTTCCCAAAGTCAGTTACCATCTTA

CACGGTGGTTACCGCATGTCAATCCCGGTCGGCATGGTTGTAGCACCAGTTCCAGTGGAT

CTCCTTCGAGCTGGACCAGTATTCGTCACTACTCTGTACGCTATACCCGCAGGACCCGTG

CCAGCGCCGTCGCCAGTTAGGTTCGTCCCGTGGTCGTCTTATCCACCGAATCCAACGGCG

CTTCAGCCATATTCAGCGCCGTACTCATCGGCACCACCGTCCCAGTACCCTGTTCCGTCA

ACTCAGTATCCGGCTGC

>Unigene0000726

AGACCCGTAAATGTGGCTAACTTTGTACTAATTCCGGTGGTTCAAGTCCCATCCTCAGAT

TTAAGGATGGTTTATCGGCCACAGATTGATAGTGTTTCAAAAACCGAACCAATTGTAATG

TGCAAATCGTTAACAAAATCATTTGAAGCTACTCCCGCGGTTCAAGTGCAGTTACAGCAA

CCGTTCTACCTGTACGATGAACGGACGCTTATACCGTTCCCAAAGTCAGTTACCATCTTA

TACGGTGGTTACC

>Unigene0000727

CCGGCTTAGTGTTAATTCCTCTTTTTGTCTCATCAATTAGGACAATGTTATCTACAAATA

ACATACACCAAGGGACTTCATCCGGCTAGTTAATCATCCATGATTAGAGCGAATAGGCAG

GGGCTCAAAGCCGACCTTTAGTGTAGTCCTACGGTGTCAGGAAACTCAATTGTTTCTTCT

TAAGGTGATCTAATAGATGGTGCCACCCCATCATACATGCCCTTTTATCACATCAATAAA

TCTTTTTGTCACCCTTTTTTTTTCTCCCACACATCTCACCAAATTTATCTCCGTATTGGA

AGATACAGAGACATATATTGTTTGCAGTCCGAGTTGTATCGTATCACAAAATACGTATCG

TGTATCATTAGATACTTATAACCATGATGCTACAATTAAACAGCCATATAGCCTGTCCCA

TTGGCATTCTAGTATTGCATGTACAAAATACTTGAATATGGAAATTCAGATGATAATAAT

ACAAGCATTCTAATTTACACGTTGTCATCCCAACTTAATTTGAACCACATAATATTTCCA

TCACGTCAGTTAGCACTTGCATTCTAAGTTTGATCAGAAAGACTCATATCCAATGCCTGA

ATCTACCTAGATATAACTTGCCTGTTAATCATACTGATGAAGTTAACCTGTGTTGTAGTA

GCCTTATGAATTCACTGTAGGGTCGACACCAGGCAAAGAACGGAAGCGTGAATATGTAGA

CAATGCAAATGGTCTTTTTTCAGCCCAGGCTGCACCTAAACCTCCACCAGCATTGTACTC

TATAACCGAGGGCATGAAAGTAGGAGGAAAGAGAACTGTTATAGTCCCACCTGAAGTTGG

TTTCGGACAAAAGGGAATGAACGAAATTCCGCCAGGGGCTACTTTTGAGCTGAATGTGGA

GCTCTTGCAAGTGCTGCCATCAACAGGAAAATAACCGATCTTATGGTACTTTGTTTAACC

ATCACTTGCATTGGTGACATTTTTCAACATTTTCTAAGAGGGCGTTTTGTTTCATAGTGA

TGAATTTTCTATTCATTAGAAAGGTGTAATG

>Unigene0000728

TATATTGAACACCCTCTTCTCTCCTCGACAAAAATCTCAACATCCTCAATGCAGTAGCTC

AATAACTGAGAATTATGCCGCTGAGCCGCCTCTGCTTCATCTTACCAGCCGACGCCGACG

AGATCCAGCCACCACCAACACCTCTGAATCCATACAAGAGAACGAGTGTCAGCCGCCGTG

TCACCACCCTCCTCCGCCGCTCCCTCCACTGGTTCTGCCACCGTCGATGGTACAACTTTC

GCCACTGCTTCCCCGACGAAGAGCACGGCTCCTCCCGGTTCGAGGACATCCACGGCATCA

ACCACAGCCCCAGCGAGCATATCGGCCGTAGCAACCCCCGCATCTTCAGCTACGCCGAAC

TCTACATCGGCACCAGCGGCTTCAGCCCCAAGGAGCTCCTCGGCAGCGGTGGATTCGGCC

GGGTGTACCGGGCCGTCCTCCCCAGCGATGGGACGGTCGTTGCTGTCAAGCGTGTCTCTG

ATCACGGCGACCGTTTCGAGAAAGCAATTGCGGCAGAGCTGGCCGCCGTCGCCCAGCTCC

GTCACCGAAACCTCGTCAGCCTCCGCGGCTGGTGCGTCCACGACCATCAGCTGTTGCTAG

TATACGACTACATGCCGAACGGTAGCCTAGATCGCCTGCTCTTCGCGCCATCCGGCGCAA

AACCAGCCCTCGACTGGGACCACCGCTGGCGCATCATCACAGGTCTCGCCGCCTCGCTGT

TCTACCTGCACGAGCAGCTCGATACACAGATAATCCATCGTGATGTGAAGGCAAGCAACG

TGATGCTCGACTCCAAGTTCAACGCTAAGCTTGGTGACTTTGGCCTGGCCCGCTGGCTGG

AGCACACCGAGACACCGGCGGCGACGCCTCCGTCGTCAATGAGATCCATGAATAACTACC

AATTCAGGTTGTCAGAGACGAGCCGGATTGGGGGCACAATTGGTTACCTCCCACCGGAGA

CCTTCCAGAAGCGCGGTGGCGCCACCGCCAAGCACGACGTGTTCAGCTTCGGCGTTGTGG

TGTTGGAAGTCGCCACTGGGAGGAAGGCGGTCGACTTGACGTACCCTGATGAAGAGATTT

TTATGCTCGACTGGGTTCGCCGGCTCTCTGACGAGGGGAAACTCTTGAACGCCGGAGATC

AGCGGTCTTTTGACGGGCCGAACCCGCTCTCTCAGCGGCTCCTCCGTCTCGGGCTCCACT

GCACGCTTCACGATCCCCAGTTCCGTCCGACGATGAAATGGGTGATTGACAAACTCACCT

CCGGCGATCTCTCCGGTGAGTTGCCTGCTCTGCCGCTGTTCCACACCCAACCACTCTACA

TCTCGCTCTCCCCTATTATCACCGCTACAAATACAGCCCATGTTAGCTCAAATTATGTCA

CCGCTCCCGGCAGCACTGTTTTCTTCACTGCTGACAGCGGCGGTGGCAGCGGCAGCGACG

GCGCTTCTGGTAGTAATAGTTGCTGTCGTTATGTATCATCCCTTCCAAAATTTGATACTC

CCAGAGAGATACCATTTAAAGAGATAATCGCCATCACCAACAACTTCTCAGAATCTCAGA

TGGTCGCCGAGCTGGACTTCGGCACCGGCTACCATGGTTACCTTGACAAGTGCATCCATG

TGTTGGTGAAGAGGTTGGGGATGAAGAGGTGCCCGGCTCTGCGAGTTCGCTTCTCCAATG

AGCTACACAACCTCGGTCGGCTTCGCCATCGCCACCTTGTTCAGCTCCGTGGCTGGTGCA

CCGAGCAGGGCGAGATGCTTGTTGTCTACGACTACTCTTCCAGCAGCCAACTTAGTCGCC

ATCTCTTCAGCCGCACTGGACCGGTGCTCTCCTGGCGCCACCGATACAACATTGTCAAAT

CGCTAGCGTCTGCCATAGTCTACCTGCATGAGGAGTGGGAGGAGCAGGTGATCCACCGCA

ACATAACCTCATCGGCTATATTGCTTGACACTGCCAACGACATGAACCCTCGGCTTGGTA

GCTTTGCACTTGCGGAGTTTCTCTCGCGTAATGATCATGATGGTCATCATGTCACCAAGA

AAAATCAAGCTCCAAGAGGTATATTCGGATATATGGCGCCCGAGTACATGGAGAGTGGGG

AGGCAACAACGATGGCTGATGTATATAGTTTTGGGGTGGTGGTGCTGGAGGTGGTGAGTG

GAGTGATGGCGGTGGATTTCCGGTGTCCGGAGGTGCTTCTGGTGAGGAGGGTTTGGGGAT

TGGAATCGAGGAGGCGGGGGCTGGAGGAGATTGGGGATCCGAGATTGGATGGAAAGTACA

ATATGACCGAGATGGTTAGACTGGTTAGGCTTGGGATGGCTTGTGCACGATCGAATCCAG

GATTGAGGCCGAGCATGAAACAGATTGTTAGCATATTGGATGGGAGCGATGAAGTGCTGG

AAAAGATGGTTTGTAAGAAAGAAGGCAGGGAGGAGATGCAGAAGAGAAATGCAGTTGCTA

TGTCCCTTGTTAGGAGAATTCAAGCCATAGGCATAAATTGAATGTTGGAGACAATGAATT

AAAGGAGTTTGTTTCATCTTTTTGGGGTGTAATTTTCTATCAAATAGTATGCATCTATTG

TGTAAAGAGAGTTGCTACTGGTTTTTTTTTTCAGGATGTTCATATTCAATATTTGATCCT

CA

>Unigene0000729

CCGCAGCTGTGCTATGGCGGAAGCGGCGGAAAGCGAACTCGCGCGCGATCTCCTTGATGT

TGGTAACAGCCTTCTCTCTCCTCCATCCGATGTAGAGGATCTCCTCAAGCTTCTCAATCT

AGCGGAGGATTTCTTGTCGCAAGTGCAACAAGCACCACCTATGTCCATGAACCTTGCACT

TCATCGTATAATGAAGGCACTTACATTGAAAGATTTTTTAAATCATTTCGATTTGGACAT

AAAAGTTGCTTTAGCGTCCTGTTTCAGTGAAATTATATATATAACAGCACCAGTTGATCC

ATATAATGAGGATTTGATGAAGGAAATCTTTCGGCTTATCGTAGATGCCTTGAAAAACAT

AGATGATTTATCCAGTCGTACATATTACAAAAAACTTTCAATTTTGGAAACCATCGCAAA

GGTCCATTCATGCGTCCTGATGCTCAATCTTGACTGTGATGATTTGATTATAGAAATGTC

CCAACAGTTCCTGAAGACTATAAGGCCAGACAATGAAGGAAGAGTCTTTTCCTTCATGGA

AACTATCATGACTGACCTACTAAATGATTGCGGAGATCAGCTTCTACCACGCCTCTTAGA

CAGTTTGTTAAAGGATATTGAGGATGGTTCACCAATTGCATGCAGATTGAGAGAGAAAGT

GATTCAAAATTGTGTTGGGAGGCTGAAACCATATTTAACTAAGATTGTCCATTCTAATGA

CACTTATTCCAATGGTTATGGTGACATCATTGCTTCCATTTGCCAAGAGAGTCATGATAC

CTCGGAACTGAATTTACCAAGTACTTCTGGTGAATGTTTGGAATCTGGAATATTGGAGCC

AGTAGTGGATTGCCTGAAAGACATGGATACTCCTACAGTTAAATCGACAACAATGATGTT

GAACGGCACTGTTCAGAGAGGGAGCTGTGATTCTGTCATATGGTCAAGTTCTGTAAAAGA

GAAGCCTGAATCTCATTATGGCATTGAGCAGTCTGACATTGCCACTATATCTGGTATTGC

TGAACTTGACAGCTTGAAATCCTCAAGAGTTGTAAACTCAGATGGCAATGTAGTTTGCTA

CATCAAGAAAAGAAGGGACCAAAGGGAGAAGTCAGCATTACAGTTAAAAAATACCACTAG

ACATTTTTATAGGCATGGTGATAAAGATTTGTCAGCGCCTAGCAGCAGGAGAGACTGTAT

AAAGGAAGCTAATTGTCAATCCGGTAAAGGAGCTTCGTATATTGATCACGAGGGAGAAAT

ATTGGCTTCCGAAGATACACGTTACCAGTCCATTGAAGAAAATCATGGCTCTGTTGGAAT

AACCTTGAGAAAGTCGTGCAAAAGTAAACATAGCATTGGTGCGGGTGAGATAAATCTTGC

GGACCTGTCCCTCGAAAATGCTGATAAAACAGTTAAACCAATTGATTCAGAAGCAGAAAA

ACTGTGCCACACAACTATAAAGAGATCATCAAGAAAAGATGTCGAAAGGAAATTATTAGA

ACAGAGAATTCAAAACACTAAGCAGCAGATACAAAATACCTTTCTCAGAACGGATCTTAC

TGGAGAGAAGATTTCAATGAATTGCGGGCAGACAGAACTTGTGGATGTTTCCCTAAAGAC

CACTGATGAAACAATTGATTCAGAAGGAAAACATCTATGTAGAAAAAATATTTGGAGATC

CCAGAAAAAAGCCACTGAAATGGAATCATCAGAACATCAGAAATCTGTAATTAAGAAGCT

GATAGGAAAACTCGTTATCATAGATGATCTAGCTGGAAGGCAGAGTTCAAAGGCACTAAA

TAAGGATCAATATAATTTGGAGGAGAGCAGTATCACAAAATCCAGGAGAAAGCGTACGAG

GGCTATTAGTGAGGCACTAAATAAGGATCAATAT

>Unigene0000730

GCGTATGAGGGCTATTAGTGAGGTACTAAATAAGGATCAATATAATTTGGAGGAGAGCAG

TATCACAAAATCCAGGAGAAAGCGTATGAGGGCTATTAGTGAGGCTTTCGAAGCAGAAAC

TGGCAAGAAGGAACTGGACGAGGCACTTGTTGGTTCAAAGATTAAAGTATGGTGGCCAGC

TGATGATGAGTTTTATGAGGGCATTGTTGATTCTTTTGACCCCGTCTCCAAGAAGCACAA

GATAGCATATTTTGATGGTGATGAGGAGATCCTACTTCTCAAAGATGAGTGCTGGGAATT

CCTTGAAGACAATACTAAACCAGATTTCCTGTGGAGGCAACAAATTGAAAATCCAGGGTC

AGAAGCTTCCCCAAATGTTTTGAATGTGGTGAATGGAAAGAACCCTATGTGGGAGCATTG

TGAGAGAGTGGCTGGGGATGCTACATGGAAGTTTAAGTGCTTACACTGCTCAAAAATTGT

TTCAAATGTTACAAGGATGAAGCACCATCTAGCAAGAACAGTGAAAAAATGTGTGTTGCC

ATGCACACACGCTTCCCAAGAAGTGGAGAAATTGTTCCGCAATATGGTTAGTGAAATAAG

GCCGAGTAAGTATGTTACCGGTTCGAATATGGTGAACGGAAAGGATCCTACTTGGGAGCA

CTGCAAGAGAGTGGGCGAGAATGCTAAATGGAAGGTGAAGTGCATCCACTGTTCGAAGAT

AGTTTCAGGTGTTACCAGGATGAAATACCATCTAGCTAGAGCGAACACGATTTCGGAGCC

ATGCACAAGCTGTCCGCGAGAAGTAGAGAAATTCTTCTTCAATCTCATTAGTCATTGAAG

CTTCGAAAAAAAATGATCATGATGCAAAGCTGCAATCGTATCCAGAAAGGTCACTGGATA

ATGATACAAGATGCTATATGAAAGTGTGAAATTGAATTTGGGTTGATAAACCGAGGTGTT

TCGGTGTATTACCAACTGAGGTGTGTCTATTTTCTTGGATTTGTTTTAGTTTTGTTTGGA

TTTGGATTTGGGTTGAGTAATTTCTTGTGGAACCATACTTGGCACCCTGAGTTCAACATA

GTTATTTATAGCACATAATGTTTGGAGGAAGCCAACTTGCTCCGAAACTTACAGGATTAT

TTTTATTTTTTTTCCAACTAAATCTTTGGTTAAAGGATCTTGAAGTTTGACTCCTGGCCT

AGCATAGTCATTTGTTATTTTAATATGGTGCTTACAGTATTATGTATTCAACTAATGTGT

TTAGACTTATAAGATTTTAATATGTAACAAATCTTTGAAGTTAATACATATAAATCTAAT

CCTCTAATTCAG

>Unigene0000731

AAAACAATAAGCCCAATACTTAAGCTGAGGAGGACATTCTGATTTCATCTCAAATCTAGG

GTTTCCGCCGTCAGTTGGATCTCCAAGCAGCCTCTTGTTAATCTCACCAAATCCCCGTCC

AGCTACGCACTTCTTCCCTCTCCATTCTAGGGTTTCAATCTGGCTATTCTTCCCCCCTCT

CCGGCATCTCTCATACTGACCCGACCTTTCAATTACTGGAACTCGCTCAGATATCGGTGA

TCTCCGCCGCCGGTGAGTTATCCGATTGCAGATGGCCGGAGGGAGCTTCCTTGCTCGACC

GATGAACTACGTCGTAGGCGATCTGGTCGTTGAGAGCCTCTCCAATAGGCCTTAAATTGC

CAAAGTGCCACTGGGTTCAGGTTCTGCCGCAGCTTTGACCCAGCTTCTACAACAAATCCT

TCGCACCAGCCACCGCACGTTCCCATGTTGTACACAGATCTTTTTATCTTGCTGGGCCTT

AAAGCAATTGATGCAACAAAAAGATTCCCCCGCCATTGGATACTGTTCTTATAAATTAAA

GTAATTGAAGCAAGGTTTTTATAAATTGAGGAATTTGGACGTCGGAGTGATTGTGCCTTA

AACTGAGATTTATTCTTAAAATATATGTAAATCTCTTTTCATGGTCGTCAAAGTTGGCAC

CTGATTAGACATCTTTGGCTGCTCGGATTTTTTGTCCTTGGTTAGTCTAGTGGTTCTTTT

TTATTATAACAGTAGATGGTTCACTATTTTGTCCAGGTTTGACTCTTTTGCATCTTTGTC

AAGAGCCTAAACTCTCCTATGTCGTTTTCAAATATTTATTACATTTAAATTTATTAGTAC

CATTAAATATATGTGTAAAGTTTCTGAACTAATTTCACCTTCGTATGGATGATA

>Unigene0000732

TTTGGTTCATTGTTCACCAAAAACAGGCTGATAGAGTTCTTTTGGTTCATTGTTCACCAA

AAACAGGCTGATAGAGTTCTTTTGGTTCATTGTTCACCAAAAATTGAGTGGGCTAGGCCT

ATAGGCTTGACCTGTCAGTTTTGGCTTGGCTCCCAGCCCAAGTGGGTTAGGCCTATAGGC

TTGACCTAATCTGACTGTCAGCTGGGTCTGTGGGTTTAATTTTTTTTCTTGAAAATCCTT

GAAACCAAGAAATGAGTCTGTTTTTAGTTATTTCTAACTTAAACTGACTGGTTTGGTTCA

TATGTTGTTTTGGCTTGGCATGTTTTTTGCACCTCCCTAGTTAATATAGAGTTTGTAAAA

CTAAACAATTGACTTTGGTCGTAGGATAACTCATATCATGACAAGTCAATCCTGCAAATC

CTTTTTTTCCTGATATTCACAAAATTCATTACTTCTTAATCTGGTCACCGTGGCTAATCT

CTTATCCTTTTGGCTCGTTCCTCTGGAACAATGGTTATTGTTATTCTCTGAATTTATAGC

TTATAATTCTTCTTGGTGACACCACTTGAATAACATACATTTTGTGGTTATATTTATATT

TGGTCTCTGTTTGTCTGACTTAGATGGGATTAATGAATTTTCGTCTTTCGACAATTTTTT

GTTGTGATATGCATACATGCACAGCTTCATTCTCTGGTTACTGTGGGTAACTTCTGTTCC

AAATTGTTATGTCACTGTGGATAACTTTTGTTCCAAATTGTTATGTTCCTTGAAGTAACA

AATTGTCTCCTTTATTTGGTGTTGATCTTGTAGAGAGGCATTTGGACGTTGGAGAACTAT

GTGATCCTCATTTGCCCAAAAACTTAATTACTCAAATTGATATTAAGCTTTTGTTTAATG

ATATTTTTTTAGGCCTTAAATTGCCAAAGTGCCACTGGGTTCAGGTTCTGCCGCAGCTTT

GACCCAGCTTCTACAACAAATCCTTCACACCAGCCACCGCACGTTCCCATGTTGTACACA

GATCGTTTTATCTTGCTGGGCCTTAAAG

>Unigene0000733

ACCTCCGCAACCTATATTTAGCCACCTCCAAAACTGCCGATTAATTTCACAAACCCCCAA

TCGAAACAGCCCCACAAATATCTCCATAAAACCCCCTCCCCCTACTCCGCACCCATGGCC

TCCGCCGCCGCCGCTGCCGTGTCTTTCCTCCTCCTCTTGTCCGCATCCGCTGCATCCGCT

GCGTGCACCTCCACGACCTTCTCCTCGAACCGCATCTTCGCCGCCTGCAATGACCTCCCC

CACCTCTCCTCCTCCCTCCACTGGACTTACACCCCCGCCACCTCCACCCTCTCCCTCGCC

TTCGTCGCCCCTCCTACGGCCCCCGCCGGATGGATATCCTGGGCGATCAACCCCGCCGGC

GCCAAGATGATCGGTTCCCAAGCGCTCATCGCTTTCCGCCAGCCCGGCGGCAAGATGACC

GTCCAGACCTTCAACGTCAGCAGCTACAACATCAAACCCTCGCCGATCGACTACCGGACG

TCAGATCTCGCGGCGGATTTCGTCGACGGGATGATGCGGATCTACGCTAAGGTGTATCTC

CCGCCGGGGATGACGACCGTGACCCAGGTGTGGCAAGTTGGGGCGTCGGTGACTGGAGGA

ATACCCGATAAGCACCCGTTCCTCCTCGATAACCTGGCGTCCATGGGCTCGCTTGATCTG

GTGCGTGGGCAGACGACGGTGGCGGCTGTTTCCGGGTTTCGGAAGCGGAATATTCACGGG

GTCCTGAATGCTGTAAGCTGGGGAGTTCTGTTGCCAATGGGAGCAATCATGGCAAGGTAT

TTAAAGGTGTTCAAGGCAGCAGATCCTGCATGGTTCTATCTTCATGTTTCATGCCAGATA

ATTGGTTACAGTGTGGGTGTTGGTGGCTGGGCGACCGGTCTTAATCTAGGCAAGAAATCT

GTGGGAGTTGTGTACGCAACTCATCGCAACATTGGGATCACTCTCTTTGCCCTCGGTACA

TTGCAGCTTTTCGCCTTGTTTCTGAGGCCAAATAAGGATCACAAATATCGGTTCTACTGG

AATATATATCATCATCTGATGGGATACACTGTCATTGTGCTCGGTATCATCAACGTCTTC

AAAGGCCTGAAGATATTGAGCCCTGAACAGAAGTGGAAGCATGCCTATATCACTATCATC

TCCATCTTAGGTGGTATTGCCGTCGTTTTGGAATGTGTCACCTGGATTATAGTTCTGAAG

AGGTCTAGCAAATCTACGAAGCGGGTCGATTCTTCGAACGGTCACCATGGTATTCAGCAG

CCATTTTCAGTTTGATCTCTGGTATACTGGGACTCAGGGATTTTTATAAATCTTTTAGTT

TATAGATAAATAGAACATGGCTTGTATCCTGGACTTGCCTGGTATTTTTTACCGTCAGTA

TTTTTTATATGTGATATTACTAAAGATGGACGCTAATAAA

>Unigene0000734

CGTTCCTCCGGGCACCTACGGCAACTATGATTCGTGTCCCTGCTATTTTGCCCTCACCAC

CCACGGCGGCGTTCGCAAGTGTCCTTGATCATCATCGGGGCGACATTTATAAGGGAGTTT

GATTGTCCAAGGCACCGATAAGAAATCTTTCGAGCGGCATAGTGTACTGTATGGAATAAA

TTTGAGGAGAAATTATTATTTTGTGAGCTTTGTATTTGTACTTCTTACATGCACTTAATA

CAATTATTTGTTGTGTTAAATGCAACTTCTTACGTATACTTAATAAAATTTCG

>Unigene0000735

TTGCCCTCACCACCCACGGCGGCGTTCGCAAGTGCCCTTGATCATTATCGGGGCGACACT

TATAAGGGAGTTTGATTGTCCAAGGCACCGATAAGAAATCTTTCAAGTGGCATAGTGTAC

TGTATGGAATAAATTTGAGGGGAAAATACTATACTGTGAGCTTTGTATTTGTACTTCTTA

CATGCACTTAATACAATGATTTGTTGTGTTAAATGCAAACTTCTTACGTATTCTAAATAA

TATTGTTTGTAATTTCTGGAGTTTTTTTTTTCCGAAT

>Unigene0000736

TAACTTTAATCAATCAGAAAGATAACATTAACTGGAAATGCTGTTTTATTGAAATATAAT

ATAATTAGCTCTGCTGGAATTAAACACAACAAGACTGAAAAGGTGTTACAAACAAAAGTG

ACACTAGATGTTATCTCCCTTAACCACTAATAATTTCTAACAATACTCCTTATTTTTAGG

TGTCCCAAGTTTCTTACCTACTCTTCTTCTCCACATTTTCGCCCTTCTTGTTCGCATCCT

CAATTGTTGCCTCCTTGTTCACATTCTCAAGTGCCTCCTCTTGTTCGCGTCCTCATTTGC

TGCCACTGAATTAATATCAGCCAACAAATTATCAGTCAACCTCTCAAAGAGCAACACGGG

TCAATCATACAGTTTAAAACGTCAAATTAGATAATAGGCTGTTTTATTTAAGCTAAATAG

TTTGTTAAATTGTTGTTAGCCCGGCGGTTCATCAGGCGAATAAAGAATTATCTTGGAGAC

AACACCGTTACTTCAC

>Unigene0000737

TTCATCGGCTATTTTTTTGAGGCAAACCAACGTTTCAACTTACTCTGTAAGGTTCTTAAG

AGGCCTTACTTCCCTACCCCTCCATGATCCCTCCGGCTGAGGAAGTATTTTTGCGGAGGA

AGTATTTTTGCTGGAATTAAAAGAAGAGGACATTGAAGCTAGACATTCCAGTGAACATTT

GATGGGAAAAAGGTGGAATGGTACAGAAAAAATTCCTGTTCGCCATTGGCATGATTCACT

TCAACAAGAGATGGGAAAAAGTATTGCTGGTGGTGAAAACCCATAATGCAGGAGCATGCC

ACCACCATTATGTTGTTCTTTGAGAGGTTGACTGATAATTTACTGATAATTTGTTGGCTG

ATAATTTACTGATAATTTGTTGGCTGATATTAATTCAGTGGCAGCAAATGAGGACGCCAA

CAAGGAGGCAGCCAGATGATGAGAACAAGGAGGGCGAAAAGATGAGAGGGCGAAAATGTG

GCTGTTGAGGATGCGAACAAGGTGAGAATGAGAACAAGGAGGGCG

>Unigene0000738

TTGAGGATGCGAACAAGGTGTGGCTGTTGAGGATGCGAACAAGGTGTGGCTGTTGAGGAT

GCGAACAAGGTGAGAATGAGAACAAGGAGGGCGAAAAGATGAGACGAAAATGCGGCTGTG

GAGGATGTGAACAAGGAGGGTGGAAATGTGGAGAAGAACTCGGGTTAGCTGTAACACCTT

TTCAGCTTGGTTATGTTTAATTTCAGTAGAGCTAATTAAATTATATTTCAGTAAAACAAC

>Unigene0000739

TGTTTCCTCCTCTGCCTGAGAGTAAATTTCGGCGTTGGAATTGTGTACATTGTCTGCATG

GAATTGATCCTTCTGCAGCTGTAGTTGTAAATGGAGCTGAAGGTCTTTGTCAGAATGATG

AGGTCAAAGGCATCAGTACTTCACTTGAAAGTGCCAGTTTGGCATCTCAGTCTGGTGAAC

AGTCATTGCTTCCCGGTTTTCAACAAATATCAGAAGAGAAAATGCTAGGAGAACAATTAC

TTGCAGCTGACAGTTCCATGAGTGTGAGCAATGTTGAATCATATTCGAAAGTATGTCCTG

CTATGAAAGAAAATGAATCTGC

>Unigene0000740

TGTTTCCTCCTCTGCCTGAGAGTAAATTTCGGCGTTGGAATTGTGAACATTGTCTGCATG

GAACTGATCCTTCTGCAGCTGTAGTTGCAAATGGAGCTGAAGGTCTTTGTCAGAATGATG

AGGTCAAATGCATCAGTACTTCACTTGAAAGTGTCAGTTTGGCGTCTCAGTCTGGTGAAC

ATTCATTGCCTCCTGGTTTTCAACAAATATCTGAAGGGAAAATGCTAGGCGAACAATTAC

TTGCAGCTGTTAGTTCCATGAGTGTGAGCAATGTTGAATCATGTTCGAAAGTATGTCTTG

CCACGAAAGAAAATGAATCTGTAGTCAGAGACCTAGCAGAAGGTCTGAACATACCCAAAG

CTAGCCATGACACCTGTGAAGTAATTCGGAATCTGGCAAGTGATATTGCTGCAACAAAAG

GCAGAGAACACTCTGGAGAAGGTACTATTGTGCTGACTACTAACACTTT

>Unigene0000741

TTAGCTACCACTATATACCCCACCCCAATTTTTTCCTCTCTGTCCCTCATCACCTTCTCC

TTGGAGCTCTCCACCCTTCTTCAAGGTATTATATAGATGAAATTTTGGTAATACATTGAA

CTTGAAGAACATGGGTTCTGGAGATTGGTTTAAGACAATTGTTAGAAGGAAGAACACAAC

ACAAGACAGGCCAAAGAAAGTTGCGGGAGTCACTGGTGAACGATCAAATGAAATCAAGTT

GAGAAATTCAGCACATAAGCGGTTCAACAAATTGGCTAATAGCACTTCTAGATATTTTGG

CATGACCATTGCAGATATAGCAGCAACTCGGATTCAAACTGCATTTCGGAGCTTCAGGGC

CAGGAAGACGCTACGTCATCTGAAAGGAGCAAAAAGATTGCAAGCTCTTGCCCAGCGACA

TTATGTGCAAAAACAGGTTTCGACCACTTTGAATCATCTCCAATCCTGGAGCAAAATACA

GATGCAGATCAGATCTCGTCGTGCTTGCATGGTTACAGAAGGTCGTATAAAACAGAAAAA

ACAAGAAAATCAATTAAAACTCGATGCTGAACTTCATGGCCTAGAGGTGGAGTGGTGCGG

AGGGTCTGATACAATGCAGGACATCCTATCGAGGATAAGTCAGAGGGAAGAAGCAACAAT

CAAGCGTGAACGTGCCATGGCATATGCGTTTTCACACCAGTGGAGAGCCAACTCTGGTCT

GAACCAGGGATATGATATTGGGAAATGCACTTGGGGATGGAGCTGGATGGAGCGGTGGAT

TGCCTCTCGACCTTGGGAGGCCAGACTTCTGGTAAAGTGTTTGAGCCCTAATAGTGGCAA

ATCGAAGCCGACAAACGAAGTTGTTAAGAAGAACAATACATCTGCCCGTAACACTAAAGA

TGCAAAGGGCTCTATAAAGATATCTCCCCGGCCAACAATAGCGAAAACAACCAGACAGGA

GCCTAAGCTGAACATCAAGAATGGATGAAAGCAGTGATAAACACAACTTTTTCCTCTCAA

TCCAAATAAAACTGATATGTACTGGATTTTCTCTTGTTTAAAATCATTGCACTGGTGCTT

AATAAGTGTGAAAAGTGAAGCAATTTGTATTTCTGGGCTTATGTTTTTGGTTGTTTGCTG

TCGATGGCAGCTAAGAATAAAGAGGGTGCTACAATTGCCATTTCTGATGTAGCAGGACTA

TTAGCTGTGTATTTGCATGTGCT

>Unigene0000742

GCGGCGGGGGATCGCAGAGGGGGAAGGAGGAGAGGCTGCCGCAGTGGACGATGCAGGAGA

CGCGGGAGTTCATCTCTATCAGGGCGGAGCTGGAGCGGGACTTCACCATGTCGAAGCGGA

ACAAGACGCTGTGGGAGGTGGTGGCTGCTCGGATGAGGGAGCGGGGGTACCGCCGGAGCC

CCGATCAGTGCAAGTGCAAGTGGAAGAATCTCGTCAATCGCTACAAGGGCAAAGAGACAT

CCGATCCAGAAATCGGCAGGCAATGCCCGTTCTTTGAAGAACTCCATGCCATATTCACAG

AACGTGAGAAGAACATGCAGCGTCTCCTCCTTGAGTCCGAATCCGGTCCGCCCCAATTGA

AGAGGAAACTAAAGAGACTCAGTGGAGATAGGTCCTCGGAGGATTTTTCCGATGATGATG

ACGAGGAAGACGAAGACAGTGAGGATGAGCGTCCCACGAGAAGTAGAAAGAAGAAGACCA

GCAAAGGTGGACAGCAGCAGCCACGAACAGCAGAGAAATTGAAGGCCACTGGGAGCATAC

ATGACTTGCTGCAGACCTTTTTCCAGCAGCAGCAGCGGATGGACATGCAATGGCGGGAGA

TGATGGAGAGTAGGGCGCAGGAGAGGATGCATTATGAGCACGAGTGGAGACAGTCTATGG

AGAAGCTGGAGAGGGAGAGGCTGATTCTGGAGCAGGCATGGCGGGAGCGGGAGGAGCAGC

GGAGGATAAGAGAAGAAAGTCGAGCCGAGAAGAGAGATGCCCTCTTGACTACTCTGTTGA

ATAAGCTGGTACAGGAAGGACTTTGATGGCGGCGAAATCGACAACTGGAGCATAGGATTG

AAGATCTCGTAGGGTTTACGTCCTGCCTTCTTCTGGCAGTTCATGGTTTGTGTTGTTTAA

AACGTTAATGATATAGAGTTTTTCCTGAAGCTAGAATTTCGTCGATCTTGTAAGTGTTGT

GGAGTTTGTCCTCTTTTTTTTTTTTTTAGAAAATATATGATCGTTGATATTATATGATTC

CTTCATTGTTTGAGTTTGACACTTTACAGTAGGTAGTTTCTTGAAGGATGGGACATGTAA

ATTTAAGTATTTTG

>Unigene0000743

CTAGATCCCTCTCCTCTCCCTCTCCTGTCACGATCTCCGAGCACATCAGCCATGGCGGAT

CAGCTCACTGACGATCAGATCTCCGAGTTTAAGGAGGCGTTCAGCCTGTTCGATAAGGAC

GGCGATGGTTGCATCACAACCAAAGAGCTCGGGACCGTGATGCGCTCCCTGGGCCAGAAC

CCAACAGAGGCAGAGCTTCAGGACATGATCAACGAGGTAGACGCTGACGGCAATGGGACC

ATCGATTTCCCAGAATTTCTCAACCTGATGGCCCGCAAAATGAAAGACACCGACTCAGAG

GAGGAGCTCAAGGAGGCCTTCAGAGTGTTTGACAAGGACCAGAATGGCTTCATCTCTGCT

GCTGAGCTCCGCCATGTTATGACCAACCTTGGCGAGAAGCTGACAGATGAGGAGGTCGAC

GAGATGATTCGTGAGGCTGACGTCGATGGCGATGGCCAGATCAACTATGAGGAGTTTGTC

AAAGTCATGATGGCCAAGTAAAATTTGACCGGTTGATTACTAGTATGATGCGATTCTCCT

TTCTCTATTACCTGGTAGTAATCTGGAATTAGATGTAGTTGTGAAATCAGAAAAGAGGAT

GATAGTATCATGTACTCTCTTGCTCTCTATGTTGGTTGTGTTTCATCTCTGTTTATCGGG

GATTGTTGCCTGTTATCCTTTCGGTGTGATTGGGATCCAAGTTCTATATTTAGTCCTGTT

TATTATTTTGTTATATTTTCTGACATTGGAAGTTTAGTGATGTTCGGAGACATGAAGTTC

TTGTGATTAATGACATGCAAACTTTGCCTTTTTGTTGGCATTGTAAATTGTATTATTCTG

GGTAGGAGTTTCTGCAAGGCACTGATTACGTAGAATCACCAATGAA

>Unigene0000744

CCCATATCATCATTTCATTTGGCTGAAATATTACATCGTGTATCATAAGATACACTTTGA

TTTATAATGACTATATATTTTTTGAGAAGATAATAAATGTATAAATAGTATATAAACAAT

TCTCTATGCATATCGATCATATAGTTGTGGTTAAAAAGTATAAACTTAAGATGTATAAAT

TGAAAGTCGAGGCCCTTACACAATATAGACTTGATTGTCTCAGTTTATAGACATTTATTA

CTCAATATAATGTATCTTGCCTTATTCATGTCTGTCTCAAGATACAAAGACTTTTGCAGG

CCGAAACATATCGTGGCTCAAGATACATATCGTGTAGGGTAAGATACTGATAAACCATCC

TACCAACCCCAAATTCTGTGAGCCAGGATCCCTGATTTTCTTAATTATGTTGGCATCCCA

ACTCGGATGAGCGGGTCACTAAGGTGGCCCCACATTACTAACCGAGAACAAATGTTTTGT

GGTGTTGCCATATTACTCTTGATTAATAATGGTAAGACTCTTGCTTTACATCCTGTTGAC

CCATCTGTGGTATGATGCACATAATGCACCTGAATGTAGCTACACTAGCTCTTAGACATG

TTCAAGCTACTGCTATGGGTCCATTGAACTTTACTATTTTGGAAAAAAAACTAGCTTGCC

TTGTTTTTAGTTTACAATGCGAGGGAGAGGAGAGGGAGAGAGATGGTCTGATGGGGCAAT

TTGGTTGTTTAACCCTTCCACCAATGTAACCTATATTCACAAATTACATAACAGCTTAAC

TGGAATTCCCGGAAAATGTTTAAATGACCGAAAAAGGCGAAAAGATAGGTATACTTGTGC

CCTTTTCCAGGCTCGGTTCCCATGTTACTACTAATTTGTACTTGAATATATCTTTTTAAT

ACTGTTCCTACAGTTATGTTTAGCACTATATGTTAATACTGTTTTATGATTACATTTGCA

TGACCTAAAAGAGTAGGTATACATTTGCACAAGGTCTGAAGAACACTTGAAACTTAACAG

GTTGCATCACAACCAAAGAGCT

>Unigene0000745

GTAGAACTCACGATTCGGTTTGAAGTACACATTCTTCTCTCCGATCATTCGTTCGAACTG

GTCAGTGCCCGCAGCTCATCTAGACAGAGATCGAAATGGGAGCGGAGCCAAGAGATCCGG

CCCCGAAGCCGCCGACCCCTTCCGGCCACCCCCCTGATCCCGACGCCTCCTCCGCCACCG

CCACCGCCGCCGATCCGCCCTCCTCCTCCATCGACTTCGGCTCCCCCTCCGCCCTCGCCC

AGATCCGCTCCCTCACTGACGTCGGCGCCATGACCCGCCTCCTTCACGAATGCATCGCCT

ACCAGCGCTCCCTCGACCTCCGCCTCGAGTCCCTCCTCTCCCAGCGCCCCGACCTCGACC

GCCACCTCTCCTCCCTCCACCGCTCCACTCACCTCCTCGACCTCGCCCGCGACGACGCCC

ACCTCCTCCTGGACTCCGTCCTATCCACCTCTTCCCTCGCCGACAACGTCTCTCGCAAGG

TCCGCGAGCTCGACCTAGCCCAATCCCGCGTCTCCACCACCCTCTCCCGCATCGACGCCA

TCGTCGAGCGCTCTCACTGCCTCGACGCCGCCCGCCGCGCCCTCGACGCCGAGGATTTCC

CCTCCGCCGCCAACGCCATCCACTCATTCCTCCAGATCGACAAGCGCTTCGATCTCGAAC

CCGGATCTGAGCGCACCCAGCTCATGGAACTGAAATCCCGCCTCGAAACCCTCGTCCGCA

GCCGCCTCGCCACCGCAATCTCCAACCGCGACCACCCTGCCGTCCTCCGCTTCATCCAAA

TCTTTCCCCCGCTCGGGATCCAGGACGAGGGGCTCCAACTCTACGTCTCCTACCTCCGCA

AGGTCATCGCCCTCCGCTCCCGTCTCGAGTTTGAGCACCTCACCGACCTTGCCACATCCG

CCACTGGACGAGATTTTATCTCCTGCCTCACTGGTCTCTTCAAGGACATTGTCCTTGCAG

TGGAGGAGAACGATGATGTTCTCCATGGCCTCAGCGGTGACGATGGCATCATCTATACAA

TCTGCGAGCTCCAGGAGGAATGCGAGTCGAGAGGCACTCAGATCCTGAAAAAGTACATGG

ATCACCGCCGGCTCGCCCGGTTAGCTTCGGAGATCAACTCTTATAGTAAGACCTTGCTAT

CAGTTGGCGCCACAGAAGGCCCTGATCCGAAGGAGGTGGAGACGTTTCTCGAGGAGATAT

TGACGCTTACCCAGCTGGGCGAGGATTACACTGAGTTCATGGTGTCGAAGATCCGCAACC

TGGGGTCCCTGAAGGATGACTTGGCCTCTCGGGCGACTACGACTTTTCGGAGTGGTAACT

TTAGCAAAGTGGTGCAGGAACTCACCGGATTCTATGTCATTCTTGAGGAGTTCTTTATGG

TGGAGAATGTGAGGAAGGCGTTGAGCATCGATGAGTATGTGTCGGACAGTATGACGACAT

CAATGGTGGATGATGTGTTCTATGTGCTGCAGAGCTGCTGCCGGAGGGCGGTCTCAACAG

CGAGTAGCAATTCGGTGCTTGCGGTGATTAGTGGGGCGATGAACCTGCTGAGCAATGAGT

ATCAGGAGGCGCTACAGAGTAAGATGAGGGAGTCGAGCTCGGGAGGGAGGCTGTTCTTGG

GGGGAGTCGGGGTGCAGAAGACCGGGGAAGAGATTGCCGTAGCTCTGAACAATGTGGATG

TTAGTGCAGAGTATGTCTTGAAGCTCCGCCTTGAAATTGAGGAGCAATGCTTGAAGATGT

TTCCTGCACCAGCCGACAGGGAGAAGGTGAAATCCTGCCTATCTGAACTCACCGAGCTCA

ACAGTGGCTTCAAGCAGCTCCTGAATGCTGGTATCGAGCAGCTGGTTGGGATGGTTACTC

CCCGCATCCGCCCGGTACTGGACAATGTGGCTACCATAAGCTACGAACTTTCCGATGCCG

AGTACGAAGCTAAAGAGGACGACGCATGGGTTGATTCGCTCCTTCATGCAGCCGAGTCAA

ATATTGTCTGGCTGCAGCCCTTGCTGACCTCCAACAATTATGACTCAGTCGTCCACCTGG

TGATTGATTTTGTTGTAAAGAGGCTTGAGGTGATCATGATGCAAAAAAGGTTCAATCAGC

TAGGTGGCCTCCAACTCGACAGGGAGGTGAGGTCGTTGGTGAATGGTTTCTCAGAGATGT

CGCAGAGACCCGTGAGGGACAAATTTGCCCGCCTCTCCCAGATGTCGACGATACTGAATT

TCGATAGGGTGTCGGAGATCTTAGATTTCTGGGGAGAGAATGCAGGGCATATGACTTGGC

TGCTCACTCCATCAGAAGTGAGAAGGGTATTGGGGCTTCGAGTTGATTTTAAGACTGAAG

CTATTGCTTCCCTTAGGTTGTGAGACAACTCAGTGTTCTGTGTAGTTGTACAACGAAACA

AAAACTAGATGATATATATGCCCTCTGTTTTCTCTTTTCATTCTGTTTAAGAGGATGTAT

CTTATTTCTCAGTACCGGCTTTGGAGGACACGACTCTGGGATTTTTGTTGATGTTGCCAA

TACATTGATATTACAGATATTAATGAAATTCATGTTTAGCAAATAATAATCGAAAACACA

GAAAAT

>Unigene0000746

CCAATGGATTCATCTCAGTTCCCTACGTTATCTTGTCCTCTAAAATTTTGAGTTCAATAT

ATACTTGCTCTCCCTCTCTCTTCTCTTCTCTTCTCCAATTATTTCACCAGATGGACTCTT

TTCATCTGAGAACTTACATTTTGGTTCTTCTACTCTCACAAGTCTCGTCATCGTCTTCGT

CATTGTCGCCTCGGTGTGACTTTCCGGCTATCTTCAATATCGGTGACTCCAATTCCGACA

CCGGTGGGATCTCAGCGGCCTTCGGCCCCATCCCTCGGCCCAATGGCCGGACCTTCTTTG

GGATGCCTGCAGGCAGAAACTGCGATGGGCGCCTCATAATCGACTTCATGGCCGAGAGCT

TGGGATTGCCTCTCCTCAGTAGTTACCTCGACTCGCTAGGCACCAACTTCACCCATGGGG

CTAACTTCGCGACAACAGCATCTACCATCATACCAGTTCATAACAAAGATATCACTCAAG

GAATCAGTCCCTTCCATTTGAACATCCAACTCAAGCAGTTCTTGCAGTTCAAGTCCCGCT

CACAGGCCATCTACACAAAAGTCGGAGGAGTGTTCAAGAATTTGCTTCCCAAAAGGGCCT

ACTTCTCTAAAGCACTTTACACATTGGATATCGGTCAGAACGATCTCACTGAGACGTATT

TCGGTAACAAAACTGTAGACGAATTCTTGCCAGAAGCCATGGCTCGAGTCACCGGTGTAG

TGAAGGTAAGTAAGCATCTGAGAAACTGATATCCGGAAAATTTGATTGAATTGGATATGA

GTGATTGTACAATTTCTTTCACAGAGTATATATGAGAAGGGAGGGAGGTATTTCTGGATC

CACAACTCAGGACCCCTTGGTTGCCTTCCCTATGTTCTGGTGCGTGGTCCTCTGACGCCG

TCTCAGTTTGATCCTGTAGGCTGTGGGATACCTTACAATGAGTTAGCCAAGCAATTCAAC

CGGGGACTTTACGACACTGTGACCCAGCTGAGGAAAAACCTAACTCTCGCGGCAATCACC

TATGTAGACATCTACTCCGCCAAGTACTCCCTCTTCACTCAGGCCAAGAAGCTCAGTAAG

TCCATACTTTCTCTTAACTGTCTATCTGCCCTTGCAAATCAATTTGGATATCCTAGGCTA

AGATTAACTTACTGCTAAGCAGTAAGCATCTTCTATACTAATTTTTCTTTTTCAGTTTCG

ATACATGAGACACACTTTTTCAAATTTTAAAAAATGAGTGATAAATCTCTGCTTTATTGT

ATGATTTCAGGATTTGAACATCCACATAGGGCGTGCTGCGCTCACGGTGGGGGAGAGTAC

AACTTCGATGACCATGTACATTGTGGAGACACGGGCGAGATTAATGGAACTATAATCCGG

ATCGGGAATTCATGCAGGAATCCACAGAAACAGATAATCTGGGATGGATATCATTATACA

GAGGCTGCTAACAAATTGGTATTCGATCAGATATCTACCGGTGCCTTCTCTGATCCCCCT

GTAACACTCAGTAT

>Unigene0000747

ATGCATTGATTTGAGTAATGCTACATAGACATCCCATATCATTCCATGATTTGTGGTATG

TGCATATATGAATTCCCGGTTTAGTTTCTCAGTGCAGTTGAGGATACTAATTCTGAAATC

AATTCGTCAATTTCCCTATGAAGTCAGTTAAGACCAAAGAAATTTTGGCAAAAATTATTT

CTTTAGCTGAGAAATGGAGTCCTTTGATCAATAATATTTGTTATTCAGATTGATGATTAA

AAAACCAAAAGTTTCCCTGTGATTCATGTCATTTACTTCTATATACTTGTTTAAAAACTT

CCAGTGGTACTCTTGAGTTATGAATTTCCGGACATACTTCAATATACAAAATTTTAACTT

TCAAGAAATGCAATTAACAGTCGGAGGAGTGTTCAAGAATT

>Unigene0000748

AATTCGTGCGCGCCGACATTAACCCTCGGATCGAAATCCGATCAATTTGTAGTTATAACA

GATAGTATCGTCTGCGGCAATGGAGGAGTTGGTGCCGATTCAGGAAGTGATGGTGGAATT

TCGTGCTGGTAAGATGTACAGTGAAGGAGCCCGGGTTGTTCCAGATACCCGCAAAGGTCT

TGTTCGCATAGGAAGGGGCGAAGAAGGATTAGTCCATTTTCAGTGGCTTGATCGAACCCA

AAATACGGTTGAAGATGATCAGATTATCTTTCCAGATGAGGCTGTTTTTGAGAAGGTGAG

TCAATCTTCAGGAAGAGTATACATATTGAAGTTCAATTCTGATGACAGGAAGTTCTTTTT

CTGGATGCAGGAGCCTAAATCTGATGGAGACCTACAAATATGCAATTCAGTAAATTATCA

CATCAATCGACCCTTAGAAGAGGAGATTGAAGATCCCCTTCAAATGCCTGAAATGTCAGA

ACATACAATTGATGGCGAGCTCTCGTCTAGAGCTGGGAACTTAGTTGATCAGACCACAGG

CGGTGAACTGGCTGGTGAAGTAACTTCTGGACCTGTTCAATTAGTAGATCTACAGAGAAT

ATTGAGAAGTATTCAACCAGGAGAAGGTGTTGAAGATCCAGATGGAGGGTTCGGGCTAGG

TGATCTATTAAGGCCTGATTTGGTCATGCCACTCATTGAGACATTGCCACTCGAACAAGG

GCTGGCATCCTACTTGCCTGAGGGTCCATGGACTCCCGAAGCTTTTCTGGACTTGCTTCA

GAGTCCACCATTCCGCCAACAAGTAGACACATTTACTCATGTGCTTCGAACAGGACAGAT

CGATCTGTCTCAGTTTGGAATCGATCCTAGTAAATACAAATTCACTGTGCTATCTTTCCT

CGAGGCCCTCGAAGACTCGGTCACCAAAGCACAAAGCAGTAGCAGCGGTGGTGGCGAGGC

AATGGATGAGAGCTAATCGATGTTTGCCATTATTTGACGTGTTTTCTTGGTATTCGTGGA

AGCTCGCAGTTGTTTTCCGGTTGCAGGTGGGTTCGAGAGGTAGGGACGTTATGAACGGAT

CATTGGACATGATGTTGTGGTTCAGACGATATTGTGGTGCGAAATTGCGATAAGAACGGA

TCATTGGACATGATGTTGTGGTTCAGACGATATTGTGGTGCGAAATTGCGATATGAACGG

ATCATTGGACATGATGTTGTGGTTTAGACGATATTGTGGTGCGAAATAGCGAGCTTATGT

ATAATCTTGCTGCAAGGATAGATATTGTACTGTTATTTTTCAGGTACTGTTATTCTTGAA

CCTTCGTATGTGGGAATACTCTCAATATTACAAATGTGGACATAATTCTATTTTCTCAAT

TCAGAGTTGT

>Unigene0000749

TCTAATGCCTTTCTCCTTCTGCGCCGGCTTCTGCACCACCGCCTCTGCCGCCGCCACGGC

GGCGGATGACAGCATACATCCCATGTCCCAATCCCGCACGCTCGACCTCACTACCTCCTC

CGCTTCCGACACCAGCACCTCCTCCGACAACCCCAGCTCCACCTCCACCGCCAGCTTCGA

CGGCAACGTCTCCCTCCGAACCCTCCCCTCCATCCCATCCGTCAACTCCCTCAACCCATC

AACCACCTCCGACTCCACCACCTTCCTCTGCCTCGCCACCATCAACTCCCTCACCGCCGC

CGCCATCACCTTCTCCACTACCCACCTCTACACCGCCACAGAATCAGCCCTCACATTCTA

CGAACTCACCGATCTCCGACAAGTCGGCTCCATCCCGATACCACCCTCCACCGGCTCAGT

CAAATCCATCCACCACGACGGAAAAAATATTTTAACCACACATCAAGACGGCCACATCCG

AGTCTGGCGAGACCACCTCATCGCCACTCTCCCCACCATCACCGACCGTCTCCTCCGCCT

CCCGTTCCCAAGCAGCTACGTCACAATCCGCCGCCACAAGCGCCTCCTCTGGATCGAGCA

CGCTGACACCGTCTCAGCTGTCACCTCTCGTAATGGCCTCGTATACTCCGTCTCCTGGGA

TAAGACATTGAAAGTCTGGCGTGACTCCGACCTCCGCTGCATAGAGTCCGTGAAAGCTCA

CGACGACGCTATCAACGCTGTCGCTGTAGCTGATGATGGTACTGTTTACACGGGTTCGGC

TGATAGAAGAATTAGGGTTTGGAGTAGAGTGGTCGGAGATCGGCGGCACAAGCTGGTGGC

TACCCTAGAAAAGCATTTGTCGGCGGTGAATGCTCTGGCTCTCAGCGAGGATGGATCGGT

GTTGTACTCGGGTGCGTGTGACCGGTCGATACTGGTGT

>Unigene0000750

GCCCGACTACCAAAAGAAATGGCTGTAGGTCAGAGCTCCAGCTGAATGGCTTCAAATTTC

AGCGGCGAAGGGCTTGCAATTGTGATTATGGGGGTCAGTGGCTCCGGCAAATCGACAGTG

GCCGAGATGCTTGCAAGGTCATTAGGTTGTTACTTCCTAGAAGCTGATGATTTCCACTCG

CAGGCTAATAAAGAGAAAATGAGTAAAGGAATTCCGCTCTCTGACGAGGATCGCATTCCT

TGGCTCGAAGCTCTTCGTGAGGCTATTCAGAAACATACATCCAGGGGCGAAATTGTAACT

CTCGCTTGTTCTGCTCTACAAAAGAAGTACCGAGATATCCTCAGGGCTGCAGACTTGTAC

TGCAATCCCAGGGATTACGCAAACTGCAATGTGAAATTTGTTTGTTTGGAGGCACCAATG

GAAGTAATTGCAGAGAGGATTAGGCTAAGGTCGATGGTAGGGGCGCATTTCATGCCTGTT

GCATTGCTACAATCGCAGTTTGATTTGCTCGAGATTGACGAGGCAGAAGGCATAATTAGG

GTGGATGGCACTGATAGCCTTCAGAATATTGTTGACAACATAGTAGCTGTATTCGAGTCT

TCTGGCAGTAAATGAAGAAGGGAATACACATATTTATAGCTAGCATAACCGATATATTTA

TTTTTAGATTGTCACATTATTTGATGATGGAAATACACATTTGTCTTATCAACATTTATT

CGCACTTGGACAAGAATGCATTGAGGATTCCATTGTATAAAGTAAGAACAAGTGAGATTA

GGGTG

>Unigene0000751

GAATTAAAACAAAAAACCGACACTCCGCCCGCTCCCACTCAAATCCAACCCCACCGCCCA

TCTCCCCCATGGCCAACAAACCAGACACCCCCCGCCGCGGCGGCGCCGCAAAATTCACCT

CCTGCCTCGCCGCCGTCGCCTTCCTCATCCTCACCGCCGCCGCCCTCGTCATCGTCTTCC

TCGTCCTCTTCCGCCCCAAGGACCCCCACATCCAAGTCGATGCCATCCAGCTCCCCTCCT

TCGCCTCCCGCAACAACACCGTCACCTTCACCTTCGCCCAGTACACCGCCGTCCGCAACC

CCAACCGCGCCGCCTTCTCCCACTACGACAGCACGCTGCAGCTGAGATACGGCGGCAGCC

AGGTGGGGTTCATGTTCATCCCGGCCGGCGAGATTGAGGCCGGGCGGACGCAGTACATGG

AGGCGAGTTTCGATGTCGGGTCGTTCCCGATGGCGGGCGGGAGCTTGGAGGTGGAGTCGA

AGATGAGGTTGACGGGGCGGGTGAAGGTGTTGAGGTTTTTTACGCATCATGTGGAGGCGA

CGGCCGGGTGTCGGGTTGGGGTGGAGGCTGGGAAGGGATCGGTTGTGGGATTTCGATGCT

GATTTGGACCTGAAAATTCCGATCTTGAATTGGGAATCGTGATCGTAAAGGTATTGGTAG

CAGAGTTGTGAAACTGCACTCTGCCGAGTTTGTTGATTGGGCTCGGCGCGAAGTGACCGA

GCTTGGCTTTGCTGTGCGAGTAATGATTGAATCATATATTTATGAGTATGCTTGCGCTAG

AAGGATTGAAAAAGGTATGCCTTGATAGTCAAAAGTTACTTTAGATAAAGATAATGGAAT

TGCTCTCTTGG

>Unigene0000752

TCCAGGAGAAGCAGCTCCTCTCGAACCCGATCAAATCATCAGTATTCCCTTCGTACCGGT

CTCTGTCCGTACGTTCCTCCCCACCGCCGGCGAGGGAGGGGGATCCAAGAGACGATGCGA

GTCTTACTCTACCTCCTCGTTGCCGCAGCTCTCCCGGCCGCTGCTCTCGCCGCCGGCGTT

ACTGCCGATGAGGCGAGGGAGCTCCGCGACGAGGTCCATGAGATGTTTTATCACGCCTTT

CATGGGTACATGCAACATGCTTTTCCTCTTGATGAACTAAGGCCTTTAACTTGTGAAGGA

GAAGATACACTAGGTGGTTATGCCCTCACTCTCATAGACTCCTTGGATACATTAGCATTA

CTTGGTGATCGTGAGCGATTTACTGCAGCTGTTGAATGGATTGTTAGTAATGTTCAATTT

GATATTAACAAAACAGTTTCAGTATTTGAGACAACTATAAGGATTCTGGGAGGTCTGCTC

TCTGCCCATCTTATTGCCAGTGATTATGCCACGGGCATGAAGATCCAGTCTTATGATGAT

CAACTCCTTCATTTGTCTGAGGATCTGGCTTTGAGATTGTTACCTGCATTTGATACTCCC

ACAGGAATTCCATTTGGATCTGTCAATCTTTTGCATGGAGTCGATGCAAGTGAAAGCAAG

ATAACATCAACGGCAGGTGGTGGTACTTTGACCTTAGAATTTGGAGTTCTCAGTCGTTTG

ACTAATAATCCAATTTTTGAGGAAGTGACAAAGAATGCAGTCCGTGGAATATGGGCCCGA

CGATCAAAAATTAACCTTGTTGGGGCTCATATTAATGTGTTCACTGGAGAATGGACCCAA

AAGGATGCTGGAATTGGCACAAGTATCGACTCTTTCTACGAATATCTTCTCAAGGCTTAT

TTGTTATTCGGAGACGAAGAGTATTTGTTTATGTTCCAAGAAGCTTATAATGCTGCCATG

CACTATCTTCACCATGATCCATGGTATGTTGAGGTGAATATGAACTCAGCTGCCACAGTG

TGGCCGTTGTTCAACAGTTTACAGGCATTTTGGCCTGGACTTCAGGTCTTAGCTGGTGAC

ATTGACCCTGCTGTGCGGACACATGCTGCCTTTTTTAGCGTCTGGAAGAAATATGGCTTC

ACCCCTGAAGGATTCAACCTTGCTACTATGAGTGTTCAGCATGGTCAGAAAAGTTATCCA

CTACGCCCTGAGTTAGTGGAGAGCACATATTGGCTATACAAAGCTACTAGAGATCCTAGG

TACCTGGATGTAGGACGGGATATGGTGGCAAGCCTCCAGTATGGTGCTCGTTGCCCTTGT

GGATATTGCCACATTGCTGATGTGGAATCTCACCAGCAGGAGGATCACATGGAGAGCTTC

TTCCTTGCTGAAACGGTCAAGTATCTTTGGCTTCTCTTTGATTTGGCTGCAGGGCCAAAC

AATATTGTTGAGAACGGGCCTTACAAGTATATCTTTAGCACAGAAGGTCACTTGCTACCG

GCATCTCCTGAAATATCTTTAGCTAGCGAACACTGCTCTTATTTTGGAGCATTTTGCAAA

GGTGATAAACGGCAGAAATATGGAATGAAAGATGTATTTGACCATCAAGATACAAATGAT

ACCCAGTTCCCTCCTAGTTGGTTTCACTCGGTTCCTCCACTCAAACTGAAAGATACATTT

TCATCGTCAGGTGTCTTTAAGCAGGGAACGTGTCCAGGATTGACACATGGGCAGAAATTC

GGCATTTCTTATGGTGACGATATCGAAAAGGATCGCGACGAACCAAGAGAACCCCCTCAA

AGACATTCCGTTGTTGTTATCTCTGATTCAAATACGGAAAATTCAAGGGATACCAAGGAA

ACTGGAAGCAACAACATTCTAGAAACAAATGAAGAAACGGAAACAATTACATATCTCCCG

GACAACAATGCAGAATCAGTTAATCAAGAAGAATCTTCGAGATAGCCGTCATCCCATCTA

TCCCGTCGGGTGTGTCTATTATTAAGACGACAGAGTTCAAGTCTCTCGATGTCTCTGAAA

TTTTCCCCATATCAGAATCAGTCGAGGCGCTCATACTGTTGTCTTTTGAGCTTGTTAACA

GTACTGTGAGCAATTTTGGCCGACTGCAGAATCTTAATATTTTGTTTTGGGGATGAGGTT

GCAGAATGGCAATACATTTTCCATATTGTAACACTGTTTAATAATAAGTTCAATTTATTG

TAAATCTACGATTAATAAAAAAGAATAATGTGATTACAATGTCCTTCATTTGG

>Unigene0000753

ATACGAGCGTTTGCCTTCAGATCCGCTAAGGTATACAAAATCGCAGATGGCGGGAGGATT

CAGGGTACTGCACCTCGTGCGGCCGTTCCTGGCAGTGCTGCCGGAGGTGGAGAGCGCGGA

TCGGCGGATCCCGTTCAGGGAGAAGGTGATCTACACAGTGATATCGCTGTTCATCTTTCT

TGTGTGCAGCCAGCTGCCGCTGTACGGGATACACTCGACGACGGGCGCAGATCCGTTCTA

CTGGATGCGTGTGATTCTCGCGTCGAACAGGGGGACGGTGATGGAGCTGGGGATTACTCC

GATTGTGACGTCGGGGCTTGTGATGCAGCTGTTGGCGGGATCGAAGATTATTGAGGTTGA

TAATAGTGTGCGTGAGGATCGGGCGCTTTTAAATGGTGCGCAAAAGTTGCTTGGCATACT

AATTGCCATTGGAGAGGCAGTTGCATACGTTTTATCTGGGATGTATGGTAGTGTCAGCCA

ACTCGGTGCTGGTAATGCTATTCTTATAATCCTGCAGCTATTCTTTGCTGGTATCATCGT

AATCTGCTTGGATGAACTTCTCCAGAAAGGTTATGGACTTGGGTCTGGTATTTCTCTCTT

CATCGCAACCAACATATGTGAAAATATCATCTGGAAGGCATTTAGCCCAACTACTATCAA

TACCGGGAGAGGTGCTGAATTTGAGGGTGCTGTTATCGCTTTATTTCATCTTCTAATAAC

TCGAACAGACAAGGTCCGAGCTCTCCGTGAGGCCTTTTACCGGCAAAATCTACCAAATGT

GACGAATCTGCTTGCTACAGTTTTGGTCTTCCTTATTGTCATTTATTTCCAAGGATTTCG

TGTTGTTCTGCCTGTTAGGTCAAAAAATGCCAGGGGACAACAGGGCTCCTATCCTATCAA

ATTATTTTATACCTCCAACATGCCCATAATCCTTCAATCTGCACTGGTTTCCAATTTATA

TTTCATTTCACAGTTGCTATACAGGAGGTACAGTGGAAATTTCCTAGTTAACTTGCTGGG

TAAGTGGAAGGAATCTGAATACTCTGGTGGCTCCATCCCTGTTGGGGGTATTGCTTACTA

CATCACAGCACCGTCAAGCTTGGCAGCGATGGCCGCCAATCCATTCCATGCACTCTTCTA

TATAGTTTTCATGCTCTCAGCTTGTGCACTTTTCTCAAAGACATGGATTGAAGTCTCTGG

ATCGTCAGCAAGAGATGTGGCTAAGCAATTAAAAGAGCAGCAAATGGTGATGCCAGGTCA

TAGGGAGTCGAATCTACAGAAGGAGTTGAACCGCTACATCCCAACTGCAGCTGCTTTTGG

CGGCATGTGTATTGGTGCTCTGACTGTACTGGCAGATTTCATGGGGGCCATTGGTTCCGG

AACTGGGATACTTCTGGCCGTGACCATTATATATCAGTATTTCGAGACATTTGAGAAGGA

GAGAGCAACTGAGCTTGGTTTCTTTGGTTTTTAGAGTCTTTTATCAGAAAGACAACTACA

AGGAGAGGCAATGTTTAGTTTTGGAGGGATGGACGAGATGTAGAGCTCTAACACATCAGG

TGTAGAATTGGAAGAGGTGTAGAATCGGAAGAGGTTTAGTTTTCTTCACAGTAGCTTCAA

AAGCTTCAAAAACTGTTAAATTATACAAGCTCAGTCTGAAGTCAAAGAGGGTGTTACTTG

AAGAGGTTCTACTGAGTTTATATTAGTTAGGTTCGAACTTGGTAGGAAGGAAGATGTTAG

CCCATATTTGGCCCGGTTACTTTAACTGCTGCTTGGCATTGTTATTTATGTTTTTTTATT

TTTGTTTTATGTATTATCTGAAGAGGTTTTTAAACTACATTGAGATCTCGGAATGGTTAA

CTATTTTGAGCTATTGTTTTTACCTAACTCATTCTCATACTGG

>Unigene0000754

CTCCACCAAAACCCTAATTCACTGTCATCCCCAGCCCTAGCTTTCGCCCAGCGAGATCCA

TCGATTGATTGAGCGAGAGTAGGAATGGCGGCTCCGTCCGGGCTCAAGCAGCGGCGCCAG

GAGTTCCCTTCTCTCTCGCTCACCGACCTCTCGCCGTCTCCATCTGCCCTGCTGCCGCAG

GAGGCGACCGGCTTCTTCGGCGCCGATCCCTTCGGCCGTCCCCAGCTGTCTTTCCAGTCA

ACCGGCGTGGCCGCCCCGGCAGTAGGATTCGATCTCCATGCTACTCAGTTGTTCAAATTA

GGCCCGGTGGACTCGCTATGCGTAAGCGAGGAATTGGGGACTGGTGATGAAGAGCTGTCC

TTTTCACGAGCATATAAAATAAACTTTAGACTGGAGGATGAGCAGAAGGCCTTTCATTCA

GAGTTTGAGCATTGGAAACATCGAGTTATTGTTGCTAGCACTGGATCTTTGGAGAATGGT

TCACTGACAACACCTAAAAGCAAATTTGATGATAAAATAGAGGCCACTTCTTCCAAGATG

TACTTCCATTACTATGGGCAATTGCTCCATCAACAGAATATGTTGCAAGATTATGTGAGG

ACAGGAACCTATTATGCTGCCGTCATTGAGAATCGTATAGACTTTCAGGGCCGTGTTGTG

GTTGATGTTGGTGCTGGTAGTGGTATTCTGTCATTATTTGCTGCCCAGGCTGGTGCGAAA

CATGTCTATGCTGTGGAGGCATCTGAAATGGCAGAATATGCACGCAGGCTAATTGCTGGA

AATCCTTCACTCAGCCAAAGAATCACAGTAATTAAAGGCAAGGTCGAGGAAGTTGAACTG

CCTGAAAAAGCAGACATTCTGATTTCTGAGCCAATGGGTACTCTATTGGTCAATGAGAGG

ATGTTGGAGACATATGTTATTGCAAGAGACCGCTTCCTTGTACCAAATGGGAAAATGTTT

CCGTCAACTGGAAGGATACATATGGCTCCATTTAGCGACGAATATCTTTTTGTTGAAGTT

GCAAATAAGGCCCTTTTTTGGCAGCAACAAAACTACTATGGTGTTGATCTTACTCCACTA

TATGGATTTGCTTTCCAGGGATATTATTCCCAGCCCGTGGTAGATGCTTTTGATCCAAGA

TTGTTGGTCTCTCCTTCTATTTTTCACACACTGGATTTTACTTCCATGAAGGAAGAAGAG

CTATACGAAATTGACATACCACTGAGTTTTATAGCCTCCGTTGGAACCAGGGTGCACGGA

CTGGCTTGCTGGTTTGATGTTTTGTTCAATGGAAGTTCTGTTCAAAGATGGCTTAGCACT

GCTCCAGGAGCTCCCACAACTCACTGGTACCAACTGCGGTGTGTCCTCTCCCAACCATTA

TATGTCATGGGCGGGCAGGAAATCACTGGGCACCTGCACCTTGTGGCGCATAGTGCTCAG

AGCTATACAATTCACTTGACTATGTCAGTGAAAATGTGGGGAGCTGGTGCCGATCAAGGA

GGAATACTACAAACTTCTTCTTGCAAATATGATCTTAAAGAGCCATACTACCGGTTATCA

CAACCACAACCATATGTGGCGGCACAGGATCAGCAGCCCCATCAACAGCTTATACAATCT

CAGGATGTGGGTGGAAATGGAACAATGTAACAATCATAGAATCAAGAATCGTTGAACTGC

TAACATCCAAGACTGAATTAAAAATGTGATTTGTTTGTAAGAGTGGTAAGCTGTTCTATG

TTCTAAACAGCAGATTTGTACTGTAAATGGTCAATTTAGCGACTGTTTGCAACTGGAATC

TGTAGATTATTTATCCAATATAATTATTGGAACTTTGTTTATCAGTGCGCACCTGGTGTG

TTAAAACGGCGGATATGTTTTTTCTTCCCGGGGTTGAATAATCAAAATTCATAGCGGATC

AGAAATTTTTGCAAAACCAAATATAATTTGGAGTTTGTCCTGATTTGACAAAAAAACTGG

ATTATTAGTAAAGAATCCAAATAAAATATTCTCTGGTGTGTTAAAACGGCGGATA

>Unigene0000755

ATATGTTTTTTCTTCCCGGGGTTGAATAATCAAAATTCATAGCGGATCAGAAATTTTTGC

AAAACCAAATATAATTTGGAGTTTGTCCTGATTTGACAAAAAAACTGGATTATTAGTAAA

GAATCCAAATAAAATATTCTCTGGTGTGTTAAAACGGCGGATATGTTTTTTCTTCCCGGG

GTTGAATAATCAAAATTCATAGCGGATCAGAAATTTTTGCAAAACCAAATATAATTTGGA

GTTTGTCCTGATTTGACAAAAAAACTGGATTATTAGTAAAGAATCCAAATAAAATATTCT

CTGGTGTGTTAAAACGGCGGATA

>Unigene0000756

CGCCGGCTCCGGCCGCCATGTCCGGATCCAAGGTACGGATTCCCCTTCTCCTCCCCAATC

TATCTAGGGCTTCAATTTCGATCCCCCCATCCTCCTAGGCTTCAATTTCGACTTCCCTTT

CCAAACCCTAACCCTAGATCTCACAGGAAACATCACTACCCGAGCTGAATCACTCAAAAC

ACTCTAGTTCTCTCCGATTCGACAGAATTTGAGATCTGAAAGATGGTTTCTTACTGAGAT

TTGGGGGTGGGGGACCTGTAATGGCCGATGAGGGAAATGAGAGCAGGCTTCGCTGGATGG

ATCTGAATCTGTACCTAGGTCTCCCTCGATCACCCCGCCGACTTGTCCTTGACGATCTTG

GCTCCGACCTCGCCCTTGGCTCTCTCCCTGCTGCTGCCGCAGCAGGCTCCGGCGACATGC

CTGCCGCAGATGCGGACATATCCGTCGTGGCGGAGTCGTCGGACCCGAACCCTCGCGACG

CCCCTTACTCCCCCACTCATGCCTCGTACAATCTCGACGAGGACAGAATCTACTCCCCTA

CTGATCCCTCTTACCACCCGGAGATGCGTGCAGCTGATGACCATCCCGGGGTAACTTACG

AACCGGTGGTGCTGGGGTTCGAGCCCCCTGGCGGGCTGTATTTGCCGTCGCAGGCTTTGC

CGACTGTCGAGGAGAATGATGAGCTTCAAATGTCGCCGTATGTTCCGATGTCTCCCTTGT

CTCCCTTGCCGCCAATGTCTCCGCCAATGATGATTGACAGGGCTTCACCGCCGCGGTCCC

GTCCGCCGCACAGGCCTTACATGCTACCAAATGTGCCTTTTCCATCAAACCAAAGGAGCT

TCGAGGACACTTACTTCGATTTTGGCCACTTGACCACTCGGGAGCCGGAAGTTGGGTCGT

CTTCGCGGGAAGATCGCATCTCGTCTCCCGAGTTTCGTTTTGCTAGGCTGATTGAAGTGA

ATGAGCGGTTGCAGCGCCGACGGTTTTGGATGTCGCCTACTCGGGCTGATTCCGGCCTGC

CCTCTTCACCTGCAACTGAACCGCTTGAGTATGGAAAACCTGAATCACCTGCTAAGCACG

TCGTGACAGAGGTTGAGGACGAGGAAACAAAAGAGAAGCTCAAGGCTGCAGCTAACTTCG

AGTGCAATATTTGCTTGGATATGGCAGTTGAGCCCGTTGTCACTACATGTGGTCATTTGT

TTTGCTGGCCGTGTATCTATCAGTGGCTGAATCTCCATTCGGATCATCGAGAATGCCCCG

TGTGTAAAGGCGAAGTTGTTGAGTCCAACATTATACCTATCTATGGGAGAGGCAACTCAG

AGTTGTGTGATGATAAGAAGCTCGAAAAGAAAGAGGAGGTACCTCTGGAGATACCCCCGA

GGCCCCGTGGGAATCGATATGAGAGCTTCAGGCAGCAGTTTCGACCCATTTCGAGGAGGT

TGGGTGAGGGGATAGCCAACTCTTGGCGGCGTCTTGTGGATCAAAGGTTACGTAACAGAA

GCAATGTGGACTCGTCCTTGGCGGGAATGTATGACATTGCTTATCAGCGAGCACCACTTA

CTCGAATGAGGGCAAGGAGAATCCAGAGGGAAGAGAGGATTTCCGAGACTCCATCTATTG

CTAGGGATAGTGGATTGCTGAGAGATGGGTCTTCGATCCCGCATGCTAGTAGTTCGAACT

TACATTTTCGAGATGGAGATCCTTGGCGAACGTATACTCTTCATAACCTTTCTACTTCCG

AGAGATTGGCAGCTATGGCTGTGGACCTAACTGGGGTTGTTGGGAGGTTTGGGAGTAGTA

GCAGCAACCTCTTTGCTGCTTCGACGTCAGCTGCCAATCTTCAGCCACCGGAGCTTCCGG

TCGTCAGTGCTCCTAATCCTGCATCTTCGGCTGCAGAACAGGCGTCTGCATCAAGTACTA

TGGCTGTTATACAGGGAGATGCCACTGCTACTGATGCATCCGTGGAACCAAACAGTGCAG

GGTCATCAAGGATCTACAGAAGAAGAGGAAGAAGCAGCGGCTCTGGTTCTTTGGATATTG

ATGGTGTTCAGCATGGAGATGCTGCTGCCACTGGTGGACCTGTGGAATCAAGTAGTGCAG

GGTCCTCTAGGATCTATAGGAGAAGAGGAAGGAGCAGCGCCTCGAGTTCTTTGGATGTAG

ATGGAGGATCTATGCATGGGCGTAAAAGAAGAAGGCTAAACTAGATATCCGTGAGCTTTG

TTGTTCAGAATAATTTGGCGTTCTTTTGTTTTCTAATGCATGTAAGTTTAATTCAGATGG

ATTTCTGTTTTAATTATCATGTCATCGACAATGTTGGATAGTTATGAGTTACTTTTTATG

TTTTATTTTCTTTGATGATTACATACTCCGTCGGACATGTATTCGAAGACATGTATGGAG

GTACTTCCGACAACCTCGTAATTGGTCTAGATTGTTTTATTATTAATTAACATTTGGGAT

GGTCATTTGTGCAGCAAGTTCAGAAGAATCTATGTATTATGTAGATGTCATCATCATCGT

TGTGTATGTGGTTTCTTCTCTGTTCAGTTTCTGTGACGGGCATGAATTGTGGAAGACAAA

CTTATCATTTGTTTGCAATTTGTGATAATTTGTTCAGTCTGACTGCTCGATCATGGATTT

TATTTCGATGAATCCATGTTTAATGTATTCATGCCATTAATATTGTTGGCTAGTAAAGAG

TTGTTTCATATGTTATGTTATCTTTGCTGTTCAGACACTCATGTATTTAGGAT

>Unigene0000757

CCCGTACGGTGGTGGGTTTTAGGTCGGAGTGGCGCTCGCCGGATTCTGATCATGATTGAC

GAGGAGGACAAAGAAACAATGCAAAAAACCCATGAGGCATCCCTAGGGTGCCTCAAGATT

AAGGCTTCTCACCATGTCTCTCAAGAAAGTGGGAATGCCATTAAGGGCTTTCCTAAACCC

GGGGATGAGCTAAACTTTCATCAGAGTAATATTGTGGAAGCAAATAGTTCATTTAACAGG

GGAGCCTTGGCTTCTCCAAATCAAGGTTATAATAGGTCCCTCTATCAGCAAGAATTTTTT

ACCATGCCAAACTACATTGATATTCAAATGCTGCGCCAGAACCAGCTATATCCGATTGAG

AACCAGTTCTATCCAATGAACTTGGAATACACTTATCCAGTTGAAAACACAATTCAATAT

CTGCCTTTCAAGATGTTTTCCCAAGCTCTCCCTCATGAAGTGCAATATCAAGAATTTCAG

TATTTTGTTGTTATTGACTTTGAAGCAACTTGTGATAAGGAAATGAACCCAAACCCTCAA

GAGATCATTGAGTTCCCATCTGTTATAGTGAACAGTGCAACCGGGCAGTTGGAAAGCTAC

TTTCAAAGATACGTGCGACCTGCCTATAATCAACATCTCACTGATTTCTGCAAAGAGCTG

ACTGGTATACAGCAAACTCAGCTCATCAGCGATGGGGCATTGCAGACTTACAGTTCTCTC

TTATGGTCACATCAAAGTTTTTACTGGATGTCACCTTCCTCAAACTTTGAAAAGGTTGAC

AGCGGTGTTTCTCTAAGCGAAGCTCTACTCATGCACGATGAGTGGCTCGAAGCGAAAGGA

ATCAAGCACAAGAATTTCGCAGTCGTGACATGGTCTAACTGGGATTGTCGTGTGATGTTA

GAATCGGAGTGCAGACTGAAAAGGATCAGAAAGCCTCCATACTTTAACAGGTGGATCAAC

CTAAAAGTTCCGTTCCAGGAGGTGTTCGGTGGCGTGCGCTGCAATCTGAAGGAGGCAGTC

CAGCTATCAGGCCTCGCATGGGAAGGCCGTGCCCACTGCGGCCTTGACGATGCCCGTAAC

ACTGCCCGCCTCCTAGCCCTCCTCATGCGCGGTGGCTTCAGGTTCTCCATAACAAATTCT

ATGATCTCGCAGTCTACTACCGACCCCACCCAGCCGCCCCAGAAGCCTAAGGAACTAATC

TGTCCCGCTGTCCAAATCCCTCACCCGTTCATCGACCCCATCTACTGCTTCTGTGGGGTG

ATGAGCATGAAGTGCATGGTCCGCAAGCCAGGGCCCACTCAAGGCAGGTGCTTCTTCGGG

TGCGGGAACTGGACTGCTGCTAGGCGCTCGCTGTGCAACTACTTTGCTTGGGCATCTCCT

TAAGAAGGATATGGTTCGATGAATCGAACCAGACTTGTGTACAAAAATGAATGGTTGATT

AGTTGGTCATGTAATTATGGGAGTAATAAGGTGTGGATCGGATCGTATGTATATCTTATG

TCGAGTAGGGTACCTCTTTGCTCATTGCCATTGGGAAGTCTGTAGGTAGAAGAGTGTCTA

ACCTGTGGAACTGACTTCTTCTTGACAGAAGAGGGGAGGGGGGCTGCTTAGGATCATGCT

TCATTAACTTAAATAATGTTGTGGATTTGGTTTCTCTTGCTTTTATTTAATGGTGTTGTG

TCAAATGTGCTTTCTCGGTGCATATATTTGGTCTTTATTTGCCTGGATTATTCGAT

>Unigene0000758

GGCCAAGAACAACCAGAGCGCCGCCGCCTCCTCCTCCTTCCAGCCATCTCCTCTGTCGGC

GAGAAGTCAAGAGGGTCCCTCGTACAAGAACTTCAAGGCTGTGAAGAAGGTGGGGGACAA

GTCTGAGGCTGTGTTTGTGAACAGGATGGAGAAGGCGACTGAGGACATCAACGAGAGCGC

GGACGCGTTCATCCGGCAGTTCAGGCAGCAGCTCCAACGTCTCGACTCCATCGAGAATTA

CAGGCAGATGCTCGCTAGAGGAGTTTAGCTCTCTCATGTTTCGTGTTTATTGGGTGTACT

TAAGCTTCGAATTAGTTTGATTTTCATATTGTCTCGTGAAATGATTTGTATGCTTGGAAC

TATAATTATACAAGATGGTATTTG

>Unigene0000759

ATGCTTGGAGCCCTAAATGAAGTTAATTCTTCAATCGACAAGTCTTTTTCAGTCATGCTT

AATGCTTGGAGCCCTAAATGAAGTCAATTCTTTTTTTCACTACCTTACTAGTATTTACAG

ATCAATATTTTACTCTTTTCATCTTGTCGGGTTTATTCTCCTCGAGCATACATTTCTCTC

ATTTTATTTTCACCCTTATTTTTCCAATTATCATACCAATCACCCTCACCGCCATAACAT

CGCTTATTATTTGTCAACCTACAATGGTTGGCCTTACATATCGAATCATTTCTAATCATT

ATGTCTATTCAGTTACGTCAATTTTAAATTCAACATTTTAAAGTTTCTACTCACAAGTAA

TCACCATAATTTCGTGTTATCATTAATAATTATAGTTAATGTTGAGTTTAGTCCCACATT

GGTTAGGCCAGACTTACAAACCAACATAAATATGATAAGGGGCTATTCCACC

>Unigene0000760

TTGGAATTTCCACACCTTCCAGAGAGAGTTATCATTCTGACTGGTATTTATAACATGCCT

TAGCCTAAAGGTAAGGTATTGATTTTTTTATTATTTCTTCATGCATCGATATTGTGTCAT

CCCCAGAATTGAGTCTTCTTTTCTCTCCCCACTCACCCGCTCCAATCGACAAGTCTTTTT

CAGTCATGCTTAATGCTTGGAGCCCTAAATGAAGTTAATTCTTCAATCGACAAGTCTTTT

TCAGTC

>Unigene0000761

CATCTGACAGAGATACATGATTCATAAGTCATTTTTGGAGAACTTTATGTTCAAAATTGG

ATTTATACTTTAATATTTTAGCACCTATTATCTTCAGATTGATGATAAGACTGAAGTTAT

TAATCATAGTATAAGAAATACTACATTTCTTGGCTATAGAAAGACCAAAATGGGGGATTT

AATATTACCTCAAGCCGAATTTGTATTAACAAAATCCATTGGTAAGTCTTTTTTTGATGT

CATATATGGCCATATTGTGCCAAACTTCTTGGATACAAACCTTATACCATATGTAAGAAA

AGAAAGGGCAAGTGCATGCGGCTTTATTGTGCACGGAAAGGTAGTACAATGGTAGACTTA

TGACCGATTGAAGGTTGCCATAATAAAGTGTAAAGAGACAGTCGACCAACACCGACAATA

TTCTAGTTCCAAGATGAAGATTGGATGGTGGTATAATTGGGAGGGATCATCTCACTTTTG

GAAATTATAGGAAATTAAGCCAAATAATTTTAGACCCCTTTGTATTCTAAAAATGTTATG

ATCCAATGCTACTTGATTGACCTAATCAATGAGATTCTGACCTCACCCATCTTCAATGAA

ACAAACTTAAACACCTTAATATGGCAACACACATTCTACAACCATAGTTTTAGAGTTACC

TTTTTTTATCATATGAGGAGCAACACAAATTGGATTAAAGAGATTGTTGACATCAAAGAC

ATTGCTACCATATGAGGTATCACTTTATGATTCCCGGGTATGTTGGCAAGGGAAATCACT

TTCAGGTAGTTCTTAGATTAGTAAGGCTGCTATGACAGAACTATGTCATCACTGAAATGA

TATGTTTCAACAATATACAAAATATTGACGTAATTTATTCAAGAACATCTTTTGTAGTTG

TGTTCCCATGACACAGAGATAATTGCCTATGATATAATTAGATAATAAATTAAGAAACAT

GGAATATGTAGTCACTGTGAGGTTAAATACCCCCACAACTCCTAGGTCAGCTCAATAGGG

GGCGAGTTTTTCTTTGAAGGCAGGAATTGATGTATCTTGTCCGGATAGTCACTTATAAAA

TCATATATGGATGGTATAGTGGCATTTCTTCAAAATTAACTTATAGCTAGTTTGGGAAAT

TCCACGTCTTTCAGAGAGTTGGGAATTGATGTATCTTGTCTAGATAGTCACTTATAAAAT

CTTATATGGATGATACATTGGTATTTCTTCAAAATTTAACCTACAATCAGTTTGGGAAAT

TCCACACCTTCTAGAGAGTTATCATTCTGACTGCTATATATAGTTCCAGACTTTTCAGAG

AGTTCATTTGTTTGGAATTTCCACACCTT

>Unigene0000762

CTTCATCAAAACCTCCTGTAACGCCACGACATATCCAACTCTCTGCTACTCCTCCCTGTC

CTCGTACGCCCGCAAAATCAAGTCCAGCCCCAAGCGGCTCGCCGACACAGCGCTCTCGCT

GAGCCTCGTCGAAGCCCGCACGATGTCCGCTGACATTAAAAATATGTCGGCAGCTGGCGG

CATGACTCGTCAAGTGCGAGCAGCGATGAAGGACTGCGTGGCGAACGTGAGAGACGCAAT

GATCGAGCTCAAAAGCTCGT

>Unigene0000763

ACTCACCATTATTATCTTAAGTCCATGACAACTCATATAATGCTTTCTCCTCTTTCTCCC

ATCTTATTCTCTCTCCTCATCCTATCCTTCTATCTACCCTCAATCAATGCAGCAAGTGTC

ACCCCTGGCCATCCATCAAATACTGACTTCATCAAAACCTCCTGCAACGCCACGACATAT

CCGACCCTCTGCTACTCCTCTCTGTCCTCCTATGCCAGCAAAATCAAGTCCAGCCCCAAG

CGGCTCGCCGACACAGCGCTCTCCGTCAGCCTCAACGGCGCCCGCTTGATGTCGGCTGAC

ATTAAAAATATGTCGGCAGCTGGAGGCATGACTCGCCAAGTGCGAGCAGCGATGAAGGAC

TGCGTGGCGAACGTGAAAGACGCAATGATGGAGCTCAAAAGCTCGTTGTTGAAGATGGGG

CATTTAGAAGGTAGGGATTTTCAGTTTCAGCTAAATACTGTGCAGACATGGGTGAGTGCT

GCTCTAACTGATGATGAGACTTGTTTGGATGGGTTTGGAGGGAGTGCTACTGATGGAGCG

GTGAAAAATGCTGTTAGGGGCCAAGTGGTGAGTGTTGCTAAGCTCACCAGCAACGCTTTG

GCTCTCATAAATTGTCTCGCTTAATTTGGAAAAATGTTTAATGTCCGTGTTTTGAGGTAG

AATATGAGGTACGCAGGATAACGTATGCGGCTGGTAGGGCCTAGTCCTGTATGGAAAATT

AGAATTTCTCCGATTCCAATTTCATGACATGAAATGGATATTTTCTTTGAAATCAATAAG

GACCCGGAAGTAAGAGAAAAATTACTCCCACTATCAAGAAAATAAACAAAAATATTTCTT

TTGAATAAGTATAATGTTTTTCT

>Unigene0000764

TTGGAGGGAGTGCTACTGATGGAGCGGTGAAGAATGCTGTTAGGGGCCGAGTGGTGAGTG

TTGCTAAGCTCACCAGCAACGCTTTGGCGCTCATCAATTGTCTGGCTTAATTTGGAATAG

TGTTTTGAGTTTCGAATCTTGAGGTAGACGATGGTATGCAGGGTAACATATGCCGTTGGT

AGTGCCTACTCTTGTATGGAGAAATTATATTTCTCCCATTCCAATTTCATGACATGAAAT

GGATATTTTCTTTGAAATCAATAAGGACCCGGAAGTAAGAGAAAAATTACTCCCACTATC

AAGAAAATAAACAAAAATATTTCTTTTGAATAAGTATAATGTTTTTCT

>Unigene0000765

GAATACGCAATGTGGACAGATCAATCTGTTAATCTGGATCTTAGAGTCGGCCCTTACCAG

TACCCCGGTTAGATCACTGGAAGATGAGTTGAATCGGCTAAGTGAAGAGAACAAGAGGCT

AATTGACATGCTGGGCAACTTGCAAGTCATTCACACTGCTCTCAGGAACCATATGATGGA

TCATATGAGCTTTGAAAAATCTGAGGTAGTTCCTATCTCGCCAGGATCGAAGAAGAGGAA

GAGAGAAAATATTGAAATGGTAACCAACAAAGAGGAGTTGAATAACAAGATTGGCGGCTC

CTATCATCAATTTGAGAATCAATCCATTACAAATTCAGGCCTGGGACTGAGCAACGAAGA

ACCCAGAGCTAAACTTTCTAGGATTTGTATTCGCACCGACCCATGGGATTCGAGTCTGGT

TGTGAAAGATGGGTACGAATGGAGGAAATATGGACAGAAGGTTATACGAGATAACCCGTC

TCCTAGAGCCTACTTTAAGTGCTCCTTTGCTCCTTCATGTCCCGTTAAAAAGAAGGTGCA

GAGGGCTGCGTATGATAGATCAATCTTGGTAGCAACCTATGAGGGAGAGCACAACCACGG

TCAACCCTGCTCGAGTACCATAGGTCCCGTCCCAAGCTTTATCTCCATCAGTCCTTCCGA

TCGCATGATCACTCTCGACTTGACGGGACCCGGATCCTTCAAGCCAGAAATTGAGGAGTA

TATTAGAGAGATCAACTCACCGCAGTTCCAACAACTAGTAATGGAACAAATGGCATCGTC

TTTGAGGGAATATCGAAGTTTTACTGCTGCTCTTTGACGAAAGATCGAAGTTTTACTGCT

GCACTTGAAACTGCAATCTCTGGGAGAATTTTAG

>Unigene0000766

CCGTCGGTTCCGAGCGCATTGTCTGTCCGCCATCGTTGTATCCATACATACCAAATCCTT

TATACCGGGCCAAATTTTTCTCTAGTTTTTTCTAGTCTTACAAGCATTGTTTTACATGTT

GTAATATTTCGTTTTTTATACGTTTTGCGAACGCGATTCTCGTCTGATCCGCTGAAAAGA

CTCGACGCACACGCCTAACCCATCTACCTCATCGACCCTGCCGAGTACGAGTACAGCGTG

GTGTCACGCCGGTACCTTATAGAAGTTCGAACGGTGTCGATAATAACCGAAAAGCCGACG

CCATGGCCGAAGTCGACGATGCTGTCATCGACGTCGTAGCGTCCGAGGTGATCAACGGTT

GTACCATCACCGGGGACAGCGACTATTCGGACAAGGCTGTGGACCCGACGTCTGTGGAGA

TGCGCGTCAAGCGCAAGGCCAAACGGCTGGCCAAGTCCCTGTCCCGGGACAGCGGCATCG

TCAACGGACATGGCGGCACCGCCACTGTGGTTCAACTCAAGAGGCGCTGGAAGAACAACA

GAAAGTCCAGGAACGGTTTCACCAAGAGAGGAGAAATCAAAAAAGGCGGTGCTGGTGGTA

AAGGCGTTTGGGGAAAGTTAGGTGAAGAAAGTGATTTGGATGCCGCTATTGATATGAAGG

ATCCTAATTATGATAGTGATTTGTTGGATGATGATAATATTGTGTTACGAGAAATCACTC

CAGAATCATCTGATGAAGACCTCAAAAATTCCATTACATTTAATATTCTTGAGTATTATG

AACATGGTGATACTGATGAAGCAGCAATGACATTAAGCGAGCTAAACATAGTTAGCAAAT

GGCACTTGATAACTCAAGTTTCCGTTGAAGTAGCTCTCGAGCACAAACCATCACAAAGAG

AGATGACATCTATCTTGTTATCTGATTTATATGGGCGTTTAATCAAGCAAAAAGAAATAG

CCCAAGGCTTTGATGTCATTTTGGGTAATCTTCCTGATCTCATTCTTGATACTCCCGATG

CTCCTATTGTTGTTGGCTGTTTCTTAGCCAGAACCATCGCGGATGACTGTCTCCCACCTA

AGATTATAGACTTCTTCAAAGAGAAAAACTATAGTGATTTAGCTAATCAAGCGTTGATAA

AAGCCCACAACTTGTTGAATATAAAACATGGATTGACTAGACTTGATAATGTTTGGGGTG

TTGGTGGTAGTTTAAGGCCAGTACAGTATCTTGTGAGACAAATGAACATGCTTTTGGATG

AATACTTATGTTCAGGTGATTTGCAAGAAGCCATTAGGTGTATTTTGGAATTGGAAGTAC

CACATTTCCATCATGAACTTGTTTATGAGGCTGTTGTTGATGTTATTGAAGCTATGAATA

CTCATACCGAAATATCTATGTGCAAGTTACTTAAAGCGTTATATGATGCTATTATTATAA

CCCCTGAAATGATGAATAAAGGTTTTGATAGAGTATTTGATGTTCTGGATGATATATCTA

TTGATGTCCCATTAGCATCTGCTGTCCTTGAACGTTTTTTGGATATGTGCATCAATGCTG

GATTTTTAGAAAGAGAAGTGTTGCGTAAAATACCAACAAGAGCATCGGCTCGTAAGCGGT

ATGTATCTGAAGGTGACGGTGGACGCCTCAAAGAAGCATAAAAACGAAGAAAAGAACCAT

TAAAAAACCTATACTCCTCTTCTCCTCTACCTCTAGAGTACAGTATTTTTTTTTCTCAAT

GTTTGATTTGATATAATTTTTTTTTGTCATTGTCATTATTTAATAAGATTTTAATTATAA

AATATAATTATTTCATTATAATTACAATTGTCCTTAGCATTACAAACTAATTTACATATT

CCAACTCTATTTTTAGTAATGTAAGTAAAGATATGTTATGCATATGTTGACAAATCTATT

ACTTAGCTGTATTATTATGCCAGCTAAGTTGTTGCTTATCAATAATAATAATAATAATAA

ATTAATATGTCATGAGTTAAGTATTCAAAATCAGCAAAATCTGTTTCATAACAATGCAAG

TAATAAGAATTAATTATTTATTATAATATTATATTCATGTTTGACATATTAGTAAATACT

GTACTCATTAGCATCATATAGGTTGAACGAATTAGAGAGTTCTTTTATATATTTAGGTTT

ACGTTAAAAAAAAAAAAACAATTTCTTGATTATAACATTTTTTATTTTTATTTTATTTTT

TTTGTATAATTCATTGGCTTATTATGAGATAGTTTATGCAAAGAATTTTTTCACTACTTT

TTCATGTAAATTTTTTTGACATTCTAAATTAACTATTTATTTGTGTTACTATGAGTGTAT

TTTCCAGATTAGTTCAACCATTTTCTAAAACATTTATAATAAAATTGTGTATTCTGTTCA

GTACAAAATTATATAATATAGATATTATGTAACATTCTTAAGCAATATTTTTTAGTATTA

TACAAATAATACATAATTCCTAGTGACCATTATGTATATCAATAAAATAACTGTAATTTA

TTATTATCAGAATAATATTAACTATATTGTACTCGAGTTAAGAATCATACTTGGCATGGA

CCAGTGTTTGTCTCCTTCACAGAGTCAAATGCCTATGTGCCTGTCATATATGAATGTCCT

CACTATAAATCAGCTATCAAGTATGGTTCTTAAATAGAGTTAGTTGCCAACATATTTATG

TTCCCTAAAAGCTGAATTGTCTGTTTAATTTTTTTTTCAAAATTAGGTGCATTTTAAAAC

ATTTTGTATGTTTGATATTTATGTTATATTAATAATATGATTTACATTTTATATTTTACT

ATTCAAAACAAAAAGCAAAAAAAAAAAATGTTATTTGATTATCAGTTTTATCAACTTATA

TAGGCTAGTGATTTTTCTTGAAATATTTTTTTATAACCAATTTAAATTTTATTTTAAAGC

ATATTATGTAACTTAAAAACATTTACTAAAATTATTTTTTGAAAGAAAAATTATGATCAG

TTTTGTAATTTATATGTATCTTTTGCTCATGGTAGCTCTGTATCTGACTTAATATTTATT

TTTCTTATTTTAGAATAAAGAAAAACTTAACTACAATAAAAGTTGA

>Unigene0000767

GAGCACGGGCAAGGTCAATTCACGAGCCATGAAGATATATTCAGAGGGTTCCAAGACAAC

ATGCTTCACATTTCTTGTCATGTTTGTTATGATCTTTATCTTCATTATGGTAGTTCTGCT

CATCCGTGTAACTTGACACACTTCAGTTTAACAAGACAGGTGTAGTAATCATCTGCCTCC

ATCACTAGTCATTCCATTGTATATTATTATTCTCTTACAATGTTTAAAACAATTTCTTAT

CCACAAACATAGATTATATACAAACTTTTCACAGGAC

>Unigene0000768

CTTCTTCCTCGAACCCTGGACTCTCGTCTCCTTGGCGATTCCCGTGCATATATCCTTTCA

AGCTCCGGCTACTCTCTCCGGCGCCAATCCGGACGCCGGTGACTCCGTCCGCACATCACT

CGGGCCGACGTCGACACCCTCCAGGGGTTCAAATGGGGATCAGCAAGACTGAAGTGAATC

TGAGAAGGTTGCTTGCAGCTGCTCCTCAGCAGCAGAACCGCACAAAACTCATACATTACA

TCACCACTTTGCGTGAACAATTGGAACAACTGGGCGCAGAGACTACATCTGATGGTTTAC

CGAGCATTTCTAAAGCCAAGTTGAACGAATATTCAGAGAACATTGAAACCCTTGCTGCTA

AATTAGTTGCTCCAGAGCCCGAACTTGAAGAAGCTGCCAATGACAAGTCAGCTGAGGAAA

ACCCTCCAAGAACAGAGCAATCTGTCTGTTCTGTAAGCCGTTCTCCTGAATTACGAAGGC

GATTTGTAGCTCAGAGAGGAGTCGAAGATAGCGGTCATAAAATTTCCGAAAAGGATTCCA

CAAAGCTTGTTAAATTAGACACATCAGCACAGGCTCACATTGAGAAACATAGAAAGCTTC

AAGAAGATTTGACAGATGAAATGGTTGTATTGGCACGGCAACTCAAGGAGAGCAGTCTTA

TGATGAACCAATCCTTGCATGACACTGAGAAGATACTTGATTCGACTGAGAAGGCAGTAG

AGGATAGCCTGGCGAGCACGGGCAAGGTCAATTCACGAGCCATGAAGATATATTCAGAGG

GTTCCAAGACAACATGCTTCACATTTCTTGTCATGTTTGTTATGATCTTTATCTTCATTA

TGGTAGTTCTGCTCATCCGTGTAACTTGACACACTTCAGTTTAACAAGACAGATACTTGA

TTCGACTGAGAAGGCAGTAGAGGTTAGCCTGGTGAGCACGGGCAAGGTCAATTCACGAGC

CATGAAGATATATTCAGAGGGTTCCAAGACAACATGCTTCACATTTCTTGTCATGTTTGT

TATGATCTTTATCTTCATTATGGTAGTTCTGCTCATCCGTGT

>Unigene0000769

CACCGATTGCGACTCATTATCCCGGTAGGTTCCGGTCACTCGCCGGTGAGCTTAACCGCC

TTCTCCGATCTGCCGTCGATGTCTTCGTCCTCGTCGCCGGCGTCGTCCTCAACCGCGGCA

CCGGCGGAACCCCATCAAAGCCCTCCATCGAATCCTGCACCCGAGACTTCCGGGAGTGCT

CAGGCATCTAATGTTCCTGGTAGCCAAAATTTGGCAAATGGTGGGCAGCAGATGTTTCCC

ATGTTCTACCCTGTAATCGGACCAGGGTCAGTTCCCGTGCAAAACTCCGAAGTAAATGAT

CATGGTGCAGGTCTCTATGCTGTTCCTTTTTCTCCATTCATGGGAACAATGGCTGGGCCT

TCTCCAAGCACACTTATACCTCTTAGATACAACATACCAACAAGGACTAGTGCCACCGGA

GCGGTCGGAGAAAATCAGGGGCAAGAAGCAAGGCAGGTGCATGGACCTCAGAGACAAGTA

GCTGTGCGGAGGTTCCAGTTCGCTATACAGCTCGACCTGTCTCTCATCCTTAAGCTGGCA

GCTGCAATCTTCTTGTTCAACCAAGACGGGTCGAGGCAGAGGCTTTTTATCCTCATATTC

TGTGCTACTATACTCTACCTCTATCAGACTGGTGCTCTAACACCACTGCTGCGTTGGATT

AAGCGAGGCGCTGCCCCTCCCCAACAGCGCCCTGCAGTTCCACCAGAGAATGCCCCTCTC

ATAGAGGGCAATGCTGGTGGTGATGATCAGAACCGTGATCTGAATGAGCCTGCTGCAGGA

GCTGAAGGAGTTCCAGCACAGCAAGTACCTGAACCTGAACCTGAGAGGGCAAACAGAATC

AATTGGTGGGGAATTGTGAGGGAGATCCAGACTGTTGTCGTTGGTTTCATCAGTTCTTTG

CTTCCAGGGTTTCATCACAACGACTAGCCCGTGATGGTTTTGACAGCCAGCCAGTCAATA

GAACGCGTGTAACAGATTCTGTTGTAAAAGAAGTTGGTGTTGTTTCGGTAGAACAAAAGA

TGTTGACTAGTTTGCTAGTTGTCTTGTCTGTGTCATTTCATACATGGTTTCCAAAGGATT

TGGTTCATATTGAGTTACAGTGCCGGTTTCAAATGGAAAACTGGCTTGAGGGCATTGTAT

GGGTCAGTTTTTGATGAATTGAAAAGCGAACCAATCAAT

>Unigene0000770

CTCCAGCTCAAAACCCCCCTTTCCCCTCCGTCTCTTCCATTCTCAACAATCGAAGCCATG

GGCACCGAATACGATTACCTCTTCAAGCTCCTCATCATCGGCGACTCCTCCGTCGGCAAG

TCCTGCCTTCTCATGCGCTTCGCTGATGATGCTTACATGGATAGCTACATCAGCACGATC

GGGGTTGACTTTAAAATAAGGACCGTGGAGCTGGATGGGAAGACGGTCAAGCTGCAGATT

TGGGATACAGCTGGACAGGAGCGCTTTAGGACCATAACCAGCAGTTATTACCGTGGTGCG

CATGGGATCATTGTTGTGTATGATGTTACTGAGATGGAGAGCTTCAACAATGTCAAGCAG

TGGCTAAACGAAATTGATAGATATGCCAATGACAGTGTGTGCAAGCTTCTTGTTGGTAAC

AAATGTGATCTGGTTGAGAACAAAGTTGTGGATTCGGTCAAAGCAAAGGCATTTGCTGAT

GAGCTTGGGATACCTTTTCTTGAGACAAGTGCAAAGGACTCTGTCAACGTGGAGAAGGCC

TTTCTAACCATGTCTGCTGAGATCAAGAAAAGAATGGCAAACCAGCCGAGCTCAAGCATG

AAGCCAGGAAATACTGTTCCGCTGAAGGGCCACCCGGTCGAACAGTCAAGCAGTTGCTGT

AGTTAGCCAGTTTCCGTTGGGTGTATGACATAGTATTGGGAGGATGTTAATGGACCCATG

ATATGGAAAAGAGGTAGGGTTGTAATGGTAATGGATGCATGGTAAAAAAAAAAAAGAAGA

AAAAGCTGGATCCAGGACAAGTTAAA

>Unigene0000771

CGTCCTGATTCCGCCGTCGCCGACGGCCTCCTCCGTCTCCTCCTCCGATCTTGACACCGA

GTCCAACAGCTCCTTCTTCCCCGACCGCAGCACCACCCTCGGAACCCTAATGGGCGTAAC

CCTCCCCGCCGCTCCTCCCCCCACCGGTGCCGTCGTCGTCCAGACGAAGCTCGAGGTGCG

AGATCGGCGGCCGCGGCGGCGGCCGGGGAGGAGCTGGTGGCGGCTCTGCAGGGACGATAT

TAGGGTTCCGACGTCGCTCGGGGACTTCCTCCAGATGGAACGGATGGGCGGCGACGAGAT

CGGCCGGTTCCTGTACGGCGAGCACGAGGCGGTGGGCGGCGGAGCATTGTTTGAGGACGG

GAGGGTGTTGCCGCCGACGGCGGCTGCGCCGCGTCAAGCGGCGGCCGGGCGCTTGCCGGT

GTTGCTAGGTGGGATCTGCAGTGGGGGTTAGGGGTAAATATGTCATTTTCATTATGCTGA

ATTTTAATTTTTTTAATATAGTTCTATGCTTTACGTTTTCTTGGTTTGTTTAAATGATTG

GATGACATGAAACAAGGGAGATATCATTGTTTAATTTCAAGACTAGTTTATGATCTATAG

AAATCTGAGAGTTC

>Unigene0000772

TCATTCCTCTTCCCGTTCCACACATACTCCCCCTCCTCTTCCTCCTCTCTTCAATCTCCG

AGGTGATGGTTTTAGTGATCAGGTTGAGGTTATGAGAATTGGGTTTTGATTGGATTGAGG

ATTCAATGGAGAAATTGTGCTGCTTCAAGTTTGCAGGTCATTCTTCAGACGAATCTGGTA

AAGGGAAGAACTACCAAGGTCAAACAAAAGCTGCTTATGGTTTTAGTCTTATAAAAGGAA

AGGCAGACCATGCTATGGAGGATTACCATGTTGCCAAATTCGTCCAGATAAATGGCCAGG

AACTCGGTCTGTTTGCCATCTTTGATGGCCATTTGGGTGATAGTGTACCTGCATATTTAC

AGAAGCATTTATTCCAAAATATCCTGAAGGAGGAAGAATTTTGGATCCACCCGGACAGAG

CCATTACAAAAGCTTATGATAAGACTGACCAGGCAATTCTTTCCCACGATCCCCAACTTG

GACGAGGTGGATCGACAGCTGTGACTGCTATTCTTGTAGATCTCAGGAAGTTGTGGATAG

CCAATGTCGGTGATTCACGAGCGGTTCTTTCTAGAGGTGGACGTGCAATACAAATAACAG

TTGATCATGAACCTGGTACTGAGCGTGGGAGTATTGAAAGAAGAGGTGGCTTTGTTTCGA

ACATGCCAGGAGATGTCCCGAGAGTCAACGGCCAATTAGCCGTTTCTCGAGCTTTCGGGG

ATACAGTCCTCAAGTCGTACTTACGGTCAGACCCAGACGTGTATTCTGAAGATATAAGTC

TTGATACAGAATTACTCATCCTCGCTAGTGACGGCCTATGGAAGGTAATGAGCAATCAAG

AGGCCGTTGAGCTCGCTTTGAGGATTAAGGACCCGCAAGCTGCTGCTAAGCAGTTAGCAT

ATGAGGCGGTGAGCAGAGGTAGTAGAGATGATATATCGTGCATAGTTATTCGATTCAAGG

CATGAAGGACTGATATGCATGGCGATATTTCCTAAACCGTTTAGGGTATCCTAACAGCAT

CTGGCTCTGGGCCACCGATTAATACTGGAATAGCTGACACACCTTCATCATCCTTATTGT

AAATGGGCTCTTGTGTGTTTAAATTTGTATGTGTCTGGTGATGATTTAGGGAGGAGATAT

ATGTTTCATAGGCACTGAAA

>Unigene0000773

AAATTAGGGTTCCAACAGAACCAAAAACCCTTTACAAATCCAGCAGAAATCAGCCGTCCC

AACCCCGCCAAGATCTCGTCTTTCATCACAAAGTTGAAAAGCAGACCCCGAGTTTGAAGC

AGAAAAGTCTAAAACATGGCGATGAGCTGCTCAAGGCTATTGGGATACATCAGTATGAGA

AAGGAGGCAGTAAGGATGGTGGGACGCAGAGACTTTTCAGCAGGCGGCAAGGCGAAGAAG

GGTGGCAAAGGTGGTGGAGGCGATGGCCCTAAATCTTCTGGTCTCAGTGAAGAGCTAAAG

AAAACCACGGTGTTTGGAGCCAATATCCTCAAGGATGGGACAGATCCCAAGATATTACCT

GATTCAGAGTACCCTGACTGGCTTTGGCACTTGCTTGACAAGCGCCCTCCATTGAGCGAA

CTTCGGCGGAAGACCGTTGATTCTCTTCCTTACGAAGACCTCAAGCGTTTTGTTAAGCTG

GACAACCGTGCAAGAATAAAAGAGAACAATGCTCTCAAGGCCAAGAACTAGTATCAAAGT

TTGAGGTATATTCTGCAACTTGATCTCTATCGAAATGCTATGCAGTTAGTAGTTAAATCG

TCATTTACGTTGGGAAATAATGAATGAATTTTGACAAAGCTAATATTTAAACTTCTGTAG

AACTATTGGCTTGAAAAAAGACGCTATCATGTTTCTCTTTGGATTTATTTAGACTGACAT

TCTTGTGCTGAAATTCCTTATAGTTTACTGGTTTTTATCA

>Unigene0000774

CCCTACAAGTTGCAACTTCTATCTTTTTTCTATGTATTCTTCTTTGATGCATGGTGGCAA

TCCTTGCATTGGTTCAAATTGTAGTGGTTGGTCATGTATTGTTTGGATCCAATCCTATGG

CAAGACGGCCTTCATAGTTTGTGGCTTCTGTGATAGCACCAAATATACTCCTTTCCCCTA

CAAGTTGCAACTTCTATCATTTTTCCACATATTGTTCTTTGATGCATGCTGGCAATCCTT

GCATTGGTTCGGATCTGCAGTGGTTGGTCATGGATTGTTTGGATTAAATCTTATGGCAAG

ATTGGCCTTCATAGTTACGTGAATCAGATGAGATGTGCATTCTCTACATCTTCGGAGTTT

GGCCTTCAAAGACATGTTGTGGTTGACATTTATTCCGTTGAGCTAGGATCTGTTATATTG

TCTTTCCCGACTTCTGCCATGTCATGTTCTTGGCAGGTCACTAGATCCTTAATTAAATTT

GTTAGAAGAGATGATAGGAGAGCAATGAGGATGAAAGTGTGGTTGGTTTCTTATTACTCA

CTAAGGGGATTTTTATAGTAGTACATAG

>Unigene0000775

AGTCGGAGAATCAGAGATGGCTCGAATGTGCTGTTAGTGAGTCGGAGAATCAGAGATGGC

TCAAATGTGAGTGTCCATAATCGTGGCTTCCGTGACAACACCAAATATACTTCCTTCCCT

ACTCCAATCTGTAGCTTCTGTCATCTTTCTATGTATTCTTCTTTGATGCATGGTGGCAAT

CCTTGCATTGGTTCAAATTGTAGTGGTTGGTCATGTATTGTTTGGATGTAATCCTATGGC

AAGATGGCCTTCATAGTTCGTGGCTTCTATGACAACACCAAATATACTCCCTTCCCCTAC

AAGTTGCAACTTCT

>Unigene0000776

AGATGAGATGTGCATTCTCTACATCTTCGGAGTTTGGCCTTCAAAGACATGTTGTGGTTG

ACATTTGTTCCGTTGAGCTAGGATCTGTTATATTGTCTTTCCCGACTTCTGCCATGTCAT

GTTCTTGGCAGGTCACTAGATCCTTAATTAAATTTGTTAGAAGAGATGATAGGAGAGCAA

TGAGGATGAAAGTGTGGTTGGTTTCTTATTACTCACTAAGGGGATTTTTATAGTAGTACA

TAG

>Unigene0000777

GGAGACCATGGTAAGGTATCCAAGGACCTTGAGGAACAAGCAGAGATGAAGAAAATGTAT

AAGCTCATCGAAGAGCACAAACTTGATGGACATATTCGATGGATATCTGCCCAGATGAAC

CGAGTCCGCAATGGGGAGCTGTATCGCTGCATTGCAGATTGCAAAGGGGTATTTGTACAG

CCTGCTTACTATGAAGCTTTCGGGCTGACTGTTGTGGAGTCCATGACTTGTGGCCTGCCC

ACATTTGCTACTTTGCACGGAGGACCTGCCGAGATCATAGTGCATGGTGTCTCAGGCTTC

CACATCGATCCCTATCAGGGTGATAAAGCCGCTGACCTGCTTGCCGATTTCTTTGAGAAA

TGTAAGGAAGATGCAACACACTGGGATAAGATCTCGCATGGAGGACTGAAACGTATCTAT

GAGAAGTATACTTGGAAGATCTACTCAGAGAGGTTGATGACTCTGGCTGGTGTATATGGA

TTCTGGAAATATGTCTCTAATCTTGATAGGCGCGAGACCAAACGTTACCTTGAGATGTTC

TATGCCCTCAAATATCGTAACTTGGCGCAATCCGTACCCTTGGCTGTTGATGGAGAGGTC

GTTGCCAATGGAACTAAATAGTTTCTGCAGCGGATGGAAAGTTGAGTTTATGATACAAAT

AAGGATCTCAGTTGGAAGTGTGTCTTCCTTCTTTTCTTGTTGGTCCATTTGACGTTTGAG

CCAGCTTTGTAGCAAACAGAGTGAACTAGTTCATGTCTTCCAATGTATTGGGATGCATAA

GGCTTAAATTTTGAGTATTCCGCAATAACAATGCAGTATTTTCTTTCTAATATCTGAAGC

ATATGCTA

>Unigene0000778

TTCTGGAAATATGTCTCTAATCTCGATAGGCGCGAGACCAAGCGTTACCTCGAGATGTTC

TATGCCCTCAAATATCGTAACTTGGCACAATCGGTACCCTTGGCTGTTGATGGAGAGATT

GCTGCCAACGGAACTAAATAGTTTTCGCATCAGATGGAAAGTTGTATTCTATTATTCAAA

TAAGGATCTCTGATGGAAGTGCGTCTTGCTTCTTTTCTTGTTGCTCCATTTGATGTTTCA

GCTGGCTTTGAGCAAACAGAGTGAACTAGTTCATGTCTTCCAATGTATTGGGAAGCATAA

GGCTTAAATTTTGAGTATTCCGCAATAACAATGCAGTATTTTCTTTCTAATATCTGAAGC

ATATGCTAGTTATACCTGCATTATCTCAAACTAATACGACAATAATTCCGGG

>Unigene0000779

TCTGAAGATGGTGTTTTTACAAACTGAATCTCATAGGTGTCATCCCGTATGATGATCATA

TCATCAATATAGAGTAGCAAAAGTACTTTTCTACGGCCTGTCGAGCGAACAAGTAGAGAA

TAGTCTTGAGAACTTTTCACAAACCCTGCCTACAACACAACCGTGTGAAAGCACTTGAAC

CAAGCTCGAGGCGCTTGTTTTAGACTATAGACGTCTCGTCGAAGACGACATACATGACCT

GAAGGAGTGGTGACACCGGGAGGTGTCATATATACGAGATCATAGAGGGCACCATATAGG

AAGACATTTTTTACATCCATTTGGTATATAGTGGCCATTGTCAGGAAGTGGTAATGGCAA

GTAGAAGAGCAATTGCCATATGAGCAACCAGAGCGAATGTCTCTTCGTAGTCAATCCCAG

GTTGTGTATAATCTCTTGCAACGAGACGAGCCTTCTTTTGTTCTACTCGACCGTTTTGTC

GGTATTTGATCTTGTAGACCCATTTGCTGCCAACAGGCGTCACATTTGGCGGACATGGTA

CAATATCCCAGGTGTTTGTTTGTGAAAAAACATTTAGCTCCTTTGTCATGGCTTATCGCC

ACACAACATTCTAAACTGCCTCCTGATATGAATGTGGTTCTTGGACACTATGGACTGGAA

AGAGAAAAGTTAGAAAGACAGGTGTAAGTAGCAACTGTCATGAATCCCGGTCGTTTTGTC

TGATGATTAAATCTTTGGGATCGTCATGGAACTATAGAATCTGGCAGAACATGTGTTGGT

GTGGTGGGGATTGGCTGATGTGGCTCTATATCAACAGGATACTTGCGCTTTAGACGTTTG

TAGACTCTATAAGATGTAGGAGGTGGAACATATAGTAGGAGGTGGAACAGGAGCATGGGT

CATTATAGGAGGGGGTGAAATTTGAGGTTCAGAGATAGGAGATGGC

>Unigene0000780

CCGTCGAGCAATGGGATCATAGCAACGATAGTCTGTGTTCGACGCTTATTCCCATAAAAA

CAATGTTGCTCGAGAGGAAAACTTGTCTCTCTCGTGAGGGGGGTAAGAGACCAAAACATC

TACACCCAAAAATGCGCAGAAGATAATCTTGGTGGTTTAGAGTATATTCAACTGTGTGGC

GTCGCGCCAGAAATAACAATAGAGGACGTGATGTTGATGAGTAGAACAATGGAGATGGTT

TCAACCTAGAAGGATTGAGGAACAAAGGCAAATGTAAGTACTCGGGCAGTGTCGAGAATA

TGACGATGCTTGCGCCCAACGATTCCATTTTGTTCGTGGGTGTGGGCATGATTGTTGAAA

AAGTGTGCCATGTGTGGAAAGAAGATTATGCATATTAGATG

>Unigene0000781

TGGATGGGAATGAATGCATTGTTAATTTATGTTCTAGTTGCCTGCAATCTCTTGCCGATT

CTAATCCAAGGGTTCTACTGGAAGGAGCCACGCAATAATATTTTACGACTGGTTGGAGTT

GGATCATAAGCAAGATGATTCTCAATAATCTTTCCCCTTCTTCTTGGAGGAGCTGGGACT

CAGTTTTGTAAATACTCAGGAGCAAGTAAATGTGCTACATCTTGCGTTTCTGTTGCAGAT

CATATAAAGAGCTGATTAGATATAAGAATATATTGTAGAATTGCACTTCATATGAAAGAA

TATCAGATTATCACGAATTCGGTTTCGTTCCCTGGTTATACTCCATGGTGCCGGCAATGC

AGCAAGAAGATGTCAACTATATAGCATCTCTTTGCATTGTGGATTTAATTTTATTTAAAT

TTTCTTCAAGCTTTGAGCCTCTAAAAATATGTATCAAATGATCTCTCTAATCTGAATTCT

GTAACCTTTTTTAATCATTAATAGCCCTGATTAGTGA

>Unigene0000782

TGGATGGGAATGAATGCATTGTTAATTTATGTTCTAGTTGCCTGCAATCTCTTGCCGATT

CTAATCCAAGGGTTCTACTGGAAGGAGCCACGCAATAATATTTTACGACTGGTTGGAGTT

GGATCATAAGCAAGATGATTCTCAATAATCTTTCCCCTTCTTCTTGGAGTAGCTGGGACT

CAGTTTTGTAAATACTCAGGAGCAAGTAAATGTGCTACATCTTGCGTTTCTGTTGCAGAT

CATATAAAGAGCTGATTAGATATAAGAATATATTGTAGAATTGCACTTCATATGAAAGAA

TATCAGATTATCACGAATTCGGTTTCGTTCCCTGGTTATACTCCATGGTGCCGGCAATGC

AGCAAGAAGATGTCAACTATATAGCATCTCTTTGCATTGTGGATTTAATTTTATTTAAAT

TTTCTTCAAGCTTTGAGCCTCTAAAAATATGTATCAAATGATCTCTCTAATCTGAATTCT

GTAACCTTTTTTAATCATTAATAGCCCTGATTAGTGA

>Unigene0000783

TGGATGGGAATGAATGCATTGTTAATTTATGTTCTAGTTGCCTGCAATCTCTTGCCGATT

CTAATCCAAGGGTTCTACTGGAAGGAGCCACGCAATAATATTTTACGACTGGTTGGAGTT

GGATGGGAATGAATGCATTGTTAATTTATGTTCTAGTTGCCTGCAATCTCTTGCCGATTC

TAATCCAAGGGTTCTACTGGAAGGAGCCACGCAATAATATTTTACGACTGGTTGGAGTTG

GAT

>Unigene0000784

GGTTGAGCCTCTCCCGTCCGTTCATACGCCCGCCCCCATCCTCTCTCTCCTCCAATGCTG

CCGAGATTCCCGGTGTTCGACTCGATCTTCTGATTCAGATCGATACATATCTCCGATCCG

ACCGCTCAGATCTTTCGCACTTTCACGATTCTCGCCGAATTCGATGGGGACGTACGAGCT

CATCAATGGCGACGAGAACGGCAAAGTAGGCGATATCGAGAAAGGATATGGCAGCAATGA

ACTCGGATCGGTTAAGGTGTGTGCTGGATCTCCGGCTGTCGTGTTCGCCGGCGACGGCGG

GCCGCCTCCGGCGGCGTCGAAGAAGAAAGAGCGGCTTGTTTCGCTTGACGTGTTTCGTGG

GATCACCGTCGCGCTCATGATCCTAGTAGATGAAGCTGGTGGCTTTTTACCCTCGATCAA

TCATTCACCATGGAATGGTGTAACCCTTGCGGACTTTGTGATGCCATTTTTCTTATTTAT

AGTAGGAGTCGCATTGGCTCTTGCGTACAAGAATGTTCCAGACCGAGTTGATGCAACGAG

AAAGGCCGTTCTTCGGGCACTGAAATTGTTTATCGTAGGACTTATTCTTCAAGGTGGCTA

TTTTCATGGTGTTCAAACTTTAACTTTCGGGGTCGATATCTTGAACATTCGATTTATGGG

TGTACTTCAGCGAATTTCGATAGCTTATTTAGTGGCAGCACTATGTCAAATCTGGCTTAG

AAATGAAGAAGATGTTGATTCTGGAGATTCCTTAATAAGGAAATACCGATATCAATTGTT

TGTGGGCTTGATAATAACAAGCATCTACTTAGCTCTCTTATATGGCCTTTATGTTCCGGA

TTGGGAGTATCAGATACCCGTGGATGGCTCTGAATCAAAGTTATTCTCGGTTACGTGTGG

AGTTAGGGGAGATACAGGTCCTGCCTGCAATGCTGTTGGCATGATAGACCGCAAAGTATT

GGGTATCCATCATCTATATAGGCGACCGATTTATGCACGGACAAAGCAATGCAGCATTAA

TTCTCCAGATACTGGTCCACTTCCAGCTGATGCTCCCTCGTGGTGTCAAGCTCCCTTCGA

TCCTGAAGGGCTCCTAAGTTCTGTCATGGCAATTGTTACATGTTTGATTGGGCTGCAATT

TGGTCATGTAATTGTACATTTCAAGGAACACAGGGATAGGTTTATTCAATGGATGATTCC

GTCCTTGTTTCTCTTGGCCCTAGCTTTTGCACTTGATTTTTGTGGGATTCATATGAACAA

GGCTCTGTACTCGTTCAGTTACACATGTGCCACAGCGGGTATAGCAGGAGCCCTGTTTAC

CGGAATATACTTACTGGTTGATGTTTATGGTTATAGGAGACCGACCATTTTGATGGAATG

GATGGGAATGAATGCATTGTTAATTTATGTTCTAGTTGCCTGCAATCTCTTGCCGATTCT

AATCCAAGGGTTCTACTGGAAGGAGCCACGCAATAATATTTTACGACTGGTTGGAGTTGG

AT

>Unigene0000785

TGGATGGGAATGAATGCATTGTTAATTTATGTTCTAGTTGCCTGCAATCTCTTGCCGATT

CTAATCCAAGGGTTCTACTGGAAGGAGCCACGCAATAATATTTTACGACTGGTTGGAGTT

GGATCATAAGCAAGATGATTCTCAATAATCTTTCCCCTTCTTCTTGGAGCTGGGACTCAG

TTTTGTAAATACTCAGGAGCAAGTAAATGTGCTACATCTTGCGTTTCTGTTGCAGATCAT

ATAAAGAGCTGATTAGATATAAGAATATATTGTAGAATTGCACTTCATATGAAAGAATAT

CAGATTATCACGAATTCGGTTTCGTTCCCTGGTTATACTCCATGGTGCCGGCAATGCAGC

AAGAAGATGTCAACTATATAGCATCTCTTTGCATTGTGGATTTAATTTTATTTAAATTTT

CTTCAAGCTTTGAGCCTCTAAAAATATGTATCAAATGATCTCTCTAATCTGAATTCTGTA

ACCTTTTTTAATCATTAATAGCCCTGATTAGTGA

>Unigene0000786

TATCTAAATTCATTTTGGAAAAAAAATGCTTTGTCAATAGTGGACCATAGGCCTATTAGT

TTCATCAACAATGCTTATAAATAGTACCAAGCTTATGGCAGCTAATTGAAGGGATGCATA

TAAGAGATTATATCCAACACACTGACCATTTTTAGATGGTAGACATTTTAGAAGGGATCT

TGGCCGCAAATAAGGTACTGGAATGGAATGCAAGGAGTAAAATGGGAAATGATGTTTTTT

TT

>Unigene0000787

TATCTAAATTCATTTTGGAAAAAAAATGCTTTGTCAATAGTGGACCATAGGCCTATTAGT

TTCATCAACAATGCTTATAAATAGTACCAAGCTTATGGCAGCTAATTGAAGGGATGCATA

TAAGAGATTATATCCAACACACTGACCATTTTTAGATGGTAGACATTTTAGAAGGGATCT

TGGCCGCAAATAAGGTACTGGAATGGAATGCAAGGAGTAAAATGGGTAATGATGTTTTTT

TTTGTCTGAGGTAGATATTGGTGAGTCTTATGAGTCATCATGTTAGC

>Unigene0000788

AACATCTTAATAGAAGACAAATTCGAAAATCTAGATCTATCATGCAAGTATTGCTTTTTG

GTGATGAAATTAATGAAGACAATTCCAAAGTCATTTTAAAATGCAAAAATATTCGTCAAA

CTGAATTTTTAGTTGACTTAAGAGAAGCAATTATGCAAGTATCTCGTCATTTTGTGTTTG

TTGATCCTAAACTTAATAATCTTTCGTTAAAACCAGACTATACCAAAGAAAGTCACTCAA

GAGACTTAGAAATATATGCAAATGTATCAAGAAATAAAAAAAGAAGAGCTAAAGCCCTTT

TAGATTTCGAAAGACACGATGATGATGAATTAGGATTTCGAAAAAATGACATTATTACAA

TTATTAGT

>Unigene0000789

TGAAGGTTCATTGACTTTCAAAAAATGCATTGAATTGAGTTTGGCTATGGAGAGTGCTAA

CAATGACATTTCAAAGATGGAACATCACGAAGCAGTTAACTACCATAAAGTTTCGAAGAA

GAAAAATACTAAGTTCCTGACCCAAGTCAAGCAATCATCATCGAATAATAGTGGCAAAAT

TGGTGGAGAATCAATAATGAAAAGTACAAAAAGAAATTGGGTATGCTATTGCTGTGGGAA

GAGTGGACATACTAAGCCATCATGTAAGTATATTAGTTATAAGTGTTCAATCTCGCCGGA

CTCCGCCC

>Unigene0000790

TGAAGGTTCATTGACTTTCAAAAAATGCATTGAATTGAGTTTGGCTATGGAGAGTGCTAA

CAATGACATTTCAAAGATGGAACATCACGAAGCAGTTAACTACCATAAAGTTTCGAAGAA

GAAAAATACTAAGTTCCTGACCCAAGTCAAGCAATCATCATCGAATAATAGTGGCAAAAT

TGGTGGAGAATCAATAATGAAAAGTACAAAAAGAAATTGGGTATGCTATTGCTGTGGGAA

GAGTGGACATACTAAGCCATCATATTTCGAAAGACACGATGATGATGAATTAGGATTTCG

AAAAAATGACATTATTACAATTATTAGT

>Unigene0000791

AACATCTTAATAGAAGACAAATTCGAAAATCTAGATCTATCATGCAAGTATTGCTTTTTG

GTGATGAAATTAATGAAGACAATTCCAAAGTCATTTTAAAATGCAAAAATATTCGTCAAA

CTGAATTTTTAGTTGACTTAAGAGAAGCAATTATGCAAGTATCTCGTCATTTTGTGTTTG

TTGATCCTAAACTTAATAATCTTTCGTTAAAACCAGACTATACCAAAGAAAGTCACTCAA

GAGACTTAGAAATATATGCAAATGTATCAAGAAATAAAAAAAGAAGAGCTAAAGCCCTTT

TAGGTAAATATTTATATTATT

>Unigene0000792

GAAATGATGTTTTTTTTTTGTCTGAGGCAGATATTGGTTAGTCTTATGAGTCATCATGTT

AGCTGGGATGTGGCATTGGGAAATATTTGGTTTTGGGAGCAAGTGGAGGAGCTTGGCGAG

GAGATGTAGCGTGACTGATAGATTCTTTATTTTAGTTAATGGGGAAGCTAAATGATTCTT

TAGAAGCTCAGGGGTTCAGAGGATCGGACCCACTTTCTCCATTGTTGTTTACAAGCATGA

TGAAACTTGTAGTAGAGTGACTCCAAAGTCTGTTAAGTTGAGCTTGTTGAAGGGCTTTCC

AATTGAGGAATGGAGACTTGTATTTCTCACCTTCAATTTGAATAGCTCATTTCCCCCCAG

AGAATGTGAAAGATCAAGTGGCAACATCAAAGGATGTTATCATTATTATCAATTTGATTT

TGTATTTGGATAGAATTGGTTGTGAGTAGGAAATTTGTCCAACTTAATAATTGGAACTGT

CATTGGGCCAAAGCAGTGGGAAGGAGTTATGACCTGTTAGAAACTTTGGGAGGGATGCTG

GAATCATGTCATGAACTTTTTGATAACTTTCACGATATGCAGCATTGGTTTGTCCGTAAG

TTTGTAATAAGATACAACAGAAAACAAGAGCAGTCTACTGTACTCACATCACGGTCTTTC

GTGGAGCTCAGTGATGATATCCAAGCAATTCAATTCATTATCTGAAATCTTGAAACTTGT

CAACCCTAAGCTTAATTTATGATCCGACATTCCCGAGGTACCTGCCTACCAGACTTGGAT

TCCTGCACCTCGTTGATACCCTGATTTCAATCTTGCTGAGGAGCGGATCCCTTCTGTCGA

CTCTGAATTCTTGATCTTACCCGCTATCCGTGGTAGAGCAGGTCATTTTTCTGAGTCCCT

GGTGGAGCAAGTCATTTTTCTGAGCCCCAATCCCTCCAGATCACTTTCTTTTCACCCAAG

ATGTGAGATCCCACTTTTAAAAGGGGTAGACGGGTGAGAACCTCAAGTTTTTACTATACC

CCAAGTATTTGGCATGGTTTAGTTCTTACCCGAACTTATCTACATTCTAGACACCAACTA

ATGGGATGATTTTGACAAATACACCTGAAGTCCACCGCATTCAGTCTTTTTTTTTTTTCC

GAATTAGCTCTTGATATTATATCGTAATTTTACACACCATTCAATAAAAAACGATTAGTA

CATAACTTTTATCCTAAGCTCGAAAGAAAGCTGTTGAATGGGATTTTGGAAAGCGAAATT

GTTAGAAATTACAGTGCCCAATTGGCTATGGAAGCATGAGATTGACAAGAAAACTCCATA

GAGAAGGTCGGTTAAAAGTAAATATGGTGATGCAGGTGGGGTTGGTTTACTGGGAGGTAA

TGGGGCTG

>Unigene0000793

GAAATGATGTTTTTTTTTTGTCTGAGGCAGATATTGGTTAGTCTTATGAGTCATCATGTT

AGCTGGGATGTGGCATTGGGAAATATTTGGTTTTGGGAGCAAATGGAGGAGCTGGGCGAG

GAGATGTAGCGTGACTGATAGATTCTTTATTTTAGTTAATGGGGAAGCTAAATGATTCTT

TAGAAGCTCAGGGGTTCAGAGGATCGGACCCACTTTCTCCATTGTTGTTTACAAGCATGA

TGAAACTTGTAGTAGAGTGACTCCAAAGTCTGTTAAGTTGAGCTTGTTGAAGGGCTTTCC

AATTGAGGAATGGAGACTTGTATTTCTCACCTTCAATTTGAATAGCTCATTTCCCCCCAG

AGAATGTGAAAGATCAAGTGGCAACATCAAAGGATGTTATCATTATTATCAATTTGATTT

TGTATTTGGATAGAATTGGTTGTGAGTAGGAAATTTGTCCAACTTAATAATTGGAACTGT

CATTGGGCCAAAGCAGTGGGAAGGAGTTATGACCTGTTAGAAACTTTGGGAGGGATGCTG

GAATCATGTCATGAACTTTTTGATAACTTTCACGATATGCAGCATTGGTTTGTCCGTAAG

TTTGTAATAAGATACAACAGAAAACAAGAGCAGTCTACTGTACTCACATCACGGTCTTTC

GTGGAGCTCAGTGATGATATCCAAGCAATTCAATTCATTATCTGAAATCTTGAAACTTGT

CAACCCTAAGCTTAATTTATGATCCGACATTCCCGAGGTACCTGCCTACCAGACTTGGAT

TCCTGCACCTCGTTGATACCCTGATTTCAATCTTGCTGAGGAGCGGATCCCTTCTGTCGA

CTCTGAATTCTTGATCTTACCCGCTATCCGTGGTAGAGCAGGTCATTTTTCTGAGTCCCT

GGTGGAGCAAGTCATTTTTCTGAGCCCCAATCCCTCCAGATCACTTTCTTTTCACCCAAG

ATGTGAGATCCCACTTTTAAAAGGGGTAGACGGGTGAGAACCTCAAGTTTTTACTATACC

CCAAGTATTTGGCATGGTTTAGTTCTTACCCGAACTTATCTACATTCTAGACACCAACTA

ATGGGATGATTTTGACAAATACACCTGAAGTCCACCGCATTCAGTCTTTTTTTTTTTTCC

GAATTAGCTCTTGATATTATATCGTAATTTTACACACCATTCAATAAAAAACGATTAGTA

CATAACTTTTATCCTAAGCTCGAAAGAAAGCTGTTGAATGGGATTTTGGAAAGCGAAATT

GTTAGAAATTACAGTGCCCAATTGGCTATGGAAGCATGAGATTGACAAGAAAACTCCATA

GAGAAGGTCGGTTAAAAGTAAATATGGTGATGCAGGTGGGGTTGGTTTACTGGGAGGTAA

TGGGGCTG

>Unigene0000794

TTTACTGGGGAGGTAATGGGGCTGAGGTGTGTATCTTTATGAAAGAGTTTATAAAGCTTA

TAGATTTCAGATGGGGGGTAAGACTGAAACTTTGGGAAGATAAGGAGAAGGCAAATAAAC

TTTTGAAGGACCAACAAAAGCATCTGGTACAAATATCAAGGAATAAAAGGAGTCTGGTGC

TACAAGTTTTATTTATTACATCGTTTTCATATTAGAAGAAACCTATTCACTTGGTAAGAG

GCTTAAAAAATTACAAGAATCGAGACAGGAGGATAGTGTCTCAAAGAAGTTGATGCGTCA

TTTTTGAAGGAAGAAGAAATCGGTGTTTAAGTTCAAATGGCAAATTCGGAACTTTTACAA

ATGATTGCTAAAAATTAATGACAGACCTGAAAGATTCCACACTTCCCTTGCTTGATGGTT

TGGCTCAAAGAGGTTCTAGCCAAAGTTAAAAAGTGAATTGGGAAATGCCCATTTACTAGA

CACCATTTACAAAGATTGTCATTTGCTAAGAAGATCATGGGATTTGTAGCAAACGGCTCT

GTTACATGGTCTGTTTGCTACAAGGTCATCGTACAGTTTGTAGAATGTCTTGATTGTGGC

AACTGAGGTATAGTTTTGATGGGCTCATGTGAAGTTGGGAAGATGGAAATAAAAGGGCGA

AATGGATATGGAGTAGAGTTCAGGACAATTCGAGGGTAAGAAATTATAAATTATTTATAT

CTATAAGCAGATTGGAGTCTGTACTAGAAGAGGGTGCAGTTGCTCTAATTTGGCATTGAG

GCCAGCATAAGGAAAATAGGGGTAACATAATTTGGGTGTTGATTTTTTTTGGTGGTTAGA

AACTTCAAACAACCAATATCTCATGGTTAACAAATGGTATACGAATGTCAATTTTTGTAC

ACTAACACGTTCTCTTTTCTGTCAATTACCAGAATGTGCAGGGAATCAAGCAGGCATGAG

ATCCATGTAGCGAAGGTTGTTGAGGATACATTCTTTCTATTCGGTGGGAGGGAGCGTGGA

GGTGTCAAAGGACTACCCGTGCCAAATTTGTATGGAATGGATAGTTCTCACCGCATCCGA

GGGAACATCAGCTTGAGGACTTGTTAGGGCTTTTATTGAGGGACTTCAGTTTGCGGAGAT

CTGGTGGTTGCATGAGCTGGAGGAACTTTCCTCCTGAGCTGGGAAACTTTTGACATGAGG

CTTGCCCTCCTTGTTTTGTATCTTGCTTTTGTTTGTACCAAAATGAAAAAAATGAAGCCT

CTCTCTATGTACCTTCATCTGTGTATAAATCTACAGTTCAATAAATATACTAATAAAAAA

GGCTCAGTACTTTGTGGCCATATGTTTTATGTAGCTTACATTATGTAAGTTGAACATGAA

CTTCTTTTGTGAGTTATTTGTTTTTGCTGATGTGTGTATTATATATGTGGAGTTAAAAGA

CATTTTAATTAGTACTTTATCTGACTTCCTCTGTTTTTATGATATCTTATCATTTGTTTA

CTAATATCTTTTCTTGTATCATACAGAGGTTAGGATTACTACACTCCCCAGCCCATGGTT

GGATTGGATCTCAAGGTGGCTCCTCTGGCCTTGTTATCAAGAAGACAATCCGGGTTGACA

TTCCTGTGGACCA

>Unigene0000795

GGCCAGGGGCACTAAATCTAGTTCGTCCGCTCCTCTTTCTCTCTTCTGCCCTCAACGTCG

GAGGCGGCGCTGATTCATCTTCTCGCCGGATCCAGTCTCTGAGAATCTACAAATGCCGAA

GATCAAGACCAGCCGCGTTCAGTACCCCACTGGATGGGAGCTTATCGAGCCCACTCTTCG

CGAACTGGAGTCGAAGATGCGCGAAGCTGAGAATGACCCACACGATGGGAAGAGGAAGTG

TGAGGGGTTGTGGCCGATCTTCAAGATCGCGCACCAGAAGAGTCGATATATATTTGACTT

GTATTATCGGAGGAAGGAGATTGATAAGGAGTTGTACGAATTTTGTCTGGATCAAGGATA

CGCGGACCGGAATTTGATAGCGAAGTGGAAGAAGCCAGGATATGAGCGCCTCTGTTGTCT

TCGATGCATACAACCACGAGATCACAATTTTGCAACCACTTGTGTCTGCAGAGTTCCGAA

ACATCTGCGGGAGGAGAAGGTGATAGAATGTGTCCACTGTGGCTGCAATGGGTGTGCCAG

TGGAGATTGATCATCTTTATTTGCAAAGTGATGATATCTATATTGTTCGCAACAACTGTT

TATGTATGACTGGTTGTATGTGTCCCGCTTCCTGGATTAATTGACTATTTCCCGTATTTG

GTCATGCCCATGGGACATGTAGTGAGCAAAGAACCTATGTATATTTTGCTGATTACAATT

ACAGAACTCTGGCCTTTAAAATGGGTCTAAGAATTGTCGTGTATCTTGAGATGGTATTGA

GATAGTGGCAGGTACATGATCTTGGTGCAAACACTACCTCTATTTAGTGTCAATCACAAT

GCAGGTGGTTTTGGTCAGTCCATTTGGAGCATGCTGT

>Unigene0000796

ATGCAGCTGTTGACCGGTTGAGGAGCCAACTTGAAGCATATATGCTGAAGAAAAGGACAG

AAGATCGTATAGGCAGACACGAGGATCTGCCAGGCTTGTCGGATGTTGAAAAACACACTC

CGATGGTTCGACACACAGCGAATAATCTCATAACTAATGGAGGCAAGGAGGTGGGAGAAC

TTGAAGACAGCGATGAAGTGGATGATGGGACGGACTCCGTGGACAGTGATTTGCATTCGA

TTGAGCTCAACGTGGACAGCAGCAGCAAAAGCTACAGCTGGAGTTACGCTACTGCGGCCA

CCAAATATGGCACTAAATCAGAGTTACTTGCAGAGGAAAAAATTGGAAGGATCATGAGCT

ACGATCACATACCAAGAAGAGGCATGGGCCTCAACAATACTACCTGTCATCTGCACGATG

AACGAGATCAAGAAATACTCTCCGGGAGCAGCAAGATACCGGAGAGTCAGGCACGGAAGC

TGGACCATGAACGATACACATCAGTGAAGGGCCTTAGGGACCAGATATTAGCCGCATCTA

TGATTTCTCTGCCAGGTGGTTTAGCAAGTCCAACACATTCACGAGATATTATCGGTCAAG

TTTGTGAAGGATTAAATATCGGGGAGATGGTGAAAGCAGCAAGGGAGAATGGACAGAGGG

CAAGAAGAAACAATACATGCTCTCAGTTCGCGACAGATCCTATTCAGTAAAATTTATCAG

TATGTTTTGTATTGTTTAGAATATCGAGTTGAGTAAATCGCTTCTCTACCAAAAGTGTGA

ATATGCTGGTAATACATGTGTAAATAGAGATAATTTGCGCACAACTATGACATTATAGAT

CATTATAATGTGTCATTAAATGAGGTTCTATTTGAGCAATAAATGTTCCTGCAT

>Unigene0000797

ATACAACCACAAGACAGAGCAGCCAGCACAGCAGCACAATCCCTCACCCGCTGTCATCTG

CTGAATAAGCTCACCATCCGAGGCCTGAACAGAGCAGTCGGAGTCCGAGCCCCCACCACA

CTCGTGTGCCCTCTCGGGCTCTGCACAGAAGGAGAGGAACCATTAAACCAATCAGGGAGT

CTTGAAGTTTTTCGTAATCTCGAAAATGGGTTGTCTCTGAATCCGTGATCTTTTCCTAAA

GTTTGTGTCTTTCTCGATGAGAGAGCTTCTCTCTTTAAATGGTAAAGAACAGAAACTTCT

AGGAAAGTTTACATAATCGTGAAGGTTTGGCCGTTGAACTCCATCCTTACAATGCCGATG

GGTGAAGCATGCAAGATCCGCAAACGGGGCTGCTCTTCCTCGTCTCCATCGACGGCGTCG

GTGCTGCAGAAATACCGGTTGAAGCGGCCGGTTGTGGTGAGGAAGAAGAGGATGAATGGT

GGGTCACCGTCGTCTGGTTCGTCTCAGTCGATACAGAATGAGGGTGGTGGCTCGCAGGCG

CCGGTTTCGGCTAGGAAGCTGGCTACTGCTCTTTGGGAGGTGATGAACAAGATCCCACCT

CCGGAGAGACAGGGAAGAGGGTTGAAGCAAGAGAGCAGGGGGAGAAACAGTGTTGTATCT

CCGGATTTGGAAAGAACGAGAATGATTCCGGTGGGTCGCCGGAAATATCAGTATGAAGAG

CTTATTCATGGAGGTATGGATCTCAGGAGTAGTGGCAGTTTAATGGAGACTGATGCACAT

TCTCAAGGTCCCATCGCCACAAATTTGGCTGTAGGAACGCGAAGCTGCTTGAAGGATCTA

CATAATAATCTCATGACATCCAAAGAAATTCTGAAATTACTTGGCCGCATTTGGGGGCTA

AAAGAAACACATAAACCTGCCAATTCCCTTGTTTGGACCCTCCGTGAAGAGGTGGATCGG

GCATGTGTTCACATTGATCAGCTCATTCAAGAACACAGAACTGACCGAAACACGCTCAAC

TTCATCAAGAAACAGTTTACAGAAGAAATGTCATCATGGAAGAGCAAAGAGGAACAAAGA

ATCAGGTATGCTCTGCGTTCTATCATGGAGGAGCTCGAGTCGGAGAAGCTATTGAGGAGG

CGATCAGAGAGAATGAACAAAAACCTGGGAATCGAGTTAGCCAGAATGAGGGCATCATTC

GTGAAAGCAGTGAAAGAGATCAAGGACGAACGAAGATCACGAGAGATAATAGAGCAGGTT

TGCAATGAATTGGTTAGGGGGATAGGAGAGGACAAGTTTAAAGTAGAAGAACTGAAGAGA

GAGTCTGCCAAGTTCCAAGAAGAGCTCGAGAAGGAGAGAGAAATGCTCCAGGTTGCTGAT

GAATTGCGTGAGGAAAGAGTTCAGATGAAGCTTTTAGAGGCGAAATATCAGTTCGAGGAG

AAAAATGCAGCTGTTGACCGGTTGAGGAATGAGCTTGAAGCTTATATGACAGCTAAAAAC

ACAAACGGGATCGTAAGTTTGTGTGGCGATTTTTCTGGCAGATTAGAGGGCGATGATCAC

AGCCAGTTGGTTCACCCAAGAAAAAGTTGCAGGGCGGTTAATCTAAGTGTTAGTGAAAGG

CATGAAGCCAGAAAACATGAAGGTAGCGATGGAGAGGGTGACGAGATGGGCTCAGAGCGT

TCGATCGAACTCCAAGAAGAACAGGAGCAGGAGAATGAGATGATTCAGATTGCTGAGTGG

CGTGAGGAAAAAAATCAGATGAAGGCATCAAAAGCCAAATCACAATTTGAGGAGAGAAAT

GCAGCTGTTGACCGGTTGAGGAATGAGCTTGAAG

>Unigene0000798

CCAGAAAACCATCTCGCCGACCCCCGAATTTGAATGAAGAAAATTCCGCCCTAATCCCCG

ACCCCATGGAGCTCTTCAAGGGCGCCCACGTCGTCCGCCTCCGCAGCCACCACGACAAGT

ACCTCATCGCCGAGCACGATTACGAGCACGTCTCCCAGGACCGCAGCGGCGCCTCCAGCG

CCGCTCGCTGGATCGTCGAGTTCGTCGAGAACGCCCCCGTCCTCCGCCTCAAGTCCACTT

ACGGCCGCTACCTGACCGCCACCAACGAGCCCTTCTTCCTCGGCGTCACCGGCCGTAAAG

TCATCCAATCCCTCCCCCGCCGCCTCGACTCCTCCGTCGAGTGGGAACCGATCCGCGAAG

GCTTCCAGGTCAAGCTCAAGACCCGCTACGGCCACTTCCTCCGCGCCAACGCTGGCCTCC

CCCCCTGGCGTAACTCCGTCACCCACGACATACCCCACCGCACCGCCACCCAGGACTGGA

TCCTCTGGGACGTCGACATCGTCGACATCCCCGCCGAGGACCCCATCCCCGTCTCCCCCA

AGCTGTCCAAGCAGGAGTCGGTGTCGTCGACGTCGTCGTCGATTCCGGGCTCGCCGCACA

GGTCGGATGGGCGGACGATCTGTTATGTGATTGCGGATGATGATGGGAACGCGGTGGATG

GGGATGAGGGGGGTTGGTTTATGTTTAAGGGAACGAGCGTGGCGGAGCTGACGAAGAAGC

TGGAGGAGGAGACGGGGATGGGGGATGTATTTGTGTGCTCGCGGAATCCGCTGAATGGGA

AGCTTTATCCGCTTCGCCTGCATCTGCCGCCGAACAATGTGACGATGAAGGTTGTGGTTG

TTCCTGCTAGTTCTAAGGCGGCGAGAAGTTTTAAGAGGACAGAGAATACTATGGAGCATG

CAAAGGACAAGGAATAAACCCATTCCCTCATTCCTCCGCACTTGGTCTGTACATTCTGCT

GCCCATATGCAAACACTAGCTCGGTACATCTTACTCATTATTTTTGATCTGGTATAGGTC

GAATGCCTAATTTCTCTCCGCTTCACTGATTTAGCTTCAGCAAGATAACTTCGAATGTCA

TCAAATTTTGTTAGATCAGTTTTTCTTTTGAACAATGTTTGCAGATCATTTGTAGATTTA

GTCTGCTAAAGATTCATAAGAACTGCATTCTTTTTGAATTCACGTTTGAGGTCCAGAGTT

TGGTTATTTTGCATTTGCAATTACATGTTGGTACTATAC

>Unigene0000799

AAAAGTGTCGGCCAGCTCCTTCACTCGATTCTCGCTCCCCCTTACCTTCCCCAATCCCTC

GCCATGCAAGCATTTCTCTCCCCTCTCCACCTCTCTCCCCCTCTTCAGCATTTCATCAAA

CACCCTCCTTGCAGGTCCTTCACAAGGCTCTCACCAATCTCCCACCCGCCCAGGCCATTT

CAGCCACACCGTGCACTCGCTCGCACTTCAATCTTGATAAGGTCAATGGCGACTGGTGGG

GATTCGGAATCGGATGGGGTGCCGAGGTCGGTCCCTGTCCATGTTGCACATGAGCTACTT

CAAGCTGGGCATCACTATCTAGATGTCAGGAGCGAGGAAGAGTTCAATGGCGGTCATGCA

GTGGGAGCTAAGAATATCCCTTACATGTTCAAACTGGGTCAAGGGATGAAAAAGAATCCC

AACTTTTTGAAGGAAGTGTCAGCTGTTTTCGGCAAGGATGATGAGATAATTGTGGGATGC

TTAAGTGGGAAAAGATCTCTCATGGCTGCAAATGAGCTTTTATCTGATGGATTTACGAGT

ATCACGGACATCCAGGGTGGCTTTTCAGCATGGGTGCAGAATGGACTTCCCACGACACAA

TGATTTGTTCTGGTCTGAGCATACTTCAAAATCTTCTGGTGAGCAAATACATTTTTGATG

AGGACAATAATCAAGGATATGCAATTTTATGGTGACTGATTCAATAATCTTCAGATCGAA

TAGGATAAACAAAAATGATGTAATCACCGGAAGAAACTTCAATTTATTAAGTCTATTTGA

ACACTTTTCTTTGTTTTCGGTATTCAGAACCCTTTTGCATCTGACCAATGTTATTATCTA

GTATCTTCT

>Unigene0000800

TTTTCGTCCTGTTCGAAATATTTTGATGATGTTAGATGATAAAATAATTAATAAAAAAAT

TTTTAATATTAATTCTAAAAATAAAATTCATAATCATATTTCCTTTAAAGAAGAAGAAAT

ATATCTTAGACACGCTCAAGAATATCCGTCTGTTTTATTGAAAAATAGTAAAATAATAGC

TGATTATGATATTAGAAGAGAAACAATTAAAAAAGAAATTAAAGACATCGCAAAAAAAGT

AAATGGATCTACGGAAATCAACTTAAAACTTTTAGAAGAAATAAATTCAACAGTAGAATC

ACCTAAGGGTGTTTTAGCTACTTTCAAAAAAAAATATATTAAATGCATACCAAATGAAAT

TCTTGTTTATATTATAGAAAAACAGCAAAAATGTTTTCCAATATACAATGAAAAAAAAAT

TTTACCATATTTTATTTTTATTACCAATATACATTCAAAAAAAGACAATCA

>Unigene0000801

GAATGTTCTGCTATTGCTAGTCATTATTTTGGGTCCAGTGTTGATATACATTCTGGAGGA

ATAGATTTGTTATTCCCACATCACGAAAATGAAGAAGCTCAATGCTGTGCTTACCATAAT

GTCGATGATTGGGTCAAGTATTGGATACATTCAGGACATTTACACGTAGGAGATAATATT

AAAATGTCAAAATCTTTAATGAACACAATCAGTGTTAAAGAACTACTCAAATCATATACT

GCGAATCAATTTCGAACATTATGTTTATTATCAAATTACAAAAATGATTTGGAATTTAAT

GAAAGTACAATGGATGTAGCTTGTGGAGTGTTAAAAAAATTTGATTGTT

>Unigene0000802

CATGGCACGTTGATGAACACCTGTTTGGCAGCGAATTCAGGATAATTGTCTTGGAATAGT

TGCAGAGCTCGCTTGGTGGCGTTTCTGAGCTCCCACTTGGTGAGTCCAGGCGAGTTTTTG

AGGTCGTTGACTTGAACAATAGTGGAGATGCCATTGGGGCTGAAATCGAGCTTTCTGATA

CTCTTCTCCAGAAATTGAACCCTCCATCGGAGAAATTTGAGGCTCTTT

>Unigene0000803

CTTTTCCCCCCGAAAGAACTGCATTTGTTACCCGTCTTGTTCCGTCACACGTCCCATCCC

CTCACCTCCTCCGCCGCCCCCGGCGGCGTCGAAATCATCTTCGCCGCAGTTCCATCAGCT

TTCCCTCGATCTCATATGTTTATCCCTAGATCTCATCTCTCTAGGGTTTAAGACGGCCGA

TCCGATGAGGTAAACGGCGGAGGAAGGAGAGAGAAAGATGCAATTAGAGTGAAGCTAGCG

AGAGAGTACAGTGAGAGAGAAGGTGAGAGAGATATTAAATGCGTTGAAACAGTCGGCAAA

TGTTCCGCCGCAGCGAGTCCGCCCTGTCCTGCTTCTATCTCTACCTGCTCTGGCTCTATT

TCCGCAAATAGCTGTCAATCCACGATCAAAATACTAGCAGATCCGAGAGACGAGACGTCA

TGCTGGATCTCAATTTCGCCTTCCCCGGCGAGGATCCCTCGCCGCCGCCACCGCCGGCCG

TCCCCGCCGGCTCCGGCGGGGAATCCGGGACATCGAACTCATCCGTCCTCAATGCTGACG

CCTCCGGCAACACCGCCGCTGCTGCAGATGCTGATTCGTCCACGACTATCCCCTCCGCCG

CTGCTGCCTTCCGGTTTGGTCTCCTCAAGAGGCCGGCGTCCGCCGATCGTGACGAGGTAG

TTGATGAGGCGGTTACTCAGCAAGCCAGCTGCTTCACTTTGCAACTCTTTCCTCCGGCGG

AGATAGCTAAGGAGGCTGGTGGCGTAGTCAGCCAGCGATGGCCGCCGGCGTTAAATTCTT

CATCGGCCAGGCCAAGATGGGCCGATCTCAGCTTCTGCCAGACTGGATTACCGTCGGAGG

CGAAGGTGTTGCCCCAGCCGCAGGTGAAGAAGAGTCGTCGGGGGCCGCGGTCGCGGAGCT

CGCAGTACCGGGGTGTCACTTTCTATCGGAGGACTGGGAGATGGGAGTCTCATATCTGGG

ATTGCGGGAAACAAGTCTACTTGGGAGGTTTTGATACTGCACATGCGGCCGCTAGAGCAT

ATGATCGTGCCGCGATTAAATTCCGTGGGGTTGATGCTGACATTAATTTCGATCTAAGTG

ACTATGATGATGATATGAATCAGATGACTAATCTCTCGAAGGAGGAGTTCGTGCACATAC

TTCGCCGGCAAAGCACCGGTTTCTCGAGGGGGAGCTCTAAATTCAGAGGGGTGACTCTTC

ATAAGTGTGGACGGTGGGAAGCTCGCATGGGCCAGTTCCTTGGCAAGAAGTACATATACC

TTGGTCTGTTCGACAGTGAAATTGAAGCTGCAAGGGCTTATGATAAGGCAGCTATCAAAT

GTAATGGAAGAGAAGCGGTCACAAATTTTGAGCCAAGTACCTACGAAGGAGAGATACTTC

ACGGGGCTGATATTGAAGCGGTTGACGTTGATCTGAACTTGAGTATCTCGCAACCTGTTG

ACCATAATCCACAGCGTTCTGACAACTCAATGGGTGTTGAATTTCTTCATGGCTCCTTCA

ATACGTCTGATGCTAGGAAATCCAGGATTAATAACACGTGTCCTCGCCTAGCTGATCAGA

GCGCACAAGTTTGGCCTGCTCCATACCCCGGTTTCTGTCCTTTAATTGAGGGAAGCGGGA

TACAGAAGATGCCCGCATCGGGCTTGCTAGTTCTCCCCAACTGGGCGTGGCAAACACATG

GCCCTACGCCACCAATGTTCTCTTCTGTTGCAGCATCATCAGGATTCGCTACAACCTCTG

TAGCAGTTACCCTTCCACCCGCCCCGCCTCCTCCCCCTAAGGCCGTCCATCACCACCACT

TCCCTCCATCTGCCTCAACCACTCATCATTACTTCAGGAAATGATAGTCAGAGAATGATA

AACTTGTTCATATATCTTATATAGTTGTATGCATGATTTATCCTATTATACAGGCATATC

TAGTGTGGGCCTCAGTCAAGGTGCAGACCTTAGGATAGAGTTAATGCCAATGCTTTTGGA

CGGAAGATGTGTTTTGTGACTTTGAAGTACAATGAATGGACTTGTCATGGCTGTGTGTAA

GAAAGAAAGGCTAGCAGCTATGTCTATAGTCAGCCTGCCTGCAGTGAGCAGTGAGCAGTG

AGCTTACAGTCTTTATGTAATTTCATTTCTTTCTTTTTGCTTTTCAGTGTGTGAATTGTT

ATATATCTGTTGAGTGGAATTCTCATATCAGATTTCGGAGTCCTTGGCATTTCAAAGCCA

ATGTTACTGTTGATCATATCATCAATATTGATTTTAGTTTTGATAATGGTTATGCTTTAA

TGGTACACGATCT

>Unigene0000804

CTGATAAACCATTACCTCAAGAAAGTGCTACTGAAAAAATAAGTAATCTTATTCATAGTT

GCACTTCTCATCAAGGGAAAATTTTATTTTTGGATGCATTTTTTCTTACTATCAAAGATG

ATTGGATGTCAATAAGTCAGCATCGTATTGATAAGTTTATGATGTTAGTGAGGCGTTGTT

TAAGACAAATGTTATTTACCTTTGTCAATTGTGATTGGGAATCGGAGTATATGGAGAAAT

TTAGTGAGGTTTTATATAGAGCTCTTAAATCATTCACATTAAGTTTGAGAACTCATTTAC

AAGAAATTTTCTTGGAAGAGCTTGCTAAAGTAAGTAGAGGAAAA

>Unigene0000805

GTCTGGCAGGGCTTCCACCACGAGGAGGTGTCCGTCGGGGAGACGAGCTCCTGTCATAAG

AGTGGCTCCGGCTCCTGCTCCTGGCCCTTCCCCTTCCACGATGTCCATAATCATCTGGGC

TTGCACTGACACTTCTGCTTCGGCGGCGGTAGTCTCTTCCACGATACTTATCTCGGCTTG

GACTAGGGCTCCGCCTCCTATAATCTCTATCCCTGTTATCCTCGCGGTACCTTGGCCTAG

GGCTACGA

>Unigene0000806

GCAGTAGATTCTAGTCCCCTCCCTGAAAATTTTACTACCATTGCCAATGAAGACTTGTGG

TTCCAGTTGAACAAAGGTTTTACCACTGATCTTGATATGCTTCAAATGACGCCCAATATG

AATCACCGTTCATTAACTGATATTTACACTAGTTTATCTTTAGATGAGGGGGCCATCATC

GCTGAGGAAAGATGCCACAATCTAGAGAGTGAGTGCAACCTCAACAAACAACAACTCGAG

GCTTCAAATCAACAATGTGCTATCCTCCAGAAAGAGTATGACCTACTGAAAACAGAGAAA

GCCTCTCTAATACAAGCAGCCTCTGTATCCAAACAACAGCTCGAGCACGTCACAGCTGAA

AAGGAAGAACTGCAGAGGAAGCTGCAGATAGAAAAGGAGAATATGCGGGAACTCAAACAC

GAAATTCGTCAGTTCAGCTTGGCGTTTATGCAGCGCGAGGGCGTATTCTCGAAGCTATAC

ATGAAATCGAGGGCAATCGTCGAGAACCTTAAAGCCCTTCGAGCTCCGGTATCTGAAATA

TATGATTGTTGAAGGTGTAGAGCTCCCGTGTACCATCGACACTATATACTTGTAGTTCAT

GTGGTAATTATCACCTTAATTATGACCAATGATATATGACTGAATTCACTTATAATACAT

TTCTATGTATGTCTATCATTATTTGTTAATGAAATATCATCGGATATTTGAGCACCAAAT

ACTGAAGTGTTTTTGGAT

>Unigene0000807

TTTGAGTTGCCAAGCAGGTTCAGCCCATATTGGCTCATGGGACAGCTATGATTGGGCGAA

TGCATCAACACGAAGAGTGATCAAGTGGGACACGAAGTGAAACCTGGAGGTTTTCCAGGT

TTCAATGTGAAGTGTGAAACATTGTGGGGGTAGGTGCACCGGTCATCTACAGATGTTTTG

TTTTGATCTTTTGGACGGCGGGGTTTTCTTGGGGTGACTTTGCCCCCGTCATACTTTATT

TCCCTAGTTAGCTACCAGCTTTTACCTCCTAGGATGTCAAAAGGGTCAGATGTTTGTATT

GTTTTTGTCAGTTATATTATTCAGATA

>Unigene0000808

GTTGCTCTCCAGGATCTGTTGTTCTTTCTCTAATGTCAGGTGCCTATAGTTCTAGTTGGA

TCATTCTTCCCGCGGATGAGTTTTACTGGTCAGCTTAATGTCTCTTTTGAATGGATGAGA

TATGTTTGGTGCAATTAGGGTGTTTGTTTTTCTTGTTTGTGGTTTTAGATGTCTATGCAT

GATTAGGGTGCAATTTATGCCGTTAAAAGCGTGACTATGCAAGATTCGGTACTGGGTAAG

CAATGATGTGCAGCTG

>Unigene0000809

CTGGTTTTAGTGGAGTAATTGCTATGACGTCAAAGGATATTGTAACCATGATGTGGTATT

GACGGTTAGGTAATATGTTAGAGAAGGGCATATAAGTGATGCAGGGAAAGAAGAAGCTTT

TGGGACTCAAATGTATTGGCTTTAAGCTTTGTGAGCACAAGGGAGTGGGAGTCTTCTAAA

GCAACAACGACTCGAGTTGGTACACACCAATATGTGGGGTCCTTAGTCATTCCTTCTATT

GACAACTCTCTC

>Unigene0000810

GAGAGTGGTTGTTTAGGTGGATGGGGTTTGGCTCTTTAAAGTTTGGACTTTTTTTAATCA

ATCAAGCTCCAAAAACATACAATTAAAATTGTTAAATCAAATATACGATAAAACTCTCAA

AGACCATGTAATTTGGAAAATGTGGGTTCGGAACCCATATTGTTCAAATCTTTTTGAAAG

AGGAAAATGACCAAGAGAGAGTCGCCAATGGGTGGGTGAGGGGTTTGTCTCTTTATAGGT

CAGACTTTTTTTTAATCAAACCCCACAAATATACAATTAATATAGTTAAATCAAATGTAT

>Unigene0000811

GGAAAACTATTGAAAGTAGCTACAACTCTAACCCATTTCAAAATATAGCAGATGGATTTC

AATCCAGAAGTTGTCAAATACAAACCTTATATGTGAGCTTTAGAGATCTCGAATGGCAGG

ATTGGATAATAGCACCAGATGGTTATGGTGCATTTTATTGCACAGGTGAGTGTAATTTCC

CGCTCAATGCTCACATGAATGCAACTAACCACGCAATTGTTCAGACATTGGTGCATTTGA

TGAGTCCTCTACAAGTCCCCAAACCATGTTGTGCTCCCACAAAAATGGCTCAACAAGCAG

TACTATTTT

>Unigene0000812

GTTTGGCATGGAGATATCGGAAGTATGCTGTCTGTATTGATGAGGAGTTATTATGGAGAT

AGAGTGTTATGCTAGAGCTCAATGCAATGAGGAGTTGATGGAGATATTAGTTGGTTGTGA

GGATATCCATGGTGGAGCATTAATTGTTAAGGAGTAGGTGTGTGTGAAGCCGTGATGTTC

TGGGCTTGATACAGAGGTCTGAGGTACGGAGTTCAATACAGAGAGATAGCTGGAAGTTAA

TTGACAGTGGAAGTTATGAAGATGCTGTGGTGTTGTGTATGCTTGACTACCATGGAGTTC

TTGAGTGGTGGAGTGTGGTGCTCCTTGTTCTTGGAGGTATTAGAGTATGGTGCTTGTTGG

CTGGAGTTAGCCTCACTGTGAGGTGTTGCTATGGTGTAGAATTAATTGAAGCTTGTGGAG

CATGAAGAGTGTGATGCTTCTGGAGCTTGCTATGATGAGGAGAATAACAGTTGCCATTAA

TGTTGTGGAGTGATGCAGATCTTGGAGTTCGAGTAGAAAGCCAAGGTGGAAGTACATGCA

GGTGTCATGGTTAGCAGTAGTGCAGGTATCTTAGGGGGCAGTGAAGCAGTGTGGTGTTTG

AGTTATCGTGAAGCTATGATGCAGAAGGATGATGCAGGGAGTTATGTAGAGCTGTAGTAG

GATGTGGTGTTTGTATTGCTATAGAGTTCCTAGTGCTTGTGAAGCTATGGAGCATCCACA

ATGGGAGAGAAAGCAATGTTATGAGTCTGGGCTTACCATGGGCAGTGAGCAGGCATAGAA

TGATAGTAGAGAATGAAACATTAAATACAAGTAACCTCTCAAAGCATTTTGGGACAGTGG

TAAGGTACATCAATGGTGAAGAACATGGCCGTGGCATCTCGGAACAATCAAGAAAGCTTG

GTACATTAGGTATTGTAGACTAAAACAAACATTTGGCATCAGATCTTATAGATAGAAATT

CTTAGTGGGTAAGAAATCCTTGGCAGCCAAGCATCCTTGGCAGGCA

>Unigene0000813

AAATACCAAGCACCACAGGTGTCACGCTAGGCCAATCACATTGTTACTATAAATTAAGCG

CTATAGGAGGCACCCCAAAAAAGTCAAGCTGAGCCTGACCCGAGCACTATTAGGAGATAA

CCCATACCCATCTATCTGAAAACGTTAAACTTAACACTACTGTGAGGCATCTCAAGTACC

ATGTTGTACTAACGACTTCTACATCTTACAACCACACGAGGCTTTTTTTTATACAAGTGT

GTGGAAGGTCACCTCTCCTATGCACCCCTTGACCCCCACTTCAGAGGTATTCGGCCTCT

>Unigene0000814

TGATTATCTCAAGGTTTACTATGGAAAGCTGTTTCCGTATGTTGATATGTTCAAATGGAT

GTCTTATGGAAATGATGGGAAACATCCAGCATGTGACCACTCCTACTTCGGCCGAAGAGA

GTTTTCTTTCACCCTGGAAAATGAAATATATCTTCGCTTCCAGTCATTCAACAATGCAGC

CGAAATGGAGAAGTCCATCAAAGAGAAGTGCCCCTTTAAAATTGATCTGGGACCTGTGTA

TAGCGTAGATCCCGCAAAACGACATGCATATGCACAGGGTGGTAGTAATGTTTTCACTCC

AGTAGAAAGAGAGCTCATTTTTGATATTGATATGACAGACTATGATGATGTTAGATACTG

CTGCTCTGGAGCTGATGTATGCTTAGATTGCTGGCCATTAATGACTGTTGCAATTAAAGT

GATGGACACTGCTCTACGAGAGGATTTTGATTTCAGTCACATTTTATGGGTTTATAGTGG

GCGTCGTGGTGTCCATTGTTGGGTATGCGATGGCCGAGCAAGAAAGCTGAGCAACGAGCA

AAGAGCGTCAATTGCTGAGTACTTTCGGGTATATAAGGGTGGAGAAAATAGCTTGAAGAA

AGTATCTTTATCAGGACTAGTTCTTCATCCTTTTCTTGCACGAGCATATACAGATGTTTT

GAAGGTCGTTTTTGAAAAGAACTTGCTCTGCAGCCAGAAACTATTTTCAACTCAAGAAAG

GTGTCAAAAGATCCTTGAACTCATTCCTGATGAATCTGTTGCTGCAGAGCTTCATGAAAA

GTGGCAAGGAAACCGAAGATCTTCCAAAGAGGACCTCAACATTGTTCGATGGGAGCAACT

GAAACATGTCGTCCAATCGGGGAAGCAAAAGGCCCAGGGGCTCCGGAGGTGTGTTGAAGA

GATTGTTTTCACTTATGCCTATCCGAGACTTGACATGGAGGTTTCAAAGCACATGAATCA

TTTATTGAAGGCACCCTTCTGTATACATCCCAAAACAGGTCGTGTTTGTGTTCCTATTAA

TCCAGACGACTGTGATTCTTTTGACCCTACAACTGTTCCAACCCTTTCTGAGCTTTTAGA

AGAGCTTAATGTTGCTGACGTGGGAGGACACTATGAAAATGAATGGGAAAAGACTTCACT

TGGCAAATACATACATTTTTTCAGATCATCATTCTTGCAGCCACTACTGAAATCGTGCAA

GGAGGACATTGAAGCTTCGTATAATGCAAAGCTCCAGCAGTCGAAGAACCCGATGAGTTG

GTAAACCAAATTTGAGCTTCTTGATTTTGAATTTCAATAGAGGTTGGATAGAATGGTCAG

GAGCTCATCGGCATGATAAACAATGGCCTCACTGTAACGAATAATTCAATTTTGAAGTGT

TGACAAAACTTTGGTTGTGATTGTTCTGTAATATCATTACTATGCTATGTAAAAAATACC

TTATATCACGCGGTCTGTCGAATTCAGCTGTCTGGACAGTATGGCTTTTTGCAAAGCT

>Unigene0000815

CTCCCACTGTTGTCTCTTCGTGAGGGAGAGGCGATATTTGCCTCTCTTTGCCTCTGCTAC

TGGTTCTCCCAAAAGTTGTCCTGATTCACAAATTAACTTTTCCCCTCCAATTGTTTTTCC

TGCTGTGGATGCTCAGAACAAACTTACTGATTTGAGATATATACAGGTGTTTGTTTAAAA

TTGAGGTTCTAGAGAAACACCCAAGTTTCTAAGAGCAATGTTTTTTCAATGTCTTTTCAT

TTTTGTAGAGCTGTTTGTCGTTATATGAC

>Unigene0000816

GCATTTTGCACTCGTGCGAAGAATTGATACCGTGCGCGCGCGCGCGAGCACTGGCAAAGG

TGGTCGAGTTGGGCGATGATACGCGTCCGGCGGCGTGTGCCGAAAGCCCGTGGCCAATGA

TCGCCGTCGTGACCCGTGACGACCACGGTAGTCGTAGAAGTGATATTATGACCACAACCG

CAGCGGCAGTAGCGACGGCGGCGGCGGGAGCGGCGGCTTGGCAGGTCACCACTCGGTCCT

CTGGTTCGCCC

>Unigene0000817

ATTTGTTCCTGGTGACTACTGGATGCCTTCAGTGTTACCTTCGGGTGGTGGGCGAGCCGG

ATCTGGCATGGAGGAGCCGCTTCTGACAGCACCTCAACCTGCATGACAACTCATTCAAAC

GCTGGAAAATAGTGAAAAGATTACCACGGAGACATCTTTTTCTCTGAAACAAATAAACCA

AAAATTCTGGGCATGCTTGAACCACATTGGCGTTTTCCCTAATTTTGATGGATCTAGGGG

GTAAATCAGCCCGAGAATACTTTTGGGAGCTATGCCAGAATCACCTAATACTGATTCCGT

TGGCAATTTGTTATACTACCTGAATTTGTTAGCCAACTG

>Unigene0000818

CTAAAATGTATAGTCAACTGAGACAAGAGTCTATGATGACTGGAAGTTTACCTATTACTG

TCCGGCATATTGAAAGTATGATTCGTATGGCTGAAGCAAATGCTAAAATGCATTTAAGAG

ACTATGTTCAAGAAGATGATGTCAACATGGCTATACGTATAATGTTAGAATCATTTATTG

AAACACAAAAATACAGTGTTATGAAAACAATGAGAAAAACATTCCAAAAATATTTATCAT

TCAAAAAAGATACCACTGAACTTTTATACTTTATTTTACATCAAATGGCGACCGATCAGT

TGACTTACATTAGAGGTATTCACGGTGTCACAGTTAACACAATTGAGATACACGAAAAAG

ATTTCAAAGACAAAGTAAAACAAATAGATATACACGACTTAAGACCATTCTTTGAAAGTA

AATTGTTCAAAAATAACAATTTTGTGTATGATGAAAAAAGACACATGATTATACAAACCT

TACTTCTTGGTGAATAAATACATAG

>Unigene0000819

GAGGTTTCCGCCGGAGGAGGTGGTGCCAGTGATGAAGCTGGGGCTGGCTTGTATGGCTCA

TATGCCGTCAAATCGGCCGGAGATGGAGGAGGTGGTGAACATATTAGAGATGATGAGGGG

TCCCATTGATAGCCAGGAGGAGGAGCTAAGCTGAGGATTCAGATGATGTTGCTGCTGCTT

CTTCAGGTTTTTCTATGGCCTTTTTCATTCAAATCATACTAGATGGCTAGCATTTGTTCT

CTGTTCTACCACAATTGGTAGGATTTGTTAGTTTTGATTT

>Unigene0000820

GGAGAAAGGTATAGTAGCAGAATTCTGCCCATGGTATCCATTCGACCCAATTATTTTGCT

GGGATCCAGCAAAACAACAAAGATATTGCTCCAAGGTTCTGTTGACAACCTCCGATTAGC

CATTGATATGGGGTGGTAACTAGAACTCATAAAGAGTTTGGTCCCTTAGAGGCAATATAA

GGCATACCAAAAATTATTGGTAAAGATCTTATCGTGGTTTCTTACTATGGAAGCTGAAAT

TCTATGTAAACACACCACATTGGTGACAAAAACTCATGCCACTGAGGTAACAGAGAAGTA

ATGCTTCATCGGCACAAAGTGCGCATATCTCGTTAGACGGTCAACTATCACCATATTGAT

GGAGTGCCCATTGGCCATAGGAAGGCCTTCTATGAAATCCATGGACATATCAATCCATGA

TTGATTAGGTATGGGAAGGGGTTGTAACAAGGCCAACTGGATTCATATAGTATATCTTGT

ATTACTGGTACACCTCAC

>Unigene0000821

ATTAAAATCGATTAATCGGTCAGGCGATTAATTTGGGCCCAAGAACCATATCATATCGAT

CTACATAAATGATTAATTGAGGATTAATCGACAATTAATCCCGATTTTAACTACTTAGGC

TTCCATGTATGATTCTTTCGAAGAGATGCCATCTCCTTATCCATAGCCTCCTTTCACTCT

TCTACATATGCACTATGCAGAGCCTACTTGTGTGTACTAGGAGTCTGATCATACCCTTCG

AGGACGGAGTCAGCACAAACTGCAAACTCCTCACA

>Unigene0000822

GGAGGGATTTCGAGAAACCCTAACCTTAAATTGAACTCAGCTGTAATACCATGTAAAATG

AAGAGAATTGGATGATCTATTAGCCCTTAGGCAAAGAATAATTATAATACAACATAGCCT

AGCCTCTCAGCTAAGTCTCATAGGCCTACAAACATTTAGTACTTGATTTCTAAGTAGTGA

TAACCACCACAACATTCTACTCCTAAACACCACAAAACCTGTTTGAGTTTGTTGTTAACA

GTTATCCAAGTTATGAGGTATGTGAGAGAGTGTTTTGTTGGAC

>Unigene0000823

GAGCCAAGGGAAAGCTCAAGTTTGTTAGTGTCGGAGGTGCTGCCTTTCACAGGCGGTGTC

GCGGTAGTGATGGAGTCGGAGGATCCTTACAGGGATTTCAGATTCTCTATGGAGGAGATA

GTGGTGGCCAATGATGTGAAGGACTTGGCTGGGATGGAGGAGATGTTGAGTTGGTACCGG

AGGCTTAATGGGACGAATCTAAATGGCTTTATAGTTGGGGCATTTGTTGATTTACTGGTT

TCTCTGTATGATCCTCACTGCCCATTTGTTTGCTCTGGTACTACTCCATCTTCTTGTACC

TCTGACGAGGGTGATAGGGTGGTATAAGAACATTTTTAAGCTGTCTGTAATACCGTACTG

CTGTTAGTGCTAACTATAACAGTTACGGGGAACAAGTCCCTGGGTATGACAAC

>Unigene0000824

GGATTCCTTGCATGATTTCTTGTGGTTTTATTTTATCTCTCATAGCGATGGATTGGATTG

TCAAGAGGAGATCCTGCTTCCATGATTTGGAGCACAACAATCTTCAACTTAACCTCCTCA

TCACCCTCTTTACCCATCTTCCCGTCCAATATTAATCTTTTTCATCAATAATCTTTTTCA

TCAAGTTCTCTTTATCAAGTTCACAAGGAAACATGATGGTATGAACCCCGACAAAGACTT

ACCAGATTAGTCCCACATAATTTAGGCATTTG

>Unigene0000825

CCCAAACTGTACTAAAATGGAAACAGTCTTTCATGCTCATCCGAGGACTCCAGATTCTTT

CCCATTTCATCACTACTTCCTCTCGACCACCAACCTATCGCATCAGAGCGCCACCCATGG

CCCCAATCCCTAGCTCCTCCCCAACCCTCACCCAGCTGCCCCAGGACCAAATCGCCTCCA

TCCTCCTCCTCCTCCCCTCGGACTCCATCATCTCCTTCTCCATGACCTGCCGCCGGTTCC

GATCGCTCGCCACCGCGGGATCCCTCTGGGAGTCCGTCTGCAGGCGGGATTGGGGCGGTA

CCTGCGTCGACGCTCTCATCACCGCCTATGATGAGAGGGCGCTGGACTGGAGGAGGCTGT

ACCGGGAGATTTCGCAGCTTTCGACTCTCGCTTGCCATCGCGTACCGAGCATGGACGGGG

CTGGGGCTGCGCCGCGGCCCAGGGCTTCACACTCGCTTAATTTTGTCTCGGATTGCCTGG

TTCTCTTCGGCGGTGGTTGTGAAGGAGGGCGGCATCTGGATGACACATGGGTGGCCTACA

TCGGAAACGGATTCAATATGAGACTAAATTGGCAGAACGTTCATTCAGGCACTCCGAGCG

GTCGTTTCGGTCATTCTTGCACTGTCATCGATGACTCTCTTCTCCTCTTTGGTGGCATCA

ACGAACATGGAGTCCGCCATAGTGATACATGGATTGGTCAAGTCATTCATGAAAGCCCCT

TCGAAGCCAAGATGTCATGGATGCTATTGGATGTGGGCAACGTCATGCCTTCACCCCGTG

GAGCCCATGCTGCATGCCATGCTGGTAACTGGAGGATAGTGCTCCACGGAGGGATTGGAC

TCTATGGTCTTCGATTAAGCGATACATGGCTGCTCGATCTATCGGATGGCCTTAAATCCG

CAAGATGGCACCAGGTCTTGAGTCCAAAACCATCGCCCCCAGCTCGTTCAGGCCACTCCC

TCACATGGATTGGAGGGAACCGCATGGTACTCTTCGGAGGAAGGGGTTTAGGCTATGAAG

TACTCAATGATGTATGGCTCCTCGACATGTTAGGAGACTACCCCGAATGGAAGGAGCTGA

AGTGTGACTCAGCTTGCGAGATGCCACTTCCACGAGTTGGTCACTCCGCAACACTGATAT

TAGGAGGGAAAATTCTGATATATGGCGGAGAAGATTCACACAGACACCGAAAAGATGACT

TTTGGGTGTTAGATGTGGGTGTATCTAAGAAAATGTGGAAGAAGCTAAGAGCTGAAGGGT

GCGGCCCGAAATCCAGGTCATTCCATAAGGCGTACACAGATCGATCAGGGCATTCCATAA

GGCGTACACAGATCGATCAGGGCGTTGTATCTTTGTGTTCGGTGGGATGGTTGACGGCGT

AATTCATCCAGGTGAAGCATATGGGTTGCGGTTTGACGGCGAGTTGTATCTCTTGGAAAT

TGTCCTTCAGTTGTAGACAACAATGATATGTACCGTTTGTAGGCATTTGATGGTTCTGTA

CCAAATAGCTATGAATCTACTATTATAAAAGAAAAATAACTATACTTGAAATAAAGTTAC

>Unigene0000826

TATGCGCAATGTGTCATTGGCGTGTCTACACCAAGTAATTAGTTGAAAGTCCAGGCCTGG

ATGATATCCGGGTCTTAAAAAAAACACTTGACATAACTATATAAAAGAAAGAAACAATAT

GCCCAATTATCACCACCTTGCACCCATGTCCAACAAAAATTATACCACAAGACCTCCCTA

AGAAAAAACAAACCAAAGACCCAATAGACCAAGTCCCATTATAAGAACACCCATAATCAT

GAGTACTACCCCACTCCCAAGATTTTGTCCATCACGGTCTACCCTCCAAATTTCGAACAA

TGTTCCATCTCACCACCCTTCATGGTGTGGCCTTACAACCGAAGTGGGTTGCCTCACCCA

CAAGTTGCACTCCTCTTCAGCCTCAACTCCTTCTATCCTTTGTTGCATCTGTTACACACA

TGTCCTCACTCACAATCCCATGAGTCGACGAATCCTGGGTATGTGTGATAGGCTGCTCCT

CCCTTCTCTTCACTAACAACCTAAGGAGTTGGCAAATCCTCTTCCTAGCACCCGATGTTG

GGTAGTCAGCCTTATGATGTTGGGCAAAAGTCCAAATGGAACGCGACCGAATGCTGTTCC

CTGGATGCGAGGATCCCAAGTTTGTGGGAGGAAAGAGGAGTGGAGTGTGAGTGGGAGGAA

AGAGGA

>Unigene0000827

TCCACCTTCTGACCCAATTGACAGTGCCCCGGGATGCCACACAGGAAGTAGAACGTTCCA

GGCCTCTTCAAAGGGATGGAGTCTGCGCCGGAGGTGTACGACGCCGCCGGAGAAGATGAG

TTGCAGGACTTAAACTGCTCTTGGTCCACTTGCAAGACATTGTGGAACTTGTTGTGGTAG

TTGAACAAGAGGGAGTCGCCGACATGGAATTTGTTGGAAGAAGCCCACTTAGCATAATCG

TAGGGGACGAGAGTGGTCCAGCCAGTAGAGTCGCCGACTTTGTGGACCGTAGCCGCCGCC

GACAGTGTGAAGAG

>Unigene0000828

TGGTAACAAATTCGACTGATTTAGATTGGTTATTTAATGAAGGACAGGCTGATCCTTATA

CTGTAGCATTAACACCAAAAATGTTTAATAGAGAGGTTTTACAAGACATTGAATTGAGTA

CTAAAGTAAATGGTATAATGTTACTAATGAATGGTACTGAAGAAGAAGTGATAAATAATG

ATAATTTACAAAATAATGGATTTAGTCCAGAAGATACATGTCCTAACCGTTATTCGGGTG

TTAGCAGTTGTCCTATGAAACCATGGAACCCTTATGGTACTGATATTCTTTTAAAAAGTT

GGAGTTTTCCCATATTTGTTGTTTACGATCAAGACACTATCAAAAATATTGTTGATTGTT

TTAAAAATCATAATTTACCTTTAAATAAACAGAATGATCAATCTTTATGTGCTCTGGAAT

TGAAGTCTCATATGTATGCTGCTGTAGATTCCAAAACATGTTTGAGACGAACAATGATGT

CTTATCAAAATATAAGACCAATTAAATTCT

>Unigene0000829

CTCCTACTATTGACATCGAGGGCTTCCATGGTCCTGCCCCAGAGCCTCTCATCAGGGACA

CGGAGCCGCCAACCGATGAGGGGTCGGCCCAGAGTCCCTCCCACGCTGAGGAGGGTTTCG

ACGACATTACTCCTGAGCGTCCTATCGGGGGCATGGTGCCCCCCGTAGAGGAGCCTGTGC

CTAAACCCCTTTTTGGTGCTGGAGAGGGGTCGCTGGAGGAGGAGATTACTGTTGAAGGGC

TACCCATCGAAGGGCCGCTAGCCGGTGAGGATGGCTCACCTGTGGATGAGGGCTTGTTCC

TGAGCCCCCGACTGGGGCGAGGTGTCTCCTATGGATAAGGAGCCGGTGGTGCCCGATTTT

TATGAGGGTCCAGAGGGCGACCACGATGTCGA

>Unigene0000830

TTGATGGCGATAAATACATTTATGAGGATTTATGCAGTTTTAAGTTCAGGATTTCTCCAG

ACTCATTTTTTCAAATTAACACATTAACTGCTGAAGTTTTGTATGATGAATTGTTCAAAT

TAATAAATCCAACAAAGACATCTACTTTGCTCGATCTGTGTTCAGGCACTGGTACAGTTA

GTATAATAGGCAGTCAACATGTACAGTCATGTGTTGGTATTGAATCTGTGGCACAAGCGG

TTAACGACGCTAAAGAGACTGCAAAATTAAATAATATACCCAATTGTGATTTTATTGAAG

GAAAAGTAGAAATGGTATTAAAGCACGTTATGGATGAATTGAGCATGGCCAGTAATTTGT

GTACAGTTTTAAATCCTGGTCGAGCAGGCGTGCATCCGAAAGTCATAAATGCTATTAGAA

ATAATAGGTTGATCAACAGTCTTGCATATGTGTCATGTAAAGCGGACAGTCCAATAACTA

TGAAAAATCTTGTTGAATTAGTTGTTAACAACAAAAAAACTCGTCCTTTTACTTTGGAAT

CGATTAAACCAGTTGATATGTTTCCTCACACAAAACACTGTGAATTAATTTTCATGTTTA

AACGTTAAATTGCA

>Unigene0000831

GGGCTAACGCATAAAAATGACGAACTCAAAAGGTTATCGTCGCGGTACCAGAGCTCTGTT

CTCACGCAAATTCCGTGAGCATGGATCCATCCCGCTGTCAACGTACATGAAGGTCTACAA

GGTTGGCGATATCGTAGACATCAAGGGTAATGGTGCTGTTCAAAAGGGTATGCCATACAA

GGTCTACCACGGAAAGACTGGCCGTGTCTTCAACGTGACACCCCACGCACTTGGAGTAAC

CGTCAACAAGCGTGTCCGTGGAAGAATCATCGCCAAGCGCATCAACATCCGTATTGAGCA

CGTGACCCACTCCAAGTGCCGTGAGGATTTCTTGAAACGTGTCAAGGAAAACGAAAAATT

GAGAAAAGAGGCCAAAATCAAGAAGACCTTTGTCCAACTCAAAAGACAACCTGCACAGCC

ATCAAAGGCACATATTGTTTCTGGACAACAGACACCAATTGTACTTGCTCCAATACCATA

TGAATTT

>Unigene0000832

TAATCATAGTACTGTATATGCTTAGTAACATTGGAGTCTTCTAATAATTGTGGGAATATG

GGTTGCCTTTTCTTATTAGTAAGAGAGTAAAAAAAAATGCCCTAAAAAAATGTCTTAAGG

TTCTCCCCATGAGTGGGGCTTATTTTGAAAAACATATACTAATGTCTTTGGAGCTAAGTT

TACTGATCCTTGACATAACTACGTGTGGCTGTTGTACCTCAAATTTTTGTTTCATCGTGT

ACCAAGATC

>Unigene0000833

TTGCAATTGCTAAACCATACCTGTAATAATTAATGAAATAATATATTTTATTATGTATTC

ATATCATAAACTGTATGTATTAATAATATTTTACTATCTATTATAATTTATAAAATAATT

TAACAGAAATACTGTACAATTTTGTTAACTTCTCCACTATGAATCTAAAATAGTAGTAAA

GATTCAAGAATGGTAACCATAGTGTAAAAAGTAAATAAAATGTTAAATGTTGGCCACGTA

ATCAAAAGAAAAATTTTAACAGAAAAATTACTCAGATGTATATTATTGTTAAGAAAAAAA

T

>Unigene0000834

ACCAGGAGGAACGTTTCCACCGCCTCCACCTGCTTGCCAGCTAATGCAAGTATTACCACC

ACCTGGTTGTTTCCATGGTCCATTTGTATCTGTAGACATGCTTATGGATATTTTCCTTAG

ACTTATACTTCCTGATAAAGCACCAATGCCGAAAGCTGATAATGGATGTGATGTGAAATT

ATTTGACACTGCTATGTCAGTGCAATGGGCGAATGAAGATACAGCACATGGTGGCTCCAA

TAAAAAAAAACGTAGAATAGGAGGACTATCTGGTAAAGGAGATGACAGTGATGAA

>Unigene0000835

AAATTACAAATAAATGGTCAAATCTTGATTAAATAACGGTTTGATGATTTAATCCCGACA

CAATTGCAATTTAAGTGACTTTACTGTTAACCTGTCCCCCTTAATGTCGGTGCTTCCCTA

TTCGTCGGACAATCCAGAACACTTTCAATGCTTACATTCGCTCTATTGTTTGTATATATA

TCTTTATTTAAATATTGAAGTAATGCATGGAAACATCCATCGTGCTCTATTTTTTTTTTT

TTTTTTTACTA

>Unigene0000836

TTTGCGGTTTTGATCAGTTCCAGCTTTCTTGCCGTTCTCATCCCTCCAGAGCGCCGCGCC

GAAGATCCGTCCGTACTTCCGCCCTAATCCCCAGTGGCCAGACCACCGCCACCTCCGCCG

GATCAAAGGTTAGCAGTTTCAAGGAAGTGACGAAACACTGTTTCACCATAATTAGGCTTG

AATCGTTGCGGATACATTCTGTTCTGACCTACTCACTGCATACTCATGGATCGTCAAGCA

CATGACTACGCGGCAGCTATGGCCTATGCTCAGCAGCAACAGGCTGGTATTCAGCAGCAA

CAACAATTTGGTTTCCACCCACAAGCACAGCAGTTCCCACAGCAAGTGCATCCCCCGCCC

TACCTGCCACCTCATCCCTCTCTTCAACAATACCCTTACCATCGCCAGATGCCTCCGCAG

CAGGTTTATCCTCACCATCACCATCCCATGCTCCATCTTCAACAGCCATCGCCTGCTTTT

GCACCCCATCTACCCCCACACCTCATGTCTCCTTCCCCTTTCACACACCCATTCGAATCA

ACCCCTCCAGTTTCTGCACCCCCTTCGGATCCCGATCTCCATAATCGAATAGACAAACTG

GTAGAATATGTTGCCAAGAATGGCCCCGAGTTTGAAGCTTTGATTCGTGAAAAGCAGCAG

GATAATCCTGCTTACAACTTTCTCTTTGGGGCTGAGGGACACAATTATTATAGATACAAG

CTTTGGCTAACCACCCGTGCGATCAGTCCAAACTTCAATCCTCCCTTTCCTCCATCTTCT

ATACCTTCGTCTCATAGTCCCACATTAAACGGCTCTCCCCTAAATCCTTCTAACATGGGT

GTAGCAGCAGGGCCTCCAACTTCCGTGTTACCGGCATCTCACCCTCCTTCCTTCCCCACC

TTTTACGATCAGCATCAACAACCGTTGCCTTTTTTAAGCCAGACTCGAACGGATTATGAG

CCTACCACCAAGGGTTTCAAAGGTCTTTCTGGGCCACTCCCATCTGATGTTGCAGCTGAA

CTTAATAATGTTCTTAGCAATCTTAACGGTACTAAAGATTCAATCAAGGGGGCTAAGACG

TGGTTTATGCAAAGGTCACCATTTGCACCGGCTCTCGCTGAAGCCCTTAGAGACAGAATG

TTTACTCTAGATGATTCTGAGAGGCAGCTCCATATCATTTTCCTTGCCAATGATATTCTT

TTCGACAGCTTGCAGCGTCGGATTAATCTGCAAGAGCTGGATAATGAGGCACATGCATTT

CAACCTGTTCTAGGATCAATGCTTGCGAGGGCATACAATAATCCAATAAACAAGGATGAG

AACCAGTCTCGGTTGGAGAGGATTTTAGATTTCTGGGCTGCAAAGGAGGTTTATCACAAG

GAAACTATTGCTAGCCTTGAGAAAGAGATGACTGGTGGACTTCCTCTGCGTTCGTCTACA

AAAAATGAGAAATCGTCTTTAACAGATCTAGGAGCTCAACAAATGCCTCCACAATTACAA

CAGAACACTGTGCCAAGCTCCTTGGATCGCCTGGAGATTCAGCCTCTAGTTCCGGCCGTT

TCATCTCAATTTGTGTCTCCTCTCATCCAGCAATTTCCATCTAACCAATTGCCAGGTAGT

ATTTATCCACAACCTGGGTTGTTGCCAATACCTTCTGTTCCTCAGACGATCACTCAACCA

CACCTACCTCATCTGTTACCTGGTTCTACGCCAATTGTCAGCGAGCAAGCCCAAGCCCCG

CCTCCATATCCTCTGTTCCCACCTGGTCTCATTCCTGGCATGGTTAGGAAGATGCAAATT

GGTAGTGGAGTTCCTTACTCCCCACTGAGCCCCCTCGACATTCCTACAGTGATCCCACCG

TCCACTGTGACGCCTTCGGAGATTCTTGACCGGGTCTCAAAGTTCTTCAAGGAGATTGGA

GAGGTGAACCCGTCGGAGGGGCCGATGAAGGCATCCCAGTCGAGCGATGACGAGTATGAA

GGGGAACCTGTAGTCCGTAAGGGAGGGGCTTGCATTCCTCCACCTGCGAATCTTCAGCAG

GCGGACCCTGAGACGGGTATGTTCTCCGATGGGACCTTTGATTGTAAGCCGGGATCTAGC

AGCTCGGGGAGGCTGGGACTGGGAGCCACTGCAGACCCCAATGAGGTGAGCCAGTACGAT

GATGTCTATTCCTCGTATCGGAAGGAGAGAAGCAGCAACTATCATACATCAATGAGTGCT

AGGGCTGGAGCGAGGTGATGAGAAAACCCTGCATCATCGTCAGGAACTTTGTTTTTCACT

TTTTCACCTTTTGCTGGGACTATTTGAGGTTATTATAGGCGAAACTCTGATGGTTTCTGG

GCGATCATTCACACAGAGATGCCAAGTTGTTTCAAGGGTCCCCAGAAGGTTTGGATCCTA

TGTCATTAGCTATTTCTGAGGTAGTTTATTGGCTGAAGGGATGTGTGTTATGTTTAGAAA

TTACTATTGATAATTTTGTTTCTGGATGATCATACCTCTAATATATGTAGCTTTTAGTAA

GTTTTCAACAGGTTTCATCTTTGGGCTGTACTGTTTAGTGTTAATCCCAGTATCGGTGAT

TCATTCCTTCCTCTGGACGAGCAATAGCAGTAAAATCATTATCCCCTCTCTCAATGTTGT

ATGGAATGCTTGCTTATATCTAGTTGTATGTATTTTGTGCATATGACGTAGCTGGTCAGA

AGATATTCAATTT

>Unigene0000837

AGATTACCCCCATATCCAGAGTCTTTTTGTTGATGAAATTGGCTCGTAGGTAGTAGAGAT

AGAGGAGATAATTACATTACTAGAAGAACCGGTAGGAATAGATGAGATGGAATCCAAAAC

TTTGACATGGCTAGATATATAAATAGTTCGTTTGCAAGTCCAAGTTGGCTGAAAACCCAA

CATGTTGGTCAATTGGGCTTTGAAATGATACCGTAACAATCAAATTAATTGGATTATTGG

GACCCAAACTACGAATGCCTGGCTA

>Unigene0000838

TTATATGGGAACAAATGAAAGGCTCCCAATTGAAGCAATGATTCAATTCATATAGCGTGT

TTGCTACATTGAGGACAAAAGAGTAATAACACCAATACCTCGTACATTTGAATGGTTATA

AAGAGAACATTGGAATGCTTAAAGTAGAAACACCACCCCGAAGAGTCTGAGTTTGATCTA

AAGAAGCAATGGAACACACATACAGAGAAATCCATTACTCAACATTGGAGTCAGCCCTGG

ACCTCTGGATTGACAATGGAGTATAAGTTTAAGACCGTTTGGATAGAGAGAAGACGGGAG

AA

>Unigene0000839

GACACCTTAGTTCGTGTATTGGATAGCCATTTAGCTGAAAAGTGGCTACATGGTTTGTAA

GTTACAACAGCCAAAACAAATTTGTTTCACGTTACATGGTTTGTAAGTTTTGTGTTTTAA

GATTGTATGTTGTTGGTCATAAACTTCACTCACATGTATACACATGATTGTGTGTAGTAT

CACATACAGGCTTGTGACTGCCAAGGTAGTGTTTGGGATGTCTACACGAGACATCTTCTT

GACTTAATTACTCTTTTGTCCGGAGGGTTGCAGTTAGAAAGCCTGTTCGGTAAGTTGCAC

ATACGTCTTGCCGGACTAGAAACCAAGAGGCAATGTGTCTGTTTTGTAACTCTGGCAAAT

CATAGCTTTTGAAATGAACCACCAAAACCTTATCCATCTAGATAGGCTGCTCAAGGGTAG

AACAGACCATTGAGAGCTCATTTCTCTCTCTCTCTGGTTTTCTAGG

>Unigene0000840

TCTATACCGATGGCTAACTGACGATGATGACGTGCTCCGTTAACACGTCACGCAGCTGTT

ACAGCAACCATCGGTAGCCGTTATCCGTTGAACAATTATGGATATCTCGGTGATTGCGAA

CGATTCTTCGCCTTCAAAAACCACCGAAAATAGCGATCGTGTAGACAACATTCAGGTTGT

GGATGATGACGAGGAGGAAGAAGAGAGTTGCACCTCCGATATAAACCGACTGAAAGTACT

TGGTGAAGATCAACTAGAACTACCCAGTGAAGATGAA

>Unigene0000841

GAGCATGCTGGGCTCCAGTAAATGGCGATGGACACCATTTCACCAAAATCAATTACCTCT

ACACTTTTTAGTCATGATTTTTCCATTTGTATCACATATGGTGTATGGGTTCGATGAGGT

CATGGTGTCATATATTGGGAATTGTTTCTGTGTAAATCATCAAGTGTAAGAAGAAACTAT

GGGACTCTGAGCCTTGCTTTAGAGAATAACAGTGGACAAACGTGTACCGTAAAACTAGGT

TTAACATTCTGACTCAAGCGAAAGCTCTCAGAATTTCACACTGTGAATCCATGTTTACAA

GCATTACAGGTGGGCCCTCAGGCCTGGTTCTACCACAGCAATGTCTTCCACTACTCA

>Unigene0000842

CCAAGATGAGCAGTATGAGGTATGATGCAGTTTGTGCTCCAGTCAAGTATAGGCAATTGT

GAGCAGCATAGGCATGATCCAAAACTAGAGCAGACACAAAGATGTGGCCAGTGGCCAGTA

GTGGTCCAGCTAGAGCAGACAAGCACAAGACACACTCTTCCCCTGTCCCTTTTTCCTCTC

ACCATCAGTACTCCCCTGGCTCGGAACAAGAGGATATTAGGGTGGCTGCAAAGAACATTA

GGGTGGCTGCAGCAGTT

>Unigene0000843

GGAATTGGATCACAGGGAGATTATTTAGCTTTACAACTTAAAGATAATCGGTTACTATTA

AATATAAATTTAGGATCTCAAATCATGACTAGTCTATCAGTTGGTAGTCTACTAGATGAT

AACATCTGGCATGAAGTTATAATAATGAGAAATAAACTTGAAGTTGTGTTTTCTGTTGAT

CGTGTCACAATTCAAGGTCGAATTAAAGGAGAATTTGAAAGACTTGATCTTAATCGTGAA

ATATATATAGGTGGTGTACCA

>Unigene0000844

ATATTTACACGTGGAAGTTTCAGTCTCGTTTACAGGAAATGATCGCTATATAATTAAATA

ATAAATCGTTTGTAATATTGTATCACAATGTGTGTACGATAGGTAATAATAATATCATAT

TGTACGAAAGTCTAATATGTATATTGTAATTATGATAATAATAACAACGACATAATATTA

TGTTCTCTTGGCAATAATAAATAATAATGAGCAATATCATGTGAACTTTTTTTTTTTTTT

ACATTGTTTCATT

>Unigene0000845

AACATTATACCGGTACACATGGACCATAAGGCCCCAGTTGGACTGAATTTTGTGCACACA

AGTCTGGTTATCTTACCCATCTTGGATTAATTATGCTTTAATTGGATTGGGATTTCCTAC

TTTCATCTATTTGTTTTTGATAAAAAGTAGGGTGAAATTAACAGGTCGATTGAATTGACC

GACCTTTGTGTGAATTGGTTTGGTGATTTGAGTATACATTATCATGTTTAATTAATTAAA

ATTAGAGAATTTGTTTACATTTAGGTTAGATCCGTCTTATTTGACTCTGAACTAATTTAG

CCAATTGTGTGATTTGTTGTTCAGAGAAACTTAA

>Unigene0000846

CCATAATGGATGTAGGAAGATTTTGTTTTCTGGACAAGTTTAGGGCATTCATTCGAGAGA

AGCTCACTGTTTTCCAAAAGGAGAACTCCAGTGCTTGGCTTGAAAAAGAAAGAGATCATA

GGAATTATAATGGGAGGATGAACGTGAAGACGTGGAGCTCGAGGAAGAATATATCCATTG

TAAAATGGAGTGCCATTTCTTGTATGTTTTTGAGGGGGAAATGGTCCGGACACTAATTCA

TACAGCTAACTTCATAACCCTGGAAAGCTAGGGTTCCTTTGT

>Unigene0000847

GCAGTCTCCGTCGAGATCCCGATCCCCCTCCCATCATGGCCTCCCTCTCCAATCACCAGA

CTACGCCGTCAAGCGATGTCCCGTACGAGCTTCGGAGCCCGCTCTCCGGCGTCCGTAAGA

ATCTCCCAGGGCCTGTGGACGTGGGACTCAAAGGCCGATCGCCGTCGAACCCCAAGATCT

CCAACGGTAACGCCGGCTACGTCCTCGAGGATGTGCCACATCTCACAGACTACATCTCCG

ATCTGCCGACCCATCTTAATCCTTTACAGGATAACCCGGCATATTCGGTCGTCAGGCAAT

ACTTTGTTAACACAGACGACAGTGTCGCCCACAGAATTGTGGTTCATAAAGACAGCCCGA

GGGGCGTCCATTTCCGGAGAGCCGGCCCTCGTCAAAAGGTTTATTTTGAGTCTGATGAGG

TGAATGCCTGCATTGTGACATGTGGAGGTCTTTGTCCAGGCCTCAATACGATAATTAGAG

AGATTGTGTGTGGCTTATCCCACATGTATGGCGTCACAAGTATACTTGGGATAGAGTGGG

GATACATGGGTTTTTACGCTCGCAATACTATTCCCTTGACACCCAAGAGCGTCAATGATA

TCCATAAGAGAGGGGGAACTGTCCTTGGGACATCACGAGGAGGTCATGACACTACAAAGA

TTGTTGATAGCATTGAGGATCGTGGGATTAATCAGGTCTATATAATTGGAGGAGATGGAT

CTCAGAAGGGAGCCTCTGTGATTTTTGAGGAAATCCAGAGGCGTGGTCTTAAAGTTGCAG

TTGCTGGCATCCCAAAGACAATAGATAATGACATTCCGGTTATCGACAAGTCTTTTGGCT

TTGATTCTGCTGTTGAAGAGGCCCAACGAGCAATTAATGCAGCCCATGTCGAGGCTGGAA

GTGTTGAGAATGGCATAGGAGTCGTCAAATTGATGGGTCGTTACAGCGGGTTCATTGCAA

TGTATGCTACCCTTGCAAGTCGAGATGTGGACTGCTGCTTGATTCCAGAATCACCCTTTT

ATCTTGAAGGGAAAGGGGGGCTTTTCGAATATATAGAAAAGCGACTTAAAGAGAACGGTC

ATATGGTTATAGTTGCTGCAGAGGGTGCAGGGCAAGAGCTTATTGCTGAAAGCATGCGGT

CTAGGGACGATGCAGATGCTTCCGGAAATAAGCAGCTACTTGATGTTGGCTACTGGTTAT

CTCAAAAGATTAAGAATTACTTTGCAGGCAAACCGATGATGCCTATAACTCTTAAATACA

TAGATCCGACATACATGATACGAGCAATTCCGAGCAATGCATCTGATAATGTCTACTGCA

CGTTGCTAGCACACAGTGCCATACATGGTGCAATGGCAGGGTACACAGGCTTCACCGTTG

GTCAAATTAATGGCAGACATGCTTATATACCATTTCATAGGATAACTGAGAAACAAAACA

AGGTTGTGACAACTGACAGGATGTGGGCAAGGCTCCTCTGCTCAACCAACCAGCCGAGCT

TCCCTAGCCCTAAAGAGGTTGAAGACTCGAAGAAAGAAGCAGAACCAGTGAATCAGAACA

TTCACAATGATGTCGAGAATGGCAGTACTGTCTGAGGTGGTGGCAGAAACTCCAACATTA

TTTGTATATGAGTGTGATTTTGTACCACTGCATATAGAAGAGGGCCTGCTACCTGTGAAA

ATCTAATCCTTATGGTTAAGGATTGGGTTTCACGGTGGTAACAATATGAGTCTTGGTAAT

GGTTTGTATATGCACATCCAGTCTATTAAGGATTTTTCCCCTCCGTCTCCAAAATGTAAA

CCCTACAATTCCAGATTAATATTTCTTTTTCACGAGGTTTTAAAGC

>Unigene0000848

ATGGCTCTCTTCTCTTGATCCTTGCACTTTGGGGCTCTGTGTTTTCAGCTTTTCTGATAC

CCTTTTGGTTGTTTTGATTCTTTTTTGCTGGAAAGATTGGAGATTTTCAGGGTGTAGAGT

GAAAAGGGTTGTGGGGGGATCTTGTGAATCTTGCAGCATGAAGTTCTATATGGGTTTGGG

TTCTAAGGTCAAGAGGCTGTTCATCCAATCTCCTGCTAAGAAGCAAGGTTTGGTTGGGGA

GGTCAAAGATGGTGGATTGGGTGTTGAGAGCAAGATGGCTGGCAAGAGCCATGATTTTGG

AAGTAAAGAAGAATCCTTCTTTGACTCACGACCATGGCTAGACTCCGATGCTGAAGATGA

TTTTTACAGCGTGAATGGAGGTGATATCAACTTACCTTGTACACTGAAATCATGCCAAGA

TTTTTGTTATGTCCTTACAAGTACATTTTGCCGTAAATGGTTTTCTCTGAACAGAGTTCA

CTCCATCCAGAGGCAACACACCAAACCATCAAACAAGCATTCGAGTCACCCCTAATCTGA

ACAAAGCCCTCTTTATCAACAAATTTCAGCAAGCCAAATCTGAATCGTCATCATCTGCGA

CCTCGCCTTCTGGTAGAAAGAAACTAGCCGAGCTTTTTCAGGAATCCTTTGAAGGAGAAC

AAATTATTAATGAAAATACTACCGAAGCCAGTACAGAGAATGGCACTCCGTGTAGCGGCA

GTGAGGCAACTCCAAGTCGGGTTTCAAAAAGGAAGAAAGAGAGGAGAGCGAGGGCCATGA

GCTGCTGCTTGCCCAGCATGGTACACAGCTTCAGTTTCAATGAGAGGAGGCAGAAGCTGA

GCCCAGCGAGCCGCAGCGCCTGAGGTGACCTCGACTCGAAGAGGTCGGAGTGTCCCTCCT

TTCCTAGAGCATATACCTGAACTCAAGGAATTAAACTTTGCTTTTGTGATCATGAAGAGC

TATGATCTGAATTGCTACATAGTTTTTTCGGAGTAATTATGAAATGTTGAAGGTTGTGTA

ATTATGTAACTTACTTTCTGGATCTATAATGGTAGGTTTTGTAAACTTCCTGAGAATTTA

TTAGGGGACACAGAATGCAACAAGCATTTGTACCTAAATGTTATGCTTTCATGCTCCCTA

TACTTTGTACTTCTTGTCCAAGGAAGAGTTATTTATAGTAATCATATGCATATTATTTGG

ATGTGGTCAATTTGTAAGGATTTCAAAGGGGC

>Unigene0000849

ATTTTCATTGTCACCCAAAATATTTTCTAAAATTTGTAATTACCCAAAAATATTTTCAGA

ATATTAGTTTGCCTCAATTTTAGTGTGAAATATTGTCTTTGGTCCATAAAAATAATTTGT

AATGAGTCCATCTTTTGGAAAATGATTTGTTTTGGTCCCGAATTTGGTAAAGTCAAGGTT

TTGGCTGGTCAGCTGAACGACCGTAAACAATGATTCGTGTGTCGAATTTAAGAGAACCAA

AGTTTTAGCGAATACTCTCACCGAGCCCCATGATGAGGGACCTACTCAATCCCTTACGTA

GTTAGTGGCCTTCTGTCCCCGTTTTGATGATTTTGTAAAATCGAACGAAACGGGCATAAC

GAGAGCTACGAGTATCCAGTTGAGGCGAGTCAGGAGGAGTTGGAATGCTCATCTACAACT

TAGTCATCTATATAACTTAGTTGACTGGCTGTACAAGATATTTGTTTCAGGAATGTTTTC

ATGAACTTTTTTTTTTCCCTTTTCCTGAATATTTGTTGCATTGAGCTGGATTACCAGATT

GCCGGATTAACATATTGTGACATAATTGCCGGATTAACAGATTGTGACATAATTGCCGGA

TTAACAGATTGTGACATAATTGAAAGGAAATGTAGTTACGAATTACATATCGCTGGAGGT

AGAAACTTTGCCTACC

>Unigene0000850

TTTATGTCACATGTTTCTCATAGGTTACTGATATTGGAATAAAAACTTAAAATATGTAGT

ATATTCATAAATACCTAAATCTTACTCTCCCATAATTTTTTGTATGGGCATGTCATGAAT

TCAATACTCACATGTCATGTCTTGGTATGATTTCTGTACTTCCTCCTAGAATTTATGGAT

CTCTTCCCTCTCCATAAAGAGGGATGGGACAAACGGAAATCCAAATTAAACATTGGTCCT

CACATTTCTATTCCTA

>Unigene0000851

ATGTACATAATACTACTATATGTTTATACTCTCGTACATACAATTCATTTTCTCTCCCCA

CCCACTCGCACACACATCACACACCTACTTTTGAACATGGACTCTAATACCAAAATCTCT

CTTCAGTCCTTTTTTTTTCTCATCAAAGACGGCTACTTCATGCAGTCAAATTGCGTGGGA

TTTCCACTTGTGGATATGAGATGAATATAAATACCTAAATCTTACACTCCTCTTTTTTTT

TGAGATGACGTGGATATCTTGTTTCGAAACACTTTTTTTTGCACATTTATGTCACATG

>Unigene0000852

ATCTATGACCCAATCTTGCTCTTTCAACTTCACATGATTAGTGACCACAAGAGCATCTAC

CTCCAACTCTTGCTTGGGAAAACTTATTGGGACATTTTTCTCATCCTTCCTAATTTTAGG

GCTGGTTATTGTGATGTGCCCTGTCGTTTCACATTGGTTATAAATTAAATGATATGGAAC

TTGGTTTGGAACTCTCCTTCCACCATAACCTCTACTTTGCTTGTAGTTGTTTCCCCTTTT

CGATGAATCAACATATCTCTTGCTTTACATTTTCCCCCCATTTTGATCTTCTTTCCATAA

ATCAATGCATTTTTACTTGTGTGGCCTTCAATGAATCACATCCATAGAGCTTTGTTTATA

ACGATTCCTTACGAGAGTAATTTACATATTTAAGGAAGATTATTGCGAGATCTT

>Unigene0000853

AAATATTATTTCAATAAGTTATGCTGTACACACACACACAAAAAACAAACACATCATTGT

AAAATCAATACATTCCTTACTCCGTTCAAAAGCTAAAAAACTATCCAGTAAACTGAATGA

AGTGTTATTTAAAATTTGTTAGATTTAATTTGCATTTACACTTAGTCAATTTTATCTAGG

TGATTATTTATCATACAATGTCTATTGATTTACTTATTAAATATTATTTTATATTTGTTC

AATTATTCTATCATTTAAACTCCAAAAGGAAAACATTAGCAGATAAAAATATAATTTAAA

AGTTCAAAAAAAACAAGCGTAT

>Unigene0000854

GGTAAGTTGACAACATGTATCCCTAAACATTTAGCTTGATTCGGACTAATCCGGACTAAT

CAAGCTACATACTTTACCGTTTTGCCATATCAAGTGTATTTATCTAGCACTGAATTCTAT

TATGACCCACATCTAAATTTGAATATGTATTATTCCAATTATTGGTGAATTTTAGCATAT

AAATTGATGCAGTAATGCACGCTTGGAAATGTGACAAGTATTAAATCCATAAAAT

>Unigene0000855

TTCTCTCCCCTACTCCCTCTACGTTCTAAAACAAAAAAAATCTACTTCAAAAATATAGAA

AAACAAAAACTTTAAAAACATCTCTTATTCTCTCCCTCTACAATCCACTAGACCCCTGCC

TTATAGCGATCCCATCTCTCGAAAACTCTCTTGTGCGAAGGGTGGTTCCTTCATCTCTCC

AGAGGATCCCCTTAAAGAATCTTAGGAAAGAGATCAAGCAATCAAATCCCTCCC

>Unigene0000856

CGTTTTAAGGTTTCTTTAGATCCTGTACGTCCAAGTGGAGTGTCTTGTATCCGTCTCTTA

TGTTGGTTAGATGATAAAAATCTACCTTGTGTACCCCCAATTAATATTATTGTTTATGAG

AATTATCCCCCTGCAAATCCAGCGTGTATAATTTCGTCCAATGACTATGGTGGCACATCA

TTTCTCATGTGTGTTAAAGATGTATTTGAATCAAGGATGATGTTGATGCCAGTCCGTTTT

ACAGTGTCTCATATACTTGACTCATGGGAAATGAGTGTTAGGCAAGCAGTGGCTCTTTTA

CTCGATAACA

>Unigene0000857

AAAGTTGGGAACCCAGGGCCTCAGAGTAAGCAGGCATGACAAGCGCCTCCAGCAAAGACT

GCACAACCTCAGGGTCCTCGTGGTCGTAGTCAGTCGGTAGTAGGCTACCATCGGCCATCA

ACGGTCGCAGCTCGAAAGCAGGAGGCTTGCCCTCACGACGGGCAAAGGGAGCGACAGTCT

CCGCCACAGGCGGTACCGGAGCGTCGGGGCTCAAGGGAGTAATTTGT

>Unigene0000858

ACTTCGGTGACCCTTCGCCAAAAAGTATAAAGCCAAAGGGTGTCACTCAAAAAATGAAAA

GTTCCATCGGATTTTCCGAAACCTTTTGAAATCATATTTTGTGTTTACACCCCAACTAAA

ATATAAATTCAAAACAAGTAAAAAATCACATTATATTTAACCAACTCTAACAGGAACTCA

AAGGGACATAATTATGTCCCTTACCGCAGTGTGGGATACTGGTTACCAAGGTGCTTGGAA

CAAGGTTAGCTTTTGTTGTAAATAGTGTTGTACGGATCTTTGAATTCTTACTGGTGTACC

GCAAACAAGTGAATTTGTGCAGCCTGGTGCTGGTGCTCCAGCTAATGAGCGTGAAGCGTG

GTGGATCAGTCGGTGGGTGCATAGCTGCCCGATGTGCGGTGAGGCCTGGGTTTGAATTGT

TTAGC

>Unigene0000859

CGTATGGCTGTTCGAAATAATTTGTCTGGTGCAGAAGAACTTTTTGTACGCAAGTTTAAC

ATGCTTTTCCAAAGTGCCAATTACGCAGATGCTGCTAAAGTAGCAGCTAATGCTCCTAAA

GGCATCCTCAGGACGCCTTCTACCATACAAAAATTCCAACAAGTTCCAACTGCTACAAAT

CAATCGTCTCCTCTACTTCAATACTTTGGAATATTACTGGATCAGGGCAAATTGAATAAG

TATGAATCTTTGGAATTATGTCGTCCAGTTCTTGCACAAGATAAAAGGCAATTATTAGAA

AAGTGGTTAAAAGAAGATAAATTGGAGTGCTCTGAAGAATTAGGAGACTTGGTCAAAACT

GCTGATATTTCTTTAGCATTGTCAGTTTATTTACGAGCTAACATTCCACCTAAGGTTGTT

CAATGTTTTGCTGAAACTGGCCAGTACCAGAAAATTATATTGTACTCTCAAA

>Unigene0000860

TACAAAATATTGAAACACATTTTTATTTCTTAAAAGAAATGTTGTATAGTGAAAGAATAA

GAGAAGTTGAAGAGAAGTTAGTTGAAGTGAGATTAGGTACAGCTAAAGAATACCGTATAC

CTTTACAAGAATTACAAGATCAAATGAAGTCTCGACTTGAAGTAGCATCTGTATTAAGAC

AATTCAAATTAACAAATTTAAAACATAAGTATGAAGCAGAAGAACAAGCAATTAGACAGA

ATTTTGAGAATAATAAAGAAATTTTATACGATACCATAAAAGCTGATTTAGAAGAAAAAA

TTCAGCGATTAGAAGAAGCCCGTAATGAAGTAGATATAGATGCCTCTCTTTGGTTAGGTC

GTAGTTTAACTCGTAGTGGACGCGGACGTGGAAGACGACCTTATGGGCATGTACAACCAA

AGCGGAAACCTGTTATTGTATCTGGACCATGCATAGTCTACAACCTTAGGGAACATGAAA

TACTTGAAGATTGGACAACAATCAAAAAGTTGTTATCATCATCAAAACGAAAAGAAAATG

CAATGCAACGATATAAACCATATGAATACAACAACAAGGTATTTCAATAAGTTCCTTTGT

TTGTATTTTAAGTTGGAACTATATACCAAAATGTAACTATTAAATTTTAATAAAGTTATT

AAACCCTTTGTATATTGATCCCTTATCTTATCCTTAACACCTTGAATTAAAATCATTCAA

TATGTGATGGATGTCTCTATTTATGTCAAATTATAAATTAAAAAACATTTTTATTCTTCT

GTACCTGACCAAATAAATATTTGAATGATTTAGAAAAGGGTTTCCATTT

>Unigene0000861

AAGTTAAAGCAGAAACTCTTCAATTAGAATTAGATCAAGCAAAAGAGCGCATTGAAGAAT

TAACAGTAGATTTAGAACTCTTAAAGGCTGAGCTGGAGAAAGGAAATGATACTGAAGAGG

GAGGAGATGGTGCCAATTCTTTTAAAGTCAAGCAACTTGAACAACAAAATTTACGTCTTA

GAGATACCCTCGTCAAATTGCGAGACTTATCCGCCCACGAAAAACACCAAACACAAAACT

TA

>Unigene0000862

ATGAGGGGGCTGGTTTTCGGCTGAAATCAAGTGGCTTGCTTAATCCAGTTTGCAGATCAA

CACTAACAATATCTTTAGAGCTTGCTTTTAAGATAATTTCTGATGTATAGGACAATTCCT

GAATCAATGGGAAATTTACAGAGTTAAAACATCAAGAAAACTATGGATTACTGTTTTATC

AACTGCTTGAGTTCTTGTTTGGCATCAGGGAAGGATCTATGTTTTTCTTTAGAGTTTTGT

GTCCAACCCAACAATACTCGTGATGTTCTTTCAGATCTGCGA

>Unigene0000863

TGCTGTAGCAGTGGACTTCAAGAATGTATGCTGGCTGGACTTGCCTGCTGCAGTTATGTT

CGAAGAATAGAATATGGCTCGCAGGGTCGATGAGGGGGCTGGTTTTCGGCTGAAATCAAG

TGGCTTGCTTAATCCAGTTTGCAGATCAACACTAACAATATCTTAGGGTCGATGAGGGGG

CTGGTTTTCGGCTGAAATCAAGTGGCTTGCTTAATCCAGTTTGCAGATCAACACTAACAA

TATCTTTAGAGCTTGCTTTTAAGATAATTTCTG

>Unigene0000864

AATAAACTGAAGCTGTCCAAGTACAAATTTTTTGTACCCCTACTAAATGTTGCACCATTC

TATTCTTCTAGATAGTAAATTTAAGCAAGATGTTTTTATCAATTATCTTCTGTGAATGAA

TGTTAGAATTGGTAGTTTATTTAATTTTTCCAATAAACTGAAGCTGTCCAAGTACAAATT

TTTTGTACCCCTACTAAATGTTGCACCATTCTATTCTTCTAGATAGTAAATTTAAGCAAG

ATGTTTTTATCAATTATCTTCTGT

>Unigene0000865

CCTATTTCCTTCTGTTTTTCATAGTTTGTTTTGTGTTTCTTCTGCATAGTTGCAGATGAA

CTGCTTGGGTCTATGGTCTGCATCAATGGAAGGGACCTGTGTTTTACTTTCAAGTTTTGT

GGCCAATCCGACAATGTTTATACTGTTCTTTCAGATCTGAGGCTGTGCGGAAGAAATTTT

CGTTTTTGTCACAGGATATGTGCAATTGGACGAGATATGTGCTCATTT

>Unigene0000866

TCAAAGATCGCCATTCTGAATGCAAATTACATGGCTAAACGTTTAGAGGAACATTACCCT

GTTCTCTTCCGTGGTGTCAATGGAACGGTTGCCCATGAATTCATCATCGACTTGAGAGGC

TTCAAGCAAACTGCTGGAATAGAGCCTGAAGATGTTGCTAAACGTCTAATGGACTATGGT

TTCCATGCACCCACCATGTCATGGCCTGTCCCTGGCACGCTCATGATCGAACCCACAGAA

AGTGAAAGCAAGGCGGAGTTGGATAGATTCTGTGACGCCCTTATTTCGATCAGAGAAGAA

ATCGCCCAGATCGAGAAAGGAAAAGCTGATATCAACAACAATGTCCTAAAGGGTGCTCCT

CATCCGCCATCTCTGCTAATGGGAGATGCATGGACAAAGCCATACTCGAGGGAATATGCA

GCTTTTCCTGCTTCCTG

>Unigene0000867

CAGGGTTCGGAATTTCGAATTATTTTTCCGGTAATTTCTGCGGTGTAGCATTATCCGGCT

TTCCCGTCGGGTGACGAGCTACAGACATGGGATTTTCATTGTGGACCCTGCTCGAGGCTT

CGCTATTGTGTCTGAACGCCATATGCATCCTGAACGAAGACAGAGTTCTGGCCAAGTATG

GCTGGTCCACGGTAAACCGAGAGTTTGAGGATTCCCCATCCACAAAAACACAGATACTGG

CACTAATGAAGTCTATTCGTACAGTCGTTAAATATCCTCTCATAATTTTCAACCTGCTGA

TCATACTTGTCAAGAGTCTCACAGGATAATAAAAATTGAATTCGTTTACAATGAAATTAT

GTTGTTTATATTTCGTGTAACTTGTTATTTGTAAAATTAATATTCTAAGTATTACACTTA

TTTATATATTTTATATTTGTATGTGTATGTTGTACAATGTGCGTATCAAAGCATTTACCA

TCTTATCATAAAT

>Unigene0000868

GAATCTTCATTCTCCATGGCAGCTGAAAGAAAGGACGAGATTTCAGGAGTCTTAGCACCC

TGCTCTCCCCATCCAAAGTCAGAGTACTCAAACGAATTGCTCCCTTGATCTGAATTAAAG

TACATGGGAGCAACATCAGAAGAAGGTGGACTGGTTTTTATTGCAACATCTGTGTTAGCA

GGGAATGAATTCATGTTGACGAGTTGATTTGTAAGCGGTTTTTCGTCCAGGAAACCCACA

GTGCTATAGTAATCTTCATCAGAGTTGCTCACGAAACCATTGTTCTGGATTTGATCTGAT

TGGGTCTTTGCCAAACTAGTTTTAACTACTGGTTCCTGAAGGTT

>Unigene0000869

GATCCTATAAAATGTTAAGACAAGGTAGATGAAGAGGAGATGGGACTTCAAAATTCTTGT

GAAATTTGTAAAAATTTGGACTATCATTAGTACATGAAAGGTTTATATGGAAGCCTGACT

TCAGAAATAAAAAATGTGAATTGTCTGAGCCTGGTGCAAATTAAACATTTAGCTGAAACT

GAAAATATATTGATTTGTCATATTTCCCTAACAGCAAAGGAAGCATGATTTATTC

>Unigene0000870

ATAGCAGACATTTCAGTTCATGAACAGCTGGCAACAACATGCAAGGACCTTTTCAGAACA

AGCATGCCTTGGTGGTTATTTGCAAAGGCAGTGGTCCTCCTCTCTTTCTCTAAGTAAAGC

AATGGCCATAGGTTAAAGAAGCAGACAAGTAAGAGGCATCTACATAGCAGCAGGGCATCT

TGGGCAGTTAAGCTCTTTATCTCCCAGAACAAGACAACAGAGGAGTAAAACACAATATTC

AAGGGGACAGTAAAACATGGAAGCCGGTAATGGTCTCCCCTGCTGTAGAGCAAGCTTTTA

G

>Unigene0000871

TCACTGATTAGTGATCACTTATCAGTGATTAGTGAGGTAATGAAGTGATCACTGATCACG

GAATCATTGGTCACACTAATTAGTGATTACTCAGTACTGAGTCATTCTTGGTTCTCCGAG

TCCACAGTCCATTCATCATGACTGCACCAACTGAATCGAATCACATACAGTCACATTGTC

ACATACAATTAAAATTTATATAAACAACCAATGCTCACAGTCACACTCACAGTGTCACAG

A

>Unigene0000872

TATTTATATTCTCTTTTCTTACCCCTATAGTGTCTATTACTTCACATTGGTCATATATCT

CATTTGTTGCATCATTAAGGCCCTGTGAAAGATGCTACAGGTAACAGAGTGTAGACATGC

TTGTATCTAAATGTTTTATCCTGTTTAGATTTAGTAAGAGAAAAGTGGAAATATCATGTT

ATGTGATTCTCTGGCTTCAAATGGGACATCAGAGCAATTCTTTTTGAGCTTGT

>Unigene0000873

TTTGATTAATTGTGGATTCTTGACTGTTTTGGAGAATCATGCTTTGCCCGAGGAGGTATG

GACCGGTAAGAAGGTTTTCTTGGCTCATCTTTAGAATTTTGACTGTACTGCATGTGTGGG

TGAATCCGGAAGAGAGATCAGAGCTTAATCCAAAGTCGAAGCGATGTGCTTTCATTGGCT

ATGGAAGCTCGAAACCCATGAGCTGGGGCTATCTGTGCTACGATCACGAGATATGGAGGA

TGTTCC

>Unigene0000874

ATGGAAAATGCTTTAGAAGCATTACGTTCACATAATATGTCTTTAACTAAAGCATCCATA

ACATTTGGAATTCCTTCTACAACGTTATGGCAGCGGGCTCATCGAGCTGGAATAGATACT

CCTAAAAAAGACGGACCTGCTAAATCTTGGAACGATGAAGCTTTAAACATTGCATTAGAC

GCTCTACGTACTGGCACTATTTCAGCAAATAAAGCTAGTAAAGCGTATGGTATACCTAGT

AGTACATTATATAAGATTGCACGGCGGGAAGGAATTAGATTAGCTGCTCCATTCAATGCG

GCACCTACTTCTTGGGGTCAGGAACAGTTAGACCAGGCATTACATGCCATACGAACAGGG

CAAACGACTGTACAACGAGCTGCAGCTGACTATGGTATACCTTCTGGTACACTATATGGT

CGGTGTAAACGTGAGAAAATTGAATTATCCCGCAGCAATCCAACCCCATGGTCTGAGGAT

GCTATGATGGAAGCTCTGGAGTCTGTACGATTAGGTCATATGAGTATTAATCAGGCAGCT

ATACATTACAATCTACCATATTCTTCATTATATGGACGTTTCAAAAGGGTGAAATATGGA

GCTGGGCATCCAGATGATGATTGTGATGATATGATTGATTCAGATACACAAAGTGAACAA

CAGCACATTCAACAACAACAACAGCAGCAGGCAAATCAGCAAGTACAAGCTACTCCAGTG

TCTCAAATAATGTTAGTTCAATACCCAGCACCACAGATTCAAATGTACCAACAACATCCA

ACTCATTCAACGACTAGTTGAGATGAAGTAAAATACTTGCAGCCATTGTGATGTGTTAGA

ACTGCAAGTATTCTCTGTAATAATTTATAAGTCTGATGGATGTACTTTTTTTTTTTTAAT

TTATCTGTGATATTTAAACAATGAAAACATTATT

>Unigene0000875

TACAAACCATGCCTGGCAAGTCTGTGTTTTGGTTCATTTTTCTTTGCATAGTCCAAGGAA

TCATAGATCATGCCAAAGCCTGTTGTCTTGCCACCACCAAAGTGAGTTCTGAAGCCAAAT

ACGAATATTACATCAGGGGTTGTCTTGTACATTTTCGCCAGCTTTTCCCTGATTTCTGTT

TTGGGGACTGTGGCCTTCCCAGGATGAAGAACATCAATCACCATCTGCTTGCGCTGAAGC

AGTCTGTTCGTCATGAATTTCCT

>Unigene0000876

GAGGGAAAATCAGAGAAGAGAGAAAATATGAGGAGAGAAAGTATAAGGAGAGAGTGGGAG

AAGTGAGAAAGGAGGCTCAACTATTTATCTCCTCTCACTTTCTCTTTCTTAGCATTTGAG

AAGTGAGAAAGGAGGCTCATGCTTTATCATCTCAATTTCTCTACTTATACTTTCTCTCTT

CACACTCCGTCTTATGACACTTACTCTCTACTCATGTTTTTTCACTCTTTGTAACCCATG

TGTCTTGTGCGTCACAGATGTTACATGCCCATTTTTTTTGCTCAAACCAGTATTAGCCTA

TTAAATTCACTCTTCTAAGCATACCTCAATAATTGTTACAAGACACCTTTATACGAAAAT

GCTTCTTGATATCAATAAGATCCGAAAATAAAAAAGATCCAAAATTAATTATTTTTAAAA

AAATTGGAGGTAAACACACAATCGAGGTAACTTATAGGGGATAACTC

>Unigene0000877

AAGAGAGAAAATTAGAGGAGAGAAAATATGAGGAAAGTAAAATAAAAGAAGAAAATGTGA

GGAGAGATAAAAAAAAGGAAAATGTGAGGGGAAATTAAGTGGAAACAAAATACATAGAGA

GGGTAAGGAGAGAAAATATGAGAGAGAAAATGAGATGAGAGGTAATGAGAGGACCGAGAA

AATTTGAGAAGAAAATATGAGTAGAGAGAAAATATGAGAAAAGAGAAAACGTGAGGAGAA

GGAATATGAGGAGAGAAAATGGAAAGAGAACACAATAGAGAAAATATGAGAGAAAATAAG

AGGAAAGAGAAAATAGGT

>Unigene0000878

AATATAATCATGACAACGTAGGGGGTAAACCCATATAAAAACTTTGATAGGGGTAAAGAC

ACAATCGAAGCAAGGGGGTAACTCGAAATTTTTATTTTATATTTCTTTGTCTTCCTGCAC

ATCCATCTTAGCATTCTCATTTTAGCTAGAATTATTTTATTAATGTGTTATTTCTTAGTT

ACCCAACATTCTGTCTTACAGATCAATGCAGGTCTTATAGTTGTCCTATAAAAT

>Unigene0000879

GAGGGAAAATCAGAGAAGAGAGAAAATATGAGGAGAGAAAGTATAAGGAGAGAGTGGGAG

AAGTGAGAAAGGAGGCTCACTATTTATCTCCTCTCACTTTTTCTCTCCTAGCATTTGAGA

AGTGAGAAAGGAGGCTCACGCTTTATCATCTCAATTTCTCTCCTAACAATTTCTCTACTT

ATACTTTCTCTCCTCACACTCTGCCTTATGACACTTACTCTCTACTCATGCTTTTTCACT

CTTCGTACCCCATGTGTCTTGTGCGTCACAG

>Unigene0000880

ACGAGGGGAGAGAGTACAAGAAGTGATAGGAGAAAGAAAAGGTGATGAGAGAAATTATGT

GGGGGGAGAGTGTATGGAGTGAGAAAGTATGAGTGTGATGAGAGACAAAAGTATGAGGAA

AGAGAAAGTAAGAAAAATGAAAATATGAGGAGAGAAAGTATGTGTAGAGAGAAAGTTTGA

AGGGAGAAGGTTGATGAGAGAGTGTCACGAGAGCAAATACGTCAAGATAGGGAAAGTGCA

AGAATTGATGAAGTGATATGTAAGGAGAGAGAAATTGTGAGGATACATAAAATGCGAGGA

TTGAGAAAATTGATATGAAATGAGAGTGAGAAGAGAAAATATGAGAAGAGGGAGAAGTGA

GAAAGGAGGCTCACTGTTTCTCTCTCCTCTCACTTTTGTCTCTCCTAACCATTTGAGAAG

TAAGAAATGAGGATCACACTCTCCTGTCATTTTCTCTCTCCTAATAATTTCTTGTTATAC

TTTCTTTCCTCACTCTTTCTAGACACTTTATCTCT

>Unigene0000881

TCCTCGTATCACATGTATCTTGTGCGTGACACATATTGCATGCTCAATAATTCACTTGAA

ACCAGTATTAGCCCATTAAATTCCCTCTTTTAAGCTTACCTCGATGATCATTATGAGACG

CTTCCACGTGAAAATGCTTCATGATACCAATATGATCCAAAAATAAGAGAAAGACCTAAA

ATTAATTATTTTAAAAAATTTGAGGACTAAAGACACAATCAAGACAACTT

>Unigene0000882

CATTGCAATTCATTTGGTCTGACTTGCCAGTAATTTTATTTTATGTCATCTAATAAAATG

ATAATTACTCAAAAGGAAAAGTACTTATTTTCGTTTTTAATTTGAATCAAAATCTACTTA

TCAGTTATTGTCGGACAAAACGTTGTGTTCCACTGTTAAAATGTCAGAAATTGAAATCTC

ATCCGGACCGGAAGATAGTGAACCAGACATTTTTTATAACATGGATATGTTTCAACAGAT

GATAAATTTATGTCGAAATAATAAAAAGTTTCCTCAGAAG

>Unigene0000883

GTTTTGACAACCATAGTGCAGGTGCTTTGCAAATTACAGTGCTGTTCGCAACTTTAAGTT

ACCTGGCTTTGGTAAGAGTCTAGCTGTCTTGAGGTGTCAGTTCTTATCAAAGAAAATGTG

TATAGGTCTTGCGAAAATACAGTGCTGCTCGCAACTTCAGGATGCTTGGGTCAAGGTTGA

TTGTGTGAGAATCTGGAGTTCTTTGGCCACTGGAAATGCTATGGGTTTTCAAAAAATAAA

TATTGACTTTCAGTGTAACAATGTATATGGTGCAAGCTAAGGACTTCTGTCAATATGGCG

AAGTACATGATACATTCTTTATCATTTGATACAATAGAACTGACGTGTGTGTTGAGCTGA

TTATTCAATAATCGTTTGAATGAAACATCGAGGGTGTTGAACT

>Unigene0000884

AGATCGGTTCTGCCCGCTTTCGTGCCCCGGAGCTCTTGTTCCGGCCAGACCTGATAGGGG

AAGAATGTGAAGGACTCCATGAGGTGCTCGTTTTTGCCATTCAAAAATCAGATATGGATC

TGAGGCGAACACTTTTCTCCAACATTGTGCTGTCTGGAGGCTCCACGCTTTTCAAAGGTT

TTGGTGATAGGCTCTTGAGTGAGGTGAAGAAACTAGCTCCAAAGGACGTCAAAATAAGGA

TATCAGCTCCTCAGGAGAGATTGTATTCTACATGGATTGGTGGCTCTATTCTGGCCTCGC

TGGATACTTTTAAGAAAATGTGGGTTTCAAAAAAGGAATATGAAGAAGATGGAGCTCGCG

CCATCCACCGAAAAACCTTCTAGCCCTGGGACCTATCCTCAGTCTCCTCTGAAGACTCAC

TT

>Unigene0000885

GGCGGTGCATGCGGTCAAATCCTGGAAGCACCAGCTAGGATTTGGATTGGGTGTTTCTAC

CGTGTTTTTTGGGCAGAAAATGTTGCCCAGCATTGCTGTAATTTTGGATTGGCGGGGTTT

CTTCTGCGTCTATGAAGAGAACATTTGTTCTGAAGAAAAAAACATATCTTCCTTTTAAAT

TTATTATTACACTTAAGTTTGTTAATTGTGTTGAATATGGTTGCAGTGATTTCAGTCCAC

TTAGAATCCTTACCGCACTCTAATTTTATTATAATATACTAAAGTTGTATATGTGCATAT

AAAT

>Unigene0000886

CCTTTAATCTCTCCATCTCTTATGAACAAAAACGTAGGCACTTCGACAACGTCCATGTCT

TTCAAAAACTGCATACAGCTCTCGTTCTCATCGCCGTTCATTCTAGCGAACACCGCCGTG

TCCATTTTCTTCGACAACTTGATCACTGTGGGATAGACTTTCACGCAAGGTCCACAATGC

TTCAACCCCACGTCCAGTACAATCAGCTTGTGATCCTCCTTGTGGTCTTGTATCAGTTTC

TCCACATCCTCCCTGCTGTGTAGCTGAACAACTCCTGAATGGCTGTCGCCGTAGTATAAT

ACGTCGCCCTCCAGCTGATCCGGCCCGATCCCTTCCTCCTCGTGGATCTTCTCCATGTTC

TTGTAGAAGCTGAAGTGAGGAACCTTTTCGATTTTCTCTCTGTTACAGAGCTCTTTGGTT

TGCTCCGATTCATCGCCCATCACCAAAATGAACTCCACATCGTTACACGTTTTGCTGAGT

GTCACCATGAACGGGTACATCCGGCTGCTCTGCGCGCTGTGTGAGGCGGCGAATTCCACC

ACAACCAGCTTGCTTTTGGCTTTCTTAAGGGCTTCATCGAACTCCTCCGCGCTGTGGACT

TGCTGGACTCGCTCATCGCCGGTGGGTTTCTTCACGCTCGGAGCAGCGGTGGCTTTGATT

GTGAAGCCGCGGCGGCGGTGGGAGAGGTGTAAATTTGAAGAGAAGGCGGTGGGTTTGA

>Unigene0000887

GTTTGCAGTAAATACAGTATGAAATACATGAATAATCCAATTCAAGATAACTTTTAAGTT

GTCAGATGCATACTGTTCTTTGTACGTATTGTTCTATGTTCATAATACATTACAACTGGA

AATTTTAATATTGACAAAAATCTTATTGAATTTCTAAGCTTTTTTTTCTTTTGTTGGTGG

TCACGGAGCGTTAATAAGATGGAGAATGGGCTGCAGACAACATGTTGGCCATGAAGCATA

CGTTTACCTCCCGCAAAGTAGTGGCGGTGGACGGCAATAATAGAATTTAAAAATGATAAA

TATTTTAAGCCCCAGAATGTTGAGGTCTAAACATAGTTAGGCAAGAAAGCACATCAAAGT

ATCACGGATGAG

>Unigene0000888

AAGGTCATCATCAGGTTTCTCACAGTCATGATGAAGCATGGCTACATAGGAGAATTCGAA

ATAGTTGATGACCACCGAGCTGGCAAAGTAGTTGTTAATCTAACTGGTAGGATAAACAAA

TGTGGAGTGATTTCACCCCGTTTTGATCTCCCTGTTAATCAGTTTGAAAACAAATGCAAT

GCCCTTCTACCATCAAGACAGTTTGGATACATTGTTCTAACAACTAGTGGAGGAATAATG

GATCATGAAGAAGCTCGAAGGAAACATCTTGGAGGCAAAATCCTAGGCTTTTTCTTT

>Unigene0000889

TCTTTTGAAGCTCCAATCAAACCTCCAATCCATCAATGGCAGTGACGTTACTAATCTCCT

CCCTCTTCCCTTCCACTCTCTCCCTCAAATACCACTTCTCTCCAAAACCCAAACCCCTCC

TCCCTATCTCAGCCACGGCATCCCGCCCCCTCCGCGCCGCCGTCATCGGCGGCGGCCCCG

CCGGTGCTTCCGCCGCCGAAGCCCTCGCCTCCGGCGGCGTCGAGACCTTCCTCATCGAAC

GCAGCCCCTCCGGCTCCAAGCCCTGCGGCGGCGCCATCCCCCTCTGCATGCTCCAAGAAT

TCTCCCTCCCCGACTCCCTCGTCGACCGACGCGTCACCCGCATGCGCATCATCTCCCCTT

CCAACCGCGCCGTCGACTTCGGCCGCTCCCTCCGCCCCGGCGAGTACATCCCCATGCTCC

GCCGCGAGGTCCTCGACGCATTCCTCCGCTCCCGCGCCGCCGACGCCGGCGCCCATCTCC

TCCCCGGCCTCTTCACCTCCCTCTCCCTCCCCACCACCCCAACCTCCCCCTACCTCATCC

GCTACACCGCCTCCCCGTCCCCCGGCGCCATCTCCGGCGTCCAGCACGAACTCCCGGTCG

ACGTCGTCATCGGCGCCGACGGCGCCAACAGCCGCGTCGCCCGCTCCGTCTCCGCCGGTG

ACTACACCACCGCAATCGCCTTCCAGGAGCGCATCCGTCTCCCCACCGAAGCCATGCAAA

CATATTCCAACTTAGCTGAGATGTATGTGGGCAATGATGTATCTCCTGACTTCTATGGCT

GGGTGTTCCCGAAGTGCGACCATGTGGCAGTCGGCACCGGGACTGTTTCAGCGAAAGCTC

GTATCAAGGAATATCAGATTTCGATAAGGGAGCGAGCCAAGCTGAAGATAGCGGGTGGTG

AGGTGGTGAAAATTGAAGCACATCCGATACCGGAGCACCC

>Unigene0000890

TTCTGAGTACACTTGCTATACGGGTATCGCCGACTTCATACCAGCTGATATTGAGACTGT

TGGGTACCGAGTGTTTTTGGGACACAAACAATACTTTGTTTCCTCCGACGTCGGTGGAGG

AAAGATGCAGTGGTATGCATTTCATAAGGAACCACCTGGTGGCACTGATGCCCCGAACGG

CAAAAAAGAAAGACTGCTCAAAATATTTGAAGGTTGGTGTGACAA

>Unigene0000891

AAGAGCAGTACAAATTTATGTACCAAAAATACACACACATTAAAATATAGTATGTGTGTA

AAGCTGGCAGAGAAATTAAACCCGGTTTATACGCACGCCTTAATACATAATACATACACA

AAATACATTAAAACATACACAAAAATAATAAATGCGGGCATAATATAATTAAAAAATATA

AACAAGAAATAAAGAAATTAAAAAGAATAAATAAAAAGAATATATACAAGGATAGACTGA

TGGGTAGCCTCCCATCTAGCGCTAAGTTTAATGTTTCCAGTCAAATGAAATTAAGCACTC

AAAGTCATACATTGATTATTTTCAAACAAAAATAAAGAATTATTACATTTACCAAAATAC

CACTGATATAACTTCTCTATCCTCTATGAAGGGGTTTCGTCAATTATTTGATTCTTTATA

TTGATGCATATAATGTCTTCTGAAGATGGTCTTTGGGCTACTCTGTAAGTATTTATTCTG

ACTTTATTATTTCCAAAAACTAAATCCATATCTATGCTTTTGAAGATTAAGTTTGCATTT

AAAGTAGACAAATTATGGTCATAATTTTTATTAACTTCTTCTTCATTATTAGTAGAAGAA

GAAGG

>Unigene0000892

CCCCTAATCTCCTCATCTCCTCCATCATCTGTATCACAGCCTCCTACTTCCTCTGCGGGG

CCTCGATAGCCGCCGACGATTCCATCCGAGCTTCCGGTCTCGGCCTGAAGGTTGCCGACA

CCCTCCGCCGCTCCGGCTGGGCCGACGAGGCCATCGTCTTCGCCCTCGCCACCCTCCCCA

TCATCGAGCTCCGCGGCGCCATCCCTGTCGGTTACTGGATGAATCTCCACCCCGTCCTCC

TAACCATCCTCTCCATTCTCGGAAACATGGTCCCGGTGCCGTTCATTGTGCTGTACTTAA

AGAGGCTGGCGGCTTTCATGGCTCAGAGGAGTAGTTCGGCGACTCGGTTCTTGGAGATGC

TGTTTGAGAGGGCGAGGGGGAAGGCGGGGCCTGTGGAGGAGTTCCAGTGGCTGGGGCTTA

TGCTGTTTGTTGCAGTGCCGTTTCCGGGGACAGGGGCGTGGACGGGGGCAATTGTGGCGT

CGGTTCTTGACATGCCGTTCTGGTCGGCCGTGTCGGCGAATTTCTTTGGGGTTGTTCTGG

CGGGGCTTTTGGTGAATTTGCTTGTTAACGTTGGATTGAAGTATGCGGTTGTGGTGGGGG

TGATTCTGTTCCTTATGTCGACGCTCATGTGGGATGTGCTGAGGAAGATGAAGAAGGCAT

GAGCTCGGAATGATGCAAGACAGAAAACTGAAATATAATTGAGCCCTGTCAGCAGATTTG

GGTAGGAATATTCCTAGAGGCCATCTTCCAGTTCCACATCGCCCTGGAGTTTAGTCTTAT

ATCAGTTAAGCCAGTCTTACAATCTAATATAGTATGTAAAATACTGCATCATTTATAATC

AATTGGTTTTA

>Unigene0000893

TACAACAAGTTTAATTTGTAAGCTCCATCACAAACTGCTTTCCCCTCCATCCCCATCCCC

ACCCCCACCTACACCTCCTCTCGCTTCTCTCCAATGGCGATCTCCTGCATCATTAGCCTA

CCAGCTACGCTGCCCCAATTCTCCCACAGAATCAACCGGGCCCAATTCTCTCAATCCAAA

CATCCAAACTTCTCCAAATTCTCACTTCTCACCCACACCAAATCCATCACCAAACCCTCC

CCTCAAATTCTCTCATCTCTTTCCCCCTCCCCCGCCC

>Unigene0000894

AAAATGATTGCAAGAAAGTTGGATTTGAATTCTTGGCACCTACTACTTGTAGAAATGCTA

TGCGTGTATTGCGTGCAATGCAACTTCGGAAACCTGTGTTGTTGGAAGGAAGTCCTGGCG

TTGGGAAAACTAGTTTAGTTGTCGCGCTGGCACAACATTCTGGACATAGTGTTGTGCGAA

TCAACCTCTCTGAACAGACAGACATGATGGATCTATTGGGTTCAGACTTGCCTTTTGAGG

GGGATAATGGAATAGAGTTTCATTGGTCTGATGGGATACTTCTGCAGGCACTGAAGCATG

GGAGTTGGATTCTGTTAGATGAACTCAATCTTGCTCCACAATCTGTTTTGGAGGGATTGA

ATTCTATCTTGGACCACCGGGCTAATATCTTTATCCCAGAACTTGGCCAAACCTTTAAGT

GTCCAGATTCATTCAGGATTTTTGCTTGTCAGAATCCTTCTGGTCAAGGTGGTGGGCGGA

AAGGTCTCCCAAAGTCATTCCTTAATCGCTTCACTAAGGTTTATGTGGATGAATTGGCTG

CTGAAGACTATCTTTCCATCTGTAAATTGCACCATCCTGCAATCCACGAAAAATTACTTC

TAAAGTTAATTTCGTTCAACAACAGCCTTTATGATGACACC

>Unigene0000895

CAGACCCTATGTTATTTGGAAAGTTGAATGAACGTATAGAATGCATTACTGAGTATTCGA

CCAAGACGGCCGAAGGATATCAAGTGGTCAATTATGGATTAGGTGGTCATTTCTCTGAAC

ACGTGGATGCATTTACAGATGGCCCTAAGTCGAATGGCAACAGATTGGTCACTATATTAT

TTTACATGACTGACGTCCCTGAAGATGGTTATACAGTATTTCCAAATAT

>Unigene0000896

TTCAAAATGTGGCTAAGCCGGGCGCCAATTAAGTCTTACGAGTTGCATTGCCATTCATAT

CTCTTTACATAAACCAAAGTTATACTATTTCCTTCCCCGAATTACTTGCTTATGAAATTC

GTATCGGTTGAGATCCCATAGGATACGCATCGTATGATGCCTATAGTGTATTCTTGCATG

GTACATTTTTAATAACTGTATATGTGCTCAGATTGTTTATGAAAAATCAA

>Unigene0000897

AAAGCTATTGCAGCAGCACCAAAACTTTCCATCTACGATTCACTTGATGAGGAAGTCATT

CAGAGTTATCTTGAATTTTCAATGACATCATTATTATTCTTCTCAATGAAGGAAGGTGCA

TGCAGTGAACAATCCAGCAGAATGACTGCTATGGACAATGCAAGTAAAAATGCCGGTGAA

ATGATTGATAAACTCACACTGACATTCAACAGAACTCGACAAGCTGTCATTACTCGAGAA

CTCATTGAAATTAT

>Unigene0000898

TCCGTGTATTTATATTGATGGGAGACTGAGATATCCTTGGCTGAGAAAGGGATGGGCACT

CTCTCTATATCCATTCTCGTCCATCCGTCCACACGCTGGAGATGAGATGATAAACCAGCG

CTCTGGTTGTAACCTCTCGTACATCACTCATTTAGGTTTAAGGATTGCAGCTATGAGCAT

GGGCACATAAATTTACGTGATGTGGGTATTTTTAGGAAGAAGCTGAGGATGAAGTCTTTG

AAAATT

>Unigene0000899

TAAACGACCAAAAATTAATCGTTTAGAACTAAGTAAAACCCTAAATATTTCACATAGCAC

ACCAAACGAATTAAAAACATCACACAATTTGGATTCAATTTTAGAAATCAACAAACTATC

TAATAGCATAAGTCAGTCACAAAATCAAAAACCCAACAATTTGTCTTCAGACAAGCCAAA

AGCTGTCAAACATTCAATGGCCGCCACACTTGTACCTATGGGTAAGTCAAAACCGACTAT

GATGACTAAGACAACAAGTTCGCGTAATCCAAAATCAGTTTATATAGACTTGGATTCTGC

TGCCGAACAGGCTCGTTTAAAGTCTACAATTCTCCAGAATAAAAATCCTGAAACGAAGAA

GACTGCTACCGTCCCATTGAGTCCAGCCATGTTGCTATCATCTTGTCCGGGTTTAAGCAT

AACACCAGTTGTGAACACAAATGATAACCGAAAACCAAATGAAACTTTATTGGCATCACA

TCATGTTCAAAACATCACAACAACTTCAAAGAATTTTAATTTTGAACAGCTCCAGCACCT

CGGTAATAGTTTGACAATTACAAAATCTGAAAAAAATACCAATAAAAAGTCCAAACCTGA

ATTAATTCTCATTGATTAGTTAAAAATCATTTAAGAATTTGATGTATTTGTGACGTTTTT

GT

>Unigene0000900

TCCAACCTGATGGAATACTTTAACACATGTTTGCGTGTTTTTACTTTAACCATCATAGTT

TTTCTTATTTAGACATACTACCAGCGGCTAGTATGGTGTATTTGAGTCTTTTTTTGGGTG

AGTTTATTTTCTTCTGCCAGTTGACCATTTTGACTTTGCAGGTCACCTCCATCTCTCAAC

TGCAGACCCCCCTCCGGTCTTGTTTGCACCGGCGAGTGGGGCGGGGTGACTCAGGGGAAA

GAAAGTAAC

>Unigene0000901

CGCGATTGGTTTGTCCGTCCACGGAGATACCATCACCAAACGCCTCTCCGGAGGCCAGAA

AAAAAGACTGTCTATCGCTTTAGAGCTAATAACCAACCCGTCAATATTGTTCCTAGACGA

GCCCACTACAGGTTTGGACAGCCAATCTTGCAGTCAGTTCGTATCATTGATGGCCGACCT

AGCCCACAATCAAAATCGTACCATGGTGTGCACATTGCACCAGCCTAGTGCTTTA

>Unigene0000902

CAGTTGTGATCGTGATGTTGTCCGTCGAATCAATTATGGACCTCCTCAAAGGGGAGGACG

ATGACGACTTTGGCTTCAAGAAGCCATAGCGGATCATCAATGGCAAGGTTGACTACTGCT

GCCAGCGGCTCCAGCAGGTTCTTTCACGGAACCGATCATTGATTGACGTGGGAGATCATG

ATCCTGTCAGACTCGAGAGGCAGTCGGATAATCCCAGTGATGATCTCGCATAACAGCTCC

AGTAGTATCCTGACCAGCGGTCCTCGGCATCGCCCGCTGCCGTCGACATCGGCCATGAAT

TTTGTCGGGTCTTCAATGAGGAAGATCACCGGGTCGCCGGCAGCGGTGCATGGCCGGAAA

CCATTCCAATCTTATTAGACTCACAAATACCCCTTGAACTCCTTGTTTTTACTGCAATTA

CGCTAGGTCTGGTTTACACTGGTTCCTGCGATGAAGAGATCGCTCAAGCCGTTATTCAT

>Unigene0000903

TGGACACAGGTTTTAGTTTCTAACAATTTTGGCGAAAGCATTATGTCAATGAGTATATGT

TTGTAATACGGCATCACATCGCCATAATATTTTTGATATTTTTGTATGAAGAATGCAAAA

ATGACGAAGTTGAACCACACATTAAAGTAACTAATAATACGAGCCGGTGAACAAGAAATA

ACGCTTGAATAGCTTAAAGAAAAATTTAAAAAAAAAGTGTCTTCATCCTAATTGTCTAAA

CGTCGACGTACAGAAGTAGTGTTTCCAGATTAGAAAAAAAGAAGGAAGG

>Unigene0000904

GCTATGCTTTTCGGAGATGATGAAAGAGCTGCCACAGAGACTGACATGCAAGCTGAAGGA

GACACCCTGATGGACGGGCTGAACACCATTGTTGATGCTGAGAATGTGGGGAGAGCTGAC

AGACTTGTGGGTACTAATTCTGCCGGGGATGCACTTGATTCCTCAGAGACTTACATGCCT

GAGATTTCGAACAATAGAAAGAAGAGAAGGAGCATGGCCGATCTTGTTATCGGAACAGAT

TTGGGGATGGATGGGCTTGGCAACGAGGAAGGTATAGCAGAGATGAATCAGGGCGAGAGA

AACGAGGTTCATGATTTGGGTGATGCTGATGGTGCTGGCATGGAGATTGAAGGTATTGTC

TCAACTCAACGTGCGAGGAAAAAGAGCAAGTATCTGTCACCTCCCTACACGAACCCGAGC

TGGAGCGATATGCAAGCGGCTTTGCACGGGGATGCTCATGCATTGGTTCCTTGGTCGCCT

AAGAAGGGAAAGGATATCACGAGCTCTTCGTTGGGATCAAATGATAAGATGAACAGGGGA

AAGGGTTCAAATGATAAGATTAGCCCTTCAGCCACATGTGCCTCTGTTGATCTGAATGTG

TTTCTTTCAGACCTACTCTCTTTAGCTGTTGATCACTACCATTTGAAGGGGAGCCGTTCG

CAGCGTACTTTCAGGATCTTCTACCAAGAATTCAGGTGCTCACAGTACCTATGTTTGGAC

TTGGGGGACGATAAAGTCCATCCAGTTGATTCAGAAACTCTAGCAAGAGGAGTAGGTGAT

CGTTCTAAAGTTAAAGCTACAAGAACGAGTAACATAGGAAAGGGTGGAGCCGTTTCTGAA

GCTGCAACTTCATTAAATTTGAACCTTGGTAAGGTGAAACATGGTAGTGCTACCCTGAAA

AAGAAGGTGACGGAGAATGAAGTTGATGGTCAAGGCCCATTGAATCTAGCTACTGGTTCT

GCGAATAGTTCACTGGGTAAGAGGAAGGCCAATGCCAGTGGCATGCCTATACCTGAGTCG

ACCAGTCGTGATCCTTTTGAATTTTCTGAAGGTGATTCGCCGAAGAAGAGGAAGAGCAAT

GGTGCAGCGTTAGCTGTAATTCCACTGAATTCTCATGATCCCAAAGGCCTCCCAGAAGGC

GGGCAGACTGTGAGCACTAGACTCACAAAAAGCAATCAGTCGGGGCAGAAGAAGGGTTCC

AAGATTAACAAAAAAAGGAAGTCTTCAGCTAGTAAAATTGGGAAGGGCTCTAGCGGAGGT

ACATCTCAGCAGAAGCGAAAGATCGAAAAAGCTCTCTCCGAATGTTCAGCTGAAATTATC

GTTACATTCTCACCTGGCTCCACTCTGCCCACCAGAGATGAAGTAGTCTCTATATTTAGC

CTCTACGGTGACTTAATCGAGCCCGAGACAGAGTTGCGTGAAGATTCTTGCAGTGTTCGT

GTTGTTTTTGCAAAAGGCAGGGATGCTGAGAAGGCCTTGAACAGTCCGACTAAGAAGACT

GCTTTCAGATCCCCGCTCACTGGTTGTCGTCTTCGTTATTTGCTAGAAACCATGCCTGGT

CCTTCTGTGTTGCAGCCTGTCAAGAAGACTGCACAGCCTGTCAAGAAGACTCCGCGTCCT

GGTCCTTCTGTGGCGCAGCCTGTCAAGAAGACTCCGAGTCCTGG

>Unigene0000905

AATTCTCAGAAACCCCTTATCTATCCAAGGGTTTCTACGCTTTGTTTCTCTACCTCTACA

ATGGAGAGGCCTGATGTCTCACAAACGCTGATCCGGCCGCCTGGGCACCAAACCCCAGCG

ACCCCTACAGCGTGTTTTCAGGGCCCGTCTCCGGTTCCGCCACCTCAGCCCCAAAACCTA

GATACGACTGCCGCCCCGTCGGCGGTTCCTGAATTAGGGCTTGACTTATCGCTGAATCTG

CCATCCCAACATATTGCAGCGACGGGGAACCTGGAGCATGAGTTTATAAATTTTGGTGAT

GGGGGAGGAGATCAGATTGGGAGTGGGATGGATGTTTTTTCCATATTTGGGGATATGGGA

GAAGCCCCCACACAGAGGGGGGTTGATTTGTTTGCTATTTTTGGGGATGAGGAGGAAGTG

GCCACGGAGAACGCCGATGCTGTTATGAATTCTGTGGAGGAAGCGGCCATGGAGAACGCT

GATGCTTTTGTGAATTCTGTGGATGGGGAAGGTGCTGTTGCGGGAAGTAGGTCGGATGCT

TTTATGCTTTTTGGGGATGAGGAAGGAGCTGCAGCGGGGAGTGGATTTGATACTGCTATG

CTTTTCGGAGATGATGA

>Unigene0000906

ATGCGGAATAAAGACCCCTCTCTCTCTAGCTCCCAGTGTTCTCCAACCGCCATCAATGGC

TATCAGCCGAGTCCTCAAATCCCTAAAGTGCAAGTCCAACTCCGTCGACGACGTCCACCA

CCCCAACTCCCCCTCCCTCCTCACAAAGCCCCTCCTCCCTCTCTCCTCCTGCTCCTCCCA

TGCCCACAGAGACGTCTACCTCTCCACAAAGCCGCCACCCTCCTCCTCCGGCCGCCGCAA

GCCACCGCCACCCCCACACTCGAAGCCCAAGTCCAAACCCAAACCACCATCCAAACTCTC

TCCTCTACCTCTTCTCCCAACCCAAGCTTTCCCCTCCCTGGAGGAGCTCCCCTCCTGCCA

CCCCTCCCGCCGCGTAGTCGAGCTCATCTTCACCTCCCGCTGGTCCCCCGGCGGCCCTCC

ATTCCGTGGAAAAATCGAAATGCTCTTCCGCGTCCACAACCCACCCCTCGCTGTCGCTCA

GTTTGAAACCCACCGTGCTGCTGTGCGCCTCCGGGGCGGCGCAGCTCGCTGCGCTGCTGA

CGGCAACGAAATGATGCGTTTCCATTGTACACACTCTTCCGGGGCTATGTATGATGCTAG

GGTTGCTACGTGCACGGTGGCGACAGCGGCGGGGAAGATGACGGAGGCTGTAAGGACGTT

TGCCGTCAGTGGTGGCGCGCATGACAGCGCTGGCGGCGGTGCGGGGCAACGGGCCGTGCT

GGTCTGCCGGGTAATTGCTGGGCGTGTCAGGAGCGAGTCGAGCGACAAGGAGTGCGAATC

AGTGAGTATAGGGATGAATGAATTGATAGTTCTGGATCAGTGTGGAGTTCTACCTTGCTT

CCTTATTATCTACAAGGTGTAATAAGGAGTTAGGGGTTTTTAATTACTCTTCTTGTGATT

TTCTTTCTTTTACTCTTATTTTCCAGTGGAGAAAGAAATTGGTTTTGAGGGTGAAGAATA

GGTAGGAATTAAAGTTTGAGTTATAAGTAGGTGGTACTCTCTTTGACCTGAAAAATAGTA

TGACAAACAATGCCAATCATTCTTTTTTTTTTCCTTTAAAGTTTCACCAAGTCCTTATAT

TAAAT

>Unigene0000907

GTCCTACCTCACCGGCGAGTTCCCCGGCGACTACGGCTGGGACACTGCCGGCCTCTCCGC

CGATCCCGAAACCTTCGCCAAGAACCGAGAGCTCGAGGTCATCCACTCCCGTTGGGCAAT

GCTCGGCGCCCTCGGCTGCGTCTTCCCCGAACTCCTATCCAAGAACGGTGTCAAGTTCGG

CGAGGCGGTCTGGTTCAAGGCCGGAGCACAAATCTTCTCGGAAGGCGGGCTGGACTACCT

AGGAAACCCTAACCTGGTGCATGCACAGAGCATCCTCGCAATCTGGGCCGTGCAGGTGGT

GCTGATGGGGTTCATTGAGGGCTACAGAATTGGCGGCGGGCCACTGGGGGAGGGACTCGA

CCCGTTGTACCCGGGTGGAGCGTTCGACCCCCTAGGGCTGGCTGATGACCCCGATGCGTT

TGCAGAGTTGAAGGTAAAGGAGCTCAAGAACGGGAGGTTGGCTATGTTTTCTATGTTTGG

GTTCTTCGTGCAGGCCATTGTGACCGGGAAGGGGCCGGTGGAGAATTTGTACGACCACCT

TGCGGATCCGGCGACCAACAATGCGTGGGCTTATGCTACCAACTTCGTTCCCGGCAAGTG

AGGTTCATGTTCATGTATCGATGTATGTGTTGTAACTTTTATTTGGGTTTGTGATTTGTA

ATGTTGACGAAGGAACTTGCTATGAAAGTGTTGTGGATGCACTTTGATATTTGCGCATGG

T

>Unigene0000908

GGGGGTATCCGCATGACGGGCTAGCAGGTTAGCCCAAGCCGAGGATGAGCGCATTTGACC

TTCAAGGGCTCTGGGCCGACCTACCGAGGCCCTGATGGCCCATTACTCGAGGAGGCAAGG

GCAAGATTTTGACTGTAACTGATAGGTTGTGTAGAGTGTGCCCATCCGACTCAAGATAGG

TGAGGAATACAGCTTAGAGGATGAACGCTTGAGGCCAACTGAGCTAGGATGACCTTATTT

CTCACAGAAGGGGC

>Unigene0000909

AACCACCCTCTTTGCCGGTCGACTAAGCGAACCCGGACCCAGTGGTTAGTGGGTCCTTGA

GAGGGGGTACCCGCGTGACGGGCTAGCAGGTTAGCCCAAGCCGAGGATGAGCGCATTTGA

CCTTCAAGGGCTCTGGGCCGACCTACCGAGGCCCTGATGGCCCATTACTCGAGGAGGCAA

GGGCAAGATTTTGACTGTAACTGATAGGTTGTGTAGAGTGTGCCCATCCGACTCAAGATA

GGTGAGGAATACAGCTTAGAGGATGAACGCTTGAGGCCAACTGAGCTAGGATGACCTTAT

TTCTCACAGAAGGGGC

>Unigene0000910

AATCTTTTGCCGGCCTCTGCCCCCTCTCTCCGCCCGCCGCCGAGGAAACAGAATGGCGGG

GAGTGGCACTACTCAACAGAGCATAAGAAAAGCTCTTGGAGCCATAAAGGATTCCACAAC

AGTCGGGTTGGCAAAAGTGAACAGTGATTATAAGGAACTGGATGTTGCAATTGTCAAGGC

CACTAATCATGTCGAACAGTTGGCAAAGGAGAAACATTTACGTGCTATTTTTCATGCTGT

TTCAGCTTCAAGACCTCGTGCAGATGTTGCTTATTGCTTAAATGCTCTTGCAAGAAGGCT

TGCGAAGACACATAATTGGGCCGTTGCCTTAAAAACATTAATCATTATACATCGTGCTTT

GAGAGAAGTTGATCCAACTTTTCGGGAAGAGCTCATCAACTATAGCAGGAGCAGAGGCCA

TATGCTGAACTTGTCCCATTTCAAGGATGACTCAAGCCCAAATGCATGGGACTATTCTGC

CTGGGTTCGGATGTATGCTCTATATTTGGAGGAGCGGTTGGAATGCTTGCGCATATTGAA

GTATGATGTGGAGACTGAACGATCTAGAACACCTGAACTCGATACTATTGACTTGCTCGA

GCAACTACCAGCTCTCCAGCAGCTTCTTTTCCGCCTGTTCTCTTGCAAGCCAGAGGGGGC

AGCTAAGTATAATAATGTGATCCAGTATGCACTTTCAATTGTTTCTGGTGAAAGTATTAA

GATATACAATGCTATAAATGGTGGCACTCTCAATTTGGTTGATAAGTTCTTTGAGATGCA

GCAGCGGCACGATGCCCTCAGGGCACTTGAAATTTATCGGAAGGCAGGGTACCAGGCAGA

GAAGTTGTCCGAGTTCTATGAAATCTGTAAGGGCCTTGATATTGGGCGGGGGCAAATTTT

TGTTAAAATTGAACAGCCACCAATTTCATTCATTTCGGCTATGGAAGAATATGTAAAGGA

TGCACCAGCTCAGCGAAATATGGTTGATGTTGAGAAAAGAGTATCCGCTCCGAAATCCAT

TTTGGCCATTGAGGATAAGAAAGCGGACAAGGTTGATGAAAATGCCGAAAGGCCAGAATC

AAATGTCATCCCAAAAGCAGCTGAAGCAACCGCACCTCAGACTGCTGAAGTCCCAGTGCA

AGCCCCAGTTACCGATCTCCTGGGATTCGAAGATTTCAGTCAGGAAGTGACACAAATGGA

ACAGAAAAATGCCCTGGCACTCGCAATTGTTACCAATGATGGTCCATCAGGGAATACCAC

AAACGCTTTTGACCTGACAACCGGGTCGACTGGGTGGGAGCTTGCGCTTGTTGGCGCACA

GAGTTCGAACGAAAGTGCTGTTGCAGACAGTAAGCTGGCTGGCGGGTTGGATAAATTGAC

ACTGGACAGTCTGTACGATGCTGCGATCGCCAGGTCGACTCCGACTGGGAACTACCACTT

GGGTCAGGTCGCACCGAATCCCTTCGAGGCTGCACAATATACCCAGGACCCTTTCTTTGC

GTCCCATACAATAGCCCCTCCGGCTAATGTGCAGATGGCAGCAATGGCGCAGCAGCAAGC

CATGCATCCAACCGGGCAGGACAGCACCAACCCGTTCGGTAACCCGTTTGGAGCGACGGG

TCAATCATATTCGTCGCATAACCCGTATACTGGTTTCATGTAGGCTGTGTCAGAGGTAGT

CTAGATTCAGCTTTAGAGATGTAGATAGACAGGGCATGTTCTGGATCGAATTGTAGTTGG

TCTTTCGTCGTCTGTCTTCTTTTTCTTGTGGTTCTCGACTGTTGGAGTCCCTTTTGTTTT

GGGGATTCTCTCAGAGTTGTTAAGAAACAAGTAAATTGGTTTAGGGAAATAATTGTTTGT

CAAGGCAGAGTTCTATAAATTGTAGTTCTTGGATGGAGTAGAATAAATTTAATAA

>Unigene0000911

TAAATTGTTGCCTCTTGTCTTTCTATTGTAAGTGTGTTGTCTTTTATGATTATATCGCCT

ATTATACATGGTTTTATATTTAAAAGGTCTTGCTACCCATTTTGGAGACAGTTGGTTCTC

ATCATAAATGTTAGATTTTATAAAGCCTTTGTTGTGACTATGAATTACTATGTGAGAATT

GGTGAATCAAAGATGTGAGATGGGAGTTTTTATCTTCTTCAAGAGGTTGCTTCCAAGTAA

TCATTTAAAAAATAAAATAAAAGCTATTGATTAATGCCAGTTGAACCGTGTCTAGTTGAA

GAATCTGCTGATATTTGCAATTTCTCGCTATTTGCTTCCATAACCATCATATGTATAACA

TTCTGATAGTTTGACCTTTTTCCCATTTAGTTTGCGAATTCTTGTATTACCATTTTTTCT

TGACGGTCTTATATTTGTCAGTTATCATGGTAGTTCCTAATTTACCTATCGCTGGGTTAG

TCCTTGAGAGAACCAATTTTTTCCCCTTATTCACAGACTGGTGCAACGGTAAGGTCGCTG

GGTTAGTCCCTGAGAGAACCAATTTTTC

>Unigene0000912

GTCTTATATTTGTCAGTTATCATGGTAGTTCCTAATTTACCTATCGCTGGGTTAGTCCTT

GAGAGAACCAATTTTTTCCCCTTATTCACAGTGGTGCAACGGTAAGGTTGCCCCATTGTG

ACCGGAGGTCACAGGTTTGAATCCAGAAACAACCTCTCCACAGGAACACGCCGTTGTGAT

GGGTTCTGTTGTGGAATCCTCGGGCACTGGGTTGCCCTAACAAGTATTTATATTCACCTT

TTTGTTTTTTCAGTATTTGAATTACTTAATGAAGAAGGTTTTCAATATGATGCAAATCTC

ACTT

>Unigene0000913

TTTTTTTTTTTAGTATTAGAGTTACTTAATGAAGAAGGTTATCAATATGATGCAAATCTC

TACTTAAGATGCTTGTGTAGGAGTTATCTCTTGCCATTTAAATGTCCGTGGAATCACAGT

TAAATTCTTCACATCATATATTGGTTTTCAGACCCATTGGTGTTTCTGAATAAATTATTC

TTTTAGGATTCTCCTTACTTATGATTAAGGTTTGGCTCTCTTATTTCAGTAACCCATTTT

ATGCATGTTTTTTATATTGTCGTTGCTCATCGGATTTTGGCCCTTTTCAAGATGGGTGGT

GCTCAAGCATGTCCATTTTATATGTTACTGGTTATGAGGCTATGCAATACTGGCT

>Unigene0000914

TTTTCTATGTTACTGGCTATGTGGCTATGCAATACTGGCTAATAAATACGGAAAATGGGT

ATAAGAAGTCAGCTGTTTCCCCCTTTGTGAACAGTATGCTCATAGTGAATAGTTCTGAGA

AGATAATCCCTTTTCCTTGTAGCATCTTGGACTCTATCTAGAATTAGACAAGCATTATTG

GAATCATGTGTAGATAACTATGTAAGTTTTTTAGTCAACTTCAGGTCACTTTATCCCTAC

TTTAGTGACTATCTCATGGTTTGTAATCCCTGGATCTGTATTACATATAAAGCCTGTCAT

TTTCTAATAAATTTTAGTCCTTTTAACTTCTTGTTTATGGTTATAGCAACTTTACCCTAA

AAGCTTAAGCTTTT

>Unigene0000915

CAGGGAACTAAAAGGGCATTACGGTAACTTCCCATCGGTTTCCGCCCCTTTCCGCGGCCC

GGCGTCAGCAAGGTGAGGGAAAAGGGGACTATTTGGACCTCTCCGGCGCTCCTCCCCGAG

AATTCGTCACCGGCACCGATCCGGACCCATCGATCAAATTCAGCTTAAGCTTCGGATCTG

AGTTTCGGGAGTCACATTGCATACACAGTATGTCTAATTACCAAGAGGAGGATGCGGATG

AAATGCCTCATGATTATGACGTAGTTGATATGGAAGATGACATTGACGGAGAATTTTATG

ATAGAGGGGTTGAAGATTCAGAATCTGACGATGAAGAATATGGTCAATTGGTGAAAACAG

ATACTACAGCTGCACAAGCCAGAAAAGGGAAAGATATTCAAGGAATACCTTGGGATAGGC

TTAATATTACAAGGAAAAAATATAGAAATACAAGGCTAGAACAATACAAGAATTATGAGA

ACTTCACCGGTTCTGGAGAACAGTCAAAAATGGAATGCAAACCAACTAAAACAGGGTCAA

TGTATTACGAGTTTTGGCGCAATACTAGGTCCGTGAAATCAGCTATTCTTCATTTTCAGC

TTAGAAATTTGGTCTGGGCTACATCAAAGCATGATGTTTACCTCATTCCCCACTCTTCTG

TTATTCATTGGTCTGCACTGAGATGTCGGAAGTCTCAAGTTCTGAATGTTTCTGGCCATG

TTGCACCCTGTGAGAAATACCCGGGGAATTTACGAGAAGGGTTTACTGAAACTCAAGTTT

GTACATTAGCAGTTAGGGATAAGTTACTTTTAGTGGGTGGATTCGAGGGTGAACTTATCT

GTAAGTATATCGATCGACCTGGAGTAAGCTTTTGTTGTCGAACAACTCATGATGACAATG

CCATCACAAATTCCTTGGAGATCTACGACAGTTCTAGCGGTGCTACACACTTCACGGCTG

CAAATAACGATTGTGGAGTTAGAGAATACGATGCTGAGAAGTTTCAACTTTGTCAGACTT

TCCGTTTTCCGTGGCCAGTAAACCACACATCGCTGAGTCCAGACGGGAGCACCCTGCTCA

TTGTGGGAGACAATCCTAAGGGGCAACTGGTGGATATTCGCCTTGGCAAGGTTACGAGAA

GCTTCCACGGTCATTACGATTACTCATTTGCGTCAGCATGGAATCCCGATGGCCGGACCT

TTGCAACCGGGAACCAGGATAAAACCTGTCGTGTTTGGGACATTCGAAACCTTTCAAAAT

CAGTCGCGGTTTTGAGAGCCAACCTTGGAGCGATTAGATCAATCCGCTACTCGTCGGATG

GTAAGTTCTTGGTGTTGGCTGAGCCAGCTGATTTCGTTCATATATACGATGTAGGGAGTG

GGTATGAGAATCGGCAGGAGTTGGATTTTTTTGGTGAGGTCTCAGGTGTGGCGATGAGCC

CTGACACGGAGGCTCTGTTTGTTGGGGTGTGGGACCGTACTTATGGTAGCCTTCTTCAGT

ACAATCTCCGTCGAAACTACTCCTATCTTGAATCTCTATTTTAAATTTGGGAGGTTTTGG

CTCGGTAAAATAAGATTGGGGATACTTGAGATGTGCATAAAGGGAGATATCTAGTCGGGT

GGATACGAGTTGCGATCAATGAAGACTTTCTTTGTAATGGAAGACTATTTAGTAATACCT

TGGTGTTTGCGAAATATTTTTATTTTCTCGTATTGTAAGGAAATGTTATTTCGTGAACTT

GTTTTATTATTTATTTTTCTGTATTTGTTGATAATGTACTACTTTTTAGGATTTTTTTGT

CTGTATG

>Unigene0000916

GCCCTCCTCTTCCCTCCCACTCCCTCCGCTCCGCCGCGACCAAATCTCAGCTCAGAATGA

AGACGAAGCAATGAGAAACTCAATCTCTGCCCTCATCCGGCGGCACCGCCGCTGCCTCCC

CTTGCCCCTTGCCAATCCCTCGCAAACCCCCCTGCGTCGCCCCCTCAACACTGGAAACCC

CCATCCGGTCCTCATCCGCCGGTTCTCGACTGGATTCACGAGCGTGCACGGCGAGAGGCC

GTCATCGGAGTACGCGAAGAGGAGGAAGGAGTCGCTGGAGAGCGAGTTCGGCACGATGCT

CGGGGTGCTGCAATCGAAGATGCTGTTTTCTTACCACCGGTTCGGGCCGTTCCTGGCGTT

GTATAGGGCAGCGATCGTGTCGTTTCAGGTGGTGAAGCTGACGATATGGCACTTCTTCCT

CCGGGATATACGAAAGCGTGCTGTGAAGTTTCGGGAGACACTTATTCGATTAGGGCCATT

CTACATCAAGCTTGGTCAAGCACTGAGTACACGGCCTGATATATTACCGAAGGTGTATTG

TCAAGAGCTTTCAAAGTTGCAGGATCAAATACCACCCTTTCCAACTCGTGTTGCTATCAA

ATCCATTGAGTCACAATTTGGCGCTCAACTTTCTGAAATTTTTTCTGATATAAGCTCGAA

ACCCATTGCAGCAGCGTCTTTGGGGCAAGTATATAAAGCTCATCTACATTCCGGGGAACT

TGTAGCTGTCAAAGTTCAGAGGCCTGGTATGGCCGTGTTGCTGACTCTTGACGCACAACT

GTTTCACATGATTGGAGGACAATTGAAGCGATTTGCGACGGCTCGGAAAGATTTGTTGGT

AGCAGTGAATGAGATGGTCAGGCACATGTTTGAAGAAATTGATTACTTATTAGAGGCACA

AAATGCTGAAAGATTTGCTTCGCTCTATGCAGTCTATCCAACTACGAGACAGGACAGTAT

CAAGGAAGGATCAGCTATCAGGGTTCCAAAAATTTATTGGAGCTTCACAAGTAAATCCAT

TCTTACTATGGAATGGATTGATGGAATCAAACTCACAGATGCCGAGCGAATGAATGAAGC

TTGTTTGAATAGGAAGCTTTTAATTGATCAGGGAGTATATTGTTCCTTGAGGCAACTTCT

TCAGGTGGGGTTTTTCCATGCTGACCCACATCCGGGAAATCTTGTTGCTTGTGATGATGG

TTCACTTGCCTACTTTGACTTTGGTATGATGGGAGATATCCCACGCCATTACCGTGTAGG

ACTCATCCAAGTGCTTGTGCACTTTGTTAACCGCGACTCGTTGGGGTTGGCAAATGACTT

CCTCTCGTTGGGATTTATTCCTGATGGGACAGATTTACAATCAGTTGCAGATGCCTTGCA

TGCATCATTTGGGGATGGAAACAGACAATCGAACGATTTCCAGGGTATAATGAACCAGTT

ATATGACGTTATGTATGAGTTCAACTTCAGCTTGCCCCCTGATTATGCACTTGTAATAAG

AGCATTGGGATCTCTAGAAGGAACTGCAAAAGCACTTGATCCGGATTTTAAAGTTGTTGA

GAGTGCATATCCTTTTGTCATAGGGCGACTTCTTGCAGACCCAAACCCGGATATGAGGAA

AATATTGAGGGAGCTTCTTATACGTAACAATGGCTCCATCCGATGGAATCGGCTTGAACG

TTTGATAGCTGCAATTTCTGAACAGTCGTCTGAATCGGTGAATTCAAATCCTACTAACCC

CACAGTAGGTGCTACAGATGCCCAAGGATGGAAATCCTTTGATATGCGCTCAGTGGTTGC

TGCCACAGAAGATCTTTTTCATTTCATCTTGTCTGAGAAAGGCCGGAGAGTTCGGGTTTT

CCTGATTCAGGACATTACCAAAGCTGTTGATGCCTTTGTGAAAGAGGAAGCTCTTACATG

CTTCTTTGAGACGCAGGTGGCAGGAGAAAGATCTGATTTCGAGAGATCTCGGGTGGTATC

CAGATTGGTGAATGGTATTCAATCATTGAGACATGCTGTTCGGGTGGCACCAGAGGTATG

GACTGCAATGTTAATACAAATGTTACTGAAAACAGAGTTCCATAGCTTTTTTCATGAAAT

ATTTTCAGTTTTGTTGAGTCACTTTGGCAAAAAGTTACCAGAAACATTCTGGCTTTGTGT

ATCTAGACTGCTCCACTGGTTGGAAAAGCAAAAAGAATTACAGGATTCATAAGTAGCAGT

GATTATCGTCAATTAATTAGTCTAAGCTAACCTACATATGAGTTGTTGAAGATTCTGGTG

GTTATTGGTTGGCAAAGATTGATATCACCTTTATATTGAAGTTATAAGAAAAACAGGTGA

GGTCGGCATGCAACATAATCTTGTTTCTTGCAGGAACTTGTTTCTCTTAT

>Unigene0000917

GAGTTCCATAGCTTTTTTTCATGAAATATTTTCAGTTTTGTTGAGTCACTTTGGCAAAAA

GTTACCAGAAACATTCTGGCTTTGTGTATCTAGACTGCTCCACTGGTTGGAAAAGCAAAA

AGAATTACAGGATTCATAAGTAGCAGTGATTATCGTCAATTAATTAGTCTAAGCTAACCT

ACATATGAGTTGTTGAAGATTCTGGTGGTTATTGGTTGGCAAAGATTGATATCACCTTTA

TATTGAAGTTATAAGAAAAACAGGTGAGGTCGGCATGCAACATAATCTTGTTTCTTGCAG

GAACTTGTTTCTCTTAT

>Unigene0000918

GTGGCCAAACAGAATATGCCGGTTTCCAGCATTGTTATTGGAATCGATTTGTACCCTATA

AAACCAGTACCTGGTTGTATATGTTTAACAGAAGATATTACAACACCTCAATGTCAGTCT

GCTCTCAGTAAAGAGCTTCAGACCTACAAAGCGGACGTTGTGTTAAACGATGGAGCTCCC

AATGTTGGTCAAAATTGGATTTATGATGCTTATTCTCAATCTTGTCTCACTTTGAGTGCT

CTGAAGTTATGTTGTCATAATCTCAGGGAAGGTGGATGGTTCATTACTAAAGTTTTCAGA

TCAAAAGATTACAATGCTCTTATGTGGGTATTTAAACAACTTTTCAAAAAAGTACATGCT

ACCAAACCCCAAGCATCACGTAGCGAGTCTGCTGAAATATTTGTTGTTTGTCAATATTAC

TTGAAACCGTCAAAACTAGATATGCGTTTCTTTAATCCAAATTATGTTTTCAAAGACTTA

GAAGTCAATCCACAAGTAAAATTGGATTCTGAAAGCAAAACTCAAAAGAAACCAAAGGCT

GAAGGTTATCCGGATAACACAACTATATTATTTAAACAATTACCAGTATCAACTTATGTT

CAAGCTAAAAACCCAGCCATAGCACTACAAGATGCATATGAAATTGTTTTAGATGATCCA

GTAATAGCTAACCATCCAAGTACAACAAATGAAATAAAAGAGTGTTGTAAAGATATTAAA

GTTTTAGGCCGAAAAGATATAAGATCACTTATGAACTGGTGGAAAGTGTTCCAAGATAAA

GAAGAGAAGGTTGAAACTGTGGATTCTGGTGATGAAAACAAAGAAAATAATGTTGAGCTA

TCAGAAGACGAAAAAGAATTGGATGAAGTTGATAAAGAAATCAAAGAGTTGCAGGATGAA

GAAAGGAGAGAATTAAAACGCAAGAAGAAGAAAACCAACAAAGAAAGAACAAAGTTACAG

ACTAAAATGAATTTAAAAATGGTGCATAAGGACGATTTGGGGCCAACTGAAGAACGTGAA

ACTGGTGTATTTCAACTGTCTAATATAAAAACAAAAAAAGCTTTAGACACTGTAATTGAT

CAAGAACCAGATATAGTTGAGTCTGAGGATTCTGATGTTGAGATTAAACCTAAAAAAGTG

AAATATGATCCAAACAATACATACCTCGACAAATCTGGAATGTTTTACCGTTCAGATGAA

AGTGCATCAGAACATGATTCATCAGATGAAGTTGATAGTGATACTGAAGGATTAGGTTTT

TCAGATAAAAATCTATCATCAAAAGCATTACCCAAAAAAAAAGTCACTTTTGCTGATCCA

AATGAAAACCATCCTCTACTTACTGATCTTGATTATCGAGATGTAAAGGACAAGCGTACT

TCTAAAGCTGAACTTTGGTTTGATAAAGATATATTTAAAAATGTAGAAAGAGAAGAAGAT

GAAGATTACGAGTTAGAAAAAATGGCTGCTGTAATTAAATCTAAAGGTGGGAAATTGTAT

AGAAGCAATTTAGATGATGACGATGATGATGATAATGAAAGTGAAAAGGGTAATTTAAAA

AAACCTAACAAAAATATAAAAGTAAACCAAAAAGATGATGCTGATGATGACGAAACTACA

GACTCCGATTCTGAATCTGAAATCAGTGAAGCTGAAGATCAAAGTGCTGCAATACCTATA

AATGAATGGGATGATAAACCTGTACTTACACCAGCAGGTAAAAAAAAGAAAAAGAGGAAT

GCTAAAGTTTTGGATGATGACGGTCTTGCTTTGGGTACAATGATGGCTTCTTCAAAAAAA

ATGAAAAGAGACATTATTGATGCTGCCTGGAATAGGTTTGCGTTCAATGATGAAGATCTA

CCCGATTGGTTTGTAGATGATGAAGCAAAACACATGCGCAAACACGTGCCTGTACCTAAG

GAACTAGTGGACGAATTTGGTAAAAGACATGAAGATATAAATATAAGACCTATTAAGAAA

GTTGGTGAAGCTAAGGCTAGAAAGAAGAAGCGTGCACTACGTCAAATGGAAAAGGCAAAG

AAGAAGTTGGAAGGTGTTGTGGAAAATACTGATGTTACAGATGCCGAAAAAGCTCGACAA

GTCAGACAGATTTACAAAAAATTGAAACAGCCTAAACAAGAAATAAAATATGTTGTATCC

AAAAAACACACGGCTGCCAAACGAGCGAAACGTCCGGCTGGTGTGAAAGGACCATACAAA

GTAGTAGATCCGAGAATGAAGAAAGATATGCGTGCTAAAATGCGAATGGACAAAAAGAAA

TCAAATAAAAAAGGTGCTAGAGCTAAGCCATTAGTTAAAACACGACGAGGTCATAAATAA

AAAATGTATTAAAAAAAGAAAGGTTTATTATAATTATTACTCTGTGACTATTATTGTTGT

TGTGTTTATTCTAATT

>Unigene0000919

CGATCTCTATTAATGGCGGACACACAGCGCGCGCCGATCAGCAAGCTCCGATGGGGAGAG

CTCGACGAGGACGACGGCGAGGACCTGGCGTACCTCCTGCCGCCGCCCATGGTGGAGGGT

CCCGACCAGAACGGGATCAAGAGGGTGATCGAGTACAAATTCGATGAGAATGATAGGAAG

GTGAAGGTGACGACGACGATCAGGGTGAGGAAGATCGCGCGGGCGCATCTGAGCAAGCGC

GCCCTCGAGCGCCGGTCGTGGGCTAAGTTTGGGGATGCTGTCAAGGATGAGCCCGGGAGT

CGGTTGACTATGGTGTCGACTGAGGAGATTTTTCTTGAGCGGCCTCGTGCCCCAGGTAGC

AAACAAGAAGAACCTTTGGCTGCTGGTGACCCATTGGCTTCTCTGGGAGGGAAAGCAGGC

GCTGTGCTGATGGTGTGCAGGACTTGTGGGAAGAAGGGCGACCACTGGACATCAAAGTGC

CCTTACAGGGATCTTGCCACTCAGAACGAGGGCTTCATGGACAAGCCGCCGACCACCGAG

ACTGCTGCTGGCGGCGCATCAGCCACTGGCAGCAAGGGAGCTTATGTACCTCCGAGCATG

AGAGCAGGTGCAGACACAAGAGGCTCTGATCCAAACAGCAGGCGTCGTAATGATGAGAAC

TCGGTTCGTGTGACCAACCTCTCCGAGGACACCCGAGAGGCAGATCTACATGAGCTCTTC

CGCACTTTTGGACCTGTGACCCGTGTCTATGTTGCTGTTGATCAGAAAACTGGGATGAGC

CGAGGGTTTGGTTTCGTCAACTTTGTGAGCAGGGAGGATGCAGAGAGAGCTATTAACAAG

CTCAATGGTTATGGTTATGATAATCTGATCCTTAGAGTTGAGTGGGCTACGCCGAGGCCC

AACTGAGATTATACTACTTGCTATCTGTCACTATTTTTGCTTGTTTTCTGTTAAGATTGT

TGGAAGTGTCTACTTTTAATGTGTGAGACTAAGAAACTATGTTTCATTCAAGTTATGGTT

ATAAGCTGGATTGCTCTTAATTCTGGTGTTTAAGAATGTCAATAAACTTTTATTAA

>Unigene0000920

TCTCCGCGTTTCCATCTCGTTTCCCCGGCACTCACTGAGCAGCAAACAGCGGCGGCGATG

GGGGCTTCGGCGTCGAGCATTACCGGCCCGGAGGGCGAGATCGGCGGCGGCGAGACGGGG

CTGGGGGACCTTCCGGAGGGCTGCGTGGCGGAGGTGATGAAGCATCTCGACCCGCCGGAG

ATCTGTCGGCTGGCGCGTGTCAGCTCGACGTTCTCGCGGGCGGCTTCGTCCGACCAGATA

TGGGGGGCGAAGCTCCCGGAGAGTTACCGGCATCTGATGAAGCGGGCGGAGAAGGAGATC

CGGCGGCCTAAGATGGAGATATATGCCCGGCTGTGTCGCCCTAATCGCTTCGACGGAGAC

GCTAAGGAGTTCTGGCTGGAGAAATGCAAGGGTGGAGTATGCGTTTTGATATCGTCAAAA

GCATTGTCAATCACCAGCATCGCTGATCGACGGCACTGGAACTACATTCCGACTGATGAA

TCAAGGTATCTTCGCTTGCTTATCTGATGAATTTGTAGCATGTATTTTATTTTTATTTAT

CTGCTCTCTACTTATGATATCTGAGTTACTAATTCTTCTGTTGATACCTTAGTTGAGCCT

ATGGTTTTTGAACAATAACAATTTCCATTATTAACGGACATCTACTATCAAAACAGAATG

GCGATGCATCACCATGTATTTCTGAACAAATTGAAGCTATTGAATTTTCAGTATCTTTTA

CTTATTTATGTGCCACTAGTTTATGATTTTCTCCTTACCTTCCAGTGTTATCTCTTTCCT

TGGATTTATATGCTTTACTCGTTTGCCAATTTTACTTTACCTCTCGAACTTATAAACCAT

CACTTGTATCTTTGTATGGTGAGCTTAATTTTATGTTTTAACACTGATAATATATTTTCT

ATGGTGGCGTAATGCATCTTCACAGATTCCTTATTTATGGACTTGACAGCTACATGGTAT

GCTTATTCTCTTGGGATATTTTAGTAGATATTTTCTGATTGTAAACCAGTTGGTTTTTTC

TTTCTGTCCAAATTCTAGATAATGACATATTCTCACAACAAAGTGGCCGAAAGTAACGAA

TGAAACATAAACTTTGCTACAGATACTTGTGTTGTTAAAGCTCGTTGATATTTCTGTGTA

TGAGATTTTCTCTGCATTATTATTAATTTTCTGTTTCACTACTTTCTTGTTATGTAGCTC

TGTAGTAGACTGGGTTCCTTGTTTACATGTAGCGGCATGAATAACACATTTTGTATGCAT

CTACACCTTGCATTGTTTCGCTTTTCCTTTCTAATCGGGGAGTCCATTGGAAGAAATGCT

GTAAGCTGGTCCCGTGCCAAAGATTTCTAAAGAGACTTTGGAACTGAAATTGACATAATA

TAAAATCAATAAAATTTTATGCGGGTATCTCCAAAACACAGCCTTGTTGATTGCGTGAAT

CAGTTTCATTAGTTCTGTTTTACCATATTGTTTTGTTTATTCTTACTAGGTGTTTATACT

GTTGAATCAGGAGGAAGTGAAGTACATGAAATATCAGTGTGGATGATTATGATGGATGTG

AATGTTTTAATAAAGCTAGTGTATGATACAATATATCGATGATAATAGAAAGACTCAACT

AACTGAAGTGACCATCTCAGAAGAGTGATTGAGAACCATATAAAGTTGAAATGTAACATC

TATCAACAACAAATATCTCGCTCCTATCCACATTCTGCTCTAGGGCAGAGAAAAATAATC

TTTATTTGGTGAGGCCTATGTAATTTCTTCTCATATATTTCATAGTCCCTTATTTTCACT

TTTAGCAAGATCAACAATAAGTCATCTCTACCACCAAACATATATAGTCTCGTAACAATA

TGACATATGTGCATGTGTCATCATGTAGTTAAACATTACATGTCTTGGTCTTTTGATTCT

TGAACTGGCATTATTATTTGAACAGAATTGACCTGTTTGTGCTCTAGACTAGTTTTATTA

GGGTTGGTTGAGAACTTGGCACAAAAACTGCTTCAGTGAACCATCTTCCCTTGAAAATAA

AATATGGTTTCCATTTCTCTTAAATAGAGGTTTGAATGCTTTGTTTTGGTATACGTTACC

GTTGGACTAATTGGTATTTGTACCTATATTGTCAACTTGTTCCTCTTTTTTTTAATCCAC

TTCCACTCCATTTTCTTCCTTTTAATGTTGGCCTTGGAGCCTTGGAGTTATATTAGATCA

CCGGCTGATCTGGTCATGGGATAGATTTGGAACAAACTCTAGACCACCTAAGACCAAATC

ATTCTCTTTTATGAGTAATGTTTATTAGCGGTCTCTGAAATACTGCATTTGTTTATGCAT

TAACAGTAATAAGAGGTTGATTGTTATCTTCTGTTCCAATGTAGAAGATGATGTACTAAT

GTAACTAACCAATTTAAAAGAAATAATGTGCCCCAATTCCTTTTGGGGCATAATGTCAGG

TTATGACCTTCTGTTGTGCTCTTTGCAGGTTTCACACAGTTGCGTACGTTCAGCAAATCT

GGTGGTTCGAGGTGGGTGGTGAAATCAAATTTCACTTGCCAGCTGGTACATACAGCCTCT

TCTTCCGATTCCATCTTGGACGCCCTTCCTCCAAGAAACTTGGTCGCCGGGTTTGGAGCA

CCGAACACATCCAAGGGTGGGATAAAAAGTCGGTGCAGTTTCAGCTATCGACCTCAAATG

GAGAGCATGTAGTCTCCGAGTGCATTTTAGACAAACCCGGGAGTTGGATTCATTACTGCG

TAGGTGATTTTGTTGTCGAGAAATGGGATGTTCTGACTGCAGTCAAGTTCTCCATGAAGC

AGATCGATTGCACACATAGTAAAGGTGGTCTTTCAATTGATTCAGTGCTAATGTATCCAA

AGGGATTTAGGCCATGGTAATTCCTAGGGCCTTTTCAATAAACCTGGAGTTGGATAAACT

CGGATGTGTCGACTACCGTAAGTTTTTGACAAGTAGATCGACTGGACACCTGCTAGTGGG

GGTCTTTCCGTTGATTCTGTGCAGTTACATCCAGAGGGATTCAGACAGTAAAAAACTGGC

ATTACTCGCTTGTATGTATTTTTTTCTAGAAAGTATTGACTATAAGGAATTTTATTCCTT

ATTGGATTTGAAAGTGTATATATATATATATATATATAT

>Unigene0000921

AATCCGCATTGGCCTGTCCAAATCCATCCAAACATCTCTTTCCCTTCCTCGATTCTCCCA

CACTCCACCAACCCCACTCTCTTGGCGCCTCCCCTCCCACGTTCTCCGCCTCCTCGCCGC

TCTGTCGGAGCTGCAATCGCCGCCGCCACCCCTACAGCACGCCGCCGTGGGTTGTCGGCA

ATTTGATCTGAGATCTAGCGCCTGGGAGTACAGTACTATTGATATGGAAGTTCTATGGTG

ATTTCATTGATAAAACAGTAATACCAGCAAGTTAAACATAGGGAAAATGGCACATACAAG

CAAGGCAGACAAGAAAGCGGCATTAGATGTTGCCTCATGGATGTTCAATGTTGTGACTTC

AGTTGGAATCATCATGGTCAATAAAGCATTAATGGCTACATATGGTTTCAGTTTTGCTAC

GACCTTAACTGGCCTGCATTTTGCCACCACAACTATTATGACCGTTGCTCTTAGATGGCT

GGGATATATTCAGCCTACCCATTTACCTTTGGCCGAACTTCTGAAGTTTGTCTTTTTTGC

AAATCTATCAATTGTTGGGATGAATGTCAGTCTAATGTGGAATTCTGTGGGATTCTACCA

GATAGCAAAGTTGTGCATGATACCAGTATCATGTCTATTGGAAGTTTTGTTGGATAAGGT

TCGTTACTCACGAGACACAAAGCTCAGCATATTGGTGGTTCTCATAGGTGTTGCAGTATG

TACCGTGACTGATGTGAGTCTTAATTCTAGAGGGTTGATAGCAGCCACCATTGCAGTTTG

GAGCACTGCTTTACAACAATACTACGTTCATTTTCTTCAACGGAAGTACTCGCTTGGGTC

ATTCAACCTCTTGGGCCATACTGCCCCAGCCCAGGCTGCATCGCTTCTGGTATTAGGACC

ATTCCTAGATTTCTGGCTGACTAACAAAAGAGTGGATGCATTCGATTATAGTGTAGTAGC

CGTGTTCTTCATCATCTTATCTTGCACGATCGCAGTCGGAACGAATCTTAGCCAGTTTAT

CTGCATCGGCAGATTCACGGCAGTGTCGTTCCAAGTCCTTGGTCACATGAAAACTATCCT

TGTTTTAGTCTTGGGATTCATCTTCTTTGGAAAAGAGGGACTCAATCTTCACGTAGTCCT

CGGTATGATCCTGGCAGTAATAGGAATGGTTTGGTACGGAAATGCATCGGCCAAGCCAGG

GGGGAAAGAGCGTCGCAGCCATTCGTTGATCACTGACAGGTCTCAGAAGCACGGAGTTTT

ATCGGACCCTGAAGAACTCGATGAAAAGGTCTAAAGACGAGATGATTTCGCATTGAATTT

TTCAACTGATTCCTCATCTCGAGGGTCCAACAAGAGAGATAGGGGGACATCGCCTGGTAA

TCGCACTCAAGCACTGAATATATTTTTCACATCTTAATGCAGCATCGATTCCCCTACTTA

AATTATGTTTTTTTAGTCACATCCATAGTTGTATTGTCTAATTGAATATGAGTTGTAGAA

TTTTGCCTTCGTCAACATTTTTTTCTTACTTGTAGACTTGTTTAGACAGATATTTCACAA

AGTTGAGGTGTTTTTTTTTCTTCTTGTCTCTATGCATTGGAAAACTTGGATGTTCTTAAC

TGACTTTGACATGATACCAAATTTATGATATTCTTCTG

>Unigene0000922

GGTTCTTTGATGCGTTTTTCGATCTGGAGCCGAGGTATTGGCATGGGTTTCTGTCGACTA

GGCTGTCGATTACGGAGCTGGCGGAGCTGAGTTTGTCACTGTTTGCTCATGCGTCTGGGA

AGAGTAGGGTGGATATTGTGACCAAGTGCCCTTTGCCTTTGGCCAGGATGATGGGCAATC

TTGCCTTACAAGCGATTTAGAAGAATGGTAATCGGTGAGTTGTCCCATATTGTATCTAAT

TTGAGATGTTAGCTCAATTGAGCAAATTTTAATTAGTTACTAGATACAATTTGGTTTTTC

TGGGACAAATGTTATATACTGCAATGCACATAGGTTTACTCAATATGTGCAGAAATATGA

TTCTTTTGCTTCAGTTTATGTAT

>Unigene0000923

AGATATCAGAATATATAATTCCCCTCCTTTTCAGCAAGCATCATACCGTCCAGCCGCCTC

CTTGATCCTCTCTTGTCCGCACCGCCGGTTCCCATCCTCATAACCCCCGCATCCTCTACC

ACTCCAGTCCAACCTCCGATGTCAACTCTTCAGTTCCCGGCGTTCCTCACCGCCGGCGAA

CTCCGCCGCCCTTCAAGGCGAACAAAGTGCAGTTCTCTCCATAGTTTTCTAGATCTCACC

CCAATCTACAAGCCCGAGCCTCTCACTATCGACATCCCATACCACGACCCTTCCTCCGCC

CATCGCTACGATGCCGCCATCATCGGCTGCGGCCCCGCCGGCCTCCGCCTCGCTGAATGC

GCCGCAGCCCGTGGGCTCCGCGTATGCTGCATCGACCCCGCCCCCCTCTCCCCCTGGCCC

AACAACTACGGCGCCTGGCTCGACGAGCTCCACCCCCTGGGCCTCGCCTCCATCTTCGAC

CATATCTGGCCCACCGCCACCGTCGCCATCGACGACAACAACATCAAGCACCTCTCACGC

CCCTATGGCCGCGTCAACCGCAATTCTCTCAAAACACTATTACTAGAAAACTGTATCACC

ACCGGAGTCAGATTTCACCCCTCCAAAGCCTGGAGCATCGAGCACGAGGAGCTCCGCTCC

TCCGTCTCCTGCTCCGACGGCAGCGCCGTCACCGCCAGCCTCGTCATCGACGCTGGCGGC

TTCAGCACCCCCTTCATCGAGTATGACACACCGAGAAACCGCCGCGGGTACCAGATCGCC

CACGGCATCCTCGCCGAGGTCAACCGCCACCCGTTCGACCTCAACCAGATGCTGCTCATG

GACTGGAGCGACGCCCACCTCGACAATGAACCGCGTCTCCGTGCTCACAACACTGCTACT

CCAACTTTTCTATATGCAATGCCATTCAACGAAAATCTAGTTTTCCTCGAGGAGACCTCG

CTGGTGAGCCGGCCAGTGCTTGACTACGGTGAGGTGAAGAAGAGAATGGTGGCGAGGCTG

CGACATTTGGGGATTAAGGTGAAGCGGGTGCTGGAGGAGGAAAAGTGTTTGATTCCAATG

GGTGGTCCGCTGCCGCGGATGCCGCAGAGGGTGATGGGGTTTGGCGGGGCAGGCGGGGTG

GTGCACCCGTCGACCGGGTACCAGATGGCGCGGGCGCTGGCACTCGCGCCGGAGTTGGCT

GAGGCTATGGCGGAGTGTTTGGGATCGACAAGGATGATAAGAGGGAAATCTATGAATGGT

AAGGTGTGGGGGA

>Unigene0000924

TTGTGTAAGCAAATGCTTGATCCGTGATTGACCTAGAATCAATTCATTAAGAGGTCATTC

TCAAATTGGTGATGTGCATTCTTAGAGGAATGCCCGGATGCCACCCTCCTTTTTTGATAT

TGAACACACAATCGTCCTTGTCACAGAGTCGGCCCTCCTATACCTTCCCAGCTTTTGCCT

ATCCAGCATATTGGCCTCTGAAAAAGAGGTTATGGAAATGTGTATACACCGTGGGAATGT

ACCTGTCTTGTCTTTTGTTGACATCGTCATTTAGATCCAAAGAGCTGATGACCACTTGGA

ACATCTTTGACGTGTGTGGCTTAGTGAGTAATCCTAAAAATTCTTTGATTAATGGTTGAT

CGTTCCCATTGGTTTGTCTACTTGTCTGGCTTCCGGCTCTTTTGCCTTTCAAGGTATTTT

TACGGTGTTGTATTTCAGAGACATGCTCATTCCCAGCCAAAGTGCCCAACAATTGCCTCA

CGCTTGAGAGGGAATGTCTCTTTGGGGATCCAAACTAGTCCGTGCCATCAGGTAATTTTA

GTGGATGCTAAGCTGCGGATTGTTGGTCAGTGACCCCAATGTAATGTCCATGACTATTGT

CGATTCAGTTGGTTGTATTGCCCATCACAGACCCACCACAACCAAGGTGTTTGAGGGGTT

CATATAATTGATGAGGGCTTCGGGAAAAAAAAAAGGAAAAAACTATTCAAATTGGCAAGC

TGGTCTAGAACGGTTCGGTTTCTATTTTTAACCCCATTTGATTCGGTTTGGGAATTAGGT

TGACCTTTTGGGTTGGTACATTTAGTCTTAGATACACCTGAGCTAGCCAAATCAAACGAA

TGTTTGTAAGGTTACATATAGCTGAGATACATTGAAAGCGTTTCACTCCCGAGGTATTGT

TCCCTATGTCACCGTTAGAGGGTACTGGAATTTGGCCACATACAAGGTAATGGTCTGAAC

CTGTCATGAAAAGGACCTTGGAGGTACTGGATATGCTCTTCGCTGGGAGCATTGCTCCCT

ATGAACCCTCCGCGAAATGAATCATAATGAAGAGAACTGAGCGGTTCAGTTTACCGGTTT

GATTCAGTTTTTGGGAATTTGGTAAACTTTCTGGTAGGTATATTTTCATAGTTACATTTG

AACTAACCAAATCAAATGAATGATTGTAATGTTACGTATGGCTGATGTACTTTGATGGCG

TTAAACAGTCTGGGCATTGGTCCCTATTAAACCGTTTACAGGAGGGTTCTGGAATTCTGC

CAGTGGACAAGGTCATGGTTTGGGCTCGTCATGAAAAGTACTTTGGAGGTACTGAATATA

CTCCTTGCTAGGAGCATTGCTCCCTGTGAATTGCCGACGTTCCATCGAGCAAGGCATTGG

TTCCTGCAAAACTCCTCAGAAATGATTTGTAATTAGTGTATTGTCGATGCTCAGCTGAGC

GAGGCTTTGGCCCCGCATTCAATGTGGAAAATTTGTGAAGCACTAGTAATACAGCTGGCT

GCTTTGATAGATTCTCTGCCATGCCAGACTTTAGAAGATGACAGTGATTCCTTGTTCTCT

CGAGGGGTCATCCAATTTCTGATGTCTGGTGACTCGTGGCAGAGAGTGGAGCTAGAGCAT

GTAGGAAGAAATGCCTGAGAGGTATGTGACCCGCTCATCTGAGTCCAGCTCCCTTCAGAG

GTGTAGAGATGGTGGG

>Unigene0000925

AGAGATGGTGGGGTACTGTGAACCTGACCCCTTTTGCCTCCCTGTTACCAGACTGAGGGA

TGACAAGTTTAGACTTTTTATGTTGTTTTTTCGGTACCCGTTTGGTCTCATTTTTCTTCA

TTAGTAGTTCGGTGGTCGGTCCTTGTTTTGAGCAGGTTTGAGATCCATTACTATGTTCTG

ATAACAAGCCTTTTTATAGCAGGCTATGAGTTGATTCCAATCGGTTTGCGATGAAAGAAA

GAGACTGAATGTTAGGGGTTTAACCCCCTTCCTATCTCTGATTGAGTTGGGTCTCCTTCA

CTTAACTGATTTTCTATCTCATCTCACTTGATCTCCCTCCACAGTATAAATTCTTTTTGT

CCCTTATGATCCCCTTCTTGCCCTTAACCCACGAAGACACAATCCAATCAGCATCTCCCA

TGGCCAATGCCCTGATGACTGTCTCCTCTGTGTACGATGTTGTCAAATACCTTCATTGCA

TGTGTCCTGAAGATGAGTGACAAAACTCCGCCATATGGCTCTCACTACAAACTGCCAGTC

CCCCCTTTTTAGCCTTCGCTAATCTTAAGAAGGTTCACTCTTGTTCGTGCCAACATCTGG

GTTATTAAACTTGTCCTGTTGAGCATTGTCAGTGCAGATCGTCCCATGGAAGGATTTTAC

CATAATGCTTATTCATTTAGTATCATGTTATCATCGGCCATTGATTTAGTAACCTCATCA

TTTATTCACCCTGAATTACTTGGTAATTTGTAGGTAATTCTTGTTGTTGACCAACTTTTT

CCTGAATCCTAATCTTGTTGATACTTAATAGTTCGAATCACCATTTTCTTCTTGTATTCT

TTTTTTTTTAATGATATTAGTTTCTGGAGTTCTGATCGCTCCCAGTTTTATGACCGGGGT

CCTGGCCTCTTGTAAGTGTGATACCAATAATGTTTAATCGTCAGTGGTTTGGAAAACCTT

GCCGCGGATTCACTCCCGTACACCATGAGCATGTAGTGCCAAATGTTTCTTTTCCCACGA

ATTACTTCAATGGTGAATTCACATTTCTATTAGAGTTCAGCTTGTTCAATATTTTATAGT

ATGACTCTCCTAAGCATAAATAGGTCCATAGGCGAGTGCTTGTCTTATGAGGTACCTTGC

AAAGAATTGTGGGGCTCTGCCTCTCTTTTCCCACCCACTATTAGTTCTGTTCCGGTGGAG

GATTCTTGTGTGCGGATTGTCCCATGACAGTGTAGAAGGATTTGAAATTTGTCAAAGTCA

ATGGCTTAAGATAAAAAGGAGTTGAGGCGGACTTTGCTCGTGGTGGTGGGCATTTCTCGG

AGATAAGTTACTTGTTATTTACCAATAATAAGAAATGGTCATGTCTACTTTATTGAGTGC

TCATGTATTGTTCTGTTATTATTTGTAGCCTTGATGTTCTCGAGATGTCAATATTCTTCT

CTTAAAATATTTATGTTACATAAATTTCCACCACATTTGTTATAACTCTCAGACTTCTAA

TTTTATCTTGCAATGGTGTTGGTCAAATTTCTGTGTTTAAAACCGAATTTATAAAATATA

CAGTTTATAACTCTAATTTAAGTACAGACTTGTGTGTTATGGCCGAGTCTAACCCACCAT

TGTTGTTGCCCTTCCGTTTAATTCTTTTTAATTTTGTAGGAGTTT

>Unigene0000926

AGAGCTGAACTCGAGGCAAAAGACAGTGGTTGGCCAACTGGAAATCAAAACTGTCCGGCT

GGAGGCAGTAGCTCGTCGAGTGGAAATCTTGGTAGTCCGACTGGCAACAACAATCAGACA

ATGGGCCTGGATAGAGGCTGGTCAGCTGGAACCTGTAGAGAAACAGTTGATCCGGCAGAC

CAAATTTAGAACAATGAACCTGGCAAGAGTACTTCGGATATCTTAGTCGGTG

>Unigene0000927

AAGTTGTGGGATCCCGAACTCGGAAAGATGGTGATCATCTAAGACGTTATCTTCAACGAA

GACCTGATGCTAAAGAAACAATACCTCATTGAAGCTGAGAAGGAAAGAGCTAAACTCGAG

GTGAAAGACAGTGGCTGGCCAGCTGGAAATCAAAGCCGTCCGGCTGGAGGCAGTAGCTCG

TCGAGTGGAAATCTTGGTAGTCCGACTGGCAACAACAATCAGACAATGGGCCTGGATAGA

GGCTGGTCAGCTGGAACCTGTAGAGAAACAGTTGATCCGGCAGACCAAATTTAGAACAAT

GAACCTGGCAAGAGTACTTCGGATATCTTAGTCGGTG

>Unigene0000928

CGGAGTCCGGCGACCTCCGAGGTTTTGGCCTTAACCATTCCCCATGCCGCCGGGCGACTA

CGACTTCATCACCAGCTTCGCCACTGCCTACGATGGCGCAGCCCTCGGCCTCGCCCTCGG

TATCGTCGCCGCCAGCTCATGGGCGAAGTATGTCGCCTCATCCTCCGCCCTCCGCCGGAT

CCACCACGCCCCCTTCTCCCGCATCTCCGACCTCCGCTCTCTCCTTGCTGACGACGAACC

CTCCCCAGCTGACGACATATGCGGAAGGCTTGTCGTCGTCCGTGGCCAGGTCCAGATCAA

TCCCGAGAGCAAGAGCCGCGCCCTAGTTTCGCTCGAGTCCGGCGAGTTAGCCGCTATTGT

GAAGCACACCGAGAAGTGTCTGTACAATGAGAGTTGGGGTATTTTCAGAAGGAGCTACCA

TCTCCAGATTCTACGAGCACTTTTCCCTAGGAAGAAGACTGAGGAGTGCTCGGGCTCATT

GAGATCAGTTCCATTCGTTCTTGCGGAAGTCAATCAACAGTCCCATTCTGCATATGTTCA

TGTCAGTTCGGACAATTCGACACATAAGCTACCTCTTACACTGGTTTATAGTCATATGCG

CCCTGTGCAGACTCCCTGGTATTCATTCATCTGGATTATTCTTGGTCACCGGTATCCAGT

TGCAATGTTGAGTGAAGAAAGTATTCTTCCAATTGGAAAGGAGATCACTGCAGTTGGCGT

CTGCAGAAACCGAGATGGGGCTCTTGAGATTAAGTCATGCATCCATTTCCCATATTTCTT

GTCTGATATGACAAAAGATGAAATTGAAGCAGATCTTACCATTAATACTAAGATTCTTTT

CTGGAGTGGTGTTGTTCTTGGAACATTTTCTATTAGTATCTTGAGCTATGCTTTCATGAG

GAACTGGCATAGGTTGAAAGGACTCTGGAACAGAAACCTAGCACAAGGATCTAATGAGGA

GCAGCCTGCAAATGATCATGATGAGGACTTGGAAGATGTGCCGGAGGGGGAGCTCTGTGT

CATCTGTTTAACAAGACGAAGGATTCATGCTTTTCTTCCCTGTGGACATCGTGTTTGCTG

CCAACCCTGTGCCATATCTGTGAAGACGGGATCTCGATCGCCATTATGCCCTGTGTGTAG

GCAGGAGATCCATTCATCTATCAGAGTTTATAATTCCTGAGGTATGTTTACTACTACAGA

TATGATACTTTGCTCGGCTCGACTCTGCTCTAGATTACCCTCTCGCAATACAAACTCTAT

CCAAGGTAGAGATTTGATGAATACCTAGTTGAAAGATTGTATATATCTCTTGCAAAGTAA

TGTGCTGTCATCTCTCAAGTGTTCTATGACTTTTAATCCTAGTTTAAAGTCTGTATATAT

CTCTTGCAAATTTGTACTGAAAACCTCACAATGGAGCTCTGGATTTGGAGTTCACCACGC

CAGCAGGATAGCACAACTGTACCCAGACATTCTTCGGGCTTTGCCGGAGTTGTGAACTTG

TAATTAAAGAAAGTTACTTGATCTTGGATATAGAATACAATATTTTTA

>Unigene0000929

CAATATTTCAGCCAAACAATACAGGCCCTATACATGTTTTGACAACCATGGTCCATTACT

GTTTTTTCCCCTTTTTTAGCATCTCTGTGCACTTCATCACTGGTCAAATCCTGAACAGTT

TATTCTTCAACTTATATGTTTCCAGTTAGGCAGGCTCGGGTCTATTTGGGTTCTTTAACC

CTGAACCTTTTTTGAGTTGACCTAGGCGTGTCAATTTTTTGTCCGAACCTACCAACCCGG

GATGTTCGAAACCAAAACAATAAGGTCCGGTTATGTCCGAATCCAAACTGGGTTAGGGCC

TAGAAAATCTCGTCCAAGGCCTAGAAAATCTTGTCCAATTCAAATTAGGGTTAGGGCAAG

GGTTGGGAAATCTGTCCCTGCCCCTGAACCCGAATCCGAATGTGTCCGAACATGAAACCA

AACACAAAAACTTTAGTATGAATTGATGTCAAACCTGCCAACCCAGGTTGCCGAACCCGA

AAAATTAGGGTCCGGACATGTCAATTAAAACTGGGTTAGGGGGTTAGGGTTTAGAGAATC

TTGCCCGATTCAAATGACGATTAGAGTTTAGGGTTGAACACTTTTGACACGTACCCGATG

TGTCCGAACACGGAATAACCCGAACCCAAAAAATTGACACTGTAGGTCAACTTTGGTAAA

TCGTATTAGGGAAAATCCAGTCATCCTGGTTTGCTGGGCCCTTATTTTCTTGTTCGAAAG

ATTTAAGTTGGCAGTTGGGATACACAACACAGCTATAAAATATATGAAATACTAAAGCAT

GTTACATACTTAAATT

>Unigene0000930

CCAACGGCGCCGAGGGTCCCGCTGCTCCGTCTCATCCTCACCCCCATTGTTCTCAGGCCC

CTCAATTCTTGACCCCTCCTCCCCTTCTCTCAATAACGAGGAGGGAGGCCCTCCCTCTCC

GTTCTCAATCTCCGATCCTCAGGGATCGCCGCCCCCGTCCCATCCCGAATGGGGCGCAAG

GGGCACTGGCTCACCGCTGTCAAGCGCGTCTTCGGTCCCGAAACCAACAAAAGCAAGTCT

CAGCATCACAAGAAGTCGAGGAGGGGATGGGGTTTCGGGGCTTGCACGAATCCGTGTATG

GCTGAAGGAAATGCTTCTCCGGTGGATATGGAGGAGATCGAGACTATGCCCTGGCCGCCG

CCCCCGCCCCCGCCCCCGCTGCCGCTGCCGCTGCAGAAAAAGGCTGTTGAGTCGGTTAGG

GTCAAGGAGGAGAGGAAGGAAGAGTTGGCGGAGGAGGAGGATGAGGAGGAGCAGAGGAAG

CATTATAAGGCGGCGGATGTGGAGGAGGAGGAGGAGGAGCAGAGGAGGCATTATAAGTTG

GAGGATGGGGAGGAGGAGCAGAGCGAGCATGTTTACTCGGTTGCTCTACCGGCGGAGGTG

GAGGTTGTGGCTGCGGAGGAGGCGTTTGAGGTGGCGAGGTTGGCGGCGGCGACGGCGAGG

TTCGAGGGGCTGTCGAGGGAGGAGGTGGGTGCAATTTTGATACAGACTGCATTTCGGGGG

TATCTGGCAAGGAGAGCTTTACGGGCCCTGAGAGGGCTAGTTAGATTGAAGTCGTTGGTT

CATGGCAGTGCTATCAAGCGTCAGACGGCAAACACCCTACATTGCATGCAAACTCTGGCG

AGGGTTCAATCACAAATTCGTTCAAGGAGAATCAGAATGACAGAGGAAAATCAGGCTTTT

CAGCGGCAGTTGCTACTGAAACGCGAGAGAGAGCTCGAGAGATTAAGGATTGGAGAGGAA

TGGGATGACAGCCTCCGATCCAAAGAGCAAATCGAGGCAAATCTCTTGAACAAGCATGAG

GCTGCTATAAGAAGGGAAAGAGCGCTGGCTTATGCGTTCTCTCATCAGTGGAAGAGCTCT

TCAAGGTCCGTGAACCCCATGTTTATGGACCCCAACAATCCGCAGTGGGGCTGGAGCTGG

TTGGAGCGCTGGATGTCCGCAAGGCCTTGGGAGAGCCGGTGTACGGCTTGTAACGACATC

GCTAGCAACAACAATGGAGCAGAGATTACTAAAACTTATGCTCGCCGCGATTCCCACTCA

GAGAGGACCTCAGCCCACAAATCAAGTCGGTCTTCCAGCCGCCAGTCTCCCGTAACTCCA

ACTTCCAAAGCATCATCTGTCACAGGTAGGATAAAGCCTGGAAGCCCGAGAAGCGGTTTA

GGGGACGACTCGAGGAGCACGATCAGCCTGAAGATGGAGTGGAACCGGAGGCATAGCGTG

GGAGGGTTCTCTATGACGGATGACGTCAGCCTTCCTAGCTACATGACACCCACGAAATCT

GCGAAGGTGAAGTCTCGATATCAGACTCTGGCTAGCGACAAATTCGAGTCCTCGGGATCC

GTTGGCTCGATGAAGAAGCGATTATCTTTCCATTTGGCAGAAAATCAAAATGTGCATTCG

CCTGTGCGGGCAAGGCGGCATTCGGTACCTCCGAAAGTCGACATTGCTTCCCTGAAAGAG

GTGGCTGTTTGAGCACGGAAATGGAGGGAAAAGTAAGCCTGCTCAAGGAGATACAAAGAT

GAGAGTAAGGGGTTGATGTATTGTACTGCAATTCATTTAAGGTTTGGGATTTGATCTATG

GGTGAAACTTAGGTTATAAGACTTGGGTTGTTGTGGGACTCTTGTTTGCAATCTTGATTA

TGGTTGGGGTTTGTTTCCACGAATTTAAGAGATTGTTGTGTATTACCTATATAAATGTGT

GTGTTGTATTTGTAAGTTTTGGGATGAGCATTTAATTACTTGGTTTTTAAAAAAAAAATT

GTTGTAAATCAAATTCGGAAGTTTGATTCATGTGAATATAAATGTTTATTTTAAAAAAAA

AATTGTTGTAAATCAAATTCGGAAGTTTGATTCATGTGAATATAAATGTTTATTTTAAAA

AAAAAATTGTTGTAA

>Unigene0000931

CCGGCGGCGTGGTGTTACCGCCCGATGTAGAGTTTGTGGATGTGAATGGCAAACCGCAAC

CCAGCGGCCAGTATCATCACTACAACAGCAACAAAATCATCAACGATAACGGTAATCGCG

GTCATGACAAGTTTCCTGGCCGTAAGTACTGACTGCGGAGGGGTCCGATTTAACTCGCGG

TCGGTGTGTTATGACTTAGGTGTACTCAATGAGTTAGGATACGTAAAATAATAATTTATT

ATATCGAATTGTGTATTATAGTAGTCTATAATGAAGGCTCTGTGTGTCGAACCGTTTTAT

CGTTAACATACATTCATAATACATTTAAATTATTTACACTGTACCTACCTATATATCATT

ATATCTAGACGGACTCCCACATCAGTGTATTGTGTAAATACGAAGTCATCATCGTTATTC

TGCATCGTTTAATATCAGTTATTTATTAAAAATATTGTTTTTTTTTTTTAATTTTTTTTG

TACATATTTTTATTCACACACATATTATATTATTATATCCTTTGTTATAAGTACGTATGA

AATATAAATATTACCATGTATTTTACTTGTCCGCCCGCTATAGAGACTCTCCGTAGAGCG

TCGTTGTGGTCGCGCGTTCTCTCTCTCTCTCTTATTTATAATATTATCTGTGTACTGGCA

GCTTATTATACCGGCATCGCATAAATAATAAATAACCTCATCGCTCTCGCAATACTCACA

TTTAATTAATGAATATATTATTTTAACGTGCAAACAGAAATTTGTTCCACATGGATTTCA

AAAGACAAAAAATATACCTATAAACATAATATATTACACAAAAAAATAATTTTAGTCAAT

ATCGTTCTTAATCTAGACGCCATTTACTGATATATTTTTCAATAAATATAATTTTTTTAT

GTTCAAGTGATATTCAGTCAATTTTTGGCGTTAATTTTAATTCGTATTAATTATTAATTA

CTATTGTATAATTTTTAGTTACTGTACCTATACCTAATAATTAATACATATAATATTATG

TTTCTATAATAAACCGTAAAGATACCAAAGTTATAAAATATTATTTTATTATACATTGTT

TTTATTTGTCCTTAATTGTATAATATTTTTGTATTAAGCCTAACTATAGATTTTGCTCTT

TATATTTTGCTGAATTATAGATGATGTTTATTTCAGATGTATTCATGTCATTTCTGT

>Unigene0000932

GGTGATCCGCGTGCGATTATAGCCAGCAATAACTCTCTTACACGTAAGAGATCCACAAAA

ATAATTCGGACTTCGTTATCGATAGTGTGTGTCTGTGCGAGTGTTGCGTGTGAACGCGAC

TGGAGCAGTGCCGCTGCGTAAAGAAGATACGAAACGTTACGATGTCGGCCAAAACGATGT

TTTACGCGTTTTTCGTCTTTGCATTGGTCGCGGTTTGGTCAGCCGCAGCTGTGCCACTGG

CCGTCGACGACTTCTCGTCGCCGGCAAATCTTAAAGAAAACCTTGAAATTGAAACTCCGT

TAGTAAGTCAAGAGCCTGAACAGCAGACGAAGGGAACGGATGCGTCAGTTCTACAAACCG

ACAGGACTGAATCATCTCCGGATGCGCAGCAGTCTAGTCAAACGGAAGAAGTGACCGTTG

GATCAGGAACTCCAGTGATTGGAACTGAACCGGGTTTATCGGAAGTTTCCAAGTCCGAAT

CTGACGTTTCGACGACAGCCACCGTCGAACAAACGGAAAGCGTCACCGGAACAAACTCGC

CTAGCCCCGTCGCAGAAACCAACTCTCCCAGCCCCATCGCCGGAACATTCCCTTCCAGTT

CCGATGCCAAACCCAACCCATCTGTCCCTGTTTCCCATCAACCATCTAGACCGAACTACT

CGGGTAGCGTGCATTCTGTGAAATTAGGACCAGGTATTCCGTTCTTTTTGGAACTGATAC

AGCCTTTCCAGCCTTTCCAGTCCTTCCGTCCGTTCCAACCTTTTCCTTCTAACAATAATG

ATCAACCATTCAGGCACCAACCGATACCGTTCGGCGCTCCCATCGATCCATTCGCAAATC

CTATGAATACTCAACCTTTCAACCAGATGGTTGAAGCATACACCAATGAGTTTAGAACTC

CCGAAGTCATAATATTAAAACCGAAAAGAAACGTCCGACTTAATACAACCGCAATGCCAC

CCATTCCGTTGCTAATGAACATCATGCTTAACGCTTTTCGGCATCATGAGCCGTCAGACA

TGTTGGCATCTTTTGACAAAATGGGCTTCCCACCAATGGAAAACTTGGGTGAAGTTCAGG

AAGGCTCAGGCAAGCCCGTGAGGAAGACTACATCCAAGACCGAAATCATTGACGGACACC

AAGTCCAAATAAACGAAACGACGGTCACCAATGGAGACGCAAATCACAAGAGTTTCTACC

ATTTCAAAGAAATTCAAGTGCTGCCCGAAGAGACGAACAAAAAAATCTCAAATGAAAAAA

TTATATCGATAGCTAATGACGATTCGGAGACGGAGAAAGTAGAGACGAACGAAATCAAAC

CTAACCCGTCTGGTACAATGCAAGCTAACGTTAACGACGACCGGAAGAACTTTATTAAAA

AGGTAGAATAACGAGATAGCAGTAGCTTCTTGTAAACGTATTAATATCAGCTATTATAGT

ATTATAATAACCTTAAGATCAATCCGTTACAAAAACGGATTGAATTAATATACCTAACGC

TTGTATAAGTTATTTTCCCAAACATCATTAACAATCCTAATATAATATACTGTTTAGATA

TTATAATATTTGTTTTTATAAAAAAATAATTTAAAATAAGTTATTAACAATATCGATATT

AAAAATCTTTTAATCGTTATTCGATCATAAAAGTATAATATTTGTATGGTTATTAATTCG

ATATTGTAATTATTATTAACAACATCATCGGCGTCGGAATAACTGAATATTTTCATTTAA

ATAATTAGGTGTTATAAAAATCAAAATGTTACAAAAACTATAAAATCAAAATATATTATT

ATATCTGTATAATATTACTTTTATATATATTTTATATTAAAAACGGTTGATTGGAATAAA

ATATTTTTGCCATAATATTCTCAAAAAGTATTATATTTTTGTAATCATTATATTTTGTAA

TACATACTATTAAAACTTTAAA

>Unigene0000933

GGGGGAGGAGTCGGTGAAGAAGGGAGAAGGATCGTTTAGGAGCCGTCCGTTTGATTGTCT

CGGTGGCGGCACTGGTGGGGAGAGAGATGATGAGGAGTCTTCTACGGCGCCGTTTGCTAG

GGTTTGAGTTTGTGCCGAAACACCACTGTTGCCGAGTCTGTGCATTGTTCGCCGAGTTTG

TGCGTCACACCCTGCTGCTAATGGCCGAGGTTTTGCGTTCTTCAGACTAGGCGTTGACTT

GAGGCGTGGCGGTGGCGGGAAATGTAAATAATTGTAATAAG

>Unigene0000934

GGGGGAGGAGTCGGTGAAGAAGGGAGAAGGATCGTTTAGGAGCCGTCCGTTTGATTGTCT

CGGTGGTGGCAATGGTGGGGGGAGAGATGATGAGGAGTCTTCCACGGCGCCGTTTGCTAG

GGTTTGAGTTTGTGCCGAAACACCACTGTTGCCGAGTCTGTGCATTGTTCGCCGAGTTTG

TGCGTCACACCCAGCTGCTAATGGCCGAGGTTTTGCGTTCTTCAGACTAGGCGTTGACTT

GAGGCGTGGCGGTGGCGGGAAATGTAAATAATTGTAATAAGGAAGGTGGAGAAAGGGGTG

G

>Unigene0000935

GGGGGAGGAGTCGGTGAAGAAGGGAGAAGGATCGTTTAGGAGCCGTCCGTTTGATTGTCT

CGGTGGCGGGGCTGGTGGGGAGAGAGATGATGAGGAGTCTTCTACGGCGCCGTTTGCTAG

GGTTTGAGTTTGTGCTGAAACACCACTATTGCCGAGTCTGTGCATTGTTCGCCGAGTTTT

TGCGTCACACCCGGCTGCTAATGGCCGAGGTGTTGTGTTTTTCAGAACAGGCGTTGACTT

GAGCGGGTGGCGGGAAATGTATATAATTGTAATAAGAAAGGTGTAGAAAGGTATGGATGG

TAGAGGAATTCCAAGGTGTTTCACGTTGGAATGTAGAAAAGAAAGGCCATTTCCACATTA

GATTTTGATACAGTCGTACTCCAATGTAATATTGTAGAGTTGAATTTTTATTAAGAAATT

CAAATTTATATAGAACTTCAGCCCGCTGATATCTCTTGATAACATAAATATTTAGT

>Unigene0000936

TACGGGGACATTTGGCAAGGGGGCGTACCCGGGATATCCTGGTGACGTGGCAGTGGACCC

GACGACGGGGGCGAGCTACAATGCGTATGGGTTATACGGGAGGAAGTACCTCTTGCCTGC

CATGTGGGACCCCACAACATCCCAGTGCTCCACGCTCGTGTAGAGAAGAGAATAAAAAGT

GTTTGCGTAGTTTGCTTGTTTTTTATTTTTCCGAATGTATATGTAAATTCTGGAAGATGG

GACTCTGACGGAAACGGAAGGCTGTGTCATTATAAATAGATGCAGTTTGATGTTTACTTA

GAAACCCTGAGCTGCTGTTTT

>Unigene0000937

GCCTATAAAACTCCCCTTCTACTCGGACGTTCGTCGCCTTCAACTTACCCCCGGTGATCG

GTGATCTCAACTCCATCCCCCGTTTCCTCGATCGCCGCCGCCGACTCCCCTCTCTCGATC

GCCGCCGGTAAGCCCCCAATCCCTCAATTCCATCAAATTCAATCCCTGATCTCCTGATCT

AGGGTTTCAGATCTCCTCTCGACCCATCAATTGATCGATCTGCCACTCGAATCCCCAACA

TGATTTCGATTAGCTCCGCCGCGATTTCTAGGGTTTTGAATTGTTAGCTGTAATTATTTT

CCGTCAACTGTTTCTCCTAATCCGATATTAAAGGACGCAGCCTAAGGGTTAATCCATGGA

TAATGGTTCTGGAATCTTTGTCAACGATGGATCGTTCATGGAGAGGTTCAAACAGATGCA

GCAAGAGAAGCAGATGAAGGAATCGGCCAAGGAGGCTAAGTCCAGCAACTCGGTGACTCC

AGTGGCTCTGCCGAAGCCTGGGGTTATCTTGAACAAGAGGCCGGCTGCTGTGGCGAAGCC

GAGTCCTCCTTCTGCATCGGGTGGGAAGCTGGCTTTCAGCTTGAAGCAGAAGTCGAAGAT

TGTGGCGGCTCCTGTTATGTTAGGGGCGGATGATGAGGATGTTGGCGAGGCTGAGACTGG

CACCGGTGATGAGCGGGCGAAGCGCCAGAAATTGGGCCGGATGGATGCTGCTCAATCGGC

GAGGCAGCAGGATGATGTTGCTCCTTCTCCCCCAAGTGATACTGCAGTGAGAATAGTTGC

GGACAAATTAGCAAGTTTTGTCTCAAAAAATGGGAGGGCCTTTGAGCAAGTTACACGTCA

AAGAAACCCTGGGGATACTCCGTTCAAGTTTCTGTATGACACAAGCTGTTCAGACTACAA

ATATTACGAGTATCGGCTTCTTGAAGAAGAAAAGGCCCTTGCACAGTCGAATGACCCTCC

AGCACTGAAAACTGCCATATCAAGCACTGCAGCTCCTAGATCGGCAAGCAGCTCTCAAAA

CAGTACTCTACAAGACGGTAATTACCAAACTCCTGCTTCAGCTTTGTATTGGGCTGATGA

GGGTAATGCATTATCAGGGAGAACCCCAGGACAAGGTGAATCCAGTGGGCCTCCATCGTC

GGATCCTATAGCAATGATGGAATATTATGTGA

>Unigene0000938

CTCAGCCAAAGCCAAGGCTAGATGGCAAGGTCGCCATCATCACCGGAGCAGCCAGTGGGA

TCGGCGAAGCTGCCGCCAGGCTCTTTGCCTCCCAAGGTGCCATAATCGTCATCGCCGATG

TCCAAGACGATCTCGGCCACTCAGTCGCTGCCTCCATCGGCCTGCCTGAGAAATGTACAT

ACAAGAACTGTGATGTCCGGGACGAGAAGCAGGTAGAGGAGCTGGTAGACTACACTCTCC

GGACTTACAACCGCGTCGACATCCTCTACTCCAACGCGGGCATCGTCAGCAGAGCCCCCA

CCATCCTCGATGCTAGCATGGAAGAATGGGACAATGTTTTTGCTGTGAATGTTCGTGGCA

CCGCCGCGGTCATCAAGCACGTCGGCCGAGCAATGGTGGCACGAGGGACCCGCGGGTCCA

TTGTGTGCACCGCGAGCGTGGCGACGGTGATGGGTGTGTGCGGGCTGGCGCCGTACACGG

CCACCAAAAGCGCCGTGCTGGGACTGGTGCGGGCGGTTGCGGGGGAGCTTGGGCCGCATG

GGATACGGGTGAACTGTGTGTCGCCATCGGTGGTGGGGACACCGATGGCGTGCAGGCTGA

GTGGGTGGAGTCAGAGCAAGGTGGAGGAGAATACGTCCTCTATTTCCAACTTGAAGGGGG

CGATGCTCAAGGTTGGGGATGTTGCGGAGGCGGCGCTCTTTCTTGCATCTGATGCGGCGG

CGTTTATCTCCGGGCACAACCTGGTGGTGGACGGAGCGATGACGGTGGTCACTCCGAAAT

TCCCAAACGAGTGATAGCATGGAAGAAAAGTAGCAGTAGTAGGAGCTACTACGTAATGAT

GTTTGTTTTCTCTGTATCTTGATCTTCTCGGATGTCTCGTGTGGACTTGTTAGAACTTAT

TCTACTCCAAGTATGGAGAATTGATGGGGAAGAGTACATCTTTGTACTAGTAAGGTTGTT

TCTTCTATAATCTTTATCATAATATAGATTTGTCC

>Unigene0000939

CTGCTTCTTGATGGACTTGCATTGTGTTGTTTCCCATTTATTTCAAATCTTTTTCCTAGC

AAGTTGAGGTGATATAGTTTCCGTACATCTGCTTCTGTACAGCCTGTTATATAAGGTTCT

GACTCCAGATTTTTCATTTTGCATGTTCTTTGGGCGATCAAAGGAAAAACATCAAATGTG

AGTAACTTGTGAACTCATCGAAGTTGATATTCTCATCCCTAACCTATGTCAACAAAAATA

CTCTCTTTGCATTAAAATTAGTACAAACTCTACAGTCCCATAATTAGGTAATATTTTTTG

TCTTCCTCGTAATATTTTTCGTTAGCTTAAGTTCATGTGCCTTATCTAATGGTACGTCAA

TATGTTCCTAGCTTTCCGCGAACATCAGCAATACGTATTAATTTTGGCCCATGCCCTTGT

ATGACTCTAATGCTATATGCAAATATTCTTTCGGGATTTCCCGATTCGAACAACTTCGCT

TGCTAAATTCTGTGCAGGTTCCGAAGAGCTTTCTTTTGCGG

>Unigene0000940

CTTGTCTGTATCAAGAACAAATTGGTGTGCCAATAGAAGACCTCTGTGGATAGAGCGAAT

TCAAAGGAGCAAGCAAGCTGCCTCCATTTCTCGCTATCTAGGGTTCTCAGAGCTCACCAA

GGATTCAATGGCGGTAACTCTGTTGACATCAATTTGCTCGAATCTCCAATCTCACCATGA

TCAGTCCCTCTTTTCTCAGGTGGGGATTTCAAGGAATCCGAGAAATCGAGGTGGCGTAGC

TCTTCGGAAAGGATCGGAATGCCCAAGCGGGAATTTGTCATTTACATATCAACGAGAGTT

GATGCAGTTTTCCTGGCCCCTACATGCAGTTAGTTCAGGTTTAGAAGCATCAATTACAGA

AAAAAAGAAGAACCACTTATCACTGGACAATGTTAAGATTGTCGTCGAGTCACGTGATGA

TGATTTTATTGATCTGAGAGTAGACTTGACTGGAGAGCAGACTCAGAAGGCGTTTGATGT

CGCCTTGGTGAATTTGGCTCGTACTGCTCCACCTATTCCAGGGTTTCGTAGAACGAAAGG

AGGAAAAACATCAAATGTTCCGAAGAGCTTTCTTTTGCGGATCCTTGGTGAGGACCGTGT

TACGAAATTCCTTATTCAAGAAATTGTGAGTACAACTGTTGGGGACTTCGTGGAACAGGA

AAAACTTGCAGTGAAGAAACAATTTAAGATAGCCCAGACAGCTGAAGAACTCAAATCCGC

ATTTACTCCAGGGAGGGAATTTGGTTTTAATGCCTCCATAGAGATTGAGAGTTCAGATCC

TGAAACAACCAGCGCAAGCCTCTCTGAGCCATCATCAACAAGTGTATGAAGTGCTCCCTT

GGTTACTGGCATTTCTGATCTTCATACGGGGCAACATGTTAGCCGTGGACGTAGACAGAG

CTCGATTTGCTTCTTGGAGTACTTACAAGCTCCACCGCCCTCTTTCTCTCGCTTGTGTGC

GTCCATTGAGTTTGTTGTTTCAGTTACGCCTAATCCTGAGTTGTGACATTGACGTCTTGG

TCATGTGTCTAATAACAAGCTTCATGACTTGATTCAATCTAGTTTTTATTGGACCATGTA

CATTTTAGTTTGGTGTCCTATACTGGTTATAAAATTGGGAAGCCTACTGCTCTCCTATTT

CCAACCG

>Unigene0000941

CATTCAATTTGCTTTGTTCTACACTGAGAACATATACACAGTAAATGTGCTGGTTCCTCT

GTCTGCTGAGATTTTTTGAATTGGTGACAAAACATTAACAAGATTGGAATTGAATTAGCT

ATATTATTTATATTGTCAGACTACATCTAGTTTTCCCACTAATCTCTACTGAACATTCCG

ATCTCATGAGTCACGAGGATGACTTACTCGAAAGGGCCGATGATAAATGATAAATAAAGG

CGATAGTGACAATGAAGTTCTTTCTACAAAGAAAACATTTAAAGCTTGTGCT

>Unigene0000942

CCTCCTGAAAATCAATCAGTCTCGTCCTTGATTTCGAGGGCTTGATTCCCGTCCGCCATT

TCCGATCTTGTCCTCCTCCTCTCAGATCTCGCCATCATCGTCGCGACGATGTACGGATTC

GAGGCGATGACGTTCAACATTCACGGCGGGTACCTGGAGGCGATCGTGCGGGGGCACCGC

TCCGGCCTCCTCACCGCCGCCGACTACAACAACCTTTGCCAGTGCGAGACGCTCGACGAC

ATCAAGATGCACCTCTCCGCCACCGAGTACGGGCCTTACCTCCAGAACGAGCCCTCTCCT

TTACATACAACCACAATTGTGGAGAAATGCACTCTTAAGTTGGTTGACGAGTATAAACAC

ATGTTATGCCAAGCCACAGAGCCGTTGTCAACATTTCTGGAGTATATAACGTATGGTCAC

ATGATTGATAATGTTGTCCTTATTGTCACGGGCACCTTGCATGAAAGAGATGTTCAGGAA

CTGTTGGAGAAGTGCCACCCTTTGGGTATGTTTGACAGTATCGCAACTCTTGCAGTGGCC

CAGAACATGCGTGAGCTTTACAGATTGGTTCTTGTGGATACACCGTTGGCTCCCTATTTC

TCCGAGTGCATCACATCAGAGGATTTGGACGACATGAATATTGAGATTATGAGGAATACT

CTCTACAAGGCATATCTCGAGGATTTCTACAAGTTCTGTAAGAAACTAGGTGGAGCAACT

GGCGAAATAATGTCTGACCTTCTTGCCTTTGAAGCTGATAGAAGAGCTGTTAATATCACC

ATAAACAGTATTGGCACTGAGTTGACTCGAGATGACCGTCGGAAACTATATTCTAGTTTT

GGTCTACTGTATCCCTATGGTCATGAAGAACTTGCTGTCTGCGAGGACTTCGATCAAGTC

CGTGGTGTGCTAGAGAAATACCCTCCTTATCAGTCAATTTTTGCGAAATTATCATATGGA

GAAAGCCAGATGCTGGACAAGGCATTCTATGAGGAGGAGGTGAAAAGACTCTGCTTGTCA

TTTGAACAGCAGTTTCATTATGCGGTTTTCTTTGCATACATGAGGCTGAGAGAGCAGGAA

ATTCGGAACTTGATGTGGATCTCCGAATGTGTTGCACAAAATCAGAAGTCCCGAGTCCAT

GACAGTGTGGTATTCATTTTCTAAATGGTTCAGCGGAGAACAGTCATTCAATTTGCTTGT

AAGTTTTTTATCCTCCTGCTTATGATTATATTATGACACGATCATGAATTGTTTGATCTT

GTGTTTGACTCAGCATTGCCAAAATAAACTATGTTGTATTCTGTAGTGTTCTACACTGAG

AACATATACACAGTAAATGTGCTGGTTCCTCTGTCTGCTGAGATTTTTTGAATTGGTGAC

AAAACATTAACAAGATTGGAATTGAATTAGCTATATTATTTATATTGTCAGACTACATCT

AGTTTTCCCACTAATCTCTACTGAACATTCCGATCTCATGAGTCACGAGGATGACTTACT

CGAAAGGGCCGATGATAAATGATAAATAAAGGCGATAGTGACAATGAAGTTCTTTCTACA

AAGAAAACATTTAAAGCTTGTGCT

>Unigene0000943

CGGAGCCCGTCCAGAACAGTCCGCTTCCATCGTCATTACCGGATCCGATACCGACCCCTT

CTCCACCTCCTGTCTCGAATATCGCCCCTCCCCAGATCGGATCTCTCCTTCTCCCTCTCC

GGCGAGGCGATGTACGGATTCGAGGCGATGACGTTCAACGTCCACAGCGGCTATCTGGAG

GCGATCGTGCGCGGCCACCGCTCCGGCCTCCTCACCGCCGCCGATTACAACAATCTCTGC

CAGTGCGAGACCCTCGACGACATCAAGATGCACCTCTCCGCCACTGAGTACGGTCCATAT

CTCCAGAACGAACCTTCTCCTTTACATACAACCACAATTGTGGAGAAATGCACTCTCAAG

TTGGTCGACGAGTATAAACATATGTTGTGCCAAGCCACCGAGCCATTGTCAACCTTTTTG

GAGTATATAACGTATGGTCACATGATCGATAATGTTGTCCTCATTGTCACTGGCACCTTG

CATGAGAGAGACGTCCAAGAACTACTAGAGAAATGCCATCCTTTGGGGATGTTTGATAGT

ATCGCAAGTCTTGCAGTGGCCCAGAACATGCGTGATCTTTACAGGTTGGTTCTCGTCGAC

ACACCATTGGCTCCCTACTTCGCAGAGTCCATCACATCAGAGGATCTTGATGACATGAAC

ATTGAAATTATGAGAAATACTCTGTACAAGGCTTATCTCGAGGATTTCTATAAATTTTGT

AAGAAACTTGGTGGAGCAACTGCTGAAATAATGTCTGACCTTCTTTCCTTTGAAGCTGAT

AGAAGAGCTGTTAATATCACCATAAACAGTATTGGCACCGAGTTGACTAGAGATGACCGT

AGGAAACTCTATTCTAGCTTTGGATTATTGTATCCTTATGGTCATGAAGAACTTGCCGTC

TCTGAGGACTTTGACCAGGTTCGTGGGGTGCTAGAGAAGTACCCTCCTTATCAGTCAATT

TTTGCCAAGTTATCTTATGGAGAAAGCCAGATGCTGGACAAGGCATTCTACGAGGAGGAG

GTGAAAAGGCTCTGTTTGGCATTTGAGCAACAGTTCCATTACGGGGTTTTCTTTGCATAC

ATGAGGCTGAGAGAGCAGGAGATACGGAACCTGATGTGGATTGCAGAATGTGTAGCACAG

AACCAGAAATCCAGAGTTCATGACAGCGTGGTCTTCATTTTCTAAATGTTTCACTGAAGT

TAGAACTAAATGCGATCCAGTCCTACAGTTTGCTTGCAAAATATTTTTCTCCTACGTAAG

CCAAGATCGTTAACGCTGTACGTTCTTGTTCGTCATGACATGGCCGAAATAATTTGTCAA

TTATCAGTCAGATGTGTTCAAAGATCTTGTGTACAGGTGCAAAGTACTTTGGTTAAGTGA

TATTCCGTTTCTTGTATTTTTCTGCGACAAGTTGGACCAAATGTGGTTAAGTTTTGTCGA

GGAACAAATAAATGTATCCAAGAAACTTCAGATATCTGTCAATTTTATTTTCTAATATTA

TCTGA

>Unigene0000944

GAAATCTGGCTTTTAATTTTGGCTTTTTTCCCGAAATCTGATTCTTTTTTGGGGTTGCCT

CAGATTCTGTTGAGCGATTATACCACTGGATCAGTCAGCCAAATCTGACTTTTTAAGTGC

AATGGTTATTGTCCGATTGCTGGACTGCTATCAAGTTTGGCTCTTCAAATTTACATGTTT

TTGTATTGGTGGTGGTGGTTTATTGTGGGCCCATTGGGATAGCCCAACAAGGATGCCCGT

AGTGGGTCCAGTATTCTTCCGCAGCACTCAAGATATAGTCTTCCGAATCTCCGATTCAGA

TCCACTTTCTTCACAACCGCTCCACTACCGAATCTATCTTCCCAAGTCAAAACGCACACG

TTGATCACCGAGTTTTCGCAGTTGCTCGCAATCTCCTTCGCGTGTGGACAACGTCGTTTG

AGGGAGACCTTGAGCGCATTAGGAGCTATAAAAGAAGATAGATCAGCTGCGGATTTCAAG

AGAGGTTTCGCGTTGAACAGGATTGAGATCATACTTGAAGCTGTGGACAGAGAGGTGGTT

GTTTGCGTAACGCCACATAACACGAGTATCGTAAGGCCAATCGCCAGCGATCCATCCATC

CATAATCCGTTGTGAATCAGGTATATTTAGATTAGATTGTTATCTTATCTAAATTGTTTC

CAACATTGGTATCGGAGGAAATAAGTTGCGTTTCATACACAATCCATTGTAATTTGTTAT

CGTGGTAAAGATGAAGGATAAACAAAACTTTTACA

>Unigene0000945

CCCAACAGGGATGTCCATAGTAAGTCCAGTATTCTTCCGCAACACTCAAGATATAGTCTT

CCGAATCTCCGATTCAGATCCACTTTCTCCACAACTGCTCCACTACCGAATCTATCTTAC

CAAGTCAAAACGCACACATTGATCACTGAGTTTTCGCAGTTGCTCGTCATCTTCTTCGCG

CGCGGACGGCGTCGTTTGAGGGAGACCTTGTACACATTAGGAGATATAAAAGAAGATAGA

TTGGCTGCGGATTTCAAGAGAGGTTTCGCGTTGAACAGGATTGAGATCGTACTTGAAGCT

GTGGACAGAGAGGTGGTTGTTTGCGTAACGCCACATAACACGAGTATCGTAAGGCCAATC

GCCAGCGATCCATCCATCCATAATCC

>Unigene0000946

TCACCACCCTTTCACCTTGCTTCATCCCTTCCCCCTCAACCCCAACCTTGGTTGGCCAAT

GTCTCTCCTCCTCACAACATCCCCCAAACATTGTGCAAATCAACACTCCCTCAACCTCAG

AAAACTCAACAAGAAACTCCTCTACACCCTCTCCACCTCTCTCCTCTCCCTCCTAGCCCT

AGCCTTCCTCGTCTACCTCATCCTCCACCCCTCCAAGCCCAAATTCTACCTCAAAGACAC

CTCCCTCAACAATTCCACCATCCACACCACCGTCGTCTCCACCAATCCTAACTCCCGCGT

CGCCGTCTACTACGACCACCTCCGCGCCTTCGCCGCCTACAAGGGAGAACAAATCACCGC

AGAGGCAGTGCTACCGCCGTTCTACCAGGGGCACGGGGAGGCTAACTTCCTCTCTGCGGT

GCTCGAAGTGGGTGATGTTTGGCAGGAGCCATTGTTAGGGTTTGAAGTGAGCCGTGAGTT

GACGGTAGGGAAGCTGATGCTTGGGCTCAAGGTGGAGGGGCAGCTGAGGTGGAAGATTGG

GACGTGGGTGTCGGCAAGGTATCGGTTTGACGTCGACTGCGTCGCTGTTATGGGGTTTGC

GGCCGGCGGCGGCGCGGCGATGAGCTTCGCGGAGGGGACTCAGTGTTCCACCACCGTGTG

AGGTGGCTCACGCTGAAATATGGTATCTGCCGAGTGTAGCAGTTTAAGCGCCGAGTGGAC

CGTTACTATCACGGCCACTTTTTGCCGAGTCTGTGTGCTAGCCTCCTAGTGTATATTCAT

GGATCAGTGCATGGATGATATTGATACCATTAGGCATTAGAGAATCGAGAGTATGCGTGG

CTGTATCTAGGGGTTTCTTTACTTTTTCCCTCACATCATAGGAGATATTTGATATACCCC

CTCTAAAAACTTATAAAGGTGGTCTTTCATTTTGGCTTTAAAAATCAATAAACAATTTGA

TTATAGTCCCCATCTTAATAGCATAAAAGACTTGGTTTCCCCTCTTAATTAATATCAGAG

TTATGTTAAGGATGTATTTTGACTTGATCTAAACAAATTGGGGAATGATGTATTTTCTTC

ATAGCACCTTTAAAACATTATAGTTCGCCTGGGATGATCATCTTCTGAATAAGGCACTGT

TTTTTATTTACAATAATGTAAGCTAATTAATTTGGTCATTCTTATGTTCTT

>Unigene0000947

AGGCCAGCTTTCATCATGGGAACCGAGGACTCCAAAGGCTCGCTGAATGACATGAATTGG

AAGACCATAGATGGTGATGGGATTAGCAGCTCAAGTGAAACGGTCGTAAAAAAGCGACTC

CCCAAGAAAATGAGACAAGTGCCTGATTACTATTTTCTTCCCCGACGATCATTACCCTCT

GCAATTGCGTTATATGGTTCCATCATAGCCGCTGGCATTGGTGCTGGAATGCTAGTGGAG

ATATGGATAAACAAGAAAGTCCGAGAGGATGGGGGTATTATCTGGGAGATGGATAAATCC

TGAAGTTACAATTCCGATTGGTAGATTGAAGATCAGGAGCCCTCAAATGCAGAGGTAAGG

AGCTACCCAGCTTCACAC

>Unigene0000948

GGTGCCTGTAGTAACTCCTGGAGAACCAAAGGGCATGGATTGTAAAATTTGGCGATACCA

ATCCCACCTCGCCTGAACCCTAGTCGCCGCCTCCAGTCGGCTAGCCCGCCGGAGATTCTC

CTAACGTCCGTCATCGCGTGCTCAGGCTAGCTTTCAACATGGGAACTGAGGACTCCAAAG

GCTCGCTAAATAACATGAATTGGAAGACATTAGAAGGTGATGGGACTAGCAGCTCAAGCG

AATCGGTTGTGAAAAAGCGACTCCCCAAGAAAATGAGACAAGTGCCTGATTACTATTTTC

TTCCCCGACGATCGTTAATCTCTGCAATTGCGTTATATGGTTCCATCATGGCCGCTGGAG

TTGGTGCTGGAATGCTAGTGGAGATATGGATAAACAAGAAAGTCCGAGAGGATGGGGGTA

TTATCTGGGAGATGGACAAATCCTGAAATTACAATTCCGATTGGTAGATTGAAGATCAGG

AGCCCTCAAATGCAGAGGTAAGGAGCTACCCAGCTTCACACAAGTGTCTTGTTCTTACCG

TGTTTTAGGCTTTTCCTTATTACTCTCTGGATAAAAACTATCATATTTGTAAGGATGGCT

GTTGAGACGTGTATTGACATATTTTCATGGAATGCAGCTATAACTTGGTTTCTCAAATTG

ACCACTTGATAAAGAAACCAAAGAAATTTTTGTTGGGCTCTAAAA

>Unigene0000949

GGTGCCTGTAGTAACTCCTGGAGAACCAAAGGGCATGGATTGTAAAATTTGGCGATACCA

ATCCCACCTCGCCTGAACCCTAGTCGCCGCCTCCAGTCGGCTAGCCCGCCGGAGATTCTC

CTAACGTCCGTCATCGCGTGCTCAGGATGCTCTCCAACCATGTGTACCTGAGTGGTGCCC

ACTGCAATTTTAAGAAGTGGAGGGCAAGTATCCTTGTACCGGTCTCACGTGGTAGAAGAA

TGAAAGCAATGTTGTATGGCCATTCAGCAAGTGATACAGATTTTTCGCAATTTTCTGCAT

GACGCCCGACTACGGACAA

>Unigene0000950

ATGTGATGTGACCCGGCTCCAACTACCACCACGTTTTTGTTAAGAAATTTTTTACTCGCG

TGTTTATTTTATTTCATATTTTTCAATTTGTTTTACTGTAATTAAACTCACAATGGGTTT

TGACCGAGGTGGCCGTGGTGGTGGACGAGGAAGAGGTGGTGGCGGCGGTGGTCGATTTTC

CGGAGGTGGTGGACGAGGTGGTTTCGGCGGTGGCGGTGGTCGTGGTGGTGGTGGCGGCAG

AGGAGGTGGTTTCCGTGGCCGAGGAGCTGGTGGTCGTGGTGGAGGTGGCCGAGGTGGTGG

TGGACGACCATCTGGAGGACCTGGTGGCTTCAAAGGCGGTAAAAAAAGTTATAATTGAGC

CTCATCGTCATGCCGGAGTCTTCATTGCACGCGGTAAAGAAGATGCACTTGTTACCAAAA

ATATGATTCCAGGAGAAACCATTTATGGTGAAAAGAGAATTAGTGTTGATCAGACTGAAG

GAGATAAAATTGAATACCGTGTATGGAATCCGTTTAGATCAAAATTAGCTGCCGCTATAT

TAGGTGGTATTGATGAAATCCATATGCCACCCGGGTCAAAAGTATTATATTTAGGTGCTG

CTAGTGGAACGACTGTATCTCATGTTTCTGATGTTGTTGGCCCAGAAGGACTTGTATATG

CAGTAGAATTCTCACATAGGTCAGGCCGTGATTTGATCAATGTTGCTAAGAAGAGAACGA

ATATCATTCCTATTATAGAAGATGCTAGACATCCACATAAATATAGAATGTTAATTGGGA

TGGTAGATACAATTTTTGCTGATGTAGCTCAACCTGATCAAGCTAGAATTGTTTCAATTA

ATGCTCAATATTTTTTAAAGAACGAAGGACACTTTGTAATATCCATTAAAGCTAATTGTA

TTGACAGTACAGCTGAACCAGCTGCTGTATTTGCACAAGAAATTGAGAAACTAAAAACAG

ATAAGCTGAAACCAACTGAACAGCTTACGCTGGAGCCTTATGAAAGAGATCACGCTGTCG

TAGTTGGTGTATTTAGACCCGTTCCAAAGGCAAAGTAAACTTCAACTAATTAACTTTTAA

TACATTAACAAGGTTTTGTACAAATACACAATGAGACCAATATTATTTTTACTTAAAAAT

AAGTATTTTATT

>Unigene0000951

ATGTGATGTGACCCGGCTCCAACTACCACCACGTTTTTGTTAAGAAATTTTTTACTCGCG

TGTTTATTTTATTTCATATTTTTCAATTTGTTTTACTGTAATTAAACTCACAATGGGTTT

TGACCGAGGTGGCCGTGGTGGTGGACGAGGAAGAGGTGGTGGCGGCGGTGGTCGATTTTC

CGGAGGTGGTGGACGAGGTGGTTTCGGCGGTGGCGGTGGTCGTGGTGGTGGTGGCGGCAG

AGGAGGTGGTTTCCGTGGCCGAGGAGCTGGTGGTCGTGGTGGAGGTGGCCGAGGTGGTGG

TGGACGACCATCTGGAGGACCTGGTGGCTTCAAAGGCGGTAAAAAAGTTATAATTGAGCC

TCATCGTCATGCCGGAGTCTTCATTGCACGCGGTAAAGAAGATGCACTTGTTACCAAAAA

TATGATTCCAGGAGAAACCATTTATGGTGAAAAGAGAATTAGTGTTGATCAGACTGAAGG

AGATAAAATTGAATACCGTGTATGGAATCCGTTTAGATCAAAATTAGCTGCCGCTATATT

AGGTGGTATTGATGAAATCCATATGCCACCCGGGTCAAAAGTATTATATTTAGGTGCTGC

TAGTGGAACGACTGTATCTCATGTTTCTGATGTTGTTGGCCCAGAAGGACTTGTATATGC

AGTAGAATTCTCACATAGGTCAGGCCGTGATTTGATCAATGTTGCTAAGAAGAGAACGAA

TATCATTCCTATTATAGAAGATGCTAGACATCCACATAAATATAGAATGTTAATTGGGAT

GGTAGATACAATTTTTGCTGATGTAGCTCAACCTGATCAAGCTAGAATTGTTTCAATTAA

TGCTCAATATTTTTTAAAGAACGAAGGACACTTTGTAATATCCATTAAAGCTAATTGTAT

TGACAGTACAGCTGAACCAGCTGCTGTATTTGCACAAGAAATTGAGAAACTAAAAACAGA

TAAGCTGAAACCAACTGAACAGCTTACGCTGGAGCCTTATGAAAGAGATCACGCTGTCGT

AGTTGGTGTATTTAGACCCGTTCCAAAGGCAAAGTAAACTTCAACTAATTAACTTTTAAT

ACATTAACAAGGTTTTGTACAAATACACAATGAGACCAATATTATTTTTACTTAAAAATA

AGTATTTTATT

>Unigene0000952

AGTTAGCCGGAGCGGCTGGCTGCCGCCGCCGCCGTGCCGCCGTGCTCCCCTGCCTCCATC

ACCACAGGCCACCCTACCACCCCCTTTAGCACCTCCTCGTTCTTCCCATTCCTCCTCCAC

CGTTCATTCCGATCAAATCATCCTCCTACCACAAATCACAAATCCCAATCATTAACCCAC

ATCCGATCCTCTACAATCCACCTATCCCAAGCCTCCTCGCACCTTCCCGCACCCTCTAGA

TAAGATCCCGACCCCCAATTTTTCATTTCTTTATGTAATTTGATAAGAATTGTGATGAAT

TATTTTTGTATTTCAAGAAACGGCGATGTCTGGCATGCATGGAGCGGGAGTAGAGGAAGC

CTCTGGATACTTAGCACGCGTGGAGAGCAGCTGGAGTAGTTGAGTGGCGCATGTGGGGAA

GGTTCTGGAGAAGACTAGACACGTGATGGAGCTTGGATTGGGTTATAAAAGGCAGTGAGG

AAGAAGTGTAAAAGGAGATCAATTTGTATGTGTTTTCAGTTCGGCAATTCTTCATTCAAG

GGGGTCTATGGTGCAAAAGTCTCTAATTTCTTCCAAGGGAGTCTATGTAGTGCATTTCAA

GGAAGAGATCAAGCAGATGAAGTCGTGTTGAATGATGATTTCAGATTGAAGCTATTGGGT

CGAAATTGTTGAAGCTGAGTTCTAGTTTTAGTTTGTTCTTGATGCTAACTATACACAGAG

TTGAGTCTAGTTCCCTTGTTCAGTGATTCTGAGTTAATACTCTAGCAGACTTGTAGTGAT

GATATGCGTTGTTCTTGATGTATAGAACTATATGACAGTCTTCATGATGTATTCATGATT

ATATAAGTTGAGACGTTTGAATTTAATTCA

>Unigene0000953

AAGTCGTGTTGAATGATGATTTCGGATTGAAGCTATTGGGTCAAAATTGTTGAAACTGAG

TTCCAGTTTTAGTTTGTTCTTGATGCTGACTATACACAGAGGTGAGTCTAGTTCCCTTGT

TCAGTGATTCTGAGTTAATACTCTACCAGACTTGTAGTGATGATATGTGTTGTTCTTGAT

GTATGGAACTGTATGACAGTCTTCATGATGTATTGGTGATTATATAAGTTGAGACGTTTG

AATTTAATTCA

>Unigene0000954

AGAAGACGAAACTGATGGAAGAATGGTGGAAGAAGACACATGGTCTTTTTGTTGAGGGAG

GCCTTATATTTGATGTGATCCAGAAATCTGTGGTTGGTGCTACTATTGGTTTTAGAGAAG

GCGTAGTCGAGTTATTTGAGTTGTTGGAGGAAAGAGGTGTACCTGTCCTAATATTTTCTG

CAGGCCTTGCTGATATTATAGAAGAGGTATTTAGGCAGAAATTACATAGGTCTTTTAAGA

ACGTCAAGATTGTGTCCAACAGGATGGCATTTGATAATAATGGTCATCTTGTAGGCTTCA

AAGGGAAGACAATTCATGTTTTGAACAAAAATGAGCACGCCTTGGATATGGCTGCTCCTG

TTCACGATGAATTGGGTGCTCCTGATGGACCAAACAATGACAATACCTCAGTGAAGAACA

GGACCAATGTGCTACTCCTTGGGGATCATATTGGAGACCTGGGCATGTCAGATGGCCTGA

ATTATGAAAACCGGATTGCTGTTGGTTTCCTGAATAACAATATAGAGGCATCTCTTGAGA

CTTATCGTGAAGCTTTCGATGTTGTTTATCTGAATGATGCATCAATGTTGGGGGTGGTGG

AGCTTGTTTCTCGACTATTCTCAACTGATCCATGATTTCGGTACTCTCCGTCAATTTACA

TGTTCAAGGACATAATATATGTTTCAGTTTTCTTTAGTAATTATGAGTAGTATGGCAGTT

TAAGGCAGGGTTATTTTGTCTTTGGTACCCAAATTTAGTAGTGTTTTTTT

>Unigene0000955

GATCATTCACTCTCCAGCCAGTTGTTGGAGTTCCTTAGAATTCTCTGATCTCCGATCATC

TCTTCTTCCCGAGCCATTTTCTAATTGCTTATTTCAGGTATTGCTTGTTTAATTTGGTTT

TCTAATTATTTCATTTCTGTGTTTCGGGTACTCAGAGTATGGATCATTGAAGCACATAGA

TGATGGACATGTCAGGACAGGATAGGAAATTTCATGAAATCCGATCGGGGTGTTTATGCA

GCCTTACCAGTAAGCTCTGCTCGAGCATACTATCCGACGCCCGCTTGCTCTCCCCACAAT

ATGCACAGTTTGTCCATTCCCGACAAATATTCCCTCATCGTCTCCTCCGTATTTTAATGA

ACACCTTGTCTACTCCCTCTACTCTAGTTTCCCACCTCGCCGGAGCGGCCCCCTCTCTGT

CACAGCCATCATGTCTCAAGATAACATCATCATCAGTGATCGTGATTTGCTGGAGATGAA

GACCGCCGCGATCCAGAAGGCCGGTCTGGGCAAGCTCCAGGTGATTGCTGATTTCGACGG

CAGCCAAGAGCAAACAAGTCACAGGTTGTTACAGCAGGGGAATGTTGAGTTTAATGAGAA

AAGACAGGCATTGTTTGATTACTATCACCTGTCGGAGTACTCTCCATCGATACCGATTGA

GGAGAAGACGA

>Unigene0000956

TTGTTTAATTTAGTTTTCTAATTATTTCGTTTCTGCGTTTCAGGTACTCGGAGCATGTAT

CATCGAAGCACACAAATGATGGACATGTTGGGACATGATAGGAAATTTCATGAAATCCGG

CCCGGGAGGTTTGTGCAGCCTCACCAGCAAGCTCTGTTCGAGCATACTATCCAACGCCCG

CTTTCTCTCTCCACGGCGCGCAAGGTTCGTCCATTCCCGGCCAACACTCCCTCACCGTCT

CCTCCGCATTTTAACGAACACCTCGTCTACATCCCTCTACTCCAGTTTCCCACCTCACCG

GAGCGGCTCCCTCTCCGTCGGAGCCATTATGTCTCAAGATGACGTTGTCGTCGGTGATCG

CGATTTGCTGGAGACGAAGACCGCCGCGATTCGGAAGGCCGGCCCGGGCAAGCTCCAGGT

GATCGCTGATTTCGACGGCACGCTAACGCGGTACTGGATCGACGGCGGCCGAGGGCAAAC

GAGTCATGGGTTGTTGCAGCAGGGGAATGTTGAGTTTGATGAGAAGAGACAGGCATTGTA

TGATTACTATCACCCGTTGGAGTACTCGCCATTGATACCGATTGAGGAGAAGACGAAGCT

GATGGAAGAGTGGTGGGAGAAGACGCATGGTCTTCTTGTTGAGGGAGGCCTTACATTTGA

TGCGATCCAGAAATCTGTGGCTGGTGCTACTATTGGTTTTAGAG

>Unigene0000957

GGGGTTTGTGCAGCCTCACCAGTAAGCTCTGCTCGAGCATAATATCCAATGCCCGCTTGC

TCTCCCCGTGGTGTGCACGGTTTATCCATTCCCGGCCAACATTCCCTCACCATCTCCTCC

GCATTTTAATGAATACCTCGTCTACATACCTCTACTCCAGTTTCTCGCCTCGCCGGAGTA

GCCCCCTCTCCATCGTAGCCATCATGTCTTAAGATGATGTCGTTGTCCGTGATCGCAATT

GCTGGAGACAAAGACCGCCGTGATCCGGAAGGCCAGCCCGAGAAAGCTCCAGTGATCGCT

GATTTTGACGGTGGCCAAGAGCAAATGAGTCACGGGTTGTTGTAGTAGGGGAATATTGAG

TTTGATGAGAAAAGGCAGGTATTGTTTTATTACTATCACCCATCGGAGTACTCACTATCG

ATTCCGATTGAGTAGAAGACAACTGATGGAAGAAGACACATGG

>Unigene0000958

TCCCCCCTCGACCAAATCAAAACTCCCCCAAAACTTAGCCCCAGCTTTGCCATTGCATGT

TGACCTCTTCCCCCTTATCTATTTGAGTTTTTACTGAAGGAATTTGGAGATCTTGATCAT

CCACTCTCCAGCCAGTCACCGGAGTTCCTTGGATCTCTCCGATCTCCGATCATCTCTTCA

TCCCGAGTCTTTTCCAATTGCTAATCTCAAGTATTGCTTGTTTAATTTAGTTTTCTAATT

ATTTCGTTTCTGCGTTTCAGGTACTCGGAGCATGTATCATCGAAGCACACAAATGATGGA

CATGTTGGGACATGATAGGAAATTTCATGAAATCCGGCCCGGGGGGTTTGTGCAGCCTCA

CCAGTAAGCTCTGCTCGAGCAACTATCCGACGCCCGCTTGCTCTCCACGCGGCATGCACG

GTTCGTCCATTCCCGGCCAACATTTCCTCATCGTCTCCTCCGCATTTTAACGAACACCTC

GTCTACATCCCTCTACTCCAGTTTCCTGCCTCGCCGGAGCGGCCCCCTCTCTGTCACAGC

CATCATGTCTCAAGATGACATCATCATCGATGATCGCAATTTGCTGGAGACGAAGATCAC

CGCGATCTGGAAGGCTGGCCCGGGCAAGCTCCAGATGATCACTGATTTCGACAGCGGTCA

AGAGCAAACGAGTCACGGTTTGTTGCAGCAGGGAAATGTTGAGTTTGATGAGAAAAGGCA

GGCATTGTTTGATTACTATCACCTGTCGGAGTACTCTCCATCGATACCGATTGAGGAGAA

GACGAAACTGATGGAAGAATGGTGGAAGAAGACACATGGTCTTTTTGTTGAGGGAGGCCT

TATATTTGATGTGATCCAGAAATCTGTGGTTGGTGCTACTATTGGTTTTAGAGAGGGTGT

TGTCACTCAAGGATATTCGGATAGCATC

>Unigene0000959

TCCCCCCTCGACCAAATCAAAACTCCCCCAAAACTTAGCCCCAGCTTCGCCATTGCATGT

TGACCTCTTCCACCTTATCTATTTGAGTTTCTACTGAAGGAATTCAGAGATCTTGATCAT

CCACTCTCCAGCCAGTCGCCGGAGTTCCTCAGATCTCTCCGATCTCTGATCATATCTTCA

TCTTGAGCCTTTTCCAATTACTAATCTCAGGTATTACTTGTTTAATTTGGTTTTCTAATT

ATTTCATTTCTGTGTTTCAGGTACTCGGAGCATGGATCATCAAAGCACACGGATAATGGA

CATGTTGTAGCAGGTGATCACTGATTTTGACAGTGGCCAAGAGCAAATGAGTCACGGGTT

GTTGTAGCAGGGGAATGTTGAGTTTGGTGGAAAAAGGCAGGCATTGTTTGATTACTATCA

CCCGTCGGAGTACTCATCATCGATACCAATTGAGGAGAAGACGAAACTGATGGAAGAAGA

CACATGGTCTTCTTATTGAGGGAGGCCTTACATTTGATGCGATCCAGAAATCTGTGGCTG

GTGCTACTATTGGTTTTAGAGAGGGCATTGTCACTCAAGGATATTCAGATAAAA

>Unigene0000960

ATTGCATGTTGACCTCTTCCCCCTTATCTATTTGAGTTTCTACTGAAGGAATTCAGAGAT

CTTGATCGTCCACTCTCCAGCCAGTCACCGAAGTTCCTCGGATCTCTCTGATCTCTGATC

ATCTCTTCATCTTGAGCCCTTTTCCAATTGCTAATCTCGGGTATTACTTATTTAATTTGG

TTTTCTAATTATTTTGTTTATGTGTTTCAGGTACTCGGAGCATGGATCATCAAAGCACAC

AGATGATGGACATGTCGGGACAGGATAGGAAATTTCATGA

>Unigene0000961

TACCCGCAAGTTGCCTCGTCAGGTTCAGGTCATATGAGGGATATATACTGGTAGATTAGA

ATAGTGTTTGCTCGCAAGACACAACAGCCAAAATACTCATTCTGGGAGAGGTCACGTGTT

CAAGTCCCTCGTTGGAATACAAGAGACAAATATGGTTCATGCGCTCGCCAAGGTTGTTGC

AGTATATCTGCTTTCTTGATTCTATCCTGGTACTGTTCCTCTGTGGAGTACTCTGCTTAT

AATGCTCTGGCCAACAAGTGCCCCCTTGCAACCAAAAGTGGTTGCAAGAAAAAAGAA

>Unigene0000962

ATCTCGACTGGGCAGAGATTGGCCCCGGAACCCTAGCCCCATTTTCATCCTACGTCACAC

CGCCATCGCCGGCGCTTCCGCCGCCCGTTTGTGGTTCGCCGACCTGCGCCACCCCGCTCG

CCCGCGGATACCTGTGCAGTCTGCCCACTCACGTTCGTGATCCCGCCAGCCAGCGGTCAC

CTTCATCCCCTCCGGCGAACAACAAGTCGGCTACCGATCTCCCGTGTAGCGGCATCACAC

ACGCCGGAGGAGTCGAGTTTCTTCGCGAGCCATTTGAGGCGGTATCGAGATTTGAGGCAT

CACTTATTTCGGATTGTGTTTTTCAGCATCGATTAAATTTGTGGGTGATTGGGGAACTAG

GGGATTGGGCTGCTTCTGTGGTCGGGGAATTCTTAATACTCGGAGTTATTGAAACCGTTG

TTGATCTGGGATTTTCTTGTGGAGAGGCGAGGAGTCGGGGAGAAGCTTGAAGTGGGATTT

TATTTTTGATGAGACTCGACCCCTAGATATCACGGGAGCCTCTATGGAGTGCTTCCTACC

TAATCGAGGTTCGCGAGGCAAGAAGTGAGCTGTAACAGGTTGGAGTTTTGGGCGGGGTTC

TCTTCTGTATTCTTAGCGGAGTCCAGAGGTATCCTGAGCCGACTCTCCTCCTGTGGTTAA

ATACCACAGGGTCAATAGTTATTTTTGGTCATATGAGGGATATATACTGGTAGATTAGAA

TAGTGTTTGCTCGCAAGACACAACAGCCAAAATACTCATTCTGGGAGAGGTCACGTGTTC

AAGTCCCTCGTTGGAATACAAGAGACAAATATGGTTCATGCGCTCGCCAAGGTTGTTGCA

GTATATCTGCTTTCTTGATTCTATCCTGGTACTGTTCCTCTGTGGAGTACTCTGCTTATA

ATGCTCTGGCCAACAAGTGCCCCCTTGCAACCAAAAGTGGTTGCAAGAAAAAAGAA

>Unigene0000963

GTCTCCAACCGCCTGAGCCCTAAACCCTAGTCGGGCGATTCGAATTCGATAGAGGGGTGG

AGAGGAGAATGGCGGAGCATCTGAGCTTGGGGATCCTCATCGACATCGTCGACGATGAAT

GGATGAGGGACACACTCCCCATAGAAGATATTCCTCTTCCGCCGACGATGGTCGTCCGAA

CTGACGAGGCCGAGGAAACACACCAGGACAAGCAACCGGATGAGGGGGATGTATGGAAGG

ATCTTGCTCTAGAGAATCCTTAGGCTCTATTGTCTAGTTAACTGCAGGATGTGACATGTA

GCTGATGCCGGCGATGAAGTAGGACATTATAGTTGAAGGCAGAATCTGAGCATTATTTGA

CTCTTATATGTTGGCATTCAGTTATTTGCAATATATGTCCTTATAAGTTGTAAGCGAAGT

ATTGGTCCGGCAGAAAACTTTGTGAGTTGTTCTTAGATTCAAGTGTTTGACGTTAGCGTC

GAAACTAAAAATTCAGTTTCTTATGCATTATTTTTCTATATTAGAGTTATCAAGTATCTA

TTTGTCACCATTAGTCATATTGTGGTCACGCTACATTCTAAGCTTT

>Unigene0000964

AGCAGTTGCAGTCGCCAATCGCTCAAGCTCTAAAACCTAGTTGAGCAATTTGAAGTAGAG

AGAGCGAGGGGGAGGAGAATTGTGGAGCATCTGAGCTCGGGGATCCTCACAGACATCGTG

GACGATGAATTGATGATGGACACACTCCCCATAGAAGATATCTCTCTTCCCCCCACGATG

GTCGCCTGAACTGACGAGGCCAAAGAAACAAACCAGGAAACTGAACCTGTGGAGGCGGAT

GTATGGAAGGATTTTGCTCTAGAGAATCCATGAGGCTCTATTGTCTAGAATGGGATCAGG

TGTGAAAGTTGGAATTGAAGGCATCATCTCCGGACAATTTTCATTTTCTGAAAGATTCTG

TGGATCATTTGACTCTTATATCTTGGCATTCAGCTTAGTTATTAAAAAAAAAAAAAAAAA

AAAAAAAAAAAAAAAAAAAAACATTGTTCTTATCAGTTACAACAGAACAAGTAACTGGTA

ACTTTTTGAACTTTTCTTAGAAAGTGGTTGTACTCTTCAGATCGGATTCAAGTGGCTGAC

ATTAGCATTTAAACCAAAATTCAGTTTCTGATTGTATTATTTTCATATATTTTAGTTAGA

TTGTCGCTTTCATGGTTCTGCAAATTACTGTATCTTTCTGAT

>Unigene0000965

AAAAAAAAAAAAAAAAAAAAAAAAAAAAAAAAAAAAAAAAAAAAAAAAAAAAAACATTGT

TCTTATCAGTTACAACAGAACAAGTAACTGGTAACTTTTTGAACTTTTCTTAGAAAGTGG

TTGTACTCTTCAGATCGGATTCAAGTGGCTGACATTAGCATTTAAACCAAAATTCAGTTT

CTGATTGTATTATTTTCATATATTTTAGTTAGATTGTCGCTTTCATGGTTCTGCAAATTA

CTGTATCTTTCTGAT

>Unigene0000966

TGTGATTCCTTGTGTGGTATTCGTACATTGAAGGTGTTTGGAGGATTGCCAGTGAGAGAT

TGCAGGTTTTCCACCAGGTGTTTGGAGGATTGCTAGTGAGAGATTGCAGGTTTCCTACCG

AGTGTTAGAGCTGATAATCAGTGAGTTCTGGGATTTTGATTACAAGTAATCCCAAATCCT

AGTTGTAGATGTTTGTGAAAATGTTCGAGAGAATCTTCATTCCGAGATGATGACCGTGCT

ATGGTCCATGGATATGATATATCTCGTTTCAATAGAAGGAGAAATTCAAGGTCTGACAAA

TGGATTGGATTGCAAG

>Unigene0000967

ATATGATCCAGGTTCTCATGCGGATGCCTCTGGCAAAGGTTGATCTTGCATTCACTTTAA

TCTTTTCTAGCATGGGGGGCTATTATCCTTGTCGAGTCATGTTCGTCGAGGCACAAAAGA

AGCTGTTGGGAGAAAATGCACAATCATGCGTAAAGTGGATTGAAGGTCATAATTTTTGAC

CCATTCATGTACTAAGACCGTGAGTATATCATTTCCCCTGCCTCTGCCTCGTAATCCG

>Unigene0000968

GGTTCTCATGAGGATGTCTCTGGCAAAGGTTGATCTTGCATTCACTTTAATCTTTTCTAG

CATGGGGGGCTATTATCCTTGTCGAGTCATGTTCGTCGAGGCACAAAAGAAGCTGTTGGG

AGAAAATGCACAATCATGCGTAAAGTGGATTGAAGGTCATAATTTTTGACCCATTCATGT

ACTAAGACCGTGAGTATATCATTTCCCCTGCCTCTGCCTCGTAATCCG

>Unigene0000969

TATTTTTTATATTATTCATAATGTCATGTGATAAGGTAATACACGAAATTAGTGGATTGA

GTAAATTATTACTATGTCGTTTGGATAATTATAATGTAATGATAAAACCTCTTATAGTCA

ATAATATAAAACTTTATATTATTGCCAAGATGACATGGATCCTCTATACGGTCACACCAA

CTGAAGAGAAACGAGTATTTTACATTTCATAATGATGTATAAGATAAAATACATAAGATG

AAATTCACTGCAAAGCCGCAAGGATGAGAGGGATCACAAGTCGGAAGTGACATAGTGCTA

TTGGGAACTCATCAAGACATAGCTCATTGATGTCACAGAGGTGTTTCACGTTGTTCGATA

GTGGACGAGGTAGGAGAAGAACGCTAGGTTATTGTTGAGCAGCTAAATGTCGGTCTTCAA

GGAAAAGAGTTGCTTCAGGGAGATTGCCAGCAGGTCACGGCTGAATGATGGGAAAAAGCT

CAGAGAAGCTAGTTGGAAACCGATCGGTAAGCTAGTTGGAAACCGATCGGTGCTCCTTAT

CAGGAGATCCTCAAGCACTTGAAGACCACTAACTAGTACCGGTTGGCGAAACCCATGGCG

GAAGATTCAGAGATGTTCAACAAGAAATATTATTGTGAGCACCATCAACAACATGGATAT

GACCCCAAGTAGTGCCAGACATTGAAGGCCCCATGGAACGATGGATGAATG

>Unigene0000970

AACTCCTTCCTTGATAACCATCCTAGGATCAACCCACAAAACAAGGGTTCGGCCAGGACG

ACGGCCACCACAATGGCCGCCGCCGACCACCCCTTCATCTCCAGCTCCCCGCCGCCGTCC

CCTCCTCCTCCGGAGCCCCTTTTCAAGAACCCCTGGCGCATCACCCCCTGCCCCGCCGCC

GCTGCCCCCACCGCCCTCACCCTCATCGGCGAGCCCCCCTCCTCCTGGCCCGCGCTCACC

TCAAACCCAAACCCTAAGCTGGAATCCCCCGATCATCCACCTCATCACCACAGCCCCTCT

CCTCCGCCCGCCGCCGCTGACAGCCCTAACACCACCCCCGATCTCTTCTCCTCCTCCGCC

TCATCTTCCTCCGATCCCTCATCCCATGACGACGCGGTGGCGGTGACAGTCGCCGCGGCG

GTGGCTGTATCCCCGGCGGTGCCGCCTCCCGCTCCGGCCCCAGTCCTACCGGTGTCCCCT

CGCAATGCTGACCAGCCCAGGATCTCCGCCTCGCACCACCGGCGCGGCAGGAACCCGAGG

TTCCAGAACCACAGGGGCGGCGGTGGCAGCAACGGCAGCCCCCGCTCCAACAATAGCTAC

GGCAATCGGGCGCCGGCTCCTGGCTGGAGGCCGAATTGGGGCGGTTTCACCCCGGTGCCA

CAGTACCCGAACTGGGGATTCTTCCCAGGCTCTGCTGTTGGTGTTGGCGTTGTTCCGCCA

GCTGATTACTTCGTGCCATATCCTCAGCATTTCAATGAGATGGTTTATCCTGATTACGGG

ATGAGTGTGGAAGCTTATGGGGCCTATTATGGGCAGCCGGCGTATGAGGTGCAGTATGCT

CCACAGATGAACCCGGCGGTTGCTGGCATTCCTTCCCCTCCGAGCCTGGGCTTTACTGTT

GATCACGAGCTTGAAGCATTGAAGACGCGGCTCATTTTCCAGATTGAATACTACTTCAGT

GACGAGAATCTGGCTAAGGATACCTTCTTGAGGAATCAGATGGATAACCAAGGATGGGTT

GATATTCTTGCGATAACCCCCTTCAAACGGGTTGTAGCTCTGGTTGATGAATTCAAGAAA

AAAGATTCCCTGGTAGAGGCAGTGAGCCTCATAAAGGATACCTTGCAGTCTTCTAATGTC

GTTGAGTTTCAAGGCAACAAGATGAGGAAGAGGATTGGATGGCATAAATGGATCATACGA

GAGACGCCATCCCCAAAACAGTTGGTGACGGGCATGCGGAGCATCAAGTTGGAGGATTAA

TTACAAAAGAAAGTCGAATGATCGAAGTCACTAAGATATCTCAAAGCGGAATGTGTCTTA

ATGTCAACTTATAAGACTTATTTACATGACTTACTCCTAAACCTTGAGGACTTGTGAAAT

TGGTCTTATCAACTAACCCTTTTTTGGAGACATGGTGCACTTGACTAAATGTTTATAATG

GTGTACATTCTTGAAGTGTTTTTATTATAATTAATTGACTT

>Unigene0000971

GACTGAGCCGAGTGCAGCGCCGCGAAACCCTAGCAATGGCGCTCGGATGGTACGGGCCGC

TGATAGATCTCTCTCGAGCATCCTCCCACCTTAACGACTTCGTTCAGCTTCTCGTCTTCG

TCCGCAGCTCCCAATCTGTCCAGATACATAGTGCTTCAAAAGGAGGGATATCATCGAGGA

CAAATATTCAGGTTGGTGATGATACAAGATCATATTTCCCTGTCTCTCTTTGGCAAAAGC

ATATGGGATCTATGATAGTTGCCGGTGATGTCATATTACTACAAAATGTCAAGATAGTAA

AATTTAGAAATGTCTTAGAGGCTACTACTATCCAGATATCTGCATTAACAGTTTTGGTAC

ATTCCTATGAGTTAAAGACCTCGAAGGGCGCCAATGAGATAGTACCACATTGCCAACTGG

GGGAAACCGCAAGAGAGAAGCTGAGGAGGGTAATTGAATGGGTGCAGCATACCGAATCTA

CATTTCAGCATCTTCAACAAGCAGACTCCTGTCAAAAAATACCGCAATCAATAAAGAATT

GGAAATCAGATGAAGAAAAGAAGTTACATCAGTGTATGTCAATCTCCGAAGTGTTAAGCA

TCACCACCTGCTGTAATGCAAGCTTTCTTGCCAGCATTAAGGAAATGCGTTTGCCTTTTA

CTCGGAGAACTGATGAAGTTGAGGAGGAACATATGTTTTCAGGGAAGAGATTATCTATGA

TGGCTGATAGTACGGTTGCAGAAGATCTTATATGCCCAGGCTGCAAGTTATGTGGTTCCT

CAATGAGTTCCAGAATCTTTGTGAATATTGGCAGTATTCAAATACGAGATCATTGTCGGA

GCAGGACAAAGCTACACTCTACTGCCCAAAGAGTTCCAAGTATCTTCATGATGTGTGCCT

GATTTACAAACCATTTTTGCTCTATGTATGGGATGAGTCAGGCGAAATTCCAGTTCTTGT

TAAGAACAACACTGCAGAAATCCTGTTCGGAAATATTTCGGCAGAGAGAGTCTATAAATG

CTTTCAGGATGAACGGCAGAATTGGTTCCCCTGTCGTAAAACGGACGAAGAATTGCATGA

TTGTGAAGTGCAGTGCAGCATGCAAAGTGAAAGCGGATATGGCAGAAAGAAGCCTGATTT

TTATGGTATTTGGTTGATTTTGATTAAATCGCTACTGCAACAGGGTAAGAACAGTCCTTT

CAGGTTTGAGATTGCAGTGAACAATGAGAAAGATTTTGATATGGGTAGATTTGAGTTAGC

GTCATTTTCGATGCCGTGCTGAGGAGTTCAACAATTGAGGTGTAAAATTCGGCTTTTAAA

AGGTTTATATAGACCTTTAGATTGGATTATGATGTCAGCAGGCAAGGTGGGAAAATGGAG

AGAATTGTATGAGAACCAAATGCGTTGTCCAATGTTGGAGTGCATGTTAAACTCATGGCT

AATTTA

>Unigene0000972

TTGAGACTAGGGTTTTGCTGCCACCACCGCCCCCATTCTCTTCATCAGTTATATCTCTAA

ATGGGGAAGTCGAGCAAGAAATCGAACGTCGAGGTCGCTGTTGCTGTCCCGGTTTCTCCG

GTGAAGCCGAAGAAAAAGAGCAAGAGGGCTCCGGAGGCGTCGCCGGAGAAGCCGGTGAGC

GCTAAGAAGCAGAAGAGAGAAGCGGTGGAGGTGGCCCCGAAAAAGCCTCTCAAGAAGAAG

CCCGAGACCAGTAGTTCCGAGGACACTAGTTCTTCGGAATCAGTGGAGGAGGTTAAGATC

CCTCCGAAGAAAGTAACCAAGCCGGCAAAGGCACCGGTTAAGAAAGAATCTAGCAGCAGC

GAAGATTCTTCTTCTGATGAGGATGTGAAGAAACCTATTGCTCTGAAGGCGCCGGCCGCC

ATCACCAAGAATGGTAGCGCACGAAAGCCCGAGAGCAGTTCTGAAAGTGAATCTGATTCG

GAAGACGAAATTGAAACTAAGGCTGCTCCAGCTGTCGTATCCAAGAAGAAGCATGAATCA

AGTGACAGTTCTGATTCAGATACTAGCTCTGATGATGAGACTGGAACTCAAACTAAGGCA

AGTATCCCGTCCAAGAAGCCAGTAGTCGCTGCTAAAAATGTTCCAGCTGCCCTATCCAAG

AAGAAGCAGGAGTCCAGTGACAGTTCTGATTCAGATAGTAGCTCCAAGGAGGAAACAGAA

ACTAAAATCAAGGCAACTAATGCATCCAAGAAGCCAACAGCTGTCCCAGTTGCATCCAAG

AAGAAGCAAGAATCAAGCGACAGTTCCGATTCGGACACCAGCTCTGAAGAGGAGATGGAA

ACTAAATCTAAGGCAGTCATGGCTTCAAAGAAGCCAACAAATGTATCCAAGAAGCCTACA

GCTGTCCCAGTTGTTGCATCCAAGAAGAAGAAAGAATCAAGCGACAGTTCCGATTCGGAC

ACCAGCTCTGAAGAGGAGATGGAAACTAAATCTAAGGCAGTCATGGCTTCAAAGAAGCCA

ACAA

>Unigene0000973

GGACACCAGCTCTGAAGAGGAGATTGAAACTAAATCTAAGGCAGTCATAGCTTCAAAGAA

GCCAGCAATTGAAGCGAAGAATGGCCCAGCTGTTGTATCCAAGGCGAAGCAAGAATCAAA

TGACAGTTCTGACTCGGATGCTTCCTCTGAGGAGGAAAATGAAACTGAAACTAAGGCAAG

TATTCCGTCCAAGAAATTGGTAACTGATGCTAAGATTGTCCCAGCTGTTGCTTCTAAGAA

GAAGCAGGAATCTAGTGACAGTTCTGATTCAGATAGCAGCTCCGAGGAGGAGACTGAAAC

TCAAGCTAAGGCTAATATCCTGTCCAAGAAGCCTACAACTGAAGCTAAGAATGTCCAGGC

TGCCGTACCAAAGAAAAAACAGGAATCAAGTGACAGTTCGGATAGTAGCTCTGAGGAGGA

GACGGACACTGAAACTAAGGCAAGCACCTTGTCGAAGAAGCCAGCAACTGGAGCAAAGAA

TGGCCCAACAGTTGTTTCCAAGAAGCAGGAATCTAGTTCCAGTTCTGATTCGGATACTAG

CTCTGATGACGAGACTGAAACTAAACCCAAGGCAAATGTCGTGCCTAAGAATATCCCAAC

TGCCCTAACCAAGAAGAAGCAGGAGTTGAGTGACAGTTCAGATTCTGATAGCAGTTCTGA

GGAGGAAACAGAAACTGAAACCAAGGCAACTGATGCTTCCAAGAAGCCAGCAACTGCAAC

TAAGATTGCCCCTGTCGTTGCATCCAAGAAGAAGCAGGAATCAAGTGACAGTTCTGATTC

TGATACTAGCTCTGAGGAGGGGAATGTTTCAGTCAAAAAGCAGCCAGTCATGGGGAAAAG

AGATCTCGAGAAGAAGCCGGAGAAAGAAGCTGCAACAAAGGAGGATGAGAGCTCCGATGA

GAGTAGCGATGAGAGTGATGAAGAACCACAAAGAAAAAAACCAAAGATTTTGTCTAGTGA

GCCAACAAGCAAGCCTAGCGCTAAAGCCATCAAGAAGGAGACTAGCAGTGATGAAGATAG

TTCTGATGAAAGTTCAGATGATTCTCCTAAAAAAACACAGCAAAATAAGAAGGATGCAAT

ACTAAAGGACACAGATGTCGATATGGTAGATGCACCATCAGCAAATGCCAATAATAAAGG

AGCTTTTAATTCTGAAAAGAAGGCGCCTTATACTCCTGCCACCCAAAGTCAGACTTCTGG

TTGTACCTTATTCATTGGCAACTTACCTTTCAAAGTTGGAGAAGACGAAGTGGCTGAGTT

CTTCAAAGAGGCAGGGGAAGTAGTTGGAATCCGATTGGCCAAAGATGAAGATGGGTTCTT

AAAGGGCTTTGGTCATGTTGAGTTCGCAACTGAAGAAGCTGCTAAAAAGGGTCTGGAACT

GGATGGTTACGAGCTATTGGGTCGTTGTGTGAAGCTTGATATGGCCCGCCCCAGGGCCCA

GTATACTCCTAGTACTGGGACACGGGGATCGTTTACCCAACATGGCGGGAGGGAGAGTAA

TTCTTACCAGAAGGGCGACAATGGTCCAAGCCAAACCATATTTGTAAAAGGTTTTGACAC

TTCCCATGACGAAGATCAGATTCGAAGCTCTCTTAAGGAGCATTTTGGAACCTGTGGAGA

GATAACTCGTTGCTCAATTCCTAAAGATTTTGAAACTGGTGCTTCCAAGGGGATGGCTTA

CATCGATTTCCAAGACCCAGGTGGTGTAACTAGTGCTTTGGAGTTGAATGGATCTGATCT

TGGCGGGTGGGCTTTACAAGTGGAGAAAGCAAGACCGAGAGTTGACAGTGGTGGGAGTGG

CGGGCGCTTTGGCGGGGGATCGGGTCGTGGCGAGCGCTCTGGTAGGGGGTTTGATAGGGG

CGGTCGTTCTAGCAGGGGCAGGGGATTTGATAGGGGTGGTCGCTCAGGCTCTGGCAGGGG

ATCCGCTCGTGGTGGGCGTGGCGGTACTCCGGTCAGCACTGGGAAGAAAATTACCTTTGG

CGATGACTGAGAT

>Unigene0000974

TTGTAGTTCTGTCTTGTCTTTCTAGAATATTGGTTTATGGTACTTCATGGATTGTTGTGT

TATTTTGTAATTCGGATTTGGCTGCTTAAGGGTTTTATGTTTTTAATGTTTCTGTGCTCT

TTAAAAACAATAGACTTGGGCTCAATAATCCAATTTGTGATTGGTTTATGCTCAATAACA

TAATCAGCTCAAGAATAATATTTTGTGATTGATTTGGGTTTATGAGACATTTTGGATAAT

CATAA

>Unigene0000975

CAAATATGTTTTTAAGCGGTGGATATTTACATTCTTGTTCGTGTCTCCTTGTAAGGTTTT

GTAGTCATCTCAGAGTAATTCTATACTTGTTCGTGTCTCGTGGTATGGTAGTCATCTCAG

AGTAATCCTATACTTGTTCGTGTCTCCCTTGTACGGTATATCTCAGTGTAATCCTATACT

TGTTCGTGCCTCCTTGTATGGTATATCTCAGTATAATCCTGGTATCTATAGAGAGAGGAA

TGTTAGTTCTTGTCCTGAATTGTGTAGCCACATCACTTTAAACTAATCAAATCAAATCAC

CAAGT

>Unigene0000976

AATCTTTGACCTCTTGACTACGATATCTCACCGAACCCCCCTCCAGCGTCACCAGTGAGG

TTCCGTTTCCAGCCACACCCTCCCTGCCGATGGCCGCATCCGTGATCGGCGGAGCAGCTC

CGAGCCCGTCGTCCTGCTGCGCTTCGCACAGAAGCGCAGCAGCTTCCTCTCCTCTCATGG

CTCTCGCTTCCAGGACCCCAGCCTCCCGAGCCTCTGTGTCGGCCCCTCAGTTAGGTCTTC

GCCTCCACTCCTCCTGGAGCGGTCTCAAGCTCGTCGGCGTCCAACCCCGTCGAAATTACC

GACAGAGAGAAAAGAAAGAGAAATGTAGAGGTAGAGTTGTTTATGCTTCTTTGTTTGGAG

TTGGAGCTCCAGAAGCCCTAGTTATTGGAGTGGTGGCTTTACTGGTCTTTGGACCTAAAG

GTCTAGCAGAGGTTGCACGGAACTTGGGGAAGACTCTACGAACTTTCCAGCCTACAATTA

GAGAGCTTCAGGAAGTGTCAAGAGAATTCAAAAGCACCCTTGAACAAGAGATAGGACTCA

ATGATGTTAAATCTTCTGTGAACAACACCTACCGACCCACCTCATTACCAAAGGAAAATC

AGCAAGCTGTTGTCGATCCAAATGGTGTGCCTACCAAGCCATATACCAGTGAAGACCTCT

TGAAAGTTACAGAAGAGCAGCTGGCTGGAGCCAGTATCCAAACAGAGCTATTTGATCCTC

AAGCATTAGATTCAACAACAACAGAAGCCTCAGCTGCAACTCCAGAAGTTAAAACTTCTG

CTGCTTCTACAGAAGGCCCTGTGACACCTGAAGTTGAAGTAAAAACTTCTGCTACTTCTC

CAGAAGGCCCTGCGACACCAGGAGTAAAAACTTCTG

>Unigene0000977

ATGGTGGAATCCTCACCGTCCCGAGAGACACCAAATCCTCCAACTCCGGCCATCGATCTC

TCGCTCGCCCTCGGCAAGTCACCCCCTCCGTCCGGCCACCGCGATACGGTGAAGCTCTTC

CCCTGCCTCTTCTGCAATAAGAAGTTCCTCAAATCCCAGGCCCTCGGCGGCCACCAGAAC

GCCCACAAGCGGGAGCGCAGCGTCGGCGGGCTGAGCTCCCACCTATACTTCTCCCCTTCG

GCTTTCCCAATTACCTCCCACTCCTGCAGGGTGCTGCCCAGCCGCTTCGACACTTTCGGG

GCTGCCCGTTTCAGCGGTGGCAGCCGCACCGACTGCGCCAGCCACTCGTCCGACAGCTGT

GAGGAGATGGTCGATATACTGAACTGGCAGAGAAGCTCCCACCAGCCTTCTCCTTCTTCC

AATGCTTCAGCTGACGAAGATGGGTCGATGCTCGACCTTTCGCTTCGGCTCTGATACGGA

GGGTTATCCTAAAAATGGTCAACTGGGTGGACGGTAAATCTGAGATATTTTTTTTGGCTT

TTTTTTTTACTTTCGATGGATGCTAGTGGTTTTATGTAATCTCTGTACTGTCCCCATTAT

TAAAATTTCACTATTTTTGTCGAGA

>Unigene0000978

ACTCACCCACCTCTTTATAATTTGGGCTCTCAGTAGAATTTCTCAGCAAGTGCATCCTAA

CTCCTTCCTTTACTTCAATGGCTCCAGCCAAGCTTCTGCTGGCCTCTTTCCTCCTGCTGT

TCATTGTCAACCTCGCCCAAACTCACCCGGGGTCCGACACTACGAAGTTGGAGCAAGGGA

CAAGAGGATCTCTTCTAGACTGTGATGCTGCGTGCGGGGTGAGGTGCAGCAAGTCCTCGA

GGCCAAACCTCTGCAACAGGGTTTGTGGGACTTGCTGTGAGAGATGTAGCTGTGTTCCTC

CGGGCACCTACGGTAACTATGATTCATGTCCTTGCTATGGCGCCCTGACTACCCACGGTG

GTCAGCGGAAATGCCCATGAAGCTGAGACGGTGGCAGAGTGCTGAAGTGTGTATGTCTAT

CTACGTATGTATAGAAGATGACTGAGTGTTGATACTGGTATGCATGTATCCATCTATGTA

GAGAAGGTGACCGAGTGTTGATCTGAAAGGGTTTGGCTGTGTTTTTAATTGGTTTAAGTT

TGTGGTCTGAGTGAAGTTGTCCACTAATAATATTGTCCAAAAGAGGAGTGTATATATATA

TATAACAGAAATAATATGATTA

>Unigene0000979

TCCGACACTAAGAAGTTGGAGCAAGGGACAAGACGATCTCTTCTAGACTGCGGTGCGGCA

TGCGGGGTAAGGTGCAGCATGTCCTCGAGGCCAAACCTCTGCATCAGGGTTTGTGAGACT

TGCTGTGGGAGGTGTAGCTGTGTTCCTCCGGGCACTTACGGTAACTATGAGTCGTGTCCT

TGCTATGGCGCCCTGACTACCCACGGTGGTCAGCGGAAGTGCCCTTGACCTGAGAAGGTC

GCAGAGTGCTAAGGTGTGTAGAGGAATCTGAGAAGGTGGCAGTGTGCTATGGTGTGTATG

TGGGTAGAGAAGATGGCTATGTATGTATGTATGAAGGGAGGGTGACTGTGTTGGTACTGG

TCTGAAAGGGTTTGGCTGTGTTTTTAATTGG

>Unigene0000980

TGATATTAATAACACTATAGACCTCCGCCCCGAAGGGGACGAAAAATGGTTTTTAGAGAA

CGAGAAGACGGTTACGCAGTTTTGCCGCAAGCTGGCTGCTGAACGCCCTCTTAAGGATAT

TCGCGATGAGTATAATTACCCCAAAAAGAAAGGTATTAAGGATGAGTGTTCAAGATTGCT

GGAGGCCTCCACTATGAAATCGCGTAGAGGCTTTGCTATTCAGCGTTTGATGAATGCAAT

GCGACAGGCTCATGCTGATGGTTGGTTTATCGTTTTTGACACTCTCACGTTGGCTGACGA

CCGATTAGAGGCGTTTTATGATAATCCCAATGCTTTGCGTGACTATTTTCGTGATATTGG

TCGTATGGTTCTTGCTGCCGAGGGTCGCAAGGCTAATGATTCACACGCCGACTGCTATCA

GTATTTTTGTGTGCCTGAGTATGGTACAGCTAATGGCCGTCTTCATTTCCATGCGGTGCA

CTTTATGCGGACACTTCCTACAGGTAGCGTTGACCCTAATTTTGGTCGTCGGGTACGCAA

TCGCCGCCAGTTAAATAGCTTGCAAAATACGTGGCCTTATGGTTACAGTATGCCCATCGC

AGTTCGCTACACGCAGGACGCTTTTTCACGTTCTGGTTGGTTGTGGCCTGTTGATGCTAA

AGGTGAGCCGCTTAAAGCTACCAGTTATATGGCTGTTGGTTTCTATGTGGCTAAATACGT

TAACAAAAAGTCAGATATGGACCTTGCTGCTAAAGGTCTAGGAGCTAAAGAATGGAACAA

CTCACTAAAAACCAAGCTGTCGCTACTTCCCAAGAAGCTGTTCAGAATCAGAATGAGCCG

CAACTTCGGGATGAAAATGCTCACAATGACAAATCTGTCCACGGAGTGCTTAATCCAACT

TACCAAGCTGGGTTACGACGCGACGCCGTTCAACCAGATATTGAAGCAGAACGCAAAAAG

AGAGATGAGATTGAGGCTGGGAAAAGTTACTGTAGCCGACGTTTTGGCGGCGCAACCTGT

GACGACAAATCTGCTCAAATTTATGCGCGCTTCGATAAAAATGATTGGCGTATCCAACCT

GCAGAGTTTTATCGCTTCCATGACGCAGAAGTTAACACTTTCGGATATTTCTGATGAGTC

GAAAAATTATCTTGATAAAGCAGGAATTACTACTGCTTGTTTACGAATTAAATCGAAGTG

GACTGCTGGCGGAAAATGAGAAAATTCGACCTATCCTTGCGCAGCTCGAGAAGCTCTTAC

TTTGCGACCTTTCGCCATCAACTAACGATTCTGTCAAAAACTGACGCGTTGGATGAGGAG

AAGTGGCTTAATATGCTTGGCACGTTCGTCAAGGACTGGTTTAGATATGAGTCACATTTT

GTTCATGGTAGAGATTCTCTTGTTGACATTTTAAAAGAGCGTGGATTACTATCTGAGTCC

GATGCTGTTCAACCACTAATAGGTAAGAAATCATGAGTCAAGTTACTGAACAATCCGTAC

GTTTCCAGACCGCTTTGGCCTCTATTAAGCTCATTCAGGCTTCTGCCGTTTTGGATTTAA

CCGAAGATGATTTCGATTTTCTGACGAGTAACAAAGTTTGGATTGCTACTGACCGCTCTC

GTGCTCGTCGCTGCGTTGAGGCTTGCGTTTATGGTACGCTGGACTTTGTAGGATACCCTC

GCTTTCCTGCTCCTGTTGAGTTTATTGCTGCCGTCATTGCTTATTATGTTCATCCCGTCA

ACATTCAAACGGCCTGTCTCATCATGGAAGGCGCTGAATTTACGGAAAACATTATTAATG

GCGTCGAGCGTCCGGTTAAAGCCGCTGAATTGTTCGCGTTTACCTTGCGTGTACGCGCAG

GAAACACTGACGTTCTTACTGACGCAGAAGAAAACGTGCGTCAAAAATTACGTGCAGAAG

GAGTGATGTAATGTCTAAAGGTAAAAAACGTTCTGGCGCTCGCCCTGGTCGTCCGCAGCC

GTTGCGAGGTACTAAAGGCAAGCGTAAAGGCGCTCGTCTTTGGTATGTAGGTGGTCAACA

ATTTTAATTGCAGGGGCTTCGGCCCCTTACTTGAGGATAAATTATGTCTAATATTCAAAC

TGGCGCCGAGCGTATGCCGCATGACCTTTCCCATCTTGGCTTCCTTGCTGGTCAGATTGG

TCGTCTTATTACCATTTCAACTACTCCGGTTATCGCTGGCGACTCCTTCGAGATGGACGC

CGTTGGCGCTCTCCGTCTTTCTCCATTGCGTCGTGGCCTTGCTATTGACTCTACTGTAGA

CATTTTTACTTTTTATGTCCCTCATCGTCACGTTTATGGTGAACAGTGGATTAAGTTCAT

GAAGGATGGTGTTAATGCCACTCCTCTCCCGACTGTTAACACTACTGGTTATATTGACCA

TGCCGCTTTTCTTGGCACGATTAACCCTGATACCAATAAAATCCCTAAGCATTTGTTTCA

GGGTTATTTGAATATCTATAACAACTATTTTAAAGCGCCGTGGATGCCTGACCGTACCGA

GGCTAACCCTAATGAGCTTAATCAAGATGATGCTCGTTATGGTTTCCGTTGCTGCCATCT

CAAAAACATTTGGACTGCTCCGCTTCCTCCTGAGACTGAGCTTTCTCGCCAAATGACGAC

TTCTACCACATCTATTGACATTATGGGTCTGCAAGCTGCTTATGCTAATTTGCATACTGA

CCAAGAACGTGATTACTTCATGCAGCGTTACCATGATGTTATTTCTTCATTTGGAGGTAA

AACCTCTTATGACGCTGACAACCGTCCTTTACTTGTCATGCGCTCTAATCTCTGGGCATC

TGGCTATGATGTTGATGGAACTGACCAAACGTCGTTAGGCCAGTTTTCTGGTCGTGTTCA

ACAGACCTATAAACATTCTGTGCCGCGTTTCTTTGTTCCTGAGCATGGCACTATGTTTAC

TCTTGCGCTTGTTCGTTTTCCGCCTACTGCGACTAAAGAGATTCAGTACCTTAACGCTAA

AGGTGCTTTGACTTATACCGATATTGCTGGCGACCCTGTTTTGTATGGCAACTTGCCGCC

GCGTGAAATTTCTATGAAGGATGTTTTCCGTTCTGGTGATTCGTCTAAGAAGTTTAAGAT

TGCTGAGGGTCAGTGGTATCGTTATGCGCCTTCGTATGTTTCTCCTGCTTATCACCTTCT

TGAAGGCTTCCCATTCATTCAGGAACCGCCTTCTGGTGATTTGCAAGAACGCGTACTTAT

TCGCCACCATGATTATGACCAGTGTTTCCAGTCCGTTCAGTTGTTGCAGTGGAATAGTCA

GGTTAAATTTAATGTGACCGTTTATCGCAATCTGCCGACCACTCGCGATTCAATCATGAC

TTCGTGATAAAAGATTGAGTGTGAGGTTATAACGCCGAAGCGGTAAAAATTTTAATTTTT

GCCGCTGAGGGGTTGACCAAGCGAAGCGCGGTAGGTTTTCTGCTTAGGAGTTTAATCATG

TTTCAGACTTTTATTTCTCGCCATAATTCAAACTTTTTTTCTGATAAGCTGGTTCTCACT

TCTGTTACTCCAGCTTCTTCGGCACCTGTTTTACAGACACCTAAAGCTACATCGTCAACG

TTATATTTTGATAGTTTGACGGTTAATGCTGGTAATGGTGGTTTTCTTCATTGCATTCAG

ATGGATACATCTGTCAACGCCGCTAATCAGGTTGTTTCTGTTGGTGCTGATATTGCTTTT

GATGCCGACCCTAAATTTTTTGCCTGTTTGGTTCGCTTTGAGTCTTCTTCGGTTCCGACT

ACCCTCCCGACTGCCTATGATGTTTATCCTTTGGATGGTCGCCATGATGGTGGTTATTAT

ACCGTCAAGGACTGTGTGACTATTGACGTCCTTCCCCGTACGCCGGGCAATAATGTTTAT

GTTGGTTTCATGGTTTGGTCTAACTTTACCGCTACTAAATGCCGCGGATTGGTTTCGCTG

AATCAGGTTATTAAAGAGATTATTTGTCTCCAGCCACTTAAGTGAGGTGATTTATGTTTG

GTGCTATTGCTGGCGGTATTGCTTCTGCTCTTGCTGGTGGCGCCATGTCTAAATTGTTTG

GAGGCGGTCAAAAAGCCGCCTCCGGTGGCATTCAAGGTGATGTGCTTGCTACCGATAACA

ATACTGTAGGCATGGGTGATGCTGGTATTAAATCTGCCATTCAAGGCTCTAATGTTCCTA

ACCCTGATGAGGCCGTCCCTAGTTTTGTTTCTGGTGCTATGGCTAAAGCTGGTAAAGGAC

TTCTTGAAGGTACGTTGCAGGCTGGCACTTCTGCCGTTTCTGATAAGTTGCTTGATTTGG

TTGGACTTGGTGGCAAGTCTGCCGCTGATAAAGGAAAGGATACTCGTGATTATCTTGCTG

CTGCATTTCCTGAGCTTAATGCTTGGGAGCGTGCTGGTGCTGATGCTTCCTCTGCTGGTA

TGGTTGACGCCGGATTTGAGAATCAAAAAGAGCTTACTAAAATGCAACTGGACAATCAGA

AAGAGATTGCCGAGATGCAAAATGAGACTCAAAAAGAGATTGCTGGCATTCAGTCGGCGA

CTTCACGCCAGAATACGAAAGACCAGGTATATGCACAAAATGAGATGCTTGCTTATCAAC

AGAAGGAGTCTACTGCTCGCGTTGCGTCTATTATGGAAAACACCAATCTTTCCAAGCAAC

AGCAGGTTTCCGAGATTATGCGCCAAATGCTTACTCAAGCTCAAACGGCTGGTCAGTATT

TTACCAATGACCAAATCAAAGAAATGACTCGCAAGGTTAGTGCTGAGGTTGACTTAGTTC

ATCAGCAAACGCAGAATCAGCGGTATGGCTCTTCTCATATTGGCGCTACTGCAAAGGATA

TTTCTAATGTCGTCACTGATGCTGCTTCTGGTGTGGTTGATATTTTTCATGGTATTGATA

AAGCTGTTGCCGATACTTGGAACAATTTCTGGAAAGACGGTAAAGCTGATGGTATTGGCT

CTAATTTGTCTAGGAAATAACCGTCAGGATTGACACCCTCCCAATTGTATGTTTTCATGC

CTCCAAATCTTGGAGGCTTTTTTATGGTTCGTTCTTATTACCCTTCTGAATGTCACGCTG

ATTATTTTGACTTTGAGCGTATCGAGGCTCTTAAACCTGCTATTGAGGCTTGTGGCATTT

CTACTCTTTCTCAATCCCCAATGCTTGGCTTCCATAAGCAGATGGATAACCGCATCAAGC

TCTTGGAAGAGATTCTGTCTTTTCGTATGCAGGGCGTTGAGTTCGATAATGGTGATATGT

ATGTTGACGGCCATAAGGCTGCTTCTGACGTTCGTGATGAGTTTGTATCTGTTACTGAGA

AGTTAATGGATGAATTGGCACAATGCTACAATGTGCTCCCCCAACTTGATATTAATAACA

CTATAGACCACCGCCCCGAAGGGGACGAAAAATGGTTTTTAGAGAACGAGAAGACGGTTA

CGCAGTTTTGCCGCAAGCTGGCTGCTGAACGCCCTCTTAAGGATATTCGCGATGAGTATA

ATTACCCCAAAAAGAAAGGTATTAAGGATGAGTGTTCAAGATTGCTGGAGGCCTCCACTA

TGAAATCGCGTAGAGGCTTTGCTATTCAGCGTTT

>Unigene0000981

CCCGCCGCCCCCAAACCCTAGCAATCAGCACCAGTAGGCGATGTCGCCGCCGGAGATCGG

GTCACCCTCGGCCGACAAGGTCGTCGCCCCGGTCGATTCCGAGCCGGCCGGCGAGATCAC

CGCCGAGCTAAAGTCCGGCTCGCTGCGGAAGCGAGGGAGGTCTGGCAAGGCCTCTGTTGC

GGAGGCTGCAAACGCCCCGCCGACTCGATCGTCGTCGAGGAAGCGGGGCAAGCCAGCGAC

AGAGGACTCCCCCGCTTGCCCTTCTCCTGCGGTCCCACCCCAGCGATCGGCGCCGAAGAA

GGGCACGAAGGGACAAATTGCCCAGAAGACGGCGGTGACCTCTTCGAGATCATCGAAGGG

GAAGGGGGTGATGGTTGAAGAAGATCCTGAGGATGATCCGATGGTTGAGGATGAGGAAGA

AGAGGGTGGAAATGCTCTG

>Unigene0000982

GAGGGTGGAAATGCTCTGCTGTCTGGAGAATCCGCAGCTCCCACTAAGAGGAAGGCGGCG

GCTCGGCTGAATGGTGCTGGGGAATCTGATGCATTGTTTGTTGGTGATCCTATACCGGAG

GAGGAAGCTGAGAAGCGCTGGCCTGATCGGTATCAGAACAAAGAAGCTGGGAATTCAAGA

TCAAGGAGGCCTGTTTCTGTCGATCCTGATGATGTGGGTGCGAAGCGGCACTACAGGCAA

GCAATGGTTGATGGTGTCTTGTACAACCTTTATGATGATGTTTATGTAAAGGCTCTTGAG

GGAGAAGATGACTATATTGGTCGCATTGTTGAGCTCTTTGAGGGAAGCGATTCTCAATTT

CATTTTACAGCACAGTGGTTTTATAGAACTGTAGATACAATTGTTCAGAAGACTTCTAAA

GACCTTGAGCCACGCAGGGTTTTTGTGTCAGAAGATAAGAATGTAAATGACCTAGATTGT

ATAGTGTCAAAGTTAAATATTGTGAAAGTCGAGCCAAATATTGATTTGGCTGCAAAAATG

AAGGCACTCCCTAAATGTGACCTTTATTTTGATATGTCTTACTCTTTGGCATATACGTCC

TTTGCAAACTTGCCCCCTGGAAATGATTATTCAAGTGGTGAGGCTTTACCATCTTTGTAC

CCTGAGGTTTCAACTTCTCTGTTGAACTCAACTGCGTCCTATACTACCCCCAAGGCTGAG

TTTACACTGCTTGATCTTTATTCTGGCTGTGGGGCCATGTCCACTGGTCTCTGCCTTGGT

GCAAGGATTTCAAATATAAACCT

>Unigene0000983

TTTTTTTTTTTTTTTTGAGAGTTGAGGCTTTTTGAGGTCTCTAATGCTCCAATATAAAAT

TAGTAGAATATGACTACTCGCAGGTGAATATTGACCTTTGATCTCTTGTAGAGATGAGTG

TGATTGCTTCTTTTTTTAATGGAGCAAACATGGTAAGTGACCTACCTCTTCTAAGAGTAG

AACAAACAGGCAAAACTTGAGGACGTGAGCCCGATGTGGTTGGTGGGGTAGAAGTTGGCG

GGGTAGAAGTGTTGCAATTGTTAGTCTGCCGAGTACTAGGTCGGATCTTGTGGGCAAAAA

TTTAATCTTATGTTATTTTAGATTTAATTGTGTGGTCACTTTTTATACGAAAACATAGTT

TTACACTTGTTAGCGTGGTGGTTGATGTTTTTGTCGTGGTATTAAATTTAATCCTCTACT

TCTAGATTTAATTGTGCGGTCTCTTTTATGTAATATTTGTTATAGGCTTTGAATTATGTG

TGTCTAGAGCAAGTGATAATGTGATATAGACCTTGACATTACTGATCATGCCACAGAGCA

GATGGCCCTAGACCACGTGGGTGGAGGTATGAAACCACAGAAACATTTATGTCCTAATGA

AACTGCCTGACTTGCTCAGTGAATCTGAGAACAATAGTACCATTTTTCGGTAAAATTCGT

AGTCTCAAAACCCGAACCATTTGGCCATGAGGTGCCCAACTGAACTAGCGTTATCAAGCA

TGAAACATTTTCCAAACATCTATGATGTTTAATTATAGCTTGACAACGAACTGTTTATAT

TTTTGTATTCAATTATGTGTATGTAAGCATACTTTTTTTATTTTCCGTTGGGTTTCAGGG

>Unigene0000984

TTTTTTTTTTTCTGATGAAATTATTTATTGGGGTCCTGTCCTGCTTGACGAAGTCAAATA

AACTCCTTACAGGGAAAAAGAAGAAGACTTAAAGTAATATTTGTATGTAAAAACAAGGGT

GTTATTCACACTTACGACCACTATCGATGCAATAAACTAGTGAGATGATGAAATTGTGGA

TGACAGTGATTGAGCACTGCCTGAGAGATGTAATGACAACTCCAAAGAATCATTTCATGT

CTGGTATATTGATTTGGAAGCTCTCTACTAGTTGAGGAGGCTAATGAAAATGTTGAAGAG

AAAGAAGCAAGATATTCATATGGTCATTATAAATATCTAGACATTGCTGAGCGCATTGAC

TAAGAACCCCCCTCCACACACACACAAATGAAAGCTGCATGGTCTTCAGTGGCCTCAGCA

CAGTTTGAAAATAAAACCCCAGCAGGCTGCCAAGTAAATCAGGCTTGCTGAAGCAGCCAG

ATGAATGGGTCATGTTGGCATCGGATCCATATTGGCCGTGTCTTGTTCTCTACAACTGCA

ATTTAGATCAGACCCTCCACATTCAAACCC

>Unigene0000985

GTGACATTTATATCTTTGACATGGGGCACATGAGTTCTATTGTCTGGGCGGAGATGGAGA

AAATGGGTGACATTTATATCTTTGACATGAGGCACATGAGTTCCCTTGTTATGCATGAAC

TGTCGGGGCATAATTTGGATGAATCCCCGTGATATATGTTGCTTGTAGATGTTAAACCTA

ATAGGGATAATTAATATTAATCTAAAATCTGATGTGGCTGAAGGGTTCAAAATTAATAGG

ACAAACGATGTAAATCATATAATAATAGGCTCTGTGTGAACCATGTTGCATGAGTGGGCA

TGCTTTTTTTTTGAGAGTTGAGGCTT

>Unigene0000986

GAATGGGAGAAGTACAATTCTTGTAACCGACATCAATGAGATAGATTTAATACTTGATTG

GTTTAATTTGGTTTAGCAAAATATTAATTTTCTTAAACATCTTACCATGTAGCACATATC

TGTTGGTTTGTTTATATATAAAAAACTGGCTAATATAGTCTTCAATATATTAAACAATAT

CTCTCTAGTGAAAATGAAAGCGCTTGAAACTGGAAGCGGTTATGAGTTAAGAGAAATGAG

CCAACGAGGATGCCAAGGAGTTAGTAATAGACACCAAGGTAAATTTGACACTAATGGGGG

AGGGTAGTTTTTTTCTGTGACGAAATTATCTATTAGGGATCTTGTCTTGCTTGACGAAGT

TAAATAAAATCCTTACGGGAAAAAAGAATACTAACTGATATTAGTATGTAAAAACAAGGG

TGTTATTCAAACTTATGAGCTAATTTCTCTTAATTTCTCAGAGCCCCGAAGCTAGAGATT

TATAGAATCTGTGCAATGAGCTAATGGGGTTGATGTGGACAGATCTAGGGAACTGTAACA

AGAATTTGCTAATGTTGGAGCAAAGGATGTCAAGAAAGCAAAATTGTAAATGTTGGCTGG

ACAGATTATACTGAAGGCTGTTTGCTGGAGCATTTGGAAGGAAGTGAAGAAATTTTTCTT

TACTCTGGCTCAGATTTTCATAGAATTGTTCTTGAAGTCTTTTCTTTTAATTGTTTGTGG

GGGTAAAAAAGCAAGGAGTTTCAAGGAATTAGGCTGGGGGATGTTATAGCAAGATGTGAT

TATGTGATTCATTGATAAGGTAACAGATTAACCGTTGCTCTC

>Unigene0000987

CTGAATGATTTTGATGTTCTACATATTTCCAGCACGTTGTGCTCTACTTGATCTCAAGAT

TTGAGGGTTTCTGACCCGGAAAGAGTAAGAGAGTCCCTTCCTTCACCTCTTTGGGGTCTA

AAGCGGTGGATAAATTATTGAGAATAACTGGGAAAAGAGGTTGACATGATGAGTTGGGTA

CTAGCTACCACTAAAGGCTTGGCAACAAAAACAAAATTCGAAGTTTTCGCAACAAAAACA

TATTTTGAAGCATTGATGAATTTTAAGTATATAAACTCAAGGGAGATTAGTTTTGTTTTG

GATATTTGTTTGGTGAAACAAAGTACCAGCAAGGGTGAACTTGTTCGCATGGGAAGTGAT

GAGAAATGGGCCAATGATCTTATCAGAGGTTTTTCAAACAAAGTTTCTTGCAAGGTGTGT

TGTATCTCATGTGTAACAATGGAGAAGATTCTGCATTTCACTTACTTTCTCAGTGTCATG

GAGCTAGAGATTTATAGAATCTGTTCAATGAGCTAATGGGGTTGAGATGGATGGATCGAG

GGAACAATATCGAGAATTTGCTGATGTTGGAGCAAAGGATGTCAAGAAAGCAAAATTGTA

ATGTTGGCTGGACAGATTATACCTAAGGCCATTTGCTGGAGCATTTGGAAGGAA

>Unigene0000988

CCATAAACAATTACATATCTCTGTTCTCATTTTGCATCGACTGCGATTCACCGTTCAATC

CAGAAGAATCCTCTTGAATTCTCTTCCCTAATCTCTCAATCCCTAATTTTACCGCATCAA

TCAGCTTCTGAAAATCTTACCCCTCAATTCGCCCCCATCTCTCAAACCCTAATACACCGG

ATTCCTCGATGGAATCGAAGGCGATCCCAGCTCAGAATCTCGATCCGCCGTCCTCCGCCG

CCAAGGTCCTCAACGCCGCCTCTCAGTTCAAGCGATGGGGCCGAAAACACCCCTTCGTCC

GGTACGGATTTCCCATGATATCCCTCACCGTCCTCGGCTCCATCGGCCTCGCCCATCTCA

TTCAAGGCAGTAAAGAGGTGACGAAGGAGAAGGATGATTTGGAGTGGGACATCATCGAAT

CGACGAAGGCACTGACGAGAACCGGGCCGACGGATGCTTACAAGCCAAAGAAGTTCTCCT

TGGATGAGGAACTTAAGGCTTTGCAAGAGAGGGTCAACATCTACAACTACGAGTACAAGA

AGATTCCCAGGCTGAATGAAAATAAGTCAGAGAAAAAATAGACTAGTCAATGTCGAAGAC

ACTATGCCGTGGATGAACACAGATTTTGAAACAAATTTTGTTGCTACAGCTCGCCTGCAA

GAAGAACCCAGGATGCCATTTTCCTCTACTTTTGCCTGTATAAAGAGATTGGGGAAATTT

GCCTAGGCACTTTGTTTTTGTCAGGAAAGAACGTAGCAAAAGTAAAGCTTGTTAAACCCT

GTGAACGTATCAGATAAATATTAGGAGTTGTGTGTGATATTATAATCTCTTTTCAGAGAT

TTTCTTCTACTCAATCGTGGGTTCTCTGTGAATGGGTCTTTTGCCAGTCTTATGACTTTA

GATTACAAGTTTATATCAGTGGGCAGGGAACAATGTGAAGGATGTGTGAAAAAGCATACG

AAGAATTATAGGCATGTGCAACAATGTGAAGAAGTATGC

>Unigene0000989

GTCGTCGCACCTCCCTTCACCAGCCTCAACGGCGGCTGCGAATCCCGATCGGCGGTGATG

TTGGCGGTGAATGCACTCGCACTAGCGCGGATGGCGATCTCAGGCGGCGGCGCTGGTGGC

GTCGGCGCTCTGGCGGGAATTGAGGGGGCCGGTGATATTGCCCTAGGCTCGTTCTGGTGG

TACATATACGCCGGAGTCTCGGCCTTTCTTGTTCTCTTTGCAGGTATCATGTCGGGGTTG

ACGCTAGGGTTGATGTCGCTCGGGCTTGTCGAGCTCGAGATCTTGCAGCGGAGTGGGACT

GATAATGAGAAGAAACAAGCAGCTATTATTCTTCCCGTTGTTCAAAAGCAGCACCAACTT

CTTGTGACTTTGCTGTTGTGTAATGCTGTTGCTATGGAGGCTCTTCCTATATATCTGGAT

AAGATATTCCATCCTGTTGTCGCTGTTGTCTTATCTGTAACCTTTGTTCTAGCTTTTGGA

GAGGTTATTCCGCAAGCAATATGTACCAGATATGGATTAGCTGTCGGCGCTAATTTTGTG

TGGCTTGTGCGCATATTAATGATCATCTGCTATCCGATTGCTTATCCTATCGGGAAGATT

CTGGATTGGGCACTTGGACATAATGAATCTGCTCTTTTCAGACGTGCTCAGTTGAAAGCT

CTTGTCTCGATTCACAGCAAAGAGGCTGGTAAAGGTGGGGAGCTAACTCATGATGAGACT

ACAATCATAAGTGGTGCACTTGATTTGACTGAAAAGACTGCCAAGGAGGCCATGACACCC

ATCGAATCGACATTCTCTTTAGATGTGAATTCAAAATTAGATTGGGAAGCAATTGGGAAG

ATTCTTTCCCGAGGCCATAGTCGTGTCCCTGTTTACTCTGGAAATGAAAAGAACATCATT

GGCCTTCTACTGGTAAAAAGTCTTCTCACAGTACGTGCCGAGACTGAGACGCCTGTTAGC

GCTGTCTCCATCCGGCGAATTCCAAGGGTTCCGGCAGATATGCCTCTGTATGACATATTG

AATGAGTTCCAAAAGGGAAGCAGTCATATGGCTGCTGTTGTGAAGTCTAAACCGAAACCC

AAAGGTATTTCATTTGCTGATGGTGGTAAGGGTGAAGAGCGCAAAGAAACTAGTGGAGAG

TCCTCCAAGTTGAAAGCTCCAATGTTATCCATAGTTGATGAAAAATCAGGAAGTGTTGTC

ATTGATGTGGATGCGTTGCAAAATAAAGAGGTTAATGGAAACAAATCAGATTCTATGAAA

AAATATGATATTGGAGCTGAGGGTAATGGAAGCACATCAGATTCTATGCAAAAATATGAT

GGTGGAGCCAATGCACCGATTCATACGCCAGCGGATATAGAGGATGGGGAGGTCATCGGT

ATCATCACTCTCGAGGATGTGTTTGAAGAACTGCTGCAGGAGGAGATAGTCGATGAGACG

GATGAGTACGTGGATGTGCACAAACGAATACGTGTTGCTGCTGCCGCGGCTGCTTCCTCA

GTTGCACGAGCTCCATCCCTTAGGAGGCTGACTGGGCAAAAGGCAGCAGGGGGGCCTCAA

AGTAGGCCAGGTTCATCCAGGGTCCTGAAGAAACCCAGTGAAGGGGATACGAATACAGCG

AGAACCCAGGGCGAGTCAGATGCTGCACCGAAGAACCAGGGAACTCTTCAGGAACCTCTT

CTAGGAAACAAGAAATAGAGCAAGATAGATGAAGGGATAATCCTTTGAGGATAATTAAGC

TATGTGGATATGTCCTCATTGGATCGGGGTTTTGTATATGTACTAGCCGAATATGGAGAC

CTGTTTCTGTTTCTTAAAGCTGTGGTAGATGTCTGCTGTACTGTGACTTGTGTGGTGCTT

ATAAGTATATTAAGATAGATATATGATATATATACTTGGTAAAATAAGCAGGTGAAGAAG

TGAGATGAAGATAAGATTAATGTGGATGATCTGGTCGATAGGGGTTTATGTTCTGGATTG

TACTTTTTCAGAGTAGGTCGAGCTCTTT

>Unigene0000990

TGGCCTTCACCGCCGCCGCCGCTTCGTCTCCGGCAATGGGCGTCGTCTTGATCGCACTCA

TGGCCTCGGTGGCTCTTTCCGATGGGGCGGTTTTCAAGGTTGGCGACACCGTCGGGTGGA

CCATCCTCGGCAGCCCCAACTACACCGCCTGGGCGGCCGGCAAAACCTTCACCGTCGGCG

ACACCGTCGTGTTCGAATACAACAAGGCATTCCATAATGTCATGGAAGTGAATAAGACGG

ACTACAAGGGATGCAACACAGCTTCGCCCCGGGTTACCTACACAACAGGCAACGACACGA

TCAAACTTAAGAGGAAGGGCCATCACTACTTCATTTGTGGCATTCCCGGTCATTGCAGCC

TCGGACAGAAGGTTGACATCAGAATTCCCAAGCTCGTGTCATCTTCGGCTTCATCTCCCT

TGCCATCGACGTCCCCATCTACTTCTCCTTCGCCCGTCCACCATGGAAGCAATGGCTCAC

GCAGTCGCAAAGGTGTTGTTTGGACATTTGCTGTGGTTGGTTTTACTGCATTTGCTGGTC

TGATAATGGCATAGGTCTCGGAGTGGCACAAGTTTATTTAGGTTTGTAGGATTATTTATG

AAAGTTTTTCTGAATGTTGGCCAAGCAATTGGTGAATCTCCTTTCTTTTTTATTTGAATG

GTTAGTTAAGGTGGTTAAGTTTTTATATTGTTGTGGGGTTAAATTCCTCTGTTTTGGGAT

GTTGGTGGGATGTTGGTGAAGAAATCAGTGAATCTTTTGTTTTGTTATTTGAATTGTTAG

TTAA

>Unigene0000991

AGCCACAAGCATCCGATATCCTCTCCTCTCACTCTTCACTGACCAGAGCCCTCGTGAAAT

CGAAACCCTCACTCCCCATTCTCGAATGGCGAAAACCTAACCCTAAATACCAACAATCCA

ACTCCCCGCCATGGCTTCGATCTCCACCTGTTTAGCATCAGCACGGCCCCGCCTCATCTC

CCTCAACCCCCACCCCAAACCCTCGCTTCTCACCCGATCGCTCGTCCCCCCATCGCTCCA

TCTCCGCCTCAAATCCCCGTCCTACAGGGTCTCAGCGTCGCCTCGGGTGATCAGAGCGAG

CGTGGATGCTCCGGCGGGGTTCAAGCCCGGCGGCGTGGAGACCGACAAGCTGCCGGCCAA

CGTGCGGAACCGGGCCATGGAGGCAGTTGATTCGTACGGCGGGAGGGTGACGATTGGAGA

CGTGGCGAGTAAGGCCGGGCTCAAGACGAACGAAGCGGAGCGAGCGCTGCAGGCGATCGC

CGCGGATACCGGCGGATTCTTGGAGGTTTCAGATGAAGGCGATGTGCTTTATGTTTTTCC

GAAGGATTATCGCACCAAGCTTGCCGCCAGATCTTTTCGAATGAAAATTGAACCTGTGCT

TGACAAAGTGAAGGTGGCTTCTGAGTATCTAATCAGGGTATCCTTTGGAACAGCACTAGT

TGTCTCAATTCTTCTCGTGTACACTGCAATTCTTGCACTTCTTTCAAGTAAAAGTGACGA

CGACGACCGTGGGAGACGCAGAGGAAGATCATTTTCCCCCGATTTAACGTTTTTCTTCCG

TCCATCTGATATATTTTGGTATTGGGATCCGTTCTACTACAGGAAGCGTCAAATGCAAAC

TAATGATGGAATGAACTTTGTTGAATCTGTTTTCTCATTTGTATTTGGAGACGGGGATCC

TAATCAAGGACTTGAAGAAGAAAGGTGGAAGCTGATAGGGCAGTATATTGCCTCAAATGG

TGGTGTTGTTACCGCAGAAGAGCTTGCTCCATATCTTGACGTATTGCCTACGGAACAGGC

TACGGATGATGAATCATATATTTTACCCGTCCTGCTACGGTTTGGTGGGCATCCAGAAAT

TGATGATGAGGGAAACATTTTGTATCAGTTCCCATCATTGCAACGGACTGCATCTAAGAG

GGTTGGTGGAAAGATGAAAAACTGGGGTGATTGGGTTGGTGGTGTTCAGCAATACCTTGA

AGAGAAGAAGTGGCTATTCAGCAAAGCCGAGACACCCCAGATGGCAATGGTCGTAGGTTT

GGGAGCACTCAATCTTTGTGGAGTTATCATTCTGGGTATCATGTCAAAGACTGCGGCAGT

TACATCCGGGGGCTTCGTTTCAGTTGTTTTAGACATTTTACCACTACTCCAGATATATGC

TGCAGCCTTCTTTGCAATACCTTTGTTTCGATATTTCCTTTTGCGTAAGACAAATGCAGA

TATCGATAGAAGGAATCTAGCAAGACAACAACGAGCTCGATCACTTGAAAGGGCAGATTC

ATCTCTCAGAAGCAAGCTGCTCAGCGCTCGAGACATGGCGCGGCGAATGGTGATCGGCTC

GGACAAAATAGTGTACAGTACTGAAAAGGATATCGCCGATCAAGACTACGAGGCTACCGA

TTGGGATAAGAGATTCAGAGAACTTGAGAGATCAGACTAATGGAGGTCGGATGCATGATC

CCAGCCTCGTACCCCTACTGATGCTTCTCCAAATCGTTATTCAAACCGTGTCCTCATTGG

GACGTTCGTGAAGCTTTCAAGCTAACAATATATATTGGATGTACCACCCTCGTTGGTGAT

TACTAGAGCCAGCTTTTCAGGTGCAGAAGGGCTCTCCAAGTTCCTATACCCTAGGATTTT

TTTTTCTTTTCCTTTCTCCTACAAAATATGTAATATACTGATTTTATGTTTTTATTCTGT

ATACATGGATAGAGGGTTATACAAGCATACCTAGGTATATAAGGTAGTTCAATGAAGTAC

CCACATCCTCCTTATATATAATGTTAGTATACGCTAATCTCTTATATGTCTCTTGATTAA

ATATGCCATCTTTGGCTTGGAGAGCATACTTCACCTTTGGTTTCTCTGCTATGAATATAG

TCTCCATATATGTAAAACAATATTTGACCGTAA

>Unigene0000992

CTATACGGCTGCCGGCCACGATTTTGGAGGTGCTATTATCAGCAGCACCGTTCCAGCTCT

GACGAATCCTGCTCAGTTTCATCTCCTCGGAAGCTCGAGCCGACAGGGGGGCGTCTCCCA

TGGAGGACCTGAAGGCCGCCCTGAGCGAGCACGTCGATCTCGTCTCCGATCTCTTCCAGA

ATTTCTCGTCGGAGCTCCGCACCGGCTTCGCCCCCGCCTACCAGAACCTCCTCGGCTTCT

TCCACGCCATCGACTGGAATGAGTCGTGGCTGATATGCTTGATAGTATGCCACGTTACTT

TGCTGTTAACAGTTGTCATCTCGAGGAAAAACGTTAACTTTCAACTATGCCTGTCCGTTT

TGACTTTTGCTGGTGTATTTCTTGCTGAGAGGCTAAATCGTTTTCTGGGGGCACACTGGA

AGAGCTTTGCTGGACAGAACTACTTTGATTCGAATGGCATATTTATCTCAGTCCTTTGGT

CTGGCCCCCTTCTTCTCATCACTATGGTGATTGTGGTCAACATTCTCTTCACACTGTGCC

AGCTAATCATAAAATGGAAAAGAGCAGAACTCAGACACCGTGCTAGACTTTCTCGTGGCA

AGCAGGAGTAGTCCCCCCTTCATGATTAATAGGTGTTCGATAGCTATTAGCATGTAGAAG

TATTGACTGTTAAGTTTTGGTGCCTAGTTTTCTCAAGTCTTTGTTAGCCGAATTGAAGGT

CTGGGGCATATTAATTTCGTCATACTTCGTATGGTCTGATTGTTATCAGATATGGGGTTT

ATTGTTTGGAGTAAGGGTGTCCCAGAAAAGGTCCTTTCAGCGCTGAAATAGATACATATA

TGAATGACCCTATGTTATGTAGTGGATATACGGTTGTCAACTAATTGTGTTGGTTTTTTG

ATTTTTGTCCTCAATATAATGT

>Unigene0000993

CAATCGATCGATGAGAACTGCAGTGCATTGGAATGAAATATCTCTTTGTGATTGTTGTTG

TTGTTCAAACGTATGGCTTCTAGATCCGGTGGCGGATCTAGGGTTTTACCGCCGGACACG

AGAAATCTCGACCGACGAACATAAGTTCTTTCTTTCTTGCAATTACTTTTCGGATTTCAA

GCAGCGTGGATCATTTGTGTTATCTTTTCATATTCGGAATAATCTGCACGAACACAACAC

AAACATGTTGAGATGTAAGCTGGTGGGAGTTGGGGCCTGTTGGTTAGGAATTGTATGAGG

ATCTTATCATTAATGCCTATGGAGCT

>Unigene0000994

CGCCGAAAGAACCGGATTCATCACCCACAAATCCCGTATTCTCTCCTCCATCCACCCCCA

AATCCGAAATCCCAATCCCCAATCCTTCCCTGACCCCTCGTCCTGATCCAATCTCCGATC

TCCGCCCCAATGGAGGCCGCCGCGCCGGCAAGGGTGCTCTCGCGGCAGCTCTCCACTGGT

TCCCTCCGCCGCAGCAGCGGCCGCCTCAGCTTCCGCCGCCAGTCCTCCCTAGACCCCCGC

CCTAGCCGCTTCGTCTTCGGCCGCCAGTCGTCGCTCGACCCCAACCGCCAGAGCCCGATT

GTTGAGGAGCTGTCGGTCCCGGATAATCTGGACTCTACGATGCAGCTTCTCTTCATGGCG

TCGCAGGGGGATCTGGATGGGGTCGAGGATATCCTTGATGATGGGGTTGACGTAAATAGT

ATTGATCTCGATGGGAGGACGGCGCTGCACATTGCGGCGTGCGAGGGACATGTGGATGTT

GTCAAGTTACTGCTCAGCTGGAAGGCGAATGTTGATGCGAGGGATCGGTGGGGAAGCACG

GCCGCTGCTGATGCCAAATACTATGGCAATCATGACGTTTACAATATCTTGAAAGCTCGA

GGAGCCAAAATTCCGAAAATCAAGAAGACTCCTATGGCTGTCTCTAATCCCCACGATGTC

CCTGAGTACGAGCTGAACCCAGGGGAGCTTCAGCTTCGGCGAGGCGATGAGGTCACAAAG

GGAACTTATCAAGTAGCTAAGTGGAATGGGACAAAGGTTGCTGTGAAAATACTAGATAAA

GAAAGCTATTCAGATCCCGATAGCATAAATGCATTCAAGAATGAGCTCACTATATTGCAG

AAGGTTCGACATCCTAATGTCGTTCAATTTGTCGGTGCTGTTACCCAGAATTTTCCCATG

ATGATTGTTTCAGAATATCATCCAAAAGGTGATCTAGAAAGTTATCTACAGAAGAAAGGA

AGACTGCAGACACATAAAGCTATTAGATTTGCTCTTGATATTGCGAGGGGAATGAACTAT

CTTCACGAGTGTAAACCCGACCCAATTATCCACTGTGATTTAAGGCCAAAGTGCCAGAAA

TATTTTGCTGGATGATGGTGGTCACTTGAAGGTTGTGGGATTTGGTTTGACGAAGTTATC

AAAAGTATCTCCAGATAAAGTCAAGTTAGCACACCCGGAAGCTCATATTGCCAATTCAAG

CATGTATATGGCACCTGAGGTTTACAAAAACGAAAGTTTTGACAAACTTGTGGATGTGTT

CTCTTTTGGTCTCATCCTTTATGAGATGATTGAAGTTTCACCATCTTTTAATCCAAGAGC

TGCTGAAGATGTTGCCAAAATGATTTGCTTGGAAGGAAGGAGACCTCCACTAAAGAACAA

ATCAAAAGGCCATTCTTGTGACTTAAAAGAGTTGATTGAAGAATGTTGGGATCCAAATCC

TGAAGTGAGGCCACCATTTTCAGAAATAATTTTGAGGTTGAATAAATTGTATGTGAATTA

TGCAAAGCAAGGTCGTTGGAAGGACACGTTTAAGCTTCCCTGGAAATAACATTTAAATGA

GTTCGCACTTGTGATGCTGTGTTTCATGTAAAAGCTGATCTCTGTACAACATCCAACATG

TTTCACATTGCTTTGTAATAGATATTGTTTACAACCTCTCTTCATGTTTGCCAACTTCAT

CTTATTCTTTGCAGGAAATAAGATGGAGAAGTCAGTTCTGGTAGAATCAACCTTGATGTT

CTGTTGCTTGTAAGAAATAAGTTTGACGTTCAGTGCTAATAATGTCAATACCATTTCTTG

TCAACAATCTGGAAGGTTTTCTTCACATAGGGAAGCTGTGTATATTAGGAGACATTTATC

TTATGTTTCCTTTTTGCTCAATTTATACCTTTTCGATCATTCTAAATCTTGTTTTCGAGT

GGGAAAAATTGTTAAGCCACATAGTAGTCAAGTAGGTTCAGACAGCTACAGAGTTGGGCT

AGGCAATGTTCTGTGCTAGCTCAGTTACATGCTGCCTGATATTGGGTCTAGTCTGTAGCC

ATAGCATTACTCTTTTCATTGATACGTTTCACCTAATCAGACTATTTTCTTGCTTGTTTA

ATGA

>Unigene0000995

TGAAGGAAGTGGGGCATGTGTTTCTTCTATTGCAATCCATGTTGGACGGAGATGCTTTGT

TGGGTTACAATCTCCTTCGATTGCAATCGTTTAGTATGTTATTTTTGTGCCTTCCGTTTC

TTGAAGTCTTGGAATTCAATTCATTCCTTGCTTGTTGTGTTGGTTTCTGGGATGTTTTTG

CTTTACTCATCTTTCTCCTTTTGCATTGAAGTTCTTTAACACTGCTTTTGTCTTTGGATT

TTGATTTCCTGGTGGTGATCGACCTTTCGGGCTCAAATGGAAAGTGGGTTAGGAATACCT

GGGGATGGAAGATGTTTGTTCAGATCTGTTGCTCATGGTGCCTGCTTAAGAGCAGGAAAA

CCATCTCCGAGTGAGAGCCTTCAGAAACAATTAGCAGATGAACTGAGAATAAAAGTTGCA

GATGAATTTGTCAGGAGGCGGTCCGACACTGAATGGTTTCTTGAAGGCGATTTTGATACA

TACATTACACAGATCAGGCAGCCTCATATTTGGGGTGGAGAGCCTGAACTGCTTATGTGC

TCACATGTTCTTCGGATGCCGATCACAGTTCATATGCATACAACAGACTCCAGCAGTCTG

AAAATTATAGCCGAATATGGACAGGAATATGGCAAGGACGATCCGATTCGAGTGCTATAT

CATGGATATGGACATTATGATGCATTACAAATGCCTCGCACGAGGTCACAGTCTAAATTG

TACTGTACTAGATAAGAAGCAGATGTATGCAGGCCAGGTTACTTATTTTCGCCTTTGCTT

GTAAGTAGCTATCAGCTACCTGAAGAATGATGGTTTCATTTAATGTGTATTCAATCATCC

ATAAATAAATATTATTCGAGGTCATGTACATGTATTTTACGTGTAACTCAAATGGAGTTA

AACGATTGTAGCATCAATTGCGATAGTGAAGATATGCAACATTGTATTATTAAATCTAGT

TGTGCTA

>Unigene0000996

GTGTTATTTCCGTGAAATTTGTGCGAATGTTATTAATACGAAAGGGAAAGGAAAATATTG

ATGTATAATTGGTGAAGGAAAGGCGCCTCTTGATTAGCTTTAATGCAGGCAATAAAGAGG

TCGATTCAAGTGGTTATGTATTAGAATGGAGTATCAAGGTTTATTCTTGTTGTACTCTTG

GGTTGGAAGGACTTTGCAAACATGGAAATCAGGAAATATGCCTAATTTATGTTTGGTTTG

ACGACGGCAAAAATATATTTTGTTTGTTCTTCTGGAAATGACAACTTTGTCGTCCAATCA

TTTTATTTGAGGAATTCAGCTAAATGTAGTGGTTTCATCGAGTCAGCTTGGCTGGTTAAA

GAAAAACTCAGTTGTCGAAGCCAATT

>Unigene0000997

CATATATCAACAGTGTGGGCTTGAAAGGAGTCCAGCATCAATAGATTTCTCAAACATGAA

TGCCATAAAGCGTTTGTTTGAGGGGCAAAAGCCGGCACTGGCGATGGTGCTGGTTCAGGT

CATCTTCACCTTTGGGACTCTCGCAGCCAAGGCCTTGTTCTCGACGGGGACCACACCCAT

GGTGTTCATCACCTATAGGCAAGCAGTAGCCATGCTGGTCATGGTTCCTGCAGCACTCCT

TATAAGAGGTCCTCGAAAGATGGCTTTGGGATTGAAAGCCTTTACACTAGTCTTTGTTGC

ATCACTTATTGGTTGCACCTTAAACCAGTACATGTATTACCAAGGACTGAAGATGACATC

CTCGTCAATGGGTTCAGCTATGAACAACCTCATCCCAGCTGTTACATTTCTCATGGCTAC

TTCTTTTGGATTAGAGAAAATAAAATTGCGGTCAATTAGAAGCATGGCTAAAATTCTCGG

AACCATCATATGTGTCGGTGGAGCAGTTTTCATGGCTTTCTACAAAGGACCAAAGCTTTT

TAATATGAATTTGAATTACCATACATTATCTACAATTCTACATTCGGTCAATGACAAATG

GATAATTGGTTTCATTTTCCTCACCGGGGCCACCTGTTGCGGTTCTCTTTGGCTTATTTT

ACAGGTACCCATTTGCAAGGATTATCTTGACCCATTGTCATTAGCAACTTGGATGTGTAT

TTTGGCAACCTTCCAATCATTCATCCTCACTTATTCCTTAGAATCAGACTGGCAAGCATG

GAAGATTACCTCAGCATTTGAGCTTATGTCAATTATATACACGGGGATCTTCGCATCTGG

TTTAACTTTCTATATGCAATCATGGGTGATATCGGTTAAGGGTCCTGTCTTCTCGGCATT

ATTTAATCCAGTCCAAATTGTCATTGCTACCGTCCTGGGTTTTATAATGCTTCATGAAGA

GCTCTACGTCGGGAGCTTGATAGGTGCTATTGCGGTTGTAAGTGGACTATATATAGTTCT

TTGGGGTAAAGCCAAGGATTTGGGGCCAAAGGGTGAGCTACAAACAGGGAATGGGATCCC

AAAGAATACAGTTTCTATGGATCCACAACTGAAGAACAGTGAGGAGGAGATTAGAAAACC

ACTACTAAATGAAGTTCTCCATGACATTGAAAGTCAAGTTAAAAGTTGAGAAATCAGTGT

CCTATGAAAAATAAGAGATGAACAATTATGTCAAGCATGTGGGGAGAGACGTTTTATGCA

TAGAAATTTTTAAATTAATTTCATTTTCGTTATCAATATTTTGACATGGACAGTTTCAGA

GTTCTGAACAGAGACCAACAATTGAATACTATTTTTAGTTTGATATTATTTGAGGAAT

>Unigene0000998

AGACCCCGGCAACATGGTCAGGGGCTCCACAATGAAAACAAACATGTGGAGAAGAACATA

CCCGGGCCAAGTGCCCCGAAAGCCCGCAGCAAAAGTAACGTAGGGGACCGCGTGCCCCAC

GGTGCCCCAACCCACTCCGGAAGGCTCGATGCCCCCAGTCCCGAGCCCACTCCACCCGGA

ACCATCGCTCCTCGCCCTCGACGAACTTCAGCCCAAGCCACGGACGACTCCCAGAAGAGG

CCGGATCCCCCCGCCTACAGGAAACCCCCCAGAAAGGCGACCACCCCCGAGCCAGGGACC

CCAAACCCCCACCTGCAAGCCTATCAGCGTATGAATGACGCCCGCCCGAACGGTGTGCCT

CCGATGGGGGGCCACACGCGCCCGCCAATGAATGGAGAGAATGACCAAAGCCAAATGGTA

CC

>Unigene0000999

AATGGTCGTCGGTAGGGGCGGTTCGTGGAATTACGCCGCGCTCGTTATCATATATGGTAT

ATTATTATGTATTAATGTATAATATGTATGTATACGCATTATGATATCTGGACGGAAGTG

CTCGGTTAATCGAGGTACTCCGGCATTATTTTTCTTTTTATAATATTATTATTATTATTA

TTATTGTAGAAGGTAAACTATACCTGTTTCGTCGAAGTCTGATTTTAAAATAGCTGGTAG

ATATAAAAGATAATTTGCAAAGTCATTTTCTTGCCCCTATCCTCCCTAATCATTGATTTA

GTGATAATCT

>Unigene0001000

TATATTACTATTTAAAGTTTAAACAACACGTTTGAATTTGTTTTATAACTTCAGTTTGAT

ATGCCTTTGTCCAACTGGTCTAAATTATACATTACCGATAATCTTAGTTATTGTTATAAA

ATTACATGCAAATTCATAGTAAATTCAAAAATAAGATAACATGAAGTTTTAACCATTTTC

ATTAATTTTTAAACTAAAAATATCTGATATTAAGTCAACCATTGGTAAATGATTTTGTTT

ATTATTAAAAATAAGTATACAACGACATTACATTTATGTTAACGTTCTAAAAGTGAAAGT

CAAGTTTCACTGACATTTTTCTTGTCTGCCCTTAAAACTAATGTGCAGTGTTTGTTGTAT

TTGGTGTTTGTGTCTTAATTTGTTTCTTTCATTAAACTTACCGGTGGAAATGTTTTTATT

GCTTAGTCATCGGATTGAGACTGAAAACTGAGACCTACACACAGTTGTTGCTGATATAAT

ATTATTATTATTATTATT

>Unigene0001001

GCCGCGCCACTCTTCTTTTTGAGGGCATCATCGTCGGCCTGAACGATGTCGGCGGCGGCG

TCAGAGGCGTCGGAGGCGATGGCGACGAAGGTGGAGGCGTCGGAGGTGACGGAGACGGAG

GCGGTGGCGGAGGCGTGGGCGAGGGAGGCTGTCCCCCGAGTGATGGAGATCGTGAGCTGC

AGTCTCTCCCAGCGCGATGTCTACTCGCTGCTCCTCGTCAGCTCCTCCTGCTACCATGCC

CTCCTTTCCCAGCCAAAGCTCTGGAAGGTCCTTGATTTGCATGAGATGAATAAAGCTGGA

GAGCGGCTGATTTCTGCACTCTCGCTGGCCAGATATCACCATATTAGGAAGATAAACCTT

GAATTTGCACAGGATATTGAAGACGAGCATCTTATTCTCCTTAGAAACAAGGGAATGGAT

TCACTTCAGGGCTTGGAGTCTCTAAATCTGAATGGCTGTCAGAAAATCTCTGATGAAGGA

CTTGAAGCTGTAACTAGTTGTTGTCCTAAACTTAAATCTTTATCGATTTATTGGAATGTA

AGGGTGACTGACTTGAGTGTAAAACATATGGCGAAGAACTGCAGGCATATTGTTCATCTA

AACTTGAGTGGCTGTAAGAATATCACAGACCAAAGTTTACATCTGATTTCTGAGAACTAT

AAAAAGTTAGAGGTACTAAATATTACTCGGTGCGTCAAATTATCGGACGACGGATTACAG

AAGATACTTTGTAAATGTCCCTTACTCCAATATTTAAATCTTTACGCCCTTTCAAGTTTT

ACTGATAAAGCTTATGAAAAAATTTCAAATCTTTTACATTTGACGTTCTTGGATCTTTGT

GGCGCCCAGAACCTATCTGATCATGGTCTTTCTTGCATAGCTACATGCAATAATTTGGTC

ACGCTAAATCTGACATGGTGTGTACGTGTCACTGATGTGGGAGTGATATCCATTGCCAAC

GGATGCAGGTCTCTCGAATTTCTCAGTTTGTTCGGAATTATTGGGGTAACTGATACTTGT

CTGGAGGCTCTGTCCAAGTTCTGCTCGAAAACTCTGACAACCCTTGATGTAAATGGTTGT

GTTGGCATAAAGAGGAGGAGCCGGGATGATTTGCTTCAGTTATTCCCACATCTGAAGTGC

TTCAAAGTGCACAGCTAAGATTATGAAAACTTCAGTACCAGGCATTTCTCCTTGTACAAT

GATAACTTACTTATTTTTATTTATTTATTTATTATTATTATTATTATTATTGTTTTTTAT

TTTAATTACTTTAATTTTTAACGGGAAGTTCGCCAAAAACAAAAAAAACCCCAAAACCCC

TCTCCGCGAGAGGAATGTTTTTTATTATTATATTTCTTTTTACGAATAATATAATATCAT

ATTTATTTGATTCAGACACAAGATTAGCATAATATACATCTTTACAATATTATTATATAT

AGTTTATAATTGGACTACTACTATAAGCATATTATACTATACATTTTTTTTATAGTCCGC

GGATTTTTTGTTTAATTGAAAACTTTTGTAAGAAGACTGTTGTAATAAAAATGT

>Unigene0001002

AGCTCCCAAGAACACCCATAAAATGGCAACTAACCCGCACATCGAAAACCGCGCACATAA

AATGTTCCAATTAAAGTAAGGGTTCCGGCGACCATTCCTGTGCTCGTCTCTCTCTGAAGC

GGCGATGAAGAGCAGAGCAAAGGGAGCGGAGAAGGAGACCCTGATCAAGCCGCCGGCCAT

CAAGAAGCCGAAGGCCGCCAAGAAAGCCGCCACCGCCGGAAGGGACGCCGCCATGCCGAA

GAAGCCACCCACCGCTTTCTTCTACTACCTGGAAGATTTTCGGAAGACCTTTCAAGAAGA

GAACCCAGATGTCAAATCAATGCGTCATGTTGGGAAGGCAGGCGGGGAGAAGTGGAGAAC

ATTGCCCTTCGAGGAAAAGGTTCAGTACTATGATATAGCTACAAAAAAGAGAGCTGAGTT

TGATAAGGCGATGGCGGCGTATACAAAGAAAAAGGAAAATGGCGAGCTTTCGGAGGGATC

GGAAGACTGCGAGTGACAAAGAACTGTGTGAAGTCCTCTGCTGAGAGTCGATCTATGCTA

ATAGAAAGAGATGTAGACTAGATTTATGAGTACATGTGTGGATTTTTATGATTGATGCAT

AGTTGTCGTTTGGGACTTTCTAATGCATAGTTTTTAATCTGTTTGTTGAATCTTTCTGTC

TGCTTGAGGAAAATTGTGTTGGGGCTAGGATGCAGTTCAGCCAAAAATATATTGTAATAT

TAGGGGTCAAGATCT

>Unigene0001003

GGAGTGCTATGCATGCACCCAAGCAACCGGCGCCCCGGCCTTCCACTCCACCAGCTGCGA

CCGCGCCCACACCCTCGAATACGCTGCCAGCGCAGGCTCCTCCCTCATTCCAATCCAAGG

CAGCGGTCTCCTTCAAATCCCACCTGCCAGACGGCCCTCTGGTCTGTTTGGCAAGGTGGT

CGATCCGCGGAGCCCGCGGGTGAAGAGTTGGAACCGGTGGCTGCTGCTTGCCCGGGGGAT

GTCGCTGGCAATTGATCCTCTCTTCTTTTATGCACTAAGCTTGGGGAGGGGTGGCGTGCT

GTGTTTGTACTTGGACTTGGAGCTGGCAGTGGTGGTGACGATGATGAGGAGCTTGGTTGA

TGTTGTTCATTTGGTGCATGTTTGGATGCAATTTAGAATGGCGTATGTGTCGAGGGAGTC

ACTTGTCGTCGGATGCGGGAAGCTGGTCTGGGATGCAAGGATGATTGCTCGTCACTATGT

CAATTCCGTGAAGGGCTTCTGGTTTGACATCTTCGTCATCCTACCGATTCCTCAGATAGC

ATTCTGGGTGTTGGTACAAAAATTGATGAGGGAGGACGAGATTAGGCTGTTGATGACAAT

ACTACTACTCGTTTTCTTGTTCCAATTTCTCCCCAAGGTATACCACGGCATCTGTGTGAT

GAGGAGAATGCAGAAGGTCACTGGCTATATTTTTGGAACAATCTGGTGGGGCTTCGCGCT

CAACCTCATAGCTTATTTCATCGCATCTCATGTAGCTGGCGGATGCTGGTATGTTCTTGC

AATCCAACGCATAGCATCATGCCTTCAACAGCAGTGCGAGCAAAACAACAATTGCGATAT

TTTGTCATTGGCCTGCTCCAAGGATGCCTGCTACCGCGTTCCATTACCATCAGGCCTGGA

TGAGTTATCTTGTCGAAACTTGACTGCATCCCTCGTAAGCCAACACAATCCAGTATGTTT

GGACAGCAGTGGACCATATCCTTATGGGATCTACAACGGGGCTCTCGCTGTCGTATCCAG

CAATTCACTTGCTGTAAAAATCCTCTACCCTATATTTTGGGGTCTAATGACCCTCAGGTA

AACTTATTTAAAAAACATAGTGCATACTATAAGTATGTATTCATCAATATTCTGCTCTTT

ATTGGTTCTACAATATCATCAGCACTTTCGGAAATGATCTTGAGCCAACAAGTAACTGGC

TAGAAGTGATATTCAGTATAATTATCACATTGTGCGGTCTAATG

>Unigene0001004

ATATATATATATCCTTTGATAATTGACTTATAATCTATGAACAATTTTCAGAATTAAAAT

ATTACGGTTCAAAAAATGAATTGCAAGGAAAGATGTGATATGAATTTAGAACAACTCAAT

CAGTGAATCACAAACAAATCGTGATTAAATCGGGATCAGTTGTGATGAAATCGATTTGGA

GATTTTTTCAGCAATTAAATTGGTTGGCGAATAAATTGGAGATTTTAAAGAATTATTGAA

TTAATCCGAGAATCCGGTAACTATGTTTTGCCATTCTTCTTCCCTAATGCCAACTCAGTC

TGGAGGAGAGAATGGTGGGCTAGGTGCACGGAGGAGATCATGGTGACTTGGTGGACTCCA

GGAAAAAGGTGAAAAAGAAATATCATGGTGACTTGGTGG

>Unigene0001005

ATATATATATATCCTTTGATAATTGACTTATAATCTATGAACAATTTTCAGAATTAAAAT

ATTACGGTTCAAAAAATGAATTGCAAGGAAAGATGTGATATGAATTTAGAACAACTCAAT

CAGTGAATCACAAACAAATCGTGATTAAATCGGGAATAATCATGATTAATTCGGGATTAG

TTGTGATGAAATCAATTTGGAGATTTTTTTCAGCAATTAAATTGGTAGGCGAATAAATTG

GAGATTTTAAAGAATTATTGAATTAATCCGAGAATCCGGTAACTATGTTTTGCCATTCTT

CTTCCCTAATGCCAACTCAGTCTGGAGGAGAGAATGGTGGGCTAGGTGCACGGAGGAGAT

CATGGTGACTTGGTGGACTCCAGGAAAAAGGTGAAAAAGAAATATCATGGTGACTTGGTG

G

>Unigene0001006

CAAGGACTTTCCCACAAGTATAATAGTTTACACTTCAGAATCTAAAAACATTTTTAAAAT

TATTATTTTTTGGTTAAAAACTAAAGACCAAAAAAAGACGTAATATAAGTCTGCAACGAT

TAAATCGGAGAATTTAGAATTAATCATGATTAAATCGAGATTAATCGTGATTAAATCGCT

TTGGAGATTTTTCTAGTAATTAAATCGGCAGGGGAATAAATCGGAGATTTTTAAAGA

>Unigene0001007

TAAATCGGAGATTTTTAAAGATTTTTTGAATAAATATGAGAATCTGGTAACTATGATTGG

GATCCCCATTCTCACAGTAGCTCTCATCATTGTACTTGGGAGAGGGCAAAGTCGTCGAAC

AAACCCTACACCTTGTCCACGGTCCAATCTCTTGAAGATGAATCGCACGGGGGAGCTTGT

GCACGGGGAGAGAGAGGAACGACAATCTAGGGGGAGGGGAAGGGAGTTTGAGAAACTCTA

ACTCTAAATCAGTCCAAGCTTTAATAC

>Unigene0001008

CCTAATGACGAAGAAAAGACGCGATTTTAAGTCTGCAACGATTAAATAAAAAAAAACGGA

ATTAATCGGGAATAATCATTATTAAATCGGGATTATATCGCTTTGGGGATTTTTTTAGTG

ATTAAATCTGCAGGTGAATAAGTCGGAGAATCTGGTAACTATACTGACAAGTGACCTTCC

ACTCAGGATTTCCCATTCACTTTGATTCATATTGTCTGTATTCCGTCAA

>Unigene0001009

GGAGCAGCTCGGAGGAGACGACATCGTTGCGAGAGATTAAGACTACCAGTTGAAGTTGTT

CTGGTTATCAGATGGCGCCACCTGCGAAAGGTTTGTTATTCTTTTAATCAATTTTTTATT

GCATAATGGCTTGTTTCCTGTCTCTGCTTTATCATCCTGGGGTTAGTTGTAAGCTTTATT

AGCATTCGAATAATGAACACAACTACATAACAAATAAGTCCTTTCTTAACTGAAAGGAAT

GTGTGTCCAAAGAAAGTGTAAGATAAATTGAGTGGCAACAAAATCAGATCCTTCAAATAT

TAATGTCTAAACAGATCTGGTATTTGTCCACGGTTTTGAAATGCTGAGCAAAGTTTCTGA

TGCTTGCCTGCTTTAAAATTAGGTCACTGTCTTTTTCAAATAAGGATTGATACTAAGATT

AAAAGTTAAACCATTGTAGCATTAATTTTTCCTATCCAGGGGGGGCAGTGAGTCCTGAAA

ACCTGGCTATCCTACCGGCACCCGACCGAAAATTCTGGGTATGGTTGCTACTAGTTTACT

CTATTATTGTTTTAGGTTGGTTTGGATAAGGAGGTATCCATATCCGATACTGGACTTAAT

ATCGGATTTTTTACCCATTGGTTTGCATAGCGATAAAATGATAATTTAGTCAATGGTTTC

AAGAGCATCCTCCAGTATCCTTGTCCGTTAGTACACGGGAGTTTTTTTTTAAAGAGCAAG

GTTGTTTCGTGTAATGTCTCTGAGTCCAATGCATGTGGGCAAGTTTCGGTCTACATGGAA

TTTTGTAGGTGACTTGAGCCTATTTTTTTTACTGTACATGGTAGCTCAAAACCAAAGTTA

CCAGTTTGGGGCCGAAGATTTAATCGGCGAGGCCTAAA

>Unigene0001010

GGAGCAGCTCGGAGGAGACGACATCGTTGCGAGAGATTAAGACTACCAGTTGAAGTTGTT

CTGGTTATCAGATGGCGCCACCTGCGAAAGTTACCTCCAAGAAGCCTGATGGCAAGGCAC

AAGCTCTGAAGGTGGCGAAGGCGGTGAAGTCTGGCGCTTCAACCCTGCAGAAGAAGGCCA

AAAAGATCCGGACTTCAGTGACTTTCCATCGCCCCAAGACACTGAAAAAGGATAGAAACC

CCAAGTACCCGAGAATCAGTGCTCCTCCTAGGAACAAGCTCGATCACTATCAGATCCTTA

AGTATCCACTGACCACCGAGTCTGCCATGAAGAAGATCGAAGACAACAACACTCTTGTTT

TCATCGTCGACATTCGTGCAGACAAGAAGAAGATCAAAGCAGCGGTTAAGAAGATGTATG

ACATTCAGACGAAGAAAGTGAACACGCTGATCAGGCCTGATGGAACAAAGAAGGCGTATG

TGAGACTGACCCCAGATTATGATGCTCTGGATGTTGCCAACAAAATCGGTATTATTTAAA

CTCAGGAGCGAACCGTGGATCAGTATCTTTTTTGTTAAATTTGTCAGTAACTTGATCTGT

TAGACACCCGGACAAGCTTTTTAGGGTTGTCGTTTTGGTTTATTAGATTCTCCGTTGAAA

TTTACTTTGTGTTGGATTTTGGGATTTTTTTTAAGGGAACAAAGCAGCTCCACCCTAGTC

TCTAAACTATTTGAAATATGGGCAATATCCCATTTTAATCACTGTGATTCATCTAAACTC

TTGTATTTGTTTTTTGTATTTTCTGTTGGTTATTGCGGAAATGCACGTCACACGTGTCCC

ATT

>Unigene0001011

GCTAGGGCTCTGCATTGATCTCCATGTCTTTCTTTTTATCTCTAGATCAATATTGGGAAT

CCCCTCCATGTAAGGGAAAGTGTGGTGTGGCCTGGCCACTGCCTCCGAACTGTCCTTGGG

TCAGTCGAGAAGTTGTAGATTTAAATTGCTTGTATGTGGAGGGTCCATGACCCATCTTGC

TAGATATCTAATTCTGGTAATGAAAACATACCTTTTTATTTGTGTTTGTTTACCGTCAGC

TACCTCCAAGAAGCCTGATGGCAAGGCACAAGCTCTGAAGGTGGCGAAGGCGGTGAAGTC

TGGC

>Unigene0001012

GCCGCCGCCGCCGCCGCCGCCGCCGCCGCCGCCGCTACCGCCGCCGCTGTCGCCGCCGCC

GCCGCTGCCGGAGCTCGTTGCAGCATAAGGACTAATTGGAGCTAATTGGCACTTGAAAAA

ATGGCCCCTCCTGCGAAAGTTGCCAAGAAGCCTGATGGCAAAGCACAAGCATTGAAAGTT

GCCAAGGCTGTGAAATCTGGTGCTTCAACCCTGCAAAAGAAGGCCAAGAAGATCCGCACA

TCAGTAACTTTTCATCGTCCCAAGACCCTAAAGAAGGATAGAAACCCCAAATACCCCAGA

ATCAGTGCCCCTCCAAGGAACAAACTAGATCACTACCAGATCCTCAAGTATCCACTGACC

ACTGAGTCTGCCATGAAAAAGATTGAAGACAACAATACTCTTGTTTTCATTGTTGACATC

CGAGCTGACAAGAAGAAGATTAAAGCTGCTGTGAAGAAGATGTATGATATTCAGACCAAA

AAAGTGAACACCCTGATCAGGCCTGATGGAACAAAGAAGGCTTATGTCCGACTGACTCCA

GATTATGATGCCCTGGATATTGCAAACAAGATTGGTATCATCTAAAGCTTTATGCCAGTT

CTGTTTTTGTCTTGATGTATGTTTTGAAGATGTTAAACATTGTTGTTGCAAACTTTGTTT

GGATTGTCATCTTTAATGCCTAAACATGGATATTTTATTTAGGTTTACTTTATGGTTTCT

ATATTGGCAGAATCTAGTGGAGTGGTGATTCCACTTATGAAATTTTAGTTGCATGCC

>Unigene0001013

TTTTAAAGATTTATTGAATAAATTGGAGAATCTGGTAACTATGCTCAAAACTACCCGATT

ATCCGATCTGATACATTTATAGATTATGGATGTCAAGTTTACACCTGATATCATTTCTTG

GGTAGGCTATGGATATAACTGATATGTGACCCCGACCATGTTGCCACCCCTAATCTTATG

CCCCAAAGTTTTCGGGTTACTTACAATGCACCCTGGAATTTGGGTTTACTTACAATGACC

CCGGGATTTTTTGAAGTAGTAATACTTGGCTCCTCTGAACATGATTTTTTTGTCAATTTT

GTCGTGCCCTACCTCGACTAGATTCTCTACAGACTCGTTTAGTTCTAAATTATCAAGACA

GTACCTTAGCAACTGAGGACTGCAATTTCTCCAGCGGGCTAGGGCTCTGCATTGATCTCC

AGCAGGCTAGGGCTCTGCATTGATCTCCAGCAGGCTAGGGCTCTGCATTGATCTC

>Unigene0001014

TTGGTTAAAAACTAAAGACTTGAGAAAATACGTGATTTAAGCCTGCAACTATTAAATCGT

AGAATCCCGAATTAATTGTGATTAAATTGGGATTAAATCGCTTTGGAGATTTTTTCGACA

ATTCAATCGGCAGGTGAATAAATCGGAGAATTTTAAAGATTTATTGAATAAATTGGAGAA

TCTGGTAACTATGGTTTTAAGGAGGAGATTTTTGGGTCTAGGACATAGAGACAAATATCC

TCAGAATTAGGATTTTGTCACCATGGAACTC

>Unigene0001015

CAAGGGCAAAACCATCGGTAGCAACCGCAATGGCAAGTACTCTAAGAAGGTGAAGACTAA

CACACCAACTCCAATTCTGACTCCGTCCGAAGAAAAAAAGCTTCTCTCTGCCCTCAGCAG

CCAGATTCTTTGCGTCCCCCGGAAATTCAAGCACTCTAAGAAGGTGAAGGATAACAAACC

GATCCCACTTCTGGCACCGTCTGAAGAAAGAAAGCTCCCCTCCATCCTCAGCTGCATGTT

TCAGGCCGCGGTAGCCAAGGTGTTATCGTGGAAGAGCGACGGGGAGGAAGCAGTGTGGAA

GAAAACCATTTTGATGGGAGACAAGTGCCGGCCGCTGGACTTCTCCGGACAGATTAATTA

TGACGCTGAGGGAAATCAGTTGCTGAAGAACTCCATGGCAGAGGAAGTAGGTTGGGGGCT

GGGTGTGGGATCATCAGTAGGGAAGAATTCATATGGGATTGTGTAATGTGTGTACCAAAT

ACTGTTTAAATTTTTCAGCGAGGTTGAACTAGGGTTTGAACATAAAAAGGATGATGAATG

AAGCTTTTCATTACGATTTCAATCAAAGTTTTGCCCTAATTGGACAGTCACTAATTGGTG

TAATCCTAGTGTTTTACTTTTATTACGATACAA

>Unigene0001016

AACTTCCTAACGGATCGCCCTTCAACCAAGGTTCATGCTGCACCTGGTGGTGGTTCCTCC

CTTGGCTACCTTTTCGGTGGCGGTAGCAACTGATGGATTACTATGCTGCTTTAGAACTGC

ACCAAGTGATTGCAGATTTCTAGTTGAAGAGTTGCTTAACCATGTAATATATATATACCT

ATGGTCGAGGCAAGAAACTTTTGCCCCTTGGTTGGTGATTTGTGTTCTCTATTTTTCTGA

AAAATCTTTTCAAGTTCTTG

>Unigene0001017

CCTTCTCCGCCTCCGCTCCCGTCGACGAGATAAACAGAGTTTAGGCTATCTCGCTTGAAC

TAAGATGGGTCGTGGAGTTAGCAGTGGAGGAGGGCAAAGCTCTCTTGACTACCTTTTTGG

TGGTGGAGAGGCTCCCACAAAGCCTGCTGCTAACCCTCCAGCTGCTGACAATGCTGGGCC

TGTTGAAAAGCCCTCTGGTGCTCCAGCTCCACTTGACAACGGCAAGCAGGTTCCTGCTGG

CATACAAGGAAACACTGCAAGGAACGCAAACAACTACTTCAGAGCTGATGGCCAGAATAC

TGGCAACTTCCTAACGGATCGCCCTTCCACCAAGGTTCATGCTGCACCTGGTGGTGGTTC

CTCCCTTGGCTACCTTTTTGGTGGCGGTAGCAACTGATGGATTACTATGCTGCTTTAGAA

CTGCACCAAGTGATTGCAGATTTCTAGTTGAAGAGTTGCTTAATCATGTAATATATACCT

AAGGCCGTGGCCAGTAACTTCTGCCCCTTGGTTGGTGATTTGTGTTCTCTATTTTTCTGA

AAAATCTTTTCAAGTTCTTGTTGTTCGGCCTATTGTTAGATCCTGATTCTGATTCGGTTT

GTCGTTGAACCAGTGGTTGTGATCTACTTTGCTTAAAGTATTTCCATTTTACCAATACTT

TACCCCGGTCACTGTTTGTCGTTGACATAGTGGTTGTGATTTACTTAGCTTAAAAGTA

>Unigene0001018

TTTGTTGCATGTAGTATGATGTCCTTATTGTATATATGTTGATGCTTCTGTTCCATCAAT

CTGATAGATAAACATAGTTTAGGTTATCTCGCTTGAACTAAGATGGGTCGTGGAGTTAGC

AGTGGAGGAGGGCAAAGCTCTCTGGACTACCTTTTTGGTGGTGGAGATGCTCCCCCAAAG

CCCGCTGCTAACCCCCCAGCTGCTGACAATGCTGGGCCTGTTGAAAAGCCCTC

>Unigene0001019

TTTTTCAACGTAACAAAATGCCAAGCAAGCAAGAGAAATATAAACACTTCACTACAATTA

TGGGGAATGGGTATGGGAGCGTGGAATGTTACTCGTGGAGAAACTAACTATGAGGGGGTA

GGATTCTCAGGATCTTGCCTAGTTGTAACAGAATACATTTCCAATAAGGAAATATAAACA

CTTCACTACGATTACTATTTTATTAGTAACACAAACATATAAGAACATTCCAAATCTATA

TCCATTTAATGCTCTTAGGTTTGGAATGTGGAGCCTATATTTGAGATACATAGTACAAGT

TTTACTAAAGGCAGCTTACAGTTTGAGGTCGGTCAATTTTTTTCTTCTGCAGGAACCAGG

CTTGCGATTTTTACGTCTTTCGCAGGACATAATAGATTCGTCTTTCGCCGGACATAATAG

ATTCGAAACCCAGGCTACTCAGCCGCTAGAGTTTGGCCATCCATTTGGACCTCTAAAACC

CAATTCTGATCTATC

>Unigene0001020

ATTTTGAAGTATTCTGCATTCTCTACCAAGAATAATCCTATGTAAAGCGTCTGTGCGCTT

TGTGCACAGCCAGTTCTCGTATCCATCATTCCAACTTGCGACTTGGGTCACCTGAAAATT

AATGAAGTTGTATTGTTACCTGATAACTACTTCCAATATACAATATTGTTTTGGGAATCT

ACTTGGCCTAAATTTTTCAACGTAACAAAATGCCAGGCAAGCAAGAGAAAAAAAGGGCAT

CCCAGTGCATGAGGCTCCTGCGACTACATGGTTCTGGGGAGTGT

>Unigene0001021

TTTGAGATACATAGTACAAGTTTTACTAAAGGCAGCTTACAGTTTGAGGTCGGTCAATTT

TTTTCTTCTGCAGGAACCAGGCTTGCGGTTTTTACGTCTTTTGCCGGACATAATAGATTC

GAAACCCAGGCTACTCAGCCGCTAGAGTTTGGCCATCCATTTGGACCTCTAAAACCCAAT

TCTGATCTATCTTTAGCACGCGAATTTCATCCGAGTATCCCATTGGCCTACTAATTTCCG

CCGTATATAGCAAATCCTGACAGTCCAGTGTCCGTGCATGTTCGCATTTTGAGAAAAGAA

AACTGTAGCATCGGATTTGGGAAAATAACTTGCAGTTCAGAGATCCTTGTCGATTCCTCA

GTACCGGTTGGTTCCTAGTCTCCTATGTATCATGATGATCTGGTATTCTTCTTACTTTCT

CCTCGTCATTGGCACAGTCGACTCGATCAAGGCCGCGAAAACCCCACATTTTGGACCGCA

TGATCTGGATCACAAATCGGAAGGTATGCACGCTTTCAGGTAACATTGCTCTGTTCCTCC

CATAAGAGACCATGCAGTAACCCATGTGTTAGCTTAGTGGGTTCTTTCCTACAGCTTGCT

CTTTTCCTTCTTAACTGTGTGTATCTCTATCGATATACCACATAATTTCTTTCCATATTT

TATGTCATGTCTGTCCATGACCAAATACTTGGAAGTAGTTACTCAAACTTCGAGAGGTTG

CAGACTCCATCCTCTGGTTAAAATTTTAGACTTAGTCCTTGGTTTTCAGTATTTTGACAT

CAATTTTGTTAAAGAAGAACGTAGAAGCAAGAAACTGAAAGTAAAATGTATTGGCAACAT

TCATATTAACAGGGAAGGTGACAGCAAGTGTCCTGAATTCTCTGCATGCTAAACTTGATT

GTGTGTGTGCCATTTGGGTTGTTATGAGAT

>Unigene0001022

GCAATCTGAGGAAGAAAAGGGAGAAGAGGCCACATGAACCACGTTTTGCTTTCATGACGG

TAAGTGAGGTCTATCATCTCGAAGAGGGATGTGGATGAAGTATGGACAGAAGACCGTCAA

GGACAGCCCTTATCCGAGGAGCTACTACCGCTGCACCACACAAAAATGTTTAGTGAAGAA

GAGGGTGGAAAGATCATTCCAAGATCCAAAGACTGTGATCACAACATATGAAGGGCAACA

CACACATCACAGCCCGGAGACTATTCGAGGGAGATCGCATATGTTAGCTTCTCTGCCAAT

GGTGCCGTCAAGCTTTCTGCAGTATCAATTGATGCAGCTGCACCCAATAGCTCGAACAGG

CAGATGAATCCGAGCATCTAAGCTCAAAGTCAGTATGATTTGTGAGGGAGAAGAAGCGCT

TCGTCTTAACGAAGAAAGGAAGAGATGGGAGAGAAGAAGCAAACACGAGCAT

>Unigene0001023

CCGCTGCACCACACAAAAATGTTCAGTGAAGAAGAGGGTGGAGAGATCATTCCAAGATCC

AAAGACTGTGATCACAACATATGAAGGCCAACACACACATCACAGCCCCGCGACTATTAG

AGGAGGATTGCATATGTTAGCTCCTCTGCCAGCGGTGTCCTCTAGCTTACTCCGATATCC

ATTGATTTAACTGCCGCAACTAGTACACAGAGATAACTCAATGTGAAAGAGACATGGGAG

AAGAAG

>Unigene0001024

ACTGTGATCACAACATATGAAGGCCAACACACACATCACAGCCCCGCGACTATTAGAGGA

GGATTGCATATGTTACCTCCTCTGCCAGCGGTGTCGTCAAGATTACTCGGATATCCATTG

ATATAACTACCGCAACTAGTACACAGAGATAACTCAATGTGAAAGAGACATGGGAGAAGA

AGATCCTCAGCGCAGAGAAACCCTAAAGAGTCAGGGAGAGAAGAAGCAAACAGGAAAATG

TGCAGCAAATTCTTTTATCAGTTTTTATGCCAGAATATGTGTTAGAGGTGTTAGCGTAGG

TGGTGCTTCCTATC

>Unigene0001025

CTCACCAGTTGAAGCTCATTGAACTCTCTCCTTGCTTACATATTTCTTCTTCGACCATCC

ACCCCCAAAATGAATAGAACATGGGGCAAGATCATCTGCCTCTTGATCGTTGCCATCGAC

ATATTAGCTGGTATTCTCGGAATCCAAGCTGAGACTGCTCAAAACAAGGGTAAGCACTTG

AGGGTATTGTTCTTTGAGTGCAAGAAGCCAGCGTATGAGGCCTACAAACTAGGGCTCGCT

GCAGCCGGGCTTCTTGCACTGGCTCATGCCATCGGTACCCTCCCCGGTAACTGTGCATAT

ATATGCTCCAAGGAAGAGCTCGAAAGTTCGTCTGCGGACAAGCAAATGGCTACACTAACC

CTAATTCTCTCATGGATTGCTCTTATAGTGGGTCTGGCATTGTTGATAATTGGGGCACTG

TCCAATTCGAAGTCGAGGGTATCCTGTGGGTTCGAGCATCACAACTTCCTGTTAGTTGGG

GGGATTTTCTGCTTCGTTCATGGCTTGCTTTGTGCGGCGTACTACGTCTCTGCAGCCATC

AACTAATGAGAGGAAGAAGGGAGAAACTTCGTACAAATCTAAAGTTTGCTCTAATTGAGG

TCATTTTGGAGGAACATGTCAGATGAACAATAATGGGGTTTGATTCTTTGTATTTCCATG

GTCTCTAAAGTAAGATATATGATGTAGATTTATACGAATAATACGTATGTTTTCAAGCTT

ATTATAGTCTTATAATGGAAATGAGACAATCCCGACAGCCCAT

>Unigene0001026

AGGTTCCAAAATGCAGATTCAAAGTTGAGTCTGAAGAAAATTCATGTCACTTATCAGAAT

GATATTTATTTGGTGACATCAAAATGATATTTATATAATTGTCTTCATAGTCATGTAGCC

ATCAGAACCCCAAGTCTGCACATGTAAATGAGATATTCATCACCCATGGACTGTCACCAC

TTTGATTTTCCACTTTTCGTGATATTGTCCTAATTGGCTCTTGTATTGTCTTCTGTATCA

AGAATTTATAGTTATCATCTCACTCGATCCTCTGCGTTTTCCTTGGTTTGCAGAACTGAT

TTTCTCTGTTGCGCTCATCTCTGTTGTAATCAAGAGCTCCCTAGTCTGCGATTGTTTTGA

GAAAGGTCGCAATTCAATTTCGAGTTGGAGGCGAACCAATTTGATTATGTTGAAAACACT

GCATACTCAATGAATTCATGATTAAATTTGACATTTAATTACAATTTCTGTGTTAACCTG

CGCTCATATCTCCTTGAATGCTCATATCTTCTCACACCATTGTTTGCTTATTAATATTGA

TGGTAATGCATGTTAGGAGCATCATCTTACTTTGATTTTATAGTTTTCTACTATACGAAT

TTCCGTATTTGTAGGTGATCTTAAATAATTTTTCACGTTGTATGTTTTTTGACTTCTCTG

ATATAATCTGTGCCCAACTGGTCGTAAGGGCTTAGGATTTATAGTTTCGTTTTATTCTCC

TTGTTAAAAAGGCAGTCCACTGTATGAGGCTCCCCCCTCTTGGGGGGGGGGGGGGGG

>Unigene0001027

GAGACTTGACCCACCTACGGAAAGGGCAAACTAGTATGAACGGATATGTGGGGATTTATG

GTTCGGTGCGGAGTGAGGGCTTTGTGGGAATGATGCGGACAATTATGTCTTAAAGTCGAG

CCAATGTGGTGCTGAACAAAGATTGACCACTCATGTGCAAATGATGCAAATATATGATTG

TTTCGAGGATTGATGAGGCGGACATGAATGCAGTCTCAAGTGCTGCGACTGTATTGAGTG

ACTGTGCTTGTTTGGCGGTACATTGATATACTGAGCCATGAGCTAGATGATTGTTGTTCG

TGCGGGTCAAAGACTCATTGGTATGTTGGGACTCTTGATGAGCAATGTGCTAATATTTCA

AAGGTCATGCATTGTAATGGATCAATGTCGAGATGTGCACATCCAGTTGAGAGCTAAACG

GCGACATGGTTCATCGTAATGTTACATTATGGGGTAATGATTTAGTATCGTTGATTGCCC

TATGATGTTATGAGGCGTCTTGGTTGGCATGGTGAGTGTGCCCTTCACTGAACAATTACA

TGAGAGCTTGTTGAAGACTTGGTTCAAGAAAAGATGAGACAATGTGCTATCACAAAGGTG

TCAAGTTCTTGGTTGCTCGGTGAAGCATCCTTAACAAATGATTGAGCATTGATCGTTGTG

ATATGTTCGGAGGCTTGAGTTGCTTGTCTACTCTCGTAAAGGGTTGTGAGTATCCAAAAC

GATGGAATGCTATGCGTCATTGCTCTCGTCCGGGTCATTTGATCCTTTAAGAAAAGGGTT

GGTCCGTGGTTTATGCATTGATTTGTATGGGTATGCACCGTGGCAAGGATAGCTATGTAA

ATTTAAGGTATGAATTGTTGGGGCAAGAGTCGCGATTTAAATTTAAGGCATGAATTGTTG

GCCCCTTGACAGGGAGCCTCTTGCACTGGGTTACACTTTTTTGTGTTGCATGGGTTTGAC

TAGAAAGAATGCAGGTTCCAAAATGCA

>Unigene0001028

TGTTTTATAACTTGCCGAGTCGACTCGAATCATCTGGTTGACTCGGCTTGTTTAGATTTC

GGATCAACCCGGCATACAAATTTAGAATGATCATAGTTCAGACTATTTTTTTTGTTTGTT

TATAAAACTCACATGATCTACTTTGTGTCGGCCAGTAGAGAACTTGATCGAATTTTGCAG

GATTTACCTTTTGAGGAGGTTGGAGAAATGGGAGTGAATAGAGGGTCGCAACATACCGGG

GCATCCTCGTAGCCTTTGCATATGTGGCAACCTGGGAAGAATCCTCCCCCTAAAGTCAAG

ACACCATTATGCCATGTGTGGCGAACCATGTTATCGAGGATGATGACATCAGATGCTTAC

CCAACATAAGCCTAATGTGGCACACTAAGAGTCCACATGTATGGGATAGAGTGCTCTGCT

>Unigene0001029

TGTGCGAGTATAGGAGGGATATTCCATCGTTTATGTCCTATCCTTGGCATTGTGCCCGAT

GGAGTTGTACAATGCAATGCTGACACCATCACATGCATGTTGTGGTTTGGGGTAATACTA

CATCGGATGATCTGGTCTAGGTTGCAAGAGGGGTATATACTTGAGATTGGACTGGGGATG

TTGTCTAAGGACTACCCTATGCGTTTTGAATCTCTATGGTGGTGTGTACGAAGCAATAGG

GGACAATTTGGCCAGAGACTTGACCCACCTACGGA

>Unigene0001030

TTCATTTGGCTTGTCCGCATGCTTATCAAAATGTAACTTCTCTTACAACAGTTTTAGGTG

TTTTTGGGTTCATCAGATTGCTCACATCAAGATGTTTACATACATATTCTATTCCCAACT

GATTTAAATGTACTCAAAGCTATTTTATTGATGAATAAAATTCTATTTTAAGGTTAAGTA

AAAAACCACTCCCCAAATTGAAACCATTAAATTATGGTTCAATGTAAAGTTGTTTGAAGA

ATTTGTTGTTAATGCTCT

>Unigene0001031

AATTGGAATGAATGGTTGTCATGTCTACTACAAATTCGGATACAAATTAAATCTATATAA

TGATAAATTAAAATATGCAACAAGGATGAACAAACAATTTGTTGAACCATCTATGATCGG

AGATAGTTTATACTTACGTACATCAATCAGAATATATAGCTGTCTAGGGAACTTCATTTG

GCTTGTCAGCTATCAGATCGCTCCAAAGGCATGCCAGGGTAAATTTTCATGTTATTGTTA

GGGTTCTATAGTACTTATCATGATCCTTTATCTCTACTGGCCTTATGGCGTCTCCATTCA

ATCCACCACTGACATCCTACGTAAGTTTTGATGGGTTAACCGACCCACACACACCCCTAC

>Unigene0001032

ACTATGATATTTAAGCTAACCTATCATGGTTGTGTAGTTTGCTTGGTAATTAGAATGATT

GGTTGTCATGTCTACTACAAATTGGGATACAAATTAAATCTATATAATGATAAAGTAAAA

TATACAACAAAGATGAACAAACAATTTGTTGAACTATCTATCATCGGAGATAGTTTATAC

TTACGTACATCAATCAGAATATATAGCTGTCTAGGGAACTTCATTTGGC

>Unigene0001033

ACTTTTTGATGGTAAAAATCCAATCAATGAAAATGCCACACGGCCTACAGGGTTTATTGG

TTTTCCATCACAGCCCATGGCTCCAATTCAGGCACCGCCTATTAATTATCCAAATATGCA

AACTATGAGTTCAATGTTAGATTCAGTTCCATTGGATTTTGAAATGCCATCTTCATTACC

TACAGTAATTGCACCTACTATGCCTAATATACCCGCACCCAAAACTCAGCCAATTCAAAA

ACCACCAGCAGCTAATATAGTCAAACCTGTTCCAAAAAACCCAATTTTTCCCAATAAGAA

CTTGGGTTTTTCAAACTTACCTGACCTTCCTAGTGTACCTACAGATATACCTATTGGTGA

TATTAAAGAAAAAGATGATGATGAAGAAACTGATTTTGATGAATTATTAAATCGATTTGA

G

>Unigene0001034

ACTTTTTGATGGTAAAAATCCAATCAATGAAAATGCCACACGGCCTACAGGGTTTATTGG

TTTTCCATCACAGCCCATGGCTCCAATTCAGGCACCGCCTATTAATTATCCAAATATGCA

AACTATGAGTTCAATGTTAGATTCAGTTCCATTGGATTTTGAAATGCCATCTTCATTACC

TACAGTAATTGCACCTACTATGCCTAATATACCCGCACCCAAAACTCAGCCGATTCAAAA

ACCACCAGCAG

>Unigene0001035

GGAGAGAGCGAACCACCATGGGCGGGTGTTTCTCCGACGTCCGGGCGGCGAAGGAGGCGG

TCGGAGTCAGCGCTCTTCAGCAGGAGGCCAACGATGCCGTCCAGAGCTTCCTCAACTCCC

GAGGCCTCCGCGGCCTTTACACCGAAATCGAGTTATCACTATCAGCATCAAAGTTACGGG

ATCGTGACATCCTTTCAAAGAGTGATCCGATGGCAATAGTATATGCAAGGAAACAAGGAG

GGATGCTAGAAGAAATTGGACGCACTGAAGTAATGATGAATTCATTGGATCCAGTATGGA

TCACAAAATTTGTAATCAATTATCAGTTTGAGATGGTTCAGACATTGGTGTTCCGTATCT

ATGACATCGACTCTGAATACCATAAGACACCTATTAGGATGTTACAATTGGATAAACAAG

ATTTTCTGGGAGAAGCTTCCTGTGTTCTGTCAGAGATTGTTACCAAGAGCAGTCGAAGTT

TAACGCTTAATCTTCAGAGCCAAAATGGGCAAGGGGGCTTACATTTTGGAACATTGACAG

TTCATGCAGAAGAAACAATTGCATCCAGAACGGTTATCGAGATGACTCTTCGTTGTTCGA

ACTTGGAAAACAAGGATGTCTTCTCTAAAAGTGATCCATTCTTAAGAATTTCCAGATTTG

TGGAGACTGGTGAGTGTATTCCCATATATAAAACAGAAGTGATCAATAACAATTTGAACC

CAGTCTGGAAGCCTGTTATTCTCACCACACAACAGTTTGGAAGCAAGGAAAACCCTCTTG

TTATTGAATGTTTCCATTTCAAGAATAATGGCCAGCATGATATCTTAGGAATGTTTCAGG

GAAGTGTGGCGGTCCTTGAAAAACTACATAGGGAGCGAGTTGGAGAAAACTTATTTGTTC

CTTCTGTCAGTGCTGACCGTAAAAAGATACATAAAGGACAAATATATGTAGATAAATTTC

TTGAAAAACCCCAGTACAGTTTTCTGGACTACATTTCCAGTGGATTTGAGCTCAATTTTA

TGGTTGCCATTGATTTTACAGCTTCTAATGGAAATCCTAACCAGTTTGATTCCTTGCACT

ATATCGACTATTCTGGCCGATTGAACTCTTATCAGCAGGCAATTGCAGGTGTTGGAGATG

TCATACAGTTTTATGATTCTGATCGTCACTTTCCTGCATGGGGATTTGGAGGAAAGCTTG

TCAATGGGTCAGTCTCTCATTGTTTCAATCTAAATGGAACTCCTGACCAGTGTGAGGTCA

CAGGAGTAGAGGGTATTATGTCTGCATATAAAATGGCCCTGCATAATGTATCCCTTTCTG

GTCCGACTTTATTTGGGACAGTGATCAATAGAGCTGCAGAAGTTGCTAGCAAGTCCCTAA

CTCATAATACGAACAAGTATTTTGTCTTGCTTATAATCACGGATGGAGTTATAACTGACA

TTCAAGAAACGAAAGATGCTATTGTAAGAGCATCTGAATTGCCCCTATCAATCCTTATTG

TTGGAGTGGGAGGTGCAGATTTTAAACAAATGGAGGTCCTGGATGCGGATACAAGAAGAT

TAGTGAGCTCCAGTGGCCGGATGGCTACACGTGATATTGTACAGTTTGTTCCAATGCGGG

AGATGCGAAGTGAGATTTCCATAGCTGAATCTCTGCTGGAGGAGCTACCCGGGCAATTTC

TGACGTACATGCGGGGCAAAGGCATCGAACCATCCTTTGCTCACCGCTAAATGGACTCTA

AGCTGAAGAATGAATTTGCACATACACTGGTTTAGCCAAATTAGTGGTGTTAGACAAAAA

TTGTCCTGTAAATGTAAGTTGTCTGAAATATGAGATTTCATTTTGGATGAATGACAGGTT

TAATGGATTATACACTCATGTGAAATATATGCAGAGTTTA

>Unigene0001036

TTGCCACTTAACATTGACGATTAAAAATGTTTTCTTCGGACGCCAATTATTCAAAACTCA

AGACTAACTTGAGACTGGCGTTAAATCGTCTAAAGTTATTGGAAAAAAAGAAAACTGAAC

TAGCTCAAAAAGCGCGTAAAGAAATTGCAGACATGGTTGCAGCGGGGAAGAGCGAACGTG

CTAAAATCAGAGTTGAACACATCATCCGCGAAGACTACTTTGTTGAAGCACTTGAAATTG

TCGAGATGTTTTGTGATGCGTTGTTATCCAGATTTGGTCTCTTACAACAATCAAAAATTT

TAGATTCTTCTCTTGCAGAATCAGTTTCCAGTTTATTATGGGTGGCTCCTCATATTCAAG

CTGATGTCAGTGAAATGAAAGTGATATCAGATCAATTAACACAAAAATTTGGTAAAAAAT

ATACTGAGGCATGCAGATCAGAAAACATGGACACTGTATCTGAAAAACTCAAGCATAAGT

TGAGTTTAAGACCACCGCCAAAGATACTCGTGGAGAAATATTTAATTGAAATTTCCAAAA

ATTATAATGTACCTTATGAACCTGATTTACAAGTCATGCAAGAATATGAACAATCTCATA

GTATAGATTCTATACTTTTTGATGGT

>Unigene0001037

GAACAAAGCTCTCCTTCAACACTAAAGGCAGTAACCATGGCAACCCCTTCCCCTGTCTTC

GAGCCCGGGAAGCACACCGTCGAGCTCGTGCACGTGAATCCCAGCTCGATGAAGTCTTCT

GATGATGTTGTTCTCCCTCCCAAACCACTGCTGATTGCTGCACCGTGTGAGAAAGGAGAA

TTCCCAACACTTCTCCTACTCCATGGGTACCTCCTCTACAACTCTTTCTACTCTCAGCTC

ATCCAGCACATCGCCTCCCACGGGTTCATTGTTGTCGTACCACAGTTATATTGTCTGGCT

GGACCAGATACTACAGAAGACATCAATTCCGCAGCAGCAACGGCAAATTGGCTGTTTGAT

GGATTGAAAGGTTTTCTCCCCGAAAATGTCCTACCAAATTTAAACGAACTCGTACTGAGC

GGGCACAGTCGAGGCGGCAAAGCTGCTTTTGCTCTAGCTTTAGGACACGCCAAGACTAAG

TTAAAATCAAAATTCTCAGCACTAATTGGTGTCGATCCCGTTGACGGATTATCCAAGTGT

TGTCATACTTGTCCTCCTATTCTCACCGGAATCCCGCACTCTTTGGAAGTTAAAATGGCC

ACTTTAGTAATTGGAGCAGGATTAAGTGAGCAGAAGAAGAATCTACTACTCCCTCCATGT

GCACCAAAGGGCCTAAATCACCAGGACTTTTATGATGAATGTTGTGCCCCAGCTTGGCAT

TTTGTTGCTAAGGATTATGGTCACATGGACATGCTAAATGATGACACCGAGGGACTGAGA

GGAAAGGCTACCTACTGCTTGTGTACTAACGGGGAATCAAGAAAACCAATGAGAATGTTT

GTAGGAGGGATCATGGTTGCTTTTATGAAGGCCTACTTACAGGGTGATACAGAAGACCTG

ACATCTATTAGAGATAATCCGGAGACTGCAAATGTTGAGTTATCAATTGTCAGTTTTTAC

GGGACAGAGTCCAGTGAAGATGTTAATGGGCACTTATCAAAGAGTTTGTTGTAGGACGGA

TTCCTACACTGTCATCGGTTGTCTGAAGAGCTCGCTCTGTATGGTTATGTAATAATGCAA

AAAGTCCATGTTGATCTTACAATTGTATGTATAACCTGTGTCCCAAGTTAAGGCCTATCC

GTATTTCGCTTTGATATCAAATA

>Unigene0001038

GAGGAGGAGGGGGCACACAGGCATCGGAAGAGCAGGAAGGAGAGGAAGAGGCGGAGGAGT

CATGCCAGGAGTGATGATTCTAGTGATGATGAAGATGAGTCCGATCGGCATCAGCGGCGC

CACAAGCGACGGGAGCATAGGCGCCACCGGAGGGAGAATAGTGGCTCGGAGGAGGATGAA

TCGAGGGCTGGAAGGGAGAAGAAGCGCTCTGAGAAGAGGCACAAGAATGAGAGGCGGAGC

TAGAAACTGGATGGTAATCATGCGTTCTAGACTAGTTCCAATATCAGTGTGACTTTGCTC

CTAATGCTACTTTGTGGATTGTGTATTCTTACTTTAAGATCTCTAGTACATTTATGCTTT

GAATACTATTGCATGGTTCTATGAACCCGGAACTTCAAGCTATTGCTTAAAGTTTGCTGT

AATCCGTAAATGGTTATCTCTTGTTACCGTGTTATTAGTATGACATATCCTTGTATGTTG

GTTAATATATGTGAAGCTTATGAGCTAGTTCAATA

>Unigene0001039

AAAACGCGCATCCCTCTCCATTCCCAAAATCCCCAATCCCTCCCACCGAAACCCTAGCTT

CGATCTCCACCCCTACCCGCCACCATGCCTGCCACCGCCGGCCGCGTCCGCATGCCGGCG

AACAACCGCGTTCACAGCAGCGCCGCCCTCCAGACCCACGGCATCTGGCAGAGCGCCATC

GGCTACGATCCCTACGCCCCCACCGCCAAAGACTCCAAGCCCTCCTCCGCCGATCACCCC

TCCCGTCCTTCTGCCGACGACGACGGTGGCGAGAACGCCTACGCCTCCTTCCAGGGGCTC

CTCGCCCTCGCCCGCATCACCGGCACCACCACCGACGACACCCGCGGCGCCTGCAAGAAG

TGCGGCCGTGTCGGCCACCTCACCTTCCAGTGCCGCAACTTCCTCAGCGTCCGGGACCAG

AGCAACAAGGAGGAGGATGCTGCCGCGGCACACGAGGCCGCTGCCGCCGTCTTCGACAAG

ATAAAGAAAGCAAATCATACAGCTGAATTTGACTCTTCGGAAGAGGAAGAGAGTGAGAGC

TCGGATTCAGACGCCGATCCGGAGATGGAGAGGATAATTGCGGCACGTCTCGGCAAGAAA

ATTGAAAAGAGAGCCAAGGATGTGTCTGGAGAAGAGAAGAGGAGACACAGCCGCAGGGGG

AGATCGAAGAGGAGGAGCCGCCATCGCTCCAGCCACAGTAGTGAGGATGACAGCGAGGGG

GAAGATAAGAAGAGGCAGAAGTTGAGGAAGAGCCATAGGGGATCTGATGACAGCGAGGAG

GAGGGGGCACACAGGCATCGGAAGAGCAGGAAGGAGAGGAAGAGGCGGAGGAGTCATGCC

AGGAGTGATGATTCTAGTGATGATGAAGATGAGTCCGATCGGCATCAGCGGCGCCACA

>Unigene0001040

TGTATGTTCCATCGACAAGAAGTAGCACCTCTAATCATCATCAATTATCATCATCTATAA

TAAGTCAAGAATATAACCGACTATCGAGCACCCTCGGACCCTCCTACATCAATATTCTCA

AGAGGCGGCACCATCGGGGATAGTCACCCATGTGTATCATCATCGCTCAGCCCCAAGGAC

ATTGTGGAATCCGTCGCCAATCTCCGTAAGATCATTAAGGACGTCGTTCAGCCTCGATAT

AGTCCTATAATTTTCCTCATCCATATATATATATATTTATTTATATATATACATATAAAT

AAATATATGTATCAAAAAATAAAAAATATATATTAT

>Unigene0001041

ATCTAATATTTCGGAGATATTTGGTGCAAAATATGTGTTTTCATTTTCCCATGCTTTTGA

TGAATTTTGCGGGTGTCGTTTCAGGTTGATGAATCTTTGTTATCGCCATCATTCACGACA

AGTTCTGATGAAGGGTGGACATCGATCGTCGGATTCGACATCATCGTCAGTGCACCTTCG

AGTTTCGTTAGCTTGTCATCTGTTGGATATTGCTGATGAGAAGCAAGGTAAACCATCACT

AATTACTCTCTCGTATAACACCCTCGATTATCGGCTCCACCATCACTCAGCCTAAGAACA

TCATGGAA

>Unigene0001042

ATCGTTAATGTACCTTCGAGTTTCAGCCCCTAGACACTCCTTGATAAGAAGCAAGGGAAA

CCACCACCAATTACTCTCTCATAGAGCACTCTCGATCATCAGCTCCACCATCGCCCAACC

CAAGAACATCATGGAATTCGTCGCAAATCCTCGTCACAAGCTCGCCAAGGATGTTGTTCA

TCTCCTCAGAACATGATGACTTAAGACGATGATTAATCATGTGCACCATCATGCATTGCA

TCATTGACATCTACGAACTTTCATGTATGTTCAATCGACAAGAAGCAACAT

>Unigene0001043

GCAACATCTTTAGCCATCATCGTCAAACGTCATCATCCACAACAAGCCAAGAATAAAGCT

GACTATCGAGCACCCTCGGATCCCTACATCAACATTGTCAAGAGGCGACACCATCGGGCA

TCATATGTTCCATTATCGCTCAAGGATGTTATCCGTCTCCAATCTCCGCAAAATCGTTAA

GGACGTCGGTCGGCCTCGTCAACTGTCACACAATATAGTTCTACATTTTTCCTTGTTCAT

ATATATAATTATTTATATATATACATATAAAT

>Unigene0001044

TATTTCAGAGACATTTGGTGCAAAGTATGTGTCGTTTTCTCATGCTTTTGATGAGTTTTG

TGAGAGTTGTTTCAGGCTGATGAATTTTTGTTAGCACCATCATTCATGACAAGTTCTGAT

GAAGGATGGACATAGATCATCGGAGTAGAAGTTATTATCATCGTCGTCAATGCACCTTCA

AGTTCAGTCCCTGGACACTCGACTGTTAACAAGCAAGAGAAAACCTCCACCAAAGAATCT

CCCGAATGGTACCTTC

>Unigene0001045

CCACCATCGCTCAGCCCAAGAACATCACGAAATTCGTCACAAATCCTCATCACAAGCTCG

CCAAGGATGTCGTTCACCTCCTCAAAACATGACGAGTTAAGACGGCGATTAACCATGCAC

ACCATCATCGCTCAACCCCAACGATGCTGTGGAATCCGTCACCAATCCCCGCAAGATCGT

CAAGGACGTCGTTCTGCCTCGTCAAATGTTACATGATATAGTTCTATTTTTTCCCTCGTT

CATATATAAATATATATATATAAATAAATATATGTATCGAAAAATAAAAAAATAATATAT

CATATTTA

>Unigene0001046

AGATATTTTGGTGCAAAGTGTGTATATGCATTTTCCCATGCTCTTGATGAGTTTTGTGAG

AGTTGTTTCAGGCTGATGAATTTTTGTTAGCACCATCATTCATGACAAGTTCTGATGAAG

GATGGACATCGATCGTCGAATTCAATATCATCATTAGTGTACCCTCGAGTTTCGTCAGCT

TATCATCTCCTGGACACTCTGTTAATGAGAAACAAGGGAAATCACTGCCAATTACTCTCT

TGTAGACCATCCTCGATCATCGGCTCTACCATCACTCAACCCAAGAATATCTCGGAATTC

GTCACAAATCCTCGTCATAAGCTCGTCAAGGATGTCGTTCACCTCAAAATAGGGTGACTT

AAGATGACGATCACCCATGTGCACCATCATCGCTCAGCCCCAAGGACGCTGTGGAATTTA

TTGCCAATCCCCGCAAGATCGTCAAGGATGTCATTCTGCCTCGTCAAATGTCACACAATA

AAGTCCTACTTTTTTCCTTGTCCATGCATATATTTATTTATATATAAAAATAAACATATG

TATCGAAAAATAAAAAAAATAATATATCATATTTATATATATAAATAAGAAGAGATTTTT

TTTTTGGAAGAAAACAA

>Unigene0001047

TTGATGAGTTTTGTGGGAGTTGTTTTAGGTCGATGAATTTTTGTTAGCACCATCATTCAT

GACAAGTTCTGATAAATGATGGACATAGATCATCAGATTGGAAGTCATTGACATCATCGT

CAGAGCACCTTTGATCATCGGCTCCACCATTGCTCAGCCCAAAAACATCATGGAATTTGT

CACAAATTCTCGTCACAAACTCGCCAAGGATGTTTACCTCCTCAAAACAAGACGACTTAA

GACGGCGATTAACCATGTGCACCATCATCATGCATTGAATCATCGACAACTACGAACTTC

CATGTATGTTCCATCGACAAGAAGTAACATCTTTAATCATCGTCAAATGTCATCATCCAC

AACAAGTCATGAATAAAGCCGACTATCAAGCACCCTCGGACCCACCTACATCAACATTGT

CAAGAGGCGGCACCATCGAGCATCATCATCTGCT

>Unigene0001048

CTCACTCCCCACCCAAACCCTAAAACTCCAAATTCGCCGTCCGCAATCATCATCGCCGGC

GGCCGAGCTAGCCAGCGTTTGCAGCCGCTCTGCTCATTCTTGGCGCCCCACAGGGTTGTG

ACTTGACCTTCTGTGCTTGCCAATCCCGTGGGAGGTTATTGCTAATGGCAGAGAGGGCTT

GCGTCAAACGTTTGCAAAAAGAATATCATGCACTTTGTAAGGAGCCGGTTCCCCAGGTTG

TTGCCCGTCCTTTGCCTAGTGATATTCTTGAGTGGCATTATGTCCTTGAAGGAAGCGAAG

GAACCCCTTTTGCAGGTGGATGTTACTATGGCAAAATTAAGTTTCCACCAGAATATCCTT

ACAAGCCTCCTAGTATCAGCATGATTACTCCCAATGGAAGATTCGCTCCTCACAAGAAAA

TTTGTTTATCAATGAGTGACTTTCACCCAGAGAGCTGGAATCCTATTTGGTCTGTATCAA

GCATTCTTACTGGACTACTTTCTTTCATGATGGACGATGGCCAAACTACTGGTAGCATCA

GGACATCAGATGATGAAAAGCGACGATTGGCCAAGGCCTCACTTGCCTACAACTGTGAGA

GCAAAAACTGTCCACATTTCAAGAAACTATACCCGGAGTATGTGGAGAAGTTGAACCAAC

AGCAAGGTCCTGAGCTGTCTGTTTCGGAGCAGACAACGCGGGGTACACACCGCAGCCTTC

CTGCTGTTGAAGGAGCCCTAATGGTTGAGGGTGAAAATGAGAGGAGGGGGGTTGCTCACA

ACAGGAGGCAAGCGAAGAAGGCGCCATTCTGGCTGCTTGTGCTTCTGGTGTCGGCTTTCG

GCCTTATGATGGCGTTGCCCCTACTCCAGATGTGATTCATTGATACAAGAGAAAGTCGTG

GTAATGTGTGGTTAGGAAAACTGTGTCCTCTGAGTTTTGTATAGTTGGTTGGTGAAAATC

TGTACGCACCCGTCTTCCAGTATCATCTTGTTGAAGAGAGTCGTGGTAATGTGACTAAGA

ATACTGTGTTCTCTGAGTTTTGTATAGTTGGTGAACATCTTTACCACACCCCCATCTTCC

AGTATCGTTGTGGTGTTAAAATGGTTTTAAGTTATTTATTACATCACTCTGGGGTATATG

TCTAGTTTTACCAAAATCTTACTGTCACGTATGTATTAAAAAGTGACTGTATTTTCTACT

TAGAGTTGGTCTTGTTATATTCCAACCCCATTACTGTAAAAAATGATCGTCCAATACTGG

AGGCATTCTTATGCCACTCTGCTGGGGAGAAAAAAAAAAGTCTTTCTTATGCCACTCTGA

TGGGGAGTTTTTCTCTATCATGGTTCTGTTAATTTCGGCATATTGGA

>Unigene0001049

GGAGAAGAATTGGATGGCTACGTGTTTCGATCGATGGGAGAAGGACCCCTTCTTCCCCGC

CGCGGAGGAGGTTCAGGAATCCGCAGACAGGATGGAATCTGTGTACCGGCGATGGATCCA

TGAGAGAAAAGACACACCATATTCGGCCGGTGAGGATGCTACTCTCACCGGTGACCTCCG

CCGAGAGCTCCACACCGCCAAATGGCAGCTGGAGGAGCTGGAGCGCGCGGTGACATCGAA

TGATGAAGAGTGCTCGGTTGGGGAGGACACGAGGTCCCGTCATAGCCAGTTTGTCGAGGC

GATTGGGAATCAGATATCAAATGTAGAGAATTCTCTGAAGGAAGCTAATGTTGAATCCTC

GGAGGGGACGGAGATGTCGAGGGTCCCCTTGGATGAAGGGGAGCGGGATGATTTGGCTCT

CTTTTTGTCAGGTTCGCTACCACCATTGCCACCGCGAGCGGTGAATCCGGTAGAGGTTGA

ATCGAAAAATGGTGCTGAGTCGTTGAAGAATCAGCGTCGGGTGCACGGGCATCGGAGGAC

GGCGAGTGCTAGTGCTGATTTTGGGGTATTGAAGATTTCGGTTTCGAATGAAGATGGGCC

GCCTCATGTTCCCCCTCCGAAGACGGCGAGCTTCTCTAACTTGACAAAGGTGTTGGAAGC

AGCGCCGAAGATGAAATGGCCGAAAAATGGTTTTAGGAAATGGAAGGGCGGGGATCAACA

TCACGAGGATGAGACTATCCCGTTACACAATCAGCAAATAAGTCAAGGACTCAATGCATG

TTATGAAAGGAGTAAGAGCTATCTTAGTAGTTGTGGTGATGGGTCCTATGATAAACAACT

TTATGGTTGGTTTGGAGTTTTTCAGAGACAACTTCAGAGGTCTCAATATCATATTCAATA

TGGTTGTACGGGTCAAGTAATTTTGTGGACCATCGGTGTTGTTCTTTTAATTGTAGTACT

ATTTGCAATGTGCGTTATTCAGAGGAGCTAGCAGTCTGATTCATCATTTATTACTAGATG

TCAAGTGAGACACAACATTGTTACCATGGAGGTTTGCAAATAGCTCCTTTCTTAAGTCAT

ACTCAAAATGTTTACCTCTATTTTCTTAAAATTTAAGGTAATTTAGTATATGGATTCCGT

GTTTTACTGGAATTTAGACTTTTGGGTCCAAGTTACAGCGATAAGCCCCAAATTTCGAAG

GTTAAGTAAAAAACATCATCCGGGGAAACAGTAAAACCCACATTGCAAGGGTAAGTAATG

ACATCTTACTGACATTAAGTTTCCTGATGTGGTGCAAATGGTTTGTCCCTAAAGCCTAAA

TTCCAAAAAAAACTTGGGACTCATTTCCTACTCAATTAGACGAATAAGGTATCACATTGG

ACTGTAAGATGATTATCTAGAATTAATTTGTTTCTTTTCTATTTTATTGGTACAGGATTG

CTTTTGCGGAGGAATGCAGCTTTTTGTGAGGGACATTGTTACTGTTTATTCATTAGGTGT

AATTAATGATGAATTCCCTGGAAGGCTTCGAGTGGTGTATTACTGTGGTGCTGAAATATG

TATAAGTTTTGATTTCTTAGATATTGTAGCATGTTTATATATGTTTTTCTCTTATCCTTT

ATTAGAGCTGTGTCATACTAAGGGTGTTGTGAGTAAAGTGTGTGGAGATAAAGCTCGAAA

GTAAAGGCAGCAAATGTTGGTATTTTCCACACAAATATATTGTAGGATGCGAGCGGTTGC

GTCAATAGTGGTGTGGAATGTCATGCTTTTGACTTGACGGTAGCATATAAT

>Unigene0001050

CGATTTCTAGGACGAGAGGTGAGAGGAGAGAGGAGAGAGAATCGAAGGGATCCCATTTTC

TCCTCCTCTGTCCTATCAATCCCCGTCAACCCCACCCCATCCCATGTCCCGGGCCGCGAT

TCACCGCCCCTGATCTTCGCAATCGTCGCCGATCGCCGCCGAAGATGAACGGGAGGGCCA

CCGACGACGCCGCCCCCTCCGCCGGGGTCTCGCCGCCACTGGAATGGAAGTTCTCGCAGG

TGTTTGGGGAGCGGACGGCCGGTGAGGAAGTTCAGGAAGTTGACATTATCTCAGCCATTG

AATTCGATAAATCCGGGGATCATCTTGCCACTGGAGATAGAGGAGGGCGTGTAGTTTTAT

TTGAAAGAACAGATGCTAGGGAGCAGGGTAGTTGGAGGGATATGAAGAGTCTAGATAGTC

CTATTAGCAGGCATCCTGAATTCCGCTACAAAACAGAGTTTCAAAGCCATGAGCCCGAGT

TTGACTACCTTAAGAGCTTGGAAATAGAGGAAAAAATCAACAAGATCCGGTGGTGCCAGA

CAGCCAATGGTGCACTATTTCTTCTCTCCACAAATGACAAAACAATCAAATTTTGGAAGG

TGCAAGAAAAGAAAGTTAAAAAAGTCTCTGAGATGAATGTGGATCCTCCACAAGCAGCAG

GAAATGGTAGTGTCGGTAGCTCAAATATAGCTAGCTCGAGAGGGCATCTTCCGAATGGTG

GATGTTCAGAGAGGCCACACAGTTATCTGAGCAGTGACTTTACATTTCCTCCTGGGGGAT

TTCCCTCGCTGCGTTTGCCTGTGGTAGCTTGCCAAGAGACAAGCCCTGTTGCTAGATGCC

GTAGAATATATGCTCATGCTCATGATTACCACATCAATTCTATTTCAAATAACAGTGATG

GCGAGACTTTTATATCGGCTGATGATTTGAGAATAAATCTCTGGAATTTGGAAATTAGCA

GCCAGAGTTTTAATATTGTTGATGTGAAGCCTGCAAATATGGAAGATTTAACCGAGGTGA

TAACGTCTGCTGAGTTCCACCCTACCCATTGCAATACGTTAGCGTACAGTAGCTCAAAGG

GCTCAATACGGCTAATTGATTTGAGGCAATCAGCTTTATGTGATAATCATTCCAAGTTGT

TCGAGGAACATGAGGCACCTGGTTCAAGGTCATTTTTCACTGAGATTATTGCATCAATTT

CAGATATAAAATTCGGGAAAGATGGAAGACACATACTTAGTCGTGATTATATGACTCTTA

AGTTGTGGGACATAAACATGGACTCTGGTCCTGTTGAAACCTTCCAGGTCCATGAATACC

TGAGGCCTAAGCTATGTGATTTATATGAAAATGATTCCATCTTTGACAAATTTGAGTGTT

GTCTGAGTGGAGATGGTTTACGGGTTGCTACTGGTTCATACAGCAATCTTTTTCGTGTCT

TTGGCCGTGTTGCTGGAAGTACCGAAGCAACGACTCTGGAAGCTAGCAAAAATCCTATGA

GGAGACAAGTCCAAGCTCCAGCAAGGCCAGCACGATCTCTTAGCAGTTTAACCCGTGTTG

TTAGGCGGGGAGCAGAAAATCAAGGAGTCGACGCGAATGGAAACTCATATGATTTCACAA

CCAAGCTGCTCCATCTTGCATGGCACCCAACTGAAAATACAATTGCCTGCGCCGCTGCAA

ACAGCTTGTACATGTATTATGCATAAAGATGGCCCCTGGATATTTATTTTTTGGTTTAAG

CAACCAGCAAGAAAGTGCAGGAAGGAGGGTGATACGTTGTGGTAGGTGGTGTAGAAGGTG

CCCTACACCACGAGGAAGAAAGACATTTGTTCCCGGGAGGGCATCTTGCTCCAATTCTTT

AGCAATAGATTTTGTATCGCTAGACATGCATGTAATGACTTGTGAATGGGGTTATAATTG

TTGTGCTAACCGTAGTTCTTTTATTCTATGATTAGGCTTTGATTGTGTTGTTATACTGAT

TATGCGAAAGATAACTAACTGCAGTATTCATATTCATTCCTTATTTATTATTATTATTAT

GATGATGATGATCC

>Unigene0001051

GGTAGCTCAGGCACCGGACTCACATCCCGCCACCGCCACACCCGCCGCTGCCGCCTCCGG

TCCTTTGAAAATCGTGAAAATTCCAAGGTAGAGCTGGTATCCTAAATTGATACTGGACCA

AAGCCAGTGACTCTGATCCAGTGTTTTTAGGGCGTGATCGAGAGATGGCGTCCGAGGAGG

GAGCAGATTCGTCGTCGAGGCTAGCATTCATGGAGATGGCATTGGAGCAGGCAAATTTTG

CTTTGGACAACCTCGAAGTTCCTATAGGGTGTGTAATTGTGGAGAATGGCAAGGTTATTG

CCTCTGGTAGCAACCGAACTACGCAGATGCGGAATGCTACCAGACATGCAGAAATGGAGG

CTATTGATGTTCTACTTAATAAGTGGCAGAAGATTGGGCTCGACCAATTGCAAGTCGCAG

AAATATTTTCAAGATGTGATCTATTTGTTACATGTGAACCATGTATAATGTGTGCGACAG

CCTTGTCCATTCTTGGAATAAGGGCGGTTTATTTTGGTTGTGCTAACGATAAATTTGGAG

GATGTGGATCAATCTTATCTCTTCATGAAATCAAATCGGAGAACTCCAGCGGTGATTTAC

AAAGAAAGGGCTTCAAATGTATAGGCGGACTTATGTCTGAGGAGGCAGTCTCTCTCTTGC

GAAGTTTCTACGACCAAGGAAATCCAAATGCTCCAAAGCCACACAGGCCTGTTCGACTCC

CACGATTGCAATCTTAGCATTTTTTCTCTGTAAGTTTTATTTCGCTGATGAAGATTAAGA

GGATATCATCAGAATACTTATATGAGATCAGCTATTCAAGATTTTTTTGTGTTACAGAAA

TCATGTTATATTGGTTACAAACTTGTGTTGAGAGGAGTA

>Unigene0001052

CCCCCGCCTCCACCCTTGACATAACCTCACTCGTCAAGAAGGACCAGATCGCCTTCTTCG

ACAAGGAGTGCTCAAAGGTCGCCGACCACATCTACCTCGGCGGCGACTCCGTCGCCCGCA

ACCGCGAGATCCTTCGTCAGCACGGCATCACGCACGTCCTCAACTGCGTCGGCTTCGTCT

GCCCGGAGTACTTCAAGTCCGATCTCACGTACAAAACCCTCTGGCTGCAGGACAACCCTG

CCGAGGACATCACCAGCATTCTCTACGATGTCTTTGACTACTTTGAGGATGTCCGCGAGA

ATGCCGGTCGAGTGTTCGTTCATTGCTGCCAGGGCGTCTCGCGGTCTACGTCCCTCGTCA

TCGCCTACCTAATGTGGCGCGAGGGTCAGAGCTTCGACGATGCATTTCAGTTTGTGAAGG

CAGCTCGTCGAATCGCCAACCCCAACATGGGCTTTGCCTGCCAGCTGCTCCAGTGCCAGA

AGCGCGTACACGCCATCCCGCTCAGCCCCAATTCGGTGCTTCGGATGTACCGGATGGCGC

CCCATTCGCCCTACGACCCGCTACACCTTGTCCCCCGGACGCTCAACGACCCCACTCCAT

CCGCTCTTGACTCGAGGGGGGCTTTCATCCTCCACATCCCCACCTTGATATATGTCTGGA

TTGGTAACAACTGCGAGTCCATCATGGAGAAGGATGCCAAAGCGGCAGCATTCCAGGTGG

TGAGGTATGAGAGGGTGCCTGGCCCTATCATCTCTGTGGAGGAAGGGCGCGAGCCGGCCG

ACTTCTGGGATGCCTTCATCAATGCACCTCTTGGATTGAACACCACTGCCCCTAAGGTTA

ACAGGGAGGACATTGATTCAGCGATCAAGATCGGTGCGGGGAAGAGGAAGGTGGATGCTT

ATGATGTTGATTTCGAGCTCTTCTACAAGGCGATCACCGGAGGTGTCGTGCCGCCGTTCC

CCTCATCGGGGCCTGGGCACGAGACGCATCTTCCGGCAAGAGAAAGCAACTGGAGTGTAT

TGAGGCGCAAGTTCTTGTCATTGGCCAGAGATGTGGATCACCGAACAAATCGAGTTCAGA

TTTTGTCCGCAGAGGCATCGACATCCCCTCATCTTTCTCCCAACTCCCTTTCATCAGACT

CGAGCATTACCTCCAAGGATAGTCTGGACTCACCTTCCTTCTCTCCCTCAACATCCTCCT

CATGGTCGCCAGGGCATCCTCCCAATCTGTCGATTACTTTGGAACCTTTAACGCGTTGCT

TGGGAGCAGCAGCTTCTTCACCATCCAAGGGTGTTTCGCGCTCTATTGCTGAGAGAAGAG

GAAGCTTTTCTCCTTTGAAGTTGCCAGCATTGGGCAAGGATGCCTCCTTACCTGGGATGG

TAAGGAATGAATCCCATACTTACCTTGTTAGTCCAAGGGAAGCATTAGAGGCGAGCAGTA

CTAGCCACATTTTGTCTAGAAATGATTCGATCAACAGTTTAGGTCGAGTTACAGAAAATA

GTTCGAGCTCCCTTTTGGAAAATGGGCGGCCATCTAGCTCGAGTGGCTCAACGTGCATTA

CTTCTGAGTCTCTAAACACTCTTGAACCCTTAGTATTATACCAATATCCAAATATGGAAA

AAGTAGCATTACATGGTAGAGAGGAGCTTGATTCAAAAACAGTGTTTCTGCTGTTGACCC

AAAATACAGGTAACAATGAAGAGCCTGGTAGGAAACTTTACTTGTGGATAGGGAGCACCT

TTGAACAGAGAAGTGGGCATACACAGCTGAAGAGCGGCAGAGTTGTAGACAAAGTTGACG

AGATTGACTGGAATCATGTAGGCCTTGAGTTTCTTGCTTCAATGAGTCTCCCAAAGGATA

CCCCAATTAAGGTTGTCAAGGAACATGAGCCAGAAGAATTCCTTGAACTGTTGAATTTGT

AGTGATGCGTCTTTCCAGCAATAGACTCTGAGAGCTCAAACTGAAATACAGCATCCTTTC

TTATTTCTCAAAGCTATGGAAATTTACTTGACATTTTGCTCAGAGCTTAAAAGTCCACAT

CTACAAGCAAGGGAGGCGCCCGAGAGTCACATAGGTGACTACTCTGTTTCTTGGCGCAAC

TGGATGTACAGTGCCTGCTCCCTGTTTGCTGAGATACTGGATCAGCAGTTAATTTAGAAA

TTAGTTTTAAATACTTTGATGTAAACAAATTTATAAACAATTTGGTCCAGTAGATGAAGA

GCTTCATCCATTTGGATGGATCACCTCACTCGTTTGGCATGTTAATGCTTATCATTTTAT

CTATATACCTAATCGTAGGTATGCACATAATCTCATTCTTGTCTGTACTAATAAGAGTTA

TTTGTTCTTGTTTTTGTTGTTGTACTGGCACTGTTGATGTTTATTAGCT

>Unigene0001053

TTGAATATTACAATGGCCGTGGTTGCTCAAGTTGCTTTAAATGATCCAATTAGTCAATTG

AAGATTGATGGATGCCTCATTTGTCCCAGCAAGTGGATACAAATTCCTGATGTCTCAGAG

GTCTGCGTGCAAAATATTGCAAAGTTTGCTGTGCAGGACTACAACCTCGTACATAAGGAG

AGTTTCAAATACCAGAGCATTAAATGTGGTTGGTTTATGGAGCTGAAGGGTGAAGACCTG

GCCTTCCGCCTCCATATTGTGGTGAAAGACTGTTTGGGACGTATCTTTGAATTGGATGTC

GTAGTTTCTGAAAAGAAAACATGTGCTGAAAAGATCAGAAAACTTGAATCGGCTAAACTT

ATTAAGAAAAACTGATCAAGCACTCGTTTGTACTT

>Unigene0001054

ACCAATGGCAGTGGTTGCTCAGATTGCTTCAAATGACCCAATCAGTCAATTGAAGATTGA

CGGATGCCTCATTTGTCCAAGCAAGTGGATACAAATTCCTAATGTCTCAGAGGTCTGCGT

GCAAAATGTTGCAAAGTTTGCTGTGGAGGACTACAACCTCGTACATAAGGAGAGTTTCAA

ATACAAGAGCATTAAATGTGGTTGGTTTATGGAGCTGAAGGGTGAAGACCTGGCCTTCCG

CCTCCATATTGTGGT

>Unigene0001055

TCCAAGGACAGCTGGTACAGTAGACAAGGGTGGCCCATGACATCTCATAGACAGTGATGG

AGTAGGTAGAGCATATTTAAGGGCAGCAATCAGGACATGGAGGTCAAGGCCAGTATAGGC

AAGACAGTGTGGCATCTCAGCATCAAAGGGGACAAGCCATGGCATACAGGTAGAGGAGAT

AGAGACTATCATGAAGACTTTAAAGGGGGTCTAGGCCGTCTAAGCACGTAAGATAGGGGA

AGGTGCTGGAAGTTTACAACTATATATATGTGAAGAGGAGTACACAGAAGCTCCAAGTCT

CCCTGTGAGCAAGTTGCATAAAGTCAAGTCTCCTTAGGTTAATCCAAGCATCTAGAAGAG

CATCAGAATAGAGAATGTATTCAATCATAGTGTTTCTTCTTCATTAGTTGCTACAGAGAT

AGAGTCCAAGCTCTTCTGTAGTTGATGTTCTTGTATTGTTGATTCAGTTGAGGTTAGACA

GCCTAGTTCTTTATCTTTAGTGAAGCTCATAGCAAGTGAATGTAGGATTGACTATCTAAG

ATTAGCATTACTTATGAATGAATGATGTAGAGATTGAAACTTCTGAAATGCAATAGTTA

>Unigene0001056

TGTTGATAGGGTTGATGCTGCACAACTATCTGCTCTGCAAATTTTGGCTGAGATTGGATA

TATTGCAAATTATCCTGTTGATAGGGTCGAAAAACTGTTAGGATATTGTTAAAAGAGAAC

TCATTACTTGCACAGAAGATTTAAGGCACACTTGACTACATCTCAAGGCACAACAAGGGC

ATGGTTCTCTAGCATAGACAGACAAGGCAATTCTCAGTTAACTTTAGAGTAAGACAATAT

GCACAGACTTGGTGATAGGTGATGGAAGACAGACATATCTCTTGAAAGGCAAGAAGACAT

AAAGGGATAGCTCGAGGCAAGAGCAGGAGGCAGGTAGAGAGTTGATGTGGGATTGGCTGA

TAGTGAAGGTTGTACAAGGGTGGCTCAGGAAGGCAAGCAGAGGAGATAGGCAGTGGATAC

ATGAGTT

>Unigene0001057

CAGTCGCACGCGTTTTTACATAACGTCGTACGCTCGCGCCCATAAGCACCGTAGTCAAAA

GTCCACGTCGTTCGGTCAAGTGCATCAGGACCGTAAATCATTAATCGGTCGTTGGTCGTT

GCGCTCTCGTCTACCCGTATTTGCCCACCGCCGACATGAAGCCGGTGAACCTAGCGGTAT

TGTTGTTCTGTTTGATCGCCCATGGACTTGGCAGACCGTCGGATAGCGATGAACGAGACG

AAATCTTAAGAGAAGCGAAGAAAGAAAAACACTTCCAACATCTGTCGCGAGCCCAACACC

GAGGATTCGCGGACAACGTAGAAGAAGCGGTTCAGGCCGTGCCATTTTACGGTAGCGGCA

AGGGCAAGTTCCTGGTGATTCATCCTTCGGGCAAGACCACGGTGGAAGACCATAAGCCAC

CGCAGTTAGCGGACGGACCCAAGGATGACGACGAGGACGTGGGACAGGAAGAGGAGACCG

GAGCTGACCCGTCACCCGCTGAGCCGCCCAATGAGTCGGTGAGCGTCAACCTGCCTCCAG

ACAACGCGTCAGTGGCCGAGGCCAAGCCCGTGGGCCTGGCGATCGCCGGCACCGGTGGCG

TGGCGTCCTCCAAGCCTGTGGCCACAGCAGTGGTCGGACCCGGTGGCCTGGCGCTGGCTA

AACCCGTGGCCACGGCCATCGCCGGCATACCTGGCGCCGAAGCGCTGGTCGGCGTGGCCA

GCAGCGGAGGCAAGAACAGGCAGAAGGCCGCGGCCGCAGCCGTTGCCGCCACACCGCAAC

CGACCAAGGCGGCAGTCAAGACCAGGAGGTCGGCCGGCCCACGCGGACCTGTCGCAAGCA

GGACCGTCGACGACACTGCCGTACCGCAGGTGTGGCCCACATACCCGTTGTACAAGATAC

CTGAGTCACACGCTTGGGGATTCATACTCAGACCTGTGTACGCTCAAAACGTCAACTGAC

GGACTAGTCAGTCAGCGCGCGACGGATATTATTAATATTATATACGACGATTATAACTTA

ATGTATAGGGCTAGGATTTATAAGCAATACAATATTATGAAAAAATACGCATTTTACTTT

AAAAACGTTCAAAATTCTATCAATTGTTAAATAGTTTCAAACAAAGGTCGAACGCAAATG

ATACAATAAAGTATTTTACAAACAGTTTTGCAAACGCCACTAGATAATTCTAGAAAAAAA

TCCACCTATTCAGTTTAGTATGAGCCAAATCATAAATAAAACTGACAATCAGTAAAGGAA

AAACATATTTTAATGCATAAAAATCCTAGCCCTAATAATGTATAATAATATTAATACGAT

ATCATAGTCCTTGCCATTCGATATTCATTACTATATTTAACGCGTCAACTTTTATTTATT

ATTTCATAATTTTATTTATACAAAGTGATTTTTTTTTTTTTTTTTTTGATATCAATAATT

TTTTTCTTTTCCGTTTTGGTGTGGGTGGATGACCCCCGCAATGCAGTATAATATAATAGT

ACTATATACACGCTTAGATACAGGAGTAGGTAATTGTAATATTATACAATATTGTGACCA

CTGGAGCGATGTATTTATGTTTTTTAGGCAAACAATATATTCATATACTCGACAGAAAAA

AATGTACTCATCAATTTATAATACGATCGACGGTCAGCGACGTATCACCATTCCTATCCT

ACTCCGGCCAATGGTTACGCCATAGGTTGGCACGAAAATGATTAATGTGTAAACCATTTG

ATATAATATTATTTTTACGTTTATATTATGCGTGCATTACAATAATTATAATAC

>Unigene0001058

GGTATGTGTGCATGGCTGGGATGGACCATCCCTCATACCGGCAATGGCAAGTATTTTGGA

CATCCAGGATGTGCAAGTTATTGTGTTATTATTATCCAAGCATGAACACATTGTCGAAGT

CCTTTTTATTCTTTTTCATCCTGAAACGAATTACGATAAAAAACAAATATGATATCATTG

CAATGGAAACCTGTCTAAACAGCTTGATGCTAAAAAGGATGAAGTATGACCACATGTGAG

TTCATCCACAAATGTATGCATCCAACTTTCTACCTGTCATGTAGGCACTTGCGTTTTTAA

GCATTTTCAGGAACAAACTGGTATGTGTGCATGGCTGGGATGGACCATCCCTCATACCGG

CAATGGCAAGTATTTTGGACATCCAGGATGTGCAAGTTATTGTGTTATTATTATCCAAGC

ATGAACACATTGTCGAAGTCCTTTTTATTCTTTTTCATCCTGAAACGAATTACGATAAAA

AACAAATATGATATCATTGCAATGGAAACCTGTCTAAACAGCTTGATGCTAAAAAGGATG

AAGTATGACCACATGTGAGTTCATCCACAAATGTATGCATCCAACTTTCTACCTGTCATG

TAGGCACTTGCGTTTTTAAGCATTTTCAGGAACAAACTGGTATGTGTGCATGGCTGGGAT

G

>Unigene0001059

GGTATGTGTGCATGGCTGGGATGGACCATCCCTCATACCGGCAATGGCAAGTATTTTGGA

CATCCAGGATGTGCAAGTTATTGTGTTATTATTATCCAAGCATGAACACATTGTCGAAGT

CCTTTTTATTCTTTTTCATCCTGAAACGAATTACGATAAAAAACAAATATGATATCATTG

CAATGGAAACCTGTCTAAACAGCTTGATGCTAAAAAGGATGAAGTATGACCACATGTGAG

TTCATCCACAAATGTATGCATCCAACTTTCTACCTGTCATGTAGGCACTTGCGTTTTTAA

GCATTTTCAGGAACAAACTGGTATGTGTGCATGGCTGGGATGAACCATCCCTCCTACCGG

CAATTGCAAGTATTTTGGACATCCTTAGTCCAGGGTGTGCATACATTTTACCAAATAAGG

GGTAAGAAATGAAGGCCTTCAACCTGAAGAGTGTCTTGTGGACAACCAATGTATATGCGG

TTCATAAAGAAAAACACTGTATCTTGGCCTGCCCAAAGTAAAGGTGAATTGTGGGATCGA

ACATATTAACTAACTATTTATCATTCTATAACTATATATATATAAAATCGTATATGCTAT

ATTTTGTTGATGTATATCAACCTTATCATCTATAGATAGAGCTGAGGATAGTCTAAGCCA

TGAGACATGTGTAGGTATCACTTGTAAGTTATATATATATATATAAATAAATAAATAAAT

AAAGATTTGTGATGGTTTTGGACAGATTA

>Unigene0001060

ATATATAAATAAATAAATAAATAAGATCAACCACACCATTCATCTTATCTTAATCTCTCT

TCCTGATGGTACCTCGTGAGAGATCTTAGTGTTCTCTCTTCCATCCCCATCGTCCATGAG

CCTTTTCCGTCTTCGATCGTACTGTTGGCGGTCTCTGAACTGAGCCGATCTAAGAGATCA

AACTTAAATATGTGATTTCTCGCCGAAGGCCCTTCATGCGTCTCCGACCTGACTAAGATC

TGAGATCTTCTGTCTGTGGTGCTTCAGATTGGGTGCCCGAGGAACCCCAAGCTGTCACTG

CTGTCATAACCTCTGTTTTCATCATCTTGTTGTCACCACCTCATCATCTTATTGTTGCCA

CCACATCCTCTGTTCTACCACCAAATTCGTGATTCGTGTTAGTGGTGAGATCTGAGGCTG

TATTTTTCATCAACATCAACCCCATCTCTCCATCTCCGGCCACTGCTGGGGCGGTTTCCG

GCAAGAGAGGTAGGAGCTGCAGTGGTTGAGGTGATATGTTGTGGATTTAAGGTCTCAAAG

TGTCGGGTTTTGGTTTGTTTTTTCGGCCCGAATCTGATCGAGTTTTTAAGTTGATTTTGT

TTTGGGTTTTATAATCCAAATTTAGATTCAGGGTCTATTTTTGGCGCCCAGATGCCAGAT

TTGAATCCGGTGCCCAGATTTGGCCGGGTTAATACAGTTTTGGCTCCAGGGGTACAAATT

TGACTTTTTTTGGGG

>Unigene0001061

TGGGGATCACCGGCTCAGGGTAAGGATATTTTGTTCTTGCTGCTTAATCTTATGTCTGTA

AGATGCTTGTGATACTTGTCTTGTTAAGTTAGATATATTCTTAATTCACGATGGATGGTA

TGATTTGATCGTTACTTCAAATTCATGAACCTATTGGTTATTGCCTCCCTATAACATCCA

CGAGAAAATAATCTGAGATAAATTTTTTTCATTTTTTTCTCATAATTTCATGTTATTATT

TTGTCACCGGTGAAATAAAAAAGGAAACTAGATATTCAGACTACTTTTGATTGGATCTTT

GGATCTGTTTGAAAGAGTAATCACCTAAGCATAAGTTTCGGAGTATTAACTTCAGAAGAA

AAAGAAATCATTGTGCTCTCTTTTTTTCTGTGTTATCTTGTTTTACTTTTCGCACTTATT

GTAACCTTACAACCAAGAGGACAATTGGAAGACACAAAAAACAATGGGGGAATCTCCAAC

AAGAATGATATTCTTTTTCTTTCCATCGTATTATCCGAACATGAATGCGAGTTATTGTCT

TTTTGTTTCTATTCCATTCCAATACAAATTCAGGTTTAGAGATGTATGATCTCGAATTGG

TGAATCCTTAGCAGGGATGGATTACACAGGAGTTCATCCATGATTCTCACCTTCTGTATG

TGA

>Unigene0001062

TGGCCTGATGATTCTCACCTTCTGTATGTGATGTGGAGTACTGGCCTGATGATTCTCACC

TTCTGTATGTGATGTGGAGTACTGGTCCGATAGTGATGTGGAGTACTGGCCTGATGATTC

TCACCTTCTGTATGTGATGTGGAGTACTGGTCCGATAGTGATGTGGAATACTGGCCCGTC

AATGGATCCAATCTCTCTGAATCCTAGTTCGATTAGTCAGATTTGGCAGAATATTTAGAT

CTGATAAGCTGATCTGAATCCAAATCTGTTATGTAGTACCTAAATTGATTGGATTCTGAT

ATAGCATAATGCAATCTGATTAAAATCCAACT

>Unigene0001063

TTCTGATATAGCATAATGCAATCTGATTAAAATCCAACTCGAATGGAAAATATAAAATAT

TGTATCTACTAAATGATTGGATTCTGATATAGCATAATGCAATCCGATTAAAATCCAACT

CAAATGAAAAATATAAAATATTGTATCTACCAAATTTCTTAAAGTAATAATTAGACTGTT

TTTAGTTTCCCTCCTTATTTTATGTTTGATTAGTTTTAACTCACTACATGAAACTCAGGG

GCTTGCTCAAAATTCGTAAAAATTAAGATAAAATGATTGATGTTTTTACTAATATTCTAA

AGCAGTTGATTTTGTAAACTTATATAATATTGTGGTAAAGTTTTAAGGTTTGTTCATTTA

ATATCGATTATATAATTAAGGATAGTAAACTATTTATTATTCGGATCTGATTATAGGATT

CGGATATCGGATTTTTTCAAACTCGGAATTGGATATGTATTTTTAGAAATCCTTCTGATT

TCAGACCGGAGTCCAATTTATCGGATCGGATTCAAATTTTAGCAAGTCCAATCCAAATCT

TATCGGTTTACAGCCCTATCTTGCAGTGGAACTATAAAAATAGTTCTTTCTATCAACATA

TAACTACCTTTTCTATGATAACATTAAATGATTTGGATATAAGAAGGATAGAACCAAAAA

GGCAACCCTTCAAACTGCAAGGGAAACATGGGTTTGAGACATGCAAGGTGCTGAATGAAG

ATTAGATTTTGCCACCCTGTTTATCCAGCAATCTACTCCTACAATAAATGAATCATGAAA

TCCCTTAACACACAAAAACTAAGGGATACTTGAACTTGGACTACTCTGCAGAGACTAGAA

TTCAAAACTTTTAGGCTTGAAAAGTATGTCTGTAATGATTTTTCCTAGTGTTTAAGGCAA

GAATGGGTGTCCAACTTACCCTTCTGAATTGGCACGAACAAGTTCTATAGCAACCAAAGG

TCTTCGTTCCTCCCGTCTCTCTTTGGCTTTGCCCACACGTTGGGTTCTATACCTTTTATC

CGAGATGTATGCATTTGCTTTTTTAAGCGTTTTCGGGAAGAAAACTGGTATGTGTGCATG

GCTGG

>Unigene0001064

TGTTATGTTTCAGTGGATGCTTATCCCACCTCGACGATGACCCAACGGCGGCGCTGCCTT

CGACTGGGGGTTGCCGGAATAAGCATAGCATAAGAAACAAGAACCGCTTCTCAACTAGCG

GGCTCGACAAGTTCTCCAAGGTGATGACAGAGCTCCATGCCAGGAGGGAGCACATCTTGG

CCATGGCCGGCTTGCCGGCTTCTGCTGTGGTGCTCTTCATGTACAACAACTCCCGCGACT

GGATTCCGATCATCGCCGGCTTGCCCAAACCACCTGCTAAGAAATCAGGTAACAAGAAGC

TACAATTGTGGCCACTACCGCCTCCACCCTCACTAGGGAGGGCAACAGATGATGTTGCGA

GACGGGCAGCGAAAGCAAGAGAGGGGAAGTCGAAGAAGAGATTCAAGTGCGCAGTTAAGG

GGAAAGGTAGGGAGCGTTTGTATTGGGGAGGGGTGATGGTGCTGATATTGGTTTGTTTGG

CGACGTTTGCTATACGTTGCACCACCATATGGTGGTGATGGCGAGCTTAAGATGAAATCC

AAGAAGGAGAAAAAGCATACGAGGCGAAAGAGAGATAAGAGGTTGTACGGAGGTGGTGCA

TCATCGACAATTTGATTATAAGTTATTTGTATTCATAATATTGAAAATAATCCAAACATA

TTGTGTATAAATTTGAGTCGGCTTTAGTTTCATGTTGAAGCTGTGAATTAATAAGTCTTT

TCAAACCTGTTAAATTTGTTTATAATGATGTATAAATTCGGTTTGAACATGCAACACTCA

TAATTAGCGATGAGGAG

>Unigene0001065

ACCCAGCCCGAATCATTCGAAGCAAACCTCTCGAGCAGCGGCGATGTCTGCTATTCCACT

GCTGCCTTGGTTCCCCGCCTTCAATCCCTCCTCCGCCGGAGTTAGTCCGGCGTACCTCAT

CACCGCGGCCTCCTCCCTCCGCTTAGGCCACCACCTCCTCAGCCGCCCTTGCAGCGGCCA

CCTCCGCCGCTTCCTCCTCCACTGCTTTCGTTACCAGGACAGTTATCATTCTGGTAAATT

ACCCAGATTCACAGATATATGGTGCCAGAGTTCAGATTTACTGAGACATTCACTTCTGGA

CACATTTGCCGATACTTTCTCAGTAGCACATTCAGCTTGCTTTTCTTTCTTTAGTGGAAA

TGGGAAGGGATCAGGAAACACAAATGTGAGGTGGGGAAATTCTCACTCAGACTCTAAGAG

AAATTCTTTGGATGGAAGGTTATGGACTAGTGTTCTTCTTGCATTCAATATCATGGTTTA

TGTCGCTCAGGTTGCAAGCCAAGGGAGGTTGATCTTATGGGGAGCTAAGGTCAACAACCT

TATTGATCAGGGACAACTATGGAGATTGGTTACATCATCCTGTCTGCATGCAAATATTGG

ACACCTTCTGGTCAATTCTTACTCATTGAACTCTATTGGTCCTACTGTGGAGAAACTTAC

TGGAGCTAGACGCTTTCTTGCAGTTTACTTCACTTCTGCAATTGCAAGAAATCAGGTTCA

GTTATGAGTTATCGGTGCTGCCAATCTCCTGCTGTTGGTGCATCTGGAGCCATTTTCGGA

TTAGTGGGAGCTTATGCAGTGTTTGTACTGAGGCACAGAAGCCTCATCGGAGGAGGGAAG

TCTGATATACAGGATATTGCACGAATGATTGCCCTAAATATGGCGATTGGGCTTCTCTCT

AATGGCATTGATAACTGGGGTCATTTAGGAGGCTTGCTGGGCGGAGCTGCCATGGCATGG

TTTCTAGGCCCAGCGTGGAGATATGATTCCCGCACTGCTGATGGAAGATCAGTGTTTGTT

GATCGGGCTCCCATTTTCAGTTTCTTAAATAGGAGAAGGTTGCAGTAGAGTAGTGTCTGG

AACAAATTATAAGGTATATCCTCTGAACACTGTATTAAATCTTCATAATTTGTAGGATTA

CAGCTTCCATAATTGTAATGCATTTTCATTTTGAAATAAATGATTATATCTTAATTTT

>Unigene0001066

GCATCTTAACTCCCCACCGACTAATCTATATTGCCTTTCTCCTTCACTCAACTCCCTTTC

ATCCATATCCATCCCCAAATCTCAATCCCTAACCCTAATTAGGGTCTCGACCGGCGCCTA

CTCTCCCTCCAGTATGGTGCTGTGAAACTTGAAATCTTGAGTAGGTTGTCATCAACTGTG

GTAAGGGGGGAGGACGTGACCATGTCGAGCAGCGGAAGCACACACTGGTGTCACCAGTGC

CGGCGAGCAGTCCGGCCACGTGGGCGGGACATCACATGCCCCATCTGTGACGGAGGCTTT

GTCCAAGAGCTTAATGAGGTTGATGGGGTTGCAAGCCCTTTCAATTTATTTGGACTCGAT

CCTGACGAAGGGCGGGACCATCGATCAGGGCTCATGGAGGTCTTGTCCTCCTTGATGAGG

CAGCGCATGGCCGGACCAAACAATCACCCAATCGATGTCAGGGGTAGACGCAATATGATT

CCTGATAATGGCGTCAGTGTCGGCTCCAGCCCTGGCCCCGGTCCTTGGCTCATCTTTCGA

GGTCAGCTTCCAGTTCACATGTCTCTTAATGATGGCCTTGAATTTCTCCATAACGGAACA

AGGAGTGGCAACTCGGGGGACGCCTTTGTTGGTGCGGGGCTTGACGAGCTGTTCGAACAG

CTGATGCGTGATGATAGGCGTGGCCCACGGCCTGCTCCACGCTCGGCTATAGACGCAATG

CCGATAGTAAAGATAAACCAGAGGCATCTTCGTGGCGACTCACACTGTCCGGTTTGTAAA

GACAAGTTTGAACTGGGATCAGAAGCACGGGAAATGCCATGCAAGCATTTGTATCACTCT

GACTGCATTGTTCCTTGGTTGGTTCAACACAATTCTTGTCCAGTCTGCCGTCATGAGTTG

CCACCATTGAGCGCTCTTGGTAATGCACGTCCGCAATCTAGCAGTCAAGATCAGAATGGT

CGTGGGCGAAACAGTGGTGGCAGCAACGGTGGTAGTAGCAGGGAAGGCGGTGGTGATAGC

CATGGGAGGCGAAATCCGTTCTCTTTCATGTGGCCTTTTCGCCCTTCGAATGGAAACTCT

AGCACAAACCAAAATACAAGCTCTACTTCTCAACAAAATCAGTCTGGGAGTAATCCGAGT

AACTCGGCCCCGTTGCATGAAGATGCCGACCAGAATCATCATATGCATTACTCTGGATGG

CCTTTCGATTATTAGGTAATCATCTTTATCCTTCATAAGCCCTACTGCAATAATAGTGAT

GTAAACAATGTGTAGCTACTATCTATTAAGTATGTCAAAGAACTACCTGTTTTTGCTTCT

GCTTGTTGAACAAATTAGATGGTGGTGTAAAGATATTTACTAAATATCTGAATGTTGCGT

TTGTTGATAGATGTGTCATGAAATCCAGAATTTTAAATAAAATACTTGTTGGAATTGTT

>Unigene0001067

TGCCATGCCTAAAGCTCTCCATTCCTTCTCCACCCCTAGCAACATGCCAGAAAAAGAAAA

CCAAGGAGAAATTCTCTGGCCGAGACTGGTTGCTAACAAGATATTCCGTTGCCCATTGGG

ATCCGATTCGTTCGTCACTGATTTTCCAGAGTTCTCATCGAGCTTCGAAAGTCCGGCATC

GGGGAAGAAGGAAACCAAAAGAAAGGTATTTGTTGGTACGTGGAATGTCGGAGGAGTGCC

ACCAACTAGCAATTTAAACTTGGATGATTGGTTGGACACAAACAACTCATATGACATCTA

TGTTCTTGGATTTCAAGAAATTGTCCCCCTCAAAGCAAAAAACATTCTCGGACGCGAAAA

GAAGAGAATCTCATTGGATTGGAACGCCCTCGTTAGGGCAACACTAAACCCTACAAGAAG

CAAAGAGCCAAAGCAAGGAGAGAAGCAAAAGGTTCATCCCCTCAAGGATGGAAACCCTAA

TTTGAATCAAGGGTTCATATGCATCATAAGCAAACAGATGGTGGGGATTCTTGTCTCAGT

TTGGGTCAGAATTGATCTCCTGCAGTACATTGAGCACCCTAGTGCATCATGCATCGGTTG

TGGCATCATGGCTTGTCTTGGAAACAAGGGTGCAGTGTCTGTTAGGTTTTTCTTGCAAGA

GACGAGCTTCTGCATCGTCTGCTGCCATTTGGCTTCAGGAGGGAGAGAAGGCGATGAGGT

ACATCGGAACTCGGATGCCATGGACATTTTCTCGAGGACGAGATTCCCACAAGTTCCTTC

TCATGATTTGCCACAGAAGATCCTCGACCATGATCGCGTAATCTTGCTCGGTGACTTGAA

CTATCGTATCTCTCTCCCAGAGGCTAAGACTCGATCGCTCGTGGAGAGGGAAGAGTGGAA

CACACTACTGGAGAAAGATCAGTTGCGGGATGAGGTGTCCGAGGGTCGAGTT

>Unigene0001068

AGTTGCGGTATGAGGTGTCCGAGGGTCGAGTTTTTGAGGGCTGGCATGAGGGGCCAATCA

CCTTCTTCCCCACATACAAATACTACCCGGACTCTGATGAATATTATGGGTGTGTTGAAG

GTAAGAAGGGAGCCAAGAGGAGAGCACCAGCATGGTGTGATCGGATTCTCTGGTACGGCA

AGGGGATGAAGCAAATCCAGTACGATCGGTCGGAGTTGAAACTCTCCGATCATCGCCCCG

TTCACGCCATTTTCATCGCCACGGTCAAGCTTCCGAGAAGCTCAAACTCCTTGGGAGGCT

TCTTCTCTTTGGATAAGTTAGGGAGTGTCACAGATTTATCTGAGTGAACTCGAGGATTAA

AGATCAATCTAAACTGTTTTGATGCCAACATCGATCACATTCTCGCTGTATCGGGTGCTC

AATTGGCTATGATTTCATGGAGATATCAAATCTAGAGAGAATGAGAGCTAGTTTTTTTTC

CCCTGGCTTTTTTTCTTGATTGATATAAACCCTAGAAGTGTAGGTGTAGCATTGCAAATT

TGTAAGTTCAAGAGGGTGATATGTTGTCTCGCGGATCTCGTTTATGTGTATATGTTCCAC

ACTTGGATGTATTGGAGTTGGGCTGGTTTGTTGATCAAGACATGAATAGATAAT

>Unigene0001069

CTTCACTGTCCTAATATTGTTTCGTTGTCCCACCTGCCCGGTTACCGATTACCGGATCTC

AAATGGACGATGACAGTGAGCTCCGCGAGCACCCACGATCGGATTCCTCCGCTGTCACCG

GCGGCGGAGACGGCCAGACGAGCATTCACATCACAGCTCTCGATGGGATTGTGAACGTCA

ACTCCCTCTTCACCCTCGCCGTCTTCATCGGCCTCGCCTGGAATCCCTCCGACCCCGCCA

CCTCCGCCGCCTCCGGCCTCCTCTACCCCGTCGTATGCGCTGCCGATAACAAGGTAGAAG

AGGATCTCGTCTCCTTCCACGTCTTCGCTTTCTCCTCGTTCCTATTTTCCAGTCTTGTCT

CCCTCTGCCTCAAACAAGCGATTCGTCTCTTCCGCCCGCCGCGCGCCCCCAAGGCGGCGC

GGATCAACCGGGCGGCGCTGCGGGTGGGAATCCTGGCATGCGCCGTTGGATCGGTGTTTG

GGTGTGGATTTCTCATGATGGCGCTAGTTAATGTTGTTCAGATCAAATTAGGGCGGCTTG

GTTGCGCCGGCTCGGTCTCCGTTGGGGCTATCGTTCCGCTTGTTACGCTTGTCCCCGCCG

CGATGATCATATATATTGGCATTGTCTTCTATGCATTCACTCGCTGATTGAGATTGCTGG

GGTTTCTCTCCAGGTATTTACTAATATTTTTCTTTTTTTGTATTTCTTGAAGAGGATAAT

AGCTGTGATTAATATTGTGTATTGAATCTGTTTGATTATTGTAAAATTATTGTAACATTT

GAGATATTGAAGCTTAAGTTGATCGATTGCTAGTGGAGGCATCTGATACCACTGAAGGAA

ATAAAGTTGGATTTAACTCTGAAGCTTAATTTGCT

>Unigene0001070

GGATCTGTGATGGACGGAAGAAGTGAGGTCTGCGAGCGCCGATCGGACTCCGGCGGCAGC

GGCGGCGGAGCTACGAGCATTCATATTACAGCACTAGACGGAATCGTCAACGTCAACTCC

CTCTTCACCCTCGCCGTCTTCATCGGCCTCGCATGGAACCCCTCCGACCCCGCCACATCC

GCCGCCGCCGGCCTCCTCTACCCCGTCGTCTGCGCCGCTGGCAACAAGGTTGAAGAAGAT

CTCGTCTCCTTCCACGTCTTCGCTTTCTCCTCGTTCCTATTTTCCAG

>Unigene0001071

TGACCGACGGAGCTCTCATTTCTAGGGTTTACTCGATTCTAGGGTTAGGGTTTACTCACC

GAGAAAGCTTCAAACTCCGATCAAAGATGGCGACCCTCCTCCTTGGCTGCTAAGTTTGTC

GACGCTTCGCCATGGACGAGGGTTACACCAGCCTCCCGTCCAGCCACCTGATCGGCTCCG

TCCCGGCCGTGATCTCCGAGGACAAGAGATTCACAGCCCCCGAAGCTTCACAATCAACGC

TGCAAACGTTCCCGCCTACGAATGGGGGTTATCA

>Unigene0001072

AACCGCTGTTGAAGAAACCGAAAGTGGCTCTCCCTTTCACCCTCCTCGATCCGTGACCGA

CGGAGCTCTCTCATTTCTAGGGTTTACTCGATTCTAGGGTTAGGGTTTACTCACCGAGAA

AGCTTCAGACTCCGATCAAAGATGGCGACCTTCCTCGTTGGCTGCTAAGTTCGTCGACGC

TTCGCCATGGACGAGGGTTACACCAGCCTCCCGTCCAGCCACCTAATCGGTTCCGTCCCG

GCCGTGATCTCCGAGGACAAGAGATTCACAGCCCCCGAAGCTTCACAATCAACGCTGCAA

ACGTTCCCGCCTACGAATGGGGGTTATCAAGCTCCTGGGACTCCATTCGTTGGAGACGAT

CAAACCACGACCAATTGGAAGGGAGTCTTTAGCATTTCGTCGTACAGTCCATATTTCAAT

GTTGATACTGACGTTGTGGTTGATAGAATCATCAGTTCAGTGTATCCCATGAATGACTTT

TCCAGAAAGATTGATGCAAATCCTGATTTGTATGGTCCTGTTTGGATCTCCACTACGCTG

GTATTCTTGCTTGCTGCCTTTGGGAACTGTGCAACTTATCTGATGCAAAAGAAGGGTGAC

CCGAGTTTGGCGTGGAACTATGATGTCAACTATGTAAATTGGGCAGCTTGTGTAATATAC

GGTTACACACTTGCTGTTCCAGCTGCATTCTACTTTTTGCTTCGGTACTTCGGATCAAAT

GTAAGCCTCATACGCCTCTGGTGTATGTGGGGCTACTCTCTGTTCATCTTTGTGCCCACT

TCTTTGCTACTGGTGATTCCGCTTGGGTTTTTACGTTGGCTCATCATAATACTTGCGGGA

TCTGCATCATCCTGGTTCATTACTTTGAACTTGAAGGCGTACACAGAGGAGAGGACTGAT

CTGACAATGTTGGTCGTCAGTGCCTTGGTCCTGCAATTTGCTCTAGCACTTTTCATCAAA

ATTTTCTTCTTCGCCTAATGTTGTATGGTTACAATGCTGGGCTGTATAGGTGAGCCTTAT

CTGCCTGTTATCCTTTGGTGGTCCTGATAATTAACTGTTTGCTCGAGTCTTTTAGAATAT

AGTAAAAGAGAAGTAAATTTACAGGCTTGTAATGAACTGCAGTTTTTGAGGGTGGAAAAA

GGATGAAAAACAAGATACGCTTTCATTCAAATTCTGTCATATATTGGTATTAATCGGGTA

ATATTCCATCGGTATACTGAGTTTTTGCATGCCTTA

>Unigene0001073

ATAAAGGGTGGAAAGAGAGACATTGAGAATGAGCTTTCCAAGTTCTCTAGAGCATCAATC

CATGGGGGAGAACACTAGTTGGCGGAATATGGGCAACCCAAGAACAGCCTCATCATCTTC

AACCTTTGATCAACACTCACAACCTTCTACTCTTCTCTCCATGAGCCCTTGGCATGATGG

AACCCAAGAGCTACCTGATTCATGGAGCCAATTGTTGCTGGGAGGATTGGCACAAGAGGG

AGACAGGTGTACCTTCAGCCCTTTCCAGTACAAGAGAATGGAAAATTGGGAGGATCAGAT

GGTGTACCCATCAGTAAGTGGGCACCAAATGGATGTCAAGCAAGAGATCTCAGAGGGTGG

ATATGGGCTTTATGGCCATAGGAGTGAAGGAATCCATGGATCCTTGCCTGTATGGGCTCA

AACCCTACAAACTTCATCACCTACCTCTTGTGTCACAACTAGTCTCAATAACAGTATGCT

GGATTTCTCAAGTAGCAGGGCTGAGAAGAGAAATCATCAAATCAATCATTCATCTGAATA

CAACAGCACAGCACCAGGTTCACCTGTTAAGAAGGCAAGGGTTCAAGCCACTTCATCACT

GCCTCTCAAGGTTAGGAAAGAGAAATTGGGAGACAGAATAACTGCCCTTCACCAACTAGT

TTCCCCATTTGGGAAGACTGACACAGCATCTGTACTGTTAGAAACCTTTGGCTACATTAG

ATTCCTACAGAATCAAATTGAGGCTTTGAGCTCTCCGTACTTGGGCAGTGGATCAATGAG

CACGAAGCAGTATGTACATGGGGAAAGGAGCAGTATATTTCCTGAAGACCCTGGTCAGTT

ACTAAATGGTGCTAAAAGGAGTAGAAATAGCCCGTCTCAACAGGATGGTGATCGCGAGCC

AAAGCAGGACTTGAAGAACAGGGGTCTGTGCCTTGTTCCAGTCTCCTTGATGTCGAACAT

CGGAAGCGACAATGCCGCAGATTACTGGCCTCCAAGTTTCGATGGCAGTTTCCAGTAGGC

ATGCATGCTAGGGTTGTAAGAGTGGATATAACAGCATGAGCAGCCACCCTGTTCAAAATG

TTTGCTGCTCATAATGCTAGGAATGCTCATGATGCTAAAGCTATACCGTCTCAACTATAT

TGTCAATGCTACTTGTATATGCGGTTAAAGTTTTTGCAACCATCTTTCAATATGTTGTAA

GTGTTAAGAGATGGTTCAATCCCTGACCTTTCATGACATAAAATAGT

>Unigene0001074

GGATTAACTCACATAGTTTGGAGCTGCCCATTTTAGGATTAACTCACATAGTTTGGAGCT

GCCCATTTTAGGATTAACTCACATAGTTTGGAGTTGGCCGCCAAATTTATCAAAATTTGA

GTTTGGGTTGGGCCAAAATTGTTATCGAAGTGTAAATTAGGTCTGCCCTTTCGAGCGAGC

AAGTTAATACTGACTGGGTAATTTAGATTAGGTCCTGTTACTTTCCAAATTATGCCTATT

TGTCTTCAAGTCTACTTTGAACTATTGTGGATCTTTTACTTTCAACTATTGTGGAACTTC

ACAGGTGGTTATTGATCATCATATACAAATGTATTTGCTTTATTTTACGAGTTCTAATTC

TAATTGTAAATCGTTTGTTAAACTCCTATGAAAATACTTTACTTCAAGATAAGTGTGTTC

TACTATTTGAATTAAGCATTGTCACGATGAGGAATCCAATCTTGAACCTCCTAATACTGT

GATGGTATCTCATGATAGGTACTGGATTTATTTTGCTTGCTTTGCGGGTCAAGATTTCGT

GTCAGTATTGATGTACATTACAAGTCCTTGGCTCTCAACTTAT

>Unigene0001075

CTCTCTCTCTCTCTCTCTCTCTCCCTCTCTCTCTCCTTCCTACAAAATCCGTCAGCAGCC

GCAGCAGACCCTCTCTCTCTCTTCTTTCTCTCTTTCATTTCTAGAGAGAGAAAGCTTTCA

AAGCTCCCATGGACGACTTCCGCTCACGATCCTACACCTACGGCGACGGCTCACTGCAGA

TGGAGCCCTACTACCGCCGCGACGGCCCGCCGCCGCCGCCGGACTTCAGGTCCTACAGCC

TCGCCGGTTACGTCGGCCCCAAGGACGCCAAGCTGAAGAAGGGGAATGGGGGATCCAAGT

CGTTGAAGGGGTGGAGCCTCAGCGACCCCGAGTTCCAGCGCAAGAAGCGTGTGGCGAGCT

ACAAGGTGTATGCGGTGGAGGGGAAGGTCAAGGGGTCGCTGCGCAAGAGCTTCAAGTGGA

TCAAGGATCGCTACTCCCAAGCTATGTATGGATGGTGGTGATTTGGTACTGGATTTATTT

TGCTTGCTTTGCGGGTCAAGATTTCGTGTCAGTGATGATGTACATTACAAGTCCTTGGCT

CTCAACTTATTGATTTCGAACGGTAAAAACTTTAAAGAATTAGAGCAAAAGAATGTGGTG

GTTTCTATACTTATTTTAAAAGTTGTACAGGCACTTATAAGATTTGTAAGTCTGTGTTGG

TTGTATCCATGAGTTCTGAGTTACTGTCAACGGACTTATTTTCCCTAATTGATGTGGTTT

TATACAAGGATTTGTTGCTTGTTTTATTAACTATGTTTTACTATTAATCTTCGAGAGACC

TAATAGAATGCTTCTAAC

>Unigene0001076

CGGTTTTTTGGGACAGAATTTGGGGACCCACATGCTAGCCAAATCAACTGAGATTAGACA

CCAGTCTCTTCCTCGTGCAAGAGGTGATCTCAACCAACAAAGCAAAATGATCCGTCTTTG

GAGTACACCAGTTAAGAAACGGAGATGGACAATGAGATCAGCTTTGGTTGAAGAAGAACA

GGGAGTTAGCCAAGGAACTCAAATTATCTTTAGCAGTAGGTTGTAGTGACCAACAATCAA

GGAAAAGGTACATGGTTTAGGATTTGCCTTATGTTTGCCTTCTTTTATTAGATGACTTTG

CCCCCTGCCCATTTTAGGATTAACTCACATAGTTTGGAGCTGCCCATTTTAGGATTAACT

CA

>Unigene0001077

AAATCAACAAAATCTCAGCGGCGAAGGCGATTTCTAGGGTTTCGATTCTCAGATCGAGGG

AGAATGGGATGCGTTTGCTCGACGACAAAGCAGGTGAAGGGGTCGCCGCCGGAGGCGGCG

TGCGATGAGGATGGGGGCCTCACGTGCGCGCGGAGTACGGGGAGGATCGGCGACTCCGGG

GAGCTCCGGCCGGTGCCGACCGGGAAGATCGGTGACTCCGGCGAGCTCCGGCGTCTGCCG

GCCGGGAAGAGCGGTCACTCGGGGGAGCTCCGGCCGCGGCCGGCGGGAAAGGTTCCAGAA

ATGAGTTCTATCTTGGGAAGGGCTGGCATTGCTAGCCTAGAGAGGGCAGTGGACGTCTTA

GATTCACTTGGAAGCAGCATGTCCAACTTGAATTCTGGCAGTGGATTTATGTCTGGATTG

ACAGCTCGAGGAAATAAAATATCGATCCTTGCTTTTGAAGTCGCTAATACAATTGCTAAG

GCTTCGAATCTTTTGAGCTCCCTTTCAGATGAAAACATCGTATTCTTGAAGGAAGAAATT

TTACGGTTAGATGGAGTAAGAGAACTGATATCTTCAGATACCAGAGAGCTCATGATCATT

GCTGCTTCTGACAAAAGGGAGGAACTTAATGTCTTTTGCGCGGAAGTAATTAGGTTTGGT

GACTTATGTAAAGACCCTGTATGGCATAATTTGGGTCGCTATTTCCAGAGATTGAGTATG

GATCTTACGCCACAGGAACACCACAGCGAAGAAGTTGAAACAACAATGCAACAACTATTG

AATTTAGCCAAGAATACTTCTGAGCTATACCATGATTTACATGCATTGGACAGATTTGAA

CAGGATTATCGGCGGAGAGCTCAGGAAGAAGAGTCACTACCTGCTTCTAGACGGGAGAGT

CTCATGATCCTACATAGTGAGTTGAAACGCCAAAGAAAACTTGTCAAGAGTTTGAAAAAG

AAATCCCTTTGGTCTAGAAATTTGGAGGAGGTTGTAGAGAAACTTGTTGATCTTGCAGTC

TTTCTACATCAAAAGATATTGGATGCATTTGGTATTACTGATACTAACTTCGCTGACCGT

GAACCAGTTCAAGGGAAAAGACTGGGTGCTTCTGGTCTTTCATTGCACTATGCTAACATC

ATAAATAAGATAGATAACATTGTGTCTCGACCACTATCTCTTCCTCCGAATATAAGGGAC

ACATTGTATCATGGATTGCCAGCAGGTGTCAAGGCAGTTTTACGTACCCGGTTGCAGTCA

AGTGATTCAAAAGAAGAGTATACCGTTGCACAGATCAAATCTGCAATGGAGAAAACACTA

AGATGGATTGTTCCCATGGCCGAAAATACTATAAAAGCACATCAAGGTTTCGGATGGGTC

GGAGAATGGGCCAATATTTGTACCGAGGACAACAACAGGAAGCTATCACCTCAGCACAAC

ATCGCCCGCATCCAGACGCTGCATCATGCCAACAAGGAGAAAACTGAAGAACACATTCTT

GAACTGGTCGTATGGCTCCACCATCTAGTGATCCAGGTGAAAAACCGGGGTTTTGGTTTC

AAGGCCTTGAAACCGATCCGAAGCCGATCCCAGAAGGATGCGCTTACACCGACAGTGCCT

AATGGTGGTTCTGGTCGAGCATTCGAGCTCTCTGAACAGGAAAGGGAGCTGTTGGGACGT

GTTAGCTCGAAGAAGATCGTGTTGGGAAGAAGCAAGAGTGAGAAGGTTCTGTCATTGAAG

GAGAAGAGGGGTAAAGACAGGCATTTGAGCAGGAGTTGCGGGAATTCTCCGATGAGTTTC

AGTGCTTCTCTGAGCTTGCATCTCAAGAGGACGAAAGTTTTGGATATATTGGATGGGTTG

GACATGTTGGGAGACTTGTCCTAGTGTGCCATTTCTTGAATAGCTCATTATTTCTTTTAT

AATTTAAATTATCTTGTATATTGTTTGCAAATCTCAGTTTTACTAGTGTTGTATATGTAC

ATATTTACTCTATACAGCCAATGTAGCAGAGATGCAGAATGAGATATTCTTTGCCAGATT

GTTTCTTTAGTTACCGACTACCA

>Unigene0001078

AAGGGAGCTGTTGGGACGTGTTACCTCGAAGAAGATCGTGTTGGGAAGAAGCAAGAGTGA

GAAGGTTCTGTCATTGAAGGAGAAGAGGGGTAAAGACAGGCATTTGAGCAGGAGTTGCGG

GAATTCTCCGATGAGTTTCAGTGCTTCTCTGAGCTTGCATCTCAAGAGGACGAAAGTTTT

GGATATATTGGATGGGTTGGACATGTTGGGAGACTTGTCCTAGTGTGCCATTTCTTGAAT

AGCTCATTATTTCTTTTATAATTTAAATTATCTTGTATATTGTTTGCAAATCTCAGTTTT

ACTAGTGTTGTATATGTACATATTTACTCTATACAGCCAATGTAGCAGAGATGCAGAATG

AGATATTCTTTGCCAGATTGTTTCTTTAGTTACCGACTACCA

>Unigene0001079

CCTCTTACTCTTGCGCATCGACCCCTTCTCCCAATTTCCAACACCCTAACAATCCCTTCC

AGCCACAGAGATCGGGAGTCAGCTGGGTCGATCCTTCTCCTCCGATGGCGATCGTATACG

CCGTGGTGGCGCGGGGGACGGTCGTCCTCGCCGAATTCGCCGCGGTCAGCGGCAACACAG

GCGCCGTGGCGCGGCGGATCCTCGAGAAGCTCCCGCCGGACGCGGATTCGCGCCTCTGCT

TCTCGCAGGATCGATACATCTTCCACGTTCTCCGTGCCGACGGCATCACCTTCCTCTGCA

TGGCCAATGACTCCTTCGGAAGGAGGGTACCTTTCTCTTACTTGGAGGACATACACATGA

GATTCATGAAAAACTATGGCAGGGTTGCCCATTCTGCTTTGGCTTATGCAATGAATGATG

AGTTCTCAAGAGTTCTGCATCAACAGATGGAATATTTCTCTAGTAATCCGAGCGCTGATA

CGCTTAACCGCGTGCGTGGTGAAGTTAGCGAGATACACACTATTATGGTGGAAAACATAG

ACAAGATATTGGAGAGAGGTGACCGCATTGCACTCCTTGTTGACAAGACTGCTACAATGC

AAGATAGCGCCTTTCATTTCAAGAAGCAATCTAAGAGGCTTAGACAAGCCCTTTGGATGA

AAAATGCCAAACTTCTGCTACTGTTAACACTCACTATAGTTCTGCTGCTGTATTTCATAA

TCGCCGCCTGCTGTGGAGGCCTCACTCTTAGATCATGCAGATCATCATGATTCAGCAATT

GGTCTCAAATTTGTGTGAGATGCACACTTATCCCCCTTCCAATGTTTATTTGCCGCCTCC

CTTGTGTGGTTTTCCTCCCCTGTGCGAATTTCCATTGGACCGTCTGCATTGATGGTGCCA

CAGATGTACTGATGTTCTCCTGGTCAAGTTTGCGTACCTTCAGAAGGCTATATGATTGTG

ATGAGGCTTTATGTATGGTGCTGTAGAAAATCAATCGATGTCGCTATATTTATACTTATT

CATAAATATGTATCTGCATTGACGCAACTATCCTTACAGGCACAGTTACTATGGTAGTAT

CAACTAGTGTGTGCTGGTGTGTTATTAATTATAGTCAGTTTCTAT

>Unigene0001080

TGCCCCAGATATCCTCCTCTTCCTGGCCACCCTGCTTGGGAAGTTGGTTCCATGCACCGA

CAGTTTGTTATCCTGTAATCTAGTTGGATTTAATAAATAATATATATACTTCATATTTAT

TTATGTTAGTGTTGAGTGTATATTTTCAGGCTCAGCGCAGAGGACTGTCTTTTGTTGAAT

GATTCTGTTTACGGTGTTAAGTCAACTATACACCTACAAAAAAAATCCATATATTGTGAT

TGTAGTTATGTTTTCTTAATAGCCTTAGTAAAATCTTGCCACCAAA

>Unigene0001081

TGCCCCAGATATCCTCCTCTTCCTGGCCACCCTGCTTGGGAAGTTGGTTCCATGCACCGA

AGAAGACAGTTTGTTATCCTGTAATTTAGTTGGATTTAATAAATAATATATATACTTCAT

ATTTATTTATGTTTAGTGTTGAGTGTATATTTCAGGCTCAACGCAGAGGACTGTCTTTTA

TTGAATGATCCTGTTTACGGTGTTAAGTCCACTATACACCTTAAAAAAATCCATATATTG

TGATTGTAGTTATGTTTTCTTAATAGCCTTGGTAAAATCTTGCCAC

>Unigene0001082

CACGCGTACCAACGACCGCGTGTCCATACGTGCGGTGTGATTGCTGAGATCCCCACCTCC

TGCTCCTCGCTCCCCATTCCCCGCTACCGCGCGCCGCCCTCCGTCGCGCTCTCCCCGCCG

GAATTGGGAACGCGGTCTCGCCGCCCCCACGCGCGCCGATGGGTTGCGCGGCGTCCATCC

CAGGCAAGAACGCGGGCCGGTTGAATAGCGTGAACAGCGAGAAGTCCCCGGTTGCCGACT

CGAAGAACCTGAAAGTTAAGTTGGTATTGTTGGGAGATTCAGGTGTTGGCAAAAGCTGTA

TTGTTCTTCGCTTTGTTCGGGGCCAGTTTGATCCCACATCAAAGGTAACTGTTGGTGCAT

CTTTCTTGTCACAAACAATAGCTTTGCAAGATTCAACAACAGTGAAGTTCGAAATATGGG

ATACTGCTGGCCAGGAGAGGTATGCTGCATTGGCACCACTATATTATCGAGGAGCAGCTC

TTGCAGTGGTTGTGTATGACATAACAAGTCCAGAATCATTCAACAAAGCACAATACTGGG

TTAAGGAACTTCAAAAACATGGTAGTCCTGATATTGTCATGGCTTTGGTGGGAAACAAAG

CCGATCTAAATGAAAGTCGGAGTGTATCTTCCCAAGATGCTGTAGAATATGCGGAGAAGA

ATGGCATGTTCTTTATTGAAACCTCAGCCAAGACAGCTGATAACATCAACCAACTCTTTG

AGGAGATTGCAAAGAGGTTGCCCCAGATATCCTCCTC

>Unigene0001083

CCTCGCCGAAACCCTAAACCGAGGGCTTCGATTGAGCTGTGAAGCTAGGGTTTAATTCCG

GATCCGATTGGACATAAATTTTATTTTCTTCTGTTATTCAATTCATTCATGTTTATGTTA

GTGCTACTGCAGAAAATCATGTGGAGAACCCTGGCTGAAGGAGACGGATTTCTACCTCAC

TGATATCTTGTCGGTTTCGCTGTTCACAACAAACAATTTTTATCGACCGTATTTGTGGTT

TCAGAATTTTTGTTGTCACTAAGGCTTTTGCAGCAATATGCCTGCTCTACAAAAGCTTTA

TGATAATTGTAAAGCCTCCTTCTCGACGAACGGACCGGTGTCCGAAGAAGCTCTTGAAAA

AGTACGATCCATTTTGGATAGGATCAGGCCGTCTGATGTTGGTCTCGAACAGGAGGCACA

GCTAGTGCGAGGATTGAAAAGCTCTACAAATGGTTTAAATGGGAAAAAAGGGCGGAATGG

GAGCGGCCAAGAGTTACCGCCGATCAAATATTTGCATATACATGAATGTGATAGCTTTTC

TATTGGAATATTTTGCCTGTCACCGTCATCTGTAATCCCACTTCATAATCATCCTGGAAT

GACTGTCCTGAGCAAGCTTTTGTACGGTTCATTGTATGTGAAATCATATGATTGGATTGA

TACAGGCAGAACCTTTGATCCATCAGAAGCGAGGCCAGCTAAACTTGTTAGGGACTGCGA

AATGTCCGCACCCTGTGGGACGACAGTTCTCTATCCTACGGTTGGTGGTAACATTCACTC

TTTCAAGGCGATAACACACTGTGCTCTGTTTGACATTCTATCGCCACCTTATTCATCAGA

GGATGGACGCCACTGTTCGTATTTTCGAAAATCCCAAAGAAAGGATCATCCTGATGAAGT

TCTCGAAGGTGTTCTCTCAAGCGGTGTAGATGCCTCTGATGTGGTTTGGTTGGAACAGTA

TCAACCCCCCGAGAGCTTTGTCATCAGGAGAGGGCCGTACAAGGGTCGAGCCATTAGAAC

TTGAGGAGTTGGGATCTTAATGCTGTTGCCTGCACTGGAAGGCAGTTTTCTGAGGGGATC

CTTTTAATTACTGTTATGGGTTTGTATTAATCAACAATAAATAGTGTTGCAGTTTGATCT

GTACATATATTTATAAAACACCATTTTAGCCAGATTTCCTAATTTTATTTGAAAGCCTTG

ACAGTGAATTTTTATTTTATAAATGACAGTCAGCTTGAGAAATTATTAAAGAAGTAACTA

TTATAGTATTGTGAGATGAATTAGTTTGTAAAATGGTATGTTAAATTTGTTATTGAGAAT

CCAAATAAATAAATATGGTATCTGGCATTAGTGAGGAATCCCACAGAATGCCAGTTTATC

>Unigene0001084

AGGTCATACTCTGATCCTCTGCGCGATGGCTGCCGTATCATCTGCCGCCATCCTCTACAG

CCTCTCCCTCCCCTCGAGGAGGAAGTCTTCGCAGCTAGAAGCATCTCAGGCAAGCCAACA

GACCACCAATGCCCCCGAGCCGCCACAGCCACAGACTCACCGGAGGTCAAGCTCTGCCAT

CAACATCCCACCAATCCTTGCAAACATCCTCCACCTCCACCTCTCCGACCACCCCGACTC

CGCTCCCCTACTCACTCCCTCTTCCATCCCCAAAATCCCCTCATGTTCCCCAAAAGAAGA

TATCTCCTCTTTCCGCAACGAGATCCATGGCCCGGCCGACTGGTCCGCCCTCCTCAACCC

CCTCCATCCCTACCTCCGCCGCGAAGTCATCAAATATGGCGAGCTCGCCCAGGCTACCTA

CGATGCCTTCGACTACAACCCCTTCTCCAAATACTGCGGCAGCTGCCTTTACGGCCATAA

TCGCCTCCTCTCCCGTCTCGGCCTAGACCGCCACGGCTACTCCGTCACCAAATACCTCTA

CGCCACCTCCCACATCGAACTCCCTTGCTGGCTCGAACGCTCCCTCCACACTGATAGCTG

GAGCCACGAGTCTAACTGGATGGGCTATGTCGCCATCAGCGGCGACGAGGAGTCACGGCG

CATCGGCTGCCGCGACATCGCTGTTGCCTGGCGCGGAACCGTCGCACCATCCGAGTGGCT

CGAGGACTTACAAGGACGCCTCGAGCCACTCGGAGCTGACCACGGGGACGCCCGTGTGGA

GCACGGCTTCCTCAGCCTCTACACTTCAAAGTCCAGCCACTCACGCTACAACAAGTCCAG

CGCGTCGGAACAGCTAATAGAGGAGCTAAAGAGGCTGGTGACCACGTATAAAGAAAAAGG

GGAGGAGGTGAGTGTGACCATCACCGGGCATAGTCTCGGGGGAGCTCTGGCGGTGCTGAG

CGCTTATGAGGCGGCAACAGAGCTCGCTGGAGTGCCAGTGAGCGTAATATCGTTTGGGGC

TCCGCGGGTGGGGAATGAGGCGTTTGCCGAGAGGCTAGAGAGGTTGGGGGTAAAGGCCCT

GCGAGTAGCGGTGAAACAGGATGTTGTTCCAAAGATGCCCGGAGTGTTGTTCAACGAGGG

GCTGAGCAGGGTGGCAGGGGTGCTTGACTGGGTGTACACGCATGTCGGGGTGGAGCTGGG

GCTTGACGTGAGGGCATCACCGTATCTGAAGAGGGCGGTGGACTTGGTGGGGTTTCATAG

TCTGGAGACTTACTTGCATCTTGTTGATGGGTACCTCAGCGAGGGCAGTGGGTTCAGGCG

GGAAAGTAAGAGGGATCCGGCGCTGGTGAATAAGGGGTGTGGCTTGTTGAGGGAGGAGAT

GAAGGTGCCGGCGTGCTGGAACCAGGCGGCGAATAAGGGACTGGTGTGTAATGCTTATGG

GAGGTGGGTGCAGCCGCAGAGGGATAGCGAGGATATACCGAGTCCGTTGAGAGACGCGCC

GATCCCATGCTCTGAACTGTGCTGAAAAAAAGTTGAATTGTACATTAAAACGTGATTATT

GTTTAGTTGGATTCTTAAGAAAGGAAGTGCTGCTACAACAATAGTAATATATCAACATAG

AATGTTGTTTCTCCTGCCATCCTTTCTTTCTGCAATGTGATGTTGAAAATTGGAAACGAA

TTGTACAATAACTGGATTTGTACATTAGAATGTGATAATTCTTAAGAAAGAAGTTCAATG

CA

>Unigene0001085

AGGTCATACTCTGATCCTCTGCGCGATGGCTGCCGTATCATCTGCCGCCATCCTCTACAG

CCTCTCCCTCCCCTCGAGGAGGAAGTCTTCGCAGCTAGAAGCATCTCAGGCAAGCCAACA

GACCACCAATGCCCCCGAGCCGCCACAGCCACAGACTCACCGGAGGTCAAGCTCTGCCAT

CAACATCCCACCAATCCTTGCAAACATCCTCCACCTCCACCTCTCCGACCATATCGACCA

CCTCCCAACCAGTCCTCTACTCATCCCAGACAATCAACAAGCGCCCGCATGGTCCCCCAA

GGAGAACATCTCCTCCCTCCGTCCCCAACTCCACGGCACAGCTGACTGGTCCCCCCTCAT

CGACCCCCTCCACCCGTGGCTCCGACGGGAAATCCTCAAATACGGCGAGTTTGCCCAGGC

CACTTACGATGCCTTCGACTTCAACCCCTTCTCCCAATTCTGCGGCAGCTGCCTCTACAG

CCGAAGCCACCTATTTGACCGTCTCGGTCTCTCTCACCATGGGTACTCCATCACGAAGTA

CCTATACGCCACCTCCCACCTCGAACTCCCCCGCTGGCTCGAGCGCTCCATCCGCGCTGA

CTCATGGAGCGACGTCTCCAACTGGATAGGCTTTGTTGCGGTCAGCGACGACAAAGAGAC

AGCTCGTATTGGTAGTCGTGACATTGTCGTGGCATGGCGCGGCACCATCGCACCGTCCGA

ATGGCTCGAGAACCTCCAGGGGAGGCTCGAGTCGCTCGGACCGGATCACGGGGACGCTCG

CGTCGAGCACGGGTTCCGCAGCATCTACACATCGAAAAGCGAGACGTCAAAATACAACAA

GTTGAGTGCATCGGAGCAGGTCATGGGGGAGGTGAGGAGGCTGGTGGAGCTGTACAGAGG

GAGGGGCGAAGAGGTGAGTTTGACAATCACGGGACACAGCATGGGGGGAGCGTTGGCAGT

ACTCAGCGCATATGAGGCAGCACTAGAGATCGCGCCAGGGGTATCGGTGAGTGTGGTGAC

GTTTGGAGGGCCACGAGTGGGGAACGAGGCATTCAAGGCAAGGTTGGAGGGGATGGGGGT

GAAAGTGCTTCGTGTGGTAGTGAAGCAGGATGTAGTGCCTAGGGTGCCAG

>Unigene0001086

TAGCTTAGTCACATGGATTCATAGAAAGTTTTCTTTCGATCCAGTTAACACATAATTGAT

AGGATATTTGATCGTTGGATAATTTGCATAATATCCGAGGAGGCACAGTTCTACGGTGGG

GCATAAACTTTCGTTTTTTTTTATGCCTTGTCGAAGCGGTCGCTTTGATTCCTTGATTGG

AGCAATCTGCTGATTCGTCTCACCTTTTGGTTTCGCCTGCAGATCCATTGCCTTTGTCCG

GTCAATAGAAGATG

>Unigene0001087

TTTTGGTTTCGCCTGCAGATCCATTGCCTTTGTCCGGGCAATAGAAGATGGAATTTTGCT

TAAAGGAAATGTCTCTGGATGAGTAGATATCTTCATTTGTAGGTAGCCTGGCTCCGTCGA

ATCAGTCGTTGGGTGGTGTGGTTACATTGCCCACTCATCATAATCTTCACTATTCTTCCC

CACTTATTTGGCTTGCATGATCAAGTGAGAAATGATATTTACTTTAAGATTATATTTAAA

ATTTGTTATATAATTCCAAAGACCAAAAACATTTGTGCAACAATTGCAAAAGCATCGCAA

ATATGTTTGTCACTTGCATCTTTAGTGTTCTTTACCTGGATCTCTCTGCTCTTGTTTTCT

TCTCTTGGCTGAAACCATAAGAAACTGACATATAACTCTCTATCCTCGGCTTCACTCGAT

ACTTGGCTACCTAAACCCACATGTAGAGATAGTTTTTCATAGTGTTAGAGGGAGGCTGGC

TGGCTGGTCCGATTGTCCTGAAGTGGGACATTTCCATCCTTAGCTCTTGAATCCTGCCCT

GCACAGAACAAGTGAAAGCAAAACAAATGGAAGAAATACATTGATGGAACGAAAGAAAAA

TCTCTTACAGGCTTAAAAGCTAGAGTGAGGCATAATGCAGGACAAAGT

>Unigene0001088

GAGGGGGAAATGGCGATATCGGCGGTGGCTTCTCTCTCCTTCCTCACCGGCGGCGCTGCC

GGCGTCGGGAGGAGCTCCCTCGCCGTCTCCAAGCCCCCGACCTGGTCTTCGTCTATGCTC

CGCAGCTCATTCCTCTCCTGCCCTTCGACTTCTCTCTCGTTCCCCTCGGCATTCTCTGGG

ATTTCTCTAGGAATCGACTTGCGTTGCAACAGTGTGATAAGACCTAAGCGTCGCCGTTCT

CTCCAGGTGACTGCAGCAGGGAAGAAACTTCTCTGCATGACTAAGAGGAATAGGTCTCGT

AAGTCACTTGCTCGGACTCATGGCTTCCGCAGACGGATGAGAACCACCAGCGGAAGAGCG

GTATTGAAGCGGAGGCGTGCCAAGGGAAGGAAAATTCTCTGCACAAAAACAAATCCAAAC

AGTGGACCACGGGGCGGTCGATAATTGAGACATCTAGCATTTGCTTTACGGAATTAGCAT

GTATTCTCCTGTTTATCTTATGATGATCGAAGTTTCTCCTTAATTTGAATCTGTTCCATT

TGTGTTTGATTAAGATGGAATGAAATTCTGTTACTGTTATTTCCATATTTTATCGTAGTT

GTAGCTTCATGCTTGCACTTATCTCGTGTAACATTCTCATGTGTGTCAATCTTACTCATG

TTT

>Unigene0001089

ACTCTTCTCCCTGTTGTGATTTCACAGACAGAGATGGCTCAACCTCTCCCCAGCGAGGAG

CTCCAGGCCCTCACCGCCGCTTTCGCAGGATGCGGAGGGTTGGGGGTGGACGAGAAAACA

ATCGTTTCGGTGCTCGAGCAATGGAAGCACCAGCCGGAGAAGAAGACCCTTTTCAGGCAA

GCCTCGTTGACTCTCTTTGATAAGCACCCCGCCGGATTCGAGCGGTGGCATGGGGATCAT

ATCATCCGGCAGCTCAAGCACGAATTTTCCCGTTTCAAGAATCTTGTATTACTGTCGACT

ATGCACCCATGGGAAAGAGATGCTCGTTGGGCTCACCATGTACTCCACAAAGCTTCTCCC

CCCACCACCCTAGTGGAGATTGCCTGCACTCGATCATCTGAGGAGCTGCTAGGCGCAAGG

AGGGCATATCAAGCTCTGTTCCACCATTCATTGGAGGAAGATGTTGCCTACCATATCAAT

GGCATTCACGCCAATATCTTGGTTGGACTAGTGAGTGCATACAGGTACGAAGGTCCTCGG

GTGAACGAGGAGATGGCAAAATCAGAGGCTAAGATAATTGGTAGCGTCATTAAGAACTCG

GGTTTGAAAAACCCTATGATGAACGATCAGGTTATAAGGATATTGACTACAAGGAGCAAA

CTTCACCTCAAGGAAACTTTCAAATACTACAAGGAAATGTACGGGAAGCCACTTGAAGAG

GATATTGGTGATGAGCCATGCTTGGTTGAAACTGTTGAATGCTTGGATTCACCAGCTACA

TATTTCAGCAAGGTAATAGACAAGGCTTTGAAGAACGCGGGAGATAAAGTCACATCAGAA

GCGCTCAACCGAGTGATTGTTTCCCGAGCTGACTCTGACATGCTGGAGATCAAAGAGAAG

TACCATAGTCTCTATGGAGTACACCTTGAAGAAATGATAGAGAAGACCACACGTGGTAAC

TACAAGGATGCACTGCTTTCCTTGATTAAAAGGAAGGACTGAGATAAACACTTGAATCCT

TTTTAAATCACATTGCGAATATATTTGCTTGTCATCATACTTCTAGTTCAATTTCTACAT

GAACATCCCAATCGTGTTACACTGCTTTTGATGTAAGAGGAATATTGTGAGACATCTATT

AATATTTTTATTATGTTAATAAAAGGTTTTATGTTTGTTTGCAAGAACTATTCGTGCATC

AGATTGAAAAAT

>Unigene0001090

GGAATCTCACCATCTCAAGAGTTAGCAGAGCAGTAGCTGCTAGCTCTTCTCCTCCCTCAG

CACCCAATCCCTGCTGGTCCAGACCTCACCAAACTCAGCCATGGCCGTGTCCATCCCCCT

TCACCACACCTTCCTCACCAGCAACCCCCTCTCTCTCCCCCACCACCAATCCAAGAAGCC

CCACCGTCTCCAATTCCTTTCATGCCGATCCTCCCTCGACAAGGACGGCAGTCACGGCAC

AGACTCAAGCAGAGCCGGGATGCAGCTGACGAAGCTGGCCATCGTGACGTTTGCAGCGGG

GGTGCTGACACTTGGGTCAGTCGGAGATGCGATGGCAGCCAAGTCCGGTGGGAGGGTCGG

CGGCCAGGCGTTCCGGTCGGCAGGGCCGAGGCCATCTGGCCCGCGGATTAACAATTCAAG

GACCAACATCTACATTAACCCCCCGGTGGCACCGCCCCTAGTGGGTGGGTACGGGTATGG

CGTGCCATTCGGCGGTTGGGGGTGGTCGCCATTCACATTTTTCGCACCTGGGCCAAGTGT

CGCCGTTGGAATTGGAGGGGGCTTTGATTTCTTTGCTGCATTCCTAGTGCTGGGTATTGT

TGCTACTGTGATCAGAAGTTTCACCAGAAACAGTTATGAAGACGAAGATGATTTCTGATG

CCCTTGTCATATTATTAAGTCTTGTTTAATGTAACGCATAGATTACTCCATTTCGATGTA

CATCATCATAGATGTATAAAGAGCACCACGTAGTAGCCATTGGAGCTTAATGTGTAAAGT

ATTTGAAATATACATACCATAATTTTGTTGATGACTGTATAATTTGAAACCAAGGTTTTA

AAATAACCCAATGCAACCTAATATTTTAGGGTACTTTTTTATCCTAGCCTTCATGTCATG

GTTTCTCTTGTCACACCAAATCAGATAT

>Unigene0001091

GTTATGCTAAACACTGTTGATCGAGGATGTAGAATTTGATTAGTATTCAGATATTCTGGA

TCACAGGTTTGAGAATATTTGGGTTTCTGTATAGCAGATTCAGATTTGGATATCATTTGT

ACCGGAAAATTGCTTGTAGTATCCGAACTAGTATGCTTCCCTAGGTGGATACATTTTTCT

ATTCCATCTTTCACCCACAAGACTTGACAATACTAGTGAGCCTTCAACTTCCATGTGTAG

AGAAAATGATCCAAGATCAAGATGAAGCAGCAAAAGAAACGGAGGAGTCAGTTGCAGCAG

ATGGCTGCTCGAGTCGATACTGTGGGCTGATACACATAAGTTGATCCAGGAGCCGGATGA

TTTCCCACCTGGGTTATTTAGCGCTGGTGACGGATCAGATAAGATTGTCTCTGTTGTTGT

ATGGTGGCATGCTTAGAAATTATTTATTGAGTATTTTGGTGAAGATTTAATATGATCAAC

AATTGCTTCTGTCAATGAAGCGGCAATA

>Unigene0001092

TGGCAAGTCATCATACCTTTCTGGTCTCTCTCTCTCTCTCTCTCTCTCTCTCTCTCTCTC

TCAGTTTTGATTGTTGAAACTGAAACAAGCAATCTGTTTCGTTGTTACGATTGAGATCAC

TCATGTTATCCATGCACACACCTACAAAGTAGCTGTACAAGTAAGTACAATAACTGAGTT

TCTGTCCATATGCAGTAGATTTCATCATCAGATGAGGTTTTTCTCTTTCTAGTATGAACA

TCTTTGCAAGGTAGTAAGTTAGGGTTTGTGGGTAGAGATCAGATTCTTGGAAGGTAGTAA

ATTAGGGTTTGTGGGTAGAGATAAGATTCATGGAAACACCCATGAGGACAGAAATTTTCA

AGGTGGCAAAATGTAAGATTCTCTTCTGGATTGCTTTTCGTTTGTCATGACTTCATGGTT

TGCTTTTCCTTGCCTTTTAAAGGAAGAGAAGGGTGGTTCTTTTCAATTAAAGATGCATGC

AGAAGAAGTAATAGATATTGGGAGGTGGTTAAAGAAAATGGTTTCAATAACTGAATCCTT

GTGAAGAATTGAGGTGCTGCCGATTTCTTGGGAGTTTATCAAACCGTACGATTTCTTTTG

TACTGTATGCATTTAAGCCAACATAAATCTACATGGTCTTTGGGATATTTAGTTTTTTTT

CAACATATGAAGCAAAGGTATTTGTAGTGCCATCCAGTTTTTGTGGTATTTCGTATCTAG

TGGTGGTATAACTGTCACTAAAAAATTTCGTTCTATTTTTGTGCTTCAAACATGTTTTAG

TTGCAGGGACAGTCTTCCCCAAAAGGTATGTTTTCCATGAAAATTTTCATGTATGAACTC

AAGCTCTGTAGAAGTCATTCCAGTTTTTGTATAATTTGCCATTCTTGTGCAATGATATGA

CTATCTAATCCTACAATATACCTCTTTCTTGTATAACGTTGAATTTGATGCCTAGATTTA

CTACTATGGTTAGTGCAGGAACTGGGAACTGTCTCCATTTTAGCATACTAGGTTGACAAT

TGTGTCTATTGAAGTTGCTCAAAGAAAATCCAAAAACATAAGTTTTTGGATATATTGAAT

TACTAGTACAATGATGCTATAAGAAACATACCAAAATTGGAGTCTTTGAGGTTATCTTAT

TAGGAAATATCACACTTCCTATTGTTAGTAATTTGTTTTAATTTCACGGTACCAGATGTT

GTTTTAATATAGCTCTCTTGCTCAAACAACATGTTTTGTAAATGAAATTTAATCACTTAA

TCCCAGAGAATAATGTATGGGGTTTGAGAGTAGTGTTAGCTTTAAAAATTCATCAAATGA

GAAGAAGCTTGAGAGAGCTTCCTTCAGTCCACTCATGGTTGCTTTTGGGATCCGAATTGG

CTGCCGATTCATTCATTCACACACTTAGTAGAGGAAGAAGCAGGAGGCCATCGTGCAAAG

AAGTCCCAGCTCCTACAGAGGAATGCATCAGGCTTAGATCATCAACTCCGTGAATGAATG

AACGGATTAACTACTCTGTGAATGAATAAATGACGCGGGAGACGATTCCATCCTATGCTT

CCCATGCTTGGACTGAAGGGAGCTCCCTCTTATTCCCAGCAATCGAGCATTCAGTTTCTT

TTCCTATCAAACAGTTTTAAGCAGTACTCATTTACCTCATTGAGTCTAAATCTCACTAGT

ACTACATCAAATACTTGACTTTCTTATTGTTTCTTTTTTGTTCTAAATTGAGTCATAAGT

TCTTACTATCTATTCCAATCTCCAATTATATAGGAGATATTGAAACCTTTGTTATATAGG

AATCCTATTGCATGCATTCTGAGCTGTTTTGTTATTTTAATTATATTTACATGATTCTTC

TCATTACTCTCTGCTACATGCAAATGTGAAGGAACTTCTGCTAGGTCACACTTGTAATGT

ATATTTATGTTAGTCAAAAGGCAAAATGCAGATCTCAGCATTGCTATGGTCTCAAGACTA

AAGGCATTTACCCTTGTATTCAGTTGGTAATATGTTAGATAAAAGAATGGATATATAATT

ATTCTTGCCTTTACCCTTATAAATTGATTGATGCTCTAGCCAATTTTTATTTGTTTTAGT

ATTTGCTATGATACATAGTGTGTTA

>Unigene0001093

CTTCTCTCTGCCGCCGTTCCCCTGTAGTCTTCTCTGGCTCCCTCTCGATTCTCCATTTCT

AGCGTTAGGGTTAGGGTTAGGGTTAGGTTTTCGAGATGGCGACGATGGAGAGCCTCATCG

GCCTCGTCAATCGCATCCAGCGCGCCTGCACCGTCCTCGGCGACTTCGGCGCCGACGACG

ACGCCTCTACCCTCCCCACTCTCTGGGAGGCGCTCCCCTCCGTCGCCGTCGTCGGC

>Unigene0001094

CTTCTCTCTGCCGCCGTTCCCCTGTAGTCTTCTCTGGCTCCCTCTCGATTCTCCATTTCT

AGCGTTAGGGTTAGGGTTAGGGTTTCGAGATGGCGACGATGGAGAGCCTCATCGGCCTCG

TCAATCGCATCCAGCGCGCCTGCACCGTCCTCGGCGACTTCGGCGCCGACGACGACGCCT

CTACCCTCCCCACTCTCTGGGAGGCGCTCCCCTCCGTCGCCGTCGTCGGC

>Unigene0001095

GCATCTGGAGTCTGTAATCAGGGCACGCATCCCAACAATCACTTCTTTGATAAACAAAAC

CATTGACGAACTTGAGTCAGAGATGGACGATCTTGGTCGTCCTGTTGCCATTGATGCAGG

GGCTCGACTGTACACAATACTGGAACTATGCCGTGCTTTTGACCGTGTATTCAAAGAGCA

TCTTGATGGAGGGCGACCTGGAGGTGACCGAATATATGGAGTTTTTGACAACCAACTCCC

AGCTGCTCTTAGAAAACTTCCGTTCGATCGGCACCTCTCTCTCCAAAATGTGAAGAAAGT

GGTATCCGAAGCTGATGGTTATCAGCCTCATCTGATTGCCCCTGAGCAAGGCTACCGCCG

TCTGATCGAAAGTTCTCTGAACTATTTCCGAGGTCCAGCTGAAGCTTCAGTTGACGCCGT

CCACCTTGTATTAAAGGAATTGGTGAGAAGGTCGATTGGCGAAACTCAGGAGCTGAGAAG

ATTTCCAACTTTCCAATCTGAATTAGCTGCCGCCGCTAACGAAGCCTTGGAGAGATTTCG

TGATGAAGGCCGTAAGACTACAGTACGGTTGGTTGATATGGAGGCATCATACTTGACAGT

GGAGTTCTTCAGAAAGTTGCCACAGGAGGTAGAGAAGGGTGGAAATCCTGCCGTGTCGAC

TGTTGATCGGTACAATGATGGTCATTTTAGGAGGATTGCTTCAAATGTGTCGTCCTATGT

AGCCATGGTGTCTGAAACTCTCAAGAACACCATCCCGAAAGCGGTGGTACACTGCCAGGT

TCGAGAGGCTAAGCGATCCCTACTAAACAACTTCTACGCGCGGTTGGGGAAGAGAGAGGG

TAATGAGCTTGGACATATGTTGGACGAGGATCCGGCTTTGATGGAGCGCAGAGTTCAGAT

GGCAAAGAGGCTAGAACTGTACAAATCGGCGAGGGACGAAATTGAGTCGGTTTCATGGTC

TAAATGAGGGTGAGATGTATAGTATTGGTTACTGAGTTGGAGATGATACGTGGCTATGAG

CATATATTGCGAGTTTCAGAGTATAGTTTTACCTGTAAAAAAACTATTAGTCTTTTCTGT

GTTACATCCACTTTTTGAACTACAGTTAGTCAGTAGGTAGCCGTACGGAATAGCTGATTC

ATCATA

>Unigene0001096

TAATTATGGGTTTACATGTTGCATAAGAAACATGACTGCTTATATGGTTTCTACATGTCA

AATATGCTGTTATTCCAGTAACTGAGCAATTAAAATTATTATTTTTTCAGAAATTAAGTT

ATTTTTGAAAAAAATCTATATTTTTTAAGAGGTGGTTAATATAGACTAAATTCATATAGA

TTATCTTAACCAGTCCAATTTTCTCATCTTCAATTGACCAGGAGTTGAAAAGATTTCCAA

C

>Unigene0001097

CGATCTAGGGTTAGGGTTTCGCCATGGCAGCCATGGAGAGCCTCATCGGCATGGTGAACC

GCATCCAGCGTGCCTGTACCGTACTCGGTGACCACGGCGGAGCCGACAGCGCACTCCCCA

CGCTCTGGGAGGCCCTCCCCTCCGTCGCCGTCGTCGGCGGCCAGAGCTCGGGGAAGTCGT

CGGTTCTCGAGAGCATCGTCGGGCGCGATTTCCTCCCCCGCGGATCAGGGATTGTGACGA

GGCGGCCGCTGGTACTACAGCTGCAGAAGATTGAGGATGGGGAGCAGGAGTATGCGGAGT

TCCTCCACACGAAGGATAGGAAGTACACCGACTTTGCGCTCGTGCGCAAGGAAATTCAAG

ATGAAACTGATAGATTAACGGGGAAATCGAGACAGATTTCTCCTGTTCCGATACATCTCA

GCATTTTCTCTCCAAAGGTTGTAAACTTGACACTGATTGATCTGCCTGGTCTGACAAAGG

TTGCTATAGAAGGGCAGCCAGAGAGTATAGTTCAAGATATTGAAACTATGGTCCATTCAT

ATGTTGACAAGCCCAACTGTATCATACTGGCAATATCACCTGCGAATCAAGATATTGCAA

CCTCTGATGCAATAAAGCTTTCTAGGGAAGTTGATCCTACAGGCGAAAGAACATTTGGTG

TTTTAACCAAGCTAGATCTTATGGATAAAGGCACAAATGCTCTTGATGTGAGCGCTTTAG

TTTGGACATAAAAAAGATACAAGCTCATATTCTTCTTTTTTATGTTCTTTTCCCTTTCAT

GTTTCTACTTGTCATACTCCCGTGTGCATACCTCTATATTCTTAAATACTGCTGACCATT

TTGTGTATAATTTTTCAAGGTCCTTGAAGGCAAATCTTACAGGTTGCTGCATCCTTGGGT

TGGAGTTGTTAACCGCTCGCAAGCAGATATAAATAGGAATATCGACATGATTGATGCTAG

GCGAAGTGAGAGGGAGTATTTTGCAACCAGTCCTGACTATTCACACTTAGCCAGTAAGAT

GGGATCTGAATATCTTGCCAAACTTTTATCAAAGCATCTGGAGTCTGTAATCAGGGCTCG

TATCCCAAGCATTACATCTTTGATAAATAAAAGCATTGACGAACTTGAATCAGAGATGAA

CCATATCGGTAGACCAATTGCTGTTGATGCTGGGGCTCAGTTATACACTATATTGGAACT

ATGCCGAGCCTTCGACAAGATTTTTAAAGAGCATTTAGATGGAGGCCGACCAGGTGGCGA

TAGAATATATGGCGTTTTTGACAATCAACTCCCTGCTGCTTTGAAGAAACTTCCATTTGA

CACACATCTATCTCAGCAAAACGTGAGGAAAATCATCTCAGAAGCAGATGGTTATCAGCC

CCATTTAATCGCTCCTGAGCAAGGTTATCGCCGTCTAATTGAACGTTCTCTCAATTATTT

CAGAGGTCCAGCTGAAGCATCAGTAGATGCCGTGCACTTTGTCTTGAAGGAATTGGTTAG

GAAATCGATAGGGGAGACTCAGGAGTTGAAAAGATTTCCAACCCTCCAGTCTGAACTGGC

AGCAGCCTCTTATGAAGCTTTGGAAAAATTCCGTGAAGATAGTCGTAAAACAGCAGTACG

TCTGGTCGACATGGAGTCTTCTTACCTAACAGTGGATTTCTTCAGGAAACTCCCTCAGGA

GGTGGAGAAGGCTGGGGCTCCCGCTGCGGCTAATGTTGATAGATATACTGATGGGCATTT

CAGAAAGATAGCATCAAATGTGTCATCATATATTGGGATGGTTTCAGATACCCTGAAACA

TACCATTCCTAAAGCTGTGGTTTATTGTCAAGTTCGTGAAGCCAAACGCTCATTGCTCAA

TCATTTCTACACGCTTGTGGGGAGGAAAGAGGGCAAGCAGCTAGCCCAGCTGTTGGATGA

GGATCCTGCGTTGATGGAACGTCGACAGCAATGTGCAAAGAGGCTGGAGTTGTACAAATC

TGCACGGGACGAAATTGATTCTGTATCCTGGGCTAGATGAGAGTAGCTGTTGGTCGTTTG

ATGTGTGTATTGCTTTGTACTATATAACAATGAGAGGAGCTTTGATTGATGTGCTGTATT

TGGCTGCGATTTCAAACATATAGTATAATATTGCCATATATTATTATCAGTTGTATTAGT

GTGTTTGCAGTCATGGAGTTGAGGTGAATATTGGGTTTACTTCTAAGTATTTAGTTTTAT

TCTTAAAATATAGATTAAATGGG

>Unigene0001098

TAATTATGGGTTTACATGTTGCATAAGAAACATGACTGCTTATATGGTTTCTACATGTCA

AATATGCTGTTATTCCAGTAACTGAGCAATTAAAATTATTATTTTTTCAGAAATTAAGTT

ATTTTTATGTTTAAGCTCGAAGCTATACTAATTATGGGTTTACATGTTGCATAAGAAACA

TGACTGCTTATATGGTTTCTACATGTCAAATATGCTGTTATTCCAGTAACTGAGCAATTA

AAATTATTATTTTTTCAGAAATTAAGTTATTTTT

>Unigene0001099

CGCGGCTTCCTCTGTTGTACCCTAGCCCTGAATCCGCCCCTGCCGCTTTTCTCCGGCGAG

CAATTGCGAGCGCGCCGCTGTCGCATCGGTAACAGTAGCCGGCGATGGCTGTGGCTGCTC

CCGGACAGCTAAACCTCGACGAGTCCCCGTCGTGGGGGTCTCGCAGCGTCGACTGTTTTG

AGAAGCTGGAGCAGATTGGGGAGGGAACTTACGGCCAAGTATACATGGCGAAGGAGAAAA

AAACAGGAGAAATTGTGGCCTTGAAGAAAATTAGAATGGACAATGAGAGAGAGGGTTTCC

CCATCACAGCAATTCGTGAAATTAAAATTCTAAAGAAGCTGCATCATGAGAATGTCATTC

AACTGAAAGAGATTGTGACATCTCCAGGTCCAGAGAGGAATGAACAAGGGAAACTAGATG

GGAACAAATATAAAGGCGGCATCTACATGGTTTTTGAATACATGGATCATGATTTGACTG

GCCTATCGGATCGTCCTGGGATGCGTTTTACAGTACCACAGATAAAGTGCTATATGAGGC

AGCTGTTAACTGGACTTCATTATTGTCATATCAATCAAGTACTCCATCGCGACATTAAAG

GATCTAATCTTCTGATTGACAACGAAGGAAATTTAAAGCTTGCTGATTTTGGCCTTGCAC

GGTCATTCGCAAATGATCACAATGGGAACTTAACTAATCGTGTGATTACGTTGTGGTATA

GACCTCCCGAGTTGCTGCTTGGAGCAACGAAATATGATCCAGCTGTTGACATGTGGTCTG

TTGGTTGTATTTTCGCAGAACTTCTGCACGGAAAGCCAATTTTGCCAGGCAAGAATGAGC

CAGAACAGCTAACCAAAATTTTCGAACTTTGTGGAACACCTGATGAGCTTAACTGGCCTG

GTGTTTCTAAGATTCCTTGGTATAACAACTTCAAGCCTTCCAGGCCTTTGAAGAGGCGCG

TGAAGGAGGTTTTTAAACATTTTGACCGTTTTGCGTTGGAGTTATTGGAAAGGATGTTAA

CGCTTGATCCGTCCCAGAGAATATCTGCACAAGCTGCACTCGATGCTGAGTACTTCTGGA

ATGATCCTCTACCGTGTGATCCTAAAAGCTTACCGAAATATGAGTCGTCACACGAGTTCC

AAACTAAGAAAAAGAGGCAACAACAGAGGCAACAAGAGGAAAATGCAAAGCGTCAGAAGC

TCCAGCACCCTCAGCCACATGCTCGGCTCCCTCCCGTCCAGACTGGGCAAGCACACCCAC

ACATGAGGCCGGGCCCCAGCCAGCCTGCCCACAATGCTCAACCTCCTGTACCCGCTGGAC

CGAGCCATCACTATGGCAAGCCGCGTGGTCCCTCAAATGCCCCAAATAGGTACCCACAGG

GTGGGAACCCAAGTGGTGGTGGATATAATCCAAATCGAGGAGGGCAGGGCGGCGGTTACA

ACAGTGGGCCGTACCCTCAGCAGGGACGTGCCCCACCCCCGTACGGTGGGAGTGGAATGC

CTGGTACTAGTGGTCCGAGAGGAGGTGGTGCTGGAGGCTACGGGGCTGGAGCTCCATCTA

ATTACCCCCAGGGTGGAGGCCCTGGCCCTTATGGTGCTCCTGGTGCTGGTCGTGGTCCGA

ACACTATGGGCGGGAATCGTAACCAGCAGCAGTATGGCGGCTGGCAGTAGCTGGAATTAG

AGTGTCTGTAAATCTGTAGTAGACACTGTCAGATTCCGACCGCCAAGCTTATTTATCTTC

TAGTTGTGTGTTTTTTAAGATTAGACTGTTCAAGATGGATGGAGGTTGGCATAGATCAAG

CTGTTTGGGGTGCCTTGTAAAGGACCTGTAAGATGTATTGTAATATCCTTCTGTTATCTG

TTTTGTGTTATGATCGACTATCATTTATTCTGGTTCCAATAATGGTGAATGTGTTGTGCT

TGT

>Unigene0001100

TTTCATACACTGTTGCCCGTGCTGGTCAGAACGGGTATCACCATCGTACTGAGATGAACA

AGAAAGTGTACAAACTTGGCAAGGCAGGAGAGGAGTCACACAAGGCTCTCACTGAATTTG

ACAGGTCATATCTTCTTTACTGCTGCTTTTCCTTGTAAATTTTCTGCATGTGCTATGATT

TTGTTTGAAACTGCTGCTTTTCCTTGTAAATATTCTGCATGTGCTAAAGCTATAGTTGAT

TTTGTTTGAAATAGGACTGAGAAGGACATAACCCC

>Unigene0001101

TTTCATACACTGTTGCCCGTGCTGGTCAGAACGGGTATCACCATCGTACTGAGATGAACA

AGAAAGTGTACAAACTTGGCAAGGCAGGAGAGGAGTCACACAAGGCTCTCACTGAATTTG

ACAGGTCATATCTTCTTTACTGCTGCTTTTCCTTGTAAATTTTCTGCATGTGCTAAAGCT

ATAGTTGATTTTGTTTGAAATAGGACTGAGAAGGACATAACCCCCATTGGTGGTTTTCCT

CATTATGGTGTTGTGAAGGATGACTACAT

>Unigene0001102

TTTCATACACTGTTGCCCGTGCTGGTCAGAACGGGTATCACCATCGTACTGAGATGAACA

AGAAAGTGTACAAACTTGGCAAGGCAGGAGAGGAGTCACACAAGGCTCTCACTGAATTTG

ACAGGACTGAGAAGGACATAACCCCCATTGGTGGTTTTCCTCATTACGGTGTTGTGAAGG

ATGACTACATTCTGATAAAGGGTTGCTGTGTTGGACCCAAAAAGAGGGTGGTTACCCTTC

GTCAGTCTCTTCTCAAGCAGACTTCTCGGCTGGCCCTTGAGGAGATCAAGCTGAAGTTCA

TTGATACGTCTTCTAAGTTCGGGCACGGGCGTTTCCAGACTACTGAGGAGAAGCAGAAGT

TTTATGGAAGACTGAAGGCCTGATTCTTGGGTTGCTTTTTTGCTTTTAGTGTTGGTATCA

CCCTGAAACTGTTTCTTTGTTTGAGTTTTGAGTTTTGAGTCTTTTGTTCTCTTTGTTTCC

AGAGACATGTTACCTGCTACAATTTTATTATGTCAGATTTTGGATTTTGTTATTCCGGAT

TTTGATCATTGTCTTTGCGATCAATAATTTAGAATCTTTGCTGGTGGTTGAGTTTATGTC

TGAGGAGAATATTA

>Unigene0001103

ACAACATCTGTTAATAAGAGCTTCAATTGTAGCGATGTGATTTGGATTCCGAGTCACTAC

AGTGAATACTGTCTCTCGGAGCAAAGCGACGCCAGCAGCTCACTGAACTCTGAGATGCTT

ATCCCTTTCTGCCGCCCCGGCGGCGACTCTAGGGTTCCTTAAATTCACGTCACCCGAGGA

GGACGAGGGCTATGTCATCCTCGCTGGGCGGCGGCGACGGTGGCGAAGGAGGCGGAGTGG

GGACGATGACGTGGCAGGAGGAGCTGGCGAGCCTCGTCGGCGACACAGGCATTCGCTACA

CGGTCGCCGGTGCCGGTGATGTGCAGCCTGATACGGCGGCGGACTTGAATGGCGGCGATC

TGCGGGGGATGCCAGTGGGGGAGTACTACGGGTTCGGGGAAGGGGCGCCGGCGGAGGAGA

GCTTCAAAGACCAGTTGAAGGGGTTCCTTGCGGCGTCGGGGGAGATGTTACTGGAGCTTG

GCAGGGGGTGCAAGGATGTAGCGCAGCAGAGTCTGATTGGAGTGGAGGAGCATTACGTGG

TGAAGAAGCTCAAGGGGCCGTGTGCGGTTGTTGGTGATCGGTTGGGGTTTCTAAACCAGT

ACCTGCCGGAGGATCGGGATCCCGTGCACTCCTGGCTGGTCGTAGTCTTGGTTTCGCTCA

TCGCGCTTACCGCACTGTATGTAAATACTGGACATCAAACTTCAGTGCAGCTACCAAAGA

AACTGTATATACATCCTCCTAGTGCTACTCGTGTCGAACTTCCAGATGGAAGATACATGG

CCTATGAGGAACATGGGGTTCCAGCAGAACAGGCTAGATTCTCTTTGATAGCTCCGCATT

CATTTCTTTCATCTCGGCTAGCAGGAATACCTGGCATCAGTGCATCTTTATTAGAGGAAT

TTGGTGTTCGTTTGGTTACCTATGATCTTCCTGGTTTTGGTGAAAGTGATCCGCATCCTT

TCAGGGATCTTAACTCATCAGCCCTGGATATACTAAACTTAGCAGATGCCGTAGGAGTTA

CGGATAAGTTCTGGATAGTGGGTTACTCAGGTGGTGGTATGCATGCATGGGCTGCTATTC

GTTATATTCCTGACCGACTTGCAGGTGCAGCCATGTTTGCTCCCATGGGCAATCCGTATG

ATTCTGGAATGACGAAGGAAGAGAGGTATAAAACCTGGGAGAAGTGGACTACAAGAAGGA

AAGTGTTGTACATTTTAGCTCGAAGGTTTCCTTCCCTTCTTCCTCGCCTCTACCACCGAA

GTTTTTTATCAGGAAAACATGGCCAGCTAGAGAAGTGGCTATCATTGTCATTAGGGAAGA

AGGATAAAGCTCTGTTAGAAGGGCCGGCTTTCAATGAGTTCTGGGAGAGGGATGTTGCAG

AATCTATTCGCCAAGGGAACACAAAACCATTTGTGGAGGAAGCTGTGTTACAAGTATCAA

ATTGGGGGTTCAGTCTGGCTGACCTTCAAGTGCAGAAGAAGCATGATGGGAACAGCATTC

TCCTTTGGCTGAAGTCCCTATACAGCCAGACTGAGCGTGAATGGGCTGGCTTTCTTGGTC

CGATACACATTTGGCAGGGAATGGATGATAGGGTGGTGCCACCATCGATGACTGAATTTG

TTCGACGGGTTGTTCCAGGTGCAACAGTACATAGACTGATCGGTGAGGGCCACTTTTCCT

ATTTCTACTTCTGTGATGAATGCCATAGACAGGTGTTCTCTACTCTTTTCGGCGTCCCTC

AAGGTCCGCTCAGCATTTCTGAGGAGAGCCTTGATCAATCAACGGAGGGTTTCTCTGAAG

AAACTTTACCCAACAACTTTACTGAGCAGGAATGAGCCTTTGTTTTTTGTTTTTTGTTTT

GTGCTCCTTCCAGTTGAAGGGATATCAGAGTATAGGGGAATTAGAGCATGGGATATATTC

TCCTATACCTTTTTTTGCCCTCGTCTTTTCCTTGATATGGTGGATAGAGTAACAAAAGAG

TGAGGCAAAGTTACAACATATGTTGTTGTATGGCTTGGAAGCCTTGGTATACAGACTCGT

GTTATCATATCAGAAAGAACTGTGAATGGAAATATCAAAGAATCTAATCAAGTTATCCCT

GTGTTTTATCGCAAACTATACATTAATCATGTGTTTGATGCTGCTGTTACGATGTGGGCT

TTTGCATAATCTAAATATGTTGCGGACACATCTTCATATATCTTCCCAGACTCTCCATAC

ATCAGAGAGTACTTCATACGTTAGAGAGTCTTATGCGAAGAAGCTATGTGATGCTTGGCG

TTCGTATACAAGATTGAAGGAAGGACAATCCCCGTCTTCATTAATTCCCTTATCTTCTGT

ACTACAGTCAATGGGACGCTGTTTGTGAAAATTTATCTTCCCGACTCATTCCAAGTGAAT

GCAGCAGTATACAAGTTCGTACTACTGTCAATAATAGTAAAATTGAACCATTCGACTTAA

ATTTTTTGTTCTTTTTGTGGCTTAATAAACTTGCTCGTATTGGTACGCATTATTACGTAT

GTTGTAATCCATTGATTTTGATAAACATGAAGTGCATTTTAAGTCTAAAATGGTTAAATG

ACCGTGTACCAATGTTTCAAATTATTGGGCTTAGTAG

>Unigene0001104

TCGTCATGGTGGCTGCTTCCTACGCCGCCGGCGTCCTCGTCGTCATCGGCCTGCTGGCGC

CATCTTGCAACGCGCAAGGGTACCGTAACGTTCTTTATGCAGGGGAAACCCTGCAACCCC

GTGAATCTCTAACCTCGCATAACTACAGCCTTATAATGCAACCAGACTGCAACTTGGTCC

TCTACCACGGCCACATCGCGATGTGGTCATCGCAAACCCACGGGCGTGACCAAAATTGC

>Unigene0001105

ATCAATAAAAATATAACCACATACGCCCTGGTCCGGAACGCACCAAACTCTGCAACATAT

ACGGCTCCACCCAAGCAAAGAAGAACTTTCCCTCGACATGCCGACAATGTCATCGTCGGC

GGCTTTGAGGATCATAATCAAAAGATTTTGCAAGATCTGCCCAATACCGGAATTACAAGG

GGATGTGTTAAATCGATTGTTGGTATGGACGGTCTCGGCAAGACAACCCTTGCTAATATG

GTTTTTAACAGCAACAAGATCAAGAGCTCTTTTAATGCTCATGCGTGGGGGTGTGTCTCA

AGACTCCACACTTGTTCATTTTTTCTGGAGTATCGGAAAACAGGTCGCAGATACGGAACT

TCAGGGATTGGGCCAATGTCAAGAATTGGACAAATCAGCGCTCAGGGAGAAGATCTGCCA

ATTCTTACTTGGAAAAAGGTCTTAAGCAGTGATAGGGAGCTGTTGGAGAGAGACACTATG

TAAAAATGCTACAGTAAGCAGCAGATAAAGTTCATTAAGCATGTACATGTACAACAATAG

CTACATTATTCTTCTTCTCATTTTTGTTATAAATAGAGAGTTTCTTTTGTTTTTTGGCAG

AGCAAGAAATACTATGAGAAGTGCTTAAGGAGCCAAGTGTGAGAGAGTTAGAGCGATCC

>Unigene0001106

ACCTAAAATAGGAAGGTATAGTGAGCATACAATTAGTTGTGTATAGTGATATGTATTATT

GTAGTATGTGTGCTAATAAGACCAATTAATATTTTTAAATATTTTCTCCCTCGTCTTAAA

TAGGTATCAAAACAATGGATCTAGTATTTCAGTTTCAGTGTCGTTGACGCACTGAATCGT

GATTTTTATCCCTAGTTATAAAATATTCTTGTGGTGTACTGTCTGTTAAGAGGAAAGAGA

GATTAAGACAAATGGTGTGTGTTGGGTGTGAAGCTGCTCTCCACCCGACAGTCAAAGGAG

ACATATTTTCTTTATTAAAAATAAGTGACCTCATTTCCTCATTGCAAGCCCTACATGTGG

TGTGGGGATGCTTTCATCTTTGACCCAGACATTCCCAAATAGTATAGGTACCCCATGTGC

TAGCATACAAATACAAAAGGCTCCAAAAGAAACACAGAAATGATAGTGTAAGAATAGTCT

TTTTCTTTCCTTTTGAAACTAATCATTTGTTTTCTAGGTCTTAAGCAGTGATAGGGAGCT

GTTGGAGAGAGACACTATGTAAAAATGCTACAGTAAGCAGCAGATAAAGTTCATTAAGCA

TGTACATGTACAACAATAGCTACATTATTCTTCTTCTCATTTTTGTTATAAATAGAGAGT

TTCTTTTGTTTTTTG

>Unigene0001107

TCCGCCTCCGCCTCCGCCTCCCCCTCCCCCAACCACTACGGCGGCAAAGTCGACTTCTCC

CTCATCGCCATCCGCGACGCCACCCCCGACGAGGTCGCCTCACTCACCCCCTCCACCCCC

GACTCAATTGTCCTCTACAAATCCTACATCAACTCCGGTCTCCATCTCCCCCTCACTCCC

CTCGTCGCCGGAATCCTCGCCTGGTTCGACATCGCCCCTTGCCAGGTGAACGCCAACTTC

TACCGCACTATCTCGGTCGCCTCCGCCGCCATCCCCCTCCTCCGCAAAGACCTCTTCCTC

ACCCCTGAGCTGTTTCTCAATTGCTACAAACTTGCTCGGAAGCGGAAGGCCGATGACGGA

ACCACCAAGCCCGAGGAAGGACCTCTGCTCAGGCTCTTCGTCCGGCCGCGCTACAACGAC

TTCGCCCCACCGGGCGGGGCCCTGCCCAGCAGCGATAAAACATGGACGCGCCATGCAATT

GTGGCTACTGGGCAATGGTTGCTGAGTGCTCACAATGTACATAATCTCCAGATCAGGAGC

TTTCCGAATCTTGAGGTTGATCTGGGGAAGCCGCCCCCGCCGATGTCCGACCAGGACGCC

GCCGCGCTCGCCCTGCTCCGGCGGGGGAAGAGTGCGAAGTTTGATAACTTTCTCAAAACG

GCCCATTGTAGGAAGAAGAAAAAGCCGTCTGAGCCGGAGCCGGAGCTGGAGAGATCAAGT

TTGATTCCGCTGCATTGGAGTAAGAAGTCCCGCAGCACAGGTCAGGGCAAGAGGTGTGTC

GGTGACAATGCTGGTTCGGGCCAAACTACCCCCAAGAGGAGGGAGTTGTTCGTCTCGTCT

TTCTACACCGAGCAAACACCAGCAATGAAGACCTACAAGAGAAAGGGATCCCCTTCAAAT

GCACGACAATCTTCGCCAAAACATCGCAAAACCGAGGCTCCAGTTGCTTCAGACGAGCAA

ACAAATATCCACACTCCACCGGGCTCTGCTAATAAGCCTACGGGAACCGACATAGTTGAA

CGTATGAGGACTTCAGCCACTACTCACCCCGAAGCTGTGCCGCTCCGACTAATTCACTCC

AAGCCTTCACCGCCTCCTCCTGAGTCTCCAGCACAACTCACAGAAAATCAGCGTGGGTCC

CGAGCAGTCCTGAACACCACTCCTATCATTCCTTTGAAGCCGGATGGGACTCCAGTGACC

CAAGCCGATTCCATCCTCGAGTGTCCCGCTGCTGCCTCTGGAGCTGCAGGCATGTTTTGC

CTCCCTCGAGATGTTGAGCTGATGGAGGGGGCCAGTCTTGACACGCTCATGTCGACCGGC

TCGATCTTCATCTCCAGGGCTATGGCTCTATTTGAAATAGGAGCAAAGCGCCTCCGGGAC

ACCCAGAACTCAAACTCTCCTTCCTCGGCTTTCGGGTTTACAGAGAAGGAGAGACTGAAG

TCTGAAGTCGAAGCGCTGAGGAAACAATGCTTGGATCTCAAAAACAAAACAGAATCCGAT

GCCTCTGAAGTCCGACAGCTCGAGTGTGGGGAAACGTTGGCCGAGGAATCCTCCCGAGCC

GGAGCCATTGCCGAACTACGTGCACAAAAAGATGAGCTATACCTGTTACTGGAGCGGTCA

GGTGTCGAGATAGCCGCAAAAAATACAGTTATACGAGAGCTCGAGGGGGTCATGGAGGAA

AATACGCAACTCAAGGCTGTCATTGAAGTTCTCAAGAAGCAATGCTCCGAGTTTGAAACT

AAAGCAGACATCGAAGCCTCGGAAGTCAGAAGGCTTGAAGACGTGCTCAGCACCGAGAGA

GCGAAGCTTGAGCTACAATTGGTGGATACAAGGAACTCGATTGCCTTCCAAACTGCAGCA

ATCACTGGCTTACATGCTGAAAAAGACGAGCTGTCACAGTTGCTGGAGCGGTGCAGACAG

GAGATTTCTTTGAAAAATTCGGTTATCAGGGAGCTTGGAGGGGTCAAGACGGAGAACGAT

GAATTAAAGTCGAAGGTTGAAGGGTTGACCAAGCAATGTGCTGAACTCAAAGCTCAAACT

GATTCCGGTACCGATCAGATTCGTCAGCTCAATGATCAGATGAATGCCGAGAGAAGCAAG

ACCGAGAAGGAGTTGGAGGAAACAAGGAGCGAGATCGCCTCTCACGTCGCAGCTATTACC

GGCCTACAAGCCGAAAAAAAACGAGCTGTCCCGTTTACTGGAGCAAGGCAGAGATGAGAT

AGCGACAAAAAACTTGGTTATCAGAGATCTTCAAGGGGTCGTCGAGGAGAGCCAGAAACT

GAAGCCAGAGTTGGAACTGCTGATGAAGCAATGCTTCGAGTTTAAAACTCAGATATACGA

TTACTCAACCGAGGTTCGACAGCTTGAAGAGCAGCTGAATACCGAGAGAGGGACAGCTGA

GACAGCGCTAGCGGAAGCTAATACGAAGGCATCTTCCCAAGCTGCAACCATTGCCGCTCT

CCAAGCCGAAAGCGATGAGAAGTCGCGTCTGCTGGAGCAGTCCAGAGAGGAGATAGCTGC

CAAAAACTCGGTTATCAGAGAACTTCAAGGCTCCATCTCGAAACTCCAAGCTAAGGTGGC

TACGTCGATGCAGAGAGAAGGGATCATCAAAAGTCTTCGTGATTCTGTCTTACTGGCTGA

GCAGCTGGCGGATAGTGATATCAAGCGAGCGAGGAAGGCTGGGTATGATGACGGTTTGGT

CGACGGCAAGAGGATTGGGTGGAAGGAGTTTGTTGAGATGCAGCAGCGAGTTCAGGCAGA

GCTCCAGAGGCCGTTACAAGAGCGATTGCGAGAATTCCAGATGCAACAGATGCAACCCCA

GAGGCCGATGTAGAATCCTGCCTTCTAGTGTTATGGTTTTAGTGCTATGGTATAACTTAT

GTGAATTATATGTTTTGGTTTCGGTTAGCTTTGAAACCTCGAATAATATGCTGGTATAGA

CAGTCTGTTTTTATGGTATCTATGCTTGTGCTGAATATGGTTTTATGTGCTGAGAAGAAG

AATGTGTTGA

>Unigene0001108

CGCACTCCCGATAGTGAAAGCAAGGATGAGGTGTCCTCTGGTTGGTAAGCTGAACCCCAG

CTGCCATTGCCTTGACCTTTGGAGGTCCTGATCACATCATGGGATCCCCGTCGCCCATTT

CCACACACAATGGAATTATCTCAGAAACCGGTCAGACTGGCTGGGTCTTGCTTCAAGCTC

AGAAAAGGAGAAGTATCCGGGGAATTCCCCAATAGGGCTATGAAGTGTTTCAATTTCTCG

AATGGAGTGAAGAAGGAAGAGGAGCCCCAGACAAGGAAGTCTGTATCAGCACGGTCAAAC

AGCACCACTTCGACTGACCGTGATGCGAGAAGATCAGGGTCTGATTTCAACTCTTTGAAT

GTTTCGGATATGAGCACTGATTCGGTAGGGAAGTCCCCGTTCCATTGTTCCTCTCAGAAG

TCTTGTGATCTCAGAGTATTCACGTTTTTAGAGCTGAAGAGTGTTACTAAGAACTTCAGC

CGTTCCCTAATGATTGGACAAGGTGGGTTTGGATGTGTTTATAGGGCCACAATCAAGAAT

TTGGAGGATCCTCCTAAAAAGATTGAAATTGCGGTCAAGCAACTGAGCCGCAGAGGCCTG

CAGGGGCATAAAGAATGGTTGACTGAAGTTAATGTCCTTGGAGTGGTTGAGCATCCAAAT

CTTGTCAAATTGGTTGGTTATTGTGCTGAAGACGATGAAAGAGGAACACAACGACTACTC

GTATATGAATATATGCTGAATGGAAGTGTACAGGATCATCTAACAAATCGGTCAACTGCC

CCACTTTCGTGGCTCACGAGAATAAGAATTGCTCTTGATGCTGCTCGTGGTTTGACATAC

CTACATGAAGAAATGGAATTCCAGATTATCTTTCGAGATTTAAAAACTTCCAACATTCTC

TTGGATGAGCATTGGAACGCAAAGTTGTCAGACTTCGGCTTGGCTAGACAAGGACCAACA

GAAGGACTGAGTCATGTTTCAACAGCAGTTGTGGGGACTATCGGCTATGCAGCCCCCGAA

TACATCCAAACCGGGCGTCTCACTGCTAAGAGTGATATATGGAGCTTTGGTGTCGTCCTC

TATGAGCTCATCACAGGCCGTCGCCCACTAGACAGAAACCGCCCAAAAAATGAACAGAAG

CTCCTGGATTGGGTCAGACCGTACGTGTCTGACGTCACAAAGATCTGCATGGTAATTGAT

CCACGTCTAGAAGGGAACTATTCTTTGAAATCTGCAATGAAACTTGCTGCCATTGCAAAC

CGATGTCTAACTAGGCAACCCAAATCTAGGCCCAAAATGAGTGAGGTGTTAGAGATGTTG

CAGGGTATTGCCGAGAGTGCTGAGACAGGATCCCCGCAGCTCCCTTTAAGGAGTTCCCCT

GTTGATGAAACCGATTCCGAAGACAACAGGATGACATTGTGCCTGAAACGGAGGATGGGA

GACTTGAAAGTGGGAGGGCTTGCATGGAGGAAATGGACACCAAAGCTTGTACGGACACAC

TGACAGTGATGATGATAACGACACAAATTCCACACTCAAAAGTGTCAGATGACAAAGTTG

AAGTTTTATTGTTTCTGAGATGAGCTTCCCTACTGGAAGAAGCAACTCGTGATCATAGAA

TGGTTAGATGGTTACGATTGTTGCTAGTTATGAGTTTTGTTTATTTGTTTATATACTGTT

TCCTTTGTTGTGATGTACAATACTACCCCTTGAATGCATGTAGATATAAGGTTTTTCCTT

TGATCAACGGCGAGAATATATGAGTTTGTAACACAAGTTGGGCATGCTGAATCCATTTTA

CTAAACTGTATAAGCTTTCTCAAATTTTACTCAATATGGTATATTATTAAGTTTGTGACT

CTTATGGCAATGCTTGTTTGAAGGCGTATCGACA

>Unigene0001109

AGTCAGGCAACAAAGCGACGATAACGTGGAGTCCAACTGTTAAAGAACAGTTAACATTTA

CGGAAAACGGCGTCAAAGGCCAATTTATAGTTCAATATGACGTAGATCATAATTCCACTC

CAAATCAAGTTCTCATCGACGATGGATATTTTGTTCATTTCTTTGCTCCGACGGATTTGA

AACCGCTTAGGACTCACGTTATATTTGTACTCGACGTTAGTGGTTCTATGGTTGGACAGA

AATTACCACAAGTAAAAGAAGCTATGGGTCAAATTTTGTCAGAAATCCATTCTGAAGATT

TTTTCACGTTGATACTTTTCTCAGATTTTGCTCAAGTTTGGACAATAAACGCTACACAAG

AAACGTCTAATCGTTGGGACGAAACTGGTAACAACTGGAAGACGAACAATAATTTTAGTT

TGGAGACATTAGGTGAAAACCGTTTCGTTTTTCCGGCAACCGAGCAGAACATACAATACG

CCAAGAAATTTATTCAAGACTTGACATCGGAAAGTAGTACAAACATGGAGGATGCCCTTA

CCAAAGCTCACTCTATCGCCAAGTTAGGAGAAACGCGTTTCAAAGATGGTGCTAACACGC

CAAAACCAATTATTGTGTTTCTTACCGATGGAGAACCGACTACAGGGATTACTGAACCCC

AGGAACTTATAAAATATGTTTCTAATACAAATGAAGAGAAGTACCCTATATTCAGTTTGG

GTTTTGGCGAAGGAGCGGATATTGATTTTCTTAAAAAACTCTCATTGAACAATACCGGTT

TCGCTAGAGTCATCTACGAAGCTTCGGACGCTTCGTTACAATTACGTAATTTCTATAAAG

AAATTTCTTCTCCTGTTCTATCTAACGTTACTTTCCAATACATTGACGCACAAGTCGACA

ACATAACTGTGACGAAAAAATCATTTAACCTGTTGTTTAAAGGAACAGAATTAGTAGTGG

CCGGAAAGTTGAAGGATGGCACATCGCCCGATTTTAACGGCACTTTAAACGCCGATTCGA

CTGAAGGAAATTTCAACCGTCCCGTTGTTGTTACGTGTTTCGATATCCCCATCATCCCTC

CCACTTCCCTTCCAACGCAGAGAAGAATTGGTCATTTAGAGAAATTATGGGCGTATTTGA

ACATACAACAATTGATGGACCAATATGAATTAAACAAGAACGAAAATTCTACGGCAAAAG

CTAAAGCACTTGAATTAGCATTAGAATATTCATTTGTTACTCCACTCACATCCTTAGTTG

TTGTTAAACCTAATGATACAACAACATCAGCGGCCCCAGAGAAAATCAAACCACTTAATG

ATAGATACAATTTTGGTTCTTTAATGGCCAGCAGTCCACTTTATCAATTAGGTCCATCTA

GCTCGGTGATGATGGCTAATAGTTTTTCACAATCATCAGCATATCCTGCTAGTATTTCAT

TAAAAGCTAGGCCAAGTCAAGTATATTCAATGGTTCCCGAAGATGTAGCTAGTTCTGCAT

CCGAAATGGTTCCTTTGTCAACTATTTCGTCAACTTTAGTTGCGGATTCAGATAATAGCC

CACCGACCTCGGTTGTAGATTCTTCTCACGTTCTAATAGAAACATCAAATTCTACATCGT

TGCATGACATAAAGGAACTAACATGGTTGAGTGATTCTATTACATTGAATGCAACTAATA

CAGTTTACCAAGTATCATTGAACACTACTGGTCAATCGTATCAAAATTGTACAACACCCG

ACCAAAGTTATGGTAATTGTAAACATTTAAAAGACTGCGTTGTGCCTTCAATTTTGAACT

CATTTGATACATATCTTTCATTATTTTGTCCAATAGAAACATTGTATGCAGGAATTTGCT

GTTTAGTGTAAATTGTAATTGTGTGAATGTCCACAAATTTCTAGGAAGCAGAATATTATT

AATATTTTTAAAGTGTTTAAGTTATTATTATTATTTATTATTGACTTATATTTTATGTAC

CTTTGTATTATCAATTAAACA

>Unigene0001110

CGCATCAAACCCCAACCCCGCGAGAGCGTTTCCACCCAAATCCGCCCTTACCCTCCGGCG

AACCCTCCGACGCCGGCGACAAATCTCCGTAAACCTCTCAGCTGATATTGTTAATTACAT

CGAGATTTATGCGGTGAATGTTCGTTTCTATGGATTTGAATGCATCACCGTTGCCTGAAG

ATGACGAGCAGCCTATTTTTGAGCACTTTGGGGATGAAATTGCTGTTGAAGACCCACAAG

AATCGGCCGTGGAGACCATGCGTAGAGAGCGTGAAGAAAGGCGCCGTCGGTTGAAAAGAG

AACACCGAGATGAAGGACCAAAGTTGACTTCACAATCAACAAAGAATGATTATTTGACTC

AGAGTAGAAACGTTAAGCCACGTGGGAGAGTCAAGGAACTTCCTCAAGGATGGTTGGATT

GCCCTTCCTATGGTCAGTGTGTGGGGTTTCTTATTCCTTCTAAGGTCCCTCTAGATGAGA

CCTTTAATGAGTCAGTCATACCTGGCAAACGATATTCTTCAAAACAAGTAATTCATCAGC

AAAAACGAGTCGGCCGAGAACTTGGTTTGGTGATTGATTTGACAAATACAACTCGTTACT

ATCAATCATCAGAATGGATTAAGCAAGGTATCAAACACGTTAAGATTGCATGCAAGGGAC

GAGATGCAGTACCAGATAACGAGGCTGTTAACATGTTTGTCCATGAGGTTATGCAGTTCC

TCTCACCCCATAAGCGTAATAAGAAGTATATTCTTGTCCATTGTACACATGGGCATAATC

GTACAGGTTTTATGATTATCCATTACCTTCTGCGTACTCAATCATTGAGTGTTACTGAGG

CAATAGATATTTTTGCCCAAGCACGTCCTCCAGGAATATATAAGCAGGAGTATATTGAAG

CACTCTATACACTTCACCATGAAAGCCCTGAAAATTTGATTTGCCCCTCGACGCCAGATT

GGAAAAGATCATCTGATCTTGATCTAAATGGTGAAGCTGTGCTGGATGATGATGGTGAGG

GTGATAGTGCTGCTCCTACTCAAGAGCAAATGGAGAACAAAATCATTACTAATAATGATA

TTTTGGGAGATGCAATACCTGATTCTCAGCAAGAATATATGCGTCATATTTGTTACCAAC

TATTTGATTTGACCCACACAGGAAGAGGTAATGCACAATTTCCAGGATCACATCCTGTTT

CTCTTAACAGGGACAATCTACAACTACTAAGGCAACGCTATTATTATGCTACATGGAAAG

CAGATGGGACCCGATACATGATGCTCATTACCACCCATGGTTGTTACTTAATTGACCGTA

ACTTTTGCTTTCGTAGGGTTCAAATGCGGTTTCCAGTGAAGAACATGAATGAGTTCCATA

ACATGACATTGATTGATGGGGAGATGATCATTGACAAAATACCAGATACTGGACTGAAGA

GAAGATATCTCGCATATGATATGGTTGCACTTAATTCACGCTCTGTTGTTAAGTTGCCCT

TCTCCGAAAGGTGGAAGCTGCTTGAGGAAGAGGTAGTCCGTCCTCGAAATAATGAGAGAA

AGCAGTTTGAGTGTGAGGGCAAATGCAATCCAGTTTACAGATACGACATGGAACCATTTG

GAGTTAGGCGAAAGGATTTTTGGTTGTTATCCACTGTTAAAAGGCTTTTAAAAGATTTCA

TCCCAAGGCTTTCACATGAATCTGACGGCCTCATTTTCCAGGGTTGGGATGATCCTTATG

TACCCCGCACCCATGAAGGTCTTTTAAAATGGAAATATCCTTCCATGAACTCTGTGGATT

TCCTGTTTGAGGTGGGAAGCGACGGCCGTCAATTACTCTATGTATACGAGAGAGGGAAAA

CAAAGCTGATGGATGGGGCCCATGTTGTTCTGGGAGAAGAATGTGAGGATGTAGAGGACA

TAGCTTCCCTCTCTGGCAAGATTATAGAATGCTCTTTGGATAAGGAGAGTGGGAGTTGGT

CGTGCATGCGCGTTAGAACGGACAAGTCGACACCGAATGATATAAATACTTACCGAAAAG

TTATGCGGAGCATCAACGACAACATTACTGAGGAAGTGTTACTGGAAGAGATAGGTGAAA

TAGTGACACTGCCGATGTATGCAGAGAGGATTGCAAAAGCTCATCAGCGCAGGAGGTAGA

ATGTGGGTAAATTTAGGGTTTCGAGTTCTGACCAGGTTCGCTTTGCCTGAGATTAGTAGT

GGAGTTAATGTGTAATTAAATATTATTTGGTAGTTTTACTGTGACTGGTTATTGCTGGCT

AACTGAAATCTTGTATAAGTCGCAATACTATGTACAAATCATATCCACCCTCGTTCATTG

TTTGTATTTCACATATAGATATCGTAGACAGGTTTGGTATTTGCTGGTTACTCCTACAGG

ACTGTAGTCAAAGGTGAGCAATTGGATT

>Unigene0001111

AACCATCAACCGACGACTCAAAGGTTAACAAAGAAAGCAAAAAAAAATCTGGAGAGAGAA

AGAGGAGAGATAGAGAGAGAGAAGAGTCCGACTCCACAACAAGTCCTTCTCCCCTTCTCT

CTCATGTCCCAGCTGGACCCTGTTATACGTGAGGAAACGGCAGCAATGAAAGAAAGAGTT

AAGAGAGACAGGAGAGAGAAAGAGAGGAGAGAGAGAGAGAAGTGTCTTCCAACCAAACAC

AAAAACCATCGCCCCCTCAGCGACCCACGACCTCGACATCATGCAGGCCCAATCCCTGTT

CCATATAGGTGCCCTTTCGGTCAGAGCCCTTACCTCCACCTTTATTTCCCCTTTCCTTCA

ACCCCACCCACCACCCAAATAGACTCTTTCGATGGTCATGAAGAAGCCGGCGAGGAGGGC

TCCCATGGAGTCAACGCCGGCCGTTCCGGCTTACCCAGTTGGGGAAGAGGCCAGGGCGAG

GCACAAGCACCAGAGTTTGTTGCTGGATTACCAGGATCTGATCAAGGAAACTGAAGCAAA

GAAGAACACATTGCTGAAGGCGAATCGAAAGAAACTTAAACTTCTTGCCGAAGTCAAGTT

TTTGCGGGGAAAATTGAAATCTTTCTCGGAAACCCCATCACTGAAAACCTCATATGTGAC

CATTAAGCAATCTGAAGCAGGAAAGCATAAAGGACCGGAGAAGCGATCAGGAGCGGGGAA

GCAAACGGGCAAGGTGACAACACCATCTACTAAACGTGTACGCATTCGAGTACATAATCA

AGTTATTTCCAGCAAACCCCATATCGAAGAGAGAAACTTCAGATCAGTAGAAGCTCCAGC

TCCAGGCACCAATGCACTGTTGGATTTGAACAAGGATGCTGAAGAATTGGAGGAGTGGCA

GTCCCTGAGAACAGATAGGTTGACGATGAGCAGCACAATGACTAATGATCTTAAGCTGCC

CCTATGTCGTGATATCGGTAACGAAGCGAGCCAATCCGGTAGAAGAAAAATTTCGTGGCA

AGATCAGTTACCTCTGAGAGTTTAGAATGCAGGAATGTAAACTTTCGGGTCTTTTATGAC

TCATCGTTGTCCAAAATCATAATCATAATCCTTGACGATAACCTTTGTGTTGCTCTGTGT

ACATGAAAATATTATATAGAAATGTTGCTTTCTTTTAATAGGTATATTACTCTTTCATGA

AAATGATACTATAATACTATAAGTTCTCTCTCTTAGATCACGTATACTA

>Unigene0001112

TTTGGGAATTTTTGAGTTTGGGTTGCTTTGTTTGTGATGGATTTTGGAGCTTTTGGGTTG

AAATCTAGATTTGGGTTTGGTGGGATCTGGGTTGGGGTTTGATTTTAGGATCTTTGTGGG

TTATTGGATTTAGATGGTGTTGTAGATTTGTATGGTTACCATCTTGGTTTTTTGGGTCTT

GGCTTGTGATATAGATTTTGATTAGTGGGTTTTTTATTTATTGTTGATTTGTTTGGTTGC

CTTCTTGCTTTTCGATTGGCTTTTAGTCAAATTTGTTTGGTTAATAAATTTGAACTTTTG

AGATCTTTGAGTTGCAATATAGATTTGGGATGTTTTTCTCTTATGTATTGGTTTTATTGT

CAATCTTGTTGGTTATTATCTTGAATTTTTAGGGGGAGTTGTAAACTACACTTGGGGATT

TTGTCTCTCGCATATTGGTCTTGGTGTCAATCTTGTCGGTTGAATTCTTGAACTTTTAGG

GTCTTTGAGTTGTAATATAGATTTGGGTTGTTGTCTCTCATGTATTGGTTTTAGAGTTTT

GAGTTGCATCTAGATTTGGAATGTTTGGATTTTTTTTGTCGGAGAGTAATGATCCTGTGT

TTACCCTAAAAGGGTTTCTTTCCTCAAATTTAGTTCTTGTTGATCATGAATCTAGGTTTT

GATCAGATCAAAGACCTAGTGTTTATCTTACCTCCTTTGTACCATGATGAGTGATGTGAT

TTCGTTGCTATTCTTTTTTTACTCCATAAAGCATCAGCACATTAGCAAATGTGCCGTTTG

CGATTGTCGACATGAACATGACACTCTAACACTCCAGCCATGCCTGTGGTATACTAGCTA

ATTTCATCATCTATTTGATTGTAGATACTATTTGGTTAGATTTATTTATCTTGTGTGAAC

TAAGGTACTTGAGAGTAAAGATCAAATATCAGTCTCATAAGGATTAAATTTGGACGATGT

>Unigene0001113

TTTGAGTTGAATCTAGATTTGGAATGTTTGGATATTTTTTTGTCGGAGAGTAATGATCCT

GTGTTTACCCTAAAAGGGTTTCTTTCCTCAAATTTAGTTCTTGTTGATCATGAATCTAGG

TTTTGATCAGATCAAAGACCTAGTGTTTATCTTACCTCCTTTGTACCATGATGAGTGATG

TGATTTCGTTGCTATTCTTTTTTTACTCCATAAAGCATCAGCACATTAGCAAATGTGCCG

TTTGCGATTGTCGACATGAACATGACACTCTAACACTCCAGCCATGCCTGTGGTATACTA

GCTAATTTCATCATCTATTTGATTGTAGATACTATTTGGTTAGATTTATTTATCTTGTGT

GAACTAAGGTACTTGAGAGTAAAGATCAAATATCAGTCTCATAAGGATTAAATTTGGACG

ATGT

>Unigene0001114

AACCATCAACCGACGACTCAAAGGTTAACAAAGAAAGCAAAAAAAAATCTGGAGAGAGAA

AGAGGAGAGATAGAGAGAGAGAAGAGTCCGACTCCACAACAAGTCCTTCTCCCCTTCTCT

CTCATGTCCCAGCTGGACCCTGTTATACGTGAGGAAACGGCAGCAATGAAAGAAAGAGTT

AAGAGAGACAGGAGAGAGAAAGAGAGGAGAGAGAGAGAGAAGTGTCTTCCAACCAAACAC

AAAAACCATCGCCCCCTCAGCGACCCACGACCTCGACATCATGCAGGCCCAATCCCTGTT

CCATATAGGTGCCCTTTCGGTCAGAGCCCTTACCTCCACCTTTATTTCCCCTTTCCTTCA

ACCCCACCCACCACCCAAATAGACTCTTTCGATGGTCATGAAGAAGCCGGCGAGGAGGGC

TCCCATGGAGTCAACGCCGGCCGTTCCGGCTTACCCAGTTGGGGAAGAGGCCAGGGCGAG

GCACAAGCACCAGAGTTTGTTGCTGGATTACCAGGATCTGATCAAGGTGAGATCTGGGTT

GAGTTTTTGATGTGTTGGATCTCGGGGGTTGTTTGATTTGGG

>Unigene0001115

AATTAAAATACACAATCCACCACAAAGATCAAAAGAAATAAATCCCCTCCTTTTTCCCTT

CTTTGTCGGCCTCCTCTTTCTTTCTCCTCTCTCTCACTGCTTTCCCCTCTCTGCAACTCC

TTGTGGATGTGATTCTCCACCATCTCTCATAGCCCCATCAACCTTTTGTCAAAAGAGAAA

GAGAGGGAGAACAACCCACCAACGATGGTTTTCTCATCAGCTCCTCTCTATCTAGATCCG

CCTGACTGGAGCCAATATCAGCAAGCTCAGCAGCATGGAAGCGGAAACGGGATCGGAAGT

GGTAGTGGGAGTGGCGGTGGCGGTGGAGCACACGAAGTTCACCAGCTCACGCAAGGGCAG

ACTCCACCGCGACCTGATGGAGGGATGCAGGTCATGGTTAGGCCTACCTCGATGGCCGAC

CGAGCCCGTCTTGCGAAGCTGCCACAGCCGGAGCAGGCGCTCAAGTGCCCCCGATGTGAT

TCACTCAACACCAAGTTCTGCTACTTCAACAACTACTCGCTCTCTCAGCCACGCCACTTT

TGCAAGACTTGCCGCCGCTACTGGACCCGCGGTGGCGCCCTCCGAAACATCCCTGTGGGC

GGAGGCTGTCGTCGCAACAAGCGGAGCAAGTCAGCTGGCAGCGGCACTCCCTCCAAGTCC

CAAACTTCTACTGATCTTGCATCCTCATCCACATCTACTATGGTAACAAATTCAAGCCTA

CCCTTAATCCATTCAGCTTCACAGTTGCCCTTCATGGCCTCTTTGCATATGCAACCTATT

TCCACCGCCGGGGGCGATTACACTTCCACCAACATTGGGCTCAACTTTGCCGGTCTCACC

CAGCCTATCGATGCCGAAAACTATCAAGTGGGCAGTTTAAGCTCGAGCGTCGGAATGGAG

CAGTGGAGGATTCAGCAGATCCAGCAGTTCCCTTTCTTCGGTGGGCTAGAGAACTCAGGT

CAGCAGCCAGTGCATGGATTATATTCATTCCATGGTGATCATCAAGGTGGTAGTGTTGCA

ACGAGTGCGGGTCTCATCTCACAGATGGCCTCGGTGAAGATGGAAGATAGCTCTCAAGGG

CTCAATTTGGGTGGGCAGTATTTGTCTGCTCAGCGGAATGATCAGTATAATTGGAGTCGT

GGAGGCAATGCAGGAGGTGATGGTGGCAACAACGGAGGCAGTGGTGGTGGAGGGTGGATG

ACTAATCTCTCCGGCTTCAACCCTTCCACATCTGGCAACATCTTGTAGTCGATATTGCAT

GCGAGGAGCTGATCAATTTTTGTGATCACAGTTAAGCCTTTTAACATGGAAAGCTAGGGT

TTCAGGGAATTGTGTTCTGAAGTTTATAGTGGAAACTTCAGAAACCCTAGAAATAGTAGC

TAGGTGTGTTGTCCGTTATTTTTAATCTAGTTGGTTTGGTATTATGAGTCTTTTCTGTAA

TGTAGTGCATGGGCTTGATATCTAACATGCACGTAGCTGCTGCTAGAAATGAGCAAGAAC

TATCCTTGCTTAGCTCATGGTTGTGTGGAATATTATTCTAGGGTTACTAAATGCAATGTT

AGTGATATTACTATCTATTCC

>Unigene0001116

CAATGAAGTGCTGCCATAAGTGCGGTTGGTTTGTGGCATGTATATGCAGATTTGTAATCT

CAGTGGCTGTAAGCAGAATCTCAATGAAGTGCTGCCATAAGTGCTCTGTTTGGGTGTGTG

ATTTAGATTTACGAAGCTAGTTTCTTTATGGCGAGGATTTGATAAAATTTGGAGGTAGTG

AGATTAAAATGATCAACTTGATGTTTTAGACTAATCTTCTTGACCAGCATTATAGTCATT

AAAATGATGTTGACATCTGTAAAGGAGAAGGTTAGTAGATGGAAAGATGAATGCT

>Unigene0001117

CATTTAGGCTCCGTATTCACTCTGGCGCTCTCGCAAACCCCTATTGAGTTCATTCTAACG

CCACCCTCGCCGGCGGTAATGACGTCGGAGCGGGAGACTGCGCCGCGGGACAGCGAGCCC

CCCGCCACGGATTCCTCCCACCAGTCAAAGTCGAAGCTCGGCGTCGAGCTCTCCCACAAC

GATACCAACCCGCCGGCCAAGGACGACGAGAGCGCGCTGCGGGACAGCGAGCCCCCAGTT

GTTGCCGCCGCCGTCGCCGAAGCCTCGGGCGACTCCACTACCCCGAACCATTCACCTCCG

AGGCCCAACCCCTTCTTCCAGATCCCGCCGGGGATGAGCTTGGGGCCCTTCCCCTCCCGC

TGCCCTCCCGTCCCCGCCCGATCCGCTGCCAAGGTCCAAAGCAAGGAGCGGACCGATGCC

GACCAGAAGCCATTCCCTGGCGCAGGACGCAAAGGAAGTGCTCCTGCGAGAGTCAAAAGG

AGACGTGGCCCGGGTGGTCTAACTAGGCTTCACGGTGTTTCACCTGAACTGCAGACCATT

GTTGGCGAGTCAATCATGCCAAGGACTCAGATCATAAGGCAGTTTTGGGCATACATTCGT

AAAAATAATCTCCAAGATCCGAATAACAAAAGGAATATCATATGCAATGATGGACTACGG

CTGGTTCTAGAGACGGATAGCACTGATATTTTCCAGATGAACAAGCTATTAGCTAAGCAT

ATCACTCCCCTAGAACCTAACAACAAGGTTGCTGTTAAAGACTCTAAAAAGTTAAAGACT

GAAAATGTCGCTAAAGCAAAACGTGATGCGGCTTCAGGCTGCATGACTCCTTCAGCTGAA

CTGGCCGAATTTTTAGGAACCAAGGAAAGGGTCCTTCAATCTGAGGCTTCAATCCGCATG

TGGAATTACATCAAAGATAACCAACTCGAGATTTCCTTCTATTCCCCGGTTATGTGTCGC

TATCATTTACTAGGATCAAGTAAGCTCAATGATATTGTGTGACTCAAAGCTTCAACGACT

CTTTGGATGTGAAAGACTTGCTGCTTCAGGGGTGTCGGAGATGTTGGCACGCCATTTGGT

TGAGAAATCATGACACAGATCCCATCAACTGCTCTCTAAGATGGTGGTGTCATGGGATTC

TCACGAGTTGTGCCCTGACCATACACATCTTCGTTCCTGCTCGGGCATAGCTTGAGTCAG

CTAATACCAGTCGAGCCCCACTAGGTACGTCACATATCACAGTCAGGTTATGACTTTTTT

ATTCCTATACCTTGTCTATGATATAAGTTATACCTGTGAATCTGATTCTTACTAGGGAAT

ATCAGACTCGAGGCATGCTCAGGACTATCACAGCTCAAGTCCCAAATCCAGATTGTCGAA

ATCCTCCAAAAGCTTTATTGTTACAATAAAATTACTGAGGTTTCACGTATCCCAAACGGT

AAATACCTGTTCAAACTTATGTTATTAATTTGTTATAGCAAGTTTTATGTACCTTCGTTT

TTTCTTAACACGGAAACTTACATGCCTGGTTGTCTTGTAATTTGTAAAATCAAAATTGAT

TTATCCCTGTGTGATATTTATAAGTGTTCGTGTCTGGGTAAAAAATTCCAAAAACTGTGA

TACGGGAGGAGAAATTGTGTCAAATTATAATTTGAAATATGAGATACGTAGTTCCCCTTT

GTAGCTCTCTGGAAATGATATACCTTCAATCAAGATGTGGTATGTTATTATAAATCTGTG

TTTGAGAGATATGACTTGTTTTTAGTAAATTTATATATTTGTTATCTTTTTTTATACATA

CTTTCTAGAAATAGCACAATGTTATTTCCTTGGATAATTTCTGAAAAACATGTGTATAAC

ACTTGGGAAAAGAAAAACAGGAGAGGGGAAATGGGTTTTATTGATTAAGTCCCTATCATA

TGAATATTTAGAGTTTGTACCGCAACAATATATTCTGTACTTAGACTTTTTCATTGACCC

AACTGATCCATATTAGGAATTAAAAGAAAAAAATGACATTCTTTAAATCTGTTATCCTAT

ACAAGAGTAGATTAATGTATCTCCGTATATGGAATGCACTTTAGATAGCACAATTGATTC

CCAGTATCAGTATGATGCTTTGGCATGCTACCTGCAAGCTCTGCTTGCATTTTTTTTTCC

TCTTAAATCAGTAAGTTGGTTGATTTCAGACTCCTAGTTGGTGGGTTATGATGCACTGAC

ACTTCCCAAAGGTGTCGTGGCGGTGTTCAACATCACTCAACACGCCTTGCCATTCTGACA

CACCACTGATGTGTCCATACTGGATAATTTTGTCAAATTTTTTAATTACCGACACTTCGT

GACACGATTTGACATTTGGTTAGATGCATTTGATTCAGTCGATGTTGCTGAGGTACTTGA

CATTCAGATTGCTCTCTTAATAATCCGAAATTGGATCTGATAGTAATTTCAAAATGTATA

TGATAATGTTGATGAAGAATGATTATTAAGAAGCAGCCATGTTTTGTTATGGACTTTGAA

CTTCCAAGCAATACCTATTATGCATG

>Unigene0001118

TACGACCCCCATAGCGAGGGCGGGGGACATACCACACTGGCGGTTGGAGGGGAGCAGTGT

ATTTTTTGTTCGGTCTTTTTACTCCTTTCTCCATACGGGAGGGGTATATGTGCCCTTGGG

CCGACAGCCTCCGGTCAGTTCGGGCACCTGTAAAGGTCTTGATGTTCGGGTGGTTGATGT

TGTAAGGGGCACTGACAACACGAGCTGTTCTATCACACCGGATACCGGGGTGGGTCGGTC

CATCACAATGCGGCCTTTTTGGAGGGCCACTCGAGCCGGCCGATCATTTTTTCATCTCCT

GCCCGGTCTCAGAGGGCATTTGGACCTTTTTCAGAACATCATTAGGGGTCTGGGGGTGCC

CTGCTTCCATTGTATAGGCTTGGACATCTTGGAGGGTCACATGTGTGGCGGGGGTACGCA

GCGTGCATGAG

>Unigene0001119

TAGGAGGACTTGTGGATGGGGCCTACTCGTCTCGGGGGAAGATTCGACATCCTATACGGT

GCCGCTCGGAGACCGGGGGCTTCGGTTGCCTCTCTTTATTCGGGATCTTGGGGCTGGTCA

ATTCCGACGAGCGGACATCTGGGGGTGGAGGGGCTAGCCCAACTTCGCTCTCTCCTGTCG

CTGCTGGCTGGTGCGGCCCCCATTGCGGGGGCGGGGGACATACCACACTGGCGGTTGGAG

GGGAGCGGTGCTTTTTCTGTCCGGTCTTTCTACTTCTTTCTCCATTCGGGGGGTGTCTGA

GCCCTTGGGCCGACAGTCTCTGGTCGGTACGAGCACCTGCGAAAGTCCTGGTATTCGGAT

GGATGATATTGCAAGGGGCTCTGACAACACGGGATGTTCTTTCGCACCGGATACCGGAGT

GGGCCGGTCCATCACAGTGCGGTCTTTGTGGAGGGCCACTCAAGACGTTCGATCATCTGT

TCATTGCCTGCCCGGTTTCTGAGGGCCTTTGGACCTTCTTCAAAACGTCACTAGGGGTCC

GGGGGCACCTTGCTACCATTGTACAGGCATGGACATCTTGGAGGGACTCGGG

>Unigene0001120

TTATCGGAGCTTCTCATCTTCGTCCTCCTGGTAAGTCCGAAACCCTAATTCCCCGTCATC

CCTCCTCCACATGAACCCTAAATCCCCCCTCCACCTACTCCCAATTCCCCCAGATTTCAC

CCCAACCTCCATCATCCCTCTCCCAGATTCAATCCCCCCCAAAATTTGGATGAAATCCAG

CCATGAATGACACATCAAATCGCCCTCGGAATCTCGGTGCGTTCTTCAGTCTCTCCTGCG

CTCTGGTACTTCTCTTCCTATTCTTCTTCAATCCCCATCCCAATTCCTTCAATCCCCGCC

GCCGGACCGTTGAGATCCCCATCACCAATTCATCGACGCCAGACGAACATGACTCCCTCA

ACAGCTCCACGGAGGCCTGCGCCGGCATAGACCGCCACTCCGGCTTCGCCACACAGTGCG

AATACCTGAGATCCAATCCACAATGCGCTGCCACTGGTGCCTTCATCAATTACCTCACCT

TCTTCTACTGCGACTGCGAGAAGTTCCGGGTCCTCGGGTACTCTGTCCTCACTCTGTGGC

TCGCTGCCCTGTTCTACCTTCTCGGCAACACCGCTGCGGATTTCTTCTGCTGTAATCTGG

CTAAGCTGTCTAACTTGTTACGACTGCCACCGACGGTGGCCGGGGTCACGCTCCTCCCGC

TGGGGAATGGAGCTCCGGACGTCTTCGCCAGTATAGCTGCGTTTGTTGGGTCGGGTGCTG

CAGAGGTGGGACTCAACAGTGTGCTGGGTGGTGCTGTGTTTGTCACTTGTGTTGTGGTCG

GGGTTGTTTCGCTGTGTGTTGCGGGGAAGGATGTGAGGATTGACAGGAAATGTTTTGTTC

GGGATATTAGCTTCTTCTTGCTGACGCTGGTGTCTCTGTCGTTGATTCTTGTTGTGGGGA

AGGTGGGTGTATGGGGATCTATCATGTTTGTGTTGATTTACATTGTGTATGCTTTCGTAG

TTGCGGCGAATGAGATTTTGAGGAAGAATGCGTGGAGGTTGAAGTTGGAAGCGGTGACGC

CGTTGCTTCCGGTGAGAGCAAGCGTCTTTTCTCAAGGGAGTCAGGACTCGATTTACAGCT

ATCTGCACGAGGAGGATCCTGAGGGCGATGGGCCACATTTGTACCCTTCGTTGCCGCAGT

GGATGTGGGCTTCGAATGTGGCTATTTACTCAAATCATTTTGTTCAGAGTGATTTGTCGG

AGAATGTAGGTCCTTTATGGGGGTGGAAGAATGAAGGAGATGGTGATGAACCTTCACTCT

CTTGTGCAAAGATCTTGTTGTTGATGGAGATGCCAATGACACTTCCGAGAAGGTTGACGA

TTCCGATTGTTGAGGAAGATAGGTGGTCGAAGGCGTATGCTGTCGCTAGTGCTGTCATGG

CTCCAGTTCTGTTGGCATTTTTGTGGAACAGCCGGCGAGGTGATGCTTCTAAGGTCACTA

TCACAGCTTTTATTGTCGGTGGTCTCATAGGCATTACTCTTGGTGCTCTTGCTTTCTTGT

TCACTAAGCATGATCGCCCTCCACGGAGATACTTGTTTCCTTGGGTTTTCGGAGGGTTTG

TAATGAGTATAGTTTGGTTTTATATCGTTGCTAATGAGCTGGTTGCTCTCTTTGTTGCAT

TGGGCGTGATATTAGGGATCAATCCGTCTATTCTTGGACTCACGGTGTTGGCATGGGGTA

ACTCGATGGGTGATTTGATGTCGAATGTTGCTTTGGCAATGAACGGTGGGGATGGTGTTC

AGATTGCTATGTCAGGATGCTATGCCGGGCCGATGTTCAACACTCTTGCTGGGTTGGGGA

GTTCCATGCTGCTAGGTGCATTGTCGACTGCCCCTACTTCCTATGTTCTTCCTGAAGATA

GTTTCCTGTTTTATACAATGGGTTTTCTTATGTCTGGACTCATTTTGGCTCTTGTTGTTT

TGCTACGGAACGACATGCGTCCTAACAAGGTGTTGGGTGCTGGCCTAATAACTCTGTATC

TTATATTCTTATTTCTTAGGGTGAGCAATGCAGTGGGTCTTGTACCATTGCCTGGTTTAC

ATTGACATGAGCTGTTATATGTTGAAATATAGTGTATATTTC

>Unigene0001121

TAACTTGAAATAGTTGCAGATGGCCACCATTGCTGCCACAAAAGAAAGAGCAACAAAGGC

ATCATAATGCAAGCAGCAACTTTGTTTTGTGGCAGCAATGGTGGCCATCTGCAACTATTT

CAAGTTAACACCAACAAGATGGTAGCACCAAGAAGACTACTCTCTAGACCAAACAGAAGA

ACAATCGTACCTCCCCAAATTGGAGCCTCCTCCACTCCTGAGACTTCCACTGTCGACTAC

AACGCCTCCTCGATTTCGTAAGTCATCTACTTAGTCGTAGTTCCTTCCTACACGACAGGA

TTATCAGATCCTATGAGTGGATAGAAAGTTAGATCGCCATTGAATCTCACTGCATGCTTT

TCACATGTCAGTGTGTTTCCTGCCGAAGCATGTGATACAATAGGCGGGGAAGCTTGTGAT

GCAGAGATGTTTCCAGAAGCAAATCCCTCGGCATATGCTAGCAGTGCGAAATTGAGAGTG

GAATCGGTGGAGGTAGACCGAGATTATCTCGAGTACGACGAGCCGAAAACGGTGTTCCCT

GGTGAGGCTTGTGATGATCTTGGAGGAGAGTTCTGTGAACCAGAGTATCAGCAAGGGGTA

TACAAGGAGAAGCCTTGACAGGATAAAATAGTTTATGTTTGTATAAATGATGAGTGTATG

AAATGAAATAAGAGATGGTTCATATGGCTTGTTTATCCTTTTCACACAATCCACATCAAT

ATGCTCCCAGAAACTTTATTCAG

>Unigene0001122

CGTGTCAATAAACAATTAACCACTAATTATATGGGACAAAGAGGGGGTCCATCCAGTAGA

ATTTACGCTTAATTGACGTTTAAACACATTTCGTGTTGCACTGTATAGAAGTAGGTGTTA

CCAAACATTGGTCTCGCGTAGCTCTGTATTTGGTATTTGAATAATACATATACTTTTTGG

GACAACATGTTCAGATAAAAATACCAAGAACACGTCATATTAAGGGAGATTAAGGT

>Unigene0001123

CTCACGTTCGCCACCCGTCTTCTGTGGAGAGAGATGCATCGGTTGCGCGTCCTCTCCCTC

GCGCGCCCCATCACCCACTGCCGAACCCTAACCTCTACCCCACCGCTCCTCCTCCCCACC

GTCGATTCTTCCGCCCCCTCCCCTCCCCCGCCGGCGCGCACCCTTGCTGACCTCCGCCGC

CGTCTCGCCGCCGAGAAGCTCTCCCTCGTCGACTTCACTGCCTACTCCGTCCCCGTGGGA

ACGAAGAAGGATCCCCTCCCGAAGCCGAAATGGATGAAGGAGACCATCCCCGGCGGGGCC

AAGTACGCTCGGCTCAAGGCCGATCTGCAGAAGCTGAATCTCAATACTGTCTGCGTCGAG

GCCAAGTGCCCTAATCTGGGGGAGTGCTGGTCGGGCGGCGAGACGGGGACGGCTACTGCT

ACTATTATGATCCTCGGGGATACCTGCACCCGCGGATGCAGATTTTGTAATGTGAAGACA

TCCCGCACTCCCCCTCCACCAGATCCCAACGAACCTTCTAATGTTGCAAAAGCTATTGCA

TCATGGGGTCTTGATTATGTAGTTATTACTAGTGTTGATCGTGACGACTTGCCTGACCAA

GGAAGCAGTCATTTTGCCGAAACAGTGCAAAAGCTAAAGGCAGCGAAGCCAGAAATTCTA

ATAGAAGCACTTGTTCCTGATTTTAGAGGTGACTCCAGCTGCGTAGAGAAAGTTGCAAAA

TCTGGTTTAGATGTTCTTGCTCACAACATTGAGACAGTTGAAGAGCTGCAGGGTATGGTA

CGAGATCACCGTGCAAATTTTAAACAATCGATTGATGTTCTTAAGATGGCGAAGACATAT

GCTCCTGCTGGCACCCTCACGAAGACTTCCATAATGTTGGGTTGTGGGGAAACACCTGAT

CAAGTGATTAGCACAATGGAGAAAGTGAGGGCTGCAGGAGTTGACGTCATGACATTTGGT

CAGTATATGAGGCCATCAAAGCGGCATATGGCAGTCTCTGAATATGTTACACCAGAAGCT

TTCGAGAAGTATAGGTTACTTGGCATGGAAATGGGTTTCCGTTATGTTGCTTCTGGCCCC

CTGGTTAGATCCTCGTACAAAGCTGGGGAATACTATATCAAATCAATGATCGACGCTGAT

CGAGCCACTACCCCTTCCACCGCAAGTATAATGCCCCAATAGTGAAAAAGATGTTCTATC

CATTCCTCATAACCTTGTAATTTATCAACCAATTGTTATAACCATTGCAACTTGAGTGAA

GTGTCTCTTCGGTTATACTTTTGCAGCTTGTTAGAGCAAGACTTCTTTGTATAATTTCCA

ACAGTGGATTTGCAATTCATGTGTTTTTGTCCAGGTTACTGACGTGTCAATAAACAATTA

ACCACTAATTATATGGGACAAAGAGGGGTCCATCCAGTAGAATTTAC

>Unigene0001124

AGGATGGTCGGAGTTTTCGACCCTCGCGGCACGAAGATGCTTACGGAGAAAGCTCATCAA

GATGCAAGCTCTCCGATGCATAGAAACCGTGTGGAGGTGGTCAAAGTGGACACACAAATA

TCGAACCGTGTGGTGAGTTCGAGCAAGAGAATTGATGGGATGCAGATGAGCTTGCTTTTA

GCGCAGGAGATGTCGAAAGGTGAAGACTCTAAGCGGATGCCATCGAATATTGTTGCGAGG

TTAATGGGGCTAGATGCGTTGCCAAACCAGCAAACCGATGTAAATTCCAAGATAAGCTCA

TATGAGGATTTTACTCTCGCAGAGGGCACACTTCAATGCAATGAGGATACGATCCCTTGC

GAGATTCAATCATGTGCTCAAGAAGGGGAATGTAAGGATGTACATGAGATGCAGCGGAAA

TCATCAAAGGGCGACAAGAACACAGCGGAAAATAAGATGAAGCTTGTTCGCCAGAAATTC

CTTGAAGCAAAATGTCTAGCCACTGATGAAAAGCTTATTCGGTCGAAAGAGTTCCAAGAT

GCGCTTGAAGTTCTGAGTTCGAATCGTGATTTGTTTGTCAAAGTTCTTGATGATCCGAAC

TCTCTGTTTTCAAAGCATCTTCAAGAGTTCCAGACACTTCAGCCTTTATCGCAGCCGAGG

CGTATCACAGTACTGAAACCAGCGAGAATGGTTGAGTCAAAAGTTGATGAACAAGTGAGA

AAACACCAACATCCGTTTGCTGAAGCTAATGGACTGGAGATGAACAAGCACCACTGGAGC

TCTGGCCTTGTTCGTCAGAGGGCTGGAAGCATGTCTCAATCCACCAGGATAGTGGTTTTG

AAGCCTAACATTGAGAATCTTCGAGAGATAAAGAAAACGGCGGTTTGCTCTGCTCCCTCG

CCAGAAATGCTCGCTGGAAGAGACTCTTGTGTAGCCATCGGATCAGGAGAAGTAGCTAGC

GACGAAGCTGCGGAATCAAGAGAAGAAGCTAAGGAGAGCACTCAACAGTATGAAGAGAGT

CTTAATAGTAATCGGACAGATGAGACCTTCCTCTCTGCAGTCTTATCGAACGGATACGTT

GGAGATGACAGCTCGCTCAATGTATCAGAGATTGAAGAAGATGGTTGCAACTTCAGTGAC

TTGGAGATTGCAACTCCAACTTCACGGCATTCTTGGTGTTCCACCAACAGGTTAGACCAT

CTTCGTTCAGTCTTGTCCTCTAGCCGTGCATCTTGTGCTGAATCATCAGTCATTAGGGAG

GCCAAGAAACGTCTTTCCGAGAGGTGTGCCATGGTCACATCAAATGGGGTTGGTCACGAA

CAAAGACAAGATCAGAGCTCGAGCACCTTGGGCGAGATGCTTGCTATTCCTGTGGTGAAG

AAAGACGAATGGAGCGATTGGCCAGCTACTTCGAGCAGTAGGCCATGTGGTGGCTATCAA

GGAACGAGTTCGAACAGTGGAACATTTGCTGAATGTAGTGGGGAGAAATCTCCTAGGAAG

TTATCAAGGACAAAATCTGATCCCATTTCGGCTCTGTTTTGTGGTGGCAATGGGGCAGAG

GATGAAGTCCCAATGTCCCTTATCAACATCCTTCCGAAATCTATACTGAAGTTGAAAAGC

GGGAAAATGTCTCTCAAAGACAAAGTTTCGAGCATGTTCTTATCGAAGATGAAGAAAGCA

GGGGGTGGGAAATTTATTCTAGGTCCCTTGGCTAGCTCAGATGAAGACTCTGCAAGAGTT

AATCATGAGAAGAAATCCGGTAGTAATGGGAACAATGGATCAAGCACTCGATTCACGGAG

AATGCTCTTTCTCTTAAGA

>Unigene0001125

GGGTCGTGGCCCTCCAAACCTCCGGCGTATCGCCCCTTACCCATACCACCAGTTCACTTG

TTCCACGCCGCCGCCGCCGCCGCCGTTGCATCACCACGGCCACCTCCTCTCCACCAGCTG

CTTTCGACACCAATTTCTCGAATCCCCCATGGATACCTCCGAAGAAGGCTACAAGCCCTC

GGGGGCTTTCGAAGAGGAGTCCAAGGACGCTCTCGTCGATATCCCCGACAATGACTCGAC

GGAGCTCTGGCTAATCCAGTGGCCCATGAAACAATTGGATCCGGCTGATTTCCACGGGAA

GAAGGTGAAGCTCAAGCTGCACGATGACGGGCAGTTGGGCGGCATCGAAAGTTCATCTGG

GAAGAGTTACGACATGGTAAGCTATGCGGCTCAAGAGCCGGATGCAACTGTCTTCATGCC

CTCTGCTTCTGGATCGAAAGTCGTTGGAAAGATTTCACGCCGAGTTTGTTTAGTTCGGTA

CCCTGACCCCGAGGAACTGGAAAAACCGAGTTTTTATAATCCCAACTTAAGCGCTATGAA

ATCAGGAGGTTCAACGAAGAAGAGCTCGCATCGTGATACATTAACTTCAACTCATAGTGT

GGGTCACCGAAGCCTTGAGTTCTCCTCGCAATCCATGAAGAAAAGACACAAGGGCAACAA

GCCACATACTGAGGGATCTGAGAAGTCTAACGAGCGATCTCACACTTCTGGAGGAGGAAG

CCAGGTCACCGATAGGTCGTTAGGGTCTGAGCAATCTCGCGGAGAGAAATTGAAGAAGAA

AAAGAAGATGAAGGCCGGGGAGTAGTTGGTTGAGTTTTATGCCATTGCTCTTACTGAAAT

ATGTGAGTCAAAATTATTCGTGTTTTGGATACCGTAACTGGAGTTTTTGCTGCCATAATA

GTTCTTCGAGAACTTGTATGATTAATGTTTTACTTCTGGTATTTGTGAAAATGTACTTGT

TCTTTTCTTCAAGTTTTGAGAGCTCGCATGTGAGAGTATGGATATTTAATATTGATATCT

GACAAAACGGTTTTATGGTGCTTATATATAATT

>Unigene0001126

CACTCACCCGTCCGTCTCTCTCGTCCGGCGGCGGCGCGGAAGCTCAGATTGCGATGGCGG

ATGAGGAACAGCAGCCTCTTAACTATGTCCACGAGACGGTACTGAAGAAGAGGAAAAACA

ATGATGAGTGGGCTCTGAAGAGGAGGGAGAGGTTGGATGCTCAGAAGCAGAAGACCAAAG

ATAAGAAGAAAGCTGCGATCAAAAGGCCTGAGCAATTTGTCAAAGAATTTCGTGACAAAG

AATTAGACTACATTCGGATGAAGAAAAGGCTAAAGCTACGAAAGACTTCACATTACGACC

TCAAGTCAAAACTGCTCTTTGTTATTCGCATTCACGGCTCGCAAGACATGCATCCTAGGT

CTAGAAAAATCCTGAATTCATTGAGGTTGAGACAAAACCTCACTGGTGTGTTCGTGAAGG

TTAATGAGGCAACCCTGAAAATGCTACTCGCCGTCGAACCATTCATCACTTATGGCTACC

CTAGTTTGAAAAGCATAAGGGATTTAGTTTTTAAGAAAGGCTGTGGGAAGATAGAAAGGC

AAAGGGTTCCACTCACAGACAACAATCTCATTGAACAGGCGCTTGGGAAGCATGGAGTTA

TCTGCATTGAAGACATTGTCCATGAAGTTGCGACATTGGGCCCCCATTTTAAAGAAGTGA

ACAGTTTCTTATGGCCATTCAAGCTAAAGATGCCAGAGAGACGGTTGCAGATGAATAAGA

AACAATTCAAAGATGGTGGCCATGTTGGAAATCGTGAAGATAAAATCAACGAGCTTATCG

ATAAGTTGAACTAGGATTTGGAGATAACATTTGTGCTTGTGTAACATCCAATCTTTTAGA

AGACATGGATTTCTATCTGGATTTGAGGGGTTGAACGCTATGTTAACTTTTTAAATTATT

AATCTTTTAAGTTTGTTTTGTATGTCTTTGACCAGCTTACACATTTGTCTGTGGAGTGCG

TTATTACCATCAGATTATG

>Unigene0001127

CACTCACCCGTCCGTCTCTCTCGTCCGGCGGCGGCGCGGAAGCTCAGGTAATCTTTCTCC

CCCAATTTTCTTCCTTGTCGCGCCACCGCCTCCACTGTCACCGCCGCCGCCGATATCTCC

TCTCGTCTCCTCTTTCCGCCTCCTCAATAGAGATCTCCTCTTCCATCTTCGTATGTCGTT

GTGCTATCAGCCGCCTCCTCCTCCTCCTCTTACCTCTGTTTCTTGAGTCCTCTTCAAATA

CATCGTCTAGTGATTCATATTGCGATGGCGGATGAGGAACAGCAGCCTCTTAACTATGTC

CACGAGACGGTACTGAAGAAGAGGAAAAACAATGATGAGTGGGCTCTGAAGAGGAGGGAG

AGG

>Unigene0001128

AGAAAAACATTATAGTGATGAAGCCCTAATTTTGACTCAAGGCCATTCATGTCAATTTAT

GTGTGTTTGGGACCCTAACTTTAGGGTTGGCAAAGTTTTGGCCGACCCAGTAAAACCAGG

TTATGGTCTTTGGTTTTGTGTCTGGACCCGAAATTCACAAAACCTAGACTAACAATTTTT

TAATAATCAACAATCACCTTAGCTGCATATAGAAGTTTAGACTCTGAGGCCTGAGTTTTG

TGTCCGGACCAGGAATCATGAGTTGACTGGTCACGATTGATGCATAAACTGCCCCACCCA

AACTGTAAGTCAGAATTCCAATTAATATGTCAATGGTTTTCCACCGGTTTGGTAAACTGA

GAATCTCAACTTAAAAGTTAGCTGTCCACTCCAGAATGTCCTGCTGTGTCCTTGCCGTTG

TTGATGGGGAAGGTTAGGGGAAAGGCTGGTAAAATTGTCTTGTTCACTCTTTTTTGATAC

TGAAGATGTTTAAACTGAAGAACTTCAAGATTTTATGGGTTAAAAAATATTTTAGCTGTA

TATAAGTGTACATAGAGACAACTGTTGTGTTATTCAAAATATATGACTTAGTCTTTGCTT

TGGATTTACAGGCGCTTGGGAAGCATGGAG

>Unigene0001129

CAAACTCTTTGTACCACTGATACAAAGCAGCAAACAACTGCTACATTAGGACTTCCTAAA

AATGAATCCACAAAAAATATTGATCTCTCTACGCTTTCATCTGTATTGGATGATGGAAAG

AATACAGAGGAAAAGTCTATTGTTGTGTCAAATGAACTAACTGAAGAAAAGTTGAAATCT

AATTCTCATCACAAACCACAAAATACAAAAATTCCATTGCAACTTG

>Unigene0001130

TACACTTCTCCGCGGCTCACTATAACAACCCCTTACCGCGTTTCTCTGCTTCTTGCATTC

TCTCACCTCCTCAACACAATACCGGAGGAGAGAGAAAGTCTTACATCCCTAAAGACTCTC

TCTTCTCCCCTCTCTCTCCTCCCTACCGCCACTCATATCAATCTCCACTGCCTCTCATAT

CAACCTCCACCATGCTCTCCCTCAACCGAGCAACCGGCTTTCTGAGCAACAAAACAGAGG

GGTTTAAGAACCGGAAGAGCCGACCGGGAGTGGTGGTGTGCTGCGGAGTACCGAGGGCGG

CGAGAGGGGTGGCGATGGAGACGGCGACGAACGGTGCTGCTGCGGTTGATCCGACGACGG

CGGCCGGTGGCGGGGTTGGGGATACCTATGGAGAGGATCGGGCCACGGAGGATCTGCCGG

TGACTCCATGGACGGTTTCTGTTGCCAGTGGCTATTCTTTGTTGAGGGATCCCCACCACA

ACAAAGGGCTTGCATTCAGCGACAAAGAGCGGGATGCCCATTACTTGACCGGCCTGCTAC

CACCAACATGTGTGTCTCAAGACCTTCAGGTAAAGAAGATGATGCACAATATTCGTCAGT

ACCAAGTCCCCTTGCAGCGTTACATGGCTATGATGGACCTTCAGGAGATGAACGAGAGAC

TATTCTACAAGCTCCTGATCGATCATGTCGAGGAGTTACTTCCAGTTGTTTACACCCCGA

CTGTCGGCGAGGCTTGCCAGAAGTATGGCAGTATTTTCAGGCGGCCGCAGGGCCTTTACA

TCAGCTTGAAAGAAAAGGGTAAGATTCTTGAAGTGTTGAAGAACTGGCCTGAGAAAACCA

TTCAGGTTATTGTGGTCACTGATGGCGAGCGAATCTTGGGGCTTGGAGATCTTGGTTGCC

AAGGAATGGGAATTCCAGTCGGCAAGCTCTCTCTTTACACTGCCTTAGGAGGAGTCCGGC

CTTCTGCATGCCTTCCAATCACTATTGATGTGGGAACTAACAATGAGAAGCTGCTGAAGG

ATGAGTTTTACATAGGGCTGAGGCAAAAGCGTGCTACTGGCCAGGAATATGCTGAACTGT

TAGAGGAGTTCATGTCCGCTGTCAAGCAAAACTATGGAGAGAAAGTTCTTATTCAGTTTG

AGGATTTTGCGAACCATAATGCATTCGAATTGCTTGCTAAGTACAGCAAATCACATCTTG

TCTTCAATGATGACATTCAGGGCACAGCTTCTGTGGTCCTTGCAGGGCTCATGGCAGCAC

TAAAGTTGGTTGGTGGAACTCTAGCAGAACACACTCTCTTGTTCCTCGGTGCTGGCGAGG

CTGGTACTGGTATTGCGGAGCTTGTAGCTCTTGAGATGTCGAAACAGACAAATGCTCCAG

TAGAAGAAACTCGCAAGAGAATCTGGCTTGTGGATTCTAAGGGCTTGATCGTTAAATCCC

GCATGGATACCCTTCAACACTTCAAGCAACCTTGGGCGCACGATCATGAACCTGTCAATA

CTCTCTTAGAGGCTGTCCAGACCATCAAGCCAACTATGCTGATCGGATCATCTGGAGTTG

GGCAAACCTTCACACAGGAGATAGTGGAGGCCATGTCCTCCTTCAATGAGAAACCACTCA

TTCTTGCGCTGTCAAACCCTACGTCACAATCTGAATGTACTGCTGAGCAAGCGTACACAT

GGAGCAAGGGTCGTGCGATTTATGCTAGTGGAAGTCCATTCGCCCCTGTTGAATATGATG

GCAAGGTTTTAGCACCTGGCCAGGCTAACAATGCGTACATTTTCCCTGGATTTGGTCTCG

GTTTGGTGATCTCTGGTGCTATTCGTGTGCATGATGACATGCTTCTTGCAGCCTCGGAAG

CTTTGGCCTCGCAGGTTTCGCAGGAGAACTTTGCTGAGGGACTTATCTATCCACCATTCA

CGAATATTAGAAAGATATCTGCTCACATTGCTGCTAAAGTAGCGGAAAAAGTCTATGAAC

TTGGTCTGGCTAGTCGTCTCCCTCGCCCGGAAAACCTGGTGAAATATGCGGAGAGCTGCA

TGTACACCCCCACCTATCGCAGTTACCGATAAATTAATTAAATATCAAGCCTCCATTTTT

GTAGTAAATAAGTAACAGTATAGACATTGTAGGTTTTTTTTAATTTATTTGGATTGGCTG

TCATTTTAACATTGGTTTTAAGTGTTGAAGGGTTTTTTTTTTTAATGAATGGGAGGCTTG

TTAAAAATAAAGTTGCATTGGTGATATTAAATGCATTTTATCAGATTTATTGTATCCAAA

TATTTTTGAGTATATAGTCTATATACCCTAGGGTACGCATATTTGCATTTCTATATACTA

CCCTTACATTTATTGATAT

>Unigene0001131

TGACAGATATATGCTCCCCAATGTAGAGGACCCTTTGAGAGGAACTCGGGTCTATGCCGA

CTGGGATGTTGCCCTTTTATGAATGAGATCATCCATCAAGCTCATATACGACCATATATT

ATGCAAACCAACATGTTAAACCGGTTCTGTTTACTGGGTCGCACAAATTGTGGTCAGACT

AAAGTCCAGATCATCGATCCGCGTTTTACAGACGCAACTATTTGACTG

>Unigene0001132

AGTTATTATTTTTATGGTTGTGGAGTTAAATTAATAATATCACATTAATAACTTACTAAT

CAAAACAATTAAACAAACTAACAGTTTTAAATTATAATATCTATTAGTTTATATTTTTTA

TTTAAAAATTAGTATTTACACTTAATGGTTTTAAAACTATCTAATAAACATAAATCTAAA

GTTATTAAAATACTACCCAAATTCATTAATTGGCTTTAAATGATATTT

>Unigene0001133

TTCTATATAGGGAAACTATATTCCACCGTCCCAAAAGTGGCAAATTGATTTCTCCCTCTC

CGCTCTCTCTTACGCGGCCTATGCCGAGATGGTGTCTTCTGAAGTTGTGGTTTATCGTGT

TAAAGAATTCTTCCATCCCATATCAGTTGCGGCACATCGCGACCTGTTTAGCCGCAGTCG

ATTACCCGTTCGTCACGCGGTCACACGCTGGTTGTCCTGATGCTATTCTGTGCTACAAGT

TTCATCGTCTCATCAAAGCC

>Unigene0001134

AGAGACTCAGAAACACCTTGACTTAAAATATTGAGAAACTACATATCATTGTTAGCTATC

ACCTTCTTCTCTCTTTATTTGTATAAAGGAAGTAGGGTTTTTGTATCAAAATGAGAAGAT

TTCTTTATGAGTCAAGTAGCTTAGCCTTCCCCTTAAGGTATGGGTTTGACGCTTGAGATC

ATCTCAGTATGACAAGAGCGGATTCTCACTACTATACTGGGAACTTCTAATATCTTTCTT

GGAAGTCATCATCATATCAAGAAGTTAGCACAAAGTAAATTTTCTATAATTCATCAAATG

GCATCATTATCGTCTTGAGTAGAACAAGAAGAATAAAGAAAGAAGAACATAACTTAGAAA

GAAGAAGGAAATGAAAGATAGCTTAAAAAAAGTTGAGGAAGACTACATGAACTTTGTATC

TAGCTT

>Unigene0001135

CGCGCTATGCATTCAATTACTATGGAGATTGTTCGTCTTTGAGTTTTTCAGTCACCTACT

CCCCTTGTTTTCGACAACAAGATTGCTATGCAACCAGAGTGATATACAGATCGTTAGCAA

CCCAATGTTTCATGAGAAGACCCAACATATTGAGATTGGCTCTTACTTCGTACTATAGTG

CTTTCTTTAGAACACCGTTCACCCTACTCACCGGTTCCAGTTTCTACTCAGTAATCTCAT

GTTTTTTGACCCCCTGCGAG

>Unigene0001136

CCTTCATAGGAAATGGAGCTTCCCACCCATATGTGGGAGATAATTTCTACTCTGGAGAAT

CCTAGTGGGCTGAGGTTTTAGGAGATACAACAGGAGTACCGCTCGATACTTATTAACCTG

GAGGAAGTACAGGTGGGTCCTAGGAGAGGAATATGAGAAGGCTAGTCAGGAGGCCGAGTC

AGAGGGTAGTCAGGTAGCTGAGTAAGGGGTTATGGTTGAGCCAGTGTTGCCCCCAAA

>Unigene0001137

CCAGGCAAGCCGCAGTTCTTACTGACTTGTACTGCCTATTTTGTGTTGTGTAGTTTTCGA

ATAATTTATGTTTAGTCAATTGTTTTTGCTCGATGCCATAGTAGAGAAAGTATTCTAGGT

GTCAAATGTAATGCAAAACTGATTGGATCTGCTAGTATCTGATACATGTTCATGTGCACA

TGTGTTCATAATTTTATGCATTTAATTGTAGCAATCTCCTTAACTTTTGCAAGATTTGTA

GGCA

>Unigene0001138

AAAATCAAATAGACTTAAGGGGAAATATGTATGATCTAGCATTTAACTGGTATGTGCAGT

TCACATCCATGCACTAATGTGTATGCCTACAGTTTCATGATACACAACGAGTCCAAGAAC

TTGGGCATTCCAAACTCAATGAAACCAACCACAACTGAGTACTACTGCATGCAAGTCTAT

ACAATCTAAAAATAGGAATGAAACCAAAGGGGAATCGTATAGATCCCACAAAC

>Unigene0001139

AAAGAACTGTATATTTCTAAGGTAAACTTTGAGTGTCAAGCTGGTTTCTCGGAGCGACAT

TGTTAAGGCTTCGTTACCAATATTAATGACTTAGAGAGAGTTGCGTAGTATTGTTGAGCT

ATTGATTGAAGCCTTAAAGGATTCAACCATAAAGATAGCTCACATAATTCAAAAACTGTT

AGAGATTTGAGAAAGAATTGGTTGACTCAGAATTAAAGCTGTTAGGAGTAATAATACAAA

ATTGTGTTCCACGAATATCATCCCCTCAATGTAACAAATTTCTGAACACCTTGATTGCAT

GGATACT

>Unigene0001140

GTCGGGAGAAGAAGCACGTCGGGGGGACCGTATGCCTGGTTAGATCAGATGTGTCGGTAT

GGAACATGAGTCGGGAGAAGAAGCACGTCGGGGGGACCGTATGCCTGGTTAGATCAGATG

TGTCGGTATGGAACATGAGTCGGGAGAAGAAGCACGTCGGGTGAACGAATGTCGGGGATT

ACTGCTTGTATGTCGAGAGAGCCGAATGTGTCTGTATGTCGGAGAGGTCCGAGCACAACG

GGGCCGTATGTCGGGAGAGATCAAAAGTATTCAATAGG

>Unigene0001141

GAGGAGAAACCTGGTGTGCTTTGGTGCCTCTCGATAATGTGTGAGGGTGGACATGTTGAT

AAAGATATACTACGAGCTGCAAGAGGGCAATGATCTGATGTTCAAAGTGGAGCCGTTAGA

GACAACCATGAAGGAGAAGCAAGTTGGCGCCAATAAGATGGCAATGAGAAAGCCTCATGG

AGAGAGGCTATGAAGAAAGAGCGTGAGTTACAGCAATGAATATGGTTAAGTTTGAACCAA

GTTCATCTGTGTTCATAGGTGACTGCTCCAGTACGTCAGTGGAGGTATTGATGGTCGAAT

TCAGCGTGACTCAGCGGAATATCAACGACATGACTTGGATGTGGGGAAAATTCCAAAAGT

CATATGTTGACAGGTGAGGAGCCTGAAAATGACTTATTGGTCATGGTCTTTCATAGTTGA

TGGCTTTGCATTTGTGACATCAATGGGAGAGGGGGGGAGAGATGTGGTACAGGAGGAAGA

ACTATGTTTTGTTTGATGTTTGATCTTGATGGAGCAGGTATGTAAACATCTGGTCATGAG

GTGGAGGCATATGGCAGGATCTCCTAATTAGCTCGGCTCAATTGGTACGGTTAGATATGG

TGATATGAGTTGATGGTAGATAGATATTTGTGTATTGGATGATAAATAAAAACAAGGAGT

TTGGTGTATGCCTTGTTTATGATTACAAATAAAAGGAGTTCACAATGTGT

>Unigene0001142

GAGAAATCTGATGTGCTTTGGTGCCTCTCGATAATGTGTGAGGGTGGACATGTTGATAAA

GATATACTGCAAGCTGCAAGAGGGCAATGATCTGATGTTCAAAGTGGAGCCGTTAGAGAC

AACCATGAAGGAGAAGCAAATTGGCGCCAATAAGATGACAATGAGAAAGCCTCATGGAGA

GAGGCTATGAAAAAAGAGCGTGAGTTACATCAATGAATATGATTAAGTATGAACCAAGTT

CATCTGTGTTCATAGATGACTGTTCCAGTACGTCAGTGGAGGTATTGATGGTCGAACTCA

GCGTGACTCAACGGAACATCAACGACATGACTTGGATGTGTGGGGAAAATTCCAAGTCAT

ATGTTGACAGGTGAGGAGCCCGAAAATGACTTATCGGTCATGGTCCTTCATAGTTGATGG

CTTTGCATTTGTGACATCGATGGGAGAGGGGGGAGAGATGTGGTACAAGAGGAAGAACCA

TGTTTTGTTTGATGTTTGATCTTGATGGAGCAGGTATGTAAACATCTGGTCATGAGGTGG

AGGCATATGGCAGGATCTCCTAATTAGCTCGGCTCAATTGGTACGGTTAGATATGGTAAT

ATGTGTTGATGGTAGATAGATATTTGTGTATTGGATGATAAATAAAAACAAGGAGTTTGG

TGTATGCCTTGTTTATGATTACAAATAAAAGGAGTTCACAATGTGT

>Unigene0001143

GGGATGGTCAGTTTAATCCGGTGTATAAGGTCTTTCAAATATATGTTATACCTGTTACTC

TGAATGTAACTGTATCGTTACCTTTGCCATTACTTCAAGGAACATACGAAACTGTAGTGA

ATGAAAAATTATTCCAAGTCGAGACAAATGGAGACAAGGATTCAGTTGCTTATAACATCT

CAATGAATCCTAGGCATGGAGTTGTCTATGTAAATGGAACAAAATCTAAATTATTTTCAC

ACAAAGATTTAAAAGATGGTAGAGTAATTTATAAGCAAACGGATTTGACTTCTGCAGCAG

ATGCA

>Unigene0001144

ATGTGTTATCCCCAAGATACCCCACCCCCTTTAATGAATCGTGAATTGTTCATGAATTAT

TAAAAAAATAAGAGAATTTTGTAGCAAGGCTAGTGTGCACTTTCGGCTGCAAATTAAGCA

AGCAGAGCCATGTGTTTGTCAGCTTGATAAATGGTTCAGCTGTGAAATGGTGAATGAAGT

TTGGTTCAGCTTTGAAATGGTGAATGAAGTTCAACACATTCTTTAAAATTGTGATTTTTA

CAAGGTACTGGAGTCTGTCCGAGGCGATTACCTATGTCCTTCCTCTGCCGTT

>Unigene0001145

CTTACCTTCCAGGTTACTTGATGGGTGAGCGAGCTCAATCGCCGATCAGTAATGTTTCTA

TCAGCGCTATGTCTCCGTTACGTGCTAATCGTAGCCATACCTCTGATGTACACACTACAT

TTTTAATGCGAAATTTTCATCATGAACTTAGCGATAAACCATCTTCCACTCCTATCTTAA

ATAGAGTCAATGATGAACCAACAAAAGCTCCAACAATTGATTTATTTGATATGCTCAATA

CTTCTGACTATAGAGAAGAACAGTGTAACAATAAAGAAAACAAACAATTTTATTCTGATA

AAAACAATCT

>Unigene0001146

GTTTGAATTTCGACCAAAGTTCTATAATGTTTCCAGACATATATATATAATATATGTAAC

CAATTTTTTTCTCGAAAACTAATAATTTAAAAGAAAATTTAATCATAATTATTAAAAAAA

AAAAGCGTCCAGGGTTTTCGTATGAATTCGTGATCAAACATTGTGATTGAATATTTTTTA

AATAAAATATATAATGTTATTAATATAATAATACATTTATAAATATATATTTTGGAAGTA

TATTTCTTAATCTATTTTAAAAGGAAAATACGTTTTATTTTTTGAGATAATGAAATATTT

TAAGTTTTTATTATGGTTTTAACACTGTTG

>Unigene0001147

TCTGGGGGCTCTGCCGCAGGTGTCGAGGGTTCCTCGTCCCCGGTGGCATCCCCTCTCGCC

GGGGTTCGGGACATCTTTTCGTCCGGCTCCACTTGTTCCCCATCGGCGGTGGGCCTGGCC

CCTGGGCCGCGCTTCCACCGGCGGTTGACCCTTGCTTCGGGCTTTCGGTCGGATTGGGGC

GGGGTGGGGGTCCAAGCTTTCTCGGACTCCTCCTCCGGTAGGACTCCCCACCTGTCC

>Unigene0001148

ATGGTGTGTGATCATGGCAAGGAGAGAAGGGTGCATTTTGGCATTACTGTCAACCGTACT

CATGGATACCTTAATTTGGTTCACATGGATATCTGGCGTCCTTCCAAGGATGCATTTGCA

TCCTTTATGGCAGGTGATCTTTCCGAAAGATCAAGCCCGTATGATGGTTCTTTGATCGTG

ACTACTCTCGCAAGTATGTGAGACTGAGGTATTGATTGACACTAACGTTGGGAGATCCAA

AGGAATTATTGGATATGATGCGATATGTGTCCTCTAATGATGGATTAGGGAAAGT

>Unigene0001149

CCCCTAGGACTTTGACTGCCAAAATGAATAGAAGGGGAAAAGGGGCATCTTTGTTTTATC

CCTTTTTGAACTCAGAAGAAAGGATTTGGTTCGTTAATCAGAACGGCTCGAGAAGCGTCC

AAACATGATTGTACCCACCCAAACCATCGGTTGCTGAAGTCCTAAGTGTGGAGACACTCA

ATGAGGAATTCCCAGTTAAGGGAGTTGAAAGCTTGTAGAAAATCAATCTTGATAGCTGTC

TTCTCCTCCTCCCCTCGAACGTGACCAATAGCCTCCTCTGTCATTGCAATAGAGTCCATG

ATCGAGCGATCGGGGAAGAATGCGGATTGAGAATCGTGGATCAGATCACCAATGACTCCT

TGAAGACGGTTGGCTAGATCCTTG

>Unigene0001150

TATAATTATAATTTTAATTAATATAAAAATAAAATTTAATCATAATAAATCAGCACCTTT

TGAATGTGGATTTGACCCATTTAATAAATCACGTATTCCATTTTCATTAAATTTTTACTT

AATTGCAATTATTTTTTTAATTTTTGATATTGAAATCTCAATTATTTTACCAATAATTTT

AAATTTCAAAATTTCAAATATATTTTATTTTAACACAATATTCATAATTTTTTTTATATT

TATAATCATTACTATTTTTTATGAATGAAAATTTGGATCAT

>Unigene0001151

TAGACCACAATATGCGTACTTACCATTTGGCGCTGGACCTAGACTATGCATTGGTATGCG

GTTCGGAATGATGCAAGTAAAAGTGGCGTTGTTTAGAATATTATCAACGTATAACATTTC

ACTGTCCAAAAATATGAAACTGCCAATAAAACTGAATCCAAAAACTATACCAGCAAATCC

TGATGGTGGTATGTTTCTTCATGTTACCAAGAGAAAAAACTAAGTAAACAGATTTTACGC

GACAATTATAGGTACTACGATTATAATTTTACTGATTGATTTAAAGTATAAAAATAATAT

AAACAAA

>Unigene0001152

ATGAACTGAGGGACTTGTTTGACCAAAAGCTACGGGCATTAGAAGATAGGCTAGGTGGCA

CTATTTTAGAGATGGCTAATACCACAGACCCAGATGGAATGTACATGAATCCTGTGCCTA

TCATGCCCGGTGACACTGGGTTTGCAAACGAGCAATTCACAGCAGAGATGAATCTCATGA

AGAACAAACTGCTGTCCCTTGAACGCCTCTGTGGACAGAAATGTCAGTCTGCA

>Unigene0001153

TGTTAAATGTGTTATATGTTGTCATGAGGGACCTTTGCCATGTCGGGTGAGCTCGCATGG

CAGGCCATCAGTTGGTCAATAAAAATTTGATTATTGTGTCGTGAGGGGGAGATCGCATGG

GTTCGTCCCCGGGTTCTCCCCCTCTGTTTTCTTGTTTTTTTTTCCTATTTATTTTATCTT

GGGAGGTCACAGTGACCACAGCTTACTCTGGAGATTTACAGTTTATCTCACATATTAATG

CAATCATTCTAATTACCCTGAAAACAAACATAATACCATCATACAACTTTATAATTTCTT

ATCTCAGATAATCTGAGCTTACATGTAATATAAAATATAATTTAAAACATATTTAGGCTT

GGTGCGAGTTTTATATTTGTTCCCAATTCGAGCAGTAGTTTAACATTACACTTGCAAATC

ACAGACACGGGCAAAATTGTATTCTCCAGCTTATTATACACTTATAATTGTCGATGTATT

CTCCAGCTTATTATACA

>Unigene0001154

AACACAAATATAATACCTATCATACAAATTTGATTGCGAATATATTTTTGAATTTCTTAT

CTCAGATAATCTGAGCTTACATGTAATATAAAGTATAATTTAAGACACATTTAGTCATGG

TGCGGGTTCCACGTTAGTTCTCAATTTGAGCATTATAACATTACACTTGCAAATCACAGA

CTTGTGCAAAGTTGTATTCTCCTGCTTATTATACACTTGCTATAATTGTCGAAATCTGTT

GCAAATATGCAAATGCTTCAAAATTTTACTCAGAAGTGGGTCCTCCAGAATAATTTCAAA

TAGAAATCACAGTCTTATGCAAAGTTTAATCTCCAGCTTAGTGTATA

>Unigene0001155

TGTTAAATGTGTTATATGTTGTCATGAGGGGCCTTTGCCAAGTCGGGTGAGCTCGCATGG

CAGGCCATCAGATGGTCAATACAAATTTGATTATTGTGTTGCGAGGGGGGATCGCATGGG

TTCGTCCCCGGGTTCCCCCCCTCTGTCTTCTTGTTATTTTTTTTGGTCTTTCTATTTATT

CTATCTCGGGAGGTTACAGTGGCAACGGCTTACTCTGGAGATTTACAGT

>Unigene0001156

CTGTTGATTATTGTTCATCATCTGCACGGCGACTATATAGGGTGATCAGTGAACGTAAAA

CTACAGTACCACACCATCACACTAAAACCATCAGTTGTGATTTTGTGAACCAACATGGAC

ACATGGTAATCGGCGCGTCATAATAGGAATAGTAAGATTCAACGACAATCACTACCGTTG

TGTAGATCTTTCCAATTAATTTTGACGTACAACCCAATTTAGAGGAAAAAAAGATACTAA

TTCAGTGACCATGAACATGAATAAT

>Unigene0001157

AAGCTACAGTTCAGATACAGCAAGAATATTCATTATCGTAATATAATTATTAGTCAACTA

TCTTATGTTGTCAAAATCCTAAAATCTTTCCTCAACTATACGGAATTGGATATATTGACA

AAGTGAAGTTTTACATACATAATACTAAACCGAGGTCCTATCACCTCGAGGTGATATAAA

AAGTATTGAATGTTGACAGACATATCAGGTACACTATGTATATGGCTATATACACTCTCA

CCTCTGATATAAGAAGT

>Unigene0001158

TCACTGCTCTATTCTCTCTACTGTTTTGAGTACCGCTGGTTTAACAAAGGAATTGAAATG

CACCAACGGTTATCCAACATTGAGAGAAACTGGCCCTATTACTTTGGATTTGGCTTACCA

CTGGCTTTCCTCACCGCAATGCAGTCCTCCTATATCATCAGTGGCTGCCTCTTTTCCATC

CTTTTCCCTCTGTTCATCATCAGCGCCAACGAAGCAAAGACACCTGGCAAAACATACCAC

TTCCAGCTCCGTCTCTTCTCCCTGGTGGTTTTCCTCAGCAACAGACTCTTCCACAAGACC

GTTTACCTTCAGTCCACCCTGAGCAGCG

>Unigene0001159

TTGTTTCTCTATCCTTTCCCCTTGATTGTGTGATTGGTGCTTGTTTCTGTGTCCTTTTCC

CTTGATTGGTCCCAATTGATGTAACCGGATCATTGGTAAACCATATTAAATCTCATGTCT

CGGAAATTGATAAGTGCTCCATTCTTGTGATTGCTTTTTGTGTAAAATCCTTGCGCTAGA

TCCGAGTTTCCATGGGGCATTTGATCTTAAGGTAGTTTTGATTCTTCCATAAATTTATTG

CATGTGTTCTTGACATAGCCCCCCTTGAACGAGTGTAGA

>Unigene0001160

CCAGAATCATGGCCGGCTGCTCAACCCAAAATCCAGAATCATGGCCGGCTGCGGACAAAT

GTGATACACGGGCTCGCGTTAGCGAGTGGCATTCCAGTCAATAGCCCACTCACCTATTTA

AGCTACTCTCCTCCTCGCCGGAGCAGCATCTAATCTCAACCCAAAATCCACAATCATGGC

CGGAAGCGGAGTTCTCACAGTGGGGCGGATGAAGCTCGGTTCGCA

>Unigene0001161

TGTATATCAATATTTTTAATTTGGACTCTAGAATGCCCCTTCCGGGGTTTGAAACATTGT

GGTGGTGGTTTGCACTAATGCCAATTGTTAAGCACCTAACAAATCCTCCCTATAGCGAGG

TTCGATCAGGGGAGGCCTGCCAAATACCAAGATCCTAACAACCCTGAACCTATCGTAGAC

GTGGGCCAAAGAGAGGCTGACTGTTAGGAGTAATACATAAATTGACACGAAACTGGTAGT

GGTACCCC

>Unigene0001162

GCCCGACCGCAAAGTACAAATTTGGGTCCGGAAATGTATAATCATGCACAAATCGTTTGC

TATCACTGATGGGGCAGTCACCGGAGAAAAATGGTGTTTACAATGGGAAATCACAAAATG

AGCCCGGGATTGATGTATGCGAACATGGAGAATCACAGAATGAATTAGGATTGATGCAAG

CTTGTGGGTATTGCATTAAATGCTCAATTACATGAATCTAAAAAGTTTGGTGCCTTTAAT

GGTGCACATGGGGAAACACAAAAAATGCATGGAAAATTCAGTTGTCATATTGAAGGGGCT

CGGGCGTTCACGGAAACTCTAAACTAGACCGCAATTGGGAAGGACCATACATTATCACAG

G

>Unigene0001163

TGTAGCATTAGAAAACACTAGGGTTGGCCGGGTTTGATACCTGGACAACAGCAAACAATT

AAATTTTCACGACAAATGATGGTAATTGAATAGGTAAGGACCATCAATGGAAATCGTAGC

ACCGTCTAACAAAAATTGTGGCTGAATTCAGGAGATGACAACATTGTTGTTCTTCCCTTT

GAAGTTCCAAAAGGAAACAGTAGAGACAACGTGGAAAGGAAACTGGAGTGGCCAAGAAAA

ATGGGACATCCTTTACGGGGAGGTTGATATAAGGATGT

>Unigene0001164

GAGAATGAATAGCAGTGGATCTCACGGAGAGAATTTTGGGATATCCCAAATCTTTTAAGT

CAATTGAATTTTATTTCAGTTCGGCCAAGTTTCTGGCCCTCTTAATAAAAGCACGCATCA

CCTCAATGCGCGAATTGCCATGACTGACCTCATATTTCCACCCTCTGCTCGGGCATTTCA

CTTGCCTTGGCTGAGTGTAGGGACTTGCAAGGGATCACAGATTGTTAAGAGCTGGCCCAC

TGAGATTCATTTACAAGA

>Unigene0001165

GGGAAATTGCAAAAGATGAAGCCTCAGCTGGATGACACTTGTTCAATGTGCAACAGTAAG

ATATCCTTATTGGATCACTTTTTCTTTCGGGGTGATAGATAAGGCTGAATGGTTCTGGAG

AATGACAGTTCATGCCACCCTCTGGGTTATATGGTAGGAAAGGAGGACACTTTTATACAG

GTTTAGAGCTTCAGGTGCTCTCATGGGGCAGAAACAGGTTCTGTGCTGCTCAACCACACT

CTCAGTCT

>Unigene0001166

ATGGAGATGGAGGGGGAGATCTCGGTTCCCTACAAGCCGGAGGTGGATCTGCAGAAGATA

AAGATGGATAGGTTTTCATTCAAGTAGAAGCATGAGCCGCTATATTTTTGCTGAGGATCT

TCAGGCACTTAAACACGAAGGAGGGCAATGGTGGGGAAGCTGAAGTTGAGGGATATGGAG

ATGGGGACTCTGTGGAGATTGACTTGAAGACCAAGGAGTTCATAGCAAAATTATATGACT

GGATAAGGCTCCAACATCTTGATTTGATGGGGAGTTGGTCAGGAGTTGTAGAGGTGAAGA

TATGAGAGAGTGCTGGGAGATTGTGTTGAGCTGTTTGTTAGTTATGTGTGGTGATGAACT

CTGAAATGTAATTGTGTTATGATGTGTGCTCTCTTCGGTGTTATATG

>Unigene0001167

GACAGATCATCGTCGGCCTCATTCCCGACGCCGGAACCATCCGTGCCAACGGTTCTGAAA

CCATCAAAGTCCCTCCCACACTCATATATGATGACATCAAGTCCACCTACAAGGACATTG

AGCCAGGCAGCATCCTTATATACAAGGTTAAGGTTGAGCTCATCGTCGACATACCGGTGA

TGGGAAGGGTCACGATTCCTGTGGAGATGGAGGGGGAGATCTCAGTGCCCTACAAGCCGG

AGGTGGATCTGCAGAATCTAAAGATAGATAAGTTTTCATTCGAGGAGAAGCTTGAGCCGC

TATGTTTCCACTGAGGATCTTCAGGCGCTTAAACACGAAGGAGCGCAATGGCGGGGAAGC

TGAAGGTGAGGGATATGGAGATGGGGACTCTGTGGAGATTGACTTGAAGACCGAGGAGTT

CATAGCGAAATTATATGACCAAATGAGGCTCCAGCATCTTGATTTGATGGGGAGTTG

>Unigene0001168

ACAAGGTCAAGGTTGAGCTCATCGTCGACATACCGGTGATGGGAAGGGTCATGATTCCTG

TGGAGATAGAGGGAGAGATCTCGGTTCCCTACAAGCCGGAGGTGGATCTGCAGAAGGAAA

AGATGGATAGGTTTTCATTCAAGGAGAAGCTTGAGCCTCTATGTTTCCGCTGAGGATCTT

CATGTGCTTGAACGCGAAGGAGGGCAATGGTGGGGAAGCTGAAGTTGAGGGATATGGAGA

TGGGGACTCTGTGGAGATTGACTTGAATACCGAGGAGTTCATAGCAAAATTATATGACTG

GATAAGGCTCCAACATCTTGATTTGATGGGGAGTTG

>Unigene0001169

AATTCACATCCCAATCCCTCTCATTGACATAAACTACCTCATCGAGAGCAAGGGAAGACA

GATCGTCGTCGGCCTCATTCTCGACGTTGGAACCATCCGTGCCCACAGTTTCGAAACCAT

CAAAGTCCATCTCACACTCATATATGATGACATCAAGTCCGCCTACAAGGATATTGAGCC

AGGCAGCATCCTTCCATACAAGGTCAAGGTTGAGCTCATCATCGACGTACCAGTGATGGG

AAGG

>Unigene0001170

GACAGATCATCGTCGGCCTCATTCCCGACGCCGGAACCATCCGCACCCACGGTTCCGAAA

CCTTCAAAGTCCCTCTCACACTCATATACGATGACATCAAGTCCGCCTAAAAGGATATTG

AGCTAGGCAGCATCCTTCCATACAAGGTCAAGGTTGAGCTCATCGTCGACATACCATTGA

TGGGAAGGGTCACGATTCCTATGGAGATGGAGGGGGAGATCTCGGTGCCCTACAAGCTGG

AGGTGGATCTACAAAAGGTAAAGATGGATAGGTTTTTGTTCGAGGAGAGGCTTGAGCTGC

TATGTTTTCGCTGAGGATCTTCAGGTGCTTAAATGCGAAGGAGGGAATGGTGGGGAAGCT

GAAGGTGAGGGATACAGAGATGGGGACTCTGTGGAGATTGACTTGAA

>Unigene0001171

AATTCACATCCCAATCCCTCTCATTGACATAAACTACCTCATCGAGAGCAAGGGAAGACA

GATCGTCGTCGGCCTCATTCTCGACGTTGGAACCATCCGTGCCCACAGTTTCGAAACCAT

CAAAGTCCATCTCACACTCATATATGATGACATCAAGTCCGCCTACAAGGATATTGAGCC

AGGCAGCATCCTTCCATACAAGGTCAAGGTTGAGCTCGTCGTCGACGTACCGGTGATGAG

AAGGGATACGATTCCTGTGGAGATAGAGGGGGAGATCTCGGTGCCCTACAAGCCGGAGGT

GGATCTGTAGAAGGTAAAGATGGATAGGTTTTCATTCGAGGAGAAGCTTGAGCCGCTATG

TTTCTGCTGAGGATCTTCAGGCGCTTGAACGTGAAGGAGGGCAATGGTGGGGATGTTGAA

GGTGAGGGATATGGAGATGGGGACTCTGTGGAGATTGACTTGAAGACCGAGGAGTTTATA

GCGAAATTATATGACCAGATGAGGCTCCAACACCTTGACTTGATGGGGAGCTGGTCAGGA

GTTGTAGAGGTGAAGATATGAGAGAGTGCTGGGAGATTGTGTTGAGCTGTTTGTTAGTTA

TGTGTGGTGATGAACTCTGAAATGTAATTGTGTTATGATGTGTGCTCTCTTCGGTGTTAT

ATG

>Unigene0001172

TGGATCGAAGGAAGAAAAGTGAGAATAACATGAGTGTTTAGACGGAACCTAGTTCAGAGC

GCGGAGAGCTAATAAGTCGATTGTCATTCGATCGATAATCGGTGCCGATCAGAGATTTCG

ACGAATGAAGATGGTGACTGTGAAGGGTCAACTGATGATCGACGGAGAGTTGAAGAGGTT

GACAACGGTGAAGGAGAGCTCTTCGGGTTTGCTTGGTGCTCATCTAAAATAGGTGTCAAC

ACCTCTTGGATAACTCCATTGAGTTGGT

>Unigene0001173

CGGGAGGAACCCAGTTGCGCATAAGAATCTGTTCGATGATTTCTTGCTCAAGATCAACAA

GGAGCTTGCATATGTGCAGTTCGAACTGCGGGGGTGCCGGAACCAGTATGATGGTAGGGT

CTTCTATGGTGTGGTTAACAATGTGTCGGATGATCAGTCCAAGATTGGGACCAAATATTC

GGCTCCGCAGCTTGCCTTCTACAAGGCCGTGGAGTGGCCTAACCGATGTGG

>Unigene0001174

GGACTTACCCACTCACACCGGACAGAAATATGACAAAAATGATTACCGAATGGCCCGATT

CGTTCAAGGAGTAAAACATATCAATCCTTCACCCGCCATCGAACTCATCCACAATGTTCC

ACCAGAACCTAAACCAGACAGGATTGTTTCTTGCAATGGCGGTGGTGGACCACTAGGACA

CCCTAAAGTCTACATCAATTTGGATAAGCCAGGAAATCATACTTGCGGATACTGTGGGCT

TC

>Unigene0001175

AACTATTAGAACTCGAGGACAATTTATTTTGAAATGGACAATGGTGTGACAACGCGTCAG

GCAAGTCAGCATTTGTGTAGGCCTAGAGCAGAACAAGTGGGCCAAGCCCTCTCATTTGAG

TCCATACTAAAAGGGTTCAATCTAAGCCCCTGTTTAGTTTATTATTTTCTCAGTTAAATA

GGATAGTGTTAGTCTATTTGTTAGAATTAGTTACCTCTGGTGCA

>Unigene0001176

GGCTTCAGTTTAGAGGGTTCAAAACCTTCGTCATCATGGACGGTCCAACCACCCGTGCAG

AGATGCTCTTGCGCGCACAGAGATATTACGCCACTGAGCCCGCTAACGCCAACATCTGGA

AGGAGAAGGAAAAAGCCGAAAACGGCTCGAAGTCCCCCGGGCACAACACGGGGGGAATAG

CTTGGCGGGCAAGAATTGGTCCCGGGCCACTGATCAGCCCGATCGATTCAGGAAACAGGA

GTTCACT

>Unigene0001177

GACGGCTCCAAGTCAAGTTTAGAGGGGGTAAGAAGGCTTCTAGGTCAGCTTGGCTAGGTG

CTTGAGAAAATGGATGGTGATGTTTGAGGAGTATGTGGCTATATATAGAGTTGAGAAGGT

GGAACTCTCGGAAGTCTCCAGAGAGAGCCCAAGTCTTCAGGATCCAAGTCTTCTAATTCA

CCCAAGTCTCTTCACTCGTCTCTGGGAAAGGACAGAAGAAGCTGATTTTGGAGCTCATGT

GGAAGATGAAGTGATATTCAGTAGAGGTTAGCAAATCTTATG

>Unigene0001178

TTACATATTATTTTGTTTTTATTATTATTTTATTGTATTCTATCGAATTATATATTCGGG

CCATGTATATTAATATTATATAATTTTATCGTGACTCTTTTTTACCGCTGTCCTCGCAAA

GTAATATAGTAACATTGGTATCGGAAAAAAACGATTCGCTTCGTGTCTCCTCCGAAACGA

TTCTAATAGTAATTTATTATATACATTCGAGTGCGCGAACCCGACTAGTTTTCACG

>Unigene0001179

ATTGCCGTCACCTCCACCACTTCTACCACCACTCCCGTTATTTCCGTCACCGCCACCGCT

TCTGCCTCCTCTCCCATTCCAGCCAGCACCACCACCGTTCTTCAGTCTACCACCAATTCC

ATTTCTAACTCCACCTCCTCCACCACCTTCTTTCCTGTTCCCTCCATTTCCATTTCCACT

TCCACCAAATCCTCTCTTTCCAGGTATTCCACCGGCATCCCATTCCGAGGGAAGTGCCTC

T

>Unigene0001180

ATTCTACATCTCCTAGTCTTTGGGCTAAATTTTTACTGGGAACTTGTTATAGTTTATGGT

TTATTCATTTACCAAGTCATATACTTCAATCTGAAGACCGTGCTACATCTACTCTACGTT

CAGCATATGAGTTATTAAATAAATTTCAAAAAACTGGTGTACATTTATTGACTGATGAGG

TTTGCTACAGGGTTATGATGCAGTTATGTGGTATTTACAATCAACCTATGCTGGCTGTAA

AACTATTACTAGTAATGAAACGTTGTGGACTTCATCCTAA

>Unigene0001181

CTGTAGCACAGGCGTTGCATGGTTTGATTTTGGACTTGCGTTACCTGAGTCTGAGTTGCC

AGAAATTTGGCCAACATGTCTTCCAAAGGTGGTTTGAAAGTTGCCTGCCCATTCTGGTTC

TGGGGATAAAGATTTTTCTGAGGTAATTTCTCCCAATTGTTGTTTTGTGGCTGATTGTGT

TAAATTGGTGGACTTGGTTCCAATTGGTTGTTGTTGTTCTGTTACTATCTGTATTGATAG

TTGTTGTTTTGACCATGGTTGGAATATTGGTTATTATTGGGTTAATAATTTGGACTATTT

TATTCTTGAAATCGAGGCGATGAGAAGTTGGGTGTATATTGATTGGCTTATTG

>Unigene0001182

GTTTATGTAATTAACTTGAGCTGTTTTTTCGAGTCCATCTTAATTTGTCATTGAGTACAT

ATGTAAGCTTATTCTTTTACGTTAGATTAGCTATATTGTTAGCTTAAATGGTTGTTTTAG

TTTAGTTGTATTGCTATTGGGTTAGATATTTATTATCCTAGTTTAGTCGTTCATGCACAT

GATAGCTTTAACCTTAAAACATGGTTCTGTTTAATGCTTCAAGAATTTAGCAATATACTA

TTATTTGTATTCTTCTTATCTAATTATTGAACGATCGTATCTGCACGTTACACATGCATA

GATATCGCTAACTTTGATTATTATCAGAAGAAAAGAAAGACAGGGAGCAC

>Unigene0001183

AAAAAATGTACAAGTTCATAGATCCCATAAACAGATCACATAAAGCTTTTGGTTCTTGCT

TTTCTTCTATATATATGATGTTTGACTTTGTAAATGTCTCAAGAAAAAAAATCCTCTATC

AATTAACTCTAGTAGACTAAATCGGTGTCCCTGACGTTCGAACAAATGTCAATGGTACAC

AAATAAACAAGAAATTAACATCAGCAATGCACATCTACAAGAAAATTATCTACTCCCAAC

TACTATTTATATAAAGTAGTGATATCACCTGCCTTGTTAGACTGCGACTGCTGGCATCCT

GATTTAACCATTCCAATTTAACTTCTTTATTATTGGAGAGGGAAATATCCTTCAGGAACA

TTCCGTGGTGTTAGCGCTCAACCCTAGACCAACACTAGCACTGGTTATGATCGGCAGGGG

TGTGATGCTCCATTCCTGTCCCTTCGGTGGAGCAGCGGCACAACTCTTAGCCGACGGAGG

TCCTATGCGGCTGATGCAAACCTGGAGGGGGCCGACCATGTTGCGACAAGGGTGATGTTC

AGATTGACAGGAAGGGTTTCTGATTGCATGTTGAGCAGGGTTCTGGGTTGGTCATGGTGT

CAAAATCTTCATCCATGGGGTTTTGGTGTTTCGCTTGATCTTGAGTAACGGTGGTCACTA

AGATTGGAGGGGTGATTCGGTCTTTGGATCAACCGTGGAACCAAAGAAGAGATGTTGATA

GGAGGCAATTGGAAACATCGGTGGAAGATTCACGTCTCATATGAATTATTCAATAGAGCA

GGTGGGATTGGCATCCCATTTGGCTGGGGATAGGGCTGGTTGGGAGTCTTTAGTGCATTT

TTTTTTT

>Unigene0001184

GTCTGCTCAATATTGATGATTCATGTATATGAAATGCTCGACTGGGACTGTTCAGTAATT

ATTTTGGCAGCAATTATTTTTATTACTTTTAGGATGTTGTTAATGGTGCAGTAAGATGCT

CACCGGTCAAAAAAGGCCCACCCTTTTGGACATCTTTTAATTTTTCGAATGACATCCTAG

ATCTTTTTCGTCCCAAACCTCTATTCTCACCGGAAACACACTGGGTTCCTGGAGTCTGGG

AAACCGACTCTCTGCTTTAAACTACCTCTAATCGGAGCTCTACAGCTGAAATTGATTCAA

ACTTTTCCACATGCCACACATGAAAAGAACAAAGATAAAAAAAATGTACAAGTTCATAGA

T

>Unigene0001185

TTAATAACTGTGATTATTAGTTACTTGTGAGTCCTGGGTGACGACTGTCTCGTCTTCTTT

TTAGTCGGATTTTTTTTATAAGAAGTAAAGGTTAGAGAGTTCAGCTGACTCGATTGAGGG

CATGAGAGCCTAGCTGATTTCTTCTGAATCCTTGTGAAATTGTTCGTCCAACTACTGATT

ATAAACTCACCGTCTGCTCAATATTGACGATTCATGTATATGAAATGCTTGACTGGGACT

GTTCAGTAATTAT

>Unigene0001186

ACCCACCTCCCTCGCCCCGTGGTCCTCCTCATCTCCTCCGACGGTTTCCGCTTCGGCTAC

CAATTCAAAGCCTCCGCCCCCAACATCCACCGCCTCATCGCCAACGGCACCGAGGCCGCC

ACCGGCCTCATCCCCGTCTTCCCCACCCTTACCTTCCCCAACCACTACTCCATTGCCACC

GGCCTATACCCGCCCTCCCATGGAATCATCAACAACCACTTCACTGACCCCGTCACCGGC

GCCGCCTTCACCATGGCCAGCCACGACCCTAGATGGTGGCTCGGGGAGCCCATCTGGGTC

ACTCTCGCCAACCAAGGCCTCCGTGCTGCCACCTACTTCTGGCCGGGTTCGGAGGTCGTC

AAGTCCTCCTGGGACTGTCCTCCAAACTTCTGCCAGCACTACGACGGCTCCATCCCATTC

GAGCAGCGCGTCGATACCGTCCTCTCCTACTTCGACCTCCCTAGCAGCGAGATACCGCTA

TTCCTCACCCTCTACTTCGAGGATCCCGATCACCAAGGCCATCAGTTCGGCCCCGACGAC

CCCGCTATCACCGATGCTATCACCCACATCGATAACATGCTCGGCAGGCTGATCGCAGGA

CTCGAAAAGCGTGGGGTCTTTGAGGATGTCACCATCATTCTATTGGGCGACCACGGGATG

GTCGGCACTTGCGATCAGAAGCTCATCTTTCTTGATGATCTATCCCCTTGGATCAAGATT

CCGCGGGAGTGGGTACAGTCGTACACTCCGTTGCTGGCGATCCGTCCACCTTCCGAGGTT

TCGCTGTCTGAAGTGGTGGCTAAAATGAATGAAGGGCTGGGTTCCGGGAAGGTTGGGAAT

GGGGATCATCTGAGGGTTTTTCTTAAGGAGGAGTTGCCGGAGAGGCTGCATTACTCGGAT

AGCGATAGGATTCCGCCGATTATCGGGATGTTGAACGAAGGGTATAAGGTGGAGCAGAAG

AAATCCAGGAGGAATGAGTGCGGGGGAGCTCATGGGTATGATAATGAGTTTTTCTCCATG

AGAACCATCTTTGTTGCCCATGGGCCTCAGTTTGAGAGGGGACGGAAAGTTCCGTCGTTT

GTGAATGTGGAGATATACAATCTCATTACTTCGATTTTGAAGATAAAGGGTGCTGCGAAT

AATGGATCAGCTTCTTTTCCGGATACGATTCTGCTTCCGAGTGCTTGAGGTGTGCGTTTT

AAAACAACGCAGGGAAGAAAAGAAAGACAGGGAGCACGATTTTGTTTCTGTGAGTATGTG

CACACAATCCTGTGTTCAGTCAGCGGTGACGATGATACACAAGTCTTTTCACTTTCGTTG

AATGTCTGTATGGGTTACTTATAAATGTCTAGCGCAGTAAGTTAAATTGCTAGCAAAGAC

CGTTGTGGTGGTCCTCCGAGCCAAAATGCACGAAGACAAATTCGTTTCAGT

>Unigene0001187

TGCCCATGGGCCTCAGTTTGAGAGGGGACGGAAAGTTCCGTCGTTTGTGAATGTGGAGAT

ATACAATCTCATTACTTCGATTTTGAAGATAAAGGGTGCTGCGAATAATGGATCAGCTTC

TTTTCCGGATACGATTCTGCTTCCGAGTGCTTGAGGTGTGCGTTTTAAAACAACGCAGGG

TTTGGCGGCACCGTGGACACTGTTCATGCTGTTTCTCCAATTTCTCACCGGCATTAAATC

TGAAGGAGGGAGATTGTGAGATGAGGGGCTGGTCTGATCAGGAAGTTGCATAGGTCTTGA

TCTTGCTACGGCAGAAGATGCCCTAGGGTTGTATCGAGGAAGTTGAGGTGTTACAACATT

TTGAGGCTCTGTGCTACTTGTAGTCTTGAGGTGCTTCAGTTGATGCAGACTTTGAGAATC

TGAAAATTGTTGGGGCTGAGTTTGAGAGGTGGGCAGAATGCCCATCCCTATAGAGCCTCG

CTGACACCGGAGGTTTGCAGATGAGGTGATGGGTCACGTTGAGCAGGTGCTGTAGTTGGT

GGAAGGAGTTAAAGATGGAGGGTAAAGAGGGGAGCAACTCAGAGGAGACACCCACGGAAG

GTGGGGGGCCTTGGTTTTTGGCTTTTAAGCGTGGCAACTACAGAGTTTGAGGGCTCTACA

GATCTGTTTAGGTCTTCATTTAAATATTTTCGTCGTCTCTCAATCTGGGGATTCTGTTTT

GGTCTTCATCCAAAGGTTTTCGTCATCTCTCACCTACCGGATGATGAGGAGGAGCTATCA

GACAAAGGCCAAACTGTTTTTGCTAGAAGTGAACAAGGAAAAGCTACATCAAGTGACATT

GTTGACTCAGCATGTTCAGCCTATTTTCGGAGGCCCTTTGCACAAGTACGATTGGACCAT

GCGGCTTCCAAATGTGTGTTAAAAGTCAAACAAATCATGCCGGAGAGCGATCAATCCCAC

TCATGTTGGGCCAATAATAATATTGGGTTATACCAATGTTGAATTCATATAGAGCGTGCT

ATTATTTGTATAACTTATTTATGCATACTTGAGTTACTAAGTTTATGTGGGATTGTTTTG

GGTG

>Unigene0001188

AGAGGAGACACCCACGGAAGATGGGGAGCCTTGGTTTTTGGCTTTTAAGGGTGACGACTA

CAGGGTTTGAGGGCTCTACAGATCTGTTTAGGTCTTCATTTAAATATTTTCGTCGTCTCT

CAATCTGGGGATTCTGTTTTGGTCTTCATCCAAAGGTTTTCGTCATCTCTCACCTACCGG

ATGATGAGGAAAAGCTATCAGACAAAGGCCAAACTGTTTTTGCTAGAAGTGAACAAGGAA

AAGGAACATCAAGTGACATTGTTGA

>Unigene0001189

TGTCGAGTCCACGGATTGGATTGCTTCAACCTTGACAAGGCGATTCGGGCTCGGCGCAGG

CCTTGGGTGGGCTGCTTTTCTCGCCTTTGGTGTAATCTCCGAACAGATCAAAACCCGCCT

CGAAGTCTCTCAAGAAGTAGCAAATACAAGAAACGTTGAAAAGGAAGAGGAAGTAGTGCT

ACCCAATGGCATAAGGTATTACGAGCTGAGAGTTGGCGGCGGCGCCACACCGAGGTCCGG

GGACTTGGTTGTGATAAACCTCAAG

>Unigene0001190

AGAACAATAAACTTTAGAACATTTTTTGATTTAGTCCCTCTATATATTGTTATTAGGCAT

TGGTCTCTTTTAATGTGTATTCGATTCATATGACTATTACATTGCTCGACCATCTATCAC

ACAGTAAGGCTTTAAGTTGTTTTTTTTCCTTGAGGCTGTTGCCCGCATGATATTACCAGT

ACATGGTCCATGTCAAGCATCCTCCCTCTCCCTTTTCCTCTCCCTACC

>Unigene0001191

CCACCACCGCTCGCGCTTCTCTCATCGAGGCTCCTATTTTGTGGGCTGGTAGACTTTGCA

TCTTCTATGCCCTTCTAAAAGCTGGTTTAGCTGGATCTCAATCTAACCCTCTTGTCTCTG

ATTTGGAGTTATCCGATGGTGGTAGTGCTGATGGGGAAAGCAGCAGTGATTTGGGGTTTT

CTAAGTGGTTGGAGAGTGTAAGAGGAAAGCCAGTGGATGAAGCAGT

>Unigene0001192

AAATTTTCCATAAGTTTGGCTAACAATAATTATAAAAGAACTTAAATTATGGTTTATTCA

GGCCATTTAAACATAAACCACTTTTTAAACCAACTACTGGAAATTATATCCAAGGCTGAC

AAATCATCTTTGTTCAGAATCATTTTTCCTATACAATGATACCTAACATTGGATTCAAAT

TTAATACATCAATTATAGTGACCTACTATATAGCAGAGCGTTACTAACTCGACCACTAAT

TTTTAAACTAGGAAAATATTTAAGACAAACTACATAGTAGTTTATAGTATTGTGTATT

>Unigene0001193

GCATGCATGATTTGAGAAAATGATGTTCAGTTTTCTTGGGCATGCTTCATTTGTAGAGCA

GCTCTTACCACATTGCCTCTTGTAATAATTCCAACCAGCTTTCCCTTGGCATCTACCACT

GGTAGCCGGCGATACTTTGTCTTAAGCAATAATCTAGCAGCATCCTCAAGATTGGTGGTT

TCTCGAACAACAAGAGGCGCCGGTGTCATCAAATCGCCGATCACCTTTCCGTTAGTTTTG

CTGAGGAGCCTTTGTACCTCATTGAATGTTTTCCAGGACCTGTCAACTTCAGGAAACATA

CTTGGATCTGTCCTTCCACCACCTGAGATGGAGTC

>Unigene0001194

ACTATGATACTAAAGTGATAACGTATAGCATAGCTTGTGCCCTTTCTTCATTTGGGCTTA

GAAGATGACTACTAAGAATTGCGCACATTGCTGATGTGACGTTCAGGTCGCGCACAACTT

TTCTTCGACAGTACAGCCGAGAGGTTGAGCGGGTTGGCTAGCGGCAAAGTTTTGCTCCCT

GTGGGTCACATGTTTGATTCCCGGTCCCTCAAATTCTAGCTAGGATACCTTCTGTCACAA

AA

>Unigene0001195

TTATGTAGCGAAACAAAAAGTCCGAGAACATTCAGAAAGAGAACTACTTATTGTTTTGGA

GTTCTGTCATATAAGCCATGGATAAGAGACTATCCAATTATATGTCATGGAATAGAGGCT

ATCCGATTGGAAGAGTAAATTATCTCTAAAGCACTATCACTCTGATTTCTACTTCTTTGG

CAGATTGGCAATCTTTCGATTGATAAACCTTTTAGATTGACAAATAGC

>Unigene0001196

TCACAGTTTACAGAATTTCACAAACAACTAAAAAGACAATACAATGTAGTGTCAACAATT

TCATTGCCACCTAAAAAAACTATTGGAAATAAGGGTGCCAATTTTGTCGAAGAACGGCGT

AAAAAACTACAAGTTTATCTAAGACAGATAATGAATTTTTTGATCACAATAAACCGAATA

TTGGCCAATTCCCCTAACAAGTATAATTTGGTTAATCTTGTACCATTTTTCAGTGAACAA

TATAACCAAAGTTATAGTGGAAAACTGTCATGGTTAAATTTAAGTTGTCTAATGGCTCAA

AATTCTGTAGTAGATTTAAA

>Unigene0001197

ATCTCTTGAAGAATGTGTGTATTTATGCTTGATTAACCATCACAAACTCTCTTTGATGTC

TGACCTCTGATGTTATTGTGACCTTCGAATGTATTGCATGATCATGAAATCTCGGAGGTG

GCAATTTTTGTTATGGTTTTCCTTGAGCACGACATTGACAAGTGGCTTCAATCGGTATTC

ATATTTATTCACTTGTGCGCTCTCCACTACACTAAACTTTATTGTATACATTATCGATTC

TTATGTTAAGATGCCTCATGGAATAATGAATGTGACCAAACA

>Unigene0001198

GGTACGGCAGTGAAAATAACCTGAAACGCAATGGCAGTTCACTTTCTATATTATCAAGTA

ATATGTCAACCATGTCACCGGTGTTTAAAAAAAATAAGGGTTTACAAGATAAACTCAATG

AACTTGTAGCATTTAGAGGTATTCTTATCAACCAGATTGATCGACTTCAAATGTATTTTG

ATACATGTTTAGCAGAATTTGCCTCTTCAAATGAAGGTGTATCAAAAAATGAAGCCTATC

AA

>Unigene0001199

AATAGCAGATTCTTATTTTACAACATTTCTGATAAAATATCCACAAAAATCCAGAGATTT

CCGGATACCCGTTAATAAAGCTGCAGCTAGCAGCTAATGAGGGTGCCAACTCCCGATGTT

CCAGCCGCCTGATAAAAAATCCCGAACCTCTCTCCCAGCTTCTCGTTGGCTTTTCAATAT

CCACCTCTCTCGGACGCGGCCGAGGCGACAGGAGAGCGAGAAGCCCTAGATTCGATCCCT

CGCTCTCGCAACCCTAGATCGTCTGCTCAATTGAGGTTAGTTCAATCCAATCCGAGCTCA

ATTTCCATTAATTCGATCATCGAGTTCTTGAAATTTGGGGAATCGAACTCAATTTCCCTT

AATTCCATCATCGAGTTCTAGAAATTTGGGGATATAATAATGAAACCCTTAAGAAGTAAA

GAGGAAGAGGAGGAAACCCTCGCCCTCTTCCCTACCCCGCCGCTCAAGAAAGACTGTTCG

ATCGGCCTCATCAAGGTCGTCTCCGCAATTGTAGTGTTCATGGCCGGCGCGGTCCTTGGT

CTCTCGTTAAGCGCGCATTTCACCGAGTACTTCAGCTCGTCGGCCGAGCTCTTCTTCCCG

CCAACTAAGTTCTTGCTGGGGACGTGCGGGAGGGGGTGCTTGAGTATCAGGAGTTTCATT

GAGCCGACGTATTTGATGCATGACATGTCCGATGATGAGCTTTTTTGGAGGGCGTCGATG

GTGCCAAAGAAGGAGGAGTATCCGTTTAAGAGAGTGCCGAAGGTGGCATTCATGTTTTTG

ACGAGGGGGCCATTGCCGTTCGTGCCGTTGTGGGAGAAGTTCTTTAAGGGGCATGAGGGG

TTGTATTCGGTATACTTGCATACGATTCCGGGGTATAAGATGAATGTGAGTGAGGTGTCG

GCGTTCTTTAACCGGCAAATCCCGAGTAAGGAGGTTCAATGGGGCTCAATATCAATGATA

GATGCCGAGAAGAGGCTCTTGGCCAATGCATTACTCGACTTCTCCAATGAACGCTTTGTT

CTCCTATCGGAAAGCTGCATACCGGTCTACGATTTCCACACGGTGTACGAGTACCTCATA

AACTCTGACAATAGCTTTGTCGACTCCTACGATGAGGATTCCCCTCGTGGGCGTGGGCGT

TACAGCAAGCGCATGCGCCCCGACATCAGGCCCTTTGATTGGCGAAAAGGGTCCCAGTGG

TTCGAGCTCAACCGAGAGGTCGCTTTAAATATTGTCGCAGAGTACAAGTACTACAACATT

TTCAGGAAACACTGCAAGCCTTCTTGCTACCCCGATGAACACTACATCCCTACATACTTA

AACATGTTCCTCGGGTCGCTAAATGCAAATCGAAGCGTAACTTGGGTTGATTGGTCGAGG

GAAGGGCCCCACCCGGCTAGATACGGCAGCGGGAGCATCACTGAAGGGTTCATTCAGTCT

ATCAGAAACAATGGCAGTTGTACTTATAACAACAAGGAGACATATATTTGCTACCTCTTT

GCGAGAAAGTTTGCTCCTAGTGCGTTGGAGCCGCTACTTAACCTATCTTCAACAGTGATG

AATTTTTGACAAGTGACAAGATGAGTGGTTGACCATTGTTTCTGGTACAACTTCAGGTTC

TGTCTCTCGATTGGATCTTGGTTGAGCTTGGATGGATGATTTTTAACTGAGCAATCGTTG

ATCACTGAGTTTGAATTTTTAGAAATTGCATCATACAAGATTTAGAAACAATGTGTAGTT

TTACAAACTTATACAACTGTCTGTGTATGACAGTATCTCAAGAGAGCTTTTCGAACTTAT

ACATGTCTGTGTATAACACTATCTCAGGAGAACTTGGAGCTGAAGGTGTATCTTCATCTG

TACTGTACTGGTAGTGAACGTGGCAATTTCGGATTTGAGTTATAAATACTTTTAGAGAAA

ATTCAGTCTTCAGAGTTCCATAAGAAATTTTGCAACTTATTGATCTCAGCCATA

>Unigene0001200

AATAGCAGATTCTTATTTTACAACATTTCTGATAAAATATCCACAAAAATCCAGAGATTT

CCGGATACCCGTTAATAAAGCTGCAGCTAGCAGCTAATGAGGGTGCCAACTCCCGATGTT

CCAGCCGCCTGATAAAAAATCCCGAACCTCTCTCCCAGCTTCTCGTTGGCTTTTCAATAT

CCACCTCTCTCGGACGCGGCCGAGGCGACAGGAGAGCGAGAAGCCCTAGATTCGATCCCT

CGCTCCCTAGATCGTCTGCTCAATTGAGGAGGTTCAATGGGGCTCAATATCAATGA

>Unigene0001201

GTGCCGATACCAGCAGATAGTCGGTGATGAGTTGCAGGAGAATTATTGTGATTGATTTAG

GCCAGCTGGAACACTAACCCCAGATTTCAGAGCGACGGAGGCTCCATAAATGGAGGGGGA

GTAATAGCAGTGGCAATAATTGACCAGTGAGGTTTCATAGCAGCCCTTGATAAGGGAACC

GTCGCTGCCGAATCCGTACGGACTCAGCTGGTGACTCTCGAGGGTGTGGATTGAGAGGAC

AAAAATGCCCTCAACT

>Unigene0001202

CTCTGGGTGTATACACACAGTTCATAAACGTTCCTTGCATACCCTTACTCGATCATAAAT

GTATCAAAATTCTTGTACCACTAACTCAAGGATTGCTTCAACCCATATAGTATTTTTTCA

GTTTGCATATCAAATCTTCCTTTCCAGAAATAATATATCCTCCAGACTATGATATATATA

TATGTATGTATATTCCTTAAGATCTTCATATTAGAATATAGTTTTGAT

>Unigene0001203

CCTCGCACTCGTTCACACGGTTGGCCAAATATTTTTAAAGACAAATCCCATCATCGTCTA

TTTGAGCGACAGCCACGTTACCATGACAACTAAATACATTTTGAGTCATTACAGAGTTAC

CAACTGATCTCGTCGACAGCGTCATTACACACGTACGCGATGTTGGTCAAAACTACTCGT

ACACACCACGTACACTGATGTACACATACACCAATACACAATTACACACAC

>Unigene0001204

TCCCATATTTTCAGGGCATGTGGGTGTACGAAATATGTGTACTGCATAAACAAAATATAG

GTCCAGTACATAGGTTGGGACAATGTAAGCAAATATTGGTATTATTGAGAATATTGTCTT

ATCAATACATGTGTGGGGTCAGTACATGATTAGGGTAATGTGTGTATACGGAGCAATGTA

TGCTTCTGGATACTTATTGGATATCATATATGTACGGATAAAGGTATGTATTTT

>Unigene0001205

AATTCTAGCGCCTTCTACTGGATTCCTAAAAACAAAAAGAAATAAATTAGAAATTTTCAA

TTCAAATCAAACAAAGAAAAGAGAAATTAGGGATCGAGAATATCTCCAGAGGCTTTCGGG

AGCTCATGGAAGGTAGGAGAAGGTGGGAGAAGGTGGTTTGGGGAGGATTTGGATGTGGAC

ATGAGAGGTGGTTGGTGATTTGGGGTTGTTGGGGAGTGAATCGGACGAATTTGAGGGTGG

AGATGAGAAGAG

>Unigene0001206

CCAATCAATTATTTCCAATAATTATGTTACATTTGAAAATATACAAGGCTTGCAAAAACA

AAACAAAAAATTACTTGAAGTCCTTCGTTCTTTATCCAAAGCAAATGACGAACAAGATAT

AAAATTAATAAAAGATTTAAAGGATGAAATTCATGTTTTAAAAGATAAATTAGAAACTCA

GACACAATTGATCAACCAACAAAAGGAAAAAATAACACAATTACAATCTTCAGAATTAAA

ACCCAAATTAACTGTACAGCTAAAT

>Unigene0001207

CCTGGCACAATGGTTAGGTTGCCCCATTGTTATCTGTATCCCACTTCCATTGTTATCTGT

ATCCCACTTCTAGCCCTCCCTGGTTCCCTGCTTTGGTGGGAGCCTCATGCACTGTGGTAC

CCTTTTCTACCCTTTTCTTTAGCCAATACATTCCATTATCCCAAGTCATTGTAATCATCA

GAGTTATTTGTCTGGAGTGTAATTTGTAACCCAAGCTTTATAGATCCCCAAATC

>Unigene0001208

ACAAGTTACATGATCCTTGTAAAACACAAAAGGTTCATTCCTTACCTTTATGTTGGCATG

TTAGATGAATATGTAGAAATCGTATTCAAAATGGGCCGGTTTAGGGATCACACCTAGTCT

TGACATTGCAGAGCCCGCTAATTCTAACATAACCATAACATAGTCCAACCATTACTCTAG

GGAAACAAAATAGAGGCTTTGATGGACACTCCAGGAGGTCATCGAGCATCATATACTCAC

CAATGAGCAGTGTGCAA

>Unigene0001209

AGTCCTGATGTTGATGTTTACTATTTCGCGGGCGTGGCATTTAGCTGCCGAGTAGGTTAG

GCCGTTGATCTCTGACCCTCCGATGATGATGTTAATGGTCTTGGTGTGTGGAGGAGGCAT

GGGCGGAATGCTGTCCCTCTTTTTCAGACGAAGCCATGGTGCCCTTCCCTTTGTCTGTCA

GGAACTGCTTGAGGTGTCCCTTGAGAAGATCCAGTATCTCGTATCTG

>Unigene0001210

ATTCCTAGTTTTGCTCCAGAAATATTTCGTCGAATTTTTTTGAAAAATCCGTTAAAACAA

AAACAATTAAAAAAAGGCTTAATGCAATTATCAGAAGAAGGTACGATACAAGTTTTTTAT

CCTTTTAATAATAATTATTTAATTTTGGGAGCTATTGGTATATTACAATTTGATGTGGTG

ATTCAACGTTTAAAAATTGAATATAAAATAGATGCAATATACGAAGAAATTA

>Unigene0001211

ACCATTTGTGACATCGTTCTTTGATCGTTACAGAGGCAGAAGAGTTCCTGTTTAACTGTC

TGAGTTGATTCTAATTACCTGGAATATGGTCATGTTGTTTCTCATAGACGATAGCAAGCA

AATTGGTACTGTCGACTCGGTTGAGCTGGAAAGATGTTCGCTGTGTTTTCTTGTACATTC

TTAAACAGCTATCACCTTGGTACATTGTTCTGTTCCCTAGACACAATCAGGTGGGATATA

TGTAGGCTGCACATTTCAAATTGCTGTAAAGTAGCGACAAATTCTGGGTGGTCGGAAGAT

AAAATCGTAAGCATG

>Unigene0001212

ACCATTTGTGACATCGTTCTTTGATCGTTACAGAGGCAGAAGAGTTCCTGTTTAACTGTC

CGAGTTGATTCTAATTAGCTGGAATATGGTCATATTGTTTCTCATAGACGACAGCAAGCA

AATTGGTACTGTCGACTCGGTTGAACTGGCAAGATGTTCGCTGTGTTTTCTTGTACATTC

TTAAACAGCTATCACCTTGGTGCATTGCTCTGTTCCCTAGACACAATCGGGTGGGATATA

TGTAGGCTGCACATTTCAAATTGCTGTAAAGTAGCGACAAATTCTGGGTCGTCGGAAGAA

AAAATCGTATGCTTGTTCTAGATTTGGAGGAAACAAATCTTCTTATATGTTTCGATCATA

AAATAAAAAGTTAT

>Unigene0001213

CAACCAAGCAGGTGACCCTAGCAGCACCGTTCCGACGCGTTCACTTCCCTCGGCCCCTTC

TCTCTCGTCTCAACGCCGTGCCCCCTACCCCGCCGCCCACCCTCCCATCGCCGTTCGTCG

CTCCGCCCCAAGCTCGAAATCCCGGCCGAAGCACCGGCGGGATCTCCCGGTCTCAGATCG

AATCGCCGAGGTCTAGAGGTGGCATCTTGACTTGTGCCTTTAGTTTGGCAAAATGGTTTC

ATTTGAGATGAATGA

>Unigene0001214

GGTAACCATGCTGGCATCCATCTAGATGAAACAGTCCGCCCCAGCTCTGAGAAGTCTAGA

CTTGATGCCTGGGTCTCCTCTCATATCAGCGGTGTCAGCCATACCCGCATCCACTCTGGT

CTTGCCACCATCAATGGCTGGATCTCTTAGTCTCAGATTGAATCGTTGAGGTCTAGAGGT

AGCATCTTGATTCATGCCTTTAGTTTGGCAAAATGGTTTCATTTGAGATGAATGACAGGA

AAAATATTGGGTTAGCCCTGATAAGGAGTAATGTTAACCATTGGAATGAGATCCACCATG

CAATTCTTCACAAAACGCAAGAACTACAAGGGTACTATCTCGTTTGGAGCTGGCTTCCTT

TTGGTTCTAATTGGATGGGCTGTCACATGCATGATCTTGGAGGCTTATGGCTCATTATTC

TCTTCAATGAGTTTTGGCCTACTTTGGCATTTTTCTTACAGAGGATTCTTATTCTTGGAT

GGTTATTTCAGCAACCATTTGTGACATCATTCTTCGACCATTATAGGAGTAAACGAATAC

CAGTATAGCTCTATCAAGCTGATACCCATGGTGTCATTACATGCCTCATAAATCCTTTGT

AAGTAAACTTAGTTCCTTAACTATTTATGTGGTGATGTGGTATGATGGCCAAAGCGAGAG

GATTTAGCAATGTGTATTGATCGTGTATTCCAAAGCCGTGGGCAAATTCATTTTCTCCTC

GACGGTTATGAAGATTGAAGACCCAATTTATGCCCATTCCTCTATTATGGCGATAGCAGT

TGAGAAGATGACTCTATAAATTAAATATTAAAGAAGGGTGGAAACATACGGTGGGTAGTG

AAGCACTTATGAATCTGCAGAAGAGACTCTTCTCGTAGAGAAGTCTTCTCAGTTATGCTT

CAGAAAATTATGGTTTCTCAGTGTTCCATTGTGAGGGAGAGTGAGGCATGCATGCATGTT

CATGGTTGTATTCAGAGGTTGAATGATATTGTAGTGAGATTGTACTATCTCTCTGTGTAA

GGTGTTGCCTACTCTCTATAAGGTTGAACTTTGTGGTTAAG

>Unigene0001215

GCCCTCCGGCAAAACTCTTCCCGTCTTATGAGATCAAATCCACTCCAAATTATAGCCCCA

GCATTACCGTTATCTACTGAGTACTTATCCACAATCAATTTATGATTTTGCTGAGGGAAA

TTAGAGTTACCACTGTTTCAATCTGACGACCAGTGTCCGGGAGTTACCAATCTCCATCAG

ATCTTCTTCTTCGGCTCGTATATATCGTCTCTCTTCAAAGGATGTTAAGAGATTCTAATT

TATCTTATTTTGTTTCAAATTTTAGATGCATGTTTGATGGATGAAGGAGTGAGGATGCAT

GGCCATTCTTTGGAAGCTCCGTCAGGGCCTCCTCCAAGGAAGCTGTTTCCTATCTCTGTA

ACATTGCCCTCGCCTATTCCCCCCAATACCTACCCCCTATAAGCTCCTCTGTGCCTCCAC

AAACCCTCCCATACTAGTGCCTGTTTAGATGAAACAGTCCACCCCAGCTCTGAGAAGTCT

ATACTTAATGACAGGGTCTCCTCTCACATCGACGACGTCAGCCACACCCGCATCTAGTCC

AGCATCCGCTCCAGTCTCACCACCGTCAATGGCCAAATCTCTCGGTCTTAGATAGAATCA

CCAAGTTCTAGAGGTGGCATCTTGATTTATACCTTTAGTTTGGCAAAATGGTTTCATTTG

AGATGAATGATAGGAAAAAGATTGGGCTAGGTCTGTTGAGGAGTAATGTTAACCATTAGA

ATGAGATCCACCATGAAATTCTTCACTAAATGCAAGAACTACAAGGGTACTATATCGTTT

GGAGCTGGCTTCTTTTTGGTTTTCATTGGATGGGCTGTCACAGGCATGATCTTAGAGGTT

TATGGCTCATTGTTCTCTTCAGTGGGTTTTGGCCTACTTAGGCAATTATCTTACAGAGGA

TTCTTGTTCTTTGATGGCTATTTCAGCAACCATTTGTGACA

>Unigene0001216

CCCTCTTTCCCTCTCCCGCTCTAGCTGCGCCTCTGCTACCGAGTTCGAACAGTACCCACT

CCATCGCAATCCGTTGCAGTATCTGCCGGATCGCTCGATCTCTTAACAGGCCGAAGAGGT

CCTCGATCAACGACATCTTCACCTGTGCCTCGGCTTTGGCAAAATGGTTTCGTTGGAGAT

GACTGACACAAAGAAGATCGGGCTCGGCCTGACAGGGTTTGGAATCTTCTTCTCATTTCT

TGGAATCATATTCTTCTTTGACAAAGGACTACTAGCCATGGGGAATATCCTCTTCATATC

AGGGGTGATGTTGACCATCGGATTCACATCAACTATGCAATTCTTCAGTAAACGCAAGAA

CTACAAGGGTACTATAGCGTTTGGGGCCGGATTCCTTTTAGTCCTCATGGGATGGGCCGT

TATAGGCATGATCTTGGAGGCTTATGGCTTCGTCGTACTCTTCAGTGGCTTTTGGCCTAC

ACTGTCGGTTTTCTTGCACAGGATTCCTGTTCTTGGCTGGCTGTTCCGGCAACCATTTGT

GACATCGTTCTTTGACTGTTATATGAGCAAACAAATACCAGTATAGCTC

>Unigene0001217

CCCCCCTTGCTCGCTCACCAGTTAACTCCTCCAGCTATCTCCGCCGCCCGCCGCCCGCCG

CCGATCCGCCCCTCCGCACCGGTGAGAACCATGGGCACTCTCGGGCGAGCGATCTACACC

GCTGGATTCTGGATCCGGGAGACCGGTCAAGCCCTAGATCGCCTCGGCAGCCGCCTCCAG

GGCAACTACTACTTCCAGGAGCAGCTATCAAGACACCGTACGCTCATGAACATATTTGAC

AAAGTCCCCAATGTTCACAAAGATGCATTTGTGGCTCCAAGTGCGTCCATCGTTGGAGAT

GTTGAAGTAGGACATGGATCATCAATTTGGTATGGATGTGTGTTGAGAGGTGATGTGAAC

AGCGTCCGTGTTGGTTCTGGGACGAACATACAAGATAACTCTCTTGTGCATGTAGCAAAA

TCCAATTTAAGTGGGAAGGTGTTGCCAACAATTATTGGCGATAATGTCACTGTTGGTCAT

AGTGCTGTCTTACATGGATGCACTGTTGAGGATGAGGCTTTCATTGGTATGGGATCGACG

ATCCTTGACGGGGTGGTGGTGGAAAAACATGGCATGGTTGCTGCTGGAGCTCTTGTACGA

CAGAATTCGAGAATCCCATGTGGGGAGGTATGGGGAGGAAATCCAGCCAAGTTCCTGAGG

AAGCTGACGGAGGAAGAGATTGCTTTCATAGCCCAGTCAGCTACGAACTATAGCAACTTA

GCCCAGGTGCATGCCACCGAGAATGCAAAGCCTCTGGACATGATCGAGTTTGAGAAGGTT

CTGCGCAAAAAGTATGCTCGCAGGGATGAGGAGTATGATTCCATGCTCGGAGTGGTCCGG

GAAGTCCCTCCAGAGCTGGTCCTTCCTGACAACGTCTTGCCGGACAAAACACCCAAGGCT

GCTATTTGAGTGTGGCTCTTAGATGGTGTCCTCTTGTTTCCCCTTGCAATAATTTTTGGC

ACGAGTTCTGTTTTAGGCTCAGACCTTGTGCCGCTCAATGTTTTTTTGGTTTTCTGGATA

TGTTTGTAATTTCAATATTGTTTCCAGTGGGATGGTAGCCAGAATGTTTCCCCTCTTTGC

ACTATATGCAGAGAGACAAGCCAAACTTGCTGGTTTTACCTTACCACGGCAGAGTTGATA

ACCGTGTTCCAATAGATTTGTATCTGTTTTCTAGTTAGAGTCCTGTACTACTTGGAAACA

ACCTACATATTGTTCTGCAACTGTAGCAGGGGAGATGGTTGCAGATTATGTAACTGCAGT

GTCTAAAAAATGGTTTCAAAACGCTTCCTACGGAAGGCTCGGAACAATCAGAATATCTTG

CAGTCCCTCAGGCCTCAACAT

>Unigene0001218

CAGAATATTTCAAGCTCGCGTCGCTTGGTTCTTCACAATCTCACCAATGATGTTTGCAGT

CATGGCCTTCCGTTATCCAATACGAGATCTCTGCTTCTGCACAGTAAATTAGAATCCGAC

TATGTGTCAAAGATCCTTCAAGATTTGAAGTTGTTGAGGGTGTTAGATATGCATCATACA

GAAGATCTACTGAGGACAGACTTGAGCTTTCCCAAGGAGATGAAGTTCATGACACACTTG

AGATATTTAAAAATATCTCGAGTTCATTTTTCTCCCTCATTAAAACT

>Unigene0001219

GTTTTGACATGTCGGATACATGATCTCGTGCGTGAGATAGGCATTGCAGAAGCTAAACAG

GATAGGTTTTTCAGCATCATTCATGGTAGAGGCAGCAAAACCACCTCGGCCGAAAATAAC

ACTAGTTTCGATCAGATTATTTCAAGCTCTCGTCGCTTGGTTCTTCAGAATCTCACGAAT

GATGTTTGCAGTCGTGGCCATCTGCTGTCCAATACGAGATCTCTGCTTCT

>Unigene0001220

GTTTTGACATGTCGGATACATGATCTCGTGCGCGAGATGGGCATTGCAGAAGCTAAACAG

GATAGGTTTTTCTGCATCATTCATAGTAGAGGCAGCGAAACCACCTCGGCTGCCAACTAT

GAAAGTAACACTAGTTTCGATCAGATTATTTCAAGCTCTCGTCGCTTGGTACTTCAGAAT

CTCACGAATGATGTTTGCAGTCGTGGCCATCTGCTGTCCAATACGAGATCTCTGCTTCTG

CAATCTGACTTCGAATCTGACTATGTGTCCAGGATCCTTCAAGGGTTGAAGTTGTTGAGG

GTGTT

>Unigene0001221

CTCCCAGATGAGCTTTGAGAAGTCGTTTGGGCTATCGCCGGTTTTTATTGAATCTACTCT

CATGGAAAATGGGGGAGTGCCTGATTCGGCACACTCGTCAATGTTGATAAAAGAAGCTAT

TCATGTTATTAGCTGTGGATATGAGGAGAAAACCGAATGGGGAAAGGAGATCGGTTGGAT

ATATGGTTCTGTCACAGAAGATATTTTAACAGGATTTAAGATGCATTGCCGCGGATGGAG

GTCGA

>Unigene0001222

TACCTTGGTTAATGTACTAAACGAACACTAGACCCTAAGACACAATGCATGTTTATTTTG

AAAGTTGTGTAATCAAGTTATATAAATTGGGTACACAAATATCTGTGTATGTTGGCCAAG

GGGATAATTAGGGGGCTGGGAAAACAATTCTGGACGTAGACACTAGCTGGTAAAATATTT

GTTTTAAATCACCCAATATTTTTCTTCTGTCAGTCGCTTGGACTAAATTTTGTTAACTTT

TGAAGCGGTTGATTATCTGATAGTACTATCATTTTTTCTT

>Unigene0001223

AGTACATTAAAAAAATATGTGATCGTTTCCAGATACAACCAAATCTTCTTGACACAATTC

CAACCAACATAATTGAGGTTAAGTTCAGCGATAAGGTGACTGTTGAATTCGGGAATGCCA

TCGATGAAAGAGATTGTCAAGAAGAACCGAAGGTCTCGTGGCCGATTGAGTCCAATCAGA

CATATTATACGCTTATAATGACAGATCCAGACAATCCCACTCGTGCTGATGCCACGAAGC

GAGAG

>Unigene0001224

CGGGAGTGAGGAGGTTGAGAAAGAGGGTAATACTGGACAGTTCCTCCTTTGGAACTTGGC

TCTGCAAGCTGATCTAGGACTTTTACTGGAGCTGCTGCCACTATTTTCTACTTGCAGTCT

TTTCCTTTTTGTTATCCTCTTTTTTATTTTTTTTTATTGCTCAAGAAATGTAAGCTTTAC

TTCCATGTTGTGGTCTCTTATGAACAACGAGATCTAATGTTTAATGA

>Unigene0001225

ACGATTATTATATACATTTTGTTTTCGCCAAATAGATTTTTTTACAATACATAAACTCGC

CATCTTACTTCCGTCTCCGGCACGACGTACGATATTACTACCATGGTGTCGTGCGCAGTG

TCAATGTTGCTGACCGCTGCGGTTTGGTGCGCATCCGCCAGTGCCCTTGCCGTCCCGGAG

ACCATGGCCGAGACCGCGTTAGCGAACGGCTCGTCGGTGACCGCAGACTGGACCACGCCG

TCTGTGATATCCACACCAGAAG

>Unigene0001226

GCTTTGCTGTAAGTACTACACGTGTTTTTATGCACTTCCTTGCAGCCATCCACGACACTT

TTCATGTATTTCCTTGCAGCCATCTTCCAGAGTGGCAGCATAGTTTGTCATGGATGAAAG

CTTCATACTTTTACGTGGACATATTGGGATGTCGTGCCAAATTGCCAGGTGTGACATTAG

ATGACAGTCGTTGGGTCTATGAAGTCAAGTACAAGCCCGATGGTACAGTCGAGCGTTGCA

AAGTGCGACA

>Unigene0001227

GGGAAGAAGAAGAGTTGTGACTTGAGCTTGTGCTGTTTTATATGCTATGAAGATAAACGG

TGGGGCGGTGGAGAGATGGCTGAGTTGGCGCGATACGGTTGCTTAGCCACTGAAATGGTG

ACGCGGTGGCGTGGTACAGCTTGGGCCATTTAAACTTGGCGCCAAACTGTTTTATAGTCT

CTGTATGAGTGGGGGAGTAACTGTTAATTCCTTTTAGTTGAGCTAGATTG

>Unigene0001228

TATATACTTCAATAAATTTCTAATTTTTTGAGGTTCAATGCAGCCGATTTGTACCTTTCA

TAATATTGGGTAACTTGTGATTTCTCTTTTAGTAATGTATAAAATTCTATCCCATCTTAA

TCTTGGGAAACAGCTGATGGGATGTCAAAAGATCTCTAAAATTTAATTTATTTTCTAGCA

TTGTGGATGATTCAACCCCAATTTTCCCCCGAGTCTGATGTTCCGGTGGTAGAATCGACT

AGCCAGCCAATCAATTAAACATCAATCCTTTTATTTATCGTTATTTAGTTACATTCATTA

GTTTTCCAAGATAGACTTACTAAAACAAAATTTAGACTGCTCTTTTAGTTTACCCAACAA

AGTCTGAGTAATAGTCGACAGACTTCACATTTCTTAATTTATTCTTTTACACATAAATTG

ATGCACATGCTGCCCAAGAAATACTGCAAATTGTATTTTTTTTCTTTTAAATACTTGCAA

TTAATAGTTAAAGATTTTATTTCAAATACGAACAACGAGCATATAGTGCATCATAT

>Unigene0001229

AAAGAGGATTTAGAGTAGACAATTGAGAGAAAAATTGAGAGGTAAGAAGATTGAGAAAGG

GCTACCTTAGGGTTAAGGTTCACTTGTTCTCGATGTCGAGCACGGGCTGAACCACAAATG

TTGTGGAGGAAGATGATTCCGATCAGGATAGTGGTGGATTCCGGCGTGGGTAGGGTCACC

ATATAACCTCACTGTCTTTCCAACAATCGATGTGTATAGATATATTTGAACAAAGAGTCG

AGAGTTTAATCTGTGTGATGTTATTACTAGTTCAATTAAACTGATTCAGTGTAACACCGT

TCAAACCAGCCTGACTTGATCTCTTAACATGAATGTATAGCCTGCCTAGCTGGCTTTTCG

ACCATGTTAATTGTCTAATTGACCGCCTAGTAACTTGTC

>Unigene0001230

AATATTAGAAGATAAAATGAGATGAGAGAAAATATGAGAGAAAATTGGAGGAGAGAAAAA

AAAGGAGAGGGAAAATAAGCGAAGAAAGAAAATAAGAGGAGATAAAATAAGAAAAGAGAA

AATCAGAGGAGAGAAAATGAGAAGAATTATAAAATATGGGAAAATGAGAAGAGAAATTAT

GAGGAGAGAATGTGAGGAGAGAATATGAGGAGCGAAATTGTAAGGAGAGAAATGAAAGGA

GAGAATATAAGGCGAAACAAAATTAAGAGGAGATGCAATATTAGGTGGGAAAATATAAGG

AGAGAATATTAGAAGATAAAATGAGATGAGAGAAAATATGAGAGAAAATTGGAGGAGAGA

AAAAAAAGGAGAGGGAAAATAAGCGAAGAAAGAAAATAAGAGGAGATAAAATAAGAAAAG

AGAAAATCAGAGGAGAGAAAATGAGAAGAATTATAAAATA

>Unigene0001231

AATATTAGAAGATAAAATGAGATGAGAGAAAATATGAGAGAAAATTGGAGGAGAGAAAAA

AAAGGAGAGGGAAAATAAGCGAAGAAAGAAAATAAGAGGAGATAAAATAAGAAAAGAGAA

AATCAGAGGAGAGAAAATGAGAAGAATTATAAAATATGGGAAAATGGGAAGAAAGGAAAT

TTGATGAGAGAAAAATTAAAGAAGAGAATTATGAGGAGAGAATGTGAGGAGAGAATATGA

GAAGAGCGAAATTGTAAGGAGAGAAATGAAAGGAGAGAATATAAGGC

>Unigene0001232

CCTACCACCAAAATTAATGGATCTGTCGCGCTCTTTATCGCCCACTGCAACGCCGCCATC

AATGTTAGCCTCCCATCGTTAGATCCGCCGGACTCCAATGTTAGGGTTTCGGCGGAACTG

GTAAGCTCATTCTACGAGTAACTTAGCATGAACCCTCTCTGCTGCATCGCCCCGGTGTCG

GTAGAGCGGGGCGCGGCCACGCCGGTTGTTCCTACGCGTGCTAAGCCGGCTGTGCCGCCT

TTGGATGCCGCGGCCCGGACGGTGTCGAGCAAGCAGCTCCTACCGTTGGATCCGGATCCG

GTGCTGGAGGAGCCGGCGGGGGAATCGGGGCCGTGTGCGGTGGCGGGGATACTGTACAAG

TGGGTGAATTATGGGAAGGGGTGGAGGTCGAGGTGGTTTGTGTTGAAGGATGGGGTGCTG

GCTTACTATAAGGTTCATGGGCCGGATA

>Unigene0001233

CTCCACCTGCCCGCTCTGCCGGAGTCCAGTCGGATTCCCCGTTCCCGTTTCACAGGGAAT

CCCAACTCCAGAGGCTGTAACTGCAGAAACAACGGAAGCTACAGTTACAATATTTTCCGA

GACGCCAAATGGGGAGCCGGCGACCTCAGTCTCCGTACCAAATGAGGAGCCGGCGACCTC

AGTCTCTGTATCTCTGGTGATTGATATACCACGGAGGGTGATGCAGGGGTTCTCATCTTC

GCCGAGTACACCGCTTCCGACTTCCTATGATTATGGGAATCCGATGATGGGAACGAAGAC

GGTGACACCAACTTCGGCCAGGCTGAGGACCCTCACTAGACTGCTAAGCAGGGGGAAGAA

GACTATGGGGGTTTCTTCAACTGCGAGTCCCAGAGAAATTGAAGATATTGAGCAGGGACA

GGGGCTATTAGTGGTAGCTGAGGGAGAGAGCCCGCGGGGAGTTACTCCCAGGATACATAT

AAATTCGTGAGCTGGAGAATGTTGTCTTCTGGCGATTATCTTGGTGGAGCAACAGATCTG

TCCGTATGGATGTAGAATCTAAATGGCTGTAGCTTATTCTTTATTTCTAATTTTTTTTTT

GGTAATTCATGTTTTGGACCTCCAAATGCATAGATTTTCCTTGGTTTATTTCTCGTTGCT

CTAATGATATATCGTAGTCTTTTATATCAA

>Unigene0001234

TTCTTCAGCTGCGAGTCCCAGAGAAATTGAAGATATTGAGCAGGGACAGGGACTGTTACA

AAGGGGAACTGTGGGGGAGAGCCGGCGGGGAGTTACTCCCAGGATTCCGAGAAATTCATG

AGCTGGAGATTGTAGAAGTTCTAATTTTGGAGTACTACCGGGGATTCCGAGATATTCATG

AGCTGGAGATGGTATTCTTCTTGAGGTTATGATTGTGGAACAACAGATCTTCGCTTAAGA

TTTAGAATCTAAATGGTTGCCACTTAATTCTTTATTTCTTCTTTTTCTTGTAAAATCTAG

TACTGGAGCTTCAGGATTGGAGATTAATCTGTATATAGATATAGTATGGTAATTCTCTTA

TGATATAACTTATTCGTTCTCTGAAAGAAAATTTTGCCTGCATTTGTTTTAATTTATTGG

CCAGTCCATACATAGAAAGAGCTGGGTATACCTCATGTAGAGGCTAAGCTTACCCTTACA

GCGAGCTTCAAAGTCTGGACTTCATTCTTCCGGCCAAGTGAAAACTGTGGACTTTTTATT

ATTATGTAATTGCCTTAGTTTCTGCA

>Unigene0001235

GCTCACGAATTTATATGTATCCTGGGAGTAACTCCCCGCGGGCTCTCTCCCTCAGCTACC

ACTAATAGCCCCTGTCCCTGCTCAATATCTTCAATTTCTCTGGGACTCGCAGTTGAAGAA

ACCCCCATAGTCTTCTTCCCCCTGCTTAGCAGTCTAGTGAGGGTCCTCAGCCTGGCCGAA

GTTGGTGTCACCGTCTTCGTTCCCATCATCGGATTCCCATAATCATAGGAAGTCGGAAGC

GGTGTACTCGGCGAAGATGAGAACCCCTGCATCACCCTCCGTGGTATATCAATCACCAGA

GATACAGAGACTGAGGTCGCCGGCTCCTCATTTGGTACGGAGACTGAGGTCGCCGGCTCC

CCATTTGGCGTCTCGGAAAATATTGTAACTGTAGCTTCCGTTGTTTCTGCAGTTACAGCC

TCTGGAGTTGGGATTCCCTGTGAAACGGGAACGGGGAATCCGACTGGACTCCGGCAGAGC

GGGCAGGTGGAGTGGGAATGAAACCACATATCAATGCACTCGAGGTGGAATCCATGGTTG

CATTTGGGCAGCATGCGGACCTCCTCGCCGTCGCTCAGCTCACAGAGGCAGACGGCACAT

TCGAGCTCCTCCTCACGATCAACTTTGAACACGGTGATAGGCAGGGAACGGAGAATGGCA

GCATCGAGACCGTGGCGGTGGAAGGTAACAGGATCGGATGCTGCGAAGATGAGGCGGGAG

CGGCGACGGCGGAGGGAGCGGTCGAGGGCCCAGACGTGCTTGGCGTAGAGGTAGAGGGTG

AGGAAGAAGAGGGCTAGTATGAAGCAAAAAATAACTGCTGCTATCATGACATTGAAGCTA

AGGCCGACAGTTGGCCCAAGGGAGTCTTCTACGTCTGTCGAGTCTTTTGCCGGCTGTGAC

ATTGTTGGACCCAATCGAGATTTCTGAAGAATTGAATCAATGAGCTCGATTGTGAATTAT

ATGGGAAGTAGAACAGGGGAACGGGAGCAGGAGAGAGAGACGAAGGAGGTGAGAACATTG

ACCAAACATGAGCCGCTGAAATTGAAGGCAGACTTTTAAACTCTCTTCAATTCTTCAGTT

TTGCTGCACATGACACTATAAGAGTCAAATAGCATGTGCTGACTTCTTATTGATGAGTGA

AAAAGAAATAACACTACCTATTTTTTTTTTTAAAAGAAGGTAGGCAACATAAAATAAAAA

AGCAAAATAAAATAGTATTAGACTATTCTAGAATGTTGTTTGTATTGGACCAAGTTCTCC

GCAAGAGCTTGATGAAGATCTGAAATGTCATTAGTGTTTCCTAGTGCTCAAAGATTGATC

TCTTGATATACAT

>Unigene0001236

TGTGTTGGGCAAGCGGGAGGATCCGTCCATATTGTCGATGCAAAGAGGAATCATGGTGAC

CTTTGCAGTTGCAGCAGTCAGATCAGGTTCTTTTTGATTGTCAGAAAATGCAACATGTGA

GTCAGAAGTTCTCAATTCATGCATGCAAATGAATAACTTAGAAGGAAAATAATTGACAAG

ATGTGATTCTTCTATGCCATGCCAACTCAGGGATCCATACGTTGACATTGGCATGGTAAT

GATTTAGTAGCTGATATTCTGCTCCTCTATTTTTTTTTCAATGTT

>Unigene0001237

AGTGTCATGTATCAGCTGAAACTATATGGGCTTCATTATCACGCGCCACCCGCTGTAGCA

CAGCCGTCCCTTCAACACCGGCCGTCGCCATAGACGTCGCCCCTGTGCCACTTCACCGTC

GCACCGAGCGCCGCCACATTCGCCTCACTCACCAACACCTCAACCACCGCCGACAACGCC

GCGACACACCATCGACACCTGCTGCCGGATCCATCCCCATCAATTCCCACTCGCCGCCTA

TCACTCCCCCAGCCTACCTCACCCCTCCCACTACATCAAATCCCATGCCTATCATCATCA

TCATCTCACCTTGAAGGTTACCAGCACCTCCATCATTAGGTGTTCGACGAATCGATTTCA

TTGCCCTCTAGGTGTTCGACGAAACGACTCAGACAATTTCCAATGCTTGGCAGAGCATTT

TTCAGCACCTTAAGGTCTCTCTCTAACATATTATTACACAATAAGCAATGTTGAATGTGG

GCTCACCACTGAAAGGAAATAAATATCTGATATTCAATGTTTAGTGAAGGGTAAACGTGA

TGGGGATCGGAGATAGCAAACCAAGTATATACAAGAATAAGACCACAATGCAACTGAAAT

TTTGATGAAGCAAACTCCTTTACTGAATTAAAGTTGAATCTTCTATTACAGCAGATGATT

CTCCAAAAGAAAAAAAAAATTCTCAACTCACTATGAAGAATAACACACAAAAGTTTTCTA

TGCAAAGATCCAAAACACACAGTTGGATTTCCAACAAAAAACTCCCCTTCTATTTATAGT

TTTTAAATTTGGAAGTTATAATTTGTTAGTTATTTCTTTTACCCGAAAGGTTGCCCAAAA

AACCCTTCCGTTAGGGTTTCAACCATTCGGGAAAGAAAAATCGCGGCTGTGTGAAGAGCC

TCCACACACGCCGGTTTTTCCAACAAAACGATGGTGTCTAAATTCATACAGTATGTTCGC

TACCTACTTTGTTTACTATATGTTCAAGGTTATGTATCGAACACTCAGTGGGACGGGTAT

AAAGCTTTACCGAAGAATTAAGTCTCTATGTATGTGGAAATTTGTGATGCACTGTTTTGG

AGGCCTCCCAAAAATTGACATCCATCTATGCCTCCACATACCACTGCTGATAAGGTGTTG

ACAGTTGAAATGCATGCAAGGTGTTCGAGCAAATGCCTGAATGTTGTGAGTTTTGTTTTG

CTTGAATGTATGTTGATCCAGGTAACTGACCTCTATCTCTCTGTACATATACTTAGTAAT

AAGCTTTATAATTTACTTGTTATAATCTATTTTCTCAATCATACTATGACATGTGCCATT

TTAGTATTGATGTTGTGATTTTATTGTTGCAGTCTCGAAATATATGTCCGTTTGGATCTG

TCAAGCGTCATGACATGCATGTCTATCACTTAATGATAGTAGAGTATCATATATAGTGTG

CTAGGCACCCAGTTCTCATGACTTCTCCATATGCGCAGATCATTGTGTTGGGCAAGCGGG

AGGATCCGTCCATATTGTCGATGCAAAGAGGAATCATGGTAGGCTCTTA

>Unigene0001238

TGAGGAGCTGAAGGAGAAGCTCGGCGGTGGCACCATCGATAACGGTGCCGTCTACGACAA

TCTAATGAAAAGCTTGATGGGGATCGGAGATAACAAACCAAATATATACAAGAATAAAAC

CACAATGCAACTGGAATTTTGATGAAGCAAGCTCCTTTACTGAATTAAAGTTGAATCTTC

TATTACAGCAGATGATTCTCTAAAAGAAAAAAAACACTCTCAAACTCACTATGAAGAAGA

ACACACAAAAGCCTTCTAT

>Unigene0001239

TGTGTTGGGCAAGCGGGAGGATCCGTCCATATTGTCGATGCAAAGAGGAATCATGTTCGA

GGATCCGGTCATATGATTGCTGCAAGAAAAATCATTGTAGGCTCTTGCTGACGGTCCATT

AACCAGGTGACCTTTGCAGTTGCAGCAGTCAGATCAGGTTCTTTTTGATTGTCAGAAAAT

GCAACATGTGAGTCAGAAGTTCTCAATTCATGCATGCAAATGAATAACTTAGAAGGAAAA

TAATTGACAAGATGTGATTCTTCTATGCCATGCCAACTCAGGGATCCATACGTTGACATT

GGCATGGTAATGATTTAGTAGCTGATATTCTGCTCCTCTATTTTTTTTTCAATGTT

>Unigene0001240

GCCCACTCTGCCGGAACCCCATCCGACTCCCTGTTCCCGTTTGCATGGGAATTCCTGCTT

CAGTGCTGGTTGCTGCAGAATCAACGTAAGCTACAATTACAACAAATAAGTTGTTTTCCG

GTACAGAGCCTCTACATCTCTGGTGACTGATATACCAAGGAGGGCGATGCAGGGGTTCTC

ATGATCCCCGAGTACACCGACTCTGACTTCTGTTGATTAAGAGGATAAGATGAGGGCGAT

GAAGACAACACCAACTTTAGCCAGTCTTGAGGACCCTGACGAGACTACTGAGCCGGTAAA

GAGGGTTACGGGGACTTCTTCAGCTGCGAGTCCCAGAGATATTGTGCACGGACCAGGGAT

GTTAAGGGGGAGCTGTGGGGAGAGCCGGCAGGGAGTAAATCGAAGAATTCCGAGGAATTC

GTCAGCTGGAAATTGTAGTACTCTTGAGATTATAATTGCTCAATATATTTCTTAATCGAC

AATAAGAAAAT

>Unigene0001241

ATGCAACCATGGATTCCACCTCGACTGCATCGATATGTGGTTCCACTCCCACTCCACCTG

CCCGCTCTGCCGGAGCCCAGTCGGACTTCCTGTTCCGGAGGAAGTTCCAGATCCGGAGCC

AGTTACCACCGCAGAATCTACAACTATAACAACTAAGTTGTTTTCCGGAACTCCGAATGC

GGAGCCGTCGAGCTCAGTCTCAGCATCACTGGTGATTGATATACCGAGGAGGGTGATGCA

GGGGTTCTCTTCGTCGCCAAGTACACCACTTCCGACCTCCGATGATTACGAGAATCAGAT

GATGGCAACGAAGACGACGACGACACCTACTTCAGCCAGGCTGAGGACGCTAACGAGACT

GCTGAGCCGGGGGAAGAGGACCATGGGGTCTTCTTCAGCTGCGAGTCCCAGAGATATT

>Unigene0001242

TATGGCTCCCCTCAGAGCAGCGGCGCCAGCGAAGCCGGAGGAGGCAAAGGGGTTTGTGGG

TGAGATGAGAGTGATGGCCATGAAGCTGCACACGAGGGACCAGGCGAGGGAGGGGGAGAA

GGCGACCGACGCACCGCCGATGGCGAAGTGGGAGCCGTCAATGGAGGGGTTCGTCAAGTT

CTTGGTGGATAGTAAGCTGGTGTTTGATACGCTTGAGGGGATCGTGAATACAGCGGCTTA

CCCTTGGTATGCTGAGTTCAGAAACACGGGCTTGGAAAGGTCTAAGGAGTTGACAAGAGA

TTTGGATTGGTTACAAGTACAGGGCCACTGTATTCCCATACCTTCTCCTCCAGGAATTTC

TTATGCTCGCTATCTGGAAGAACTGTCTGAAAAGGATTCTCAGGCATTCATCTGCCACTT

CTATAACATATATTTTGCTCATACTGCTGGTGGTAGAATGATTGGTAGAAAGGTGGCTGA

GAAGATACTGAACAAAAAAGAGCTTGAATTTTACAAGTGGGACGGGCAGCTATCTGAACT

ATTGCAGAATGTGAGAGACAAGATCAACCTAGTTGCTAGTAGCTGGTCAAGGGAAGAGAA

GGATCATTGCTTGGAGGAGACAGAGAAATCATTCAAGTACTCTGGGGAGATACTTCGTCT

GATACTGTCTTAATTTATACGAATCCTCCTTGATAAAGAAAATTGTACATAATGTTTACT

GTAAATCATCTTCAGCATAACAATTGCCTGGAGATAATATCTGTATAAATCCTTTTAAGA

CTTTTATGAAGTT

>Unigene0001243

GATGTTCCTGCGCGGACGTCCCATCAACCTGTACGCGCCGACTGCTGAGATCGCGACCTA

CGACATCACTAAAGTGGCCACCGCTCCTTCGTCCAAGCTAAAACTCGATTGGGTTTATGG

GTACAGAGGAAAAGACTGCCGTTCCAACTTATACTTGTTGCCCACCGGTGAAATGGTATA

CTTTGTGGCCTCCGTGGCTGTACTGTACAACGTCCAAGAACAAAGTCAGAGACATTATTT

GGGACATACGGACGACATCAAATGCTTGACCATTCATCCCAACAAGCTGTTGGTAGCTAC

TGGCCAGACTGCTGGACACGATAACCACGAAGGAAAACCTCATATTCGAGTGTGGAATTC

AGTCAGTTTAGCAACAGTGGTTGTGCTGGGTTTAGGAGAATTTGAACGGTCTATCAGCTG

TCTGTCGTTTTCTAAAGCGGACGGTGGCAGTCTTTTGTGTGCCATCGACGAGGGAAACGA

TCACAACATATCCGTGTGGGACTGGCAAAAGAACGAAAAGGGTCACAAGATCACGGAGAC

TAAGTGTTCTATGGATACCGTTGTGGCTGCGGAATTCCACCCACTGGAACGAAACCAAAT

CGTAACCTGTGGTAAGGGCCACATCAGTTTCTGGACACTGGACGCCGGCGGTACTCTGTA

CAAGCGCAACGGAATATTCGAGACTACTCGCGATCGTCCCAAGTACGTGACGTGCTTGGC

GTTCACGCAAAATGGAGACGTCTTGTCTGGAGACTCAAACGGATCCGTTGTCATTTGGGG

ACGTGGAACAAATAACATCCTAAAAGTCGCACGAAATGTGCATGAAGGATCAGTGTTTTC

CATCTGTGTGTTGAAAGAGGGCAGCATTATAACCGGAGGTGGCAAAGACGGTAAACTGAT

CCAGTTTGACACTTCGATGCATCCCACTGGATATGAAGCACAAATAGCTGAACATTTGGG

TGGTATACGTACAGTATCAGAAGGTCGTGGATCACAATTGCTAGTCGGATCAACCCGCAA

TTGTATTTTGATCGGTTCTCTGTCGATGGGCTTCAGCCCTGCTGTCCTGGGCCATACCGA

TGAACTGTGGGCGTTATGTCCTCATCCTACTTTAGCTCAATTTCTTACAGCCGGATTCGA

TCGCATTGTCCAACTCTGGGACTCGTTGTCTCGCTCTGTCGTATGGAGTAAAGACATTGC

AGAACAAGCACAGTCGGCTGCATTCTCTATTGATGGTCGAATAATCATTGTTGGTTGCGT

TTCTGGAAAATGGATCGTTATGAACTCTGAGACACGAGAGATTTATTCGACTCACATCGA

TGGAAATGAACCAATACAGGTTGCAAAGTTCTCACCGGACGGTCAACTACTTGCACTTGG

TTCCAGGGACAATTACATCTATATATATCAGGTCTCAGATGGTGCCAGAAATTACACTCG

TTTAGGACGTTGCACTGGCCATTCAAGCTTTATCACTCATTTGGATTGGTCAAAAGATAA

CGAGCACCTTCGCAGTAACTCTGGAGACTACGAGCTGTTATATTGGAAAGTGAAAGTTTG

TCGTCAAGTCACTCACAGCACTCAACTTAGAGACACTGTATGGGCATCCAACTCTTGTGT

CTTGACATTCTCCTCCATCGGAATTTGGCCTGAAGGAGCTGATGGCACTGACGTTAACGC

ATGTGAACGATCAAATGGATCTAAGCTTATGGCTACGGGAGACGACTTTGGTAAAGTGAA

ACTGTACTCATACCCGGTCAAACAACCTAAGTCGCTGTGTCACGTGTATGGAGGCCATAG

CAGCCACGTGACAAACGTGACTTTCTTGCATGACGACTCCAGACTGATATCGATTGGTGG

CAAAGACACCAGTGTCCTACAATGGGAAATCATATAAAAGAAGAGCGTTAATT

>Unigene0001244

CACCTCTCTCTCTCTAGCTCTCTCTCTCCGATGTCGGCTAACCCCTTCACCGCCGACCTC

CCCGGCGCTCCGACCGCCACCGGCGTCGGCCTCGGCTACGGCATCGCCATCGCTGTCGGC

ATCCTGGTGTTGGTGTCCACCATCATGCTCGCTTCCTATGTCTGCGTCCGCGTGAAGGGC

CGCCACCGCGGCGATCATCTCCCCACCACAGCTACAACCTTAGCAGCACCGTCTTTCTCA

GTTGGCCTCGATGGCCCGACCATCGACTCCTTCCCTAAGTTCATCTACGGTGCAGCGGCT

GCACCACCGAAGCCGGTTACTGGTTTATGTCCAATTTGTTTGTCTGAGTACCAAGATGGG

GACGTGATTAGGGTGGTGCCGGATTGCGAGCACAGCTTCCATGTGGCTTGTGTTGACGAG

TGGCTGCGGGTGAATCCGACGTGTCCGCTGTGTAGGAACTCGCCGGTGCCGTCGGCGGCG

GCGACTCCGGTGGCGACGCCACTGTCGGAGCTCATCCCGCTGGCTATTCATGCCAGGTGA

AGGTTGGTGCCGAGTTTGTGATCCGTCTAGACTTTTTGTTAGGTTTTGTGTCTTGTTGTT

CGGTTTTGCTCAGCCGAGTTCGTGTATTGTGCATGTTTTACTATACAATCTTAGACACTG

TTAGACCAGTCTATGTATCGTCCCAAACAGCTGGAGCCCCCCGTCCGCATTCAACTCTCC

GGGCCACAGCTCCTGAGATGAT

>Unigene0001245

TGCCGCTGCTCTCTCAATCCCCCCCACCCCACCTCTTCTCCACTCGGCATCGCTCTCCCT

GCCTCATCCCCTTCCCGATCCTACCCCGCAAAACCCTAGCCACTCGCCCTCTCCTCCTCC

GCCGCCGCGAGCCGCCCCGCGCATTCCCCCTCAAAGACGAAGATCTCTCCTCCCTCACCT

CCTCCTCCGCCCCCTCCGCCAATGGCCCCACTCCCCACCTCCCCGCCGCCCGCTCCTACG

AGAGCGACCTCGCCTCCCTCACCATTGTCGGAGCCGCCTCCGCCGATCAAGCCCTCACAG

CCGCCGCCGCCGACGGCGGCGCCCTAGCCGACGAGCACCTCGCCAACGGCTCCCCCACCA

TGGTCGTCGAGACCGTTTTCCCCGGCGCCCCCGACGAGCACAGCACCGTCTCCACTCGCC

TGTTTTTACCGACTGATAAGGTGAAGGACAAGGCCAGGAAGCTGCGGAGGAGCATACTAT

CTAGCAGAGGTTCCATGCCCGAGAATATACTTGCCATGACATTCCGCCAAGTCGTAATTC

ATCATCTCTGGAGCTTTAAATTGGTGTTTTACTCCGCTGGGGCGGAGAGGGATATGACGG

ATCTCGCCAGCCCGAGAACGACCCTGGCGGAGTTTACTGTTAGCTCGTCGGACCGGGATC

TCCTTTCTGCTCTTGCTGAAGTTGTCTTCTCTTGCGCTCTTGAGAGCACTGCAAGGGAGC

ATGGTGGTGGTTGGTTTCAGAAGCAGAGGAGGATTACTTCGAGAGATTCCGCTGTTTGTT

TGGGTAGAATATCGGAAGGCGAGATAGTGGAGAGTGCGAGGAGGCAAGCTGGGAAGTTTG

ATGCGGTGAAGGGGGAGATGGTGAATAGGAAGAGGAGAGGGAGGCATCGGTGGTGGGTGG

TGCCAAATTATACGAGGCTGGATAGAGCTGGGGGAGCTGGATTCAGTGATTGGACAAATG

AGTTTGTACCTGCATACAGGCTGCAGATTGACAGCGGTGCATATAAGGATGTGGAATTTG

AAGGTTGGAAGAAATTGGATGGTAGTAGGTGGGAAGTCCTCCTAACCCACTTCCAAATGG

TTGAGTTGGCGAACATTCTTGACATGTATTACGAAGACCTGCACACAATTCCTGACAAAC

AACTTTCATGTGGCATGCTTACAGAAACTTCAAATATTTCGACCAATAAGTTTTCTTCTT

TGAAAGTGTTATTTGTCACAATTGCCGGTGGATGCATTCTTATTTCTGTCAGCATTCTTG

CTCAAGTTTGTTGGCCCCATCTCCTCAGGGCTGGAAGAGCTTCAGGACAGAGTAGTCTTG

TTTCATCATCTGGAGTTGATTGCTGCCTTCTCAAGTCTACTGAAGACACCAAGCTTGAAG

CTTTGTCTGTCTCGGTTATTCAGACGATCAAAGATGATCTCAATTGGTCTGGAAACGTAA

TGTTTGATAAGAATATTGGTGCTTGGACTGGTATCCTTCCTAGTTACTTGAAAGATGAGG

ATTCCGTTTCTGAGAATGCTACTAGCAACATAAAACCTGCAACAAATTTCAACGGCGAAG

ATCTACAAAGGCATGAGGAATTATATGTTGAAAGAGCTCATTTGGGAAAAGATAATAGGG

ATTTGCCGACAGATGCACAGAATATTGCAAGTTATCAGGTGGTTTTGTCAGGTGATGGCA

AGATAATTGGGTTTCAACCAACAAGTCGATTAGCTGTCAACCATTGGGCTTCTAATCCCC

TAGCAAGGGTTTTGTACGCAGGGAAAAAGCTCTCTCCGGGTTTTTGGGAGCCGAGCCTCA

AGATTCCGCGACCAAGTGAAGTAGTTATACTAGAACTATTGATATCATTGGATCCTGAAT

CATATTTCGCATTGGCTCGAGTTGAAAAGTAGTCACTAACATCCACCTCTAGTCTCCTAT

GCGGCTCAACACTGTACATAGGAATTAGTATTTCTTGGTGTGTTTTATGTGTAGTTTTGT

CTTTGAAATTTTCAATTGATATTTTAGTTGGCAGCTCATTAAGTGTGGTCTACATGGAAA

TTTATTGGGGTCTATTATTATGTTGTGTATCTCCGGTCTCAGATGTGATGTGTAGGATAA

CTTTAGGTTCAAGTCACATTTTTGAAGTGTTTATATTTAATTCACCTGTTTGATGTAAAG

CCA

>Unigene0001246

CCAGTGCCCGTCAGCGTCCCAGTCGACCGCCCATACCCGGTGCACGTACCCGTCGAGAAA

CACGTCCCGTACCCAGTTGAGAAGCCAGTGCCTTACCCAGTCAAGGTGCCAGTCGACCGC

CCATACCCGGTAACAGTCGAGAAGCACATACCGTATCCAGTGGATAAGCCCGTCCCGTAC

CCGGTCGTCAAGCACGTCCCTTACCCGGTTAAAGTTCCTGTGAAAGTCGAAGTCCCCGTA

CCGTACCCGGTCAAGAGTCACGATGACCATTACGAACATCACGACGACCACCACAGCAGC

TATTAACTGGACGGATACAGAATTGATAAGCATTTATCGCATCAAAACGGTCTTATGAAA

CCGGAAAATGGTTACCACTAATTTGTTAAGTACTTTTTTATTTTTTATTTTTTACTTTGT

AAATATTAAAATATTTTTATAATATAAATATACTTCCTATGTTATTTATCTTTAAACAAA

ATTAATTTTAATTATATAACGGAATTAAAAAAAAAAATCTCTCAACTTTGACTGTTACCG

ACTAAGATCTTTTTTTTATGACCTTAAATTAACTTTTATTTTTGTATATATTAAACTTTT

ACCCCCAACCCCCTGTCAACCTGTACCATAATCAGCTATATTCTCCTCTTTATATAGCGA

CGGGAAAAAAAAGCTATACAAAGGAAACTGTCGAGGGACGACGACAATATTATATGAAAT

GATGTATAATGCAGTATGAATAAAGTTTATTTTCTTTGAAAAAAAAGCTATACAAAGGAA

>Unigene0001247

ATTGCTGTTGTCGTCGTCGTCGTCGTCGTCGTCGTTATACGTATCACTTGCACACCACCA

CAACCATGAGATCGACTTTGTTCGCGGTGTGCGCTATTGCGCTCTGCGCTCACTGTCTGG

CCGCAGACGACAAGTCGGCCAAGAAGGAAGCCGTCGACGGCAAGACCCAAGGCAAGAGGA

GCTTGTTCGACACCGGCTACGGTTTCGGCGGAGTGTCCGCTGACAGTTACGGACACGCCG

ACATCCTGTCCGCCGGTTACGGACACGGACTCGTCTCGGCCAGCGGTTACGGCGCCGGTT

ACGCCGACTTCGCCGGTCACGACGCGTACGCCGCACACTCGTACCAACATCACGTGCCAG

TCAAGACCATCACCAAGAGCGTCGGTCTCCCATACCCCGTCGAGGTGGCCAAGCCATACC

CATACCCAGTCAAGGTTGCCGTACCCGTGGACCGCCCTTACGCCGTAGACGTACCCCAGC

CGTACGCCGTACACGTCGCCAAACCATACCCAGTCAAGGTTCCAGTACCTCACCCATACC

CAGTCGTCAAGGAAGTGCCAGTGCCCGTCAAAGTACCAGTCGACCGTCCATACCCCGTGC

ACGTTGACAAGCCATACCCAGTGTACGTCGAGAAGCAAGTGCCATACCCGGTAGAGAAGC

ACGTCCCAGTGCCCGTCAGCGTCCCAGTCGACCGCCCATACCCGGTGCACGTACCCGTCG

AGAAGATCGTACCGTACCCAGTCGAGAAGGCCGTCCACTACCCGGTCAAGGTACCCGTCG

ACCGCCCATACCCGGTGCCAGTCGAGAAGCACATCCCATACCCAGTTGAGAGGGCCGTGC

CATACCCCGTCGAGAAGCAAATCCCGTACCCAGTCAAAGTGCCCTACAAAGTGCCCGTCG

CTGTTCCATACCCGGTCAAGGCGTACCAACCAGACCACTACGAACACCACGACTACCACC

ACAGCAGCTCCGGATACTAATCTGACTCCTTCGTAAGCAGCTAGTCACCATCACCATCGC

CCTTCCCCCCACACATTACTACCCCTCGCCACCCCCCAAAGTTACCAGATATTAACTCGC

GCCCAATTTATATGTATAATAATAAGATAATGCCATCATCCTTCTTTTTTTTTTCGTTGA

GCCAAATCAAATTCGCCAGATCGAATAGGTCTGTAACGAGCTAACGCTATAATCAAAAAA

AAAAATCAAGTCGCGAATCGTATCGATAATAATATTATTATTATATCATCATAGTGTTCG

TGTTCAATATCTAATACCTACAATTCTTAGGGAGAAAAACGAAAAAACCAAAAAAAGATC

TCGATAGTTTTCACCTCACAGTTGTATCAACTCTGTTGGGCCGTTGCAATATTATATTTT

AATACGATTGATAGTTTAATTCGCACGCGACGTGAATGTAATAATATAACATTATATGAA

CGGACGTAATATCAGCGCGTGTTATTCGTTTAAGCTCAATATTATAATATGTATACATTA

TTATTTTTTTTTTTTTTTAATTACATTTTTTATTAATATACGTTATTTTTCTATGTTTAA

CAATATGTAAAAATACAATACATTATATATAGAAATTGTGACGAATTTAATAAACCA

>Unigene0001248

CCACCGGATATCACCCGCGGCCGCCGGCGATCCTGCCGGATATCACCGGCGGCCGCCGGC

GGGAGATCGCGGCGGCGGCGGCCGCCTTGCTGAGCTCACCCACCGTCATAACAGGTGATA

GCCACCCCTCGCATATTTGAAATGCCTTCGCCTAATTCAAGTCTTGTATTTGAAGATCCA

ATATTAAATTATTTTAAGGTTGAGCCAGATAATCAGTGGGTTAGAACAACAGATTTTACT

CCTAATT

>Unigene0001249

GTGGGAGATCGCCGGCGGCCGCCGGTGGGAGATCGCCGGCGGCCGCCGGTGGGAGATCGC

CGGCGGCCGCCTTGCTGAGCTCATCCACTGTCATAACAGACCTCACCCTAACAAGTGTTC

CTGGAGTGACTTGGATGGAGACTTGTACTTTTTTAGTTGGGATTCTGCACTCATTACTCC

TCGCCAAGTCCCGTCTATGGAATATACCGCGGCATCAGCCGAGACTATGATGCTACTATT

GAGG

>Unigene0001250

TCTGGGGAGATCCCCCCCCCCTTCCTCGAATATATAAATACCTCATTATTCAGTTTTCTT

CCTGTTAATCATACAATTTGTCTTGATAAGTAAAAACACCAAGGAGTAAGAAGATATGCA

CCCAAGTCTCAATGTTGAAGAATAATTCATGTCACACACAATTGCTTCTGTGTTTGCTGT

CCCGCATTTATATACAACTGAGTGTTTGTGGTTTCCAACTTTAGGTGATAGCCGTGCCTC

GCATATTTCAAATGCCTATGCACAATTCAAATCTTGTATTTGAAGATCCAAAATTTAATT

ATTTTAAGGTTGAGCCAGATGATCAGTGGGTTAGAACAACGGATTTTACCTCTAATCGTA

GCATTGGGCAATCGTCTGCAATTTGTTTGATGGTACTGGACAGCTGCAATCTTCAAACAT

CCGTTAGTACTTTGTTTACTATAAGAACGATAATGGACCTTTCTAGTTGTAGTTTTGCTC

TTCTTACTCTCGCAGTTTGGATTTTGTCCCCATTGTCGGACCCAGACCTGGGATTGATGT

GCCTTACAAAATACTCTTCAAGATAAATCATATGGTCCAAAATGGGACTCTCATGGGACA

AACACTTGATGATGAAT

>Unigene0001251

GTGGGAGATCGCCGGCGGCCGCCGGTGGGAGATCGCCGGCGGCCGCCGGTGGGAGATCGC

CGGCGGCCGCCTTGCTGAGCTCATCTACTGTCATAACAGGTGATAGCCGCCCCTCACATA

TTTGAAATGCCTTCGCCTAATTCAAGTCTTGTATTTGAAGATCCAATATTAAATTATTTT

AAGGTTGAGCCAGATAATCAGTGGGTTAGAACAACAGATTTTACTCCTAATT

>Unigene0001252

CACATATTTGAAATGCCTTCGCCCAATTCAAGTCTTGTTTTTGAAAATCCAAAATCTAAT

TATTTTAGGGCTGAGCCAGGTGATCAGTGGGTTAGAACAATGTATTTTATTCCGAATTGT

AGCATTGGAAAATCGTCTGCAATTTGTTTGAAGGTACTAGACAGCTGCAATCTTCCAAAC

ATCTGTTAGTACTTCGTTTACTATAAGAACGATAATGGACCTTTCTATTTGGAGTCTGGC

TCTTCGTACTCTTGCAGTTTGGATCTTGACCCCATTATTGGACCCAGACCAGGGATGAAT

G

>Unigene0001253

CCACCGGATATCACCCGCGGCCGCCGGCGATCCTGCCGGATATCACCGGCGGCCGCCGGC

GGGAGATCGCGGCGGCGGCGGCCGCCTTGCTGAGCTCACCCACCGTCATAACAGACCTCA

CCCTAACGAGTGTTCTTGGAGTGACTTGGATGGAGACTTGTACTTTTTTAGTTGGGATTT

TGCACTCATTACTCCTCGCCTAGTCCCGTCTATGGAATATACCGCGGCATCAGCCGAGAC

TATGATGCTACTATTGAGG

>Unigene0001254

CCCAAAGCAGAAACCCTAGCCGCCTAAACCCCCGCACATCCCTCACAAAAACCCTAATCC

CCAAGCCCCAAACCCTAGCCGAGATCTAGCCATGGGCAGCGACAGCGAGACTGAGAAATC

AACTAAGGACAAGAAGAAGATCGCCGCCCTCGCCCCCATCGCCAAGCCCCTCGCCGGCAA

GAAGCTCTGCAAGCGCACCCTCAAGCTCGTCAGAAGAGCTGCTGAGGGCAAGTGCATGAA

ACGTGGAGTGAAGGAGGTGGTCAAGAGCATCCGGCGCCAGCAAAAGGGATTGTGCGTGAT

AGCTGGCAATATATCTCCGATCGATTGTATCACACATATCCCCATCTTATGTGAGGAGTC

AGATATTCCGTACATCTACGTCCCCTCAAAAGAGGATTTGGGAGCTGCTGGGGCGACGAA

GAGGCCTACTAGCTGTGTGCTGATAATGACAAAGCCGGCCAAAGGGGAATTAGACCCAGA

GGTGAAGGAGAAATTGCAGGCTGACTACGATCAGGTCGTAACAGAAGTGAAGCAACTGGC

ATCAACTCTTTTCTGAAGACCTAGATGGGGCCTGTTAGTTTCACAAGGTCATGTTCAGGA

TTTATTTGCCTTATATGTCTTATATTTTAGCAGGAATTGAGGCCTTCATTGGTTATTCTG

TACTGCACGACATTGAGATATATTCGTAATGATAGTACTAGCCATCAGGCATGGGACGCA

TGATCAATTTCTACTTAAATTTTCATCATTTGAGTTGATTTTTTTGTTTTTGATTTTGTT

TTGTCTATGGTTTTGTGATTAAGTCACCATAGGCTGCTACATACAGGCCCCCTTTTAT

>Unigene0001255

TCCGCCACCGTCTAGGGCGGCGCCGGTAGCAGTGTTCCACAGCTGGTGGAAGATGGTTGC

CCCGTTGATAAACCCTAATCCTGTAGTGTACAAGCGAAGAGAGGGTCAGGCCCGGAAGCC

ATCGGCGGTCACAGATGAGTACGCCGTGGAGCTCATCGATCAACAAGAAATTTTTGATCA

TATTAGAGATATAAGGGACCCAGAACACCCACATTCACTGGAAGAATTAAACGTGGTCAC

CGAAGATTCGATTGAAGTCAATGACAAACAAAGTTATGTTAGGGTTACATTTACGCCTAC

AATGAAGCATTGTGATTTTTCAACTCTTATAGGACTTTGTTTACGTGTAAAGCTTTTGCG

CAGTCTTCCTTCTCGATTTAAGGTGGACATCAGGGTTGCTCCTGGAAGTCATGTGTCTGA

ATCTGCAGTGAATAAGCAGTTGAACGATAAAGAGCGAATATCAGCTGCTTTGGAAAGACC

CTACCTTGCACAAACGATCGAGGGATGCTTAGCACCGACATTTGTTTGATGCATCACAAG

GGAGGAGAAGACTTGGATCAACTTGTGGGCTAGCTCGAGTATATTGATAATTGGTTTGCC

GTCCTTGATATTTAGGATTTTATGTTATTGCATTAAATGCTAACCTGTTCGAAATCAAAG

ATATTTCCCTATATGTTTCCATGTTAAAGCTATATGACGTATTACTGCATCGTATATTCT

TATTTATCAAAGTGC

>Unigene0001256

TGAAACAATTTCCCTATCACATGTTTGAACAAGCTCTCAAATCTCTCTTTCACATCAAAT

CAAATCTCTCTCAAATCTCATTCCAACTCTCTCAAATCTCATTCTCATCAAATGGGCTGC

ACCAAGTAGGTTGCCTACGTACCTCTGTCAGAAGGATCAAGCCTGCCGTAGTTCATCTCC

TTCGGCTGCACCACTCTGACCGTGGTTGCCTACGTACCCTGTGCCCGGAATCAAGCTGTC

GTAGTTTGTCCTTCGGCTGCACCACTCTGAC

>Unigene0001257

TGGGTTGTTGCTCTCGCCACTCTCAGCATCATTTCAGCAGCTATCTTTTCATGTGCTGAT

GGAGCTTCCAAGGGCGAAGGCACGAAGGAGCCGCATATCTACGATGGTGGTGGTAGTTTT

TCAGCAGTGTGGGGCAGCTTGCGGCGCATAAGGCAGTTGGTTGAAATCTAAGTCTGTTGG

TGAGGGGGATGAGGTCCTTTCTTGTGACCTAAATTTCATCAGCGAGGTCATTTGTATTTT

CCACTACGATATCTCAATGTTAATTATTTGAAAAATATGTTGCTGTTGTGATTCGCTAAC

CTTCTATAGAGTTTTCAACCA

>Unigene0001258

TTCTGAGTGACGCAGGAGCAGGCGGTGGTGGTGTTCTGCTGTTATGGGTTGTTGCTCTCG

CCACTCTCAGCATCATTTCAGCAGCTATCTTTTCGTGTGCTGATGGAGCTTCCAAAATCA

AAGGGTCGACGGAGCCGCATATCTACGATGCTGGTAGTAGTTTTTCAGCAGAGTGTGGAG

CGGCTTGCGGCGCATAAGGCAGTAGGGTGAAATCTAAGTCTGTTGGTGAGGGGGATGAGG

TCCTTTCTTGTGACCTAAATTTCATCAGCGAGGTCATTTGTATTTTCCACTACGATATCT

CAATGTTAATTATTTGAAAAATATGTTGCTGTTGTGATTCGCTAACCTTCTATAGAGTTT

TCAACCA

>Unigene0001259

GTTCTGAGTGAGGCAGGAGGAGGTGGTGGTGGTGTTCTGCTGTTATGGGTTGTTGCTCTC

GCCACTATTAGCATCATTTCAGCAGCTATCTTTTCGTGTGCTGATGGAGCTTCCAAGGGC

ACAGGGTCGACGGAGCGGCATATCTACGAGGGCGGTAGTAGTTTTTCAGCAGAGTGTGGA

GCGGCTTGCGGCGCATAAAGCAGTAGGGAGACATCTAAGTCTGTTGGTGAGGGAGATGAG

GTCCTTTCTTGTGGCCTAAATTTCATCACCGAGGCCATTTGTATCTCCTGTTCCTATATC

TCAATGTTATTTCTCTGAAAATTCTGTTTCTCTTGCGATTCGCTAACCTTCTGTAGAGTT

TTCAACAG

>Unigene0001260

TGGGTTGTTGCTCTCGCCACTCTCAGCATCATTTCAGCAGCTATCTTTTCGTGTGCTGAT

GGAGCTTCCAAGGGCAAAGGGTCGACGGAGCCGCATATCTACGAGGGCGGTAGTAGTTTT

TCAGCAGAGTGTGGAGCGGCTTGCGGCGCATAAGGCAGTAGGGAGACATCTAAGTCTGTT

GGTGAGGGAGATGAGGTCCTTTCTTGTGGCCTAAATTTCATCACCGAGACCATTTGTATT

TCCTGTTACTATATCTCA

>Unigene0001261

AGGGTGCTGAGCGAGGCAGGAGGAGGTGATGGCAGTGTTCTGCTGTTATGGGTTGTTGCT

CTTGCCAATCTCAGCATCATCTTGGCAGCTATCTTTTTGTGTGCTGGTGGAGTTTCCAAG

GGCAAAGGCTCGACGGAGCCGCATATCTACAATGGTGGTGGTAGTTTTTCGGCAGAGTGT

GGGGCAACTTGCGGTGCATAAGGCAGTTGGTTGAAATTTAAGTCTGTTGGTGAGGGAGAT

AAAGTCCGTTCTTTTGACCTAAATTTCATAAGCCAGATGATTTGTATTTCCAACTATGAG

AATTCAATGTTAAAATGGTTTTTGTAGCTAACCTTTTGTAGAGTTTTCAATAATCATTAA

AAACTGCAGCACACTAGAGA

>Unigene0001262

CACCCGATGGTTCAGGCGATGACAATGAGAACACCGAGGAGCATTTCCCATATGATAAGT

AGGAGGCGTAGGAGTTGCCAGAAGAGGTAGCTCGGTGGTGGCTGGAGGAGCAACGTCATA

GTTGGTTCGTGCAGTGATGCTTTGCTGGAGTGTTTCCTAATGACAGACGGCCACAATCGC

CTCCGAGAAGGATGAGAACTCCCTCTGGCTAAAGAGTTAAGTAGCAAGG

>Unigene0001263

TGCAATAGAAAGTTTCATCCACAGTGTGAACATGAGGGAAACAAGTACAAGTGTTCACAA

TAGTTAGCAGTGAAAGTCATGTGAAAAGATGCAACAAAGTCAAATATCTAGGGAATGAGT

GTATCAGAAGGTGGAGCCGAGTCATTCGGAGTATTAGTGATGGAGCCGAGTCTAGAGAAG

CTAGAGGATTGAAGTGTATCAATCGTTGGAGATGGAGCATGAGTCGTTGCATGTGGAATA

ATTATAATGAGTATATGGGCAACAATAGTGAAGAAGATATTTCCGTCCGGCCGAGAGTGG

CGCCCACTTCGACCCCCTCGGCTAGCAT

>Unigene0001264

TTTGGGCAACAAGAGCAAATATCTCTTCATAGTCGATCCCGTGTTGTTGAGTAAATCCAT

GTGCGATAAGGCGAGCCTTCCATTGCTCCAACTAGTCGTCAGGTCAATACTTGACTTTAT

AGACCCATTTGTTGTCGACATGCGTCACACCTGGAGAATGGAGAACATCTCAGGTATGCG

TATGTGAGAATGCTCGAAGCTCCTCTGTCATGTCCTGATGCCACTAACACCCTGAACCTC

TGTCAAGAACCAGTGCCAACAGTTCTACCAGAGCATGGATAATACCTAGCCTAAGAAGTA

GAAGAACAATGTAACCATTAGTCCCAACAAAGGGCGTTGACAGAGGTCAAGTTCAGATAA

GAGTGTGGTGTAATAAGCACAGTAGAAAAGATCAACCGTTCAGTCTGATCGGTGGCACTA

GTAATTGTTCCACACTGAGTGATGTGCAGATATGATCCATTCGTAGTGTGGAGATGAGAT

AATGGAGTAGAAGTATCTACGGTGTTTAGCAAAGAAGCACCACGGGGCAAATGATAAGCG

TCATTGGAATAAAAAATCCAAGGAACATGTCTACTAGAAGTTGAAGCAGAACCATGCCGA

GTACCGGTGATAAAGCGTAGAGAGCTGAAATTGAGTCCATAGAAGCTGGAGGGCTGAAGT

ATAGTATTCGCTGGAGATGTAGCATAAGATGC

>Unigene0001265

CTGCGTGATATGCAGATGAGACCCATCCGCAGAATGAAGACGAGGTAACGATATATAAGT

ATCTATAGTGCGAAGTAAAGAAGCATCACATGTCATATGATAAGATGCATCAAAATAAAA

AATTCAAGGAGCTTTTGTACCAGAGGGGAATGAAAAAGGACACGGAAATGCGGGGCCAGG

GGAAATGTCATTGGTGATGTAACCTGGAGGCATGACGAGTTCCCGATATTGACGTTGAAA

CTCAGTAATTTGAAGTTGTATTGTGAGATCCGGAGATGAGGTGGAGGTAGTTGAGGGTGG

TGTAGCAGCTGTTGTGGTTGATGTAGTTGTATAACTTTGTATATGAGAGTGACGTCCGTG

CCCGCGTCCCCCATCGCGGCCTTGGTCTCGCCAAGTTGAAGCTCATGGTGGAGGATCCGG

TAAGTCTCCACCCGATGGTTCAGGCGAT

>Unigene0001266

TGAGGTAATAGAGTAGAAGTATTTAAAGTGCTAAGTAAAGAAGCATCACATGTCATATGA

TAAGATGCACCGAAATAAAAAAATCCAAGGAGCTTTTGTAACAGAGAGGAATGAAGAGGG

AGACAGGAACGTGGGGCCGGGGGAAATGTCACTGGTGATGTAGCCTGGAGTCATGGTGAG

TTCTCGATATTGACGCTGAAGCTCAGCAATCTGAAGCTGCATTGTGAGATCTGGAGACGA

GGCGGAAGTAGTTGAGGTGGTGCAGCAGCTGTTGTGGCCGATGTAGTCGTATGGCCTTGT

ATGTGAGAGCGACGTCCGTGCCTGCGTCCCCTATTGCGGTCTCGTCCTCGCCCAGGTTGA

AGCTTAGGGTGGAGGATCCAGCAAGCCTCCACCCGATGGTTCAGGCGATGACAGTGAGAA

CATCGAGC

>Unigene0001267

CTACTAGAATATCATTTCCTTTGTGATTTAATTTTAATATAATAATATTTAATGTGTATA

TAATATTACAGTTTTATGAATAACACTTTTGGTTCAAAGAGTCGGGTCATACGAATGAGG

TTGTGCGATCGTATTTAGCACCTCCCTGATAAGAGGTGCGTTGTCACGCACCAAGATTTT

GACCCTACCCAACACTTAAAAGACTTTTTCATCCATGAAGTCAATTTTGATTAAGAACCT

TTAAGAAATATTTTAGAGTATCTTCTAGATGGTCCTTGTTGGATATTATGTTATCGAGGG

TTTACTTTTATTC

>Unigene0001268

TCGAGGGTTTACTTTTATTCTAAGCATTTTTAGGTTAGAGAATATTTTAGGTATTTTCAA

TAAATAAATGTAATTAATATATATAAGTAGGTGAAGAACTCTAAAGTGTCCCTAATCATT

GGAATGAGGACAAGTTCCATCATTATAAGGAGGGGAGCTCATCCACTCTAACCATTCAAG

AGCGGATTAATCCAAATATTTCTGAGCAAAGTTCTCCTTCTTTGTATGTCTAAATTATAA

TATATATTTTTTGTGTGGTCTCTAGATTTCCGTAAACGGATTTTTACATTCTTTTTGCCG

TCAAAGGCAGCCTTTCTATACAGTCACTTCCGTGACAGATAACTTTGTTGGGTCCCTTGT

CTTGGTCGTTAGGCCTGACATGAATTTGCTTCTCCTTTCAGCTTACTTGTGGTTGAAACA

GCTGATCGCCCAGGATTACTGGTTGACCTTGTCAAGAAT

>Unigene0001269

TTTCAATAAATAAATGTAATTAATATATATAAGTAGGTGAAGAACTCTAAAGTGTCCCTA

ATCATTGGAATGAGGACAAGTTCCATCATTATAAGGAGGGGAGCTCATCCACTCTAACCA

TTCAAGAGCGGATTAATCCAAATATTCCTTAGCAAAGTTCTCCTTTTTTGTATGTCTAAA

TTATAATATATATTTTTTGTGTGGTCTCTAAATATCCATGAAAGGATTTTTACATTCTTT

TTGCCTTCAAAGGCAGCCTTCCCATACAGTCACTTCCGTGATAGATGACTTTGTTGGGTC

CCTTGTCTTGGTCGTTAGGCCTGACATGAATTTGCTTCTCCTTTCAGCTTACTTGTGGTT

GAAACAGCTGATCGCCCAGGATTACTGGTTGACCTTGTCAAGAAT

>Unigene0001270

GAGTTGAAACTAAGTCCAGAGAAGCTGGAAGGCCGAAGATGTAGCATAAGATGCGGCATG

TAGTGGAGTCGTAGTAACCGATCGAGTAGGAATGATGGAGATAGATGTGTGAGTAATTTG

TTTTAATACATATATAGTACTTGTTATCATACCTGCTGTAGGACCTGTTCCTGCTACATG

ATTTTACATTACTACGACAATATACATTACTACGACAACAATATAGTTGACTGCCACAAT

ATAGTTGACTAAAAAAAAGAGGAAGGGAGGGGGAGGGGAGCTCGTTCATGGGAAAGATGA

ACTGCGTGGGGAGCTCGTGCACAGAGAGGATGAACCACACGTTATTTATGCTTGATTTGG

TCCAAAAACAAGAACTCACAAGTTGTTGCCATGTGACACTTTCGGTCTAAAAACTGGAAC

TCATAGCTTACTGCCATGTGGGACTAGACAAAAACAAACAACACTTCCAACAACCTCAAA

ATCCTGAGGTCCTTCCGTCTTTCAGAAAACACTGTATGTATGCAAGGAAAAACACCTTGA

ATTTGTGCGCAGAATACTGTATTTCGTCAAAATTCTTGAACCGAAAATAAATCTAAAAAT

CTATCTCGCCTATCAAATCTTCACCCATGTTTGGCATGCCACATGGTAGGCATCTAGAGC

TCCCAGATTTTGGGCCTCGTCCATCATGAACATTGAATTTTTTGATTGCTTATGCATCAA

ACCCTCCAAATTACTTGGTGTGGATTTAAGGTGAGCTCAAGGA

>Unigene0001271

TCTGAGATACCTCATGTTTGAAAGCTTCACAACTATGATACATACACAACTTCAGGCCCA

AATTAAGGCGTTAAGGTTAGACTGTGCTTGTGACTACCTGTCTGTTGTCATGACATGCAT

ACTTTTTTCCCTCTCAAGGTACTATTTTTCAACAATCTTGCCAGCACACTGATGAAGAAA

ATGGTATCGCTGAGCGTAAACATCTCCACATCCTTAACACTACTTGTGCCCTTCTACTTT

CTGCTTTCGGTTCTCCCACTTTTTGGGATGTGGCTGTTCTCACCGTTTTTCTCATCAACA

TCACCTCTATCGGTGTTCGTGATGCCACAACTCGACAATAGACATAGAAGAAACATGAGC

AGAAATCAGAGACACAAGAGCAATAACGGAAGGAACATACAAGTAATCCAAATGATATAG

AATCCTTGTTGTGATCCTGTACAACACAACAATGGACAAGAAAGAAACAGTCAATCCCAA

TATGCAGAGACATCCAACAAAAATCAA

>Unigene0001272

ACCTCCAATGGTTTGTTTGATCTCGTTCATTCTGACATCTGGTACCGCTCCTATTACTTC

TTTCTCTTGCTATCGTTACTATGTGAGCTTTATTGATGATTATTCTAGATATACTTGGGT

ATACCTTCTTAACCAGCGATTTGAGATACCTCATGTCTATGAAAACTTCACAACTATGAT

ACATACACAACTTCAGGTCCAGATTAAGGCGTTAAGGTCAGACTGTGCTTGTGACAACCC

GTCTGTTGACACGGTTCTCCCACTTTTTGGGGTGTGGCTGTTCTCACCGTTTTTC

>Unigene0001273

TGAAGCTCAACAATCTGAAGTTACATAGTAAGATTCGGAGATGAGGTGGAGGTAAGATCC

AGAGATGAGGTGGAGGCAGTTGAAGTTGGTGCAACAGTTGTTGGGGCCGTTGTAGTCGTA

GAGGCATGTATGTGAGAGCGGTGTCCACGTCTGCATCCCCTGTTGCGGCCTCATCCTCGC

CTAGGTCTGGAGACACTTTGAAGCTTAGGGTGGAGGAGCCAACAAGTCTCCACCCGATGG

TTCAGGTGATGATAGTGAGAACACCAAGGAGCAAATTCCGTAGGATAAGTAGGAGGCGCC

AGAGTCATTAGAAGATGTAGTTCGGTGGTGGCCGGGGGAGCAACGATGTATGGCAAACGA

TCGCAATCACCTCTGAGAGGGACGAGAACTCCCTCTGGCTGAAGAGTTGAGTGACAAGAG

CGTTATGCTAGGGCATGAAGGCGAAGAGTTGGTAGACTTCCTGATGTTCGCTCCATGCTG

CCCAATCGTCGTGAGTAGAACCAACACGAGGCTGCATTGATCGTAGCTTGTTCCAGCGCA

ACTGTAAGTGGCCAATGTGCTTGATGATGGGAGCTCCATCACGTCGAGTAGTCTACATTT

GGCGATAGAGGTGAGCGACCAGGGCTCCAGAGCGCTGCTTCAAGTGCTTTGAGAGGCGAG

TCCACATTACCTGAGCAGTGGAGAGCATGCCGATTTCTAGGACAATTCCCTCGCCTAAAC

ACCGGATCGACCATTCCATGACCCAGTAGTCTTCTGTGTGTCAGGTGGTGAGGGCAGCGA

GGGAGTTGGCTGTCAGGAGAGTAGTCGTCGTAGGAGTTGCCGCATCAGAGGAGTCGTCTA

CGACCGCAGTGGAGCTGGTAGTGTTGGGAGGGGTTGGGCGTGAGGTGCTCAAGAAGGTTG

TGAGCGCGGAGGGTGCTGTGCATTGTCAG

>Unigene0001274

AGAATGTTGACAGAGATTAAGTTTAGGCAATATCATGGTGCAACAAATACTGTAGATAAG

CCAACTGGCTAGTTGGATCGGTGGCTCTAGTAATTGTTCCATACTGTGTGATGTGCGGAT

GAGACTTATCTGCAGTGTAAAGACGAGGTAACAGAGTAGAAGTATCTATAATGCTGAGTA

AATAAGCATCACAAGCCATATGATAAGAGGCACCAGAATAAAAAACCCAAGAAGCGTGTG

TACTAGAGGGGAATGAAGAGGGAGA

>Unigene0001275

CGAGTCTCAGAAGAGAGCTCGCAGAAACAAACCCTGCAACTGCTTTCAAAGCTTTCGTGC

TCCTCGCTCGATTCATCGAAGGGAATGGAGAACGAAGACTCTGGCAGTCCGAGTTGGGGA

GCTTCTTTTCTGATGCAGACGACGGAGGATGTGGCGAGAGCGGTGGCTGCGGCGGCGGCG

TCGGCGGCGACTGTGCGGTCGCCGCGGCCGTCGGTGGTTTACTCGTCGAGCGATGATAAC

GGGAACAGCCCGATTCAGAGGTTGCAGAATCAGGTGAGTCGGGTGTTTAAGGGTTTATCG

CCGGCGCCGGAGGTGAAGAAGAGGGCGTACAATCCGGAGATTCTTACCAGTCAGAAGCGG

CAGTGGGCGAAGTTTCAGTTGCAGTCTTTGGATCAGAGGCCTGTAAGAGAAGATTCAAGA

CTTTTTGAGAGCATGGTAGTTGTTGGACTTCATCCCAACACTGATGTCCAAACACTTCTA

CAGCAGATACTGGAGAAAAGTGACGAAGATACCAAAAGGTCGAGGAACTTGCTGCATTTT

AATAACCAAGTTCATGCTGAAGTGAATCTCGAACCGCAGGTTTTATTTGTATATCCTCCT

GGGAAACAATTACCTCTCAAATACAAGGATCTGCTTTCTTTCTGCTTTCCTGGAGGACTG

GAGGTTAATGCTGTGGAAAGAACCCCTTCGTTAAGTGAGCTGAACGAAATACTTCTTGGA

CAGGAAAAGCTTAAGCAAAGTGACCTGTCTTTTGTATTTCGTTTACAGGTGGCTGATGAT

TCGACCTTATATGGATGCTGTGTTTTAGTTGAAGAAATAGTACAAAAGCCTTCAAATTTG

ATTTCAATGCTTTCGAAAGAGCAACCATTTGTTTCTTCTCTAAGCCGTTATATAGTAACA

ACACCTCGATGCTATTGCATCCTATCAAGGCTTCCATTTTTTGAGTTGCATTTTGGCTTA

TTGAGAAGCATTTTCACTGAAGAAAGGTTTGAGCGGCTAACAAAAGGCATTGACATGCTA

AATTTGCTTTCCCAAGAAGAAATTCAGGAAGGTTTTGGAAAAAAAGACATCCCGGAAGAC

ATTACGAAGGACAAATCTAATAGCACTATTCCAGTGCCGCTATTAGCTGAGAATCTAGTA

GGTCTGACAGAACAATCCAATGTGAATCCAGAAGCTTCTCCTCAGAAACACGATGGTAGT

TCCATAGAAAACCAACAAGTTGAAGTTAGTGAAGATCCCAATGCTGTCATAAATAATGGA

GAATGCAGAAATTCAATTCCTAATGGTAAACATGACCCTGTTGAAGATGCTGAAACAGAT

AAACAATCTGAAAGCCGTTTTCCAGATACTACCTTGCCACTTTTCTGGCATCATCAGTCT

GAAAGCTCTGAATCCTCTGCCAGCTCCCAGGATTCTCCACGTGAAGAAAGGAACTTTAGG

GAAGATATTGATGGAGAACAAGAAGAGCCTTCATCCTCTGGGCAGGAAGATTCCACCCGT

CACAATGACATTCTGAAATGGGCCAAGGCGAATAGTCATGGATCACTGCAACTAATTTGT

GAGTATTACCTATTACAATTACCCCCAAGAGGTTCAGTGCTTACATTTCAGCCTTTGGAG

CACCTACATCCCTTGGAATTTCATAGACCGGGTGAAACAGTTCTCCATATTGCTGGTTCC

ACCATTGATGTTAGATCTTGCAGTACAAGCTTAGAAGTGGCTGAGGCGCATACTGCACTT

TTAGCAGAGGAGGAAGCTACTGCTTTATCTGTTTGGGCAGTTGCATGCATATGTGGCAAC

CTGAGGCTTGAGAATGTGATGATGATGTTTGCTGGGGCTCTTCTGGAGAAACAGATTGTT

GTTGTCTGCTCAAATTTGGGACTCTTGTCTGCTTCAGTTCTGTCAATTATTCCCCTAATA

CGTCCGTACCAATGGCAGAGCCTACTAATGCCAGTCTTGCCGAATAACATGTCGGATTTT

TTGGATGCACCTGTCCCATACATTGTAGGCATAAAGAATAAGACTGCCGAGGTACAGTCG

AAGTTATCAAATGTGATTCTCATTGATGCAAACAAAAACCAGGTGAAGTCGGCTTCAATG

CCACAATTGCCTCAACATAAAGAGTTATTGTCCTCTTTAAGTCCTTATCATGCAACACTG

GTTGGTGAAAGTTATCTCGCCAGGAAAAGGCCAGTTTATGAATGCACCGATGTGCAGGTT

GATGCTGCTAAAGGTTTTCTTGCAGTTCTTAGATCCTATCTTGATTCTCTTTGCTCGAAT

TTACGATCTCATACTATCACAAATGTTCAATCCAACGATGACAAGGTCTCCTTGCTTTTG

AGAGAGAGTTTTATTGATTCTTTTCCTTACCGTGACCGGCCCTTTATGAAGCTTTTTGTG

GACACCCAATTGTTCTCTGTACAAACAGACCTAGTTCTCTCTTACTATCAAAAGGATTAG

AGCTGCCATTCTGGTGGTTAAGGTACATGCAAATTGGATCATGAAACTGATACATGTAAT

TATGTGTTTATGTTAGTCAATAGATTTATATTATATGGGAATCAGTTTCAATACTGCTTT

TTTTTTTTTTCATTTTCTTTGTTTTGGTCGTTCATATTGTAAATTAAGATACTGATATTT

GGACTGTATCCTACCCAGAAGTTGTAACTGTAGTTAATGGGTTTGTAGGAATTATTTTTA

TGCCAAGTTAAAAACTTCAAATATGTTGTACTTGAACATATGGAAATCATTTTTTTTTTT

AA

>Unigene0001276

CATTATCTTCCGAGAACCTATACCCTCACACACAGCGACATCACCTCCCAGATCACTCTC

GCCATCTCCCACACAGTCAACATAGCTCAGATAAAGGGCTGGTACAGCAGGCTTCAACGC

GATGAGGTGGTGGCAGAGTGGAAGAAGGTAAAGGGGGAGACATCCCTCCATGTCCACTGC

CATATAAGTGGCGGCCACTTCTTCCTCGACTTCATCGCCGGTCTCCGCTACTACATCTTT

GCCAAGGAGCTCCCAGTCGTGCTAAAAGCTTTCATGCACGGTGACTCATCACTCTTTCAG

CAGTACCCGGAGCTGGAGGAGGCCCCGGTGTGGGTCTACTTCCAATCCAACCTACCTGAG

TTCAACCGGGTTGAGCATTGGGGGCCGCTCTGCGACGCTACAGGTAGAGCTCACCCGGCG

GTGCAGCCAGAGAGATGTGATGGCCACTGTGATTGCTGTTTTCCATCCCCTAGCCTGAAC

CAGTGGCCACAAGGTGTGCGGGAGGAGCAGAATATAGAGGGGCAATTGGAGTAACGAGGC

AATAAAAAATTTGTACAGTAGTTCGATTTTTTTTTTTCTTTAGAATTGGGGAGGTCTACA

GATATCAGTAGGTGTTGGAAGCTAATTGTTTGTGTGATATAAAACGATTTCAAATTTA

>Unigene0001277

AGAAATGACAACTTTGTTGTTTTTCTTCCTGTGTTTTCTAATGTCTCGTACTCAGTGTTC

TGCAATGGTTGACAATGTTGAGTTTGGGATAAAACGACGGAATATGACACTTTTTGCAGT

GTATTTGCGAACATGTTCTTTTCTCACATGAATTTGTTTTTTTCCACACAGACGATTTTT

TTAACCACAAACATGGTGTATTCTGGTAGGTTAAGGACTTCACAA

>Unigene0001278

TTAAGGACTTCACAATTTGAGGTATATTTGCTGTTGCTTGTTGGCACGTAGTTGCTTGTT

GGTACGTGGATCTGGGTTGACTGTAGTGCCACATGGCAACATGCTGTAGGCTCTTACCTT

GTGCTGGAAAGAAATTTGTTTTTTTTTGTCCATAAAGTCGGTGTTTTCATAGACCTACGG

TTTTTACCAGACATGGTGTATTCTGGAACGCGAAGGACTTCGCAATTTGAGTTGTATTTA

CCGTTGTTATCATGTGGATTTAGCGGTTTGGTGGGTTGAGTGCGGTGCCACATGGGAACA

TGCCCTAGGTTATGGGTTTGGGGCTGAGTGATTGATTCAAGGTTGATTGGGTAGAAAGAA

AATGGGATGCACGGAAAGATCTGAT

>Unigene0001279

TTTTTTTTTGTCCATAGAGTCGGTGTTTTCATAGACCTACGGTTTTTACCAGACATGGTG

TGTTCTGGAACGCGAAGGACTTCGCAATTTGAGTTGTATTTACAGTTGCTATGATGTGGA

TTTAGCGGTTTGGTGGGTTGAGTGCAGTGCCACATGGCAACATGCCCTAGGTTATGGGTT

TGGGGCTGAGTGATTGATTCAAGGTTGATTGGGTAGAAAGAAAATGGGATGCACGGAAAG

ATCTGAT

>Unigene0001280

AAAGAAATAAAACCCCCATTTATCTAATTTTCAATCCAAGACCCTGCCTTAAAAGGGCAC

CATTTAAGATTTCTGCAAAATATGTTTGTGGTATCTCCATCATTTTTCCTTTAATAGGTA

AACCCTCATTTATCTACGGTATTCGTTACTTGTCCTATTTGATATTGTGATGGGGGATCA

CTGGATCAAATCCATAAACAGCCTCCTCCTGATGCAGGGGTAAGACTATATACATCCGAC

TCTCCCAGTATCCCGTAGTGGCGGGGGTGT

>Unigene0001281

AGACTTTTTTAGTGAACCTCGGGATTAAAACAGAATGGGTTTTACATTTTGACTGAATTA

GACTTTTTTAGTGAACCTCGGGCTTAAAACAGAATGGGTCCTGTATTATTCTGGCTGTTT

TGACAAACCGTGGTATAAACCTTCAATGTGTCCCTTATTCTAAACTCTTGTTTTTTGATG

TGCACACTAATATGTGTTTTTTCAGAGATAGTTCCTTGTCTTGGTGATCTTAAGCTCCAC

ATCTATTGATCCAACACCAGATCACCCCTACTGTAACGACGAGTCTCTACTGCCCAGCTT

GCAGTATTGGGAAGCATGAGCTTGTTGATGATATCAGGGAGGTTAAGAGGCATAGTGGAT

GATGAATCATTCACATGACTCTCATGTAGTATTGCTCTGAAGTCTTGAGCTAGATGGCTT

TTGCAACTAGGTCAA

>Unigene0001282

CACCTGCAAACCAGAAGTGATGGCTGATTACATCCATGGAGGGATACTTGAGAGCTGGTG

GAATTCGTCAAACATCAATAGCTTCGACGCCATGGATGAGTCCAATCCGCAGAACTCCGG

TTTCCTCTTATCGCCCACTTCCATCATGGACTGGAACCAAACTTTGCTGTGACCCTTCTT

GTTAATTCCTCATTTTATTTTTGGTTGATATTTAGTATTAAATTCTCTCATCTGATGAGG

CTCTTCTTATCTTAGAAGTTGTGGTAGGGATGAGACTAGCTTGCATCCCGTGCTTCAAGA

AGATCGAAGCTCGAAACCAGTTTTCCAGCACGAACCGTCAATGGATTCAAAACTCGCTGA

AACTCCTTTCAACAACTTCCAGTTGATTCCTGATTCTTATAGAAACCCTACAAATATGTT

TGAAGGGCTGTTTGAATCCAATATTGATTTTCATTGTTCAAATGGTAACAACACACCTGC

AAAATATGGACCTGTAGGCTGAGAAATCTCTCAGTTCTGCAATCAATGGCAACTCACATC

CAATGTTTCAGCGGCAACTTCTAGTGAAGTGAACACAATGTTTCATTCTTCGCCTTCCAC

CCAATTTGCAGCTCCGACAGTTTCACAACAAACCAATTGGAGTAACCCTATCAACAAGAC

AAATGCGGGTGGAGTTAGAAACTCAAGTTCTTCCTTCTCG

>Unigene0001283

TCCTTCTCGGTGAGAAAAGTCGACAGCAGTGAGCCTTTGTTGAAGAAACCCCGCATCGAC

ATGCCATCATCATTGCCGGCCTTTAAGGTTAGAAAAGAAAAGCTTGTTGACAGAGTCGCC

GCACTCCAGCAACTAGTTTCACCTTTCGGAAAGACTGACACCGCATCAGTCCTCCACGAG

ACAATTGATCATATTAAGCTCCTCCATGATCAAGTTACTGTTTTGAGCCACGGATATCCA

AAGACCGGGTATCCGATACACCATCAACGGAATCTTCACCAGTACAAGGAGGACAAGGAA

CCGAAAGAGGACCTGAGGAGCCGTGGGTTGTGTCTGGTTCCGATCACGAGCATATCCCCC

GTAACTAGCAACACTTCTTCAAATTTATGGACTTCCGCTTTTCTAGGAATCTACAGGTAG

TGAGGAGCTCACGGAGAAGTTTGC

>Unigene0001284

CCAATAATGACAACACACCTGCGAAATATGGACCTTTAAGCCAAGAAATCTCTCAGTTCT

GCAATCAATGGCAACTCACATCCAATGGTTCAGAGGCTACTGCTAGTGAAGTGAACACAA

TGTTTCGTTCTTCGCCTGCCACCCAATTTGCTGCTCCGACAGTTTCACAACAAACCAATT

GGAGTAACTCTATCAACAAGTCACATGCAAGCGGAGTTCGAAACTCAAGTTCTTCCTTCT

CG

>Unigene0001285

ACCTGAGGAGCCGTGGGTTGTGTTTGGTTCCGATCACGAGGGCATCCCCCATAACGAGCG

ACACTTCCTCAAATTTATGGACTTCAACTTTTCTAGGAATATAAAGGTAGAGAGGAGCTG

ATGGAGAAGTTTGCAAGGCTGGACAAGGATCAGAGGCTTCGATGACAAAAAATTCAGTGA

AATATTATAACATTTTGACGTTGGTAGGGTCAATAAGATAAATGAAGCTAATGACAAAAG

AATCAGAAAAAGTGAAAGTGGGAAACATTTTGGTTTAGTGTTGTACATATTTTGTATTCA

TGTTTTGATC

>Unigene0001286

GTGTTTCTTCCACTCAGCTCTATCAGTTCCCAACAAGAAATCCGAAGCTACACCAGCGGC

TCGGCTGTTCCTTCTCCAATGGCCAGTAGAGTATGCAACCCCTTCTTCCCACCCCCGTTC

GCACCCCTCTTCCGCCGGACAGCACTAAAAGAGCTGACAATTGGTTCCAAACTATCAAAA

CATTGCTTCAATAGGCTGTGAGGACTACCGCTGGTTCTCAGCACATGTCGTGTTTGTAGC

TTAAAGGACGGCTCCTGAAGATTGATGGCCTGATAGCAAATCCAGTGAGCTCTCATCATC

GTTTACAGTATCTAAGACGGGAGCGGCGAGCGGGGGCATCTCTTGTTGATCAAGTTTCAA

GCCAACCAGTTTCGAGAGTAGTTTGCAGAATGATATCTGCTACGCCTAGCTGCTCTTTCC

ACCTCTATGTCGACTCCCAGCGTGGTACTGATATGTCGGTCGCTGCGAGATGTCCATGTA

CTGATATATTGATCACCACGAGCGGTCTCTGTGTTGCAGTTACTAAGTTGTTAACTGCGA

TTGCTGATGTGCAAATGATGATATATGGTTATTGGATGGGGCCTGAGATTGAGGATGGCT

GGGGATATGTCGAGGCTCTTGTTTATGGGAGCTTTTGATTAAGCTAGACTATCAGTTTTG

TAATTTTTTTGAAACGTCTGTGGTGCAATTTTTCTGTCTGCTTACTGCCTAGACTGCTGT

TATCCCTGTGATGTAGTTTGGTAGATAGTTCACTGCAGGATTTTGGTAATGGTCCGTCCT

CGTTCAAGTAGGTTTTGGAATTGAGCAAAATGATTACTAAAATAAAATAAAATATTGGAG

TTCAGCAAAAAAATAAAGAAAAAAAAGAAA

>Unigene0001287

ACGAAGCTTCCCTCAGTTGGTAATCGGATTGGCCTCATAGAACGCTAAGAGCTCGAATAT

GCTTGTGTAACATAGTAACTAAAATAATATTTCTGACCCTAGTGTCATTCTGAATCTTTC

TTCTCCTTATGGTTGTAGAACTAATTTCGGAGCAAATATTTATGTATCCTTATTTTTCAT

AAATAATTTTGTGAGCTGCCCTCTCGCTAATTTCTTAATTGAAAAAAAAAAAAAAAAAAC

ATACACACACACACACACACTTGATATAACTGAAAATAA

>Unigene0001288

ACGAAGCTTCCCTCAGTTGGTAATCGGATTGGCCTCATAGAACGCTAAGAGCTCGAATAT

GCTTGTGTAACATAGTAACTAAAATAATTTTTTTGACCCTAGTGTCATTCTGAATCTTTC

TTCTCCTTATGGTTGTAGAACTACTTTCGGAGCAAATATTTATATATCCTTACTTTTCAT

AAATAATTTGTGAGCTGCCCTCTCACTAATTTGTTAATTCAAAAAAATAACCAAC

>Unigene0001289

GAGGTGACGCATGCATGAGGGCGCCGAGGATGATACCAAGCGCCACCGAGCTTCAGGAAG

CTGGAGTGCAATTGAAGGCCAAGGAGAAGGCCAGGTATTGTTAATATCTTATTTTTCGCT

TTTCCATTTTTACAAAGATTACGGATTTGTCCCAATTTGGATAGGACTTATAGAAAATAT

TTATTTGGTCAACAAATCAAGATGAACATCTTGTGAAATTTTTGAAACATAAATTTACTC

CCCCCAACCAATCTTGCCTCAGTTGGTAGCCTGATTGGCCCCAAGGACCCAAGAGATTGA

ATTCCAAAAAAAAAAACTAAAATAATATTTTTGACACTATTGTCATTCTGAGCATTTCTT

CTCCTTATGGTTGTAGA

>Unigene0001290

AAAAAAAAAAAAAAAAAAAAAAATCTGAACCTATCTGAGTCGAGTCTCGCCCGACCGAAT

CCCACCGCCCCCGCGCGAGGTCGGCAATGGCGAGAGCCCTCGCAGCACCTCCTCGCCGGC

GCGCCCCCGCTCTCCTTCCTCTGTTTCGTATCACTCTCCGCTACCTATGGTCGCCGTCCT

CCGATAACGATCCTGTCGCCGCCGCTGCCACCAATAGTTCCACCGCCTCAAACACGCGGC

CTCAGCAGAAGAAGCTCCATCGCCTTGCGTCCGTAATTGATGCTGTCAATGAGCGGAAGC

TCCCTCCTGAGCTTCGTGATCGTGCCAACGCCATCAGATCAGAAACTGATATAGTGAATG

TTGTGGAGCAAAGAATATGGCGCTCCATGGAAGAAGGGCATTTCGAGAACCTACCAGGAA

AGGGGAAACCCCTTGATCTAAGCACGAACCCTCATGCGGATCCTGCTGAAAATACCTTGT

ACAGAATCCTTTCGAAAAATGGTTGTGCTCCGGAGTGGGTTGAGCTGAATAAGGAGATCC

GGTCCAGGATCACAGAGTGGAGGTCAGCCCTTAAGAAAAATTGGGCAAACAGAACGGATC

ATGACAACTCCAAATGGTTAGAGGGCTCTGAGGCCCTTCAGGTACAAATGAACGAGATAA

ATAGGAAGGTACTGCGCTACAATCTTATCGTGCCCTTCGGCCGCCAGATGTTCGGGCTGA

AGTGGGAGAAAGAATTAGCCAAGTTGGAGGAGTGACTATCGAAACACTGCTTAACTTACC

TCTTGGAGAGGTGTTGTATTGTAGTTTGATAAAATTTGTTTTGTTCCATGTTTGGGTTGT

TAACACATGATTTCCATATATGGCTTGTGAAGAATTCTGGTGTAGATTCACTGTATAAAT

CCAATATCTATGCTCCACTGAATTTTCCACATTTTAGGAGAAAACCCTTAGCTGTTACTA

TTTTAACCATGACATACTTTGAGGTTTAATATGTTTCACCAGTTCAGT

>Unigene0001291

CAACAGGCAACATCAGAAGGTCTCCTCATGAGAGCTTTCACCTGCCTGGCTTGAGAGTTC

CTGATGCTTAATTCTTCCTTGGATGTCTGAGAGCCCTATTATTGAAACTCACCTTTTGAG

CAACAATATGTACTCTAAAATATTTTTATATGGTTGGGTTCAAATTTTAGTTTCAATCTG

AGTACAATGTTTAATTTTAGTTTCTTATTATCCTACAAAAAGTTGTTTTTTAAAAAGTGT

ACTCTGTTTATTTGGATGTGGTTTCGAGTCTACCATATGTTTACTCTTGTTTTTTATTAT

GAAATCATCTTGC

>Unigene0001292

TGTTAGTTTTTTTATTATTATTATCATTGTAGTCAGTATAATTTGAACTAATCCCAATTC

TCTAGCAATAATTATGATGATTTTACTGATTGTCTGTTGAATATAGTAGTATTTATATAC

CAATAGATGTTGGATTGCTGTATTTTATGAAGTTTTCAGTCATTTATCATTAATGATAAA

TAAGTGAGATACTATGTATAAAGTGAGGTACCATTAAGTATTGTCTTCCAAGTTCTCTTC

CTTTTAACTATAAAGTTAATTTTGTTGTTAGTTTCCTGGCTAAATGAGATGGTTGAGCTA

ACCTGATAGAGGTTTGAGTTCTGTTGAGAGGGTTCAATGATGACAGATTCATAGGTCTGT

TTCTCTAAACATTCGCAGTGACCGCCTGAGAGCTTTCGCCTTTCTGGCTTGAGAGCTCCT

GATGTTCAATTCTTCCTTGGATGTCTGAGAGCCCTTTTGTTGATACTTAAAAGTACTCAC

>Unigene0001293

GAAGAAGGAAACTACTGCAACCTCCACTGGCATGTGCGAGAAGCTCTTCAACGCCTTCAA

TCCAACTCTCCGACCCCTCCGGCGCCTCACCTTTCGCCGCCCAGACCCCGAACCAACCAG

CCAGACGACTACCTTGTCTCTACCAGATCATCCTTCATTGAATGACCCCGCAAAACAACT

ATGTTCTCCAACGATACCACAAGCAGCAGCACCTCCTGACAACAAACCATATCACACCAC

ACCACCATCGAAGGCTTCAGTGCAGCTCAATGCCACCAAGGCTGAGCCAGCTGTGCCGCC

GAAGCCAACTAGCACTACTACAACCATTCAGGAAATTAAGGTGAGTGTAATGCCTTCACC

AGCACCACCACATGCTCTGCAGAAGGAAACCGTTAAGCCGCAACCACCGACGGTGGCATT

GCCCAAGCCGCAACCTCCTCCGGTAGCTCCAGCGCCCAAGAAGACTATTAATGACAAGTT

TGAAGAATATATCAGCCGTGCAAGGGCTAAAATGAGAACTACTGATTCCAGTGTTGGAAG

GGCACCGACAGCCGTGCAAGGGCTAAAATAAGGACTACTGACTCTATTGTTGGAAGGGCA

CGGACAGCTTGATTGAAGAAGAAGATAAATAAAAGTGAGTAAGTATTATTCCTGTTTCGT

CCTAGAAATGGGAGGAATAAACATGTTTGATTATAGTATTAATTTGATGTATCTGATCTC

TTTGTAAGAATTATGAGGTGCTGAAATTAATTAGCCCATGTTAACCATTATTTATTGTAT

GAAATATAATTTATCTTTAGTATC

>Unigene0001294

GCGGCGGCGGCGGCGGCGGCAACGGCGGCGGCGATCTTGGCATCGATTCCTGCCACCATT

TCCCCCTCGATTCCATCCGAAAGGTAAATATTCGATGCCGAATAACACTCATTTCTTCTT

TTCAAATAATATTTCGTAGATTCTGAGAAAATTCCATTTTGCTTCCTCTCTTGTTTTCCT

CTGTTCTATCGGCAGCTCTAGCTTTGTTCGCAGTTGCACGTGCTGGTGATCGAAAATCAT

CCGACAAATCCAAAGGAGAAGAAAATCATGGTCGATCCGGTGAACAACG

>Unigene0001295

AGCTGGTGCCTAACATGGACAAGCAAACGAACACGGCGGACATGCTAGATTTGGCAGTCG

AATACATTAAGGATCTGCAGGGACAAGTCAAGGTACAGGTCCAAACATTATAGATTAGTA

CCAAATTTCTGTTTAAAGTAACTTCAGTAAGCTAGTTAGTAGGCTGGAGGAGTTGATTTT

TTCTAGTTAATCTATATAACAAATGACACAATTGTGGATGCCCTACAAGAGTGATAAGCT

TTAGTCAAAGTGGAAAGAAGATTGGATAGATGGAATCCCCTATGAGCTAAAACTAGTGTT

TGCTTTTGAAATGACTCTTCAAATTGAATTAAGATATTGATGTGTTTAAGACATTGATGA

ATACTTATGCATATATGTACTATTTGCTACATTGGATTTGAATCTATGCTAAAATCCCTG

ATTAAATTATCAATTCACAACTTTTGTGACCTTGTCGAGAGTACAATTGTTAAATAACTA

GTCAGATGGACAGTTAATCTAGTTTCCGTCAGCTAGTAGTCATCCTGTCATGAAGGGAAA

GTAGGCCCCTTGCGGTTCTGGAGGAGAAATCTCCAAACCTCAAAATCTTTGATGGTCTAA

TTTCACATA

>Unigene0001296

CCCCAATTGAAAAATCCTTAAATCCGGTTCCTTGGCTTCTGTGCTTCGAGAGACGACGAA

GGAAATACCATTACAATAAGAGAAAAAAAATATTCAATCGAGATCGAGAGAGTAGGGGGC

CAGAGAACCATCGAGATCCATTGTCCCTCGTTGATGGCGAGTGGAGTGAGTAGGAGACTT

CCAGGAGAGATAAGCGAGCCGTGAAAGCCATCACCACTTTCTTTGGTTCTTCTCCGACCT

TTCTTGGTATCATCTTCTTCATTCACTGGTCCAGCTTTCTTTCTTTATTAAATATTTTCT

GAGCTTAGAAAGTTCATCAGAGCGGCGGAGAGATAAAGTGAGCTATGTACGGATCGACTC

CGCTATCCAACGATCTGAACCTCCCGTATCCGGCGGCGGCAGTGGCCGACCGCCACCAGC

GTCACCAACAGCAGATGAGCTCCGGGCTGCTGCGGTACCGGTCGGCGCCGAGCACGCTGC

TCGGCGATTTGTGCGAGGATTTCCTCCCGGTCCGGCCGTCCAGCCCGGAGACGGAGACGT

TGTTTGCGCGGTTTCTGGGGCCGGATCTCCGCGGCGAGATTGAGGATAAGCATGACGAGA

GGAGATCTCACTTTGCTCCTCCGCCGCCACCGCCGCCGCCGACGACGACCGCAGCGATGG

AGGCCGGAGATGTGGCTGCTGCAGCGGCAGCTGCTCATCAGCAGATGTATTACCACTCCC

AGCAGCAGCAGATGCCGAGCCAGAGCTCGGCTGAAGGCTCGTACCGGATGGCAGTGGAGG

CAGAGCAGATGAAGACCGGCGGCGGCGGCGGCGGCGGCAACTGCTCCAATCTTATCCGGC

AAAGCAGCTCGCCGGCTGGTCTCTTCTCTCACTTGAACGTAGAAAATGGGTATGGTATGA

TGAGGGGAATTGGTAACGGCGGCTACAAAAATGGTGATGGGTACTTGGGGGAAGGGTGTG

GGCCGGCGGCGAGCAGATTGAAGAGGCAGATAAGCTTCTCGTCGAGGCAGAACTCCGCGA

CATCAGGCCTGATGTCACAGATATCGGAGATGGGGAGCGAGGGGATGAGCCCCGGGAATG

AGGAGAGCAGCCAGTGTTATGGTCCGGGTGGGTTCCCGACGGGGTCGTGGGACGACTCTG

TGCTCTCCGACAGCTACTCCGGCCTCAAGAGGTCCATTGACGGGAAGTTGGTCAATGGTA

TAAATCAATCTGAAGCTCAGAATGGAGGCCTTGGATCATCGGACATGGCGACCATCGAGA

AACTTCTCCAATTTCAGGACTCGGTCCCCTGCAAGATCCGAGCCAAGCGGGGGTGTGCCA

CTCACCCCCGGAGCATTGCTGAGAGGGTACGGAGGACACGGATCAGTGAGAGGATGAGGA

AGCTGCAGGAGCTGGTGCCTAACATGGACAAGCAAACGAACACGGCGGACATGCTAGATT

TGGCAGTCGAATACATTAAGGATCTGCAGGGACAAGTCAAGACGCTTGCGGACGGCAGGG

CGAGCTGCACATGTTCGTCTAGCAAGCCGAAGCCATACTCGAAACCAGTGTGAACTTGCT

ATACAAGAATCACAGAGCAGTTAGAAGCCTAAGTTCTTGTTTGAGGTGGCTGTAGAAGGT

CGCGGAGATGGCTGCTGGTGAGGCAGACAAGAATTGTATAGCAATGATTTACTAGAAGTT

ATACTTGCCGTTATGCTGTATGTAAGTCCTACTTGTTTAATTAATTACTGTCGATCTATT

ATCAAAGCAAAGTCGTGCAAGACTTGTTTCGGATATGAACATGGTTTTAGAGCCAAAAAT

GGGTCAGCATGAGGAATAGGATGTTGAGAATTAGATGCAAAGATGGCCTCTTTCCTCTGT

TGGCCAGTGGTTAAGTATGTGCTCCTTTCTCTAAAATAGGAAGTCCAAAAATAGTAACTT

TTGGTTCTGAAAATTTTGGTTGTCCTATCAGTCCTTATCCCTGTACCTATCATCTCTACC

TTGTGTGCAGGGGTAGCAGATTTGCACTTATATGAAA

>Unigene0001297

TACGTGACTAATCTTAGCCCTTTAACTACGCATCTTCCAGTGTATTGGAAATTCCAGTGT

CTATAAATGTGTTAAATTAGTCTCTTTGACGCCAAATTTGTTTATTAAAAGTTCGAGAGT

TGCTCGATGTGTTAATATATAATTCACATAGGAAATATATGTATACCTTATCTTACGATA

GACGATACACCTTAAGATAGACCTTATGACACTGTTTTTTTGGAGAAACCGATACACGAT

ACACCTTTCGATACAATATGCTTTTGGAGAAACCGATACATGATACACTTTGCAACATGA

TAACCTTGCCTATACAGATGTATTTGTGAGATTGTAATTATTTTCCCTCTTTTTTTTCTT

TTGTCGAGCATAATGACTAGGTGACGGGCCTCAACTTACTTCAATTTGAAGGAAATTGAG

TAAAATGTGTATTGTATTTATTTTGTTTTTGTTAATCCGAACTCTCATGTTTAAAAGAAA

TAGTGTCATGTATAAAATTGTTGAAACATGCCTTTCATGCATTTATCTTAGTAGAGCATT

CCATAGCTATCTTATGAATGTTGTTCAAATCCATATTTTGTCCATGTGGACATTAAACAA

AAATTATAAGTCTTAACATCCCATACGTCTTGCTATCTTTGTGTGCTTTGATTGTGATAA

TATAGTCTTAATATCTTTCAAAGCTAATTGAAATCTGTATGTTACTTATAATCCTTTAAC

AGACGCTTGCGGACGACAGGGCGAGCTGCACATGTTCGTCTAGCAAGCCGAAGCCATACT

CGAAACCAGTGTGAACTTGCTATACAAGAATCACAGAGCAGTTAGAAGCCTAAGTTCTTG

TTTGAGGTGGCTGTAGAAGGTCGCGGAGATGGCTGCTGGTGAGGCAGA

>Unigene0001298

GCGGCGGCGGCGGCGGCGGCAACGGCGGCGGCGATCTTGGCATCGATTCCTGCCACCATT

TCCCCCTCGATTCCATCCGAAAGCTCTAGCTTTGTTCGCAGTTGCACGTGCTGGTGATCG

AAAATCATCCGACAAATCCAAAGGAGAAGAAAATCATGGTCGATCCGGTGAACAACGAAA

TGGCGATGGCGACTCCGCTGTGGCTCAAGCCGCTGCTGAAGACGAGCTTCTTCGTCCAAT

GCACTGCCCATGCCGACGTCCACAAGAGCGAATGCAATCATTACTGCCTGGATTGCATGG

GCGAAGCAATATGCCCTTATTGTGTTGCAGAGCACAAGGATCATCATGTTGTTCAGATTC

GGAGATCGTCCTACCACAATGTGATCAGGGTTTCCGAAGTCACGAAATTCATCGACATTT

CTTGTGTCCAGACCTACATTATAAACAGTGCGAAGATCGTCTTCCTCAATGAGCGGCCGC

AGCCGCGTCCGGGCAAGGGGGTTACCAATACCTGTGAAATTTGCTGCCGCAGCTTGCTCG

ATTCCTTCAGGTTCTGCTCTCTTGGTTGCAAGCTGGGAGGCATGAAACGTGGAGACCTTG

AGCTCACATTTGCTATTCGGGGCAAACATGGTCGGGAGTGGCTCCACGGTATGTCGGAGT

CTGATGAGTCATCTTCGACGCCACGGAAGATCCAGAAGACATCGGCCTTTGGCCGGACTG

TCGAGCTGCCAGTGTGGAAGTGGTCGGATGAGGACGGTGGCGGCAGGGCGACTGGCGGCA

TATCTCCGGCGACACCACCTATCGTCAGCTACCGGACATCCAGAAGGAAGGGCATTCCCC

ATCGAGCTCCATTCTAGAAAGAAAAAAGAAAGAAGTAGATTACGGTAAAAAGCTTCTAAA

TAGTCTGTGAAAATAGGTAGGAAGTTATGGATAGTGTGAATAAATGGAGAAATATTCTCC

ATGTCTGTAAATATGGGAAAGCATATGAGGATGGATTAGATACATAGAAACTTGGAAAAG

AAACCCTAGTTTTGTTATCATAAGAACTTCAACTTTGTAGATAGATGATATAAGATGGAG

TGGTTTGCTTTATATGTTTATTCTTTATATATTAGTCTCTGTAGATTGTTAGTTTTGTAC

ATAGTTATGTAAGTACATGAAATTTTTATATGTCAGTTATGGTATCATGCTTCCAATGTA

TTGAATAA

>Unigene0001299

TATCATCCAGTGGCGCAGCGTGAGGAAAAAACCCAAAGAGCGAATCCGAGTGCGAGTGGT

CGAGGGCGGCGGAGGAGAGGAGCAACCACGTCTCCGAACCTCGGCCGCGCGATAATGAGG

GACAGAGGGGAGAACACGACACCGCCGCCATCCCCTTCCCTCCTCATCACATCCTAGACT

CGCCGATGTCGGACTCCGCCTCGCTTACCGGGATGGAATCCCTCGACGTCGAGGACTTCC

CGCGGGTGGGGGATGCGGGCGATCAGTTCGCGGAGGTGTTCGAGGTTGCGCAGAGGGGAA

ACCAGGCGTTCCGGGAGGGCAGATTTGAGGAGGCAATCACTTGCTATTCTAAAGCTCAAC

TTTTAAAGCCTGGTGATCCTATTATCCTTAGCAACCGCAGTGCTGCATTCTGTAGGATTA

GCAAACTTCTGAGAGACAGGTCAGCATCTGAATCTGAATATGAACCATTGAATGGTCTGG

ATCCTACAACACATGCTGAGCTTGCACTTAAGGATGCTGAGAAAGTGATACATACTCGGA

GTAATTTGCCCAGATCGTATTTATTGAAGGCCACTGCTCTTATTCTGCTTGAACAATATG

AGGATGCACGTGGAGCACTTCTTTCAGGCCTTCAACTCGATCCTTTTAGCCGTCTCCTTC

GGACTTACCTTCAGGACTTGGAGAAGATTTCAACTGGTAAACTTAAGAAGATAATACAAC

GAACAGATGACTTCGAATGTACCCTGTGCCTGAAGTTGTTGTATGAACCGGTCACTACTC

CTTGTGGACATTCTTTTTGCCGTTCTTGTCTTCTGCAGTCCATGGACCATGGTAATAAGT

GTCCTATGTGCCGGACAATCCTATTTATCAGTCCCAGGACATACCCTATCAGTGTGACAT

TGAACAACATAATACAGAAAAATTTTCCTGAGGAATACGCGGAGAGGAAATCTGAGCAAG

AGAGTTTTACACTCCTAAGTGTGGATATGATACCTCTTTTCGTCATGGATGTGGTGCTTC

CGTGTCAAAAATTGTCGCTGAATATATTTGAACCTCGCTATAGGCTGATGGTAAGAAGGG

TAATGGAAGGAAATCGTCGGATGGGAATGGTTGGTATAGATTCCGCAACAGGATCTGTTT

CTGATTATGCTTGTGAAGTGGAGATTCTTGAGTGTGAACCACTTCCTGATGGACGATTTT

ATTTGGAGGTTGAAGGTCGTCGTAGGTTTCGTATTCTTCGATCTTGGGATCAGGATGGGT

ATCGGGTTGCAGAAGTTCAATGGATTCAGGATTTGCACCCACCAGAAGGGTCACAAGAGA

GAGATGATTTACAACAGATGGTTAGTGGGGCAGCGGAATTGGCTCAAACATGGATAAGGC

ATGTGCGAGAATCTGCACGAATTGGTAGAAGACGGCATTTGGAACTTCAGCCTGAAGGCA

TGCCGGGACCACATGATCCCGAGCGATTCAGTTACTGGCTTGTGAACCTGTTGAAATTAA

GGCCTTCTCAGAGGGTCGATCTATTGCGTCTAAGGGATACCCGGGAGAGAATATCTCGGG

GGCTTTCCTTTCTGAGAGCTGAAGAACAAGGCTGCCGAGTACAATAACAGGGATGGTCTC

CCATAAATGTTGTTAATTCATCATTATATAATTGTAAATGCATTCTTTATTTTTGAAGAT

AATACGTACTTTTAAGGTCCATCCAGTCAATAATTATAAGTGACGGAAGGAGGATGCCCC

TTTATTGTAGTGAAAGTAAAATATAAATATAGAATACACATCTTTGTCATGATCTCTGGA

TTTTATTATTGGACAAGCAACAAATTGAGTAAC

>Unigene0001300

TTCCACCATGACCCTCGCCGGAGCTTTGCCTGCTGTGCCTCTCTCCCCCTCCATCACCTC

TAATCGAGCCCCAATTCACACCATAGATTCCACTCAACTTTCTCTCTCTGAATCCCTAAC

CAATTTCAAGCATTACCCAACCAATCCATCGGAATGATCTCCTTTTCACCATGGATCTGA

CAACTTCATCTTCTCCTGCAAGCCCCTCACCTTCGAGCACTACCCAATCCTCATCTAGGA

TTCCTCTCTCTCACCCCTTCTCTTTCCTTGGATCTCTGATTCAAATTGGAACTAATTTAG

ATCTTATCTTCTTGCAGGTTAATGGAAGGTAGTAGGGAATTGTGGGTAGAATCTGAAGCT

CTAGGCAAGAGCAACAGATAAGTCTAGCAAAGAGGATTCATATGGAGCACTTCTTTGAGG

CCTTCTACTTTATCCTTTTAGCC

>Unigene0001301

TTCCACCATGACCCTCGCCGGAGCTTTGCCTGCTGTGCCTCTCTCCCCCTCCATCACCTC

TAATCGAGCCCCAATTCACACCATAGATTCCACTCAACTTTCTCTCTCTGAATCCCTAAC

CAATTTCAAGCATTACCCAACCAATCCATCGGAATGATCTCCTTTTCACCATGGATCTGA

CAACTTCATCTTCTCCTGCAAGCCCCTCACCTTCGAGCACTACCCAATCCTCATCTAGGA

TTCCTCTCTCTCACCCCTTCTCTTTCCTTGGATCTCTGATTCAAATTGGAACTAATTTAG

ATCTTATCTTCTTGCAGGTTAATGGAAGGTAGTAGGGAATTGTGGGTAGAATCTGAAGCT

CTAGGCAAGAGCAACAGATAAGTCTAGCAAAGAGGATTCATATGGAGCACTTCTTTGAGG

CCTTCTACTTGATCCTTTTAGCCGTCTCCTTCAGACTTACCTTCAGAATTTAGAGAAGAT

TTCAACTGGTAAAATTTCTGTATTATGTAGGATTAGAAAACTTCTGAGAGACAAATCAAC

ATTTGAATCTGAATATGAACCATTGAATGGTCTGGATCCTATAACACATGCCGAGCTTAC

ACTTAAGGATGCTAAGAAAGTGATACATAATC

>Unigene0001302

CTTTCCTCCGCCGCCGCCTCCGCCTCCTCCTCCTCCTCCCTCAATCCCCTTTCGTTCTAT

CTCTAGAAATTGAGTTTCTCTCTAGAATCTAGGGTTTACTACATGGGGAAGCGGCGAAGG

GGTGAAGTGGGATTACGATGAGCTGTTGCTGCAGCGGAGGAGGTGCCGATCAAAGCTGCT

GCCGTTCTCATACGCATCTGCTGCTCCGATGGACGCCACGCCGGGATCTGTCGGGACCGT

CTCCAGCCTCTCGCTGAGGCTGGGGCAAGCCGTCTTCTCCACCGCCTCCCTCCTCTTCAT

GTCCGTCGGAGTCGAGTTCTACAGCTACACCGCCTTCTGCTTTCTAGTGACAATCATGGG

CCTGGTTATTCCGTGGAGTGCGACACTTGGAATCGTAGATATATACTCTATCATTGTTGG

ATGCCCTCTACGGCTTCCTGGTGTTATGGTGATTGTAGTTGTGGGTGATTGGGTGCTATC

TATACTTTCGTTGGCAGCAGCTTGTTCGACTGCTAGTGTTATAGATCTACTCCATGATGA

CGGGTCTTACTGCCCTCCCAGGTTCTGTAGCAGATACCAGCTATCTGCAGGGATGGCATT

TTTGTCTTGGTTTCTAACTGCTTCCTCCTCTCTTTTTAATCTATGGCTCGTGGCTTCATG

GTAACTGGGCCTGCTCTGCTTATTCTACTTTCATAGAATCTGTTAGATAATAATATCAAA

ACCTACAATATTCCAAAGGGGTATAGATTGTTTATATTAGCAACTCAATTCTGGTGGCAG

ATATTCTCTGTTTTTCATCTATATTAGCACAGAATCATTACAATCTTCTTGGCATTGGTT

TTAAAGGGGTATAGTTTGTTTATATTAGCAACTCAATTATGGTCATGACCGGCAGATATT

CTTTCTGTTTTTCATCTATATTAGCA

>Unigene0001303

AGTGCTTTTCATAGCTACATAAACAACCATCTTGACTTCAACCCTCTCTCTCTCTCCTCT

CATGATGGCGGAGAGCTCCAGCTTCGGCCCTTTCATGGAGCTCTTGGAGTCCGGCTACGA

TGTCACCAAACGAGAAGGAACAGATGAAGAGTATCTCCTCTGCAACCTCCTCTGTCCTCC

AAGCTCATCGAACATGCTCTGCTTCGGCGCAGATCATGCACAGAAATCCAGCGACTCGTC

AATCTCCACCATCGCCTCTTCGCCGCCCTCCATCTCCACCAGCTCCGAAGCCCGGGCGAG

AAGAGGACCGAAAGCAACCGCCGCCGCCGCCGAAGCAGTTAAGAAGACTAAGCCGATGGG

TTCTGCTCGCCAAGTGAAAGCCAAGAGCAGCAGGAGGAAGATCGGTGATCAGATCGCGGC

TTTGCAGCAGCTCGTCTCCCCCTTCGGCAAGACAGATACTGCCTCTGTTCTTCATGAGGC

ACAGTGCTACATCCGATTCCTTCACGAACAGGTTCAGGTTCTGAGCTCTCCTTACCTTGA

ACACCTGCCGACCTCTGTGCATGACATTGGAACAGGACGAAGATGGAACGATCTCCGGAG

CCGGGGGCTTTGCCTTGTCCCCATATCCTGCACCGAGCACATGGCCAACAGCAGCATCAA

TGGTGCTGATCTCTTCTGCTCTCCCAGCTCGAACCCCTAGGCCCCAAACTCCTCCCCTCC

TTTTTGGGTTCTGTTTCATTTCTCTTTTTAGATCAAACTTTTTGGGTTCCTGCTCTGCAG

AGCTGCTGCTTCCATGATGTTTTTGGGGGAGTGAAGCAGAGGGAACAGTTCAGTTTATAA

AAGGAAAGCTAGTTTGTAGACAATCGTATACTATGTTACTTTTTAATACCTAAGCTTGTC

TGTTTGGTACTAGGAAAAAGATGGGCATGTGATCAATTGTGTTAATATGGTTGAGTATCT

TAAAATTGAGGTGAACAAATTGTATTAATCTGGTTGAGTATCCTGAAATTGAGGTGAACA

AAGTAACGAAGTGGGGTCT

>Unigene0001304

CAGCCACCCCAGCCGAGCATCTCCTCCGTCTCCCCTTCTTTTAGATCTCACCACTCATCA

ACCATCAACAAAGAGAGTGAGAGAGAAGAGCATTGCTCCATCATCTCTCTTCCTCACCTA

GTGTTCTCCTTTCTTTATTTACCTCTCTAGCCAGGAGATTCCCCGTGGGTTTCTGATGAC

AAGATCCTGGATGGACGCCCACAACTGGATGCTCGACCAGAGAAAGAGCCGCGTCTGGAC

CGAGCACGATAACAAGCTGTTCGAAGATGCCCTGGCCTACGTCGACGAGAACTCGCCGGT

GCGGTGGGAGAAGGTCGCCGAGATGATCCCCGGCAAGAGCCCCGCCGATGTCCTCTCTCA

CTACCATGATCTAGTGGAAGATGTCACCCATATCGAAGAGGGCCTCATCCCCTTTCCCGG

CTACTCCTCTTTCATGCTGGACTGGGAGGGCGGGCATGTGTACGAGGGTTTCCGGCACGG

CTTTTGCGGCGGCGGTGGGAGGCGGGCCGGTGGCCGGGCTGCCGATCACGAGAGAAAGAA

GGGGGTCCCCTGGACCGAAGAGGAGCACAGGTATTGGTATTGCCATCCTAGTTCAATTAT

ACTGTATTGTCTTTCACATCCATTCATAAACTGAGGCTAATTATATTTTCTCTCTTTTGA

ATTTGTTGTGCTTGCAGGTTATTTCTGATGGGTCTGAATAAATATGGTAAAGGAGACTGG

AGGAACATTTCCCGCAACTTTGTTGCCTCCAGAACCCCTACTCAGGTAGCTAGCCACGCC

CAGAAGTACTTCATCAGGCAAAAATCGGGTAGCAAGGACAAGCGAAGATCCAGCATACAT

GACATCACCACTGTCAACCTCCAAGAGGCGAGGCCAGCCTCTCCTTCCCAGCAGTTATCG

ATGCAGTCTACATCGACCAATGCTCCCGGTACATCGGACCAATTCTCCAACATGGTCGAT

GCGATTCAACGCAACGAGGTCGTTGGTCTATTCTCGCAGGCGCCTTATGGGATCAACTCT

TACATAATGAAGTCGCAGGCGCAGCATTTGCATAGGAGTGCGCTGCATAATTCTATGGTT

GGGCAGCATATGTTGTTTCAGATGCATTAGAATGATAGTTTGGATCATTCATATTGATAT

ATTAGAGATTCAGGCCGAGAAGTAGTGTTTCTCCCACATATTTTATATATCAGAGCTTTT

GGGCTTATGTTGATTTATCTTGTCTGTATATTTTTACCGCTATCATTAATAGCACTCCAT

GATTCGGTGTAGCAAAGAAAATGATTTGTATTTGATGGTTGAAGTTAGCTTAGAATGTTT

TATTTCCTAGTTTGATGTGACTCATTGGCCTCGATTTGATCGTGCAA

>Unigene0001305

CTCTCATGCAAATAGAGTTGTGAGTCAGCTCATTACCGATCTGCACACTAATTACCATGT

TGTTATTCTCCGTATTCTGAAGTATTGGTGAGGCATAGTCACTCACTCCATGTTCTTCCC

TACTACATCATGACTTAAGCTTCATGCTTATTCTGATGTTGATTGGGTTTGCGGTCCTAT

CACAAAGAATGCTACTCCTGATTTCATATATACCTTGGAGACTCTGATATCTTGGATGAG

TAAGTAG

>Unigene0001306

CTAGAAATACAGTGCTCTAGGAGTGGACCAAGTATATTGAGATAGATGGTCACTACATTT

GCCGGTCGCTATTTTTCCAAATCGTACACCTAACGTACGTCTTCGTTGGTAGATTGCCGA

CTTCTTCACCAAAGCTCACATGACTCACCGATTTCAATTTATTGTGTGCAAACTCATGTT

TGATCAACCATGAGTTTGTATAGGTGTATTGACGTACAAAGACAACTCTAGATGTAGCGT

AGTGTATATCTTATCCGAGAGGGTATTTATATTGGTGTGTACATAGCACAATAAGTGTGT

GAGTATCACTTATATACTTCTATAAATATCTCCTTATTAATTAATGAGTAATAAGAAACT

AACCTCATTTTCATCCTCATAACTTCTTCTCTTTTATCTTTTATCAATAATTATGTGACC

AAAATCAATCTAATCAAACATATCCATTATCTTTTATGTCCGGATTTCTTTAGTCTTTTT

AGTCGACCCGAAAATCGACACAAATAAGTCCGTATCAAAAATAGAACCCTCTGCTACAAT

TCAGGCATGTTTGTAACCAGTAGACAGGAAACAGGCAATACTAATTAATGTTTTCAAGAT

TTCTTTGTTTACTTTAGTACTCCTAAATTGCATAATTAATTTTGGTCTCTCTTTTTATCT

GATTATTGACTTTGCTCCTGTTTTCATTTTTGAAAGTGTTTGTCCTTTCTGAAAACAAAA

ATCTTTTCCTTGATAAACAAACCTCAGAAGAACGTAAAATTCTCATTTAATATCAACAAT

ATTAATTATGTTCTTGTACTGCTAATATTTGCGTCTGAAAAGGGCGACGCTACACACTCA

TAGTGGGAGCAAACGAGAGACGTTGGAGTAGGGTTCGGAACCAGCTCAAAGTTGTTGCAG

ACTCCTTCAGAAT

>Unigene0001307

ATCGGGCCGAATAAAAAACTGCACCACCATCATTCTCTGGCTACAGAGAAATGGCAACGG

TGCCGAGCTGGGTGTCGACACTTCCCAATCAGTTATGTGTGCCCAATTTGAATGCTAACA

CTCGTTCGATAGCACCCACATCATTTGTAATCAAGGGAAGCTCGAGAGAAACGTCTGTGT

ATGAGAAATATTGCTTGACGAGAAGAATGGTATTATTAGGAGTAGGGGGATTGGCTAGTA

GTTTGATCCCCACAAGCTTGTCATTTGCTGGAGAAGTTCCGAAGAATTATGAAGCTTTTG

TAGACATTTTAGATGGATATTCATATTATTACCCCTCAGATTGGAGGGACTTCGACTTTA

GAGGGCACGATTCAGCATTCAAAGATCGTTTCGCAGCATTGCAAAATGTTAGAGTCAGTT

TTATTCCCACTAATAAGAAAGACATTCGTGATATGGGACCCATGGAAGAGGTTATTTTTG

ATATGGTAAAAAATGTCTTTGCTGCACCTACTCAAATACCAACAGTGTATAACATGCAAG

AGCGTACCTTTGACGGAAAGAACTACTGGACCTTTGAGTACGAACTTGCTACTCAAAGTT

TTGCTCGTACTGCTTTTGCAACAATAGCAATTGGCAATGGGCGACGCTACACACTCATAG

TGGGAGCAAACGAGAGACGTTGGAGTAGGGTTCGGAACCAGCTCAAAGTTGTTGCAGACT

CCTTCAGAATGTTGGACATATGATTTCCGAAACTCAGTGGGGGCAAATATGTAATTTCAG

TCTATTCATATATATATATATATATGAATTTGTTTTACGAAGAGGATATAGTCATGTATT

AATCCTAAAATTTTGAAAGTAATTTGTGTTCCGTCCACACCCTTTTCTAAAAAA

>Unigene0001308

GAGATTTTGGAAGCCAAATGTTTAAAAAAATGCAGTTGCTCTTTTGGTCGATTGTCAGAA

ATTTGAACTTGGGGACCTGGTTTCATGTTTTAGCTCTGTCATCTGATTGGCGCCCATATA

TTTTCTACAAACTTAATATTTGAACTCAAAATAACCTGCAAATCACAGGCATCAAACCTG

TAACCATTTTCTTCTGTACCGGAATACCCCTCTGCCCCAGTCATGGGTGGATAGTGGATT

ATAACAATTCTAGAAGGGTAATATCATCTATGTAGAAAACCACATATTGAGTGGTATATG

AAAGATGATTTCAAAAGTAAAATGATTAGTAATTTGACCAACAGCCTGTGAGAGTTGTTA

AAATTTAAGTTAGGGACATACAACCATGGTTTGAAGTTTTGGTTTCGGACCCAACCGAAA

CTGAAATGCTTTGGCCGAAACAGATGAACCAGGCCGGATCTGCAATAGGACAATCAATGT

CTGAAACCATCTGAAACAGACCTTGGTTTTCATTTTGGTTTTCATTCTGTTTCGATTGAA

GCATATCGACCAACCCAAACCGCTGCAGTAGCCCTGAAACACCCAGGCCGAAACCGAACT

AGTTCGGGTGAAACATTGAACCATACCACCATTTCTTTGGAGTCCCTACTTGAGAACTAG

GCAACCCTAGTTATCAAAAACATGATTTATGGGTTAGGATTGCCCAGTATGGAGTCTACA

GACCATTGGGAATGGACAGGTTGGGATTGTGCCAATAATCCGAGCTTCTTTGGAGTTATC

AATATAAAAATGAGGCTACCAGTTGGAAGTTTTAGCTGTACCCCCACCGTCCTAGTTTTA

GACCTCTTTCAATTGCCATCAATTTTCAAATTCCTTGACCCATATAAGTGGTCTGAATTC

CAAGGCATGCCTTACTGTTAAAATTGCATAGTTATGTTGGTGGTAGTTCAGTTTTGGAGA

TGTGTGTTATTTATTCGTATTATGACAAATTTTGAGTCTTACATCTACTTTCCTTGATGT

GCTTAATTATTTTCAATATGTATGTAATTACATAGAATGTTCAAGACAATTTCTCATAGA

TTTCTGTGCAATTCTCCTAAGTATCATCTACCAAAAAATGCAACCTTGGCCAACATTTCT

TAATAAATACTCTATAGAGGTCTTGGGTTCA

>Unigene0001309

AGTTATCTATCAATATGAAAATGAGGCTAGTAGTTGGAAGTTTCAGCTGTACCCCTACTG

TCCAACTTTTAGACCTCTTTCAATTGCCATCAATTTTCAAATTCCTTGACCCATGTAAGT

GGTCTGAATTCTAAGGCATGACTTACTGTTAAAATGTAAAATTGCATTGTTATGTTGGTG

GTAGTTCAATTTTGGAGATGCGTGTTATTTATTCGTATTATGACAAATTTTGAGTCTTAC

ATCTACTTTCCTTGATGTGCTTAATTATTTTCAATATGTATGTAATTACATAGAATGTTC

AAGACAATTTCTCATAGATTTCTGTGCAATTCTCCTAAGTATCATCTACCAAAAAATGCA

ACCTTGGCCAACATTTCTTAATAAATACTCTATAGAGGTCTTGGGTTCA

>Unigene0001310

ACACTGCATGGGAGCTCGATTATCGCTTCCAGTTGATGTTCTAGATACAGCCATCTGCCT

ATATGCTGTTTAACATACATAGACTGATACAAATAACGGGTTGCATCTCTTCAGACACAG

AGGCCGGTTCGCTGCAATGGAAGTTCTCTTCTAATATCTTTTCAGCTCAGTGCTGCAAAG

AATGAATGGATTCACATCAACATTTGTGAAGTTATCTTTACACCAACTTGACAAGATACC

CAGCAAACAATCCTCCGCGGTTAGTTTCAGATTTTGCCATATTTATTACTACCTGCATCA

TGTGATATTCTGCATCAAACCTAGCTGAATTACTTGTTATATATTATTCATTTTTTGAAG

ACTATATTGTGCTTTACTGGACTAAAGAATGCCCAGATCCAACTAGAACCTAGAATGAGC

CTCTACTATTCAGCAGAGGGAGATGTCGAAAACCACCAAATCTCCTGTGTCTGGTTTGTG

TAAATAAAATTTGTTGTAATCAGATTTACAGGGTTACTATCTTTGATT

>Unigene0001311

CTTTTCCGGGAGATGCAGCGAGCAAACGTGAGACCTGATGAGATCGTGCTGGTCAGCGTG

CTCTCTGCTTGTGCCCGTCGTGGCGCACTCGGGCTAGGGAGGTGGATTCATGACTACATA

AAGAAGAATGACGTTCGGGCGGATGTCTATGTAGGGAACTCTTTAATTGACATGTACGCC

AAGTGCGGATGTGTCGATGAGGCAATGGGCGTTTTCAAGGAGATGAAGGAGAAGGATACG

CTGTCGTGGAACTTGATTATCTTAGGATTGGCGAACAATGGTTATGTTGAGAGTGCATTA

GAGGTGTTCTCTCGCATGCTGAGGGAAGGGTTCGCACCCAATGATGTGACCTTTCTTGGA

GTACTTATTGCATGTGCTCATAATGGATTGGTTGAGAAGGGGCTCAGAGTTTTCGAGAGC

ATGAACGAAGTTCATCAAGTGGAGCCAACGATGAAACACTTTGGATGTGTGGTTGACCTC

CTTGGGCGTGCTGGCGAGCTGGAAAAAGCGCATAAATTCATCAAAGAAATGCCTGTAAAA

CCGGACCAAGGGGTCTGGAGGATAATGCTTGGAGCTTGCAAAATGCATGGCAATGTTGTT

CTAGCTGAGATTGCGACCCGAGAGCTTGTTGAGTTGAATTGTGGGGACAGTGGGAACTAT

GTGCTTTTGTCGAACACATATGCAGCTGCAAACCGATGGAATGAAGCGATGAAAGTGAGA

GAGATGATGATAGATACTGTGCAGAAACCGCCCGGTTCAAGCTCAATTGAAGTTAGCTGC

ACAGGGGATGAGTTCAGAGATGCAGAAATTCCTCCAAGAAAGAATCTGCTCGCTTTTGCG

TAACATATTCAGCTCTTTTTCCTGTTGTCCACAGAAGAAATCCCCTTCAAAAGAAAACAA

ATCAAAGGGATTTTCGCTTAACACTGCATGGGAGCTCGATTATTGCTTCCAGTTGATGTT

CTAGATACAGCCATCTGCCTATATGCTGTTTAACATACATAGACTGATACAAATAACGGG

TTGCATCTCTTCAGACACAGAGGCCGGTTCGCTGCAATGGAAGTTCTCTTCTAATATCTT

TTCAGCTCAGTGCTGCAAAGAATGAATGGATTCACATCAACATTTGTGAAGTTATCTTTA

CACCAACTTGACAAGATACCCAGCAAACAATCCTCCGCGGTTAGTTTCAGATTTTGCCAT

ATTTATTACTACCTGCATCATGTGATATTCTGCATCAAACCTAGCTGAATTACTTGTTAT

ATATTATTCATTTTTTGAAGACTATATTGTGCTTTACTGGACTAAAGAATGCCCAGATCC

AACTAGAACCTAGAATGAGCCTCTACTATTCAGCAGAGGGAGATGTCGAAAACCACCAAA

TCTCCTGTGTCTGGTTTGTGTAAATAAAATTTGTTGTAATCAGATTTACAGGGTTACTAT

CTTTGATT

>Unigene0001312

CACAGCAGTACAATCTACAGCTGTGAAGTCGGGGATGGTGGCGGACGCCTATGTTTCAAA

TGCTCTCATCCACCTATACTCAGCTTTTGGAGAACTGGATTGTGCACGGTTGGTGTTCAA

TGAAATGCCGGTGAGGGACTTGGTATCGTGGAATTCACTCATTTGTGGGTTGAGCCAGAA

GGGGGCTTTGCGAGAGGTGCTGGCGGTTTTTGATTCAATGCGGGCGGTGGAGGTGCGAGC

GGATGCAGTGACGATGGTAAAGCTCGTGTTGGCTTGTAGTTTGTTGGGCGAGTGGCAGTT

GGTGGAATCGATGGTCGCGTATATAGAGGAGAATGGTATAGAGATGGATGTTTACCTCGG

GAACACTCTGGTCGACTGCTATGGCCGGCGGGGTTTGACCGAACTGGCGGAGAGGGTGTT

CAAAGGGATGAAAGTGAGAAACATGGTCACATTCAATGTCATGATCAC

>Unigene0001313

GTCAAAGTGAACGAGTTCAAACGAACCATGAGAGAGATTAGATGATTGAAAATGTCAAAA

TAGTAAACTTAGTCATTGTGCAACCGACACAAGTTGTACTCTAATTAGTGATGATGCAAC

CTAAATTGTCAGACTCGGTGAGCAACTAAAGTCTTGGGCTTGATATATGCCCTAAAAGTT

GATGCTACAACTTAACAGTAGACGTAGAAGAAACAAGAGGGAAACTTGAGACACATGATG

AAGAACGAAAAAAATGCAAATAATGCAAGCAATTCAAATAAT

>Unigene0001314

CGAACCATGAGAGATAGATTAGATGATTGAAAATGTCAAAATAGTAAACTTAGCCATTGT

GCAACCGACACAAGTTGTACTCTAATTAGTGATGATGCAACCTAAATTGTCAGACTCGGT

GAGCAACTAAAGTCTTGGGCTTGATATATGCCCTAAAAGTTGATGCTACAACTTAACAGT

AGACGTAGAAGAAACAAGAGGGAAACTTGAGACACATGATGAAGAACGAAAAAAATGCAA

ATAATGCAAGCAATTCAAATAAT

>Unigene0001315

TGACTGCTTCTCACGCCCATGAGGAGGTTAGAGAATAAAACATCGACTCCCAAAGACACA

AAGAAAATCATAGTGCGGGGTGAATGAGTAAACACGACTATGAGGCATGGCAACACCAAG

GATGTCGAAAGGTGTGATGTTGATGAGAAGCACAACATGTTATCAGCCTCGGCCCAGAAA

GTAGTAGGAACGAAATCATAAAGTAGGACATGAATGGTGTTAACTATGCCATGGTGCTTC

CGCAACACCATTTTGCTCGAGGTGTGCATGCAAAACTGTTGAAAGTGTGCAGTGAGAGGA

GAGAAAAGTACATATGTTAAAAGACTA

>Unigene0001316

CGCTGTGAGGAAGAGATGAGAACAAAACATCTACACCCAAAGACACAAAATAGGTCATAA

TGCGGAGGGTCAGATTATACAAGACTATGAGTTGTACCACCAATGAGAATGCAGGAAGGT

GTGATGTTGATGAGAAGCACAACATTGAGAATAACTTCGGTCCAAAAAGTAGAGTAACAA

AAGCAAAAAGTAGGACATGAGTTGTATCAAGAATATGACGGTGCATGC

>Unigene0001317

AAACTTGCCTAGGCCCTAAAACCCTAACCATAAACCTAACCCGATCGAGATCGGGAGGAT

GGATCACATGGCGGCAGCACAGGAAGAGCGGATCGTATCCGATCGCATGCGGAGAAAGCT

CGACGAGGTGAACGTAGCAGCGCAGAGACAGCTCTCGCCTGTCCAAGACCACATCAATTT

CACTCTCCAGCAAGCATACTTCAAGTGTGCGTATGACTGCTTCGACAGGCGGCGGCGACA

GGAGGAGATCAACAACTGTGTGGAGCATTGCAGCGTCCCTGTTCTCACTGCCAACAATGT

AGTTGAGACCGAGATGGCTAAGTTTCAGGAACGGATGAATCGCTCTTTAATGGTGTGCCA

AGACAAATTCGAAGCCGGAAAACTCCAACAGATAAAGACAGGAGCTATGAATGATTTGGA

GTCGTGCGTTAATCAGGCTGTTGAGGACAGCTTAAAGGTCCTCCCTCACATCGTTGATCA

CTTGAAGGCATCCCTTTCTTTCAACTGACTTATTGAGATCCTAATAGCTTTAGACATTAT

AGCCAGAGAGAATGTGATCGAAACCCAACATCTGATTGATGTCTGAACCAATCTCTTTGA

TATGGAGGCTCATCGTGCTGGAAACTTTTGTTTAACTTACATCCAACATTGGTTGCTGGA

AACTTTTGAGTCAATTTGTTGAATGTTCAATAATGTGGTCTTCATATTGATGAATTTATC

CCATCATTTAGTGAACTAGTGGCCATATGTTCTCTGAAATCCCCAGAAGAGAGCTGACCT

GTTGTGTTTCAAAGGTGCCCACGCTGAAACTGACAAGGCTTAATGCCAAACCAAGCTATT

TGAATCTCTCTCTCGGTACATGTTCGCCAATTATGTTGTTCTGTTCATTTGTACGGATAA

CCCAAGACAATCTTGGACAATTTGTTCGAAAATGTCAGCCAATCAAGTACGATTAGTTAC

CTGTTTTGATTGTTTCATTCTTG

>Unigene0001318

GTGACGACAGAGTGATGTTCTTCGTCATTGGTAATGGTGAGGGGGGCTGATCCTTTGTTG

AGGCGGTGACGGGTACGGAGAAGAACTCCGTGGCGAGGGCACACCCTTGCTGATTGGAAT

TTCAGTGGCCATCTAGATGGGACTTCTCGCTGGCACTGGGGAGGTGAGTGTGGCTTTGTC

GCCCCAAGGCGTGATGGCCGAGGGTATTGGAGCTTCCTGTGAAGCTAAGTCGGAAAAGGA

GCGTGGCATGAAGGCTGTTGCGAGCTGCATGAGGCGGTGAGAGCAACAAGGTGACCTCCT

CTTGTTACAG

>Unigene0001319

ACCTCCTCTTGTTACAGAGGCTGGAACAGCCGTATGCCTAGCTTCTGCTATTGCTGCTGT

TGTGTGCGAAGGAGTCGTCTCCCTGCTTTATCCGAGGTGGCTCTATCGGTGGGTGCGAGC

TGCATGAGGCGGTGAGAGCAACAAGGTGACCTCCTCTTGTTACAGAGGCTGGAACAGCCG

TATGCCTAGCTTCTGCTATTGCTGCTGTTGTGTGCGAAGGAGTCGTCTCCTGCTTTATCC

GAGGTGGCTCTATCGGTGGGTGCGAGACCTTTATAGTCAGTTGCTGATGGAGAGGGGTTT

AATCAGGGAGATCTGTGGTGGATTGAGGGGTAGGAACGGTGAGGAATCAAGGAGATCTAG

CTCGTTGGAAATGATTATCCTGATAGCCCGAGGTTAGTCGCTTACACTAAAGTTTGGTGG

AAATTGTAGACTGCATGCAACACTACTCCCGCAATATGCATGACTGTATGTAGCTTTACA

AAGATGGAGAACTTAATACTTGACTAGTGAAGTTTTTTTTTGCTTTCAATCTTTTAATGA

TAGCTTTCTTCATATGCGGAG

>Unigene0001320

ACCTCCTCTTGTTACAGAGGCTGGAACAGCCGTATGCCTAGCTTCTGCTATTGCTGCTGT

TGTGTGCGAAGGAGTCGTCTCCCTGCTTTATCCGAGGTGGCTCTATCGGTGGGTGCGAGA

CCTTTATAGTCAGTTGCTGATGGAGAGGGGTTTAATCAGGGAGATCTGTGGTGGATTGAG

GGGTAGGAACGGTGAGGAATCAAGGAGATCTAGCTCGTTGGAAATGATTATCCTGATAGC

CCGAGGTTAGTCGCTTACACTAAAGTTTGGTGGAAATTGTAGACTGCATGCAACACTACT

CCCGCAATATGCATGACTGTATGTAGCTTTACAAAGATGGAGAACTTAATACTTGACTAG

TGAAGTTTTTTTTTGCTTTCAATCTTTTAATGATAGCTTTCTTCATATGCGGAG

>Unigene0001321

ACCGGGTCAACCACTCCATGACCGCTAAGCGGCAAGAGTGGTTACTGCCATCGAGGGAGT

GGTTACGGAGTTAGCTGCTGGATCAGAAGAACTCCCTGACGTTGATGAGGTTGCTGGTAG

AGTGGGACATGAGGAGGTTAGGTGCTCAAGAATATAGTACGCCCGGAGAGTGTTGTGCAC

AGTTTGGGACCATGGTCCCCTTGTGAGGTGTCAGAGAGTATGGAGGGAAGAAGGAAAGTT

TTGGATACCTTCGTGCTGAGGGTAAGAGATAAGAGCTTTGCTGGTGAGATGTGGAGGAAG

AGAGGATCACAACGGTGCGATCGGATCGAAAATGGAGATCGAGATTGTCCAGAAAAGGGT

TCAAGGAGATGAGTTTTTTTTTT

>Unigene0001322

TGCGCCAGTTGGTGAGAGTGACGAGGTATTCTTTCGTTGAAATGTCGACTATGGAAGTAG

CTGTTGGATCAGAGATGCTTTCTGGTGTTGATGAGCTAACTGTTGGTGGGGTGGGACCTT

TGGAGGTCAGGTGCTCAAGAAGATTGTGTGCTCGGAGAGTGTTGCGCACAGTTTGGGACC

AAGGGTACTCACTGTGAGGCCTCCGAGAGAAGTATGGAGGGAAGAGGAAATGTTTTGGTT

ACCTCCATACTGAGGACAAGAGATA

>Unigene0001323

GTCAGATGCTCCAGAAGATTGCGGTTGCGCACTGTTTGGGACTAAGGCCCCCACTGTGAG

GCTTCGGAGAGAAGCATGGAGGGAAGAGGAAAGGTTTTGGGGACCTCCATGTAGCCGGTG

CGGAGTGGAGGAGAAGGGGATCGCGCTGATACGATCGGATAAAAAAAAACGGAGAACTGG

AGTGCCCGAAGATGAAGTTGTAAGGAAGTGTCCCTATTTTTTGTTCTTTTTTTTTTAATT

AATGAACAATGC

>Unigene0001324

AAGTGGCACTATCAAGTATGTTCAGTTTTTTTCGTCTTTCACAATTTGGATCAATATTGA

CCCTTTGATGAGATGTTAAAATGGCTGTGATTCAGCAGAAGGTAAACAGGAGGGGTATCT

TTATATATTTTTACACCTTAAGGGGTGATTTTGGGAACCAAAAAGCGAGGATTAGTGTAT

ATTTAGGATAATTCATATCCTACAAGAGACATATTTGAAGAAATTCTACCCTTCCTGCAC

AGAGGGGCAGTAGGGTCTTTTTTGAATTCCTGGGATGCGGTGGCATGGTTGTTTTCAGTG

ACTTCTGAGGTCATCTTATCATTTTGCTTCAGGTGAGGTTAACGCCAAACACAGTAACTT

TTGTGTCTAAACTTTGTAGGGAAGAGTTGCAGCCCCTTTGTATAAGGTGTCTTTCAATAC

CTGCGATGCCACCCTAATTCAATACTTCAATTAAATAATAACCCAAATATTCACCAAAAC

TAATAAGTTATACATGTCCCTGTCCTAACTAACCGAGACTTTGCCCGACTAGTTATATTA

ATATTTCTCAAAAGAGCATCCACAAAGGCAGGGGCTACGCAGTCTCACCTATGAGACCAA

AAGCATTTGTTCCTCCGGATAAGTATGTGTACTCATCACGCGAAACCAACGCCTGGTGAT

CCATGCACACAAAATAAAATATTTTGTAAGGCATATCCGCATGGTCATCCCGGCCGTCCG

AGCCCTGAGACCTTTGTTTAATGATACAAATAGTGCCCAAACGTTTTAAATGTCGGCACT

GGTAGGGGTTGCTACAATTGACCAGATATAGCACTCAGTTGGTGGTTTGAGACGAGTTTA

GCAAGTCCACCCCGCTGATGGGGGTGGTTTTACGGGGTTTGGGGCTACATATACCGAATT

GCCCTCCTCATTATGGATGGTTTAAGTGAATGAATGACAATGTTCCACTATCCAAAGAAA

TTACAGGAATAACCGTATAAGTGCATACCTGAAACAATGTTCTGCTACCTCACATTTAAA

AGTTAAATATACATAGTTGTTCAAAAATCCATGTATCATTGTGGTTACCAATATCTTGAA

CGAGTTATACCTTCCGTGATTGTTCAAAAGTGCAATATTTGTGAAAGAGGATTCTTCGAG

TGTTGTGGTGTCTTCACGGGTATGTACTTGTAGAATTATATCCCAATGGTAATTCACTCC

ATATATTTACTTTTTTTTTATGTTTCATGGCACTATATAACCTTTTGTTGGTCTGGGTTT

CCACTATGCATAAATTGTGTAGTTAAATTTTCTGAATTAAAATGTTACTGTAGAGCAAAT

CTAATAATAAATAAGCGTAAAACGGATAAGGGTTGACGGGTCAAGATGACTCAATCCTAA

CCCATTTATTGAACTGGTCAAGTATCCTTAATCCTAACATGGTTGACCCTAACTATTTCC

TGTTTAACCCATTTAATTTAAAATAATTTTAAATAATAAATTTGAAACTAAAATAATCAA

TTTTTTATTTCTCCAATAAATTCTTTTGTAACCTAAAAAGGGTTATTATACGGCACATGA

TTTAATTTAACCTCTGGAGTGGAGAGGATAAGAAAAATTGTGATCAATGAATTTAGTTGC

AAAATCTAGAGTGGAGAGGATAATAAGTTAACATGTGATGTTAGGCGGATTTGGCGGATT

AGTGTTAGGTTTAGCGGTTTAAAATCCCATAAATGCCCCTCACCTTAGAGATTGGTCCTT

TTGTCTTTGAAATGGATCATACATCGGTTAGTTATTGGTACTTGAACACCTAATTGAGCT

AATTCTTTTTCGGGACCAACAGATAAAAGATGCATATGCCCTCAAGTTCTTTGAGGACAA

CTCGAGCCGCCTACCTGAATGGTGCAACAAAGATGACGACATCAAGCTTCCGTTCTGCCA

AATTAAGGGAAGATACAGGATGGAATTGCCCGAGTACAACACAGTGGAGCCATACTCACA

CATGAATGAGAGATGTCCGTCTCTGCCTC

>Unigene0001325

CCCCCATAATATTCTGGTCATCTCCTCCCCTCCTTAAAAATATCAATCCATCCCGCAACA

ACACCATAAAATATCTATAAAGTCTCTCAATCCCCCACTTTCACTCTTCTCACATGGATC

TCCTCATGATGCGGTCTTCTCTCAAAGCCCTCTTCCTCCCTCTCCTCCTCATACTCTCTC

TTCTTCTACTCTTTCACAGCTTGAATTCGCCTTGGCAGGGCGATTATAAGAGGAATTTTG

TGATCCATCCGGTAGATCTGCTCCCACTGCTGCCGCGGTGGGCGGCGTTGCCGGTCCTGC

GGTCACTTCACAGCGCCGTGGATCTGCTTCCAACGTTCATCGGGACGGCTTCATCGCCGA

ATACAACCCTCCACTGGAAGGGCGCGTGCTTCTATAACACAACGGCATGGATGGAGTTTC

ATAATGAGACAGGATCTCGCTATGGGGGCGGCACCCTTCATATCAAGATGGCCAATGCTC

ATAGTTGGACATGTATGGATCTCTATGTTTTTGCAACTCCATACCGAGTGACATGGGACT

ACTACTTTCTGGCTCGAGAACATACCCTTAATTTCAAGGAGTGGGAGGGGGAAGCTGAAT

TTGAATATGTGAAAAAGAAAGGTGTCTCAATCTTCCTCATGCAATCAGGGATGTTAGGAA

CTCTTCGGGCTCTATGGGATATTTTTCCTCTGTTTACAAATACAGGATGGGGTGAAAATT

CAAATCTTGCTTTCCTGAAGAATCATATGGGTGCATCATTTGAGGAACGGCCACAACCAT

GGTTTTCAAATATCAGTATTGATGATATTCATTCTGGAGATTTCTTAGCTCTATCAAAAA

TCCGTGGGCGGTGGGGTGGTTTTGAGACTTTAGAGAAGTGGGTCACTGGAGCTTATGCTG

GTCATACTGCTGTCTTCCTAAAGGATTCCGAAGGAAAGCTTTGGGTTGGAGAGTCGGGAC

ATGAAAATGAAGAGGGAGAAGATATTATTGCTGTGCTTCCGTGGAATGAGTGGTGGGATT

TTGAGCTGACCAAAGATGACTCAAATCCTCAAATTGCAATCCTTCCTCTGCATCCAGATA

TTCGAGCAAAATTTAACGAAACTGCAGCTTGGGAATATGCCAGAAGCATGTCAGGGAAGC

CCTATGGTTATCATAATATGATATTCAGTTGGATAGACACTATAAGAGGGAACTATCCTC

CACCATTGGATGCACATTTGGTTGCTTCTGTTATGACCATGTGGAGTAAAATGCAGCCAG

ATTATGCTACTAACATGTGGAACGAGGCCCTGAACAAACGTCTAGGCACCCAGGGTCTTG

ATCTATCTGACATTATAGTTGAATCTGAGAAGCAGGGTTTATCTTTTGAAAAACTGCTTA

CGATTCCCGAGCGAGATGATTGGATTTATTCAGATGGAAAATCAACCTCTTGCGTTGCTT

TCATTCTTGAGATGTACAAGGAATCAGGGCTCTTTGATCCGATCGGCAACTCCATACAAG

TCACTGAATTCACTATAAAAGATGCATATGCCCTCAAGTTCTTTGAGGACAACTCGAGCC

GCCTACCTGAATGGTGCAACAAAGATGACGACATCAAGCTTCCGTTCTGCCAAATTAAGG

GAAGATACAGGATGGAATTGCCCGAGTACAACACAGTGGAGCCATACTCACACATGAATG

AGAGATGTCCGTCTCTGCCTCCGAAGTATATCAGGCCGGAAGGTTGCTGAAGACGTTCTT

CCTTGGGGCTAGTATAAATATACAAGGGCCCACAATTGGAGCCAGAAACATGAGTTTGGG

AAATAGCTGTACATTTAAGTGTAACCACCATCTGTTGGATAAAGGAGTAGAAATGCATGT

ATATATGTGTAGTTGGAAAGATTTGATGTGGTTCATCTCCAGTTAGTTTGTTGATATTTG

TCAAATCACAAAAATAAATTCATGAACTGTAGTAGGATTTGTTGTTAACAGTAACTTTAC

CAAGCAATATAACATTGTTAGGTGTCTCGGATCTGCGTTTGTGTTAACTTAAAAAGGTGT

TGTGAAACTGTGACACAAACTTATTTGTACCTTGTGTTGGTGATTCATTTCGGGGTGAAC

CATGTGTATACT

>Unigene0001326

CCTCCCCCCAAAACCCCAAGAGGGTCCTCCCGACCGCCGGCGATATCTCGTTTCCGGCGG

AGGCGGCGATGTTCGACCCCTCCTGTCCTCGACTGCCGCTCCTCAGGCAACGCTCCCTCC

CGAACGCCGATATTCGCCGCCGCCGCCGCGAGGGAACCTGGCACCGCCGCAAGGAGCTCC

CACGCGCTAGATGCGGCACCGTCATCCGTAGCGTCACTGATGATGACTTCGATGAGCTCC

GCGGGTGCATAGAGCTGGGATTCTCGT

>Unigene0001327

ACGACACAGTGCTCGATAATCCGGAGGAGGCGAAGCGGCTGTCGCAGCTCCTGCCGGCGC

TGGATGTGTTCTACGACGTCCGACGGAGCTTCAGCGATGTGGCGGCCGGATCATCGTCGT

CGACTGATGGATCGCCGAAGGCTAGCCCGATCCTCTCTTCAGGTATCTGTTCTTTTTTTT

CTTTCTTTTTGAATTTGATTAGTCCATCATCAGATTTTAGGATTACCGGATCGATGTTCG

ATAGAATTGATGTATGTAAGAGTTTGCCTAAGGTTTTCTTCGGTACTGATTTGAGATTGC

CCTCAATTTCTACCGAAATTAGGATTT

>Unigene0001328

GGGTCCTCTCGACCGACCGCCGGCGGTATCTCGTTGCCGGCGGAGGCGGCGATGTTCGAC

CCCTCCTGTCCTCGACTGCCGCTCCTCAGGCAACGCTCCCTCCCGAACGCCGATATTCGC

CGCCGCCGCCGCGAGGAAACCTGGCACCGCCACAAGGAGCTCCACCGCGCTAGGTGCCGC

ACCGGCATCCGTAGCGTCACTGATGATGACTTCGATGAGCTCCGCGGGTGCATAGAGCTG

GGATTCTCGTTCGACGACACAGTGCTCGATAATCCG

>Unigene0001329

ACAAATCCCTCTAACAGAGAAGAGGATGGGTGTGTTGGATTATGTTTCTGAGCTCTGCTC

CGTCCCCCACAATCACAACAAGCTCAAGAAGAAAAAGCAGCTCCAGACGGTGGAGATAAA

GGTGAGGATAGACTGCGAGGGCTGCGTGCGGAAGGTGAAAAAAGCAGTGGAGGGAATGAA

AGGGGTTAGCTCGATCGACGTGGATCCGAAGCAGCACAAGTTGACAGTGATTGGGTATGT

GGAACCCAACAAGGTGCTCCGGAGGGTGCAGTGGAAGACGGGGAAGAAGGCGGAGCTGTG

GCCGTACGTGCCGTACGATGTGGTGCCGCACCCGTATGCGCCTGGGGCGTATGACAAGAA

GGCGCCGCCGGGGTATGTTCGGAATGTGGTGGAGGACCCGGACGTGGGGCAGCTGGCGCG

GGCGAGCTCGACCGAGGTGAAGTACACCACTGCCTTCAGTGATGATAATCCCAATTCCTG

TTCGGTGATGTGATGTGAGTTTCTGGTTTGTCCTTTGAGTAGGATAAGGGGACATGCCAG

CTTAGAATAAGGTTAGTAGAATGGTTGTATCAAGTCTGTAGATAAAGAGTCGGTCTTTGT

ATATTTTTGTTGTCATTTGGGTTCTTTGTTCTTCAAATCTATTTGGTGTCTTGAGTCTTC

AATCTATAAATCCCCAGGAACATTTTATATCTTACTATAATTATTTTGGGTGTCTTTGTA

ATTTTACTGTCAAAATAAATATGGGTGATTATATGAGCTTTATTTGATCGAAGATATTTT

CA

>Unigene0001330

CCGAGCGATTAAAACCATTTTGGAAAAGACTGTGAACCGTGCTAGGTCCGATTGGTCGGA

CAAACTCACTGATGCACTTTGGGCATACAGGACAGCATATAAAACACCAATTGGCATGTC

ACCCTACAGATTAGTCTATGGGAAATCCTGTCACTTGCCCGTTGAGATTGAACATAAAGC

CTACTGGGCCGTCAAAAGCATAAACCATAACTACGAACAATCTGGAAAAAAGAGACTACT

AGATATAAGTGAGCTTGAAGAGATTAGAGATAGCGCCTATGACTGCTCTAGGCGTTATAA

GGAGCGAATGAAAGAGATTCATGATCGATACATCAAACCCAAGAATTT

>Unigene0001331

CCTATCTCTCACATTTTTTCTAAATCAAACTCCATGCGGTTTCTCTCACCATTTCTCTTT

CCACATTTTTTTCTCTCACTGTCTCTCCTTGTGATTGCCTCTCTCACTGTTTCACTTTCT

TTTAATGTTTTTCACTATTTTCTTTTTGTAAATGTTTTTCTCTTTGTGCTTTCTCTCTTA

TAATTTCTCTCTCCGTTCCGAAACTCAACATTTTCCCTACTCTACCAACCATCCTTTAAA

TTTGTTTCTCCAAACCACCATTTTCATTTGTTCTCTCACCGATTCCTTCTCAGCATTTTC

TGTCTAGTATCTCTCCCCACGTTTATCTGTCTCTATCTCGAATTCCCGACATAGCGTTGT

CACTATCGCACGTCTTATCTCTCTTAGCGTTTCTCTCTTTGCTCACATTTTCTCTGTCTG

TGTTATGAAAATTCCACATTTCCCTATCTAACAATCACCCTTGATATCTCTCTCCCACAA

CCACCTGCACCATTCGTTCTTCTTAAAGCATCCCAAACTACCATCCTCTCATGATCTGCA

TATGCAGGCCTTAACCTTATTTCTCTCATACTCGACCTTATTTTCTCCTCTCTCATCGTC

TTCATCGAAACCTTGATGCAATGAAGGCCTCTCCTATATGGTGTCAACCATGACCTCCTT

CATGAGAAGCCGATGTGGGTTTTTGCCATGGTATCTCCCATCAATCCCCTTCTTCACTAC

GTGATTTTGTGGGCTTTAGTTGTACTAGACCTGTGGTGGGTAGTCTCAAGGTGTGCTTCC

ATTGTGTGTTGGAACTGCTACTAATGATTTTCTGTCATGGACTCGTGGTGGTATGATAGA

TCAGGTTTTGTATCACACGATGCAAAATAGGTATAAACATAAGATGAGGAAGAGTTGTTC

GGTGAAACTACTTTCGAGAGAGTATCGGCTTATCGAAGCAGAGAAGGATCAAATCCCGTG

GAAGTAAAAGGTGGAGAGGTTTTCTCAGTAAGATGTTATTGTAGAGGTCGACTGATCTGT

GGTGATATTTGGTTGTTGGGCCTACAGTGCTTTTCGCCCTTCCCGCTATCTGCTACATGG

ATGTGAGGTTGGACATTCTTCTCTCCCGCTATTACAATACACAACATATTAAAGTATATC

CCTGATTTCCGTGTCATCAATCAATTCATTTTTCTTCTATCTAGTTATACACCAATTGAG

TTCTCCTGGCTACAACCCTTTGTTGAAGGGTTGTGTATGTTGGGCAGTCACATCCCTTTG

TCCATCAAACCCTATATGTTTTAAGCTTTTGGCTTCTCTATATATCCAAACGATTTCTAG

ACCCAACTCTGAAGAGAACTAAGGCCCTCCACAACCGAGCTGCACGGCACGGCACAACAC

CACTGACTGATCCCCTTGATTGCACAACCTCTCAATACAGGAAAGTTGAGATGCAACCGA

TGATAGATTTAGTAATGGTGAAGGTCATGAAGTCGTTGGCTGTGGTAGTCATAGAAGTAG

TTTCATTGTTGCCATTCAAAAATTTTATGCAATTCTTTCC

>Unigene0001332

CCTATCTCTCACATTTTTTCTAAATCAAACTCCATGCGGTTTCTCTCACCATTTCTCTTT

CCACATTTTTTTCTCTCACTGTCTCTCCTTGTGATTGCCTCTCTCACTGTTTCACTTTCT

TTTAATGTTTTTCACTATTTTCTTTTTGTAAATGTTTTTCTCTTTGTGCTTTCTCTCTTA

TAATTTCTCTCTCCGTTCCGAAACTCAACATTTTCCCTACTCTACCAACCATCCTTTAAA

TTTGTTTCTCCAAACCAACATTTTCATTTGTTCT

>Unigene0001333

TTTACAATTCAGCTCAATTCCCGATCTCCTCCGCTTTCAAAACCCTAACCCCTCCCCCAA

TTTTTTACTCAATTTCTAGGGTTTCCACCTCGAGGTGGAGAGATTCCCAGAAATTTCAAC

CCTCACGTAAACAATTACGCAGATTTCAATGGCGGATCACTTATTTTTAACCCTCTGTCA

GTCCCTCGATCCAACAAACAATGGAGCTCCTCCTCCTCCTCCTCTTCGTCTTCTCTTTCG

GCGTGGTTCGGTCCATCGATCCGCCGCTCTCACCCAAGGGTTTGAACTACGAAGTGGCGG

CGTTGATGTCGGTGAAGAGCAAGATGAGGGATGACACGGGGGTGATGGGCGGATGGGACA

TCAATTCTGTGGATCCGTGTACATGGACGATGGTTGGTTGCTCGGCGGACGGCTTCGTTG

TCTCCTTGGATATGGGGAGCAATAGATTGGCGGGGACGTTGTCTCCCAGCATTGGCAATT

TGAGCCACCTTCAGACAATGCTATTGCAGAATAACGAAATATCTGGTCCTATACCGGCTG

ATATTGGGAAGCTATCTAAGTTGAAGACTCTTGACCTCTCTAATAACCAGTTAGATGGAG

AAATTCCAAGATCTCTTGGTCTTCTGACTCAATTAGGATACTTGCGTCTCGACAGGAATA

ATTTGTCCGGACAAATCCCTGTAGCAGTTGCTAATCTTGCAGGCCTTTCTTTCTTAGATT

TATCATTCAACAATCTCAGTGGTCCAACTCCAAAGATCCTCGCTAATGGCTATAGTGTTG

CAGGAAATAAGTTCCTTTGCAATTCATCTACCTTACATGGTTGTGCAAATGTCCCTTTGC

CGGTGAATGAAACGACCACGCTTGGAAAAACCAGCAACCACCCATTAGCAATGATCGTTT

CCTTAACTGTTGGGTGTATCTTGACGATTTCTTTGCTGTCAATTCTCTATTGGCTATACC

ACTGCAGATGGCGTCTACTCTTTGCTTCTGATAATCGAGATTATGATTTTGAAATGGGAC

ATCTGAAGAAGTTCTCATTTCGGGAACTGAAGATAGCGACTGACAATTTCAATTCCAAGA

ACATCTTAGGCCAGGGTGGATTTGGAGTGGTTTACAAGGGCCACCTTAGGAATGGGACTC

TGATAGCCGTAAAGCGACTGAAAGACCCTATTGTAAGCGGGGAGGTTCAATTCCAGACAG

AAGTAGAGATGATTGGTTTAGCATTGCATAGGAACCTCCTACGGTTATATGGATTTTGCA

TGGCACCAAATGAGAGGCTGCTGATATATCCTTATATGCCAAATGGGAGTGTTGCTGATC

GGTTTAGAGATCGTAACAGTGACAAAGCATCACTGGATTGGAGCAAACGAATGCGGATTG

CACTTGGAGCTGCTCGAGGGCTACTATACTTGCATGAACAGTGCAACCCGAAGATCATTC

ACAGGGATGTTAAGGCTGCAAACATTCTGCTTGATGAGAGTTTTGAAGCAGTTGTTGGAG

ATTTTGGGTTGGCAAAACTTTTAGATCAGAGAGAGTCCCATGTCACAACTGCTGTGCGTG

GTACTGTAGGACACATTGCGCCAGAGTATCTATCTACTGGACAGTCGTCAGAGAAGACAG

ATGTTTATGGCTTCGGAATTCTATTATTACAACTGATAACAGGGCAGAAATCTTTTGGTT

CCACTGTTGGTCAAGTTCAAAAGGGAACACTCCTGGACTCGGTCAGGGTTTTACAAGAGG

AGAAGCGACTCGAAATGATGGTCGATAGAGACCTCAATGGCCTCTTTAGAATGGACGAAC

TAGAGAATGCCGCCGGAGTAGTATTGTCTTGCACTCAGCCGAGTCCGACCCTCCGGCCAA

AGATGTCTGAAGTCGTGAAGTCGCTGGAATCTGCAATCAGACCAGAGCAGGTAGAAGAAT

CACACGTACAAATAGCTTCTGGAAAAACTTGGAGCTTCTCTAGAAGTTACGATGATGGTA

ACGAAGCTTCATCGTTTATCATCGAAGCAATCGAGCTTTCAGGGCCAAGATGATGATGAC

CACCTTCTTA

>Unigene0001334

AGATGATGATGACCACCTTCTTACACAGAATCGGTTTTGCTTGTAGTACTGTTGCTGGTT

GATTGTCCAGTCTCTTTTTGCTGATAGAAGATACAACTGTAAATAGCCAGTGATACCTTG

ATGTATCTTGCTGGTAAACTCTTTCAAGGTTAAAGTCACTTCTTAATTTTGTCAATCGAT

GGTAGCCAAGATGTTTCTGGAATATTCATAACATGTACAGGGAC

>Unigene0001335

GGAAAGCCGGCGAACCTCTCCTCCCCCCACCACCGCCGATCATCGCCGCCACCGGCGATC

ACCGGAGCTAATGGCTGACCCCTCCGCCTCCTCACCCGCTTTCCCGTCCGCCTCCTCACC

CACCTTCCCTTCGAAGAAGGCCCAATTTCTCCCCCGCTCCCTCACCTACCACCACCACCA

CCAGCGCCGCCTCCCTGTCAAGCACCTCTGGCCCCACTTCGTCGCCGATGACCCCAAGCG

CGCCGCTCCCGCCCTCCGCGCCGTCCTCTACACCACGTCCATCCGCTCCATCCGCCGTAC

GCACCAAGACTGCGCCAACGTCCGCTCCATCCTCCGCGGCCTCCGCGTCGCCGTTGACGA

GCGCGACGTATCCATGGACGCTGCCTACCGCCGTGAGCTCCAGTCCCTCCTCGGCAAGCA

GCCCCTTTCCCTCCCCCACCTCTTCGTCGCCGGCAAACACATCGGCGGCGCCGATGAGAT

CCGTCAGCTCCACGAGGCCGGCGAACTCATGAAGATCCTCGACGGCGTTCCGGTACAGGA

TCCCGGCTACGTCTGCGCGGTATGCGGCGGCGCCCGCTTCATCCCCTGCTCCCCCTGCAG

GGGCAGCCGCAAGGTGTTCGTCGAGGAGGAGGGGAGGATGCGGCGGTGCGAGGAGTGCAA

CGAGAACGGATTGGTACGGTGCCCTAACTGCTGCTCCTATTGATGATGATGAATTAGGAC

TCCGGATAGATGTATAATTAGGGTTTCGCGATATTGGCGGATTGGATTTCGATGGAGATT

TGGATTAAGGTAGTATTTGTACAAATTATAGAATTAAAATATAATCAGATTGGGCCCAAT

TAGGGTTATCCGAGCTGGTATTGTAGATTGTGATGCCGTCGGATTTAGATATGGTTTGCA

AGAATAAAACATGCGAGTATATTTATATTAAAGTTTGGAATTGCCGACATATTGTGTGTT

CAGATGGTTGGATCTTCATAAGGGAAGCTTTGGTTTTTTGTGTTGTGTAGTTACGAAATC

TTTGATTTGGGTGCTTGGTATGTTGGTTGGATCTGCTTTTAGTTGTTATTATGCTTTTGG

TGTTATTTGTATGATGACAGTTATGATCTTGCTAAATAAGATGTCAGTTAT

>Unigene0001336

TCCACAAATACACTATTCCACAGTTCTCAGTCATCCTGCCATCGGCCTCCAAACCACCGG

GAGCGCACGGCACCAAAAATGCTCAAGTTCTTGTCGAAAGTGCAGATCCAGTTCAACGCC

CTGGATCCCCGGACGGCGTCGTGTCTGGAATTCCTGGCGCAGTGCACGTCGCGCAAGGCC

AAGGAGTCGAACCCCAGCTGCCAGTTGACGCTGCAGCGTCGCACCGATGATCACCCGCCG

CAGATCACCGTCACCTACGTCAATGGCGTTGAGGAGGTCATCGACGCCACCTCCGTCCCG

GCCCAGCACATCCGCAACCGCATCCTCGAGCGCGGCCAGCTCCTTGAGACTGAGCAGATG

TTCCGCGAGGCCGGCGAGCCCTGGCCGGTCCTCATTCCTGAGGAGGAGATAAACCAGAAA

TTCCCCGGTACCAAGCCTAAAAAAGCAGAAGAAAGGAGCCAGTAACCAAGCCTGGCATCT

ATCATTCCCGCAGTTGAGGTGTCGAGATGAACATGAGACTCTTTGCCATTTTAGTTTTGG

CCAAGGAATGATGGGTTAATGAATTTTAGTTATATTTATGAATAAGATGTTCAAAATGAT

GGAGAGAAGGAATGGCACGGATGCAACTCTGATTATTTTTGGTTTCTACTCTCATCTACC

TTTTTTTGTTCTAACACTTCCAGTAACTGTAATGGCTCGTTCGCCCTACATATAGTTGGT

CCAGGGCATTAGACTCCATTGCACAACTTCCCCTGAAATATTGTACCATATATTCAAGTC

AATCACATTTTTCTGGAGTATCATGTAGTGCTAAATTTCAGCATGATTTAAGGTTGGTTG

CTTAGTTGATTTGAGTAA

>Unigene0001337

CGGCGCGTTATCAATTTTTTCCGCGCGTTCTCTCGCCGTCCACACGCAACAATATTCCGA

ATCGGAGCAGCTGCAGGTGCGGCTTACAAGTGCGGTCCACGACGATATCGGCAATATTAT

ACTTCGGTCACATAACGCCCAAATATCGATTTCCAGTTGGCCGGGAAACCGACATAAAAT

TTAAGCTAAATTTCCGAATCAGTTTTTAAAACAAGAAAAATGGGAGAAAGACGTGGCACT

AAAGCATTAGAACTATGGTG

>Unigene0001338

CGGCGCGTTATCAATTTTTTCCGCGCGTTCTCTCGCCGTCCACACGCAACAATATTCCGA

ATCGGAGCAGCTGCAGGTGCGGCTTACAAGTGCGGTCCACGACGATATCGGCAATATTAT

ACTTCGGTCACATAACGCCCAAATATCGATTTCCAGTTGGCCGGGAAACCGACATAAAAT

TTAAGCTAAATTTCCGAATCAGTTTTTAAAACAAGAAAAATGGGAGAAAGACGTGGCACT

AAAGCGTTAGAACTATGGTGTAAGCGAGTGACTCAAGGATATGATGGAGTGTCCATAGAA

AATATGTCTACTTCGTGGAAAGACGGACTTGCATTTTGTGCTCTTATTCATCATTTTAGA

CCTGATTTGATTGATTATAATAGTTTAGACAAAAATGATATTTACGGTAACAATGAGTTA

GCTTTTCGAGTTGCTGAAAATCACTTGGGCATTCCTGCACTTTTAGATCCAGAAGACATG

GTTTGCGTCGAAATACCAGATAGATTATCGATATTAACATACTTATCACAGTTCTACCAA

ACTTTCTCATCACAAGCTACAACGCCTGTGAAAACATCACCTGAATCGCCAACCACAGTT

GAACGGCCCAGAACTAACACAGGATCCGGTCAAAATGCTCCAACAAAGTTGGGCGCAGGC

GTTGGCGGTTGTAGAGTGTGCAACCAGTCCGTGTTCCTAGCCCAACGTCTGCTCATCTCT

GGTCACCTATTCCACCGGACGTGTTTCAAGTGCGCCCGGTGCTCCGCCCTGCTGAGCGTT

GCGAACTATTACGAGACGCAGGACGGCAAGTATTGCTGCGAAACGTGCCCGGACGAAGAG

CGGACGGTGTCGCCGACCGTGTCGTCGACGACGGCGGCTACGACGACGCCACAGTCGCAG

CAACCGTCGTTGGTCGGCGATCGCCGTGCGCTATTCGAAACGCTGGCCGCCGCCGATTGT

ACAGACGACGTGAAAGCGTCGAAAAAGGCGCAGCGGCCGGTCACACTCAAAATGGTCGAT

CCTCTGACGACCACTACCGCCGCCGCCGCCGCTGCCGAGAAGTCTGCTGTCACGTGCAGC

GACGACAACGACAGCGGCGTAGTGGTGGACAGCGACAAGAAAGAGTCGAGTGACGCAAAA

ACCTCGGCAAGCGTCACCGGCAGTCGCGAAACCCTTTCCGACGTAGTGGCGGTGGTTGCC

GACGAGGATGGCGAATCGGCGTTGCCGCCACCGTCGTCGGCGGACGATCGAGTGACGGAC

AATGCTACTGCTGCTGTTGTCGTATCCGACCAATTGCCCGACGTCGGTGGTCCGCCCGTC

GTCGTCGAGTCGTCACCTCCTACTCTGCCGTTGTCGGACGATTCAAAACGACCCGACGAC

GAAATAGTGGTCGAACCACTAAAACACGTTCAGATCCCGCCGATATCGGATGATCTGAAA

CAAGTCCTCGAACTTCCTCCGGCTAGTAAAGCCCCGGCCGAAGAGCAGCTTGAATCCCCG

GAAGATTACGAGATAAAAACCCTATCCAACAAGCTGCTTAAAATGGATTATTACGAGAGT

AATAAAGAGGTACCGGTTCCTAGTCCTAGAAAAAGAATTAAATCTCCGATAAAAACCAAT

TACCCTGAAAATTTAAATCCTTTCGACGACGAAGAGGACGATGGTAACAATAACAAGAAT

GATAGCACAAATCCGTTCGGATCCGATTTCGAAGACGAAGAACTTTCAGTTAAGAAAAAC

GTTAGTTTGAATCCGTTTTCGAGCGATGAAGATGACAACGAGCCTGTTTCTCAGCCCCCA

TTACCAAAACCTAGGACGGTTTTGTCTCCTATATTGAAACCACGATATCAAACCCATCGT

AAGCAAAGTAGCGCTTCGAGTATATCTTCCTATTCAAATACTCCTCGAAAAAAGAAACCA

GCTCCACCACCTCCAGTATCAAGACTATCTCTTGGAAACATTGAATACTCAAATAATATG

CATATGTCGTCTCCTAACAGTTTAGAAGACTCCAGTTGTAGTGGAAGTGAACCATCATCA

ACCACTAACCAGGAACGATACAGATTACACAAGAGCACACATGGGAAATGGAAAAGAAAG

AAAGGACCAGCTCCTCAGTGTCCAATACCACAAAGAAGACGAATTAAACCTATGCCTATG

GAAGATATTAAACAAGAGTTGTTAGATATTGAAATAAAACAGCAAGAGCTTGAGAGACAA

GGAGTTAAATTAGAAAAAGATATAAGATTAAAGTGTAATGAAGATTTGAACAATGTTGAT

GTAGAAGAAATGGTGTTACAATTGTTTGAATTAGTTAATGAAAAAAATGAATTGTTCAGA

AAACATGCTGAACTTATGTACCTGCGAAAGCAACATAACTTTGAAGAAGAACATGCTGAA

TTAGAATACCAAATACGGGTATTAATGTCTAAGCCTGACCACACTAAAACAGATACAGAA

AAAGCACGAGAAGAAGAACTTATCCGAAGATTGGTTGAAGTTGTTGAAAGGCGCAATGAA

ATTGTTGAAAGTTTAGAAAAAGATAGACTGCGCGAAGCCGAAGAAGATCGTAGTGTGTTC

AATCAAATTGGAACTTTTGCTCAAGGTTCAAAAAATATCCATCGTATAGTTTACTAGTAT

AAAAATATGTTTGACATCATTAGTGGTTTGCGGAATGACGTTTTGTTCGTTTTTTAAGAT

GAAACTTTGCCATGTATCAAACCAGTAAATACCACAGTTATGGAAGGCAAAAATAACTTT

TCCAAAAGACTGTTGAAGAATATCAAAAAGGTAAAATCAAAAAAAAGATAAACAGGAAGT

TGCATCACCATCTCAATCGCCTTCAAATTCCTCACATAGTAAAGGCACTTTGAAAAAGTT

TATGACTCTAGGTCATTCGTCTAAGAAATTGTTGAGGCCTAAATCCAGTTAGAACCATTG

TTGTGTTAAAATTGTGAACTGAAGAATTATTGTAATATATTATTATAAGAAGTTACAATT

GATTTAATAAAAATTATATCTTTTAAGATTGTACATTTTACTTTTCTCTGCAATTGTAAT

TTTTTGAAATGTTATGTAAACTGGCAACGATTTGTTAATTTTTTATTATTAAATATTTTT

GTTGCAATTTAAAGAGCCATGTAGTCGTTATCAATTGTTTTATTTTGTGGCATTGCTTAA

ATAATTGAACATATCAAATACTCAATCATATGAATTGTTAGATTTTCATGTGTATAATAT

TTATGTAACTATCAATTTTGTACTTAAGTATTACTTAACATTAAATAAGTTAAATTATTA

TACCAATTATATTTAATAAGTAATAACCCATTTACATGACATATACTCATAAACCTAGAA

TATGAGCCTATTTATATTTATTGTTTAAATATGTTAATTTATGTTTAAATTTGTTTCTAA

TTAA

>Unigene0001339

AGAGTTGAAACCAAATTGAGTTGATATAACATTCGCATATGTTCACAAAACATTCGTACA

TCACATTAACAGCAAACGCTCTTCTTCTAACCGAGCTTGGACAGGACATCGTTGAGGGTC

GACACTGTCTGAGCATAATACTTCTCTGCTTCTGAGCTGCTCTTGATCTTTGCTGCATAG

TCCAAATTGTTGATGTCTTGGAAGAGCTTTCCTGTGAGGTCCTTAAGGGCTTGCTTCTCT

TCCTTAGGCTTGGCAGATATGATTGTCTTGAGATCATAACGGAGATACTCCGCCTTGAGA

CGAAGGTCGTCTCGAACGAACGGCCACGCCTTCTTGTCAATCGATCCTTTTACATTGATG

ATATCCTTGGCTGATTCTTTCGCCCTGGCTACGGCCAATTCTGGACTTTGTGGTTGCAGG

AAGAATCGCTCCTTTAAGGGCAGGTCCAAGTCTCGTGGCTGGTCCGAGTTCAAAGTCCCA

GGGAGTCCACCGGAGGGAGGGGGCGGGGGACCAAGCTTGATGGGCTTAGCATCAGCGAGA

ACGGCTTGAACGAACGAGGCAGAGGTGACACCGGCCGCGACCAAGCCAAGAACGGCCCTG

CGGCTGGTCTCTGGCTCAGCAGGCACTTGCTGAGCTCTGATCATCAACCCAGACCGGGGG

ACGGAGGACCTGCTGCTGCTGCTGCCGCCGCCAGCCACACTCAATCGGGTAGAGCCGCTA

ATTTGAAGGCTACCTTCCAACACAGCTTGAGAGGAACCACATAGCCCTGCCATTGACGCC

ATAGCTTATGATCAGCTACCTTCTT

>Unigene0001340

CGTTGAGGGTTGACACGGTCTGAGCGTAATACTTCTCTGCTTCTGAGCTGCTCTTGATCT

TTGCTGCATAATCCAAGTTGTTGATGTCTTGGAAGAGCTTTCCTGTGAGGTCCTTAAGGG

CTTGCTTCTCTTCCTTAGGCTTGGCAGAAATGATTGTCTTGAGATCATAACGGAGGTACT

CTGCTTTGAGACGAAGGTCATCGCGGACGAATGGCCAAGCCTTTTTGTCTATTGATTCCT

TTACATTGATGATGTCCTTGGCTGACTCTTTCGCCCTCGCCACCGCCATCTCTGGGCTTT

GTGGTTGCAGGAAGAACCTATCTTTAAGTGGCAGATCCAAGTCCCTTGCCTGGTCTGAGT

TCAGAGTCCCAGGGAGTCCACCGGAGGGAGGGGGCGGGGGGCCGAGCTTGATGGGCTTAG

CATC

>Unigene0001341

GAGAAGATGTGGATTTTTGTGAAGGGATTCATTGTTTAGTTGTGAAGTTTGGGTTTGAAC

TTGATCTGTATGTCTACACTTCTTTACTGACAATGTATGCCACATCGTCAAGTGTTTTGA

CAGCTTGCTCGGTTGGAGAAGATGTGGATTTTTGTGAAGGGATTCATTGTTTAGTTGTGA

AGTTTGGGTTTGAACTTGATCTGTATGTCTACACTTCTTTACTGACAATGTATGCC

>Unigene0001342

TCCCCCTCCCCCACCTCACTCTCGCCGGATCGCCGTCTTCGTAGCACCCGCCACCTCGCC

GCCGCTAGCCGAAACCTTCCTCCACATCCGTCGCGTCCACGTCGCTCGCACCTTCCGCCA

CCGCACTCTGCCCCGTCCTCTCCGGCGATCCGCGAGATCTGCCGACCAGACTCGGCCGTC

CGTGCTGCCACCGCCCCTGCTGTCGCAAGTATCGCCATCACCCGGCCAATCATGCTGCCT

GGATCATCTTCGCCGCCTTGTCCTCATTTCCTCTTCAATCAGATGTGAGTCGGCAGCTGA

GTTCCCAATCTGGCAGCCTCATCTCATCCCAATCCGGCAGACTCATCTCATCGCCGCACC

ATTAAGCTCTCCACAACCACCTCTCTCTCCGCCTGCATCCCCAACAATGGAGCGCGCCCG

CAAGCTTCCCCTCCCCACTGAAATTTACCCCCAAATCTAACCAGGTTCACCGTCCCCTCC

ATACTCAAATCCTGCACCACCCGCCTTGACCTTGCCCTCGGCAAGCGCATCCACGCCTTC

CTCATCTAATCCACACTCCACTTCGACCCCTTCATCGCAAACGCAAATGCTGTTCTCTCG

CCAACGCCCACAACGTGTTCGACCAATTGTCCCACCGAGACCCCACTGTCTGGAACATCA

TTGTTGCCACCTACTTTCACCACCGCCGCCCAATCCATACTCTCTCACTCTTCTGCTACA

TGTACCTCGCCAGCGTCAAGCCCGACGCCCACTCTCTCAGCAATGTCAGCATCAGTAGCC

ACGCAACTATCAACTTCATCAAGCAAGCTCACGCTTACATTGTTGCTAATGGATTCTATA

GTGACCCTTTTCTGGATATGTTTGCCCGCATGAGGACAGAAGGTGGGCAGCCGTAGGCTG

CCACCTTTTCAAGTGTTTTGACAGCTTGCTCGGTTGGAGAAGATGTGGATTTTTGTGAAG

GGATTCATTGTTTAGTTGTGAAGTTTGGGTTTGAACTTGATCTGTATGTCTACACTTCTT

TACTGACAATGTATGCC

>Unigene0001343

AATCATATGCGAGTCCGCAGCTGAGTTCCCAATCCGGCAGTCTCATCTCATCGCGGCCAC

CATTAAGCTCTCCACAACCACCTCTCTCTCCGCCTGCATCCCCAACAATGAAGCGCGCCC

GCAAGCTTCCCCTCACCGCTGAAATTTACCCCCAAATCTAACCAGGTTCACCGTCCCCTC

CGTACTAAAAGCCTGTACCACCCGCCTCGACCTCGCCCTCGGCAAGCACATCCACACCTT

CCTCATCCAGTCCACACTCCACTCCGACCCCTTCATCGCCGCCTCCCTC

>Unigene0001344

GTGGATTTGCTCATCACTTCAAAGCTGGTGCACTCAATGTTCTTCTTCGTGTGTCAAGCA

CCATCAGCAATTCTCCTTTCATATTAGTGTTAGATTGCGACATGTACATGAACAACCTGG

ACTCAGCTAGACAGGCCATGTGTTTCCACCTCGATCCAAATCTGGCGCCTTCACTCGCCT

TTGTTCAGTTCCCCCAGTGTTTTAACAACGTCAGCAAGAATGATATCTATTCTGCTGAAT

TGAGAAACTTTTTCAAGATTCTTTGGAAGGGCTGTGATGGATTAAAAGGGCCAATATTAT

CTGGTACCGGATTCTACATAAAGAGGAATGCACTCTATGGAGTTAAACCTGATCTCACTA

CATTGAAAGAACTTTGTTCCGCTGGCAACTCC

>Unigene0001345

AGGTCCAATATTATCTGGTACTGGCTTTTACATAAAAAGGGATGCACTCTATGGAGTTAA

ACCTGGTCTTACATCATTGGAAGAGCGAAAGAATACATTACGTAGCTCGAATGGGTCTAT

TGCATCATTGAGTCAAAATACAGTGAGCAGATACACAACCATATCGTCATCAGAGGTTAC

TCGAATGGCTTCATGTACATATGAAATTGATACTAAGTGGGGAGAACAGATAGGGTTTTT

GT

>Unigene0001346

AGGTCCAATATTATCTGGTACTGGCTTTTACATAAAAAGGGATGCACTCTATGGAGTTAA

ACCTGGTCTTACATCATTGGAAGGTGGTACAACAATTATATTAGTGTAATATTACATTGT

TAAGGCCAATTTGGATTTTATTGTAGTGAGATTCTCGACTGATGTCTCCCCGAGAGTGCT

CGGAGAGCTCTAAATCCTATTTGGATACCCTGATGATTTGATAAATTGGGACTTAATTTC

AAATCTCAAATGACTGCGGAGTGCCTAACATCTGAAGCTCCGATTGGCGTTTTTTGCCTT

ATATTCTATACTCATATTTTGATTTAAGATTTACTATAGCAGGAAGTCCAAAACTTATGT

TGGGTTCTTCGACCATCTTTTGAAGAGCGAAGATATATGAAGTCCATGTTTGACGCTTAT

GATCAGAAGATCATAAACTTATCCAAATCCAAACCTAAGAAACAAAAACAATCTCTGATA

ATTCATTTTACGTGTCCATTGCTCAAAACTATAATTTCAAGACACTTGTTCTGTTTGTCC

AGAGACGTGTATTGCATTTCTCTTGTATAGTCTTAATAGTTTTTCGTATTAATGATTGAT

AATCAACTTTTAGCATCCATGCTTTGTTCTCTTTTGCTGATATAGAACTGTGTAAATTAA

TTTTCTTTTAACTAATTTAAAATTCATTTTCATGATGCATTCTAGTTTTTATTAGTATGC

AACGGTGAAGTTGTCATGTTTTATTTTAAGAAATATTTTATATTCATTCTCTCTTTATTT

ACAATAAGATATCATCCTTATTAATTATTACATAAGTTTTCTTGATACATTGACTTATAC

AAAATTGGATGTTTTCTTTACAATGATGTTTCACTGGCCACTTAGTAAAGGATAAGATTA

TTTAGGAATTTTAATAGG

>Unigene0001347

TGTCCGTTTTCCGTCTATATTGATGTTTTCTGACCCAAATGAGTTCTCAAGGACTCAACG

TCTTGTTATTGCTTAAAAATGAGTTTCTACACTGATTGAACGTTCCACCTGCATCACAGA

GCCAAAGAATGCATTCCGTAGCTCGAATGGGACTATTGCATCATTGCGTCAAAACTCAGT

GAGCAAATACACAACCATATCGTCATCAGAGGTTACTCGAATGGCTTCATGTACATATGA

AATTGATACTAAGT

>Unigene0001348

TTTCCACCTCCTCCCCCTCCCCGTATTTCAATCTCTCCCACAATTCTCATCGCCTCTCAC

GCCCATTAACCCGCCAACACAGGCTCTCGCAGCGGATCTGGGACCTGATGGCGACGACGA

AGAGACCAAGGAGTGGATCCGGTGGTGCCGCGGATCTGAAGAGAGCGTCGGAGATCGTCA

TGGTGCTGTCGGCGTTGGGGGAGATACGCGGCGGCCGTGATCCGACAGCCGCGGAGAAGG

CGCTGGTGTCGGAGGGTAGGGAACTGCTCGTTGGGTTGTGCGAGCAGGTGAAGCCCAAGG

ATCTGTTCTCGAGAGAAGCAGTCAAGTTCGTCGTCGAGGACTTCGGGCTCGATCCGAGAT

TCCGGTCGCCGAAAATTTCCATTGCAGATAAGCTAATGCTCACCAAGAAAAAGATGGAGG

AATCCGAGGAACGACTCAGATTGAATGTACCATCCACAAGGCCATCTTTTTTAGCGCAAG

TTGGAGGTCATCGAGCTGAGTATGCGCTAAGCAAAGCCTCATCAACTTCAGTCCTCAGCC

AAGAAAGTAATACTTTCCAGTCCTCCAATCAACTGACAATAAGTCATGGTATGCAGGCTT

CTGTTGCACATGCTGCACCAGAAAGCCTGCTCATTGGGCACCCATCTTCAAGAAGCTTAA

CTTATGTGCATGCAGATTCGGACATTACCATCCATAATGACATTGCAAATAAAGTTGAGA

TGGTCTTGCAGCAAAAAGTTTCTGATCAATCTATCTGTACTCTACCATCAATTGACTTCA

TGAACAGACCCCTAAGTTGCCAAGTTTGCATGATTATTATCAATGATGGGGAAAGTTTAC

TAGTATGTGATACTTGTGAGAAAGGAACTCATCTGAAATGTCTTCGGCCTTTTGCTAATG

GAATCACTTCTAAAACTGTCTGGCATTGTCCAAAATGTGTGAAAGCAAGTAATGGCAAGT

CTTTCCCTCCAAAATATGGCCCGGTTATAAGGTTGAGTGGTACGTCCAAGGCTTCTTTGA

ACACGCTAGTGTACCCAGTGAAAAGACGAGATCATTCAGTTGCAACGGTAAACAAAAAAT

TAGTATCTAACGAGAAATCAGCTTCATTATATGCTTCAAACATGATGAGCACAGTTGATT

GGGGAGATGACATTCTTGAAGTTATAGGAGAGAAGGTTTTCTATATGTCATGCTGCATTA

ATGGAACATCTCACAAACTTCAAGATCATGTTCTTGTTTCTTGTAACAGTCGGAAATTAT

TCCCCTCCAAACTACAGTCTCTGTGGGAGGACAAGAAAACTGGGTCAAAGTGGGCTACTG

TGGATCCATATTACCTTCCCTGCGACCTTCCAGAGGCTGTCAAGCGACCATCTACCCCTG

AGAAAAGTGAGGTATATGCTTCTTCAGATAAGAGGATCACCATATTGGCTGGCTCGATTC

ATGGTCCTTGTGGAGTCTTAACTTTAGACAAGTTAAGGGAAGAAAGTGATAAGACTCAAT

TAGGAGATACAAATAGAGGTCTACGTCCAACTTTCATGTGCAGATGGTTATATGATGAAT

CGAAGGGTGTCTTTCAATCTGTCACCAATTAACTTAGTGTTTCTATTTTGGACATCAACA

TCTAAATCACAATCAGTTAGTCATCTTTGGTTATCTTTTGAAGCTTTAAGTTATTAGGTG

CGAACATGTTGATAGAAAGATAGGAAGGCTTGACACCAATTTTGTACCATATGTGTAGTT

GTCGAGGTTAAACCAATCTGACAACCTTACGTAGTTGTGCTTATTATATCT

>Unigene0001349

AAAAAGCTCCAATCACCTCTCCCGTCTCCAGCCCCAAACACTCTCCGTCAACAAAGTCCG

AGCAGCCGGTCCGCGCCCAGCGCTGGACGATCGCGGCGGATTGATCCGCCCCAGCCTCCG

TCATTCCATATTCGATGCCGCCCGCCGTCGTGCCCGCGCCGCCGACTGATATCTCCTCCT

CCGCGCCGCCTGCGGTGATCTCGGCCTCCGCCAGCCCGTCTTCTTCGCTGCCGGAGGGGA

TCTTCTCCCTGCCGCCCACAGTCATCTCCTCCTCCACGCCGTCCGCTGTCATCTTCGCCT

CTGTGTGGATCTTCTCCTTCGCACCTCCTGCCGGGCTGTCATCTCCGCCTCTGGGCCGCT

CTTCGGGATCTCCTCCTTCGCACCTACTGCCGGGATCTTCGCCTCCGCGCCGCCGCGGGG

AGGCTTTCATCTCCTCCTCTTAGCCTCTACTGTAGGTTGCTCTAGTGTGACATCTTTTCC

TGCCTCGCTTGGCCCACGACATTCCTCTTCCCGTCTCGACTAATGAGATCTCCTCCTCCA

ATGCCATGGCGACCGTCAACTCGTCCCAATGGCAGCCAACAAATCGAGTGTGATGTCTTT

TTCGCCTTTCTCAAAGGGAGAGGAAATATAAGGAGTGAGGAATTATTGCCTCTTACGACG

ATCTTCTCCCGCAGGATCAGATATCTCAATTTTGACATTCTATCGTAAATGTAAATATCT

TTTTATTCTGCTAGGTCTAATCTCCATGAGAATATATGGATCAATGTTGGATACAATGTT

GTTAGTGTTACG

>Unigene0001350

CCGCGCCGCCGACTGATATCTCCTCCTCCGCGCCGCCTGCGGGGATCTCGGCCTCCGCCA

GCCCGTCTTCTTCGCTGTCGGAGGGAATCTTCTCCGTGCCGCCCACAGTCATCTCCTCCT

CCATGCCGTCCGCTGTCATCTTTGCCTCTGTGTGGATCTCCTCCTTCGCACCTCCTGCCG

GGCTGTCATCTCCGCCTCTGGGCCGCTCTTCGGGATCTCCTCCTTGGCACCTCCTGCCGG

GCTCTTCGCCTCCGCGCCGCCGCGGGGAGGCCGTCATCTCCTCCTCCTAGCCTCTACTGT

AGGTTGCTCTAGTGTGACATCTTTTCCTGCCTCGTTTAGCCCACGACATTCCCCTTCCCG

TCTCGACTAATAAGATCTCCTCCTCCAATGCCATG

>Unigene0001351

TCCACACACAACTAATGCTGTTCTAACTCCCCTCTTTTTCTTTTCCTCCCTTTTTTCCCC

ACCCAACTCAACTTCCCCTCATTACCCCCTTTTATTATTCCGCCACCACCATCACCTCCC

CTTCTTCATCACCACCCCCATCTCTGTTAGCTCACAAAAGGTGTCACCTTTCTCCATCAA

TCAATGGAAATCCATGAGCTCCCCTCCCCAAACATCTCCACCATCAGCAACAGCGGCCCG

CCGCCGTCTCCTCTCACTCCCAAGTCCATCCCCCGCTCCGACGCCAACCCGTACCCAACC

ACTTTCATTCAGGCCGATACCACCTCTTTCAAGCACGTAGTCCAAATGCTCACCGGCTCC

GCGGAGACGGCTGCCAAGACCGCTGCTGCCGCGGCGGCTCCGCCGCCGACTGCCCGCCTC

CCAGGCCCCAAGAAGCCGGCGTTCAAGCTCTACGAGCGCCGCGGAAGTTTCCGGCATCTG

AAAATGATCAGCCCGCTCCTCCCGGCGTTCCTCAGCTCCAACCCTGGATCTCCAGCCGGC

GGACCCTCACCGCGGAAGCAGCCGGAGATCCTGTCTCCTAGCATACTCGACCTCCCGTCA

CTTGTGTTGAGTCCCGTGACGCCGCTCATCGCCGATCCCTTCAACCGCTGCTCACCGAGG

CCGCCGAACTCAGCGTCGTCATCGACGATGTCGGAGGAGGACAGGGCCATCGCCGAGAAG

GGTTTCTACCTCCACCCGTCGCCGCGTGCGACCACTCCCCGGGAGGCGGAGCCGCCGCGG

CTGCTGCCGCTATTCCCGGTGACGTCGCCGAGAGCAGCCGCTCCGTTCACCGGCCATTGA

GGTAAAATGATGCCTAATTGTTGAGCTTTTATGTGACTTCGCCTTGGAGCAAGATTGGGC

TTGTCAACCCATTGATGGCAAAGCTAGTAGCTTTTGTGCTTCAATCTTACAATTTAGGAC

TTGGAACTCTGATGATCAAGGTGTAGATCTTTTCACTTCCATCAATAGACTGGAATATCT

TCAATGGGAGAAATAAAACAAGGGATTGATGCTAGCCAGATGATGTTAAATATTTAGGTT

TATCATAAAATAATTTCAAATTTTGTTTTAGTGTTAGTAATTATGTTTGAATTGGTTTTA

AAATTTGAGATGTGAAGTGTATTGGTTTGAAATCTAAAT

>Unigene0001352

AAAAAGTCTTATTCAACATGGTCATTTTATGTCGGATGAAATCAATGAGAAGATCCAAAT

TCTCGAACAAAGACATCATTTCATGACCGAAACTTGGGAAAAACGTAAGGTCTTGTACGA

TCAAAATTTGGACACACAGCTGTTTAAACGTGATGCGGACCTACTGGAGTCTTGGATACA

AACCCGAGAACCTGTATTAAACGATGACAAGCTGGGTGACTCTATATGGCAGGTCGAAGA

ACTCATCAGGAAACACGAAGATTTCGAAAAGACAATCGAAGCGCAGGAAGAGAAGTTCAA

TACGCTAAAAAGGATTACTCTGTTGGAATTAGCGTTTAAAGAACAACTGGAGACCGAAGA

GCGGGCTCGAGTTGCGGAAAAAGAACGACTGGAACGAGAGCGTCTGGAAGCACGCAAACA

ACGCGAAGTGCAACGTATTACAGACGAGAGAAAGAAAGAAGGTGATCGGTGGAAGGTCAA

TAACGCCGGCGAGACGATGAATGGTGACGATGACGAATTCGACCCTAACATGAGGCTCGT

GGTTCCCGGATCACCAGCATTAAACAACAGTGGTAATTTCTCTTCAATTTTCGGAAATCG

TATGAAAAAAGACGTAAAACGGGCTGAAAGTATGAAAGCGGACGTATACAAGAAACCTAA

ACGAACGCCCAGTTTTACCACTCGCAGAAGAACTCAAAGTTTCCGAAAACTTCAAAAATT

GGAAAACGTAGACCAACTGCCCCCGGTCGAAATACAAGGGTTCTTGGACAGAAAACACGA

ATTACAAAGTAGTGGGAAAAAAGCAGCGGTCAGGTCGTGGAAAACTTTCTATACGGTGTT

ATGCGGTCAACTATTGTGCTTCTTCAAAGATCAAGAGGATTTCGTGTCCAGTAAAGCGGC

AACGTCGCCAATCATTGTATTCAAAGCGAAATGCGAAAAAGCGTTCGACTACACGAAACG

AAAACACGTGTTCAGGCTATGCTGCACGGACGGTTCAGAGTTCCTCTTCTTGGCCGACAC

GTCCAAGGAAATGGAAGAGTGGGTCAACAAGATCACGTTCCACGCGCAACTTCCTCCAAG

CCTACAGCTTCTGAGCTACGACGACAACCAAAAGGGTTCGCCACGATCAGACTTACAGAC

TCCAGTTCCGTCAACGGTCATCAGCACAAGTTCGAGCGCGGCCACGACGCCGGAAACTGA

ACGCAAGCCCCTTGCACCATTGCCGCCGGTTGTAAATATACGGAAAGACAGCCAACTCCG

TCGGCCCATACCCGTACGACCTCCTTCGGTGGACAACATACCGTACCAAAACAATCACTC

GGCCGTCGACGCTACCGATTTCGGTACCAAAGCATCGCCGTCCGGATACCCCGCTCGGCC

GCATTCATATTACTCAAACGGCAACAATGACTGGTCCAAGGACTCAATCCACCCCAAGTC

ACCAGACGTCGCCCCGCCACCGTTGCCGACCACCGCGCCACCAATCAAATCGTCTAACAG

AGAAACATGGACTTCAGACTACAGACAGAGTGCCGTGTACGCAAATATCGACGTGCTCAA

GCAGCCACCTCCAGTGCCAAACCGATCAGCCACGTTGCCGGTGACGACGAGCAGCAGTCA

GCGGGCCAGCGAGAGTTCCAGCGAGAGCGAACACCAGTCGCCTATGGGCCGTAAAGACAA

GAGGTCCAGCGTTCTCTCCAGTCTTTTCCGCAAAAAAAAGGCCACCAATCTGTAAACACG

ATGTACCTACTGACTGCAGATGATGGCGCCTCGTCAATATATTATTATAATTAATATAAT

CAATATTTTCCTATAGATTATGTACACAATATCCATACAATTATCTATCCGCCATAATAT

GTACGTGTCGTAGATACATAGATCCAAATATTATTATTGTTTTTACACGCATATTATGTA

CCATAATAGTCTTAATAGTGTCGGAACAGGAGTGAAAACCCGTGTTAATGGTTTAAAAAA

TGTCAGTTGCTTTCGACAAAACGTCGAGAGACTTCGTTTTCAAAAAATTCGAGCAAAAAT

AATGATCGAAAAGCGTACGTTTCAAAACGTCTCGTAACTATTTCCGACTTTAATAGTAGT

CAGCTGTATATATCACCTATATATTATAATACATAGGAATTATTGTATTTATATTTTTCA

ACTTCATGATTAGAACGTGACACGAACTTCAACCTTCCTCGTCCCCTCCTACGGTATATA

ACATTACAATTTAAATCGACCGTATTACAATATAAATGTGTTTGTGTATTACTATATAAT

ATACTTATTTCGTATTCGTATAATATTAAATCAGTGTGCCATTCATTGTATAACTATATC

ATAATATGCTCTGTAAACGCGTTAGAGAAAAGATCCCACACCGAAACTACGGTGCAGTCA

ATGTTGTATGTTATTATGTTATGAGTTAGATTTTATGGATACCTATATGTGTGTGATTTT

TTTGTTTAATTTTAATTCGTCGCCAAACGACATACATTTTTATTATATATTTAGATAGAT

CATAGTTTTACCGTTGTGTACCATGTATTTATTAAGGTATGCCTACCAATTTCATAGTAG

TCTGAGGTTTTTATTGGTTTTGTTTCTGAAAAATTCGGCTAAAACGAGAATAGACCATTC

CGCCATTGAATGATATATCTATATTTTTATAGGATTTTTTTTTTAAAATTTTAATTGACA

AACTTGTGTTATTATATTGGTATTGTACTTATATGTTTATGGTCATTTAATGTGTAATGT

TTTTTACTACAGCATGTTTAAGTAAATTATGCCATTATAGAAATTATAGATATATTTTAT

TCCTCCGTAGATTTTAATAATTCTGTTTTTCTATCATGTATAAATATTTTTAAACGTTTT

ATTATGTATTTTTGTCTATTATTGTTATAATATGTTTAATCTTTATGAAATTATACAATT

ATTTTTATTACTATCAAAGCGATCATATATAATATATGTTATTATTTATTATTGCTGTCC

ATATTATAATATGGCATCCAAATATGC

>Unigene0001353

GTTTTCGACCGAACTGCCGATGAAACCATCAGCTGGATTCAAGAAAAAGGCGGGTTATTG

GCTGCAGAAGATTACGGCCATGATTTAGATACAATACAAACTTTAATCCAAAAGCATCAA

GGTTTTGAAGCAGACCTCGCGGCAGTCAAAGAACAAGTTGAATGCGTCGTACAAGAAGCT

GGTCGGTTGTCAGCCATGTTTCCAGATGCCAAAGATCACATCGAAGTGAAACAGGAAGAA

G

>Unigene0001354

ATGAAAAAACTTGAAGGAATAGAAAGAGAACTAAACACATTCCAACACACTCTTGAAAGA

CTAGCAAAATTAAGTAGGAACCTAGTAAATAGATCACATTTTGATTCAGAAAATATTTTA

CGAAAACAGACTGATATAGAAGAAGTTTACACAGAGCTCTGTACATTAAATAAAAGAAGA

GCAACTAGATTAGTAGAGAGTCGAAAATTCTATAAGTTTATACGAGAAGCTGAAGATGTT

GCCGAATGGATAGGAGATCAAACAACAGTAGCTGCTTCTGAAGATGT

>Unigene0001355

GCTGCTTCAGAAGATTATGGTCGAGATGTAGAGCATGTGGAACTTTTAATCCAAGCATTT

GAAAGCTTTATGAGTGGTTTAAATGGCAGTGAAGGACGTATCTCGAGTTGCATAAACATT

GGTCATAAGTTACTTGAAGAAAATAATCCTGAACAAGATCAGATTAGAATAAGATTAGAA

GACACTCAGCAACTTTGGGATGATTTGAAAGAATTAGCAAATGCCAGACAAGAGGCTTTG

ATGGGTGCTAAACAAGTGC

>Unigene0001356

GAAGAAGCAGTGGACGCTTGGACTACATTATTAGAAAACGCCAGTCAAAGAAAAAGCAAG

CTGAACCAAGCAGAACACTTGCAGTCTTACTTCGATCAGTATAGACATTTAATTGCATGG

ATAAACGAAACTATTGCAAAAATCACGTCTCCCATGCTCAGTCAAGAGGTGAGTGGTGCA

GAAATGCTCATACTACGACACCAAGAGTACAACGCCGAGATTAATAGTCGATCTGAAGTG

TTGGGCGAGTTCTATCAAACCGGAAAAAGTCTTATTCAACATG

>Unigene0001357

TATTCCTTTTGCATCCATCCTCGCTTCCCTTCCTCTTTTCGGCCACCAAGCCACCACTGC

CTCAACCCCGAAATCTGCACACCCCCCTCCATGATCCCCATCACTCACAATCAACAATCC

CCCTCTCAAAATTCGATCTCGTCCCCGTTCTGAGACATGATTCAGCTCCTATTTCTCGTG

CTCTTCGCGGAGGGCGCGGTGGCGTTCCTCCTGATGGTGGAGATCGGGCCGCTGCGGGAG

CTAGCGGTGAGGGGGCTGGATCAGCTCAAGACCGGCAAGGGCTCGGCGACGGTGAAGACG

CTGGCGATCACATTGTCGGTGATCCTCCTTTCAAGTATTACGAGCATTCTCAAGATCCAG

AACCGGGGGATCAAGATGGGGACGGTCACGCCGATGGATCAGGTGCTATGGAGGACTCAT

CTGCTCGAGGCTTCACTCATCGGCTTCAGTCTTTTCCTCGCATTGGTTATTGACCGCTTG

CACTATTATCTCCGTGAATTAATAACATTGAAGGAAGTTGCCAACTCTTCAAAAGAAGAA

ATTGAGAAACTTGAGAAAGAGCATGTACATTTTAAGGAAATGGAAGGAAAAACTTGTAAC

GAATTGAGAAAACTCCAGGAGGAAGTGGGTTGCTTGACGGAGAAACTGCAGAATGTCAAG

TTAGAATGTGAAGGAAAGGAGAAGAAAACACAGTCTGCTGAAGCACATGTTGCTTCCCTT

CAGAAACAAATGGAGGAGCTGCTTCTGGAGTACGACCGATTGTTGGAGGATAACCAAGTA

CTGCAGACCCAGGCCCTATCATTCAGAAGCTAAGCGCTTTACATGTGTGCTATGTAACAA

CTTTTCTCGAAAAGTGACCGTGACGTGCTCTGTTCATTTTCGAGATGTTTCTGATTGTCG

AATTTCTCCTGTGTTTTTGTAGGTGGGCTTTTATTGGTCTCACTTTTCCCCATCCTTTGG

AATGATTACCAAGTACATGTAATATTTAACAGATTTTTTAATCTCTGTAAGGTATTTAGC

CAGCGGTTTTGGTTTTGGTTGTAGACTGTACAAAGCTATGTACGGCCTGATTATCAGGCT

CCTCGTTTGAGTGTTTTCACATGACAATGACAGTTTTACGTGAGTTTGTTAAATATTATT

ATTGATGCTATGATGTTGA

>Unigene0001358

GTCTCCATCATCTCCCGCCTTGACAGCTTCCGAGAGCTCTTAGCCACCGCCCTCCTCTCC

CGTCAATGGCGAACCTTCTGGACCCTCTCCCCAAACATCAATTTTGATTTTTCCGAAGAA

CAACACAGACCCGATGTTGTACTCAGCCATCTCCTCTCCTGTCACCGTCGACACTTATCC

ACCTGCCGGATTTCACACCTTGCTTACTCCGGCAAGATGGTCAGTTGGTTTCCTCCTGAC

AAACTGGACTCGCAGTGGATCACCCATCTCACCCTCAAGAGAATCACCAACCTCACCCTT

TCCTATAACCCTAGTTTCAACACTGATCCATCTGAAATCCTCACCATCCCCTCCCTCGAG

GAACTGACATTGTCCGACTATTACTTGGATGCCTGGCAGCCTCCTACCCAAGGCATCCTT

CTCCCACGCCTCCGCTCCCTCCGTCTCAAGCGTGTCTCGCTACTCGAACAAGCCCTCTCC

ACCCTCATTGCCGGAGCTCCTCTCCTTGAACGGCTTGGCCTGTCTAATTGCTACTCGTAT

CAGCATGTACGGATCCATTCACAGGTCCTCAAGGTACTGGAGATCGACCGTCAGAAGTCA

GTCATCACACTAGATATCGATACACCCACTGTCACCACTCTGGTTTTACATGGCTGGATC

AGATACCTTAAGATCAATTGCCCCAGCCTCTCTTCTCTAAGCACCATCTGCGTTTACGCT

GAAGACCATGTTGAAGACAAACAAGACAAGCTTATTAGGAGGTGGCGGAGATTGGAAATT

CTTAAAATAGATGATATGGAGATACTTGAGCTGATTTGCGGATTTGATGCCAGATGCAAA

GAGGATATTCCTTCACCATTCTCCGACGTCAGAAACAACCCTAGCTGCTTTGCAGGATTA

>Unigene0001359

CACCTCTCCATGCAGCTCCCTCTTCGGGCTCTGCACCTCACCACTGCCCTCCCTGCCCCT

CCTCTCCCACCCACCCTCTCCATCCCTCCACATATCCTCCATCCACAAGCGTAAACCCAT

CCTCAGCCTCCCCTCAAATCCCCAGCCTTCACACCTCAGCAAGCTCTCCCAACTTCTCCA

ATGTGGCGCCCTTCTAACATCTATGAACGCGCCGGCCGCGCTGGCGGTGACCGGGAGCAA

CAATGAGAATGAGGACCTGGTGATGACGTTGGTAGGGTGGGGGATTGTGGCGGTAATATA

TTTGTTTGTGACGCCGCCGATTCTTTTCAATTGGATGAGGTTGAGGTGGTACAAGAGGAA

GTTTTTCGAGATGTACTTCCAATTTTTATGCGTCTTCCTCTTCTTCCCAGGGATGTTGCT

TTGGGCGCCATTTATCAACTTCAGGAAACTTCCAAGAGATCCCACAATGAAAAATCCTTG

GGATACTCCAAAAGATGGTACCCCTCTGTACAAGCCAAGAGGATATCCTCAGTGATTGTG

TGATGGAAAGGGGTTTGATGTTTATGGGTAAAAGATATAATTAATTATTCATAACCTTTG

ATACTTAATGATCCATTTCTATGTATGATTTTAGCAATGTTGCTAACATAAAAAATTGCA

AGATTTAGTTGGGTATAAAGCAACATTAAAAATTGCAAGATTTAGTTGGGTATATGATAA

GGCTATAAATATATAGACACGACATACTGTTGTGCTTTCTACTCCTCAATTGTTATTTAT

AATCTTTGATATTTGAT

>Unigene0001360

AAATTGCCCCCCCGAGAGCAAAATGCTTAGTAGATCTGAATTAAATGCAGAGACATCATG

GTATAGCCAGGATTATGGATACTGTTCAAGTCCACTGAAAAACCTCCAGGTGTGGTGTCA

AGGAAAGCTGAGTGTAGAAATGTCCCCTGAGCTTGGAGGAAAGTGGTTGATAGGAGGCCC

CGAAGGATTTCAAAAAGAGTTTCCCATCATTGCTGTCCTGCGTCCCTTTGTTTCCAAAAT

AGGCCCATATGTAGTCAAGCAAAATCACATCCAAGAGCTGCTGACCAGCCCTGTGAGATC

ATTGAAGAAGGTGGTGCTGTCCCTGTCCACTGCCAACATTTCATCCATCAGACCACACTG

TGCTCCCTTCCTGTCCACCCTCTACACTGGCTGGCTGGCCTGGCTTCACAGCCGCTCCAT

GCAGGAGGCCCGCACCAGGAGAGACCTTCTGGCCACAGCCCTGGGAGGAGGAGGTGCTGG

GCTGGGAGTGCTCAACAGCATGAACGCTGAGGTCTTGGCCAACAAGTTGGAGGCTGTCAC

TTCAGGTGTGCAGGGCCTCCTCAACCCCTTGAATTCATCCCTGACCAGCCTGGGTATGGG

GCAGTGGCTTGTGTCTGAGGTGCTGCCTACGTGGGAACACATAAGTGAGAAAGACCACCA

GGTGCTGTTGCAAGCGCTGGGAATCGAGCAAAATAATGTCTCCCTTGCTCTTAGTTGCAT

CCAGGCCCAGATGTGGGTGCAGAGTGTCATTGCAGGTATCCTGAGGGATGGCGACAACGG

CATCCTCCCCACTGAGATCCGAAAGATCGTGTGGGACGCAGCCACGGAGAAGGAGCGGCA

GCTCCAAGCCTGGTGGAGGCTGGTGAACTTCACCCACGACCAGGTCCTCAACTCAGTCAT

CGCCCACGTCCTCACCGTGGTGGAAGCCCGCATCGAAAAGGTCTACCCCATCGTGGCCCT

GGGGATTAACACAAATGGGTCTGTGGTCTATCCGCTGGACCACCGGATGTGGGCAAGAGT

GTCAGACAGAAAATGGCAATCTGTAGATTTGGAAGCCTGCATTTTGGAACAGGGGCTGGG

ATTCATATGCGAAGATGATGCCCTTAAGGCAAGTGATGTTTGCTTTGATACCAGTGAGGG

GGTGTGCCATTTTGAGATCAATCCTCGCAGCAATAACAAAACCATGTTAGTTTACGTAGG

GAAAGGGTGTGTGTGCTTTAGAACAATGTGTAAATACGTGCAAATTAATGACATCTACAA

TCAGACCGTATTCAATGATTCAAATACGTGTGCTTGCAATGTAGCTATTATTAGAGGCTG

TGATTTTGTGTATAAGCCACCAGTTTTTACTAGCCAATTACTAATCAGAAACTATAGCCT

GTATCGTAGTATAACCCCGACCCCAATCGGTATGGATTTGTCGCTAGTAAAAGAAATGTT

AGAGCATGCAAATTTACAACAGCTTTTAGAAAACGCTAAGGCAGAAGCTAAAAAGATCCT

AATAACTGTACATCATGATGGTAATGTTATAAAGCAGATAGTGGAACGGATTAAGAGGGT

GGGAGAGCACCACTGGTGGGAAATTTTCTTTGGCTGGTCCCCAACAGCGACCGGCATCTT

CAACGCGCTCCTGCATCCTGTTGTAATCATACTGCTAATGCAAATGTGTGTGTGCTTTGC

CATGGTTGCTACTTGTTACTGGATAAGGCAGGTGAGGCTCTGCATTGAGAACCAGCTGAA

AGGACTGGGGATGGCCAAGCGCCTCCTACCATAGTCAAGGGCAAGACTGGGTTGGCCTCT

GTGTTTGCCACCAAGAAGAACCTTTAGCTCCGCTGTTGGCTGCAACCCAGTAGGCGTTGA

GCGGTGGGGACACAAGCCATACCAGACCTTGATGCGAGGGGGCTGCCTCTGCTGATACAA

GAGGACCATCCAGGAGCAGAGGGCAGGCCGTGCAGCCTGCAGGTGGTGTGAACCACAGAA

TGGTTGAGGTTGGAAGGGGCCTCTTGAGGTACCTGGCCCAATTTGCCTATTCAGGCAGGG

TTACCTAGAGCAGATTATCTGGGACTGTATCCAGCTGGGTTTTGACTCTCCCTGCAGTAG

AGACTCCACAACTTCTCTGGGCAACCTGTTCCAGTGCTTGACTACTCTCCCAGTAAGAAA

GTAAGAGTAACTTCTCTCTGAATATCTCACTCCCAAATACTAATGACAAAAAGCTGCTTT

TGTGTGATTTTAAGACTCCTTAAGCAGAGGAGAAGAGGTTAGGGAAAGCCAAGGACAGTG

CTTCTGGAAGGGTACAGAGGCAAATGTAACACCTGAGTGCAGAAAAGCAGACACTGAAGA

TGGCAGAGATGTTATCAGCCTCAGAGACTTGGAAAGCTGCTAGAGGAGCAGAACAAATGA

TCGTGTGATCACCTTGGACTTAAAGGAGCTGAATTCACATGGACCTTTGCAGCAACCTTC

CCTGGGTTCCCAAGACTCCCAAGACGTCTGGCCAGCGGATTGGTGAGATCTTGTTCTTGG

AGCCTGGACATCACTGCTTTGCTTCGCTTAGTTGTGCATGTAACCAATAAA

>Unigene0001361

AATAGCTCACTGGTCAAGTCGGCCTGCGCGGAAGATGTAACGGGGCTCAAACTATGCACC

GAAGCTGCGGCAGTAAAAATTTATTTTTTTACTGGGTAGGGGAGCGTTCTGTAAGCCATC

GAAGATATATTGTAAAGTATATTGGAGGTATCAGAAGTGCGAATGCTGACATGAGTAACG

ATAAAGCAGGTGAAAAACCTGCTCGCCGAAAAACCAAGGTTTCCTGTCCAACGTTAATCG

GGGCAGGGTAAGCCGACCCCTAAGGCGAGGCTGTAAAGCGTAGTCGATGGATAACAGGTT

AATATTCCTGTGCTTAGTTTTATTGCGAGGGGGGGACGAAGAAGGTTAGGTTATCCAGGT

GACGGTTATCCTGGTTTAAGTGTGTAGGTATGATAATTAGGAAAATCCGGTTATCTTTAA

TCTGAGGCACGATGACGAGTCATCAAGGTGACGAAGTAACTAACACCACGCTTCCAAGAA

AAGCCTCTAAGCTTCAGATAAAGCTAAATCGTACCCAAAACCGACACAGGTGGTTAGGTA

GAGAATACTAAGGCGCTTGAGAGAACCCAGGTGAAGGAACTAGGCAAAATAGTGCCGTAA

CTTCGGGAGAAGGCACGCTAATTTAAGTGAAAGGATTTTCTCCTTTAGCTAAAGTTAGTC

GAAGATACCAGCTGGCTGCAACTGTTTATTAAAAACACAGCACTGTGCAAACACGAAAGT

GGAAGTATACGGTGTGACGCCTGCCCGGTGCCGGAAGGTTAATCGAAGGAGTTAAAGGAA

ACTTGAAGCTCTGAACTGAAGCCCCGGTAAACGGCGGCCGTAACTATAACGGTCCTAA

>Unigene0001362

AACCTCATCCGAGGGCGACGCACAGGAACGGCTCACAGAAGATGAAGTTGAACTCCCTTC

CTCCAGCTGCAAGCCTCCGCACACTCGGTGCCTTCCGGCAGGCGACCCGATCCCTTCCAG

CACCATGGGCTCCGCATGGCCCGGTCTTGACAAAAATCGGGGGCCCGAACTTGACCGTGG

GAGGGTCGAGACAGGTTCAGATGGTGGAGTCGCGTGATCTCTCCTGACAGTATGGCTGCC

CCCAACAGGATTGCCTCCCCCAGTAACAAGTCGTGGCAGGCCGGTCACCCGAGCGCTATG

CAGCCCAATCCTCCGACTCTTCACTTGATCGTGCGGCCGCACCACTCGGTCTCTCGGTCT

CACTAGGATTCTGAGCAGACCGTCTCGAGCTCGGTCTGAGAGGACGCATCCCGGGAGAAG

GATGAAGAGCGGCGGACCCACCCTTGATTTCAACGAGTAGCCCGACCAACCATGTATAGC

TCGGATTGAACCTCGGCTAAGAGGATGGATGGATGAGAGCAGCCCGCTCTCCTCCAACAT

CTGATCGCACAGGAACCAGGATAAGTTCTGCACTCCGGGTGGTCCGAGCTTGACAAAAGC

CACCGTCCGGGCAGGCAAAAGGCCAAGTGCAGGCGAAAAGGCAAGTCCACGTCGTCGGCT

GGCCCCCTCTCCCCCTCCAAACTTGCCCCAAACAGGACAAACGCTGCCCTCCAGATGGTC

CGATCTTGACAAAGGTCGAATTACGGGACGACATAAAAGCATCGAGGCAGGCGTAGGATG

ACCTCGATCCGCCCTCATTTGGCCTGAGCCCAGGGCTCAGGTCTGACCAAGGCAGTTAGC

AAGGCACAACTAAAGCTTTCATGAAAGGGAAAGAATGGCCAGCACCTCATTTTTATAGGC

CGACGTCTGAAG

>Unigene0001363

TCTTGACAAAAATTGAGGGCCCAACCTTGATGACCGTGGGAGGGCCGAGACAGGTTCAGA

CGGTGGAGTTGCGCAATCTCTCCCGACAGTATGGCCGCCCCCAACATGATCGCCTCCCCA

ATAATAAGTCGTGGCAGGCCGGTCACTCGACCGCTATATGGCCCGATCTTCCGACTCTTC

ACTTGATCGTGCTGTCGCACCACTTGGACTCTCGGTCTCACTAGGATTTCGAGCAGACTG

TCTCGAGCTCGGCCTGGAAGGATGCACCCCGGGAGAAGGATGAAGAGCGACGGGTCCACC

CTCAATTTCAACAAGTAGCCCGACTAACCGTGCATGGCTCGGATTGAACCTCGGCCAAGA

GGACGGGCGGATGAGAGCAGCCCGCTCTCCTCCGTCATTCGATCGCACATAAACCAGGTT

AGGTTCCGCACTCCGGGAGGTCCGAGCTTGACAAAAGTCACAGTCCGAGCAGGCGAGAGC

CCAAGCGAAGGCGAAAAGGCGAGCCCATGTCATCGGCTGGCCCCTTCTCCCCCTCCAACA

TCACTCCAAACGGGACAAACGCCGTCCTCCAGATGGTCCGGTCTTGACAAAGGTCGAATT

GC

>Unigene0001364

TCTTGACAAAAATTGAGGGCCCAACCTTGATGACCGTGGGAGGGCCGAGACAGGTTCAGA

CGGTGGAGTTGCGCAATCTCTCCCGACAGTATGGCCGCCCCCAACATGATCGCCTCCCCA

ATAATAAGTCGTGGCAGGCCGGTCACTCGACCGCTATATGGCCCGATCTTCCGACTCTTC

ACTTGATCGTGCTGTCGCACCACTTGGACTCTCGGTCTCACTAGGATTTCGAGCAGACTG

TCTCGAGCTCGGCCTGGAAGGATGCACCCCGGGAAAAGGATGAAGAGCGGCGG

>Unigene0001365

ACACTAGAACAACACCTCCCCGCTACTAACAGAATCCGGCGGGAGGCCACATCAGCTCAC

ATGACCTACCTCCTCTCGAGCCATGAGCATCCCGCGTGGGACTGCCATAGTCGACCAGCT

AGGCCACCGCCTCGGAGGCCAGTTGTCGGCCCCTCTACTCCAACTAAGCCTAGTCCACTT

GAAAAACACGTAGCCCGGAAGCGCGAACCAAAAGCTTTGCTCCTTGAGACAAGCATTACG

TACTTACCCGGCAAGAAGCAGGAGGCCACTAATGAGCCCGGCCCAAAAGCCCCACTGCCA

CATACGCACGACGCCCCGATGGGCCGGGCCCA

>Unigene0001366

CTTACCTGCGGGTTGAGCATGTCCGCCCCGCCTGCGAGTCGAGCAAGTCCTCCCTGCTCG

CGGGTCGAGCGAGCCACCGGAGGGCTCGTCGGCTGAGAGCCCGCCGATCCATAGCCCCAC

GTCGCTGTCCTATTGCCATTAATGCACGTCTCTCCAGTCACGCTGGAAGTGACTGGCACC

ACCCGCCGTCACTCCCCAGGTAACGTACGTCACTCTCCGACTATTCTGGTGGTTGTCCCC

GTCGAACAGTAGGCATCGCTCA

>Unigene0001367

AAAGGGAAAGAATAGCCAGCACCTCCTTTTTATAGGCCGACGTCTGAAGAAAAAGCCAAA

GGGAAACTGTACCGGGGGCCCCCTTGGCCAGACGCGCCAGTCGATGTGACCGGACCCAGC

CAACGACAAGTCTTATCCCACGCCGATCTTCCGGGATTTGTCACAGAAGAGACAATGCAT

CATTTCCAAAGAGAGGGGCCCAGTCCATGACCAGTCAACACTAGA

>Unigene0001368

TCGGAGTCCCTCCCCTTCATCCAGCGCTTCCACGGCAAGACGATTGTCGTCAAGTACGGC

GGCGCAGCCATGAAATCCCCCGATCTTCAATCCTCCGTCATCAACGATCTCGTCCTCCTC

TCCTGCGTCGGTCTCCGCCCCGTACTCGTCCACGGCGGCGGCCCCGAGATCAACGCCTGG

CTCCTCCGCCTCGGCGTCGAGCCCCGCTTCCACAACGGCCTCCGGGTCACCGACGCCCTC

GCCATGGAGGTCGTCGAGATGGTCCTCGTAGGCAAAGTCAACAAATCGCTCGTCTCACTC

ATCAACGTCGCCGGCGCCACTGCCGTCGGCCTCTGCGGAAAGGACGCTCACCTCATCACC

GCCCGCCCATCCCCCGATTCCGCCACCCTCGGCTTTGTCGGGGAGATCGCTAAGGTAGAC

CCCACCGTCCTCCTCCCCATCATTGCCGATGGGCACATTCCTGTCATTGCATCGGTCGCA

GCTGATGAACATGGGCAGGGGTATAATGTGAATGCTGACACTGTGGCGGGTGAGATTGCG

GCAGCTGTAGGGGCGGAGAAGTTGATTCTGCTGACAGATGTGGCGGGGATTCTGGGGGAC

AGGAATGACCCCGGGAGCCTTGTGAGGGAAATTGATGTCAGCGGGGTGAGGAAGATGATG

GAGGAGGGGAGGATCGCTGGGGGGATGATACCAAAGGTGGAGTGCTGTGCGAGGACGCTG

GCGCAGGGGGTCAAGACCGCTAGCATTATTGATGGGCGGGTGCCGCACTCACTGCTGCTA

GAGATTCTTACGGATGAAGGCGTTGGGACGATGATCACTGGGTGAGTTTTGATCACCCGG

TGAGTTTGCTATTGGTGGAGGTGAGTTGGGTGAGAATCATTGTATTAGTAGTATCAGCAT

TCTGTTGATTAATTTAGTAGGTTGGATTTCTAGGATGTAAGGTAATGTTGATTAATTTAG

TAGGTTAGATTTGTAGGATGTAAAGTACGGTAGCTTAAGGCAGTAGCAATTTTCTATTTA

TGTTAAATTTTGAGATTGGTTTAGTTTATTACTGTAGGAAA

>Unigene0001369

GAGCTGATTTCGATCAGGTCCAGGTAGTTTTGTGCCTAAGTACCTCCAACTATTATACCT

CCCCAGCGAATTTTCGGTGAAAAACTGGTTATCGATTGGGAGTTTAGGGGTTTGGATCCC

AAACCCTAACCCTCTTCGCCTCGCCCCCCATCGCATCTCCCTCAAAGCCTCCATCCCCGT

GACCTCAGCCTCTGTCCCCGCCTCGTCGGCGGGCAAAAACTGCGTCGAGATCCTCTCGGA

GTCCCTCCCCTTCATCCAACGCTTCCACGACAAGATGATCGTCGTCAATACGACGGCGCA

GCCATGAAATCCCCAGATCTCCAATCCTACGTCATCAACGACCTCGTCCTCCTTTCCTGC

GTTGGTCTCCGCCCCGTACTCGTCCA

>Unigene0001370

TCGACACTCCCTGTCGACTCAGCCATTTCGACCAACAGCTGGGAGCAGCATAGCAAATCT

GAGCAGGAAAAATGTCCGTTGCAAAACCCTCGGCCGCTCCCCACCTCTCCGGCCTCTTCC

TCTCCAAACCCCTCCGCATCCCAAACCCTAACCCTCTCCGCCTCGCCCCCCGCCGCCTCT

CCCTCAAAGCCTCCATTCCCGCGACCACGGTCTCTGTCCCCGCATCGTCGGCGGGCAAAA

ACCGCGTCGAGATCCTCTCGGAGTCCCTCCCCTTCATCCAGCGCTTCCACGGCAAGACGA

TTGTCGTCAAGTACGGCGGCGCAGCCATGAAATCCCCCGATCTTCAATCCTCCGTCATCA

ACGATCTCGTCCTCCTCTCCTGCGTCGGTCTCCGCCCCGTCCTCGTGCACGGCGGTGGCC

CCGAGATCAACTCCTGGCTCCTCCGCCTCGGCGTCGAGCCCCGCTTCCACAACGGCCTCC

GCGTCACCGACGCCCTCACCATGGAGGTCGTCGAGATGGTTCTCGCCGGCAAGGTCAACA

AGTCGCTCGTCTCACTCATCAACGTCGCCGGCGCCACCGCCGTCGGCCTCTGCGGCAAAG

ACGCTCACCTGATCACCGCCCGTCCTTCTCCCGACTCCGCTGCCCTCGGCTTTGTCGGGG

AAATCGCGAAGGTCGACCCCACCGTGCTCCTCCCCATCATTGCCGATGGGCACATTCCTG

TCATTGCATCGGTCGCCGCCGACAAGCGGGGTCAGGCGTATAATGTTAATGCTGACACTG

TTGCTGGAGAGATTGCTGCGGCTGTAGGGGCGGAGAAATTGATTCTGTTGACGGATGTTG

CGGGGATTCTTGGGGATAGGAATGATCCCGGTAGCCTTGTGAGGGAGATTGATGTAGGTG

GGGTGAGGAAGATGATGGAGGATGGAAGGATTGCTGGGGGGATGATTCCGAAGGTGGATT

GCTGTGCGAGGTCGCTGGCACAGGGGGTCAAGACTGCTAGCATTATTGATGGGCGGGTGC

CACATTCACTGCTGCTGGAGATTCTTACGGATGAGGGCGTTGGGACGATGATCACCGGGT

GAGTTTGCTGGTAGTGGTGATGAGCTGGGTGAGCACTGTTGTATTAGTAGTATCAGCATT

CAGTTGAATAAATTAAGTAGCTGAGTTTCCAGGGCTGTAGCATAGTGTAGTCCCTTATTT

ATGGTATCATGCTTTGAACTTTGAGGTCGATAATGTAGTCTATTACTGT

>Unigene0001371

ACGATACCAGCGGCCCAACAGTGATCATCTCCATCCCTTGGATGCTCGAGAGCACCTCAT

TTTCCAGCTCCTGAAACGTGTTTACAAGAACCTTCGGCTTGCATGCTGCTTCCCTGTCTA

GTATCTGAAACATATCTTTGAAGACGGGAGCTACTAAGGCGTGTTTCGGATTAGCGTCGA

AATTGGTAGAGGTCAGATAGGAAGGCAAGTCTCGGATCTGGAGTGGGGGAAGCCCAGGTA

GCGC

>Unigene0001372

TGCATGCTGCTTCCCTGTCTAGTGTCTCAAACATATATTTGAAGCCAGGAACGACAAAGG

CATATTGCGGATTAGCGTCGAAATTGGTAGAGGTCAGAAAGGAAGGCAAGTCTCGAATCT

GGAGTGGGGGCAGCCCGGGTAGCGCCACACTAAAGGAGGGATCATTCTGGTGTGCTCTGA

TGATATCAGCGTATCCATGGAAGTAGGCGTAGTACACGGCGAAGACGGCAGCAGGCTGGA

TCCAGAAGAGGACGGAGGGGATGCCAAGGTCTCTGGC

>Unigene0001373

TAGTCTCCATCTTCCGGCGACTGGCAATCCACGTCTCCAGCGATCGGCGGGCCCTCTCCT

CCATATCGGCGGCGACTGGCAATCCACCGTCTCCGGCGACCGGCGCGCCCTCACCTCCGA

GAATTTGAAGTCAGGCCTGCTGATAGAAGATGGTCAATGCAATCAAGGGTCTATTCATCG

AGTGTGACATACCAATGGCGCAGTTCATTGTCAACATGAATGCAGCGTTGCCATCGTCGC

AAAGGTTCATAGTGCAGCAGCTCGACCCAACACACTTTTTCGTGCAACCTCATGTAGCGG

AAATGATTCGCAGCAAGATATCGGAGTTCAGAGACCAGAACACTTATGAGAAGCCAGCAT

GATAATACTTTCGGATCTGGCCGCCTGCTCGGGGCTTCTTTCAATGTTTGCGCCCTCTGT

TTTCGACAATGATACATGAGACTCTCTCTGCAACATAATGTATGTTATGGAAACTAACTA

GTTATGGATGTAAAATGGATATAGAGTTTGCGCTACTTTGTTGCTTTTGGGTCGATTACA

GTAGTTGGTACATGATATTGCGTCTATGAATTCAGTTTTATCAATTTACTGCCAATGTGA

CCGTTCAGATATGCAATTCAGATCCAGTTCAATGAATTAATGGGTCTGTTGAGA

>Unigene0001374

TCCAGACACTTAAAAATGTGCTCCTACACAGAATCCAATTGGATCACGACACACATAATA

ATTGGCTCTTGTATATAATGTCAAATGAAGAATTGTGACATTTCTTTAGTAGATTTCTGG

ATTTCTCAAGATATATTTCAACATTTCGGGATTTGTTTTTATACGACTAACATAGACACC

AGGGAATGGGTGCTAGAGGCTCGGCTCAAGAAGAGGTCGCAGGTTCAAGTATGCAGGGGA

GAAAGTTAAATAATCGAACCACCAAGTAGTTTGCAGGGAATATCCACCAAGTAGGACGAA

TAAGAACATAAAACTTAAAGTCCATTATATCTTGTATAACCATCTCTGTGTCTATTTTAA

TGAGATTTTATTTTTATCATGGGGATTCTGGCATTTCAAGCTGTAGACCTGTAGTGAAGA

GGTGGTACTACTGGGCTAAGGCCTATTATTTATTTGTATTCGTCTAGAATGCTCCATAAT

TATTATACTTTTTTTCCATGGTAGGAGTTCCAAGACTTTACTCCATTGTCAAGGTTGTCA

AAACAAGAATCGGACTTGTATCGTTTGTCCAACAATTTCGTATTGTAACATAAGATGCAT

TTCAGATTTGAAAATTTTATGCATGATACATAAATATATTTTTATACAGACTTGTCATAT

ATTTTAGTTTAAAATGAGAAAGCATACA

>Unigene0001375

TGATACATAAATATATTTTTATACAGACTTGTCATATATTTTAGTTTAAAATGAGAAAAC

ATACAATTTTTAAAACCAAAATATCATGCTTTGAGTGTATCACTTGATATACACGTACTA

CCCTTATAATGTCTTGTCTTATAAGCTACAAAGATATGTATCATTTGCCAGTTGAAACAT

ATCATATTGTATGATACGTGCCGTGTACTGTAGCATACTCACAACCATGTCAATGATACT

CTGCTCAATGTTACTATA

>Unigene0001376

AAATGAGAAAGCATACAATTTTTAAAACCAAAATATCATGCTTTGAGTGTATCACTTAGC

ATACAATTTTTAAAACCAAAATATCATGCTTTGAGTGTATCACTTGATATACACGTACTA

CCCTTATAATGTCTTGTCTTATAAGCTACAAAGATATGTATCATTTGCCAGTTGAAACAT

ATCATATTGTATGATACGTGCCGTGTACTGTAGCATACTCACAACCATGTCAATGATACT

CTGCTCAATGTTACTATA

>Unigene0001377

CGACATGTTATAATTGGCTCTTGTATATAATATCAAATGAAGAATTTTGACATTTCTTTA

GTAGATTTCTGGATGTCTCAAGATGTATTTCAACATTTCGGGATTTGTTTTTATACGGCT

AACATAGACATCGAGGTATGGGTGCTAGAGGCTCGGCTCAAGAAGAGGTCGCAGGTTCAA

GTATGCAGGGGAGAAAGTCAAATAATCTAACCACCAAGTAGTTTCCAGGGAATATCCACC

AAG

>Unigene0001378

GCCATTAGATAAAACAGTGAACGGAGAAAATTCCAAAAGCATACATTGATAGTAATAGAA

GAAGAGAAGCTAGAGGTTCTTGGATAAGACTTGAACTGGAGAGATAAACAGTACTCAGGA

TGTCAGACCTTATACCAATAGTCAAGATTGAGGAGCTCTCTGTGAGAAACGGAACAAAGA

ACAGTTTCAGATAGCATATCCAGACACTTAAAAATGTGCTCCCACACAGTATCCAATTGG

ATCACGACAT

>Unigene0001379

AACGCCACCGGCACCGAATTACGGCACTGGTCCGACATGTTAGCGTCGCCGAGACGTCCG

ATCGCGCAGTGGCACACGCTGAAGGATCCGGACGACGACGCGAAAAACTAAAAGAACGTT

TACACATAAGAACCGACCGAACGACAGAACATCGCCGACGGTCACCGTCATCGTTTAACC

ATACGCTCGTCTCAATCGCGGATATTATAATATAATAATATTAAAAAAAAAAAAAAACAG

AAACCATTCCTCGAAACAACACGCGATTCGGAATCTATAAAATACAAATAGACGCCTATA

ATACCCGAACACGGGTACAACGTACACTATAAAATAATATCGAAATAGACAATTATTACT

GTCCAAACGAGGAAACGCGCAATTTCGTGTATTGTTATATTTTCGCATAAACATATTAAA

ATATAGGTACGACATCTGTCCAAGTGTCATTATACGATATAATATATACGTACTTCGTGT

CCGGCGTGTGATCATATCATCGATATTTCTCGTTCCTTGTTTTCACGCGGACTGCAGCGA

TTTTAATAATATTATATTGTGTAATGATTACAGTTGTCGTCAATCCGTATAATATTCATC

GTTATACTATAACATAACCCCGAAAAATTATTTACCGTTCATAATACGTTACTATACCAC

GAACTCGATTCGTGATTAATACCAAATAACACTTACCGGTCATAAACGTTTACTATGTTA

TAAAATATACGTTCATAGATACACGTCCGAGCATGGCCTAAGATATACATAATATATTAT

AATATGTATATTTTAGTTCATACTTCCTGGTTACCCGATGATCGCCGTTTATCGTACAGA

CTCCGAATTTAGAGAGATATTATTGTACCTACGCAAAAACAAACGCCGTCTTATTGTGAT

GCGTATTATTCAAACATAATTCAGTGATATAGGCGGGGAGGCCGAGGAAGCTGCACCTTT

CCCAGCAATTTCCCGAAAATTAAAAAACGCTTATCTAAAAAAAAAAAAAAACGAGTAACC

GTGTAATAAAATATTGTCGTTTTTATGAAGGTTACAGCAAAAGTAAAAAATGTCATAAAT

CCACGGCATCGGCCTACTAGAGATCCCAGTTAAGCATCCCCTGTTTAATAAATTATCAGC

CCCCTCCTCCAAAAACAAAATTCTAGCAGATCATTCGTGTAATTATGTATTGTACCGTTT

GTACTCGAGAGCGATACGAAATTATTGACGATACCGAACAAAAACTAAAACTAACGATAC

ATAACTGTAATATTACAAAATACGTCATATAATATTATAATATACACCGATGTCTTCTCT

GCGTTATTATAATTATATAGGTAATAATATTGCCGGTAATAATAATATATAATGTGTTGC

CTATATATCAACGCCCTAACAGGTAATATATTATACATAATAGGTAGGTATTCTCATCTT

TTTTCGTTTTATTTTCTCTTCTCTTCTTGTCTTGGCGGAAAACCTACTTAAAATAAAAAA

AAAAATAACATTACATTAATAAATATATTATTATATATTATATATAAATTATAATATTAT

CTTCGTTAACCCCCCCGTGGGTGTTGTTCTATAATTTATACTAAGCCACTGTTATTCGCT

TCATGTAAACATTATTTAGTTTTAATACACCTACCTACCTATACCTAGTTCAGAAAATAA

TACCATCG

>Unigene0001380

ATTTCTCCCCCCTTTGTCCATCCCAATCTCAACCCAACCACACACTCGCCTGCTGCCCTC

CATCGTCCGGCCGTCCAAGAGGACACCACCTGCGCCGTCGCGCGTCGCCACCGTCTCCTC

CGTCTCCACTACAGCCCCTAATGCGGCAGATCTGGAAAGGAGGAAGCTCGAGCTCCTGCA

AGCCGTGCAGGATACACAGCGCGGCCTCACCACCACCCCTGACCGCCGCTCCGCCATTGA

AGAGGCCCTAGTGTGCGTGGAGAGTTACGATGCAGGGTCGACGGTCGACCTCAGCAAGCT

GGACGGTACCTGGCGGCTGAACTACACCTCTGCCTCTGATGTGCTCATCCTCTTCGAGGC

CGCCGCTCGGCTCCCCTTCCTTCAGGTGGGTCAAATTTTTCAGAAATTTGAATGTCGAGA

CAAGTCCGCTGGAGGAATTGTCCGCAATGTTGTTCGATGGAGCATTCCGACCTTATTGGA

GGAACAAGATGGTGCAACATTAATGGTTTCTGCAGAATTTTCTGTCCTATCTAAGCGCAA

CATATATCTCAAGTTTGAAGAGGTTGCTGTTGAAAATATCAAGATTAGTGAGGAGCTCCA

AGCCTTTATAGCTCCAGCTTTACTCCCCCGTTCATTTTTAAGCCTACAGATTTTGCAGTT

CCTTAGAACTTTCAGAGCTCAAGTTCCCGTGAGAGGACGTGAAAGGCGATCGCCTGGAGG

AGTGTATTACCTTTCGTATTTGGACCGGAATATGCTTTTGGGCCGTTCGGTTGGTGGTGG

TGGTGTGTTTGTTTTTACACGCGCCCAATCTCTTACGTGATTGCTTTGAAGTGACACAGC

TTCAACCCTTTGGGAGACAATGGCTGGTATCGGTAAGAACTCCAATGATGTACACGATAT

CATGCTAACAAACTCGTATAACCCTTCAGAAATCTGGATAATTCTGCTGCGCTAAACCTG

ATTTATATCAGATTTTTGTATGTTTCAGATAAATGTGTGGCCATATTTTGAAAGATCATC

TATATTGTATTTATCAAAATATTAGTTTAGAATCCGCACATGTGCAGGCCGAATTAACAA

TATGTTGATGTACATAAGCAATCGTAGTTGTAGCTAAGGATGTTTTGTTTAGGTGTGTAT

AGTCTCGTGTCTGAATATCACCGATATACTACTATAAGTTACTGTATGTTGAACATCGTG

T

>Unigene0001381

TAGCAACTATATAATAAATCAAAAACTATACCAGAGGAGGATATTAGAAACTTATCTGAA

TGCGTCAATGACGGAGAACCCCTGGAGATCGAGATCGTGTCCGTCCAACCGCCCACTGAA

CGATCTCGATCCTTAGGGCTTACGGTGGATTTAGGACGATAGAGTATCCAACAATCTTCT

CGCTGGGACCGCTTATCTGATGCCTGAGTTCGGGTTTCCGGAGAAGTTTCAGACTTCTTG

AAACCTGTTGTCTGCTCCTGACCTCTGATCCTGTGGGGGGGAAGAAGAAGAAGCCCTAGA

CTCCGAAGCGGAACCCCCTTCTTTTACCCTCGAGCCCTCCTTTATAGGCCTTCCCGTTTC

TTGAGAGGAATCCGGGATTAGTTCTATCTTCTGACACTTCTGTTAGTCCCATTTTCGTTT

CTTGCTTTATCTGTTGGTTCCCACTGAGAGAGGTAACCTCATGCAAAACTGATTCCGTTT

GAATCCTTATCTGCTGGGCCGGCGGGATGAATTAACTGATTGATTCACTTGGGATTTGCC

ACGTGAAAATCCGGTGTTGGCCTTTAAGGAAATAGTCCTGAACCCTTTTCTCATGCTGTG

ACCGTGTGCTCAGCCCAAACCTGCCGGTTTATGCTTAATTGCCGGGTCCTGCCACATGTT

CGGGCTTAGCTCGAGGCTCTGCGGGCTAAACCCGCTAAAATTACCTGGGTATGGGTTAGT

CTCGTAATTCCTGGCAACCCGGAATTTATGGTGCTTTCAGCTCTCTTCTATTTATAAGCA

TTAAAATATCTTTGAATTTGGGACCAGATTCCACCTTGCTGATTGGTCAAGTCAACATTA

AATGCTCAAGCCTATAAAATAAGTCCTCATGTCTCAAAAACTGTCAAAATCCCATTTTGC

AGATTCTCTATGTTTATTCCCATCATATCCGACAACTAATCATACCCTATGATTTTCTCA

ACAAATCTCATAATTTATTCATATTTCCATGGAACAAACCGAGAGAATCCCACATATTAC

ATGAGCAAGGACACAATTATTCCAACCGCCACTAAGCTTACCAATATAATCATGGATCAC

CCATGAATCATATCTATGCTCTCTCTTATTTCCTACTAAAATCTATGACTCTAACATGAC

TTATCCTTGACAATTATCCTATCCTTGATGAAGTAACTGTTATCCTCCTACTATATGAAT

TGTGCTGAATGTTGAAATGACCAACATGAAAACGGGATGGGATGATCCATGAGCTTAAAG

AAGATTGGTCAATACTTGATTTCCTCTTCATTCAAATTAATCGAGCAAATCGGGGGCAAT

GTAAACCTCCACTTTTTCCTTGATCATCCCATCGCTACAATAAAACATGAAAGGCATTAC

AAGTCTACCCAAATACTCAAGATACATATAAAATGAGTCTACATGTTGAGTTAGTCCTAA

TCTTTATTGAATCCGACCCAAACTAAAGGGGTATGATCTTTCTGTCAAAAACCAACAAAA

TCTTAAGATCAGATACCACCTGTCCTCACAGTTGGAATCTATCCCTTCAACAAGTTAGTA

TGCCATGAAGCTTCGAGATTAACCATAATAGAGGGAAATCTGGCTCTGAGGATATTCACT

ACAGGTTACATGGTACTTATCTATAACCCTAAAGGGGATCCGAGATAAAGAATCCAAGGA

TCAACTCAGGAATCACGAATACTCAAGTCTTCTCCCAGCAGAGCCCTAGTTTAATCTCCA

TTAGAGATTAAAGAATGATTACCCTCGGAGACTCTTGGCTACTTCCGGCGGGGTGGTCGG

GCGGAAGTGACATACTCAACTCTCTCTATTCCCTATTAAGTTCATCTTCAAATAGGTCAT

AAGTTTCCTAGTATTTTACTGTATTTACCATATATCTTGTTCATAAAACACGTGTTCAT

>Unigene0001382

TCATATCTATGCTCTTTCTTATTTCTTACTAAAATCTATGACTCTAACATGACTTATCCT

TGACAATTATCCTATCCTTGATGAAGTAACTGTTATCCTCCTACTATATGAATTGTGCTG

AATGTTGAAATGACCAACATGAAAACGGGATGGGATGATCCATGAGCTTAAAGAAGATTG

GTCAATACTTGATTTCCTCTTCATTCAAATTAATCGAGCAAATCGGGGGCAATGTAAACC

TCCACTTTTTCCTTGATCATCCCATCGCTACAATAAAACATGAAAGGCATTACAAGTCTA

CCCAAATACTCAAGATACATATAAAATGAGTCTACATGTTGAGTTAGTCCTAATCTTTAT

TGAATCCGACCCAAACTAAAGGGGTATGATCTTTCTGTCAAAAACCAACAAAATCTTAAG

ATCAGATACCACCTGTCCTCACAGTTGGAATCTATCCCTTCAACAAGTTAGTATGCCATG

AAGCTTCGAGATTAACCATAATAGAGGGAAATCTGGCTCTGAGGATATTCACTACAGGTT

ACATGGTACTTATCTATAACCCTAAAGGGGATCCGAGATAAAGAATCCAAGGATCAACTC

AGGAATCACGAATACTCAAGTCTTCTCCCAGCAGAGCCCTAGTTTAATCTCCATTAGAGA

TTAAAGAATGATTACCCTCGGAGACTCTTGGCTACTTCCGGCGGGGTGGTCGGGCGGAAG

TGACATACTCAACTCTCTCTATTCCCTATTAAGTTCATCTTCAAATAGGTCATAAGTTTC

CTAGTATTTTACTGTATTTACCATATATCTTGTTCATAAAACACGTGTTCAT

>Unigene0001383

TAAAACACGTGTTCATCCCCAAGCCAAAGTGAAGATCTTGAAGAGATTCATGACATTCTT

CACCTTGTACAACAAACTTATAGCCCAAAGTCCAGCCATAAGTGTTCCTACACAGCTACG

CTAGAAAGGATGAACCTAAACGTGAAAATGTATTATATGTCACTGGGATTATCAAGTGAT

TCACACAAGGTTCCAGGATATATTACTCAGTAGATCTCATTGATATGTTTAACCATCCTT

GTCATAGATAATATGCATTTATCATGCTTAACTACAATGCGTGTTACAAGTTCCTGTCAT

GTAAATATTGTGCTATTGTATACGCATGAGTCCACCTTGTCAATCATACAGTGTAGGGCA

TTGTGCTATTATATGCGCA

>Unigene0001384

GGCATTCCCTCATGGCGGCTCTCAACTGGAGGAAGGAGGCGGACGACAACCTCAAGCGCC

TTCATTCTCTCCTCTTCGGCGCCGACCTCGCCATCGACGCCGGAAATCCTGCCGCCGCTC

GCACCCTCGGCCTCCGCCTCCTAGGCTTCCTCGACTCCCGTTCCCAAACCCCTGTCGATG

CCGCCTACATCGCCCCAATCCGCGCTGAGGTCTCCTCCAAGGTTGCCGCCGCCTCCCGTG

CCCTCGCCGCCGACTCCGATCGCGAGGCGTTTGAACAAGCACAAAGAGATGTAGGCCCTA

TTTTTGTGAAGAGAGCAGACATTGACATAGAGAAGATTAAACAATCAAAATACTTCCGAG

ATTTTCTACAAAAAACCAGAGATGTTACTGAGCCATTGGATTCCAGCGTGTCATCATATG

CCAATCTATTCTCTGTTCGGGATTCTGCTATTCAGGGGCAGCAACCACCTCAGGCAAGTC

AACTGGATAGGGAACACGAGAAATTGAGTGTTAGGGCTTCAAAACTCATGACACAGGCAA

AGTTGGCATCAATGTATGGTAGTAAGGGCACAAGTAATACTTCTTACAAGAAAATGCTGA

GCTCTGTAAGTAGCTTCCCCGTGGAGTGCATTGATGTTGAAAATGAAAGCATGAACAACC

AAAATTATCGGAAAGCCAATTCTGATCCTGTCATTTTGCAAGTTGAGGACGAAGAAAAAC

ATCACAGGATCCCTCTGAAAACAAAACGTCGTCAATCAGCGTTTACCAGCCCCATATGTG

AAAGTCTAAAATCTCCATCAAGCACTGAGGAAGCAAATACAGATAGTACTGGCAATGGAT

TTGTAACTGCCAGAACAAAGCTGGAAATGGATTCTAGACAAAAGAATGGTCTTATGGGTC

CTCCTAGTACTTCTGTGTCCCCACAAAGTGATTATACAGGCAACATGCGAAGCTATAGCT

CGAGGGCTGGTGGTATTTCTCGACGTGGTTTGCGTGGTAATTTTGTCCCTCCTATCAGAT

CTAATGGAGGGAGTACATGTAATATGTCCAACTCAAGGGCTACGGGGAAGTGCGATGACT

CATTAGAAGATTCAACGAGGAAATGTCTGGAGCTGTTGTGTGGCCCTGATGGAGAGCTTC

CTGAAAAGCTGAGGAATCTGGAACCTCGCCTTATTGAACATATCAGTAATGAGATAATGG

AAAAGGACCCCAATGTGCGGTGGGACGACATCGCTGGTTTGGAACATGCAAAGAAGTGTG

TAACAGAGATGGTTATCTGGCCTTTACTACGTCCTGATATATTTCGTGGTTGTCGTTCCC

CAGGAAGAGGTTTGCTTCTATTTGGTCCTCCGGGCACCGGCAAAACCATGATTGGAAAAG

CCATTGCCGGAGAGGCAAAAGCTACGTTCTTTTACATCTCTGCCAGTTCATTGACAAGCA

AGTGGATTGGTGAGGGTGAAAAGCTAGTCAGGGCACTTTTTGGGGTTGCCAGCTGTCGTC

AACCTGCGGTAATATTTGTGGATGAAATAGACTCCCTATTGTCCCAGCGCAAGTCAGATG

GGGAGCATGAATCTAGCAGGAGACTGAAAACACAGTTTCTAATTGAGATGGAAGGATTTG

ATAGCGGAAGCGAACAAGTTCTACTAATAGGAGCTACTAACAGGCCTCAGGAGCTTGATG

AAGCTGCACGCAGACGACTTACTAAGCGATTGTATATACCTCTCCCTTCACAAGAAGCAA

GAGCTTGGATAATACGTAACCTTTTAGAGAAGGATGGTCTATTCAAGCTTTCAGAGGAGG

ATATTGCTTCCATATGCAAGATAACTGAAGGTTATTCGGGGTCTGATATGAAGAACCTTG

TGAAAGATGCATCAATGGGTCCTCTTAGAGAGGCGTTGAGGCAGGGAATCCAGATTACTC

AACTTAAAAAGGAGGACATGAGACGAGTTACAGTTGAGGACTTCTCAAGTGCATTGCAGG

AAGTGAGGCCTTCTGTTTCCTCGAGTGAATTAGGTACCTACGAGACGTGGAACCAACTTT

ACGGGAGCTTATCAATCTGAGGGTTGTCAATTCATTTTTATTGGGACTTCAGCCAGACTC

TTTTTTAGGTGCACTTATTTTTTACTATTGACGAATAGTTTTAATGTTGTACTCTAGTAC

TTCGTTAACGTCTTTTGTATGACTTGGTCTTCTGGGTTCATCATATCGACAGTTTTCCCG

CTTTATTTTCTGTTGTGTGTTTTTGATGAGGTAGATTCTGACGTCCATGAATTTGTAATC

TTATAATGTGAATCCATCCTTTTACCTGTAACTTTTGACACTGATTTAATGAGGCA

>Unigene0001385

TGTACCAATCGCGGTTAAAAAATAAGGGCTCCTTTTCAGATCCCATTCTCCTTGTTCGGT

AGCGCATCCGTGCTCCGATCATCAGCTCCCTTTCTCGATCCAGACCTCCGTGCCCTCCGC

CGGAGGCCGATGGCGCTGATCGAATTTCCAGTCGACCACCGGTCACCGGATCCAGTAGCC

GTCCAACTTGCCGAGCAGATTAAAATGTCGGAAGGGAAAGCTGATTGGAATTTAACGAAA

GACATTTTTTTCGTTGAATTATTGGCTAATGAATGTCGCGCCCTTCGTAAACCCATTGGT

GGACTGACAAAGGCGGCATGGAGAACGATCGTGCGGGAGTTCACTGC

>Unigene0001386

TCAAAGCCCCGGCGTCCATCAAAGCTCCAGTGCAGGTCAAAAGCTCACGAGGAACCGAAA

CCTGATTGGAATCTGACGAAAGATCTTCTTTTCGTCGAGTTACTGGCGAATGAATGTCGT

ATTCTTCGTAAACCTGTTGGTGGTCTGACGAAGGATGCATGGCAAACGATCGTGCGGGAG

TTCACTGCGAAATATGGGTCATTCTATCAGAGGGACAAACTCATCAATCGGAAGCGGACG

TTAATAGAGCATTATAAAATGCTGAAGCATTTATTAGAAAGTTCAAGTGGCTTTGAATGG

GATGGTGACAAACTCAGAGCCTCTGAGAGTGCCTGGGAAGAAATGATTGCTAAGACACCG

GGAGTACGGAGCTATAGGGATAAATCATTCCCTCCTTACGAGAGTCTCTGTACGATCTTC

CAAGAAGGGGCTGCAGACGAGCCAGCCTCATTGGCCGGTACAGAGGTTGTAGATGTTGCA

GAGGCGATCGGGAGCTCCTCCTCGGACCGTCTACCTACCTCCCCTCCCTCTCGAACCAGG

CTCCGACAACTCTCTATGACTCCGACGAGTAATGTGACTCCTCGAGTGTCGTCAAAGCCC

CGGCGTCCATCAAAGCTCCAGTGCAGGTCAAAAGCTCACGAGGAACCGAAACCTGATTGG

AATCTGACGAAAGATCTTCTTTTCGTCGAGTTACTGGCGAATGAATGTCGTATTCTTCGT

AAACCTGTTGGTGGTCTGACGAAGGATGCATGGCAAACGATCGTGCGGGAGTTCACTGCG

AAGTACGGGTCATTCTATCAGAAGGACAAGCTCATCAACAGGAAGCGGACATTGTTGGAT

CACTATAAAATGCTGAAGTATTTAATCGAAAGTTCGAATGCCTTCAAATGGGATGGTAAC

AAACTAACAGCCTCCGAGAGTGCATGGGAGGAAATGATTGCGAAAACGCCAGGTGCGCGA

AGCTATCGCGACAAATCATTCCCTCCTTACGATAGTCTTTGCACAATCTTCGAAGGGGAG

GTTGCCGATGGTCGTGAGCTCTTGGAGGCGAATGGGAGCTCCGATCGTTCGAACGATGAC

CCTAGGGAGTTGTTACCAGAACGGGCTGAGTCCAGTGACTCTAGCGAGGAGCGTGTTCCA

GAACCTCTCCCTCTCAAGCGGGCCCGATCGAATGATTCGGGAAGTTCCCTGAGGGAGAAG

AAAAGGAGACAGAGAGACACAGATCGAGATGCGATAGCAAGGATTGCTTCTGCGGTTGAA

AAGTTCACCGAGACATACGCTGAGACTTCTAGACACTGCCACTTTCGTGAAGTCTTGACT

GCGCTGGACGAGACTCCTGAACTTAACGAGGGGGACTATTCGAGAGCTGTTACGGTGCTG

GCTTCGGATCCGAAGCTGGGGGATGCGTACGTTCTTCTGCCTGCGCGACGCCGTGTAGCT

TGGCTGCGGTCTCTTTTCTGTTGATGTGTTATGGTGGTGTGGTTGCATTATATTAGATTA

TCGGTATAGTAATTGGTTGACTCCTTAGACTTATGTTGTCATGTAATATGTGATAGTAGG

AGATGTTATTGCTATGATGAACTTGAAGACTATGTTATGTGTGATGTGCATTGTACAGTA

TGGGGCGGATTTGATTTTATGTTGCAGATTATATTGTTAAAATTTGGATGCCCGTTTCGT

CTCAAGAACATCCTTCTTGATGGCGATCCCTGTTAAGTTGAAGTAGTAGCAGATGTGTTA

TAT

>Unigene0001387

CAAATTTTATCTTTCAGGATAGTCACGTTAATATTAAATTAGATATTAATATTATTATAA

AAATCTATAAGGTTATTACAATAGAGTTTGTAATTCACTTTAATATAATATTATATGCTA

ATTTTTTCATAAAAGAGTGTATCGTAAATTTTCAAAATTCTATTTAAAAAACCCGGTTCT

CCTGTCCACAGTACAATTCCTCCCTCTATGTACACCTTATGGAGAGACACAACTACGTCA

ACAAAAATCAAATTTTACACGGGACCAGGAGAACTAAATCTGCTAATTTGGCAAATGACT

GAAATTATCCCCACCAACCACGAGTGGTTAGTCGTCATCGTTAATGGACTCGGATACCAC

CGAGGGCTTGTTAGAAGCGTACTAATTCATCGGATGGATTATATTTACTATACCCACATT

GAACTATTAATAATCCTGCCGCTGATCAACCGCCACATACGAGCCGCAGCACTGCTCCCA

ATTCCTCCCCCTAAACCATATCCTCGCGCATCCGATTATCCCCATGGCAGTGCCCCTCAA

CCCTGCCACCACCGGCGTCCGCTCCAGCGTCTGAGTTGCCAACAAAGCACTCATCATAAG

TACACA

>Unigene0001388

TGATTTTCTGTATCACCCTAACGACAAACCCTCTCGCTCTCTCTCAGCCCCCATTGAATT

CATGGCGACCCGGCCACCAGCGGCGGCGGCCACCACAATTAGGGCTTCGGACATGGAGAA

GCTGAACCTCGAGCAACTGAAAGCCCTAAAAGAACAGACGGATCTCGAGGTCAATCTCCT

TCAGGATAGCCTCAACAAGATCCGGACCGCCGCCACCCGACTCGAGATAGCCTCCACCGC

CCTCCACGATCTCTCGCTCCGCCCCCAAGGGAAGAAGCTGCTGGTGCCGCTAACGGCGTC

GCTGTACGTTGCCGGGACTCTCGATGATGCGGAGAAGGTGCTTGTTGATGTCGGGACGGG

TTATTTCATCGAGAAAACCATGGCTGAAGGCAAGGATTACTGTGAACGAAAAATCAATTT

GCTTAAATCCAATTATGAAGAGCTTTTTGAGGTTACTTCGAAGAAGAAAAGCATGGCAGA

TGAAGCGGGATTGATATTACAAGCAAAACTGAGGCTCGCATCTGCAAGCTCGTGAGGATT

GAAATCTCACAACTGTTCTTAGAATCTTGTCAGAAATCGGAGGCCGGATTGTGTGGGTTG

GACATTTGGAGTGAATTAGAGCATGTTGAATTCTTTTAGTAGGACATGATGTACAATTAC

AAGCCAAACCTATAATATTCCGCTGCTTAGTTGTGTGCTCATTTAGCATTTTAAATATAA

ACTAATTAATGAAGGATTTGTGTTGAGTTTGATTTGATTCCTCTTTGAAGGATGCCATAT

GTTTCTGACATATTCAGATGGACTATTGCCTCTCATTTTGTTGGTAATCAGGGTAATTAT

>Unigene0001389

AATTAGTTTGGGAAATTTTTTGGAGGAAATTAGAAAGGGGGTTACATGGAAGAATATGAA

TTTGTGTCCACAACCGGGTTTCCTGTGCCACTTAAGCGACTGGTCCTAGGACTGGTTTGT

TACCTTGGGCGTAAATAAGGGGAAATCATGTTCTTTTGTAAAATAAGTTATTTAACTGAT

ACTTTAAATGAAATTTGCCCCATATATTTTGACTTCAATTATATTTGCCGATGTAACCTC

CTACAGAGTAATTGGCAAAGCCTATTGTTGTAAGTCCTGCCTCACGAACAACTTCTTGAT

GTTACATGTTGTATGCATCAAGCAGTTCATCATTTACCTGCGAAACTGTCTAACTGGTAT

TCAACTCAGTGTTATCAGAATCTCACGAATC

>Unigene0001390

ATATTTATGCATTCTCTGCATGAGCATATTTCACAACTATGAATCCCAAAAGATATTTGT

CGAAGTAAAGGAGTCGGGCTTGATATTTAAGTGTCAACTGTCAAAGAGCGATAGTCAAAT

TCAACATTGACACGTTATTTGTCAAAGAACCTGCGTTATATATAGCACTGAACCTTTATC

AGCTGAAGCCTCGACTCTGAGAGTTGGATTGGATGTGGATCCTAGGAAAAGATGCATGCT

TTGGCTTCT

>Unigene0001391

CCGCCACCAAGTTGCAAACCGCCGCCCACTGTGTGGCTTTCTCTCGCTGTCTGACAGCAA

CTGTCTTCCCTCTCCACGTATATCTCTCTCATCACCGATCCAGCAATCCACTTCGAATTA

GTGCACGAAAGCATCGGCATCGCGGACGTGGCCAGGGTTGGGCCTTTTATTTCTAGACAC

CAGAAGAAATTCCTTTCTCTGCCTCGTTTATAGGCATAAATGTCCAAGAGTTCGACACGC

CAACTACGAAGATGAGCAATGAGGACAGTCAGAGGTTGGGTGAGAATATCCTGACAAATG

GGGATCTCTATGTCGGGAATTTCGATAAAGTACTTCCTCATGGTATGGGAAAATATATAT

GGTCAGATGGAACCTGCTATGAGGGTGAATGGGAGAAAGGCAGAATGATGGGAAGAGGAC

AGGTATCCTGGCCATCGGGTGCCAAGTATGAAGGTGACTTCTCTGGAGGTTTCTTACATG

GATCTGGCACTTTGTCTGGACTTGATGGTTCTACCTATACAGGGTCCTGGAGGATGAACC

TACAGCATGGGCTGGGAACCAAAGTTTATTCTAATTCAGATGTTTATGAGGGTCTTTGGA

ACGAAGGACTGCAAGAAGGTATTGGAAAGTACACATGGAGCAATGGAAATACCTACATTG

GGAATTGGAAGGCTGGAAAAATGACTGGTCGAGGAGTTATGAAGTGGGTGAACGGTGATA

TGTTTGATGGTGTCTGGTTAGAAGGATTAGAACATGGCTCTGGTTGTTACAAGTTTGGTG

ATGGAGCATATTATATTGGAACATGGAGCAGGGGGCTAAAGGATGGGCGGGGAATTTTTT

GGCCAGCTGGAAACAAACTTCCGTATCCGCATGAACATTACGAGTCCATACTTTATAGTG

ATGAGGCAAATAGTATATGTCCTGATAATTTGATAAAATCTGGAGGAATCAGGACAAGTT

CAAAAAGGAGCATATTCGATTTATGGAATATTAGCAGTTTTCTTGAACATTCTGGGCGGA

TATCACATATGACGGCATCACTCCGTGGGTTTTGGGATCTTCATGATTCTGGCAGAATAC

CGTTACACAACAGTTCCCTTTCACTGTCCTCTTTACCAGATGGTCAACACGAGCATGAAA

ATGGTAGTGTTTTGGTTTTTGAAAGAGAGTACATGCAAGGGGTGTTAGTAGTGGAGAGAC

TGAGAAATTGTAACCCCGGGACACCACACAAAACCAAAACCAAATGGTGCCCGGCGCTTA

GGACAAAACATTCAAAGGGTCGTGGAGAAATTATTTACAAAGGTCACAAGAGTTATTATC

TGATGCTGAACTTGCAACTTGGAATTAGGTATACTGTGGGAAAAATCACTCCTGTTCCAA

TGCGGGAAGTTCGTTCCTCTGATTTTGGTCCTCGAGCTAGAATACAAATGTACTTTCCGA

GCAAAGGTTCACAGTATACACCTCCCCACCACTCGATTGATTTCTTTTGGAAGGACTACT

GTCCAATGGTTTTCAGGCGAAAACACCCGAGTTGAGCTGGTCAATTCGGTCCGGTTCCAT

CAGTTTCTACACAGCCCTTGCTTGCAAATCGGAATCTCCGGGAAATGTTTAAGATAGATG

CCGCAGATTACATGATGTCTGTGTGTGGTGGCGAGAGTTTGAAGGAACTTCCTTCTCCAG

GGAAAAGTGGCAGCATCTTTTATCTTTCACAAGATGAACGATTTGTCATAAAGACACTTA

GGAAACATGAGCTTAAGGTTCTACTGAGGATGCTTCCCAACTATTATAATCATGTTGGTG

CGCATGACAATACACTCATAACAAAGTTTTTTGGTGTCCATCGGATTACACTAAAATCTA

GACGCAAGGTACGATTTGTAGTGATGGGAAATATGTTCTGCACAGAACTGCGTATACACC

GTCGATATGATTTAAAAGGTTCTACTCAAGGAAGGTTAACGAGCAGGGACAAGATTAATG

AGAATACCACTTTAAAAGATCTTGATCTGTCATATGTCTTCCGTTTGGAGAAATCCTGGC

GGGAATCTCTTTTTAGGCAAATCTCTCTAGATTGTATGTTCCTAGAATCAGAATGTATAA

TAGATTATAGTATGCTATTGGGATTGCATTTTAGAGCTCCTGAGCATTTGAAGCCTCCCT

TGGAACCTGGAAGTACACCTGAAACTAGTTCAATTTTACTAAATGACATTGATGTGTCTT

CACAAGGTGATACAACTATTACTCCAAAGGGTTTGCTCCTAGTTGCGCATGAACCAGGGT

CTGCGAGTATGCCAGGTTCACACATTAGAGGTAGCACATTGAGAGCATCTACAGCTGGTG

ATGCGGAAGTTGACCTTCTTTTGCCAGGTACCAGAAGATTGCGGGTGCAACTAGGAGTGA

ACATGCCTGCTCAAGCACACCTGAAGCTTCTTCACGACAAAGGCCCCGACTCGACCGAAG

TCGATCTCTTTGAGGTGTATGATGTTGTTCTCTATCTCGGGATCATCGACATTTTACAAG

AGTACAACATCAAAAAGAAAACTGAGCACGCATACAAGTCTCTCATGTTCGATCCCCTCT

CAATTTCGGCTGTTGAACCGAATTTGTATTCGAAACGGTTTATCAGTTTCTTGGAGAAGG

TTTTCCCAGAAGAAATCTGAGGTTGTCTAGAGTATTTGTATCTTGTAAAAGCGCTCTGTC

AAATAGTTCATAAGATAGCCCTTATTTATTTATTTATTCGTTCACAATAATATTATAACT

TGTGATTGTAGGTCATTACATTCCAACGTAATAAATTTCAATATTTTCTTAAAACAAATT

GTAACGGTCGAAAAAATGTGTATCATTAAGGGTGTTGGGCCCATAGTTGTAACCTTCACA

CGCCAGTAACCAGTATGACTGTGGCGTCTGTTATGGTATGTTTATGATGTTGGCTTGGTT

G

>Unigene0001392

CCCCTCCTCCGCCCCTTCGCCCTCCCCCTCCGCCGCCTCGATCCCGCCCGCCTCCGCCTT

TCCACCACCCGCCGTAGCCTCCTTCCCCTCGCCGTCCTCAACAACGACGAATCGCAATTA

GATATCAATGTGGATAACAATACCGAAGAGGCACATGCACACGCAGAAGAGAACCATGCA

CACGCCGAAGAGGTATGGAAGAAGTCACTCGAAACCTTCAAAGAGGAGGCGCTCAAGCTG

AAAGATAAATCACACGAAGCGTACAATGTTTACTCCGAGAAAGCTATGGTGGCACTGCTG

GAGGCATCAGAGAAGCTCAAAATCGAGGCGGAGAAGGCGAGGCGGGATTTGGGGAAGCTG

TCAAAGGAACTTAGCGAGGAGGGGAAGGTGTATCTTGATGCGGCGGCGAAGAATTCGCCG

GATGAGGTGAAGGATATCGTTGAGACTTTTGCGTCGGTGGATGACTTGAAGCATGTTTCG

GTGGTTCGGGATTTTTATGTTGGGATTCCTTATGGTGGTTTGCTTGTTCTTGGAGGGTTT

CTTTCGTTTATGGTGACAGGAAGCACATCGGCCATCCGATTTGGCGTTATTCTTGGAAGT

GCGATTTTGGCTTTGGGTGTATTGAGCTTGAGATCGTGGAAGAGGGGGGAACATTCTCCC

CTCCTTTTGAAAGGGCAAACCGCAATTGCAACAATTCTCTTTATAAGGGAGTGGCGGGTA

TTTTCTCGGGTGCACTCATTTTCATCTATTTTAAAGACCCTTGTCAGCGGAGCAGTTTTA

GCGTTCTACGTATATAGGATTCTGGTGGACGGAGGTGACAAAGGTCCAAGCACTGAACAG

GCCGCAGAGTAGCGGGCTGTCGAGGCACCAGTAGTTCGGATTTTAAAATCTGAAAATCGT

GGCTTGGCGGCTGACGAACATTTCCTGAAAATGAGAGTCATGCATCTGTCAAGATCGGTT

TCTTCTTTTATGTTTTCATTTTATTGGCCTTGCTTCGGTTTGTATTTGATTCTTTTAGAA

TCTATCAAAGAGATTTCTGGTCATATTTAACCTGCTGTTATTTATTTTTATGAATAATGT

GACTCTTCTGGCATTAATACAGGGCTGTAGGGTTTTCGAAGATTCGATTCATTTCTT

>Unigene0001393

CGCTCGCGAAAGTGAATTCGATTTTATCATTTCATAATATTACTCTACTATTATTATTTT

TTTTTTGTCGAGTGATTTACACGTCGCCGTATCTCATACATCATCACCACTATTAAATTT

TTTTTAGACGCGGTCCTCGTCAAGATGAAGATCTATCTACTCGCTTCCGTCTTGCTCATC

GCCGCATATCAAGTTCACGGCCAGAGAGAAGAGTTCAAGTGCCCGGACGACTATGGTTTC

TACCCACACAGCTCATCGTGTGACAAGTACTGGAAGTGCGACAACAACGTGGCCGAGCTT

AAGACTTGCGGCAACGGTTTGGCTTTCGATGACACCGACCCCAAGTACCTGAAAGAAAAC

TGTGATTACCTACACAACGTTGACTGCGGTGCTAGATCACAATTGGAACCTGCCATCAGC

ACACCTCACTGCCCAAGATTATACGGAATCTTCCCAGACGATGTCAAGTGCGATACCTTC

TGGAACTGTTGGGGAGGTGAAGCTAGCAGATACCAATGTTCCCCGGGTCTGGCTTACGAT

CGCGAATCCAGAGTGTGCATGTGGGCCGACCAAGTACCTGAATGCAAAGTTGAAGAAGTA

GCAAACGGTTTCAACTGCCCAGCCGCCGGAGAAGTATTGGCTTCCATTGGATCTTTCAGC

AGACACGCTCACCCAGACGACTGCAGAAAATACTACATCTGCTTGGAAGGCACACCCAGA

GAATACGGTTGCCCGATCGGCACAGTTTTCAAGATCGGTGATTCCGACGGCAGTGGAAGC

TGCGAGAACCCCGAGGACGTTCCCGGATGTGAGGACTACTACGGTGACTTGGATCTGAAG

GCGATCAGGAAAAGCGAACTATTATCCGGGCTAGACAACAAGTCGAAACAATCATCGTCG

GCCGAGTCCAAGCCAGCACCAGCAGCACCCAAGGCACAGCCACAGAGGCAACAACAACAA

TCCAACAGGCCACAACCCCAACAACAGCAGTCCAACAGACCACAACCCCAACAACTCCCC

GTCTCCTCGCAGTCGTCCAAAGACTACGAAGACGCTTAGACAACTACAGCGACGTAACTC

GCCCGCGGTGACGGACACACATACACAAACACGCAAACACACTGACTTTTTGAGTTTTTT

TTTTATATAGAACTTACGGTTTTTGAATTGCGTGAGCACCGAATTTTCTCTCCCCAACCC

CTATACTACTTACTACCCCCGCCCGACGTATATAAAATTAACGATTTATGTATAGAGGAT

TTATATACAGGTGCTCACACAATTTAATACGTTTTTTTTTTTTTTTAATTATTATACGTA

ATATTATTTTGTTTCCTTTTTTTTATACGCTTTTTTTTTTTTTTAATCGACGTCGTACTC

GAATTCGATCGACTGTTCCGTCCGTTCCCCTAATCCCCTGTGTAATTTATCCCTGTTCCC

ATGATTAATTGTAAACAGTTACGGTTAACACATCAATGCGACGGTGGCAGCAGCTGCAAC

CGCAAATTGCTACTTTTAATACTGCCGCTGGACATTCCTATCACCAATACAGACACGTTT

TTGTTTATATGTTTTCGTATTTAAGTGCAAAAATCAAAAAAAAAAAATATATTTAACTAC

CCGAATTTTAATTCAAAACAAAATCAATTCAATATGTGCATTTATTATGTATTAAGACCC

CAACCGTTACGTTTTAGTTAGGTAATCCACATTGTCCCGTTCACAATATATTGCGTTTTT

TT

>Unigene0001394

CGTCAGCCCCCAAACCCTAAAACTCCCACCTTTCCCCTCAGATCCGCCCCTCCCCACTGA

TTCCAGGTCAGATCTCGCCGAATCACCCCCCAACCTCGACCTCCCTTCACGAATCTCGAG

ATTTAGGGTTTTTTTCCTCACGAATCTCGAGATTTAGGGATCTTTGATACGATGCCCTCG

ATCCCGCCCTCCGACGACTCGATCCAGATCCGAGAGGTCTGGGCCAGCA

>Unigene0001395

CCAAACCCTAACCCCCGCCCCACCTGGGCCGACTCCCCGGTCTCCGCCTACATGCGCTAC

CTCGAAACCTCCCTCCAGCTCTCCCCCGCTCCGCCGCCGCCGCCGTCGCTGCCCTCGCCC

GGCTTCCCGTCCCCCACCGCCTTCCTCAACCTGCTCTCACCTCGGTCACCGTACCCGCTC

CTGTCGCCGGGGTTCCAGCACCCTCCGCCGCTCTCCCCTAACTTTTCCTTCTCTCCGATG

ATGTCGTCCCAGCAGGGGATCCTCGGACCGGGACCGCTGTCCCCGGGGCTCCGTTACCCG

CTGTCGCCTTCCGGGTTCCTCTCGAGCCCACGCTGGAGGGATATGTGAAGACTGCTGCAA

CAATGGTACAGAATGGCACTTCCTGTTTTTTTTTTTTCACTCGTCTTACTTTTATAAAAT

AGCTGAAATGTAATTACTATCTGCTAAGCAAGGAAGAGATCACAATATGGAGATTAGTAA

TTGTTTTTCTGGAGGAAAACTGAAGTTGTAATTTAGGCGAAGTAGTTGCGTTTTGTACCT

GTGAACTGATGGGTGATCTTGTAAATCTACTGCGAAATCCGTGTGTTACTTCTTTTTTTT

CCAGTTAATATATTGGTTTTTTTAGTTCAGTTTTTGTTTTGGACCTCCTCTCTGCTGCTA

CAATTTTGATGTTATCTATAAGCAATAATTGCGATGGACTTGATAAATTTC

>Unigene0001396

GGGAGAGTAAATAAGAGGAGAATGAATATGGTTGGGAAGAGAAAATATGAGGCGATGGAA

AATACAGGGAGAGTGAATTGTGATGCGAGAAACTATTTAGATAAACAAAATGAGAGGAGA

GAATATGCTAGGAGTGAGAAAAAATGTAAAAAAAGTGAGAAAATGAGAGGAGAAAAATGT

GTGGGGAGAGAAAATGAGGTGAGAGAAAATGAGAAAATCCGAGCAGAGAAAATAAGAGGA

GAAAAAAATAAGTGGCGATAAAATAAGATGAGAAAATCGGAGGAAACTCTGAGG

>Unigene0001397

ATCCTCAATGCCGAGGAAGAAGGTCAACCTAGCGTTGATGGCCAATGACACCACCCGCAA

GGCTTCCTTCGCGAAGTGCTGCAAAGGTTTGATGAAGAAGATCACCGAGCTTACCTCCGT

CTGCCATGTCCCGGCCTGCACAATCATTTTCGCGCCGCACGAGCACAAACCAGATGTCTG

GCCGTCAATGGACGGCGCAATGGAAGTTCTCCGCCGGTTCAAAAGCCTCCCCAAGATGAT

GCAGTCCAAAAAGATGACGAAGCGGGAACGCTTCCTACAAGAACAGGTGGAGAACGTCCA

TGAACAGCTTCGCAGGCAGGAGAGAGAGAACGACGAGCTTCGGCTCAAACTCCTCCTGTA

CGAAGACGTCGACGGCCGTAGCCATCATAAGGTCGGTATACAGGATATTCCAGGCCTCCC

CAGTTTGCTGGAGATGGAGATGAAGGGTCTGAAGGAGCAGATAGACATGGTTCAGCAGTA

CATCGCCAAGTCTTTGGAGATGGCCGCCGCTACCGAAGTTGTCGAGAATTTCTTACTAGA

CTATGCTGAGATAGATGACAACATGCCCATTGTTGGGCAGGAGATGATACCAAAGCCCAA

TTTAACCAACAGCTGCAGCAACTGGCGGAAAACCGCATTACTGATGGCTGCTGCCGCCGA

ACCAAAGAACTACTGGCTCAATTTTGCAAAAGTGGTGCTGCAAAACCAGAACTGTAGGGT

GAAAGATGAGTATTTTCCTTTTGATGGGAAGGAGATGATGCAGCAGCCATATGTCATGGA

TGGCAGATCAATGTCTGCAATAGCTTCTGATGTGCCCGTGGCCCATATACCTAAAGAAAA

TTACTGGCTCAATTTTGCCAAAGAGGTGCTGCAAAACAGGAATTCTGGGGTCACTGACGA

GTTCAAGATCTCTGATGGGCTGGAGAGGATGCAGAAGCTATATGTTATGAACCATGGCAG

AACTCTACCTCCAAAGGGTAATGACTGGCTCAATTTTGCAAAAGAAGTTCTGCAAAGCCA

GGTTTGTGGGTGGACAGAAGGGTCCATGACCTCGGGAGGGCATGATATATCTGTTTCTCA

AGTTGCTGTGGCTGCTGAACCAAGGGACATTGACGGTCTTGATGTAGCAAATGAGGTGCA

GAAGGTCCTGAACTGTGGGGAGGCTAATGAGTTCATAAACTCCTGTAGGCGGGAAATGAT

GCAGCAGCCATATCACTTAGACCATGGCAGCACCTGCATTGGAACTGATTCCCATGTATA

GTGAAGATCATTTTAGTTTGTGTTTATGTAAATATTAAATCATTCAGGGGCATTGTTTTG

CTAAATGTTCACTCCTTGTTGATGTGTTATGTTTGGTTGGATTGAGAGAAAACAGAAATT

TTCAGTGAAATGATTTGGTG

>Unigene0001398

TTTTAAATGGATGAGGCTATGGTTATCCTTTTTTAGTAATAATGTAAATGGTTTTAAATG

GATGAGGCTATGATTATCCTTTTTTTAGTAATAATGTAAATGGATAAAATTTGTTAGAAT

GCGTTGATTACTTTTGAATGGACTGAATTAGAACATGTTTTATCTCTCTTGTGAACATAA

ACTCATTTACTTAAATCATTATATTTTAAATTGATAGTCACAAATACACAATTTTTATTT

ATGCTTATTTATGA

>Unigene0001399

TTTCAGGTGACCTGGACTAGCCAACCCACCTTACTCGAAGCTGACTAGCTTTGAACGGGT

TTGGGTTAGATATTCATATGCACAAATCATTTTCAGGTTAAGCCGCTTATCAAGCTGGTC

GGTTTCGATTTATGCTTCCGATCCACAAGTCGATCCACAAAACTTGATAAATAAATCAAT

TTATAAAACAATTATCTTTCATTTGTCAATCACAATTCACATAGATCACCTATACTATTC

CACTACCCACAGTATTTGAAAGCTTTGCTTCTTTGCATTGGATGTCGTCATTGATGATGC

AACGATTGTCAT

>Unigene0001400

TGAGCGTGTTGGGCCATGATTATGGGTTCAGATCATTTCCGGGTGATCCGTCACATGACA

TGTTTTAAACAGGTTAGGGTTAGAGCTCCACAGATTCAGATGACCTGAATGCATGAGCTG

ACTCGAAATTGACCCGTTTAATAAGGATTTGGATCAAAACTTTACATGTTCAAATCAATT

TTCGGTGATTTAAATTCGTTCCGACCCAAATTATCTCGCTTTGAACAAGAATGGAGTCCT

ACATGCATTAAGATCAATTTCGGATGATCGAGACCCACCAACCCAATATGAAGCTGACAT

GCTTTAAACATATTTGGATCCGACCTCCATAGGTTCGGATCATTTTCAGGGATCATTTTC

AGGTGACCTGGACTAGCCAGCCCACCTT

>Unigene0001401

CCATTGACAAATTTATAAATGCCTATAGACAAAAAAAAACCCCTAAAGTATTTAATGATA

ACATGATAAACTTCGGATTGTCAATTCAAATCTAGAATACTAAATGGGTCAAATGAGACG

GGTTACATTAACCCGTTTATATGCTGGTTAGTTTTGACTTTGAGTATGTTGTTGGATTGA

GACTGGACCGACAAGACCAATTTACTAGGTTAGGGTTTGTTGTTGGGAGACCAAAAATTA

CTGAATAGGCCCCTTAAGGACCTTAGGTTTGTGTTGGTTGACCTATAAGCCTAGTTAACG

CTAAGAAGCTTAGAACTTATAAGCATTACTATGTTTTGCCTCTCCATAGATTTGAATCCT

CATCCATCCTCCCCGCTTTATTCACAATATTCGGCTCCTGGTAGTGCTCTTTTCCCTCAT

TCATCATTGATCTGATGCCTAACATTAGGGGTGGCAATGGGGCAGAGCGAGATGATTTTT

CTCATCTTCATCTCCATTCCTAACATTAGGGGTGGCCTGATCCCCTCTTTAAACGAGATG

GAGATATTGCCTTCGTTCCGAATATGGATGAGGATCATCCTTGCCCCACCCCCCCTAAAT

AATGTGTTAGTCTTCCCTTAAAAGATTTAAATATGTTATTATATATTTACCATTTTGAAA

ACATAAATGAAGATCCAACTTAGTTGTGGAGGTGAATGTGATAACTATTCAGTTTC

>Unigene0001402

CTATTCAGTTTCACATTTAATCTTAGAAGTGATGATTTATAAAATAATATTTTCAAATAG

CAAATGAACTTAATGAGACTGGTTCGAGATGAGGACCTTTATCATATCCCCATCCCTTTA

CCGAATCTAGGATAAACCTTGTGATTCCTAATCTCCGCCTCATCAGGCGATGGTTGGGGA

CCCATCCTCATGGGATTTTGCCATCCCAATCCAACTTACCTTTGACCTATGGATCTACGG

CTCCCACAGGCGCTTGACACCTCCATTAATCCACTACCTAGACTAGCATTGATGTATTGA

TTACTTGCTCAAGCACTCCTCCCTATCATTCATTTTAATGTTGTTGCCCAGCCTACCCTC

AAATTGTACTCGTCCATCTAGTATTTAATGAATGTGAGTAATCAAAGATAATGTATCATC

CCTAATTTTCGTGACTTCAC

>Unigene0001403

ATGGAAGCCAATATTGCAAACACAAAACAATCCCCTCTTCTTTCTGTTTGGTAGAACTAG

CATTTAATCGACATTGAGGCTCAACAAAAAAAACACTTTAATTTTGTATTCTAAAAGCTT

CCTCACCCACCCAATACACGCTTCAATTTATATCTGAGACCGACTTGTCAAATCTTTTTA

GAACAGAGGAGATAATACTGAGAGAACATGATGTAAGATTTATTTTCTAAGTCGCGGTCT

AGAGGCAACATTCTGGATTTTAAATG

>Unigene0001404

TATGTTGTTTGTTGATTTATATTTGTGGTTGGATATTCCTAATGCTTAATATGATCATAG

TTAAACCTTTTAACTTAAAACTCTCTTGTAACTGCTTAGGTTAAAACCAACCAACTATTC

GGTGGTAATTTTTGTCCAGAGAATTATAAATCAGTAAATTAGAACTCGTTTATGTTAGAA

GGCTCAGAATATGTGCTAAGAATAGGTGATGATAAAAATGATGGAAATCTCATTTAAAAC

AAGAAGATACAAAGAGAAGCAAAAGAAACTCGCCTTGGTCAACAAGTCTG

>Unigene0001405

GCGGCAATTTGACCAGTATTGGAGGAGCTAAGTCAAACCAAAACATTTCAGCTGAAACTG

ATAATTTACTTTAGAGTTTCGGCTAAAAAGACCAAAACGTGTATTGGTTTTCGCTAAAAC

CCCCCAAATGGTCGCAACTGCTGAAACTGAAACACTGCATCCAAAACAGCAAACCATAGC

TTTATCACGTGGTGGTAGATTTGTATTTTTGTTTTACCTCACAGCAAAAGGCAGTTATCC

TGTCTGCTGTCTGCACAAATATTCACTCGATCATGATTAGAATTTTGATCTTCCCTTATC

TACTAATATAAAAAACAAGCGAGTACTCATGCATTCGAAGCACGGGACAGTCGGGGATAG

AACTCTGGTGTGGCGGGAGAGGGAGGTGTCAGCCTGTACCTTGGACAGTGCTAGCCAGTC

GATGCTTTGTGTGGGTGCCCAAGACCTTGACACAGGCTTCATAGCAGGGCATAGAATTGC

TCATGGGAGTGTGGGATCAAGTGTGTGCGGAATAGTTAGGATGTCGAAATTGCACTCACC

CCTTCCAGGGGTATTTATCTTTTTGGGTGTTAGCCCCTGAATTCATGTTTACATT

>Unigene0001406

CTTCCCTTATCTACTAATATAAACAACAAGCGAGTACCCATGCATTCGAAGCACGGGACA

GTTGGGGATAGAACTCTGGTGAGGCGGGAGAGGGAGGTGTCAGCCTGTACCTTGGACAGT

GCTAGCCAGTCGATGCTTTGTGTGGGTGCCCAAGACCTTGACACAGGCTTCATAGCAGGG

CATAGAATTGCTCATGGGAGTGTGGGATCAAGTGTGTGCGGAATAGTTAGGATGTCGAAA

TTGCACTCACCCCTTCCAGGGGTATTTATCTTTTTGGGTGTTAGCCCCTGAATTCATGTT

TACATT

>Unigene0001407

ACTTTGTTTCAATGGAGTCTGGGATGAAGGCTGGTATGGATGGGACTTCCGGTAGCCGAG

CCATTTCTGCTCCGGCTCCGCAGTTCACTCACTCCCTGCTCCGTGTTGCTGCCTTTCTTG

CGACGATGGTTGCAGCGATTGTGATGGCAGTGGCAAAGCAGACGGAGATTGTCAGCGTCG

TCATCGCTCCCACCTTGCCGCCGTTGCCGATTCCGGTGACGGCCAAGGTCAGCTACTCGT

CCGCCTACGTATTCTTCATTGTCACCAATGGCATTGTATGTGGCTACTCCATCCTCTCGC

TAGTAATCTCATTTGCAAACAAGGGACGCAGACAGGGCCTGGCGTTACTGATCACCGTAA

TGGACATCGCCGTGTTGTCCCTCCTCTTTTCTTCCCTCGGGGCAGCAGGGGCCGTCGGAG

TTGTGGGGGAGCAAGGGATTACTAAAGTAGGATGGAACAAGGTGTGCGATGTGTATGGTA

AGTTCTGCTCGCACATAATCGCATCGGTCGTCATCTCGTTCTTGGGCACGATTTTCTATC

TCTCTCTGGTTCTCTCGACAGTCATCAGCCTTCATAGGATGCTTCAGTAGGGTCTGAGCA

TCGGCAAATGTTTACATTCTACATCTTCTGTCAAGGTTTCTTGTTCTACTGAACTATTAC

TGTCATGTTATTTTCATGAATGTATTGTGTTTGGGCATGATTCTGTGTCTCTCTGGATCT

CTCAACAGTCATTAGCAAATGTTTACATTTTACATCTTCTTTCGTGGTTTCTTGTTCTAC

TGAACTAT

>Unigene0001408

AAGCTACGTCAATCGATATTAAAGCTACAACTGTGATCGAGGGTGGTAAGAAGAAGGTAA

CTAAAAAGAAAACAATTAAAACCGTGAAAGACGGTGTTGAGAATGTTGAGGTCATAGAAT

CTAAACCAGAAATTACCGAGATTTTCGATGATACCAATTCATTATTACATCCCAATTTGT

ATGATTTTGAGAAAACTACACATATTCCTGATATTCTTCATCCTCAAAAAACCATTACAC

CTTCACAACATGACACTTCACACACAAACGATTTTTCATTACTAGATGATATACATTCTA

CA

>Unigene0001409

CCATTGCCCGAAATCGAAGAGTTGAAACCTGAAGAGGCTACTCTTATCCCTGACGAAGCT

ACGTCAATCGATATTAAAACTACAACTGTGATCGAGGGTGGTAAGAAGAAGGTAACTAAA

AAGAAAACAATCAAAACCGTGAAAGACGGTGTTGAGAATGTTGAGGTCATAGAATCTAAA

CCAGAAATTACCGAGATTGTTGATGAGGAGACTAAACCATTGCCCGAAAT

>Unigene0001410

ATTACTCTTACTTGCATCTACTTCATTACATACAAGGGTATTATGATTGGTCAAACATGA

ATATCTTTGAAAGAAATTCATTACTACGTACAAGGGTATTTATAAGATTTAAAAGGGGAC

ATAGTTATTGTAGCTTTGGGTGCTGCAGCTATATCTACAGCTGCAGCTCATAATGCTACA

TTGTACTTATCCTTTACAGTAACACGCCCCCTCAAACTCAAGGTGGGTCGAACAAACATG

A

>Unigene0001411

GGGATAATAGGGAGGGTAAGATGTATGCAGCCTTACCCCTGCATGCAGGGAGGTTGTTTC

CGAATTTGAGCCCATGACCTCCAGATCACACGGCGGCAACCTTTACCATTGCCATTGCGC

CATAATTGAATGAAAAGATTACAAGAAAAAAAAAGTAGAGGAAGAATAAAAATAAGAAGC

AGAGAAAAGGAGGAAGTGAAGAGAAGTGAAAAAGGAGAAGAAGAGGAAAAATGAAAAATC

AGAAAAAGATTTGTTTAAAAAAGGTAACCTAGTGCATGGGGCTTTTACCACTACGGGGTA

CTAGGAAGGTCAGATGTACGCAGCCTTACCCTTGCATGTAGGGAGGGACTGATTCCGGAT

TTGAACCCATGATCCCAAGGTCACACGGTGGCAACCTTTATCGTTGTGCTAGGGTCACCC

TCTTTGTTAAAGATTACATAAAAAGTAAGTAGAAGGAAAAAAGGAAGCGGAAAAGGGGAA

GTGAAGAGAAAGGAAAAAGGAAGGAGAAAGAAGAAGCAACATGAAAAGGGAAAAGAAGAG

AAAATGACTAAAATCTGAATTTCACCTAGAAGAACAACTCAATGGAATGATGCCTATTTC

GCTAGCTTCTCCAGTTACGCGG

>Unigene0001412

AGGCTCCCACCACTACGGGGTACTGGGAAGGGTCAGATGTACGCAGCCTTACCCGTGCGT

GTAGGGAGGCTGTTTCCGGATTAATAAATACTCTACATTCACTGATTCTTAAAGAGAAAA

GAGGCCTGTTCGAAGAGGGGTCACAGCATACAAGCGTTGATACTTCTGCTTCTGTGCTTC

CATTTCCCACTTAATTACTGCTTTGGTTTCTAAGACGAGCTATTAACGAACTATCATCTT

AAGAGTACTACGTTTATAGGAACCCTGGTCCTGTTACAACAATATGTCAACTTGAGTGAA

AGACTGATATGTGACATTATGCTTGCTCCATGCTGCAATTAAGGGTTGTTACCATTATTC

TTCAACCACGATCACTTGCACTTCAAAATTCCATAAATTCTGACGTCATAAGTGAAGCCT

ACCATGTTTGAATCCCAGTCTGGATTATTTACATGTTCCTTGTGGATTGAGATTATTCTC

ACAATCTCATATGGACGAACCTGCAAATATGGGTTAGTGGCTGCTTCCATGTACTTCCAA

CACTGAAGGGCATGACTGATAAGTGGGGAAAATTAAAATAAATTGAGAGGTATGTTGCTC

CTTGTATGATTAATGATTCAGTGACAAGCCAATAGACAAGGTAATGCGACTAGTAATGAA

TCTTTCCCAAATATCACTAGTACATAGACTTCACATTCTTTTTGTTTTCATATTTTTTGT

ATGGCAGTTACACTTCATGTCCCTTCATATTATTTTGTTCTACATATTTCTCCTTTCAAT

AAGGGCGTGTTTGGCAAGCCACTTTCCTTCAGAATACCTAAGGTGAGAAAAGTACAGTTT

TTCTCATGTTTGGAAAAGGTCAGGAAGTTAGCTCAACTTCAGAAGACAGATGTTTATTTT

TGTCTGAACTCAAGATGTCTGTGCTCACATCATCGGAAAACAAGGGTCATATTCTAGGAT

CAGAATAAGCATTATACCAATTTTTTTTTTTAATGATTGCAAGTATTATAAACGGATGGA

CTGTGATTGCAACAAGTGTTTGTCAGGAGACCGGTGGTGGACTAAAGAAGTCGATTGCA

>Unigene0001413

AGGCAAAACATGAAACTCTGATTCAGTTAATCAGATAAACTCTGATTCGGTTAATCAGAT

AAAATGAATTATCTGGACCGTACAGATAAAAGAATGGTTACAGACAATCTGTTATCCGCC

TGTTATTATCCGATTTGTTATTATCTGTACAAATAAAATGAATTATCCAATATGTTATTA

TCCGTCTATTATCCAGTCCGCGTTACGGACAATCCAAACAGAAAAACAGTAGTCGGGTTC

TGTTATCTCTTCGTATATTACAGATAAATCCAATACCAGTCTGTTCATTCTGTTATCTAT

GCCGCCGTATGTTTTGTTCTGTTATCTATTTCGTCGTACGTTTCTCACAATTCTCCAACT

CGTACATAATTTGATAAGATCTCAAATTTTGGACAAACAGAAGAAGAGTCATCCTGTAGA

AGTATCCTGTGGCATCAAGTCGGCTGCACCATTTTGACCATGGCTGCTTACGTACTCTCT

GACCAGGATCACGCCGTCGTAGTTCTTCCAGATTCTTCATATAGTCTCCACCTCACTCCA

ATTGCCCCGAAGGGCCATGCCCTCTTGGGGAACTCAACAAGGGCCAATACAGAACAAGTC

CAAACAGTGTTGGAACTTACTACTTGTCACGACCTTAGTTAACATGTCTGCTGGGTTCTT

ATTAGTGTGCACCTTCATAAAAGAAACATCTCCTTCCTCTATGACATCC

>Unigene0001414

CAAGCAATCTCCGGTAACTGCCCTTCACCCCTACACCCCCAGCTGAGATCTCCCGCCCTC

CTCCAATGCCCGACATCGAGGCCGCTGCCGGAGACCCCGTCAGCCCCCGGCCGCCGACTC

CGCCGCCGCTTCCCCCCGTCCCCCGGCGGAGCTCCGACTCCTTCCTCATCCTCTGCCGGT

GCTTCGCCGCCCTCACCGCCCTCACCGCTCTCCTCTGCGTCGTCGCCAACGTCCTTTCCG

CTGTTCGGTCTTTCCGCAATGGATCCGATATCTTCGGCGGGATCTTCCGGTGCTACGCGG

TCGTGCTGGCGCTCTTTGTGGCTGTCGCAGAGACTGAGTGGGGGTTCATCATGAAGTTCT

GGAAGGTGTTGGAGTACTGGGTGGGCAGGGGAATGCTGCAAATCTTTGTTGCGGTGATGA

CAAGAGCATTCCCGGATATATATAGGGAGAGGAGGGATCTGGTATTGCTTCAGGAGCTCT

CCAGCTATTTGCTGCTTTTCTGCGGCGTGATCTATGTGATCTCGGGTGTGCTATGCATCG

GGATCTTGAAGCGTGCACGGCAGGACAAGGCAGTTTCGAGGGAGCAAGCGGCTAAAGATC

TAATGGAACTCCATCGTCGCCGGGAAGAACTCGAGGCACTGTTAGTCCCTGAAAGGGATT

GAGAACATGGTCGGCGATAAATCTGGTTTAAGCATGAAAGGTCTCTTCGACATAATCTAG

GGAACTGGTTCCTCTTACCCAGCGGGACTACCAGACTCCATGAGCTCTGTCGGTTTGATT

GATTCCGTTTTTGCTATGAGAACTGCGGCCTTCGAAATAATGTATTTGTGTAAATAAAGG

TATTATATCTTTCGAGAAATGTTGTATGCTTGATTACCACGGCTAGTTTCGATATTTAGA

ATGTTTCTTTCTGTTCCTATACTAGCTCAGCTGCTTCGATGATCGTGACATTGGTGATTG

ATTTATAATTCTTGAGCT

>Unigene0001415

CGGCCCTGTGGTTTTCGCCGTTTTTATTTCGATGTAAAGTCGATTGTCTTCGAGGAGCGT

CTCGCAAACGCCCTACCATCATCTAAGCTCGGCAATCGCGTTTGGAGCTAGTCACCGTCG

GTTTCCAGTGTGGAATCATGGCCAACTCCGTGCGCAAGATTTGCTCGAAAATCGGAAACA

GCCATCAGTACTTGTTGGCCACGGCCAAAACGTCAGCGTACGGTGGTCATGTCGTGTCGG

ACTCCGTGCAACAGCACTTGGCCCGACCACAGGTCGAAACAACGTGCAAAATCAATTATG

CCGAAAATTTGTTCCGTCAGTCACAACCGTTGCTCGACTACATTGTTCGACCCGTCAAAA

ACAAACAATCTTCCGAAATGGCTGTTGAATGGTTTAACTCTGGGTTTAACAGACACACAA

ATCCCAAAACCGATGTTTGTCACAGCACCGCTTTGAAACAAGACAGTCCTACCAAGTCTG

ACGAAAACATCAGTTCGTCAGATTCTAAAGGTTTACCAAGTGTAGATCAGCTAGAACATG

TTTATAATAGGTTGGGAGAAGATTTACCATTACTGTTCAAACAAAATTTAGACTATGCAA

TATACCATCCCAAATTGGTATTTGAGAATAACATTAGAGGAACAAAGAGTGTAGGCTTAG

AATATTTTATGTCAACAATTTCTATACTACGTTTGATGGGTCATTTAAAGTATGCTTTTG

TAAAATTCGATATTTTAAAAATAACCAAACATCCAGAAGATGGAACGATTAAAATACGTT

GGAGAATCAAAGGTATATCAGGAATGAAAGTGTTCTTAAAGTTTTGGAAATTCAAACTAT

GGAATTTGGAACAAGTCATTAAAGAAAATGTAGAATCATGGCATGATGGTTTTTCAATTT

TCTATTTAGGATCTGATGGTTTGGTTCATAAGCTTACTGCAGACAAAATGCAACCATATA

ACGACCCAGTAATAGAGGAAAAAAATCCACTAGCGACAAAATTGGCCCTCTTTTTAGGAC

TATCACCTCAGTTGGGTAATTTGGATTGCTTGGTTTCATTTGAGTCTGAACAGACAAATG

AATTACTGAAGATGGTTATTATGTCTTTAGAAGATGTCATATAATGTCATTAGAAACAAC

ATTTAAAAATACTTCGTGCCCTGTTAAAAAAATATCCATTATTTTTATACCACGTGGAAA

CTATTATTTAATAATTTTTGTGTGGCCGTTGTATATTTGTTGTTTTGTTAATTTATGAAG

AAATGTTTGTTGAATGTAAAATAGTTAAGCAAGTAGATAGTCAATTAAATTTAACACCAT

TAATGTTGTTGTGATGAAAACACATGATTGCTTATAGAATTACAAAATACTATAGATAAG

CATTGTGCAATTGACTTTGAATAAATCAAACAT

>Unigene0001416

ACAGAGTCCAAACGGAGTCCTGCAGGACTGAGCTGCAAGGGAGTAGCCTAGGTGGTGGTG

CTGGTGGTGAAGGTGAGAAGAGCGGAGCTCCTGCGGCCGTCGACGGCGCCCTTGACTCAT

CCCCCAGATCTGGAAGCTGTTGAGAAAGTATTCTTCTTATGATGCTTGAAGGAATGTAGC

TTCCCATGCATGTAGCAACAAATGTAAATGGCAACTAGGCAGTTTTATGGAATGTATTAA

ATGTTGTATGGGGAAGGAAATTCAACACATATCTGCAGTCAAGGATTCAAACTGTGTAGT

TCCTGAGCGACATAATGACCATGCATTTAATCCTTCTGAATTCTCTGGATCTGATGAAGC

GACAGAAGGTATACTTGACAGTCAAAGTCTTCATGGGCATAAAGAACATGAAGTAAAAGG

TTGCACTTTGGACGGTCCTGTTGAGGTCTCCTTGTCCTGTAAAGATGAAGAAGGGCCTCA

TATTGAAACCCTAACTATGGGTGCTGGCAATCTGGAGAAGAAAAAAGTGAGCCGTCCAGT

GCAGAAGCGGACTGATCATATGAACTTAGAATCTACTGTGAAACCAGCATCAAAATCTGC

AAATGCACGGCCAAAGCACACCATTCCTCAACCATTTGCTCTTGCAACAGAAAAACGTGC

TTCTGGTGGACCCCGTCTATTTGTTGCTAATAATGCTGGAAATGGAGACAGAACTGCTCA

ACCACAGAACACTTATAAGAAGAATAACTTGCCTCCGACATCATTGAAGCCTCTGGAACC

ACATGACGGGAAACATATCGATGATGAAGATGTTTGCTCCATTGCTTCATCAACTGCAAC

ATCCATGAGAGCTTTCAAAAGCAAGTTTACTACGGGCTCAGCCCCAACTTTTAGAAGCAG

TGAGCGGGCAGAGAAGCGAAGGGAGTTTTACTCAAAGTTAGAAGAAAAACAACAAGCTCT

GGAAGCAGAGAAAAACCAGTGTGAAGTTAGGACTAAGGAAGAACGGGATGCTGCTTTAAA

GCAACTGAGGAAAAGTTTGACTTTCAAAGCGACACCTATGCCAACGTTCTATCACGAGGG

ACCTCCTCCTAAAGTTGAGCTCAAAAAGCTGCCCACTACTCGTGCTAAATCACCAAAATT

CGGTCGAAGGAAGAGTTGTGGCGATACAATGAATTCATCCCCTGGAGAGCAGAAAATTGG

ATTCCGCACAAGGACGACCCGTCACAGCCTGGGTACTATCAACGCAGACACCAACAAGGT

GCAGGTGCAGGTGCAGGTGCAAGACGGGGCGAAAATTGGAACCTCGGCTTGTAAGGACAA

AGACCGGCTCGAGCCAGTAAAGGAGTCTAAGTCAAATCATCAGAAGGTAATTGAGCAGAC

TACTATGCAATGATGACTCAGTTTGGCAATTCAAATTTCAAATCCTAGTCGAGTATATTT

TCCTCCTAAATCAACTGAGTTGTTTGTCACTGGCTTGATTTGTCCCGAAAGATAATGTCT

CCGATTTTTAGAACTTTGTTGCTAGGATTCGCCTTGCATGATTGTGTAAATTTTGTAAGT

TGTATAGGTCTCTGTTGCCTGGCTGGAGATATAAGAACTGGTTGCTGTCTGTTGCCTTGT

AGGAGTTTATCGAAACTTTATGGAGAGCTATCCTTTTGTGTTTGGAAGACCGGGTTTTTG

>Unigene0001417

AGCAACAACAAGAACAACAGGACAAAGCAACAGCAAGAACAACAGGACGATGGAACTGGA

CAAAGCAACAACAAGAACAACAGGACAAAGCAACAGCAAGAACAACAGGACGATGGAACT

GGACAAAGCAACAACAAGAACAACAGGACGATGGCTTCTACGGTGTTTGAGGCTAATTTG

TCGCATGGGTTGTGAGGCTATTGCTGCCGTTTATCCCCTCGGTTGCTTTTATGTGTTGGA

AAGCAGAGCAAATCCTGAGGACAGGAGTCATCAGCATTCCAATTCTTATACCTATCACCT

CCACCACTGTTTGACCAAAACGCCGTATCCAGCTGCAAGCATACCAAATGGGACCCCACC

ATAAGGGTCATAGGAACCAAATACTGCAGTCCCGCTCCCTGACTGCCTGTAGAAATCCGG

CCACACATCGACTAAAATGGCAGTAAGGCATCCAGGCCAGACAGAATCATACCTTAATCG

AGATCAAATACCCG

>Unigene0001418

AACCATAGCATGGGTTGCTGCCCCTCCCATGCACTGCCTCTTCCCTGGCTTTTTCCTTCC

ACCACCCCTTTCTTTCCTAAGATTATTTATTTCGTCATATTTGTTTTTTTAGTTCTCTCG

ATTATTTATTTCATCATATTTGTTTTTTCAGTTCTCTCTTAGAAATGTAAATTTGTTTTT

TTAGTTCTCTCGATTATTTATTTCATCATATTTGTTTTTTCAGTTCTCTCTTAGAAATGT

AAATTTGTTTTTCCAGTTCTCTTCACTAGTACATTTCTAGTATATAGATAGCATTTCAGA

CAAACTAATTCATAATGTTTTATTTGTTAGATTGTATCAGGACATGCAACAAATTGTGCA

AATTGTGCCTGCGAACTGGACAAAGCAACAGCAAGAACAAC

>Unigene0001419

CATGTGAATAATAGATCGGCCCACAGGAAGGCTATTATTTGACCGAGATTAAGTTTGCAC

TAGATATCCTGAATATAACATACTAATTATCTTGTGAGTGTTCTAATTAATCTTTTGTAA

GTTCTCTGTCCAGTAATGAATAACCCCAACATTACTACCCAAATCAACTATATCCCAATG

CTAAGTGGTACAAATTTTAAAAACATGGAAAAGGGAACATAAGGGAGTACATTATGGAAA

T

>Unigene0001420

ACCGCCTCTATGGTCCCCCGCTTCCCCCTGCCGCTCCGCATCCCCTCTGCACCGACCACC

TCCAAAGCCACCGTAGAGCGCTGAGGGACCACGACGCCTTCACCAATCCCCTTCCTTCAC

CTCCTCTACCATGCAAACCAAGGTCCACCTCGATCCTCTCCCATCCACCAACTCTGGCAG

CTTTCGACACCGCCAGTGAGCTCCGCCAAGTATGTCTCTCTCCCCCTTCAATCTAATTTT

GTACTAATTCGGCTAATTGCGTGTGTAGGTGTGCAGTTGGGTGCTAATTGGCTTAGATTT

GATGGGGATTAGATGAAGCTGAGCTCGGATTAGGGAGTTGGTGATGGAGTGAAGAAGGAT

TGGGCCCAAGGTATAGTGTGAAGGCTCGGGATTGGCTATTACTTGAGGGTTTGAATCAAG

ATTTACTGGGAGTTGGTACAAGTTAGGCTCGAGATTGGGATAAGCTTAGAAGAGGACAAG

AGTTAGCTAGAGCTGGAGATGAAGGTGCTGTGTGGATTGATGACCTGGCTGGTTCTGTTA

TAAAAGGGGCACTGATGCATAGAAGGAGAGAGCTCTGTGAGGGGATGAATTGATGAAGCT

CTGCCAATAATGAAGCTGAAGAGATGAGTCCCTGCCCAGTACCCAATTCAATACAATTCA

TATTCAAGATTTTCATTCCAAGTTCACTTCTAGCTTGTATTCTCAGTCTGTAAGTCTTGG

TAATCAATTAGAGTGTACTTAGATTCTCTGTGTAGTTCTTGAATGATTTCATTTCCACGT

TCAATATTAAGCGAGATTAGGACCTGAATTAATGTTCTTGGCAGAATGCCATCTCTGGCA

GTAGAGTATTTACTTTCTTTTACACAGTGAATT

>Unigene0001421

TTGTCCTCAGCATGGCCATTTTGACAACCTTCTCCTCTTGAGCTCTTAGGATCCGGGACT

GCATCTGAGCCACACTCATTAGAACAGGCTTCTTGATGGACTTACCCAAAGCGGCCGCCT

CGACCTTGTGAATCCTATCTTCGAGACATTGGAGGCTCATTGGTGACTTTCTTCGCAGCT

ACGACAGTGAGGATGAAAGAGTCGGAGGTTTGCAATAGCCCTCTCTTGACCAGTATGCGC

CTTCTTCGTGGCCATCGCAAGGGAATACACCACCCGTTGCCGAAGAGCACGTGTTTCTAT

AGCGGTCATCACTGCCGCAACAACTAGCTCTCTGACTATGGAATTGTCAACTTCATTAGC

TTTACAACCAGCGGCGACTTCGGATCCCGAACACTGT

>Unigene0001422

TCGGATCCCGAACACTGTTGTTTCGCCATCATTTCATCCATAAATGCCCCCCTGTTAACA

TTGACAACCTGCAAGGACACAAGCTCCTCAGCAACACTTTTGGCCTCAAATTCCTTGGTT

GCTAGCAACGTCTCAAGATCAGCGACTCGCTGCAACTGAGCTTGATCTTCAGCAATGGCT

GAGGGCATTATCTGAGAAACAAGTCGTAAAGAAATGAGACAAGATTCATA

>Unigene0001423

AAATTCATCAAACTTGACCGCCTGTAATACTTTTGATCATCACCTTTTGAGGATTTTTGA

GACTCAACAATTTTGAGGGCGGCTGCTCTAAGGCAGGAGGCAAGGGTTGAATCAACTTCG

GACCAATTCAGTCCCAACATATGTTCAACTTCTTTAGCAACACCCAACTTTCTGTCGGCT

TTGAGTTCACTGGTATAGGCATCAATGGTGACCTGTTCATGGATTGCAAT

>Unigene0001424

ACCGCAGCTTCGCCGGCTCCCGGCGGGGGTCAGGAGCTCTCGAACCCCCCGACCGACGGC

ATCACCAACCTCCGCTTTTCGAACCACAGCGACAACCTCCTCGTCTCCTCCTGGGACAAG

TGTGTCAGGCTGTATGATGCTAGCGCGAACGTGCTGAAGGGGGAGTTCTGGCACGGGGAC

CCCGTGCTGGATTGCTGCTTTCACGATGACTCGTCGGGGTTCAGCGCTGGTGCGGATCAC

ACGGTCCGGAGATATGACTTCACTTCTGGCAAGGAGGATATCTTGGGACGGCATGATGCT

CCAGTTCGCTGTATTGAATATTCTTATGCAGCAGGCCAAGTGATAACTGGCAGTTGGGAC

AAAACACTGAAGTGTTGGGATCCTAGAAGTGCTAGTGGGCTTGATCATTCACTTGTTGGG

ACATACCCACAACCTGAACGTGTTTATTCTCTTTCTATAGTTGGCCATCGGTTAGTGGTT

GCTACTGCAGGGAGGCATGTAAACGTCTATGATTTGCGGTATATGTCTGAACCTAAACAA

AAGAGGGAATCTTCGTTGAAATACCAAACGAGATGTGTATGTTGTTATCCCAATGGGACA

GGTTTTGCTCTTGGCTCTGTGGAAGGTCGAGTTGCGATGGAGTTCTTTGATTTATCGGAG

TCCGGACAAGCTAAGAAGTATGCATTCAAGTGTCATCGAAAATCCGAAGCTGGGAAGGAC

ACTGTTTATCCGGTCAACGCAATTGCTTTCCATCCCATCTATGGTACATTTGCCACTGGG

GGCTGCGATGGGTACGTCAGTGTCTGGGATGGCAACAACAAAAAAAGATTGTTTCAGTAT

TCGAAATACCCAGCCAGTATTGCAGCTCTATCCTTCAGCAGAGATGGGCGCCTCCTTGCG

GTGGCATCAAGTTACACATTTGAAGAGGGAGACATACCCCATGACCCCGATGCCATCTTT

GTTCGTGATGTCAATGAAGGTGAAGTGAGGCCAAAGCCTAAAGCACTGCCTACGTCTCCA

TGACTTACATGGAGAGTTTGCTTTGTGTGTCGCATTTTGTTCAGAAGGTGGAACTATCCT

CCAAAGTTTGGTTGATTCACAATATAGGGTTGTCTTTCATTAGTGGGCTGAGTGCCAAAG

TTTCCAAGATTGGGAGTTGGCATCATTACATATAGTTAAAGGGAAATCTTATTGTATTAG

CTTGAGAGAACGAGCAAGTCTGGATGGTTGAGAGATTTACTGTGAAGTAGTGGTTATTCC

CACCCCAATGTTGCAAATGTTTTGTGGATACTATTCTAGTTATGAAAGGGGA

>Unigene0001425

CCGAGTCCGTGCATCTCCAGCGGCGATGGACGGCGCCGGCTCCGACGATACCTGCTACTA

CTCCATCCTTCAGATCCGCAAGAACGCCACCGCGGCGGACGTTCGCGCTGCTTACCGCAA

GCTCGCCCTGAAGTGGCATCCAGATCGGTGGGCGAGGGACGCGAAGGCCGCCGGGGAGGC

GAACCGGCGGTTCCAGCGGATCCAGGAAGCGTACTCCGTTCTCTCCGACGAGGCGGAGCG

GGCGATGTACGACGCAGGGCTCTTTGATCCTTTCCACGATGACGACGAAGAAGACGAAGT

TGATATCACTGACTTCGTGCAGGAGCTGCGTTCGATGATGGACAGCATGAAATCAGAGAA

ACCGGCCAGTTTGGAGGAGCTGAGGCAGATGTGGGCGGATATCGCTGGCAGCGGGAAAGG

GCAGCAGCAGGCGCAGGCGACAGCAACGGCAACGACAACCAAGGGGATGCGTGCAGCCCC

TGCACAGGGGGGTTTCGGTGGGAACCACTTCGGGTGGTAGGGTGGGAGATTTAGGATTAG

TAGTATTAAAAAAAAAA

>Unigene0001426

CCATCCACATACTCAACCGTCGGCACATCTCTCTCTCTCTCTCCCATGTATCCACCAAAG

CCAACTGGTCAATACTACCCAACTGCATCGCCGGTAGATGCGGCGCCACCGCCGTCGATG

GGGATTCCAGTGGGTGCTATCAATCCCAATAATTACCAGCAGTATCCGGCTGCCTTTCAG

ACACAAACTCGGGCCTCCGTCCCATGGTCCAGCGGACTCTGTGACTGCACCGACGATTGC

GGCAACTGTTGCATGACTTGTTGCTGCCCCTGCATCACATTCGGCAGGATCGCTGAAATC

GTCGACCAAGGCAGCACCTCTTGTGGGGCGAGTGGCGCGTTGTATGGGCTGTTGGAGTAC

TTTACTTGCTGTCATTGGCTGTATTCATGCATGTACAGATCGAAGTTGAGGGCGCAATAC

TCCCTCGCTGAATCCCCCTGCAATGACTGCTTGGTTCACTGCTGCTGCGAGGTCTGTGCG

TTGTGTCAGGAGTATCGGGAGCTTAAGACCCGCGGGTTCGATATGACCATAGGATGGCAT

GGAAATATGGAGAGGCAAGCGCGTGGGGCGGCCTTTCAGCCGCCCATGCAGGGCGGCATG

ACCCGTTAAAGTCTCGGTGATCGATAATTTACGAAGGATAATATTTATTTATTTTATAAT

GTTTGTTTGAGTGTTTGGAAATTGTTGTATTTGTGAAAAATAATGTGGAAGGTCCTCGTT

GAAATTGATGACGCTATATATTTAAATGTTTCGATTGTACTGCAGCTTAAGTTTAAGTTC

GATATTCGTTGAATGCCGTTTGAGA

>Unigene0001427

CCATCCACATACTCAACCGTCGGCACATCTCTCTCTCTCTCTCCCATGTATCCACCAAAG

CCAACTGGTCAATACTATCCATCAGCACCGCTGGTAGGTGCAGCGCCACCGCCGTCGATG

GGGATTCCAGTGGGTGCTACCAATCCCAATAATTACCAGCAGTATCCGGCTGCCTTTCAG

ACGCAAGATCGGGGTCCCGTCGCATGGTCCAGCGGACTCTTAGACTGCACCGACGACTGT

GGCAACTGTTGCATGACTTGCTTCTGCCCCTGCATCACATTCGGCAGGATCGCTGAAATT

GTCGACAAAGGCACCACCTCTTGTGCGGCGAGTGGCGCTTTGTATGGGCTGTTGGAGTAC

TTCACTTGCTGTCATTGGCTATATTCATGCATGTACAGATCGAA

>Unigene0001428

TACTGACTGAGGTCCGGGGTCGGTAACTGGCGTTTTAGTTGAGACTCGGGAGGAAATACC

CTAGTGCCGGAATACCCCGGACGCCTCCGCCGTCGTCTTTCTCGCCAGAGCCGATCAAAT

TCACCGGAGCAGAGCCGATCATCATGCCAGGGGAGAGGGAAGAGAAGGTGTCCCTTGAGT

TGACGGACGAGGTTCTCAAAAGCATGGAGGTCGGCCTGGTCTTCAGAGATTACAATGGTA

GAATCAGTTCGATGGATTTTCACAGAAGTGAAAAGTTTATGGTAACTGCCAGTGACGATG

AATCCATTCGCCTCTATGATTTCCAGAATGCAATGTGGTTGAAGACCATCAATAGCAAAA

AATATGGAGTTGATCTCGTTTCTTTCACTGATCACCCAACAACAGTTATCTACTCTTCAA

AAAATGGTTGGGATGAATCATTGCGGCTTCTTTCTCTTAATGATAACAAGTACTTGCGGT

ACTTCAAAGGCCATCATGATAGGGTTGTTTCTCTCTCGTGGTGTCCTTGGAAAGATTACT

TCATCTCTGGTTCTCTTGATCGAACTGTCTTATTATGGGATCAGCGAGCTGAAAAGTCTC

AGGGCCTATTACGTGTGCAAGGGCGGCCTGCAGTCTCTTATGATGACCAAGGAATGGTGT

TTGCTATCGCATTCGGAGGATATATACGAATGTTTGATACACGAATGTATGAAAAGGGTC

CCTTTGATAACTTTTCTGTTGGGGGCGACTTTTCTGAAACCAATGTAGTAAAATTCAGCA

ATGATGGCAGGAAGATGCTCTTGACAACTATGGGTGGCCATATTCATGTGCTCGACTCCT

TCCGTGGCACCATGATATCCACGTTCACTGTGAAGCCAGTTTCAAGCAATGCAACCCTGG

AGGCTTCTTTCAGTCCTGATGGAGCATTTGTTGTATCAGGTTCTGGCGACGGTTGTGTTT

ATGGCTGCGATGTCACTCGTGGAACACTTGCTTATTTGGGTAAAACTGATACGGAGGCAC

CACCGATCAAGTGGGCTCCAGGAAGTCTCCTGTTTGCATCCGGATCATCTGAATTGTCAT

TTTGGATTCCAGACTTATCTAAGTTGGGGGCTTTTGGTGGCATGAAGTAGAGCTTCCAAA

GGGAAAAGACTATGGGGATCATTGCTAAGGTTACATATCATTGAATTCAATGGCGAAGTC

ACACTAGGATCAACTTACAAGTTGATGGTTTTCTGACTCGCCGTTTTCATGGAAGACAGA

TGAAGAATCTGTCCACATTCAAAAGATTAGGATTTGTAACAAACACTACCGCAAGGTGAA

GGATGCAAATTTATCGTGGTTTAGCTCTCTTTTATTGTGTCCCCCCAGCAAGCAACTTTT

ATTTAATGTGACATGGTATTTAATGTGACATGGTATGTATATTTTTATATACAATGCAAC

CATATGGTACGAAATGCGCCAGGCAAAGCAATCTTTATTTATTGTGACATGGTATGCATA

TTTTTATATAATTTGCAACCACATGATATAAAATGATTAGACAAAACAAGTTTTATTTAT

TGTGATATGG

>Unigene0001429

ACATTGTTGGATATCCTCGTATGGGCCCGAAGAGAGAGCTCAAGTTTGCTTTGGAGTCAT

TTTGGGATGGCAAGAGCAGCGCCGATGATCTACAAAAGGTTGCTGCAGACCTTAGATCTT

CCATCTGGAAGCAAATGTCCGATGCCGGGATCAAGTACATCCCGAGCAACACCTTTTCGT

ACTACGATCAAATACTCGATACCACTGCAATGCTCGGTGCCGTCCCCGAAAGATATGGTT

GGACTGGTGGAGAAATCGGGTTCGACACATTCTTTTCTATGGCTAGAGGGAATGCTACAC

TACCTGCTATGGAGATGACCAAGTGGTTCGACACCAACTACCATTTCATTGTTCCTGAAT

TGGGCCCTAACACCGCCTTCACATATGCTTCTCACAAGGCAGTGTCTGAGTACAAGGAGG

CAAAGGCGCTTGGCATTGATACCATTCCAGTCCTTATTGGACCTGTTTCGTATCTGTTGG

TTTCAAAGCCTGCTAAAGGAGTTGAAAAGACATTTTCTCCTCTGTCACTCCTAGAAAAAG

TTCTGCCTGTATACAAGGAAGTCATTGCTGAGTTGAAAGCAGCCGGTGCTTCATGGATTC

AGTTTGATGAGCCCACTCTTGTGCTTGATCTTGAGTCTCACCAGTTGGAAGCATTCACCA

AGGCCTATTCGGAACTTGAATCATCATTCCTGGGTTTGAACACTCTTATCGAGACCTACT

TTGCCGATGTTCCTGCCGAGGCATACAAGACTATCACAAGCTTGAAGGGAATCTCTGGTT

TTGGATTTGATCTTGTCCGTGGAACCAAGACCCTTGACCTTATCCAGAGTGACTCCTTTC

CATCCGATAAATTCCTTTTTGCTGGACTTGTTGATGGGAGGAATATCTGGGCTAACGATC

TCGCTGCTTCCCTCAGTACCCTTGAATCCCTGGAGGCAATTGTTGGAAAAGACAAGCTTG

TTGTATCGACATCTTGTTCTCTCATGCACACTGCTGTCGATCTTGTAAACGAGACAAAGT

TGGACAGTGAAATCAAATCTTGGTTGGCTTTCGCTGCACAGAAGGTTGTGGAAGTCAATG

CATTGGCTAAGGCATTGGCTGGTGAGAAGGATGAGGCTTTCTTTTCAGAAAATGCAGCTG

CTCTGGCATCGAGGAAGTCATCTCCTAGGGTGAACAATGAAGAAGTGCAGAAAGATGCAA

CTGCTTTGAAGGGATCTGACCACC

>Unigene0001430

TCAACCAATTCGCATTCCACCCCGAGGTTTATGCGAGTCTCACTCCAACAATCACAGATC

GTCAGTGAACATTCCAACAAGCAAAAACATGTTCTTCCGCCCCGGCGGAGGCGGCGGCGC

TGCGGCTGCCGCCGTGCGCTCTATTCTGGTGCGCCACTTCTCGAGGAGTCGCGCCAAGGA

CGTGCGGCGTATCAACCCTAAGGTCCCGAAAGAGGAGGCCAAGGAAATCTCCAGCAATAT

CTACGATATCATCAAGGCTCAAGGCCCGCTCACTGTCTCCAATGCCTGGGACCACGTCAA

GGATGGCGGTGTGAACGGGTTGACCAGCAAAACTCACATGAAGATCATGTTGAAGTGGAT

GTGGGGAAAGCAGATGTTGAAGCTATTCTGCACTAAAATTGGTTCAAACAAAAAATTCAT

GTATACCACCCTGCCTGAAGACCCTAAAACTGTAGCCCAAGGTAGCTCGGCAGCAGCGAC

GCCTCTGGCTTCAGAGCCCCCAAAGCTCGGGAAAAAGAAGACCAAAATGCTGGCGATAAA

GAATAAGAAGTTGCTAGAGGCACAGGCGCGCCAGTCTTGATTATGCACAACCAATCACCT

CTGTCCCTTTCGTAATGAAATTCTCTGGAATTGTTTTGTTTTCGGAGCCGTGAACTATTG

TTGAATGGGTGCTGCTGCAAATTTGAGCCATCTATTTTGGTGAAATGTATCTGGGAAGCA

AATCAAAAGAGAAGTGGCTGTGTATTTCAACGGTTTTGGTTTGAGCATTTCAGTTCCGTC

TTAGCCTCTGTATCACGCGTCATAATTTTCTTGCCGCATTGACATACCTTGCCTCACCAA

CATCAGGCACCAATCTTAACATATTTGTTCAACCCAATTGCAACATATTTGTACAACTCA

ACAACTTGCTTTCTCACTCTAAGTAATTGGAGCAACATGTATGGGAAGAAGAGAGGAGAG

>Unigene0001431

AGACAACTAAGAAAATGATCGACATTCAGTTTATCTTGATCCTCATCTTGCTACTTGGGC

TGGGGTCCTGCGAGGACCTGCAGACCAAGTGCTCGCCGGAGCTGACAAAGCTGATGGCAT

GCCTGAACTACGCCACCGCCCAGGCTGATGCCCCGACCGGTGAGTGTTGCACCTCAGTGG

CGGGCATACGGGGGAGTGACCCCGAATGCCTATGCTACATCATTCAGCAAGCCAGCAAGG

GAGGTGCTACTACGAAGAGCCTCGGCCTGCAGATATCCCGCCTCCTCCAGCTTCCCCAGG

CATGTAAGCTGACTAATGCCAGCGTTAACGACTGCCCTAAGCTTTTAGGCTTGTCACCGA

GCTCCCCGGACAATGCCATCTTCGCCAATAGTACGAAGGCTACTACTACTACTGCTTCCC

CAACCACCGGGTCAACAACGGTGGAGCCTAAATCATTTGGGGTCATGCAACAGATTCAAC

TTGGATGTACCATTGCCATCACTGCCGCGTCTGCTATATTCATCTCTGCGTAGAAGCATA

AACTTGGCTCAGAGAGCTTTTTAGTTGCCAGTTATGGTTTTATGTATGATCTTGCTTTGA

ATTTCACTTCAAGTTTGTTCACAGAGTTCTAAATTTCGCTTCAAGTTTGTCCACAGAGTT

CTCATTATGAATGCGATGGCATCTTTAGTTTTGCCTCAAATGTGACTTGTGATGTGTTTA

CGAATATCTAAATATGTATCACAAGAGCCACTGATGTCGGCTCAGTTGGTAGGCAACAGT

TTAAATCCCGAGAAAAAA

>Unigene0001432

TGCCAGCGTTAACGACTGCCCTAGTAAGTTTTCCTACAACTAAACCATAAAAAGTGTAAT

TTTTATCTACTTTTATCTTACTCTTCTTGATTCTGATTTTGAGTACAGAGCTTTTAGGCT

TGTCACCGAGCTCCCCGGACAATGCCATCTTCGCCAATAGTACGAAGGGTAAGGCCTGCA

AAAATTTCTTTTATTGCCAAAATGAAGAGTTTTGTTATCTAAATTACCATACTCAATTCT

GCTGCAGCTACTACTACTACTGCTTCCCC

>Unigene0001433

AAACCCTCTCCCCTCCCTCCACCCCTCTGACGAAGATGGCTTCTCTGCCAATCCCTGCAA

GCTCTCTCACCACCCAATTCACCTCCAAACCCTTCTCTCTCTTCTCCCCCAAACCCATCT

CCTCTTCCTCTCTCCTCTCTTTCCCCAAACCCACCACGATCCACCTCTCCAGGAGGCAAC

GGAGCACCCCCCTACTCGCCGCCGCCGAATCCCTCGAGACCCCCGACGTCGTTCCTGATG

TCACCGCCGAGGCAGAGCCGCAAGTTGCAGATGAGCAGGCCGAAGAAAAGGAGTTGCAGG

TGGTGGTGAAGGGAATGGAGAAGCCGCGGCTGGTGCTGCGCTTCATCTGGATGGAGAAGA

ATATTGGTCTGGCGCTGGACCAGGTGATCCCGGGGCACGGGACTATCCCGCTCAGCCCCT

ACTACTGGTGGCCGCGCAAGGATGCATGGGAGGAGCTTAAGGCGAAGCTGGAGGAGAAGC

CATGGATCTCCCAGAAGCGCATGATTATCCTACTCAATCAGGCCACTGATATCATCAACC

TCTGGCAGCAAAGTGGCGCAGCCGCCTTGTAACAGAGTTTGACTTGACCCAAGTATGTAC

TGTACCATTGTTATTCTAGAGCTGTAATTTTGTTGTTCAAGCAGACCCTACTGTTGTCTT

AGTGGAATTTCTCTCAAGTTACAAAGTTTATCACAATCACACAACATTAGGAAGCTATCT

CCCTGTTTATTCTTGTATGTTGTGATCAACTGAATTGTCTTCATCTAAATATATTGCACT

GTCA

>Unigene0001434

GTTAGTGTTTGAGTTCAAGTTGCAGATGGTAGTAATTCTAAGCCACTGAGGATTAGACCA

AGGCTTCTGTGATACTAAAGGAAGAAAAATAATGGTGCTCAAAGTTGAAGTATACCCAAT

TCTGATGCTATAGAGCCACGAGTTAGGCTGGATTCCCTCCTCCACACCCAGAGACAGATG

ACTCAATGCAATTTTCTCTCAGACATTTCATCAAATACCGAATGAGCGTTCCTTGACAAC

ATTGAAGCGAACCAGAAGAGCAACTCGAATTTCCCAAATCAAGTGAATCACACAAGAAAA

CCTATCCCCCGAACGATCTTGACCATGATGCTCTTCGGCATCATTGACACCGGCTGCAGC

AGGCTGTACTTTCCGTGCAACACCATGCCCAGGTTATTGATGCTCAATAATCTCTCCTCG

CGCGTCCAGAAATTGGATCCGGTGACAGAAGATGTTGAACCCAAGTGTGTTAATGTGGTC

TTGGTGATGCCGCCATTGCCGGAGGTCATTGTGTGTGTTGATGTTGGTCTTGGTGATG

>Unigene0001435

GACAATACTTTCTAATTCTTCCCGTTTCATTTGATTTAATTTCTAAATCCGTCCGGTTTC

TTTTTCTCATATTTGTATTGTGTTTTTTGTTGTTGTTTCTCTCTTTATTTTTTTTTCCAT

CATCTAAGGGTGAATTTTTTCCGACAACAATAAGTTGATTTGGGAGATTTTTAGTTCTTT

TATTTCAGTATTTGACTGGATTGTTGTTTGTTTGTTTAGCCTCTTATTATTTCCTTTG

>Unigene0001436

TGCAGGACTCCTCCAGCTCTTCATTCATGGCGGCGGCTAGGGTTAGGGTTTGCGCAGCGG

TGAGGATCGTGATGGCAGGTTTGGATCGAGAAAGATCGGAGACCAGCAAGACATCGGGTA

AGAGATTTCCTTGCTCAATCCCTCGATTCCCTTCTTAGTGATCTAATCGATCTGGTTTTG

GTCTGTGTTGCGAGGTTGAGAGGAGAACAGTGAAGTCGATGCCGGAGTCGGGGTAGAGGA

CGACGAAGCTGGCCGGAGTATACTTCATCTGTTTCACTTTGATCAAAGCTTGTACCCACT

TATCCATGGCTGCTAAGGATTCAAAGCTCGACGAAGTAGATCGGAGGAGAACTCGATGGT

GCAAAGACGTAGATCGGAGGGAATCTCGACAGCGTTGTGAATCTGCACAAAGAGGCAGAT

CGGTGGGAGCTCGGCGTCGGGTGTCAGTCGGCAAGGCTGACCGGGAGCCGTAACTGCAAT

CAGTAAGGTAATGCTTGAACATGTACTGAAGCTTCCGAACCCGGGAATGCAGAGCAAACT

GCTGTTCAACCCACTTC

>Unigene0001437

GAGGGAATCTCGATAGCGTTGTGAATCTGCACAAAGAGGCAGATCGGTGGGAGCTCGGCG

TCGGGTGTCAGTCGGCAAGGCTGACCGGGAGCCGTAACTGCAATCAGTAAGGTATCCTTC

TCCGATTTCAATGTAGCATTTTTGATCTCGGATCTCTTCTCATCAGTAGCTCAAGCCATG

TCTGTATGCTGGTGGTGCTCAAGAACATGAGTCTGTGCTTCAGAAATTCCAACATATGGT

GGAGTTGTTGATAAAGAAACATACTGCCGAGTTGCTCTAGAACTATGACCCGGCTTGAAG

GGATGCACAAAGTGCCCTACCCTCTGCAGACTATCGGAATTAAGCCTAGAAGCCCTAATT

GTTGCAGAAGAAACCTGAACCTGATCAGGATTACCAGTATCACTTCGTGTCAGCCTCAGG

CTTCCTCTCCGGTGACCCCTTCAAGTCCCATCCAACTTTCTTGGGTGCAACAGCAACCTT

CATCAA

>Unigene0001438

CCCTTGTTGGAGGTCATTGAGCGTGTTGATGTTGGTCTTGGTGATGCCTCCCTTGTTGGA

GGTCATTGAGCGTGTTGATGTTGGTCTTGGTGATGCCGCCCTTGTCGGAGGTGACAATGA

CGACATGAACTCTGGCTTGTTGTTCTACGGGAGGGCGGCAGGGTGTGCGATCGCGGGGCG

GCGGAGGGGAATGAGCAGGTCAAGTATTGAAGGACGCAGAGTGTGGCAGTGTGGATGAAC

TCGTTGGGCTCTGGAGGTGGTTATGGAGGTTATGGCGGGTATCTCGCGATAGATGAGGAG

CTTTTGGATGGTGTGGTCTTTGGTGGTGATGAAGAGTGGGTTGTTATGCCTACAATGGTT

TTTCTCCACCAAAGATTTATTCTAAGTGGGCCACCATATTCTTGATATCACCAGCAAGAT

CTTCACAACCATGCATGCTCTTGTGCTGGTCGCGGT

>Unigene0001439

TCTCCCCTCTTCTTTCGCCGACGCTGCTCGTGACCTCGCCGGAATCTTCATCGCTCCGTT

TGTGTTGCAGTAAAATCAAGGTTGAGCAATGGGGAAGAGGAAGTCCAGGGCGAAGCCAGC

TCCTAAGAAGCGAATGGACAAGCTTGATACAGTTTTTTGTTGTCCCTTCTGCAACCATGG

GAGCAGTGTTGAGTGTCGCATTGATATGAAGAATCTGATTGGAGAGGCCCAATGCAGGAT

TTGCCAAGAAAGTTTCAGCACTACAGTCACAGGGCTGACGGAGGCTATAGACATATATAG

CGAATGGATAGACGAGTGTGAGAAGGTCAATACAGTCGAAGATGAATATTAGTTTCGCAG

CCTTGTTGGCTGGGTAGTCACATGATGCATGTTGGCCATCTTTGTCGCTTGGTTGGAGTG

ACTTATATAGGCGTTGTGTTGTTTTAAAGTTTGTGTGCAGCGAGGCAACTTAAAATATTT

CTGGTTTCTGTGTGAAGCTTTTGTTAGTCTGAGAAATTGAGCAGAACATCTTGAATTCTG

AAATTTGAGTAATGCAGTATACTATTGATATAAAAATGACTCAGATTTTTTTTCTTAAAT

AATCATGTGCCGGTAATATATATTCATTACCATTATTCA

>Unigene0001440

AAAAAAAAAAACACTGGAAAAACAATAAGAGAGAACACTTCAGAGTTTGCCACCCGTTAC

GGCAACACTCCGACGCTCGTCCCCCTTCGTAGCCGTTATCAGTGAGTTGTGCTTGAATCA

TGGGGAAGAGGAAGTCCAGAGCGAAGCCAGCTCCCAAGAAGCGCATGGACAAACTTGATA

CTGTCTTTTGTTGCCCTTTCTGCAACCATGGGAGCAGTGTTGAGTGTCGCATTGACATGA

AGAATCTGATTGGCGAGGCGGTGTGCAGGATTTGTCAAGAAAACTTCAGCATGACAGTCA

CAGCGCTAACAGAGCCGATCGACATATACAGTGAATGGATCGATGAATGCGAGAAGGTGA

ACACTGTCGAAGATGATGAAGAATAAGCTTCGGTTGGTCGTTGGTTCAGTTTCGATGTGT

TAGACTACTGTGTATCTTCGACTAGTTGGACCTGAGTGGATGTGTAAACCTGTTGTATGG

TGGTAGTTTGTGTTGGTACTGTATGGATCTATAGCGGTGTATCATGTCAGTCGAATTTGA

ACATGCGATGCTGCATTATTATCACTAGAAGGTTATGTTAATATGTTGGTGGCATGATGG

TGTTCTTGTTGATGTTTGGT

>Unigene0001441

CAAATTTAGCAACCACAACCATGTCCATGGTGAAGCAAGTCTTGCTCTACGGCTACCGTC

TCAGCCCCTACTGTGCCATCGTCGACCACGCCCTCCGTCTCAAGAGCATCCCGTACCAGT

ACATCGACGAGGATCTCAGTCACAAGAGCCCGACCCTCCTAAAGCTGAATCCAGTTTATC

GCAAGGTGCCCGTCCTCGTCGTCGACGGGAAAGCGATATCCGAATCACTCATAATCATGG

AATTTATCGACGAGACATGGAAGGAGAACAGCCCTCCCCTCCTACCCAGGGAGCCGTACC

GGAGGGCGAAAGCTCGCTTCTGGGCCGACTACATCTACAAGAAGTTGGTACCGGTGATCT

CGGTGATGCGGCGTGCTCGAGGGGAGGCGTTGGCGACGGCGACGGCGACGGAGGAGTTCA

TTGATGTGTTGAAGACGCTGGAGCGGGGAATCAAGGAGGAGTTGTGGAGAGGGGACGGAC

CGTTTATAAACGGGGACAAGCCTGGGATCATTGATGTGATTTTGGGATCGTGTCAGTATC

AGTTTCGTGTGCTGGGTGAGATTGCAGGTGTGGAGCTTCTTGAGAAGGAGAGGACGCCGA

TTCTGTGTTCGATAGTGGCGGCGTATGCGGAGCTGGAGGTGCTGAAGGATGCAACGGTGG

CACCGGAGAAGCTGCTGGAGAAGATTCAGGCTGCGAGGAGGACGGCTTGAGGGGTGTTAT

ATTAAGAGTTTTGGTCGTTATGCGATGAACTAGCGTTCATATTGCAATGGGATACACGGC

TTTGATTTTGTATCTGTGAGTTACTTGGAAAAGTTTAGTGCTTGGAATTAAAAAAATGGT

TTGTCATGTAATCAAGATAACAATTTTAACATTT

>Unigene0001442

ACCGTTTAAGTTGAAGTGACTCTGTTGGCACCCCTTTGCTGGCGAAGTCACGGCGTGGCC

GGAGATCTGAATGCCACGATGTTGAATCCCTCCTCTCACATAGCAATACCTTGGTGAGAG

AAACAGGTCAGGGTAGCTGAAATGGGGCAGGCGCTGCGGCGAGCTACGGGGAGGCTCCAG

AGCTCGAGACCACCGGATCACCCGCCGGTGATGGCCAGGAAGCCGGAGCTCCGCCCGCCT

CCTCCTCCGGTCCCGGCTTCGCACGACGTCCAGGACCGGCTCGATGCCCCCAAATCTGAG

GATAGCTCGAGAATAAAAAATGATCTAGGTGTCGTCCTTGAAGAGCGTGATCCAGGATAT

GATGCTATGCTAAACAAGATAGTTGGTAGAATCAGTACTAAGCCTGGAGGAAAACTTGAA

ATGGGCGAGGCATTCATAGTAGAGAAGTATAATAGGCCCATGCCGAAGCTACGGAGCACC

AAAGCAGAGACTGCGGAACACAAAAAAGAATCCGTGCCCCCAGGAACGCTCTCGGTCGTC

CAACTGCAACAAATCATTCGTCTACACCAGGGCAAGTCCGACGAACACCAAGCTCCAATG

GCGATTCCTGATATCGCAAGGAGATTCAACCTTGATGCCGCTCAAGTGGAGAAGATTGTG

CAGTTCGTTTCATTGCTTCCGGAAGAGCAGAAGAACAGTGAAACCTAATGTTTGACGTCA

TGTTTTACTCGTTATCGTGTTTAGTTAGAACAGTCAAAACTAATGTTTGACGTTATGGTT

TACTTGTTTTCTTTTTTAGTTTGGGTGGCCAGTGACTACTGGCGCTGGTGTTCTTGTAGC

GTTGTCATGTTGGTATGCCAATTTTCCAGTGGCAGAGGAAAGAAGAGGGATCAGAATATG

AGTGTTGAGAGGCTATCAATTCAATAAGCCGATCAGATTTTGTATT

>Unigene0001443

GTGCCAGAAGTGAAGAACGAGTCATTCAGCTGTTTCGTGTGCTGAATAAAATGTTTGAAA

AGCACAAGCAGACTAGCAAGCGCCATCTTGCTATCTATACACCAATCATAATACCTGTTT

GGTCACAGGATATGCATTGCACCTATGAATTTCATCTCCTCTTGTTCATAAAACTTGGAA

TTAGCAATACTGTGCTTTATTTTTATCTGGATTATCGTGCTAAATGATGGGGGTGTTTTG

ACACAGGTTCGACTGGTTGAGGATGATCTGATGTATATAGCACATTTGTTCTGGTTTATG

AATTTAGCTGTGTGCGGAACAACAAAGAGGCGGATATGCCGATCACTCTCTTCAAAGAGC

AACTCAACCAAGCTTTCTCTGTGCCTATGTCACCTGAAGCGGTGGCAGAGCTCCGTCTAC

AAGCGTATAATAAAATAACAAAGACTATTGTTCCTGATAACATCTTCTCTCAGTACACGT

ACAAGATACTCCCGAGTGGCAACCATTTATGGGCGTTCAAGAAACAGTTGGCCATTCAGC

TAGCACTTACATGCTTCGTGTCTTACATGCTTCAGATAGGTGATCGTTCTCCGAACAAAA

TTTTATTCTCCAAGAACACTGGCAGAATATTCCAAGCTGATTTCCATCCAGTTTACGATG

CAAATGGGATGGTTGTATTCAATGAGCCGGTTCCTTTTAGGCTGACAAGAAACATGCAAG

CTTTCCTCTCCCACTTTGGAGTGGATGGTCTTCTTGTCTCTGCCATGTGTGCCGCTGCAC

AATCTGTGGCTTCACCCAAGACGAGTTTCTCTTTTTTTTCTTATGCTTTTTCGTAACCAG

ACGTGCATGATAAGTGATTTTTTTTTATTGAATTATGAAATTTTTACTGACATGGTGATA

AATACTCCATGAACTTGCAGCAAAGTGAGCACATATGACATGAGCTGGGCATGTTCTTCC

GAGATGAGTTATTATGTTGGCCTTGTAGAAGGCCGTTCGGGGCTCCGTCTGCTCCAGTTA

CTGCATGCGGGATAAACCCGTCAGACTTGGAGCAGCAAGTGAGAGTAAATGTGAATCATG

TTATTGCTCGTATTAAAGGAATATCACCACAGGAGTTTTATGAAGAGGATGATAATAGGA

CAGGCCTGCCACAATCTGTTCAAAGAGGTGCTGTGGAATTGGTTGATGCAGCACTGTCTC

CTAGAAATATATGCATGATGGATCCAACTTGGCATCCTTGGTTCTAGATACTTCCATAGA

AAGAGGGGTTAAAAAGAACACTTGGTTGAAAAAAAGAGTACTTCTTGATGTGCCTTATCC

TTGATATATTTGAGTGCTTAGTCAGTGTCATTCCCATCTTCGTTACTAGTGGTAGAAAAG

AGATTTATCC

>Unigene0001444

GCGACAAGGCACCGACGACAAACCAAAGCAACACTCCCAACACAGCTAGACCCCGAGGCC

GAGAAAATGGTCGGGTGTGAGAGCGCAGCCGGACCACAGTGCGCCGCCGCCGGTCACCGC

CCCATCCTCTGCTCACTGCCGCCCTCCAATTTCAGACAGCTGTGGCAGAACAACAAGGCG

AGGAAGAGGTGGCCCGCCGGAATTTCTGCTAGACTCGGCGGGGACGATTCTGATCCGCTT

CTCCAAGCTGCCCTTTCAGCCGCTTCCCTTCGTTTTCAAGAAACCCTCCGCCCAGATCCT

CTCTTCCTCGATAAATTTGCTGGATGCTTTCTTTCACTCAATAACAGTCCTAATGATGTG

GAAGAGCGCCACCTGGTAGTTCCGATAAATGCTTCTCCTCCATGCCACTATAGTCTGGCG

ACTAAATTCATCGATGACAAGTTGCATAGTTTGATGAGCAGCAATGAGGATCTCAGGCAG

ATTGTCTTGCTGACAGGCGGTATGGATACACGCCCGTATAGGCTGAGCTGGCTGCGCTCG

TGTGTTATATATGATTTATCTCCTGGGAGTGTCTTCAAAGTAGCATCAGAAAGGCTTGCA

GGTATCGGAGCTAAAATTTCAAAAACCTGCTTGCTAGTGCATGTTCCTATAGAGTCCTCC

GACTTGCAAGCAATTTTGTGTAAGAAAGGCTTTAATGGCAGCAGACCAAGTATATGGGTA

CTACAGGGGTTACCGCTGATGACTTTTGCAAGTTTGAAAGACATTCTGTATATCATCAGC

AGTTTAGCCATGAAAGGAAGCATTGTTATAGGAGAATTACCCGGATGGTTGCTCAGGACT

GAGTTTGGAAACACGTTTTCCATACAAGAACGATTGGAAAAACTGTTTATGAGCAATGCC

TTTCAAGTTGATTTAGTTGACTATGATGAAGTTGCGAGAAGTCTGCACCTGGATACCCTG

CAAGGAGAGAATGATAATGTCCTTTTTATTGCAAAACAGCTCCGCCTATCTGATGCTCAG

ATGGAGAGCTGGTGGACACATCTGGAGAGGTTAGATGAAGAGGCAGATGAAGAAGGTTTT

GAAGAGTTGTAGCTTCTTCTTTCTTTAGACTTTTCATGTTAAACCAAATTCCCTCCCAAA

CTTGAAAGCTGCTAAGGAGTTCAATGAGTGTGGAATATTACTGGGAAATACATTTTATAG

AAAGGGGAAACTTGATATTTATGCC

>Unigene0001445

AAGTGTAGCACCAGGGTCTCACGCTGCTCATCCTATCCTTCTCTCATCTGCAGAGTCCCT

AAACCCTAACCCTAGAAATGAATCTCGCCACCGCCGTCTACCCCCACCCTCTCCACCTCC

TCCCTCTCCCCGCCACCTCCTCCCGATCCTTCCTCCCCCTCCGCCGCCGCACCGCGGCCC

CGATCCGATGCTCAATGTCGCAGATCCACAATTACGGCACGGTTGACTTCGAGCGGAAGC

CGGCCCTCAACTGGAGCTCTCTCTACCGCCGGATTGCCATGACCCAGGACCCCGCCGCCA

GCCCAGGCTCGGTGCTGGACCGCTGGGAGGAGGAGGAGCGGCGTCTCAATAAGTGGGCCC

TCTGCCGCATCGCCAAAGAGCTCCGCAAGTTTCGCCGCTTCAAGCTCGCTCTCGAGGTTT

ATGATTGGATGTCAGCTCAAGGTGATCGGTTTGTATTTACTTCCAGTGACATGGCAATCA

AGTTGGATCTGATTGCCAAAGTATATGATATTACTGAGGCCGAAAAACACTTCTCAATGC

TTGATGATGCCTTGAAGGACAAACGCACATATGGAGCTCTTCTGAATGTTTATGGTCATG

CGAAGATGAAAGAAAAAGCAGAGGCCACTCTTGAGACAATGAGAAACAATGGGTATGCAA

CCGAAGCACTTCCTTTCAATGTGATGATGACTCTTTATATGAATGTTGGAGAGCATCAGA

AAGTTATTTCTATCATAAACGAGATGAAGGAAAAGGATGTGAGACTTGATGTTTTCTCCT

ACAATATCTGGATAACAAATTGTGCAGCCATGGGACAAACAGAGGAGATGGAACGAGTAG

TGGGGCTGATGACTTCGGATAGCAGTATCAATGCTAACTGGACCACTTACACCACACTGG

CAACCATGTACATTAAATTAGGCGATTATGAGAAGGCATATAGTTGCTTACAGGATGCTG

AGATTAGGATGACAGGAAGGGGTAGGATACCTTTTAATTATCTCCTTGGGCTTTATAGCA

GCATTGGAAAGAAGGATGAGGTCTATCGGGTATGGGAATGCTACAAAACAAAATTTCCTA

GCATCTTGAACCTGGGATACCAGTCAATGATATCATCTTTAATCAAACTGGGAGATATTG

AAGGAGCTGAGGCGATCTATGAAGAATGGATGTCCACCGCAGCAAACTATGACCCAAGGA

TCTGCAATCTCATGTTGCGTTACTATGCAGGGGAAGGGCTTGTGGATAAGGCTAAGGGTG

TTCTTGACAACTTCTTAGATAAAGGAGGGAAGCCGAAACCGAGCACATGGGAAATTCTTA

CAGATGGCTATATCAAGGTCAAGCAAATCCACAATGCATTGTCATGTGTGAAAGAAGCGG

CATCAAATGACATTGTGCAGAACTGGAGGCCAAACTCTACAAATATAGCAAACTTTCTCG

CATTATGTAAAGAACAGAATGACATGGCTGGTGCGGATATGTTGATGGACTTCCTACGTG

ACACAGGATGTGAAAGGGAGGAGTATAAGGCATTGTTTAACATGCATTTTGGGGTCGTTG

GTGTGCAAAGATGAACATTAGGGTGATGGACACACTAATATATTTTGACTTCTTGTGTTC

TGCCCAAAATCAGAATGTCTGGCAACAGAAGGAGGTGGATTGGTGTGCCCTTGTACTACT

AGAGTTTTTTCTTACCAGTTATTCTTAGGCCATGTGTGAGTGGGTTACTGGATAATACAG

TTCAAATAACCGGAATACTGCAAGCTTAGACAAAGATATATCAGAATGATTTTTATTAGG

ATTTCCTCTTGTTTCACGGGCTCTGAAATTCCAGTTTTCCATGTTCCACTAAATGATGCC

AGAGGTTTTCTGCAGAGAATACATGGGACATAATCACATGGCTCTTCTGTATAGAGGTGA

TGCTCTGTTCTAAGACTGGCCAAACGCTAATTTCAAAATTAACTTTTTATGTTAATTATG

TGATGGTGGAATGCTTTGATATTTATGAACTGAGATTGCTCTGACAAATTTCCATCGCTA

GAAGGAATGTGATCCAGCTTGCCCTTTACCATTGAGTTATGATTCCTCCGGTGGTTCATA

TTTCTTATACCATTATAGCTTATTTTTCCTGTGCTTGGTAAGTCTACCACTCCCGTACCT

TTTGTATATGTTTGGTTTATATGGTTAACTAATGTACTACCTCTGACCTAATAATTCCGT

AAGTACTTGAGAATTCAATTTCATTCTTGTGTGATGCATGCTACGAGGGAGGAGGAAGTT

ACGAGAAGGACCTTATTGAGGAAGGATTGTTGATTCTGTATATTTGATTTTGAATGTGAC

AGAAAGACTGACATATAGTCAATT

>Unigene0001446

TTTAAGAGGAAGGAGGAATCCAGAATCGTTACACACTTGAGTGGAGTGAGCATGGCGACG

GCTGTCAGGCCGTTTCTCAGGGCCAGACTGTGCAGGAGCATTTCGTCCACGACGGAGGCG

GCGCCAGGCCCACCCCGGCTGCTGAGATCCGGCACCGGTTTGGATCTGGTGGGGGCGAAG

GAGTACGAGGACTACCGGAGGTCTCTGTATGGGGAGATCACCCATAAAGCCATCCTTGTC

GACGCCGTCGGCACTTTGTTTGTTCCGTCCCAACCCATGGCCCAGATCTACAGGCAGATC

GGAGAGAAATATGGGGTGAATTATTCAGAGAATGAGATACTGAATCGGTACCGCCGGGCT

TATGAGCAACCCTGGGGTGCTTCGCGGCTGAGATACGTACAAGATGGGAGACCCTTTTGG

CAATTCATAGTCAGTTCTTCTACAGGGTGTTCTGACTCTCGGTATTTTGAAGAGCTATAT

CAATATTATATGACTGATAAGGCTTGGCACCTCTGTGATCCAGATGCAGAAAGAGTTTTT

AGGGCCTTGAGGAAAGCTGGAGTCAAAGCAGCTGTGGTCTCAAACTTTGACACCCGGTTG

CGGCCTCTTATGCAGGCTCTGAAATGTGATCAATGGTTTGATGCTGTTGCAGTATCAGCT

GAGGTAGCAGCAGAAAAGCCCAACCCTATTATATTTTTGAAGGCCTGTGAATTGTTGGGA

GTGAAACCTGAGGATGCAGTGCATATAGGCGATGATCGCAGGAATGATGTTTGGGGTGCT

CGGGATGCAGGTTGTGATGCATGGCTTTGGGGCAGTGATGTCCACTCTTTTAAGGAGGTT

GCTGAAAGGATTGGGGTCCAAGTATAAAGAAGGCTCTCATCCATCCTGGAAAAACGAATC

TTTCATGAAAGCCGCCGAAAAATTCTAAGTGTTTGAATCCTGCCTGCCCTGTATATAGTA

TCAAGTGTAAGCTGTATATTAATGGATATGTCTGAGCTGTATATTGTTGGATAATAAATA

AATAGTAGACATGCATGTGACTTTTTATTTGCTCTTCATTTTACCATACCAAATAAAATT

GTGTGATCTGTTCCCTCTTGGGA

>Unigene0001447

CCTGCTTTCTCTTTCTCTCACCTTTTCATTCCAATAAAAAACTCCCCCCTGAATCATATT

TAATTACCCAAGTGTTGGATTACTGTTGGACGTGGTGATGATTGCCCATATGATAGTGAG

GTTTAGTGTGTGAGAGAGCTGGGATCAAAACAGAGCTCGCCATTAATTTAAGCTCCCAAC

CTCACTGTAAATACCTCTGTGTTCCGCTCATTTATCATACTAGTCATTTGATGCTAATCT

GCTGTGAGTTTCTGGTTGTGCAGAGGGACTGCAATCAGAAATAGGGAAAGAATTGAATTG

GAAGGGATGAAGAGATCTAACCAGGACAGCTACGGCGGGGGTGGCAGCATGTACTCCACT

GGAAAGGGCTGCATGATGGAGGAGCAGCCGGACGCCGGTGTGGATGAGCTTCTGGCGGCG

TTGGGTTATAAGGTGCGCTCCTCCGAGATGGCTGACGTGGCGCAGAAGCTGGAGCAGCTC

GAGATGGCGATGGGCGGCAGCGCTGCCATCCCGGATGACGCTCTCATCACCCACCTCGCC

GCAGACACCGTCCACTATAATCCATCAGATCTGTCCTCTTGGCTGGAAAGCATGTTGACC

GAGCTGAACACCCCGCCATCGCCGCTGCCGGAGCTGCACGCGCAGCTGTACCAACAGTTC

GACCTCCCGCCGGCCCCGCCGGCATCGTCATTTATTGACCCCGCCGAGTCCACCATCACC

ACCGCTGACTATGAGCTGAAGCCGGTCTACGGACCGGAGCAGCCGACTACGAAGAGGATG

CGGACCTCGTCCCCGTCCTCCTCATCCGGCGGATCGGTCGTCTCGACGGAGGCGAGCCCG

CCGCCGCCACCGCTGGTGGCGGCGGACCCGCAGGAGGCCGGGATCCGCCTCATCCACGCC

CTGATGGCCTGCGCTGAGGCCGTGCAAGAGGATAACCTGAAGGCCGCTGATGTTCTGGTG

AAGCAGATCCCGGCGCTGGCGGCATCGCAGGTCGGATCGATGCGTAAGGTCGCCGGTTAC

TTCGCCGAAGCCCTCGCCCGCCGGATCTACCGCCTCCACCCGCAGCAGGACTGCTCTCTG

GACTCTGCCTTCGCCGACATCCTCCACATGCACTTCTACGAGTCATGCCCTTATCTCAAG

TTCGCCCACTTCACCGCAAATCAAGCTATCCTAGAAGCCTTCGCTGGCTGCCGCCGCGTG

CACGTGATCGATTTCGGAATGAAGCAGGGAATGCAGTGGCCGGCGCTGATGCAGGCGCTG

GCGCTACGCCCAGGGGGGCCACCGACGTTCCGCCTGACCGGGATCGGCCCGCCGCAGGCG

GACAACACCGACGCGCTGCAGCAGGTCGGATGGAAGCTGGCGCAGCTGGCCGATGCGATT

CATGTCGACTTCGAGTATCGTGGATTTGTAGCCTCCAGCCTCGCCGATCTTGAGCCGTAC

ATGCTGGAGGTTCCGACTCCTGCTGGGGAGGAGCCGGAGGCTGTCGCTGTCAATTCGGTG

CTGGAGCTGCACCGGCTCCTCGGCCGGCAGGGTGCGCTGGAGAAGGTGCTGGCGACCGTC

CGCGCTGTAAAACCGCGGATTGTGACTGTGGTGGAACAAGAAGCAAATCATAATAGCTTG

CAATTCGTGGAGCGGTTCACTGAGTCGCTCCACTACTACTCTACCATGTTCGATTCACTG

GAGGGCGGAGTGACAGCGGGGGTGGAGCAGCAGCAGGACCAGCTGATGTCGGAGGTCTAC

CTTGGGCGGCAGATCTGTAATGTCGTCGCGTGCGAGGGGGCGGAGCGGACCGAGCGGCAC

GAGACGCTGGTGCAGTGGCGTGCACGGATGGGGATTGCCGGATTCGAGCCGGTGCACTTG

GGCTCGAATGCGTTCAAGCAAGCGAGCACGCTGCTCGCGCTGTTCGCTGGCGGGGAAGGT

TATCGTGTGGAGGAGAAGGAAGGGTGTCTGACGCTGGGGTGGCACACGCGTCCGCTCATC

GCCACCTCCGCCTGGCGTGCCGCCGGAGCCGGCAACGGGGACTCGGTCTCCGGTTGATGG

GCCCACTGCGGTTGAATCATGGTTATCCTTGCTCCGGGATTAAATTTCAGTTTTTAACTT

ATTGGAAATTTTATTTTTCAGTATCTGTACTTCTAATCTCGCTTTTGAATTATGATAAAT

ATGAATATGTTGGGAATAAGGGCGAGAGAGGGATGTTTTTATTTGAAATGTAATCTGGTT

ACTGTTGTTGGTTTTACACTTGGTTGCCGTGCATCCTGTAGTGGAACAGTGATGCTGGAC

AGAGAAACATTGGGTCCCGCCCGCCTGAAATGCAGTGGCCGGAGCCTGTGGTCACGCGGC

CTTCTCTTATGATGGATTACTTTGTCAAATTTTGTAGCCTGGAGTAGGCCAGTTTATTTG

TCACTCA

>Unigene0001448

TCCGAGCCAACCTGACCAACACCGCCATGGCCGACGCCCTCCAAGCTCTCCCCACAGTCC

AAGGTCTCGATGAACTCCACAACTTACACTCCCCAACTCAAGTGAAAGCACAAGCAGCCG

GCACACATGCCTATCCCACAAGGAGTCATTCTACGAGGAAACAGAACAAGAGAATGAAGA

CCACCCAAGAGGAAGTGGAACATCAAGATGTTGATGAAGTTGTATCTTCAGCTGAGGTTT

CGGGTGAACAATTTCATTATACTCCAGAAACAGGAGGTCTCGTTGATCTGAGTACCAGCG

ATTTAGTACTTTGCGCTAAAAACCTCATTACAATGGAGGAGACGATGCAATCAGAACCGG

TGACAACAAATGAACTCAATCTAGATCCTACTTCTGACAAAGTGAGTGGTGCTGCTAC

>Unigene0001449

TGAGCTTTTGATCGAATGCTCTGCTGAGCTCTCGTCTGGACAAAGAGAAGAGGTACCCTC

GGTAGATATTGAAATATTCGAGGAGAGGACACCATCAGAACCGGTGACAAGATCTAGCCA

CCTAATTGAACTCAACCTTGATCCTACTCCAGACAAGGAGACTGCTGCTGCAAACTTCAA

ACTTCAACATTGCAGCCAGGGTGCTAAGAGCGTGAAAACCGAGGTTGAAGAATTCAAGCT

GCAATCTCCAGAGATTAGGACCAGATTGGAT

>Unigene0001450

AGACGCTTGCTTGCAGTTTGTACTTTTGAAAACGTACAACGTACAAATCGCCGTCCGTCT

TGAGCCCATCACGTTATTATTATCATCATGAAGGTCTCTTACACAACCACCGTCCTGTTG

TTAGCCGTCGTGGCAGCCGCTACGTCGACGGAAGTTCAAAAATCCACCGCCGCTGCCGCT

CCGTCGCCAGTACAATCACCAACGCCTTTCCCTGGCCAAGGAAGCGTTCCGTACCCGGCA

CAGAGCTATCAATTCACGTCTGACTACTCCGGACAGTTGGCTAAATCTTCGTTCGGACCT

AAGGCCGCCTTACCATCGTCTGCTTTCGCCGCTTACCAACCGCACCAGCAACAACAGCAC

TACAATTTTGGCCCATACCAACAGTACTATCCGGCTTCCGGATACCCCGCCGCCGGACCG

TACCCGTCCGCGTACTCTACACAACCGTTGGCCGGCGCCGCTTACCCGACTCCGTACTCG

GCTTACCCGTACCCGGCTCAACCGTACCACGGTAGCTCTTACTCGCCCAATCCGGCTGCC

TTCCCGTCGTACTACGGAGGTCAATTCGGCGTCCCGTCCGCCGCTCAATACAACGGTCAC

TACTCGGCCGCCGCTCCATACAACGGTCACTACTCGGCCGCCTCGCCGACCGTCACTCCG

TACTCATCGCTGCCTTCCGCTTACCAACAACAATCGCTACAGCACTCGCCGTCGTTCTAT

AACAGCGGTGCAGCCGGCCAACAGTACGGCCAGTCCCAACAGTACCTCTCCCAACAGCAG

TACCCGTCCCAACAGTACCAATCTTTCTACGCAAACTCGCCGGCCGCCCCCGCCGCCGCC

GCCACCACCTCTGATTCGGCCAAATCTTCAACTGAGAGGAAATAAAAACGATTATGATCA

TCATCACGGATACCCTGCCACAAGATTTTTGACTTATTATTTCACTACTTAGGATATATA

CGTGTATATTATATTATTATTTATTCTGCTTCACTTATTGCCATATTATATGATCAAATT

AAGCATAATAATATACTTAACGTAATTATAAAAACTGTTTTATAGTCATTGTTATAATAT

TATAATACTGGTTCTGTACGGCATAGCTTGTCATTGCGATTTACTTCTCAAACTCAGTTA

AATGTAAAGTGCAACGATTGTTTGTGCACAGTTATTTGCATTGTTTTGACCAAACATAAA

ATACGAAACTAAAATTATATTCAATCACAAAAACTAGTTTGTTTTCTAC

>Unigene0001451

TTCTTGCTCTATCATGTAGAGAGCAGATTAGAGAGTAGAACTGGAACAATTTGTAAATCT

TCTGGAACTGCAGGGTTGATGAATCATCCGTAGGCAGCCGGTGGTCGCATATGAAAACCA

GTACCGAACTTCTTTCTCTACTGGGTAGAAAGCAGAGTAGTGGGTAGAATGGGACTCGTT

TGTGAATCCTCTGGAACTGCAGGGTCGATTTATTTATTTATTTTTCAAATAATGT

>Unigene0001452

CCACTAATTCAATGGGAAAGGTAATTTCAATTCTTCCCTCCTAAACCAGAGGGAGGGGGA

GCATTCAGTATTAGTATTATCCCTAACATCCAAATGTTGATTGTTGCAGCAAAAGATAGT

AGTGAGAGTCACCATGGAGGATGGAAAGAGACGCTCCAAAGCTCTCAAAATAGCCGTCGG

TCTGCCCGGAGTCATCTCGGCTGCGCTGGATGGGGAGAAGATTGTGGTGGTCGGAGATGG

GATCGACTCTGTGAAGTTGACTGTAGTGCTCCGGAGGAAGATGGGATTTGCGGAGCTGGT

TAGCGTGGCTTCCGCAGAGGAGAAGAAAGAGGGAAAAAAAGAGGAGAAGAAGAAGGCTGA

AGGTGGTGAGGCCAAGAGCTTGCAGTGGGTGATGCATCCGTATGAGACTGTGGTCTGGCC

ACAATACCAGTCCAGCATGGGGCAGCCGGTGGTGGGATATGAGGACCAGAATGAACCTTC

TTGCTCTATCATGTAGAGAGCTGATTAGTGAGCAGAACTAAAACCATTTGTAAATCCTCT

GGAACCGGCAATCCTTTTTCCCCTAATAATCTTGGTTTTCTTCCATGATTTTATGAATGC

TGGGTAAAATTTCGTGAGTAAATGTTATTTAGTGAAATTTATGTGGCATAAATTTTCACG

ATATTTGCTGGGTATTATTCTGTTGTGTACAAATTTGGTATTATAACTTACTCCAACTTG

CTAAT

>Unigene0001453

TACTCCTCCGTTCACGTATGTGTATAAAACTATCATAGTATCTCTCAGCTCATCTCATTC

CTATACCGCGAAGCCCGAAGCCACTAATTCAATGGGAAAGCAAAAGATAGTAGTGAGAGT

CACCATGGAGGATGCAAAGAGGCGCTCCAAAGCTCTCATGATAGCCGTCGGCCAGCCCGG

AGTCATCTCAGCTGAGCTGGACGGGGAGAAAATTGTCGTCGTCGGAGAAGGGATCGACTC

TGTGAAGTTAACAGTAGTACTCCGGAGGAAGATGGGCTTTGCGGATCTGGTGAGTGTAGC

CGCCGTAGTTGAGAAAAAAGACGAGAAGAAGGATAAAGGAGTTGAGGCCAAGAGCTTGCA

GTCGGTAATGAATCCGTATCCGAATGTGTTCTGGCCACAATACCAATACACCATGGAGCA

GCCGGTGGTCGCATATGAAAATCAG

>Unigene0001454

AGTGAGAGTCACCATGGAGGATGCAAAGAGGCGCTCCAAAGCTCTCATGATAGCCGTCGG

CCAGCCCGGAGTCATCTCGGCAGAGCTGGACGGGGAGAAAATTGTCGTCGTCGGAGAAGG

GATCGACTCTGTGAAGTTAACAGTAGTACTCAGGAGGAAGATGGGCTTTGCGGATCTGGT

GAGTGTAGCTGCCGTAGATGAAAAAAAAGACGAGAAGAAGGATAAAGGAGTTGAGGCCAA

TAGCTTGCAGTCGGTAATGACTCCGTATCCGAATGTGTTCTGGCCACAATACAAATACAC

GATGGAGCAGCCGGTGGCCGCATATGAAAATCAGTACGAACCTTCTTGCTCTATTATGTA

GAGAGCAGATTAGAGCGTAGAACTGGAACAATTTGTAAAT

>Unigene0001455

CTCCTCTCTTCTATCTCGCCGCCTCGCCTCCCCCGATCCTCTCCTCCCCTATACACACAG

ACTCACCGGGGGGGAGTCCTTCTCTTCTCGCCGCCGCCGCCAGCTCATCCCCGCCGCCTC

TAAGGAGGGGATTAGTAGTTTTGGTGAGTTTGATAAGTTGGCAATGACTGGAGGTGTAGT

CGAGGATCAAGGGACAGGTGGAAGCTCGTCGTGCATTGAGGGGATGTCGAAC

>Unigene0001456

ATCGCGTTGGGAAAGCGTTTGTTGTTTGATTTTGTTGTCTCAGAGGGGATTAGTAGTTTT

GGTGAGTTTGATAAGTAGGCAATGACTGGAGGTGTAGTCGAGGATCAAGGGACAGGTGGA

AGCTCGTCGTGCATTGAGGGGATGTCGAACGAGCAGCGTTGCCACTCTGCCGATTCGCTT

GCAGAGTGGCAGTCTCGTGAGCAGGTTGAGAATGGCACACCCTCGACGT

>Unigene0001457

ATGTTGAGGAGCAAGATGAAGGTTATATAAAGGCACCCCATTCTTCTTCTCTCTTCTATC

TCGCCGCGGGTCGCCGCCTCGCCTCCCCCGATCCTCTCCTCCTCTCTACACACAGACTCA

CCGGGGGAGAGCCCTTCTCTTCCCGCCGCCGTCGCCAGCTCATCCCCACCGCCTCTAAGG

AGGGGATTAGTTGTTTTGGTCAGTTTGATAAGTAGGCAATGACTGGAGGTGTAGTCGAGG

ATCACGGGACGGGTGGAAGCTCGC

>Unigene0001458

AGATATACCATCAGAGCTAGGAAAATTGGTTATGCTGACAACCATGAATCTCTCACATAA

TATTCTAACAGGTATTGTTCCATTATCTCTTGGAAGAATGGTATTATTGATATTAATTGA

TTTGTCATACAATAATCTCCCACATAATATTCTAACAGGTGTTGTGCCATCATCTCTAGG

AAGAATGATATCATTGATATCGATCGATTTATCATACAACAAGCTAGAGGGGCCCATCCC

TACAACCAAACTATTTCAAAATGCTCCACTACAG

>Unigene0001459

ACAGGTGTTGTGCCATCATCTCTTGGAAGAATGTCATCATTGATATTAATCGATTTGTCA

TACAATAAGCTAGAGGGGCCACTCCCTAAAGCCGGAATATTTCAGAATGCTCCACTACAG

TGGTTTGTCCAAAACAAAGATTTATGTGGTGAAGTTTCTGGTCTCCCTCCATGTCTTCCA

ACACAAAAAAAATAATAGTAACTCAAGAAGAGATCGCTCCATTCTACTAATTATCCTTCC

TATTATAGGAAGCTTACTTGTTCTATCTTTACTCATTATTATAGTGTGTTATCGGAAAAG

GAAATATACCGAAGATGATGGAAATCAAGTTATAGTTGGGGATTTATTTTCTACCATGAA

CTTCAATGGCAAGGCTGCATATGACTTCATC

>Unigene0001460

ACTATTCCAGCACGCTCCACTTCAGTGGTTTGTCCCAAACAAAGATTTGTGTGGGGAAGT

TTCTGGCCTTCCACCATGTCTTCCAACACAAAAAAATAATAGTAACTCAAGAAGATTTCC

CTCCGTTCTATGGATTATTCTTCCTATTATAGGAAGCTTACTTATTTTCATTTTACTTGT

TGTAATGGGATTTTTCTGGAAAAGAAAACATATCGAAGTGGATGAAAATCAAGTTGTAGT

TGGGGATTTATTTTCTGCCCTGAACTTTGATGGCAAGGCTGCATATGACTTC

>Unigene0001461

TGGAAATATGGTAGCATTGATGTCAATTGACTTGTCATACAACAAGCTAGAGGGGCCCCT

TCCAAAATCCAGACTATTTCATATTGCTCCACTACAGTGGTTTGTTTCAAACAAAGATTT

ATGTGGTGAAGTTGTTGGCCTTCCTCCATGTCTTCCAATGCAAAAAATCAATCATCACTC

TAGAAGAAGTCGCTCCATTCTACTGATTGTCCTTCCTATTTTAGGAAGCTTAATTATTTT

ATCTTTACTTGTTGCTATGGTGTGTTGCCGGAAAAGAAAACATACCAGAGAGGATGTAAA

TCAAGTTGTAGTTGG

>Unigene0001462

TGTCCCTGGCTACCTATGCCCCACTCTCCCTCCGGCCCCATGCTCCTCTCCTCCCTCCGC

CCCCAAACTCCGACGATCCCTCGCCGGAATCCGGCGCTCTCTCGCCGGAATACGGCGCTT

TCTCGCGGGAAATCGATCGTGCCCGTCAATTCCTCCCTCGAGACCCACGGGAACCTGTCT

CACGTCTCCCATGGGTCCACTCACCCGGCCCTCTTCTCACATTCCGCTGCTCCGGCTCCG

GTTTGCAATTTAGGGGTTCGCCGTGATCCAGATCTGGGGTTCTCTTCCTTGCTGTTCGTC

ATCTCCATGGTTTTGGGTTCATTTTTCTCATTGGTGATCGTTTCACTCCCCGCCTTGATT

TCTTTCAGAAGGTTGGCAGTTTCTATGGAAAAACTATCTAAAGTAGTGAACGAAGAAGTG

CCTGGAACCCTGTCTTCGCTAAAACTGTCGGGTTTGGAGATGAATGACTTGACCAGCCAA

CTCGATGAACTAAGGCAAGGAATATCAGGAACTAAATAAGGGAAGAAAGGCTGAGCAAAA

CGTTCTAGATTTAATGGAGAAATAAATAGTTCACCAGGAAACAGACCATTTTACTAGCTT

GAACTATTGGACATAGTACCAGACGACAGGATTAAGAAATGATTTAGGTACCTCCATTAT

GTGTTTCCTTATCTGGTTTTTTCAGTCACTTTTGACTTGTGAGTTTATCAATTTCAATGC

TTGTTTACGATATAAGAAGTTGCATATTTGTTTGTGTCACAAGTTAATGTTCATTTGTCT

TGAAAAAAT

>Unigene0001463

GATGAAGGGTTTTTGGAGTACCTTGAGGATATTATAGGAACAATTCAATATGTGGAGAAG

ATTGAAGAAGCATCTAAAGAGCTTGAGGTTCTGAATGAAAAACGTTCTGGAGTAGTCCAA

ATGGTTAAGCTAGCTGAGAAAGAAAGAGACGGCTTGGAGAATGTCAAGAATGAAGCAGAG

ACTTATATGGTTAAGGAATTAGTTCTCTTAAAGTGGCAAGAGAAGGCAACCAAATTAGAT

TCTGAGGACGCTGCTTCCCATGTTGCCGAGACTCAAGATAATGTTTCCAACCTAGAACAA

AGTCTCAAAGCTGAAAAGGAGAAGATTAGTCAAAACGTCAAAACATTGAAGGAACTAGAG

GCTGTGCACAACAAATATTTGAAAAGACAAGAGGAACTGGACAATGAAATGCGAACCTGC

AAAGATCAGTTCAAGGAATTTGAGAGACAGGATGTAAAACATCGAGAAGATTTGAAACAC

TTGAAGCAAAAAATAAAGAAAGTTGAAGAGAAGCTTGAAAAGGATTCTTCCAAGATTGCT

GAAATTGAAAAGGATAATGAAGAATCATCAATTTTGATTCCGAAGCTTGAAGAAGAAATC

CCCAAACTGCAACAACGGAAACTGGATGAAGAAAAAGTTCTAGAAGAACTCAAACTGAAG

TCTAGAGATGAAACTGAGAGGTTCCGTTCTGAGCTTGCCGAAGTCCGTACTGAGTTGGAG

CCCTGGGAAACTCAACTGATAGATCATAAAGGAAAGCTTGATGTTGCACGTGCAGAAAGT

AAGCTCTTGAAAGAAAAGCATACTGCCGGTCGAGTAGCTTTTGAGGATGCTCAGCACCAA

ATTGATGATTTAGTAGAAAAGATAAAAAAGAAGAATGAATATAAAGATGAGATTCAAACA

AAAATCACGAAGAACAAGTCTGAGGCATCAGAAGCTCGTAAAGTGGACGAAGAATGCAGG

AAGAAGCAAGAATCGCTTGCCCCACTTGAACAAGCAGCCAGGCAGAAAGTCACTGAGATG

TTATCAATTTCAGAGTCAGAAAAGAGTCAGAGTTCTGTTATAAAAGCAATACTGCGAGCT

AAGGAATCAAAGGAAATAGATGGAATTTATGGTCGTTTGGGGGATCTCGGTGCTATTGAC

GCAAAATATGATGTTGCCGTATCAACAGCATGTTCTGGACTAGACTATATTTTAGTGGAG

ACAACAACTGCAGCACAAGCTTGTGTCGAGTTACT

>Unigene0001464

GATGAAGGGTTTTTGGAGTACCTTGAGGATATTATAGGAACAATTCAATATGTGGAGAAG

ATTGAAGAAGCATCTAAAGAGCTTGAGGTTCTGAATGAAAAACGTTCTGGAGTAGTCCAA

ATGGTTAAGCTAGCTGAGAAAGAAAGAGACGGCTTGGAGAATGTCAAGAATGAAGCAGAG

ACTTATATGGTTAAGGAATTAGTTCTCTTAAAGTGGCAAGAGAAGGCAACCAAATTAGAT

TCTGAGGACGCTGCTTCCCATGTTGCCGAGACTCAAGATAATGTTTCCAACCTAGAACAA

AGTCTCAAAGCTGAAAAGGAGAAGATTAGTCAAAACGTCAAAACATTGAAGGAACTAGAG

GCTGTGCACAACAAATATTTGAAAAGACAAGAGGAACTGGACAATGAAATGCGAACCTGC

AAAGATCAGTTCAAGGAATTTGAGAGACAGGATGTAAAACATCGAGAAGATTTGAAACAC

TTGAAGCAAAAAATAAAGAAAGTTGAAGAGAAGCTTGAAAAGGATTCTTCCAAGATTGCT

GAAATTGAAAAGGATAATGAAGAATCATCAATTTTGATTCCGAAGCTTGAAGAAGAAATC

CCCAAACTGCAACAACGGAAACTGGATGAAGAAAAAGTTCTAGAAGAACTCAAACTGAAG

TCTAGAGATGAAACTGAGAGGTTCCGTTCTGAGCTCGCTGAAGTCCGTACTGAGTTGGAG

CCCTGGGAAACTCAACTGATAGATCATAAAGGAAA

>Unigene0001465

AAGAGTATTACAATCAATCCAGGGAGTTTTGTAGAATGCGTGGAAGGTGCTTAGGTTGCC

TGGATTGATCTGCAGTGGTAAGATGTTGCGCATCTCGTGCGTTGATAGTCTGTATTTGTC

ATATAAAATGTTACCTTATTACACCCATATTTGTGTAAACTGGGATGAAAGTATAGTGTC

TGACAATTGATCAACTATCTTAGTTTTCATCAGTCTACTTACATGTTGG

>Unigene0001466

CAAGCTTGTGTCGAGTTACTTCGGAGAAAGAACCTGGGTGTTGCAACTTTCATGATATTG

GAAAAACAAATGGACTATATGAGCAAGATGAAGGAGAAAGTAAAAACCCCTGAAGGTGTC

CCACGTCTCTTTGATCTAGTCCGGGTCAAGGATGAAAAGTTGAAGTTGGCCTTCTTTGCT

GCTCTGGGAAACACTGTTGTAGCAAATGATCTTGATCAGGCTACTAGGATAGCATATAGT

GCGGATAAAGAATTCAGACGTGTAGTTACACTTGATGGTGCACTTTTTGAGAAGTCAGGT

ACAATGAGTGGTGGTGGAGGCAAGCCCCGTGGTGGCAGGATGGGAACTTCTATAAAAGAA

AGTGTTTCGGGAGAAGCTGTTGCAATTGCTAAGGAAGAGCATGCTAAGCTGGAGGAGCAG

ATGAACATCTTACGACAGAAGATAAATGATGCCACAAAGCGTTATCAGGCATCTGAAAGA

GCTGAAGCCCACTTGGAAATGGAACTGGCAAAATGTCAGAAAGAGATTGACAGTTTGAAC

ACACAACACAGTTATATTGAAAAGCAACTTGGTTCTCTGAAAGCTGCATCACAGCCAAAA

AAAGATGAACTGAGCAGACTAAAGGAGCTGGACTCAACAATATCTATTGAGGAAAAAGAG

TTGGAGAAGCTCACCAAGTGCTCTAGTAAACTCAAAGAACAGGCTTCTGAGCTTCAAAAG

AAAATAGAGAATGCAGGTGGTGAAACACTGAAAAAGCAGAAGTCGAAAGTCACAAAATTG

CAATCTGATATTGATAAAGCTAGTACTGAAATCAACAGACACAAAGTTAAGATGGCGACG

GGCCAGAAAATGCTTAAAAAGTTGACCAAGGGGATTGAAGAGTCGAACAAAGAGAAAGAA

AAACTAGTTAATGAGAAGGAGAAAATGGCAGCTGGCTTTAAGGATATAGAGCAAAAGGCA

TTATTGGTTCAAGAAAACTATAAAAAAACTCAAGAGCTCATAGATAAACACAAGGATGTT

CTTGACGAAACAAAGGCAGAATACAACAAATTGAAGAAAACAATGGATGAGCTACGGGCT

GCTGAGGTTGATATAGATTATAAACTGCAAGACATGAAGAAGCTTTCAAAGGAATGGGAA

ATGAAGGTAAAGGTGCTCAACAAAAGGCTTGAAGATGTGAAGGTGGAACTTAACAAGCAC

ATGGACCAACTTCAGAAAGATGCGATTGATCCTGAAAAACTTCAAGCTGCTTTGAGCGAT

GCTTCTTTGACTGATGCTTGTGATATGAAAAGAGCAAAGGAGATGGTGGTGTTGTTAGAA

GCTCAGCTGAAAGAAATGAATCCGAACCTAGATTCAATCGCTGAATATCGAGCAAAAGCT

CTCCTGTACACTGAGCGTGTCGATGAACTTAATGCTGCCACCCAAGAGCGTGATGATCTT

AAAAAGCACTATGATGGTTTGAGAAAGAAAAGGTTGGATGAGTTCATGGCTGGATTCAAT

GTTATATCCCTAAAGTTGAAGGAGATGTATCAGATGATCACACTCGGGGGTGATGCTGAA

CTGGAACTCGTTGATTCTTTGGATCCTTTTTCTGAAGGGGTTGTGTTCAGTGTGAGGCCG

CCAAAGAAGAGCTGGAAGAACATTGCTAATTTATCTGGTGGTGAGAAGACGCTCAGCTCG

TTAGCGCTTGTGTTTGCTCTTCATCATTATAAGCCCACTCCGTTGTATGTTATGGATGAG

ATAGATGCTGCATTGGATTTCAAGAACGTCTCTATTGTGGGCCATTATGTCAAGGAGCGC

ACGAAAGATGCCCAGTTTATAATCATCAGTTTGAGAAACAACATGTTTGAGCTTGCAGAC

CGCCTTGTCGGCATTTACAAGACTGACAACTGCACTAAGAGTATTACAATCAATCCTGGG

AGTTTTGTAGAATGCGTGGAAGGTGCTTAGGTTGCCTGGATTGATCTGCAGTGGTAAGAT

GTTGCGCATCTCGTGCGTTGATAGTCTGTATTTGTCATATAAAATGTTACCTTATTACAC

CCATATTTGTGTAAACTGGGATGAAAGTATAGTGTCTGACAATTGATCAACTATCTTAGT

TTTCATCAGTCTACTTACATGTTGG

>Unigene0001467

CCGCATCATCGAGACCCCAAGTCCGACCAGCGCTCCCAAAAGCCAACCATCCCACCGCCT

ACGTTTCCACGCCATCTGGCCCCGCCTTTATTCTGTGCACACCTCCATTTTCTCATGGGT

TTAAAGCAACACCCAGCCCGCTCGTGATCTCAACTGCTCCCCTTCAATTCACACCCATCC

CTAAAATCCTAATTCCCTCTCATGCCTCCACCCCCCAATCATAGCACCCATGCCGTCTCC

CCTCCGCAACGGTGACTTCGGCGCCGGCAGCCCTTTCCGTCACCACCCCTTCCGCTCCAC

CGT

>Unigene0001468

CCGCATCATCGAGACCCCAAGTCCGACCAGCGCTCCCAAAAGCCAACCATCCCACCGCCT

ACGTTTCCACGCCATCTGGCCCCGCCTTTATTCTGTGCACACCTCCATTTTCTCATGGGT

TTAAAGCAACACCCAGCCCGCTCGTGATCTCAACTGCTCCCCTTCAATTCACACCCATCC

CTAAAATCCTAATTCCCTCTCATGCCTCCACCCCCCAATCATAGCACCCATGCCGTCTCC

CCTCCGCAACGGTGACTTCGGCGCCGGCAGCCCTTTCCGCCACCACCCCTTCCGCTCCAC

CGTCTCCGAGCAGAAGCTCCGCCGCCTCAACACGCTCATCCTCCTCCTCCGTCTCGCCGC

CTTCTGCTTCACCCTCGCCGCGGCTATCTTCATGGGGACCGACTCCTCCCGCTCCGGCTC

CTGGATTGACTTTTTCTCATTCAGGTTTATGCTTGCAGCGAATGCTATAGTAGCACTGTA

CTCACTGTTTGAGATGGGCGCTTCGATCTTTGAGATCCAGAGAGGCGTCACGCTGCTGCC

GGAGACACTGCAGTTGTGGTTCGACTTCAGTCATGATCAGGTGTTCGCCTACCTGCTGCT

GGCGGCGGAGGCGGCGGGGGCGTGGGAGGCGCGGGAGCTCCGGCGGGGGCGGCGGGCTTG

CAGTGGAGCTTTCTGTGTGCAGGCCGATATTTCGGTCGCGTTGGGGTTTGCAGGTTTCGG

GTTCATTGCGGTATCTGGTTTGATGTCGGGGTACCGATTGGCGTGCTACAGCATTACCGG

TTCTCGATTCAACGTGTGAATGGGAGCCGTATTGCTAGGGAGGGGCCGGGTTTGGTGAGG

GAGGCCTACCCTTGGTGTCGTGAACTGCACGCTTTGCACGTGTCTAGATAAGATAGGGCA

TAGTACACAAGCAAGGTACCATAGGGGGAGAACGTAAGTCAAAAGTGACTTTTTAATTTG

AGATATTTTGTTATTCTGTTTCTCAGTGATTGTAATGTGGCCTCCCAAAAGTTACCCACT

CCATTAGGTGGAAGTGGGTGAACTACCAAAATTATTTACCGGTTTTTTTAGCTTTAAGAT

TTGACTGTCATAGTTATATAGCCAATTAATAATATCGGGTGATGGTTCCAACAACTTTAT

AATCGTA

>Unigene0001469

ATATGGAAGGCCGGCTTCAACTACGACGAGCGTCTCATTGGATTCATCCGAGGCTACCGC

AGGGTCTTCTATCAAGGGAGCACGGACCACAGGGGGACGCCGGACTTCCCTGGCCGGACA

GTCACTCTACAGCCACACCCTCACGAAGTCTGCTGGGGAGCTGCCTATAAACTTTCTGGA

GAGGAAGATGCGAAGATCGCGCTATCATATCTAGAAGTAAGGGAGAAGCAATATGACACA

AAGGTTTTTACTGATTTTTTCACTGACCCTGCTTCTACAAACCCAGCCATCAATGGAGTA

ATGGTATACATAGCCTCGCCTAATAAGGAAGCTAATCCGAACTACTTGGGACCGGCATCG

CTTGAAGAAATTGCCAATCAAATTATTCGTGCTGAAGGTCCATCCGGTCCCAACCGCGAC

TACCTTTTCCAGCTTGAAGATGCCTTGAATCAAATGGGATGTGAAGACTCGCATGTCAGA

GACCTTGCCAATGAAGTTAGAAGAATCCTTCAGAGTCGGTCGGAGTAAGTGATTAAGCAA

ATTCAGAGTTTCGAGTCTGGATATTTATATTACAAACAAAGCATGGTGATTAATCCTGCA

TTGCAAATGCCTATTCATCCATTTTTATGAACCTTTGCTTGGGCTACTGTCATGAATTTG

AAATCTTTATAAAGCGAGAGATAGGTTTGTGATTACATTATTCCAGAAGAATC

>Unigene0001470

TTAACTCGCCGTCCCCCGGATACATTTCACACCCCAACACCGCCACCTCCGCACAAACAA

AAAATCCCGCCGCTTCTCTCGCCCGCAAGATCTACCGCTAGGGTTTCGCTCTTACAAGAT

CGTTCCCACCATGCCGGCCACGCCTCCGTCCGGCTACGGTTCGATCCCCACCTCGACGCC

GCCGTCGCCGCCGCCACTGCCGGCGGCACCGCCCTCTACCTCCATGCCGATCGAGTTCAT

CTCCCGCGCCAAGGATCGCGGACGCGCTCTCATCGCCACCCGCCGCCCCTGGCGCGAGCT

CGCCGACCCCTCCTCCTTCGCCCGCCCCTACGGCTACCGCGAAGCCATGGCTCGCGTCCG

CCGCAACCTCGCCTACTTCCGCGTAAACTACGCGATCATCATCCTTTTTATTCTCTTCCT

CAGCCTGCTCTGGCACCCGATCTCCATGATCGTCTTCATTGTTGTCTTCATCGCCTGGTT

CTTTCTCTACTTCTTCCGCGATGAGCCGATCGTTCTGTTCCACCGCGTGATCGATGATCG

GATTGTGCTGGCTATTCTCTCTGTCATTACTGTTGTAGCTTTGGTGTTCACTGGGGTTGG

GTTGAATGTGCTGGTCTCGCTTATTGTTGGAGTTGTTATCGTTGGAGTTCATGCGGCGTT

CAAGGCCACGGAGGATCTGATGGATGATCAGGAGGCGGGGGATGGGGGCTTGCTATCGTT

TGCCAGTAGCTCAGAGTCGGGCGGACGTTATGTCAGGTAGTGGGGGTTTTTGTATGGTAT

GGGTGTAGTTCGAGCAAATGCCTGCTTGGATTTCTGTGGAGGCAGCTTTGTTGACATTGC

ATCATCTAAATAAAGGTTGTGTTTCTTTCTCTGAATCTGGTTTACAATGTTTGTTTGTTC

ATAGACTCTCTGTAAATTTTATGATCAAATTGTCGAATCGCTTCTACTTCAGCATTGAAT

GATTTACATATACGAAATCACA

>Unigene0001471

TTCCCGGCGCCGGCGGACAGCGCCAGTACGGTGGCTCCACCTCCGCCCGGCTCATCTCAT

TACGCTCCCGCGGCGCCGCCGGGATCGGGCCGAGGTGTTCGCCGGTGTACGGACCGGACA

GCTCGCCGGCGGTGTGGGGCCCGCCGGAAGGCCTCCGAGCTTTCTCATTCGACGAGCTGA

AGGCGGCGACGAGGGGGTTTAGTCGAGCGTTGGTGATCGGGGAGGGGGGTTTCGGTTGCG

TCTACAGGGGCGTTGTTAACGGCTCCAGCGACGGAACGGCAGCGGCGGTCGATGTTGCGG

TTAAGCAGATGAGTCGCAGTGGATTACAGGGGCACAAGGAATGGATTACAGAAGTTAATA

TCCTGGGGGTAGTGAAACATCCCAATCTTGTCAAGTTGGTCGGTTATTGTGCTGAAGACG

ATGAAAGGGGTATCCAAAGGCTTTTGGTGTATGAGTTCATGCCAAATAAAAGTTTGGAGG

ACCATTTGTTGGCTCGTGTTTCATCAAACCTCTCATGGACATTGAGATTAAAGATTGCGT

GTGATGCTGCACGTGGTCTAGCATACCTACATGAGGAAATGGATTTTCAGTTAATATTTC

GCGATTTTAAAACATCCAATGTTCTGCTAGATGAGGACTTCAATGCAAAGCTCTCGGACT

TTGGGTTTGCCAGACATGGACCGGCAACGGGAGTTGGACATATTTCAACATCAGTAAGTT

CAATGTTGTTGGAACTGTAGGCTATGCAGCTCCAGAGTATGTCCAAACTGGAAGGCTGAC

GACCAAGAGCGACGTATGGAGCTTCGGTGTTGTCCTCTACGAGCTCATAACGGGAAGACG

ATCCATCGACCGGAATCTTCCAAGAACTGAGCAGAGCCTCCTTGAGTGGGTGAGACCTTA

TGCGACCGACCCCAAGAAACTCCATAGAATTATTGACCCGAGGCTTGAAGGAGAGTGTTG

TAAGAGATCTGTTCGGAAGCTCGTTGCCCTTGCAAACAAATGCCTCATGAAGCAGCCCAA

GCTTCGACCTAAGATGAGTCAGGTGGTTGAGATGCTAGACGAGATCATCGGGATGTCGAG

TGGGACGGGTAGTGAGACTTCTACTGTAGCTACATCGATTCCAGATGAATCTTTCAAAGG

GACGGATATACCGAAAGAAAGAGTAGCTTGTCAAAGACGGGTTTTCGATTTCAGAGAAGT

GATCAACCTGAGGAACAGGTCTGTGGGGAGGTTGGAGAGATTATTGGGACCGGGGTCTAT

AGGGAAGCTGGCAAGGACATGGGGTTACGACTAGATATTGTTAATAGATAATCCGATGAC

GTGTATGCTTTCATTTTTTTTTATCGACAGACCATGACTGTGGCGCTAGAGGCTCAATTC

AAGGTGATGTAGCGAGTTCAGGTATTTTTATCGACGGACTATGACAGTGGCGCTAGAGGC

TCAACTCAAGGTGAGGTAGTGAGTTCAGGTTGAAGGTAACGAAGATGTAGAATAACAAGG

TTAGAGAAGTTCGATCCATGGGGGTTTATTTCGGGATTCAAACAAGGGTCCTCTACCATG

CTCTGAGAGAGGGAATAACAAGGAAGCTATTGCAATTGGTTTTTGTGAGTTTGTATACAC

AAGGATGAAATAAGTTGTTTAGTAGATATGAATGATTGTACTAGTGGTATAACAGACTAA

TTTTCTCTAATCTAAAGATGTGTTTA

>Unigene0001472

ATTTATTCGTTCGAATTCACTTCCAAGATGAAGACATCATATTTTTGCGTTCTCTTAATG

TCGGTATTGGTAGTCCAGATTTCATCTGAGGAGATTTCTGAGCTTGATAAAATGGCAATG

AGCTTGATACATTCTCTGGTTGACGTTGTAGAAATACCATATAGGAAACTTGAAAATGTT

TTAAATCCATCTGTAGCTAGTGAAAAAAAAGAACCATTTTTAAAAGATATCCTAGCCAAA

CTTAATCGGAAGAAAGAAGAAATCGAAAAGACCTACTTGGAATCACTAACACCGGAAGAA

AGAGAATTGGAAAAGAAGAAATTTATCGAATATGCCAATAATTCGAAAAGAATAGCTAAC

TCAATTCGGGACACTAGTAAAGCAGCCAGGTTGATGATAAATACATATTTCAACAACCTA

CCAAAAAATGTCCAGAACAAGTTAATTGATTTGGGAAAAGACCTTAATAATATTTTACAT

AAAATAAAGGCATAATCCAAAAACGAATCTCCTGATGAAGAAAATAAATAACTCTACGTG

ATCAAAATTATTTGTAAGAACCAACAAATCATGTCGTCAATATTATAGAATCATTTTAAA

CTACCTGCGACTAATGATTATAAATTCATATCAATAATTTTGTTTTATTTATTTAACTAG

GTACATGCATTTTCAACTAACCTTCTTTGTCGTTTATAATGAACTCAAACATTAAAACAA

TACCACTAAAATATTGTAATTTGTAATATGTATGTAGTTTATTAATACATATTTATAATT

ATAAC

>Unigene0001473

AAAAAGAACCATTTTTAAAAGATGTCCTAGCCAAACTTAATCAGAAAAAAGAAGAAATCG

AAAAGACCTACTTGGAATCACTATCACCGGAATTAAGAGAATTCAAAAAGAAGAAATTAA

TCGAGTATGCCAATAATTCGCAAAGAATAGCTAACTCAATTCGGGACACTAGTAAAGCAG

CCAGGTTGATGATAAATACATATTTCAATACCCTACCAAAAAATGTCCAGAACAAAATAA

TTGATTTGGGAAAAGATCTTAATAATATTTTACATAAAATAAAGGCATAATTCAAAAATG

AATCTCCTGCTGAAGAAAATACATAACTCATCGTGATCAAAATTCAAAAGTATTTGTAAG

AACCAACAAATCATGTCGTCAATATTATAGAATCTTTTTAAACTAGGTACCTATACCACC

TACGACTAATGATTATGGATTCATAATTTTATTTTTTATTATTGTTTTATTTATTTAACT

ATGTATATTTTCATCTAACCTTCTTCATCGTTTATAATGAACTCAAAAC

>Unigene0001474

TCGCAGTACCGGTATCATCGCCCAGTTCAACCAACCAGATCGGAAACCCAACCTCGTTCT

CTCCGCCCAGCTCGATCATTTCAAGACCGCCCTCTCGTTCTAAACACTCCCTCATCTCAG

CTCAACCGGAACCAACCATGGCGGCCCTCGCGATCTCCTTTACCCCCTCCAGGGACCTCC

ACCTCCCCTCGCTCTCGGGTCGCCCCATGAAGCCATTGGAGCAATCTCTTGCAAACTTTA

GCCCATTGAATTTGTCTCTTGGGACCATTCGGCCCAAGAAAGCAAATATCTCAAAATCTG

AAAGGCGACTCATTGTTCGAGCTAGCTACAGTGATGGAGGAAGGCAAGGTGGTTCAAGTA

TCTTCATTGGGGCCTTCGTGTTGGGAGGAATAATAGTGGGTACACTAGGGTGCGTTTATG

CACCTCAGATCAGCAGAGCACTTGCTGGGGCTGATAAGAAAGATCTGATGAGGAAGTTGC

CAAAATTTATCTATGATGAGGAAAAGGCTTTGGAGAAGACGAGGAAGATTCTTGCAGAAA

AGATCGAGCAACTGAACTCTGCCATCGATGATGTTTCTTCTCAGCTGCGAACCGACGATG

ATGGTGTAGATGGAGTTTCTGTGCCCTCTGTCGACGAAGCCACAGTCTAGGGTTATCCCT

AGCATGTGTCTCTGCTCTATATGAAGCTTTTGATGTTATCCTATTTTCCGTATTAGAAAA

CCATTTTTGTTTGTTCGTAGGATCTGGGATGTTTTTTCCTTGTATGTATCTGTTTCATAT

ATGCTTCAAATAATTTAAATAATCCCCAGAAGCCGGTAGTTTGGAAGGGAAACAAGTATG

AATATGAAAACTGGTGAAAAGTTGGAACTCTGATTACATGAAGAAATCCATCCTTTTGTT

TCTATTTGCATTCTTGGGTTCCACATTTCAATTGGACCTACATTGGC

>Unigene0001475

TTTCCAGCCGCCTCGGCGGTGGATCCGGGGCCGTCCCTCACCGGCGGAGCCAGATGGCTC

CGTTTTCTCCTAGCTGCCGCCCGAGGATTTGCTCGGATCTTGTGTCTGCTCTATCGATGC

GTGCTCCGTCTCTGCCGACGATGGTCCCTGCGATATGTTGAGTTGGATTGCTAGAGCTAT

ATGCAGCTTGATCTCTCGAGTGTCCGGGTCACGTGCAACATGGAGGCCAAACATCCCGAC

TGAAGTTCTCTCCAATATTTTGTCGAGACTTCCGGCTGCGTCACTCCAGAGATTTAAATC

TGTGAGCAAGGAATGGATAGAAATCATCTCCGACCCTTACCTTGCAGAACTGCATCTGGC

TAATCAACCATTGGAATTTGAGCTTATCTACATATTTAACATGGTCAGATCATTCTTCTT

GGAAAGTCCTGAAGAAAATTGCTTCAAAAAGAGGAAGATACCAGCAAAGATGATAGTTTA

CGATACATGCAAAGGTTTAGTACTTTTTGGGAAGGTGAATGGTATGTACATTGTTAATCC

CATCACAGGATACACTCTTAACCTTCCTCCATTCCCTCAATATGATCATTCCGTTGTTAA

AGCAAGCATTGGACCTCTTATTTATGCAAGAATTGGATACGATTCTTCCCTCAAAAAGTA

CAAAGTGGTCGGTGTTCATCAGACGAAATGTCATATTATTACACTGCAGGAGCAAGGATG

TAGCGCATGGAGGGAGTTGGACATGCCACCCCTTATTAGCATGATGGCAAATTGTAGCCC

AATTTTTGTAGAGGGATGCTTGCATTGGATCAACCCGACAGGAATAATCTCCTTGGATAT

CAATCTAGAGAAGTTCTTTGAAAGAACAGAACTTCCCCGCAGTCCCCTTTTAGAGGACAC

GGCGCCCCGTTTCCTTGAATGGGATGGGTGTCTTGCTGTCATACCCAAGTACCAATGGGA

ACCATCTTATTGTATCTTCATCTTGAGGGACTCGATGTGGACAGAGCTGGAGCTCGACGT

TACATTCTTCGAGCACTTGTGCCCTTCAGAGGAATTTGGTGAGACCCCTCCGTACCCAAT

TGCAAGCTTGAAAAATGGTCAGCTAATTATATTCAAGAAATTAGGACACAGAAAGAAATT

TGGACGCAGAAAAATTCGTCCACCTCTACCTGATGACGGCCGCTTCTTCGCATACCATCT

GCAGGACCAAGTAATGACACCCTTTTCAGCGGTGGCGCCTCAGGGAGAAGAAATATTCAG

CATGAATGGCTACTTCTGGAATGGCTCAATCATTCCCCATGTCAACTCTTTAATTTCTTG

CTGATACAATTCAATAACCATATTTATACTCGGCTACTAGAGTGTCTTAATTTTTATGTA

GTACAATTTTTCATGTGAATATTTTTTTTTTCCTTTCTCATATATCCAGTTGTTTGGAAT

ATTAGATCATATTTATGTATCTGGCAATGTTCTTTCACTTGAATAATTTTTAATGGTGAA

G

>Unigene0001476

CGGTTTCCACGGATCTGATCACCGCAGATATAAGATATCAGGGCGCCGCTCTGCCGATCA

ATTCACCAATTGATCAACGATGTCGGGCTTGATGGAGAAGGCGAAGGAGTTCGTGGCGGA

CAAGATCGCCCATGTGAAGAAGCCGGAGGCGAGCGTCTCCGACGTCTCCTTCAAGAGCGT

CACCCGCGATTCCGTCCTTCTGCACGCTGAGGTTTCGGTGGATAATCCTTATTCCCACCG

CCTGCCGATCTGCGAGCTCTCCTACACTCTCAAGAGCGCCGACAGGGTGGTGGCATCAGG

GACAATGGCCGACCCCGGGTGGATTGAGGCGTCGGGGCTGACAAAGCTGGAACTGCCTGT

GAAGCTGCTGCACAGCTTCCTCATCAGCCTGATGAGGGACCTCGGACGGGACTGGGATAT

CGACTACACGCTGGATATTGGCCTCACCATCGACCTCCCGATTATCGGCAACTTCACCAT

TCCCATCTCTCAGTCCGGCGAGTTGAAGCTTCCCACTTTCTCCGACCTCTTCTGAGTTCG

GATGATCAACTCAAGAGGGATAATAATAAAAGGCATGATCTTAATCAAGGTTGTGTGTAA

ATGTAGGGCAAGTATGGGGACGAGCCCGAGTGTTGTAAGTGGCTGAATGTTGTAATAGAT

TACTCGTTTAAATTAATAAATAGGAATTAGGA

>Unigene0001477

TTGAATACATTATACTTTTTAATTTTGTTAAATAATATGATACGATTAGGATATAATATA

TAATATTATTATTATGTGTATTCAATATTCTAGGTTAGTATTATAGTAAGGCAGGTATTA

TTATGCGTGTATTTTTGACTTGTACAAGTATACGCCAAGTACGCGTATCGTATATTTTCA

AAGTTTAAATAAGATTTTTGCTCATCGTCATCAGTCAGTCATACGACACGTCATTATCTC

ATAAATAATGAAAGCATTTTTCATTATTGTTTATTTATTTATTTTTTTTTTTTAGAATCG

TTACTGATATTGTTATTTAGTATTACTAATTTATAACATAGTTGTACGTATACGTATATA

TTACATTTAATATATTATATTTATATAGTTTTTAGTGTTATAATAGAATCATTGTTGTTG

TTGAGAATATTGTTACCTATAATACCTATGAAGTAGGATAATATTTAATAATGTTGACGA

CGGTGTTTATTTTGTATAGTGTTGTTGCCGATGTTCAATACCTTTTTTTTAAAAATTTAA

TTTACACACGTCACACATTTACAAGACTCATATTTTTATTATA

>Unigene0001478

ACGACGACGACGACGACGACGATGACGACGATGACGACGACGACGACGACGACGATGACG

ACGATGACGACGACGACGACGACGACGATTCCGGCGAGACCAGAACGGTAGCCAAAGGCC

GAGCGCGTGCGATGGCTAGCAGCCCAGTGACCTTGCCATCCTCGTACGTGGCCAAGTATA

ACAGGTTCGTAGACAACATACTGGGCCGGATCAACAAGGTGTTGAGGAAGAACTACGACC

CGGTCAACGTACGCCTGCAAACACCCACACAGACCACCGACCATAAGAAGAAGGGTACCA

CCTCTCAAAAACCAAAAACCAAACCCAAAGTGGAATCCAGGGAAAGCGTTCAGCAGACGG

AAGTGTCGAAAAATGGCACTGTACCAGCAGCTCCGGTGACTGAAAAGTCGGCGGCCACTG

TGTCACAACATCCGACTGCAGTAAACGGCACATCTGCTCGTTCACAGCGCAAGCACCACA

AACACCACCATAGAACGTCAAAGGCGCAAAACAACAAACCAAAAGTCACCAAAAACAAGC

CCGCTGCCAAGCCCCATAGTCCACCCAGAGCCAGAGCCACCATGTACGGATTGAGCAGTC

TGCGCCGGGACGGAGACGTTACCGTGAACGTGATGAATACTCACACGACAGTAAAGACCA

AGTTTTTGGTTGGCCCTCTAACGTTGAAAGTCGAAAAAGAGTTTGGACGAGGTGCCAAAA

AGGAGCTGAGATCGGCGACAGCCACCACAGGCGAACTGGTCGGCCGACTTAACTTGCGAG

TGCTTACCGGCGGCGCGGCCACGTTGCACTCCATCCGCGTCCACCAACCGAAACAAATTG

ACAGCCAGGACGACCACGACAAGACTAGGGAGTACGTGTGGAGACGGTCCAACATGATCG

CCCACCTCGTGTCGCAGAAACTGACGTACGCCACGAAGTCCATGTTGCAACCGGTCCCGA

ATAAATCCGCGGCCTCTAAACCATAGTAGAAGTGGAGTTCAACCGTACTCAATGGCTGAT

TAAGTGTTAAGATATTTTCACTATTTATTAATTTATTTATGATATGAATATATTTATGTA

TTTATATATATATATTATATACATTTATAGATATTAATTTATTAATTTATTTATTTATCA

AAATACATAATAATGTATTGTATT

>Unigene0001479

TTTAAGAGTTGACTGAATAGTTGGAGAGTCGTGCAACTGCTGCAGTGTGTTCGGTTTGCT

GATTCGCGTGCGTTTACCATCAAATATTCCGTGCGTGCGTGCCATTACATAGAGTGTTGC

GAGTTATAATTTTTTTTTCACCGATTCGCGACTGCTGTTCGGATCGTAAGCCGAAAATCA

ATTCCGTGGAGCGGAACTTACTGACATCTAGAAGACGTTTTTATTAATACATTTCAAAGA

TGATAAAATGGGTGCTGATCGTGAGTTTGGGGTGCTGCGCGCTGATGTACAGCGCCAGCT

CATCTCCCATTGTTAGCTCGACATCCGAACCGGAACAAGGACACCATAACGCAAACGCAG

CCGTACCCGAGACAGCCGACAAGCTGGTGGCCACGTCGCCAGACAGCGGAAGGCAGTCCG

CCGAAACGTCCGCCGTACAACCGGCCCCTGTTTCCGAATCGGTTCCGGCGGCTCAGCCGT

CTGCACCTGCCGCCGTTGCGCCGGAAGCGTCCACAGACGAGGACGATGACGATGACGACG

ACGATGATGACGTGGTCGACTTGGACATCACCGGAGACGATGACGATGACGACGACGAAG

AAGAGGAAGACGATGATGACGACGACGACGACGATGATGACGACGAAGAGGAAGAAGATG

TTGTACCACAACCAGCCAGCGCAAACGCCGCCAATGGTGCCGCTGATCCAGCGACTGCCG

CTTCCGGATCGACTTCTGGTACCGCTGCCACAGCAGCCCCTGCAGCCCCGAGTTCCGGTA

GTGAAGACGACGATGATGATGACGAAGACGAAGACGATGATTACTTCGACAGATTCTTCG

AAGATATCCTTGGAGGTTGATCGAACCCGATCGACCGCGGGAGACTAACAATTACAGAAA

CATAACATCAAGATACGACAATGTGTACATAAAAAAAATATCTATGTATACACATTTTTA

CATTGTATATGACATGTGAAAAACAACATCGAGAAACTCATCTCACTACTTAACGGACTC

GATCAAAAACACTTTGATATACAAAAAAAAAAAAACCAACAACAACAACAACAACAACAA

CATAGATTTAAGAATGTAAAAACAAAACTTATTTATTTAAAGTTTTTATTTATTTATTTA

TTTATTTATTAATTAATAATTTATTTTAGATATTAGAAATTCAATATTTCAACGCTATAA

TATTACACCTATAATTTATTTTTATACACATATACCTATATAATAATATATAAAAATATA

TATTTATTTACGACAATTTGTTACCAAACAACAACTACTCGAATAACGAATACATACATA

CATATATATAAATATATATTATTATAGTGTGTCATTTAATAACAGTCAAAACAAAATTAT

GTAAATTCGTGTTCAATATAGAGCGTAAGATTTATTATATTAATATCTTTGAGTGTCATA

ACGAATTATTATTTATTTATTTTTTTTTTTTTTTATTAAAAATTTAAAAAAAAACATGGC

TGTGATAGCAAATATT

>Unigene0001480

TCGCCCACCTCGTGTCGCGGAAACTGACGTACGCCACGAAGTCCATGTTGCAACCGGTCC

CGAATAAATCCGCGGCCTCTAAACCATAGTAGAAGTGGAGTTCAACCGTACTCAATGGCT

GATTAAGTGTTAAGATATTTTCACTATTTATTAATTTATTTATGATATGAATATATTTAT

GTATTTATATATATATATTATATACATTTATAGATATTAATTTATTAATTTATTTATTTA

TCAAAATACATAATAATGTATTGTATT

>Unigene0001481

GGAGTCGAGTGTGCAATAAATCAGCAAAGAATACGACATTTATATTTGTACAATATAGTA

TAATATACAATATTATTATAATATATTATATTATAATATAGTGTCACGTGTTCTCGCCGA

TGATATTTTTTTTCTTATTATTTTGTCGTCGTCGAATTTATTTTAATTGTTACATGTAAC

GTCATTACAATCATTATTCTCGTATATTATAATAATATATATATATATGTATACATATAT

TATATATTATATTTAATGCGCGTA

>Unigene0001482

CTAATCGAGCTTCGGTGTGCGAAGAAGCAAAATCTGTGTACTGAGAGAACCACCATTGCC

GATCAGCTCTACTGCTAAAAAGCTCCAATCTTGCAATGGACAACCCCTTCAGCCTCAAGG

TGGGGCAGGTCTTCACTGGCATCGGCGCCGGCTGCGGCGTCGGCATCGGCGTTGGCCAGC

CCATACCCTTCGGTTCAATACCAGCGCTGCAGCAAGTTTTGAATGCAACAAGAGGCGCAA

CAGATCCTTTTATTGGTGTTACAAGATATTTTAATGGTTATATGAAAAGGTTGGGACTGA

AGCATATCGAAGCTGGTGTTGGATGTGGGGTTGGTGTAGGCCATGGTTTTGGAGTAGGAC

TGGCCTTGAAGCCTCGGGTGATTCATCAGATGCAATCATCTGTTGGGGAAGCAGTGGCTA

AGATTATGACGAAGCTAGGAATCACACCGAGTTTATCTTCTGTCCAAAGTGGCATACCTG

GGCCTTTACCAAGCAGCAATGGCGTGCTAACCGAGACACATGGTAGAAATGCACAGACCT

CAACCAATAGTTTCATGGATCGGTTATCTGATATTACAGGAAGCACGATTCGGCAGTCAA

CTTCTGAAAGTGTCACTCGTAAACCTATGCAAAATCATGCATCAACGGAAAGCCCATCTG

ATGTGTCACTTGGAACGAGCTCTGAAAAGGTTATCGACACGTTCTTGAAGAATCCACTTT

TCAATAATGAGGAGGAACTGGAGCTTAATGAATTGGCTGGAAATTTGCGCTCCGAGAATA

ATCTGCTTCAAGTGTTACTGAGGCACCAACAAGCCATTGAGGAACTGGTGGAGGAGAATC

AGAAGCTGCGTCAAGTAATAGTGGAAGATTTGAAAGTTTCAGCAAGCAAGCTCCAAGCAA

ACAATGGGAACAAAACCAAGTTCATCTACCATTGTTCCGACTGCTTCGAATGCCGTCGAA

GAAATCGAAAAGCAGGAAGATAACATGGTCTACTCGAAATCAATAGTTTCATCACATTCT

TGACCATAAATGGGAAATCATAAACCTAGAAGGCGAGACGATCTTGTTTTTCAAGGAAGA

TTGAAGTGCATGTCCTCAAAATCTATAAGAGGATTGTTGCTCTCCCGAGTTATGTGTACC

TTCTAGGCATTGAGTTTTATTTGGAGAATGTCATCAAAGTTGCGTAACATTTAGTTCTCA

TAACTCTTCTAGTCATTGAGTTTTATTTGGAGAATGTCATCAAAGTTGCATAACATTTAG

TTCTGATCCAAGATTTTGTTTTCTACCTTTGTGCCTCATATTTAAAG

>Unigene0001483

TTTTAACAACAAGAGCTAGCAATGGCCTGCATGAATCCATTGGTCCTTGTTCTCATTGTT

GCCCCTCTTGCGATGTTAGCAGAGAGTCAAGTCTCTCAGAAGGATCTTAGCTTGGATCTT

GGTGGCCTCGGAGGCATCGGACTTGGTCTTGGTGCGGGCATTGGTGTTGGGATAGGAGGT

GGTAGTGGTAGTGCCTCTGGTTCTGGTTCTGGATCAGGTTCGGGCTCGGGCTCTGGTTCT

GGATCGGGATCCGGT

>Unigene0001484

AGAAACAAGAACTAGAGATGGCTTGCATGAAACCATGAATACTTGTTCTTCTTGTTACCT

CCCTTGCAATGTTAGCTGAGAGCCGGGTATCTCGGATGGAGCTTGGCTTGGATCTTGGTG

GTGATGGGGGGCTCGGGGTAGGCGTGGGTACCGGTGTTGGTATCGGGCTAGGAGGTGGTA

GTGGTAGTGTCTCCGGTTCGGGCTCGGGTTCTGGTTCTAGCTCGGGCTCAGCATCCGGTT

CGGGTTCTGGCTCAGGAGCAGGTTCGTATGCTGGTTCTGGTGCTGGATCCTCTGCTGGGT

CAGGAGCTGGCTCAGGTTCAGGATCCGGTGCAGGCTTAGGCTCAGGGCAGGGGCAGGGCT

CAGGTTCGGGACATGGCGAGGGTTCGGGAAGAGGCCAGGGTTCAGAGTCAGGGTATGGTG

AAGGACATGGATATGGTTCTGGTTACGGTAGTGGCCATGGCAAGTGAGGATAATCCATGT

GATAATATGTACTATGGTATGAACCATCTTCTATGTATGGAGACAAGGAAGTCGTGAAAA

ATAATTTTATCTCTTGGTTCTTGCTTTTGCTTATCTCTACCCTACCCTATCCTACTTTTT

GGCTTCAAAATCTAA

>Unigene0001485

TAGTGCCTCTGGTTCTGGTTCTGGTTCTGGATCAGGGTCGGGCTCGGGCTCGGGCTCTGG

ATCTGGATCAGGATCCGGTTCAGGTTCTGGATCAGGATCGGGATCCGGTTCAGGATCAGG

ATCCGGATCGGGTTCAGGTTCAGGAGCAGGATCATATGCTGGTTCTGGTGCTGGATCCTC

TGCTGGGTCAGGAGCTGGCTCAGGCGCGGGATCTTCTGCAGGTTCCGGAGCGGGTTCAGG

GCAGGGTCAGGGTTCAGGTTCGGGAAGGGGTCAGGGCTCAGGATCAGGTTATGGCGAAGG

CCGCGGTTACGGTTCGGGCTACGGTAGTGGCCATGGGAAATAAGGATAATACATGTAATG

TTATGTATTATTGTATTCACCATTTTTGTGTGTAAGGAAATGGTGAAGAATAAATATTTT

GTCTTGTGTTTCTTGATTCGC

>Unigene0001486

TATGCTGGTTCTGGTGCTGGATCCTCTGCTGGGTCAGGAGCTGGCTCAGGCGCGGGATCT

TCTGCAGGTTCCGGTGCGGGTTCAGGGCAGGGTCAGGGTTCAGGATCAGGTTATGGTGAA

GGCCGCGGTTACGGTTCGGGCTACGGTAGTGGCCATGGGAAATGAGGATAATACATGTAA

TATTATGTATTATTGTATTCACCATTTTTGTGTATAAACAAGGAAATGGTGAAGAATGAA

TATTGTCTTGTGTTTCTTGATTCGC

>Unigene0001487

CATCTCTCCCATCATGTTTGAATCTTGATCTCAAACCCTAATCCGATGCTGTCCGTCCCA

ACATTCTCTCCCTCCTCCAACCCCGCCCTCGTAATCCGACAATCCCTTGTCACCTTCCAC

CCCTCCCCTCTCCGGGCTCTAGCTCCGCCATGGCGGCGGTTGCGGAGGGGAGGAGCTGCG

GTGACCGCCTTCAAGTCCCCCTCGGCTGAGCTATCGGCGGCGGATGAGGAGTGGCTGAAG

AAGCTACCAGAGAGGAAGAAGCCGCTGTATTCTCACAGCCTGCCGTGCATAGAGGCCTGG

TTGAAGAGCCTAGGGTTTAGGCAGTCTCGCGAGGACCGCGCTGCCTGGCTTATTGAGAAG

CCCGACTGGCATGCTCAGCTTTCCCTTGATGTTACCGATCTATACATCAGATACTTGAAG

AGCGGGCCCGGTAACCTGGAGAAGGACGTGGAGAGGAGATTCAGCTACGCGTTGAGCAGA

GAAGATATTGAGAATGCGATACAAGGTGGACCATGAAAGCAAAGAGATGTGAAGTGTACA

AATATTTCCGGCCAATCAGAAAAATGTACTGATGCAAACTCTTTTTGTATCCAAAATCCT

TTGAAGAACTGAATGAACTTTGTTTGAGTTTTGCATTTTTAATGTTAAGTTACTTTATAT

CTGAGCTTATTTACTAGGTCGATTTGCTGGGGACATGATGAGTTTCATCATAGTGATGAC

AATGGTGCTTTGACGTTTTCGAATGTGTCTTAAACGTTAAGAATGAATAGATATTGAATT

G

>Unigene0001488

GGTAACCTGGAGAAGGACGTGGAGAGGAGATTCAGCTACGCGTTGAGCAGAGAAGATATT

GAGAATGCGATACAAGGTGGACCATGAAAGCAAAGAGAGCTGAAGTGTACAAATATTTCC

GGCCAATCAGAAAAATGTACTGATGCTAACTCTTTATGTATCCAAAATCCTTTCAAGAAC

TGAATGAACTTTGTTTGAGTTTTTGCATTTTTGATGTTAAGTTACTTTATATCTCAGTTT

ATTTACTAGGTCGATTTGCTGGGGAAATGATGAGTTTCATCGTAGTGA

>Unigene0001489

TTCCTCTCTCGCTCTCCCTCTCGATGGTGATGACGGTTGTCCCTCCACGGCGGTCGATCC

GGGACGCCGTGCTGCACTCCGTGCTGACATACAACGCCGGCGGCCACCGTCACAACTACC

CCCCGAAGAGGTGTTCCTGTGGCGGCGCCGACGAGCCAGCAGCCGCTCCTTGGGGGAGGG

AGGAGGACGGCGACGTGGAGATGACTCAGATCGGCGCTGAGCGTCCGAAGAACGTCCTCA

TACTCATGAGCGACACCGGCGGCGGTCACCGTGCCTCCGCCGAGGCAATACGGGACGCCT

TTCGGCTTGAGTTCGGCGACGATTACCGGGTGTTTGTGAAAGATTTGGGGAAAGAGTATG

CAGGATGGCCTCTGAATAGCATGGAGAGATCCTACAAGTTCATGGTCAAGCACGTCCAGC

TCTGGAAGGTGGCGTTCCATAGCACCTCTCCTCGCTGGGTCCATTGCGCCTATCTCGCAG

CCCTCGCCGGCTTCTACGCCAAAAATGTGGAGGCTGGCCTTAAAAAGTACAAGCCGGACA

TAATCATAAGTGTGCATCCCCTTATGCAGCACATCCCTCTATTGGTACTTAAATGGCAGA

GACTACAGAAGAGGGTTGTTTTCGCGACCGTAATCACAGACCTCAACACCTGTCACCGGT

CTTGGTTCCACAATGGTGTGAATAGATGCTACTGCCCCTCAGAGGAGGTGTCTAAGAGAG

CTCTGCTTGATGGTCTGGAACCTTCCCAAATTCGTGTCTTCGGTCTGCCGATTCGCCCGT

CTTTCAGTCGAGCGGTTATCAACAAGGATGAGTTGAAAGAAGAGTTGGAGATGGAACCTC

ATCTACCTGCAGTCCTATTAATGGGAGGCGGCGAAGGAATGGGACCAGTGAAGAAAACTG

CAAAGGCCATTGAAGAATCACTATATGATGAAGAATTAAACCGACCGATTGGGCAGATAA

TAATTATATGTGGACGTAACCAGATTCTACGATCTGCCTTACAATCGATTGAATGGAAGG

TCCCTGTGAAGGTAAGAGGATTCGAAACGCAGATGGAAAAGTGGATGGGAGCTTGTGATT

GTATCATAACGAAGGCAGGGCCAGGTACAATTGCAGAGGCTCTGATAAGGGGGCTTCCGA

TTATCCTCAACGACTTCATCCCCGGACAGGAAAAAGGTAATGTATCTTATGTCGTGGACA

ATGGAGCTGGAGTCTTCTCTCAAAGGCCGAAGGAAACAGCTGCACTAGTCGCAAGATGGT

TCGGCCCTGAAGCGGAGGAGATGAAGAAGATCTCGCAAAATGCGCTGAAACTGGCTCAAC

CTGGTGCGGTTTTCGATATTGTTCGAGACATCGATGAGCTTGCTCGACAGCATGGACCGA

AGGCCAGAATCTCCTACCCTCTCATCTCGCCATTCTCCTACCCCATTTAGGAGCCACTGA

TGAATGCCTCTCATCATAGGCTGAATTGTAGGACTGGGATAGCATTGACAATGTACAGCT

GAGTTTTTCATTCTTTGTTGCAGTGGCCTGTGTGTATAGATCTAGATAGAGTGGGGACTA

TAAAGGCCTTCGGTGGTCTATCATTTTGTTGCAAAACTAAATTTGCACCTGTAGTTGTGG

GCTTGTGGTTGAATAATATCTTTTAAGCAATTGTTTAAAAAAATGACCAATCTGTTTTTT

CAAGACAAACTTTTCTTAGAATCGACACAGCGTGTACGGTTGTAACATGATGTCAGGTGA

AGAGAAGTACAAGAAAGTCCAATTGATACCTGGTATCTTGTTGTTGACATAAGACTGGGA

ATCTAGTGCTAGACTGTAATAGAGTA

>Unigene0001490

CATCACCTCCTCCTCCTCCCTCTTCATCCCCCACCCACTTGAATGCTCACCAAGAAGGAA

CTTTACCTCACTCACCGTCTCATCATCAAAGGCCAACGCGAAAAACCCCGCCCTCCTCAT

CCGCTGCACGAGCCGCGCCCCTGACTCTTGCCTCTCGATCCTCCTCGCGCCCTCGGAGCC

GATTATGCTCTCAATCTTGATCCCCACATCGGCCTCGTACTCCATCCTTTGTGGGCTGTT

CTTGGGCAAGAACGTCTCCAGTGCATCAAATGGTATCCATAAATAGTTGAAACAGGCCAT

GATTCTTGAGGTGAGGCTCGGGGAATCCAAATCAGCATCCTCGTCGGTGATTGTGACTAT

GCATGGATTGAGCTTCCTGATCATCTCCAAGAACCCATCTCTTGTAATTCGATGCTCGTG

TTGGCTTTGTGTAAAGGGTAACCTAGATAACTCCGGATTGGTGGCATTCAGAGAGAGATC

CTCGGCAATTGGCTCATCAGGCAAGTACCGGAGCCAGCTCTGGCAGTTGATTACTACAGC

TTCTCCATCTCTGAGTCCTAAAGATCTTGAATCAAATTGAATTGCTGCCAATTGTTCAAG

AAAGCTAAGAGGCTTATCATAACCATTGCCAGACTTGAGCACGTTGAACTCGAATGGTAC

TTCTCTTAACTTAGCGAAGTTGGCTAGACGGAGTCCGACTTCTTCGATTGAGACAGTGAG

CGAGGGAGGGACTGGTGGCCTTGCAGAGGGGACAGTGATGCGGAGGCAGGGAGCTCCCTC

AGCTCGTTTGGCTAAAGCATCGATGAGGGTCGGCCACTGCATGCAGTGGGTGATGCTGAA

ATCAATTACATGGATCCTTGCCTGCCCCCTTACAGCAGCAAGTATGGCACCATTGGATGC

GCAGAAGCCGAATCGATGCCATGGAGTGAGGTCTACATAATCTGCGAGCTCAGTGACTGT

CATCCGCCGTGAAGACGAGTGGGTTGAGCCTGGGATTGGCATGTTTGTGGCACATAGTCT

CGCAGCCCGGGAGAGAAGAGCACTGAGTAAACAAGAAGCAAGCCTTTGGTTGGGGTCCCC

GTGTGGGGAAGCAACGTTGTTGAGAACCCACATGACCTGCTGAGCTAGGGTTACATCGTT

GCTCTCGAGAGCGCTGGCGCAGTGAAGGAGGAGCTTCTCGATGCAGGCCCCATCTAGGTT

TCCGAGGCATCCCTGAAACAAGGTGGAGTGGAGGGAGATGGCAGTGTTGGGATTTCCGCT

GAGCTCTGCCTTCATTGTGTCCGGTAAAGGGGGTCGAAGATGAAAATTAGGGTTTCTGGC

AAGGGTCACGTGCATGGGAGGGTCTAGTTAGTTGGCATAAATGCCACGTGGCTAGTGTGG

GTTGGTGGAGATGGATTGGAAGGGCTAGGGTTTTATCCTGTGAGGTATGGGTAGCTTTGG

AGTTCTCAAAGAGATAGGATTTCTTCTTTAACTTTTTGTAGAACAGAGAGGAGAGAGAGA

GAGAAGGGGGAGTACAGGAGGGCTGGGGGATTTGTACAATTGAGATGTGGTTGTTTATAT

AGGAAGCTCAACAAAGCCTTTTAAGTTA

>Unigene0001491

AGTGGAGGGAGATGGCAGTGTTTGGATTTCCGCTGAGCTCTGCCTTCATTGTGTCCGGTA

AAGGGGGTCGAAGATGAAAATTAGGGTTTCTGGCAAGGGTCACGTGCATGGGAGGGTCTA

GTTAGTTGGCATAAATGCCACGTGGCTAGTGTGGGTTGGTGGAGATGGATTGGAAGGGCT

AGGGTTTTATCCTGTGAGGTATGGGTAGCTTTGGAGTTCTCAAAGAGATAGGATTTCTTC

TTTAACTTTTTGTAGAACAGAGAGGAGAGAGAGAGAGAAGGGGGAGTACAGGAGGGCTGG

GGGATTTGTACAATTGAGATGTGGTTGTTTATATAGGAAGCTCAACAAAGCCTTTTAAGT

TA

>Unigene0001492

ATGAATGGTAAATGACGCCCCGTCAGACTGAAGAAGAGGGCTCAGGAAAGATTCACATTT

AGACGATCAATCCGTCCGTTTGCTCATACATGGGGATCCTGTACGACACATACGAATGGT

AAATAACGGACCCGTCAGACCAAAGAAGAGTGGGCAGAGGAAAGACACACATGAGCACAT

TTTGACAATTATCATCAATCCGCTTGTCCATAAATGTGGAGCCAGCACGGCATATTAAGA

TGTTAAAGTTGATTATCAGATGATTGGTCCTGCTCTTTATCTAGGGCTCCAAACTGATCT

GATTCGGATCAGATGTGCTGAAATCCTGATCTTTAT

>Unigene0001493

TAAGCTGTGAACAGAAAAAAGAATAAAAACTTCAACCATTTCCGTCCTTGCGCTTCCTTC

CCCAAACAGGAAACGCCGATGAATTGAAATCTCTGTCCGTCGCAGCTGCTGATGCTGGCG

GTTCAGATCCGCGCCGAGACTCCACCGCCCCCATCCCGACCGGCGCCGCTGGCTCCCCGC

TTCATGAGATCCGGCCGGAGCCCCGGCGCTGGCAAGTGCCGCACGCCGGAGCCGCTCGAC

TTCTTCATCTGGACTGTCGAGGATGTTGGTTTGTGGCTGGAGGAGATCAATCTTGGTAGT

TATCGGCTGGTTTTCCAAGCTAATGGTGTCAATGGGGAGTATTTAGAGAGTTTATCCATG

TTCACAACTGAACAAATCCTTCGGTTTATTAGGCAATGCCACATGAAGTGGGGAGACTTT

ATCACTCTTTGCAAGGAACTAAGGCGTATTAAAGATTTTATCTTCTGGCCCGAATTGGTA

GCTCTGCACAGGAGGGAGTAATGTTGGTTGAGGTCCAAAAGGTAGGGCACCAAAGGATAA

AAGCAGGACATGAGTAGTTATGGGTGTATGTGGCGATGTTTCCTCGTAGTAATTTTATTA

TGTTCATGATTGTGTGGATATGAATGGTAAATGACGGCCCCGTCAGACTGAAGAAGAGGG

CTCAGGAAAGATTCACATTTAGACGATCAATCCGTCCGTTTGCTCATACATGGGGATCCT

GTACGACACATACGAATGGTAAATAACGGACCCGTCAGACCAAAGAAGAGTGGGCAGAGG

AAAGACACACATGAGCACATTTTGACAATTATCATCAATCCGCTTGTCCATAAATGTGGA

GCCAGCACGGCATATTAAGATGTTAAAGTTGATTATCAGATGATTGGTCCTGCTCTTTAT

CTAGGGCTCCAAACTGATCTGATTCGGATCAGATGTGCTGAAATCCTGATCTTTAT

>Unigene0001494

ACACGGGTTAAATCTGGGTTAAATGGTCAGATAACAATTAAATCCCAATTTGGCGATTTA

ATCATGATTTAGTTACAATTTAATCGTGATTTGCAGTTAGTGCTTGTATCCCACTTTTCA

TGGATAATTCACTCTTCGCCAAACAAACCACCGCCTCTCAGCACTAGCATTCTCTCATAC

TGTTTGTTTTTATGACTTTTATTTGAATATTTGGTGATTTTCATGATTTTACCGTGAATC

ATGATAATTTGATCTCGCAGATTTTAT

>Unigene0001495

ATTTTTTGACTCCAAGTAGAGCCTCCAAAATAAGATAAAGTCGTAAACCCTCCCATTTCA

GTTGTTTTGGCCATATCTCTTGAACTCAAATGCATCTGGCATACCGTTTATTCAAAATTC

CATATACAAGAGATTTGAGACTAGTAATTCCCCCAATTTTTTGACTCAACCAAGTAGGAA

TAAATTTATATTTTTAGGTTTGTTTCCATTTTGGTAATATCATGTAATATTTAATCATTA

TTTTGCATATAAGCAATTGGGATTAAGGTTTCTTCGCCTCTCAAGTAATTGGGATTAAGG

TTTGGTAACCAGTGCACTGAAACGTTTCAGTTCGGTTGTGTACAAAATTGGATCTGAAAC

TTCAAAACATGGTCAAGGGGCAGCAAAACCCCACATTGTT

>Unigene0001496

GCCTCTCAAGTAATTGGGATTAATGTTTGGTAACCAGTGCACCAAAACGTTTCAGCTCGG

TTGTGTCCAAAATTGGAGCTGAAACTTCAAAACATGGGCAGCAAAACCCCACATTGTTGG

GAGCCTCTTTCAACTTGCACTCCGTTGATTGGGAGCCTCTTTCAACTTGCACTCTGTTGA

TTTTATATCTTCTATTTGTTATATTTATTTTTTACTCCAAAATTTTCTAATTTTGGATAT

TGTTACACTCTTAAGGTTGTTGAGTGTTAATAATCATCAGTTTGTCCTATTTTATACTAG

AGTGTTCATGGGTTGGTTCGGGTTTATTTCTTACCTAAATCGGAACCATCTTTATTTGGT

TCTGAGATTTTGGAAGAGAACCAGAGTGAGAGCCGACCTGATCTGTCCCAGCGGTTTTTG

GGTTGGTTTGGATCTGTTCTTGGTTCCCCGGTTGATTCTGAAACTTTATATGCATCCAAT

ATGTCTTATGTCAAACTATAAACAAGCTAAAAGTAGAAATAGTTGATAATTTGTTATTCA

ATCCTCCATTGTAAAATTATGTATCAATGTTATTATGATATAGTTCGGTTATTAGTATTT

TGGATCGCTTTGCTGCACAAAATGTTTTCAAGCTTCAAAATGCCTTGGCGCTCTTATTGC

CACCTAGTCGCAAATTGTGTCTATTGTGTTCAATCACAGTTAATGTTTCAAATAGAGGAC

ACAATTTTGTTATTGCTGCCACTTTTGTGGTAGCTGAGTCCTAGCTAAATTGTGGCCACT

TTTAATTTGGACTTCTTTTTTGAAGTCCAAAATTGAAGGTCACGTTTGGCGGCAAACATA

TCCCAAGTTGATGCCAAATGATATTCTTTGGATGCTCTATACAACATTGTGAACATGAGT

TGAGCTTGCCGCTCAAATTGACTTCATGCATTAAGAATCCTTCCCTTCTCAGGGTCACCC

ATCAGCTAGCGGCCTCAATTTTGCTGAGGATGCCCC

>Unigene0001497

TGAGAGGGAGAAGAATTCTGGAAGTTAATACAGATCACCCCATCATCAAGGACTTGAATG

TAAGTTTTAGGGAACAAAATATTTGCTGTTAGATTAAGTATCGAGGTTTACCACTCTAGT

TTCTGGTTTACTGAGGTTACCACTCTATTTGCTGTTAGATTAATTTCACTCTAGTTTTTG

CTTAGAATTGAGTTCTTTGTTATAGCAATATCATTATTGTAAAAAATTAGCACTGCCTGC

ACATAATCTGCTGGCTGAATTCAAGTTATAAGTTATGTATTTACCAAAATAGTAACTCAC

TAAAATTTCCTCCCATCTTCATCCTTGTTCTGCTTTTTACTTATTGGGAGACCTGTCTAT

AAGATCGAAAGTATCCTAACAAACATATAGACACCAGCTTTGTCAGAGGACCATTCAATT

CTTTAAAGACCAACCACCTCTTGAAGACTTTGATATAGGATCAGCAAAGGAAATTAAATG

GAACTGTCTCAGCGATAAGTGAACTGCTCTCTCTGTTTGTGTGTGCGTGAACTGATATCA

CGGTAGAGAGACACCAAAGCCTCTCTCTCACACACAACTGATATCATGATGGAGGGACAC

CAACGATAACAGAGGCCCAGATCCTTCCCCAGCCTCACTTGTGCCTGCCTTTCGAGCCGA

TAGAGTTCTGGTTACTGGGCTGCTTACCTCTGCCAGCAGCTTTGTACCTTCCACTCCACC

CCAAAGTTATCTCTAGAAATCCCAAGAATCAAGCCCAATTGTCAATCTGGGTCTCATTGG

TCATATGCTGTTAATTTGGTTCTATAGCCAGTAGGCCCAATCAGTAGCGGCACCAGAGTT

GTAATTGAAGCAGATCGGCTCAATTATTACTTGGTCATCCCTCTTGTTCCAGCTTCCTCT

GTCAAGCGAATCAGTTTTCTCATGCTTTACAGTTCTCTGCTGGTCTATTCATTTTTATTG

TTGCAAATAATTATTCAATTCTATTTTTATGTGTTCATGAGACTGATGACACTGTCATGG

GTAAACAAACTGATGGAGGAGGGGT

>Unigene0001498

GATGCAGCTTTATCCTGTGAGCTGGGGATGCGAGCGCTGGCATTAGCTGTTGGCAGCGAT

TTCGAATCTGGTTCAGACACTGATACACATGACAAAACTGATACAAGTAAAGATGGTGCT

CTTCAACAGGGTGGCAACCTTGTGATAAAGCTTTTAGAGAGTGAAGATATACAAGATTTC

AGTAAGTTCTGCAAACCGCGTTTCAAGAGAGCATCATGGTTGAGGCCCAAGGCAACTAGA

TCATGTTCGAGAGAGATTTATTTGATATGCCAAGGTTTGCGATAGTATCTCAGCACTATC

AGATTCATTAGAAGAAATATTTTGCAACAATATTTTGAAATATTTTCCCTTGCTCTTATA

AAAAAACTTGTAGCCTTTGTTACTATTGATAATAGTAGTCTGGAAAGTTTATAAAATGTT

ACTTCTGCCAAATCTTTTCGTTCATTACTTTCTA

>Unigene0001499

TAATTACCCATTGTAGTGTCCTGGTGTCTGCACGGGCTCAAATCCCGTCTCCCTCGTTCC

AAGCGGCGCCGCCGCCGCCGCCGTCGACGACGACTATCTTTTCTCATTGAGCTGAGGGGA

GCCGATGAGTGGGGCTAGGGCGCCGGATTTCTTCTACCGCGAGGCCCAGCGCCTCGGCTA

CGTCGCCAGATCCGCCTTCAAGTTACTTCAGATGCAGAAGCAGTACAAATTGATCACCCC

CGGCGCCTCCGTTCTCGATCTCGGTTGCGCTCCCGGCGCCTGGCTTCAGGTGGCTTGCCA

GAGCCTGGGATCAGTGGAAAAGGGTGGGGCGGTGGTGGGTATTGACTTGAAGAAGGTGAA

GGTTCCGTCTCAATGCGATGCGAGGGTGAGGACGGTTTGTGCTGATGTTATGAGTCTCTT

GAAGGAGCAAGCTAAGGCAATGTCTCCGCAGGGGAACGGTTTCTCGGTGATACTATCGGA

TATGTGTCCAACAGTGTCTGGAATCACAACAAAAGATGCAGCTTTATCCTGTGAGCT

>Unigene0001500

CCCTTATCAGCACCTCTGCTGCCGTGATTATCAATGGAAAAGGGTTTCGATCGGGTTTCT

CCACCTCTCCTATATCCGATTTGATGATTGGAAGGAATAATGGGGGCGATAGTATTGCAG

TCGAGATTGCTGCCGCTGGACGGAGAATGCCTCGAGAGGCATCAGATCCATTCGATCAGT

CATCAACGCCGGAGCCTGAGACCGTGGACTGCTCCAATTTTGCGAGATACGAGTTGTCAG

ATGAGAAGATACAGATGGAGAACACCATTTGCTTTGGACACCGATGCTTCTCCCGAACCT

GACGGCCAAGATAGTGTCGAAGGAGACAATGCAGGGCTTGGCAGGACGAGACTGGGCATA

ATCGTCCGTGCGGCCGCCAGGCGGCTCTTAGAGAAGCTGAACGCTGCCAGAAAGAACTTC

CCGATGAAGATCTTTCTCCTCCTCCTGGGTTTCTACACTGCCAACGCTCTGGCGACGATT

CTGGGACAGACTGGCGATTGGGACGTTCTGGTTGCAGGGGTTGTGGTGGCTGCCATTGAG

GGGATCGGCATGCTCATGTATAGAAAGCCATTCAGTGCGCCTGTTGGTCGATTCAAGTCA

TTGATTGTTATGGTCAATTATTGGAAAGCAGGCGTTTGCTTGGGTCTGTTTGTCGATGCC

TTTAAATTGGGAAGCTAGTGACAAAATGAGGAATGAATTTTGATTTAATAAAATATCTGT

GATGTCCATACATAATTTTTGTCTAGTCTGTACCGAGTTTGCATAATAGTCCAACATGGC

TATATATCTTTTTCTTGTGGCCTGACTTGATGATGTTGACCTCGCGCTATGATTATTGTT

CTAAATTTTCAGTTTAGTGTAAGGAAACATAACAA

>Unigene0001501

TGGCGCTTTTGTCGCACTGGCTCCGGCGGGGTGTGGCGGGGTTGGTGCAGGGGTATAATC

TGGCGATGGTGCTGGCGGCGGCGATGCTGAATTTGTTGTATTTTGAGCCGAAGGCCACGA

AGGTGATGTTTGAGAGGATGAAGATGGAGAAAGAGGAGGGGAGGGGGCAGGATGTAGCGG

ATGTGGTGCTGGAGACGCCGGTTGTGGCTGCGACGACTACGGCAGGGACACAGACGGCGA

CGTTTGTGTCGGCGAGGTCGGTGACGATGGACCAAGAGGCGGTGAAGAACAGGATGGCAA

AGCTAAGCAGGAGGCTGAAGAGGCTGAATACTTATTCTTCGTTTCTTAACATTTTGGCTC

TCATGGGGCTGACTTGGCATTTGGTACATCTTGCTCGTTGCATGCAGGTGAGCTGTTGAG

AGGGTTAGGGTTAGTTGGTGGATGTTGTTTGTGTGTGCTTTGTAGATTGGGAGCTTACCT

TTGTTTGTGTTTATACTTGCTTTGTAGTTTAACTTGTATGCTGATGAATCAAACTCGGGC

TGTTTAACTTAAGTTGTTTTCTACATAATGTGACGTACCAGTAATACTTGTTGGTAGAGA

ATAAG

>Unigene0001502

ACTCAGCCAAAATGATGAACCTCCTCGCCTTGGCTCTCATCACCTCCACCCTCGCCACCG

CCGGTGTTTTCTCCCCGCCGGCGCCGCCTCCCAACCCCCAAGACCCCGTCCTCCGAGAAG

GCCACCGCCTCATCGTCGTCGAGTACGAGCGCCAACTCCCCTCCGATCATCCCACCACCG

ACAACATAATCGACGAAACCAAGCACAAATTCCGAGAAGCCGCCGCTGCCCTCCCCAACC

TCGGCCAGGGCCTCTCCACTCCCCCGGCCACCCATGACGACAGCCTCAAAGACAAGATCT

GCGACGCCTACGGCAACTGCAAGGACAAGGTCTCCTCCCTCCTCGGCAAGGGCAAGGGCA

AAGCCTCCGTCGCCAAGGACGCCGTCACCGACAAGCTCTCCGAAAAAGCTAACAAGCTCG

AATCCGGCGCAAAGGATGCCGTTCACATGGTAAATGAAAAGATCTCTGAGAAAACAATGG

ATGCCAAAGCTGCTTTTGATGAAGCCAAAGATGCTGTCAAGGATAAGGTATCCGAGAAGG

CAAGCCAACTGCAACAGGGCGCCAAAGATGCCGCCGACAACATCTCCACTGCTGCTGCAA

CCGCCGTCTCCACTGCGGAGGCAGCGGCAGCCAACCTGACTGACATCATCAAGCGTGCTA

GAGAAGTTGCGTACGACATGGTGAAGTACGCCGGGGCGCCGGTGCCACTGAAGACAGCGG

CAGCGGTGGCACAACTACTGGCGACGGCGATGGCATATGGGGCGAGTATCTGGGTGACGT

TTGTGTCTAGCCATGTGCTGGCGAGGGCGCTGCCGCGGCAGCAGTTCGGGGTTGTGCAGA

GCAGGTTGTATCCGGTGTATTTCCGGACGGTGGCTTACAGTGTTGGGGTGGCGCTTTTG

>Unigene0001503

AGGAAACAGAAAGACAAAAAAACTCTCCCCCTCCACCATCTCCTCAATTCCCAATTCAAT

TCGCCGCCGCCCCGCCGCCGACTGCCGCCTCAGTCTCCGATTGCCGCGGCCTCTGATCAT

GTCGACCGATGCTGACGACGACTTCCTCTCCTCCATGAACTCGGAGGACGAAGACGATCG

CTACCTCAGCGACGCCGACGCCGACGCCGACGAAGACAGCTTCGACGATTCCGCCCCCTA

TAGCCGCCAAGAGAAATCCCACTGGTCCCTCTCCTCGGTCATTACAAGAGAATCTCTCCT

GGCAGCGCAGAATGAGGACATACGGAAGGTGATGGAGCTGTTTGCCCTGAGAGAGCAGCA

CGCCAGGACGTTGCTTATTCATTACCGGTGGGATGTCGAGAGGATATTTGAGTTGCTTGA

GCAGAAGGGGAAAGAGAAGTTGTTCTTGGAGGCGGGGGTGACTGTTGTTGAGAGGAAAGG

TTGTGCGTTTTCGAGCTCTTCGGACTTGGTTATTTGCAATATCTGTTTTGAGGAAGTACC

TGTGGGTGGGATGACTGAGATGGATTGCGGGCATTACTACTGTAATGATTAAGATTGAAT

TCTGAGTTCCAAAATTCAGACTTGTCCATGTTGGAGATAGGATGGTCCACATCGCAGATA

TAGGGCAGTCTTCTTTTCAGATAACTATGGCCTGCAGAGTGGGAATTGAAAAACTCGGGT

CTTTTTGTTTTCTCCGTAAGTCCCCCTGCCTGCGCATACGGGCGCCTTAGAGGAAGGCGA

TTTCGTTGGTGGTTCTGAACCATTCACCTCTACATCTGTCAGTCACCAGCTTCTTGGAGG

GGCTGAAATGCAATCTTAGGCCTTTGTGTGTTGTCTGGTTGAAGTTCTGGTTCATCGATT

TTAACAGATATGTTCATACTTTTTCTTCTCAAACATCGTGCCATATATTTATTTTTTTAC

CTGCCTGATTGATAACACATCTAAATTTCTTATTTCTCAGGTTGGACGGAGCATTTTATA

ATCAAGATAAAAGACGGTCAGAGTCGACGAATTAGATGTATGACCCTCAAGTGTAATGCT

ATTTGTGATGAAGCAATTGTCAGGAGTTTAGTTAGTGCAAGGCATCCTGATATTGCAGAT

TCCTTTGATCGATTTCTACTCGAATCTTATATCGATGACAATAACAGTGTCAAATGGTGC

CCCAGTGTACCACACTGTGGAAATGCGATAAGGATTAAGGGTGATGTTTATTCTGAGGTG

GCCTGTGCATGTGGAGTACAGTTTTGTTTCAATTGCTTGTCAGAAGCTCATTCACCTTGT

TCTTGCCAAATGTGGCAGCTCTGGGTCAAGAAGTGCCAGAGTGAATCAGAGAATGTGAGT

TGGATGACGAATAACACGAAGCCTTGCCCAAAGTGTCACAAACTTGTTGAAAAGAATGGA

GGCTGCAACCTTGTTGTGTGTCTCTGTGGACAAGCATTTTGTTGGTTGTGTGCTGGTGCT

ACTGGAAGAATGCATACACGGTCGCGTATTGATGGCCACAGCTGTGGACGCTTCAAACTA

GACCAGGTAAAGACGACAGAACACGCATTACAAGACCATTACCGCTACATGCACTATCAC

AACCGTTACAAGGCTCATACTGAATCTATGGAAAAGGAAAACAATCTCAAGAAAAGCATC

CAACATAAGATAGCCATCTCAAAGATGAAGTTCAACAGAATGAAGGATTTCACGTGGGTA

ACGAATGGGCTGAGTAGGCTCTTCAGATCAAGGCGCGTACTTTCGTACTCCTATCCTTTT

GCATACTACATGTTTGGCGACGACCTGTTCAAGGATGAGATGACACACGAAGAGAGGGAA

ATAAAGCAGAATCTATTCGAGGATCAGCAGCAGCAGCTGGAATCAAATGTCGAGAAGCTT

TCTCTGTTCCTCGATGAGAATTTTTATAGTTCGTCAGAAGACGAGGTTATGTTCACAATG

AGGAATGTTGTTAATCTTTCGAGTGTCGTTGATAACATTTGCAAGCAGATGTACGAATGT

ATCGAGAATGAGTTATTGTATCCTCTCGAAACTGCAACTCATAATATCGCTCCATACAAA

TCCAATGGTCTTGAACGGGCGTCTGATCTCTCTGTTTGTCAGGAATCCGTACAGAGTAAC

GATCTACCAGGAAGACATTCTGGGCTCAAACGTTACTTGCCTACGAGTTCTGATGAGAGT

GAGAACTCTTCTCAGAAGCAGTCTCGTGTGGGGGCTGTGTCTATTAATCTTAACATGCCT

GACAGAGGCGATGGAGAAGAGCTATAAATATGTTCCATAGGCGCATATGTTGCAAGTGTA

CAGCGTCGGTTACTGGTGAGGTTATAAATAAATTAATAGGGTTGAATTTTTTTTGCTCAT

ATAACTTGTTGAAGCTTTTTCGGTACAGAATGGCCGGAAATAGAAACATAGGACGGTATA

TTTTGCTATAACTTATACTTGTCTAGGAATTTTGTGGCGTGTGTGTATATATATATATAT

ATATATATATATATATAT

>Unigene0001504

AGGGCCACGTTTGCAGCGTAAGTTGTACATTCTACAGACTAGAGCATGCATCTCAGGCTT

GTTTTGAGTGCTTCCGCGCGGGTATGTTGTAGGCGGGGTTTGTGAAAAGTTCTCGAGACT

TCTCTCTGTTTGCTCCACGATACGCGTCAGAGTGCTTCTACTCTATTCTGTTGACATAAT

CATCACGGGAGATGACGCCTACGATATTCAGTTTGTGAAGCACCATCTTTAATGATAGTT

TTAGATGAAGGAACTAGGCCTTCTTCACTACTATCTCGGTCTTGAAATCTCACAAAAGTA

CATTTTGGATATTTTGGAGCACGTAGACATGGTTGATGAGAATACTGGGATACTCTACAT

CATAACAT

>Unigene0001505

GACCGATTGAGGGTGAGCCTCTTGTGGATCCTACTCGCTATAGATACCTTGTCGGCCAGC

TTGTCATTCTCTGCATCACTAGGCCGACCATCTCTCACACAATTGACATTATGAGTCAGT

TTGTTACTGTTCTGCACACTAATCACTACGCCGCTCTTCTCCGGATCCTGAGGTATTTGT

GAGGCATAGTGACTCGCTCTCTATTGTTTCTTGATACTTCACGACTTGAGCTTTGAGCTT

ACATTATTGTTGATCGGGCTTGTGACCCTACTATGAGGAAGTCCACTATTGACTTTTGCA

TCTTCTTGG

>Unigene0001506

ACTCTACATCATAACATAAAGCTGAGACCGATTGAGGTTGAGCCTCTTGTGGATCCTACT

CGCTATAGATACCTTGTCGGCCAGCTTGTCAATCTCTGCTTCACTAGGCCGACCATCTCT

CACACAATTGACATTATGAGTCAGTTTGTTACTGTTCTGCACACTAATCACTACGCCGCT

CTTCTCCGGATCCTGAGGTATTTGTGAGGCATAGTGACTCGCTCTCTATTGTTTCTTGAT

ACTTCACGACTTGAGCTTTGAGCTTACATTATTGTTGATCGGGCTTGTGACCCTACTATG

AGGAAGTCCACTATTGACTTTTGCATCTTCTTGG

>Unigene0001507

TGTCAAGTACTACTAAAGAAATTGAACATCTCAGTGTTGATATGAGCATCCAAGTACACT

CACCCAACTCCTATTTATTGTGACAATAAAAGCACTATCAATGTAGCAAACAATTTAGTC

TTTCAGGAATGCTCCAAGCACATTGAGATCAATGCTTATTACGTCCGCCAATAATTACAG

CTCAAAGTCATACAACTACCTTATATTTCTACTAATGCTCAAATTGACTATTTTTTTTAA

ACAAAAGCTCACCCCATATCTTGTTTTCAATTCTTTGTAAGCACACTCATATTCGACCCA

CC

>Unigene0001508

TGTCAAGTACTACTAAAGAAATTGAACATCTCAGTGTTGATATGAGCATCCAAGTACACT

CACCCAACTCCTATTTATTGTGACAATAAAAGCACCATCAAAGTAGCAAACAATTTAGTC

TTTCAGGAATGCTCCAAGCACATTGAGATCAATGCTCATTACGTCCGCCAATAATTACAG

CTCAAAGTCATACAACTACCTTATATTTCTACTAATGCTCAAATTGACTATTTTTTTTAA

ACAAAAGCTCACCCCATATCTTGTTTTCAATTCTTTGTAAGCACACTCATATTCGACCCA

CC

>Unigene0001509

ACCACTAACTTTTGCATCTTCCTACGAGATCCACTGATTTCTTAGAAGAGCAAGAAGCAT

GATAAAGTTCACTCTCCAACACTGAATTAGAATATCATGCCATGTTAAGTGCTACCAAAG

AGATTGTACATCTTAGATGACTCGTTACTAATATTGGCGTTCCTGTACAGTCACCCACTC

CTGTACTGTGACAATCAAAGTGCTATCAAAATAGCAAGTAATCATGTCTTTCAAGAACGC

ACCACACATTGAGATTGACGCTCATTACGT

>Unigene0001510

ATTTCAGATGAAAGAAGAGTTTTGGGCCCTATTTGTTATTTTCTTGGACTAAATCTCATA

GAGCTCTCAGAGAATTATTCTCTCTAGCAAAAATATATCTTTGATATCTTGGAGAGTATA

ATTGATTGATGTGATGATTGTGTACACTTCTTTTCGGTAGAATGTAAAGTCGAGATTAAC

GGATGGCGAACCTATTGTTGATCTTACTCGCTATAGATACCTTGTTGGTCAGCTTGTCTA

CCTTTGTATTATTAGTTTAGACATCTCTTATTTACTTGGGATTGTGAGTATATTTTGCTC

CTTGTATTGATCACTCTATTATCCTTCGAATCCTGTATATCTATGAGGTATTGTAAAACG

CTCTCTGTTCCTTGCTAACTCATGTCTTGAGCATAGAGCTTACATCGACACTAATTGGGC

TGGCGATCCCATTATGAGAAAATCCACTATCGGTGTTTGTATTTTTCTATGTGACTCATT

GATTTCTTGGAGGAGCAAGAAGCAATGCATAGTTTTCACTCTTCAGTATTAAAGTAGAAT

GTCATGTTATGTCAAGTACTACTAAAGAAAT

>Unigene0001511

CGTTAGTCGTCCTCCTCCTCCACCTCCACCTTCGTCTCCATCTCCATTCCTCTCCAACCT

TTTCGATCAATCCAGAAAAACCAAATCAAACATGGAGCACAGAATGAAGCGCACACGGAT

CAAAGCCCCCACCCCTGAGATCTAAGACCCCTCCTCTCCGATCGCTTCCCCTCGGATCCG

CCACCGCTCCGGATCATGTCGAGCTACGGCGAGTTCAACCAGAGGATCGACTACGTCTTC

AAGGTGGTGCTGATCGGCG

>Unigene0001512

CAAGGTGGTGCTGATCGGCGACTCGGCCGTCGGGAAGTCGCAGCTCCTCGCCCGATTCTC

CCGCAACGAGTTCAGCCTCGACTCCAAGGCCACCATCGGGGTTGAGTTCCAGACCAGGAC

GATCGCCATCGATCACAAAACTATTAAGGCGCAGATTTGGGATACTGCTGGCCAGGAGAG

GTATCGGGCAGTGACGAGTGCTTACTACAGAGGAGCAGTCGGGGCGATGCTAGTCTACGA

CATGACCAAGCGCCAGTCATTTGATCATATGGCAAGGTGGCTGGAGGAGCTACGGGGACA

CGCTGACAAGAACATCGTGATCATGCTGATCGGGAATAAGTCGGACTTGGGGACCCTCCG

CCAGGTCCCCACCGAGGATGCCAAGGAGTTTGCTGAGCGCGAGAATCTCTTCTTCATGGA

AACCTCCGCTCTCGAGGCCACCAACGTCGAGCCCGCCTTCCTGACCGTCCTAACCGAGGT

CTACCGGATCGTCAGCAAGAAGTCGCTGGTCGCTAATGGTGATCCCGACTCAGGGGGTAA

CTCGGCCCTACTGAAGGGTACTAAGATTGTTGTTCCAGGACCGGAGCCACCAGCAAAGGC

TGCATGCTGTATGTCCGGGTAGCTGGCACAGACTGCCTTAGTCAGGCCCGAGGGATATGA

CTTTTGTTTCTCTTGAATCTGGGAAGTATTATTTTTCTGACAATTTTTCTGATTTTTTTC

ATTTGTTGCTACTGGATTTGCATAGGTAGCACCTGGTTTGAATTGTTCCAGGCTTTGTAT

ATGAATTTTTCCCATTTCCTGTAATTTAGTGAGAACACATGGAATGGTTTCAAAGAGATA

AAACAGTTGATTTCTTTTCAGTTATTTGGATGCTTTGTTATGC

>Unigene0001513

CACCCAAACACTCCCAACCTATATTTGCATCGCCGACCTCTCCCCCCTTTCCGCCGCAGT

CGCCTCCTCCGCTCATCCCAAGAGGGCTCGCCGCTCTAGCCCCCCTTCGCTCATCCCCCC

AAATCCGGTCGCCGGAGCCCGGCGGCGGAGTCGGCGGAGCAAATGGCGTACCGCGCCGAC

GACGACTACGACTACCTCTTCAAGGTGGTGCTGATCGGCGACTCTGGCGTCGGGAAATCG

AACCTCCTCTCCCGCTTCACGCGTAACGAGTTCAGTCTGGAGTCGAAGTCCACGATCGGC

GTCGAGTTCGCCACCCGCAGCATCCGCGTCGACGACAAGGTCGTCAAGGCCCAGATTTGG

GACACTGCTGGGCAAGAAAGATACCGTGCGATCACAAGTGCGTATTACCGAGGCGCTGTG

GGTGCGCTCATTGTCTACGATGTCACACGTCACGTCACATTCGAGAACGTTGAGAGATGG

TTGAAGGAGTTGAGAGATCACACCGACAACAACATTGTGATCATGCT

>Unigene0001514

AACAACATTGTGATCATGCTCGTGGGGAACAAGGCCGATTTGCGCCACCTGAGAGCTGTA

TCGACGGAAGACGCCAAAGCCTTTGCTGAGAAAGAGAGCACTTTCTTCATGGAGACATCG

GCCCTCGAGTCATTGAATGTGGAGAATGCCTTCACTGAAGTTCTGACACAGATCTACCGT

GTCGTGAGCAGGAAGGCCCTTGATATAGGTGAGGACTCAACCACTCTACCGAAGGGACAG

ACCATCAATGTGGGGTCGAAGGATGATGTTTCGGCGGTGAAGAAGGTGGGTTGTTGCTCG

GCGTAGATCTAGTACATGTAATTTGGAGGCATTGGTTTTTATTCTGGACTTTTAAGTTTT

TGGACTCTGTGTGTGTGAGAAGTTTGTATTTTATGTTAAACTTTTTTTGAAGGGAAGTTT

GGAATCATCAGTGTCACAGGTTTATGTTAGGGCATGTTGTTATCTTCTATATGCAGTTTA

TATCTAAGTAGAATATCAATTTTTTTTTGCTGGTGGTTCTTGATGTCCTATGATTCATGT

TTG

>Unigene0001515

CACCAAACACCACTCCACCCATCTCCATCTTCCAAGACAAGCAAATCCTCGCCCCCTTCA

AATCCCTCCCCTCCAATCCTCCCCACTCACAATTTCACCCCCATGTCCAATCCCGACCTC

GAGCGCGGCGCCAAGCCTGCCCGCCGCCCCGGCCCATACGGCCCCGCCGCCAGCAACGGT

GGCGCCTTCTACTACGATGGCGGCGCGGTGGCGGAGCGCGGCTGGACGTCGTGGCTGGTG

CCCCTGTTCGTACTGGCGAACGTCGCTATGTTCATCGTGGTGATGTTTGTCAACAACTGC

CCCGGTCATACCAGCCGCTTCGGGTCATGCACAGCCAGGTTTCTTCATCGATTCTCGTTT

CAGCCGCTGAGGGAGAACCCGCTCTTGGGTCCTTCTTCCAATACATTGGAGAAGTTGGGA

GCACTTGAATGGAACAAGGTGGTCCATGAGCATCAAGGATGGAGACTTGTGTCCTGTATT

TGGTTGCATGCTGGTGTTGTGCATTTGCTGTTAAATATGCTTAGCTTGGTTTTCATTGGC

ATTCGTCTTGAGCAGCAATTTGGATTCATGCGAATTGGGGTAATATACTTGGTATCAGGG

CTTGGTGGTAGCATCCTCTCATCGCTGTTCATTAGAAACAGTATATCCGTTGGTGCGTCC

GGTGCATTGTTTGGTCTTCTTGGAGCAATGCTCTCGGAACTTATCATAAACTGGACTATC

TACAGTAATAAGGCCGCAGCTCTCTTTACTCTTCTGGTTATCATTGTTATAAACCTGGCT

GTTGGAATACTACCACATGTCGACAACTACGCCCACATTGGAGGGTTCTTGACGGGGTTT

CTCCTTGGATTCATTTTCTTGATCCGACCACAGTTTGCTTGGTTGGAACGCCATAATTTG

CCCCCGGCAAGTCAGGTCAAGTCGAAGCACAAGACGTACCAGTACATACTGTTGGTGATT

GCCCTGCTTTTGCTTATTGCTGGATTGGCAACTGGTTTGGTAATGCTGCTCCGAGGGGAG

AACGGGAACGATCACTGTCATTGGTGCCACTATCTGAGCTGTGTACCGACATCAAGGTGG

AGCTGCGGAAACTGAGAAGAATGCATTTTTCACATAGCCGGTAGAAAAGAATGAATGAGA

ACATATTACGTATTATTATTCCAGTGTGGCATCATACATTTATTCGTTTGTACATTCTGA

AGATTGGGCTTTTCTTGGGGG

>Unigene0001516

AAAAGTGTGAACACACGTTGTCTCCACTCACCTACTCCATCCCCACTTCTCTTTCCTTCT

AATTCTCCTGGCCTTATCTCCTCAACTTCAGCTCTCATCAAGCAGCAACAGTCACAGGAT

GGATCCACCTTCTTCCCAAACAATGTCCACTGATGACACTCAGGACAAGATCATCGACGA

CTGTTGCTCCTGCTGCTATTACTGTGCTGACAGCTGCTTGGATACGTACTGCTGCTCTAG

CTGCTAGCGTCTCTGTTCTTCTCTCAATCTTGGCTGTTGTGACAGAATTATATTATTCAG

GTGATGTTTGGACTTTGGATTGTGCTTGCAAGATTAGTTTATGCATTTTAGACGAGTTTT

CTTTATGATCTCGGCAAACTGAAAACTAATAAGAGGAATTTAAGTT

>Unigene0001517

AACGACGGAGTCGATGGAGTTCGATCCCCCGACAAGCAACCACTCCCCTCATCATCATCG

CCGTCTGCGCTTGCAAGCGACGTCGACCCCCCAACAGCTCCGGCCATTTACTCTCTCCTC

CTCTACAGCAAGATATTCCATTCTCACTTGAGATCTCTGGCGATGGGGGCGGCGGTGGGT

GTGTTTCTCCTCCTGCTGACCGCCGTCTGCGGGGTGGTGGCGCAGATTGATAGCTGCGGA

GGCATGGATCCGAAGGCTTTTCTCGCGGCGCCGTTTAATGAGACCGTGTTCACCTGTCGC

CCAATGTGGAACAACTTCTTTCTCAGGTACTACCAGGATAATGATAATGTTTTGAGCATT

GTCTTGTCCACTGTATATACAACCGGGTGGGTTGGAATGGGATTCTCAAAGGATGGGATG

ATGGTTGGATCCAGTGCGATGGTCGGCTGGATTGGTAAAACAGGTCTAGCCCATATCAAA

CAATTTGATCTACGTGGTCAAACTAGTTCACAAGTTGTAGTAGATCAGGGACAACTGCAA

TCCACTGGTGCTTCACCAGCAGTGGTTCTTTATGGTGCCGACATCTTCTTGGCATTCCAA

TTGAAATTTTCGGCCCCTGTTACCCGGCAGAACCTTTTGTTTGCTATTGGGACTGCTATT

CCAGTTAATAATGTACTGCAAAAACATACCGACAAAGTATCTATTTCCTTTGATTTTTCA

GCAGGTACTACTTCTGCTTCATCCTATCCATATCAGCTTAAAAGGTCACACGGAGCACTT

GCCATATTTGGGTGGGGTGTGCTTCTACCAATAGGTGCCCTAGTTGCAAGATATTGTAAG

AGATGGGATCCTTTATGGTATTACCTTCATGTTTCCATCCAGCTTATAGGATTTATCATT

GGGCTTGCTGCTGTGGTGGCTGGAATTTCGCTTTACGATAAACTGCATGCCGATATCTCA

ACTCACAGGGGACTCGGCATTTTCATATTTGTTCTAGGAATCCTTCAGGTGATAGCTTTT

TTCCTACGACCCGACACGGACTCCAAGATCCGTAGATACTGGAATTGGTACCACCACTGG

TCAGGGAGGTTCGTTCTCTTCCTTGCAGTGGTGAACATATTTCTTGGCATTCACGCCGCA

GATGCAGGGACTTCTTGGAAGGTCGTCTATGGTATTAATCTTGCAGTCCTTTTATTGGCA

ACTATTATCCTAGAAGTTCTCTTGTGGACGAGATGGTCGAGGAAAACTGTTACGACTACT

ACTACAGCATTGTGAATACCACCTGATTATTTTGCTCTCAGTAAGGCATAGTTTCTCTTG

CATCCGTGTTGAATAGATTTCTTTTATGTATTTTTTGACAATTATGTTTTCCAACCAGCA

CTTGCGATACATTGTTGATTTTCTCAGTTTTCTCTTTTCCTCAACATTGTTGATTGGAGT

TTAATTTGTTGTCCTGCGAAAAGATTTTCGTTTGAATTAGTTGCATATGTAGATGTCACA

GTTTTCCTGTATGATATTTATCTCAAATAGTTGCATATGTAGAT

>Unigene0001518

TCTGTTCACTTCTACTGCTTGCGAGCTCTGGAGATGTCGAGTGAGATATCGAGCTCGTTT

TACGGGAGACTGGATCCACCGGTGCCGGAGGTGTGGCCGTTCCCGATCCCGCCGTCTCCG

ACGATCGCCGCCTGGGCCGGCGACCGGGAAGCGGAGTTGATCCTCTCGGAGCAGAGCGGC

CGGAAGAAGCGGAGGGTCTCCGCGGCCCCGACGATGTCGGAGGACGATTCCTCCAAGCTC

GCCTCCACCAGCAGCGGCAATGATCCGACAGTTTGTGAAGTTAAAAGACCAAGAAGCAGC

AGGAATTTGAAAGCTGAGGCTGAGGCAAGTTCAGGTGTCTGTAATAAGCCAATAAAGAAA

AATGCTCAACCATCCGAAGCACCTAAACCAGATTTTATCCATGTGAGAGCAAGAAGGGGT

CAAGCTACTGATAACCACAGCCTTGCAGAGAGAGCTAGGAGAGAAAAGATAAGCGAACGT

ATGAAAATTCTTCAAGGTCTGGTCCCTGGATGTAATAAGGTGATTGGCAAAGCATCGGTC

CTTGATGAGATTATAAATTACATCCAAGCATTACAGAATCAGGTTGAGTTCCTCTCCATG

AAGCTCGAAACTGTTAATTCCCTAATGGATATTGGCATTGAAGGGTTCCCTCCAAAAGAT

AGAGGGCCAGAGGATTCATATGGAAATCTGAATTTCACAACAATGATGTTGCAACATCCT

TCACCATCTGAAGAGAGAGAACCAGAGAGGTATTCCCTAAATTAAATTTGAAATTTGTTT

TTTTTTCACTTATCTAGATTCAAGATGCTACCTTGATTGTTCATGGTGTTGTGTTGTACT

CATGGGCTTGTTGATGCCAAGTTCAGACTGATGTTATGAGTTAGTAATGATGTGTAGAAA

AAGGAACCGTTTTCTGTAACCCCTATGTTCCTGAAGGGAACTTTCTGTTGTTGGGAAAGA

GATGATGAGCTACCCCCCTTATGGTCTTTTTGATGCTGGTCTAATAAGCTAATGGAACAA

AGTCAATAGCTTTGTCTATTATTTGAGTAATTGTAAAGGTATTTAAATTTACTCCTTGGT

TTCTTTGACAATATGTTACCGGTTATTG

>Unigene0001519

CTCTCTCTCTCTCTCTCTCTCTCCCCATTAGTGGCGATGGCCGGCTCCATCCTCGCAATG

GCGCTCGCTGTCCTCACCGCAGTCGCCGTCGCTCAGTCCCCGGGAGCCGCGCCCACCGTC

TCTCCTCCGAAGCTGGCCCCACTATCGCCTCCGACTGTCGCCCCCATCTTGGCGCCGACT

CCATCCCCCGTCAGGCCACCCACCACTACCCCCGCTCCGGCCCCCGCCGGCAAATCGCCA

TCCTCCGCCCCGGCTCCATCTTTACCTGCTCCGGCATCCCCGCCTGCTCCTATTGCCTTT

CCTCCCTCGATTGCCGGTCCTCCTGCGGACCCCCCCACCACACCGGAAGGATCCAGCGCT

GCTACCCTGGCCGCTGCTAGCTGGATCGTTGTCGCCGGCGCAATCGCTGTTGCCTGTGCC

TTCTAGTTTGTTGACACAGGCGGCTCTACATGGTGCTCACTCGCCTCCGACGATTACGTG

GATTCATTTTCACCGGATTAAGATTGTATTGTTTATGATCTTTTATAATTAATAGTTGTT

TATGATTTGTATAAATATTTGAGGTTGGCTTGACATGACATGGTATAGTTTTCCAATATT

TTTGGGTTACAT

>Unigene0001520

AGCCTATATAGATGGTTCTCCTTGCTTGCTTCAACAAAATCTTCCAGTTGGAGCATACAA

ATCTCTTCCTCCAAATTTTCATAAAGAAAAATCATCTTTATATCTATTTATTGGTGTAGA

TCCTCAACAGTCACAATGTTAAGTGTTGTTCTGATTGTATTAAGCTTCATAATTTGGGAT

AAAATCCCAGTGAAACTAATACTTGATCTCTGCTAAAAATCTTTCACTACTAGTCTTACC

TTATATTACAAACTTCCATCGGGCACCGTCAACTGGTATACTTACCTATTATAGAGTGCC

TTATTGCCTACTAGAAGATTACCATAATCTCATATAT

>Unigene0001521

CTTCCTCAGAGCTTGGTTCCCCCATACAAACTATTTTAGCCTATACAGACGGTTCTCCCT

GTTTGCTTCAGCAAAATCTTCCAGTTAGAGCATGTAAATCTCTTCCTCCAAATTATCATA

AAGAAAAGTTATCTCTATATCTATTTATTGGTGTAGATCCTCAATAGCCATAATGATAAC

CCTTATTCTGATTGTATTAAGCTTCATAATTTGGGATAAAATCCCTATGAAATTAATACT

TGATCTCTGTTAAAAATATTTCACTACTAGTCTTACCTTATATTACAAACTTCCATCGGG

CTCCTTCACCTGGTATACTTACCTATTATAGA

>Unigene0001522

TCTCTACTTCATAGAAACTGTGTGTCTCGTGATCATTGCATCTGTAACGAAACTTATCAC

CTCTATAACTAATAAAACTATAATATCTTAACTTAGGATCAAGCTTGGACTTTTTTTATC

AAACTCACCAACATATATCTAGTGCGACCAAGCTCTTATTGGTGAGAAAGAGATATATCT

TTGCCCATCTTATACTTTTTCAGAAAATCTCATATCTAATGTAATTTTAGGTCTATGATT

AATAAAGTATATTACAGTGTTTATCATTTCAGGCCAAAATAGTTTTGGTAGACCTAATGG

AGACTTATACTCCAGATATGCTCCAATATAATATAATTCATCTACTCTATTATGTCGTTC

TACTGTGGTTTGTCGGGAACCATCTTTTGTATACAAATCTCATTTGTAACATAATAGCTC

TCAAATTCGGCACTACAATACTCTCCTCTATTATCGGACTTGAGATACTTCAACTTAGCA

CCAATTTATTTTTCTACAACAAG

>Unigene0001523

TGCTGGGAAGCAGCTGGAGGACGGTCGCACACTTGCTGATTACAACATTCAGAAGGAGTC

GACGCTGCACCTTGTCCTCCGTCTCCGTGGTGGCCAGTAATTTCTCTATTCTGATCCTTG

TTGGTTACTTCTCTGATCTGATGCGTGGTTTTGGTTCTCCTTTCTTATGTTCATCTGTGG

TTTGGTAGGTAAGGTCTTGTGACTTGTCCTTTTAAGTTTGGTAGGTAAGGTCTTGTGACT

TGTCCTTTTGAGTACTCTTTGGTTGAATAATAAGTTTGAGATTTGGTTACTGTTGAAATT

GCACAAGTACTTTGAGATCTGTACACTGTTGAAATTGCACTCTATATTTACCTTGTAGTG

CGGTACAGTATATCAATGTCC

>Unigene0001524

TGCTGGGAAGCAGCTGGAGGACGGTCGCACACTTGCTGATTACAACATTCAGAAGGAGTC

GACGCTGCACCTTGTCCTCCGTCTCCGTGGTGGCCAGTAATTTCTCTATTCTGATCCTTG

TTGGTTACTTCTCTGATCTGATGCTTGGTTTTGGGCCTCCTTTCTTATGTTCATCTGTGG

TTTGGTGGTTAAGGTCTTCTGACTTGTCCTTTTAAGTACTCTTTGGTTGAATAATAAGTT

TGAGATTTGGTTAGTGTTGAAATTGCAAAAGTACTTTGAGATCTGTACACTGTTGAAATT

GCACT

>Unigene0001525

GTTCTTTCCCCTTGTGCGGCTTCTCTCTCACTTGAGGGTGAGCACGCTGTTCCCCTACTC

ACAAATCCCCCCCCCACCCAAGCAACCGCCATCATCGTCTTCGTCCCAGGGAGTCCTCAG

AACCTATAAGAAGCAACTATTAAGGGATTCAGATTGTTTTCAGTCTTTGGTGCGGAACAA

TGGCAGCTGGTCCTGGACTTGAGTCACTTGTTGATCAGAATATATCAATCATCACAAATG

ATGGACGTAATATTGTGGGAATTCTGAAAGGCTTTGACCAGGCTACCAATATCATTCTCG

ATGAATCACATGAGCGGGTTTACTCTACAAGGGAAGGTGTACAACAAATAGTTCTTGGGC

TGTACATTATCAGAGGGGATAACATAAGTGTTGTGGGGGAAATAGATGAAGAGCTTGACG

CAGAGTTGGATTTAGATAAACTGAGGGCGCATCCCCTCAAGCCTGTGATTCACTGACCCA

CAAGCTGTGAAAGTCTCTCCAGAGACCAAAATATGCCCTCGGTTTCTTATGATAGTAGTA

GAAGAGAAAACGTGCAACTAGATGGGGCTTATTTCAGAAACCATTGTCTTATGATATAAA

GATTTAGCAAAAGGTTCGGTTAGCAGATATGACTGTAGCTTTTAGACATTGCAAAATTGG

AGTATCAAATTCTAGGACTCATTTTATGGGGAATATGCGGACTTTGGTGATCTCTTGACA

TCAGAGTTTAGCTGTTTACCTGATTTGTGGGTAAGGAGACGGCCAGCGAAGGGGAAAAGT

TAGCTGAGCCCTATCTAAAATGTGATGAAAACAGATTAAAAAAAAAAAAAATTACTTCTG

GTATTCTTCTTGTTTATGAATAACTGTCTATCTGGTATTTTATCTTGGTTGAAGAATAGC

TGTTATGATGAATGGATTCTAGGATGTTCATATCCCAGATTATGTTAAATCCTTAATGGA

GCAATGATCGGACCCAAAATAAC

>Unigene0001526

ACGGAGCATCATCTCCAAGATAGATCCGGTCGAAATTATCGACTACCATCCCCTTTGCAT

GAAAGGAAGTACCTGAATCCACGATCCATGAAACTGAGTCTTTGACCAAAGTTAACACCA

TAGCATTATCCTCCGCTTTTAGATCAACCACGATTGTAGTTTTCTTACCCTTCGGATCCA

GAACTGAGCTTTGCTTCCCTTGAGCCTTTGTTGACCAACAAACTCTAGCAAAGTGACCAG

GCTTCC

>Unigene0001527

CCCGCCACGAGCCACCACAAGAGCCTTGCTTGAAATCCACTATAAGTCTCCGTAGTAAAA

GTAGTCATGTCCCACCGAGATGAGATTTCGCTTCAAATTCAGAACATCACCTCCTTCGAC

CACAATCTTGAGCCATTTGCCAACACCAACTCATAGTCTCCCTTCCCATCGATATCACAC

AGAGTATTATCTCAGAGATAGACTCGACCGAAGTCGCCCTCCCGGAAGTTACACAACCGG

CCGACACTCGTCACATGAAAGAAAACTCCTGAATCCACGATCCAAGATTTTGAGTCTTTG

GCCAACATTAGCACCATGGCATCATCCTGCATCCTCTGCTCCACCATAGTCTTCTTGCCT

>Unigene0001528

CCCGCCACGAGCCACCACAAGAGCCTTGCTTGAAATCCACTATAAGTCTCCGTAGTAAAA

GTAGTCATGTCCCACCGAGATGAGATTTCGCTTCAAATTCAGAACATCACCTCCTTCGAC

CACAATCTTGAGCCATTTGCCAACACCAACTCATAGTCTCCCTTCCCATCGATATCACAC

AGAGTATTATCTCAGAGATAGACTCGACCGAAGTCGCCCTCCCGGAAGTTACACAACCGG

CCGACACTCGTCACATGAAAGAAAACTCCTGAATCCACGATCCAAGATTTTGAGTCTTTG

GCCAACACTAGCACCATGGCATCATCCTACATCCTCCGCTCCACCATAGTCTTCTTGCCT

TTTAGATCCAGAACCCGGTTCTTCTCTTCTTGGGCCTTCA

>Unigene0001529

CAGTCTCTAGCAAAGTGACCAGGCTTCCCACATGTGTAGTACTCTCCCTCGCTTTTGCTC

TTTGATCGCGACTTGCTACGATGGTGATGTCCTAAGCGATGTGCGAGAGACCTATCATCT

CTTCCCCTCGGTCTTCCCCTTATTTCAACACTCAAAGCGCCATGACTCACTGATGCATCG

AGCCCGTAGAAGCTCTCAGTGAGTCTTTCCCAATGATAACTGAGACCATGTCTCCAAAGT

TTAATTCATTCCTGCAATTAGCTGTACTAAGAGTCATCAAGAAGTTCTAATCCTTTAGGA

GTCATGAAAACAATGCAAGGATGTGCTCAATGAACTTGA

>Unigene0001530

TCTCCATACCAATAAGATCTCTGGCACAATCCCCTACGAAATAGGAAGCCTAGTGAATCT

AACTACTTTGGATATCTCACATAACCTTCTAACAGGTACCATCCACTCCAACTTTGGAAA

CCTCACAAGACTTGAACACTTCGGTCTCGCCCAAAACGAGTTATCAGGCATTCCTCGAAA

TTTGAATCAATGCACAAGTTTAATTCGGGTCCGCCTTGATGGCAACCGACTTAAAGGGAA

CATCTACAATC

>Unigene0001531

TCTCGATGACCTCAACTTCTCTGCCATCCCATCTCTCATCGTACTCAATCTCCGCCGCAA

CCACCTCTCCGGTTCTATCCAGCCCAGCATCGGCACACTTTCTGTGCTCACCTCTCTCAA

CCTCGCCCGCAACCATCTCTCTGGCAGTCTTCCAACTTCTCTAGCCAATCTCCAGAAGAT

AAGAGATGTTGATCTCGCTTTCAATAGCTTGATAGGTGAGTTGCCTCCTGAAATCTTTGC

TAACTGGACCCATCTTCGCATTCTGGGCCTATTCCAAAATCAGCTCTCTGGTTCAATACC

TGCAGAGATAGGCCAACTTGGTAGCCTAAACTGGTTGGGGTTATCCTACAACAATCTCAC

CGGCCCAATCCCTCCATCTCTCTCTAATTTAACCGAGCTAAGAGTTTTAGCCTTGGG

>Unigene0001532

TAATGAACCTAACCGACTTATGGTCATCACACAATTTTCTCACGGGCTCTATCCCTTCCA

GCTTAGGAAACCTAGTGAACCTTGACGCGTTGGTGATCTCAAATAATAGTCTCACAGGCT

CTATCCCTTCCAGCTTAGGAAACCTAACCATTCAAATAATGCTTCTCCATGATAACATGC

TGTCGGGCACCATCCCACTTGAAATAGGAAGAAACCTTCAAATACTTGATATATCGGAAA

ACTACTTCTCCGGTTCTTTGCCAGCTCATATTTGTAAGGGAGGAGCTCTTCAAATGCTAT

CAGCAGTAAACAGTCATCTTGATGGTCATGTACCAAGGAGTTTGAGAGAATGCACAAGCT

TAGTTCGGGTGCGCTTGGATGGGAACTTGCTCACCGGAGACATC

>Unigene0001533

ATCTCAAATAATAGTCTCACAGGTTCTATCCCTTCCAGCTTAGGAAACCTAACCATTCAA

ATAATGCTTCTCCATGATAACATGCTGTCGGGCACCATCCCACTTGAAATAGGAAGAAAC

CTTCAAATACTTGATATATCGGAAAACTACTTCTCCGGTTCTTTGCCAGCTCATATTTGT

AAGGGAGGAGCTCTTCAAATGCTATCAGCAGTAAACAGTCATCTTGATGGTCATGTACCA

AGGAGTTTGAGAGAATGCACAAGCTTAGTTCGGGTGCGCTTGGATGGGAACTTGCTCACC

GGAGACATC

>Unigene0001534

TCTCGATGACCTCAACTTCTCTGCCATCCCATCTCTCATCGTACTCAATCTCCGCCGCAA

CCACCTCTCCGGTTCTATCCAGCCCAGCATCGGCACACTTTCTGTGCTCACCTCTCTCAA

CCTCGCCCGCAACCATCTCTCTGGCAGTCTTCCAACTTCTCTAGCCAATCTCCAGAAGAT

AAGAGATGTTGATCTCGCTTTCAATAGCTTGATAGGTGAGTTGCCTCCTGAAATCTTTGC

TAACTGGACCCATCTTCGCATTCTGGGCCTATTCCAAAATCAGCTCTCTGGTTCAATACC

TTCAGAGGTAGGCCAACTTGGTAGCCTAAACTATTTGGTTTTATCCTCCAACAATCTCAC

AGGCCCCATTCCTCTGTCCCTTTTTAATTTAACCAACCTCGGATTTTTATTTCTGGATGA

TAACAACTTGTCTGGCCATATTCCCTCGGAACTAGGAATGCTTAACCGGCTAGTTTGGCT

GAAGTTGTCTGGAAATCTTTTCAATGGTACGATCCCACGAGAAATTGGAAACCTAATGAG

CCTAAGCTATCTGGATATCTCAAACAATCTTCTCTCCGGCTCCATTCCTTCTGGTTTAGG

AAATTTGACAAGTCTTAACACGTTTCTCCTCTATACCAATTTTCTCTCTGGCACAATCCC

ATGCGAAATAGGTAGCCTGGTGAACCTAATCTACTTGGAGTTTCTGGACAATCTTCTAAC

CGGCTCTATTCCTTCCTGCCTAGGAAACTTGACCGGTATTAAGCTGATGTATCTCTCAGG

GAATAACTTATCCGGCACAATACCACATGAATTAGGAAATATGATGACCTTAACCGACTT

ACGTTGCACAAACAATCTTCTCAGCGGTGTGATTCCTTCCACTTTAGGAAATCTTACTAA

TCTTAACATTTTGGTTCTCTATAACAATCTTCTCAGCGGTGTGATCCCTTCCACTTTAGG

AAACCTTACTAATCTTAACTTTTTGTCTCTCTTTACCAATAGGATCTCTGGCGCAATCCC

ATACGAAATAGGAAGCCTAGTGAACCTAACTAAGATGGATATCTCAAATAACCTTCTAAC

GGGTACAATCCCCTCCAACTTTGGAAACCTCACAAGACTTG

>Unigene0001535

AAATTGGAAACCTAATGAGCCTAAGCTATCTGGATATCTCAAACAATCTTCTCTCCGGCT

CCATTCCTTCTGGTTTAGGAAATTTGACAAGTCTTAACATGTTTTATCTCGATACCAATT

TTCTCTCTGGCACAATCCCATGCGAAATAGGTAGCCTGGCGAGCCTAAGCTTGTTGGACC

TTTCAGGCAATCTTCTAACCGGCTCTATTCCTTCCTGCCTAGGAAACTTAACTAATATTA

AGAAGTTGTATCTCAGTGAAAATAGCTTATCTGGCACAATACCACATGAATTAGGAAACC

T

>Unigene0001536

ATTTGACAAGTATTAACGCTTTGTATCTCTTTAAAAATAAAATATCTGGCACAATACCAC

ATGAATTAGGAAACCTTGTGACCCTAACCCAGCTGGATATCTCAGACAATCTTATCAGTG

GTGTAATTCCACCTCATATAGGAAACCATACCAATCTTAACCTTTTGAATCTACATACCA

ATAAGATCATTGGCACAATCCCACATTTATTAGGAAACCTAGTAACCCTAACAGACATGG

AGATCTCAGGCAACCTTCTCACCGGCTATATTCCTTCCAGTTTAGGAAACCTTGTGAACC

TAAAGATTTTGATAGTCGCAAACAATCTTCTCACCGGCTCTATTCCTTCCAGCTTAGGAA

ACTTGACTAATATTAACCATTTGTATCTCTTTGAAAATAGCATATCTGGCACAATACCAC

ATGAATTAGGAAACCTTACTAATCTTAACATTCTGCTTCTCTATACCAATCAGATCTTTG

GCACAATCCCA

>Unigene0001537

CGCGCCTAACAAATCTGGATATCTCTGACAATCTTCTCACCGGCTCGATCCCTTCTAATT

TAGGGAATATGACACGTCTTTATCTCTTGGATCTCCGTAATAATATGCTTTCCGGCACAA

TCCCATACGAAATAGGAAATCTAGCGAACCTATTCATGTTTGACATTGCAAACAATCTTA

TCACCGGATCTATTCCTACCAGCTTAGGAAACTTGACGAGTATTAACACTTTGAGTTTCT

TTAAAAATAACACATCTGGCGCAATACCACATGAATTAGGAAATCTTATGACTTTAACCC

AAGTAGATATTTCGGCCAATCTTCTCAGCGGTGTGATACCTCCCCATATAGGAAACCTTA

CAAATCTTAAAATTTTGTATCTCCATACCAATAAGATCTCTGACACAATCCCACATTTAT

TAGGAAGCCTAGAAGCCCTAACAGACATGGAGATCTCAAACAATCTTCTCACCGGCTCTA

TTCCTTCCAGTTTTGGAAACCTTGTGAACCTAATGTTTTTTATTGTCTCAAACAATCTTC

TCACTGGCTCTATTCCTTCCGGCTTAGGAAACTTGACTAGTATAAACTATTTGCTTCTCT

ATAAAAATAGCATATCTGGCACAATACCACATGA

>Unigene0001538

GAAACCTTACTAATATTAATATGTTGCTTCTCCATACTAATAAGATATTTGGCACAATCC

CACAAGAAATAGGAAATCTAACAAGCCTAACCGATTTGGAGTTTGGATTAAATCTTTTTA

GTCGCTCTATTCCTTCCAGCTTTGGAAACCTAGCGAACCTAACATTTTTGATGATCTCAT

ACAATCTTCTCACGGGTTCTATCCCTTCCAGCTTCCGAAATCTGACAGCCCTTCAGGAAT

TGTTGATCACCGGGAACATATTGTCGGGCACCATCCCACTTGAAATAGGAAGAAACCTTA

TTAGACTTGATATGTCATACAATAATTTTTCCGGTTCCTTACCAACCGATCTTTGTAAGG

GAGGGTCTCTTCAATAC

>Unigene0001539

GGAGAAGAACCGGACAATGGGCGGAGGCGACGGAGCCCAGGAGAGGAAGGGCACCGCCAC

CGCCACGCCAGAGGCGGTGGTACCGTCCTCGGCCTCGGCCGCCGCCTCGCCGGCGTCGGT

GGTGCCGTTCTCCTCCGCCGCGATCGATATCTCTCTCCCGCTGCCCGAAATGAAGCCTCA

CATCGTAGATCTGTGCAAGGATCTGTTCAAGAAATGGTCATGTCTCGATGGTTCTTGTTT

CTCGATAGAGACGGTTTCTGGCGGTATCACGAATCTATTGCTGAAGGTATCCGTGAGAGA

CGAAAATGACAATACATCTTGCCTGACCGTGCGATTGTATGGCCCAAACACCGACCTCAT

AATTGATCGGAAGCGAGAATTGCAGGCCCTACCATACTTGTCAGCTGCAGGGTTTGGCGC

GGAACTACTTGGAGTATTTGAAAATGGGATGGTTCAAGCATTCCTCAATGCTCGTACGCT

TTTGCCATCAGACATGAGCAACCCTAAGATAGCTGCTGAAATTGCATCGCAGCTCCGCAA

GTTCCATCAGGTGGTCATACCTGGTTCCAGAGAACCGCAACTGTGGAATGACATTTTCAA

GTTCTTTGACAAAGCTGCGGCCTTAAGCTTTGGTGACAATGAGAAGCAAGCCAAGTACGA

AAAAATTTCATTTCAAGAAATCCGCCAAGAAATTATTGCACTTAAGGACTTGTCCGATCG

TCTAAATGCTCCGGTGGTATTCTCCCACAATGATTTACTTTCTGGAAACTTAATGCGGAA

TGATGAGGAAGAGAAACTCTACTTCATTGATTTCGAGTATGGATCATACAGTTACAGAGG

CTTCGACATTGCAAATCATTTCAATGAATATGCAGGGTTCGACTGTGACTATAACTTGTA

TCCTGACAAGGATGCACAATATCATTTTTTCAGAAATTATTTGCAATCTGAGAAGCCACA

TGAGGTGCCTGACAAGGATCTGGAAGCCCTTTATGTCGAAACAAGTACTTTTAGGCTAGC

CTCACATATTTACTGGGCTCTGTGGGCTCTTATCCAGGCGAAGGTCTCCCCTATCGACTT

CGATTATCTCTGCTATTTCTACCTTCGGTACAACGAGTATACGAAACAGAAGGAGACCTG

CTTCGCATTGGCACAAAAGTACCTCTCTGCCTCACTTGCTGAATGATATCGAATTACAAG

GATTGCATACCTGTGTATTGTACACTACTTTTTTTGAGAATAGAGGCGACCGTCTCACTT

ACGGTCGCATTAAATTTGTTTGGATTTTATTATGAATAGCATGTATGTGTAACACCTCTT

GTATTATCGACTAGTCGTTCCTGAGGCCATGGAGGCTCAATGAAGATTGGTGCGGATGAT

TGATGCGCTCAAGCTTGTCTTCATGGTCCATAGACGAGATTATTTATCTCTAGTTATACC

CTTATTTTTATAGGAGTATCTCTACTAGAGTGTAATGTGGAGATGACATTCAAATACATT

TTTGGGTTTTTTTGTTGTAATTGATGAATAAATCCAATACTTGTTTTCTTATATTGTTCT

GAAGAACCTTTTGATTTATTATCTCAACTTCATCGAAATTGAATCATGTCACAGAAAA

>Unigene0001540

CTGACCAATGTTGAATCTTCTTGACCGCCCTTTGTCCATGTCTAGATAATTTCATAAGTT

ACTACTTTATTCTGTATATCTTTAAGTATTGTAAATTTTTAATCCTATAATTGCACTGCC

ATTAATGGTTATTCTGACAACACATCTGCCTCTTCCTTCCTATATAAGTAACATTGCCCT

TAAATTTAAGTAAAGTTGAATAATCTTGTAATTTAAGTAAAGTTGAATATTCTTGTTGAC

AAGGAGCGATTGCCTGCCTAAAAGACTATATGCTATAGGGCACCTCTAGAATTTTTGTCT

TCTATATGGTTCTATTTGAATCTCGTAACTATTTTATAAACTCACATTATATTTTCATAC

ATTAAGGACAAATTTTCTTT

>Unigene0001541

TGGAGAGAGATGAGAAAGAAAGAGAGATGGAGAGAGAGGGAGATGAAGATTGGAGAGATG

AGTTGGTGGGGTAATCCGTGGTGGCAGAGCCGCAGTGACGAGCGGCGGCGGCCGTGATTG

TGGCTCACGGCTGCGAGGGATGGAACTTCAGTGAACCTGGGCAGCGATCCCAGGTGAGCC

TTCCCCGAATTGCTGTAGATTTGTGAGTTTTTTTTTTTACCCTTCTTCTTCACTTCTACG

GAAACCCTAAATCCAAAATCACCACTTTGGCAGCCATCCTTTATCATAATTAGAGCATAG

ATAATGAGTAAGTTCGCTGATAAGCTGCAAGAGAGGGTGGCATCAATTACATCCATGGTT

TGTAATGAATTTTGTTAGTTGGGAACCATTTGGAATTATGCTTGAATACTCACGAGACAA

TTTTCACAAGTGTAAT

>Unigene0001542

AAAAGAGTATACATGAGATCCCAAGAGAGTGGGTGGTGCATGAGCCTGAGAAAGTGGGCG

ATTCAAGAGCTTAAAGAGGAGAGAGATGAGGATAAGGGAGTCAGCAGCGAGGGATCAAAC

AAGAGATATGGGAGCCCAAATGGTAGGCAATACAGGAGTACAAGAGTGAACAAGAACGCT

AGAGAGTGTCACTGTGTGTGCTAGAGTACCATAGAGTATAATTGGATGCGTGGGAGTACT

AGAGAGTGCTGCTCTGCATGTTAGATTATTAGAGAGCATTATCCTGATTATGGGTCACCA

TAGAATGAGAGCTTGAGTGAGCGGATGGTACAAGAGTTTGGAGAGCTTAAGATACGAACG

ATACAAGAGTTTTAAGAGCGAGTGATATGAGGGATTAAATATGAGAACTCGGCTTGAATG

CCAAGATGAGTGAGAACTTGTGTGAAAGCACTAGAGTGAGACTGAATATTAGAGCATGCA

TTTGAGAGTACAAACACATGAGTGCTAGAACCTATGTGTGTTAGAATGCATGGGCAAGAG

ATGGTGTGCAAATGCATCAGATCATGTAGGAGTCTGATTAGGGGGCACGTAGAGAGTGGT

CTGATTACTAGATGATCAGAGAGTTGGAGTCTAATAACAAGCTAATTGAAGAGCATGAGA

TAAATTACGGGCTAGTTAGAGAGCGTGTATCTGATTATGGGCCAATCATAGAGTGTGGGA

TCGATTACGAGATAATTAGAGAACATGGGATTGATTATAGGCCAATTAGAGAGCATTAAA

CAATTACATGTCAGTTAGAGAGTACGAGCTCGATTATGAGACAACTAGAGACTTTTTGAG

TATGAACTCATATTGTGGGTGATATGGGAGCTGAGAGTGAGCATGAGCCCAAGTCCGAGG

GTGTGCGTAAGCACACTAGAGCAAAAACTCGTGTGACTAAGTGAGAGTTCGAACAATTGA

GTAAAGGACATGATATGACCCCTGGGGTATCAAGTCGATTATAGACTGGTAGGGCATAAG

CCCAATGACCCATGATATAGTGTATGCCAAGAAATAATTGGTTGTGATGGCCATATACAA

CCATTGTACTTTCTGGAGAAGAATACCCCTAAAGGACAACTAATGTTTACTTATCTCGCT

AAGAGATCAGAGTGTTTATGTATTAGTGTCTGTGTGTTACTCTGGGACTTCTAGAGTTAT

TAAGATAACTCCGACTTCGATTAGAAGTATTGAGAGTATGGTTTCCTAATAAAGAACATG

TGATCATTTGAGGATTTGATAGATGTTGTCAATGGTGAAAATCTAAAGTGATTAAATATA

CTAAATAGAGGAAGATATGATAGATCTGAGGAGTGTTTAAGGTGTGAGATTTTGGGTTGC

TAAAATAAGGACCTTGAGTGGGTCAAGATGTGATCTCGTATAGTACCTGAGGGTCTCTCC

TTTTTGATTCAGGAAAGGAAAGAGTTGACTTGCTTCTTCATACCCGTCCGGGGATGTGTC

AGGATGATGTGGCAATTAGGTCTTGAAGAATCAAACCCGAGTTTATGAGGTTTTGAGTGT

TTGGCTCAACGGAGAGCGAGTGATCTAGACTCGAGTACGGTAGACGTTCTTTCTTTACTA

TCTCATCGTGTTGTCGTTACTTGAGCACTCGCTCTGGTATGGTGGTACTTTGTTCCCTTA

AAAATCTATTCCATATCAGTGGGACTAGTAGGTCTACCATATCAGTGGGACTAGTATATT

CGTACTCAATTTATGAGGTACATGGATAATCTTAATTTATTATTATGAGGTTACGGTGCC

AAATTTTCCTGTTTAAAGATACAGAGAAGTATGGTTGGTTGAGGTCAACTATATGCCCAG

TAGCTCGATACCGCAGTGCTATCAGGTACCGGATTTTCCTTAGGGTTTTCAGGACCGGGG

TGTTTGGGGTATAGGGGACACACCTATCAGAGGTGTGTGGTATGTAGTTGGGTGAACCAA

GTGAAGGGAAATTATAGGATTACATTTATGATGATAGTAGTTATATATTATACTTGCCAT

TTCGCAAAACTATTATCACACTTGCCTGCATGATAACTAATGCCTTCGAGAACTAATGTC

TTCAAGAACAAATATCTTTGGGAGTTAATATCTTTGGGAACTATTACTTTATCTGTATGA

CAAAGTTGTATGATGATGGTACAATTTCATTGTATCATTCATTATGTCATCACAGGGTAA

TGTACCGATTCATGATATCAGTTTGTAGCATACTTAATATGGTAGGTTGTGGTAGTGTTC

TTATTGGTAGCATAACCGTTAGCGTATGTGATTGGTAACTGGAATATACCTATGTATTAT

TGTAAGATACCATCTATAGTTGTAGCCTACTTCCTAATAGAGATTGAGTACTTACCAGGA

CATTATAGTTCTGTACGATTAAATACAATTGTACACACTTGTTAATAATTGTAGATTCTT

TACCAATTTCTTGTTGAATTATCTCAAGCTTACTTATTCATTCTGTACACTTATTTACTA

TTTTTATTTGCTAGGGTAAAGTACTTACTGTTATGATTACTGGTATTTTTACTACTTTAA

TACATATAATGTTTAATAAGATAAATAATGTTGTTAGTGTTCTATTGTTCGGTATTTGAT

AGAATATCGAACAATGGAGTATGTGGTTGAGTGTTTGCTAATGCTCATACTAGCCTATGT

GTTTATCTTAGTTTTGGGGTGAAGATTCTCCCAACCTGGATATCCTCAGCCCCAATATTG

TTTGACGTGTTTCATGCAGAAGTATAGTTAGAGGGTACTTACGATCGCAAGTTGCAAAGT

GGGTCTATCTGGGTAGAGTTGTTTTTGTTGAGATATTGTACCATGAGTTTGTGTATATTG

AGTTATATAACACTCACAAAGTTAATTATTCTTATATGTAACTTTGTTGAGTTATAAATA

TTAGTTTTGTGAAAATTTATTATATTGAATTCAGTTATGGTTTACTCCTTATATTTGTAT

GTTTTTAAAAAAACATGTGATTTCGACGATTAATAATTTATGTAGCGAGTCTTCTGATGG

AGCATGATAAACTTTTTGGTATAAAAAAT

>Unigene0001543

CCTATAAAGTGCATAACGCTCAGCTGCATTCCCTACCTCCTAAACGGTGCATAGCAACAG

CGGTCTCTTTTCCCAGTTTTTGGGGTTTCGGACTCGCACAACTCCTCTAGGGTTTTCCAT

TTTCGGACGGACCCAAAGTCCGGCGAGAGAGGGATAAGTGACAAGGAAGCCATGTCTTTG

GGGTATGCGGAGAAGCTCTCCTACCGGGAAGATGTCGGCGAAGTGGGGATGTCGGAGATT

TTCGACTCGCCTCAAGTTTTGCAGGAGAAGATTAAAGAACTTGCCACTATGATACGGGAG

AGTAAAAGTTTGGTGGCATTTACTGGGGCAGGAATTTCAACTTCTTGTGGCATTCCTGAT

TTTCGAGGACCCAAGGGAGTTTGGACTCTCCAGCGTGAGGGTAAGGGAGTGCCTGAAGCA

TCCATACCATTTGATCGTGCAATGCCAACGTTGACTCATATGGCTTTAGTTGAACTAGAG

AGAGCTGGTATTCTGAAATTTGTCATCAGCCAGAATGTAGACAGCCTTCATCTTCGATCC

GGAATACCGAGGGATAAACTTTCTGAATTGCATGGGAACTCATTCAGAGAAATTTGCCCT

TCTTGTGGATTAGAATATGTACGGGATTTTGAAATTGAGACAATTGGAATGAAGAAAACA

TCAAGGCATTGTTCTGATTCAAATTGTAGAAAAAAACTTAAAGATACAGTTCTTGATTGG

GAGGATGCTTTACCGCCAAAGGAGATGGTTCCTGCTGAGAATCATTGTAGAGCTGCTGAT

CTAGTACTATGTTTAGGGACCAGTTTGCAGATCACACCCGCATGCAACTTACCTCTAAAG

TCAATCCGTGGTGGTGGAAAGATTGTGATTATTAATCTTCAGCGGACTCCTAAGGACAAA

AAAGCAAGTTTAGTGATCCGCGGGCTGGTTGATAAGGTAATTGCTGGTGTTATGGGCATC

CTTAATCTTCGGATTCCTCCTTACATTCGCGTTGATTTCGTCCAACTCAGTCTCTCTTAT

TCATCACCAAAGAAAAATTATGTCAAGTGGATCGTTAGGATTGCAAGTATTCATGGAACG

AAAGCTCCACTACCATTTGTCAAATCAGTGGAGATCTCATTTTCGGAGAGGCCGGACCTA

AAAGCTGCCTTCCTACAAAAGCAACCCTTTTACTTGAAAAGGGAGACGGCAAGAAAGAGG

CCATTTAAAATGATACTCAAATTCAACTTAGGGGATGGTTGTGGCTGTCCATGCACATCA

ATTGAGTGGGAAATCGATTTCCAGGCGAAAAAAGATAGCTTCGTCCTTGATAAAGAAGAA

GTGGTCCGAAAGCTGAGAGATGCTGCAGATGAAGAATCTCGCTGTGGGCATACTGCTGTG

ATACAGCGGCAGGCATTTCCCAGGGCAGAAACCACTGTTCATGCAATGGTGACGAACATG

ATCAGGTACGACAATCCAGATGCACACAAGATTCTTTCCAACAACTTCATTGATGATGTC

GCCGGCCATGCACTCAAGATGAGCCCGAAAAACAGTTTTAACGAGACAAAAAGAGGCAAT

GAAGCTGTGAGTGATAGGGAAGCATCCCCAAAGAAGCCTAAGTTGACTGAAGCTGCACCT

CCAGGTCCTGATATATTTCGCTAGTACTATGTACAGAGGGATCAGTCCCAGGTCCTGATA

TATTTCGCTAGTACTATGTACAGAGGGATCGACTGATAATTACTGGCACCGTTTAGTTTA

GCAACGGTATACATGGGCGTACTGGGTGAGTTTTGCATATATCGCACCCCGTTTTTGTGG

CATACAAAGGGGAAAACTACGCAGGGGTCTCTGTACGACCGTTTTTGTGTTATTTTAATG

CTTTCTTACTTGCTTGTAGTTCAGCGGTAAAATAGTGCACGGGTTTGACTGCACGCCCCA

AGTGAAATATCAT

>Unigene0001544

CTCCCGCCACCAACCTACCCTTTCATGGAAACGGCGGCTGAGCACAGCAGCGTCGCGGTG

GTGCTGGTGCCGCTGCCGGCGCAGGGCCACCTCAACCAGCACCTCCACATCGCCCGCCTC

CTCCTCTCCCGCCCCGGCATCTCCGTCCACTACGCCAGCACCGCCGCCCACACCCGCCAA

GCCCGCATGCGCAGCACCGCCGCCACCAACACAAAAATCCATTTCCACGACCTCCCCGTC

CGACCCTTCGCCACCCCTCCCCCGGACCCCACCGCCCACGTCAAGTTCCCCTCCCACCTC

CAACCGCTCTTCGATGCCACCGCCGACATGGAGCTCCCCCTCGCCAACCTCCTCCGTTCT

CTCTCTGCCCATCACCGTCGCGTCGTTCTTATCCACGATGGCTCCATGTCCTTCGTCGCC

GCTGCCGCCGTCTCCGTCGTCAACGTCGAGACATTCATGTTCGACAGCATCTCTGCTTAC

GCCGCCATGTCATTCCTCGACAGCTGCGCTACCTCTTTCTCTTTCGAAGGCTGCTTTACG

GCGGAGTTTGCCGCCTTCGTCCGCCGCCAGCGGCGGCTCCGGGAGGCCACCTCCGGCCAT

CTCATCAACACATGTCGAGCTACGGAGGGCCGGTTCATCGACATGCTGGCCCAGCAGACC

GGCTGGAAGGACCGGTTCAACTTCGCAATTGGCCCGCTGAACCCGATTCAGGTAGACCGG

TCGGGGCACCCATGTATGGAGTGGCTGGACCGGCAGCCGGATGACTCGGTTGTCTATGTG

TCATTCGGGACCATGTCATCGCTGCCAGATGAGCAGGTGCGCCAGCTGGCATCCGGGCTG

GCTGACAGCTGGCAGAGATTTATTTGGGTCGTGCGGCACGC

>Unigene0001545

CCGGTGTGCGGCGCTTCGAGCTCGGTTTGGTCTGGTAAGTCTATTTACCCTCTAAATTAG

GGTTAGGGTTTTGATTTTGATTTTATGTTTTCAGATCTGATTAGATCTCTTGATCAATTC

ATTTTTAATTTTAGTTTTAAGTTTGTGTTGTGTAGATGATAAATGTTGTTGTTATTTTGT

ATTTTTAATGTCGGTTTATGAATTGAGAACTGATTTAGTTGGATCATTTATTGATTATAA

TATTTTGTTGAAATTAGATATTTACTATTGTGGATTATACTTTTGTTTTAAAAATTGTTG

AAACTCAACTAGATCATGGTGATTTAATTAGAATTTTTTGCGGAAAAGTGGAGAAAAAAA

AAATGTTTTGAGGTAGGTAGGATGGATAATTTTGTTACTAACGTAGTTCTTACTATAAGA

GTCGCAGCCGGTAGTATCTTTGCTAGATTTTTCCACATTATTCCCTACACGATATACTTC

ATTCTATTGACTAAAGCGAGACAGTTATTTGTAGGCACTATTCAATCAAGGAAGTGAAAT

G

>Unigene0001546

CCGGTGTGCGGCGCTTCGAGCTCGGTTTGGTCTGGTAAGTCTATTTACCCTCTAAATTAG

GGTTAGGGTTTTGATTTTGATTTTATGTTTTCAGATCTGATTAGATCTCTTGATCAATTC

ATTTTTAATTTTAGTTTTAAGTTTGTGTTGTGTAGATGATAAATGTTGTTGTTATTTTGT

ATTTTTAATGTCGGTTTATGAATTGAGAACTGATTTAGTTGGATCATTTATTGATTATAA

TATTTTGTTGAAATTAGATATTTACTATTGTGGATTATACTTCTCTTTTATAAATTGTTG

AAACTCAACTAGATCATGGTGATTTAATTAGAATTTTTTGCGGAAAAGTGGAGAAAAAAA

ATGTTTTGAGGTAGGTAGAATGGATAATTTTGTTACTAACGTAGTTCTTACTATAAGAGT

CGCAGCCGGTAGTATCTTTGCTAGATTTTTTTCACATTATTCCCTACAC

>Unigene0001547

CGTTCTGATAAAGACAACCACGACCTCTGTGCCTCCTTCACTTGCGATCCTTGTGGCCCT

GAAAACCAAACTAGTCGTTTTCCGTGATGTGGCAGTTTGAGAGCGACGTGGAGGCGCTGC

CGGCTGCCTCGTGATGCTCGCTCTTGGTGTGGTCGCCCTCGTCGTCACTCGGACTCTCTC

ACGGGGCAAAGCTTTGGAGTCTCTTGTTGACTGGAGCTCTTCTTCCGGAAATCAGCTATG

ATACAAACTGGGAGAAATTTTTGCTAAGAGGGCCGTGTACTCCTGTGCAATGGAGTCTGA

CTACGGTACACTTGATGAATCATTGAACTCTGGCAGGAAGATCATCATGCACCCGCTCTA

CCTTCCTCGCTCACTGCCGTGGCTCGACCTGAAAGTCTTCTATGTGAGGGTAAGCAACAT

TGAGGCCGATGACTCGATGCCCGAGAATCTCACCCTTAACCATGTGCCACTCACCCCGGA

TACCATCATCGAAATGAATGGCCGAAGAAGCAGCATTTACTCAGATTGGGTTTCTTCTGT

GCTTAGGAGGGACAGGGTTGACAGGACATCAGAAGAGGTCACTTTCGTGAGCACTGATAG

TATAAGGATTAACGGGAGTGTGAGGTTCGAGGTCTGTCATCGGGACGATATGCTGCTTTG

TGGGGTGCTGGAGTTGTGTGATGCGAATGGCTATATTGGAGAAGCAAAAAACCATGGAAA

GAAATGGAGCATGAAATGCCAACAGGTGAAATTGGATGGTACATGCTTCTTGAAGGGCAA

GCAACATTTGAGCACGGAGTCGGCCTCACCGACAGTAGAGGTTTATGTTGCTGCATGTTT

CTCAGGAACTCCTATTATCTTGACTAAGACTCTCCAGCCAGGATTTCGGAAGAAACACCC

GATGAAGTTGGTGCTAGATTCAATTCCTGAGCATGAGACATCACTACAACAGAAGCTGTT

CTCTGAGGATGATCTACAGTCATCAGAATACCAAGCCTACAAACGAGACACCGATGACAT

TGATTACAGCAATATGTATTCGAGAGAAGAATACATAGAACGAGAGGATGGAGAGCTTTC

ATGGTTCAACGCAGGCGTAAGGGTGGGTGTCGGTATCGGCCTAGGCATCTGCCTTGGTGT

TGGAATTGGTGTTGGCGTGCTGGTTCGGACTTATCAAGCGACAACAAATAACTTCAGGCG

ACGACTCATTTGATCCTGAACCAACTTGTCTTGTAATCGTCAAGAAAACTCTGGAAATTT

TGCCTCTCCCTGTGCATAAGGCAGCTTAACATGTCCATGCTGTGAAATGCTGCACACTTC

TGGCTTCCTTGGTGTGTTGTATTTCTTCTTTTCTTTTGCTTGTGGAGCCTGTTGTTTGTT

TAACAATAAGACTGGGATCATCTGAGGCTGTGGCAGTATAATGGTATTTTTCTCTTTGTA

CCGATGGATCATGGATGTGTTTCTTTATATTTTCAACA

>Unigene0001548

CCAATTCCTCAGTGGTGAGCGAGCGGCAGCTAGGGTTTCGGCGGTCCAGAACTTCGTGCG

AGTTCCGTACATGCTCATCCCCTCTCGAACTTCGTGCGAGCGGCAGCTTCTTTTTCACCG

TGATTCTGTATCGCCGATTCTAAGGACTTCTTTGAAATGCGAGCTTGATCTTATGGTGTT

TTTTCAGTGGCGAGGGGTGGCTAGGGATTTGGAGGTGGTGATTCAAGAAATTCCTGCTGA

GGGAGGACAGGGAAGCTCCGCCGAGGTGAAGCAAAGGAACGCGAAACTCGGGGAAGCATT

TGGAGTGATCAAGACTGAAGCTGAAGCATCCGGATAGCTCAAGCATGAAGAGGACTCGGG

AAAGATCGGGAAGTCTATAGAAACATCAAGAAAATGATTGGATAGCAAGCAATAGAGATC

CGGATTCGATGATTCTGGATGATGGAGATAACAAGCAATCGGAGGCTATATAAAGGAGGC

ATTGGCAGCTGATGAAGGAATCTTGGAGGTCAGCTTCTCTTTGAGGGAGAGCTTCTCTTC

GAGGGAGAGCCAGGGTGTGTTTCTGGTTAGTCTGCGAGTTCTTTTTCTTTGATTAAGAGC

TAGGTTCTCAGTTTGTTCTTTGAGTGATGATGTGAGATTGTGAATTGAAGTTCTATCTTG

AGAGTTTGGGAGCTGATTCTGATCTTCAGTTCTCTGTGAATTGATGTCAATTGAGTCAAG

TCTGTGTGTGAGAT

>Unigene0001549

TGTGAGATTGTGAATTGAAGTTCTATCTTGAGAGTTTGGGAGCTGATTCTGATCTTCAGT

TCTCTGTGAATTGATGTCAATTGAGTCAAGTCTGTGTGTGAGATAGTTCATAGTTCTCAG

TCCTTTCTTCAATTGTGAGCTATGTTTGAAGTCTAGTCTTGAGTTGCTGAAAGCTTGTCC

TCTCTTTGATTCCTAGTTCTATGAGTGTTGATGTTGAGAGATCGTGATTCTGTGTGTGAA

TGTAATTGAGTTGAGAGTTTATATTGAGAGAAAAAGGCAAGTGTAATGAAGTTCAACTGT

GAAGGCTGAGATCTTCTTCAAGTCTGTAAGTACTTTAGTGCA

>Unigene0001550

TTTAATCTGAGTTGTTTTCCTCTTGAAGATTGAACCTCTTTTATCATTGAGCATTTATTT

AGGGTTTTAATCTGAGTTGTTGTCTTCTCGTAGATTGAACCTCAATGCTATTTACTCTCT

GGCCGACGTTGACCATGTTATTTTGGGTATATAGTGTATTTCAAAATTTATTAGGCTACG

TGGAGCAATTCATGTAATTACGAGGAGAAATTCAAAAATGTTTAAAAATATTTCTCTTAG

TTTTTAATCAAGACAACCAAATTTAGGGATTGAAGTTGTTGAAAAGAAAGAGGATCCATC

GTGAGCTCATTCATGATCATGGTGTCTTTGTTTATATATACCAATGGCCTAATTTTGGCC

AATTTTTTAATGACTTAATTTATTCCTCCATTGTTGTCCTCTTGTACTTTATATCCAACT

TTCTTATCCACTAGTCCTATAAGGTTACGTAATAGAGAAAATTTAAATTAAACGATGGAG

AGGCTAATTCTTTTTGGTAATTTTGAATTAGGCTCCAAAACTTAGATGTATTTTGTGATC

GCCGCTTGTACATACATTTATTTGACTTTTCTTCCTAAGCTTTAACGAAGAATTTAACGA

AGCTTTACAAATTTGAAATAGTATTTTGGTATGTGTCATGTATAGAAAGATAAAATAATA

TAGAAGCTCATAAAGATGAGTTTTACGGAAATATTTAGGTAAAGGGTAAAATAAAAATTA

GGTCACTGATGCCGTGATCTTAGTTTGCGGGCTTAGGAAAGATCTTTTATTTTTTCAATA

ATAAAATATAATAAGAGAAACAATACTGAGGACAAAGGTGGGATTTGATCAATATAGAGG

CAAAGTGAAAAAGAAAAATCTGGTGTGCTAATGCAAACCGAGATCTTAACTAAATAAAAA

GCCCTACTTTTCCAGAAAAAAGAAGAGGCTATATCTAAATAAAGAAAACAAATTGAATTG

CTCATCTTACTTTTGAGAGATATTGATAGTCTCACGTCACAAAATGTACAAGAAGAGAGA

CGGACATTTATCT

>Unigene0001551

ATCAATTATCGAGAATTTAGATTCATTATTATGTCTTTTATTCTATAAATTAAAAAAAAA

AATCATATTTCTATAATAGCAATTTAGCATGAACCTGTCCCCTCCCTCCTCAAGAACTGA

TATTTCTTTGATTTTAGATTTTGTAATCTCTGGTATGAGGGTATTCTTTCACACTCTTTC

AAGGGTGGTTGCTTCAAGCCATACCTCTTGGTTCTCTTCATCTTTACCTCTTTTATCATT

GGGCAGTTATTTAGGGTTTTAATCTGAGTTGTTTTTCTCTTGAAGATTGAACCTCTTTT

>Unigene0001552

AAACTCCGGCGACGCTCTCTCCGCCGGCGACGCTCTCTCCTCCGGCGAGACAATGGTGTA

CCACTCTAGCTTTGTCGACGAAGATGGTATCACCAAAGCTTGTGGTTGTCCACTTCTTCC

TCTAAAAAGTCACATCAGGGGTCCCGCTCCAGTCTCAGATCCAGAGAAAATCGACATTGT

TGATGAAGCAATAACATTCTTCCGGGCAAATGTCTTCTTTAGAAACTTCGACATTCAAAG

CTCAGCGGACAAGCTTCTCATATACCTGACACTTTACATCAATGTGGCATTGAAGCGACT

TGATGGTTGCAGAACACTGGCTGTAGGGACCAAAGCAATCATCAACCTGGGCCTGGAGGA

GGTTCCGGTTCCTGGAGAAGCAGGGTTTCCCTTCCCAGGTCTCTTCGTCCTTCCTCAGTC

GCAGAAAGAAGCAGAGCTATTCCGGGATTATTTGAAGCAGATAAGGGAGGAGACTAGTGG

GAGACTCCTGAGCTGCGCTTACCGGCCTAATGGAACTCCAAATAAATGGTGGTTAGCATT

CGCTAAGAGAAAATTCATGAACACCATTGTTCAAGAATCTGGCAGCTAGCAAGAAACGAT

GCTAACTGGAGATGGAGATTGTGGTACATATGTCCTTGCCCATATTTTCTGTAGTCCTCT

GTCTCTAATTCCGCCTAATAAACTGAAAAAGTACTCTTTTGATCATTTCACACGCACCAG

AGTTGTCTAGTCACTTGTGGTCAGACAGTTTGGTATTTCCTTTTGATTTTTGTGACACTT

TTATATATGATTTGGTGTGGTCGATTATATGAACTGAATTTTTCATTGCCTAACTCAAAC

GTATTATTTGCTTACCAGATTCTTCTTCCAACATTTTGCCATGTATTG

>Unigene0001553

GGAACTAGAAGGAGTTGTCTCTGAGGTATCTACATCTACTCCTCAAGTACAACTAGAAAA

AAATATTACTGAGGATGGGATTTTGTGTTCGATAATATCATCCCTGTCTGTGAGAGCAGC

AGTAAGGACTTGTGTTTTATCCACTAGATGGAGACACATCTGGACCAATCTCTCTAAATT

AAGATTTGACACTTGGATGCTTGATTCTCCTACTCGTCGCGCATTACAAGATATGAGTCT

CACCACAGAAGAAAAATCTGAATTACTAATGAGAAAGACGGAACAATTCATCCGAACCGC

AGATTCAGTCCTGCAGCAGTACCGAGGAGTCAGAGTAGAGGGATTTTTTGTCAGCTATTA

CCTCACTAAAGCCCACTCTCACCATCTTGATAAATGGGTCAGTTTTGCAGTTGCATCAAA

AGCTAAAATTCTTGCTCTCCGACTGCACAATCCGTATCTCAGAGGAGATGAGAACGAGCT

ATATAATTTTCCATGCCAGCT

>Unigene0001554

CAGGGATAGAACCGACGCCTTGCGTCAAATCCGGCGGTTACGGAGCGGCGGTGTCCTAAT

AAGGAAAAGCCATTCGAGTCGGACAACAGTTCCTCCTCTTCATCTTTGATTTATAAATAG

ATCTCTGCAGGTACGAAATTCAAGTTCTTTTTCTTAACAATTCCATCTTTCCTTTCAAAT

CCGACCAAATAATGGCGAAGAAGTCCAGATCTAAACAGAAAAAGGGGGGATCTCGATCTG

GTTGCAAGATTATTACCCAGGATTCTGATAGCGAAATTCTTAACGAAATTGTTACCCAGG

ATTCATGCCAGATAATTATCCAGGATTCTGATAACGCAATTACTACCCAAGATTCTGATA

ACCAAGTTACTCGGGATTATGAGAGTTTGAGTTTTGATTGTTTTAACGAGGATGGGATCC

TCTCTTTGATATTATCTAAACTGTCCTTGAAAGAAGCAGTAAGGACTTGTGTTCTATCTG

GCAGATGGAACCACGTCTGGAGAAATCTCCCAATGTTGAATTTTAACGTGGAAAACATAT

TTGATGAGGAGACTCTAGAACGCTATGAAACTATAGAACACTATGATAGCTTTCTCACGG

AGGTGGACAAATTTGTTTTACTGTTGCTAAATACTGAGTACTTCATTCAAACCGTAAATT

CAATCTTGCAACAACATCAAGGCCATGTTAGAGTTGATACAATTAATATTATTTATTTCC

TTTATAAAGCACACTCTCTCTTTCTTGATGAATTGGTCCGCTTTGCAGTTACATCAAAAG

TTAGAGTTCTTTGTCTGGATCTTCGCCAGCCTCACTCTACATTTGAGGATGAATTCGATG

AGGATGAATTTGAGGATATATTTGAGGATATATTTAGGGATTTCGAGCTGTATGATTTTC

CATTGCACCTACTAACCTCATCCTCTATAAAGGAACTATATCTTGTTTGTTGTACTTTGA

AGCCACCCTACGAATGTCTCAACCGCCTTTTAGTTCTGTCTATGTCAGATGTGGGTGACA

CAGGAGAGGACATTAACCATCTAGTGTCTCACTGTACTTCTCTTCTGAGACTCGT

>Unigene0001555

GAGAAAGAAATTCCGAATTTGGCGCCGCCCCGATCTGTTTCGTCCGGGATGGAAAGGTAC

GAAAATCTATCTAATGGGGAAAAAGTTGAGATCCAAGCCAAAAAATAGGGGATCTACAAC

TAGACGACAGATTAACAGCCAGGGTTGTGTAGGCCAGGTTATTACTCAGGAATCTGAAGG

CGGGATTATTACCCAGGATTATGAACCCAAGAATATTTTTCAGGATTCTGAATCCCAGAT

TATTACCCACTGCTGTGAAGTCCAGATGACTACCCAGGGTTTTGAATGTATTAACGAGGA

CGGAATCCTCTGCTTGATAATATCATCACTGCCTATTAAAGAAGCAGTAAGGACTAGTGT

TCTATCTCACAGATGGAAGCACATCTGGAAATCTCTCCCCATATTAAACTTTGATATTGA

AACTGTACTTGATGACAAGACTCTAGCTCTTTGTGAAGGATTTCTGACTGCAAAAGATAA

ATGTTATTTACTGTTGGTGAAGACGGACCAATTCATTCAATCCATAGATTCAATCCTGCA

GCAACATCAAGGCCGTGGTAGCGTTAGAGTAGAGAGTTTTGTTGTCCGG

>Unigene0001556

GAGATAAGAGGGATACAGGCGGCTGATCGGCGTCGGTAGGGATTGGTAATTCTAGTCGGA

CATCAATTCCCCCTCTTCTTTTATTTATAGATTGATCTCTCCAGGTACGAATATCGAGTT

CTTTCTCTCGGCAACTCCACTTTTTCTTACAATCCGACGAATATGGGGAAAAAGTTAAGA

TCTAAAAAGAAAAATAAGAAATCTCGATCTGGTGAATGCCAGATTATTATCCAGAATCAA

AAGGAGATTAGTATCCAGGATTCTGATAGCGCCTTTATTACTCAGGATTCTAATACTCAG

GGTCAATCTGAGATTATTACACAGAATTCTGATAGCGACTTTATTATCCAGAATTCTGAT

AGCAACTTTATTACCCAGGATCAATTTGAGATTATTACCCAGGATTTTGATACCGAAATT

GTTATTCAGGAATCTGAAAGCCAGATTGTTATCCACGGTAGTGAAAATTCGAGTTTTGAT

TGTTTAAATGAGGACGGGATCCTCTCTTTGATATTATCTTCACTGAGTTTGAAAGAAGCA

TCAAGGACTTGTGTTCTATCTCGCAGATGGAATCACATATGGAGATATCTCCCAGTATTA

ATTTTTAATGTGGAAAACATACTTGATGCGGAGACTATAGAACTCTATGAAAGCTTCCTC

ACGGGGAAAGATAAATGTTATTTACTGTTGGAGAAAACCGAGTACTTCATTCAAACAGTA

AATTCAATCGTGCAACAACATCAGGATCATGTTAGAGTTGATACCATTCATATTATTTAT

TTCCTTTATAAAGCACACTCTGATTATCTTGATGCATGGGTCCACTTTGCGGTCGCATCA

AAAGCTA

>Unigene0001557

GATTATTACCCAGGATTTTGATAACGAAATTGTTATTCAGGAAGCTGAAAGCCAAATTGT

TACCCAGGGTAGTGAAAGTTCGAGTTTTGATTGTTTAAATGAGGACGGGATCCTCTATTT

GATATTATCTTCACTGAGCTTGAAAGAAGCATCAAGAACTTGTGTTCTATCTCGCAGATG

GAATCACATATGGAGATATCTCCCAGTATTAATTTTTAATGTGGAAAACATACTTGATGC

G

>Unigene0001558

GTATTTCCTTCATAAAGCACACTCTCACTATCTTGATGCATGGGTCCGCTTTGCAGTTGC

ATCCAAAGCTAAAGTTCTTTATCTCCATCTGTGCCAGCCTCACTCATTCCGGAATATAGA

GATGTATGATTTTCCATTACACCTGCTACCCTCATCCTCTATAGAGGAACTATATCTTGT

TGCCTGCACTTTGAAGCCACCCTACCCATCTGATTTTCTCAACCGCCTTAGAATACTAGC

TATGTATGACGTGGGTGACACAGGCGATGA

>Unigene0001559

CGGCGTTGGTAACGATCGGGAATTCTAGTCGGACATCAATTCCCCCTCTTCTTTTATTTA

TAGATTGATCTCCCCAGGTACGATTATCAAGCTCTTTCTCTCGACAAGTCCACCTTTCCT

TGTAGATCCGAAGCATAATGGGGAAAAAGTTAAGATCTAAAAAGAAAAATAAGAAATCTC

GATCTGGTTGCAAGATTAATGAGTGTCAGATTATTACTCAGAATCAAAAGGAGATTATTA

CCCAGGATTCTGATAGCGACTTCATTACCCAGGATTCTGATATCCAGTGTCAATCTGAGA

TTATTACCCAGGATTTTGATACCGAAATTGTTATTCAGGAATCTGAAAGCCAAATTGTTA

CCCAGAGTAGTGAAAGTTCGAGTTTTGATTATTTAAATGAGGACGGGATTCTCTATTTGA

TATTATCTTCACT

>Unigene0001560

TACCCGTCCCAGTCTGGCGGGCTATCTCAGGCTCCGCAATCTCCACCTCCCCACGATTCC

ATTTTCTCTCCTCCGTCTGCTCTCATCATCACCACAAAATCCCTCAAACCCTAAACTCCC

ACTCGCTGTGTTCTGGGACCTCGACAACAAACCTCCTAACTCCATCCCCCCTTTTGACGC

CGCCGTCCGCCTCAACCTCGTCGCCACCTCCCTCGGTCCCGTCCGCTTCACCGTCGCCTA

CGCCAACCCCCACGCCTTCCGCCACGTTCCCGCTCCCGTCCTCGCCGCCCGCGCCCGCCG

CCTCGCCTCCCCTCAGCCCGCCGTCTGCCGCGTCTGCGGCCGCAACTTCTACAACCACTC

CAAGCTCCTCCACCACTTCCGCCAGATCCACGAGCGGGAAAATGCCAAGCGCCTCAGCCG

CATCGAGTCCGCCCGCGGCGCCCGCCGCGTCGACCTCGCTGCCAAGCTTTCCCTTAAAAT

CAACAAGTACAGGAAGGCGGCGCAGGATGTGCTGGTACCCCGCTCTGGGTATGGGCTCGC

TGACGAGCTCCGGCGGGCCGGAGTTGTGGTCCGGGAGGTGGAGGACCGGCCGGAGTCTGC

GGACCGTGCGCTTCGGGCGCATATGATGGAGACGATGGATAGGGGGAGGGTGGGTTGTAT

AATGTTGGTGTCTGATGATG

>Unigene0001561

CACTCCCACCGAGATTCCAAGTCTCCGTCGTCGCAATGGGCGAGGTGAAGCTCTATGGGG

GCATGATTCCTGCGATCTCCATGGTTCACCATGCCCTCAAGCTCAAGGGCGTCCAATACG

TACATGCCGAAGAGGAACTCGAGTTCGATCCGGTCACCGATAAGCTGCCGGTTCTTGTCA

TGGACGGCAAGGCGATCGCCGGTTCGTCGGCCATCCTCGAGTTCATCGACGAGATGTGGA

AGGATAACGAGCCTCTTCTGCTACCGGTTGATCTCTTCCAGAGGGCTAGGGTTCGATTTT

GGGCTGATTTCATCTACCAGAAGTTGATCCCTGATGGCGATTTGATCATGAGATCACCAA

TAGAGGTACAAGAGAAAACAGTGAAAGAATTCACCGAGCATTTGAGCACACTGGAGGATG

GTATTAAGAAACAGTTTCCGAGAAGGGGTCCTTTCATCAACGGGGTGAGCCCTGGCCTGC

TTGACGTGATCATGGGGTCGAGCTATGTCTTTTTTAAGATTCTCGGAGAGATGACTGGAA

AGGAGCTGCTGGACGAGGAGAGGATACCGGCCTTGTATGAATGTATGATGGCATATACAG

GCCTTGAAATGGTGAAAGATTTGACAAAACCTCATATTAAAATGCTTTCGCGGATCCTAA

CTCTGAGAAAGAGGGCATTGTTGGCTGTTACAGGTTCTACGCCTCAGAGTTAATTTTTCT

CAAATAATTACTCGAGAATAATTACTCGAGTGTATATACGTATGCTTCTCCTTGTGGTGT

TTAATTATTAGACCTATAAATATATTATAATGGCTTCAAAGCATTTGTTTGAGCTTTGAT

CTCGTAAACCATTTTGAATTTTTGTGATTAACAGAGTGACACATAGTTTTGTAATTAACA

GGTCTGTGTGATGTTTTTGTTTACACTGTTTTTGCATCATGGTCTTTGGAAATTAATG

>Unigene0001562

TTTGGGTTCTCATGGTTTGACCATTTTATAGGAGAGAATAATGTGATAAACCCAAAAAAA

ACTGTTGCGGTTTCGGTTTTTCTCCCGCCAAAATAGGAGATAAAACCCTAATTAGGAAAA

CTACTGATGCTGTGGTTTCTCTCCCGCCAAATTACGCTCCCGCCAACTGCTTTGGTTTAC

CTCTCCCACTAAAATAGGATAGGAAAACTAATCTCTCAACCCACTGCTTTCCCACAATTA

TTCACCTCAGATTTAGTTTGGATAAAACT

>Unigene0001563

TTTGGGTTCTCATGGTTTGACCATTTTATAGGAGAGAATAATGTGATAAACCCAAAAAAA

ACTGTTGCGGTTTCGGTTTTTCTCCCGCCAAAATAGGAGATAAAACCCTAATTAGGAAAA

CTACTGATGCTGTGGTTTCTCTCCCGCCAAATTACGCTCCCGCCAACTGCTTTGGTTTAC

CTCTCCCACTAAAATAGGATAGGAAAACTAATCTCTCAACCCACTGCTTTCCCACAATTA

TTCACCTCGGATTTAGTTTGGATAAAACTAAAGACGAAATACCCGTTCCATCTTTAACCT

TTAATCTTTATCTCGGGCTGTACCCTCAGGTTGCCTAGGTTGCCTACGTATCCCCTGCCC

TGCCCTGGAATCAAGCCCGCCGTAGTTCGCTATAATCCCCTGAAAGAATAACTCAATCAT

GATGCCTTGCACCTGTATAAGAGCCATACAATGCTGATGCTTCTCGTTCGGAACAACTTT

CGTGTTGATCTTCACTAACTTGATATCGCCTTCTTCAATAACTTCCTGGATGAAGTGGTA

GTGCACATCTACGTGTTTGGTCCTGACATGGTGCACATGGTCTCTGGAAAGATAGATTGC

ATTTTAACTGTCGCAGAACACTTTCATCACGTCTTGGTACAGGCCTAGATCATAAAGTAT

GCCCTGTAGCAAGATTGCTTCCTTCACGGCTTCCGTAGCCGCTATGTACTCGGCCTCCGT

TGATAAAGCAACTGTTGACTGCATGATAGACCTTCAACATATGGTTCACCGGCCATAGTA

TAAACGTAAGTCTTTCACCGAAACCCGTTTCTCAAAAATCAGCCCCACTGAAGTAGTATC

GTGTAGATATCCGAGTATCCATTTCACTGCCTGTCAGTGCTCTTTTCCCGGGTCGTGCAT

AAACCTACTGACACCACTGACTGCTCGGGAAATATCTGGCCTCGTACAGACCATGACATA

CATGAGCCTGCCAACTACACTCGCATATGGTACTGACTTTATGTACAATTTTTCTTCATG

TGACTTTGGACAATATTGAGTACTTAATTTAAAATGATGAACTAAATGTGTACTCACTGA

CTTGGTTGCTTCATTGATGCTGAACTTTTGAATGATCTTCTTGATATAAGCTGTTTGAGA

GAGTTTGACTCTCCCTCTTTTTGTATCCCAAGATATTTCCATCCCTAGTATCGTCTTTAC

CTCTCCTAAGTCCATCTCAAATTCTGATGATAGCTGCAAATTCAGTTTCTCAATTTCTGA

GTTGTTCCTAGAAGCAATCAACATGTCATCCACAATAAGAATATGGTTGCCAATTATAAT

CTGCATATCCAAGTCTGGAGATCACTTAAATCAGTAAATGAAACTTCTCATATATTTTGA

TAAACCCGGACACTCACGTAATTGTTAGTTTTTCTTTTAATTTTTCCCTTTCTTTCTTTC

TATTTTCTTCATGAGTTTCATGATTATTATAATTTAACATGTGTATTTTCATATTACTAC

TCTCTAAGAATTTTCACTAGCATTAACCGTGCAACCACTGAACAAAAGTACCGCCTTTGG

AATTTCACTAACATTACTACTCTCTTAGCTTAGAATCACAAATCAACCGTGGTTGGCCAC

ACACTGCTGCATTCTAACAGGCTCCAACTGTTCCAAATCAATCCACTGCATTCTTAACCT

GCTGCGTCAAATAAAATCTGATACGTTGGGACGCACATATTTGGAGGCATTCTAATAGGT

CAAGTCTCCCGTTAAGAGACGGATTATGTTAAATGATAACTCTGGTAAGACAGATAACAC

TCTGAACTCCAATGATGCTGAAAGGTTTATGATAGTAGTTTGGAGCAAGGGGGTCACTAC

TTATGACCCTTCCCTAAAGAAAGAGTTCGTGTTGGTTGATATGGCTTAGAAGCCAAGAAC

AAATGATACCTTGTTCTTCAAGATAAAGCAGGAGAAGGCCATAAAGATTATAAATTCCAA

AGGTAACCCTCCTATTGTGATTTATTATCTATTTTGATTTTTTTTCTTTTGATAAATCAT

GACATATTCTGACTCTTACTTCCTAAATTCGCGTAGTGAGAATTGTCGAGTTGTTGACGA

TCTACATCATTATCCAATCCAAGGACAATGAGGTCAATATTGGTAACACTAAAGGTCTAA

TCTACTCCAAATGAGAGAAGGATAAGGAGTAGGATGTTGGATATATAGCTCGTATTTCGG

CGATAGATGTATTGGTGGAAGATAAAATGTAGTTTAATACATGGGTTTTTTGGTAGTAAC

ATTAATGTGCATACATCTCTTTTGAATGCTTTCGCGTTATGAGCTGTGTGTGCATCCAAT

GCCCCGAAAACCTAAGGGTTCCTCCGTAAGGTTCGTCTACG

>Unigene0001564

TTCATTCTTCATAAGAGATTGTATCTCTTCTTTCATAGCGTTCTTCCAATCTTGATTTTC

TAAACACTGAATAGCTTGAGAATAACTAGATGGAATATCATCACATACCTCATTTACCGG

GAGAGCATATGCCACCATTGTTTCAATCAACCTGTCTCGCTTTTTGATATTCCGCTTGGG

TCTTCTTTCTGCAATAGATTCTGAAATCTGCTCTTGATTCGGAGTGTTTATAGGTGCTAT

ATCATCATCTTCTGGCTACAGATTTTCTA

>Unigene0001565

GCATAATCAGAATCAACATATCCTGAACAAAAGTCTTCCATCGAACCCTGTTTCTCGAAA

ATCAGCCCCACTGAAGTAGTACCATGTAGATACCTTAGTATCCATTTCACAGCTTGCCAG

TGCTCCTTTCCCGGATCATGCATATACCTGCTAACAATACTGACTGCCTGGGAAATATCT

GGCCTTGTGCATACCATGGCATACATGAGACTGCCAACTGCACTCGCATATGGTATCGAC

TTCATGTATTCTTTTTCTTCCTGTGACTTTGGACAACATTGAGTACTTAATTTAAAGTGT

TGAGCTAAAGGAGTACTCACTGACTTGGATGCTTCACCGATGCCGAACTTTTGGAGGATC

TTCTTGATATAAGCTGTTTGAGAGAGTTTG

>Unigene0001566

TTATCATTCCCAATCTCCCTCACCGATCTCATACCCCCAGCACAAACGGTTAGCTAGGGT

TAGGGTTTCCAGCCATGGCGATGCGGGCCTTCTACAACGAGATCAAGGCGCTGCGGGTGC

AGGATCTCCCGAAGCACCTGAAGCCGAAGATGACATGGGATCACATCAAGAAGACGACCG

ACCGCGCCGTCGATAGATACATCGACAAGTACATCGAGACCAGCTCCGTCGAGCCGCTCT

TCCATGTCTGTATCGGCGGCATGATCTTCTCCTACCTCGTCGCTCTCCCCGAGGAGCGCC

GCCACCTCGAGCACCAGCAGCAGATCGCCGCTGGCGGCGGCGCCGGACACCACTGAGGCC

GGTGTGTTCATCTATTAGGATTGAAATTCTCTGCGGTTGATTTGGGAGTGGATAACCAAC

TGCCAGAGACATGATTTCCATTTGATTCGCACTCCTGAATAAATTCAGGTTATATGTGGA

ATGCGGAACTCTGCTGTTTTTGCAAAATTATATCATACAATTTTCCATCCGTCTTTTAAT

ACGGATTTGGATAGGGAATTCTTATTTTTCTTCTTCTTTGTAATCTATTTTGTTGAATAT

GAATCCTTTAATCTCCTCCC

>Unigene0001567

AAGCTTAAAGATTTGGAGCAGGGGACACAGATCCATCGTTCCATCCAAGCTAGCGGGATA

GTGCCTGATCTGAAACTTGAAAATGCACTGATTGATATGTATACCAGCTGTGGGAACATG

AATGCTGCCGAGGCACTATTCGAGAGCATGAGTGTTAGGGATAGAATATCTTGGACTGCA

ATGGTAGTTGGATTTGCTAATCTGGGGCAGATTGATAATGCGCGTGCAGTTTTTGATCAA

ATGCCTGAAAGGGATGCTATATCGTGGACAGCCATGATCGATGGCTACGTGCGAAGTAAC

CGCTTCAAGGATGCTCTCGAGATATTTCGTGGGATGCAAGGTGCAAGGATCAGGCCGGAC

GAATTCACCATTGTCAGCGTGCTCGCAGCTTGTGCGCAGTTGGGAGCGTTGGGTGTAGGA

GAATGGGTAAGAGTTTACATGGACCGCAATAAGATCAAGGCTGATATCTTTGTGGAAAAT

GCTCTAATAGACATGTATTGTAAGTGTGGGAGCATGGAAAGTGCGGCAGCTGTATTCCGT

GGTATGAATCGGAGAGACAAGTTCACCTGGACTGCCATGATCCTAGGTTTCGCTGTCAAT

GGCCATGGCGAACAAGCTCTTGAATTGTTCTCGAAAATGTTGGGAGCTTCTGTGAGACCT

GATGAGGTAACCTTCGTCGGAGTCCTTTCAGCCTGCACACATGCAGGTTTGGTGGACAAA

GGAAGGAAATTCTTTACTGATATGATTACTTCTCATGGAATCATGCCCAATGTTTTACAC

TACGGGTGCATGGTCGATCTCCTTGGCCGAGCAGGGCAGCTTACAGAAGCATTGGAGACC

ATACATAACATGCCAATGAGACCGAATTCTGCAGTTTGGGGAGCTCTCCTTGGTGCTTGC

AAAGCTCACAGGAATGTGGAGATGGCTGAGTTGGCTGCTAAGCATCTCCTTGAGCTGGAG

CCTAAGGATAGTGCAGCTTACGTGCTTTGTCCAATATATATGCCAAGTGTAATAAATGGG

AAGAGGTGCGCAAGGTGAGAGAGATGATGATGGAGAGGGGGATCAGGAAGATGCCCGGGT

GTAGCCTGATAGAAATGAATGGTGTTGTTCATGAGTTTGTCGCTGGTGATATATCTCATC

CAAGATGTGATGAGATTTACAACAAATTAATGGAGATTAGCAGTGAATTAAAACTTGCTG

GCTATGCATCTGATACTTCAGAGGTCTTTCTTGATGTAAGCGAGGAAGAAAAAGAAAAAT

CAATTTATTGTCACAGCGAGAAGTTGGCGA

>Unigene0001568

CTCGCCCTCTCTGTCACACACAAAGCCAGCCAGTCATGGAGACGAATCCGAACTACAACA

GGGGAGAAGTCGACGAGCCCCCAATCGACGAGCCCTACAGCATCAACTTCATCCCCTCTG

AGCTCTTCCTCAAATTCCGGAAGGAGCTCAAGGGTCTACGCGTCGGTATCAATCTCGAGT

TCTCTAATTTCGAGGGAAATGACTACCGGGCGAAGATGGTGATGAAGCCGCAGACTCCCG

ACCGGAGGTGGAAGTTCATCTATGAGCCGTTGCAGGGGGATATACGCCTGCTGTCCAAGA

AGATTCCCATAACTCAGTATCTCAATCTCCAGGTTGGTATAGGTCATAATTTCCAATGCA

ATGTTACTGGTTGGAAATGGAAGGTTTCTACTTGCCTAGGCGGAGATGGTGTTTCTCAAA

TCAAGAACAAAACATCTATTGGCCTGTTCCCAGGTTTTGATTTGCGGATTGGCTGGAGGG

CAGAGTACGTCCTTCCCGAGATCCATGGGGCCGTTGGCACTGGCGAACCCATGATTGACA

TGAATTACGGACGCTTGCAAGCATCGATCGACAGAGTGGAGGCCATCCTAACGCATAAGA

GTTACTGATTCCAGGTATTTGAATCTACAGTCTGAAAAAGTGCCAGAGATAAACGACAAT

GGGCGACTCACCACCAGGTTCGGCCTTTGTTGGCACAAAAACCCAATGGTTCATGTACAT

ATTCAAGCTCTCTTTTATACCGTAGCTGACTGAAGAATTACAGATATGATAAGTTAGATT

TCTTCAGCTTTAAGTTAGATTTTCTATCGTGTTCAGTTCTCAATTCTGGGGTGGAAATGA

ATTTTTCAAACCCATGTCAAAGGGATTTGAGAAATATTTTGACTTCTAAGTATAGTTCTT

TCAAGTAGACATCAACATTTATGACCATAATATCGTGATCTCATTACCATTTAATGCT

>Unigene0001569

TTGTATCAGCTCAATTGTTCCAACAAAACCCAATGCCACGTCTACTGCTTACAGACACCG

CGACTCCACCTGCTGCTCCTCCCGGTGGAGGCCTCTACAACAGCAGCAGCAGCGGTGATG

TTGTCTCTAACGACGGTGGAGGCCTCTACAACAGCAGCAGCAGCGGTGATGTTGTCTCTA

ACGACAAAATGTTGATTGTCCTCGTGGCTCTTCTCTGCCTACTGACATGCGTGTTGGGGA

TGAATTCGATTTTGCGCGCCTGTCGCCGCCGGTGGATGACTGACAAGGCTGCCAAGGAGG

GGCTCCAAAAGCAGGTATTACGAAAGATACCGGTGGAGGTGTATACGACAGGGGTGGAGA

TACTGGGCACGGATTGCGCGATATGTCTGGATGAGTTTGCCGATGGGGAGAAAGTCAGGG

TACTGCCAAACTGCAACCATGGGTTCCACGTCCGGTGCATTGATGCATGGCTGGCGGCGC

ATTCTTCATGCCCGACCTGCCGCCACTCGCTGGTCGGCTGTGGAAGTAGTGAGGGCGGCG

ACGGGTGGTGTCCGGTTGCTGTGGTGATTTAGGAGTTCAATTTTTTTTTTTTTTTAATCC

TGAAATATATTAGAGGTATACTCCCGACATCTATCAGTTTTAAATACCTTCCCAATATTT

CCAGATTGATTCTGGATATATCTTTTAAAAAATTTAAAAATCTCAAAAGCTTCAGATTTT

TGTTTCAAAAAGTAGACACAAGTCTTTTTACTATAGTCATTAATGAAAGTAATAAAATCA

TGATTACCTCCATTTTACACGGTCTCAATAGACTCACAATATCTGAGTACACAAGTTCCA

ATTAC

>Unigene0001570

AATATAATTGGGAGTCACCTCGCGAAAGTGCTACCAGCAAGAGCGGCGTCTGAAAAAGCA

TAGCTTCCGGCTTTGCTCTCCTTCCCCGAAAGGAATGGGAATTTCGGGATCCGCTTCGAG

TTGCGATGCCGCGACAGCCGCTGAGACCGCAACGATCTCTGATTCGCCCCTCTCTTCCTC

CCCCGCACACCACCAAGCATCCTCAGATTCCAACATGAACTTCAGCGAGCGCGCCCTATC

GGCTGCTGGCGCCGCCGTGCTCTCCGCCGTCCTCGTCAACCCCCTCGACGTTGCCAAGAC

GAGATTGCAGGCGCAGGCCGCGAGACCGCCCGGTCACCCTACGGAGCAGGGTCGGTTCGG

AGGGCGAATGATGTCCATTGGCCCGAACAGGTACAAGGGGACATTGGATGTTTTCTACAA

AGTCACTAGACAAGAAGGGTTTACTAGATTATGGAGAGGCACAAATGCAGGCTTAGCATT

AGCTGTGCCCACTGTAGGGATATACTTGCCTTGTTATGACATATTCCGCAACTGGATCGA

GGAATACACAAACCACAATGCTCCAAAATTGACACCATATGCTCCCTTAATCGCAGGTTC

AGTTGCACGGTCAATTGCATGTATAGTTTGCTCTCCTATTGAGCTAGCAAGAACTCGAAT

GCAGGCATACCGAACCGGAGCAAAGCCCCTGGGAATGTGGAAAACACTACTTGGTGTTGT

ATCACCACTCAGGAGTACAAGTAACCATCAAAATTTTCAAAGTTATTGTCTTCTATGGAC

GGGTATTGGTGCACAACTTGCCCGTGATGTGCCATTTTCTGCTATTTGCTGGTCTACACT

TGAGCCAATTCGAAGAAGATTATTGGCACTAGCTGGTGATGAAAGCAATGCTGCTAGTGT

TTTAGGGGCCAATTTCTCAGCTGGTTTCATTGCAGGCAGCCTTGCTGCTGCCTCTACATG

TCCCCTTGATGTTGCAAAAACTAGAAGACAGATCGAGAAAGATACAACAAGGGTTCTAAA

CATGTCGACAAGGCTGACGTTAGTTGAAGTTTGGAGGGATGGGGGATTAAAAGGACTCTT

CACCGGTGTAGGTCCGCGGGTTGGGCGTGCGGGCCCTTCAGTTGGAATTGTTGTTTCCTT

TTATGAAGTGGTCAAGTATGTTCTTCACCAAAGACATTTAAATTCTTGACAAAAGAGTAG

CAGAGTATGCATAGAAAAGGAGTCAATGCTGAAAATAGGCATATTTACCGCTAAATCCTT

TGTGAAGTTGGTATAACTCGAATAAGATGCTCGGCAATTTTGTGAGGGAGTACAATTTTT

ATGTAGCTCAAAGGCAACAATTCGTACTTTCTTCAGACTTTTTATATACTTTATTTACTT

CCCTTTCTGATGGTCGGTAATAACAT

>Unigene0001571

TGACGTTAGTTGAAGTTTGGAGGGATGGGGGGTTAAAAGGGCTCTTCACCGGTGTTGGTC

CGCGGGTTGGGCGTGCGGGCCCTTCAGTTGGAATTGTTGTTTCCTTTTATGAAGTGGTCA

AGTATGTTCTTCACCAAAGACATTCAAATTCTTGACAAAAGAGTAGCTGAGTATGCATAA

AAAAGGACTGCATGTCAATGCTGAAAATAGGTACATTTACCGCTAAATCCTTTGTGAAGT

TGGTATAACTCGAATAAGATGCTCGGCAATTTTGTGAGGGAGTACAATTTTTATGTAGCT

CAAAGGCAACAATTCGTGACTTTCTTCGGACTTTTTATATACTTTATTTAC

>Unigene0001572

TTGTTTATGTTTAATTCACTTTTAGAAGCAATCAACGTGTCGTCAACATAGAGAAACAAA

TATATAAAACTTCTATTTATCAATTTTTGAAAATATACACATTAATCATGATCACTATTG

ATATAGCATTTCTTTAATATAAAAACATCAAACAGTTTGTACTAGTATCTCAGGTAGTGT

TTTTTTCCATACAATGACCTCTTCAACTTGTCTACGAGGTATTCTTTTTCA

>Unigene0001573

AATAATCTTTCTTTCTTTAGGATCCAAGAGCTTATTACCTTTTTTATTCTAGATTTGAAC

GCTATAAACATAGCCTTTTTCGAACGTGGGCCAAGCTTATCTTCATACACATGATAGTAA

GCAACATAATTGAATACTCGAAACCGATCATAGTCCTTCGCAATATTACCTACTCATATT

TTCATAGATGTTTTACCCTCGAGTGCTGACAGGGTAGCTGATTCACCAAATGAGCAACAT

AAGTAAGGGACTCGGCCAAAAATATCTTTGTCCAACCAGAATTAGAGAGCATACACCACA

CT

>Unigene0001574

TTGTATCGAACGCCGTCTTCTGCTCCTTCATTTTTGTGTAGTCCTATTTGCAACCAACCG

ATCTCTTATCTTTTGATATACATGAGATCCCATGTTTCATTTTTTGAAAAGATTTTATTT

CCTCTTCCATTATATTTTACTAATTTTATTTTTTTTACTAATAATTGATTGATAATACAT

TATATGAATATCTTCACTATCTTAAATTACTGGGAGAGCATAAGCTATCATTGTTTCAAA

AAAGCTTTCATGTTTCTTTATATTTTTTTCGGTCTACTTTTTGTGATTTTTTCTTGAACC

TGAACATGACTTGGAGTACTTGTCGACACATCATCAACATTATCGTGCAAGTTACCTACC

TTATAAGTCTCCATTGCTTGATATATCCATGGGAGCATTATCAGTATGACTTTCCCTCTG

AATTTCCTTAAGACTTACT

>Unigene0001575

ATAAAAGAGACCCTCCATTGCTCCCCACCTCCACCTCATGTGGCCGTCCTGGCGCAAGCT

CCCCCGCGGCAAACCTCCCGCCGCCGCTGCCTCCGCCTCCCCTACGCCGTCCCTTACCCG

CTCCTCCCTTTCCTTCAAAGACCTCCAATCCCTCCTTTCCGACGACGATGACTCCTCCCC

CGCCGCCTCTCCCAACCCTAACCCTAACCCCTCGCCGAAACCCGGCCTCTGCCACCGCTC

CTGCTCCGCCTCCTCCGCCCTCCGCTCCTGGCGCATCCGCTCCCAGCCCAGCTCCCCGCC

CGACCCCTCTTCCTCCGCCGACGCCCACCGCATCGTCCTCTACTTCACCACCCTCCGTGC

CGTCCGCAAGACCTTTGAGGACTGCCGCTCCGTCCGCACCATCCTCCACGCCCTCCGCGT

CGCCGTCGACGAGCGCG

>Unigene0001576

AATCCGCTCCAATTCCCCACCGCCGATGTGGTCGTGGGGCAAGGTCCGCCGCGCCGCCGC

TGCCGCCGCCGCCTCCGGCGGTGGGGCCACCGCCGCCACCTCCCCCCGTCTCGCCCACTC

CTTATCCTTCAAGGACGTTGACGCCCTCGTCCGCGCCGACGACCCCGACGCCACCAAGTC

CGCCGTCTTCCACCGCGTCCGCCTCACCTCCACCCCCTCCCGCGCCTTCAAGTCCCGTCA

AGCTCCCGCAGACAACCGCGTCGTGATCTACTTCACCTCGCTCCGCATCGTCCGCAAGAC

CTCCGAGGACTGCAAAGCCGTCCGCTCGATCTTCCACGGATTCCGCGTCGCCGTCGACGA

GCGCGACCTCTCCATGGATAGCTCCTTCCTCACCGAGCTCAAGTCTCTCCTCGGCCGCAA

GCAGCTCTCCCTCCCGCAGATCTTCATCGGAGGCCGGTACGTCGGCGGTGCCGACGAGAT

CCGCCAGCTGCATGAGTGCGGCGAGCTGAAGAGGCTGATCGAGGGGATGCCCATGGTGTC

AGCGACTTGCCCTGGCTGCGGGGGAGTCAGATTCGTCTTGTGCTCGTGTTGCAATGGATC

GCATAAGTTCTACTCCGACAAGGGCGGTGGGTTCCGGACCTGCGGAGCTTGCAATGAGAA

CGGGCTGGTGCGGTGCGGGGAGTGCTGCGGACTCGGTGCGGCGGATGGGTTTTGATGGTA

TTCTGCCTTGCTTGAGGTTAGTTTGGTAGGGATAGAGGAGTAATGATAACGCAGTTCATA

GAAATTAGGATTATGATTTTTGGTTTGGATGGATTTGGTAGCGTTGATCGGAGATAACTG

AAGAGGCAGCCCTTTTTTTTTTTTTCTTAATTAAATTTTTGTGATGGGCAACAAGGTCAA

TGGGTGGATCAATGTGTTTTCTTGAGTAAATATAATTCATTGATTCGTCTGAAATTGAAG

TGTACAATTAATATGTTGAAATTGAAGTGTAGCTAGATAAGTGAATTTTATTA

>Unigene0001577

CCGCGTCGCCGTCGACGAGCGCGATGTCTCCATGGACTCATCCTTCCTTGCCGAGCTCAA

GATCCTCCTCGGACGCCGCCAGCTCTCGCTCCCGAAGGTCTTCATCGGCGGCCGGTACGT

CGGCGGCGCCGACGAGATCCGCTACCTGCACGAATCCGGTGAGCTGAAACGGATCGTCGA

AGCGTTCCCGGTGGCCGCCGCCGACGTCTGCAACGCCTGCGGCGGCGCGGGATTCGTGCT

CTGCGAGCATTGCAGTGGGAGCCACAAGCGGTATACTGAGAAGTCCGGATTCAGGACTTG

CACGGCGTGCAATGAGAACGGGCTTGTTCGGTGCTCTGAGTGCTCGCTTCCGGCGCTGTG

ATCGAAGGCGAGAGATGGACGAGAATTGATCCATCGACGCCGACGAATGTGCATTCGAGA

TTCAGAACGGTTTGAATTTCTTGATTGATTTCGGGTTCAATTATTGGAGTTTACTTCGTT

GGTATTTGATTAGGTGATAGAGGATAGTGGGAGGTTTTTTTCTCCTCTTTTTGTGTGAAT

TTTATCCCATGCATAAATTGTGATAAGTAGTATATACAAGCTTAATTATTTATTTTTGTG

TTGTTTCATCAGATAGATTGAGGGGTTAGGACCTCGTATAGATTACTTGTAACATTTGTG

AAAAGAATATTAATACAAAAAAAA

>Unigene0001578

TTGCACAAACAGGAGCAGGGCTCGGGAGCGATAGCATGGCCGCCGCCGCTCGAGCCAATT

GCCTCCTCGTCACCGGCCCTCCGGGAGTGGGGAAGACGACGCTGGTGATGCGGGTGGTGG

AGATGCTCCAGCGATCTCACCCCAATCTCAACATTCGGGGCTTCTACACCCGGGAGATCC

GAGAGGGGAATGAGAGGGTCGGATTCGAGGTGGTCACCTTGGACGGCAGATGCGGGCGAC

TGGCTTCCACTACAGTTTCAAGTGCAGAGTCTCACAGATGGCCTACTGTTGGCAAATATA

AAGTCGATCTCACATCATTTGAATCATTGGCATTGCCCGAATTGCAGGTAAAAGAGGGTA

TAGATCTTTTCATTATTGATGAAGTGGGTAAAATGGAACTGTACAGTTCATCATTTTTCC

CTGCTGTTAAGAGAGTCCTTGAATCCAATATCCCTCTCTTGGCATCAATTCCTATCCCAA

AGTACGGCCGAGATATTCCTGCAGTTGTGCAGTTACGAGATCATCCAGGTGCAGAGGTGT

TTACACTTAATCGAGGAAACCGAGATTCTATCAAAGAGCATGTCTATGCCCTGCTGTTAA

ACCTACTGCAAAAGCACTGATAAGATTTGCAGCCAAGAAAGCTGATCGTTGCTACATTCC

TTGCATTCTCAGATTCATTCATTCTGTTCGGTATGAATTCGCGGCTGATTTCTGGTCCGA

GCTGATGGGAGTGTTTAGTTAAATTTCGTCGATATTCTGATGGAGAGAGATGCATTCGGA

GTGTTTAGTTAAATTTCGTCGATATACTGATTGATTTGGAGAGAGATGCATTCTAAAGAA

TAGAAGAACCGAACAGTTTAGCCAGACCCAATCTGCTTTGTGCCAAATCGACTGGCCATC

TCCGCACAAATTTTGCAGAAAATTCGGATCCCTATGGACTGTTTGAGTTTTTAATTGAAA

AATTGGTAAGCATTAAGTCTTTTTTTAATTGAAAAGCTTATAAGTATTTTGTCTAACTAC

AAATGATGTATAAAACTGATGGAACTCATTAAGTTTTTT

>Unigene0001579

ATGCATTCGGAGTGTTTAGTTAAGTTTCGTCGATATACTGATTGATTTGGAGAGAGATGC

ATTCTAAAGAATAGAAGAACCGAACAGTTTAGCCAGACCCAATCTGCTTTGTGCCAAATC

GACTGGCCATCTCCGCACAAATTTTGCAGAAAATTCGGATCCCTATGGACTGTTTGAGTT

TTTAATTGAAAAATTGGTAAGCATTAAGTCTTTTTTTAATTGAAAAGCTTATAAGTATTT

TGTCTAACTACAAATGATGTATAAAACTGATGGAACTCATTAAGTTTTTT

>Unigene0001580

AATCTGCAGCTGCACCGCCTAGCCGGAATGATAAGGGGAAGGGGGTATTTATGGAATCTG

CAGCTGCACCGCCTAGCCGGAATGATAAGGGGAAGGGGTTATTTATGGAATCTGCATCTC

ATAGCACTGAGTTGCCTGTTAAAATAGATCTAACTGGAGAGCAACGTGCATATGGCCCTT

CATCTACTTCGTTGCCTTCATTCGGGAGGTTCATGAGAAAATCTAAGGATGAATTGTCGG

ACTCTAAAGTTTTGGACTCGACTGCCAAAAATGCCATAAAGTCTGCTGCCCTTGCTGCCG

AATTCGAGTATGAAGATGAGTACGATGATTCGTTTGATGATCTTGGTCTGAATGTCGCGG

AATCAGGATTTGAAGAAGCAGAGAACCTGAATGATAGAATTGGGTCAGACACCAGTGGCC

CCCAAAATTCTAGTTCGAAATGGGGCTCACAGAAGAAGCCGCAATTCTTTGTCAAGGATG

GTAAAAACTACAGTTACAAAGTTGCTGGCTCAGTTGCTGTAGCTAATTTTCGAGATGCAG

CTGTTCTAAATCAATCTCAGAGAGATATGATCCATGGTCTCGGACGTGGAGGCAACATAC

CTCAAGGGGTCGTTAGACGGCCCATGGACTCAGAACAGCAGGTTGACGAGTCTCCTGACG

AAGATGAAAATCCAGCAAGAGGAAACTCGAGTTCTCGAGGCAGGGGAGGAGGAGGAAGAA

GAGGAGGAAGGAATCACTATAGGAAGGACAGAGCAATGAAGAAACACATGTCAGGATTAA

GCGGCTATTAGAAGCCTCTAGTTATGTTACCCATTTGGAATGTGTGATTGGAGTATTGGG

CTCCTGTTTATACTTGGTTCATCTTTTTGCTTGGTTCTACAAAAGGGTATCTAGTTTTGG

ATTTTGTTGCATCATTGGGTGATTTGAAGGTCGGTAGATTGTGTACATCAACTTTTTGAA

CATGTCTATATGTTAACATGAACAATTATATACCAGAATATTGAAGCCCATATATTTTTG

GAATATCTGGTACATCACATTGTTGCTGATCAGTTTCTGTGTTCTTTTTCGAATAAGCAC

ATATTTTGAAGATGTTTGTA

>Unigene0001581

CGGCCTCTCCCCAGTGCAGCCTCGCTGGCGCCCTCCCCATCATCTTGCTAGCGCCCTCTC

CAACCTCATTGGTATCATCATCGTCCACGAATCCAGCACATCATAAGCAGCAGACTCTCT

GTCTACCGAGACCCACCTCTTCGCCTACTGAGACCCGACGGAACCCAGTTGGCACCATCC

CCAGCAGCTTCAACGACACCACCATCACCGACATCCTCCGCCTCTGGATCGATGACGATG

GGTCCGATCTATCCCATTCGTCACCCTTCGGTCCCAACCACCCTTTATCTC

>Unigene0001582

ACGGTAGCATCAACGGCAAGGTCCTCACACGCTATCGGCCTCTCCCCAGTGCGGCCTCGC

TGGCGCCCTCCCCATCATCTTGCTAGCGCCCTCTCCAACCTCATTGGTATCATCATCGTC

CACGAATCCAGCACATCATAAGCAGCAGACTCTCTGTCTACCGAGACCCACCTCTTCGCC

TACTGAGACCCGACGGAACCCAGTTGGCACCATCCCCAGCAGCTTCAACGACACCACCAT

CACCGACATCCTCCGCCTCTGGATCGATGACGATGGGTCCGATCTATCCCATTCGTCACC

CTTCGGTCCCAACCACCCTTTATCTC

>Unigene0001583

TCCCTTCCTACCACTGCCTTCCTATGCTCAGCCTCACCAATGCTCCTCCTTAGCCTCAAT

CTCCTCTCTAGGGTTTCGTCCCCAATCCAAAACCCTAGACGTTATTCCCTCCCCCCGGTC

AAAACCCTAGCCAGACGCTCCAATGCCCTCTCTGCATCCTAAATTTCCAATTCTGTGCTC

CACAGCACTAGCTGCGATGACCTTGTCTGCGACAGCAGGATTATCCCGATGCTGATGAGC

ACGGTAGCATCAATGGCAAGGTCCTCACACGCTATCGGCCTCTCCCCAGTGCGGCCTCGC

TGGCGCCCTCCCCATC

>Unigene0001584

GTGTTGGCAACAATGGAAGCTTGCACTTTGGGATTTTGAAATTGGATGACAACATCATCT

CTACAAATATTTGCTTCTTCTGTAGCAGTGATATCGTTTCCCCCTATCCTTTTCAGTGTA

TTCTGAAGCCTCTTGTCATCTGTAGTTGTGGTCTTGTGAACCACCATCTTCTTCCTGCAC

ACACTACCCTTTTCACCGGTGCGAACTGCACAAGCTATATTGATGAGCTTCTCTGTTCCA

TTATTGCCGGAGATGAAGTGTGGTTGGGACCGAAGGGCGACGAATGGGATAGATCGGACC

CATCGTCATCGATCTAGAGGCGGAGGATGTCGGTGATGGTGGGGTCGTTGAAGCTGCTGG

TGATGGTGTCAACTGGGTTCCGACGGGTCTCAGTAGGCGGAGAGGCGGGTCTCGGTAGAC

AGAGAGGCTGCTGCTTATGATGTGCTGGATTCGGGGACGATGATCATCCCAGTGAGGTTG

GAGAGGGCGCTAGCAAGATGATGGGGAGGGCGCCAGCGAGGTCGCACTGGGGAGAGGCCG

ATAGCGTGTGAGGACCTTGCCGTTGATGCTACCGTGCTCATCAGCATCGGGAAAATCCTG

CTGTCGCAGACAAGTGTTAGACCTCTAATCTTGTACTCAACCAAAGAATTAGTGGTTAGT

GGATTAGTGGGCAGAGAATTAGTGGTTAGTTGGCTCCTCACGTTCTCACTTGACGTGAGA

ACGTGGAACCGGTAATCCCTCGTACTAAACCTATATATAAGTGTAATCACATTTTGTTTT

GAATTGAAGAGGAAGCAGCAGAGAGTTCAGTTACCAAGTTCAGTTCTGCTGAACATTTTG

AGTTCAGTTACATTTCTTCCCCTCAATTTCGGTCCCAGTTGTGAAGAGTGTAATTCCGGT

GGCAGCACATACACAGAGGCAGCTGTTCAATCTCGACAGTGAGCTGAGTTCGTAGTAGCC

AGGAGGGCAGCAGCCATTTCATTGCAAAGTTCCAGTGTACAGTGAGTTCATTTGTTTTAT

TTTGCTATTTTAATTTGTCTCCCCTGCGTTGCGTTGCAATTTCCAGTTCCTCTCCTTCCT

CCGATCTCCAGCAAAGAAGGCACTCAACCTTGCAGCCCCTTCCTCCTCCACCTTGGTCCA

CAACAAGGTCATCGCAACTAGTGCTGCGGAGCACAGAATTGGAAATTTAGGATGCAGAGG

GGGCATTGGAGCGTCTGGCTAGGGTTTTGACCGGGGTGAGGGAATAACGTCTAAGGTTTT

GGATTGGGGACGAAACCCTAGAGAGGAGATTGAGGCTAAGGAGGAGCATTGGTGAGGCTG

AGGTTTT

>Unigene0001585

TCGGGTCTCAGTAGGCGAAGAGGTGGGTCTCGGTAGACAGAGAGTCTGCTGCTTATGATG

TGCTGGATTCGTGGACGATGATGATACCAATGAGGTTGGAGAGGGCGCTAGCAAGATGAT

GGGGAGGGCGCCAGCGAGGCCGCACTGGGGAGAGGCCGATAGCGTGTGAGGACCTTGCCG

TTGATGCTACCGTGCTCATCAGCATCGGGATAATCCTGCTGTCGCAGACAAGGTCATCGC

AGCTAGTGCTGTGGAGCACAGAATTGGAAATTTAGGATGCAGAGGGGGCATTGGAGCGTC

TGGCTAGGGTTTTGACCGGGG

>Unigene0001586

GTCCGGTCTGCCGGCGGTGGAGGAGTACAGTTGCTACGACATCCTTCCGTTGTCACCGTG

AAAGGATCGGCGTTGCCGAGGATCTCATTTTCATGGTTCAAGCACAAATCACAGTTGAAC

AAACAGAAAACTTGTGTTGTAACACTAACAGCGCTGAGGTCAAGGAGCCGGCCATTGCAC

CACCTGCTTACACGCTAGGCGTCTACAACTACAGTCTAGACTCGTGGCACCGCATAGACA

TGTCTGCTGCACCGGTGCCCATGTTTGCACAGTGTGCCGTCGGTGGCGGCAGCCAGGTTG

TGTTGGCAGGCGGGTGGGATCCGAACACCTTCAATCCGATTGCCGAAGTAAGGTTAATTG

ACCTCGCTACCGGGACGTGGCGGATGGGGTCGGCGATGCCTACTGCGC

>Unigene0001587

ACAAATTAAATCCTTTTTTTTGTATCGGCGCACCTTCCACCATAAAATGCCCAACTCCCT

TTGTACTTCCCGTTCAAAATCCTCACATCCAGTTTCCCTCAAAATCCCACAGAGTGAGAG

ATGGCTCAGTGCTTGATTCCTGGCTTGCCAGACGACATAGCACTCGACTGCCTCGCCCGG

GTGCCGCATCGGTTCCTCCCCAGCCTCCGTCCGGTCTGCCGGCGGTGGAGG

>Unigene0001588

ACCTCGCTACCGGGACGTGGCGGCGGGGGTCGGCGATGGCTACGGCGCGGTCTTTCTTCG

CCTGTGCTGGGGCTGGTGGGCGGGTTTACGTTGCCGGTGGGCACGATGGGCTGAAGAATG

CGCTGAGATCCGCGGAGGTGTATGATTTTGAGAGTGATGGGTGGACGGCGCTGCAGGAGA

TGGAGGATGAGAGAGATGAATGTAGAGGAGTGGTGGCAGGGGGAAAGTTTTGGGCGGTGA

GCGGATATGGGACGGAGAGGCAGGGGCAGTTTGATACGACGGTGGAGGTGTATGATGCGG

AGGTAGGGGCGTGGAGCAAGGAAGATGGGATAGTGGGGGATGACGGCAACGGCGCCACAG

CCTCCTCCGCCGCATTCTTTGCGGTGGTGAGGGGAGGCGAAGAGAGGGTGTGGTGCGTGG

ACTGTGGGAGGAGGGAGGTGAGGGAGTACGAGGTAAAAGGGTGGAGAAAGGTAGCAGCAG

TGCCGGAGAGGTTGCGGTCAAGTGTTTCCGCGGCGGTGATGGGAGGAGGGGAGAGGGTGA

TGGTGA

>Unigene0001589

TAGCATCGTTTCAACGCCACAAAATATAATTGAATGCATACTAGACTTGCCTTCAGCGAC

AAACACAGCAGCATTGTACCAAAAGGATCCAAGAATGTAGACAATTGACTCATAGGTCCT

CCGGCGCAGCCAGGAATATATTGTAGCCCGGGCACACAATACTCAATAATTAGAAGAACT

CCTAATATACAGGAATATCTACTATCATCTAGCCTATTTGTTCCTAAAAGATGTTTTTAT

C

>Unigene0001590

TAATATACCGAGGAATATCTACTATCATCTAGCTTGTTTGTTCCCAAAAGCTGTTTTTAT

CAACAACGTTTCAACACGACAGATTAAAACTGAAGGCAAGTCTAGTATGCATTTTAAATA

TGGGTGGGGTGTACGTAAATTCAGATTGAGCTTTGTGGGTGTTTTGAATTCCCAATACTC

TATAACTGCAGTTGAACGCAAAGCAATTTGCTTTTATTGTGAGATGAATTTCACTTGTCC

CTCAATAGATGTGGCATATTTGTCTTTCCCACTAAACTCTTTTTGGCTTTTGACTTTCCT

CCAAGTGTTTTTATAAACTATCCTTTATTTTA

>Unigene0001591

CTTTTATTGTGAGATGATTTTCACTTGTCACTCAAAAGATGTGGCATATTTGTCTTTCCC

GCCAAGTCTTTGTGGCTTTTGTTTTTCCTGCTAAAGATGTGGCATATACTATCTTGCATG

GCTTCACCTTGATGGGCAGTCTAAGCCTTTGATTTAGTAAATACAGGTGGAAAGCCCATG

CAGACCCAGTGTAGGGCTGCTGCCCTTGGAGCAGCGCATGTGATGTGCACAGGAAACGGC

CGCGGGCCGGACGCCTTCAGCGGCAAGCACAGCAGCATTGCCCTAAAAGGATTCATGAAT

GTAGACAATCGGCTCATAGGCCCTCCGGCATAGCTAGGAATATATTGTAGACCAGAGATA

CAATATTTAATAATTAGAAAAACACCTAATATACAGGAATATCTACTTTCATTTAAGCCC

ATTTGTTCCTAAAAGCTGTTTTCATCAACAACGTTTCAACACCACAAAATAAAATTGAAG

GCAA

>Unigene0001592

GCCTATTTGTTTCTAAAAGATGTTTTTATCTACAGCATTTCAACGCCACAAAATAAAATT

GAAGGCAAGTCTAGTATGCGTTTTAAATATGGTTGGGGTGTACGTTAATTCAGACTGAGC

TTTGTGGGTGTGTCGAATTCCCAATACTCTATGACTGCAGTTGAACTCAAAGCAATTTAC

TTTTATTGTGAGATGATTTTCACTTGTCCCTCAAAAGATAGTGGTATATTTGTCTTCCCC

GCTAAAGTATTTGTGGCTTTTGTCTTTCCTGCTAGTGACGTGGC

>Unigene0001593

ATACAATATCATGATATCTGGGTACTCTGGAAAGGGATATATCTCTCGAGCATTTGAATC

ATTCCGTGTTGTGGTGGATTCAGGTATTGAACCGGATCACTTTACCATGGTAGCTCTTCT

CATGTGTTGCAAGCAGCTGAAAAGTTTGCCTTTGGGGAAATCGATTCATGGATGGATTGT

AAGGAGATTCTCAACTTGTGGATGCGGCTTGGTTTTAAGTAATGCTCTTCTGGACATGTA

TGCCAAATGTGAGGAAATGGAGATTGCTTTGCGGATTTTTAATGTAGTTAATGAGAAAGA

CTGCTTCTCATGGAACATAATTATTGCTGGATTTGCAAATATCGGCAAATTGGACCAGGC

TTTAAGGTTTTTCACGGAAGCTCCTAGCAAGGATGTGGTTTCATGGAACTCTATGCTTGC

TGGGTATGCCCAGAAGGGGGACTGCAGAACAGTAATGCACTTGTTTCAGAGAATGAATGG

GGATAATGTTAAACCTGATAAGGTGACAGCTGTTTCTCTGGTTTCTGTTGCTGCGGAGAT

GGGAGTTCTGAATCATGGGAGGTGTGCACATGGTTGGGTGGTGAAGGCACATGGGTACTC

AGATTGTTTCTTAGGATCTGCAGTAATAGACATGTATTGCAAGTGTGGAAGCATTGGGAA

AGCCCTAGTTGTTTTGAAGTTGATTGCTGCCAGGGATGTTACAACTTGGACAGCCATGAT

CACAGGGCTTGCTTTTCATGGTCATGGAATAAAAGCTTTAGAACTGTTCTCTAAAATGCA

GGAAGATGGTTTAAAGCCAAACTCAGTGACCTTGATTGCGATTCTTACCGCTTGTAGTCA

TTCTGGCTTAGTGTCTGAGGGTTACAGAATTTTTGAGGAAATGAGAGAAAGCTACGGTGT

TGAACCACAAGTAGAGCACTACAGTTGTCTTGTTGATCTTCTTGCGCGATCAGGGAGGCT

TAACGAGGCACTGCATGTGATTGAAAGGATGCCATTGAAGCCGAGTAAGTCAATGTGGGG

AGCCATTTTAAGTGCTAGTAGAAGCCCAGGAAATGTTAAGCTTGCAGAGACTGCTTTGGA

GGAACTTCAAAAGCTGGAACCCGAAGAAGACGGTGGCTATGTTCTGATGTCCAATGTGTA

TGCTGCTCATGGGAGATGGGGCTCCTCTGACAATATCAGAGAAAATATGGGACACAATGG

AGTGGCGAAGATTGCGGGTTGCAGTAGTGTTGTTACTGATGGGGTGGTCCATGACTTTGT

TGCTTCAGAGAAGAAACATCCAAGATGGGGTGAGATTTATTTAACATTATGCGACTTGCA

TAGAGAAATGACGCCAGTTGCCGATGCACCTTAACCTTTTGCAGCAATAAT

>Unigene0001594

CCCCGCCTTCTGCCACCTTGCCTTTGACTCCTCCGTCTCCGTCCCCCTTCGAGCCCACTC

TATCCCTCGATCTCCGCCTGCCCTGATGACTTAGCCCGCGGGCTCGAACGCCCCTTGCTC

ACGGCGCCCGCGCCCTTGAAGCAGCCGTCCGTGGCGGCGGTCGGATCCCTAGAGTCAGAT

CGTGATCTTCGACTTCCTCGCCCGCCGCCATACTCCTCGACTCCGTCCACCACCTCCACA

AGATCCTCCGCGGCATCAATGGCGACCACCGCCATCGCCGCCACAGTAGTTCGCGCCGTA

CTTCATGCCCGTCCCCTGCATCTGCGGCTTCGTCTGGATATCGGAGGATCAACCCATCCT

CCGCCCGCCGGATCGGCGATCCCGCACTTCGCTGAGTGCCAGGGACGCCGCCAGCCGTGC

CTTCCCCTCGCCGTCTGTGCCATTGTCCGCTCCCTCTTCAGCCCCACCGTGGCGCTGGCG

AGTGGCGGCCGGAGTGCCGCCCTCCTCCGCAAGCTCTCCCTCCCTGCTCAGGACTCCCTC

TAGTCCGGACTCATCGCAACCACCGACAACCCTCCGCAAGCAGTACCCGATCAAATTCAC

CGGCCGTCAGTGCCGC

>Unigene0001595

CGGTGCCTTTAGTATCGTCCCCATAAAATCGAAGAAGATAAGCTGATTCAGAAACACACA

GCATCCGCAGCTCAGCTCCATGGCCCAAGCTCTAGCCGCCCCCTGCGTCCCGGTCTCACT

CCTAGATGCGACTGGTGCGACCAAATCGGTACGAGCAGCCGCCCTGCCGCGACTTGGGAA

CTCGTCCAAGATTCCGGGGCTCACATCTTCGTTCAAGTGCGGTTCTCCTCTTTTGTCTGT

GGTAAAATCCAGGAAAAGCAAACTGGCATGCCAGGCATATTCACTTTCTATCAAGTGTGA

GCAGAGCGCGAAGGAGAGTAATGGATTGGACCTATGGCTAGGTCGTTTTGCGATGGTTGG

ATTTGCATCGGCAATTACCGTTGAAATTGCAACTGGGAAGGGACTTCTGGAGAATTTCGG

GCTTGTAACACCGATACCGACACTAGCTCTTGCTGTTACAGCATTGGTTGGAGTTCTGAC

GGCATTCTTCATCTTCCAGTCTGCTTCTCAAGATTGATCACTATGTACTTGGAATGCAGA

GGTCCATCAGCTATTATATTTTCTCAAAAGCAAATGTAACAACAGTATGGATACAACATG

TAACCATTGAAAAAGTTATTTTCACTACTTTCGCGTCCAAAATTGCTCAGATAAAATGGG

ATGAGTTTTTGTGTAATGAGTTTTTGTGTAATGTGTTTGAGGAACAAGCAATATTTGTCC

ATTTTCGATATACGGATGCTTCTAGATTGTTTACTGCATAGACTGATGCAAGCTGAATTA

TGCGTAGACTGATACAAGCTGAATTATGAAG

>Unigene0001596

ATTCTCTGTTGTTGATCCAGCAAAACATTTGTCGCGACCAAATCCAGTCAAGCTTCCCAT

TTTCGGGAGAAACTCTCTCGAAATGAACAACGCGCAGACGCAATTTCTCGATCAATCTAT

GCGATTTGTGTAGCTGGATTGAGGAAGCTGATGGATAGCACCGCACAAGATCCCTCAAAT

GGCGATGGAGATGCGAGGAGGGAGAGATCGCTCGAGCCGTCTAGTTCAACCCTGGGTAGT

GGCTACCGTCTGTTCGACCGGCAGCGGTCGATTCATCAGATAATCGGAGGCGGTAAAGCT

GCCGATGTCATCCTTTGGAAACGTCGACGAATTTCTTTTACTGTTATAATTGTTGCTACT

ATTGCATGGCTTGTATATGAGCGATCAGGATTATCGTTTCTGACAATTTCGTCAGATGTG

CTGCTGATATTAATTGTAATCCGGTTTGTTCGAGCTAATGGTGCTGTTCTTCTAAATAAA

CAAATTAGGCCACTACCAGAATTGGTATTATCAGAGGAAATGGTTAACAGTGTCGCGGCT

TCGTTTCGTGTTAAAGTTAACAATGTGTTGTTGATGGCACATGACATCACTCTTGGCAAG

GACTTCAGGTTATTTTTCCAGGTTGTGGTACTTCTTTGGATCTTGTCAGTCGTTGGTAGT

TTCTTCTCATTTTTCACTCTTGCATACATAGGAACAATAGCGGCGATCACTGTTCCAGCT

CTGTACAATAAATATGAAGAACGAGTCGACCGGTATGCTGGGCTGGTTCACCGGAAGTTC

TCCAAGCATTACAAGGTGGTAGATGAAAATGTTATCCGGAGAATCCCGGCTCGCATTTCC

AACGACAAGGATGCATAATCTCTCAAGAGAGCTTCCATCCTTTTCCCGGGTTGATTCCAT

GAGAACTTGGTATACTATAAGCAGTAGACACTGTAGTGATTGTTGCACTTAAAACTCCAG

TTTCATATAATGTTCACCCTTTTGTTCGATCACTTGGTTAGTTATTCTTTTTATAATTTT

TTTGGAATAATTATTGTTTACTGCTTTTCTCCCTGATCGGTTGTATCCTCTTTTAACAAC

TTTTTTATTATGTCTTCTATAATAA

>Unigene0001597

GATCAAAGTGTTTTCGTTGATTGAGATCTGTTGTATTTTATGATTTTGAGGTCAATCTGT

TGTGCTTGTAGTCGTTGTATGGTTAATTATGTGTTAGTTATGCTCATGATCTACAGATTC

TTCATTATTTGGAAGAACTTAACTTCTAGCTTTTTTTTACAATGAAAATTGTGCTATATA

TTATAATTTTTGGTAAAAACTATAGCAATGTATTTAGCTATATTTTTTTTTGTAACTTTG

CTTTTAATTGGATTTTAAGCTTAGTTCATGTGAGACATAATTTTATAACTCTAGATCGAA

ACAAAGCCATTCTACGGTAATAACTCAATTGTAGTATAAGTGTATTGAAATCGGCATCAG

TGACAATTAATTTCTTAGGTTATTGAATCTCATTTTTTTGGTATAAGAAGCTATTGATTG

TGGCGTGCCTACATTTTGATTCACATAGCTATTAATTGGTAATAGATTCATATTTTTTGG

TTGGCTGATCTTGAATTTGATAATAGTAAACTAATATTGATATCCTATGGCATATAGTCA

TGCATGATAATCTTTTAGTGGTTGTCTATCAATTGAAAAACTGTATTGCAAATTGATCTG

GCAATAGAATGATGTTGGTAATTGTATTATACATTCCTAGTACATGATTCACTCATAAGT

ATCATACCTAAATTTTATTAGGTGCACATTGTCTGATTTGATTGTTTACTCAAGTGACTT

AATTATTTATAATTAAGTGAATGTTGTATGCTTTAGTAATACCAACAGTGAAGGTGTATA

CAAATATACTATAATATATAGTTTGTTCTGGTCTTCGGGAGCAATTGAATAACCCAAACA

TCTTTCTTTTACGTGTATGTGTAGGTTGCCAAAGATCCATCTGGCAAACAGATAAATGCT

CTTGAGCAGCACATTAAGAACCTCCTCAGCCCATCCACCCCCTTCTTCTTTAACACCCTG

TACGACCCTTATCGCGAGGGTGCAGATTTCGTCCGTGGCTATCCTTTCAGTCTCCGGGAA

GGAGTTCCAACAGCCGTTTCTCACGGCCTTTGGCTCAACATTCCTGATTATGATGCCCCC

ACCCAACTTGTCAAGCCTCTGGAAAGGAATAGCAGGTATGTTGATGCGGTTCTCACAATC

CCCAAGGGAACCTTGTTCCCCATGTGCGGGATGAACTTGGGATTCAACCGGGAGCTTATC

GGCCCTGCAATGTATTTTGGGCTTATGGGTGATGGTCAGCCGATTGGGCGTTACGACGAT

ATGTGGGCGGGATGGTGCATCAAGGTGATTTGTGATCACTTGGGATGGGGAGTGAAGACA

GGCCTTCCCTACATTTGGCACAGCAAAGCGAGCAATCCATTTGTGAACCTCAAAAAGGAG

TACAATGGTATTTACTGGCAAGAGGAGCTGATCCCCTTCTTCCAGTCAGCGACTCTGTCG

AAGGAGTGCACGACCGTCCAGAAGTGCTACATTGAGCTCTCCAAGCAGGTGAAGGAGAAG

CTCGGTAAGGTGGACCCTTACTTCACCAAGCTAGCAGATGCCATGCTCACCTGGATCGAG

GCTTGGGATGAGCTCAACCCATCGGTGAGTGCTGCTACACCCGAAGCAGCCAATGATGCA

GCCAAGGCCAAGTAGGATACCCTTAGTTTGCATTTCATCATTATCTCGTTCTTTTTGGTA

TACCCTCAGTTCGCATTTCGTCATTACCCCTTTCTTTTTGATATAATGCTATTGGACTTC

TAGACTATTGAATGTAACTTTTTTTCTTGGATAATCATTTTATGAACTCTTTTGTAGTTC

GATTAATGCGTTCTTCTTTATTTGAGGTCTTAATAATATCCTGT

>Unigene0001598

CTCTGTTGGGAAAGTTACTCCTCAGCAACTTGAGAAAAGGTTGAGGATTCTGACGCCTGT

TGCAATATATGATGTGAGGACTGCATAGTTCTGACAGCAGCGATGCTTCTATCTGGTACG

AGTTAATTGTTTATATCATTTTGGATACTTGGGTCTTGGTATATGCTTGTTATCTTGGAA

AATAGGAAGTTAGAAGCTATCCTTATAAGTGAGCTATTATGGTTTTTAAAGATTTTTTTC

AATATGGCATGGTTTCTATTTTGGGTTGCTATAATTTGAACTTGAAAGTGTTATAAACCT

GATGTTTCATCTAGTGTATGACTATTTGCTTTAGTTTATACGTTTTAGCTAT

>Unigene0001599

CTCTGTTGGGAAAGTTACTCCTCAGCAACTTGAGAAAAGGTTGAGGATTCTGACGCCTGT

TGCAATATATGATGTGAGGACTGCATAGTTCTGACAGCAGCGATGCTTCTATCTGGTACG

AGTTAATTGTTTATATCATTTTGGATACTCAGGTCTTGGCATACTTGTTATCTTGGAAAA

TAGGAAGTTAGAAGCTATCCTTATAAGTGAAAGCTATTATGGTTTCTTAAGATTTTATTC

AATATGGCATGGTTTCTATTTTGGGTTGCTATAATTTGAACTTGAAAGTGTTATAAACCT

GATGTTTCATCTAGTGTATGACTATTTGCTTTAGTTTATACGTTTTAGCTAT

>Unigene0001600

TCCACCACCCACTAAAAGTAGGGTTTATCCCCAAATCCCTTTCCCCTTCTCACAAATTCT

CAATCTGCACTCAATTCCTCCGATTATCGCTGTCCATGGCTCGCCCCCTCACCCCCGGTG

ACCGACCTACGGCCGCCGGCTTCGCCATCATCAAATCTCGATCTCTGCATCCAAGCGTTC

TCAGGAGTTCATCTCCGAGACCGTCAAGCGCTCCCAAGAGATCTCGATC

>Unigene0001601

TCCACCACCCACTAAAAGTAGGGTTTATCCCCAAATCCCTTTCCCCTTCTCACAAATTCT

CAATCTGCACTCAATTCCTCCGATTATCGCTGTCCATGGCTCGCCCCCTCACCCCCGGTG

ACCGACCTACGGCCGCCGGCTTCGCCATCATCAGATCTCGATCTCTGCATCCAAGCGTTC

TCAGGAGTTCATCTCCGAGACCGTCAAGCGCTCCCAAGAGATCTCGATC

>Unigene0001602

TCCACCACCCACTAAAAGTAGGGTTTATCCCCAAATCCCTTTCCCCTTCTCACAAATTCT

CAATCTGCACTCAATTCCTCCGATTATCGCTGTCCATGGCTCGCCCCCTCACCCCCGGTG

ACCGACCTACGGCCGCCGGCTTCGCCATCATCAAATCTCGAGATCTCTTGGGAGCCAAGA

AGGAGCGCTCCCAAGAGATCTCGATCTCTGCATCCAAGCGTTCTCAGGAGTTCATCTCCG

AGACCGTCAAGCGCTCCCAAGAGATCTCGATCTCGGCTGCCGAGCGCTCCCAGGAGTTCA

TCTCCGAGACCGCCAAGAAATCCCGTGATCTCGCCGCC

>Unigene0001603

TCCACCACCCACTAAAAGTAGGGTTTATCCCCAAATCCCTTTCCCCTTCTCACAAATTCT

CAATCTGCACTCAATTCCTCCGATTATCGCTGTCCATGGCTCGCCCCCTCACCCCCGGCG

ACCGATCTACGGCCGCCGGCTTCGCCGCCATCAAATCATCCAAGCTCGCGGCGGCGGAGT

CCGACTCCACCCTCCTCAGCTCCCGCTGCGCCGATCTCGAAGCCAAGCTCAAAGAGGAGA

AGGAATGTTTCGACATCCTAGATGCAAACTTCTCCGAGCTGGAGCTCAGAAAGTTCGCAG

CCGAAAAAGAGGTGGAAAAGCACAAGCGAGTTTCGGATCAGCTCCAGGACCAGCTGGACC

GGGCCACAGAAGACATCACCCTGCTCAGGGCGAGGGACCAGCGAGCCCAGGAGAGGATTG

ATTTTTTACTGGAGGAGCTCCGGCAGAAGGAGCAGGAGTTGGCTGCCAAATTCGTAGAGC

TCAAGGTGGAGAATCAGGACTTAAAGTGCGCGAAGCGGCGGGCGCAGGAAGAGGCCGAGC

AGTGGAAGCGGAAGTACTGTGAGGTCCAGGTCCGGTTGTTGGAGCTTTTCGATGAGAATC

TTGCTTTTCGGGCGAAGACTGGTGTGGTGGGGGAGAAAGAGGAGGGCGCTCGCCCACATT

GTGTCATTGAGGTTAGTGATGAGGACGGAGGGGGAAAGGAGTCGAGAGCTGATGGTGTTG

AAATGACAAGTCCTCGCCGCACTCCAGTTACTAGCGGAGGAGGAGACAATAGTAAGAACG

GGGATACGGTCCCGATTAAGCTGAAGAGGAAGAGAGAGTCTGATGCGATCAAGAAAATTC

CTGAATCCAATGGCTCTGTTGGGAAAGT

>Unigene0001604

TCGTCCTCGCGACGTCTCTCGTCACCGTGCCTCGAGACAAAGCTCGTCACCATCGCCTAC

AGTCCATCATCACCGTCATAGATGACCGGCGTCTTCCTCCTTATTGACATCGTCGCCTCA

TCTCCAACCGGCAATCACGATAACAGCTTCTACAGCTATGACGGCAAATAGTTTCCTTCG

CGCCCTCGCCTTCTAACGATTCCGCCTTCCCGGACTCTGTGCCCGAAGCCCGATATGGTT

GATTAGCCAACGAAGACAACGACAGCCTCGAGGTTCACCCAGCCTTCGTCGAGCACCGC

>Unigene0001605

TGGGTGAACCTCGAGGCTGTCGTTGTCTTCGTTGGCTAATCAACCATATCGGGCATCGGG

CACAGAGTCCGGGAAGGCGGAATCGTTAGAAGGCGAGGGGGCGAAGGAAACTGTTTGCCG

TCATAGCTGTAGAAGCTGTTATCATGATCGCCGGTTGGAGATGAGGCGACGATGTCAATA

AGGAGGAAGACGCCGGTCATCGAGGACGGTGATGATGGACTGAAGGCGGTGGTGACGGGC

TTTGTCTTGAGGCACGGTGACGAGAGACGTCGCGAGGACGAAGGCGACTGAATTCGGTTT

CGCTAGTGAGGAGAGATCGCGTAGTGGCTCGATCCAGTCTGGTCCTTCAGCGATCCAATC

GCCTCCTTGATCATATCAAGGTAGGCAGGGTGGAGAGCTCGCTTTTTCTCAGTGGAGGTC

CCCTCTTTGGCCTTCACATCCTTGGCCAGCTTCGCTGCCAGTGCGTCACAGGAGGAGAGG

ATGAGAACCATGACGGGAGTCCCGACTGTGGTGGAGGCTAACTCAGTGGACGTCATTTCG

ACAGAGAGGAAAGAGGGATCCGTGGTTGCAGTGGTGGTTGAGGGCAAAAATGTCACAGCA

GGTCATCCGAGAAGTGTGTTAATAAAGCT

>Unigene0001606

TGTAGCATTCTTTGTAATGAGATGTTGACATGATATTTGAGCACTTAAATGAGATTGTTT

GATTTTGTGGTTGGAAAATATAAGATTAAATACTGATTTCTCTTATTGTTCCATTCACAA

GACTTGGAGAACAAAATTGTTGAACATGTGATAAGAATGTTTATTCAGTCGTGTTTGACA

GAACAGGAATGGACGAATCCAAAACCGTTTCAGAATGGCTGAAGAGTTGTATTAGAAAAT

AAAAAAAAGTTACAAATAATCATGAAAATAAGGAAAAACAGAATGTGACTAATATAATTG

ATTAGTGCTCATTTATGATGCACTATATTGAAGAAAAACAAGTTGAAACAACAGGAGCAT

AAAAAATTTAAGAAACAATTGGTTGTTGACTTGTAGCACATGTGTGTGGTCGTTAGGATT

TCACGAGACTTTATTTATGTCATAAGCATCATTTTTTAAGTACCCCTGAGGATTACCCCT

GAGGAACATATCCAA

>Unigene0001607

TTTAATTTCCATAACTTTATTATTTTTATAATTTTATCTTAACTCGTTGTTAAGGTTATT

ATGTGTCGTGAGTACTACCGTTACAATCCCACAACGCTATCGAAGAGTTTTGTTGTTACT

TTATTAGTATAATACGTAACGTGTTTTCGTTACTGGATCCGGCAAAGGTAAATGGTAAAG

GTCCGGGTTTTCTCCAGACGCGCTCTCGACGATGTTATCCAAAACATTCGCCGATCGTTC

GTAGTTGTGCGGTTTCGGTGTGAACGGAAGAGCTTGGGCATCGTTGTCTATAGATAAATT

GATCAGAAGAAGACTTAACCATGTATTTGTATAATATGACATTACAGCGTGGTAGCGGCA

TTACGCATGCTATCCATGGTAATTTCAGTGGTGGAAAAGTACAGGAAATTTTAGTATCTC

GTGGAAAATCTTTAGAGCTTATGAGACCTGATACAAATACTGGCAAAGTCCATACATTGC

TAACTGTTGAGGTATTTGGAATTATTCGTGCTTTGATGAGTTTTAGGCAATCTGGCGGCA

CTAAAGATTACATTGTTGTTGGTTCTGATTCTGGAAGAATTGTTATATTGGAATATTTGC

CTTCAAAAAATATCTTGGATAAAGTGCATCAAGAAACGTTTGGAAAGAGTGGATGTAGAC

GAATTGTTCCTGGACAATATTTGGCTATTGATCCAAAAGGAAGAGCTATAATGATTGGTG

CTGTAGAAAAGCAAAAATTAGTATACATTCCTAACAGAGATGCTGAAGCACGACTTACAA

TTTCATCCCCTTTAGAAGCTAATAAAAGTAATACACTTGTGTACCACATGGTTGGAATCG

ATGTAGCTTTTGACAATCCAATTTTTGCCTGCCTTGAAATTGACTATGAGGAAGTTGATG

CAGATCCAACTGGAGAAGCTGTTCAAAATACGAGGCAAACTCTTACATTTTATGAAGTTG

ATTTTGGTTTAAATCATGTAGTTCGTAAATATAGTGAACCTTTAGAAGAACATGCTAATT

CTCTTATTACTGTTCCTGGTGATAAAGATGGACCTGGTGGTGTACTGATATGTTCAGAAA

ATTATATTACCTATAAAAATCAAGGAGAACAACCAGACATACGATGTCCAATACCACGTC

GACGTAATGATTTGGATGATCCTGAAAGAGGAATGATCTTAGTGTGTAGTGCTACACACA

AAACTAAATCTATGTTTTTCTTCTTGGTTCAGACAGAACAAGGCGATGTTTTCAAAGTGA

CATTAGAAACCAATGAAGAATTTGTGACAGAAATTAGACTGAAATATTTTGATACTGTTC

CAGTAGCTTCTGCTATGTGTGTTCTAAAAACTGGGTTTTTATTTGTAGCATCAGAGTTTG

GTAACCATTACCTGTATCAGATTGCAAATTTGGGCGATGATGATGATGAGCCAGAGTTTA

GCTCAGCAATGCCTCTCGAGGAAGGCGATACATTTTTTTTTGCTCCAAGAATATTGAGAA

ATTTAGTATTAGTTGATGAAATGGATTCATTATCACCCATAATGGCTTGTCAAGTTGCTG

ATTTAGCTGCTGAAGATACCCCACAATTATATATGGCTTGCGGTCGAGGTTCAAGATCTA

CCTTAAGAGTTTTAAGGCACGGTCTTGAAGTTTCTGAAATGGCAGTTTCCGAGTTACCTG

GAAGTCCCAATGCAGTATGGACAGTGAAAAGAAGAGCAGATGAAAATTTTGATGCCTATA

TCATTGTGTCTTTCTCAAATGCCACTCTAGTGTTGTCTATTGGAGAAACTGTGGAAGAAG

TTTCCGATTCAGGTTTCTTAGGAACTACGCCAACTCTATCTTGTTCTCCATTAGGTGATG

ATGCTGTTGTACAAATTTATCCTAATGGTGTGCGCCATATTCGTTCTGACAAAAGAATGC

ATGATTGGAAAGCCCCTGAAAAAAAGAAGATCGTAAAGTGTGCAGCTAATCAACGACAAG

TTGTAATTGCCCTTGGTGGTGGTGAACTTGTATATTTTGAAATGGATCCGACTGGACATT

TAAATGAACATAAAGACCGTAAAGAAATGAATTCTGATGTCCTTTGTATGGCATTAGCAA

ATGCCCCTTCAGGAGAACAGATGTCACGCTTTTTAGCTGTTGGTTTGACTGATGAAACAG

TCCGTATCATTTCTTTAGATACTACAGATTGTTTAGTACAATTAAAAATGCAAGCGATAC

CAGCTATGCCAGAATCATTATGTATTGTAGAAATGGGTGCTAGTGATGGTGGTTCTTCTG

ATGAACCAGGAATGAATTCACTATCAATGTTGTATTTGAATATTGGTTTACAAAATGGTG

TATTACTTCGTACAGTCCTGGATGGTGTTACTGGTGAAATGGCTGATACACGCGCACGTT

ACTTAGGAGGCAAACCTGTAAAACTTTTTAAAATCAGAACCCGAGGAAATGAAGCCGTAC

TTGCCATGTCAAGTAGATCATGGCTTAGTTATTACTACCAAAATCGTTTTCATCTAACCC

CGTTGTCATATGAAAGTTTAGAATATGCATCTGGTTTCTCTTCTGAACAGTGTCCCGAAG

GTATTGTTGCTATTTCTGGTAATACCTTGCGCATTTTGGCCCTTGAAAAATTGGGTGCTG

TTTTCAACCAAGTTTCATTCCCTGTTGAATATACACCCAGAAAATTTGTTATTCACCCTG

ATTCTGCTCATTTAATTGTGGTGGAAACTGAACACAATGCTTATACTGAGCAAACCAAAA

AACAGAGGCGAATTCAAATGGCAGAAGAAATGCAAGAAGCTGCTGGAGAGGAAGAACAAG

AATTAGCGAAGGAAATGGCTGATGCTTTTTTGAATGAAGATTTACCAGAAAATATTTTTG

GTGCTCCCAAAGCTGGTCCTGGAATGTGGGCATCATTGGTCAGATTGTTTAATCCAGTTC

AAGGCATAACAGAAGCTGTTTATAGGTTCCCTCAAAATGAAGCAGTTATGAGTGTTGCTC

TAGTACGTTTTTCGAGTTATCCGGATTCACAGTTTGTTCTTTTTGGTGTTGCAAATGAAC

TTCATTTGAACCCAAGACATGTTACTTGTGGATATATCTACACTTACAAAGTTAACCCTA

CATGTACTTCTTTGGAATTGGTTCACAAGACCACAGTTGATAATGTACCAACTGCAATAT

GTGGATTCCAGGGACGTGTGATAATTGGTATTGGTAGAATTTTACGACTTTATGACATTG

GTAAAAAAAAACTTTTACGGAAATGTGAAAATAAACAAATACCATTATTAATAATTGGAA

TTCGTGTTATGGGGTGTAGAATATATGTGAGTGATGTTCAAGAAAGTGTGTATATGGTAC

GATATAAACGTAATGAAAATCAGTTAATTATTTTTGCTGATGATACACAACCTAGATATA

TTACTGCAATGGAAATACTTGATTATAATACAGTTGCTACAGCTGACAAATGTGGAAATA

TTTCAGTGGTCCGGCTAGCATCATCAATATCTGATGAAGTTGATGATGATCCAACAGGTA

ATAAAAGCTTGTGGGATCGTGGTTTACTTAATGGAGCCTCTCAAAAAGCTGATTTCATTG

TGAATTTCCATGTTGGAGAAATCTGTACTTCAATACAAAAAGCTACATTGATACCTGGTG

GTTCTGAATCTCTTGTTTATGCTACGGTGACTGGTACTATTGGTGTTCTCGTACCATTTA

CTGCACACGAAGAACAAGACTTTTTTCAGCATCTTGAAATGCATATGAGATCTGAGAACC

CACCACTTTGTGGAAGAGATCATTTATCATATCGATCTTATTATTTCCCTGTAAAAAATG

TTTGTGATGGTGATCTGTGTGAGCAATATAATTCTATCGATATTGCCAAACAGAAAAGCA

TTGCAGCAGATCTCGACAAAACTCCTGCCGAAGTGTCTAAAAGACTTGAAGATATTAGGA

CAAGATACGCATTTTAAATTATTACCTACCTTAATTTTTTGTAATTATATTTATTAAAAA

AAAATTTATATGTAATTAGCTATATTTTTATCTAATAAGTTATTCAGGTCAAAATAACAA

AATGTCTAACTTTGTACAAAGAAGTTCCTGGTCAAAGCACTGTGTCATGTTGAATGTTTT

TTATGTGTAATAGTGTATAGTATAATATCTAATTATAAGACCATGGTATTGTTGTAGCAT

CATAGCATCATGTATTATAATAATTTCACCAAATAGTGTTTTAA

>Unigene0001608

GCGCCCACCAGTTGGTTGTTCTGAGATCACCCCTGTAACCCTAATCCCCACCATCACGCC

CGCCGGTTCGTTGATCAGCGATATCCTCGGCCATGGTGACCACGTCTCTCGTGGTGGTTA

TTGGCTGTCTTGTTGCGGGAATTCTTGCTTTACTTATCTTACTGGGTCACCTGTGTATGA

AGTTACGGAATCCCGTCGGCTGAAATTCTGAAGGGCCGTACTGCATCCAGAATTCGGCCG

TGCCTCTCCGCCTCCTAACAGGCATGCCTCTCCAAGTCATGAGAGAGGCAACCTCCGATT

TCTGCACAGCCAGCATCCTGGGCCGTGGTGGTTCTGCGACAGTTTACAAGGGGCATATCA

ATGGAGATCCAGTTGCCCTTAAACGACTGAATCGGGGCTTCCGAGGACAAGAGGTAATTA

AGACTTTTGAGTCTGAGTTGGCCGTCCTCTCCAATCTAAATCATAAGAATCTTGTCTCTA

TCTTGGGCTTTTGTTTTGAGGAAGGCGAGATGATTTTGGTTTACGAGTACATGCCTTGCG

GGACTCTGAGCGACTATATCTTTAACCGGGCTCAAGCACCAGAATCTGCATCATATTTGG

ATTGGAAAACCCGAGTTGCAGTTGCCCTTGATATTGCCAGGGGGCTCGATTATCTTCATC

GGATGGCCCTGCCTAAAAGCTTAATCCACAGGGATTTAAAGCCATCAAACATACTTTTAG

GATACAAGATGAACGCCAAAATCGGCGACTTCGGCCTTACCAGACTGGCCCCAGGAGGCG

AAGCATCCTCAGTTCATACTAGGCTAGGGGGTACATTTGGCTACATCGCGCCAGAGTGTG

CACGTAAGACTTCTCTTTAACTAATTTAAATTTACTGACGTTGGATTCGCTTAGCACTTT

TACATTTACGGAATTTACATTTATTTAACAGTAACAGCTCGTGTGAAATTTTATTTGTTG

ACATTTGTTTAACATTTTGCTTAAGCATTTGAAAATTTATTAACAGTAA

>Unigene0001609

ACTCGTCTGCGAGCACACTCGGCCTCTTCCCCAAAATAGAAACCCCGTTCTAGAGGTTTT

GGCGCCAACCACCTGAACCCTCATCTCGACAAAGAGATCGAGCTCCCCAATGGCGTCGCC

GACCTCTCCGATGGACATCGACGATGCCGCTCCACCGGCGCCGTCCCTCAACAAGGGCAA

GTCCGTCGTCGTCCCCGCCGACGGCAAGTCCGCCCCCTGGGTCGAGAAGTACCGCCCCCA

ATCCCTCGCCGACGTCGCGGCCCACCGCGACATCATCGACACCATCGATCGGCTGACGAG

TGAGAACCGGCTGCCGCATTTATTGCTGTACGGACCACCGGGGACGGGGAAGACTTCGAC

GATCCTCGCGGTGGCGCGGAAGCTCTACGGGGCGCAGTACCAGCGGATGATTTTGGAGCT

CAATGCCTCTGATGATAGGGGGATTGAGATCGTCCGGCAGGAGATCCAGAACTTCGCCAG

CGCCCGCAGCCTCTCCTTTGGTGCAAAATCTGCTGTGAAGTTGGTCCTGCTGGATGAGGC

TGATGCCATGACTAAGGATGCTCAATTTGCGTTGCGTAGAGTGATAGAGAAGCATACAAA

GAGCACAAGGTTTGCACTTATTTGCAATCATGTCAATAAAATTATTCCGGCACTACAGTC

GAGATGCACTCGGTTCCGGTTTGCTCCACTTGATGCTTTTGACATTAAAGAACGCCTTCA

ACATGTGATAAATTCTGAAGGGCTTGATGTGTCTGAGAGTGGACTTGCTGCTCTTGTTCG

ATTGAGCAATGGTGATATGAGAAAGGCTCTGAACATATTGCAGTCAACACACATGGCTTC

CCCACAGATAACTGAAGAAGCTGTTTATCTCTGTACCGGAAACCCGATGCCTAAAGATGT

TGAGCAGATTGCCTTTTGGCTTCTCAACGAGTCTTTTACAACTGCTTTTAACTATATATC

TGATATGAAAATGAGAAAAGGACTGGCCGTGGTAGATATCATAAGGGAGGTTACTATGTT

TGTTTTTAAGATCAAAATGCCATCAGAAGTCCGAGTTAAGCTAATCAATGATTTGGCCGA

TATTGAGTATAGGTTGAGTTTTGGGTGCAATGATAAGTTGCAGCTGGGAGCGCTCATCTC

TACTTTCACAACTGCCAGGTGTGCTCTGGTTGCCGCAGCCAAGTAAAATCTAAATGTTGA

GATGTATGTGCCCCGCATTGTCTTGAACTGTCAGCATGTGGGGTTCAAGTTGGTATGATG

AAATACTGGTTTTGTGTACTGTAATTTGGTAGCGACAAATCTTAGAGAAGTTTGAGATGG

AGAGCTGCAAGCTTTCTTTGACTTTGGATGTCAAATCATCTGGATTGCAGTTTATCATTC

TTAACCAAATTGTCTCAATATTTGCTTTATTAACTCCAAGCCTTGAGACTTTGGCTGATG

A

>Unigene0001610

AACCCCCTGAACCCTTGTCTCGACAAAGAGATCGACCTCCCCAATGGCGTCGCCGACCTC

TCCGATGGACATCGACGCCGCAGCTCCACCGGCGCCGTCCCTCAACAAGGGCAAGTTCGT

CGCAGTCCCTGCCGACGACGATTCCTTCCCCTGGGTCGAGAAGTACCGCCCCCAATCCCT

CGCCGACGTCGCGGCCCACCGCGACATCATCGACACCATAACAGGTAGTGTGACTACAAA

GGCCGATGTGTACAGCTATGGCATAATTCTGATGCAACTAATAACAGGCAGGGATGCAAT

TAAACCGCTTGAAGACGATGACATCACCTTAGTGGAATGGTTCAG

>Unigene0001611

TGGGCCGACGTCCACCCTAGCCCTGGTTGGCTCCCCGGACCCTCCTCCGCCCGCGAGACC

GAAGCCCTCAACAGAGCCCTGCAGGTCGCCCTCTACGGCGATACCTCCTCCTCCTACACC

GCTGCCGCCGTTTCCTCCTCCGTCAACTCCGAGCCAACCATCTCCACCTCCACTTCGGAC

TTCGCCCCACTCCGATCTTCAAGTCCGACGGCTCCACCGAGGATAAAGAAGCGCAAGTCC

CGAGCCAAGAGCCCCAGAGAGGTAACCTACATCACCGCCAATCCGGCCAACTTCCGTCGC

CTCGTCCAAGAGGCGACTGGGTTGCAGGTCGGAATGATGATGAAACCAGCGCCACAGCCG

CCGGTGGGTGCACCAGCAGCTGCGGCGCCGCCGACCCCGGCTTTTTTTCTTCAGCCGGGA

GGCGGCGGCCGGCTCGCTATGTGCAGCGGAGCCGGTGACTGAGGAGGGGCAGAGGATAGA

GTTTGACAGGTTTCAGAACTGCTATTTTCCTACACTGGACTCGTGGACTGTGATATGAGT

GGTCGGTGAAGGAGATTTTGTATAGTCAAGGGCTCAGTATTTCAGAAAGTTAGTTATTTG

CGAAGGGTGCGAACCATTAAATTGGATTAAGCTGCTCTTTGTTTTTTATTAATTTTTCTT

GAATGCATTATCATGGTAATTTGTTGTTTTAAATTCTGCGTTTATACGAATTATTTGTAC

GAATTTGATAGGAAT

>Unigene0001612

TTCTCAACAAATCCTCATCGCTCATTACCCTACAAAACCCCCTCACATCCAACCACTCCA

CAGCCGTTCCTTATCCTGTTATCCAGATATCTTACCCCTACTTTTTCTTTCTTAAAAAGA

GTGCGGGAGAACTGAATCCCCTCACCTCACTCCTCTCATCCCTCTACCTCCTGCCCCGTT

TAGGGTTCCAGCAAACCCTAATCACAATAATCATTTTTTTGCTTTATATTTTATCTTCCG

ATCTACCAATTCAAACATTCTTGAACTCTTAGAGTTTGCTCTTTGCTCTTGCTTTTTACA

TGGGACTGTCTGAGAAGCATCAAGTATCCGAACAGGAAGGAATGGTGTGGGTTATCGGCG

GAACTCCACTCAGGCCGATTAAGACGAAGAATGGCAGTGTCGAAGATGTAGAAAAAGAAA

GCATGGAGATAATGGTGATGACTCCGAAGAAAAAGGAAGCGAGGATACCCGAGAGACTGC

CATGCCCTGCAGCGCCAAAGAAGCAGAGGCCTTTGCTGAGGTGCAACCCCGATGAGGTTC

AGTTCTTCTTCGTGCCAGACTTGAATTCTGTGTTTGTTCCTCGTGGAAAGGGAAGCTTAG

TGTAGTCCAGTAATGGAGGTGCAAGTTTATGTACAAAGTTGTTGATGTATCTTTAGTTCA

CCTAGAGTAAGATCATATTGTTATTCCAGTTTTACTATTTATTCAGGGA

>Unigene0001613

CATGAAGGCCTTACCTCCACCTCCTCTCTCACAACCATTCACCTTCTCCTCACCGCCGAA

AGACGGCATCAGCCCTAGCATCCTCCTCATCATTGTTATCTTAGCTGTCATCTTCTTCAT

CTCGGGCCTCCTCCACCTCCTCGTCAGATACCTAATGAGGCCCGTCGCCAGAGACCCAGA

TGAGATAAGTGCGACAGCGATGGAGGGCCAGCTGCAGCAGCTCTTCCATCTCCATGACGC

CGGGGTGGATCAAACCTTCATCGACGCCCTCCCCGTCTTCCTCTACAAAGCCATCACCGG

CCTGAAAGACCCCTTCGACTGCGCCGTATGCCTCTGTGAGTTCGCCGGAGACGACAAGCT

GAGGCTGCTCCCCAAGTGCAGCCATGCCTTCCATCTCGACTGCATTGACAGATGGCTCCT

CTCCCACTCCACCTGCCCCCTCTGCCGGACCGGCCTCCTCTCCGACTTCACCTCTAACTG

CAGCCCTCCTCTGTGGTTTGCTCTCCAGTCCACAGCCGCCAGCGAGAGCTCGCGAGAGAT

CTCGCTGGCACCCGAGGACGAGAATGCACTGGCATATACAGATGTGGTCGCAGTCAGACT

CGGCAAAATCAAGAATATGGACGTTGGTAGCAGCGACGGCAAGCTTGACGGACGGAGGTG

TCACTCCATGGGCTCGTATGAGTACGTCATGGACGAGGGCTCTCTGCAGGTTGCAATCAA

ACAACGCAAGGCGACTAAGAAGGTAGCTATGTCTGAGTATGACTGCCGGTCAAGGAGAGG

TTTGGGTACTAGGGTTGAATTACCGGATGCGAGGATAACCGGGAGAAAGGAGAGTTTTTC

GGTTTCGAAGATATGGATGAGGTCGAGGAGATCAGCGATGGCGACGGCAACGGCGACAGA

GAGTTCGAGGAGGGCGGTGTCGTTCAGGTTGCCGTTGCAGAGGCAGGCGAGTGCGGGGTT

GGAGAAGAGTGGGAGTGAGGTGGATTTGGATGTGGAGATGGGGAGCTGCAATGAGAGTGT

TGTGTCTGAGGCTCCTTCATTGAGTAGAAGGACTTTGCTTTGGATCATGGGGAGGCAGAG

CAAGGTTGGGAGTTTTATTTGAATTAGGTTTGGATATAGTTGCTTGTTCATTGAAGCTCT

GTATGAGACAAATTAATAAACACAATATACA

>Unigene0001614

TCAGTCCAGACTTCCGCACCCAGAACCCTCTACACCACCTCATCACTCACCACCGAATTC

ACAGAAGCCGCAAAAGGGGAGGAGGAGGGAGATCGCCCGCAGCATCAGCAGCCATGAGTG

CCATGAAATTCTGCCGCGAGTGCAACAACATCCTGTACCCGAAGGAGGACAGGGAACAGA

AGATTCTCCTCTTCGCCTGCCGCAACTGCGATCACCAGGAGGTTGCTGATGACAATTGTG

TCTATAGAAATGAGATACATCACTCCATCGGAGAGCGCACACAAGTCCTACAAGATGTTG

CAGGTGACCCCACGCTTCCTCGCACTAAGAGTGTTCAGTGTGCTGTGTGCAAACATCCAG

AAGCTGTATTCTTTCAGGCTACTGCAAGGGGGGAAGAAGGTATGACACTGTTCTTTGTCT

GCTGCAACCCAAGCTGCGGGCACAGATGGAGAGACTAGCGCTATGACGCCGAATCCAAAC

CTGAATCGTGTCGGACATTATGTTGAGAAGCTTTTTGCACCTTGAACAATATTGCTTTAT

AAAATTTTAAGTTTACTGATGGTAACTACTTGGAATATACCCCTTGTTTTTGTATGTGGT

AGCCATCTGACTAGACAATCAGATATAGCCTTTAAAGTTGTGTCGGGCTTCTTATTGACT

ATTAGATATAGCCGGTTTTTTTATTTTTTATTTTTACCTTTTTAAAGTTGTTTGGGGATC

CTTATTTAAAAATAGTCATGACACACCTTATGTTGCCCCCAAGGTTTTATAATACTTAGG

GTGTTATGTTGAT

>Unigene0001615

TCTTACCCGACTCAATCATTCTCCGTGATTTTTCTAATTCTTTTCGATTATCATCAGAGC

GATCGATCTCGGCCGCCGCTGCCGCTGCCGGAACTCGATTCCAGTCGCTTCTCCAATCCA

ACACGAATTCGTTGAATCCTGACATGTACCGGAGCCTGCCACCGGCCCCCACACGGGGCG

GCCCGCCGTCGGACATCGGCGGCGACGACGGCGTGGTGACACTCGATCAGATACCGAGAT

GGAGCGACACGGATCACTGGTCCTTATACGGGACCGGGGAGCCGTCGTCTCCCTTTTCCT

ACTTCTCCGACCCGCTGGCGTCGCCGTCGGGAGCCGATGCCGGTCCCGTGTCGAGGTTCC

CAGTCGACCGAGAGATCAATTCAAAGGTTTGTCTGTGGCGTGGCGATCCGTGGAATCTTG

AGGTTGATGCGGTAGTGAATTCTACTAATGAGCTATTGGATGAAGCAAATAGTAGCCCTG

GTTTGCATGAGGCGGCTGGCCCGGGGCTCGCCGAGGAATGCGCTACACTGGGTGGATGTC

GGACAGGAATGGCGAAAATGACTAATGCATATGGTCTTGCCGCTAGGAAAGTCATCCATA

CTGTAGGTCCTAAATATGCTGTCAAGTATCATACTGCTGCGGAGACTGCTCTGAGTCATT

GCTATCGTTCTTGTTTGGAGATTCTTATTGAAAATGAACTTCAAAGCATTGCCATTGGCT

GCATATACAAAGAGGCCAAAGGTTATCCTCGTGAACCAGCTGCTCATGTCGCCATCAGGA

CTGTCAGGCGATTTCTGGAAAAGCAGAAAGATAAAATTACTGCAGTTGTTTTTTGTACTA

CGACATCTACTGATACTGAGATTTATAAAAGATTGCTCCCACTTTACTTCCCGAGGAATA

AGCTTGAAGAGGAGGTAGCTATGTCAAAACTTCCAGCTGATGTTGGGGATGAGAATGGCG

CGACAACAATCGATGAGCGGAAAATCAGAATAAAACCTTTACCTGCAGTATCTATTAACG

TACCGAAAGCTTTGACAACTCAATCAGATGTTCCTCTCAGAGATGCTGGACCGCCAGCGA

GACGGACTTCTTTCCGTTTGGACTCGTATTTGGATCCTGCATTCATGTCCTTAGCAAAAG

ACCCCGACCAGAAAAGGAAGGAGCAGTGGCAAATGGCTGCTAAAGCACAAAATAGCTTTC

ATTTTGCTAAGTTGCTTGGATTTGGTGATCTTGGTGGGCCTCCTTTATCTGCAGCTGAGG

AATATTCACTTCATTCGAGATATCTTGCTAAAGCAAATTCTCTCAATCTTTCAGATATTG

CTGAAATGAAAATTCTTTATCGCGGCGGAGAGGACAGTGAAGGACGTCCTGTAATGGTGG

TTGTAGGAGCTCACTTTCTATTAAGATGTCTTGATTTGCAGCGTTTCATTCTATATGTCA

TCAAGGAGTTTGAACCCTTGATTCAGAAGCCTTATACGATTGTCTACTTTCACTCTGCAG

CATCTTTACAAGTGCAACCTGATCTTGGATGGATGAGGCGATTACAACAGATTCTTGGTC

GGAAGCACCAGCGAAATTTACATGCAATATATGTGCTCCATCCTACTTTTGGTCTGAAGG

CGGCCATCCTTGCTTTGCAGCTTTTAGTAGATGGACAAGCATGGCGAAAAGTCGTGTATG

TCGATCGTCTCCTCCAACTGTTCAAATATGTCCCTCGTGAGCAGTTGACCATTCCCGACT

TTGTCTTCCAGCATGACCTTGAAGTCAATGGAGGGAAGGGACTTATTGTTGATCCGAGAA

CTAAGTATATATATCACCGCCCCTCAACATGATCTGCTGACTGCTTGTGTTGAATTTTGT

TTTTAGTGTCAATGATCCTGTTGTTGATCGTTGAGTGTTCTTTGTAGAAGTCCGAAGTAT

TTCTCTGTAATTCTTTGAATTATCTTTTTCATTATGTTCTATATATGGAAATGAAATTTT

TTGTTTTAACTCCGTTTTACAAGGATCTGTATAATTCCTGAGAAACACTTTCATGCGGGG

AGTGTTGTAGAAATGATTATCGTTGCCATAGAGTTTTGTAGTTGTAAAATGTGTAAATAT

TGTGGAAGTGTCCAATGGTAATTATATGGCAGTTGTAATTTGGAGCGGATAGTTTATTTA

GTGAGGGTGAATATGCTGAAATGGTTTGCAGTTATACTTACCAGGTTTATATGATGTATT

ATTAAGGACAAACATATAAAA

>Unigene0001616

AGGGATGGCATTCAGTGGTGTGCGAACATGGCAGAATCTCGGAATTACGGTGGACTTCCT

GCTTGGTTGTGGCAAACTGAAGGGATGGCATTCAGTGGTGTGCTAACATGGCAGAATCAC

TTGGTATAGACGTGCCATGGATTATGTGCCAACAACCCAATGCACCTCAACCAATGATCA

ACACTCGCAATGGATTCTATTGCGATAACTTTACCCCCTAAAAATGCTACTAGCCCAAAG

ATATGGACTGAAAATTGGACTGGTTGGTTCAAAGCATGGGATGAACCTGATCATCATAGA

TCTACTGAAGATTTAGCTTATTCAGTGACATGCCTCTTCCAATCCAACGACACATTTCAG

AACTATTACATGTATCATGGAGGAACTAATTTTGCTCGTACCAGTGGTGGTCCATATATT

GCAACATCTTATGATTATGATGCTCCTCTTGATGAGTATGGTAATATAAGGCAACCCAAG

TGGGGTCATTTGAAGCAACTCCATGCAGCTATTAAGATGTTGGAGACAGTTCTTGTTGAT

GGACAGACAGAGGGAATACGCCGATATTGGCAATGGAGTATCGATAACAAAGTTCTCTGC

TGATGGTGTTGTTCCGGGTTGCTTCTTGACAAACACAAACACCACCACTAAATGATTAAC

ACTGCTAAGGTGAGCACGCAAACTTTTGTGATGGTGAAGAAGCCAAACACCGCTGAGAAT

GAGCCTGAAACGCTGTCTTGGTCATGGAGGCCTGAGAACTTGGGAGCTCCCCTGCAAGGC

CTGGGTGCTACCTTTTCTACAATCAAGCTTTTAGATCAGATTCATGTCACCCTGCAAGGC

CTGTGGTGTTGTGAGGCACGGTGGAGATCGGCGGGGTTCTGAGGGTGATTGCTTGTTCCT

TGATTGTTGATGAAGACCAGGGCAAAGAGATGCAGACGGCAAGAGCGGGATGATTTTGTT

AGAGGTTGACCGTGTTTTCCGGCAACCTCTTTCCGATGACCAAGCTCGTGACCACTTCGT

TCAAGATCCTGATTTGTCGGATCCGGAAGAGTGATTGCTTGTTCCTTGATTGT

>Unigene0001617

GTGATTGCTTGTTCCTTGATTGTTGATGAAGACCAGGGCAAAGAGATGCAGACGGCAAGA

GCGGGATGATTTTGTTAGAGGTTGACCGTGTTTTCCGGCAACCTCTTTCCGATGACCAAG

CTCGTGACCACTTCGTTCAAGATCCTGATTTGTCGGATCCGGAAGAGTGATTGCTTGTTC

CTTGATTGTTGATGAAGACCAGGGCAAAGAGATGCAGACGGCAAGAGCGGGATGATTTTG

TTAGAGGTTGACCGTGTTTTCCGGCAACCTCTTTCCGATGACCAAGCTCGTGACCACTTC

GTTCAAGATCCTGATTTGTCGGATCCGGAAGAGTGATTGCTTGTTCCTTGATTGT

>Unigene0001618

CGGTTACCTTCGTCTCCCCCCATCCACTAGTAGATGATCTGGCTGTAGCCCTCAGACAGT

GAAACAAGTCCCTGCTTGTCTGCTTCCCCCTCTTTTCTTTTTTCACTGCCTACATTCCCC

ATTATTGTCTTTGGGTTTGGGGGGCAGCCTCTGTCCTATCCCTTGGCATCCAGGAGACAC

CAGATTTTGTTTCAACTCATCATCAGGAGGAGATGGGATCTTGGGTTGCAAAGATTTGAG

CCTTCTTGGTCCCTTGGGAGACTGATTCTTAGTCATCAGAATGAGGGATTTCCCATCTTG

TTTCGGCGAGAGCGGCGTCCAGGTCGCCGATGCTGCCTCCTCGAGCAGCGGTGGCAAGAC

TGCTCAGAATCTGGTCACTTGCCTCTACCAGACGCAGCTCAGCGGGCGGCCTTGTGTGAT

CAACATCACATGGAGCAAGAACCTGATGGGGCAAGGGCTCAGCATTGGGGTTGATGACTC

TGCCAATCAGAGCCTCTGCAAGGTGGACATAAAGCCATGGTTGTTCTCCAAGCGGAAGGG

TTCCAAGAGCTTGGAAGTGGATTGCAGCAAGGTGGATGTTTTCTGGGACCTCTCGGCCGC

AAAGTTTGGATCCGGGCCAGAGCCTTTGGGGGGCTACTACGTTGCAGTGCTCTTTGATCT

TCAGATGGTTCTTCTTCTCGGTGATCTTACCAAGGAAGCATATCGCAAAACGAGTTCATA

CCCCTCTCCGTTGAATGCGGTGTTCATCTCCAAGAGGGAGCACATTTTCGGAAAGAAGGT

CTACTGCACGAAAGCTCAGTTCTTTGAAAGTGGGCATGTTCATGACATTGCAATTGAGTG

CGAGACGAATGGGCTCAGAGACCCCGTCCTTGAGATTCGCATAGACAAGAAGCGGGTGAT

GCAAGTGAAGCGGCTCACATGGAAATTTCGTGGAAACCAGACTATCCTAGTTGAAGGGAT

CCCTGTGGAGGTGTTTTGGGATGTGCACAATTGGCTTTTTGGCTCGCTGGCCGGAAATGC

GGTCTTCATGTTCCAGACTTGCTTATCGTCCGAGAAATTGTTGCCATGGTCTTGCTCGCA

GATACTCAGGGAATCCCAGTTAAGAGCTCTTGGGTTTTCATTAGTTTTGCATGCGTGGAA

GAATGAGTAGGTGTATGGCTAAATGCCTCAAAGATGTTATTTTACTCATCTTCTTTTTCG

AGTGCTAATACATTCAGTGAGTGATTTGTGTGCCCAGGGCTTCTTCCATTTGTTTTCCCC

TATACCTAGTTATAAATTTTATCTGTTTGAATAGATTGGACAATCCAAGTCTTCTTATTG

GTCATTGTATATGATTTTGTATCCAGAATCTGTTTTTTT

>Unigene0001619

TTACATGGCACCTGAGGTCCTCAATGGCAACCCCTATAACAGAAAGTGTGATGTTTATAG

CTTCGGCATTTGCTTATGGGAGATATACTGTTGTGATATGCCTTATCCTGATCTTAGTTT

CTCAGAGATCACTTCTGCAGTCGTGCGACAGAACTTGAGGCCTGAGATACCGCGTTGTTG

TCCAAACTCTCTCGCCATTGTGATGAAACGGTGCTGGGATGCGAACCCCGACAGACGGCC

TGAGATGGATGAGGTAGTGACTATGTTGGAGGCGATCGACACATCCAAGGGTGGTGGCAT

GATCCCGCCAGATGAGTCGCAAGGATGCATTCCATGTTTTCGCAGGAACCGTGGTCCCTG

AGAAGGCCAATTCTCTCTAATGGTAGATATTTATTTGACTGACGCACTATCATGGATGAT

GGATATATGTTTTTCAACGAGTAGGATTGTGCGTTAAGACTTGTGGTGGTATGGCCAGTA

TATTTGTCGGCAAACCACCCAGCTCTTTCGACGTACAAAATTGTGTAAATGGTAGCTTCA

TGTTTATTTGATAATTGGCAAGTCTTGACTGGTTTATCACCTGAATTGTATTGTTCAACT

ATTTATTTTGATATATCTTTATG

>Unigene0001620

CAGCCTCACTCCTTTTGAGAACCAACCCACACAGTTATCATCTAGGAAACAATGAAGGAG

GGGGGCGGCGCCGGTGACGGCTTCGTCAGGGCCGATCAGATCGATCTCAAGAACCTCGAC

GAGCAGCTGGAGCGCCACCTCAGCCGCGCACTCACTATAGGGAAGAAGGCGGCCGACGAA

GACGCCGTAACGGCAGCCGCCAAGAGTCGGAGGAAGAGGGAGGAATGGGAGATCGATCCG

TCAAAGTTAATCATCAAGGGCCTCATTGCCCGCGGCACATTTGGGACTGTTCATCGCGGT

ACCTATGATGGGCAGGATGTTGCAGTCAAATTGCTCGACTATGGAGAAGAAGGAAATAGG

ACTGACGCTGAAATTGCCTCACTCAGGGCTGCTTTTACTCAAGAAGTATCTGTTTGGCAT

AAGCTCGATCATCCTAATGTAACTAAGTTTATTGGAGCTACTATGGGCGCTGCAGATCTA

AATATTCAAACTGACAATGGACCTCTTGGCATGCCGAATAATGTTTGTTGTGTTGTCGTC

GAGTATCTTCTTGGGGGTGCACTGAAAACATATTTGATAAAAAACCGGAGAAAGAAGCTA

ACATTTAAAGTAGTTGTTCAACTGGCTCTTGATCTTGCAAGAGGATTGAGTTATCTTCAT

TCACAGAAGATAGTGCATCGAGATGTGAAGACAGAGAATATGCTTTTGGATAAGAATAGA

ACTCTGAAAATTGCTGATTTTGGTGTTGCTCGTGTTGAAGCTTCAAATCCTAATGACATG

ACAGGCGAGACAGGGACCCTTGGTTACATGGCACCTGAGGTCCTCAACGGCAACCCATAT

AATCGAAAATGCGATGTTTACAGTTTTGGCATATGCTTATGGGAGATATATTGCTGCGAT

ATGCCGTATCCTGACCTCAGTTTCTCAGAGATCACTTCTGCGGTTGTTCGACAGAACTTG

AGACCTGAGATACCACGTTGCTGCCCAAGCTCAGTCGCTAACGTTATGAAGCGATGCTGG

GACGCAAATCCTGATAGGCGGCCTGAGATGGACGAGGTGGTGGCGATGTTGGAGGCGATT

GACACTTCTAAAGGAGGAGGTATGATTCCACCTGATCAGTCTCAAGGATGTCTGGTGTGC

TTTCGCAAGCGTCGAGGACCTTAGGCTAATGAATGAAGTACTTGTGGTGCTATGACAACT

GTGTTGATTTGAGTTCGATTGCCGGTTGATGTTTGTCGACAGTGTAAAGATTATTGTACT

AGCAGATTAGTCGATTGTAGTATTGATGGGATTCGGCTACATTGTAAGATTGTAGTGCGG

TATGTCATATTAACTTCTTTGTAAATGATAAACATAAAGTTTTATATATATATATATATA

TA

>Unigene0001621

GGGTCGAGTGGGTCAGGCAGACACGTTGTGGAGAAGCAACCCATGTGCTCACATGCAACA

TCGAGAGGGGAGAGGAGGATGAGAGAAGCTGGTACAGATTTTTGTACAACTTGCCAGCAA

TAAAAGCAGCTTCAACCTCAACACACTTTTCGCCCTTCTGCTTTCTCCTCTTGCCGTCGT

CATCATAGAGACGATGGAGGGAGGCGATGGATTCGTCCGGGCAGACCAAATTGACCTGCG

GAGCCTAGACGAGCAGCTCGAGCGCCACCTCACCCGCGTCTGGACCTTGGAGAAGAAGAA

GGAGGCCAGAGAACGGCAGAGGGAGGAGTGGGAGATCGATACATCCAAGTTGATCATCAA

AGGCCTCATCGCACGCGGCACCTTCGGCACAGTCCACCGCGGGGTCTATGGCTCTCAGGA

CGTTGCAGTGAAATTGCTGGACTGGGGGGAAGAAAATAGAACAGAAGCTGAAATTGTTTC

ACTTCGGGCAGCCTTTTCTCAGGAAGTTTCTGTTTGGCATAAGCTTGATCATCCTAATGT

AACTAAGTTT

>Unigene0001622

TCCGACCAGCCACTCATTCCGACCGATCTCTCTGCCTCGCGCCGCCTCACTCCGGTAAAC

CTCCCCCTCCCATCAGATCAGATCATCCCAAAAATCAGTCCACAGATACACCTCATCTCC

TACCCCTAACTAATTCATGATTCTATTAGGTAAATCTAGAGATCTCGATCTCCTGTTCTT

CAGTCTTCATCGGAAGCAGCCATCCGAAGCTACAGATCTCCGATCATCTCTTCATCTCAA

GCCTTATGTGTTAATCTCAGGTATTAATTTGTAATTTTTAGATTTTCTAATTTGATTTTT

ATTTCCTCATTTCAGGTGTTCTCCAGAGTATAGACTGTGTAATGGAGATCTCGAGCATGT

CGGAGCCGTCAAAAAACACGTTTCCTTATTTTCATTATGCCAGAGCCGACAAAGGTGGTG

CGAATCTATGGGGTGGTGCAAACACTACCACACATCACCAGGGTTGAGCACGAAGGAGTG

GTTCCAGGGAAAATTCAGGCACATTATTGTTTATGTCCAGTCAAGTTTGAGCAGAACTCT

GCTCTTAATAGGCTGGAGTGCAGACATGTGTTCCGTCGGGTTGTCTGGAGAAGTCCAGTA

TGTTTGAAATTTATGTTATATGGAAGCAAGGATGCATGTTTCCCCAACTGCTTCCATTCT

TGCCGGTGCCCCTATACCTCCCCCATCTTCTCCTCCTCCAATTAAATGCATGATGTGTTA

ATTTCTCGAGCTTGTATCCGTCATCTATGGCGTCTGTTGCGGACTCTGACAAATTACAAT

CCAGAGAAGTGTATATTGTTGGTATTACACACACCATTAGGCGGCTTTCTAGGTTCACTA

CCTTCTTTGACTACCACTAAATTTGGTTTTATTGCTATTGCATGTGTTCTTAAGAGGGCT

AATGTTGATACAACACTTGTACAAAAGGTCTATGTTGGTAATGTTTTGAGTGCTAATTTG

GGGCAGGCTCCAGCACGACAGGCTATATTAGGTGCTGGAATCCCGAATTCAGTGATTTGA

ACCACAATCAACAAAATTTGTGCTTCTGTGATGAAGGCTACAATTATTGCAGCATAGAGT

ATTCAGTTGGGTATTAATGATATTGTTGTTGCTGGTGGCATGAAAAGCATGTTAAATGCT

CCTAAATACCTGGCAGAAGCTAAAAAAGGTTCTCGATTTGGGAATGATACTATTGTTGAT

GGTATGCTCAAAGATGGCTTGTGGGATGTATATAATGATTTTGCCATGGGGATGTGCGCT

GAGATATGTTCTGATGACCATTCAATCACAAGAGAAGAACAGGATTCCTATGCTATTCAG

AGCAATGAACGGGGAATTGCTGCCCGAAATGGTGGTGCCTTCACATGGGAGATAGTTCCG

GTTGAAATTTCTTCAGGTAGAGGAAGACCACCTGTTATTGTTGACAGGGATGAAGGCTTA

GAAAAATTTGATCCGACAAAACTAAGGAAGCTCCATCTAAGTTTTAAAGTAAATGGTGGT

AATGTTATAGCCAACAATGCTTCTAGTATCAGTGATGGTGCTGCTGCATTAATATTAGTG

AGGGGAGATGCATAGAAGCAGAAGCAGCAGGGCTGGGAGGATATGGGATTCGTTGCTTGT

GTATTGAAGCTACCCGGAAGACACGGTCTGCATTTGACATAAGGAACGGGTGGAATACCG

GGCCTATAATAGTTTGGCACAATAATTACGGGCATTTCAATTCTATAAAAAAGACAAAGC

TCTCACAGAACAAAAGGTGGACAGCAGAGCACTAGTTCAGGATTGTGGATGAGAATTCTT

TTATAGTTTCTTGTGGTGGCTTAGGCCTTAGATCTTCAGCTAGTGTGTCCCATTATTGAG

AGGATTATTAAGTGTTGTGAGGATTATTTCTGTTGAGATAGTTTTGTGGTGAGGTCCTAC

GATTTTCTTGTAATTTGTTGTTGGTAAGCATTGGTGTGGGTTATTGAGAGAGAATGGTTC

AGTGAGTGTTATAAGGAGAAGTACTCCCCATGTGACATACGGGGATAGTCTCTGTGAAGA

CATTCTTGAATTGATCAACTCTCTGTAAGCTCTTGGTTTATGATATTAATGAAATAGAGA

A

>Unigene0001623

TGTGACAGAGATAGACGTCCCCTGACCGCCGAGTCCCGACAGTGTCACAGAGACCATAAT

TTCCATCTTTCTTCGACCAGAAAATCAGAAGAGGGCTCCCCCATCTCCTCCTCATCCGAT

TAAACGCAGGTTGTGTTGATTTCTCGAGCTCGTATCCATCATCTATGGCGCATGCTGCGG

CCTCCGACAAATTACAACCCAGAGATGTGTGTATTGTTGGTGTTGCACGCACACCAATGG

GCGGCTTGCTTGGTTCACTATCTTCTTTGTCTGCCACCAAACTTGGTTCTATTGCTATCG

GATGTGCTCTCAAGAGAGCTAATGTTGATGCAGCACTTGTACAAGAGGTCTATTTTGGTA

ATGTTTTGAGTGCTAATTTGGGGCAGGCTCCAGCGCGACAGGCTGCGTTAGGTGCTGGAA

TCCCGAATTCTGCGATCTGCACCACAATCAACAAAGTTTGTGCTTCTGGGATGAAGGCTA

CAATGATCGCAGCACAGAGTATTCAGTTGGGCATTCATGATGTTGTTGTTGCTGGTGGCA

TGGAAAGCATGTCAAATGCCCCTAAATACCTGGCAGAAGCTAGAAAAGGTTCTCGATTTG

GGAATGATACTATTGTTGATGGTATGCTCAAAGATGGCTTGTGGGATGTATATAATGATT

TTGCCATGGGGATGTGCGCTGAGATATGTTCTGATGACCATTCAATCACAAGAGAAGAAC

AGGATTCCTATGCTATTCAGAGCAATGAACGGGGAATTGCTGCCCGAAATGGTGGTGCCT

TCACATGGGAGATAGTTCCGGTTGAAATTTCTTCAGGTAGAGGAAGACCACCTGTTATTG

TTGACAGGGATGAAGGCTTAGAAAAATTTGATCCGACAAAACTAAGGAAGCTCCGCCCAA

GTTTTAAAGGAAATGGTGGTACTGTTACAGCTGGCAATGCTTCTAGTATCAGTGATGGCG

CTGCTGCATTAGTATTAGTGAGTGGAGAGAAGGCTCTTGAACTAGGATTGCACGTTATCG

CAAAGATTAGAGGCTATGCTGATGCAGAGCAGGCCCCTGAGCTATTTACAACGACGCCAG

CACTTGCTATACCGAAGGCTATTGCGAACGCTGGGTTGGAGGCTTCACAAGTTGATTATT

ATGAAATAAATGAAGCTTTTGCTGTTGTGGCCCTAGCCAATCAGAAGATTCTTGGTCTTT

CTCCTGAAAAACTTAATGTGCACGGTGGAGCAGTTTCCCTGGGTCATCCTATAGGCTGCA

GCGGAGCTCGCATTCTGGTCACTCTGTTGGGGGTTCTTCGATACAGAAATGGCAAGATCG

GAGTTGCTAGCATTTGCAATGGTGGCGGGGGAGCTTCTGCACTCGTACTACAACTCATGC

CTCTTGCTGGTAAAGCGCGATCGGCATTATGATAGAAATGTATTTATGCTGGAAATGGTG

CTCAAGTCGAGTTTTCAAGCAGTAATAATGTGCTGATCTTTCCCGGACTTCTATTTTGTA

ATTTTTATTTATAGATTGGAAATCGTGTAGTATGAGAGATTTGATGAATAATGTGTATTG

CTTCGCAAGTTATAGTAACTAAGTGTAAATTGTCGAAGTACAAATAAATGAAAGAAAAAT

GGTTTTATTAGTAATGTGTAATTTTCAAT

>Unigene0001624

CAAATGGTTGAGAAGCGATCGCTCGTCTCCTCGATCTGGACTCGCCATCGCGAACGAGAT

CTCGGCCTCGTCACTTGGAGCCTCGGCAATGGCGTCGCTCCTCTCTCCGTCCACCGCACT

CTTCTCCCCCTGTGTTTCTCGCTCATCTTCAAGGAGATTCCCAACTCGGAGTTCACTCAT

CAAGGCTGTCAGCTCCGCCGAGCCGGACAATGACAAGGCCGTCCAATCCGCCGGTAAAGC

CCCGTTGGCCTCCACTGATTCCCCTGCTGCTGTCCGTCCCAAGAAGCCCGTCTACTCAAT

GAAGAAAGGGCAGATTGTACGGGTAGATAAGGAGAAGTACCTGAACAGTATAAACTACCT

GTCTGTTGGTCATCCACCATATTACAAAGGCTTGGACTACATATATGAAGACCGTGGTGA

GGTGCTGGACATACGTATTTTTGAGACCGGAGAGTATGCTCTGATTGCTTGGGTTGGAAT

CCCAACTGCACCTGCATGGTTACCAACAGACATGCTCATCAAGTCGGAAAAACTCGACTA

CGAGAGAATCTGATCATGCCAAAGATTGATCGATTGCTATTCTTCAAGCTGTGTGAAGTC

ACAGAGCAAGGAGAATCCGTGGAGCCTAATGATGTAGACCATCCTGTTTTGCAACCATTC

TCCGTATGCCCTTTCTCCGATGCCAAAGTAAGCATGTTTTTCGTATATCTGAAAGTCTGA

AAGTGATGCGATGAAATTGTAGTCGATATCAATGTTTCAAAAGTTATTCTATATTGTAAC

ATTATATCTGTCACCAAACTGATATCGATCACCATCGAATCCCAATTTTTGAAGGAAAGA

CTTCAACCTATCTCGTCACCAAACTGATATCGATCAC

>Unigene0001625

GTCTCCCACGCATCTGCTTCTCGTCGATCTCTTTTCCAATTCCCCCTTCCCAATCTCTCC

GGTCACCATTGGAGCTCCCGAGGATCAACAGAGCTCAATCTAGGGTTTCTCCACTCGAAT

TCAGCTGGCATTGATCTCCAGGGAGTGGACAACTTCATGAGTAGAGGACTGGTCTGCATT

GATTAATTATACATTACTGATCCATGGGCGAGTGGGTTATTGGAGCATTTATAAACATAA

TAGGGAGTATCGCTATAAATTTTGGGACAAACCTTCTAAAATTGGGCCATGAACAGCGAG

AAAGACTTTCTGTGCTTAGCAGCGATGAAGCAAATGGCAAGCTTATTCTGAAGCCCATCA

TACACTTCCAAACTTGGAGAGTTGGCATTCTCTTCTTTGCTCTTGGGAATTGCCTCAACT

TTGTTTCCTTTGCATATGCCGCACAGTCACTTCTTGCAGCTCTTGGGTCAGTTCAGTTTG

TTTCAAATATTGCGTTTGCCTACTTCGTCCTGAGCAAGACAGTTACCGTCAAAGTTATGG

TAGCCACAACCTTTATTGTTTTAGGCAACATCTTCCTTGTTTCTTTTGGCAATCACCAGT

CACCTGTTTACACACCAGAGCAGTTGACTGAAAAGTTCAGCAATCTCGTCTTTCTGCTTT

ATTGCCTGACTTTAGTGTTGGTAGTAGCTGTCAATCAGTATATGTACCGGAAAGGGGAAG

CTTATGTCTCAGTTCCCGGGCACGAGTTCACTTCGTATTGGCGCACATTGCTGCCATTTT

CCTATGCCACTGTTTCTGGTGCTGTAGGGTCATGCTCTGTGCTGTTTGCAAAATCTTTGT

CTAACATGTTAAGACTGACTATGGGCAGCAATTATCAGCTCCATAGCTGGTTTACATATT

CCATGCTTCTATTGTTTTTTAGCACCGCAGGATTTTGGATGGCAAGGTTGAACGAAGGAT

TGTCTCTGTTTGATGCAATTCTCATCGTCCCCATGTTCCAAATTGCATGGACCTTCTTCT

CTATTTGTACAGGATTTATATATTTTCAGGAATACCAAGTATTTGATACATTAAGGATAA

TGATGTTCCTTCTAGGTATGACATTTGTATTCGTTGGCATTTCGTTGTTAGCACCTGATG

ATTCTAAAGGTAATGACACAAAGGATTCTTCTTGTTTGTCTCCATCTGCAGTTGAAAGCC

TTGGAACAGACATAAACAGGGTTGTAACATCAGAAGAGAATGAAATAAAAGATCTGAATT

CTTTTATACATTCAATGGCGACAAAGGCAAAGTCTCTTATAGCAAAGACAAAGGCCATGT

GTTCTTTCTCCTTGGGGTTAGGCGAGGAGTCGCTAAACGCATCTTCGGTACTCGTAATGC

CCATGGTATCTTCAAGGACAACTGGGTTCAGAGGAGCCAATTTTAATCGTGCGAAGTTCA

TTCCTTTGAGGAATACTGATTGGAGCAATCCCTCGATTGATGAAGACGACGATGATGATG

GAGAAATGAGAGACACAAGATCGTTACTTCTATAGTCCCCTTTGTATAGGTGATGTAATT

ATTTTTTTGCTTGTTGGCTTGAAGAGTATACTATAAATTGATTTTCGGACCACGGTGTAC

AGATAATTCTTTATTTTTATCATACATAGTATCGGGATTTGATTTGTAAAGATGATGTAT

GTTTTTCACTTCCTGTTTCTGAGTTATGTGTTAGATTTACTTACAGGAATTTCCTCGAAT

GAAT

>Unigene0001626

CCCATTTTCAGTAAAAAATATTCTTCACATCTTTCTCACCCAAACAATGGCGCGGTGCAT

TTTGCTCTGTGCAGCATTGCTGGTCTTAGCCGCCACCAGCTCCGCTCAGGGCCCTGCAGC

CGCCCCGACCACCACTCCAGCCACCGCCGCCCCACCAGTTGCCACCACCACCCCTGCCCC

ACCCGTTGCCACCACGGCACCCGCTGCCGCTCCCACGAAACCCACTACCCCTGCCCCCGC

TGCTGCCCCTGTGAAAACCACGACTCCCACCCCGGCTGCCGCCCCCGCGAAAACCACCAC

CCCCGCACCGGCCGCTGCCCCCACCACACCGGTTGCCCCAGTCTCCACTCCACCTGCGGT

TACCCCGGTTGTCCTCCCCACGCCAACCGCTGCACCGGTGTCCTCTCCTCCATCCCCTGC

CCCTGTCAGCTCCCCGCCCGTGTCGGTACCGACAATATCTCCACCAGTGCCGGCGCCGTT

GGCTGCCACTGTAACCCCCGCCACCGCCCCGTCGAAGCCTGCTGACGTCCCGGCCCCGGC

GGCAAGCAAGGGAAAGAAGAAGAAGAAGACAAAGGCTGGCGCCCCCGCTCCCTCCACCGC

TAGCCCGCCGGCTCCGGCCACCCCCTCACCGGCCGGATCTGAAGGTCCGTCCCCCACCGC

CACCGCCAATGACGAAGCGAGTGGCGTGGCGAAGAATGTAGCTGGAGGGTTGGTGCTGGT

TGCTGCTATGATGTTCGGATTGTTCAACATGCTGATCTAGTTCGAATGAGGTCACATTGT

TACATATTGGCTCGACTGATGCTGCCACTTGTTGTTTTGTTTGATCGATTGGTGTTCTCG

GTGGTAGAAGGATAGCGGGATTATACATTGAGCTTGGTGGATTTATTTTGTCTGAAGGGA

TTGTATGAGACTGTTTTCTATTATTTTATTGAATGATTTACTTTTTGCTTTATGCGTCTG

TGATTGGAGCATCTTTGTGTCTATTTCTGCTGTCGCTTTTGTACGATAGCTGTTCCTGGT

ATGATAGTTGGTCCTTTGCTTAAATTTTTCAATCAGACAAATAATT

>Unigene0001627

GGGACCCCTACTATAGAGAGAGAAACACAACCTGTGCCGCCATTTTTTTGTGAAGCAACT

CATTTCAAACACACTAAAAGGAGAGAGAAATAGAGAGATTTTCTAGTTTGTCATCTGCTC

GGATCTCACTTAGTTCCAGCATCTACATGATGGAAGGAAGGGAGTTTGAGAGGACAGTGA

CGTCAGTGCCACAAACCGGCGGTGACGACGGTGCTGGCGGCGGGGTCAAGGATTCCCCCT

CCTCGACCCCGTCGAGCACTCCGTCACCGCCAGCTGCTTCCGCTGCCGCCCCCACCTTCA

TCGTCGGGCTTGCGGCATCAGTCCCGCCGGCGACAGGCTTGGCCGCGGCGACGGGCACGG

AGCTCGGGATGGCCCCCATGGGGATGATGACAGGTAGCGGCAGCAGCGGCGAGAATCTCG

CCAAGAAGAAGCGCGGGAGGCCCCGGAAGTATGGCCCTGACAGCATGGCTCTCGCCCTCT

GCCCGACCTCCCCGGACTCCTCCGGAACCAAACGGGGTAGAGGGCGGCCACCGGGCTGCG

GCCCGCGCCAGATTCTCGCCACTCTAGGAGACTGGTTTGCATTCACAGCCGGAGGAAACT

TCACACCCCATGTGGTTACTGTGGCAACAGGCGAGGATGTTACTGCGAGGATTCTATCCT

TTTCTCAGAAGGGGCCGAGGTCTATATGTATTCTTTCTGCAAATGGGGCAATCTCTAATG

TTACTCTGCGCCAGCCGGGTTCTTCTGGTGGTACCTTGACATATGAGGGTCGCTTTGAGA

TACTATCTTTGTCTGGATCCTTCACCATTGCTGAAAATGGAAGCGCACGTAGCAGTAGAG

CTGGTGGGATAAGTGTGTCATTAGCTGGCCCGGATGGTCGTGTGGTTGGTGGAGGTGTTG

CTGGCTCATTACTGGCTGCTAGTCCAATTCAAGTAATAGTTGGGAGTTTCATCCCAAACA

CACTCAAGGAGCATAAGAAGAAGTCTAATCAAGGACCTTCAACCTGCCCTCCCATTCAAA

CGAGTGCAAGCCCTTTGATGTCTGCTAGACCAATATCCCAGGCAAATCCAGATGATGACT

GTGAAACTCCCAACTCGGCAACAACATTGGCAGCACAAAACCACGGTGATGCTGCAAACA

GAGGGAGCAATCACCCAGATCCGAACTCTGCCTCTTTCAGGGCTCCTGTTTGGAAGGGAA

TGCAGCAGTCCCAGCCGGATCACAAACCGTCCCCAGACATCAACAGGACTTCGGGTTTCC

AGGGGCCACAGCCGCTTTCAGAGCACAGGCCGCAGCCCCTTTCAGAGCACCGGCCGTCCC

CAGACATTAACCGAGCCCCTGGTTTACAGGGCCTGCAACCCCTTTCGGAGCACAGGCCGC

AGCCCCTTTCAGAACACAGGCCGCCCCCAGACATCAACTGGGCCCCTGGTTTGCAGGGCC

CACAGCCACTTTCGGAGCACAGGCCGCAGCCACTTTCAGAACACAGGCCGTCCTCAGACA

TCAACAGGGCTCCTGGTTGGCAGGGGATGCAGCCCCTGTCCGAACACAGGCCTCAGCCTA

TTTCAGAGCATAAGCCGTCCCCAGGCATCAATAGACTGCAGCCCCTTTCGGAGCACAGGC

TGTCGCCAGACATCAACAGGCCTCCTATCTGGCAGGGGCTGCAGTCATCTCAGGAACGTA

AACCGTCACCAGATATCAACAGGGCTCCCAGTTGGCAGGGGCCGCAGCCTGTATCCGAGC

ACAAACCGTATGTGGACACCAACAAGGCTCCTGGTTGGCAGGGTCTGCAGTCCCCACCGG

AGCATGCGCCCTCCCCTGACATCAACAGGGCTCCTGGTTGGCAGGGGCTACAGTCCCTGC

CGAAGCGCAAAACGTCTCCAGACATCAACAAGGCTCCTAGTTGGCAGCTGATGCAGTGCC

CGTCTGAGCGCAAACAATCTCCCGACATCAACATCTGCTTGCCCCGGGAATAACCTGAAG

GTTTATGATTTGACTTCTAACACCGTTCCTGTTCTAACATTTGGAGTGGTTTTTCTGCTA

GTTGTTTTATTTGAGGCTTAGAGTATGTTTGTTTGCAAGACAAAAGAGTGTAAAATCTCG

TTTATTATGCCAATTAATTGTTGTACTTGTTCTAAGTTATTTAAACT

>Unigene0001628

CACAGAGAGCGAGATCATGAATGCCCTACGCTTGCTGACCCGAGCCGCCGCCGCTTCCCG

CGGCCGCCCCGCCGCCGCGGTCCGCGCCTTCGCCACTGATCTGCAGGCGGAGACGGTCGG

GGATCCCTCGTTCATCGAGGCGTGGAAGAAGGTGGCCCCAAACCTAGATCCGCCTAAGAC

TCCGCTCTCCTTCATGAAGCCGCGCCCGCCGACGCCGTCCTCCATCCCCACCAAGCTCAC

CGTCAACTTCGTCCTGCCGTACCAATCCGAGATCGCCGGAAAGGAGGTTGATATGGTCAT

CATCCCGGCAACAACTGGTCATATGGGTGTTTTGCCTGGACATGTTGCCACCATAGCAGA

GCTCAAGCCAGGGGTTCTTTCTGTGCATGAAGGAAACGATGTGTCAAAGTACTTTATCAG

CAGTGGGTTTGCATTTATCCATGCAAACTCTTTTGCTGACATTGTAGCTGTGGAGGCCGT

TCCTCTGGACCGCATTGATCCCAGTCTTGTCCAGAAGGGGCTTGCCGACTTCACCCAGAA

GCTCAACTCAGCCTCAACTGATTTGGAGAAGGCTGAGGCCCAGATCGGGGTGGATGTTCA

CAGTGCCCTTAATGCTGCCCTAACAGGTTAAGTTGACATGGCTGAAAAGGGGTTGTAATA

AAATGTCCGACATTCTTGTTACCTAATTCATGCTTTTGTAGTAGCAGTTGTTTTTCTTGC

AGATGTTTCGAAATTCGGAAACTTTAAATGGCTAAGTTAAGCTGCAGCCTTGTTCTTTGG

TGTGTGATTTTTTCAGTTCCAAGTATATGCTACAGAAGAGTGTTTGATTCTCATGAGAAT

GTTTTTTCAATAACAAACGTGTTAATCTGTTCTAATCCTTTGGTGAGAGGCATTTGTGTT

GTGTATATTGATTTGTATGT

>Unigene0001629

TCCCCCTCTTTCTCTCTCCAGATTTGGATCCTCTGTTCCCCATCCCCAAACCCTAAGGAG

ACGAGCTTGACCCATTTTCTCTCTCCTCCCTCTCTCCACAAGAAGTGGATATGTTGGGGG

CGGCGAGCCTGAGAGCCAGTGTCATGGAGACCACCTGCGGTTCTCTGTTCAGGGAGCTGG

AGCGCATATGGGAGGATATTGGCGAGACGGAGGAGGAGATGGATAAGATGTTGTTAGAGA

TTGAGAGGGAGTGCCTGCAAGTGTACCGGAGGAAGGTGGAGGAAGCGGGCAAGGCGAGGG

CGCGGCTTCATCAGTTGGTGGTGGCGCAGGAAGCGGAGTTTGCGGCGCTTGTTGCTACGC

TTGGTGAACATGGTCTCCAATTGCAGACAGAGAAAAAGCGATCATTAAAGGAGCAGCTTG

CTTTTGTGACACCCCTGTTGGAAGACCTAAGGGCAAAGAAAGATGACCGCATCAAACAGT

TTGCTGATATATGGTCAAATATTGAGAAGATCAGGGGAGAGGTAACGGGATGTAATAATC

AGAGTGAAGCATTTGATTGTGTGGCTGCTATGGAGGAACATGATCTTTCAATAAGAAAGC

TTAACAAATATCAGGCGGAGCTTCGCAACCTCCAAAAGGACAAGTCTGAACGCCTTCATA

AGGTTATGGAGTATGTGAACGAGGTTCATTCTTTATGCGGTATCCTCGGTGAGGACTTTG

GAAGGACAGTGTCTGAGGTACATCCCAGTTTGCAGGATCCTGGTTTGGGGCCATGCACAA

ATATCAGTGACAGTACACTTGAAGGTCTTTCTCATGCCATCCAGAAATTAAAAGCAGAAA

AGAAGATCCGGTTTCAGAAATTACGGGAAACTGTCGAATCACTACTCGAACTGTGGAGCT

TGTTGGATTCACCCGAGGAAGAAAAGAAGACTTTTGGAAAATTAACATGCATTGTCGGAA

CACCTGAGCAGGACGCTGCACAGTCTGGTTTATTATCATTTGAAACAATCAAACAGGCAG

AGGCTGAGGTGGAGCGGTTGTCAAAGCTAAAAGCCGGCAAAATGAAGGAGATTGTTCTAA

AGAAACGGGCGGAATTGGAAGATACCTGCAGACAAACGCACATTGAACCTGATATGAGCA

CAGCACCTGAGAAATCCATTGCATTAATAGATTCTGGTCTTGTGGATCCTTCTGAACTTC

TAGCTAATATTGAGACACAAATAGCAAAAGTAAAAGAAGAGTCTCTAGCGAGAAAAGAAA

TCATGGACCAAGTAAACAAATGGCTAGCAGCTTGTGAAGAAGAGAAATGGCTTGAAGAAT

ATGACCAAGATGAGAACAGGTACAGTGCCGGAAGAGGGGCGCACTTGAGTCTAAAACGTG

CGGAGAAGGCACGGCTGACTATCACCAAAATTCCAGCTATTATTGAGAATTTGAAGAGCA

GAACATATGCATGGGAGAGGGAGAAAAAAATGCCATTCCTATTTGATGGGATCCGCTTGG

TAGACATTATAGAAGACTACAAACTGTCAAGACAACAAAAGGAAGAGGAGAAAAAGCGAT

ACAGGGATCAGAAGAAGCTCGAAAATCTACTACTTACTGAGAAAGAAGCTGTTTTTGGGT

CCAAACCTAGTCCAAAAAGATGCTTCTCCAATAAGAAGGCAAATGGCTATCATGCACATG

GAAATGGCAATGGTTTTACAACTCCCAAGCCTCGTCGCGTTTCTTCTGGCTGTGTCACCC

CCGAGCTTCTCACACCGCGCTCGTATTCTTGTCACAAAACTGGGCATTTTAAGGAGACGA

GACGGTTGTCAACTGCGCCATTGAACTTCGTTGCTCTACCAAAAGACGACAATGGCTCTT

CCTTTGCATCTGTTTGTGGTTCAGAACCTGGGTCTCCGCCTGAAGCTAGATGCAGAATTC

AGTTGGATGAAATCTATTCTGGGACTAAAGACCAATGCAGGAGCCGAGGTTCGGGGGAAT

GATTGCCTCGGAATTTAAATCTTGTAGCGCCTAGGCGACAATAGTTTACATGTCAATAGA

TTTGGCAATTACAGTGTGTAGATACAGAATCTAACTAGAGCCTTCTGAACTAGATGAGAC

TCTTTTTCTCACTTAAGAAGCAAGATGGTGTAGCTTGTACTGCTATTTTCATGTTTATAG

TTTTAGCCTGCTACCTGTATTTACTCGATAGTACAATTCACATTGTGTACTGTTTGTACG

TCTTTTCTCAATGGCCTAACTAATATTTGATTTTTCGTAGGGAT

>Unigene0001630

CTCATAAACTCCGGACCTTCACAGAGTCTTCAAATGGAGATTTACATGGACGAGAAGTGG

AAGCTATCCAAGAAAGGCAGCAGCAGAAGCAGCAGTCGGAGGGTCGCCGAGGGCGGCGGG

ACAAGTGGCTCTTTGCTGAGGAAGTCTGCCAGTGCAAGGGAGCCACGGGCGGCTCCTCGG

TCATTTTCGAGTCGGTGTGCAGGCCTGGTTAAGCAGCAGAGGGCTCGCTTCTACATCATG

CGCCGCTGTGTCACGATGCTTGTTTGCTGGCGGGACTATCCTTGACCGCTATCAGTTTGA

TGAGAATTAGAGGGGTTTTTTTTCTTGTATATATTATTTTGCGACAGTTTTGGGTTAGTA

ATGGTGGGGATGTTTTGGGGGATATGTAATGTTTCAGTACTGTGAAGTATCAGTATGAGA

AGAGAAGATGATGATAAATGAAAGA

>Unigene0001631

AGAGGAAGAGAGGGAAGATAAGAGAGAAAATAAGGAAAGAGTAGAGAAAATAAGGGAAGA

GGAGCAAGAAAGTGGAAGGGAAGAGGAGAGTGGAAGGAAGAGGAGAGGGAAAATGGAAGG

ATAACACCCCGATGCATTTTTCTTCAGCTGATGATATGTTTTAGTTTCCCCCAATGTGCC

AATGTATGTTGATGATGATCTTTAATGCACTTGCTGGTTTTTTGTGA

>Unigene0001632

GAAAATAAGGAAGAGGAGAGAAAGTGGAATGATGACACCCCCGATGCACTTTTCTTCAGC

TGATGATGATATGTTTCAGCCTCCCCAAGTGTGCTAATGTATGTTGATCTTTTGACGTAC

TTGCTTGTTCTTGGTGATGACTTGGAGGTCAATGATGCACATGGTTGCTTGATGGTGATC

GGTGTTTGACAACTTCTTAGAAGCTGATGGTGCCAAATAAAATTGTTGACAAAGGTAAGA

CAGTATGTGTGCATGGCATGTTAAGGATGAGGAGATGTTGATAACGGTGATTGTTGATGA

GTTTTCGAGGAACGTAGCGGCACCAATGAGTGTTCTTGGAGTTGTCGTCAGACCTACATG

ATATGCACAAAAGTGTGTCAGAAGCATGAAAAAGTTATCGTGTTCAATTATGTACAGTTA

AATTGATGGAACTACAATTGTGAGATGTA

>Unigene0001633

GAAAATAAGGAAGAGGAGAGAAAAATAAAGGAAGAGAAATAAGGAAAGAGGAGAGAAAAT

AAAGGGAAGAGGAGAGAAATAAAGGAAGAGGAGAGAATAAAGGAAGAGAAAATAAGAAAG

AAGAAAAAAAATAAGGAAGAGGAGAGAAAATAAGGAAGAGGAGAGAAAGTGGAATGATGA

CACCCCCGATGCACTTTTCTTCAGCTGATGATGATATGTTTCAGCCTCCCCAAGTGTGCT

AATGTATGTTGATCTTTTGACGTACTTGCTTGTTCTTGGTGATGACTTGGAGGTCAATGA

TGCACATGGTTGCTCGATGGTGATCGGTGTTCGACGACTTCATAGAAGCTGATAGTGCCA

TATGAAATTGTTGACAAAGGTGAAACAATATGTGTGCATGACATGTTGATGACGAGGGGA

TGCTGACAGTGGTGATTGTTAATGAGTTATCGAGGAACGTAGTGGTACCAATGGGTGTTC

TTGGAGTTGTCGTCAGA

>Unigene0001634

GAAAATAAGGAAGAGGAGAGAAAAATAAAGGAAGAGAAATAAGGAAAGAGGAGAGAAAAT

AAAGGGAAGAGGAGAGAAATAAAGGAAGAGGAGAGAATAAAGGAAGAGAAAATAAGAAAG

AAGAAAAAAAATAAGGAAGAGGAGAGAAAATAAGGAAGAGGAGAGAAAAATAAAGGAAGA

GAAATAAGGAAAGAGGAGAGAAAATAAAGGAAGATGAGAGAGGGAAGAGGAGAAAATAAA

GAAAGAGTAGAGAGAAAGAGGGAAGAGGAGAGAAAGTAAGGAAAGAGGAGAGAACATAAA

AGAGGAGAGAGAAAGAGAGGGAAGAGAGAATAAAGAAAAAAGAGAGAATGAAGGAAGAGG

AGAGAGAAAATGGAATGATGATACCCCTGTACATTTTTCTTTAGTTGATGATGTTTATAC

GTTTTAGTCTCCCCAAGTGTGCCAATGTATGATGATGATCTTTGACACACTTGCTGGTTC

TTGGTGATGACTTGGAGGTTAATGATGCACATGGTTGCTTGATGGTAATCGGTGTTCGGC

GACTTCATGAAAGCTGATGGTGTCAGATGAAATTGTCGACAAAGATGAGGCAATATGTGT

GCATGACATGTTG

>Unigene0001635

GGTTGCTCGATGGTGATCGGTGTTCGACGACTTCATAAAAGGTGATGGTGCCAAATGAAA

TTATCGACAAAGATGAGGCAGTGTGTGTGCATGGCATGTTGATGACGAGGGGATGCTGAC

AATAGTGATTGTTGATGAGTTATCGAGGAACGTAGTGGTACCAATGGGTGTTCTTGGAGT

TGTCGTCAGACCTACATGATATGCACAAAAGTGTGTCAGAAGCATGAAAAAGTTATCGTG

TTCAATTATGTACAGTTAAATTGATGGAACTACAATTGTGAGATGTA

>Unigene0001636

CTCTCTCTCTCTCTCTCTCTCTGCAGATGAGTCCTACGATGTCAGTGGTCATGCTGGTTA

CCCTACTAATCTTAGTCACCACAGCTCAAGGGATCAGGATGGATGAACAATCCTTAGTTG

CCTTCCAGAAAAAAATTAATGACAAAGGTTTGATCAAGGGAGCAGCTGGAGATGGAGATA

ATGCTGCTATATGCAGAGATGGTGAGCATTGTTCAGGAAGCCCTAGAAAGCTCATGGCCA

AGAGAATGAGCTTGGAGGGGATCAACGAGGACGCACAAAGACATCGATCTGGGAAAGAGG

GCATCAAGGGCAAGGAATTCCACACTGAGGTTACCCCGGAGGTGTTCCCCGACATGATAG

ATATAGAAGGGATGGATTATTCACCTGCGAAGAGAAAGCCCCCCATTCATAACTGAGTGA

TGAGGGAAGGCGCTAGCTAGAACTAAAACAAAGAATCAAAATCAATAGAATAGTGTTAGA

GGCGGACATCATTCCATGAAATGGTGCAGTTTTTTGGGGCTATAGTACCCTTTTTGTGCA

GATACTATATTTATAAATTTTATATGAACAAGTTGATGATAAGCTACTTGATCACCTTGC

AACTTATATGGGGACCAATTACACATTTGATTCCTAAGTGATTGTTGTGCGG

>Unigene0001637

AATGTGCTGCCTCATATGAAGGCCGTACTGTCTCACCTTTTTCCCCAACAGGACAGTATC

ACTGTGAAAAGTAAAGCTTCTAAAGATTTACCAATTAATCGTATGCAGAAAAAATATGTT

CAGCGTGACATGAAAATTCTAGATAGCACCCATCGAAAGGATATTTCCTGTATTAAAAGA

GGAAGGCACATCAAATTAATGAAGAACAGTTGTACCAAATGCAGTTTCCCAAGGGAAAAG

TGACCAAGAGGCGAAGAACAATGCGGACATGGGATAAGGGGCTGTAGATTACTTGTTACA

GGCCACTCAAAACTTAAAGGATGGTGATGTCACGGGTATAGTTAAGCATCACGGAAATGA

AGGATCGGAAGCTGTAGATAAACTTATTCAGCAAAACTCCCAG

>Unigene0001638

ACGTCTTCTCTCCTTCTCCCCCACGCCTCTTTCCCCTCACATGGCTGTGCTCTCCTGCTG

CGATCGATTCCCCGTCGTCTCCTCCAATCCAATCTTGAACCTCTGTAGCCTCGAATTCAC

CCCTGACGTCGATCGCGCTTGGTCCCGTTTCTGACGCTTTGATTTGGTGCTGTGAAGCTT

CCAGAATGGGGGGAAAGCGCGCCCAGCGGAAGGGTGCGACGGCTGATCAGCTTGACAGCA

GCGATGATGCGGATAGTGTGAGTTCTACTTCGACGGCCATGTCGGACTTGACGGTGGCCA

ACGTGGGGGAGACGGAGAAGGTGAATTCCTTGGATTTTACTCTGGAGAAGTGCTTGGATG

CACTTTATGAGAAGAGGGGATCTACGAGGGAGAAGGCCTTGTCGACGCTTATTGATGCCT

TTGAGGGTCATATTCTTCTTACATTTGTGGAGAACAAATTTATTACTTTGTTACATCAAT

ACATTAGCTCGGTCAAGCGAGGGTCCACCAAGGAGGCTTGTTTAGCTTCTCGTGCCATTG

GTCTGTTGGCTTTAACTGTTGGATTTGGAGACAATGCACATGAGATAATGGAAGAGGCAG

TACTGCCACTTTCACAAGCCCTCAAATCAGGATCCGAATCACTCAAGAAATCACATGTAC

TTTACTGCTTAGCTATTATCACATTTGTTGGTGCCAATGATCTTGAAGAAAAGGAGAAAT

CAATGAAAATTATGTGGGATATGATCCATCCAAAGGCGGGTTCCAATGTTGGAACTACGA

ATAAGCTGGCTTCTGCTGTATTAGCAGCAGCCATATCTGCGTGGTCTTTTATTCTGACCA

CGTTCAGTGGTTGGAGACTCGATCATAATAATTGGAAGGAGTCAATTTCATTCCTTTCTA

CTCTGCTTGAAAGAGAAGATCGAGCTGTTCGCATTGCTGCTGGTGAAGCGATAGCACTTA

TTTTTGAGATAGGAAAATTAGACACACACTCAAAAGAGGAGAGTGACATTGAAAGTTCGG

GACCCGAGGGCTTAAAACCCAAAGGATTTGAATATGTTCATTCATTGAAGGGTAAAATAT

TAACTCAGGTAAGAAATCTTTCTTCCGAGGCGGGGGGAAAAGGCACTGCCAAGAAAGATC

TCAACAATCAGAAGGACTTGTTTCAGGATATTTTGACGTTTCTTGAGACAGGTGATTGTC

CTGAATTGTCAATGAAAATTTCTAATAAAGGTAGCATTTTGACAGTGTCTTCATGGACAC

AGTCAATACAGTTGAACTTTTTGAAGCACTTTCTTGGAAGTGGCTTTCTCAAGCACATGC

AAGAGAATGAACTGCTTCATGATGTTTTTAACTTCACACCAGAGAAGCAAGGAAAACATT

CAGTCCAAGACAAGAGGATGTTTAGGTCACCAAATTCTATACTGAACAAAGAGAGAACAC

AAATTCGGAACAAGCACAGGATGTTAGCTCAGGATAGGAACCAAGGCCACTTCGCCATGG

ACGAGTCAGAAGAATAGATGTTTGATAGCAGGAGGGGCGGGAGGATACTGGATCAGATAA

GCATAATTTTCTATCCTTTAGCTTGATATTTGCATTACTGTGTCAGTGAATGGAGATTGG

AGAACTAATTATGTATTACATTATTATTTTGGTAAACATATTTTGTGTAATCATAATCAT

GCACGATAAAGGTTATGTTTATGATATTTAATTTTATTGAGTGGTTATCTGGTATTGGGC

TCCATTAGTCGT

>Unigene0001639

TGACTGCAACAACTCATCTCCTCCTTCCTCTCTGTAGTGTTTTGAAACCCTAGATCCACC

GCCCGCCACCAATTGCGACTGGGTAGGATTGCTCCTCTGCAGGAGGGTATGAATGGAGTC

GTGTTAAGGTTGCGGCTGCGATAAATGTTTGAGGGAGAGGCGCCAAGGTACTTTTGCTTA

GTCACGAGGAACGATCATGCGATTGGCGAGAGGTTGAGGCAGCTACGTACTGTGGGGTGG

TGCGGTTCCGACCGTAGCTCGAGGAAGATCGAGCAGAGGGGTGATGTTGAAAGGAGTGGG

GAGAGATCGCCGGTGAAAAAGCTATAGGTTTTCATAGTGGGACAGAGGGAACTAGCATGC

ACTCCTCCCGGGTTTCTGACTTCGGGGCTATTGATCAATCTATTGGCTTCCGAATTGAGG

ATGCTGCTGACCTGAGCAGAAATACTGTTCTCCATCCAAGATCTAGTGGCCTGGCTATTG

TTTCTGATACTCTACAATTTAGTAGATTCAGTAAGCCTCTAGCTCCTGATATAAGTTCAT

CTAGAGTTGGTTCTGTGGCATTACACCAAGAGAGAACAAGGCAGCCAAACCTGGTATCAC

GTAGTAACCTTGAGAACTGGGAAGAGCCCATAATGGCTGATGGCAGTCCTATGACTGATA

CATCAACAGACGCAGAAGCCGACGAGAAGAATCAAATGTTTGAGGTAGGCCAAGTAGCTA

CAGCGATGGTATCTGATTCTAGTGATAGATCAAGAGAAAGAACAGGGGACCAGAGGTCTC

TTCGTCGACTTGCTCAGAACCGTGAAGCTGCGAGAAAGAGCCGTTTGAGAAAAAAGGCAT

ACGTCCAACAACTGGAAAGCAGCAGGCTGAAACTAACTCAGCTTGAGCAGGAACTCCAAC

GAGCTCGACAGCAGGGCACCTTTATTTCAAGTTCAGGAGATCAAACTCATTCAATGAGTG

GAAATGGAGCTTTGGCATTTGACGCTGAATACTTACGGTGGGTAGAAGATCAAAACCGAC

AGATCAATGATCTGAGAGCTGCAGTGAACTCACATGCAAGTGACACTGAGCTTTGCACCA

TCATTGATGGTATTATGGCACACTATGAGGAAGTATTCAAGCTTAAGAGTGCTGCATCCA

AGGCAGATATTTTTCACATGTTATCAGGGATGTGGAAGACACCTGCAGAGAGGTGCTTTC

TGTGGATGGGGGGTTTCCGGTCGTCTGAACTTCTCAAGCTACTCGTGAATCAATTAGAAC

CTCTTACCGACCAGCAGTTGGTAGGAATTTCAAACCTCCAGCAGTCCTCCCAGCAGGCGG

AGGATGCTCTTTCTCAAGGGATGGAAGCACTTCAGCAATCGTTAGCAGAAACGTTGGCCT

CTGGCTCTCTTCGGCCCTCAGGGTCGTCTGGTAATGTCGCTAATTACATGGGTCAGATGG

CTATGGCGATGGGAAAGCTCGGGACCCTCGAGAACTTCCTCCGCCAGGCCGACAACTTGA

GATTGCAAACTCTGCAGCAGATGCGACGCACACTCACTACTCGCCAATCTGCCCGAGCTC

TCCTCGCAATAAGTGACTACTTCTCACGTCTGCGCGCCTTGAGCTCTCTTTGGCTAGCTC

GTCCACGGGAATGAGTTGATTTGGCCTATTCTTGGACGCCCGAAGGATCGTACCAGAATA

ATGCATGTTCTCGCTCATCGATGAAGATCAGTCAGGTTTACGTATTTCCTATGGTGATCG

GCTCCAGAAAAATTCGGAATGCTCAACCCCTTGTACTTGTTGTACTTGTAAATCATGTTT

ATGAGTAGGATTTGTGTACCAATTTGTTGTGTTTTCAGCTTAGGTTACACCAGATGTGTA

TGTTTTGCACTATCAGGCTTATTAGACTGTTTTCGAGAAATTATCAACTTTACAAGCATT

CACTGTCATTGTATTTATCTTAATGAACAATTGTGGTTGCAAAAGATTATAATTATTTTG

GTTTCCCCCTTAGAATGTGTTA

>Unigene0001640

TCATAACCGCCGCCCGCTCATTCCCATCCCCATCTCTATCTAGGGTTAGGGTTTCATGTC

TGCCCCGTACTCCCCTCCCACCACCGTTGCCCTCCGCCGCTGCCGCTGTCTCTGTTGCCG

CTGCTTCTGCTGCTGCTGCCGCCCGTCCGTCCTCTAACCCCACCGGTTACGCGTCCGATC

CCCGCCATGGATCTCACCCACCCCCAGTCCAACCTCACCCTCGGCTTCACCTCCCACCGC

GCCGACTCCACCGCCCCCCAGAGCCCCCGATCCATCCCCCTCCAGCTCCAGCTCCTCGAT

CAACAGTCGGAAAACCACAACCACCGCACCGATGACGCCGACGACTCCGAGGACGGACAC

CGCGAAGAAGAATTCAGCATCCTCGGCTACACGATGTGCCTCAAGCGCCGCGCCGAGCGC

TCCTCCTCCTGCTCCTCATCCTCCTCCCTCCACCCCGCCAAGCGCGCCGCCCCAGATCCC

GACCTCGAGGCGCGGCGCGCCCGTGTCCGCTCCTGGGGGAACCAGTCGCTCGCCGCTGCA

GACCCGGACCTTTTCGAGATCATGGAGAAGGAGAAGCGGCGCCAGGTTTCGGGGATTGAG

CTGATTGCTTCTGAGAATTTCGTGTGCCGGGCTTCACTCGAGGCTCTCGGCAGCCATTTG

ACTAACAAATACTCCGAGGGCCTCCCGGGTGCTCGCTACTATGCGGGAAACCAGCATATT

GATCAGATCGAGCGGCTTTGCTTTGCGCGTGCGCTTGCAGCTTTCGATCTTGATGGCGAG

TGCTGGGGGGTTAATGTGCAGCCGTATTCGTGTACTTCGGCTAACTTTGCGGTGTACACT

GGGCTTCTGTTACCGAAGGATCGGATTATGGGGCTGGACTCGCCGTCGGGTGGGCATGTT

AGCCATGGGTACTATACGCCGAGTGGGAAGAAAATATCAGGGGCGTCGATTTTTTTTGAG

AACTTGTCGTATAAGGTGAATCCGAATACTGGGTACATTGACTATGATGAACTTGAGAAG

AGGGCCATGGATTTCCGCCCGAAGATATTGATTTGCGGGGGTAGCTCGTATCCCAGGGAA

TGGGACTATGCACGGTTTAAGCAGATTGCAGACAAATGTGGGGCTGTACTGATGTGTGAT

ATGGCTCAAATTAGTGGGCTTGTGGCGGCTAAGGAGTGCCTTAGTCCATTTGATTATTGT

GATATAGTTACATCAACAACTCACAAGAGCCTCCGAGGTCCTAGGGGTGGTATAATCTTT

TTTAGAAAAGGAAGAAAGATAAGGAAGCGGGGTCTCTCCCTATGTCAAACAGATGAGAGT

GATCTATATGATTTTGAGGATAAAATAAATTTCGCCGTTTTCCCATCAATGCAAGGCGGA

CCTCATAACAACCATATTGCTGCTTTGGCTGTGGCTCTAAAGCAAGTGGCTACTCCTGAA

TACAAAGTATATATACAGCAAGTCAAGAAAAATGCTCAAGCATTGGCTTTTGCATTACTT

AGGAGGAAATGTAAATTGATCACTGGGGGTACAGACAACCATATGCTACTTTGGGATCTA

AGAACTTTTGGCTTGACAGGTAAAAACTTTGACAAGGTCTGTGAGGCTTGCCACATTACT

CTGAATAAGACTCCTGTATACGGTGACAATGGTAGTATATCACCTGGAGGAGTGAGGATA

GGTACTCCTGCCATGACTACCAGAGGTTGCCTGGAGGGTGACTTTGAGACTATTGCCGAT

TTCCTTCTCCGGGCTGTTCAGATTGCAAGCAATGTACAGAAGGAACATGGTAAGTTGCAA

AAGGAGTTCCTTAAGGGTATGCATAATAACAAGGATGTTGTGGAGCTTCGGAGCCTTGTG

GAGAACTTTGCTTCTCAGTTCGCAATGCCAGGATTTGACATATGAGACTCCTGAATCCCC

TCAAATTTGAGGTTCATTGCTGCAGAGCCAATCTCAACCCGAAAATATCCTTGGCTTGTA

AAGTTCGAAGCCAGATTATGTATGCAGCGCTGAACTGAATTTCCAATTGTTTTGTAGATA

TAGGAATATCCTTTCTTCTTTTACTTCATCGTACATTGTCAGAAAGTATACACTTTGTAA

TACTAAATTATTGTTAAGTTTAAAGCTACAGTATTTTTTTCCAGTCTCAACT

>Unigene0001641

CAGCCAAAAAGGCATGTAGATGCACCGGGTATGAAGAGTGATAAAATTATAGTTAGTGTT

GGAGCTAGAGGAAGGGTTGACTTAAATTGACATGGAAGGATGTAGTAAAAAAAAAAAAGG

ATCTGGTAAGAATGAATGGAACTAAGCATATTTCTCTTTACAGAGCCTGATGGGGAAATA

TGATTCATTTAGCCGACCCCAAGTAATTGGGATTCCGGCTTGGTTGTTTGGTTTGGTGTT

TAGTATTTTGAGGTAACGACTGTCTGGGATAATATTATCCTTTTTTTCCCTCTCCAAGCA

TGTAGATCAATCTCCCATGCAATTCCTTTTTGTAGGTTTATATTCTAGAAATTCTAGTAA

GTCTTCCCATTTTATGTAGTCTTCCCATTTTATGTAGTTAATTAGTAATATCACCCTTGT

TGGTAATTAGTGTTATACCCCCTAGAGCGAGTTTCAAGGTACTCAAATTTTTTTGAAACA

ATATTTTATTGGATATGTAATCCTAAACAAAAATTTACTTCATGAATTGAAATAAATTCA

TCTGGCAATAGCAACAAGTGATGTTTTAGCTATTATTTTTTTGATTGAACTATGTTCTGA

AGCATAAATCAAACCCTATTACCCCTTTGTTGATTTCTGTAAAATGTTATCTTTTTCTTT

GTCTTGACTGGTGGCCTTACAAATCCTTATTATAGGTTTATATGGCAACCCTAGTTTTTA

ATTGAGTATCAACTGTCAACATTCTTTACTTTTTGAAAAAAAAGAAGTGCCAACATGTGT

TGCAATTCTGTCCTCTTACGACTTCTATATTAACTCTAATATATAACCCCAATCTCCTAT

CTTCAAAAATGACAAACATTTTAATTAGCAACTAACCTTGTTTCTCACTAACATTTAAAG

GCCAAACTTGATTTTCAAGGTTATCCTAATTGAAAGCACATATAAATTCATTCTCTATAC

CCCCCTTCAGTATTGGTTCCCACTTTTCTCTGGTTTCAATTGATTCTCTTCTACTTTATG

CAGACATTGTACTTTATACTAATATGAAGAGCATGAACTTTATCCATAGATATTGCTTGT

TAGTCTTGCTGCTACATTCAGATTCTGTCAATACAACTTTCTCCCATGTGGATACAAAAG

AGCCCCCAAATTAGTTTTTGGAGGTGTGACTGAAGGTGTTTGTGTGACCATAATATTGGT

ATCTAGTAAAAAGGCCATGGATACTGTTTAACATTTTTTTGGGGTTTTATTCTAATGTAA

TTTCAGTTTCTTTCGTAAGAAAACCCAAGTTTCAATTTTCTTTCTTGTCATGCCTAGAC

>Unigene0001642

TCCTTCAAGCTCAAATTCAATGGCTGCGGAGCCGGCGATAGATCTGCTGCGGCGGTTCGT

TCCGGCAATATGCCGGGACGAGGTGTCAGGTACCGCCGGAGGCCGCGGCGGCACCGGCGC

CGACGGCGACGCCGAGGCTATTGAGCTCAGTTTGGACCTTTCGCTTGGAGGGGAGAGGAG

GGAGAAGAAGCTGGTGAGATCATCGACTGTGGCTTCCGTTGGGCTTACCATGTTTCCTGA

TGAGGAGTTGAGGGGTGTGGGGAGGACGTGTTCGCTGCCCACGGAGGTGGAGGAGGAGGT

GCGGAAGAGGAAGGAGATTCAGGGGATGAAGAGGATGGAGGCGAAGAGGAGGAGGTTGGA

GAAGAAGGAGTCGAGATCAGGATCGGGTTCGGGGTCACAAGGGAGCGCCTCGTCGGTATT

ATCAGATCTCGAGAGCCTGCCGTCTCAAGGGAGCAACTCTAAAGAGGTTAAGAGCCCATC

GCCTGTGCTTGGTCAAGCCAACCACAAGGCAACAACCGTTGTGGCGGGTAAGTGTGTCAA

GGCTTCCAGTGAGGCGGACGACCCCTCGACAAAAGCTATGGCAGCAAGAGACCAGGACAT

GGAGAAGAAAATGCTGGAGTCAATGCCATGTGTGTCAACTCGAGGCGACGGGCCAAACGG

GAAGAGAATCCATGGCTTCCTCTACAAGTACGGCAAATGCGAGGAGGTGAGGATTTTGTG

TGTCTGCCATGGTATCTTCCTCACACCAGCTGAGTTCGTGAAGCACGCTGGTGGAGGGGA

TGTCGCGAATCCTCTTCGCCACATTGTTGTGAGCTCGCCCTCTCCATCAGCTCTCTTGTG

ACAATGGTGGTTGTCAGGAATCAACCTGTGTGTTGATCATGTATACTACAAGTAATTATC

GATACAGATTACTTGACGATGCTTAGGCGAAGCCAATGCATTAGGTTTGGAATCTTGTTT

ACTGTAAACACTTTTAAAGAGCTCAATTGTTGGTAAATTCCAAAGTTTTAGATGTCAGAA

GAGTTTAATTTGGCAAGTAGAATTGATTTAATGTTGGAATGA

>Unigene0001643

AGACAATGTTGACAGTCTCTCATGATTATTTACTTGATAGCTGGATGTTGGATGCAGACA

CTTCGTATACACTTCGTATCATCTGTGTCAGGTGCGGGAATGGTTCACCAATTATCGAGA

GGTTAGGAGAAGCTCGGTCGTTCGATTGTTTTGGATGAGTCCATGTCAGATTATTAGTGT

TGGTATCGTAAGATTTCAGCTAGTAGAATGTCTATGGAGAAAGCTAACAAAGGTATGCCA

TGTTCCGACCCTTTCTAGAGGGTTGATTTTTCTTGGAGTACTTGAGACACAGGGCTAGAG

GAACATTGGTGAGAATGACTACAGTATGTGTACTTTGGTGACCGACTGTTTATGTCAGTG

GAATGGCATGCAAATCTCTATTTCCTTCTAGGTAGTATTGTGGAAGGTAGAGCTATGGTT

TCTTTTATTATTGGTGATGAGCAGATGTGTGGACTTAATTTTGGCACATGTGGCTTGGGC

ATGTAAAGTGAAAGTGGGCCGCGCACATTGAGCATGCGGGGCTTGTTTGGCAATGACAAG

TTGGCAAAGCTGGACTTTTGTGAGAGTTGTGTCTATGGGAGGCAGGGTGAGTTTCAGTGG

TGCTACTCGTGATGCCGCATGGATATCTCTAGAATTGCATCCAAGAGGGTTCGTGGTACT

TAGTGTCGTTTACTGATGACTTCAACAAGAGAGTTTGGGTGTATACCATGAAGCACAGAA

CTGAAGTCACGAATATCTTCATGAAGTGGAAAACTATGGTTGAGAAGCAAACGTGCAGGG

TGATCAAGATTGTGAGGTCCAACAACGGCGATGATCCCCCTAAAGGAGTTTTGTTATCGG

GATGGCATTCGAAGGTATTCCAGGGTGAAGACGACGCGATAGAACAGGATTGCAAAGCGG

CTGAATACAATTGGAGAAAGCACGGTGCATGCTGTCCAATGCTGGTTTGAGCAGGTTTTG

GTGTTCTTTATTCTCCAAGACACCTGTGATGGGATGAGGCCCGGTGTCGTGACCTGGAGT

GGGAGGCACGAAGTAGTTTGCGTAGGAGGCGGTGAATCAGAGGCTCTGCTCTGCGATGAG

AGAGACAAGTCAGGGACAGCTGGTGGGATTGGTGGACATGGGATAAAATGAATTTGGTAG

CGTATAAAAAGAGGTGTACAGGGGAAGTAACCATCTGAGTGCGTGGCTTTCTCAAGGAAT

TGTGACCGAAGAAAACTTGACCTTTGTGGATTGACGTCGGGAGGATGACGTGGATAGTTG

TAGATTGTGCGGTTGCCGTTTAGGGTTCCGGTCTTTTAGCAGGTGTCGAACTAACATGTG

TTCTATGCCCTACGCCTCCCATCTCAGCAGTCACACCTATACCAACACAGACTATTTGAC

ACATATGAGAATGTCAACCTTTTCTATCATACTCTATGGCTTTTGCTACTTGCCAATGAA

AACCTCCCAGAGGTGAAAAAAACAGGCTGTTGTATTTGGGGTTGGTCTTTGCCATCTCCC

TGGGCGCATGATCACACTCGGTAGCTTCTCCGAATTGCCAATGAAGACCTCAAAGAGATG

TTAGACCATGATGTTGTTCTCGCCGCTGGTCCTCGCCATCTTGGCAGGAACACAATCTTG

ATTACGCTCCATAGCTTCTCCACACGCCGATAAAGATCTCAGGGAGATGACAGACCATGT

TATACTTGGGGTTGTCCTTCTCCTGGGCACACGATCTTGATCGTACTCTGTTACTTCTTC

GACTTGGCGACGAAGATCTCGAAGTTTACACTCCGGGGCTGGTCCTCACTGTCTCAAACC

TATGCCAATGCCGAGAAAGATGATGAGTGAAATTTTCTCTATTGGTGCTGACTCTTAGTA

TCATCAGACATTTGTTTCCTTCATCTATTATCAGAACACCAATATAACTTCCCTTTAAAC

CTAATCTTACATTCAAAGTTTCCAAGCCAAATTTCCAACCAAAAGCTGGAACCACTACTA

GATCCCTAAGGGTGTTGCGTGGTTTGTGTCTTTTGTGAGTATGGCATCTTCCATTACTCT

TCTCCCATCGGCTTGCAATATCCAAGCCATCACCATCGTTACTCTATACCCTCATGCTCC

ATAGTCGTAAACACAAATTTCTTCACCTAATGATCTCATTATTGATAAATTAATTTTGGA

CCTTTAGAATTTAGATACTCCTCACAAACTGATTTTAGTTTATCTGAATTTTATTAGAAT

TTTCATACCTAATGAATTAGAATAGAAAGGGATTTCCATAGCAGCCGAATAATGTTGGAG

GCTTGACATAGTTTGTTAATTTAACTCACACATCATGCGAGCTCTATTTATAACATTTAA

TTTATTATTTAATGTTAAAGTTTTTTTAATACATTTGTCTATTTTCTTAATACTCTAATT

TAGCGTGCATATTATGTGTGGGTTCAATATTAGCCCGTTTGGAGGGTT

>Unigene0001644

CGTTTGGAGGGTTTCATGATAGACTAGGTTTGGTTATGAATAAGTTTCCATGATGTTGCG

GCTTACGAGCAAGAATGTGTAACAAAACTACACAAGCTCCTTGTTGTTTGTCATTTCCGT

GCTATGGTACTATCAAATCACTTCTTTTCTTTGAAGAAGAAAACTGCCAAATATCTTGTA

TCATGTAATGTTGAGAATGATGCCCATGGAAACATATTACACCATAAATACTAAAATTCC

TGTAAGATTCTTAACCTAAGGTATTTGTTTTGTATTAATTGACTCTCAATATATGCAGAT

CTGTGCTCTTGGCATTTTCAATATATTTTAGCCATGTCTCCTGTCTTCTTATTTCCTTCG

AACCTCTTATCTTTATTCTTTGTTTTTCTTGCTGTCGATATAATACCATAGTTCTGAATT

CTGATCTCACATTTTGGTGACATTGATCAGGAAGTCAATTTTGTGAGGCATATGCTGTAG

TGTTTCAATAAACTGGTTATGGAATCCATTTTCTCTATTGATTCGTAAACTATCTAGCAA

ATTTATCTGAATGCAAGGATGCCATCCCCGCATTATCAAATAGTATTCGGATGTCTAACT

TGCCTTGATATCTGTTTACTTGATTGTTTATTTTAATTGTGGCTACATATATTCCATTTT

GTAACCTTTTCTTTTTCAGTTATCACCTATGTCTCAAGTTAAGGATGATTGGCTGGTAAT

GGATAAACATTTACTTTGGAACCAGTTGTTATGTATGAGTAAATAGTATCGTACTCTGGA

ATTAGCAGCTTCAATCTCCCTGAATTATTACCATCCAGACAAATACTCTATGAATTACAG

CAGCTTTTATATATGCCATTAATTTTCTTTTGTGAAACTAATCATTTTTTGAAGAAGTAT

AAGAATAATAGTTTTCCCTAATGCTTAATACTTGATCAGCTCGGGATATTATATGCCAGG

TACCATTGTCTTGGCAAAAGGAGTTATGCTTCCCTCGAATATTTTTCAGGTAAGTCAGTT

AGGTTTTCCTTCTCTGGAAGGGAGGGGCACTTCTTTGTTTGTTCACAAAGGAGTGGACTT

CTTCGGGCGCAGTTTAACTGAAGTTGGGCTAAAAGAAGAGGATGCAACAAATGATATGTT

AGTAATTCTTTCAGATGTATGGCTAGATAAGAAAGAGGTATGGGAAAACCTGGAAGTGCT

CTTCAGTTGCTATAATAGTTCCGAGAAAATTCCAGCTTTGTTTGTTTTAATGGGAAATTT

CTTGTCTTGCTCGCTTTCTCCTGAGTCTAGTTTGAGAAACCAGTTCGAAGAACTGGGGAA

AGTCGTTGGAGCTTACCCAAAAATAAAAGAGAAAAGCCGGTTTCTGTTTATTCCTGGTCC

TGATGACCCAGGTTCCTCAAAAGTTTTGCCACGCCGTCCGTTGGCAAATGATTTATTTGA

AGGATTTCGAAAGCACATGACGCCCCCTGATGTCCGCAGCAACCCTTGCAGGGTTAAGTT

CAACAAAAGAGAGATGGTTATTTTCAGACATGATATGGTTCAAAAAATGTTAAAGTCCTG

CTTGATCCCTCCTCCGAAGAGCAGTACTGATTTTGAAAATCTTGTAGCGACAATTACGCA

ACAACGCCATCTCTGTCCAATACCAGTGGAGGAACAACCTCTTTTCATAAACTATGATCA

TACCCTTCAGTTATATCCATCTCCACACACGATAATTTTGGGTGACACATATAAGTCAGA

ATATATTGAGTTTGGGGGAATAACATGTTTTAACACGGGATCTTTCGCTAGAGGTGGATC

ATATTCAACATATTCTACATGCAGTGACAAGGTCGAGTTGCGAATCATCGGAGGTGGCAG

TGGCATGTTCTGAGCACATTGAAGAAAAATGTAATGGTTTGTTCTGACTGAAAAGAGGTT

GATGATGCTCCTCCGTGACAGCCGGTAAATGGTAGCAGTGGATTAAAATCTTTTGGTCGC

CCTCCTTATATGTAAGTTGGGTTCTAGCGTGATTGTAACCAGACTCTCACACTTCATCTT

TTGGTCGCCCTCCTTATATGTAAGTTGGGTTCTAGCGTGATTGTAACCAGAC

>Unigene0001645

TGGGAGTAGACACATTTTCTCTGAATTATTTCTTTCACAAAATCCACTCTCACCACCTTG

ATTCTTGGATTGACTTCGCTGTTGAATCAAAAACTAAATCACTTGTACTCAACCTTGGGA

TATGTTTCCGAACTAGTAGGAATAAGCCACAGCTATATGAGTTTCCATGTCATATGCTAG

GATCATCATCTATACAATTCTTACTCCTTTCGTATTGTGAACTGAAACCGCACCATGAAT

TTGAGGGTTTCCGCCACCTTAGGACTCTTTGCCTCTCCCGAGTAAATGTTACTGAAGAGG

GAATCAGACGCTTACTATCAAACTGTTCTACTCTCCAGGATCTCACAGTTGAGAAGTGTC

AAAAATTGTTGACACTAAGCATTTCTCACCCTCTAAGTCAACTGACATTTCTAGAAGTAC

AATGGTGCAGCAAGTTGACGAAAATAGAAATCAGCGCCATCAATCTCGATTTTTTAGCCT

ACAGTGGCCCTTCGGTAGAGTTCATTTTAAGAGAATCTTCACGTCTTAAAAAAGCAACAA

TTTATTTTGAATCCTACAACATGGACTATGCTCTCACTATGTTATCCAACAATCTGCGTT

GTCTCGAGGATCTGGTGTTTGAAATAGATGGCAATTCAGAGGGGGGACTCCGTTTGGCGA

GAAATTCCAGTCGATTTATTCGTCTGAGGCATTTGACCTGGCAACTTTCATATACATTAC

AAAATAGTAAGGGGCACCAATGTTTAGCTTCACTCCTACGTGTGTCTCCCTTTTTG

>Unigene0001646

CAGTTTGACCTTGCCAACATCCTTCATGCTAAGAACTATTCTCTCATAGATCCAGAAGAA

TATACTGCTGAGGACCGTTATGTCCTAGGAGAAGAATATGCTGCTGAAATGAGGGACAAG

TTCATCGCATCTGTCAATTCTGTCTTGCACCAACATCCCCGTGTGGGAGTAGACACATTT

TCTCTGGATTATTTCTTTCACAAAATCCACTCTCACCACCTTGATTCTTGGGTCAACTTC

GCTGTTGAATCAAAAACTAAATCACTTGTACTCAACCTTGGGATATGTTTCCGAACTAGT

AGGAATAAGCCACAGCTATAT

>Unigene0001647

CGGGAACGTTCATCGTCGCCAGCGAGCCTACGCCGACGGGAGTCTCCCTCACTCTCTCAC

CGATGGACAAGATCTTCCCACCATCTACCTTCGTAGAGATGATACTTGTGTTTAAGCAGG

GTGGTGAAGATGTTGTATATAAAGTAAGAGAAGCTCTCGCCAAGGCCCTCGTCCCCTACC

ACCCCTTAGCAGGGAGGATGGTCAGGTCGGAGAGTGGAGAGATGGAGGTG

>Unigene0001648

AGGCATGGCTGTGACCCTTCAGGTCACCAAATTCTCATGCGGCGGATTTGTCATTGGCTA

CGTGTATGACCACGCTGTCGCTGACGGTGTCGGAATGGCACAATTCATGGTCGCAATATC

CGACCTGGCAAGAGGTCTCGACGCTCCCACCATACAGCCCATCTGGTCCAGGGACTCCAT

CCCCGTCCCACCAAAACTAGCAACATCCAAGACATCTCCACGTGGCCCGCCACCCCCGTT

CCCTACAGCTCGCAACAAATACTTCCACGTCGACATCTCAGCAGACCGGATCAGCGAGCT

CAAAACCCGGTTCAAGAGCCTGATGCCGATGTTACCATCGCCATCCCCGCCAATGTTCGC

CACCACTTTGTCGACAATGGCTACTACGGGAACTGCCTGTATCTACTGATGGTCACCGTC

GA

>Unigene0001649

GAGGAAGTTGGCAATTTTGGTCGAAAATTGGACGAAAGGCGTGGATATGAGATTCGGGAG

CAATTTGATATACATGACCGATTGGTCGAGGTTGGGGTTCGATGGGGTGGACTTCGGGTG

GGGGAAGCCCGACTATATTGCGCCATTGCTCGATAACCACCCTGAGGTGGTCGCTTACAT

TGTTAGATCACCGACTCCGAAGAAGAGTGTTAGGTTGATCACACAATGTGTTGTGGAGGA

GAACTTGGCAACTTTACAAGATGAGATTGTGAAGGCTTTTGAAATCTGAGTGTTTAGAAT

TTGTTATTGGGTTTATATGATTTTGTTGATTATTAAGTGATGTATTATTATGAATAATTG

TTTATTTCTTTGGTTGATCGCACAATGTGTTGTGGAGGGGAACTTGGCAACATTACAATA

TGAGATTGTGAAG

>Unigene0001650

CCATTGCTCGATAACCACCCTGGGGTGGTCGCTTACATTGTTAGATCGCCGACTCCGAAG

AAAGGTGTGCGGTTGATCACACAATGTGTTGTGGAGGAGAACTTGGCAACTTTACAAGAT

GAGATTGTGAAGGCTTTTGAAATCTGAGTGTTTAGAATTTGTTATTGGGTTTATATGATT

TTGTTGATTATTAAGTGATGTATTATTATGAATAATTGTTTATTTCTTTGGTTGATCGCA

CAATGTGTTGTGGAGGGGAACTTGGCAACATTACAATATGAGATTGTGAAG

>Unigene0001651

TCACTTGCCAGGTAAATCTTCCGAAACTGTCAATTTGTTGGAATTTCAGTAGGCAGGCAC

AGTTTACACACAACATGGAGATTCAATGATATATTCGAATATCATATTAGCCGTCCAAGT

TGTATACCGTATTCTCATCAAGTGTTCGTTTTCTCACCTATTTGGATTACATGTCAATTG

TTCCTTTTGAACGCCTTTCTGAATGGCCTACCCCATTTTGACACCCAGAGCCGTTTAAGA

CTATGCATGTACCTATACACCCCCACTTTGCGGGTCAAATAGGGACTCGAGGATCTAATA

TTTGAATTTATAGACTGACATTGGGGATGGTCTTAAATGGCTGTGTTTAAGCGCAAGGGT

TTGAAAACGCATATTGTATTGCTTACTCTCATATTGTGGATATTAGCAT

>Unigene0001652

TATCAAACTCTTTCTACAAAAGGGAGAATGCTTTACTTCTTGTTCTACCTCTTGGTCTGC

TCAACCAAAAATCAAAAAGACCATCTCTATTTATTGAAAATAGAGGCTATGTTGCTTCAA

GTTTTCTTTATTTGCTGCAGGTTTTTGTTTTTCTTTTCTGCTGTATCTTTGACTGGCACC

TAGTCTACCTTGGTTGCGGTAGACTGTAGATACTACTTCCTCTCTCCGTATACATAAACA

GAAATCCCAACAAAATAATAGCTGCCATATCGGTTTTGGCCCACTAAATTTGTCCATTTT

TTAGCCGGTAAAAAGTACACGAGTACACTATTCCATGTTATTTCTACTGTACTGTGCTCT

TTTCTCTAACAACAATATCATGGATCTACTGTTGATTCGTTGTTGCAGCTGTACTGTGTT

CCTCCCTCCAACAATAATGATGTTTCTTTATTGGGCTCGAGCTAAGGGTGCAATAATCGG

TTCAAGAACTCTTTCCTCAACAAGAATGTTGTGATCTACCGTTGTTACGACTGCACTGTG

CTTCTCCTCACTCCAACAATAATGATGTATATTTACCAGGCTTGAGCTAAAGGTGCGGTA

ATCAGGTCAAAAACTCTTTCCTCAACAACAATGTCATGATCTACCGTTGTTACAGCTGCA

TTGTGCTTCTCCTCACTCCAACAATAATGATATATATTTACTAGGCTCGAACTAAGGGTA

ATTACTTAATACTAGGCTCGAACTAAGGGTGCAGTGATCAGGTCAAAAACTCTATCCTAA

ACAACAATGTTGTGATCTACCGTTGTTACAGCTGTACTGTGTTTCTCCTCTCTCTAACAA

TTATGATGTACTCAAGCTAAGGATGCAGCAATCAAGTCAAAAGCTCCACCCACCAACAAC

AATGTCATAAATCTACCGTTATTGCAGGTATAATGTACTCCTCGCTCCAACAATAATGAT

ATACATTTATTGGGCTCAAGCTGAGGGTGCAGCAACCGGTTGAAAGGCTTCTAATCGTGA

AATTCTCTCATCGGCTTGGGCGCTATGGACTACAGCCTTGCTCGGGCTATTGGTATACGT

GTATGGAGCTTATGGTGTCTTAAATTCCAATTAGGATTGTTGAAAAAACTGTTAGTCAAA

ATGCCCTGTGATAAACATGGAGTCATCGTGGATGGAAATTCGACGTTGCCTCTTCTTATT

CTTTGCGATAAAAGAGAAAGCTGCATTTCTCTCTTGGTTATGATTCTGTTTGCGGACGTC

GGGATTATGATCAGTGGGGTCCCAAGTAGTTTATTTATTGTGCCATATATATTTGATGAA

GATAATGTTTGTTCGTTTTTGTGATCCTTAATATGTTGGCATCACAACGGAGTCAAAATC

AGGGA

>Unigene0001653

TTTTACTCTAAAATTCCCCTCTCTCGGTCAGCTCTGCATCAAGGTTGAAAATGGCAATCG

AATCCGTGGCGTCGAAGTTCCTGCGCCCGCGCAAGATCCCTCAGTCGACGGACATCGCCG

CCGCCGCCACATGGGGCATCGCCGCTGGCACCGCCGCCCTCTGGCTCGTCCAGCCTTTTG

ATTGGATTAAGAAGCAGTTTTTCGAAAAGCCCCAGCCAGAAGAGTAGCAATCTCGGAGTT

CGTCAAAGATTTTGCGCTGTTTGGTTTTCAGTAATAACACGACAATTATTTCTTTTCCTT

ATGTTACTTTGTTTCTCCTTAACAGGAGACGAACCATTTGAATGTTTCTCAGTAGTTTGT

TGAATGTGGTCCTTTTAAAGAAGGAAAGTGGCATGAGATTTTCTGATTGATAATAGGATT

TGCATTTTTAGTTTCTGGGTTATCTCATAACTAATATTTTTTTGAATGTAATCTCAAAAC

TAATATTATTTTCGTGTAAAAGATTTCAAACTGCAGTTCATTTTG

>Unigene0001654

GAAGCAAAGGTTAGAAGGATGGGTGCCCCTACAGCAGGCTACCGGGTGGTAGCCCCAGCA

GAATTTGACCTGCCCAGTCTGTCACGGAGTTTTCACAGGGGCAACTCCTCGAAAAAAATG

CCTGACGGATCGGACAGAGTTTCCTAATCCGAGATGTTTTCCCGAATCCAAGTGTTTCAG

ATACCGTCAACTTCCAGAGGCATGCATTCGTGGTAATCTAACCGATTAGGGTGGTGGGGG

AGGTTCTATCCTATTAATAAAGGATTTGTGGACTCTCGTTCGTACTTAATAATATGTGGT

CTTCTCCTTTGAATAGTGGCTTTGAGATCGTTGGTACTTAATAATATTTGGTCTTCTCCA

TACTCTCTTTGTATGCCTAGTTCGTACTTGATAATATTTGGTCTTCTCCATACTCTCTTT

GTATGCCTAGTTCGTACTTGATAATATTTGGTCTTCTCCATATTGTCTTTGTATGCCTAA

TGAATGCATTAACTGATAATATATGTTATTTACCCTGTTGGATTGTCAATTTATTATAAC

TCCTTATTTTGACTAAACGAATCCTTTTAGTATATTCGGAATCTTATTTTTGTTTTACAC

TGTGTGAAAGAAATGAACATGTTAGTTTGTGAGATGCATTGGAATTAATTCTGTCTTCAC

GAAATAAGTCCTAATCACG

>Unigene0001655

ATACAAGCGTGGAAGCCCATTTGAGCTATTTGAGCGCGAAGGTGATATGACCCTGGCGAG

GAGAACAGAGACTTTGCTGATAAGGTTGACACCCGACCATGAAGATACTCAACCCTGCCA

GTGGGAGGCCTAGCGAGGTCGATGGTAGAAGTCCGTCGATCCCCAAATAGTTGCTTGGTG

TAGTAGGTAGTAGCTGAACACTGAAGCCACTAAATGCAGTAGTAGAGTTTTGAAGCAAAG

AGTGTTGCCTGAGAACGAGATGGAAGCAAGGGTGCTCCATCCAAGAGCCGCCATAAAAAG

TAGACTGTGTACAAGGGCAACAAAACCCATAATTATTTCAGAAGCAAAGGT

>Unigene0001656

CATTTGGTTCCAGAACAGGAGGGCCAGGTGGAAGACAAAGCAGCTAGAGAAGGACTATGG

AGTCCTGAAGAAGCAGTTTGATGGCATCAAAGCTGAAAATGATTCTCTACAAGCCCATAA

CAAGAAGCTTCAAGCTGAGATCTTGGCACTTAAAGGGAGAGAAGGATCAGAGCTCATCAA

CCTCAACAAGGAAACCGAAGCTTCATGCAGCAACAGAAGTGAGAACAGCTCTGAGATCAA

CCTGGATGTCTCAAGAGCTTCAGCCAATGAGAGCCCGCTGCATCCTCATCAAAGCAGACC

ATTCTTTCCATCAATGAAGACACCAAACATGGTCCAACTTCTCCAGCCCTCCAGATCAGA

GCTCCAGTGTCACAAGATTGAGCACGGCGTACCGGATGAGAGTTTCAGCAACCTGTTATG

CAACATGGAGGATCAGTCTGCCTTCTGGCCGTGGTCGGATCAACACAATTTCCATTAATC

TATTTCATTCCGTCGTTATCGTGGTTATCGCACTACCCAAATTGCTCTCTATATTTGGAC

TAGGTAATTTTTGTAATATCTTGCATTGAAGGTAAGTGAGGTAAAGCATGAGTTTGGGAC

TTATTTCGCGGCTATTGTTCAGGAGTACAATCTTCAAATTTGAGTTTGCTTGGGAAGGGA

GCTCTACCAGATAAAATAAATGCTCCTGATGTAT

>Unigene0001657

CATTTGGTTCCAGAACAGGAGGGCCAGGTGGAAGACAAAGCAGCTAGAGAAGGACTATGG

AGTCCTGAAGAAGCAGTTTGATGGCATCAAAGCTGAAAATGATTCTCTACAAGCCCATAA

CAAGAAGCTTCAAGCTGAGATCTTGGCACTTAAAGGGAGAGAAGGATCAGAGCTCATCAA

CCTCAATAAGGAAACTGAAGCTTCATGCAGCAACAGAAGTGAGAACAGCTCTGAGATCAA

CCTGGATGTCTCAAGAGCTTCAGCCAATGAGAGCCCGCTGCATCCTCATCAAAGCAGACC

ATTCTTTCCATCAATGAAGACACCAAACATGGTCCAACTTCTCCAGCCCTCCAGATCAGA

GCTCCAGTGTCACAAGATTGAGCACGGCGTACCGGATGAGAGTTTCAGCAACCTGTTATG

CAACATGGAGGATCAGTCTGCCTTCTGGCCGTGGTCGGATCAACACAATTTCCATTAATC

TATTTCATTCCGTCGTTATCGTGGTTATCGCACTACCCAAATTGCTCTCTATATTTGGAC

TAGGTAATTTTTGTAATATCTTGCATTGAAGGTAAGTGAGGTAAAGCATGAGTTTGGGAC

TTATTTCGCGGCTATTGTTCAGGAGTACAATCTTCAAATTTGAGTTTGCTTGGGAAGGGA

GCTCTACCAGATAAAATAAATGCTCCTGATGTAT

>Unigene0001658

CATTCCCCTCCATCCCATCCTATCCCTCTCACAAACACCACCTCTCCCTTTCATCATGGC

ACCTCTCAATCATCACCAAAACACCCTGCTGGCTTAAGCCCCTACAAGCTTCTTCACCTA

TGGGCTGCAATGGCATGGCCTCATCACCCTTCTTCTCTCCAAACTTCATGCTTCAGATGC

AGAGCACCTCTCATGAAGAAGAGCAGCCATCTCTCATTCCCCTCTCCCCCCTCCTCCCAT

CCAACCAACAAGACTTCCGAGGTATGGCTACCATGTTGGGGAGGAGATCTATGTCCTTCT

CTGGGATCGAGCCCACCGAAGACATGACTGGTGGAGATGAAGATCTCTCCGATGACTGCT

CGCAGGCCGGAGAGAAAAAAAGGAGACTAAACATGGAGCAGGTGAGGACACTGGAGAAGA

ATTTCGAGCTCGGAAACAAACTGGACCCAGAGAGAAAAATGCAGCTCGCGAGGGCTCTAG

GGTTGCAGCCAAGACAGATAGCCATTTGGTTCCAGAACAGGAGGGCAAGATGGAAGACAA

AGACTCTAGAGAAGGACTATGAGCTCCTCAAGAAGCAGTTTGAGGCCAT

>Unigene0001659

ACTGCTCGCAGGCGGGGGAGAAGAAGAGGAGGCTCAGCATGGAGCAGGTGAGGACACTGG

AGAAGAACTTTGAACTTGGTAACAAGCTGGAGCCAGAGAGGAAACTGCAGCTAGCCAGGG

CCCTTGGATTGCAGCCCAGGCAGATAGCCATTTGGTTCCAGAACAGGAGGGCAAGATGGA

AGACAAAGACTCTAGAGAAGGACTATGAGCTCCTCAAGAAGCAGTTTGAGGCCATCAAGT

CAGAGAATGAAACCCTACAATCCCAGAACAAGAAGCTTCAGGCTGAGATCTTGGGTCTTA

GAGGGAGGGAAGCACCGGAGCCCTTCAATCTCAACAAAGAAACTGAGGGTTCATGCAGCA

ATAGGAGCGAGAACAGCTCTGAGATAAACTTGGACATCTCAAGAACATCAGCAATTGAGA

GCCCCCTGCATCCCCATCAGAGCCTTTCATTCTTTCCGCCCGTTAGGCAACCAGGTCTAG

TCCAACTCCTCCAAAGCTCTTCCAGATCAGATCTTCAATGCCCGAAGATGGAGCACGGTG

TACCGGATGAAAGTTTCAGCAATCTGCTGTGCAGCATGGAGGATCAATCTGCCTTCTGGC

AGTGGCCAGATCAACACAATTTCCATTGAGCTGATGGTTCCTATGGTGATGAATTCTCCT

CCGTTCTTTAGGCGAAAAAGGTTGTTTATGGGGCCTCCATGACAGTCAGGTTTTCGGATC

ATTGCCTGCAGAGAACAATTCTACATGTATTTGAAGCGGCCATGGGCAGGGAACTACTAA

GGGAATAAAGAGTATGTCATGTTTGTAGATTTATGAGCCACACTATTTATTTTGTTTCCC

TTCATTATTAAATATGTGAAATAATATTATGAAATCAAGCCAATGCTAGATGAT

>Unigene0001660

CGGTCGCCGAGAAAATAGGAGGGGCACAAGGTTAGGGGAGACGGCGTGATCATTGGGTCA

AGTCTGTGTTGGCGGGCAGCAAGAAGAGAAGAAGGTTCCATTTTTATAACGGACAGTGGA

GAGAGAGAAGAAAATTCTTTGGACAAGGGAGATAGGAATGAAATTTGAAGTTTCAAATCC

AACCGGCATAATTGCAGCAATTTCAAACTGGAACCAAAAGAAAATCGGGTTTAGGAATGT

CCGGTCAGTCTCCTCATTTTAGGTGTGAGACTTGAGTGTATTGGATGGCCCATGTTGGAT

TTGTCTAATCTAGGTTGGCCAAATAGCAGATTTGGGAGAATTTTGTTTGACGACGGAGGA

AATGACTTGAGTGTATTGGATGGCCCATGTTGGATTTGTCTAATCTAGGTTGGCCAAATA

GCAGATTTGGAAGAATTTTGTTTGACAACGGAGGAAATGACATTAAAGAAATAAGATTTA

TAAGTACCATTAAGCGGTGGGTGCTATTATAGCTGTATACCTAGTCTAAAGATAGCTAAA

TAAAAAGGCGACGTAACATTCTCGCAAGATATATGCAGAAAGGTACAATCACACCTAGGT

T

>Unigene0001661

AAAGGTACAATCACACCTAGGTTCGAATTCTTTGCATTCACGCAAGATATATGCAGAAAG

GTATAATCACACCTAGGTTCTAATTCTTTACATTACCGGTAAAAAGGGGAATGTGGTTTT

CACCATCATCTTTTGGGGAGAAAAGAGTGGCACCTGACATATGTGACTCTTTTCTTCTTA

TGTAGTTTAAAATCCTTCTATTCCTCCTTTGACTCCATCGCATCAACGATACCTACGAGA

AAAGGGTTCTCGTCACGCCAACATCCAAGAGAGGGGGACTCAAAAGAGAACTAAGGGAGG

AGCCTTTTCCCACAAAACGACTCCATCTTCAAGGTCAAATTCAATGATGGGCTTCGATTC

GGCGAGAGATCCTAATCACTTTCAATTGGTATTAGAGCAGGAGCTCACTTGGATCTTTGC

CCTCAACAACAATCAAGCTACAACACTCAAGCCTCAACAGTCTGGATGTCTCGGGAATCC

CCATTATAATGATTCTTGAGGGCACCAAGTAGAGAAGGCGACAAATCGGCCCTTGCATAG

GATATATAATTCAATCATGGCCCTCTCCGAGAAGCAAAAGGGTAACATAACTAATTGTTG

ACGATATTGACATGGAGAATAATATTCTTCAGATGAGAAGATGCCACACTTGATGTCGCT

TTTGAATTATACGCATTGTCTTTGGATTAACTAGTGTCAA

>Unigene0001662

TGTAGCAGCACCTCCACTCTCCTCTCTCCATCACAAAGATGAAGATTTTAGGGTTAGGGT

TTCTCCTCCTGCTCCACCCATTGTCAATCACAGGTCAAGAAGAACCATCATCAATCTCAG

TCTCTCTCAGCCCCACAGACCTCCACACAGTCGCCGCCAGCATCCTCGCGGCAGAAACCT

GGCTCAGATCCCATGTCCTGGCCCACTTCCCCTCCACCAATATCTCCACCATCATAATAG

GCAGCGGCATCCCCTGCAGCAGCTCCCAGTGGAGCTCAGTCCTTTCCTCAACGAAGAACC

TCCACCACTCCCTTCACCGGTGGGGACTGGAGGGAGAGATCAAGCTTTCAGTTCCCCTCT

CCCCTGACTGCCACCCAGATGCCCTCAGACCCATCACCAGGAAGCTGTTCCAGTTTGCCC

CAGTGCCGGACGGCTACTTCTCATTTGCTCCTGATGGGTCCCCAATTGTCATCCCTGCCA

ACCCGCCGGAGGCCACGGTGATCCCGCAGCCGCCAACTGCGTCCCCTGCCAACCCGCTAC

TGCCGCCGCCGTCGCCGTCGCCAACTGTGATCCCCCCGCCGGCGCCTTGCCCGGCGCCGG

GAGCAGGGGATGGGCAGAAGGGGGGACTCTGGTGTGTGGCGAAGCCGTCTGTGCCGGATG

AGAAGCTGCAGGAGGCGATGGACTATGCTTGTGGGGAGGGAGGGGCTGACTGTGGGGAGA

TCAAGCCGGATGGGAAGTGCTATTATCCAGATAATTTGGTGGCTCATGCTTCTTATGCTT

TTAATAACTATTGGCAGAAGACAAAGATGGCCGGAGGTAGCTGCAGCTTCGACGGGACAG

CCCTGCTCATCAATTCTGATCCTAGTTTTCTCCAATGCCAATTCATGCTTAGTTGAGGGG

ATGTTGTTTCAACATGGTATCTAGGGAATACATGGGTACTTTTGCAGGGGTACTCCTGTG

TCGAATTCTGTAGGCAGCTGAAGAGTTGATTAGAGCTTCGAAGTAATATAGTCTTTGTTT

TATTAGATGTTTTTTATGGATATTTTTGTGGCCGCGATTTTCTTTGAAGTTCTAAGTAGG

GAATGATAATGTATTTCAGGTTCCGATCCTGAAGGAGCAAATCAGCATCAAAACTCAAAT

TTGTGATTGAGAAAACTTAGGCTTGTGATTGCATGAGTGATTATGCCTAGAAATAAGATT

GTCGCATCTGCTTCTTGGTGTAAGTTAAATTTGAGAGGATGACATTGTCGTGAGATTATT

>Unigene0001663

GACCAAGACAGAGACACAGACCGATATGTGCATGCATTGGCTTCAAGTGCGCACGCACAA

AGCCGTGTGCGCGTGCACCAATGTCTGGTGCGTGCACATTTTCCTGTGCTGCATCAAACC

GGCCTCCAAATCACTGTGAATCGCTGAAATCGAGCCACATATCAACAGATGTAACTAATA

AGCCAACGATACATTTGCTTAAAAGTGGTATTTTGTTTCAATTCTTTAAATATCATAATG

CCTCTTCTTATTGTAACTTTTTAAGGAAAAGAATCACAAAAAATAAATTCAGGTTGTATC

CATTATTCCGGTAAAAATATGGTGTTTTTAAATTGGGGACAACACGTGGAGTAGCATGGA

AAAGACACAGTATGAAAGGTGATTATATTTTCACACCACTAACTTATCTGAACACACCAC

TTTTGTGCTACAAGTTTGTCCGCTATCATTTATCCATTATGTTCTCACATATCGGTACAT

ATCAATTCTCACACCTCCACAAAAACCCACCCATCTCACACAGTTCACTTCCAACCCAAC

CCCACACAATCTAAGGAGCTCTCTACTTATCTTGTACCCCAAGTCCTCTATTGTTTGGTG

TTAATGTAAAATAAAAAATCCAAATTTTGTGTGATGGGAGGATGTTTATCACTTTCATGA

AAAGAGTAGCAACAACAAAAAAACAGGATAAGTTAATACCTCAAAATTTTGAAAAGAATA

ATGATGGAATTAGGACTTGAAAGTATGTCCGAATTTGAGAAATCCGTCCGAAAATGGATT

ATCCTTTAATGATTTTTCGGCCAAATTGGTTTTGTCCGCATTTGAGTAATCCGGATGTCA

TCCGGATTTAATGAATCCGGCCAAATTGATTTTAGTTAGATTTTTAATTTCGTCAGGAAA

ACTAATTATATTATGAACTTAAAACCAACAGAAGTCGGTACTGAAATCATCTGTATTATC

ACGGTAGTTGATACCAACATGTCAGCATTTCTACAACCGTACACTGTGGTTACTTTTAAT

ATAGTGTTGGCATCAGATTACATTTTTTATCGAGTTACAAGTTGTAGACACCATCAACAA

AAGATATGGGCGTTGTTAGTAGGATTTATAAAATAAACAAAGGTTCGAGACGTTGGTATC

GGTCGATATGGTTTTTGGGTTCCAACCCCAAAAATCATATCATATCGGCCTACACCTGAT

ATATGACCCCAAAAAT

>Unigene0001664

AGCCCAGCCCACCTGGTGGCGGCCACGAGATCCCTACCCTCCCCGCTTCCCGACAAAGGC

ATATATAAAAATCCTAGCCTCTCCCGATCAGCCCTTTCTGGATCGAGATCAGCTCCGTCT

AACAAGGGTAGGTTAATTTAGCACCAATGACTAGTTTACAACTAGCAACTGCTGAAGTTT

GCGACGCAAACCCCCACCTGATCCTAAGTGGCGAGCTCCGTGCCTTTCAACCTGTTTTCC

AAATTTATGGCCGACGACAGGTCTTCTCCGGACCAGTTGTCACTCTCAAGGTGTTCGAGG

ACAATGTCCTCGTGCGCGACTTCCTCGAGGAGAAAGGAGGTGGCCGCGTTTTGGTCGTCG

ACGGAGGTGGAAGCATGCGCTGTGCAATACTAGGCGGCAATCCCGTTCAGCAAGCACAAA

ACAATGGATGGGCTGGCATAGTGGTAAATGGTTGCATAAGGGATGTTGACGAGATCAACG

ATTGTGATATCGGTGTGAGGGCCCTGGCATCGCATCCAATGAAAGCTAGTAAGAAGGGGA

TTGGGGAGAAGCATGTCCCGGTGAATATCGCTGGCACGAGGATTTGTGACGGTGAGTGGC

TTTATGCGGATACTGACGGAATTCTCGTTTCACGGTCTGAGCTGTCCGTGTGAGCCTCTG

AGCCTACGATGGCTTTGTATTCGTATCGATAGTCGATCTATGTCATGCTCCATTGACATA

CATAGCTTTTTGTTGTATTTGTATCGAAGTCAATGACTTTGTAATGGTATTAATAATGTT

CCTTGTGTGCTTGGCAATGATGATTTTATATGTTTGAGTTACTACAATGATTGCAATTTT

ATTGAAGATATAATTGTTCTGA

>Unigene0001665

CCGACGAAGGGATCTAATCCCTTTCCCCTCAATTTCGTCCAATTTCCTTCCATCTCAATC

AAGGATTTCCATTAACGTCTCTCGTTATCTTTGTATCTTCGATTACTGTCGGGCTCGGCA

GGTGGAGGATCGGGGGATGGGAGCTGATCTTTAGCATCAAGGACAGCAACTACCATGATT

CATCAAGAGTCCGAAGAAACAATGGAGAAAGTGCTTGAGGAATCGGTAGGTTGCCTTAAT

GCTAAAGATGTTCACTGCAACTCAAAAGATAGCACGGATGTTATTGAGGAGTTACCTGAG

AAAAAAGATGAAGCAAGTGCCAATCCTAATGGTTCTCTGGAATCGGACAACTCATTTAAC

AGGGAGACTTTTGTGCCTCTAAATCAAGGCTATAATCGACCCTTTTACAACCAAGAATGT

AGAGCATGGCCAAAGTTTCATCCTGATTTTGAAAAGCTTCGAGCGTGCCATACATTTGAA

ACCCATTTTTATCCGATGTACAGGGATCCCTATATTGTCGAAAATAGAAACTATATGCCA

TTGAAGATGTATTCTCAAGGATATCCACATGAATTTCAATTTCAAGAAATTGAGTATTTC

GTGGTAATTGACTTTGAAGCAACATGTGACAAGGATAAGAATCCACATCCGCAGGAGATA

ATTGAATTTCCATCTGTTCTAGTTAACAGTGTTACTGGACAACTAGAAGCTTCCTTTCAG

ACATATGTGCGGCCTTCATATCATCAACATCTGAGTGAGTTCTGCAAGGAGCTCACTGGC

ATACAGCAAATTCAGGTGGACAGAGGCGTGCCTCTAAGTGAAGCTTTACTCATGCATGAC

AGATGGCTTGAAGAGAAAGGGATTAAGTACAAAAACTTCGCTGTTGTGACATGGACTAAT

TGGGATTGCCGCGTGATGTTGGAATCCGAATGCAGATTCAAGAGAATTAGAAAGCCTCCC

TACTTCAATAGGTGGATCAACTTAAAGATGCCCTTCCAAGAGGTGTTCAGTGGTGTACAC

TGCAAACTGAAAGATGCAGTCAAGCTGGCTGGACTGACATGGGAGGGCCGCCCACATTGT

GGCCTTGATGACGCCCGCAACACCGCTCGCCTCCTTGTCGTCCTGATGAACCGAGGCTAC

AGGTTCTCGATAACCAACTCACTTGTGTGGCATCCACTAGATCGTCCGCCAGTATGTCCG

CTGCCCAACCGCCCCATTCATTCGCCACACCACTCCCATAGGCCGAAATCGCCACCGTGG

CCTGCGTTCCAGTTCCATCCTCTGCGGGACCCAACTGACAGGGACAGGTGCCCCTTCTGC

TTCTGTGGAGTCGAAAGCAGTAGGTGTGTTATCCGCAAACCTGGCCCGATGCAGGGCAGG

TGCTTCTTCGGGTGTGGGAACTGGACAGCCGCAAGGCGTGCCATTTGTAACTACTTTATG

TGGTCGTATCCATGACGAGACGAGCATCTGATGCTTGGATCGACTCTGAGGGATGAGAGC

TTGTTGCTTCATATATATGTAACAGAGTCTGTAGGTAGTGAGTGTTCTAATCAGTGAAGC

TGACTACTTTGTTAAAGAGACATTGGTATTTATTAAATAATCTGCGAGAACTGGATTTTT

TTTCTCCTGTATTTATCTATGGATATATCTATTGAAATGCTTGATTGGG

>Unigene0001666

CCAAATCACGCTATGCTCTGCGGTTGCAACAATAATAACTGTCTTGAAACGACCATTTGC

ATTTTAAAGAAGAACATTGAGCAAATGAGTATCTGCTATCTCAGACAAGAGGTGTTGAGT

GTTCCTTGAACAGGATCTTGAAGGGCCACCGGCTTGTCAGGAGAGAAGATAGCATTCCAT

TTGGTCAGTGCTAATGATGCCCACAGGCTAGGAGCCTCAATCGAAGATGTGCGCATGAGT

TGTTGCAGAGTCACCGACTGGGCATTAGATTCGACGAAGGCCGACAGGGCAGAATGGAAA

GCTAAGAGAATGTTTAAATCTCCGAGACGAGAGGGAGATGGATCGATTGACATTGCTCGG

AGTTAAACCCAGAGTTCCAAATATCCGAGAGAGCCTCCTTCATAGCTTGATCAAAATTCC

TAATTGCGTTAGGTATGCATGATGGGGGGACAAGTGTGGAGCGCAAAATTAAACTTAGGG

TGCCGACAACTAGTACGAAGGACAAGCTGCATATGTGGATCATAAAGTGCCAAAAGGTGC

TGAAGAGTGCCCCTGATATCCGAGACACAATGTGAAACAAAGGACTCAACATCCAAAATT

CGAGATGATCGACCACTGCTTATGATGAGTCCCCCAAGAAGACCAATTATTGAGGCGCAA

ACACACTCGATTGAGTTAGAGAAGTCCATCAAGAGGTTGGTGTAGTGCTCGGCGACCAAA

TCTCATATTTGGCCTCATTTAGCCGAAGACCGTACAATGGAGCAATTTTCTAGATGAAAT

GAAAAGGCCCTTAACAGATCCGGTCTCTTAATGATAATAATCCCATCGTCCAACTACCAA

GAATTCAAGTCTAGATTGGTCACAATGGCTTGTAGAGCCAGGAGCAAGGGCTGAAGGGTA

ATGACAAAAGGAGCCGCCCCAAGGGATCACTTGCTGAACACCTGCAGCACTAGAAATTTC

AAAGTCACCGAAATACATAGTAGGTTGACCAATATAAATATACTTCACCCATCGACTAAG

TCAAAGAAAGTGATTGCCGACACTTGTAAAAACCTTATCACGAGATATCGTGTTGAAGGC

ATTTGCGAAATCAAATTGGAGAAGGTCGCGTG

>Unigene0001667

TCGACTAAGTCCAGGAAAGTGATTGCCGACACTTGTAAAAACCTTATCACGAGATATCGT

GTTGAAGGCATTTGCGAAATCAAATTTGAGAAGGTCGCGTGACGCATCCCCACCAAGTTT

CTCTACAGGTTGGTTGACTGCATGAACAATTGCCTCAACACTAAGAGTGATGATGATGCC

AACTTGAATAGGGGAGAGTTACTCTTCAGCTTGCTCCTGGAATCGGAAAATAAACCTTCC

CACTTTTGAGTAGACCTTGACCTCAAAAGGTTGAAATGGTATGTAATGGCCTTTCTGGGG

CCCAGAATGACGGAATAGGAAGAAACAGCCAGAATGGGTTTCAAAACGGAACGATGGGAA

AACAGGATACTGTTCTCATTCCATTCCGGCGAACCATGACTTAAACATTGAATCTAGGAT

GTCTTTTATT

>Unigene0001668

CTTAGAGCAAGATGGGCAGATTGAGCCAAATTGAAGAGCATATCCGAAGCAGGTTGTGTC

TATAAACAAGATGAGAGCCTCTTGATAAATAGTATCATGGTCACCTGCCGTGTTAGAAAA

TGTCTGACATCCACAAGCCGTACAACTCTGGGCTTAGAAAAGAGGGGATGTCCTATACAA

TAAATAAGTTTTTTGAAAAGTAGTTGAGTTAGTCCAAGATGTGGGATCAGGAGAGCAGTC

ATCCACATCGTACTATGCTCTGCAGTTGCAGTAGTAATGATAGTCTTAAAATGAGGACCT

GCCTTTTGGAGAAGAGCTTTGAGCCAATAGGTATTTGCTATCTCAGACAAGAGGTATTGT

TGATTTCTTGAAGCAAGAAGATCTTGCAGAACCGCCGGCTTATCAGGAAAGAAAATAGCA

TTCCACTTTATCAGAGTCAGGGATGTCTACAGGCTAGGAACCCCGGCCTACGATTTATGC

ATAAGTTGTTCTTGTAGAGTCACTGACTGGGCAGTAGATCCGGCGAATATTGACATGA

>Unigene0001669

ACGGTGCAAACCGCTCGGGCAGCACTCTCAAAACCCCGGCACAACCACCGCTGTGCCGAG

CGGCAATGCGGACACGCCGCGACCGTAACCTAACAAAGCCGGCTCCGGCGCCGGATCTCC

CTCCCCAAACCTCAGATCAGATTTTCGATGACCTCCAGTGGAGTGATTTAAGCTTCACGA

AGGAAGACATGGGTGAATGGAGTCAGATTCAGATGAATACCGAAGACAAGGAAGAAGAAG

TTAAAGACGCAGAGGCGAAAGTTCCTTTGAAGGAAGAGGAAGCAGAGAATATCATTACTG

TACCGGTCGAGCTAGCTATGGATGTCAGCTTCCATTTCACGACTGAAAAAGTATTCGATT

CAAGAGAAACTGTGATCCAGTGGTGCCAAGATGTTGGGAGAGAGTTGAAGATGGTTGTTA

CCGTTGTTAGGAACGATAAGCCATACAAAGGGAGAAGAGGGAGGTTGGTACTGGGTTGCG

AGAGGCGGGGGATAGACCGCAGCCGCCGAAAGAGTAGTGCGAACAAGACAAAACCCCGTC

AGAATTTGGGTGCAAGCAAATGCAATTGCCCTTTTGAGTTGAAGGGTGAAGCGCTTGATG

GAGACAAATGGAAGTTGGAGGTTAGTTGTGGTTTACATAACCATGAACTACAAGAAGCAT

TGGCTGAGGATGCTCGTGATGAAGAGAAGAAAGTTCTTGTTGATGCGACAACTATGCCGG

TGGAGGCAGTCGTGTCTCTTGAATGCCAGGATGCTGAGAGTTTCTCCAAGGCAATTGTCA

GGGTGGGGGATGCAGATGGAAAAGATGTGACGTCTTTGCAAGATTCTCAATCATGTCCTC

AAGCTAGTGGTCAGGACCAGCTATATGAACGGCCGCCACCCTATAAGCATATTGAGAGGA

ATGAATTCTCATACAGAAAGCACAAGAAGCAGAAAGAGGAGGACATTGTTATTTGTGAAT

GCCGGTATGATGCCGATGATCCGGATAGCGCATGTGGGGATGAGTGTTTGAATGTTTTAA

CTAGCACCGAGTGCACACCTAGTCGCTGTCCTTGTGGCAACTACTGCAAAAATCAGAAGT

TTCAGAAATGTGAGTATGTGAGGACAAAATTGTTCAAAAGTGAAGGTCGTGGATGGGGTC

TTCTTGCTGAAGAAAATATTAAGGCTGGGCAGTTCGTTATCGAGTATTGTGGTGAAGTCA

TATCCTGCAAGGAAGCGAAACGGAGGTCTCAAACTTATGAAGCTCAAGGATTGAAGGATG

CATACATTATTTACCTCAACGCAAATGAATCTATTGATGCCACCAGAAAAGGGAGCCTTG

CTCGGTTCATTAATCATTCGTGTCAACCTAATTGTGAAACGAGGAAGTGGACGGTGCTGG

GAGAAATAAGAGTTGGTATATTTGCGAAGCATGACATTCCTGTTGGTACAGAATTAGCAT

ATGATTACAACTTCGAATGGTATGGTGGTGCTAAGGTCCGTTGCCTTTGTGGTTCACCTG

GTTGCTCAGAATTTCTTGGTGCTAAATCTCGTGGTTTCCAGGAGGCAACTTATCTCTGGG

AAGATGATGATGACAGATACTCAATTGAGAATGTCCCTCTGTACGATTCTGAAGACGATG

AACCCATATCAAAGTTACTCAAGACAGCTGACTCACCAAATAACACCCAAATGGTTGAAA

TGGAACATAATGAGCTCGTGTTGTCTTGTTCAATGCCAATTATTGTAGACCCACTGAACT

CAGTCCCGATGGAAATTGATGAAGTTAATAATGAGCAGACAGAAGACACTAGTATGCTTA

CACTTGATACTGAGCAAAACTATACACCAAAAAGTGCAACAGCATCTCAGATCCAAAGTA

ATGGTGCTTGCCCGGTCTATCAATTAGAATCAAATCCTCTCTCCAGAATCACATCTGATT

GTATGAATGGAAGATCAAAAAGCCATGCTCGCAAGCAAGTCAACATGAAGCAAGATGCTG

ACCACATAATTTCAAAGGAAGGTCGCGAAGAAATACTCGCTTGTCAGGAAAGGAAAAATC

AGTCCGCCACTGAGCTTGCTTTATTGTACGACGAAATACAGCCAGCAATTGTAGAGCATG

AGCGGGACAGCCAAGACAATGTAGACATTGCTGTCGCAGAGAAATGGATTTTCGCCCACT

GTGACTATCTGAAAAAAGATTTCGACTACTCGTGTACAAATATGAGGAACACCGCATGCC

CCTCCAGAAGAGCTCAAGATGGCATCGCCAGTCAAGGAGATGATATTTCTGACGACGGAA

TTATGCAATGAAGTGATCCCGGCGAATGCCATTTCTCCATTGATCAGCAAATCGGATTAT

TGGCTGTTTACTAAATTTGTCATTTAGTTTTTAACATGACAAGTCCTGGAGATTTCTGAT

TATTGGTTTGGATCAAACTGTTATTTTATGTAGATTACGTAATGCAGAGAGGAGAAATGT

GCTCCACCAATCAAAGATGCGCATATCAGGTCTCGAAGGCAACACAAATATATTGTATCA

ACTATGCCATTTAAAACGGGGAAGGCAAAATTGTCGTGAAAGGAGAGAAAACTCAATATT

AAAGATAATAGCAATTGTACAAAATGACAACAAAGTCACTCAACTTGTATGTGACATATT

TCGATATTTAAGCATAAAAATATATTTGTTGTGCTAATTTTAATGAGTTATCTATTGCAC

TTGTACATTGTGTATCCGAATATGATGTCGGTGAGCA

>Unigene0001670

AATCCTGAATACGAAGAGCTGATTTTGAGTGCTGATATTGAAAGGAAATGTGCAACATAG

GATGCTTAATGCCCAAGAAGCTTGTTGCGTCCCTCCACCTACCATATGCGTACCATAGTA

CAGGTTATGAGGAAATGTTACGTATTACAATGCCATAGCAATAAAGCGTATAGTGATTCT

GAGAATGCACTAGTTCGTCGATCTTAAGCAGCATTGAAGGTTATGGGAGGGGGTGTACCA

GAATCTCCGGCCAGTCGTTGCGAAGGAGTTCAATGGGGTGTGGGAGCGAAGGATCAGAAG

CATGAACGGACAAGGAAGAAGGCCTATACAAGGATAAGTGAAGTGCCAGCTGTGACCACT

CCAGCATGTTTAGATGTGTGACATCTTTGGGTTCTGGTATATGCTAACACTTTGGACGGC

ATTACTATGGAAGAGGAGTAATGACCATAGCAGCACATGAAGATGACAACATTCGGAAGA

CTGCTTTTATGGTTGATTGAGGGTCTGGACAGTCTTTGAGCACGAGGATAATATCTATCA

GCTACCCGGTGCATTATGTATAGCTGCTCAACAACTCGCTAAGTTGAATGCTGTAGTGCC

TGCAAGAAATGACGATCCTGACTGCATAACTAGACTCTTTACGAGCGGTAACACTGTGGA

GTATGCTAAAGTTATGCTCAACAAGGGGCGCGCTCTTCCGATCGGATCACTTGTGATGAC

CTCATTTCAAGTAATCTGGGTGGGGATTCAAAAATGATGAGATGGCTTGTGCATTCTGAC

AAAGGTCATTTTCAGGAGAAGTATGAAAAGGCAAGTCAAGTTGCTAGTGATGCAGTTAGG

AGTATAAGAGCAGTAAGCTCTTTTACTGCTGAAGACAAGGTGATGGAGCTTTACATGAAG

AAATGCAAAGGCCTAATGACTACAAGCATCAAGAATGGTTTAGTTAGTAGAATGGGTTTT

GGGCTCTCCTTCTTTATTCTGTATTCTGTCTATGCA

>Unigene0001671

AGTTGCTAGTGATGCAGTTGGCAGTATAAGAACAGTGGCCTCATTTACTGCTGAAGACAA

GGTGATGGAGCTTTACAAGAAGAAATGCAAAGGCCTTGTGGCTACAAGCATCCAGAATGG

TTTAGTTAGTGGAATAGGTTTTGGGCTCTCCTTCCTTATTCTGTATTCTGTCTATGCAAC

AAGCTTCTATGCGGGAGCTCGTCTCGTACAGGACGGCAAGACTACATTCACGGGTGTTTT

TCGTGTTTTCTTTTCTCTCAGCTTTGCAGCAGTAGGAATTTCTCAGTATAGCAACCTAGG

TCCTGATGCTAAGAAAGCAAAGTCTGCTACAGCTTCAGTATTTTCAATTCTTGATCGCAT

GTCAAGTATCGATTCTAGTGATAAATCGGGGATTAATCTTGAGACGGTGAGTGGAAATAT

TGAATTTCGGCATGTAAGCTTCAAGTATCCAACAAGGCCAGATGTCCTGATTTTCCAAGA

CCTCTGCTTAGCTATTCAATCAGGAAAGACTGTTGCTCTGGTTGGCGAGAGCGGGAGTGG

CAAATCGACTGTAGTGTCACTTCTCCAGAGATTTTACGACCCCGATTCAGGTCATATTTT

GCTAGATGGAGTCGAGATACAAAAGCTGAATTTAAGGTGGCTGAGGCAGCAGATGGGTCT

GGTAAGCCAGGAACCAGTTTTGTTCAATGATACCATCCGGGCGAACATAGCTTACGGAAA

AGGCGGGGATGCTACTGAGGCTGAGATTCTGGCGGCAGCGGAGTTGGCAAATGCTCACGA

GTTCATAAGTGGGTTACAGCAGGGTTATGATACTTCAGTTGGGGAAAGAGGGATCCAACT

GTCTGGTGGTCAGAAGCAACGAGTCGCCATCGCCCGTGCTATCATAAAAGAGCCTAAGAT

CATGTTACTCGATGAAGCTACGAGTGCGCTGGACTCTGAATCCGAGCGAGTTGTTCAGGA

AGCGTTGGACCGAGTGATGTTGAGCAGGACGACGATTGTTATTGCTCACCGATTATCGAC

AGTTAGGAATGCGGATTTGATTGGTGTAATTAGAAATGGAATGATC

>Unigene0001672

GGTCCTATGTTGGCTTGGATGGATGTAGTTAAAAAAGGATATGTCAATATTGAATGTAAC

TGAACATAAAGTTCCTGACAGCCGAATTGAGAAATATGATTTATGTTGCCAATACCAAGT

AATTGGGATTGTTTGGGTTGAACCAAGAATTGAGGACTGTCCCTTACCCATTTACCTGAA

CCGGGTGATATGTTTATCAAATTATATCAATTCATGTATAGTATCAGTTCCAATGAATGC

AGGGTACTAAATTTAGATAATTGTTTTTGCTTTGAAATTAATAGCAAATTGCAACCAC

>Unigene0001673

GCATTCACTTAAGAGTAACAACTCCCTAAAGGTAATGTCCAAAGAATCTATGATTATATA

TTCGTACGGTTTTTGATTTATGTCCTCATTACAGATAATTTAATAGAACTTTATTCCCCT

TTTAATTTTATTGGAGTTGTATCCCTAACATATTTTAGGGCCAAGAGCAGGTTCCACTTT

GGATGATAATTAATTTAACCCAACCCCATAGCTTGGGTTTGTGCACGGATAGGGTCTGTT

CTGGTTGATGTCCTACTATCGAACTAAACTCACCATGCACACCCATAAATGTGTCAGCAT

TCACTTAA

>Unigene0001674

GTTTTTGCTTTGAAATTAATAGCAGATTGCGACCACTTTGGTAAATAGGAATATTTGGAA

TCGAGAACTAAAGTGACTGAAACTTTAGTTTTAGGGCCAAGAGCAGGATCCACTTTGGAT

GAGAATTAATTTAAACCAACCCCATAACTAATGGTTTGTGTTTGTGCACGGATAGGGTCT

GTTCTGGTTGATGTCCTACTATCGAACTAAACTCACCATGCACACCCATAAATGTGGCAG

CATTCACTTA

>Unigene0001675

GTGCCATGGTTCTCATTAAGCTGTGTGGATTCTTATTCTGAAGATTTGGAGCTGAAGATC

GTGTGCAAATTCTCATGCTATTGTGGAAAAACTCCTATCTGTGTTGGTTGATGCTTTTTG

CTAAATATCTGTGAACCAATATCCGTTTTTGGGTTACCTAATGTCGTTCTTGATGGTTCA

AGGGTGATAAAGTTAGGATCATTTACTGATTATTGTAAATTTGATAGAAGATTGATTGTT

CTCTGCATTCTAACTGTTGATCCTTCAATACCTGTTCGAGAAGTATTTCTCTCTCTGTCT

CTCTCTTTTTGTTATTT

>Unigene0001676

CTCGTCACATTATCGTCCGGCCGTGTGCATAACACATCAAACGTCGTATACGCGCGATTC

GTGATGGATCATCGTCGTTCAACTACAACGTGGTGGTGCGCCTTGGCCGTGATGGCGTGC

ACCGTTGCAGCGTGTACTGGCGCCAACATACCGCCGTCGTCTACCGTCACGGACTTGCTA

TGGACCGGACTGAATGGCGTCATCCAGCCGAGGAGCGGACTCAAAGCCGACTCGAAACAA

GTGCCGGAGAAACGGTGCGAGTGCCAAAGTCCGGAGCTCAAGTCGTGCGTGTGCTGCTTG

AGAATGAGTCTACCTTTCTTGGACTTGACCACGTCTCCAGGGTGTGTCAAGTTCAAATAC

GTGCCAGACATCGAGTCGATAGCTGTGAACGTGACTTACGGTAAAGGCAACGTCCAGAAC

GGCGTCATTAAGGGAAATAATCCAGAACCTGTGTGTATGGACGTGCTGGTCGGATTCGGT

CAACTGTGCGCTCGATTCGTGGGTAACTCATCTACGCCAAAAGACTTCGACGGTTGTTTA

AAGTTAGAGTCCACCTTATTAAACGAAGTGCAAAACTCAATTCCCATGGGATGCTTTAAA

ATGGACCAAAATCGATTAATTTTCAACTCTACAGTAATCGATATACCGGAAGAGCAGTCC

AACAATAGCACAGAATCGGGTGAAGAAGAGAGTGAAGAAGAAGGCGAAGAAGGTGTAGAC

CCCAGCGAAGAGGCGTTAATACAGGCATTTGGAGAGACGGCCGTGCAGGGTGTCACTTGG

CTGGTCCAAGCCATGGGACTGAACCTTAACCAATCCAACAATACCGGAGCCACCACTGCC

TCAAACGGTGTAACTGGCCAACAACCACCTACTCCTCCGAATGTTTAAATAGAAATATGA

CACCTGCAAACTAACATATTATTGTTTTTATAATTTTGATTTTTTAAGGCCACACCTATG

TGACCTCATCAACCCAACGTCTATAAACCTATATTTATTTGTATTATTATTATTATTATT

ATAGCATCTGCTGCTGCTATAATTCAAACTGACAACACAAATATATGCCAT

>Unigene0001677

GATGAGCCCGAGCACTGCCGATCTGGAGGCGCAGCGGATCCTGGAGGAGTCCTCGGTGGG

GATCGGCAGCCGGGCGAAGGGGCCGGCAGGTCTAGGGTTCCAGATGCCGCTCCACTATCC

GAGGTACAGCAAATCCGATTACGAGACCATGCCGGAGTGGAAGCTCGACCGTCTGCTTGC

TCAGTACGGCCTGCCCGTCTCCGGTGATGTTGATGTGAAGCGGAGCTTCGCCATGGGGGC

GTTTCTGTGGCCTGCCGATTGCCCCAAAACAGAGCAGAGCATGTCCAATTTTCATGCTGC

AGCATCTTACATCAATTTGACGTCAGTACTTGTGATTTTGGTCATGCTGCAGCATCTTAC

ATCAATTTGACGTCAGTACTTGTGATTTTGTTTTTCTTCAAGTAGTTGTAGGTGAAGAGC

AGAGGATGTA

>Unigene0001678

CCTTAGGATGCAGATTGCTTGGGAGAGATCCCAGGGCTTGCTTGAAGTTGTCACAGGAGG

CAAGAGGTTCAGAATACACCAAAGGACATTGAGAAGATCTTCAGTGTGTCGTACGTGTTA

GTCGTTTGGAGCTTGATATATGTCATAGTCTGCGTAATGCCAAACATTGCCCATTCAGTA

GGTGTCGTGAGTCGATTCATGAAGAATACAAAGTGTCATGACTGGATCTTGAGTCAAATA

GTCAAAGGCACTTCAGGGTATTCTCTGCAGTTTGGTGGTGCGGACATCCGGTTGTAAGAA

TTTATGGTTGTAGACATGGTTGGTGTTATAAATGAGACAAAAAGTACGATAATGTATATG

TTCACCCTTGGTTGCGGAGTTGTGATTTGAGTTTGCAAGCTGCAGAAGTAGTGTGTTGCT

TTCAGTTACAGAAGCGATGTTGCAGCCACACATTTGAGTAAACAGATGATATTGTTGAAG

GTCCTCTTTGGCAAGTTAGGGTTCGAACGGCATCGGCTGTTTAGTAATAGCCAAAACGGT

GTCCACCTGGCGAAGAGATAAAAGCACATTGGTATTTGCTACTGTTTTATATGATCGCTT

ATGGAGAGAATTTTTTGAGAAAATCCACACAAG

>Unigene0001679

TTTTTGAGAAAATCCACACAAGTATTGGCTCGACAGGTATAATGACCAAGACAGTGTCCC

AGAGAAGTTGAAGTATGTTGCTTTTGTGGGCTTTCGGTGATTGTCAATGAAGGTCGCGGC

AGGAGATGGAGACTCATTGGTTGGATCTCCAAGTGCTAGATTTGTAGCCCAACTTCAAGC

CAGATTTGTAGCCCAACTTCAAGAAAAAGAATCATTCATGAGCGATTATCTTCCTAAGAG

CCTTGGCGGGCGAAACTGATAAAGATTTAGCGCATGTTTTCACTCCAATCTAGAGAGAGA

GCCAAGCAAGACCAAAACTGATAAAGATTTAGCGCATGTTTTCACTCCAATCTAGAGAGA

GAGCCAAGCAAGACCAATCTAGAGAGGAAGACTCACTGGGACGTGCTCGCGCCAACCGTG

TGATCTAGTTACTCTCGTTTTACAGC

>Unigene0001680

GAAACCTTAGATTCATTACATGCGCTTTATGAGAATTTGAAGCTTGATAATCTCCGGAAG

AGGGATATTGAGCTTCTTGTCGTTTTGTTATATAATGTTGCAGCTTCTTTGGGCGAGACA

AGTTATGTGGACTATTATATTCGTGACTTCCCCTGCCTATTTCTTGAAGTTCATTCATTT

CATCCTAATGTTGCTCCAAAATCTCCGCCATGCCTGTTTCGATGGCTTGAAAATTGTCTG

CGGAATGGCTGCCATTCAGTTAATGCTAATGACCTTCCATCTCTAGTATGTAATGACAGC

AGTCACGTACTCAGCTGGGCCAGGAAAATAGTGTCCTTTTACAGCCTACTACTAGGCGGG

GAAAGAATAGGTAGGAAGCTCTCTTCTGGAGTTCTCTGTGATATTGCCAAGGGATCAGCT

AGTACACCTGAGGAAGTAACTGTCCTGGCCATGGTTGCTGAAAAATTTGGTCGTCAACAA

TTGGACTTACTTCCTGTTGGTGTATCACTTCCTTTACGCCACGCATTGGATAGATGCCGA

GAATGTCCTCCAAATAACTGGCCTGCAGCTGCTTATGTTCTAGTTGGCCGTGAAGATTTA

GCAATGGCATGCTTCGGATCTTTGAGGAAAGGAAATGGTTCTCGGAACAGTGTGAATTTG

ACTTCTATATCTGTTCCGTATATGCTTCATTTGCATCCAGTGACTACTGCTACACCAATT

TCTGACACCACGGGTCTGAATAGTGTGAAATCAGAGGATACAGATTCCGTCTTTAGGGCG

GAAGATGGCATGGAACACATGTTCAACTCAGGGACACAATTGCGTTACGGCCGCGATTTG

CGGTTGAATGAGGCCAGACGGCTCTTATCCTCAGCAAAACCTGTGGCTATCCAGTCAACT

GTTAATCCTAGTGGTTCTGAGCAGGACCTTCAACAGCATCAATTGTGGAATCTCGCACAG

CGGACTACTGCTCTCCCTGTTGGCCGTGGAGCGTTTACTCTAGCTACCACTTTCACTCTT

TTGACAGAGGCACTTGCTGTTCCAAAACTTGTTTTGGCAGGCCGATTGCCTGCACAACAA

AATGCAATGGTGAATCTAGATCCAAATATTAGAAGTATCTCTGAACTTAGATCTTGGCCG

GAGTTTCACAATGGTGTTGCTGCTGGATTGAGGCTGGCACCTTCTCAGGGGAAAATGTCA

AGAACTTGGATACACTATAACAAACCTGAGGAGCCAAATTATTCTCATGCTGGTCTTCTT

CTTGCCCTAGGCTTGCATGGTCATTTACGTGTTCTAACAATTACTGATGTGTACCGTTAT

CTTCAAGAGCAACATGAGATTACTACTGTTGGAATATTGCTTGGGGTTGCGGCATCATAT

AGAGGAACGATGCACCCAGCAATATCAAAGATGCTTTACCTTCATGTTGCTTCTCGACAT

CCATCAAATTTTGCAGAAGTAGAATTACCAACACTACTCCAGTCTGCTGCTCTAATGGGC

CTCGGTCTTCTTTATGAAGGATCAGCACACCCATTAACTATGAAGACTCTTCTGGGTGAA

ATTGGCCGCAGAAGTGGTGGCGATAATGTTCTTGAAAGAGAAGGTTATGCGGTTGCTGCA

GGATCTGCTTTAGGACTTGTTGCCTTAGGGCGAGGCAAAGATGCTTTTGGTTTTGTTGAA

TCTTTTGTTGATCGGCTTTTCCAGTATGTTGGCGGCAAAGAGAATTCCAATGAAAGATGT

TCAAACATGGGGCAAATAAATAATGATCATAACCACAGTTTTGGGCAGATGATGGATGGG

GCACAGGTTAATGTTGATGTAACTGCTCCCGGGGCTACAATAGCATTAGCTCTAATATTT

TTGAAGACGGAATCAGAAGAAATGGCATCTAGACTTTACATTCCAGTCACGTACTATGAT

TTACAATATGTGAGGCCAGATTTTATAATGCTCCGTATTATTGCTCGGAATCTCATAATG

TGGAGCAGAATTTGCCCATCTCGTGATTGGATTGAATCACAGATTCCTGAAATTATCAGA

GTTGGGACCTTGAGCATAGGAGACGGAGAAAATGACGCGGATGAATTTGATGCCGAAGCG

CTTGTTCAGGCATATGTTAATATTGTTGCTGGCGCCTGCATTTCACTTGGGTTGAAGTAT

GCCGGTACAAGAAATGGTGATGCCCAAGTGTTACTTTATGACTATGCTGTTTATTTTCTT

AACGAGATAAAGCATGTGACTATTTCAAGTGTGGCTTCCATGCCAAAAGGATTATCTTGT

TATGTAGATCGTGGTACCTTGGAAATTTCACTGCACCTTATTGTTCTCTCTCTCTGCGTG

GTTATGGCAGGCTCTGGACATCTTCAAACTTTTAGATTGTTGAGATATCTGAGATCCCGA

AGTTCTTTAGAAGGCCATATGAATTATGGAATCCAGATGATTGTGAGCTTGGCTATTGGA

TTCTTGTTTCTAGGGGCAGGGATGCAGACCTTTTCGACTGGAAACAGTGCCATTGCTGCA

TTGTTGATTACTCTTTATCCGCGTTTGCCTACTGGACCAAGTGACAACCGTTGCCAACTT

CAGGCATTCAGGCATTTATATGTTATTGCTTCTGAATCTCGTCGGGTACAAACAGTAGAC

GTTGATACAGGTCTACCAGTCTATGCTCCCCTTGAAGTAACCATTGCGGAAACAGAAAAT

TATGCTGAGACTAGCTTCTGTGAAGTTACTCCGTGTATTCTGCCAGAACGCTCTGTGCTG

AAGAGTGTTAGAGTTTGTGGACCAAGATATTGGCCTCAGACCATACAGCCTCCAACTGAA

GATAAATCCTGGTGGAAATCTGGAGACCGAGGGGATCTTTTCAATGGTGGTGTCCTATAT

ATAAAGCGAAAAGTTGGATCATGTTCTTATGTGGACGATCCAGTTGGCTCTCAGTCTTTA

CTTTCCCGGGCAATGCATAAGGTTTGCAACCACTCTGAGCCTGAATCGTCCAAGGTCGAT

CAGTTGATCAGTACTTATTCAGCTGATCCAAGTTTAATAGCTTTTGCACAACTTTGCTGT

GGCTCCTCCTGGAACAACAGGTCTGATACCAACTTCCAGGAATTTTGCTCCCAAGTGCTA

TTTGAGTGTGTGAGCAAAGACAAACCAGCCCTCCTACAGGTGTATCTTTCACTATACACA

ATGATCGAGTCGATGTCAGAGCAAGTGAAAAGCGGCCACCTTGCTTTTCACGATTCACTT

TTCCTTTCTAGTTTGAAGCTAGCATTAGCCTACAACGAGTCTCTGATCAGTGGCAAGCTT

GCATGTAACGGAGGTATCATTAAATCTAGTTTTATTGGATCTCTTGGGAAACGTGTTGAA

GAGATCCTGAGATGTTTGCAGACGCTGAGTGACAATTGTCTCGACTATTTCAATAAGGGG

AAGTGGCCCAGTGACCTCCATCACAAGAATGGTAAGATGGAAGCGATACATCTTTCTTGG

TATCTTCAGTGGTATGGTATACCACCTCCCCATGTAGTGATGTCGGCAATGGAGAGGATT

AAGTCCAAGTTCCCTGTCTCCTCGCCCACTGTTCCTCTGTTACGATTGTTGCTTCCGACA

ACTCATGCAAGAGCAATAGCTGAGATTGACAAATTGCGGCTTGAGTTGGTAAGGTGACAA

TGAACAGAACTTTCTATTTTGGAGAAGGCCATCCATGATATAAGCTTGATCATGAAACAA

GTAGTCTGGTATACAAGGTTTAGGAAGAACTGAGATTATGAAAGTAGCTAATTTTAATAG

TGATTATCTTTTTATTTATTTTTTTCTCTAAAATTATATCGGTCACAGAACACTTTGATT

GTCCTTTGAATGATAATTGTATAAGAGTATATGTAAAGTTTAGCTAATTGTAATAATGAT

TATCTTTTTATTTAACACTTGGATTGTGCTTTGAATGATAATCTTATAAGAGTATATGGA

AAGTTTGTAACTTATGTACTGTTATTT

>Unigene0001681

TTTAATAAAACTCCAAAAAAATCATGTTGAATAGAAAGTCCATAGCAGATCAAGGAGCAT

GTTACTGCTTCGTCACCTTTTGGAACATTGTTTTATAGATGTGTTAGTTGATCAATGCCA

TTGTCGTTGTGCGGTTCAATGAGGCTCTACCGCCAATTCTGACGGCGCTCAAGGTGCTGG

ATAACTCGATCTGACTAGTACTTGAGGTCGCTCAGCATTTGAGGGAAAATATGGTCCGGA

CCATCGCCATGGATATGACGGAGGGACTTGTTCGCAGACAGAGGGTGCTT

>Unigene0001682

GAAACCTTAGATTCATTACATGCGCTTTATGAGAATTTGAAGCTTGATAATCTCCGGAAG

AGATGTGTTAGTTGATCAATGCCATTGTCGTTGTGCGGTTCAATGAGGCTCTACCGCCAA

TTCTGACGGCGCTCAAGGTGCTGGATAACTCGATCTGACTAGTACTTGAGGTCGCTCAGC

ATTTGAGGGAAAATATGGTCCGGACCATCGCCATGGATATGACGGAGGGACTTGTTCGCA

GACAGAGGGTGCTT

>Unigene0001683

TTGTCCTTTGAATGATAATTGTAACTGAGATTATGAAAGTAGCTAATTTTAATAGTGATT

ATCTTTTTATTTATTTTTTTCTCTAAAATTATATCGGTCACAGAACACTTTGATTGTCCT

TTGAATGATAATTGTATAAGAGTATATGTAAAGTTTAGCTAATTGTAATAATGATTATCT

TTTTATTTAACACTTGGATTGTGCTTTGAATGATAATCTTATAAGAGTATATGGAAAGTT

TGTAACTTATGTACTGTTATTT

>Unigene0001684

ATGGAAGGGACATCTGTACGCTCCAGATGAGCAATGGCCACCGTAAGCCGACCATTAGAC

ATGAACAGTGAGACAATAGAGACATCTATGCAGAGGTCACGACCACATGAAGGGAGAAGG

ATATCAGTCGGCCTATTCGTAAAGTCATAAAAAAGATGTCTCTTCCTTGGAGCAAGACGG

GAAGATCGAGCCAAATTGTAGAGCACATCCCGAAGCAGGTTGTGTCTGCGAACAAGACGG

GAGCCTTCTTGGCAAAT

>Unigene0001685

TCCGGCACCGAAGCCACCTGTCTCTCCATGATGAAAGGCATGAAAAACTGATTATTGTCT

TCACATCTCTTCTCATACTTGTTAATCTTTCATTGTTCTGCTATCTGAGCCGCCGTTCCT

CGACCCTGATTAAGGTCAGGAGACATGGACGGTGAGACGATAGAGACATCTATGCAGAGG

TTACGACCATCTGCCCATGAAGGGAGAAAGATGTTGGCCGACCTATCCATAGAGCCATAA

AAAAAATGTCTCTTCCTTCCTTGGAGCAAGGCGGGCAAATCGAGCCGAAGTGAAGAGCGC

ATCCCGAAGCAGGTTGTGTCTGCAAACAAGACGAAAGCTTCCTTCACAGACAACATTGTG

GTCACCCGTCGTATCCTAAAAGGCCTGACGGCCACACGTCATACAGCTCCGGGGCTCAGA

AAAGAGGGGATGTCTCGTACGATACAACAAAGCTTTCGGAAAGCCGTTGAAGACATAGTT

AGTCCAAGGTGTGGGATCGGATGAGCAG

>Unigene0001686

TCCGGCACCGAAGCCACCTGTCTCTCCATGATGAAAGGCATGAAAAACTGATTATTGTCT

TCACATCTCTTCTCATACTTGTTAATCTTTCATTGTTCTGCTATCTGAGCCGCCGTTCCT

CGACCCTGATTAAGGTCAGGAGACATGGACGGTGAGACGATAGAGACATCTATGCAGAGG

TTACGACCATCTGCCCATGAAGGGAGAAAGATGTTGGCCGACCTATCCATAGAGCCATAA

AAAAATGTCTCTTCCTTCCTTGGAGCAAGG

>Unigene0001687

TGAGCTTTGAGCCTACACTGATGTCGATTGGGTTGGCGAGTACCACGAGGAAGTCCACCA

TTGGCTTCTGCATATTTCTCTTAGAGACTCGTTGATCGTTGATCTTTTGGAAGAGCAAGT

AGCAAAACAATTTCACTATCTAGCACTAAGGCTGAGTATTTGAGTATCACTCTCTCCTAC

TATCAAGGAGATCATTATCCATATTGCAAGCAATCCAATATTCCATGAGCGGATCAAACT

TATCGAGATTGATGCTCACTATGTTTGCTAGTCGCTACAACTCAAGATTGTGCAACTATC

CTACGCCTCCACTATCGCTCAGGTTGCTGACTTCTTCACCAAGTCTCACCAGA

>Unigene0001688

ATATGGTTAAAATATCAGATCCAGATGTAGCTTCAAAATACAACATCATCAGTTTACCAT

CTTTAGTATATTTTAGGAAGAGGGTACCTGAAATATTTGACGGAGATTTAAGTGACGAAG

ACAAAGTATTATCGTGGTTAACTTCACAGGATGTGTTTGAAATAAAAAATGAAATTGATG

AGGTAAACAAGAAAATGCTAACGAAATTACTTAATGAAAATGAATTCGTGACGGTGTTTT

TCTACGAAAATGATGTACCCGAAAGTGCTGAAGTGCTGTCTAGGCTAGAGACCATAGACG

ACGAAACGAACAACATGGACATCACTTTTGTAAAAATGGCTGATGCTCGTTATGCACGCA

AATGGGGCGTTACTAAGCTTCCAGCCATAGTATACTTCCGAAATAAGTTTCCAAGTGTAT

ACAGAGGAAGTTTGGACACCGAACTCGAGGTGCTCGAGTGGTTGAAGAAAACTCGTTTCC

GTGAACCAGAGCTTAATCTCTTCATGTACGTGATGATCACAATATCTTTGGCGTTCGTAC

TTTACACGGTACTGCTCG

>Unigene0001689

GCGGTATATTTTTATAAACAAGCATGCCATGTTTGCGATCAAATTTTAGAAGATTTAGAA

CGAGTAGATGATGAGTGCGATTTATATGGCATACATATGGTAAAAATTCAAGATCCACAA

TTAGCTAAAAGATATTCAATTAAGACATTCCCAGCACTTGTATACTTTAGAAATGGAAAT

CCTCTACTATATGATGGTGATTTACAAAATGAGGAATCAGTTCTCGAATGGTTAATAGAT

GACGACAATCGTGAACTCGCAGATGAAATTGAAGAGGTAAACAGTC

>Unigene0001690

TGCATCTCCAACTACTGACGAAGAGAAGGAATATATGTCACAAATTCCTTATGCAAGTGT

TGTTGGTTCTTTGATGTATGCTATGGTGTGCACTCGACCTGATATTGCTTATGCAGTGAG

CATGGTGAGTAGATACATGGCAAATCCAGGAAAGGAGCACTGGCAGGCAGTCAAATGGAT

CTTAAGATATCTTTATGGCACGTTAAACAGAGGAATCATCTTTGAGAAAGGAGCTACAGC

TATGGTATATGGTTTCGTTGACTCAGATTATGCTGGAGATGTCGATAGACGCCGATCTAC

TACAGGTTAT

>Unigene0001691

GAAAAAAATAATTTTAAGTTTATAAACAATACTTTGTGGCCGTGTGTAAATATACGATCA

ATATACGTAATTTAACAACGATCCTTAAAAATGTTTTGATAAAAGAAGTCTTAGGTTATT

TGGGTATTGCAAATAAAATTGTTCGTGTATATCATCGTGATATACTGTAATTTAACCTAT

AGGTACCTATTACCGCGTGTTCTTAAGTACCTAGATAATATGATATGATTTAGCAGTAAG

AAACCTATATAGCCTATAATATCGTTATATTTCCAACTAATTCGAGACAAAAATGTTCGA

CGTCGATCATAAATCAAAATTCATAATATATAC

>Unigene0001692

GAAAAAAATAATTTTAAGTTTATAAACAATACTTTGTGGCCGTGTGTAAATATACGATCA

ATATACGTAATTTAACAACGATCCTTAAAAATGTTTTGATAAAAGAAGTCTTAGGTTATT

TGGGTATTGCAAATAAAATTGTTCGTGTATATCATCGTGATATACTGTAATTTAACCTAT

AGGTACCTATTACCGCGTGTTCTTAAGTACCTAGATAATATGATGATTTAGCAGTAAGAA

ACCTATATAGCCTATAATATCGTTATATTTCCAACTAATTCGAGACAAAAATGTTCGACG

TCGATCATAAATCAAAATTCATAATATATAC

>Unigene0001693

AAAATTCATAATATATACTCAAAACTTGACTCAATATTCTTTAAATAGTAATGTATGCTT

GATTTCAAAAATCAAAACATTGTGAGTGTGACAAATATTTAGATATTATATGCATACCAC

AATTCAGTACCTAACGCTTTTCGGATCGATTGTCGGAAGTGGGACCACCCACCTGTATTG

GGCAAATACCTTCCGCGCGCATTGGTAAAAAATAAAATGAATCGTTTACTTTTGGGCCAT

CTAATTCACTTATAAATCTGTTAATCTGTTTTAACGCCTCGATTTTCGCCACTGATTTTG

TTACAAGTCTTGACACGAACAAACGCATTTCCAAACTTAATCATGCACTTGGTCACATCG

ACTAGTCGTCATTTTTGATTTTGATGGCAAATAAAATACCCTCTCATATTTTTCACATTT

CTTTTTTTACTATTAAAAACAACCACAACATAATACTACACCGTAATAAAATTAATACTT

AATAGTTTTATACGTATATGTGACACTGGCACACAACTGTTGTAATGAAAAATTTGTATG

TAAAGGATATTTTTTTGTTTTTACAACTAACCCATATTGTGGATAAAGTATAGTCATTTA

TTTATATTTGTATTATTATTATTATTATTATTTTTTTTTTAATTTTAATTATTATTTAGT

GCAATGAAATTGAGTTCCTT

>Unigene0001694

AAAAAAAGGCTATTGGGGTAATGCAAATTTGTGGCTACTCTTTTAGGAGAGTAAATCAAA

CTTTCCCAGAAATTATCTCATTTTGCCTCTCTACTCTTATTTTATTTCTCCTCCCATTTT

CTCTCACCTCAAATATGAGGAGAGAAATAAGGAACTAGATGAGAGATAAAATGTGTGGAT

AGAGAAAATTTGAGAAGAGAGTAAAATGATCGGAAAGAGAAATTACTCTGGATAAAGAAT

TGTTTGG

>Unigene0001695

AATGTTTTGTGCTTGGTGTTACCTTTTGAAGATGTTAGTGATATTTTATTTTCTTCACCC

AAGGGCTATGTGCGTATGATGACTTAACTCGGATTCCCCTTCAGGACATCTAACTGGATG

ATGCAAAAACGAGTTTCTGATGCACACTGATAGGCATATTTTGTTTCTTTTAGTATATTA

AAACTATAATTTAATTTGAATCTCTTAACATAACAGATAATAAGAAAATTTTGTCCTGAG

TTATTTTCTTTTTTGTAATCTCTGTTTCACAGCTTGAA

>Unigene0001696

CTGCATTTATTGACTTTGCCTCATGTCATGTTAAACCGGTGATTTTTTTTTTTTCACGAC

ATGTGCACATAATCTGGCCATTGTGGATGACTTCTATGGCATATTTACTCATTCTTTAAG

TAACAGATTGATTCTTCTCGGTATCTGGTGTTGATCTTGTAACATGAAGCATTTGGAGAA

CATCATGATCTTCGTGTGCACAAGAACTCAATTACAAACAAATTATAGACTTATTGTTGA

ATGTGTATCTTCGTGTGCACAAAAAGAACTTAATTACAAACAAATTATAGACTTATTGTT

GAATGTGTATCTTCGTGTGCACAAAAAGAACTTAATTACAAACAAATTATAGACTT

>Unigene0001697

ATTGCCTCCGTCCAACAACTACAGATACCTGCTCACGGTGGTCGATCGATTCACTCGTTG

GCCGGAAGCTTGGCCACTCGAGAACGTATCAGCTCACGCTGTCGCACAGATGCTAACCAC

GAACTGGATCGCTCGATTCGGTGTTCCCGACACCATCACGACCGACCAAGGGAGACAGTT

CGAATCCGAGTTGTTACGCTCACTCTTTTCGACATTCGGTATACAACACTCGAGGACGTC

GCCTTACCACCCTCAGTCGAACGGCATGGTCGAGCGTTTTCATCGAACGCTCAAGTCTGC

GCTTACCGCGCACGAATCGTCGCAGTGGTCAACGAAACTGCCGATCGTACTACTCGCGCT

GCGCAACACCGTGAAATCCGGTTTCGACCTAACGCCAGCTGAATTGGTGTACGGTGCGAC

GCTTCGACTTCCCGGCGAGATGTTCCACGCCGCACCCGCAGAGCCGCAGCCGCCCGAGTT

GGTAGCTACGCTCCGAGATTACATGGCCCAACTTCGTTTCACGCCCGGTACGAACCACAA

CACGCGACGTGCTGCTTTCGTACCGAGTGACCTGAACCACGTCAGTCACGTCTTCGTGCG

AGTCGACGCCGTCAAGCCGCCTCTCCAGCC

>Unigene0001698

GCCGCCTCTCCAGCCACGATACGAGGGACCTTTTGCCGTCGTAGAACGCCGTGAAAAAAC

ATTCCAGGTTCAGCGACATCGATCAACGACGTGGGTATCAATCGACCGACTCAAGCCAGC

ATTCATCGCCCGTGAAGACCCGACCGCCGAACATTCGTACGCCGCGTTTTCGCTCGACGA

CCCCCCGAAAAAACGTGTCCGTTTTTCTCTTTCCGAGGGGGAGTAATGTGGTGATTACGA

CGTGTTATCACCACTTACGCTAGCGCGTCCGTCGTATGATTATTGTCGTCACTATTTTGT

TTTCCGTTTCTAACGTCTGTGTATTTTTATTGTTTATGTTGTAAACGTTTATTTGTTATA

TTGTATTATTTTTTTGTATCGTCTTTGATCGCGCAACGACGCGCCATATTAATTTTTTTC

TCCCGCCTCGCGCGCTAACGTTTTCGCCGTTACGGGCTCCAAGCTTTTGCTCGCTCGCCT

CGAGTTGTCACTTTTTCGTCGCAATTCGACACGACGCGTATAACCCGAAGTCCGCTCGCC

TTTTTATTTGTAACTCGTCACGAGATTTACGTTTCGCTCGTACGACATATTTTTTTGTAT

TATTTGTCACGCCGACTATCGTCCGTTTCACCTTTATATTTACGTTTTTATACCAACTAA

TTATTTTGTTGTGTCGAAATAAAATTTCGCCTACTACGTTGTTTTTTCTAAATAAAAGCA

TTTGTCTTATTTTCTTTCTAACTTATACCTTCTCCTG

>Unigene0001699

GCCGAGTGTTTAACGCGCTACATGGACTAGCGCACGGCGGTAACCGCGCCACGCTGCGCC

TGATCAAGGCCCGATATTGCTGGCCGGACATGCACAAAGACATCACGCGATGGACTCGAT

GCTGCGAACCATGTCAACGCGCAAAGGTAAACAAACACGTCGTTTCTCCGATCACGCCTT

TCGCGCCGCCCGAACGACGTTTTGGTCACGTTCACGTCGATCTCGTCGGACCATTGCCTC

CGTCCAACA

>Unigene0001700

GCCTCTATGATTTACAATCCGACAGGATCCTTCAAGGGTTGAAGTTGTTGAGGGTGCTAG

ATATTCATAATATACGAGATCTATGGAAAAACTTCGGCTCTCCCAAGGAGATCAAATTCA

CGACACACTTGAGATATCTAAAAATAGTAGGAGCTAATCCTCTCCCATTAAAACTTCTGT

CTTCAGTAGGAAAGCTTAGCAATTTGCAGACTATAGACCTTCACGATAGCTTTCAAATTT

CGGAGTTGCCAAAAGAATTCGGGGAGCTTTCTAATTTGAGACACCTATTCCTGCCGAATA

CAATCAATGTGAGTTCTGCACCATTTACTGCTACAAAAAAGTTGCAGACCCTCCTCAGCA

TATGCTGTGATCAACAGTGGA

>Unigene0001701

GTGGTTTTGTGCCGCAGGGGCTAAATACAACGACTGGACCCGCGTCTGTCAAGCGAACCC

AGGGTCGGGACGGCTGAGAGAGCTCTGTCGTCTCTGCGCGGAGTACAGTAGCGCAGACTG

AAGCTAGGGGAAAGCCATGGCCGAAGAATCGAAAGAGCCCGGGGACGGCGCCGCCACTAA

CATCTACAACGTCGAAGCCGCCGAGATCCTCGCGAACGAGGCTCAGCTTCTTTCGATCGC

CGAGGCGTCGCCGATCTACGAGCAGCTCCTCACGACCTTCCCCACCGCTGCAAAATACTG

GAAGCAATATGTGGAGGCATACATGGCTGTAAACAATGATGAGGCTACCAAACAGATATT

CAGTCGATGCTTGCTGAATTGTCTTCAGGTCTCTCTTTGGCGCTGTTATATACGTTTCAT

CAGGAAAGCCAATGATAGAAAGGGAGCAGAGGGTGTAGAAGAAACTAAGAAAGCTTTCGA

GTTTATGCTGAATTATGTAGGGGCTGACATCTCATCTGGTCCTGTTTGGATGGAGTACAT

TGCCTTCCTGAAGTCCATGCCGGTTACATCTGCTCAGGAAGAGTCACAACGCATGACTTC

TGTGCGGAAGGTATACCAAAAGGCAATTGTTACCCCAACCCATCACGTCGAACAGCTTTG

GAGAGACTATGAAAACTTTGAGAACTCTGTTAGCCGTGCACTGGCAAAAGGTCTAGTATC

TGAATATCAGCCGAAGTTCAACAGTGCCAGGGCTGTCTACAGAGAACTGAAGAAATATAT

CGATGAAATTGATTGGAATATGCTTGCCATTCCACCAACTGGCTCCCACAAGGAAGAGCA

GCAGTGTATGGCATGGAAAAAAACTATTGGCCTTTGAAAAAGCAAACCCACAAAGGATTG

ACTCATCATCTTCCAACAGACGTATTACTTTCACCTATGAACAGAGTCTGATGTATCTGT

ATCACTATCCTGATCTATGGTATGATTATGCATCATGGCATGCAAATAGTGGTTCTCTGG

AGTCTGCAGTCAAAATTTTTCAGCGTGCCTTGAAAGCTCTTCCCGATTCCGAGTTGCTAA

AGTATGCTTTTGCCGAGTTGGAGGAATCTCGTGGATCAATTCAGCCTGCCAAGAGAATAT

ATGAGAGTCTTCTGGTTAACAATCCAAATGCAACATCTCTAGCACACATTCAGTTCATTC

GGTTTTTAAGGAGAAATGAAGGTGTTGAAGCAGCTCGCAAGTACTTCTTAGATGCCCGAA

AGTCGCCAAGTTGCACATATCATGTATTTGTTGCTTATGCTATGATGGCATTTTGTCTTG

ATAAAGATCCAAAGGTTGCACACAATGTTTTTGAAGCAGGACTAAAGAGATTCATGCATG

AACCAGGTTACATTCTTGAATATGCAGATTTCTTGTGTCGTCTAAACGATGATAGAAACG

TTCGGGCTTTATTTGAGCGAGCATTAAGTTTGCTTCCCCCAGAAGAATCTGTGGAGGTAT

GGAAAAGATTTACTCAATTTGAGCAGACGTATGGAGATTTGGCAAGCATCTTAAAGGTTG

AACAAAGAAGGAAGGAAGCTCTATCTAGGACAGGAGAAGAAAGTACACCCGCATCGGATA

GTACTTTGTATGATGTCGTCTCACGTTATAGTTTCATGGATCTTTGGCCGTGTTCATCTA

AAGACCTAAATCATTTGGCTAGACAAGAGTGGCTCGCGAAGAACATAAACAAGAAAGTTG

ACAAACCAACTCTATTGAATGGAGTTGATCTCGTAGATAAGGGTTCCATTGGACAAATGA

ACAGCTCGAAATTATCCGCACTATCTGCAAAAGTCGTTTATCCAGACACTTCTCGGATGG

TTATTTATGATCCTAGGCAAGCACTAGGACCCCAGATTCCATTGGTCCCCAGCTCAAATA

TCCCAGCCGCCTTAGTTGGGGGTGGAAATACGAAAGTGGTTGATGAGATATTAAAAGTAA

TGTCACCCGCATTGGTGGCTTTTATTACGCACCTGCCTGCTGTTGAAGGGCCATCCCCAG

ATGCAGATGTGGTTTTGTCTGTATTGCTTCAGAGCACCATACCAACTGGGCAAACTGTGA

AATCAACTTCTGTACCAGCTGGCCAAGGTCCCAGCACAAGCGATCTTTCGGGCTCAAGCA

GATTAGTACCAAACGGGTCGTCACAGAGACCTAGGGAAAGTCAATCTGGGAAGAGAAAGG

AGCTTGAGAGGCAAGAAGATGAGGAATCAGTGAGCGTACATAGCCGGCCATTGCCACGAG

ATGTTTTCCGAATTCGTCAGATTCATAAAACTCGTGGAGTGAACAGTTCGCAGACGGGTT

CAGCTGCATCTGGAGGGAGTGCATTTTCTGGGGAGCAATCTGTTAGCACCGAATAATAGT

ATTTATGCTCCCAATTCAGGATACATGGTGATTATTTATACTGTTCTCTGTTTATCGTAT

TTAGTAAACCTTTTTCAGTGTTGTTACTTGTAAATGTAATGTATTAGTAGCCTTTCTTTG

GATTGTTTGTATGATGAGTGTTGTTACTTGTTTGGATAATTTGACCAACGCTC

>Unigene0001702

CACTATTGCTCAACTTTGGAACAAACCTTTAATACCTGTTAACCATTGTATTGCACGTAT

CCTTTAAAAGCATTATATATTTTTATTTAATACAAATTATTAGCTTTCAACCTTAATGAT

CTTGACTAGACATAGAAATGGGACGCTTAATAACTGGAAGTGACAACCCTACTGTTTTAT

ATGTTAGTGGAGGAAATACTCAAGTCATAGCTTATTCCGGGAATTGTTATAGAATATTTG

GTGAAACAATTGATATTGCTGTTGGAAATTGTTTAGACCGATTTGCAAGAGTTTTAAAAT

TATCCAATGATCCTAGTCCTGGATACAATATTGAACAAATGGCTAAAAATGGTAAAAAAT

ATCTTAAATTACCTTATGTGGTAAAAGGTATGGATGTTTCATTCTCTGGTATATTATCTT

ATATAGAAGAAAAAGCACAAACATTGTTGTCATCTGGTGAATATACTCCTGAAGATTTGT

GTTTTTCACTTCAAGAAACTCTTTTTGCAATGTTAATAGAAACAACAGAAAGAGCAATGT

CTCACTGCCAGTCAAAAGAAGTACTAATAGTTGGAGGTGTTGGTTGTAATGAAAGATTAC

AAGAAATGATGAAAATTATGTGTGATGAAAGGAATGCAATATTATATGCTACTGATGAAA

GATTTTGTATAGACAATGGAGTAATGATAGCTCATACAGGAACTTTAATGCATAATAGTG

GATATAAGACTACATGGGAAGATACATTTTGCACGCAAAGGTTTAGGACAGATGAAGTTG

AAGTTACATGGAGGTCATGAAAATGTGTTAAAGCTTTTTTTTATATATATACACTACACA

ATATCTTATATTACACCAATAACCAAAGTATTCTTGCTAAAACACAAACCAATAAA

>Unigene0001703

CAAAGTTACAAACACAGACAAAAGTACATTTAAATACACTTTACTTCAAAACAATGGTTA

TTAGCATAGGATTTGAAGGTAGTGCAAATAAACTAGCAATTGGAATTGTTAAAGATGGTG

AAGTGCTGGCGAATTGTAGAAGAACATACATAACTCCACCTGGTGAAGGATTTTTACCCC

GAGAAACAGCGACACATCACCAAAATAATATTGTATCATTATTAGAAGAAACAATCAGAA

CATCTGGCATACAGCCGGAACAGATTGATATTGTATGTTTTACAAAAGGTCCTGGAATTG

GTGCTCCTTTGGTTAGTGTTGCTGCTGTGGCACGCACTATTGCTCAACTTTGGAACA

>Unigene0001704

TCCCCATTGAGATGTTATCTCCGCAGTATATGAGTCTCAAGGATTTGAAAAGGAGAATAC

CTTTCTACACGATAACCTAAAGGATGACCATGACATTATAATTAAATAATATATAATAAA

AATTGACCATATCACCCTTAATTTTCTTCGGCTACCATAGTGTGTATATACCTAGTATAC

CTTTGTTATGAATCCCTCAAGATAGATAATGAGAAGTTCATTGTCACTATCTATAGTCAT

CACTATAAAATCAATATTCTTGTCATTATCGATGCCCTTGGCATATCGCTGGTCATCCCC

TCTGATTGTACACTTTGAGGAGCAACAACTCCATTTTCATTGAGGACATCACCTTCAAAC

ATATATGTACCCATAATAGGTTCATTCGCACTTATAAATTTTAACCTTCTTTTTGGTTGT

TAGCATAAATTTAGTTTTTTAACACTTGGGACCATGACAACCACCAACAAAAGTGGTCTT

TCTTGGCTAAGTCACTCCACATTACCTCTAATGATTAATGAAATTTCCTTTTTCAGTTGA

TCTTCCATTCCATAATCAAGTGGAAGGCCATCGTCGCTATGAGCACTTCCACTTGTGAGT

TTAAGTCTATTGTTCTCCCTTATATGATCACTAATATTCTCCAGTACCTCTGTCATCATA

TGTCTCCAAAAGACTCATTGGAGGAGATGAACAATTTCATTGAGAAGGACCAACAAAAGA

ATTCTCTGTACCAGAACAAAATTATTGCCCATAATCATAAGGATTACCAACATACATCTT

CTCCACTTTCTGTAATTAGAACAACTTTCTTAGCTTTCCATTTGTTTTAAAGTGATTGTG

GAAGGGTTCCTAAAGAAACTTTTGGGTTATGATTATGATAATGCCCAAATAGAGCCTTAT

ATTTACTAATCAAACTCTCTTTTTGAAAAAAATTCCTAATCACCCCTTGGACTCCTCTTC

GGATAAAAAGGCAGGAACTTAGCTAACTTCCAGGTCTACGAGTTATAAGTATTTGGAATA

ACTGCTATGTATGAAATATAAGAGAACATGTTTTGATTTTATAATAGTGGGATTAAGTTA

TATTAGCATCATGAAAAATATCCAACAGATAAATCAAAAGATAAAAGATAAATGAAAGAT

AAAATAAAAGGAAAA

>Unigene0001705

ATCTTGATGATGTCCCTAACTCCACTAGACCAGCAAGATCTTTTGAAGACATTCCCATTC

TCTCCTCTTTTCGGAAAATTCTGTTCCTTTCCTCCCAAATCTTCCACATGATGGCACGAG

GGATTTTATTTCAAATCCATGGTGGAATAACTGTTCCCAAATTGACTCCATATTTTATTG

GAGAAATCATGGTGCAAGAACAGACGATCAGGATCTTCCACTACTTGTTCTCATGGAGTG

TAGACCA

>Unigene0001706

TTCTTGCCTACTGGCCTTACTACGGTTCAAGAATCATGCCTCCAAAATGGTGGGGGTCCA

TGATGTGACTTTATTGTGATTACAAAAATAATGACAATGAGGGTAAAAAATTACTACTAT

GCAACTTACGTTTTGGCATTTTGATATAGTGACACATTCTATATTCTATATAAGATGTCA

TATAATGATTACCCTTTTTATACCTATGGCATTGGCAATTCGGCTCTTCATGGCTGTCAA

CCAG

>Unigene0001707

ATTGTGCCGTGCGGGGCTCTTTGTATTAAATCAAGATGTCTTCAGGAAAGGCTTTCATTG

GAAAACCAGCCCCTGATTTTACTGCCACAGCTGTAATGCCAGATGGGCAATTCAAAGATA

TCAAGCTCTCTGACTATAGAGGAAAATATGTTGTGTTCTTCTTCTACCCTCTGGACTTCA

CTTTTGTTTGCCCAACTGAAATTATTGCGTACAGTGACAGAGCTGATGAATTCAAGAAAA

TTAACTGTGAAATAATTGGAGCTTCTGTTGACTCTCACTTCTGTCACCTTGCCTGGATCA

ACACTCCTAAGAAACAAGGTGGTTTGGGCACTATGAAAATCCCACTGGTTTCTGACACAA

AGCGTGTTATTGCCAAAGACTACGGAGTACTTAAAGAGGATGAAGGTATTGCATACAGGG

GTCTGTTCATAATTGATGAGAAGGGGATCTTGAGGCAGATAACAATCAACGATCTTCCTG

TTGGCCGTTCTGTTGATGAGACCCTCAGGCTTGTGCAGGCCTTCCAGTTTACAGATAAAC

ATGGAGAAGTGTGCCCAGCTGGCTGGAAGCCCGGCAGTGACACAATCAAGCCTGATGTTC

AGAAAAGCAAAGAGTATTTCTCCAAGCAGAAATAAACTTTCATTCTGAGTGGAAAGTAGT

ATGCCTTACATAAAGAGCAAGTACCAGTTACCAAACTTTGACTAAATCATTGCATTTGAG

TCAAGGGATCATGCTATAGGTATAAACAGTAACTTTTTTTTTACTTGAAGGAAAGTTTGT

CCTGTTGACAGTAACTTCATAGAAAACCCTAATGATCAATATCTGTACATCATGGCCTAT

TGCCTTTAGTGACAATGCAGTGTATGTGGTTAGCCATGTGTTGCATATAGAAAATGGTAT

CCTTTTGTAACTATTAAACT

>Unigene0001708

GAAATATGTCTCTTTATTACATCTTCGTAGGTATTTGGTTGTTCTTGTTATACAAGCATA

AACCTTGGAGATATTTCTGAGCATGAGTCCAAGTTAAGGAAGTATTCCTTTGTTTGATAT

AAAATTAAGTAGTTTAGATACATATGCTACAATCACAAGGGTAATAAAACCTATATATGT

CGTGACACCATATTTAATGAAGATGTGCTTTATAGAGATTGGGGCAAGCAGGTTGAATAT

GAGAAACCTATTTGTGTAG

>Unigene0001709

TGCTTGGTGGCCTCGAGTTGCGATCATCTTGCTGGTGGCGGGACCATTTGCCTGTAGACT

TTCCAATTTAGACCAGAGCAAGATGTGACAACTTCTGGCACCGAGGTGGTGATCTATAGG

TGAACTTCTGACTGGGCCTGCAAAAAGTGGTAGCCTCCCGAGAGTTGGGAGGTACCACTC

TCCGTGTTCAACTCAGGATTATGAAGGGGGAAAATTGGGATGTCGGACAAAAAA

>Unigene0001710

GAGCGGAGCCCCCGCCGCCACCGCAAGGAATCCTTGCCGCTGCCGCCGCATGGAGTTGCC

GCCGCTGCTGAGGGCACCCTCTGCGGGAGGTCTGACCGTTATTTCCGGCGGTCTCCGACG

CCGGAGACGAAGATGTTTTGTTCTCAACTTTTTTTTCGTTTGTTTTTCTTTTCCGACTTA

GAGAAGAGATCTCAGATCCAGAGGGAGGGTTAGAGAATTCTTGAGAAACCCTAACTCTAG

ACCCGCCTCTGCTTTGATACCATGAGAGAAATAAAATCCATTGATTTTCTCCACATCATT

ACA

>Unigene0001711

CGTCGCCGCTAAGGGCAGCCTTTGTCGTTGTTGCCAGAGAAGGGTTGGTGGCCGAGGTTT

GACTGCACCGTCGTCGGGAGTCGCCTCCGCCACTGCTGGCGGAGGAGAGGCTGCTTCGGG

AGAGATGCAACCCTCGAGAATCAACACTGCTATAAGGAGTTGCCGCTGAGGCTGTCGGCG

ACTGGCGGCACTGAAGATGAAGAATGTATATTTCCTTATTTTTTTTTCTGGCTTAGAGGA

GGGATCACAAGTTCAGAGGGAGGGATAGAGAATTCTTGAGAAACCCTA

>Unigene0001712

GACCTGCTCCAAACCCTTCAAAGCCGAAAAAACGTTATTCCATCATTGGTGCCTCACTAG

GCGTCCAAGTCCATCTATGACAAAAGTTTGACCAAGTCCAACCATTGATACCTCCCTAGA

CCCTCAACCAATCCTCGGCCAAAATCCGGCTAGTCTCGATGTTGGCACCTTGACACGCAC

CCCCAACCTATTATTGGGTTAAACTTGGTAAAAAATCATTGCCTCAGAAGATGCTAAATC

ATTCGAAACTGAAAAAAATCGTTTAAGTATTCAAAATTATCGTCTACCTTCTATAAGGTG

CATGATGCCGAATC

>Unigene0001713

TGTTGACCCATAGTTGGCTGCCTGGAGCTTTCATTTCGGAAGCATATTAACACATTTCAG

CCAGAAAGGAAGGGGAAAAAGGTCGAAACTGCAAAATTGAGTGTCCGGTGTAAATTTGTG

TAAGGCGCACAACCCGATCTATAAGGATTGTGAAACCCGCAAAAATGTGTTGGGGGCAAT

TTATACGGCCCGACAGTCAAATTTTATTTTTTTCGGGTTATTAAGTTGAACATGGAAATG

TTGTAGGACTGTT

>Unigene0001714

TTCTCGCCTGGACCAGCGTGATTCAACGCGATGTACCCCCCAGTCCGCCGCCCTCTTCGC

GCACGACGGCTTCGATCACGCCTCGACCTACTGTAGGAGACTGGCGGGGTTCTGCCAGCG

TGTCCGAAGCTCGAAGTTCGAGTGCTTCAACGGAAGCCAAGGGAACCATTAGGGATGAGT

TTGACTTAGATGGCTCAGTAGGGCATACTTGGAGGATTGCAAACTATATCACATTAACCG

AATTTGCCCCTCGCTCAATCCGCATAACACGGTTGTGATTATTTTCTGGTA

>Unigene0001715

CCTCAGATTTCACCTGCCACCCCCCGTCTGATCGCCGATGTACCCCCAGTCGCCGTCCTC

GTCGCGCACGACGGCTTCGATCACTCCTCGACCTACTGTAGGAGACTGGCGGGGTTCTGC

CGGTGTGCTCGAAGCTCGAGTGCTTCTACGGAAGCCAAGATGTATTGAAAATCGGAGTGA

ATTGAGGGGACTTCCGGGATCTGGAAAAATGGGTTGTTGAGGTTGTGTGGAGGGATACCT

AAAGCCCCTTGCCTGGTAAGGGTTCTAAAAATGCAAATTCACTAAGAACGTGTGTGATTC

CAGAGCATGGACTTCTGTGGCAGTATGGTATCTTCTCCCTCACGTTTT

>Unigene0001716

CTCTGCTTTTATGGTGTGTGGGTATCCTGCAATACCAGCCTTGAGAAAGACGTTCAAAAG

ATATGGTGAAATTCTTAGAGGTTGTGTGGAGGGATAACTAAAGCCCCTTGCCTGGTACGG

GTTCTAAAAAAGCAAATTCACTAAGACCGTGTGTGATTCCAGAGCATGGACTTCTGTGGC

AGTATGGTATCATCTCGCTCACGTTTTAAGTTAGTTTGGTATCTTCTTGTGGCTTGAGTT

CCTGTCTCTGAATTTAGTCAAGATTAGTTTCATCCGTACTTTTTAACAAAGGTTGTTTTG

ATGTGATGAGGACTAGAAGACCACCACATCTA

>Unigene0001717

AGGGAACCGTTAGGGATGAGTTTGACTTGGATGGCTCAGTAGGGCATACTTTGAGGATTG

CAAACTATATCACATCAACTGAATTTGCCCCTCGCTCAATCCCAATGATACAGTTGTGAT

TGTTTTCTGGTACATCAATGTGGATAACGCTCAATCCCAATGATACGGTTGTGATTATTT

TCTGGTACATCAATGTGGATAAATGGTTGTTTGGGAAGTTTTATGGCGGGCCTCCGCTTT

TATGGTGGGTGGTATGTACCCTTTGCAAATTTTCACTGTTGCTACTATGGTACCATCAGA

TCTTTTCCTCAACTCTTATTGTAGCATCT

>Unigene0001718

CCATTCCAACGAGTAGTTATTTACCTACCAAATATGTGTGCAATGGAACCAAACAAACAC

CATACTTCTTCCTTATGTCCTTAAGATTATGTTTGAACCTATCACAATGCCCACTTCCAC

GTTAAGCAAGAAAATCAGGTCATCGAATGTGCCCTCTATGTATACAGATGGTTTGTTTTG

CTACTTCAGTTTCTCAGGATATTTTTGAATGATTCCAGCAGACTTGTCCACATTAGACAT

GTATTACTTAATTACTCAGCTGAAATAATTTTCTTATTAAACTCTTTCAAATGTATGGTC

ATCTCATGATAACTAAATTTTATTGCACAGTCAATTGGAGGATGTTCAATGCCAAAAATT

GTGTGTATAATAGTTTCAACCTAAATAAAATAGTATTCCTCAACTTTTCCTTGCCAAAAT

TGTGTGATGCACTTACTGGCTTTTCTATGGTTTGTTTGATTCAGATATCAAGTTTGTGAC

GTCGACCAAAGTAGCATGGTTAGGACCGGGATTGGGATTGGTGTTCCATATGCCAACTGG

TTACGGAACTAGACAAACATAGTAGATGTGATGGCGCACACCTGTTACAAAAATAGCAGC

AGAGCCTGGCCGATGTCTCAGAGGGGAGTGCTCCCATTTGAGGCTCTGTTCCCCAAACTC

GTACCCTATAATGGTGGTGATGATGCATCTCTCCATCTACCCGCCGTATTCTCCTCCC

>Unigene0001719

CCTTCCCTCAGATTTCACCTGCCACCCCTCGTCGACTCCGATTAATTCCGATCGGTCCCC

GTTTCTCGCCTGGACCAGCGTGAGTCGACGCCGATGTACCCCCAGTCCGCCGCCCTCGTC

GCGCACGACGTCTTCGATCACGCCTCGACCTACTGTAGGAGACTGGCGGGGTTCTGCCAG

CGTGTCCGAAGCTCGAGTGCTTTTAAGGAAGCCGAGGGAACCATTAGGGATGAGTTTGAC

TTAGATGGCTCAGTAGGGCATACTTGGAGGATTGCAAACTATATCACATTAACCGAATTT

GCCCCTCGCTCAATCCGCATAACACGGTTGTGATTATTTTCTGGTACATCAATGTGGATA

AATGGTTGTTTAGGAAGTTTTATGACGGGCCTCCGCTTTTATGGTGTGTGGTATGTACCC

TTTGCAAATTTTCACTGTTGCTACTATGGTACCATCAGATCTTTTCCTCAACTCTTATTG

TAGCATCT

>Unigene0001720

CTCTAATCCTCTGCCTCTACAATCCTTGCCTTCCTTTGCCTCAACCAGATGGCCACCCTC

TCCCCATACCCCTGCAACATTTACCGCCATGCTGCTGGCAAGATTATTTGACATCGAGGC

CTTGAGCGATAACGGAAAGATCAAGATGATCTTATCGTTGTACTATGATGCTTGCACGAT

GGGAGGGATTGCCAATTACGGGCTCACTCACACAACATCAATACTGCTGGATCCAGTCTG

ATATAGTATGCAGATTGACCTAGCCAAGAACAAGATCTAGTATGGGTTTTATTTTCTGAA

GATATCTTGTCTGGGTTCGGTAAACAACTGAAAGAATAGGGTACCAAAGGTTTTGTACG

>Unigene0001721

CACACAATCTGAAGGAATGGGAGTGGAAGGCAATTGACATGTAGCTTCTGTAAATCTGAA

TCAGAGTCTTTTATGTGTCTTTGTAGAAGAAAAAAGTAGCTATAAATGCCTAATAGGGAC

AATGTAAAACACTATGCCTCCAATATGAAAGATACTATTTCTGACCAATTAGATACTATA

TAAAGAATGCCAGACACGGGTAGTTATTTACCTACTAAATATGTGTGCAATGGAACCAAA

GCAACACCATACTTCTTCCTTATGTCCTTAAGATTATGCTTGAACCTATCACAACACCCA

CTTCCACGTTAAGCAAGCAAATCAGGTCATCGAATGCGTACTCTATGTATAGGGACAGAT

GGTTTGTTTTGCTACTT

>Unigene0001722

CCCTCATCTCCATTCTCCGCCGCTGATCTCCGCCGCTGCCGGAGGAAGAGGAGAAGATGC

CTCTCTACGATTGCATGCTCCTGATGAAGCCTAAGGTGACGAAGGAGGGGATAATGGATC

TCGTGTCCCGGGTGAGCCGGCGTGTCTACCAGCGCAACGGCGTCATCACCGACATGAAGT

CCTTCGGAACCATCGATCTGGGCTACGGCATCAAGAAGCTCGACGGCCGCCACTTTAAGG

GTCAGTTGATGCAGATGACTGTAATGGTTCCGCCCAGCTTTCCTAAGGAAATTCAATACT

TGAACAAGGAAGACCTGCTTCTGCGCTGGCTGGTGGTGAAGCACAGGGATGTCAAATATG

GCAACGAATCCTTATACGAGGACGAAGGTCGGGATGAGCTGCAAATGTTCCATCCCAAAG

ATGGACCGCTCGCTGAAAATGGGGACGATGACGATGATAGTGTCGAGGAAGATGATGGTG

ATGAGGAGTACGATCTGGAGTAAGTAGTAGCATGCTTGATTTTTTAGTTTTGGTATGTGA

TGTTAAGCTCTGAGTCAGATATAAAATGTTGAAGTTTGGTGGAGTTGCATAAAGTTCAGA

TAGGTTTTGACATGCTTTATCTTTGTATTTCGTTTAAACGTGTCCAATGTCTCAAATTAC

ATCTCCAGTTACTCCTTTCTGACATTTTCTCCTCAACAAATAGCAATCATTCTAAGGATA

TACACCATT

>Unigene0001723

GCAACAATGAGAACTTATAGGCTAGGAGGACAAAGACTTTTAAAAAGATTAAAAGCCATC

ACTCTATTTTTATTTCAGTCACATCCTCGAAAGCATGGGAGAAAGAAAGATGCTTAAGAA

ACAGCGTAGCGTAAATTATAAGATATAAAGTGAGAACTGAGCAGGAAGTCTGCCACGAGC

TAGATTACCAAATAACTTCCATTAGTCCTATAATGAGAGCAAGCCCCATGTATAGCACGT

ACCTCATCAGTTGATTGTAGGGGCTGTCTAAGCCATCAACCCAATAAATTCTATTCTATG

TATCCACTGTCCTGGTTCGACGGAAAGCATCGTTGGAACCTCCCTTTCTTGCATTCGTTC

CCACATCGCTAAGCGGCACTCTGTATTTGTATTTACATATCTTTCCAACGCGAAGTAAGG

TGCTTTATTT

>Unigene0001724

GATATATCCAACAAGCGGTAACCTTTATTTTTCTTTCCCCTGCTGAGATCATAGATATAA

CAGAGTTCTTCATTTTTCGTTCTTCTATAAATCTTGAAATATAAGCTGCTATATTTCGTC

ACGAGGGCAATAATCAGGGGTCCTGAGTGTTATCGTTGAGTCCTGTTCGGCAAATGTTCG

GAGGAATAATGCAATTGGTTCGTCACTTCGCCTCCTTCATTTGCTCCAAGTACAAAACCA

AATGCTATAACTATCGGGTCCACCGTCTAACCTAACTTGGCGGCATCCGGTTTTGTAACT

GGAGCAATGAGGAGTCATTCATGAAACTCTGGCATTACTTTCATCCTTCTGCAGCAGCAT

CTAATCTTGTTCCCCCTTGGCATCATCCATTCATATCCGTTGGATACTTTTTTGCCTTGT

TTTTAGAGGTAGTTTACTCAAAAAATCTCATTGAGATTTCTTTTGTTTCGTGCTTTTCAA

ATTTCTGATGTTGTAGTAGTTGTAGTACTGACCCTCGACATTGCCGGTTTGCCCCGGGAT

TTCAACAAGTGTAGTTGCATAGTGACAGACTATGTGGAAATGAGTTAAGCATGAGCACTT

TTCCAGAGGTGTCGTGCGGCGGTGTCGGAC

>Unigene0001725

CGAATTGGATATGAAAGTGGTGAGACTCTGTCTCCGCGAAGTCTCTTTCAAATAAGAATA

GCTGGGGTAGGGCGCTAACGAGCAGGAAAGTATGAAACTCAATAAATTGCTCGACCGTAG

GAAGAGGTTCAAAAGGTTGTAGAGTCGACTCGTTCCGTTTTATAGGGGTTGCTACGAGTG

GAATCTCTGTTTAATTTTTTTTGTCCAATGGAACCATGTTTTTTAGAGGCATCCTCCCCT

CAATCCAAGTAAAGTAGACTGATCGTCCAATGCAATGGAGCTAGCTAAGATTCTTAAGTG

TCTCGATTGATCCAATCCGTACAAGGCATGATGCCTGCGCGTCGAACTCCGAGTCTTTAC

GCCGAGTAAACCTAATAAAATAACATATTATAATTTTCCTCGTCAAGCCAAGCGGGAATA

TTCAAATAAGATTGCTTACCCTAACTTGTATGCTTATTATTCCTTAGATAGGCATAAATA

AAGTAAGTTTACTCATTGCCCGACCAAGCCAATCATGCTATCCTTACCTTCCAACCTTCT

ATTCCGCTTCTGCAGCAGCATCTAATCTTG

>Unigene0001726

CACGGATGGTAAGCCTGGAGCCCCATCCATACTCCTCATAGAAATTTCTGTGCGGATTCC

ACCATAGAAGTGCTCTGCTCCCTCACCCCTCTCGACGGAGCAGGAGCCGGAGCCGATCCG

TACATCCTCTACGCTCCGCCCGCGTGGAAATTTTATGCGGGCTGTCGCTGTGGCAGGAAC

TGAACCCTAGCTTACTACCTGCCGATCCGATGGCGGCATCATTCCCCTGGATTTGATGCA

CTGGAAGCTTCATTTGAGGTATGGAATTGTCTGGTTCTTCATTAGATAATGGTTCTGCGG

GAAATGCCGAAGGATCCTTGAGCGGGAAACC

>Unigene0001727

CACGGATGGTAAGCCTGGAGCCCCATCCATACTCCTCATAGAAATTTCTGTGCGGATTCC

ACCATAGAAGTGCTCTGCTCCCTCACCCCTCTCGACGGAGCAGGAGCCGGAGCCGATCCG

TACATCCTCTACGCTCCGCCCGCGTGGAAATTTTATGCGGGCTGTCGCTGTCGCAGGAAC

TGAACCCTAGCTTACTACCTGCCGATCCGATGGCGGCATCATTCCCCTGGATTTGATGCA

CTGGAAGCTTCATTTGAGGTATGGAATTGTCTGGTTCTTCATTAGATAATGGTTCTGCGG

GAAATGCCGAAGGATCCTTGAGCGGGAAACC

>Unigene0001728

CACGGATGGTAAGCCTGGAGCCCCATCCATACTCCTCATAGAAATTTCTGTGCGGATTCC

ACCATAGAAGTGCTCTGCTCCCTCACCCCTCTCGACGGAGCAGGAGCCGGAGCCGATCCG

TACATCCTCTACGCTCCGCCCGCGTGGAAATTTTATGCGGGCTTTCGCTGTCGCAGGAAC

TGAACCCTAGCTTACTACCTGCCGATCCGATGGCGGCATCATTCCCCTGGATTTGATGCA

CTGGAAGCTTCATTTGAGGTATGGAATTGTCTGGTTCTTCATTAGATAATGGTTCTGCGG

GAAATGCCGAAGGATCCTTGAGCGGGAAACC

>Unigene0001729

CACGGATGGTAAGCCTGGAGCCCCATCCATACTCCTCATAGAAATTTCTGTGCGGATTCC

ACCATAGAAGTGCTCTGCTCCCTCACCCCTCTCGACGGAGCAGGAGCCGGAGCCGATCCG

TACATCCTCTACGCTCCGCCCGCGTGGAGATTTTATGCGGGCTGTCGCTGTCGCAGGAAC

TGAACCCTAGCTTACTACCTGCCGATCCGATGGCGGCATCATTCCCCTGGATTTGATGCA

CTGGAAGCTTCATTTGAGGTATGGAATTGTCTGGTTCTTCATTAGATAATGGTTCTGCGG

GAAATGCCGAAGGATCCTTGAGCGGGAAACC

>Unigene0001730

GGGATCTCTTTTGGAGATTTAATGGTGCTATAGGATCAGATTATTAGCTGAAAAATAACT

TGTGTTGATCAAGGCCAGGATTGTTTATTCACTGATGGCAAACCACTTAAGTTTTCTGGT

TCACTCAGTTTTATGGATTGTTCATTTTGTAATTTGTTCTCAATTTTATACTTTAGTAAA

TATTTTTATCTTTTCAAAATAGTAAATGGTTAAGATCAATTGTCTAGTTTCTGTTTAATT

>Unigene0001731

AATTTCTCTCTTACCCTTGAAGACGTCGAATGCGCCTTCACATAAAAACCTTTCAACAAT

TGATTTCGTAATTTAGTCGAAAAATAAGTTGGTCTGCTTCTATCCTGTTGGAAATGGTGT

GGTGCAATCTAGTCCCTCCAAAAATCAATCCCTTTATTTGTTAGTTCTGAAAAAAAACTT

TTACCCCCAAGGGATAGATTGTTGAGATTTCTTCTGAAGACTCCAATAGTCTGGGCCTTT

TGTGGGAATGGAGAGTAAAGTTTATATTATT

>Unigene0001732

TACTGTTCTTTGTTCTGCATTGTGACAGTTTTCCGAATGATTGAGCATATGTTACAATTT

TGTGTGTAGTTCAGTATTATAAAGCATTTACATGTTTTATTCTTGAATGTTGCAAATAAG

TGAACAAAACATTAAACGTGTAAGCTCATGTTATCTCTTATATTCCTATTGCCAATTCCG

TTCTGTTCCTTTAGAGCTATGAACTTCATTCTATTTGTAGATTCTCTACCTCTCTACAAT

ACAATTGTTGACTACCACTAAAAGCAGAGTGGCACAATGTTGTGGAGGCCGACACATATT

GGAGTTCATCTGCTACCAGTATTGCTTCCTCCAATGAATTTTGCC

>Unigene0001733

GTTTATATTATTTTTGCATTCTTCATTTGTTAGAGAGATTTGGGAGGTGTTAGTTCCAAT

TTTCCGATCTAGTTAGCCTTGGAGAATATTAGCAAAAGGGCCATGTTCAAGTTTGGAAGT

AAAAGAGGTCAATTACTCAATCTAAGTGCAATACAATAGAATATTTGGTTGGCTAGAGTG

GAGGAAAATAGATATTAGAAACCAATTTTTTTATGTGATCTCTCAACTATGTAGGAACCA

CATGCATCCTTGTT

>Unigene0001734

GACGCTTTCGAGAAGTTTTTGCAAGAACTTCCTCTATCTCGCAGCCGCGCAATCATGGTA

ATTCCGGCACATCGTTTGTAGTTATTCTCTGTAGTATGCATGTATTGTTCTGCAAAGATT

TATGCTTACTGTCTGCGATTATGTTGCTGAATCTCATGAACTGGCAAATTCATGGATAAT

AGGTGTGAAGGAATATTGTGGATGCCATTGTTTCATAGAATTTGTGAGTGTAGTTGGTTT

GCATGGCAACCCATGCAATTTATGCTATCATCGGCACAAATTCCCTAGAGATGGATGGCT

TTGTGGTTGAACAACTTTTTTAACTGAAATATCATGATTAGTACATTTTATAATCCAATG

GTTTAATTGTTGATGCACTTGTTAGTTATAACCCCTTGTCGCCCCTTCTGTTATGTTACC

ACTCTATGGTTTATTCCCTTAAGCCCAGG

>Unigene0001735

TTTCAGCACCTCTCAATGGCCTCCGTTCTGTACGGAGCCGCCGCCATCCTCATCCCGGGC

CGCCGCATCTCGCCGGCGGTCAGCTCACCCCCCGTCGTTTCTCTCACCAGGAGGAGATCG

ACGGTTAGGGCGGCGGAAACCGACGCCAATACTGACGCAGTTAAGCCTACGGTGCCGGAT

AAAGGTCCGGCGGGCTCGAGCTTCAACCAGCTGCTTGGCATCAAGGGGGCGTCCCAGGAG

TCGGATAAATGGAAGATTCGTCTTCAACTTACAAAACCTGTTACTTGGCCTCCTTTGGTG

TGGGGAGTAGTCTGTGGAGCTGCTGCTTCTGGAAACTTCCATTGGAACATTGAAGATGTT

TCTAAATCTATTGTTTGCATGATTATGTCGGGACCATGTCTTACAGGCTATACACAGACA

CTGAATGATTGGTATGACCGGGATATTGATGCAATTAATGAGCCCTATCGCCCTATTCCT

TCAGGAGCTATAACTGAGGGCGAGGTTATAACCCAAATCTGGGTGCTTCTTCTAGCAGGC

CTTGGCTTGGGAGCTTTACTAGATGTGTGGGCAGGGCACAACTTTCCTATTGTTTTTTAT

CTGGCTGTGGGAGGATCTTTGCTTTCTTATATTTATTCTGCTCCACCCTTGAAGCTCAAG

CAATTTGGATGGATTGGGAATTTTGCTTTGGGGGCAAGCTACATCAGCTTACCATGGTGG

GCAGGTCAGGCTCTATTTGGGAATCTCACCCCTGATGTAGTTGTTCTCACACTCTTGTAC

AGCATTGCTGGGCTTGGGATTGCTATTGTAAATGATTTTAAAAGTATTGAAGGGGACAAA

GCTTTGGGACTGCAGTCTCTTCCGGTGGCATTCGGTATTGATACCGCCAAATGGATATGC

GTAGGGGCAATCGATATTACTCAGATATCAGTTGCAGGCTACCTACTTGGTGCTGGGAAA

CCACTTTATGCACTCGCTTTACTTGGATTGATCATTCCACAAGTTATTTTTCAGTTCCAG

TATCTATTAAAAGACCCAGTTAAATATGATGTAAAATACCAGGCCAGTGCGCAGCCATTC

TTGATTTTGGGTCTCTTAGTGACGGCCTTGGCTATTAGCCATTGATACAGAGACTTACCG

TATTCTACTCCGATTTATATGTTGATCGACTCGGGGTGAGGGAGCGCAACAATCTGGAAT

GATGAAGATTTAGGTAGATGTATCATTGGAATGTTTTGGTTTTGGCCTTGTTTTTTCTTT

TGTTTTATGCCTGTAAATGTCACATCTATGATTGTTGTACCCCTACATATCTGGCGGTGT

ACTGAGACTTGCGTTACAGGCTATTTGAAATCTTTTGCAAGATAATCTAGGTTGAGGCAT

TCCTCCAATTTTGATTTGGATCTGGTGACGTGGGGAAGAATACATTGGAAGAGCTAAATC

TGCAGATATTAAATGTGTGCTAACATGATTACAATCAAACATTTAACATTGGCAAGTTTG

TTTTCTTTACAGTTTGTCTGACATACTTGTAACATTTGTGGTTCTTTTTGGTTCGTCGCT

ACGACTAA

>Unigene0001736

TTTCAGCACCTCTCAATGGCCTCCGTTCTGTACGGAGCCGCCGCCATCCTCATCCCGGGC

CGCCGCATCTCGCCGGCGGTCAGCTCACCCCCCGTCGTTTCTCTCACCAGGAGGAGATCG

ACGGTTAGGGCGGCGGAAACCGACGCCAATACTGACGCAGTTAAGCCTACGGTGCCGGAT

AAAGGTCCGGCGGGCTCGAGCTTCAACCAGCTGCTTGGCATCAAGGGGGCGTCCCAGGAG

TCGGATAAATGGAAGATTCGTCTTCAACTTACAAAACCTGTTACTTGGCCTCCTTTGGTG

TGGGGAGTAGTCTGTGGAGCTGCTGCTTCTGGAAACTTCCATTGGAACATTGAAGATGTT

TCTAAATCTATTGTTTGCATGATTATGTCGGGACCATGTCTTACAGGCTATACACAGACA

CTGAATGATTGGTATGACCGGGATATTGATGCAATTAATGAGCCCTATCGCCCTATTCCT

TCAGGAGCTATAACTGAGGGCGAGGTTATAACCCAAATCTGGGTGCTTCTTCTAGCAGGC

CTTGGCTTGGGAGCTTTACTAGATGTGTGGGCAGGGCACAACTTTCCTATTGTTTTTTAT

CTGGCTGTGGGAGGATCTTTGCTTTCTTATATTTATTCTGCTCCACCCTTGAAGCTCAAG

CAATTTGGATGGATTGGGAATTTTGCTTTGGGGGCAAGCTACATCAGCTTACCATGGTAA

AATTGTTTGTGCTGCAGCATTCTTGAGAAGTTCATGTATTGTTATGCTGGAAGTGTGTAA

CACAATATTAATTAAGAAAGCTACACTGTGCTAATTTCTCCTGCTGATTCAAAT

>Unigene0001737

AACGTACTGATGTTTCCGAACCAGTTGTTGGTGAAACACTGTCTGAAAGTGAATCAAGTA

GTTGCACAACAGCTGAAGAAGAAGAAGATGGATGGGAATCTGGTCAGTGATTTATTCAAA

ACATAATTAGCACATTAAAAAATAAGAATTAGTGAAAAAATAATTTTATTATTCTTTTAA

AATATTTCTCATTTAGGCTATCGCTTTAACTACCTGTCTCATTAGGCTTGTGGAAAATAT

TAGTATAATTTACACATGAACAAACTGTCACTTGTGTATCGTTTTGTAACTATTATTTTC

CCTTGTATATACTTTAATTTTTAAAATGAACATTTTCTTTATATAAAAATCAAATTTGAT

ATAATTAATATTTTTATTCTTGTTTGAACGTTTTAGTAAAACGCTATCATCCCTAATTAT

TGTTGTGTAGTATTTTTTTAGAATAATGATTGGTGAAGGCCGCTTTATTCTCTCCCTTAG

GCTTTCGTAAACCTGTCGCTTGAGCACAATACACCTGATGGCGTAGATGCAAATGTGATT

GTGTTTCCGGTGGTTCTAGGCGAGTCGACGTATCTGTAAGATATTGGGAGTGCCACAAAC

AGAAGACGAACAACATAAACGTATTTTTATATGTATATGTATATCTATATATGTATATAA

TAACTGCAAGTTAATGTTTTTTTTTAAGTTAAAAATTTATATAGATTATAACAATTATTA

TAGTCTGGATTTTAAAAACTCCTCCATAATTAGGCATACACTCGCCTATTTTATTATTTT

TATAGTACAAAGAAAATAAAGTTTTAATTCTGATAACTTTATTAAAAATTTATTTTTTAT

TTTCGTGTTCATCTGTTAATTTTTATTTATATGTCTTTTTTTTTAAGTCTTCCAATGTTG

CCATTGAA

>Unigene0001738

TTTTCATTTCATTTTCTTTTTCCCCTCTTCTGTTGCATTGTCCCTTTTCATCTCTGGAGC

GTCCAGTCATAAATTATCACTCTAATTCATCATACAAATAGACATAATGTTAATTTTATT

GATCACTTATAAAATGTGTTGTTGATGTATAAACTACCTATACATAATTACTCTGGACTT

GACTATAAGTCTAGTATATACAATGCACATAACTAACCATAAACAGCAGGGCCATGGACG

AACAATCTACTCGTCTGGTGCATAGCTCGACAATTCGCACCAGAATGGCCATTCCCAACA

TCAAAATGACTAATCCAATTAAAAAACTGGTCAGTAAACAAAGAAAGCGTTATACTGAAG

ATGGGTTCAACCTCGATTTGACTTACATTCTACACAATGTGATTGCAATGGGATTTCCTG

CAGAAAATTTAGAAGGTGTAGTTTACCGTAATCATATAGATGATGTAAGCAGACTCCTCG

ATCAGAAACATCCTAATCATTATAAAATATATAATCTTTGTTCCGAGCGATCTTATGATT

CTCGGAAATTCCACCAGCGTGTAGCTACCTACCCATTTCAAGATCATAATCCACCTAGTA

TTGAGGTTATTAAACCTTTTTGTGAAGATGTTCATCAATGGCTTGATAAAGACAGCAGAA

ATGTAGCTGCTGTACATTGTAAAGCTGGAAAGGGCCGGACAGGTGTAATGATATGTTGCT

ATCTATTACATAGTCTACAATGTGTATCAGCAGAAGATGCTCTCAATTATTATGGCCAAA

AGAGAACGTTTGATAAAAAGGGTGTTACTATTCCCAGTCAAAGGAGATATGTCAACTATT

ATGCATTATTAGTTGAAAAAAATTTAAATTACAAGCCTGTGGTATTGACAGTGAAGGAAA

TAAAATTAGATCCAGTGCCTAACATTTTTAATTCGGGGCAAACATGTATCCGTTTAGTAA

TATCAGAATGGATAAATGGAGATAGTTCAAAAATCAGAAAAGTATATTCATCTGATGTAC

AAGATATGCGGCGTAATGCTTCTGATGTAACAATTCGTTTAACACAAATGACCCAAGTCC

TTGGAGATGTAAAAGTAGAATGTTACAACAAACATAAAATGAAGAAAAAGGAAAAACTAT

TTCATTTCTGGTTTAATACTTTTTTCGTTTGTGAACCAGCTGTTACTGAAAATGGTAATA

GAGAAAATAAACATTGTGGCCAGCAAGATAAAACTGTTCATTCAAATGGTATGGATGATT

CAACTAACTTTAGATTTCCAAATAATCAAATGCGTACACACTCTGTTGGTCATGTGGGTG

GAAGTGATAATCGCCTGTTGACATTAAACATGAACAAATGGGAATTGGATGGCCATAAAG

ATAAACAAAATAAACTTTTTAGCGCGGACTTCCATGTAAGTTTATTATTTGAACGTACTG

ATGTTTCCGAACCAGTTGTTGGTGAAACACTGTCTGAAAGTGAATCAAGTAGTTGCACAA

CAGCTGAAGAAGAAGAAGATGGATGGGAATCTGGCTTTCGTAAACCTGTCGCTTGAGCAC

AATACACCTGATGGCGTAGATGCAAATGTGATTGTGTTTCCGGTGGTTCTAGGCGAGTCG

ACGTATCTGTAAGATATTGGGAGTGCCACAAACAGAAGACGAACAACATAAACGTATTTT

TATATGTATATGTATATCTATATATGTATATAATAACTGCAAGTTAATGTTTTTTTTTAA

GTTAAAAATTTATATAGATTATAACAATTATTATAGTCTGGATTTTAAAAACTCCTCCAT

AATTAGGCATACACTCGCCTATTTTATTATTTTTATAGTACAAAGAAAATAAAGTTTTAA

TTCTGATAACTTTATTAAAAATTTATTTTTTATTTTCGTGTTCATCTGTTAATTTTTATT

TATATGTCTTTTTTTTTAAGTCTTCCAATGTTGCCATTGAA

>Unigene0001739

GTTAAATAAAAAAGCGGCGTTAGATGTAGCAGCTCAACAAAGTGTTACTGTTACTAAAGT

ATTAGACCCAGAAATTGATTTGGGTACACTTCTGTGTTTCGATTCAAATGATTTTGATAC

TCAACAACTCAGATCAAATCAAGAGGCATATTTAACAAATGAAACTAGGAATAACGTGCA

ATTACTGATCAACAAATTATTTGAATTGCCTATGAAAAATGAAGATGGTTATATGTATGT

AGATTTACCAGAACCTAGATATAATTTACCCCGTGAAAAACCATTACCAACACCTAAACC

ACTCACTAAATGGCAGAAGTTTGCTATTGAAAAGGGAATCAAGAAAACACCTAAACCAAA

AGCAACATGGGATGAAATTCTACAGAAATGGATACCAACTTATGGTTACAAAAAAGCTGC

AGCAGACAAAGAAAAAGATTGGATATTGGAAGTGCCTGGCAATGCTGCTGATCCAAATGT

TGATTTATTTGCTCAAAAACGAGAAGCAAAAAAAGAGCGTGTTGCTAAAAATGAATATCA

AAGGTTACGAAATATAGCTGCTTCACGTAAAATAAAAGTACCTTCTGTTGGATTACCACC

TTTAGAAGGAACATTACACTCAAATCAGTTGAAAACAGCTGTTGATGTAGCTAAAATCTC

AACTGCATCAGTTGGTAAATTCCAGCCAAAAATAAGCAAGGAAATAATATCAACTAAAAA

AAATATACCTGGGTTAAAGAAAGATAAAATCCCAGCTAAGAGTATGCAAGAAGAAAAATC

AAGTAGTCTTCAAATATTAGAATCAATTAACAACAAAAAACCAAAATTCAACTTGGAAAA

AGCTGCTAATAAAGCTATTCATGCAGAAGAAGTCGAGAGAGCTCGAGAGAAAAAAGAAGG

GGGTGATAAAAAGAAAGGAGGACGTTCACGCAGCAAAGGATCATTTAAAAGACCTACTGC

CAATACTGGCAAGCGTTTGGGAAAACAAAAAGGTGGACGTAAACGTCGATAATATCACAA

TTTTATA

>Unigene0001740

TTTGCAACTGTACACAATTTCTAATTCAAAGTTCCATTATTTTACGAGTTGGTTTGTCAG

AAAAGACTGCTTGCTGCCAAGGGAAATGAACATAATGTAGTTTGAGACATGAATATTGAT

GTTCACCCAGGGAAGTGATGTATACGGCTTGCTTTTATCCGTATGTATACTTTGATCCAT

GTATGTGACTCTAAAATATCATACATACTGAAATATTACTATGCTTTTGTGTCTGCCTAT

CTGATATAATGTAGCTATATGCTGAAATGTACATGAAAATATTTGAGAAAGTTTGCTTTC

ATTTTTTTTT

>Unigene0001741

AACAAGATCTATTCAGATTGGATGAACTCCAAATAGATTTCAGAGCCAACTCGTCGAGCC

CGAGCCTCACTACTTAAAAAAAGGGACAACAACTTCGCAGCATCTTCTAACAACCCACTT

CAAGGACACTCCAGCACACATCACTACGAAGACAAGCCAGTAACCATCAACTTCAGACGA

CATCGGAAACCGCCCAATATCACCTAAACAATTTCTCTGAGTTGGAGCCATCTCAGAAAG

AGATGCTGAACACACAACACCACCAGCAGACTTGTTGGCGAGTCGGATGATTTACTGGCG

AGTCGGATGGCAATCTCCTTGAACTTCGGGGACTTGCTGGCGAGTCGGATTTGCTGGCGA

GTCGGATGGCCAACAAGCTCCTAAAAATCGGACATTACAAGAAAATTGAAGAGCCACAGA

CCATCTACAGAGTTCTTCCATCTATCCACACTGAAGACAATGGAGCCTACGATCCCATGA

TCTTCTCAATCGG

>Unigene0001742

AACAAGATCTATTCAGATTGGATGAACTCCAAATAGATTTCAGAGCCAACTCGTCGAGCC

CGAGCCTCACTACTTAAAAAAAGGGACAACAACTTCGCAGCATCTTCTAACAACCCACTT

CAAGGACACTCCAGCACACATCACTACGAAGACAAGCCAGTAACCATCAACTTCAGACGA

CATCGGAAACCGCCCAATATCACCTGAACAATTTCCCTGAGAATCCATATGTA

>Unigene0001743

TATTGTTTTATGTTATCTCGAATCGATTGTAATTACTTTTCGCAAGTGTGGATTACATAC

ATGTTTGCCTCAATTGACGTTGTCGAATGACTAGAAATGGTGTTCAATTCAATATGTCCA

GCAATAAAGCTTGTCGAATAATAGTAATGGGAATTGCTGTTTTAATTTGTGTAGCTGGAT

CATAAAAGTGATAGCTTTCAATTTGGGAGCTGATTGTGTTTGTTTCATCACTTGTTCGTC

AGACTCGAACATAGCCCTCTTGTAAGAAGAAAGATGCCCAGGGACTCTATGTGAGTCCTT

CCCAATTTCATGACTTACCTCTGTATTTGGGGGAAAAAAGTCCTATCATCACACTC

>Unigene0001744

GGTTGTCCCAGCCCCATCTCTTTACCCACCTCAAACCGCCACCAGTCAGGCCGCCGCCCC

TTCAACCTCTGAGAACAGTCTCTTCCGGCCCCTATCGTCGCCGCCGCCGCCGCCGCCTAT

CGTCGCCGCCGGCGCCATTTTCTTGCAGCAGGGAGGAGAAGGGGAACGAGAAGAAGGAGA

GTGGGACGAGATAGGGTACGAGAATAAGGAGAGGGGGAGGAGCCGGAGCGCCTTGAGGAG

CGGATTCGTTGAGGATGACAGAGTTCAAGATCGAATTTATAACTGAAGATTGTCTGCAAC

CATTCATGAGGGGGAGATTAAAGAAATGCTCAAAAGAGATACTTGAAAAAACTTTTCCCA

ATCTGGGCTATGAGATTTTGGTGTTAGAGACGTCAGATGCAATACAATATCATGAAAATC

TACTTCGATTATCCCATGAAAATATATTGAGATCAGTAGCTGTTTTTGACGTACCTAATA

CTAAATTCTCAGTTCTCTGTTTCGAGGATTGTTTTAGCAGTATATCTGAACTTTCTTTAC

TGTTGTTTGGCGATGGTCCAAAAAATGTACCATGCGATATTTCCCCTATATTTCGAAAGC

TTATATTCCAAATTGCCAAGGCAATTCACCTATTGCATACTAGGAAACAGTATCATGGAT

ATTTGAACAATGAGTCAACATTTCTTAGTAATCTGGGTGGAACTTGTGTTGTGAAACTTG

CCAGTATTCGAGGTCTTTTAAATACTGGTCAGTCCGGTGAAAAGGTTTATGATGAAGATT

GGCATTCTTTAGGTAGCATGATTGAAGACATTTTTAAAAAGAATGACAGAATTCCAGCTT

ACTCTGTTCAGAGCTACATTGACCACTTAAAAGGTGCAAATCCATGGCGCTTCGTGTTCA

ATCATCCAATCTGGTTGTCAACATCCCGCAAAATTAGCTTAATTTTTGAGATGGAAGAAG

CCCGGCATAGTGTGAATGCATCGAGCCTGAAAAAATCATACTGGCCGAATGACGATCGTC

TCCAGGAATTTTTCAAGGCAGAACCTCAGAGGAAATACACCTGTCATGTTCTGGATTTAG

TTAGACTGTTGAAAAATAAATATTGCCATCCTCAATTTGACAATACTTCATTCAAAGATT

TCAATGGGAATTTCATTTTGTTTTTGGAAGAGATTGAGAAGAGGAATCCTGAGCTCTGGA

CAGAATTGCTGAAAGAGACACAAATTGATCTTAACATTGAGGTGCCCGGGGTGCACCAAG

AGTACACTGAATGGTATGAATCTGTTAATTTTGATGGAGAAATTGGGAATCTAACTCTAG

ACTCCTCTTCCACCTCCAAGGGCGTAACTTCCTCTCCCTCCCAAGATACATGTGTCTCTT

GCCAAGTTTTCACTGCCTGATTTCACGAGTTTTAGCTTCTTCTTCATCCATGTGACTGCT

TGATTTCACAAGTTTAGCTTGGGCTGATTTGTTTAGCTGTGTCTACCGGCGTTGACCATT

GAGCTACTTGGCTGCTTTCGTTGTGACTATTGAGAACATGAGGTAGTTAGGCTGGATAGT

TGTAACGGCAGGTGTTGACTATTGAGATAGTTGTGCTCGGATACTCTTAGGTTTGTTTGG

CTGTGACTACTGGCGCTGACCATTAAGATACTGTTTAGTCAGATTGGTTTCCATTTCAGA

TTAGTTGATTCCACTAAAATTCTTAATCAATCTATTCTTTATTGATATGAACATACTGTG

GACAGTGGTTTTGGCTGTTTTGTTGGCGTTGGTGTTGACTGTTGAGATACTAGGAATTTT

GGCTGT

>Unigene0001745

AAGCTAATGGTTTGACATCGGCAGAAGACATTTGCGTGGATAGAAGTGTTGGTATAAAGC

GGCCTAAACATTCAGGAGACGAAGGACCTTCTATGGCTGAGTTAAGTGGCTACAATGCCA

AGAGGCAGGAATTCGATCCCGAATATGATAACGATGCAGAGCAGTCACTGACTGAGATGG

AATTCAAAGAAAGTGACACCGAGGCTGAGCGTGAACTGAAGCTGAGAGTTCTTCGAATTT

ATTTATCAAGGCTTGATGAGAGGAAACGGAGGAAGGACTTCATCCTGGAAAGGAATTTAC

TTGAACC

>Unigene0001746

TGTGCTGTTAACATATTTAATGGACACTACGATTCACAGCTCAACAATGTCAGCCGTCTG

TTAGGGTTCCGACGAGCCCCGTTCCAGGCCCGGCGAGAGAGCGATGGGGCGCTCCCGCGC

CGTCACCCACACGACCGACGATGATGCCGGCCATAGGTCAAAGAGGAGGAGGGTGGCTTC

AACTGGCGAAACTTTGGAAAATGTGGCATCAGGTCCAGGAACAAGTGAAGGGAAAAAGGC

TTCATACCATTGCAATTATTGCAACAGGGATATATCAGGAAAGATACGCATTAAATGCAC

TAAGTGCCCTGATTTTGATTTATGTGTGGAGTGCTTCTCTGTGGGTGTTGAAATTACCCC

CCACAAGAGCAACCATCCTTACAGGGTTATGGACAACTTGTCATTTCCACTTATTTGTTC

AGATTGGAATGCAGATGAGGAAATACTACTTTTGGAGGGAATTGAAATGTATGGAATGGG

CAACTGGGTGGAAATAGCTGAGCATGTAGGTACCAAGAGTAAGGTCCAATGCATTGACCA

TTATATGACAGCATACATAAACTCCTCTTGCTATCCTCTTCCGGATATGTCTCGTGTTAA

TGGGAAGAATAGAAAGGAACTTCTAGCTATGGCAGCTAGAATGCAAGGCGAAGGAAAGAA

AGGACTTTCAGGGGTTGGTGATCTTACACCAAAAGAGGAGTCTCCATTCTCACCTTCAAG

GGTCAAGGCTGAAGATTTAAGTGGAGAGCCTCTAGCTGGACGATCACCCTCAAGCATAAT

TGCCGGAGTCAGTAAGAAGGCGTCAACCATGGGGCAATTCAAGGACCGTTCTCAAGGATG

TAAACTGGATGGTTTGACATCGTCTGAAGACGTTCACTTGGATAGAAGTGTTGGCATAAA

AAGGCCTAAACATTCAGGGGACGAAGGGCCTTCCATGACCGAGTTAAGTGGTTACAATGC

CAAGAGGCAGGAATTTGATCCCGAATATGATAATGATGCAGAACAGTCACTGACTGAGAT

GGAATTCAAGGAAAATGACACGGAAGCTGAGCGTGAACTAAAGCTGCGTGTCCTTCGAAT

TTATTTATCAAGGCTTGATGAGAGGAAACGGAGGAAGGACTTTATCCTGGAAAGAAACTT

ACTTACTCCACACCCAATGGAGAAGGAACTATCAAGTGAAGACAGGGAAGTGTTCCAGCG

TTTCAAGGTTTTCATGCGTTTTCAATCTCAGGAAGAGCATGAGAATTTACTGAAGAATAT

CATTGAGGAGCGTAAAATTCGAAGGAGAATTGAAGAGCTTAAGGAAAGCCGTGCTGCTGG

TTGCCGTACACTGGCCGAAGCAAAGTTGTTTACTGAGCAGAAAAGGAAGAAGGAGATGGA

GTTGAGTGCAGAGAAAGCCAGGGAAAGTGGGCAGATTTTGGTCAAAGGGACGGAGTCTGG

CGGTCGGGTTAACAGCACAAGGCCCTTTGATGAGTGGGATGTCACCGGTCTTCCTGGTGC

TGAGTTATTAAGCGAAGCTGAGCGACTGCTCTGCTGCCAGAACAGACTGCTGCCGAGTCA

TTATCTGAAGATGCAGGAGATATTGGTGCAAGAAATATTCAAGGGCTCGCTGGTGAATCG

ATCTGACGCTCATCGTTTATTTAAGGTGGATCCAAGCAAGGTGGACAGGGTTTACAAGAC

GATCATAAAGAAGTTGGGCATGCATGATGGCTTAATTGATGCTTGAGTTTTAGATGTTAT

TTAATGAGCCCGTAAAATATTTTTGGAGTTGCAGCGATAAAACATGGAATATATTCTAAG

TCTGTAGTATAGATTGATGGACAAAAGTGACACTTTTGTGGATCTTTTGAGGTTAAATCC

CGAGATATTTAATTTCAAGTTGTATTTGGGTGTCACGAACCTCTTTGATCGTGTTGTGAG

TACCAGTGATGTTTACATCAACCATTAACACAACGT

>Unigene0001747

GGTTTTCATGCGTTTTCAATCTCCGGAAGAGCATGACAATTTACTGAAGAATATGATCGA

GGAGCATAAAATTCGAAGGAGAATCGAAGAGCTTAAGGAAAGCCGTGCTGATGGTTGCCG

TACGCTGGCTGAAGCAAAGTTGTATACTGAGCGGAAACGGAAGAAAGAGATGGGCTTGGG

CGCACAGAAAACTGTTAATTGTCAGCTTAACAAAAGTCCAATTGATGAGTGGGATGTGAC

CGGTTTTACTGGAGCTGAATTACTGAGCGAAGCTGAGCGACGACTTTGCTGTCAGAACAG

ACTGCTGCCGAGTCATTATCTAAAGATGCAGAAGATAATGGTGGAAGAAATATTCAAGGG

GTCGCTGATAAATCGATCTGACGCTCATCGTTTATTCATGGTGGATCCAAGCAAGGTGGA

TAGGGTTTATGAAACGGTGATAAAGAAGTTGGGCTTGCGGGATGGCGTAATTGATGCTGC

TTCACATTGCTCCCTCAAGTAATAAATGTTTGTTTAGCCTTTAGAAATTTTCTTGTCAAT

TTTATTGGTATATAAGTGGGTTAAATATTAGCTGTTAAAACTTTCAACTAGATTTTATGC

ACCATGTTTTTCAAAGCAAATATAAA

>Unigene0001748

TTCCGAATGAGAAGCACAAGCACTGTATGAGACTTATATAGGCAATCCCTTCGTGGATTT

CAGCGAATTACGACGGACTTGATTCCAGGGTGAGGGATACGTAGGCAACCTTCGGGTGCA

ATCCGAGATAGATGAAGATAATGATGTTGCCGACTGGAGTTTAATACTAAATCCGAGGTG

AAGATTGTTGGATTGGCGAGACAAAATCAAAGAAATCAAGACTAATCAGTGTTTCATGCG

CACACATTTCT

>Unigene0001749

TTCCGAATGAGAAGCACAAGCACTGTATGAGACTTATATAGGCAATCCCTTCGTGGATTT

CAGCGAATTACGACGGACTTGATTCCAGGGTGAGGGATACGTAGGCAACCTTCGGGTGCA

ATCCGAGATAGATGAAGATAATGATGTTACCGACTCGAGTCTAATACTAAATCTGAGGTG

AAGATTTTTGGACTGGCGAGGCAAAATCAAAGAAATCAAGACTAATCAGTGTTTCATGCG

CACACATTTCTTTAGAATGCGGGCGCATTTCGAAATACGCACACAGCTTGTCACATTT

>Unigene0001750

GTTGAATTTGCCCAAATGTTACTATCTGATGATGCTGAACCGCTTGCTAATATTAATCAA

ATTGTGGATATATTTATCGAGCAAAGCATGGTTCAACAATGCACTAAATTCCTTTTAGAA

GCATTAAAACATAACCGTGAAGCTGAAGGCCCATTGCAAACACGTTTGCTTGAAATGAAT

TTAATATCTGCCCCACAAGTTGCTGACGCTATACTTGGTAACCAAATGTTTACTCATTAT

GATCGCGCTCATGTTGCCCAACTGTGTGAAAAAGCTGGACTTTTACAAAGAGCTCTGGAA

CATTATACAGACTTATATGATATTAAAAGAGCAGTTGTACATACACAGTTATTAAATCCT

GAATGGTTAATTGGTTACTTTGGATCCTTAAGTGTTGAAGATTCTTTGGAATGTTTAAAA

GCTATGTTAACTAATAACATAAGGCAAAACCTTCAAATTTGTGTTAAGATTGCAACCAAA

TACCACGAACAATTGACAACTAAGGCATTAATTGATTTATTTGAGTCATTTAAAAGTTAT

GAAGGATTATTTTACTTCTTAGGATCCATTGTTAACTTCAGTCAAGACCCAGAAGTTCAT

TTTAAATACATTTCAGCTGCTTGCAAAACTGGACAAATTAAGGAAGTGGAGCGTATATGT

CGGGAATCTACGTGTTATAATGCAGAAAGAGTTAAGAATTTCTTAAAAGAAGCTAAGCTT

ACTGATCAATTACCATTGATCATTGTGTGTGATCGATTTAATTTCGTACATGATCTTGTG

CTGTATTTATATAGAAATAACTTACAAAAATATATTGAGATATATGTCCAAAAAGTGAAC

CCTTCTCGTCTCCCAGTTGTGGTCGGCGGACTCCTTGATGTAGACTGTTCAGAAGACATA

ATCAAAAATCTTATACAAGTTGTGAAAGGAGAATTCTCTACAGATGAATTAGTAGAAGAA

GTGGAGAAGCGTAACCGTCTTAAACTTTTGTCGTCTTGGTTAGAATTACGTGTTCATGAT

GGTAGTGAAGAACCTGCAACTCATAATGCTCTTGCTAAGATTTATATTGACAGCAACAAC

AATCCAGAAAGATACTTAAAAGAAAATAAGTTCTATGATAGCCGTGTGGTTGGCAAGTAT

TGTGAAAAGCGTGATCCACATTTAGCATGCGTTGCTTATGAACGGGGAAAATGTGATTTG

GAATTGATAAATGTTTGCAATGAAAATAGTCTTTTCAAATCTGAAGCTAGATATTTGGTG

AGACGTCGTAATCCAGAGTTGTGGCTGGAAGTATTGAATGAAAATAATGTCTATCGACGT

CCACTTATTGATCAAGTTGTACAAACAGCTTTGTCTGAAACTCAAGATCCTGAAGATATA

TCAGTGACTGTAAAAGCTTTCATGACTGCTGATTTGCCCAATGAATTGATTGAGTTACTC

GAAAAGATTGTATTTGATAACTCATTATTCAGCTCCCATAGAAACTTGCAAAATCTATTA

ATTTTAACAGCTGTTAAAGCTGACAGAACTCGTGTTATGGATTATATTAATCGTTTAGAC

AACTATGATGCTCCTGATATTGCATCTATTGCTATTAACAATGAGTTATTTGAAGAAGCT

TTTGCTATATTTAAGAAATTTAACGTTAATCAATCTGCTATTCAAGTACTTATTGATAAT

GTGAAAAATCTTGATCGCGCTTATGAATTTGCTGAACGTTGTAATGAACCAGGGGTTTGG

AGTTTATTGGCAAAATCTCAACTGCAAAGTATGTTTGTTAAAGAAGCTATTGACTCGTTT

ATTAAAGCTGATGATCCAAGTGCGTACATGGATGTCGTTCAAACTGCACACAAAACAGGA

AGTTGGGAAGATCTTGTACGTTATTTACAAATGTCTCGTAAGAAGGCTCGTGAATCTTAT

ATTGAAAGCGAATTAATCTATGCTTATGCCAAAACAAATCGCCTAGCAGACCTTGAAGAA

TTTATTTCCGGACCAAATCATGCTGATATTCAAAAGATCGGTGACAGATGTTTTGAAGAC

AAAATGTATGAACCAGCTAAACTACTGTATAACAATGTCTCAAACTTTGCACGCTTAGCT

ATTACACTTGTGCATCTTAAAGAATATCAGGGTGCTGTTGACAGTGCCCGTAAAGCAAAT

AGTACAAGAACATGGAAAGAAGTGTGTTTTGCTTGTGTTGATAATAACGAATTTAGATTA

GCACAAATGTGTGGTCTGCATATCGTTGTTCATGCTGATGAATTAGAAGACTTAATCAAC

TACTACCAAGATCGTGGTTATTTTGAAGAACTTATTAATCTTTTAGAAGCAGCTTTGGGA

TTGGAACGTGCACATATGGGAATGTTTACTGAATTGGCTATTTTATATTCCAAGTATAAG

CCCGCTAAGATGAAGGAGCATTTAGAACCTTTCTGGTCTCGTGTTAATATTC

>Unigene0001751

GTTGAATTTGCCCAAATGTTACTATCTGATGATGCTGAACCGCTTGCTAATATTAATCAA

ATTGTGGATATATTTATCGAGCAAAGCATGGTTCAACAATGCACTAAATTCCTTTTAGAA

GCATTAAAACATAACCGTGAAGCTGAAGGCCCATTGCAAACACGTTTGCTTGAAATGAAT

TTAATATCTGCCCCACAAGTTGCTGACGCTATACTTGGTAACCAAATGTTTACTCATTAT

GATCGCGCTCATGTTGCCCAACTGTGTGAAAAAGCTGGACTTTTACAAAGAGCTCTGGAA

CATTATACAGACTTATATGATATTAAAAGAGCAGTTGTACATACACAGTTATTAAATCCT

GAATGGTTAATTGGTTACTTTGGATCCTTAAGTGTTGAAGATTCTTTGGAATGTTTAAAA

GCTATGTTAACTAATAACATAAGGCAAAACCTTCAAATTTGTGTTAAGATTGCAACCAAA

TACCACGAACAATTGACAACTAAGGCATTAATTGATTTATTTGAGTCATTTAAAAGTTAT

GAAGGATTATTTTACTTCTTAGGATCCATTGTTAACTTCAGTCAAGACCCAGAAGTTCAT

TTTAAATACATTTCAGCTGCTTGCAAAACTGGACAAATTAAGGAAGTGGAGCGTATATGT

CGGGAATCTACGTGTTATAATGCAGAAAGAGTTAAGAATTTCTTAAAAGAAGCTAAGCTT

ACTGATCAATTACCATTGATCATTGTGTGTGATCGATTTAATTTCGTACATGATCTTGTG

CTGTATTTATATAGAAATAACTTACAAAAATATATTGAGATATATGTCCAAAAAGTGAAC

CCTTCTCGTCTCCCAGTTGTGGTCGGCGGACTCCTTGATGTAGACTGTTCAGAAGACATA

ATCAAAAATCTTATACAAGTTGTGAAAGGAGAATTCTCTACAGATGAATTAGTAGAAGAA

GTGGAGAAGCGTAACCGTCTTAAACTTTTGTCGTCTTGGTTAGAATTACGTGTTCATGAT

GGTAGTGAAGAACCTGCAACTCATAATGCTCTTGCTAAGATTTATATTGACAGCAACAAC

AATCCAGAAAGATACTTAAAAGAAAATAAGTTCTATGATAGCCGTGTGGTTGGCAAGTAT

TGTGAAAAGCGTGATCCACATTTAGCATGCGTTGCTTATGAACGGGGAAAATGTGATTTG

GAATTGATAAATGTTTGCAATGAAAATAGTCTTTTCAAATCTGAAGCTAGATATTTGGTG

AGACGTCGTAATCCAGAGTTGTGGCTGGAAGTATTGAATGAAAATAATGTCTATCGACGT

CCACTTATTGATCAAGTTGTACAAACAGCTTTGTCTGAAACTCAAGATCCTGAAGATATA

TCAGTGACTGTAAAAGCTTTCATGACTGCTGATTTGCCCAATGAATTGATTGAGTTACTC

GAAAAGATTGTATTTGATAACTCATTATTCAGCTCCCATAGAAACTTGCAAAATCTATTA

ATTTTAACAGCTGTTAAAGCTGACAGAACTCGTGTTATGGATTATATTAATCGTTTAGAC

AACTATGATGCTCCTGATATTGCATCTATTGCTATTAACAATGAGTTATTTGAAGAAGCT

TTTGCTATATTTAAGAAATTTAACGTTAATCAATCTGCTATTCAAGTACTTATTGATAAT

GTGAAAAATCTTGATCGCGCTTATGAATTTGCTGAACGTTGTAATGAACCAGGGGTTTGG

AGTTTATTGGCAAAATCTCAACTGCAAAGTATGTTTGTTAAAGAAGCTATTGACTCGTTT

ATTAAAGCTGATGATCCAAGTGCGTACATGGATGTCGTTCAAACTGCACACAAAACAGGA

AGTTGGGAAGATCTTGTACGTTATTTACAAATGTCTCGTAAGAAGGCTCGTGAATCTTAT

ATTGAAAGCGAATTAATCTATGCTTATGCCAAAACAAATCGCCTAGCAGACCTTGAAGAA

TTTATTTCCGGACCAAATCATGCTGATATTCAAAAGATCGGTGACAGATGTTTTGAAGAC

AAAATGTATGAACCAGCTAAACTACTGTATAACAATGTCTCAAACTTTGCACGCTTAGCT

ATTACACTTGTGCATCTTAAAGAATATCAGGGTGCTGTTGACAGTGCCCGTAAAGCAAAT

AGTACAAGAACATGGAAAGAAGTGTGTTTTGCTTGTGTTGATAATAACGAATTTAGATTA

GCACAAATGTGTGGTCTGCATATCGTTGTTCATGCTGATGAATTAGAAGACTTAATCAAC

TACTACCAAGATCGTGGTTATTTTGAAGAACTTATTAATCTTTTAGAAGCAGCTTTGGGA

TTGGAACGTGCACATATGGGAATGTTTACTGAATTGGCTATTTTATATTCCAAGTATAAG

CCCGCTAAGATGAAGGAGCATTTAGAACTTTTCTGGTCTCGTGTTAATATTCCTAAAGTG

TTACGAGCGGCTGAACAAGCACATTTGTGGTCAGAGTTAGTATTTTTATATGATAAATAT

GAAGAGTATGATAATGCGGTATTAGCCATGATGAATCATCCAACTGAAGCATGGCGAGAG

AGTCATTTTAAAGACATTATTACAAAAGTTGCTAATTTAGAATTATACTACAAAGCAATA

CAATTTTATCTCGATTATAAACCGCTTCTGCTCAACGACTTATTGCTAGTGTTGACGCCA

CGAATGGACCACACTAGAGGTGTCGCATATTTCACTAAGACAAATCATCTGCAATTAGTC

AAAACATACTTGCGTTCTGTGCAATCTTTAAATAATAAAGCTATAAATGAAGCACTTAAT

AACTTACTGATTGATGAGGAAGACTATCAAGGTCTACGAACATCTATTGATGCTTTTGAT

AACTTTGACACTATCGCACTTGCCCAGAAACTCGAAAAACATGAACTTACCGAGTTCAGA

CGCATAGCTGCTTACTTGTACAAAGGAAACAACAGGTGGAAGCAGAGTGTGGAATTGTGC

AAGAAAGACAGATTATATAAAGATGCAATGGAATATGCGGCAGAATCAAAAAATTCAGAA

GTAGCTGAAGAATTGGTTACGTGGTTCTTAGAACGTGGAAACTATGATTGTTTTGCAGCT

GCTTTGTTTCAATGTTATGATCTTGTGCACCCAGATGTTGTATTGGAACTAGCATGGAGG

CATAAAATCATGGACTTTGCTATGCCTTATCTTATTCAAGTCACTAGAGAATATGTTGCC

AAGGTAGATAAATTAGAAGAAGCCGAATCCAAGCGATTGGAAGAACACAAAGAAGAAGAC

ATTAAACCAATGATGATACCTGAACCACAATTAATGTTGACTGCTGGTCCATCAGTTATG

GGTAATATGTACTCGCCGTCCTATCAAGGAACTCCTCCTTCACAGCAACCTTATGTACCC

CCAAGTGGAGCCCCATATGCAACTTCAAATACTTTACCCTATCAGGGCTATGGCATGTAA

TGTGCCTATTAAATACCCTTTTCCTTTATAGCAGTATTTTATAAGAAAACAATGATGGGC

TTTGCTATATTCTGATAGAAAATTTACTACCCAGGTATAAAATATTTATATTTTATAAAT

AATCAATTTGAAAATCATTCCAAAAAAATTATCTAAATAGGCTTTTGAAAAAACGTGTTC

TCATCATTGTTAGATAATATTAAATAGTTAATGTTAATACTTGTTAAAAAATATTACTTG

TTTAAATTATTCAACTTCACTTCGTAACTTATGATTTATTTGCGTATACATTTTTAATAT

TGATTTCACGTTAATTTTTATAATTTATTTTATTTATAATCGTATTATTATTATTATTAT

TATTATTATAAAACATTATTATTATTATTATTATTATTATTATTATAAAACATTATTATT

ATTATTATTATTAT

>Unigene0001752

CCTGTAAACCTTACAATTAGCACAGTAAGAGATCGAGCAACTGTCTTCTTAGACCAGGTC

ATAGTGAGTATAATATCTCGCAACTATGAAAATAAACCAGTGAGCTTGAATATAAACAGT

AAAGTTCAAAAGCTAAGCATTCTAATAGAAAATCAGGGAAGGTTAATTAATTACAGAAAT

TCTTTAGTAGACAAAAAGGGTATTTTTGAACCCGTAAAATTGGGTAACCATGTACTGGGT

CCGTGGAAGATGATAGCTTATCCTCTGAATGAAACGTCATGGTTTTCGACCATAGAACCA

CGCAAATCCGTCGTTTTACCGGCATTTTATAAGACAACATTTACGTTACCAGAAAACTTA

ACAAAACCTTTAGATACGTTTTTGGACCCTACTGGTTGGAAAAAGGGCGTCGCTTTTGTA

AACGGGATGAATATTGGTCGATATTGGCCTTCGTGTGGTCCACAAATTACTCTATACATT

CCCGCTTTATTTTTAATTCCTTATCCTGGACTAAACAACATCATAATGTTAGAATTGGAA

GGTGTTCCTGAGAACTTGTCAATATCGTTAGTTGACAAACCTAATCTAAATG

>Unigene0001753

TTTTGAACTATTATTAGCTTTGGGGGATGGTCCCTCTGTGGAGGGGTACTTTGGTACTTT

TGCCCCTCCCAATCACATTATTATGCTCAACAGTGAAGGTTTGTTTCGTTCTGGTGTTCT

GATTTATTACCGTTTTCCATTATAATCTATGGCTCAGCATCCCAAGCTTCTGAGAAGGCA

TGAATATGTAGTATCTATGGAGCCATTCCTATTTTTTTTACTATCGTCGAAATCCAGACC

CAAATACAGGTAAGCATTAAATGATCTTCAAATATGTACATCCTGTCTTGGTTCTGGGTG

GCTAAGTTAGGTTGTCTATCTACAATTGGACTATTATGATACTCAGTGGACTCGTGCATG

CCACACTCACATTTAGCTGTTTTAGTGTTGAATAGTCTGACATCTTGACATTCCTGTTGG

CAT

>Unigene0001754

GTAATTCTTGATCCACATCCTTCTTTTTTGCCATAGAAATAATCGAATCGTAAGTTTCAG

TTGTTCGCTAAGTGGGCTCTAATTATGCACGAAAAATGTCAGATTGCTACCCGGCGTGTG

TTCTAAGTTTGTATTGGCAAATATCTCTTTAAATAGTTGTACTAGCAGCAGATGCATAGA

TAAGATAGACAAAACAAATCTAGCCTTTTGAACTATCTGTCTGTTGTTATACTTTTATCA

TTTGTGTCCAATTTGATGATTTTTCCTATATCTTTCTTATCCTACAGTGTTGCACAGTGC

TATTAGTGATCATCGCGGTGCTGGCCATTCTTATTATCCTCCAACAACACTGAGTATTAA

AGCTAAAAGAATACATGCTTTGATATTTTTACAATTTGGTCAGGACAATCCGAACTGCAA

ATTCTTGTATTCTAGATAACAAAATGTGCTTATGTTCTCATATATATGTATCATTTTGAG

GATG

>Unigene0001755

GCCATTGGAACTGTCAAGGACACGCCACAGCTCCGCAACAATCTGGAAGAAGCGAGGAAG

CATGTTGAAGAGCTGGTAAATGAGATCTCTGAGAAACTCAAACATATCACAGATGCCAAT

CAAAATAGCTATCCTTCAGAGAAAATAGCTGTTGGGAAGCTAGAGAGAGACTTCAAGGCA

CTCCTCCAGGACTTCCGCACAGTACAGGAGGTCAAATCTGCCAAACTCAAGGCGCTGGCA

TCGGTTCCCTTGCCTTCCAGAGCTCCCTCTACTTTTGTTGCTACCCTTCCCACTATTGTG

ATTTACACAGAATCTCAAGAAGAAAACCAGACCGAAAGCCTGGCTGTAACTGCCAGAATC

TTTCGACTCAATACTGCTCTAGCCGGGCTCAGGCGGATGGTTAAAGCCATGGGAACTTCT

AAGGACACACCAGAGCTTCGTCAGAAGCTTGAAGAATCACGGAAGCACATTGAAGAACTG

ATGCAAGAAACTACAGACAAACTCAAATATCTCACCAATACAAACAGAAATAACTCTCAG

GCACAAAGGACAATGAACAGTAAGTTAGCAAGAGACTTAGAGGTAGTCCTCGAGGACTTC

CACAAAGTACAAAATCTTGCAGCAAAGCAATACATAGCCCTGGCAATACCTCCTTCCGTT

ACTGTTCCCATACCCACAAGGATGGCACCAAAGAAAAAGAAATGGCTAACAAAGATGAAG

AAAGTAATTTTCTCCTCCAAAATAAAAAATGGTAGCTCTAGTAGAAGTGGTGAGTCTCGT

TCAAGTGTTGGATTCGGAAACTTGGATTCCCAAACAGATGCTACAACAGTGGATACAGAG

GATAACGCTCTCCTGTGGAACCAAAAGAGGTGCGAAATACAGACTTATACCGAGGTAGAC

TTCAATGAAGCAACAATAAAGGAAAATGAGAAAGGGATCAAGAATATCCAACAACAAGTA

GAAGAAATAAATGGAATCTACAAGAACTTAAGCCTCGTAATTCATGAGTCTACTTTATTA

GATGTTGAGAAAATATCAACTCCAATACCCCAGGTTAAAAAACAATTCATACAGAATTCC

CAAGCAATATGGTGTTGCACAGTGCTATTAGTGATCATCGCGGTGCTGGCCATTCTTATT

ATCCTCCAACAACACTGAGTATTAAAGCTAAAAGAATACATGCTTTGATATTTTTACAAT

TTGGTCAGGACAATCCGAACTGCAAATTCTTGTATTCTAGATAACAAAATGTGCTTATGT

TCTCATATATATGTATCATTTTGAGGATG

>Unigene0001756

CAGATAAGAGCAGTTACGGATTTGTCATATTTTAGCATATAAGTAATTATTTTACCCATT

TTAATGAGTATGGCAAACGAGGCGTCTGACCAAAACTATGATGTTATTATTTTACACATT

TTAAAGAGTATGGTAAGGGAGTCATCCTGACCAAATCCGCCCAGGTGTCCTCCATTTACA

CCCAACTGTCATAACAATAATAGCATCATGGTCACACTACGCTAGTGACATAGACTTACT

ACTTTATAAC

>Unigene0001757

GCTATAGTTTGTCTCCTACTCGTCGTTCTCGCTTCTCGGCACGGGCAACTTGATGGATGT

GAAGAATATCTTTCTTCATGGTACACTTTGAAGTCGTTCACATGACACTTTCATTAGGCG

TCACCACTCCTTTCGGTTATGTTCGTTGCTTGCGTTATGTTGTCTACGAATTGAAGTAGA

CGTCTCGAGCTTGGTTTAAGTGCTTTCGGGCGACTGTCTTGCAGACGAGGTTTGTGGAGA

GTTTTTAAGACTTCTCTTTGTTTGTTTGCTCCAC

>Unigene0001758

GCTATAGTTTGTCTCCTACTCGTCGTTGTCACTTCTCGGCACGGGCAACTTGATGGATGT

GAAGAATATCTTTCTTCATGGTACACTTTGAAGTCGTTCACATGACACTTTCATTAGGCG

TCACCACTCCTTTCGGTTATGTTCGTTGCTTGCGTTATGTTGTCTACGAATTGAAGTAGA

CGTCTCGAGCTTGGTTTAAGTGCTTTCGGGCGACTGTCTTGCAGACGAGGTTTGTGGAGA

GTTTTTAAGACTTCTCTTTGTTTGTTTGCTCCAC

>Unigene0001759

AAGCGCATCAGATTTGCACTTCCCCAGCGTGTCTGCAAACCAGAGGTACCTCCGCCACCA

CTCGAGCTCATCCTGGATGTACTCGAGTGCATGACGCATGCTCGAGCGCTCCAAACTCGT

GTTCCAGGCCTGATTTCAAGCACACTCCGCCTCAAACAATTCTTCCCTTTTCTTCTTCTC

CATAACGGCTACACCTCCTGGAGATTCCCTACCTACTCTGCAGCGGGAAAGGGATCAAGC

TGGCCATATTTCTCCATGACGGAGAAGCGAGTTGACAGAT

>Unigene0001760

GTCGTGCCCCTCATAGTACCAATCTTGAAGAACAGTAAATTCACAGTTATGATAGGATCA

GACAAGTTAGCATGTTGATTGCTTAGGCAGTCGTGCCCCTCATAGTACCAATCTTGAAGA

ACAGTAAATGCATAGCTCTGATAGGATCCAGTGCTTTCGCAGTCAAGTTGGCATACCTAA

GTCATTAATGGTTAAGTAAAACTTTAAACAACATAAAGGTACACTTGGAAAGGTGATGTC

TTTTTTACTTCATAGATGTGCAATGATTGTCCCTTCCATTTTT

>Unigene0001761

GTTTGGCTTTGTTTCCTTCCGCAATCAACAGGTCTGTCTAATATCTATAGCCGGAATGTA

GGGTGCCTAGTTAACAAAGTTGGCCATCATTGTGAATCGGTGCTTTTGATCTTATTTAGC

CTTTTATACCTACCAGAACAGCAATCCTTTTCTGCAGATCAATAATTTTGCTTATGAACT

ACTTGATGGTTTTGGAGACACGAGAACATAATAATTACTGAATATGATGGCCAGGCTCCC

TTGGTGTGTGCTCGTCTGTTAGTGTAACTATAAAGGCTCACTTGTAATAGGGGAGACCCA

TGCTACTTTTCCAGAACCTTAGGCTGGCCGCTGGTGTGCCTCATTTCCCTTGAGGCCTGA

AAGCGTTGTTTTCTCCAAGGTTATAGTCTCATGTCTGCCCAGAATAAGATCCATCAACTG

ACGTGAAGGTTTAAAACTTGCTATGTTTTTCCAAATTATCCACCAAAATTCCTGTTAGGC

GCTCCTGCCGTGCTTCAGTCGTTGGTTTCTGAAAATAACCCTGAACCAAGGCGTCACCCC

CCCTCACTTCTTTTTCTGTTAAATCTTCCCCCTTTTAAAATCTGTAACCAGAAACCTAAT

TTGATCACCTCAAACCTTTTGCCTATGAGTTGATCGAAACTCTTAACTCTAATACCATGA

ACTTGATTTTGAAACCGAAAACCCATCTCTTCTTAGCCAGCATTATATGTGCAACACGGG

TTAGGTTTCCTTGTTTTCTAGGAAAATCTTGGAACTTGAAGATGGGATTAGGCAATCGCT

TGAGGGCCATACTGGTACATGCTGACTTCTTTGGTTGCCCTTCTAGTAGTAACCTTGAAT

CTCCACTAGTTTATGTTAAAATATAGCTTATACCCTTTTTAAAATATGACTCCCTTTGAA

TTCTGTAACCTGGGGATCAGTTTGATAACCTTAAACCGGTGGTCTACAATTTTATCGAAA

CTCTTAACTCTCTAATCCTTGAGTCTGATCTTGAATCCGAAGCTTCTCTTCCCATCCAGC

ATTATTTGTTGACCCGAGGATCAGACAAATTTGCATGTTGACAGCTTAGGCTGCTCTGCC

CTTCATAGTACCAATCTTGAAGAACA

>Unigene0001762

TATTGAAACACTTACGGGTACAGCATTTGAAATTACTGTGTGCCCAACCGATTATATAAG

TTCATTGAAGTCACGAATACAACGTGTTGAAGGTATACCAGTCAACCAACAACATTTGCT

CCTAGGGGAAAAAATATTATCTGACCATACAACTATAGCTAGTAATAATCTTCACGATGG

CTCAACATTGCGATTGGTATTATCCTTACAAGGTGGGCCCATTGGCACAAGTCGTAGAAT

GTTACCCTTGGACAATGAAACTATTCGACAATTGGTTAATCTAAATAAAAAAGAAATATT

AGAGGATGCACCTCCAGGTAGTAAGCTTGCAGTCTTAATATTTCGCG

>Unigene0001763

AGGGAGAAGTTACCAAAAATTTAATATGTTAATTCAAAACTCAGAATACATATTATTTCT

TATTTGTAGATGTAATTTAATAGGTACTTGTGACTCACACAACCTTTAATGATTGTCTGC

ATATTGAATTATAAATAATTATTAAAATGCCTCGTTGACCCATTTTTCCGTAACTATGCA

ATACTTGTTGAGTAATTTAACTATTTAATAATTGACTCAAACATTGTTGTTATAGAAATG

GTTTTTAAAGAGCTTTTTACGTGTTCTAACACCAACTTACTAATATAGTTGTGTAATGTT

GACTATCTTCCATTTAAGCAATTTATTTAACTTATTATAAGATACTACTCATAAAATAAT

AATTATAATAATACAATTATAGCATTTTTGGTTGTTAGAATTAAAGTACGGGTAAAAATG

TAATT

>Unigene0001764

CTAGACATACTGGTAGTACTTCCAGCATTGACAGTATTTCAGAATCGGGTACTGATATTG

ATGTGTCCAGCACAGATTGGTCTAGACGTCCTAGTAGTTCATCATTGGTGTTATCCCATA

ATATCCACATAAATGTTTCTAGACAAGCATCACCGACTGAACAAATGACTGCTATGTCCC

TATCCAATAATACGGGATTGGGAGCTATACATAGAATACTGAATAGATCAGACATGCCTA

TTCAACTTGATGATGATGAAGAACCTATGGAATCAGAAAGTAATAGAGTGGAAAAAAATC

TCCAGCTATTGTACCTGAAGATCTGAATCAGACTGTACCTAATCAAGTGGTAGAGAAAAA

GAAAAAACGGTGCGCTCAATGCAATAAAAAACTAACTATTAGCGCTCAGTATACATGTCG

ATGTGGGCTTCTTTTTTGTCCAGTACATAGGTACTCAGAATCGCACAATTGCTCCTATGA

TTATCAAAAGAATGGTCGCAACTCAATTGAAAAGAACAACCCTTTAGTTGTTAGGG

>Unigene0001765

GTGAAGTTATTAACTTACTTCGTGTAATTGAAAATAGTGATGGTTCTTTAACAAATTTAA

AGGAATCCTCTAGAAAATTATCTACAGTAGAATTACCAAAAAAGAAAAAATCTAAGCCAT

CCGTTGAAAATAATACGACATATCTTAAGATGATGCTGTTGAAACATAATTTAGAAACTC

TTTCTATTTCATCAAAATCTAAGTTAAAAAGAACAAAACCTGAGCATCGTAAATCAGCTT

CAGCACCAGAAAAATCACAAATGAAGACTGAAAAAGATAGTAGCAAACTACATACATGGA

GATTACCTATTGTTCAATCAACTCCTGTTATGGGGAAGTATGTTCGTAGTGCTGAATTTT

CAAGAATACCCGTTTTGCCGACTATTGCAAGTACTACATGCTCGGAAAAGAATTCAAAAT

CAACTACAACTAGACAACTTTGCCATATCATGAATGAACCTAGACATACTGG

>Unigene0001766

GCATGCAGCTGGTATGGTTGTTGACGGTGCGGATGCGGGTGGTGCTGGTCTGATGCATGC

ACCTGATATGATTGTTGGCAGTGAGGCAGTGGGTGTTGATTTGGTGAATGCGGCTGGGAT

GGGCGTTGGCTTCGCGGGTGTTGTTGAAGGTGTCGCTGCAGATGCTGGTTTGGTCGATGC

AGCTGGAATGTATGATCCTCAGGTTGGTGTGGCGGGTGGTACTGGCACAGTGGATGCTGC

TATGCAGCATCACATAGATCGTGTCAGTGAAATCATCGAAAGCGAGGATGTAGGTGCGGT

TTTAGATGATTTGCCTCAGCAGGAGGCGGTGGCTGTTGGACCAATGAACCTTGACTTACT

GTCTGTAAATCAACCACCTCAGTGAGGTTCTGTATTTGGTGAGGCTCCTTCGCTGTTGCT

GTGCTTGATGCAAATATAATTTTTCTTCAGACAATTGTGAATTCTGATCTGAGGTTTAGA

CTCTCCAAGGATTTTTTTTTTTTTTTAAAGAATCATTTATTCACTTTGCTCTTTTATGTT

TGCTGCGCGCTGAACGATGCAAATCATCGTGGTATTGCAGTCCGAGAGATATTATATGGT

AAATTGCGTAAACTTTGTTCATTGGATTATATTAAGATGTTATGTTGGTAACTCACTTCA

TTCTGTAATCCTGCCATGGAAATTGAAATCCAATTTGGGATT

>Unigene0001767

TGCAGCATCACATAGATCGTGTCAGTGAAATCATCGAAAGCGAGGACGAATGCGTGGTTT

TAGATGATTTGCCTCAGCAGGAGGCGGTGGCTGTCGGACCAATCAGCCTTGAGGTTCTGC

CTATAAATCAACCACCTCAGTGAGGAAATTGTTTAGTTTGTCCACAGTGAAAATTTTAGT

CCCTCTTAATCAGATTTCTTGGCTTTTCTCAAGGCCTTTTTTTGAGATAGTTATTTCAAG

ACCTTGCATCCAGTGTTGTCAGAATCTTACTATTCACGATCGGAGGCGGCTTTCCTGTCC

AGTGGGGGGTATTTGCCCTCTATAAAATAGAAC

>Unigene0001768

TTCTGTATTTGGTGAGGCTCCTTTGCTGTTGCTGTGCTTGATGCAAATATAATTTTTCTT

CGGACAATTGTGAATTCTGATCTGAGGTTTAGACTCTCCAAGGATTTTTTTTTTTTTTTA

AAGAATCATTTATTCACTTTGCTCTTTTATGTTTGCTGCGCGCTGAACGATGCAAATCAT

CGTGGTATTGCAGTCCGAGAGATATTATATGGTAAATTGCGTAAACTTTGTTCATTGGAT

TATATTAAGATGTTATGTTGGTAACTCACTTCATTCTGTAATCCTGCCATGGAAATTGAA

ATCCAATTTGGGATT

>Unigene0001769

GGGTTAGGGTTTGCCCCCAATTCACCGCCAGGAGATGGAGTTATCCGGTGGCGTCTTGTC

GAGGAGGTCCATCCTCGCCGCTCTCCGCCATCCTCGCCGCGGCATCTTACTGGAGATGGG

GCTGTGAAGTCAGGACATCGATATATGTTTCTCTTGTACACCTCTTCCCATCCATCCTTT

CCCTCTCTCCACCTTGTGGCTATTCATTTACAGAAATTTGCAGAGTCGGACATATCATTG

CTACGGGTGCTCCTCCACCTTTTCTCCTCATACCCCCAACCACGTCTTACCTCAACATTC

TTCTCAACGAGCAGTACATACTCACTTTACTGCCTGGTGGCTGCTTTCCCTTATTCGTCT

ATGTTGTTCTGGGTCATGAGATTGAGAGGGTCCGGGCTGGGCTTCATCTTCTCCCTCTAG

AGGGCGCTGGTTTAGTGGATCCAGAGGGGAATTTTGTTCACCAGAACGAGGTGGTGGGTG

CTGATTTGGTGGATGCGGCTGGAGTCGGTATGGCAGGTGGTGGTCTGGGGCATGCAGCTG

GTATGATTGTTGATGGTGCGATGGGTGGTGCTGGTCCGGGGCATGCAGC

>Unigene0001770

CCCAAATGGCTCGTTAGAGAGAGAGAGAGAGGGGAGGGGGAAATCCATGGTTAGGGTTAG

GGACTGCCCCCAATTAGGGTTAGGGTTTGCCTCCAATTTCACCGCCAGGAGATGGAATTG

TCCGGTGGCGTCTTGTCGAGGAGGTCCACGGCATCCTCACCGCTCTCCGCCATCCTCGCC

GTGGCATCTTACTGGAGATGGAGCTGTGAAGTCAATGGAGCTTCAACGGAGTTTGAAGGC

GATGAGACACTTGGGGCTTCATCCTCATCTTCTCGTTCTCCATCTCCATCGATCTCAGCT

ATTCAGAATTCAAATGCAGAGTTGTGATCGACGGGGATTTGCTGATCCAACACTTGCTTC

TGACATATTAGAGAGAGAGACACTCCCCCACCTGATGCCGAGTGGGGCACTAAGGCGCAC

CTGAGCGACTTTGGAGGATATACTCCCCCATGTGGTGCCATCAGGCGAGATAGCCAGTAG

GCCAAA

>Unigene0001771

AAGAGGCAATTGTATGGATCTAGCTGGGAATGCCTTAATTACTTACAAAGAGTTGGACCA

AATACGTCACTTGTTTATGAGGGGTATATTATCTTCAGATGCTGTAAGACCTTTTGCACT

GGGTTGCCCTTTTATGCTGCAAGACCTGTCCCGAATGGCCCATTTGGTTGCAGTGCTGTA

ACGGCCTGGGATGTCTTTTTTGTTCTCACATCCTGGGAGGTATTATGGGTTGTTGCATGG

CTGTTATGTTGTATGAAAGCC

>Unigene0001772

CATTATCGACTATAGTTTCTGGAAGGCAGACTTGTAAACATTGTCTCTTTCTTACAGCAC

AATATGACTTATGGCTGTGATCGTGATGTATACTTTTAAACTTAGACAATGAGAAACATT

ATCAACTAAAAAATTATTTATATTGACAGATATGTATTTATCAATTTTTATATCATCATA

TCATTGTATTTTATTTATGAACATATACTTAATCATTTCATTGTTATCAAACTGATCAAT

TATATTAATTTTTGATTTATGTTTTTTTTATGTTTTTTTTAAGTGTAAAGTCACCACAAA

CATGATTTGATAACCACTGTTGAAACCACCAACTTGGACATTGACATGTTTTGGTGATTG

GAATCTGTGATACTTTGGAATAACTAGCTATTTTATATTATGCTAAATGTGTGTAACCAA

ACGAATAAGATTCTAATGCAATATTAGTTAATAAGTGCCTACTTATTTAATTTTTTTTTT

AAGTTTTTAATTTATTATATGATTGTACTATGTTGTACCTACCAATAAAGAAATAAAAT

>Unigene0001773

CATTATCGACTATAGTTTCTGGAAGGCAGACTTGTAAACATTGTCTCTTTCTTACAGCAC

AATATGACTTATGGCTGTGATCGTGATGTATACTTTTAAACTTAGACAATGAGAAACATT

ATCAACTAAAAAATTATTTATATTGACAGATATGTATTTATCAATTTTTATATCATCATT

GTATTTTATTTATGAACATATACTTAATCATTTCATTGTTATCAAACTGATCAATTATAT

TAATTTTTGATTTATGTTTTTTTTATGTTTTTTTTAAGTGTAAAGTCACCACAAACATGA

TTTGATAACCACTGTTGAAACCACCAACTTGGACATTGACATGTTTTGGTGATTGGAATC

TGTGATACTTTGGAATAACTAGCTATTTTATATTATGCTAAATGTGTGTAACCAAACGAA

TAAGATTCTAATGCAATATTAGTTAATAAGTGCCTACTTATTTAATTTTTTTTTTAAGTT

TTTAATTTATTATATGATTGTACTATGTTGTACCTACCAATAAAGAAATAAAAT

>Unigene0001774

CATTATCGACTATAGTTTCTGGAAGGCAGACTTGTAAACATTGTCTCTTTCTTACAGCAC

AATATGACTTATGGCTGTGATCGTGATGTATACTTTTAAACTTAGACAATGAGAAACATT

ATCGACTATAGTTTCTGGAAGGCAGACTTGTAAACATTGTCTCTTTCTTACAGCACAATA

TGACTTATGGCTGTGATCGTGATGTATACTTTTAAACTTAGACAATGAGAAACATTATC

>Unigene0001775

GTTGACAGTTTTTCATTTTGGCTCGCCCGAAATCGGTAGACTACCACAAGAAGCGGTTTT

GGATTTCTGATTTCTATTCGATCCGATCAAGTTGACTATACACGAGATCCAATCGAGAGA

AAATTTTGAGCCTAAACCAACAGCTACCTTTTTCCCCGACCAAATACGATATATATACGA

CAATAAAGCAACTCGTTTTCGAATCGCAACGTACCAAGCGCATTATTTATCAGAAATATT

ATTTAAAAAAGTATCAACAAAGTCTCGTTAAAAAATGGCTTTAGAAAAAATCGCCATGAC

CCAGCGAGCGATGGTTGAAGAACAACAAGAACGAATTGGAAACCAAAAGTGGACTCAATA

CTTAGCTGCCTTTATTGCGACGATTGGAGGGTTCATCGCGGGTACTGCCCTAGCATGGAC

AGCTCCAGCCGGTCCAATGATGGAAAACAATCAGTATCCGTTCATTATTTCAAGTGAGAG

TCTCGCGTGGATAGGAGCGTGTATGCCACTTGGTGCCATGCTTGGATGTCCAGTTACCGC

TGGCCTAGTCGATAAATTGGGACGTAAAAACATGATGTTAATGTTGTGCATTCCAACACT

TATTGGTTGGGCAATGATAATATGGGCGGAATCTGTGGCATGGATCTGTGCGGGCAGACT

TTTGACTGGGTTCGCCAGTGGATCGTTGTCTGTAATTGTACCTCTGTATACTTCTGAAAT

AGCCGAAAATGAAATTCGTGGTACTTTGGGTACATACTTCCAGTTACAAGTCACCGCAGG

AATTTTATTCACCTATATCGTAGGATCTTATTTCAATATATTTAGTTTGACTCTTATTTG

TGCTATTATTCCAATCGTATACGTGGCTCTGATGATTTTAATTCCCGAAAGCCCAAACTT

CCATCTCATGAAAGGAAACGTCGAAAAAGCTCGTTTATCTTTGAGATATTTCCGCGGACC

ATATGGCACAGTGGACCAAGAATTGTCTATAATGCAAGACTCGTTGGCTAAGACTGAAAG

GGAAAGAGTTCCGATAATGGAAGCATTCCAAACAACTCCAGCTAAACGCGGTTTGTTTAT

TGGTCTTGGAGTTATGCTTTTGCAACAATTTTCTGGATGCAATGCTGTCATATTCTACGC

TACATATATCTTTAAAGAATCTGGAAGCGCAATGGAACCAAATATGTCCACAATTATCGT

TGGTGTTATGTCGGTAATTGCCACATATATCTCTACACTGATCGTTGACAGATTAGGAAG

GAAAATTCTCCTTTTGGGCTCAATTATTGTTATGGGAATTTGTACGTTGTTAATTGGCGG

TTTCTTCTTCTTGAAGGCTAACGAATATAATGTATCATCAATTGGATTTATTCCACTTGT

GTCCATGTGTGCTTTCATAGTTCTGTTCTCTCTTGGTTTTGGTCCTATCCCATGGATGTT

GATTGGTGAAATCTTCCCGGCACAAATCAAAGGTACCGCTTGCTCGATCGCCTGTATGGC

CAACTGGTTTTTCGCTTTTATCGTCACAAAATTCTTTTCGTCGTTGGTATCTGCCATTCA

TATATACAATACATTCTGGTTATTCACATTTTTCTCCGTGTTGGGTACGTTCTTCGTAAT

TTGCATTGTTCCTGAGACCAAAGGCAAGTCGATGGATGAAATTCAAGAAATGCTCGGCGG

AGGCAACGATCTCACACCACCAACACATGCCAATGTTTCCATCGACACCAAAGGAAAATA

CTAAATTAGACCTTACTAAAGGATTAGAAACACGTTCCATCTCTACCTTCCCGAATAGTA

TTAACATGATTGTATTATTGAATCAATAATATAAATCGTTAATTTATACAAATGAATTGA

TAATTGCATCTGTTGAAAGATGTTACATTTTTATAGACAATTATTTATTGAATAATCATT

AGTGAAATCATTATCGACTATAGTTTCTGGAAGGCAGACTTGTAAACATTGTCTCTTTCT

TACAGCACAATATGACTTATGGCTGTGATCGTGATGTATACTTTTAAACTTAGACAATGA

GAAACATTATC

>Unigene0001776

TCAATGACCCACAATCTTATTCAGACTCGCAGCGATCTGCAAGAACCTAGATCCATCCAG

GTTGTCAAGGTATTTACGACTGCAGCATCTGCAAATGACGAAGGGATGGACAGCCACGGG

CATTCTTTTACCAGAAATAACAAGTCTGGGATTCAGGTCGATGACCCACTATATTATTCA

GACTCGCAGAACGCTGAGAAAACCTCTCCCAAGAATGGAGTTATAGTACGCGGGGTGGAT

GAGCTTTCTTTTTATTGGCAGCGATCTTATTTGAAGCTCTCCGGGTGTGGATTCGCAAAT

TTTTCATGAGTATGTCAGAGTAACGATATGCACGGGCTTGGAGTTAGGAGCAAAGAGCCG

AGGTGATAGTGTTCGGTCCAGGTGTGTTCGGCCCTTACGGACATGCGTTCACGACCGGCT

CTTTATCAACGAACATGATCCAAAAATCCCCATTGTCCCGGTGTGTTTCTTCTTCCCGAT

GTTCACATCTGGTCTCTCCCCGATGTACGGTTCCTAACGCATCCGGCAATCCCGACAACG

GATCCTCAACATGCCCAGGCTTCCTCCATCATTTTCGGCATCGGCAGCATCATCAGCGAC

ATTCAACGACAAGTTCATCGACATCATTGGCCACAACAACAATAGCAGATTTCTCAGCAC

CCACAAACCGTTCTTGGTTTCTCGGCAGCCATATGTTTGTATGTATGTGCCGCATTTTCA

CCCATTGGTGACCACAAGGGCTTTGGT

>Unigene0001777

CCCTACAGTTAATCAAGTCCTTGCTGGATCCGCACCTCAGCCGCCAAGCCTGCATTAATG

CCACTGTTGTCGGATCAACGCCTTCGATTTCGAGCAATGCCCACCTCGACATCCAAGCTG

CAACCCAGCTCCTACATCATCGTCAACAGCTCCCCCACCCATGCATTCATCTACAACTAT

GGTGTCCCCTCACTGACGGATGACGCAACTATCGTGGCAGTTCCGGACCCTTCCTGTCAT

GCTCATCACATCCTCGTCTGGCGTTCAAGGTATCGATATCATAAGAATTATCTTAGGGAG

AAATCAAGGAGGGTAATAGACTTCGGCAGAACCACTTCATCATTGAATGGAAGGAGCCTT

TCCAACACAATTGGATTCTTGAGCTGCAGAAGCCAACAGCAAAGGCAGCAGCAAGATGCA

AGGATCTGGACC

>Unigene0001778

GCAATTTCGAAGGGTCCGTTATTGGTGCTCAAAGCTATCAGGTTTCGGACTTTAGTGATG

GGGAAATGATTGTTCTGGTTGCCTGTTGTGCTCTCTGATGCAGAACATGATCGATGATTC

ACCACGGTTGCGAGGCTCTCTACCAGAATGCATGCCCTTGAATAAGCTATTACACGCAGC

CAATCTGTGAATTCAGGGGTTTTGATCATTCCATGTGGTATTTAGATGGAAGTTTACATG

ATCAGTTGTAGAAACCACATTCTGATATTGTCCGTCTGGTGCGTACAAAGCACAGATCAG

CAGGTATGTATATGCAATTAACAGATACATATATTTTTAGTTCTTGGCTTTGTAGGAGTT

AGT

>Unigene0001779

AAATGGTGTCGAAACTCAGCGGCTAGTGCGTGTGTTATTAGAAGGACATGTTACGGTTTT

AATGTTTAGTTGGAGCTTTTCATCATTTATTTAACTGCTTCGGATTTTAGAGCTTTCCAG

ATTGATCTTTACGGAAATTCTTGTATCAATGCCCTTACAACAGTTGCCATCTTCATCAGC

TATCAGGTTTCGGACTTGAGTGATGGGGAAATGATTGTTCTGGTTGCCTGTTGTGCTCTC

TGATGCAGAACATGATCGATGATTCACCACGGTTGCGAGGCTCTCTACCAGAATGCATGC

CCTTGAATAAGCTATTACACGCAGCCAATCTGTGAATTCAGGGGTTTTGATCATTCCATG

TGGTATTTAGATGGAAGTTTACATGATCAGTTGTAGAAACCACATTCTGATATTGTCCGT

CTGGTGCGTACAAAGCACAGATCAGCAGGTATGTATATGCAATTAACAGATACATATATT

TTTAGTTCTTGGCTTTGTAGGAGTTAGT

>Unigene0001780

AAATGGTGTCGAAACTCAGCGGCTAGTGCGTGTGTTATTAGAAGGACATGTTACGGTTTT

AATGTTTAGTTGGAGCTTTTCATCATTTATTTAACTGCTTCGGATTTTAGAGCTTTCCAG

ATTGATCTTTACGGAAATTCTTGTATCAATGCCCTTACAACAGTTGCCATCTTCATCAGC

TATCAGGTTTCGGACTTGAGTGATGGTGAAATGATTGTTCTGGTTGCCTGTTGTGCTCTC

TGATGCAGAGCATGATCGATGAT

>Unigene0001781

TATGGAGGGTATAATGTTTGTAATTGGATCCATACGTGATAACATTGAGAGAGGAATTTT

ATCTAAGATGTTTATACATTTACTAGGAAAGTGATGACCAGGGTCTGCAATCTAAAGACC

CTGCAAACTTTAGAATGAAAAGTATACTCACACTTGAGCAGGCAGAGTAAAGCATGTCAC

ATACTCGAATACTAATTTATAATCAATTTAGTCATTTATAGATGAAGGCACTCAAATGCA

TTATAGAACAACGATCACACATCAT

>Unigene0001782

TATGGAGGGTATAATGTTTGTAATTGGATCCATACGTGATAACATTGAGAGAGGAATTTT

ATCTAAGATGTTTATACATTTACTAGGAAAGTGATGACCAGGGTCTGCAATCTAAAGACC

CTGCAAACTTTAGAATGAAAAGTATACTCACACTTGAGCAGGCAGAGTAAAGCATGTCAC

ATACTCGAATACTAATTCATAATCAATTTAGTCATTTATAGATGAAGGCACTCAAATGCA

TTATAGAACAATGATCACACATCATGTTTCATATACATGGAGAAATGTTTATATGCACTT

TGAATTTAAATTATTAAGATATCTCACATACCTGATTACTTTTAGCATTTCTAGTGATAC

TTAAGACTCTTGATGTATTTACCTACCTTTTCTGTTCTCTACTTTGTAGTTAAATATCTT

TTCCTCT

>Unigene0001783

GTTGCAGTCTGGAGTTTGACAATTCTCATTTTCTTTTATGAAGAGCTACAATCAACTTGA

TCTTTTTGTCGTCGTCGCTACGTAGGCAAATTTTTTTTACGACTACAGGCAACTTCCGAA

TAGTTAGTTGAGCTGTGAAAGCTTGGGAAACCCATTGAGAAGCTTGAAGGTAAAGGCAAG

GGCAAGGGCAAAGGCAAAGACTAAAATTATCAGATGCAGATTGGAGCCGAAG

>Unigene0001784

ATTGGAGCCGAAGAACAAAAAGGACAGAATGTTTCGGCAGGCTAAACCCTCTCTGTTGAG

GCAGGGATTTCATCCCTGCAGATTTTCACCAGGCGAATATCTCAAAGGCGAAATGATTTT

CGTCTTGTGGGTTCTCCTTTTGGGGCTAATGGGTCACCTTGAAGATTTTTTTTATTGTTG

GGACAACCATTAGGAAGTCAAGTCAAGAGTGAGCAAGTCAAGAGTGAGCATAAAGCCTCT

CTTGAAGATCTACTAGATATAGAAAAAGACGAGCTTCGTACAAACCTTCCTTGAAGATGT

AGAAAAGTGAAGCTTTGAGATGAAGATTAAATATTGGCAATAACTTAAGAAGATGGTTTG

AAA

>Unigene0001785

GAATGTACAAGTATTCATAAAGATATGACGATGTTTGCGCTTGAGAACACCATTATGCTC

ATGGGTGCGAGAACAAAATTATTGAAAGAGAGTATCATGAGACTGAAAAAAGAATACACG

AGGCATGATAAATACTCATGCGCACAGTCAGACCAAAAAATCTTGATTTGAGTGTGAAAT

GTAGTCTGAACCATAGCAGTAATGCCTCATAAGCACGGAGAACCTTCGATCGAAGACAAA

GGAAGTACTCCCTAAGTGTAAACCGTCAAGGAAGCTCATTTGTAGCGTAAGTCAAGCTGA

CGAGGCATGATAAATACTCACACACACAATCAGACCAAAAAAATCTTGATCCGAGTGTGA

AATGTAGTCTGAACC

>Unigene0001786

CAATGGTGGGATCAGGATATGAAATGATATTAGAGCGACGAAAAGCTAAAGAATTCAAAA

AAAGACACATAGCTCAAGGAAATAATAATGAAAGTGATTCAAAAGTAGAATTTACTTTGG

ATAAAATGCATGCCATTGAAAAGATGCATAATATGAAGGGTTGCGGAAATGATGACGGAA

CCTTAGAAGACCAAGACATGTTATTCAAAGTCAGCCGAAACGAACAAATTCTATTAGCTT

TTTCGGCTGTGAGCAATGGCCGAAAAATTTTACATTGTGGACCGGCGCCAGCTGAATCGT

TAACTTGTGTACACGGACTTCGATTTTTAAGTTTGGCTTGGGTTATAATGGTGCATACGT

ACTTACAAGTGTTTTCAATTGCAGAAAACAAGACGTTACGGACATTGACCGAGAGGAACT

TTATGTTCCAAACAGTGAGCAACGCAACATTTTCCGTAGATACTTTCTTTTTTATCAGTG

GTTTATTGGTTACATATTTGTATTATAAGACGACCAACAAAAAAGACGGGCCAGAAGTAG

CTGTATCTCGAACTCCGAAATGTAGTTCTTCTATTCTGAAAAATGATACAATTAAATTTT

TAAAACTGCTGGGTTATAGATTTATCAGATTGACGCCAGCGTACATGGTTGTCCTATTGA

TGGCGGAGGTGTCTAGCCGATGGCTTCGAAACATTTCCGTATTTGAACCAGAGAACAACA

ACCACATAAGTTGTGCTAATTATTGGTGGAGAAATATGCTATACATAAACAGCTTGTATC

CCAGAAGCGAAATGTGCATGTTGTGGAGTTGGTATATGTCCAACGATACGCAATTCTATG

TTTTAGCAGTCATACTACTTCTCGTTTCTGCCAGACATTTCAAAGGAGCAGTTATTGGAT

TGGTGGCGTTCTTAATCGGATCATGGGTCGTCACAATATTCATTGCAGTACATTACAATT

TTAGAGCAAGGATAGAGGAACCATTTGGATTATTTGATCAGCTATATGATAAACCATGGT

CTAGACTTGGACCTTATTTATTCGGCATGTTCGCTGGATGGTTTTTGTTTGTTACTAAAT

GCAAATTGAAAATGTCCTTCCTAACGGTCGCTATTGGATGGTTCCTGTCGCTGTTCAGCT

TGGCGTACTTGGTGTACGGACTTCACGTAGTGCACTTGGACCTGGCTGGTTCCGCTCTGT

ATGCATCCGTAGGACATTCGGCTTGGGGTGCCGCACTCGCATGGATCGTGATCGCCTGCT

GCACTGGTCACGGGGGTTGTGTTAACTCAGCGTTATCATGTAGACTGCTTCAACCCCTTA

GCCGTCTGACGTATTGCGCCTATCTGGTACACCCCATCATCATGAC

>Unigene0001787

GTGTAGAGGGGGAAGTGAGGAGATAACAAGGGTGATTGGATCGGGAATCGAAGGTCTGAT

GTGTCAGGAAAGCCTCCGAGAAGAACAGGCTATGCTGCTAATTTATTTTTGATTTTTTTA

TAGGTCGGTACTACTCGGGTCTGCTCAAACAGACCTAAAAATGATCAAAATAGAGAAGGG

AAGCATATGGAGGGACCTTTTGTAGATCCCTAATTTGGAGGAGCAAACCTAAAATGGGG

>Unigene0001788

ACTTTATTGTTTATCTGTTTCTATTTTTTTTATTGTAACCATCTTTGTGGTTGAAAAGAC

TGATCTGGATCGAACCTTATACGTGTTGGATCGTTCTACATCTACCTTTTGATAATTATG

AGGGTAGGGGAAGTTCACAAAATTGCCTCCTAAGGTGGAGGAGATTAATCCCTCTACTTT

TATGCATGTAGACTAGTAGAGAGTAAAAAACAAATAAAAAAGTTGGCCTTTGTTTCTCTC

TCTTCAAGGAGTTTTAGTTGACTACAG

>Unigene0001789

TGTGTATATGGTACATATATGTATGGGTTTCTTCCCTTTTTCCTTGTACTTTAGTTGAGT

TGATTTGGCTATGGTTATACTTTGCTTGCATTTTACTTCCTTCTGCATTAGTTGTGTTGA

CTCGGCTATGGTTCATAAATAATTTGCGTGGAAGATAGGAGATTACAGTGTGGTTTACAT

CACCTTTTGGCTTTCACTCAGATTCATCTCACAGCTGTGTCTAGTTTCCTCCTATCAGAG

TTTCTTAGATTGTATGTGGGTCTCTGATTTTTGAAATGGGCTTACTGATTCACTGTACGG

GTGATAGTGGTTGTGGTCAATATTTGAATAGTTTGGTCAATACTTAGTTTTCGTGCTTGT

CTAGTTTCAGCATAATATTTGACAGTTTCAAGATTGAAGTAATGAATATCACCATGTCTG

TGGAGGATCAACTAATG

>Unigene0001790

TAAATCGAAGATTTTAAAAAATTTATTGAATAAATCGGAGAATCTGGTAACTGTGTATAT

GGTACATATATGTATGGGTTTCTTCCCTTTTTCCTTGTACTTTAGTTGAGTTGATTTGGC

TATGGTTTTACTTTGCTTGCATTTTACTTCTGCATTAGTTGTGTTGATTCGGCTATGGTT

CATAAATAATTTGCGTGGAAGATAGGAGATTACAGTGTGGTTTACATCACCTTTTGGCTT

TCACTCAGATTCATCTCACAGCTGTGTCTAGTTTCCTCCTATCAGAGTTTCTTAGATTGT

ATGTGGGTCTCTGATTTTTGAAATGGGCTTACTGATTCACTGTACGGGTGATAGTGGTTG

TGGTCAATATTTGAATAGTTTGGTCAATACTTAGTTTTCGTGCTTGTCTAGTTTCAGCAT

AATATTTGACAGTTTCAAGATTGAAGTAATGAATATCACCATGTCTGTGGAGGATCAACT

AATG

>Unigene0001791

GGCGATGAAGGGAGGACAGCTCTGGCCGCGTCTGTTCCGGCGATGGCGGCTGAAGGCGCC

GCCCGCTTCGGTGACCGATCCAGCTCTTCAGCCGATCGCACTTATGTCGCACTCGCGCGA

CGGTGCCGAGCACAATAGCTTTTTATGGAAGTCTCGTTCTCTCTTAAGATCCGCGCTTCA

GGCTAAGGCTGATCATCTGAGAAAGTAAGTTCTCTTCCCTCCCGAGTATTCATGCCGACG

GATTACATCGCAAGCATTTTTTCGTTGATACGCTGTGTTTTCTTAAATTTCCGAGTCTGT

ATCTCGGCCGGTTTGAGATACATAGTATCGATCCAGAATATGATTAATGATTATGGATAT

GCCACTAGCTGTTGTAATTCCTACAACATCCTACTCTTTCAGATGATTGTATGCGTGGTG

AGGTCTCTGTTGTAATCTTTGATTTGATTGGGATATTA

>Unigene0001792

TGCTATTGCCGGGTCTAAGCTTATAAAAAGGATTCGGTTGATGTCGTTTGACAAGGCTGT

TCATATGGAGGTTGGGTGGTTTGACAAACCTGAGAATTCCAGTGCAGCAGTTGGGGCGAG

ACTTTCATCAGATGCTGCAACAGTTCGGAATCTTGTTGGTGATGCACTGGCACTACTTGT

TCAGAACGTCTCAACTCTCATAGCCGGTCTGGTTATTTCTTTTGTTGCATGCTGGCAGCT

GGCTCTAATCATATTGTTAATGATACCGGTGATGGGTCTTAGCGGCTGGATCCAAATGAA

ATTCACGAAGGGCTTCAAT

>Unigene0001793

GGTGAAGGTTCGGGTTTCTTCTTATCAAATTTGCAGTGTTAACTCGGACTAAGAATACTT

AAAATTGCCCACTGCAGTAGAAATTCCAGACACTATACCAAAGGAATTCAGTGCTTTGGA

GCCGCCACAAAGACAAAGAGGTACCTCACAGCCATCTTGCTTACCTAAACAAGCCAGAGC

TTCTAGTTATCCTGCTGGATGTAATTCCCACAATAGCTAGTGGTTTGATTCCCGTTTTCG

AGGCACTTTTGGGCACTATAATCA

>Unigene0001794

GGCACTTTTGGGCACTATAATCAACACACTCAACGAGCCACCCCAAAAGCTTTGAAATGA

TTCAAATTTTTGGTCACTAATGTTATTGGTCTTTGCTTTCGCAACCATTGTGTCCTTTCC

AACTAGAACATACTTCTTTGCTGTTGCTGGGTCTAAGCCGATAAAAAGGATTCGGTTGAT

GTCATTTGACAAGGCCATTTATGGAGATTGAGTTGTTTGACAACCCTGAGAATTCGAGTG

GAGCAGTTGGGGCGAGGCTTTCGACAGATGCTTCAACAGTTCGGAGTCTTGTTGGTGATG

CGCTAGCATTTCTTGTTCAGAACATCTCAACTCTCATTGTCGGTCTGGTTATTTCTTTTG

TTGCATGCTGGCAGCTGGCGCTAATAATATTGTTATTGATACAGGTGGTGGGCCTTAGCG

GTTGGATCCAGATTAAATTCACGAAGGGCTTCAATTTAGATGCTAAGGTTTGGCCATACA

ATCCAAATTGGAACATTAGATATTCATGATTGCTTTAGTTTACTGTGTCCTAAAATTGAA

AAAGTCACATGTTTCCTTAACTTTCTTTCTTATGTGTTACAAGTACTATAATGTAATTGT

TTTTGTTGATAGAATCTTTGCAAATATTTTCTACGTAAGTTACAGGATTATGTACATATA

TTTTGTGAAATCATTAGCTATCCCCAACCAGAATTAAAAATGTTTATGCACAGCAGTAGA

GTCTAAAGAACTAAATAGATGGAGAGACCCCCGGTCTATAAAAGAAAAGCAAGAGCCGAA

GTAATTCTGGATCCACGAGTCCCATCCATCACTAAGGCCCAAAGAGCGAACAAATTCAGA

ATGTTTGAGGTGTTCATCTCAGTCGAGTCTTTGTTATGAAAGATGTGACTATTATGTTCT

TGCAAAAGATTGCAAAGAGAGTCTGCTGGCACCACATCTCAAGGATGGAGACTACACTGA

AGACTTCTCTTTTGGCTTGTCTCCCAAACCAAGAAGGAAGACAAAGGCAGTCTAATCTCA

CTTTTTACCAGAGCCGTCATCCAGACCCGGTGAGAAAATGGACAAAAGAACGAGGTGTCT

AGCAGATTTTTCCGAAGAATGACAAATTCTGCAGGAGGTATCACCCTCCCAACCAAAGAT

GCATTTGCCAACAATTGTCTGTTCTTCCGATGACGGAACAACCAATTGAAGATTTTGACC

TTGAAAAGGATGGGACCTTTCCAGATGAAGCAGGAATATGGACAGCGAATAAGACCGTCA

TTCAACAAGTCGCAAAGAGAATGAACCGAAAAGGCTCCATTAGAAGTAAGCTTTGACAAA

TGAGAATCTATGCAGGGTCAAGTGCAACAGAGAGCTCAGATGAAGTGTGCAACAGAGAGC

TGAGATAAAGCGCACACCTCACGTCTCTTAGATTACGAGGCAGTCTGCCGGAAATTTAAG

TCTCCAACGACTGTTCTTGAAGTTAAACTGGGAAGCAATATCACAATCTTTCTCAAAAGC

TGTACCTGGAAGCCAGGTGGAGTCACCAACACTAGCATCAAGCCAGAATCAAATTGCTTC

CCTTTGGACACCGAAAAGATCTGCATGTACGAAAGCTCTCCATAACGCATTGTAATCCCC

TTCCA

>Unigene0001795

GATGAAGAATGTGGACAAAGAATTTGTACTTTGAACGTACCTATTGTGCATGTGTCTATT

ACCTAATTTTGACTGGTGTACTGTTTATGGATTATGGAGTGGTGATTTATGCCATACATA

AGAGTAGGCTGTCATATGGAATGAAAATTCCAAAAATTCCGATATCTGAACTGAAGTTTT

CTTACTTTGGCCCCTCAGGCGGAGTTCTGAATTCTGAAAAATCCGATTCAAAAAATTTCT

GAAAAAAGTTGGAACGGAATCGAAACCCTTTATATATTTTGAAATTCTGATTTTTCTGAT

TAATAATTTCAGAGAAGTACATATTTTCAACTCCAAAAATGTCTTGACTCT

>Unigene0001796

CTTCCACCATACCCCTCATCCCTCCTTTCATCTCCTTCCTCACCAACTGCCCCGATCGTG

CCACCCTCCACTCCACTCCCTTGACTTTGATCTCTGTCTGCTCGACAAGAAACTCTATAA

GGCGTTGAGGGGGCTTGCTGGGAACGGCAGGGGTAATTGGTTGAAATGCTTGTGCCCCAG

CAGCCGACTTCATGTAATGGCGGCTGCCTGGGGAATTTGTGGATGGCATGAGGGGGTGCG

GGAGAGGGGCGGTGGTTGCCGGTAGTGAGAGAGGAGGTTGTTAAGATGCCGCAGATGAGA

GGTGGGTTGTTGAGGATGATCACAGAGATGATGAGGTAGCAGAGAAGCGATTGAGATC

>Unigene0001797

TCGTTCCTCCTTCCATCTCCTTCCTCACCAACTGCCCCGATCGTGCCACCTTACACTCCA

CTCCCTCGCCTTTGATCTCTGTCTTCCCGACAAAAAACTCTACAAGGCGTTGAGGGGGCT

TGCTGGGAACGGCAGGGGTAATTGGTTGAAATGCTTTGCCCCAGCAGCCAACTTCATGTA

ATGGCGGCTGCACGGGGAATTTGTGGATGGCATGAGGGGGTGCGGGAGAGGGGCGGTGGT

TGCCGGTAGTGGGAGAGGAGGTTGTTAAGGTGCCGCAGATGAGAGGTGGGTTG

>Unigene0001798

ATTGATAGAGAACCAATTAGATACAGACATCTTTAGTAGAAATATTAGGTAGCACCAATT

AGGAGGGTCTCATTTATTTATGGAGTTTAACAGTGGGGATGGACCTAAAATAACAAAAAT

AAAAGTTGTTAGAAAAGGTACGGAAGAGCTTGAAGCGAACAAAAATGATTCGGAATTAGA

GAGAATGTCAAGGCATGATTTTGATTTGGTATTGGTAAGACGATCCTTGGTAGTTTGCCG

AGGCTAATGGTTATTGTTATAATTGTTGTGCCAGTAGAAGATGGTGCCATCATAATCCTC

TATTGAAATTAGGGTGAAGATGTTGTATAAAACAGATAACTTCCTTTTTTATTTTTCTGT

TTGTATGTGGTGGTGGTAGTACTTAGCACCAGGTTATCTGATTTACCCTTCAATTTCAGC

AAAAAAACTTAAGCAGATTTTTTT

>Unigene0001799

CAATACCCCAACCGCTTTGACACACCTTGATAAGGAAACTCGTGTGTTAGGCCACATTAC

TTCTCATACCCCCTCAACTCAAATTCATGACCCCACACTCTCCATACTGCCATCATAACC

ACCCATTATGGAGAAGACCTGTCAAATCATTGGCGATACCATACCCCTAAGTAGATGAAT

CCCTAACCTCCCACTAGAACCTCAACCCCTTTGGATTCCATACCAAAAAATATAGGGTTG

AGGAGGCGGCTCTTGCCACTTGCCCCTCTGCCATCCTTCCCTGCACTTCCAGATGTCATC

CTTCCCTGCCATCCTTGTTATGTCGACGAGCTCTCTACT

>Unigene0001800

ATAACCACCCATTACAGAGAAGTCCTATCAAATCACTGTCGATACCACATCCCCAAGTGG

ATGAATCCCTAACCTCCCACTAGAACCTCAACCTCTTCGGATGCCATGCCTTAAAAGGAT

AGGGTAGATGAGTCGGTTCTCGCCACATGCCCCTCTACCTCTATTCTCTAGTTACTTGCA

AGCAGATACAAAATTGGTTTCTTTCTTCATCTAATATCGATGCATCTCTCCCTTGAATCT

GTGGATGCAGACATACCTCCAAACCAATTAAATTTGTCTTTTTTAT

>Unigene0001801

TCAACACCACCTACTTCACGTTAGAACAACTGTAACCAACCACTATACTACACGGACATA

CATCGATATCATCACCACAATAATCACCTTTCAAGAGGCAAAGACTTCAACACTTTACGG

TCTCCCCGCTCCAATCTGACCATCGTTGGTCAAGATTGAGAACCAGGGAGCCACTATACA

CTAAAGATCCATCCAAAAGGAAATGTTGTCTCAGATCCTTCTATGCCCTGCTTAAAGGCC

GAC

>Unigene0001802

GCAGATGATATGGCATTTTTTTTTACGACAGGGGGTATCCGGACGGACCCCAACTAGTCT

TTCCGGGTAACATTGCGTGAGATTCAAGGAAAAGATGAAACTTGGGTGGGGATTCACAGG

ACAATATAGTAGTTCGAATTCGGCCTGATACATCTGCTGCCATGTGTACTTTCATATCCA

AGTCGAGAGGCGGATCAAGGACTTCTTTAGTTGATAAGTTTGGGGGAGGGATTGAGATCA

TTGGTGTGCCATTTCTTATGGTAGATTCCGAACTTC

>Unigene0001803

GCAGTCCTATTAGTCAATCCATGATGTTAGACGAACTAGCCATCGATTATTCCGAGTACT

TAAAGGTCAACAATGAAAATGAGGTTAGCGCTCTAAAAGATGTAGTTGAAGACATGCTCA

CCAGATTGGAAGAGTTTCAGACATTTATGGAAATGGTCCGTGCCCTACGAATGGAAAGTA

CTATGATGCATTATGATTCAATAAAAGCAATGAAGTCCAAGGTTACTGAACTCACAAACT

CTGTAAACAAATTAGAAAAGCTGGTTAATAAAGTTGGTGAAGATGTAGAATTGGTTGATC

AACAACTAACTGAAGCTGAGGCTTGTATGCCTATTAATAAGGATGGTCCACTAAATACAA

TTCTGAAACCTTTTTTTAAAAAACAAGAAGACCATTCTTCTAAACCATTGCCTGTCTATG

AACCACCAGTAATTTTTAAGTCTGACGATTACTTTCTCGCAACAGAAGCAGAAGATGCAC

TTAAAATAATTGAAAATGATACTACCACCGATAATTAACTGAAAAAAATTAATACTTCTT

TTTATTTTTAAAATCATGTATGA

>Unigene0001804

CCATAACTACTATAAACTGATCAGTCATCACCATGGTTCGGCCGCAACAGAGATATCGTG

GAGTCCGACAACGACACTGGGGCTCCTGGGTCTCGGAAATCCGCCACCCTTTACTGTAAG

TCCTCTGCCAATCTCCTCTCGATATTCTGTTTCAGTGCCCTCATTATCAATCATTGTCTT

TATGTTTTCAGGAAGACGAGGATCTGGCTGGGCACGTTCGAGACAGCCGAAGATGCAGCC

AGAGCCTACGATGAGGCAGCGAGGCTGATGTGTGGCCAATTGGCGAGAACCAACTTCCCT

CATGACACGAATGCCTTACCGTCATTAGGGTTCAAATACTTGTCGGCGAAGCTAATGGAG

AAGCTGCACCAGTGTAACTTGGCATCACAACAAGGTGGTGAAGGGCGACAAAGTTCAGTG

GTGATCGATAGTCCGTCGGTGACTGATGGGGAGAAGGAAATGCAGTATTTGGAAGAGGAT

AACATCAAGCAGATGATTGAGGAGTTACTGGATTCTGGGCTTTCTGTGGAGTTATGTTCT

ACTGCTGTGTAGTCTTGGGTGGAGGGGTTTTCTTACTTACTACCCCAACTAGCGGCTTAT

TTATGAGTTACTCCCAAGCTACAGTATTTAGACTTGTACCTGGTTTAGTTTTCATGGTGC

TAAAATTAATTAGAAAGTACAAAATATATATTAGAGATAGAAGAAACCAATACAAGTCCT

TC

>Unigene0001805

ATGGGTTTATTACATTAGAACATGAAGTATCTTGTCTTGACGTTTGTTCATTTCAAGATC

AATTTTCTAAAACTATAAGTTTGGTTGCTGTTGGTTTATGGATGGACATATCTGTGAAAG

TACTTCAACTTCCTGACTTTGTAGAACTTGTTCGAGAACCTTTGGGCGAAGAAATCATAC

CTCGATCAATATTAATGGTAACATTTGAAAATATTGACTATTTATTGTGTGCGTTAGGAG

ATGGAAGCTTATGTTATTTTCATTTGAACCCTGAAAATGGGGTATTAAGTGATAAGCGCA

AGGTCAATCTTGGTACCCAACCTACTTTAATCAGAAAATTCCAATCACTCAATACTACAA

GTGTATTTGCATGTTCAGATCACCCAACTGTAATATATTCATCAAATAATAAATTGATTT

TCTCAAATGTTAATCTGCGCAAAGTTAACCACATGTGTTCACTTAATACTAAAAGTTATC

CAGATAGTTTGGCTATGGCAACTGACACTTCTATTATTATTGGTACTATTGATGAGATGC

AAAAACTACATATTCGTACTATTCCTCTCGGAGAAGCACCTAGACGAATTGCACATCAAG

AGTCAAGCAAATCATTTGGTGTAATAACAATGAGAATAGATGTTCATGAAGGAATAAATT

TAGTCCCTGCTAGACCTAGCGCTAGTACCACTGCTCAGAATACTTCTGGTGCAATTAACA

GTCGTTTACCAAATAGTACATCCGTAGCTAATAGTAATCAGGGACCATTAAGTCTTGGA

>Unigene0001806

ATGGTCTTGAAGTTGAAATTCATAATATGCTTGTACTAGATCAAAACACCTTTGAAGTAT

TGCATGCACATCAGTTAAATTCAAATGAATATGCACTAAGTATCATATCAGCAAAATTGG

GTGATGATCCAGCCCCTTATTATATATTGGGAACTGCCGTTGTAAACCCTGAAGACCAAG

ATCCCAAATTGGGACGCATACTTATATTTAATTGGGATGATTCTTCATCAAAACTTACAC

AAATAAGTGAGAAAGAAGTAAAAGGTGCATGTTATGCTATGGCTGAATTTAATGGAAAAC

TTTTAGCTGCAGTTAATTGTACGGTTCGATTATTTGAATGGACTGCTGAAAAAGAATTAA

GACTTGAGTGCAGTCACTTCAATAATATTGTAGCACTATTTGTGAAAACTAAAGGAGACT

TTATTGTTTGCGGTGATTTAATGAGATCATTAACATTGTTACAATACAAAACTATGGAAG

GCAGTTTTGATGAAATAGCTAGAGATTACAATCCAAAATGGTCAACAGCCATAGAAATTA

TTGATGATGATGTATTCTTAGGCGCAGAAAATGATAAAAACTTATTTATTATACACAAAG

ACAGCACATTGGCAAGTGATGAAGCCCGACATCAACTTCAAGAAATTGGTCAATTCCACT

TGGGTGATCTAGTAAATGTATTCAGGCATGGATCATTAGTAATGCAGCATTTCACTGATA

CTCATGTATCAGTTCAAGGAGGTATTCTTTATGGTACTTGCAGTGGAGCTCTAGGGTTGG

TAACGCAGTTGACTCCCAAAATGTATGAATTTCTGACCGATTTGGAATTAAGTTTAGCTA

CAGTAGTTAAAGGCGTAGGAAAAATTAAACATCAACTTTGGCGAAGCTATCATACTGAAA

CAAGGATTGAGCCTTCCGAAGCATTTGTGGATGGAGATTTGATTGAAAGCTTTTTAGATT

TATCTAAAAAAAATATGTTAGCAGTTGTTAATTCACTTCAGGGATCCTACGATCACGATT

TCAAGAAAATTCCAAAAGATACAAAGTTAACTTTAGATGATGTCATTAAACTGGTTGAAG

ATTTAACCAGAATACATTAATTGATAATTAATTTCACATTTTATGTACAAATCATGTATT

ATACCTTCCTTTGATGATCTAGTTTATTCATTTAAATAATATTACTACAAATAGTTTTTT

CATAAGTTAATATATTTTTTACTTAATTTGATGGCCCTATTTTATGCTATATCTTAAAAA

ATAAACAATCTATTTATA

>Unigene0001807

GGGGGAGAGGTTGTTAAACTGATGTTTGATCTGGTGAGGTACTTAGATAGTAGGGAGAGG

TCATTGTACTGATGTTCCAAGGGGAGGAGCCTGGTTGGTTGTTTGGATATCCATTCCTCT

AAAATTCTCATTTCTTAACCTTCACCTATTAGCCTGGTAAATGGCATAGTTGTGTAAAGC

CTTTCGTGAACTAGTTCTAGTTATAGAACCCGGTTCCCATGTATGGTTAAAACACATTAT

TCGGAGATTTACCTGCCACTATTTGAGCTTCCATCTGTTGCAGTTTGGCATTTTACACTT

TCCAATTTTTGAAAAGTTAATCGATCAA

>Unigene0001808

GAAACAATTTGTTTACTTGTAGATCTGGTAATTCTTTCATTCATTCTTTGGCCTGCCTAA

AGAGAGTTTGGATTGTAGCTTCCTCTTTTGGCCTTCACATCTGGAAGGGATGATCTTGTG

AGGTCCACCTCCACACAAAGTACCGTATGAAAAATATCGGTAGCAATCCGCAAGATTAGT

GCCAAGGGTCTTGTTTGAGTGTTACCAACAAAGTAAACCGTAAGGTTCTTCCATAAAGAA

GAAGAAGAAAACACTCATTAAGCCACAAGACATGCAATTAGGGTACTCCAACCAGGGAGG

TGTCTTCAAATGTTCCAAGGGGAGGAGCCTGGTTGGTTGTTTGGATATCCATTCCTCTAA

AATTCTCATGTCTTAACCTTCACCGATTAGCCTGGTAAAGGCATAGTTGTATAAAGCCTT

TCGTGAACTAGTTC

>Unigene0001809

GATGAGGACGACCAATGAGGAGAGATCGAGATGTGGACAAGGACAAATCCAATCACACAG

GTCTGTTGCTCCAATTGCCAGTCTTTGCCGCTCCGATCGCCAACCCCCTCTGCTCCAATC

ATGAACTACCAGTCTGCCACCGTTCTGCTCCGAGGTCGCCTCTGCTCCCTATCGCAGATT

ATCTCGGTTCCATCATTGGATAACCATTTCAACTTTCCGACAATCCCCTTTTAGTTAAGG

TTTAAGTTGGTGTTATGGTAC

>Unigene0001810

GATTTACAATTGAAAGCAAACTGAAGTCACACATCAAGGCTCACCAACAAAAGAACATGG

CTGTGAAAGATACCCCGATGACAAACAAATTCAATAATGTGCCAAAGACTCATTCTGCTG

CCAATTATCGGTCTGCTTCTGTTGCACGCCCGTATGCCTGTCCGTATGAAGGATGTAGTA

AAGCTTACATTCATGAGTACAAATTAAATCTCCACTTGAGAAACACACATGCTGGCAATG

ACAGCAAGTCCTCTCTGGTCACCACCAACCACGCCGTTGCTCAAGCAATTGATCGAGATG

CTCACTGTGCCAATCTCAAGAGAAGCAATCCCATGACGACTCATATGATGCCTCCTCCAA

AGCTCCCAAACAGAATGGGCTCGAGTTTCAATGCTGTCAACAAGCAATGGCCAGCAGATG

AGATGTATGAGGATGACAGCAATGGATATTATGAGAATGCTGAAGTGGGC

>Unigene0001811

GCTGCCGTCCATGGGTATAGAAAACATGCTGCCGTCCATGGGGAAAGGCAGTATGTTTGC

CAATATGAAGGTTGTGGAAAGAGATTCATGGACAGTTCCAAGTTGAAGAGACATTTCCTA

ACTCACACAGGAGAAAAACCTTTTAAATGCTCTTTTGAAGGCTGTGGTAAGCCCTTCGCC

TTGGATTTCAACTTAAAAGTGCACATGAAAACACATTGTCCGGAGAACTATCATGTTTGT

TCCTACCCAGAATGCGGGAAAAGGTTTACACATGAAAGCAAATTGAATTCACACATCAAG

GCTCATCAACAAAAGAACATGGCTGCGAAAAATACCCCAGCGGCAACAAAACACAATAAT

GCGCCAAAGATTCAGGCTACCAATTATCGGTCGTCTTCTGTTGCACGCCCGTATGCCTGT

CCATATGAAGGATGTAGTAAAGCTTACATTCATGAGTACAAGTTAAACCTCCACTTGAGA

AGCACACATGCTGTAGAAGATCCCAGCAAGTGCACTCTGGCCAGCACCGATCATGCCATT

GCTCAAGCGATTGATCAAGGTGTTCACCGTGCCAACCTTGAAAGAATCAATCCCGTCACA

ACTCATATGATGCCACCCCCTAAGCTCCCGAACAGAATGGGCTCGAGTTTCAATGCTGTC

AACAAGCAGTGGCCAGCCAATGAGATGTATGAGGATGACAGTGAGGGATGTTATGAGAAT

GCTGAAGTGGGCCTCTGGAGATACCAAGGCGAGAATGAGGATGAAGACGAGGGGACAGAG

GATGAAGACTAATTTTTCTTGATGGTCTATTCAAAGAAAGAAAAATAATAGAAAAAGAAC

TGTGCAGTTTGGACGATAATCCCCAACAATAACTCGGATGTTTGAAACACACAATAGTTG

AGCACTGAGCAGGATAGTTTACTTGTTTATAATGCATAGAGGTGGTTTGTTCAGGGGTTT

TGCTGTTTATATGTTATAATACATATTCACATTCATTCTTTAATTTTACTAATAGCAAAT

TTTAGATGA

>Unigene0001812

CGGGAATGAGGATGACGATGAAGGGACAGAGGATGAAGACTAAATTTTCTTGATGTTCTG

TCTATTTCAAAGACAGAAAACCTGTGCAGTTCGGACGATAATCCCCAACAATAATTCGGA

TGTTTCAAACACACATTAGTATAGCACAGAGCAGAATAGATTACTTGTTTATAATGCATA

GAGGTGGTTTGTTCAGTAGAGTGGCGTTTTACTGTTTATATGTTAG

>Unigene0001813

ATCGGAATTATCGACGGGGATTCTGGCAGATCGCCGAGGATGTTCCTCTGAGCTCCACCC

TCGTTCTTCCCCCAACTTGCCTTGACCCGACTAGGAATCCCCTAGCTCGTAAAGAGACAA

CTCATAGGCTGATTCCCCTCGTTACTCTCAGCGCCAAACTGAGATAATCATTTGCTACTA

CTGTTTCAGATTTCATTACCAGGCCTAATGGTTGTATGCTAGATCTTTCAGAAACCCTAT

GGTAGCATCAGACTGAGTTCGAAAAAAAAAAATG

>Unigene0001814

TATACCCTAAAACCACGCCCACAAACATGCGAGTTCACGTGAATCATGACAAGCATATTA

CTGCTCTCATTTATTTGATTTCTACAAATTATATTAAGTACATCTTTACCAACTCTCACC

CAATTCGGTGGAACATCTGACTTTATGGGCTAGATATACTCATTAAAACATGATTTTAAC

AATTTTGATGGTCATCTGATTCTCACAATGAACTGGCATGGAGGTAGTCAAAGTA

>Unigene0001815

AAAGGTAGCCATTTCTCCGCACAGTTATTTAAGCAGCGAAACACATGTTGTTTTCTTGTA

ACAAAAGATCAGTTCAGAATAAATGAATCCTAGAATGTCCAAATACAAAGATTGAAAATG

TCCAAGAGTTCAAAGAAGTGAATATTGAATAGGTACAGCATCATAGTTACTGAAAAAGGT

GAAGATTGAATAGGTACAGTATACCGTAAGGATGCCATTTCTCACAATTATGCAAAGGGA

ACACAATGTAATGGTGTGGGAT

>Unigene0001816

AAAGGTAGCCATTTCTCCGCACAGTTATTTAAGCAGCGAAACACATGTTTTCTTGTAACA

AAAGATCAGTTCAGAATAAATGAATCCTAGAATGTCCAAATACAAAGATTGAAAATGTCC

AAGAGTTCAAAGAAGTGAATATTGAATAGGTACAGCATCATAGTTACTGAAAAAGGTGAA

GATTGAATAGGTACAGTATACCGTAAGGATGCCATTTCTCACAATTATGCAAAGGGAACA

CAATGTAATGGTGTGGGAT

>Unigene0001817

TTTCCTTTATGATTACTTTTTGGGTGATTTAGTGTGTTTAATCCCCCAAACGGATAAAGA

TACTGGTCTAAGAAAAAGTAGATAGCTCCGAATTAAGTCTCTGATACAAATTGGAACAAA

AAAGATACATAACATATGGACATCTGATTTATAAAAGGTAGCCATTTCTCTGTACAGTTA

TTTAAGCAGCGAAACACATGTTGTTTTCTTGTAACAAAAGATCAGTTCAGAATAAATGAA

TCCTAGAATGTCCAAATACAAAGATTGAAAATGTCCAAGAGTTCAAAGAAGTGAATATTG

AATAGGTACAGCATCATAGTTACTGAAAAAGGTGAAGATTGAATAGGTACAGTATACCGT

AAGGATGCCATTTCTCACAATTATGCAAAGGGAACACAATGTAATGGTGTGGGAT

>Unigene0001818

CCTTTACGTGCACTGTCCGCTTTGACACAGAGGTGGAGCTGGCATATTACAACCATGGTG

GAATCCTACCATACGCCATCAGGAACCTGATCAATGCCAAGCAATAGATATTTATTCTAA

GCGTCGAGGATGAAACAGACGCAATTCAACTTGTATTACTTTGTTCAATTTGTGCCTTGA

GGCATGCACTTGAGATATAGCTTCTTGGGTTTTTCTCTTCGTGCCCAAGAGAAGAGATTA

AGACTGGAAATAAATACGATGAGGGAGGTGTCTCAGTGTGATTTCTGAGATGAGTTTTGA

TTAAAGCATATTAAAGAGAAACATTGTTTTATCTATCCCGTCATTTCTTTGTTTTCTAAT

GGTTTTAAATTTTGTGTAGCACAGTCTAATAGGAAGGTCCCATCAATATTTCGGAGTATT

CGTGGAATAT

>Unigene0001819

ACGAGGACTGCGTGAAAATACTCAGACGGTGCAAGGAGGCGATACCAGCCAGGGAGGAAG

GAGGTAAGGTGATTATAATCGATACGGTGATGAGATCGGTAAATTCGCATCCCAAATCGC

TGGAAACGCAGCTTTTCTGGGACATAATTATGATGGTGACGTTCAATAGTGCTGAGAGGG

GAGAAGACGAGTGGAGCAAGATCTTTGCTGATGCAGGGTTCTCCGACTACAAGATAACTC

CTACTATGGGCCTTCGTTCAATTATTGAACTCTATCCATGATTACAAGATAGGTCCCACT

ATGTGCTTCCGTTTAATCATTGAGCTCTATCTGTAGTTCTTAGATATGCTGTGTAACTAT

ATTGTTGTAGGCAATTTATGTTGATT

>Unigene0001820

TCCCGAGAAGGAAACACGGAAAGATGAAGTGCCTGTAATAAATCTTGATTCTGATGATGA

AGATGGATATCCTCAGGCAGGAGTCAATTCCACTTGTACTCTGAAGAATCCTCATGCGCC

AGCATCTTTTGAGTCTCTGTTGAAGATACAAGCTAACTTATCAGATGACAGTATCTCGAA

CCAGCTAGTACTGTATCCACAAGGATTTACCAGTTTGAATTTTGATACCCCTGTTGTTGG

GGAAAGGCCTTCTGTTCAGTATGAAAAAGTTGTATTAAGGGTGAATGAGAAACCATCATC

AATTCAATATCAAAAGGTGGTCTTAAATAATGGGACTGAGCAGGAGCAGACTCCACTCCC

TCTTCAGAAGGAGCCTGC

>Unigene0001821

CCATGCCGTTATGAGTCATTCATGTACTCCTCTGCATCTAGCTGTAGAATCGGTGCAGGA

CTCCGGATATAAGGTTATGGTAGGTAACCAAATTTAGATGCAGATGGTAGCAGAATAGTA

CAGTTCAGTGAGCTTTGGCACAATGAGAGAGATCATCTTAGATTTGTGCTTTGACGAGCT

TCGGCATACTACAATGGTGTTTGATAAAATGCCCCACAAGGACTTGTTCTCATGGAATAC

CACGATAGACGGCTTCATGAAGTGTGGGAGTTGGGATCTCGCTAGCAACTTGATTGAGGA

AGTCAGTGGCCAAGGCCAGGTTGTTACATGGACCTCAATAATCTATGATATTTTTCTTAA

TAGCCTCCCAAACGAAGCCCTAGAGATGTTCCACAAGATAAGTTCTCTTACTTCAATGCT

CAGTGATATGTACTCCAAGTTTGGACTAATATCTGATGCTCACATCATTTTTTTAGTAGC

GTGTGCAATCAAAAATGGGTGGGTGATTGAAACTCAATGATCAATGGTCTTATTTTCCAT

GATATTGGCCATCAAGTATTATAGAATGAGATGAATATTGGTAGATGTGATGGGTACATT

TTTGAAGGTAAGGAGTGGGATTTCTACAGCAGAAAATTCAGAAGAAGAAGGGAAAGGCTA

GTGCTGCTGCGTAAGTATAAGGCTTTATATTTTGTTCCTGCTTTGATATTAAACTAATTA

TATTTTAAATATAGCAAGTTGAAATTCCAAGTGGACATCAAAATTTTGATTTGGTTTGAA

TTGAATTGAAGCTTTTTTGGAATCTCTGAGTTATTTTTTTAGCATTAATACCATGCTTCT

AACAAGAGATGGCAGCACGGTTCGAGACTATATTCATGTAATGGACTTGGCTGATGGTCA

CTTGGCAGCTCTTGAAAAGCTTTTAGCTACATAAAATATTGGTTGTGTTACTTATAAATT

AGGGACCGTTTACAGTACATCAGTTCTTGAGATGGTGCAACAATATTGATATTAGCTAGT

GGTGAGAAGGCTCTTGAACTAGGACTGTAAGTGATTGCAAAGATCAAAAGCTTTGGTGAT

GCAGCTCAGGCCCCCGAGCTAGTTTTTAATAGCACCA

>Unigene0001822

CCGACCACCTCTTCCGATCACCGCCACCCCGACCCTCAGGTACATGTTCTATCTTTCCTT

TGTCCATTTTATTCTAATTAATTTGTATATTTTGTAGGTTTCAGTTTGATTTCAAGGATT

GGCTAATGGTGTCTATGGATATGGTGCTGATTTTTTTGCTGACCGATGAAGAGGAGCAAT

AGAGGTGGTGGTGAGTCTGACCTCCATGCCGTTATGAGGTCCGACGACCTTCGCACCGTC

GATTCCATCTTCATCTCCAACCCTCTGACTATTAACTCCCGTGACAGTCATTCATGTACT

CCTCTGCATCTAGCTGTAGAATCGGTGCAGGACTCCGGATATAAGGTTATGGTAGGTAAC

CAAATTTAGATGCAGATGGTAGCAGAATAGTACAGTTCAGTGAGCTTTGGCACAATGAGA

GAGATCATCTTAGATTTGTGCTTTGACGAGCTTCGGCATACTACAATGGTGTTTGATAAA

ATGCCCCACAAGGACTTGTTCTCATGGAATACCACGATAGACGGCTTCATGAAGTGTGGG

AGTTGGGATCTCGCTAGCAACTTGATTGAGGAAGTCAGTGGCCAAGGCCAGGTTGTTACA

TGGACCTCAATAATCTATGATATTTTTCTTAATAGCCTCCCAAACGAAGCCCTAGAGATG

TTCCACAAGATAAGTTCTCTTACTTCAATGCTCAGTGATATGTACTCCAAGTTTGGACTA

ATATCTGATGCTCACATCATTTTTTTAGTAGCGTGTGCAATCAAAAATGGGTGGGTGATT

GAAACTCAATGATCAATGGTCTTATTTTCCATGATATTGGCCATCAAGTATTATAGAATG

AGATGAATATTGGTAGATGTGATGGGTACATTTTTGAAGGTAAGGAGTGGGATTTCTACA

GCAGAAAATTCAGAAGAAGAAGGGAAAGGCTAGTGCTGCTGCGTAAGTATAAGGCTTTAT

ATTTTGTTCCTGCTTTGATATTAAACTAATTATATTTTAAATATAGCAAGTTGAAATTCC

AAGTGGACATCAAAATTTTGATTTGGTTTGAATTGAATTGAAGCTTTTTTGGAATCTCTG

AGTTATTTTTTTAGCATTAATACCATGCTTCTAACAAGAGATGGCAGCACGGTTCGAGAC

TATATTCATGTAATGGACTTGGCTGATGGTCACTTGGCAGCTCTTGAAAAGCTTTTAGCT

ACATAAAATATTGGTTGTGTTACTTATAAATTAGGGACCGTTTACAGTACATCAGTTCTT

GAGATGGTGCAACAATATTGATATTAGCTAGTGGTGAGAAGGCTCTTGAACTAGGACTGT

AAGTGATTGCAAAGATCAAAAGCTTTGGTGATGCAGCTCAGGCCCCCGAGCTAGTTTTTA

ATAGCACCA

>Unigene0001823

AGATGGGAATGGGAATTGGCGGAGCCTGAGCTCTGCCGCAATTTCAGATGGGAATGGGAA

TTGGCGGAGCCTGAGCTCTGCCGCAATTTCAGATGGGAATTGGCGGAGAGACAATGATCG

TGCCCTGAGGATAGTTAAAACGCCAACAAGAAGATGCTCTCTTCGAAATTGGAGAAATGC

AAGAATAGCGTGTCGAGAAAGTACGATGAAGTGGAACGTGGACAGGTACAGGGACAACAC

CGGCATGAGTTTAGTGGTGGGAAAGGGCAATGTTGAGACTGTCACGATGAGAATGGGCAA

AAATGACATTGCTCGAAACTTGATGGCAGCGGGCGGAAGTACTACGCCGACCAAGCAGAT

CATTCAGGATTGAAGAACTGGCATGAGGCAGAAAATTAGAAGAGGCCATCAAGGCTGTAC

GAGTTATCCAGACCTCCCTCAAGGTGGCGGATAAGATATGAATGGTGAGCCCAGAGCGCG

TGCATCACCACGATCCTCATTGCTAGAGCCCAAGTTATTCGGAGCTGGCTTCATGAGAGT

CTACTTGGCAATATGATC

>Unigene0001824

TTTCTGCCATGACATCTCCTTCCTCATCCGAAGTTTTCTTTTGTGAGCTCTCTCCCTCAC

ACAGAGGAAAGAAGGAATACGAGTGTTATCCCTTGGATGAAGACTATGCAGATGGACATG

TTCATGATTCCCTCCGGCCTGGCGGTTATGGTCGTCTACCACGGATGGCTCCTCTTCACC

ATATTGCGCTACCCCAAACGCACCGTCATCGGCCTCAACTCCCTTGCCCGCCAGCGCTGG

GTCACCATCATGATGGCCGATCCCTCGAAGAACGGCATCCTCGCCGTGCAGACACTACGT

AACAACATAATGGCGTCGACTGTGCTTGGTACGACAGCAATCACCCTCACTTCCCTCATC

AGCGTCTTCGCCGGCGTCACCAGTCCGCCTAGCTCGCAGATCGACCGTATCAACCCTCCA

GATTCGTCTTTCAAGTTCTTCACCATCTCCGTCTGCTTCTTCATCGCTTTCCTCTGCCAC

GTGCAGTCCATCCGATACTACGCTCACGTTAGCTTCCTGGTCACCGTGGCATCAACTGGT

GGCGTCTCCACTGCTTACGTGGCAAGAAGTCTGAATAGGGGGACTACCTTTTGGTCCATT

GGGCTGCGGACCTTCCACGTCTCATTTACTCTGTTTCTGTGGATTTTTGGGCCGATACCT

ATGTTTGTGTGCTGTGTGTTGATGTGTGGGGTGTTGTATTTCTTGGATTTCACTAATGAG

TGTACAACGGAGTCTTCTAATGGGGAGAACGACTACTATGTTCCTGTTTAGAACATTCAA

TTGATCATACTTTCTCCTCTTTTTCTTTCCTT

>Unigene0001825

GAGATTACTTTTAAAAGATGCTGAGAATTGTGTATCTATGCTAGAAGCTAAAGTTGAAAA

AACGGGTCACACTGCTATTCAATATAATCGAACTTTTAACCTGCGTCGTGATAGATTTAC

AAAGCTGCAACAAGATACAAATAAAAAAATTGTAGATTCAAAGACTGCTAATGATAAATC

TAACTTATACAAGCCTTCAAGCCAAAATTTAAGTTCATCTTTGTCAAGAGTTGACTGTGG

ACGTTATAGTTTAAGAACATCTAGAGTACCTCAACCAAATTCATCACCATTGTTGTCTGC

TCGTAAAAATTTATCTTCAAAAAAAAAATGGAACTCGAGTATACTTTGCAACAAAGTTCA

AACTCCCAATAAAGAAACAGAATTGGAAAATTGGAAGCGACGTAAAAACTATGACCCATT

AAAAGCGGCTGCGCAAGGAAAGAAAAAAGATCAACAAAAATTGTCATCATCTTCCAAGGA

AAAAAGTGTTGAAAATTCTGGTAATCCGGTATTGAGGTCAGCATCTTTTCATGGCCGAGA

TGGGTTTTCTCAAGAGGATGACAGTGATGGTTGGGAACAAAAGAGTTTACATTTCTATGA

TCATGTTGATGATTGGCATAAATTACCACAACCACCTGCTACATCGCGGGATCATTCACC

CCCACCATCCAAAAATGAAAATCTTTCTTCATCAGCAAATTCATCACTGGCCATGCTGCC

TACTCTAATATCGGCACATAATGGCTCACCTACATTTTTGCATAAATCATTGTCTGCATT

TAATAGTTCTACAAGCTCTTCTCTTGATGTCAGTTCAATGCCAGATGCTTTATTTGATGA

TAAAGATTCGTTGGATATGGTTCAGTTATCACATAAAGCCAAAAAACGTGGTATTGAACT

TATGAAAAGAATAAGGAAAGAGTTACCAGAGTATGACGATATAATACTTACTAGGGGAAT

TGCAAGTATAGATATGATGGACGATTCGTTACATATTCCAATGAAATCTTGGGGTATGTC

AGTCTCGGCAACTAATATTAACTGTGTCGATAATATATTCAGAATACTTGAAAACGTTTT

ATTCAGAGACAGTGATAG

>Unigene0001826

ACCGCTTATCAGTGTTTTCTAAGATTCGTCATGAACCGACTTACCAAGCCAACTGCACAT

GCGATATCCGATCAAGTGCAAACCATAGCGTACATCAAAGAACCCACAATACTTGTATAA

GGAATCTGAGACATATACTCCCTTTTTTCTTCAGTGGAGGAAGATGCCATCGATGAGAGT

TTGAAGTGTGCATCCAGTACTCACCGGTTTGACCGTCTTCATGTCGAACCGA

>Unigene0001827

CCAAACCCGAATCCGACAAACTACCACTCCACTGCCTCCCTTACTCCTTCCCCCGCCGCC

GCAGCCAGCCGATCGCCTCCGGCGATGAGGCCGCTCCCCATCTCCTCCGTCGCCTCTCTC

CTCATCCTACTCGTCATCTCACAATCCCTTATCCACCCTCTCGTCGCCGCCGTTTCCGAG

GAGGATTTCACGGAGGAGCTCCTGCTTCGGCCCTTGCCCGACCGGAAGCTTCTCTCGCAC

TTCCACTTCCAGACCCGCGTCCCTCCATGCGACGCCAATGGCCGCCACCACACCGTCTTC

CCCAAAGCCATCGCCCAGCTGGTTCGGAAGTTTCAAATCAAGGAACTAGAGTTGTCTTTC

ACACAAGGTCGCTGGAACTATGAGAGCTGGGGTGGGTCAGATCCAATGTCCAGCAGCCAT

GCTAAGCCCCCCGGTGTAGAGCTGTGGGCCATTCTTGACCTCCCAACTAATGAAATCGAC

ATTAATTGGAAAAATCTTACTCATGCACTTTCAGGCCTATTTAGTTCTTCCATCAACTTT

ATGGAGTCTTCTACTGCTTATTCTGCACCTGGTTGGGGATTTCGTTCGAATTCAAGTAAC

ATAAGATATGGTGCATTGCCCCGTGAAGCAGTTTGCACAGAGAATCTCACTCCTTGGTTA

AAACTTCTACCTTGTCGAGATAAAGCTGGTCTAGCTTCTCTACTAGATAGGCCATCTATT

TGTAAAAGCTATTATCTTTGTCAAAGGTTGCGACTGCTCTCAAACAAATTAACTGGGATA

GTTCTTGAACAGACCCTGACTGTTGTGATTAAACCCAGTTTCCCCAGAAACAGCCTAGTT

TCAAAAAATGGGTTCCTTCAGCCAAGTTGGTCTGTTAGTTCAGTTTTCAAAAATAAGGTG

ACAGGACAGTGTATTCTCAGTAAAGCAAGCCGAATTTTCTTTGAACTAGAGAAAGGCTTA

GTTGCCGAGTTAGATAAACTGGGATCTGGAGATTATTGGAGCAATTCAGTTCTTGAATTG

TCTGTAGCCCCAGACAGAGTTATCAGAGAGTTGAATGCATTGAAAGGTCAAAGTAGTTCT

ATTCTTTTTGAGTATGAAATTGTTAACTTCAGCAATGATGATCCGTTGGATGTTGGCATC

ACATGGAAGCTCCCCCTGTTATGGTCTGGTTCCCGTGCATTATTCCACACTAGCAGATTT

TTGATGGGAAGTGGTAATGAGAGAGGTTCAATTGCCATCTCATGGCAATCGACTGGATCA

GATGAATTATTTGTGAACAGATCACAGGGGTGTACAATTCAGGCGGTTGTTTTCCAGGTT

GTTCCGTGGTATGTCAAAGTTTATTATCATACACTAGAATTCTTGTTGGATGGGAAGCCT

CAGAATATCTCAGATGTTTTTGAGAAGATACACATCTCCCCTTCAGAAGACAAGCTGTCG

CCTGGAACAATGGAAGCTAGTCTCAAGTTTCCTTGCAATATGAACTCAGCTGTACTTACT

TTAGATTTTGACAAGGGATTCTTGCATATTGACGAGTATCCTCCGGATGCTAATCAGGGA

TTTGACATTCCTTCAGCTCTTGTTAGTTTTCCCAGTTTCCATTCAAGCAGACACTATGTT

ATGGATAACTCCCCAAATCTATCTCCTCTGCTGGATAAATTAGAGGAAGGAGATATTGTG

AAATCATACACGGAGGTGTTGCTTGTGCCTTTGACCACTCCTGATTTTAGTATGCCATAT

AATGTCATCACCTTCACATGTACGGTGTTAGCACTATACTTTGGATCACTTCTCAATGTC

CTGCGAAGAAGAGTTGGGGAGGAAGAAAGATTACTCAAAGCAAAAGCAAAGCCTGGGTTG

ATTCCCCTGATCCTATATAAGTTTTCTTCCAAATTAAAGGGACGAACCACCAAATCAGCG

GAGTCATCGCCACCATCTTCTGCATCACCATCTATACTTGGACATAAGGTATTGCTCAAG

GTCATATTTGTAGCAGTGGTAGCGGTAACATGGAATTACTTTTTTTAATGATAGCTGAGT

AAAAAATAGATGAAATATATTTTTCAGTATAATACAATCCTATGCAATGCAGAGCTGATT

TTGTAACGTATTCAGAGGGACTATTTTCATAAACAGTTTTAGTAAACATCAATAAATTAT

ATCTCAGTCCAGATAGCAGTATCTGGGGCTG

>Unigene0001828

CTGAGTTTTAAGGAATCCTAATACCTAGAGTTATCCGGAAAAGATAAATAAAATACTTGG

GCTAAGCCCATTTATTCTAATGTGACTAGTTGGGCCTTGCCCATTTTGTGCTCTAAGCTT

TCATCTTGCTGAGCAATTTTTTTGGTTCCTCATTGGTCTTCTAGCAATGGCATCTAGAAG

CCTTCTTCATCACCATCAATGTGAGCGTTCAATCTCTCGAGGGACAAATTGAAGAAGCAT

AATACTCCCCCTTTGA

>Unigene0001829

CGTGCGACTTTACCCTCAGGACGCAGCGAGCCACAGCAATTTGGGAGCTATACTTCACAT

CAACAACAAATTAGACGAGGCAGAACGTTCGTATAAAAACGCGTTACGACTGCAGCCGGA

CGACGCGACCACGTTGGCAAATCTTCGGAAACTCAAAAACGTCCATCACAATCATCGGCA

CACGGTTAAATGAACGATATCCACCATCTGTAAAATTATAAAGGTTCATCCCAGCCGTGG

TCATCATATCGTCGCCAATTTCCTACAATACATTATAAATCATATTATTATATTTATTTC

GTTATTTTCCATTGAACACTTATGCTTTTTCGTATCTTTTTGTTTTGTAACAATATTTTA

TGATGTTAATTCTTTCTTTCCTTTTTTTTTTTTTTCAAAACAATACTCGTTGACATTATT

AACAATATTTTATGATGTTAAT

>Unigene0001830

GATTGTGAACTAGAGCAACTAGGAAATCTGCTACAAGTTTTGGATACTATTGAAGTCAGA

CATCTTGAGGATGGATTAGTGTCGTCAGCTGGAAAAAGAAGTAGACACTATCCTAGTATA

AATAGGGCACATTGTCCACTAGGAAAAGCTATTGTAACAAGAAATTAATATCAAGGATTT

ACTATTTTACTAGTACATATTTGAAGGATTAACAGTTAATGTTAGAAATTGTTATGGAGG

CAGGAATCCAAGGTAGTCTAG

>Unigene0001831

ACTCCTCGGTACGGAAGCTCCTTCCGTCTCTTCTCCCTGGAGCCCCAACCCTCCGTCGTC

TCCTCCCCCCTATAGCCATGTATCTTTCTCTCTCACAAAGAAAGAGAAGGAGGACGCAGC

CGGATTGGTCCGCCCTCCCGCTCGATTGCTTAGAAGAGATCTCGAGGAGGCTTCTCAGTT

GCGACGACTATTTCCACTTCCGCTCCGTCTGCTCGACCTGGCGTTCAGCGGCACCCAAGA

GGTCGCGTCTCCCTTTGCTCCTAGTCATGACAACAACCAG

>Unigene0001832

TTGTTTTTTTTTGGGCCGTGCACCAAAGGGATACAAGATTGTAGTCTTCTCAATATGGGG

CTTTGTTTGATGAGTTTATAGTTATTGATGTCCGGCCTTTATTTATCCTGAGGTCGAGTT

AATGATGGGACCACATAATGAGACCACTTGCATAATATATTGTCATGATTTTAACAAGGA

ATTAAATTATGAGATTTTGTTTTCCAGGACATTCCCCACTTGATGGGAATCAGTTGAAGC

AATCGGTGGCCCATACTTGTGTACCCTACCGGTAGCTACCCAAACATATCTATAACCAAG

CTTTAACTTGTATTTTGTGACTTCGACAACATAGGAAGTTCGACATGTTGGTATCTGTTT

TACTCCCGGCCTGAAGTCATGGACAAAGTTGTCCAAAGAGGATAGTTCGGAAAGTTCGAA

GAAGGGATAAAC

>Unigene0001833

AATAATTGATCATAAGTGAAATATTATTTTTATATTATTCACTTCAGAAAATAAACCACC

ATAGTGATGAACAAAAATAAGTAAACAATAAACATCGATCTATCCAAAACTAGAGCCAGG

TAATACCATTGACTTTCTTTCAGTTGAGGAGGTTTTGATCTATCTTCAATTGATTCGTCT

AAAAGCTTGTCCTCGTCATCAGTCTGTAATAAAATGTAAACCATGTAGAATTAATT

>Unigene0001834

AGCGAGCGAGCGCGGATCTCGGTCACACAGCCGCCGCCGCCGCCGCCTGCGCCTTTATAA

GTGGGTGCCGCCGAGCCCCGCCACGGTCAGCGCGCTGCCTCCTCCTCAGAGCCGTCCGCG

AGTGGACCCCAGCGCCGCGCCGCTCCCGCCCTTCCTCCTCCGCTGCCGCCATGGGCCTCA

CCATCTCCTCGCTCTTCTCGCGCCTCTTCGGCAAGAAGCAGATGCGCATCCTC

>Unigene0001835

GCAGGTCAACAGGTGATTCTTAATTTCCCCCCTTTCTTGCCACAACATATGTATGTTTTC

TCAAGTTTGATGCAAAATAGCTCCAACTTCAGTTGTATTTGGTCATGGCTTTGTTTATCC

TGTTTATAAAAACAAAAATCAAGTAATTTACACTTTATTTTATAGAAATTGCCATAAATT

ATTGTTTTCTTCAGACATCTTCCTCTTGGTAAATGATGTATAATGACATCTTATCTTCAC

ACCAATACAGTTGTTCACGAGCAAACTTGCAGTCGCAACCACATTATGTTTTAGAGTATG

GTTAAATTGGTTTTCTGGGAGAG

>Unigene0001836

ATATGAGCTTAAAAGATTCTAAGGGAATATCAGCCAAACCATTAAATAATTTCAGAGAAT

TGCTTTTACAAGGCAATAAGTCTGAAGCTTTAGAATTTGCTGTATTAAATCAAGACTGGG

GATTTGCATTTATATTAGCATTATATATGGATTCTAAGACATTAAATTATGTTAGATCTA

AATATTTTCAAACAATTCCACAAAATGACACTCTACAAACATTCTTCCAACTTAGTAGTG

GCAATATTCCATCATGCACAACTTGTTGTACGG

>Unigene0001837

GAACTCCGACACCGGCTCGCCTCAGAGCGGCGGTGCAGTTCTAACCCGGCCGAAGTACAA

CAGCACACTAACATTCCTCCGATTAGGAATCGACGGCAACCTCCGCCTGATCACATACAA

CGACAAAGTCGATTGGGGCCCGTCGGAGATTTCGTTCACACTCTTCGATAGGGATTCAAC

TCGGGAGAACGAATGCCAGTGGCCAGAGCGGTGCGGACAGTTCGGGTTGTGCGAGGACAA

CCAGTGCGTAGCCTGTCCAACAGAGAACGGACTGGCGGGATGGAGCAAGAGCTGCGCGCC

GAAGAAGGTAAGTTCCTGCGATCCCAAAAGCTTCCATTACTATAAACTAGTCGGTGTGGA

TCATTTCTT

>Unigene0001838

AGTCCGAGTCCCAACGACACAGCCTAAACCGGTCCCAGTTGCTCCCTTTCGCGGCCCGCT

CGCTCCCTGCTCCGGCGACCAGCCTCTCCGGTGATCATGCTCTCCGGCGACATCCCTCCG

AACCAGACCATCTACGTCAAGAACCTCAACGAGAAGGTCAAGAAAGAAGAACTCAAGAGA

TCCCTTTATGCACTGTTTTCCCAATATGGAAGAATTTTAGATGTGGTAGCTCTGAAGACA

GCCAAACTTCGAGGACAAGCATGGGTGGTATTTAGTGAAGTTACAGCTGCCAGTACTGCA

GTTCGACAAATGCAGAACTTTCCGTTTTATGATAAACCAATGAGGATACAGTATGCAAAA

ACAAAATCAGATTGTGTTGCGAAAGAGGATGGTACATATGCTCCAAGGGAGAAGAAGAAG

AAGCAAGAAGAAAAAGCTGCTGAAAAGAAACGCAAGGCCGAGGACACACAACAATCTGGT

CAAGCTGCAAATCGTTCAAATGGACAGAATAATGGAGGGCCTCCTGCCCATGCTGCTTCT

CGTCAAGGAAAATCAAGTTCGCAGGAAGCTGTTGCACCAAACAACATCCTCTTCATCCAG

AACTTGCCTGACGATACAACAGGAATGATGCTGCAAATCTTATTCCAGCAATACCCTGGT

TTTAGAGAGGTCCGCATGATCGACGCAAGGCCTGGGATCGCCTTTGTGGAGTTCGGAGAC

GACTCACAGGCATCGGTTGCCATGTTAGCTCTTCAGGGTTTCAAGATTACCGCTCACAAC

CCAATGGCTATTTCCTATGCAAAGAAATAGTATTCTTCATGAGGATTTTGTACTTTGTGA

GACTGATCCTCGGTTAGTTGGGACTGAAGGTTTGGATTGCACTGCCGTTCTTAAACATTT

TGTTATCAGAATTTCTTGCAAGATTGGAGAGTTGCAACATTTTACTGTATTAATTTAAGA

AGCTAATGAAAAACCTGGTGATGACAATGGTTTTGCTATATATATACCGTGG

>Unigene0001839

CGCGGCTCGGCGCGGCTCCGCGCGTGTGGCGCGCGCCTCCAGCCCGGCGCGGGCGAGGCC

GCGGGGGGCGCCGGGGGGGAACCTTCCCCCTTCTGTTCGGGCCGCCTCCGTTCCCGCGGG

GGCGGCCCGTTCGGGGGACGGGCCCGCCGGCCCCCGGCGCCGCTGTCCGACCGGGGCGGA

CTGCGCTCAGTGCGCCCCGACCGCGCGGCGCCGCCGGGCCGGGCTCGGGCCACGCCAGGG

CGCCCGGGGTCCGCGGCGACGTCGGCTACCCACCCGACCCGTCTTG

>Unigene0001840

CTCATCTTGAAGTATTCCTTCTGTTCTTAATTCTCTTCCGTCGAAGAGGAAGACAAAGTT

CAATATGGCATCTCGTGTTCTCCTCCTGCTTCTGCTGGCTCATGTGGTTATGGCTTCTGA

TCCAAGCCAACTGCAGGACTTCTGCGTCGCGGATCCAAACTCGCAGACTTTCGTGAATGG

CTTGACCTGCAAGGATCCGAGAGTCGCTCAGCCAGATGACTTTTTCTTCCCTGGCCTTCA

CCTCCCAGGAGACACCAACAATCCTCAAGGCTCAAAAGTGACCCCAGTCAATGTAGTCCA

AATCCCAGGCCTCAACACCCTCGGTATATCGCTGGTCCGCATCGACTTCGCCCCTGGTGG

CCTCAACCCTCCTCATACTCACCCGCGGGCCACCGAGATTCTTTTTGTGCTCGAAGGCAG

GCTCACTGTCGGTTTTGTCACCTCCAGCACCGGAAACCGTCTGTTCACCAAGATCCTCAA

CATGGGTGATGTCTTTGTGTTCCCTCAGGGGTTAGTTCACTTCCAGTTCAACAAAGACCA

CAAGAATGCAGTAGCATTTGCAGGTTTGAGCAGCCAGAATCCTGGAGTTATCACCATTGC

TAATGCGGTTTTCGGATCAAAGCCGCCGATTTCTGATAGTTTGCTGGCCAAGGCATTTCA

GACTAATACTGCAACCATTGATGGCATCCAGGCTCGATTTGCTTAGGTTTATTGATGTTT

TATTTGTTTGCTACAGTTTCAATGTATCTGCATGTAAT

>Unigene0001841

CCAAGGTTAATGAGAGATCGCAAACCATTCCAATTGCGAAAAACATTAATCGTTTACAAT

TTACTGCAAGTCATATTTTCCACCTGGATATTTTACGAAGCATGGGATGGTGCATGGGGT

AATGGTTACAGTCTTCGATGCCAACCTGTTGATTATTCAACATCCCCAAGCGCTATGAGA

GTGGCTCGTGGATGCTGGTGGTACTTCTTTTCAAAATTCACGGAATTCTTCGATACCTTC

TTTTTCGTTATGCGAAAGAAATACGATCAAGTATCTACCCTGCACGTCATACACCACGGA

ATCATGCCGATGAGCGTGTGGTTTGGCGTTAAGTTCACACCCGGCGG

>Unigene0001842

CATAGCTCAGAAGACCTATCAGCATGTAATGAATGGGAAAACCATCAAAAAACTAGACAT

ATAAGTAAAATTGTTGAGTATTTTGAGGAAAAACAGAGTCAACGGGAGACACAACCTTCC

ATACGACCAAGACGATCAGGTCTTGTATCTGGAGTCTTACGACGAGCAACAAACACTACT

AGTCCAAATGTCTGTGGCAAACGTATTTTAGTTTGTGAAGGAGCTGTAAGGTCAAAACTA

AAGCTTTTTGATAGAAAATAAAATTTCTATTCAGGGTGCTACTTACCATTAAAATTTTTT

TTTATTATATTTTTTTTTATGGTTGGTTGTTTTCCAAAATTTGTTTCATCTAGTCATAGG

TACTACAAAATGCCAAAACAGTATAATGTATATTAAAACCATAACAATAAT

>Unigene0001843

CTTTCCTTTCTCTTCTTCCACTCTTCTTTTTCTTATCCTTTCCCTTTTCTCTTTTCCCTC

TTCTTTCTCTCCTTTCCTTTCTCTCTTCTTCCACTCTCTTTCTTCTCTCTCTTCTTCCTC

TCTCCTCTTTCTCTCTCCTCCTTTCCTTTCCCTTTTCCCTCCTCTCTTCTTCCTCTCTCC

TCCAATCCCTTCTCTCTTCTTCCTCTCTTCTTCCTCTCTTCTTAATTTCTCTGCTCTCTT

CTTCCTCACTTCTTTATTTCTCTCCTCTCCTTTCTTCTTTCTCTT

>Unigene0001844

ATTTTTAATCCAAAATATACATCGGCCCAGAGTTATAATATGACAACCGGCCCACCGGGA

ACTTTCCCGGTATCCCGGTGGGCCAATCCGCCGCTGAGTCTACAGCTAGTGAGTAACAAA

AAGGAATAAGATGCAAGATAAATAACATATTATTGTAGGTATCAAAAATATTGAAAGTTT

TACTAGGTATCACTTTAAGTTTATGTGTAACCTAGGTGGTATACTGATTACTGGAGACTT

GTGATAATGA

>Unigene0001845

AAAAGTAGAAATTATCTAATTTGTTTTAGTTTTATAGTAGATCAATACAGACCTATATTT

CTTCCAATCAGAAATGAAATTTGAACAGCTTTTTTAGTACCTTCGTATACAAAATGTTAT

TTGTGATGGATGTTCCTTATGTTAAATGTCTATTTTACTTGATTTTTCTCTTTCACCGCT

ACTTCTCAGTGGAATCCACTAAGATACTATTATCTCTCTGTGTATCTGCCACTGTTC

>Unigene0001846

AGTGCAGCCGTTGGGATCTGGCGCCCATGTCTCCGCTGCAGATGGCAATCGCCGTCGCCG

GAAGCCACCGCCTAGGCTGCCAGAGAGTTCGTCCGAGATGGGGATCTGGCCGGAACTGAT

TGGATTAAGGCCGGATTCCACCTGATTTGGTTGGAAACTGCAGATTCCTCCCAAAAAGCT

TCGGATTTTGTCAGATTTGGCCAGAAATCTCCGAATCTCGCCCAAAAGTGTTCTGGATCC

CAACAAATGTTGTGGAGGAGTTTGCATTGGAGAGGCTCGCCAAAAATAAAGAGGAGAGG

>Unigene0001847

AAATCTTTCTACTTCTTGCTGCTGAAGGCGGCTGGCCTGTGCAGTTGAGCTGCACGGGCT

TTCTACTTGTCATCTACTATGGAACGAGGGCGGATACCACAAGGATTCTACGTGTTTTAA

GCAAGTTAAAAACATGCGGTATGTTTTCAAATACAAGCTTTGCGTTAAGTGACTTTAATG

CCCAAGGAATTGTTAATTCTCATCCAATCACTAGTATACTTTTTGTATTCTGGTAATGGG

TTCAACCTAAG

>Unigene0001848

GTCCCAGCGCTGCGTCTCCGTTGCGGCCTTAGGCCTCGTCCTTTTCTCAGAGCCCGAAGG

TGGGTGCCTGCTCCTTCCCTCCCCGCCGAGGACCCTGCGGCTTTTCCCTATGCCCTGTAG

CTCTCCTCCTTTCTGGACTCTGCGCGGCCGCCGCTCCGGCGCCCCTCCTCCCGTGTGAAG

CGCTTCCCGCTCCGGGCCGCGCCGCCTCCAAATTCTCCTCCCTTCCCTCGGGTTTCTTCT

CCTCAGCCGCCGCCGCCCCTCGGGGGGCTACCGCCCGCCATGAGCTACGGGCGCCCTCCG

CCAGACGTGGAGGGCATGACGTCCCTCAAGGTGGACAACCTGACCTACCGGACGTCCCCG

GACACTCTCCGGAGGGTTTTCGAGAAGTACGGCCGCGTGGGCGACGTCTACATCCCCCGG

GACCGCTACACCAAGGAGAGCCGCGGGTTCGCATTCGTCCGTTTCCACGACAAACGCGAC

GCCGAGGACGCGATGGACGCCATGGACGGGGCCGTGCTGGATGGCCGCGAGCTCCGCGTG

CAGATGGCCCGCTACGGCCGCCCCCCGGACTCGCACCACAGCCGCCGCGGCCCTCCGCCC

CGCCGCTACGGGAGCAGCGGTTACGGCCGCCGCAGCCGCAGCCCTAGGAGAC

>Unigene0001849

TCTCACGACAAACCCGACCCATAGGGCCCGGCCCAGATCTCGACGCCGTCGGCGGCGGCG

CCGCCCCTTCCTCTTCCTCACGACCCCACAGCTCCCCCACGGCGAGCTCCCCGCCGGCGG

CGTTGAGGCCGTCCGATCGTCCACTCCGCCCGCTGGCGGCATTCCTTTCTGATTCGAATT

GCCCTCTGCTAGGGTTTTGTGTCAAGGGCGTGACTCATTCGCCCAGGAATAGCGAGGAGC

TCATTTACTCAGGAAAATTGAAGACTGGCTGAATATAGAGACTATACTTTCTTTCCGGAA

GGTTTGCACATTAGAGCATTTTTCACAACTATGGGCTCGCTCAGTGTCCAGGAAGAAGGA

CCTGATAACTTTTTCTGAGACGAATTTTGAACTCCGACTGGGCTACACTTGTGCAACAGT

ACTCCTGTTAAGAGAATATTATCAGATGCCTTAAAGAGAATAGTACAATTAGAATGTTAT

CCCTTGTACATGCATACATTTCCAAATCAATAACCACTTGAAGGTTTTCAGATTTCAAAG

TTCCCGTTTAGGCACCCTTAAATATCTTAAAGCTCCTGGGGTCTACTCATCACTACCTGG

CAAAAAGGTCCTTTAGAAGTGGAGGAAATACTGTTAAACTTGTATACACTCATGTTTATT

>Unigene0001850

TAGTACAAGGAGAATGTTATCCCTGGGAGATGCATACCTTTCAGGTTTTTAGACTTCAGA

GTTCCCGTTTGGCCACTCTTTAAGGAGCTTAAACTCCTGGGGTCTACTCCTCACTACCGG

CAAAAAGATTCTGTGTCTCTCCACGACTCTCTCCTAGGCTCCTTGTTGAGGACACCATGA

TGCTCTAATGAGCATTCAGGTGGTTTCTTATTTGAAAGATTCACATAGACCTGGCATTTA

GACCTTATTATAGCAA

>Unigene0001851

CACGACCCCACAGCTCCCCCACGGCGAGCTCCCCGCCGGCGGCGTTGAGGCCGTCCGATC

GTCCACTCCGCCCGCTGGCGACATTCCTTTCTGATTCGAATTGCCCTCTGCTAGGGTTTT

GGATGAAGGGCCTGAACTTTTTCCGTGACTAATCTTGAACTCCGACTGGGCCACACTTCT

GGTGAGGGAATGTTATCAGATGCTTTTATGAGAATAGTACAAGGAGAATGTTATCCTCTT

GCAGATGCATACTTTTCCAAATCAAGCACCACTTGAAGAATTTCAGACTTCAAAGTTCCC

GTCTAACAACCCTTAAAGATCTTAAACTCCTGGGGTCTACTTCTCACTACTTGGCAAAGA

GGTTGGTTAGAAGTGGAGGAAATGATGTTAAACCTATCTATACTCATGTTTATTTTAGTC

CAAAGTACTATCTTAAAGTTCCCATTTTGAGCTTTGTGGCGGCATGTTTCAGTTTTTGGG

CAGCTTTATGCAGATTCTGTGCGTCTCCACAATTGTATTTTCTCCTGGGCTCCTTGTTAA

GGACACCATGATGCTCGAATGAGCATTCAGGTGGTTTCTTATTTGAAAGGTTTTCATAGA

TTTTGACATTTAGGCCATATTATACTTAAAAAAGTAACACTAACACTAGTTGGGAGGATG

AG

>Unigene0001852

GCCCAGCGGACCCCCAGAATAGTTGTAATACCCCTTGCTCGGCATTTACTTGTTGTACAC

CACAAGCCCCGTAATACCACAAAGCAGAGCTGTGATCTTTAGCTTCCCCTCTCCGTCGAA

AAAGCTTCGGTGAAGGTCTCTCCAATGGCCGCCGCGGCCGCTGCTCGCTTGCTGCAGAGG

CGACTCACCTCCCTCCTCCGCCAGGACCACTCCTTCTCCCCCATCCCCCAAGCAGGGTTC

ACACAACCGGCCTGGAAACCAGCAGCTTTTAGAACTCTCCATTCGCAGACAGGATCTGTT

AATGGAGCTGATCATAATAGTATGGACACTGAAGGAGCTGATGTGAGTTCAGAAACTACC

AAAATCTGCAGAGATGGTCCTCATATCCCTACCGAGTTTCCTGAAATGACGAAGTCACAT

GCTGATGAGTTCGATTTGAAGCACAAAGCTCTTTCTAATCTGAAAACATCCGGGAGGCAC

AATCTTGCCATGATTTTCACCTGCAAGGTCTGTGAGACGAGATCCATGAAGACCATCTGC

CGAGAATCTTATGAGAAAGGGGTGGTGGTGGTTCGTTGCGGAGGTTGCGATAACCTCCAT

CTAATTGCGGATCGACTTGGTTGGTTCGGAAATCCGGCGAGTGTGGAGGACTTCTTGGCA

GCACGTGGAGAAGGGGTGACAAAAGGTTCGGTGGACACGCTAAACCTGACATTGAATGAT

GTGGCTGGGGCTGGAATCCATCATGAATCTTCTGAGGCTATGAACTGAGCATTTTTACAT

CTGTGGTTGTCTCGAGGTATAGAAACGCAAAATCGGTCTTATTGTTGATCATGGGTATTC

ATTCTTGAAATTTTCCGAGCACTTAGTCTTCGCATATCGATGCTATTTTAAATTTATAAT

CTGCTCGAGGATAAGATACCTGGACAAAAGATTGCTGTTTTAAATATATGTTCTGCTTGA

GTTCATAAGTTTTATTATCTATGGTCTTTATTTATTGCA

>Unigene0001853

TATATCACAATAGTTATTATATTATGTATCCTTTTTTACGCCTTTTCTTTATTATTTTTT

TACTACATTATATTTTCTACATTTTTTTTTTATGACTAACAAAAATAATTTTGTTAAGTA

CAAAACCAAAAATCCAAAAGATGTTCTAAACTACTATAACGAATTAACACAAAACAAACA

ACAAAAGTGTTCATAATTTCAGAGCGATTTCGAGAGGTTAACGTATTATTCTTTAATACT

TATAAATAAAAAATATTAAAAAGAATTTGTAAATAAAATTTGTTCTCATCATGTGTTTTT

ATACTAGAAAAACATTCAAAAGTTGTTATTTAATGTTTTGTATTTTTGCATGCATACTAG

AATAGAATGTCTTAACATTTAGATAAATCGAACAAAAATTTATTTTTTTTTTTTTACCTT

TTTTAAACTCTTATATTATGTTCATTTAATTATTATTATGATAACTATTATTAATTTTAT

TTGTAT

>Unigene0001854

CCCAAATGATCCCTAACCTGACGAATCAGATCGGCCTAGCAGCTCCTGGGGAGCGGTCTG

TCGGAGATAGGGATGTCCAGATAGGGCATAGGGAGTGCCCCAGGGAGCACTCGTAGGACA

ATGCCAAGCCTGTGGCCCTTGTCACATCGCGCCCCGGGTAGAAGAGCTGCGTCTTTGCAT

AGTTGAGCTTGAGGCTGGATGCCCGTCCAAAACAAGACGAGGAAATGTAGG

>Unigene0001855

GTCCCGGATAAAAGACTATATTTTAGTGATAGATTATCTGTGATAAAATATTAATTGACG

AATGAACGGAAAAAAAATTACGTGGAACTCCACAATTATGACGAAATTATATTTCTGGCA

AATACAATGAATTCTCATATTATCCGTTTATATGATAAAAAAAAATATACTATTTTATTA

TATAATTTTAATAATTATTATCGCCGTTAAAAAAATAACATAACCTCTCCATCGATTTCT

TTTCTTTCCTATCGCTATACATCGAATACGCGTTTTCGCAGCGCACTAGTATCATAATAA

TACACTTAAAACTAAATATATATTATATACATATATACAATATTGTGTACATTAACTGAT

TTGATATTATATAAATATATAAAATAAAAAATGTGTATATGACTAAATCTAATACGTATA

ATACTTATACGTTATATTATATGTATATTATATACGTATGCA

>Unigene0001856

AGATGACAACACCAAGTTTTTTCACATGGTGGCGAATGGGAGAAGGAAACACAATCTGAT

TTCGGAACTGAAAATTGGCGAGAGTACTCGGAGCTCTCAGATGGAGATCATCAGGGAGGT

GACGAGACGCTTCAGAGCTGCTGCGTTGCGTGTTCAGCTGATAATTAGTTATGATCAGAC

TTGATTGAGAACATATCCTATTGGGATGTTAGTCTGGATGCCTTCATGATGGAAGTGAGG

GAGGTGGTCTTTGGG

>Unigene0001857

TTGAACCGTATCCAATGAGAGATACTATGCAGCATTTTTGTGAAAAACATTTAGATAAAA

TTAAAGCTTACATGGAAAAGGTTTCAGTTAGAATTCCTCCTCCTGCTAAATGCACAATTG

AGGAAAGAAGAGCGAAAAAGACGGCAAAACTACATTTTGCTTGTGAAGGTCGAGGTGAAC

ATTGCCTCTATAATCGTGCTTTATTTACTGTACGTACACGACATCCTCGTGTTTGGATTC

ACCTTATGTTTCTTGCTCTTCAAGCAAGATCATCGTCAGCACTTAGTTCACGTCATCCAT

CTGTCAGTGCCTTAAAAAACTGCTGGGATATTCTCAAGTGTGAAACTAGGCCATTTCTGA

CATTAGTAACCTCTGCATTTCCATGCTCAAAAGATCAAGATGCTGTCTTAAATGAACTAC

GTAACAGTGGTTACTTTGATGTATTTGAACATTG

>Unigene0001858

CAGATGCTTAAAAGAAGAAATGTTAGCACTTTTCCAGATGATGCCACTTCTCCCCTTCAG

GAAAATAGGAGCAATCAGGTTTCTGCACACTGGTCTGTTGAAGAAATAGTCAAAGGAGTT

AATAGCAATAATATGGAGCTTCAATTGCAGGCTACTCAAGCTGCCAGGAAACTCCTTTCA

AGAGAGAAGCAGCCCCCCATAGACAACATAATTCAGGCTGGTTTGATTCCAAAATTTGTC

TCCTTCTTGAGTAGAGCAGACTGTAGTCCTATTCAATTTGAATCTGCCTGGGCGCTTACC

AACATTGCTTCTGGCACATCAGAGCAAACCAAGGCGGTAGTAGATGGTGGGGCTATTCCA

GCCTTTATTTCACTTCTGGCCTCTCCTCACATTCACATCAGTGAACAAGCTGTGTGGGC

>Unigene0001859

AAGGCTGTAAATATCTGAGTGTATGTGTATGTGCGTACTATTGTGTTTGTTTCTAAAGAA

TCTTCTAAAACCCAATAATAACTGAACTCAAATTTGTCAGGGTGAAGTGCTGTCTGAAGA

TGAAAATGTTTTTCCTATTCAACAAGCAAAAAATCCAGGATTGTCTCTGATCATTTCCCT

GTCAAGAACATTCCATTCTTAATAAGTGCACTTTGAAGGTAAACTTTCCTGTGTAGTCCT

CCAGGTTGTTGAAAGGCTTCTGTGAGGACAGTCTACAACTCAAATTTCAGAAAAAGCCCA

CCAAGAAATACTTTGGTCACCTGCTTTTCCAGCTCTTAATGCAGGTAGATTTAGTGATGT

GCATAGGTGGAAGTAGGATAGCTCCACGCTCCTAGAACCAGCAACCACACTCTTGCCCAC

AGCAATATTCATGTCCTGCTGTTCACATGCAGTCTCATTAGCATCATATAAAACCCAGTT

GGCTGTCACTTGCAGATTTAAGCA

>Unigene0001860

TGTAAAGATTTATGCTCGAAATCCAATATTTTGCTAAGAATGTGAACTCAAGTGCAGCTC

ATTGTTAGCTTCAGTATAAAAATCTGTACAGATTGTGTATAGGTAATGTGGTTCTTTGTA

GGGAGACCTCTATTTCTAAAGTAAGTAGTAGCTGAGTGCTGCTACAGTTCAGTTTTAAAA

GAAGAAGTTGTGGAATGTTTCTGCATATTTAATGGTTTTGTTGAAA

>Unigene0001861

AGGGCCAGTTAAATGTTACTGAAATCGTGATGAGTAGGATGAATGATATGAATCTATCAG

TCGTCATCTCTAAATATAACACTATTGGAAATCCTCGAGAGTGGTTCATAGACATTGGTG

CTACACCTCATATCTGTGCTGTCAGGGCTATGTTCTACGATTACCAAGTAGTGGAAGGAG

AAAAGTTATTTATGGGGAACTTTTTTACATCAAATATAGAGGAAAAAGGCAAGATCATTC

TG

>Unigene0001862

TAATATTTTATAAGTTTGATTTAATTTTATTAGAATTTAAGTTTAAAATTTTAAGAGTTT

TAATTTTATTTTTTAGAATTTGATTTTTTTTTGAATTTAATAATTTTTTATTTTCTATAA

AATATTTAATTTCTATATTTAATATTTTTTTTTTTAGAATAATATGAAATCTATTATTTT

TAAGGATTAATTTTTTTTTGAATAATTTTCTTTTTATTGGATTTTTTTCTTTAAAATTAT

TTGAAACTGGTTG

>Unigene0001863

AGTAATACACTTAAATCTGGAGGTAGTTAATCAATGGAGTGAGCTGGCCTGGATGTGAGG

CGAGGACATGTTGTGTATGAGCTTTGGAGAGATGTATAATTGTTTTTTCCCCTTCTACTT

AATGATTTAGTGACAATTTCTGCCGAATTGTTGGTTTAATTAGAGTCGTTGAGTCTCTTA

TACATGTCTGCATGTAAAGTAATTGTTAAATGATTGGAGTATATTCTTAATTCTTTTCCA

TCTGTAGATGGATAAGAGGTGCTAATAGTTGGTATCCAACTCAGTTTTGAGGAGAGAACT

TTGGTGTACTCTGCGTCTTTTGTATGACTTGTCTTTTGATTTCTCAAAGATTAATTTTT

>Unigene0001864

AGGGGCCTCGACCCTTGACATTGTTTTCAAAAGTTTGAGCCGACGCTTGAGACATATCAG

ATTTGAGCATGAGTAGCGAATTTGCTCGAGTAATTGTTGTGCTCAAGCGTTCTAGACTGA

TTTCTAAGTTGATACTGATGCCCTCACCACCTTTGTCGCATCAAAATCAACTTTTGCTAG

TGTATAATTGCAATCTTTGTCCTTCATCATATGCATCAATTCCCCGTATTTGTAGTTTAT

GTTGCAATTAAGCATATAATTTATGACACTG

>Unigene0001865

CCTTGACATTGCTTTCAAAAGTTTGAGCTGACGCTTGAGACATATCAGATTTGAGCATGA

GTAGCGAATTTGCTCGAGTAATTGTTGTGCTCAAGCGTTCTAGACTGATTTCTTCGTTGA

TACTGATGCCCTCACCACCTTTGTCGTATCAATATCAACTTTTGCTAGTGTATAATTGCA

ATCTTTGTCCTTCATCATATGCATCAATTCCCCGTATTTGTAGTTTATGTTGCAATTACG

CATATAATTTATGACACTGACATCATA

>Unigene0001866

CTCTCTTACCATTAGGAATCGGAGCCAAGTGGAGACTCAGGCGAGCTGGTCCCTTGCTCG

CCACCCCCATCGTCTCCATCTCTTCCGCAGCGATTAGGGTTTCGTCTATCCAAGCTCCGG

ATCCAATCATCGGCTGGCAAGCATGGGAATCATGTTCTCCCAGATGTTCTCCTCCCTCTT

CGGCAACCGGGAGGCTCGGATCCTCGTGCTAGGCCTCGACAACGCCGGAAAAACTACAAT

CCTCTATCGGCTTCAGATGGGGGAAGTAGTCTCCACCATTCCAACAATCGGGTTTAATGT

GGAGACCGTGCAATACAACAATATCAAGTTTCAAGTCTGGGATCTGGGTGGACAGACAAG

CATCAGGCCATATTGGAGATGCTATTTTCCAAACACTCAAGCTATTATATATGTTGTTGA

TTCTAGTGACACTGATAGATTGGTAACTGCAAAGGATGAGTTTCATGCTATCTTGGAGGA

GGAAGAATTAAAACGTGCTGTTGTACTCATATATGCAAACAAGCAGGATCTCCCGGGTGC

AATCGATGATGCTGCAATAACAGAATCCTTGGAACTTCACAAGATCAAAAGCCGTCAATG

GGCTATTTTTAAAACTTCAGCTATTAAAGGAGAAGGAATCTTCGAGGGCTTGGATTGGCT

GAGTAATACACTTAAATCTGGAGGTGGCTGATCTATTGGAGTATAAACTTGGGAAATATC

TTATCATTTCATGAGTTGGCGATAGATAATACTGAAATGTTCTCTAATTAGAGCCATCCA

GTCCCATACATTTCCGATGTAAGGTGTAGCTTAAACGACTGCGATACATGTATCTGTAGG

TTGTTAAAACGTTGGATCCGCTCTACCCAGCTTGGTCTCAAGACGACTCAGCCTTATGGA

TTTCTCTGATTTTCCATCATATATACTGAAGGTATTCGTGTTCAACGCTCGGTGCAATAT

TAGGTGCTCATGGCAAAAGGCACATCCATTTTCATGAGCAAGAGGTGAAGTCTCCTTTGG

TTAACATGGACCAGACTCCAGAAGAGTCATTGTCTTACCTCAAAATAGGACCAGATATAA

ACCACTCGCATCGTTCCTTGCTTGAGGGTAATGTGTCACTCGAGCGCTTGTGTATGTTGT

GCTCGAGGGGCCTCGACCCTTGACATTGCTTTCAAAAGTTTAAGCGTCGCGTATGTTGTG

CTCGAGGGGCCTCGACCCTTGACATTGCTTTCAAAAGTTTAAG

>Unigene0001867

ACGGAGCTAGTGGTAACCTCTCGTTATATCCGACGTCGGGGAAAGACGTGAGTCTGAACT

TCGACGCGGATACTCGATGCACCACAATTAGGGACGTAAAGTGGAGACATAATTCGGCGA

TCCGAAAAAACGTCAACGTTTCATTTCAGGCGGACACATCGAATGAATTAAGTTGGAAGG

AACCAAAATTATCATCTCTAGGACAAGTATAAATAATATATTTCCCCATGCGCGC

>Unigene0001868

TGAAGAGGAAGAGCTCAATGATCTCATCGCAACTGGTTTTGAAGAAGCACCTGTAGATAA

AGAAAAGCGGAATCAACTTCATCAGAAGTGGCTTGAACAGCAGGACACAGCTGCAACAGA

CAATGTTCTGCAGAGGCTTAAGGCTCGCCATAAACAGAGAGAGCCAGCACTTGCTCTTGA

GGATGATGATCAGGAAAGTGAGGATGGTTCTGAAGATGAAGGATCTTA

>Unigene0001869

TTGCAAGGGTTTCTATTTCTGGCCGAACCGGCCAATCCGTGTACGATTTGTTCTGAGCCT

TTTAAAATTGAGCCTTGCCCTCTCCAATTTGCATTTTAGGCCTAGGTTTTATTTTATTAT

AGGGATGGCATTTCATAGTTAAATTCAGGGCGGCACTAACGTGTTTCTTTTTACCTCCCA

AGAAATGGGTTTGATTTGTAATTAAATTATGGTTTTGAAATCTTATAG

>Unigene0001870

CGACCACCCTACTATCTATAGGTAATCTCAACTACAGTGCTATAGGGGCCACCACCGTTC

TTTCCTCCTCACCTATAAAACCAGCAATCCTGGCCCAGCTCAGGGGTCTTCTCCATCACA

CAGTGCTTCATCTCCATATAGGCACAACTCCGGGCTCCCTCCTCTCCAACGCATCCGTGC

CAAGCTCCGTTGTGAGCTCTCACTGAGCTTCTACGTCGCCAAGCTCGCCGAGCACA

>Unigene0001871

TTACACCTCAAAGAAGTTGTCTATGTTCCTATGTCAGAAGAACAAAATTAACATATTGTA

GTTTACTTCTTGAGAAAAGTTAGATTAGATTAGATTGATCTCCACATCCAACAATCTCCC

ACTTGGAGTCGATCTGATATATTGAGGACATAAATTCATCACAATCATCTCCCTTCATAA

TGTTGATCTTTAACACCTTGGTGTAGACCAATTGCCTCGACGGTGGTGGTCTTCAACATC

TTAGTGAAGACCAACTGAAGCTACACATAACTTTAATTTCTCTGATGATAGAATCTTCAT

CAACATGTCTATGATAATTAATCTATCTAACAGTAAATTCTTCAATATGTCAGTAGGA

>Unigene0001872

ACAACATCGAGCGGTCGTCTATCACGCTTTTCCGACGTTGTGCTTGATCGCGATGACTTG

CCTCGTGGCGACGATCACCTGTTCGAGACCGACTACGACGATCGGCGACTCGTCCGTCGC

ACGCGACCGGAAGTCGGCCGAAGGAAGTTTTGCGATCGGAACTGCCCGCGGCACCGATCT

CCACCACGTCAGACACCGTCGGTACATCATCTTGACGCCCGTGCGGCA

>Unigene0001873

TTGGCTTCGAGTAAATGTCGTCAGAAACGTGCCAATATAGAGATGCAGCAGAAACTACCG

TGAGAGAGCGTAGCAACACCGGAGTTTAGTAGGTTTCTACGTCTCCTTTGCCCTTCATGA

GAATCGGGCTCCTAGCCATGACATGAATGGGAGGAAGCTGATATATAGAGGAAGTGATTT

GTTGTCAATTTGGTAAACATAACCAAATATTGTGCCACAATCATTTAGAGGTCTTTTGAT

AGAGATGTGTTAAAAATTGTTCGATAATTACCAAGAAATATTTTGAA

>Unigene0001874

GCAAGAGGGCGCTTTGTTTATTTTTTGGAAATGACAAAAAATTAGGAATTGTGATTTGTC

TTTAGTATATGCACTTGTAGAGAGGCGGTGGGATACTACACATACCTTCCACTAGGGTTG

TATTAAAAACTGATCTAACTCAGGAAACTGTCCAACTGTCCAAACCAGGCTAAATCGATG

GTTCGGTTTTTATACTTTTCCGGTTCGGTTTTGGATTACTCAATCCGATA

>Unigene0001875

TATCAGTCTCTCTTGATACCATAACAAGAGAGATGAGTTGAGTTGAGGATGAAATTGTGG

TTAGATTCTCATTATTGATTAGGGGATATTTATAGTACAGGGTGATATTCACACAATCAA

GTGCTATTTACACCACAGTAACACCCTTTTTAAGCTACACATACCATTGACTACGTACAT

TTAACATATCAGCTGGGTTGGATGCTACACTAACATATCTACGGTTCTAACTGGTGTTTC

TGCGTTATAAGTTGTTCATGAGGCCAATCAGATAGAAATAAAAAATATATGAAATGAGAT

GTAACATGCTAATACCATTCAGCAAAACCAAAAATGTATCCTGCTAGCAAATA

>Unigene0001876

TTGCTCGACAATCCACCGGATTGAGTCCGTAACTCATGTCTCCCCGCTTGATGATTCGCT

ATTCGATTGGAGCTCACAACTCTCCTCCGATGTTTCTCGCCATTGTGCATCGCTGCACTC

TCTCGACAACAGTACTTGTATAAGAGCTCGTAATCGTCTAGGATTCTCATCATCATGCGT

TGCTACATGCGCTCGACAATCCGCTACCGGGATATGAGCTCGGGACTCCTTTTCCTTGTT

CAATGATCTGCTGTCAGCATTGGAGTCTGGGAATCACATCGACCATGGCCCTCTTTGGTG

CATCACTGCACTGGATCGATAATACATTGTCTGGTTTGAAGCATATGACTCTTGCCTCCT

CTCAGTGATCCGATCACCACCAACTTGTCGAGTTGCCGGATCGAAGCATGAAACTTTTGT

TGCCCGACT

>Unigene0001877

TTCATTCACATCGAAATCCTTAATGGGTTCTCTCCCAAAGTCAGTGCCGGAGGAATTGAT

CAGCGTCGACGTACACGCTGCTTATGACCTCATCAACACCGGCAAACACCGCTACTTGGA

CGTCAGGACGGTAGAGGAATTCAGTAAAGGTCACCCTGTTGAAGATGCCATTAACATTCC

TTACATGTTCTTCACCCCGGAAGGGAGGATAAAAAACCCAACATTTTTGGAGGAGGTCGC

TTCAGTTTTAAACAAGGATGAACACATAGTAGTGGGATGCCAAAGTGGAGTGCGGTCTCT

CCACGCAGCAGTGGATCTTCTTGACACTGGTTTTAAGGATATAAAGAATATGGCGGGTGG

CTATGCCATGTGGGTAGAGAATGGATACCTTGCGAAAAAGCACGGAGAATCTGAAGTAAA

GCACGGAGAAATTGAAGTGAAAAAGCATGAAGAAACTGAAGTGAAAAAGCAGGAAGAACT

GAAGTGATCATGCTTCAGTCAGAGTTTCAGTGCTACTAGTATGCATATATTTCGAAGGAA

TAAAAGCAACACTAAGTTTGTCCATAGAAATAAT

>Unigene0001878

AGAATTTCTCAATTTCTCTTCTCTCTCATGGGGTTTGATGGATTTCGTTTCTGGAATACT

CTTCTCCTACTACTGCTGCTTGTCTGCAGGTCAGTGCCGGAGGAGTTGATCAACGTGGAC

GTACACGCTGCTTATGACCTCATCAACACCGGCAAACACCGCTACTTGGACGTCAGGACG

GTGGAGGAATTCAGTAAAGGTCACCCTGTTGAAGATGCCATTAACATTCCTTACATGCTC

TTCACCCCGGAAGGGAGAATAAAAAACCCAAAATTTTTGGA

>Unigene0001879

AAAGCACGGAGAAACTGAAGTGAAAAAGCATGAAGAACTGAAGTGACCCTGCTTCAGTCA

GTGTTTCAGTGTTACTAGTATGCATATATTTCGAAGGAATAAAAGCAACACTAAGTTTGT

CCATAGAAATAATCATCTTGCTCTAAGGGACTACATTGAATTTTCTAACAAGTTTGTATA

TGGTACCGTTGACTCAATGAAATTCTGACGTCTCAGTGTGTATTTGGAAACAAGTGTAGT

AGGGCATGCCCTTGTCTGATATATGATTTTTTTAAT

>Unigene0001880

GGAGTACTCTTCTCCTACTACTGCTGCCTGTCTGCAGGTCAGTACCGGAGGAGGTGATCA

GCGTCGACGTACACGCTGCTTATGACCTCATCAACACCGGCAAACACCGCTACTTGGACG

TCAGGACGGTGGATGAATTCAGTAAAGGTCACCCTGCCGAGAATGCCATTAACATTCCTT

ACGTGTTCTTCACCCCGGAAGGGAGGATAAAAAACCCAAAATTTTTGGAGGAGGTTGCCT

CAGTTTTAAACAAGGATGAACA

>Unigene0001881

GTGTCACACTAAAACGAAGTGTTCCACTCTTCGTTTTTTCCCGAAACCTTTTGAAATCAA

ATTTTGTGTTTACACCCCAACAAAATATAAATTCGAAACAAGTATAAAAAGGCGTTATAT

TTAACCAAATTCCAACAAAATGTGCTTCTTCTCCGGTAACAATGTGTCCCTATCTTTTTC

CTGTGTCGACTTCCTGAGATACCATAAATGATAGTTTGTGAGCTAATGACATCTTAAACA

GAGCAATGGTAGTGACATGCAAAAAAAAAAAAGAAAAATTTGTTGAGCTACAGATTGAGA

CATGTTCCTAGCCCGGAGGGAATCGGTTGGGCTGATTTCTACATGCTCACGCTACCGATA

TGGGAGCTCATGAATACTAGCGAAAAGAAGATTGATTTATCTTCTCACCCTGTTGTTGTG

GACACTCTCTTTATAAAATACAAAGCAGTGTTTAGTACAATGTCAGGTTTTCTTTCATGT

TTCGGGTAGGGTTTCTCTCTACCACATCCCTCGAAATACTTATCAGGTATATAACTCCAT

GTACCCGGAATTT

>Unigene0001882

CGCCGTTTGGGGCCGCCATGAACGTGGTGTGGGCCGCCGTCAAGGCGTACGTGCCGGAGG

ACAGCCCGTGCGTGGAAGCTATCATGAAGCGCCGCGGCGATCGGGCTGCGGGGGGCTCGT

CTTCCGGTGCCGGAAACGGGACGGCGGACGGCGGGGACGCTGAGCGGAACCGGTTGCAGG

GTGGCAGCAAACCGGACACGAACCCGTTCCGGACCACGTGCAACTCGGAACTGTCGTCGT

ACGGTGGCGTGGAGAAACGCGACGGCGGCGTGGACGGCGACGGCGTCATCAAGAGGACGC

CGCAAGCGTTTAGCATGGACGACGGTAGC

>Unigene0001883

TATACCCTCAACCTCGCCGGCGATTAACAGGACTTAAATAGAAAAATCCCCATCACCGGC

CGCCCCGTCTCCGTCTCCAGCCGGCTAGACTTTATCCACCCCCACCATCTCCAATGTCTC

CCCCTTCGCCATCCGCTCCCCTCCTCTCCTCACCCGATGGGCTGCGTCCTCCCCAAACCC

TCAAACAAATTACCGCCGCGCGGTCTCGACAGGATGAAGCTCGAGAGCGACTATCTCCCA

AGCAGCGAGTTCGCGGGGCCCACCAAGCGCCCGTCCGGCCGGATC

>Unigene0001884

GTGGAGGGTTGCGTTAGGATGCTGACAACCAGCGAAAAACATCGTCAGGTCACTCATGAT

ATGAATCCTAATAAGACAATAAAAAATGATAGGACATTTCCTAGAAGCCACGTGTTGTGC

CTGCCTCCGAGTGCTTCGAAACATAGATAAGGGGTATTGTGCTTGCCATCGAGTACAATG

AAGAATATGCTAAGGGCATTGTAACTGCCTCGGGGTACTTTGATAAATAGGTAAGGAGCG

TTGTATTGATACTCAGATGCCTTGAAAAATAGGCAAGGGTTCTCATACT

>Unigene0001885

TTTCTATTTATGTAGACTGTAAAATGTAAAAATATAAATACATATATACATAAATTATAT

ATTATATAATAATAATTAATTAAATTAATATTATTTAGCTATATTATTGTATGGTATGAA

TGATGTTTTGTTGATCTCAGTTATGTTTTGTAGATAACGATTGTGTTGAATATTGGGAAG

GAATTTTGGTTGTAATATTTTTCTTTGTGATTTGATAGTGAGGGCACTAGTTTTTTAAGG

TTATATTTTTTTCGTTTTAGTTATGCAGAAATAAAAAATAAATCACAGTTAAAAATAATA

TT

>Unigene0001886

TTAAATTCATTTAAAGATGAACCAAAACTCTATGTTGAAGCTCAAGAAGGTGATCCATTT

AGACTTGCTTGTCATCCTCCAACTGGATGGCCTAAGCCAAATGTATATTGGCTGATAAGG

AATAAAGCAGGTGGCTTAACAACTGTTAACAACACTAGAATGACATTAGATCCAGAAGGT

AATCTCTGGTTTTCAAATGTAACTAGACATGATACTAGTGATGGATCTACATATTTTTGT

TACGCTGAATCAT

>Unigene0001887

CATATATGTGGTCGTCTACAGCTGCACTGGGAATGATTGATGTATTAACGCTCAGAGAAT

GTTCACTGGATTCTACAAATTATTGTTTTACACAAATTCGTGAAAATGCGTGTAAAGCAA

GTTATGGCATTTGTAATATAAAAGTGAATGGTTATTACATAGAAATGCTTTTATGCACTA

TCATTGGAATAATTTGGTATTTTGCTGTTAGAAATACGCTAAAAAAATTTCAAACTTTGG

ATTCTTCTCATTGGCTGGTTAATATGAAAAGAACAGCAACGGCCAACAAAGAATGAGTAA

GAATAATGGGATATGTTACCATAACTGTTGTGACTTTTTGGACGTAACCACTTCGGCATG

TAAATTTGGTGACATCTGTTTTGGCTTTGGACTTAAGGAATATTTATTTTGATATTTAAA

ATGAATTTGATAACTTAAAAA

>Unigene0001888

GAGAGCAATAGGTCTATCAGTTCTTCGCCTCCATGCTAGAATACTCAACTATTTAGCTTG

AAAGAGTTCCCATGGTTCTCCGAGGCAATTGCGATTGTCGGCCACAAAGAGGTACTCCAG

CAAACTGTCATCGAATGGGTTACCTACAAAGCCCCTCTTGTCTCCACTGACCTTTCCTTC

TTGGCAACGCCCGCTTTGATTACAGGGTCTGCTCCTTGATGCAGTCACTGCCATCGTCTG

GATCATCGAGTTGAGATATGCAGGTTACTTCACCACGAGCTCCTGCCTCAACCGA

>Unigene0001889

GCCTCATAACAGTTTTCGGGGTTGCCTCTGCAAAACGAAGAACTTGAGTTAATTTGGTTG

ACGGTATAACTTATGCATTTGTTGATCATGGCATTTTCTTTTTCTCGGTATACTCACTCT

CCGCTCCTCCGAGCTGATTCACTGCCTCTATGAAACGCTCATGCAGCTCAGGTGTCCATT

TTAATCGAGGTTTAGCATCAGTCGAAAGCACCAATCCTGAATCTCCTGGAATATTTGCTC

CGTGTAAAAGCAAATGCCTCTCTGGGGAAAAGGGTGCCCTAGAAAACATGTTATCTCCTT

GGTTGTGCTGGTGATGATACATTTATGCCTCTGAAAGGACATAAGGTGGATATTAGCCGA

AATACACTGACAA

>Unigene0001890

GTTAACATTGAATACGAGTTGCAGGAGGAGAGCCTAGAACTGAACGACGTGGTCATCATG

ATCCCTTTACCGTCTGGTGTTGGTGCGCCGGTGATTGGGGAGATCGATGGCGAGTATCGC

CATGACAGCCGGAGAAATCTCCTTGAGTGGTGCCTGCCAGTGATAGATGCCAAAAACAAG

AGTGGCAGCCTGGAGTTCAGCATAGCAGGGCAGCCAAATGACTTCTTTCCAGTACAAGTC

TCCTTCATCTCCAAAAAGAACTATTGCAA

>Unigene0001891

GTGATGAGTCTTGTTGTTGGTGGTGTTTCTTGTAAATATGTTGTCTCCAAAACTGATCAT

TCATTTTTATAAAGAAGAATATTTAACAGATTTTCTTGTAACAATCCAACAATAACATAC

TTGTTAGATATTTGACTGCTCCCCGTGGACCCATTATTCATGTTGGATATGAAGCTTGGC

TTGGTAAATGAGTACTAGGTATTGAATGCTTGTTGAATTCCATAATTTGCTACAAATTTG

AATATTCCAAACATGTTTGGTTAATGGTTATAACTGGTTTGCAGTGTGG

>Unigene0001892

ACCCTCTCCTCTCCACCACTAATCGGAGATTGATCTCCACCTCCTCCCGCCCAATCTGCC

ACCACTGGTAGAGCTCTGGTGGCCGAAATCCCTCTCTTCCTCTCAAGATCTTGTCTAAAA

TTTACCCCCAAGTCTGCCTTAGTCCCATGAGTAAATCCCCCCAATCAGTTTTGGTTTTCC

CTGATAAAATCAGGAGATTTGATAGATTTCCATTCCAACCTCCATCTTGATCAACGGTGA

CCTCAATTTCTACCTCCGGCAGCCCCAGCCATCCAAGCCTTCCTACCAACCCCTCACCTC

TTCTCTTTTTGATTAATTTGAATGATGACTAACTATGGATATCTTTGCAGGAACATGGAA

GCTCGAACCAAAGAAGGAAGGTGGGATTGGAGATAGGATCAAGCATATAGAGGAAAATAG

ACCTCCAAGGATTACTAGCAGTGACCCTGGCAGAGCATGGGCATTACAAAAGCAATGATG

AAGTACATGAAAATGGTAAAGGTAAGCAATAGCAGTATTGGCATAAAGTGATGAAGCAGT

GTAGGCATGGACAGCCAAGAAGAGCAAGTAAGTGGTCCAAGGCATAGATGGTGGCAGTTG

ATTTTATGAAGTCTAGGCAGTGACAGTATGCAAGGATAACCTCTCTCTCAACCTCTTTGT

GTAGGCAATCCAGTAAGCATGGGCCAAGGTATTAAAGCACTAAGGCAATCCAACAAGCCA

AGAGATTAGCTTCTACTTCGGCTACACAATGATGTTCTGCCTTGGTTTGGGAATACTATG

CAGTGTTATAATTTGTTTGAACTCATCAATCATATAGTAGTGACACAAGGTTTAAAAGGC

TTTTGGAAGGGGAATTTTGTCAACATTCTTCGTACTACACCATTTAAGTCCGATAATTTT

TATGCATACGATACATATAGGAAACAACTTCTCAAATTATCTGGCAACAAAGAAGAAACT

GGCATTACTGCTACTATGTTGTGTATTCCAATAGATACTATCCGAACAAAGATGGTTGCA

CCTGGAGGGGAAGCATTGGGAGGTGTTATTGGTGTTTTCCGTCACATGGTTCAAAC

>Unigene0001893

CTTGTTACACTCACCAAATCCAAGAATCTCTAACCCATGTGCTCCCTCATTCAGATGCTC

CCCTGAAAACTCCGCCGAATCCCGCCACCACCATCTCGTCAACATGCGCGATTCCCTATT

CGCAGAACCTATTCGTCATCGCCGAGAACCTCCCCCAAAACCTTAACCCTAGAAATCCAA

ATCTCCGGCTCCCGCCCCCTCCCATCACGATGCAGCAGCGGCCTGAAATCCAGTTTCGCG

CCTCCTTCCCCACCCGCGCCCTAGCCTTGACCGCCGAGGCTGCGGCGCC

>Unigene0001894

AGAAGCCGAAGGTTAGAGGGCGGGGAGCGAGGGCTAGTGCGGTGAATACTACTAAGCATT

TGTGGGCTGGGGCTGTCGCCGCCATGGTCTCGAGAACTTTTGTTGCCCCCCTTGAGAGGC

TAAAGTTGGAGTACATTGTGCGTGGAGAGCAGAGGAATCTGTTTGAACTCATCAATCATA

TAGCAGCGACACAAGGTTTAAAAGGCTTTTGGAAGGGGAATTTTGTCAACATTCTTCGTA

CTGCACCATTTAAGTCCATCAATTTCTATGCATACGATACATATAGGAAACAACTTCTCA

AATTATCTGGCAACAAAGAAGCAACAAACTTTGAAACATTTCTTGCTGGCGCTGCAGCTG

GCATTACTGCTACTATGCTGTGTATTCCAATGGACACTATCCGAACAAAGATGGTTGCAC

CTGGAGGGGAAGCATTGGGAGGTGTTATTGGTGTTTTCCGTCACATGGTTCAAACAGAAG

GGTTCTTTTCCCTTTACAAGGGCTTAGTTCCCTCAGTTATTAGTATGGCACCTTCAGGTG

CAGTTTTCTATGGCGTGTATGATATACTGAAGTCAGCTTATCTGCATTCTCCCGAAGGAA

GGACTAGGATATTCCAGATGAACAAACAAGAAGGTAATGCCTTAGATCATCTGGAGTTGG

GTCCCATGAGGACCTTACTCTATGGAGCCATTGCTGGTGCTTGTGCTGAGGCAACCACAT

ATCCATTTGAAGTAGTCAGAAGACAGCTTCAAATGCAAGTTCAAGCAACAAGGTTAAATG

CTTTTGCAACTTGTGCCAAGATAGTTGAACAAGGTGGTGTTCCTGCACTTTATGCTGGTC

TGATTCCTAGTTTATTGCAGGTTTTGCCTTCTGCTGCCATTAGTTACTTTGTGTACGAGT

TCATGAAGATAGTATTGGAAGTTAAATGAATAGAACCTCCAGCCAGTCTTACAACTAACT

TCCATTTCAGTGTACCCCCTGATTGCTTAATAAGGACTATAGAAACTCTGTACTTGATCC

CAATTGTCGCGACTATTGCAAGATTGATTTCTCAGCCCGAGGCAAGGCTAAAGTGTTGTA

TACATTGGAAGACTTCTGAGCCTGAGGCAAGGCTAAAGCATTGTACACAGACTCAAACAC

CAGATTGATTTACATACGTTGTCATCATCAGACTGATTCATTACCAAATCACTCACACTG

CTGATATGAATGTGGGATAAAAAGCCTATACAAGTTTTTGCGACCTAGATTTAAATCCTG

ATTTCGATCTTGGCCTGTATTCTTTATGTTGTGAGAGTAAGTCAGACACAAATTCTGTGA

AAATTTAATTTTGCAGGCGGACTCATCAAATATTCTGTTTGTGCACCTATCTTTTGAGGG

CCATGTGAAATAGATCAGAGGTTTCATTTTTGTGTTCT

>Unigene0001895

GTAGCCTTTATGTGTAAATGTAAGCGACACCATGGGCGACCACATTTTCTGTTGAAGGCG

GACGTTTTTTTGTCTAATATGCACGGACTCTAAGGTGACCACATTATTCGTCGAAAGCTG

CTATTTCATGTCTATGTTCGCGACGTGGAGGCAACCGTGTTTAGTGGTTGCCTTGTGTTG

ATGTGCATGTGTTGTCGTTAATGACGCATGGAACCCCATGACGCCAATGTGGGCATTGT

>Unigene0001896

TGGAAAAAGTCGAGGATGGTTTCGTTCATATAAGACCAGAAAGGAACGATTTACCGAGAT

CTATCACAATCAATGAGAAAGATCTATGCAAGCATTTTAAAGTGGGACAACATGTGAAAG

TCATATCCGATGTCCAAAAAGGTGTAACGGGTATGGTGCTAAAAGTTGAAGGTCGTGTGT

TGGTCATCTTATCAGACATAACGAAGAAAGATATTCGTGTTTTCG

>Unigene0001897

CTGTCCGTGAGTAGAGCTTCTTAGTCTCCGGTGGCCGGGCCATTCCTATTCAGCTCATTA

ACTGAAATTCCGAGTCTCTATTCAAAAGTGGGTCCGGAATTTTAGTTACCGCTCGGCTCC

CAGGAGCATCAGTAGGATTCACAGGGTTAATCACTGAAATTATCTCTGTGCACTAAAATT

ACCGTTCGGCTCTCAGGAGCATCAGTCAGCATGAACGTTTCCGAGGAATTGGGTCTTTTT

T

>Unigene0001898

CTGGTCTGGGAGCCAATTTTAAGGCTGTTGAATTGGACACTGAGAGTGATGGAAGTGAGA

TTCAAGCAGCACTTGCTCAGTGGACTGGACAGCGGACCGTCCCCAATGTGTTCATAGGTG

GGAAGCACATTGGTGGCTGTGATGCAACAGTAGCACTGAATAGTAAAGGGGGACTGGTTC

CTCTCCTGGCTGAAGCAGGAGCGACTGCCAAAGTCTCTGCTTAGAAG

>Unigene0001899

GGCTCTAAAGATTTATACATTGATAGAAGAAGATAAGCTAACTCTTGATGAATTCAAAAA

ATGTCCATTACATCCAAAAAATGAAGACGAAGATGCAATTAATTGGATATTTATTGTTGA

TTCTTTGAACTATTGTTTTTTTGATCCCAATAATAAATCCCATTGGAGTGTTACTTGGAA

AAATGAAAAACATACAGGATATTTTGGTTTATGTGCTGCTATTAACAGA

>Unigene0001900

GGATTCTTCAAGCCCAGTTCTGTTGGAATCTCATCAACTGGATCAAAATGCGTGGCAGTT

CAAGTTGGGGAAACTCATGCGGAGAAGAATAAACGCTTGAACAGACCGATGTCACCACAT

CTGACCATCTACAAACCTCAGTTGACATCGATGTTGTCGATAACTCACAGAGGCACTGGA

ATGGCTCTGTCAGCTTACGCTTTCGGCATTGCAGCAGCTGGACTCTGGTAT

>Unigene0001901

TTGCTCTTACGCTGTCATCCTTGTCATCCTTATTTTCAAGTTTTCTTTTAATTCTTTTCT

TCTGGTTTGCTTCCGCAATTCTGCAAAAATGGGCTTCCTGACTTGGGCGGTTGTTTCGGT

AGTTGGCGGTGCGGTCGCCATGACTGGAGTGGGCTTACCCATTGCGGGTTTACTAGGATT

TTCAGCGGCTGGACCCGTTGCTGGGTCCTTAGCGGCAGGGGCCCAGGCGTACGTGGGAAA

CGTCGCAGCTGGCTCAGTCTTTGCAGCAGTTCAAGCATTAGCCATGGCCGCTCCAACGCC

TTGAAACGGTATTGCATGCTGTCTGACTGGAAAAGGGAGTATATTTTAAATTTCAGCAGA

TTTTAGTCATGTACCTGCCATC

>Unigene0001902

ATCATCGAATGGGAAGAATCATCAACACTTAGGCTGTGGAACTGGTATATTTTCCTTTAA

GCAATTTGAGGGTGGCCTGCCCAACAGGCACAAATTATAGTCAGGAGAAATGGGTTAGGG

AGAGCGAAATCAAAGTTAAAGGAGACTTGTAAGATGTTATGGATGGAGTCCATAGGTGAA

TAGCACAAAAAATTGGGACGGAAGCTTGGTTCTGTGTTTTGGAGCTCCCTTTTAATGCTC

AGTCAGAGGGAATGGGTAGGAGACTTGGACAGAGTTGGGAACGCTGGTGGAAATG

>Unigene0001903

GTTCTTTCCCCACTTCTTGCCCTCAAACTTCTGGATTTTCATCTTGTGATGTGAACTAAT

GTGGCCCTGATTCATAGTAACCACATCCACAATCGGCTCTGCATCCATGTTACTAGATAC

ATCCTTGATGGCCTCCATCTCGACCTTAATTCAACAAAGAGAGTAGAACCTTGCTAGGAT

CTCGTCACAAACTTAAATAGGTAAAGGGAAATGTCATTGTGATATTTACCTTTCCGGTTC

TCTAACCCAGTCTTCAATCTTTACACAAATAACACCATGATCATGAATCCCGACGCTCTG

ATAT

>Unigene0001904

GAGTCACCGCATCCACTACGAGCTCGCAGGTCTATAATAATAGGCACCTATCAGGACGAG

CGCTGCGGCCATTTCTGACAAGTACAGTCTTTCGGTCGCAGGATAAGCACATAAACATAT

CAAGATGGGTAAGGGTTACGATAGCGACGAAAATACCGTAGAAGAATGTCCACTGGCCGA

CGGACTGCGATTAGTGGCCAAACAGGAGTTGCGCGAGGATGATGTTATCAGGACACATTC

GTTGCGAGCAATGCGCAACTGGATCAGGAAACACCCGGACATCGTTAACTGTCGCACAGA

TGCACCGTTCCTGTTACGATTTTTAAGAACGAAAAAATTCAGCGTTCCCATGGCACAAGA

CATGCTGGAACGGTACTTGGTCATCAGACAACTGTATCCACAGTGGTTCTCCAAACTGGA

TGTCGACGACCCGGTCATATCCGACATATTAGACAGCGGATACCTGGTACCGTTGCCGGG

CCGAGATGAATTTGGCAGGTGTGTAATGTTCTCGTGTGCCGGCCGTTTCGATCCATACAA

GTACACGTCAGCGGATATGGCCCGTGTGCACGCGCTTATCGGCGAGAGCCTTATGGACGA

CCCCGAAAACCAGATCAAAGGGTACAGTTATGTCAACGACGAGTCCGGATTGCTGATGGG

ACACATGACGCTGTGGTCGTTTTCAGATCTAAAACGCATCATACGGTGTTTGCAAAGTTC

AACGCCAATGAGACACAAGTCCACGTACTTCATAAACGTCCCTAATTTCGCAGACAAGTT

TTTCACGGCCACGGTTTCATTTTTAAACTCCAAACTCAAGAGCCGAGTAAAGTTCTTCAG

CGGTCAAAAAGACATACAGAAATTCATCGACCCCAAATATCTACCAAAAGAGTACGGTGG

TGAAATACCTTTGGCCACAATGATAGTTGAATTGAAGAAGAAACTCAGAGCCAAACGTGA

AATGTTAATGGCTCTAGACGACATGAAAATTAACATTAATGGAAAAGGAAAAATAATTAG

TGAATTCGACGACATGCAAAAAGAACTAACCGGATCATTCAGGAAATTGGAAGTCGATTG

ATGTGTATTTTTATTAATTTTAATTTTTTATTTTATTGTTACCATAATATTATTGTATGT

CCATTACCACCATTCGTGAAGATTTTATTTGTTTTATAATTTTTAGTAATATAATTTTCT

TTTAATAAAGAACCATTATAGGAATATCAACAAATTTCATAAAATTTTATTGTCTTAATT

TTGCTGTTAAA

>Unigene0001905

CAATTCAAGGGTTTAGTGATTCAGCTGTGGTGAAAGGATCAATTGGATATGTTGCTCCAG

GTAAATTTTTTGTAAGTTATTTTAGTTTTACATTTTTTATCCAGTGCACATGACAATCAG

TTTTAAGCTGCAAGAAAGCCGAGGTTTTTAGCTTGACGCTACCTCGAGTTCAGTTTACTC

AAACAATTAAGTATAATCTAATGTATGTTGTATTGACTGTACGTTGCGGTCACGGTCTGA

ATTGATATGCTACTGATCCGACAACCAAGTATCGACCGAATTGGTCGTGTATCAGCTGTG

TCAGCCTGGTATTGGCTGGTATAGGTTTCGTATCGGTCCAAATGAGCCGGAATCTGCCTG

TATCGGGTGGTATAGGTTTTGTATCGGCCCAAATGAGCCGGTATTCGTCCGTATCAGCCG

GTATGAGTCCATATCATTTCAGTATTGGCTTGTAAATCCCATATAGGTTTGTACTGGTTA

TATATTTCTAGCTTTTCTTTTTTCTAATTCAACTTTTTTATATGTACAGAAGTTCTCTTA

TTTATAAATCAACTGATCTGTCGTGACTGATACTGTGACGGCCGCATCAGACTACGATGT

ATCATTTGGCCGATTGCTCGATATCATGACTAATACCGCGACAGCAAACCTTATTACAAT

TCAACAAAATAATGAATTTCAACTTGGGATGAATTGGTGGTTTCTGATTCTGCCATTGTT

AGCATATGTAACATTCATTACACTGTTTAAACTAATGAATCGTTATCCGGAATTCAGCTT

ATAGTGATTAGATGATTATGATCTGTCTTCTTTTGAGCTCCGGTAATTTTTAGCTCTAAT

CATTATTTGAAATTCAGAATATGGGATGGGTGACCGACTCTCAACACGTGGAGATGTATA

TAGCTATGGGATTCTTCT

>Unigene0001906

ATAAACGTCATAAGAATAAAGATATAAATAATGATGATGGTGGCAAGCATAAAAAGAATA

AACGTCATAAGAATAAAGATTTAAATAATGATGACGATGGCAAGCATAAACAGAATAAAC

GTCATAAGAATAAAGATATAAATAATGATGATGGTGGCAAGCATAAAAAGAATAAACGTC

ATAAAAATAAAGATTTAAATGATAATGATAATGGAAAGCATAAAAAGAATAATGGTCATA

AGAAAAATAAAACAATTGACGATGATAATGATAATGGTGGGCATAGAAAGAAAAGTCATA

AAAAAACATATAATAAAGTTGATGATGATATTGTAAGTAAGCATAAAAAGCATAAAAGTA

ATAAAAAAAAAGATAGAAATTATAATGATGTCGAATTATTACAAAGTAAAATAACTGATA

ATAAAAAGAAGAAAAAGATAAAAGAAAACGTATATTCAACCGAGGAAAGTAACGATGTTA

ACTATAGTTCTAGCATTCAAATAAACAGTGAAAATAGTGATTCTGTAAAAAGATTAAAAA

CGACAAGTAGTAAATCAAAAATTTCTAAAAAAACTAATTCAACTAAAAGCAAAAAATCTC

CGCCTAAAAAGAAGTCAAAATATGTAAAACATTCCAAAACTATAA

>Unigene0001907

GATAAGAAAACTACTGCTCTGAAGCAATTACAAGGTCATATCTGGAAAGATGGATATGAA

AGTGGTCTTCTAAAAGGTCATTTATATGAAGATGTATTACCTGTTTTAAATAAGCTAACT

GACTTGGGTAAAAAAATATACACTTACTCTTCTGGGAGTACAGAAGCTCAAGAGTATTTA

TTTCAATATTCCATGTATGGAGATGTGTCTAGTATATTTTTAAAGTATTTTGATACTAAA

ATGGGTCCAAAAGGCTCGGAAACCAGTTATATAAATATTGCTAATGAGATAAATGTAAAC

TGTAGTGATATTTTATTTTTAACTGACGTAGTTGCTGAAGCAGAAGCAGCTGTAAAGGCC

GGCTGTAATTCAATTCTTTTGGCAAGACCTGGTAATGCTCCTTTGGATTCTGAGAAAAGT

TCAAAGTTTAGGATTATAAAGACATTAGATGAATTATTAAAAATTGAGTATGTTGTACCT

AAATACATATAATACTTAAAATACATTTTGATAACCTTAAAATTGTAAAATCATAAATAC

ATCTCATTGATTATAAAATATGTTATATTTATATTTTATTAATAATTATTCTTATGATAT

GCTAGAAAAT

>Unigene0001908

GCGGATTTGAAACTGGATTACCTGGATCTGTACCTCATGCACTGGCCCCATGCCTTTGAA

CGAGGGGACAATCTCTTCCCAAAGAACCCTGATGGCACGATGCGTTATGATTACACTGAC

TACAAGGATACCTGGAAGGCTATGGAGAAGCTGGTAGAAAAAGGTCTTGCGAAAGCCATT

GGGCTGTCAAACTTCAATAGTCGTCAGATTGATGATGTATTAAGTGTGGCTACTGTCAAA

CCAGCTGTGCTCCAGGTAGAATGCCACCCTTACCTAGCCCAGAACGAGCTGATAGCTCAC

TGCCAGAAACGAGGACTGGTTGTTACTGCCTACAGTCCCCTCGGTTCTCCTGATCGCATG

TGGAAACACCCAGATGAGCCTGTGCTTCTAGAGGAACCTGGGATCAAAAAACTGGCAGAA

A

>Unigene0001909

CGGTCCGAGATGGCGTCGGTCCTCTACCTCCCCCTTGCACCCTACACCCATTCCAGCCAC

CGGAGCGCTGCCCTCAGGCGGGACCCTTTACAGCGTCCCAGCATACCCGCGTCTACGCAG

GTAAGCCACGCTCTTCGGCAGAGTCGGAGTCTCAGAACAAGGTGCTCCGAGTGGCATGGT

ACGGAGAGCTCCTTGGGATTGCCGCCTCATTCTTTGGGCCAGCTGCTCCGGCAGAGGGTG

TTGTTGCAGATGATGGGGGTCGGAAGTAGTGGGTC

>Unigene0001910

TGATGTAGTTTTCAATGAGGATGTGCTACTCAAAGATTGGGACAAAGTTGTTGTTGACAC

TGATGGTGATTCAGAAAGATCATTGTAGTTGCAAAATAAAGGTACTCAGATAGAAGTGCA

GTCTCAGGAGGAGACTCTCTCACCTTTACCACCATCATCTCCGGCAAGACTGAGGAGGTC

TGAAAGAATTACCAGTCCACCTAACTGGTATGTATTTGGTGATTATTTGTTGTTGACTGT

GAATGGAGAGCCAAAAAGCTATCAAGAAGCCTGCAACGGTGACTCCAGGTTCGAGTGGGA

GTTAGCCATGAATGATGAA

>Unigene0001911

TCCACTACAACCTGCCTAGATGGTTCTTCGTGCAGTGCTCTTCCTTGACATGTTTTGAAG

CTTCATTTGGTATGATGTTGAAGAGAAGAAAGATCATCAACGAGGACTAAATTTCTGGTT

TGTGTATATTTTGATGTATAACCTTGAGTCTTCTTGTACTGGTATATGAGGTTTGTACTT

AAAAATGAATTTTAATAGTCTGGATCAATGTTGTCAGGATTGGAATCG

>Unigene0001912

GTCTTTAATTCCAAGGCATAACTTGTATTCATGCGCTTCATATTAGCCTGTAGGTCGCTC

CCAGCAATATAGCCATCCCTACCCTATCACGTCAATCCCACGAGCCTCTTATCTATCTCG

TTCGATCACGGCGGGGGAGCAAGTCAAAATAGAAAAACTCACATTTGGTTTAGGGATAAT

CAGGCTCGAACTGATGACTTCCACCACGTCAAGATGACACTCTACCGCTGAGTTATATCC

CTTCCCGACCCCATCGAGAAATAGAACTAACTAATCCTAAGGCAAGGGGTCGAGAAACTC

AACGCCACTATTCCTGAACAACTTGGAGCCGGGCCTTCTTTTCGCGCTATTATGGATAGT

CAAATAATAGGAAAATTTGGATTCAATTGTCAACTGCTCCTATCGGAAATAGGATTGACT

ACGGATTCGAGCCATAGCACATGTTTCATAAAATCCGTACGATTTTCTCGATCTAAATCG

AGCAGGTTTGACATGAAGAAGATTTTGTTCAGCATGTTCTATT

>Unigene0001913

AGGAACGCAGACCATGCAGGATCCAAACAATATTGATCAACGGAGCAACATCGTCAGGGC

TCCTTGCGCAGATAGGGCTCCTGGAGAAGACCATTCCGGCGAGATCTCGTCACTCATGTG

ACTCAGGCAAAACTTCACTTCAGCCCCACCAGGATCGTCCATATGGGCCGTCTGGGGGAA

ACAGAACAAGTGATGGGACTCATGCAGGACTAGTCTGCACCGACAACTGGTGGGTCAC

>Unigene0001914

TCTACGAACTGATGGGCAAATTCGCCGAACCGTGGCTCAAGGATGGCACGACGGTGGCCG

CGCACGTAGATTTCGTGTTCCAGAAAATGGACCTTAACCGAGACGGGGTGGTGTCATTCG

ACGAGTTTATGGAAACTTGTAAAAATGACGAGAACATAACCCGTGCAATGATCTCAATGT

AGACAAGAGACATCGTAAATATAGGTGATCCGTATGATACCGTGGATGTCGTTTGAAAAT

AAAATTATACCCGAAAAACCGATGGGCAACTACGGTTGGGCAATGTAAAATCGACAGACA

TCCGAACGATAAATAGTAGT

>Unigene0001915

AACTCATAAAGCTCCCGGCGGAGAACCTTGCCGCGATGGCAAGCACCTCCTCCTCCCCTT

CCATCGTCTGCGCATCCTTCAATCAGGACAATAGTTACTTTTCGATCGGGACCAAGGATG

GGTTCAAAATATTTGATGCGCGGAGCGGAAGATTGTGCTATGAGAGAGTTATTGGTGCAT

TTAGCATTGTGGAAATGCTGTTTAGCTCTAGTCTACTAGCTGTTGTTGGAGCAGGGGAGC

AGCCATTGTTATCTCCTCGCCGACTTTGTCTATTTAATACTGTTACTGGAACTGCACTTA

GAGAACTCAACTTTTTGACAACAATCCTGGCCGTTCGATTTAACAGAAAAAGGCTTATTG

TAATATTGCAGGACAGGACATTTATCTATGACTTGAACAGTCTGTCTATCATTGACACAA

TAGATACTGTGCCTAACAACAAAGGACTATGTGCATTTTCTCCTACATCCGAAGGGTGCT

ATTTGGCTCTTCCGGCAAGTACATCTAAAGGATCTGTATTGGTTTACAATACCATGGAGC

TACAATCACTTTGTCAGATAGATGCTCACCGTTCACCATTAGCTGCAATAGTCTTCTCTT

CCAACGGAATGTACCTGGCAACAGCTTCTGAACAAGGAACTATAATTAGAGTGCATCTTG

TTTCACAAGCAACTAAGGTGAATAGGGAAGGCTATATATACCCATGTGCATGGATCTCTG

TGGGGAGAGCGTGGATTCCATTGCGTCATATAGTTTCAGAAGAGGAACATACCCATCAAC

CATTTATTCTTTATCCTTTGGTCCATCTGAGGAACTTCCAGATATCCTTCTTGCTACAAG

CTCATCAGGCTCTTTGCATGTCTTTCTTCTCGGATCTATGATAGATCAGAGAAGCAAAAA

ACCCTATGCGCTCTTGAGTTCAATGATTCCTGACTCGGTCAATGATGCATTTGATCCTGC

TCATCATGTTCATCATGATGTGGTCCCTACAGGAGTTAAAAGTTATGTGACCGTCTACAG

TGCTGAAAAAGTTCCAAATACTTCAACAGTTTCTAACTTCCGGGCATCTGTTTTTATCAT

TACACACAATGGATATTTCCGTGAGTATATCTTGAGTGTTACGAAGTTGAGCGAATCCTC

GTGGAGCTTGGAGCGCGAAGTCAACCTGCTGGAAACTAGCATTGGATAATCAGTAAAAAT

CCGGAGTAAATCTCATTTCCCACATGATCACACACCATCTCCTGTTCAGAGATCTGAGCG

AACATGGGCTAAAATAAAAAAAGCAGCATAATAGATTTATTTAAATGTATTATAGACGTC

TTGGATAACAATCTTTAACATTATTTACATGTATTGTAGACATCTTGGTATAACAATCTT

TATAATAATAATAATGTATGATATCTTTTGAAAGGTGGCTGGTATGTACATGTTTCAGCC

AACTAGTGAAATGTATATCATTCTTCGGCGTCTTGTAAGTGACAGTCAATAAATGG

>Unigene0001916

GAAACGACCGATATAGAGGAGAGAAGAGAGCGTGTGGGGAGGAATCAACAGTGAGTCTGA

GTCCCAACAAACAAATCAACGCCTTCAGGACGAGGATAGCTTTGGTTGGGCGATGGGCGG

AGGAGCGTCGAGGTGGAGGACGGCCGGAAAGCGGCTCTGCTTCGCCTGTAGCGCCGCTGC

AGAATCGCCGCCGCCGCCTCTTTCGTTGAAAGCTAATTCCAGCTCCGTTGTTTCAAAGAA

TGTGGAAATTGTGGACGTTACAGAGGATGCCGGGCGGAACTGCCGAGGTAGCAAGAATAT

TTGTGCGATATGCCTGGACTCTCTAAACAACGATGATACTGGTGACGGTGAAGGAGAGGA

AAGAATCTACACAGCCCAATGTGCCCACTCCTTCCACTTCCTCTGCATTGCCTCCAACCT

CCGTCATGGCAACTACACCTGCCCTATCTGCAGAGCCTACTGGTCCCACCTCCCCCACCA

CCTCACCGCCTTCTCCCGTCCCCACTCCAGCTCCGATCCTGTCCTCCGCATCCTCGATGA

CTCCATCGCCTCCTCCCGTGTCAACCGCCGCTCCTCCCACCGCTCCGCCACCCGCTATGA

TGACGACGACCCCGTCGCTGACACCACTACCCCCTCACACCCCAGACTCCACCTTTCCCT

CTCAAGATCAGTCCTCTCTGTGAAACTCTCTCACCAGCAAGCAGCAGACGTGGTCCTCGT

CGTCAGCCCCAATGGACCCCACCTGAGGCTCCTCAAGCAATCTATGGCAGTTGTCGTCTT

CTCGCTGCGTGCCATCGACCGACTCGCCATAGTCACTTGTTCAGCCGCCACCAGCCGTGC

ATTGCCACTACGGCGCATGACACCAGACGGCAAGCGCCTTGCTCTACACATCATCGACCG

CCTCTTCTACGTCCACGACACCGACCCGATTGAAGGGATCAAGAAAGGGGTCAAGATACT

GGAGGACAGGATGCACCGCAACCCACTTTCATACATCATCCACATCTCAGACAACCCCAC

ACGTGGTTGTGAAGTTGAATCCCCTGTCACGATCCATCGGTTCTATGTCTGGAACGGGCT

GGTGATGCACGAGTTCGAGGATTTCTTGTTGAGGATGCTTGGAGGTGCGGTGAAAGAGGT

CCAGCTGAGGGTAGCTGAGGAAAGGGGATTGGTGAGGTTGGGGGAACTCAGGGTAGGAGA

GGAGAAGAGAGTGCCTGTGGACCTGATGCGAGAGTCGGGGTACATCTGCATCAGCTACAG

ATACATTGAAGGGGCCGGGGTTGAAGAGAGGGTGACAACCGAAGAAGTGGCTGTCAGGAT

CGGAGAGAAATCAGAGTGCCCAGAAGATGGATCGTGCGAGGAAGGAGGAGGGAGGAGCAG

CAGCAGCAGTGTCGAAAGGTGGGATTACCTCGACCCCTTCATGGCTAGAAGAAGATGGGC

AAAACGTTTACATGGATACAAGACCTGACACCTATACTTATCATAAGTGCAATTTTCAGT

TTACTTGTTGTATGTTCAAGTTGTTGTAAGGTTTTTTATTCTGAGATCAAACAGCAGTGT

GAAAACAACTTGAAAGGCTTAAATTAATATAAACTTAGATAA

>Unigene0001917

CAATTTTGGAAAAATTTGCGTGGAAAATAAGGATTCTGGTGTGGAAATATAATTTACCCC

CAACTCGAGCAGAACTCCGAAAACGATTCGGATAAAGAATTTGCTGCCAACTGAGAGAGA

CGAGTACCCCTCTGCCCATAAATCCATCTCAGAGTTCTCGAAACCCTAACCAGATTGCCG

AATGAGTATTTCCAGGGAAGTTGTGTCTTTGTGGATGCTTTAGAAGTGTCAGCTCGATGC

CTTCATGCTTCATTTGTAGGATATGCTTAGTTCTGGATTCTACTCTCTCTTCTAGTTCCT

GCATTTACCACAGGAAGCTCCATTGGCATGATGACCTCTTTGGTGCATGATTCATCTGAT

GGTAACGGACCTGATGATCAGCAACAGCAGATTGAACCTGATAACCAAAGTCAAGGTCAT

GAAAAGGGGGACTTTCAACCTGGCACAGCAGTTCAAAATCCGGATTATGCAATACCTTCC

AATCACCTTGAAATGGGACATACTATGGCTCCAACGGCTTACCCCTATATGGACCCTTAC

TATGGAAGCATGATTGCATATGCTGGTCAACCTGCGATCCATCCACACTTGATGGGAGTC

CTTCAACCTGCGGTGCCTCTCCCCTCTGATGCGGTTGAAGAGCCTGTGTATGTTAATGCA

AAGCAATACCATGGCATTATGAGGCGCCGACAGTCTCGTGCGAAAGCCGATGCAGAAAAT

AAATTGATAAAATGCAGAAAGCCATATCTACATGAATCACGTCACTTGCATGCTCTAAAA

AGAGCGAGGGGCGATGGTGGTCGCTTCCTCAATTCCAAGCCGGAGAGCAATCAGCAAGAA

GGCGATCCCTCCAATGACCAGAATGTTCCATCTTTGGAGAATCCATCCTTAAAGGATAAT

GAGCATGCATCGGTCGCTTTGATTAATGGTACGATCATGGGGATTGAAGGTTTGGGCTCC

GGAAAACATAGCTTGGATGGAGAATCGCCATCCAAGGGGGTGGCTAAATGAATATAGTGA

TAAAAGATGTTTTATGTACAGTTGTTTATCTGTGTTAGACTGTTGCCAGTAATAGATTGG

TAATAATAGGTGTAAGTATTATGTCTGAGTTGGTTGTGTTAAGAAAGTCTATGGTTCATT

ATGTTGTATAGTAGTCAATAAGAGCTATTTAGTTGTTTCTGTTTAAGTACTGTGAGATTT

ATTGTTGGCAGTGACATTCGTTGCAGA

>Unigene0001918

TCGTAAGCTCCTCAATGGCTCCAACATGGACAGCATCCGCCGTGAGGTGCAGATCTTGCA

CCACCTCACCGGCCACCGCAACATCGTCGAGCTTAAAGGGGTGTACGAGGACAGGAAGGC

GGTTCACCTCGTGATGGAGCTGTGCGAGGGCGGGGAGCTGTTTGATAGAATCATTGAGAA

GGGGTGTTACTCGGAGCGGGCGGCGGCGGGGTTGTGTAGGGAGATTGCGACGGTCGTGCA

TAATTGCCATTCGATGAGGGTGATGCA

>Unigene0001919

CATCCTCGGCCGCGGCCAGTTCGGTGTCACTCACCTCGTCGCCCACCGCCATACTGGCGA

GCACCTCGCCTGCAAGTCGATCCACACCCGCAAGCTCGTCCGCCTCTCCGACATTGACGA

CGTCCGCCGTGAGGTGCAGATCCTGCACCACCTCACCGGCCACCGCAACATCGTCGAGCT

AAGGGGGGTGTACGAGGACCGGCAGTCGGTCCATCTCGTGATGGAGCTGTGCGAGGGCGG

GGAGCTGTTTGATAGAATCATTGAGAAGGGGTGTTACTCGGAGCGGGCGGCGGCGGGGTT

GTGTAGGGAGATTGCGACGGTCGTGCATAATTGCCATTCGATGAGGGTGATGCA

>Unigene0001920

GGCTCATGTCCCCCCAATAGTCGCCATTATTAACGGCCCACTGTATCAGCCTTGCGGATT

CCTGTATCCCATTTATCTCTCCTACTGGTCGGACTTCAGAGTACCATAATACAGCACCAA

CGATGGGGAACAGCAACCGCTCGCCCACTCCGTCCACCTACCCCATACCGGCCGCCGCCG

CCGGTCCTCACCGGCCCTCGCCCGCCACCTCCTCCCCCGCCCTCGGCCGCGTCTTCCACC

GCCCCATCGATGACGTCCGCGACACCTACGCCTTCGGCCCCATCCTCGGCCGCGGCCAGT

TCGGC

>Unigene0001921

ATGGCCGACGTCCGTCGCGAGGTGCAGATCATGCACCACCTCACCGGCCACAGCAACATT

GTCGAGCTAAGGGGGGCCTACGAGGACCGGCACTCGGTCCACCTCGTGATGGAGCTGTGT

GAGTGTGGGGAGCTGTTCGATCGGATCATTGCGAGGGGGTGTTTCACGGAGCAGCAGGCG

GCGGGGTTGTGTCGGCAGATTGCGACGGCTGTGCATAGTTGCCATTCGATGAGGGTGATG

CATAGGGATCTTAAACCGGAGAACTTCCTGTGTTTGGGGAAGGAGGAAGATTCGCCTCTC

AAGGCCATTGATTTCGGTCTCTCTGTCTTCTTCAAGCCAGACTTCGGAGTTCCAACACCA

GCGATGGGAAACGCCAACTGCACGCACTCCGAGTCCAATGATCGCCAGCCTGCCACCACC

CCCGCCGCCCGCCGTCTCCCCCCTCCGCCCACATCGACCGCCCCTGCCCTCGGCCGCGTC

TTCCGCCGCCCCATCGAAGACATCCGCGACACCTTCACCCTCGGCCCCATCCTTGGCCGC

GGTCAGTTCGGCATCACCTACCTCGTTACCCACCGCCACACAGGCGAGCGCCTCGCTTGC

AAGTCGATCCCCACCCGCAAGCTCCTCCGCCGCGCCGATATGGCCGACGTCCGTCGCGAG

GTGCAGATCATGCACCACCTCACCGGCCACAGCAACATTGTCGAGCTAAGGGGGGCCTAC

GAGGACCGGCACTCGGTCCACCTCGTGATGGAGCTGTGTGAGTGTGGGGAGCTGTTCGAT

CGGATCATTGCGAGGGGGTGTTTCACGGAGCAGCAGGCGGCGGGGTTGTGTCGGCAGATT

GCGACGGCTGTGCATAGTTGCCATTCGATGAGGGTGATGCATAGAGATCTTAAGCCAGAG

AATTTTCTGTTTCTTAGTAAGGAGGAGGATTCGCCTCTCAAGGCCACGGATTTTGGTCTC

TCAGTTTTCTTCAAGCCTGGAGATGTATTTAGAGACATTGTTGGAAGTGCATATTATGTT

GCTCCAGAGGTATTGCGACGAGACTATGGGATCGAAGCAGACATATGGAGCGTGGGAGTA

ATACTCTACATGCTTCTTTCTGGTGTCCCTCCTTTTTGGGGAGAGAATAATCAGGCTATA

TTTGATGCTATTTTGCGGGGAGATGTTGATTTTGAATCTGATCCTTGGCCTTCCATTTCT

AGTGGTGCAAAAGAGTTGTTGAAGAAGATATTCAGGGAAGATCCAAAAGAGCGGCCTTCT

GCAACCGAAATTCTGAACCATCCATGGGTTAGAGAAGATGGGGAGGCGTCTGATAAGCCA

ATTGACATTACAGTACTAAATAGAATGAAACAGTTTGTGGCTATGAACAAGCTTAAAAAA

ATAGCTTTGAAGGTGATTGCGGAGAATCTTTCAGAAGAAGAAATCATGGGTTTGAAAGAA

ATTTTCATATCCATGGACATTGATAAAAGTGGAACAATTACATTTGAAGAACTAAAAAAT

GGCCTATCAACGTTGGGTTCCAAGCTTTCTGAATCTGAAGTTAGGCAATTGATGGAGGCG

GCTGATGTGGATGGGAATGGGATCATCGATTATGTTGAATTTATTACATCTACAATGCAC

GTGAATAGAATGGAAAGGGAAGATCGGCTATACAAAGCATTTGAGTATTTTGACAAGGAC

AATAGCGGATACATCACAATGGAGGAACTGGAGCAAGCTCTGAAACAGTATAACATCACT

GATGTTGCAACAATCAAAGATATCATTGCAGAAGTTGATACAGACCATGATGGAAAAATT

AATTACGAAGAGTTCGTGGCCATGATGCGGAAAGGCAACCCTGCAACTTCAGTACAATGG

CAAAATTAGAAATTGTTGGCGGAACTGATCTGGCTGGCCAGACTTTTGCAGCATTCGTCT

TCATGGGTTGCCCCAATCTTGTGTATTTGATGGTGTTGTGAATTGTTTTATTTAGATCTC

CGCTTTCAACTATATAGCTTTTAGAATTGTTTTTCAGTATATAATCTTTTTATCAGTAAT

TGCTCTGAGTTGTGAGTGGTAACGAGCGATTATAATCTGC

>Unigene0001922

TGGTTCCTTCTCGTTCCCCAGATAACCAATCAAAACCCTAACCCTAACCCTAACCCTAAT

CCTAACTCGCCCAAATTCTCTAAATCACTCTTCTCACTCCCCACCTCAAACCCTAGAGAC

GCTATCCCTTCCCTTCTCCTGAACCAGTTCCTAACTACCAGGAATGGTTGAGAACGGAGC

TCTCGCGGCGGTCCTCTTCATTTTAGTCACAGTCGGAGCCACCGATCCCGCCGCCGAAGG

CGCCGGAGCAGACAAGCAACAAAAGGTCTCGCCGCCTCCGATTCCGGTTCTTTTACCGAA

TCCACCTCCGCCAGTGCCTCTCAAGCCGGAGTTGAATCAAGCTGATAAGGGTCAGCTTGA

GTCCGGTGGGTTGGCGACCGTGCCTCCGCCACCTAGCGGAGCTGGCAAAATTGACGAAGG

AAAGAAAGGAGGTGACATTTCTAGCAGAGATACAAAGTCGGAGGTAGGGTGTGAGGCAGT

GGCTGAGAAGTGTCATGCTTCGGAGTTGGTTGCTTGCCTTCAATATTCAGGAGATGCGTC

TAAGGAATTATTCCTATTAGTTCAGAATGAAGGGAAGGATTCTTTGATTGTGAATATCAA

AACATCAATAAATAGTGATACCAACAAGCTACAAGTAGCCAGACACAGTGCTGAAAAGGT

TGGCAAGCATCCTAACAAATGCCACGGATGTCAAAGAGAGTATAAGAATTACTCTGAATG

CTGGAAAAGAGGATTGCATTATTGAAATTAAGCCATCTGTTCCTGATTGGAAGCGCTCCC

AACAGTTCCTTGCTTATGCTACTAACTTGACTCCAATTCATGGGCTGTATTTCCTGTTCA

TTATCGTATTTATCGTTGTTGGGGCATGGACTTTCTGCAAGTTAAAGAGGATGTCGAGGA

GGAGTGATTCAGGAATTCCGTATCAGCAGCTCGAGATGGGGACCCAGCTGCAATCTAGAT

CAGTAGTTGCAGATACAAACACTGCAGTTGGCTGGGATGAGAGCTGGGGTGATGACTGGG

ATGAGGAAGAAGGGGTGGGGGGACCTTCAGAGAAGCATTCTACCGCAAATGTTTCGTCAG

ATGGCCTCCGTTCTAAGCCTCCCAATAAGGATGGATGGGATGTCGATTGGGATGATTAAA

AACTGTCAATGAGCAAGCAACGACTATTTATTCCACAAGGTTAAGAGTCATGAAGATGTT

GAAGTCAATTCTGTTTTTGTTGGCATAGAAAAAAGAATTAGCTTTTTCGGATAAAGCATT

GTGCAAATGGAATACCCCCTATACACATAATGATTTGGTATTGAGCCTGAATCTGCGGAA

TACATTTTTAACTACTAAATATTTGGAGACAAACATTGAAGGTTGCGAGCGACGCTCTGC

TTTATACAAGACCTAGCATCCTCAAATGCCGTTAGAAATTATGTATCAGGCAGATTCTGA

AGCTGTGTTGTACATGCCTGGTGAGTTTTCCCTCCCTAGTTGCAGACAATGGATTTTTGA

TTTCATGGGAGCTTAATGGTTTCTTATTTTGTCTGTCTTATCATGGAATATTCTGTATAT

TCTCGCTCTTGCTCTTTAATGACCACAATTAGGTTGATCACTGGCTTCAAGAGTTGCAAG

TATAGGGCTGTTTATCTCTGATCAGTGTGTTGATCATTATCACCCGAAGAGAAAATATAC

TCCTCCACCCGTCAAGGATCGTGTCAGAACTACCGAATGTGATTGGGTTGGTGAATTTAT

TTGAAAGGTTAAAATTGTTAAAAC

>Unigene0001923

TGGGCTTTTCTGGTTGTGGGACAAATGCGGTTGGCCCACATTTGGGGAATTCTCTGTAAC

ACTGAATCAACAATTTAAAGTGCTTCTCTTGTATATTCTTGATGAATCAACATATATGCA

TAGATGTAGGTGTAAGACTTTTGTGGGTTTGCTGGATCTATCACCGGATTGTGCCCGGTA

GTGAGATACATACAAGGGAAGTCATTCTTTGATCACGTACATTGGGCTTATATGGACGAC

CATTTGGTGGTGGGTGACGTAGACAGTCTCCACCACAAGGTTTCTCCTCTAAGCCGCCTC

CAACAACAAAGTCGATGGGAACTCATTTCTTACACGTGAATCAGATTAGGGGTAAAGCAG

GGTCACGAGGGATTTATAGCAGAAGGGGAGAGGGAAGTTTGTACCCGGCCATCACTTCTC

TTTCCATGAGCTTTCTAATTGAAACCTAAGTAACTTTTGCTATACCCGGCCATCACTTCT

CTTTCCATGAGCTTTCTAATTGAAACCTAAGTAACTTTTGCTATACCCGGCCATCACTTC

TCTTTC

>Unigene0001924

GAAGAGGCGTCTAAAGTAAAACAAGAAGCATAGTTGTTGGACGAATTGGGGAGACTTTTG

AGTCGGTGTCAGGGCTCATCAAACCACATCTTTAAGTATTACAAACTTAGAACGAAGAGC

AGTAGGAACATGAGAGACAATATCATGTCTACGACTAACCAGCTGGTGAGATTGGTGCCA

GGGCTCATCAAACCACATCTTAAGTATTGCAAACTCATAACAAAGAGTAGTAGGAACATG

AGAGACAATATCTGGTCCGCGACCAACAAGCTGGTGAGATTAGTAGAGCGATGTGTTCCA

CGGCCGTGACGCTGAGCACCCTTTAGCTCAGGATGAAGTTGCCAACATGACTCCACATGA

TGACCCTCTTGGTGATAGGTAGTGTAGTGTAGTGACGGAGGACAACATGATGATGAGAAG

CATGAGCATCCATCAGCAGCAGGAGCCCTTTTCG

>Unigene0001925

GGATATATAAGAGTACAAAGAAAACAAGAGGAGAGAAAATATAATTACAAATAAAATGAG

ATTAGTGAGAAAATGAGAGGAGAGAGGAAATGAGAGGACAAAAAAAATATGAGGAGAGAA

AATATAAGTACAAATAAAATGGGATAAGTGAGAAATGACAGGAGAGAAAATATAAGGAGA

GAAAAATATAAGGAAAGATAAAAAATGAGTAGAGATAAAGGAGAGATAAAACGAGCGGAG

AGAAAAAAAAGAGACGATATAAAACAAAACAAAATATAATATGGATACTAGTTGTAGTTA

TATTACTACAAAAATATTTAAATGAGAGAATGGGAAAAAAATCAATTGTAAAACTAGATT

GAAGAGGAAGACACTGCAGAATTTTGATTTTTTACTCATTAATCTCAGCTGCTTATATA

>Unigene0001926

GGAGATAAAAAAATGAGGAGAGATTAAATGGAGAAAAAATGAGAGGAAAAAGAAATTATG

AGGTAGGGAAACAAGAGAAGAAAATTAGATGCAATAAATAATGAGAAGACAGTAAATATG

AGGGGAGAGAAGATGAGAAAAACAAAAAAAAAATATTTGGAGAGAATATGAGAGGGGAGA

GAAAAGTATGATGAGAGAAAAATAACAAAGAAAATGTGAGGAGAGTGAAAATATGAAGAA

AAATTAAGAGGTTGAATAAAATGTGACGATAATATATAAGAGTACAGAGAAAATAAGAGG

AGGGAAAATATAATTACAAATAAAATGAGATAATTGAGAAAATGAGAGGAGACGAAATGA

GAGGAGAGAAAATATTAGGAAAAATAACAAGGGAGTAAAGAGAAAA

>Unigene0001927

GAAAGATAACAAGGGAGTAAAGATAAAATCTGAGGAAAGAGTATATGAAAGGAGATAAAA

AAAATGAGGAGAGAGAACATAAAATAAGAACAATGAGAAGAAAAAGAAATTATGAAGAAG

GAAACAAAATAAAATAAGGAAATAAGATGGCATAGATAATATGAAGACGGTAAATATGAG

GGGAGGGAAGATGAGAGAAGCAAAAAAAAAAATGTTCGAAGAGAATATGAGAACAAAGAG

AAAAATATGAGTAACAAGGGAATATGAAGAGAGAAATTAAGAGGATGAAGAAAATATGAC

GAGAATATATAATAGTACATAAAAAACAAGAGGAGAGAAAATATA

>Unigene0001928

TTAGGGTGCTCTGATACCAAATTGTAAAACCAGATTGAAGAGGAAGATACTGTAGAATTC

TTATTATTTTATTCATTAATCTCAGCTGCTTATATAAGCATAGAGAAGGGATTGTTGGAT

AATAACCCCACTATTAAAGCTTGCCTAAAGATTAGGACAACTTTGACTAATTTGATATCC

CCACTATTAAAGCATGTCTAAAGGCTAGGACAACTTTGACTAATTCTCTAGACTAATTCT

AACCAGACTTAACATTTGGTTCTCAACAGCTTCAAATGGCGTCAATACAAATTTTGCGTT

ACTTGTCCAGTTTTGGACTCTTTTATACCAAAAAATTGTAAAAGCTTAAAAACATTTCTA

ACACGGAAAAAGGCTGACAAACAATTTGAGCTACACAAGCATGCTAGTCGAATCATAGTT

TTACTAAAGTTCCTTCGGCAAAAATTGACGACATGCTGTTTTAACATTATCGAGTTTCAA

TGTGTCGCCACTTGGTATGTTGGTTGCCTACAGATCTCTTCTAGGTAGTACATCAGATAC

AATAACTACTTATATTTCTATTGTAAACTTTTAATCACTTAAGTTGACTGTACTTCTGTA

AGACTGTCTCTTAAAGAGAATTTACTCTCTTT

>Unigene0001929

TATTAAAGCATGCCTAAAGACTATGACAACTTTGACTAATTCTAACCAGACTTAACATTT

GGTTCTCAACACTCCTCAAGATGGGTGATATATATCAATCATCCCCATCTTGTTATATAG

TTTAGAAAGACTCAAGGACCTAAGACCCTTAGTAAGACCATCTGCGACTTGTTCTTCTGA

GCAAACATGATCAAGCTTTAATATCCCGTTCTCTAGCTTCTCTTTGATGAAGAACCG

>Unigene0001930

TTGACTAATTCTCTAGACTAATTCTAACCAGACTTAACATTTGGTTCTTAACATTCTCCC

TCAAGATGGACGATAGATATCATTCATCCCATCTTGTTACATAGTTTTAGAAAGACTCAA

GGAGCTAAGACCCTTCGTAAGACCATCTGCCATTTGTTCTTCTGAGTGAACATGATTAAG

CCTCAATATCCTGCTCTTTAGCTTTACTTTGACGAAGAACCATTCTATCTCGATATGCTT

TGTTCTGTTATGCTGAACTAGATTGTTAGCTATACTCAGTGCAGCTTGAACTCAGAAGGT

GTAATGGTGCTCCTTGATCGAACTTAAATTGTCACGAGAACTTTTACTCACAACTCTGAC

ACTCCCAGAGCAATAGTTCATAGACTGTTTCTTACTTCTCTAGGAGACTAGGATACCCCC

TACAAAGACACAGTACCCTGAGGTGAACCTTCTGTCATTCATGCAGCTTGTCCAATATGC

ATTGCAGTATCCTTCAGCACCCGGGTGTCCATTATTTTTGAACCATAGCCCTCTTCCAGA

AGCACCCTCGGAATAAAGAAAATTGCATTCATGTGGCCCTTCTTTGGATCATGCATGTAG

TAGCTTACTACACT

>Unigene0001931

AGCTGCTTATATAAGCATAGAGGTGGGGTTGTAGGATAATAACCCCACTATTAAAGCATG

CCTAAAGACTAGGACAACTTTGACAAATTTGGTAACCCCACTATTAAAGCATGCCTAAAG

ACTAGGACAACTTTGACTTATTCTCTAGACTAATTCTAACAGCTAATTCCAACACTAATT

CTAACCAAACTTAACATTTGGTTCTCAATAATATCCAACGTCAACTCTTTTGCCCGTGTG

AGGATCCTGAATAAACAAAACACAAGAATTTGTCAAGCCAACAACACAAGGACAATCGAT

AGAAATAAGAGTAAGCACCTGTGGTGCCACATCACAAAAAAATGATCAAACATCCTGATG

AATTGGTAGCACTAGTGATTGTGCCGTACTAAGAAATGCACACATGTTACCCATTTGCAG

TGTTTATCTTGAG

>Unigene0001932

CCTCGTCATCCCTCAGTCCAACGTATTGGATCTCCGCTAACACCGGCCCCTCGACGCACA

CGCCCAAATCGATCTTCAAAAACTCCGCATCCCACCTCCAATCCGTCCCCATCCCCTCCC

ATGGCAAAGCTCCGCCAGCCACCGCCGCCACCGGCGGCGTCTACGGCCAAGCAAACCCTA

GACTCCTACACAATCAAAGGAACCACCAAATCCATCCACCCCGGCGACAGCGTGCTGATG

CGGCCGCCAGAGCCGGGGAAGCTGCCGTACGTGGCGCGGGTGGAGACGATCGAGGCGGAC

CGGCGCGGGGTACGGGTCAAGGTGCGGTGGTATTACAGGCCGGAGGAGTCAATCGGCGGG

CGGCGGCGGTTTCACGGGTCGAAGGAGCTGTTTCTGTCGGATCACTATGATTTTCAGAGC

GCTGATACAATTGAGGGGAAATGCACAGTTCATACATTTAAAGAGTATACTGGGCTTTCG

GCTGTTGGTGATGGCGATTTCTTCTCGAGATTTCAGTATAAATCGGCGAATGGAACCTTC

ATACCCGATCGAATTGCTGTGTTTTGCAAGTGTGAGATGCCTTATAATCCAGATGATCTC

ATGATCCAGTGTGAGGACTGTTTGGAATGGTATCATCCTTCTTGTATAGATATGACCATT

GAGGAAGCTAAGAAAATTGAACATTTCTTTTGTCAAAGTTGTTCCTCTGAGAAAGAAAAG

AAGATGCCAAATTCTGAAAAGGCATCAAAAGAAAGTAGTTCCTTTGAGAAAGAAGAGAAG

AAACCAAATTCTGAAAAGACATCAAGAGAATCAGATGTAAAGATTGCGGGTGATCCAAAG

AGTCGCTACTGATGACAGATTGTCAAAAGTCTCCAAGAAAAGAAACAAAGAAAGCTGAAA

AATTAGAAAATAGTAATGGGTTAAAGAACAAGTGTGGTTGAGAACTCTGTTATTATTATC

TTGAATAGATTTCTAGCTGATTAGCAAAGATGTGAATTCAAAGCGCCGGAGAAAGTGATA

TCAGAGGCAAAGACAGACCTTCAGAGTTGTATAGACAGGAAAGTAGGTAAATTAAGTTCA

GCGAGAAATGGTGGTTTGTGAACTGTATATAGGTCAGGAAAGTAGTCTCTTGAGGAAAAA

AAAAAAAAAAAGATTGTGGGTAATCAGGTCCTATCCATCCTATGTCTGTGTCCGGAATTA

AAGAACATTAAATTGGGTTCCAGTGACTATGTTGATGAATGTGTTTGTGCCGTATATTTG

CTTGCTTGTGATTGATTTCAGAAAATTAATTGATGGTAGTATTCCACAATAATTTTACGC

CAGAAATTTATGAGAAGATTGTTTTGATTTGATTGCTTTTTAACTCCTCAATTTGTCAGA

TGAAACTTCTGTTTGACGTC

>Unigene0001933

CCTCGTCATCCCTCAGTCCAACGTATTGGATCTCCGCTAACACCGGCCCCTCGACGCACA

CGCCCAAATCGATCTTCAAAAACTCCGCATCCCACCTCCAATCCGTCCCCATCCCCTCCC

ATGGCAAAGCTCCGCCAGCCACCGCCACCACTGACAACATCTACGGCGAAGCAAACCCTA

GACTCCTACACAATCAAAGGAACCACCAAATCCATCCACTCCGGCGACAGCGTGCTGATG

CGACCGCCAAAGCCGGGGAAGCTGCGTATGTGGCACGGGTGGAGACGATCGAGGCGGACC

GGCACGGGGTACGTGTCAAGGTGCGGTGGTATTACAGGTCGGAGGAGTCAATCGGCGGGC

AGCGGCGGTTTCACGGGTCGAAGGAGCTGTTTCTGTCGGATCACTATGATTTTCAGAGCG

CTGATACAATTGAGGGGAAATGCACAGTTCATACATTTAAAGAGTATACTGGGCTTTCGG

CTGTTGGTGATGGCGATTTCTTCTCGAGATTTCAGTATAAATCG

>Unigene0001934

ATTTATAGGAATTTTCTGATGTGCTTCTCTTTAATCTGTAGATTCTTTATCTATTTTTAG

TAATCTTAGCTGCTTTTCGCTCAGATCATTCGTCTCTGCTGCGCTGCTCCGCCAGAAGAT

CAGATCCAGATGGACAGGGAGCAGCGGAATCAGAGGAGGGCTTCGGCGGAGCCCGATCTC

TTCCTCCAGTGGGGGAACCGCAAGCGGCTCCGGTGCGTCAAGGCGCGGGATGACGGCGGC

GGTGGTGGCGGTGGCGGCGGCGGTCAGCGCCGCACAGCTACCCGGGTCAATCGCCGCATC

CTCCGGGGGGTCGAGAAGGATCAGCATCTCTCATCGCCGCCACTGCCACCGCCGCCTCCT

CCTCAGCCGACACCATCCCAAAACCATAGGTGATAACCGGAATTTTAGTCTTAGGATTGT

TTGCTGTTGAATCTGAATACCGATCTCTTGTTTCTTCCGCAGGGCGTCGGAGTCGGCGGG

GAGTGAGAGCCGGAAGCCGACGAGATCGGTGTCATCATCGCCGGAGAAGGAAGATCGGTT

CTACACGACGAGGGGGTCTGCCGCGGTGGCGGCGGCTGTTTATGAGGAGAATAACGGCAA

CGGAGTGGGGGACGAGAAAGGCGTCGCTGCGCTTCCGAGATTCTTCCTTTCCTTGTCGAA

CAAGGAGAAGGAGGAGGATTTCATGGCGATGAAGGGCTGCAAGCTGCCGCAGAGACCAAA

GAAGCGGGCGAAGTTTATACAGAAATCTATACTTCTGGTTAGCCCGGGGGCGTGGCTGAC

GGATCTATCGCAGGAGAGGTACGAGGTTAGGGAGAAGAAGAGCTCGAAAAAGAGGCGGAG

GGGATTGAAGGCTATGAGCATGGAGAGCGACTCAGAGTGATGGAATTGGAGGTTATGTTC

AACGGAAACCCTGTTTTAATGAACAGAATGCAATGGTGGTTCGGGTCTATAGATTTTGGA

ATTTTTGTATATACATCTTAACTGAGAAATTTTGGGTCCCTGGTTTTCTTTTAATTTTAA

TGGAGTTTATCTGGAAAACTATTTGAGCTATGTTGTCATGGTTATTGTTTATTATATCTT

TTCTGGCAAACAAAAGGTATGTGAAGTGCGAACAAAGAAAATAAAGGCATGAATGATAGT

TGTTCAAAATTGAGAACCTACCTCTCATACCAAGCTAACCAAACTTTTATGTTAGTTGGG

TTGGTGTGTGGTCAGATAGTGGGCTTCTTTCCATCGGTGCAACTATATGATTATCATTTG

TGCTTTCAGGTCCTGTTGTTGCTTTCTTAATTACCATTTTTTTGTGGAATAATGAAAGTA

TTTGGGGTGGCCTGTTTCATTCATTTCTTTTTCTAGAATGGGTTAGAGAGGAACTTGGTG

GGATTACCTTG

>Unigene0001935

GGGAGAAGAAGAGCTCGAAAAAGGTATATTTCTTCGTGTTTTGCTGTTTATTTTTAGAGC

TTGCAGTTGATTGAATACTTTCCATTCGGTTTAATGCAATATGGGAACACAAATGGTGTA

GATTTGTTGGATATTTTGATGGTGAATGATTAATTTGGTGATTTTTTTTCCATTCATATG

ACAAGACATGGGAACTTGGTCTGTGTAGATTTATTGGCTATGTTGATGGTCTGAATGAGT

TGTAAAATCATTTTTGTTCGGTTTGACCCAATTTGGAGTCTTGGATAGAGTAGAGTTGTT

GGCTGTTTTGTTGGTTGATAAATGGTTGTTTTATCTTTTTCCCCTTTGGTTTGATGCCAT

TTGGGGACTCCGACAGTATACATCTGGTGGATATTTTGATGGTCGATGAATGATTTATTT

GATCTTTTGATTTGGTTTTACACTACCTAGGAGCAACGAGGGTAGATTTGTCAGTTAATG

GTTGATTCTTCCATTTCATTGATTTTGGTGGTTTTGTTTGTGATGTAATAATTTGTTTGA

TGTGTGAATCTTTTGCAGAGGCGGAGGGGATTGAAGGCT

>Unigene0001936

GCAACTATATGATTATCATTTGTTTGTGCTTTCAGGTCCTGTTTGTTGCAACTAGCCTTA

GCAGTTAATAGGGGTTTCTTAATTATAGTTTTTTTGGAATAGTGAAAATATTGGGGGTAT

CCTGTTTCATGCATTTTTTTTGCTAGAATTGGTTAGAAAGGAACTTGGTGGGATTACCTT

GTTTTATGTATATGTTTGGTATTCTTTTTTGTGGAATATTGAAGATATTTGGGACATCCT

ATTTCCTGCATATGTTATCAAGAATTCACATAATTTATTAGAAGACAAATTGGGAAATTA

CCTTATTTTCTGTTTGGGTGTGCAACTTAAATATTTGTTTTGCCGTTTTTATGTTTTGTT

GCTTTAAGGACCGGTTAACTTGTATAGAATGGACCTAATCATAATGAATCTGAAACCCTT

AAAGATAACTTATTTTAGTTCCCACTTTCCATTCTCAATTTTGACCCTTTCCTGCTTGCA

TGCTAATTTCTATCCTCAAAATATCTGAAATGGAAGTGTATGGAAACAATTTGAAATTGT

TTCTAACATGGCTAACAGAAATTTAGTTAAAAAAATAAGTAAACTTGAAAAGAATATTAT

AAAATGCCATTTCTTATTAGGTGCAGTTGTATGATTCCAACACAGCAGTATGAACACTGT

AG

>Unigene0001937

GGTGCCAATGATAACATGGCCTCGCTTCGGGGATCAGTTTGTGAACGAGAAGCTGGTGAT

TGAAATACTGAGAGTCGGTATATCGCTCGGGGTGACAGTGCCACACTTTTATGGAGTTGA

TGGTGAGGAGGTGGTGGTCAAAAAGGATGAGGTGGAGAAGGCAGTGGCAAAGCTTATGGA

TGGAGGGAAAGAAGGGGAAGAGAGGAGAGAAAGGGCGATGAAGTTGGGTGAGAAG

>Unigene0001938

CTCGCTTTGGGGATCGGTTTGTGAACGAGAAGCTGGTGGTTGAAATACTGAGAGTTGGTA

TATCGCTCAGGGTGACAGTGCCACACTTTTATGGAGTTGATGGTGAGGAGGTGGTGGTCA

AAAAGGATGAGGTGGAGAAGGCAGTGGCAAAGCTTATGGATGGAGGGGAAGAGAGGAGAG

AAAGGGCGATGAAGTTGGGTGAGAAGTCTAGGAAGGCAATGGAAGAAGGGG

>Unigene0001939

GTGGGGGGTTTTGTGACGCACTGTGGGTGGAACTCCCTATTGGAAGCTGTGTCGGCAGGG

GTGCCAATGATAACATGGCCTTGTTTTGCAGATCAGATCGTGAACGAGAATCGGGTGGTT

GAAGTGCCGAGAGTCGGTATAACGCTTGGGGTGACAGTACCTTACTTTTATGGATTTAGT

GAAGAGGAGATCGTGGTGAAGAAGGATGAGATAGAGAAGGCAGTGGCATTGTTTATGGTT

TTGGGGGGGAGCACGACGAAGTTGAGTGAGAAGGCTAGAAAGGCAATAGAAGAAGGGGGG

TCATCTTACGAGAACTTGAATGACTTGATCCGATATGTTTGCAAAATTGAACGAGCAAAT

TAGTCATGATT

>Unigene0001940

GGAACTCCCTATTGGAAGCTGTGTCGGCAGGGGTGCCAATGATAACATGGCCTCGTTTTG

CAGATCAGTTCGTGAATGAGAAGTTAGTGGTTAACGTACTGAAGGTCGGTATATCACTCG

GGGTAACGGTGCCATATTATTATGGAGTTAATAGTGAGGAGGAGGAAGTGATAAAAATGG

ATAAGTTGGAGAAGGCTGTGACAAAGCTTATGGATGGAGGGAATGAAGGAGAAGAGAGGA

GAGAAAGGGCGATCAAGTTGGGTGAGAATGCTAGGAAGGCAATGAAAGAAGGGGGGTCAT

CTTATGAAAACTTGAATGGCTTGATTCGATATGTTCGTGAAATCGAACGAGCAAATTAGT

CATGATTTAATGTTTGAAATGAAAGGGCACAACCTTGCTTGTATTAACTTATGGGCAAAT

TAGTCATGGTTTAATATTTCAAGTGAAAGGGCACAACCTTGTATGTACTACCTTTGAATT

CTTGAATGTTTTTCGAACAATTAATTGTCTTTTATGCATATAATTCCATATACTACATTT

GTATGTTGGGACCACATTATT

>Unigene0001941

CCGCAGGTGGTTATACTGTCTCACCCCGCCGTGGGGGGTTTTGTGACGCACTGTGGGTGG

AACTCCCTATTGGAAGCTGTGTCGGCAGGGGTGCCAATGATAACATGGCCTTGTTTTGCA

GATCAGTTCGTGAACGAGAAGCTGGTGGTTGAAGTGCTGAGGGTCGGTACAACGCTCGGG

GTGACCGTGCCATACTTTTATGGAGTTAGTGAGGTGGAGGTGGTGGTGAAAAAAGATGAG

GTAGAGAAGGCAGTGGCAAAACTTATGGATGGAGAGGAGGAAGGAGAGCAGAGAAGAGAA

AGAGCGATGATGTTGAGTGAGAAGGCTAGGAAGGCAATGGAAAAAAGGGGGTCATCTTAT

GAGAACATGAATGACTTAATCAGGTATGTTTGCGAAACCGAACAAGCAAAGTTGTCATGA

TTTAATGTTTGTAGTGAAGGGGCACAACCTTGCAAGTATTACCTTACAAGCAAATTAGTC

ATGATTTAATGTTTGAAATGAAAGGGCACAA

>Unigene0001942

GAGGTGATAAAAATGGAAAAGGTGGAGAAGGCTGTGACAAAGCTTATGGATGGAGGGAGT

GAAGGAGAAAAGAGGAGAGAAAGGGCGATAAAGTTGGGTGAGAAGGCTAGGAAGGCAATG

GAAGAAGGGGGGTCATCTTATGAAAACTTGAATGGCTTGATCCGATATGTTTGTGAAATC

GAACGCACAAATTAGTCATGATTTAATGTTTGAAATGAAAGGGCACAATCTTGCATGTAT

TAACTTATGGGAAAATTAGTCATGATTTAATGTTTCAAGTGAAAGGGCACAACCT

>Unigene0001943

GGAAGCTGTGTCGGCAGGGGTGCTAATGATAACATGGCCTCACTATGCAGATCAGTTCGT

CAATGAAAAGTTAGTAGTTGATGTACTGAAAGTCGGTATATCACTCGGGGTGACGGTGCC

ACATTATTATGGAGTTGATACGGAGGAGGAGGAGGTGATAAAAATGGAAAAGTTGGAGAA

GGCTGTGACAAAGCTTATGGATGGAGGGAGTGAAGGAGAAAAGAGGAGAGAAAGGGC

>Unigene0001944

TTCTCAACTTCCCGAGGAGAGAGACGATGGCGGAGCCGGTGACGACGGAGTGGCGGTGCG

AGCGAAACGACGGCAAGCGGTGGCGGTGCAAGAAGCGGAGGAAGGACGACGATACCCTCT

GCGAGCACCATTACGCCGAGAAGATGGCCAAGTGTGGCCGAGGTGCCTTTACCAGCAAGT

CGCGCCGTCACCGGGGTGGCAAAGCGGCCATCTCCCCCCATAAGCCAATGGATCCCCCAG

TGGAGGGTTTCGGTCGGGATGAGAAGGATGATAGCTATCCTCCAGGGTTCGGTCCATCCA

CAAGTGACGGAGAGGTATCTATCGATAAGACATCCACTGAGGGGACATGTTTGATCGTCG

GTCCAAATGAAAGGGATCCAGTGGTCGATTCGACCAACATGGTGGAAGAGCTCTGTATCT

CCAGGATGTCTAAAGCAGCAAAGAAAATGACTTCAAATTCCAACCTTTGTTCAGTGGTTC

AAGAGAAGAACAAGGGTCCTGAGAACAAGTTATATCAGAGTAAAGTGGGCCCGGTGTGGT

GTCACCAGTGTCACATGAGTGACAAGGGGAGGGCCGTGTCTTGCATGTATTGCAAGAAGA

AACAATATTGTGGGCGATGCATGACCCGCTGGTATCCTCACCTCTCTGAGATGGAATTTT

CAAAGAAATGTCCGTTATGTCGTAACAATTGCAATTGCAAAGCATGCTTACGAAATGGAA

TTACCAAGCCTGCATTTATTATAAAGAAGTCCGACATAGCAAGGCATTCTTATTATGTGC

TACATATGTTAGTTCCATGGCTGAAAGAATTACGCAAAGATCGAATGATAGAGAAAGAAT

TTGAGGCTACAATTAAGGGTTTGGAACCAACTCAATTGAAGTTACAACAAGTTGAACACC

AGAAGGATGACTGTGTGCACTGCAACAATTGCAGAGCTTCTATTTTTGATTTTCACAGAA

GCTGCCCTAATTGTTCATATGCCTTGTGCCTCATTTGCTGTCGCGAGCTTCTTCGAGGCC

TCCTACCTGGGAGAGAACGAACCATCACTTATGGAAACTGGAAAATAAGTGACAATGGCA

ACATTACTTGTCCACCAAAGGAAGTTGGTGGTTGTGGCAGTTCATTTTTGGACCTGAAGT

GCATTATATCCGAGGAACACATTTTGGGATTAGAACAGAGAGCTACTGCAATTGTCGGTG

ACAATT

>Unigene0001945

CCCGTCACGCTCTCGCAATCTCTAGCGCTCCGAAAACCCTCTCGGGGATTGAAAGCGATA

GTCGGAACTCGGATCGATTCGTGCTTCTAGCCGGAGACGATGCCCAAGCAGAAGTCGCTG

CAGCCGCCGCCGCCTCCCGATGAGAAGCGGTGCAACCGCAACGACGGAAAGGGGTGGCGC

TGCCCTAATCCCAAGAGGGATGACCACAACTTCTGCGACCATCATTTTGAGCAAAGAATG

CGCTCTGCTTCGAAAGCAAGCAGAGGGGGTGGCTCGCGCAGCGGAAGAGGACGCCGCCGG

CGGGAGCGGGTGGACGGCGCTGCCACCGCCGGCGGGAGCGGGCAGACGCCGAGGGCGAAG

CGATTGAAGATTTTGGAAGATGGAAGTGAGGATGAGAAGCCCAAGAAGGAAGCTGTTTAT

GTTAGGAGGGGGAAAGCTCTGAATGTGCCGAAGAAGCGGAAGAGGGATTTGGAGTCTATT

TCCAGTAGCGAGGATGAGGAATTGATGGCTAAGGTTGGTGGTGGAAAGGTGAAAGAGTTG

ATCTTGCAGCCCAGGGAGAAAAGGAAGCGGAATCCCACTATCGTCGATCCGCCGAGCAGC

GATGAAGAAGCTGGATTTCTCGCAAAGGTCTTTAAAGCGGCGAAGACGCGCAAGAGGGAG

CAATTGGGAAAGGGGAAGAGGGTGAAGAGGGAGGATTCAATTGATACCATCTCTGATAAT

GATTATGCTCAAGAGAAGGATAAGGTTGCTAAGAGGAAGCGTTTGATGGGGAAAGATTCA

CTCATGTGCCATCAGTGCTTTTCGAGTCAATGGAAGGTGGTCCAGTGTACGAACTGCAAG

AGGAAGCGATATTGCAGGAAGTGTATTAATCGATGGTATCCTGATTTATCAGGGACTGAA

ATTGCAAATAAGTGCCCTGTTTGCCGCAACATTTGCAATTGCAAAGCGTGCTTACGGAAG

AAAGGTCGAGTTGCCAAGCCTCCAGAAAAGAAGTCACAGTCTGACATTTTAAGACATAGT

TATCATGTTTTACATTCATTGCTTCCATGGCTGAAAGAGGAACGCCGGACTCAAATGATG

GAGAAGGTTGTTGAGGCTAGGATTAAGGGTTTGGAACCAACTGAGTTGGAGCTGCCAATG

GCTGTATGTGGTGAAGGTGAACGTGTGTACTGCAACAATTGCCGGGCCTCTTTTATTGAT

TTTCACAGAAGCTGCCCTAGTTGTTCATATGACCTGTGCCTTACTTGTTGTCGTGAGCTT

>Unigene0001946

ATTGTTACTTCCAGCAATGGACCATCAATTATAACTTTGAAAGTCAAGAGAGTTGATTGT

TACTTTTGCCGGTAGACTGGAATGGGAGATTCCTAGTTTCAATTATGACTTCTAGTCAGG

AGAGCTGATCGTTAATTCTACCAGCTAGATAAACCAGAATGAGAGGTTTTTGGGATCAAT

TACAACTTCAAAAGTCAAGAGAGCTGATTGTTACTTTTACGAAGCTTGCCAAATTACTTC

CCAACATAAAGTATAAAGTTGTTGAAAAGATTGATGACAAACATAGTACATCAATCTTAT

TGTATTCTCTTTCCACCAAAGTATGAAAAT

>Unigene0001947

ACTGGCTGCCATTCTGTAGAGAGAAGAGGATGCTCCAGAGGTCACCGGAGGTTTACTTTG

CCTCCGACGAGGGTGGGGAATCCGATGAGATGAAGAGTCAGGATTGGCGATTGTATCCGC

ACAAAAGTCAGGATTGGCGATTGTATCCGCACAAAACTGGTCAGTGGCACCAGTTGTATC

ATGAGTGAATTGGCCTATGGTTTGTCCTCTGGCGCGAACATTATTTGGAATATTAACTCA

CAATTGGCCAAAATAATAATTGAAATCTGACCCTTCAGATTTAGAAAATTAGAGTACACG

GACAGGAATGGGAGATTTGGGTTCAATTATAACCTTAAAAATATGAGAGAGCTGATTGTT

ATCTGCTTCAAAAGTCAAGCTAGCTGATTGTTACTTCCAGCAATGGACC

>Unigene0001948

CCAAGCCTCCCTCATGTGCAGGAACAGGACGGCCACTCTGGCCTCCACCCCCAAGCCCCG

CCCTTCCCCACGCGTCTCCGACACTTCCACCGCCACCAGCCACGGCACCTCCACCACGTC

TACCTCCACCACCACCGCCTCCTTCTCCTCCGACTCCCTCCGCGACTCCCTCCGTGACCT

CGCCATCCTCTACCCCTTCCCCGAACTCTCCGCCGCCACCAACAATCTCCTCACCCCCCG

CTGCTCCCTCCGCGGCTCCCCCGCCATCGTCCTCCACCGCCGCCTCCGCGCCGACCCCGC

CTCCATCCCCTCCCGCCTGGCAACCTTCTGCCGCAGCCAGCACACCAGCATAGCCCGCCT

CCTTGGCGCCTCCATCCCCGGCGACGGCCGCATCTTCCTCGTCTACGAACAAATCCCCGG

CGCCAATCTCTCCGACTGCCTCCGCAGCCCACACAACCCCGGCTTCACCTCCCTCTCCAC

CTGGAAGTCCCGCATCCAGATAGCCGCCGACCTCGCACAAGGCTTGGAGTACATCCACCA

CCACTCCGGCGGTCAACGTAGCAACGTGCATAACCGGATCAAAAGCTCCGGCATCATTGT

CACCGAACCGGGCTTTCGTGCCAAGATATGTGATTTCGGGGCTGTTGAGTTGTCAGGCGA

GCTGACAGAGACTGAAACAGCGAGAGGGGTGAGGATAGAGGGGACGAGGGGATACATGGC

CCCGGAGGTGCTGGCTGGCGAGCGGGTATCGCAGCGATCAGACGTTTACGCCTTCGGCGT

GCTTATACTGGAGCTGCTGTCGGGGGCTGAGCCGGTGAGGTACCGGTTTGTGGATAAGAG

GTTAGAGATGGTGGCGGTGGTGGAGACGGCGAGG

>Unigene0001949

GGTTTACTTTGCCTCTGACGAGGGTGGGGAATCCGATGGGATGAAGGTATGTTACACATG

AACTTCCCTTTTTCCGTTCTTGTTTTGAAAAGTTGTGTTTTTTTTTCCCAATAATAGGTG

ATATTTAAGGCATTTCCCAATTTAAATAATATAATAATATAAAAAATGTCTATTTATAGT

AACTTTTCTTAATTTAGCAATAGTCGGGCCATCGGGAATAATGATAGTTTTGTAAGTGCC

CACTTGCATGGCATAGACTTCGTGGCTGTCATACATGAAATTATAGGGGATTATGGGTCA

CCTATTTTTGTATCTTTGTCCCAGAGTCAGGATTGGCGATTGCATCCGAACAAAACTGAT

GTCAGCGACATCAGTTGTATCGTGAGTGATAGGCCTATGGTTTGTCCTCTGGCGCCATCC

TTATTTAGAATATTAACTCACAATTGGTCAAAATAATAAATGAAATCTAATCCTTCAGAT

TTAGAAAATTAGAGTACACGGACAGAAATGGGAGATTTCAGTTCAATTATAACTTTAAAA

GTTTAGAGTGTTGATTTTTACTCCTACTTATGGGAGATTCCCGGGTTCAATTATAGCTTC

AAAGGTCAGGTAAGCTGATTGTTACTTCTAGCAACGGACCGTCAGTTATACTTTTGAAAG

TCAATAGCCAGCAACGGATCGTCAATTACATCTTTGAATTATATTTTTGAAAGTCAAAAG

AGTTGATTGTTACTTTAACCGGTAGATTGGTATGGGAGA

>Unigene0001950

GAGCTGATTGTTCCTTTTCCTTACGGAAAGTTGTTGAGAAGATTGATGACAAATATAATG

CATCAATCTTATTGAATTCTCTTTTCACGAAAGTATGAGAATTGCCGTGCCTCAATAATT

GTAAATATATATATTATCCATTACACTACTAATAAAACTTATCAAGAATGATTCATCATC

CCCTATCCCTCTTAGTCTCAAAAGTCAAGATTGGTTATTGTATCTGAATAAGGGTGGTGT

TAGCTTTTGTATCGTTAGCAATGGGCCAATGGTTTGTCATCTGATGCCAACCTTATTTAG

AATATTTTGTGATGAGTAGAGTGACGCAGCCCCTAATAGAGAATAAAACTAATATTGACC

AGTTCTCACTCTTCACCAGTTCTATTGTAATACATTGTACCCATTTGATTCTCCATTGTA

CATGCTATTTATAGCCTGTAAACTTTAATCAAAGATGTACCAATTATTCATTACTTCATA

CCATCTTATATAGTATCAGAGAGGAGTATCTGCAGAGACAAAGAGGCTGAGTAGCTACTG

AACCCCTGCCATCCTCTACTCCTTTCCTGAACTCTCCGCTGCCACCAACAATCTCCTCAC

CCCCCGCTGCTCCCTCCGCGGCTCCCCCGCCATCGTCCTCCACCGCCGCCTCCG

>Unigene0001951

GAATCAACCGCCCTAGTCACAGACGAGCATCTTAAGAAGACAATTGCTTACAACATTTGA

TGAAGTCCACCAAATATGCGTAAAGAAAAATGAGATCGAAATGAAAAGAACATATCCTTC

GGATCCAAAAACAGAAGAAGAACAAAGTCAATGGCCTAAAAGCAAAGTTTGATTTTGTGA

TGAACTGAACCAGGGAGAAGGAGGCCTTCAATGCCTACCTAGCGGACATCGAACAAGGCG

GTGATTTTGGGGTGTGCTAAAGATGAAGAGCATTTGTAGTATAGGGATTGATCCCAAATG

GACGCCATGATCAAAGCTAAAAAAATGGCTATCGTATCAAGAATTGCAGCCTCTCATAGT

TCTAAGGAATCTAGTTGAAGCTCAGGACAAGACTGAGTTACTTGTAATTTTTCTATTTTT

TGTCCAAGCATTAGGCTTTATAACTTTTTTTTTTTCTGAATTATGAATGAAGTATTATTT

TGCCTTCCCATATTTGTATATTTACTTTCTCATTATCTGAATGATTAACTTTAGTGTTTT

ATGAATAATGTTTTTCTTGTTTCCATTCTTCTTTCCACAAGTTCAAGCATCATTCGGGAA

CTTTCTCAGAATCGCACAACAATTTTCATAGGTCATAAAGACTTCAAAATTTAAACCACA

GTAAATCAAATTAACAAAAGTCACAGTATGTGGTTGGCAATCAACCAAAAGCAGACAGTT

CGCATCAAAACAAACTAACTTAATCACAACTTAAATTATAAAAAATATTCAGTTTTCATT

CTGAATCATGTACTCAATAAAAATTTTCATTAAGAAGTACAACATAGTAACACCGGAATG

GCACCAAAGCATAAGCCCAAATATATTATGAACTCCTTCCAAAGTAGTTCCGAATCACAA

TTTGAATAAAACAGATAAACAATTTTCTATAATGTTCAAACACTTAAAAGTTTTATAAGT

TTTATAATGTCAATGATGATTTTTCTTGAGTTAGTGGACATTCCACAGGTCCAGAAGCAT

CTCATCTCCTATCCCAACAAATTTGTAAGCATCCGCGATAATATTTCGGTAATGATGAAG

GATTCATGTCACTTTGGATACATATTGTTTTTTGGACTTTCCATGTATCTCAACATGTAG

TCTCCTACTTGAATCTTTTTCGGTCGATGGTGTTTGTAATATGAGTGAGGATTTGGCCGT

TGTGATCAAGATTCACCGGTGAAGCAGTGCAAGGTGTTCAATTGTGGGAGAGCTCAGCGG

ATTGAGGATTCTGATCATGGAGATTGTTCCTCACATTGGTCTACCACATTGATTAGGGGT

AATTCTTTGTTGATTGTTGTTTGGATTATAATTGCAATCTTGTACTCTTCAATTATCAAT

GAAATGCATCTGAGGTCGCCGTTCGTGGAAGTTGGCATTGCATAGGCCGAATCATATAAA

TCTTGTGTGTTGTTTACTTTCTATACTTGGTGTGTTGCTCGTGTTTGTTGCTTGTTGAGG

ATTCTCACACCCTTTGCTAGATCGAAGGCCTCAGGTGATTCCTATCAAGTTGGTACTGGA

TAATTGTGTTACAACTGGTATCAATGGGGTGAATTTGCCAACATGATGTTGGGTATGGAG

CTTGAGCTCGTATCATGTATTCTCATAAATTTATATAATTTTGAGATCTTTTACAAAAAA

AATTAGGCTATTAATGAAAAAAAAGATGATAAGAAAATCATACATATGCTTCAGAGAAGG

GAGATCGAATGTGCCTGGTCCAGGACAAAATACTCAGGGAGCAACACCGTCTTATGACGG

AAGACAAAAGTATGCACGAGGAGAGGATACAGTAATGTTCAGTCACTTATGGTGGCCTCC

CTATCCTCTAAAGAAATCCATTCTCGTAGAAAAGATTTTGAGTAAGTTTTGAGAGTTGCC

CAAATCAAGGTTCGAGCACTTGCGGCTCAAAATGAAGAGAAACCTGCCAAGGAGTTGCTC

CACCTCCGAGGAGGTCAACATCAACATCAGGGAGACCATGCTTAAGCTGTCCAAATTTTC

AGACAAATGGTTAAAAAAGTTGAGCAATATGAGGAAAAAAGAATTGTATTTGGTGGGTAT

TCTTGCTTCGGCGGAGCTTACCGAATCAACCGCCCTAGTCACAGACGAGCATCTTAAGAA

GACAATTGCTTACAACATTTGATGAAGTCCACCAAATATGCGTAAAGAAAAATGAGATCG

AAATGAAAAGAAC

>Unigene0001952

TCTGATGGATGCGGGAAGAGTGCACAAGGCAGTACTGATTACTGCAAGGCGCATGGGGGA

GGTAAGCGGTGCTCATGGGGCCAAGGGGAACCAGATTTGGGCATCGGTGGCCAATGCGAC

AGGTTTGCTAGGGGAAAAACTGGCCTATGTGCTGCACATGGTGCTCTGGTGCAAGACCAA

TGTGTTCACGGCGGGGCATTACTTGGAACTATCACTACTCAAAACCCAATAGCGAGGAAA

GACGAGAAGATGAAAGGGATTACCTTTGAAGAAGATGTATACTTTAATATAGGGAATGGT

AGACAGAGCCCCATCGGGTGGAACAATATGTATCACTTGACACCGATCCAGGCTAGTATG

GTTTCTCTTCCAGAAGGTAGAGTGCATGGGGGCAGTCTGTTGGCTATGATGGCTGCGGGT

TTAAGCATTGAGGGCCACTCTGGAAGCCGTGCCGAAGGTGGTACCTCAGAGGAGCGTTCA

CCATACAAAACGGTACTCGACTGGGTGTAAAGGCAAGCAGTGTAGCCTTCTTCATTCTGT

GAGGTCTGTCTGGTCTGGTCATGGCATACAATGGTCCTTTGAGAGCTCTGTCTTCGACTG

TTCTAGTCTGTCATTACTCCTGGGTGTGGAAACTGAGGGAAGCAAACTATATCCATAACT

TGGTCCTGATCCATTTCATACCCCTTGTACTATGAATGAGGGGAAATAAGAGACTGGCAA

AGTTAGTGCGTCGTGTTATTTGTGCTTTTCTTAGTTTGTTTGTGTGTTAGTTGAATAAAT

ATGTAGATTGTGTGAATATGTGGTCAGTTTGAATGCTTATGTCACCCTTGTCTGTCTGCA

TATCAATATTTCTTCGTGTAATTTGCTTTGTTTTTTTTTGTATTTATCCATGACCTAAGA

CTGTTTCGGCTACAATCTTTTAT

>Unigene0001953

CACCCACCCATTGTTTCATCACTGCCCTGCAGCTCCATATTACTCTCAACTCACACCATT

CCTTCCCACCAACCTTAACCCCGCGACAAGATTCACTCATGGCCGCAGACTTGTCTCCGA

CACTATGGATCGGCGCTGCAGTGGTAATGGGTCTCGGAGGTTGGTTCTATGCAGCCATCC

AGCCACCAGAACCCCTGCTCTGTGGCTCCGAGGGCGGACCGCCAGTTACGGCCCCGAGGA

TCAGGCTCAGCGACGGCCGGTTTCTCGCCTACGCAGAGACCGGCGTCCCTAAGGATCTAG

CCCGGTTTAAGATTGTCTTCTGCCACGGCTTCAGCAATACCAGGCTTGA

>Unigene0001954

GGCTTCAGCAATACCAGGCTTGACGCCTTCATAGCCTCCCCTGAACTCACAGAAGAGCTA

GGTGTATACATGGTAGCATACGACCGAGCAGGATATGGGGAGAGCGATCCAAATCGTCGA

CGGTCGCTGAAGAGCGAGGCATCAGATATCGAAGAGCTTGCCGGTGCACTGAAGTTAGGA

CCCAAGTTCTACTTAATTGGCTTCTCTTTGGGAGGCCACTCTGTCTGGGCATCCCTCAAG

TTCATTCCCGGAAGACTGGCCGGAGCTGTCCTGGTAGCACCCGTGATAAACTACCGATGG

CCGGGCTTCCCAAAAAACCTCTCGCGGGAGTTCTACGCCAAACAAGATGTGGGAGATCAG

TGGGCTTTGAGAGTCGCATACTATACCCCATCGCTGCTATACTGGTGGATGAAGCAGTCT

TGGCTGCCATCCTCGACTGTCATCAAAGGCACTTCCTACCTTGCGAACCCCCTGGACGTT

GAGCTCCGTGACTACCTTATGTCCTCTGGAGCTTTTTTAAAGCGGAAGAAACTGGCCACT

CAGCAAGGCATTCAGGAGTCGTACTACCGGGACATGAC

>Unigene0001955

TGGAGCTTTTTTAAAGCGGAAGAGGCTGGCCACTCAGCAAGGCATTCAGGAGTCGTACTA

CCGGGACATGACAGTGATGTTTGGGAAGTGGGAGTTTGATCCAATGGACCTCCCGCCGGC

ATCCTTCCCGGTCCACATCTGGCAAGGTGATGAAGACGGGCTGGTTCCTGTTGAGCTTCA

GCGTTATATATGCAGCCGTCTCTGCTGGATCAAGTACCATGAGCTTCCTGCTCATGGCCA

TTACCTGTCACTGGTGCCAGGTTTCGTCGATGCCGTTCTTAGGACCCTGCTCGTTGAACA

TTCTACAAAGTAAGAAATTTTTAGTAGGCAAAGTGTTCGCAGTAAATGACTTTTTTTTTT

CTTTTGTTGCAGTTCGTTATCTTTACTCTTAAATATTTTGTACTTTGCTTCTCGTAAGTT

TTCGCATAACCCATGACCCATTTCAAGTTCATGATAAAAGAG

>Unigene0001956

TTTCTTCTTTCGGTCCCCCGTCACAGGAAGTTGGATGATAAGGATGAATTCCTCAGTGGA

CGACATATTCTCTCTCAGCTACAGAATCGCATGATAATGCGCTGAATCAAGCTGAAATCT

ATTGGGACGGAAGGAATCGTTGGATCTTTACCTCGCTATCGATCGCTACTAAAGATAAGT

CATTGCGGTACAATTGAAATGGATACCCAAAGGCTTAAAAGCAGTTACCATAGAGACAAT

ACTAAGCTAGCATCCCGCAACAACACAAGGACTAGTTATAAACCACTCCCGAACACTTTA

TCAGTTGCTCCTGCAGTATCAAGAGAACACAATATCAATGTGCACGAAGTTACACATTTC

TTTCCTTCTCCAGCTCTTTCTTGTACAGAGAGCCTCCCTGTTGTTGGTCATATTTCTTAT

ATGACCATTCCTGAAACAAGAAATGAATACTTGGAATCTTCATGTAAAGAGTCTTCAACA

TCCAAGGTCATTCAGGATGTGCATATTTCAGCGAGATTGATGGAAGAATTTATGGAATTA

GCTAGAGCTAATACAAACAATGACTTGGAGACTTGCGGAATACTTGGTGCTTTCCTTGAG

AATCGGATTTTCTTCATAACTACTCTTATAGTACCTAAGCAAGAATCAAGTTCCAATTCA

GTATGACAAATTCTAGTTAATTCTAATTTTCATTTTTCATAACTCTTATTCAATAAATGT

ATCAATGGAGAAACTCAGTCATGTTCCAAGCAATAATTTTTTCGACAGTGCCAGGCGATA

AACGAAGAGGAAATTTACGCCATACAAGAGGAGCGGTCTCTTTTTACAGCAGGATGGATT

CATACACACCCTTCCCAGACTTGTTTCCTCTCATCAATAGACCTGCACACTCAATATTCT

TACCAGGTGATGTTACCGGAGGCAGTTGCTATAGTGATGGCTCCAACGGATCCGACTAGG

AGCTATGGAATATTTCGGCTAACTGACCCTGGCGGCATGACTGTCCTGAAACACTGCAAA

GAGAAGGGATTCCACCCCCACCGAGAAACTGCTGATGGTACTCCGATTTTCGAGAGTTGC

TCCAATGTTTACATCAATCCAAACTTGAGGTTCGAGATTATTGATTTACGCACTTCTTCC

TCGTGAATATAGTTACGACTTCAGCATAAGATGTACATATATAAATGCATATAGACAAAA

GCATAAGATGTACATATATAAATGCATATAGACAAAAGCATAAGATGTACATATATAAA

>Unigene0001957

TGAGCCTAAAGGTGGACCCGAGTAAGCTTTTGCTTTATACCATTTATAATAGTTGATGAT

TCTAACTTAAAGTATTTATCCTTCTTGAGTTCAGAATAATTAATGATTCTGATTTAAGGT

AACAATTCTCCTTGGGCTCATGAGCCTAAAGGTGGACCCGAGTAAGCTTTTGCTTTATAC

CATTTATAATAGTTGATGATTCTAACTTAAAGTATTTATCCTTCTTGAGTTCAGAATAAT

TAATGA

>Unigene0001958

TGAGCCTAAAGGTGGACCCGAGTAAGCTTTTGCTTTATACCATTTATAATAGTTGATGAT

TCTAACTTAAAGTATTTATCCTTCTTGAGTTCAGAATAATTAATGATTCTGATTTAAGGT

AACAATCCTCCTTGGGCTCGTGAGCCTAAAGGTGGACCCGAGTAAGCTTTTGCTTTATAC

CATTTATAATAGTTGATGATTCTAACTTAAAGTATTTATCCTTCTTGAGTTCAGAATAAT

TAATGA

>Unigene0001959

TGAGCCTAAAGGTGGACCCGAGTAAGCTTTTGCTTTATACCATTTATAATAGTTGATGAT

TCTAACTTAAAGTATTTATCCTTCTTGAGTTCAGAATAATTAATGACTCTGATTTAAGGT

AACAATTCTTCTTGGGCTCCTGAGCCTAAAGGTGGACCCGGGTAAGCTTGTTGGACCCGA

GTAAGCTTTTGCTTATATCATCTAAAATAGCTCGTGAATCTTGAGCTGGACCCACGCTTA

TATTATTTAAAATAGTTCGTGAATCTAAAGTTAGATCCGAGCAAGCGTTTGCTTATATCA

TTTAGAATCTGATAGTCACATGTCCAAACCTCAAATCAAAAACCTGACTCTGACTCAGAA

CCTAAATTTTGTGCATGGTCGGATTTTCTGTCTTAGGCTTTCTATGCAGAAAAGTAAGCT

GTGCCGGATAGAGATGTATTAGATCATATATTTTGATTAAAAAAATATGAAAATATAAAA

CATATAACGACATGATTGGGGTAAGGATATCTCATTTGTACCGATAACCTTATCAGGTAG

GGTACAGTACCCGACCAGTTCCCATCTCTTATTTTGAGCTGCCCCTATTCGAGAATGTTA

ATCTCTATTCTGCTTAGATTTCATATTATCTCAGTTAAAAGTAAATAAATCAAATCCTAA

TGGCTGAGACCATATCATAATGTGAATGATGTGCTGGAAAATTTCTAATTTTTTAAAAGT

AATTTCAGCTTCTGTAATTTATAATATATCTTAATGATACGGCACCATTTGGCAAATAAA

TACATATATTTGTATCTTCCGATACATACAAGGATCAATTAAGATATATTATATTGGGTA

ATATTTGTGTATAAATCGAGAAACTTTAACAAAATATACGATACAAGTCTCCGACTTGGT

GTGGAAACCCTAATTTCGTGCAGTGGAGAAATCACAAATAATTCACAATTAGAGTTAACC

ACAAACTAAAAGAAAAAGATAACACATATATTTAATATGGTTTAATCCAAGTTAACCTAT

GTCTGTGGAAGGAGGATTTTTATTATAATAATAGAGGAAAGGGGAGAGAAACGATGATGC

TCAACAACTATAAATAGAGAGTTCTTCTCTTCTCTCTCAAACTCCTCTACATAAAGGGAA

AGCGCAAATTGGAGAATGTTTTTCGCTCTTGTTTTCTCTCACTTCTTTGCTCTACTCAAA

ACAATAAAACCACCTATCTATTTATACACAAAAATCAGCGAAGGCTACTGTAGCAATTTC

GACTGCGGCATTATTCACTTTACAACGTCATTGTTCACTTTTCAGCTGTTGCACAATTCA

CCACTATTTACTTTTCACTGTTCACTTACGGGTTGACTATTGTATATTTCAGTAAAAAAC

CACTGGTTATGCATTGTAGTTAAAATACTGCTGATCCCTCTAACAATTTTCCACATCAAC

AAGCTTTTGTGAAACAAAAAATGAGTAGATTACTATGAGCGACTCACACTCGTCCATTCA

AGATGACACACGTTCCGCCCGATTAGCCTGAAAAGCTCTCAATCTTACTGTACCTCCTCG

AGGTTACCTACGTACCCACTATAGGGATCAAGTCGATAGTAGTTCGCTAATGCCCCAAGG

GCCAATCGCCAAACTGAGAGGATGTGAGTCAAATTCTTGTAGTAAGAGAACTTTAATACT

GGCATGACCTTGATTAACATGTCTGCGTGATTATCATTTATGACAATTTTATTTAATAGG

ATGTCGTCCTCTTCAATGAGCTTGTGGACAAAGTTATATCTGATATCGATATGCTTCATG

ATGCTCGATACGATATTATGGAGAAATAATATGAATGATATTCTGGAGAAATGATACGGT

CGACAAATAGTATTGACGATCATGTTGAAGATAATGCTCTGCTATTATAATTTAACTAAT

TATTAGCAAAGTAGATTATGCTCTGTATATTAAATTAGTACTATACACATTAATTTGTTA

TGGTAATTGAATTTGTATCATTGGCCAATTTCCTCCGACAAAATTTTGGTCCATCTGACA

GCCATCTGATATGCCACGTCAACCGGTTGGACATTGATAAATCTGAGTAATGCAACATTT

ACATGGTAACTCGCAGATAATTAATTTGCGTTTGTTTATTCTTTTTAATGTCAATGGTAC

TTACTATTGGGTTTTTTTTTTTTTTTAATTCTTTTATCCTCTTAGCCCTGTGCCT

>Unigene0001960

TATTCAGTCTCCTCCACAACAGTGAAACCGTGCGGCGCTGCTCCTTCGGCGGCGGCTTCA

AGAGAGCGACCTCCGGCGAAGATCTGCGATCTCCGTTCAATCCCAGTTGGTCATCATGCA

GGCTAGTGACAGGTTCAACATCAATTCCCAGCTTGAACATCTTCAGGCTAAATATGTAGG

AACAGGGCATGCCGATATGAACAGATTTGAATGGGCAGTGAACATTCAACGTGATAGCTA

TGCTTCCTATGTTGGACACTACCCCATGCTGGCATATTTCGCAATTGCAGAGAATGAGTC

AATTGGAAGAGAACGATACAACTTTATGCAGAAAATGTTGCTACCATGTGGAAACCCTCC

TGAGAAAGAAGATGATTGACTTGTTCCAATCTTATTACCTCCTTGAGAATGGCTTTTGAG

CATGTAGTCAAGAAATATCTCAATTCTATGATACCGCCGAACAATTTATTTCTAAGTTAG

ACCAGTTGTCGGAGCTGCTAGTTGTTATGGTATGTACCATACTAAATGTGTTCTTTGGAA

TGATATGTGAGCTAGTCGTAACCATGAAGGCCTTTCACAACTCGCTTCCTCACATATTCT

AGTATGTTATATCAATTGGTCTGGTTGCTAGTGTGAAGTATCCTAATTTTCTCTGCCAAT

TTCTATTATTAATGTGATTAGTGATTGTGTAATTACAT

>Unigene0001961

CCTACGAAATCTCCATCTTGCTCCAATGGCGACGGCGACGCTGCAGCAAGTCTGCTCTGC

CCCTCTCGCCCGCGCCCTCCGCTCCAGCGCCGCCTCTCTCACGGTCCCCGTCCGTAGTGC

CCAATCCGCTACCTCCCGTGTCCTCCTCGGCATGTCCGAGCACGACCTCCAGCAGCTCGC

CGTCGAGTTCGGTCAGCAAAGCTACAGAGGAAGGCAGCTACACCGGCTTCTGTACAAGAC

CAAGGCCAAGGAAATCCAAGAATTCAGTCACTTGCCGCAGGCGTTCCGGGATCTTCTTCG

GGATGCGGGGTGGCGGGTTGGGAGGTCGCCCGTGCATCATTCCGTTGCTTCCGCTGATGG

GACTGTTAAGCTACTTCTAAAGTTGGAAGACAACAGGGTGATTGAAACTGTAGGGATACC

GGTGGAAGACGATAAAGGCTCCTTTCGTCTAACTGCCTGTGTGTCATCACAAGTAGGCTG

TCCCTTGCGCTGCTCATTTTGTGCGACTGGCAAAGGAGGGTACTCAAGGAACCTCCAACC

ACATGAGATTATCGAACAGGTATTGTCTATAGAGGAAATTTTCAAACATAGGGTGACAAA

TGTTGTGTTTATGGGAATGGGAGAGCCAATGTTGAACCTGAAGTCAGTACTGGAAGCACA

CCGATGCTTGAACAAGGATATTGAAATTGGGCAAAGGATGATGACAATATCCACAGTGGG

AGTTCCAAACACAATCAAGATGTTGGCGTCACACAAACTCCAATCAACATTGGCTGTCAG

CTTGCATGCGCCAAACCAGAAACTCCGTGAAGCAATTGTTCCCAGTGCTAAGTCCTATCC

TTTGAACGCGATTATGGAGGACTGCAGAGACTATTTCCTCCAAACTGGACGCAGAGTGTC

CTTTGAGTATACACTTTTAGCTGGGGTCAATGATGCGCAGGAGCATGCTGTAGAACTGGC

AGAGTTGCTCCATCGGTGTGGCGGCGGTTACCACGTTAATTTGATACCATTCAATCCAAT

TGATGGATCTGAATATCGAAGGCCTTACAAGAAAGCGGTGCTAGCTTTCGCTTCTGCACT

GCAGTCTCGCCGGATAACCGTCAGTGTTCGACAAACACGGGGGCTTGACGCAGGTGCAGC

GTGTGGGCAGCTGAAGAACGAATTTCAGAAGAACCCTCTACCTGCGGTGGTATCAACCAA

GCCCGAACCCTCTCCCACATTGGATGCTGTACCTTTGGCATGAGTAACCTATTACCCTCG

CGATGGTATTTTTGAATAGGCACTAGGATGTGTTAGGGGTTGCTATTGACCTGCTATTGA

TGTGTCAGATCACAGCAAACCTCAACCGAAACAAGATGAGTTCTATCAGCGAAGAGGAGG

TGTCTGCAGGCGAGAAAAGATCCTGAGATGTTATTGTGTTTTCTTGTTGACAGGTAAAGC

TTACTGTTAGGCATATAAGAAATAGCATCCATACAGGACACACAATGCATGAAAAACATT

TGATCGTTATACTACAAGCCATTTGGTACTCTTGCAGAGAGAAATTTTTGGTGATCTTAA

TATTATTTATGATTAAGAGTTGTTCTTTGGTTTTAAATAGTATCAAATGACACA

>Unigene0001962

GAAAAAAAAAGGAGGGCAGGAAAGGGTAAGGTAGCAAAATAGAACAAAAGGCTCTCATCA

ACAGCTCTTCTGCTTTCTTCTCCCAAACCTCATCCAGAGAATCAGAAGAGAGAAAAGCTT

CTTTCAAAGATCCAAGTTGTGAGAGAATGAATACAGGATCATTCAGCTCTCTTGTGCAAC

AAATTGAGGGTGGTCAAAATTGGTGGAACATGAGCAACATGAGGATGCAGACTTATGAGC

AGTCCTTTCTTCTACCCGCTTCTTCACCATCGTCATCGTTGTCATCATCATCTTCTTCAT

CGGTACTGACTCAGTATCCCGCAGCTTCACATCTTGTTCCGACGAATACTTGGCATGAAA

ACCAAGATCTCCCTGAGTCATGGAGGCAACTCTTGATGGAAGGACTGGGGGGTGAGGATG

AGAGGTGCTGTTATAGTACTCCTTTCCAAGCTAAGCAAATGGATAATTCAGAGACTCATC

AGTTTGTCTACCCATCAGTGAGTGTACATTTGGGTGATGTGAAGCCGGACATCTCGCAAT

TTCCACCAGCTACTCCTAATATATCTTGTATCACGACTAATCTCAACAACAACATATTGG

ACTTCTCGCATAACAATCAAGAAAGCAACCACAATCAACAAGATTATTCATCTGAGTGCA

ACAACACAACTGGTTCTGCTTTCAAAAAGGCTAGGGTTCAAGCCTCCCAAACACTACCAA

CTTTCAAGGCGAGGAAGGAGAAGTTGGGAGATAGAATAACAGCTCTCCACCAGATAGTTT

CTCCATTTGGGAAGACTGACACAGCTTCTGTCCTGCTAGAAGCCATTGGCTACATCAGAT

TCCTGCAGGCTCAGATTGAGGTTTTCTCTTTCCCTCTCACTCTCCATTGTTCTCTCACCT

GAAACAACTTGATGGCCTGAGAGCACGCAGTTAAAGCTGTTGCAAAGGCGGTGGTGTTTT

ATGAACATCCGTTTGTGAATGGTGTTTTTTTTGTTGTCATGTGTTGTTCTAGTAGTTATG

CCCTTCCTCATCCTTTTGTTTTTTCCTAGGCTCTGAGCACACCGTACTTGGGCGGTGGAT

CTAATGCTAACATGAGTGAGTCTGATGTCAAGGATGAGCCAAAGAGGGACCTGAGGAGTA

GAGGGCTTTGCCTTGTTCCAGTATCCTGTACAATGCGAGTTGGTAGCCACAATGGGGCGG

ATTACTAGGCTCCGACTTTTGGAGGAGGCTTCCGGTAGGTACATTCACTGTTCGTGGGAT

GTTCAGGCTCTACAGGTAGATATAACATCATGAGCAGTCACTCTGTTAAAATCCAAGGCT

GATTGCTCATGATGCTATGCACCAGCCTCGGATAGGAGAACCTATACACCAATATACATG

CTTTCTTAGTTCAAAAACCTCTGTTGTATATCTTGTTTTAATGTTGCTACTAATCTTCAT

GCTTGTTCTTATGTGTATTTTGATACTAAATCGACATATTCAGCAGTGATCA

>Unigene0001963

GTCCAGAGAATTATACCAGATAAGGAGAAAATTTCAATCACTGCGAAGAACAAAACCCAA

ATGGCCATCGATTTGGGCATCAATCTCTCCTCTCCAGCATCCGTTACGCTGAATTTTACC

GTTCAGAGCTACTGTTCAGGCAGGGTTGGCCATGGGTCCTCAGTTGCAGTTACTTGTCAA

AGCAAGTTGTGGACAGATGGTGCCCGTTGGTCGAGAAAGGTTCCCTTTGGATTGGGAATG

AGAGAGACTTGTTATAAGGTACTGAGATGCACTGGGTCATCATCTTCTGAGGGAAGCTCG

GAGGATCCAAAGTCTTCAGCTAAAGAGAAGGTACCATTCGGATACTCAAGAAAAGATGTC

TTGCTGATAGGTCTTGGAGTGACCTTACTAGGCTACGGTTTGAAAAGCGGATTGGAGTTT

GTTGGAGTTGATCCTTTGAAAGCAGGCAATGCTGTTCAGTTGATTCTTGTCTTGGGCCTG

ACAGTTGGATGGATTTCCACATACATATTTAGAGTCTCAAATAAAGATATGACTTATGCT

CAGCAGTTAAAAGCCTATGAGGTCAAAGTCATGGAGAAGCGGTTGGAGGGCCTTACAGAA

GCCGAACTAGCAGTTTTGATGGAGGAAGTTGAGGAGGAGAAGCGTATATCCAGTGGCGAA

CAAGCCTGACTAGAGATCACCGTTTCGGGGATGCAGGGATCGTTTGTATAAACACCTCAG

TTGAGAGAGCTTACGACAAGACTTTGTGCATACAGCTCATACAAAGGATAACCAGTTCAT

GTAAACTTTGATACATATGTTTTTGGCGTATTCATAAGTATATCAGAGATAAAAGCCTGA

ATTTATTAGTTATGTACCACCAATTTGTTGTTTCTCAAGTGCCAATCAAGAGAGAGTTTA

TCTCACTAGCCTACCACTAGATATTAACACTCTCTCTCAAACGTAGGATGATTCAAGCTG

TTGGAGCAAGGGTGTGGAGTCATTCACACCATCACGAAGAACTATGGATGACTTGATTCC

GAGACACGGATATGTAGGCAATCAGGAAGGTGCAGTCTGTGAGTCAATCAGAGACATGGA

GCAGAAAAATGTGCCCGCATTATTGAGTAATGCGCGCGTATATTATAATGCGCCTGTATC

TGGAGGTTCAGTGTGCGCATTGTGAAGGACTCTCAGAAGGTGCTCTCGGGTTATGGAGTG

TGCACGCATTCCTGCAGAATGTGTGCGCAATCGCGTCGGGAAATACGTGTGCATAACATT

GTATTGCATGTGCAATTAAGGCGTCGATTCAGACATATTTTTTCATCCGGCAACAGACAA

CTGGTGTGTCTAATACCAAAACGAAGAATGATGCTTCAGAGAGACGCATAAATGTCCGTT

GATGGATAGGAGTATCAACCGGATGTGTGTAACTCCGAAATCCTTCACCTATTTAGGGAA

GGGTATTTTATGGGGTTGATGTACTGTGATTTTGGATTGGCATTAGAGTATATTATTTCT

TCTTAAGCCTTAGAGAATTCAGAGAGTTCTTTGAGAGTTATTGCTGTAGTTGAATCTCTA

TTTTAAG

>Unigene0001964

TTCAGAGGTATTATATGGCAGATACAAAGTCAGTTTCCACTCCGCTCGCAGGTCACTTCA

AACTCTTTAGCAGTCAGTCATCAATACATCGAAGGAGTACACACATATGGTAGGCATACC

ATATTCCTCAGTAGTCGGAAGCCTGATGTACGTCTTGATATGTACTAGACCGGATATTGC

CCAAGCAGTGGGAGTTGTTAGTCGGTTTATGAACAATCCTGGCAAAGAATATTGACGAGC

AGTGAAGTGGATTTTTAGATATATGAGAGGAACCAATCGTTATGCTCTTCACCTTGGTGG

ACAAAATACAAATATATAGGGTTTTGTTGATGCAGACACGACAGGTGCTACAGACCGAAG

GAAGAGCACTACAGGTTATGTGTTCAGGATTGATTCTGGAGCAGTTTCATGGTCTTTCAA

ACTCTAGAAGATAGTGGCTCTATCGACCACTGAGGCAGAGTATATAGTAGTGACAAAGGT

AAACAAGGAGATGATCTGGATGAAAGAGTTTCTTAATGAATTGGGCGTGAAGCAGGAAGA

ATATCCATTGTTCTGTGATAGCTAGAGTGCGATGGATCTTGCTAAGAACGTAGTTTTTCA

TTCAAGGACTAAACACATCGACATCCGCTATCACTATATTCGAGAGTTGATCCGGAGAGA

TGAAGCTTCAGAAGATTCATACAGATGAGAATCCGACGGATATGCTTACTAAGCGTGTGT

CGCCAGACAAATTGAGGTTGTGTGCAACCTCTTTGGGACTTCATTGAAGACGGAGGATGC

TACGACAATCGAACACTTTCGGAGAAAGCAGTTTGCAGAGGTCTGACATGATCGCCTCCA

AGTGGGAGATTGTTGGAGCAGAGGTGTGGAGTCATTCACACCATCATGAAGAATTACGGA

TGACTTGATTCCGAGGTTTGGATACGTAAGCAACCAGGTTGGTACAATCAGTAAATTAGT

TAGTCAGTGACAGCAGCAAATGCGCCCGCATTGTTGTGTAATGCGCGCGCATAATCATAA

TGTGCCCGCAGTTGGAGATCCTGCGTGCGCATTGTGAAGGACTCTCAGAAGGTGCTCTCG

GATTTTATGGAGCGTGCGCGCATCCTTTTAGAATGCATGCGCAATCGCGTTGGTAAATGC

ACGCACATAGCTTCGTATTGCGTGCACAATTAAGACGTCATTTCCAACAGTCTTTTCCAT

CCGGCAATAGACAACTGGCGTTCGAAGAAGTCGCTTTAGAGGGACACAGAAATGTCCGTT

GATGAGGGAAGCAGCGTCTGTTGATGGATAAGGAGTATCCACCGGATGTGTAACTCATAA

ATCTTTATCCTATTTAAAGGAAGGGGTTTCAGAGAGTTAAAACACTGTGAGTTTGGATTG

GCATAGAGTAGTTTCTTTCTTCTTAAGCTTTAGAGAGTTCAGAGAGTTTCTTTGAGAGAT

CAATTGCTGTAATTGAATCTTTGTTTCAAGATTTAATAAAGGAGTTTGTTAGCAAGTGTG

CTACTGTTTTTATTTATTTTTATTCTTGTTGTGTCATTATTCTGATCCCAACAAGTGGTA

TCAGAGCCTTGATTCAAGAACAATGGCAGAAGACATCAGATTCAAGATCAAAAAGTTCGA

TGGTACGAACTTCACTTATTGGCGAATGCAAATGGAAGGCTCCTATCCGTCGATCTCACC

GTCACCAAGAAGGCCCTTAGTCTCTCCGGCAGTGACTTCACCATCACCGATGTGAACGAC

AGGGTGTT

>Unigene0001965

TCATGTATTTTGAGGTTGAGTAGCTATACGGCGTTCTATTCCACAGCAGAAGTTTGCTGT

TGGGTTCTTGTGGCACCACTTGGTTTTTTTGAGCTATGTAGTCTTGTCTTTGAAATTCGA

TGATATCTTATTTATACTCTGGGATCCTATAATGGTATATACTTGATCACTTATTGCTGA

TATGCAGGCAGCTTAACAAAACTTAGCTCCCTCACTTTTCTAACTCCGATGTTTGCTTCA

GTATTTGGGTT

>Unigene0001966

GGCGTGGGGGTCTATACTGGTGTTTGGAGCTGTGGATGCTGCTTGCTTTCAGGGGTTTCT

TGCTGAGGGCTTGCAGAGGACATCGGCTGGATTGGGTAGTGTAATCATTGATTCACAGCC

TTTGACAGTATCGGTGCTTGCATTTTTATTGTTTGATGAAACAATAGGTGCAATTGGAGC

CGCTGGGCTTGTGCTTGGTGTTATTGGGCTTCTACTTCTTGAGATTCCAGCACTTTCATT

TGATGGAAGCAATTCAACAATATGGGGCAGTGGGGAATGGTGGATGCTTCTTGCCGCTCA

AAGCATGGCTGTTGGAACTGTCATGTTCCGTTGGGTGTCCAAGTACTCTGATCCTGTCAT

GGCAACCGGATGGCACATGGTCATTGGTGGGCTACCTCTGTTAGTAATATCTGTGCTCAA

TCACGACCCAGCCATAAGCGGGAGTCTTAAGGAGCTTACATCCAGTGACCTCTTGGGACT

TCTCTACACCTCAGTTTTTGGAAGTGCGGTCAGCTATGGAGTATATTTTTACAGTGCAAC

AAAAGGCAGCTTAACAAAACTTAGCTCCCTCACTTTTCTAACTCCGATGTTTGCTTCAGT

ATTTGGGTTCTTATATCTCGGCGAGACATTCACTCCTGTGCAATTGGTCGGTGCCTTAGT

AACTGTGGCAGCTATTTATATGGTCAACTACCGTGAGATTGTCAATAAAGCGTGAAAGAT

ACGTTGGATCTATCTTCTTAACAATCACGCCCAGTGGCTTGTTCTTAGGAGCAATATAAG

CAGAGTTATTGGATTGGGTTGAATTAATTTCATTGGAATTGAAATGCATACGAGAAAGTT

AGTACTGTATGAAGGCCTCATTTCTGTTATCATTGTTTACTTCGACTGAATAATCAAAGC

AATTTAGCGAGGCCTTTGGTAGGTGGTTGTCTGAGGTATTCTAATGGTCGTCTCACATTT

GGATGATTATTCGCCTCAGTATTATTTTGTAGTACTAATATTACATGGAATGTGCTTGGA

ATTATTTTGCGCTTGAGAGTGTACACATTGTGGTTATGTATATGATTAATTAAATATTCT

GTTTAAATATTTTAATATCAGGCTTTGTT

>Unigene0001967

AAATATCGTAGCCAATGTCAACCTCCTCCATCCTCCGACTCCCCAATTTTCTATTCTCTC

CCCCACCAACCTCCCTCCATGTCTCCGCCCTCCGCCTCCGATTTCGCTTTTCCCATTCCC

TCAAAACCAGACCATCCATCCACTGCACCACCAAGCCCTCCACCAACTCCCCAATGCCAG

ATCTCGACGTTGACTGCATCGGCACCGGTGCCGACACCTCCGAAC

>Unigene0001968

GCCCCATTGACAACACTACCTCTCCCGGATGTGACCCCCCTCTACCCCTCGTGGTTGAGT

CTTACTCAATGTTGTGGCTTGGAGTTCATAATTACTTACTCTCCTGTGTTCAAGATTTTC

TTACTACGCTTCATGATGACCATGATCCGGTTCCTATCGTGGGTCAATTGAGGACCTGAG

TGGTATCATACCATGACAAGGAGTTTCAAGTGTTTGTGCGTACTCAAACCTGGGATGTTG

TGCCTCTTCCTTCTGAAGTTGCGATGTTTAGATGTCTTTTGGCAAGGCCAAGCATCGACC

TGGTTGGTTTTGTTGAACTTTGAAAAGGCTCGCCTAGTTGCTCGTGGCTTTCTTAGTAGT

ATGGGGTTGACTATGGGGATATCTTTGCCCCAGTTGCTCAGATGACTACGATATGCACTT

TGCTTGTCATTTTACCGGTCGTTAGTAGCCCTTATTTAGATCGATGTGAAGAATGTGTTT

TTTCATTGTGTTCTCTTTGAGGTTGTTTGCATACGACCAACTTTTGGCTTCATTATTATG

CCTCAACACGTATGTTATTTTACCGGTCGTATGTTATCTTCATCAAGCTATTTATGGGCT

CAAACTAGTCCCTCCTACATGGTTTGAGTGTTTTTATAGTGTGGTGTTGCGGAGTGGTTT

TT

>Unigene0001969

GGTTTTTTTTTTTTTTTATGGTGGTCATGATCGTTCATTGTTTGTTATATATGAAAGATA

TGATTATCATTGGCGATGATATTGCGGGCATTTTCTCTTTGAAGCGCCTTTTTCAGACGA

AGAACTTGGGAGACGTATGATACTTTCTTGGTCTGGAGATTGCTTAGAGCTCTCGCGGCG

TACTTTTATCTCAACAGAAGTACACAATGAATATCCATGTTAGTGTTGCGCTTTCTGATT

CTAGAGTTGTGTAAACCACTCACAACTGAATGTGTTGCTTCCTACTGATGGTTACTTCCG

TATACTACACAGTACATGCAGATTGCGTGTCAACTCATTTACCTGTGCATCACTAGACAC

GGTATTGCCAATGTTGTCGGTGTTATAAGGTCGTTACTGTCCGCTCCTGTGTACACTATG

GCACACTTCGAAGGATTCTTTACTGATTGCTTGGAATGGAACTCATTTGTTGTTCTTGCC

TTAGTCCTCTCCTTCGGAGTTGTGCGCCTTATGTCATCTGGGCTGCTGATACTGCTATTC

GATGATTCACATCTGGATACTGTTTGTTCCTTGGTGATTCTCCTCTTTTGTCTTTTTTTT

GTTGGGCTAACCCTACTTGTGACCACGATAGAAACATCTATGAATCCCCCCTTACCCCCA

TCTCACCAGACCTGGTATCAAACCTGGGCCATGTATAGCGTGA

>Unigene0001970

CAGTTGCGCAAGCTCAACCTCCTCAACTCACGGGCTATTGGCCGCGATAAACCCTATCGC

CGAGTTCGTTCGCTCCAGGCCAAGCTCAGCTTCTCCTCGCCCGGTCTCGGCGTAGCCCTA

CTCTCGGCTTTGCCGACTGGCGCTCCTCCCCCAGCCTCCGTCGAGCTCCCTCCGCCGCCG

CCGCCGCCGTCGTCTCGCCCCGCTGGTCCCGACGCCCGACACAGCACGCAAGTTGGAGCT

GCTCGCGAATTCTGAGTGTTTTTGTAGATCAATTACCTCTGACAATGAAAGTCAAACAAG

CAAATGCAGGAGAGCTCACAAATTTTGAGGTTCTTGACTTCTTGCGGACTCGTGGTGCGA

GCACTGATCCTTTGGGATGTCTTGGTGCTGTTGCACCATCAGAGTGCAAGGTTTTTGAGT

ATCTTGTTAAGACTGCTGCTTGTGACCAATCAAAGGAAGCTCTTGACGAGTTTTTGAGGA

GATGTGAAAAGTTCAAGCTTGCAAAAGCTGAGAAACTCAACATTATCAACCTGAGGCCTT

CCTCAGAAGCAGAAATCTATGCGATTGTATACAACTGTGATAAGCGTCTAGTTGTGGATG

AAAATAATGGGATAAATGAAGTTCAAGAGTTGATGGATTTGGTGGCTGAGGTTTTGCCGC

CACCTCCAGAGAAGTCTGAAGAAGAAGCAATGCCTGAAGAAGAGGCGGGGGATGAAGGGG

CCGGGGATGAAGAAGAGGCAGGGGATGAGGAAGCAATGGAGGGCTAGGCGGCAATGCTTG

GTTAAAGGTACAAACGAGTATGAATACTAAAAGGTACAAACGAGAATGAATACTAAAGAG

CAATGCATGGGAGGTATTTTTTTATTTTACAATAACTTAAGTTAAGTTTGTTCGATCTAT

GAGTATAATGTTAACAGATGACTTCGAGTGGATATAT

>Unigene0001971

AGAACCTCCCGCCGCCCAAAGCCGCTGTCGAGGGAGCATCGCCTCCGCCGCCGCCGCCGC

CGCCGGACGTCCTGCCAGAGATTCTCCGCCACTCTATCCCAATTCGGGGCGTCTCGCCGG

TGCCCGACTCCTCCCTCTCCGGCTCCTCAAAATGGATCGTCAGCGGCTCGCCTGACACCG

CCTCCGCCAGGGTCTCTCGCGACGTCCTCAACCCTATCAGTGCCTTCGTACGGATGCCGC

AGGCCACCTTCGGCCCGAAAAGGTGGCAGCTGCCCAATGAGCAACCAACTGTCTTGGCTT

CAACAGCAAATGAGTTGCGGCGTGATAGGTATCCGCCTGCTGACCCTGAGAAGTTGAAAG

CCCTAGCAGTAGGTTACTTGCAACTTGGTAAGGCATTTGCTATTGCTACGGCAATAGTCT

TTGGAGGTGCTACATTTGTGTTCGCATACACTGCCCGTAAACTGCAGATGACTAGTACTG

AAGACATCAGAAGCAAAGGAAGGGACCTCATCCAACCTCGAGTAGATATGGTGCAAGAGA

AAATGTCTCCGCTACGAACCTGGGTTGAGAATGTATCGCAGAAATGGCACTTTGAGAGTG

GCAAAGTAGATGAGAAGTCTATTGTCAAGGAGCTTTCGAAAACTCTTGGTGCTCGAACAT

CCAATTGATGCATTCTGGTTTGTCTTTAACGAGATCTGTATATACACTCGTGTACATTTT

ACATTAATACTTAGCTACCATAATTTGATGCATTCTGGTTTGTCTTTTAAGGAGATGTGT

GTATACACTCATGTATATTGTACATTAATACTTTAGTTACTATAATTATGGACCTTATTC

TGGAATGAGGTTTATGAATGTATTTTTGTTGTACTGAAACTATACAGTTAATTAAAAAAC

AAATATTGTAAACGACATTAATGTATTTGACTAGAGAT

>Unigene0001972

GTGCTCTCCAGAGGGTTTGTTATTAATAAAAGATTATGAAAGATGGCGTGAGGAGGCAAA

AAAAGCATATGACCCTTCAACAGCTGACAAGGCTCTGTCAGTGAAGAGTAATCCATTTTG

TGAACTTTTGAGTCTGCCAGATAGGTGGCCACGAGGACTTGACTGTGATTATCTAGATTC

AACCAACATGCTTCGTGAGTTTTGGCACAAATCTCAGAGTGCTGTGATTATTGATGATCA

GGAGCGAATTGTTAAATCCATCCTATTCATATTAAGTTTAATGTGTGACACTTGGCGTCC

TTTTCTCATCATTACAACTCTTGAGCTCCTTTCATTATGGGAAGCTGAGTTCGCTCGTCT

GGTGCCATCCATTAATTTGGTTGTTTACAATGGGAATAAAAACACTCGAAAGTTTATTCA

GAATCTAGAATTTTATGATGATGGTGGCTGCAGAATATTTCAAGTGCTTCTGTCACATCC

CAAAGCTATTGTTGAGGACCTTGATTCTTTTGAATGGATTACCTGGGAAGCTGTTATAGT

TGATGAGTGTCAACAACCAAAGGTTTCTACTCGTTTTGAACAACTCAAACTACTTTCGTC

TGATTTCA

>Unigene0001973

GTGCTCTCCAGAGGGTTTGTTATTAATAAAAGATTATGAAAGATGGCGTGAGGAGGCAAA

AAAAGCATATGACCCTTCAACAGCTGACAAGGCTCTGTCAGTGAAGAGTAATCCATTTTG

TGAACTTTTGAGTCTGCCAGATAGGTGGCCACGAGGACTTGACTGTGATTATCTAGATTC

AACCAACATGCTTCGTGAGTTTTGGCACAAATCTCAGAGTGCTGTGATTATTGATGATCA

GGAGCGAATTGTTAAATCCATCCTATTCATATTAAGTTTAATGTGTGACACTTGGCGTCC

TTTTCTCATCATTACAACTCTTGAGCTCCTTTCATTATGGGAAGCTGAGTTCGCTCCTCT

GGTGCCATCCATTA

>Unigene0001974

AGTTTCAATCTGTAATCCAGGATGACGACTCTGATGTTGGCAGTAAGAATCCAAAGCTGC

GAAAAACGACTCAAGTTTCAATCTGTAATTAAGGATGACGACTCTGATGTCGGTAGTAAG

AATATGGAGAACTCATTGGAGTATTGGAGCCCTAAAGGCCACAGTTCAGACTCCTCTCCT

ATACCTCAGAAGAGGGAGTCGGAGGACGGAGTAACTTGCAACGGTTTTGCCATGGAGAGC

AGGAACAACAATGATGGGGCTTGCCAAAATTATATCTCAAGAGTCATACTCGACACAGTC

GA

>Unigene0001975

CCTCCAGCCGGACGTACGGAAAGAACGCTGTTTGCGGGCGGCGGTTCACTGAAACCCCTT

CCCCCGGCGGCTCGAAACCCTAGACTTCGATCTCCTTCCAGCGGCCGGAAACCCTAGAAT

CCGCTTCATGGCGTGCTTATCGATCAAACAATTTTCGTGGGGTCTCACCGGCGGCCTTCC

CGTCCGACGTAGATCGCCGCGGCCGCCAGCCATCACATCCTTCTCTATGAAAAAATGTAT

GTCGAAGGGCCTCCATATCTCCTCCTTCGCAAAGGGCTTCTCCTCCGCCAAGGCGAAGAA

TCGAGGCTTCGACTGTTCGTGCTCGGCAGAGTCGACCCCGGTCGGAGACAAGAACACGGA

GCTATCTGGGGAGTCGACCCCGGCCGTCCACGAGAACACGGAGCTATTGGATTTTCTTTT

CTTTAAACCCGAACCTTACAGTCTCCCTAGATGGAACTTGAAAGTTTTCTTTACAGCCTT

CTGCTACGGCGCCTACACCATCCACAGCAAGATGTTACTCAACAGTTTTCCCTGCCCATG

GCTCCTCTCCACGGCCTACGTCGCCCTCGGATTCCTGACTTTCGCCTATATCTCTGGCGA

TCTACCAATAGACTCCGGCTTCTGGAAGTCTCTCTTTCCGGTAGCGGTTGCACACACAAT

TGGGCTTGCAGCAGAAATGGTAAGCTTGTCAAAGTTGCCGGCAGATTCGACCCACATCAT

CAGGTGTGCCGAGCCGGTGTTCACTGTGCTGCTGTCTATGCTTTTCGGGGGCCAGTCTTT

CCCCTTGCCTGTCTATCTCTCGCTTCTTCCTATAGTTGCAGGTTGTGCCCTTGCAGTCTC

CTCAGACCTCAACTTCAACATGATCGGTTTTATCACTGCGATGATAGCGAACCTTGGATT

TGCATTTACAAATTTTCTTGCAGGGAAAGGGATGAAGGAGGAGGGAGTGAAAGTAACACC

TCTCCAATATTATGGCTGTCTCTCCTTGTTGTCTTTCTTGATTCTTGCACCATTTTCCTT

TGCAATCGAGGGACCCCAGAGGTGGGCTGCAGGTTGGAAGATGGCGGCTTTGAAGGATGG

CGCTGATGCAATAGTAATGCTAATAGGGTCGATTGGCATGCACTACTATTTCTACAATGA

GGAGGTCTACCGGTTCTTTCCCACTATTCAGGCGTTCCCATTTCTTACAGTCAACACTGC

AAAAAAGATATTTGTTATTTTCTCTTCCATCATATTTTTATGCACGCCTGTTCAGACGAC

GAGTCTAATTGGAGCTATCATTGTGATCTGTGGGATCTTCCTCTACTCTCGGGAAATGTG

CAAGATATCAAGAATTCAAACCTGGTATTTGTAGACAGTTATTTTTTTCTTCCTATTTTT

TGCTGCTAGACATTAACAGGACCTGGATTTTGTGAGAGCAGATAATTATTGTTATATTGT

TAAGTACCCAACTTTATTCTTTTAAGTTGAGAAGTTTAGTATGAAATTA

>Unigene0001976

CTGCAAAAAAGATATTTGTTATTGTCTCTTCCATCATATTTTTAGGTACGCCTATACCAA

CTACGAGTCTACTTGGAGCTGCCAGTGCCATCCTTGGGATCTTTCTCTATTCTCGGGAAA

TGTGCAAGATTTCAAGAATCCAAAGCTGGTACCTGTAGAAAGTTCTTCATTTTCTTCATG

TCTTTTGCTGCAAGACAATTGGATAAGATGAGTAGTGTTTTATAAAAG

>Unigene0001977

CATTCAATGGTCGGAAAATTTCAAGGTTTGGCAGATTCTATACAAGGTTATCTTGATTAG

TTTGGATCATATTGATGTTCTTGACGATGATTGATTATTGGTTGATAAGGGGAAGATGCC

ACTATCTTCAGATGCAGACAAGTGGGATCCACCCGCGAATACTCATAGCTCAACGAAGTT

GCTTACATCAGAGGAAATTACGCAGATGAAGAAGTTGGAATAGAAAGCTTGTAGTATGAT

CCGATTGTGCCTGACTCACAATGTTGCGTATCACTTTGTGGATGAGACTACTTTGAGAGG

TGTTGTTGGCACAATTCTTACACTCGGACGCAATAAAACTTCAAAATAAACCACTTGACA

CATGTCCATACACCTATTTGTCACATCTTCACGACAACCGACTGAATTCATTGAACCATT

CAAAACACTGTCAATATCCGTGTATTTGTAATGATTTCTTGTGAAGTGTTCAGGACTTAG

AAAATCACCGAGTCACATAATGAACCGATGACAAGTCTGATGAAGAAGGCAACCAACTTG

ACGATCGACGAAGTGCATCGGGAGAACTGGACCATCGTGGGAAAATTGAAGATTCGTAGT

GCTTGGGAACCATCATCGACGCTCGACCATCAGTATTCTCCATCGCCACTTGCACCCCAT

TGTCGCAAACCATCGTCATCCCCCACATCCATTTTCCTCACCCCATTTTTCCCATTTTCC

AAATTTCCGCTTTTTGTAAATTACCACCCCTACCACCCCCATCTAAACCTTGTGATATTT

ATAAAATAAAATAAATTATATAATAAAAAATTATTATATGTTA

>Unigene0001978

GGGTGTCTATCTTGGTTCCTAATCTTGGAGAGGGCTTAATCAAGGTGATCAAGATCTGTC

TGAGAAGCATCACGAGGATCGATTACCGGGGAGCTCATCGGTTCAGTATTCCGATCGTGG

GAGGTGGATCATCACATCGATCATTCATATCGATTGGGGGTAATCCTTTTACATGATTGT

ATGTCATGTTGTTTGGGCTGTAATCGTATTTGTGTTGTCTTCGATTATTAATGAGAAACA

TTTGAGGTTGCCGTCCGTGGAAGTATGCATTGCTTGGCTGAATCATGTAAATCCTTGTGT

TTACTTTCTGTACTTGGTTTGTTGTTGCTTGTTCGTGCTTGTTGTGTTTGCATTTGGGTG

TTCTCACATGCTTTGCTAGATCGACGAACTGTGATGATTCACATCAAGTTGGTATTGTTG

TCGAATTTAGTTACATTGGTATCTGGATCTTACTTGTGAGTTACAACAAGTGGTATCA

>Unigene0001979

ATCAGTCTTGAATATGTGCACATTATGGAGTTGGTGTTTATGCATAAAATCATGTGTGCG

CGCATGTTGGCTAGGTCCTGTGAATTTAGAAGATGCGATTAGTGGGCGCGCATATGCTGA

AATGCGTGCACATACCCATTCAATGTGTGCGCATATTGACTGATTCCAAGATGTTCAAGA

TGCATGTTTTAATCTTTGCTGTGGATGCCCTTGGTAATGTTCTATGAGCACCCATGTTGT

TCCTTGCTACTATATAATGATATTTGGGTTTCAAGAATAATAATCTCATGTATCTCAAAT

CTTCTATCTCTTCCATCTCAAGTGGGAGTTAGAGAGCTTTGTGACAGAGAGGAGTTGCTA

GAGAAACATAGCAAGGTATTCGATTGCGGGAGAGCTCATCGGATTGAGGATTCAGATCAT

CACATCGATTGAGTTGATCTTTGGATTGATTGTATTTCTTGTTGATTGAGTTGTAATCGT

ACTGTTCTCTTTTATTATCCATGAGAAGCATATGAGGTCATCATTCCTTGAAAGTAGGTA

TTGTATTGGCCGAACCACGTAAATCATGTGTGTAATTTATGCACTTGATGTGTTGTTTGT

TCGTACTTGTGTTGTTTTTTTGGGTGTTCTCACATTTTTTGCTAGATCGACGAACTGTGA

>Unigene0001980

GGGAAGGTTCAACAAGTCTTGGAGAGAGAGGGTTTGACCAAGGTGATCAATATTTGTTAG

AGAAAGAATGTAAAGGTTATCAATTATGGGATAGCGCATCGATTTGTGGTTTCCAATCAT

GGACGTGGGTCATCACATTGATCATCATCATTATTTGAGAGTAATCATTATTTGATTGTA

TGTTATGTTGATTGAGTTGTAATCGTACTTACGTTGTCTTCGATTATCAATGATAA

>Unigene0001981

GTTTAAGTGGCTGTGAAAGTGTTTTGTGATAGTCAGAGTGCCATCTATCTAGCGAAGAAC

CATGTTCACTCAGCTAGAACAAAACATATTGATGTTTGTTATTATCACCTAAGGGATGCT

ATTGAAGATGGCTTGGTGGCCCTCGTGAAGGGATACGAGTGAGAATCTTGCGGACATGCT

GACCAAAGTAGTACCAATGATCAAGTTCGAGCATTGTCTCGACTTGATCCAAATCTCTCC

TTTGAGGTGGGCACTTTGGGGCGTTGGCGGTGATGGATGACAATGCGATTGATGCTTCTG

GCGGAGGATACTTATCTGCGTCTGAAATGCTCACCAAGGTGGAGATTGTTGTGAATGTGA

CTCGTATTTTGGTACTGGAGACGAAGAT

>Unigene0001982

TATACGTGGGGGCTCGTGACACTTCCAATAAGAAAGAAGCCGATCAGATGCAGATTGATT

TTCGCAATCAAAGATGATGTTTCAGACTGCAAAGTTTGTAGATGTATACACTATAAGGCC

AGATTGGTGGCAAAAGGATATGCACAAAGGGAATGTGTGGACTACAATAGTATTCTCTTT

GGTGGTGAACACCTCGATCCATGCCCTTCTTATTAGTGGCGCAATTTGACTTGGAGCTTG

AGCAACTGGATGTCAAGAATGCATTCCTGTATCGAGCCTCGGAGGAAGACATGTTGTTAG

AAAGGAAGATACAGTGCAATCTGAAGAAGTCTTTGTATGGCTTGAATCAAGATTTGAGGC

TGTGGTATAAGAGATCTGATACCTTCATGATTGAACAAGGGTATCAAAAGAGTTTGCATG

ATGTTTGTATTTACACAAGGAAGTCCAAGGATGGATATTTGGTATATCTCTTACTGTATG

TTGATAACATGCTTATTGCTGTAAAGAGCACGAGGGAGATTGATGAGTTGAAGTCGAAGC

TGGGCAAAGAGTTTGAGATGAAAGATCTAGGTTCAACGAAGAAGATATTGGTATGGAGAT

TGTCAGAGGAAGGGAGAATGCTAGGCTAAGGCGAATGTTGTCTCGGCGGGAGTACTTGGA

GAGGATGGTACGTCGATATGAATGGATGCAACCAAACTTGTCAGCACTCCACCCGCTCCA

CACTTCCAGATCTCGGGAAAGACATCTCCGATTACTGACGAGGACATGGAGTGTATGTCA

CAGACTCCCTATGTAAGTACTATTGGTTCCTTGATGTATGTCATGGTATGTACGCGATCG

GATATTGCTCAAGCAGTGAGCATGGTGAGTCGGTATATGACGAATTCGGGGAAGGAGTAC

TAACATGTCGTCAAGTGGATTTTGTGATACCTTCGTGGTTCATTGGACATAGGTTTGATC

TTTGAGAGGGTATTGATGCGATGGTCCAAGGGTTCGTTGATTCTGATTATGCTGGAGATC

TCGATAGACATCGATCCATGATGCGATATTGTTTCATGTTGGCGGGAGGTTCGATTAGCG

GGAAGTCTTAGGTGCAGTCTACTATTGCTCTA

>Unigene0001983

AAATGCTCACCAAGGTGGAGATTATTGTGAATATGGCTCGTATTTCGGCATTGGAGGCAA

AAGGTGAAGCCGCAAAACTGTTCGACTCATAGCTAGCTGGTGGCAACAATATTGTGCGTA

TCGGATCCGGATGTGTGCGCATTATGGGGTCGATGCGTGCGTATGAAACCATGGTGCGTG

CATATTGGCAAGATCATGCGAACCTGCATGATACGGCGAGCGGGTGCAGGCGCATCTGGA

GATGGTGAACACATATGCAATGATGCATGCAGCTGCGTGCACATTCCGTCTGTTTC

>Unigene0001984

TCTGACTTCGTTGATTAGTTGGCGATTTGGGTTTTGCTCAGGTGGTTGTGATACTGTCAT

GTGATAGTCAGAGTGCCATCTATTTGGCGAAGAACCATGGTCACTCAGCTAGGAAGAAGC

ATATTAATGTTTTCCATCATCACTTGAGGGAAGTTGTCGAAGATGACTTGATGACTCTGG

TGAAGGTTGATACATGAGAATGATTTTGGTGTAGGTTAATACACGAGATAATCATGCGGA

CATGCTGACCAAGGTGGTACCGAGGATCAAGTTCGACGATTGCCTCGACTTGATCCAAAT

ATCTCATTGTTAATACCCTTCGGGGCATGAGTGATGATTGATGATGATGCAATTTATGTT

TGTGGCAGAGGAGACTTGTGCTTAGCTGAAATGCTCACCAAAGTAGATAACAATAGATAT

TGTTATGAATGTGGCTCAGATTTCAATATTAGAGGCGAAAACAAAAGGCACGAAGCACGT

ACGATGCATGT

>Unigene0001985

AGTTCGAGCATTACCTTGACAACTTGATCCACATCTCTCATTGTTGATGCCCTTCGGGGC

GGCGACAGCGACCGATGATGATGTAATTGATACTTGTGGCCGAGGAGACATGCGTCTGAC

CGAAATGCTCACCAAGGTGGAGATTGTTGTGAATATGGCTCGTATTCTGACATTAGAGAT

GAAGGCGATACCGTTTGACGTATTGCTTGCTGGTGACGCTATTAATGCACGCGCATTGGA

TCTGGATGTGTGCGCATTATAGAGCCGGTGTGCACGCATGATACTTTGGTGCGTGCGCAT

GTTGGCCATGTTCTGCGAACCCAAGAGTTGCGAACCCAAGAGTTGCAGCGAGCGGGCATG

GGCGCATCTCGGGTTGGTACGTGCGCATATGCATTGATGCACACATTCTCAGGAGGTGTG

CTGTCGTGGCCATTTTCTGGTGTAGGGCTTGCGCGCAGAATAACTTGACACTAGTCGGGG

TATTCTACGTCGGAAAGATTTTAATATACGAGGTTCGATGCAAGTCTCTCCAAAGTGGGT

GCTCCACCGGACAGTTTGGAAAGTTGGGGTGAAAAATTGGGCAATGCTCTGCAGGACTAG

TAGGAGGGCAGTTGACAGTCCCGACACATGAATCTCGACTCCCCCATCATCTGTGT

>Unigene0001986

TCAGAAGGGATGAACTAACGATTCATGTTACCTTTGACGGTCTCTGTCTCTCACTCTCTA

TGGCCACATCAGAGGACCCCGTCGAGTCCATCCTCGCCCAGGCCGCCGACCTCTGCGCCC

TCGAACAGATCGCCGCCCTCAACGTGGCTCACCTCCCAGGCTCCATCCTCCCCACCGACC

TCGAAACCCGCTTCCGAAAGCTCGCATCCTTCCCCCCTCCACCAGTTGGCCTCCGCCGCC

TCCGCACCGACACCGACATCCCCGCGATCCCCAACCAATTCGATACCAATCCATCGCCTC

CCACCGTATCCCCGCTGGCTGATCATCAGGTTTCAAAATCCGAGCCCTCCTTGCCTCCTA

CTTCTGCAAAACCAATCAGCATCAATGACTCAGATCCCAGATCAAGGTCTGGGATGATCA

GTTCCCCCA

>Unigene0001987

TCCAGAATTGATGAACCAACGATTCATGTACCTTAGACTCTCTCTCTCTCTCCATCTCCA

TGGCCACACCAGAGGACCCCATCGAGTCCATCCTAGCCCAGGCCGCCGACCTCTGCGCCC

TCGAACAGATCGCCGCCCTCAACGTGGCTCACCTCCCAGGCTCCATCCTCCCCACCGACA

TCGAAACCCGCTACCGAAAGCTCGCATCCTTTCACCCTCCATCAGTTGGCCACCGCCCTC

TCCGCCGCCTCCGCACCGACACCAACATCCCCGCGATCCCCAACCAATTCGATACCAATC

CATCGCCTCCCACCGTATCCCCGC

>Unigene0001988

ATACTTGCTGATCTCAAAGACGAGGAGAGGAAGCTGAAGAAGATGATCAAAGAGCAGGAA

AAGGTGAATCGAGAGACGGAGAACGTGTTGGGGTTGGTAAAGCAGGCTTCAGTGAGATTG

GTCCTGTCCCATGAGCTGCAAAGCGAAGAAGATGAAGAGTTCTGATGATGGTTGAGAAAT

GTTAGTACCGGTCTGTTTACCTTCTTCACAATAGTCTGGAGTTCTTGAGAGGAAAAGTTT

CATAATAGTCTGGAGTTCTTGAGAGGAAAAGTTTCATAATAGTCTGGAGTTCTTGAGAGG

>Unigene0001989

CCCTCCTTGCCTCCTACTTCTGCAAAACCAATCAGCATCAATGACTCAGATCCCAGATCA

AGGTCTGGGATGATCAGTTCCCCCACGGATTCTTCTCCGTCCCCACGGGAGAAGGTGGGG

TGCTTTGGATGCTACCCGAAGCGGAGGCAGAGGAAGAAGAGCGTTGATGATGGGTATTGG

GAGACTAACGATGCGATACTTGCTGATCTCAAAGACGAGGAGAGGAAACTGAAGAAGATG

ATCAAGGAGCAGGAGAAGGTGAATCGGGAGACGGAGAAGGTGCTGGGGTTGGTGAAGCAG

GCTTCGGCGAGATTGGTCCTGTCCCATGAGCTGCAGAGCGAAGAAGATGAAGAGTTATGA

TTATTGTTGAGAAATGTTAGTCCTAGTCTGTTTACCTTCTTCATCATAGTCTGGAGTTCT

TGAGAGGA

>Unigene0001990

CAGGCTTCGGCGAGATTGGTCCTATCCCATGAGCTGCAAAGCGAAGAAGATGAAGAGTTC

TGATGATGGTCGAGAAATGTTAGTACCGGTCCTTCTTCATCATAGACCAGAGTTCTTGAG

AATAAATGTTTTAGAAATTGAAGAAATATATTTTCTATTCTCTAATATTTTGATAAATTT

ATTAAAGAAGAATGTGAGGAAAAGTTGTCTTTATATTTTTCATTT

>Unigene0001991

CAAGCCTGAACACGATGGGTATGTCGAATTATACTTTATTCAGCAAATCACTGAATTGTT

CGGGGTCGAAGACTGGATTCATTAGAGCTTGAGACCGAAGGCTGGGTTCATTAGAGCTTG

AGACCGAAGGCTGGGTTCAATATAGCCGGATGAAATATTTCAACTGAAGAATAGTTAGTT

ATGAAATGTTTTAGCTGAAGAATAGTTTGTCCATATTCATGGAAGGTCCTATTCGGGGAA

GCCTGCCGAAGACTCGATGAAGATCATATTTGGCAAAGTCTGTCGAAGGACTTGCCTAAG

GCCCGATAAAGATCGGAGGTGGTAATAAAACATATTCGACAACAAAGCTTGTCGATGGAC

TTGCGGAAGACCCAATGAAGCATCGACAAGCGGAAGACCCAATGAAGATCCCGGGTTGTT

GAAGACCCGCTAAAGATTCGGTTTGACTAAAATTATG

>Unigene0001992

CCCTCATTGCCCTGGCCGTTGACCTCTACGCCCCCGAACAGATCGCAGCGCTTAACATGG

CTTACCTCCCAGATCCCATCCTCGCCACCGACCTCGAAACCTGCTACCGCAAACTCCCAT

CCTTCGCCTCTCCACCAGCTAACCACCGTCCTTTCCACCACCTCCGCCCCGACACCAACA

TCCCCACAATCCCCATCCAATTCGAGACCAACGGTTCAGATCCCAAATCAAGATCCGGGA

TCATTCGATATTCTTCATCCCCACCGGAGAAGGTGGGATGCTTTGGGTGCTACCCGAAGA

GGAGGCAGAGGAAGAAGAGCCTTGATGATGGATATTGGGGAGCCAATGATGGGATTATTG

TTGATCTCAAAGGTGAGGAGAGCAAGCTGAAGAACATGATGAAGGAGAGAAGAGCAAGCT

GAAGAACGTGATGAAGGAGCAGGAAATGGTGAATCGGAAGACTAGGGTTGCAGAAGCAGG

CTTCAGTGAGACTGATCGTGCGCGGTGAGCCGCAGAGTGAAGATGGCGAAGAGATCTGAT

CATTTGTAGATAAGTATTAGTACTATTTTGTTAGGCTTCTTCGTTGTACTCCAGGATTGG

GAAAAGCTTAAGAATATGGTATTTTCTAATTGCTTATTATTTGATAAATTTATTAGAATA

GCATGTGGCGAGAATA

>Unigene0001993

GGTTGGTGTGTCCTTCTGCCACCTCTCAAGTTTACAACTGTGAAAAATTCTCCCAACCAC

ATTGGATGCATCAGTGCTCTTATATTCCCTTGACGGGGTCTGATCTCACTCCATGCTTCA

CACTTCTCTCAACTCCCCTCACCAGAGAACTCTGTCTGAGACCCTCATTGCCCTGGCCGT

TGACCTCTACGCCCTCGAACAGATCGCAGCCCTTAACATGGCTTACCTCCCGGACCCCAT

CCTCGCCACCGACCTCGAAACCTGCTACCGCAAACTCCCATCCTTCGCCTCTCCACCGGC

AAACCACCGTCATTTCTACCGCCTCCGCACCGACACCAACATCCCCACAATCCCCATCCA

ATTCGAGACCAACGGTTCAGATCCCAGATCAAGATCTGGGATCATCAGCACTCCCGTAGA

TTCTTCGTCCCCACCGGAGAAGGTGGGATGCTTTGGATGCTACCTGAAGAGGAGGCAGAG

GAAGAAGAGCCTTGATGATGGGCATTGGGGAACCAATAATGGGATTATTGCTGATCTCAA

ATGTGAGGAGAGCAAGCTGAAGAAGATGAAGGAGCAGGAAAAGGTGAATCGGAAGACTGG

GGTTGCTGAATCAGGATTCTGCGAAACTGGTCGTGCCCGGTGAGCTGCAGAGTGAAGATG

GTGAAGAGATCTGATCATGGTCGGCAAGTATTAGTACTATTTTGTTTGGCTTCTTCGTTG

TACTCCGGGATTGGGAAAAGCTTAAGAATATGGTATTTTCTAATTGTTCTTATTTGATAA

ATTTATCAGAATAGTATGCGATGAAAGTAGTAAATTATTT

>Unigene0001994

CATCTGGAACAGCCTCACTCCACATTCAAGGTAGAGCTGTATGATTTTCCGTTGCACCTG

CTAGAATCATCCTCTATAGAGGAACTGTATCTTGTTAGCTGCACTTTGAAGCCACCATAC

GAATTTGATTGTCTCAACCGCCTTACAATACTAGCTATGTGGTTCCCCGGTGACATTCGG

GAGGATATCAACCATCTAGTGTCTCACTGTTCTTCTCTTCGGAGACTAGATGTGATGGGT

TCCAAAGAATTAGTGATTCTAAGAATTCCTCACTCTCTAAGTCAATTGCGTAAACTCACT

GTAGAAAAATGTGATCACTTGAAGGAAAT

>Unigene0001995

CATCTGGAACAGCCTCACTCCACATTCAAGGTAGAGCTGTATGATTTTCCGTTGCACCTG

CTAGAATCATCCTCTATAGAGGAACTGTATCTTGTTAGCTGCACTTTGAAGCCACCATAC

GAATTTGATTGTCTCAACCGCCTTACAATACTAGCTATGTGGTTCCCCGGTGACATTCGG

GAGGATATCAACCATCTAGTGTCTCACTGTTCTTCTCTTCGGAGACTAGATGTGGTGGGT

TCCAAAGAATTAGTGATTCTAAGAATTCCTCACTCTCTAAGTCAATTGCGTAAACTCACT

GTAGAAAAATGTGATCACTTGAAGGAAAT

>Unigene0001996

ACCAATTCATTCAATCCATAGATTCAATCCTGCAGCAACATCAAGGCCGTGGTAGCGTTA

GAGTAGAGAGTTTTGTTGTCCGGTATTTTCTTTACAAGCCACACTCTCATTATCTTGATG

AATGGGTCCGCTTTGTGGTTGCATCAAAAACTAAAATGCTTTATCTCCATCTGGAACAAC

TTTTCTCTTATTTTAATGTAGAGCTATATGATTTTCCGTTTCACCTACTAGGGTCATCCT

GTATAGAGGAATTATATCTTGCGTACTGCAGTGTGAAGCCACCCTACCAATTTGATGGTC

TCAATCACCTTAAAATACTGGCTCTGTGGCACACCGATGCCACTGCAGAGGATATCAACT

GTATTCTGTCCAACTGTTCTACTATCCAGAAACTGGGTGTGGTTGGCTCAAAAGAGTTGA

CGATTCTAAGCATTCCTCACTCTCTGAGTCAGCTGTATCATTTCGCCGTAAAAGAATGTG

ATCACTTGATGAAAATAGTGATTGATGCCATAAATATTAGCTATATGTGCTATGAGGGAG

TTCCTGTGGATTTCGTATTAAGGCGACCTTCATGCGTAAAAAAAGCAAAGATCTATTTCA

AAACTGAAGGGAAAGACCATGATCTCAACTTTTATCTCACTAAACTAGCAAAAGATCTGC

CCCATCTCCAGTTTCTAAACTTGAATGTTACTGGTTTTTCTAAGGTGGAATATTCGTCTA

AATGCTCCCCAAGCTTCATTCATCTAAAGCGTTTGGATTGGGAATGCCCGCAATGGAGTG

TTGGTGTTAAGATGAGCCTTCTTCATTTAGATACTCTCTTGGATGCTGCTCCC

>Unigene0001997

CTCGGCGCGGCCTCGGGGTTTTGTACGGAAAACGGATCCAAAGAGAAGAATAAATTCTCG

ATTTGGCCTGGGCAGCCGAGTCCAAATCGGAGGCGTTACTGAGAGGCAGCGTTGCAGATT

TTGGAAGATCAAATACAATCGGACGAAGTCCTCATCTTTTATCAGTAGTTTATCGGATAT

CACTTTGTTTCTTCTCCAGATTTGAAGTTTCTTGGTAGATTTGAAGAATCTGATGGGAAA

AAAATTGAGACCGAAGCAAAAAAGTAGGAGATCTAGATCTAGACGACAGATTAGTAGGCA

AAATTTTGACGGCCAGATTATTATCCGGGATTGTGAATCTAACATTATTACCGAGGATTG

TGAAGCTCAGATTATTACCCAAGATTGTGAATCTGAGTTTATAACTGAGAATTGCGCAGG

CGAGATTATTCGGCAGGGTTGTGAAGGCGAGATTATTCGGCAGGGTTGTGAAGGCGAGGG

CCATATTAATCGCCATAGTTTCGATTGTCTTAACGAAGACGGGATACTCTGTTCCATACT

CTCCCAACTGCCACTTAAAGAAGTGGTAAGGACTTGTGTTCTATCTCGCAGATGGAGGCA

CATCTGGATGTATCTCCCCATATTAAACTTTGATCATTACAGCATACTTGATGATGAGAC

TCTAGAACACTATAAAAGATTTCGCACTGGGAAAGACAAATGTTTTTTACTACTTGGAAA

GACAGATCAGTTCATTCAAAATGTACATTCAATCCTGCAGCAACACTCATGCCATGTTAG

AGTAGAGGAATTTCTTATCAAGTATTTCCTTTTCAAGCCCCACTCCCACCATTTGGATGC

ATGGGTTAGCTTTGTGGTTGCATCAAAAACTAAAAAGCTTTCTCTCTATTTGCAACAACC

TCACTCTAAATTCAGTGTAGAGCTGTATGATTTTCCGTTTCACTTGCTACAATCATCCTC

TATAGAGGAATTATATCTTGCGTTTTGCACTTTGAAGCCACCCAACCAATTTGATGGCCT

CAATCATCTTAAAAATCTATATGTATGGGGTGTCAGTGCCACTGGAGAGGATATAAACCG

TCTATTATCCAACTGTTCTAGTCTTCAAAGATTTGGTGTAATGATCTCCAAAGAATTAGT

GATACTAAACATTCCTCACTCTCTTAGCCAATTGGATCGTCTCGTCATAATTGAATGTGA

TAACTTGACAAAAATAGAGATCAATGCTAGAAATCTCAACTATATGGGCTATGACGGAGC

TCCAGTGGATTTTGTATTAAGACAACCTTCATGCCTCAAAGAAGCACGAATCTACTTCAA

AACTAAAGGGAACGACAACCTCAATTTTTATCTTATTAAACTAGCAAAAGAACTCCCTCA

TCTCCAGTTTTTAGCCTTGAATATTAGTGATGTTTCCGAGGTGGCAAATTTGCCTCAATG

CTCCGCAAGCTTCATTCATCTAAAGCTTTTGAATTGGGATTGCCCGAAATGGAGTGTAGG

TGTCAAGATGAATCTTCCTCATTTGGATATGCTCTTGGATGCTGCTCCTTTCCTAGAAGA

ACTGAAATTGCGTCTTTCAACATGTGGGGTTGAAGAAGCACCGCCAAGAGTGACGAAGAT

AAAGAAGAAGTTACCTGTACATCCTCGTCCTCGTCCCCATCTGAAGAGTATTGAAATTAG

TGGCTTTCATTTCGAGACGTGGCAAGTTCAGATTGTACTGCATCTTCTTAAAAGTGCCAT

CGGACTCGAGTCAATGACATTAGACGAGGCCCAAGAAATTAGTGAGCCGGGCCAGTTGCC

TGTGAAAGAAATAAGTGAGAAGGGCCAGAAGCCTGTGAAAGAAATCCGCGAGACGGATAT

GAAAAGTCTTATCCAACAAGTTCCACCGAATGTTAAATTAATCATTTTGTAAAGTCATAT

ATTGCACTTCTCTCTCCTATTTCTTAGAGCTAGGGTGCACTTGATTATAATGATGAAATG

AAACTAAGCGTTGAATAGTAATATTTTCCGATATCTATTT

>Unigene0001998

CCAAAAAATGCTGCAAAATTGGTTCCAGCAGGGGAAGAAACTGAACTTCTTGTTGGTTTG

CATAATGAAGGTGAATCAGCGTTGAATGTTCTTGCTATTACTGCTAGCCTTCACTTTCCT

TATGATCATCGTATGCTCGTCCAAAATCTGACTACTCAGGAGTATTTCAAGGCTTCAGTG

CCTTTATCTGCTCAGGCTTCATTCGCTTATGCTTTTGCCGTCAGCAAATACTTGCAGCCT

GGCTCCTTTGATTTAGTGGGTACCATAGTTTATGAAATTGATCAACAACCGTACCAGAGT

GTCTTTTACAATGGTACCATTGAAGTTGTTGAGGTTGGAGGGCTTTTCAGCGTTGAAACT

GTTTTCCTCGTTACCCTTGGAGTTGCCCTCTTTGTCCTTCTAATATTATGGGGAAACAGT

CAGCTGCAGAACTACTCAAAGAAAACCAAGAAGGCTCCCAAGGTTGAAGTAGGAACCCGG

GCAGTTGATGCTAACATGGATGAGTGGTTGGCGGGAACTGCATATACTCAATCCCAGTCG

ATCAAGTCGAAGAAGAAAAAGTAGAGGGTTTCGCGGTTGTCCCTTGTTCGGTTTTGTAGA

TCTGGAGAACTGAGGAACCCCATAGATAACAATCCTGGATGCATGGAATACGTCTAAAAC

AGTTTTAGTTCTCTTTCATCTATCTTTTCTAATGTTTCAGAATTTTGATGAAAATGAACA

ACTGGCTATGTCACGACTTCTAGGGAGCTTGTTCAACATAGTTACCATGTTTGAGACTTG

CAGAACTGCTTAGGATCCATATTTTTGCTTTATGTTATCATCTGAAGGAAACATGTACTC

CATGCAATCTGCGCTTCTCACAAGTACAAGATTTCTATCCTGTCCCTATTTCTTTAAGCA

CCAATAGGATTCAAA

>Unigene0001999

CACCGCTTGGCCATATCTCCACACCGACGTGCCGCCCTGGTTCTTCTCCTTCCTCCCGCT

CAGTCGCCCCTCTCGCGCCATGGAACCTCGCTCGTTCTTCTCGCTCTTCGTCGCTTTCCT

CCTCATCGCTTCTCCGATCCTCACAGTTGTATGTCAGTCGGTAGAAGATGAAAGTGTAGC

TGAAATGTTTGAAGGAGGTGATTTAGGAATTGTTGGTGATGATAGCCAAGAATTTGGTGA

TGGAACTTATAGCGCAGCCCCTGGAGTACATACAGTGTGCATTTTTCCAAAAAATGCTGC

AAAATTGGT

>Unigene0002000

ATGGTCACACTCTCTGAGAAGAACTTGCAGGCCAAGATGGATTTCTTTCTGGTAGAAGCC

AGATGTAGCATTTCCTACATTTCCTCCCGTCCGAACTTGCTATTCTCTAGCTTGGAGAAG

AGACTAAGGCCACGCCTCCGTGTTATGCATCTTCTAGAATCGAAGGGTTTATCTGTCCAA

GGGAAAGGGTTCTTTACTAAGATGAAGATGCCCGATGATGAGTTTGTGAAGAGATATATA

GTTCCACACAAGGATGCAATTCCAGAGCTTTATGACATTTTTTTGGCTGATTGTGCTTCT

TGATCCCAATTAATATTTAGGACAGTTGAAGTATGGGTTTTTATTTATATCCAAATAGGT

AGTGTACCATGTGCTAATGTTAAGAGTCTCACAACAGATTCAATAACAGGGGCCATGTGT

CAAACAATTGTTTGCAGCAAGTGAAGATCTGAGTGGACAGCTTTACCAGTATTGAGCTTT

TGTCCTAGTGTTTGAAACAGAACAGAAAGTGTTTCATGTTGCTATTATCTGTAGTTTGAA

TCAAGTTGACACCTTGCTCTTATATGTTGAGCGGTCTACTCTAATATGTTGATCCAACAG

TTAACAATGAAATGATCCTGCTACAAGAGTCCTTTTATGTTATGTTATGGAGATGTGAGG

TACCTGTGAATGCAATCGAAAATT

>Unigene0002001

AGAGATCAGGCTGGAGACGCTTTAAACCCTAGAAGTCTATACGGTCAGTGTTGATCAAAT

TTAGGTTTCCGGCGGACGGAGGGGAGGCCTGCGGCGGCTCCCTTGAATCAAACAAGTAAG

CTTTCTCTTCTCTTCTCTGATTCATGACCACCATGTTACTTCTTCTTGCAAAGAGGGAGC

TCTGTTCCTTGCTCCTTCCCCTTTTTGCTGCACATTACCACCCTCTTCTCCCCCACTGTT

TCTCTTCATCTGCGACCCAAACCCCCAAATTCACCATAGTTGATTACCTCATCACCAATT

TTGGCTTCTCCCCTGATAAGGCTGCCAGGGCCTCCAAACCCATTGCCCACCTCAAATCGC

CGGACAAGCCAGAATCCGTCCGCCGCTTTCTCAACCAACAGGGCTTCTCTGAGGCTGAAA

TCAAGAAATTCATCACATGGTTCCCCCAAATCCTCTATTCCAGTCCTGAAAATACCATAG

ATCCCAATCTCCGCTCCCTCCGCAACACGGGTCTCTCCACCTCTGCCCTTACTCAGATCA

TCCTCTCCAACCCTGCTGCCCTCGTCCTCCGGTCCGCTGTGCCCAGGATCATGTTCTGGT

TGGATTTCCTTGAAGGCGACGAGGCAGCTCTTGTCAAGGCCTTCAAGAATAGCAGCGGCC

TTGTCACCCATGACATTAACCGAATAATTG

>Unigene0002002

AGAGATCAGGCTGGAGACGCTTTAAACCCTAGAAGTCTATACGGTCAGTGTTGATCAAAT

TTAGGTTTCCGGCGGACGGAGGGGAGGCCTGCGGCGGCTCCCTTGAATCAAACAAAGGTC

GAAGAACACAAGATAATGAATCCGTGTGAACATGTCTATGTTCGTGTAAAGATCATGTTT

TCGTTTGATGCCACGTCTTTTCCAAGGGATGCAGAGCTCCACCCTAGCAGGACTTTGTGC

TACACTGTTCCTGGGAAGCAGGCTGGTCGTTCCTGTTGCATGGTAGGTTGTGGCGATGGC

CATCACAGGACTATGCCTTGCATCTTTGTGGGGGCAATGAGACACGGATTTTGAACATGA

GCTTGGTCCCTCTGGGTCCGGAAGAAAAGCGGCGAGATGAGTTCCGTACAGAAATAGCAG

AAACAGCGGCGGCCGAGTTGAGATTTATTTTTTGACCTGTTCGCACATTCATCAGTCTCT

TTACTTGCGATTCTCGGTGGCTGGAGCTTTGAATTTTACAGCAATTCCTCCTCATAGTCC

CCTGTATATTTTTACCAACTTCATAATGAAAAAAAATAAGAGGATTGTTATCTTTCCTAA

GATGGAAATAAATGAATGGGCTCTGGGTAAGTTGAAA

>Unigene0002003

TGGCCATCACAGGACTATGCCTTAGCATCTTTCTGGGGTAATGAGACACGGATTTTGAGC

ATGAGCTTGGTCCCTCTGGGTCTGGAAGCAAAAGCGGGGGAGATGAGTTCCGTACAGAAA

TTGCAGAAACAGTAGCGGCAGGGTTGAGATTTATTTTTTGACTTGTTCGCACATACATCA

GTCTCTTTACTTGCGATTCTCGGTGGCTGGAGCTTTGAATTTTACAGCAATTCCTCCTCA

TAGTTCCCTGTATATTTTTACCAA

>Unigene0002004

TCTAAACCCTAGAACGGTCAGTGTTCATCAAATTTTGATTTCCGGCGGACGGAGGGCATG

GCTGTGGCGGCGGCGGTGGTGGCGGTGGAGCCATGAACAACCACCCTTGAATCCCACAAG

TAAGCTTTCTCTTGTCTTCTCTGATTCATCATCACCATGTTGCTTCTACTCGCAAAGAGA

AAAACTTGTTCTGTGATTCTCTCCAATTTTGCTGCACATCACAACTCTCTTATCCCCCGC

TGCTTCTCTTCATCTGCGATACAAAATTCACATCCCCAAACCCCCAATTTCATAATAGCT

GATTACCTCATCACCAATTTTGGCTTCTCCCCTGATAAGGCTGCTAGGGTGTCCAAACCC

ATCTCCCACCTCAAATCTCCCGAAAAGCCCAATTCCGTCCGCCACTTCCTCAACCAACAG

GGCTTCTCTGAGGCCGAAATAAAAAAACTCATCACATGGTTTCCCCATATCCTCTCTTCT

AGTTTGGAAAACACTTTAGATCCCAATCTCCGCGCTCTTCGTAACTTGGGCCTCTCCACC

TCAACCCTTACCCAGATCATCCTCTTCAGCCCCGCTGCATTACTACTCCGGTCCGCTGTA

CCCAGGATCAAATTCTGGATTGATTTCTTTGAGAGTGACGAGGATGCGCTTGTCAAGGCT

TTCAAGCTTAACAGTGGCCTCGTCGGCCATGACATTAGCCGGATAATTTCCCCAAAGATA

TCACTGTTACGGAGCCAGGGACTCTCCATCCAGGATATCCGAAAGATGAGTTGCTGTGTT

GCGGGTCTCTTCACACTCAGTATCACTGAGATGGAGGACAGGCTGAAACGTACAGAGGAG

CTGGGATTCCAGTCTGAGTCGAAGATGTTTCGTGTCGGATTGGAAGCCATGGTGCGCATT

GATAGACAACTATTCCAGAAGAAGATGGACGTTCTGAAGAGCTTTGGGTGGTCGGAAGAC

AAGTTCCTTACCGCAGTACGGAAATCTCCCAGGATGGTACTACTCTCCCAGGAGAACTTG

CGGGTCAAGATGCATTTCTTTCTGGTAGAAGCCAGATGTAGCATTTCCTACATCACTTCC

CATCCAGATCTGCTGCCTCATAGCCTGGAAAAGAGATTAAGGCCGCGCCTCCTTGTTCTG

CATCTTCTAGAATCAAAGGGTTTATCTATTCAAGGGCAAGGATTCTTTACTACTATGAAA

TTGTCTGATGCCAAGTTTGTAGAAAGATATATAGTCCCACACAGGGATGAGATTCCGGAA

CTTGGTGACATGTATGCCTGATTGCGCTTTGTGATCCTAGTTATGTATCACAACAGATTC

AACAACAATCATTATGTTGCTTACAGAAAGTAAAGGTTTGAGCATCTCGCTGTACCGGTA

TGAACTTCTGTAGCAGTGTTTAAATTAGAACACAAAAGTGTCTGATGTTGGTATGTCCAA

CTATTATTTCTAGTTAATTAGTCAAACTGAAAAGTTCCAGTAATATGTTGATTCATCTAC

TCTACTGGTATTTGTTTTTTTTGAAGTTTATTTAATTTGAAAAAGTTTAACAGTCCAGCT

ACATGAAGATGTTAGTCATCTGTGAATTCAAT

>Unigene0002005

CTATGCCACGCTTTCGGACCCAGCCAAGCGCACCGACTACAACCGGATGAGAGCTCTACG

TAAAGAGATATATGATGGTATATTATTTATGGTGATAGCTCTATTTGCCAATTGAATTGT

AACCTTATATCGGAGAAGAATCTTTCTCAAGCTTGGTTGTTAGAATGTCTGTTTCAAACT

TATTACGGTGGATGTTGATAATGAGACGTCCGATGGCTCACTACATTGCTCTACATTCCC

TTTCCATCTCCTATGGGGGAGAAGTATTAGCTTTTGTAAGCTCTGTAATTATATTTTTGG

GATTTTTTTCTTTTTAATTTTGGGACATGTATTCATCCCTCCCCCTCTCTCTCTCTCTCT

CGATCCAACAGACTTAGACGAGCAGGTACATGTCCTTCAGAGCCATGGACAAGCACATGC

TCTTCAGATCCATGGCTGGACCTGCACAATTTATGAATAAAAGCTGGACTCCATAATATT

TGTGAATAAGTGAATCTGTGGAGTCAGATGATTCTTGAATAAAGAAGAAAGTGAGAAACA

TCTCTACGAGGGTTGTTGGACAATGCTAAAGGATTCTCCAAAGTGTACGGTTGATGTCAT

CTCTTATCCTACAGGTGGTATAACCAAGAGTCATCATATAACTGTAAGCAGTGTGGTAAC

TTCAGGTTGGAGAAAGGGAGGAGGATTCTGACACACAAATCTACCTCAAGAGTCAAAATA

AATACTGAGAGCATATACAAATCTCTTCTTGAAACATCATAAGAGAGAAAGAGAACTTTG

ACAATTGAAGAACATAGTACTGGAGTCATAATCGATTCACAACCAAGACACTTCAAACAA

TGAAATCTTTTTTTACAAAACTTGAACAGTTAGATGATCCAAGTGCCCAATTTGTTTGA

>Unigene0002006

CCCAAAAACCGTTGACAAGATTCCCTTTTCGGTCCCCCAATCTGTTATTTATCGGCGCGT

GTTTGTTGTTCTGATACACCCTGTAAGAATGACTCTTCAGGACTAAAATCCAATCTGGGC

ACCCTCCCAAACACCAGAAGATCGCATTTTGGGAGATGGGTAGGTGGGAATTCGATCATT

GAGCGCCTACTTAGATCCTATTTTGAGGTTTCAGGGCCAAAATTGAATTGGGTTGCAATT

TGAATGGCTCAAGATGGAGGATTTCAGCATGTGTTCTTGCTTTCCAGAATGCATGAATTG

GATTGTTGTACCGTTACAATGAAGTAGTTGATACCATTTGCTTGGAACATGTGATTCCTA

AGCATCATGGGCAAT

>Unigene0002007

CCCAAAAACCGTTGACAAGATTCCCTTTTCGGTCCCCCAATCTGTTATTTATCGGCGCGT

GTTTGTTGTTCTGATACACCCTGTAAGAATGACTCTTCAGGACTAAAATCCAATCTGGGC

ACCCTCCCAAACACCAGAAGATCGCATTTTGGGAGATGGGTAGGTGGGAATTCGATCATT

GAGCGCCTACTTAGATCCTATTTTGAGGTTTCAGGGCCAAAATTGAATTGGGTTGCAATT

TGAATGGCTCAAGATGGAGGATTTCAGCATGTGTTCTTGCTTTCCAGAATGCATGAATTG

GATTGTTGTGCCGTTATAATGAAGTAGTTGATTCCATTTGCTTGGAACATGGGATTCCTA

AGCATCATGGGCAATTCCTTCGGGTGTTCGGCATCCGGGGAGCGGCTAGTGTCCGCGGCG

AGAGATGGGGATGTTCAGGAAGCGAAGGCTCTCTTGGAGTACAATCCGCGGCTTGCTAGA

TACTCTACGTTCGGAGTTCGGAATTCGCCGCTACATTACTCCGCAGCACAGGGACACCAT

GAGATTGTTTCGCTTTTGCTTGAGTCCGGGGTTGATATTAATCTGAGGAATTTCAGAGGG

CAGACTGCTTTGATGCAAGCATGTCAGTTTGGCCATTGGGAGGTTGTTCAGACACTCATG

CTTTTCAAAGCTAATATTCACAGAACGGATTATCTCAATGGTGGGACTGCTCTTCACTTT

GCAGCTCTAAATGGTCACACTCGTTGCATTCGTTTGCTTCTCGTTGATTATGTGCCTAGT

GAACCAAACTTTTGGAGTATCATGAGGAGCAGTTTGGATGCAGAAGGTTCATTGCCAGAT

TTTGACGAGGGTGCTCTATCTAGCCTGGTTAACAGACGGGCGGATGGAGGCATCACTGCG

CTCCATATGGCGGCACTAAATGGGCATGCCGGCAGTGTACAGCTACTCTTGGACTTGGGA

GCTTCTGTTTCAGATGTTACTATAGAAGACGGGACAACAATTGACCTAATAGGCGCAGAA

AGTACACCTCTTCATTATGCTGCTTGTGGTGGCAATGCAGCATGTTGTCAGGTTCTTATT

GCGAGAGGGGCTAGTTTGACTGCCGTGAACGCCAATGGTTGGACACCTTTGATGGTAGCT

CGTTCGTGGCATAGAAATTGGCTGGAGGGCATACTAAGCAGGCAGCCAGAGGGACGAATA

CATATTCTTCCTTCCCCTTATCTATCTCTTCCTCTTATGAGCATAATGAAAATTGCTAGA

GAATGTGGTTGGAGAAGCACAAATCTGTCACCTGCATGTGTTGATCAATGTGCTGTTTGC

TTGGAAACAAGATGTGCAGTAGCTGCAGGAGGTTGCGGCCACGAGTTTTGCACCAGGTGT

GCACTCTACTTGTGTTCCACCAACAGCAGCACTACTGCAACGGCTGCCCCCCCAGGCTCG

ATACCTTGCCCTCTCTGCCGTCATCCAATCGTCTCATTCATCCAGCTTCCAGGCATGAGC

CCAATCAGGGGTCCCAGAACGAGCCTGTCCTTGTCACTGTGCGCTGCATGCCCGATCGTA

AGTTCCGAGCAGGCCACCTCGATGGCCACTCAGCTTTGCAAGCCAGTTGACTTCAACTGT

TCCCGCATCCCGCCTCTGGGCTCCTCTTCCTTCCGATCGCTTAGCTGCCAGAGGTTCCCA

TCGATGAAACTCAACTCGACACTATGTATGGGAGATCCTGAGACGAGTCGGTGCCTCATT

AGGTGTTCGAGGTCGGCGTTACAACGGTCGTCGTCGCATGTGGAGAGTAGCAGTAGGAGG

TCATGGCTATTTGCCTTCAGCCAATATGCCACTACTAGTGGCAATAGCTAGCCTTCTCTG

TTATTTTTTTTGGTAACTTTTTTGTATAATTCTTTTGTTCCCTCATTTGTGTCACCTGTT

TGGACTTTTCCTTTTATGGATTTCGACATGAGTATTGCAACAATTTGTTGATCAGGTGGT

GTATGATATATCCATGTTATATGAGTTGTTAGAGACTACTTGTGTAGATATGTATGTTTT

GTTTTGATATATATA

>Unigene0002008

AGCGGTCGTTCCGTTCTGGATGGTTCCTCTGGTGCACACGACTGCCCAAATCTGCTAGAA

AAGTCCTTTGTAGGTTCTATATATGAAATACAGCTGGCATTCGCTTTTGCTCAAGCCACA

TATTTGCTATTTTCTGAATCCTTTGGTTAGGCCCGATATTCTTAGTTAGTCCACTCTCCA

ACGACATAAACTTTTGGTAAAGTGGTTGCATGTATAAGTGCACTATGTTTCAAAAATTGG

TCATGTACTGAAATCTCCCTCCTTCCATACTCATTCCCTGCGTGTCCAGAAGGCACCACC

ATGGCATTCCTGCCTCTGTGGGTCCTTGTACTTCCGACTTTATCCGCATGTAACGGGCTT

GTGATGGGGGTGCTAAGCCAAGTTGTTAATGTACTGAAGGGGACATCAAGACAT

>Unigene0002009

ATAATTACACTTCATTTAGGCTGTCCTAAAGAAGCCTACACACAATGTAGCATGCTCCAT

AACAGTTAGCTTTCCACGTTCCTGAGCTCGTTTAGCATACTACACCGATCCACAATCATG

GTAACTTCCATTTTGAGCCATGAGACACCCTAGATCCTATCTTATATGTAAGTAAATATT

TTTTTTAAGATAGTTGCAAATAAAATATTGCCGGAACAGTAGATTATTTGTATTCCTTCA

AGACATGCCATCCCCAAAAGTAATATCTTTCTAATTCAACTACAGTTTTGAGAATAGTTG

ATTTATTGCTTGTAATTTTACT

>Unigene0002010

GGTCTCTTAGTTAGTATGCCAAGTCACCAGTTCCACATAACCATAGTTACCAGAAAAATA

TCAAAAAGAGCTCACACTAAAAGAGTTATGATATGCTTTTCTTCTTTTGTATCATTGTGT

AGACGAGAGTTTTGGGACAACAGACGGGTCTCTTAGTTAGTATGCCAAGTCACCAGTTCC

ACATAACCATAGTTACCAGAAAAATATCAAAAAGAGCTCACACCAAAAGAGTTATGATAT

GCTTTTCACATACCATAGTTACCAGAAAAAGGTATTTTAGTGAACCACAGTTTGAAACAC

TGTTGCTGACCGCTACACTAAATATTAGCTGTTGAAACGA

>Unigene0002011

ACCAATGTTGTGATGTCCAACCTATAAGAGTCCCTTTGTTAATATCAACTCAAGTGGAGA

AGACTTCACACCAAAAGAGTATGATATGCTTTTCTTCTTTTGCTCACAAGAAATGTGGTG

ATATTTCATTGTGTAGATTGAGAGTTTTGGGACAACAGACGGGTCTCTTAGTTAGTATGC

CAAGTCACCAGTTCCACATAACCATAGTTACCAGAAAAATATCAAAAAGAGCTCACAC

>Unigene0002012

GGGATGTCCAACCTATAAGAGGATTCTGTTTTTTTTTTCTCAATATCAACTCGGGTGGTG

AAGAGTTCACACCAAAAGAGTTTGATTAGTATGTTTTTCTTCTTTTGTTTCTGTTAGTAT

TGTGTGTAGATGAGAGTTTTAGAACAGACAGGTACCAGAAAAAGGTATTTTAGATAACCA

CAGTTTAGAACACTGTTGCTGACCCCTACACTAAAAATTAGCTGTTGAAATGAGGGATAT

AAAATGACTGCACC

>Unigene0002013

GGGATGTCCAACCTATAAGAGGATTCTGTTTTTTTTTTCTCAATATCAACTCGGGTGGTG

AAGAGTTCACACCAAAAGAGTTTAATTAGTATGCTTTTCTTCTTTTGTTTCTGTTATTAT

TGTGTGTAGATGAGAAATTTAGAACAGACATGTCTCAATTTGTATGCCAAGTCACTCGTT

CCACATACCATAGTTACCAGAAAATGGTATTTTAGATAACCACAGTTTGGAACACTGTTG

CTGACCACTACACTAAAAATTAGCTGTTGA

>Unigene0002014

TCCCATGATTTCATCCAGAAATCATGGGTGACAGTGGTTGTGCATAAAATGTTGCATTCT

ATCCGTCTCCCCATAACAGGACATCTTTTTTATTTTATTTTATGGTATGGTATAATTCCG

CCCTACTATGCCCTGATAAAGCAATAGGTCCAGACAGGTTTCCCTTGGGTGTCATCAAGA

AATCCTGGGATTTTACGAAAGTAGAAATTATGCAAATGTTCGAGGAGTTCCATAAAACCA

ACATGTTAGATTGGAGTCTGAGGGCTACACTTATTGTGTTGATCCCCCCAAAAAAGATCA

TTCCTGGATTGGAGACTACGGACCAATAAGCTTACTAAACAACATATATATATATATATA

TAATTTTGGCAAAAGTTCTTTCAGCCAGGTTGAAGCCGAATTTGAAGAACATTATTGGTA

CAAGGAGCTTTTATCAAAGAAAGACAGATTTTAGATGGTGTTCTGATTGCAAATGAAGCA

GCTACTAGCAGGAAGAAGTTTAATGTAAACCTGGATTTCTCTTCAAAGC

>Unigene0002015

GACTCGTCCGATCCGCCGCTCCCCGCTACTGCCCGGCGGGATGCCAGATGCCTCGTGCAT

TTCAAATCATGTGCCTCTATCACAAAATGATCGTACGTTACCTACATCTACAATACCAGA

ACAAACAACAAGATACTATTCATGTGACACTGATAAATGAAACTTGACAAGAGGATAGTG

CTGCAAAGCGGTCGTTCCGTTCTGGATGGTATGAGTTTTTTGTTAGTATATATATATAAA

TAATTTTGAGCACTCATAAATTTATTCCAGTCATCAATACATATC

>Unigene0002016

GCTAAACTATGGTTGCTGTCTAGCTCTTGGTTTTGGAAAGATGAACCAGATAGCCAACGA

GGACATTCACTTCCATCAAACAAAATTCTTGGCTAGGCTCGTGCACTTGATCAAAGTTAT

GTCAAAATTCAAAGATCCTTATAGTGCTGTGCGGCGTGAATGATTCTGCATCCCATCACA

CTACACTTAAAGATCTGTTTAGACGAGGGATGACAATGACTTCGACACACA

>Unigene0002017

AGGGATGCCAATGACTTCGACACGCAAACAGGAAGGTGGAACCGAGCGCTTGGAATGCGG

AATCCAACATTCCAGCTCAACCAAACCAATAAGTTTCTTTCTGAATGTCTTTATATCCTG

AAAAACTGTTCCTATAATATATATGCTGATTACAATAGATATGCCTACAAATATTTACAC

GTGCGACTTAATTAAACTTCATTTAGACCAGGATGTCTTAAAGAAGCCTACCCATGATGT

AGCATGATCCCTAATAGTAATTTTTTGGCCTTCCTGATGCCGCTCAGCATACTACACCAA

TCCACATTCATGGTAACTTGCATTTTGAGCCATTTGGCACCCTAGATTCTGTCTCATATG

TAAGTAAATATATTTCATTCAGATAGACAACTGCAAAAAAATATTGCATGATCAGTAGAT

ATTTGTTTTCCTTCCTGTCATGCCATGCCAAAATGTAACATCTTTCATATTCAACTACAG

GACCGAGAGCCGTTGATTTATTGTTTGTAATTTTACTTAATAGATACTTTCCTC

>Unigene0002018

GACTCGTCCGATCCGCCGCTCCCCGCTACTGCCCGGCGGGATGCCAGATGCCTCGTGCAT

TTCAAATCATGTGCCTCTATCACAAAATGATGATATGTTGGTACCTACATCTACAATACC

AGAACAAACAAGATACAATTCATGTGACACTGATAAATGAAACTGGACAAGAGGATAGTG

CTGCAAAGTGGTTGTTCCGTTCTGGATGTCAAAATCCAAAGATTCTTGTAGTGCTGTGCG

GCGTGAATGATTCTGCATCCCATCACACTACACTTAAAAACCATCTGTTTAGACGAGGGA

TGACA

>Unigene0002019

TGCCAGATGCCTCGTGCATTTCAAATCATGTGCCTCTATCACAAAATGATCGTACGTTAC

CTACATCTACAATACCAGAACAAACAACAAGATACTATCCATGTGACACTGATAAATGAA

ACTTGACAAGAGGATAGTGCTGCAAAGCGGTCGTTCCGTTCTGGATGGTATGAGTTTTTT

GTTAGTATATATATATAAATAATTTTGAGCACTCATAAATTTATTCCAGTCATCAATACA

TATC

>Unigene0002020

GAATAATATGTACATGCACATTAAAATAGATCCGGCTAAAAAAAACTTTAGGCTTGGTGC

TTAATGACACTTCATTTAGACTATATGCTGTCCTAAAGAAGCCTTCCCACAATGTAGCTT

GCTGCCTAACCGTTGACTTTCCACGTTCCTCGGCCCGTTCAGCATACTACACTGATGTAC

AATCGTTGTAACTTGTATTTTCAGCCATGAGGCACCCTAGATATATTATATGTAAGTAAA

TATATTTCGT

>Unigene0002021

TGCCGATTATATGTGGGCAATCTTGACCAAAAAATCACAGAGACTCATATAATAAAGATG

TTTTCTCCCTTCGGAACGATTGTATCCGAGGATTTCTTGTGGCACACCTGTGGTCAGAAG

CGTGGCCAACCGCGTGGTTATGTGTTTATCAAGTATAGTAGCAAGGAGTGAGAATGCAGG

AAGCTCAACTGGCCAAGACGAAGATGAACGGGCGGTTGGCTTGTGGGCGCCCTTTGGTGG

TTCGTTTTGCTAGTGAGAAGTATCCGGCAGACTTAACCGGCCTCCTCCCGTCTTCCTCTA

ATGCCAAAAGGCCAAGCATTGCGAGTAGCCTCTCAGGGCGAGAAATGAACCGGAGTGCGA

AGATTGCAGCGATAAAGGGTAAGCTGAAATCTTTGGAAGAACAGGGATGCAGTGTGAAGA

AGCAAAGGGTAACAGAAGCAACTTCTGGCCTTGCGAATCATTTTTCTAATTGCTAAGATT

GTCGCTAATGCCAAACTTCTTCCATCAAGGTGGAACACTGTAGTGGCTGATTTCTGGTTC

TTGTTGCGTAAATTGGCCTTGCGGATAACTAAAATGTATGTCAGTTTTTCATATTGTTTT

TATGGGTGTACACTGTGATCGTATGGAAAAACAATTGGAGAAGAAATATCTATTGGATCA

GGACCATGTACACTGTCAGAAGAGGGATCATTGGATGCACTTCCAAGTATAACACCATTT

TTTTCGGAGA

>Unigene0002022

GCTCCTTAAGTGACGAAGACGAAGCTGATGAGGAGAAGAAAGAGAGGAGGAAGAAGAAGG

GGTTGAAGGAGAAGCTGAATGGTGATTATAAGGCGGAGGATACGGTGATGGTGACACAGG

AGGCGGTGAACGTAGAGGAGCCTGACGACAGTACCATCGTGGTGGAGAAGGTGGTGACTG

AGGCAGTAGTGGAGACACCGACCGTGCTGAAGGAGGAGAAGGAGGAGTGTCTGGA

>Unigene0002023

ATTATACAGAGTTTCAAGCCAAAGTGAGATCTCTCACCCTTACATTCAAGCTTCCTGATC

TTTATCCATGGCCGAGGAACACAACCATACTGTTGAGACCTCCGAGGTGGAGGACCGAGG

CCTGATGGACTTTGTGAGCAACAAGGAGGAGAAGGCTGACGAGGAGGTTCTTCTCGCCGG

AGTCGAGAATTTACAGGTAAAAGATGAGAAGCTAGAGGACGAGAAGAAGCACACTCTTCT

CGACAAGCTCCACCGTTCCGACGGCTCCGGTTCAAGCTCATTAAGTGACGAGGCGGATAC

GGTAGTGCTGACGCAGAAGAAGGTGGTCGTAGAGGAGCTTGACGACAGTACCATCGCGGT

GGATAAGGTGGAGACTGAGGCAGTAGTGGCGACACCGAAGGAGCTGGGGGAGGAGGAGAA

GGAGGAGTGTCTAGAGGAGATCATGGAGAAACTCCAAGAGCGCAAGAAGATTTTTGAGGA

AGGAGCGGTGGAGTCCATTACGACGGAGGAAAAGGAAGGGGTATTAAAGTCCACTACGCC

GCCACCGCCTGCTCTGGTGGAGGAGAAGGAGGAGTGTCTGGA

>Unigene0002024

CTCACATCTTTAACTGATAGTATGTTTTGACCTAATTGTGCTTGTACAGTTAAGTGACGA

GGCGGATACGGTAGTGCTGACGCAGAAGAAGGTGGTCGTAGAGGAGCTTGACGACAGTAC

CATCGCGGTGGATAAGGTGGAGACTGAGGCAGTAGTGGCGACACCGAAGGAGCTGGGGGA

GGAGGAGAAGGAGGAGTGTCTAGAGGAGATCATGGAGAAACTCCAAGAGCGCAAGAAGAT

TTTTGAGGAAGGAGCGGTGGAGTCCATTACGACGGAGGAAAAGGAAGGGGTATTAAAGTC

CACTACGCCGCCACCGCCTGCTCTGGTGGAGGAGAAGGAGGAGTGTCTGGA

>Unigene0002025

GTTGTATTTTGGGGGTATTTTTTTCCATTTTACAAACTTTGGGTTGGCTGTTCTGCTCTT

TTTGAGGAGTGGTTGGTTGCTTTCTTGGGTCCATTAGAACTCTTACTTTATCATCAAGAG

TAAAGCGGGTTTTAAGCCCAAATTCTTATTTTTGACCAAAGCCCACCCATAGCGGATAAT

ATGCAAAATGCACTCAATAGCTATTCGGAGTGGTTTCCATTTGCTATTTAGCTTTTGTGT

GCTTTGTCTTGGTCCGTGTTTCTTCTGATTTATTCCGTTGCTTTTCTTATCTTTCCCCTT

TTTGTTTGGGTTTGATTATGGTTAGTCCTTGTATATATTTTTACACATGCATTGCTTCCT

TTTCTTTTTTTTTCAATCGAGCTCTTA

>Unigene0002026

GGAGAAGGAGGAGTGTCTGGAGGAGATGATGGAGAAACTCCAAGAGCACAAGAAGACTCT

GGAGGAAGGAGCGGTAGAGTCCATTGCGCCGGAGGTAAAGGAAGGGGCCATAGAGTCCAC

TACACCGCCACCGCCTGCTCTGGCGGCGGAGCCTGAGGTGGAGAAGGATGAGTGTGAGGA

TGAGACGATGGAGAAACTCCAAGATCACAAGAAGACTCCTGAGGAGGGAGCGGCAGAGTC

CATTGCGTCGCCACCGCTGGCTCCGGTGGGGGAGCCTGAGGTGGAGAAAAAGAGTTTTCT

TGCGAAGATCAAAGAGAAACTCCCCGGATATCAGAAGAGCAGCGGAGAAGAGGGGGCGAA

GGCTGCTGCAGCAAGCGAGTAGAAGAAGAATGGCAGCGAGATTGCAGTTGTTTTGATGTT

GTTTTGTTGTGTTTATGGGCTATTTGATCCCATGGCGTTGATGTTTCGTTTGTTTTATTT

TCTTGGTTTTGGTTTTGTATTCTTTGTAATGGTGTTTATGTTTTGTTTGTTGTATTTTGG

GGGTATTTTTTCCATTTTACAAACTTTGGGTTGGCTGTTCTGCTCTTTTTGAGGAGTGGT

TGGTTGCTTTCTTGGGTCCATTAGAACTCTTACTTTATCATCAAGAGTAAAGCGGGTTTT

AAGCCCAAATTCTTATTTTTGACCAAAGCCCACCCATAGCGGATAATATGCAAAATGCAC

TCAATAGCTATTCGGAGTGGTTTCCATTTGCTATTTAGCTTTTGTGTGCTTTGTCTTGGT

CCGTGTTTCTTCTGATTTATTCCGTTGCTTTTCTTATCTTTCCCCTTTTTGTTTGGGTTT

GATTATGGTTAGTCCTTGTATATATTTTTACACATGCATTGCTTCCTTTTCTTTTTTTTT

CAATCGAGCTCTTA

>Unigene0002027

CGGGACGAAGGGGATGATGAGAGAGAAAACAGGAGACTGATCTCCTTCTCCTTCTCCCTC

ATATGGGTCGTCTTCTTTCTCAACTCTTCTTCCCGTTCTTGTTCTGTCTCTACGCCCTTC

TCCGGTGGCCAACTGCCGTCAATGGCGCTGACGCCTTGGACCCTCCCCGGACTCAGATCC

CACGCCATCTCTATCATTCCAGCCTTAACAGTGGCACTCTGTTGAAAGAAGTTGAGGCTT

TGGTGGGTCGTCATCCAGATAAATTGACTATGGAGACAATGCGAGCAGGAAACAAGGGTT

ATGTTGCTGAGATAGCAGTGGTTACATACAATTATGTGAAAGGAAAGATAAAAGATAACA

AGAAATCTCGGGTCCTTTTAAGCTTTGGACAGCACGGGAGAGAACTTGTTACCTCTGAGG

TTGCCTTGCATCTCCTTTCTGTTTTGGCTGGAGAGCAAAGAGTCGTTGGCATGGACCCGA

CAAACACAAGTACTATGTTGAACAATCTTGTGATAAAGGTGGTACCGATGGAAAACTTGA

ATGGCCGCAAACTTGTTGAAGCTGGAGACCTCTGTGAAAGGAGGAATGGAAGAGGAGTTG

ATCTGAACAGAAATTGGAGCGTCGATTGGGGTAAGAAGGAAAAGGACTATGACCCATATG

AAGAAAATCCTGGAAGCTCTCCTTTTAGTGAACCTGAGGCTCAGATAATGAGGAAGCTCT

CGAAGTCGTTTAGTCCACATATATGGGTGAATGTACACTCTGGAATGGAGGCTCTGTTCA

TGCCATATGACCACAAAAATGAAACGCCAGATGGGCTCACGTCAGAGTTGATGAAATCGA

CTTTAGAGGACCTGAACCGTCACCACTTTCAAGACCGATGCTTAGTTGGACATGGCGGGG

GCTCTGTCGGGTATCTAGCGCATGGGACAACAACAGATTACATGCACGACATAGCTAAAG

TACCGATGTCTTTCACATTTGAGATCTATGGAGACCTATTAGCATCTTCGAAGGACTGCT

TTAAAATGTTTAATCCTGTCGACGAGGTTGCTTTCAAGGGGGTTATTGAAAGCTGGTCGG

CTGCGTTTCTCGAGATTTTTGAGACGGCTCCTGGACGGTTGGAGCGAGCGAGCAAGTTGG

TCGGTGCTGAAGAGGACAGGTGGGTTTCTTCGAAGGGCAGGAAAGTTGAGGGGCTCGAGC

TAGGAATGCAAGAGCTGAGGACTTATTTTAGGTTATTTATGCTATCGTCGGTTCTACTGA

TGTTCATGTTCTGTTCGAGGATATCGAGGAATAAATATAAGCCTGCTCTGGGACTTTGAT

TTTTGTGTATTTGGAAAATATATTTACATTTTTTATTGTTGTAACATTCCTTTTATAATT

TGTGTGGACTAAAGAAAGTTTCAT

>Unigene0002028

GGATCTACTACACTGCTAACAATCCAATCACAACAAACCAACACAACCAATCCAGAACAA

CAATCAAAATACAAAACACAAGATTTACATGATTCGGTCAAGCGACCTACGTCCATGTGG

TGAAGGGGAAGAGTTCCACTAATCAAGCTAAGAGCAGTCTATATGATATAGAGCTCGATT

CATAGAATATCAAAGATCTTTGATGGTGACTAACCTCTCTGTGTAGAACCCCCGCGAACA

AGCTAGCTTCGTAAGCTTCAATCCAAGGGGGTCAAAGATTGCTCTCAACCAGAAGGCACC

GACACACAATGCATAGTGCTTCACACCGGAGCACGCTAGCAACAACTGGTCCAATAAGTA

GAGCCACAAATGTCTC

>Unigene0002029

CTATCAATGACAAAATAGAAGCCAACCCTCCATCCAAACTGAACATATATGTGGTAGAAT

TTCAAAACTTTTCTGAATGACCAAATAGAAGCTTCTTCGAATGACCAAATATAGGTTGAT

ACTCCACCCAATTGAACACGCTTGTAGTGGACCTTCGAAACTTATCTTCGAAACTTATCT

GAATGGTCAAATAAAGGTGATTCTCCGCCCAAACTGAACACATACGGTAGATTTTTATAA

CTTTTCCGA

>Unigene0002030

GTTCACCCCACTGTGAGGGGGCCATATCATCATATGCAAAGAGACCGCGAATCAAGTCAT

TCCATTCCCCACATAGTTCACCCCACTGTGAGGGGGCCATATCATCATATGCAAAGAGAC

CACGAATCAAGTCCAAGCAGGAATGGATCTTGATCTCCCAACCAACCTTGGTGAGTGGAA

TAATTAGAACTTCTTTGACAATGTGAAACCCGCTTTATGTGGTGTTGACAAATTTGGACT

GCCGAATGACAACACTGTAACTACTGAAGCTATCCAACTGACCTTCGCGAGATCCAAATG

CAACTCCAAGATTTTCAAGCGACGTATAGCCGAACATAAATCGCTCCATATGGTGTTGAT

GAATAGGAATTATCGAATGACAATACTGCGACTATCCAACTGACCTCCATGAGATCCAAA

CACAACTCCAAGATTTTCAAGCGATGTGTAGTTGAACATAAATCGTTCCATGTGGTGTTG

ATGAATCGGAATTGTCGAATGACAACACTGCGGCTACCCAACTGACCTCCAAGAGTTTCA

AACACAACTCCAAGACTTTCAAGTGAAGCACGAAGTCTGACAAAAGTTTGAACCTCCACC

TAAAAACATATCACGCCCGTGGTAGACTTTTAAAACTTTTTTGAATGGCCAAATAGAAAT

GGACACTCCATCCAAACTGAGCACCCCTGCGGTAGACCTTCATAACTTCTATCAATGACA

AAATAGAAGCCAACCCTCCATCCAAACTGAACATA

>Unigene0002031

TAACACCAACTTACACATGCACAACTCAACTTACACACACAACCTTGAAATTTTGTGCAC

GCAAAATTTCTATGCTTTCTCTTGTGTGAAAAACATTTCCCTGAAACGATCTACAACAAG

CCTCCACCTCGGTGAAGGAATTGTCGGACAAATCTGAGCTGATATAAGCACATCAAAGTA

CATTAATAGCGACTCAACATAAGGTACTGCACCTGAAAGATTGCCTATGTATCCACTCGC

TCATGGAATCATACCCCACATTGTTCACCCCACTGTGAGGGGGCCATATCATCATATGCA

AAGAGACCACGAATCAAGTCCAAGCAGGAATAGATCTTGATCTCCCAA

>Unigene0002032

TCCACGTTAACAACCGAGGTGGCGAGATATTACATTATCGTCTGCCGAATACCGTTGTCG

CGCCCGCCGCTTGCACAGTAATTAATAATAATAATATATATTATATTATATATACACATA

TATTGCTACGCACACACACGCGCGCACGCATCGAGACCACGTAATTAATTAATAATATTT

AATAGTATTTTACCGTTGTCGGTGTGCCCGAGCCCGTTGTCCACACAGTCGTCCGACCTC

CAACGAATTTGAAGCAATCATGGCCGATTTAGATAATTTTTTTGCAAAGAAAGACCGTAA

GAAAACCAAAGGCCAGAAATTCGCGACCTCCTCGGATAATATGGCTACCGGACAAGAAGA

ATTACAAAAAAAACAAGACAAACAGAAAAAAGAAAGATCTGAAAGGTCGCTCACGATTCA

AAGTAATTACGCGACTGAAAACGACAACAAAAGTTCTTCCAAAATGTTGGAAGAAGAATG

GAATGAATACCAAGAAGAAATAAAAGACTATACAACACTTAAGATGCAAACTTTGACAGC

TGATTGTCCATCTAGCTTTACGGATAACAACGATAAAGATGATGAAATAGAATTTGAAGA

AAATGAAGCAGGAGAAATGGTACCAAAACGAAAAAATGCAGGACCTTGGAAATTAACTGA

ACCTGAACAACCTCCTCCTCCACCACCAAAAGCCGCTCCTCCAGTAGTAGCTGAAGTACA

GAAAAAAGTATATGTTCCACCAGCTCAACGTAACAATTTTCAAGGTGGACATAGAAGCGC

ACCAAGAAGTAAAGCAACTAGCTTAGATGTTAAAGATGAAGAAATGTTTCCAACACTTCA

AGGCAATAATAGTACTAAAAAAAGTGTAGCTCCTGCTGCTAGAAAAGTTGAAACTCCAGC

ATGGGCCAAGTATCAATAATTAATAATTATGATGAATATGCACATAAATATTATCTTTTA

AAAAAATTTCTAAAAAAAGAACGCATCATTAAATTATTTTTTAGTAAATGTTCATAAAAT

ATTTTCTACAATGATTATCACAATTCATAATAGAATTATGTTTTTCATTCATTAGTCTTA

TCGAAAATTAAAAC

>Unigene0002033

CTGTGATCAAATTTCTATAAATATCATAATATTTTTAAGTGATGTACTTTTTTGTTAATT

TCATTTATTATAATTATGAGCTGTAGATTTGTATACAAATTGATATCAACCAAAACTATT

CCTATAATTTTGAGAACATATTAACATGCAATTTATTTATATCATATTTATAATTGCATT

ATAAAAATTATGCAGGCACAACTCATTAGATATCTTAATATATTATACACATGTTATATT

ACGTAATGCTATGTTAATTATGTTGAAATTGTCTAATTCAAGTGCCTAAATTGTTTTGAG

TTGTGTATCATTAAAAACTTACAAAAATATATCATAGTATAAGTATTAAATTTATTTTGT

TGATATAATGTAACATAAAATTATCAAGGTAATTACTAATTACATTATGTCCTGATTAAT

ATATTTTTTGAAAAAACTTTTCATTTTAAAAACCCAAGAATTAATACTAATATAATGCAT

GTTTGATA

>Unigene0002034

CCAAATCGACCACCCTCCGAGGGAGCTCGAGGACGACATTTTCCCCCAAATTGAATTCCC

GCTGCTCGAGACGGCGAGAAGGATCGATCGATAGCGAGCAAGATGTATCATCCATCCCGA

GGTGGCGTTCGAGGCGGCCGAGATCAATTCAAGTGGGATGATGTCAAGGTTGATAAGCAT

CGGGAGAATTATATAGGTCATAGTCTTATGGCCCCTGTTGGTAGATGGCAGAAAGGTAAG

GATCTACATTGGTATGCTCGAGACAAAAAGGCTGCAGCTTTAGATGCAGAGGCAGCCAAA

GAGGAGATAAAAAGGATCAAGGAAGAGGAAGAACAGGCAATTCGCGAGGCTCTTGGTCTG

GCCCCCAAGCGTGCTACCCGACCTCAAGGCAACCGATTGGACAAGCAGGAATATGCTGAT

CTCATCAAGAGAGGATCTACAGCCGAGGATTTAGGTGCGGGCCATGCCGAAGCAGCACAA

GTTCAAGGACTAGGCCTATACAAGATGCCTCGTGATCAAGGAGAGTCACGCGCCCTACCA

AACAAACTGAAGCAAGTGATGCCCGGTAAGGAGGAAAAGGGTTCGTCGATTGCTGAAGAA

GTCTCTGGAGAAGATGATGATTCTAGGAAGAAGAATAGGAGACACGAGGAAAGGAGAGGC

GAGAAGGAGAAGAAATATGAGAAGCATGAGAAGAAAAGGTCTCATGATTCGGATGACAGG

CGGAAACATCAGAGGGAGGAGAAGAGGCCTCATGATTCGGACAAGCGGAAGCACGGTAGG

GAGAAGAGGCGGCGGCATGATTCTGATTCTGATTGAAGTGTTCGAGCAGTTCTTGTTATG

TATCATACCTTGCTCTGAACTCGTTGACACTGAGATGTTGAGAATTGGTGTTTAAGCTGT

TGAAATGCTTTTGTATCGTACCAGTATCTTCCTGGTGACAATTCCGATTTGTGGATTTCT

GGACTTGATTCGTCATAGGATTAGTAGTTGGTCAGGAAGTGCCAATTAAAGAGCCAGAGA

TTCATTCAGGCACTACCTCTTTTGCTGCGTTGTGTCACTTCTTTGGCAGAAAATAAGTTG

TGATATTACATATTATGTCTATTAATGGTAGAACTGAGCACAACACATAGACATGTAAAA

CTGAGCAGCAGCAGTTTTTAATTATT

>Unigene0002035

TGGAAGATGAAGGGGTTTGTATCGTACGTGCACAGCATGGTACGATCACTGTTAAGCAGC

GTGATTGTGCCATGCTGTGTGCGTCCGTTGCACCTCCCTTCCATTGCATGCTCTGTTTTA

CGTGCTACATCTCTTCAAAGATTACGATAATCTGGCGTCTTCCAACTCTGTGTTCTGGGG

AATTAAAGTTAATAGAGTTAGAGATTTTGAATTCATTATTTGGAATAATGAAAACTCGTA

ACGAAAATACATTTATCTGAAAAAGGAAGTGTCCTATACTGTTTAAGAAATCAAAGCATC

ATGAAAACTGGCGATTTCGTTTTAGCTCCTAGTATATTTATTTGTAATTAAAGTTGAGGA

GGTTTCCTTATGGTAGCTTAATGATGTAAAGTCACTTACGAATGTTTTTGGAAATATAAT

TA

>Unigene0002036

AAATATTCTAAGATATGATTGTGTGAAGAATAAAGCCGTAGCAAGCTGTACAGTCTGTAA

GAATGGCAATATGGATAACCGTCAGAATGATTTTACACCATCTTCATCTAACTTATCTTC

TTATAGATCTTGATTCTGGAAGATCTTCGTTGAACATTCACAATTCCGTTGCATCATCTG

GCAAGATCTACAACAGGTTCACAGAAAAGCAATTTATATTCTGATGTAGATGGGACATCA

ATTGTGATTGATACAGATAATGCCGAGACCAGTTCCCAGCCACCAAGTCCTATTGTGTCT

TCTGAAAAAGGGGCATCCGTTGTGATCGATCGGGCTGACAGTAGTTCTCATTCACCAAGT

CTTGTTGTGCATTCTGAAAAATGTATTTTTATTATTGCTTAATTATTTTTCGTAATTTTA

TCTTACTTTATTTTCTTCTCACTTAAATTAGCCTGGACCCTTATATTATATCAAAATTTA

ACATGAATATGTTTGTTACGGGTAATTTTCATTACCCAGG

>Unigene0002037

ATCTGGCAAGGTAAATATGCATCAGACAAACTCTAGATATGAATCTTCCTGACTTCGGTA

TCTTGGTGGAAAATTTCACAAAATGAACACACTAATATCAAATTTACGTTGCATTTGATC

TGCTCTACATGTGTCTAAAAGATCTACAACAGGTTCACAGAAAAGCAATTTATATTCTGA

TGTAGATGGGACATCAATTGTGATTGATAGAGATAATGCCGAGACCAGTTCCCAGCCACC

AAGTCCTATTGTGTCTTCTGAAAAAGGGGCATCCGTTGTGATCGATCGGGCTGACAGTAG

TTCTCATTCACCAAGTC

>Unigene0002038

GAATAAAGCCGTAGCAAGCCGTACAGTCTGTAAGAATGGCAATATGGATAACCATCAGAA

TGATTTTACACAATCTTCATCTAACCATGTTGATTCTGGAAGATCTTTGGTGAACATGCA

CAATTCCGTTGCATCATCTGGCAAGGAGTATAAAAGATCTACAACAGGTTCACAGAAAAG

CAATTTATATTCTGATGTAGATGGGACATCAGTTGTGATTGATAGAGACAATGCTGAGAC

TTGTTCTCATCCACCAAGTCCTATTGTGCCTTATGAAAAAGGGGCATCAGTTGTGATCGA

TCGTGATAA

>Unigene0002039

GTTGGGAGAAAATGTTCACAGGCCTCAGTTAAGATCTCGTTCAAAGGTAATAATATCATT

ATTTTTTGATATTATTTACTCCCTGGCTCTGCTCGGAGCTTATTGAAGCAATGCAATAGG

CAGTGGAGGAAATATTCTAAGATATGATTGTGCGAAGAATAAAGCCGTAGCAAGCCGTAC

AGTCTGTAAGAATGACAATATGGACAACCGTCGGAATGATTTTACACGATCTTCATCTAA

CCGTGAGATGTCATTTTGTTAGTTGGACCATACAAATTCTTAAGATGACCGGAGAGTATA

GGAATGATGTTT

>Unigene0002040

ACCCACCATCGAGCTAGGGTTTCTTAATCCCCACCGACGATGGGCGAGACATCAATCCGC

CAACCCCTCCACCGCCTCTCCCCAGCTCCGACGATGGTGCAACCGGCAGCCTACGCTTCG

CCTCCGATCCCCACGACAAGTCTGCCCCTCTGCTGCTGGTGAAGCTCGAGTTTGAGGACC

CCCACGAAGACGAGCCCGACACCCCCCCCGTCCTCTCCATGCGCCCTTCTCAATCTCATC

CATATTCGATCTCGTTCCCTGATTCAATTATGTGAAGGATCCTGCTTTGGAGAGATTGAT

CCTGTTGCCACTGATATTGAGGTCCCAGTCCCGCTTCGTGTGCTTGACTCACATGGTGCT

GCTGCTCGAGAAGCTCATCATGTGCTGCCACACGTCTCCTTGCCATGTATAAAGACGGAT

TCTGCACGATTGGGCAAGCTGAACCTATTCACGTTCAATGCACTTGATCAAGGTACATGC

ACTTGATCAAGATACATGGTAATCGGGGTAACTTCTCGGAAATGATAAGCGTGTTTGAGG

AGTTGAGGGCGAGGGGGTT

>Unigene0002041

AAAGGAAGGAAGGAACCGAACAACCCCACTCTCCCTCATCCCCTTCCTTCCCTCCAAAAT

GCTCTACCCCACCACCACCCTCCTCCTCCTTCTCCTCCACCTCTACACACCATCTTCGGC

TTCAGCAGTCATTGTCGGAGCTCACCGGCGACATGCCGTCTCCCACCACCAACCCCCGGG

CTTCCCCACCACTCAAACATCCTGCGACTTTTTCACCGGAAGCTGGGTTAGGGACGACTC

CTACCCTCTCTACCAGTTCAGTAACTGCCCGATCATTGACCCACAATTCGACTGTCAGCT

TTACGGCAGGCCGGACTCTGGCTACCTCCGTTACCGGTGGCGGCCGGCCGACTGCGAGCT

GCCAAGGTGAGAATTGAATATAAAACTATGTGCCATATAAGTAGCAGTGACGTTTTCTGC

TGATAGGTGTGTTTGTGGATATAGGTTTGATGGAGTTGATTTTCTGACGAGGATGCGAGG

GAAGACAATCATGTTCGTCGGAGACTCACTCGGGAGGAATCAATGGGAGTCGCTCATCTG

CATGGTTCATGCTGCGGCGGCGCAGTCGGCAACTCAGATGATTAGAGGGGACCCTCTCTC

GACCTACAAGTTTCTGGAATATGGAGTCTCTCTCTCCTTTTATCGTGCTCCTTACTTAGT

AGACATCGATATTATTGAAGGGAAGCGAATTTTACGACTTGACGATATTTCTGCAAATGC

CAACATGTGGAAGACCGCCGACATCCTCATCTTCAACTCTGGCCACTGGTGGACTCACAA

TGGTGCTCTACAAGGATGGGACTATATGGGGGATGATGGAGCTTACTATGCTGATATGAA

TCGGCTGGTCGCTTTTCAGAGGGCGCTGAGCACGTGGGCCAATTGGGTTGATGTGAACAT

CGACAGGGTCCGGACTCGGGTGTTCTTCCAGTCTATTTCCCCGACCCATTACAACCCTAA

CGAGTGGAATGGCGCGGTATCAAAGAACTGCTACGGCGAGACTTCCCCGGTGACTGGCCG

GAACTACACGGGAACTTATCCCGACCAAATGCGGATCCTGCAAGCCGTGGTGAAGTCGAT

GCGGAGCCCCACCTACATGTTGGACATCACCACCTTGTCGGAGCTCCGAAAGGACGGCCA

CCCCTCCATCTACAGCGGCGATCTCACCCCCGAGCAGAGGTCGAACCCCGACCGGTCAGC

CGACTGCAGTCACTGGTGCCTGCCCGGGGTCCCAGATACTTGGAACCAGCTCTTCTATGC

TGCTCTTGTCTCCTGACAACGAAAGAGTTGTCTTGTGTATCAGGATTTATGCTCCTCTTG

TTTCACGAGGAAGGAAGAGTGGCCTTGTGTATATGGATTTTTAAGCATTTATATTGTATT

TATTGGTGTTGAGCAATATTGGAATTAATAGATAAGTGTACAACTTGATCACTTGGTGGT

TTACCATAAATCTAGCTGCACACTGACTTTTGTACGCTTGCTCTCTTTTTTGGAAACTCA

CCTTCCCATACAATAATTGTC

>Unigene0002042

ACAAATTTTAGCATACTTTTAATGGTCAACACTTCTAATTTTAAAATTGATTTCCCGAAC

TGATGTGCCACACAATGTCCTCAACTTGACATACTTGCAAGAGCAGATCACAATGCCTAG

CATAATTCTTACTTTTTATAATAAAAAATATCATGTGTGAGTGTCCAAAAGAGTCAGTTG

TAATTGAAATATATGTTAAATTGTAATAAGAGAACAAATTTTAGCATACTTTTAATGGTC

AACACTTCTAATTTTAAAATTGATTTCCCGAACTGATGTGCCACACAATGTCCTCAACTT

GACATACTTGCAAGAGCAGATCACAATGCCTAGCATAATTCTTA

>Unigene0002043

TTTTTCCCGGGCTTTTAAACCGGGCCATTATAAATACGGGGTGCACGCGCTCAACAGCAT

TCAGTATCCGACCGGCTTTTCATCCGACGACACTTCAGCGGCTCTTTGCTTTTTTCTACT

ATCCGTGTATACCGCGACCGTGTTCTGCCAGCAGCTCGCGCACATACTACGATATTACCG

AAAGATATCGCCGTCGCCGAAGTGTTTCGATTAAATTGAATTTTTTTATTTTTTTTTTTG

AAAATTTTTTTGAAAACTTTCTCCACCGCCGCAGCAACGATGGGTTTGGATCCGACTGGA

CTGTACTGAGTACGCTTGACGCCGTAGCTCTACACACGACGACAATCTACCTGTATCCGT

CGCAATCGCACCCAACGGTCTTGGGCATAACGCATTCGAAAAGGAACCGAGAATTTTTTT

TAAAATTCAATCTCAACTTTCGTATTTCGGAACGCATCCACAGCAAAAAATTAACATTAT

GTGCGGAGGAGGACCAAAACTCGACCCAACTGGCCTCTACTAACCAGTGTAGATGCACTT

CACAGCCAACAGCCACACACAATTCAACATTATGTTTAAATCAGGTTTACGAAAAAACAC

GAATTCCAAGATCATGGCCCTAAAAGTCAGCACCTTTTACCACGGCACAATTAGGCATCT

GTACTGGCCTCAAACAACAGTTCGCTGGAATCAAGATTTTGTATGAAGACAATAAATTCT

GTTCAATTTCCTCATATAAGACTCGAGGAGGACGATGAATGATGATATTCACAACAGAAT

ATTATTATTATCATTATTATTTTTTGACTATTTAAAATCTCAAAATAGTTTTATTGTTAA

ATTTGTCTGTTTTTAAGATTTTCGTTTCCTCCTCGCTTTTTTTTTATTATTATCATCTGG

GTTCACATTAGTTTCATTGGGAATATGGACATCTGTATTAGTTAGCTGATCATAGTCCCC

GTGGAATAGGGTTTGTGTATTTATTTATTTATTTTTATTATTATTATGAATACGTTCCAA

GCTTGAAAAATATTTTTAAATCTTTCTTAAATCACATTGGATTTTAATTAAAATAATGTT

GCTTATTGTTAATTATTTAAACATTAACAATATATTAATTTCAGTCTTATGTTATTTATG

AACGATAAATATTATTATTTTTATTATTATACTATCATTATTATTTTTTTTTTTTTATAT

ATAATGGAATTGAACTTATATTGTATTCATCAAAATGATTAACAACAAACATTAGAACAC

TATGAAACAAAAAAAAAAAAAAAATCACGTGTATTAATACGTAGGCTTAAAAGACTGACT

TAACTCTGGACTTCTATAAAATAAAGTTTTTGTTTCCAATTCTAATAATGTATAGTCTCA

GGAACTATGTTTTTTCTATTTTTATAATATTAATCTTCTCCGTATTT

>Unigene0002044

TTGTCCCAAATTCTCCCTCTCCCCCCTTCATCCGAAAGGAAGCCCTAATCTCTCCATCGA

TCAAAAAGGAAATCCCAAATTCACGTTAGAGGTCTGCAGATACTCGGAATACTCAAGCGG

ATCTCGACGATGGTGGCGTCGGGCTCATGGGCGACGGCGTTGACGAGGATCTCACCATAC

ACGTTCGCGTCCATCGGGATTGCAATCTCCATCGGGGTCTCCGTCCTCGGGGCTGCTTGG

GGAATCTACATTACCGGGAGTAGCTTGATAGGTGCGGCCATCAAAGCTCCGAGGATTACC

TCTAAGAATCTCATCAGTGTTATTTTCTGCGAGGCTGTTGCTATATATGGTGTCATTGTA

GCAATCATACTGCAGACGAAGCTAGAAAGTGTCCCATCATCTCAGCTATATGCAGCAGAA

TCCATGAGGGCTGGATATGCTATCTTTGCATCCGGGATTATTGTGGGTTTCGCAAATCTT

GTCTGTGGGCTCTGCGTGGGGGTTATTGGAAGCAGCTGTGCACTCTCTGATGCTCAGAAC

TCGTCACTTTTTGTAAAGATTCTAGTGATTGAAATCTTCGGCAGTGCGCTTGGGTTGTTC

GGAGTTATCGTAGGCATAATTATGTCAGCTCAAGCAACATGGCCGGCAAAGGCATAGAGG

TCGGTTCAGTAAACCATGCGTAATTTCTAATCTCTTTTGCTCTGGTCTAAATGAGGTTAA

TGTATGTAGTTTTATCTTAAATCTTTCTCTTTGTATCAACTTGTTTCTATCATATGCTCC

CCCTCATACCTGTTTGAGTAATTAATTTAGTTGTAGTGTGGGGCGACTTTTTGAGATACA

TTCTTTCTTGCTTTAGAATATAAAGT

>Unigene0002045

ATCTGGATCAAGCCACCTGATTGGGATTTCCTCGGACCCCATCGATCCTTCAACCGAGAG

AGAGAATCCACATCCTCTTCGCCTTCGATCGAGGAGATTCGACCTGATCGCAGTAGCAAT

GGTGGCTTCGAGCTCGTGGTCGACGGCGCTGATGCGGATCTCGCCCTACACGTTCGCCTC

GATTGGGATCGCGATCTCGATCGGCGTCTCCGTCCTCGGCGCTGCTTGGGGAATTTACAT

AACGGGGAGCAGCTTGATTGGTGCGGCGATCAAAGCTCCGAGGATCACTTCTAAGAATCT

CATCAGTGTTATTTTCTGCGAGGCTGTTGCCATATACGGCGTTATTGTTGCAATCATATT

ACAGACGAAGTTAGAAAGTGTGCCAGCATCTCAGCTATATGCTGCCGAGTCTATGAGGGC

CGGATATGCAATCTTTGCTTCCGGGATCATTGTGGGTTTCGCTAACCTTGTATGTGGGCT

CTGTGTGGGAGTTATTGGAAGCAGCTGCGCGCTCTCTGATGCTCAGAACTCCTCTCTCTT

TGTTAAGATTCTGGTAATTGAAATCTTCGGCAGCGCGCTGGGGTTGTTTGGAGTGATTGT

AGGGATAATTATGTCTGCTCAAGCAACATGGCCGTCTAAAGCATGAGATTGTATCTGTTT

CTTGAGCCACACAAAAGCCTTATCAACTGTTGTGGCATCGAATTGCAAATTCATGTAGTT

TTTGTCGGATCTCGTTCGCGGTACCAAATTCTTCATATTATAGTCCTGCTTAATCATGAA

TAATTGTTTTCGGGACGGATATGTCTCTTAAGCACATCTCGGGCTATGAAGGGTTGAAAT

ATACCGCTTTCATATATTTTATGCGAGCACGTTTAACTCTTATTTTGTTCATATTTGTGT

TTGTGGTTGTTCCCATACAGCATTAGAGTCAAATTTTCCTTGATGTCTGTGAATGTAAAC

CCACTGGGAATCTGATACTTCATGGTATTGATATGAAATGCTGTATATTGCGTGGTGTCT

GTCTGCTCATTATGCACTATTTT

>Unigene0002046

GAGGGAGGAGGATAAGATCGGTATCGTAATTTTGCTTCAGCTGGTAAGAATCGAGCCCTT

GTCCCCTGTAGCATGCTTTCCCATCGGATCCCCACAGCACAATATATAGGGTTTTCGAGC

CCCCCCCCCAACCTCAGTTAGGTCGTGCTCTCATGATTTCTTCTCAAAGATTTCGTGATT

ATGTACAAAATTTAAGCGGGACCTCCCTATCTTGAGTCACTCACATGCAGCAACAAAAGC

ACCTTTAGTTATTTAAGGTAGGGCGTAACAATTTTATCGCTCGATTTCAGGCGGTCCAAA

TTAAATGAAGTACTTAGAATTTCTTATTCTTGCATTTTCAATCTTTAACGATGAAATTGC

ACTAGATTGTATTGAATCAAC

>Unigene0002047

TACTTTTAATGGTCAACACTTCTAGTTTTAAAATTGATTTTCTAAATTTATACTTCAGGC

TTAGGTTATAGATGTGTCTAAAAACATTTTATATCATGTATATATACTCGGTGGAATTGA

AACATGGTTCATTTAATATAACTTTTAGCGACCCTAATTTCTTTCTTGAAGATTACTTGA

TCCAAAGATTACAAACTATGATTTTACAAATGAAATTATTTTCCTTAATTTAGTTATCAT

TTTTTATTCTTTCTTATATTAGGAGGTGGGATCTTTGGCCTCTTATCATGGTTCCTGTCT

TATAGTCTACCTAGGCGGTGTACTTCGGCCTCTTATATATTATCAGATTATCGGTGTGAG

AATTGGAATATTTTTCATTAATTTTTTCTTTATTTTGTTTTCTTTCTTCGAATGCGTGCC

TGACAGTTTAAGCATCTATCTCTACTGTTTCCACTTCATGAAGATTTCTCTCAAATCATC

TTAAATCTTATGGTACGTACCCTCAAACTTCTAGAGAAATCAACAACAAAAGATACAAAG

AAGGCCATCTCCCAAAGATGTGTTCGTGCAGGGATCCCAAATATGCATTTGAACCAAATT

AAAGGTTTCATGAGTATTGTGGACGACGACACCGAGCTTTACCCTTATCTTCTTGCCAAG

ATCACAATGCTCACGTGTTTCCAAATTGGAGGCCAAAGCACCAACCAAAAGTCCCCGTCC

CGTAAGAGAATTAAAGACTCATCATCCGTATGTCCCAAGCACGTATGTCACAATCTGGTA

GTATCGACAACCGAATATGATATCTTCTCCTCTCCTGTAAATGTCCTCCCCTTCGGATAG

TACAAGCTACCACGTCGCGTCCCCTTCATCAAGACCATTGCACCATGTATCGCTTTTAGA

ATTCCATTACTGATAGATATTTTGTAGCCAACAAACTCTAAAGCACCACTAGAAATCAAA

ATTTTCTTCATGGTAGGAATGTTAAGGTGGAACGTAGCAAACATCCCTCAACTCTGTAAT

CATCTCATCGAATATCTTGATACAAACAATATCAATCCCAAGTGTGCTGCATGTATGGTC

ATTGGCCGTCAAAACTGCATCTGAATCCAACTTTTAGAAG

>Unigene0002048

AAAAAAAACTCCATGTTGGAACAAATATGATCGAAATATGTTAGGTAGCGCATGTATCCA

AAACTATCGTGACTCGACAAAATAACTGTGGATGAAGTGGATAACAACGAAAAATCTGAA

ACCAGATAATCATCATCAACCTTTTTGTGTGCAATGTCGACTGTGGAAGACTTCTGCTCT

GCGTACTTGGCTTTAAACTTCGGGCAGTCCTTCCAGTGCCCTAACTTACGACAAAATGCA

CATTCATCTCTTCCCGATTCTCAACTTCGATCGGGATCTCATATCATTGGACATGCTTCA

ACTGTGAGATCTACCTCTAGCTGTCAGCACCTCAAATTTCGATCTCTCAGTGGACGCTAC

ATCCTGGTTTTTGAGATCCTTGTTAATAAGATTTGTAACCACATCATCATACTCTATAGT

ATCCTTGTTGGCAATCAACGAGTCACACCAATTTTTGTACTCTAGCGGTAGAGAATTCAA

CAAAATTATTGCTGTGTGGTGGACATCAGTCTTCCCATCAACACTGTACAAGTCTGACAA

CAATTTGGCCTAGTTCATATGTTCAATCACGGTCGTCGTCTTGCTCATCTTGAAGGTGTA

AAGCCTCTGTAGCAATAATGTAGCAATAATCGACTCTCGTGACTCTTGGAGTTGTATCTA

TATCCCTGAGAATATTCCATGTTGATCTTGCACTAATTTCAAACATTATATGATACTTGA

TATCTTGAGTTAGACATAAGCGTATGATGTCATAAGCCGTCCTGTTCATCTTTTTCCAAA

GAGCCTCGTCGATGTAATCTGAAAGTGTCTTTTTTAAGAGTATCGTTCATATTTTATGCG

ACTAGTGCATCCTTACTTCGCATTTCCACATCCCCAAATTGTTAGTACCGATGAATTTGA

CAACGTTGATCTTGTGATGATTTGCAACTGACTCAAATGGTGTAGCAACGGGCCCGAGTT

CGCATCTTTCTTCAGTCCGATCTTGGACTCGACACCGGCGACTTCTCGCCCAGTATCTTC

TCCGCTTCAAACATGGTTTAAATGCGATGCTTGTTGTAGATAAAACTCTTCCACACCGAA

GTCGAATCTCGAAGCTCTGATACCACTTGTGCCCACAACGGAAGTCCGATCACACAAGAA

CCCAAATAATCCAGTTACAAACCATACTCAACACACATCATCATATCAGATCACAAAACA

TAGATGCATACATATGCAAATAAAGGGGAAGAAATTTTATTGGAAATCGCAGAAGAAGAT

TGCAACGATGTGTAGAAACCATCCACACCGGAAAACCTGAAGCCTACCCTTGTCTTCTCT

GCTCCCGAACTGATGTAGACGTTTGAGGCGGTCTTCACTCTCACCGGCCGGCTCTGCTTC

AACAACACTATGGCTTTGCTTCTTCTTCCCTCCAGCTGGGCTCTGCGGCTCTAGTTTCCT

TCTCTCGTCTTCTCCGCTCCCGAACTGATGTAGACGTCCAAGGCTTCTTGCACTCTCGCT

GGCCGGCTCTGCTCAACGGCTGTGCTCCAACAACACTGCAGCTGTGCTTCTTCTTCCCGC

TATCTGGGCTCTGCAGCTCTAATTGCCTTTCCTCGAAGTCTCTGTGGCTGGCTTGGACAT

GCTGCTGCCTACTTAGATCTGTTCTGGTTCTCTTTTAATTCCAAGATTTTCCAGCATATC

CTGATTGAACAGGGTTACGAATAAAAACAGATACAATAGATAAACTCTAACTGCAGAGAG

TTTATCCTACCAACACTCACGATAAACTCTAACTGCATAGAGTTTGTCCCACCAACATTC

AACGTGCCACATAATGTAAAATCTCCGAGCGCCTCCTTCAACATAAACCACTGCCACCTT

TCACTTGCTAGCTGACATAAAAAGAAAGGTTTTCATCACTTTAGTCTTATTCTCTATAGC

TTAGTTGATTTTGGTCCTCTTTTCTATAGATTGAGTTTAGTACTCTAATTCTTTCAAGTG

ATAATTTTTTTGAGCAAAATATCTTTTTGAGCTTGAAAGATCTACTGCTGTGAAAGTAAG

AAAAATTAGGAGGGTCGTATTGGTATCAATAATAATTGCGATATTTCATAATGCCAGTAC

AGCCTTGGTATTGATTTTTCTTAGTTTCATGGTTGTAGAACTTTAAGTTAGCGTAGCGGT

ATTTGCATGCCTCTTTTAGAGTGGTGATGAGGACATGTTATGCGAGATGTGCCGACTAAC

TGACCCCCTGAACCAAAAATGTTTTTCGAATATTTTGAGCACATCCAAAAATAATTACGT

AAATAAGACTGAGGCCAAATATGCTATTCCAAATGGTAACTAAGGTAAAATCCTAAAAAT

AAGTGATGGTCAATGAGTCTTTCTCCGAGGCACAAAGAACACAAATTATTGTGACATATT

AATACTCTGCAATCTGTCTTTAATAAATAAAAGTCAGCCCCTGACATCCCACACATGAAG

AAATTGACGGTCAATTGAGTCTTCCTCTGGGACACAAGTTAACTGTAAGTCCCAATTTTG

AATAACCCAGAAGAGTGACGGATATAAATGTTGCGTCCGATACAACACTTGCGATAAATA

TGGTAAACAAGATGTGTATAAATGTGAATAACCCAGAAGAGTGATAGATCCACCTTGATC

AAACATGCATACTGTTATCCTGTGTTTGTACCTTTTCAACCCCAGTTCTCCCTGTTTTGG

GGGCTTTGGGTTTGTTGTATTATGGATCACACAAACAAAAACACGTCCATCACTTTTTCG

TATAGCCTGTTCATTGTTTGAAGTCCATATATTCATTTCATTTGTATTTGAGACACACTT

GTATGTGAATTTAACCTATGCCCTATATTCCTAGAATTTTTAAATGCCACTCTCTTTTAC

ATATCCTCCCAGTGTTATTTTCTGCGAGGCTGT

>Unigene0002049

TACTTTTAATGGTCAACACTTCTAGTTTTAAAATTGATTTTCTAAATTTATACTTCAGGC

TTAGGTTATAGATGTGTCTAAAAACATTTTATATCATGTATATATACTCGGTGGAATTGA

AACATGGTTCATTTAATATAACTTTTAGCGACCCTAATTTCTTTCTTGAAGATTACTTGA

TCCAAAGATTACAAACTATGATTTTACAAATGAAATTATTTTCCTTAATTTAGTTATCAT

TTTTTATTCTTTCTTATATTAGGAGGTGGGATCTTTGGCCTCTTATCATGGTTCCTGTCT

TATAGTCTACCTAGGCGGTGTACTTCGGCCTCTTATATATTATCAGATTATCGGTGTGAG

AATTGGAATATTTTTCATTAATTTTTTCTTTATTTTGTTTTCTTTCTTCGAATGCGTGCC

TGACAGTTTAAGCATCTATCTCTACTGTTTCCACTTCATGAAGATTTCTCTCAAATCATC

TTAAATCTTATGGTACGTACCCTCAAACTTCTAGAGAAATCATCAACAAAAGATACAAAG

>Unigene0002050

GCTCACAGAAATCAGAGTCGCTCTGCTCTGCTCCTTTCTCCTCCGAGCATCGGAGCCCGC

GACCCTCTCAGCGGTCTACCATGAAGGGAGAGCCCGGCCACGGCGGCGCCGAGGCCGGGC

CCAGCTCTCGGCCGGCGCCCGCGCCGTCACGGCGGTTCCCGCCAGCGGCGCAGCCGGAGA

TCATGAGAGCGGCGGAGAAGGACGATAACTACGCCGCCTACGTCCACGAGGCCTGCCGTG

ACGCCTTCCGCCACTTATTCGGTACTAGGGTTGCTGTTGCTTACCAGAACGAGATAAAAC

TATTAGGCCAGACACTTTATTATATTCTGACCACAGGCTCAGGGCAGCAAACCCTGGGAG

AAGAGTACTGCGACATAATACAGGTTGCAAATTCTTATGGCCTTCCACCAACACCTGCAA

GACGTGCCCTTTTCATTCTATACCAGACAACAGTTCCATATATTGCAGAAAGAATCAGCT

CAAGGATTGCTTCTCGTGGCATGATCTTGGCCGACTCCCAGTTTGAGGAGCTCTATGGAA

GCAACCATCCTAGAGGGAACCAAGTACAATCTTCTGTTGTTAGTGAAAGCTTGTCTTCAA

CAGTTGCACCTGCATCAGCTATGTTAAAGTTGAGAGAGAAGTTCAATGGTTTATGGTTGT

GGGCAGTTCAGAAATGGCCTTCGGTACTTCCATTTGCACGTGAGATTATACAGTTGGCTC

TTCGCACAAATCTTATGTTCTTCTACTTTGAAGGTTTGTACTATCATATATCAAAGCGTG

CAGCAGGCATTCACTATGTATTCATTGGAAAGCCGTTAAACCAAAGACCTAGATACCAAA

TCTTGGGTGTTTTCCTTTTAATACAGCTATGTATTCTTGGTGCTGAAGGACTGCGCAGAA

GTAATTTATCTTCAATTGCTAGTTCTGTTCGTCAGACATCTCCTGGGAGACATCTTTCTA

CAGGGCGTGATCTACCTATTTTAAATGAAGATGGCCATCTGATTACAAATACTAACCTAC

CTAAAGGGGATTGGACATCTGATTCCAAGGCTGTTCAAGAGCCCCAACAATCAGGTGGCA

CGGGTAAATGTACACTCTGCCTGAGTCCTCGCCAACACCCTACAGCCACGCCCTGTGGCC

ATGTTTTTTGCTGGAGCTGCATTATGGGATGGTGCAATGAAAAACCCGAATGTCCTCTCT

GTCGTACGCCCGTAACTCATTCCAGTTTAGTTTGTATATATCATTCTGACTTCTAGAGGA

CTTCAACTCCACATATACACAATTCAAGTTGGGTGAGAGGATTTCACGCCCCGTCAAACA

CCAAATATACAAGTAATTACTTCAGAATAAGGTAAAGCCTGTTTGACCTGAACCACTTCA

AGAAGACCGATGATGAAAAATCCTAACCATGGTTCGAGTGCTGGGGAATGATTTCCTGTT

TCATGGTCTGTTGATTGCATAATTTACCACTGTGCCATTGGGAGACCTAGTCGTAGAGTA

ACTTTTGATATTGTTGTATCATCAATAATCGATTAACAGGGCTCTGGCCGATGCATAGCT

GTAAATGTTTTGTGGTGATTTGGTTGTTACCGTAGAAAATGTCTTTTTAGTATGATATAT

AGTTTTCAGAGTTCCTCTTTTGGAGGCTG

>Unigene0002051

GCTCACAGAAATCAGAGTCGCTCTGCTCTGCTCCTTTCTCCTCCGAGCATCGGAGCCCGC

GACCCTCTCAGCGGTCTACCATGAAGGGAGAGCCCGGCCACGGCGGCGCCGAGGCCGGGC

CCAGCTCTCGGCCGGCGCCCGCGCCGTCACGGCGGTTCCCGCCAGCGGCGCAGCCGGAGA

TCATGAGAGCGGCGGAGAAGGACTATAACTACGCCGCCTACGTCCAC

>Unigene0002052

ATGAGAAGAGAGTGGGTTGGTGATCGGTCTAAAAGAACCCACAAAATCTCCAGGGATCCC

ATTATAAGCCTAAATTTAGATGGCTTCATGTCTTGCCGCCTTAAATGATATTGCGACCTA

AGGCAACTGCTCCTTACCGGATGGCATGGGCTCTTTGTTAGTGTCTATATAGATAAATAG

ATAGAGAGAGCCCGCGTGCGTATGCTTATACGTATTGTCTGGAGTTCATATCATGACAGA

TAGAGAGAGCCTGCGTGTGTATGCTTATATGTATATGTATTGTGCCTGGAGTTCATATCA

TGATATTGAGAGCGTGCGTGTGTACGCTTATATGTATTGTGCCTGGAGTTCATATCATGA

TATTGAGAGCGTG

>Unigene0002053

ACACAAGGTAGGAAGCTACGAAGTACAAATCGGTCCTTCCTCTCATTGTTGGTCCATAAT

GTTATATGGATAGTTCCACTGGGAATACTCTCTCGTAGCTTTTATGGGAACAGTTGTTGT

GTTGGAACTCTAGCCAAATCCCAGTCATTATCAGATAAAACATCGAAGTTACCTCAAAAA

CTAAAGATTCACCAATTTTCGAGCCGGAGGAGAAGGCGAAGGCGATGGTCATCAAGCCCA

GAGACAATGGAGAACAACGTTAGTATATCCCATTCTCTTGAGGCTCACCTGTCTTCAGAG

CCAGCTACAATCACTGAAGAGAAGTTATCAGAGGAACATGTGAACAATTCTGCATTTGTC

AACCAAGCTGCTATAGTTTGGAATGACATGAGAAGAGAGTGGGTTGGTGATCGGTCTAAA

AGAACCCACAAAATCTCCAGGGATCCCATTATAAGCTGGTGCACAACCTACGATGATTTA

CTTTCTAATAGCCAACGATTTCCGAAGCCAATCCCTTTATCGGAGATGGTAGATTTCTTA

GTAGACATTTGGCATGAAGAAGGGCTTTATGATTAGAGAGCGATTCCGTCTTTGCAGCAG

TAAAATTGTTTCATTTGTCTCCTTGCCAAACCCATCGGACTATGGGTATAAAGACATTTG

TATCAAAATATAATTCATTACTTGAGATCTGAGCCTTTCGTTACGGAACTGCTTTTATCG

GCTTGGCATTAGTGTGTTGTAAAGAAACTATAATGACATGTACATCTGCGACAACACTAA

AATACCTGGTTATGTTCTTAACAACCCACCTCCTTATAAGTCATGTTCCCTATGTTGCAA

CACACCACTATATCATTAAGGAACTTAGTGGCATAAAGTTCTCCGAAATCTGATACAT

>Unigene0002054

TATGAACTCCAGGCACAATATGTATACATATAAGCATAAACACGCAGGCTCTCTCTATCT

GTCATGATATGAACTCCAGACACAATACGTATAAGCGTACGCACGCGGGCTCTCTCTATC

CATTTATCTATATAGACACTAACAAAGAGCCCATGCCATCCGGTAAGGAGCAGTTGCCTT

AGGTCGCAATATCATTTAAGGCTTATAATGGGATCCCTGGAGATTTTGTGGGTTCTTTTA

GACC

>Unigene0002055

TTGTCCTCGGGAAAGATCTTCCCCCCTTCTTCCTCTTCCCAGTGACCCTTTCAAGGTCGC

GACTTGATGTTCACATCCGGCTCCTTGGCCCAAATCACCATCATGGTCGTCGTCCAATCC

CCCAAATAGTTCCACCATCGATCAGCCCACCACAGTTTTTTGTGGAGATAAGGAAAATCC

ACATTAATGGATTGATTCTCTAGTAAAAGATGGATGATGGAGAAGTAGACCTTTCAAGCC

ACCTAAGATTCTTGAACCCGGACTCGCCGAGTAGCTTTGATGAGTTCTTGATGAACACAA

GAACATGCACACATACCCACACTTGCAACCCTCCGGGGCCGGCGGCGGCCGCCCACACCC

ATACATGTTACCACACACATACCCAAGTGCTCGCCACGAGCGAAGGGGAGGGGGATGACG

AAGCGGCGAAAAAGCCTCGAAAACCGTTGGGGAATAGGGAGGCGGTGCGGAAGTACCGGG

AGAAGAAGAAGGCTCATACGGCTTATTTGGAGGAAGAAGTGAAGAAATTGCGCCTCATGA

ATCAGCAGCTCATGAGTAGATTGCAGGGGCAGGCGGCGCTTGAGGCTGAGGCAGTTCGAT

TGAGGAGCCTGCTGGTGGATTTTCGAGCGAAAATTGATGGGGAATTGGGCGCTTACCCTT

TTCAGAAGCAGTGTGGCACTCCTAATTTTCAGTGTAATGACGGTATCCAACTTGCTGGGG

TTGCGGGGGTGGCCGGATGGCAGGGGGGCTGCATACCTCCGGTTGTGGATTGTCAGGTTA

GACCGAATGAGAATGCTAGGCAGGATTTGGAAATTACCGAAACTATTAACTCAATGGCTG

TCAACCAGAGCTTGGGTTCGACTACTTCTCGAGCTGAATGAAACATCAAGATATGCGGCC

TTCAAAGGGTTTTTGCCATCTTGATTTTCAAGTGCTGTTGGAATCACCTTTCAAACTGCT

ATGCTGCAAATGGTAATAACACTTGCAGTGAAGTTGTTTTTATACTAGGTACAGGTAGAG

ATGATAAGCCCTTTTTACAAGAATAATGTTTATAATTTGAAGGTTTCCTCAATATTTTGT

TATTGAAAGATCTAGATGTACATAAGAGGTGCAGCTTCCTAGTATGCATATATGCACATT

ATACTTCAATGCAGAATTATCCGCATGTAGTTTGACAGAAAATATGTTCAAGTTTGTTTG

CTCAGTTTTCATATGGAAATGAATCATGTATGCAAGTGTCATTTGAGTTCCTTGTGATGT

CACAAAGTTTTCGCAGGGTAAGTATATGATCATCA

>Unigene0002056

CTTTACTCATATTCCATCCGCCCCCACCTACCCCTTCCTCTCCGTTCTCCGTCCTCAGAT

CCAGCGGCTGACACCGGGGCTCTGTTTGGCCTCTGCCCTCCCCAGGGTTTAAGGCCTCGG

AGTCCCCGTCGCCAATGGAATCCAAATCCGCCCGCCGCTTCAAGATCGGCAGGCAGTCGT

CGCTCAAGCCGGACAACGCCGTCGACGAGGAAGACTCTGACGGGATCGAGATCCCCGATG

ACATGGACATCAACTTCCGTCTCATGTGCCTCGCCAGCGAGGGAGACGCCGAGGGGATCG

AGGAGTTGCTCGACTCGGGAGCCGACGTCAATTTCAAGGATATCGACAGCCGGACGGCGC

TGCATGTGGCGGCGTGTCAGGGGAGACCGGCTGTTGTTGAGGTTTTGTTGCGGAAGGGGG

CGAAGGTCGATCCGGAGGATCGATGGGGGACCACGCCTCTTGTTGATGCCATACACTATA

AAAATGATGAAGTGGTCAAGCTTTTGGAGAAATATGGGGCTAGAGTCCAGATTGTTCCCA

TGCATGTCAAAAGTGCGCGTCAGGTCCCAGAATATGAGATTGACCCAAGTGAATTTGACT

TCAGCAACAGTATAAACATCACAAAGGGAACATTTCGTGTGGCCACATGGCGTGGAACTC

AAGTTGCCGTTAAAACGCTCGGTGAAGATGTGATCACTAATGAGGATAAAGTAAGGGCAT

TTAGGGACGAGCTTGCCTTGCTTCAGCAAATACGGCATCCAAATGTGGTTCAGTTTTTGG

GTGCTGTCACACAGAGTAGTCCAATGATGATCGTGACAGAATATCTGCCGAAGGGTGATC

TTAGCGCATTCTTGAAACGGAAGGGAGCTTTGAAACCATCGTTGGCAGTGAGATTTGCCC

TTGATATTGCGAGAGGACTGAGTTACTTGCATGAGCATAAGCCTGCTATTATTCATCGTG

ATCTTGAGCCTTCGAATATATTGCGGGATGATTCTGGGCATTTGAAAGTTGCCGACTTTG

GTGTTAGCAAGATGCTAAAAGTAAAAACTATGAGAGAGGATACACCATTGACATGTCACG

ACACTGCTTGCAGATATGTGGCTCCAGAGGTCTTCAGAAATGAAGAATATGATACAAAAG

TGGATGTATTTTCATTTTCTTTAATTTTACAAGAGATGATTGAAGGATGCCCACCCTTTA

CTACAAAACAAGATGTCGACGTTCCAAAAGCATATGCTTCTAAAGAGAGGCCACCATTTA

GAGCTCCACTAAAGCTCTATTCACATGGATTAAAAGAGTTGATCGAGCATTGCTGGAGTG

AAAATCCTGCTGATAGACCAACATTCAGAGATATACTCGATCAGTTAACCTGTATTCAAA

ATCAGATTGCTCATAAGAAGCGTTGGACGGTGAGACCTCTAAAATGCTTCCAAAATCTTG

AGGCTATATGGAAGCGGGATAGTTCCAGTTCCAGCCGATCATCACGTTCCTCCCATTCTA

CGCGGTGATTTCCACTGATAATTTTTCTTGTACGAGTAAGTTTTTGAAGATCAAGGAATC

ACAACCAGTAAGAAGAAGATAAATCCATTGTGGCGTGGCGGCTATTACCCGATTAGGGTT

ATAAATTTTTGCAGCTGTTTGGTTTATCTACGGGGATTTGATTGCTTCTATTGAGTCTAT

TACAGGTTGATTCTGCAAGTGCAATGTGTACCTTCTCTTGATCCCAGCAAGCTACTTCGC

CTGGAAATTTACATAACACAAGTTCGTTTGGAGTTGACATTTTCATACAATGTTGTACTC

TGTATATAGGTATTTATAGAAAACTATTTATGCTACTCTAGTCTGTAACCATTAAAACTT

ATGCTTGCTATTTATTGTCTGTGCCTGGGTATATTTTTCATCTGTTGTAACTGTAAGTTG

CGAGGTATGTGCCTGGGTATATTTTTCTCTGTTGTAACTGTAAGTTGCGAGGTATGAGCC

TGGGTATATTTTTCATCTGTTGTAACTGTAAGTTGTAACTGTAAGTGGTGAGGTATGTGT

CATTATATTAATATTTCGGCCATTATAAAGGATCTGATTATAATATTTCGGCC

>Unigene0002057

AAGAGAGCATCCCCACCTATTGCCTTCGCCGACCGCCGCCCCCTTCACCGAACCAGCAGT

CTCCTCCTACACCACCGCCGTTCATCGGGTTCCTTCTCCTCCCGGCTAACCCTAGCCCGC

CGGCCTCACCTAATCCGATATAATTCTTCGAATTGCTAAGAGGAACGATGAAGAGAAGGC

CCGGAATTGCTGGATTAAAGACAGCCGCTGCTGCTCGGGATCAATATCGACTGGTAGGAG

AAAATGTCGCCAAAATTCGAACAGATCTGATGAAAGAACAACTCGAGACATTCAAATCTC

AGTTGGAAGACTTCGCACGCAAGCACAAGAATGACATCCGTAAAAACCCAACATTCAGAT

CACAGTTTCATGAGATGTGTGCTAAAGTTGGAGTTGATCCCTTAGCCTCTAATAAAGGAT

TTTGGGCAGAGCTGTTGGGGATTGGTGATTTTTACTATGAACTTGGAGTACAGATTGTTG

ATATTTGCTTGGCAACAAGACCACACAATGGAGGTTTGATCGACTTGCATGAGCTCTGCA

GCCAGCTTTGTCAAAATCGGAAAGTTGCTCGTGAATCAATATCTGATGATGATTGTTTGC

GTGCTATAAGTAAGCTGAAGGTCCTGGGCAGTGGTTATGAAGTTATATCTGTTGGGAGAA

AGAAGCTTATTCGATCGGTTCCTACTGAGTTGAACAGAGACCACAATGAAATTTTGGAAC

TGGCTCAGGCTCAAGGGTATGTGACCGAGCAAGAGGTCGAGAAAAAAACTTTCATGGTCT

TCAGGCCGTGCGATTGATGCCCTCAACACGCTAATAGAAGGTGGGCTTGCCATGATTGAT

GATGGTCACAGAGACGGGAAGCGACGGTATTGGTTCCCTTGTGTCGGCACTACCATCTTT

ATTACCTCTGTAGCTGAGGGACCAAAGGTTTAGGTCACGAAGATCTGTTGATACCATGTT

GGTCGTGCAGAATCTCGTCATCAATGGCATGGGGAAGACAGTATTAGCATTGTAAAATGA

TTTGGAAGAGTACATAAAATATATAGCTGATTATACTACCTCCATGTGATTTGCCCCTTA

TTTCAGTGAATTAGAGATTTGTAACTTTTCTTATCCCTTTCCCCCCAAAGGACTGACAAA

AATATATATTGATATGATATATGTATATTGAGCCGGTGACATCTATAAGATCACCCGCGA

AAGTCAACTTTAAGCTCCTACTTATATACTCAAATTATCGAT

>Unigene0002058

AGACAATGGCCGCCCGATTGGCGGCCCCAGTCTCCATCTCCTCCCACTTCTGCTCGCCCT

GCCGGAAAGCCTCACTCCTCCGCACCATGGCCTCTTCCTCCTCCGCTCCTCTCAATCTCG

AAATCATCGGCGCCGGCCGCCACTCGCTCCTCGAATTCACATCCCTACAGAAACCCTACA

AAACCTTCCCCATCATCGGATGGAACCGCCACGTCGAGACGATTTTCGCCTCCTTCTTCC

GCTCCCTTCCGCGCGTCCCCTTCCGCCGTGAGTGCCTGCGGACGCCGGACGGTGGGGCTG

TGGCGATCGATTGGGTCTCCGGCGATGATCGGCGCTTACCTGCTGATTCCCCGGTGCTCA

TCTTACTACCTGGTCTCACAGGAGGGAGTGGTGATACTTACGTGAGACATATGCTCGTAA

GAGCTAGAAATAGAGGATGGCGTGTCATCGTATTCAATAGTCGTGGCTGTGCGGACAGTC

CTGTTACGACTCCTCAGGTGCTGAAGCATTGAACATTCTGTTGCTGATGCCATCAGAATT

TTGTCGAAGATCTTGTCTATGCTGAAGTTTTTTCTCCTCTCTACATATTTCTGATACCAA

TAAACCAAGTTTTATTCCGCTTCGTTTACACAAGATCTATCTCAAGTTGTTAGTCATGTC

TCATCATGCTACACCGTGGCGAAGATATACGCGGTTGGCTGGTCTCTTGGAGCAAATATT

CTAGTACGATATTTGGGTCAGGAATCTCATCATTGTCCTCTTTCTGGTGCAGTATCCTTG

TGTAACCCCTTCGATTTGGTCATTGCAGACGAGGACTTTCATAAGGGTTTCAATAACATA

TATGACAAAGCTCTTGCTAGATCTCTTCGTAAAATCTTTAACAAGCATGCACTATTATTT

GAGGATCTTCACGGTGAATATAACATCCCTTTGGCGGCCACTGCCAAGACTGTCAGGGAC

TTCGATCAAGGATTGACACGCGTATCATTTGGTTTCAAGTCCGTGGATGATTATTATTTC

AACTCCAGCAGCTCAAATTCCATTCCAAACGTGCAGATACCTCTGTTATGTATACAGGCT

GCCAATGATCCAATTGCTCCATCGAGGGGAATTCCTCGCAAAGAAATAAAGGAAAACCCG

CATTGTAGCCTGATAGTCACTCCACAAGGCGGCCATCTCGGGTGGGTGGCAGGGGATGAA

GCTCCGTTCGGAGCTCCTTGGACTGATCCTGTAGTAATGGAATTTCTGGAGCATCTCCAT

AAAGATAAAAATACCGAGAGTGCCAAGAATACATGCCTCAACCAGCCGGACGGTGTTCAA

CAGTCGTCACCCCTGCTAGAATTACATATACAATAAGACGAATTTGCACCATTCTTGCCA

CCTCAACGCCATATTAATGGTCAAAAAGGAAGAGGCGGTCGAGGTGTATAAACACCATTT

ATTCATTTTTTTTTCATGTATGTCCACCATTGCTTTGTATACAGTCGGTTATGTATTTGC

TAATGACTACTGTTGCTCGTCAGGGTGGCTACTTCAAGTTGTCGTTGTGCTCCTTTTCCG

TCAAACACTCTTGTGCATTCTCACCTGTAACATTTAGAATGGTATTGGTTAATATGACTC

TTCCGCCAAACACTCTTGTGCATTCTCACCTGTAACATTTAGAATGGTATTGGTTAATAT

>Unigene0002059

CAAACACTCTTGTGCATTCTCACCTGTAACATTTAGAATGGTATTGGTTAATATGACTCT

TCCGCCAAACACTTGTGCATTCTCACCTATAACGTTTATAATGGTATTGGTTAATATGAC

GGATATTTTGATGACTTGAGTTATTTGTCAGATTCGGTGAGTTCTAGTGCACATTTTGAT

TGATAATTTATATTAGTATGTCTCTACTCATTGTTCTATTATGTATCCTCTTAAAATCAG

CACATTGATATGTTA

>Unigene0002060

GATCCCCTCCTCTTCCATCTTAAAACACCAAATCCATGTCCTATCCCGCAGAGGGAGGGA

TCTTTCTTCTTCTACCTTAAAGATTCCTCAATCTCATTAATCCCGAAAGCACAGCATGGC

ATCAGTAAAGATCAGCACCTCCAAAGGACAAGCTTGGTTTTGCACGACAGGATTGCCGAG

CGACGCCATCATCGAAGTCGCCGAAATGAGCTTCCATCTCCACAAGTTTCCATTGATGTC

CAAGAGCCGGAAGCTCCACCAGTTGATAACCAAGCTGGAGCAGGGCCCCGGCTCACCGTC

AGCATCCCAAGAAGAGCTGAAGGAAGTCGAGCCGCCTCACATCATCCTCCCAAACTTCCC

CGGCGGCACCGAGACCTTCGAGACTGCCGCTAAATTCTGCTACGGCGTAAAGATAGATCT

CACGCCATGGAATGCCGTCCCACTCCGCTGCGCCGCCGAATACCTCGACATGGCCGATGA

CTACGCCGAGGACAACCTCATCTCTCGCACCGAGCGCTTTCTCTCCCAGTCCATCCTCCG

CGGACTTGGCGAGTCGATACAAGCCCTCAAGTCCTGCGAGGATGTCCTCCAGCTCGCCGA

AGAGCTCGGCATCGTCCAGCGCTGCATCGACTCCATCGCCGCCCGCGCCGACTCAGCCAA

CTCATGCTCACTCTTCGGCTGGCCGGTGAGCGAGGTCGCCGCCGTCAGCAGTAGCGGCGA

CCACCGCCCTGTGTTGTGGAACGGGATTGATACTGGGGCGCGGCTGCGCCGCAGCAGAGC

GAGATCATCCTTCTCTGCCCCGGGCGGCGCTGCTGCCGCGGTGCCGCCCGGACCCTGGCA

GGAGGACCTCACGGTCCTCACCCTCCCGATCTACAAGCGGGTAATCGCCGCCATGAGGGC

GGCATATCCGAGATCGGATCTACTCGAGGGCTCGCTCGTATCCTACGCCAAGCGCTCGTT

CCCTGGGCTCTCACGCTCCAGCCGGAAGCCATCGTCCGCCCCTATAGCGACAGAGGCGGA

GCAGCGAGACCTGCTGGAAACTCTGATCCTTAATCTCCCGCCGCTGGAGACCGACGGCCG

CCGACGCGGCGGCGTCCCCATCCTGACAGCGAAGTTCCTCGCCGGACTGCTCCGAACAGT

TAACATACTCCGGGCTTCGGAGGCCTCCAGAAAAGCCCTAGAGCAAAGACTAGCATTTCA

GTTAGAGGAAGCCACCGTCGACGATCTACTCTTGCCAAGCTACTCATACACCGTCGAAAC

CCTCTATGATGTCGATTACGCCGAGAGAGTCTTACGATACTATCTCGAAATTCCATTTCA

AGACAACGGAGAAGGTTTGGTGAAAGTTGGGAAGTTGATTGATGGATATCTAGCTGAGAT

AGCCTCCGATGCTAACTTGACCGTGGAGAAGTTTTGCGATCTAGCACTTGCAATTCCTGA

GGGAGCCCGGGTTTACGACGATGGATTGTATCGTGCTGTGGATGTATATTTGAAGGCGCA

TCCTTTGTTGGTGGAGGAGGAGAGGGAGAAGATTTGCGGGGTGATGGACTGCCAGAAGCT

TACACTGGAGGCTTGCACACATGCCGCACAAAATGAGAGAATGCCGCTGCGGGCGGTGGT

GCAGGTGCTATTTTTTGAGCAGCTGCAGCTTAGGCAGGCGATTGCGGGGACGGTGGAGGC

GGCGGCGGCGGCGCCAGCGCCGGCGGAGGCGGGCGGGACGTGGCGGGCGGCAGTGAGGGA

GAATCAGGTGCTGAGGATTGATATGGATAGTATGAGGAACCGGGTGCAGGAACTGGAGAG

AGATTGTAGTAGTATGAGGAGGAAGGTGGAGAGGCTGGACGGCAGGGCGGCGTCGGTGGT

GCCGCCCCCGGGCGGCGGGTGGAGGAAATTTGGGTGTAAATTTAAAACGCAGGTGTGTGA

CTCGCACGAGCGGGCGGTGGTGGAGGCTAGGAGGAACAGGCCAAATGAGAGGAGCCTGTG

ATATTTTGACACGTGGATTTTGTTTCTAAATTATGGAATGTGTTGGGTCTTGGGGTATGT

TGATGTTGTCGAGAGTTGGTGGACATGTAACTTATTACACGAGGAAAGAATGATATGGAG

CTGTTGGTTGAAGGAATTGAAACGTTGACACCCACCGAAACAAAGTTGTCAATGCCTATC

AACGGAATTTAAGTAGGAGGGGATGAAGATGGGGCAACTGTTTTTGTATGGATTTGTATA

ACAAATTGAAT

>Unigene0002061

ACCTTGTGGAGATGGAAGCTCATTTCGGCGACTTCGATGATGGCGTCGCTCGGCAATCCT

GTCGTGCAAAACCTGTAGAGAGGGTGAGATTGACATTGATCCATGGAGCTTCCAATGGAA

AGTGAGCAATTGAAAGAGGGATTGTTGTACCAAGCTTGTCCTTTGGAGGTGCTGATCTTT

ACTGATGCCATGCTGTGCTTTCGGGATTAATGAGATTGAGGAATCTTTAAGGTAGAAGAA

GAAAAATCCCTTCCT

>Unigene0002062

TTTTCACCTTCGTCGTCGAAGGACAAATCGAAGAAAGCGCCGCCGGCAGCAGCGGCGCCG

AAGAAGCCGCCGGCTAGTGGGTCTGGGAAGAGGCGGGGACCGGCGCCGTCGGCGCACGAG

CGGCATTACACGGCGAACAGGGCGGTGTCGGAGGAGATGCGGAAACGGACGTTTCTGCCG

TACCGGCAGGGGCTGCTTGGGTGCCTGGGGTTCGGTTCACGGAGTTATGGTGCCGTCAAC

GGCTTCGCCCGGGCCTTGAACCCGGTTTATACGAGGTAATGATAGGAGGTTTTGTATAGT

TTTTATTGGTATAATTATGGTCGTTATTTTTTGTGGCCGTTGGATCGGCTTGATCACGGA

CTACATTATTAGTAAGTATGTAGTGTGAGTTTATTGTATTGTTTTGATTAATTTGTTTGT

TCTACAGTTGTCAATCTCAAGATTTGGGAGTTGTGGGGTTTTAATAAGCTTCCATAATTT

GAGTAGATAATTGATTGTCTTATTTGCTCACTTCATGTGGCATCAACGTGACATGTTGGG

ATAGCATTTGTGTTTAGGTAGTGTTAAGTTTGGGCTGGTAAAAGTGTGATATATGGAAAA

ATTGATGGATGAAACTGTAAATATTACAGTTAAGAGGATGATTGGACCACGAGGGCTATG

CTGCCATACGCTGAATATGTACTGGTTACTGAATATCCTAACTGGCCGACGTCAGCGAAC

AAATTTGTCAGTACTGACAGAGCACTATAATACAGACCTGCTAGTTGAGTCTCGGGTCCC

ACTGGTGATGTATTGGGCACAAGAAACAGTGAGCGAGCTGGCTGTGGGTCCCGGGAGTGG

TTGAGAAGGGTACATGGAGACGCAGACGGGTGTGGTTATGGGTCACTATCCATTGACGAC

TTTGGAGATAGTTGTACCGTCGATCTGAGCAATGAGTTATCTGGTCCCCTTCTTAGCATA

GATGGGCCTGTAATGCATCGTATCATTTTTAAAAGATTTTTTTTTTAGAAAAACAAATAT

ATTAAATTATGTCAGAGCTACTTGTATGTATAATACGTCCAAATTTTAACCATCCCGTTT

GTGCGAACTTATCCGGAAGAAAGTA

>Unigene0002063

CCATCCCTCCTCTCCTCAACCAATGGAGCTCGAAAACCCTAACAATGGCGACTTCATCGG

CGACGACCTCGACAGCGCCTGCTCCACACCCTACACCAGCGCCCCGTCCAGCCCTGGCCG

CGGCGCCAGCCGCGGCTACTTCTTCAGCGCCCCCGCTAGCCCAATGCACTACATACTCAC

CTCCACGCCCTTCTCCGTCGCTGCCGCCTCCTCATCCGACGCCCCCCTCTCGGGCTCCAG

CTCTTACGACTTCGACTTCTCGGCGCCCTGCCCCGCCGCCTCTGTGGCGGCCGGATCCAT

GTCGACCGCTGACGAGCTATTCCTCAACGGCCAGATCCGACCGATGAAGCTTTCGCCGCG

CGGCCATCGGCCGCGCCCTCTCACGCCGTTGCTTGATGTCGATACTGAGGATGAAGACAT

CGAAGAGTCAGAGGTGGAAATGGTGAGGGGGCGGGATCTTAGGACTCGTAGCCGATCCAT

GCACCGGCGGACTAGATCCATGTCGCCGATGCGACACTCGGTGTGGCGAGAGGATGAAGA

TGAACGTAAAGATGAAGATCTCGAAGTAGAGCTGGATTTGAAGCAAGCTGAAGACA

>Unigene0002064

GGAGGAAGAAGGCAAGAACACCATGGAAGATGGAAGTGGTGGCTCAGAAATAATGGCGGT

TGATTGATCGGTTTTGTCAGCTTTCGATGTAAAACCCCTTTGTAGCTGAACATATCTGAA

GGCTCGAAACATCCAAGTTGCGTGTAATGGTTGAATGAACTTTAGAACTTCAGAATTTTC

GTAAATGAAACTGTTTAAAAAGAAAAAAATATTTAAGATTGCACCTGTTCATTCAGATAG

TC

>Unigene0002065

GCTCTAACTTAGTTTCCCCAAATTTCGCTATCTCCCCCAACCTTTGAAACCCTAGCCCCG

CTCTGTTCCTCCGATCAGTCGTCATCAACCAGCCGCCCCGCAGCGCTCCTACTATCGGCG

ATCCCCAATTCGAGATCTGTGAGCTCCAGTGCACCGATAATCGCTTCCCTCAGCTGAAGT

GATGTTAGCTAAATCGAGAAAAGTTTCCGGGAGAGGAGAAGAGATGGCCGCACATCATGC

CTTTGGCCCCGAAGACGAGGTTATAATCAAACACCGGCTCTTGACTCGCACCACAACCAC

AAGGGGCGAACCGCCACTGAAGAAGCTTCACAAGAAGTTCCTCTCATTCGCCCTTGAAGT

CGAAAAGGACACAGATAACTACACCGATTGCGATAGACTCTACAAGGCATTTCTCCAAGA

GATGGCCACTTTCGAGCTGCCACTCCTCAAGAGTAAAGCTGTTGTCGATGCGAATCTCCG

AGAGAAGGCAGGCTTTGATGAAATGCAGGAGGGGATTAATCGGCAGATATTTCAGGCGAA

GGTCGATATCGAGGATCTGAAGAAGCAGCTCGAGGCAAGCAAGATTGAGCGGAAGCACAA

GGAGGAATGTGAGGCGATTAGGAAACTGATTGCTCTCCAACCTCCGAGGTCAGAAACACA

GAAGAGTATCACCGAGCTCGAGAGGGAGATCGCAGCCTTGGAGTCTGAGAATACCTTGTG

TGCGAAGACATTGGATTTACGGAAGAAGCAGTTTGCTCTTCTCTTGCATGTGGTGGATGA

ACTGCAGAACATCATTGAGGATGAAGAGAAGGGCTTAGCGGATGAGTTGAGGACAATCAC

GGAGGAAGAAGGCAAGAACACCCTGGAAGATGGAAGTGGTGGCTCAGAAATAATGGCGGT

TGATTGATCGGTTTTGTCAGCTTTCGATGTAAAACCCCTTTGTAGCTGAACATATTTAAA

GGCTCGAAACATCCAAGTTGCGTGTAATGGTTGAATGAACTTTAGAACTTCAGAATTTTC

GTAAATGAAACTGTTTAAAAAGAAAAAAATATTTAAGATTGCACCTGTTCATTCAGATAG

TC

>Unigene0002066

GGTATCCTGGTTACCAGAGTGCTCTTAATTGGCGGCGGGAGAAGTCCAGGGCTACCTTGT

TGGTGAGGTACAAGGTAACTCTGAGGCAACCCTTGGAACTTACAAAGGAGACTCTACTAG

GGGAGAATGTCGATATTGAGGATCTGAAGAAGCAGTTTGCTCTTCTCTTGCATGTGGTGG

ATGAACTGCAGAACACCATTGAGGATGAAGAGAAGGGTTTGGCAGATTAGTTGAGGCCTA

TCACGTGCTCATTATGGTGTTGAAGCCACGACCTAGCCATGTCATCCACCCGTCACCCCT

GGGACTCCTTCAACCCATGCCATCTACGTCGCCACTTAGA

>Unigene0002067

GGAGGAAGAAGGCAAGAACACCATGGAAGATGGAAGTGGTGGCTCAGAAATAATGGCGGT

TGATTGATCGGTTTTGTCAGCTTTCGATGTAAAACCCCTTTGTAGCTGAAGGCTCGAAAC

ATCCAAGCTGCGTGTAATGGTTGAATGAACTTTAGAACTTCAGAATTTTCGTAAATGAAA

CTGTTTAAAAAGAAAAAAATATTTAAGATTGCACCTGTTCATTCAGATAGTC

>Unigene0002068

CTGACATGGGCCCCCTTCTCCTCCTTCCCCTCCTCAAAACAACAAACCCCCCCTTCGTCT

CCCAGTCCTTCAAAGGCAAGCGAGCAAAAGTAGACAGCGATTGCGAGATCCATCATCACC

CCATTCACCGGATCTCCCGCCAATCATGCCCCCTCCCCCATAGCTCCCTCGCCGGAAATG

GGGGAGACCGGAGCGCCGGCGATGGCCCCGCTTACCGTCGACCGCGGCGAGGAGCG

>Unigene0002069

CTGACATGGGCCCCCTTCTCCTCCTTCCCCTCCTCAAAACAACAAACCCCCCCTTCGTCT

CCCAGTCCTTCAAAGGCAAGCGAGCAAAAGTAGACAGCGATTGCGAGATCCATCATCACC

CCATTCACCGGATCTCCCGCCAATCATGCCCCCTCCTCCATAGCTCCCTCGCCGGAAATG

GGGGAGACCGGAGCGCCGGCGATGGCGCCGCTCACCGTCGACCGCGGCGAGGAGCGCTGG

CGCCACTTCGACAACTCCGTCAACGCCGTCTCCTTCGGCTTCGTCGCCACCGCGATACTC

ATTTTCATGTTCCTCGTCATGGCCGTATTCGAGCGATTCCTCCGCCCCCGCTCCTCTCTA

GCCACGCAGCTCGCCGCCGCTGGCGGCGGCGGGTTGCGGCGGCGGAGGGGCGGATTCGAT

GTCGAGGCCAGGGGTGTCGCCGGGAAACTGGATTACCCTTCCCCCAAGATGTCAATCTAC

GCCAAAGGGGTCTCAGTACTGATGCCTGGTTATGATATACCAACATTCATCGCACATCCT

GCCCCTGCACCCTGCCTGCCCGAGAGAATATCATGGCCGACGCATGAACAGAACCTGACC

GTTGCCTCTGCACCAAATCCAAGTTAAACATCTGTGTGGCAGCCTCGACTCACCCTACTT

CGGATTCTTAACTTTTTGCAAGTTGAGCCAGCTGCGCACTGAAGCTTCTCATCCTGTACC

CATTTTTCTTGTTCAGGCTTAGTCTGGCCCCTCAGCAATCTGGAAGATGCCCAGAGGCAT

CAGAGGAAGAACATTAGTGAATGTGTACAGTTTTGACGTACTGATTAAGCTGTCCAAATT

ATTGCCACCCCCGGTAGCAACCGGCGCGGTGGCTAAACTATTAACGAATGCGTTTTTCTC

TTGTATAAATATTTCTGATTCATCTCTTGTTTCTGCTTGAGATTGCTATGGATGGGCCAA

AATAGCGCA

>Unigene0002070

CCATTTCTCCCTCCGTTCTCTTACCCACCGCAGTCATCGCGATCGTCAACCGATGTCGCT

CCCCAACGGCGCAGACCTCACCGCCCCCCTCCTCGGTCGTAGCGTTGACGTCATCATCGA

CGTCAACGGTACGGACCGAGGTGGCGCCGAGGGGGCGGCGCCGGAGAACCCGTACAGGTT

CCTCGGCGCTGAGGCGCCGCCGCCGATCCGGCCACCGAGTACTGTGGATCCGTTCCGAAA

TCACACGCCGGTGGTGGCCGGGGCGTACGAGTGGTGTAAGATGGTGGTGACGGTGCCGTT

GGCGTTGGTTAGGCTGGTGGTGTTCGGATTGGCGATTATGGTGGGATTTGTGGTGACGAA

GCTGGCGCTGTTCGGGTGGAAGGATATGCAGAGTCCGATGCCGCGGTGGCGGTCGAGGAT

CATGTGGGTTACTCGTCTGTGTTCGCGGTGCATCTTATTCTCGTTCGGCTATCACTGGAT

AAGGCGAATCGGACGACCGGCTTCCCGAGAGATAGCTCCCATAGTTGTATCTAATCATGT

ATCATTTATAGAGCCTATTTATTTTTTCTATGAACTTTTCCCCACTATTGTTGCTTCAGA

GTCGCATGACGCCATGCCTTTCGTTGGAACCATAATCAGAGCTATGCAGGTTATTTATGT

TGATAGGTTCTCAGCGCAGTCCAGAAAACATGCCGTCAATGAAATAAAGGGCTTAACTTC

AGAGAAAGGCTTCGTGCAATAGCTTTTCTCGTCTTCTATTGTTTCCCGAGGGTACCACTA

CAAATGGGAGGGTCCTTATTTCATTTCAACTGGGCGCATTCATTCCTGGCGTTCCTGTTC

AACCTGTTGTTGTTCGCTATCCCTATGTACATTTTGATCATTCATGGGGTAATGTTTCAT

TGGCGACGCTTATGTTCAGAATGTTCACTCAGTTCCACAATTTCATGGAGGTGGAGTATC

TTCCTATCATTGTGCCTCGTGAGAGCAAAAAAGTAAATGCTGTTCGTTTTTCTGAGCAGA

CCAGCTATGCCATGGCGAGAGCCCTTAATGTCGTTCAGACAAAGCATTCTTATGGTGATG

TAATGCTTCTGACCAGAGCATCACAACTGACGAAGGACAAGTGTTCAAATTACATGGTGG

AACTGGCATCTGAAAGTGCGTTCGGCATTAGTACTTCTGAAGCCATGGAACTTTTGGATA

AGTTTTTAGCTATGAGTCCAGATTCCAATGGACGGGTTAACATTCTCAATTTTCAGAATG

CTTTTGGATTGAACTTGAGTTCTCTCTCTGAGAAGATATTTCGGTACTTGGATGTGGAAA

AGAAGGGAGCGATTACATTTCGTCAGTTTGTACTCGTTTCAGCTTACATTCGGAAGCAGC

CATTATTCGCGAGAGCATGCGACACGGCTTTTAGTATCATCAACAAAACCGAAACGGGAT

GCATCACCATGAAACAGCTAATCGACATCTTTCCCTCAACAGTAAACGAGGAAATAATGT

GTTGTGTTTTCGGTTTGATGGATGTTGATAACGATGGAGCCGTCAGCCGGGAGGATTTCT

CGACATTCCTCCACAAAAATCCACTTTTGATTGCACTTTTTGCCGAGATATTATCAGAGG

AGTCGACATGATGTTGGGGGTGTAGAAATGATATATATGATCGACAATAGCAGCGGCCAT

ACCATCATGGTTCTTTTTATTAGTAACTAGATGGTAACTTGTCAGTTTAAGGGTGTCGGC

GAAATTTTGTGAGTGATACATAATATTTATGTTTTAAGGTGTATTAATATAAAATGCGTT

TGTGTGCATTGCTTTTGTTGTAAACATAGGGTGCAACCACTGCTGTGGAGAAATTATGGT

CAGGTATCTTTCATGGTTCTTTAGTAATAACATTTATCAAAATTG

>Unigene0002071

CGGGCACGATTCGGTGTGTGGATCTAAACTCCCCCATTTTCTCCCCAGTCCGGAAGTACC

AAACCCTAGCCGCCTTGCCGTCAGTCCCCTCGCCGCCGCTGCTGTCCGGACTCATCGACC

GGATTCTCTCCTCTCCTCCACCAGATCGTCGAAGGATCAGAGATGCCGTTAGGGTTGATT

GCTGGGCTAGCCAGGGCAATGCGGAGGAAGCGAACTTCATCTTTAATTATATTGTCCTCG

AAAAGAGCCCCCCGTAATTATTACAAGGGAAAGAACTGCAAACCGACTGGTTTCCACACT

CGCAAAGGTGGCTATGTTCTCGTGGATGAGAAGCTGCCCCGTTATATCGTACCAGATTTG

ACTGACTTCAAGCTGAAGCCTTACGTGTCTCAGTGTGCGAGGGATGCAACTGCCACCTCT

GCGTCTAGTGAGGCTGCCAGTGCTGCTAGTTAGCCTACAAGTTTCATGCCTCCTCCTTTC

GCCCAGTGAGGCCGCCGGTGCTCTTTCTTTTTTACTTTGCTGCAGCTTCAGTTATCGTAG

GTTAGACTGTTCAACTCGTCTGTATTAGTCTATGGAAAAGTGTTAACATTGAATGTCAAA

TCTTAGTCCAGAGTGAAGACTGAACGATGGCTGTTGGACATCGACATTCTAGATGCAATT

TTGCCCAACTGGACCACCAAGACATTCAATTCCATTAATTTGAATCCGTGGACACTTAAT

CAAATGTGTTATGCGGGGCTATTTACTCG

>Unigene0002072

GGGTGGAGATCCAAGCTCCCCATTTTCTCCGCGGGCCGGAAGGACCAAACCCTAGCCGTC

CTCGCCGTGCTGTCGCCGTCCGGACTCGTCGACCGGATTCTCTCCTCGCCTCCGCCAGGT

GCTCTAACATTTTCTCCCCGGGCCGGAAGGACCAAACCCTAGCCGTCCTTGCCGTCAGTC

CCCTCGCCGCCGCTGGATTCTCTCCTCGCTTCCACCAGACCGTCCAAGGATCAGAGATGC

CGTTAGGGTTGATTACTGGGCTAGCAAGGGCAATGCGGAGGAAGCGTACTTCATCTTTGA

TTATATTGTCCTCAAAAAGAGCCCCCCGTAATTATTACAAGGGAAAGAACTGCAAACCGA

CTGGTTTCCACACTCGCAAAGGTGGCTATGTTCTCGTGGATGAGAAGCTGCCCCGGTATA

TCGTACCAGATTTGACCGACTTCAAGCTGAAGCCTTACGTGTCTCAGTGTGCGAGGGATG

TAACTGCCACCTCTGCGGCCAGTGAGGCTGACAGTGCTACTGGTTAATCGACGAGTTTCG

TAGCTCTTCTTTAACTTTGCTGCAGCTTCAGTTGTCGTAGTTAAAATACTGTTCTACTTG

GCTGTATTAGTCTGTGAAAAAGTGTGAACATTGAATGCCAAATCTTAGTCCAAATTAAAG

ACTGAATGGTGGAAATTGATAAATTCTCTGATATGTAAACGGAAATGAATATACTATTTC

AGTGCCGTTAATAAATTTTGCTCAAGCTCCCCGTCCTTTCTCCTTCAAGAAATGAAAAGG

>Unigene0002073

TTTAACTTCCTTCGCTCACTGCACATCGGGGACTAAACTGCCGGCAATGTCGCTACAGAT

CTCCCTCTCCCCTTCTCCGACAATCCGCTCATCTCTCGTCCCGTCCACAGCCTACCCGAG

TCGAGCCTTTGCCGTTCAAGATCGGCGAGCGGCCGGCGGGGTCGCTAGGGTTTCGGTGAA

TTCCAGGAGGGGACTGGCGGCAGTGGTGAGATCGAGCTTGGACATATCGACCGGCGTGGT

CGTCACTGTGGGGACGGTGACGGAGGTGAATAAGGACACGTTCTGGCCCATCGTGAAGGC

CGCCGGGGATAAGGCGGTTGTTCTCGACATGTATACTCAGTGGTGCGGCCCTTGTAAGGT

GATTGCCCCTAAATATCAAGAATTATCAGAGAAACACCTCGATGTTGTGTTTCTCAAGCT

TGACTGCAATCAAGCAAACAAGCCTTTGGCAAAAGAGCTAGGACTAAGGGTGGTTCCAAC

CTTCAAGATTCTCAAGGACAACAAAGTCGTAAAAGAAGTAACAGGGGCCAAATTCGACGA

TCTAGTCGTCGCCATCGAGACGGTAAAATCAAGCTGATAAGTTGATCTCGCGACAGGCAC

AATATTCTGAAAATTTGTGTACATTCTATGCGGTATTAGTGTAAATTTGATATATTATCA

TGCCATGATCCCTTCATCAGATGTCAATATATACTTACATCAGTTAAGGATGCAGAAAAT

CCTGAATGCATTTTATAAACTCGTTCTACTTGTCGAAATTGGTATTGTGTTGAATTTCTT

GCACACCACCATATGAACAAGT

>Unigene0002074

AATAAGTTTTCCCGAATTTGTTAGTCCTAATTTAGGTAAATTAATGCAAAACTTGTTCAC

AAGAAATGGTGCAGCATATATTTGAGACTCTACATTTTTAGGTAAATCAATGCAAAACTT

GTTCACAAGAAATGATGCAGCATATATTTGAGACTCTACATTTATGTAAAAGGCTATTGA

TAGATAAATGCAGCTTAAGCCTTTAGTAAATAGGCAGGTTTGTGTTGGAGTCAGATTTGG

TCAAAATCGAATTGATTACATAAACACACAGAACCTGACTACATAGATACAGAGACAAAA

TAACACTAAATAATGCAAATAGCGAACAAAGAAGAGAAACAAAAGCATACTACACATATT

CATGTAGTTCGACACCACCTATGACCTTCTCAACCATGTCGCATAACAGCCCTGTTGTGT

CATATTTTTGGTTGAGAATATAATCTTATACCATCTGTAATGACCATCCCTCCAGTTACA

ATGATTATTTTTGGGAAAAACCTGATAGCAAAACCATGCAGATTAGACCCAAAGCGGATA

ATATAATCTCATGCGTGTCATTACACCAGTCCATATTTATTCTCTCAAGACTTCTATCAT

AATTCAAAATAAAGAGTTTGACACTAGTCTTACACAATTATTCCTAAGAGACTCGGTTGA

CTGGCATCTAAAAAGTGACGGCAACCTTCTTCAAGCTAAATGGTCTGGGTAACTAACTTT

AGTCCCAAGCTACCAACTTGTAGTCTTGGTCGACTAATTTGGTCGACTAATTGGCCGTTC

CAGAAATTCATTTGGAACTTCCTACTACTGCTGTTGCTACCTAAGAACCAATATAATTAT

CCCGTGCCTTAATTCGTTAGGTCTCTCTTCAACTTTATCCCAAGTTCTTGCAGACCAATA

TCCCTAGACTCACTTCAACTTTATCCCAAGCTCTTGCAGACCAATATCCCTAGAATCACT

GCCATCCTGGTCAATACCATTTGCATTCTCTGACTAGGAAATAGTAGGGTAACCCAAAAA

TGTGAGCCATTATGGTTGGTACCCCTGATGCTAATAAAATGAATGAAGCCTTCATGCACT

GGACTCCTCGGGAAACAAGCAAGAACATTGTCTAAGCTGGCGTGAGGCATACACAAAGGC

ATACAAGCCAGACAGACAACTCTCTGTTCCTTGAGTGTGCTTGATCCTTTTGACAAGATT

AAGATTGGGATCTAGGAATACATGCTGATTAGTGAATTGTAACAATTACAAGGAGCATGA

TAGTGAGGCTTTTAATTAAAGCAAAAAGATAGACATTTTACAGACAGTGTTGGATTGAAG

TTCAACTTGGTTACAACAATCTATTTTCAGGTGTACGTGAACGATCAATTCCTGAACTGG

GACCCTGAAAATCGGATCAAAGTCCGTATCATCTCAGCAAGGGCCTACCATTCTCTGTTC

ATGCATAACATGTAAGTGTAAAAGCAGTTCATTTTTACCCATCAATTCTGTGTATCTCAT

GGTGTATACGAATTTCCCCAGATAAGTGATTCTATTACTGTTATGTTCTTTTAAATTAAT

TATAGTATTAGTTGTAATTGTGCATAGTAAATACGTGACTCCATTACTTTTTAAATCAAT

GGGGACACATAAAAGTAGGTAAAGAAATTGTGCATACAATGACTGTGTGGGTGACAGGTG

TATTCGACCCACACCTGAAGAACTGGAGGATTTTGGTACTCCGGACTTCACAATATACAA

CGCAGGACAGTTCCCGTGTAACCGTTACACACATTACATGACTTCATCCACCAGCATAGA

TCTTAATCTTGCTAGAAGGGAAATGGTTATCCT

>Unigene0002075

GCAAATTTCGAATTTGGCGAAATTCGATTCCCGGGCGATCGTGGCGCCATTTTTGATCTC

TCACCCAATCTCACCCAGTTCTCCCCTAACACCTGTACCGAGTCTCTCTCGAAGATTTGG

CGTCATCTCACCCTAATTTCTCAAAATTAGGGTTTCGGAGGATTCTGCGATGACGAGCTG

AACTCGGAGCTGATTTTGAGTTACACCGACGCTAAAGAATCAAGCTCATAGAAGATACTA

AGTTTCCATGAAAATAGAGCAACAGAATGCTACTACAGTTACATCTGCGAAAAGCGAAGT

AGATTTAGGACTTGACTATACCAAATCTGGACCATGTCCTATGAAAGTTGCCAAGCAAAA

AATGGCAGGGGAGTTTGATATAAAATTAGGTCGGGATGTCATGGCTTCATCAGATCATTC

ACTGGATTTGGATATTCAGGTTTCAGGAACTAGCTCCCTGGAATCAGTGCCTGCAATTCC

TGTATTTCGACATTCAAAATCTCCACTTTCTTGTGAGAAACTTAAGCTACAGTTGTATCC

TATAGATGAACTTACTCGTGTCCGGTTGGAGAAGGATGGGCATAACCCTTATCTCGAGCT

TACTCTTGGACCCCAAAAGAAAATATCCTCCATAGTGAAGCACCTCAATAGCAAGTGGGG

AATCTTGAATACTGTAGGTGAGCTCATGCTCTTTCCTTGTGATGCTAGAATTGAAAATCT

GTATAGGTGCAGAAGATGGACTTTGAAGGACTCTGATGTCAAAACAGCTGATGTGTATGT

ATCCATGGGTCGCCCTTCTGTTTTTCAATTAAGATATGGATGGTTCTCTATTCTTGAGAC

AAAGGCAAGTGCTACATCTCTGCCATCTTTCCATCTTGAGGATATTAACCAAAGTAAAGA

AAATCTGAAGATCAATGCCAAAGATGTGAGTGAGTTTCCATTTAGTGTAATCAAACAAGA

ACGCGGACCAGTCTGTGTTGATGACTTGGACAATAAAGTTGCATTCACACCCATTGTACT

CGACAAAACAGTTCAAGCACCGGATAATCTGAGAACTGGTACTCGTGAGATCTGGACCGA

TAGTCTGTCTAACATAAGCATGGGAGCGCTGTTCTCTGAAGCATCAGCGATACCCGATGG

CAACTGTGCGCCTCTACAGAATCTCTCAAACTTTCAACAGATTCCTATTACCTGTGATTC

ATTCGACTCTGCTATTGCTGGCTATATGTCACGTTATGAAGCACCAAATTCATCGAGTCA

CACATCAAACTTCTGGGATTCTGAGGTAACATGCCAGGCATTTCCGATACAAAAGACATC

AGCTGATCAGAAGACAGGGACCAATCCAATTAGACTTCGTGATATGCTTAACCTGAATGC

CGAGCATAAACTGTACGATGATCAAAAGACTTCCAAATGCAATCTTCAGCTCCATACAGA

AGGCAATGCTGCCCCGATCAGGACTGATGTGCACTCTCACACTCAGAACAATTCTCGTAA

GAGAACTATTGCTGATATGCAACATGATACAGAAGGTTCACATTATGAAGTTAGCGGTTT

GGAGAGTGGAGATATCTATAAGCCTGGTATATTGCACAAGTTTCTCTAAAGCATTTCACC

GGTGAATACATCATCATTTTTGTCCGTCAATTGTGAGGATTATAGATGTGTTATAATTAT

ATTCTTCATTTGGTTTGGTTCTTGATATTGGTTGGCTTGAGGTTATTCAGCGTCACAGGG

GGAAAGAATTCCAGAGGTATTTTTCTGATTCTTGATACATTTCGATGTTCCAGTCATTTG

AAGCAGCAAATTTTGAAACCTTGGTATAGGAATTTTATTGAGTAATTCACTCAAAAGGAG

ATTTGTCTTCTGAACCATTTATGATGTAAGAAAAAGGGGAATGTTGACTCTTAATGTTGA

GTAGCAATAACATCTTTGTTGCTTGA

>Unigene0002076

AAAAACCCTGGTCTCCCATCAGTTTGAGCTCCATGGCCTCTTCGACTGCTTCCAACTTCT

TCTTCCTCCTCCTCGTTGCTGCATTTTTCTTGGCAGAATCTACTGATGGATTTGTTCTAC

GCAAATCGAGGAAGGCCCACCTCCAGTTCTTCGTGCATGTCATAACCACCGGCCCTAACG

CCACAACAGCAGTTGTGGCGGGGCTCAATAAAACATCTTCCACCTTCGGTAACATAGACG

TGTACGATAATATACTGAAACAGGGAGTGGATGTGGGCTCTGAGGAGATCGGACGGATAC

AGGGAATCGATGCACAGGCATCACTGGAATGGCCAGCGGTGGCGGCCGTGTACAACTTTG

TGTTCACCAGCGGGGCATACAACGGAAGCACTCTGGCGATGATGGGGTACCATGACTTGA

GAGAGCAGAGATGGGAGCAAAGTGTGATTGCAGGGAGCGGTGAGTTGAGGATGGCTCGAG

GCTACGCTATCGCGCAACTCGTCAGTTTCACTCCGGCATACATTGTCATAAACTTCAACG

CCTATCTTGTGCATCATTAGAGAAAGAATTCATTATCTTTGACGCTATCGTATTTAATTT

TGTTGTTCAAATACTTGCTATGTAATAGGCACGATAGTTTTTTTATAATTATAATTGCAA

TTATAAAGATTT

>Unigene0002077

GAAATCCTCATCTCCCATCCAGTTCAAGCTCCATGGCCTCTTCAACGGCTTCCAACTTCT

TCTTCCTTCTCCTCGTTTCTGCATTCTTCTTGGCAGGATCTACTGATGGATTCGTTCTAC

GCAAATCGAGGAAGGCCCACCTCCAGTTCTTCGTGCATGTCATAACCACCGGCCCCAACG

GCACAACAGCCGTTGTGGCGGGGCTCAATAGAACATCTTCCACCTTCGGTAATATAGACG

TGTACGATAACATACTGAAACAGGGTGTGGATGTGGGCTCTGAGGAGATCGGACGGATAC

AGGGAATTGATGTGCAGACATCACTGCGACCGCCAACAGTGTACAACTTTGTGTTCACCA

GCGGGACATATAACGGAAGCACTCTG

>Unigene0002078

AAGAAGCCATCACCGTCACTCACCCCCTACATTGCCCCTCTTTCTCAAACCCATTTCTGA

TCTGCGATGAGGAAGCACGGCTGGCAGCTCCCATACCACCCTCTTCAGCTGGTGGCGATT

TCTGTTTTTCTAGCGTTAGGGTTCGCCTTCTATGTTTTCTCTCTCCCCTTTGTCGGGACG

AAGCTGCTGCAGGACATTGTTCTAGTTCTCTATACTCCTCTGGTTACATGTGTCTTCTGT

CTATACATCTGGTGTGCTGCAACAGATCCTTGCGACTCAGGAATTCTCAGGACAAAGAGG

AATGCCGGTATAGAAGATGGTGGAAAGCAGAATTGTCCTAAAATTTCTGCTCAACATGGT

GCACAGGGACAATCTTCACAGCAAATTAGTGAAGATGGCATGTTCTATTGCAGTATTTGT

GAGGTTGAGGTGCAAAACAAAAGTAAACACTGTAGGGTTTGTGATAAATGTGTTGATGGT

TTCGATCATCACTGCAGGTGGATCAACAACTGTGTAGGAAGAAAGAACTACCTAAAATTC

TTCATTCTCATGATTTCTGCTATTCTATTGCTTATTCTACAATGGTCCGTCGGAATCCTT

GTGATGTTGCGATGTTTCTTTGAGAGGAAGCGGTTCTCTATGGAGATCACGTTGAAATTG

GGAAGCAGTTTTTCTTTTGTGCCATTTGTTATTGTGGAGGTCATTTGCAGTGGTTTGGCG

ATGTTTGCCACTTTACCTCTTTGCCAACTTTTCTTCTTCCATGTTCTCCTGATAAAAAAG

GGAATCAGCACCTACGATTTTATCATAGCAGTGAGAGAGCAAGAACAGCTAGGTGGTGTT

GGTCATCAAAGCCCTCTGATGTCCTCAGCGAGTTCACTTACTGGATTTAGCATTGCAAGT

TCTTTCAATGCTTTTCACCATGGTGCATGGTGCACTCCTCCAAGATTATTTCTTGAAGAT

CAGTTTGATGTTATTCCCCCAGAAGCTGGAATTTCTACGTTGGCGAAGCGGTCAGTGCCA

GATACATCATCAAAGAAAAAGACTGTGGCAACTAATCCCTGGACCCTAGCACGACTAGAT

GCAGACGAAATCTCAAAAGCAGCGGCACAGGTAAGAAAAAAGTCGAGAATCCTACACCCA

GTCGAGAGACGAGCAATACCACTCGGGCAAGAAAGCGATGCCAACTTCCTCAGTGGCAGC

GGCTCGATGGTTCCAAGACCCGCTAACCAGCGCCTCCCTAACAAAAGAAGCAGAGCACCA

ATGGATCTCTCTCTCGAACCGCCTGCAAAGATTTCCACTAGTAGTACTGACAATGAGACC

AATTTTGCTCTTGAGACATCCACTTTCGCCCCTCTGCAACTTGAAGCGAGGAGTGCCTTC

AGGTCAATCCCTGGAGTTGTCACATTTTCTCCTGATAGCAGTCTGGATTCCCCCGACCTC

CGGTATCGCGATTCTTCTTCTGGAGCAGAAGAAGGCGTCCTGAGGTCCCTGACTTCTGCA

GCTGAGCATACGGCGGTCCCTTTCCCAAGATCGAACAGTGATGGGTACGAAGCGTCTGGG

GGTGAAGATAGTGACCGTGTTCCATCGAGAATTGTGCACAGATCGTCAGACTGGAGTAAC

CTGCTCGTCAGTACGAGTCAGGATGGTCTGTTGAACAAGTTGAAGGCGTCGTACTCGGGA

GGATTGCAGGCTGATATGTGAAAGTAAGAGGCAAAATTTGATGTTTTGTTGCTTATGTCG

ATCGAATTGGACAGTGAATACAGAAAGAATAATTTTCTTTGTAATGTGTTGTCTTGTATA

ATCGACTAGTCTAGCTTGGATAGT

>Unigene0002079

GGTGGTCAAGAGAGATCTGGTGACTCCACCAAAACATCTCGAAATAAAAACAAAATAAGT

AGCGTGAGAGCGATCGTGCCGCTCTTAAATCGATCCCGCTGCATTATTTCGCTGAATCTG

GATCGGGATCGGGATCATGCAACTGCTGTAATTGCTGTTCTTCCGGATCTTAGATAAAAT

GGCCCATTAGAGCTTTTTGGAGAATGAGCAAATAATGGCAGCTGCGGAAGCAAGGACTGC

TTGGCAGCGAACTGCTAATCGTTGCTTTGTGCAGGAAGATGCTAAGAGGGCTCCGAAATT

AGCCTGCTGCCCATCATCCTGTGTGCAGCAGTATGAGCAGGGCAATATCAATCCAACAAA

TGGACCTGACCACCCTGCTCCCAATTTCATACCTTTAAACTGGTATCCCACACATTCAAA

CCTACCGCCCGACACAAAATGGTGGCTCCAGCTACAGCCAAATTTCGGGTACCAGAAGGA

CCTCACATGCGAACAAGTGAACTGTTTAGAAGATGAGCTCGATGAGCCGAAATCCGAAAA

TACAAACCCCACGTTGAATCAACCCGAGCCACATCTTGGTGGTCCTACAAAAAGAAAGAA

CGACTACCCTTCCGAGTCCCCGCGAATGATCTCCACAGCTTTTATGAAGAATGAATCTAT

CAAGAGAGGTGAAGATGTGAAGACAAAAACTAGTAACTCACAACAAACCCTCAAGCATAA

GGTTGATATGGATGATTACTTTTACCATGATGCGGAGCTTATGGATTGGAAATCCGAGAA

AACTTCTCTCGACATGGAGACACCTTGGGCAGGAGGTAACAAGTGCGAACCCTGGTGGCG

TGTTGTTGATAAAGATGAGTTGGCTTCGTTGGTCGCCCAGAAATCGCAACAACATATTGA

GAATTGTGATCTTCCCAAACCATCGCAAACTAGCAAGGATCCGTTTGCTTGCCTTGAAAA

TTTAGACGGTAGCAGAATTTTCTCAGCATCTGGTGGCCGGAAGTTACATACTGGCATGTG

CAATCCCATTGATTATGCGCCACATAGTTCTACTTCTGGTAGCTTTGATGAAAAACATTG

GTATTCCAGTGAGCTAGGTTGTTCACCGCACTATACTGAGAAACTCGCCAGCGCCACCCA

CACTTATGCGACCACAAATACCGATCCACCCGATAACCAGCCCACGGCGAAGAATGATCC

AAGCCGAGCTCAACTCCTTGAAGCCCTGCGCCACTCCCAAACACGAGCTCGGGAAGCTGA

AATGGCTGCTGAAAAAGCCTACAATGAGAAGGAACACATCGTCAAACTCCTCTTCCGCCA

AGCATCCCACCTCTTCGCCTACAAGCAATGGCTACACATGTTACAGCTGGAAAACCTCTA

CCACCAGTTCACAAGCAAAGATGGTCAGATACCGATTCTCTTTCCCGCCCTCCCTTGGAT

GCCATTGAAGGGAAAACCGTCAAAAAAGGGGAAGAAACACAAGAAATGTCGATTTTGCAG

GTATGCGGTCGCCTTTGCAGTTGGGTTGGGTCTGGCTGGTGCTGGCTTGCTCCTGGGCTG

GACACTCGGGTGGCTGCTGCCTGCTTTTTAGATTGCCTCACCAATGGAGGCGATACTGCA

ACAGGTGTGAATCGAATGGTTCGGCTGATTTGATCGGAATTTGTTTTTCGGTAATGTGTA

TATGTATGTAGTTTGTGGTTTGTACATGTCTTGAGTTGTGGATGAAAGTAAATTGTATAT

ATGTGTATAGAGGATGATAGAAGTTGTAGTCCTCGGATCTTTTGCAGTGTTCGTGCAACA

TTATTGTGATCAACCTTGTTTATAACTTATGAATATATTTTTTATAGCAAAGTTGT

>Unigene0002080

CGAGTCCCCTGCGCCGAGTCCCACCTCCGGCTCGGCGGCGCTAGTGTCGCCAGCCGCCGC

TGCTGCCGCCGTCATGATCTGGTCCACCGTCGTGCTATTCGGATCATTGCGTCACTGAAG

GGGATCTGGCGCTCACTGCTTCGGATATCAGATCTGCGTCTTCAGAGTATCTAGTATATA

GATTTGCTGTTTTAGGAGTGTTTTGTGTGTTTAGATCTGAGAGGATTTTGTGTTTGGAGT

TGGGGACTCAGATTATGGGTATTCTTTTATATGATATTTGGCTATGTGGACTTTTATCAA

TATAAATTATGGTGTT

>Unigene0002081

CCACCGGAGCCGGAGGAGCTCAACATCCATGGACTCCAAGCTCAACTTCGTCTTCATCGT

GGCTCTCGCTTTCGCGTGCCTTGCTTGCAAGGTCGCCGCGGATGTCGAGTCCCCTGCGCC

GAGTCCCACCTCCGGCTCGGCGGCGCTAGTGTCGCCGCCCGCCGCCGCCGCCGCCGTCAT

GATCTGGTCCACCGTCGTGCTACTCGGATCATTGCGTCACTGAAGGGGATCTGGCAGGAT

TTCAGATCTGCGTCTTCAGAGTATCTAGTATATAGATGTGCTGTTTTAGTGTTTTCTGTC

TTTAGATCTGAGAGGATTTTGTGTTTGGGGTTGGGGACTCAGATTATGGGCATTCATTTA

TATGATATTTGGCTATGTGGACTTTTATCAATATAAATTATGGTGTT

>Unigene0002082

GAGTCAATGTGGGGTGAGGCCGGGGCTGAAGACCGCGGTTCCGTATGATTACTTGGATAC

AAGCGAAGGGAGTGGATGGGATCAGTTCTTGAAAAGGGTGGATGAGGAGCAGAGAGCCCA

GGATGTGGAAGAAAGTGGTGTTTAGATAGTGTAAGTTGTTGCATGTTGTGCTTCTTATCT

GAATCTATGATTCATCAAGTTCTTGCTAGGTTAATAGTTGGTATTGTGTGCTATTGTTTT

CCAACTTAAAACTTATGCTTATGCAGAGTTTGAACTTGTTGATTTCTAGTTTACTGTTTT

TAGATTACCGAAGTAATACTTAGA

>Unigene0002083

CATAAACACAGGTATTGGCTGAAGGGCTATTAAAGATACTCTCCACTAAAGATCAGATCT

GTACTTGGAGACTGATGCATATCCGGCGAGCTCGGGGGTGAGGAGATGCGCTGCTCAGCC

TCCCGAGATTCTCTTTGGTGATAAGTCATGTTACACATTTTTACTCCTGTGCGAGATGAA

TTTCTGGTGCGAGAATTCACAGCTTCACTTCTATTTTACACTTTCAGCAGAAAACCAGTC

ACAGTTTCACTTGTAGTAGAAGACCTTTGCCAGATGCGGAAAATGTAGAGGAAGGGGATG

GTTGAGTAACTTGTATGTTAATGTGTAGCTATGACACCATATATAGAGGAACACCCCATA

ACCGATATG

>Unigene0002084

GAGTCAATGTGGGGTGAGGCCGGGGCTGAAGACCGCGGTTCCGTATGATTACTTGGATAC

AAGCGAAGGGAGTGGATGGGATCAGTTCTTGAAAAGGGTGGATGAGGAGCAGAGAGCCCA

GGATGTGGAAGAAAGTAGTGTTTAGATAGTATACGCCGGGTGGCGCGGTGGCACTAACGT

CGATGTCCGACATTTTTGGGAGACCTTAAGGGTGGAGAGGAGGCAATATGTGTGACATTA

ATCATGAAGAACTTGACATAGTCATTGAGTTTCAGGGTGAGCTAGAGGCTACCTGGTTGC

CAACGGTGAATTCTGACGTAGAGCCTCTTGAATACCCCTACGGAGGAGCTGTAAGCATCC

CGTTGGGAGTGGAGCTAGGAGCCTTT

>Unigene0002085

GACCCTTGTCTGCTCAGAGAGAGAGAGAGAGAGAGGGGGAGCATGGGAAGACCACCTTGC

TGTGACAAAGTAGGGGTGAAGAAAGGGCCATGGACTCCAGAGGAGGATATTGTTTTAGTT

TCTTACATTCAAGAACATGGTCCTGGGAATTGGAGGGCTGTTCCTACTAACACAGGCCTA

ATGAGATGCAGCAAGAGTTGCAGGCTTAGATGGACAAATTACTTGAGGCCAGGGATCAAG

CGCGGCAACTTCACTGATCATGAGGAGAAGCTTATAATTCATCTCCAAGCTCTTCTCGGC

AATAGGTGGGCTGCAATAGCTTCCTATCTCCCAGAGAGAACAGATAATGACATCAAGAAC

TACTGGAAC

>Unigene0002086

CCATGCTCAATTTATACTCCTGAATCATGAATTTGGTTGCAGACAAAGTAATGTCCACTA

TCCAATCCCGCTTTTGTGTAACCTAAATTTCTGAAGGGAGACCAAATTTTGTTTATATAT

GAATAAGGAGATTTTGTTTTGGATTATACGGCACTATCTAACAAAATGTCCTTTGATTTT

GTTTTTTTGTTTCCTCTTTTGTTTGTGATACAATCTAGGTGGGCAGCTATAGCTTCTTA

>Unigene0002087

AAGAGAAGTTACAGAACAGCCTATGAGCCTGTGAGGAGGGAGGAGAGGTGGAGGTGGCTG

GCTAGTCATGGGGAGGCCTCCGTGCTGCGATAAGGTTGGCATCAAGAAGGGCCCTTGGAC

ACCGGAGGAAGATATCATCCTCGTCTCCTATATCCAAGAGCATGGTCCTGGAAACTGGAG

ATCAGTCCCTACAAATACTGGTCTGATGAGGTGTAGCAAGAGCTGTAGGCTGAGATGGAC

TAACTATCTCAGGCCCGGAATCAAGCGCGGAAACTTTACCCCACACGAAGAAGGGATGAT

CATTCATCTCCAGTCTCTGCTAGGCAACAGGTGGGCAGCTATAGCTTCTTACCTTCCTCA

GCGAACGGACAATGACATCAAGAACTACTGGAACACACACCTCAAGAAGAAGATCAAGAA

GTACCAAGCCGCGGTCGAAAGCTTCGGCAATTCTCGATCAGCCTCATGCCATCAAGAGCT

TCTGTCCAGAGGCTATAGCAGTGAGCCCTCCATGCACTACCCCTCACTCCCGAGCTTCAA

CAATAGCTCCTCCTCTGCCACCTACGCCTCCAGTACCGAGAACATCTCGAGGCTCCTTGA

GGGGTGGATGAGGTCATCCCCAAAGCCCGGTCTCAACCTCACTCAAGACAAACAGCAGCC

ATTCAATAACAGCTCCAGCAACAGCAGCAATGTTAAGGGTGACAATGAAAGCTACCCACC

GCTTCCCCATGGAGATTTCGACTCGTTCCTTTCTTTCGAGAATGCATGGGAGAGGTCGTC

AGCTGACTCTACCCTTCACGGGTCTGATCACTCTGAGGCTAAGGATAACTTAGAGAGCCA

GCAGCCTCCCATATCATTGCTCGAGAAGTGGCTTCTTGATGAGGGCACAGGACTACATGT

GGAGAACCTCATGCAGCTCTCTGTTGATTGCAGTTCCCAGACAATGTTCTAAGTCTTTTC

CAAAAATGACTTAATTACTATTGTTCATGTTATCTAATGAGCGCGGAGACCAGCTCTAAT

CAAGAACTAGTTCTCAAATGTAATTAGTGACCAGTGCTTTAATATTTATCGAAATAATAT

CAATTTGTCTGGA

>Unigene0002088

ACCCCACACGAAGAAGGGATGATCATTCATCTCCAGTCTCTGCTAGGCAACAGGTATTCC

ACATATTCAGCCCATGCTCAATTTATACTCCTGAATCATGAATTTGGTTGCAGACAAAGT

AATATCCACTATACAATCCAGCTTTTGTGTAACCTAAATTTCTGAAGGGAGACCAAATTT

TGTTTATATATGAATAAGGAGATTTTGTTTTGGATTATACGGCACTATCTAACAAAATGT

CCTTTGATTTTGTTTTTTTGTTTCCTCTTTTGTTTGTGATACAATCTAGGTGGGCAGCTA

TAGCTTCTTA

>Unigene0002089

TCTTTATGCCCAAACCCCACTGCCTATCCCCACTCGTGACATCCTCACCCATTCAAACAC

GCCCGCCGGCATCCTTCAACCTCACTCCCTGCGGGACCCAGCCGACCCTTACACTTCCGC

CCCCAATTTCAAAATCCCCTACAGATCGCCGGCGCAAAACCCTCGCTCCCGCAGCCCCGA

CCGACCCTCCGCCATGGCCGCAACTCATCTAGATCTCGACCTTGTGGTCTCCGGCCCGTC

GGCCTTCGGCACCGGGGAGTACGCTTTTGCTGATTCCGGGAACCTCGAGCACTGCGCGAA

GTACTTGAATCAGACGCTGGTGACATTCGGCTTTCCGTCTTCTTTGGATCTCTTTGCTAC

GGATCCGGTGTCGATTGCACGGACGTGCAATTGTATATACTCGTTACTTCAGCAGCGGCA

GCGGGATGTCGAAGCTAGGGAATCTGCGAATGAGCTGAGACAGAGATTGCAGTCTGACAT

ATCAAGATTGGAGGCTAAAGTTGAAAGGTTTGAAGCACAATTAGCTGCGAAAGATCGAGA

TTTAGCCACCTTAACCCGAAAGGAGGCTAAAGCTGCATCAGCTTTCAAAGCGCAGATTGA

GAAATTACAACAGGAACGAGATGAATTTCAAAGAATGGTCATTGGTAACCAGCAAGTGCG

CACCCAACAAATACATGAATCAAAGAAAAAGGAGAAGGAATACATAAAGCTACAGGAGAG

GCTTAACCAAGTGTTAGTGGAGAAAAAGAAGGAATCTCGTTCAGGCATGGAGATCATGAA

TTTGCTGCAGAAAGAAGGACGCCCACGTGGAACATGGAATGGAAAGAAGGCTGATAGCGA

TTTCTATAAAATGATTGTTGATGCATATGAACTGAAGCAGCGAGAACTGGTGGCAGAGAA

TGCTGATTTGCGGGCATTATTACGTTCAATGCAGATGGATATGCGGGATTTCTTGAATGC

TCCAAATGGGCTTCCCCAACCATCTCTGGCTGTCTCTGAAAACCAGGATATGGGGTCGCC

TCAGTCCCCTTTAGGTGGCAGGACGGATGTCTTCGACCTGCCTTTTCATATGGCTAGAGA

TCAAATTGAAGAGAGCTTACGAACTAAAATGGCTTCAATTAAGTCACGGATGGGTCAGCT

TCAAGATGCGCAAAAAGGTGCAGAGGTGACCTCTGAGGCAACCGAGAGAGAGCTTGAGCT

TGAAGCTCAACTTGTCGAAGCTCGAAGCATCATCCAAGAACAGAAAACGGAAATAAACAT

ATGGAGGTGGCTTTGAAGCAATTGGAGGGTTTTCAACCGTACTTACGGATAAGGAAACAT

TTGTTTAGTGGGCTTTGAAGCTTTCAGTGGTATTTGAGTTCTCTAAGTAACAACGCAATA

TTCTGGAGAGAGAAAAGACCGATTCTAGTGTGGAGGCCGACATCCTGGCAATGGGGGCAA

GCAAAGAAAGCTTTAGTGCAGTTACTTGTGACTGGCTTGTTCTTAATTGGAGATCGCTGC

TAGGAGCGTATGTGGTTGTTTGCTGCAAACCGTCTCTTTTTGCTTCAATAATGTCCAAAC

ATATTGTGAAGTCCGAGAAACCAAGGAGATTGAGTGGACATCTCGAAGCTGAGAGGGAAC

CGATCCATCTATCTTCTACTGAGGCAGTGTAAGTTGAATGGCTAATGCCATACTGATGAT

AGCCTGCATGGATCTGTAGACTACTCTATAGCATCAGAAAGTAAACGAGGCTTCATCTAT

GTAACATTTGTAGGTATACTTGGATTCAATGGAGGATTCTAGCGGGTGATTCAGAAACTC

AGTTGAATCCCCAGGTTTAGTTGTGTATGTAATTTTAGTGAGTTTAAACATTTCTTTTAC

ATCTGTTGCAGGATTTGATTTTTTTCTTCCCTATTTC

>Unigene0002090

ATCGAGGGAGAATAGACTAGGATGGTTTGGGCAGATTATAAATATGTATTTTACTATTAT

ATGCATAGTGTCATATCATACTACATTTAATTTTATTAATAAATTATAGTACTGTAACTT

AGTTTTGGACAGATCTCTGAGGACTCATTAGACTTTTCCTTTAACCTTGCCAGATAATCA

ATTTGAGATTGGGTGTTGGAACATGGTATTTCTGTGAGGTTTACATGTGTGGCTGTCTGT

TTATACTTGACTAGGTTGTGGGCTATTGTATTCTTCACTAATACTATTTGTTTATATTAA

ACAGTTCGCTTAGTAAGTTCTAATTTAAAGATCATCTTTTAGTTAATGTTACTTCTAGAT

GTGAGTATATAATATACTGGATAGGTAGAGGGATTATTTAAGTGAACTAAACATCTGACT

TACAAAATTGTCCTGGTTTTATTATGCTTGCAATTCGTTGGATTTTGTGCACACATGATT

ACTTAGATTTCACATGACACATTGATGTTTACGTATATGCAGGCTTCAATAATGTCCAAA

CA

>Unigene0002091

ATCGAGGGAGAATAGACTAGGATGGTTTGGGCAGATTATAAATATGTATTTTACTATTAT

ATGCATAGTGTCATATCATACTACATTTAATTTTATTAATAAATTATAGTACTGTAACTT

AGTTTTGGACAGATCTCTGAGGACTCATTAGACTTTTCCTTTAACCTTGCCAGATAATCA

ATTTGAGATTGGGTGTTGGAACATGGTATTTCTGTGAGGTTTACATGTATGGCTGTCTGT

TTATACTTG

>Unigene0002092

ATGATGTTGTTGAAAAGACTACACTGCCGCACAAAACTTACCGAGTCCTTGACATTTTCG

AAGATGAGCCTTTAGAAGATGAAATGGATGATCACTTGTTGCTGCCACAAGCCAGTGTGG

GCCAGAAAAGGAGAAGAGAACCAGTTAAACCTGCACCCCTGAATATTAAAGATCCGCGCT

TGACGCAATTTGGGAGATTGCTGAAAACCATGCCAAGTTCAAGGAGATCACGCCTTTTAG

CATGCATTCCTCAGGAGGCAACCCCAAAAGCATCCCAAAGTCAGAGGAGTGGATATTCTG

GTTACAAGCAGCCACGGTTGTATTAGGACAAGGAGAGTTAAGAGGACTGGGGACAGCAAG

GAAGCTTCTATGATATATTGCTGGAATCAAAATCTATCCTCCCTAGTACTATGTGTTGTT

TGTCCATAGATTGGTCTTGTGTAAAACACCTCTACTGGAGCTATAGAACAGTTCTTACT

>Unigene0002093

CAATTAATGCCCTGTTCTAAGGATATGTACCCAGATGATTTCCTGGTGTCAGTGCTTGAT

TGCATAGAAATCCCAGATGATTCCCAGGTGTCGGTGCTTGATTGGATAGACATCCCAGAT

GATTCCCTGTTAGTCCTTGATTGCATAGACATCCCAGATGATTCCCTGGTGTCGGTGCTT

GATCACATGGACACGCGCCTCGTTTCATATTCCAGTAGCAGCTTAGGCAAAACACCTGAA

AAAAATAGAAATCCAAGTGCCTGTGAAGTTGTTTCTTCAGAATCTCATGTGGTCATGGCT

GACATAGATCATCAGGGAAGTCCTTGCGAAGCAGATGATGGGACTGAAAAGACCACAAGG

CTTAACAAACCTGATAACATTGTCCCTAAGTTCTCAGAGGCTTATACATATCATGGTGCA

AGTCCTAGCCAAGCAGATGCTGCTGATAAAAGGTCCGTTATACAATATGCATCTGATAAT

ATCACCAATACCTCCCAAGTGGCACTAAGTTATCACAGTGCCAGTCCTTTCGAACCAGAT

GATGTTGCTGAAAAGACTACACCGCTACACAAAACTTTCCGAGTCCTCGACATTTTCGAA

GATGAGCCTTT

>Unigene0002094

CTGGTTGATCAGACCTTTAGAGGCCGCCTTAGAAGCCAGCTGCGGTGTTCTCGGTGTGGT

CACTGCTCAGACTCATTCGAGCGCCTACTTGATATTAGCCTAGAGATTGATGATGTGGAC

TCATTGATAGATGCTCTGTGTTCTTTCACGAAGGTGGAGAAGGTTGAAGATGCCGTCTGT

GGAGGCTGCAAGGCCCAAGGGATTATGGAGAAGGAATTGAAGCTCGA

>Unigene0002095

CTAGAGAGAGAAAGGGGAAGATAAAAGATGGGAGGGAGGAGCCCTACGCTCTAAAAGCAT

CGGCGGCGTGCGGAAAAGGAGTCCCAGATCGACAGACCGAGCGAGATTTCGTTGGATCCG

TCCCCCACTCGGCTCGATTAGCCGCGGATCTCGATCCGCCCTTCTCTCCGGCGGTGCTCC

AGATCTAGGGTTTGGGGGCCGATTGCAATGGCGGCCGTGTTTCCGGGACAAGTTAGCGCT

TCTCAGGTGGGGAGCTACTTCGTGACGCAATATTATCACATACTGCAGCAGCAGCCGAAT

CTGGTGTATCAGTTCTATACCGATGCAAGCACCATGGTGCGGATTGACGGGGCGACGTCG

GACACCGCGAACGGGATGATGCAAATCGACTCGCTCATTATGACCCTTAACTTTACTGGG

ATAGAGATCAAAACAGCTCATTTTGTCGATTCTTGGGAGGGAGGTGTCCTGGTAGTGATT

TCTGGTTTTGTGCAAACCAAGGAGTATGCTGGCACAAGAAAGTTCGTACAAACTATTTTT

CTTGCCCCTCAAGAGAAAGGATACTTTGTTCTGAACGACGTCTTACACTTGCTTGACGAG

GAGCAAGTACATCAGTATCCTGCTGCCATAGTAACTCATGGCAATTATGAGAGCAACTTG

GTTGCACCAAATCAAATTCCAGAGCCAGGTTCTGACTATATGGCTGGAGAAGAGGTCCAG

GCCAGGGATTACGTGGATCCTGCTTATGCTGAAGAAAGTGACGCTGTTGATAACTATGGC

ATCCCTGAGCAGCAGGAACAAGGCTATAATGTTCCTGAGCAGCATCAAGTTTATGGTATA

CCCGAGCCACAACAAGTTTATGGTATCCCTGAGCCACAACAAGTTTATGGTATTCCTGAG

CCGCAGCAGCACATCTATAATAACATCCCTGAGGCACAACAAGTCTCTGAACTTGATAAC

AGAGTAGACGAAGCTACTGTAGAAGATACCATTGCCTCATTCCAAAGTGTGGTGAATGTG

ACCCGGGATCCACCTCCTGCTCCTGCAGAGGAACTGGTGGGGGAACAACCCAAGCAAACC

TATGCGTCGATATTGCGATTGGCAAAGGCACAATCTAGTAACTCTGTACCTCAAGTTGCT

CCTGTGAGCAAGGCGATTCCAGTGACTTCAGACTGGAGTCAGGCATCTCAGTCAGTTCCC

CAGCAGTCTTACCCTCCCCAGCAGGCTTACCCTGCATATTCGGCAGTGCCAGAGAAATCC

AACTCAGAAGCCGTGGAGGAAGCTTATGCTCTAGAAGATGAAGGCGAAGAAAAATCTGTT

TATGTTGGAAATGTTCCCGCATCCCTCACAGAATCCGACCTTGAGCAAGAGTTCAAAGAC

TTTGGGAGGCTCAAGTCTGATGGTGTTGCCATCAGGAGCCGCAAGGACTCGGGTGGTTAT

TATGCCTTTGTCGAATTCGAGGATGTTACTGGTGTAAGAAATGCACTGAAGGCCTCTCCA

TTAATTATAAAAGGCTGGCAGATATATGTAGAGGAGAGAAGACCTAACAGCAGCATGTCT

CGGGGAGGAAGACGGGGCAGAGGAAGGGGTGGTTATGGGTCAGAGGCCCCGAGGGGGCGC

TTCGGCGGGCGCAGCTACGGAAGGGGCAGTGCTCAGGACACCGACAGGGAATACAACAGT

CGGACGAGGGCGAATGGAGTCCAGCGAGTTCCGCGCCAGGAGAGGGGCATTCTGGGCAAT

CACGTGTCGCGAAACGGGCAGTACTCGTCTGATAACTAGCTTCTATGGGTCTTCAGATCT

TCCTCCAGATCCGGGGCATCTTTACTCGCCCTTGCATAATATGGTCGCCTTTAGTTTTGT

GGGTCTGATTACATAGCTGTTTTGGTTTAGTTAATTTCTGGTTGCTCGGACTATCTTTTT

AAGTGGCCTTAGAGTTTATGTTTTGGCGTTCTGATGCGACAGTGCAGTTGGAATGGGCCT

CTGGATCCTAAAATTTTGGTTGGTTCTCGTGTTATCAGTGTTTGGGAGAGTACATGTTTG

GTTGGATTGCAAAAATCGTTTGGGTGCTATAATCATATATAATATGTTCAGTTTGCAATC

AGTTGGTTTGTATCCGGT

>Unigene0002096

TCTTTGCTCGCCCTTGCATAATACGGTAGCCTTTAGTTTTGTGGGTCTGATTACATAGCT

GTTTTGGTTTAGTTAATTTCTGGTTGCTCGGACTATCTTTTTAAGTGGCCTTAGAGTTTA

TGTTTTGGCGTTCTGATGCGACAGTGCAGTTGGAATGGGCCTCTGGATCCTAAAATTTTG

GTTGGTTCTCGTGTTATCAGTGTTTGGGAGAGTACATGTTTGGTTGGATTGCAAAAATCG

TTTGGGTGCTATAATCATATATAATATGTTCAGTTTGCAATCAGTTGGTTTGTATCCGGT

>Unigene0002097

TGGCTTCACACACGACTTCAATATAAATCTCTGATTCTCTCCTCTTCTCTGCATCTCAAT

TGTTTGAGAAATGGGAAATTGTCTCGCACCTCTTGTGAAATCTCACAGCTCTCCGAGCAC

CGGCTCTTCAAGCCCAGGGCGGTCGAAGACGAGCAGCAGCAACAGCAGCGCCAACACCAC

CGGGAAGCTATCGAGCACCAGCACCAGCACCTCCGGGGAGTCTGCAGCCAGCGGTCACGA

GGTTTATCCTGAGGGGCGAATTCTGGTGGTTCCGAACCTCCGTGTCTTCACTTTCACTGA

ATTGAAGAGTGCTACTAAGAATTTCAAGCCTGATACGATCCTCGGGGAAGGCGGTTTCGG

GAGGGTTTATAAGGGATGGATTGATGAGAAGGGTTCGAAAAGCGGGAGCGGGACGGTGGT

TGCTGTCAAGAAACTGAACCCAGAGAGCATGCAGGGGTTTGAGGAATGGCAGTGTGAAAT

CAATTTTCTCGGACGGCTCTCGCATCCCAACTTAGTCAAGCTCTTGGGTTATTGCTGGGA

GGACAGGGAGCTACTCTTAGTCTATGAGTTTATGGCAAGAGGGAGCTTAGAGAATCACCT

CTTCAAGAAATCTGTCCAATCACTCTCATGGAGTTTAAGGATAAAGATAGCCATCCAAGC

AGCTAAAGGTCTTGCATTTTTACATGCATCAGAGAAGAAAATAATCTACAGGGACTTCAA

GGCTTCAAATATCCTGCTCGACTCAAACTACAATACAAAGCTCTCCGATTTCGGGCTAGC

AAAGAATGGACCAACTGGTGCAGACTCACATGTCACCACACGGATTATCGGTACCAACGG

GTATGCAGCTCCCGAGTACGTCGCTACTGGTCATCTATATGTGAAGAGTGATGTGTATGG

TTTTGGAGTCGTTTTGCTAGAAATGATTTCTGGTCAGCGTGCTATCGATCCAAATCGTCC

GAGTGGTCAACAAAAACTAGTAGAGTGGTCTAAGCCATACCTAGAAGATCGCAGAAAACT

GAACCGTCTGATGGACCCCCGTCTGGAGGGACAATATCAATCAAAATGTGCTTTTCAGGC

AGCCCAGCTCAGTCTAAGGTGCTTGGCTGAGGAACCGAAGAACCGGCCATCTATGAAGGA

AGTCGTAGAAACTCTAGAGAAGATCGCGAACAGCAATGTCAAGATCTCTCAGAATCGACA

ACCACTAGTTCATCCAAGGCGCGGAAATGTTGGTAGTGAGGCTCGGGCGAGATGATAGAG

AACAATAGTGACGCTGGCTCTGGTTCGATTGCCTGCCACTTTGTAGATCAGACAACTTAT

TTTTTATATGATCCTAGTTGATGATTTTGATATCACAAGCTATTCATGTGTTTCAAATGT

GATAATTATTTGTACTAATATTGGTGAAGACACTTGGATTATAAGAAAAGTTCGTGTTTT

TTTAGAGAGATTGGTGTACTAAAGCTCTATCATTAATCAACCATAGCCAGTAAGTTAAAT

TGAATTGCA

>Unigene0002098

CCACGTAGCCCAGACCCCAGTATTTACTGCTCCTTCACCTCCTCGTGCCGTCGGTCCTCT

CGACTGAAGCGGCAGCAAGAGCTATGTCGAAAAAGGTTCACCTCACTGGAGTCCTGAAAT

CCCATGCCCAGAGCATCTACGAGACCTTGCAGATTCTTGACAAACCCGCTGCCGAGAAGG

TCGATTGGTCGGAGATCACCAAACTCGGCGATCAAATATCCAAACAAGCAACAGTAGCCG

GAATGCTCTGGAGTGGAGAGACACCTGATGTTAAACAAGCTGAAGAAAACATGAGTGCAT

ACTGCAATGCGCTTCACGGATTCATTTTAATTTGCCATGGCAGTACGGCGGGTGCTGGGC

CTACTCTCCATGCTAGTATCCGTGCTTCTGCAAAACAAGTTGTCGATCACAGCCTCTCGT

TGTTGACGCGAGCTATTAACTCCTACGACTCGCTCAATTCTGGTGAAAGATCATTGATTC

CACAACTATCAGGATCAGTTTGGGAAGCATGTGATGCTCTTAAGAAGGTGCCCACTACAA

ATTGTGTCGCTGTTGGGCGAGCGATAACTCAGGTAGCGGTCTCCGTCAAAGATGTCCTGC

GGGAGATGAGAGAACTCAAGCTTTCTGTTCAAACCGATTCAGTAGTTGGTACTGGAGATG

GGGCGCATGGATCGAAAGAAGATGATTCTAGTGATGATGGTGACCTGAGTGAAGATCTTT

CCCCTGAGGAGATGGGCGTTGCTCAGTTAGTGATCACTGTGATATCGGATACATTACTCG

TTTTCAAAGAGTTAATCCGCTTTATTTCGATGAGCTTGCGCAAGGACTTGGATCACGAGC

CCGGTTATGTTGATTCGATGGAGAGATTACTGAGCTACTGTGAGGATGTGGGAGTGAGTG

TGAATGATTTAGGGGCTTGTGTTTACCCGCCACAAGAAATCTCTCAGATGACGTCGATTG

CGAAGAAGATGCATCTTGTAGCTGACAATATACAGATGCAGGTGGGGTTGTTTGGTGGGT

CGCCTGAGGGTTTACTGAGGGGTATTGAAGGGTTAAAGAGTTCATTGGGGAAACTGGAGT

CTGGTTTGAATGATGGTCTGACATCTCAAATGGAAATGCTTGCTGTGTAGGATGTGTACA

AAGGCAACCCAGATAAGTGTCTTGTCTCAACTGCTGAGTTTCTTATTCGCATTTGTTAAG

TACGGGTATCAGATTGTTGGACATTTTTTTGCAGGTCAGAATCTTCTTGTACAAGTATTT

TACCTGTCAGATTTTACTAATATGTTAGTTTCTTATTGGCATTTGTTAAGTACGGGAATC

AGCAATTAGTTTCTTATTGGCATTTGTAAAGTACGGGAATCAGATTGCTGGACATTTTTT

TTCAGGTCAGAATCTTCTTGTATAG

>Unigene0002099

ATGTTCACAGCTTCCATTTAGCTATAATTAGGTATAATCTCTTCTCTCATCTTAGAGTAT

TCTATTCTTATCCATTCTACTTATTTGCTATGAAGATGGAGCACAAGGCCTTCATGCCTG

TGAAGACCACCGAGCAGGTCTTTGTCACCTGCAAGCCCGTCCAGCGCCAGATCGGCGACT

CACAGACTCGCACGGTCCGCATCTTCTGTAACGACCCCGATGCCACCGACTCCTCCGGCG

ATGAGGGCGACCGCCGCCGCGTTGTCCGCTATGTGCACGAGATCCGCCTCGAGCCCAGCT

CAACCCGGTCGGAGCGGTCACTTGCAAAAGTGAAACCGGGAAAGAAGAAGCCGGCGGGTG

CTGGAGTTGGCGCCGGCGCTGCTCCGGCGAGCACGAAGTTCCGCGGCGTGAGACGCCGGC

CGTGGGGGAAGTACGCGGCGGAGATCCGGGATCCCCAGCGGCGGGTGCGGATTTGGCTCG

GGACTTACGACACGGCCGAGGAGGCGGCGAAGGTCTACGACACGGCAGCGCTGCAGCTCC

GGGGGCCGGACGCCACCACTAACTTCGCCGTGGGCGAAGTCGAAAGTAATGTTTGTGAAG

CTGCGCCTCCTCCTCTGGCCAAAAATAGCTCGTTCGGTTCTCTGTCAGCTGTTGATGAGT

CTGGAGACGAGTTCAATAAACTCTCGTCGCCGACCTCCGTTCTCCGAGGCTTCAACACCA

CCACCACCATGGCAGAGGAGAAGCCGGCCTTTGCGCAACATATATATGTAGAATCAACTT

ACGAAGATGAGGCGAAGCCGGAGTTCGCTCATCAATTGTTTGATGAAATGACTTGCAATG

AATATGAGGCGTATGAGCAAAAGCCAAATTTGGGGCATATGGAATATGAGAATACACTCG

AGATGGAATATGAGAATAAACTGGAGTTGGCGAACCAGGAGTTTGATGAGATGGCGGATA

TGCTTGACCATGAGGCCTCGGGGTCGAGGGATACCATGTTTTCTACCTATTGGAGCGAGA

ATGCATCTGCCTGGGAGGCGAATGAGTTCATTGATGCCTCGGAGCCGGTCACATGGGAGC

CGAACGACTTTCTTGATCCCTCGGCGGTCAGCTTGTTTGAGGAAGCTGATATGCTATCTA

TCAATTGGAATGATTCCATTGTATCAGATACAAGATTGTTCGAGGAAGCAGACTTCTTGA

ATGCTGATTCAAATGGCGCTTTGGAACCGAGGTTGTTCGATGATTCAGGTTTATTGACGA

CGGATTTGAGTAATTATGGTCTCGGATTATCTTCGGATGACTCGAAGTGGCAGCGTGAAT

GGCAAGTGGATGAGTTGTT

>Unigene0002100

GATTCAGGTTTATTGACGACGGATTTGAGTAATTATGGTCTCGGATTATCTTCGGATGAC

TCGAAGTGGCAGCGTGAATGGCAAGTGGATGAGTTGTTTGAAGATATTGGAGACTTGTTT

CCGATTGAGCCGATGAGAGTATGAAATGTGGTTGGTTGTTTCCGCTCTAGGTCTATTAAA

TCGGCCAATTTTGTAGCGGGTTTTTGAATTTGTTTATTTGTAGTTTGAATGTGAAGTGAG

ACAAAAGCTGGCTTTGGGCCGGTGATGAGTGCTTTATTGACTAATTTGAAACTTGTGCGG

CTTTAATGTGAACAATTTTCTTGGGAAT

>Unigene0002101

TCCCCATACTAGCCCGCTGCTCTTTCCCCTTCTCGTTCTGCTCAATCCTAGGGTTTCGAA

ACATCGAAATTCAATTCAAAATCCCAATAAAATCGACATCACGATCAATTCGAAATCCCA

ACAAATCGACATCTCCCCAAAGTAATCCCTCCCCTCAAATTCTCGCCCCACAGTCCCCCA

AATTCGCTGTTCGATCATGGCGTCATCGTCCCCATCGAGCTCCCTCTCCGAGCCGGCGAT

CATCGAGCAAGTAATCACCGAATTCTTCGCCAAATCCCTCCACATCATCCTCGAGTCCCG

CTCCCCCTTCGAATCCTCTCGCAACTACGCCGCCACCGCCGCCGATCCCCTCGGCTCCTC

CTCCCCCTCCTCCACCTCCTCCTCCTCTTCCACCGGCCGGCAGCGCGACAAGTGGTTCAA

TCTCGCCCTCCGCGACTGCCCCGCCGCCCTCGAGAACTTCGACCTCTGGCGGCAGAGCAA

CCTCGACCCCCTCGTCATCGACGTCATCCTTGCCCGCGCCGCCCCCGCCGGCTGCCTCGT

CCGAACTCTCTCCGCCAAGGACCCCCTCTGGCCTTCGTCGCAGGATGATTTCATCTCCGA

GTCCAGAAGCAATAAGATCGTGGAGAGGTGGGTGGTGCAGTATGATGCCAGAAAGAAATC

TATTGATAGAGAATTGAACTCCAGCAGTGCTGCCGCCGGCGGCAGCGGTGGCGGAAGCTC

CCACACATCCGAAATCCCAGCTCAGTACAAGAAGACGTACAAGCGAACAATCATACTGCT

GCGGTCACTGTACGCCACCGTCCGGCTTCTGCCGGCGTACAGAGTCTTCCGAGCTATTAA

CTCATCCGGCCAAATCCGTCCGATTAGCCTCTCACATCGGATCTCTTCGTTCGTAGAGCC

GTTCACTCGTGCGGAGGACGCTGAGATGCGGCAGTTCAGCTTTGCCGCGGTGGATACACC

GTGCGGGAGGTTGTCTCTCTCGGTGTCGTACATTCCGGCGCTGGAGGATGTGAATTCTCA

ACCCTCACCACCATTGTCGACAGAGTTGATAACTGATTATGTTGGGAGTCCTACGACTGA

TCCGTTCAAGAAGTTTACACGAAGGCATAGTTGGAGCATTGACCGTGCGGGGATGCCGCT

GGTGTCTCCCTCCCCGTCGCATGCTTACTCCGATTCGCTCCCTTTGAGGTATACCCCTCC

AGATCATAGTGATAGCGAGTTAGGTGCTCGTAGAGTAAATGCAGGTTTTGATGAGAATTG

GCCGTCACCGCCGTTTTCGGTATCGCCTACACCTTCGCCACCGGTTCATTTTCGCGGTGG

GCACACACCAAATAATCTTTTACGGGCAGAGAGTGCGCCAGTTAGCATTCCATTGCCCCA

GCTCGGGAAGAGCGGCGGACTGGTTAACTATGGCCTTCCACCAACATCTTCTCGCAAGGT

TACAAAGACTGGGTTTTCCTCTTATGCAGATGCGTCCCCTGATGGTAAGCCGAGAATGAA

GAAAGAATCAATGAGGTTTCTAGAGTACCAATCTGACTCATCTCTACAGAAGGTGTTGTC

CATAGGGAGAGAAGACATTGCTAGTTTATCTGGACTTTGCAGTTCACCACATATCCCGTT

TTCTAGGAGCCCTAGTAGGTTGACTTTCTTAGATGAATTTGATGATCCTGAGTTCACATA

TCCATTTGCTGTAGATGATGTGGATTTAACTGATACAGGCAACAGGATTAAGCCTTATGA

TGCCAAAGGTCATGAGAGAGGAAATTCCGACAACAGAGAGTTATTACTAGACAAAACGTC

TCCAGATGCTGCTATTGGTGCCCTTGTAAGAATGTTCAAGGCTGCACCACCACTGCAGCG

AGATCTTTCCAACTCGGTCAGTAATATGCAAGTATCCATAGATGAAATTTGGAATGACAG

AGTCCTTGATGACAAAGGCATGGACATCAGGGTTGAAAAACTTGATAACGGTGGACCAAA

TGTGGAAATTGCAGCTTCTGGGCTCTTCAAGTCAAAGACTGCTGCAGATGCATTTGTAGA

GCTGAGGCATTACAAAGAGTTGAAGGAGCAATTACTCAGACAAGGTGGTTTGCCGTCATT

AGGAACGACTGAGCACTGAAATATGCAGCTGAAGGCCCCTAAGAGTTGTAAATATCTTCA

TTGTTCATCTACATGTTCATAGTGTTCTATTTTACTTAAAGCAGCCGAAATGGTATATAC

GACATGAGGTATGTCTGGTATTTCCTTTTAACATTCAGGCACTACGCCTTTCTATATATG

TATATAGCATGAAATCAAGTACAAGTGTCAGTGAATCCATAGCAAGGTTGTCGGTATTGC

TGTTTGCTTCATGAAGATATGTGAACCAATATCTTTGTTTTCAAATTCACACTTCGTAGC

AATTCT

>Unigene0002102

GTTTCTTGTTTTTGGAGTGTTGGATGCAGTGGAGTAGAAGCTTGGCTTGCAGGTTCTCTC

TCTTTACTTGCTCTTCTTGTTCTTTTGTCGTCCTCAGAATTAATATCTTATGTCGTTGGG

GTATTTTCGTCATGAAATGTGGTTAGGGGTATTTTCGTCATGAAATTTTTGGATAATTTG

ATTGGGTGAATCCATTTGAAATTTTGAGTGGGAGATACTTAGAGTAGGATGTCATGTTTT

TTATTTTTGTTGAGTATAACACACTGTCTGCAAGAACAATATCTCATGAAAATTGGCATT

GAATCAATAATG

>Unigene0002103

GGAGGCGAGCGATTTTACGATATTGAGTGTTGAAGAACCACCGGTGTTGTTGGTGACGGC

GGAGGAGGACGACTCGATGGAGGCGAGGTGGAAGGCGATTATGCAAGGAGGGGCGGCGCC

GGTGGTGAGGTTCACCCTGCCGGAGACGGCGGCCGGTGAGGGGTTGGTGAGGGAGGCGTC

GGAGATTGGTAAGGAGGATTTGAATCAGAGGTTTGATGCGTTCATCAAGAAGAATTATGA

GCAGATCCGGGTCGCGACTGCACTCCGAGACGGCTAGTTGTGATTAGTAGTTATTTATAG

TTTTGAAGATGCAATCTCCAGCTTCTGTTGTGTGTTTCTTGTTTTTGGAGTGTTGGAGGC

AGTGGAGAAGAAGCTTGGCTTGCAGGTCTTTTATTGTTTTCAAATTCTGGAGCTGGAATT

GGAGCATAAGAGTGTACTAGCTATGGTTTTACGGGTAATTTCCACATATTTTACCCTTTG

TGTCATTTAGCTTACCTTTTAGCATAGTTGCTTCGTGGATGACCTGGTTTTTTTCTTATT

GGTCATGATATGGTTTCATACTACAATAACCTGTGTGTAATTAATAACTTGTGTGTAATT

AACTATCAGTTGGAACTGATTCAGAGTTGTGATGATCTTTCACTTAATGTCTAATTCGAG

TTAAATCCAC

>Unigene0002104

AAGATAGCATCGCTGGAGATCCCACAGACGGCTGCCGTTGAAGATTTTGCAGAATCTGAG

GAATTGATCCCGAATAACTACCCAGAATTCGACAGATCTATGTCAGAGCAGAATTTTGTT

GATGATACCGAATTAACTTCTCTCGAAATTGAGAAGAACGATTTTACGATAATGAGTGTT

AAAGAACCACCGGTGGTGTTGGTGGCGGCGGAGGAGGACGACTCGATG

>Unigene0002105

TCCGCCAACCATGGCGCCTCTTTCCTAATCCCCACCGTAACCGCCGCCGCCGCCGGTCAC

TCCTTCTTAATCCTCCCCCGCCGCTCCAATTTCATCATGTTAGGCTTTAAGGCGTCCGAT

CTGGGCAACCGGCCTCGAAAGCCCGGCCTCGGCATCGCGGCTAGAGCAGCCACCGTCATC

ACCGGGGTCGTCTCCGCTGTCGTCGTTGCGAGAACCGCCGTCCCCTACTCCGCCGCCGTG

ATAGCCACCGCAATCCCCGTCGTACGCGCCTGGATGGCCCCGCCGTTCCTCTTCGCCGCC

ATCCACTTCATCATCCTCACCATCTGGAAGCTCTCCGAGAACAAGCATCAGTACGATCCT

TCTCCTCCTCTGAACGATGATAAGCCCCTGGCCAAGATAGCATCGCTGGAGATCCCACAG

ACGGCTGCCGTTGAAGATTTTGCAGAATCTGAGGTAATAATCTCAAATAACTACCCAGAT

TTCGGCAGATCTATGTCAGGGCAGGATTCTGGTGTTGATATCAAATTAGCTTCTCTCGAA

ATTGAGGCGAGCGATTTTAGGATAATGAGTGTTGAAGAACCACCGGTGGTGTTGGAGACG

GCGGAGGAGGATGACTCGATGGAGGCGAGCGATTTTACGATAATGAGTGTTGAAGAACCA

CCGGTGGTGTTGGTGACGACGGAGGAGGATGACTCGACGGAGGCGAGCGATTTTACGATA

TTGAGTGTTGAAGAACCACCGGTGTTGTTGGTGACGGCGGAGGAGGACGACTCGATGGAG

GCGAGGTGGAAGGCGATTATGCA

>Unigene0002106

AAGGCGATTATGCAGGGAGGGGCGGCGCCGGTGGTGAGGTTCACGCTGCCGGAGGAGGCA

GCCGGTGGGGGGTTGATGAGCGAGGCATCGGAGGTTGGTCAGGAGGATTTGAATAAAAGG

TTTGATGCATTCATCCAGAAGAATTACGAGCAGATCCGGATGGTGACTGCACTTGGAGAC

ATCTAGTTGTGATCAGTAGTTTGACTGCACTTGGGGACGAATAACTAGTTGTGATTAGTG

GTAGTTGATATTAGTTTGGTAGATGATTTTTTTTTTTTTAAAGTGTTGGGGGCCTTTTTT

TCTTTTACCTTAAAGGTGTTAATTTTGTTTTATACTACAAAAACTTTATGTAAAAAAAAA

AAAAAAAA

>Unigene0002107

TCCTCAAAACCGCCGACCATGGCGTCCCTTTCTTAATCCCCACCGCCGCCGCCGATGATC

ACTCCGGCCAATCCAATCATGTTAGGCTTTAAGGAGTCCGATCTGGGGCACCGGTCTCGA

AAGCTGGACCTTGTCACCGCTGCTAGAGTCGCCAGCATCATCGCCGGCGTCGCCTCCGTC

GTCGTCGTCGCGAGAACCGCCGTCCCCTACTCTGCTGCCTTAATTTCCTCCGCAATACCC

GTAGTACGCGCCTGGATGGCCCCGCCGTTCCTCTTCGCCGCCATCCACTTCATCATCCTC

ATCATTTGGAAACTCTCCGAGAACAAGCATCATGAGGATGCTCCTCCTCTGGACGATGAT

GAGCCACCGGCCAAGATAACATCACCGGAGATCCCACAGACGGCTGCCAGTGAAGATTTT

GCAGAATCTGAGGTAATAATCTCAAATAACTACCCAGATTTCGGCAGATCTATGTCAGGG

CAGGATTCTGGTGTTG

>Unigene0002108

AGTACTGAGGAGCTCAGACCCCCGTTCCCTCTTGCGTCTCTCTCTGCACCCCTCCACCCA

CTTCGTCAACCCTTGACTCAATCAGACCCGAAATGGGGGCAGCGCCGGAGCCCCGATCCG

CCGCGGGCGGCACCGACGATGGCGGCGCCCGCCTCCTCGGCGCGCTGGGCGACTCCAACT

GCTGGCCCCCTGGCGTCCGCTTCCACCCTACCGACGAGGAACTAGTCCTTTACTACCTCA

AGCGCAAGGTCTGCCGCCGCCGCATCCGCCTCCCGATGATCGGCGACGTCGACGTCTACA

AGTGTGAGCCCTGGGAGCTCCCAGATAAATCGGTGCTGCGGAGCGGCGACAAGCAGTGGT

ACTTCTTCAGCCCTAGGGATCGGAAGTACCCGAACGGATCGAGAGCCAACCGGACGACGC

GGCGCGGCTACTGGAAGGCTACAGGCAAGGATCGCACCATCTCACACAATTCCCGGCCGG

CGGGGTATAAGAAGACGCTGGTATACCACCAAGGCCGCGCCCCGAAGGGGCAGCGGACGG

ATTGGGTGATGTATGAGTACACTATGGATGATGAGATCCTCGCCTGCTTCAACAACGTGC

AGGACTACTATGCGCTGTACAAGGTGTTTAGGAAGAGCGGGCCGGGCCCTAAGAACGGGG

AGCAGTATGGGGCGCCGTTCAGAGATGAGGTGTGGAATGATGATAGTGATGATGCCGATG

ATAGTTTGGCAATTCAGGAAGTGAGACAGGAGGATGCAATTGAGGAAGTGAGACATGAGG

ATGCAATTCAGGAAGTGAGACAGGAGGATGCAATTGAGGAAGTGAGACATGAGGATGCAA

TTCAGGAAGTGAGACAGGA

>Unigene0002109

TTCAGGAAGTGAGACAGGAGGATGTTATTCAGGAAGTGAGACAGGAGGCTGGTAGTGTAC

ATTCACGTGGAGTGCTACTCGATGACACACCCATTTTTAATGCTGGTGATCTGGAAGATT

TTTTTAAGGAGATTACCAATGAGGAAGTTGTTCCACTGCAGACCCGGTTTCCTGCTTATG

ATCCAAAGGTTGATGTTGGATCAGAATTTGGTAGCCATGACGTGGGGCCATCCTATGCCA

ACTCTAGCATTACCAGGCAAAGTGATGATGTGTGGTGCAAGCAAGACAGTCTAGGTACAA

ACTTTCAACTTCTGCAGTCAACTCCTACGTACGTCCAAGCTATCGAAACAACTGAGGTAA

CTTCTTTTGATGAGTGCTACGATCTGGGTCAAGCTGTAGCAGCTGAAGAGTTTATGGAGA

TGAAAGATTTCAATTTCCCACCTGTTTGGGGTATGGATGGTGATGGTATCAAGCACGAGG

ATGGCATCCCTGACTCTAATGAATTTTATGATCCTTGTGATTATTTTGATCCTGCTACAG

TTTTGGCTGAAACAATGGGTTCTCTTGATGCATTGGCCGAGCATTCAAGGTTGTACCTCG

AAGGCTTCAATGAGTATGGGACACAAAACCATGATCCTGGCATGACCAGTGGGCTCTGGA

CACACGAGCAGCAGCCCTTCAATGTGTCCTCATCTATGGTGTCAAAGGAAGTCGAAATGC

CACCACAGTCGTCAGGTGCTTATCAGTTTGTGTTTGAGTTCTCTTAGCATTTATGTCATC

GAGCATTTGTAGCATGCCACTGTTTTCCCTTAATTCTCTGTGATTTCCGATACAATGTAG

GTGTTATTTTTAGTAATAATCTGTCGATTCTCGGCGGTGACACTCAAGCGCAAATTGCTG

GAACTGAGTCAGACTCATGGTTCAACTCGGCTGTCTCTGCGTTGTTGGGTTCAATACCTA

GCCCTCCTGCTATAGCCTCAGAGAATGCCTTGATTACTAGGGCACTTGAACGGGTTGTGA

GCTTTAAGGCAACAGGGCAGGTTGTTGTTGAAGGGCAGAACTCTACTGCTGCTGTTGGGA

GGAGGCCAAGAGGCCGTAACAGCGGTTTCGTTTTTATCTCGTTTCTGGTAGGTTTGGGTG

CCATCTTATGGGTGGTGACTATTGGAATTGCTATAAAGATGATCAAGAGTTTGTGGGGAA

GATTTGTAACTTCATAGTATGCTTATATGAACATATAATGTGGCAAATTGCACCAACTTT

GTTGGTTTTTTGTCCTGCTATATATGTACCGCATATTGTCATTGTTCATGCATAGTATAT

ATGTGAACTGTTAGTATTTTTGACGGAATCTATG

>Unigene0002110

GACATAACAAACTCTCACATGCCATGAAAAGACGGAAGAAATGGAAAGCATTGCGACAAA

CCAAAGCTCATCATCTACTACACTCATACATATCCAGGGGATGCTAAAGGCACTACGATG

GGATAACACTCGAAGAAGCAGCAGCAGCACCACCACCACCGGCAGTTTTAGCTCTGCGAC

GACGTGACGGGGCAGCTAACCCGGCACGTCGGGCACGACCGCCGGGCGGCGAGCCATCGG

TCGACGCACTGGACGTGGAATCCATGGTTGCAGTCCGGCAGTACCCGAATTCTGTCCCCC

TCCGCAAAGTCAGCAAGGCAAACGGCACACTCCGCCTCTGCTCCGGTGAACTTCGTCTCT

GCTGAGAATAATAAGGTTGGGAGTGTGATTGCTTCCTTTCTTGCTCCGGCCTGCTTCTCG

GGGTCTTCGGCCAGGCCTCGGCTGCGGCGAGCGCAGAGGAGGAAGCGAATGGCAACGTTG

ATGGCCAGGCCCAAGAGGAGGCCGCACAAGAGGACGATGACGGTGATGACAGTGTTTGTG

GTGAAGTCTCCGGCGTTTGAATAGGGACCCCATTTGTTCTCGGCAACCGGCATGGTTGGA

GAGAGAGAGAGAGAAGCTTTATGAGAGTTTTACCACGAATCTAATGAGGCTGGAATGTAA

CATTTGGGCAGTGTCGTTTAGTTCTGGCTAAATTGGAGGGGAAAGACGGACCAGAATGTT

CTCACAAATAATTGAAGCTCCAGGGAGCATCAATAACTGCGGGCATCACATATCCACGGG

CCTCTTTAATGTGGCAAAGGGATGCCATCGACTGTACCCACATTTTT

>Unigene0002111

AAAATAATCTCTTTTATGTGATAGCAAAGTAAAAGATTGAGTCTTTAATGTATATAGTTC

TCTGTTGGGGGAGGTATGACATGGGTGGTTCCTTGAGATCCAGGTAACCCAGATGGAAAG

CCCATCATTCAACTTTTCTTGTTGGGCTCTCTTTCCTGTGATCCATCTCCACCTTTACTA

GGTTCTTCCTCCCTACCCATCTTATATATTTCACTTATCTTGCCTTTCCACAATTGCTGC

CCTCCAAGGTGAATTATTGGAACCCCTCGGGTAGTGGTAGATCCCGGTGAATTGTTCCGA

AAAGCTGCTAATCAGATGGTGATGAGGAGGAGGAGGAAGAAGGTGTGGTGTAGCAATGTG

AGCTCTTATGATTACTGATGTTGGTACACCGTTTAGGATTGGCAATTCACTCTGTGGTGG

ATTGATTGGGGTCGCTTCAGTTCACACGATGGCTGATGATGGAGATGTCGATGATTTGGA

GATGAGGTCGATACTGGCGAGTAGCGATGATGATAGTAGTAGCATTGCTCGAGCAATGGA

CCCCGGAGCCTCGTCAGGGGCTTTGAGGGAGGTTATTGCAATGGAAGCTATTGGAGAAGG

TGTGGAGGATTGTGATTTGAGTATAGCGCCTTCTGTCGAGCTGACGATTCAACAGAGTGA

CGTTGGTGCAAGCGGTGGTCGCTCAGTAATGGACTATGTACCACTTTGGGGATGCGTAAC

GATGTGCGGGATGAGGCCGGAGATGGAGGATGCGGTGGTGACTGCCCCGTGGTTTTATCG

AATACCACTTGAATTGTTGGATGGCGTGAGCACTGGAACGAGCTACATGCCGGCCCATTT

CTTTGGAGTCTATGATGGTCATGGTGGTGCTCAGGTTGCTAACTATTGTCAAGATCGTCT

CCATGTTGCGTTGGTGGAGGAAATCGAAAATGTGGTTAATGGTTTGGAAGGTACGACCGA

AGGTTCGCAGAAACTATGGGAGAGAATATTCACTAATTGCTTTGTCAAAGTGGATGATGA

GGTTGGGGGAAAGGTACACCGAAGCACTGGTGGAAACACAGGCGATACAGTTCAACCTTC

CTTAGTTCAATCAGAACCCGTTGCACCGGACACCGTAGGATCCACTGCAGTGGTTTCTGT

GATTTGCTCCTCTCACATCATCGTTGCCAACTGTGGAGATTCGAGGGCAGTTCTCTGTCG

TGGCAAGCAAGCTGTGCCTCTGTCAGTTGATCACAAACCAGATAGAGAAGATGAATATGA

AAGGATTGAAGCAGCCGGGGGCAAGGTTATTCAGTGGAATGGGTACCGTGTTTTTGGTGT

GCTTGCAATGTCAAGATCAATAGGAGACAGATACTTGAAACCATGGATTATTCCCGAACC

AGAAGTCACGATCATTACACGGACCGGAGATGATGAATGCCTTATACTAGCCAGTGATGG

CCTATGGGATGTCATGTCAAATGAGGAGGTATGTGAAGTAGCTCGAAAGCGAATTCTGTT

CTGGCACAGAAAGAACGGCGCTGTGTCTAATTCCCCACACACGGGCGAAACCTCTGATCC

AGCAGCTCAAGCGGCTGCAGATTATCTATCGAAGCTCGCTCTCCGAAAAGGAACCAAGGA

CAACATCACTGTCATCGTCGTGGACCTGAAGGCACAGCGGAAGCTCAAGAATAGAACTCA

CCCGAGTAGCTTAGATGCAGTTTGATTACAGATGAAAGTTTATTTTAGCCTTTATTCAAT

AGCCTTTAATATATAGCCCACATTCTTTTTAAAAAATTGGGCTGTATGTAAGATTATATG

TTGTAAATGTACTATATAATCACTCGAATTAAAAGGATTTATCGTATGATGCCAAACATG

GTAATGTCTGTAAAACATATTATGTGGGCTTATGGGCATTTGTAGTTTAGTTGGAGCTCT

AAGGATGCTATCATGTGTATCATTAGCATGATAATTAAATTTTATTGTGTATTTATACAA

TTTGTCGATGGTCTATGTTATGTAATTTTTTCC

>Unigene0002112

CACCTCCCCCAACCGACACCTGTCTCTCTCTCTCTCCTCTCTCTCCCACTGCTGTCCTCC

ATCTACTCACCACCGAGCAGTAGCAGCAGCACCACCACCACCACCCAGGAAGCTCAGCTC

TCCATCACCATCATCACGCCACTGGTAATTCCTCCCCCCTTTCTCCTCTTTGTTACTGCC

AACTGTTTTGCCCAACATACATCCCCCCTCACCAAGGGGGGTGATGAGAGAGAGTCAGTG

AGAGTTCAGAGGTTAGACAGTAGAGACCTCCACTACTCTCTCTCTCTCTAATGGGTAACT

CAAAATAATCTCTTTT

>Unigene0002113

CACCTCCCCCAACCGACACCTGTCTCTCTCTCTCTCCTCTCTCTCCCACTGCTGTCCTCC

ATCTACTCACCACCGAGCAGCAGCAGCAGCACCACCACCACCCAGGAAGCTCAGCTCTCC

ATCACCATCATCACGCCACTGGTAATTCCTCCCCCCTTTCTCCTCTTTGTTACTGCCAAC

TGTTTTGCCCAACATACATCCCCCCTCACCAAGGGGGGTGATGAGAGAGAGTCAGTGAGA

GTTCAGAGGTTAGACAGTAGAGACCTCCACTACTCTCTCTCTCTCTAATGGGTAACTCAA

AATAATCTCTTTT

>Unigene0002114

CTCGTCGGAGCGGCGATCGCAAGATGTCATCAAAAGGTGATTCCGGTGGTGGCGCCGCCG

CGGCCTTCGCGGAGATAGTGGTGGTGCGGCACGGCGAGACCACCTGGAACGCTTCCAGGA

TCATCCAGGGACACTTGAACTCTGAATTAAATGACATTGGAAGACAACAAGCAATTGCGG

TGGCTGGTCGGCTAGCCAAGGAACCTAAATGGACAGCCATATATGCGTCTGACTTGAAGC

GAGCTGCTGAGACTGCGGAAATAATAGCTAATAGTTGTCATTTGCCTGAGGTATTAGTGG

ATCCAACTTTGAGAGAGAGACATCTTGGGGATCTTCAGGGTCTGACATTACAGGAAGCTG

CAAAAACAAAGCCTCAGGCTTACAAGAGTTTTTCATCTTCCAAAACCGATCAGGAAATCC

CAGGTGGCGGAGAAAGTCTTGACCAGTTTTATGACCGATGCACCTCTTCCTTGGAAAGAA

TAGTGAAGAAGCACATAGGGGAAAGGGTAATAGTGGTCACCCATGGAGGTGTCGTGAGAC

AGCTGTACGAGCGTGCCTGCCCAATGGGATCATTTAATAAGAAAATTGAAAATACGTCGG

TCAATATTTTTCATATTTCGGAAAGCGGTGACAAATGGATCATCAAGACATGGGGAGACA

TCAGCCATCTTCGAGATATAGGAGTACTTGACAATGCTTTTGGTGGTGACAGAACATCTG

GGTAGCTTACTCAGAATCCGATGCGCCCAGTAGTGTTTATCGTAGGTCTGGTATTTATCA

TTTAAACATGTGAACTCGTGGACTTTTTAGACCTTTATGAGCTCATAACCCGTCCATAAT

TTCTGAACAACTGGCTTGATAATAATCTTCTTATAATTATTGAATCATGTTGCAAATTTG

GTCAAATTTGGTCTAAAGTTATTAATATTGGATTTTGTTCTGAGCACGATCCATTTGAGT

TCTAATCTTGTGAA

>Unigene0002115

TCAGCTGCAGCATCAGACTAGCGCGTGTATTACCGGAACCAACCAAAAGAGCCGCAGGCC

TTTCCCAATAAACTATTCCCCTCCCAACTCTCATATGCAGCCCATCTCCTCTTCCCCTCC

ATCCCCAAATCCTACATAAGCCCATCCTTTATAGCAAAGCCATCTCTGCAACCCTGCAAG

TATTGAGTTCCCATTCTCAGGGAAAGCTCTGCCATTAGATCAAAAACCATCTTGAGAGAA

ACTCTCTCGATGGACAATTTGGGCTGGGAGAACACACAAATTCAGCAGTGCTTATGGAGC

AATCAGCTCAACAGTATATGGGAAGCAAACACCAATCATCCAAGTTCACCAATCATCCAA

ACAGAACTCGATGGAGCGCTGCTGAGTAGGGGCCTAGAGCTGCAGGGGATTCAATCATGC

GAGACGACTTCAAATTTCGATAGCCAGGGCCAAATGAGTGAAATGCTAGCTCCGGCTTTC

GACTCGATGTACAACAGTTTGGAGCTGGAGTTGCTTCAGCGACAAGAGCTAATGAGATTG

GCAGCAGCATCGGAGAATTATGCAGCTTCCGATGTTGCGTCCACTCCATATATAGCCTCA

ATTGGATTGCATGAATTCAACAGCAATGAAGTAGAAGGATATTACTCGAACCCAGACGAG

TCCGCATCACAAGGATCATCAAATCCTATCCCGGTGTCAAGAGCTGAGCTTAACAACAAG

CGAAAGTTGGAGGATTATGCTAGTTCTTCAACTTCGGAGCTAGGGTTTCAAATCAACTTC

GAGAATCCATCTAATTTCAAGAAATCGAGATCGGTAGCTGAAGCATCGTGCATCGATTTC

CGAAAGGAGAGCTCCATCTACGAGCCCGATACGGAGGCGATTGCGCAGGTGAAGGAGATG

ATCTATCGCGCTGCGGCTCTGAGACCAGTGAGCTTAGCTGAAGAAGTCACAGTGGAAGCA

AAGCCTAAAAGGAAGAATGTGAAGATATCGAGTGAGCCGCAGACAGTCGCTGCGAGGCAT

CGGAGGGAGAGGATAAGTGAGAGGTTGAGGGTGCTTCAGAAACTGGTTCCTGGTGGGAAC

AAGATGGACACTGCATCGATGCTTGACGAGGCTGCCAATTATCTCAAGTTCCTTAAATCT

CAGGTCAGTGTCCTAGAAACCCTAGGTACAAGACCTCCCACATATCAGTCTTTTCCCATG

CAGACCCTTTTCCCCAACAATTTTGATGCTCAAAGGTTGCATCAAACTTTCGATGAAACT

ATACCGAATATTTGATTTGTGGCATAAACACTTGCTCCACTTTATTGTTCATCGATTTCT

ACCGCAATTTCATGAGATTTGAGTCGACCTTTCAATGTAATGAGAATAATGCTAGACATC

GCAATTGGTACCCACATTTTGTATCATGGAGTACGAAAATCAGATGATTTCATTATTATT

GGGACCACAACAACAATGATATGAGTCACTTGAATTTGTATCTATTGTGATACCAAATTT

GCAATGTGTAGAATTATTCTTTGTATTACTTCTAAGCTGAAAAATAGTAGTTGTTGTGGG

AATTGATGAGCAATCTAGATGAGTAAGTAGTTTATCCAATTTCGGAGTATTTGTTATAGT

TTTATAGAAATTTGATGTGATCAAGTTGCATTGAGCTCTTTCCTCACCAAGAGCCCTAAT

TCAAGCCATCTCAACAAAAGTTGTAATGCAAATAAGCCCCTTAGCACTCGTCTTACAATC

ACCCAATTCTTATTTTGCCCTACTTTTCTCCCTAATCAAAACCCTAATTTCAAACATATG

AGATAAAAAAGTTTAACCTTTTGATGTTCACTTGGCCACCTTTTAACCATGCAAAAGCCT

CTTTCTATCCCCATAAAGCAGCTGTTCAAAGACTATCATCACCACCTTTAAACCCTAATC

CCTTTTCAACAGTCCTCTTCCCCATGCAAACCCTAATTCAAAGAAAATCCTCATCATAGT

CTCATGATGGCAAACACCTCTGAACTGCAGCAGTGCAAAAGCTATCCACAGAGAGAAAGT

TGGAGGAAAGGAATCATTCAGCCATTGTTAAGAGTGCTTCAGAGCTTGCTTTCTAGGGTT

TTGCTGTGAGTTTGAGGATCTCAGTGCTGAAAATAGAATATTTTAAGGCACCCAATGTGA

GGGTCGACATTCCCTTTGGGGATTGTGGGTGTGTTGCAGTAGCCACTGTTCCCTTCATTC

GAGCCACGCGTGCCATTGGGAAGGCCACCCCTACATGTTGGTTAGCACGTGTGGTGAAAG

AAAGGATTCTCTCACTCTCTCACCTGAAGGATGAGGTGGGTTCTTGGTTTCAATGTAACA

AACTTTGTGTTTGATTGGATGAGGATGATGTGAGATGGCAAGTGGCCATGGTGTCTGTTG

GGAACTTTGTATTCTTTTTTT

>Unigene0002116

CCGCCAAGCAACAGAGCTTGCGCCGCCAAACGGGAAACGAAAAGAGAGCGAATTGGGAAT

CGGGAAACTTGGATCGCCGGCCGCCATGGAGATGTCACAAGAGGACTTCGATCGCATCCT

CTCCTACGACCAAGCTCGCCGCGATTCCGAGGCGCTGTACGCCGTCAATCCCGACGACGC

CGACAATCTCACCCGATGGGGGGAGGCGCTTCTCGAGCTATCACATGTCCAAGACGCCGA

CTGTTGCGTGGAGATGATAGAAGATGCTGTTTCGAAGCTGGAGAAGGCATTGCTGGTGGA

CCCGACAAAGCACGAGACGCTCTGGTGTCTCGGGAACGCACACACCTCCCGCGGGTTCTG

CACGGCGGAACATGATCTGGCCAAAGTCTATTTCGGATTTGCTGCTCAATGCTACGAGAA

TGCGTTGCAGGAGGATCCTGGAAACAAGATGTATGCCAAGTCATTGGAAATGGCTCTTAA

GGGACCAGAGCTGCATCTAGAGCTCCAGAGACAATCAAGAAGTCAGCAGGGTTCTGTTGG

TGGATCATCCGTATCAAATGCCAAGGAATCGAAGGAGCAAAGTGACGACAGCCTCATGTA

TGACATTTTCGGATGGGTAATTCTTGCAGTTGGATTAATTGCCTGTGTTGCGATGTCCAG

TTCTCAGGCCCCTCCACCCTCCCCCAGGTGAGAGATTATCAAAAGATTATTGGATTGGAT

GAAGTTTTGGTGGCAGCATTTGGAGAAAAACTGCTGCAATACAGGACCCTTTATTTAGAA

ATCTTTTCTATTAGATTGATTAGTCCTTGCAATTTATTTTGTTGAAATTTATAATTTATA

ATGGACACAAGCTTATAGTTTTGGTTTCTAGACAGTGTTTGTTGACAATAAGTTGGTAGA

ATTAAACATT

>Unigene0002117

CGGAGTGCCTCCCGATCCCTCCTCTCCCCCCGAGGAGGCGCTGCCCGCGTCAAGGTCTCC

GCCCGCGCCGCCGCAGCTGCCGAATCCGTGGATTCGGCCTACTATGCGGGGCTCAAGAGG

TGCTTGGAGGATCCGTACGATCCTGTGTCGAATCCCGACGGTGTTATTCAGCTCGGATTG

GCGGAGAATCAGTTATCTCTGGATCTGATCGGCGAGTGGCTTACGGCGGCGGATTTGAAG

GGATTATTGGTGGACGGGGAGCAGAGTGGAGGCGGGGTTGGGATCGGCGGCATTGTGACA

TATCAACCACATGATGGGCTGATGGATTTGAAGATTGCTTTGGCTGGTTTCATGAGTCGA

GTCATGCGGGGGTCAGTGTCATTTGAGCCGTCGCAGATCATATTACTGGCCGGTGCGACT

CCTGCAATTGAGACATTAAGCTTCTGTCTAGCAGACCCTGAAAATGCATTCCTTGTTCCT

TCACCATATTACTCGGGATTTGACAGGGATATCAAGTTGCGGACAGGTATAGAGTTGATA

CCTGTTCCATGTCGAAGTACCGACAACTTCAGCCTAAACATTGATGCTCTTAAAAGAGCA

TATGAACAGGCTAAGAAGCGAGGCGTGACAGTCCGTGCTGTTCTTATCACAAACCCCTCC

AACCCTTTGGGCAGTATACTGAACCGGGATAAACTGTACGATCTTCTGGACTTTGTCACA

GAGAAGAACATCCATCTGATTTCCGATGAAATATATGCTGGTTCAGTCTACGGAAGTGAT

GAGTTTGTGAGCATCGCAGAGGTTGTAGAGACGGAAAACTTTGACAGAAACAGGGTCCAC

ATAATATACGGCCTGTCAAAAGACCTCTCTCTTCCCGGCTTTCGGGTGGGAGCTATCTAC

TCCTACAACGAAAGTGTCCTGGCAGCCGCTTCGAAATTCGCAAGGTTCTCTTCAGTATCG

GTTCCTACGCAACGCCTGCTTGTGTCTATGCTCACAGATACCAAATTCATCACTAAATAT

ACAAAGACTAACAATGCGAGGCTCCAAAGAATGTATGGATTATTTGTGGAAGGGCTGAAT

CAGCTAGGCATCGACTGTGTTAGTAGCAGTGGGGGTTTTTACTGTTGGGCAGATATGAGC

AAGTTTATACGGTCCTACAGTGAGAAAGGGGAGCTTGAGCTATGGGATAAACTTCTTAAC

ATAAGTAAGATAAACATAACACCGGGATCATCGTGTCACTGTATTGAACCTGGATGGTTC

AGGTGCTGTTTTACAACACTGACCGAGACGGATGTGCCTATAGTCATTGAGCGGATCAGG

AGAGTCACTGAAAAGTAATTAAGTTCATCATTAGGTGCAACTTATATAGTCGCAAACAAC

ATCTTTTGACATTAAGCTGAAAACAGGCAGGTTGCTGTTGGCATTGGAAAGGTACAAATT

GACATAACACTGAAATATCCATTCTACATTCTAAGATGACTTGGCTGAAGCGAGCATTGT

GCCTTCAGAATTCAGAGGAGTCCCTGCCAACTCCTTGGCTGCTGTTGAGAAATGCAATAT

GTTTACTGTTGATGGTTTTGAAGAAAAGGCAGAGAAGTTGCTGGCTCTGAAGGAAAAGCT

AAATAGATTCTTCTGAATGAACTCTCGCAGTCTCTGGGAAAAAGAATAAGCATGCATAAT

GTGAGCATCATATCCAATAGTAGGCAGTTATGTAACTAGAAATTGCTGGTGCTTGTTGAA

ACTCTTGTTCTGGAGTATTACTATGTTCTCTCCTTACTAGATTGTATCCATGGTTTGTGT

GAAAGTTGTCGAAGCAGAGTTTTATGTGTTAGATGATATCAATCACTCTGTTCCGATTTT

GTTCTATCCAGATGGCGACTCATGAACATTTATTTCAAGTCTGTCATGCAAAATTCCGCA

ACACAACAAATAATGTACGTGCATGCTTGTAAGTCCTATTTTATTTAT

>Unigene0002118

CCAGCCAACACAGGTTCATAGTTAAAATATACCAGCTTTGATCTTTAGACCTATTTGAAA

CATGGTGTATGTTTTACAGTCGTGAAATGAGAATTTATCTGAGATTTATGAGTTTAATCG

GTGGAATTCCTGAACAGAATTTGTCAGCTAGGTGATGATGCGTATTTTTGGATGTCGTTT

TAATTAATGGGACTTGAAGTTTGATATGTTTGTACTTTGTTAATTTTGTGAGGTGTTTTT

TGGAGGCATGAATCAGTCTTTAGGTATCAGGATTTAGAGATCGATGAAATATTGGAGGTC

ATTTTCTCTAGTGGTATTTTGGTAATCTCGGTGCTCTCTACTGTTGTTTTGATAATTGTG

CTTCAGGTTAGCTCTTCGGGTTGGTTGCTCTATCAAGCATGTAGGCTATTACTATGGAAG

GTAGGTAAAGCTGGAAGCCAAGTTCGCTAAAGTTGGGAGGACATTTTCAAGTTTTGGCTA

TTACATTTTTTATGAGTTAATTCATGTATACGCTGCCACCCATCCCAATCTGATTGTCTT

GTGTGTTTTATCTGGCAGACATTGAGGAGCTAATCCCAT

>Unigene0002119

TGTGCTAATTCATAACGAACGGTTGCCTCTTGGTGCCCGCTTGCCCGGATGTAAGCATTT

CTCTTGGTTGGAAGGGTTTTGCGGGGAGACTTGCTGGAATTAGGCACAGTGTTCTGCCCG

CTTGATCGGGCATTGGTCTTGGTGGATTTGGATGATGCTTTTGGGGCCCGGCCCATCAGA

AGGCCACGTGCTAGAAGCGTGACCGTAGATTTCTGACAGGACACATCAAGTTGATTACTG

TTTTATGATTTTATTTTCTCCGCCTTCCATATATGTGCTAATTCATAATGTTCATAACAA

CTATTGTGATGAGATACAAGGAGTTTTTTATTTTAACTATCTTACATTTACTCTTTTGTA

AGCGTAATAAAATAAGCAAACTGGAGGATGCTGTAGGCGTGCACAAATTTTCAGCTGTCC

AAACATGCATCAATATCCATCCAATGTGCGTGAAGACGGCACCTCGCTGAACGAGTTCTT

CCTAGGCTTAAATTGATGTTTGAACTATGCTGTGAAAATATTGGTGGGAGGAGAAAATTA

TTTCTTTGTAGACTTTGAAAATATTGTTGTGCCAATAACTTCATTCCAACAAAAAAGAGC

TCAATGTTAATGAAACTAGCAGTGTAACATTTGATTTTCTTCTTCAAGTTGTGTTATCAC

TTGAACTGTCCTAAATTAATATCACATTCCATTTTT

>Unigene0002120

GAGAGGTAGTTTAGAGCTTGTGTAGTGTCCAAAAGTTATACTTGGACCTCTGAAGAGGGT

TTATATAGATGTTCGAGTTGATCATGGGGCCTAACCCGGGATTCATGCACATGGTGGTGG

CTTGTTGTCATGCTGCAAATACTAGCGTGGTTGGAGGATGCGTGGCCTGAGGAGGTTGGG

AGTCATGCACATTTAATGTTGCATGTGTCCCCAGGAAAATTGAGAGTTGTGTGCAGCTAG

TGGGCCGATCATTGGGCGGTGTATTTAATGCAGCATGTGCTCTTGGAGAGATTGGGTGGT

ATGCAAATTTAATGTGGCATGTGGAGATCTGGAATTGTGTTAGACTCGTCGGACCTATGT

GAAGGTGTGGCAATGAACAAGTCCAAGCATGTGCCTAAAAGGTCGGATGTTGTTTGCGAC

CAGGCTTGGTGGTGGTTGGTCGCAGGCTTGATTGCGTGTTGCTCTCTTGTGATGCCTTAC

TCGGAGATTGGCCTTGAGTGGATGTGTCTTGGGTTGGGATTTTGCTTGAGCGAAAGTCTG

ATCGTTCGGGTCATGTGGGTGTGCCCGCTTGATTGGGTTCTCTTGGTGCTTGCTTTCCCT

GATGTAAGCCTTCTTACTTGGAGGGGCTTTGTAAGGAGACTTGCTCGACTTGGGCGTAAT

GTTTTGCCCTCTTGATCGGATAGGGCCTTGGTGGATTTGGATGATGCTCTTAGGGCTTGG

TCCATCAGAAGGTCATGTGCTAGGAGCGTGACCGAGGATTTTTGGTCATGTACATCAGGT

AACTACATGCTATTTTTCTATTTTTTTAATATCTTAATGATTGCAATGATGAATTTGGGA

AGTATATTGATTGGGGGCATTTTGTG

>Unigene0002121

GATTCTTGTCGAGTAAACAAACTCATCTCAATAGAGCATCAGATACCAAAAAGCGCCACC

GCACTGAGCTTTCGCGCCGCCGTCCACACTTCGCTCGGCGGAGATCGGATTATGGAGGAG

AAGCCGCAGCCCGTGCCCGTGCTCTACTGCGGCGTGTTCGGCCTCCCGGTGGAGTACTGC

GATTTCGGCCCCGATTTTGAGAAATGCATGCCCTGCCGCCACGCCCCGACCTCTACCCCT

ATCTCGTCAAAGGTGAGCCTTCGATGTCTCTCCCGCATCTCGATCGGTGCTTATGATTTT

GTGATCGAGATGCGAACGAGGGCGATGCTGACAAGGTGCTGTGTCTGTTCTTTATTGTTT

TCCTAAAAAAAAAAAAAGAAAAAGGAAAAGAAAAAGAAAAATTCAAGAATGAACACAGTT

GACTTGATCCATTAGTAACACACAAGAATAGATGATCTGCTCCAAAAACACTGAAAAATT

AGGTTTCCTCCCTCCACGAGGAAACCTTCAGCTGTTACCTGTTGAATTCGTTGAATTGCA

AATGAATGGTGGTTACTTCTGTGACAACTGCCGTCGGAGGAGACATTTTGTTCCGGCACT

GAAACCACCTGTCGTCTCAACGATGAAAGAAATGAAAAAGAGATTACGTTCATTGAAACC

ACCTGTCGTCTCAACGATGAAAGAAATGAAAAAGAGATTAC

>Unigene0002122

TACTCCTCTCCTCTCCCTATCTCGTGCCTTTGTTTCCCACTGTCTTCTCCCTTGCTGTGC

TCGCCCCCTCCCTCCTCTCAGGGCGTTTATCAACCGGAGGCGACGAAAGAGATGGGTGGA

GTGACGTCGACCATTGCGGCAAAGCTCGCCTTCTTCCCACCGACCCCACCATCGTACACC

ATCGCCATTGACCGGCGGAACGGGAGGCTCGTGATGCCCGAGGTCCACCTGTCAACGCAC

GAGGACGTTGCCGTCCTCCAGCTTCGTGCCCGGACGGGAAACGACATCGTCGCCGCTTAC

GTGCGGCATCCTCGGGCCACCATCACCCTTCTCTATTCCCATGGCAACGCCGCAGACCTC

GGCCAGATGCTCGGCCTCTTCGTCCAGCTCAGCTCTCGGCTCGGTCTCAATCTCATGGGG

TATGACTACTCTGGTTATGGGCAATCCACAGGGAAGCCATCTGAGGATAGTACGTATGCT

GACATAGAGACAGCATATCAATGCCTCAAGGAAGATTATGGAGCAAGAGAGGAAGATATC

GTTTTATATGGTCAGTCTGTTGGAAGTGGTCCAACTGTTGATCTGGCCTCCCGTTTACCG

AATTTGAGAGCTGTGATTTTACATAGCCCCATCCTTTCTGGACTAAGAGTGCTATATCCA

GTCAAACAAACATATTGGTTCGACATTTTCAAGAACATTGACAAAATTGGCCTGGTTTCA

TGTCCGATTTTAGTCATTCATGTTTTGCGAAATCTCCAAAGCCGCCAGATTACAAGTGAC

AGATTCATATCGTTATCCTTCTCACTCCTTATCCTCCATGCAGTTTGTTGGTGCAAATTA

CTTCCTAATTGATTCCAGGGTACATCAGATGAAGTCGTTGATTATTCCCATGGAAAGCAA

CTCTGGGAACTTAGCAAAGTTAAGTTCCAACCACTTTGGCTGGAGGGCGGTGGTCACTGC

AATCTTGAGATGTACTCCGAGTACATGAAACACCTGAAGAAATTTATTTCAGCCATTACT

GAAGGAAGGGCTGCCAGTTGAGGAATGACGGCAGTCGAACTGAAAATGAAAGAAAGAAAG

AAAGCTGAGCAATTACAGGAACCATATGGCTGTAGATACAGAAGTGATAAGTGCCAAATA

GCCGTGACAAATAGAATTCGTACGTCATGTTCATACTGTTGAGGTATATATAATCTTGGT

GACAAATTCCAAATTATCCAGAAAGATGGAAATTGTACTTCTTAAGGGACTGGTCTTTTG

CTGGGAAGCAGAACCAAAAACTTTGTTGCATCTTCTGAGTGAATGGATTGGTTAGTTTTC

AAA

>Unigene0002123

GGAAACGACATCGTCGCCGCTTATGTGCGGCATCCTCGGGCCGCCAGCACTCTTCTCTAT

TCCCATGGCAACGCCGCAGACCTCGGCCAGATGCTCGGCCTCTTCGTCCAGCTCAGCTTT

CGGCTCGGTGTCAATCTCATGGGGTAATTTCCTCTTTCTATATCGATTACTCTACCTTCT

TAAATGTTCCCAAATTGATTGATCTGGTATTCAATTATATTGTCAAGGTGATGGCTGCCT

TTTCTCTTG

>Unigene0002124

CCTCTCATTAAGTCGTGGATGAAGCCCTTTGCCAAGACTGCATTGACCGGTCAAAATGGA

CAATTGCATGTAAGGTTCGCCTACTTGATGAATGAAAGAACATGAGAAAGGCTTCTCAAA

AAGGAAAAATTCCACGAAAGCCCACTACAGTACAGTTGAAGTCAGCGACTTAGTTAGCCT

AGCCTCGCCATTTCTTTTTAGAAAGCGAAGACGACGACTTAGATAAAAG

>Unigene0002125

ATTGCTCGGTGGAGTATCCTTGATGAAGGATTCAGTTTTGATCATTGTAGCATGTTCGGT

AGTATGGGATGCTTGTGTGCTCTTGTGCAACGTTGCAGGTATCCAAACGGATGGAATGTT

ATGTGTCATTGTGCTAGTTCGGGTCATGAATCGTTTCATGAGAGGGCTGATCCGTGGTGT

GTGCAGTGGTATGCACGTGTGCGCATCATGGATGCTACACAAGGGCCAGGAGTTCAAAAA

TCCAAGGATGATTCGCCGTCCCCCAGGACACAATGCCCAACAAATAGGTCC

>Unigene0002126

TTTTAACACAAAACAAGGATGGAAGTGTCTTATATACATACTTGTTGTTTTCTTTTCCTA

TGTAGTATGGCGTTAGCCTAAGAGGTTATGCAATTCCTTACTCTTTTAGAGTAAGCATTA

TGCAATTCGTCACTTATAGATAAAACAAACGACAATATCTATATATGTCACTTCCGTACT

CTGTCTGACTAAAACCGTTGCGGCCTTTACCGTTCATGGTAAGTAACAGGATTCGTTAAT

GCACCATCTTCAAAACTAGGGATACGGAGTTTTTCGAGAAAAGGTCCCATCCTTCAATAT

CATGATTGGGTCGACCGGGTCAGGCCCGATCATGAGAGAATAGAAAATTTAAAACGTACA

TTGCTGTTCCAGCGGAAATACTTGGTTTAATTCTACCACTTCTACTAGGAGTAGCCTTTT

TAGTGCTAGCTGAACGTAAAGTAATGGCTTTTGTGCAACGTCGAAAGGGTCCTGATGTAG

TGGGATCGTTCGGATTGTTACAACCTATAGCAGATGGTTTGAAATTGATTCTAAAAGAAC

CTATTTCACCAAGTAGTGCTAATTTCTTCCTTTTTCGAATGGCTCCAGTGGCTACATTTA

TGTTAAGTCTGGTCGCTTGGGCCGTTGTACCTTTTGATTATGGTATGGTATTGTCAGATT

TGAACATAGGGCTACTTTATTTGTTTGCCATATCTTCGCTAGGTGTTTATGGAATTATTA

TAGCAGGTTGGTCTAGTAATTAGGGGGGCGGCCGTTCGGTCGCCTATGATAGACGGACCA

ATTGGTCAAAAATGGGTTTGTGCCGCAGGTGTTGAACGATCTACTCTACACAGGTGTGTG

GGCTTACAGGGCTAGGGCTTTCACCGCCTTTCTTTCATTCATCGGCCCTCTAGTCGGTCA

CTTTTCCGGGCCTAAGTTAAGTGGAAGTCATAAAAAATAGATGTTTCTCTTCGCACCTAA

GATCAAGAGGAAAGGTAGCTTGTCAAGCTGGCCTAGATATTAAAAATTATTTCATCTTCG

ATTCCTGCTCTGCTGTTCTTATTTGTCAATGCTACTAAGGTAAGGTTCGCCTACTTGATG

AATGAAAGAACATGAGAAAGGCTTCTCAAAAAGGAAAAAGGCCACGAAAGCCCACTACAG

TACAGTCGAAGTCAGCGGCTGAGTTAGCCTAGCCTCGCCATTTCTTTTTAGAAAGCGAAG

ACGACGACTTAGATAAAAGAGCGTCGGTGCGTAGTTCCACTACACAAAGTAACTTAGCTC

GACTAACAGAGAGGCGAGGGGGAAGACCCTACATAGAAGAACTGCTGTTCGCTTATTAAG

GTATAACCTTTCGCTCCCCGGGGCAAAGCCGCTTCGCACCACTGATAGACTACTAAAAAT

GAAATTCCAAAGGCGTTTGTTTCGCAAGCCTTTGGAATTGTCAGTAAACTAAGTAGGGCG

AACAGCCCCCTGCGAATCCGTAACTCTGAGAGCATGCCGCAAAAAAAGGATGGTCCCCTA

TGCATTGAATTCTTTACGGAACGGAAAGAATTCAAGTACCTTACCCCCATCATGGTGAAC

CTCTCCTTGTGATCGGGATGAGGTAGATGCCTCCCAGCCGGGGGCGGATCGAATCGGAGT

TTCCTTAGGTAGCCACCGACCTACAGTTATCCTTAAACTTCAGTGCTTGGTGGAGAAGAA

GCGAACAAAGGTACGCTCGCTTGCTGTCTTGTTCTCTGCCGCGGACTGGGATCGCTCGCC

AGCTAGGCCCTCTCGAAGAAAGTTGGAGCAACATTGTATGAGAGAGAGCATATTACCCAT

CTTCGGGGACAAGGGGCGGAACGACCTCTCGATCTACTTACTGCAGCCCAGGACGGGCGT

CGTCGTCTAGGCGTTCCCTGTTGCTCTCCCCTACGCCTAGGACGTCGTCTGGGACAAGAG

CCATAGTTAGTTGCTGTTTCCATTTGGTTTTTTCTCGTTGTTGATACCGGCAAGACCCAG

CCAGATGATGATGTCTGCTGGTTGGTAGTGAGAGGACTCTTAGTACCCGCAAAAAAGGGA

CGCTACAATCATTTGGAATTTAGTTTCTTGCTCTCCCTTAGCAGCGGGAAAGGAGTCTAT

CTATCTGCCTAGCTTTGGTCGATTTCCCCCAACGCAATTCAAAAACGGAAATTCCACGAA

TCCCTGAGGTGGATAAGTCCGACGACTCAGCAGCAGTGCGGAATTGACTTTATCGTCCGG

TGGATCTGATCTAGAGTTAGGGGGCAATACATACCAAAAGGTTCGAAGAAGAAGAAGAAG

GACCGCGCATAAGAGTATATAACCAACCCACCTCACTCCGCTCGCCTATTTATTCACATT

CGTGAAGCGGCTGGATGCATAAGGGAAGTCACGAGCACAGAAGAGCTTTATAGTATGCCC

TAAAGGGAAAGTCTCTCCTTCAGCTAGAATGTCAGGAAATTTAAAGAAGCGCGGGGTCAA

ATGGCATCGAAGATAGTCGTCATCGACGAGAAAGTGTCGGACTTGGCGAACGAGCTATTG

CCGCGGGAGAGGATCACGGGCCAAGAACGTAAATTAAATGATATCTATACATTGTATCTT

ATGAGTCACTAAAGTGACTCCCCTCATTCCCTTTTATGCTCGACATCAGGCAGTTGACTT

CGTCGCGATCACACTTGCGATACACCCGGGCGTTCCGAAAGCTGTACAAGATTTTGTTAT

TGTTGCGTAATGAAAGGGATCAAAGTGAAGTTATAGTAGCTCTGACTCATATACTTGCCC

ATGTCATGACGAGTCGTCGACAACATCGAGGCTTTTTCTCGACTCTCTTGTCCGTAGGCT

GTGGGATGTAAAGATCTTTAAGCCCGCTTCTAGATACATTATCTGCTTG

>Unigene0002127

CAATTCATCAGACAGAGTGGGCCCATTTCCAATTTCATCCCGAATAAACAAGAAAAAGAT

TCAATCTTTCGATCTCAGAGTCAAACCTAATCAAGTTTTGGGGAACCCTTTTGAGGTTTT

CCATACAAATTGCCCACCAAAATCTCAGATCAAGATTGGGTCTGTTGTATGAAAAGGTCT

GCAACACAGGGAGATGAGTAGCCGATACTACGAGTATGAAAAGCCTGCCCCAGGTAATGT

AAAGAAGTTAAAGGACCTATTGCTTAAAAGTGAGAATCACATCTGTGCAGATTGCAGCGC

TCCAGATCCCAAATGGGCGTCAGCTAATATTGGAGTATTTATATGCACAAAATGCAGTAG

CGCTCATAGACGACTCGGTACACACATCTCGAAGGTTTTATCAGTGACATTAGATGAATG

GTCTGATGATGAAATTGAATCTATGATTGAGGTCGGTGGCAATTCCTCTGCAAATTCAAT

CTATGAGGCCTATTTTCCTAATGGCATTGTGAAACCTAATCCAGACTCGAGCAACGAGGC

ACGCACAAAGTTTATCAGGTCTAAATATGAGCTGCAAGAATTTTTAAAACCAAGCTTACG

AATCATCTCTTCGAAAAGCTCTTTCAAATCTTGTAATTCAGGAAAGGATGCTGGTAGTGG

CTTCGACACAGACAGTTCAAATAAGACGACTAATGTTGGAAAAGCCAATGGCAAGTCATT

CAAGTCTCTTTTTTCTTTTTGGGACCAGAGCAGTCACATGGCATCTTCTATGTTCGAGGT

ATCTGCGGTTATCGGAAATTTGAAGGTCAAAGTGATCAGAGGCATAAACTTAGCGATCAG

GGATATGCTAACTAGTGATCCTTATGTTGTTTTGACTCTTGGGCAGCAGAAGGTCCAAAC

TACAATAATAAAAAGTAATTTGAATCCTATATGGAACGAGGAGCTTAAACTAGCGATTCC

TCAACGTTATGGTGCACTGAAACTGCAAGTGTACGACTACGACACATTCTCAGCAGACGA

CATAATGGGCGAGGCTGAGATCGACCTACAGCCGATGATAACCGCCGCTATGGCATTCGG

AGACGTCTTAATGTTGGGCAACATGCAGATCGGGAAGTGGTTGAAAACGAGCGACAACGC

ACTCATCAAGGACAGCACTGTCAACATCCTCGACGGGAAGGTGAAGCAGGAGGTGTTTCT

AAAGCTACAGAACGTGGAGTCGGGCGAGATCGAATTAGAGCTTGAATGGATCCCTCTTGA

CTGAGGGATATGATGGCGACTCCTCATTGTGATTTCTTGAGCCTATATTGAAATGTTTCT

TTTTTCTTTTTCTTCCCTTTGTTTCTGTATTGTAAAGAAAATGTAATGTGTGATTCTTTC

TACATAAGTTGGTACTTGAATATATGAGATTATTCATGTGATCCAGATTGTGAGATCAAC

TCGAATATCCTAGTGTTGCCAAAATATACTTGTGAATTATCTCGGGATTGTGCCAAATAT

GGTGATGGAATTTGAATTTTGTTTTAATCATTCGTATGA

>Unigene0002128

ATTGCAAGTGGTGGAATACAGAACCCTAGCTACCCTTTTCTCTATTCAGCTAAATCCCCA

TCGGAAACCATAACCCTCTTCCCCCTCCCACCATGGCCGCCCAGGGCATCCGACTCAACC

ACATCGCCCGAGAGACCGCCGACGTCAACCGCCTCGCCGCCTTCTACCAGGACGTCCTAG

GGTTCAGCCGCATCGAAACCCCCAATTTCGGCGATTTCGAAGTCGTCTGGCTCCAGCTCC

CGCCCTTCTTCTCCCTCCATCTGATCGAGCGGAACACGCAATCGAAGCTCCCCGAGGGCC

CGTACAGCTCAGCGGCGGCGGTGGCGGATCCTAAGGGGCTTGCGAGGGGGCACCATTTAG

CGTTCGGTGTGAGCAATTATGATGAATTTGTCAAGGGTTTGAAGGAGAGAGGGCTGGAGG

TGTTCGAGAAGACGCAGCCGGATGGGAAGACGAGGCAGGTCTTCTTCTTCGATCCCGATG

GGAATGGCCTGGAGGTAGGGAGCTGGGGTGGCCCTTAGCTTCTC

>Unigene0002129

TAGGAGCTCCAGCTACAGGCACAGCTTTATGTATTACGGTTTAAGCAGAACTTGTTATGT

TATAAGCTTTGTTGAACTATCTTATGTTTAGTTGTTGGTAATTTGTGGGCCTTTAGTCCC

ACGTGGATGATAGTTGTAGTCCCATGTGGATGATAGTTGTAGGTACATACTTTTATAGGT

GATAGTCCTTAAAAGATCAATAATTGTAAGTGGGCTTAATAGGGGGTTTTATGAGAACTC

TTTTTAGACCTATTAGTGAGCTATGATGTATATTAAATAATCATAGATTGGCATGGGGCT

CTGGAGACCTAGTTAAAATTCGGAATATATTCC

>Unigene0002130

AAGACGAGGCAGGTCTTCTTCTTCGATCCCGATGGGAATGGCCTGGAGGTAGGGAGCTGG

GGTGGCCCTTAGCTTCTCAGAGAAAGCGGAACTGGTATGTATGCATAAATAATGGGGAGC

GGTGTTATGTTTCTAGGAGCTCCAGCTACAGGCACAGCTTTATGTATTACGGTTTAAGCA

GAACTTGTTATGTTATAAGCGTTGTTGAACTATCTTATGTTAAGTTGTTGGTAATTTGTG

GGCCTTTAGTCCCACGTGGATGATAGTTGTAGGTACACACTTTTATAAGTGATAGTCCTT

AAAAAATCAATAATTGGAAGTGGGCTTA

>Unigene0002131

CTTCCTTCAGTCGTGTATCGTGCAATTATTACATTGGAGCCCAACCTCGTGGTAGATTGT

GTTTCACTTAGAGATGGTAAGAATCGAGCTTTGATTGCTAAAATCGTCAATGGAAACTGA

AGACTCTTGAGTGTTAAGAGGCGTGTCTTTGATCGTTAAAACCGTCAATGAAAACTGGAG

ACTTTTGAGCGTTAAGAGACATATCTCAGATGGGACTGATAGACTTGTCAACTC

>Unigene0002132

CGGAGATTGAGCTCGATGGGGGGAAGATGTATAGGCAGCGGGGGTGCTGGGAGGATATTT

GCCATGCGATTCTCGATGCACATCGGTTGATTTATATTGTCGGGTGGTCGATTTATGACA

AAGTAAGGCTGGTGAGGGAGGCGGCGCTGCCGAAGGGGGTGCCGGACACGTTGGGGGAAC

TGCTCAAGTACAAGTCTGAGGAGGGGGTGAGGGTGTGCTTGCTTGTTTGGGATGATAAGA

CCTCGCATGATAAATTGTTCCTCAAGACGAGAGGAGTAATGCAAACTCATGATGAAGAAA

CTAGGAAGTTTTTCAAGCATTCTTCTGTAATTTGTGCTATGTCACCTCGATACGGGAGCA

ACAAGCTTAGCATTTTCAAGCAACAGGTCGTGGGGACCCTATTTACTCATCATCAGAAGT

GTCTACTGGTTGATACCCCAACTTCTTCAAACATGCGAAAGATTACTGCTTTTATTGGAG

GTCTTGACCTTTGTGATGGCCGTTATGATACACCACAACACAGACTTTTTCGTGATCTCA

ATACTGTCTTTTTAAATGATATTCATAATCCCACATTTCCTGGAGGAACTAAAGGTCCAA

GACAACCATGGCATGATTTGCATTGCAAAATCGAAGGTCCTGCTGCATATGATGTCCTTA

AGAATTTTGAGCAGCGTTGGCGTAAGGCAACAAGACATTGGGCATTTAAACAAGGATTTA

AAAGAGTATCACATTGGCGAGATGATGCCCTAATTAAACTAGATCGCATCTCGTGGATAG

TCACTCCTTCAGCGGCTGTTCCACCTGATGATCCGAGCATACGTGTCTCCAAAGAAGAAG

ATTCTGAAAACTGGAATGTTCAGGTTTTCCGCTCTATCGACTCTGGTTCTGTAAAAGGAT

TTCCCACACTTGTTCATGATGCTGAGGCAGAGAACCTTGTTTGTGGAAAGGATGTGGTAA

TTGATAAAAGCATTCACACAGCTTATGTAAAAGCAATCAGATCTGCCCAAGACTTCATAT

ATATCGAAAACCAGTACTTTATTGGTTCTTCTTATGGCTGGCCCTCATACAAAAGTGCAG

AAGCTGGTAATCTTATTCCCATGGAACTGACCCTAAAGATTGTTAGTAAAATTAAAGCCA

GAAAGAGATTTGCTGTGTACATAGTCATACCAATGTGGCCTGAAGGACCGCCCACTGCTG

CCCCTACACAAGAAATTCTCTTTTGGCAGGGTCAAACAATGAAAATGATGTATGAAATCA

TTGGGAAAGAGCTTCAATCCATGAAATTTGAGAATGCACATCCACGGGATTACCTAAATT

TCTATTGTCTTGGAAATCGAGAAGAACTACCCAAGAATGTGTTGCAGCCAATGGATTTAC

CGTCAGATAAAAGTACAGAGGTTTTGTCTCAAAAGTACCGGAGATTCATGATTTATGTTC

ATTCAAAAGGTATGATAGTAGATGATGAGTATGTGCTTTTGGGGTCTGCTAATATTAATC

AGAGATCGCTAGATGGTTCAAGGGATACTGAAATAGCCATGGGAGCATACCAACCTCATC

ACACATGGGCGAAGAAACAGAGACATCCACATGGCCAGGTATATGGATACAGGATGTCAT

TGTGGGCAGAACATCTTGGGTCGGTGGATCCTCGGATCAAAGAGCCCCACACTTTAGAGT

GTGTGAGCTTTGTGAATCAGATCGCTGAAGACAACTGGTCCAGGTACACAACTGACAGAG

TTATTTCGTTGAACGGACACCTTATCAAGTATCCAATCATGGTGGATATCGACGGGAAGG

TGACCGCCCTGCCTGGCCATGAGACATTTCCTGATGTTGGTGGCAAGGTTTTAGGAGAGC

ATTCTACTGCCCTTCCTGATGAGCTGACAATGTAGCATACATAATCTGTTAAGTTATACA

TTTTATTCAACATTGTAGAGTTATATTATTTCCTTCCTTCAGTCGTGTATCGTGCTATTA

TTACATTGGAGCCCAACCTCGTGGTAGATTGTGTTTCACTTAGAGATGGTAAGAATCGAG

CTTTGATTGCTAAAATCGTCAATGGAAACTGAAGACTCTTGAGTGTTAAGAGGCGTGTCT

TTGATCGTTAAAACCGTCAATGAAAACTGGAGACTTTTGAGCGTTAAGAGACATATCTCA

GATGGGACTGATAGACTTGTCAACTC

>Unigene0002133

TAGTGAAAGCTCTTCTAACCAAGAAACCTAGAGTTTTGACCAAGACTGATAAGAAAGGGC

AAACTGAACTTCATATGGCAGTTAAGGGAAGGAGTCCAGAGATAGTTGAGGTGATTCTTG

GATGTGAAATCAGTAAGGTTAACATGGTGGATACCAAGGGCAACACAGCATTGCATATAG

CAGCTAGGAAGGGCCGGACAAAATAAACCTCATCTATCGAGACTGGATCGATAACTCATT

CTTCACTCACTTCCCTATAAAGAATGTTGGAGTTTAGTTACAAGCAAAGGAGTCCAAGGG

ACCAGATCAATTGAAATAGGCAGGAAGCTCGAGTGAGGTACATCAATGGGCATTACTCTC

TCTAGCCATGGGCTTGTTCGGTGGCAACACAGATATCTGTGCAGTGAGGCAAGGCTATGA

GCAGCTCGAGATCAAGAAGCGTCAGGGCATGGCACAAGGTTAGAGAAGGGCAAGG

>Unigene0002134

ATCTCGGATCTGACCTCTAATATGTGTAAACCGTGTAAATACTGCCCTTGCTGCTGCACT

TGCTCAGATTGTGTGGAGGTGCTTGAATGGATCACAGATCAGTAGAATGTGATGTCTTCT

GATATTGGAGAACACGACTGTCGAATACCGAATTCAAACCGCAAAAATGATTAGAACTGA

GTAGTGTTGTTCTTAGATATATCTTTCTGACAAACTATTTCAGTTTTAATCGTTTACGGG

GACATTCTCTAAATCTTGTATATACATCCAGGGAAATTAAATTTTCACTTTAGTGTTGAA

T

>Unigene0002135

TTGAGAGAGTTGCTGTCGAAGCAGAATCAGGCCGGGGAGACTGCTCTGTTTGTTGCTGCA

GAGTACGGGTATGTTGATTTGGTGAATGAGATGATTATGTATCATGAGCCGGAAACTGCT

GCTATAAGGGCCAAGAATGGGTATGATGCTTTGCATATTGCTGCCAAGCAAGGGGATGTA

GATATCGTCAAAGAGCTTCTAAATTCCCTCCCTGAACTCTCCATGACTGTGGACTTATCA

AACACCACAGCATTACACACTGCTGCAGCACAAGGTCATATAGAGGTGGTAAATCTCCTC

TTAGAAGCTGATGGAAATCTGGCACTTATTGCGAGGAGCAATGGTAAGACAGCCCTGCAT

TCTGCTGCGAGGAATGGGCATTTGGAAGTAGTGAAAGCTCTTCTAACCAAGGAACCTGGA

GTTTTGACCAGAACCGATAAGAAAGGGCAAACTGGACTTCATATGGCCGTCAAGGGAACG

AGTCTAGAGCTAGTTGAGGTGATTCTCGGATGTGAACTCAATATGGTTAATATGGTTGAT

ACCAAGGGCAACACAGCACTGCATGTAGCAGCTAGGAAGGGCCGGACACAGATCGTAAAG

AGACTACTAGAGGTTGCGGAGATAGACACCAAAGCCATCAATAAATCTGGAGAGACTGCT

CTCGATGCTGCTGAGAAAATGGGAAACTCAGATGTGGCTTTAGTATTAGTGGAGCATGGT

GTCCCGAGCGGAAGGACCATCAGACCCCCTAATCCCGTCCGTGAACTAAAACAGACAGTC

AGTGACATAAAGCATGATGTCCACACTCAACTTGAACACACCAATCAAACTCGTATGCGA

GTCCAAGGGATCGCAAAAAGGCTCAGCAAGCTCCATGCCGAGGGACTCAACAATGCAATC

AACTCCACCACTGTAGTTGCAGTTCTCATTGCCACCAGTGCATTTGCAGCCATCTTCACT

GTTCCAGGCCAATACCTTGATTCCGAGGACCTCGCCCCAGGCCTCTCCTTGGGAGAAGCA

TATATCTCAAGTGACATACCTTTCATGATCTTCTTCGTCTTTGATTCAGTGGCTCTCTTC

ATATCCTTAGCGGTTGTGGTTGTTCAGACTTCTCTGGTGGTTATCGAGAGCAAGGCAAAG

AAGAAGATGATGATGATTATCAACAAGTTTATGTGGCTTTGTTGTGTGCTGATTAGTGTT

GCATTTCTTGCTCTTTCTTTCATTGTAGTTGGGGATCAAGAGAAGTGGTTGGCTATTGGA

GTGACGATTATCGGGACTGTGATACTATC

>Unigene0002136

GCATCATGTTATATTGGGTGATTTTGCACAGGATGGAAGCTAAAAAAATACGGAACATCA

GACGAGCATCACTCAGCAGATCGAGGTCATGGTCTGTCTCTCATGTTTCGGATGTGGATT

TGTTGAACAATAAGCGAATGTATGCAATTTGAGATCGATGGTAGCTCTCTGTCTGCTGCA

TCTCGGATCTGACCTCTAATATATGCTAACCGTGTAAACAATGCCCTTGTTCCTGCACTT

GATCAGGTTGTATGGAGGTGCTTGAATGGATCACAGCTCAATAGATTGAGATGTCTTCTG

ATATCGGAGAACACAAGTGTCGAACACTGAATTCAAACAGCGAAAATGATTAGAACGGAG

GAACGTTGACTTCGGATATATCTTTCTGACAAACTATTTCAGTTTTAATCGTTTACGGGG

ACATTCTCTAAATCTTGTATATACATCCAGGGAAATTAAATTTTCACTTTAGTGTTGAAT

>Unigene0002137

GGCCTGTGTAAGGTCGGGCGGTCCAGGCAAGCGATCAAACAGTTGGAGATCATGCTTGGT

AAGGGTTTGAATCCCACCTCCACGACTCTCAACATTCTCCTTGAACTCCTTTGTAACCAG

GCAAGTGCGTGGGAGGTGAAATGTTTGCTTGAGAGGAGTGGCGAGTTGGGATTGAGATTT

GATGTTGTTAGCTACAACACTGTGATGTGCAGGCTCAGCGAGGACAGGAATTGGATCTCA

GTACTTAAGCTGTTCACGGATATGATGAAGAAAGGGATTTATCCCGATACTAGGACATTT

AACATCGTGATCAACAGTCTTTGTAAAGGTAGGCAGTTCTACCGGGCCAAATGTATATTC

AACTGCAAGGGGTTCACTGCTGATATTGTGACGTACAACACATTAATCCACGGGTTTTAT

GCAGTGAGGAAGACAAAG

>Unigene0002138

GCCCCCGCCGTACCCATACCTCCGCCACCGCCGTTACCGCCGCCGACGACCAGATCTTCG

CCGACGAGCTCCTCGCCAAGCTCTCCGACGCCGCCCGTCACATCACTCCCCCATCCCCTG

CTCCTCCCCCTGGTGCTCGCCCACTCCTCCCCGACCGTCGCCACTACAACCTCCTCATAA

AGGCCTTCAGCTGCGCCGGCCTCGACGACGAGGTCCTCCGCCTCTACAACGAGATGCGCC

GCTCCGCCGACTGCAAGCCCAACCTCCCCTGCTACACTACCGTCATCGACGCCCTCGTCT

CTGCCCGCCGCCTCCGCGAAGCCGAGTCCGTCTACGAGGACATGATCAAGGCCCGCATCG

TCCCCGACGTCACCGTCTGCACCGTCATGGTAAAGATCTACTCCGACTCAATAAATTGCT

TCGATTCTGCTGTCCAAGCCTGCCGCTCGATCGAGCGGCGCGGGTGCCGTCCCGATGTCG

TCACGTATTCAATGCTCATCGCCTGGCTCTGCAAGGCTGGGCGGCTGGGCGATGCAAAGA

GAGTCCTGGATCAAATGCTGGAGGGGAAATGCGCCCCGAATACCCAGACA

>Unigene0002139

CCGAATACCCAGACATACACCCCGATCCTCCAGTATTACTGCTCCCATGGCAAGATTAGA

GATGTGAGGCGACTTCTCGAGTCTATGCGTCGCTCCGGCTGCAGGCCGGACACCGTGGCC

TACAACATCTTCATCCAAGCCCTCTGCAAGATAGGGAGCTTCGGCGAGATTGAGAATCTC

CTTTTAGATGACTGGGAACCCAGTGCGGTTACATATAACATTTATATCAATGGCCTGTGT

AAGG

>Unigene0002140

ACCAAGATGAAGCACTTCCAGCCCAGATGAAACTCCGCAGCTGCTTGATTTTACTACCAC

ATGCAATGTCCTACCCTCCGGGGCTCACACCACATTGGTCCATGGGTTTTTGGGTGGTGG

GGTAAAAGAAGGAGCTTTGCCTTCTGCTTTTGAAGACGGGTATGCAGAAGATTCTCCCGG

AAACAGTCACTTCGCTTGTGATTGTTGATCACATTTGTGGGGATTGGAGGTCAAAGGAGG

CGATTGAGTTAGATCTCTAGAAGAGGACTGCAGGATTTGGTTCACATCTGGCTGATTGAT

TGGTCACGAGCAGGGGGAACATGGAGAATTGCTAAGGCTCTCCGAGGAAATGCCTGAGAG

GGATTTTTGATGGCCTCTTATTCGAATTTTTGATAAAGGCAGCATGTAGGAAGGGTTATT

GTGAAAGCTTAGAGAAGGATAAAGTCTGTTTTGTTCTTGGCGAGATACTTGGAGTTGGAT

AACTCATCTTTAATCATTGTTCTTTGGATGTGAATATGTTTCTCATTGGAGAGATGAGCT

TGCATCAGCCACTGGAGTTGTAATTGGTTGTTGGGTCTGTTTCTGGGTGATGAGGAGACT

AGTCGAGGTGAAACACTCCGAAAGGTTATGGTTTCCTTTCTGCCCCCTCGTTGTAAAGAT

GAAGACTTGGTTTAAATTCACGGGCTTCTCAGACTATCAATGGTTTCTAGCAGTTCCTGT

GTTGATAACGAGAAGTCTCTCTTCAGAGGCCTTCACGATAAGTGGTTTTTGGTTGCAAAA

TGCATCAAGGTCAACCCACCCAGTATTATACTTCTTTAACCATGAATGACGATGCAATCC

AGTTAATAACAGCAGATTCCATATTATGGGATCATGAGAAATAAATGGAGATGCAAATTG

CAAGTTGAGATGATCTTTTTCATAACAAGTATCGGAGAGCTGTGGTTTTGTATACCATCG

ACACTGGTGAAGTAACTTAATATTTATGGTTTCTGTTGAGTTACTATTTGTTATCCAAGA

ATGCAAATAAGTTTTGACATTACAAACTTCGAAGAAATTACGTCACAAGTTAGTGCAATT

TTGTATTTGTTTCCATCATAACTGGTGTGTTATGGTGGCATTATACCAAGAATTCATATA

CCGAGACATTGTATTCATTCAAAGAGAGAACGGAAAATAATACATGAACTGTTGGAATTC

TTTTAAGAGTTCCATAGCGAAACGTTGTATATTTAAAAAATTAAATAATGCA

>Unigene0002141

CTCTCCCTCCTTTCTGTAAAGCCGCCACCCACGCTCGCCAACACAGGAGAGAGAAACAAG

ACAGAGAGAGGAGAGAGAAACTGCTTGCTCCCTAGGATAACCGACGGAGAATGAGCCGCT

GCTCCACGTGAGCACCAGATCCGTGATGGCAATCGTCGACGACAAAGCTGCTCCGGCGTG

GGGACTCTGCCGGATGCCCTTCTGGCAGGGGGGCGGTGGCACTGCCGCCGCCGCCGCAGG

CTCCTCATCGTCGAATTCATCATCCTCGCTGACACATCACCAACACTACAACCCTCACTA

CCCCGGTCAGGCGAACATGGAGGGATCGAACCCGCAGCTCGGCGGATCTAACGGAAGTTC

GGTTTCATCAGTGGCACGGGCGCTGCTGCCGACGCGCCGGAGGCTGCGGCTAGATCCCGC

TACCAAGCTCTACTTCCCATACGAACCTGGCAAGCAGGTTAGAAGTGCAATCCGGATTAA

GAATAGCAGCAAGTCTCATGTAGCATTTAAGTTTCAAACCACGGCACCAAAGAGTTGTTT

TATGCGCCCTCCTGGTACTATACTTGCTCCTGGAGAGAGTCTAATAGCAACTGTTTTCAA

ATTTGTGGAGCATCCGGAGAATAACGAGAAAGAGGACAAAAAGTGTAAGGTTAAGTTTAA

GATTGTGAGCCTGAAGGTTAAAGGGCCTATGGAATATATTCCAGAATTGTTTGATGAGCA

AAAGGATCACGTTTCAGTTGAACAAATTCTGCGGGTTGTATTCTTGGACATGGAACGCCC

TTGCCCCCAATTGGAAAAGCTGAAGCGCCAGCTGGCCGAAGCTGAGGCAGCTGTTGAGGC

ACGGAAGAAACCTCCTGAAGATAGTGGTCCTCGGATTGTTGGCGAGGGGCTTGTCATCGA

TGAATGGAAAGAACGGAGGGAGAGATACCTTGCTCGGCAACAAGTTGATGGAGTCGACTC

AGTGTGAAGATTCGTGTGTTCTTACTGTGAGCCTTTCTTGTTACGTCATTTACTTGTGTA

GTGTGTTGAGAAAAAGTATTGCATTTGAGAACAATAATGTGCTCAAGGAGCATGATATTT

AATAATTTGTGGATTTGTGAAGCCTATTTTAGGAATATGTCCTAGATGTAAATAAACTTG

GATTTTTTTTTTTGCTTATATGGGTTAATATTTTGTACTTTGAAAGGCACAGATTGCGAT

ATTATTTGTACAGTGGAGCAACGGAGATCACACAGGTGGTCTGCTGGGTGTTGTTTTGT

>Unigene0002142

AACTTCATCACTCCCTTCGGTAAAGTCGCCGCGGCCGCGCACAAAATTCCCCTCCGTCGA

GCTACTCCGCCTACTCCTCGGCGACATACTCGAGGCCAGTAATCGTCGCGTTCTCTGTCT

CTCTTGATTGAGGCCGATGAAACCGTCGTGCTGCCGATCCCCGACGAGGACACTGTGACG

ACTGACAGCCGGCCGTGGGGGCTCTGCCGGATGCCGTTCTGGCAGGGATCCGCCCCCTCC

TCCGGCGGAGGAGGAGGGGGAGGCGGATCATCGACGAATTCGTCGTCTTCGTTGACTCAC

CACCAGCACTACCCGGGTCAAGTGCAGGCAGAGGGGTCTAACTCCCAGCGGCAGGGGCCA

CCGGGCGGATCTGTCTCATCGGTGGCGTGGTCGCTGCTGCCGACGCGCCGCAGGCTGCGA

CTGGATCCGGCTACTAAGCTGTACTTCCCATATGAACCTGGTAAGCAGGCCAGAAGTGCA

ATCAGAATAAAGAATATCAGCAAGTCTCATGTAGCTTTTAAGTTCCAAACAACTGAGCCA

AAAAGCTGCTTTATGCGCCCTCCTGGTGCGATTGTTGCTCCTGGCGAGAGTCTAATAGCA

ACTGTTTTCAAATTTGTGGAGCATCCGGAGAATAATGAGAAGCAAGACCAGAAAAGCAAG

GTTAAATTTAAGATCATGAGTCTGAAGGTGAATGGGCCTATGGAATATGTACCGGAACTG

TTTGAGGAGCAAAAAGATCAAACTGCGGTTGAGCAAATTCTGAGGGTTGTATTCTTGGAT

GTGGATCGCCCCTCCCCTCAAATGGAGAAACTGAAACGCCAGCTGGCTGAGGCAGAGGCA

GCAGTCGAGGCACGCAAGAAACCTCCTGAAGATACTGGTCCTCGGATCGTTGGCGAGGGG

CTTGTCATTGACGAATGGAAAGAACGGAGGGAAAGATACCTTGCTCGGCAGCAGGTTGAA

GGTGTTGACTCGGTTTAAACTGTAACCCGATGCTGGTACTTACTGTAAGCCTTTCTTTTT

TTTGCTTTGTTATGAAATGTTTTACATAAGTAGTAGCAAAAATCTGTGGTTATCCACGGC

CCCTTTTATATTCTTGTCTGCGAAGGCGTGTGTTTAGAGTTTCAATGGGAAAGAATAACA

TGCTTAATTAGCATGAATCTTTCAGATTTGTGTAACTCGATTTAAGATTGAATCCTACTT

GTAAATAAATCGTATATTGTTAATTAATGTATAATACAATTTACTTGAGAAGGTGC

>Unigene0002143

CCCGTTTCCGCCGAGCTCTGACACCACCCACCACCGCCGACCACCGCCCCCATAAAACGC

TATCGTCCACCGCCGATTCATTTGATGCTAACGCGATGGGCTCGCTACTCTCGTGCATCT

CCGACACCGAACCTCGCCCCTCTCCACCGTCCCGCCGCCGCGCGCCGCCGGGCGCCAAGT

CCCGCCGTGAAACCCTAACCCGCGAGGAGCACGCCATGGCGGCGGCGGCCGCGATGCTTC

TGCAGCAGAACGGCGTCTCGCTGCCGTTCGACCGCTCGTCGTCGCTTCGGTATCCGCCGC

CGGCGGCGGGGCAGAAGAAGCAGGCGCTGCCGCGGAGCTCGAGTTCTCGTGCCCGATCGC

TGACTGATCCTCTCGTGCAGCCGCAGCAGCTGGTAAATCAGGACTTGAAGCTTGATGACC

TGGAGACCAACCATTTTGTTCTTGTTCACGGGGGTGGTTTCGGTGCTTGGTGCTGGTATA

AAACTATAGCACATCTTGAAGATTGTGGATTCAAGGTCAGTGCGATAGACCTCTCTGGTG

CTGGAATTGATTCATCTAATACTAACAGCATCACAAGTCTAGCCGAATATGTGAAGCCTC

TTACCAATTTCCTTGAGAAGCTCAATGACGGGGATAAGTTAATTCTGGTTGGGCATGATT

TCGGTGGCACATGCATATCGTATGCTATGGAAATGTTCCCATCAAAGGTAGCCAAAGCTA

TTTTCATCTCTGCAGCTATGCTGGCAAATGGCCAGAGTACTCTCGATATGTTCTCTCAAC

AGGCCGGCACTGATTATCTGATGGGCCAGGCTCAGATTTTTGTATATGCGAATGGGAAGG

ATTCTCCCCCAACTTCCATTGATCTCGACAGATCATTGCTAAAAGAGTTGCTGTTTAATC

AGAGTCCTGTAAAGGATGTTGCACTGGCATCGGTTTCCATGAGGTCGATTCCTTTTGCGC

CGGTACTTGAGAAGCTCTCGCTCACCAATGATAACTATGGCTCAGTTAGGCGATTCTACA

TCGAGACCGGTGATGACAATGCGGTACCTCTCTCTCTCCAGCAGAGCATGTGCAATCTGA

ATCCGCCGGAAAAAGTTTTCCAGTTGAAAGGATCTGATCACTCACCGTTCTTCTCGAAGC

CTCAGGCTCTGCACAAGCTACTCGTTGAGATTGCGAGGATTCCCTCACCCCAACTTGAAT

GAGGTTCCAAATACTCAACTCAACTCAGGCAATACAATCCTGCACTAACTATGCCAAGGA

TATCATACATACATAATAGATGGGAATCAAGTTAAGACTAGTGCTTGTGCGCAACAATCA

AAGTGCGAAAATAGCTATTAACCCACTTGGATAGTGTTGTGTTACATTTTGAAAAAAATG

TGTGCATACAGGCATACAAATAATGTAGCATCAGATTATTTTTCCTCTTTGTAATAATGG

ATTCCATTTCACCTTCAGCCTTCGGATGATATGAGGTAAATTGCTGTTTTCTTTGAATTG

TGAGAGAGCTTCTCTGGTATTGTG

>Unigene0002144

TAATTACTCCAATAAAATCCCAGCTCTTGTTCCGTTCTTGAGCCTAACAATGGCGTCGTC

TCTGACGGTGCTCAGCGGAGTCCCGAAGCGGAAGGACTCGACGGTGACACGGGAGGACGA

CCAGCTCATCATCACCCCGCTCGGCGCCGGCAACGAGGTTGGCCGCTCATGCGTCCACAT

GTCCTACAGAGGAAAAAACATCATGTTCGACTGCGGGATCCATCCGGCGTACTCGGGGAT

GGCGGCGCTGCCGTATTTCGACGAGATCGACCCGTCCGCCATCGATGTGCTCCTTGTTAC

CCACTTTCACCTTGATCATGCTGCATCTCTTCCCTACTTTCTCGAGAAGACGAATTTCAA

GGGTCGGGTTTTCATGACCCATGCAACAAAAGCTATATACAGGCTACTTCTATCTGATTA

TGTCAAAGTGAGCAAAGTTTCTGTTGAAGATATGTTGTTTGATGAGCAAGACATTCTTCA

TTCCATGGACAAGATTGAGGTTATTGACTTCCATCAAACACTGGAAGTGAGTGGAATCCG

ATTTTGGTGCTATACTGCTGGTCATGTCCTGGGGGCTGCCATGTTCATGGTTGATATTGC

TGGAGTCCGAGTTCTCTACACTGGTGATTACTCCAGAGAGGAGGATCGCCACCTCCGTGC

TGCTGAAACCCCACAGTTCTCGCCTGACATATGCATTATTGAGTCTACCTATGGCGTCCA

GCTTCACCAGTCCCGTCATGTCCGTGAAAAGCGTTTTACGCAGGTCATCCATGAAACTCT

CGACCAAGGAGGACGTGTGCTAATCCCAGCTTTTGCCCTTGGCAGAGCCCAGGAGCTTCT

CCTCATCCTGGATGAGTACTGGTCCGAACACCCCGAGCTCCATAACATTCCTATCTACTA

TGCCTCCCCTCTGGCTAAGAGGTGCATGGCTGTTTACCAGACCTATATAAATTCCATGAA

TGAGAGGATCCGGACCCAGTTTGCCAATTCAAACCCGTTTGATTTTAAGCACATCTCCCC

GTTGAAGAGCATAGAGAACTTCGATGACGTCGGTCCATCTGTGGTGATGGCGAGTCCAAG

CGGGCTACAAAGCGGCCTTTCGAGACAACTATTTGATAAGTGGTGCTCAGATAAAAGGAA

TTCTTGTGTGATTCCAGGGTATGTTGTGGAAGGGACACTTGCCAAGACTATCATCAACGA

GCCCAATGAAGTCACCCTCATGAATGGTCTCGTCGCTCCACTCAACATGAAGGTGTACTA

CATATCTTTCTCTGCTCACGCAGATTCTGCACAGACATGCGCGTTTTTGAACGAGCTGAT

GCCACCCAATGTAATCCTTGTCCATGGAGAAGCAAATGAGATGGGAAGGCTGAAGCAGAA

GCTTATCACTCAGTTCACTGGTACGAATACAAAAATCATGTCCCCGAAGAACTGCCAATC

AGTTGAGATGTACTTCAGTTCTGAGAAAATGGCCAAGACAATCGGGAAACTCGCTGAGAA

GACGCCGGAAGTTGGGGAGAATGTCAGCGGCTTACTTGTAAAGAAAGGCTTCACATATCA

GATAATGGCACCTGACGACCTCCATGTTTTCTCACAGCTATCCACTGCAAACATCACCCA

GAGGATCTCCATCCCATATTCTGGTGCATTCGGGGTCATAAAACATAGGTTGAAACAAAT

ATATGAGAGTGTGGAACCTCCGACTGAGGAGCCTGATGTCCCTACATTGATTGTGCATGA

AAGGGTGGTGATAAGGCAGGAGTCGGAGAACTATGTGACAATGCAGTGGCCATCTGATCC

GATCAGTGATATGGTTTCCGACTCCATTGTTGCGATGATCCTGAACATAAGCAGGGAAGG

ACCGAAGGTGACTGCAGTTGAAGAGACCGACAAGACAGTGGAGACAGAGAAGACGGCCCA

GAAAGTGATCTATGCTCTGTTAGTGTCCATGTTTGGTGATGTGAAGGTTGGAGAGGAAGG

AAAACTGGTGGTGACTGTCGACGGTGTTGTAGCACGCGTGGATGGTAAGAACGGTGAGGT

GGAATGTGATAATGAAAGTCTCAAGGAGAGAGTGAAGACGGCCTTCCAACGTATACAAAG

TGCTGTTAGACCAATTCCACTCTCAGCATCATGAGATGACTAATCCATGTACTTCTAGCT

AAACATGTTGAGCGTTTGCCAAAAGAGAGTTTCTACAAGGCGTCTATGAGGTACTCTCGG

TTTAGTCTTTGATTTTACTGGTAAGGCACTCTCTTTTAACTTAGTCTTTGATTTTGTTGG

TAAGGTACTCTCTCTTAACCTACTTAATCTCAACTGTTTGTGTGAACTTTAAACTTTGAT

CTGATGGTTCAGTTGTATCATTCCCTCCATGAAGGAAGAGGGGAAAAAACCTTACTGGGC

ATTTTCTTTTAGGGATGTTATTCCTTCCGTGTTTCAAAGCATTTTTTTTTTACATTACAC

TTTCTCAGTAACTATCAGTCATATATTTGAACTAAATGTTAAACAAAGTACCATACTTCT

TTTATTGGTTTCAAAAG

>Unigene0002145

AGAACTTTCTAGGTGTCCCTCCTTGTCTTTTGCAATCAACTTATTACTGATCTGTTGAGC

AGCCTACGATGGCATCGTCCACGACGATCATCAATGGAGCCCTAAAGCGGCGACAATCGA

CCGTAACGCGAGAGGGAGACGAGCTCATCATCACCCCGTTGGGCGCCGGCAACGAGGTTG

GCCGGTCCTGCATCCACATGTCCTTCAGAGGAAAGAACGTCATGTTCGACTGTGGGATTC

ACCCGGGGTACTCTGGGATGGCGGCGCTACCGTATTTCGACGAGATAGACCCGTCTGCCA

TTGACGTGCTCCTTGTTACCCATTTTCATCTTGATCATGTTGCATCTCTTCCATACTTAC

TGGAGAAGACAAATTTCAAAGGACGGGTTTTCATGACACATGCAACAAAGGCCATATACA

AGCTGCTTTTATCTGATTATGTCAAAGTGAGCAAAGTTTCTGTTGAGGATATGTTATTTG

ATGAGGAGGATATCCTTCGTTCAATGGACAAGATTGAGGTTATTTTGTTCAATCAGACTC

TGGTAGTGAATGGAATACAATTTACGTGCTATCCTGCTGGACATGTCCTGGGAGCTGCCA

TGTTCATGGTTGATATATCTGGAGTCAGGGTTCTCTACACAGGTGATTACTCCCGAGAGG

AGGATCGCCACCTCCCTGCTGCTGAAACCCCGCATGTCTCTCCTGACATATGCATTGTTG

AATCTACATATGGTGTCCAGCTGCACCAATCCCGAGATATCCGTGAGAAGCGCTTTACAG

AAGTCATCCACCGAACTCTTAACCAAGGAGGGCGTGTGCTCATTCCAGCGTTCGCTCTAG

GCAGAGCCCAGGAGCTCCTCCTCATCCTAGAAGAGTATTGGTCTAAGCACCCTGAGCTCG

AAAACTTTCCTATCTACTATGCCTCCCCTCTGGCTAAGAGGTGCATGGCGTTTTACCAGA

CTTATATAAATGCCATGAATAAGGGGATCCAGGTCCAGTTCGCCAAGTCGAATCCATTTG

ATTTCAAATACATCTTCCCATTGATGAG

>Unigene0002146

CGGCGCTCAAGTTGTCGATCATAAATGGCTTGTGGATTCAGTGGCAGCTTTACGTTTGGA

ACCGGCATGATGATGTTGATGATGATGTTGATAGTTTGCAGCTCGAGGGTTGCTTTCGTC

TTATCGGCGCTCGAGTTGTCGACCATAGATGGCTTGTTGATTCAGTGGCAGTTTTATGTA

TGGTAGTTTGCAGCTCAAGGCTTGCTCTCGTTTTATCGGCGCTCAAGTCGTCGATCATAA

ATGGCTTGTGGATTCAGTTGCAGCTTTTCGTTTGGAACCGGTATGATGATGTTGATAGTT

TGCAGCTCAAGGATGCTCTCGTCTCGTTTGCTAATGCTTTTTTATTTTATTTTGTTTTGA

ATTCAATACCGCAGACGGGACTAATAGGATTTGTCTTTCACATATGGCTATGGTGGTGGG

AGTTTTTTTTTTTTTTTTCCTAATCAGAGA

>Unigene0002147

CTCTCCAATCTTCGAATCCCGTCACCGCCGCTTCCGCGTCCCATCTAGGGCTCAGTTAGC

TTTTGGATGAAGTCACATGCCTCTGATGTATAAGGTCCCTTACTTTTGAATCTGTGCATC

ACTGTAGAGATACCATTCATCCACAAAGAATCCGAGCAAGAATTATATGAACTCTAATCA

CAATGCCAAAGCAGAATGTGTCACCCCAGTTTGCTGTTCCTTCAGAGAAAGGGACACCAG

ATTGTTGCAGATCATCAAATACTAACACACCGCCTTTCGAAACTGAAGTAAATGATCGAG

ATTCGAAGAGACAAAAACTGAATGCTGAGCATGGAAAGCGTAGTGTCACGCAATTGGATG

CTTCAGATCATGCGGACACCAATGCAGCTAATCAATGTGCATTTTGCCATTCGTGTAGGA

CCACTGAGTTGACTGGTCCACTGCTACATTATTATGGTAATGGAAAACCGGTAAAGGTAG

AGGAAGCAGACCAGACTACGGTCATACATGTTCATGATCAATGTGTTGACTGGACACCCC

GAGTTTATTTTGATGGTGAAACTGCGATGAATTTAAAGGCTGAAGTGTCTCGTGCTTCGA

AGCTTAGGTGCAGCGATTGTGGGCAGAAAGGGGCAGCTCTCGGTTGCTTTTATAAGACTT

GCAGGAGAAGTTACCATATCCCATGTGCATTCGGGATTCTGCAGTGCCGGTGGGATTTAG

AAAATTATCATGTGCTTTGTCCTCAACATTCTTCTAAAGAATTGCCATGTGATAAATCCA

AGAGTCAAAAGAAGATTGAGGTTAACAATTCTGGACCGTCTCAAAGAGATTGTTCTGAAA

CTCCATCTGAAGTGCCCTTTTCTTCTAAAGAACCACGGGAAGCGCACTGGACAGCTGCAC

CTCATCTCACAAATGTATGGATTTTGTGTGGTTCTGCTCTCTCGAAAGAGGACAAGGTGA

TCTTGAGAAATTTGTGTATTTTATTCTGTGGATATAAATTTTAGCTTGAATCTTAGTAAT

ACAATGCTAGTCTCTGCATTTCCCATTTTGCCCTTTCTATTTTTGAATGTAAAAATCTAC

TCCATGCTCCATTTTTTGAGTGAACTACAGGACCTTCTGGACAATTTTGCAAACTTAACC

GGAGCAACTGTGACCGAACTATGGAGACAAAATGTGACCCATGTCATTGCATCCACTGAT

GAGCGTGGTGCCTGTTTCAGAACTTTCAAGATCCTCATGGCTACATTGCGTGGGAAATGG

GTTCTATCAATAGACTGGATTAAAGCTTGTATCGAGGCTAAGCATCCTGTTGAAGAGAAA

CACTATGAAATAGGTCTTGACATTCATGGTTGTGTTGATGGACCCAAGAAAGGAAGACTC

AGGGCAATTAAAAAGGAACCGAAAATTTTCGCAGGGCTCAAATTCTATTTCAGCGGCTAT

TTCAAACTTGCATACAAGGGGTCCTTTGAGAGCCTTGTTGCAGCCGCTGGCGGGACCCTC

ATGACCAAAGAGGACTTACTAACCGCCCAAGATTCCCGCAAGAGCCGCTCCCCACAGACA

TCGTATGTGATTTATAATTCAGACTCCCCACAGGATTTCAGCTCAAGAGATGCTAATGAG

GTGGTGATGAGAAGGTACGAGGAGGCCAAAGCACTTGCAGGGAGGATCGGTGCTCGAGTT

GTCGATCATAAATGGCTTGT

>Unigene0002148

GCAAGACAGCATCATTGCCCCCCCCTCAACTCTAGGGTTCAGGAAATACGGATCCTATTG

GTCGGAAAGATATTCGGAAGATTGACCAGGGTACGGAGGTCTCTATTAACGGAATGGAAC

CGATCCCTAGCCCTCCAGCTGGTTGCAGCATTGATGAAGATAACGGCAACAATGATCAAG

ATTCTGGAAGATTACCATGTACTGTTGATAGCAAATCAAGCTCAGCAAGACAAGAGGGAT

CCAAGAGAAGGAAATTGAATAGTGAGTTTGGAAGACACCATATAATCAAGCCTTTGAGTT

CTCCAACAAAATGTGCATTTTGTCACTGTTTTAGAATCACTGAGGGTTCTGGTCCAATGG

TACATTATCGGAATGGGAAACCAATAACCGTGGACGAAAAGAATCAGACTAATGTCATTC

ATGCTCACAGGAAGTGCATTAACTGGTGTCCGAAAGTCTACTTCGATGGTGAGACTGCAA

TTAATTTAGAAAAAGAATTGTATCGTGCTTCAAGGCTTAAATGCAGCAAATGTGGTCTGA

AGGGAGCAGCACTTGGTTGCTTTTATAAGCATTGTCGGAAAACTTTCCATGTTCCATGCG

CATATACGACTTCTGGTTGTCGATGGGATTGTGTACATCTTCATTTGCTCTGTCCTTCCC

ACACTTCAGAAGAATTGCCATGTGATAAATCCAAGAGTGGGAAGAAGTTTGAGACCAATG

ATATTTTGTCTCCTCAACTGTATCATTCGATTCCTCAACTTTCAATTAGCACGCCATTCG

ACATCATCTACTCAGACATTCTTATTTACTTTGCTTTTTCTAATCTTGTTTTCTCGTTAT

CGTGTTAGACGCAGATCTGATGCTTTACTGAGTGAATTGCAGTGTAACCAGTCCTCAGCG

GGTGCACCACACGCAGCAAGTGGATGGATTTTATGTGGATTTGATCTGTCTAAATACGAG

AATGAACTTTTGGTGAAGTTTGCAATGCTGACTGGTGCATCCGTGACCGAGCAATGGGAG

AGAAATGTGACCCATCTCATCACATCCATTGATGAGTTTGGTGCCTGCAGTAGAACTTTC

AAGGTCCTCATGGCTACTTTGAAGGGAATATGGGTCCTAACAGTAGACTGGGTTATAGAT

TACATGGAGGCCAAAGAGCCTGTTGCTGAGGAGCCTTATGAAGTCGTTCTTGATATTCAT

GGTTTTGCTGATGGCCCCAAGAAAGGGAGACTTAGAGCAATGAACAAGGGATCAAAGCTC

TTTTCTGGATTGAAGTTCTATCTTATCGGCCAATTCCCGGACCTTCTCAAAGGCTTCATT

GAGGAACTTGTTTTCGCTGCTGGTGGGACGATCTTGAACAGAGATGACTTCTTAAATCAG

CGGGACTCTGATACTTCTTATGTTGTTTACAACGAGGATCCACCGGAGGAATGCATCCCA

GTTGATATTGCTGATCTTTTAAAGAAGAGGTTTGAGAGGGCTAAAGCTTTTGCAGCTAAG

AAGGGCGCTTGGGTTATATGTCACAAATGGCTTTGGGATGCAATCGCTGCTTCCAGTCTG

GAAATTTCATAGAAGAAGAGGGTTCATATTTTACTTTGTTTGTAGTAATCATTTGCAACT

TAGTGGTGAACAAAGTACAGTGTTTTTATTATTAGCCCATTCTGATAATTATTCATATTT

TATCAGCTTTGGTATTGTTGCTTCCAGTCTGGTCTTTAC

>Unigene0002149

TAACCCCTTTTTAGATATAGTTATCCAAACCATGTGTGTAGTGACTGCTCTCACAGATAA

TCAAGTTTAGAAGAAACACCTCTATGGTTAAACTTTCTTTTATTAAAGTATCTGGATGAG

TTTACTTGAAGTGTGCGAATTTTAAGGTATTTGTGGGCCACATGTTACATGTTGGCTGAA

TGCAAGAAGGTGATGTGTATTTTAGTATGTCGGCCTCTAAAGCATTAATACTCCATTGGT

GGTACCTGAATCTGTATACATGCAGTACTTCTGAATAGCTATCCTTGATCACTGCATATT

CGGGAATCATGGATACTCTCGGTTT

>Unigene0002150

TTTTTAGTAACTAAAATTTGGGGATTTTAGGATCCAGCCTGTTCAGTTACACATTCCTTT

ACGTGGGGAAATTGGAGCACTAATTCTCGCAATTGGAGATCTGATTGAGAATCCTAGTCA

ATGGTGCATCAGATACTCTCTGCTCCTCTCTCTGTGACCTGACTCTTCCTTTCGCATCAT

GGACAACGATGGCTGAGACCGTGAGCAAGTGAAGCAGAGTCCACCCTCAACCCCCGTGGC

CGCCTCCAATTAATGCCTAGTGCCTACTATGTTGTCATTGCCTTTGTGAATCGCAAATCT

TTGCCCCCTTTTTGATTGCTGCTGTGAACCCGGTTCCAAGCCGAATATGATCCCTGTTCT

GATTATCTCTCTTCAGAGCACATATATATAGTGGTAGTGTGGTACCTATCTGAATTTGAA

AATTGCA

>Unigene0002151

TATTTTGTTAACTTTCAAGTACTCATGGATATTGTATATGATATTCTCTACTCTTCTTAA

TTGCTTTTATTATTGTTCTATATGTGGAATTGTGGATTAATGAATATTATATATATGAGA

ATGAAAGAATATAAGAATTTATATTCTAAATTATAATTTATGACATATATAAAATAATAA

TATATTACTTTATTTCCATATCCCTGATTTATTTTATCCTGAGTTTTTGAGATTTTGTTG

TATCCCTGAATCTGTGCAGTATATTATCCGGGCATCCGTGCTTCATAATTTTAGCCCATA

GTACAACAATCCTTGAAAGATGTAATTCTGAATTAGATTTCATGGCACTAATCATTCGTA

GATCTTGCTTTGAAGTTTCAAGTGATTCATTAGTATATATTGCTTTGAAGTTTCATGTGA

TTCCATTGACAGCAATCATGGGTGTGGAGATAC

>Unigene0002152

GGCGGTGGATGTACATAAAGCGATCATGGCTCCTTGGTAGACTCTGGGCAAGAGGGAGGT

GGAAGAGAACATGGGTAGAATTGCAAGTTAGCTGAAAGAAGGGCTTATGTGCTAAGGGAG

AAAATCACAGGGAAGTAAAGTGAGCTAATAAATACGACATGTGCACAAGTACAAAACAAA

GTGAGCTATACATGTGCCTCAGTACAAAACATGTAAACCACATGGATGTAAATTGTAATT

AACCCTGAAAGTTAAGGCTAAATAATCCAAAAGACAAAAATGAGTGGGATCCAACTGGGA

TTAAGGCTTGGTTTGATTTAACTGGAGACCCTTACCAAAGGGAAGCATGCACCCAATCCT

TTGTGCTACCACCTATACTTCGTATAGGGCTGTTGTACCTGGGTACTTAGCTGATATGAT

ACTT

>Unigene0002153

GGCGGTGGATGTACATAAAGCGATCATGGCTCCTTGGTAGACTCTGGGCAAGAGGGAGGT

GGAAGAGAACATGGGTAGAATTGCAAGTTAGCTGAAAGAAGGGCTTATGTGCTAAGGGAG

AAAATCACAGGGAAGTAAAGTGAGCTAATAAATACGACATGTGCACAAGTACAAAACAAA

GTGAGCTATACATGTGCCTCAGTACAAAACATGTAAACCACATGGATGTAAATTGTAATT

AACCCTGAAAGTTAAGGTCAAATAATCCAAAAGACAAAAATGAGTGGGATCCAACTGGGA

TTAAGGCTTGGTTTGATTTAACTGGAGACCCTTACCAAAGGGAAGCATGCACCCAATCCT

TTGTGCTACCACCTATACTTCGTATAGGGCTGTTGTACCTGGGTACTTAGCTGATATGAT

ACTT

>Unigene0002154

ACCACCGCAAAACATGAATCGGGGGTCCCCAATTTCCATCAGAGTAGAAATAGGTGAGAG

AAAGAGATAAATTCCCATTTAGATCTCCCCTCTCTCCCCTCTGGAACTCCTGGCCCCCTC

GAGCTCGCTCCATTAATAGCTTGCTCGGCGTCCGATCTGAGGTATCTATCGGAGCAATCA

TGGGTGTGGAGATACTGGGGGCTGATTCCAAACCGGAACTGGCTAAGGTGGTTAAGGAGG

ATGGAACTTCTATTTCTGAGACGGGCAGCAAGGCAAAATCAAATTCAGAACAGGGGGTTG

AGGTACCAATCCAATTTGGCTTGGTGGATAGTTCACATGGAGTAAATGGAGAGTCCAAGG

AAACGGGCCACCTGGAAACGTCTAATTTACCAAAGGATGCCGTTGATGAATGGCCTGCAC

CGAAACAAACCCATCACTTCTATTTTGTTAGGTTTCGGTCTTACGAAGACCCAAAGCTTA

AAACAAAGATTGAACAAGTCGACAGGGACATTCAGAAGAAGAATCAAGCAAGGTCTCAGC

TTATTGAGGCGTTGAGGGCAAAACGGTCTGAAAGGGCGAACTTGATTTCGCAACTAAAGC

CGTTGACCGCTGAGGACAAAGAATACAGGACGGCCATTGATGGTAAAAGGAAGGAAATGG

AACCTCTTCACCATGCTTTGGGGCAGCTACGTAGTGTAAATAATGCAACAAGGGAGAAGG

GTGTAGGTCTATGCTCCTCAGAAGAGGAACTTGATGCCCTGATCTATAGCTTGAATTACC

GCATACAACATGAAAGTAACTCGTTGGTAGAGGAGAAAAAGTTACTCAAGGAAATTAAGC

AACTTGAAGGAAGTAGAGCTAAGGTTAAATCCAATGTTGCTGAGAAGGTCAAGATTCAGG

AAACCCATGGAGAGAAAGAAGCTTTACAAGACAGAGTTAAACTAATTGGTGGTGATATGG

ATGAAGTCAGGAAGGAGAAACAAGCTGTTAGGGGTAAAATAAAGCTTTTAGAGGATGAGC

TTAACGTTGTAATCGATGAAATCAGAACCTTGGAGGAACAATTGGATGCTATAACTAAGA

AAAGGGAGGAGACGTATGCATCCCTTAATGAACTGAGAAAAGGGCGTGATGAAGCGAATG

TGCGGTTCTACCAAAACCGATCACTACTGAACAGTGCCAGGGATCTCGCTGCAAAGAAAG

ACATAGCGGCACTTGGAGAGCTTCACTTCAACGAGGTAGAGACATTCATGTCAGAATGGA

GCAGCAGTAAAGTTTTCAGAGATGATTATGAGAGGAGGATACTGTCATCACTCGACAGTA

GGCAGCTGAGCAGGGATGGACGAATGAGGAATCCCGATGAAAAGCCGATTGTCGCTCCAC

AGCCAGTCATCGAACAGGCAACTGTACCAACAAAAGTAACTCCAAAGCAAACAAAGGAAG

AGGCCCTACCCCCTCCTGTCTCTGCTCCCAAGATTCAAGAAGAGATCGGTAAATCTACTG

AAGTAGCTCCGAGAGCAAAACCTGGTCGCTCAGACGACACCGGAGAAACTTATGAGATTG

GAAAGTCCAAGGAGCTGTCAAAACCTGATGCTGCTAAGTTGAAGGAAATGAAAAGAGAGG

AGGAAATTGCTAAAGCAAAATTAGCGCTTGAGCGTAAGAAGAAGTTAGCCGAGAAAGCAT

CAGCAAAAGCAGCAGCAAAAGCTCAGAAGGACGCTGAGAAGAAGATGAAGGAAAAAGAGA

AGCGAGCTAAGAAGAAGGCTGGCAATAGCGAACCTGCTACTCCGGCCGATCAGACTGAGG

AGACGGATGCAAAGTCTTCAGAGCCAGAAGAAGCTGAAGTGAAACTAGAAGCTCCGGTTG

CAGCAGCCAAAGAAAAGAAGGAGAAAGTGAAGTACAGAGGGCGGGCAAGAGCTCCGGATC

CACTTCCGAAGGTTATCCTCAAGAGAAAAAAGGCGCAGTCGTATTGGTCGTGGGCTGCTC

CTGCTGCGGCGGTCTTGGCCGTGGTTGTCGCTGCACTTGTCTACTACTTTGCTGGGAGAA

TCTGAATGGATATTGGCAAGGAGATGAGCGACATGGATCAATTTTGCAGTTGAGGATAAT

ACTGGTATAAGTGGACTTAAATTAATGGATCTTTATATCCTGCTTTCGAACACCAAATGT

TTTGTTGCTTGGTTGATGACTGTATGCCATAATTATATCTTGTAGCATGTTCACGTTTAT

TTTGTTAAATGTCTGCAGTTGCACTTTAAAACAAATTTCCATCTAAAAGAGTGAACACAT

TTCGATTACTATGAAACACATTGTATTGCACGTTCTCATATAATGGCTGAAGCCCAGATG

TTGGGGCAGGTTA

>Unigene0002155

CAGCCACCACACCACAGGCCCTACAGATCTCGATCTCAACACAGGAAGGAGGAGGATGTC

GTCCTCGTTCGACACCTTCAGCAACGACGGCGAGGAGGCCCCCCGCGGCTCGCCGCAGCC

CTTCAACGACGACGGTGACTTCCTCGGCTACCAATCCCAGCGCTACGAGTCCTACTCCAG

CTTCCCCGAGGACGACGCCAAGGACCCGATCTCCGATCCCATCCCCTCTTCCGGCTTCGG

CGGCGACTTCGGCGAGGAGATCCCCGTCCATCACCACGGCTCCAGCGCGCCAGCTTCGCC

GGAGGGTTATGGCGGGTACGGGGATCCGTTCTCGATGCCGGAGAGCAACGGGAAGGCGTA

CGGGGAGGAGGAGAATGGGGGGATCTTTGCGTCGGACGGGCCGGTGCTGCCGGACCCCAG

CGAGATGCGGGAGGAGGGGTTCGCCCTAAGGGAATGGCGCCGCCAAAATGCCCTTCATCT

GGAGGAGAAAGAAAGGAAGGAGAAGGAGTTGCGGAATCAGATCATTGCTGAAGCTGACGA

GTACAAGAGGGCTTTCTTTGAGAAGAGGAAGCTTAACTGCGAAACCAACAAGTCCAACAA

CAGAGAAAGAGAGAAGCTCTATCTGGCCAACCAGGTGAAGTTTCATGCAGAAGCAGATAA

GCAGTACTGGAAAGCAATTGCAGAGCTTGTACCCCACGAGATTGTTGGCCTTGAGAAGAA

GAGAGGGAAGAAAGATCAGGAGAAGAAGCCATCAATTACAGTCATTCAGGGACCCAAGCC

AGGCAAGCCTACTGATCTTTCGAGGATGCGTCAGTTACTGGTGAAGCTGAAACACACTCC

CCCTCCGCACATGAAGCCTCCGCCACCTCCTGCCAAGGAAACCCCTGCCGCAGCTAAAGT

CGGAGCCGCTGCAAAAGACGGTGCAAAGGATGGAAAGAAACCTGCATCACCTGCAAAAGA

AGCTGCTGCAGCTAGTGCCCCTTCTACTTCGCCACCCAAGGAAGGAGCTCCCCCTGCAGC

GCCTGAAGGCCAGGCACCCGCGGTACCAGAACCAGAGGCAGCTCCTGCTAATGAATGAAC

TGGACAGCGTGTCGTCATTTCTTTCCATCCCGTAATGATTAGTAGTATCTCAGGCCATAT

TTGTTACGGAGAATAAAGACTGATCTTTGTAGTAAAGCTGAAGTCTCCTCTAGCAGAGGG

AGTCTCTCAGAAATTTGATTCCACAGTATTGTTTATTGTGTTGTTTTTTGGTTTGTTGAG

GATGGTTATTAACATACTTGATCTATTCTTTTGAGATGTACCTGTAGAGAAAACCAACTC

TGAATTTCTTGGTTACCCTAACATGTATCTATTATTGGATCCTGTGTGTACCTGTTTGTA

TGTGGGCGGTTCTTGATAACATGGTTCAGGGGAACATGCTGCACAAACT

>Unigene0002156

AGTTGTGGATGCTCTGATGGTGGATGATAGCTCTCAAATGCCGGCCAGTGCTGACTGTTC

GAACTCCACTAAAATTTCATTACAGAGGTCCATTCTTCACCCGAGTGAAAGAGAGCGAGA

TGGAGCAGCCGGCACTGACCGTGACGAACGCTGAGAAGCTGGTGGCGGCGGCGATGGCCG

GCCGCGACGCCTCCCACGACGCCGCCCACGCCTTCAGGGTCCGGGACCTCGCCCTCTCCC

TCGCCAAGGAGGAGGGTCTTGAGGACCACAGACTAGAAATTGTGGAACTTGCTGCTCTTC

TTCACGATATTGGAGATTACAAGTACACCAAGGATGGTACCGAGGACACCAAAGTTGTCG

AGAAGTTTCTGGAGGAGGAGGGGTTAGATGAGAGCATGAAGGAAAGGATATTGTGCATCA

TAAAAGGAATGGGTTTCAAAAATGAAGTGGCACGTATATCATGTGATGATTCATCATTGG

AGTTTGGGGTTGTGCAAGATGCTGACCGTCTTGATGCGATTGGTGCCATTGGAATTGCTC

GCTGTTTTACATATGGTGGAAGTAAGAACCACATACTGCATGATCCCAAGCTATTACCAC

GACAAGAGTTGTCAAAGCAGAAATATATGGACAAGAATGAGGAGCAGAGTTCCATTAACC

ATTTCCATGAGAAGCTCTTCAAGTTGAAGGATTTGATGAAAACAAAGGCTGGTAAAAGAA

GGGCCGAGAAAAGGCACAAATTCATGCAGGATTTTGTAGCTGAATTCTATGAGGAATGGA

GTGGTAGAGCTTGAACCCTGCTTCGTCCTCTCCGCTGTGAAACCTATTTGTAGTAGAAGA

ACATATTGAAAATGAGTACTTCTGATTATGTAAAGTTTCATAAAAATCATTGTGTTCAAT

CTCATTAAACTGTGTGATGTAAAAGTTAAACCCATGATTTATCTTTTGCAAATCTGGACG

GATTACATTCTCAAAAACAAAATGTAATGTGATTTGTGATGTTTTATTTTTTTACTTTTA

CTGTTGAGTACCGCTGCTAATTCT

>Unigene0002157

TGGCATACTTTGGCCGAGCCCGATAGTTCTGCTCACTTCCGCCGATCAAGCCAAGTTCTC

CGGCGATCCCCTCCACCAGGTAACCCTAACCCCCTCTCTGTTCCGATAACTGCTTTGTTC

ACACCCTCCCATCATCATTCATCGATCCGTCAAACTGCCACCATTTTCACTGAGGCATTT

CGTCGAGCACCCACAGTACACCAACTCAACGGGTGGGCAATGCTGCCGCCGCCTGGAAAG

TCTTTCACATGATTATGCTTTGCAGCTTCGAATTTGGAGCTTTGGCTGTAGAAATTATGC

TAATCTCTTCGTAGAGATCTCTCGGGACAAGAGTCTGGTTACTACCAAGATGTCCTTAAT

CAGTTTTTGGTCAGGGAGCTTCCATCGATTAAGTAAAAACATTGGAGACCATTGCGTTGG

GTTGCTTAAGTTTCATACTGGATCTACTCGTGGAATATCTGCTAATGCAAGGAGAAGTAG

CTGCAGAACTTTAACAACAAATTTGGAAGAAAGATTTCATAAACCCTATATCAGTGAACC

AGGAATCGTTAATTCGGCTCTTTTTGGTGCGAATGTTGGGTCAGCAAGAACATTGAATGC

TACTGAATCAGATGTCTGCGGAGTTCTGGATTTTCAGTTATACAATTCCCTACAAAATAT

CCTTGAAAACAGAATCTTTGGCGGTTCTTGTGCTTATATTGTCTTTGACCTTGAAACCAC

TGGGTTCTCCTATTCTGACAGAATAATTGAGGTTGCAGCACGGGATCTTTTTGGAGGACA

GAATAGTACAATTGAATCCTTGATTAATCCTGGGAAGCCTGTCCTAAATTCAGAGATTCA

TCACATTAGCAGTCACATGGTTAACAATCCTGAAATTCCAAGGTTTGAAGAATTCATTCC

AATATTTTTGCAATTCATACGGAGCCGCCAGACGGATGGAAAGCGAGTTGCATTAATGGC

TCATAATGCAAAAGCATTTGATGCGCGTGTCTTGGTCAGGGAATTCCGTCGTTGTTCTGT

TGAGTTTCCTTCAGACATTGTGTTTATAGACACACTTCCTGTTACACGAAAGTTGGAGAT

GTTGGATGGATCGAAGAAAAGATCGCTCAAGGCATTAGCTGAGTATTACAATATCCCTTT

AGTCGGCCAAGCCCACCGAGCAATGCAGGATGTGATAAGATTGATTGAAATAATCAAGAG

AGTAACTTTTGAGCTAAAGATACCTCTCCATGAACTCGAAAAGTTAGCCTTTCCCGCACC

TCAGGTAAAAAGTATAATAAAACCGGGACCCAAAACCTTTTATTCGAAGAAAGTACTAAG

GCTCTGATTTGTGGCACTTATCTTGCTTCTATTTATTGTTTTTTTCTTCATAAGAAAATT

CCTCATTGAGAGCTATCTTCTGATACTGCAATAGTTCGTGTTGTACTGCAAGGTTATTAT

AAATATTATTGGTGTTAAACTTTTAAA

>Unigene0002158

TTGATCTTCATCTTTACAAACCATATTTCAAGGCTATAGAGACAGATCCATATGAATTTC

CATGCCAGCTGCTTGGGTCATCTTGTATAGAGAAATTGACCCTTTCCTGTTGCGCTTTGA

AACCGCCCTGTAAATTTGACGGTCTCCGCCGCCTTAAAGAATTATGCCTGTTCTATGTAA

ATATCAACGACGAGGGTATCAACCATCTCTTGCATAATTGTCACAATCTTGAATCTCTAA

CTGTGTGGAACTCCCCACAGTTATTGACACTGAGTATCGATAATCTAAGCCAATTTCAAA

GTTTGGAAGCTATAGACTGCAACCACTTGAGAGAAATTAAAGTTAATGCCGTAAATTTCA

ACCGACTGCGTTACCGGGGAGCTCCGATACATATCATAGTTAGACAAGGTTCGCAGCTTA

TAGATGCAAAAATCTATTTAAGAACCGAACAAAAGCAAGAAAATCTAGACTTTTATCTTG

ATAAATTAACAAGGGGACTTCCTCGTCTTGAGTCTCTAACCATACTCATTACTGGTGTTT

CCAAGGTGGGCAATTTCCCAAAAGTCTCCAATAGCTTCACTCATCTAACGTGTTTGGAGT

GGGTATGGACGGCCGGTGTTAGAATGAGCCTTCCTTTTCTACGGACACTCTTGGATGCCT

CTCCCTTTCTGGAGAAATTGAAATTGCATCTTTCGGCAGGAGTGGTAGAACCACCACCTC

CAAGGCTAAGTGAACTAAGCGAGATGCTACCCAAACATCCCCGTGCACATCTAAAGAAAG

TGGACATTTTTTATTTTTATTTCGACCAGTGGCAAATTGAGATTGTGTTGTATCTTCTTA

ACAATGCTACTGAGCTCGAGTCAATGACAATAGAGAAGGGCCATGATCTTTCTACGGAAA

TCAATGAGATGGACATACAAAATCTTACTCAACAAGTCCCGGCAAATGTCAAATTAGTGA

TTTTGTGATTTTCTTGAAGGGCCGTGAACCTACTCAACAAGTCCCGGCAAATGTCAAATT

AGTGATTTTGTGATTTTCTTGAAGGGCCGTGAACCTTCAGAAATCAATGAGACCAATGAG

AAGGACATACAAAATCTTACTCAAC

>Unigene0002159

GACAATTGAAATTAGCGACTTTTACTGTGAAAAATGGCAGATTGAGATTGCACTATATGT

TCTTAGCAATGCTATCGAACTTGAGTCGATGACGATTGAGAAGGGCTGTCCATCTTTCAC

AGAAATCCAGGAGAGGGACATGGAGAATCTTAGCCAAGAAGTTCCAGCAAATGTCAAATT

AGTGATTTTGTAATTATCTTCTTCACTTTTCCCTTTCAACAATTTTTGTATTTATTTATT

ATTTTTTCTGAATTTATCAGGCCATGATAATACGAGCATGTTGATATTTGTAGTAAGCCA

ATGAATTGAAATTAATATGCTTCCTTCCGAAAAAATACTTTTTGGATGGTCTGTACTAAA

CAAGGACTAACATCAAATTACCATGA

>Unigene0002160

TGTGGTGTCACTGAGCAGTGTGTGCCATTACAAAGTGCCCTAAATTCCGGAGACCAGCAG

CCAGCTCCGCTCCGGAATCCCCTCCCGATCGCGATGCCGGGCCCCGGCCCGCACATGATG

TATACGCTCGGCGCGGGAGTCGGGCTGATGCGGCTCACCAATGGCCGATTCGGGCCCCAT

CATTGCCTCTTCTACGCCGCCAACGCTTTCCTCGGCCCCGATCTCGGCTCCTTCGCTGAG

TGGCTCTCCTCCGCCCTCGGCCTCGGCGGCTCGATCGGATCGCTCGCCATGTCCCTCGTT

CACCACCCCTTCTACTATCCCCTGTTGCTAGGGATTCCGCTCGCAGTTTTCTATTCGTGG

TTGTCCGCCGTCCTCCGCCGCGGGATTCTCGACTCCGCATCGGTGGTTCCGCTGAGACGA

CGACAATGCTTTATGTTGGTGGCTGCGGGATCCCTGTCTCACTTTTTCTTGGATCACCTG

TTTGAGGAAAATGGCCATTCTTCAATGTATTCTTGGATATTGAGCACAGGTTGGTGGAAG

AGTGTGGCTCCAATCAACCCAGATGCGGTTGTTGTAATTGGATTCTTATGTACTTGTTTA

GTTGGAGGCTTTATATATATCAACAGATTCAATGATGGGAAGTCTATCATAACTAAATCG

AACCAATCCCTGAGGCTTATCATGGTTATAGCAAGCCTGTATTGCATGTGGTGCACCATC

CAGATATACTTGCGTAATCCTCCCCAGCCAGCTATTGGTGAAGAAGCAGATCTTGGAGTT

CTCATCTTTCTTGGTATCTATTTTTTCCTCCCTCATAGCTTGTGTATTATGTCAATGAAC

CAGAAAGATTATGGTGACATGCCGTCAGATCAATTACCACTGTAACAGCACTTCATGCAT

TCTTAGCTGAACACCCCATGATTATTGGGGCTTGGGAATCTGTACTTGGGATTTTCACTA

CAAGACATGCTTATTGTATTGTTGGTTACAGACATACAAATATATAGATAAAGCAGCAAC

AAAATTGATGCCAAGCTAAATGTACGAAAGTTTCACTGAAGTTACATACTCAAATTCATG

GTTACCGCAGCAGCTGGTGGACTCATCTCTGCAATATGTAAATCATTTATGTTGTAATTA

GAGATTCATTTCCCTTTCTGCATTCTGGATTCCGCTAGCATAGATGTTGAAGACTTTGAG

ATCCAAATTCTCCCACTGGTAGATATGGCTATATTAGTAACAACACTTTCATATATGGCT

GTCTTAAGTGGTTAGATGTACCTATATACTTCTGTCTTGACACAATGAACCCTTAAAAAT

CCAGGTATGACCTTTTCCTTTTTACCTGCGATGCAGATTGTCTAATAT

>Unigene0002161

CTATATACTCCTCCGGAAAAGAGAAATATTTGCATGTGTTAAATGTTTCGAGAGATTGAA

GTATTTATTTGCTTCCCATTAGGAAGCTCTGAAACTGGCATGGATCCTCATACATCTCCA

ACTTTTTAATAAAAATAGAAGATAGATCAGTAGATTTGAGTTTGATTACTGTTCAGAAGT

TTCTCCCACAGATCCGGTTTTTTGTTTCAGAGAATTCAAATCTGTCCTCTGGAATATGAT

GGGTGGGAATTAAGAGGGCCTCTTTTCTGGCAGAATTTCCATTTGTGAACAGAGAATAAA

AGAAGAATTTAGATAAAATAATTTATACTTTCTGAATCTAATATGTGTGTATCTAATTGA

ACAGCCGCAGATCAATCCATAAGAA

>Unigene0002162

TCCATTAGGTCCCCCTCTTTCTTGATCCATTTGCTATCTCTACTACTCCATTTTCCACCT

TGCCGGCTAGCGCCGTGCTCATACCGTTTCTCGTTTTTCCATTTGTTTGGTTTGTGTGAT

TGATTGAAAGAACAAATAATGGACAGAAGGTTTAATAGCGACTCGCCAGTGTATGAGCCG

GAGGATGTGGGGATTTCGCCGGAGACGAGTTCACCAAGTCCGCGTAGCGGTGATCTGAAG

GCGGTGCATCAGTTCTCCTCGACCTCCTCCCCCTCCCCTAAGAGAAGCCGCAGATCAATC

CATAAGAAAGTGGTCACAGTACCAATCTCAGAGGTTGACAGCTCACGGCCCCGGATCACC

GGAGAGGGGGCACCACCGTCGGACTCATGGGCGTGGAGGAAGTACGGCCAAAAGCCCATC

AAAGGCTCGCCATACCCAAGGGGTTACTACAGGTGTAGCAGTTCAAAGGGTTGCCCAGCA

AGAAAGCAGGTGGAGCGAAGCAGCTCAGATCCAACCATGCTGGTTGTCACCTACTCCTGC

GAGCACAACCACTCCTGGCCGCTACCGAAGAGCCATCACCGCTCCACTGCTGTCATTCTC

AACACTTCAATTCCTTCACCGGTGTTGAATGAGGAAGAGCCTGAGGCGGTGTTGGTGGTT

CCGGTATCCGAGCCTGCGGTGGACAAGTTCTTGATTGGCGAGGAGTTGATGCGGGATGAG

TACTACTGGTTCTCTGATGTCGGGTCGCCATCGGCGACATCGACTGCGGGATCGACTGAC

GACCTGTTGCTGGGGTCGATTGTTGTGTCTGCTGATATTGCCGCAATGTCGTTTTCTGCG

GAGTGCGACGAGCTGGGACGGTGGGAGGAGGAGGAGGACTCGTTGTTTGCGGGACTGGGG

GAGCTGCCGGAGTGCTCTGTGGTGCTCCGGCGGGGGTTTGTTGATGGTCGGAGGTGTGGC

CTGGCAGCGTCGTTGTGATTGGCAGGGAGTGGCACTAATGTTACATAAATTTGATTTTTA

AATTTTAATAAAGTTGAATCACTTTTGTTGTGTTTATAACTAATTTAGAGCAATAGTCCG

TATTGCCGGTCTCCGTCGGCAACAACCAACAGG

>Unigene0002163

CCCAAACTACTACTTCCTGAAACCTTTGACTATAGCAGGACAACTGAGTACCTGTGCGCT

CGAATGTACGAGCAGAGAGAGTCCTGCGCCTGGCAACTGGTGTCCACTCCAAGTCGGGGG

AGAGCTATTCATCAGTTTGAGTGAGAAGGCTTCAGTCTTTAGTTTAGGTGGCATACTTCT

GGCCCAGATATGTGGGCTGATAGTGCCACACCAAAACCGTATGTCTGTGTGGGCTGGTAG

GCTTTTGGACTGGCAGTAATTTCCCTATAAAATTTGTGTGGTGGTGGATTACTTATGACA

TGCAGCCTCTACTTGCTGTTGGTGTAGGTTAC

>Unigene0002164

GACATTTCGCCCGCCTATGGCGAAGGTTTCAGATCTGCCAACACAGCTGTCAACAAGTAC

GGGCTACGTCGGACGCTAGATCATATACGCTATACGGGGAATTTCCCTATGTAGGATGTA

CCCTTTATTGTGTATGGTGGAGTTCGATGTTAATAAGATTGGGATGATCGAATTGTGATG

AAAATTTAGATCCCATCTGGTTGTTCATCAGAATGAGTACAATGGTAATAAGCAATTGTA

ATGGGTAACTCAAAAGTGAAGTTCACTTTTGTGCATATGATAAATTGGTATAGCAGGATG

AAATTGGTTATTCAGTGTTAAA

>Unigene0002165

CCTCCCTCACCTCCCCTACACATTTCCCCCTAGCTTTCCAAGATGCCTTGTGCTCATCTC

TCCCGTTCTTCTCCCCCCTTTTTATCCCCATCCCCGCTTTATCCCCTCCTTTCCTCCTCC

GTTCCTTTCCCATCATCAACTCGGGCCGTTCTTTACCTCTCCCCGGAGCTCCATTCACGC

TTCAAATGGGCTCTTCGGCTACCGAGTATCTCTCCCAGTCCTGCGTTTACGGTCACACAA

CTTCATCATACCCCGAGAAAAAATCCCGCTTCATGAAGTGGCTTAGCAAGATTTTCAAAG

GAACATGCCAGGAAGAATCGAGTGGACGTCGACCACGAGTTATGGAGGAGGAGAACATGT

TTCGGCGTGGACCAAATAGATCTGTGGTAATGTATTATTATTCTTTGATATAGTGGAATT

TATTTTGTCCAATTCATTTTACGGAACTGCATTTGTTACTGTCCCATGACTTTCGTTGGC

CTCATTACATAAGGATAAACGCCAATTAGATTTGACACAAATAGCATCATCAACTAACTT

TTTGATAATTGATGTATTTATTTTCACTCGTCTGTTGTGTGAGATTGGTTTTTGTAAGTT

ATTTATAGGATGACCAGTCAAAAGCCGAGAAAGAAGATTTGGATCATGCAATTGCACTTT

CTCTTGCTGAGGATTCCAAGAAGCCGAATGGCTACAAAGGGCACGACCATAATGACGAAG

ATCTTTCTAGAGCTATGCAAGAAAGTTTGAACTTGTCCTCCTTTCGTCCGTACAACCCTG

TACAGTTTTTACCAAGGGGATATAGAGTTTGTGGTGGGTGCCATTGCGAAATAGGCTATG

GTCATTATTTGAGCTGCATGGGAACATTTTGGCACCCACAGTGCTTTTGCTGTTTCAGTT

GTAGTCAGCCCATCCGCGAAACTGAGTTTTCTTTATCAGGGAGTCAACCGTACCACAAGT

CGTGCTATAAGGAGTTGCATCACCCCAAATGTGATGTATGCCACCAATTTATCCCGACAA

ATAGGGCTGGTTTGATCGAATATAGAGCTCACCCATTTTGGGGACAAAAGTATTGTCCTT

CACATGAACATGACAATACACCTCGTTGCTGTAGCTGTGAGCGAATGGAGGTTTGAATGA

TTGATGGAATGTTTTCGTCTCTCTTCTTTTCAACTATGATCCAGTTTTGGTGATGTTAAG

ATGCATCCACTCTATCGATATCATATGGCTTCCTTATAACATGCCTTTTTTATTGTAGTC

GAGAAAGACAAGGTACATGGCTTTAGGAGATGGCCGCAGCTTATGTGTAGAATGTCTGGA

CTCTGCTGTCATGGATACTGGGGACTGTCAACCGCTTTACCATTCAATCCGAGACTATTA

TGAAGGAATGAACATGAAAATAGATCAACAGATTCCCATGCTTCTAGTTGAAAGACAAGC

ACTTAACGAAGCCATGGAAGGAGAAAAGGATGGCCACCACCACATGCCCGAGACAAGAGG

TTTATGTCTTTCTGAGGAGCAGACTGTTAGCAGTATACTTAAGAAGCCAAGAATCAGTGC

ACACCGAATCTTAGACATGAGATCTTGTCCACAGAAGTTAACTCGCAGATGTGAAGTCAC

AGCAATTCTTGTTTTATATGGGCTTCCTAGGCTACTAACAGGTTCCATTCTTGCCCATGA

GTTAATGCATGGCTGGTTACGCCTCAAAGGTTATCGCAATCTAAGCCCGGACGTGGAAGA

AGGTATATGCCAAGTCCTCTCTTACATGTGGCTCGAATCTGAGGTTATGCCAGGCTCAAC

GAGCATGCCATCATCATCTTCTCACGCTCCTTCATCCTCGTCCTCTGCATTTCCGTCGTC

CAAAAAAGTAGCAACATCGACGATCGAGAAGAAGCTTGGGGAGTTCTTCATGCATCAAAT

TGCTCACGACATTTCGCCCGCCTATGGCGAAGGTTTCAGATCTGCCAACACAGCTGTCAA

CAAGTACGGGCTACGTCGGACGCTAGATCATATACGCTATACGGGGAA

>Unigene0002166

GAAGACCAACACTTGTACAGTGTGTTAAAGTAGTGTATCTGTGTACTGTCTTTTATTCAA

GATGCCTGGTTTTGGAAGGATGAGGGAGCATGCAAATCAAGGAGATGAAAGTAGAGCAAG

CTGAGGAGAAAAACTTCAAATAGATGTCTCCGATTCTTGAAGGATAACAAGGGGTTAGAA

TGGAATAAATTATTTATAGACAAGTATTTTCATTCATGCTTGAACAGTGAACCCTGCTGG

TCCCAAACAAGATTGTAGTACCCAAACTACTACTTCCTGA

>Unigene0002167

TTGGGTGGAGATGGGGTGTTCGGGCACTTTGAGCTTAGGTCCCTTTGTTGGCTTGGGTGG

AGATGGGGTGTTCGGGCACTTTGAGCTTAGGTCCCTTTGTTGGCTTTATGTCGAGTCTTT

GTGACAACTGTTTCTTCTTTCTTGGTTTAGTTAAATGGTCGACTGCTGAAGAGATGGGTT

GGTATTCTCGATATCTGGTTGAAGACATCGGACAATCTATATACTCTGGAAATGTTATTT

TGTTCGTGGTTTTCCTTTTCGGG

>Unigene0002168

CCATTAGACGTTCTGAACGGTAGGTAGTATATTCAATTTTACAAATATCGACCATTTCAC

CTCTTTAGACTTCCAAAGGCTTTTAAGTAGAATAGCCCAAAATAAGTGTAATTGTGTGAT

ACCGACAGGTGTTTCTTTTTTATTGAGTTTTATTCTGTGTAGTGATACAAGATGGCAATG

GAAGTGACACAAATACTATTGAATGCACAAGCAATAGATGCTGCAGTCCGGAAGAATGCA

GAAGATAGCTTGAAAAGCTTTCAGGAAGAGAACCTCCCATCTTTCTTGCTATCTCTTTCC

AGTGAGTTAGCAAACAATGAGAAACCCGTGGACACACGTAAATTAGCTGGCTTGATCCTG

AAAAACACTCTGGATGCTAAGGAGCAACATAGAAAAGACGTACTCGCCCAAAAATGGTTG

ACTCTAGACTATCTCGATACATCTGTGAAGCCACAGGTCAAAGAGTGCTTGTTGCATACC

CTTTCTTCCCCTGTTCCCGAGGCTCGGTCTACTGCTTCTCAAGTCATTGCTAAGATTGCC

GGTATCGAATTACCTCACAAGCAGTGGCCCGAACTTATTGAAACTCTTTTGTCCAACATC

CACCAGCTTCAGCCCCATGTCAAGCAGGCGACCCTTGAAACTCTTGGCTATTTATGCGAG

GAAGTTTCATCACAAGCACTTGAGCTAGACCAGGTGAACAAGATGCTGACTGCAGTTGTT

CAGGGAATGAACGCTTCCGAGACGAACAATGATGTCAGGCTGGCTGCGACAAGAGCGCTG

CATAATGCTTTGGGATTTGCACAGGTCAATTTTTCACATGACTTAGAGCGGGATTATATA

ATGCGAGTTATCTGTGAAGCGACATTGTCCTCCGATGTGAAGATAAGACAGGCTGCTTTC

GAGTGTCTCGTGTCAATATCCTCTATTTACTATGAGAAGCTCGCATCGTACATGGAGGAC

ATCTTTTATATCACGGGAAAAGCTGTGAGAGTAGATGAGGAGCCGGTTGCACTTCAAGCT

ATCGAATTTTGGAGCTCGGTGTGTGATGAGGAAATCGACATATTAGATGAGTATGGAGGT

GAATTTACTGCTGATTCCGGGATCCCGTGTTTCAATTTCATCAAGCAGGCGCTTCCAACC

CTGGTTCCTATGCTATTGGAGACACTCCTTAAGCAGGAGGAGGATCAAGATCAAGATGAA

GGGGTGTGGAATATCGCAATGGCTGGTGGAACATGTTTGGGTTTGGTCGCACGAACTGTG

GGGGATGAAATCGTCCCCCTTGTGATGCCTTTCATCGAAGAAAACATAACTAAGCCTGAT

TGGAGACAGAGAGAAGCAGCTACATATGCGTTTGGTTCAATTTTGGAGGGGCCGTCCCCT

GACAAGTTGGTTCCTATCGTCAATGTTGCCTTGAACTTAATGCTCGCTACTTTGTCGAAT

GACCCCAACAACCATGTTAAGGACACCACTGCATGGACTCTCGGTCGAGTGTTCGAATTT

CTCCATGGCTCGACTATGGAGATACCTGTCATAACCCATGAAAATTGCCATCAGATCCTT

ACAGTCTTACTCACAAGCATGAAAGATGTCCCTAATGTTGCAGAGAAGGCATGTGGTGCT

CTTTATTTCCTCGCTCAGGGTTATGAGGATGTAGCACATTTAGCATCACCATTATCCCCT

TTCTTCCATGATATTGTTCAGGCTCTTCTTAATGTGACCCATAGGGAAGATGCAGGAGAG

TCCCATCTGCGTATCGCAGCATATGAGTCTCTGAATGAGGTAGTAAGGAGTTCGACTGAT

GAGACAACACCGATTGTGGTGCAATTGATCCCGGTGATCATGATGGAGCTCCATCAAACC

TTCGAATCACAGTCGTTAGAGAAGCAGAATGAAGTGCAGGGTCTTCTATGCGGTTGCCTA

CAAGTAATTATCCAGAAACTCGGAGCATCAGAGTCGATGAAATACGCATGTATGCAGTAC

GCAGACCAGATGATGGAGCTTTTCCTAAGGGTTTTCACGTGCAGAAACGCAACAGTTCAC

GAAGAGGCTATGCTTGCTATTGGTGCCCTAGCATATGCGACTGGGCCAAATTTCTTGAAA

TACATGCAAGGGTTTTATCCATGTCTGGAAGTTGGTCTTCAGAACTACGAGGAATATCAG

GTTTGTGCTATCACTGTCGGTGTGGTTGGTGATGTATGTAGGGCATTGGAGGACAAGATA

CTTCCGTTCTGCGATGGGATAATGACACATCTTCTCAAGGACTTGTCGAGTAATCAGCTG

CACCGATCTGTTAAGCCCCCGATATTCTCGTCCTTCGGTGATATCGCGTTGGCTATCGGG

GAGAAATTCGAGAAATACTTGGTTTATGCAATGCCTATGCTTCAGAGTGCAGCAGAGCTG

TCTGCTCATGCGGCAGCTTCAGACGATGAAATCACTGATTACACCAACCAGCTCAGGAGT

GGAATCTTGGAAGCCTACTCGGGGATATTCCAGGGATTTAAGAGCTCGCCAAAGACACAT

CTGTTGATGCCATATGCACCGAACATTCTTCGGTTCCTCGACAGTTTGTATATGGTGAAG

GATATAGACGACACGGTGACGAAAACTGCAATTGGAGTTCTCGGAGACCTGGCTGATACG

CTTGGTACCAACTGTGCTTCTTTGATCGACCAGTCGGTGTCGTGCAGAGAATTTCTTGAA

GAGTGTTTGTCGTCAGATGATCATTGGATCAAGGAGTCGGCTGATTGGGCTAGGTTTGCT

GTCAGCCGAGCTGTGTCTGGTTGATTTTGTAATGTAGGCGTGTTTGTACATGCATTTGGG

TTGTATTGAGTCTGGTTGAGGATTGTATTCATGCATTGGTCATCTTCATTGTTGCGGAGA

GGATTTTGTTTTGGGTGGAGATGGGGTGGTCGGGCACTTTGAGCTTAGGTCCCTTTGTTG

GCTTGGGTGGAGATGGGGTGTTCGGGCACTTTGAGCTTAGGTCCCTTTGTTGGCTTTATG

TTCAGTCTTTGTGACAACTGTTTCTTCTTTCTTGGTTTAGTTAAATGGTCGACTGCTGAA

GAGATGGGTTGGTATTCTCGATATCTGGTTGAAGACATCGGACAATCTATATACTCTGGA

AATGTTATTTTGTTCGTGGTTTTCCTTTTCGGG

>Unigene0002169

GACAACTGTTTCTTCTTTCTTGGTTTAGTTAAATGGTCGACTGCTGAAGAGATGGGTTGG

TATTCTCGATATCTGGTTGAAGACATCGGACAATCTATATACTCTGGAAATGTTATTTTG

TTCGTGGTTTTCCTTTTCGGGGTTTTTCTGATGCCGCTGCATTGGTTGTCTATTATGGTA

CTCTACCTTACTGTATATACTTTTGGAGTGGTTTTTTAAGTTGAATTCGGATATCATGTT

GCATGGAAGTTATTTGGTAGTTGGTATTGGTGTTTGATCAGATGTTAAATCTTTTGTCAT

GTATGAATTTGTGAATCTACTCTGTTGTATTTGGATTTAATGTTGCATTGGAGA

>Unigene0002170

AATGTAGGCGTGTTTGTACATGCATTTGGGTTGTATTGAGTCTGGTTGAGGATTGTATTC

ATGCATTGGTCATCTTCATTGTTGCGGAGAGAATTTTGTTTTGGGTGGAGATGGGGTGTT

CGGGCACTTTGAGCTTAGGTCCTTTTGTTGGCTTGGGTGGAGATGGGGTGTTCGGGCACT

TTGAGCTTAGGTCCCTTTGTTGGTTTTATGTCGAGTCTCTGCGACAACTGTTTCTTCTTT

CTTGGTTTAGTTAAATGGTCGACTGCTGAAGAGATGGGTTGGTATTCTCGATATCTGGTT

GAAGACATCGGACAATCTATATACTCTGGAAATGTTATTTTGTTCGTGGTTTTCCTTTTC

GGG

>Unigene0002171

CCCCCAAATTCGATTCAATTTCTATCTACAATTGATGGGGCAGATCCGACTCAAGCTGAT

TTTGGTTTTACATGTGTTCAATTAATGCTTAGAGTTTTCTCAAATTACTGTATGACTTTA

TCGGGGGTTGCCATTGATCTTTTATTGGAGGCGGAGGATGACGTCGGGCGCCGCCGTAAG

AAGCTCCTCTCCGGAGTGCTCAAGTTCTTTGAGGACGATGCCGCTGTCGCCGATATCAGA

ACCGAAGGTACC

>Unigene0002172

TCCCTTCCAGCTTTCCCCATATTCCCCTTTACGACCCCCATCCCCACCGTCAACTTCCAT

TTCCAAGCTCTCTCTCTCTCTCCTTTCCAAGCAGCCAAGCTCTGCTTCATGACACACAAG

TTCTACTGCATGACGATGAGGATCCATATCGACTGCGATGGTTGCTACCGCAAAGTAAAG

AGAGCTCTCCTCAAGATCCAAGAGCTTGAGAGCCATTTGATAGAGAAGAAGAATGGTAAG

GTGAGTGTGTCTGGGGCTTTCGACCCGCAAGATGTTGCCATAAA

>Unigene0002173

AGCTTGAGAGCCATTTGATAGAGAAGAAGAATGGTAAGGTGAGTGTGTCTGGGGCTTTCG

ACCCGCAAGATGTTGCCATAAAGATAAGAAGGATGACGAACCGCCGCGTCGAGATTTTGG

AGATCAAGGAGGCTGAAGAGGTACCTCCATGAACCAGAGAGAGAAGACCATGTTGGCCAT

GGACGAATTATCGCTTGGATCTTGATGTGTGTATGTGTGCGTCGGTAAATCGACTAAGTT

GTTTACATGTTTTTCTCGTATCTGGTTGAAGGTTAAAAGAGAATAACCCTCTATGTTCAA

GTATGTTATGAACGGTCAAATTATATTGAGCAGGGAGATCCGAAATGAGTATAATAAACG

GTCATATTATATCAACTCAGTTTACTC

>Unigene0002174

AAAAAACAGCCCTTCGACAATCTCAAGGGCCCATCGATGCTCTCGATGCACCAAACCCCC

GACCTTCAAATCCCCTCCTCGTCCAACACGGCACCTCTCTTGTGATCGGTGATGGCTTTC

TCCTCCGCGAGATGCCCCTCCTCTTCCCGTTTTGCCCCTCTGCCGACAACCCAGAAGGCG

ACTGCCTGCTCCCAGTCCCTTCATCCGATCCCGAAGACCGTCTTCAGGAAGTGGGTCTGC

GCCGATTCGCACAAGAATCGGAAGTGGAACGTTGTGCGGATGCAAGATGCCTCTGCAACT

GTACCCGTGCCATCTGCAAAAGTTCCACAGGTGAACATGAAGGTTGAAAATGGCCCGTTA

ACCACTCTGCCTTCAGATAAATATCAAAACGTTCCAGTTCTTGATACTGCTACAGAGCCT

ACAGTTAGCATTACCATTGTTGGTGCATCTGGAGACCTTGCGAAGAAGAAGATATTTCCT

GCACTGTTTGCACTTTATTATGAAGATTGCCTCCCAAAGCATTTTACGATTTTTGGTTAT

GCTAGGAGCAAGATGACAGATGCTGAATTGAGAAACATGGTGAGCAGAACACTAACTTGC

AGAATCGATAAGAGGGAAAATTGTAATGACAAGATGGATGAGTTTTTACGTCGATGCTTC

TATCATTCAGGTCAATATGACTCCGAGGAAAATTTTGCAGAGCTAGACAAAAAGTTGAAA

GAACACGAGGATGGGAAGGTCTCAAACCGCTTATTCTATTTATCAATACCTCCAAACATA

TTCATAGATGTGGTAAAATGTGCTAGCGTGTCCGCTTCTTCTGCAAATGGCTGGACAAGG

GTGATAGTCGAAAAACCATTTGGTCGGGACTCCGAATCTTCTGCTGCTTTGACAAAAAGT

CTGAAGCAGTACCTAGCGGAGGATCAAATTTTCAGAATTGACCACTACTTGGGGAAGGAA

CTAGTTGAGAACTTGTCCGTTCTTCGCTTCTCAAATCTTGTCTTTGAACCTTTGTGGACA

AGGCAATATATAAGGAATGTACAGTTGATTTTTTCTGAAGATTTTGGCACCGAAGGACGT

GGAGGGTACTTTGATAGCTATGGTATCATCAGAGACATTATGCAGAATCATCTGCTTCAA

ATTTTAGCCTTATTTGCCATGGAAACACCTGTCAGCTTGGATGCGGAGGACATAAGAAAT

GAAAAGGTGAAAGTTCTACGTAGTATGAAGCCCTTGCTACTGGAAAATGTTGTGACAGGA

CAGTACAAAGCCAATAGAAAGGGTGACATTTCACACCCTGGATACACGGATGATAAAACT

GTACCCAAAGACAGTCTGACTCCAACATTTGCTGCAGCTGCCCTTTTTATAGACAATGCA

AGGTGGGATGGCGTGCCTTTCCTTATGAAAGCTGGGAAAGCATTGCATACTAGAAGAACA

GAGATTAGGGTACAGTTCAGACATGTCCCTGGCAATCTATACAAAAGGAATTTTGGAACG

GACCTTGATCGAGCTACAAATGAGCTTGTGATACGGGTACAGCCTGATGAAGCTATTTAC

TTGAAGATAAACAATAAGATCCCTGGTTTGGGAATGAGGTTGGATCGCAGCAATCTGAAT

CTTCACTATTCAGCAAGATACTCGAAAGAGATACCGGATGCATATGAGCGGCTTCTGCTG

GACGCAATTGAAGGAGAGCGACGGCTTTTCATTCGTTCTGATGAGCTGGATGCTGCATGG

GCGCTCTTCACCCCTCTGCTGAAAGAATTGGAAGAGAAGAAAATTGCTCCAGAGCTCTAT

CCTTACGGGAGCCGTGGACCCGTAGGTGCGCACTATCTCGCAGCCAAGTACAAGGTCCGA

TGGGGCGATCTCAGTTCCGATCATTACAAAGCTGATCCCTAGTAGCTTGTCGATTTATGT

TGTTGTGTCGACAACTGTAGACCGAGATCATTACAAAGCTGATCCCTAGTAGCTTGTCGA

TTTATGTTGTTGTGTCGACAACTGTAGACCCCTGGTGAGTTAGACTGGAGCTGAAAATTC

ACAAGGTTAGGAGTTGGATGATTTGCTAGTCGTTGAAGTGTGTTGTTATTGTTTGAGTTT

TGAATTGCACGAGAGTATGATAGTGAGTAATGGGTTGAGGATGGAAGGCATTTATGTTTA

TCTTTCTGGTTTGATGGTGACTTTACCTGGTCTGTTATTGCCACGGATGGGCGACATTAT

TGAAATAAATGTTATTGATGTGTTGGGTTGGCAGGGGATATGATATACATTCTCTCTCGT

ATTTATTTAAATGTTATAATTTT

>Unigene0002175

ATGCGAGAGAGAAAAATGTGAGCTTCATTCAATCCCCCCATTTTATTTCTGGAGCTCTGT

TTTTAACCCTAAAATCTCTGACCAAGAATGAGGATAGAGGTGCTAGCCCTCTCGCTGCCT

TCTCCTCAGTGGGAGAGGTTATCATCGTCCCATCAAGCTAGCACTCGTGGCACGGCAGAA

ATGGGTGCTACCATGGAGCTTAAACCATCTAGCTTCATCACCAAACCATCTCTGCCGGCA

AGGAATATTTTGCCAAGATCTTGTGTGAGGCATGGGAGAGTGGAAGGTTGGGTCAATCCA

TGTAGTGGGAAGAGGCCATTGTCGGTGAAATGGGTGGACTCGTCGTTGCCGACTAGGAGG

CGGGTAAGGGAAAGGTTTGCGTCGACTACGGAGGCTGTGGTGGCATCTGAAGCTCCGGAG

GTGCCGGCTTTTGTTGTTGGGAATGAGAAGGTTGGGGTCTTGTTGCTTAATCTCGGAGGC

CCGGAGACACTTGAAGATGTGCAGCCGTTCTTGTTCAATCTTTTTTTCTGATCCAGACAT

TATTCGACTTCCTAGAATGTTCCGGTTTCTCCAAATGCCTTTGGCACAATTTATCTCTGT

TGCCAGAGCCCCCAAAAGCAGAGAAGGCTATTCTTCCATTGGCGGTGGTTCTCCTCTCCG

GCAGATAACTGATGCACAGGGAGAAGCATTGAGACAGGCACTCTGTGCTAAAAACATCCC

TGCGAAGGTATATGTTGGAATGCGTTATTGGCATCCTTTCACTGAAGAAGCTATTGAGCA

GATTAAACGGGATGGAATAACAAAACTTGTTGTGCTTCCACTATATCCCCAGTTCTCAAT

ATCTACTAGTGGTTCAAGCCTCCGTTTGTTAGAGAGTATATTCAGGGAGGACGAGTATTT

GGTTAACATGCAACATACTGTTATACCTTCATGGTACCAGCGTGAAGGGTATATCAAGGC

TATGGCAAATTTAACAGAAGAAAAGTTGAAAGAATTTGAACGTTCCGAGGAGGTTATGAT

ATTTTTTAGTGCTCATGGAGTACCACTGGCATATGTGGAAGAAGCTGGTGACCCATACAA

AGCGGAGATGGAGGAATGCGTAGACCTGATAATGGAAGAGCTAGAAAGACGGGGAATCAC

TAATGCATACACCCTTGCTTACCAGAGCCGAGTTGGTCCGGTGGAATGGTTACGTCCCTA

CACTGATGAAACCATTGTAGAGTTGGGGAAAAGAGGAGTCCGAAGCCTTCTTGCTGTCCC

CATTAGTTTTGTCAGTGAACACATTGAAACTCTGGAAGAAATTGATGTGGAGTACAAAGA

GTTGGCTCTAAAATCTGGCATAAGGAAGTGGGGAAGGGTTCCTGCTTTAGGTTGTGAACC

GACATTTATCACAGACCTTGCTGATGCAGTAATTGAAAGTCTTCCTTATGTTGGAGCAAT

GGCGGTTTCAAATCTCGAGGCTCGTCAGGTAATAACTATTACCTGAATCACCAACTTTAA

GGTGAAAGAACTCTTTTCACCAGATTCGTAGTTAATACAAACTTACGATACATGATATGG

TAGGGGACCGTTACAATTTTTTACGATACACTTAAGAGTAGCCTATCTTGTGATGCACGA

TACAATATTTGTGGTATACTACACAATACAAACAATACATGCTCATATTGTGTAGCATAT

CACACATCTCATTATCCACATGTGTGGCATATGCTCATTTAAGTCAGATTTCAGAGCATT

TCATGTCACTTCATAATCTAAATGTTTATATATATTTATATTTATTTATATATATATATA

TATATATATAAATTTAATATACATGTTGGATATATTTTTATATAACATATCTTATACTAC

GCGATACGAGGGTGAGCGAAACACGATAAGATACACATTTTCAAAACCTTGACCAGATTA

ATTTAGTGTATGCATCATCGATTTGGTTATATGTTATGCGTAACCTGAATATTATTTTAA

ATGGAAAAGAACACAACGTAATGGGCTTCAATATAAATCATTATGCAGGTCATATACATG

ATTTCACCCTGAACATGATTTTAAATGGAAAAATTAAAACATTAATGGAAAAGGTAAAAA

GAAAGATATTGCACTTAGGTGTTACCCAGAACACCATCAATACTAATTTTAACTGAGAAC

ATAATAGAAAAGAAAAATAATTATACACATGGTTATTAAAAACTTTCGATACGCGATACA

TATCATACAATACTGGGACAATACAAATCAAAATATGTATCGTCCTGGCAGCGTATCGAA

AAAATGTATCACGTATTGTAAGATACGTATCTTATTGGAAAAAAAACATGTAACACTGCT

AGGCCCGCATGACACCACCGATATTCCAGATTTTTCACCCAGCCTCACTCTTCTCCCTTG

GACATGATTAAAAAAAAAAAAGTAGTTATATGGAATCAATGACCATGATGTTTGTGTACT

TCTATTATTTGCATATTTATGCATGTTTAATATATTTATATATTAATATATAAAATGTGG

CAAACCTTATTCTACACGATACGTTACGATACATGTATCATGAAATGGGATGACCGATAC

ACGATACAATACACATCTTAATAATCTTGAATATACATAAAAGATAAGAAGTTTCAATTC

CAAATCTCTCGACGCCCATCCATTAACTTATGTTACGTCATTTAACCACTACTCTGGTTA

ATGTTAGAACTGAAATTAATGCACTACCAGTCGATCTTCTTTCTTTCTATTTTGCAATGA

AGTTACTTTATATTTTAATACGGTGGTACTCTATTTTGGTTACTGAGGGAAGGAACCAAA

GAAAACAGAGGAGGACCTTCTTTTCGAACCCTATGGAAATTGGAATAATTTTAAAGGCTT

CGATATCTTGCAGTCCCTTGTCCCCCTCGGAAGTGTTGAAGAGCTATTGGCAGCATACGA

TTCAAAGCGACGACAACTTCCCCCTCCGATAATGGTGTGGGAATGGGGTTGGACTAAAAG

TGCTGAAACCTGGAACGGCAGAGCTGCCATGTTAGCTGTGTTTGTGCTTCTGATCCTAGA

AGTAACTACTGGTCAGGGGTTGCTTCATCAGTGGGGCATCCTTCCATTGTTTCGATGAGT

CGAAGATTTCGCCTCTCTTCCACGCGGCAAATGAAGCTACTAGTGAAGAAGATACTAATG

CCTTATATAGATCATGTTGTTATTGCTGACAACTTGTAAGCTTGCTGGCTGCGGTTGTAC

AATTGCCTTTTTCGGCTAAATCAGTGGCGATACTAGTGTATATTGACATTCGGGAAAATG

TATACTTTTGTTATAAATGTGGTTTTGGATTAAAATGAATTCGACTAAATTACGCGGCGG

ATTTAACAAAAAATGTCAGTTGTGCAACCTTGTTTATCAGCTCTCTGAGCACATTTCAGG

AATGGATGATGTGGTTTCTCTTTTGTTGAATAA

>Unigene0002176

GGGAGTAATTTGAAACCATTCAAAGATAATTCTGGGGATGAACCTGAAGATGAAATCAAA

CGAGATACCAAGGTTATGGTGAACACTGATTCAAACGAATCCATTAATGAGTATGTGGAT

ACGAACCCTGTCAAGGCCAATAAGGACCGTACGGATGAATCAGAAACTAAGAAAAATACT

GGCAATCAAGAAAGTGCCCAAGACAACATCAAGGAAAAGAATTACGAAGGAAAGATCAAG

TATCAACTTTCCAGTGAGCAATCCAGAAGCTCAGAATATAATTCAAAAGAGCAAAAACAG

TCTGGTATGGAGAAAGAGCCCGCGCAAAAGGGTAACTCAGATGTTAATACAATGCAGGA

>Unigene0002177

CCATCTCCCGCCCCAACGACTCCAAGGGTATTTACGTCATTCCAAAACGTCCGGCTCGTC

CGCTTCGACAAGCAGCGCGGTTCACGGCCCCATCCAACACCCTCGAGATCCGCTAGCATC

ATTCGCGCGCCGGGGTCGGAACCCTCACGCTCCCGCGAGCGAGATTACAGCTCCAATAAG

AGATACCATTTGAGGCTTGAATATATGGTAGAAAATGGCATCTGAAAGGAACCCAAGAAT

GGATACTAGGAGGTCCTCAACGTATTGCTCAACGACAACCCTCGTTGTGTTTGTTGCTCT

TTGCTTGGTCGGGGTTTGGATGATAACATCATCAACCATAACTCCTTTGGACCTGTTTTC

ATCCGAAAACAAATCTGATGTCAAAGAACAGACTTCCGGGACCGATTCCAAGACTTTTGA

GGATGGTTTGGGGGATGTAGAAGACAATATGAGAGAGGATACCAATACTAAGACAAGCAC

ATATGTATATGAACCTAATAAGGAGAATTTGAAGGACGAGAATCTTGTGAAGGTCAATGA

GGAACGACTGAATGAGTCAGATGATACATCGAAAATGAAGACAGATTCTGACAATCAGGA

AGTTAACCAAGACAACATCAAGGAAAAGGATTCTGAGGAAACCATAAAAGATGAACAGGG

AAAGAGCATAGATCAAAATTTTGCTGAACAAGAAAATAATGAGAAAACTAAAATCTCAGA

AAATAGTTCGAATGAAACTGGAGCTGATGAGCAACAACAATTCGATAATGAGAATACAAA

TGGGAATCCGGTGAAGGACAATGGTATGAATGAGGTGTTTCCTGATGGGGCTCAGACAGA

GCTTTTGAATGAGACTGGTACTCAGAATTCATCATGGTCGACACAAGTTTCAGAGTCAAA

GAATGAGAAGGAATTGCAAGCATCTTCATCATCAAAGGGACAAATGGTCGAATACAGCTG

GAAACTTTGCAATGTCGCCGCTGGAGCAGACTATATTCCTTGCCTCGACAATGAGCAAGC

TATCAGGAAACTTCCAAGCACAAAACACTATGAACACCGAGAGAGGCATTGCCCCGAAGA

GGGCCCAACCTGCCTTGTTGGTTATCCAGATGGATACAGACAGCCGATCCAGTGGCCTTT

TAGCAGGGACAAGGTATGGTATAAGAATGTCCCTCATACGAAGCTTGTGGCTTATAAGGG

ACATCAGAATTGGGTGAAAGTTTCTGGAGAATACCTCACTTTTCCTGGTGGTGGAACTCA

GTTCAAGAATGGTGCACTCCATTACATTGATAAAATACAGGAGACATTGCCTGATTTAGC

ATGGGGAAAACGAAGCCGTGTAGTACTAGATGTTGGATGCGGAGTGGCTAGTTTTGGAGG

CTACCTCTTTGAAAGAGATGTGCTTACAATGTCTTTTGCTCCAAAGGACGAGCATGAAGC

CCAAGTACAGTTTGCTCTGGAAAGAGGAATTCCAGCTCTATCTGCTGTCATGGGAACGAA

AAGACTCCCTTTCCCTAGCTGGGTTTTTGATGTTGTTCACTGTGCTCGTTGTAGGGTTCC

ATGGCATATTGAAGGTACCTCTTGAAGTTTTTTCTGTTCTTTTCCTTTTCAGGTGTGACT

TTCCGTATCATGGTGGGAGTTTGCAGGAGGTATGCTTTTATTGGAGTTGAACCGGTTGTT

ACGGCCTGGGGGTTACTTTGTGTGGTCTGCTACTCCCGTCTATCGAAAGACTGAAGAAGA

TGTCAGCATATGGAATGCCATGACTGCCCTGACAAAATCCATGTGCTGGAAAGTCGTTGC

CAAATCTAAGGATCAACTAAACCAAGTCGGTTTTGCAATCTATAGAAAGCCTTCAGACAA

CAAATGCTACGAGAGAAGAAAAGAAAACAGCCCTCCCCTCTGCCATGAATCTGATGATCC

AAATGCAGCCTGGAATGTTCCCCTGCAAGCATGTATGCACAAGGTGCCTGTAGATCCTTT

AATGCGCGGGTCACAATGGCCAGAACAATGGCCATTGAGGTTGGAGAAAACACCTTACTG

GCTCATCAGCTCCCAGATTGGGGTTTATGGTAAACCTGCCCCTGAAGACTTTCAGGCAGA

CTATGAACACTGGAAACATGTTGTAAGCAAGTCATATCTGAATGGAATGGGAATTAACTG

GTCTAGCGTAAGAAATGTCATGGACATGAGTTCTGTATATGGAGGTTTTGCTGCCGCTTT

GCGAGATATGAAGGTGTGGGTCATGAATATCGTCGCGGTTGATTCGCCAGACACCCTTCC

TATTATCTATGAGCGCGGGCTATTTGGGATGTATCATGATTGGTGTGAATCATTCAGCAC

CTACCCTAGATCGTATGATCTTCTTCATGCGGACCATCTTTTCTCCAAGCTCAAGAAGAG

GTGCAAATTGTTACCTGTGATTGCTGAGGTGGATCGGATCCTGAGACTAGATGGTAAACT

GATTGTAAGAGACAATGCAGAGACGATTGGTGAGATCGAGAGCATGGCAAAATCCTTGCA

CTGGGATATCAGGTTTACCTACTCAAAGGATAACGAGGCATTGTTGTGTGTTCAGAAGAC

AATGTGGCGGCCTGAAGAGTTAGAATCCAGTACGTAGGCCATGACTTTGGATCACGACTA

AGAATTTTTTTCCAGCGACTCAGTATGGTATTTTTCTTTCTCATTCCTCCCATAAATTAG

TTCTTCTGGACAAGAAGGAGAATGTGAGGAAATGCATCACATGTTGATTGTAGGAATATT

CTGTTTTTGAATCAATGGAGGGTCAATTCATA

>Unigene0002178

TCGCCGTTGACAATGCAGACTCCCCTGACTCCACCGTCTTCGTCATCCGCGCCCGCAACC

GCATCGGCCTCCTCCAGGTGATCACCCGCGTCTTCAAGGTCCTCGGCCTCCGCATCGAGC

GCGCAACTGTCGAATTCGAAGGCGACTTCTTCGTCAAGCGCTTCCTCGTCGTTGATTCAC

ACGGGGCGAAGATCGAGGATCCGGAGAGCCTCGCACGGATCGAGTTCGCGCTCCGCGATG

CGGTCGAAGGGGCTGCTGGCCCCGCCGCGGCGGCGGATTCGAGTGGGCTTGTTGTGCGGC

GGGCGGGGTTGGTGGCGGCGTCTGGGGAGGGAAGGAGTAAGGTGGAGAGGATGTTTCATT

TGGCGGATGGGTTTTTGAAGAACGATCCGGTGAGTCTGCAGAAGGATATTATGGATCATG

TTGAGTACACTGTTGCGAGATCTAGGTTCAGCTTCGACGATTTTGAGGCTTATCAGGCAC

TGTCACACAGCGTAAGAGACCGTCTGATTGAGAGATGGCATGATACCCAGCTCTACTTTA

AGAGGAAAGACCCAAAGAGACTTTATTTCCTATCGCTGGAGTTTCTGATGGGGCGTTCAT

TATCAAATAGTGTTATTAATTTAGGCATAAGGGACCACTATGCAGAAGCTTTAAACCAAC

TTGGCTTTGAGTTTGAAGTATTGGCTGAGCAGGAGGGGGATGCAGCTCTAGGTAATGGTG

GTCTGGCTCGACTTTCTGCATGTCAGATGGATTCCTTAGCTACAATGGACTTACCTGCTA

TGGGTTATGGGCTTCGTTATCAGTATGGCTTATTTCGCCAGGTCATATTGGATGGATTTC

AACATGAACAACCAGACTATTGGTTGAATTTTGGTAATCCTTGGGAGATAGAGCGCGTTC

ATGTTTCCTATGCTGTGAAGTTCTATGGCACTGTTGAAGAAAAGCAATCAAACGGCAAGA

AAAGTAAAGTATGGATCCCCGGAGAAATGGTTGAGGCTGTTGCTTATGACAACCCAATAC

CTGGTTATGGGACAAGAAATACTATCAATCTCCGTCTATGGGCAGCGAAACCCAGTGGTC

ACTATGATATGGAATCTTTTGACACTGGAGATTATATTAATGAAGTTATCAGCAGACAGA

GAGCGGAGACCATTAGTAGCGTACTGTATCCTGATGACCGTACTTACCAGGGGAAGGAAT

TGCGGTTGAAGCAACAATATTTTTTTGTATCAGCCTCCCTTCAAGACATCATTCGTAGAT

ATAAAGATTCCCACAACAACTTTGATCAGTTCCCTGATAAGGTGGCCTTGCAACTAAATG

ATACTCATCCTTCTCTCGCCATTCCTGAGATAATGAGGGTGCTTGTTGATGAAGAAAATC

TGAACTGGAATCAAGCATGGGATATTGTGTGCAAAATATTTTCCTTCACTAGCCACACTG

TAGCTCCTGAAGGACTTGAGAGAATCCCGGTCGACCTACTTGGTAGCCTTCTTCCGCGAC

ATCTACAAATAATATATGATATAAACTTCTTATTCATGGAGGAGTTGAAAAAGCATTTTG

GTGTTGATTTCAATCGACTTTCTCGTATGTCAATTGTTGAAGAAGGTGCTGTCAAGAATA

TTCGAATGGCGAATCTAGCACTTGTTTGTTGTCGTACTGTAAATGGTGTGTCTCGTGTTC

ATTTGGATACCTTGAAAACAAAAGTATTCAAGGACTTTTTTGAGCTTTGGCCTGAAAAAT

TCCAATACAAGACAAATGGTGTTACTCAGCGTCGGTGGATTGTTGTTAGCAATCCTAATT

TATGTGATCTTATCTCAAAATGGCTTGGGACGGATGCTTGGATACGTGATGTAGACCTTC

TAATGGGATTACGAGATCATGTCGAGGATGCAAAACTTCAACAAGAATGGAAAATGGTCC

GAAGGGTCAACAAAATTAGGCTTGCAGAGTTCATTGAAACAATGACGGGCATGAAGGTTA

GCCCAGATGCATTGTTTGATGTGCATGTCAAGCGAATACATGAGTATAAAAGACAATTGC

TAAATATACTCAGTATCATCCATCGCTATGATTGTATAAAGAACATGGATAAAAAAGACA

GGAAAAAAGTTGTTCCTCGGGTGTGCATAATTGGTGGAAAGGCTGCACCCGGTTATGAAA

TGGCAAAGATGATAATTAAACTGTGTCATGCTGTTGGAGAAGTGATCAACAATGATTCTG

ATATTGGTGATCTCTTGAAGCTGGTTTATATTCCTGATTACAATGTCTCTGTAGCTGAAC

TAGTGATACCTGGAAGCGATCTTTCGCAGCACATCAGCACAGCAGGCCATGAAGCATCTG

GCACTAGCAGCATGAAGTTTTTGATGAATGGATGCCTACTTTTGGCTACAGCTGATGGCG

CCACAATTGAAATAATTGAAGAAGTTGGAGAGGAAAATATGTTTATATTTGGTGCCAAAC

TACAAGAAGTTGCATCATTGCGAGAAAAAGGCCAGAGTCGTAAAGTGCCACTTCAGTTCA

CACGGGTTGTCAGAATGGTTAAAGAAGGCTACTTTGGTTATCAAGACTACTTCAAACCTT

TATGCGACTCCATTGAGCAAGGTGGTGACTTCTATCTTCTAGGATATGATTTTGCGAGCT

ACCTCGAAGCCCAGGCCGCAGCAGACAAAGCTTTTGTTGATCAAGAGAGGTGGACTAAGA

TGAGCATTCTTAGTACTGCAAGTTCTGGAAGGTTCAGCAGCGATCGAACTATCGCGGAGT

ACGCAGAACAGTCGTGGGGAATCGAGCCTTGTAGATGCCCATTTTGATTGTACCTACTTA

AAAGCCCATGATCAACTGAGAGAATGCTGCTGTATGCTTGTATTTTTTTGTGTTAATTTA

TTATGTCACACACAAGTAGGCTTTATCGATTTGTTTATTGAAGTTGTAACATTATTGTCA

TAAATTTATATAGCTTTTGAGGCATTTTGAGGTAAACCCATGATTTGATAAGAAATTTTC

ACTGAATAAGTACAAATTGATATGGGAATAAATGTTTTTCTGCTTTGCAAGATTAGCTTT

GCTA

>Unigene0002179

TGCACAAACGATGCCATTTTGAGCTGCTCTTCACCCTCATCAATGGCAACTCTTGGCCAC

TCTCTAACCCTAATTTTGCCCAATCCCCGGTCAGGATGCTTGAAATGCTCTGCCCTAACC

AGTAGAACTTTCTGCAGCCCAAGTTCCACTTATCCCAGCTCTCGGCGACCATCTCCGACC

CACTTGAAACTGAACCCTCTCACCACCGTCCGAATGAGCGCCGCCGTCGAGGGCGGCGAT

GACGAGACGAGGCAGGCAAAGGAGATGGCGGCGGCCCGGAAGAGATGGGAAGCTCTGATT

AGGGAGCAGAAAGTGAAAGTTCTCACACCAAAAGAAGCTGGTTATGCGATTCAGCTTTCG

TACAAGACTCTCCTTGACGTTCGACCATCACCAGAGCGTAACAAGGCATGGGTTAAAGGC

TCGGTGTGGATACCCATTTTTGATGTAGACAACGCGAACGACATTGGAACTCTGCCCAAA

AAGATCACTAACTTTGTAATGGGAGGATGGTGGAGTGGGAGTCCTGCATTAGCGTATGAT

AAGAATTTCATATCAAAAGTTGAGGAAAAGTTTTCAAAAGATAGTGACCTTATTGTAGTG

TGCCAAAAAGGACTACGATCTCTTGCTGCTTGTGAGCAACTCTACAATGCTGGTTTCACC

AATCTGTTCTGGGTACAAGGAGGTCTGGAAGCAGCTGAAGAGGAGGATTTTCCACGCGAA

GGCTCTCAGCCCTTCAAGCTTGCTGCAATTGGCGGAGTTTCTGAGTTCTTAGGCTGGACT

GACCAACAACGCGCAATAGCTGCCAAGGAAGGATGGGCTTATAGATTAACCTTCACTGGT

CGTCTGCTTGGACTTGTTATACTTGCAGATGCTTTGCTTCTTGGGGCTCAACGACTCGGT

CCTTTACTTCAAGAACTGAGATCTCATTAGCAACAATCGTTTCATCGCTTTGCACCTAGC

GCTAATGTTATCATCTTATGAGTTAGTAAGTTTTTCAAATTTTTATTTGATGGTTATTTT

TGATTATCTTCTATTGAGTTTAAATTTTATTGTTGAGTTTGTAAAACAGATGCAGAACTT

CAACTTTCAAAGGTTGTTTTGTGAGTTCATTCACACTTTTAATGGGTACATAGGGGAAGA

AGTATGATATAGTTGTTTT

>Unigene0002180

TATGAAATGTATTTTATATCTCTGGTGTCATAGTAGCCCCTATGTGTCTACACGGTGGTA

CCCTCCTAGCTTAGGTTGGACATCTTTAGGAGCCAAACCGCAGAGGGAACTCTAATCAGT

CTCATTGCTAGGGAGGTAAGCTCCGAAAACCCTAGTGAAGATAGGGGTAACCCCTACCGG

GCGGCGCGGCGGCGGGTTTCAGGCAACGAGGCGGGGCGATCTTAGGCGTCTAAGCGATAC

CGGTGAACTCAGGGTACCTAGCATCCTAGAAGGACAATATCTAACCCCAAAACATCGACA

ATGGATCATGGATCACCGGGTTCTCAGCGGTACAGAAAGAAAACCCGCAAATTCAGTGAA

GATGTGCTTATTAGTCTACCAAAGAACATCATCGATGCCATTCTTATTCGTTTACCGCTA

CGGGATGCTGTTCGGACAAGTATCTTATCACATGCTTGGAGGTTTGCATGGACGGATATT

TCAGAAATTGTCATAGATAGGGCCAATTTCCCCCGTTATGAAAGCCGATCCGTAATGGAA

TATGATAGTAAAATTGTTAGTATTGTGGATCAAGTTTTTTTACTTCATCAAGGCCCAATT

CACAAATTCGAACTTTCTGTCGATTTTAGGAACTGCAAACATATTGACAGGTGGATACTT

TTTTTATCGAGAAATGGCACTAAGAAAATCACTATAATTCCTTCATTTGTTGAAAGATTT

GAAGTCCCCTCTTGTCTGTTCTCATGTGAGGGAATTACCCATCTGACATTAAGCTACTGT

AGATTGGTCCTTCCTGCTTTGTTCATTGGTTTTAACAATATCAAAACACTAGTTCTCCAA

AACATCAGCTGCTCAGAATACGATCTTCATGTTTTGATTTCCAGCTGCCCCCTTTTGAAT

CGACTAACACTCGAAGGTGTTCATGGTGTTAGCCGACTATATCTTTCTGCACAAAATCTT

GTGACCTTTAGTGTTGATTGTTCCTTGGGCAATATTTATTTGGTGGAAACCCCAGTTGTT

ACTGATATTTGCATCACATATTTTATTCGAGATCAATACCTGAAGGTTGCAAAAGGGGCT

ACATGTGAATTAGTTAAGATTCTTGGTTGTCTACCTAGTATCTCGAGGATGACTTTGAAT

TGTGCAGCTTTACAGGTTCTCTCAGCAGGTAATATCCCTTCGAAGCTTCCCATGACTAGT

GAGCTATTATATTGGTGCGCCGATATAAATTTTGATGATGTGCAACAAATGTTAGCTGCT

CTATGCATCTTTAGAAACTCCCCACATTTGCAGAAGGTTCATTTATCTGCCTACTCAGAC

GAGAATAACATGGTGATGCTATCTACTGAGGACTTCTGGAAGGCACAAGAATACTTTAAT

GGCTTGTTTAGTGAACTCAAAACTCTAATCGTATCATGCTTCAGTGGGACAGATACTGAA

CTTAGATTTGTAGAACTTGTTCTTTCCAGTGCTCCAGTTCTTCAGATGGTACAAATTGAG

CCACGAGAACCTATAGATGATGGGTACAACATTTCAAGAAAGTTGAATCGGTTTCGAAGA

CTTTCATCAAAAGCTGAAGTGGACATTACTGAATAAATACTTATGTCGTCACACATGCAA

GGATAATCAGCAACCAAATGGGTGTTTTACTAGTTGCATGTTGAGAATCAATGTATACAC

CATTATTACTATTTTTGTAGTAGTACTTTTGGGGTTTATGTATATTGAAATAGTACATGT

TTTAGGGATCCTGTATCAACTGCAACTTTCTGATGTTTAAGATTAGAATTTTGTTATCTT

TTGTCTAAAT

>Unigene0002181

TCCAAAATTCCAACAAATGTTGAATGTCATTTATCACGTCTCATTTAGGTTTAGCAACAT

AGAAAATAAGCTTACTTAGATAGTTTTATTAATGAACATGCAAATTTTGCCATAGCTTCC

CTGATTATGTTGCACACATTTTTTCCATGGTCATCTCCATTAGCTAAGGTCATGACTAGT

GAAGGAAGATTTGTTGTTAAGAATAACCTAAGTCCCGCAATGGTTATTTAATGCTATAAG

ACCGGATGAGATGGAT

>Unigene0002182

ATAAGACCGGATGAGATGGATAAACATTAACATGCTAATAATGTTTGCACATTTCTCTAA

ATTTTCAGCCAGATGGTTATCCACCTTATTGGAGCGAGCATACCTACATAATTAAAGTTA

GATGGTGTTAAATCTAATTGTTTAATAGGCTTATCTGGTTATCCCAAAAATGACACTATT

AAGTATATGGCAATTTTTGTCCTCTTACGTAAATTCAATTTATGTGTAATTCAATCCATC

GAAACAACATGAGAAAATTTAAATAATTTTGGAAAATGTGCGCATTTCAGGTATATTTCG

ATTAGGGCTGTAGTATAAAAATAAGTTACATGAGAGAACGTGTTAAACTGCTCCCAATAG

CAAGGGGTAGCTTGGAATTTTGGAAAGATCCAACCCTCCCCATGCACTTGCCAGGGACTG

AGCACGCACGAGGGAAGTCTCCTCTCTCTTTGTGGTGCTCTTTATGTCGCCCCTCTTAGC

GGCATCTTCTTTCTTGCTGCGGCTTGATTCCCTCTCTCCCATCTCATTGAGGTGTTATGA

GCCTGTGGGCTCCAGGGCACTAATACTCACTAGTCTCTTTAAGAAACAAGTCCGGGAAAA

GACAATATTAAGTTTTTTTTCCAATCTTTTCGTATTTCTTTTTGTTTTCAGTTTTAGTAT

AAAATAT

>Unigene0002183

ATCCAATCAATTGATTAGATAAAATACAAGATTTATGTGTTTGATCAATCGACTTATATT

CATGGGGCGAGGGATAAGACTTTCACTAATCAATCAAAGAGCAGTTTCATATAAGTTTAG

ATCTTAGTTCCTAGAATCTTAAGACCTTGGATGATGGCTAACATTTATGCAGATTTCCAG

CAAACAAGCAAGCTCCATGCCTACACATATCTCCAACAAATAAGTAAACATTACACCTTC

AGATTTCTCTATGTCATTAGGCCCCCTCAATTGCTACACGGACTAAGATGGGCCACAAGA

CTGCTCTCTACTAAAAGGCACTAAGACACTTTACATAATACAACACATTGGAGCACCAAA

GTAAACTAATAGCTAATCTAATAAGTTGAGCCATAGATGTCTCCTCAAATTTTCCTCTTG

AAGTCCCAAAGCATTCTTCAACCTTTCATAGCATCTTCATGAGGTTAGACAAGTCTAGTT

TTTCTAAAATT

>Unigene0002184

CGAGGGGTAAGACTTTCACTAACCAATCAAAGAGCAGTTTCATATAAGTACAGATCATGG

TTCCTACAATCTCAAGATCTCAAATGATGGCTAACATTTATGCAGATCTCCAACGAACAA

GCAAGGTCCACACCTACACATATCTCCCGCAAATAAGTAAACTTTACACCTTCAGATTTT

TCTATGCCATTAGGCCCCCTCAATGGCTACACGGGCTAAGATGGGCCACAAGACTGCTCT

CTACTAAAAGACACTAAGACATTTTACATAGTACAACACATCGGAGCACCAAAGCAAACT

AATAGCTAATCTAATAAGTAGAGTCATAGATGTCTCCTCAGATTTTCCTCTTGAAGCCCC

AATGCATTCTTCGACCATCCATAGCATTTTCATGAGGTTAGATAAGTCTAGTATTTCCAA

AATTTTAGTTGTTTGCCGAGGGCATAATTTAGCCATCCATAATCCGATAAATACAATATT

GGTATTAGAATAGAGTGTCTCAAAATATTTTATAAGTCTTTAAACTTGCAAATCTTCAAT

AAATGACTTCTAGGGTCTCATAATATATTTTACAGCATCAAATCTGAAAACATAGCTTGT

CTTTCTCTTAGCTAAAATCACACTTCTCCAAAAATCCAACAAATGTCGAGTGTCATTTAT

CATGTCTCATTTAGGTTTAGCCAACATAGAAAATAAGCTAAGATAGTTTTATTAATGAAA

ATGCAGATTTTGCCATAGGTTCCCTGATGAATTATGTTATACACATTTTTTCCGTGGTCG

TCTCCATTAGCTAAGGTCATGACTAGTGAAGGAAGATTTGTTGTTAAGAATAACCTAAGT

CCCGCAATGGTTATTTAATGAGATAGATAAATATTAACACATTAATAATGTTTGCACATT

TCTATGAATTTTCAGTCAGATGATTATCCACCTAATCGGAGTGAGCATGCCTACATAATT

AAAGTTAGATGGTGTTAAATCTAGTTGTTTAATAGGCTTACCTGGTTATCCTGAAAATGA

CACTATTAAGTATATGTCAAATTTTGTCCTTTTATGTAAATTCAATTTATGTGTAATTCA

ATCCATCGAAATAGTAGGGGCAAATTTAAATAATTTTGGAATATGTGCATTTCGGGTATA

TTTCAGATTGGGCTGTAGTATAAAAATAAGTTACATGAGAGAACGTGTTAAACTGCTCCA

AATAGGGAGGGGTAGCTTGGGAAATTTGGAAAGATCCAACCCTCTCCATGCACTTGCCCG

GGACTGAACACGCACGAGGGAAGTCTCCTCTCTCTTTGTGGTGCTCTTTATGTCGCCCCT

CTTAGTAGCATCTTCTCTCTTGTTGCGGCTTGCTCCCCTATCTCCCATTCGTTGAGGCAT

GATATGAGCCTGTGGGCTCAAATGCACTACTACTCGCTAGTCTCTTTAAGAAAGAAGTCC

GGAAAAAAAGACAACATTAAGTATTTTTCCAATCTTTTCATTTTTTTTCAGTTTGTGTAA

TATATATATTCCGCCTAGCGTGCATCTGCAAAAGGTTTTCTCATGAACAAGGAAACCTAA

ATTTTATGTATTTGCATTTTTTATTTGAAAATCAAGTGTCTTGATGTCTTCAACAAAGAC

ACACTGCTCTGGGGTACTTAAGAGATTAATTGGTTACCTTTGTAGGTAAATGTATACTTG

TAATACACTAGATCATGGTTGAAAAGGCTAAAATTCTCCAGAAGGGATCTACTTATGTCT

TATGTCCTGCATGCATGCGCTTCTGTTCCACAAATCTCAACCTATTCCATTACTCTCTTG

CGCCCATGCAATGAATGCACGAGTACTCACTACTGATGGATACAGTTAAGTTGAGCCTAC

AAAAGTATGGTCCAATAAGGGTCAGATATATGTAGACCATTTATAACCATTTTTCTATAC

ATATTCTATAATTTCCAACAATATAAATATACATATTCTGTAATTTCCAACCATATAAAT

GCAAATAACCTTATGACCTAATCTGTTACCGCAGGTATACAGCCAGCCCATATACGCCAA

ATTTGAAGAATGGATT

>Unigene0002185

ACTGCGGGCTATGGAGCAAACGAGCGGTGCAACGGAAAACCAAAAGCAACTGGCCAATAA

ATAGGTTCTGATCCTATTCAACAATGACGGATCTACTTCTCAACACTCACAACAAATCAG

TCACAAATTATCAATGCAACAATCACATACTATCAACACAGAGAACACATGATTTACGTG

GTTTGGTCAATCGACCTATGTTCACAGGGCGAGGGGAAAGATTTTGACTAATCAATCAAA

GAACAGTTATTTGGTTTTGAGAATTTCAGGATCATAGATGATCACCAACCTCTATGCAAA

TCTCCAAGAATAAGCGAGCTCTACACCTATACAAATCAACAATGAACAAGCAAGTTCTAC

ACTTTCAGATTTATCCATGTTACACTTATCACAGACGAGGGCTCCATAGGGCTCCATACA

AATATGGTCCCACGAAAGTCTCAAAGTGTCTTTTAGCTTCCGTAGCAATTTTACGAGGTT

AAACCAA

>Unigene0002186

CTCAATTTTTCCAGATTTTTCAGTTCCAGAGGCCATAAATCAGTCATATGAAGTCTGATA

TACACAATATTGGTATTGAAATTGTGCATCTTGAAGTCCTTTACAAGTCTCTAAACTTAT

AAACCTTCGAAAAATGACTCCATGAGTCCCAAATTTCATTTACAACAAAGAACTAAAAAA

CATAGCTTCTTTTTCTGTTAGCTGAAAATACACTTCCCAAACAAAAGTCCAACAGTATGC

ACATTAATATTTTGACTGAAGTTTCCCCATTATTTCGGGGCTTGGTGGACACCATTCTCC

TAGACTGCTCAATTCCCTCAATCTCATGCTCAATTCCCTCAATCTCATGGTTCTCATGGG

CCTTGCTTTGTTTATGATTCATCCCCACCAAGGATGTGGAGTTATGGAGAGAGGATTCCC

ATGCCTCAACTCTCACGAAGACCTAAACACATTATGGGAGATACAGTAGCCAATAACAAT

AAGACCTGTTCAACAATGTACC

>Unigene0002187

GCTTGCAGTCATGCGGGGATGGTCGATGAGGGGCTGTGGGTTTTTGACATGATGGATGGT

TGTGGAGTTGAGAAGAGGGAAATACACTATGCTTGTGTGGTGGACATGTTGGGCCGGGCG

GGGAAGCTCAAGGAAGCTATGAATTTTATTCAAACTATGCCGTTTGTGCCGGGGAGGGAT

GTGTGGGGTGCATTGCTGGGTGCTTGCAAGATTTACAATAATGTGGAGCTTGCGGAGGTG

GCGGCGGAGAGGCTGTTTGTTCTCGATCCCGAGAATGCGGGACGGTACTCTGCATTGGCT

AAGATATACGAGTATGTG

>Unigene0002188

GCTAAGATATATGAGTATGTGGGGAGGCGAGAGGATGCGGCGGGGGTGAGGAAGGCGATG

AAGGATAGAGGTGTGAGGAAGCTATTGGGTTATAGTATGATTGATGTTGATGGTGAAGCT

CGAATGTTCGGAGTGGAATCCGAGGTGGATCCAATGGCAGATGAGATCTATGCTGTCCTA

GAACTACTGCACCAGGCTGCGGAGGAGGAAGGTTAGTCAGATTGCTAATCCTCTCCATTG

ATATGAGTTTTGATTAATTAAATTTTGCCTCAGTTTATTTTGACTGTTGTT

>Unigene0002189

CTCACTGGGCACGCAGGAGATTGCCGTCGCCGGAGGACACAATACCATCGCCGGAGGATA

TTTGGAGCCGAGGATGCCGCCGCCGCCGCCGGACATTATCACCGGCCGCCGCCGCTGTCG

CCGGGGTAGAAGCTTCCGGCAGCAGAGGCCCAAATCCTTCTGAATCTGAGCACAGAGACC

TCCTGAGGGACTCCCTAGCCTCCGATTTTGGCCGGAAAGAGAGTTGGATTTCCTTTCAAT

TCCCATGCAAGCTCGCACCACCCTTCATAGAATCCATTCCCCTCCAACCTCACTCCTCCA

ATCCCTCCAATCCCTCTCCAACACACCCCTTCCCCCCCTCAGCTCTTACCACTACACCCA

CCTGCTCAAATCCTGCACAAACCCCCAAAACCTAACCCTAATCCACGCCCAAATCATCAC

CTCCGGCCACCACTGCAACCCCTTCCTCACCACCAACCTCATCAGCAGCTACGCTGCCCT

CGGCGGCCCATCCATCCACCACGCTCGTCAACTATTCGACCATTCACCCCAGAGAGACAC

ATTTCTATGGAACATCATGATCCGAGCCTACGCCATCTCCGGTCCCCCCACTGATGCGCT

AGCCATTTACAGCCACATGAAACAACAAACCTCAATACCCTCCAATTGCTACACCTACAC

ATTCGTCCTCAAGGCCTGCGCTGCACTAGCCGACCACCGCACTGGCTGTGCAGTGCATGC

TCACACTGTCAGATCCGAACTTCACAATACAATATTCGTCGGCAATGCGCTCGTTGCCTT

CTACTGCAAGTGCAGCGAGATGAAGGCTGCATATACTGTGTTTGACAGTATGCCAGTGAA

GGACATTGTTAGCTGGAACTCCTTAATTGCGGGGTGCGTGCAAAATTCCCGTGCAGCGGA

AGCTATCGCCACCCTCCACCAAATGCTGCATGCTGCGGTTACCCCGGACCGGGCTACGCT

CGTCTCCATCCTCCCTGCTTGCACTGCACTGGCGGGGATGTGGGCGCACTCGTGTATCAT

TAAG

>Unigene0002190

TCCATTCCCCTCCAACCTCACTCTTCCAATCCCTCAAATCCTTCTCCAACACACGCCTTC

CCCCCCTCAACTCCTACCACTACACCCACCTGCTCAAATCCTGCACAACCCCCCAAAACC

TAACCCTAATCCACGCCCAAATCATCACCGCCGGCCACCACCACAACCCCTTTCTCACCA

CCAACCTCATCAGCAGCTACACCGCCCTCGGCGGCCCATCCATCCACCACGCTCGCCAAC

TGTTCGACCATTCGTCCCAGAGAGACACATTTCTATGGAACATCATGATCCGAGCCTACG

CCACCTCCGGTCCCCCCACTGATGCCCTAGCCATTTACAGCCACATGAAACAACAAACCT

CAATACCCTCCAATTGCTACACCTACACATTCGTCCTCAAGGCCTGCGCTGCACTAGCCG

ACCACCGCACTGGCCGAGTGGTGCATGCTCACACTGTCAGATCCGAACTTCACAATACAA

TATTCGTCGGCAATGCGCTCGTTGCCTTC

>Unigene0002191

TTCAAGGAATTTTACAACAAATATACAACAAAGAAAACCCTCCGAAGCTCTTGGCCTTTA

AAGCACTCCACATCAATGTTGAACCCATTCCAATGAATTGGCCTTCAAAGCAAGCTTTAT

CAAACGATACCTATTTCAAGTCCTTGGCCTTCAAGGCATTGCATAACCAATGTAATTCTC

TTCAAGCCTCAAGCCCACATTCCACAAATGGTAAAGGGAAATTCACAGAACCCATGCTGG

TATACAACACATTTATGTTATCCTTTCCAGATTTCTGCATCCTTGTTTTGGTCTCTTTGG

ACTCATCTGCTGCATTTCATGAGAGTAGAAAGTCAACATTTCCCTTGACGGGGTTGGGGG

TGTGGGTGAGTGGTGGTTAAACCATAATGTGCATCTGTACTATACTTTTTACTGATTAAG

GCGGCCTTTCTTGCACTCAAACT

>Unigene0002192

TTTTGTTATTGATTTTTAATCAGTTCAATATACTATTCTTTGGGCTAGTCCAACTTACCT

GTGTGGGCACTGCTCTTGGTGGTGCCCTCTCTTTGAAGCTTCAATGTGGTCAAAACCACC

CAAATTTCCAAATTAACCACGAGAATTTTCACAAGCAGAATTTCAACTTTTCTGACTATC

CAATCTCCTTTACGTATTAATCACAAATTGATTACAATATCAAAAACAGAATGAACCCAA

ATTGATCACCGTCGGTAAATCGGCCCAAAAAATTCAACCTTGTTGCGAAGCTCCTCCACT

TGTTGCCTTCAGCTCTATCAAGAATCTCCTTTACTTCATGAGTCTTCAAACTTTTACTAA

TTGAACCTCTTGCAATGCTC

>Unigene0002193

CCGACAAACCGCCCCGCCCCCGGCGCCACCTCCCGGCCGCGGCCGAGGTCACTCCGGAAA

CCCTAGTACCGGAGAAGTGGAAGGAGGTGGCGGAGAAGCTCAATTATACCAAGAAGGAGA

GACGTATCATTGCCCGACAGGACAAGTTTCAGCCCCGCCGGAAGCTGACACCCGTGCCGG

ATGCGGGGGAGTATAGGGCGTACAGGGAGATGAAGATGGGTCAGCTGAAACCTGTGGTCC

TTGATCGACCTGGTCAGCTTCCTTCTCAGAAGGATGAGGGGATATTTATCGAGGAGATGA

AGGAGCCCCCGGAGAGGGGGCGGGCGGCTCCGAGGAATCCTAGGGTGGGGTTAGGGGCTG

GGACCATGGAGGATATTGCAGATTTTTTCAATAGTGATGATTATACTCCGGGGGTGTCAA

TTGAGGAGAAGAAGCTACAAGGTCAGAAGCTGTTTACACAGGAAGAAAAAACTCTCTTAA

ATAAACGGATACCTGATCTAGCAGTTGCTACTTCTAATAAGTGGATGCCTGTTCACACTC

TTGCTGCGTCAGGGGAGTTTTATTTATTGGATATGTTACTGAAGCACAATGTTGATATCA

ATGGCATCGATAAGGATGGCTTCTCAGCAATTCATAGAGCTGTCATTGGTAAGAAACAGG

CAATCATCAACTACCTGCTGAGGAACTCAGTAAATCCTTTCATACGTGATAAAGAAGGGG

CTACCTTGCTTCATTATGCAGTCCAAACGGCATCAACCCAGACTATAAAGATTCTGTTGT

TATACAATGTCGACATAAATCTTGCAGATGATGATGGATGGACCCCATTGCATCTTGCTG

TGCAAACACAACGAACAGATATTGTTAGACTTTTACTAATAAAAGGAGCTGACAGAACTC

TGAAAAGTCAAGATGGATTAACACCCTTGGATTTGTGCTTGTATTCTGGCCATGGTATGC

GAACTTATGAGCTCATCAAATTACTGAAAGAGCTGCCAAAACCTAAATTGCATCCCTGAA

AGGGCAATTCAGATGAATATTTGCAGTGTTCAAGTTTTGTTGTATATCCACTATTTCGGC

ATGCCTCTGGATAGGTCACACCTTCGTGGTCTATGGAGACAATTGTGAATATCAATATGT

ATGAGCGAAAATTTTGATGCTCAAGATTCACAATTTGATAACATCCAATCATATAATCTG

CTGTTGTCGCTTGATATGATAAGCGGTTCGACCAAGACGTATGAGTCTGGAACTGGAACT

GTGATAATGCCTCTCTCTCTCTCTCTCTCTCTC

>Unigene0002194

AGGATGCCCATTTATGAGATGAAGTAAGGGATTGGATTCGAGGAGTTTCAGTTGGGGAAT

GGAGATAAGTGGGGACGGATTTATAGGGAGGGGGGCTGAGAGATCCGAGACGTGCGGTGA

CGGAAGTAGTTGTGGGGTAAGAGTAGTGTAACGGTGACGAAAGATGGGTCCACCGATCCC

CGCGACACGGAGTTTCCGCCCCCCTTTTAAAAGAAAAACTAAGGAAAGCAGTAGGAGCAC

ATATGGTAATCGCTGTCTGGTCTTTTAATGTTTGTCTAATGTACTTGTGTAAGAAATATT

TGGGTTTCCCGTCTGGCTCCTATATAAGAGGATTGGATGTCCATAAGACATTTAATGAAT

ATT

>Unigene0002195

AGGATGCCCATTTATGAGATGAAGTAAGGGATTGGATTCGAGGAGTTTCAGTTGGGGAAT

GGAGATAAGTGGGGACGGATTTATAGGGAGGGGGGCTGAGAGATCCGAGACGTGCGGTGA

CGGAAGTAGTTGTGGGGTAAGAGTAGTGTAACGGTGACGCAAGATGGATCCACCGATCCC

CGCGACACGGAGTTTCCGCCCCCCTTTTAAAAGAAAAACTAAGGAAAGCAGTAGGAGCAC

ATATGGTAATCGCTGTCTGGTCTTTTAATGTTTGTCTAATGTACTTGTGTAAGAAATATT

TGGGTTTCCCGTCTGGCTCCTATATAAGAGGATTGGATGTCCATAAGACATTTAATGAAT

ATT

>Unigene0002196

CCCACAACTACTTCCGTCACCGCACGTCTCGGATCTCTCAGCCCCCCTCCCTATAAATCC

GTCCCCACTTATCTCCATTCCCCAACTGAAACTCCTCGAATCCAATCCCTTACTTCATCT

CATAAATGGGCATCCTCCGATCCTTCCTCCACCGCCGCTCCCAACCCCCATCTCCGTCGC

TGGCCACCCCGTCGGCCCCATCATCCCCACCTCTGCCGGCCGGCGAGCTACAGCGTGTCT

TTAACAAGTTCGACTCCAACGGCGACGGCAAGATCTCCTCCTCCGAGCTCGCTGCCATCT

TCGACAGCCTCGGCCAGCCGGCAACACCTGACGAACTCCATCGTATGATGGAGGAGGCTG

ACGCTGACGGCGACGGCTTCATCAGCCTCGACGAGTTCATCGACCTCAACACCGCTCGTG

TCGATCCAGAGGCAGCGCTCGAAGACCTCCGCCACGCGTTCTCCGTGTTCGATGTTGACC

GCAACGGATCGATCTCGGCGGAGGAGCTGGCGCGAGTGTTGCGGGGGATCGGGGAGGCGG

CGTCGGTGGCGCAGTGTAGGAAGATGATCAAGGGCGTGGATCGGGACGGGGATGGGATGA

TCAGCTTCGAGGAGTTCAAGGACATGATGCAGGCGTCAGGGCTCGGGTCGGCGATCGCCG

CCGCCGTCACCGCCGGCGACAAGATCTGAGTTAGTCCTTGTTCTCAGCTTCTATCTCCTT

TTCAATATAGCTACTACATTTGCGTTTCTTTCTATAGAATCTTGATTTCCGGGTCTGGAG

AACTGTACATAATTGGAGGTTTCTTGAAATCAAAGAATCGCTATAGATGAATGATTGCGT

TATAGATGAATGATTGTGTGGCGATATTAATTTTGCAGGATGGTTGATTAATTTTGTGAT

TGATTTCGAGATTGGGACGCGTCCTCTCTTTTGAGTGAACGAGGAGGTCGGCAGAACTCG

TTGTCGGTGGGACCCATGTTCTCTTTGAGTTGGTTGAGAAATCACATGATTATTGCCAAC

TGGGAGGAGGACATTCTACTGAGTACAGGGGCCGGCTTGATTGGCCAATGGGTGGCCGGG

CATGTTAAAGCTCCTGTATTCTCTGATAATTCAATTAATATAATTTCTGGTCAATCCTGT

TATTGGGAATTTTGTGTCCTAGTTGCAGGCTAGCCTGCATGATTTAAGGACTTTATGAAT

CTATTCTATGTAATAACAACACAATCCTATTTTGAATATTTAAGTGG

>Unigene0002197

AGGGGAGAGGTGGGGAGAGAGCTGGATGCAATCATGGCGGCTCACGAGGGGGAGTTAATG

GGAGCTGGAGGAGCCAAATCCAGTTGTGGGTTTTGATTTTTCTCTCAAGAGGAGTGAGAA

AGCGCCATTCTTTGCATACCCTTTGGTGAGGATGGTAGAGGAGTGCTGTTTCTTCAACAA

GGAGACAGTGGTAAAGCCTGTGAGGAAATCGCCGCCGGTGTTGGGCAGGATTGTGCTACT

GATCAGTGCGGTCTCCATTGCGTACATCTGCTTGTTCTGTTTTAGACAGACGGGCTTCCG

CGGGAGTCATATATCCGCAAGGGTTGAGCTAATGGAAAGGGCTTGTATGCATCATCGAGT

CCCGCTGACTGAGATCCCTTACTTACACTATCCCGAACCCACTACTTTTAACAGGAATGA

ATGTGGATGTGCTCCTGTTCGGTTCTTTGCAATCTTGTCGATGCAGAGGTCGGGAAGTGG

GTGGTTTGAGACTTTACTCAATAGCCATGTTAACATTAGCTCGAATGGGGAGATATTCTC

CGTGAAGGAGAGGAGGAGGAATATTTCGACTATCATTGAGACGATGGATAAAGTGTACAA

TCTGGACTGGTACAGTAGTGCGTCGAAAAATGAGTGCACGGCTGCCGCTGGCTTCAAGTG

GATGCTTAATCAGGGTCTCATGGAGCATCACAAAGAAATTGTCGAATATTTCAATCGTAG

AAATGTTTATGCCATATTCCTCTTTAGAAGGAATTTACTCCGTCGGTTGGTCTCACTCCT

AGCAAATGATCACGATCGAGGTGCCAAACAATTGAATGGCACACATAAAGCTCATGTACA

CTCCAAAAATGAGGCAAATGTACTCGCAAAATACAAACCAATTATCAATGCTACACTACT

GATTCCCACTCTAGGACTCGCGGAAAAGTTGGCTGCAGATGCTTTGGAGTCGTTTAAAAG

TTCCCGCCACATTCTTCTCTACTATGAGGATCTTATTCAGAACCGTACGAAGCTGAAGGA

AGTCCAGAAGTTTCTTGGTGTACCTCAGAGAAAGCTCATCAGTCGTCACGTAAAGATACA

CACAAGACCATTGTCCGAACAAGTCGCAAACTGGGGCGAAATCCAAAGCGCCCTTCAAGG

AACTCAATACGAGAGCTTCTTGAATGCAGACTATGAAATGTAGAATGAAATCGTTGTGTG

CCAGCAGCTGTAGATAATTTCACACATACTCTTTCTCCTCACCAAGCCGGAAAATTTTGG

TTGCAAGAATCATGCTTCGGTGCTAATAGATTCGTCACAGAGTTCTCTTGAATTGCCTTT

TAACCGCAGTCAATGTATGAAAGTTGGCCGCGCGCATCTTCTTTCAAAAGGTTCACTTGT

TTCTTACTCGCCTTTCTTTCACTTTGTTATTTTGTTTGTCTTATACAGAACTACACATTG

CTGTTTTATTGTTAGCTCGAGTAAATATTTTCTAGGGGTTGGTTAATGGTAGAACTTT

>Unigene0002198

AGTTCTGGCCTGGGAGGGACCAACGCAGCGGTGTTGTGTCCTAATCTCCTGTTCAGCCCC

GCCGCTCATCCCTATTCCTCCCTCCCCCGCCGCATCCGGCCGGCCGGCGCACCGTCTCCG

ATCAAGATCGCCGCCGACTCCGGTGAGCTCGTCGGCGCTCAATCGGAGATCAGCCCAGCG

GATCTCACATGTTATCGATCTCGCAAGGTTTATTTTTGTGATGATTGGAAGCCTATCGGC

TTCTCAGCCGAGGCCGGAGTATCAGAACCAAAGTCAGCCGAAGTAGAAAGTATAGTTCCG

GTGGTTTTCCAGTGCGGGTGCTCAGAGTTGGCGGAGGTCCCCGAATGAGAAGCTTCATTG

GCGTTATATAGTGCAGGGGAGGAAGGGTTGCATCCGTCAGACGTCTTTCTTCCTCGGCGA

AGGCTTCTGCTGAGTTGGAAAGGTTGTTTCCTTTCGGTAATGACTGGCTTGCCTGAGAGA

GATCTTGCTGCTAGCTTAGAGGAAAAGTCCGAACACAATAGATCCTCAAAGATTTCCTTG

TTGTCTAGCTTATTGGCATCCACTTTCTCCATCTGTGAGTCACATCCAGACTCGTCTTCT

TGTGAAAGGAAGGTAAATAAATCAACAAGCTATGGCTGGACGGCAGCTGTGAAGAAAGTT

TTGACTATTGGCTCGATGAGGAGGTTCCAGGAGCGTCTCTTAGGGACAAGTAAAACCGAC

ATTTCAAGCTCAACCAGTGACATATGGCTTCTTGGGTCATGCTATAAAGTTCTACGTGAG

GATGCATCAAATGCTGGTAATGGCCTTGATGAGTTTTTACAAGATTTCTCATCGAGAATA

TGGATAACGTATCGTAAAGGCTTTGGAGCCATTCCTGAGTCAAAGTTGACTAGTGATGCC

AACTGGGGTTGCATGATCAGAAGCAGTCAGATGCTTGTGGCCCAGGCATTGCTTTTTCAT

CGTTTGGGGAGATCTTGGAGGAAATCCGCTCAAAAGCCATATAATCCAGAATATGTCGAA

ATCTTACATCTTTTTGGTGATACGGAGGCATGTGCCTTTTCCATACACAATCTGCTTCAG

GCAGGAAAGGATTATGGCTTGGTTGCTGGATCATGGTTGGGCCCGTATGCCATGTGTCGC

ACTTGGGAAACTCTTGCCCGCACTCACAGAGAACGTGCTGACTTTGTCAATGGAAATGAA

ACCTTGCCCATGGCAGTGTACATTGTTTCAGGTGATGAAGACGGGGAGCGAGGTGGAGCA

CCGGTTGTCTGTATCGACACTGCGGCTAGTCTCTCTTCTGATTTTAGCAAGGGAAAATCG

ACATGGGCACCTATACTTCTATTAGTTCCTTTGGTTCTTGGGCTAGAAAAAATCAACCCT

CGGTACATCCCATTGTTATTGGAGACATTCACATTTCCACAAAGCCTGGGCATTCTGGGT

GGGAAACCTGGGGCCTCAACTTACATTGTTGGTGTACAAGATGAGAAGGCATTGTATCTG

GATCCTCATGAAATCCAGCCGGTGGTCCATATTAAAAGCGATGACTTGGAGGCCGACACT

TCATCGTATCACTGCAGTACGATTCGGCACATGCAACTCGAATTGATAGACTCCTCCTTG

GCCATTGGCTTTTATTGCCGCGACGAAGACGATTTCAAAGATTTCTGTTCACGAGCTTCG

AAGCTGGGCGACATATCGAACGGGGCACCGTTGTTTACGGTGACCCAGTCTCTTCGACAC

ACGAGTTCGATACACCATGAAGACGGTTTGCATGGTACATGCGACATTGTGGGGGGCGAT

GCTTTCGGCATAGATAACTTGAGCGACAATGACGGTCTAGCAAATGAAGATGATTGGCAA

ATCTTATAGAGCATTTCAGGGCCTGTACATGTAAACGGAAGGAGAAAGTAATTTAATTAT

GCATAGAACTAGGTGGCTGGATTGCAGCAGTAACTGTCTTGAGTGATTTGAAATGTAAAA

AATATTTATTTACTAGAGTCGCACTTATTTGCTATTTGTCTGGAACCCTCTCGAGTCTTA

TTATCTGTTTGCTGTCACAATACCTTCACAAAATTTGATTAATTTTCACTCCATGA

>Unigene0002199

CCGGCCGTTGCACCGTCTCCGATCTTGATCGCCGCGGACTCCGGTGAGCTCGTCGGCGCT

CAATCGGAGATCAGCCAAGCGGATCTCACATGTTATCGGTCTCGCAAGATCAAGTCTTTC

CACCTCCGCCTCTCCCTCGCTGGAAGCGCCGCCGACCGCCATGTCCCGGAGGCGTTGACT

CTCCTCTACCTTGGCACCCCGCTGGAGATCAACGACGTGCCGTTCTCGGAGATTGAAGTC

TGTGTGTTTGGAGAGAG

>Unigene0002200

CAAACAGAAAATCACAAACTCCCAAATCAACGATTCAATGTATCTTCAATCGCCGGAATC

CGTCTCCTCCGCTCCCAATCGTAAGCCCCCTCTTCAAACCCTAACCCTCTCCCCTTCTCC

TCCCTTTTTCTAGGGTTTCTAATTGGAAATTCATCGATTTCTAGCGCCTATGCTGCCACC

GCGTGCCGCAGCCGCGGCCACCCAGCCGGCTCTTCTCAACCTCAGCGACCTCGTCCCCAC

CCTGAAGCCCGAGGGCGCCGGCGTCGATCCGGCGGTTCGGAAGCGCCTCTGGCTCCAGGT

CCGCCGAAGCCTCCTCTCCCTCCGCGACACCATCACCTCCGAGTGGGAGATCCACCGCTC

GCGGCCGGAGGAGCTCGACTTCGCCCGCATTGACGATGAGATGATTCGCGCGCGGCGGGA

CGCCGCCGAGTCGATCTTCAACATGGACAAGTTCCTGGCCACCGCGGGGAGGATTGTTGC

CAGTCTCAAGAAACTGGCGGCGAAGGGCGGGGCTGTGAAGAGAAGCTGGGAGGTGGCGAT

TGTGTCGAATGGGCCGCCGCTCCCGCCGCCGGCTGCGGCGGCGCCTGTTGAGGAGATGTG

CGGGTTGAAGAAGGTGAGGAGGGAACTGGATCAGGTGAAGATGTCGAGGGATAAGCTGTT

GCAGGAGCTTACTTTGCTTGCTGGGAAGAAGAAGAGATTGGAGGAGGAGTTTGTTAAATT

GAGGGAGGAGCTCAAGACCACAAAGCAGGAATTGTTCGAGGGATACTGATCTGAGTTTGC

TAACTTACATGTGATTGTATAGAGGTATGTGAATTTTGGTTTTTTTGGGTTTCTCTTGTC

ATGAATTGTTGTTGAACCATGCTTGAAGTTGAATCTTGGGTGTTTCATTTTCGGATATAC

CAGATCTTAGAGTTCGATTTGCAAAACAGAGACAAAAATGGCAATTGTTTTTAAAACAAA

CTAGGCATCAA

>Unigene0002201

CAGGAATTGTTCGAGGGATACTGATCTGAGTTTGCTAACTTACATGTGATTGTATAGAGG

TATGTGAATTTTGGTTTTTTTGGGTTTTTCTTATCATGAATTGTTGTTGAACCATGCTTG

AAGTTGAATCTTGGGTGTTTCATTTTCGGATATACCAGATCTTAGAGTTCGATTTGCAAA

ACAGAGACAAAAATGGCAATTGTTTTTAAAACAAACTAGGCATCAA

>Unigene0002202

CCAATGTCCTCAACGTTCCACAGCCCCTCCGATCTTGACCAGTGCGTCACCGCCGCTGTC

AAGGCGGCGGAGGCGCGCGGCGACCCGCCGCTCGTGCGAGCCGTGGAAGCTTGCCACCGT

GTGTGGGATGCCGGGCCGGGCAAGCCCAGCCTGGAGCTCTGCCGCATCCTCGTCTCCAGC

CTCTGCTTCGAGAACAACACGGCGCCGTTCTGGAAGCTCCTCGACCAGGGTCTTGCTCTC

CGGCTGCTCTCGCCGATCCAGACCCTTGCGCTCCTCACCCCCAGGGTTGTACCAAACCGG

CGGGCGCAGCCGGAGGCTTACAGGCTGTACCTGGACCTGTTGAGTCGGTATGTGTTCACG

TCGCCATCAGTGGAGCTCGATTCTTGCAGAGACGTGATAACAAAATCTGTAGACGAGGCT

CTGCAGCTTTCAAATACATTTGCAATTCACAAGATGGATTTTGGACAGGCAGTTGTTCTC

TTTGTTTTAAGTGTCCTCACTAGTTTAATTGACGCTACATTGGAGGATTCGAGTCTTCAA

TCGAAATCTACAGACAGACGTGGCAATGCATATGCTAGCGAAGCCCGCCAAGTTACTAAC

ACAGATCTTAGAAGGAATTCTAATGATAACCGGCATGAACATCGTGAGCAGCTTCGTAGA

ACTAATTATCTTACTGCTATTGAGGCAATGGAAAAAGTTACTGCAAATGAGAAGACCAGA

GTATTGCTTCGGCTCATTTGTCTAAACATGCCTGAGAACTTCCGCAGCCTTCTGCAGAAA

CTGCACTTAATTGAAGCTAATAAATCAACATCACAGAGTCTAGTAGCTGCCAATCATGTA

TTGGCGAAATTGCTTAATAACATAGAAACATCTGTGGATGGGGAGTACCAGTTAAGTAAG

TGTCAGCTACTTGGTGTTCTGGTTGATGCTGGGTCCTGTGGCTGGACTCCCTGTCAAAGT

TACGGAGCTGGAAGGGCTGCATGTTGGATTCCTTTTGATATTTTTATGGAGAATGCAATG

GATGGAAAATTTCTTCATGCTCTATCCCCTATTGAAATACTAACAGAATTAACCAAGACC

CTTCAAGTGATCAACAAGGCCAGCTGGCAGGAAACGTTTCAAGCTCTCTGGGTTTCAGCA

CTTCGACTCATACAACGAGATCGTGACCCATGGGAAGGACCTGTACCCCACCTTGATACT

CGTTTATGCATGTTGTTGTCTATTGTCCCCCTGGCAATAGTTCCTGTTCTTAAGGAGGAT

AATAAGTTGGTTTCTGTGACGAACAATGATTTATCAGGAGGTGCAAGAGCTGCCCCTGAG

CATGGCATGGAAGGAAATAAATTTGCTCCAAGAAGTCATGGACTAATTTCCTCTCTTCAG

GTATTAGGACAGTTTTCCGGGCTTCTATCTCCTCCTCCCTCGGTGGTGAATGCAGCTAAT

ATTGCTGCTACCAAAGCAGTAGCTTTCATGTGTAGATTCAAGACGGGAAGTGGCGGTCTT

AACGTCATTGGTCCCAATGATGCTTCTGTTAAAGCTGTTGGGAACATGCTGCATCTTATA

GTTGAAGCCTGTATTGCAAGAAATTTGATTGATACATCTGCTTACTTTTGGCCGGGTTAT

GTTGTTCCCTTTGCACCATCAAAATCTTCCCGGTTAGTTCAAGAGTCTCCTTGGTCAACC

TTTATGGCAGGAGCTCAATTGTCCGGTTCACTTAGAGATATTCTTTTGGCTACTCCCGCC

TCAAGCATAGCGGAAGTTGAGAAGTTATACCAGATTGCTCTCAGCGGATCTGAAGACGAA

AGGTTAGCTGCTGCTAAAATTCTATGTGGCGCATCTCTTGTTCGTGGCTGGAATATTCAG

GAGCATGTGGTGCATGTTGTTGTCAAGCTACTATCTCCTCCCACACCTCTTCATTGTCCA

GGAAATGGAGCAGGCAATAACTTAATTAGTTACATGCCCATGATAAGTGCTATTTTGTTT

GGAATTTCCTGCATCGACATTGTGCATATCCTTTCTTTATATGGCATGGTTCCAGATTTT

GCAGCAGCCCTGATGCCTCTATGTGAGATTTATGGGTCACAATCTCCACCATCAAATTAC

AGATCAAACACTTCCGATGAAACGTCCATTTATGCGGTCTTCTCGTGTGCATTTCTCTGC

CTGCTTCGCTTGTGGAAATTTTATAAACTCCCAGAGGAACACTCTGTTGCAGGCCGTGGA

GGCACTGTTAAGCCAGAGTTAACTCTTGATTATCTCCTGCTAATGCGCAACAGTCGCATT

GCTTTGCGAAATTCTGCAGCTATGGATAGCACCCACACCATTACAGACTCAATCAATCAC

TCATCAAGTCAGCCTGTGTATATTCACTCATTTCCAAAGCTACGAGCTTGGTATGTTCAA

AATCAAGCTTGCATAGCCTCAACGCTCTCTGGTCCTAGTAATAAAAATCCTGTGCACCAG

GTTGCCAATAAGATATTGAGTATGATATGTCGGAAAATGACTCAAGTAGGGACTGTTTCT

GGTAATTTATCATCAAATTCTGGTAGTAGCATCAGTGGTTCACCTATAAGCCCAACAGAT

GATGCTTATCAGAGGCCAAAACTTCCTGCATGGGAAATTTTGGAAGCCATTCCATTTGTT

CTTGAAGCGATTTTAACTGCATGTGCACATGGGAGGCTTTCATCGCGGGATTTAACTACA

GGCCTTCGGGATCTTGTTGATTTCCTGCCTGCTTCTCTTGCTACTATAGTAAGTTACTTC

TCAGCAGAAATCACTCGAGGAATATGGAGACCGGTTTCCATGAATGGAACTGACTGGCCC

AGCCCTGCTGTTAATCTTCAGTCAATTGAGTCTGAAATAAAAGAAGTTCTTGCTTCTGCT

GGGGTTCATGTACCTAGCTGTTGGACAGGTGATATGACTCCGATGCTTCCATTGCCAATG

GCGGCCCTCGTAAGCTTGACCATAACATTTAAACTTGACAGGAGTTTGGAATATGTCCAT

GGGGTTGTCGGTCAGGCGCTGGAGAATTGTGCAACAGGCTGCTCATGGCCGAGCATGCCC

ATCATAGGAGCCCTGTGGACCCAGAAGGTCCCCCGATGGCATGATTTCATTGTCCTCTCA

TGTGCCCGTGCACCCTTCACTCAGGACAAAGACGCAATAGTCCAGCTGATCAGGAGTTGC

TTCTCCTCCTTCCTCGGGCGCTCGATCAACGGCGGTTCTCATCTAATGGCCAAAAGTGGC

GTAAATGGGTTGTTGGGTGAGGTCCTATCTGACAACGGGCTGCGCCTGCCTGTTGCGCCA

GGCTTCCTGTACCTGCGCACATGCCGGGCATTCCACGACCCTCAATTCCTGAATGGTCTT

ATCCTCAAGCATGTCACTGAGTGGGCACATGAACTGACAACTGGATGGTCTTGTGGTGGA

CCCACACAGCTTCGATCGGGTCGTATGTCACTTGCTGAAGCGACCGCTAGAGTGCGTGAG

GTGGCAACTTTGGGGTCCAGCTTGCTGTGTATTGCTGGTGGAATCCAGCTGGTGCAGGTG

CTGTATCAGGAGACACTGCCGACTGCCCTTCTATCCGAAAGGGAAATTAAGCAGGGTAGT

GCAGGGCTTGCGAGCAACATTCTGGAGGGATATGCAATGGCATATATGCTGATTTTATCT

GGATCTTTTGTGTGGGGAATTGGGAACACCTCGCCAGCCTACACGTCAGTTTTCTCATCG

AGGCGGGCCCGGGTGATGGGGATGCACATGGATTTTGTGGCGGGGGTAGTAGACGGAAAT

GTATTACTTGGGTGTGATCCAGCAACTTGGAAGGCTTATGTCTCGTGCTTAATTGGCTTG

CTTGTGAGCTATGTTCCAGCCTGGCTGACAGAGGTTAAACAAGAGACCTTGCGGAAACTA

GCTAGCGGGCTTAGAGGGTGGCATGAATGCGAGCTTGCGCTCTCGTTACTCGAGCGAGGA

GGCCCAGAGGCTATGGAGGCTATTGTCGAATCGGTGTTATGATATCTCGTGTTTCTACAG

CTGCACTGCAGCCGTCATTTCTAAATATCAGTGACACTTGGGTGACGGTATCTGGATTGG

CTTCGGGAGGTGGATCTCATTGCTCTTATTTGATACATTGGAGTGAGTTTCAGCAAACTG

AAAGTAAATATTTTATGGTGAGGCACTTTGCATACTAACTCTAGGCCATATATAGTTGAG

TTGTTTATGCAAACAGGGAAAAATTTGTATAATCCCTATGCTATATATTTCTTATAAATT

TTTACTTTTAATGTAGGCTCTTAATGTAGGCATTCAACGTGTTCTCTTGTGACTGATTTT

GTTTTAACATATATTTTT

>Unigene0002203

GCTGCCGAAGCCAAATCCTCCATCGCCGCCGATGGCGATATCCCAATCTCAGGCGGTCAA

GCGCCTCAACACCTCCTCCGGCCGCCGCCGCTTCGTCTTCAAGAGTTTCTCCCAGCGAGT

CGAGGAGATCGAGATCGACGTCTTCCGCAGCCTCGATCCCGTCAAAGCCGAGCCCTCCGC

CGGCTCATCCTTCTTCCGTGACGCCCTCCTGCAGTGGAGAGAGCTGAACACCGCGGAGGA

CTTCATATCGGTTTATGATGAGCTGTTACCGATCGTCCAGACGTTGCCACAGATTTTGTT

GCACCGGGAGAGGATATTTACAGCACTGCTCGGGAGGCTGAATATGGGAGCTAAACTGTC

GTTAGAGCCGATACTGATGTTGGTTGCTGCTCTTTCCAGAGATATCCTAGAGGAGTTTCT

GCCTTTTTTGCAGAGGTGCACCGATTCTTTGGTTGATTTACTCAACACTGGTGCTGATCG

CGATCCCGAGATCCTGGAACAGATCTTTACATCATGGTCATATATCATGATGCATCTGCA

AAAGTACCTTACAAAGGATGTCGTTAATGTGCTCAAAATTACAACCCGTTTAAGGTATTT

TCCAAAGGATTATGTTCAAGAGTTCATGGCTGAAACCATTTCTTTTGTGTTAAGGAATTC

ATCAGTTAACCAACTTAGAAAAGGTATTAGAAAGGTAATATCTGAAGCCGCGAGAAGTTC

ATCTTCTATTGGGAAAATTGGGGCTACTGCATTACTCTGGCATGTCTCAAGAGGGGTTTC

TTCAAGGCTTCACTCGAGAGCTGTAAATGTGTTGCAGTTGTTGATGGACAAATCCACATT

TAGGGTTAGTGATAAATTTAACCAAGGC

>Unigene0002204

TAAATTTAACCAAGGCTCGGAAACTGTTCTTCAGGTTATAACTGCTGTTCTTCATAGAAT

ATGCAAGGAAGTTGATCAGAAAGAATTGGATTTGGTATACAATTGCTTACACGAAGAAAT

CATTAGTTGCATCAGTGATGGTTGCTTGGTGCATCTCAGTCGATTATTGCATCTTCTAAC

TTCTACCGTACACCATGGTAGTAAGAGCAATGCTATAAAGAGCCAAAGAATGATTGAACT

AGTCAAAGCACTTGTACAGGCTTATATTATGCCTAAGGACACTATGGAGTCAGAAGATCA

TTATCCTGAAGTTATAAATAGAGTTTTGGAGTTGATGCTCTGTCTTCTAGACCCCTTCCT

TGATTTGTTTGATTTGACAGTTATTTCAATGCTCTATGCTCCTGCATTTAAGTTAAGAAA

CTCCAGTGTGCTCATGTTTATTAAAGGACTATTGCAAAAGGGTCCTCACATCACTCATAT

CTTCAAAACTCCCATAATAAGTGCCACAAATAATTTAGTTGAAGCTTCACCTGAAGAAAC

TATTGGCTTAATTCTGACATTCTTCGAAAGACAAGGGAAGCGACAATCATGTGAGGCCCT

ACATGGAGTCCCAGAAGATAATATAATGACACTGCAGAAATTTTGTAATGATGAACTCTC

TTATTGGTTCAAGAAGCTGAGAGATATCACAGCCAACCCTCCCTCGGATATGCAAATTAG

CGATTCCGAAATGGCTATGACATGGGGTGTATTATGCTGCTATCCTCAGGTTTGTTATCG

ACAAGAGAACATACCAATTGTAACAGATATAGTAATCACTCTCGATCAGCTTTTGGGAAA

TGAACATGACAACATTGCAACTCTCCCTAAGTCTACCTGGCAAAGTCTTCTTGGTGCTGC

ACTTACGGCATATCATAAGTTGCTGTTGGCGGGAAAGGGTGGATTTTCACAAGTGCCATT

CTTTTTGCGTCTTGCAAAGAGACACAAGTCATCTCCACAAATATTGTTTGCTGTAGCTGA

ATTCTTGGATTCTGTTCTTGGGCCATTGATTGAAGCTGATGCCACTCCAGTTACTTTCCC

TGAACTTGATGTTCAAGATGCTGTCGATTCAATCAGTATATTTTCAGATAATTTGGGTCT

TCCAGATAAATCTATTCGGGCTTCTACTTTAAGAATATTGTCTCATTATGCACCTTTAAA

ACTGGAGCCATCGACAAGTGATGAGCCCCCTTCTAAGAAATTGAAGACAGAAGAATCTGG

ATCTTCCAATGAGGATTCTCAAAGCATAAATGTTATTGAGCTCCTTCTGTCGGTTGAAAA

AACACCTGTTTCAATCGCTTCAAGTCGTAAATTCGTTATTCTACTCTCTAGACTGCAAAT

GGGCATTACTACTGCAAGGATCCATAATGGCTATTTACCCCTTGTATTAAGTGGAATTAT

TGGCATCTTACATAATCGGTTTGGTCATTTGTGGGAGCCAGCACTGGAGTGCTTGAGTAC

TTTAATAAGCAAATATAAGGAGCTAGTTTGGGAGAAGTTTGTTCACTATCTTGGAAATTA

CCAGTTGAAGTTTCTGTATCCTGATGACCAGCTGGTCAATTTGAAAGCTGAAGGTCTTCT

ACCAACAACCTTGAGTGACCGCTTTGACGTGTTTCTAGCATCTGAAATCGACTGCACACC

GTGTATGACCTTGACAACTTTGTTGCTTCAAGCACTTCAGAAAATCCCTGAGATTGCAGA

GTCTTGTTCTCGGCAACTTGTTCCTTTATTTTTTGAGTTTTTGGGCTACACTGGTTCTGA

TATCGGAAGCTGCCATAAGTGTAAGGGGAAG

>Unigene0002205

GGGACTAATGTTGATCCGAATACTGCAGTTGAGGAAAACTGTGAGGAAGGTTACTATAAA

ACTACTTCTAACAGAATTATGCTTTGTTCAGGAAATGGAAATTGGATACCAGATCAGGAT

AAATTATGTTTGAAAAAATGTCCACCTATGATTTCGGATAGCTTAGATTTGGATTGTGTT

TTTAACGGTAAAAGAGAAAATTGTTCAAACCCATCAGTACCAGGTACTATATTAACACCC

AAATGTAAAGTCACGCACACTGTACCAAATGGACAAATAGAGACTCCAATCAAATTACGT

TGTCAACAAGATGGGAATTGGAGTGATCGACTTTATACGTGTATTCCATATTGTGGACGA

CCATATACTCCTAACCAAGCATTGATTTTAGAGGGTGTTGAAGCTGTATATGGATCCGCA

CCTTGGAATGTTGGAGTATATCGAAAAAACAATAAAAATAGTTTTGACTTAATATGTGGA

GGATCACTGATTGCTCCAAATTTAGTTGTATCTGCTGCTCATTGTTTTTGGCAAGAAAGG

TTGATAGATAGAATATTAATAAACGATGGTACTTATAAAATTGCTGTTGGAAAGTATAAG

AGCGACATTGCAATCAAGGACAATGCATTTACTCAGATTGTTGATGTTGGATTGATTTAT

TTGAAGGAGGGCTATTATGGTGCTTCCGGATATCATGCTGAAGATTTAGCCATTCTTGTG

TTACCAATCAAAATTACAATGAGCAATGTAGTTTCTCCTGTTTGCATGGACTGGTCTAAA

AGATATTCCATTTCAAATGGATCTATTGGAAAAGTTGTCGGCTGGGGAAAAACAGAAAAA

ATGGTAGAAAGTTCAGTTTTATTAGAAGCTGACCTACCATATATTAATCGTCAGACGTGT

CGGAATATGTATCAAAACGGATTTCAAAACTTTGTTACTCTTGATAAATTCTGTGCTGGT

ACTAAAGGTGGACAAGGAGTTCATCAAGGCGATAGTGGTGCTGGATTAACCTTTGTACAT

AGCTCTTTACATTTTTTAACTGGAATTGTAAGTGTAAAGGATGCAAACACAAATGATTCC

ATAGCAGTTTTTACAGATGTTAGTCG

>Unigene0002206

CGGAAGTGTTGAATCTTTTAGCTGCCATAAGTGTAAGGGGAAGGAGTGGAGGTCTGTGCT

CAAGGAGTGGCTGAACTTGTTGAGGCTGATGCGTAATGCTAGGTCTTTGTATCGAAGCAA

AGATCTCAAAGAAGTTATGATAAACAGGTTACTTGATGAAATTGATCCTGATATACAGTT

AAAAGTTCTGGATTGCTTACTAAATTGGAGGGATGACTACCTTGTCACATACAATCAACA

TCTTAAAAATCTGGTAATGTCGAAGAACCTTCGTGAGGAGCTCACAACTTGGGCTATTTC

AAAAGAGTCCCAGATTATTCAAGAAGATCATAGGAATGTTCTAATTCCTATTATTATCCG

TATTCTGACTCCAAGAGTCAGGAAGTTAAAAGCACTTACTTCACACAAGCATGGCGGTGT

GAACCATAGAAGGGCTGTTCTTTACTATTTGGCACAACTTGATATAGACGAGCTTCAACT

CTTCTTTTCGTTACTTCTGAAGCCATTACTTCCCAGCGTACTTGAAGTCGACGTTCTTGA

CAGGTCAGATCATGGATATAGCAACCTTTCAGACAATTCCATTTCATCCAATCTTGTGAA

GTGTTCGACCTTAATATCCATTGCAAAGCTGTCCTGGAAGAAGAGACACGGATTTTTGCG

TGTTGTTGAAGATATCTTGAGAACTTTTGACGAATTTCACATCAAAATATTTCTAAACCC

GTTGTTGGGAATTGTTGTTCGTTTATTGGAAAGCTGCACATTGAACATAAATAGTGAAGG

CACAAGTTTACATAACACTGGGGGTGATTATTCTCCTGGTGACTCAGAGATGTGTACTTC

TGTTAAGCAATTTAAGGATCTACGATCTTTATGCCTGAAGATAATCTCTATTGCATTGGA

TAAGTACGAAAGTCATGATTTTGGTTCTGACTTCTGGGATATGTTCTTTATGTCTGTAAA

GCCTTTGATAATCAGCTTCAAGCAGGAGGGTTCCAGTAGTGAAAGGCCAAGTTCTCTTTT

TTCGTGTTTTGTTGTGATGAGTCGAAGTCAAAATCTTGTTTCACTTTTAGCCAGGGAGGA

AACTCTTGTTCCTTCCATTTTTTCAATCCTTTCAGTGAGGACTGCGTCTGAGGCTATCAT

TAGTTCGGTTCTGAACTTTGTGGAGAATCTATTGATTTTAGATAACAATTCGGACTGCAT

TGAGGATGATTCACTAAAAAGACTTTTGCTACCTCACCTAGAGACTCTTCTCCAGAGTTT

CCATGGGCTTTTCCGCAAAGAGAATCACAGAAAATCAAAGCTGGGGCCTGGAAAGATTGA

ATTGAGAATATTTAAACTCTTAGTGAAATACATAAGTAATCCTTTGGCAGCTGGGCAGTT

CCTTGATATAGTACTTCCATTGTTCAAAAAGAAGTCTATAAATTCAGACGAGTGCTTGGA

AGGTTTGTGTGTGATCAAGGGTATACTGCCAGTTGTAAGTAAGGAAGCAACTGTAAAAGT

TCTTAGTGCTATGAATATGCTTCTTGATTCAGTTGGGTTAGATCTACGACTGTCCATTTG

CGATATTCTTGATGGGCTTGCAGCAAAAGATCCGTGTTTGGCATTCTTGGCTAAACTTGT

ACGAGAATTAAATGCCGTATCTTCCTCAGACATTGATGAGCTTGATTACGACAAAAGATC

CGACGCTTATAATGTGATTCAACCACAGTTGTTTTCTCAGCTCAGAGTAGACCATGCAGT

TGTCATACTGTCTCATTGTGTTTATGACATGTCTTCTGAAGAATTGATCTTTCGGCAAAG

TGCATCTAGGTCATTGCAATCTTTCATTGAATTTGCCTCGTCAATTTTAAGTAGGGACGT

GAAGGATAGCCAAGAGATGCTGTCACATGATCGGTCAGGCTTAGTGACAACAAATCTAGT

TCCAGATACTGTTGATAATAGTACCACTTGGTCTAATTCCTGCATTCAGCAGATAATTAA

AAGAACTTTTCTGCAAAATATGGGAAAAGCTATGGGCAAGGATATTTCCATTCAGAAGGA

GTGGATTGCCCTGCTACGTGATATGGTTTATAATCTTCATGGAGTTGCATCCGTAAATTC

GTTTAGGCCTTTGTGCAGTGAGGATGCAGAAGTTGATTTTTTCAACAATATTCTTCACTT

GCAGATACATCGAAGAAGAAGGGCATTGATACGTTTCAAAAATCTCATTGGTGCTGGTAA

CTTTGCAGAGAGCATCACCATGAAAGTATTTGTTCCGTTGTTCTACAATATGCTATTTGA

CGTGAAAGATGGAAAAGGGGAGCATTTAAGAAACACATGCGTAGAAGCACTTGCTTCGAT

AACAGGTCAAATGAAGTGGGAATCATACAATACTTTCCTAAAGAGATGCTTCCGTGAGGT

GACTCTTAGGCCAGACAAACAGAGAATACTGTTACGGCTTATATGTGCAGTCTTGGATTC

ATTTCATTTTTACGATGAGAGTTCCAGCCAAAATTTCAAAGATGCTGAAGGTGAAGGTCC

TAATTATGGCAATATTGAAGGTGATTTCAGTAGCACATTGCCTACTACTTGGTCTGCATC

AAATGTCCCCTCTGAGATACAGGTCTATCTGCAAAAGGCAGTCCTCCCACAGATACATAA

ATTGTTGACATCAGATTCTGAGAAAGTTAATGTGAATGTTAGCCTTACAGCTCTCAAATT

ACTCAAGTTACTGCCAGTAGAAACATTGGAATCACAACTTCCAACACTTGTCCACCGCAT

TTGCAATTTTCTGAAAAACCGGTTGGAAAGCATACGTGATGAAGCAAGATCTGCTATTGC

TGCATGTCTTAAGGAGCTTGGGCTGGAATACTTGCAATTTATTGTTAAAGTTCTGCGAGG

AATATTGAAGAGAGGATACGAATTGCATGTTTTGGGGTATACCCTCAATTTTATATTATC

TAAAACAATTAATGATTCTGTAATTGGGAAGCTCGATTACTGTTTGGAGGAACTTCTCTC

CGTAGCTGAGAATGATATTTTTGGAGAAGTTGCTGAGCAGAAGGAAGTCGATAAAATTGC

TTCCAAAATGAAAGAAACGAGGAAGAGCAAGTCTTTTGAGACTCTGAAGCTGATTGCTCA

AAGCGTTACATTCAGAACGCATGCTTTGAAACTACTTTCACCTATCAATACGCATCTTCG

GAAACATCTTACACCAAAGGCCAAAGCAAAGCTTGAGAAGATGCTACATCACATTGCCTT

GGGAATTGAATGCAATCCTTCCTTGCAGCCTACTGAACTGTTCATTTTTGTGTACAGTCT

CATTCAGGATAGTATTACTGATAAAGATTCGATAACCGAAAAGAATTCACATGTGAAAGA

GACACCAATGACCGGTGATCTTGGGGTTAACGAAAAGACCATTAGTGAAGTGACAAACAG

AAGAGACCGTTTATCTTACTATGTTCACCAGTCACAGAATTCTTATCTTGTTGCTGTGTT

TGCCCTTAGAGTGTTGCATAATCGTTTGAAGAATACAAAGCTCGACAAAAAAGACGAGCA

ATTACTATCAATGTTAGATCCCTTTGTCAAACTGTTGGGAGATTGCTTGAATTCGAAGTA

TGAAGATGTTCTGTCAGCTGCTCTCAGATGTCTTGCACCACTGTTAAAACTGCCCTTGCC

TTCTCTTGAAGCTCAGGCTGATAAAATTAAGATCTTACTTCTAGAGATTGCCCAGAGATC

TGGAAATGCAGGCAGCCCACTGCTTCAATCTTGTTTAAAATTGCTCACTGTGTTACTGCA

GAGCACTAGAATCTCTCTTTCAAATGATGAACTGCATGTTCTTATTCAGTTTCCCCTGTT

TGTTGATCTCCAAAACAATCCTTCCTCCGATGCCCTTTCTTTATTGAAATCAATAGTTGC

CCGTAAGCTGGTTGTCCATGAAATCTACGATGTAGTTATGCAAGTGGCTGAATTGATGGT

AACAAGCCAGTCAGAACCTATACGAAAAAAGTGCAGTAAAATTCTTTTGCAGTTCTTACT

TGATTATCGTCTTTCAGATAAACGCTTGCAACAGCACATGGATTTCTTACTTTCTAATTT

AAGCTATGAACATTCCACTGGAAGAGAGTCAGTGCTTGAAATGCTTCATGCTATTCTCAT

CAAATTTCCAAAAAGTGTTGTAGATAGTCAGGCACAAACATTTTTTCTCCATTTAGTAGT

AGCATTGGCCAATGATCGGGACCAGAAAGTTCAATCTTTGGTGGCTACCGCGATAAAAGA

GTTGATTGCACGCGCTAGTCAGAATGTGCTACATCCAGTTCTTGAATACAGCCTTCAATG

GTATACGGGTGAGAAGCAACATTTGTGGAGTGCCGCAGCACAGGTTCTGAGTTTGTTGGT

TGAAGTATTGAAAAAGGGATTCCAAAGGCATATTGAGAACACACTGAGAGTTGCAAAAGG

TATACTTGAACTATCAGTCGATGCGTCTACCAATCAAGTAGCTGTCAGTGATAATGAACG

TGGAATCCCATATTGGAAAGAGGCTTATTACACTCTTGTTTTACTGGAGAAGATGCTAAT

TCACTTTCCCGATCTATACTTTGACAAGAACCTTGAGGAGGCATGGGAAATGGTTGTGAA

GCTTTTGTTGCATCCACACATGTGGTTGCGGAACATAACTGCCCGTCTGGTGGCCTTATA

TTTTGCTGCTGCATCAGAGACTAATACAGTTCAGCATGAGAGGTTGGACTTGGGAACAAT

GTTTCTATTCAATCCAAGGAGACTTTTTGCAATTGCAGTTTCGTTCGTGAACCAGCTCCG

GACACCTCCAGTTGATGACGCACCAAGAAACCTCATAACTCAGAATCTTGTGTTTTCTAT

CTATGGTTTGCACTCTTTTACAAAACCGAGTAATTGGGTCGATTGTGATCCTAGTGAACA

AGGCTCCTATCTCGAAGCTTTTAACATGTTTGGCTCAAGAAAAGCAAAGTGTGCTTTCCT

TCTTTACTCAAATGATACAACTCGAGGGGATAAAACAGATGGTCATCCAGGACCTCAGTT

GTTGCTAGTCGTACCCCTACTTAGGAAGATGGGAAAGCTTGCATTACAGACAGAGGATAT

CCAGATGAAAATAGTTTTTGATTGCTTCAAGATGATTTCATCACAAATTGGCTCTGAAGG

TTGCCAGTTGTATGCTATACATATGTTGCATCCAGTGTACAAAGTCTGCGAAGGATTTGC

GGGGAGGGTCATCAGTGATGAAATAAAACATTTAGCAGAAGAGGTGCGGGACAGCATACG

GAATGTATTGGGGGTTGATAAATTTGTGCAGGTGTACAACGTGATTCGGAAGGAGCTCAA

GGAGAAAAGAGACAGGAGAAAAGAGGAGCAGAAACTGACCGCTGTTGTCGATCCTATGCG

GCATGCAAAGAGGAAGTTGCGTGCTGCGGCCAAACATCGAGCCCACAAGAAGAGGAAGAT

AACAACCTTCAAGATGGGGAAATGGCAGAGGTAGCACTGCCACGTGTCCACTAACAATTG

TCAATGTCCAGTTTTCATACTTTCTTTGTACTGGTTGACTTTCCTAGAGTTGGGAGCCAT

ATTATGGCAGCAAATTTTGACATGGATTGAATACTTTTGAGTGATTTAGTAACGATTGGT

GATTTATCTGTGGGGTTGGGTGTGTATACGTACCTTTTTAAGATTAGTATTTATATGGGG

CCTGGGGGCCACTGTATTGGTTGATATGCCTCTTGTTGCAAATTGCGCATGGGTCGGGAC

AACCACCTTTGAAGTGAATCCTCCCTGCTTATGCATTTGCCAATTATGTTTGGATGCAGA

CATCAAAAAGAAGATATCAATGAACTCTATAAAAGCCCCAGGAGTTA

>Unigene0002207

TATAAGCGGAGTCGGAGCAGAGCAGAGCAGGGCGGTGGAGGTGCTTGTTTATTATCTTTC

CAGATCCGATGGGAGGTCTCTGACCCCCGACATTAGCTGGGAAGTGAAAAATCTGTATCT

CAAGAACTTCTACAATCCCATTTCTGGGCTCCACCGATGAGGGGAAGACTTTTCTGAATT

ATGCTTTAGAGCTTCTGATAAGCATGGCATGCCAAGCTGTGTATATTCCAGATCTGATGG

GGAGTCATGTCGACAAATGTTTCAGTTCTTTCTTGAGATCAAGATTTAGGCGACCCTCAA

ACTTGCATTCTAGAATCAAAACAAGAAACCTGGGAATCCTTTATGAATGCTTTTTGAGTT

TGACTCCATCTCGTCCTCAGGCAACGACAAAGAGAGAGACCGCCTTGAAATGCTCTCCTT

CAGGAAACAATCAGATTGAATCAAATCATCTCGTTGAGCTTCCCCTCTTTCCACTTCCTT

TGGTTCTTTTTCCTGGCGCAGTCCTTCCTCTTCAGATCTTCGAGTTCAGGTATCGTATCA

TGATGCACACTCTTCTTCAGACAGATCTGCGGTTCGGCCTCATCTATAGAGACAACTCGA

ACGGGATGGCAGATGTTGGATGTGTGGGAGAGGTAGCCAAGCATGAGCGGCTTGTGGACG

AGCGATTCTTCATCATATGCAAGGGACAAGAGAGATTCCGAGTATTAGATCTTGTCAGGA

CGAAGCCTTATCTGGTGGCAGAGGTTGAGTGGTTGGAAGACCGGCCTTCGATGAGTGGCA

AACAAGACCTCGAATCACTCGCTTTGGAGGTGGAGACGTGCATGAAGGAAGTGGTCAGGC

TCTCCAACAAGTTGAATGGGAAGCCAGAGAAAGATGCCATTGATCTGCGGAAGAACCTCT

TTCCGACTCCGTTCTCGTTCTTTGTTGGGAGCACGTTTGAGGGCGCTCCACGAGAGCAGC

AAGCGTTGCTGGAGTTGGAGGATACGATGTTGCGGCTGAAAAGGGAGAGGGAGACCCTCA

AGAGCAGCCTCGGTTACTTAGCAGCTGCGTCTGCAGTTAATGATGCATTCGAGTTCTCTT

CTACGGATGAAAACTAGCTGTGATGTTTAGAAATCGGAAATTTGTTAGTTTGAGGCGGCC

GCACCTGTATATAAACAATGTCTGGATCTTTTCCTCGCAATTTGGGTTTCCATGTATAAA

TTGAGGTGTTATTTTGCTTAGTTTCTAGAATGGAATG

>Unigene0002208

ATCTCATACATCAATTGAACTGAGATTGGAAATCTTCAGGCCCGCAGATGAAGAAGATAT

TCGCGTCTCTCCTCGGAAGTCCGAGAAGCTCCGAAAGATCCCACCACCAATCACAGCTCA

AGGAAACAAGAGAATCAACAGATGACCCACAAAGTAGCACTACCAGCACGGGTGGCCTCG

AGAACGAAACAACTAAAGATTGTGTGACCACCGATTATGAAAATATCATATGGAACCCTT

GCTTTGATGATGGCCTGAGTAATTGGTGCGGCCGAGGATGCAAGATTCTTCTACATAATA

GCTTGGGAGACGGTACGATACTTCCAGTAAATGGAAACTTTTTCGCTTCTGCAACGAACC

GTCAACATAGTTGGAATGGAATCCAGCAGGAGATCACTGGCAGGGTGCAGAGAAAGATTG

CATATGAGGTCACTGCAGTAGCTCGAATATTGGGGAATGTGAACACTGCTGATGTGCAGG

TGACTCTGTGGGTTAAAGCTCCAAGTGGCCGTGAGCAGTACATCAAAGTTGCTAAAATGC

AGGTATCCAGCGAGGAATGGGTGGAGTTACATGGGAAGTTTTTTCTGAACAGTCATTTTA

CAAAAGCTGTCATCTCTCTAGAAGGGCCAACTTTGGGTACTGATATCCTTGTCAATAGCT

TAGTAGTCAAACGTATGAAGAAAGCTCCGCGTACCCCTCCTCCCAATACCGATAATGCTT

TGTTTGGAGTTAACATAATCGGTAACGCCAATCTCAAGAATGGACTGAGCCAGTGGAGTC

CTTTTGGTTCATGCAATCTCCGGATATGCTCCGGTTCACCAAAGATTTTACCTCCTGAGG

CTGGTGATTCCCTTCCTAATCATGATCCACTAAGTGGTCTCTACATTTTCGCATCTAATC

GCACCGAAATCTGGATGGGAGCGTCGCAAACAATAACAGACAAGCTGCAGCTGCACTTGA

CATACAAAGTTGTAGCTTGGGTTTCAGTTGGCCAAGGTGCAATCGGACCTGAAAGTATCG

ACATTACACTTGCTATCGACAAACAGTGGGTAAACGCAGGACATGTACAAGTTACCGATG

GTAACTGGCATGGAGTCGGTGGATCCTTCAGAATTGATACGAAACCTTCCAAAGTTGTTG

TTTATGTTTCAGGTCCCTCGCCAGGTGTTGATCTGATGGTCGCTGGACTCCACATTTTCC

CCATTGACAGAAAGGCGCGATTCAAGTATCTAAAAGAGAAAACCGACAAGGTACGAAAGT

GTGACATTATCCTTAAATTCCCAGACAGAGATACCCTCAACATGTCCGTGAAAGTTCGAC

AAATGCAGAACAGCTTCCCGTTAGGTTCATGCTTCAACAGGTCAACCATCGATAACGAGG

AATTTGTCGATTTCCTCGTGAAGAACTTCAACTGGGCAGTGTTCGGGAATGAGCTAAAGT

GGTACCATACCGAGTCACTGCAAGGGAAGTATAACTACCAGGATGCTGACGAGCTGCTGG

ATTTCTGCGAACGACATGGGATGCAGACTAGGGGCCACTGTATCTTCTGGGAAGTGGAGG

ATGCGGTGCAACCGTGGATACGAACACTGAATCGGGATGATTTGATGGCGGCAGTCGATA

ACCGTCTCAGAAGCCTCCTGACTAGGTACAAAGGTAGGCTTAGGCACTATGATGTGAACA

ATGAGATGTTGCATGGCTCTTTCTTTCGAGATAGGCTCGGGGAAGACATCCGAGCGTACA

TGTTCAGAGAAGCGAAGTCGCTCGATCCTTCTGCCATTCTTTTTGTGAACGACTACAATG

TAGAAGAGGGATATGATATGAAGTCTAC

>Unigene0002209

CCGGAAATGTACATTGAACAAATCCTTGATTTACAAGAGCGAGGGGCGGAAGTGGGGGGT

ATCGGTCTACAGTGCCATATCACTAATCCGGTTGGCGAGGTCTTGTGTGCTGTATTAGAT

AAGTTGGCGATACTGGGACTTCCGATCTGGTTTACTGAACTGGATGTGGTGGCGGAAGAT

GAGTACGATCGAGCTGATCACCTGGAAATCGTTCTTCGGGAAGCATATGCGCATCCTGCG

GTGGGGGGAGTGATGGTGTGGGGGTTTTGGGAATTGTTCATGTGGAGGGAGAACTCGCAC

TTGGTTGATGCTGATGGTGAAGTTAATGAGGCGGGTAGGAGGTTGCTCGCTTTGAAGAGA

GAGTGGATGTCAGAGGCTGAAGGGGCTGTCGATGCAGTCGGGGAGTTCAGGTTTAGAGGG

TATGAAGGGACATACACCATTGAAGTAACTGCACCTTGGAAGAGATCAGTGCAGACATTT

GTTGTTGAGAAGGGGAGATCTCCAGTGGTTTTAAACATAAACATATAGGATTTACATGAT

TGCATACCGGGACTTTGTTATATGATTTGATTTTGTAATCGACCTGGATAATGGTTACTG

TAAGCTTGTATATTATTTTTG

>Unigene0002210

ATCAATACTCCAGAACAGAGACCGTTCTTTTCAAATGAAAGTGAATGATGGCTTCAACCT

AAGCTCAGCTGCTGTGATGGGCCAATCTTCCATTTCTCTGCAGGGTTCGGCTATTGGGAC

TCGGTCAATGTCAACAATCAAAGGAAGGAGCATGCGGAGTAAGGTGGAGTCGAGAATGCG

GAGAGAAACTGGGAAAACTATGAGAGAGATAAGGCGTGCTAAAAAGTTACGGAAGAAGTT

GATGACAGACGAAGAGAGGCTAATATACAACTTAAGAAGAGCCAAGAAAAAAGTGGCGCT

ACTCCTGCAAAAACTCAAGAAATACGAGCTACCAGAGCTACCCGCTCCTCGACACGATCC

CGAACTACTCACAACCGAACAACTTCAGGCTTTCAGGAAAATCGGCTTCAAAAACAGGAA

CTACGTCCCTGTTGGAGTTCGTGGAGTTTTTGGAGGTGTCGTCCAAAATATGCATCTCCA

CTGGAAGTTTCACGAGACGGTGCAAGTTTGTTGCGATAACTTCCCTAAGGAAAAAATCAA

AGAAATGGCGACCATGCTTGCAAGATTAAGTGGTGGCATCGTGGTTAATGTACACAATGT

CAAAACGATCATCATGTTTCGTGGGAGAAACTATCGACAGCCCAAGAATTTGATTCCGAT

AAACACGCTTACTAAAAGGAAGGCATTATTCAAGGCCCGATTCGAGCAAGCTCTTGATTC

TCAGAAGCTAAACATAAAGAAGATCGAGCAACAACTACGACGAACAGGGGTCAACCCCGA

GGACCCAGTTGCTATGGCCAGCATCCAGAGGGTTGCTTCCACATTCTTCCGTGCGATCGA

TGAAAAGCAAGGCACTCCATACGTCTTCCATGGTGATAGAAAACCAACAAGCGAGAGTTA

CGAGACCGACTACACTACGAAGGAACCAGACTACCCGCCTTCTGATGACAGCGACCAGGA

GGAGCTTGATCGTTTCATTGCAGAAATTGAGGACGCAGCGGACCATGAGTGGGCGGAGGA

GGAAGCAGCGGAGAGAGAAGAAGCATCCAAGATACGGTATTGGAAGAGAGAAGAAGTCGG

GATGAACAGGAGTTCACAAGATGTTAGAGGCCGTGAAAGAGAGCATAGTATGGATGGTAA

GCAACGAGCCACTAATATAAGGAAATGGGACCGCAACGACGATATCTCAGAAGATATCTC

AGGAGAATCGGAACAAGAATTGGATTCATCTTATGAAATGCGTGTTTCACCGGATATCCA

TGACGAGTATTTCGAGGATGATGACAACAATCAAGGTGTGGAGAAGCGATATTCGAAAGT

GAGAACAGGAGGGAAATCGGCTCAAGTGATGACAAGATCTAACAGAGATATGCATGATGA

TCCTGATGGCATGGCATTGGTTTCAGACGATGATGGTGGAGACACTCGAGAGATTTCGAA

AGGCGAGGTTTACGATTTCCTCGGTAGTGACGATGATGAGAGTGATAATGGTAGGAGTTA

TGGTGGTGCGTCTGAGTTTGAAAACTTTGATGAAGACGATATAGACCGCCTGACCCCTTT

CGTAAATAATGCTTACAATTACAT

>Unigene0002211

CATCGCATAGGTTCTTGTTCGACGGTGAGAGTTCAGGTCTGCTCGGTCCTGGGAAGAGCC

GCTGGCCGTTTGTCAGTGGCTACGGTTTTGTGACATGAATATATTGTGCGCAGCGGAAGC

AAAAAATTCAAATTTCTTTTTCTAGATGATGCTCAATTCAATCAACTCAAAATAGCAAAT

AAGACTGTCAATCACAAGTTCTCCAACATCAACTATCACATTTCAAATC

>Unigene0002212

CATCGCATAGGTTCTTGTTCGACGGTGAGAGTTCAGGTCTGCTCGGTCCTGGGAAGAGCC

GCTGGCCGTTTGTCAGTGGCTACGGTTTTGTGACATGAATATATTGTGCGCAGCGGAAGC

AAAAAATTCAAATTTCTTTTTCTAGATGATGCTCAATTCAATCAACTCAAAATAGCAAAT

AAGACTGTCAATCACAAGTTCCCCAATATCAACTATCACATGCTTGAACAAGCTCTAATC

TAAAGGGAT

>Unigene0002213

CTGAATTTAGCATTCTGAGTTAAAGAAAATATATAAATAGATTACTGATTAAGGCTATCT

GAAAAATAAACAAGTTCGGTGCCGTGCAGTCTAAGTTGCTGCACTTATTTAAGAGAATTA

AATACGGTGTTCTGCAGTTTTGCTGCTATATTTATTCTGAAACAAGATAAAATCAATTTT

TAGGGGATGCCAGAATCTGCACCTCTTTCTCTGATTCTTTTTCTGCTGCTGTGACTTCCT

TGATCTTGTGCTTCTTGTTCGCTGTACCTGTTTTCCTGATCTGCGAGGCTGCTATGCTGC

ACTCCATCGTCCCAACTGGATCGGTATATCCGGTCAGATAGTTTCAGTGTGACTGGTAAC

TACCATATGTGACTGACACAGGTACAGTTTGACCCTTCTAATGGATCAGAACTTTTTTCC

TGCTCAGGTCAGCATTCACCTGGGTTTGTTATTAAGTTTGTTTGAAGGCGGGATATATAT

TTTCTAGTCATAATTTCTTGCACACAACTTAGCTACAAGTTTGGTATTCAAAGTTATAGT

TGGTGGACGAATGTAGCTCATATATATATATAGTCCGACTCTTTAATAGTGGTCAGTCAA

CATTCATCGGTTGTAGATTTAGAGATAGGTAGACGTGGTTTGCATAGTTGAGCTGATGGG

GCTGATTCGAGCTTGTTGAATTCGATACTTGTCGATCAGATTGGGGCGGGCGTTGGTCTT

ACATGTATGGGCGACCTTGTTGCCCCCCACCCCACTGAGACTTTCAGGAGTTAATGTTTC

ATACTTCTACCTATAATTGTGTGTGAACCGGATGACATAGGCACAATTCGGCCCAATTGA

TCGGAACTAATTTTCTACTTCGACTAGTTCTAATCCACTCTCTCGATATGGTAGAGGCAT

GGTTACAACATGCTTTAAGAGTAATATTTTGTTGTGTCGGATTTGTTTCGGTGTCTTGTT

TTTATAGTCGGATTGTGATGTAGATGTAATTACGTTGATGTTAGTGAATTGGCTAAATGT

TGTGTCGGATTGAGTCTGGCGGTGGGCTTTATAGCCAGATTACGATGCTATGGAAACTGT

TGACAAGGATAACTTCGTACATCATAGAGGTATAAGTTTTTGTTTTTGTTTTTGGTATGA

CGTAGGTGGAGACTAGTTTTGCTTGATACCATGCAATTCAACACTTTCTCAGAGTTTCGA

ACTTTCCTGCATTAAGAGTTAGGCTACACTGGTACTCCACTTAAAGTGATGAGCAATGTG

ATTAGTTGACATATAGCATGGACAACTTTCTGGTAGCCCTCAGATGTTAGGCATGAGATC

TGCTCTTTCTGACCCTTTCAGACTTAAAGCCATACTGACCTCTAAAAGAGCCAAGCTCCT

TTTCTGTTGTGCCTCATCAGGAACTCATGTAATTGGTAGTCTCATATATTTACCACAAAT

AGCGTTGTCAGGGCCTTTAGGTAATGTTGGATCATGAGTTTTTATGGGATTGTCCCCATA

CGGTGCCTATGATGCTTGGGCGTAGGGGGTGATCATCCGGAGGTAGGTGTAGCCTTATTC

CCCTAAGTGATGGCTTCCTCGTGTTGTAGCGACCGAGGATATGTTTCCTGTGTCGTAGCT

ACCGAGGAGGGGTTTTCGATTTATACACCGAGTGGTAGCATACTTCCTAGCATTTCTCAG

AGATGGATGGGCGATCACATCGCAATGATGCTAGTTCCTTCGTCTCCTTAATAGATCATG

GTTCGGTTTGACACTAGAGATTGGGGCATCCTTTTCCAACCCTGCTGCTCTTGCGAAGAT

TCTTAGGTTCTCCCATAGGTGCTTTGTACTATATGATGACCGAATTTGGTAAGGGAGGCT

CGACAATCTCGATGAGATCTCTGTTGTGCACAGCGGAAGCAAAAATTTCTTTTTCTAGAT

GATGCAATTCAATCAACTCAAGATAGCAAATAAGACTGTCAATCA

>Unigene0002214

ATTCCTTTCTTGTATACGATGTGTCCTAGAAACTTCTCGGACGACACACTGAATAGACAT

TTCTCTATATTTAACCATAGTCGGAAGATACGTAGCCGATCGAAGGCTTCTTCCAGATTG

CGTATATAATATTCTTTCCGGGTACTCTTTATTAGTAGGTCGTCTATGTACATCCTCACG

GTCTTTCTTAGCATCCCTTGGAAGATCTTGTTTACTAGTCACTGGAAAGTCGCTCTAGCG

TTCTTGAGTCCGTTCTTGAGTCCGAAGGGCATGACTGTATAACAGTACA

>Unigene0002215

CGCTTATGTCCTACGATCGAGTCTACCAACTGACAAATTCTGGGCAACAGGAAACTATTT

TTAGGACAAGCCTTATTTAGATATGTAAAGTCCACACAACCCCTCTACTTTCCGTTCTTC

TTTGAGACTACCACTATGTTGGCGATCCACACTGGGTAGTGGGATTCTTTGATGAATACG

ACTTTTAGTAACTTCTAGACTTTTGTCTTGACTATGACGGCCTTTTCATCTGACAGGGGA

ATGTGTGCCTGTGCTTTTGGCTTCGTCCTTTTTTAATGTTCGGTAAGTGCGTGATGACAT

CCCGGCCGATCCCCGAAATGTTCTCCACATTCCACGCTAATACATACAAGTTGCTAAAAA

AGGCTACTAATGCCTCTTGATCTCTATCGGTTAACGATTCTCCTACGTACAATACTCGTT

CTGGGTTGTCTGGATTTATCTGTACTAGTACCACATTTTTGACCTATGGCTTGCATTCGG

AGTTCGCCTCGTTTTCAGTATTCTACGTCAGCATGCAGATGCTTAATTAGTATTTAGGTT

TCTAGATTTATTTATATGTTTACTGTTTTAGCTTCTGAATTTATAGTTATACTATACA

>Unigene0002216

CTAAGGTAGCCGGCTTCATTGTCGGATTTTAGTGGAATATTGCTAAGGTAGCCGGCTTCA

TTGTCGGATTTTAGTGGAATATTGCTAATCTGGATCTTTGATAACGATAAGATGTATGCC

ACTTCCACTAATATAAGATAGCTGTCTTAATTGTCGGAGTTTAGTGGAATATTGCTATTC

TGGATCTTTGATGACGATAAGATGTATCTCACTTCCACAGATAACGTGGTCTAGTGCAAC

CACATAAGATAAAAATAAAATTCATATTTTTTAACTTGTTATCCTCTCCTCAATTCTAAA

TGGTCCAACCCTACATTTGTGCACCTAAATTCATTGATAATGGTTTTTCTTACTTTTCAG

AGAATAGATTAAATTTTAATATTCTTTTGTAACACACAACAATATTTATTAAAGAAGTTT

GTCTGAATACATGCATGTTCGGGGAGTGGTTGGTGCACAAGCCTTGATGTTATGTAATAT

TTGAGGCCGGTTTGGTTTTGGTTGCGGTTTATCCACTTTTGTCGCAACTTATTTGATTGT

TGATGCAGTTTTTCACTTAGTTGCTTCTGTTCGGGTTCAATCCCTTGACATCTGCCTAAT

GTATGCACCTATGTCTAATGTGTTGACTGCCGGGACCCTTACAAACAAATAAAAGAGTTA

TTTATCGAAGAAATAAAAAATGATTATTCAATTTTAGATTAGTTATTTAACAATTTTTAA

ACCATGTAGGTTAAATGGGCTAGGGCTAGGTTGCCTCGTAGGGTTAAAATTGAGATAGTT

CACTCATTTAAATTGGTTAGGATTATGGTTGAGTCATCTTGCATGGAACCCTAACTCTAA

CCTTTTTGTTGCCCTAAGTGAAGTGTATTGATAGTCGTGTGTGTAATTAGATTTATAATG

TGACAATGGTGTAGTGACTGAACTGGTTATGAGTGTCCACAAAAGTGTTGCTAAAACTAC

TTTTAACTTGGATACTTGGGGACAAAGTGCTCCAACAGTTGTGTTAACCTAGCAAATGAC

TCACAAAATGAGATTTCTCTCTCCTAAAACCAACAAGGTGCATGTGCATAATAGCAAGTC

TTTTTGACAAATTGTTGGGGTGGAAAGATGGATGATAAGCCACCATCAAGCATTCTTATA

CTTTGAATTATGACCCTCAATCATATAACACTTTATATTATCTCTCATGATATTTGAAAT

TTAACTTTCCCCATAAAGATTATAACAGCGTACATATTCTAAATAACATTTTTTGGGGAA

ACAAAATTGAAAAGTTCTAGGTCTAAATTGATTTTGCACTTGTGATAAAGGTTTATGTAG

AAAAAAATAGTGATGTCTGCAGAATTCAAATATTAACATCTCATATAGCAGACTTTTTAG

CAATCTTGTATCAAATTTATAATTTTGTACCAATTTTACATAAAAGTTACCAACATAGTA

GCAAAATCTCAGCAATACTGATGTGGATGCTTTTCAGTATGAAAAAGAGAGAAACTACAT

ACAAAAACTACACCCACGAAAAAGAAACTGCCAACAAAAACCACGTCCTTCGGCATTTAT

TGGAAAGATATATAGAATGAAATAGACAAATATGGTATTTAAAAGCATCCAGGAAGGGCC

GGGCTATTTTCAACCCGCCGGTCTTGTAATTGAGACCAGAAGTATGCAGTCCACTGGGTA

CCTTACGACGGTAGAAAATCTCACCAGGCTTTCTAAAAGTGGACGAGGTAATGGTTGAAG

AGGAGGTGAATTTGAAACTTTGCCGTAAACCATTCGGCTCTCAGAAAGCTTTATGTGTAT

GAACATACTGATGACTTTTAGAAGCTTCTTAGGGAGTAAAAATAGAAGAGGAGGTGAATT

TGAAATATTGACCTAAAGCCGGCTGCTCTCAGAAAACTTGGCAGTGCATACCTGGTCTTT

TGCCTTACCTGGTCTTTTCTAAATGCCTTACGTCTTCTAAAATTTTGGCCAACATAATCT

GAAAATGCTGCTGCCCCCGCTTTTAGAGAGCCAATCAGAAGGGGCCATCAAGGTTTGTGA

AAATGGTTTGATAAGAGCGAAAATGACAGCCCCTATTCCAATCCAGACGGCCGGAACTTC

TGGAAAGCTGTTCCCTGTCCGTTCTCATTCTGACAAACCTGTTACCTCTACACGCAGAGA

GGTTCCAGATTCGAATCCGTGACCTCCATGTCACATGGCAGCAATTTTTATCATTGCGCC

AGGTTCACCCTATATTGTTCTTAAAATTTAAAACAAGTGTCTAGTAGCAGGGAGGCTGCA

CTCGGGTGGTTTAAAAGTGATTCCATGGCACTACCTTTTGGATACCGAGTCTCATGTCAA

GCATAATGACAG

>Unigene0002217

GCTAAGGTAGCCGGCTTCATTGTTGGATTTTAGTGGAACGGGGCAAGGGCATCCACATAA

GGCCGGCTTCATTGTCGGATTTAAGTGGAACGGGGGCAAGGGCATCCACATGAGGTAGCC

GGTTTCATTGTCGGATTTTAGTGGAACGGGGTACGAGCATGCACATAAGGTAGCCGGCTT

TATTGTCGGATTTTAGTGGAATATTGCTAAGGTAGCCGGCTTCATTGTTGGATTTTAGTG

GAATATTGCTAATCTGGATCTTTGATAACGATAAGATGTATGCCACTTCCACTAATATAA

GATAGCTGTCTTAGATGTCGGAGTTTAGTGGAATCTTGCTAATCTGGATCTTTGATGACG

ATAAG

>Unigene0002218

ACCTACCCATCCCACCTTATCCGGCAGTCCAAAATAAACTTCTCATCAGACGGCTGGTGC

CTGCCTCACGAAAATCTTTCCTCGATCTCCACTCCCAGACCCATGGCGACGAAATCCGGC

CCTGAGGACGCGCCGTCCAAGACGGCGGCGCTAGACACGCCGCTCCACACGCTAGGCTTC

GAGATCGACGCCGTCTCCGCTGCCGAAGTCACCGGCCGGCTGAAGGTGACCGAGCTCTGC

TGCCAGCCGTTCAAGGTGCTTCACGGCGGCGTCTCCGCCCTCATAGCGGAGGCGTTAGGG

AGCATCGGCGCGCACGTCGCCTCCGGCTTCCGCCGTGTCGCGGGCATACAGCTCAGCATC

AACCACCACCGCTCAGCGCCGATCGGCGACCACGTCTACGCCCGCGCCTCGCCTGTACAC

GTCGGCAGAACGACTCAGGTATGGGAAGTGAACCTTTGGAGGACGGATCCATCGACATCG

GAAAGGGGACCCTTATTAGCTTCATCACGGGTCACTCTTCTAACTAACCTGCCTGTACCA

GAGAGCTCCAAGACTGCAGAAGAGACTCTAAAGAAGTACGCCAAGCTATGAGCTGCATTA

TCGGAACTGCTGTACTTCCACATGTAATCCTAGTATCAATGATTTGTATTAGTGGGAAGT

TATTTGATATGATTATTTTACTACAGTGTAATTTACCTGATTCACTACC

>Unigene0002219

GCGCCTCGCCTGTACACGTCGGCAGAACGACTCAGGTAACCGAGTTATTTTGTCGCTACG

TTGAAGCTGCTAATTAGTTGGGGTCAATTGGATGTTAGTCCTGCTTGTTTACCTATTTTT

GGATTCAGAGTCTTTTAAGTCCTAACAATCTGAGTTCGAAATAGTAGAGCACTAGATTTA

AGAATCATAAATGCTACACGCTTGACCTGCAATTTTAATTCCAAGAATG

>Unigene0002220

TCATATGTAAAACCCTACCCATAATCTTCCCCTGTTTCCGAATCCCTGTTTGAGCAATTC

AAACCCCAACTTGAATCTGGATTCTCTGTCATGAAGCTCGTGGTGCACGTCATCGAAGCC

CGCAACCTCGCGGCATCGGACCCCACCGGGTTGCCAGAGCCCTACGTGAGGCTACAGCTC

GGTAAGCAGCGGTTGAAGACGAAGGTGGTCAAGAAGAGCTCAACCCCCTTTTGGGATGAA

GAGTTCAGCTTCTGGGTCGCCGACCTCGGCGAGGAGCTCGTCGTGTCGGTGATAGATGAG

GAGAGATGTTCGAGTTCGACTGTGCAAGTTAAGGTACCTCTGTCGAGGATCCTTGATGAG

GGGAAGCGGTCGCTGGAGACGGCGTGGTATCGATTGCAGCCGAAGAATAAGAGGAGCAAG

AATAAGGATTGCGGAGAAATTTGTCTCGCCATGTTTTTGTCCCAAAAGATTAGTTCCTCT

GAAGAAACACCCCTGACCTCACCTTTTTTCTCTGATGGCCCTGACTCGAATTCCGACAAG

TCATCTGAACTTACAAGAGAATTGTCTTCAATGACTTCCCATGATACAGTGGACTTCTCT

GCTGTATCAGAAGTTGAAAATGCTGAACCAATTAAACAAGAAAAAAAAAGTACTCCGTCA

TTTGTTGACCACTTCTTTAAGATATTTTCGGGGAAAAAGGCAGAAGTTGAAGTTACAACC

GATAGAGATCTTGATTTTGCAGATTCCATTGTGACATCAGGGGAGCTTGAGTCAGTTGAA

GAGCAAGTTGATGATGTTGGTTCTAACTTGACATTTGATGACTTATTGAAAATTATGGAG

TCAAAGGATCAAGGAGATGAGATGCCTGAGAGTCTACCCGGTGGAGTACTTCTTAACCAG

TCTTACGTTGTAGCACTGGGTGATCTCAACTCTCTTCTGTTTTCGCCGAGTTCAAGTTTT

TTGCAATCATTGGCGGATCTTCAAGGAACTACAGGTCTTCAAACTGAACCCTGGAAACTT

GAAAATGATGGAGTGAGCTTGAAAAGAGTGGTTACGTACACGAAGGCTGCAACTAAATTA

GTCAAAGCTGTAAAGGCTACAGAGGAGCAGATTTATATGAAGGCTGATGGAAAAGCCTAC

GCAGTTTTAGCAAGTGTAAAAACTCCAGATGTTCCCTTTGGTAGCAGTTTTAAAACAGAG

GTTCTCTATTGTATAATGCAAGGGCCCGAATTGTCTTCTGAAGACCAATCTTCTCGTCTA

ATAATATCTTGGCGAATGAATTTTCTTCAGAGTACCATGATGAAAGGTATGATCGAAAAT

GGAGCAAGACAGGGACTGAAAGACAGCTATGCTCAGTTTGCGGACCTGCTATCTCAGAGT

GTCAACCCCGTCGATATAAATGACTCTGTGTCCAGTAGAGAGCAGATCTTAGCCTCTGTA

CAGAAGGAGCAGGAGTCTGACTGGAGGCTGGCGTTTCGTTTTTTCGGAAACTTTACTGTT

ATAAGCGCTCTTTTTGTTGGATTATATGTTCTGACCCACATCTTGCTTGCAAAGCCAAAC

CAGATTCAGGGTCTAGAGTTTGTTGGCTTAGATTTGCCAGATTCTATTGGAGAAGTTGTG

GTCTGTGGGATTTTGGTTCTTCAAGGGGAACGCCTTCTAAATCTGATAGCACGGTTTTTG

CAGGCGAGGAAGCAAAGAGGCAGTGACCATGGAGTCAAATCACAAGGGGATGGATGGCGG

CTAACTGTTGCCTTAATTGACGGAAGTAACTTAGCTGCAGTGGATACATCTAGTTATTCT

GATCCTTATGTAGTTTTCACCTGCAACGGTAAAACAAAAACAAGCTCAATCAAGTTCCAT

ATATTGGATCCCCAGTGGAATGAAATATTTGAGTTTGATGCTATGGATGATCCTCCATCT

GTATTGTATGTTGATGTCTATGATTTTGATGGACCTTTTGAAGAGCCTGCCTCCCTTGGC

CACGCTGAAGTGAACTTTGTGAAATCAAATTTATCAGAGCTAGCAGACATATGGATTCCT

CTTCAGGGAACATTAGCTCAGGCATGTCAGTCTAAGTTGCACTTGAGGATTTTCTTGAAT

AATACACAGGGTACTGCAGTTGTTACAGAATATCTGACTAAGATGGAGAAAGAGGTTGGG

AAAAAGATAAATTTGCGGTCCCCACAAACAAATTCAACATTCCAGAAAGTCTTTGACCTT

CCACCAGAAGAATTTCTTATCAATGATTTCACATGTCATTTGAAGAGGAAAATGCCTTTG

CAGCCCCTCCAAAACTAGATTCAAGGGCTTTGATCGCTCCAGTTTTCCTAGGGACAACGG

TGAACAAAACGATTCATGTAATTACGGGGGTGCTGCCGACCAAAAGCATGCACTATGCTA

TTGAGGACCTATTACATTGTTGAGAGACCATGGCCGGCTATTTTTATCTCCAAGAGTATT

TGGATATTATACAAATCCTTTTGGGCATAAAACCAAATTTTACTTCCTTTGGGAAGACAT

TGAGGAGATCCAAGTACGTCCACCAACTCTATCATCAATGGGTAGTCCATCTATTGTGAT

TATACTCCGTCAGGGTAGAGGTTTGGATGCAAAGCATGGAGCAAAAGCTCTAGATCGTGA

TGGTAGACTCAAATTTCATTTTCAGTCATTTGTTTCTTACAATGTTGCACACAGGACAAT

AATGGCACTGTGGAAAGCAAGATCGTTGAGCACGGAACAGAAAGTGCAATTAGTTGAAGA

AGAATCTGGAGAAAAAGTTCTCCATTCCGAAGGGAGTGGATCTTTTCTTGGCACGGAGGA

TGTCAAACTTACAGAGGTTTTCTTGGCAACTGTTCCTCTTGCTTTACACTCACTAATGGA

GCTGTTCGAAGGAGGTCCTTTGGAGCGTAGAGTGATGGAGAAAAGTGGTTTTGGCGACTA

TTCTACCACACCATGGGAAGCAGATAATGCCGATGTTTTGCAAAGGCAAGTTAGTTACAA

ATTAAACAAGATGAAGAAATCTAATGTATGTGAAGTGACGAGCACTCAGCAGAAATCTTC

ACTTCCTGGTAGAACTGGGTGGGTTATTCAAGAGGTGATACTGCTCCACAAGGCCTTACT

AGGCGATAACTTCAATCTTCAGTTCAGATACCAGATAGAAGATCTCCGTTCCGGGTCCAA

TGCATGCAGTGTACAAGTATCTGTTGGAATTGATTGGCTAAGAAGCTCCAAACACCAGAA

GAGGATCACGAAAAATGTGATGTCCAATTCATCTACTCGTGTGAAGGAGATGTTCCGTCA

AGCCGAAAAGGAGCTCACAAGAGGAAAGTAGAACCGTCTCTTGCATCTGGCAAACAATGA

AGTTGACAATTGTAAATAGATACACACTAAGAGAACAAGTTCCTGATACCATTGGAGTAT

TGAGCATCTTAAACACAAAGGAGTTTTCAGCCGAAGCACAAGTCAAGTGGAAAGAAATTG

TGCTCCCATACTTATTTTTCTGCCCCTGTTAGTCTGTGTCTTCAGTTTTCTGCAGCTTCG

ATTATGTGGTTAAACTCGGACCTCCAAGATTTATGATAGATAATCAACTGCATTTCAACA

GGATGAAACAGACCACTGAACACCGCTGTTGAAGAGGTTTAAAATTGGCCACTTTTGAGC

TAAAATTGGTCTGTTCTAACTCTGACCTTGTAAAACAGTGAGGAAGTATTTTTACTTTTG

CTTCAAACCATATCCTGAGAGAGAAATACAAAAACGAAGCATGCATGTTGGTAAACAGGC

TCTCCAAGTTCTGCTTCTGTTATATTCATCTTTACGTCAAT

>Unigene0002221

GCTATGCGAATGATGAACGAACCAACAGCCCCTTCATTCCCCTTACTGCCTCCGCCACCG

ACTCGGATCGATCACTGCTGATATTGGGGTCGGCGAATTCGTCCTCAGAGCTAGAAGCTG

GTCTGATTTGATCCGCTTGGCCTCGCCGTTGACTTGGATCATCACTACTGATCTCTATGG

CTATGGGGGCCGGCGAATTCATCCCCAGAGCTTGACGCTGGTCCGTGTTGATCAAAATTC

GGCGCCATGTTCGAGGCTCATGTTTTGCATCTGCTCCGGAAATATCTAGGGGAATATGTT

GAGGGACTCTCAACTGAAGCCCTTAGAATAAGCGTCTGGCAAGGTGATGTTGTTCTCAAA

GACTTGAAATTGAAGGCTGAAGCACTGAATTCTTTGAAGCTTCCTGTCACAGTAAAAGCT

GGTTTTGTTGGCACTATAACTCTAAAGGTTCCCTGGAAGAGACTTGGCAAAGAACCTGTG

ATTGTTCTCATCGACCGTGTGTTTATTCTCGCTCATCCTGCTCCTGATGGACAAACTCTC

AAGGAGAAAGACAGAGAAAAAATTTTTGATGCAAAACTGCAACAAATTGAGGAGGCAGAG

TCTGCAGCCCTTGAGGCGGCATCCAGGAGATCAAAAGAACCGGCTGGAAATTCATGGTTG

GGCTCTCTTATTGGGACTATAATTGGGAATCTTAAAGTCACCATTAGCAATGTGCATGTC

AGATATGAGGACTCAGTGAGTAATCCAGGTCATCCTTTTTGTTCTGGAGTGACTTTATTA

AAGTTAGCAGCCGTTACAATGGATGAGCAGGGTAATGAAACCTTTGACACTAGTGGAGCT

CTAGACAAGCTGCGTAAGTCTTTACAACTCCAGAGA

>Unigene0002222

TAGGAACTGAACCGGCTGGAAATTCATGGTTGGGCTCTCTTATTGGGACTATAATTGGGA

ATCTTAAAGTCACCATTAGCAATGTGCATGTCAGATATGAGGACTCAGTGAGTAATCCAG

GTCATCCTTTTTGTTCTGGAGTGACTTTATTAAAGTTAGCAGCCGTTACAATGGATGAGC

AGGGTAATGAAACCTTTGACACTAGTGGAGCTCTAGACAAGCTGCGTAAGTCTTTACAAC

TCCAGAGA

>Unigene0002223

ATTGGTCAGGCTGCTGCTCGAATCATTAATATTCATCAGACAGAGGTTGTCTGCGGTAGA

TTTGAACAACTTCATTTCACCACGAAGTTGTATAACAAAACCATTGGTTGTGACGTATCT

TTGAAGTTCTACGGCTTATCTTCCCCTGAAGGTTCCCTTGCTCAGAGTTTCATCAGTGAG

AAAAAAACCAATGCTTTGGAAGCTAGTTTTGTGCATGCACCTGTCGGTGAGGATGTGGAT

TGGCGGCTCTCAGCAGCAATTGCTCCATGTCATGCTACAATCCTGATAGAAAGCTACAAT

CGGTTGGTGGAGTTCATGAAACGTAGTAATGCTGTTAGCCCTACAGTGGCAATGGAAACT

GCAACTGCTTTGCAGATGAAGATAGAACAAGTGACCCGCAGAGCGCAAGAACAGTTTCAG

ATGGTATTGGAGGAACAAAGCAGCTTTGCACTTGACATTGATATTGATGCTCCAAAAGTA

AAGATTCCTTTGGTGGGCATTGAATCTACTTTCCAACATAGCCAGTTTCTTCTAGACTTT

GGCCATTTCACATTGCAAACCAGAGAAGGACAGCTGAATGAGCAAAGGCAGAGTCTATAT

TCTCGTTTTTATATAACAGGAAGGGATATGACTGCTTTTTTTATGGATGGTATTTCTGAA

GTTAACTGTCAAGTATCAGCGTCACTTGATTCACAATATGTTGATCCTTTCTATTCCCTT

CTTGATCGCTGTGGTATGTCAGTCATAGTTGACCAGATTAAAATACCCCATCCAA

>Unigene0002224

ATTGGTCAGGCTGCTGCTCGAATCATTAATATTCATCAGACAGAGGTTGTCTGCGGTAGA

TTTGAACAACTTCATTTCACCACGAAGTTGTATAACAAAACCATTGGTTGTGACGTATCT

TTGAAGTTCTACGGCTTATCTTCCCCTGAAGGTTCCCTTGCTCAGAGTTTCATCAGTGAG

AAAAAAACCAATGCTTTGGAAGCTAGTTTTGTGCATGCACCTGTCGGTGAGGATGTGGAT

TGGCGGCTCTCAGCAGCAATTGCTCCATGTCATGCTACAATCCTGATAGAAAGCTACAAT

CGGTTGGTGGAGTTCATGAAACGTAGTAATGCTGTTAGCCCTACAGTGGCAGTGTAAACT

GTAACTGCTTTGCAGATGAAGT

>Unigene0002225

CAGATTAAAATACCCCATCCAAGTTATCCTTCAACCCGTGTTTCTGTTCAAGTTCCCAAT

CTTGGTATTCATTTGTCACCAGAAAGATATTGCCGAATCATGGAATTGCTTAATATATTC

TATGGCTCAGAAGAAATTAGTGATCAGAATACTAATGGACATCTCCAAACTGATCACGTG

CCCTGGCATCCTGCTGATCTAGCCACTGTTTCTAGGATCCTAGTTTGGAAGGGTATTGGC

CATTCTTTAGCTCAGTGGCATCCTTGTTTTCTTGTTTTGTCTGGACTATATCTTTATGTT

TTGGAATCTGAATCCTCCCAGAACTATCAAAGGTGTTGCAGCATGGCTGGCCGTCAGGTA

TTTGAAGTTTCTCCTACAAGTGTTGGTGGGTCGCTTTCCACAGTTGCTGTAAGTTCAAGG

GGATTTGATGTTCAGAAGGCTTTGGAATCAACCAGTACAGTGATCATCGAATTTCGAGAT

GAAGAGGAAAAGACTGCTTGGGTTAAAGCGCTTGTGCAAGCAACTTATAGGGCTTCGGCT

CCTCCTAAAATTGATATACTGGGTGAATCAGTCAATCGGTTGCCAGAATCTGTTGGAGCT

TCCCCGAGTAATTTGGGATCGGCTGATCTTGTTATTAATGGAACACTTTTGGAAACAAAG

GTGCTGATATATGGAAAATTGGACCGTACACATGAAAATATTGAGGAAGCATTAATTCTC

GAGCTCCTTGGCAGCGGGGGAAAGGTAAACATTGTCCAATCTGGTGGTGGTTTGATTGTA

AAAACAAAACTGCACTCCTTAAAAATAAAAGATGAACTTCAAGGGCGTCTATCTATGTCT

CCACAATATCTTGTTTGCTCTGTGCTGAATGATAATAATAACGTTGGTGGCTCTCCTTCC

ATTGATGTTAATGAAAATGAGTTGCATAGTTTTCCACTGGAGGAAGATGATTGTTTCAAA

GATGCAATGCAAGATTTCATGTCAACTCCGGATCATAGCTTTCGTTCGGACTTGATTTCT

AATTTAGCAGCTAATCCTGATACATGTGAGCAATATATTGAATATAGTGATGCTCTAAAT

CCCGACAAGGACCAAGCTAAAGCAAAGAATGAAATATTCTATGAAGCCACAGATGAGAAT

ATATCTGATTTTGTGGCTGTTACATTTTTGACAAGGAACTCTGAGTCACCTCTATATGAT

GGTATTGATACACAGCTGAGCATCTGTATGTCAGCACTGGAATTTTTTTGTAATAGGCCA

ACTCTTGTGGCCTTAATTGATTTTGGTCTTGATTTAACCCTAGCGAGTTCGAGAGTTAGC

CGCGTAAATGATACGAATATCCCACTATCGGATACCACAGTTACGGGAACGAAGGAAGAA

AATGGCCGTACTTTAATTAAGGGGTTGCTTGGTTATGGAAAAGGACGTGCAGTTTTCAAT

CTGAGCATGGACGTTGACAGCGTTTGTGTGTTTCTCAATAAAGAGGATGGTTCTCAGCTT

GCAAAATTTGTGCAAGAAAGTTTTCTCTTGGATCTAAAGGTCCATCCTAGCTCTATTTCT

GTTGATAGTACTTTAGGAAATATGCGGCTCCGTGATATGTCTCTTGAGCCAGATAATTGC

TGGGGCTGGCTTTGTGACACACGCAATCAAGGGACTGAATCTCTGATAAAGTTCACCTTT

CAGTCCTACAGTGTTGAAGATGATGATTATGAAGGATATGATTATAGTTTGAGTGGCCGA

CTTTCTGCTGTCCGCATAGTTTTCCTTTATCGGTTTGTACAGGAGATTACCTCTTATTTT

ATGGAGCTAGCTTCTCCGCACACTGAAGAAGCAATTAAGCTTGTTGATAAAGTTGGAGGC

ATCGAATGGCTAATTGAAAAATATGAGATTGAGGGTGCTGCTGCAGTAAAACTGGATCTC

TCATTGGATACTCCAATTATTATCATTCCAAAGAATTCAATGAGTGGAGACTTTATGCAA

CTTGATCTAGGACAATTACGAGTCAAGAACCATTTTAGTTGGCATGGTTGCCGAGAGAGT

GATCCTTCAGCTGTACATCTTGATATACTTCATGCTGAGTTACACGGGGTCAACATGGCC

ATAGGAATCAATGGTCTTCTTGGAAAACCAATGGTGCGAGAGTGTCTAGGTTTTAATATT

CAAGTACGCCGCAGTTTGAGAGATGTTTTCCGTAAAGTTCCAACTCTTGCTCTTGAAGTC

CAGGTCGGTGTGTTACATGGCATTATGTCAGACAAAGAATACAATGTTATATTAAATTGT

TTATACATGAATCTATATGAACCACCAAGACTTCCTCCAAGCTTTCGTGGCAATGCTGCT

GACATGAAGGACTCAATACGTATGCTTGCTGACAAGGTCAACCTTAGTGGTCAAGTCTTA

CTTTCCCGAACTGTTCTTGTTGTTGCTGTGGAGGTTCACCATGCTTTGCTTCAACTGCAT

ATTGGCCTTGAGGAGTCTCCTTTAGCCCAGATTGCTCTTGAAGGTCTATGGGTATCATAC

CGGAGTTCTTCGTTATCTGAAATGGATGTATATTTAACTATTCCCAAATTCTCCATTCTG

GACATACGTCCTGATACTAAACCTGAAATGCGGTTGATGCTTGGTTCATATACTGATATT

TCTAAAACTAGTATATCTAAAAATTCTGATTCTACTGGGGTGTCTCTGGTCTCCTCTGTG

GATTATGTTCCTGCCAAGAATCTAGAAGACACAACTGATATGGAGGTGTCCAACTTGACC

ATGCTAATAATGGATTATAGATGGCGCTCGTCTTTTCAATCCTTCGTGATTCGAATCCAG

CAGCCTCGCGTCCTTGTGGTTCTTGACTTTATTCTTGCAGTTGTTGAATTTTTTGTGCCT

GCTTTAGGCACGATAACAGGCAGGGAGGAAACCATGAATCCAAAGAATGACCCTTTAACA

AACTCTAATAACCTAGTACTATCAGAGTCCGTTTACAAACAGAAAGATGATATTGTACGC

CTATCCCCAAGAAGACAGTTAATTGTGGATGGTTGTGGCATTGATGAGTTCACATATGAT

GGTTGTGGAGGTACATTATCCCTGAGCGATGAGTTTGATTTCAAAGGGCATTCTTATTCT

GGAACAATTATTATTATTGGTCGTGGTAAGAAGTTGCGCTTCAAGAATGTTAAGATAGAG

AATGGTAAACTATTGAGGAAATGTACGTACCTGAGCAATGAAAGCAGCTACTCCATCTCT

GTGGATGACGGTGTTGAGATTTCTTTATGGGATAGTTGTAGTTCTGAAATGAATAAGGAG

TGCTTGGAACCGAGTAATGAATGCACCGATGGAATGACTGACCCAATCACAGGTTATGAT

GCTGCGGCTAGTCCTATACAAAGCTTTACATTCGAAGCTCAGGTTGTCTCCCCCGAATTC

ACCTTTTATGATTGTTCAAAATTATCCTTGGACCATTCGTCACATATTGAGAAGCTTCTC

CGTGCGAAGATGGATATTAGCTTCATGTATGCTTGGAAGGAAAATGACACTTGGGCCCGC

TCTCTTGTGAAAGACCTAACTATTGAGACTGGCTCAGGCCTTGTTGTTCTTGAGCCATTG

GATATTCAAGGAGGATATACTTCTGTTAGGGATAAAACAAATATATCACTTATATCAACT

GATATATGTATCCACCTTTCTTTGAGTGTTGCTTCTCTCATACTTCAATTGCAAAATCAA

GCTTCTGCAGCATTGCAATTTGGAAATGCTAATCCATTGGCTTCATGCACTAACTTTAAG

AGGATTTGGGTATCATCAAAAGGGCATTTCCCTGGTTACAATCTCACATTTTGGAGGCCT

CAAGCTCCTCCCAATTATGCTATATTAGGTGATTGTGTGACATCAAGACCAATTCCTCCT

TCACAGGTTGTAGTAGCAGTTAGTAACATGTATGGCCGTGTACGAAAGCCTCTCGGCTTC

AAACTCGTTGGTTCGTTATCAAATTTCCAAGAACTGGGTGATGAGAGTCAATCAAAGAGT

GACATTGACTGTTCCATATGGATCCCAATTGCCCCACCAGGCTACACTGCATTAGGGTGT

GTAACACATGTTGGAAATCAGCCACCACCATGTCACATTGTTTATTGCCTTAGATCTGAT

CTTGTGACATCAAGTGCCTTTTCGGATTGCTCATTCTATGTGCCATCTAATCCGAGGAGT

ACATCGGGGTTCAGCATATGGCATGTTGACAATAGTGTTGGTTCATATTACGCACATACG

TCTGTGGATTGCCCTCCTATCATTGGTTGTTTTGACTTGCACCAGATTCTTAGGCGCAAT

CCTGATTGTTGTCTGTCTGTCAGCAATCAATCCAGCTCAAATAACCCTGTTGACAATTCT

CAGCCCGAAGGTTACCATAGTAGTGTATCCTCTGGGTGGGACATTTTGAGATCTTTGTCC

AGGAGTAATTATTTTATGTCCACCCCTCATTTTGAAAGGATATGGTGGGAAAAAGGCTGT

GATGTTAGGAGACCAATTTCAGTATGGAGACCGTTACCACGCCCAGGCTTTGCTGTTCTT

GGTGATTGTATAACAGAAGGGTTAGAACCACCTGCCCTAGGTTTAGTCTTTAAGTGTGAT

GACTCAGTTATTTCGGCAAAGCCGGTGCAGTTCACCAAAGTTGCACATATAATAAGAAAA

GGGCTTGATGAAGCTTTCTTCTGGTACCCAATTCCTCCTCCAGGTTATGCTTCCTTGGGA

TGTGTAGTTACGAAAACTGATGAGGCTCCAAGTAAGGATCTCTTTTGTTGTCCAAGGATA

GATCTTGTAAATCAAACGAACATTTCAGAGGAACCGATATCTAGATCTTCCAGTTCAAGG

GGTTCAAACTGCTGGAGCATCTGGAAAGTTGAAAATCAGGCATATACCTTTCTTGCAAGA

TCTGATATTAGAAAACCTTCGAGCCGTTTAGCATACAGTATCAGTGACTATGTCAAACCT

CGAACACGTGAAAATGTAACTGCAGAAATGAAATTAGGATTTTTTTCCCTCACCATCTTG

GACAATTTGTTTGGTATGATGACACCACTTTTCGACACAACCATAACAAACATAAGTCTT

GCAACACATGGGCGTCTTGAAGCAATGAATGCAGTACTTATCTGTTCAATTGCTGCATCC

ACATTCAATCGTCAATTAGAAGCATGGGAACCTATTATTGAACCATTTGATGGGATATTC

AAGGTTGAAACATATGATGCTAGTGAACAACCTCCATCAAAAATTGGGAAAAGAGTTCGC

ATTGCTGCAACGTCTACTGTAAATCTTAACGTCAGTGCTGCCAATCTTGAAACTCTTACT

GAAGCTATGGTATCATGGGGTAGACAAGTTGATCTAGAGAAAAAATCATCAAATAAAAGC

GAGGAAGAAGATGAGTCTTTTAGACATAATGATGATTTGACATATTCTGCATTGGAAGAA

GATGATTGTCAGAGAGTGGTTGTTGAAAATAAACTTGGATGTGATATTTATTTAAGAAAA

ACTGAGGAAATTTCCCAATCTATTGAATTGTTACAGCATGACAACCAGACCTCCCTTTTG

ATGGCATCTCCAAGGTTCTCAGATAGATTAAATATAGTTTCTAAATCCCGTGATACACGC

TACTATGTTGCTGTCCAAATCTTTGAATCCAAGGGACTACCAATCTTGGATGATGGTAAT

AGCCATGATTACTTCTGTGCTTTGCGATTACTAAGTGATAGCAATGCATCAGATCAGCGT

AAGCTTTTTCCGCAGAGTGCAAGGACTCGTTGTGTGAAACCTCTAATTACAAAGGCAAAC

GGCTTGGATGAGGGCTATGCTAAGTGGAATGAACTTTTCATATTTGAAATTCCTAAAAAG

GGGTTGGCAAATTTGGATGTTGAAGTCACAAACCTTGCTTCAAAAGCTGGGAAAGGTGAG

GTCATAGGTGCATTATGTCTTCCCATTGGTAGTACTGCTACTACACTGAAACGGGCTGCT

TCCATAAGGATACTACAGCAAGCAGCTGATGTTCAGCATTTTTCATCATATCCCCTAAAA

AGAAAGGTTGAGTTACCTACTAATAACGGCAGTAAATACTCTGGTTCATTGGTAATCTCT

ACATCTTATTTTGAACGGAACGTTGATTCAATTTTTCAAAGGGGAGTAGATAACTCAACA

ATATCAGATAGAGATGATAGTTTCTGGATTGGGCTTGGTCCTGATGGTCCATGGGAGTGC

ATTTCTTCAGTTCTGCCACTTTCAGTTGTTCCAAAATCTCTTAACAAAAATCCTTTTGCC

TTTGAAATTGTCATGAAAAGTGGCAAGAAGCATGCTATCCTTAGGAGTCTTGGGGTTGTT

ATTAATGACTCTGATATCCGGCTCGAAGTATCTATATGCCCTGCTTCCATGCTTATCAGC

CCTCTCTCGAACAAGGGACCAAATAGTCCTGCTGTAGTGAGTGAAGAGATCTTTGAGAAT

CAGAGATACCAACCAATTTCTGGTTGGGGAAACAAGTCATCTGGTTCTCATGATAATGAC

TCAGGACGTTGGAGTACACGGGACTTTGTTTACTCATCCAAGGATTTCTTTGAGCCTCCC

CTTCCTCCTGGTTGGCGATGGACCTCTGCATGGAAAATTGAGAGGTCTCAATTAGTGGAT

AGTGATGGATGGGCATATGGGACTGATTTTCAGAGCTTAAAATGGCCCCCAACTTCTACA

AGGTCATCGTCAAAGTCACCGCTAGATTTTGTGAGGCGGAGACGTTGGATTCGTACAAGG

CAACAGTTACCCAAGGAAAACATTGATAGCATGAGGAATGTCGTTGCTATTGTTAATCCA

GATTCCTCTGCCATTTTACCATGGACAAGTATGGCCAAGAATATGGAATTCTGCTTGCAA

ATTCGTCCTTATACTGAAAGTTCTGATAAAGCATACACTTGGGGTCAAACTGTCACTCTG

TCTTCTAGCAAGGATCAAGCAATCGGTCAGCAAGCTTCACGTGCTACACAAAGCTTTGTG

AAAAATACAGATACTGTAAATTCTTTACTGAAATTGAATCAGCTGGAGAAGAAAGATTTA

CTTTTATGTTGTAATTCTATGATTAGTACCAAACAGTGTTTCTGGCTCAGTATAGGCACC

GATGCTTCAGTTCTCCATACAGAATTAAATAATCCTATTTATGACTGGAAAATATCTATC

AATTCAACTCTCCGATTGGAAAATAAGCTTCCTTATCAAGCGGAATATGCTATATGGGAG

AGAACAGTTGAAGGGAATATGGCAGAACGACAGCACGGTATTATTTCACCAGCTGGATGT

GCATTTATTAATTCAGCAGATCTACGGAAACCTATCTACTTAACGCTGTTTGTTCAAGGA

GGCTGGTTTTTAGAAAAGGATTCTATTCTGATCATGGATCTATTAGGCCTTAATCATGTG

TCTTCCTTCTGGATGGTTCAGCGACATAGTAACAGAAAATTGCGTGTGAGTGTTGAGCAT

GACATGGGTGGAACAGATGCTGCTCCCAAAATAGTTAGGTTGTTCGTTCCGTACTGGATC

AGTAATGACTTGACAATACCTTTGTCGTACCGTATTGTTGAAGTTGAGCCATTGGAGAAT

CAGGAGACTGATTCTCTCTCTATGTCTAGAGCTGTTAAATCCGCTAAGTTTACCTTGAAA

AATAGCTCAAAGTCATTGGATAGGAAGCTTAGCTCAAGAAGAAACACTCAGATATTTGAA

ATGATTGAGGACGTAAATCCAAAAGTTATGTTATCTCCCCAATATATTAACCGTAGTGGA

ACTTCTTCAACACAATCCCGCAATGATACCATTTCTTCGTTGCGTGTGGGGATTTCTGTA

GCTGTGCGTCATTCAGAATATTACAGCCCTGGTATTTCTCTTCTTGAGCTTGAAAGGAAG

GAGCGAATTGATGTCAAAGCATTTGCATCTGATGGATCATATTACAAGCTGTCAGCACAA

CTAAAAATGGCCTCTAATAGAACAAAGGTTATTCATTTTCTACCGCAGATTTTGTTTATC

AATAGGATTGGACGCAGTCTATCGTTATCACAATATGATACCAAGTTAGAAGAGTGGCTT

CATCCAGCTGATCCTCCAAAACAACTCAAGTGGCAGTCTGGTGCTAGTAATGAATTCATA

AGGTTGCGGTTGGATGGATACAAATGGAGTGCTCCATTTAGCATACAGAATGAGGGTGTG

ATGTGTATTTGCATGAGGAGTGATAAACAAAATGGCCAGATATATTTGAGAGTAGAAGTA

AGGAGTGGAGCAAAAATTTCACGCCATGAGGTTCTATTTCATCTTCTTTCGTTGTCAAGC

CCTTACAGGATTGAAAATCGTTCGCTGTTTGTACCAATATGGTTCCGCCAAATAGATAGT

TCTGATGACTCTTGGCGGTGTCTCCCGCCTAATGCTGCTGCTTCATTTTTCTGGGAAGAC

ATTGGTAGAAAACGGCTTTTGGAGGTCTTGGTAGATGGAAACGACCCTTCAAGATCTGAG

AAGTATAACATCGATGAGGTTATGGATCATCAACCAGTGCAGTCATCTAGTAAACCTATT

AGGGCTCTACGTTTAACAGTTCTTAAAGAGGGGAAGACCCAGGTTGGCCGGATTAGTGAC

TGGATGCCGGACAATGAATCTTCTGCAGTTCCAACTAAAGAAGTTGCATTACCTATTTTT

AAAGAAAGTGATCACGGGCAATCATCTTCTCACTTGGAAAGTGAATTTCATGTTACATTT

GAGCTTACAGAGTTTGGCATATCCATCATTGATCATATGCCAGAGGAAATTATATACCTC

TCTGTGCAGAGCCTCTTACTCTCTTATTCATCTGGATTGGGCTCTGGGATCAGTAGATTC

AAGCTACGAATGCATTCGATTCAAGTAGATAATCAATTACCTTTTAGTCCAATGCCAGTT

CTCTTTAGACCACAGAGGGTGGGGGATGAGCTGGAGTATATACTTAAGTTTTCCATGACC

ATGCAAACAAATGACTCCCTTGATTTCTGGGTTTACCCATATCTTGGCTTGCAGGGTCCT

GATAATTCTGCATTTTTGATCAATATTCATGAACCAATTGTTTGGCGTCTTCATGAGATG

TTTCAACGAGTTCAACTCAATTCTGTGTTTCACTCTCCAGCAACTGCAGTGTCAGTAGAC

CCAACAATAAAGATAGGGTTGCTTAACATCTCTGAAATCCGATTCAAATTTTCGATGGCT

ATGTCACCAGCTCAACGACCAAGAGGTGTTCTTGGGTTCTGGTCATCTTTGATGACTGCA

TTAGGAAACACTGAGCATATGCCTGTGCGAATTACTCAGAGATTTCATGAAGAACTATGC

ATGCGTCAAAGTGCTCTTATTAGTACTGCAATATCAAATATTCAAAAAGATATTCTCAGT

CAACCTCTTCAACTGCTATCCGGTGTAGACATTCTTGGAAATGCAAGTAGTGCGCTTAGT

AACATGAGCAAAGGCGTTGCAGCATTATCCATGGACAAGAAATTCATTCAGAGTCGACAG

AAACAGGATAGCAAAGGTGTTGAAGACATCGGTGATGTAATTCGAGAGGGAGGAGGGGCT

TTTGCAAAGGGACTTTTTAGAGGAGTCACTGGCATATTAACAAAGCCTCTCGAAGGAGCA

AAATCTTCTGGTGTTGAAGGTTTTGTACAAGGTGTCGGTAAAGGAATTATTGGTGCTGCT

GCTCAGCCTGTGAGTGGGGTACTAGATCTTCTATCTAAAACCACAGAAGGTGCAAATGCT

GTGAGAATGAAGATAGCATCTGCTATCACATCAGAAGAACAGTTACTCCGTAGACGTCTT

CCTCGAGTAATTGGTGGGGATAATTTGTTGCAGCCTTATGACGAACACAAAGCAACAGGA

CAGGCTATCTTACAGCTGGCCGAATCAGGTGCCTTTTTTAGCCAAGGTGATCTCTTTAAG

GTTAGAGGGAAGTTCGCATTTTCTGATGCGTATGAAAATCATTTTCTACTTCCGAAGGGA

AAGATTCTTCTAGTCACTCATAGAAGAACTCTACTGTTGCAACAACCATCAAATATCATG

GGTCAGCGGAAATTTAATCCCGCTAGGGATCCATGTTCAGTATTATGGGATATTTTGTGG

GATGACCTAGTGACCATGGAGTTAACAGTCGGAAAGAAAGACAGCCCAGGATCATCTCCG

TCACGCCTTGTCTTGTACCTTCAGAGTAGATCAATTGAATCGAAGGAAAATGCTCGCATC

ATAAAGTGCAACCGTGGCTCCAATCAAGCTAGTGAAATATATTCATCAATTGAGCTAGCA

CTGAAAACTTACGGGCCACATGCATCAAAGGACATTCACAAAAGGAAGGTACCAAGACCA

ATTACCCCCTGTATCAATAATGTTGAATCTCCCGAAGTTTTTGGTATTTGGGGCATTCAA

GGACAGGTGCAAGATCCTCTGAATTCGGCATTTGGTGCAATTCTTTCTCCCAACAACCCA

GACTTTTAACTAAGAAATTCTGGATCCAAACAGATTTCTGCAGTGTGCATTGCCATGCCT

CGTAGACAATTATCCTCAGATGTGGTCCTATAATCATATGTACGGCTGAATGTTGTAAAT

AAAATTTTGTTTTCTATAAGCTGTCTTTTTAGCCTCTTCGAAATGGGCCTTTGTGGTAAG

ATGAATGCTTGTGAATCTCAGAATGAACCAACCTAGCTTAACTGATGTTTAGA

>Unigene0002226

TTGAGAAGGCTTCTCTTGTCTTAAGTGATGTTTCATTAACTATTTCTGAGGCTCAGTATT

ATGATGGCATAAAACTCATAGAAACAATTTCAAGGTATAAGACCCGAGTTGATGTTTCAC

ACCTAAGGCCTGTCATCCCTGTGTCTGAAGATCCTCATGCATGGTGGCGCTATGCTGTAT

TGGCTGGTTTGCAGCAGAAAAAATTATGCTACTGGTTCTCCTGGGAAAGAATTAGGCATC

TTTGCCAGCTTCGTCGTCACTATGTTCAGTTGTATGCGGCTTTATTGCAACATGCATCCA

ATGTGGAACTATCTGAAATAAGGCAGATTGAGAAAATCCTTGATTCAAAAGTAATTATAT

TATGGAGATTACTTGCACATGCTAAGGTTGAATCCGTAAAATCTAAGG

>Unigene0002227

AACTCCAGAGACTAGCTGTATATCACGACTCTGACTGCGCTCCTTGGAAACTTGATAAGA

GGTGGGAAGATTTGAATCCTGCTGAATGGAGTGAGATTTTTCAAGATGGAATTGACGAAC

CTGCTAGCTACGGAGCAGTCTCTTTGGGCAAAGTGACTCACAGATACCTAGTATCGCCTA

TAAATGGAGTCCTTAAATATCACCGTCTTGGAAAAAAAGAAAGACAAGATCCTGATATT

>Unigene0002228

AGGGCAATCTGAAAAGAAGCTGGTGGTCTTTTGGATGGCGCACCACATCAGAAGACCCTT

CTGTTGGAAATAGTTCAACTGACTCACAACTGGTGGAAGAAGAGAGCTTAACAAAGGAAG

AATGGCAAGCAATTGATAAAGTATTAAGTTACCAGGCTGATGAGGACTCCACTCATCTTT

TGGGAAAGGATTTCCAGAATATGATACAATTCTTGATAGATGTG

>Unigene0002229

CCGAGAGAGAACAGGGGAGAGAAGGACCCTTTTGGCTTCGATTTGGAGCAGCAGGAGAAT

CCCAGCGGACAAAACCACCATAGATGCTTCCTGATATCGTGTGCATGGAAATTCATACAT

TTTGAGATAGGGATCATACATGAAGATGGATGCATGGAGCTCCAACAGGAAAGCTCCAAG

CTTGGGGCGCTGTCCACAAACTCGAGGACTCTTTCATCTTCATCCTCAGCATTTGTCTCT

GCAAGTCAGTCTCCTTTCTTCTCTCCACGATCACCGCAATCTCGCCAGACCGAACCATCC

CCGCCGGACATTTCAAACATCACCAATGGTCTTCTTTTAACCGGGAACCCTCTTCCCTCT

AGTAATCTTTTCAAGCAAGATGAATCGAACAATACCGGATTTGTGGCTTCCGATGTCTCT

CCGAAATCAAGTTTCTGTGCTTCTAGTAGTGTTACGACCCCCGGAACAGTTTTCAGAAGC

TCAGAGTTCGTTTCGCCTTATAATGGAATCTGCAGCCATTCTAATTACAGTCATGGTCAC

ACAGTACAAAAGGAGAAACAGAAGAGATTGGGGAGAAGCCAGGGGAAGTTTTCCGTAACT

CGGCCTTCTTCGGTTTCTTCCACCAGCCGGTTGAAGAGCTATGATGTGTATATAGGTTTC

CATGGCAGGAAGAGAGCGTTGCTAAGATTTTCGAATTGGCTCCGTGCAGAGTTGGAACTT

CAGGGTATCAGTTGTTTCGCATCAGATAGAGCTCGGTGCAGGAACTCCCGGAGCCATGAC

AATGTTGAGAGAATAATGAATGCCTCTACCTTTGGGGTTGTAATCCTGACAAAGAAGTCG

TTTGGTAATCCTTACACAATAGAGGAGCTCAGGAATTTCTTGGGCAAGAAAAATTTGTGT

CCGATTTTCTTTGATTTGGGAGCTGGTGACTGTCTTGCTAGGGATATAATAGAGAGAATG

GGTGAACCGTGGGAACGGCATGGTGGTGAGTTGTGGACTCAGTACGGAGGATTGGAGAAG

GAATGGCGAGGAGCGATTGATGGTCTCTCGCAGGCTGTAGACTGGCAGATGGAGGCAATC

GATGGAAACTGGAGGGACTGTATACAGAGGGCGGTAATTCAGTTGGCTACAAGATTAGGA

AGGAGAAGCGTGGTGGATAGAGTAAACACTTGGAAGGAAAGGGTCGACAGTGAGGAATTC

CCATTTCCTCGAAATGAGGATTTTGTCGGGCGGAAGAGAGAGCTTTCTCAATTGGAGCTT

ATTTTATTTGGAGACGTTAGTGGGGATGGGGAACGAGAGTACTTTGAACTCAAGACGAGT

CATAGGAGAAAGAATCTGTTGAATGAAAACACTAGAGATCGGCAATCGGAGAGCAGCAGC

AGAAGGAAAGAGCCAGTTCTATGGAAGGAGTCCGAGAAGGAAATTGAGATGCAGAGGGTT

GGGAGTCCCCATCGACAGTTTCGCTCTCTAAGGTCGAAGAACGGAGGGAAGAATTGTAGG

AGGAAAAGTGCCTTGAAGCTACTGTATGGGAAGGGTATTGCTTGTGTTTCGGGGGATTCG

GGTATTGGCAAGACTGAATTGGTTCTGGAATTCGCCTACCGGTTCTCACAACGATACAAG

ATGGTTTTATGGATCGGAGGCGAGACGAGATATATAAGGCAAAACTATCTGAACTTGCGG

ACATCTCTTGAAGTTGATCTTAGCATTGACAATCACTCGCTTGAACGAGGAAGGGTCAAG

AGTTTTGAAGAGCAGGAAGAAGTCGCCATTGCAAGGGTGAGGAAAGAGCTCATGCGCGAC

ATCCCGTTTCTCGTCGTGATAGATAATCTCGAGAATGAAAAGGACTGGTGGGACCGGAAA

GCGTTGATGGATCTTCTTCCACGCTTTGGCGGAGAAGCACATTTCATAATAACGACTCGC

CTTCCTCGGGTGATGAACTTGGAGCCGATGAAGCTTTCTTACTTGTCTGGCATGGAAGCT

ATGTCCTTGATGAAGGGAGGGATAATGGATTATCCAATTGTAGAAATTGATGCTCTCAGG

CTCATCGAGGAGAAACTCGGTAGGCTTACTCTAGGCCTTGGGATTGTAGGTTCTATCCTT

TCCGAGATACCGATAACTCCGAGTCGCCTACTTGAAACTATAAAGAGAATGCCCACTAGA

GAATTCCCTTGGAGCGATCGAGAAGTTGTTACGCTGAGACGACATGCATTTCTCATCCAG

CTCTTAGATGTATGCCTTTCGATTTTCGACCATGCAGATGGGCCGAGGAGTCTGGCGACC

AGAATGCTTCAGGTGAGTGGTTGGTTTGCTCCTGCTGCAGTTCCGGTATCGCTTTTGGCT

TTAGCTGCACGCAAGGTTCCCGATAAGCATCATCGTACACTTTTCTGGAAGAAATGCATG

CGAGCAATTACCTGTAGATTCACGAGATCACATATTAAGAGATCTGAAGCTGAAGCAGCG

TCTATGCTGGTGAGATTTGGGATCGCAAGGAGTAGCGGGAAACCTGATTGTATACATTTT

CATGAAATTATCAAGCTATATGCTCGGAAACGAGGAGCAACCCGGGTTGCTCAACCGATG

GTTCAGGCAGTTTATCTCCGAGGTTCCATTTCTCTATGTTCAGAACATCTATGGGCAGCT

TCATTCTTAATGCTCGGTTTTGGATCGGATCCTGTTGTTGTTGAGCCTAAGCCGTCAGAG

TTAATGTACTTCGTGAAGCATGTGGTTTTGCCTCTCGCCATTCACACTTTCATTAATTTC

TCTCGGTGCAACGCTGCTTTGGAACTCTTACGCCTCTGCACGGACGCATTGGAAATATCT

GCGGAATCTCTGGTTTCACGAGCTGAGAAATGGTTTGATAGATCATTCTTCTGTCTGAGA

CCGATTCAGTCGGATGCCCAGTACACTTATCTCTGGCAGGAGCTGGCACTCTTGAGAGCG

ACCGTCTTGGAAACGAGGGCGAAGCTAATGCTAAGAGGCGGAAAATATGACATTGCGGAC

GACCTCATTCGGAAGACGATATTCATCCGAACATCTATATGTGGTGAGCATCATGCGGAT

ACCATGTCGGCTCGAGAAACACTCGGTAAAATTACAAGGCTTCTTACAAATGTCCATGTT

AGTTGATTTTGATGTAGTTTCTCCACAGTTCGTCATATATTCTTTCATGTGGTTTCATGT

TACTATCATAGAAATGCCCTAGTATCAATAACAAGTAAATTACTGAAAATAATCTTTAGT

CATGTTTTCGCCTTTGTTCGAATCAATGCAGTTTTGTATCCTGTAGAAATATATGCAGTT

TTGTATCTTGT

>Unigene0002230

GGGGAAATCCCTTCGTAGTCAGTTAAAAAGTCAAATACCATCAGTCCGTTACATCATTAT

TGATGTGTGGAGGCATATTTTACAAATTCAAGCCACCATTGGCAAACATTTATATACCAT

ATGTAGCCTTCATGATGTATATCCTGAGTTATGTGGTGGTTAGGTACATGACATGTTGAC

CCTTGGATTTGTTGTTTTTTAGCGGTAAATTATTTCACACCGTTGAAGTGTTATGTATTG

GGGTCGCTTCAGAACA

>Unigene0002231

ATGTTCTACCTCGATCATGTCTTACCAGTGAAGAATTTGAATAACATATTTACTTTGTTT

TTATGGCAGTTAAAAAGTCAAATACCATCAGTCCGTTACATCATTATTGATGTGTGGAGG

CATATTTTACAAATTCAAGCCACCATTGGCAAACATTTATATACCATATGTAGCCTTCAT

GATGTATATCCTGAGTTATGTGGTGGTTAGGTACATGACATGTTGACCCTTGGATTTGTT

GTTTTTTAGCGGTAAATTATTTCACACCGTTGAAGTGTTATGTATTGGGGTCGCTTCAGA

ACA

>Unigene0002232

CCCTGTCGTCTTCGCGGGTGTGCCCTAGGCCTTCCAGATTCTTCCCCCAATCCGCGTCCC

CGCTCGCTCCGCGGCGCCACCAGTCGCCGGCTATCCGCCGCCCGCTATCCGCCGCCCGCA

CTCGATCGGTTGCTACGCCTCTTCCCGGTTATCGGAGTCTGTGAATAGATACCCGCAATC

CCCAGATCTGAGCCCTTGTTGCCACCTTGAATATTTTGGGGGAATGGCTAGCAATTCACA

ATCTTCAGGAGCACAGTTTAGACCTGTGGCTCCAACTCAACAAGTGCATCAATTTGCTCC

TGCAACCTCCCAGCAATTCCGACCTATTGGGCAAGGCGTGCCTGGATCAAATGTTGGGAT

GCCTTCTGGTCAAACTCACATCCCTCATTTTTCTCAAACGACACAACATTTTCCTCCAAG

ACCAGGTCAACCCGCTCAGGTTCCACCTTCATCCCAAGCGTTTCAGATATCGTACAACCA

GCCCAACCGGCCAACTGCGCCTGGCTCCTTACAACCCCAACAAAATGCTCAGATCCCAAA

CAATGGTCTGCCTAGTTTGGGTGGAATGGGTTTTGGACTTTCTTCGTCTTATACATTTGC

AACATCTTATGGCCAACCACCTAATAGTATCAATGCTCCACCCCAGTACCAACAAGCATC

CCAAATTCATGCTCCGATGATGGCAGGAGGGCAGCCTTGGTCTATGCCTGGCGCACAAAG

CGTACCTCCTGTCACCCCGTTGATGCAGGCTCCATCTGCCCCGTCTCCTATTACAATTGC

ACAACCTAATTCCATTGAACATAGCTCTTCTGATTGGCAAGAGCACAGAGCTGCTGATGG

GAAAAGGTACTATTATAATAAGAAAACAAGGCAGTCTAGTTGGGAGAAGCCCGTGGAGTT

GATGACACCAATTGAGATGTCTTATGGCACACCTGGACCAAAACCAACTGCTCTATATGG

TCATTATGAAGGCGGGTTTCAGTATTCTCAAGACAGAGGGCTGATGCGTCGACTGAATGG

AAGGAATTTACCACGACAGAGGGTCGAAAATATTATTATAACAAAGTCACAAAGCAATCC

AAATGGACAATTCCTGATGATCTTAAGTTAGCTCGTGAACAAGCAGAGAAAGCTGCAGCT

CAACCTGAAGTAACGGAAACCAGGGCAGCTTTTGTTGCCCCAGTCGCTTCGACTGGTCTT

CCTGAGATGCCTTCTTTGGCCACATCAAACATTTTGGTTCCTTCAACAGTATCTGCAGCA

GCATCAAGCCCTGTTCAAGGGACAACTGTATATCCTATTTTTCCTGTTGTTGCTTCAGAA

CCATTATCTGTTGGTACTATGGTTTCTACTGGAATTCCCATCAGCCCTGTTGGGACACCA

AATGCCACTGGTTCGTCAAACCTTAACCTGCCGGTTATCCCCTTTTCTGCTCCTATCGCT

GCAAATGAGGCTCTTTCTTCAGTTTCGATGGATGGCACAACTATAACCCATGTCAATCAT

GACAGTTTATCTGTGCCAAATGATGCAAGTGGGCCTGATGGAGCTTCTGCTGTGGACCTG

GAGGAAGCCAAAAGGGGAATGGCGGTGGCTGGTAAAATCAATGCGACTCAAATAGAGGAG

AAAACAGCTGATGAGGAACCTTTTATTTATGCTAATAAATTGGAGGCTAAGAATGCCTTC

AAATCACTTCTGGAGTCGGTAAATGCTGAATCTGATTGGTCTTGGGAGCAGGCTATGAGA

GTTATTATTAATGACAAGAGATATGGTGCTTTGAAGACACTTGGAGAGAGGAAGCAAGCA

TTTAATGAGTACCTTGGCCAACGAAAAAAACAAGAGATTGAGGAAAGACGTGTTAAACAA

AAGAAAGCACGGGAAGATTTCACTAGAATGCTAGAGGAGTGCCAGGAACTGACATCATCG

ACTCGATGGAGTAAAGCTATCACGATGTTCGAAGACGATGAGAGGTTTAGTGCTGTTGAG

CGGCAACGAGATCGAGAAGATCTGTTTGAGACATATCTTGTGGATCTCCAGAAGAAGGAA

AAAGCGAAGGCTGCTGAGGAGCAAAAGCGAAATAAATTGGATTTCAGAGCTTATCTTGAG

TCATGTGAATTTATTAAGGTAAATAGCCAATGGAGAAAACTTCAGGATAGGTTAGAGGAT

GATGAAAGATGCACCCGTCTGGAAAAAATTGATCGCCTGGAGATTTTTCAGGAGTATATA

CATGATCTTGAAGAAAAGGAGGAGGAAAAGAAAAAGATTGAAAAGGAACACATCAGACGC

GCAGAGCGCAAAAACCGTGAAGAGTTCCGCAAGTTGTTAGATGAACACGTTGGTGCTGGC

ATTCTTACGGCTAAAACTCACTGGCGTGATTATTGTACCAAGGTCAAGGATTCACCACCG

TATCTGGCTGTATCATCAAACACATCAGGTTCAACGCCAAAAGATCTATTTGATGATATT

GCAGAGGAACTCGAGAAACAGTACCATGATGACAAGGCCCAAGTTAAGGAAGCAATGAAG

ACCGACAAAATTGTTCTCTCATTAACATGGACAGTTGAAGACCTAAAGGCCGCTGTTACG

AATGAAGACAGTCTGAAAGGAATATCTGAGATAAACTTGAAGCTTATTTTTGGCGAGTTA

CTTCAAAGACTGCGAGAAAAGGAGGAAAAGGAACGTCAAAGGCAACTAGATCTTGCTGAT

GATTTCGCTGATTATATTCATTCAATCAGAGAAATAACTGCGTCATCAACATGGGAGGAT

TGCAAACAGCTTTTTGAAGATAGCCAAGAGTACAGGTCCATCAGTGAAGAGAGTGCTGCT

CGGAAGATTTTTGAAGGGTATGTTGGTCAATTGCAAGAAAAGGTGAAAGAGAAGGAACGT

AGAAGAGATGAAGAGAAGGCTAAGAAAGAAAAAGAGAGAGAAGAGAAGGAGAAGAAGAAG

GAGAAAGAGAGAAAGGAAAAGGAGAGAGAACGGGAGAAGGAAAAGGAAAAGGCGAAGGAT

CGATCCAGAAGGGATGAAACAGAGGTTGCTATTGTTGATTCAGTAGATAGCCATGGCTCA

AAGGACAGGAAAAAGGAACGATCTAGAAAGGATGAAATTGAGGTTGCCACTGTTGATACC

ATAGAAGGTAGCCATGGCTCGAAGGACCGGAAAAAGGAAAAGGACAAAGATAGAAAGTAT

CGGAAGCGTCATCACAGCACAGCAGATGATGGAAGCTCCGAAAAAGATGAAAAGGAATCT

AAGAAGTCGCGCAGGCATAGCAGTGACCGAAAGAAATCTAGGAAGCATTACACAACTGAC

TCAGACAGCGAAGGTCGGCACAAGAGGCATAAGAAGGATAGAGATGCGTACCGGAGAAAT

GGTGCTTACGAGGAGCTTGAAGACGGGGAGCTTGGAGAAGATGGGGAAATCCCTTCGTAG

TCAGTTTATCAAGTGATTTCACCAAGGAGGCATATCTACAAATCAGGTGGTTTCATTCCA

TCTGCTGCCAGCATTCATTCTTGTAGCATATTTGAAATGATGCCCCATTGATTAGATATT

TTTTAGAGGTAAAAGATTTTACACCATCGAAGTTATATATTATTAGTGTCGCTTCAGAAC

ATATTCTGTTGAGACCAACGTACAGAATTTTGCTTCTATAACCTTAATTTGACCGTGTGT

GAAAGTTAAGCCATGCCATAAAGTGAAAAGAAAATCACAAATTTGGTTTACCCGAATAGT

CTTGAACTTTGGCATGTCGTAATATTGCCAGCCCTTTGGGAACCATGGATAAGGAAGTAA

AATCTCAGCCTGAAAAAATGAGGTTTGTTTCGTTACCATGGTTAAGCATTTAAAATTTAA

TACATGATCAAACCACTACGGAAGAAGTGCATCGGACAGCTGAGGAGCGAGTCCCGCGCC

CAAGCGGGGCGCGGGA

>Unigene0002233

GCAGGAACCTGGCTGGATTATTACCCATGTTAAAGAAAGTGATCGTATGGAATATTTTTA

TAATGGTCTTGTAACTGGGCTAATAGGAGTCTGTGCATATGACTTTAGGCCTTTTGGAGG

TATCGCAAATTAATGTATGCAACACCTCTGGCTAGTTACTAGGTAGGTTTGAGTATTTCG

TCTGGATTTATCAAAAACATCTCATAAGTTTTGATGAGTCAAACACCTAAATCATTCTTC

TGATGGTTCCATCAAAGAAGTCTCATTAGTTAGAGACTAACCAAAATGGGCCCCTCATTA

AATATGAGGATTCTGGCCTCACCAAATTGGTCACATTGTGTTATTGTGTTATGTGTATAT

AGAGTTCAGGAGTCAACTTGTATTGAATTTGAGGGGCAAGTGAAAAATAGGCGTCTTATG

TGTATAGAGCTCAGGAGTCGAATTGAATTGAATTGAATTTGAGGAGCGAGTAAAAAATAG

ACCTCTAGTGAATGAATGCATTTTATTGGGTTTGGAGGTACTTCTGATGAAAACTATTGG

TGTGAGTCTCTCTTCAAAGTGGTTCATCAAAGTAGGTTTGATATTGTTAAACCATTTTTG

ATTGATTTAAAGGTGACATTTATCTCTTAATAAGTTAGTTTACAGACATTGTATTCTCCG

TTTGCTGCGAATGCTCACATTTTGATGCAAAATTATCTTTTTTGAGTTTTCAAAATCATG

TCGATTTGATGACTCGAATGTTGTACGTGTTAATATTTTTTCCATGTCAGTAAAATTGAT

ATATTTTATTGCAAGTCTCCGATCCATCTGTGGGATGCTGCTCTTGGCCATGTCCTTCGT

CGGATTTTTACTGATTCCACTGCAGCACTTGTCCAGCATGCTCCGTGTGATGACTTAAAC

ATACTCTTTTGTCTTGTCAACTCTAATATCTCTTGCCTAGATGTATGTCATTACATTTAC

TACTTTTGTAGCCTACTGTAATATCACACGTTTTTTTTTGTCTCTTACTGATAATGGTGA

AGTTTCTGGTGCAAGAGATTACCTCTTGTCCTCTTACTTGCAAATTAAGAGAAAAAAATG

GAAAATTAGTAGTAGCCTCCAATAAATGTTGAAGAAAATGTAAATAGAGGAAATTAATTA

TGTGTTAGAATACGCGAGCTGGTACTTGGTCTTGGGACAGTAACTAAAGGATCCAATGAC

CTGACGTCCCTAAACTTTTAGCAAGAAAGATCTGTAAGGATGCAATGACCGCTAGAAAGA

TCCGCAAAGGCAAGAAGACTAGTA

>Unigene0002234

CTGTAATATCACACGTTTTTTTTGTCTCTTACTGATAATGGTGAAGTTTCTGGTGCAAGA

GATTACCTCTTGTCCTCTTACTTGCAAATTAAGAGAAAAAAATGGAAAATTAGTAGTAGC

CTCCAATAAATGTTGAAGAAAATGTAAATAGAGGAAATTAATTATGTGTTAGAATACGCG

AGCTGGTACTTGGTCTTGGGACAGTAACTAAAGGATCCAATGACCTGACGTCCCTAAACT

TTTAGCAAGAAAGATCTGTAAGGATGCAATGACCGCTAGAAAGATCCGCAAAGGCAAGAA

GACTAGTA

>Unigene0002235

TCTGGAATGCACTGGTTTCTGATCAGTTTTGGTCCTAGCCGAAATGCCCTTATTTTTGGC

AGTTTAAGATTTTTTTTTTTTGTTGACAAAAATCATTTCCAGTTTCAGCCAAAACACGAA

TGTTACAGTAAATATTGAGTTTCACCGAAATGTTAATATAAATGTCCAGTTTCGGCCAAA

ACGGACACATCTCGTTTATATCATTTAGAACTTTCCTGATAAAAAAAAAGGACAACCCAG

TGCACAAGGCTCTCACCATTATGGGCTACACAGCCTTACCCCTTGTATACAAAAGGCTGT

TTTCAGATTTTAACCCGTGACCATAAGAACTTTCCTTTTATATATATATATTTGAATACT

AATTTATT

>Unigene0002236

ATTTAAGTTTAGACAGATGTAACGTAAATGAGGCAAACTCAATTCTGACAAAAATCAGAT

AAACAAAATAAAAAAAATGTACACCCCCAACCCACCCACACATTTTATGTAGCATCTGTC

ACGTCAGGGTCCTCTTTTTTCTATCTATCATCTATAGGAGTTTTGTGAGAAACAACCGCG

GTACTATTCAAACATTGTACGCTGTGTGTTTATCCGTTTCGGCGAAAAATAAAACAAACG

TACGGCGAAACAGTGTTTGAATAAACGAAACCAACAAACGTACGGAACTATCTGTACGGC

GAAACAGTGTTTCAATAAACGAAACCAACAAACAGTGTTTATCCGAAACTACAAAGCAGT

TGGGTTTCGTTGCTATTTAACCAGAA

>Unigene0002237

TGCAAGGATTGGACGAGTGTGCTTCCGCGCTGTCCGGGTTTTTCCCAACAATTGTGGGTT

TTTCCCAACAAGTTTCGAATATTTTAAATACAAAAGACTCTCCTGCATGGTAAGTATGAT

TTGATCGGGTGATTTCAGGACTGCTTTGGACTAAATTTTGCCTCCTTGGAGATTTGTCTA

GTAGATTGAAAATGAAGCCTACTTTTATTAGAACTGGACTGAGTGGTTGTCCTAATAAAT

GAACTAATTTTTAGTTGAATATCTTCTCTTATACCTTTCATGTGGACGTAAAAGGGCAAC

CCAGTGCACAAGGCTCCCACTAATATGGGGTACTCCATGTGGATGTGTTGCTCACTATTC

AACTAATAAGCACAGACATGTAACACAACTATGGCACGACACCGATACACCGACATATCT

CAAAGATACTAGGTCATGACATTGTCCAGACATAGTTATAAATAAATCAATATAGCTGAA

TTTACATATCAAATTAAATTAAGCTTCCGATATAAGGGCATGTCACAACTCCATAAAAAA

ATTGCATCATAATAGGCAAAGTCTGATAATAAACACATGCACAATAGAAAGTGCAGGGAA

AAAATCAAAGTTCAGATCTACAATCTATCCTCATTCTTCATAAAAATCAGTGACTTACTT

ACAAAACATTAAGAAGTCCAAATTTGAGTAATCAAGTGAGTCACTCTAAATCTCAAGTAA

CTCATTTTGAATGAATCAAATGCATATAGCCTATCACTAACCACTACAACTCAAAAACTA

GGTGAGTGTTAATAATGATGTCGATCCATCCCCATCATATATGGCACCATCCTCACATAT

TAGATGGAAATGATGTGGTTGTCAGATATTAAACTCTGGACCTTCTTAGAAATCCCTCAG

CGCTGACAGCATACTACTGACCACTACAGCCAAAGGTTAGTTTGGGGGAGAGGATTCAAA

TCAACAAATACCCGTCGATGTCCATAAAGTGTCGATGAATTCCGGGTCAATACGTTGTTT

TCGGAAGCATGTCGGTTGAGTGTTGGAGTTTTGTGTCGGAAACCGCAACAACACCTCTAC

CGAATTCCACCCTCTGTGCTTCAATTATGTGCTTTTGACTGCATAGTTCGTAGGTATGAA

TTTATCCTGAGCAGATTGTTTTTTGGGGCATTTGTCCTCTGCAGAATTAACCCACATATT

T

>Unigene0002238

TCGCTCAGAGTGCATCTGTTCAGGTAATTTTGATGCCTTTTTAGTTAATTCATGTACGAA

AATATGCATGTTAATTGATTCATATCGGAGTTCTTAGCTAATTGATGTCTTCTTAGTCCT

ATCACTATGGAGTTCTTACTATTTCCTTGTTCGGTGACAAATGGGACTTGGATAATAAAA

ACCTCTCAGGATTCTGAAGGTAGAAGTCTGGTTAATACACTATACTCATCAATCTTTAAC

CGATACCTAAAATTCCATCAAAATGTGCTGTTACCGTCCAAAGTTGATTTACATAGCTTC

TCAACTTCGGCTCTCTAGATTTCGATACCTCAG

>Unigene0002239

TCTTCTTAGTCCTATCACTATGGAGTTCTTACTATTTCCTTGTTCGGTGACAAATGGGAC

TCGGATTATAAAAACCTCTCAGGATTCTGAAGGTAGAAGTCTGGTTAATACACTATACTC

ATCAATCTTTAACCAATACCTAAAATTCCATCAAAAAGTGCTGTTACCATCCAAAGTTGA

TTTACATAGCTTCTCAACTTCGGCTCTCTAGATTTCGATACCTCAGATTGAAAAAAATCT

TGATACCAGTGATGTTTGTATTTAAGTTTA

>Unigene0002240

GTTTATATGTTTTCCTTTCAGTACACCCTAGTTTTTCGTTAATGAGGACTTCTATCTGCT

GTTGAACTCTAACAACAGTTTGGTTGCCCGATTTCCTGCTTCAGATCCTACTATTTGGTT

TTGCGATAGTCTGGTGATATTGGAGCTGTTACTTTGGTGGGTGATTGGGGTGGTGGCAAG

ATTCATGGAGTTGTTACAGATTGAGAATGAATTGCCACAGAGGCAATTCCGGAAACAGCT

TGCGGCTACTGTTAGGGATATTCAGTGGAGCTATGCAATCTTCTGGGCTCTCTCAACCAA

GCAACAAGGGGTTTTAGTGTGGAAGGATGGATATTATAATGGTGAGATAAAGACTAGAAA

AACAACTCAAGCAGTTGAGTTTGAAGATGATGAAATAGGTCTGCAGAGGAGTGAGCAGTT

GAGAGAATTATATGGGTCTCTTTCTTTCGGTGATAGTAATCATCAAATGAAAAGGCCTTC

GGCTTCGTTGTCACCTGAAGATTTGACAGATATGGAGTGGTATTACGTGGTTTGCATGTC

CTTTACCTACCGTCCAGGAGAATGGTTGCCAGGAAAAACTTTAGCGAGTAATCAACATAT

CTGGATGAGTAATGCTCCCTCTGCAGATACCGAACTATTCTCACGGACTCTTCTAGCAAA

GGTTTTGTTTTCTTATTAAGTTCTTATTGTGTCTTTGTTCCCTGATTTTTGAGCAAACAT

CAGAAACTGATTATAGTCTCTTATATATATGACTTATCTCTGATCGCTTGATTGATCGCC

TCACACAATCGCTCAGAGTGCATCTGTTCAGACGGTAGTATGCTTTCCATTCATGGGTGG

TGCTCTAGAGCTTGGTACAAGTGAACTGGTTTTGGAGGATCCATCCCTTATACAGCATGT

AAAAACTTGTCTCCTGGAGAATCCAACTCCTGTTTACTCCCAGACATCTATATCAAGTTC

TCCCGTGACAGGAAATGGGGAAGATAATATGTTCCCAAATCTCAATCCTGAATTAGTAGA

CACAATTTTTTTGGATAACCATATTCTAATGACTGAATGTCAAACCCCAGTGGAGAGTTA

TAACCCAGGTTTGCCTTTTGCACTTCCTTCGCCTGCCCCACCAGAAGAAGCTGGGCTAAT

CCAGGACAAGTTTGATGAACTTTGTGAAGAATTAAAATTCGGTTCTCCTGACCACAGCTC

GAATATCTTCTGTCCAAATCAACACGCTGACAACACTCAAATAATAGAGGGGCTAAATGT

AGCATCTCTAGATCAGGGTAACCTGCTTATGGATAATGACTTAACTGCCTTTTGTGGTTC

TTTGGAGGGAGGTGGCTGTATATCCGAGACATTTCTAAATCCTCGATCGGTTCCATCTTC

CCCCAAGGGTGAAAGAGTACAGAATAATGCAGTTGATTATCTTCGAGAAGGGGATTTCAC

CAAGCTTGTCTCCCCAGATCTCAATGGCAGTGACTCTCATTATACTAGAACACTTTCTGA

TATTTTAAGTAACTCGAAGCAACTGACATCAATTCCATACTTCTGGACTAACTCTTATGA

ATCCAGCTTTTCCACATGGAAGAGTGACTTGAGTTTTCCTGAACTACTCGGTAATACGCA

TCAAAAGCTGTTGAAGAAGATTTTGATGGATGATGTATGGATGTATAGTGATCGATCATT

GAAGCCCCAAGAAGACGACAGACTTAAGAACAAATTTCCCAAAACAGATGTGGATGATGC

CAGTGCAAGTCATGTAATATCAGAAAGGAGGAGAAGAGAGAAGCTGAATGAGAAGTTCCT

TGTTCTCAAATCACTGGTTCCCTCTATTACCAAGGTTGACAAGGCATCTATCCTCGGTGA

TACAATAGAGTATCTCAAAGAGCTTCAGAGAAGAGTCGAAGAGTTGGAATCTTGCAGAAA

ACTTGTAGATCATGATCCAAAAGGAAAGCGGAAACATTTAGATGTTACAGAGAGGACATC

TGACAATTATGGCAGCAACAAGATTGCTAATTGCAAGAGAGCTTCTGCGGGAAAGAGGAA

GGCATGTGTCATCGAGGAAGCAGAGACTGAACACCAATGGATATTAATGAAGGACGGTCC

AGTTCATGTCAATGTTACAACAACAGATAAAGAGGCTATAGTGGAGCTGCACTGCCCGTG

GCGAGACTGCTTGCTGCTCAAGATAGTAGAAGCCATAAGCAATTTACATTTGGACGCACA

TTCCGTGCAGTCATCCATAACTGAAGGGATCCTTGCATTGACACTAAGAGCCAAGCATAG

GCGTTCGGTCGTAACATCAACAGCCATAATCAGGAGAGCCCTTCAGAGCGTTATAAGTAA

ATGTGTTGATCAGGAGAGCACTTCAAAGCGTCGTAAGTAAATGTGTATACAAGTTATTCG

TCCGATGTTCGTGCATATCTGAGTTATCATGTAACGATGACACCACTGCTACTTGGCTTT

TCCCTCAAAAAGCTACAAAAATGGTTATGTAAATCGATGATCGAGAAAGAAAGACCCAGT

ACTCGACTATTTAACTCATCTTGTCAGTTGTTGGGAGAGACTTGTGACCTCAAACTGTCT

GGAATTTTCAAGCATGTAAATAGACATTACTAGTTGTCTTTTTATCAATATGTCACTTAA

ACAACTTGTATATTCACTTCGAAATTGATTATGTTAGGAATATAGATGCTGGAGTCAGGT

TAGAATTCGATTCTGATCTAGTCGGACTTTAATATGATTTATCCAAT

>Unigene0002241

TAAGTAAATGTGTATACAAGTTATTCGTCCGATGTCCGTGCGTATCTGAGTTATCATGTA

ACGATGACGCCACTGCTACTTGGCTTTTCCCTCAAAAAGCTACAAAAATGGTTATGTAAA

TCGATGATCGAGAAAGAAAGCCCCAGTACTCGACTATTTAACCCATCTTGTCAATTGTTG

GGAGAGACTTGTTACCTCAAACTGTCTGGAATTTTCAAGCATGTAAATAGGCATTACTAG

TTGTCTTTTTATCAATATGTCACTTAAACAA

>Unigene0002242

ATTGTTTAAAAAATTAAGTTCCTTTGAAACTGTTATACGAAAATTCCATTTGGTTTGAAA

TATAATGTCATCATTTTCAGTCAATTACAGTTGCTGTAGTTTTATTAATTTTGTTGTGTG

TGCTGACATTTTTAGTTTCATGTAATCTGAAATTACATCCCTCATCTGCCTCTTCAAATA

TGTCTTGCATGTCGAATTGTGCCCAAATTGTCATTTATATGATTGATTATTTTACAAATA

TTTTATATGCCACAGCATGAAAAAGCATGACTCGT

>Unigene0002243

CCCAACCAAACCCTAGCCGCTCTCTCAGTTCGATCGGCCCCGCTCCGTCGCCGCCCGTCG

CCGCCGGATCAGTAAGCCTATCTCTCTTCTCCTCCCTTAACCTCCTGCTCTGCCGCCACC

AGCGTCTGCAATTTGTTGATCGACAGTATTGAGTTAGCTTCTAGCTTTCTGTGTTTTCCT

GCTGTTAATTTCGCCGGCGATCGCCGGCCTTAGTTCGTCAACTGAACACTATGGGAGGCT

CGAGGTCTCAGGCCAATAAGGCCCACAAGAGCCGATTTGCGTCTAAATCGTCGCGGCAGG

TTCACCGGACCTCCAACACTGATAATGGTAGAATTGCAAAGCCGGATAAGAGTTCCCTCA

AGGGCGCTCGTGCTGCTCGGCTCCAGCGCAGCAAAGTGATCCGTAGTCAGAAACGTGCTG

CTTTATTGAAGGAGAAGAGGGCCTCTAGTGGACCATCAAGCTCTCCTCGTGTCATTGTTC

TTTTTGACCTTTCTTCTTCTGTAAACTTGAACTTGCTTGTGAGGGATCTTTTAACAGTCT

CATTAGGAGAGAATGGTGGAGATATTTCTTCAGCAACGGTTGCTTCTCCTGTTTACAAAC

TTAGAACTACGGTCTTGCAAGCACCATATGGAGACCTATTGGCCTGCATGGAAATGGCAA

AGGTTGCTGATCTGATTGCCTTTGTAGCATCAGCCAATTCACTGTTTGATGGATGTGAAT

CCAACAATCTCATCGACTCATTTGGAATGCAGTGTCTCTCTTTTTTCAGAGCTATTGGTT

TGCCAAGTACCGCTGTCCTGATAAGGGATCTTCCTACAGACAATAAAGGGAAACAAAATT

TGAAAAAGATGGCGGAGACTTGTCTGGCTTCTGAATTGCCAGAGGATTGCAAATTCCATC

TAGCAGATACCAAAGACGATTTGCGTAAGTTCATGTGGCTTTTCAAGGAGCAACACCTTT

CAGTACCTCATTGGAGAAGCCAACGACCTTATCTTATGTCCCAAGAGGTTCGTGTGGAAC

CTGAAGGCAACAATGGTGGAAATTGTACTTTGCTTCTGTCTGGATATGTGCGTGCCCATA

ATCTTTCTGTTAATCAACTGGTTCATGTTTCTGGTGCTGGAGATTTTCAGATAGGCAAGA

TAGATGTCCTCAAGGATCCATTTCCGTTGGTTGAGAGGAGATCCAATTCAATGGATATGG

AAAATGGGCAGAACACAGAGGTTATCCGCACCTTACTTCCAGACCCCTTGAACCAGGAGC

CTTTGGTTGTCGAAAATGCACCTGACCCTCTTGCTGGGGAACAAACTTGGCCAACAGAAG

CAGATATGGCTGAAGCGGAAGCAAATTTTAAAGTGTCAAAGCAAAAAAGAAAGAAGCTTC

CTCGAGGCACTTCAGACTACCAGGCTGCTTGGATTGTGGATGATTCAGACAATGAGGACC

CTGTCAGAAGTGAGGAAGAAGGTGATGGTATGGTATTGGATGGCGAACATAATCCAGTTA

AAAATGAATTTGAGCACTCAGACAGCGATGAATTCCCAGAAACTATGGAAACTTTTGATG

AAGAAACAGAGCCTGATACTGAGATGCATGATGAAGAGAACATGACCAAGGAGCAGATAG

AGGCAGAGATCAAGAAGATCAAAGATGCCATAGCTGATGATGAAGAGTTTCCGGATGAAG

TTGATACACCAATAGATGTGCCAGCCAAAAAGCGATTTGCCAAGTACAGGGGACTAAAGT

CGTTTAGGACTTCATCATGGGATCCTAAAGAATCTTTGCCACCAGAATATTCAAGAATTT

TTGCTTTTGATAATTTTTCAAGAACACAGAAACATGCCATATCTAAAGTTTTAGCTATTG

ATCAAGGGAGTGCTGATGAGTGTGTTTCAGTTGGTCAGTATGTCAGGCTCCATATAGTGC

ATGTGCCTGTTAATGTTGCTTCAAAAATTTGCCTTTTGTCCGAGAAATTACCTGTGGTAG

CATGCGGCCTTCTACAACATGAATCAAAGATGTCTGTGCTTCACTTCAGCATGAAAAAGC

ATGACTCGTATGAAGCTCCTATCAAATCGAAAGATACACTTGTTTTCCATGCAGGTTTTC

GTCAGTTTGTTGCAAGGCCTTTATTTTCGTCTGATAATATAAATTCTGATAAGCACAAGT

TGGAAAGGTTTCTACATCCGGGTCGCTTTTCAATAGCATCTGTCTATGCCCCTATCTGTT

TCCCTCCACTGCCTTTGATTGCCATGAAATGTAATCCTGGAGAGCCCCCTTCTGTTGCGG

CGATTGGTTCATTAAGAAGTGTAGATCCTGACAGGATTATTCTTAAGAAGATCATTTTAA

CAGGTTATCCCCAGAGAGTGTCCAAGTTAAAGGCCACTGTTCGGTTCATGTTTCACAGCC

CTGACGATGTTAGATGGTTTAAGCCTGTGGATGTCTGGACGAAGTGCGGTCGTAGGGGTC

GAGTAAAGGAACCTGTTGGCACTCATGGTGTCATGAAGTGTGTATTCAATGGAGTCGTAC

CACAACACGATACCGTATGCATGAGCTTGTTCAAGAGGGTTTATGCTAAATGGCCGGAAC

AACGGTTTCCGATATGAGTTGTAGGCGATTGTTGTCGTCGGCATGGTTTTATCGTGCTGT

TGGGAGTGAAGCAATGGTTCAGAATATGGTACTCGTATATGGTGGTCGCTAGAGCAGCGG

GTACTCCGTATATGGATAGCTACCAAGTTATTGGTAAAATTTTGACTTGTTAGAGATTGA

GTTATATATTTATTTTGACTTTTAGAGATGGGCACTCTGTATATGGATAGTTACCAAGTT

ATTGGTAAATTTTTGACTCGTCAGAGATTGAGTTATGTATTTATTTTGACTTGTAGAGAC

GGGGGCGAAAATTGAGTTACATATTTATTTTGACTTGTTTGCAATATATATAATTTATAT

GAAACTTATAGATGCATATGTATAGGCATCATATTTTGATTGTCTAATTATTTGACGGTA

AATACTCTTAGCAGATA

>Unigene0002244

AAGATGCCATAGCTGATGATGAAGGTATAAGTATTAGTGTTGTACATATTATGTTTTTTA

TCTTTTATTTTTTTGATTTGGATATACCACAACGGCATTGTACGCTCTATATAACCCTAA

CCATCAAGTTTTGTCTGTTGTAGATGGGGTTAGCCTTATGATATCCCGTTTGCCACTCAC

AACTGACCTTCTACCTACGGCCCTCCGTGTGCCCAGTTCTTGTCAATTGTAAAATTTTAT

ACACCATTGTGTTCTTGTTGACGGGGAATGATCAGATGGCTGTGATCATAGGAGCAAATT

AAGATGGGAAATCTGCCCCGAATCACTGTTCGATCGGTAAATCTTGTTTAATGAAGGCAA

TGTCTTTGAATTGTTAATGTTCGCTAGAGAGCGTGATGGCCAACTAGAAGTTATTTTGGA

AGGGTGTCTGCTATGGTTACGGTTGATTTCAACTGACTGGGTTCGATTCATATCCGAAAG

ATCCACCAGAATAAGAACCCAGGCCATAGAGACTCTGGGGAGGTTTTATAACTGGACATG

CATACGTTGCACCCAAGTTAGCCGACGAAGATTGTTTAGTGTGTAAGTTTAACCCATGGC

ACTCGATGGCTTCTCCTTGTTCGCTGTGAACAATTTTTCTACACAACATCCTCATCCTCA

GAGTTGAGGACGAACTCTTCACAACTAGTGTGGAGGATGCAAGGCGAGGCCTAGGTATGT

ACTCTAGCAAGTTGGCCTTGCCGAATGAGCCATACCAATCCAGGTGTCCAATTTTAGCTC

GCAAAGCCATCTGAAG

>Unigene0002245

CAACTTCGAGCGGCGAGGAGGCGGTTAAAGGAGCAACCCGCCAAGCTAAAGAGACGAGTG

CACAAAGGTATTCGTTCTACACAAGGTGCGGGTTGTCGAGGACCCAACCATGGAGGGACT

GATGTAACTCGCTCGCAGGACATGACGCACAAATTTTGGCTCGAGGGGCGATTAAACAAC

AAGATTGTGTATGCGACGAAACTTGAGCAAGGAGCTACCTTAGGAGCATCGTCACACGAG

GTGTTACGATACTGCGATGATGACGTCTGCGGATTGGGTGGGAGAGTTTGTTACGGACCA

AGGTTTTGGTTCCACCCAACCCCTTTAATTCCTTCCCTCATCCATGAGTCCATCTAGCCT

AGGAGTTTTGATTTCTCTAAGGAATATTCTACGTATCTTCTACGGTTCTCATGTTCTAAA

TTTCTCTAGGGTAGAGAATATTCCATAGATCTTCTAGAATGCTGTAAATGTTAAATGTAA

ATGGTGTAAAGAGTAGAATTATGTGGACTC

>Unigene0002246

AGAAATGAAAAAGAGATTACGTTGTTTATACTGCCTCTTATTATTGTTTGTCTTCCATTG

CTTTGCTATCTCAGCTGCCTGTCACGGATAAAAGTTATGGTTCCACCCAATCCTTTTGAA

TTCTCATCCTTTCATGAGTTCACTTAAACTAGGAGGCTCTAGAATTCTCTATGAAATATT

CTAGAGTATCTCTTATGGTTTACATGGGTTTTCTAGAGTTCTTTAGAAAAAGATATTCTA

GGAATATTCTAGAATCTTGTAATTATTACATGTGAATATTGTAAAGAGTAAAAGTGGGTG

GAGGGATCTAAAAATTTCCTAACCATTGGAATGTGGATAAGTGACACAATCCTAATGAGT

CTTTGAAAGACTCTAGACCCCATTTGAGTTGTATATAAGGAGAATGGCTCTTTTGCTTCA

ATCCATCCAAAGGGATCAATCAAGGAATACAAACAAGTTTCCTACTCTCTAGAGTGCTCT

CCTCTCCTCTTTCTTCTCCACACTTAGAGCGAGTGTGAGTGTTTGGCTGGATAGGCATCG

CGGAGGGTGTTCTAGCGCCGCACGGGCTCTGTGGGGAGGTAGAATAGTTGGTCTGTGACA

AGTGGTATCAGAGCCGAGTTCGGAGAGCATTATGGAGTGGAAAATTGCTATGGAGTCCCA

CGTTGTCGTCAACCCTGAGCATGGTGGTCCAAGCAAACCACCCCGGTCCAGGAGGGACAA

GAGCCGGGATGTGCATAGCGTTCCGACTTAGGTTCGCTCGAGAAGCGAGTGGGCCTCATG

GAGGAGGATGCAAAGGGCACGACAAGGCGTGTTGAGAACTTGGTGGAGTTTGTGCAGGGA

TTGGAGGGAGATGTGCTCGCCTCC

>Unigene0002247

CAAGGGCGCACAGGCACTCGCCCGAGCAGAGTTGACGTTGCACAGGGGGCAAGTAGGTTA

CCCAAGCAACACTACGAGGGGCGGGTGCGTGGAGACATTCGTTCCATAGTATGAGTCGAT

GAGGATCAAACCATGGAAGCACCAAGGCTACTTGCTCATAAGGGCATGACACGCAGGCTT

TGGATCAATGAGCGACTAAGCAGCGGAGGATGCGGCGAAGGGGAAACTCATGCAAAGAGC

TACTCGGGGACATCGTGGCATGAGGTGCTGTTGTACCGCGATGAGGACGTATGCGGATTG

GGTGGGGGAGTTTGTCACGGACCAAGGTTACGATTCCACTCAATCCCTTTGACTCACCTT

TTTATTGAGTTCACTTGGCCTAAGAGTCTTTAGAATTCTCTATGGAATATTCTAGGTATT

TTCTATGGTTCATATGGATATTCTAGAGTTCTCTAGGAAAGGGATATTCTAGGGATCTTT

TAGAAAGTTGTAATTATCACATGTGAATATTGTAAAGAGTAGAATTAGGTTGTTGGATCT

AGAGAATTTCCAACTGTTTGGATGTGGACAAGTTGCATAATCCTAATGGGCCTTTGGAAG

ACACCTTTGAGTTGTATATAAGGAGACGAGCTCCTTTGCTCCCATCCATCCCAGGATCGA

AATAAGGAATGCAAGCAAGTTTCCTACTTTTTTGTCCGCTCTTCTTTCTTCACCCCCTTC

TCCATACTTGGAGTGAGTGTAAGTGTTTGGCTGGCTTCACATCACGGAGGGTGTTCTAGT

ACCACACAGAACTCTGCACGTAGGCAGAATAATTGGTCCGTGACAGTTGGGTTTTATTTG

TGCTCTCCAAAAAGTGCAGTTCCGCAGGCAGAGAGCTGGAGTTTGCTCTCAATAACCCTA

ACAATTTTGTGAATCTGTGGACTGCAGCTGGAGAGGAAATTTAAAGCCGATTGGCATTTT

GGCTGAGACTATCCGCTGATCCCGCCTGGGAACCGGATTTGGCCATAAGTGAATGACCCT

GTCACTGTAGGTGGCCCTAGCTGAGGGTAACCCACCCCAGCTGAAATGAGCACCCCCACC

TTTTTTTAATAAATAGTATAAACACATTAATATAATTACAAATATAGCATAAACATACGA

A

>Unigene0002248

ATTAAAAACATCTTATTTATATGCAAGAATGTGCATATAACAACCCATTAATGAAATTGC

CTCATTAGCATGACTGAATTCGGAGGAATGAATGGTATGCAGAGTGAGAGCGTGTGCAAA

TGATTCATGGGAGATGTGAACAATAGAAGAGAGGCGGACATCTGACAACAGTCTGCACAC

AAATGACTTCACCAACAGGGAGGCTCTGGATGGCGGAAGTCACAGACCGGCTGGTGGACC

GCATTGATGCTACGATGGAGTGCAGGGCATTGGAGACCACCACAAGGAGGCTAGAGGACC

AGACAGATGTTGCGGCGGAGTATAGGGTGACACAGGAAGTGTTGTCGGTGGTTGAAATGG

TGGGAGAAACGTCCCTAGGCGGAAATGGAGAAGGACGATGTGTGAAGTTGTTTTTGGCGA

AGCATGGTTTTATACATTAATAATGTACCTATTGGGAAGGTAACAAGAAATGAGTTGAAA

CTGCCTCAAAATGAATATTTGGGACGCTCTGGTGAACACCAACCAAATCTTAAGTGATGA

AATGGAGAAGTATCACCGATCGGGCTTGGCTCAGTTGGTAAGCGGACACGTCTCGTGCTC

GCACTCTGGATAAAGGTCAAGAGTTCGAAATCCAAAGAGAGCACTATTGGTAGGGGATTA

CCTTCACGCTTCAGTGCCCCGAATGGAGATTAGCCAAGGTGCGCACAAGCCGGCCAAGAT

AATTCCGATTAAAAATAACCAAAAAAAAGGAAAAAAAAA

>Unigene0002249

CCCTTAAAGTATGATGCTCATTAGGTTAGCGCTAGCAGTAGCCTGCAACACCGCTAGTAG

AGGCTGTCAGAGTGAGAGCGTGTGCAATCGATTCATGGGAGATGTGAACAATAGAAGAGA

GGCGGACATCTGACAACAGTCTGCACACAAATGACTTCACCAACAGGGAGGCTCTGGATG

GCAGAAGTCACAGACCGGCCGGTGGACCGCATCGATGCTATGATGGAGCGCAGGGCATTG

GAGACCACCACAAGGAGGCTAGAGGACCAGGCAGATGTTGCGGCGGAGTACAGGGTGACA

TAGTACGTGTTGTCGGTGGTTGAAATGGAGGGAGAAATGTCCCTAGGCGGAAATGGAGAA

GGACGATGTGTGAAGTTGTTTTTGG

>Unigene0002250

GCCTGATGAAGGACAGGGAAATTGAGTTTCCTTATTGCTCAAGCTCAAGGGCATGGAAAT

TGAGTTTGAATCCATTTAGTATTTCAAATGGATGCTCTACCTCGACTTATCGCTCAAGGT

GTATATAGAGTTTCTGAAACCTTTAAAGTTGCTTAATATTTCGGATCTAATATACAAAGG

CTGTATATTATTTAAATCTGTGAAATGATATAGGGAGTTCTAAATCTGTGAAAGGATATA

GAGAGTTTTTCTGATTTTTGGCGCGAACGTGGGTGAAGCACTACTGCCATGAAAAGAGTT

ATCCGAAGGCAATGTAATCACATTGGGGGACAATCCCAGATTTGTCCTGTTGCTCCTTGC

TGGGATCGTCCTTCCTTCACCCCAGCCAGCATATTGCATTGAGGGCAACAACCGCAACTG

CCAGAGGACCGAAATGACTGAAAAGGTCCGTGCGTTGGGCACTATCCGGAATTTGATATG

GCTGCACGGTCCTTTGTTTGGGCGACCCCAACCCAAATGATAGTGGGAGACACTCTCAGG

GTATCCTGGCACATGGCACATGGGATGCATAGGAGAAGTATTTGTAGGTGGACTCCGTTG

GCTGTTGTCTTTTCCACAGAGGGGCCATGGCTGTGAGAAACTCCAAATGAGTAGTTTCAC

GTTCACGGAATGAGAAGTAGTTTCACATTCATGGTATGTCTTATACTTGAATAAGGATTA

CTCTCAAGCAGATTTTATTGTAATTCTTTTAGTGTATTGGGTATTA

>Unigene0002251

AAAGAAAAAAAATGGGGAGAACTATTTTGAAGGGGTGGTGTTTTGCATTTTGAAAGTAAA

AAAAATAAACAAAATTAGAAAGAGAAGGCCAAAAAGGTTTTTTGGCCGGAAGGATATCCG

GCACATGAACTGAGTTGAAACTGTGTGTGTCAAAACAAATACTTGGGGACGCTTTAGTGA

ACACCAACCAAATTTTAAGTAATGAAATGGCGAATTATCTTGAAGGTGCGTTGTTTTGAG

TTTTGAAAGATAAAGAAATTAACAAAAACTAGAAAAGAAAAATGAACAAAGACTAGAAAG

AAAAAGGTCAAAAACCCTTTTTGGCCGAAAGGATGAATGTGATTAAAAACGCTAGGCTAT

TATTATTTATAACTACTATAGATCCCGCAACATCACCGCAGATGATGTGGCGCGGCATGA

ATATCAACGAGTAGTAAGCAAAATATATTTAAATGATTTCTGTTTGTTGCTTAGGCTTAT

TTTCAAGGTCTTAGAAAATTATAAAGGTAGTCGAACCCTTATAGGTCCAAAATTATAATA

TAACTAAGCAGAATCGATCTAACAGAATAAAATGGGTGAGAATTAGTCTTTGCAAACATC

CGAAAACTAAAAATAAAACTAAGAATATAGTTGAAAATATTTTTGTTATATTATCTATTA

TAAAAGTCAGTAGCAGACCAATATGATTGGGGTATTAGAATTGTCTTATTTGGTTCACAT

GTTGTTGGTTATGTTTGGTTTA

>Unigene0002252

AGGTTGTTATTGGGATATACTCCAGGTTTAGATTGCTAGTGTATAAAGATATTTATGAGG

CTATTTATATTGAACCTTTAGGCTGGATTATATCATTTTTTTTTCCCATGGAATCATGAG

AGGCTTTGACGACAACAGGGTGGATCGTCGTCTTCCCATATTTGATGCAGATCTCCTCGA

TGTCTATATCTGCCTTGTCTATATCTGCCTCTTATCTGGAATTATTCCAGATCACCTCAA

CGTCCGGATCAACAGATTTGGATTTTGATGTACTCGAGGTCCGAATGTTCATCAGCTCCA

TCCCCTCCCCACAATTAGAACTACACCATACAATGGATCTTGTCAGATTTTTAGAGAATT

TGGACCAGTTTCTCATATTTCCTTGACATTCGGATCTGCCGATCTAGATCTACTTAGTCC

GCACCTTCGTCGACTCTGTCAACTCCCAACAATCAGATCTGATCCATACATTAGATCTCT

TCAGTTTTCCATCGGTGTTGGACCCATTTCCCGGCCGTTTTTGGTCCAATTTTCAAGTGG

ATATGGTCCAATTTTTTTACCGAATTAGCAGCCATAACTGATCCGGATCCATCCTATTCT

TATCACCAATGTTTATCTTCGCCGACTGCCCTCAACATTCATGTCGGCCTACCTCTCAAC

GTCTATCTTCTCTAGCAATCATCGATGTCTATAGTCTTCGTTAGGCCTCCATGCTCAATT

TGTCCTCCGAGTCAATATAGGCTTCCGTATTGTGTCAGTACTCCAATGCCATCGAGTTTC

TCCTGTGTTATCAACACCAAGTTTCTTTCTTTGGGATGAGGTGGAAGATTACCACGACAT

GAGGTTTATCCGTTTTATGGATCACCACATAATGGATCATGAGGTGGAAGACCACCATGA

GATGAGGGTACCCACGTTCATGCACCATCACCGATGTTATTTTTCCTGCGACTTCCCTAC

CTCGATGACTTCTTCTCTTCGGGTTTGATAACATGGATCTCAACACCCTCATGGCGTTTA

TCATTAGTTTGGTTCTCATTGATGAGTCATATTTATCATGTGAGTGATATTTTTACCCAT

TAGTGATCACATGTATCTTTTTTATGAGTTCAAGATGAAAGTATTATATCGGTGATCTCC

AATTTGATGTGGTGTTAGGATTGTAGATATTGGGAGTAGGTTGACATGCGGATATACTTG

GGTTGTTATTATGATAGACTTAAAGTTTAGATTACTAAGTCTATCAAGCCATTCATGTGG

CTAATTATATTGAATCTTTTACTGTGACTATATCACATTTTCTCCATTTCTACTCTAAAC

TTAAGAATCAAGACCTTGATAGGAAATCACATGACACAAAGAGCCTTTCCGGGTGGTTAT

CGACAATGAGGTAAGAATTTGAGACCTTCCATTGGCTGATTTGTGCTTTGTCTACACAAA

TTATAGAAGATAATTTTTTCAGGCATTTTGGACTACTCAACCCCCCCCCCCAAAAAAAAT

AAATAAATAAAAAAATAAAATAAAAAATAAGGAAAAATTAAAGATGGAATTTTATTTGGA

GTACTAGAAATATAGACAAACAATAAACTCTACTCCCAATAAACCAAAATAATATTATCT

AGGATCGTATTAGTTTTGGGAACCCAACCAAGCCTTAATCCCAATTACTTAGGTCAGCTA

TGTGAATCCTAAATCCTATTTCTCCATTCAGCTCTGTCAAAGGCA

>Unigene0002253

ACGATGGCAAAATAAAGTTCTAATAACCTAACCAAACCAACCAAGCCTTAATCCCAATTA

GTTTGGGTTGGCTTGAACTAAAGTTCTAATAACATAATACTAAAATAAATTAAAACCGAG

GTCATATCACCAACACGAATGTGGATGGACGTATTAGACACACTATATATGTTTATACCA

AGAATCTAAGTTTACTAGGGTGCTGTATGCATGGAGTGTATGTTTATACTAAGAATCTAA

GATTACTAGGGTGCTGTATGCATGGATGCTGATGTAAAGTAGTTCCACTTAATATTTTTA

TGCTCAAACATTACAAACTTTGGATATGTATTGGATACACCATCACAATCGAAGACAGAT

GTGTTGCATACATACCAGCTTAGTGTCCAACAAGTATTGTATCAGATATTATGTAATACA

TGTGTATAACACCCCGTATATGAAGTATCTGAGATATATGGGTTGCACAGACAGTCACAA

ATACATGGTGCAAGTTCGGATACAATACGATACACCCACATGCCAAATTTTATGAACTTG

GGACCTTTGATATGGCCAGTATACTTTAATACTATAATATAATTATTTTGTAAAGATCCA

TCTCCTCCAAAATCCAAAATCTGTACTCATATCATTTGATATTGGATGTAAAATTTAAAT

TGACTCCTA

>Unigene0002254

TGAGAACCAGGGCCTTCCACACCTCATGGATCTCGCCCTCCAATAACATCACATCCTGCC

ATCCTCAGAAACCACTGGTACGCAATGGCCACAGCCTCAGCAGCTCCGATAGCCAGCCAG

CTGAGGAGCAGCCTGCTCAGCTCCACCACAAAGACCCTCCTTGCCACTCCCAGGGGCATT

TCGGGAACTCCCCTCAGAGACCTTCACTCCAAGAGGAGAACTTCCCTCACAGTCGTAGCT

GTCAGTGCAGAGAAGGTAAGTATGGTGTGATTCTTCTCCATATATAGGTCTGTTGAGCTT

CAATGTCGGCCGATGTATGGTGTGGTTCTTCTCCATCTATAGGTCTGTTGAGGCTCAGTG

TCGGCCGATACGACATGGTCGACTCCGGTTATGAAATGTTTTTCAGTTCCACTTTAGGGT

TACCAGAAGTTCTCTAGAGTTAATGGTACATTGTGTTTACATGGCAGCCAACCACCTTCC

AAGTTGTCCAACCCATCAATGGCGACCCCTTCATCGGCAGCCTCGAGACTCCGGTCACCT

CTAGCCCCCTGATCGCCTGGTACCTCTCCAACCTTCCTGCCTACCGCACCGCTGTCAGCC

CACTCCTCCGCGGCATCGAGGTGGGTCTCGCTCACGGCTTTCTTCTCGTTGGTCCCTTTG

TAAAGGCTGGGCCGCTGCGCAACACAGAGTATGCTGGCGCAGCTGGGTCTCTAGCTGCGG

GAGGGCTGGTGGTGATCCTTAGCATTGTTCTCACCATGTACGGCGTGGCCTCGTTCAAGG

AGGGGGAACCATCAATTGCGCCATCACTGACACTGACCGGGAGGAAGAAGGTGGCTGACA

AGCTGCAGACGGCGGAGGGTTGGGCGCAGTTCTCAGGCGGGTTCTTCTTTGGGGGAGTGT

CTGGGGTTATCTGGGCTTACTTCCTGCTCTATGGTCTTAACCTCCCATACTTCGTCAAGT

AGAGTGTCTGTTTAAATGATTGTCCCGTGAGAGTTGTACTTGTATTTTCAGAAAGTTTGT

AATTAAACAGACTCTTATCAGTATCGATTATGCAAACACCTCTTTAACTATGGCAATTTA

TCTAAATTGAGAAGATTTTGTAATACGGAGCCCAGGGATGAAGCCTCACAGACCCCCGCG

AAACATCAGCCCTGACGGGAGCTGTTTCAGCCTGGAGCATAAGTTACACAGGGATGAAGC

CTCACAGACCC

>Unigene0002255

TATTTGTGACTGTCTGTGCAACCCATATATCTCAGATACTTCATATACGGGGCGTTATAC

ACATGTATTACATAATATCTGATACAATACTTGTTGGACACTAAGCTGGTATGTATGCAA

CACATCTGTCTTCGATTGTGATGGTGTATCCAATACATATCCAAAGTTTGTAATGTTTGA

GCATAAAAATATTAAGTGGAACTACTTTACATCAGCATCCATGCATACAGCACCCTAGTA

ATCTTACATT

>Unigene0002256

ACATCCTCTATCCTCTTATGAGAAGCAGAGACCACCTCTCCCATCTCCTCCGCTCCACCC

CTTCCCTCCAAACCCTAGCCCAGCTACACTCCCTCCTCCTCCGCACCGGCCTCCTCACCT

CTCCGGACTGCTTCTTCGCCACCCACCTCACCGCCTCCTACGCTGCCCTCCTCTCCCCCT

CCCACGCCCTCCATCTGTTCGACGAAATTCGCCATCCAACCACCTTTCTCTACAATGCTG

TCCTCCGCGCTCTCTCCCGCTCCCACCAATGGCCCCAAGCCCTCCACCTCTTCCGCCGCA

TGACCCACTTCCCTAATTCCCGCCCTGACCCCTTCACCCTTCCCATCGCCCTCCGGGCCT

GCACGGCCCTATCCGCCCTCGCAAACGGCTGCGCAGTCCACGCACTATCCATA

>Unigene0002257

CAGTCCACGCACTATCCGTAAAATCCCCCAATGCCAGCTCCGACATGTTTGTTGGGGCAG

CGCTTATCGGTTTGTACTCAAAATGTGGCGACATTAATGATGCACGCCGTGTGCTTGATG

GATTTGTTGAGCCGGATGTCGTGCTGTGGACCTCGGTCGTATCCGCGTACCAGCAGAATG

GGTATCCGGTCGAGGCAGTATCGATGTTTCGCAGAATGGTGGTTGGGAGACTTGTGGTGC

CTGAGCCGGTGACGGTGGTGAGCCTGGTCGCGGCTATCGGGGAGATGGGGGGACTTCGCG

AAGGGAAATGCTGTCATGGGTTTATGGTGAGGATGGGGTTTGATTCTGATTTGTCACTGG

GGAATTCAATTTTGAATTTGTATGCCAAGATGGGCGCGGTCGAATATGGGCTGAAATTGT

TTGAGGGTATGGGATACAGAGATGTGATCACTTGGAGCTGTATGGTGGCCTGTTATGCTC

GAAACGGGATGCCTGAAAATGCCTTGGGTGTCTATAAATGGATGGTTGATGTGGGGGTCG

AACCGAACTCAGTCACTGTGATCAGTGCACTGCAGGCTTGTTCAGAGTCTCTTGATCTGG

AACAAGGTCGGAGGCTGCATGATTTTGTTATAGGAAAAGGGTTTGAGATGGAGCTGTCGG

TTTCGACTGCCTTGATCGATATGTACATGAAGTGTTTGCGGTATGGCGAGGCCATGGATA

TATTTCGCAGGATGCCTCAGATAGATGTTGTCTCGTGGGCTGTGGTGATAAGTGGCTATG

CTGGAAATGGTTTCCCCAGTGAGTCACTGCGATTGTTCAAGCGCATGTTATTCGAGCGAG

CGAGCGTCGATGCAGTGGTGCTGGTGAAGGTTGTGTCTGCGTGTTCACAGCTTGGAGTTC

TTCGTCAAGCCAGCTGCATTCATGGCTATTTGGTAGGGTGTGGCTTCAATGGCAAGATTT

TTGTGGGTGCTGCGCTAGTGGACTTGTACTGCAAGTGTGGCAGCCTTGACAGTGCCATTA

GAGTGTTCGAAAGTGCCAATGAGAAGGATGTGGTGCTATGGAGTTCGATGATCGCGGGGT

ATGGAATTCACGGCCTTGGTAAAGAAGCAATTGCAGCATTTGAACACATGGCCGAGTCTT

CGATCAGGCCTAACAACATCACCTTCATTTCAGTCTTGTCTGCATGCAGTCATGCCGGTT

TGGTTGTTGAGGGGAGAATGATTTTCGACAGCATGAGTCGTGTATATGGGGTTATGCCTG

ATTCCGAGCATTGTGGTATCATGGTCGATATCCTCGGTCGATCCGGTGAATTGGATGAGG

CTATGAGGATAATACAGGGAATGTCTGTCCCGGTTGGCCCTCATGTGTGGTGTTCTTTGC

TTGCTGGCTGCAGGACACACCATAATATTGAAATGGGCGAGTTGGCGGCGATGAAATTGC

TTAAACTTGAGTCTGGACACGCTGGATACTACAATCTCCTGTCAAACATATATGCTTCTG

ATGCTAGATGGGATGATGTGGCTGATATTCGACGTGCGATGAAGAAAAGGGGAGTGAGAA

AAATTCCTGGACATAGCTCAGTTGAGGTTGGTAGTGAGGTATATACTTTTTTGGCTGGTG

AGAGATTGCATGAAGAATTAAGGAGTATTTATGAGATATTGGAGAAACTAGAGGTGGAGA

TGATAGAGGAAGGCTATATTCGTGAGAGATACAAAGAAGTATTTATTGAGGTTTGTTCAT

AGGAAATCTCATGACGAGGAATGATTTATTAAGATCCAATATAGAATGGCGGAACTTCGG

TATCTCGAAGGAGATAGAGTAAAATATTTGGAGTGGATACACCGACTTGATGATGGGTTT

GACAAACTCACTACTTGCGGAGGTAAAATTCAAGATGTTAAATTGCGTCTACTGCTATGA

>Unigene0002258

GAAGATTGTGGAGATGGAGAAACTAAGGGCCAAGTTTGAGAATGTTGTGATTGCGATTCA

TGAAAGGGCTACCTTTGATGCCTACGTAAGTGGGCTTGGGGCGGACAAGAAGTTGGGTGC

CGCCCAAAAAGCTGAGCGCCTACCGGGTTGAAGTAAATGTAAGCATTGTCAACGTGCCAA

AGCCATCATCGTCAAGATTGTTGGATCCCACTCTTCGAAGGGGATAATTAAGAGTACTAT

CATCGGTTAGGTAGTCTTGTTTTCACACAAGTTGTATCATGCGTTTGTTGATCATCTTGT

GAGGTCTGACTATATTTGTTTTGTTTGTGTGTAACATTATT

>Unigene0002259

TACCGGCGATCTTCAGCGATCTGGATTCCGGATGAGGATGGAGAAGCAGTTCAGTATTGC

GGAGTTGATTTATGATTTGAAGATTTACGGGGTCTATGTGATTTATACAGGAGGCAACGA

GGGAGCTTGCTTGTGAGCCAAGTTCGGTGCGCCTGACCGCTCCAAGGAATGCAAAGTCAT

ACAAGATTGTCGCCGAAACACGCATCACACCTTGGAGTGCACCGAAGCCCAGTAAATCAA

CACCCTTGACACCTGCCCTAGCTAGCTCCGCGGTGCATCGGGTCGTTCCTGAGTTCGTCG

TGCCTGTCATTCCGACGACTCTGAAGCCCAAAGATGTCTTACTGATCCCTATCGCAGATG

CTACCTAGGTTCCAAGCCATCGAAGTCCTTGCCTGGAGGACCCACTCCCAAGCTCTTGAA

TTTTCAAGAGTTGTTATCCGGTCCAGTGGATATGAAGTTTGATTGTATCCGAGTATCTCA

ACCCGGAGCCTGTGGATCAAGACATGCCAAGCCAAATCATCAATCTTGACGAACTGTTTG

GTTAAGTCCAAGCCCAGCCACAAAGATCAGGTAGATCCTCTCTCTCAGGGTGAGTAATGT

TGAGGCGTTTGTTGCTGCTGATACGACACTGCTTCATCGGGTGTCACAACAATGAGGAAT

GGGGCATTGCACTATGGTGATGCACTGGAGTCTTCTCAAGCTCATTCAATATCAGTGCCC

CTAATTCTTGGGTTTAGTGAAAGCCCGATGCACCGTTCTGCCGTCCTCATGTGGCAACTT

GTTGAGATAATGCCTTCGCCCATAGCCAAAGTTGAAGCGCAAATGCAGCAGCTCACCGCC

CTTAAGGCACAACTGGCTGTGAAGGAAGTTGATACGGAGAGTGTTGTCAAAGAACTGGTT

ATCCTGTAGGGCATTGGTGCCAACAAAAAAAGTTCTAATAGAGGAGATGACGACGGAACA

ACAACGCTTGAGATCAGCCGAAGCGCCAAAGCGGAAGATGCTCACCAATGTGATAAAGGA

ATAATATTGCTTGGGTGATGACCCCACGGAGATGCATACCCTTCGATAGCGAGTAGTATA

GGCCCTTATCATTTGCCCTAAAGTGGTTAGGGTTGTTGAGGAGAGGGGGTTGGCAACCTC

TGCCACCTGCATAATCAAATTATCACATAGTTGTTTGCCCTTTACGGGCAATTGGCTAAG

AATATTGTGCAAGGAAAGAAAACACAGACCCTCACCACCCTAGCCGCTCAAAGAGTAACC

AACGTGCCCCGCTTGCCTAAAATAGAGGATTTAAAGGGCCGAGACGGTCACCTTGGTTGA

TTCGTCACATTGGGTAAATCAACCGAGGTGTCCGCTCCAATGAGCACAACTGAGATGGAG

GCTCGAACCCATTGGGTCTAAAGATGAGAAGATTGTGGAGATGGAGAAACTAAGGGCCAA

GTTTGAGAATGTTGTGATTGCGATTCATGAAAGGGCTACCTTTGATGCCTACGTAAGTGG

GCTTG

>Unigene0002260

GACTCTGAAGCCCAAAGATGTCTTACTGATCCCTATCGCAGATGCTACCTAGGTATGAGT

GTGCCACCACGGGTTTGTAATGACTCTGAAGCCCAAAGATGTCTTACTGATCCCTATCGC

AGATGCTACCTAGGTTCCAAGCCATTGAAGTCCTTGGTCTGGAGGACCCACTCCCAAGCC

CGTAAATTTTAAGAGTTATCCAGTCCAGTGGATATGAAGTTTGATTGTATCCGAGTATCT

CAACCCAGTATTTGAGCTAATTGCTTTTGAGT

>Unigene0002261

ACAGACCCTCACCACCCTAGCCGCTCAAAGAGTAACCAACGTGCCCCTCCGCTGCCTAAA

AAAGAGGATTTAAAGGGTCGAGACCGTCACTTTGGTTGATTTGTCACCTTGGGTAAATCA

ACCAAGGTATCCGCTCCAATTAGCACAACTAAGATGGAGGCTCGAACCCATCGGGTCTAA

AGATGTGAAGATTGTGGAGATGGAGAAACTAAGGGCCAAGTTTGAGAATGTTGTGATTGC

GATTCATGAAAGGGCTACCTTTGATGCCTACGTAAGTGGGCTTGAGACGGACAAGAAGTT

GGGTGTCGCCCAAAAAGCTGAGCACCTACCGGGCTGAAGTAAAGTAAGCATTGTCAACGT

GCCAAAGCCATCATCGTCAAGATTGTTGGATCCCACTCTTCGAAGGGGAT

>Unigene0002262

CATATCTAAGATTCATTGGTGCATCTTATGATACAGTATATGGGGTTAGCCGATACACGA

TACCATACGTGTTTTCAAAACCTTGGTTATAAGCTCATTGTGAAGTATACCACTACCCCT

TCTCATGCTATTCCTTTATGACACTCAGTAATGAAGGAATAGCTGCGAGACTCAAGACTA

GACATGATCAAGCTACTTACCTCCAAATTCTGAAGCTGCGAGACGGACATTGAGAGTTCC

TACAGTGGCATTCGCATCACCTTCCAGCTGCTGAAAAGTTGCCAAATCTCCGTACCAGTG

CCAGCATATATCTGTAAAGGCATATGCAGTACATGAGCAGTTATTCAGACAAGCTGAGAC

ACATTCTGCCTCTCTCCCAATATCTCGTTTGATTTCATAATTTGCCGGGAATTTCACATG

ATCAGGAACTGATATGAACCCATCTGGCTTCCCTCCACTCAAGCATTGTAGACTACTTCT

GCGCAGACAACCGCCTCCTGTTTCTCCTTTGCCCCAATCAGCCGATGATCCTTGTTGGAA

TCCCCTTAAGCAGTGACAATAGGGACCCTGAAATCTCTGGCTGCACACGCTAAAAGAGCC

ACATAGATTGAAAGAATCACACCCATAAAATGGTAGGGAGAGATATTCTAGCCAGGCATG

GTATGAGTCATCCCATGCCAGAAAAGAGAGAAGCCCTGTATGATCAATCAGAAACCTTAT

GTACCTAGAGTAAGAATTAACTTGGCTCAGAGTTAAGAACTCGTGACCACCCCCCATTGA

GACATAGCTGACATTGTAATTGGGCCTATCATAATCTATAGAGTTCGGAAGCCTTCTGAA

CCCCAAACCACTCCATAGCCCAGAATCCCAGTATACGTGACTTTGTTGCCACAGGAAGAA

CTGCTTGGTGCCGTTGGGATTAGCCCCAAAAGAGAAGTCCCCCATAAACGGGTCGTCACT

GCTTTTCCATGAGGTGAGCAATCGATTCTCGTTGCCTACCATGCCAAGTAACATGCCTGG

TAGTAAATTGTCAGTAGGATGATCGAAACTCTGCCACAGTATCTTACCTTCAGCGTCCCT

CGACACAAGATTTCCAGTATCCAAGATGGTAGCAGTTGTTGCTGTCGAGGAGTCAGAGGT

GAAGATGTAGTAGGAGACACCTCTGTTGCCATCCAAGACCATCATATTTCCATCACCGGT

AAATGTCAGCTTGCCAGAGGTATTGACGAGGGGCCAGTCCCTGTTGGCCACCCACACCAC

AGTCTGTATTGTGATGTTATGGTACCATATCCCGACGTAGCGGTTACCGTTGCCAGGGTT

GAAGAAGCCGAGTTCGAACGTACCGTTTGCAGAAACCAGTGTCTCTCCATCGCTGATTGA

TTGTTGTGGCAACAAGGTGTCTGCTGCTGATGCGGAGAGTATCAAAATATAAAGAAGTTT

TGATGTAAGACTCCAGTTCATCTGCTGAGAAGTGGTGAA

>Unigene0002263

AGCAACTCTCTTCTCTCTCTCCGCTTCAGATTCTAATATGATCGGCAGAAATTCTTGATT

CTTTTGTTCACGACTTCGCAGCAGATGAAGTGGAATTTTACATCTAAACTTCTTCATTTC

TTGATACTCTCCGCATCAGCAGCAGACACCTTGTTGCCGCAACAATCAATCAGCGATGGA

GAGACTCTGGTTTCTGCAAACGGTACGTTCGAACTCGGCTTCTTCAACCCTGGCAACAGT

AACCGCTACGTCGGGATATGGTACCATAACATCACAATACAGACTGTGGTGTGGGTGGCC

AACAGGGACTGGCCCCTCGTCAATACCTCTGGCAAGCTGACCTTTACCGGTGATGGTAAT

CTGATGGTCTTGGATGGCAACAGAGATGTTTCCTTCATCTTCACCTCTGGCTCCTCCACA

GCAACAACTGCTACCATATTGGGTACTGGAAATCTTGTATTGAGGGACGCTGAAGGTAAG

ATCTTGTGGCAGAGTTTCGATTATCCTACTGACAATTTTCTGCCCGGCATGTTACTTGGC

ATGGTAGGCGACGAGAATCGATTGCTCACCTCATGGAAAAGCAGTGACGACCCATCTATG

GGGGACTTCTCTTTCGGGGCTAATCCCAACGGCACCAAGCAGTTCTTCCTGTGGCAACAA

AGTCATGTATACTGGGATTCTGGGCTATGGAGTGGTTTTGGGTTCAGAAGGCTTCCGAAC

CTGATAGATTATGATAAGCCCAATTACAATGTCAGCTATGTCTCAATGGGGGGTGGTCAC

GAGTTCTTAACGCTGACCCAAGTTAATTCTTACTCTAGGTACATAAGGTTTCTGATTGAT

CATACAGGGCTTCTCTCTTTTCTGGCATGGGATGACTCATACCATGCTTGGCTAGAGTAT

CTCTCCCTACCATTTTATGGGTGTGATACTTTCAATCTATGCGGCTCCTTTAGCGTGTGC

AGCGAGAGATTTCAGGGTCCCTACTGTCACTGCTTAAGGGGATTCCAACAAGGATCATCG

GCTGATTGGGGCAGAGGAGAAACAGGAGGCGGTTGTCTTCGCAGAAGTAGTCTACAATGC

TTGAGTGGAGGGAAGCCAGATGGGTTCATATCAGTTCCTGATCATGTGAAATTCCCAGCA

AATTATGA

>Unigene0002264

ATAGGTGATACGTTTTCCGTGAATGAGATAACAAGATGCATTTATGTAGGGCTACTATGT

GTTCAAGAAAATGCTGCTGATAGGCCAACGATGACCGAGGTTATTACAATGCTTAGTACT

GAAAGTGCGCCTTCGACCGATCCTAAACAACCTGCTTTCTACACTATGAGAATCCAGACC

AAAACAGATCTTCCACAGAAAACTAGTTCTGCATACAATTTGACTGTTACGTCTTTAGAT

GGTCGATAGTACTCAATTGTTTGTATGCCTCACTAAGTATAATGAGGATAAAAATAGGCA

TTAAACTTTGATAACGTTAAGAATTGTGGTGCATGTACTCTTATGGGAGCTTCCTGACAA

AGGAATCATATGGTTGTACATACATCAGGAAGATATCTTGAAATTAAAGAATTGAAAGAA

AGCTTAAGACTCCAT

>Unigene0002265

AGGAGAAACAGGAGGCGGTTGTCTTCGCAGAAGTAGTCTACAATGCTTGAGTGGAGGGAA

GCCAGATGGGTTCATATCAGTTCCTGATCATGTGAAATTCCCAGCAAATTATGAAATCAA

ACGAGAAGTTGGGAGAGAGGCAGAATGTGTTTCAGCTTGTCTGAATAACTGCTCATGTAC

TGCATATGCCTTTACAGATATATGTTGGCACTGGTACGGAGATTTGGCAACTCTTCAGCA

GCTGGACAGTGATGCAAATGTCACTGTAGGAACTCTCAATGTCCGTCTTGCAGCTTCAGA

ATTTGGAGGAGATAACAAGAAGCTGAAGATAATCGTTTTAGTGACGGCTTTATTTGGTTC

ACTTTCTTTTATCTTAATTGGGAGTTTTGTATGGTGGAGAAGAAGCAAAGGGAGAGAGAA

GCGGAGTCAAAAGCAATCCATACTACTATTAGGTGCAGAATTAGCCAAAAGGCTTGGAGA

AAATGACAAGAGGACCCCAGAGTTTAGATTATTTGACTTTTCCCGCATAACAGAGGCCAC

TGATACATTCTCAAATGCAAACAAGCTTGGAGAGGGTGGCTTTGGACCTGTTTATAAGGG

TAAATTATCAGAGGGGCAAGAAATCGCTGTAAAAAGACTCTCCGCGCGATCAGGTCAAGG

ACTAGAAGAATTTAAGAACGAGATTCTACTGATTGCCAAGCTTCAACATAGAAATTTAGT

GAGGCTTTTAGGTTGCTGCATTCAAAGAGAAGAGAAGATTCTAATCTATGAATACATGCA

AAATAAAAGCTTGGATTTCTTCCTTTTTGATCAAGAAAGAGGAGCACAACTAGACTGGAG

TAGCCGCTTCCGCATCATTGAAGGAATAGCTCAAGGCTTAATTTATCTGCACAAACACTC

CAGACTTCAAATTATCCACAGAGATCTGAAAGCGAGTAATGTCCTTCTCGATGCAGATAT

GAACCCTAAGATATCTGATTTTGGCTTAGCCAGGATTTTTAATCCAAAAGAAACAGTGGT

GAATACAAATCGGGTTGTCGGCACATATGGCTACATGTCACCTGAGTATGTTGCCGAGGG

TATTTTCTCGGTCAAGTCTGATGTTTTCAGCTTCGGTGTCCTAATCTTAGAGATTGTAAG

TGGAAAGAGAAATACTGGCCATCATGAATACAAGAACTCCTTTAACCTTGTTGGATATGC

ATGGGAGTTGTGGAAAGAGGGAAAGTGGTTGGATCTGATGGATCCCTTACTACGTGATAC

AGTTTTAATGAATGAGGTAACAAGATGCATTCACGTAGGGCTTCTGTGTGTCCAAGAAAA

TGCTGTTGATAGACCGACAATGACGGATGTCATTTCTATGATTAGTAGTGAAACTCCACC

TTCAACAGATCCTAAGCAGCCTGCTTTCTTCACTATGAGAACGAGCAAAACAGACCTTCC

ACAGAAAGCAAGTTCCCTTAACAATGTGACTGTCACGACTCTAGATGGTCGATAGTTCTT

CTAAGCTTGTTTGTCATAGTTTGGATATTAATTAGACAAACAATCATCTTTTATATATTT

TTTGGAAGTTCAAGAAATGTTCAAGAAAGCTTGAGAACTCTATTGATGAAGTGAGGCTTG

T

>Unigene0002266

AAGCTTGGATTTCTTCCTTTTTGGTTGTCAAACACTCAACTGTAACAATTTCCGATCAAC

ATTTTCTGGTTATGTGCTCTAACTTAAGTACTCTGATTTCTAGATCGAGAAAGAAGAGCA

CAACTAGGCTGGAGTAGACGATTTCACATCATTGAAGGGATAGCTCAAGGCTTAGTTTAT

CTACACAAACACTCTAGACTACAAATTATCCACAGGGATCTGAAAGCAAGTAATGTCCTC

CTGGATGCTGATATGAATCCTAAGATATCTGATTTCGGCTTGGCTAGGATTTTTAACCCG

AACGAAGAAGTGACAAATACAATCCGAGTTGTCGGCACATATGGTTACATGCCTCCTGAG

TACGTTTCTGAGGGCCTTTTCTCGATCAAGTCAGACGTATTCAGCTTCGGTGTCCTACTT

CTCGAGATTGTAACTGGAAAAAGGAACTCTGGCCTTCATCAATACAAAAACTCCTTTAAC

CTTGTTGGATATGCATGGGAGCTGTGGAAAGAGGGAAAGTCGTTGGATCTGATGGATCCC

TTACCAGGTGATACTTTTTTAACGAACGAGGTAACAAGATGCATTCATGTAGGGCTCCTC

TGTGTCCAAGAAAATGCTGCTGATAGGCCGAACATGACGGATGTGATTTCTATGCTTAGT

AGTGAAACTGCACCTTCGACCGAACCTAAGCAACCTGCATTCTTCAGAATGAGAACTCCA

ACCCAAGCAGATCTTCCAGAGAAAACAGGTTCTCTTAACAATGTGACTGTCACGTCTCTA

GATGGCCGATAATATTTCAAA

>Unigene0002267

CCGAATAAAAGCTTGGATTTCTTCGTTTTTGACAGAGAAAATGGAGCACAACTAGACTGG

GTTAGACGCTTTCACATTATTGAAGGAATAGCACAAGGCCTCATTTATTTGCATAAACAC

TCTAGACTGCAAATTATCCACAGGGATTTGAAAGCTAGTAATGTCCTCCTGGATGCAGAC

ATGAACCCTAAGATATCTGATTTCGGCTTGGCTAGGATTTTTAATCCAAGAGAAACAATG

GCCAATACAATCCGAGTTGCCGGCACATATGGTTACATGCCTCCAGAGTATGTTAATGAG

GGCCTTTTCTCGGTCAAGTCTGATGTTTTCAGCTTTGGTGTCCTACTTCTAGAGATTATA

AGCGGGAAGAGGAACACTAGCTTTCAACGACACAACAATTCCTTTAACCTTGTTGGATAT

GCTTGGGAGTTGTGGAAAGAGGGAAA

>Unigene0002268

ATATTTCAAAGTTTGTAAGTCATAGTGATGGATTTTATGAAGAAACAATCATTTATGATA

ATTGGGATAGTGCTTAGAAAGGTCTGCATTTATTCTAAAAGGATTTTCATTGTGATGAAA

TCAAATGATTGTAGATATATTTGGAAATTAAAGAGTTGCTAAGAAAGATTGAGAACTCCA

TGAACGATGAAACGAGGCTGGACAAGTTTACAATGATTTTTTTATCCAAGAGAATTAGTA

TTTTGTTTAGTATATG

>Unigene0002269

AGGTAGGGAACGGAGACACCGGAGACATTCTCGAACTTCTCGTGATAAGCATCAAAGTTG

TTTGACTTCTTCCGGCCGACACCGCAGAAACCCCCTTATCAGCACGGAGGCGAAGGAGAA

GGGTGCAGGAGGGGAAGAATCCGGCGGAGGCTGAAAGAAGAATATCATCATATACGTTGA

GGGATCCGGTGACGAGGCGCTGGACAGAAGCGGGGATGCCGATGAGGGAGGA

>Unigene0002270

AGGGTGATGAAGATCTGGTTGAACGCTTGCACCAAATCAGGTTTCATGATAAAAGAATGA

TCTATCTTTACCTCGTCACAAATCTCACTCCCTATCGATTTCTCGCAATTACTGTCATCA

TGTTGTACTCTCAAGTTCAGAAGCTTCATCCTGCAATTCACCAGAAACTCTGGCAGCCGC

CGAATCTACCACTTCAACATTAACATCAGCTTCCTCAGTCAGCTTCCTCAATTACATGAA

TCTCCGCAGCCACAACAAACTTGTTGTCAACGGAGCATTCCAGGTCCAAGTTACAGCAAT

TTCCAACCTCTTATGATATCATGTGAACTCAAAATCTCCTTCGGGTTCTAAATCAGAGCC

ATCATCAGCTCCTACAGAGGCCATCTGAAACTCAACTACCCTTTCTGTGGCTGAAGGGGC

GGCACAATCAGAGCGCTCCTCACCATCATTAACCCCATCAGAGGCCATCCGAACCCCAAT

TTCTCCTTCTAGGGAGCAATTGGCGGCACAATCAAAGCCCTCAACTTCTGAAATCCCTAT

CAGGTTAATCTCAACTGCGCCCCAAGATGGCTCAAGGATCTCCAAAAACCCGCTCTCAAG

CCCGACACCGCAGATCCTTCTCCCCGGATTCGCTCTGCTTCATCCATGATAACAGCGGGG

TTGTTGCCGCCTCGGACTGCCGAGAAGCAGAGCAGTCCTCGCACATCTCGGTTGCAGCAG

CCAGCCGGCGGTGGCGGGAGCAGTAGGTTGAGATGGACACCACGGCGGCACGGATGGCGC

AGAGGGTGACACGGTCGAGGAGGGAGCAGTGGAGGCAGGGTGGGTGGAGGCCTAAGAGGG

CAGCAAATTTGGCGATGAGGATGTTCTCTCCAAACAGCCCCTCTACTTCGAAGCCTCGCC

CAGCATTTGCAAAAGATTCATGAACAAACTGCCGCACCTCAGCAGAAGCCACAACCCATC

TCTTCACCATCTCAACCAACAATGTCAGAGCACTGTCCATGTTCCCTTCAAGGCAAAAAT

CTGCAACCAAAAATCCATAACTACTCGAATCCTCCCACAGACCTCTCTCTGGCAATATGC

TAAAAATCTCCACTGCATGCCTGAGGAATCCTGCATTCACCAACTCATCAAAGACCTTGT

TGTAGAATCCCAAATTAATCTTCGGACCACTATCACCACTATTCACCATCTTATCAAAAT

AATCCAATGCATCCTGAACTCTCCCTTCTTTTAAGAATCCATCAATTAGGAACCCGTAAG

TGGCAGCAGCAGCAGTGACCGAAATGGGACGCATTTCAGCGAACAGCTTCTCTGCCTCAG

CGATCATCCCTTGCTCACAAAGCTTCCCGAAGATGTTATTGAAGCAACCGACGTCCATCA

TGCACAGCCTCCTACCCTCCCGGTGGAACACCTCGTTGGCCTCGGCGTAATTCCCCTGAT

TGACGGAGGAGGGGGAGAAGGGGGCATTGGCGATGATGTGGCGGAAGATGACACTGGCTG

TCTCGACATGGCCGGTGTCGCAGTGGGTGTTGAAGGAGACGACGTTGGAGACGAAGTTGG

ACTGGGTGAAGAAGTAGGCATGGAGGGCGCAGGCGTCATCAAGGCGGCCGGCGCGGAGCA

TAGAGGTCATGTCGGCGTTGCAGGTGAAGACGGTGGGGCGGGTGGAGGAGAAGACGACGT

GGCGGGCGGTGGCGGAGGCGGCGTCGAGTTCGCCGGCGCGGTTGAGGGACTGGACGCGGT

TGTGGAAG

>Unigene0002271

AGGGTGATGAAGATCTGGTTGAACGCTTGCACCAAATCAGGTTTCATGATAAAAGAATGA

TCTATCTTTACCTCGTCACAAATCTCACTCCCTATCGATTTCTCGCAATTACTGTCATCA

TGTTGTACTCTCAAGTTCAGAAGCTTCATCCTGCAATTCACCAGAAACTCTGGCAGCCGC

CGAATCTACCACTTCAACATTAACATCAGCTTCCTCAGTCAGCTTCCTCAATTACATGAA

TCTCCGCAGCCACAACAAACTTGTTGTCAACGGAGCATTCCAGGTCCAAGTTACAGCAAT

TTCCAACCTCTTATGATATCATGTGAACTCAAAATCTCCTTCGGGTTCTAAATCAGAGCC

ATCATCAGCTCCTACAGAGGCCATCTGAAACTCAACTACCCTTTCTGTGGTTGAAGGGGC

GGCACAATCAGAGCAATCATTAACCCCATCAGAGGCCATCCGAACCCCAATTTCTCCTTC

TAGGGAGCAATTGGCGGCACAATCAAAGC

>Unigene0002272

GGAGGAGAAGACTCCCGAGACACCGAGTTCTGTTGATGGAATCCATGCCTTGCATAAGAG

GTTTTATTTTGATAGGAGAGAGTCGAGTGTGGAGTCTCAAGATGGAAGTATTGCTAGTGA

GGTTGAAAGTAGCGAGATCTTGACTGCAGATCGACTTAAAGCTGCTTTGAATGCTGAGCG

CAAGGCCCTCAGCGCATTGTATGCTGAATTGGAAGAGGAGAGGAGTGCTTCTGCAATTGC

TGCCAACCAAACTATGGCGATGATAACTAGGCTTCAGCAAGAGAAGGCCGAAATGCAGAT

GGAAGCATTGCAGTATCAGAGGATGATGGAGGAACAATCGGAGTATGATCAGGAAGCTCT

GCAGCTTCTAAATGAGCTCATGTCGAAGAGAGAGAAAGAGAAACATGACCTAGAGAAGGA

GCTAGAAGTGTATCGCAATAAGGTCCATCTTTACGAGGCCAAAGAGAGGAGAAAGAAAAA

AAGGAGTAATGGTAATGCTATGAGCACAACCTCTTCTCCATCATCGAGTGCTGATGACAG

CGACGACATATCTTTTGATTACCATGAAGATGATGATTGCTTCAAGAGTCTCAAGGACAG

CAATCAAAATACTCCAACTGATGATGTTTTGGGATCTAGAGAAATGCCAAGGGAGTCAAA

TTATCTGTATACACTCGATACTTCCTTGGCTGATTTTGAGGAAGAGAGGCTCTCCCTACT

AGAGCATCTCAAGACATTAGAAAAGCAGCTCTTCATGTTGAATGGCAAAGAGGGATCCAA

CGATCGAGAAAGTATGGAGCATGAAAATGGTCACGAATTATTAAATGGTTTCTCAAATGA

TTTCGGTTCCAATGGAAAGCTTCATGATGATGAGAGAAGCACATCTTATCAAGGAAAGAG

CCTTCTTCCTCTCTTCGATGCCATTAGTATGGAAAATGGGGATGAGCAAAGCACCGTACC

ACACCAGATGGATGATAATAAGAAGCTTGCGATCACCCAAGAGATCGACGGTGTATATGA

GAGGCTACGAGCTCTTGAGTCTGATAAAATGTTTCTCAAGCACTGTATTAGCTCCTTGAA

GAAAGGCGATAATGGGATACATCTTCTTCAAGAGATCCTGCTGCATCTTCGGGATCTCAG

AAATGCGGAACTCCAAGTGAGGAATACTTCTGATGGCTTTGGTCCTCTCTCGGACTAGTT

GGCACAAACTCAAGATGGAGGATTACTGGATGTCGATGGTGGCTTGAAGTTCGATTATGT

AGTCTGAGAGTTGCTGATGGATGGATTGAGGTTCTATTCCATGTGAAACATTAGAATCAA

TGATGGTGGCGATGGATGCCTGGCCAGTGGTACCACAGAAGGCCTATTTGGTGATTTTTT

CTTTTTTTCTTTTTTTCATTTTTAATGTTTGTTGGTTGGTTTAGTTTGTTGTCTGCAGAC

CTTTGAAGGTGTCTGTATTGCAAAGTCCCTTGTTTTTCTGGGGCTGTTCTTTGTTTCTTT

TGCTTTGCCTACCTTTGGTGGGGGCAGAGGGGGTGGTGGGTCTGCCATTCTGATGATGTT

TGTGTGTGGGGTAGAGATCATGGCTTGCAAAGCTGGACAACTCCATTGCCAGCTCCAGGG

>Unigene0002273

GATAAGAGTCTGATGACGCTTGATGATTCCTTCGCTGATTTTGAGGAAGAGAGGCTCTCA

ATACTAGAGCGACTCAAGGCATTGGAAGAGAAACTTTTTATGTTGGATGATGATGATGAT

ACTTCCAGTGATCCAGAAACTTCTGATCATTTCATGGTAGAGAATGGTCATGCACTAAAT

GGGGATTATGAGGGTGAAATTGGTTTTATGAATGGTTTCTCACATAATTTGCATGGAACT

GGAAAGCTTTTTGTCGAGAGGAGAAACTCGAGTTGTAGAGGAAAGAAGCTTCTTCCTCTC

TTTGATGCCACTAGTATCCAAAAGGAAGGGGTGCTATGTACCATGCCAGAGGAAACTGAT

GCAGCATCTGAACCTGTTTCCAAGTCAGTTCGCAAGAAGCTTGCAATTGCTGAGGAGGTC

GATAATGTTTACGAGAGGTTGCAAGTTCTCGAGGCTGATAGAGAGTTTCTTAAGCACTGT

ATTAGCTCCGTGAAAAAAGGGGACAAGGGTATTGATCTTCTTCAAGAGATTTTACAGCAT

CTTCGGGATCTGAGGAATATTGAGCTCCGAGCGAGGAATGCCGGTGGTTCTCATGCATCG

CTTTCAGACTAAATATCACAAACTCAAAGATGGAGGAGCTTAATTGAGTTCTCGGCGGTT

CAAAGTGACTCAACAACCCACGGATGGCTTCATTTGCGCGGAATAAAAGGTTCACAATAC

ATACGTTAGCCTAATATGTTCATGTGTAAGCAGGCCAGGAGGAAGGTGGCCCTGGTTCTT

TTTGTTGGTCCGTCGGTTTGGCCTGTTGTGAGCCAACAGACCATGTATGATGCTTGTAGT

CTGCCTTCCAGACCCCGGGGTTTTGTGGGCTTTTCTTTTTTCTTTCTCTTTCCTTTGGGG

GTCCCCTCACCTTTCTCTAAGGCAAGGTTGGTGGGGGGCTGAGAGAAGGGTGGTAGGTCA

TCCACCCATATCATGAAGTGGTAGAAATTATGGCTTGCAAAGCTGGACAACTCCATTGCC

AGCTCCAGGGCCCAACTCCATTGCCAGCTCCAGGGCCAGTTATACAAAGGTAAAGTTTAA

GTTCTTTGTTTTTCTTTTCTTCTTTGATTTTCTTTTTAGTATGGTAAAGTGGTAGGTGTA

TGTATGTTTTTTTGAATATATGTGTTTAGTATAT

>Unigene0002274

ACCTCACCACCTTCCCCCTCTTCTCTGACCAGGAACTTTTCTCCACCCCCTTTCCCCACA

CCCCTTTCCTCCCTCCCTCCACCGCCGCCCAGCAGCGCCGCCCCAAATCAGCCTCTAAAT

CCGTCCCACCGACAAAATTCCCACCCAATTCAACACCCATCACCCCCAAATCCATCGAAC

TAGCAACTCAAATCAATGGCCGCCAACAAATTCGCCACCCTCCTCCACCGCAACACCCAC

AAAACCGCCGCGATCCTCGTCTACACCCTCCTCGAATGGACCCTCATCGCCCTTCTCCTC

CTCAACGCCCTCTTCTCCTTCCTCATCGCCAAATTCGCCGCCTTCTTCGGCCTCCACCCG

CCCTGCCTCCTCTGCTCCCACCTCGACCGCAGCAGCTCCTACCGCGACACCCTCTGCGCC

GTCCACGCCGCCGAGGTCTCCATCTCAACCTACTGCTCCCGCCACCGCCGGCTGGCTGCT

GCAACCGAGATGTGCGAGGACTGCTCTGCTTCTCGGCAGTCCGAGGCGGCAACAACCCCG

CTGTTATCATGGATGAAGCGGAGCGAATCCGGGGAGAAGGATCTGCGGTGCTCGTGCTGC

GGTGTCGGACTTGAGAGCGGGTTTTTGGAGATCCTTGAGCCCTCCTGGGGCGCAATTGAG

ATTAACCCGCTAGGGAATTTCGCAGCTGAGGGCTTTGATTGTGCAGCCAATTGCTCCCCA

GAAGGAGAAATTGGGGTTCGGATGGTCTCTGAAGGGGTTGAGAATGGCGGGGAGCTCTCT

GATTGTGTCGCCCCTTCAGCCCCAGGAAGGGAAATTGAGTTTCAGATGGTCTCTGAAGGA

GCTGATGATGGCTTTGATTTGGAACCGGAAGGAGAATTTGAGATTCACATGGAATCAGAA

GGGGTTGGGGATTACTGTAATTTGGACCTTGAAAGGGAATTTGTGGTTAAGATGGTTGAT

GATGCTTGTTTACAAGTGTTTGACAACTCCTTTGATGGCGATGAGGGCCGCCTGGTTCCG

GTGGAGTTGATTGATTCAGCTACGGTGGCTGCTGGAAACACTGTTAAACTTGTCAGAGAA

GATGATGCAGAGCTTGTGAAGCCGTTGATAGAATTTATGGACATAGACATTCCTCGGCCT

AAGAGAGATGCGTTTACCATGACGGATGGAGATAATGGTGCAGAGCTTTGGAATGATCAT

GCCGAGAGAGATTGTGCCGTGCCGATTGTGGAGGATATAGAGATGGGGATTCCTTGTATT

GTTGAAGTGATTGGTATAATTGAAGATCATCCTGATGAGTCTGTTGGAAGTTGCTCGGTT

GAGAATAAGTTTGTTGAGGCTGCGGAGATTCATGTAATTGAGGAAGCTGCTGTTGATGTT

GAAGGGGTAGAATCCGCAGCAGCCAGAGTTTCCGGTGAATTGCAAGGTGAACCTTCTGAA

CTTGAGAGTACAACCATTGATGCTGATGAGAGTAATTGCGAGATATCGATAGGGAGTGAG

ATCTGCGACGAGGAAAAGATAGACCGTTTTCATATTGAGGAACCAGATTTGGTGCCAATT

GGCATAGAGGCAGAGTGTTCAGAAGATTTAATTGAAACAATTGATAAATGTGAAGCAATT

GAAACTGAAAAGGCTGATCCTAGATTTGTTGAGACCGGGGAGGTTCATGTAATTGAGAAA

GATAGTGTTGATGTTGAAGTGGTAGAATCCGCAGTTGCCGGAGATTCCGGGGAAGCGGAG

GGTGTAGCTTCTGAACTTGAAAGTAAAGCCATTGATGCTGATGAGAGTAATTGCGAGATA

TCGATAGGGAGTGAGATCTGCGATGAGGAAAAGATAGACCACTCTCATGTCGAGACGGAA

ATTGGCATAGAGTGTTCAGAAGATTTCATTGAAAAAACTGATAAATTCGAAGCAATTGAA

ACTGAAAAGGCCGATACAATTGTGAAAGGTCTGGATTTGTCATCTCTAGGTCAAGAGCTT

AATGATGCCGAGGAGGAGAAGACTCCCGAGACACCAAGTTCTGTTGACGGAATCAATGCC

ATGCACAAGAGGTTTTACTTTGATAGGAGAGAGTCGCTTAATGATGCTGAGGAGGAGAAG

ACTCCCGAGAC

>Unigene0002275

AAGGGGTTGAGGATGGCGGGGAGTGATTGTGCCGCCCCTTCAGCCCCTAAAAGGGAAATT

GAGTTTCATATGGTCTCTGAAGGAGCTGATGATGGCTTTGATTTGGAACCGGAAGGAGAA

TTTGAGATTCACATGGTATCAGAAGGGGTTGGGGATTGCTGTAATTTGGACCTGGAAAGA

GAATTTGTGTTTAAGATGGTCGATGATGCTTGTTTACAAGTGTT

>Unigene0002276

CTGCTTCAGACTTCGCCGGAGATGGCGCCGGAGTAAGGCTTTTAGGCTGAGATCGTATAG

CTTCTTCATTCTTCGGTGCATAAAGATGTTAAAGAGAGGATTCAGAACAAGAGAGAAGCC

GAGTTGTGGAAGAAGCAGTAGCAAGAGGGAATTGTTGGTGGCGAAGGGAGAGCAAGGTCG

ACTGCGGTCATGTGTGAGATCCAACTCCTTCCACGCCGAAGCCATAGCTGACTGTCTGGA

ATTCATCAAGAGGAATTCGGTTTCCGGGGTTGAGGATGAGTCAGTCAGGCAAGGATGAGG

GTTCTTTCAGGCCCCAGAATATTGTTTTTCTCTGTCTTGTTTGCATGTTGTAGTTTGTGT

TCGCCTCTTCTGCCAGTGTGAAGGAGTTTTAGGTAATGGAAGATAGAAGGTTGGAACCCA

TCTTGTTTTGTTCTTCTTGTAAAATAGAAATTTATATATGGGCGAATGAAGAGATTGTTT

TTTTTTCAAAATTTGAATTGCTTAGGAATAGTACGACAATTTCATGTTTACTGAATTTT

>Unigene0002277

TCTTCAGATCTAGGGTTTCCCCAAAGCAGTCGTCAATCTGGTCGCTCTCTCCTACCACCC

GATTGTCTCCCCTAGGGCCCTTCACAGCATGTTGGCGAAATCCCGGCACCTGTCTCCATG

CCGCTATCGCCTCCGACGGCCTAGAGCCCGTATCATTCGGTATCCTCTCCCACTCCATCG

ATCAGATGGAAGGATCGCGCTCCACGAACCCGGTTACGGTTTTCGATTTTTATGGTTTCA

ATCCCTAAATTATC

>Unigene0002278

GAAATCTCCACCGACATTCGATCAAATCCCTCGCCGGCCGGCGATCAATTCCTCCCTTAT

CAGGGTTACGCCCCAGCCGGCCAAGATGCAGCGCCCCCCTCCCCCCGACTACTCCCTCAA

AGAGACCTCCCCTTTCCTCGGCGGCGGCACTGCTGCTGGCGACAAGATGACCACAACCTA

CGACCTCGTCGAGCAGATGCAGTACCTCTACGTCCGCGTCGTCAAGGCCAAAGATCTCCC

CGCCAAGGACCTCACCGGCAGTCTCGACCCCTACGTCGAGGTCCGCCTCGGCAACTACAA

GGGCACCACTCGTCACTTCGAGAAACGCACAAACCCTGAGTTCAACCAAGTCTTCGCCTT

CTCCAAGGACCGCATCCAGTCCACCACCGTCGAGGTGATCGTCAAAGACAAAGATTTCGT

GAAAGACGACTTCGTCGGCCGCGTCTCCTTCGACCTCAATGAGGTCCCCCGCCGCGTCCC

CCCGGACAGCCCGCTGGCGCCGCAGTGGTACCGGCTGGAGGACCGGCGGGGCGACAAGGT

CCGCGGCGAGATTATGCTCGCCGTCTGGATGGGGACACAGGCCGATGAGGCATTCCCGGA

GGCCTGGCACTCCGACGCTGCCGCCATCGCTGCCGAGGGCGCTAACAGCATAAAATCAAA

GGTATACCTCACACCCAAGCTCTGGTACCTCCGTGTCAACGTCATCGAAGCGCAGGATTT

GGTTCCGTCTGACCGGGGCCGGTTCCCGGAGGTTTACGTTAAGGCGATCCTCGGGAATCA

AGCTCTGCGGACGCGGGTGTCACCGATGAGAACAATTAATCCAATGTGGAATGAGGATTT

GTTGTTCGTGGCTGCCGAGCCTTTTGAGGAGCACCTGGTGCTCAGTGTTGAAGATAGAGT

CGCGACTAACAAGGATGAGGTTTTGGGTCAGGCGGTCATTCCGTTGCAGAATGTTGGCAG

GAGGCTGGACTACAAGCCAGTGAACTCCACCTGGTACAATCTCGAGAGGCATATGTCGGC

AGCTGTGGCTGCTGAAGGCGACCAGAAGAAGAAGGAGGTGAAATTCTCTAGCCGAATTCA

CCTTCGTATCTGTCTGGACGGCGGGTACCATGTGCTCGATGAATCCACCCACTATAGCAG

CGACCTCCGCCCGACGGCGAAGCAGCTGTGGAAGCCGAGCATTGGCGTGCTGGAGCTCGG

TGTTCTGTCTGCCCAGGGCCTGCTGCCGATGAAGACGAAGGACAGTCGCGGCACCACCGA

TGCGTACTGTGTGGCGAAGTACGGCCAGAAGTGGGTGCGTACGAGGACCATCATCGACAG

CTTCACTCCCAAGTGGAATGAACAGTACACTTGGGAGGTCTACGACCCCTGCACCGTCAT

CACCATCGGGGTTTTTGACAACCACCACTTGAATAGCGGCGGCGTAGCCACGAGGGATGT

GAGGATTGGGAAGGTTCGAATTCGACTTTCGACCCTGGAGACTGATAGGGTGTATACGCA

TTCCTACCCTCTCATTATGCTCCTTCCCTCCGGGGTGAAGAAGATGGGTGAGGTCCAATT

GGCTGTGAGATTCACCAGCTCTTCTCTGCTCAACATGATGTATATATATTCCCAGCCGCT

GCTCCCCAAAATGCACTACCTCTACCCGCTTTCGGTGATGCAGCTCGAAAATCTCCGGCA

CCAGGCTACCCAGATAGTGTCTGCGCGGCTGAGCCGCGCCGAGCCCCCGCTGAGGAAGGA

GGTGGTGGAGTACCTGCTCGACGTGGATTCACATATGTGGAGCATGAGGAAGAGCAAGGC

GAACTTCTTCAGGATCATGGGGGTGCTCTCCGGCTTCTTCGCCGTTGGAAAATGGCTCGA

ACAGGTCTGCAAATGGAAGAACCCGGTGACTACTGTGCTCATCCACATCCTCTTTGTGAT

TCTGGTTCTCTATCCTGAGCTGATACTACCTACCATATTCCTCTACCTGTTCTTGATTGG

GATTTGGTACTACCGATGGCGTCCACGCCAGCCACCGCACATGGACACGAGGCTGTCACA

CGCGGAGACCGCACACCCGGATGAGCTTGATGAGGAATTTGATACGTTTCCGACGAGCCG

TCCGTCGGACATTGTGAGGATGAGGTATGATCGTCTGAGGAGTGTGGCTGGAAAGATACA

AACTGTGGTGGGGGACTTGGCGACGCAGGGGGAGAGGCTACAGTCACTGTTGAGCTGGAG

GGATCCGAGGGCGACAGCATTGTTTGTGGTGTTCTGTTTGATTGCGGCCATTGTGCTGTA

TGTTACGCCCTTCCGCATGGTGATATTGCTTGCGGGGTTGTATGCGCTGAGGCACCCGAG

GTTTCGCGGGAAGTTGCCGTCTGTGCCACTGAACTTCTTTAGAAGGTTGCCGGCGAGGTC

TGATAGCATGCTTTGATGAGAGGGGATGAGTATCTGTTGGTCTGTGAAGGTCTGATAGCT

TGCTTTGATGAGAGGGGATGAGTAACTGTTGGTCTGTGAAGGGAGGAATCATACTTGGTG

GCAAGATGATGGTTGTGGCGCCTATCCAGTGAGTAATCTTCGTGGTGAGCAACATGCATT

ATGTATGATATTTATATTCGAACTAGTTCTTTTTACTGTATTTTTTTTTTTTGAATATCA

CCTTTTTTTTTTCTTTTAGTTTCATTGCTTCATTACTATATTCTGTGTATTTGGGGTGAG

CTATGTGTATAATCTTTACATTCTGTGTCTGCACCGTGTGATGAAC

>Unigene0002279

CTAGTCACCAATCCCGATAACCCCTCCCACCACTTCAACGGCATTCATCTATGGAAATCC

GGAGAAGCAGCCGGAGGAGATGAATCGAAACAATTCGAAGCTGCAATCCCACAATGCTGC

ATTCCGGATGCTCGGACACTACTACAGCCAAGTGTCGATTTGCAGCGCGCTGCTAAGTGC

CACTAGCGGGCCATGAAGTTGAATCCGGACAATGCTGAAACCGGAGGTGCCGGATAAGTC

TCCGAGAGCATTTTGGGCGTTTCGGAGTTTGAGATACTTGCTGGTGCATTAAAATAAATG

GACTGAGGCAGTGCAGAGTGCCATCCGCGGCTATCCGGCTTGTGCCGACCCGTGGGAAGC

GCTCCGCCTGGCTAACTTGGGCAGCTATGAAGTCATTCGGCCAAGCTATTGAATTGGAAG

ACTCAAAAATATTTTCTTTGAATGAGGGTGGAAATATAATGTTGCTGCTCAGTTCTTGTA

GATAAGGTGTTGAGCAATTTCGTATTGTTTCGAAAATTGCGTCTCGTAATGTCTCCGCTC

ATTTTGGACTTACATCTTGCTTGCTTGCATGCATGGTCA

>Unigene0002280

CCCTTGATCTTCAGATCTAGGGTTTCCCTGTGGACGTTGCTCTGCGAGGCGTGTATGCTG

CCTTTACTTCCTACCGGTCTGCCCCCTCTAGTCACTGGTGTCGTCCTCGACCTTCTCGTA

CCAGCACCGGAGATGGCTCCCCACCCGCCGTTGTTGCCGACGACGGTCCCGAGAAAGAAG

AGCCGGAGAAGAAGACCCATAAACCGACAGCGAAGGAGCCGAGGGTCTTCTTCTCCGTGG

GGCAGGAGAAGAAGCTGCCACCAACGGGGCTATCAGCACCCAAAATAGACTCTGCGCAGG

GCGCCCCGCCACCAGGATTTATGTGATTTTGTGTAAACCGCAGAAAGCTGATCAAACCAT

TAGTCTACGAGCCCTCCTATCGCCGGCGAACTCCAGTCCCACTGCCTCCACGCTCTCTTA

TTCTAGGGTTTCG

>Unigene0002281

TCCCAACTCTCTCCCGTCCTCTGATCTTCAGATCTAGGGTTTCCCCCAAAGCAGTCGTCA

ATCTGGTCGCTCTCTCCTACCACCCGATTGTCTCCCCTAGGGCCCTTCACAGCATGTTGG

CGAAATCCCGGCACCTGTCTCCATGCCGCTATCGCCTCCGACGGCCTAGAGCCCGTATCA

TTCGGTATCCTCTCCCACTCCATCGATCAGATGGAAGGATCGCGCTCCACGAACCCGGTT

ACGGTTTTCGATTTTTATGGTTTCAATCCCTAAATTATC

>Unigene0002282

AGGAGACGAATCAAAACAATTTGAGGAACGAGCAGCTGAACACTTCAACGGCGTGAACAA

TTCGGCGAAGCTACTGAATTGGAAGACTCAGAAGTATTTTCGTTGATAGAGGGTGGAAAT

ATAATGTTGCTTCTCAGTTCCTGTAGATAAGGAATTGAGCAATTTCTTCTTGTTTCGAAA

ATTGCGTCTCGTAATGCCTCCGCTCACTTTGGACTTACTTTTTGTCTGCTTGCATGGTCA

AGGGAATGTGTGAACTCTGAG

>Unigene0002283

CGCTAGTGTTCACTCCAATGATTCATTCATCCATGCAAGCACCTCTGAACTGTTGAGAGG

CCAACAACACAGCCATGAAGCTGGCGGTGGAAGTGGTGGAGGCCAGCGACCTCATGCCAA

AAGACGGCCGCGGTACGGCCAGCCCCTTCGTCGAGGTCGAGTTCGACGGCCAGCGTCACC

GTACCGAGACCCGCCCCCGAGAGCTCAACCCCGTCTGGAATGATACCCTCATCTTTGACG

TGGCCGACCCTGCCAACCTCCCCGACCGCACGATCGACGTCTCCGTCTACCACGATAAAT

CCTCCTCAATCCCCAGCACCCACCACAGCCGCAATTTCCTTGGCCGGGTGCGAATTTCCG

GCGCCGCCGTCGCCCCATCCCCCACCGACGCCCCCCTCCAGCGCTGCCCGTTGGATAAGC

GCGGCCTCTTCTCCCATATCCGCGGTGATATCGCCCTCCGCGTCTACTCCCTCCCAGACC

CCTCCCCCGCCGCACAAGCGCCGGCTGAAGCTGGCGCCCCACCCGCCGCTGCCGCCGACG

GCGACAACGGCGGTGGTCCCGAGAAGGCGGAGCCGAAGAAGAAGATCCATAAACCGGTCG

CGGAGGAGCCGAGGGTCTTCTTCTCCGTGGGGCAGGAGAAGAAACCGCCACCGGCGGGGC

CGGCAGCTCCCAAAATAGACCGGGCGCAGGCCGCCCCGCCACCGGCGGCAATGATGCAGA

TGCCGGGGGCTCGACCCCCACAACCCGACTACGGGCTGGTGGAGACGAGCCCCCCTCTAG

CCGGCCGGCTCGGCTACCGGAGCAGTGACAAGATGGCTTCCACCTACGACCTGGTGGAGC

CGATGCAATTCCTGTACGTCAATGTAGTGAAAGCTCGGGACCTCCCGTCGATGGATCTCA

CCGGCAGCCTCGACCCCTACGTGGAGGTGAAGCTGGGGAACTACAAGGGTGTCACCAAGG

CGTTAGAGAAGAATCAAAACCCGATGTGGCGGCAGGTGTTTGCTTTCTCCAAAGACCGCC

TCCAGGCAAATCTGCTCGAGGTGACTGTAAAGGATAAGGATGTGGTGAAGGATGACTTCG

TCGGCAGGATACTGTTCGACCTCACCGACGTCCCGCTTCGAGTCCCGCCAGACAGCCCGC

TAGCGCCGCAGTGGTACCGGCTGGAGGACCAGAGAGGCAATAAGAGCAACCGCGGCGAGA

TCATGCTTGCAGTCTGGATGGGGACTCAGGCAGATGAGTCCTTCCCGGAGGCCTGGCATT

CGGATGCACATTCCGTCACACTGGAAGGACTGAATAATACTCGCTCCAAGGTCTACTTCT

CACCAAAGCTCACCTACCTCCGGGTGCACGTCATCGAGGCCCAGGATCTCGTCCCATCAG

ACAATTCCCGGCCACCGGACACCTATGTTAAAGTGCAGCTCGGTGGCCAGCTGCGTCGCA

CCCGCCCCTCACCCTCCCGGTCCATCAACCCGGGCTGGAACGAGGAGCTGATGCTCATCG

CCTCCGAGCCATTAGACGAGCCCCTCGTCATTACGGTCGAGGACCACATCGGCCCTGGCA

AGGATGAACCACTCGGGCGGCTCGTCCTCCCAGTGTCAACTGTGATGAAGCGCACCGACC

AACCAAAGCAAGTCGAAGCCCGTTGGTACAGCCTCAGCAAACTATCTGCCTCCTCTGACG

AGGGAGAGAGGAAGAAAGAAATCAAGTTTTCCAGCAAGATCCACCTCCGGGTCTTCCTTG

ACACCGGCTACCACGTCCTCGATGAATCAACACACTACAGCAGTGACTTTCGCCCGGCCA

ACAAGAACCTCCGGAAGCCCAGCATTGGGATACTCGAGCTTGGCATCCTCAGCGCCCGCA

ACCTGACAGCAATGAAGGCCAAGGACGGCCGTGCCACCGACCCTTACTGTGTTGCTAAGT

ACGGCCCGAAGTGGGTACGCACACGAACACTCCTCAACACCCTTGCCCCGCGATGGAACG

AGCAGTACACCTGGGAGGTGTTCGATCCCTGCACCGTTGTCACCATCGGCGTCTTCGACA

ACTGCCACATTACCGGCAGCAGCGGGAATTCCGACTCTGCCAGAGACCAGCGGATAGGCA

AGGTGCGAATCAGGCTCTCTACACTTGAGGCCGACCGAATCTACACCCACTACTACCCAC

TGCTCGTCCTCCAGCCATCGGGCCTGAAAAAGACCGGCGAGCTCCACCTCGCCGTCCGTT

TCACCTGCACGGCATGGGTCAACATGGTGACCCTCTATGCCAGGCCACTGCTCCCCAAGA

TGCACTACGTCCTCCCGATCCCGGTGCTGCAATTGGATTACCTCCGCCACCAGGCAATGC

AGATAGTCGGGGCTCGGCTCACCCGGGCTGAGCCACCACTCCGGCGAGAGGTGGTGGAGT

TCATGCTTGATGTAGATTCCCACATGTGGAGTATCCGGCGGAGCAAGGCTAACTTCTACC

GCATCACCGCCGTCCTCTCTGGTGCAGCTGCTATCGCCAAATGGTTTGACGGTGTTCGGA

ATTGGAGAAATCCAGTGACAACTATACTAGTCCATGTGCTGTTCCTTATCCTGGTGTGCT

ACCCTGAGCTGATCCTCCCCACTGTCTTCCTCTACTTGTTCATGATCGGCATCTGGAATT

ACCGGCTGCGCCCCCGCCACCCGCCACACATGGACACAAAACTTTCCCATGCTGAGTTTG

TGCACCCGGATGAGCTCGATGAGGAGTTTGACACTTTCCCAACAAGTAGGGCAGCCGATT

TGGTACGGATGAGGTATGATCGGCTGCGTAGCGTGGCTGGGCGTGTGCAGACGGTGGTGG

GGGACATGGCAACGCAGGGGGAGCGGGCACTGTCATTGCTGAGCTGGAGAGACCCCAGGG

CAACAGCCATCTTCATACTCTTTGCGCTGATCTTCGCAGTCTTCCTCTATGTCACACCAT

TCCAGGTCATTGCTGTGCTGGCTGGACTGTACCTGCTGCGCCACCCAAGGTTCCGCACCA

GAATGCCCTCTGTACCGTTCAACTTCTACAGGAGATTGCCAGCCAAGTCTGATATGCTTC

TCTAAGCTGAGATCCGAGTTTGAGATATCATCCTGTAATTGCCCTTTAGTTGCTTCTTAA

TCTGCTGTGGATATACAATCAGAGTGTATTCTAGGCCCCATAAATAAAAGTTGTAATCTT

TTTGGGATGCATTTTGTGAATCACGCCATCTCTGGTTCCGATGATAATGATTGGAATCAG

CCTCTGTTGTGTGATCACTGACTGTGGTGGAAACTTCTCAAATCTTGGTGAGCCCAGAGC

AGCTTTCTATCGATGATATTCTTTGCCTGCAAATATTAACTGGATATCAGATAATTATTG

AAGTGGTTGGTTGGACCATCATGCTACCTGTAACATGAAGCCAAAACAGAGACCACACAC

TCAGTAGTTGATCATAACAAGGAGATGCTCAGACAGGAGACAAAGCAATAGGCAAAATAT

TTAATGTTTCACTTGTAAATAAT

>Unigene0002284

AGGAGACGAATCAAAACAATTTGAGGAACGAGCAGCTGAACACTTCAACGGCGTGAACAA

TTCGGCGAAGCTACTGAATTGGAAGACTCAGAAGTATTTTCGTTGATAGAGGGTGGAAAT

ATAATGTTGCTTCTCAGTTCCTGTAGATAAGGAATTGAGCAATTTCTTCTTGTTTCGAAA

ATTGCGTCTCGTAATGTCTCCGCTCATTTTGGACTTACATCTTGCTTGCTTGCATGCATG

GTCA

>Unigene0002285

TATCCCTTTAAAACCCGCTCCCAATCTCTTCACTCTCTCGCCCCTGATTTTCAGATCTAG

GGGTTTCCCCGCAGAGTCACGATCGATCCGCAAAGGTGCGGATCCTGTTCTGCGAGGCGT

ATGTGCCGCCGTTACTTCCTGCCGGTGTGTCCCCCTTCCAGGGGCCGGTGCCGTCCTCGA

CCTCCTTGCACCAGCGCCGGAGCTGGCTCCCCACCCGCTGCTTACGTTACTTCCTGCCGG

TGCGCCCCTTCCAGCCGCCGGTGCCGTCCTTGTCCTACTTGCACCAGCGCCGGAGATGGC

ACCCCACCTGCTGCAGTCGGTCCCGAGCGGGAGAAGAAGACCCATAAACCGATAGCGGAG

GAGCCAAGGGTCTTCTTCTCTGTGGGCCCAGGGAAAAAGCCGCCACCGACGGGGCTAGCA

GTGCCCAAAATAGACCGGGCGCAGGGCGCCCCGCCACCAGG

>Unigene0002286

GGCGGTGGGTCAACTCGCTTCTGTCAGGAGAGGCTTCACACTCCTTTAAGCCGTATCTAG

CTTGGAGGCCGAAGAGTCGTCACCCTGGAATGGATACGTTGTCACCTAGGATAGGATTTT

TAATTGATCTTTTCCATTACATACCACCAGAATTGCATGTGCTGAAAACATCCATGAACT

CCCAAACAAGTTCAGTTTAAGTGACTATGTATGATGCACACTTCTACATTCTATTTTCAG

ATAGAAGCGCACACGCACTCTCACTTACTACTATTAATACTGCTTATCATGCTACATAAA

TTATTTAACATTGTCCAATACAAAGTCCTTGATGCTGTTAGCTTTTATTACTGATATTTG

AACTAACCACTACACTCACAAAAGCCAGGTTCG

>Unigene0002287

AAGAAACTCTTGTTTGCTGAAGACCGAAAAGTTACTCTCAAACAAATTGATGAGTTTTTG

GTTAACAAAATTGCTCCAAATGAGCTTGTTGATGAGATAAAGGTACATATCCTCTTCAGA

ATTTCATTTTAAATGATGTGGCACAAATTCCATGTGAGACCTAGTTCGGGACAAACGTAT

ATTGATCTTGTGTAGAAAACTCAAAAATAAGTAATCTTAATACGTAATAGCAAACAAGAG

ATTAAAATTATCAACTCAAAAATCTATATCAACCGGAGATCAGGAATAGAGCCTTATTTA

TCATTTGTATGAATAGATAAATATGAGGACATCTTTCTTAGAATAAAAAAATCCCACACA

ATACAATAGTTATGCATGGATCATATTTTCATTCCAAAGATTAAAAGTAGGGATGATTAC

AGTTACCTTGCTGTTGTTTACTTGTTTTGCACTTAGGCCCATGTAGTTTCTTTTTTTTCT

AAATTTACTTCCGGTTGTTTTCTCGGTTAGCACTTAAGCCCTTCTGTCGGCCTATACGAT

TAAATATCTTTTAAAATGACAATTTTCGTGTCTTTTCTCTTCCTCCTCCAGAGTCCACTA

AGACACCATGATCTCTTCTGCATACCAATCACTGTTCTCTCCTTATGGCCCAGTGGGTGT

TGAGGAGCAGAGGAAGGAAGAATGACAAGAGTATAATATACAAGGACTTAAGGTTGAACG

CTGACAGAAGGGCTTATCAACTAACAGAAAGAACCACGTGGAAGTAAAGTGAGCAAAAAA

ATACCAACCATAGGGATATAAATTGTAATTAACCCTCATATTTATGGAGAAAGAATTTTT

TTAGCAATCATTTTTCCTATCTATATAATGGTATTTGTCATACAATCACCATTAAAAAAT

TATTCTCAAGGTAGGTGTTTGTATGTGAAACCAAATGGAACTTCTTATTGATTGTCTTTC

ACAGAAATTGAAGGATGTCTCGTCGGATTTCAGTCTATTTAGTAATAAATGAAATCATTT

CTTCCATATATAATTATCAAGATACATAAGACGATCCCTGTTGATTTTTTGTTGCTGACA

TTGCACACACTATCCGGTTTATGGAAAGGTTCATATTGCTATAGTTCAATCATGATGAAA

TGAGACCTCTCTGGAGGTTATGAAACACACTTTGTTCTGGAGCATCAATTGACGTTGGAT

CGAGCATTTAATATCCTACTGTCGTGAGTAGCACTTTGGTTTTCTAAATATTATTGGCGG

AACTTAATTCTTTTATAAGTGCTGAAAATAAATCAATTTGATTATGACTTACGAGCTATG

GAAATTGATATGACAATGACATTGATGTTAGATGATTGGCAAGGTCCTTCTACCAGGATC

AAATACCCCGACAGCCAGTCTACCCGCTTCAGCGGAAGTAGCTCCAGT

>Unigene0002288

ACTGCCAATGGCTTTAAAGCTGCATTTGCAATAAAGGATGGCGCAGAGGGACCTCGAGGA

TGGCTGAATAGCGGCCTTCCTTGGACAGCGTCTAAAAAGATTCTAACACTTGATTTTGAT

GGTTTAAAGGATACAGTCACGAGCATACTCGGAGAGGGAGAATATGGACTGCCTATTACT

CTTGGACTAGCAGTTGCTACGGGCTTAGGAGTAGTTGCGTTCTCTGAGATAGAGACTGTG

CTGCAACTTTTGGGGTCTGCTGCAATTATTCAATTTGTTACAAAGAAACTCTTGTTTGCT

GAAGACCGAAAAGTTACTCTCAAACAAATTGATGAGTTTTTGGTTAACAAAATTGCTCCA

TATGAGCTTGTTGATGAGATAAAGATGATTGGCAAGGTCCTTCTACCAGGATCAAATACC

CCGACAGCCAGTCTACCCGCTTCAGCGGAAGTAGCTCCAGTAACCTCTGCATCTCCTACT

GAAGAACCCAAAGTTGATGCTACTCCAGACGTAAATACTGAGGTGAAGGTGGAATCCGCT

TCCGATCAACCAATGGTAAACTCAGTCCCAAAAGCAGAAGTCATCGAAGAATCAAAATCT

GTGCCGCACAGAGCACTTTCGCCTTATCCATGCTATCCAGACTTCAAGCCTCCAACTTCG

CCCACGCCCTCTCCGCCTTAGAAGTTCATGGATGTTTCTGATTTAAGCTTGCTCGGATCT

TCTTGCACTGATACAGGCCAAGCACTCAACTTTTCAAGAAGTCTAACATCGCGGTGGTGT

CTTGAGTCATGTTGCATAAATTCCAGGCAATATGTTGTCCATTGTTTGTTTGTATGTCGA

TGATCGAACTTGCCATTTTCGCCCGTTTCTGTTTACGAAGGATGAGTCATGTAGGCTTTG

CACTCTATACTCTTATTTTTTTTGTTTTGAGAATGTAGTTTTTTTGCAGACCAAATCAAA

TATCTTGTACAAATAGAATTATGGCAATGCGTTAACCATAC

>Unigene0002289

TTCAGTGAAGGAGAAGCTCACCAAACCCACAAAATCCATGGAAATCAATCCCAGTTCTCT

GATTTCTTGACAACCCATTTCTCATTTACCTCCGAGGACCTCAGAATTATGCGATGGAGG

TCCTCAATGCAGCAAGCTTGACACCAATTTCACTCCAAACGAACAAGAGAGCACAACCAA

GAAAGCTCTCACCATCGATACCTCTGAACCCTTCATCTTCTCCACGCCCAGGCTTTGTCA

AGCTCATCTCACAGGGTTTTACCGGAGGTTTAGCTGCTCTTTCGGCGGCTCTGACCAATC

ATGGTTCCGCGAAAGCCCTGACATATGAAGAAGCTCTCGGACAGGCTAGAGGGATCTCTG

TCCCATCTGTTGGCTCAGCCGATTTTGACGTCGGCGGTTTCCTTGATGTGTTCTCCAATT

TTGTATCGGAGAATCCTCTGGTGATCGGCGGTGGGGCTTTGGTCGTGGTGGTGCCACTGG

TGCTGTCCCAAATTCTGAAGAAGCCGAGGGCTTGGGGGGTTGAGTCGGCGAAAAATGCTT

TTGCGAAGCTGGCAGAAGATGGTAGTGCTCAGTTACTTGATATAAGGGAAGGAAAGGATT

TGAAGGAGGTGGGTGGTCCGGATTTGAAGAGCTTGAAGAAGAAGGCGGTTTCGGTAGTGT

ATAGAGGGGAGGACAAGCAGGGGTTCTTGAAGAAGCTGGGACTGAAATTCAAGGATCCCT

CAAGTACCACGTTGTTCATTCTTGACAAGTTCGACGGAAACTCTGAACTAGTGGCAGAAT

TGGTTACTGCCAATGGCTTTAAAGCTGCATTTGCAATAAAGGATGGCGCAGAGGGACCTC

GAGGATGGCTGAATAGCGGCCTTCCTTGGACAG

>Unigene0002290

TGGGTAATGAGAAACGCAACCACACATACACATTTCATCCTCAAATCTCTCCTCCTTTGT

CATCACCATTCCACAGAAAACTGAACCGGTAATCACATGTAGTTTCAATTTCAAGATCGA

AGCATGTTTTAGAAAAATGGCCATGTGCCATGAAAGTCAGGTAGTTCAGCCAATGAATAA

TAAAGAACGGCATTTCTCCAAGGTTTTTTTCCCCAAGTCAGTGCTACTGAAATAAAAACA

GTTTTTTTTTTTTTTAATTATTTTTAACCATGCCTTATTCAAAATTTCTTGGCTACAACT

ATATAGCTAATACAAACTTATGATACTTGTTATGATACACGATATAGTACATCTGATATT

TTTTTTACAATACTTTATAAGAGCAACGTATC

>Unigene0002291

CTAACAATTTTTTGTGTAGCAAAAAAAAGTGTCTTACGATACACAATATGTTGCGATTCA

CAAGCGTATGCATTTTTCATAACATCGGGTACAACTACAAGCGGTGGATTACCTTTTTTC

AAGAAGTCCAATCTGGTCCAATTCTATCATTCCGCCAAACTGCTTGATTGTTGATTAAAT

TCGTCTCTGTAATAATTTCCACTTAGCAGTCCTTAAAAAAACCACTTATTTGTTATCTAA

ACAAGTAATAGTCTTACTGTCTGAACTAATTGAAAGAAAACCTTCTTTCAATTAATCAAA

AATAATGCCACACGGTTCAGTATTTCGATTTATTTGGGAACTAAAGTGATTTTGGATTTT

TTTTTTCCATTTTGCCTCGAGTCCTTTATATTAAAGAATTGGGGAAATTTGACTTACACC

CCAAATAGATATGCATGATTTGCATTACAACTTTTTTTATTTGATTTACCCCCTCCCTCC

TCCGGGAGGTCACTTAAAATGGTTCTCTACAGATGAGTATGTCCTTGTTAATGATTTGTC

TTAATATTGTGGTAGTAGGATGTTATGTTGTATTGAAAACTCTGTTGGCATAATTTTTGA

TTTTTTTTGGGAACTATGATCTTTTTTCAAAGCATGATTAGGGTGTTAACTCTAACATAA

AAGACATGTAGGGAGGATAGCACAATTTTGGTGCATCTATTAGGGACCTAAAGACTCCTA

TGATTAGATATCATTTTTATAGAGCAAATTTGGTTTGTATGCCTCAGATTTACCCTGTAT

CAGTGGTGCTGGCTAA

>Unigene0002292

TCCGATACTTGGTTTCTGCACAACATGCTATAAAATATATATATGATATGTGACTGTTTC

ATGTACGATATCATACGTCTCATTATCTCATGGGTGTGCTGTATGCGCTTTTAATCTGTC

TTTAAATTTTATTTTGACCCTTCCATGATTTTAAAGGGTTTTACTTTTATATATAAGATA

TATGTCTCATTATCTCATGGGTGTGCTGTATGCGCTTTTAATCTATCTTTAAATTTTATT

TTGACCCTTCCATGATTTTAAAGGGTTTTAC

>Unigene0002293

AACATGTGATTGAGAGAAGTTTGACAGTTTCTGTTGTCACAAATGGCTAGTAAATGGACA

ATGTGTAGTACTTCTAGTTTCAAATTTCTGCCGCCCAATGTAATGTGTCTATGATTTTTT

TGGAAGCGCCTATCCATCAAGTGTACCTTGTTCGAAGGCCTTAACACATTCAAGTGGATG

GTGTTGAAGAAGAGCTTACCAATTATACATGATGAGGATTTTTGTATGTTGAATTTGTCG

TTTGGTTCCAACCATCTGTCTGAAGGTAAAATCTCTGATGCTACTCTTGTTTTCCTTGAG

GAGTTGGTGTCTGCAATATAATTAGGATACTTCAGTTTTTACTCTTACTTGAAACTATAA

CCTCTTGAAACTATAACCTCTTGATTAGACGCTTAGAACAAGATGTTGTTAAGCAACATG

ATTCAGTTTCAAGGTTTTAAACTCAGTTGGTACCTGATTTTTCTGAATATAAAATTAGAT

GTGTACTTTACAATCATGATTTGAAGGTGTGTCCAAATTGAAGGGTAAAACACCTCCCTT

GCTAGTTCACGTTTTTCTTTAAAAGAACCGAATTTACTGATGTATTATTTTTTTAGGTTA

TAATGAGTTTTAGGCACTTGTAAACACCTGTTTAACCCTAGTTATGTCGCAATTGCTACC

CACTTGTTGCAAAATGTATGGGTTTTTCTGTCTGGATAAATTCTAACAAGACTTTCCCTG

TTTGTATGATCGACATGAGACAATTACTGCCGATTACCTACCTTGGTTTATGCTGAAGGT

GCCAAGTTGGAATTGGTTTTCTTTCATAATGCAGATGCAGCAAACCTTTCACCAGGTGTC

AACTTTACAAAATCTTCCATTCTCCACTGATCTACTGAAGAGGGGCTATGAGGCTTTAAC

CCTTGCATTGCAGTATAGTTGGCTCCAGTAAAGCATGCACATCCATCTGGTGGATACAAT

CAGCAAAACTTCCGCCCTCGGG

>Unigene0002294

GTCTCATTATCTCATGGGTGTGCTGTATGCGCTTTTAATCTATCTTTAAATTTTATTTTG

GCCCATCCATGATTTTAATGGGTTTTACTTTTATATAAAAGATCTATGTGTTTTTAAACT

TATTTATAATATTGAATTTAATCTTATCTCGCATCCTAACTGCTAGCTCTTTTGGTGTGG

AGTGGTTAGTAACCTGGTATCAGAGCTTGGTATCTGGAGAGGTCCCAGATTCGAGTCTTT

CCATCCATGGCTTGAATATTTCACCATCTCGTGCCTGCCTAGTTCACCTGCTATGGTCAT

ATCAATGTTATCTATCACCCCGATTCGTGCCTGTTTAGTTCTAGTTTTTGGGGTGTAGTG

GTTAGTAATAGTATACAAATCTAACAATTTTTTGTGTAGCAAA

>Unigene0002295

AGATCGAAGCATGTTTTAGAAAACTGGCCATGTGCCATGAAAGTCGGGTAGTTCAGCAAA

TGAATAACAAAGAAAGGCATTTCTCCAAGGTTTTTTCCCCAAGTTAGTGCTATTGAAATA

AAAACAGTTTTTTTACTTTTATATATAAGATATATGTCTCATTATCTCATGGGTGTGCGG

TATGCTCTTTTAATCTATCTTTAAATTTTATTTTGGCCTATCCATGATTTTAAAGGGTTT

TACTTTTATATAAAAGATCTTATGTGTTTATAAACTTATTTATAATATTGAATTTAATCT

TATCTCGCATCCTAACTGCTAGCTCTTTTGGTGTGTAGTGGTTAGTAATCTGGTATCAGA

GCTCGGTATCTGGAGAGGTCCCAGATTCGAGTCTTTCCATCCATGGCTTGAATATTTCAC

CATCTCGTGCCTGCCTAGTTCACCTGCTATGGTCATATCAATGTTATGTATCACCCCGAT

TCGTGCTTGTCTAGTTCTAGTTTTTGGGTTGTAGTGGTTAGTAATAGTATACAAATCTAA

CAATTTTTTGTGTAGCAAAGTGTCTTACGATACACAATATGTTGCGATTCACAAGCGTAT

GCATTTTTTATAACATTGGGTACAACTACAAGCGGTGGATTACCTTTTTTCAAGAAGTCC

AATCTGGTCCAATTCTA

>Unigene0002296

GCTGGCAGCAAATTGGCAGCGAATCCCAGGACAACACCAATGCTGTCATCCAAGAGGGCG

CCCAATTCGCAAGCCGCCTCATCGGAATCAAAGAGAACGCCAAATTTCAAAACAAGGACA

AGATCAGCAACCAGTCACCAGGCCTCAAGCTTGTAGTCGATCAAGCCAAGCGCTGCCACA

AAGTTAAAAATGTGTACGTCTGGCATGCCATGGCCGGATACTGGGGCGGCGTCAATCCTG

CCGCGGAAGGAATGGAGCACTACGACAGC

>Unigene0002297

ACAGCACCCCATGCTTCCACAAATCCCTCCCCTCCCCTTTCTACATTAAACCCCCCTCCT

CTCCCCCTCTCCGTACCTTCTCTTCCCGCATTCGCCAAATCAAGGGATCAAGCTTTATCA

GCTGCTCCGAAGTAAAGAAGAATCAGGACAGGATGACTATTTCCTCAATTGTCAATATCT

CCGGCAGCAATCTCACCGTTTGTGGCCGCACTGTGCTGTCCAACGTTCCAGACGCTGTTG

TCTCCACTCCCGCCGCCTGCAGCCCCGTCGATGGCATCTTCCTTGGAGCGGAGTTTCCCC

AAGCTGCCTCCCGCCATGTCGTCTCCCTCGGCACATTCAGGGATATGAGGTTCATGGCAA

GTTTCCGATTCAAGCTATGGTGGA

>Unigene0002298

TGTCAAGTTGTGTTCAACCATGAATTCAAATAAGTCCAGAGATAGTGATGTTCACTCTGA

TTTACTTATCCCTCTGAGCAAGAACAGGGCACCAGCAGCACTGGTGATCTTAAGGGTCCG

GGGTTGTGGGAGGTTTGGAGCTTACTCTTCTCAAAGTCCACTGAAGTGCACCTTGGATTC

CCTGGAAGTGGAGTTCAGTTATGACCCAGGCAGTGGGCTGCTAACGTTAAACATGCCAGT

GCCGGAGGAAGAAATGTATAGATGGTCCCTCGAGATTCAAATCTAAGGGGACGAATATTT

GTAATTTTAATTGAGTCATTGCTGTTGGTGCTTCAGAGTCATTTGAAGTAGCATGTAATG

TTAGATTAATGTTGCTTTAATTTCCAAGTGAGTAAGGAAATTTACCCCATGTATCAGAAT

AAGGCATTTGCATCT

>Unigene0002299

TGGTTGCTCCATCTATGTCAGTGATAAGCCCGGGCATCACAACTTCGAACTACTGAAGAA

GCTGGTTCTTCCTGACGGCTCGGTCCTGAGGGCCCAACTCCCCGGCCGGCCTACTCGTGA

TAGTCTTTTCAACGACCCTGCCCGTGATGGTTCCAGTTTGCTAAAGATATGGAATATGAA

CAAGTGCACTGGTGTGGTGGCAGTGTTCAACTGCCAGGGAGCTGGATGGTGCAAAGAGAC

AAAGAAGACTCGAATTCACAACACAGCTCCAGGCACTCTCACTAGCTCTGTTACTGCTAC

TGATGTTGATAGCATCGCCCAAATTGCTGGCACTGACTGGGATGGCCAGTCCATCGTGTA

TGCCTACCGATCAGGGGAGGTGATTCAGCTGCCGAAGGGAGCCGCAATGCCTGTCACCCT

CAAAGTCCTTGAGTATGAGATCTTCCACTTCTCTCCACTGAAGGAGATCGCAAGAAGTGT

GTCATTTGCGCCAATCGGCCTCCTCGAC

>Unigene0002300

CGTGCCTCTGACGATTTCTACCCACGAGACCCAGCATCACACACCATCCACATCTCCTCC

GTCGCCTACAACAGTCTCTTCCTCGGTGAGTTTATGCTGCCCGATTGGGATATGTTTCAC

AGTCTCCACCCCGCTGCTGAGTACCATGGTGCAGCAAGAGCTGTTGGTGGTTGCTCCATC

TATGTCAGGTATTTCTTTCCCCCCAGACTCGAACCAAAGATGTTTTAATTTCGTACAAAT

TTGATCCTTAATCAGTCTGCATCTGGTTAAGATTGCCGCCGTGTGACTAGAATGTACGGA

CTCGAATCCAGATACATCTGACCCTCCCCGTTATCCCACATTGGTGGGAGGCTCGTGAAC

TAGGTTGCCCTTTTTTAATCAGTCGCATCTGGTATGAATGCTCTCTAAATTCTTCTGTGC

TGTGTTTGTGATTATTTCAGTGACAAGCCGGGGCATCACAACTTCGAGCTACTGAGGAAG

CTGGTTCTCCCAGA

>Unigene0002301

CGTGCCTCTGACGATTTCTACCCACGAGACCCAGCATCACACACCATCCACATCTCCTCC

GTCGCCTACAACAGTCTCTTCCTCGGTGAGTTTATGCTGCCCGATTGGGATATGTTTCAC

AGTCTCCACCCCGCTGCTGAGTACCATGGTGCAGCAAGAGCTGTTGGTGGTTGCTCCATC

TATGTCAGTGACAAGCCGGGGCATCACAACTTCGAGCTACTGAGGAAGCTGGTTCTCCCA

GATGGTTCTGTCCTGAGAGCCCAACTCCCTGG

>Unigene0002302

CGTGCCTCTGACGATTTCTACCCACGAGACCCAGCATCACACACCATCCACATCTCCTCC

GTCGCCTACAACAGTCTCTTCCTCGGTGAGTTTATGCTGCCCGATTGGGATATGTTTCAC

AGTCTCCACCCCGCTGCTGAGTACCATGGTGCAGCAAGAGCTGTTGGTGGTTGCTCCATC

TATGTCAGTGACAAGCCGGGGCATCACAACTTCGAGCTACTGAGGAAGCTGGTTCTCCCA

GATGGTTCCGTCCTGAGAACCCAACTCCCTGGCCGGCCAACCCTTGATAGCCTGTTCAAT

GACCCAGCTCGTGATGGCACAAGTTTGCTGAAGATATGGAATATAAACAAGTGCACTGGC

TTGGTGGGAGTATTTAACTGCCA

>Unigene0002303

ATCACAAACTCGTCGTTTAGTGCTGGCGCGGAATATAAAGATTGAGGATTCGGCGCTGTC

TCCTCTCATTCTGAGTCGAAAGAGAGAGAAACAAGCTTCCCAAGAGGGATTCTCTTTGGA

TTGGTTATTCACAATATTTTGTAAGTTGTTAGATTGATACTGCTAGAATGATGTAAATTT

CAATAACTTAATCCTGTTATAGCATTTTGTTGAACACTAATTTTTGAGAAATAATCACTA

ATCAGTTTGTGTTGAGAGAGCGACATCAGCCGACGAAGATGACAGTGACACCAAAGATCA

GTGTGGACGATAATAATCTCGTCATCCAGGGAAAGAAGATTCTCACGGGCGTCCCAGAGA

ACATCGTATTGACTCCGAGCTCTGCCGTCGGCCTCGTACCTGGTGCCTTCATCGGCGCTA

CTGCTCCCGACAACAAGAGCCACCATGTCTTTCCCTTGGGAACCCTTGAGGGGCTCAGGT

TTATTTGCTGTTTCCGATTCAAGCTATGGTGGATGACACAGCGGATGGGGACGAGCGGGC

GGGAGGTGCCGCTCGAGACCCAGTTCTTGCTGGTGGAGAGCAAGGACAGTATAGGCACAG

ATGACTCCGTTGCTGTCTACACAGTTTTCCTCCCACTACTAGAGGGGCCCTTCCGTGCCG

CTCTACAGGGCAATGAACAGGACCAAATTGAAATTTGTCTTGAGAGCGGTGACAAGGCCA

TCCAGACAAACCAAGCTCTGAATATGGTTTACATGCACTCTGGATCGAACCCCTTTGAAG

TCATCAGAGAGGCAATCAAGGTGGTCGAGCAACATATGCAGACATTTCACCACCGCGAAA

AGAAAAGGCTTCCTTCTTTTGTTGACTGGTTTGGGTGGTGCTCATGGGATGCTTTCTACA

CCGATGTCACAGCAGAGGGCATTGAACAGGGCCTCAACAGCCTAGCTGTAGGTGGCACTC

CCCCTCGGTTTCTCATCATCGATGATGGCTGGCAGCAAATTGGCGACGAGAAAAACAATC

AGTCTGACACTTCTGTTGTGCAAGAAGGTGCACAGTTTGCCAGCCGTCTTGTTGGGATTA

AAGAGAATACCAAATTCCAAAACAAGAACGAGGGCGACAACCAGCCACCGGGCCTCAAGC

TCGTTGTCGACCAAGCCAAGCGCCACCACAAAGTTAAAAGTGTCTACATCTGGCACGCCA

TGGCTGGCTACTGGGGAGGCGTGAAGCCTGCTGCAGAAGGAATGGAGCACTACGACAGCT

CCCTGGCCTACCCGGTCAGTTCCCCCGGCGTGATGGGCAATCAGCCAGACATCGTCATGG

ACAGCCTCGCCGTCCATGGCCTTGGCCTCGTCCACCCGAAGAAAGCGTTCAACTTCTATG

ACGAGCTCCACCGCTACCTCGCCTCCTGTGGCATCGATGGTGTCAAGGTGGACGTCCAGA

ACATAATCGAGACCCTCGGATCCGGCCTTGGCGGTCGTGTTAATCTCACTCGCGCTTACC

AACAGGCCCTAGAAGCCTCTGTGGCTCAGAACTTCCCTGACAATGGCTGCATCTCCTGTA

TGTGCCATAACAATGACATGCTCTACAGCGCGAAGCAAACTGCAGTAGTCCGTGCCTCTG

ACGATTTCTACCCACGAGACCCAGCATCACACACCATCCACATCTCCTCCGTCGCCTACA

ACAGTCTCTTCCTCGGTGAGTTTATGCTGCCCGATTGGGATATGTTTCACAGTCTCCACC

CCGCTGCTGAGTACCATGGTGCAGCAAGAGCTGTTGGTGGTTGCTCCATCTATGTCAG

>Unigene0002304

CCCAACTCCCTGGCCGGCCAACCCTTGATAGCCTGTTCAATGACCCAGCTCGTGATGGCA

CAAGTTTGCTGAAGATATGGAATATAAACAAGTGCACTGGCTTGGTGGGAGTATTTAACT

GCCAAGGTGCTGGGTGGTGCAAGGAGACGAAGAAGACTCGGATTCACGACATAGCTCCAG

GCACTCTCACTAGCTCAGTTACTGCTCAAGATGTTGACAGTATCAGTAAACTCACTGGTG

CAGATTGGGATGGCCAGTCAATCGTCTACGCCTACAGATCAGGAGAGGTTATTAGGCTGC

CGAAGGGAGATGCATTGCCTATCACCCTCAAAGTCCTTGAATATGAGATATTCCACTTCT

CTCCCTTGAAGAAGATCACAACCAGTGTATCTTTTGCACCTATCGGCCTCCTCGACATGT

TCAATTCAGGTGGAGCTGTTGAATTTTTTGACGTCAAGTTGTGTTCCATCATGAATCCAG

ACTGTGATGATCTATCGAGCCCTCTGAGCAAGAACAGGGATGATCTATTGAGCCCTCTGA

GCAAGAACAGGGCACCGGCAGCAGAGGTGGTTCTGAGAGTTCGAGGTTGTGGAAGGTTCG

GGGCTTACTCTTCTATAAGGCCGCTCAAGTGCTCTTTGGATTCCCAGAAAGTTGAGTTTA

GCTATGAAGCAGACTGCGGGCTGCTGACAGTGAACATCCCGGTTCCGGAGGAAGAGATGT

ATAGATGGACTCTCGAGATTCAAATCTAGGTGGGTGATTGTTGTTATTTTCTTGTTGGTG

ATTCAGAGTGATTTGAAATAGTATGTAATGTTAGATTGATGTTGCTTTAATTTTCAGCTG

AGCAAGGAAATTTGCCCCTTGTTCCAAAATAAGGCTTTTGCTCGAATATTTTTCTTGGGA

CTCCATGGGCAAGTAACTGCAATTCTGTTTGCTACTTGCAACCCACTATAATAAAATACA

AATATGTGA

>Unigene0002305

CGTGCCTCTGACGATTTCTACCCACGAGACCCAGCATCACACACCATCCACATCTCCTCC

GTCGCCTACAACAGTCTCTTCCTCGGTGAGTTTATGCTGCCCGATTGGGATATGTTTCAC

AGTCTCCACCCCGCTGCTGAGTACCATGGTGCAGCAAGAGCTGTTGGTGGTTGCTCCATC

TATGTCAGTGACAAGCCGGGGCATCACAACTTCGAGCTACTGAGGAAGCTGGTTCTCCCA

GATGGTTCCGTCCTGAGAGCCCAACTCCCTGGCCGGCCAACCCTTGATAGCCTGTTCAAT

GACCCAGCTCGTGATGGCACAAG

>Unigene0002306

AAGCCTGCTGCAGAAGGAATGGAGCACTACGACAGCTCCCTTGCATACCCTGTCAGCTCC

CCAGGCGTGATGGGCAATCAGCCTGACATCGTCATGGACAGCCTGGCCGTCCACGGCCTT

GGCCTTGTCCACCCAAAGAAAGTTTTCGACTTCTATGACGAGCTCCACCACTACCTCGCC

TCCTGTGGTATTGATGGTGTCAAGGTGGATGTCCAAAATATAATTGAGACCCTCGGGGCT

GGCCTTGGCGGTCGTGTGAATCTCACTCGTGCTTATCACCAGGCCCTCGAGGCGTCTGTT

GCCCGCAACTTCCCTGACAATGGCTGCATCTCCTGTATGTGCCACAACACTGACATGCTC

TACAGCACGAAGCAGACTGCAGTCGTCCGTGCCTCCGATGATTTTTATCCTCGTGATCCA

GCATCACACACAATCCACGTCTCCTCTGTCGCCTACAACAGCCTCTTCCTTGGAGAATTT

ATGATGCCCGATTGGGATATGTTCCACAGTCTCCACCCTACTGCTGAGTACCATGGTGCA

GCGAGATCCGTTGGTGGTTGCTCCATCTATGT

>Unigene0002307

GAGAAAATGTGATGAGAGAATATTTAGATAAAAAATGAGGAGTGAATAAAATAGGAGAGA

AAATGTGATGAGAGAATATTTAGATAAAAAATGAGGAGTGAATAGAATAGGAGAGAAGAA

TATGAGGAGAGAAAATGTGTGGAGAGGGAATATGAGGAGAGAAAATGAGTGAAAAATTCA

GAGAGAACAAATGATGTGATGAAAAATGTTTGGAGAAAGAAAATAAGAGGAGATGAAATA

AGATAAGAGAAAATCGGAGGAGAGAGAAAATGAGAAGAGAAATAAAATATCAAAAGAGAA

ATGGGGAG

>Unigene0002308

AAATGTCAATAAATGAGAAGAGAAATGTGAGGAGAGTAGATGAGAGGGGAGAATGAGAAA

TAAAATGAGGAGAGAATGTGAAGATAGAGAATTAGTGAGGGAGGGGGAAGATGAGGAGAG

AAAACGACAAGAGAAAAATGTGAGGTGAGTAGATGAGTGAAGAGAAAATTTGAGAGAATG

TGATGTGAGAGTAAATTAGTGAGAAGAGACAAAAATGAGGAGAGAAATGTGAGGAGAGAG

TAGATGAGCGGGGAGAACGAAAAGAAAATGAGAGGAGAGAATGTGAAGATAGAGAATTAG

TGAGGGAAGGGGAAGATGAGAGAAAATGACAAGAGAAAAAAATGTGCGGTGAGTAGATGA

GTGAAGAGAA

>Unigene0002309

TAGGAAAGAGAACATTATAGAGAAAAATATGAAGAGAGAAAAATATTGTAAGAAAGAAAT

AACCAGGAGATAAAATATAAAGAGGCAAAATTTGAGAAAGAAAATGAAGAGAGAACACGT

GAAAAGAAAATGTAAGGAGGAAAAAATGAGGATAGAGAAAATGAAAGGAAACAAGAAATT

AAGAGGAAAGAGAACTTAGGAGAGAAAATGAGAGGACAAAGAGAATGTGAGTAGAGTAAA

TACGAGGAGAGAAAATGGGAGGAAATAAGAAGTATGAGTAACATGAAAAATATGAAGAGA

AATAAGAGGGAGAAAATATGAAGAATTTTATGAGTACCGACAAAGGAGAACCGAGATTCG

AGGAAGAAAATGTGTCATTTTC

>Unigene0002310

GAAATGTGAGGGAGTGAAGATGAGTCAGAAAACGACAAGAGAGAATAAGTTGAGAGAAAA

TGACTAGAGAGAAATGTGAGGAGAGAATATGGAAGTAAGTTGAAAGGAAATGACACGAGA

GAGAAAATTGTGAGGAAAGAAAATATGAAAGAAATATCATGAGAGAAATTGTGAGTAGAG

AGTGTGAGGAGAGAATTTAAGTAGAGAGTGTGAGGAGAGAATGTGATGTTTGAGAAAATG

TAAGGAGACAGTAGATGAGAAGAGAGAAAATGATGGGAGACAATTTGATTGGAGGAATTT

TGAGAAAAAAATTAGAGTGATAAATTTCTCTCCCATTTCGTCTTTCACCGTAGTCTTTTT

TTCGTCACATTCTCTCTTCACACATCAACTTTCCTCACACATGTACTTACATCAACTTTC

ATCATACATCAACTTTCCTCGCACTTTCTCCTTAAATTTTCACCACACATCATCATATTT

TCTCCACCAATTCTTCTCTCCTGTCATTTGCTTTCTTCTCATATTTTCCTCGGTTTTCTC

GCTTCATACTCTTTCCTAGCATTTCTTCACTTCACATTCTCTATCCTCTTATTTTCTCAC

TCCTAATGTTTTTTCATTCAGCTTCTCCCTTGTCAGTCATCATATTTTCTCTCCTCTTTT

TCCCCCTCCTTATTTCCTCTCCTCTCTCATTTGTCTCTTATTTTCTCTCATAATATTCTC

CTTTCCTAATATTCTCCTTGCTCGTTACATTCTCGGTACTTATATTTTTTCCTGTCGTAA

TTTCTCCCCTCCTACAGCTGGAATATTTTTGGACAGAAATTTAGGAGGGGTTTTACATAT

TGGCAAGAGAAACGCTATACTGCAAGTTTAACTCCTGGAAGGATAACATTACCCTGTTTT

GAATTCCATATTAAAGCACAATGGTTACTCTCACCTAGTTTTGTAACACATGGTGAAGAA

CAGTCCTACTTGTATACCATTTCCTGGTTTTCCAGATCAAAAGAATTATAA

>Unigene0002311

TGTGATGTGAGAGTAAATTAGTGAGAAGAGACAAAAATGAGGAGAGAAATGTGAGTAGAT

GAGAGGGGAGAACGAGAAAGAAAATGAGAGGAGAGAAAGTGAAGATAGAGAATTAGTGAG

GGAGGGAATATGAGGAGAGAAAATGTCAATAAATGAGAAGAGAAATGTGAGGAGAGTAGA

TGAGAGGGGAGAATGAGAAATAAAATGAGGAGAGAATGTGAAGATAGAG

>Unigene0002312

TAGCAAATATCAATGTGCGCTTAGAAGAGTGAATTTAATGGGTTATACCAGTCTTCAGTG

AAAAATTGGGCACGCAACATGTGTCCAAAGAACAATATGCGGTACTTCTTCGACTTTTAA

TGAGAAATAAAAAAATTTGGATTTATGAAAAACATATAAGAGTGTTCGATTATGTTTGTT

CTTTAACATAAAAACATATTTGTTCTTTAACATAAAACTACAAAATATGAAGACTTGATA

TATTGTTCTTTAATATAGAACACAAAAATACGAAATATCGTTATCCTTCTATGTGAGGAA

AGAGAAAGTGAGAGGAGAGTGTGAGGGGAGAGAAAAAGTATAAGGAGAGAGAGAGTGAAG

CAAGAGAAA

>Unigene0002313

GAGAGAATATGAGGTGAGAAAATGAGAGGACTAAGAAAATCAGAGGAGAGAATAAATGAG

CAGAGAAAATATGAGATGGAAAATGAGAAGATAAAGAAAATAAGAGGAGATAAAATCGGA

GTAGAGAGAAAATCGGAGGAGAGAATATGAGAGAAAAATGGCAAGAGAAATTTTGAGGAG

AGAAAAATTAAAGAGAATATGATGAGAGAGAATGAGATGAGAGAATATGAGAAGAGGGGA

ATTTCAAGGTGAGAACGAGGAGAGAATGTGAGGAGAGAAAATTAAGTAGAGCTAAAATAT

AAGGATGGATAGGATAATGAAAGAGAAAATGGGGTGAGAGAAAATATTAGGAGGGAAAAA

TGAGACAGGAGAGAATGAGAGGAGAGACTAATAGAAGAGAAAATATAAGTAGAGAAAATA

TGAAAGAAAATAAAAGAGAAAAATAAGATGAGAGAAAATATGATGATAGAGAATGTCGGG

GGAGAATATTACAAGAGAGAAAATAAGAAGAAAAAATATTAGGAAAGAAAATGTGAGGAA

AGAGAAAGTGAGAGGAGAGTAATTATGAGGGGTGAAAAATATTTGGAGAAAAAAATTACG

AGGATATAGAAA

>Unigene0002314

TGAGAGGACAAAGAGAATGTGAGGGGAGAAAATACGATGAGCGAGGAGATGAGAGAAAAT

ATTTAGAGAGAGACAATGAGAGGGGAGGGAAAAATATGATGAGAAAATAACATAGAGAAT

GTGAGGAGAGAAAAAATGTTACTAACATGAAAATATGAAGAAAGCAAATAAGACGAGGGA

GAAAAAGTGAAGATAATATATAAAAGTAGAAAGAAAATGAGACGAGGAAAAAATGAAGGA

AAAAAATTATG

>Unigene0002315

TTCATTACCCCTATAACTTGTCTTGATTACTCATCAATTTTTTAAAATGTTAATTTTGGG

TCTTTTTTTATTTTTGGGTCTCATTTGTATAACAAAGCATTTTCACGCAGAGACATATCA

TAGCAAATATCAATGTGCGCTTAGAAGAGTGAATTTAATGGGTTAATACCGGTCTTCAGT

GAAAAATTGGGCATGCAACATGTGTCCAAAGAACAATATGCGGTACTTCTTTGACTTTTA

CTGAGAAATAAAAAATTTGGATTAATGAAAAACATATAAGAGTGTTTGATTATGTTTGTT

CTTTAACATA

>Unigene0002316

TGCAAGTCCAAGTTACCTATTCTAGTCTCAAAAATATTTGACATACTTGCAAAACCAATT

TGAATAAACAAATGAAAAGGCATTATTAAAATTAGAAAATAAAAATCAAATTTCAAACTG

TTATTAAGGATAGTTTGAATTATATTGGTGTCAGATCCGAAACATTGCTAAACGGATACG

AAACCATTTCAAACTGACTGCAGATTTTGGTATTAGGTGTCTGGTAAGAACCTAGTCTGC

CGTCAGGAAATCTCGGTAAAATATGGGCTCTTAGGACTAAATCTTAGAAAATAATTTGAG

TTTGGCATCCTGATATGGAGTTGTTACGAGTTCATTGTTTTCACGACAACACAATGACAC

CAAAATCACAATGATGTAACAATAATATCATGAGTCCACATTAAAAGCCACCAAAACAAC

CACTTTGGACATACGATTTAAAAGGCTCCAAAATACTTATAAAAATATAGTTTTATAAAA

GTATATAGCTTTATTTTTCAAATAAATAAACATAGAAGCCAATTGAGGTTCGAAACTCAA

GACCAATCATGGACAATATAGTGTTGTGCCTCACCTCCACAAGTTAAGTGAGGGCATGAC

ATTCTGCTATCATAGCAGGCGATGTGTGGTGTGGAAACAACAATCTTGTGACAACAATAT

GTCATATGTCGAGAATCACCAAAAAAGAGACAAATTATGATACACATATACTGTTAATAA

CATCACTATTTAAATCATGAGAAACATTTTATAAACAATTTGTTGAGACAAAAACTGAAG

GATGATTTTGAGTTACTCGCCTATAGGTTGTTTGGATTGTGTCTTTACCTTCTATTAAAG

TTTTTATTTGGT

>Unigene0002317

CCCGGTGAGGCCGGCGGCGGCGCCATCGCCGACTTGGTATATGGAGCGTACAATCCATGT

AAGTTCAGGCTACAGCTCATGACCCTTTATTTGGAAGGTTATTTAGGTACACAGATAATA

GGAGATATTTAACATGCAAGTCCAAGTTACCTATTCTGGCCTCAAAAATATTTGACATAT

TTGCAAAACCAATTTGGATAAACAAATGAAGAGGCATTATTAAAATTAGAAAAT

>Unigene0002318

TTCGGGCCAAGCACATAGCGTGCAATAGCCCAACCTCCTTGATCCTTGAAGCTAAATTTA

ATGCTAAACCTCAATGCATAACCAACGTTCTGATACCAAGTATCGAGATCAAACACTGTC

TTCAATCGGTTCTAAACCCAAATGTCAGGGAAGGATGCCTTTTCCTTCTCTGGATGTCGT

CAGCACAACTCCGAAGCCTGATGTGGGTCAATGTACGGTGAGGAGTCGATGCTAACGATG

AAGCTCTGTTGGAGTTCAACATGAACAAATAATCCTCCACCCGAATCTCCCTGTGCAGAA

GCGATAGCTCAAGATCTTAGAAACTCATGACGAAAAACAAACACAAAGACAAAAGAGAGA

AGAAGCACACAATCACAACACA

>Unigene0002319

TTCTCGAAGCTCTTATATCAATTATCACAGATCGGACACAATCGACAATTTCGATGTCAA

ATGTCGGGGCCGAAACGTGGTGAGGAACAATGTCGGCGATAGAGCTCCACGATACTCCAA

CCTGAACAAACAATCCGCCACCCGAATCACCCACAACGAAAATGATACCTCAGGATCTGG

GAAACCCATGACGGAAAACAAGCACACAGATAATAGGGAGGAGAAGCACAATCACAACAC

AACGATCTTGTGTGTTTCACCTAACTTGTTCCTACGTCTATGGTCACCACAACAATCCAC

TAGAATGAAGTACAATGGAGGAGATCTCAGATCTCTACGACACACCCTCCTCACCTCTCA

ATTACAATCTCTTTATATGTGGACGATCTCTCTTGATTAGAGGATTTCTGTGGTGTTGTT

TTATAATGAAGGTCCAATGCCTATTTAACCCGATTTGGTTTCTTGATTGCCAAGGAATTT

GTCTTCCTCTCCGAGGAAAATTCCAATTACATGTTAATGTATCAATCCTTGCTTGCTAAG

GAACACACCTTGCACTCCAAAGAATCCGGTTGGACTGAATCACAGTTATAGCAAGTTGAA

TTCAACAATTCCTATCACTGGTGCGCACATGTATCAATGGTGTGCACGTGATCACACAGC

TTAGAATTTCGTCACTACCGTATTAGCGCCCACATGCAATCCGTATGCACCGTTGCTCAC

AGTCGCGCTCCCCTACTTGTACATTCCGCTTGCTCCTAAGTGCGCCGTATCTTCTCCGGT

AACTCCTGCACCTCTTGTGCTTCTCGTTAACATCAACAAACTCAACGATCACAACAATGA

GTAATGGATCAAGCACCTTCACTTGCATCATTTACTCCCAGAGCAACATCGGATATCTTC

CGAGTCATTGAACCTCCCGAAAGCTGCCTTCATACCTTGCAATGGCAGAGGAATCAAGTC

AGGCGTAGTTCTCCATAGGGGAGGAGAGACACATGGTTCGGGCTCCACGAACCAACATCG

ATATGGGTCTGATATTTACCAACCATGCCTACATCTTTAATTATCAAGCTTTTGACCTAT

ATCCATCTCCACCATTCTTGGCCACCCCTTCTCCCCATTCTTGTTATCATCTCCCATAAC

CACGCAAATTTGTGTGTTATCTTAGAATTAGAAAAAAGAAAGCCTTAATTTCTTTGTTTT

TTGTTTGGATTAATGAACTTTTACCTCAATCGAGAAATCCACTTTCTAGTTGACTTCTTT

TAGTTCCACTTGAGCTGCTGTATGATTGTTTAGTTATTTAGATAGTCTGGTATGATTAAA

AAAATGAAATCAAAATATATGAATTGTATTTTTTTGTTTTTCAAATATATGAATTGTACT

CTATTTGAATTTTTTTTTACAATTCATATATTTTGATTTTATTTTTTTATCATAGCAAAC

TACCATTTGTTTTACAGATGTTTTCATTGGAAGCCGAAAACTAAACCAAGGAACCAATGT

TATGCTCGCAAACTGGCGTGTGCAAAACATCAATTGTGTCGATGCTCTCTTGTACACGCC

ACCATATGAATGTTGTCGCAATGACTCTTGCCATCTCAATTACCCGTGGATTCAATTTTT

CATGTCAACTCATCCGCAATTCTCCCCAAAAATTCGATAGCCCTCCCAAAAAATGGGCCT

GCGAAAAAATCAACCTCCACCTCTGTCCACATGGATCCTCCGTCCTACATGGAACATCTA

TAGCGTCCAAGGTTTCAAATAGCTAAAGGTGGTCCGATGCATTTTGACCTCCCCGAGGCG

AGGGGGTATTGAGGCGTAAGCTTCAAAGTGATTTGAGGTGTTTTCATTAGAAATGTACAA

GGTTTCAAAATGCT

>Unigene0002320

GTGTGCCATCTTTAAGATGCACATCGCCAAAGTTGCCCTCAACATAGTTCAACAATTTCG

AAACATCTCGTGGTGTGAAATGGTGCGTCTGAGTCCACATGCTTGAGAGAGTGCAAATCC

ACCATCCAAAGGCCTGGACTTTGAATCATATATCGCTTTCTTGGTCTCCTTGTCGTCGTT

AATCCTTTGTAATTGGCAATATCTAGCAAAGTGTCCCAACTCGTCATAATTGAAGCATCC

ATCTTTGCCTTTACCACCATGTCTTGATTTGCCTTGGTGCATTCACAACTTGGACCGACA

CCCTTGTTTTTGCTCTATCTCATGTTGGTTCATCGATCCTTTACATTTGGTGTCTTGGAC

ATTGCCATCACCAATGTCATACACCACTTTGCACGCCCAACCCTTCATAATACAGTGTCC

ATCATTGGTGCCCCCGACCTTGCCATCACTACCAACTTGTGTCTTTTTTTTGCCCAATTC

TCCCGCGTATAATGCCCAATCTTCGCACAATTGTAACACTTATCTTTGCGGTGAGATCTT

GGCTTACTTCTGTGCTTTCTCGGCCTAGATCGATCTCGGATCTTTGCTTCATTTATTCGT

TGAGCTCTTCCTTCAACACCAATAAGCATTGCGCTCTCGGATGACTCGGCTTCATCCGAG

GAATCTCTCTTTCACTTGTCCACCTAGAATACTTCTTAGAAGACTCATCAACAAACACCT

TCTCGAGGACTTTTCCTCCAAGAGCATGGACGCCACCGTTGAAGAAGGAGAAATGGATCA

ACTCTAGTGCTTTACAGTCAAGGACATTTCAATCATTTTTGTATGCCCTCCGGCTTGCTC

TCGCCAGGAACCAAATGGAGATCCATCACATTCAAGAAATCCTCAATTTGCATTCGTCAA

AACCTGTGTTTATGGTTCCCATTTGATTAATGCACAATGAACTCGACCACTCCTCGGAAA

TTCCATCAAGCACCATCCTTGCATCAACTTCAGGTCAAACGCGTAGCTTGTAATAGCCCA

AATAGATGAACACACTCAACTACCTATTGGGAATTATGATGAACACCTTTCGTGCAAACA

AGAACAGACCAAATGCTTAGTTTGCAATAATTCTTACAAGATCATCCACAACTCGACTAA

CATAGAGGAATTGCGTCGAACACCCTGTGTGTAGTAGGTTCGGGAGAAGCCCAGAGCTTG

CAATAGCCTTGAGTTTGCTTTGATCATTTAGCTTTGATTCTCGAAGCTCTTATATCAATT

>Unigene0002321

GATCTTGATTTGCCCGAGTGCATCCTCAACTTGGACCAACACCCATTTTTTTCTCTCGTG

TTGTGTTCACTGAGCCTTTGCCATCATGCACCACTTTGCACACCCAACACCTCACAGTAG

AGTGTCCATCATTGGTGCCCCGGACCCTGCCGTCACCAACAATGTGTGCCTTTGGTGAGA

TCTTGGCTTGCTTCGGTGCTTTCTTGGCATAGATCGTCGGGCCCTTGCTACATTCTTCCT

TTGAGCTCTTCCTTCAACATCAACAAGCGAAGTACTTTCAGCTAACTCGGCTTCATCCGA

GGAATCTTTGCAGGACTCATCCACCCGGAACATCTCCTTGGAGTTAGAAGATTCATCACC

AAAGACCTTCTCAAGTGACTCCAACAAATCTCGCGAGGATGTCTACGAACATGGACGCCA

CCATCGAAGAGGGAAAAACGGATCAGCTCTAGTACTTCTCGATCAAGAACCTTCCCATCA

TCTTCATGCATGCCCTCCGGCTTGCTCTCGCCAAGAACCAAGTGAAGATCCATCACATAC

AAGAGATCTTTCATGTGCATCCGCCAAAACCTGTAGTCTCGGCCTTCAAACTTCTCAATC

TCAAGATTTTTGTCTTCCTCTGTCATGGCTCCCACTTGATTAGTGCACAGTGAACTCGAC

CTCTCCTCAGGAATTCCATCTAGCACCTTCTCTACTAGTACCTTCTCTGCTCCAACTCTG

GGCCAAGCGCGTAGCTTGCAATAAGCCTAAATAGATGAACAACATACTCATCCACTGTTT

GGGAATCATGTTGAACACTTTCCGTACAACCAAAGACGGGCCAAACACGTAGCTTGCAAT

AGCCCTCACAAGATGATCACCAACTCCAATCTTGTAGAGGAATTGCGTTGAACACCCTGC

GTGTTGTTGGTTCGGGCCAAGCACGTAGTTTACATTACCTAACCTCCTTGATCCTCGAAG

TTGAATTTGATGCGAAGCTCGATGCAGAACTAGCGCTCTGATACCCGAATGTTGGAAAGG

ACGCCTTCCTCTTCGGATACCGTCTACAGCCCCGAAGCCTGATGTCGTGGGTCGATGCAC

GGTGAGGAGTCGATGCGGATGATGAAACTTCGATGGAGTCCATCGCGAACAAATAATCCT

CCACATGAATCATCCGATGTGGAAGCAACAACTCAGGATCTTGAAACTTCTTAATAAAAA

ACAAGTTCAAAGATAAAGATAAATGAGAGAAGAAGCACACAATCACAATACAAAAATCTT

AGATAGTTCACCCCTCATTGGGCTATGTCCACGACTGTCGCTATCAATCTGACTTGCAAT

CAAAGAAGATGTACAATGGGGAGATCTCGGATCTCAACACTCATAGACTTCTCTCACCTC

TCCTATT

>Unigene0002322

GTGTGCCATCTTTAAGATGCACATCGCCAAAGTTGCCCTCAACATAGTTCAACAATTTCG

AAACATCTCGTGGTGTGAAATGGTGCGTCTGAGTCCACATGCTTGAGAGAGTGCAAATCC

ACCATCCGAAGACCCAGCCTCCGAATTATTTACTGCATTCTTGGACTCTTTGTTGTCGTT

CATCCTCGACAACCAACAATTTCTAGCAAAGTGCCCCAACTTGCCACAATTGAAGCTTCC

ACCTTTCGTTCATCCTCGACAACCAACAATCTCTAGCAAAGTGCC

>Unigene0002323

ATACAAAAATCTTAGATAGTTCACCCCTCATTGGGCTATGTCCACGACTGTCGCTATCAA

TCTGACTTGCAATCAAAGAAGATGTACAATGGGGAGATCTCGGATCTCAACACTCACAGA

CTTCTCTCACCTCTCCTATTACAATATCTCAATGTGAGTGATTTCTCTCTTGTCAGTGGT

GATTTACAGTGAAGGTCGAAGGCCTAATTTCTACAATTCATTACTTGCTCACCAAGGTCT

T

>Unigene0002324

TACGTGTCTTCTAGAGGCAAGTATCTTTTATTTTATTTTTCAGATTGTTTCTGCAATTTC

TTATGAAATTTAGAAGTCTGGCCATAATCGGGTTTGTTTTGATCGGGTCGGGTTGGATAT

TATCCTATAAAAGGGACCCTTGTAGGAGTGTTCTTTTTAGTTAACGATGAATTAAAAAAT

TGGTTGCATGTGTTTTGAGGGCTTGACTCTGATTGCTTCTTCTTCCTCTGTTCATCACCC

AAAACTCTAATCCAGATTAGGGTTTCATCAATATGGATAATCCAATTACTCATCCGCTGG

TCTTGTGTATACTTCACCCCTCTGCTCCCATGATTTTTATAATTAGCCATC

>Unigene0002325

CCTCGATACACCAGTATCGGTCTACCCCCAGTCCTAGGGCTCACAGTGGTCGGCTCATCA

ATCCCGCCTCCCTCTGCATCCCACCCCCAAATCCGAAGAAGCGATGGATCCGCAGCAGCC

GGAGCACGAGGTCTACGGCGCCGACATCCCCTACGCGGACGACGAGATGGCTGCCGCCGA

CGAGATCGCCGCCGCCGTGGACCCCTCCTCCTCCTCCCCCAAGCCCGAGAACATCGAGGA

CATGAAGAAGCGGCTCAAGGAGATCGAGGAAGAGGCGGCCGCTCTCCGCGAGATGCAGGC

GAAGGTCGAGAAGGAGATGGGCGCCGTCCAAGATTCTTCTAGTGACACTGCAACCCAAGC

TGAGAAAGAGGAAGTGGATTCTCGTTCAATCTATGTCGGAAATGTGGATTACTCATGTAC

CCCAGAAGAAGTCCAACAACATTTTCAGTCTTGTGGGACTGTGAACAGAATAACGATTTT

AACAGACAAGTTTGGCCAACCCAAGGGATTTGCATATGTTGAGTTTGTTGAAGTTGAATC

TGTCCAGAATGCTCTTTTACTCAATGAGTCAGAGTTGCATGGTCGTCAGTTGAAGATTTC

AGCTAAAAGGACTAACGTCCCAGGTATGAAACAGTACCGTGGACGGCGACCTAATCTATA

CATGGGATTTCAATCTCGAAGACCGTACATGCCTCCCCCTTATTTTTCTCCTTACGGTTA

TGGCAAGGTCCCCAGATTCAGGCGGCCGATGCGCTACAGGCCATACTACTGAGTGTGCAT

ATCTAGATGGGTTTGCCTCGTATGTTTGAAAACATTCTCATGTGTGCATTTCAGTATCTT

GATACCATTCAGATACCTTGTATTATTTATCGACAAAAGGGCGACTTAATCTCCAAACCA

GTAGGGCAGTGGTTCTGAATTTGATGAGGCTATCAAGTAATGAGCTATTCTAGGATTTAT

CATGATTTATATGTTGTATGGATTGAGAAAATATTTGACAGTGTCTATCGTCGACAAATG

TTTACATA

>Unigene0002326

GTTAAGTTGTCCTCCCCGCATTTTTTTTACTCAGCATAATTGCGAAGACTATTATAAGTC

TTGTTTTATTCATGACATGGGTTTACAAGCCTTTTGAAGTTCTTTTTTTTCCTTTCGTTT

TTTAATATTTGCACTAAAGCATAATTGTGAAATCTGTTAAAAGTGGTAAGTTTCTCCGTA

TAAATAAAAAAGTTCTACATTTTAAAAGAAGTCTCTTTTTTTTAAGGGGGCTGGGGGGTG

GGTGAA

>Unigene0002327

TTCCGCCGCCGCCGAGACGCTGACCACGACGGTTTATTGTTGCCGGACGCCGACCGACGT

TTTCGTCCCGAACCCGTCGATTCCGTTATCGCGCCCGATAGAATATCGCGTCGTCCACTT

GGCGGTTGATACCGACGACACGGATGTCCATCGTCTGAGAGGAATTGAAATCACTCGCGA

ACGTGGCGCGATCGCTGTAAAATCAAACTGTTCAACTGTCGTCCCATTGATGTGGTGACG

TCGATCGAGATGACCGAAAACCGTAATGCTACAGTAGTTGAGTCTGCACTTCAACGTTTG

GCCTAAACAACATTCGCTGCCCTCTGTTACCGGTCCGTTGTAAATGTTCACTGATAATCA

ATCACTATGGGAAAGCTGCTTTCCAAAATTTTTGGTAATAAAGAGATGAGAATACTTATG

CTTGGTTTGGATGCAGCTGGAAAAACAACTATCTTGTACAAATTGAAATTGGGTCAATCT

GTAACTACAATACCAACAGTTGGCTTTAATGTAGAAACAGTTATTTATAAAAACGTCAAA

ATTAATGTTTGGGACGTTGGAGGTCAAGACAAAATACGTCCACTATGGCGACACTACTAC

ACAGGCACACAAGGTTTGATCTTTGTAGTGGATTGTGCAGATCGTGATCGTATAGATGAA

GCTAAGCAAGAACTACATCGAATAATAAATGACAGAGAGATGAGGGATGCAATCATTTTA

ATATTTGCCAACAAACAAGACTTGACTAATGCAATGAAACCACATGAAATACAAGAAAAA

CTTGGACTAACAAGAATACGGGATAGAAATTGGTATGTTCAACCATCGTGTGCTACCACA

GGAGATGGATTACATGAAGGCCTTGCTTGGCTCACATCAAACTTTAAACAATGATACCAA

AGTATAACACAATATTATACTTTAAATATCATTATAATTTTTTTAAGGAAGCTTAGGACT

AAAAGAAATTCCAAACGTACAAATTGAGTACATTACTTGTTAAATATATATAAAAATGAA

CATCATTATTTAAACTAAGATGTCCATTTTAATAATAACTGCGCTGAGAGGACTGGCCTA

ATAAAATACTGAATTATTCACAACTGTTTAAATATTATAAATATAATTTGTAATATGTAC

TGAACAAAATTGTCATAATATGCTAAAACTGCCTAACTATCCTTTTGATGTGTATATTTG

TAAATTGATTCCCGATCATTATCATTTATCAGTATACATATTAGCTTAAACAATAAAATG

TAAGCAATATTATTTATATATACATATATAAATATATATGTATGTAAATTAACTAATAAC

GATATAATATTTGTTCTCGAGTTTAGAATATTTTATCTAGGCCTGTAAATTAGTATATTT

AATATGTACTAAGTATCATAATATTTTATACATAAATACAGTTCATAATAATTAGGATTT

TATTAAAAAAAAATTGTAATAATATTGTTTCATGCGTGGAAGAATCCTATTTTTTATTTT

TATTGTTATTTATTATTTTGTACAAAATAT

>Unigene0002328

CAGAGTTGGAGAGAGTAATAGTTGATGAAAGAGATAAGTTCTTAACTCATAGTTTACAAT

GCTGTGCTGGATTTGTAATTGATAAAGCTGGAAATAAGCCAAATACATTGAATTCAAGAC

CTCAAGTTGTTGTTGGCGTAGTTGGATTAGGTCATGTTGCTGGTATTAAAAAGTATTGGG

AAAAAATTACTGATGAGCAAATTGCAGAACTTATTATAATTCCGCCTCAATCAAGAAGTA

GCAAAGTTATTAAAGTTGTCATCAAGGTTTCTGCCTATGGTTTACTTTTATGGGGTGTTT

CAAAGATCCCTGGTGTAAGATCATTATCAAAAATGGCTTATGATGCAGTTACTTCTACAT

ATGTTCAAGGAAAATTTATAAAATTACAATAAACTATCAAAAATCAATATTCCGTTATAT

GTATATTATATTTTATGTCATAAATAATAAATAGCAAATACAATTATACAAATAAATATA

TTTAATAATGTATAGCAATAATCAATGCGAAGATAATTATGATCATATTGATATTTCACT

TGTATATTCTTCAACAAACATTCCAGGACCTGGCTGTAATATGAGCGATTTAAACAGCTG

TCCCAATCAAATGTGTAACTGTACTGATTTGTGCCAAGCACCAAGCTGTGAATGCTTTGG

TACAGCTGGTTATAATAATTATGATAATTTTGGTAAACTAATTTCAGTTAATAGGTTAAT

TTATGAATGTAATGATTTATGTAAGTGCACATCATGTCAAAATCGAATTGTTCAACTTGG

ACCCAGAATTGGCTTGCGCATTTCTCACTGCAGCAAAGGCTATGGATTATATACCGATAT

CGCTATAGAAAAAGGACAATTTGTTTGTGAATATGCTGGTGAAATAATTGATTTAACTGA

AGCACAGAGACGTTCAAATGCAGATAATGAAAATTATATATTTGTGATAAACGAACATTT

TTCTGGTCATATAACCACAACCGTAATTGACGCCACAGTTATTGGAAATATTGGTCGATA

TATTAATCATAGCTGTGAGCCCAATTGCATGATTGTGCCAGTACGAGTTGATTCATTAAT

TCCAAGGCTAGCAATTTTTGCAATAAAAGATGTTCAGTGTAATGAAGAAATAACATATCA

TTACGGAGGTGAAGGATCTCACTCTCAAAATAATTTGTCTGATGTTAAATGCTTATGTGA

ATCATTTAGTTGTAACAGATTTTTACCTCGTAAAACAGGGTTATTTTAAAATATATGTAA

TCAGTGTATATATTATACTTAATAATAATTAAATGTTAGAATAAAATAATTATAATTATT

GTTTAAACATTTTTAATGAAAAAAAAAAAATTTCCATCATTTATGTTTAATTTTTAATTT

TAAATCATATACTACTGATCAGGGGCATGCTCAGCTTTTTCCAATTGGGGGGTGATGAGT

GGATGAACTGTTAATTGTTTTTGTTGATAGTCATCATAGACACGAAATGTATAAATGCAT

AAGAAAAAAAGTTCATGGGGGGATATGACACCCTCATCCCCTCTGTGAGCACGTCCCTGC

TACTGATTGGATAATTTTTTATGTACTTATACTTCTTATAGTAAAATTAATTTACTTATA

>Unigene0002329

GTTAAGGATCGGATTAAGGGGTGGGGATTGCATGCGGGAGAGGTGATCCGCAGAGGGGAA

TTTGTTTGTGAATATGCTGATACTCGTACGAGACAAAAGGTTGTCACTGACCGTGAGTTC

TTGACTACTGAAGAAGCTAGAAGGCGGCAACAAATATATGATGAGCTTGCTCATGTTGGT

AGGCTTTACCCTGCACTTTTGGTTGTGAGAGAGCATCTTCCATCTGGCAAAGCATGCCTG

AGGGTGAACATTGATGCGACTAAAATTGGGAATGTTGCTCGTTTCATCAACCATTCATGT

GACGGTGGCAATTTATCGCTTGTCCTGGTGAGGAACTATGGATCATTGCTTCCGAGACTG

TGTTTCTTTGCTGCTTCAGATATACCTGAAGGCAAAGAACTCACCTTTAGTTATGGAGAT

GCCAGGCTTAGACAAAATGGCCTTCCTTGTGTCTGTGGCAGCAGTCTTTGTTTGGGCATG

CTGCCATCAGAAGAAACTTGATGCCATAAATTTGAACGTGATTACTCAGAACTTCAATTC

AGTTGCTAGCAGGCTAAGCTGTTATTGGACATGTCACTGCAAGATCGAGTCATTGACTTA

GTACCTAGTGACATCATGGTTATTGGTTGTTCAGAAGCTCTTGATCACGACCACAGAGTA

CTTGCTGAGTAGGTACGTTCTTTGGTAAAGAATGTGCTTTTCTTTTCTTTTTGGTGACAG

ATATCCTCTTTTTCAAAAGAAGGATTTGTTTAAATGTCTCAGTTGATAGAAAAGAGTAAA

AAATGTAATCAAGGTTAGTAATAGTTGTCTTGAAGT

>Unigene0002330

GGCTCTGATCAACCCTAGATATTTTTGACATAATGTATGTTGATTGATGTTAACATAGAT

AATCTTTCTTATAAATCGTGTTTAATACGCGAATTTCCTTTGTTTCAGGGAAGTTCGAGA

TTAATCTTGGAGAAGAGTACTTGTAGAGAACGATGCATGACTTGGGTTTGAGCGAGAATC

TGCTAAAGTGTATCATGGACACATACAAGAAGTCAAAGAAGGGAAAAGTGAAGAAAATCG

GAAGATATGGTCACTAATCGACAAACCTCCCAGTTCAAAAAGAGGCGCATGTATTACATT

AATGCGCACACATTCACTATCTAGACGTGTGCATTCTGTTAAAGGTTGTGAAAGTTATCA

AGTAG

>Unigene0002331

AGGAAAGAAGGCAGTTGGGTGCAAGTGGGTCTTCACATTGAAGCAGAGCCCAGAAGGGCG

TATTGAGCGCTACAAGGCATGGTTGGCTGCGAAGGGGTACAGCCAAGCGTATGACATTGG

CTATAATGAGATCTTTGCACCAGTGGTGAAGATGAGCACAATAGGACGCTAGTTTCCCTG

GCAGTGAAGGGTGGGTGGAAGCCACACCAACTCGATGTGAAGAATGACTTTCTTCATGGT

GACTTGTTGGAGGAGGTGTATATGGAGATACCACCAGGTTTCGGTACAAACCAAACAGTT

GGTAAGGTGTGCAGGCTCAGAAAGTTAATTTATGGTTGAAGTAGTTTCCTCGAGCCTGGT

TTGATAGGTTTGGAAGGGCTATGGTAGGCATGAGATATCAGCAGACCATATTGTTCTTTC

GGCAACAAGGAGGTCACACTACGATACTTG

>Unigene0002332

GGTATGATTCCTCCTCCTCCCGTGACACTAAGGACTTCTCGCAGCAATGCGGGAGTTCCC

CCTGATCGATATGGTTTTCCACATGATATTGCCCAGTTTGTCTCTTACTCTCACATATCA

CCTGTACATGGAGCATTCATTGCATCTTTGGACATTGTCTCTATCCCTAAGTGTTGGCAG

GTTGCTAAGGAGGATCCAAAGTGGAAAGCAGCCATGTAGGAAGAACTTGGGGTTCTCGAT

AAAAGCAGAACATAGGAGATAGTGTCACTTCCAGTAGGAAAGAAGGCAGTTGGGTGCA

>Unigene0002333

GTCTCTATACCCAAGTGTTGGCAGGTAGCTAAGAAGGATCCTAAGTGGAAGGCTGTCATG

TAAGAACTTGGAGCACTCGACAAAAACATGACATAGGAGATAGTGCCACTAACAGGGGGG

AAGAAGGCTGTTGGGTGCAAGTGGGTCTTCACAGTGAAGCGGAGCCCAGAAGGGCGCATT

GAGCGCTACAAGGCACGGTTGATAGCGAAGGGGTACAGTCAGATGTATGGCATTGACTAT

GATGAGACCTTTGCACC

>Unigene0002334

CACAGGTGATATGATTCCTCTCTTCTCCTCCCACTCCCATGACATTAAGGAGGACTTCTC

GCAGCAATGCGGGACATCCTACTGATCGTTATGGTTTCTCACATGACATTGCCCAGTTTA

TCTCATACTCTAACATATCACCTGTACATGGAGCATTCATTGCATCTTTGGACACTGTCT

CTATACCCAAGTGTTGGCAGGTAGCTAAGGAGGATCCAAAGTGGAAAGCAGCCATGTAGG

AAGAACTTGGGGTTCTCGATAAAAGCAGAACATAGGAGATAGTGTCACTTCCAGTAGGAA

AGAAGGCAGTTGGGTGCAAGTGGGTCTTCAC

>Unigene0002335

GGCGAAGATGAGCACAGTAAGGACGCTGGTTTCCCTGGCAGTGAATGGTGGGTGGAAGCT

ACACCAACTCGACGTGAAGAATGCCTTTCTTCATGGTTGTTTCCTTTGAGATGACACTTT

CCCTGGTTACCACACTCCGCCAAATTACGTTTGCAGATTCCACCATGATGTTTATGACCT

CAAACAAGCTTGAGCCAGGTTTGAGTATTTTCGTATTGTTGTCTTACATACTGT

>Unigene0002336

GAGATTGTAGTTGGCAGCGTTTTGAGTTTATAGGACACGATTACATTTATGAGTACTATG

ATTTTTGACTGCATTGCTCAGTGGATAAACTTTACATGAGTCAGAACATTGGGCTTTGTG

TATCATTATGTAATTGTTGGTGGTTTGTTCTACCAGGTTAAAGATGGTTTCTTGTTGTTG

CAAAATGTCTTGTCATTGTTCTGGCTTATTTTCAGTTGGGATTGTTGA

>Unigene0002337

AGTTGGGACATTGATAAGCATATTGCCTAAACTTTTTCTGAGAATAAAGTTGGCAGCATT

TTGAGTTTATAGGACATGATAACATTTATAAGTTCCATGATTTTTGACTGTATTGCTGAG

TGGATAAACTTTACATGAGTCAGAACATTGGGCTTTATGTATCGTTATGTAATTATTGGT

GGTTCTACCAGGTTAAAGATGGTTTCTTGTTGTTGCAAGATGTCTTGTCATTGTTCTGGT

TTATTTTCAGTTGGGATTGTTGACATGTTATCTTGTCATGTTGCTTGTCATGATGGGTAC

CTTGGATGTTTGACTTTTGAAAGTCATTGAAGGGCTGTTGGATTGTTTGCGACTTGGCTT

ATATTGAACTTC

>Unigene0002338

TTAGTAGATTAATTGATGTGAGTGTTCTCATGTTTTAATTTTGGGCTATTTTGAAGGAGG

AGAGCTCTTTTGCTGAGCATTTGAAGTTTTGACATGTCATCCTGTTTGACTTAAATAAGT

TTTTTGACTTGGTCCTGCTGACAACTTTTTTCTAATATTTTATTGGTAATGGACAACTGC

ATTGTTGGATGGTTTAGTTTGCTGACACCCCTTTCTATTCAATATTTGAATGGAAATGGG

CATGTGTCATGTTGGTTTGTAGGAGTTAGTTTGGGTGTCAGTATGATTTGTTGGGTTCCT

TTGCATTGGTCCTACTATGTTTTGAGTTCATTTGGATACGGTATATTGAGTTTAGTCTTC

AATTTTTACTTGGGTGTAACCCTTGACGGCGTCTTTGCAGGGCTATTGGTTAATGTTAGA

GGAACGGTTTATCTTTGCTTGTGTTGGTGCATCTGGGGAGAGAGGAATGCATGGGTTTTC

AGGGGC

>Unigene0002339

TTGATTACAGGGAAAGTTGGGGGCCAATTTCAAGAAGTTAAAAAAAGTTAGTGTTTGGGC

ATAATTCGGTTTGGGTTTTAATTGATATGTCATTGGTAACTTTAATAAATTGGCCGGATT

TATGGTATGGTTCATTATCATTTTGAGTAACGTGAGGACAAGGTCCTCTTATTATGATTG

TTATAATATTTAGCTTTCTAATTATTTTTTTATGGACCACCTAAACAAATGTTATTGATA

ATGAACCAAAACTAATGTTAATGATGTGATACAAATTGAATCAAATGTTTTTGACTTGTT

TTTGACTTATTTAACTATTTTATTTTCTTTGTACAGTGGGATGAGTTAGAGATAGTTTTT

GTTACCTTTAAGAAATGAGTGAGATTTGGGTTTGAGGTTTTGATGTGTTAACCTGCTTTC

ACACTATGTGGGTCTGACCTTGTTGGCACCACCTATATCTAATATTTATGTTTACTGAAA

AATGTGCCGATAAATGTGCTCTTAGATGGTTCACCTATCACTATGTTTTGTTGGGGAACA

CTTTGTAATGGACAAATAA

>Unigene0002340

TATAAGTACCATGAATTTTGAATGTTTTGCTGAGTGGATAAACTTTACATGAGTCAGAAC

ATTGGGCTTTATGTATCATTATGTAATTATTGGTGGTTTGTTCTACCAGGTTAAAGATGG

TTTCTTGTTGTTGCAAGATGTCTTGTCATTGTTCTGTTGGATTGTTGACGTGTTATCTTG

TCATGTTGCTTGTCATGATGGATACCTTGGATGTTTGACTTTTGAAAGTCATTGAAGGGC

TGTTGGATTGTTTGCAACTTGGCATCTATTGAACTTCCTTTTATGGAGGATTTATGGGAG

TAGCTTCACTCATGTTATTGATTACAGGGAAAGTTGGGGGCCAATTTCAAGAAGTTAAAA

TAAAGTTAGTGTTTGGGCATAATTTGGTTTGGGTTTTAATTGGTATGTCATTGGTAACTT

TAATAAATTGGCCGGATTTATGGTATGGTTCATTATCATTTGGAGTAACGTGAGGACAAG

GCCCTCTTATTATGATTGTTATAAT

>Unigene0002341

TCTTCCTTTCTCTATTAATTAACCCATCCTCTATCTTCTCCTCAATACTCGCCCCCACCC

CCCTTCTCGTCGCCGTGGCCTCCACCGGCCAGTATCCATCACTGCCGCCGTACCTTGGAT

ATTTGACTTTTGAAAGTCATTGAAGGGCTGTTGGATTGTTTGCGACTTGGCTTATATTGA

ACTTCATTTTATGGAGGATTTATGGGAGTAGCTTCACTCATGTTATTGATTACAGGATAA

GTTGGGGCCAATTTCATTAAGTGAAAAGTTAGTTTGGGTTTGGGCACAATTGGGTTTGGG

TTTTAATTGGTATGTCATTGGTAACTTTAATAA

>Unigene0002342

TTTGGAACTTTTAAGAAATGTGTGATATTTGGGTTTAAGGTTTTGATGTGTTCTGCTTTC

ACACTGTACTGCTCTGACCTTGATGGCACCTAACGTTTACTGAAAAAATGTGGTCTTAGA

TGGTACACCTATCACTATGTTTTGTTGTGGTACACTTTGTAATGGACAATTAATGAGTCG

AGTTTAACAATTTGGTTTACTTAGTAGATTAATTGATGCGTGTGTTCTCATGTTATAGTT

TTGGGCTATTGACACCACTTCTTTCTAATATTTTATTGGTAATGGACAACTGCATTGTTG

GATGGT

>Unigene0002343

CACCCTGTAACCTCACCATTATCTGAACTCCGCCATGATGCAACCACCGCCCTCCTCCTC

CTCTCTCTCCCACTCAATTAAATTCCCCCCTTCAAAACTGCCTTCCCATCCCCTCTTACC

CCGCAAGCTCCTCCACCGCCGCCTCCTCGCCAAACCGGCTGCTCCCTCCGCCCCCCTCGC

CGGCGGCGACACCTCCGCTACATCCACCGCCGATGCCGCCCTCCTCTCCCTCCTCCGCTC

CCGCGACACCGATGCCGCCTTCGCCGCCTACTCCCGCCTCCCCCACCTCCCCGGCCCCGT

CCCCCTCAGTCGCCTCCTCTCCCAGCTCTCCTACCAGAACACTCCCGCCGCCCTCGCCCG

CGCCCAGTCCCTCCTCCGCCGCGTCCGCGACGCCCGCGAGCTCCGCCGCCTCGATGCCAA

CTCCCTAGGCCTTCTCGCCGCCACTGCCGCCCGCCACGGCGCCGCCCCCTACGCCCTCTC

CGTCCTCCGCTCCATGCTCCGCTCCGGCTACCTCCCCCACGTCAAAGCCTGGAGCGCCGT

CGCCGCCCGCCTAGCTTCCGCCCCAGACGACGGCCCCACGGAAGCCCTCCGCCTCTTCGA

CTCCGTCATCCACCGCATCCGCCGCGCCGGCGCTGTCGCCTCCGACGCCCGCCCCGACAC

CGCCGCATTCAACGCGGCCCTCAACGCCTGCGCCAACATCGGCGACATCCCTCGCTTCCG

CCAAATACTAGAAGAAATGCCCGAGTTCAACGCCACCCCAGACGTCCTCACATTCAACAT

AATGATCAAGCTCTGCGCGAGATCCAACCGCAAAGACCTCCTCGTATCAACATTAGAGCT

AATAATATTCCGAGGTCTCGTCCCTTGCATCACCACCTTCCATTCGCTCGTCGCAGCCTA

CGTCGGCTTCAGTGATCTCGATACCGCTGAGAAGCTTGTACAAGCAATGCGGGAAAGCAG

AAATGACATCTGTGCTGTTCTCCGTGAATGCAATGGAGATAACACAATTGTTCGACCAGA

CAACGGAACTGAAGCTCATGATGTACTCGAAAACCTTGTGAATTACAACCACCGTAATAA

CCCGCCACCGCTGCTGCCTAAGATGCACACGCCCGACTGCCGGATGTACACAACACTGAT

GAAGGGGTACCTGAAAGAAGGCCGCTCGGACGATGTTGTCCGCATGATCCGAGCAATGCA

GAGGGAGGCGGACCCAGCCAGCCATCCTGACCATGTGACTTACACTACTGTGATCACTGC

GCTCGTCAAGGCTGGTGCTATGGACCGGGCACACCGCGTGCTTGCTGAGATGGATCAGGC

TGGAGTGCCCGCTAACCGCGTCACCTACAATGTTCTGCTCAAGGGCTATTGCCACCAGCT

GCAGATCGGCAAGGCGAAGGAGTTGATACGAGATATGGCAGTGGATAAGGGGATCGAACC

CAATGTGGTCTCGTACAACATACTTATCGATGGTTGTATACTCGTCGACGACAGCGCAGG

AGCACTGGCCTTCTTCAACGAAATGAGGGAGCGTGGGATTGCACCGTCTAAGGTCAGCTA

CACAACACTGATGAAGGCGTTTGCGTTGTCTGGACAACCAAGGCTGGCCCATAAAGTGTT

TGATGAAATGGGAAAGGACCCGAGAGTGAAGGTGGATAGGGCGGCTTGGAACATGTTGAT

TGAAGGGTATTGTAGGTTGGGATTGGTAGAGGAAGCGAAGAAGGTTGTCGAGAGGATGAA

GGAGGATGGATTCCAGCCGGATATCGCCACCTATGGCAGCCTTGCGAACGGGATTGCACT

GGCAAGGAAGCCGGGCGAGGCTCTCCTGTTGTGGAATGAGGTGAAGGAGAGGTGCGCCGC

GTCGCCACCGCTTAAGCCAGATGAAGGGTTGCTTGATGCTCTTGCGGATGTATGTGTGAG

GGCTGCTTTCTTCAGGAAAGCACTCGAGATAGTGGCCTGCATGGAGGAGTTTGGGATATC

GCCGAACAAGACCAAGTACAAGAGGATTTACGTTGAGATGCACTCGAGAATGTTTACGAG

CAAACATGCTTCGCAAGCGAGGCAGCTGAGGAGGACGGAGAGGAAGAGGGCGGCTGAGGC

GTTCAAGTTTTGGCTGGGACTCCCCAACTCGTATTACGGGAGTGAGTGGAGACTGGAACC

GGTAGAAGGGGGTGACTAGGTCGCTCTCAGAGCGTTGAAGATTGTATACATGACCAGTTA

CCTAACAATTTAGCGTTCAGGCTGAAGTGATATTTATCATGGTATCAGAGGGGCTGAGTT

ACTAAGTGAATTCAGGAGGTAGACAATGTTGTGAATCTAAACTGGTTATTTGTTGGTTTG

AATTTTGATCTAGTGAAATACATATTGTTTGGGCATCTCATAATCCCTGGTGTATCTAAC

TGTGTAGAAATGCATAAGGGAATATCGATTCAGGTAATTTATTTCCTGGGAAAATTACAG

AAGTGGATATTTGATGATTTCTAGCGAGTTGCCTGGTCCCGTCAGTAAAGCGTAAAGGTA

ATCGCCACCGTTCTGACGCTCTAAGAGTAGGAGGTGGAGCTCGGGCCACTTTTCACATGC

TTTAGCTATGATACCTGTAGCTGTGTGTTTTAGACCAATGGTTGTGTTCGTTGGACACTT

AAAAAGATTCTGGATTCAACATGAACTAAGACCTCTTAGAGATTTATTGCCTATCATCGA

GAGCTGAAGACTGAGAGCACGTATGAGATTTACTGCCAGCAACGATCAAAGCAGTGGTAC

TTTTTCTTTCAGATAAAATCCTGTCATCGAGAGCCAAAGAATGGGAGCACTATTAGAAGT

ACTTATCTGCTTTTTATAGGTTGATTTCTTTACATTCATCTCAATTTATATGTTAATTGT

CATAGTCGGCCAACACTTGTTGGTATTAGCAC

>Unigene0002344

AGTTCGTGCAAATTGGGCCCAAAATTCCCCACATGGTTGCGGAATCAGACGAGTATATCC

AGGCTAGATCTGTCAGGAACAGGCCTTTCTGACACAATCCCTAGTTGGTTCTGGGAGTTA

TGTCTGTCAGACTTAAATATGAATCTGTCTCGTAACCAATTGAAGGGGGAGCTCCCAAGA

TCAATAAACTGCTTCTTCATATTTGGTCTCGATCTGAGCTACAACGGATTTGAAGGGCTG

ATACCAAATTTGGATCCAATGTTGGACTTCGTCGATCTTTCTTACAACAATTTCTCCGGG

CCTATTCCTAAGTCTCTAGCAGGTGGGGTAGACTATCTATCAAGATTACTTCTATCACAC

AATAGGCTCAATGGCAACATTCCATCGTCTATTTGTGAGGCAAATCACTTGCAAGTGGTT

AATCTCTCCTACAATGAGCTCTCTGGAGAACTCCCTGATTGTTGGGCCAACTCTTCGTCA

AGCCTTGGGGTTGTCGACTTTTCAGGTAATAGATTGTCAGGGGGAATCCCTAGCTCAATT

GGAAACCTAAATCAACTGAACTCCCTCGATTTGTCAATAAACGAACTATCGGGAAGCATT

CCTCACAGCCTAGAATCTTTGAACTTTTTAGCCATCTTGAACTTGTCCAACAACAATTTG

TCAGGACCAATTCCAACAGGAGGCCAATTTTCAAAATTTCCCCCGTCATCTTATAGCAAC

AATATTGATCTATGTGGACCTCCTCTACAAAAGAAATGTTGAGATGGATAGGTCTTCTGA

TGAACATGGTGATGATGATAGTGACGAAGACATCTTTTAAAGCCCCTGAGATCTATGCCG

CTTGCACCAACTACGAGGACGTCACTAACTCAATGCCATATCATTATAGATGGAATATCA

CTATTGTGTCTTTTTTTGTATTTATTTCCTAAGTTTGGTGTTGTACGTTATGTTGTTGTA

TTTGCTTTCTTTTAGTCTCACATCAACTACGATGAGGAGACGCTTGAGTTCATGCTCATA

TTTTAGGGCACCCTTGTGTACTGATTAATCATTTGATAAATAAATTGAGTT

>Unigene0002345

GATCTATGTGGACCTCCTCTACAGAAGAAATGTTGAGATGGATAGGCCTTCTGATGAACA

TGGTGATAATGATAGTGACGAAGACATCTTCCGAAGCCCCTGAGATCTATACCGCTTGCG

CCAACTACGAGGATGCCACTAACTCACTATCAGATAATTATAGATAGGATATCACTGTTG

TGTCATTTTTTGTATTTATTTCCTAAGTTTTGTGTTGTACGTTATGTTGTTGTATTTGCT

TTCTTTTAGTCTCACATCAACTACAATGAGGAGACGCTTGAGTTCGTGCTCATATTTTAG

GGCACCCTTGTGTACCGGTTAATCATTTAATAAATGAATTAAGTTT

>Unigene0002346

GATCTATGTGGACCTCCTCTACAAAAGAAATGTTGAGATGGATAGGTCTTCTGATGAACA

TGGTGATGATGATAGTGATGAAGACATCTTCCGAAGCCCCCGAGATCTATGCCGCTTGCG

CCAACTACGAGGAAGCCACTAACTCACTGCCAGATCATTATAGATGGGATATCACTGTTG

TGTCATTTTTTGTATTTATTTCCCAAGTTTTGTGTCATACGTTTTGTTGTTGTATTTGCT

TTCTTTTAGTCTCACATCAACTACGATGAGGAGATGCTTGAGTTCGTGCTCATATTTT

>Unigene0002347

TAGTCGATGCACATCCACAAGCTCTTATCATGTTTCTTTAAGAATAACACAAGGTCACCA

TAAGGCACCTTCGAATTGCGAACTAGTCCCGCCTTCAACAACTCATCCAACTTTTTTCGA

AGTTCTACCAACTCCGGTGGTGACATTTGATAAGGCTGTTGGGAAGGGGGCTTTGTGTCA

GGTTCTAACTTGATCATGTAGTCTACAACTCGCTTGGGTGACAATGCCTTGGGTAGTTTA

TGGGGCATGACATTCTTGAACTCCTTGAGCACTTTTGTCATGTCATTGGGAAGAGAGTTG

TTGTTGAAGACCATATCCTTCAAGGGCAATGTATCAACAAATGTAGGCTCTCCCTTCTTT

ATTTGTTAGGCTGAAATCTGTTCCATG

>Unigene0002348

GCCATCATGGTTTGACATCATCTGATATATACATTGTTGCTACCCCTTTTGAGGTTGCAT

TCTGAAGTATTGCTCTAAATCAAAGAGAGTTCTTTTGTTCCGTCATAGGTTCGTGGCTTG

GTGGCTCTTACCCAATCCGACCCCATGGCAAGATGTTGATCTCCATGATGTGGCTGGTTT

GCTGTACCAACTGCCTGTGTGAGTACTGTCACTTGTGTAGGCAGCTAATTTAGTTGCTCC

TGTATGGTCCTGATTGAGTTTCTGGATTCATCATTAAAGAATCAATAGTGTCAGAAAGTT

CTTGATTGCTAGCCTCAAGGGCTGTTATCAGATTCAAACTCTCAATGAGTTGTACTTTGA

GCCGGGTCAACTGGGCGGCCATGTCGAGTTTTTCTTTGTGGCTAGTTTGGTCGTGTCTCG

GAGTGTTCTGAAACAGGTGTCTCGCCATGCACAACACCACTCCCTTCTTTGTTTCCCAAC

ATCGTCTAGGACTATAGTCGATCAAGAGTCATAAATGGTTTGACCTGCTCTGATACCAAC

TGTCACGAACTTAACCAAACACTCTCGATATTCCGTGTGGCACCTTGTGTTATAATGTAA

GGGTTTTCAATAATGCCAATAGCTGACATGTTACCTTGGTGCAATGTTCTAACCCAACCG

TGATTGTAAGGTCAGCTTTGTACAAGGTCGTGGCTTGTTCTCCTGTGTTCGTATGAGTGA

GATTCTCGCACCCAATGTTCACAATTGCGTAACACAACACTTCAAGCCATTCCCAAGAAG

CATACAATAGAGTTTGGCATGATGATAGGAAATATTGTATTCATTTTTCAATGATTGCAC

AATATAATTCCTTGACTACAACAACCATTGAATTGATCGAGCCAACGACTAGCTGCGCAC

TATGTTGGTCTGAGTATTGGAGGGCAAAATGAATACCAAACCACTATAGTGATGGCTCAC

TTGATCTGACTGTGCAACCCTCACTACCCATTAGTCGCACTGCTCCATTTTACACCCCTA

CGGTCCAACGTGTTTGGTGGAACAACCAAAATTTTGG

>Unigene0002349

CCTTCGCCTGACCAGCAATAGGCCGCACCTCAGAGTTCACTGCCTTCATCCGACTCGAGT

CCTTTTCAATCTTCAACCCGAGTCGTCGCGCCTCGCGATCAGAGATGAAGTTATGAGTCG

CACCAGTGTCCAGCATGGCACGGGTCGACCTACCATTGAGCATGATGTCAACATACATAA

GCTCATTGGCTTTCTGTTCCCTTCTCTTCTCCCCCACTTGGCCTTTGAGGGCATTCAGAA

GCCGCAACGCACCCATGCGCATCTCCTCTGTACACACCTCATCATCATCAACACTCTCCG

TAGCCGCTGTCTTCGTTTGCAGGGCATGGAGGGCATTTTTGTGGGGGCATTCAGAAATCC

AATGTGGCCCATGACATATGTAGCACCCCCTTTTCGGGCCCTTCGTCGCTTGACCCTTCT

TCGGGGCTTCCCTCTTTTCATTCATGGCGACGGCTTTCCCCTTGTTGCCTGCTTGGGGTC

GTGGGGCCGGCGACACGGACTTGTTCTTGGCACTCGATTGGCTGTCACGAGTGTCATAGT

CGTTCAAGCGCTCCACCATTGCAATGGCGGAAGATCTTGCACTCTTCTATGCAGCCCAGT

TTGGGTCCATAGTTTCAACCCATCCAGGAAGCTGA

>Unigene0002350

TCTGTTCCATGTTCTTTTCTGTCGGCTTTCATGTAGCAGGAACAATGTAAGATAGTTTCC

TTATCATGCAGATCGCAACAAGAAAAGGCATTGGGATCACTCTTGCGATTTGCAGAAACC

CTTTTTTCTAGAAAACATGGGAATCATTCAAGGGGACGACCTTCAAATTGATTTTGCCCT

TCTAAGACCCCACCTTGATCGGCATGCCTTTTGCCCGACCAACAATAAGTCGTGCCTCAG

GTTCACTGCCTTCATTCGATGTCCTTTTTAAGCCTCAACTCAAGTCGTCGTGCATTGTGT

TATTAGATGAAGTTACGAGTCGCACTAGTGTCCACCTTGGCACGGGTTGACATATCGTTG

AGCATGTTGTCAATGTACATGAGCTCATTGGCCTTCAATTCCTTTCTCTTCTCCCCCACT

TGGCCTTTGAGGGCATTCATAAGTCGCAATGTGCCCATGGGCGTCTCCTCTGGATACTTA

TCTACATCACTTTTTATTGGCGTCTTCATTTGTTGGGCTTGGAGGGCTTGCTTGTGGGGG

GCATTCAGACATCCAATGTGGGTCATGACATAAGTAGCGCCCCGTTTTTGGTCCCTTTGT

CGCCTAACCCTTCTTTGGGGCTTCTTTTCATTCATGTGGACAACTTTCTCCTTGTTGCAT

GCATGGTGTTGCGGAGTTGGTGACATGAGCTTCTTGGTGTTGGTTTGGCTGTCTTGGTTG

TCGTGGTTGTTGAAAGTCATTCAAGTGCTATGTCGTAATGGCGGAAGCAAGATCTTGCAC

TCTTTTGTGCAGCTCAGTTTGGGCCCAGGGCTTTAACCCATTCACGAATCTAAAGAGCTT

GTTCTTCTCTGTCATGTCCCTAATGTCCATCATCAGTACAGAGAGTTGCTTTACGTACTC

CTGGATTGGCCTAGTTTGTTGCAAACTTCTCAACTTTCGTTGAGTCAAAAACTCCACATT

CTCGGGCATATATTGTGCCTTTAGCTTTTACTTCAAGTTTTCTTAGGTCGTGATGTTGGC

CAGGCCTTGATGAATGTCCTCATATCGGGCACGCCATCATGGTTTGACATCATCTGATAT

ATACATTGTTGCTACCCCTTTTGAGGTTGCAATCTGAAGTATTGCTCTAAA

>Unigene0002351

CCATCCAGGAAGCTGAAGAGCTTGTCATTCTCTGCCATATCCCTAATGTCAAGCATCAGG

GCAGAGAATTCCTTTACATACTCCCGTATTGGCCCAGTCTGCTGCAATCTCCTCAACTTC

CGTCGAGCCAAGAACTCCACGTTCTCGGACAGAAATTGTGCTTTCAGCTTTCACTTCAAG

TCTTCCCAAGTCGCGATGTTGGCCAAGCCTTGGCGAATGTCCTCATATCGAGTACACCAC

CATAGTTTGGCATCACCCGACAGATACATTGTGGCCATCGTGACTTTCATTTCTTCCCCT

TCCGGGCGTGCCACCCGAAAGTATTGCTCAAGGTCGAAGAGGAAGTTCTCAAGCTCCTTT

GTATCTCGAGCTCCGCCACAGGTCCGCGGCTCGGGGATTCGCACCCGACCTGGCCCCATG

GCAAGATGGTGATCTCCAGGATGGCGGCTGGTTCGCCGCACCAACTGCCTGCGTGAGTAT

CGTCACTTGCGCGGACAGTTCATTCAGCTGCTCATGCAAGGTTCTGACCGAGTCCCTGAA

TTCATTATTCAAAGAATCAACAGTGTCAGAAAGCTCTTGGTTGCTAGCCTCAAGGATTGC

CACCCGATCCAAGCTCTCAGCAAGCTGTGCTTCGAGCCGGG

>Unigene0002352

AAATATATGTGATTTTTTTATGAAATTTATGCTTTGAGTCGGTAAGTTTGAGAAGTTGTT

TTTTTGTTGGCATGATTTATATTCTTCGATGGGTTTTTATATAGGATACGAGACTACAAA

ATCCCACGTATCACAGTTCTTTTCCTTTTTTATAGGTTTTTATGGGATACACTGTAGGCA

CGCAGGCCTGATTTGTTGGCAAATAAGTTGCAATAAAACTGCAGGGTCTGAGATTCAAAT

CGTGATCTAAGTCCCTTTTTGAGAGGG

>Unigene0002353

TGTTTGAAATTCTTCGATGGGTAGTTGGTGAGGGGATCTACAGATTCTTTTTTGTGTGGT

TCTGCTAGAATATTAATGATGTTGTTTGAAGTTGTATCTGCCAAATCCCACAGCTCTCTC

CCGCTATTTACTAGGTTTATATAGGATACGAGACTACAAAATCCCACGTATCACAGTTCT

TTCCCTTTTTTATAGGTTTTTATAGGATACACTGTAGGCACGCAGGCCTGATTTGTTGGC

AAATAAGTTGCAATAAAACTGCAGGGTCTGAGATACAAATCGTGATCTAAGTCCCTTTTT

GAGAGGGTATGATTTGTTGATTCTATTGATTATTTAATGTTCCAACAGTAGTAATATATT

TATTTTAACTTTTCAATTCAGATTAATTGAGCTGCTGTGATTCACCATAAACGAATCTCA

ATTGTTCTTGCATTGATCTGACTGTTTATGCTTTTATTAATTATAAGTCCTTTTTAAATC

TCTTCTTATAATAGGAGAAGGAGATAGAGTAAGAGAGGAATGAACGAGGTGGAGAGAGAA

CATGCATGATAGATAGGTGGGGAGGACAGAGTTGTCAAATTTTGTGGTACCATAATTTAT

TAATTTTCATTCTTTCAATTTTCATTTTTTTAGGGCATGTTTTGTTTCTTTTTAAAGATT

TTTTTATTTATTTTAAAAACTTGGCAAGGGATAGTTTGATTTTGTTTTACATTTTTGCAC

AATAAGTGTACATCATTCTAAAATACTTTGTTGCTCGTATAATTTATTTTTTCTTAATTT

CATCAATTGATAAATGAAGTGCTGAGAAAAAAAAAATATAATGGATACATAATTTGTCCT

AATTGAAAGATATTTTTAAGTTATTTAAATTTGGTATATATGTCAAGACATCTAATGGTG

GATATGTTAGCCTGATTGATTATTTTCGAGATGGAGGATACAAAATCATTATGAACAATA

ATAAGAATATCAAAATAACAAAAATCTTACTAATAACGGAATTTTTGGTCGCAGGTCTAG

CCCACCCCCAGTACTCAGGTAACTGAGTAGTTCCTTGCTTCTAACACTTATAAATAAATA

TTAACTATTTTAAATTAATAGATATATATTTTTTTGTAGTAACTCAATAATATTTGAATA

TATATATATATATTATTTATTTATTAATAATTAAAATTATATAAATAGGCCTATTTTTTA

TTTTGTCTTGGTTGCATAAGCATTTGGACCAATATGTCTTTATCATATTCATTGTATTTC

TCTTTGATGCTTTACTCTTCGTTAATCATTTATAGCATTATTCTATAATACATCTGTCAA

CATATTTTGTTTGTAGTTTGATGAGCAACAATTGAACTTCCTGGAAAGAGAGCAAACCTA

ATTTTTTTAAGTGTCTTTACATTATCTTGTACTTATTTTTAGAATATATGCCACCCTCCT

TTTATTGGTACTCTTAATTCGAAGATTCTAGATATGAGAGCAAGAGGAGGAAGATACAAT

TGTTGCTCTTATTGGTGATAAATTTTCTAATTCTTTTTATTATGTGTCATATAACATTTA

ATTTTAACTTGTTATTGTACATTAAATTATAAGGTCCCATTAACTTTTGAAATGTTTGAA

AATGAAGTTATTGAATCTAGTTTTGCACTATTAGAGAACCCAAGCTTGTCCTGATTTTAA

GAACTACCTCTCCTTTTATATCTTCCCCCCCCCCCCAACGTAACTTTTGAAAATGAAGTT

ATTGATTCTAGTTTGGCAGTATCAGACCAATTTCAAGAACTACCTCTCCATTTGCTAATC

TCTTCACCTCTTAGATCTATTATGTATTTGAGGATGACATAAAAGCTGTGTTAATATGTT

TTTGTCTAAGGTAGAGTGTTGGAAACACATGTTTTTGTCTAAGGTAGAGTG

>Unigene0002354

TGGAGGGATCGGCTGGAAGGTCCCTGTGTCCTTTGAGCTGCTTCCCCTTCCATGACACAG

AAACTTATGTGATTCTTTTATGAAATTTATGCTTTGAGATTTTTAGAGAAATATATGTGA

TTCGTTTATGAAATTTATGCTTTGAGTCGGTAAGTTTGAGAAGTTTAATATATGTGATTT

TTTATGAAATTTATGCTTTGAGTCGGTAAGTTTGAGAATTTTTTTTTGTTGGCATGATTT

ATATTCTTCCATAAATAGTTGGTGAGGGGATTTACAGATTTTTTTTGTGTGGTTCATTTG

AATT

>Unigene0002355

GAGAAGTTTAATATATGTGATTTTTTATGAAATTTATGCTTTGAGTCGGTAAGTTCGAGA

AGTTTAATATATGTGATTTTTTATGAAATTTATGCTTTGAGTCGGTAAGTTTGAGAAGTT

TTTTTTGTTGGCATGATTTATATTCTTCCATAAATAGTTGGTGAGGGGATTTACAGATTT

TTTTTGTGTGGTTCATTTGAATTTTAATGATGATGTTTGAAATTCTTCGATGGGTA

>Unigene0002356

CCACCGGATCCAGTCCCTTCCTAGGGCTCCGATCGGAAATCCCAGGGTTTCAACCGATCG

ATCGACCGAGCGGGAAACGCAAACCCTCGCTGCCGAGATCTCCGCAAACCCTAACCCTAG

CCTTCGCTCCGGCGATGGAGCTGCAGAACACGGTGAAGGAGGCCCTGAGCGCCCTCTACC

ACCACCCCGACGACGGCGTCCGCTCCCGGGCTGACCGCTGGCTCCAGGATTTTCAGCACA

CCATCGACGCCTGGCAGGTCTCAGATAATTTACTTCATGATGATAGCAGCAACATAGAAA

CTCTCATCTTTTGTTCTCAGACTCTTAGAAGCAAGGTGCAACGGGATTTTGAAGAGCTAC

CTTCTGAAGCATTTGGACCTTTGCGCGATTCTTTATATAAATTGCTAAAGAGATTCAACA

AAGGCCCTCCTAAAGTTAGAACACAGATTAGCATTGCAGTTGCTGCATTGGCCGTGCATG

TGTCTGTAGAAAAATGGGGAGGTGGTGGCATTGTGAACTGGTTGAGTGATGAAATGAGAT

CTCATCCTGAATATATCCCTGTTTTCCTAGAGGTTCTCACCGTCTTACCACAGGAAGTTT

TTAGCTACAAGATAGCTGCTCGTCCTGAAAGACGCCGCCAATTTGAGAAGGAACTCGCCC

TATCAGCCGAAGTTGCTATTGGTCTGTTGTCTGCTTGTTTGGGTTTTGATGAATTGAAAG

AGCAGATCCTGGATGCATTTGCTTCATGGCTTCGTCTGAACCATAGGTTCCCTGCAGCTA

TTCTTGCTTCCCATCCGCTGGTGCATGCAGCCTTGTCATGCTTAAATTCTGAGGATCATC

TGGAGGGAGCTGTCAATGTTATATCAGAACTGATACACTATACTGTGGCTGGGAACTCAG

ATGGTTTGCCTTCACAGATGCCTCTAATTCAGGCTGTTGTTCCCCATGTTATGGGCCTTA

AGGAGCAACTTAAAGATTCTTCTAAGGATGAAGAAGACGTAAAGGCCATTGCTCGTTTAT

TTGCAGACATGGGTGAGTCATACGTCGAGTTGATTGCAACAGGTTCGGATGATTCTCTGC

ATATTGTACAAGCATTGCTTGAAGTTGCTTCTCACCCTGAATATGACATTTCTTCTATGA

CATATAACTTCTGGCACCACCTCCAAGTTCACCTGAATAGAAGGGAGTCTTATGCATCAT

ATGGATCTGAGGCAGCCATTGAGACAGAGAGGAACCGGAGGCTACAAGTTTTCCGCTCAC

CATTTGAATTACTAGTTTCTTTCGTTAGTTCCCGTGTTGAATATCCGCAGGACTTCCTGA

ACCTCTCTGAAGAGGACCACAGGGATCTCAAGCAGACCAGATACGCGGTCAGTGATGTAC

TAGTGGATGCAACTGAAGTTTTAGGAGGCGAGTCAACACTTAAAATTCTCTTCAAGACCT

TGGTACAGGCAATAGAAAAGTGCAGAAATGAGGGCCAGTGGCAGCCTGTAGAAGCAGCTC

TCCTTTGTATTCAGGCAATCGCTAAGTCAGTTTCTTGTCAAGAAACAGAGGTTATGCCCC

AGGTTATGATGTTGCTTCCGAAGCTACCAGATCAGCCTCAACTTTTACAGACAGTTTGCT

CCACCATTGGGGCATATTCGAAATGGATTAATTGTGTGCCAGATGAACTTTCAATCCTGC

CTCCACTGGTTGATATTCTCACAAAGGGCATGGGTAATTCTGAAGACACTGCATCAGCTG

CAGTTCTTGCATTCAAGTACATATGTGAAGATTGTAGCAAAAAATTTTATGGATCACTGG

ACGGCCTTTTCCATATCTATCATATAGCACTCAACAGTAGTGGGAGCTATAAAATATCTG

CTGATGATTCGATGCATTTAGTTGAAGCGTTGAGTGTCGTTATAACGGAAATTCCAACAG

AACATGCTGAAAAAGCTTTAGACTTGCTCTGCTTGCCTGCTGTTGGTACTTTACAGGAAA

TAACTAATCAAGGCAGTGCATCCTTGAAAGAAATACCTGCACGGCAGCTTACGGTTCATA

TTGACAGACTTGCATGCATTTTTAGAAATGTAAGCCTTCCAGAAGTTGTAGCAGCAGAAA

TTCAGAAAATATGGCCAATTTTAAAATCCATCTTCGACAATCGGGCATGGGACATGAGGA

CTATGGAATCATTATGCCGCGCTTGCAAGTATGCAGTCAAAACTTCTGGAAGGTTCATGG

GGGTCACAATCGGGGCAATGCTGGAAGAGGTTCAACTTTTATACCAGCAACACGATCAGC

CTTGTTTCCTTTATCTATCCAGTGAAGTTATAAAGATATTTGGGTCTGATCCATCTTGTG

CAAATTACCTTAAAGTTCTGATCGAAGCCCTTTTTGGCCATACCATTAAACTTCTTATGA

CCATCAATGATTTTACAGCCAGGCCGGATGTAGCAGATGACTGTTTTTTACTAGCTTCCA

GATGTATGCGCTACTGTCCTGATTTATTTGTTCCTTCCACCATATTTTCTTCATTAGTTG

ACTGTGCATTGATTGGAGCTACTATACAGCACAGAGATGCCTGCAAATCCATATTGACGT

TCTTATCTGATGTCTTCGATCTCGTAAACTCTTCTGCCGGAGAGAAGTACCGGCATATTA

CAAACAGTGTGCTACTTCCCCGAGGCGCCACTCTTACCAGAATTCTGATCGCCTCCTTCA

CTGGAGCACTTCCATCATCTCGAGTAGACGAGATAGCGTATGTGCTACTAGCGTTGACCA

GAGCTTACCACATCAAGGTGTTGGAATGGGCCAAGGGAAGCATCTCGTTGATACCACGTA

CTGCCTTAACAGAGGTTGAAATTTCGAGCTTCCTTAAAGCTCTATCAGATGCGGCTTTAG

GGAATCCAGGTTCGGATTTGACGAATACACTTGAGGAGCTGTCGGAGGTTTGCCGACGCA

ATAAATCCGTCCAAGATATTGTTCAGGGGGCCTTGAGGCCTCTCGACTTGAACTTCACCA

TAGTTTCCTGATTCGTAAAAGAACTGGAGAGGCAGTCATAGTTTGATTGAAGCGACTTTC

CTCGTGCTGGCTATATATTGCTCAGTTATTTACATGATAAGGGTGGTGGGCAGTGAATGT

TTGTTTATATAGGTATTTGCTGCATCACCCAATCTTATAGAGATGCGACTTATTTGAACA

GCAATTCTTTGATGAAATTTTCAATCAGTATATAGTACGCGTGTCATCTGTCTTATTATT

GCTTTCGTGAGAGTAATTTTTCTTGCAAATCATTTTGTTAAGGTTTTTGGTTAATATTTT

TTCAAATAGATATTGAAATA

>Unigene0002357

CACCGCGGAGATCCGCGACGCAACTTCGAGAGGCGGCAGTGCCGCAACGGGATCACTCAC

CCCCCGCCGGTATGGATCTTCTGCCGAAGGAGACGCCGGCGCTGGCTCGCCGCCAGGGGT

TCCGGTCGCTGAAGCTGGTGAGCGTTCCCATGGACGAGCCGCTCTCCGAGTCACCGGTCG

GCGTCGCGTACGGCGTGCTCGACAACGGCCTCTCCTACTACGTCCGCGCCAATCCGAAAC

CGCGGATGCGCGCCGCCCTCGCCCTCGCCGTCAAGGTCGGCTCAGTTTTGGAAGCAGAGC

ATGAGCGCGGGGTCGCTCACATTGTTGAACATCTTGCATTCAGCGCCACGAAGAAATTTA

CAAACCATGATATCATTAAGTTTCTGGAAAGCATTGGGGCAGAATTTGGTGCTTGTCAAA

ATGCTTTGACTTCTTCAGATGAAACCATATACGAGTTATTTGTTCCTGTAGACAAACCTG

GACTTCTATCCCAGGCCATATCAGTTTTGGCAGAATTCAGCTCCGAGATACGCGTTTCCA

CGGAGGATCTAGAGAAAGAACGAGGTGCTGTTTTAGAAGAGTACAGAGGAGGGCGCAATG

CTTCAGGACGGATGCAGGATGCGCACTGGGTTTTAATGTTTGAAGGTTCAAAGTATGCAG

AGCGTCTACCAATTGGGTTGGAAAAGGTGATTCGGACAGTTACTCCTGAGACTGTGAAGC

AGTTTTATCAGAAATGGTACCATTTACACAACATGGCTGTTGTTGCTGTTGGGGATTTTG

GTGATACTGAGAGTGTAGTTGACTTAATAAGGACCCATTTTGGGCAGAAAGTTCCTCTGT

TTAATTCTCCCCCAGTCATACCGGATTTCCCTGTTCCATCTCACAATGAACCTCGCTTCT

CCTGTTTTGTCGAGTCAGAAGCTGCTGGCTCAGCAGTGGTGATCAGTTGCAAGATTCCGG

TTGATGAGATGAGGACTGTAAAGGATTACAGGGATTCCCTTGCAGAGGCTATGTTTCATT

GTGCTCTAAACCAAAGATTTTTTAAAATATCCCGTAGAAAAGATCCACCTTACTTTTCTT

GCTCGTCGGCTGCAGATGCTCTTGTTCGTCCTGTAAAGGCTTATATAATGACCTCAAGTT

GCCGAGAAAGAGGGACAGTTGAGGCCCTTGAATCAATGCTGATGGAGGTTGCCAGGGTTC

GGGTTCATGGTTTCTCCGAGCGTGAGATCTCCATTGTACGTGCTTTAATGATGTCTGAAA

TTGAATCTGCTTATTTAGAGCGTGATCAAATGCAATCCACATCTCTAAGAGATGAGTATC

TGCAACATTTTTTCCGCAAGGAACCTGTTGTTGGGGTAGAGTATGAAGCACAACTACAGA

AAACTATCTTACCTCATATTTCTTCATCCGAGGTATCAAAATTTGCTGTGAACTTCTGTA

CGACATGCAGTTGTGTAATAAAGATAGTCGAACCTCGATCTCAAGCTACTTTAGAAGACT

TAAAAGCTGTTGTGTCCAAGGTTAATTCTTTGGAGGGAAAAGAAAGTGTTCATCCCTGGG

AAGATGAACTTGTTCCGGAAGAAATTGTTATCCTCAAGCCAAACCCTGGGTGTATTGTAC

AACAATCAGAGTTCCCTAGCATTGGTGCTACCGAGTTACTTCTTTCAAATGGTATGAGGG

TGTGCTATAAATCCACCGACTTCTTGGATGATCAGGTCATTTTCACCGGATTTGCATATG

GTGGTTTGTCCGAATTATCAGAAGATGAATATTTTTCATGCTCAATGGGGTCAACCATAT

CTGGTGAAATTGGTGTATTTGGTTACAAACCATCAGTGCTTATGGATATGCTTGCTGGTA

AAAGAGCTGAAGTCAGTACTAAAGTTGGGGCATACATGAGAACCTTTTCTGGCGATTGTT

CACCCTCTGACCTTGAGACTGCATTGCAGCTTGTCTACCAACTATTTACCTCAAATGTGG

AGCCGAGAGATGAAGAAGTAAAAATAGTGATGCAGATGGCAGAAGAAGCTATAAATGCTC

AAGAAAGGGATCCATACAATGCATTTGCAAATCGTGTACGGGAACTAAATTATGGGAACT

CGTATTTTTTCCGGCCAATTCATATTGGTGACCTGAAGAAAGTGGATCCCATAAGAGCTT

GCGGATACTTCAGTGATTGTTTTAAGGACCCTTCAACTTTTACTGTTGTGATAGTTGGGA

ACATCAACCTTACAGTATCAATTCCTCTAATTCTGCAATACTTGGGTGGAATACAAAGGC

CCTCTGAGCCTGTTTTTCATTTCAATCGTGATGACTTAAAGGGTCTGCCGTTCAAGTTTC

CTGAAACCATTATTAGAGAGGTTGTGCGGAGTCCTATGGTGGAAGCACAGTGCTCTGTCC

AGCTAGCCTTTCCTGTGGTGCTGAAGAACACATCAATGATGGAAGAGATTCACTTTGTTG

GATTTCTGAGTAAACTTCTGGAGACGAGAATTATGCAAGTTCTCCGGTTCAAACATGGGC

AGATATACTCGGTGGGTGTTTCTGTCTTTCTTGGTGGTAATAAACCTTCAAGAACTGGAG

ATGTCCGTGGTGATGTAAGTGTAAATTTTTCATGTGACCCAGACATCTCAGCTAAACTTG

TAGATCTAGCCTTGGAGGAGATAATATTTCTTCAAGATCAAGGTCCGTCAGATCGGGATG

TCTCAGCTGTCCTTGAAATTGAACAGAGAGCGCATGAAAACGGATTACAGGAAAATTATA

ACTGGTTGGATCGAATTCTGCGTAGCTATCAATCACGATCCTATTTTGGTGATCTGAGTG

CTTCATTTGAGATTCAAGATGAAGGACGCATAAAAGTCAGAAATTCTTTAACGCCATCTA

CAGTACTGTTGGCATTACGAAGAATATTACCTTTTCCATGCAGAAAGCAGTACACTGTTG

TCATTCTTATGCCCCATATATCACGGTTGAGGTTATTGAAGTCTTTACTTCTGTTGAAAT

TCAATGGATTTAGCAGAGATGCAAAGATTATGGCTGGCACAGCTGGTGCAGTGGTGCTAG

CAGTCAGTTTATGGAGATATTCACGAGGTACATTGAAGCCGTAAGATTCTGCCTATACCC

GTGGAAAAAGGGCATGGCGGGTGGTTGATGTACAATACTGCCAAATATTGCCATGCTGAA

TCCAGCGAAGATAGGGGGAAGCTATTGATTTAGCAAAATTTTGTCAATCAGGGAATTGAG

CGAATGCTCTTGAAAGTAAGCACTTCCACTGTTTCGGTATTCATACGAATTGTTTCAGGA

GAAGACACCCTGATGGCTTGAAGACGTGGAATTTGGAATTCCTCTCCCGCTGACAATGTA

CAAATATGCAAATGCAGATCGTTCAATTAATGAAAAGAAAAAATTAATGTATTTTTGTGC

CTATCATGCAATTATTCGAATTGCTCTGCTGTAATTTTGAAGCTATTATATATTATATTA

TATTATTAACCAATTGATCCAAGCATCGTGAATATCCATCAGTAGACTTGAAGCTTCAAA

CCAGAAGCTAAATCTTAACCAAGTTTGAATGTTATAATTTTGCAATTGATCCAAG

>Unigene0002358

TTTCGATCCAACTGGTTATGGAGCGGCAATGGGAAGGATGGGAGCTGGGTTTGGTGGTTT

TCCTGGAGGTCCTGCAAACAATCCGTTTCCTGGAATGATGCCTAATTTCCCCCCAGTGGC

GCCTCATGTCAATCCAGCTTTCTTTGCTAGGGGGATGCCGCCTGGCAGTGTTGGAATGTG

GCCTGATCCCAGTATGGGTGGATGGGGAGGGGAAGATCAATCAAGCTATGGTGAGGATGC

AACATCAGATCAACACTATGGAGAAGGAAGCCATGGTAAGGATAGGGTCTCCGATCGGGA

TCGGTCAGGTGTTATGGATAGAAGGCATGATAGGGATAAAGATATGGGTCCCGGGCAAGA

TTGGCCTGAGAGAAGGAACCATGAAGCGAGAGACATGAACCGTGAAAGAGATCGAGATTG

GGAGAGGGAGAGGGAAAGAGAGAGGGATAGGGATAGGGAAAGAGACAGGGAGAAGGATAG

AGAGAGGGACAGGGAGAGAGAAAAAGACAGGGAGAGGGAACGAGATAGGTACAGAGATGA

CCGAGACAGGCATGGCGACCATCACAGGCACAGGGATCGCGACCAAGGGCGTGATGAACG

GTCAAGGGCTCGTAGCAACTCTCGGGACATTGAACATGGGAAGAGGCGGCGTCCATCTGA

ATGAAGTCTGTCCTCTGTAGCTGTCTTGGCATTGAGTTGCTTGCAGGGGTCTGTTGTAGC

CACTGGGGTCACAGCGGGATGTGTTTGGCTACAGATTCAGGCCCCTATTATTGTGGACGC

CAATCTCCTCTTTTGAGCTTTGAACTATTTTGGGTGCCATCTGCAACCCTTGAACTTAAC

CCCTATTTAATACTTGCCACTTTCATTTTGGAACTTCTTGAATGTATCAACTTTTATGTA

TTGGATGTCTGAATGCAGTGTAAGATACTATCTTGAAGACTGAAATGGTCTTTGTAAAAT

ACCCGGGGATTCATTACTTGCCCCTTGTTTGGTAAAGGGTATCACATCCCCGGATGAGCG

GAGTCCTGTTTTGATTGTTTTGAGCTGAATTGACTCTGCGAAGACATCCTCATACCTGAA

AAATGCTGATACGAGCAACACAGCTTGAAGGCTGGAGTCGCTTATCCATTTTTGAGTTAA

TATTAGTGCCCTTCATTTTTTGTTGATGACTTCCCTGTTACCCCATGTTGTAATGCTTTG

TAATGATTTTTAGAGTTTAGTTTTCTATCCATAGAGGGAGTCAAAAGCTGTTTCAAGCCA

TGTAATGCCTTCCAACTTCTGTTTTTCACCCACCTGTATTAGGCTTTAGGCATTGTAATG

CTTTTTAAAATTTAGTTTCCCCTCCATGTTGGGGGTTAAAAGCTGTATTAGGCCGTGTAA

CTCTTTCTGAGGGTTAGATGTATTCTTTTTGTTGGCCAAATGATTCTTTGTATCATAGGT

ATACATTGTAATCACAATCCAATTATTAATATAGTGTTGT

>Unigene0002359

TTCAAGACTAGGGTTTCGCCTCCCGTCACGCAAACCCTAGCCGGCGATCACCACCATGGA

GGGCGGCGGCTTCCACCACAACGAGGCGATATCCGCCGTTCAGGACGAGGATCAGTACTA

TGGCGAGGACGACGACTACGACGACCTCTACAACGACGTCAACGTCGGCGAGGGCTTCCT

CAACTCCCTCCCCCCCAACAACGGCGGCTTCATCGAGGAGCAGCCTCGCAACGAGCCTCC

TCCTCCACCGCTGCCGCTCGCACCCCGGCCGCCTCTCCCTGTACCGGAGCCGATGATCCC

CGGGGTCGCCGGTGAGCCCAAGGCGGAGCATCCCGTCACCGGGTTCCCCGAGGCCGGCTT

CCGGGGAGCTATCGAACCGGCCCCGCGACCGCCGGTGATGCTGGGGCAGCCCCCTATCAG

GGACGCCCAGATCCATGACGGTAGGGACATGTACCCGAGGCAGGGCAGTGGCTATGGGGT

AGGGGGGAGTGTTAATGGCAGTGGAGGGCCTGGTGGGACTACTCTCTTCGTCGGGGAGTT

GCACTGGTGGACGACGGATGCTGACGTTGAGGCAGAGCTGTGCAAGTATGGACATGTGAA

GGAGGTGAGGTTCTTTGATGAGAAGGCTAGCGGGAAATCGAAGGGATACTGCCAGGTTGA

TTTCTATGACGCGGCAGCGGCGGCTGCCTGCAAGGAAGGGATGAACGGGCATGTCTTTAA

TGGTCGTCCATGTGTTGTGGCGGTGGCGGCGCCTCATATTGTTCGCAGGATGGGTGAGGC

GCAGGTGAACAAGAATAGGAACCAGTTGATGACGGCACAACCTCAACCGCCGATCGAGCA

GAAGGGGAGAGGTGGGGGTGGACCTCCTATGGGTGGAAACTATGGCGGCGGCCGCGGTGG

TGATGGCGGCGGCCGTGGTGGTGGCGGGAGAGGTTGGGATAGGGGTGGAATGGGTGGAAT

GGGTGGAAGAGGGCCTATGGGGAATATGAGGAACAGAATGGGGCCAATGGGTGGCAGAGG

TATGATTGGGAATGGCGGTGGAATTGTCGCCCCACCACCACCAGTTATGCACCCCGGGGC

TATGATGCCTAATTTCGATCCAACTGGTTATGGAGCGGCAATGGG

>Unigene0002360

GCGCCCCTCTAGGGTTTACCCCCCGCTCAAATCCGACGCTGCGAGCCCGCATCTCCACTC

CCCCACCAGGGTTTTGGATCCACAGATCCCGGGCTGACAGTTTCAGAATTTCCAGGCTCT

ATTCGAACTCATCACTCCAAAATCTAACAAATCAGCACCTATCGCGCTTGAAGTTAAAAT

CTTGTGGAATTCTAGGGTTTTTACAGATTCTTGATTCTTGCAGGAGATGTACGGGCAAGG

GAGCTATGCTCCTCAGTACCGCCAGGGGCCGCCGGCCCCTCCACCCATGTACCAGCAGCC

GCCGCCGATCGGCCCGCCGCAGCCGATGCCGTACCAGCAAGCCCCGCCGCCAGGACCTCC

GCGTCCGCCGGTCTACCAGCACGGCCCGCTCCCTCCCCCGCCGGCGCCTCCGGTGAACAT

GGGGCAGCCCTACATGCACCCACCCCCACCCCCCCACCAGCCTTCCTATGCTTCTTATCC

TCCACCGCCACCGCAACAGATGTCAATGCCACCGCCCCGGATGCTACCACCGCCGCCACC

GCCCCCTCAAGGGCAGGTTATGTACCGCCCCCCGCACATGTTTACACCTCCTCCTCCGCC

TCCGTCTAGCTTTATGTCGCCGTATCCATCTTTTACGCCGGTCGGGGAAGCACCATTGCC

TCCGCTTCCGGCTTCTCCTCCGCCGCTTCCGCCTTCGTCGCCCCCACCGCCTCCGCCGCC

GCCACCGCCGCCTCCGGCTACAGCATGCTCGCCGGATGTGCCTGATGTTGAAGAGGAGCT

AAACCATCCTACTTCACCACCACCGAGGCCGGATGATGAAGTTGTTCATACTATTGAGGA

ATTTTGTAGGTTAATTGCAGATAATGGGGCGGAGTTTGAGAACTTTGCTACTAAGAAGGA

GGCTGAGAATCCTGATTTTGCATTCTTGTTTGGAGGGCCACCCGGGAGTGCTGAAGCTAT

CGGGCATGAGTATTATCAGTGGATGAAAAGGAATTATAAATTTGTAGAGAAGTCACAGAA

TGATGTACACGATGGGCCGAGGACAGGAGACAAGGATGGTGCCTCACAATCCTCTGACCC

GGAAGATGAGGAAACGGCGGCTTCACCTGCAGTATCTGATATGGATATGGAGGATGATGT

CACCCCTGACGGAGACAGTGGATTTGACAATGTGACTGAAATATCAACCAGGGAGCCAGT

ATCTGTAAGCAACAAAACGGATAGTGTTAAAGAGCCTTCGCATGTACTTCGAAGCACAGC

GGAGAAGCTTGTCTTACCACCGGGGAGTGTTTCTGAAGATGATGTCAGCCCCAATGGACA

TTGGGGCATTGATGCTGTCAGAAATAGGTCAGCCAGAGACATGGTACCTGGAAGAAATGA

CATGGATGCTGAGCCTTTGCATGAATCTGGGAGTACAATGGAGCTTGTGCAAGAAGTAGC

TGTATCTGGCACCTTAACTTGCCCAGACTCGTTGCCACTTCTGCAGGATGGTAGAGGTGA

CAAGGGTTCTGCATTTACTGAAGAAGACATTGTGCATCAACCGGCGGTGACATCCCTTTC

AGTTTCAAATATACAGCAGGCTTCATTGGCTATTGATGGGGGCAAAGTCGAGCATATATC

TAGGGTTTTTGTTAAAGACCGGAGCCCATTTCGATTAATTCAAGGTTATGTCTCGGATGA

TAGCGATGAAGATATTAAGATGGAGACTGTTGAGGACATCACAACTGGCAGGGATCTGTC

AAAGGCAGATGATAATGTTGAACTTGAGAAGGCCTCGTTGACTGAAGTTGGACAAAAAGT

TTCAACTGGTCTAGGTCAAGCCACGTCCGTGGTGCCAGAAGAAGCTGAACTGCTCTCTGA

TTCTTCTCAGAAGACATCTGTTTCACCCAGTATTGTCTGTCCTAGTCGCTTTGATCCCTC

AGTTGATACTGCAGGAACATCCGAACGTCTTCAGAGCATTAAAATTGTTAACCAAGTCAA

AAAACTTAACGTTGAAGAAGATGTACAGGTCACTGCCAAGCCTGATGTGGATGAGTTTGG

ACGGTTGGTAAGGAAAGGTTCCAGTGACAGTGATTCGGATGGCCGGGTTGACATTCAGAG

TGGTAAGAGGGGGAGGAGTTTGAGCCGCAGTCGGTCTCCTCATGGAAACAAATGGAGGCG

GACGAGCCGCAGTCCTAGGGGGAGAGGCAAGCGAAGCACGTCTCCCAGGTCGCCGGGGCC

GAGCAGCAGGTCTCCACCAGCTTCTAGGCGGATTAATGCGTTTGCAAGACGGGATCGGGA

TCAGCCTCCGGAATGTTTCAACTTTCTTCGAGGCAGGTGCTTCCGTGGACCATCCTGCCG

ATTTGTCCACAATGAGTTTGGTCGGCAAGGGAGCAGGCAGCAACAATATCAGGAATATCT

TCAAGATCGTGAAAACTATGATGTGCATGTTGATGGTCCAGTTTCTGGAGATATCTCTAT

TGACAAAAAGATGGATATTAAGGAATGCAATGATATTCCACAAAAGGAAGACAACTCGTC

AGAGATGCAAGCCTCCAAGGATCTGTCTGAAACCAGCATCAAGACTTCAAGTGATGGCAT

TCTTGGAGAGAGAGATGCATCCCACTCTGTTCTAGAGGGCGCAGAACCTCCTGTAAACCA

AGAGGTTGGTGAAATAGAAGTCCCTGATCAGGTTTCTAAAGAGGTAATATCTGAAGTGCA

AGGGCCACAGGTTGAAAATGTAGAGAAAACATCTGAACTTCCTGCTGAAGAGGAAGTGAA

GTCAGTGCCATTGCCAGAGTCTTTTCAGCTTGGGCAAGCTGAGAGTTTGCCAATTCAATC

TGCTGAGAAGAGCCAGACCCTTCCTGACGTAATGTCTGTGCAACAAACAAACTCCAGTAG

AGAAACATATTTTCAGCTTACTCAACCCAGTTCGGTCTCTACGTCGACTCCAGAGCAATC

ACAAAATGTTACTCAATATACTCGTATGGAAGATGATATGCAAGCTGTTCAAGCTAACCA

GGCCACATCTATCCAAAATCAGATGCCAGAAGGTCAACCATATCCATCCCAAGCTCCAGT

AAGTGCAGTATATCCTGACCAGACCCAGCCATTTTCTAATAATGCCTCTATACAACAACC

TTCTGATCAATCTGTGCCTCCTAGAGAATTTCTGCAACCTAGTCTCTTGGCCTCAGGTCC

TAGTCTTCTGCCATCTCAGTTGCTACCGCCTGGACCTAATGGACAACCCCCACCCAGTTA

TCCTGGAGCTAATGCAAGTTCCTTTTCCATGCAAAATTCAAGTGAGTTTCTTCCACCACC

TGTTCCAAGTCACCAACCTCAGCACCCCCACTCACATACATTTCCTTCCCATGGGCCTAT

AACCGGTGACTATAATCCTTCAAAAGTTCATCCTCCAAACCCTGCCTGGTCTAATCTCCC

TCCACCACCACCACGACCACAAAATGTAAGTTCTGGTCCTGGATGGCCTTCCTTTCCACT

CTCGGATCCTTCCCTTCCGCAATTTCAGCAGAATGCCTTACTTCCCAGGAGTGACTTTAA

TCCTGCAATGAGGTCTATTCCCCCTGGAGAACTGCATCGTGTTCAGGTACCTGATCTCCG

TCGGTTCTCATCGATGGAGACACAACATCCGCTGCATATTGATGAATTTAAGCAGATGTC

TTTACCTGTGAGAAACCAGCAGGATCAACCATTTGCCAGAGATGGCCGATATCTGCAGCC

TCCAATGACTGGGCCTGACTCTCAGAGGGACTTTCACATGCATCATCAACCTTTTATTCG

GGAAGATGTTAGAACACCGTTACCTGGCAACGGTTTCGGGCACCCCCAGTCTCTGCCGTT

TCCAAGAGAGCAGCTTCCAAATAGACCATATTCTTTCGCAGAGGACAATCTGCCTCCAAG

GTTTCTTCCAAGGGAACAGTTTCAGAATATGCACGATTTTCAGCGTCAACCTCAATTTGG

CCACCAGCAGTCTACCAACAGTAATGTTTCTCTGAATTATCATACACCAGGAATGGTTGA

TGCCCCTATCACAAGATACTCATCCAGCTTTCTTGAGAGTAACTTGGCACCTCATCCTTC

TGGCTTACCTAAAATTCCAATCTCATCACACTACAATCCCTATGCATCAACTTTCGAGCA

GACACCTGCAAGCTTTAAGTTTGGTCCCAGTGTTCCTGGACGAGAGAATGATGCCAGCTT

TATTAACAGATATGAACCAGGACATGTTTCAGTCGGTGAGACTGCTTCAAGAATCACGGC

TTCACCACCCAATTATAGTAAATCCACCGAACAGTTTATACCAAGAACTGGCGTCAATCC

TCCTGATGCCCCTGCAGAGGTGTTACCTATGATACAAAAGCAGACCGTGAAGGATTCAGC

TTCTGGAGATGCATATGATCCACTGTTTGACAGCATCGAACCATCTTCTAAAATTTTCAA

CAAGGAGGATAATGCTAAAGAAAATGTGGATATTCTTGTTGGTGAGAACAGTAAGCAGAA

AGAGGTCATGACTGTTGAACCTAAGTCGGAAGCTGATGAATTAGGTGAGGTGGCGACAGA

TGCAGAAGTGGGTGCTGTGGAGAATGGGAGCCCGATTCTTGTAGATGGTAAGGACTGGAG

CCCCCCTGGAGTTCCAGCTGATTTGATAAATGTTGCCGGGGGTGAGATTGAGATTGATCA

AGTCCGATCTCCAGGAAAGAGTAAAAAGACCAAAGATTCCAGGTCGATGAAGCTTTTCAA

GATTGCTCTTGCAGATTTTGTTAAGGAAGTTCTCAAGCCGTCGTGGCGCCAGGGAAACAT

GAGTAAAGAGGCATTCAAGACTATTGTGAAAAAAACAGTGGAAAAAGTTTCTGGAGGAAT

GCCAAGTCACCAGGTTCCCAAATCACAAGCGAAGATCAACCAATATGTCGAGTCATCACA

GCGGAAGCTGACCAAACTGGTGATGGGATATGTTGACAAATACGTCAAGGCATAGATTAT

GGTGGATTGTCGTGGTGTAGTATCTTAGACTGGTTTTATTTCGTCTTGTTTGATGATTCT

CCCTGAGATTTGGAGACCTGTTGATGCAAGGAAGTCTGATAGCAAGAGGACCAGGGCAAG

AACCCCTCATCCACTGAACATGCATATCTGTTTCGAAAGATCAGGGACTGGTTATTTGCA

ACTCTGTCGAAGCAACAGAAATGTGTAAACTCATTTCTCTTCTGGGTGATTGACTTTTTG

CCTGTTCCTTGCATAATTATCAATTGATAGCCTTTTGTAAATTGTTCGTTCTATACTTTT

TATCTGACTAGATTAAGCTCTTTTCCCGTCCCCACAAGGGATCCCCCATAACATGAATTC

ATTGTGTGCCCATATTTTCTAATTTAGGCATAATGACTTGTATGCTTCTTTAGTTGACTA

TATGTATGATGGTACACGGA

>Unigene0002361

CCCCAAGCTCAAAGGGGACTCCTTGAAATCCCTAAATCCATCAAAAGAATCGATCTCGAT

ACCAAATCCCCCAATCTCGAGGGGTCAGTGGGGAGGTGATGGCACTGGATAGTTCTACCG

CCAAAAACTTGACACTATTACCGGCATATCTGAAGGGGCGCGAGAAAGAGGTGAAGGGAT

TGAGTTCTGTGAGTCCCATGATGGTGGATTGTTCCGATGCCGTCGAGGGGTTAAGGAAAC

AGTCGAAGCGGAAGAGGGCTTCGAGTGATGGGAATGCGATGAAACGTGACAAGGAAGTTG

TGATTGCAGAGTGGAATTGGGAGCTTAATGAGCTGTTTCAACTATATAAGGAAGTTTCAG

GTGTCAAGTTGGGTTTAGAAGGGAGTTCTGGTGCTTTGAAGAATGACGTTGCATGCTTGC

TAGAGGAAAGCAGCCTTCCTTTTTCGAAGTTGGTGGAAGAGATTTATGAGAAGCTCAAGG

TGAGAGAGGGTGTTATGTTGTCTAGTTTAAGAAGTATTGTGCTGGATGTTGGGGTGAGGG

TGATGTATGGGATCACAGACCCAGAAGCCGACGTGCTCGAAGATGTTTCGGAGAAGCGAT

TATGGTGCTGGGAGGCAAGAGAGATGAAAGTGTTACCAAAGATTGAACGCGGTATTTTGA

CTGCTCGACGCACTATTCGGAAAAAAATTCATGAGAGGATTACTAATATTTTAGAAATGA

CGGCTACGTTATCAGTTGCAGAAAGCCATGAAAGTTGGACAAATGATACAGTGAAGACAG

CTTCAGTGAAGCTTGGGAAAACTCCAAATGGGAAAGAGATCCGTGCATTGTTGGAAAAAT

TAAAACAAAAGAATGATGCTAACATTGTTGCAAAAGAAGCCAAGTTGAAAGAGGAAGAAA

AGAACAGAATAGAGCACCAGATTCAGAAGGATAAAGGCCAAAGAGAACTCGGCCGGCTGA

GTGAGGAGTTTGACAAAGAAGAAATGCCTCACGAGAAAGCAGTTGAATGCAGCACACCAA

AGAGGCAACAAAAAGAAGCCGAGAGGAATCAGCGGCGCCGGGAAAAAGAAGAAATTGAAG

CGAAAAAGCAGAAAGCCATAAGAAAGCAAGCATCACTTATGGAACGTTTGTTTAAAAGTA

AACCGAACATTAATAATCCAGACAAACCAGAGCGAGGATCATCCTCAAATGATCCATTGT

CTGACTCAGGCAAAAACGGCGAGGTGGCTAATGCAACTACTACATCGATGGACTGTTTTT

TCTCTGAGCAAGGGATCTTGACTATTGAGGACCTCCAAAAGTCAAATGTTGTTGGTTGGC

ATAAGTTGCGTCATTGGAACAGATCGTGCCGGTGGGGTGTTCGGCGCAATCCTAAGATTC

AGTTGATTAAGGAGCTAAAGTTGCAGCAATCATCTTCTGAAGCTCAGCTTTCTGAGAAGG

GTTCCAGTTCAAACACGGGACATGTCGCTTCCAAGAGAAAACTGATGCAGTTTGACAAGA

GCCACAGGCCTGCATATTATGGTACCTGGCACAAGAAAAGCGATGTAGTGGGGCCACTAC

ACCCTTTTAAGAAGGACCCCACTTTGGATTATGATATTGATAGTGATGAGGAATGGGAGG

AGGAGGATCCTGGTGAAAGCCTTTCTGATTGCGACGATGACAAGGAAGAGAGTTCAGATG

AAGAAAATTTAGAGGGGGAAGATGAAAATGAAGACGGTTTTTTTGTTTCTGATGGTTTTC

CCTTAGAAGACGAGGTATTCCTTGTTGATGTTACTTCAGATGCCATACAGGAAGAGGCCA

GATACTCCTCTTATAACCCACAGCTTGAAAATGAGGAGTTCAAACAATCACTACGGTTTC

AGATGTATCTTCACAATTTGACGGAGCAAGCTCTTCGAAAGGACCAGCCATTGGTTATAT

CGAATCTTATGCATGAGAAATCTGAATTGGCGATGGCCAAAGATCTCAATGGGATAGCTA

AACTCGAAATGATGTGCCTGCAAGCTCTCCGCATGCAGGCTTCCCCAGGTGGTTCTGTTA

TAGATATTCCAACAAGTCAGTACTCATCAAGTGAGGATCAACAAGTCTGTCAATCCCAGT

CCAGAAAGAGCAGCACACCCAAAGCTATTGGAGTATCAATCCCAGATTCTTACTTGCCAG

AATTTGTGCGGCGAATCCAGTCAAACTCCAGTGGGATAAGTAAGATAGTTGAATCACTGC

ACAGCAAGTTCCCGGCTATTCCAAAGTCGCAATTAAACAATTATGTGCGGGAGATATCAG

ATTTTGTGGATAACCGGTGGCAGGTTAAGAATGAAATTCTAAGCAGGTTTCATATGAACT

CGCCAGTTTTAGTCTTCGGAGTAGATATATCAGAGTATAGCTACTCAAATGAGGATCAAC

AAGTCTGGCAATCCCAAAGCCTAACAGCTACGGCAACATCTATTCCGGATTCTGACTTAC

CAGAATATGTGCGGTGTATCCTAGCAAACTCCAGTGGGATCAGTAAGATTGTAGAATCAT

TGCACAGAAAGTTTCCGGGCGCTCCAAAGTTGCAATTGAACAATTGGGTGCGGGAGATAT

CATTTTTTGGGGACAATCGGTGGCAGGTTAAGAGGGATATTCTGAGCAGGATGGGTCTAT

TGAACTCGCCAGACAAGTGTGGGAAGGTGAAAGGTATAGGGATGTATTTGTCGAAGTGCT

CTTCAGCTCCCAAAAGTAAGACTGCCGATGACAGCCCAGGTTCTTCTTTGCAGCCGTGCT

CTAAAATGGAAACTCATCATGGTGACAAGACCCATATCCTGCAGACCGATAAGCAGCTTA

CTTCTTTACTATGAGTCTCTTGTCAGTTGTGTTAGTTTGTTTTTCCTCTGCTTAGTTTTG

GTCTATATGCAGTTCTCCCTGTTTTGAGTTCTGTTTCTTTGGAAGATCTAGCCTGTGTTG

GGATATAGATAGAGGTGCATGTGTAAATATTGGATTAAACTAGTGTAAATTAAAAGGCTT

TT

>Unigene0002362

TCTCCATCTCCTTCTTCCTCCTAAGCCCTCACCTTCATTGACCTAATCAAGAGGAAGAGA

GAGAACATGGTCACCTCAACCTCTTCGTCATCCTCCTGCTCTTCGATCAATTCTTTCGAC

GACCTATCCTTCACCGCCGCCAGCAAACCCGTCAAGTTCCTCTGCAGCCATGGAGGCAAG

ATCCTTCCCCGTTACCCCGACGGAAAGCTTCGCTACGTTGGCGGTGAAACCAGAGTTCTC

GCCGTACCCCGATCCATTCCCTTCTCAGGTATACACCAACCATCTTTATTTGGAAAGTAA

TCAGAGTCCAACCGGGACCACTATCTTAATTTTCTAAAAATGGTTTGATTCAGATCTGCA

AGCCCAGATGATGAAGCTATGCGGATCAGAGTCACCGGTGATCCTGCGCTGCCAGCTCCC

GACCGAAGAGCTCGACGACGCGCTGGTCTCCATCACCTCAGACGAGGACCTGGCCAATCT

CTTCGAGGAGTACGACCTCGCTGGCCGGGATCGGCTGAAGATCCGTGCCTTCTTATTCCC

ACAACCAACGAATTCTCATGCTTCTTCTTCAAGTCCGCCACCGCCGGTGAGACGGGAATA

CACCGCCCGGCCGCCGCTCCCCGTGGTGGCACGCCGCTGCGTCCACCAGATAACTCGCCA

GCACCAAACTATGCGGAACCGATTGAGATAACCATGGACTTTTGTCTATGTAAACAGAGG

ACTGTAAACGATAATAGTATAATTAAAAATATATATATCTAAAATGGGTGAAAATTAGCA

AATAAATTGCTGATTTTCTGCACTCCGGTTTGATGGACGTCACCCATTGTTTCAATCCAA

TATCCCATTTGTTGTCTGTTGATGTTGTCCATCCTTTTGCATTCTCTCTCCCATTCACTT

CTGTGGTTTATCCGTTAAATCTGTTCTGATCCATCCGTGACTACAGTTTGTTTTTCCGTT

ACTGTCCGTGACTTGTGTTCGGTGACAGTCTGTTAGTTCATTCCGTTACCCATCCGTGTC

AATGTGTGTTCGTTCTGTTATCCGTCCGTGACCTTCATTCCGTGACGGTGATTTTTATTC

GGTGAGTATCCGTTCCTTTGTTCCGTGTCTACTTTGTTATGATATCGTTTTCTTTATACA

TTGATTTGTGTGGACTAGAGGGGCTGCACTCATCCTTTTCTTTGTCGGGACGCCTCGTTT

GGTTCGATGCTGGTCCCGTTGTGGGACCGATTGATATTTTTTTTCGGTTTGCTTTGCTTT

CAAGATTCGTTTTATGTGTTTATGCCTTTCGTGACTTTTTCTTTGGTCTGCAGGTCAATT

GGAGGGACTGCTTGTCTAGTGGAGCAGAGAATCAGAATCAAAACTTGCCGACTTACATAT

GGAAGAATTAGATTATGAACCAATTAAGAAACAGGACAAATACTATTGAGTAAACTATTA

ACTTTGACCGAAAAATTAATTTGGAAAAACTAATGAGGTCTAAAAAAAAAAAATCAATAA

ACAAATAGATTGGACAAATGCTAACAATTTGAGCCAAAAGTTTAAACCTCATAGATGTAG

GCAAACCCAACAAGGTTCATTAATAAAAATTGGCAAAATAATAAAAATTGGTAAAATGAA

ATTTATCGGGTAGATAAAACAATTTTATATAAAATTAATCTTGAAGATTTTTAAATTACA

AGATCATTTAACATAGGTAAAATTACTTGATTTTGAAATTATTCTATAAAATTAACCGTT

GATTTTGAAATTATTCTATAAAATTAACCGTTTTTTGTCTTACAGTAATTAGGTAATAAT

TCGTAATATGTTCACTATGACAC

>Unigene0002363

AGCCCTAGGGTTCTTCAGCTCAAGCTCAATCACCTCTCCCATATTTTACCCATTTCTACC

TCATCATCACACAAGCAACGAATAATAAGGCCGCATCCACCACACCCCTTCACATGGGGG

AGCTCTGAGCTCTGATTTCACAAATCTCCTACCTCGCCCCCCATTCCCCTAAATTTTCGA

TCCCTAGCTGCTAGATTCGCATTCATGGAGGCGAGCGCCGGCCTGGTCGCCGGCTCCCAC

AACCGGAACGAGCTCGTCGTCATCCGGCGAGACGGGGAATCCGGCCCAAAACCGCTGCAG

CAGCTCAGCGGTCAGATTTGCCAGATTTGCGGCGACGATGTCGGGCTCACCGCCGACGGC

GAGCTGTTCGTCGCCTGCAATGAATGCGCCTTCCCGGTGTGCCGCACCTGCTATGGGTAC

GAGCGCAAAGAGGGGAACCAGGTCTGCCCCCAGTGCAAGACCCGGTACAAGAGGCTCAAA

GGATGTGCGCGAGTCGCCGGAGACGATGAAGAGGATGACACCGATGATCTTGAGAATGAG

TTCAGTTTCACCGGGAGGGAGAGAAATGATATGCAGTACATGGCGGAGGCTATGCTGCAG

GGGCACATGAGGTATGGTCGCGGCAGCGATCTTGATATGCCGCAGGTGGTTCATACAATG

CCTCAAGTTCCGCTGCTCACCAATGGCCAGATGGTTGATGATATCCCGCCGGAGCAGCAT

GCTCTTGTGCCAGCCTTCATGGGCGGGGGAGGGAAGAGGATTCACCCGCTTCCGTTCTCA

GATAGTGTTCCAGTTCAACCCAGATCCATGGATCCATCAAAGGATCTTGCTGCTTATGGC

TACGGGAGCGTGGCGTGGAAGGAGAGGATGGAGCATTGGAAGCAGAAGCAAGATAAATTG

CAGGTGATGAAAAGTGAGAATGGTGGAAAAGATTGGGACAATGACGGTGATGGCCCCGAC

TTGCCACTAATGGATGAAGGAAGACAGCCCTTATCAAGGAAAATGCCACTTCCGTCTAGC

CAAATCAACCCTTACAGAATGATTATCATGGTCCGTCTAGTGGTTGTTTGTTTCTTCTTC

CACTACCGACTATTACATCCAGTGAACGATGCATTTGGCTTGTGGCTTGTGTCTGTTATA

TGCGAAGTCTGGTTTGCAGTTTCGTGGATTCTCGACCAGTTTCCAAAGTGGCTGCCAATT

GAAAGGGAAACTTATCTCGACAGACTGTCGTTAAGGTATGAGAAGGAAGGCCAGCCTTCA

CGACTGGCTCCGGTAGACTTTTTCGTGAGTACAGTCGACCCACTGAAGGAACCCCCTTTG

GTCACAGCGAACACTGTACTCTCCATTCTAGCGGTGGATTATCCAGTTGACAAAGTTTCT

TGCTACGTGTCTGATGATGGTGCTGCAATGCTGACATTTGAAGCATTATCAGAGACATCC

GAGTTCGCGAAGAAATGGGTACCTTTTTGCAAGAAGTTCAATATCGAGCCGCGTGCTCCA

GAGTTTTATTTCCAGCAAAAGATGGATTATCTCAAGGACAAGGTCGTAGCTTCTTTTGTG

AAAGAAAGGAGAGCGATGAAGAGGGAGTACGAAGAGTTCAAGGTCCGAATCAATGCTCTT

GTGGCGAAAGCTCAAAAAGTCCCTGAAGAAGGATGGACGATGCAGGATGGAACTCCGTGG

CCTGGGAATAACGTCCGAGATCACCCCGGGATGATTCAGGTATTTTTGGGCCAAAGCGGC

GGCCATGATTTGGACGGAAATGAGCTTCCACGCTTGGTTTATGTCTCGAGAGAAAAAAGA

CCTGGGTTCAATCACCACAAGAAGGCTGGTGCTATGAACGCCTTGGTTCGAGTATCTTCT

GTACTCACGAACGCACCTTATCTGTTGAACTTGGATTGCGATCATTACATTAACAATAGT

AAGGCGCTTAGAGAGGCAATGTGCTTCATGATGGACCCTCTAGTAGGAAAGAAAGTGTGC

TATGTCCAATTCCCCCAGAGGTTCGATGGGATTGATCGACATGATCGATATGCAAATCGG

AACATTGTTTTCTTTGATATTAACATGAAGGGTCTTGATGGCATCCAAGGGCCAATTTAT

GTCGGCACGGGATGTGTGTTTCGAAGGCAAGCACTCTTTGGTTTTGATGCTCCAAAGACG

AAGAAGCCACCCACGAGGACTTGCAACTGCTGGCCAAAGTGGTGCTGCTGTGGATGTTGT

TTCTCATCTAAGAGGAAGAAGAAGACTACCAAGAGTAAGCAAGAAAAGAAGAGGAGGAGC

TTCGGAAGAGGAGACAATGGTGCGCCGGTCTTTGCTCTGGAAGGGATCGAAGAGGGCATT

GAAGGTATTGAGAGCGAAAAAGCGATGGCCTTGTCCGAACAGAAGTTGGAGAAAAAGTTT

GGGCAGTCTCCCGTTTTTGTTGCGTCAACACTGCTTGAGAACGGTGGCACACTGAAAACT

GCTTCTCCAGCATCACTACTAAAGGAAGCCATCCATGTGATTAGCTGCGGTTACGAAGAC

AAGACTGACTGGGGAAAAGAGGTTGGATGGATCTATGGTTCCGTGACAGAAGATATATTG

ACAGGCTTCAAGATGCATTGCCATGGGTGGCGGTCGATCTACTGCATTCCCGAGAGACCC

GCATTCAAGGGTTCTGCGCCGCTCAATCTCTCCGATCGTCTTCACCAGGTTCTTCGATGG

GCTCTTGGTTCAGTTGAAATTTTCTTGAGCAAGCATTGCCCTCTGTGGTATGGGTATGGG

GGCGGCCTCAAGTGGTTGGAGCGGTTATCATACATAAATGCTACTGTGTATCCCTGGACA

TCGATTCCACTGCTCGCTTACTGTACTCTGCCTGCCGTTTGCTTGCTGACTGGGAAATTC

ATCACTCCAGAGCTTAGTAACATTGCCAGTTTATGGTTTTTGTCGCTCTTCATCTGCATC

TTTGCTACTAGCATACTGGAAATGAGATGGAGCGGCATTGGAATGGACGACTGGTGGAGG

AATGAGCAGTTTTGGGTTATCGGTGGTGTTTCTTCGCATCTCTTTGC

>Unigene0002364

AGAAGAGAGACAGAAGCAGAAGGGCTGCCTGCCAGCCATTTCTCACAGAGACCAACGGAA

GTTGGTAGCTGCCGGAATTGGTAGATCTGGTGGTCCGGGAGTTTTTGATCGCTGATCGGA

ATGGAGGCGTGCGCCGGCCTCGTCGCCGGCTCCCATAACCGGAACGAGCTTGTCGTTATC

CGCCAGGAAGGGGAATCCGGGCCGCGGCCGCTGCAGCATCTCAGCGGCCAGATTTGCCAG

ATTTGCGGGGATGATGTCGGGCTCACCCGCGACGGGGAGCTTTTTGTCGCATGCAACGAA

TGTGCCTTCCCGGTGTGCCGGACTTGCTATGAGTACGAGAGGAGTGAGGGTAATCAGGTC

TGCCCGCAGTGTAAGACCAGGTTCAAGCGGCTCAAGGGGTGTGCGAGAGTTGCCGGAGAT

GATGAAGAAGACGATGTCGATGATCTGGAGAATGAGTTTAGTTTTAAGGGGAGGGAGAGG

CATGATATGCAGTACATGGCTGAGGCGATGCTGCAGGGGCAGATGAGGTACGGGCGCGGG

AGCGATGTCGACATGCCGCAAGTTGTTCAGACGATACCGAGTGTTCCGCTGCTCACGAAT

GGCCAGATGGTTGATGATATTCCGCCGGAGCAGCATGCTTTGGTGCCGGCTTTCATGGGA

GGGGGAGGGAAGAGGATTCACCCTCTTCCGTTCTCAGATCCCGGCCTTCCTGTGAACTCG

AGATCCATGGATCCATCCAAGGATCTTGCTGCTTATGGATATGGGAGTGTGGCGTGGAAG

GAGAGGATGGAACAATGGAAGCAGAAGCAGGAAAAAATGCAGGTAACGAGGAATGAAAAT

GGTGGTAGAGATTGGGACAATGATGGTGATGATCCGGAAGTGCCATTAATGGATGAAGGT

AGACAACCATTGTCAAGAAAGCTGCCTCTCCCTTCTAGTCAAATCAATCCCTATAGAATG

ATCATCATGATTCGGCTAGTAGTTGTTGGATTTTTCTTTCACTACCGGGTCTTACATCCC

GTCAATGACGCATACCCATTGTGGCTTATTTCTGTCATCTGTGAAATATGGTTTGCCGTT

TCGTGGATTCTTGATCAGTTTCCAAAGTGGCTGCCAATTGAAAGGGAAACTTATCTCGAC

AGACTGTCGTTAAGGTATGAGAAGGAAGGCCAGCCTTCACGACTGGCTCCGGTAGACTTT

TTCGTGAGTACAGTCGACCCACTGAAGGAACCCCCTTTGGTCACAGCGAACACTGTACTC

TCCATTCTAGCGGTGGATTATCCAGT

>Unigene0002365

CTGGAGAGCGAGGGTTTCGAATTCAGGGGGCGAACGAGCGCTGATAGCGGGATTGATGCT

GGCGGCGGCGGTGGCGGACCACGGCAGCTCGGCGGTCTCCGGCTTCTAGGGTTTTACTCT

CCCACAGCGGCAACGGCGGCGGCGACATCTCCGGATGTATTTGTTTCCATTCTCTGTGAC

ACACTTGTGCAATTGCATTACACTAAACAAATGCTGGCAGTGGTGCTATTAAGATTAAAA

TTAAAGTTGTTACCAAAGTTTACTCGGGATCCTTGTGGCAGTTACTGAAATCGCAAACCA

GAAGCCAAATTCTCAAGGAAATCATTGCTTTATTGTGTCTGCAAGTTTCTATGATTATTT

CTATTTATTTATAAAGAAATTGATGATACCCTCAGTTTGTGTTGAGGACCGGTTGATCAA

AGTTTTCAGTTACCTATACACTATCTAAAACAGCATGAGCCTTGGAGATTTCTCTACAGT

TATTGAAATGAAGGATGGGCTTTCAACTCTTTGAGAGTCGAGGAGCTAGTATCTATGATG

CAAAAATAGAAAGATTGTA

>Unigene0002366

TGATGCAAAAACAGAAAGATTGCGGTATGAATATGGTTGATGGTGCAACTAGGCAATGGT

CAACCATTGCGGGAACCTTAGCAGCGACAGAGAACACTGATTGTCTAAATCATTTTGTTA

AATTAAATGGGCCAACCTTTCTCAATCAATGGCTTTTGGAATGCCAGAACAGTGATAAAA

GTGTCAGTGAAAATTCGATAGAGGGATTGATAACTGTGTTACTCACGACTCTCGAGAAGC

TCCCACTTGATAGAGAGGAATTGATTCTTTCTGGAATTGGGGTCACAGTTGAACATCTCC

TTGGTCACAGAAATGCTAATATCAAGGAAAAGGCAAAGACATTAATGGAGAAATGGAATT

ATACAAGGACAATTAGTTGCGATGATTTGAACAATGAAGACACTTCTTTGAACAATGAAG

GCACTTCGAACAATCTGCCTAAATCTGAAGCTGATGTAAACACGACCGAGGAAGTCCACT

CAACACATCCAGTTCTCAGCATTTCTCCAGGCCTAGGGATTGACGAAGCAAGTTGTGAAG

CAGAAATAACGTGTGCTAGTCCTCAATTAGATACCACTAAAGGTGTGGAGAAATCCATGT

CAGAACAACTTAAACATGCGACCTTATCGAAACCGTTGGGTGAAAATGCAACTTTAGGAG

ATGTGAATGCTTTCTGTTCGTCTACCATTTCGGGTTCTTGTCCGGAGAAGTCTGTTACAG

AAGAGTCTCCTGGTTATAGGGGAACCGACAACGATCAGCGAGATATTTCGGTTCTTAGAG

AAGTCTCTGGTGGCATGGAGGAGACACAGGTGGAGGCAAATATGAAGGGAGGCAGTCCAT

CCAAATCAAGTCGAGAAGAATCTTGCAACGGATCATCTTCTTCTTCTGATCTATCCGCTT

CTTCACCGTGCAGTTCAGTGGAAGATTCGAGAGGTTTTATGTTCCCTAGTGGTTCACCAC

GAGCGGAGGAAATTGATCAGATGGATAATAACTCGGGACTGGGACTTGAATACGAGGAGA

TAGATGCTTTAGAAATGGCACGTCAAGTTGCTTTGGAAGTGGAACGAGAAGTTGTAGACT

ATCGAGAACCATTTTGCAGCTCTCCTGAGGTCGAATCAGGAGATTCTGCAGATATCCATA

GCTCTCCGAAAGACAAGCAAAATCAACTGTTGACTGACAATATTATTAGTAACATGTTAC

TGGTTGAGAAGGATCCTTCTGATGATGCCTCTTCTCCCAAAAATCATGAGGCTCTAGTTC

CAGAGAAAACTATGCCTGATCCTCGAGAACACGAACAAGTTCCGGAACCACAAGGACCAG

CTTTTAAACTTGACAAGAAACCACTCGACTTTGATCTTAATGATGATGTATGCACCGAGG

GACCTAATTGCTCAACGAATCCCATTCCTATTTCCACTGCCAACTTATCAGCACCTAAAG

CCGTCGTTGCAACTTCCAGAGGTGCTCTATGTCTGCCGATTAAGCCACTTCAGTTTAATG

GTGAAATGGGATGGAAGGGCTCCGCTGCTACTAGTGCTTTCCGACCGGCTTCTTCTTGTA

TAAAGACTTGTCCTGGCCTGAGTCAGAAACAGAACTTCCTTGAAATTGATTTGAATGTGG

CTGATACTGAAAATGATGTGGCAATTGATCCATTATCAGTTAAACAAATGCCTCTTTCTC

CCAACCTGCCTTCGGTTGAGTCATCTGTAGAAGTGAGTTCTAGACGAGCAGAGCGGCTTA

AGCTGGACCTTAATCTTAACCGCCTGGGGGATGAGGACATATCTACAAACCAGTCTCCAT

ACTGGAGGCTTCATCACCAGAATGGGGACCAAGGCCTGTCTTCTGCTTCATCATCGTCCT

CACAGCAGCACTCAATGAGAGACATCGACTTGAATGACAATCCATGTTTTATAGAGGCAT

GTGGTTCTCGTAACCTTGATAACTCGTCTTCCAAGGCCTCAACGACTACCTACGGGAGTT

CGAAGTTGAATGATCCTGGCATCACTATTATGGGTTCAAGGCTGAATGATCCGGGCATCA

GTATCATGAGTTCAAGGTTGAATGATTCTAGCATCAATATCATGGGTTCGAGATTGAATG

ATCCAGGCATCACTATCATGGGTTCAAGAATGGCCGTGGATAGGAAGGACTACATCAACC

AAGCTCACCAACCCTTTATGGGCAATGGAATGCACATGGAGACTATGACGACTACTAGGC

CAGTACTGCCTTACCCTCATATGCCGACTTTCTACGGGTATAATGGACATCCAGTGGGGC

CAACCATGCCTATGCACCCATCGTATTATGGACCGCCAGGTATTCCTTACATGGTCGATC

CTAGAGGAGCTACCTTCATTCCACAGATGTTGGGTCCTGTGGGTCTGAATGGAGCACACT

ATGCGAGACCACCTTTCATTATGAGTTTAACTGGCGCACCCTCGGGTATGAATGGAGTTA

TGCCTTCGAGATCTGGTTGGGATTTGAATTCTGGCATTGCATCGATAGATGGCGGATCTC

GAGACGGGGGGAGCTTTAAGCAACCGTTCATGCACCGTCACAATGGTCTCATGGAAGAGA

GCTTCAAGCAACCGTTCACACATGGTCACATAGAGGAGCAAGCAAGAATTTCGTCGCAGT

CGTCTAGTCCGAGCATGTCGGTGAAAAGAAAAGAGCCAGACTGCGGGTGGGAGTCTTACG

CACTTGGTTACAAACATATGACATCAAGGCAATAGTTTCTCATATCCGAGCATTTATGTG

GTGTTACAGAAATTCAGAAAATGATGGGCCACATGAACTATGTGGGATAACTTGAGATAA

GAATGAATAACTATGGCGGTAACAGATAGTATGATGTGAGGTTTTGGCTGAAAGTTGTTA

GGCTTGAATGATTTTGTGTCTTGTGTCTTTTTTTTGTCGGCTGATTTGTTATAGAGAGGA

TTTGCAGCTGATAAAAGCTGGGATAATGTAAATCTGTTATAAATATTCTAACTCAGTGAA

GGAATTAGTGTCTGTGATGAACGCGCATATGGCTCCCGTTTCTTAATGATGGTGACGGTA

GAACATGAAAACGTATCCGGTCTCATTTTGTTGGTTGTTGATAGTTGGTAAAAATGATGT

CCTAATTAGATAGTATTGTAAGCA

>Unigene0002367

ATATCTGCAGAATCTCCTGATTCGACCTCAGGAGAGCTGCAAAATGGTTCTCGATAGTCT

ACAACTTCTCGTTCCACTTCCAAAGCAACTTGACGTGCCATTTCTAAAGCATCTATTTCC

TCGTATTCAAGTCCCAGTCCCGAGTTATTATCCATCTGATCAATTTCCTCCGCTCGTGGT

GAACCACTAGGGAACATAAAACCTCTCGAATCTTCCACTGAACTGCACGGTGAAGAAGCG

GATAGATCAGAAGAAGAAGATGATCCGTTGCAAGATTCTTCTCGAC

>Unigene0002368

TCCAAAGCCCCATAAAACCCCCGTCACCGCGCATCTCGTCACCCATTGCTCCACCTCTTC

CGCCGTGACTGCCACCACCGGCGCCGGTCGCCTCATCTCCTCCGCCTCTCTCCGCCGCCA

GTCGCCCCATCTCCTCCGCCTATCTCGAAGGTCAGTTCTCTCAACCGCCGATCCTGTCTA

TTTTTCAATTATCGAATCCACATTTCAATCCAGATCTGCTCAAAACCCTAGATCCCGTCC

AGATCCGGCGAGATCCGCCGCCGTCGCCGGCGAAGATGATCTCCCCGGCCCGATCCTCAA

TCTGGCTCGCCCTCGCCGTCCTCATTATCAGATCCGGCGACGCCTTCTACCTCCCTGGCA

GCTACCCCCACAAGTACCTCGTCGGCGATCCC

>Unigene0002369

GGTCAACTCCCTCACCTCCATCGAGACCGAGCTCCCCTTCAGCTACTACAGCCTCCCCTA

CTGCCAACCCCACGGCGGCGTCAAGAAGAGCGCCGAGAACCTCGGCGAGCTTCTCATGGG

CGACCAGATCGACAACTCTCCCTACCTCTTCCACGTCAACGTCAACGAATCCCTCTTTCT

CTGCACCACTCCTCCCCTCTCCGATCACAACGCCACTCTCCTCAAGCAGCGCTCCCGCGA

CCTCTACCAGATCAACATGATCCTCGACAACCTCCCCGTCCGCCGCTTCACCCAGCAGAA

CGGCGTCGTCATCCAGTGGACCGGCTTCCCTGTCGGCTACACCCCCGATCCCGCCGGTAA

CGGGAACGACTACATCATCAATCACCTCAAGTTTAAGGTCTTCATCCACGAGTACGAGGG

CCGTAGCATGGAGATACTCGACGAGAGCCTCTCCGTCATCCCGCAGCCGGACGACAAACC

CACCCCCGGGTACGAGATCGTCGGGTTCGAGGTCGTCCCCTGCAGCGTCAAGCGAGATCC

TGCTAAGACATCGAAGATGAACATGTATGACAAGATTGAACCCGTCGCCTGCCCGACCGA

GCTTGATAAATGCCAGCTGATCCGCGAGCAGGAGCGGATCACCTTCACTTATGAGGTGGA

GTTCGTGAAGAGTGACATCCGCTGGCCGTCGCGGTGGGATGCTTATCTGAAGATGGACGG

TGCGAAGGTGCATTGGTTTTCGATCATGAATTCGCTGATGGTTATCTTTTTCTTGGCGGG

GATTGTGTTTGTGATTTTCTTGAGGACTGTGAGGAGGGATTTGACAAGGTACGAGGAACT

GGATAAGGAGGCGCAGGCACAGATGAATGAAGAGCTCTCTGGTTGGAAGCTTGTTGTGGG

GGATGTTTTTCGGGAGCCGGCCTGTGCGAAGTTGCTTTGTGTGATGATTGGGGACGGTGT

TCAGATCACAGGAATGGCGGTGGTGACGATTGTGTTTGCGGCTTTTGGATTCATGTCTCC

GGCTTCACGGGGGATGCTTTTGACAGGGATGATTCTGCTGTACTTGTTTCTTGGGATTGC

GGCTGGGTATGTCGGGGTTCGGCTGTGGAGGACTATCAAAGGCAGCTCCGAAGGGTGGAG

ATCAGTGTCCTGGTTGGCAGCTTGTTTCTTCCCCGGCATTGTGTTTGTGATCCTCACTGT

ACTGAACTTCATTCTCTGGGGGAGCAAGAGCACAGGGGCAATCCCAATCTCTCTGTATTT

CAAGCTGTTGTCGCTGTGGTTTTGCATCTCTGTTCCTCTGACCCTCCTCGGAGGGTACTT

TGGCACCAGGGCAGGAGAAATTAAGTTCCCTGTTAGAACTAACCAGATCCCTAGAGAGAT

CCCAGCTCGAAAATACCCTTCATGGCTGCTTGTTCTCGGTGCCGGAACTCTCCCCTTTGG

CACCCTTTTCATTGAGCTCTTCTTCATCCTTTCCAGCATCTGGCTTGGGAGGTTTTATTA

TGTGTTTGGGTTCCTGCTGGTGGTCCTTCTATTGCTTGTTACTGTCTGTGCTGAGGTTTC

CGTGGTTCTGACCTATATGCAGCTTTGTGTTGAGGACTGGAGGTGGTGGTGGAAGGCTTT

CTTTGCTTCTGGTTCGGTTTCCCTGTATGTGTTCCTGTACTCCATCAATTACCTGGTGTT

CGACCTGAGGAGCCTGAGCGGACCCGTATCAGCTATTCTCTACCTCGGATACTCACTCAT

CATGGCTTTTGCTATTATGTTGTCTACTGGCACCATTGGGTTCCTGATTTCGTTCTATTT

CGTACATTACCTCTTCTCATCCGTCAAGATCGACTAGAAGTCAGCTCAGTTCAAGACATT

TTGAGCATATTACAGTAACTGGATAGAAAGTTCTTGATTCAGTGTTCAAGACGTACACAT

TTGAAGTTTAAGTTGATCATAATGATGGATGCATGCCCTAAAGACATTTTCAATAGTGGA

TAGTTTCTCTGATGGCTGCAATTTGTGATGGTTCACTGTTTTTTTCCGGCTCTGACTAAT

CTCCAAGGCATGTTTGGTATGCTTATATTACCTTTTCCGATTCCTTGTTTTCAATGTGTG

TTTTAGATCTATTTTGCTTAAGAATTGCTAGTTTGGACTTGTAACTAGAGGAGCAGTTTT

GTGTGTCTCAACTGGTTTCTTTTGGGTGCTAAGATCATGAAGATTGTTCAGTCAGTTAAT

ATGTACTCTGCTTAATAACTTTGAGAGTGAAATCAGTAGTTACAATCCTTTTGATAGCAG

TGATGAGTGGTTTCCCTACCGCATGATTTTTCTTT

>Unigene0002370

CGCCGATAAATCAAAAACAGCATACCGGCATCTCTCATACATTTGACCCCCCATCCTCAT

CGATCGTTCCCCAATCCGGCCAATCAGGTACGCCCCCAAACCCTAGATCCGCCCCGATCC

TGCCGAGATGCGATCGATCTCGCTGATTCCGGCGATCTCGCCGCTCCTCCTTCTCCTCCT

CGCCACCCCTTCCTCCGCCTTCTACCTCCCCGGCAGCTACATGCACACCTACGCAACCGG

CGAACCGATCTCCGTCAAGGTCAACTCCCTCACCTCCATCGACAC

>Unigene0002371

CGCCGATAAATCAAAAACAGCATACCGGCATCTCTCATACATTTGACCCCCCATCCTCAT

CGATCGTTCCCCAATCCGGCCAATCAGGTACGCCCCCAAACCCTAGATCCGCCCCGATCC

GGCCGAAATGCGATCGATCTCGCTGATTCCGGCGATCTCGCCGCTCCTCCTTCTCTTCCT

CCTCCTCGCCACCCCTTCCTCCGCCTTCTACCTCCCCGGCAGCTA

>Unigene0002372

TTCTACCTCCCCGGCAGCTACCCTCACAAGTACCTCGTCGGCGATCCCCTCTCCGTAAAG

GTGAACTCCCTCACCTCCATCGACACCGAGATGCCCTTCAGCTACTACAGCCTTCCCTTC

TGCCGCCCCCAGGAGGGCATCAAGGACAGCGCGGAGAACCTCGGCGAGCTCCTCATGGGC

GATCGCATTGAAAACTCGCCCTACCGCTTCAAGATGTTTACAAACGAGTCTGACATTCTC

CTCTGCCGCACGAACGCACTCTCCACCGACGAATTCAACCTCCTGAAGAAGCGGATCGAC

GAGATGTATCAGGTGAATCCGATTCTCGACAATCTCCCGGCGATCCGGTACACGAAGAAG

GACGAGTATTTCCTCCGGTGGACCGGTTATCCTGTCGGCATTCGAGTCGGGGATGTTTAC

TATGTGTTCAACCACCTCAAGTTCACTGTTCTTGTGCATAAGTATGAGGAGGCGAATGTT

GCGAGGGTGATGGGCACCGGAGATGCCGCCGATGTCATCCCGAATTTGGAAAAAACCGGA

TCCGGGCCTCCCGGGTATATGGTTGTGGGATTCGAGGTTGTTCCATGTAGCTTTCAGCAC

GATACCGAGTCGATCAAGAATCTCAAGATGTACTCCAAGTATCCGGCGAAGATACAGTGT

GATCCGACTACAGTGGCTATGGCTGCGAAGGAGAAGCAGCCACTCGTCTTCACTTACGAG

GTATCGTTTGTGGAAAGTGATATCAAGTGGCCGTCTCGATGGGATGCTTATCTGAAGATG

GAGGGGGCGAAAGTTCACTGGTTCTCGATTCTCAATTCCATTATGGTGATAATCTTCCTA

GCTGGTATTGTGCTTGTAATTTTCTTGAGGACGGTGAGGAGGGATCTGACACGGTACGAG

GAACTTGATAAGGAGGCACAGGCACAGATGAACGAGGAGCTTTCGGGGTGGAAGCTTGTG

GTTGCTGATGTTTTCAGGGCTCCTAGTAATCCAACGCTGCTTTGTGTACTTGTGGGCGAC

GGTGTTCAGATTCTCGGCATGGCGGTTGTGACTATCATGTTTGCAGCTCTTGGGTTCATG

TCGCCAGCGTCTCGTGGGACTCTGGTCACTGGTATGCTGTTCTTCTACCTCATTCTTGGA

ATTGCTGCTGGTTATGCTGGTGTTAGGCTATGGAAGACCATTAGATGTGGGGATCATTCT

GGGTGGAAATCGGTGTCGTGGAAGGTTGCTTGCTTCTTTCCGGGGATTGCATTTCTGATA

CTGACAGTTCTGAATTTTCTCTTGTGGGGGAGTCATAGTACCGGGGCGATTCCAATTTCG

CTATACATTATTCTACTCATGCTCTGGTTCTGCATTTCGGTGCCTCTTACTCTTGTCGGT

GGATTCCTTGGAGCAAAAGCGCCACATCTCGAGTATCCTGTCCGAACTAATCAAATCCCT

CGTGAAATTCCTCCGCAAAAATATCCGTCATGGCTGTTGGTTCTTGGGGCGGGCACTCTA

CCGTTCGGCACCCTCTTCATCGAACTTTTCTTTATCATGTCGAGCATTTGGATGGGCCGA

GTGTACTATGTGTTTGGTTTTCTCTTTGTCGTGTTGATTCTTCTTGTCCTTGTTTGTGCT

GAGGTCTCGATAGTTCTCACTTACATGCACCTTTGTGTGGAGGATTGGAAGTGGTGGTGG

AAGGCCTTCTTCTCATCCGGGTCGGTGGCTTTGTACATCTTCATGTACTCGGTCAACTAT

CTTGTCTTTGATCTCAAGAGTTTGAGTGGACCCGTATCCGCTACGCTTTATCTTGGCTAT

TCTCTCCTCATGGTCATTGCCATCATGCTGGCCACTGGAACAATTGGGTTCATTTCTTCC

TTCTTGTTCGTGCACTACCTCTTCTCGTCAGTTAAACTAGATTGAGCTACATAAGAAGCT

TCCTGACCTGGAGCATTATCAAAGATCTGATGGAAATATGCAAAATGGTATGACAATCAT

CTTAGTTTCATTTGGCTCTTGTTGTTTGGGATTCAATAGAAGAAAATAATACATGGAATA

TGAGTTTTAATCATTTTTTTTAGCTAGAACTGCATAATATGTAATAGTTGGATCTTGTCA

AGCTTTTGTAATCTTTCGGTTAGGACAGAATAATTTTATTTTTCTAATGCCTACTTGTAC

TGTTGGTTAAGAAAAAAAATGTTCGTTGCATCTAGTTTATG

>Unigene0002373

CGCCGATAAATCAAAAACAGCATACCGGCATCTCTCATACATTTGACCCCCCATCCTCAT

CGATCGTTCCCCAATCCGGCCAATCAGGTACGCCCCCAAACCCTAGATCCGCCCCGATCC

GGCCGAGATGCGATCGATCTCGCTGATTCCGGCGATCTCGCCGCTCCTCCTTCTCTTCCT

CCTCCTCGCCACCCCTTCCTCCGCCTTCTACCTCCCCGGCAGCTA

>Unigene0002374

CCTCTTAAAACTTTTGGTAACATGTATTTGATAAGTGTAAAAAAGTATATATGATTTTAA

TTGTTGGCTGTAATGCAGTCAACACATGTCTCAATAAAATTGAAGTTTGGTATTTCGGTG

TTTAATTTGATTAAATTATACTTTATTTTTTGCTCTTATATGGTCGAGCAATGCTATTGA

CGCCACGGTATATGAAACCACTGTTCCATTTTCTCTTGACCTTTATGATTGCCACACAAC

TTCAGGACTCTTGTATTTTTATACCTTATGCAAGCCTTGTTCATATGCGTAGGCTATATT

GGTGCAAGGACCTTTTTGACCTCAAAGAAAAACATGCTCTTCAATCTTGTCTTGAAGG

>Unigene0002375

GGTTACTGTTAGCTGTCTGAGACTGCTCAGGTTGGAGAATTCAAAAAAGGTAACTGTGAG

CTCTGGGAATAATAAGAAAACTGAATAGTAATGGATGAGCCAAGGTAGAATTTCATACGA

TTGATGTCTTGCCTGAGGCCTTGGAATTAAAAAGGATATCACGAGTCATGATGGATGTTA

ACATATTCTTCGGCTTTCAATGATTTTGGATCGAGCGAAGGGTAGGTTTTTACCATCCGA

AGAGTTATGAGTAAGCTGAAGTGTTCGTCTTTTGCCCCAAGGCCCTAGAACATGTGAATT

TTTGGTCATTGCATGATGAAAGATCTCCCAGGAGTAAAATGTGAAAGAATTATTTTCTTT

GTATACTTGTATTGATGCCTTTTCTGTCTTATACTCTGGTAGATGCGCAAATCAAAGTGG

AATGGTTGGACTTAATAGTTATCTTGTTTTCCTCTTAAAACTTTCGGTAACATGTATTTG

ATAAGTGTAAAAAAGTATATATGATTTTAATTGTTGGCTGTAATGCAGTCAACACATGTC

TCAATAAAATTGAAGTTTGGTATTTCGGTGTTTAATTTGATTAAATTATACTTTATTTTT

TGCTCTTATATGGTCGAGCAATGCTATTGACGCCACGGTATATGAAACCACTGTTCCATT

TTCTCTTGACCTTTATGATTGCCACACAACTTCAGGACTCTTGTATTTTTATACCTTATG

CAAGCCTTGTTCATATGCGTAGGCTATATTGGTGCAAGGACCTTTTTGACCTCAAAGAAA

AACATGCTCTTCAATCTTGTCTTGAAGG

>Unigene0002376

CCATTTCCCCACCTCATCTCAATTGATGGCGCTCTCTCGAATCCCTCACCCTTCCGCCGC

CGACACCGGCGGCCACCACCACCCCCCTCTCTACCACCACCACACCGCCGCCGCCGATCA

CAATCCTTACCCGCCGTACCCCCGGAAGCACCGCCCCCGCCCCTTCCCCACCACCGACGC

CGACGCCGACACCGTCCCCCCGGCCAAGCGGCGGAAGCCCACCGCTCCCGGCTCGTGGCC

GCAGGACACCGAGAAATGGCACCGCTATCCCGCCTCCAGCGACGCGCCGTCAACGAGCAG

CAGCGTTCCCCTCCCCCCGTTAGCTTCAAGATCCGAGGAGGAAGCGGCGTTTATGTCGAG

GGCTGAGATCGAGCGCTGCTCGCCGTCGCGGAAGGATGGAATCGATTCGCTGCTGGAGGC

GCAGCTGCGGTACTCGTACTGCGCTTACCTCCAGAGCCTGGGACTCCGGCTCCAGATGCC

GCAGACTACGATTGGGACTGCGATGGTGCTTTGTCACCGGTTCTTCGTCCGGAGATCACA

TGCTTGTCATGACAGATTCTTAGTTGCAACAGCAGCTCTATTCCTAGCTTCCAAATCTGA

GGAGACACCATGCCTTTTGAACACTGTACTCAGGACTTCTTGTGAAGTTTGCCAGGCAGA

GAACCTTGCTTTCCATCCATATTTGCTTTCCAATGACTGGTTCGAACAATATCGTGAGCG

CGTCACTGAGGCTGAACAAATGATACTGACCACATTGGATTTTGAACTCGACGTACATCA

CCCATATGTTCCTCTTACAACAGTTCTTAACAAATTCGGCCTTTCTCAGACTGCTCTGCT

CAATCTTGCATGGAACTTTGTCACTGAAGGGGTCTTCAAGAGAATAATGTGCTCACATTT

ATGAGTTCTTCTTAGCTGGAGGATTGATATGCTGTGCATGGAAAGCATTTTTTATACGTC

TGAAGTTGAGCAACTAGCAAGATATGATATATATGAATATTTATGTATATCCAACATTTG

TTCTGATTACTGAATAAACCTTAAAATTTGCAAC

>Unigene0002377

TATATGTTGGCACTACATACCTACATACCTATAACCTTTCTTTTAACGGAAGATTCCTTC

CGGTGAATTTTGATTTGTGAAAGCCAAAAGATTCTCCCTGCCCCTGGTCCATCCTTTTCA

TTTGGTGTAGCCGAATAACATTTGCAAACTTGTCACTCTCTGTCTCCCTGAACATCTTTG

TGTTTCATGTTGAGGCTTTTATCTTTCCTATTAATTTTACAATCTGAACTTGATTTTCAC

GACACTTGTTGGTCATGACTTCTAAAACTACATATATTCCTTTTTGGTTCGAACTAAATC

GTTCACCATTTTTAGCGAGAACCTTTACAGTTTAGTTCTCTCTCTCTTTTTATATTGCCT

GTTTGCTCTTCCGGTTTTACTTGACATTGTGTATAATCTGGAACCACATAATCTTCGTCT

ACCACTTTCACCTCAGATTGTAAGGGAACTGCCAGCAGGTCTCCGAACTTATGCTTCAAT

AAACAAAAACCTAGTCTACAGCTACTTTTTTACCCATACTCTAGTCTAATACATATAACC

ATAGATCACAACAATCTCAGACTAGATGAACAAGTCTTGTTTTAAACACAATTTTTATAG

AGCGATTACTACATGAACTTAACCCTTAATATTGAAAAATTGATTTAAAATGATACTTGA

TGATTTATAAACATTAGAAAGGTTTAAACAATTCTAAAACTTAATGCACTAGTTCTGGCA

CTTGAGACCTCACTGGCTGTTGTGAAGCATCAGACCTCTTAGGTTTCGTTATCCCAAAAG

CATATTTGGTATCATTCAATATTGTGGAGTTGATGCATTTTTGAAATCAAATAGGCAGTT

ACTTTTGGAATTATTTTACGCATAAAAGAGGCGACCCTGGCGCAATAGTAAAGGTTGTTG

CCTTGTGACCTGGAGGTCATGGGTTCAAATCCAGAAACAGCCTCCCTGTATGCAGGAGTA

AGGTTGTGTACATCCGAACCTCCCAGTACCCTGCTGTGCACTGGGTTGCCCTTTATAAAA

GTGGCGTTATTTTATTCATTAGGCTTTTTCGTTATTTCTTATCTTTTCCCAATTTTTCCC

AAAATCATTTTTCCATTTTTTTTTCGTTTGCCAATTTTTTTTTTTGGACTAATGCTTTTG

ACATTTTGAAAGTTTATTTTTTAACTATCACGGGCCATGTTAAGTTCCTTGTTACCATTA

TCTGGTGATGGGAGTACAATTTACTCCGAAAGGACCCAAACAACACACATGTGTTCTGTT

CATTATGATTAGAAATTCTACAACCATTTCAACATATTTTTGCATTGTTATATCCTGCAA

ACACTAAATGGGTTGTTATATCTATATATTGCAAACAATTATTATTATTGAGATTTAATT

CTATTCCGAGAAATCTTTTTTCTTCTTTTGGGTTTGGTAGTGCGGCAGTTGAAAGGGCCT

GTTTTGTTTACTGCATGCAGTATTTGGGTGTAGGGCGTTCCTAGGATCCCTCTATCTTGT

GCACCAAAGGATATGTCAACGGAGTCACCTGATATTTTATCAGAAGCACAACCTATTCAT

ATTTTAATCAACTTGCTCTTTCAGTGCATGATTTGAAGTTCAGCAGAGCTTCTGCATTGT

CGTGTGTTAGCCTGTATAGTATATATAAATAGTTGTGCCTTTAATAAACAGAAGATACAA

ATACAAAAGGGATAAGCAAATTCTCTTGTAAGTGTAGAGAGTTTCTGTTTCTGATTGAAT

GCCTTTCTTCCTCAAATGCCCTGGAGCGTAGTTGGGTTCAATAAATTGATGCATAGTTCC

CCTGGCACCTCCGCACTGCTATAAATTAGCTCTTTGCCAGCATGTGAAGGTTCTTGGATA

GGATATCAGGCAGGCAGCTTCTTTGATTGTTGGTAAGGAGAACAATTAGATAGCGATTCG

TCTACTTTGCTGTATGTTGTTCCTATAATGCTTAAGTAATGTATTTTTTATCGAGTTTGG

AACTAATACTGCCTAAACTGAGTTAGGAAGTAACTTATATTACAACATCTGTCCTATTTA

TGATTGCAGCTGATGAATGGTTCGGCTATTATTTGTCCCCTCTTAATCGTTAACCTTGCT

TCCACTAAACATGAATAACCGGATGTTATGTTATGGCAGACTTCGGACTTCACTTTGGCT

TCAGTTTAAGCCCAACCAAATTGCTGCTGGCGCTGTTTTCCTTGCTGCTAAGTTCTTAAG

TTTTGATCTTGCTTTCTATCCGAGCATCTGGCATGAGTTCCATACAACACCATGGGTACT

TCAAGAGGTTGTTCAACAGCTAATGGAGATCTTCTAGGACGGGAGTTACTTGTAATGTTA

CCTGTTACATGAAAGGTTGGTTTGTTAGTTATGAAACAAAGTTGTCAGTACTGAAATGAG

TTATACTGCTTGCCGTAATGTTCCATATGTAGAGTATGCTGCTTTATATTTTATCTCTAA

AGATATACCAAAGTGGATACAAGTCCATACCTTCAAATAAGTGTTGTGCTGGTTTTGGTC

TTGACTCTCATTTTTTGAGTTGTTGTG

>Unigene0002378

GGGCGATGGAGTTATTTATTTATCGACTCACTACTTTTGCCAGCGAACCATCATAGTTCC

GTAATGATGTATATACTTACCATGATTGAATAAGTCTATGGATTCCAAAATTGGTGACTA

CTGTAAACATTTTGTATGAGGTATATATTTAATCAAAGATTTCAATTGATTAATAAACGT

TCAAATGAATTCAGTAGTTATGTCAAGAGAGACCCCTGCTATTGCTAAAAGCATCAAACT

TGTGAG

>Unigene0002379

TAGGTCAATTAAGGATAAAATGTTGGAACCTCATGGGTAAGGATAAGGTAATGGAATTGA

TAGATAAATTCATAGACCTAGGTGGGATAAGGGAATTGATAAAGGGAATAGAGATTATGA

TAGAGATCATACTGAGAAACATAAGAATTCCGTACGGGTACAACTCTTATTTGAACGAAG

TGCACAAAATGCGATCTTTTTTGTCTAATAGAACAAACACCTCCTTCGGACCCTTAATTG

AGTCGGTCAAGATAAAATCTGTTTATCAAAGTGCTTCTCTGATTGCTCAAGACATCTCTT

TTCAACTGAGGAATAAT

>Unigene0002380

CAGAGATATCTTAGAGGAGTTTCTACTTTTTTTTGCAGAGGTACACTGATTCTTTGGTTG

AGAAACATAAGAATTTCGTACGGGTACAACTCTTATTTGAACGAAGTGCACAAAATGCGG

TCGTTTTTGTCAAATAGAACAAACACCTCCTTCGGACCCTTAATTGAGTCGGTCAAGATA

AAATCTGTTTATCAAAGTGCTTCTCTGATTGCTCAAGACATCTCTTTTCAACTGAGGAAT

AAT

>Unigene0002381

TCACATCCTCCCACCAAAAATATGGATTAGATTCCTTGATTCTCCTATCATCAGAAAAGC

CAAGAACAATCAGCCATATCTAGTAAAAGAATATCTCTGAAGTGTGAGATTTTCCTTTTG

ACATCTGCCTGGTAATCAAACTCTCCATTTTTCCATTCAAACCTATGAGCTGTGGGAGAA

AGTGGCACACTAATTATTCAATTTGGTGGGGTTCTCTAATGAGGAAGAGAATCCAGCAAA

GGGGGAGATGGGTGTGGGGAAACAGCAAGAGAACAATGGCTATGCTTTTCTTTTTTCTCT

GCTTTTGAGACCCAAATTTGAACTGTTTTGCTTTACTCTCTCTAGTGCATCAAAGATTCC

TTTCCTTTCAAGTCAAAGAAGTTGCATAAGCACTTAAAAGAGAGGAGTCATGAAGAAGAA

GTGAGTACCAAACTTCCAGAATTGCAGGAAATGGCTAATCAAGTTGATCAGCAAAGCGCG

GTTGTATCGGTGGAAGCTAAGCCACGACTCAGGTGGACACCGCAACTTCACGGGCGGTTC

GTCGATGCAGTTTCGCTTCTTGGAGGAGCAGAGAAAGCAACACCGAAGGCGGTGATGCGA

GTAATGGGAGTTTCTGGGCTGACTCTATACCATCTGAAGAGCCATCTTCAGAAATTCAGG

ATGGCAAAGAGCCAATATCTGAACGCGAACGTGAACATCCCACATAACGACGAGGGACAG

AATTGTGTGTTAGTTGATTTAAGGAAATCAGATACGGAAACTGCCTATTCTGAAATTGAT

CAGAGGAAATCAGACACAGAAACTGCCTGTTCTGAAATTGATCAGACCAAATCTCCTTTG

AATGTGTAAGTTCTGATTATAATGTGACGCGAGATTCAATATTGGGAAAAATCTTTAATA

GTAGAGGTTGACTGTCTGAAGAGCCATCAACTGTCAACTGTTTCAGGCCCATGCTACAGA

TGCAAATTGAGGTGCAGAGAAAGTTGGAAGAACACATTAAGGTCCAGGAACATCTTCAGC

TCCGGATTGAGGCACAAGGAAAATACTTGCAGTCTGTTCTAAGAAAGGCGCAAGAAACAA

TTGCTGGTTATAGCTCGAGTTCTGCGGATGTGGTGGTTGGCAATGCAGGCGAGAATTCTG

AAATGGTCTCTGTGATGGACATTGAGTCCATGAGTTCTACTATCAATATACGGAGCTTCA

CAGCGAAGCGCGCGCAACAAGATGACAGCTCGACTGATAGTTGCCTGACTTCTACAGAGA

GGCAGGAGGTGCAGAATGTGAAGAGGGTTAAACTAGGAAGTGGATCTTCTTACATTTGGG

GTGAAGAGTCTAATGTGGACGATGTGATCAATTCAAATCGCAGCAATGCAATTCAGTTGC

AAGACATTGATCAGGCTGGTGAGAAGAAGAGACCTCGGCTTGAGATGGGATATGGTTTCG

AGCAGCGACAACAACTTGATCTCAATAGATAAGCAAGTTAACAATTTCTTGTGTATGCTT

GCATTTGGGTTGAGAATTTCACTTGTTCTGAGGATCAGAAATTTATGGTTCGAGTTACGA

TAAGGCATTTGTTGATGTTCATGTAACACTGCATCAAATGTTTAGATGCTAGAGAGTTTG

GTTATGGCTAAGTTAATTTAGGGAAGTATCAAAAGAGCTAGACAAAGGGCCAACTTGGAC

ACAGGATCTGATGGTGATGAGCTTTTGGCTACTTGCAATTGACCCAATCAAGTTGTAGGA

AGAGTTTATTGTCCATTGTAGCAATTTATAATATAAATATGAAATGTATTCCTCATGTTG

TTTAAAGTAGTAGTGAAGT

>Unigene0002382

CTAGCTTCTTGACCTTTTTTTAGTCTAATAGTTTTCGGTCTTATAGTCAACTTCTACGAA

CCCAAGCATCTATACTGAGACTGGCATTGACTGTAACGCTTCAGACCTTAGCCTAAGTAC

AAGAGAGGCGCTGTCTAAGTACCAACCAAAGCCACAGATCCTCTTAGGAAGATAACCATC

ATATGAACTCCTCCGAAACGCTCCGCCACCCCAATCCTTCCATGCATCGCGGACAGGTGG

GAGCGGCTAGATATA

>Unigene0002383

GACATAAGTAAGAATAAGAAAGAGAAGAGATAATATTAAGGGTCCGAAGGACGGGTATTT

GTTCCATTTTTGAAACAGGAGTCGTTTTGTCTTTTTCTGTCTTCTTCCCTTGATCCAGGT

GAACATACCAATACAACGTATTCGCGAGCGCACCTAACGATGACTTTCGTCAGTAGTTTG

AACCTGGACTAGAAACCCCTACAGTGTAAGCCGTGAGTACTGCAAAGCAAAGGGACTCTG

CAGGGATAGAAGTTGCTCTTCTCGGACCTGACCAATCTGAAAGACACATCTGCGTGGCGA

CTCCACTGGGGAGAGTTGAAGTCTGTGGCTTATCTCAACTGGCTGGCCCACTAGGAATAA

CCCACTCCGTGGACTAAATCATCAACTGGCGACCTAGCTTCTTGACCTTTTTTTAGTCTA

ATAGTTTTCGGTCTTATAGTCAACTTCTACGAACCCAAGCATCTATACTGAGACTGGCAT

TGACTGTAACG

>Unigene0002384

ATGAACTCCTCCGAAACGCTCCGCCACCCCAATCCTTCCATGCATCGCGGACAGGTGGGA

GCGGCTAGATATAGCTTACAATAAACTTTCAGTGATCAGATTAGGCACTTCAATGGAGGT

AGATGCATTTGACCGACCGCGAGGACTATGTTGAGTGGTATGCGTCAGTGTCCACAGTAA

GAAAAACCTCCTTTCATTCTTGTGTTCCCATTGACCAGGAGCTTGATTCGAGGACAGAGA

GAAGATGGTTCGGCTGTGGCCACACTGATTGATTCGCGGAGGAAGAATAATGTAGCTTCA

GCTGTGGCCACACTAAAACCGAACCAGCAGTTTGGTACTGTCATGAAGAAAGATGTTGGC

TCACTTGTACCGCACAAAAGCTAAAACTACAGCCAAATTCTGTGAGTAACAAAGATGTAG

ATTTAGATGAGGCGAAGAAAGATGTTGGCTCACTTGTACCCGCACAAAAGCTAAAACCAC

AGCCAAATTTTGTGAGTAACAAAGATGTAGATTTAGATGAGGCCGAATGGAAAATGAACT

TACAGCTCAATTCGGGCAGTA

>Unigene0002385

CTAGGCATCGCGGAGGTGTTCTAGCGCCGCACGGGCTCTGCGCGTAGGCAAAAAGGTTGG

TCCGTGACATGTGGCTGAACTGAGAACGAACAAGCATCTCAGTTCAGGGAAGAAGACTGG

CAGTTTGACTGTGGTCAAAGGAAAACCAAACAAGCAGCTCGATTCAGAGACGAAGGAAGG

CGTGAGGCCAGTAGCAGTCAACAGAACCGAATCAGCTGCTCAGTTCGGGAATGAAGAAAG

ATGTGGGCA

>Unigene0002386

CTCTTTTCAACTGAGGAATAATAAAAGATCATTTCATTCCATTTTTAGTCAAATAGTGAA

AGATATTCCTTTCATAATGCCAAAAGGGGTTCCGGGGATACGTATTTGTTGTTCAGGTCG

ATTAGGAGGTGCGGAAATAGCTAGAACAGAATGCGGAAAGTTTGGAAAAACATCTTGTAA

TGTATTTAACCATAAAATCGATTATGCTTCTGCGGAAGTCTCTACTCGTTACGGAATTTC

AGGTGTCAAAGTGCGGATCTCATATACTCAAAATCAGAGGGGACGTGCTATATCCGAAAC

GTACGAAATATAGTAAATCTAGTAAAGGCAGATGTAGTAGGGGTTGCGAACCGGACGGTA

CACAACTTGGTTTTGGAAGATATGGCATTAAAAGTTGTAGGGCTGGTCGTCTTTCATATC

GAGCCATTGAAGCAGCGCGTCGGGCTATAATCGGACACTTCCATCGTGCAATGAGCGGAC

AATTCCGAAGAAATGGTAAGATATGGGTAAGAGTTTTCGCTGATCTTCCTATTACGGGGA

AACCTACAGAAGTGAGAATGGGAAGAGGAAAAGGAAATCCTACGGGTTGGATTGCTCGTG

TGTCCACGGGACAAATCCTATTTGAAATGGATGGTGTGAGTTTGTCAAATGCTCGACAAG

CCGCTACATTAGCGGCGCATAAACTATGTTTGTCAACCAAGTTTCTTCAGTGGTCGTAAC

GTAATTGGTTAGTGGGGGCCGGGACTCTAATTTGGTGAAGTGTTTGTTCCTGAACGATGG

AAGTGGAAAGACAACTCGAGGCCTTTTTTTATTTTTCATCGCCGAAATTGTAGGACTCAC

CTTCTTGTTCCAGGCGTACGACCACGTGACGGTGTCACTTTTCCGGCCCGGTAAAGTGAC

ATAAGTAAGAAT

>Unigene0002387

AAACGAGAATCATTTCGACCCCATACAACTTATCAATGTTTTGGTTTTATTTTTCTTTCC

TTGGTATACCACAGGGATATAACTCATTACTCACTCCTCTAATGAGATGATGAGGAAATG

GTTTTATTTTTCTTTCCATCATATGTACGACAGAGATATAACTCGTTATTCATTATTTTC

TAATAGAATGATGATGAATTGTTGTTCGTGAGTGTAGTAGAACCATCAAATCAAC

>Unigene0002388

AGAGACGCACTTCAGAAGATGGGTGCTCCTCTCTCATCCTGGCCCTGGGAGAATCTCCGA

GTTTACAAGTATCTCTTGTATGGGCCACTAGTAGGGAAGGTCGTATACTCTAGAGCATGG

GAGGGTGGGAGCTCCGATGCATGGTGCTTTCACATCCTGTTGCTCTGTGCGCTTAGGGGT

TTGGTTCACCAGCTCTGGTGCAGCTTCAGCAACATGCTGTTCCTCACCAGGCGGCGGAGG

ATAATACAGGGTGGAGTTGATTATCAGCAGATAGATAAGGAGTGGGATTGGGACAATTTT

CTGATTTTGCATGCTTTGATTGGAGCAATGACTTGTTACTTCTTCCCATCAATCAAGGTG

CTCCCAAAATGGGACCTCAGCGGGGCTGTTGCAGCTCTTGTGATCCATGTTGGGGTGTCC

GAGCCTCTGTTCTACTTGGCTCACAGGCTATTCCACACACGCCAAATGTTTGCCGACTAT

CACTACCTCCACCACTCCACCCACGTGCCAACGCCTTTCACAGCTGGATTTGCGACTCCT

CTGGAGCACATGGTCTTGACTGTAGTGATGATCGCACCCCTTGTCGGAGCTGCGACGATC

GGGTCGGGATCGGCTGGACTGATATATGGCTACATCTTGCTCTTTGATTTCTTGAGGTGT

ATTGGGCACTGCAATGTGGAGGTGTTTTCTTATAGGATGTTTGAGGCCTTCCCTCTCCTC

AGATACCTCATCTACACACCAACGTACCATAGCCTTCACCATACTGAGAAAAACACAAAC

TATTGCCTATTTATGCCTCTTTTTGATGCCTTGGGAGGAACAATCCATCCAAAGTCTTGG

GAGTTACAGAAAGAGATAAGTTCAGGAAAGAATGAACGAGTCCCAGATTTTGTGTTCCTA

GCACACGTTGTGGACGTGATGTCGAGTTTACACGTACATTTCGTGTTCCGTACCTTTGCT

TCGACTCCCTTCAGAATCCGTCTATGGTTGTTGCCTTTGTGGCCGGGAGCTTTTATCGTT

ATGCTCATGATGTGGGCATGGTCCAAGACTTTCCTGGTTGCCTTCTACAACATCCGTGGG

CGCCACCATCAGATTTGGGTGGTTCCCCGATACGGCTTCCAGTACTTCTTACCCTTTGCA

AAAGACAGCATCAATGAGCAGATAGAGCTGGCAATCCTGAGAGCAGATAAGATGGGGGTG

AAGGTTCTCAGCCTTGCAGCACTTAATAAGGTTTGCTACCTCATGTAGCCATACGAATTT

TCTCAGTTCCCTCTTTTTCAGGGTTTCCCTACTAACATCAGAGAAAGTAAAGGCAGTATG

GCATCACATAAATGGTTCTTTATAGTCCCATTGCACCATTGCTTCTTCGTCTCCAATGTC

AAACCTCTAACAACAATGTTCATATTCCTTTTTATTTTCCTTTGATTTCGCTCTTTTTTC

CGAGTGAGGTTTGGCCGAGAATCTTAACCATATTCTGGTGCTCCTCCAGGCTCGAATTGG

AGGCCCAGGAGAATTCAGGGTATGTGTTTGAATTGGCATATATGAGCCCATACAATCAGA

ATAAGAGTGGGAGTCGTTTTTCAGTTTTACAAACTATCAATTTTGATAACTTATATTTGC

TTCATAGTTAATCAAGTTAGGCAATTATGCATGCTAGTTCCTTAGCAAACTTTGGGTTAG

AGTTATACAAGTGACCGAAAAGCTTAGTATCTTCAGGAACCCACAAGTTAAAGTTGTATA

ATCTGCAGTCCCTTTTAAAATCAATAAAAAGTTCTGAATTAAGGATACAGGTGCGCGATT

ACTGTTTCAATTTTTGGGATGAAATTAAAATAAACAACCTATATTGTTTGAAATTTAATT

GATATCTTTTTTGGAAGATCCGGAAAATTCCTTTAGAAAAGTATGATTTATTCAAAAACA

GATATGAGTAGAAATATTTTGAGATTTCGATATTCTTAACAAAAATGTGCAAATTTAAAT

TTGGTGTGATTTAGAGATTTTCCTTAAAAAAAAAAAATCAATGGTACCATGGAAG

>Unigene0002389

TAGAGTTCCAACAGTATCTGGTTCAAGTAACCAAGTATCAAGCTGCTCAGAATTGCAAGA

CATGGATTGTTGGCAAGTGGATAAGTCCGATGGAGCAACTCTGGGCACCGCCTGGCACAC

ACTTCCATCAGTTTGTTGTCCCTCCGATCCTTGGCTTTAGAAGAGACTGCACCTACGGAA

AGCTCGCTGCAATGAAGCTGCCTCAGGATGTACAAGGGTTAGGCATGTGTGAGTACACAT

TGGAGCGAGGAGTAGTTCACGCTTGCCATGCCGGTGGGGTGGTGCACTCCTTGGAAGGAT

GGACACATAATGAGGTCGGTGCCATCGACGTTGATCTTATCGATGTTGTGTGGGAAGCAG

CAATGAAGCATGGCCTATCACCAGTATGAAACGAGAATCATTTCGACCCCAAGCAGCTTA

TCAATGTTTTGGTTTTATTTTTCTTTCCTTGGTATACCACAGGGATATAACTCATTACTC

ACTATTCTCTAACGAGATGATGAGGAAATGGTTTTATTTTTCTTTCCATCATATGTACGA

CAGAGATATAACTCG

>Unigene0002390

GTTCAAGTAACCAAGTATCAAGCTGCTCAGAATTGCAAGGTTTGAATGATCAGAGATTTT

AACATCAATATCAATCCGAACTTTCATTTATTTTGGTTGCTATTCATCCCGGCTTTGAAG

TGAAAGATGAAAATATTATTTTTTAATACAAGCAACCTAGATGAAAATAAAGATGTCAAT

AAATAGACTGAAAATTCCTCTCGACCAATAAGTGTGACTTTTTGATATGAATATGAAAAT

GAAGTATGTATGGTCTGGATAATGATTCGTATTTATTTGTTTGAAATTCCAGACATGGAT

TGTTGGCAAGTGG

>Unigene0002391

TTTTATGCTCATTCTTAAAAGGATAGTTAGGTATCCAAATAACCTATTAACTTTTCGAGT

TCTAAATACACGTAATAAATACTTTTAAGTTGTAACAACCAAACTTGGCTTTGAAAACCT

CAACAATTCAAAAGATAATTCAAGAGTTAGAATGGCCCAAAATGGCAACGTGAAAAAGGG

CCGAAAAATCATTGTTTGATTACATGTTATGTCATTATTCCAAACATAATCAAGGGGTGT

GAACATGATTGATCTTAAACTATGAACCCTAAACCACAAACACAATT

>Unigene0002392

CTCTCTAATCTCATCGCCTGATCTCTCTCTCTCTCTCTCTCAATGTTGAAGTCGAAGTGG

AAGAAGGTGCCTGTCAGGATAATTGTGGACAAACAGAGGAAGAACGTGATATGCGTGGAA

TGCAAGAATGATTTCGTCGACATCCTATTCAGCTTCCTGACACTTCCACTCGGGACCATC

AACATACTCCTCGGCAAGAAATTTCCCAAGAACGGCTTCTATGAATTGTATGAAAGGATC

ATGAATCTTGATCGTGTTTACCTAGAGACAGAAGCCTGCAAGAACATGATGTTCCAGCCA

AAAAGCAAGTCTAGTATGTTTTTGGTAACTAATAATCTTGAAGTGACACCCTTAGATGGT

TCAAAGATGCAAGCAGCTCTCAACAATTATCGGAATTCCAGACTCTTCCACACAATTGAT

GTAGAGGTTGGTCGTGAGGAGATTTTTTATTTGCTAGAAAGATGTCTTATTTCTAAGAGC

CCCTTATCGGATGTATTCCTGCGGCCCAAGTACACAAAGTTATTGAGATTCTTTGTGAGA

CCTCGGCTGCCAAAGACCTCTTTATCAGATGTTCTTTTATGGTCGAAGTTTGCCGGAGTT

TACTACGTTGAGAAACTAATGCGAACAACACCTGAATTGGATAACAAGAGACACTCAAAT

AACATAGGAATGAAGTTGCTTCTAAACGACTATGAAGTGTATTCCGAAGGCAGCTATAAC

AGCTTCTTAACCCTCCCGATTGGTTTCATTATGAAACTCCTTGATGATAAGAGGAGGTCT

TTAGTTGATGTTTTCACCAACCTGCACAGAAGCGTGGAAGCCTTCTGTAACGTTGCTGAA

AAGAATGGTGACCCGAGAATTGGAGGCTCTGAAAATGTTGAAATTTTCTATGATGTCCCA

CAATGCTTTTAGTCATGATGTCAACAATTGGCTGAAGGAATGCTAAGAACTCTGGTTGTA

TTAAGTTACTATTTCATACTTGGGTGTTTTCTATATGAGAATTTCCTTTGGGAATTCAAA

TGCAGACAAGTGTCTTAATCTTTAATTGATGCCACGATGTCAACCAATTCGTGATGAGCA

AGAATTTTTGTACCA

>Unigene0002393

CATGATCGCCGGATACATGAGAGCTGGTGACATAACCCATGCAGTCTCCATCTTCCAATC

CATGCCTCATCGGGACCGCGTCTCATGGACCGCCCTCCTAAACGGCTGCGCCAACAACGG

CCAGACCGAGGATGCGCTATACCTTTTTCAGGATATGCTCACAGACAATGTAGCCCCAGA

TTACGTGACAATCATCACCGTAATCGCCGCCTGCACCGACCTCGGCACGCTCGGTCTCGG

CCTCTGGGTACATTGCTATGCTATGCGGCAAGGGTTCGGACGCAATATCCGAATTGGGAA

TTCGCTGATTGACATGTATGCGCGGTGTGGCCGCCCCGAATTTGCCTTTCAAGTGTTTGA

TGGTATGAGGGAGAGAACTCGGGTGACATGGAATTCAATGGTGGTGGGGTTTGCAATCAA

TGGTTGCTCGAGCGAGGCTCTCGAGCTGTTTTCATCGATGCAGGTTGCGGGTATCAAGCC

TGATGGGGTTAGCTTCACCGGCGCTCTCACGGCCTGCACGCATGCTGGATTGGTTGAGGA

GGGGCTGGGGTACTACAATGTGATGCGGAAAGAGTGTAGGATTCATCCGAGGATTGAGCA

TTATGGATGTGTGGTGGATTTGCTCGGACGGGCAGGGCGGTTGGAGGATGCGATGCGGGT

GGTGGAGAGCATGCCGATGGAGCCGAATGAGGTCGTGTTGGGGTCGCTTCTTGCGGCTTG

TAGGATGCACGGGGATGTGGGTATGGCAGAGAGGGTAATGGGGATTGTTGTTGAATTGGA

GCCTGATTGTGATTCAAACTATGTTTTGTTGTCAAATGTGTATGCTGCGATTGGGGAGTG

GGGCGAGGTCGGGGAAGTGAGGAACAGGATGAAGGCTCGGGGGATTCGAAAGAATCCTGG

GTTCAGTGCGGTGGAGGTGGACTGTGCCTTCCATGAATTTGTTGCTGGGGACCGAGCGCA

CCCACAGAAGGATGATGTTTACGATATGCTTGAGCTGCTCCGGTTCGAGATGAAGCTATA

TGGCTATGATCTTTTAATGTCACCTGGTAGTGACTGATGAAAGAAGCAATTTGTATGTTT

TATAAGGATCGTTGAACTGAATTGGAAAGACATGATAGCATTGAGCAATCTTAGTGGTTA

CACCCGATAAACGAAAGACTTGTACTTCAGTGAGCTGAGCAATTCGATTCTCTGGGACTG

TGGTTAGAGCTTGGATCCTGTCATTCATTGGCATTTGATGAATTGACATATTGCCCTTGA

TTGAGAACAAATTTGAACAACACGAAACAAGGTATGTATGGAACTAATGGGGATATTAGA

AAATAC

>Unigene0002394

GCTCGCCACGCCCGCGCCAACAACCTCCCCGCCGCCGCCGCTACCCTCGCTACCATGCTC

GCCTCCTCCGCCCTCCCCAACGACATCACCCTCCTCTCGGCCTGCGCCGCCTTCCCCTCC

TCCCACGCCGCGCTCCCCCTCTCCCGCCTGCTCCATACCTTTACCCTCAAACTCGGCTAC

TCCACCAACATCCCCATCGCCACCACCCTTCTTGATGCCTACGCCAAATCCAACCTCCCC

CATCTCGCCCGCCAGCTGTTCGACACAATGCCTCACATGAATTCAGTCACTTACAACATC

ATGATCGCCGGATACATGAGAGCTGGTGACATAACCCATGCAGTCTCCATCTTCCAATCC

ATGCCTCATCGGGACCGCGTCTCATGGACCACCCTCCTAAACGGCTGCGCCAACAACGGC

CAGACCGAGGATGCGCTATACCTTTTTCAGGATATGCTCACAGACAATGTAGCCCCAGAT

TACGTGACCTTCATCACCGTAATTGCCGCCTGCACCGACCTCGGTGTGCTCGGTCTTGGC

CTCTGGGTACATTGCTGTGCTATGCGGCAAGGGTTCTAACGCAATGTCCGAATTGGGAAT

TCGCTGATT

>Unigene0002395

CTATATGGCTATGATCTCTTAATATCACCCGATAGTGACTGATGAAAGAAGCCCGATAGT

GACTGATGAAAGAAGCAATTGGTATGTTTTACGAGCATCGTTGAACTGAATTGGAAAGAC

ATGATAGCATTGAGCAATCTTAGTGGTTACACCCGATAATCAAAGGACTTGTACTTTGGT

GAGCTGAGGAATTCGATTCTCTGCGACTGTAGTTAGAGCCCGGACCCTATCATTCTTGGC

>Unigene0002396

GATTGGGGAGTGGGGCGAGGTCGGGGAAGTGAGGAACAGGATGAAGGCTCGGGGGATTCG

AAAGAATCCTGGGTTCAGTGCAGTGCGGTGGAGGTGGACTGTGCCTTCCATGAATTTGTT

GCTGGGGACCGTAATTGTAAACAGGGATTCAAGCTAAAATAAGCCACCGGTTTTGAGAGA

GGGAGTGGCCAACTTGAAAACGTTACATTCTCAGTTCGATTAAGAACGGTTACCAGGGGA

CATAAAAACCCAACCTCCGCGTGCGTTACTTGCTAATCAATCTTTAAACCGTTTTCTCCC

TCTTTATATACAAATCTCATCGCCTGGTCTTGCATACCCCTATCTCTCTCTAATCTCATC

GCCTGATCTTGCATACCCCTCTCTCTCTCTAATCTCATCGCCTG

>Unigene0002397

GTGGGGTTTGTGATCAATGATTGCTCGAGCGAGGCTCTCGAGCTGTTTTCATCGATGCAG

GGTATGGGTATCAAGCCTGATGGGGTTAGTTTCACTGGTGTTCTCACGGCCTGCACGCAT

GCCGGATTGGTTGAGGAAGGGCTGGGGTACTACAATGTGAAGTGAAAAGAGTGTAGGATT

GGTCCGAGGATTCCACATTATGGATGCGTGGTGGATTTGCTCGGGCAAGGAGGTTGGAGG

ATACGATGTGGGTGGTGGAGACCATGCCGATGGAGCCGAATGAGGTCGTGTTGGGGTCGC

TTCTTGCGG

>Unigene0002398

CCACCCTCTCACTCGTCCTCCCCCCTCCCGCCTCTCCACCACCCTCCCTGCCGCCGCCCA

GCGGCCTTCCTCCCTTCTCAGGCGATCGACGGAACTATCACCTCCGCCGCCGCGCCGACG

ACCCCACCGTCATCACATGGTCCTCCACCGTCGCTCGCCACGCCCGCGCCAACAACCTCC

CCGCCGCCGCCGCTACCCTCGCCACCATGCTCGCCGCCTCCGCCCTCCCCAACGACATCA

CCCTCCTCACCCTCCTCTCGGCCTGCGCCGACTTCCCCTCCTCCCCCGCCGCGCTCCCCC

TCTCCCGCC

>Unigene0002399

ACTGTTGGAAGTATTGTATATTCTGATTTAAGAGTGATACTTTCAAGCCAATTATTTTTT

CAAAATGTAGGGATCCAGATTTGAGATCATCAGCCCATTGGATGAAAATATGAAAAACCT

GGTTTCTGTGGTTGTCAAAAAGATTTAGAAATTTGATTGAGGTTGATGGAGATAAGGACG

AGCTGTTATTGTTGGAGTAACATGTGAGCGAAGATTGTGCTTGCAAATCTATAGAACCGG

ATACCTACTACTGAAGCGGAGCAGAGTTGCTAAAGAACTTCCTAAACA

>Unigene0002400

CAGAAAAGGAGAAACAAGTTGATTCTGAACATACACATACAAGATTATACATCTCAGGTG

CTGGGAGCAGTTTTATTATTTAATGCTGAATAAGGTAAATCCTGAACCCTGCCCTACATA

TTCTATTGTATCACTCTGTTACAGGATGAATCAAACCAGCCCTCTCCTATACGATCTAGC

AAGTGACAAGATACATACCATATGGCATGAGATGATACTGGTTAGTATTCTGACACTTTG

CATTGAGCTCCCTATTGAAGCCTTCTATATTTTAAATTTCAGAGTAAGTGCCATATCAAG

CTGTATAGATTGCTTGTCTGGTTGGTTAATGATGTTGCCATCTTGTTTATGTTGTTATTC

CAACGTCTTACAAAATTTTCTGGGTCACTTGATACGTGTGACAAAACTTATAGGAAATAA

AGCAGTTCTTTAAAGGAAAGCCTACAGAGGATTGTAAATGTATAGAAGTAACAAGATATT

GTAATATTTGAGGCAGTGATAGAATTGTCACAATCTATTATTTGAGGCAGGACTATACCA

AACCCTTTTACTCAGCTATTGAGAAGTATCTTTGCTCGAGGAAGCAAACAGTCAAGTTAG

CCTTG

>Unigene0002401

TGTGGCCGGCAAGGATGGTAGTTCCTTCTCTGTTACCATCCTTGGAGGCTCCAGGGTGAT

AAAAGCTCATGGGTGTGATGTTCGGGTGCGCCAGACTTGGCAGGACGGACGATGGGTTAT

GACTGACAATGTAGGCATGAAATCAGATGTTCAAGTTATTGACAATCCGTCAAAATTAAG

GAAGTATTTCCTCCAAAGAACAAAATCGTGCATTGAAGCAAAGAAACAAACAAGCAGAAA

GATGAGAGCTACTGGAGGTATTGGAAGGGGGCAGCCGTGGAATGTGAAGGCTCCATATTC

TATACCAGAAAAGGTAGCTGCTGGCGCTTCACCACATATAATGCTTGCAACTGCGTTACC

TTTACTGGATATGGATAGGAAATTAAATGCTCAGTATAATCCTGCCAGTACCGCCTGTTC

C

>Unigene0002402

GGGGCGAAAAGCGATCGAGCGGAGCTTGGGGAGCAAGTTTTCGAGCTTTTGTTTCAAACA

TGAAGTACAGGAAGGGGTCCAAGGTTGAGGTGCTCAACACCAAGGATCCAACCTCGCCGG

AGTCCTGGCGGCCTGCGGAGATCGTCTCAGGTAACGGGCATACTTATCGAGTCATGTATG

ATGACTTTTCACCCGACAGCTTGACCGCGGATAGAGTGCCCAGAAAGGACTTAAGGCCTT

GCCCTCCTCTGGTTGTGGGCCTGAGGACTTGGCATGAAGGTGATCATGTCGAGGTTTTCG

AGCACAACTCATG

>Unigene0002403

ACACCGACGCCTTCGGCGTGGCTGGTGGATTTATAAAGTTAAACAAGGAGGAACAAATCA

TAGGCGGCGTGCGATCAGAGAACGGGGACGGGGTGCAACAACAGATTGAGGAGCTTAGGG

AGCGTTTACTGAACATCCGCTTCTTCACTTCCGTTGACGATCGACTCAGAGGTTTTTGAG

CTTTTGTTTCAAACATGAAGTACAGGAAGGGGTCCATGGTTGAGGTGCTCAACAACATGG

AT

>Unigene0002404

TATTTGAGATCATCAGCCCATTGGAGGAAAATTTGAAAAACCTGGAATCTGAACTTGTCA

AAAAGTAAGTATTGCAGTGATTGGCTGGCTAGTCCAGTTGTACAATTGCACAAAGGACTC

TGGGACCACTCAGGGATCCATATTTGAGATCATCAGCCCATTGGAGGAAAATTTGAAAAA

CCTGGAATCTGAACTTGTCAAAAAGTAAGTATTGCAGTGATTGGCTGGCTAGTCCAGTTG

TACAATTGCACAAAGGAC

>Unigene0002405

ACACCGACGCCTTCGGCGTGGCTGGTGGATTTATAAAGTTAAACAAGGAGGAACAAATCA

TAGGCGGCGTGCGATCAGAGAACGGGGACGGGGTGCAACAACAGATTGAGGAGCTTAGGG

AGCGTTTACTGAACATCCGCTTCTTCACCTCCGTTGACGATCGACTCAGAGGTTTTTGAG

CTTTTGTTTCAAACATGAAGTACAGGAAGGGGTCCATGGTTGAGGTGCTCAACAACATGG

AT

>Unigene0002406

TTGAGTGGGAAAACGAAGGCTGTCTTTGTGCGTGAGAGATTCAGAGAAAAGTGGCAACAA

GCGAGCAATTAAGAGGAATTCCAGTTTTCAAATCAACGATCAATCAAGAGGTTTCCGAGC

TTTGGATTTGAATCTCTACAAACATGAAGTACAGAAAGGGGTCCAAGATTGAGGTGCTCA

ACACGATGGATCCGACCTCACCGGAGTCCTGGCGGCCCGCGGAGATCGTCTCAGGTAACG

GGCATACTTATCGAGTCATGTATGATGACTTTTCACCCGACAGCTTGACCGCGGTTAGAG

TGCCCAGAAAGGACTTAAGGCCTTGCCC

>Unigene0002407

TGAGATCAATTTCATTCTAATTTGATTGCAATCTCCACCAGGTTTTTGAGCTTTTGTTTC

AAACATGAGGTACAGGAAGGGGTCCATGGTTGAGGTTCTCAACACCATGGATCCGACCTC

ACGGGAATCCTGGCTGCCCGCGGAGATCGTCTCAGGTAACGGGCATACATATCGTGTCAT

GTACGATGGCTTTTTACCCGAGCTTGATCGCTAGAGTGCCTAGAAAGGACTTATGGCCTT

GCCCTCCTCTGGTCATGGGCCTGAAGACTTGGCATGAAGGTGATCATGTTGAGATTTTTG

AGGGTAACTCCTGGAAGCCAGGGGTTGTGGCTGGAAAGGATGGTAGTTGCATCTCTGTCA

CCATACTTGGATGCTCCATGGAGATCAAAGCTCATGAATGTGATGTTCGAGTGCCGCAGA

CTTGGCAGGACGGCCGATGGATTATGACTGACAAGGTGGACTCGAATTCTGACATTCATA

TCATTGACAACTCCTCAAAATTAAGAGGCATGAAGAAACAAATCAAGAGATGTGAGCAGA

CCAGCAGGAAGATGATAGCTATTGGTGAATACGGAAGAGTGAAGAGGCGGAAGGCTAAGG

CTCCATATCCTATACCAGAAAAGGTAGATAGGGGCAGGAAATTATTTGCTCAACACAATC

CTGCCAATATTACTTGTTCCGTCGTTGATAGTTGTAGTGGTAACAACACCCCATATTTAG

ACAGTCATAATAATGAAGTTGAATCTTCATGTGAGTTGGGAAGAAGTCAATCTCTTCCCA

GACTAGGAAGTCCTGGCGAAGAAACGCACCTGTTAGAGTTGTATGCTTACCGGCCAACCA

TCTTAGCATTTCATGCGTCAGGTCTGTTAACTTGGGAGAAAGAATCACTGTTGACAGACC

TCCGCCATTTGCTCCACATATCAAATGATGATCATTTATTGGAGCTACGGCGTTTAAAGT

CTGCCAATGCTTAAGTTTCCTTTCCTTTTTATTTTGTTTACAATGTTCCATGAGTTGTTA

GGAAGAACAAACTGTTCAAAAATTATCAAAAGTGGGAAATACTTGGCAAGATTTTTGTAT

GGTATGAGTCTTTAAGCAAA

>Unigene0002408

AGATAATACATCTCAGATGCTGGGGTCAATTCTATTATTTAATGGTGAATAAGGTAAATC

CTGAACCTGCCCAACATGTTTGTATTGTATCATTCTGTTACCTGTTGATTCTAGCTGAGC

CTTGAACAAGAAGATTGCTCCAATACGACCTCGCCTGTGACAATGTACATACCTTGTGGC

ATGAGATGATACTGAACGTTGGGCAACTAAGAAATGAGCTGAAATGGGGATGCTTAGGAT

CAATACTGGAAGACTAGGAAAGAATAGTATTAAAAATAAACATGTATTCTTGAAAAGGAT

GGGGCAACACTAATTGATGACAAGTTGAGGAGAATAATATGGTTTGGTCACATACGAGGA

CAAGTTGTTGCATTGATTGGGAAGAGTGACAGGACACTTATATACTGTGCGAGTTCTCCA

TAAAAATTGTGAGAGCAGGTCTGAATAGGCTTTAGATTCCAGGGTGGGATTATCACAAGA

GATTTCTGGGTTGTTGGAGACACGGATGTCACTCAGTTAGGATTCCGAGCTAGCTGAATG

GATTAGGAAGAGGGCCTCGTGCTATATGCTGACCATTTTGGAAGGGAGGGGATGCTAGAA

AGTGTGAGGCAAAGATGCGGAGGAGCAGTGGTATCAAGAATGTGACGGAGCTTGCATTCA

GCAACACCTGTACAAGACTTTCTTGAGCAGCCTTTTCAAAAATAATCTGGAGCTGGGACC

AGCAGCTGGAGAAGCTTAGAGTTGTACCTTTGTGTGTATTTCTGGAAGTTTGGCCTTCTC

TTCTTGAACTGTAACTCTGTTTATGTTTTCTGATTCTCTTTTATGTTTTTGTATGACTTC

GTATAAGTTTCCCCTTTTATTAAT

>Unigene0002409

TCGAAGAGTTCGAGTTCTTCGAGATACCTTGTCAAGAATTCCCAAGTTGTTGTCAGGATG

TCAAGTCCCCTTAACAGGAACACTTGCAAAACTTCAAGCGCCCAATTTCTTGTCCCCTGA

AGAGGAACACTTCCAAGTTGTCACGATGGCAAGTCCTCTTAAGAGGAACACTTCCAAGTT

GTTGAATTGGACGCAAAACTTCAATCGTGCAAGTTGTACGTGGTAGAAAATGAAGTAGAA

TCATCATTATACTGCAGTCTGGAGAGCTGGAGGAAGAGTTGTTCTTTTCTGTCCCCTTTC

GTAAATTGTATATCTTATATTTCTCGTGAGGCGACCTGTTTTTGTTAAATG

>Unigene0002410

ACCAGAAAAGGAGAAACAAGTTGGATGTATCCTACATATATGAGATAATACAACTCAGGT

GCTGGGGTCAGTTCTATTATTTAATGGTGAATAAGGTAAATCCTGAACACTGCCCTACAT

ATTGTATTCTATCACTATGTTACATGTTGTTTCAAGCTAAGCCTTGAACACGAAGATTGC

TCCTGTATGAGCTAGCCCGTGATGTGATACCTACCATAGGGCATGCAATGACACTGG

>Unigene0002411

TATTTGAGATCATCAGCCCATTGAACGGAAATTTGAGCTTGTCATAAAGTAAGTTTGCAT

TTAGAGGTTGTTGGCCTTTACATAGATTTCATTTACATTACTTATCGCATGGGTATTTAT

ATTTAGTAGGAGGACAGTTTCTTTACGGATTATCGGAAGTAGTATTAGAAACTGTCTCGG

ACAATTTCGTGATGGGTTAACTACCCAAAAGGTTGGTATAAATATCTACCTATATCCCGA

TAAAAAATAATGATAAATTCCCTTTCTTCTCCCTTCTCTGCAAGAACATTTAAGCCGTGG

AACAGTGGAAGATTTTCATTTCCATGATTTCTCCCCGGATCAATGCTGCACGAGGAATA

>Unigene0002412

CCTCTACATTGGAGGTATTTCTCCACAATCAACATTACATCCTGGTTTTAATATTGACCA

TGTGGCTCCACTTCTGAGGGGTTAGATATTTGTACTCGTTCACATGTGATCAATAGAGGT

GTATCTATTGCTAGAGTTCATCTCTCGAAGAGTTCGAGTTCTTCGAGGTATTGAGGGTCA

ATCGGTCAATCTGAGTTTGACACCAATTGAGAATCTTCCACTCCAGAAGTTAATTTGCTT

ATCTGTCTATGTTTTGTATTTTCAAGATACCTTGTCAAAAATTCCCAAGTTGTTGTCAGG

ATGTCAAGTCCCCTTAA

>Unigene0002413

ACACCGACGCCTTCGGCGTGGCTGGTGGATTTATAAAGTTAAACAAGGAGGAACAAATCA

TAGGCGGCGTGCGATCAGAGAACGGGGACGGGGTGCAACAACAGATTGAGGAGCTTAGGG

AGCGTTTACTGAACATCCGCTTCCTCACTTCCGTTGACGATCGACTCAGAGGTTTTTGAG

CTTTTGTTTCAAACATGAAGTACAGGAAGGGGTCCATGGTTGAGGTGCTCAACAACATGG

ATTCGACCTCACGGGAATCCTGGCGG

>Unigene0002414

TTGAGGAAGAGAGAGAGGGAGAGAACGAGGAGAGAAAATAATTATATTATACAAAATATT

TACGAATTTTCGATCCAAATGAAAATCAGAGGCGCTCACAATTGCTGATCCTGATCGAGA

TCTCCGCTCGCAATTCGCCGGGATCTGCATCAGGTTCACCGAGCTCCGGTGATCTCCGTC

TAAGGAATTTCGGTCAAATTGCCCGAGATCGTTCAGTTTGAGATAAGGAGCTTTGATCAA

AGCGAAAAAGGTTTGCAGATTGTTTTGCTGAATTTCTTGTTGTCAAAAGTCTTTGAGATA

TGGTGGTGCACAAGAGTGACGAATATGGATCGAGTGGCGACCGGGTCCCTCGGATGCCGC

ATGTTCCAAGATCAGCAAAGGGTAAGCGTTCGGTCAGAAAGAAGTCCGAGGACAATGAGA

TGTGTGCATTTGACATTCTTGCTACCGTAGCTGGGAAGTTGCTGGAGACTGAGAATTCTG

TAACCCCATTTAGCTCACCCGGAACATCTAACCCTAATTCCGCAAAAGTTAAGAAGGAGC

TATCGAATGAGGGAAAGTCACCCGTAGCTAAAGAGTTAGACTATAGGAGTTGTAACAATG

GTGCTTTGGGATCTGATGGCCTGGGTATCTATAATGCAAATGGTCATTTGATGACTTCTA

GTACACCTTCAGGATGTTCATCTGTTTTTGTAAAGCCTGATACATCAGAGAAGAATGCAT

TTGCCAGGAAGTCGCTGGTTAGTGACAAAAGGGTTGAGGTCGGACATGCTCTAGGGACAG

TTCTTCAGAAATGCAGCATAAATAGGCGCTCACCTGATTCCATGTATTGTCTCCAGGCAC

TGCTGCAAGCTGAAGTGGGAATGTCTGGAAACCTGTTACAACGCAATGTATCAGGCATGT

GTAGTATGGAGGATGCGATGGATGTAGATGCCAAACCACCTGCTTTGGTCAGCTCAGAGA

GCAGCGCGGAGGTTCCTCCTGGTGGGGTTCGCATGTCTAACTGCTTCGTTTCGAAGCACA

GGGAACATTTGGAAGATGTAGTAATTAGAGATCGTAATGATGATGACAACTCTTCTGGGT

GCACTCATCCGAGCACCATGCCTTCAAAGGCATTTAGGCCTCAACGTGTAGGAGACCGTA

GGATTAGGAAGTTGCTGGGATCCAAGTACTGGAAAGTAGCTCCAACAACTTTTAAGGACC

GGGAGCTCTCTGATGCTGAGGTGAAGCCGGTCTATCGTAGCAGGAGAATGTGTTATACAC

GACAAAGGACACAGAGAAGCACATTCAAGAGGGTGAAGCAGTTTGAGCACCGCACAATAT

CAACATCTGATGGTGGAATGTGTAGGGAAGTTATATCGGACTTGCCTCGTAAGGGCAGCA

TCAAGTTAGAAGCAAGTGAATCACATGCAACACTACATGGAGCCAATGGGGTATCTTCTT

CAACTTCAGGTCAAAAAACTTCTTATGGGGAGGAAGATCTACATGTTCAATTCAGTATCA

AGTCTTTCAAGGTGCCCGAACTCTTCATTGAGATTCCTGAATCTGCCACTGTTGGCTCGC

TGAAGAGGACTGTGATGGAGGCTGTCAATGCAATACTTGGTAGTGGACTCCGAGTTGGAG

TTCATCTCCAAGGAAAGAAGGTAAGGGATGACAACAAAACCCTTGCCCAAGCTGGAATAT

CTCATGATGAGAAACTTGAGAATTTGGGGTTTACATTGGAGCTGAATCCTACACCAGCTC

CACCATTGTGTGTCAACCCCGAAGATAAACCTATCCCTTTTCCATCTGATGCTACTGAGC

ACCTAACAAGGATCTCAACTATGGTCCCCCCTACAGATCAAGGGGCATCCAGTGCCATCT

TGCAGCCACCATTAGTTGCTGAAGTGAACTGCCCTGAGAGTGATCACGATTCGGTTCACT

CTACACCAGATGTGTCATCACTTGATAAAACAGATATTTACGCAAATGCTCTGGTTCCAG

TTCCTGCCGCGAGCGCTGAGCCACTGGATGTGGTTCCTCTGCGCAAGTCACGTAGGTCGG

AGCTTGTGCAGCGTCGGATCAGGAGGCCATTTTCTGTTACTGAAGTGGAAGCTCTTGTTC

AAGCAGTCGAGAAGCTCGGGACTGGAAGATGGCGTGATGTAAAAATTAGGGCGTTTGACA

ATGCCAAGCACCGAACATACGTCGATCTTAAGGACAAGTGGAAGACTTTGGTGCACACTG

CTAGGATATCCCCCCAGCAGAGGAGGGGAGAGCCGGTTCCCCAGAACCTCCTGGACCGGG

TTCTCGCAGCCCATGCCTTCTGGACTCAGCAGCAGGCCAAGCTCCAGATTAAGTCGCCTC

TGACTGAAGCTTGCCTTCTCCTTTGATACATAGGCTAAATTCTTTTGACTTTTCAGTTGA

TCCATGTAAAAATTTCCGGAAGGATCGCAAAACAGGGAAAACAGTTTAAAGGGAAAAAAA

GAAAAAAATTGTGTAATAGCCTCTCACATACAGCTTTGATATGTGTAAGCCAGGTCTCTC

AGTCCTTGTGTTCTCGGAGAGGAGAGGGTTCTTTTGATGATATTGTACATGGTGATGATG

ATGGTGATAAGGTGGTCTAAGGCGAATAAATTTGCTAACATAATTCATACAATGCTCGCC

GTTTGGTTTGGCGTCCTGATTGGGAAAACTGAGATTCATTCATTCTTTGAAGAAGTTATT

GCGTTATGGATAAGAAAAGAAAGTGAAAAGGAATGTGATTTTTTTTGTTTTTGGTTAATT

AGGATTGTGGGAGCAGTAAATTATTTGTTGGGATGTAAGTTTAATAAATGATTTTCTTTT

TCTTTCTGTACCAAACATGTGTAAGATTTGATTCAACTTATCATAGATTTAAACCCAACA

TAGGTGATTAATGGGATATTTTA

>Unigene0002415

CTACCAGTTTTATTGTGTTTAGACAACCATGTTAAAAGCACAAAATTTTGGTTTTATAAA

ATAAAGGAGAATAAGTGGTCGGCATGGATGGTGATAACTTTGTTAGGAATTGTGATTGTG

ACGGTGGTGGAGGTTACTTAAAAGTATGTGGAACTAAATTCTGATTTGGTGGTTGTGGTG

ATGCAGGACAAGTGGAAGACTTTGGTGCACACTGCTAGGATATCCCCCCAGCAGAGGAGG

GGAGAGCCGGTTCCCCAGAACCTCCT

>Unigene0002416

TAGTTGGAAATGGAGTCACTGACCCACAATTTGATGGCGATGCTCTTGTTCCGTTCGCTC

ATGGAATGGGTCTTTTATCAGATGAGCTGTTTCAGGAAGTTAATACTGCCTGTGGGGGAA

GCTACTGGAATCCAACAAATGAGAGCTGCGAAGAGAAGCTTGATAGAGTTGATTGGGAGC

TTAAGAACCTAAATATATATGACATTCTCGAGCCGTGCTACCACAGCCCTCAAATTAGAG

AAGTGTCTGATAATGAGAGGTTGCCATCCAGTTTCCGAAGGCTGGGTGAGACTGATAAGC

CTCTACCTGTAAGGAAAAGGATGTTTGGACGTTCTTGGCCTTTAAGAGCTCCAGTAAGAG

ATGGACGTGTTCCCACTTGGCCGGAGCTCGGAAGCCGTTCAGTTCCATGCACGGATGATG

AAGTTGCAACTATATGGTTGAACAATGAAGAAGTGAGAAATGCAATTCATGCCCAACCGG

AAAGTAAGCTAGGCTCATGGGAGTTATGTACAGGAAACATCAATTTTCATCACGATGCTG

GAAGCATGATTAAATATCACAAGAGCCTCACTGGTCGAGGTTACCGTGCACTCATATTGA

GTGGGGACCATGACTTGTGTGTGCCGTATACGGGCAGTGAAGCATGGACCAGATCAATTG

GATATCCTATTATTGATGCATGGAGACCTTGGTATTTTGATGACCAAGTTGCTGGATATA

CCCAAGGATACGCTAACAACCTCACCTTTCTCACCGTAAAGGGATCTGGACATACTGTTC

CTGAGTACAAGCCCAGGGAAGCATTGGCTTTGTTCAGCCGTTTTCTAGCCGGTGGAGCCA

TCTAAATGTTGTCGGAAAATTGTTTTTCCGTGATTTCAGAAATAAATTAAATTAGATCCT

TTGTTTTTTATCCTTAGGAATCTTGGTATTACACCGTGGTACTTGATTTTGGATCCTGTG

GTCCTTGGTGGCCGGATCGGTACTCATGCTAGCTCTAATGAAAATTGAGTTCAGCAGCAT

TTTGATTACATATCGTACGATCTAATGTGTTTTAAAATGCTTACATATCATATGATCTAA

TGTGTTTTAGGATCCATTAAGAAACTTTTGGGATTTCTAAAAATCATTTGTGTATATCCC

TTTAG

>Unigene0002417

ACCATGATTTTTCTTATAGTTTATGCTTGAGCATGTGTGCATCCTTTGAATGTTGTGATC

AGTGTTCTAAAAGGCGGCATATGTATCCGCCTAGGTTCAAACATTGACTACTCTGTATTC

CTTTTCTTTCTTCATACCTATATTTACTCGATACGTAAGCTAATGTTTGAAGCCTATAGT

TTCTTCTCTTATATGTGAGGAACCATGGTTTATACCTCGCACAACCAGTTGCTATTATCT

CTTATAT

>Unigene0002418

CCCATCCAGCGCAGCCATCGTTGATGATCGCCTCCTTTCTGTTTCTTCCCCCATCCAGCG

CAGCCATCGTTGTATCATCACCCCAACGCCTCACGTTCACCTTTCTCCCTAATAAGCTTC

CCCTTCATCTGGCCCTGATTCTCTATCATATATAAATACTCCTTTCACCCATCAATCTCT

TTTCTTCATCCCTCACCATGTCCCGTCTCACCCTCCTCCTCGCTCTGCTTCTCCTCTTCC

TCCCCTCCATCGCCGCAGCACCGGAGAGCGATATCATCACCCACCTCCCCGGCTTCAAAG

GCACTTTCCCATCCAAACACTACTCAGGCTACGTGACGGTGGACGAGGATCATGGGAGAA

ACTTGTTTTACTACTTCGTGGTCTCCGAAGGGAATCCTTCCAAGGACCCTGTGGTTCTCT

GGCTCAATGGCGGCCCCGGGTGCTCCAGCTTCGACGGGTTCGTTTATGAGCATGGTCCGT

TCAATTTTGAAGTTGGAAGTTCGCCGGGAAGCTTGCCGCAGCTGCATCTGAATCCTTATA

GCTGGTCTAAAGTTTCTAACATTATCTATTTGGACTCCCCGGCTGGTGTTGGGATGTCGT

ACTCGGCCAATAAGACCGATTACACTACTGGGGATAACAAGACTGCATTGGATTCACACA

AGTTCCTCTTGAAGTGGTTTGAGCTATATCCAGAGTTCCTCGCAAACCCTTTTTTTATAG

CCGGTGAATCATATGCTGGCGTTTACGTTCCGACTCTTGCTTATGAGGTTGCTGGAGGAA

TTGAAAGTGGTATAAAACCTACACTAAACTTTAAGGGATAC

>Unigene0002419

TCCACCTAAAACCATCAATAAAACATTTCAGATCCACTCCCTCGATTTCAACAGAACCCC

AGGGTTCTTCCCCGAGGACCTGCTCCTCCCCATCGCCGTCGTCCGATCCGCCGCCCCGTC

TGCCGTCGCCACCGATCCCTCTCAGGTCAGCCCCCTCGATACTCCTCTACTAGCAATCGA

GCTTTTCTCCATCTAAATCCCGGTTTCTCTCCATTTCTAGGTCAGTATACATGAGAGGAG

GGTTGGTGTCGACTTCGTCTAGGATTAGATAATAGTTTTGAGCTACAGATGGGTCGCGGC

AAGAGAAACCCTAGCAATCGTCCTTCACCGATCGCTATCAAGTCAGCCGATACACCATCG

GCGAACGGCGATCTCAAATCGGTTCCGCCGCCAACGGCGGCGGACCTGAAGGCGGAGTGC

GACCGCGCGCTCACCGCCATCCGCCGTGGCAACCACACCAAAGCCCTCCGGCTCATGAAG

GATGCATCCTCCCGCTTTGAATCCTTCCCGATCTTCCATCGCGTCCACGGTACTGTCGCC

GTCAAGGTGGCCTCAATCCTTGATGACCCCGCCGCCAAGCTTCGCCACCTCCGTCTCGCG

CTCGACTCGTCCCGCCGTGCAGCAGCCCTTTCTCCTTCGTCCATAGAGTTTGCGCACTTC

CACGCATCCCTCCTCTACGAGGCGGCTACCGAAGCCCGCGGATATGAGGAGGTCGTGCAT

GAGTGTGAGCGTGCCCTTGCGGTAGAAGACCCGATTGACCCTGCCCGTGAGGTCCTCCAT

GATGAAGCCCCTACTCCACGGCTCGCAACCGCCGAGGCCCGCATTGCCCATATGCACGGG

GAGTTGCGGAGCCTCATCCAGAAGGCAAACATCGCCTCCATCTCCTCTTGGATGAAGACG

CTTGGCGGCAACACTACTATCCCCGGCGAGGAGAAGTTCCGCCTCATTCCGATGCGCCGA

CTCACTGAGGACCCCATGGAGGTGTGCATTGTGCCCGCCGCCCCTCGCCGGCCTAATGAG

ATTAAGAAGGCAACAAAGACACCTGAGGAGCGACGGCAAGAGATTGAGGTTCGTGTGGCT

GCTGCACGACTCATCCAGCAGAATTCAGGCGCTGACTCCGTCAATTCTATCAATGAAGAG

GGCGCCGACTCTCCGAATACCTCACCGACACTCTCGTCCTCCTCTGCGGCCCAGCGCCTG

GCTGAGCGTCGCAAGCTGAACAACTCCCGGAAGCTCACCTCCTCGGTTGATCGGATGGAT

CAGGTTCGGTCATACTGGAACTCCATGTCCGCAGAGCAGAAGCAGGCCTTCCTTGCGGTG

AGCATTGCGGATCTTCGGGCATATTATGCTCCTTCGCCGAAGGATTCTCTTGCATCGGAT

GTGCTCTCCGAGGCTCTGTCGTTTGTGGCGGACCATGAGGCGTGGGAGTTTTGGTCCTGC

TGTCGGTGCGGTGAGAAGTTCACCAACTCCAACTTCCACACGCATCACATTGTGCAGGAG

CACATGGAGACCCTCTCGCCCAAGTTGCAGTCGGTGCTGCCGCAGGAAGTCGATGGACAG

TGGGTGGAGATGCTCGTCAATGGCAGCTGGAAGCCCATTGACGTGGATGCAGCTATGTAT

CTGCTTGGCGAGGAACAGCTCAAGTTTCAATCTGTAATCAAGGATGACGACTCTGAAGTT

GGTAGCAAGGATAAGGATAGCTTATCGGAGTACTGGAGTCCTAAAGGCAACAGTTCGGAC

TCCTCCCCTTCACCCCAGAAGAGGGAATTGGAGGACGGAGTAACTTGCAATGGGTTTGCC

ATGGAGAGCAGGAACAACAACGACGGGGCTTCCAACTTTGAATGTGTGTCTCGAAAATGG

CCTCTGTCCGATGATGTGGAGAGAATCAAGCTTCTGGAGAGGATTCAGAACATCTTTCAG

TCGCTCGTCAAGCATAAGAGTCTCTCAGTTAGCCATCTTAACAAGGTGATCCAGTTTGCC

ATGGATGAGATCCAGGGTTCGCCCTTTTCAGCGATACTGCTGACCCATGCACTTGACCAG

TCACCACTGTGTATCTGCTTCTTGGGCACCCCGCAGCTTCGGAAAGTCTTCAATTTCTTG

CAGGAGCTCTGGCAGTCATGCGGAGTAGGAAGATACTCGGAAAAGGACAGCATGACCGGT

GATGTTAATGGTGCTGGGCAGGTGAAAGAACTCGTAGAGGGTGTCACCTTTGAACATGGC

TCTTCGAGTCTCCTCTTGGATGGGCGCGTATTTGGTGAAAAAAGTGGTTCCTCCATCAAT

GAAGTAACCGATAACCTGCCCAATGCGAATGCTATTGTCTCATGGTTGTTTAGCGGCCCA

ACCAGCACAGACCAACTATCGGCATGGATCCGCATGAGAGATGAGAGATCACAGCAGGGC

TTGGAGATCCTTCAGAATCTCGAGAAAGAGTTCTACCTTTTGCAGAGTATGTGTGAGAGG

AAGTCCGAGCACTTGAGCTATGATGAGGCCTTGCAGGCTGTTGAGAGCCTCTGCGTAGAA

GAACTTAAGAGAAGGGAGCATGCTACAAAGTCCACAACTCAGAGTTACGAGGCTATTCTG

AGGAAGCGTCAGGAGGAGCTTATGGAGCGAGATAATGAACCGATATTCATCAGCAGCAGA

TTTGAGTTGGATGCGGTATCTAATGTGCTTAAGGAAGCACAAACTTTGAAAGTGCCTCAT

TTCGGGTATGATGAGACGTTATCGGGTGTAACATCTCGCTTTTGTGAATTGGAGAACATT

GAAGATGATGAATGGAGAGTTAACGACTGCTTTCAGCAAGCAGATAGTTGCATTGAGTTG

GCGATGCAGAGACAGAAGGATCAGTTATTAGTGGAGCTTAACAAGATTGATGCAAGGATC

ATGCGGAATGTCACTGGCATGCAGCAGTTGGAACTCAAGCTTGGACCCATCTCTGCCTTT

GATTATCGAAAAGTTATTTTGCCTCTTGTAAAGTCTTTTCTGCGGATGCATTTGGAGGAT

CTAGTTGACAAGGATGCCACGGAGAAGTCTGATGCTGCAAGAGAGGCCTTTTTAGCGGAA

CTTGCCCGTGATTCTAAGAAGACTATCAATAAGGATGGCGAATTGAAACAAATGCACGAG

AAATCTAAGGATAAGAAAAAGAACAAGGACAGAAAAATCAAGGATGTGAAGGTTGCGGGC

TGTAATGATCCTCTTATCTCACATCAGGAAACAATAGAACAGTCTAAAATTCCTGCCAAT

GGCATTCTTACTGAATCTGAGCCTATCTCAAGTGATGATTATTTGAAACAAGAAGAATTT

AGACGCAAGGTTGAGCTTGAGGCAGAAGAAAGAAAGCTTGAAGAAACTCTGGAGTATCAG

AGACGGATTGAAGACGAGGCAAAACAAAAGCACCTTGCTGAACTGCTTAAGAGTGGGACT

GGAAATTCTCCAGATAATTTTGGAGATGAAACTTATGTTACTGGTTCCGGCTTAAGTGCA

GATTGTCTGGGTCCAAGGGACATGCTAAGTTACTACAGTCAGACAACCTTGCGTGATAAC

ATTTCTCCAGATATTATTGCAAATGGAATAACTTTTGGCGATTTTTACTTCTCTGAACCT

GTCATGCGGAAGAACCATGAAAACATGGAATTTGACCGGCCAAAAGTTGCATTTGCCAGA

CAGCATTTGGTTCAAAATGTAGAAAAACAGCAGCCGGTGGATAACCCCATGGATATGGCA

CATGAACCTCGTACCGTTGAAGTTCATCCTGTAGGTCAGACCATCAGGATGGCAAACAGC

TCACCGGGCATCCAAGTAAATGGTGTAAAGAGAATTGAAGCCTCTCCAAAGTCTTCCAGT

GGCTCTCTTTCGAAGAAAAAGCGGTCAAATAGCCAGTCACATTCTAAAAAAATGCAAGGA

GATGATGGTAGTAAGACACTACACGAATCACGTGCTGGGGATGATGATGAAGAAGAAAGG

TTCCGGGAAGACCTCAAGAAAGCTGTACTTAATAGCCTTGACGATTTGGAAGCACATAAA

AATGCATCAATTACTCCAGTTTCAAGGCTATATCAAGATTTAGATGTTTCTGATGCTTTA

AGTAATGGGCATGCAACTATTTCAAGTGGAAAAGATGTATTCGGAACAGGCCTGAAAAAT

GCAGTTGGTGAATACAATTGCTTTCTAAATGTTATTATCCAGTCATTGTGGCATTTAAGA

AGATTCCGTGATGAATTTCTGAGGGCATCTGTGATGCACTCACATGTCGGTAATCCCTGT

GTTATCTGTGCTTTATATGATATCTTCATTGCTTTGAGCAAAGCAACAGAGGATAGTCAG

GGCGAAGCTGTTTCACCTACTTCTCTGAGGATTGCTTTGAGTAACCTTTATCCTGATAGC

AATTTTTTCCAGGAGGCACAGATGAATGATGCTTCTGAGGTATTAGCAGTTATCTTTGAA

TGCTTGCATCTGTCCTTCACATCTTCTGGCGATTGCGATACTGAATCAGATGGGAGTAAC

TGTGGGGGCACATGGGATTGTTTAAGTGCTACCTGTATAGCACATACCTTTTTTGGAATG

AACATATTTGAACGGATGAACTGTTACATTTGTAGTCTAGAATCCAGGCACATGAAATAT

ACTTCATTTTTCCATAACGTCAATGCAAATGCACTCCGAACAATGAAGATGACATATGCA

GATAGCTCATTTGATGAGATTTTGAAGATGGTGGAGATGAATCATCAGTTAGCTTGTGAT

GCAGAAGCTGGTGGCTGTGGAAAGCCCAACTATATTCATCATATTCTTTCTTCTGCACCA

CATGTTTTTACAATTGTTTTGGGTTGGCAGAATATGAATGAAAGTGTTGAAGATATATCT

GCGACACTGGCGGCCATTACCACTGAAGTGGATATCAGGGTCTTTTACCGTGGTCTGGAT

CAAGGGAGCAAGTATTCCCTCATTTCATTGGTCTGTTATTATGGGCAACACTATCACTGT

TTTGCGTTCGAGGACGACCGATGGGTCATGTATGACGACCAGACTGTCAAGGTTGTTGGT

GATTGGAACGATGTTCTTTCTTCATGTGAGAAGGGTCATTTGCAACCCCAAGTTCTTTTC

TTCGAGGCTACCAGTTAGTTTCTTTTACAAGAAATTCACCCGAAAATAGGTATCGAGGAT

TAAATCACCACCAAGTACTGAAATGTGAAAAGATAAATTGGTCGGCATATTCAGTTTGGA

CATTGGAACCAGAAAGACGGGGAAATAATTTCGTAGTGACGAGATCAATCCCTTCAATAA

AGCTGCAGGATCAATGAGCTCAAATCTATACTTATCTGGAGTCATCAAAGGTGATTTTCA

GGATCGGGTGAAGTATATTGGAGTTCGATACGATTTTTCGTTAGCAGATACTGGGTCTCT

ATCTCCGAGGTGTGCATAGAATTTTCCTCGCCCTATTGACCTCATCGTAGTTTATTCTGA

TTCTTATAGACATCTCGAGGATATGCGAAGATAAATCAGTTCTATGGTTTATATTTTGTC

GAATTACCGTAAGATCGTGGTGGTTAGTTATTGGGAGTTTGGGCTAATTTTCCGATGTAT

AGAATCATGTGCCTCTATCTAGGTTGAGGTATGCAAATTATGATGATGAAAAGGCTCATA

GCACTGTCACCTGCATTCTAATGGATTTGTTTTTATTTATGGGTATTGAACTTAGACCTT

GTTACTCAAGAATATTTTGTTTTTTTACTTCTTCGGTCCATTTTCTGCTACTCCGTAGTT

TGGAGGGGGGAATTTTTCTGAATTTATGTTTCTGAATTATAATGCTTTATTGGTAGTCTT

TTAAGGATGTTGTGTTTCTTTATTCATATTGGTGGCAATAAGAATTATATGTTGTGTTTG

TGTAAA

>Unigene0002420

TCCACCTAAAACCATCAATAAAACATTTCAGATCCACTCCCTCGATTTCAACAGAACCCC

AGGGTTCTTCCCCGAGGACCTGCTCCTCCCCATCGCCGTCGTCCGATCCGCCGCCCCGTC

TGCCGTCGCCACCGATCCCTCTCAGGTCAGTATACATGAGAGGAGGGTTGGTGTCGACTT

CGTCTAGGATTAGATAATAGTTTTCAGCTACAGATGGGTCGCGGCAAG

>Unigene0002421

AGCAAGTATTCCCTCATTTCATTGAACAATTTTCAGAAATTGCAAGTTGGCTCGTGTGGT

AAAAAGCCTTCCTGTGACCTTCCGGTACCGAGGGTCAAGTTGGTGAAAAAGGCATCCAAA

GCAGTAACATAGTTGTCAAGAGGTCTTTAAGGATATCTTAACTTTTAATGTGGTTGGGGA

CAGCAACATGAAATTCTCCAACGTACAACAGCTGCTGATTGGATATCATGTATATTGTCT

CCCTCCTTTTTTTTATTCTTTATTTTTAAT

>Unigene0002422

TTCGTGCCCAGCCCGACCAACTTCTCGGTGTCGCGAGAGCCTACTAACATTCTGTACCCT

GGGGAAACCCTAAAAGCAACCGAATCACTCACATGGGGGACTTTCCAGTTCAAAATGAAC

ACAGATTGCAACCTTGTGCTCTATGACCAGTTCAACTCTGTGTGGCACACCAATACCTAT

GGCAAAGGCACCGGCTGCCACTTAGCCATGGGGACGGATGGGAGCCTCTCCGTCT

>Unigene0002423

GACGGATGGGAGCCTCTCCGTCTATGACCACCGTAACACCCGTTTGTGGGGTGCTTATTT

CGAGGGCCCGGTAGGGAAGTATGTCCTGATCCTAACGAAGCACCGGAAAATCGCCGTCTA

TGGAAGGGATATCTGGTCCACCAACTGGGACGGGGTAGAATCCAGCAAAGGAAATATCTC

TATGGTGACCAACTAGTGATGGATTTGGGACGAGGGTGCGTACAAGATCTAGTGCTCGGC

AGTTGCAGTCTTTCTTTAATTTCGTTTCAGCTAACGTGTGAATCTGTAGCTCAATTACC

>Unigene0002424

TTAATATGTTTTGTGGGCACCCTTATGAGCACTACTCTTGCTCTCGTGACAGAGGACGGA

AACGCAGCTGCTTGGATCATAGGTTGGGACTCAAGGCTATTCGCTGCTGTGCACTCTGTA

AGATTTAACTCAGGGATTGTTAAATTGTAATTAATTTGGATATGGATGGATAATTAATTA

AGTCATTTGTTTTCCGGGCAAATTATGCACTGGGGTCGCTTATTATGTGCAAGGAGTAGT

CATGAAAGAAAGCGATGCAGTTTTTGTTACCGTTTTCAATCCACTCTGCATGATCATTGT

TCTGTTTCTGGGATTCATCATTCTTGCCGCGGTCGTCACTCTTGGAAGATTGCTTGGTAT

GATCATCATTGTTATTGGACTCTACTTCTATATATGGGGCAAAAGAAAAAATTATGTGAC

TCCAACATTGAATGCTAACACGAAAATAGCACTTGAGCTACCCAAGGCGAAGAACGTAGA

TGCTGAAAATGGTGAAAATTTAGGACCAATCATAGAGGATGAAAATGATAAAAATTTAGC

AACTGGCAAAGACGCCATTGTAGTCGTGGGCATCCCTACAACTGTTTCAGAAGTCACTGT

GAAAGTTGATGGCGACTGGGTATCGACAAAACACTAATATAGTTGTC

>Unigene0002425

CCAATGCATTTAAGCCGTCCACTCCCTCCCCCTCCCCCCGCATAGGGTTTCGAGAATGTG

GGGCGCGACGGAGGCTGCGTTCTCGCGGTCGAGCTCGTACCGCGAGGGCGGCGAGGAGGA

GGAGGAGGAGGCACTGCGGTGGGCCGCGCTCGAGCGGCTCCCGACGTTTGCGCGGCTGCG

GCGGGGGTTTCTCAGGGGACACGCCGGCGAGTATGAGGAGATCGAGGTGGCGAGGCTGGC

TCCCGATGACCGGAGCGCGGTCATCGAGCGGCTGCTCGGGGATTCCGGCGATGCTGAGGG

GTTTTTCCTGCGAATGCGGCAGCGGTTCGACGCTGTCAAGCTGGAATTCCCGAAAATTGA

GGTTCGATTTCAGGACCTGAAGGCTGATGCATATGTCCAGGTCGGCAGCAGAGCGCTGCC

CACCATCCCCAATTACATTTTTAACATGACAGAGGCCTTCATGAGGCAGTTGAAGATTTT

CCCTGGCAGAAGATATCAGTTGTCAATACTGGATAATATTAGCGGGATAATTCGACCTTC

TAGGATGACATTGCTTTTGGGTCCCCCGAGCTCTGGAAAGACAACTTTACTCTTGGCTCT

TGCTGGACGGCTGGCTTCCAATTTGAAGGTCACTGGGAGCATTACTTATAACGGGCACAG

GCTAAATGAATTTGTTCCTCAAAGAACATCAGCTTATGTTAGTCAGCAGGACTGGCATGC

TGCAGAGATGACAGTCAGAGAAACCATGGAATTTTCAGGACGATGTCAAGGCACTGGAAT

AAAATATGATATGCTTATGGAACTTACAAGAAGAGAGAAGGATGCAGGCATTAAACCTGA

TGAGGATCTTGATTTGTTTATGAAGGCATTTGCTTTGGAGGGAATGCAAATAAGCTTAGT

TGTCGAGTATATACTGAAGATTTTAGGGCTGGACCTCTGCGCGGACACACCAGTGGGGGA

TGAGATGATTAAAGGGATCTCAGGCGGACAGAAAAAGCGCCTTACCACAGGTGAACTACT

CGTTGGCCCGGCTCGAGTTTTATTTATGGATGAGATATCTACTGGGCTTGACAGTTCAAC

TACTTATCAAATCATCAAGTATCTTAGACATTCAACTCATGCACTTGATGCGACCACGAT

TATATCTTTGTTACAACCAGATCCTGAAACTTACGAGTTATTTGATGATATCATTCTCAT

ATCTGAAGGCCAAATTGTTTATGAGGGACCTCGTGATGATGCTGTAAACTTCTTTGCTGC

CATGGGCTTTAGTTGTCCGGAGAGGAAAAACATGGCGGACTTTTTGCAGGAAGTTACATC

TAGGAAAGACCAACAGCAGTATTGGAGCGCTCTGGATTGTCCATATCAATTCATACCCGT

TTCGAAGTTTGCTGAAGCTTTCAAATCATTTCATGTTGGTAAAAGATTGTCCGAAGAATT

AGGTGTTCCTTTTAATCGTCTTCACAACCATCCTGCAGCCCTCTCAACGTCTAACTATGG

TGTTAAAAGGTTTGAGCTTTTGAAGACGAACTTTGGGTGGCAGTTGCTATTGATGAAACG

AAATGCATTTGTGTATATCTTCAAATTTATTCAGCTTCTACTTGTCGCTCTGATTACAAT

GACGGTTTTCTTTCGGACAACAATGCACCATGACACAGTAAATGATGGATATATCTTTCT

CGGGTCACTGTATTTTGGAACAATAATGATTCTGTTCAATGGGTTTACAGAAGTTCCATT

GTTGGTAACAAAACTTCCAGTGCTTTACAAGCATAGGGATTTGCACTTTTATCCACCCTG

GGCATATACACTTTCTTCTTGGGTTTTAAGTATTCCGACTTCACTTATAGAGTCTGCAAT

ATGGGTGGTAGTTACATACTATGTGATTGGCTATGATCCAGCAATTACCAGATTTCTTGG

GCAACTCTTATTGTTTTTCTTTTTGCACCAAATGTCGTTGGCTCTTTTCCGCGTGATAGC

ATCCTTGGCTAGAAATATGATAGTTGCCAACACCTTTGGATCTTTTGCAATGTTGATTGT

CATGCTTCTTGGAGGATATATTATTTCGAGAGATAGCATCCCAAGTTGGTGGATATGGGG

ATACTGGATATCTCCATTGATGTATGCGCAGAATGCTGCTTCGGTCAATGAGTTTCTTGG

ACATTCTTGGGATAAGAAAGTTGGAAAGCAGAATGTGTCGCTAGGTGAGGCCATACTAAG

AGAACATGGTTTATTTACTGAAAGTTACTGGTTTTGGATTGGTGTTGCTGCCTTATTTGG

CTACACAGTCATATTAAACATCCTATTCAGCTTGTTCTTGACTTACCTCGATCCACTGGG

GAGGCAGCAAGCCGTTGTTTCAAAACATGATCTCAGAGAGAGGGATCGGGTAAAGAAAGG

GGAGAATGCAGTTGTTGAGCTGAGATCCTATCTACGTTCAAGCACTCGATCAGGGAAAAA

AATTTGGGAACGTAAGGGAATGGTATTACCATTTCAGCCACTTTCCATGTCCTTTACTAA

TATCAATTACTACGTGGATGTGCCTGTGGAACTTAAACAAAAGGGCATCTTGGAGGATCG

ACTGCAGTTGTTGGTCAATGTCACTGGAGCATTTAGGCCTGGAGTACTCACAGCACTTGT

TGGTGTTAGTGGTGCTGGCAAAACTACCCTCATGGATGTTTTAGCTGGTAGGAAGACTGG

GGGGCTCATAGAGGGGACTATATCTATTTCCGGTTATCCAAAAATCCAAGAAACTTTTGC

TAGGGTCTCTGGTTACTGTGAGCAGAATGATGTCCATTCTCCTTGCGTCACAGTTATCGA

ATCTTTGCTGTACTCAGCATGGCTCCGTTTACCTTCTCATGTTGACTTGGAGACTCGGAG

GGCTTTTGTAGAAGAGGTGATGGATCTTGTTGAGCTCACTTCACTCAGCGGCGCACTTGT

TGGCCTGCCAGGGGTCAATGGATTGTCCACAGAGCAGCGCAAAAGGTTGACTATTGCTGT

GGAGCTCGTTGCAAACCCTTCTATCGTGTTTATGGATGAACCGACGTCTGGCTTGGATGC

CAGATCTGCAGCCATTGTGATGAGAACTGTACGGAACATTGTGGACACGGGGCGAACAAT

TGTGTGCACAATTCATCAACCAAGCATAGACATTTTTGAGTCTTTCGATGAGCTGTTGTT

CATGAAACGTGGCGGAGAACTTATATATGCTGGTCCACTAGGGCCCAAATCTTGCTATTT

AACTGAATTTTTCGAGGCAATTCCTGGAGTGCCTAAAATCAAAGATGGTTATAATCCTGC

TGCATGGATGCTTGAGGTGACATCAAGTAACATGGAAAATCGTCTTGGTGTGGACTTTGC

TGATATTTATCGTGGATCTGAACTGTACCAACACAATAAAGACCTGGTTGAAAACTTAAG

CAGACCAAGCAATGAATCTAAAGAGCTAAGCTTTCCAACCAAGTATTCCCGGTCATTTCT

CACCCAATATATTGCTTGCCTCTGGAAACAGAATCTATCATATTGGAGGAATCCTCAATA

TACAGCTGTTCGTTTTTTCTATACTGTCATCATATCTTTGATGTTCGGAACAATCTGTTG

GGGATTTGGTTCCAAAAGGGAGACCCAACAGGATATCTTCAATGCTATGGGATCCCTGTA

TGCTGCTGTCCTATTCATTGGAATTACCAATGCCTCTGCCGTTCAACCTGTCGTTTCCAT

CGAAAGATTTGTTTCATACCGAGAGAGAGCTGCTGGGATGTACTCAGCATTACCATTTGC

ATTTGCCCAGGTTTCTATTGAGTTCCCATATGTGTTTGTACAGGGACTTATTTATGGCAC

CATATTCTATTCTATGGGCTCTTTTGAATGGACAATGTCAAAGTTTTTGTGGTATACATT

CTTTACATATTTCACTTTACTATACTTCACATTTTTCGGCATGATGACAATTGCAATTAC

GCCAAATCATAATGTGGCTCCAATCATTGCTGCACCATTCTACACACTGTGGAATCTCTT

TAGTGGATTCATGATTACACACAAGAAATTACCAGGATGGTGGAGATGGTATTACTGGGC

CGACCCGATATCCTGGAGTCTGTATGGCCTTCTGACCTCACAATTCGGGGACATAAAAAA

GCCCATGAAGCTCGCTGATGGCCAATCGAAAGCTGTAGGATTATTTCTCGAGGAGTACTT

TGGATTCAGGCACGACTTCTTAGGTGTCGTGGGAATGATGGTCATATTCTTCTGTATGGT

ATTTGCCGTCATTTTTGCTCTCGCGATCAAGTACCTCAACTTTCAGAGGAGATGAATGCT

AGAGTGCTTTTTGTCCAAATCCAAATTCATTCAGTCCCGTCACCTTGGATCTCAACAAAC

TATTATCAGAGGCAAGTCGGATCCAAACAATGTAGTATCAAGGTGATCGATTGCGAGGTA

TTTAACATATTCGTAATCAATGATTGGAGTTGTTGATCTGGTTCTAATGGGTGTAATGGC

CCAGAGAAGGTTGAAAAGGTGTAGAGTGAAGACATGCATTATGGCAACAATGGTTCGATT

ATTATTTCTCAGGTGGCTCCCAACACGGGTGTCTTGTTTTTTTTTTATAAGCAGTCTCGG

TGAGTTATAGATAATTGACTCTAGGGTGTCTTTTCGTACTACAATATATGCAACAAGATG

AAGAACTATGTTAATGGAGACTTCAAGAATGTATGTCTTGGTGATGAGAATCTTGCAACA

TAATAGGTAGAGAAGATGCACAATTAAATTTGTGCAATGAGATAATCGTGAGATGAAAGA

TGTGAGATGTCCCAAGTTTTTGTAAGAGTCATCTTAGTGGGATAACTTGCTGATTCATGT

ATGACAACCTCGTTCACTATAGACTCGTGAAATATATCTAGTGGGATG

>Unigene0002426

GCATCCTTGGCAAGCACTAGACAATTCAACTAACAATTCAACTCACTCCTCTCACTGTCT

CTCTCATACAAACTACTAGCTAGCTCCTTCTATTTCTTCTTAGATAACAACCGTCTTTTA

AAAACTGAAATTGCTTATGGAATTGCTTTCGAGGATAAGAAACCTTAAGCCATACGCTCT

GATGGTGTTTCTGCAGTTTGGGTTTGCAGGGATTCATATCATCTCGGTTGCTACCATAAA

ATCAGGCATGAGTCACTATGTGCTACTCGTCTGCCGCAACATTGTTGGTGTCGCTGTGGT

GGCTCCGTTTGCGCTCTGGTTCGAAAGTAAAACAAGACCCAAGATGACAATAGCTGTCTT

CCTCATGATTAAGACAATAGCGCTCATGGAATAAGTATACTAATACTCAATATTTTACAA

AGCCCTATAATTGAGTTCATCTGGTCCAAGGGAGGTAGCCACACCGATGCTGCCCATGAA

GGAGCCAACTGGCTTATGGGCACAATGGCCCTTCTTGCCAGTGCCGTTTGTTGCTCGGGC

TTCATCTTTTTACAATCAAA

>Unigene0002427

TGGTGCGGACATGTTGCAATTTTTTTTTTTAATGGCGGTTGTGCCGACAATTTATTTTTA

ATTTACAATTTGGAGGATAATTTGAGATAAAAATGTTAATTACAAGGTTAATCACCATTA

ATTGTAATTAGTGGGGGATAAGGGTCACCATCCTTGTAATTACATGGATGTTCATACTGG

CAGGTCAGTTTTTTCTCACTCTGAACATAAAGAGGCATATTATTGCAGTATATTCTCCTC

CGTTCCCTGGAACCGTTCTTTGAGAACTGGTAGTGTTGGT

>Unigene0002428

AATTTAATATTGTTCGGGTTAAGCCGCCCTTAGGGGTTTGGGGGGTATGATAGTAGCAGT

AAGTATTCAATTTAGTAATGAGCGGTCCCGGAAGATGTATAATGATTTATTGGATTTTTA

AAATTTTGATAATTTTAGAATCATCTGAAGTTTTGACACTAAGCATTGGGGTTTACTTTT

CCCCTGGGGTTTACTTTTCCCTTCTATTGATGTATATAGGTAATAGTGTAGTGTGTAACC

TAGTGAAGAGGGTTTATGTGTTGGTGTGTATTTAGTAGTGTGAATATTATTGATGTATTA

TTATAAATATATCCGGTAATGCAGAATTATTCATACTTTCATTTTCAAATCTCTCTTCTA

TTTCATGGTATGAGGATTCGTCTTGAGTGAAGATTTTTCGTGCTCCTTTCCTCTCCCTTC

AGGTGGTCATTGTTGTGTTCGTGACAAAGTGGTGTAATGTTCCCTCTTCGATCCCCTTAA

CCGCCCAATGATGTCATTGTTTACTATATCCATCACTTCAGTTAGCCTTTACTCACTTTT

AGGCCTCTAAAGAGTTGATCATGTTGATTAAGATATTGGATATTGTGTTTTTTTTTAGGG

ATGATTGGCAAAAAAAACTCTGGTGAAACCAACTTTTGGGGTGGTGGCTGTGATGACCTC

TATATTTCACCAACTTTTGGGGTGGTGGCTGTGATGACCTCTATATTTCACCAACTTTTG

GGGTGGTGGCTG

>Unigene0002429

TGGTGCGGACATGTTGCAATTTTTTTTTTTAATGGCGGTTGTGCCGACAATTTATTTTTA

ATTTACAATTTGGAGGATAATTTGAGATAAAAATGTTAATTACGAGGATAATCACGATTA

ATTGTAATTAGTGGGGGATAAGGGTCACCATCCTTGTAATTACATGGATGTTCATACTGA

CAGGTCACCTTCGTAGTCAATATGCCTCTGTTGTGAAATCTAGTGTTGGTTGAAATCTAG

AATGAATATCAGTGTATACCTATTGTGGGTTGATAGGAAACCTTAAAAGCCAAAGTCACT

TTTGCATGTCGAGCCAAACGTTTTTGTCCAATCTAATGTGCCCTATGGCATTGTCAGATA

GCAAAAAGTGTATTTCTGATTGTTCCTAATCACTTACTAGTTTTATGGTATTCTGGTATT

CTGGAGTGTTCGAACTTATGTTTGAACTTTACTTATTTGTAAAGCTAGTCTCTGTATTCC

TATTTTACAAAGATTGTGTACGCCTGGAGGCTGCAAACAAAGTACAATTTTATATTTTGT

TATTTCGAATCTCCTGTCAAACTATCCATTCGTTTTTCACCCAAATGATGTTAGATGCAT

CACCTAACCAATTAACTGAAGATAGGCATCACCCAACCAGTCGCCCATTGTTGGCTTAGG

GTAGTCTTGTTATGAGATTGTATTTTGGTGGTTATGAAAGGCTTTTTCAAACTGAGCCTA

ACTAAGTGTTTCACAATATAGGACCAATGCTTTCTAGTTCGCTTGTCGTCGGAGAAGATT

GGGCCTATTTGACAAATTTGCTTGCCTCCCTCTCCATGGAATGGAGGTTGGAGGGAAATG

GCCGACAATTCTTTGTGTTTTGGTCTGGCTATTCTCATCTCTTTGGACCTCTTCAATAAC

ATTAGTCTTAGCATAAGTAGGCCATGAATCTGCATTGTCCCTGTTGTCTTGGCTTTAAAC

CCAAATTCATGGACTTTTTTGCCTCCTTGAGACAACGTGCTTCCTAATATACGATGCATT

TAGTGATTGTTCGGGTTAAGCCCCACTTAGGGGTTTGTGGGGTATGATAGCAACAATAAG

TATTCAATTTAATATTGTTCGGGTTAAGCCGCCCTTAGGGGTTTGGGGGGTATGATAGTA

GCAGTAAGTATTCAATTTAGTATTGTTCGGGTTAAGGCCCCCTTGGGGGTTTGGGGGGTA

TGATAGCAGTAAGTATTCAATTTAGT

>Unigene0002430

GGATGGCTGGGGATGTGTGGTAGTGTGGAATGTTATTCTTTCCTGGTTGCATCTCACTTT

GAATTTTTCTGAAATCTCTAGTTGAGATTCTTGTACAGGTGGACGGACAGGGGTTTTGGA

TGCTGACATTCTGGGCTCATTTCCAACTATGTTGACTTTTAAGTTTTTGGATGTTGTTTT

CTCTTGTAAATAGCTATTTGGAGGTAATGGATATTTCTATATTGGACAAGTTGCGAGTTA

ATCGGTTGGACAAGATGGT

>Unigene0002431

CTGTGATGGGGTGCGTAACTGTAATAACCTTTTGTGTTGGTCTTTTTCTCTCCGCCAAAG

GCGGCCACTACGTGCAATTGATTGCACGGACTTTCAATTTGTATATGGTTGATCCAAGGA

CTATGGGGCTTCAGTTTTTGTTTGTAATGTCTTCCAGCCGAGTGTTGTTTACTATGTATA

CAATATTATGTATATAGAAATTTACTTTCAAAATCATTGAACACAAATCATAACTTATGT

GTAGTATAATATAAATTTTGTGAGTTATATCTTTGTTCAATGTTATATATATATATATAT

AAAATTTATCTACATTTTTCAGAGTAGTATTATTTTAGAGCCTAAA

>Unigene0002432

GGATGGCTGGGGATGTGTGGTAGTGTGGAATGTTATTCTTTCCTGGTTGCATCTCACTTT

GAATTTTTCTGAAATCTCTAGTTGAGATTCTTGTACAGGTGGACGGACAGGTGTTTTGGA

TGCTGACATTCTGGGCTCATTTCCAACTATGTTGACTTCTAAGTTTTTGGATGTTGTCTT

CTCTCTTGTAAATAGCTATTTGGAGGTAATGGATATTTCTATATTGGACAAGTTGTGAGT

TAATCGGTATCTGTGGCTATTGG

>Unigene0002433

AATAGCTATTTGGAGGCAATGATAAAAAATGCTCCCCAAGATATTTCTATATTGGACAAG

TTGAGAGTTCATAATAATCTGTCTATCAGACAAGATGGTGCGATGGTGCGGCCGTGTTGT

TGACTTTGCATGACAGGGTGATTGAGCAAGGTTACTTTGCATGGCAGTCCGGTCTCTTTT

TATGTTGTGAATGTTTCTAAACCCTTCTAATCATTTATTTTTTCTTTTGCAATATGCAGC

AATTTTGTCCTGACAGTTTACCAAATTTTTGATTGTTCAATCTTTCTGTCAAGCGGTCTC

TTAGCTGCTTAAAAGATGTTTCAATTTAATATTTTTATTATTTAAGTTCTATCTGCTTAA

AAGATATCCCTCAACTTAATATTTTATTATTTAAGCTCTATCTGAGTTGGAAATCTGATG

GTAATTTGGATCGGTATTTTAATTCTTATCTCCTCAAACAGCGGTGGTATTACTTTCAGG

ATATGGAGGTGATGGGTTGTGATTGATGCGTGGTATTTTATGTGGTGTTAATACTTTTAT

TCGCAAGATGGGTGGCCTGCAAAGAAGCGAGTGAATATGATGACAAATAAATTATATTTA

TCTGAATATGAAATGATCCTGTCATGTTTGGTAAGTAATTTTATTAGTCATTTAAGTCCG

TAACATATTTTGACCATTACTTTTGACTCTAGAACAGTCATCTGAAATATTTTAATATTG

TTATGAGAATTTTTTGGTTTAGACAAAAGGGATCTGAAATTGGCTGTATCTAATGGTGGT

ATCTAATGAGGGGGTCGTTGTTTCCCAATTTCCAAGCTGTCCTTTTACTTAACATTGCAG

TTGTGTCGTTAACATGATGTTTCTCCTAATGTCATGATGTTTCACAGTTACATTAATCCA

GAAAAATACATAAACGTGGCCTGGTAAAATTAAAATACCAGCGAACAATATATGCCTGCC

CCAATTATCTAAGCAGTTTAAAAAATATCCATTCTTGTTATTTTGTTATTGCAATGTGAC

TTTTACCGCTCAGCAATTTTAGAGAAAGAAATCTAAACCTTCAGATTGAAGATAATGAGT

CCCGGAGATAATATATATTATGTGCTAATATTGATAGCTATGACTTGTTGCAATCAATAA

ATACTTCAAATGGATATGAGGGGCTTCCAAAATTTCTAAAGAAAATGGCTGAAGACCACA

CGAAATTGGGGTAATAAGATTTATGAAAGAATATATGTAATTCTTGAATTCTTGGGGAAT

GCTTTTGGAATGTGCAATCCATTAAGTAATCTCTGCCTTCAGTACATATTTATACCTGCT

TATAAATCTGATGACCTGAAGA

>Unigene0002434

ATGACCTGAAGATAAGATGAAAGTAACTAAGCAATTTTTTCTGTCTCTCGAAGTAACATG

GTGGGAACACTGAGCAATGTTATTTTTCGAGATGTTTTGTTAAAGCTTAAATTTTTTCAA

GGCTATTAATTATTCTGTAAGGAAGTATTTAAATCTATATCAGTAATTTATGTAATTCCA

ATTTTTCGACATACTCTAATTATAGAATCTTTAAATGTTTTTCGGAACTTAGTTCTTTTA

AGGTGGAAAATTACACTTAAAATTATTGGATTAAGTATTGATATTTTGTATTGTTATCCA

GTTGAGCATTGTCCGTATGTATGTATGTACATTTTTCAGAGTAGAATTATTTTAGAGTCT

AAAGGAAACATGAAAGGAATAATCAGTAATGTTTAATTTTTAAAATTTTGGATTTAAATA

AAATATATTCTTCTAAGCTATTAATTATTTTATGTGCAAGTATTTAAAGCTATATCAATA

TTTTATGTAATTTCAATTTTACAACATAATCTAAATGTAGAATCTTATTGGATAAAAATG

ATGATTCAGATTTAAATGTTTCCAAAGTTATTGTAAGGTGAAAAATTCCACATAAAACCT

TGTTCACTTGTGTGTGCGGCCTAATAATTTGATCTAATTTTCATTTGTATTAGTATCTTG

TTATTTTTGGCATATGCAAGTTACAATTTCAAATTTTTTTAAAATTTTAATTAAAGTTTG

CATGGGAAGCAGGATTTGGCTGTGAGCCTCGTTCATTGATCCTATTAATGAACATCTTCA

TCTCGTAAGTATTGCTTCATGCAGACATGTTTTCAACTAAGTCACCACTCCCTCGACAAC

CTATTATGTAGAGACACCACATGTGGCTGGATACTGAATGACCTTGCCATCACTATATTT

TTAGAACCGACATTTGTTCAATAATTATCAAGAAAGTACTTTTTGTCCTGGTTTGTAGAT

TTATTGTTTAGTTTTCACCGTGAAATATGATGCATAAGAATCCCAGATGCAAACTTTGAC

ATGAAAGTTTGGTTGTGATCCATATATAATCTTCTCTGCCCACAAAAATCCACACTTGCA

ACTTTAGAAATGACCTTGCCATCACTCTATTCAGAACTGATATGGGGTCAATAATTATCA

AGAAAATACTTTTGTTCTGGCTTGTAGATTTATTGTTTAGTTTTCAGCATGAAATATGCT

GCATAAGAATCCCAGATGCAAACTTTGACATGAAAGTTTGGTTGTGATCCATAT

>Unigene0002435

TTTACAACATAATCTAAATGTAGAATCTTATTGGATAAAAATGATGATTCAGATTAAATG

TTTCCAAAGTTATTGTAAGGTGAAAAATTCCACATAAACCTTGTTCACTTGTGTGTGGGG

CCTAATAATTTGATCTAATTTTCATTTGTATTAATATCTAGTTATTTTTGGCATATGCGA

GTTACAATTTCAATTTTTTTTTAAATTTTAATTGAAGTTTGCATGTGAAGCAGGATTTGG

CTGTGAGTAAATGCCTCGTTCATTGATCCTATTAATGAACATCCTCATCTCCTAAGTATT

GCTCCATGCTGACATGTTTTCAACTAAGTCACCACTCCCTCGACAACCTATTATGTAGAG

ACACCACATGTAGAGACACCATGATACACCTATCTATTGGTGGCCGGAGACTGAATGACC

TTGCCATCACTCTATTTTTAGAACCGGCATGGGTTCAATAATTATCAAGAAAGGACTTTT

TGTCCTGATCTGTAGATTTATTGTTCAGTTTTCAGCGTGAAATATGCCGCATAAGAATCC

CAGATGCAAACTTTGACATGAAAGTTTGGTTGTGATCCATATGTAGTCTTCTCTGCCCAC

AAAAATCCCCACTTGCAACTTTATAAATGACCTTACCATCACTCTATTCAGAACTGATAT

GGGGTCAATAATTATCAAGAAAATACTTTTTGTTCTGGCCTGTAGATTTATTGTTTAGTT

TTCAGCATGAAATATGTTGCATAAGAATCCCAGATGCAAATTTTGACGTGATAATAATTA

TGATGATCATATGATAGGAGTTATAGAGCAGCTAATAAT

>Unigene0002436

GTGGTTTGGTATTATTTCCTTTTTTATAAACTCTTTTAGTTGTGACTTCAACCAACCTTG

ATATTCTTATTGATATTCTTGATATTCTTAAAGATCTGTAATGGCCGCCATAGCCTATGG

ATCCATTTACAAGATGGCTCTGATCCAGAAAATTTATTGGTAGTTCATTTGCATAAATCA

GATTCTTGAATCTAATCTGGGTTTTTGTTTATTTTAAAATGCCTTTTGTTGAATTTTTTG

AGTTACAATACTTATCT

>Unigene0002437

TTTTTGAGTTACAATACTTATCTTGTAAAGAACCAACTTTCGATCGTTTTTGCCCTTTTG

TTTCACTATATAAGGGAGCTTCTTGTCAATTTCCAGTGTGGCCAACTTGGAACAAATATT

TTGTGCCCAGATGTTGCTTAACCTTGCTTACCAAGGTGCAGATTGGGTTATGGGTGAAAG

GGCACAACTTCAATTGTATTGAGGGGAATGAGAGAGAATTTCTGCCAGGGTGAGGGGCCC

GACAGGGTTATGATGATTATGGTGCGGAGATGTTGCAATGTTTTTTTTAATGGCGGTTGT

GCCAGCAATTTATTTTTAATTGACAATTTGTAAGACAATTTGAGATAAAAATGTTAATTA

>Unigene0002438

ACTCTTTGCCTCAAGATGCATTAAGGTGAGTCTGAACAATTAGCATGTTAGGTGGGTTTT

ATTTTCTTGCATTGACATTCATGTCATATTTCAGGATGTATGTTGTATCGCCCCCACCGC

TCGTCTCATGATTATTAGGTGGTTCCCACCAATTACCTTGGGATTGGGTCATGAAGGTAC

CACCATTTTTTCTTGGATACATAGCAGGCGGGCCCAATCGTTTGTCTCATGATACAAACT

ATGTGGATCCCACTAATTGCTTCGGGATGCGTACCGGATGGGTCTCACCAAATATATACC

TCAAAGTCTCACATATAAGCCCAAAAGTATATATTCCATCATCCAACTCACTTATGTAAA

GTTGCTCAATAGGCAAAGAGTGTTGTCGAGCAACTTTTTTAAGAATTGAACAAAACAATA

ATGCACCAAATATTTTTTCACGATTACATCATTGATCCTTTCCC

>Unigene0002439

CACATCCCAGGACAAATGATGAGCCATGACACCGCTTTTAAATTATTTTAGAGTTTATGC

TCCCACCAATTGTGTCTGAATACATGTAATGTTGATCCTACCAATTACCTGAAGATGTAT

ACCATTGTCTGAAGATATTTGTCAAGTCGATCCCTTTACTTAAAAGGAGATATAAAAAAC

ATAATCACATGATTCATAGTGAATTTTGTAACAGGGGTAATTGTGCCCCTATTGATCTTA

AGGATAAAATTGTTCTTTAATAATTATCTTTTAAATAATAGTCGATGCACATTTATATAA

ACTAAAAAAACCACATAGCAAAGCCAATTTC

>Unigene0002440

CAACTTAACATGGTGTCAAAGCAATCAATTTTTAGCTGTGATTCTTGATGTGCCCCACAA

AATACACGTGGTTATGCATGAGGGAGCGTGTTGAAAAGCATGTGTGCTCAGTCCCATATC

GAGGAGCTCATCTTATTGACTTGTTTATAAATATAAATATAAATATAAATATAAATATAA

AGAGCCCCACTACTCTCAGCTATCTAGTTTTGAGTTTGATGTCTCGATTGTTAACATGGT

ATTAAAGCCCAGATCCAGGCCTTACAGTGACCACCAAGCCTTCCA

>Unigene0002441

ATATAAACTAAAAAAACCACATAACAAAGCCAATTTCTAACTAAATCTTAATTAGGGGCT

AGATTCAAAACGGAATAACCTTGTAAATTGTCTTTATATCAATTGTATTTGAATTACTGT

ATTATGTTTTCGGTTGAGTGGATGAGTACAGAGTAGTCATAGCCATTGTGCGGCATGTAC

AATTACTCCAACAAGCGAACATGAGTTGTGCACGGTATACATGGGGTCGACCCTATTGGG

TTATAAGTATCTATCCATCCTTGATCCACCATAGCACCACAAGCCTCTCTTTAGAATGGT

TGAAGACCTGTCCATTACCACCACCCCGTCTATCTGTTCAAGGCCTCTCTGTATCTATGG

AGATGAGACTTTGCAGCGATTAGGAAGCCTTCTGGCTGAGAAAAATAGCCTGTCAGTCAA

TAAAATATATAAATCTATTGTCTGAACTTTTTTTTTTAGTTTCAAGGTGAAATATGGTTT

TTTTTTCAGGATACTTCATTAATAAAAAGAAACCAAATTAGATATAAAATACTTTGTTAA

AATGATGTTTTCTTGGTCTTCTCAGCACGTTAAAAATAATGTACACTGTTCATACATATC

TATAATACACTTTTATGTTCTAATTTAATATACGCACAAACAATACCTATAAAGTTCTTA

TGTTAATTATTAAGCAGAGAACATGTCTGAGATAACACATCCTATTTGTTCCTTAGGCTT

TGATGTTAGGGTTTGTTAGTGTCAACGTATTGTTAGGAGAGTTAGTATGTCAGTTGGGGT

ATGGTGGTGTTAGTCATCCTAAATTATTTTTAAGTTCCAACCCTATATTAAGGGATGTAA

ACGTGATAATGGATATCAGAAAGTCCTGAAAGTATTTCCTCTCATCTTCTATTCCA

>Unigene0002442

GCGGGAACTGCGTCAAAAAGATATCTTGCTGCCACAATTTCCCTGCAATGTGCCTAGCCC

ATGGCCTTGTATCCAAAACACATGTCCCGTTGTCCTTATATGCTTGGAATGGATTGATGA

GTTATTTGAAAAATTTATTGGACGTGTTATCATTGGATTACGTATGAACGGATTATGTGT

CGGATGTGTTATTATCGAATTATGCGGCGGATGTGTTATTATTGAATTATGCATAATCAA

ATTTGTGTCATTATTGGATATGTTATTATTGTCCAATTTGTAAAGAAATTATTTGACTGA

TGAAGAGAATGTTGTCGACATTATTTTTGTATGTTGAGCAACTTTACCATTGCGGGTT

>Unigene0002443

ATTGCGGGTTGACCGGTGGAACGCATGATTTGGAGCTTACATATAAGTTGTTGAGGTGTA

TGTTTGATGAGACTCATCTTGTATGCATCCTGAGACAATTGGTGGGACCCAACTGACATG

CATATTAAGACAATGATGGAACCTCCTTACCACGTATCCTGAGGTAATTGGTGGTGCCTA

GTGAAGACATCCATCGACAAACAATGAGATACATCTGACACATCCCAGGGCAAATGATGA

GCCATGACA

>Unigene0002444

ATGCCCGCTTCGTGGAAATGGTGGAGGCAAGACATCTCATTGATTTCTTCCTGCATAGCC

ATTTCTTTGATGCAAACTGCACAAGTTGGCAGTATCTTCGTCAATAAGAATAACTTCAAG

TAGTTTGTTGAACTCGACTTTTCCCGCTTGTTGGTGAATATCGTCGTTGGGGCTCTGCCT

GTTGAGACATGTATTTCTGATCCACATATCATCAATGTGAGGTGATGTGACATTGATGAG

ATTCAACTTGAGCACCAACTTTGCACCATTTTTTAATGGCGATGATATACACAGTGACAC

CGATTGTAGGTGGTGGA

>Unigene0002445

TATCTTCGTCAATAAGAATAACTTCAAGTAGTTTGTTGAACTCGACTTTTCCCGCTTGCT

GGTGAATATCGTCGTTGGAGCTTTGCCTGTTGAGACATGTATTTCTGATCCACATATCAT

CAATGTGAGGTGATGTGACATTGATGAGATTCAACTTGAGCACCAACTTTGCACCATTTT

TTAATGACAATGATATACACAGTGACGCCGATTCTAGGTGGTGGACGATGTTGCAAGTGG

TACACACATGAGAGTTTCCTCTCCTAATCTTCACCGACGACTAACATAAAAAATGAGTGT

G

>Unigene0002446

TGGAAGCCCTCTTCCTCAGCCATCGATGTCTTCCTGAACTTCCTCACAGGAAATGACTCT

ATATCCACACCCTCTTCGTCTTAGAATTCCTATTTGTTTGGGAACCTCTTGGTGTGTTGG

TGTGTGCCATCTCCAGTGTCTATGAGCAGATTGACGCTGGTCCACTTGACAGTGCCCTAC

TTGTTCAAATTAAGGCCATATGTGAGGCGCTGATGACACCCTTTATAACATGTTCAGACT

AGTGGAGTGTGAAATGCTTGCGTGATCGGGTGCTGCTAACAGTCAGTCATGGTGGAAAAT

CTATTTGCACCACTACTTCGCCGCCTCCAGCACCAGCGGCCCTGCTGCCAAGCGTCATCA

CCACTGCTGCTGTCGCCGGAGTCGCCCCTATCATCCATCTCACCAACTAAAAAGGGAAGG

GGATGTTTCTCAAATTGAGCTCGAACATAATGATGAGTTGGCTGAGTAAGGAGCTTCCAG

TAGATAGCATGGATGTCTTTGATGTTTACGGTAATACGTTGACAGTTTTAGTGGGAATAT

A

>Unigene0002447

GGAGCATCACAATTCTCAGCCCGGGTAGAAGATTCAAGCGTCAACACTCTTGCCTTGCAG

GTTGCCTTTATTGGCCTCGACAGTGAGGCATGGCCTTCTATGCCGCCCTTGACGACTGCT

GCTAGTCCGACGCAGTGGAAGCCCTCTTCCTCAGCCATCGATGTCTTCCTGAACTTCCTG

ACAGGAAATGACTGTATATCTTTGCAATGGCGGCTGGATCCACACCCTCTTCGTCTCAGA

ATTCCTATTTGTTTGGGAACCTCTTGGTGTGTTGGTGTGCCCCATCTCCAGTGTCTATGA

GCAGATTGACGCTGTTCCACTTGATAGTGCCCTACTTGTTCAAATTAAGGCCATATGTGA

GGCGCTGATGACACCCTTTATAACATGTTCAGACTAGTGGAGTGTGAAATGCTTGCGTGA

TCGGGTGCTGCTAACAGTCAGTCATGGTGGAAAATCTATTTGCAC

>Unigene0002448

ATTTCACTTCCTGGCCACTCCCCGCCTCCTCCATCCCTCTCCCCCACCATGCAAACCCAA

CTCTACCCTGCGCTCCACCTCCAGTTCCTCCCCTCTTGCTCCGTCCAACGCCCTCACCTC

TCCTTCATCACTCTCCTGCGCCCTCCTCCCCGCCGCCCCACCCGAGTCGGCTGCTCCGCC

TCCGCTGGCAGCGCCGCCGGCAAACCTCGGAGGCCATATCCGTTCGATGAAATCGAACCG

CGGTGGCAGCGTTACTGGGAGGAGAACAAGACGTTCCGCACCCCCGACGAAGTTGACACC

TCCAAGCCCAAATGCTATATCCTCGACATGTTCCCCTATCCCAGTGGAGCTGGATTACAT

GTTGGCCATCCACTTGGCTATACTGCCACTGACATTTTATCAAGATTCAAGCGCATGAAA

GGTTTCAATGTTTTACATCCAATGGGATGGGATGCCTTTGGGCTTCCTGCTGAACAATAT

GCTATTGAGACAGGGACCCATCCGAAGTTGACCACAATGCGGAATATTGCACGCTTCCGC

TCTCAGCTCAAATCATTAGGCTTTTCCTATGACTGGGAGCGCGAAATTTCCACTACAGAA

CCCGATTATTACAAATGGACACAATGGATTTTTCTTCAGTTGTTGAAGAAAGGACTTGCA

TACCAGGCCGAAGTACCTGTTAATTGGTGTCCTGCCCTTGGAACTGTTTTAGCTAATGAA

GAGGTGGTGAATGGTGTCAGTGAGAGAGGGGGTCATCCAGTTATAAGAAAGCCAATGCGT

CAATGGATGCTCAAGATAACTGATTATGCTGACCGTCTTCTTGAAGATTTGGATGATATT

GACTGGCCTGAAAGTATCAAAGAAATGCAACGCAACTGGATAGGCAAATCTGAAGGTGCT

GAATTCGAATTTGACATACTTGATCTGCATGGACACGAACTAGGTGTTAAGCTGTCTGTT

TATACAACCAGACCGGACACCATCTTCGGTGCAACGTATCTTGTTGTGGCACCTGAGCAT

ATCTTATTATCATCATTAGTATCCGAGACCCAACGCAAATGTGTAGAGGAATACAGAGAG

CTTGCATCAAGAAAAAGTGAACTTGAGCGAACTGATCTCCAAAAGGACAAAACCGGAGTG

TTTAGTGGTTCGTATGCTAAGAATCCAGCTAGTGGAGAAGTCATTCCCATCTGGGTTGCA

GATTATGTATTAGGAAGTTATGGCACTGGAGCAATTATGGCTGTACCCGCACATGATTCC

CGTGACTATGAATTTGCCATAAAATATAACATTCCAGTTATTAGGGTTGTTGAACCATTT

GATAAAAGCTGTGATCTTAGTGAACCATATTTATACGATGGTATTACAACGAATTCATCT

AATTCATTATCTGGGCTGAATATCAATGGCTTGCCGTGCAAAGAAGCTGCATCAAAAGTC

ATAGATTGGCTTGAGCGTATGGGCAAGGGAAAGAGAAAGGTGAACTATAAATTGAGGGAT

TGGCTTTTTGCTAGACAACGCTATTGGGGTGAACCTTTTCCAGTGATTTACCTCGATGAT

ACTGGTGAAATGGTCTCACTTGCGGAAAGTGAACTGCCTCTAACTCTTCCTGAATTGGAT

GACTTCACTCCTACTGGGACGGGGGAACCCCCATTGGCAAAAGCAACATCTTGGATTAAA

ACCAAGGACCCTCTATCTGGGAGATCTGCAACACGAGAAACAAGTACAATGCCCCAATGG

GCTGGATCCTGCTGGTACTATTTGAGATTTATGGATCCGAAAAATTCAAGTACATTGGTT

GATAAAGCTAAAGAAAGGTATTGGAGCCCAGTGGACATTTATGTTGGTGGCGCTGAGCAT

TCTGTCCTGCATTTACTCTATGCTAGATTTTGGCACAAGGTCCTCTATGATATAGGTGTT

GTTTCGACCAAGGAGCCATTCAAATGCCTCATAAATCAAGGGTTAATACTTGGAGAGGTT

GAGTACATGGCCTGCAAAGACAAAGATGGCAATTTGATTTCTGCAGATTCTGCTGGTATG

CCAAGTGATCATTATCAGGAAAGGATACCTGTTGAAAAGGTGATAAAAGTCGGTGATTAT

CATGTCTTGAAGGATAATCCTGATATCCATCTGATTGCTCGGGCCTACAAAATGAGCAAA

AGTAGAGGAAATGTCATCAATCCTGATGATGTTGTTGCTGAGTATGGGGCAGATTCTCTT

CGCTTATATGAAATGTTCATGGGACCACTGAGAGACTCTAAGACATGGGCTACTGGTGGG

ATTGAAGGGGTACATCGATTTCTAGGAAGAACTTGGCGGCTGATAGTTGGTGCTCCTTTG

CCTGATGGATCATATCTAGATGGAAGCATTGCCATTGATGTTGAACCTACACTAGAACAA

CTCCGTGCTCTTCACAAATGTATCGAGAAGGTCTCAGAAGAAATTCAAGAAACAAGATTC

AACACAGGGATTTCTGCTATGATGGAGTTCGTCAATGCTGCATACAAGTGGGAGACCCAT

CCAAAATCAATCCTTGAGGCATTTGTTTTGTTGCTTTCTCCTTTTGCACCTCACATGGCT

GAAGAACTATGGTCTCGACTTGGACATACATCTTCCTTGGCATATGAGTGTTTTCCAGAG

GCTAACGATGAGTACTTGAAAGATACAAGCATCGTGTTGCCCGTCCAGATAAATGGGAAG

ACTAGGGGAACCATTAAGATTGATGAAGCATGCTCCGCCGATGATGCACTCAAATTGGCA

TCCTTAGATTCCAAATTGTCGAAGTATCTGCTTGGGAAGACAGTCAGAAAAAGTATTTAT

GTTCCCGGAAGGATCTTGAATGTTATCTTGGACCAGCAGAAGGTTCCTTCATAAATCTTT

TTATCTTATAGCTAATTACAATTTTTGTGCATTGTTTGATGTGTTCTTCAAATTGCTCTA

TAATGCAAATCCATTCTTTCATTTGTTTTATCCCAATAGAAAGTATTCTTTGTAACATCA

CAGAGGTAGGAGTAAAATAATATACCATGTCTACACGATTTTTTAAACTTTCCAGTATTT

AGTTTCAACCTTGTTTGT

>Unigene0002449

TCCTTGTTGGCCTCTGGGTTGTTCTTGTACCCGGCCGTTACATTTGGTCCACGGATGCAT

ACCTCGCCTGGCACGCCGGGCGGGCGGACACTTCCATCCTCACCGAGCAGTGCCATCTCC

ATCCCAACCGGCCGCCCCACGGACCCAGGCCTATGCGGCCCATCCTCTGGCAATGGATTC

GTCGCCATCATATGCGTCGCCTCCGTCATCGCATACGCCTCCAGCACCGGCGCCCCAAAC

GCCGCCTCCAGCCGCTCGAGTATC

>Unigene0002450

TCCTTGTTGGCCTCTGGGTTGTTCTTGTACCCGGCCGTCACATTTAGTCCACGGACGCAC

ACCTCGCCTGGCACTCCAGGCGGGCGGACACTTCCATCCTCACCGAGCAGTGCCATCTCC

ATCCCAACCGGCCGCCCCACGGACCCAGGCCTATGCGGCCCATCCTCTGGCAATGGATTC

GTCGCCATCATATGCGTCGCCTCCGTCATCGCATACGCCTCCAGCACCGGCGCCCCAAAC

GCCGCCTCCAGCCGCTCGAGTATC

>Unigene0002451

TTTTAGCAAAAACATACTCTCACAATGGAGAACCAAACCCTCACCGGAATCCTCGCCAGC

GCCGCCGCCGCCTACCCCTTCCGTCCCGCCCTCTCGGTTCCCGGCCGCCTCTCCCTCACC

CACTACCAACTCCTCCAACTCGTCGACTACGCCGCCTCCCGTCTAGCCGCAGCCGGCGTC

CGCCCCGGCGACGTCGTCGCCCTGTCTTTCCCCAACAGCCCTGACTTCGTCATTGCCTTC

CTCGCCGTCATCCGCGCCCGTGCCGTCGCTGCCCCCCTCAACCTTGCCTACACCCAGGAG

GAGTTTGACTTCTACCTCTCCGACTCCGACGCCACCCTCCTTCTCACCAACCCCGAGGGC

AACCCCGCCGCCGAGGCCGCTGCTGCCAAGCTTCACCTCCGCCACGCCACCGCTTCTCTC

ACCAAAGATGAATTGATCTTTTCACTCACCAACTCCGACCCAGGACTAGCTGAGCTTACC

ACAAATATGTCCTCCGACATCGCTCTCTTCCTGCACACCTCTGGCACAACCAGCCGGCCG

AAGGGAGTCCCGCTCACCCAGCTTAATCTCGCCGCCTCCGTCAACAACATAAAATCAGCT

TACCGGCTCAGCTCAACCGACTCCACTGTCATCGTCCTCCCTCTCTTCCACGTCCATGGC

CTCCTCGCCGGGCTCCTCTCCTCCCTCGCCGCTGGCGCTGCCGTGTCACTGCCAGCCTCT

GGCCGGTTCTCCGCCTCCACCTTCTGGGCTGACATCCGCACCGCCGGCGCCACCTGGTAC

ACCGCCGTGCCGACAATTCACCAGATTCTCCTCGACCGGCACACGACCAAACCCGAAGAA

AGCTACCCGAAGCTCCGGTTCATCCGGAGCTGCAGCGCGTCGCTGGCGCCGGTGATACTC

GAGCGGCTGGAGGCAGCGTTTGGGGCTCCGGTACTGGAGGCGTATGCTATGACGGAGGCA

ACACATTTGATGGCGACGAATCCGTTGCCGGAGGATGGGCCGCATAGGCCGGGGTCCGTG

GGGCGGCCGGTGGGGATGGAGATGGCACTGCTCGGTGAGGATGGAAGTGTCCGCCCGCCC

GGCGTGCCAGGCGAGGTATGCATCCGTGGACCAAATGTAACGGCCGGGTACAAGAACAAC

CCAGAGGCCAACAAGGATGCATTTGCGTTCAATTGGTTTCACACCGGCGATATTGGGATC

ATTGACAGTGATGGCTACCTACACCTTGTTGGCCGGATCAAAGAGCTTATTAACCGTGGA

GGGGAGAAGATATCGCCGATAGAAGTTGATGCGGTGCTGCTGTCGCACCCGGACATAGCT

CAGGGGGTGTCCTTCGGTGTCCCTGACAACAAGTACGGCGAAGAGATAAATTGTGCGGTG

ATACCGAGGGAAGGGGCGGAGATAGGGGAGGAGGAGGTGATGAGGCATTGTAGGAAGAAC

TTAGCGGC

>Unigene0002452

TTCGGTGTCCCTGACAACAAGTATGGCGAGGAGATAAATTGTGCGGTGATACCGAGGGAA

GGGGCGGAGATAGGGGAGGAGGAGGTGATGAGGCATTGTAGGAAGAACTTAGCGGCATTT

AAGGTGCCGAAGAAGGTGTTTATCACAGACTCGCTGCCAAAAACGGCAACTGGGAAGATC

CAGCGCCGTATTGTGGCTGAGAATTTCTTGGTACCGATGGCGAATGCGACGAAGGTTGGG

GCATAATATTGCGTGTCTTGCTTTTTAAGAAGGTTGTGGTAAATCAGCTGCAGAGCAATG

AATTTAAATTTAATTTATCCAAAAATCAAAAAGTTGTTTTAGTAAATGTATTGAGTAAGA

TTTTGTTTTATTTGAC

>Unigene0002453

GATGACATCGCTATACTTTGTTTGGCAAAGGTCCCTCGACGGTACCATCAAATCCTTAGG

TGTGTCTCGAGGAGATGGAGAGCTTTATTGTGCAGTGAAGCATGGCTATCTTGTCGCCAA

AAGAACAATCTACAGGAAACATGGATTTATGCCATGTGTAGAGACAAATCTGGAGGAAAT

TGCTATTTTGTATTGGATCCTAAAAGACGACGTTGGAAGTCCCTGCATGATGTCCCTCCT

CAATGTGTAAAGAAAGAGGGCATGTCCTTTGAAGCTTCAGGAAATAAGTTATATCTCTTG

GGAGGTTGCAGTTGGCGAAAAGATGCTACAGACGAAGTATACTGTTTTGATGCTTGTAGA

AACAAGTGGGATGTAGTTCCTCCATTGCCAACAGCTAGGTGCTATTTTGCCTCGACAGTT

CTGGATAATAAACTCTATGTTGCTGGTGGGCATGGGATGGATTCACTTACTCCAAAATCA

TGGGACGTGTATGATTTGTTCTCCAACAGCTCAACTACCGAGAAGGATCCAACAAAGTTT

CACCATGCCATTGTTAAGTGTGTCGCTTTGGATGATAAAATTTATACTATGCACATGTCT

GAGGATAATCTGAGGTTCCGTGCGGGGGTTTACGATCCACTACCTGGGACATGGCATGAG

CTGGAAAATGAATCAGCTAGAATAGCGCAATGCTGTGGTAGGCCTACGGTCACGGTGGGT

GGAACTCTGTACATATTAGAGGAAACTATCGGGACGAGGCTTATGATGTGGGAGAAGGAG

AGCAAGGAATGGGTGGCACTGGCTAGGCTGTCGCAGCATCTCACAAGGCCACCTTGCCAC

TTGTGGCAATCGGAAGGAGTATTTTCCGTATAGGGAAAGGGCTGAACACCATGGTTATCA

ATTTGGATCATGTGGCAAAGAATGCGAGGATGCTGGTAAATGCTTTAATTGTTCCGGACT

TCAATTTGGACGTGACTGTCATTAGTTGCAAGACAATCACGATCTAAAATGTGTTCTCGT

ATTTGTTCTGTTCTTGTGTTTGGAAATTCACCATGAAATTCGTTTGGTTAGAGACAACCA

GAAGGAAACTTATGTATTTGGGTGGATGTTTAATTGATATAAATTTACATCACAGTGCTT

ACATTTCATCTACTTTCACTTTAATCCTAGTAGGGGATGACTGCAATAACTTATC

>Unigene0002454

GTACAAGCAGGTAGAGTTGAACGGACCATTGATACGTGGTCTTCCCGATGACATCGCTAT

ACTTTGTTTGGCAAAGGTCCCTCGATGGTACCATCAAAGCCTTAGGTGTGTCTCGAGGAG

ATGGAGAGCTTTATTGTGCAGTGAAGTGTGGCTTTCTTGTCGCCAAAAGAACGATCTACA

GGAAACATGGATTTATGCCATGTGTAGAGACAAATCTAGAGGAAATTGCTATTTCGTATT

GGATCCCAAAAGACGACGTTGGAAGCCCCTGCATGATGTCCCTCCTCAATGTGTAAAGAA

AGAAGGCATGTCCTTTGAAGCTTCAGGAAATAAGTTATATCTCTTGGGAGGTTGCAGTTG

GCAAAAGGATGCTACAGATGAAGTATACTGCTTTGATGCTTGTAGAAACAAGTGGGATGT

AGTTCCTCCATTGCCAACAGCTAGGTGCTATTTTGCCTCGACAGTTCTGGATAATAAACT

CTATGTTGCTGGTGGGCATGGGATGGATTCACTTACTCCAAAATCATGGGATGTGTACGA

TTTGTTCTCCAACAGTTCAACTACCGAGAAGGATCCAACAAAGTTTCACCATGCCATTGT

TAAGTGTGTCGCTCTGGATGACAAAATTTATACTATGCACATGTCTGAGGATAATCTGAG

GTTCCGTGCGGGGGTCTACGATCCACTATCTGGGACTTGGCATGAGCTGGAAAATGAATC

AGCTAGAATAACACGATGCTGTGGTAGGCCTACGGTCGCGGTGGGTGGAACTCTGTACAT

ATTAGAGGAAACTATTGGGATGAGGCTTATGATGTGGGAGAAGGAGAGCAAGGAATGGGT

GGCACTGGGTAGGCTGTCACAGCAGCTCACAAGGCCACCTTGCCAACTTGTGGCGATCGG

AAGGAGTATTTTCCTTGTAGGGAAAGGGCTGAATACCATGGTTATCAATTTGGATCAAGT

GGCAGAGAATGCAAGGATGCTGGTAAGTTCTTCTATCGTTCCGGACTTCAATTTGGATGT

GACTGTCATTAGTTGCAAGACAATCACGATCTAAAATGTGTTCTTGTATTTGTTCTGTTC

TTCTGTTTGCAAATTCACCATGAAATTTGTTTGGTTAGACACAACCCGAAGGAGACATGT

ACTTGGGTGGATGTTTAATTGATATAAATTTACATCACAGTGCTTACATTTCATCTACTT

TCACTTTAATCCTAGTAGGGGATGACTGCAATAACTTATC

>Unigene0002455

CGGGCCCCTCCACCGTCTCCCCAGCTCCGCCCTTCCGTGCCAGAAATTGGGAGCGATCTC

TCAATCCCCACTTACAGATCCGGAATTTGGAGCCGGACAGCATCGCGCATCTAATCAATC

GCTATATCCTGAAATCGGATCCAAAGAGGAGTTAGGGAATCATGAGAGTTATGGAGGGTT

CATCTGAGTACAAGCAGGTAGAGTTGAACGGACCATTGATACGTGGTCTTCCCGATGACA

TCGCTATACTTTGTTT

>Unigene0002456

GCTCAGAAAGAAAATATCCACACTGACGGATTTAGAAGAGCATTCACTGTGGATGGCACG

TTCATGGAAAATTTTTGGATGGAAGGAGATGATTTCCAAGAATGATGTCTTCCAAATAAC

GGAGCCCTGAAGCCAGTTGAGGAAATCTCTCTTATCTAGTTTCGGCCTGAATATGTCATG

ACACTTGTTCCAATTTTGAGGATGGGGGTGTTACTCGTTTGGTAGATACTTCAATGAGAC

TCATGTACTTGTTTTC

>Unigene0002457

AGAGTTTTGGTATCCATGCTCAGCGCATCTTTGTTAAGTTTGCAGAAGTTTGGAGGAACC

GGCTACTCACAACCAATTCTCCAAGCTTGATTGAAATTGGCAATATGATAATTGAGTACA

AGGATACCATTGAGGTGGTCAATGATGGGCCAGTAACCTACAGGCTTACCATGTTAGATT

CTCAGAACAAAGGATACAGAAATATAGTACATTTAATCTTTACACCGACATATTTTGTGA

TTGAAATCCCAAAGGACGGGCATATATTTGTTACAGGCTCAGGCAAAGGGCCAGCTATCA

ATGCAATGATGGAGAAAAATATGGTGC

>Unigene0002458

GGTGTTCACATCGTGCGCTTTCCCTGCCCTTCCATCTCCGTCCCCTCAGATCCGCCACCA

CACAGTCCTCTCCTCCTCTCCAATGCGTTTTCTTTGGCTCGGTTAATGCGTTCCAGATCA

AGGAGTCGGCAGGAATAAGGTGGCCCACGCCGGATCAACGAGGTGCCTCGCCGCTGCCGG

AGATACCGGTGATGGAGAAGAAGAGGCCCGAGATCCTTCCCCCGAAGATAAAGGAGTGCT

CCCAAAGGAGTAAGTGATGCTTCAATCAAAGAGCTCTGC

>Unigene0002459

TTCTCCAAGCTTGATTGAAATTGACAATATGATAATTGAGTACAAGGATACCATTGAGGT

GGTCAATGATGGGCCAGTAACTACAGGCTTACCATGTTAGATTCTCGGAACAAAGGATAC

AGAAATATAGTACATTTAATCTTTACACCGGCATATTTTGTGATTGAAATCCCATAGGAC

GGGCATATATTTGTTACAGGCTCAGGCAAAGGGCCAGCTATCAATGCAATGATGGAGAAA

AATATGGTGC

>Unigene0002460

CGGCCAGCACGTTAGCTCGAAGCCATTCATCGACTATGCAACATAACACCAGAACCCCTT

CCAAGCGGCGAACCACCGTCGTTTCCACTCTCTTCCCTCTCGATATTCATCTACTATCCA

CCCACCAAATCAATCAAATTCCTCCTCCCCCCCCCCCCAAAATGGATCTCCGAGTCTCCG

TCGCCAACCAAACCGACTTCGGCCTCCAGCTCGCCCGTCGCGTGGCATCGACCTCCGCCG

CCGACTCCAACCTCGCCCTCTCGCCGGTCTCTATCCACGCTGTCCTCGGCCTCCTCGCCG

CCGGATCCCAGGGCGCCACCCTTGACCAGCTCCTCTCCGTCCTCCGCGCGGGCGCCGCAG

CCGATCTGAACCAGCTCTCCTCCCAGGTTGTCTCCCTTGTCCTCGCCGACGGCTCGGCCA

GCGGCGGGCCGAAGATTTGCTTCTCGAATGGCGTCTGGGTGGATGCATCGATGTCGTTGA

AGGAGGCTTTCAAGGCGATCGCGAACAAGGACTATAAGGCTGAGGCCAAGGCTGTTGATT

TCCAATCCAAGGCCGATGAGGTGACGTCTGAAGTGAACTCGTGGGTAGAGAAAGGGACAG

CAGGCCTTATCAAGGAGCTTCTTCCTGCCGGATCTGTCAACCAAAACACCAGGCTTGTTT

TGGGCAATGCTTTATACTACAAAGGTGCTTGGGAGAACAAGTTTGATGCATCTAAAACAA

AAGAATCCGAGTTTCACCTACTGAATGGAAGCTCTGTTCAAGCTCCCTTCATGTCGACTA

AGGAGAAGCAGTTCATCTCTGCCTACGATGGTTTCAAAGTCCTGAAGCTTCCGTACAAGA

AAGGTGAGGACAAGAGGCAGTTCTCCATGTATATATTTCTCCCCGATGCTCGAGATGGGT

TGTGGAATCTTCAAGAGAAGCTGAGTTCTGAATCAGGGTTCCTAAACCACCATTGTCCAA

GGCAAAAGGTCGCTGTCGGTACTTTCAAGCTTCCCCGCTTCAAGATCTCATTCGGGTTTG

AAGCATCTGAAGTCCTGAAAGGTTTAGGTCTGATACTGCCCTTCACCGGTGGCGGCCTGA

CAGAGTTGGTGGATTCTCCGCTGGGTGGTAAGCTTTATGTGTCTTCAGTTTTCCACAAAT

CTGTTGTCGAAGTTAACGAGGAAGGAACTGAAGCAGCTGCTGGTACTGCTGCTGTGCTTT

TGATGAGGTCGTTGCCTATGGATCCGTTGGACTTTGAGGCGGATCACCCGTTTGTGTTTG

TGATCAGAGAAGACACTACTGGAGTGGTGTTGTTCACAGGGCATCTGCTGAATCCATCCA

TAACCGAGTAATTTCTCTGATGGTAACAGAGTTCTGTAACATAACGCTTGACCTTTATGG

AGAGTGTGTTGTTTCATCTTGCAGTTTGGCCTCAATGTACTAGTTGTGTTAGAACATATT

TTCTGTATTGGTTTTCTTGTTGCCCATATGTTGTTCTATTCTATGTTTGGGCTTCAGTAT

TGCTTATAACAGAATATTCTAATGTTTGCTAATTATACTATTCTTCTGTGTTGTACCGGA

TATTTCTGTGTTGCACTAGTTTATATTATGGGTGTACGTTTGTAATAGAATATTTTTTTT

TTGGCTACTGTGG

>Unigene0002461

CTAAGATTCTCGTAATAACTTTGCTATATTGTTTGCTAGTTGTATTGCTACATATTTTCT

GTACTGGATTTTTTGGTGTCCGAGTGTTGTGAAATGTCAAGAAGTTCCAATCTATATTTG

GGTTCACTGATCTTGCGGTAAAAGCAAAGTGTTGCTTGTAGCACAATGTATATCTGTAAA

TTTCCATATATGTTGTATGTATGCTATGTTGTCCAAATGTTGGAGTTGTTATTCTCTATA

TTAAAGGTTTAATGGAAAGTAAAGAAAA

>Unigene0002462

TGCTTTATTCAATTTCCTCCATAAATCCCCTTTCGAACCATCATTGTCTTGTCCAAAAGA

TGGAAGCCTCCGATCTCTCCCTCGCCGGTCAAACCGACTTCTCCCTCCAGCTCGCGCACC

TTGTGGCCTCGACGTCCCCCGCCGATTCGAACCTCGCCCTCTCGCCGCTATTAATCCACG

CGGCCCTCAGCCTCATCGCCGCCGGATCGAAAGGTGCCACACTCCACCAACTCGCCTCAG

TCCTCCGGGCGGCAGATGAAACTGATCTGCACAGCCTCTCCTCCAATCTCGCCTCCTTCG

TCCTTGCCGATGACTCACTTAGCGGTGGTCCCCGGATCTTATTCTCCAGTGGTGTCTGGG

TGGACGCATCGCTGTCGCTGAAGTCGTCATTCAGAGAGATTGCTAACTCGGCCTACAAAG

CTGAGGCTAAGGCCGTCGATTTCCAAACCAAGGCCAATGAGGTGAAATGTGAAGTTAACT

CGTGGGTAGAGAATGCGACAGCTGGTCTTATCAAGGAGCTTCTGCCTGCTGGATCCGTCG

ACCGTAACACTAGGCTGGTTTTGTGTAATGCTTTGTACTACAAAGGAGTTTGGGAGGAGA

AGTTTGATGCGTCTAAAACCAAAGAATCTGAATTCCACTTACTCAATGGAAGCTCTATTA

AAGTTCCTTTCATGACAAGCAGAAA

>Unigene0002463

AAAGGAGTTTGGGAGGAGAAGTTTGATGCGTCTAAAACCAAAGAATCTGAATTCCACTTA

CTCAATGGAAGCTCTATTAAAGTTCCTTTCATGACAAGCAGAAAGGAGCAATACCTTTCA

GAATATGATGGTTTCAAAGTCCTGACACTCCGGTACAAGCAGGGTGACGATAGTAGGCAC

TTCTCCATGAATATTTTTCTACCGGATGCTAAAGATGGGTTGTCGAATTTACATGAGAGG

ATGAGTTCTAAATCAAGGTTCCTGGACCTCCATTCTCCATGGAGAAAGGTGAAGGTGGGT

AAATTCAAGCTACCCAAGTTCAAGATTTCATTTGGGTTTGAAGCAACTAAAGTTTTGAAA

GATCTAGGCCTGATACTGCCCTTCACATGCGATGGAGATCTGACGGGGATGGTGGATTTA

CCTGTTCACGTGTCTTCAATTTATCACAAATCTGTGGTCGAAGTTAATGAAGAAGGAACT

GAAGCAGCAGCTGCTACTGCGGTTGTTATGATGCCAATGTGTCTTACTAGGGATCCCGAT

CCTGTGAACTTCGAAGCTGATCACCCCTTCTTCTTTGTGATCAAAGACGACGTAACTGGA

GCGGTATTGTTCGTGGGGGATCTGCTGAATCCATTAACTGGATAATGTTCATTGCAACTA

CAATAATTGTCTGCCCTAACTGGATACTTTCTCATCTTACCGTTTGGACTTCCTAAGATT

CTCGTAATAACTTTGTTATATTGTTTGCTAGTTGTATTGCTCTATAGTTTCTGTACTGGA

TTTTTTTGGTGTCCGAGTGTTGTGGTGAAATGTCACGAAGTTCCAATCCATATTTCGTTT

CACTGATCTTCCAA

>Unigene0002464

GACTCCAACCTCGCCCTCTCGCCGCTCTCGATCCACACAGCCCTCAGCCTCCTCGCCGCC

GGATCGAAGGGCGCCACACTCCACCAACTCGCCTCAGTCCTCCGGGCGGGAGAAGAAACC

GATCTGCAAAGCCTCTCCTCCAATCTCGCCTCCTTCGTTCTCGCGGATGGTACACCAAGC

GGTGGGCCCCGGATCTTGTTCACAAATGGTGTCTGGGTGGATGCATCGCTATCGCTGAAG

TCCTCGTTCAAAGAGATCGCTAAATCGGCCTACAAAGCTGAGGCTAAGGCCGTCGATTTC

CAAACCAAGGCCAATGAGGTGATATGTGAAATCAACTCGTGGGTAGAGAATGCCACAACT

GGTCTTATCAAGGAGCTCCTTCCTGCCAGATCTGTCGACCGTGACACTAGGCTGATTTTG

TGTAATGCTTTGTACTACAAAGGAGTTTGGGAGGACGAGTTTGATGCATCTGAAACCAAA

GAATCTGAATTCCACCTACTTACTGGGAGCTCTGTTATAGTTCCTTTCATGACAAGTAAA

AATATGCAATACCTCTCTGAATATGATGGCTTCAAAGTTCTAAGGCTCCCGTACAAGCAA

GATGAGGATATGAGGCAGTTCTCGATGAATATTTTTCTACCGGATGCTCAAGATGGGTTG

TGGAGTTTACATGAGAAGCTGAGTTCTGGATCAGGTTTCCTAAATAAGCATTGTCCATGG

AGAAAGATGGAGGTTGGTAAATTTAAGCTCCCCAAGTTCAACATTTCATTTGGGTTTGAA

GCATCCAGAGTCTTGAAAGATTTAGGCCTGGTACTTCCCTTCACCAGGGCCGCAGACCTG

ACAGGGATGGTGGATTTGCCCGTTTATGTGTCTTCAGTTTATCACAAATCAGCGGTCGAA

GTTAATGAGGAAGGAACTGAAGCAGCAGCAGCTACTGCTGTTGTTATATCTATGTATTGT

TCAATCAGTGATTCTGATTCTGATCCTATAGACTTCGAAGCTGATCACCCCTTCGTCTTT

GTGATCAAAGAAGACACCACTGGAGTGGTATTGTTTGTCGGGCATGTGCTGAATCCATAG

AGAGCATAATGTTCAAAATAATATTATGATTCTGATGGGATCATTGTGATATCGAACTAC

AGTAATTTTCTGCTTGGTCATCTAAGGTTCTTGTAATAATGGTCGTCTAAGGTTCTTGTA

ATAACATCCTATATTGTTCCAATCCATATTGTTTACTAG

>Unigene0002465

AACCAAGGTGCTAGAGGGCAAAGGCTATCCATGTACAAAGCTATTGGCCCACCGATGGGT

AGCTTGGAGATTGAGACATATTCCAAGAAGCAACACAACTACGTGAAGATAATTTTACGA

CAGATAGCAGATGGAATTAAGATGAATATAGGGTATAATATTGATACATGATGTAGTTAT

AGCTATAACAAATGTACTCAATAACTCGAAATTGGTACGTAGTCTTATTTTTTTAAATAT

GGTGAGTTGGTATGGTAGATAAATTCATGTAACAAATAATTTTCGAATAACGGGATAGTT

CATTCTCTGATGTTGAAGGCCAATGAGGTGAAATGTGAAGTTAACTCGTGGGTAGAGAAT

GCGACAGCTGGTCTTATCGAGGAGCTTCTTCCTACTGGATCC

>Unigene0002466

AAGCGAACTTGTTCAGAAGTATGTGGGAGAAGGGGCTCGAATGGTTCGCGAACTATTTCA

GATGGCTCGCTCGAAGAAGGCTTGCATCGTCTTTTTTGACGAAGTTGATGCTATTGGTGG

GGCCCGCTTTGATGATGGGGTTGGAGGCGACAATGAAGTCCAACGTACTATGCTTGAGAT

CGTGAACCAACTCGACGGCTTTGATGCCAGAGGAAACATCAAAGTTTTGATGGCAACCAA

TAGACCCGATACATTGGACCCTGCCTTACTGCGGCCTGGACGGTTGGATCGGAAGGTTGA

GTTTGGGCTGCCTGATCTGGAGAGCCGGACACAGATCTTCAAGATCCACACTAGGACAAT

GAACTGCGAAAGGGACGTGCGCTTCGAACTTCTCGCCCGACTCTGCCCCAATTCGACCGG

AGCGGACATACGCAGTGTTTGTACTGAAGCGGGCATGTATGCTATTCGGGCACGGAGGAA

GACTGTTACTGAAAAGGATTTCCTCGATGCTGTCAACAAGGTCATCAAGGGATACCAGAA

GTTCAGTGCCACCCCGAAATATATGGTTTACAACTGAAAATTCTCCGCAGGATTGTAAGA

GTTGATGAGAGTTTATAAATGCAAGATTCATGTGTGCTATCTTATGTTTTATGGGAACCG

GAATTTGGTTAAACATTCACACGGTTACTGGTTTAGATGAATGTAATCAATTGGTTTTGT

GAAGCATACAACTATTTGAACAGTTAATTGCTGGTGAGCATACTGGGTTTATCAAGTATT

TGATTTGTTAATTGTTTATAAGCACAGTAACCATACAATATGTTACGGGCTTATGAAGCA

AACTCATCATTGTGTTACATATTACCTTTAGTACCCTGTAGT

>Unigene0002467

CTTAACCTCGTCTCCACCTCCACTCTCACCGCCGCTGCTCCGAAACCCTAATCTTACTGA

AGATTTCGCCATGGCCCCCGATCCCGATGAAGTTCTGAACGAGAAGAACCCGCCGCCGCT

CGACGAGGACGACATCGCGCTCTTGAAAACCTACGGGCTGGGGCCTTATTCCATCACGAT

TAAGAGCACGGAGAAGGAGATCAAAGAGATGTCGAAGAGGATTAATGATCTCTGCGGAAT

TAAAGAATCTGATACTGGATTAGCTGCACCGAGTCAGTGGGATTTAGTCTCCGATAAGCA

AATGATGCAGGAAGAGCAGCCTCTTCAGGTTGCAAGGTGCACTAAGATTATCAATCCAAA

CACGGAAGATGCTAAATATGTAATAAATGTTAAACAGATAGCCAAGTTCGTTGTTGGCCT

AGGTGACAAAGTCTCTCCTACCGATATAGAAGAAGGAATGCGGGTCGGAGCTGATAGAAC

TAAATATCAAATTCAGATTCCGTTGCCCCCAAAGATTGATCCAAGTGTTACAATGATGAC

GGTAGAAGAGAAGCCCGATGTCACATACAATGATGTCGGTGGGTGCAAGGAACAAATTGA

AAAGATGCGTGAAGTTGTAGAGCTTCCCATGCTTCACCCAGAAAAATTTGTTAAGCTAGG

TATTGACCCTCCTAAAGGAGTTCTATGTTATGGCCCACCGGGAACCGGCAAGACCCTTCT

TGCCAGAGCTGTAGCGAACCGAACCGATGCCTGCTTCATTCGTGTCATTGGAAGCGAACT

TGTTCAGAAGTATGTTGGAGAAGGGGCTCGGATGGTTCGAGAACTGTTTCAGATGGCACG

TTCAAAGAAAGCCTGTATTGTGTTCTTTGATGAAGTGGATGCAATCGGCGGTGCTCGCTT

TGACGATGGCGTTGGAGGTGACAATGAAGTGCAGCGCACTATGCTTGAGATAGTAAATCA

GCTTGATGGTTTTGATGCTAGAGGAAACATCAAAGTTCTGATGGCTACTAATAGGCCTGA

TACTCTGGACCCTGCTCTACTGCGCCCTGGACGATTGGACCGCAAAGTTGAGTTTGGTCT

TCCTGATCTAGAAAGCCGGACACAGATCTTTAAGATACACACAAAGACGATGAACTGTGA

AAGGGACATTCGGTTCGAACTTCTCGCCCGTCTCTGCCCCAATTCCACTGGAGCCGACAT

AAGGAGCGTATGTACGGAGGCTGGTATGTTTGCAATCAGAGCTCGAAGGAAGACGGTGAC

TGAGAAAGACTTTCTTGATGCGGTAAACAAGGTGATCAAGGGCTACCAGAAATTTAGTGC

CACCCCGAAGTATATGGTTTACAACTAACGACTCTGCAGGTGAGGTCAGAACTGCTAGTT

TTTATATTGCGAGGTGTCATCTGACGTTTTGTTGGATCTATGTAATGTGTATGTGGTAGG

AGAGGATATGGATTTGATAGAAAGACTAGCGCTTATTCAGATGATTGTTCTGTTGGATCG

AGGTATATGATGTTTTATTGGAAGTTATATGTCTCTTGATTAGTGTCAGATGACGCTTTG

TTGGATCCAATTGTTTTATCTAAAACCTTTTACTCTATCTGAAGCTGAGTTATACCCTAG

ATGCTGAACTTATACTCTGTATCTAAGGCTACACTACACCTTCCCTCTGTAAACTGAACT

TGTATTTTTCTTCAGATTCTATATGTATATTGCATATGGTCATATGCCCTATTTGCCGAA

TATGAAGTTGTAGTATGTGTTTTCCCCATAAT

>Unigene0002468

CGGTTGGTATTTTGGTGCTCGGTGCCCTTAAAAATCGGCACCGACAAATCCAAATCTCGC

AGTTGTTGCCCCTCGCCCGCAGTCGATCCGAAATCCTACTCCGATCTCGTCATGGCCCCC

GATCATGAAGAAGTTCTTGACGAGAAGAACCCACCGCCGCTCGATGAGGACGACATAGCG

CTCCTTAAAACTTATGGATTGGGGCCGTATTCTGTCACGATTAAGATGAGGGAGAAGGAG

ATTAAAGAGATGTCTAAGAAAGTCAACGATCTTTGCGGAATTAAAGAATCTGACACCGGT

TTAGCTGCACCGAGTCAGTGGGATTTAGTCTCTGACAAGCAAATGATGCAAGAAGAGCAA

CCACTTCAGGTTGCAAGGTGTACTAAAATTATCAGCCCAAACACAGAAGAGGCAAAATAT

GTGATAAATGTCAAACAAATCGCCAAGTTTGTCGTTGGCTTAGGCGACAAAGTCTCTCCT

ACAGATATTGAAGAAGGGATGCGTGTGGGAGTTGATAGAAACAAATATCAAATTCAGATA

CCCCTGCCCCCAAAGATTGATCCCAGTGTTACAATGATGACCGTAGAAGAGAAGCCTGAC

GTCACATACAATGATGTTGGTGGATGCAAGGAGCAAATAGAGAAGATGCGAGAAGTTGTT

GAGCTGCCAATGTTGCATCCCGAAAAGTTTGTGAAACTCGGCATTGATCCTCCTAAAGGA

GTCCTGTGTTACGGCCCACCAGGTACCGGTAAAACTTTGTTGGCTAGGGCAGTAGCCAAT

AGGACTGACGCATGTTTCATTCGGGTCATCGGAAGCGAACTTGTTCAGAAGTATGTTGGA

GAAGGGGCTCGGATGGTTCG

>Unigene0002469

GTCAGATGACGCTTTGTTGGATCCAATTGTTTTATCCAAACCTTTTACTCTATCTGAAGC

TGAGTTATACCCTAGATGCTGAACTTATACTCTGTATCTAAGGCTACACTACACCTTCCC

TCTGTACTGAAACTGAACCTGTATTTTTCTTCAGATTCTATATGTATACTGCATATGGTC

ATATGCCCTATTTGCCGAATATGAAGTTGTAGTATGTGTTTTCCCCATAAT

>Unigene0002470

CAGTAATGACGTCATTTGATAACTCGTCTTTTGCGTATCTCGCAAAATACGAAAGAAGCT

GGTTTATATTCTCCTTCCTCCTTGTGAAATAGTAGCTAATTTATGCAGTAGTAGAGGAGA

GGAGATAATAATATGTTGCTAAATAAAGAACAAACCGGAACTCTCAGTTTACGTCAATGT

GTTTGACGATCAGAGCTTCCAGTTATCGTTGTAGTGATAAGTTCTCTCGTGTCCAGAAGT

TGACCTGGGA

>Unigene0002471

CAGTAATGACGTCATTTGATAACTCGTCTTTTGCGTATCTCGCAAAAGACGAAAGAAGCT

GGTTTATATTCTCCTTCCTCGTGAAATAGTAGCTAATTTATGCAGTAGTAGAGAAGGAGA

GGAAATAATATGTTGCTAAATAAAGAACGAACCGGAACTCCCAGTTTACGTCAATGTGTT

TGATGATCAGAGCTTCCAGTTATCGTTGTAGTGATAAGTTCTTCCGTGTCCAGAAGTTGA

CCTGGGAGGTCGAACCCACACTCAGGTGGTAGGGGTCTAAATCTCCTTCTCTCTGGGGAG

AAGTCGATTACCCTACTCTG

>Unigene0002472

CTGGAGATTGTTGAAAATCAGTTTGGAAATTTTGGTTCAGTTCCCACCTGTTATTTTGAG

AAAAAACATTTTCGATACAATGTAAAGTGTCAGGGTTATTGATAGGCATTTGATTTTGGT

CGAAATAGTTGTCATTGTGCGGGTAGGAAAAATTGTTTTGAAATAATAGACATTGAAAAG

CATAATGGTCAATTCCCTGACAGTACTTACAACAAATTTGATTGTCTGAGTTATT

>Unigene0002473

CAAATTTGATTGTCTGAGTTATTTTAGAAAAAACGCCTTCATTTTGGTGATAAAAATTTT

AGTTTTGAAATGAAGGACAATCGGAGGTGTAATGGTCGTAATTCTGACAATTGTCACATG

AAAATTGTTGGTTATAATTGGTATATATGTTGATAGTATAACAACCAGAAAGTGTGTAGA

TCGAAAATATAGATAGAAAAAATACAGATAATCTTAAGATTGCTAAGTGTATTTGGTTAT

CTATGAGATCTTTATATCTCACTAATTGAATAGTTAAATTAACTATTCCGATAAAATAGC

AGGCAACAACCTGACCTAACACCTAGCTTTTCTAGCAATTGACTTGACTCCCCCAGGTGG

ACAGGGAGCTTCTCGAAGGCGCTTACACACCTGTGCTTCTAGTACT

>Unigene0002474

ACTTCACGTCATCTATCCTATAAGTTAAAGTTCAATAAACAAGATATTTTTAATTGGTAG

TGTTACATTAAAGGTTTTAGTGTATTTTGATTTATTTTGTATAATCTGAGAAAATAAAGC

GGTGGTTTGAATTTGTTAGGTGATTATTTAATGAAACATATCTTAGTTACATTATCCTTC

TTTTTAATATAAAATTTGAAAGTTCAAACTTACAAACATGACAAATTGCTCTTATAAGTG

TTTACTTTGAATGCTTTCAAGTTCCAGATCTCATGTTTGCGTTCTTTCTCTTTTATATTA

TCTGTAGTTCAATACCTT

>Unigene0002475

TCCATTTCTTGATTTTGCGTGTAAGCTCTTTGATTTCAGTCTTGATAGTTATTTTCCTTA

AAATACTCTCTCGGGTTTTGAGAATTTTGATTTTGGTCAAATTCCGACGAGTGTTGAAAA

CCGTTTTGTTGATAATTATTTGTGGGTTCCCAACATGTATTTTGTGAAAAAATATTTTAC

TCAGAAGAATCTCCCGTGTTTTGAAAATGTTGATTTTGGTCAATTTTCAGAGTTTGTTGA

AAACCAGTT

>Unigene0002476

CACAGGAAAATTGTTAGTTGTTATAATTGGTATACATGATGATAATATAATAACCAAATA

GTGTGTAAATTGGAAATGAAGACAGTCAAAATCAAATACAGATAACCTTAAGATTGTTAA

GTGTATTTGGCTATTCATAAGATTTTAATATCTCACTAATCGGACAATAAACTCCGATAA

AATAATAGATAACAGCCTGACCTAGCTAACACCTAGCTCTTCTAACAATTGACTTGACTC

TCCCAGATGGACATGAAGCTTCCCGAAGGTGTTTGCGTACCTGTCCACCTAGTACTGACC

ATGCACAGACTAGGGAGTAAGTGGATCGCTATTGGAGCTAGAAATGTTACTGGAGTAGTG

CTGAAACTGGGATTTTCTAGAGCTTCCTCGTATATATTTGAGTTCTGGGAATGCAGTATG

AGGATTGTTCGTGGGCTGCAGATAGGTGAAAAATTCCTACCTGCGTCGGGTGAGCCCTTA

AGCGAGTTTTAAAATGACTATCCACAATGCTTCACTTCACGTCATCTATCCAATGAGTTA

AGTTTAATATACAAGATAATTTTAATTTCTATAGTTACACTAAAAGTTTTAGTGTATTTT

GATTTATCTTGTATAATCTGAGAAAATAGAGCGGTGGTTTGAGTTTGGTAGGTGATTAAT

TAATGAAGCATATCTTAATTACTCTATCCTTACTTCTTTTTATTCTAGAAATTAAAAATT

CAAACTTTCAAACATGACAGATTGCCTTGGTGAGTGTATTTGTTTTGAATGCTTTCAAGT

TCCAGATCTCATGTTTGCGTTCTTTCTCTTTT

>Unigene0002477

TTTTTGCTGAGCTGGACACGAATTCTAGCCGTGGCGGATTTTGGTGTCAACACTAACTCC

GTGGTGTGTGTTTCCAGTTGTGTGTTATATGATTCGAGAAAGAAATTATATGAATCGAGG

TCAAGTGTGTATTTCAGAAGGTGGTATATGTTCAACCTCTTATTCTATATCAATAATTAT

AAATTCTTTCTGGGTCTCTGTGAGAAATTCTTGGTTCGGTGTTTGTATATGGACAAGTAG

TGATTCCTCCCAAATAGAAATATTTGGATTCGGAAGTTCAATATCCTTCTCCAGAATGTC

TCCAATGGAAGGGTTAAGATTAAATTTCTCGTGTTCCATTTTCAGTTGAGTTGTGATAGG

GTCAGGTTTGCTCAATGAAAAATGGGTTGAAATTGGCAGTTCTTCCAAATTAATATGTTG

GGTTTCTAATCCAAGTGTATTCTTCCTCAAAATCTTCAAATTGGTCTCAAGATGACTTCC

CAACAGTAGATGTGTAATCAATAGTGAAATTTTCAAGATTGGCAATTGGCTGATCATAAA

CTATGGTCTGTTGGCTGTGGGTATTTTACCAATTTTTCTTCCGCCAGCAATTTAGCATCA

AGATTCTTTAGTTGTTGGGCCTGGGTTTCCTGGAGTTGGTTTATCATTTGAATACTTTGG

AGTAATGTATCCTCCAAAGATAACTTGGGAGACTGATGATTCAGGATAAATTGGGTGGCA

TGAAAGATTTTGAGGGTCCATTTCTTGATTTTG

>Unigene0002478

CAATTCCATTCCTCTCCTCCGATCTATCTCTTCAAACATCTCAAATTTATTGATTCTTAA

TCCAGATCCACCTCTTCAAACATTAAAACTTAATTGATCTTTCCCAACTTCAACCATGGC

GATGGAGGCAAAGCAGATTGCAGCGCACATCAAGAAATGGCTCTTCCTCACAATCAGAGC

AACTTACAGATCAGCTCGCCAGCACCCAATTCTGTGGGGAGTGGTGATCGCGGTGTATCT

CTTCTACAGGTGCTTCCCTTCCTTCTTTGCTCTTTTAATTTCTTCGTCACCAGTCATCGC

CTGCACCACCGTCCTCCTCGGGCTCCTACTGAGCTACGGCGAGCCCAATCTCCCGGAAAT

TGAGCCCCCAGTTGAAGATTTGTTTACACAGCAAGCCGAATCGCTTAAATCGGAACCCCC

GGTGGATGATGTGGTCCTTGACGACGACAGGAGCTTAGCTGCAGAGGCTCATCTTGATGC

GGGAGTGGTGGAGGGAGATGCAGAGGTAGAGGAGAGGGAATTCATGCCAGAGAGTATAGT

TAACCATCTTAAGGTGAGAATTGAGAAGCCGGCTGTGGATGAGAGCCGATTCGAGTCCTC

TCTAGGTTCGCCGTGGCTGACTGTCGGCATTGACGATGTGGCGTCGGAATCGGAGTCGTC

GGACGGGGCGGAAAGCTCGTCCCCCGATGCTTCGATGGCGGACATTATTCCAATGCTTGA

GGAGCTCCACCCGCTACTAGACTCCGAGCCTTTGCAGACTGCAGATAATGAATCCGATGC

CGGCAGTGCGGAGGAGGAGCAGCAAGAAGAAGAGGAGGAAGTACACGCTGATAACGGAGA

TAAGGCGGTGGAGTGGACGGCAGATGATCAGAAGAATGCCATGGATCTGGGGATTTCGGA

CATGGAGAGGAATAAGAGGCTGGAGAGCTTGATTGCGAAGAGGAGAGCAAGGAAGGATCT

GCGATTTGCAGCAGCGGAGAGAAATTTGATAGACCTCGATGATTTATCGGAGTTCCAAAT

TCCGGCGATTTTTGCACCGAGGCAGAACCCATTTGATCTTCCTTACGATGATACAATTCC

GCTATCAGCTCCCTCTGTGATGCAGCCGAGGAAGAACCCTTTTGATCTTAATTTTGATCA

AGTTGGTCAGAGCAGCAGTTACAGTCACACGACCGAGAGCTTGGTTCATGGGAAGCTTCC

GCCACCCCCGGTTCCACCGCGGGAGAGTTTCTTCGCGAGGCACGAGAGCTTCAGCTATGG

AGTGCAATATCCGGGGGATAGCAAGACGGAGAAATCGAACAGTAGGTTGAAGCCGTTTTT

TGTTGTGGAGAGGGAGGGCGAAGCCGTGTTTGCCACTTACCAGAGGCAATTGAGTGAGAT

GAGTGACTCCAAGTTGAGCTCGATTCCTGATTCTGATGCAGTTTCATCGGTTGTGGACGA

GGAGCATCAGAATGAAGTTTTAGAAGAGCTGGATAATGAGGTGTTATCTCCTGAGTATCG

GCTTGCTAAATCTTCTGAAGAAGAGAATAGATTGCTCGAGGAAGTTGATTCAGTTCTTGA

TCATAAAACTAGCGGAGGTAATGTAGGTGAACGAGTCGGGATTGATTCAACTACTTTTAA

CCCTGAAGAGGCTCAACTGTCAGACAATGCTTTGCCAGTGGAAGAGGAAATCAGGGAGAG

AGAGCTTCATTCATCCATCTTGGATACATCCGGATCAGAAACTATGGAAGATAAATATGA

TGGTTCCTCGTTGAAGGCAGCTATGAAGGTTTCCGAAGAAAACACCTTTGAAGAATTGGT

TGATTTTGAGTCAGGAATGGCCAACTCCTTAAATGTTGCAGATGATACCATGCATTCACT

TCCTGCAGGTTCCAGTTCTACTGATGAGGAGAGCGTTATTGACAGTGCGCTCGTACACCC

TGTTTATGATTTGAGCCCATCATCTACCAGAAAATCCCTCTCTAATGTTTCCAGTTTTGA

TGAAGCATTGCTCGACGCTGGCTTGCAGGCACATATTGGAGTTGTAGGTTCAACTTCGAA

TCTACTTGAGAGGGATATTTCCACAAATCAAGTTTCCAAATCAGCCAGTGAGGATACTGA

CTTGGCATCTGCAATTGGAGGACCCGGGCAAGCATTGCTTGATTTGTCTTCCACGAACAA

AAATGAAGCTGAATTTGGCACTGTAGCTGAACAAAATATACATCCCTTGAGCTTGTCATC

AGTCGATACCAAATCAATCAATAATAGTCTGATTGATGTGGACGGGAGTTTTGTACTTGA

AGGAGGCGAGACAAGGGATGCTTCAACTCCTTTTGATCTGTTGACTTCATCTATATCAGA

GAAAACACTAATATCAATGGAGAAATATCCCGTGGAAGCATCCTCTCAAGCTGGTAGTGC

AGACTCCCAGACCGGTAGTCGGTCGAACTTGCCTTCAATTCCTGAGGAGAACATGGAAGA

TTATAAGCCGGAAGAAGAACATGGGAGCATGGCAAGCCTTGCTGGAGTCGCTGGTTTAAC

TGGCTTGCAGATTATTGAAGAGCCTGATGTTGGTTTATCTGATATTGAAGAGCCTGATGT

TGGTTTATCTGATATTGAAGAGCCTGATGTTGGTT

>Unigene0002479

GGCTTCTGGGTACAGTAATATCCTATCTTCCAATGCTCGGAGAGACATCAACAAGGTAGC

CTCAGTAGATTAACTTAAAGTAGGTTTACAAATATCTTTTTCATAGTTCTTCTCTTTGTT

CCCCTAAAAAGGGCAACCCAGTGCACAAGGCTCCCGCCAATGTGGGGTACTGGGGAGGGT

CAGATTTACGCAGCTTACCCGTGCATGCAGAGACTGTTTCTGAATTTGAACCTGTGACCT

CCAGGAGGTCACATGGCCGCAACCTTTACCGTTGCACCAGGGTCACCCTCCGAAATGTCT

TTGTTTCGTTACATCTCATAAATTTCTAACTGCAGGTTGTTTTTGTCGTGTTTACCCCAT

CGATGGTATTTGTGAGCCTAGCAAAAACCG

>Unigene0002480

GTCCAAATTCTTCTCCCTTTTGACCTAAATCTGCTTCCTATCTTCAATTCACTTCGAATT

ATGTTTCTTCATGCTCTTGTATGGTCAAATCTAGGGTTTATAACTGCAAATTTGTGTTTC

AACAAATTGATTGATTTGATATATTTAATTCTCGTGTAGTCAAATCTAAGGTTTCAAGTT

GCAGCAATCTGTGATTTGTGTCTCAACAAATTGGTTGATTTGATAGATTTCATGCCCTTG

TACTTAAATCTAGGGTTTCAAGTGGCTGGCGGCAAACTGTGATCTGTGTCTCAACAAATT

GTAATCGAATCTGGGCTTTTAAGTGGTAGCAAACTGTGATTTGCGTCTCAACAAATTGTA

GTCGAATCTAGGAATTTATGTGGCAGCAACTGTGATTTGTGCCGGTAGCAAACTGTGATA

TGGGTCTCTACAAAATTTTAAAAAATCACCGAGTGTGGCATGGACTTTATGACGTTTACC

TTGTGGCTGTCATAAGATATAGAACAATTCCAAACCCTAGCCACAAGACCTTTGCAATTA

TTGATGACACTAAAGAGTCCATGATTATGCTCATTAGGTCTCTTGATCAAACTCATTACA

ATGATAATTTGATAGACATCATCCCACACCATAAATAGCCCTGTGTAGTTGCTTCATACT

TCTGCCTCCATAGCGGTCCTAAGTACCAACAAAGTCTATAAACAGCAAACCGATAACTAT

ACTCCCAATATTCGTCAATTAGCACCAGCAACATAAATTTTGCACCAGCCAAAAGGCGAC

AATTCCAATGCAAAAAAATAATTTGCCGAAGGGGATTTGTTGGAGCTACCACACGATCTA

TCAAACCGCTCTTTGAGAATTTGTGGGTAACAAATTGTGTCCATTGGGCCCCTTTGTTGG

AGTACTTTGTGTATAATGCTTTACCACTAGGCCTCTAGAATACCACATGTACATGAATAC

CTCATAATCAAGTGTATTATGATACTTCTCAAAAATGTGTTAAAACAAGTGCACACCCGT

GTTACTTTCCACCTTTCTCTACCTATTCCTCCACCTCTCTTTTCTCTCTCCACTCTACCT

CTCTCGTAGCATGAGCATAGAAATTGAGAGGCTTTCATTGATTATTGCAAAACGACTACG

TCTTTACTACACATACAATGTAACATCTCGATTAATTAGTTCTTATCTTTGATAAAGTTA

TTGTTGGCTCTATTCTAAAAGAAAAGACTATAAGCTAGTTGAAAAGACCATTCTAATATC

AAGGTAACTTCTTAAAGTCCTTTTATGCTATTCCTTATTCTCAATTATAATACCTCACTG

AATACTTAATAAATCTTGAATCTTGTAGTCCGGACACCTTTTGGTATGGTTCCCCTTATT

TCTCATTTATATTACCTTACCAAATAAAGGGCAACTTAGTGCACAAGGCTCCCACCAATG

TGGGTTACTGGGAAGGGTCAGATGTACGTAGCCTTACTCCTTTAAGTATTATCTTGCTAA

ATACTTCATAAAATCTTGTAGTCCAAACACCTTTCAGTATATAAATATTCAAATACACTA

CCCATGTATTATAACTCAAATATTTAGACCCCCTACTGGAATTTGAACACTGTGTAGCGG

CTTTTACTCCCTTTTATTTGAAATCCTAGAAGTCTCCTCTAAAGCCATGGCATAATATCA

GTCAGTGTTTACTGTCACGGTCACATTTTTTTTGTTAAATGGCACCTTTTTTTTTTGGTA

ATTGGAGGAGTTGAACCCGGGTGGTGCCGGAGTTTGATGTGCGGTTGTACCACTGGACTA

GCCAGCCAACCACTTAAATTGCACATTTTTTTTTT

>Unigene0002481

ATTACACTTATTTGTGTTGTATTGGCAGTGTTATTGTTGTGTCTGCGAGATAGTTGCACC

GTGTTCACGTTGGATGGGAATCAATGGTCACTGTCACCTAACAAATAAGATACCCACGAG

GGACTAACGGCAAAGAAACTAACCATGAGGGTTAAAAATCACTTTTCTACGTGAATTTCT

CCGAAGAAAGTATGAAGACAATTTTTTGAATGTTCGGTTAGACGTATTATGTTAGTCTAG

AAATGTTTCGTTAAGAATTTTGAATTTTGGTTTGATTTTCAAGAAGGAAATTTTTTAGAT

CTGATGAGACATTGTTCTGAATTGTGGTTTCAGTGTAAAGAAGGAATATATCTGGATTTT

CCAATTGAAATAGTTGGCACTAGCCGAAATTCCATGAAGAATTAGAAAAAGTTGGGCTTT

CGCAAGATGCATAAAAGTGGTGGTGTGAATCCTGGAATGATTAGTCAGGCATTACAGACA

ATACCATTTTCACTAGAGGAACTTAAACAATATTTATGCAACATTCTATCTCCACACATT

CCAAC

>Unigene0002482

TTTTCTGTAGAGTTGTTGTGTCGCATCAAGATGGATGAGGTCATATTCAGAATGGATCAG

GTGTCACCACCTCAGCCGCCCTACCAAGTCAATGACGGTCCAGACGGGGTGTCATGGTCT

AAGGCTTGGTTAGTAAATTATGAAAAATTAACAGGGCCTGAAATAGGCAAAGTGTGTGTT

TGGGTTTTATTAAAAAATTTATGACCGGATGAAGTTTGCACTTCTGATCCCCTCCATTGT

TACAGCTATCAAGACTGACTACTGCTAAGCGTCCTAAAAAATCGTCAAAGGTCAATAACT

TGTTTCATAACAAGGCTTTGTTAAACATGGATGCGGGTGATAGCAATGAAAATCCTCTGT

TAGTAGAAATTGTTGATGATGATAAAGACTGCT

>Unigene0002483

TCCAGACGGGGTGTCATACAACTTTTCTGTAGAGTTGTTGTGTCGCATCAAGATGGATGA

GGTCATATTCAGAATGGATCAGGTGTCACCACCTCAGCCGCCCTACCAAGTCAATGGCGG

TCCAGACGGGGTGTCATGTTCTAAGGCTGCGTTAGTATATTATGAAAAATTAACAGGATC

TGAAATAAGCAAACTATCAAGACTGACTACTGCTAAGCGTCCTAAAAAATCGTCAAAGGT

CAATAAC

>Unigene0002484

GGATGAAGTTTGCACTTCTGATCCCCTCCATTGTTACTGCTATCAAGACTGACTATTACT

AAGCGTCCTAAAAAATCCTCAAAGGTCAATAACATGTTTCATAACAAGGCTTTGTTAAAC

ATGGATGCGGGTGAGAGCGAAGAAAATCCTCTGTTAGTGAAAAGTGTTGATGATGATAAA

GACTGCTATGTGATTGATGCCATTAATTTCACCCGTAAACTAACATTTGATTATACGGAT

GATGTTCTCTTGTTGCAGAGAAAGGATCGATTGCGTTGAGGGATTTCCCACATCCCAGAC

ATCTTTGTGGGTGCTT

>Unigene0002485

TAAAGTGACATATCAGTTAAAATATCAATTTGGCTTTTATTTAGTTAGGATGCAAGGGAT

TCAGCAAGCTCCTATTGACGTGGGATGTGAGAAAGAGTTTTTCATCGAGAATGTAGGAAG

TATATTGCTGCCACCACTGCTGCAAATAACTTTTCTGTAGAATTGTTGTGTCTCATCAAG

ATGGATGAGGTCATATTCAGAATGGATCAGGTGTCACCACCTCAGCCGCCCAACCAAGTC

AATGACGGTCCAGACGGGGTGTCA

>Unigene0002486

TACCGCGACCAACATGAACTCGGGATATTTCCTTGACTTCGAGCCCTGGACTTTCAAATT

CCCCTTCGAATTGAGAAAACTTATCTTCTTCCCTATCCAGTTTCACAACGGGACCAAAAA

ACCCTTTGCTTTCAAGTTTCAGCAAGACGAGAGAACGAGAGGCCAGGATTACACCGTCCA

GCCGCCCGAGGGAGTTGTGTCTCCACTATCCATATGCAAAGTCATTGTTAGGATGCATGG

TATTCAGCAAGCCCCTGTTGACAT

>Unigene0002487

TTTCCTGTCCAGTTTCACAACAGGACCAAAAAACCCTTTGCTTTCAAGTTTCTGCCAGAC

GAGGGAACGAGTGGCATGGAGTACACCGTCCAGCCGCTCGAGGGAGTTGTGTCTCCACTA

TCCATATGTTTACCTTCCTGTTAAAGTGACATATCAGTTAAAATATCAATTTGGCTTTTA

TTTAGTTAGGATGCAAGGGATTCAGCAAGCCCCTGTTGACGTGGGATGTGAGAGAGTTTT

TCAACGAGAATGTGAGGAAGTATATTG

>Unigene0002488

ATCTTCGGTGGCGTTTAGAATAAATTTGATTAAACATTTTTTATATCCAGCTACTGATCA

GAGAAAATTGGGTCGCCTACCGCCACCAACATGAGCTCGGGATATTCCCTTGACTTCGAG

CCCTGGACTTTCAAATTCCCCTTCGAATTGAGAAAACTTATCTTCTTCCCTATCCAGTTT

CACAACGGGACTGAAAAACCCTTTGCTTTCAAGTTTCAGCAAGACGAGAGAACGAGAGGC

AAGGAATACAACGTCCAGCCGCCCAAGGGAGTTGTGTCTGCAATATCCATATGCAAAGTC

ATTGGTCAATTTTTATTCAACCTGCTATTCTTTTGTTTACCTTGCTGTTAAAGTGACATA

TCAGTTAAAATATCAATTTGGCTTTTATTTAGTTAGGATGCATGGGATTCAGCAAGCCTC

TGTTGACGTGGGATGTGAGAGAGAGTTTTTCGTCGAGAATGTGAGGAAGTATATTGCTGC

CACCACTGCTGCAAATAGCTTTTCTGTAGAGTTGTTGTGTCGCATCAAGATGGATGAGGT

CATATTCAGAATGGATCAGGTGTCA

>Unigene0002489

TATCAGCGACCAAGCACTTGCGACTTGTTGCCACCACCGGTGTTACGCTCCGATTTTCAG

TCGGCGGAAACAGCCCTCCGTCGCCGCCGCTGAAAAATCTAGGTTTCCGCCCGTGCCGTC

CGAGCTCTAGGGTTTACAGTGAAGTCCATCGCCATGGTCGACCCCCGGGTGAAGATTGGG

CTCTTGATTCCTTTTCATGACAAGCCTATGAAAGATGCTTCGATCTCAAACATACCTATT

TACGAAGGAGCCAATGTTGTCGGCCGCGATAATCTCACCGCACCTGACAAGAGGGTCAGC

CGCAAGCAAATCAGCTTGCATGCGTTGGGCGATGGATCTGCTGAAGTTGTTGTGGAAGGA

TCAAATCCGGTTATTGTCAAATCTGGTGATCGGAGGAAGAAATTATGTTCCGGAGATAGA

GTTGGGTTAGTTCATGAAGACGTTATCGAGTTGATTCCGGGGAATCACCCGTATAAATAT

GTAATGACCGTTGGCGATCATACATTGTCGTCGGAAACAAATGTTGGAATGTCGAGTAAA

GAAAGACGACATAGTGAAGAGGATGCATCTGCTGTCAAAAGGAGCCGACAGATTCTTGAT

GATGAAGCTTTTGCAAGGTCTTTGCAGGCATGGGAAGAAAACAGCTCTGGGCATGATAAC

GCTGTAGAGACTACATCTGGTGTTTCTCGGTCAGACCCAGCTACTGATTTAGGTTCTTCT

GAAGCTGTGGGGGCTTTTCGCCATTTCAATGTGCCTAAAGACAGGCTATCCTATATGTTT

CGACTTATGCGAGTCCAAGGTATTCCAGCACAGGCAAATTCTTCTTCAGTTGCTATTCGG

GATGTGGTGCAGGGGAATATTCTTGTTGCTATCCTTTCAAATTACATGGTGGACATTGAT

TGGTTGATGACCTCATGCCCTGATTTGAGGAAGATTCCTCGTGTGCTTGTTGTGCATGGA

GAAAGTGGGGGTACCGTAGACCATCTGAAGAAGAGCGTACCAGCAAATTGGGTCCTGCAC

AAACCACCACTTCCTATATCGTATGGAACACATCATTCAAAGGCTATGCTTCTCGTATAT

CCACAAGGCTTGCGTGTTGTTGTACATACAGCAAATTTAATCCATGTCGATTGGAATAAC

AAAACCCAAGGCTTATGGATGCAAGATTTCCCTTGGAAAGATCCAAAGGACTCGAAAGGA

AGTTTTTTTGAGAACGACTTAGTTGATTATCTCAACGCACTAAAGTGGCCCGAGTTCAGA

GCTAATCTTCCTATGATGGGAAGTGTCAACATCAACGCATCATTCTTTAAGAAGTTTGAT

TATAGCAGCGCGAAGGTAAGGCTCATTGCATCTGTTCCTGGTTATCATATGGGTCCTAAT

CTTAAGAAGTGGGGCCATATGAAACTACGAACTGTGCTTCAAGAGTGTATATTCGATAAA

GAGTTCTACAAGTCTCCCCTTATTTACCAGTTTTCTTCCCTTGGTTCGTTGGATGAGAAA

TGGTTGGCTGAGTTAGCATTGTCTATGTCATCGGGTGTTACAGCTGATAAATCACCTCTG

GGTTTAGGGAAACCACTTATCATTTGGCCAACCGTGGAGGATGTTAGATGCTCATTGGAG

GGCTATGCAGCTGGTAATGCCGTGCCAAGCCCACAAAAGAATGTAGAGAAAGAATTTTTG

AAAAAATATTGGGCAAGATGGAAGGCAAATCATGTAGCCCGTTGCCGTGCCATGCCTCAT

ATAAAGACATTCACTCGATACAACGGTCAAAATCTTGCATGGTTCCTGTTAACATCAGCA

AATCTGAGCAAAGCTGCATGGGGTGCACTCCAAAAGAACAATTCACAGCTAATGATACGT

TCTTATGAGTTGGGAGTGCTCTTCTTACCATCTAACACTGAGGACCATGCAACTGAGTTT

TCATGCACGGAAAAGTGTAGTTTGAAACAGGGTGAACACAAATTACCGGAAACAGAAGGG

CGCAAAACAAAGCTGGCGACTCTTTGTTGGAAAGGAACAAGTGATCCATCAACAGAAGTG

ATCCGGCTCCCCGTACCATATCAGCTCCCTCCTCAGCCCTATACATCTGAAGATGTTCCT

TGGTCATGGGACAGGAGGTATACAAAGAAAGACGTATATGGTCAGGTATGGCCGCGTCAT

GTGCAGTTGTACACCGACCAGGACTCCTGAAGATAACATTGATCTGGAAGAGACTCTGCA

ATTGCGTTTTCTGCAGTAGCAAGCAAGATTGTATGAGTTTATTCCTCTCAGAGCAGCCAA

TATGTTGCAAATTGTGGTTTGTTTTAGAACCTTCTTATGATTTGATCTGAATAGAGAGTA

ACAGCTTTCCTTATTTCATTAGTAAATTTACATTCTGAAGTACTCTATTCTTCCCTTTTG

AGGGGTATTTATAGAGTACAGAGATTTGTGGTGAAGTAATCTGCAAAATATTTATACTTT

TAACTAATCTAACTTTTACAATGACCTGTACGTA

>Unigene0002490

CTCCCCGTACCGTATCAGCTCCCTCCTCAGCCCTATACATCTGAAGATGTTCCTTGGTCA

TGGGACAGGAGGTATACAAAGAAAGACGTATATGGTCAGGTATGGCCGCGTCATGTGCAG

TTGTACACCGACCAGGACTCCTGAAGATAACCTTGATCTGGAAAAGACTCTGCAATTGTG

TTTTCTGCAGTAGCAAACCTTGATCTGGAAGAGACTCTGCAATTGTGTTATCTGCAGTAG

CAAGCAAGATTGTATGAGTTTATTCCTCTCAGAGCAGCCAATATGTTGCAAATTGTGGTT

TGTTTTAGAACCTTCTCATGATTTGATCTGA

>Unigene0002491

CGTTGGTATCCCGCCACCCTCACACAATAATCGAATCAATCCGCAGAACCAACGGCTGCC

AACAAATCTGGCTCCCCCTAGCGGCAACGAACCGCCGTCATCCGTCCGCTGGGACGAAAA

AAAGTTGGCAGCCTATTAATTGCCCCCTTATCTCATTCTCAACTTGCAGTCTTTTCGTCT

TTCCCTCTTTGAATTCAACAGCCCAGCAGAAGCAGTTGGGCTGCCCCCTCTCGGCGTGAC

ACCTGAAGATTATCAAGACTAATTGGTACGATAATCAGAAGGCAAGAACTCAAGCATCAA

GGTAAGCAGGCAACGGTTTCTTGAAGATGTTGCCTGGCACATCTGGAGAGTCGAGCGGAA

ATGCGAAGATGGATTTCAACAATATGGAAGTAATAGATGATGACGCATATCGGGGTTCAC

CAGAGAGGGAAGCTCTTGAAAAGTGGATGGCATTTCCGACGGTAGATTCTCGAGAGAGTC

AGTTCAATGATGAAATATATGAGGAGTCAAATGGAGTTGGAAGCAGCAGAATGATGGTTG

AAAGGGCTGCAGAGTGGGGGTTGGTGGTTAAGTCGGAAAATGGAGACCCTGGTAGCCGTA

CTGTGGGAGTAAGGTCATCTGGGGAGAGGCAAAAGCCATCCATGGATGGAAATAAGTTTA

TGAGAAATTCGGATGAGTCGAATTCTTCGATGGAAGTCAACTTACCCAGGGTGTCCCAAG

ATCTGAAGGATGCATTGTCGACTCTGCAGCAGACGTTTGTTGTGTCAGATGCATCGAGGC

CAGACTGTCCGATAATGTATGCTAGTGCTGGGTTTTTCAGCATGACTGGTTATTCAACAA

AGGAAGTTATTGGAAGAAACTGCCGTTTTCTTCAGGGACCGGAAACAGATCAGGTGGAAC

TTGGGAAAATAAGAGAAGCTGTAAAAACAGGTAAGAGCTACTGTGGCAGGATCTTAAACT

ACAAGAAGAATGGCACGCCTTTCTGGAACCTTCTAACGATAACACCCATACGTGATGGTA

CTGGAAAGGTCATTAAGTACATAGGAATGCAGGTTGAGGTTAGTAAGTACACTGAAGGAT

TAAATGATAAAACTATGCGGCCAAATGCACTGCCTGTCTCCCTGATCCGTTATGATGCAC

GACAGAAGGAGAAGGCAATGTCCTCTATAACAGAAGTTGTCCAGACTGTGAAACATCCTC

GTTCCCACTCACAACCAGCGGTTATTGAGGATGTTTTGAAGCTTGATGAGCATGAAAATT

TCCATACCGATTCTCCTTTACGAAATCCTGTGGACACGAGAAGTTCTAAACCACCTGGAA

GACCAACTGCCCAAGTAGATTCCAAAATTGAGTCGACAAAATCGAGCATGAGAAAATCGG

GTCGTCTTTCATTAATGGGATTTAAAGGAAGAAAACAAAGTACAGCTGAAAGAAGTGAAC

CCATAATCGAACCAGAGATTTTAATGACCAGAGCTGTAGAACGCACAGAGAGCTGGGATC

ATGTGGAAAGAGAAAAAGATATACGGCAAGGAATTGATTTAGCAACAACACTGGAACGCA

TTGAAAAGAATTTTGTGATCACAGATCCTAGACTACCTGATAACCCCATAATATATGCGT

CAGACAGCTTTCTTGAACTGACAGAGTATTCACGAGAAGAAATTTTAGGAAGAAACTGTC

GGTTTCTTCAAGGTGCTGATACAGATCAAACCACTGTTTCGAAAATAAGAGATGCTATCC

GAGACCAAAAGGAAATCACTGTTCAGCTGATTAATTATACAAAAAGCGGAAAGAAATTTT

GGAATTTATTCCACTTGCAACCTATGCGTGACCAGAAGGGCGAACTTCAATACTTTATTG

GTGTGCAACTAGATGGAAGTGATCATCTTGAACCACTCCGGAGTCGTCTCTCAGAGAGGA

CAGAAATAAAAAGTGCTAAACTGGTTAAAGCTACAGCTGAGAATGTAGACGAGGCTGTTA

GAGAGCTTCCTGATGCTAACTCGAGACCAGAGGATTTGTGGGCAATCCATTCACATCATG

TCTCTCCAAAGCCCCACAAAAGGAATAATTCTTCTTGGATAGCCATAGAGAAGATTACTG

GAAAAGGTGACAATATTGGGTTACAGAATTTCAAACCAATAAGACCACTGGGATGTGGTG

ATACCGGAAGTGTACATTTGGTGGAACTGCAAGGTACAGATGAGCTATTTGCGATGAAGG

CAATGGACAAATCAGTAATGTTGAATCGCAATAAGGTTCATCGAGCATGCATAGAAAGGG

AGATTTATTCGCTATTGGATCATCCGTTTCTCCCAACTCTGTACACTTCATTTCAGACCA

CAACACATGTGTGTTTGATAACAGACTTTTGCCCCGGAGGAGAGCTATTTGCATTGCTAG

ACAAGCAACCTTTGAAGATTTTTAAAGAGGACGCAGCCAGGTTTTATGCTGCTGAAGTTG

TAATTGGACTGGAGTACCTTCATTGTCTTGGAATAATATATCGCGATCTGAAACCTGAAA

ATATTTTACTCCAAAAGGACGGGCACGTTGTACTTACCGACTTTGATCTATCATTTCTGA

CATCTTGCAAACCTCAGGTATTAAAACAGGCATTGCCGAAGAAGCCAAGAAAGTCAAAAG

ATCAGCCTCTTCCAACCTTTGTCGCAGAACCAAGTACACAGTCAAATTCATTCGTAGGAA

CAGAAGAGTACATAGCTCCTGAAATCATCATTGGAGCAGCCCATAGCAGTGCCATTGATT

GGTGGGCCCTTGGAATATTGCTTTATGAGATGCTATATGGCCGAACACCTTTCCGAGGGA

AGAATAGACAAAAGACGTTTGCTAATATTCTGCACAAGGATCTGACTTTTCCAAGTAGCA

TCCCAGTGAGCCTTGCAGCGAGGCAATTAATCCATTCGTTGTTGCATAGGGACCCTGTTA

GCCGTTTGGGATCAAGAACCGGTGCCAACGAAATCAAACAACATCCTTTCTTCCGTGAGA

TTAATTGGCCCCTCATTCGTTGCATGGCTCCTCCTCAGCTTGATGTACCACTAGAGCTAA

TCGGTAAAGATACAGAATCCAAACCTAACGATGATCAATTGGATGATGAGGAAATGCTTG

TTCAGAGCCTACCAACATTTTAAGGTGACTGGCACGGTGACGGTGTTAATTGCATTATTT

CCTTGTCCCAGTGGTTATTGCCCTTGATTAGAGGTGTCACATCCAAGATAGGCTAAAGCA

GAGAAGATACAGTGGCGATACATATGAACTACTATATCCTCGAGCGTTAAATGCAATAAA

ATCATCAAATCGCAAGTGAAATGCAATCATGATTTTGAAATAAAATAAGCAGATCGGTCA

TAAAGATGGGTGTCACCTTCTAAAGAAAGAGCAACATATGTGAGAATATAACTCATCTTT

CTTTCTGTATAGAGTTAATGTGTATTACTTTTGTTACTCCGTTGCAATAAAATTCTGATT

TATGATTATGTAAAATAAGTTGTATCATTGTTATTACCGTGTTTCAACCAGCCAATAATT

AAGAGATTTCTCAAGCATTGAACAGAACTGACATACTGTAGGGAATTGAGCGTTCTTGTT

TAGATAAATGAAGC

>Unigene0002492

TGTTTATATGAGTTCTCTTCGAGTTAAAAAATAGTGTTGGCCAGTTTTCAAAATTACAGA

TCCAATGGGTGAATTTCTTAGGAGTTGAAGATGATAAAACCTATATTAGAGCCATACACT

ATAAGTTGGTTGATATCCATATAAATAATAGCTAAAAGAAACTTACCGTAATATCCATAT

ACTGGCTTGTCTTTTGTATCTCGCGTTTCTTTTTCATATTTTCTATTCTAGTGTATTGGA

TAAATATTATATAACGCATGAATGAGACATTACTCACCGTGAAGCAAAATTTTACTTATT

TAACTATAATATTATTGACGTTTCACTGCAGGCTCCTCCTCAGCTTGATGTACCACTAGA

GCTAATCGGTAAAGATACAGAATCCAAACCTAACGATGATCAATTGGATGATGAGGAAAT

GCTTGTTCAGAGCCTACCAACATTTTAAGGTGACTGGCACGGTGACGGTGTTAATTGCAT

TATTTCCTT

>Unigene0002493

ACTTTTCCAAGTAGCATCCCAGTAAGAAATATGTGTTGTTACGAATCACAATATCTCCCT

ATTATTGTTTTTGCATATATTTAGTTATCTTTTCATTTTTTAACTGAGAAGAAATTGTGG

TGTCATCTATTTTCTTCTCTGACAAAATGGAAATGTTTAAAAATTAAAGAACTATTCAAC

ATTCAATGAAAATACTATGGTTTTGTACTCAACATTCAATGAATATACTATGGAATTGTA

CGAAGAT

>Unigene0002494

AATTCCTGATCTCCGTCGTCCCTTGCCTCAGTCTCTCTCCTCGATTCGACACCCCGCTCA

CCGTACAAGGCTCGAATCCTTCGTCTCCTCCCCCTCAATCTCGATCCCTTCTCGTGCGAC

CTCAATCCCTCTCCAGGGTATGTATTCTGAGATCGTTTAACTACCCTATTCTAAAAGAGG

GAAAATTCTGTCTTAAGATGGCATTAGCTTCTTCAGTTGGGCCTTTCTCTCCCAATCAAC

ATAGGGTTTGGGAAGATCCATCTTTCATCAAGTGGAGAAAGCGAGATGCTCATGCGCCGT

TGCGCTCTCATGATACCGTCGAAAGTTCACTTAGATATTGGTCTGAGCGTAGCAAAGTGA

ACTTTCTGCTTTCTAGCTCAGCCTCATGGGATGATGCCGCTGTATCTGATGCTCTTGAGA

GTGCGGCTTCATGGGTCAAGGACCTACCATATGTTCAGTCATTGAGTGGGCATTGGAAAT

TCTACTTGTCCCCCACTCCTGCAAATGTTCCTTCAAATTTTTATGACAATACTTTTGATG

ATTCAGTTTGGGAAGCATTGCCAGTGCCTTCGAATTGGCAGATGCATGGGTTTGATCGGC

CCATATATACAAATACTCTATATCCTTTTGCTATCAATCCACCATATGTCGATTCAAACA

ATCCTACTGGCTGCTACAGGAAATATTTTCGCATTCCAATAGGATGGGAAGGTCGTCGAA

TCTTGCTGCATTTTGAGGCTGTTGATTCTGCATTCTTTGTGTGGGTAAATGGAGTCCTTG

TTGGCTACAGTCAGGATAGTAGACTTCCAGCTGAGTTTGAAATTACAGACAACTGCCATC

CTTGTTCTTCGGACATGGACAATGTTCTCGCTGTTCAGGTCTTCAGATGGAGTGATGGTA

CTTATTTAGAAGATCAAGATCATTGGTGGCTCTCTGGTATTCACAGAGATGTGCTTCTTC

TAGCAAAGCCACAGGTGTTCATTACAGATTATTTCTTCAAATCTAATTTGGATGAAAATT

TTCTGACAGCAAATGTCCAGGTCGAAGTTATACTTGACGTGCCACAAGAGAAATCTGGTG

ATGTTAATCTCGCAAACTTCAGCATGGATGCTACATTGTATGATAACACCTGGTGGCATG

ACTCTGCGGATGGAAGCACTGATTTAAGTGCTTATAGTTTGGTGCATATGAAGCCCATGC

CACCACGTAGTGGGGTTCTTGGGTTTCACGGGCATCATTTTGAGGGGAGATTGGACAGAC

CAAAACTTTGGTCTAGCGAGAATCCTAATTTATACACCCTTGTTGTCATCCTTAAAGATG

CATCTGGGAAGGTGCTCGATTGTGAATCATGCCAAGTAGGAATACGGCAGATCTCTCGTG

CTCCAAAGCAGATGCTTGTCAACGGTCTTCCTGTAGTAATACGGGGTGTTAATAGGCATG

AGCATCATCCGCGAGTTGGAAAGACAAATTTGGAAGCATGTCTAGTTAAGGATTTGGTTT

TGATGAAACAAAACAATATTAATGCCGTGCGAAACAGCCATTATCCACAACATCCAAGAT

GGTACGAGTTGTGTGATCTTTTCGGTCTCTACATGATAGATGAAGCCAATATCGAAACAC

ATGGTTTTGATTACTGTGGTGATTTCAAGCATCCTACGCTAGAGCCTAGCTGGGCGAGTT

CCATGCTAGATCGTGTCATTGGCATGGTGGAGAGGGACAAGAACCATGCTTGTATTATTG

CATGGTCTTTGGGAAATGAATCTGGATATGGACCTAACCATTCTGCTCTTGCTGGTTGGA

TTCGAGGAAAGGACTCCTCGAGGCTATTGCATTATGAAGGTGGTGGATCAAGAACTTCAT

CAACAGATATCATATGCCCCATGTACATGCGTGTATGGGATATGGTGAAAATTGCTGCAG

AACCAGATGAAAATAGGCCCCTTATTCTTTGCGAGTACTCACACGCAATGGGAAATAGTA

ATGGAAACATTCACAAGTACTGGAAAGCAATTGATAGCACTTTTGGACTCCAAGGAGGTT

TCATTTGGGACTGGGCTGATCAGGGTTTACTGAAAGAGGGCAAAGATGGATGTAAACATT

GGGCCTACGGAGGTGATTTTGGGGATACACCCAATGACTTGAATTTCTGTCTGAATGGCC

TCATATGGCCAGACCGAACACCTCATCCTGCACTCTATGAGGTTAAATATCTTTATCAAC

CCATAAAGACTTTTCTAACAGAAAGCAGTGTTAAGATAGTTAATAAACTATTTTTCGAAT

CAACACAAGAATTAGACTTCAATTGGTTTCTTCAAGGAGATGGATGCAACCTGGGATCCG

GTATACTTAATCTCCCACTCATAGAACCACAAAGTAGTTATGACATTGATATGGAATCGT

CTCCATGGAGTTCTCTCTGGAAATCATGTTCAGCAACGGAAGTGTTTTTAACAATAACTT

CAAAGCTCAAGCATTCAACACGATGGGCCGAAGGTGGTCATGTCCTTTCATCGACACAAC

TATGCTTAACTGCCAAAAGCTCCGGACCTCATGTAATCAAATTGATCAACGATACCACTT

TGGGTTGTGAGCATGTTGGGGACACTATAACATTAAGCAAGAAAGATGTTTGGCAAATCA

AGTTCAACACTTGCACGGGAACAGTTCATAGCTGGATGGTTGATGGATATCAATTGATGC

CCAAGGGTATATTTCCCTGCTTTTGGAGGGCACCGACTGATAACGACAAAGGAGGAGGTC

TAAATAGTTATGCCACTAAGTGGAGTGCTGCCAGTCTTAATAATCTGTCGCTTCATGCCG

ACCACTGCTTCATTCAGAAACAGACAGACCATGTTGTCCAAGTGACCACAGTCTATTTTG

CTGTTCCTGGGGATGTCTGTGATGAATCACAGATAAGTAGTTCAGCTCTTTTTAAGGTTG

ATGTGAGTTACTGGATCTATGAATCTGGTGATATGATAATTGAGTACTTAGTAAGTCCTA

AGGATGGTCTTCCTCCTCTACCTCGTGTTGGGATTGAGTTTCATGTGGACCTATCATTGG

AGCATACTACATGGTATGGGAAAGGACCCTTTGAGTGTTATCCAGATCGTAAAGAAGCTG

CCCATGTTGGCATATATGATAGCAATGTAGCCGATTTGCATGTGCCGTACATTGTTCCTG

GTGAATGTTCGGGCCGAGCAGATGTCAGATGGGTAGCATTTCAAGACAAGAAAGGATATG

GTTTGTTTAGTTCTGCATATGGTACATCTCCACCAATGCAAATGAATGCAAGTTTCTATA

GTACTGCAGAGTTGGACAAGGTAACTCACAACGAGGACCTGGTCAAAGGTGACAAAATTG

AGGTTCATCTTGACCACAAGCACATGGGTATTGGTGGAGACGATAGCTGGTCACCGTGCG

TTCACGAGGAGTACCTTGTGCCTCCAGTCCCTTACTCATTCTCGATAAGAATGTGTCCGA

TATTCCCATCAACTTGTTGCACGGATCTCTACCGATCTCAGCTCCCCCAGTGATATCGAA

GCGATCGATGTAAACACATCGTCTTGCACTATCGAACAGA

>Unigene0002495

CAAACACATTTTCTTCTTCAAATCACTTCTTTGTCACTCGGTATTTTCTGAATTCTTTGT

GAGGGATTTCAGAGGAACTAATACCTGGTGCCCAAGTATTCATCTGGGTTGAAGTTGAAA

CCAATACACATTTTTGGTTTATAGTGGAAGTGAATGTTCTCTGTACTTTTGCCAACTACT

GCACAATGTCCAGGGACTAAAGATCTTGCAGGGTTGAAGTTGTGGTAACATGAGTTTCGA

TGTTGATAGCTTAGGACATGCATTGGGTTTTGGGGAGATCAAGTTTATGGTATTGGTAAC

TAATCTG

>Unigene0002496

TCGCCTTCCACCGCCGTCGCTCGCCGGATCCAAAGATGAACAACTTCGACCACCGGTACA

GTGATCCCAGCTCCTTCCGCGATCGGAGAAGCGATCTGATGCCTCCGCCGCACATGCCAC

ACTTCCCGCTGAGCGGCGGCGCAGCCGCCGGATTCGGCCGCAGCGGAGCTGCTCCCTTCG

GGGGGACGGGGCCCGCGCCGGCTTTCCCTCCGCCTAGTATTGCCGCTCCTTATTCTTCCG

TCGGAGACAGATCCGGGAGGGGACCTGGTGGTCGAGGAGCCGATTCTGGAAGGGGCCGTG

GCGGCGGGAGAGGTTTTGATGCCGGCCGTGGCGGCGGGGCAAGTTTTGATACCGGCCGTG

GCGGCGGAAGGGATACCGGCCGTGGCGGTGGACGTGGCGGCGGATTCGGCGGAGGAAGGA

GCTTTGATGGAGGTCGCGGTGGTGGAAGAGGATTTGATGGAGGGCGCGGTGGAAGAGGGG

ATAGAGGTTTTGACGGTGGGAGGCGGGGTGGACGAGGTGGCGGCGGTGGGTTTGCGAAGC

CGCGCGATGATTTGGATAACATTGCCCTCCCGAAACAGAATTTCCAAGGAATTCTTCCTT

TTGAGAAGAATTTTTATGTGGAGAGTCCTGCCGTGCAGGCTATGACGGAGCAGGAGGTTA

TGGATTACCGCCTGAAGCGCGATATTACTGTTGAGGGACGTGATGTGCCGAGACCAGTTC

GGTCATTTCAAGAAGCTAACTTCCCAGATTACTGTCTCCAGGTAATAGCACAAAGTGGAT

TTGTGGAACCAACATCCATCCAATCCCAAGGGTGGCCCATGGCCTTGAAGGGTAGAGATC

TAATTGGTATTGCAGAGACTGGTTCCGGGAAGACACTGGCATATCTATTGCCAGGTTTGG

TGCATGTTAATGCACAACCTCGCTTGGCCTATGGGGATGGTCCTATTGTTCTGGTCTTGG

CACCTACGAGAGAACTTGCTGTTCAGATACAAGAACAAGCTGTGAAGTTTGGAGCACGTT

TTAAGGTCCGGAGCACGTGTATCTATGGGGGAGCCCCGAAAGGGCCTCAGATTCGTGATC

TTAAAAGAGGGGTTGAAATTGTTATTGCAACGCCTGGTCGGCTGATTGATATGTTGGAGG

CTGGCCACACAAACTTGCGAAGGGTGACCTATTTGGTATTGGATGAGGCTGATCGCATGC

TGGACATGGGGTTTGAGCCTCAGATAAGAAAAATTCTCTCACAAATCCGGCCTGACCGAC

AGACATTGTATTGGAGTGCCACATGGCCTAAAGAAGTCGAATCTTTGGCTAGAAAGTTCT

TACAAAATCCTTACAAGGTAATAATAGGATCCCCTGACCTTAAGGCTAATCAGTCTATAA

ACCAAATTGTTGAAGTTGTGTCGGAGCATGAGAAGTACCCCAGGTTGATCAAACTTTTGA

CAGAATTGATGGATGGGAGCAAGATTCTGATATTTCTTGAAACTAAGAAAGGATGTGATC

AGGTTACTAGGCAACTGAGAATGGATGGATGGCCCGCTCTGTCAATTCATGGGGATAAAG

CCCAACATGAAAGGGATTGGGTTTTGTCTGAGTTTAGAAGTGGCAATAGCCCTATAATGA

CTGCTACTGATGTTGCTGCCCGTGGTCTTGGTAGGACCATTTTGTCACAAATATATCCGT

CTCGTTTTCTAGTTCTCAAGGGTTCTCAAGGATGACAGGTCAGTGGCTGCTTCTAGTTTC

TGAATGACTGAATTTGTGGATTGGTGGGCGACAGAGGTGAGACCGGAACTAAGGTAGTGT

AGTTTGTTCTGTTTACCTCTTATGAGTGTAAGCACTGGCAGTGTTACCAGGGCATGATTT

GTGCTTTGGTAGAAATCTTGAGCAGTCTTTTAAGTTTCTGTGGATGTTCTGCCATTGGTA

TATACAGGATAACCAAGGTAGTGAAGCTTGTTCTGTTTACCTCTTATGAGTGTAAGCACT

GGCAGTGTTACCAGGGCATGATTTGTGCTTTGGTAGAAATCTTGAGC

>Unigene0002497

GTGTGCACATACCACTTGTACTCGTATAAATACCCTTTTGTGGGCAATGAGGAACTCAAC

CATACAAACTCATTTTTTCCTCAATTCTCTCTTCTCCTCCTTTGTCACGTTTAATTACAA

ATATAGCTGATTGGACTCCGTACCTCTGTATATTGGTAAACTTGTGCTCACCTCTTAGTA

AAATCAAATTGTTGGAAGTATAACTGTTTGGTAATTACATTGTAAGTTCTCCATTAGGCA

CAGTGTCTTAATCATTTAATCAGACTAGAGCGAGGCATGAATTTCTAAGTCAGTGATATA

GAATAATATACTGAATTCCTTTTTTTATGCAAGCAGTCATTCCCGGACTAACTACTAGTT

CACTACTTGCCA

>Unigene0002498

TGTGGGCAAATGAGAAACAACACAAACACATTTCTTCCTCAAATCTCTCCTCCTTTGTCG

TCTTGTTACTCAGATTACACTCCAGAGTTCTATTGGATATTTCTTTTCCTCACTCCAGAG

TTCTATTGGATATTTCTTTTCCTCAAATCTCTCCTCCTTTGTCATCTTGTTACTCAGATT

ACACTCCAGAGTTCTATTGGATATTTCTTTTGAAGATATTGAATACAAGATACCACAAAA

TGATCAAGATAGTACATCTCACAAGATGGATAGTGTTTTGTTCTTCGGTTCCTTGGAAGT

TTATACATGTCTTCCCTGTATCATCTGATCACTATCAACTAATGTTTCTATGCCTCCTTG

CAGGAGGA

>Unigene0002499

TTGTGGAGTTTAAATAATAATGGTGTGTGTGCACATAGCAGTTTTACTCCTATAAATATA

CCCTTTGTGGGCAATGAGGAACTCAACCACACAAACACATTTTCTTCCTCAAATCTGTCT

CCTCCTTTGTCATTACGATTTAAAGATAAAAGTTTATCTTGGGTATTCAAACACTTCAAT

GTCTTCATACTGTTTCTTCCATGCACAACAAGAAATTTCTGGAGCTTGCCAGAGTACA

>Unigene0002500

TCCTATAAATACCCCTTTGTGGGTAATGAGGAACTCAACCACACAAACACATTTTCTTCC

TCAAATCCCTCCTCTGTCATTACGATTGAAAAATAAAAGTTTTAACTTGGGTATTCAAAC

ACTTCAATGTCTTCATACCGTTCCCATGCACAACAAGAAATTTCTGGAGCTTGCCAGAGT

ACAGTTTACCAGATATTATTGATCATTATTTGTACTCTTGCTTGTTATCCATCTCGCCAA

AAGTCCCATGCGCCCTGATTTTTATCTCTGCTAAAGTAAATGGGTCCTTTGTGTCCGTCC

TTTCCTCCATGCAGCTGTTGTTCGGCTTTTGAGATCTCGGGACAAAGGCCAATGGGTTGA

CTACCAACACTCTCCCTCCCACTCTCATCGTATGTAGGTAGGAAAGGTCAGACTACAAAT

AGTTGATTCATACAATAGGATAGCTGCTAACAACTATGAATGGACGAGGATGTAAGAGTC

TAGATGTGATGACATCACCAAGTACTATTGTAAGTGGTAAAACTATAAAATACCTCGTGT

AGTTCTTTGCACTGCTTCATGATAAGTTTATCTAGTTGTCTTGGGTAGAATATACATTAG

GAAGACAAATCCTATGGCTAAGACCATAT

>Unigene0002501

GTAATGGTGTGTGTGCACATAGCACTTGTATTCCTATAAATACCCCTTTGTGGGCAATGA

GGAACTCAACCACACAAACATTTTTTTCCTCAAATCTCTCTTCTCCTTTGTCACTACCTA

TGGTTTTGTCTGATCTTATTTGGGAAGGCAGTGATTCAAATTTTATTATTATAAAAAAGT

CATTGTATTCTTTATTGTTTTTTTTCCGAGTCTTTCCTTGCAGGAGCCTTAGAGGAGCAA

GCCGAATTCCATGCCAAAGATTTGGTGGGGCAGAAAACAGTCCTGGCTAGAAGTCTGGAT

GAGCAATGCGGCTCCCAAGCACACATTTTTGTCAGACTGCACGAGAGCTGGGAATGGAAT

TGTGGAAAGAGCTATTTTTATTTTTTATATGCCACTTTTAGTGTTCAACTTATTTTTTTA

TTCGGTGTTAAGTTTCTTTCAAATTGGTTGGCGAAGTCTTATTCATGAATTAGTGTATTA

AATCATTGAG

>Unigene0002502

TGTGGGCAAATGAGAAACAACACAAACACATTTCTTCCTCAAATCTCTCCTCTGTCATCT

TGTTACTCAGATTACACTCCAGAGTTCTATTGGATATTTCTTTTGAAGATATTGAATACA

AGATACCACAAAATGATCAAGATAGTACATCTCACAAGATGGATAGTGTTTTGTTCTTCG

GTTCCTTGGAAGTTTATACATGTCTTCCCTGTATCATCTGATCACTATCAACTAATGTTT

CTATGCCTCCTTGCAGGAGGA

>Unigene0002503

TGTTCTGTTTACCTCTTATGAGTGCAAGCAATGGCCCTGTTTACCAGGGTGTGTTTTGTG

CTTTGGTAGAAGTCCTGAGCAGTCTTAAGCTCCTATGGATGATCTGCATTGGTTATATTT

AGGATTTTTTGGTGTTTAGTTTGGTGTGTCGCCTCCACCCAGCCCAACCCAACCCATCCA

CCTATAACTGGATACAGTGGCGTTTGGCTGGTCTTAACCTGATCTCTTGTTGTCTGAATC

CCCTCTTCTTTTTGTAGAGATTCTTATGAACAATAATATTTCTAGTAGCCTCCTGGATGT

ATATTTCAATACAAGATTAATATCAAAGTCTTCTGTATAAAAAGTGGTCCATCGGCGCAT

TTTCTAGAAGGACCATGGACTAAACTTGGAAATTTTATGCAAGTGTCCCTCAAATTGTAA

TGAACAATTCCATCTTGAGCTTTACCTGATACTTGGTATCCATCAATTGACTCCTTGATC

TCACAGCTTTGGAATGAATGCATATCCAAATACACGATTTGGTATTGATAATGCTCTACT

ACAAAGCAAAAAAACTTTATTGTATTTCATGTTTGGGTTAAATAAATGTACCAAACAGTT

TTTTGAGCTATTAAAATTTTTCCATTTAAGCTACCGGTCCAAGGTTGACTTTTAGTTCCT

GTTAACCTACATGTACAACTCTAGGTGTAGGAGTGGTGATAGTATAGCCAAGGAGATGTT

TGTGGGGTGTCAATAGTAATGGTGTGTGTGCACATAACAGTTGGTACTCCTATGAAGATC

CCTTTGTGGGCA

>Unigene0002504

ACTATCAAGCATGTTACAATGCTCAAAGCTTAATCCTTCGCTAAGGATGCTCCATCGAGC

AATTGAGAACCCGACACCTCTTTGCCAGCACATTGCCTCACCACAACTTGACCCGAGCAA

GTTTTTAATAAGCTCCCGTGCTACTGATTAGCATACTTCCCTTTCAGTCAAATAAGACAC

CCCATAACATCTTGCAGGATAACCAACGATACTGCATCTTGATCCCCGACAT

>Unigene0002505

TTGCGCACGGGCCTGCACTGATCTGTTATAATGCAGGAGACCTTGGAAACTTAAGCATGC

CACTCCGCCGAGAGTCTTGACATGCCAACAAGTCTTTGGCCCAGATGAACGAGAATGCAT

CTCGCCTGCTACCCACGTGCTAATGTACTACCAGTCCAACACGGCCACTCAGCACTGCTG

CGGCGCCTGAGACATGCATCAAACTCTCGCCCGCCACACTGTTTCTCTGCATAATCATGT

CCTTGCATCCTTTGCACGTGATTCTCCAATCATTTTTCAATACCACACCGGCTCAATACA

TTGGCCCGACTTCAAAGCACAATACTCAACATCATCTGCAAAG

>Unigene0002506

TTGATCCCCGACATAGCACAACCGTCTTGCACCATTTGCATATGACATCTCTCTTCTCAG

ACAAGTACAATGAAACTTGTTGCTAGTTAGCTCATTTGGACGACAAATCGTGCCAAACAC

TTTGTGCGTTCATCCCCGCAATGAACTGTTACATGAAGCCTTACGAAACAGTAGCACCCC

ACTCTGTCTAGAGTCGTGACATACCAATGAGTCATTGACCCTCACGAACAAGAGTTAGCC

TTGCCTGTGGCTCATATGTCAATGTACCTCCAGTCCCTGAGCATACACTAAGTCCGCCAC

ATCAATCCTCTACACAATCATGTCCTCACATCCTTTGCATATAAATATCCCATTCT

>Unigene0002507

TTGTATAAGAATTATTGAATTTAAGTTTGTGTTATCACAATTGGTATCAGTCCAAATTTG

AATTGGCATCCTTGATAAACCTTACCAATTTTCCTCAAATCCAGAAGGATTCTTGTGCTC

AGGTGAACGTAACTTGGACTGGGTCTGATAGCCAATCTTGCACAAAGCGAAATTTTTTAC

CCAGCACATGTCTGATCGGTGGTCTCGCCTAGGCAAGAGTTTCTGCCTCGCCAAAATTGA

ATAATGTTAGTTGATTTTGGATTCGGGTTCAACTCGGTTTGGGGCTGTCTGCATGTTTCC

AACAATATGTGATGTTTTATTAATATGTTGTATGCTTTATTTCGGCTTGTTGTATGCTCC

CAGATCATGATCGCAAAACAAATTTCTCAAGAGAGGAAATGAGATGTGCAAAATATCCTA

ATTATATATATAT

>Unigene0002508

CTGTAGCAATGTCGGAGGGCGCAACGGAGCGATGGTGTGCGGTGACCGGCGGGAGGGGGT

TCGCCGCCCGGCACCTCGTCGAGATGCTAATCCGCGCCGGCGAATATTCCGTCAGGGTGG

CGGATCTGGGGCCCTCCATCAAGCTCGACCCCTACGAGGAGGACGGCGCCCTCGGTCACG

CCCTCCGCTCCGGCCGAGCCCTCTACGTCGCTGCCGATCTCCGCAACAAGGCCCAGGTCG

TCAAGGCATTTGAAGGGGTGGAAGTTGTTTACCACATGGCTGCCCCTGATTCATCCATTA

ACAACTACCAGCTTCATTATTCGGTCAATGTTGAAGGAACCAAGAATGTCATTGATGTGT

GTGTTGAATGCAAAGTTAAGAGGCTTATATATACTAGCTCTCCTAGTGTTGTCTTTGACG

GCGTTCATGGAATTATTAATGGGGAGGAATCAATGCCGTATCCTGATAAGTTAAATGATT

CATATTCAGAGACTAAAGCAGAAGGAGAAAAACTAATAATGAAGGCAAATGGTAGGGAAG

GGATTTTGACATGTTGTATTCGTCCTAGTAGCATATTTGGCCCTGGCGATAAGTTGCTTG

TGCCGTCTCTTGTTGCTGCTGCAAGGGCTGGGAAATCCAAGTTCATTATTGGTGATGGCA

ACAACATGTATGACTTTACGTATGTTGAGAATGTTGTACATGCCCATATATGTGCTGATA

GAGTTCTTGCCTCTGGCATGGATGCAGAAAAAGCAGCAGGACAGGCTTATTTCATTACAA

ATATGGAACCAATAAAATTTTGGGAGTTCATGTCACTGATCCTTGAAGGCCTTGGATATG

AAAGGCCAAGAATAAAAATTCCTGTCGCCGTTATCATGCCCATTGCTCATCTGGTAGAGC

GGATATACAAGGTTTTCTCTCATTATGGGATGAGCGTACCACAGCTGACGCCTTCTAGAG

TTCGACTTCTCTCATGCACCAGAACCTTCAATTGTTCAAAAGCTAGGAAACTTCTTGGTT

ATGAACCTATAGTAACACTCGAGGAAGGCCTGAAGAAGACTATTGAGTGGTATTCCCACT

TGAGAGCTGTGCAGAAAAGCAGAACATCTAAAGCTTCTCTCTTCCTTGGAAGTGGAAATA

TTGGCCGCACCCTTCTCTGGAAAGATACGAAGCAGACCCTCACAACATTGCTGTTTTTGA

CTACTATTTATTATTATCTATTTGCATCTGGATATACCATCATAACAGCAATGGCAAAGC

TTCTCTCTCTGGCTGCACTTTTCTTGTTTGTCCATGGCATGTTACCGACAGAAATGTTTG

GCTACAAAGTTGAGAAAATACCACCTTCATACTTCCACTTTTCCGAAGAGAAGGCTCAAC

TTCTTACTTTTTCGGTCACTTCTTCGTGGAATTCTTCAGTTGGCATACTGAGATCTCTTT

GCAAGGGGAAGGATTGGGCACTATTTTTCAAGGTAGCTACGTATCTTTTGATTCTCAGCT

TTCTTGGTGCCATGTCAATGCAGACAGTTTACAAAATAGGTATTTTTGTAGGCTTCTTCG

CCTTTTCTGCTTATGAGAAATGGGAGGATGAAATTGACAGTCTATTTAATGATGCATGCA

CCTACATTCTGAAATTGAAATCTAAAGTTACCCAAAAGTTGAAAAGACACTAATGATATA

TCTTACACAGTAATATCAAATTCTTTCAACACCAAAAGGGGCATCCCTGGTTTATGCCAT

CATCAGCCCGGTATCTCAGGCTAGTTTTCGTGTGTATTACTTACATTACATCTAGTGTTG

ATCTTGATAGACCATATTTATCTGCATTGGGTATATGTGTCTGAGAATATTGTTGTATAC

AATTGCAGAATGTGGAGAATTTTAACTCCAAATTGTGTACTTCTGATGCCTGATCTTCCT

TAACGATCCATGTATGGATTTCAGGACTCACGATGAGCTTCTACTTTCTTCTGTTACATG

TTTAATCTGGCTTGAGCTTCTACTTTCTTCTGTTACATGTTTAATCTGGCTACTTGCGTG

GCCTTTTTTTGTGTCAGTTTTAGTAACTATTATATTTTAGACTATAAATGAAGTGGCCAT

TGTATGAAACTCTTTAATTTGGATTAAAGTTTGTATTAGTTTCTTCTACTTTGTCTTCTA

CAGATTTCAGATATTGGATATTCAATTTGTGTTTCTCCAGTTTGGAGATGCTATAAGATA

>Unigene0002509

TTTATCACCCACATCGTGGACGCTCTCACAGGTCTTGAGATCTCCGCCAAGAGTGTACTG

AGATCTCCAGCGCAAACACTAAGATCGGCAGGCTACCGAATCGTCAGCAGCAATGACGGT

GGCTGGAACGGAGCGGTGGTGTGCGGTGACCGGCGGGAGGGGATTCGCCGCCCGTCACCT

CGTCAAGATGCTGCTTCGCGCCGGCGAGTACTCCGTCCGTATCGCCGATCTCGGCCCCAC

CATCAAGCTCGATCCCTACGAGGAGGACGACGCTCTCGGCCGCGGCCTCTGCTCCGGCCG

AGCCCTCTACGTCTCCGCCGATCTCCGCAACAAGGCCCAGGTCGTCAAGGCATTTGAAGG

GGTGGAAGTTGTTTACCACATGGCTGCACCTGATTCATCCATCAACAATTACCAGCTCCA

TTATTCAGTCAATGTTGAAGGAACCAAGAATGTCATTGATGTGTGTATTGAATGCAAAGT

TAAGAGACTTATCTATACTAGCTCTCCTAGTGTTGTCTTTGACGG

>Unigene0002510

AATCAGTCAGGCACAACTAAATGAACTAGGGAATTTATGAATTTGTATAAGAATTATTGA

ATTTAAGTTTGTATTATTACAATTGGTATCAGTCCAAATTTGAATTGGCATCCTTGATAA

ACCTTACCAATTTTCCTCAAATCCAGAAGGATTCTTGTGCTCAGGTGAACGTAACTTGGA

CTGGGTCTGATAGCCAATCTTGCACAAAGCGAAATTTTTTACCCAGCACATGTCTGATCG

GTGGTCTCGCCTAGGCAAGAGTTTCTGCCTCGCCAAAATTGAATAATGTTAGTTGATTTT

GGATTCGGGTTCAACTCGGTTTGGGGCTGTCTGCATGTTTCCAACAATATGTGATGTTTT

ATTAATATGTTGTATGCTTTATTTCGGCTTGTTGTATGCTCCCAGATCATGATCGCAAAA

CAAATTTCTCAAGAGAGGAAATGAGATGTGCAAAATATCCTAATTATATATATAT

>Unigene0002511

CCTTTCAATAGAGGGAGTGGGAGTGGAGTCAGTGATCTTCTCGAGTTTGAACCCTTCAAT

AGAGGGAGAGGGGGTGGAGTTGGAGATCTTCCAGAGTTTGAACCTTTCAATAGAGGGAGT

GGGAGTGGAGTCAGTGATCTTCTCGAGTTTGAACCCTTCAATAGAGGGAGAGGGGGTGGA

GTTGGAGATCTTCCAGAGTTTGAACCTTTCAATAGAGGGAGAGGAGGAGAAGATACTCTT

CAATTTGAGCAGTTCATGCCCAATAGAGGAAGAGGGGGAGAAGATCATCTCGGCGTCGAG

CAGCGCCTCACTCCCCCGCCTTCCTTCTATACCGCTGACTATCCACGGTCCACTATGGAG

GTTAATGAGGAGACTCGAGATAATCGTGGCTATGCTGAGAATAACCTTTTGGGCAGTAGA

GGGAAGAGGGTGCCTTCTGGAACACCTAAGAAGGAGAAGCCCAATGTTGCGTCCCGGATA

CAAAGTGGAAAGTCGGTTTGGGAACGGATTGAAGAGAGGCCTGCTTTCCTCCCATACACA

GGGAATCACCAGTTGGAGAGTGATGCTCTTGATTTGTCGTTTAATTCTGATGCCTTCATG

GCTAAGGTGGTAGAGTCGTCGGTCCCAGTTGGCCCTAGCTCCGAGGTGAAGGTTATACCT

GCATTGAACCCGACAGGCATTAAGAAAAAGATTAAGAAGAAGGATGTAATGAAGAGGACG

CGAGTTGTCATTCCGATTGTTACAACTTCTAAAAGTCCCACATCTGCCAAAGGAGCACAG

GGGGCAACTGCAGTGGGAAAATCATCAGAAATAATTGCTCTTTCTGGTAAAACTGTTAGT

AATGGCAGCTCACAGTCATGTCTCAAGACACCTCTAAAAACTGCTTCGGGTTCGGTTGGG

AAGAAAGCAGCTAAGAAATCTGCTAAGACAGTTGTTATGAAGGAAGATGGCATCACTGTT

GATGCTTTGGAAGAAGATGCAAAAGTAAAGGAGGAATCTAGAAAGTATGCAGATGCGTTA

CTATCCGAAATCAGTACACAGCTGTTCCAGCACGGATCATTGGTGGTGGCAACACCGAAA

GAGAAGATAGCTGGGGAACAATCTGAGGAGCCCGGTAGGTCAGCAAGGACTGGTGATGTT

AAGGATATTAGTGAGGAAAGATTTGTGACTCCTGATGGGAAGCCTGGTTCAAACTGTGTA

GATCCTGTATGTTCTGTAACAGGAGTTGTGGATAATGATACTACGAAGATCTCTGATCAA

ACAGGTGATGTAGCTGTCACATCAAATGAAGATTCCACTGCAGGGGGGTCCTGTAAAACT

ATTGATTTGGTAGACAATGAAGTTGCTGTTGAGTATATAGAACGATCTCCACAAACTGGG

GAGATCACTGTGTGCACTGAGCTCTCTCTGACTCCTGCCTATGAGGCACAAGTTGAGATG

CCATTTGGTTCTGTGACTTTCCTCGGCAGTTGTAGTACCCCCAAAGGAAAGAGGGGAAAA

AGGAAAAGGGAAGTCGAAGAGAAACATCAACAGTTTGCAACTGGCAAAGTATCAGTGTTG

AGTGATAGTAGTTTTGACTTAAATGTGAATGGTACGGCAACTGTTCTGACAGCTGAAATG

ATGGATGTTGTTCTCGATCAAAGGCAGCATGATGGACCCAAGGATGTTGACGGACCCAAG

GATTATGAATATAATGGCATCGACCTGCAGGTGAGCAATGAGCCCAAGTCTGGTTATGCA

AGGCTTGGGCCAGATTACTTGGGGGTTTCTATGATTGATTCTGTTGTCAAACAGAAAGTA

GACTCTTTGACAGCTGTATTCAAAGTGCGAGAACATTCATTTGATACCCAAAATAACCTG

CCTTTCAACAGTTCGTTTGTATCAGAAGAGAACAATGAGGTGAGATCAAATAAATTCATG

CCACCTCAGCTCCTGCCGAAAGTGCAATCTTTGTTACCATATCAGCCTAAAAAACAAATG

CTTGTGGATAAAAAAATAGTGGGTCAAGAACCTGTTAGGAAAACCAATGCAGCTTCTGGT

GTTACTAGAGCTGTTCCTATTCGTGTAACTTCAGCAGGTCCTTCCAAAGTGCCCACCCGA

TCAAGTCATAGTGTGAGAAACAAGACATGGCACCGAAATGAGACCCCTTCTTCTGAATCA

GTTTCACATGGAAATTTGGAAGGTCCAGGTGAACAATCACAAAAGAAGTTTGGCAGGCCT

CCAAATTCTTATATCCGGAAAGGTAACAGCCTTATTAGAAAGCCGGCTCCTGATGTCCAT

CCTCCAAAGCAAGCTTATGATCTTGATATCTCAAGTAAAACAAATAGAGATGCAATGAAG

AGAAGCATATGTGACGAGATTGAGGTGAAGAAAACAGATACTACCATTATTTCTAATCTC

CCTTTTGAAAGACCCAAAACCCCCCCTCTGCCTCCTAGTACCAAGCTGGCGAACTGCGCT

ATGAATCTACCGAGTGAATATCCTTATCCCATATCAGAGGAACCAAATCCAGAAATAGAT

GCTCAAACTCGCTTGGTACAAGCTGAAAACCAAGCTATTGATTTGCTTTCTGGTACACCG

GAGAATCAGAATATTACAAGTGGCAGAAAATCTGAACCTTCAAATTTAAAGAAGATGGTA

TATGTGAAACCCAAGTCAAATCAGCTGGTTGCTGCTCCTAGGCCTGAAGCGTGTACCCAA

ATTCTGGCTTCTTTTCCATCGTCGGATCATTACTACAGAAGAAAAAAGAACCAGCTAGTT

CGGAATGCCTCAAAGACTGATATTCGGCAAAAACACAATGCAGTAAGCACTTTTGGTAAT

TCTATTTCAGATAATCAAAGGATCTCTACAGTTTCCAACATGAAGCGCAGCACGGCAACT

AAATTTATGAAGAGGCTAGATAAAGTACTACAGAAGACTTATAAACACCCAAAATGTTCT

CGAGTCTGGACATTGAGTGGGGAAATGCCAAATGCGAAAGGTGTTTCATCATCAAACCGT

TGGAAGGTTCTGCCACACCTTTTACCTTGGAAGAGGACAATCTACTCAAGGAAATTTAAT

CGTAATAACATGACAGTGCCAAGACGTTCACTATCACTGTTTAGGTGAAGTATTACTAGT

GACCTTTCATGCTTATATCTACTTCTACTGATCCTTCTCTTGCTTTTAATTTTTTTATCA

TTTTTTGGCCTTTGCCTGCTTGCTTATTCATGAAGTAGTTGAACTCTTAATTTTCCACTT

TCTGCTTCCAGTAAAAAATTGCAGCATACGAGGTTACGGAATACAATTTACAAAGTCTCA

ACTGATGGTTTCTCTCTTCGAAAAGCTGGGGTAGTAAGCATTGGGGGATCTAGTCTGAAA

TGGTCAAAATCCATTGAGAAACATTCAAAGAAAGCGAGCAAGGAAGCAACACTTGCAGTC

GCCGAAGCTGAAAGGAAGAAAAGAGAGACACATGTAACTACATCCCTTGGTGCCAAGGGA

AAGAGTGGGAGAGCTTTACCTCGTGAACGCATTTTTCGTATTGGTTCAGCTCGATACAAA

ATGGAGGCATCCAGGCGGTCTTTAGTTCGGATTCCAGATGAGCGATCGTCAAGTGCTGTG

GATCTGAAATCTGGAAACAACAGTCAGACACCATTTGTCCCCAGGAGGTTATTAATTGGA

AATGACGAGTATATTCACGTAGGCAATGGTAACAAACTTGTTAGAGACCCCAAGAAACTG

GTTCGCATCTTGGCAACTGAGAAAGTTCGATGGAGTTTACACACGGCTAGATTGCGCTTG

GCCAAAAAACAGCAGTATTGTCAGTTCTTTACTCGATTTGGCAAGTGCAATAAGAATGGG

GGCAAATGCCCGTATATCCATGACCCTGCAAAGATTGCTATCTGCACTAAATTTGTAAGA

GGATTATGTTCCAATACTAACTGCAAGTTAACTCATAAGGTCCTTCCAGAGAGGATGCCG

GATTGTTCATACTATCTACAAGCACTATGTACCAATATAAACTGTCCTTATAGACATGTA

CATGTGAATCCCAAGGCTTCTGTCTGTGAAGACTTCCTCAAGGGTTATTGCGCTGATGGT

GATGAGTGTCTCAAGAAGCACAGTTATGTATGCCCTCCTTTTGAAGCAACAGGAAAGTGT

CTGGAAGGATCTAAATGCAAACTCCATCACCCCAAGCCTAAAAGCAAACCCAAGAAAAAG

AAAGTCCCCATTGTCCAAAGCAGCAGTCATAGGCGTTATTTCGGTTCTAGTGTTCTTGAG

GCTGGTGAGCCTTTAATAGCTGCCTCGGGCCAACATGTTATGGAAAATGTGGGGGACTTT

TTTTATTCGGATGGGCAGTGTACTGATTATATCCCTCTAGATATTGGTAGTGAAGGCGAT

GTTGAAGTTGATGATCTGATGGACTTTGAGCAGACTCTGTGCGACTCTGAATTCTCTGAA

ATGCAGGAAAATGATCTTGATGCCCTCATTAAGCCGGTCTTAATCATGAAGGCTAATTTG

ACAAATCCATGTTCTGGTTCTTAATATGGAGTATCATCCAAGAAAGGAACTTCTGTTGAG

GTCGAATTTTTTTTTTTTTTTTTTTAAGCTCCTGTTATCTTTCTATATTTCCATTTTCAG

GAGGGTTAGGGGGTGGATTTCTCTTACAGCAATGTCAGGGGCATGACAGAAGTGGCAGGT

CTTGTTACCCTGGAGGTCAGAGGGTTTTTCTTGAACGAGTTGTACGGTTCTCTCTAGCTA

CATTTATCCAGGGATGCTGGTCTGGTTGAAGGAAAATCTGAGATATCTGTGA

>Unigene0002512

AGGAAAATCTGAGATATCTGTGATAGTAATTTTGAAGATGCATATTTCTCTGAAGGCACC

TTTAAGGCGGCTTCAGTATTGTGTATTAGTAGATTTGTGTAGGGATTGATTGGGGCATAT

CTGAGAAGAGCTTATTAATTTAGGCCATGTTATATGTTGGAAGCTTGAAGAAGGATATAG

ATTGCCAGAGATGATGCGCAGAACAAGTTTATGCTTTCGCTGCGGAGATGGAAGGTGGTG

ACTTCTGTTTTGCAGGGGTAGGTTAAATACTGTAGGTTAGGTATACTGGGTCTTTGTGCT

GTTTCATTCTTTTACTATGTTAGATGTTTCTCTGTCTCGTGTAACTGTACAGTATGGAAA

CAATTGTGTTAAAGGTTAATGTACTTCCATTTCTGTTTTGTCGA

>Unigene0002513

TTTTATACATTCTTTTTCTTACATGGATTAGTGTAGTGCTGTAAACTGGTCTGATTTGTA

TGCCAACCTTTTAGGTTAGAGCTAACTAATCTTGGTCTTGTTTTCCTCTAGTCTGTCTTG

GTTTTTTCTTGTTGCATCAGATGTTTTGAAGACAGCTAGAGTTTGTGTCTGTTTGATTCC

TGTTGAGACTTGCTTTTGTTTGAAGGTGAATTTTGCGTATTTGAACAAAATGCATGTATG

TTGTGGCACTTGGGATTTAATGTCAGCTGAAATATCAATAATCAT

>Unigene0002514

TACCTTCATCTGTATGCCATGAACTCGAACGAGAAGGGATTTATTCATCTGTGAGCCATG

AACTTGAACAAAAAGGGATTTATATGGCTGTATGTTATACCATATGTTGTCTCACTTGGC

TTCTCGTAGTCTGATCTCAGAGTTTCTACTCATGTTCCTCACTTGAACAAGAAGGGATTT

ATATGGCTGCATGTTGATAGTCCATATGTTGTCTCACTTGGCTTCTTGTAGTCTGCTCTC

AGTTTCTACACATGTTCTATCTTATCCCTTGGGTTTTTTGTTACATCAGTTAAGCATGTA

TGTGGCTTTTCTGAAATGTTCCCTCTACTCATCCTAAACTGGTTCATTTGTCCCCATACT

GTGTATGGTCTAAGAAAAATTAGCCAACAAACAAGTATGTTTGCAGCCATTCGTACTATC

AAACACCAATGACCGTGCTCTGACCACTTGGTTCTCTTCAATATAAACAATACTTCCATC

TGTGCAAACACTATTTTCTATTGGGATTGTAACAATGATAAGATTATGCAACGTAATTTA

TTGTTGTGGTCAAGTTGATCCATTCATTTCAGTGACTCTGAGTAACTACCGTACTCCCCA

CTCCTCATTTGAGGCATATCAGTTTACCAGGTAATTTGATTCAGTGGATTGCCTTGATTT

TGTGGGAAGATTTTTCATTGTGACTCTATAGTGTTGTGGCTCAAATCTTTGACATTCTCT

TAATGTTTGTTGTAAAACTGTGTTCATTGGTCTTCCCAGATAACAAAATTTTAGGGTGTT

TATTTTATTTCACACTTGCAGACTTGTTCTCCAAGATATGATCTTCAAAATAGAGGTTAG

TCTGTATCCAGTTTGGTGTGTATAGTCTGATGAAATCTCTCTCTTCTTTTAATCCGATTG

AAGCAGTTTTCCTTCTTTTTTTTCATTGTTTAATATTGTCCATTTCTTATTTAGGCTTTA

TTTTTGTTACTTTTTCTTTTATAACATTTTCTAGTCTGAAAAGGCTTTTTTTTACTTCAT

ATTTTAATTTATTGGATATGTTTTTATTGAGATACATTTTGATTTGAGTTGAAATTGTGT

TTTAATCTATTTTATTTTATTACCAGCTTGTTTTCTTTCTTTTATTGTGGACATATTTTG

TAGCAGTACCAGGACTGTATCTGTTTGATGTGTGTTTATTTGCACTTTTTGACCGGACGT

TGGTCCTGGGAAATTTTATGTGAAGGTGGGCAGTTGGGATTCATTCTCTGTTGAAATATC

AACAATCATCTTCTCTATGTATATGTGTGCTTTCTTTATAAGTTTATTTTATACATTCTT

TTTCTTACATGGATTAGTGTAGTGCTGTAAACTGGTCTGATTTGTATGCCAACCTTTTAG

GTTAGAGCTAACTAATCTTGGTCTTGTTTTCCTCTAGTCT

>Unigene0002515

CCACTCGTTTGTGGCGAATCAGTTTATCATGTAATTTGTTTCAGTGGATGGCCTTGATTT

TGTGGGAAGATTTTTCATTGTGACTGTAGTGTTGTGGCTCAAATCCTTAACATTCTCTTA

ATGTTTGTTGTAAAACTGGGTCTTCCCAGATCACAATAGTGAAGCACTAGGGTGCCATTT

TTAGTTTCTTGTGAGTTTCCAGGTTAAGTAAATTTTAGGTGGTTTATTTTATTTCACACT

TACAGATTTGTTCTCCAAGATACAATCTTCAAAATAGTGGGAAGTCTGGATCCAGTTTGG

TGTGTATAGTCTGATGAAATCTCTCTCTTCTTTTAATCCGATTGAAGCAGTTTTCCTTCT

TTTTTT

>Unigene0002516

AGTATTAACTGTTATATATTGAATCAACTTAAACCTGCCAAAGATAATCGACCGATTGGT

ACTCGTTACCTGTAATACCTGCTTCATTCCTAATATGAATTAACTATGGGATATTTGACT

ACTGCAATTACTCGCTCTTGTTTATAGTAGTACCTGCTTTATACTTAATTTGCATTAACT

ATGGGATGCTCTACTATTTCATGCCTACTACAGGCCTTTGACCAATTCACTATTGTTACT

ATGATTACCTTCATCTGTATGCCATGAA

>Unigene0002517

TTTTGTAGCAGTACCAGGACTGTGTATCAGTTTGATGTGTGTTTAGATGCTCTTTTTGAC

TGGATTTTAGTCCTGGGAAATTTAATGCGAAGGTGGGCAGTTGGGATTCATTCTCTGTTG

AAATATCAACAATCATCTTCTCTATATATCTGTGTGCTTTCTTTGATAGTTTTTTCTTTT

ATACATTCTTTTTCTTACATGGATTAGTGTAGTGCTGTAAACTGGTCTGATTTGTATGCC

AACCTTTTAGGTTAGAGCTAACTAATCTTGGTCTTGTTTTCCTCTAGTCT

>Unigene0002518

GCAGCTGAAGATTCTCTTGTGAAATATTGTTATCTTATTATTACTCCATTATAGTTTTGG

CCTAGATGATATTTTGTGATTGTGATTGCACAATGTTTCTTAAATGTTTTAGACAATTGA

TCACCCACATTGGTGTAGCTATGGGTTCATTTGAATTATGGTCTGTTTTTTTTTTACCTT

GGTTATTTTCCTTTTTGTAGACAGTTTACTAGTTTTAGGATAGCTTTATTATCTTTAATG

TTTCTGTCTGATTTGGTAAGATATATTCTGTTTCATTCTCCTTCGCTTATTATGT

>Unigene0002519

AAAGTGATTGTGAAGGTGGGCAGTTGGGATATATTTGCAGCTGAAGATTCTCTTGTGAAA

TATTGTTATCTTATTATTACTCCATTATAGTTTTGGCCTAGATGATATTTTGTGACACTG

AGGTTATTGTGATTGCACAATGTTCCTTAAATGTTGAAGACAAGTGATCACCCACATTGA

TGTAGCTATGGGTGAATTTGAATTGTGGTCTGATTTTTTTTGAATAATTATTATTGTTAA

GACTACTTGTTGGTGCTATTTTTTTTTACCTTGGTTATTTTCCTTTTTGTAGACAGGTAT

ACTAGTTTTAGGATAGCTTTATTTTAAAATTCGTATCTTTCATGTTTCTGTCTGATTTGG

TAAGTTATATTCTGTTTCAGTTTTCCTTTGCTTATTATGTATTTTATGTTGCTACAGGCT

ACCTTTGATTGCCTTATGAAGACTTATGG

>Unigene0002520

TTTTATACATTCTTTTTCTTACATGGATTAGTGTAGTGCTGTAAACTGGTCTGATTTGTA

TGCCAACCTTTTAGGTTAGAGCTAACTAATCTTGGTCTTGTTTTCCTCTAGTCTTGGTTT

TTTCTTGTTGCATCAGATGTTTTGAAGACAGATGCAGTTTGTGTCTGTTTGATTCCTGTT

GAGACTTGCTTTTGTTTGAAGGTGAGTTCTGTGAGTTTGAACAAAATGTATGTATGTTGG

CACTTGGGATTTAATGTCAGCTGAAATATCAATAATCAT

>Unigene0002521

TTTTATACATTCTTTTTCTTACATGGATTAGTGTAGTGCTGTAAACTGGTCTGATTTGTA

TGCCAACCTTTTAGGTTAGAGCTAACTAATCTTGGTCTTGTTTTCCTCTAGTCTTGGTTT

TTTCTTGTTGCATCAGATGTTTTGAAGAAAGATAGAGTTTGTGTCTGTTTGATTCCTGTT

GAGACTTGCTTTTGTTTGAAGGTGATTTTTGCGAATTTGAACAAAATGCATGTATGTTGT

GGCACTTGGGATTTAATGTCAGCTGAAATATCAATAATCATTTTCTCC

>Unigene0002522

ACCCTTCAGGTCATCGATTTCTCCTCCAACTCTCTCGCCGGCCCCATTCCTTCCCATCTC

CCCTCCACCCTGGTCGAGCTCACCCTCTTCGACAACCTCCTCTCCGGCTCCATCCCCGAC

TTGGGTCATCTGACCCAACTCCAAATCTTGGATCTCGGCTCCAATATCCTTACTGGCCCA

TCCCCGCAAGCCTCTGCAACTGCAATTCCCTATTTGCTCTAGGCCTCAATAACAATAACC

TCACTGGCACAATCCCCCAATGCATTGGTGAATTGGGTATTCTGGAAGTCTTCCAAGCTT

ACACCAATGGACTGTCGGGCTCGCTGCCGGAATCGTTGTGGAGCTTGACGGGTTTGAAGA

CACTGGACCTGAGCGAGAACCAGCTCTCAGGTACTCTGTCTCCGGAAATTGGGAACTTCT

CCCATCTCGAAATCCTTCAACTTTTCAAGAACCAGTTTACCGGGCACATTCCAATAGAGC

TGGGCCAATGCACAAATCTCACTCTATTAAATATCTATAGCAATATATTTACCGGTAGCA

TCCCTCCTCGGCTCGGGGATCTGAAAAAGTTACAGGCCTTGAGGCTGTACGACAATCGAT

TGTCTTCACCAATCCCATCCTCGCTATCCCGATGCCAATCATTGATCTCTCGGGGTCTTT

CTCTGAACAAGATAACCGGGACAATACCATCAGAACTGGGGTCACTGAACTCCCTAAAGG

TACTAACACTTCACCAAAACAGCTTGACTGGGAGCAATTCTACCTCATTGATGAACCTGA

CAAACTTGGTCTATTTATCATTGAGCTTCAATTCACTGGCAGGGCCTCTTCCAGCCAATT

TGGGATCTCTATATAAATTGGAGAAACTGATCATAAACTTCAACTACTTCGAGGGTTCCA

TAC

>Unigene0002523

CTACTCCTACTCCTCTCCTCCATATTACCCACAATCTTGTCGTCATCAAATTCCCAAGTC

GAAGCTTTGTTGGCCTTCAAATCCTCTATATCTGAGGACCCTTTCGGCACATTATCAAAC

TGGACCACCACCATCCACCACTGCAACTGGTCCGGCATCTCTTGCGATCCATCGACCAAC

TCCGTCGTCTCCATCACTCTCATCGAAAAGCAGCTCAAGGGCACCATCTCCACCGCCCTC

ACCAACATCTACACCCTTCAGGTTATCGACTTCTCCTCCAACTCTCTCACCGGC

>Unigene0002524

CGGGCTCGGAATGCTAGGGAATTTGACATTCTTTTCAATAGGCGCAAACCAACTATCAGG

AGCAATTCCGGAAGACATCTTCAATTGCAGTAGTCTCACTACTCTAAACATGGCTGGGAG

CAATTTCACCGGGTCTCTGAGCCCACGGATCAGCCAGCTAGCCAGTCTGAGAAACTTAGC

TTTGCAGTACAATTTGTTTAAGGGATTGATTCCAGCAGAAGTTGGCAACCTCAGCATGCT

ATCAAGTTTATCACTTGGTGCAAATAAATTTGAAGGTGAGATTCCTGCCGAGATACTTAA

GCTATCGTTGCTTCAAGCACTGTCCCTCCATGACAATC

>Unigene0002525

CATACAACCAGCTCACAGGGCCGGTTCCTCAATCATTGATAGCTGGTATGAAGAACATCC

AGATATTCCTGAATCTTTCAAACAACTTACTTGTGGGATCCATCCCAGAAGAGATTGGAG

GGCTGGAGATGGTGCAAGCTATTGATATTTCAAACAATAATCTCAATGGAAGTATTCCTT

CGACTCTACAGAGCTGCAAGAACTTGTACTCCCTCGATTTGTCAGCCAATCAACTCTTCG

GAGAGCTGCCCGGTATCATATTTCAGAAGCTTGATTTGCTTACAAGTTTGAATCTCTCAA

ACAATGAGCTGAACGGGAGGATTCCGGAGGAGTTGGGCAGGGTTACAAACTTGCAAGAAC

TCAATCTTTCCTTCAATAAGTTTGAAGGGCCAATTCCCCAGGGTGGCGTGTTTAGGGACC

TAAAATATTCGAGTCTTGAAGGAAATCCGGCTCTTTGTGGGTCAAAATCATTCTCTCCAT

GCAAGATTAAAAGGCGGACCTTTCAGAAAAAGATGCTCATAATTGTTTTGGTATTATCAT

TTGTATCTTTGCTCTTGCTAGTGGCTTTAATATTGTTGATCAAGTATAAACAACAT

>Unigene0002526

TGGACGGGTAACTAGCCGACGAGGTTGAAAGACAATTAGTCTTGGGGTATACGTATTTAG

AGGCATTTCTCTTGGAATCTTTGGTACAGAAGATTTCTTGATTTGAGCTCAGTATTGCGC

AGAGAAATTAGCTAATTCATGCCACAATTGTATTCCGTCTTTATAAAGATAACTTTTTGT

TGTTGAGTAAATCCAGACATCAATTAAATTGGTTTTGGAGTACACATGAATCAAATCTTT

TTGTACTAAATTGACTTTATCACGTTGAACCTG

>Unigene0002527

CTTCCCGGATGTTGGGCATTCACATACCAAATCATGGAAGCAATCCATTGATATCATCAG

CATTGATCCAAGGCACCATTGGTTATATGGCACCAGGTGATTTTTTGTTGCTCTTTATTG

ATATTTGTCATTCACATTGTTTTTTCTATAAAAAAATTGGCTGGAAGAAACTGTAGATAG

CAATTGCGTTTTCAATTAGAATTACAGGTGATTCTAATGATTTTATTATGGATTCATAAT

AGATCTTGTATACTTACAATA

>Unigene0002528

GCTGATGGAGTTGTTCACCAAGAAAAGACCAACAGAGTTGATCGGAGAGACTGGCGAGCC

GCAAACGCTGCAAGAGTTTGTGGGTAATGCACTTTGTGGTGGGATTGAGAGTGTTCTGCG

TATATTGGACAGTGATATGCCGCTTGCGACTGGGATCGAAGAAGGGAAAATCGTGAGAAT

TCTGCAATTGGCAGTGTCATGTACCCTCTTCACCGCCGAGGAGCGTCCAGAAATGGAAGG

AGTTTTGTCCTCATTGTTGAAACTGAGCAGGGATGGAATGGTGCCTGCCACTGCTTGTGA

TGGAAAAGATCATGGACGGGTAACTAGCCGACGAG

>Unigene0002529

GGACATAGCTACTGATTTCTTCAGTGAAGGCAATGTGATTGGAAGTAGCAGTTTGAGCAC

GGTCTACAAAGGCAAGTTTGAAGGTGATGGGATGATGATCTTAGCTGTGAAGAAGCTGAA

CCTGAATCAGTTCCCGGTGGAGTCTGATAAGTGTTTTAATACAGAGCTCAGAATATTGAG

CCGGTTGAGACACAGAAACCTCGTCAAGGTTGTGGGTTATGGTTGGGAAGGAAGAAAGCT

CAAAGCCTTAGCTTTGGAGTTTATGGAGAATGGCAACTTGGATAGCATTATACATGGTAA

CAGAGAGGATGAGGATCGATTTAGATGGACTAATTTCTATGAGAGGTTGAGGGTCTGCAT

CTCAATTGCCAATGGTCTCGTTTACCTACACTCGGGATATGATTTCCCCATTGTTCATTG

TGACCTCAAGCCCTCCAACATCCTCATGGATCGAGATTGGGAGGCTCGTGTGAGCGATTT

TGGGACTTCCCGGATGTTGGGCATTCACCTACCAAACAACGGAAGCAATCCATCGATATC

ATCGGCATTGATCCAAGGCACCATTGGTTATATGGCACCAGAGTTTGCATATATGAGGGT

GGTTACCACAAAAGTGGATGTTTTCAGTTTTGGAATTCTGCTGATGGAGTTGTTCATCAA

GAGAAGACCAACAGAGTTGA

>Unigene0002530

ATTCTAGGGAGGCTTGATCTTGCCGACCAAAGTCCGTTTCAGCTCGGTCTGCAAGTGGTG

GAGATTTGTTGCAGCAGACAACCCAGGCATAAAAAAAACATTTTAGCTTGATACCCTGTG

AAGATCCGGCACACCTTATCGAAACTCTTCTAAGCACATACTGGAGATCATGGACATGGG

CAGTTTTCATTGGCGGCTATGAAGGATGGCTGGTATTGCTGATATGTAATACATTTCTTA

GGATACTTGATATGTTCTTATTGAAACAGGGTTGCCCCTCATCTCGCTCCCGCCTCCTCC

TCATGTTCGGGATGGTGATTTCGCAATAAAGGTAGTTGTGGGAAACAGACGACTCTGGTT

AACATATCCTATTCTGGCGGGATTGTTAACAGAACACGGAGTACTGGATTTTCTGTTTGG

AGATCCGTGATTAACAGTATTCCCTATTTCTGTGGGAAAGAGAAAACTAAACAGAACAAG

GTTACGTTTTATTTTGTTTTGGGTTTAT

>Unigene0002531

TTATGTAGTAAAAGATGGGAAATCAAATAAAGAGAAACAATGGTGTAAGAGGGTGTGACA

TCGAATTGGCGTAAGAGGGAAACATCAATGACGAGGGGGGATATGTACCAAGGTTAACCC

ACAAATCATGAGTAAATCGTAATAACATCGGATTAAGTCATCAAATTGTAATTTAATTTT

ATCATTTAGAGGGCAACCCTAGCGTAACGGCAAAGGTTATCGCCTGTGTTTGAATCCGGA

AACAGCCTCCCTGCATGCACGGGCAAGGCTGTCC

>Unigene0002532

ATTGGATATGGACATGACTTTAATTACCAGTTACATTGCTATGTTCTGAAGGCTTGTTGC

AAACCAAAGTGGACTGTTCGGTAACGTTGATATCAGTGCTATCCCTCTCCCAAAGGTAAA

GTGTCAAATGACTGATATGTGGAATATTTTTTCTGTTCATAGATCATATAATTAGGAGCA

CCCACATTCCATCTCGACTCTTGGGGGACGTGGTGCATTTTCACATCACATTTATACCTC

ACAAAATATATAATATGTCCAGTTCACAATCTCGTCTTCTAACTTATTATGGGTTAAGTA

TACGTTCAGGACCTGCCTGCTGGCTGGAGTGTCCGGTGATAGCGGACAACTGTTGCCGCT

ACCGGCGGGAATATTGAGATCATAGGGTTGGCATGCATTGTGTCCGTCGAGTGAGGTCCA

TCCAAGATGGGAAAACGGCTTTTGGGGCTTGCTCGTCAGTTGGTGAGGCCGGCAGCGATG

GGCGTCAACAACCACATCTTCGGGCAAGTGCAGATTTAGAGGTTTTGAGGGTTGTATCTA

GCGGTTTGGGTTGCGGTTTGGGTTGAAAGATCAAATTGGTGAGGTCGAAATATTACCTTA

AAAAGTTATTGTGTATCAAAAAATATCATACCTAAAAATCAGGGTTAGGTTACGTAGTAA

AAGATGGGAAATCAAATAAAGAGAAGTAGTAAAAGATGGGAAATCAAATAAAGAGAAATA

ATGGTGTAAGAGGGTGTGAGCATCGAATTGATGAAAAAGGGAAGCATCAATGACGAGAGG

GTATATGAACCAACGTTAACCCACAAATCTTGAGTAAATCGCAATAACATTGGATTAAGC

CGTCAAATTGTAATTTAATTTTAATTTGATCATTTAGAGGGCAACCCAGCATAACGGTAA

AGGTTACTGTCTGTGTTTGAATCCGGAAACCACCTCCTTGTACACAGGATAAGGCTGCGT

ACATTTGACCCTCCCCAGTACCCTGCAGTGATAAGAATGTACCCTCCCCAGTACCCTGCA

GTG

>Unigene0002533

AAGTGTCAAATGACTGATATGTGGAATATTTTTTCTGTTCATAGATCATATAATTAGGAG

CACCCACATTCCATCTTAGCTCTTGGCGGGCGTGGTGCATTTTCACATTATATTTGTACC

TCACAACATATATAATATGTCCAGTACACAATCTCATCTGCTAACTTATGATGGGTTAAA

TATACGACCAGGACCTGCATGCTGGCTGGAGTGTCCGGTGATAGCGGACAACTGTTGCCG

CTACCAGCGGGTATCTTGAGTTCATAGGGTTGGCATGCATTGTGTCCGTCGAGTGAGGTC

CATCCAAGATGGGAAAACGGCTTTTGGGGCTTCTTGTTGGTTGGCAATACCGGCAGCGAT

GGGCGTCAACCGCC

>Unigene0002534

TTTGGGTTGAAAGATCAAATTGGTGAGGTCGAAATATTACCTTAAAAAATTATTGTGTAT

CAGAAAATATCGTACCTAAAAATCAGGGTTAGGTTATGTAGTAAAAGATGGGAAATCAAA

TAAAGAGAAACAATGGTGTAAAAGGGTGTGAACATCGAATTGATGAAAAAGGGAAGCATC

AATGACGAGAGGGTATATGAACCAACGTTAACCCACAAATCTTGAGTAAATCGCAATAAC

ATTGGATTAAGCCGTCAAATTGTAATTTAATTTTAATTTGATCATTTAGAGGGCAACC

>Unigene0002535

TGCTGGCTGGAGTGTCCGGTGATAGCGGACAACTGTTGCCGCTACCAGCGGGAATCTTGA

GATCATAGGGTTGGCATGCATTGTGTCCGTCGAGCGTGGTCCATCCCAAGATGGGAAAAC

AGCTTTTGGGGCTTGCTCGTTGGTTGGCGAGGCCGGCAGCGATAGGCGTCAACTGCCACA

GCTTCGGGCAACATATTCTGGTGCAGATTTAGAGATTTTGGGGGTTGTAGCTAGCGGT

>Unigene0002536

TCCCCTTCTATTTATTTTGTTGTTGAGAAGTACAGCTACCTGAGAAAATTTTCAATCGCT

TAGACATTTTTTTGCAGATGTCTGAAGAATCATTTAAGATGCAGTTACATCTGGGTCATG

GTTTTCCCAAGATGAGAGAAGAACCTTCTAGGATTCAAACAATCCTAAATATGAATGACA

GGAAGCCTACTTTTGATCACCAATCTGAGAGTGCTTTGAATCAGTTGGTGCTTTATAGAC

CATATACAAGTGATGTAAATCAAGACATGTCTACAGTTCTTAACCCCTGCAGGGATCGTA

CCAGATCCTTAGCATCTAATACTTCGAGCAGAGTGTTACCATCTGTTGGTCAGTTCACTG

TGCAGTGTGCAACATGTCTTAAATGGAGGTTTGTACCAACTAAAGAGAAATATGAAGAAA

TACGTGAACATATCTTGCAAGCACCTTTTGTGTGTGACCGAGCCCGTGAATGGCGTCCTG

ATATATCCTGTGCTCATCCGGCTGACATTTATCAGGATGACAGTAGACTCTGGGCTATCG

ATAAACCTAGCATTGCTCAACCTCCTCCTGGATGGGAAAGATTACTTAGGATAAGAGGTG

AAGGGAGTACGAGGTTTGCTGATGTATACTACTCGACCCCAACTGGAAGGAAACTGCGTT

CAATGCCCGACGTTGAAAAGTACTTGTTTGAGAATCCAGAGTACATCAGGAATGGGGTGA

ACTTATCCCAATTTTCATTTCAGATACCAAAACCTATTCAAGAGAACTATGTAAGGAAAC

GTGGTGCACGGCCAACAAATCTATATGTTGGTGATGCTCCAGAACCCCTTATACCTTGTG

AAGTCAATCCACTATCTTGGGCGGCCCCTCCTACGCATACAGAACTGGAACTTGGCGGAC

CTGGATTGTCGACTAATTACTTTGTGCCGCCTTTTACTGAACAGCCACCTTTTGCTGATC

AGCTAGGATTGTCGACTCATTACTTTGTGCCGCCTGTTGCTGAACAGCCACCTTTTACCG

AGCAGCCACCTGCCAAGAAGAGAGCAGTGAAGCGAGGACCCAAATCTATGCCGTTGGTTC

ATGACCACCAGGTTATCATCTTGGATTAGCAGGTGACCAATGCCTTCCTCTTGCAAGCTT

ATACATGCTCGAGGTTACCGCTGTGATTGTTAATACTGAATTTTGGTATTTTATCGTAGA

AACTGAAATTATGTCTAGAAGCATCTGTACAGTCATGTCCTAGGAACACTTGGAAGGATT

TTAAATTGTGCGGAAAATGTTTTCAGTGTCTTGTTTTCTTTTTTCTTTTTGGAATGGAAA

CTATGCATAATATTAATTAGTGCGGATAATGTTATTTTTTATGTGGATGATGGTT

>Unigene0002537

AAACAGAGTCTCGGAGGGGCAAGATCGGAACCCTCCCTCTCCCTGACCTCCGATCCGACA

CCCCCTCCCATACGCTACGAAAACGGAGGCGAGAGAGCCGAGGTACCGGAGCTCCTACTC

CTCCTCGGATCCCTCACAAGGCTTCCCGGACTCGTTATACAACTCTTTTCTAAATTGGAT

TTGGACTATTTCAACTAGGAGTCGAATTGATGTTAGTGTAACCCAACCTGCTATTAAGGC

AGCAGAAAGATAGAATAATTGCTATTGAAGTACATGCAAAATCCTTATGGCTTCTGCGAA

CGGGCTATCGCCAGGTCATCTGAAGCAGCCTCTCTTCTCCTTCGGTGTCATATCTGACGT

CCAATATGCCGACATCCCCGATGGCCATTCATTCCTCGGTATCCCCAGATATTACCGACA

CAGTATCCATGTCCTGCAGAGGGCAGTCACCACTTGGAATGATCACAAGAAACACAAGTT

CGTAATCAACTTTGGTGACATAGTTGATGGGTTCTGCCCCAAAGACAATTCGCTATCGGC

AGTGCAGAAAGTGGTTGGAGAATTCGACAAATTCAACGGTCCCGTGTATCACATGATCGG

GAACCACTGCCTCTACAATCTCCCACGCAGCAAACTAATTACGCTTCTCAAGATCCCTTG

TTCCCGTGGATGTGCATACTATGACTTCTCCCCAAGCCCCGAGTTTCGGTTTGTTGTCTT

AGATGCCTACGATATTAGTGTCATCGGTTGGCCACAGGACCACCGGAACTCATTGGAGGC

CATTGAGTTCTTGCGTGAAAAGAATCCTAATGCTGACAAGAACAGCCCGGTGGGTCTGCT

GGGGCTCGAGAAGCGGTTCTTGATGTTCAATGGTGCAGTTGGGAAGGAACAGTTGGAGTG

GCTCGATGGGAAGCTTCGAGATTCGACGAAGCGAGAGCAGAAGGTGATTATATGTTGCCA

CCTTCCCTTAGACCCCAACGCAGCAGCGCCAGAGGCACTTTTATGGAACTACGGCGAGGT

GACCGATGTGATACATCGGTACAGGTGCGTGAAGGCTTGTCTGGCCGGCCATGACCATAA

AGGCGGTTATTCGGTTGATTCACATGGTATTTATCACCGAGTTCTCGAGGCTGCTTTGGA

GTGCCCTCCTGGGAGAGATGCCTTTGGTCATGTAGATGTCTACTACGATCGTTTGTTCCT

CTCGGGTACTGATAGAATGCCAAGTACTGAGATGATTTTCGGTTCTTAACTGACATATAG

CTAATCTGTTTGATATTTATAATTCTGTTTCTTACCTGGGTATTATGGACTATTTATGAC

ATCATAATTTAAAGAAGTAAAACAAAATATATCTTTCTG

>Unigene0002538

GGATCCCTCACAAGTGAGTACGCCCTTGATCTCTGCCCGCGCCCGCCGCGCTCTCCGAGT

CCGGTGGCGCTGGCCTACGTGCTCACCAAGTCCGGCGGCGCCGGCCACTGCGCTCACCAA

GTCCGGCGGTGCCGGCCTTTGCGCTCAAAAAGTCCGGCGGCGCTGATTTGTTGATGTGAA

ATTCGATGCGATCGTTGTTTGATTTTCAGTAGGATCCTAAAATTAAGCCCTAGAATGAGT

TTTACCGCCGAATCTGGTTTGGTAGCCGCAATATTTTCAATTTTTGTTGATTTATTTGGA

GATCTAGTTTTATTTTCTGGGGAAATATCATGCATGTGCTTGTTTCAGCTACATAGTTTA

ATATTGCACTGGTTTGTGTTGATGTTTAGTTGATTGCTCTT

>Unigene0002539

TCACATATGGGAGACACATCACCCGAGGCGAACAGGGAAACGGGATCCCCACAAAATTCA

AATCGAACTCCGGAGAAGCCACTTAGTATTCGTGAATTTGATGCAGAAAATGGTGACGAT

AAATCTGCTGAGTGTACTCCGAATCAGCTGGTGCTTTATACGCCTGATGAAAGGGATATG

AATCAAAAAACAAATGAGGCATCCACTCAAGTTGTCAATCGTGCTTCATCATTCCATAGT

CCCTTGCCCCGAAATCATCCTAGCCGAGCTTTAATGCCCATAGGGTCATTTACAGTCCAG

TGTGCCTCGTGTTTCAAGTGGAGGCTTATTCCGACACAAGCAAAGTATGAAGAAATAAGA

GAGACAATTTTGCAACAGCCCTTTGTGTGTGATCGAGCTAGGGACTGGCGCCCTGATATA

ACCTGTGATGATCCCCCAGATATCTCTCAGGATGGTAGTAGACTCTGGGCTATCGATAAA

CCCAACATTGCTCAAACGCCTCCTGGGTGGGAAAGGGTGCTCAAGATCAGAGGTGAAGGA

AGCACCAAATTCGCAGATGTGTATTATGTTGCACCATCCGGAAAGAGATTCCGTTCAACG

ATCGAGGTCGAAAAGTACCTATTTGAAAATCCAGAGTACATTAGACAAGGGGTAACTTTA

TCTCAGTTTTCTTTCCAAACCCCAAGGCCTTTGCAAGAGAATTATTTAAGAAAGCGTCCT

CCACGTGTGATGAATGGAAATGGGGGTGCTGGTTCGGGAACTCCTCCTGAACCTGTTGAT

GTGAATCCGTTGTCTTGGGCGGAACCCGCCACTCGGCATGATATGCTCCTTGGTGAGTCG

ACACCACCTGTGAGGAAAAGAAAGAAGGTGCCGAAGCCGCCACCCAAGGAAATGAGCAGT

GGCCCATTGTTCAATCTGCCACGGAACTTCTTGGAAACTCAACAAGACGGTCGTGCTTCT

CCGTATGAGTTGTGAGCTGCCGTGCCACAGGTTTGCATACATGACATTAGGTACGTTGTG

GATTTCGTGAGATCTCTTAAAAGACAATGTGTATCTGAGATGTCTGCTTTGGAATTTTAC

TAAAACTTGGATGTAAGTTATATATGGTAGGGCAGTAGAGCCAGACTCTTAAATGTTTCT

TAGTACTAAAGCTAATCCCCAGCCGGTTGTTATCTTGTGTTTTGCTGAGCTATATGGTGA

ATTACTTTAAAAAACCCGAGGACACTGTTCCAGTTATTTTGATTTGCAGTCCCCAAAAGG

TACACACCATCTACGGTTGTCGATACATATCAAATATGTAATTTGATGTCCTAACTGGAT

TTGCCGCCGCGCT

>Unigene0002540

TTGTTGATGTGAAATTCGATGCGATCGTTGTTTGATTTTTAGTAGGATCCTAAAATTAAG

CCCTAGAATGAGTATTACCGCCGAATCTGGTTTGGTAGCCGCAATATTTTCAACTTTTGT

TGATTTATTTGGAGATATAGTTCTATTTTCTGGGGAAATATCATGCTTGTGCTTGTATCA

GCTACATAGTTTAATCTTGCACTGGTTTGTTTTGATGTTTAGTTGATTGCTCTT

>Unigene0002541

CCACTGCGCTCACCAAGTCCGGCGGCGCCGACTTGTTGATGTGAAATTCGATGCGATCGT

TGTTTGATTTTTTAGTAGGATCCTAAAATTAAGCCCTAGAATGAGTATTACCGCCGGATC

TGGTTTGGTAGCCGCAATGTTTTCAATTTTTGTTGATTTATTTGGAGATCTAGTTTTATT

TTCTGGGGAAATATCATGCATGTGCTTGTTTCAGCTACATAGTTTAATATTGCACTGGTT

TGTGTTGATGTTTAGTTGATTGCTCTTTTTCTAATTTTTGATTTGTATCTTTGCCTTTGT

AAAACATTTGCTAGAGTAGTGGTGGCATTCTGTGC

>Unigene0002542

CAATTTCACCGACCGAGATTGGCTGAACACATCGTTTCTGTTCTACGACGAGGACGCTCA

GCTGGTGAGGGTCAAGGTCAGTGACTGCCTCGACGCCAGCAAGATGCGTTACACCTATCA

GAAGGTCGACGTACCATGGTTGACTTCACGGCCGACGTCGGCGACGGCGAATAAGAGAAC

CCTCTTGACAAAGAAGCCCGCCACAACAACTTCCACGACCGTCAAGTTTCCTCTGGTGCT

GAAGAAAGC

>Unigene0002543

ACCACGGCAACATCGACCGCATCTGGGACGTCTGGCGTGGACTAGGAGGCAAGCGGACCA

ACTTCACCGATCAAGATTGGCTCAACACATCGTTCCTGTTCTACGATGAAAATGCTCAAC

TGGTGAAGGTCAAGGTCAGTGACTGCCTCGACGCCAGCAAGATGCGTTACACCTATCAGA

AGGTCGACGTACCATGGTTGACTTCACGGCCGACGTCGGCAACGGCGAATAAGAGGACCC

TCTTGACAAAGAAACCCGCCACAACAACTTCGACGGCCGTCAAATTTCCTCTGGTGCTGA

AGAAAGCTGTTAGCATAAACGTGAAGAGGGAGAGGGTGTCGAGGAGCGGGAACGAGAAGG

AGAAGGAGGAGGAGGTG

>Unigene0002544

CCCCGCCCCCTCCCGATTTCCAACAGAAGGGTAAGGTCTTCGTCGGCAACCTGCCGCTCT

GGATAAAAAAACCGGACATCGCCGAGTTCTTCCGCCAGTTTGGGCCGGTCAAGAACGTGG

TCCTCATCAAGGGCCACGATGACCCGGAACGGAACCTCGGATACTGCTTTGTTATCTACG

GTGGTCCGACTGCGGAAGAGGCGGCTGCGCGGGCGGTGGAGCTCGATGGCGCGGAGTTTC

ACGGCCGGCCGCTGACGGTGAAGTTGGACGATGGGAGGCGGTTGCGGACCCGGGCGGATG

AGAGGGCGCGGTGGGTGTCGGGGGAGGGGAAGAGAGAGTATAGGTCAAAGTGGCATGAGG

AGAGGGATATGGACTGTCAGAAGTTTAGGAAGGTGATTGAGACGGAGCCGGAGAATTGGC

AGGCTGTGGTCTCAGCATTCGAGAGGATTCCAAAGCCGTCCCGAAGGGAGTTTGGATTGA

TGGTTAACTATTATGGAAGGAGGGGGGATAAGCATCATGCTCGTGCTACATTTGAGAATA

TGAGAGCACGAGGAATAGAGCCGAGTTCATATGTGTATACCAACCTGATTCACGCTTATG

CAGTTGCTAGAGATATGCGAGGAGCTATGTCTTGCTTTGAAGAAATGAAAGAAGAAGGAA

TTGAGTCAACTGTGGTGACATATAGCATCCTTATTGCTGGCTTTGCTAATATTGGTGATT

CTGAATCCGCAGATAATTTGTTTAAGGAGGCAAAACGGGAGCTCACATTGTTGAATGCCA

TTATATATAGCAACATAATATATGCTCATTGCCAAACTGGTAACATGATTCGAGCTGAAG

ACTTAGTGAGGGAAATGGAAGAGGAAGGGATTGATGCACCTATCGATTTATATCACACTA

TGATGGATGGTTACACAAATATTCGTAACGAGGAAAAATGCTTGATAGTGTTTGACAGAT

TGAAGTACTGAAGATTCATCAATAACCACCTATTTCTTCAGACTTCTTGACCCTTTCTTT

TACGTCACCCCCTTACTGCCACTTCAACCATTCAAAGATTGCATCAATGAATATCAGCTT

GGGAAGCAGCTGCTTATGTGCCTTTGGGGATTGATGATCAGTAAATTCAGCCAACACAAG

ATTTGTTTCTCTCACATTTCCATCACCATGGCTTTATCTCCACCCCGGAATGCGGTTTCA

CACCTTCAATTATCAGTTATGGGTGTCTGATAAATTTGTACACTAAGATTGGGAAGGTAT

GTAAAGCCCTTGAGATCAGCAAAACAATGGAAGCATTTGGCACAAAACATAATAGCAAAA

CTTACTCAATGTTAATTAACGGATTCATCCAGTTGAATGATTATGCAAATGCTTTTAGTA

TTTTCGAGGAAATGCTGAAATCAGGGTTGAAACCCGATGCTGTCCTTTATAACATTATAA

TCAACGCTTTCTGTAAAATGGGCAATATCGACCGAGCAATCCGCATGGTTGATGAGATGC

AGAGAGAGAGGCACCGACCTTCACCTCGAACATTCAGGCCCATCATATATGGCTTTGCTG

AAGCTGGAGAGATGAAAAGAGCTTTGGAGATTTTTGATCTGATGAGGCGTGTTGGATGTG

TACCAACTGTAGAGACTTATAATGTTCTTGTTCTTGGTCTTGTACGAAAGCATCAGATGG

AGAAGGCTGTTTCAATCATTGATAAAATGTCTCTAGCGGGAATCAATCCAAATGAGCATA

CTTACACGACTATCATGGAGGGCTATGCTGCTGTGGGTGATACGGGTAAAGCTTTTGAGT

ACTTCACAAAAATTAAGGAGGGAGGTCTTAAGCTTGATGTATTTACCTATGAAGCATTAC

TCAAGGCATGCTGCAAGTCAGGTCGAATGCAAAGTGCTTTAGCAGTCACAAGGGAAATGA

GTTCTCGTAAGATACCCAGGAATACATTTGTCTATAACATACTAATTGATGGATGGGCTC

GAAGGGGTGATGTATGGGAGGCTGCTGATTTGATGCAACAAATGAAACAGGATGGAGTTT

TACCTGACATCCATACTTACACTTCATTCATTAATGCATGCTGCAAGGCCGGTGATATGC

TGAGAGCTACAAAGATGCTTGATGAAATGAAAGCTTCTGGTGTAAAGCCTAACATAAAAA

CATATACCACTTTGATTAGAGGATGGGCAAGGGCTTCGCTCCCTGAAAAAGCTTTGAGGT

CTTTTGAAGAGATGAAATTAGCAGGACTGAAGCCTGACAAAGCTGCGTACCATTGCTTAA

TGACTTCTCTTCTCTCAAGGGCAACCATCGCCGAAGAGTACCTCAGCTTAGGGATTTTAG

GTGTTTGCAAAGAAATGGTCGAACGCGAGTTAACCGTCGATATGGGAACTGCAATTCACT

GGTCCAAGAGTCTCCGCAAGATCGAGAGAACAGGTGGTGAACTCACTGAAGCACTCCAGA

AAACTTTTCCTCCAGCATGGAACTCACATGAATTACCAGAGGGAACCTCTGAGCAAATGA

GCAGTAGTGATGGCAATGTTGGTGTCTGTAGCGAGGACGATGACATTAGTATTGACATAA

TAAATTAATAGACAACAATTTGAGTTAATATCAGTTGATTCAGATGTTAATCTCAGAAAC

CCTGATTTGAATTAGGGTCTCAGTTCCATTCGTTGCTTATCAAAATTTGAAGATTGTCAA

AGTTCGATCATATAAGCATCAGTCTGAGCAGGTATGTGCCTCAAGGCGTCAAAATGTGAA

GCTGAAGGTTTTACTGATTTGTATACAAGTGGGGGAGTTGCGGGTGCTTCTATCCACGAT

GGCCCTTCAATGGAGCGGAGCTTATCATGTACGTATCTTTTCTGTATACTAATGAGAATT

AATGGGCATTGAAATTCTGTTGGGGCTTGTAGATTAATTGTAGGGGTGGGAGGTTACTAA

TTATACTTGAAGGCAACCCCAGATTTGAATTTGAAAAATATCACCTGCACTCTTTTCATC

CATAAAGGATCAATTAAAGGATGTTCCTGAATGTTCTTAATGGCAGTGATGAATTCTCAT

TCAAGTTGGATTGGCATCAACACGTCAACCTCCATGGTAAATGCTCACATATCTCAATGG

ATTTCATACCACTATTGAATTTTAATTATCAGATCCTCTCTGTAAAATGACAAGAAAATT

AGATTATCTTAATTAATCAGTAGTTATTCATGCATATAGAGATTAGATGTTTATGTGTGG

ATTTGTATCTATTATGATATTTTCAAAACAGCCATCTTTGTTATAAAGTTTGTGTTTCTA

TGTTATCAAGATACATATATCACTATTACTATGTGATAGATTA

>Unigene0002545

GAGGGAGAGGGTGTCGAGGAGTGCGGATGAAAAGGAGAAGGAGGAGGAGGTGCTGGTGGT

GGAGGGGATTGAGGTTGAGAAGGGCGTGTATACCAAGTTCGACGTTTACATCAACGGCCC

GGCAGAGGGGGTGACGCCGGCAGCGAGTGAATTTGCCGGGAGCTTTGTGAGCGTGCCGCA

CCGGCACCAACACTCGGGTAAGTTGAAGACGTCATTGAAGCTGGGAATTACTGATTTGTT

GGAGGATCTACAGGCCGAGGGGGATGAGAGCATTGTTGTCACGCTGGTCCCGCGCAGTGG

GAAGGGGAAGATCAAGGTTGGGGGGATCTCTTTTGAGTTTGCCTCGTAGGTTTACTTACA

CTCCCCGATAGCCTTTCTATTAAATAAAAACCTTCCAATATTTTAAAATAAACTTTGCAA

ATCTGTATACAGAGAAGAGGTAAATTCTCTGTCCAAATAAGCCTGTTTGGCTCTGTAAGT

TAAAATCATGTTGTATTGTGTGTTTTTTCCTCTTAAAGTTAACTTTGCTTATTGGGTGAA

ATTCCTTAGCTGGTGTTGTAATGGATAATAAAAGGGCTATTAGGTGTTCTATTCTTCTCT

GGTGTACATGGCCCAATTTGGATTTACTTTAAAAGTTTTAAATTATTATTTTATTTTCGT

CTTA

>Unigene0002546

CTCAACTCTCAGTTTCTCTTCTACGACGAGAACGCTGACGTGGTGAAGGTCACCATCAAG

GACTGCCTGGACATGAGTAAGTTGCGTTACTGTTACCAGAAGGTAAGCAACCCATGGATC

AACTCACGACCAAGCCCGGCTGTCACCGTGAAGAAGAGAACCGCCACCGTCAAGAAGCCC

CCCACAGCCGGTGAAATCAAGTTCCCAATAATACTGGACAAGCCGGTGAGTGTGACGGTG

AGTAGGAAGCGGCCGTCAAGAAGTCAG

>Unigene0002547

CCAACAATGGCAGCCTTCCCATGCAACTCTTTCCCCTTGGCTTCGAGGGCACTTCCCATC

GTCTCTCTCAAGAGACCCAAACCACGCCCTACCATTACCTGTAAAGCCAACGGCGCCGAC

GATAAGCTCGATCGCCGTGACATCCTCATCGGTCTCGGCGGCCTTTATGGCCTCGGCACC

TCCCTCCCAGCCCTCAGCCTACCCATCGAAGCACCAGACCTCTCCCAGTGCGGCGAACCC

GACCTCCCCACTGGCGCCAGGCCCATCAAATGCTGCCCACCCCCCTCCTCTAAAGTCAAA

GACTTCAAGTTCCCCTCCCCATCCGCCCCACTCCGCATCCGTCCCCCCGCACACGCCGTC

GATGCCGAATACATTTCTAAGTACCAAGAGGCCGTATACAAGATGAAGCTACTCGAGCCT

ACCGATCCCCGTAGCTTCAGCCAGCAGGCAAGGGTTCACTGCGCCTACTGCGACGGCGCC

TACAATCAGGTGGGGTTCGAGGACTTGGACCTGCAGGTCCACTTCTCATGGCTCTTCTTT

CCCTTTCACCGCTTCTACCTCCATTTTTATGAGAGGATCCTCGGAAGCCTCATCGGCGAT

GACACCTTCGGGCTGCCGTTCTGGAACTGGGACCACCCCGCCGGAATGACACTACCTCTT

ATGTACACAGACAAAAATAGTCCACTGTACGATACTCTACGGGATGTCAACCACACGCCA
[truncated: 52,881,123 more chars]
